# Different classes of small RNAs are essential for head regeneration in the planarian *Dugesia japonica*

**DOI:** 10.1186/s12864-020-07234-1

**Published:** 2020-12-07

**Authors:** Zhonghong Cao, David Rosenkranz, Suge Wu, Hongjin Liu, Qiuxiang Pang, Xiufang Zhang, Baohua Liu, Bosheng Zhao

**Affiliations:** 1 School of Life Sciences, Shandong University of Technology, 266 Xincun Western Road, Zibo, 255049 People’s Republic of China; 2 Institute of Organismic and Molecular Evolution (iOME), Anthropology, Anselm-Franz-von-Bentzel-Weg 7, Johannes Gutenberg University, 55099 Mainz, Germany

**Keywords:** *Dugesia japonica*, Head regeneration, Micro RNAs, Piwi-interacting RNAs, tRNA fragments, miR-124

## Abstract

**Background:**

Planarians reliably regenerate all body parts after injury, including a fully functional head and central nervous system. But until now, the expression dynamics and functional role of miRNAs and other small RNAs during the process of head regeneration are not well understood. Furthermore, little is known about the evolutionary conservation of the relevant small RNAs pathways, rendering it difficult to assess whether insights from planarians will apply to other taxa.

**Results:**

In this study, we applied high throughput sequencing to identify miRNAs, tRNA fragments and piRNAs that are dynamically expressed during head regeneration in *Dugesia japonica*. We further show that knockdown of selected small RNAs, including three novel Dugesia-specific miRNAs, during head regeneration induces severe defects including abnormally small-sized eyes, cyclopia and complete absence of eyes.

**Conclusions:**

Our findings suggest that a complex pool of small RNAs takes part in the process of head regeneration in *Dugesia japonica* and provide novel insights into global small RNA expression profiles and expression changes in response to head amputation. Our study reveals the evolutionary conserved role of miR-124 and brings further promising candidate small RNAs into play that might unveil new avenues for inducing restorative programs in non-regenerative organisms via small RNA mimics based therapies.

## Background

The limited regenerative capabilities of most vertebrates including humans, particularly regarding damage to the central nervous system (CNS), call for effective therapies that foster the replacement or healing of wounded tissues. Therefore it is imperative to understand the molecular mechanisms of regeneration and signal networks that induce and promote this complex process.

Planarian flatworms possess an extensive potential of regeneration and are one of the few animal species that can easily regenerate their head after decapitation including the complete neoformation of a functional brain within 7 days [1–4]. Despite their relatively simple morphology, planarians have a highly structured CNS featuring a true brain consisting of a large number of different neuronal cell types [5, 6], a well-defined adult stem cell population comprising roughly 30% of all CNS cells and a clear anterior-posterior (A/P) polarity that is maintained during regeneration [7–9]. Moreover, planarians share more genes with vertebrates than other popular model organisms such as *Drosophila melanogaster* or *Caenorhabditis elegans* do [10], and many genes expressed in the planarian CNS are highly conserved in humans [11].

Recent work has shown that a planarian reaches three main milestones to restore its head. First, it must determine that it is missing a head rather than a tail. Second, the anterior pole must be formed at the anterior tip. Third, the missing tissues must be reconstructed [12]. This process involves two systems, i) Pluripotent neoblasts that can generate new cell types and ii) muscle cells that provide positional instructions during the regeneration process. The subepidermal planarian muscle tissue is a major source of the positional information that orchestrates tissue turnover and regeneration programs [13, 14]. During regeneration, *wnt1* is expressed in the posterior pole [14–16] and knockdown of Wnt signaling results in animals that regenerate heads at all blastemas, while animals with constitutively active Wnt signaling regenerate tails rather than heads [17–19], and the polarized activation of *notum* in muscle cells at anterior-facing wounds in turn steers Wnt function [15, 16]. In addition to Wnt signaling, the hedgehog (Hh) signaling pathway represents another essential regulator during head regeneration, and animals with defective Hh signaling show severe A/P patterning defects, completely fail to regenerate heads, or ectopically regenerate tails at anterior-facing wounds [20, 21]. The anterior regeneration pole is formed by a cluster of collagen^+^ cells which co-express *notum*, *follistatin* (*fst*) genes and the transcription factors *foxD* and *zic1,* and knockdown of these anteriorly expressed genes results in impaired head regeneration, yet without induction of ectopic posterior markers at anterior-facing blastemas [22–24]. Finally, a number of other factors such as CHD4, p53, and MEX3, coe, lhx1/5–1, pitx, klf, and pax3/7 have been shown to be required for head regeneration and regeneration of multiple neuron subtypes [4, 25–27]. However, the factors that regulate the spatiotemporal expression of these genes, which are crucial for the proper patterning of the planarian head, are not known.

Micro RNAs (miRNAs) are small, non-coding RNAs that act in post-transcriptional gene regulation and play important roles in virtually all biological processes including stem cell self-renewal, proliferation and differentiation [28, 29] and a number of studies have shown that miRNAs are critical regulators of regeneration [30–33]. Contrasting their functional importance, our knowledge of miRNA expression patterns and function during head regeneration in planarians is far from being complete [34–36]. In addition, our current understanding is based on experiments in the planarian *Schmidtea mediterranea* (*S. mediterranea*), presuming but not having any evidence for an evolutionary conservation. However, finding evolutionary conserved mechanisms is vital when the long-term objective of research that uses animal model systems is to gain insights that in the end are applicable to humans. The planarian *Dugesia japonica* (*D. japonica*) possess equally impressive capacities to reliably regenerate a head including a functional brain within days, and, although the genus *Dugesia* represents the closest known relative to the genus *Schmidtea*, both taxons have evolved independently for at least the last 43 million years [37]. Hence, mechanisms that are not conserved across these two planarian species will, apart from being interesting in an evolutionary context, likely have no implications for human therapeutics.

In this study we monitor small RNA (sRNA) expression profiles during head regeneration in *D. japonica* applying state-of-the-art high throughput sequencing technologies. We identify homologous and *Dugesia*-specific miRNA genes and provide a detailed analysis of the major sRNA classes in *D. japonica*. We describe the dynamic sRNA expression patterns during head regeneration and compare the observed patterns with that of *S. mediterranea* with the aim of identifying conserved regulatory regimes. Finally, we validate the functional importance of selected upregulated sRNAs including miRNAs and tRNA derived fragments (tRFs) by demonstrating that their knockdown in head regenerating animals severely impairs regeneration, resulting in eye-less heads, cyclopia and other phenotypic defects.

## Results

### Detection of 4 novel *D. japonica* miRNA genes

For each library, 8.6 to 14.7 million reads were successfully mapped to the genome of *D. japonica*. Based on our small RNA transcriptome data we identified 36 miRNA genes with ShortStack [39], 32 of which have homologs in the planarian *S. mediterranea* while the remaining four miRNA genes lack sequence homology to other annotated miRNA genes in miRBase, thus representing either novel miRNA genes aquired on the lineage of *D. japonica*, or alternatively ancestral miRNA genes that were lost in *S. mediterranea* (dja-miR-novel-1/− 2/− 3/− 4, Supplementary Table S1). We checked for a significant enrichment of specific GO terms assigned to the putative targets of the novel miRNAs and found that dja-miR-novel-1 targets (*n* = 205) are particularly enriched for genes involved in apoptosis and regulation of JNK cascade. Dja-mir-novel-2 targets (*n* = 136) are enriched for genes involved in membrane organization and dja-mir-novel-3 targets (*n* = 105) show an enrichment for genes connected to photoreception. For dja-mir-novel-4 we did not observe any enrichment, possibly due to the high number of predicted targets (*n* = 715, Supplementary Table S2).

### miRNAs, tRFs and piRNAs represent the major sRNA fractions in *D. japonica*

**Fig. 1:**
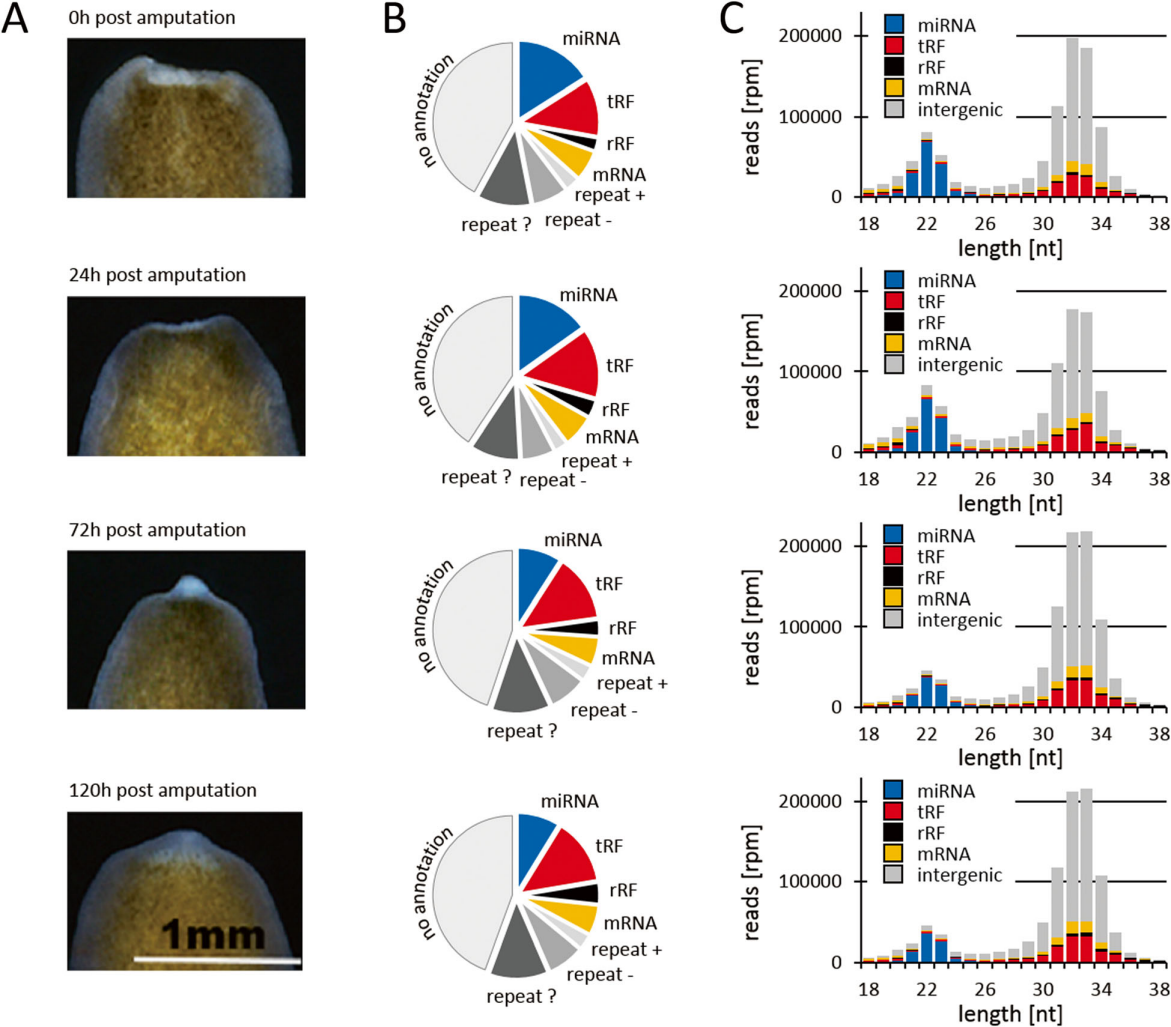
sRNAs expressed in the course of head regeneration. **a** Progression regeneration after head amputation. **b** Fractions of different sRNA classes. tRF: tRNA fragments, rRF: rRNA fragments, repeat +: repetitive sequence in sense orientation, repeat -: repetitive sequence in antisense orientation, repeat?: repetitive sequence with unknown orientation (from unclassified repeats). **c** Sequence read length distribution of different sRNA classes

Generally, the annotation of small RNAs using the unitas annotation pipeline [41] yielded similar fractions of small RNA classes across the different time points of sampling after head amputation (Fig. 1a and b, Supplementary Table S3). While 41–45% of the mapped reads could not be assigned to any coding- or non-coding RNA class, 20–23% of the reads mapped to repetitive sequences of the genome. Further 12–14% of the reads represented fragments of tRNAs. miRNAs made up 9–16% of the mapped reads (Fig. 1b). The sequence read length profiles revealed two distinct peaks around 22 nt and 32/33 nt. As expected, we found that miRNAs represent the main fraction of reads within the size range of 20 nt to 24 nt. However, most sRNAs across all libraries fell in the size range of 30 nt to 34 nt, including the majority of tRFs, most of which derive from the 5′ end of mature tRNAs. In addition and even considerably exceeding the number of tRFs, sRNAs derived from intergenic regions of the genome make up the large proportion of 30-34 nt sized sRNAs (Fig. 1c, Supplementary Table S4).

**Fig. 2:**
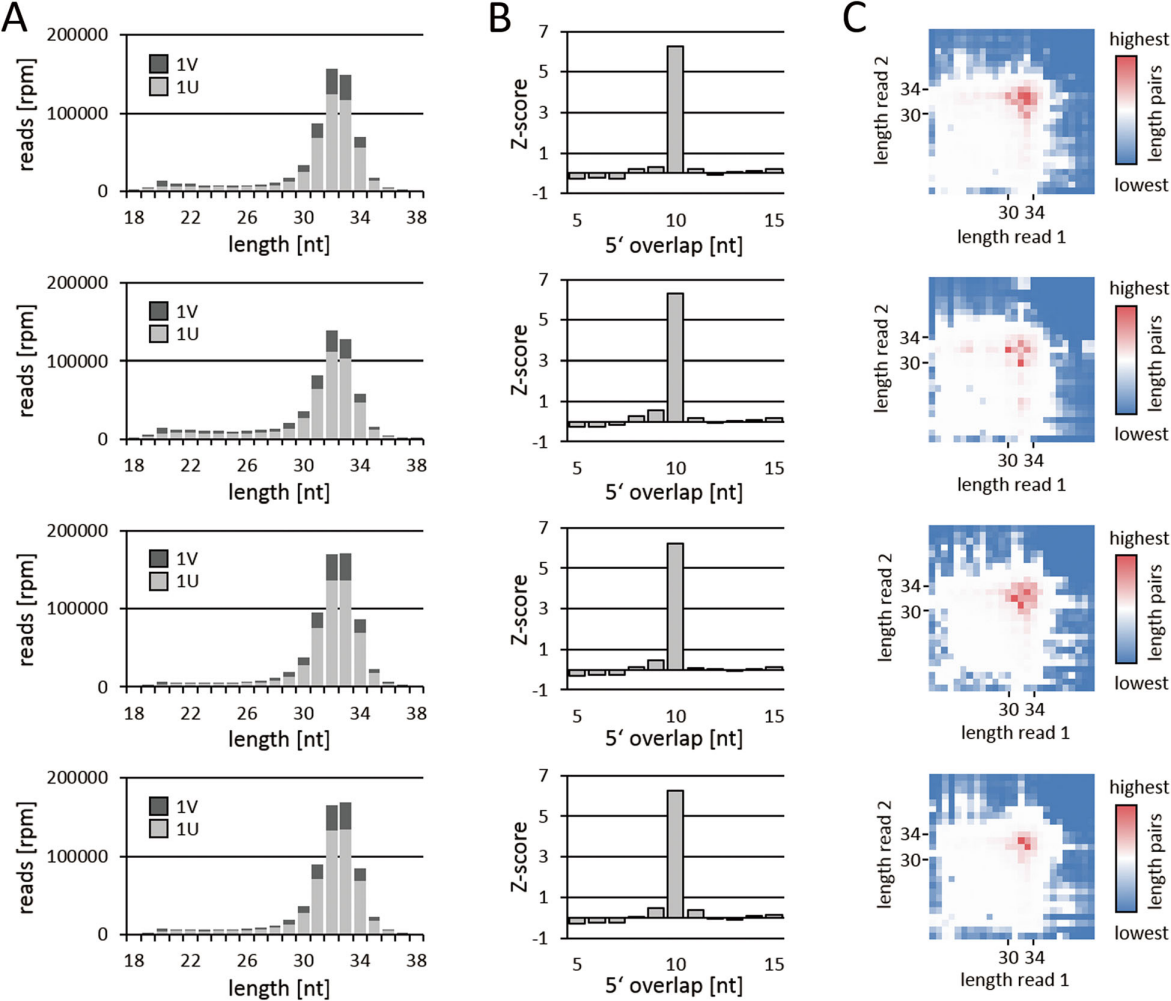
Characterization of putative piRNAs. The four rows represent the different sampling time points 0 h, 24 h, 72 h and 120 h post amputation from top to bottom. **a** Share of reads with 5′ U (1 U) within the fraction of reads that did not match any other class of coding or non-coding RNA. **b** Z-scores for different 5′ overlaps of mapped sequence reads. **c** Ping-pong-matrices show the most frequent length combinations of two sRNA reads that form a ping-pong pair (10 nt 5′ overlap)

Based on previously published results we assumed that this fraction represented piRNAs and we checked for typical piRNA characteristics [45, 46]. First, we noted a clear bias (77–78%) for uridine at the 5′ end (1 U) which is typical for primary piRNAs and distinguished this population from the other annotated sRNAs in *D. japonica* (Fig. 2a, Supplementary Table S5). In addition, considering the sub-fraction of repeat derived sequence reads, we observed a clear bias for sequences antisense to repeats (2.4–2.5-fold) suggesting a role in transposon silencing (Supplementary Table S6). Next we analyzed those reads that mapped to complementary strands of the genome and found a significant enrichment for 10 nt 5′ overlaps (ping-pong signature), implicating the presence of primary and secondary piRNAs (ping-pong piRNAs, Fig. 2b, Supplementary Table S7). To verify that the observed ping-pong signature is generated by 30-34 nt sRNAs, we checked the size of sequence reads that form ping-pong pairs and indeed found that the majority of ping-pong pairs combines sequence reads with a length between 30 nt and 34 nt (Fig. 2c, Supplementary Table S8). Finally, we used proTRAC to identify genomic piRNA clusters which in total yielded 283 distinct genomic loci that, while making up only 0.16% of the *D. japonica* genome, comprise on average 5% of the putative piRNAs which is very similar to findings regarding piRNA clustering in *S. mediterranea* (Friedländer et al. 2009, Supplementary Table S9). Together these results strongly support our assumption that the fraction of intergenic 30-35 nt sequence reads represents genuine piRNAs and we will bona fide refer to these sRNAs as piRNAs in the following. Noteworthy, given the fact that most predicted clusters are less than 10 kb in size with the largest cluster reaching 20 kb, we cannot rule out the possibility that many of the predicted piRNA clusters in fact represent dispersed piRNA producing transposon copies. Therefor we will use the term piRNA cluster in sense of a piRNA producing locus in the following.

### Dynamic sRNA expression patterns during head regeneration

**Fig. 3:**
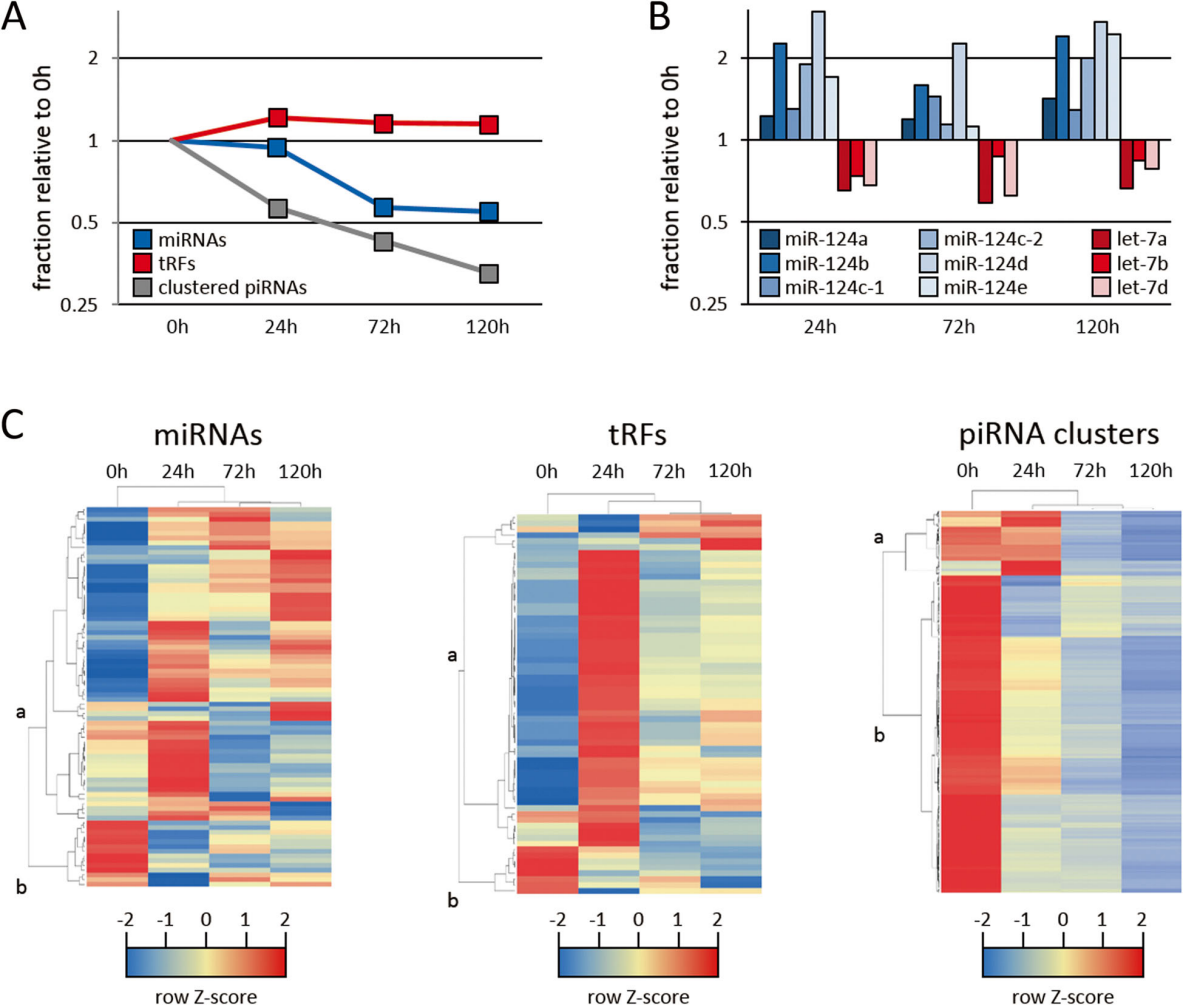
Dynamic expression of sRNAs during head regeneration. **a** Expression changes of different sRNA classes in the course of regeneration. **b** Upregulation of mir-124 family and downregulation of let-7 miRNAs in regenerating animals. **c** Columns in heatmaps represent different sampling time points. Rows represent miRNA genes, source tRNAs and piRNA cluster loci, respectively

We found that head amputation induced a global shift regarding the relative abundance of miRNA, tRFs and clustered piRNAs (Fig. 3a). While the relative abundance of miRNAs substantially decreases from 16.0% at the time of amputation to 8.8% 120 h post amputation, the abundance of tRFs increases moderately from 11.8% to 13.6%. At the same time, although the overall fraction of putative piRNAs remains constant, the fraction of piRNAs arising from piRNA clusters drops from 8.9% to 2.9% (Fig. 3a). Since miRNAs, tRFs and piRNAs arise from different and largely independent pathways, we wanted to know whether the changes in their abundance are due to general effects regarding the particular biogenesis pathway, or alternatively are caused by more complex alterations in the composition of each small RNA pool, possibly representing a directed response to head amputation. In the first case we would expect all sRNAs of a particular class to show roughly the same degree of up- or down-regulation, while in the latter case each individual sequence would exhibit its individual expression course. In favor of the latter alternative, the expression profile for each sRNA class reveals that not only the overall abundance is subject to changes during head regeneration, but also the respective sequence composition (Supplementary Table S10 and S11). Regarding miRNAs, we observed a consistent up-regulation of miR-124 family members following head amputation, while let-7a, let7b and let7d become less abundant (Fig. 3b). Similarly, different tRFs and piRNA clusters show contrary changes regarding their relative expression. Noteworthily, the hierarchical clustering pattern for the different sampling time points (0 h, 24 h, 72 h and 120 h post amputation) reveals that the global miRNA-, tRF- and piRNA cluster expression profile in regenerating animals (24 h, 72 h and 120 h post amputation) is more similar to each other compared to that observed in animals directly after amputation (0 h, Fig. 3c). In each case we can distinguish two groups of sRNAs based on hierarchical clustering which we will refer to as group-a and group-b. While group-a sRNAs predominantly show an increased expression in regenerating animals, particularly in the early phase of regeneration 24 h post amputation, group-b sRNAs are less abundant following head amputation. As we would expect small RNAs that are involved in the process of head regeneration to be upregulated in regenerating animals, we assumed group-a sRNAs to be critical for regeneration. To gain support for this assumption, we performed antisense oligo-DNA mediated knockdown experiments focusing on selected sRNAs.

### Knockdown of different sRNAs induces severe regeneration defects

**Fig. 4:**
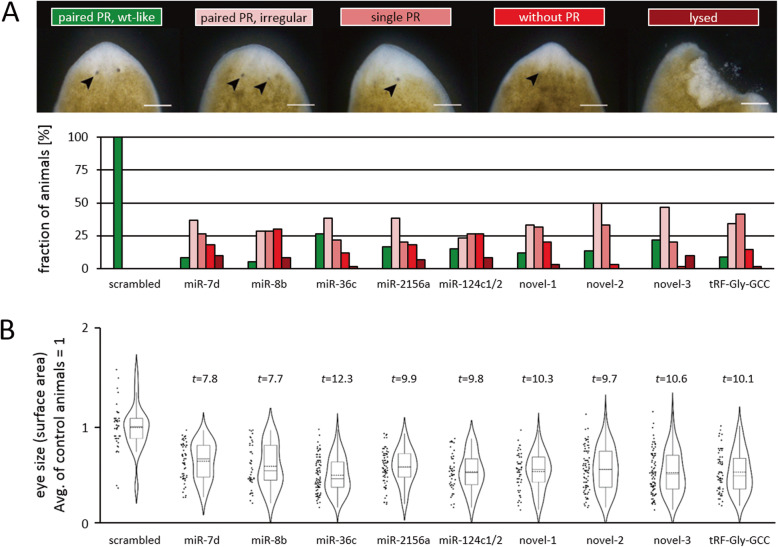
Knockdown of different sRNAs leads to impaired regeneration. **a** Fraction of observed phenotypic defects during head regeneration per knockdown condition. PR = photoreceptors. Paired PR were considered irregular if PR size and/or distance to each other was beyond the range observed in control animals. Paired PR were also considered irregular if they lacked the surrounding white region. wt: wild type, **b** Significantly reduced eye size in each knockdown condition. Solid lines within boxes indicate median values, dotted lines within boxes indicate mean values. *t*-values are derived from a *t*-test for two independent means (2-tailed hypothesis) comparing scrambled versus sRNA in question. Scale bars: 250 μm. Corresponding *p*-values are < 0.0001 in each case

To check whether the observed upregulation of specific small RNAs following head amputation is either a symptomatic consequence, or alternatively orchestrates the process of head regeneration, we performed knockdown experiments for selected small RNAs. Strikingly, while control animals transfected with scrambled oligomeric DNA regenerated normal heads and photoreceptors (PR) throughout, the vast majority of animals treated with 400 μM anti-sRNA oligomeric DNA showed various types of PR defects, including the complete absence of PRs and cyclopia. In addition, even when the animals regenerated two PRs, these were often small and/or exhibited merely the light capturing pigment cells while lacking the white region around the pigment cells. A smaller number of animals showed lesions in the head region and subsequently lysed (Fig. 4a).

Whenever animals regenerated two PRs, we measured PR size (surface area) and the PR distance to each other, relative to the head diameter. For each knockdown condition, we found that PRs were significantly smaller (*p* < 0.0001) and reached only 52–66% of the average surface area of control animals (Fig. 4b). Regarding the PR distance to each other we did not notice a significant shift of the mean distance compared to control animals, but, however, found that the variance was increased which frequently resulted in PRs with an exceptionally high or low distance to each other.

To check if regeneration-associated genes are potentially targeted by those eight miRNAs whose knock down resulted in impaired regeneration, we predicted target sites on the entirety of *D. japonica* mRNAs annotated with maker [47] using miranda [44]. We then compared the number of target sites on regeneration-associated genes (foxD, wnt1, beta-catenin-1, APC, NOTUM, CHD4, coe, pitx, patched) with the number of total mRNA target sites. We repeated this procedure with a control set including the eight most abundant miRNAs (bantam-a, miR-13, miR-17b, let-7a, miR-1c, lin-4, miR-281, let-7b) that did not show enrichment after head amputation. However, although we identified 33 putative target sites of phenotype-associated miRNAs on regeneration-associated genes, we found no evidence that these miRNAs target regeneration-associated genes more frequently than other genes, compared to the control set of miRNAs.

## Discussion

Owing to their outstanding regenerative capabilities, planarians represent an important model organism to study molecular pathways connected to the process of regeneration. However, to assess whether findings from the widely used model *S. mediterranea* are likely applicable to other animals or not is difficult, since we often lack information on the evolutionary conservation of molecular pathways. As a first attempt to close this gap and to extend the currently available data [48, 49], we analyzed the changes in sRNA expression in response to head amputation in *D. japonica*. We show that while the miRNA fraction as a whole shrinks after head amputation, the expression of specific miRNAs increases. Knockdown of these miRNAs induces severe impairments of regeneration, demonstrating the functional importance of these miRNAs for the process of head regeneration.

Recently, it has been shown that the miR-124 family is crucial for regeneration of the brain and visual system in the planarian *S. mediterranea*. Remarkably, knockdown of *Dugesia* orthologs dja-miR-124c1 and dja-miR-124c2 resulted in similar phenotypes including head lesions, absence of eyes and cyclopia, suggesting a deeply conserved role of this miRNA in the process of regeneration. Interestingly, miR-124 is also highly expressed in human brain tissues where it functions as a master regulator of neurogenesis [50]. Further, experiments in Parkinson’s Disease mouse models revealed that injection of miR-124 alleviated neurodegeneration and promoted neurogenesis [50, 51]. Given the deeply conserved role of miR-124 in regeneration and neurogenesis across distantly related species like planarians and humans, a systematic functional examination of other small RNAs involved in planarian head regeneration appears promising, all the more, as regenerative therapies that base on treatment with miRNA mimics show encouraging results in the mouse model [52, 53].

Surprisingly, all of our knockdown experiments yielded very similar results regarding both the quality and quantity of phenotypes. Since these sRNAs lack obvious 5′ homology and thus likely target different sets of genes, we presume that they act critically in very early stages of regeneration, where any kind of dysregulation results in similar final defects.

Noteworthily, we show that sRNAs that are critical for planarian head regeneration not only include miRNAs, but also small RNAs with yet widely unknown functional potential such as tRFs. A number of recent studies has demonstrated that fragments of mature tRNAs can be more than pure degradation products, being involved in processes such as transposon regulation, global translational repression, sequence specific gene regulation, response to environmental stress and transgenerational epigenetics of metabolic disorders [54–63]. Interestingly, specific tRFs, including fragments of tRNA-Gly-GCC, have been linked to neuro-developmental disorders by inducing a cellular stress response in mice [64]. By showing that 5′ tRF-Gly-GCC is upregulated in regenerating *D. japonica* animals, and that knockdown of 5′ tRF-Gly-GCC induces impaired head regeneration, we add yet another functional dimension to the biology of tRNA derived fragments.

Regarding a possible involvement of the piRNA pathway in regeneration, we found that the expression of piRNA clusters is greatly reduced upon head amputation. PIWI proteins represent markers for somatic stem cells in deep-branching metazoans [65, 66] and it was recently shown that a nuclear PIWI is required for cell differentiation in *D. japonica* by silencing transpsons [46]. We thus speculate that the downregulation of piRNA clusters reflects a shift from stem cell self-renewal in pre-amputation individuals, towards cell proliferation and differentiation in regenerating animals, possibly yielding a regeneration-specific population of piRNAs that is loaded onto PIWI proteins in regenerating animals. In fact, evidence for functional roles of the piRNA pathway in neurons of different invertebrate species exists [67].

## Conclusions

In summary, our study confirms the deeply conserved role of miR-124 in the process of regeneration. It further reports a number of sRNAs, including 5′ tRF-Gly-GCC, whose knockdown produces pursuant phenotypic defects, rendering these sRNAs attractive candidates for further functional investigations regarding their role during regeneration in different taxa including mammals.

## Methods

### Housing conditions and regeneration experiments

Head regeneration experiments were carried out with an asexual monophyletic strain of *D. japonica* which was originally obtained from Boshan, Shandong province, China. The animals were maintained at 21 °C in mountain spring water and fed with beef liver paste once of week ad libitum. Animals were starved for 1 week prior to all experiments. Twenty animals were divided equally into four groups, each group include five animals. Each animal was decapitated just behind the auricle, and transferred into fresh water to regenerate. Different regeneration group animals were collected at 0 h, 24 h, 72 h, and 120 h after amputation, collected animals were homogenized in TRIzol (Invitrogen) immediately. Every five animals constitute a mixed pool of RNA library for RNA sequencing. All experiments involving animals in this study were approved by the Animal Welfare Committee of the Shandong University of Technology.

### RNA extraction, library preparation and sequencing

Total RNA was extracted with TRIzol (Invitrogen) according to the manufacturer’s instructions. Washed RNA was dissolved in 20 μl nuclease-free water and the concentration was determined using the NANODROP 2000C system (Thermo scientific).

Illumina’s TruSeq RNA Preparation Kit was used to purify 15-40 nt small RNAs from total RNA. Size selection and quality control were performed by BGI, Hong Kong. Libraries for each time point were sequenced on an Illumina HiSeq4000 sequencer (single-end, 50 bp). The raw reads were filtered according to the following criteria: 1) more than four bases had a quality score below 10 or more than six bases had a quality score below 13. 2) Reads containing homo-polymers, such as poly-A. 3) Reads with 5′ adapter contaminants or without 3′ adapter sequence. 4) Reads without insert tag. 5) Reads with a length below 18 nt. Sequence data can be accessed at NCBI’s Sequence Read Archive under the accession PRJNA549868.

### Genome annotation

In order to obtain a comprehensive set of reference sequences allowing to classify our small RNAs, we annotated the genome of *D. japonica* [38] (assembly Dj Genome ver1.0) using tRNAscan for tRNA annotation (Supplementary Table S12), rnammer for rRNA annotation (Supplementary Table S13), maker for prediction of mRNA including UTRs (Supplementary Data S1), and RepeatMasker as well as WindowMasker for the annotation of repetitive DNA (Supplementary Table S14). tRNAscan v.1.3.1 and rnammer v.1.2 were run with default parameters. For mRNA annotation with the maker pipeline we used available nucleotide and peptide sequences from *S. mediterranea* and *D. japonica* obtained from NCBI nucleotide and protein database. We conducted RepeatMasker v.4.0.7 annotation using *S. mediterranea* and ancestral repeat sequences using the option -slow and the cross_match algorithm. Non-redundant repeats with a minimum size of 50 bp annotated with WindowMasker v.1.0.0 were combined to a merged repeat annotation. For de novo prediction of miRNA genes we merged sRNA data from the different sampling time points and ran ShortStack [39] allowing 2 mismatches. The predicted miRNA precursor sequences were aligned to annotated miRBase stem-loop sequences with blastn and miRNA names were assigned according to the best given an E-value was below 10^− 6^. Otherwise the miRNA gene was assumed to be novel.

### Small RNA mapping and annotation

Clean sequence reads were mapped to the genome of *D. japonica* with SeqMap [40] allowing up to 3 mismatches. Subsequently we filtered the best hits for each sequence in terms of mismatch counts, allowing no more than one internal mismatch and two non-template 3′ nucleotides using the custom Perl script SeqWrap.pl. The obtained map files were used as input for sRNA annotation with unitas [41] based on the obtained reference sequences (see above) using the options -slow -pp. The amount of small RNAs derived from repetitive DNA was calculated using the custom Perl script RMvsMAP.pl. piRNA clusters were predicted separately for each dataset with proTRAC v.2.4.2 with the option -pimax 35 to account for the presence of piRNAs > 32 nt. The predicted clusters were merged as described in Jehn et al. [42].

### Differential expression analysis

Heatmaps for differential expression of miRNAs, tRNA fragments and piRNA clusters were created with heatmapper [43] applying clustering of both dimensions based on average linkage and using the Pearson distance measurement method. Colors refer to row Z-scores. Only miRNAs with at least 100 reads per million miRNAs, and tRFs with at least 100 reads per million tRFs were taken into account.

### GO term enrichment analysis

To check if miRNAs that are upregulated following head amputation, thus possibly playing a role in the process of head regeneration, we selected miRNAs that were found to be at least 2-fold upregulated upon amputation (*n* = 5) while having a minimum average expression of 100 reads per million miRNA reads. For each miRNA we chose the most abundant isoform and predicted putative targets of these five miRNAs in combination as well as separately using miranda [44] with default settings, searching for target sites aligning to the 3′ UTRs of predicted *D. japonica* mRNAs. We applied the same strategy to determine putative pathways regulated by the four novel *D. japonica* miRNAs. To assign GO terms to the de novo predicted mRNAs we used blastn to align predicted coding sequences of *D. japonica* to coding sequences of *S. mediterranea* for which GO term annotations are available [38]. We assumed coding sequences to be homologous if the alignments yielded E-values ≤10^− 6^. We applied Chi-square tests with strict Bonferroni correction of alpha-error to identify GO terms that were enriched in the set of predicted target genes.

### Knockdown of selected small RNAs

Based on de novo miRNA annotation and differential expression analysis of sequenced small RNAs, we selected a set of candidate small RNAs for knockdown experiments in regenerating animals (dja-mir-novel1/2/3, mir-124c-1/c-2, mir-2156a, mir-36c, mir-7d, mir-8b, and 5′-tRF-Gly-GCC). *Dugesia japonica* planarians were retroauricular amputated. Head-less individuals were collected in 24-well plates (20–30 individuals per well, bottom area 1.9cm^2^). Each antisense oligomeric DNA solution adjusted to the 5uM concentration was added to each well, collected animals were washed with approximately 100ul solution, then discard this solution and added 300ul new solution in each well (final concentration was 5uM). The treated animals were cultured at 21 °C for 7 days and the medium was changed by fresh antisense oligomeric DNA solution on the third day after decapitated. Phenotypes were recorded at 7th day after amputation and each experiment was repeated three times. Paired photoreceptors of regenerating animals were considered irregular when i) the surface area of both photoreceptors differed by more than 33% and/or ii) the distance between both photoreceptors relative to the head diameter differed by more than 33% from to the scrambled control average.

## Supplementary Information

**Additional file 1: Supplemental_Data_S1.** The reference sequences for mRNAs (including UTRs).

**Additional file 2: Supplemental_Table_S1.** The identified 36 miRNA genes with ShortStack.

**Additional file 3: Supplemental_Table_S2.** The enrichment of specific GO terms assigned to the putative targets of the novel miRNAs.

**Additional file 4: Supplemental_Table_S3.** The annotation results of small RNAs using the unitas annotation pipeline.

**Additional file 5: Supplemental_Table_S4.** The length distribution of small RNAs.

**Additional file 6: Supplemental_Table_S5.** Share of reads with 5′ U (1 U) within the fraction of reads that did not match any other class of coding or non-coding RNA.

**Additional file 7: Supplemental_Table_S6.** The sub-fraction of repeat derived sequence reads.

**Additional file 8: Supplemental_Table_S7.** The number of read pairs with 10 nt 5′ overlap.

**Additional file 9: Supplemental_Table_S8.** The size of sequence reads that form ping-pong pairs.

**Additional file 10: Supplemental_Table_S9.** The genomic piRNA clusters.

**Additional file 11: Supplemental_Table_S10.** The expression profile of miRNAs.

**Additional file 12: Supplemental_Table_S11.** The expression profile of tRFs.

**Additional file 13: Supplemental_Table_S12.** The reference sequences for tRNA.

**Additional file 14: Supplemental_Table_S13.** The reference sequences for rRNA.

**Additional file 15: Supplemental_Table_S14.** RepeatMasker as well as WindowMasker for the annotation of repetitive DNA.

## Data Availability

The small RNA-seq data generated in this study have been submitted to the NCBI BioProject database (http://www.ncbi.nlm.nih.gov/bioproject/) under accession number PRJNA549868.
