# Supplementary material for: Different classes of small RNAs are essential for head regeneration in the planarian Dugesia japonica
Source: BMC Genomics. 2020 Dec 7;21:876. doi: 10.1186/s12864-020-07234-1 (PMC7722302; doi:10.1186/s12864-020-07234-1)
Supplement: Supplementary file 1 — Additional file 1: Supplemental_Data_S1. The reference sequences for mRNAs (including UTRs). [file 12864_2020_7234_MOESM1_ESM.pdf]

>DjScaffold1560:43141-50421 strand:+
tttcatataattttaaataagttacaaaaagtatattgtataacccatttaataaagttt
gtatgaaaaacatctgttactaacttacaccagtcgtacgacggatgaaaataacttaca
aaaacattaaatatccaagatctccagtacacaataatagttcttaaagaattatttttt
tctgacggttgttaattacataattctatgcattatcatctgctgttcaatacaacaaat
ccaaattaaaatcagttctcgaattttaaagtaacgaattattctaaaaacgacaaataa
attacaaaaacagttttaaaattaatttttgtaaaattcaattttcaattttcagtttcc
ggaaattgttaaagattctagtggtgaagagattcacataacatattccaaacaaagtac
cggtcctgatcgacaaacctggggtggcaaaatagaatttctttgttcatgtgttgcaAT
GGCTGTCGGATTGGGAAATGTTTGGAGATTTCCATATCTTTGTTATAAAAATGGTGGTGG
AGCGTTTCTAGTTCCTTATTTTTCTATGCTCTTCCTTGTAGGATTACCATTATTTTTTAT
GGAGTTAGCATTTGGTCAGTTTGCTAGTCTCGGTCCAATTACTGTTTGGTCTATTAGTCC
ATTATTTAAAGGTATTGGGTACGGAATGGTACTGACATCTTTCATTTTGTCCACATATTA
TAATGTTATAATAGCTCAAAGTTTATATTATTTATTTGCTAGTCTCAATTCTGTTTTGCC
TTGGATGTATTGTGATAAAAATATTAAAAATAGTTCATGTTTTGATTCAACA
>DjScaffold1560:99595-107202 strand:+
TTTTCGCGGGGTTTTACTAGACCGACCACTCTAGACTTATTTATTAAAAAATCAGCGCAT
CTAGATGAAACCCCAACATTATTATTATTATTATCATTATTATTATTATTATCATCTTCA
GGTATCAATAGATTCAGGAAAGACATTGCTCTAATGACCACCAAAAGGCCAAATTGGTAT
TGGAGAATAATGTGGATGTATATATCACCTGCTGTGATTACATTTTTGATAATATTTTAT
TTTATAACGGGAGACACGCTTAAATATGGCTCATATTCATATCCAAAACCTTTTATTACT
TTATCTCATATAATTGCTCTTTTCCCATTAATATTTGTTCCAGCAGTCTTTTTGTATAGA
TATTGCAAAGAAGGTGGATGGATTATTTTACGAGAAGTGATAAAACCAAAATCTGAATGG
GGTCCAGCAGATGAAGAAAATAGACAGGTTTTTATTAGTAGTATCATGCGAATGGATGAA
CAATCAAACGCCATTTCTGGAAGTAAGGAAGCGAAAATGCCTTCCCCAGATAATATGTCT
ACTCCGTTGGGTGACTCGTTTTTCCAAAGTAAAAGGAGTCTACTATCAAAATTCAGTGAA
GTAGCAGCAAAAGAAAATTCTAAAAACACTTGTCTAAAAGACTGTTAAtatcaaatatcc
aatatttcattggtgatttttataaattttagtagccgatcttttataagttcaaatagt
gtgatttgattgttattttttgtgatttttgttgtaattcatttgtaaaattatctccc
aatactcgcataaaac
>DjScaffold1843:21880-34533 strand:+
aattttttgaatctcgaattgccataattttaagggaatatagttgtttctccaatcta
ctctggagtattaattaatatcacgacaattgtttataattaattataaacagtttataa
ttaattataaacagtttataatagtgaataatttgcagtattttctattttacacacatt
aataaacagattgaaagaaagtaatgctgccataaaagcaaaaacaaatatttccgactt
agaaaatcgagtgataaaccttcgttcaaataatgccccaatcccaagcactgaagacat
agagaaacgtttacataatctcagaggactaccagtcaataatccaactattaataaaag
caatccatcacaattttatgtgaaacctaaagccgagcaagaccaaatttctcaattact
attgcaaATGCAAGATGAAGTAAAGATTGGTGAAAAAGTTCAAGACACAAATAAAATTCA
AAATGAAAATATAGAAAAAAGATTATTAGATTTAAAAGATACAAAATATCCTCAAGACGT
TAGAATAAAGCAAAACAATGATTATCTCAATGCTGATAAAGTTTTAGAACAAATCTTAGA
TGAAATAAAAATAGAAGGAAAATTTCCATCGGATTCTCCAAATGTTTTAGCTTTAAATCA
ATATGAAGAATTGCCTTATTGTTGTTTATGTACCAAGGATGCCATTTGGCGTTGTCACGG
CTGTGATGAAGATTTATATTGTGATTCTTGCTACAAAAAATGCCACAAGAAGAGTGAATA
TGGACCTCATCCACACGAAGAATTTAAATCAGTT
>DjScaffold2477:5262-7812 strand:+
agaaaagataaaagcaattaaataatatgaacaacctaaggcttacaaattattacagaa
aatttattaaagattcggatttcgatattgataatacgacgagtgttccaaatccaacta
acaataataataataataataataataataatgaaagggtaaatttaatatttatga
aataataatttttgtcatattaatttgtttaaatttggttttaaatatttggaaataatt
ttaacaatcagaaatgtataatcaattatctattgcagaaactattcagtgaccttcgaa
aaggcattcagaccATGACGGTGCATGGCACTACAAAAGGGTTCATTAAAATAAATGAAA
AATGTGAACCAATTATTTGGGATGCTAAATGGAACCTAACATTTGCATTGGCTTGTTATA
CTGATAATAACCAAGGGAAAGCATTATTTTTCGGAGAAGAATGGTATTTTTGGTATATCA
TGAACGATAAAGACAAAGTTAACATTCGCAAGACCTTTGTTGATTGGCTGTCTCCAAATA
TTTCCAATCCAAAATTGGTCAATTCTGAACATTTTAAAACAGCTTATGATGTTCGTCAAG
CACTAAATGAAGGAGATATTGTAGATTATTTCACATATGTTGCTTATCCTGATGAAGTCA

TTGACGAGATAAAATCTTTACTACTGAATGGTAAAATATCTCTGGTTTACGATCATTTCA
ATAATGATATACTTAATGAAAGTGTAAGAATTTCAAATGAGAAAATATTAGATATGTTTA
ATATTATACCAGATACAATATATTTACCACTGGTAAGGATAAAAACTATACAATGAAGG
AAGAAGACCTTAACGACGAATTTCTTGATTTGAAAGTGAGATTTCGTAAACTTATTGATA
ATTATGATTATCTAATATCAAATGGACCAAGTGTGTTGAGTTTATACAATAAAATGTCAC
GAAAAATGATTAAAAATGCCAAACTTGATGACAGCATAATAAATCTTTTAAAGAAATATG
ACAGCTTGATTAGTTACATCTGTCCATGTAAAGACGATCCTGCAGTTGATGCAACAGATC
GAGCTATACTCAGTATTTTCGGATTATTAGCAAAAGGTCAAGGTTTAGTTAATTCAAATT
ATAAGCTACCATGTATTGATCTGTTTCCTGGTGATTTCAAATTTCCACCAAAAACTGAAG
AGATAACTTTAAACGTAATTGGTGAAATTGCTTACACTCGTCTTCCTATTGGCTATTATC
TTGCAGCCGGTGAAACCTTAAAATATTCAATTGAAAAATCAACAGGTATTTGGAGCATGA
GTGTTGGTTGTCAATCTGATATTTTGAATGAAAACAGGCCAGAATGGTATCGATATCCTG
ATATTTATGTTGATCTTGTACCATTAAATGCTGAAGGAACAATATTCTCTCCATCTGGAG
GAGCACTGTGGTTAGTAAATACAAATGAAAACTCCACCATTACCATCAAATTTTCAAATG
TTGTACCAACTCCATTTTTCAATACATTTGATGAAACAGCAATATCTGATTGGAACAACA
AACAACGAAATAATCCAGGACTTTGGGCACAAATAGTTGGAGAAGATATGATTTTCTGTG
TGAAAAGTGAGAAAGTCAGACAGTTTGATGGTAACTATTTGAAAACATTGATGGAAAGTT
GGGATAGAATCGTGAGGAATATTCATGTATTTGCAGGATTAGATTGGAGAAAATATAGAC
CAACTCAAACTATTCTTGATATCATGACATCGGCTGGTTCTGCACATGCTGGATATCCAA
TTAGCGCTTATGAAAATTGGTTTGCAAATACATTTGATAAAGTTTATTTAAAAAGAGAAG
GCAGTTGGGGAATGTGTCATGAAATAGGACATAATGTACAATCTGGTATTTGGACTATGT
CTACTGTTGGTGAAGTTACAAATAATATTTTTTGTCTATTTATTTTTAATAAAAATTGGA
AACTTCCGTACCGCGGATTAAATAACGAGTCATTTCAAGAAGAATACAATGAAGTCAAAA
GTTATTTGGATGGTGGTGCTCAATTTAATTTTGGGTCTGGAAGTCCATTTTTAGCGCTCG
CGATTTATACACAAATGGCAAATAATTTTGGTTTTGGATTTTATAGAAAAGTATTTAGTT
ATTATATGGACCATCTAGAGTTAAAAGCAGACAGTGATCAAGAAAAATACGATTTATGGG
CCAATATTACAAGTAGTATTACTGGTCAAAATTTAGTACCGATATTTGATTTTTGGAATT
TTCCAATAACTGATGAAATGAAAGAGAAACTCTCAACTTTGCCATGTTTTATTCCAGATG
ATTTCATGATGAAAATTAGCCAAACTCGAGAAGAATATATTTTGAAAAAGTATCCGAATT
GTCATAGAAAATCTACATTTAATGAAGATTTTGATCCATCAAGCTTCACAATTGGACACC
GATCACAATTCATAAGAAAATATTAActaaatgcaaactataagtgtgataaatatttat
tt
>DjScaffold2477:79329-87921 strand:+
TTAAAAGAAATACGTGAAAAAGGAGGAAGGGAAAGTGAGAGGGTCATAGAATTATGGAAT
GCATGTGTTAGAGATTTATCTTATAAATTAGGAGACGAAATTTGGATCATTTACGAGCAA
GTATTCATTTCAGCTTTGGATACTGCTGAAACTCAAATCGCCAATGAATGCTTTGATGCA
CTTAAAGTAAAGTTTAAGGAATCATCTCGAATTAGACGTTTAAATGCAATGCTACTTGAA
TATGAAGAGAAATTCGATAAAGCAAGGAATACGTACGAAAATCTTCTTAAAGAAGATCCA
ACAAATGTTCTAACAATAAAAAGATTTATTGCAATGTTGAAAGCTCAAGGTAAACCGATT
GAGGCCATCAAAGAATTAAATAAATATCTGGAAAAATTCATGAGCGATTATGAAGCCTGG
GGAGAACTATGTAATCTGTATTTATCTGAATGTGACTATAAGAAAGCAGCATTTTGCATG
GAAGAATTAATTTTATCCAATCCTCATAATCATCTTTATCATCAAATTTACGCCGAAATT
AAATATACCGAAGGTGGCATTGAAAACCTAGAAATAGCCAGATCTTATTTTGCAGAAGCG
TGCAAATTAAACGAGAAAAACATGCGAGCCCTTTTAGGATTACATCTTACCTCTTTGAAT
CTTGCTCAAAACAATAAAGTTTCAAAAGAATTGAAGAAAGAGTGTCAACAATCAGCTACT
TGGGCAGCTGCAATTATTACTAAGTTATATGAATCAGTAACTGTGGATATTGATTCGGTT
TCGGAGGTGGGATCAGAACTAACTAATTTAATCGACAGATTGGAAATCACCTCTGGCAAC
AGTGATTGAaagatgcgtgacaatactagtctcatcaattttttgtgtccctcgtataa
taattaaattaaattacttcactgttaaaaatgaatgttgcacattttttcgatatgaaa
cactttattattttcacttttgaatttatgttagtattttttcttggaccttttaaaagt
tcataaaaaattatttggattttataaatattgaataacttgtattctgagttgctcctgt
tttattaattcttaaatgttttcatgaatccaataatatattgatttcctagaaaattga
atgctttgatgcacttaaagtaaagtttaaggaatcatctcgaattagacgtttaaatgc
aATGCTACTTGAATATGAAGAGAAATTCGATAAAGCAAGGAATACGTACGAAAATCTTCT
TAAAGAAGATCCAACAAATGTTCTAACAATAAAAAGATTTATTGCAATGTTGAAAGCTCA
AGGTAAACCGATTGAGGCCATCAAAGAATTAAATAAATATCTGGAAAAATTCATGAGCGA
TTATGAAGCCTGGGGAGAACTATGTAATCTGTATTTATCTGAATGTGACTATAAGAAAGC

AGCATTTTGCATGGAAGAATTAATTTTATCCAATCCTCATAATCATCTTTATCATCAAAT
TTACGCCGAAATTAAATATACCGAAGGTGGCATTGAAAACCTAGAAATAGCCAGATCTTA
TTTTGCAGAAGCGTGCAAATTAAACGAGAAAAACATGCGAGCCCTTTTAGGATTACATCT
TACCTCTTTGAATCTTGCTCAAAACAATAAAGTTTCAAAAGAATTGAAGAAAGAGTGTCA
ACAATCAGCTACTTGGGCAGCTGCAATTATTACTAAGTTATATGAATCAGTAACTGTGGA
TATTGATTCGGTTTCGGAGGTGGGATCAGAACTAACTAATTTAATCGACAGATTGGAAAT
CACCTCTGGCAACAGTGATTGAaagatgcgtgacaatactagtctcatcaattttttgt
gtccctcgtataataattaaattaaattacttcactgttaaaaatgaatgttgcacattt
tttcgatatgaaacactttattattttcacttttgaatttatgttagtattttttcttgg
acctttttaaaagttcataaaaattatttggattttataaatattgaataacttgtattct
gagttgctcctgttttattaatt
>DjScaffold3552:41290-44651 strand:+
tagttacgtattttgtttaaatttttgattaaaattgaagtattaagaaatgactctagg
tagaccagaagctgctttagctaaagccaaagatttaataaatgtgtcaaaactaaatac
ggcaatagaaattttaagtgaatgtatgaaagcgcagaaaaaacaaaaagcatgggttaa
aacccacgaagANNGTTATGATGCTTTTGGCACAATTATGTGTTGATAATAAAAAAAGTC
TTCAGTTTAAAGAAATTATTCATCAGTATAAAAACATGACTTTTCAGATGGTTCCTAAAT
CGCTAGAGGATGTTGTTAAGTATTATTTATCTCTTGCAAAGCAACGAACAGAAGAAGCAA
GAGATCAAGCTAAAATACTTGTTCCGGATATTGATGATTTAGATAATCCGGATACTCCAG
AAAATCTGATGTTATCCGCAATTAATGCCGATAGCGAACAAGAACGTCAAGATAGAGCAA
TTTTGACTCCATGGTTAAAATTTTTGTGGGAATCATATAGACAGTGTATGGAATTATTGA
GAAATAATGTGAAATTCGAACGACTTTATCATGATATTGCTCAAGAAACTTTTGAATTCT
GTCAGAAATATGATCGAAAAGCTGAATTTCGTAAATTATCGGAAATATTAAGAAATCACA
TGTCGAAATTTCAACAGAGTTCTAATACAGTTGGAAATACTCCTCAAGCTCAAACACCAG
AAACTTGGGTTTTGCATTTAGAAACTCGTCTGAAAATGTTGGATTTTGCATTGAAATTCG
AATTATACAACGAAGCGTTCAAAGCAGTCGAAGACGTTTGGAATTTACTAAATACGTCGA
ATATATCTGCTAAACCGAGAATTCTAGCCAATTACTATTTGAAAACGTCTTATGTGTTTT
TCGAATCTGGAATTTCGTATTTGTTTCATGCAGCCGCTCTGCATAAATTATTTTTCTACA
GTCGAGAAAATAAAAAGAACATGTCGCAAACGGAACTTGAACAAATAGCTCTACAATTGA
TTATTGCTACTCTAGCTGTTCCGTTGCCGAATAACAGAGAACCGATCGATCCATTGTTAG
AATCGGGAGAAATCACAAATCCCGTTAAACAGAAAACTTTATCTATGTTACTAAATTTAC
CAGCACCTCCAACCAGGGCGAGTTTAATCAAAGACATTCAAGATAATCATATTTACAATT
TAGTGAGTTCTGAAATAGCAAATTTCTTCAAAATTCTCGAATCGGATTATAAACCGTTAT
CAATGGCAAAAAATGCGCCGGTTATTTTGAAATGGTTACGAGAACATCCGGAATTGAATG
TTTATTCGGATTTAATTCAAAATATTGTCGTTAGCAAAACGATAATTCAAATCGGAAAGG
TTTATAAAACAATTAAAATCAAAGAATTTGAAAAGCTTTGTCCGTATGTTGATTCGCTGA
AATTACAGACGATTGTGTTCAATTTGATTCGTAATTTGGAATTACCAATCAGAATCGACC
ATAAATCTGGAATTATCGAATTTGATGTTTACACTGACCTTGGAATCAGTCAGAGAAACG
ATACGAATCTGATTTCCCAGGAAATCAATGGAATCAGCACATTAGCAAGACAGTTGTCGA
AATTTTCAATTGCGATTTATGAAGTCGCGGAACATATCGGTTATGTGAATGATGAAAAAC
AACATAATAGAGATTGGACTCGTCAGTGTTATATAAAGAAAATCAAAGAATCTCATCAAT
TAATGCTTAACAGAAAGAATTTGATTGAAACTCGAAAAGAAGAAATCGAAAGAGTAGAAG
CGGAAAACCAGGAAAAATTAAACGATCAAATGCGTATTCAAGATGAGAAACGACGTTTAG
AAGAAAAGAATAGATTATTAAAGGAGCAAGAACAATTAGCAAAGAAGAAAATGGAAGAGC
AAAAGGAACAGCAGCAGAAGAAAATGATAAAAGCGAAAATCAATTATTTGAAAAGTAGTG
ATGCTGGCAATAAGGTTTTGAAAAACTTTACGGAGAAAGAATTGGAAGAAATGGACGATA
TAAAGCTGGTTAAAATGCAATGTGACGAAAAGAATCGAATAAAAGAAGAGGAAGAAGAGA
AGTTTAAAAAATTGGAACAAAAAGTCGATTATTTCCAAAGAGCTTTACGATTGGAAGAAA
TTCCTCGAATTAATCAAGTTCAATTGGAATTCTATGAGAAAGCTAAGAAAATGTTTGACG
CTAAAGAAGAGAAACGACGAAACGATTCTCGCGAGTTGCATTTGATGAGATTGAAAGATA
AAGAATTGTGTTTGAGAATCGAATCAGATATCGAGAATTATATTCGATCGATCAATCACG
AGTCAGAGGAGAGTTACGAGAAATTATTGAATGAATGGGAAGAGAGAAATAAGGAAAAGA
AACGAGTTTATCGAGAGAAAAGAATGGCAGAATTACTGGAAGAAAAGAAACAAGAAGATG
AAATTATTAAACAAAAAGAATGGGAAGAAGAAAATGAGAGGATAGAAAGAGAAAAGAAAG
ACAAAATTGAAGCACCAAAAGCGGCTTACATTCCTCCTCATTTGAGAAGGAAAGTTCAAG
AAACCATTCCAGTTACACCCGTAAGTTCCAACAAGTCCACTGTTAGCATCATGAAGAGTT
CTGGTACGCCTCCCAGTTTAGAGAGGAACAAAAAACCAATTGAATCAGAACAAGGATGGA

GTCGAGTATCAAAAAAATAGatgataataataacaaataataattttgtatttcacgtgc
ggtgtttcaataaattgattcatttatatttgtttttatgtatttgtgttaattatgaa
atagagatggttggt
>DjScaffold3552:63023-80222 strand:+
atacattacaacATGCATGGACCATGGAATACGAAACTTATCAATTTAATTGCTAATACT
CTACGAAAGAATGATCGAATAACCTTTAAAATGAAATTGGAAATTATTCAAGCTCTTTCT
GAGGATAAGACATCATTAAAGAAGCCTGGAAGCAATCAGCAAACACCAATTATTCGTCGT
TTACTTCTGACAAAGGATAATGACGATAATAATGATATTCCAATAATTAGAAATCATCAA
GAAGTTAAACAAAGATCCAATTATCAGTCGACAATAAATTCTCAAAAATCTCCTAGGTCT
GTAAAAAAAAATAAAAGAATTTGATTCTATTCAGTGTGAATTAAGTGATAATTTATCTAAA
TCATTTGGAAAACTCGATCAAATAGAAATAGTTGATAAAGATCATAAGAGACGTGGATCA
ATGAATAATGAAATTGTTATTGAAAACTCAAATTTTCAGACGATTAAGGAAATGTTTAGT
TTGGAAAATAAAGTTGATGTTGAAGATGATGAACTTCAACTTATTTCACGTAAAACAATA
TCAACTCCAAAAGAATTTGTATCTATGCAATATAATATACCAAAAGATGAGGTTATCAAA
TTGGCAAATAGCAAAAAGAAGAAATTAGTAACTGTTCCTAAAGTTGTTCCTCTTCTAGGA
ATATCAATCGAAGGAGGGATAAACACTCCTTATCCATTAGCAAGAATAACTAAAATTGAG
CCTAACAGCTGTGTTGGTCTTAATGGCGAAGTAATACAAGGAGATATTATTCTCGAAGTT
GATGGGATGCCGTTAACTGGTATGGAACACAATAAAATAGCTCAAAAAATAGCTGAAGCA
TTTAAAAACACATCTGCGGATTCGATAAAATTTTTATTGATGCAAACAAATATTGATTTA
CAACAGATATCAGATAATATTGATTATCAAAGCACTTAC
>DjScaffold3902:9571-18128 strand:+
taaagtgtaattaaatttaccaatatttataatagtgacaaactaaaatatcatcgcgca
cttttcaaattcacaattagaaaaATGCGGGTTGCTGTAATAATTTTGTCAATTTTGTAT
TATGTATCTGCAATTGTACAAGTCTCATTAAAACAGTGTGAAATTTCCAAAGCATGTTTT
AAAGCTTCTGAATCATGTACAGAAGACCAATGTGATTTTATAATAACCTGGAAGCCCAAA
AATTACCAAAACAAAAACTATTTAGAGATAGAAATGACAGGAACGGTTTTGTCAACTGGG
GGTTATATTGCAATAGGATTTTCAGAAGACAAATTAATGGGAGACGATGGGGTATTGGCA
TGCTATACTAATCCAGGAACTGCTGACTTTAGTATTGTTGCCGGATTTAATGAAAAATCT
CAAAGAAACAATAAATTTTTAGCAAATTCTCAATTGGATAAAGACTTTCTTGTCAAATCA
GGAAGCAAATATGGTGGAAATTTTAATAGAGATATAAAAAGAATTTCTTGTCGGTTTTTA
CGCTTAATGACTCCAGAGAAAAACATTGATAAACTTAAAGATCTGAGTCAAGGGAAAAAG
TATTATCTACTTTTAGCAAAAGGACCTGATGTCAGTATCAATGGATTAAATAAACATTTT
CCTGGTTCTAATGTCATAAGTCATATACCCATTGATTTCACCAGTGGTTCCTATAATACA
GTTATTGGATCATCGAAAGTAAATTCTCCAGTAGCAAAAGCGCATGGTATTCTCATGATA
ATTGCATGGATTTCTTGTTGTGCGATTGCATCATTCATTGCTCGATATTATAAAGATGTT
TGGTTTCGAAAACCGATTAAGGAAGGAAACTGGTTTAATCCTCATCGAGGTTTACAAGGC
TGTGGATTTGTTTTAATAATAATCGCACTAATATTAATAATAATTCATGTTGGAGGTTAT
TCAAAACTGGAAGATTTTCCTTCGAAAGTCCATCCGATATTGGGGTTTATCGTTATTATT
CTATTTGTGATTAATCCATTTGTTGCTCTTTGTCGATGTCAGATACAACATCGAAATCGT
CCAATTTTCAATTGGATTCATTTTATCATTGGTATTTTAGCTTACATTCTTAGTATTCCA
ACCATTATGATCGGCCTAAAACTACCTGGAGCTGGTCTTGCTTTATCTCAGTTAAATTTT
CCTCAATGGATTGTGTTTATATTTGTAATTTGTCAATTAATTCTTGAAATTATTCTAGAA
ATTCACAGCTGTATGACATACAGAGGAAATAAAGACAAAAGACACAAACATGAATTGGAT
CAAGTCGAATATGAAAGAGCTTTACAACACAATTTAAAACCGTCAGACGTTCCAAAACCG
CCTCGAACTAAAGGACGGAATTTTAAGTTGTTAATTCTGGGATTCTACATAACTCTATGT
ATTGTAGTAACACTGGCGCTCTGTATTATAATCATAGTTTATTGAacctcaatgatttat
tggctttataacacttttgtaatatttgaacattgacaatttttagttataattaatttt
ttattttattatactataaagttaatgtaataataataataataataataatctgatgtc
atgatttgataa
>DjScaffold5344:22962-30363 strand:+
CATAATTCGAGCAAGTATTTAATCAATTCAAATGAGAATTATTTGACAATATTGAAACAA
GATGAAAAAAATTCATAATGTAATTTGTTATGGAGAGAAAGATGATCACGATAAATTATAT
AACAACATGGAATATAACTGGATAAATTCAAAATTTTCATTGGTTGAATTATCGGTATAT
CAAATTAAAGAAACACCAATAAATACTTATCAAATATTATTTATTTTATTATTGATTCTA
TTTCTGGTTTCATTTGGTCTTAATATATATGAAATATATCAAAAGATTTTCAATAACAGA
CAGAGCAATTCCACTCCTCAGTCTATTTCTATAAATTCTCAGCAAAAATCT
>DjScaffold5003:2922-7192 strand:+

AATGTTATCTCAGAAATAGAAATTCCATTATCACTGGCAACTGTCGGTGGGAAAATTGAA
GTGAAAGGATTGTATGATAACTTTAAAATCGATATTCCTTCTGGTACGAATAGTGGTGAT
ACTATAACAATACCAAACAAAGGTATTAAAATAATCAGTCAATCAAATTATGGATCTCAT
ATTTTCACCATAAAATTAGTTACACCGAAACATTTGAGTAGTTTTCAAAGAGCACTCATA
GAAGCATTTTCAGAATCAGAGACAACACATATTCCATTTGAATTGCGAAAAAGTTGAaaa
atttgttttcaagaaactataaggaaatatttaaattttaacgattttcaggttcgtcct
cagaagttgaacacattaaaaacctacttcttactttcacaaaatttggtcattcaccac
agggcagtacaaatagtgcaagtaatcaaacatattcagacatgcagtcaaatggtttaa
cagaattcattaaagatgggtggaattatctgaaaaaaatctttcgatgattattcagaa
taattttttaatatcgaataaacc
>DjScaffold5003:28118-28658 strand:+
aatttaaaaattatttttcaattaaaattttaagtttaatgattttattcattatttaaa
aaacaaacatttaattgtattttcaggatgaaaaaatgaaaattcttacaatgagtgttc
aactaagacaggtaaaaatgaaaattgtttcaaataaacaaaactttgaattctttaatt
ttctattaatcaaatagatttggaaaaacaaatatctcagatggaatcccaaagaatatg
gtaatataaaatcgatatatttacctcatagtaaaatgtggttaccagatattgttttgt
ttaataagtaacattgtttgtttattcaacaaatcaatattttatagtgaatgttctttt
tattataaacaatagtgccgatgggaaatatgaaacacaatatcctaacaaacctattct
atattacaacgggttaattgattggaaaagtccggcaatatttcagagtacctgcgaaat
cgatgttagatattttccatatgatgaacagatatgctatttaaatttcggctcatggac
atataataaagaacaagtaaaactgagacatatcaaacaattagaagcatttattgggAT
GAATTATTTACAACCCGATAGTATTCCATGGAAAGAATCATTAAATATTTCGAAACTTAA
GCATTTTTCGAATGGTGCATTTCTAACGAACTATCACAAAAGCGGAGAATGGGATTTAAT
GGGCACACCAGCAATCCTATCTGGAACGGAAAATATCGATACTCCATATGACTATATCAA
ATTTGGAGTTAAAATACGACGCAAGAAAATGTTTTATAATATCAATTTAATTTTCCCTAT
GGTGGGATTGAGTATTATGGCAGCACTCAGTTTTTATTTGCCAGTGAAAAGTGGAGAGAA
AATCGATTTATCTACTTTGATTCTTTTCGCAATCGCTGTGTTTTTTGTGCTTTTAAGTGA
GTCCGTTCCACCTTCATCACTTGTCACACCGATAATTAATCAATATTTGATAACAACGTT
TTTATTGACATCTCTCTCAGTAATCAGTACAGTTTTCGTTTTGAATATCCACTTTCGAAC
TCATTTGACAGATGAATGTATTCCACAATTTATCAGAAGATTTTTACTGAAGTTTTTACC
GAAAATTTTAATTTTAAAATTACCACCGAAATATGAATATGTGGATGTTTTAAAGCAAGA
ATTTTTGAAGCACAGAAAGAAAACATATTTTATTCGAGATTCATTTTCAAGTGATTCAAC
TACACAATTTTTGAGAAAAGATTTCGATAGGTGTATCCAATTTTTTACAAATAATAGAGA
AAAAAAGTTAAATAAAATTGCTGAAATGATTTCTGAGCCCTCTGAAAATGGAGATGATAG
TGAAATTTTAAATTTGGTAaatttaaaaattatttttcaattaaaattttaagtttaatg
attttattcattatttaaaaaacaaacatttaattgtattttcaggatgaaaaaatgaaa
attcttacaatgagtgttcaactaagacaggtaaaaatgaaaattgtttcaaataaacaa
aactttgaattctttaattttctattaatcaaatagatttggaaaaacaaatatctcaga
tggaatcccaaagaatatggtaatataaaatcgatatatttacctcatagtaaaATGTGG
TTACCAGATATTGTTTTGTTTAATAATGCCGATGGGAAATATGAAACACAATATCCTAAC
AAACCTATTCTATATTACAACGGGTTAATTGATTGGAAAAGTCCGGCAATATTTCAGAGT
ACCTGCGAAATCGATGTTAGATATTTTCCATATGATGAACAGATATGCTATTTAAATTTC
GGCTCATGGACA
>DjScaffold3823:27222-34041 strand:+
attacataccaaaaattgaacttcgtacatcaattaaaaaaattttataactaaattatt
ttgcataATGTATTCCGAAAAGGATCCAATATTATTTGATAAAGATTATAAAGAAATAAG
CAAGAAAAAGTTAAATGTACTAAAGATACCGTTAATAATAGCATGTGTATGCATAATCGT
ATTGTTGTTAATGCTGATAATTCTAGCAATTATCATATCGCTCAAATATGTAAAGAATGA
ATGTTTGTTGCCAATTAGTAACTCTACTTCACAAGGTTTGTATATGCATTCTGCAATTGC
TTCCGATGCAGTACCATGTGCAGCGGTTTCTAATTATATTATGTCTGTTAAAGGTGGGAA
TGCTGTTGATGGGGCTATTGCTGGTTTGCTCTGTACATGTTCAGTTAACTTTCACAGTTG
TGGGATCGGAGGTGGATCCTTTGTAATGATTTACAACAAAACTACCAAAGAAGTTCATGC
ATACGATATAAGAGAAGAAGCTCCTGGACAAGCAACAGAAAACATGTTTGCTAACCATGT
TTCATCTATTACAGGTGGTAAATCTATTGCTGTAATGGGATTGGTTTCTGGTTATTACGA
AATTCATAAAAGATTTGGAAAATTGGAATGGAAAGTATTATTCGAACCATCTATCGACAT
ATTAACGAATGGGTTTTATGTTTCAAAAAGTATACAAAGCGGAATTGATTATTTTTTTAA
AACTGCAACAGCATCTGAATTTTCAGAATTGAGAAAAGTATTAACCGATTCCACAACTGG

AAAACCGAAAGTAATTGGTGACATCATAAAAATCCCAAAACTTGCAAATACTATGAGAAT
TTTATCTAATGAACCATATGCTATGAAAAATGGATCATTAGTAGATAAAATGATTGCTGA
AATCTCAGCAGAAGGTGGAATTATTACCAAAGATGATTTCCTCAAATATGAAATGAAAGA
ACGAAAACCAATTTCAGTAAATTTGGGAAACAATAATACTCTCTACACATTGCCTCCTCC
ATCTAGCGGGGTCCTTGTTGCGTTTAGTCTCAATGTTTTATCGAATTATAATTTCGATGT
CAATTTCCTTGATTCTTGTAAAAACACCGAATTGTTTTTTCATAGACTTGTTGAAACAAT
GAAATTTGCATTTGCTAAGAGGTCAGCTCTCGGCGATATGGATTTTGTTAATGTTTCAGC
ATTAGTTAAAAATTTGACATCTGATTCTTATTCTGCGTGGATCAAGTCATTAATTACTGA
CGATAAGACCCATCCGATTTCATATTATCATCCAAGTTTTGAATTACAACAAGATCATGG
TACTTCTCATTTCAGTTTAGTTAGTCCTGATGGAGATGCCGTTTCGGTAACATCCACGAT
TAATTATCTTTTTGGTTCTCTAATAATGGGAAATGAAACTGGTATCGTTTATAATGATGA
AATGGATGACTTTTCTTCACCAAATCAAACTAATATTTACAATTTACCACCGTCACCAAG
CAATTTTATAAAGCCATATAAACGACCTATGTCGTCTATGTCTCCATTTATTATTGTTGA
CAAAAACGGACAAGTAGTATTAGTTCTTGGAGCCTCGGGAGGAAGTAAAATACTTTCAAG
CACAACACAAGTTGCTGTTCTTGCTAGATTGTTCAATAAAACGATGAAAGAAGCGATTGA
CATTCCACGAGTTCATCATCAATTATTGCCTGATGTAATTACCGTTGAAATGAACTTTCC
TGATAATATTATCAAAAGTTTGGAGGAAAGAGGTCATAAAGTAGAGAAAAATTCAGATAA
AGCAGTCTGTCAAGGTATCAAAAAAATCTGAAAATGCACGTTTTTGGCAATGTTATGCTGA
CCATAGGAAAATGGGATTGTGTGATGGTTCATAAatatattactgattattttaactaaa
acgatttgtccactaaaagtattttaaaaaaaaacttatctttttagtagtttcattcat
tgcaccttttttaattacgctattcttccaaagtgaaaatacattttcgactgtgttcag
gaaattgaaataagtaagaagatatttaatgttttaatctgaaatttctgaatcatcaaa
taatcctctgtaatggtgagttctggtattgtccatgatgaatatttgatttaatatgcc
attttctctacaatcagattttatctctcacaaagactccttgaagcggtcgccattgac
agtcttcttatgagccttatggtaaatcatactatatttgttaatggtatgtacaacaga
aatatttttacttctgacaacagacacactaatgaaagcggattatccacttttactttg
acaacaaattggtcttgtcaaaaccacaaatccaatctcatgtagaaaaatcaagttttt
attgtcattacttgttttaaaagtctgatagttagaggcataaacctttctttcattaat
tgtagcttcacattttcaccatacaggcaataatgtgactctttttagaatatagtagaa
ttaacatagtgacttgtcaattgcacttttggatacttcagtactaaagttgtccggtta
gaaaactggactaatgtctcagtaccttcttggttcacaatggtaagcagtgctttgtta
gcttctaaacttagtttagaatgcctataaacctcgcgtttgaatgcaaaatctcacctg
acttcaagtatctttaacaatactattaactgtttcatagttttggtcattttatgccgg
aatagaagataccgtatatcattctaatgtccttgatattattctttctctgtaatattg
gcaacattcatacacacacagaaattgaagaaatagtcataatgtgaaatagaaatttaa
gataaatttgttttaaaaaagattgtttcagttaaaattgtatttttaaataaatgagaa
aaccacaaaagtttgggataattacattaaaattaaattagatttgagaagattaaactg
gattaaaggactggattaggcgagttggagtttatgcatcttactccatatcttgctcat
tctgagattctcagaacactgtcatagaaaatttaaaataatattattgaataattagaa
tgaagttATGACGTTTTTCATTTTTGTGAAAGGTGGTAAATCTATTGCTGTAATGGGATT
GGTTTCTGGTTATTACGAAATTCATAAAAGATTTGGAAAATTGGAATGGAAAGTATTATT
CGAACCATCTATCGACATATTAACGAATGGGTTTTATGTTTCAAAAAGTATACAAAGCGG
AATTGATTATTTTTTTAAAACTGCAACAGCATCTGAATTTTCAGAATTGAGAAAAGTATT
AACCGATTCCACAACTGGAAAACCGAAAGTAATTGGTGACATCATAAAAATCCCAAAACT
TGCAAATACTATGAGAATTTTATCTAATGAACCATATGCTATGAAAAATGGATCATTAGT
AGATAAAATGATTGCTGAAATCTCAGCAGAAGGTGGAATTATTACCAAAGATGATTTCCT
CAAATATGAAATGAAAGAACGAAAACCAATTTCAGTAAATTTGGGAAACAATAATACTCT
CTACACATTGCCTCCTCCATCTAGCGGGGTCCTTGTTGCGTTTAGTCTCAATGTTTTATC
GAATTATAATTTCGATGTCAATTTCCTTGATTCTTGTAAAAACACCGAATTGTTTTTTCA
TAGACTTGTTGAAACAATGAAATTTGCATTTGCTAAGAGGTCAGCTCTCGGCGATATGGA
TTTTGTTAATGTTTCAGCATTAGTTAAAAATTTGACATCTGATTCTTATTCTGCGTGGAT
CAAGTCATTAATTACTGACGATAAGACCCATCCGATTTCATATTATCATCCAAGTTTTGA
ATTACAACAAGATCATGGTACTTCTCATTTCAGTTTAGTTAGTCCTGATGGAGATGCCGT
TTCGGTAACATCCACGATTAATTATCTTTTTGGTTCTCTAATAATGGGAAATGAAACTGG
TATCGTTTATAATGATGAAATGGATGACTTTTCTTCACCAAATCAAACTAATATTTACAA
TTTACCACCGTCACCAAGCAATTTTATAAAGCCATATAAACGACCTATGTCGTCTATGTC
TCCATTTATTATTGTTGACAAAAACGGACAAGTAGTATTAGTTCTTGGAGCCTCGGGAGG

AAGTAAAATACTTTCAAGCACAACACAAGTTGCTGTTCTTGCTAGATTGTTCAATAAAAC
GATGAAAGAAGCGATTGACATTCCACGAGTTCATCATCAATTATTGCCTGATGTAATTAC
CGTTGAAATGAACTTTCCTGATAATATTATCAAAAGTTTGGAGGAAAGAGGTCATAAAGT
AGAGAAAAATTCAGATAAAGCAGTCTGTCAAGGTATCAAAAAATCTGAAAATGCACGTTT
TTGGCAATGTTATGCTGACCATAGGAAAATGGGATTGTGTGATGGTTCATAAatatatta
ctgattattttaactaaaacgatttgtccactaaaagtattttaaaaaaaaacttatctt
tttagtaattaactaatatttaagcttttgtaatcagttaatctgtATGCCTCGAGTAGC
TCAAAGTGGAAAACTGGATATAAAAAAGTTAAATGTACTAAAGATACCGTTAATAATAGC
ATGTGTATGCATAATCGTATTGTTGTTAATGCTGATAATTCTAGCAATTATCATATCGCT
CAAATATGTAAAGAATGAATGTTTGTTGCCAATTAGTAACTCTACTTCACAAGGTTTGTA
TATGCATTCTGCAATTGCTTCCGATGCAGTACCATGTGCAGCGGTTTCTAATTATATTAT
GTCTGTTAAAGGTGGGAATGCTGTTGATGGGGCTATTGCTGGTTTGCTCTGTACATGTTC
AGTTAACTTTCACAGTTGTGGGATCGGAGGTGGATCCTTTGTAATGATTTACAACAAAAC
TACCAAAGAAGTTCATGCATACGATATAAGAGAAGAAGCTCCTGGACAAGCAACAGAAAA
CATGTTTGCTAACCATGTTTCATCT
>DjScaffold83:15545-20803 strand:+
ttttttaataataattgacttaggaaggattttgcaattcatattgattactgttttgga
caaatattttatcgatttattcaatattgattactctatcaactaaattacaagaaaatt
gaagagatttttgttgaatattttaacgaattttgtgaaacaaattaatcaatatcaaat
caaataaattgataggaaatcatccaaaaaatttgattgtctattaatatttattgagtg
attatattctttaaataaataATGGATCCCGAAGAAAGACAACGACAAAGAGAAGCACGC
AGACGAGCGCGACAAGAAAAAACAGAGGAGCCTGACGAAAATGTAAATGAATTCTCAAAT
GGACCAGCAAATACAGATGCTGAGGAATCGAGAGAAAGAGAAGAAGCTGAAAACCGTGAA
AGACAAGAACGACAACAGCGAGAAGATAGGGAAAGGCAAGAAAGAGAAGATAATTTAAGA
AAGGGAAGAGAGCGAGAAGAGGCAAATAGATTGAGAGAAGAAGAAGAAAAATTGCTTGAA
AACCAAAAGCGTAAAGAAGAATCAAGATTGCAAAAAGAAGCACAGGAAAAAGAATCAATG
AAAAAGAAAAAACCAGAAAAAAAAACGTAAAGGTTTAGGAGGCTTGTCTCCAGAGAAAAAA
AGAATGCTCAAACAATTGATCATGCAAAAAGCTGCTGAAGAAATGAAGGCAGAAGCTAAA
AAGAGGCAAGAAGAAAAGGAAAACTATTTAAAAACAGTTGTACCAAGTCTAGAGCTTGAT
GCAACTACAATAGATGAAATAACTAAAATCGTTAAGAAATATTATGATAAAATGTGCAAT
TTAGAGGAAGAAAAATATGACTGGGAATTAAAGCTTCAGAATTTGGATTTTGAAATCAAC
GAATTAACACTGAAGGTAAACAATGCGAAAGGAAAATTGTGAgtttattctttatttgat
aattaaatattatttatgaaaacaatttatgagaaaataacacacaagtcaaataataat
aaaatggtttttattattaaattatattcatgttaataagaaaaatattagctgaaacgt
ttatctgaaaaacatgttatgtgatttaataaatgtggtcataattcgatttttgtagat
ggctgcctttcaattctaacataatatatttcattatcagaatattta
>DjScaffold24:36721-52059 strand:+
AATTTTTCTCAGTTAGTTTGTGATGTGCCACATGATAAGAATCTTTCTTTTCCACTGCAA
TTTTCACGTCCAAATCAGTTTAATAAAATTAATGATATGGACACAAAAATCACTAAATTA
CCTAATGGATTAAGAGTTGCATCACAAAATCAATTCGGAGTTCAATGCACTCTTGGAATA
ATGGTTAAATGTGCATCAAGATATGAAGCCAGACATATAAGTGGATTATCGCATTTTATT
GAAAAATTAGGTTTTCAGACCACAGAAGAATTTCAAAGTAAAAATGATTTACAAATTGTT
CTTGATGAAAGTGGTGCAATTTCGGATTGCCAATTGGCTAGAGATTTGATAATATATGGA
ATATCTGGTGAAATATCGAAATTGGAGAAATTAGTTAAACTGTTGGGTGAAGTAGCATTG
AGGCCGAGAATCACACATGATGAAGTGGAAGTAGCGAGGCAAGTTATTGAACATGAACAC
AGAGCAATGCGAATAATTCCCCCGCCAGATAACTTGATGCAAGATCTAATTCATCAAGCA
TCTTTCAGAAATAATACATTAGGTCTTCCTAGATACACACCACTCAACAATATAGATGTT
ATTACTCGATCAGATATCATAAATTTTATGGCTTTAAATTTTCGACCAGAATCTATGATT
TTAATTGGCGTAGGAGTTGATCATGACGATATGGTTAATTATGCAGACAAATACTTTGTT
CCTAATTCTGATTTTCTAATCAACGATCCAGATATAAATAAGGATATTTTGCAGCGTGGT
CTTGATCTTTCAGTTCCTCAGATGACCGGTGGAGAGGTTTTAGTGGAGCAAGATTTATCT
CAGTATCATGATCCAATGCCAGAATTTGCTCATGTTTCATTAGCATTGGAATCTTGCGGA
GTTAATGATCCACAATTTGTACATGCCTGTTTACTGCAAATGTTAATGGGTGGTGGAGGT
TCATTTTCGGCAGGTGGTCCGGGTAAGGGAATGTTTTCCCGTCTCTACAGTGAAGTCCTG
CACCAATACAATTGGGTGAATTCTGTTGTTGCTTTAAATCATTCATATAGTGACACTGGG
CTTTTTACCATTACTGGCAGCTCTGTACCCGGTAATCTTGATACTCTCACATTTGTTCTC
GCGAAACAGTTAAAAAATATGGGTAAACCAATAGATAAACAAGATTTTGCTAGAGCTAAG

TGCCAATTGAAATCTATGCTTTTGATGAATTTGGAATCACGCCCAGTTGTCTTTGAAGAT
ATAGGTCGACAAGTTATTTATCAAGGTCACAGAAATTCAGCTGATTATTGGGTAGAACAA
ATCGGTAAGCTATGAaaagccaagtg
>DjScaffold357:359-4145 strand:+
GGTCAAGTGGGTGGTGAATTGCCATACTTTATTGGAAAACAGTATGGATCTGGTTGGTTG
CAAACAATTGGAAGATATGCTTTGCCGATTCTTAAACGTATTGGTTCATTTGGTATGAAA
ACTGCTCAGGATGTGCTGGCAAAAAACGGAAAAATACTACCAACATTAAAATCAAATGCA
TTAGCTGAACTTGGAAATGTAGTGTCTTCATTACCGACTGGAGTAACCAATATGATACCG
AAAGTAGCAGACATGTTGCCAACAAGCATAACTAGCAAGATTCCGAATAGTATAATTAAT
ATTATAACAAAACAACCTACAAAAGAACCTGAAAAACAAAATAAAAAACGTAAGAAATCA
AAATCATCAGGTATAAACAAACGAATGAGAGGACATGGAACTATATTCGATTAAtgattt
gcggaaaaactgaattgtgtatttttgaccgaccatctccccaagcagt
>DjScaffold442:14023-14599 strand:+
tttcagtatttatattattaacaaaaacgacattatttttgcaccaacctaataataata
atggtttttaagatcagtgagtaaagtatttttgtaggaatccctttatcattgtaagga
aaaattgtcattatttctcacaaataattcgaattttatttttgggtgttatttatatta
gaattgataatctaaataacaatgattatttatctcattttctttattaaacacattaaa
gtagaaactaataatattgttgaattcgaagataattgttaattttatgaatatttaatt
gtaggttaaagagtcaatcgagtattacgttattaagaatgacattctaactgattttgt
aaatgatgacggcatagttttattacgtaaagatgctgttttgaatattgaagattgtaa
cgaagatttttctttgattattgataaaagagttcgctttcaatataataatgaagacga
aattggagtattaaaatggaaaggaaaaattgatggtttaaaatgtattggtgtagatcc
acataacgaaaaattgatttcagactggataatccatttcaatccaaatgcatattggac
gatcatttgtttagatttttggaaaacaaaggaatttctataATGTTTGAAATCGAAGA
TCCTTTCAATTATGATATTTTTACTTATTTAAAAAAGAAAACATTTGTAAAAATTAAAGG
TGATGAAGGCACCATAACATCTTATTTTACTGATAAAAAGAATCTTTTTTATGAAATTAA
AATGGATGATAACGAAACATTTGTTATTGCTAATGGTAATGACGTTAAATGTGGTGCTGT
AGCAAAGAAAGAAAATAAATCTTTCGTAACAATGAGAAGTCCGGATGATCATGGTGTTCT
AACTAAATTTAATATTAATGATTATGTTGAAGTTAAACCCGATTCTGAAAATAAATTACA
AAAATCATGTTTTGGCTTTCTTGTTTGGATGGGTTTTGATGGTGATAGGTCCTTAAAGTT
TTTGATAAATTTGGAATATCCTCATGAACTTGCTTCTAGTGGAACTTTTTATGGCCAAAA
CCTGCATGATATTAAAAATAAACATGGTTTGTTAGTCTATCCAGATTGCTTAATTCCATT
CGGATATGTTCCAGGCAAAGAGAGTCAAGCTAGTTTTCAAGAATCATCTTGTGTTGATGA
TTACCAAATTCCCCGTTCAAACATTACGTATGGTGAAAATAAAGGAATTCAAGGAGATAA
AAATTCATGTTACATGGATACATCATTATTTGCAATGTTTACATTCCAAGAATTATTCGA
TAATCTTTTAATTGTTCCTAAGGATGAAAGATATGAAATTGTAAACATTCTTCGATCAAA
AATTATTTTTCCTTTGAGGAAGCATGAATATGTTAGCTACAATAGAGTTAGTGAATTTAG
AGAAATTCTGGGGAAATTATATCCTGAAAAAAAGTTTTTACATAGTGAAAAAGACCCAGA
AGAACTATTAGAAACTTTATTTCATCTGTTAACACCACGGGATCCTTTACTTAAAACATA
TGATATAAAATCTAAAAACTGTCAAAATTGTTTCATGACTCAATTATTTTACACGAATAA
AAATAATTCAGGTGTTACAACAATTAAAGAAATGCTTAATCGAACATTTTTGGAAAGCGG
TATTCTGATAGATAAGCCACCAAAATGTTTAATTTTACAACTACCCCGCCATGGTACACA
ATTTAAACAATATTCAGAAGTTGAAATTTCATCGACATTAGACATAACTGATTTATTACG
TTGTGGAGACCACAAATGTTCCCAGTGTATTAACAAAGGCACGATTTTGTGTCTTACGTG
TTCTGAAGAAAAGCAAAACTTTACCTATTTTTGTAGTGATCACAAAACACATTGTACACA
TCCTGGTAAAAATGAGTTTGTCGATGATAAATTTCCTTTTTATAAAGGGAAGATTAAGTT
AGAATTGTTCGCAATATTGTGCATTGAAAAGACACATTATGTAGCTTATGTAAAAACTGG
TGAAGTTCTTAATAAATGGATATATTATGATAGCATGGCTACTTCTGAAAAAAATGTTCT
GAAACCATTAATTCGTGTTACTTCAATAGGAACATATTTTTCAAAGAATGAATATCATTC
AGTTGTCGACGAAGATCTCATTAGAATTAAAAAATTTGCGTTTGTATGTTTCTATAAAAA
TCCAGCATTGGAATATTATTTTAAAGATTTTAATCACGTTAACCAATAAaattcatctga
gtaatcacatttgaactatgcgttgcctttctgattttgtaattgcttaaagtataaaat
ggataaacaactttttttaattcaatcctataattaaatttattttgctttaactatta
aaacaaaattaatattattaatttaatactattatcaaataaaattggtcaattaatcat
tgattaagattgatcatttaaaggataaattaaaattttaaaatatgtttgaataataat
tatatgaatagcctagtggagtatgtttgtttttcacgcataaataaacatactctact
gggctattcatataactattattcaaacattttaaaattttaatttatccttaaaatgat

caatcttaattaatgattaattggctctatattattcataaatatgatattaaaataatt
ttattttagATGAATTCCTCGTATGAAAAAAATAATGTTATCATTATTCCATCCAAGGTT
AAGAATATCCAATGTATTGATTCAAATATAATAGGAAAATATATTGTTTTTCAAACTGAA
TCTTCAACTTATAAAGGTACTGTGAAATGGTATGGTCAAATTAATGATACGAGAGCTTTT
GGTATTGAAACAGATCCAATTGAAATGGGTACTAATGGTGAATTTAATAATAAAAAAATC
TTTGATTGTAAAAAAGAATGTGGAATATTAATTAAAGAACCTGTCAAATATTTGGAAACC
GATGCATTACATGGTTTTGTTAATTATAATGAATTCAAAGTTGATAAAAAAATTTACAAAT
TCCAAGTCTCAATTGGTCTATGTTAATTTTAAAGACGAATGTAGAATCGGCAGAATTTTA
AATGGAGGATTTATTAAGGAAATAATTTGTTACCAAATTAAAATAGTTAAAGAGTCAATC
GAGTATTACGTTATTAAGAATGACATTCTAACTGATTTTGTAAATGATGACGGCATAGTT
TTATTACGTAAAGATGCTGTTTTGAATATTGAAGATTGTAACGAAGATTTTTCTTTGATT
ATTGATAAAAGAGTTCGCTTTCAATATAATAATGAAGACGAAATTGGAGTATTAAAATGG
AAAGGAAAAAATTGATGGTTTAAAATGTATTGGTGTAGATCCACATAACGAAAAATTGATT
TCAGACTGGATAATCCATTTCAATCCAAATGCATATTGGACGATCATTTGTTTAGATTTT
TTGGAAAACAAAGGAATTTCTATAATGTTTGAAATCGAAGATCCTTTCAATTATGATATT
TTTACTTATTTAAAAAAGAAAACATTTGTAAAAATTAAAGGTGATGAAGGCACCATAACA
TCTTATTTTACTGATAAAAAGAATCTTTTTTATGAAATTAAAATGGATGATAACGAAACA
TTTGTTATTGCTAATGGTAATGACGTTAAATGTGGTGCTGTAGCAAAGAAAGAAAATAAA
TCTTTCGTAACAATGAGAAGTCCGGATGATCATGGTGTTCTAACTAAATTTAATATTAAT
GATTATGTTGAAGTTAAACCCGATTCTGAAAATAAATTACAAAAATCATGTTTTGGCTTT
CTTGTTTGGATGGGTTTTGATGGTGATAGGTCCTTAAAGTTTTTGATAAATTTGGAATAT
CCTCATGAACTTGCTTCTAGTGGAACTTTTTATGGCCAAAACCTGCATGATATTAAAAAT
AAACATGGTTTGTTAGTCTATCCAGATTGCTTAATTCCATTCGGATATGTTCCAGGTTAT
TGTAAAATCTTTATTGTATAAcctattgaccattattatcgaaaatttgttgccatctct
ctgtaaatatcaagattccctttcataatatttatgattttgttcagtaaaaaatgttt
caagatacattttgataatattatctggagttatgatattaaaataattttattttagAT
GAATTCCTCGTATGAAAAAAATAATGTTATCATTATTCCATCCAAGGTTAAGAATATCCA
ATGTATTGATTCAAATATAATAGGAAAATATATTGTTTTTCAAACTGAATCTTCAACTTA
TAAAGGTACTGTGAAATGGTATGGTCAAATTAATGATACGAGAGCTTTTGGTATTGAAAC
AGATCCAATTGAAATGGGTACTAATGGTGAATTTAATAATAAAAAAATCTTTGATTGTAA
AAAAGAATGTGGAATATTAATTAAAGAACCTGTCAAATATTTGGAAACCGATGCATTACA
TGGTTTTGTTAATTATAATGAATTCAAAGTTGATAAAAAAATTTACAAATTCCAAGTCTCA
ATTGGTCTATGTTAATTTTAAAGACGAATGTAGAATCGGCAGAATTTTAAATGGAGGATT
TATTAAGGAAATAATTTGTTACCAAATTAAAATAGTTAAAGAGTCAATCGAGTATTACGT
TATTAAGAATGACATTCTAACTGATTTTGTAAATGATGACGGCATAGTTTTATTACGTAA
AGATGCTGTTTTGAATATTGAAGATTGTAACGAAGATTTTTCTTTGATTATTGATAAAAG
AGTTCGCTTTCAATATAATAATGAAGACGAAATTGGAGTATTAAAATGGAAAGGAAAAAT
TGATGGTTTAAAATGTATTGGTGTAGATCCACATAACGAAAAATTGATTTCAGACTGGAT
AATCCATTTCAATCCAAATGCATATTGGACGATCATTTGTTTAGATTTTTTGGAAAACAA
AGGAATTTCTATAATGTTTGAAATCGAAGATCCTTTCAATTATGATATTTTTACTTATTT
AAAAAAGAAAACATTTGTAAAAATTAAAGGTGATGAAGGCACCATAACATCTTATTTTAC
TGATAAAAAGAATCTTTTTTATGAAATTAAAATGGATGATAACGAAACATTTGTTATTGC
TAATGGTAATGACGTTAAATGTGGTGCTGTAGCAAAGAAAGAAAATAAATGTTTGAAATT
ACGATTACTGACTTATTAAttaaattgaattcagctttcgtaacaatgagaagtccggat
gatcatggtgttctaactaaatttaatattaatgattatgttgaagttaaacccgattct
gaaaataaattacaaaaatcatatgatattaaaataattttattttagATGAATTCCTCG
TATGAAAAAAATAATGTTATCATTATTCCATCCAAGGTTAAGAATATCCAATGTATTGAT
TCAAATATAATAGGAAAATATATTGTTTTTCAAACTGAATCTTCAACTTATAAAGGTACT
GTGAAATGGTATGGTCAAATTAATGATACGAGAGCTTTTGGTATTGAAACAGATCCAATT
GAAATGGGTACTAATGGTGAATTTAATAATAAAAAAATCTTTGATTGTAAAAAAGAATGT
GGAATATTAATTAAAGAACCTGTCAAATATTTGGAAACCGATGCATTACATGGTTTTGTT
AATTATAATGAATTCAAAGTTGATAAAAAAATTTACAAATTCCAAGTCTCAATTGGTCTAT
GTTAATTTTAAAGACGAATGTAGAATCGGCAGAATTTTAAATGGAGGATTTATTAAGGAA
ATAATTTGTTACCAAATTAAAATAGTAAGTATG
>DjScaffold442:72832-78205 strand:+
TATAGATTTAATGAATCTATCAGAAACCCATGCTACCTTCTGAATTTTCAAGATATCAAT
TTATTATTAGATTGTCCTGTAGATTTTAGTGTTTTACAAAATTTTCTTCCAAGACATGCT

CATTTAGAAGATGGAGCATCCAATTTGCCAACCTTAGGCATTAAATCAAATGAAATTTTA
CAAAATTTAGGTGGTTGTACATATTTTAATTCAAATTTCAAAATTGGATTTCTTCGAAAA
TCTAAACTCAAAGAAATCTATCCCGGTAGAATTGATGCAATTTTAATATCAAATTCATCA
ACCCTCCTTGGTTTACCGATTATAACGGAAAACGGTTCCTTTAAAGGGAAAATATTTGCA
ACTGAACCAACTATACAATTTGCAAAAATTATGATGATCGATATCATATCGAATATCAAG
GGTTTAGCTAAAACCGGTAAAACATTGGATGTTAATTCACCAGAATTTCTTCGATTAGAC
GGTTCCTCATCGTTCAACACTGCCGATATGTCGTCTTGGCACGATATTTACTCAGACAAA
GCTGTTCAATCTTGTCTCTCAAGAATACAAAGAATTTCTTTCAATGAACCTTTGAACTTG
TTTGGACTGTTGGAAATTCGTGGAATCTGTTCTGGTTATTCTATTGGCAGTTGTAATTGG
TTGATCAGCAGCAAATACGAAAAGATTTCTTACATTTCTCATTCGTCTGGATTGGTTTCT
CATGCTGCACCACTCGACACCTCCAATTTCAAAGATGTTGACGTCATGATTATCGGAACT
GCGGTGCAGAGTTTTGCTGCCCCGATTGAGAAGTTGTGCGATGAATTATGCGGGAAAATC
TGTGATACGATCAAATCTGGGGGAAATGTGCTCATTCCGATCACTTGTTGTGGACTGTTC
TTCGATTTGATCGAGAAAATCAGCTCAGAATTGATGCAGAGACGACTCGAGCGCACACAG
ATGTTCATTTTGTCGAGTCAGGCCAAGACGAGTTTGGCGTATGCAAATATTTACGGAGAA
TGGCTCGATAGACAACGAGAGAACCAGCTGTACACTGCAGATTACCCGTTCATGTTCGAT
AAGTTGATCAAATCAAATGTGCTCATTCCTTGTGATGGAATCAGTGGAGATTTCAGTGAA
AAGTATCGTACTCCTTCCATAGTTTTCGCCACTCATCCGAGTTTGAGAATGGGTCCAGTT
GTTCATTTGTTACGATTGTGGGGATCCAATCCATTGAACGGGCTGTTCCTAGTTGATATT
GAGAACTACACATCATCCAGCAAAAAAGATAAATTAGAAAGACTTCTTGGAAGTTTTAAA
CCTTTATCAATGGCAGTTCATTTCTTTCCATTAGATACCGGAATTAACGTCAACGAAATA
TTACCCTTGATTGAGCAAATGTCATTTCCAACTAAAGCTCTCGTGTTACCCGTTGAATGC
ACGAGAAACAAGATTTTAATGAAAGGAACAGAATCTCTTAGGAAATTCGAATTGAAACAT
GAAAAATTTGTCCAAATAGTATTTCCTGATAATAATTCAACTCTTGAAGATATTATTATC
AGTCCTGCAATAGCTGATCATTTGAAACCGATTATTGTCGATGACGACACTAATTTGGCA
TTGTTCAATGGGAAAATTTCATTGAAGGATGGTCAATACGAAATTGTACCGAGTGTTAAT
ACATCGTCTTGTCCGTCGAAAACGAGATTATTATTTGGAAACTTGGATCCTAATCAATTC
ATTGATGAATTGAAATTACAGGGGCTGACTGATGCTAAATTGGAGGTTGTTGAGGATGGC
AAGAGATATTTAGTTTATTTCAACGAAGAGGATAGTCTGATTCAATTGAATGATAATTCT
ACTCACGTTGTTTGTCAAAATGAGGAAATGCGCAAGAAAATCCAGGATGCACTTGTTGCT
TGTTTTTCTGTTTTGTCCTTGTAAaacttgtgaaataaatcaataaatgttcatctgact
tgttatatagatttaatgaatctatcagaaacccatgctaccttctgaattttcaagata
tcaatttattattagattgtcctgtagattttagtgttttacaaaattttcttccaagac
atgctcatttagaagatggagcatccaatttgccaaccttaggcattaaatcaaatgaaa
ttttacaaaatttaggtggttgtacatattttaattcaaatttcaaaattggatttcttc
gaaaatctaaactcaaagaaatctatcccggtagaattgatgcaattttaatatcaaatt
catcaaccctccttggtttaccgattataacggaaaacggttcctttaaagggaaaatat
ttgcaactgaaccaactatacaatttgcaaaaattatgatgatcgatatcatatcgaata
tcaagggtttagctaaaaccggtaaaacattggatgttaattcaccagaatttcttcgat
tagacggttcctcatcgttcaacactgccgatatgtcgtcttggcacgatatttactcag
acaaagctgttcaatcttgtctctcaagaatacaaagaatttctttcaatgaacctttga
acttgtttggactgttggaaattcgtggaatctgttctggttattctattggcagttgta
attggttgatcagcagcaaatacgaaaagatttcttacatttctcattcgtctggattgg
tttctcatgctgcaccactcgacacctccaatttcaaagatgttgacgtcatgattatcg
gaactgcggtgcagagttttgctgccccgattgagaagttgtgcgATGAATTATGCGGGA
AAATCTGTTGGATTTTGCTTTGTTTTTGTTTGCTTATTTGATACATACACACACACGTAC
GCAGGTGATACGATCAAATCTGGGGGAAATGTGCTCATTCCGATCACTTGTTGTGGACTG
TTCTTCGATTTGATCGAGAAAATCAGCTCAGAATTGATGCAGAGACGACTCGAGCGCACA
CAGATGTTCATTTTGTCGAGTCAGGCCAAGACGAGTTTGGCGTATGCAAATATTTACGGA
GAATGGCTCGATAGACAACGAGAGAACCAGCTGTACACTGCAGATTACCCGTTCATGTTC
GATAAGTTGATCAAATCAAATGTGCTCATTCCTTGTGATGGAATCAGTGGAGATTTCAGT
GAAAAGTATCGTACTCCTTCCATAGTTTTCGCCACTCATCCGAGTTTGAGAATGGGTCCA
GTTGTTCATTTGTTACGATTGTGGGGATCCAATCCATTGAACGGGCTGTTCCTAGTTGAT
ATTGAGAACTACACATCATCCAGCAAAAAAGATAAATTAGAAAGACTTCTTGGAAGTTTT
AAACCTTTATCAATGGCAGTTCATTTCTTTCCATTAGATACCGGAATTAACGTCAACGAA
ATATTACCCTTGATTGAGCAAATGTCATTTCCAACTAAAGCTCTCGTGTTACCCGTTGAA
TGCACGAGAAACAAGATTTTAATGAAAGGAACAGAATCTCTTAGGAAATTCGAATTGAAA

CATGAAAAATTTGTCCAAATAGTATTTCCTGATAATAATTCAACTCTTGAAGATATTATT
ATCAGTCCTGCAATAGCTGATCATTTGAAACCGATTATTGTCGATGACGACACTAATTTG
GCATTGTTCAATGGGAAAATTTCATTGAAGGATGGTCAATACGAAATTGTACCGAGTGTT
AATACATCGTCTTGTCCGTCGAAAACGAGATTATTATTTGGAAACTTGGATCCTAATCAA
TTCATTGATGAATTGAAATTACAGGGGCTGACTGATGCTAAATTGGAGGTTGTTGAGGAT
GGCAAGAGATATTTAGTTTATTTCAACGAAGAGGATAGTCTGATTCAATTGAATGATAAT
TCTACTCACGTTGTTTGTCAAAATGAGGAAATGCGCAAGAAAATCCAGGATGCACTTGTT
GCTTGTTTTTCTGTTTTGTCCTTGTAAaacttgtgaaataaatcaataaatgttcatctg
acttgtta
>DjScaffold2211:28094-31192 strand:+
aaaattttccaaatatttttgtaaaattatttagaatttaataaaattataaactcaatt
aatttcaatttttaatgaatttctatttgaaaaccgataattataatttttagcatgaaa
tgaattattgacgtttcatttaaataccctaaagaaaaattatttttaaattatagattc
tccagtggtttctgataaagattctctagtggaagaaattccattacaaactgaagaatc
gatttcaagaggaacttcgaattattccaccttcacacgaaagcgaccagtacacctatc
aaattcattacaaagaaagaaacgtcaatcactaATGAGTCCACAATCGATTCCTAATTT
AGATTCTGATGATTTATTAACTCCTGATTTTGCTATGACTCAATCAATGTATGAACCTTT
AGAAAAACGAAAACAACAAGCAAAAATGGAACAGAATTCTAGATTGTTAAATTTATTAGC
ATCTGAAAAAATATCTCAAGCGGCCAAAGAACGAAAACTCAAAGAGATGAAAAAACGAGC
AATAGAACAGAGAAAGAGAAATGAAGAAAAACAAAAATTGGCTGAAGAAAGAAGATTGCG
AATGTTAGCCGATCAAGAAGCTAAGAAAAAGAGAATTCTTGAGAAAATACACCTACCAAG
TCCAATGACAGGCAGTTGCTATGGAAACATGAATAGAAGTATAATTAAACAAAGAGTTTC
TGTAGGAAATGGCTTTATAGATAAACGACCGTTGTCGTCCACAGGAAAGGTATTCGGATT
CGGATCAAGTGCAAAAAGAGAAGTCTGTGAAAGACCTTCTCAAAACAATTTTAAAAATAA
AGATAGCGATTTTGGAAATAAAAGAAGTGATCAATCAATTTCACGAATGTGTACTAGTCA
AATAATTACTAATTCAAATTATAATAAAGAGAGAAAATCATTTCTACCGGCGAAAGGTAT
TTAAaaagataattaatcaattttaaaaaatagaaatttattattaaatgaaaattttaa
attttaattttaatgtgaaaaagtataattttttgtcaattaaatttgttgctgtaaaat
acttgaaatttttattatacataaaaatattaaagaaattatttttgattcttttcaaat
tgttaacatttcctattcaaaaaactttaaaaaattaattaaaaaaaattaattaaaaaaa
ttaattaaaaatattaattaaaaaaattaattaaaaaaaggaattcaacaaatgaattta
acaaatgaatttaaaaatggatttaaaaattgatgtaaaaatttgaagttattaaattca
aaaataaatgatatttaataa
>DjScaffold2592:40523-43249 strand:+
ATGAATATTGGTCAAATTGGCAATACTGTTGGACGACTGTGGAGGAATTTATCAGATGAT
GATAAGGAAATATTCAATCAGGAATATAAAGATGACATGACCAAATATGAAAATGAATTA
CGAGTTTATTATAAATCAACTGTCTATTTGGAATGGTTGAAGAAAATTGAAGAAAATAAT
CAAGCAAAAATTAAAGAAAGTCCAAAAAAACAAATTGTGCAGTTAGAAAAGATGGTTGTG
GATAAAGAAGCTGTTTCAGAAAGTAGTTATTTGCAAGCAGATGTAGAGTCCGATGAGGAA
GTTGACACCTTTGATATCAAAATGGAATCTTCCAATCGATTTTATCGAAATCATGGTCTA
ATGTTGGAATTATTTTCCGATACTCGTGTGCCTAGTAATTTTAAAAATATCACCGAAGAT
CAATCCAGTGTAAATATTCAATCTCAAATCGATGAAATTGAATCTCACATGACAGAAATT
CACAATCTTCACTTTAATCGCATCAACGAAATTAATCAGAAAAGCGAAAATTTTCAGAAT
TTGATGAAATTAGAAAACGAAAAAATGAAAAGTTTAATTACATCTCCAAAACTTTGTGAT
TGAttaattaatgcaattacgtaatattcaatgtgcctatgttt
>DjScaffold3397:40826-49578 strand:+
gagaaatttctcagtatagatactgggaattttATGCAACATGTAGTCGTTGAAGGTAAT
GTCATAATAAAAGGTCGTCAAGTTCCAATTGAAACGCCCGTAAATAAAATATCAGCAATG
GCGTACAAAAATAATGTTGTTATAATTGGTGATTCCGATAGTAGCGCTCTTTTCTGGAAT
TTTAAAACATTAACATCAGTTGCTGTTCAAGTTAATCTTGGGAAAATCAATCGAATAACT
TTCGATAATGTCACATTTGGTCAGAATAAACATGTTATTATGCTTTGTGCCGATGGAATT
TCGACATGGGATTGGAAACAATGTACTGTTGTTAAAACAATAAAATGGGGCAAAGATATC
AATAACCGTATTAATGGAGTTGATTGGCTAGATGATGGTCACACTTTAGCTCTTTTGGCA
GCATCAGATATCAGAATTGTCAGAGCAACTTCTTTGGAGACATCATGTTCTGTGGTTAAT
ATTGATGATTTTTCAGGTTAGcgaaatatttctcgttttttttaatgcttatttttgtta
acatcatctctaataattgatattttctgaaaattgagaatctctcattcacgatactgt
taatttgattgtcaaagatgtttgttttctcataatgtaattgagagaaccaactatatt

ttagttagagatatccattataaatacaataaatttatttcaa
>DjScaffold3397:66065-67031 strand:+
TTTGTCCTGAAACATTATTTACAACATCAACCTTGGAGATCAGAATTTTCTGTTTATCCT
TCACTATGGCAATCAGAAGAAGATATTATTAAAGATCCAAAACTAAGAAAAGTTTTTGAA
CAATTGCATAATTATCAATTCCTGGAATCTTTCAAGCATGTTTCTATATCTAGTGCGCAA
AATCTAGCCACTAGATGTTATTTAACTGCGGTTTTATTTGGTGATGATTATGAACTGAAA
TTTTGGCTGGTTGTTCTCAAAGTATTTTTTCCTGATAAGTATGAAACTTGTCTTCCTTGG
GAAATTTTGTGGTCAACTGAAATTTACCGAGCTTATCAAAAATCAAGGTTATGGAGTTTA
GAAAGGAAAAGAACATCTCACAAACAGACCCAAAAATGCGCTGAAGCTTTTATTATGCTA
GGAGATCTAGATAGAGCTGTCCGATTGCTCTTGGAAACTCCTCCTTCTAATATAAATTAT
GTATCAGATCTTCAATTGGCTTGTTTGTTGGCTAATCAATCTAATGAAACCACAAGACCA
AATAAAGGAGCAACTGTTGGCGGAGATTCGACAGAATCTAATTTAAATGCACATCACAGC
ACCGTGAAATTGGTGGCAACACAGTTTATTTCAAATAATTGTTTGAAAGAAGGTGTGCAA
TTATTGTGCGCTATTAACAAGCAGTCTGACGCATGTCGGTATTTAGAGACTTATCATGAA
TGGAAAACTGCAATATGGTTAGCAAAGGTAAGTCCATTATTATTTGAATTTTTTATATTT
AATATAATTAATTTGTGAgattaattaaaataattaaaattttctaatatacttggtaat
t
>DjScaffold2720:17706-20479 strand:+
gttacttATGAATAACGATTATTTTTATCCTGAAGATGTTGAGCAACGAAATGTTATTGA
CAGACTTGCTAATTTTGTAGCCAAAAATGGAGATGAATTTGAATTAATGACAAAACAAAA
ACAGCAAGGAAATTCGATGTTTGAATTTTTATTTGGTGGAACAAATAGCGATTATTATCA
GTACAGATTATCTGAGGAAAGACGAGTTGTGGAAAGTGAAAGTCATTTGCATCATAACGC
GTCAGAGTTTTTAGAAAATAAATCTACCACATCTTTCACCCCATCTAATCAATTTGCACA
TGGCTTTGATTTCACACAATCTGACTATTTCCAAAATTTCGACGTTTTAAATGAAATGAA
TAAGCGATTATCTGATTTGAAGATATCCCTGAATGCTTTACAAGGAGAACATTATGCGAA
ATTGAATGACAGCCAACAGAACTTGACAACAGAATCAATTACAATTAATGAAGAAAAAGT
TATGAAATGTTTGAATACAGTCAACGTATATCGATTCAATAAAATCAAAGAGTGTTGTAA
GGATTATAATATTAAATTTGAAAATATGGAAAATACCATACAATTTTTAATTACCAGTTG
TACTAAAGACAATATATGTAATGGAAAGAACTTCGTTATCGAAAATATGGTAAAAGACAG
TAAAACATCACAGTTTATATGTGAATATCTTCTTGCAATTGTTTCAAAATCTAGTTTATT
TAACGATAGATTGCATATAATATATTTTATAAATGATCTACTTCACTATTGTAAAAGAAA
AAATGTGCCCATTTTATTGCAAGCGATCGAATCAGTTATAGTCCTTATTTACTCCATGTG
TGTTTCTATGGCCGACGAAGAAGGAAAAAGTAAATTAAATAAAGTGTTATCTCTTTGGGA
ATCCAATTCTTATGTTAAAATTGATATATTGAATTCAATGCGAACATCAACCGACAATCC
CTCAATGTATATTGATGAATGGCAGGAAAAAGAAATAGCGTCTATGAAGCGGGATGAAGA
TGATGGGAATGTCCCGAATAATAATATATTGGCAGAAGAATCTATTTCTAGAATATTCTC
CGACGCAAAGATGAAATTAAGCAATTTGGAAGCAAACCACAATCAGTTAAAGTATGAGTT
GAAAATTGAAGAAGAGAATATACAATTGGAAATTTATCAACTTGAAAGAGATATTAAATT
AGCTGAAGCGGAAGTACGAAAAACTCCTCGAGAGACGAGTCATAATGACTATACAGATAA
TGATTATTCGGTTCAGTTAATGACTAATTATGATTTGTCATTTCAAATGCCAGATATAAC
TCGACCGCCTCCTGGATTTGGAATGTGTGAGTTGCCAGTAATAAATGTCGAACCTCCTTT
TGTGCCTCCCATGATTCAGCAACCAGAAGTTGAACAACGTCCTGAACGACCCCCGATACC
ATATTGGAGATTACCTGCTGCTGTAATGGTTGCACATATAAAATTATCTCAATACGATTA
TGAACCTATTGATTCTAATGCAATTCAATTACCACAATTACGACCGCCTTCAAAGCGACT
AATTGACATAACAAATACGTTCTATCAGCCTCCTAGTCACGAGAGACCCAGGGATACTGA
AGGATGGGAAAAATTAGGTTTACATGAGTTCTACAAAACTAAAGAAATGGCGAAAAACGG
CGAACTACCGAATGATTTTATTAATGTCTTACATGAGAAGCAAAATGAAGATGATTTCAA
CGATTTAGTTCCAATGAATGAAAGATTAATGGATACCGATTATCGTCATATGGAAATGAA
TGAAATTAACGATTTCAGAACATTTTCCGATATGAGTTGCTCGAATTCCGAATATAGTTT
TCATCATACTCCATCACAATATGGATTATCTCCACCCCATTGTAGCCCGCGCATTAATAA
CCAAAGATCTTTGTTGGGATCACCTTGGGAAGAATATCGTCATCCCACATTTACCAATAA
TCCAGATATGCCACCAATGCGAAGTATGAATTTTAGTTCCAACAGCCGAATGATACCGAG
CTTGTTAGGTGTCCCTCCTCGTCATATGCCGCCCAAAGTCACACCATTGATGAGTCTTGA
TATATCGAATAATTTCAAAAGACATCCAAATCCAAGGACAAAACGAAACAATTATTCACG
AAACATCGATGGTGTATCGTATAATGATAGAAATAGAAACCGACATCGATACGTTTCTAG
ATCTCGATCACCGTATAATAAATCAACGTCTCGATCTAGATCCAGATCAAGATCATCGAG
GTACTCCCGAAGTCGCTCACGAACATACTCAAGATCCCGATCATATTCAAGATCTAGATC

ACAATCTTCACGTTCTTCTCGAAGTAGTGATAAACGTCCTTATAAAGGAGGTTTTATTGC
GAGGAAAAGCCGTTCTAGGTCGTTCAGTAGATCTCGCAGTGCGTCCAGGTCTCCTCAACT
GAGACCGGAATACGATACTTCTCACAATCAGTTTAATGCAAATCAGTGGACTGATCGATT
TAATTAAaaatatatttccatgttaatactttttaaaaataaaattgatgttatg
>DjScaffold817:92648-93205 strand:+
ggatatttacatctccccatgagcataagtcaaatggtttagcagagcgcgcaattataa
ctgttagagatatgatagtaacatccttgaaatctggaggagaggagaattgagttttca
tttaattgcacgatatcaaaggtgacaaaattctctccgATGGAAGTTATCTATGGAAGG
AAAATAAATATTCATTCAACGAAAGAATTCAATAGTAATCATAAAGAAGTTCTGGAAAAG
TCCGAAAGTAACCTAAACGAAGCTTCGAAAAAAATGATGAATCAAGAAGGAAACCGAGTT
GACAGGGAATTTAATGTTGGAGATAAAGTTCTGGTAAAGAGAGATCCGGCAAAACGAAAG
AAAATGATTATATATATATATATGATGGGCCTTTCGAAATTATGGAATTCCTATCACCTA
TAAagtaagattgcagaattcagaaggttcaaaatacagacgtatagaatggctgaaaag
gtgttaacaatctggaaaagacggataagttggaaataaatgtgaccttgaa
>DjScaffold1320:11930-30450 strand:+
aattttagATGGATTTTGAAATAAATCCTAACTATTTGGACTGTATTAATGATTCACCGC
AATTTAGAAAAAATATAAATTTATTATTTTCAAAAATTGTTAACTTTCAAGATGTGATAA
CAGAAATATCTGAAAGTTTCAGCCTATCCATAAAATCTGGAATTGCATTTTCTTGCCAAT
ATCAAATATTTTGTTCAAAAATCGAAAAATTGCACAAGTTATATCCTGATAAAAATAGTT
TTCAAAGTGACATTGATAAAATCTTAAAAGTATTTTCACAATTTAAGGAACACTTTAAAT
TACTTATGGAAAATTTTCATATGATCCAGAAAGATTTCAAATCGTTTCAGGATAATTTCA
AAGAAATCGAAGCTTGTAAGAAAACCTTTCAAAAATCGACTGAAAATTTGGAAAGTTGTC
AGCTGAAATGTGCCAGTTGTCCAAAATCGAAAGTTAATGAGTGTTTAGAAGTTGACAAGC
AATGTAACACTGCACGTAAAGAATTCACCGAGTCATCATTAACTTATTTTACAAAGATTG
AAAAATATGATATTTACAAGCAAAGTATTTTCTCAAATGCTTGTATTAAAATAATTGAGA
AGAAACGAGAATATTATGAAGTGGGTGATGATTTCATGAAAAAATTAAATGTATGGTCAG
ACGATTTAAGAAAAAGCAAAGGAAAGTATGATGTCAAATATCAAGGGAAATGTGATGAAA
CCAAAGAATTAATGAAATATTTCACGAATCAGTCTTCATATCAAAAAGAAAACACGTCAA
GAAGTGTAGTTATAGAAGGATATCTTTATAAATATCCCCGGAAGTCTTGGCAAAAATGGC
AACGAAGATGGTTCTGTGTAATGAGCAATCAGTTGTTGTCATGTAAACGTAATGAAGACT
CTTTCAGTATAGTAGAGGACGATCTACGGATTTGTACAGCAAAAGAATTAGTCGATAATG
ATAGAAGGTTTTGTTTTGAATTGATTGGATTGGATCATACACACGTGCTGCAAGCAGACG
ATGAAGAAGCACGGAAACGATGGATTTCGTGTTTTCAAAACGGGATGAGCTCCCAAATGG
GTACATTAGGCAGCAAAGATCAACAAAGCGGAGGACGAAGTGTATCTGCTATCGCTATAG
GAAGTGATAGTAATTCAGACAATAACAGTAATTGCAAACGACGAATATCGTCACAATACG
GTGGACGCTCAACTAGGAGACATTCCAATCAGACGCAAATCAACAATGGTGGCGGATCAA
ATCGGAGTGAAGACAGAAAAACAAAATTATTATCAAATGTACTGAATGTACGAGGTAACA
ATTTATGTTGTGATTGTAATGATGGCAAACCGAGATGGGCAAGCTCGAGTCTTGGAATTA
CTCTATGTACAGAATGTTCAGGATCACATCGCTCATTGGGGGTCCACATTTCCAAGGTTC
GATCACTTACCCTGGATAACTGGGAACCGGAAATGCTCTGTGTCATGTTTATGTTGGGCA
ACGAATTAGTCAATCGGATATTTGAAAGTGACAAACAAGCTATTGTAAATGCGGCACAGA
GCACCACTAATAGAATTGTTAAACCCGATTCAAACACAGACATGACAATAAGAAAGGAAT
GGACACGTGCCAAATGGGAAAAAAAGCAATTCGTTCAATCAAATGTAAAAGAATTACTTT
CAGATTTGGAGTGTTATATACGTGAAAATATATCAGTGTTTTCAGGTGTACTTCAAGATG
GTGGTTATCCATTCACATCCAGTTCAGATCTCCTTAGATATGGTCTTGAAATAGATTGTT
TACCGTTGATGATGTTAGCTTTGGCTCTTAACGCTAGTCCAAATGGAGATGAAAGTGAAG
ATTTTATTCCCCTTCACTTAGCTGCATCTACGGATTTTTGGCCAGGTTGTGAATTCCTCA
TTCTAAATGGTGCTAAGATCGATATCGTTGATAAAAACAACCAGACTGCATTGCACAAAG
CTTGCCAGAATGAAAACGTTCTCGTTGTATGGCTGCTCCTCAAAAGAGGGGCTCGTCATA
ATATTCGAGACAATAATGAAAATGAAGTACTGCAAATCGCTATGAACACAACAAATGCCA
ATACAGTAACTCTATTGAGGCTGCAAATGTTAAACGAGAATACAAAAGTAAGTAGTTTAA
ATTGAaaccttaaaatatttgtttatgaataactttttctatctgaatagatagattaaa
aaattgtaatagattctttattcgtagttattattcatttgtttagctaattttacatga
ttgagggcagcaggttatcacgaggaaatgtagcattgatacgaaattgaatttattaag
tgtaaatatttagtcagcacattctattttgtaaattattccaatattt
>DjScaffold1320:50764-53875 strand:+
tcttaatgaaatttctattttagggttggaagaagaggaataggagaaatttatctaaaa

agtctaatgtcaacataaatgaagatttatgcattttgtgtgatattaaaagtgatgaac
cttatttaggtcaattaataaaacaatcaggaattgctcttcatgaaaattgtttatttt
tcagtccagttttgcatcaagaatttgatccatctcttaaaagaaaaggctatctacttg
ggtttagactctctgacatagataaagaagtgaagcgaattagccgtttgaaatgttttg
tgtgtaaaaagaaaaagggtggaatcggttgttgcATGGGTGCATGCAAACGGTCATTTC
ATTTGTCTTGTGGACTTAAGATGAATGCAGTTGGGATATTTACGGATTCGTTTACGTTTT
ACTGTAACGAGCACGCTGAACACCAGGAATTAACAGAACAAATTGCAATGTGTGATAAAA
TAGTTCCTGAATGTGCCGCTTGTTTAGAATCAATAGTGGATGAGGAGAATACAAGTGGGA
CAGATATGACCGATGAGCAGCGGTGGAAAATGTTTATTAGTCACATTGGAAACCGACAAG
TTATTCACGGCAAGTGCTGCAATCGAGTCTGGTTGCACAGGACATGTATGATAAAATATG
GTTTATCTGCAGGTTTGCATCATGTAAAATGCCCAAATTGTTCCAACAAATCGATATTTC
TTGATACTTGCATGAAATTTGGAATCTGGGTGCCTGATAGAGACGCTTCATGGGAAATGG
AACCGGGTGCATTTCAGGACATGTATACCGATCAACCGCCAATAGATGATAATAATCACT
CTGTTAATGAAGAAGTTGTGGTTATTCCAGATTCTGCCCTGTCTTCGCTTCCGCCTCCGC
AACCTGGTGAAATCGAGGACATCAACGATGAGAATCAACAACCAATAATCATTGATGATT
CAAATATTTCTCCCACAAATACTCACAGAATAAATCAATCAGATATCCCTTCCCCTTCCA
TAATTCCAAAGGAACCAATTTCATCGACAAGTAGAAAGAGGACTATTCAACGAAGTTCTT
CTCTAGGAGGAAATCAAATTCCAATAAAAAGACGCCGACGTTCAAATGAAATCAAACCAA
TGAAAACGGTACCACTTGTTACTCGTAATTGTTCAAAATGTTCACGTTCGTTGGTTTGCT
TCAGCGTAAACGACAGTGACTGCGGTAATAATAAACCTCTCTTGGTGTCTTCAAATAATA
ACAATAATAATCAGGTAAGTCAGTGAttgactttagaatttatattgtatatttactaat
gttgctataataacttattttgtgctataaaaacttattttgtgctaaaatcaattattt
tttgtaatattcaattattttgtgctatcttaatgaaatttctattttagggttggaaga
agaggaataggagaaatttatctaaaaagtctaatgtcaacataaatgaagatttatgca
ttttgtgtgatattaaaagtgatgaaccttatttaggtcaattaataaaacaatcaggaa
ttgctcttcatgaaaattgtttattttcagtccagttttgcatcaagaatttgatccat
ctcttaaaagaaaaggctatctacttgggtttagactctctgacatagataaagaagtga
agcgaattagccgtttgaaatgttttgtgtgtaaaaagaaaaagggtggaatcggttgtt
gcATGGGTGCATGCAAACGGTCATTTCATTTGTCTTGTGGACTTAAGATGAATGCAGTTG
GGATATTTACGGATTCGTTTACGTTTTACTGTAACGAGCACGCTGAACACCAGGAATTAA
CAGAACAAATTGCAATGTGTGATAAAATAGTTCCTGAATGTGCCGCTTGTTTAGAATCAA
TAGTGGATGAGGAGAATACAAGTGGGACAGATATGACCGATGAGCAGCGGTGGAAAATGT
TTATTAGTCACATTGGAAACCGACAAGTTATTCACGGCAAGTGCTGCAATCGAGTCTGGT
TGCACAGGACATGTATGATAAAATATGGTTTATCTGCAGGTTTGCATCATGTAAAATGCC
CAAATTGTTCCAACAAATCGATATTTCTTGATACTTGCATGAAATTTGGAATCTGGGTGC
CTGATAGAGACGCTTCATGGGAAATGGAACCGGGTGCATTTCAGGACATGTATACCGATC
AACCGCCAATAGATGATAATAATCACTCTGTTAATGAAGAAGTTGTGGTTATTCCAGATT
CTGCCCTGTCTTCGCTTCCGCCTCCGCAACCTGGTGAAATCGAGGACATCAACGATGAGA
ATCAACAACCAATAATCATTGATGATTCAAATATTTCTCCCACAAATACTCACAGAATAA
ATCAATCAGATATCCGTATTTTCTTAACTCAATCTCAGCGAATATTATCATTGAATCCAG
CTTCCCCTTCCATAATTCCAAAGGAACCAATTTCATCGACAAGTAGAAAGAGGACTATTC
AACGAAGTTCTTCTCTAGGAGGAAATCAAATTCCAATAAAAAGACGCCGACGTTCAAATG
AAATCAAACCAATGAAAACGGTACCACTTGTTACTCGTAATTGTTCAAAATGTTCACGTT
CGTTGGTTTGCTTCAGCGTAAACGACAGTGACTGCGGTAATAATAAACCTCTCTTGGTGT
CTTCAAATAATAACAATAATAATCAGGTAAGTCAGTGAttgactttagaatttatattgt
atatttactaatgttgctataataacttattttgtgctataaaaacttattttgtgctaa
aatcaattattttttgtaatattcaattattttgtgcta
>DjScaffold1320:77354-78039 strand:+
ccaaacgattcgatgttaaaaatttgaaatcaagaATGTGGTCGATTCTGAAAGATCGTA
TTGGCGCGGTTGATATAAAAACGGTGCAGGAAAAGAAATATCCAGATCTGCATATATCTA
CGAAAAAATGTTTTTTTTCTGATCTATTGCTCGCCTTGAAAGGTGAACTGCCTGCTCAAC
AATTGAAAGATCTGAGTGTACCGATCTTGTTGACCTGTCTTTTGCATTTGGCCAATGAAA
CAGGCTTGGATATTGGGAACACAGATGACTACAATGATCTGGTTATTGGACAAGGATACT
CAGAGTATGATATTGAACAAATCAATGAATTCGGTGTTGGAAATGGTTTGATCAATAACT
CACGCAAAAGGAACTCAGCAGGTTGCTCTGGTGAAACCAATCGTCGTAAAAAGAAAATTA
GTTTTCTTTCTGAAGATGCTGACGATAACGATGACGATAACGATGACGATGACGATGACG
ACGAATAAtaagattgttgattgactgattgttttgtttgagaatatagatagataatta

tgattctaacattcagttgtactcatttgtatctcagattcctgaatatgtgctgaatat
gtgctgaatatgtgcgtttaatagattgtattgatgtcttga
>DjScaffold5400:18363-23011 strand:+
TTTAACTTGCCAAGAGAAGTCATTAAATGGTTACAGTGTTTAGATTTATCTAAGAAAATT
CAAAATCCGAAATGGGACATTTCAAATGGGGTACTTGTAGGTGAAATATTTAACAGATAT
TACATAAAAGAATTAAATTCGGCGGTTTTTAACAGTGGTCAGTCGATTGATTCGAAATTG
AAAAATTGGTATTTAATTGAAAATTTTATCAAAAAGTATAAACTAAACATTCCAGAAAGT
TTAATTTACGGAACTATTTATATGAAAGAAGGAGCGGCAATTTTACTATTGCGACAAATC
CATCAAGTTTTGACAAATAAAGGTGAATCTTTATCAGATGTAGACTTGTCACTGTTCAAT
GATTCTCTCTATCAACAAGGATTACCACTTCATGCAAGATCAACAGCGGCCGCTTCTATT
AAAAATAATCTTAGTATTCATGAATTGGCTGCTGAACCGTCAATTAATTTAATAAAACAA
AAGGCCCAGCACCTGATATTAGATCATCGCATGAAGAGAGAATCAGATCGCAATTGTTAT
CCTGAAAGATTTCAAGTTCACAAAAGAATAGGAGAAAAATGTGCAAGATATGCACCAATG
GAAGACACAGCACAAAGAAAGAAAAGTGTGACAACTGAAAAAGAATTCCAAAATTCACAA
GTCAATAACTATGGATTGATCCGAGATATTTCTGTTAATCAACCATCGAAAATTGAAGCA
AGATAAcggacgaatgttatctaatttataaataaaacaatgattttcattttcgttaat
aaatgtttcaccgattgttgatactaaatgtaattaa
>DjScaffold4765:37510-45954 strand:+
agagatctttctataattgtgacaaattataaacaaagttattgattaaagttgctattt
gtttATGTCTGAACATCAGAAAAAACTTCTTATAAAATGTGGTACAGTAATAAATCATGA
TTACACAATTGAAGCTGATGTTCTAATTGAAAATGGTAAAATAATAAACATAGCAAATAA
TATTGAAGATTCGTCAGCTGAAACCATTAATGCTACTGGATGTTTTGTTATGCCAGGAGG
TGTAGATGCAGAAACATATTTTGCAGTTGAACGAGGTGGGGTTAAAAGTCCGGAGGATTA
TTTCTCTGGAACCAAAGCAGCCATTTATGGTGGCACAACTACCGTCATTAATTGTTTGGA
AATATTAAAGGATGAATCCGTGAAAGAAAAATATGAATCAGAAAAGGAAATTGTTTCAAA
GGAAATTGTTTGTAATTTGGGATTCCAATTATGTTTACAAAGCATTAGCGATTCCGTAAA
AACACAATTTCAAGAACTATTGCAGTCCAAATCTGTAGTTCCACTAGTTCGTTTATCAAC
AGTAGATAAAGGATGTCATTGTGTCAAAATTGATGATGGTGATTTGTATGACGCTCTCTG
TTGGTGTAGAGATAATCATGTTCTGCCCACTGTTCATGCAGAGAATGATCAAATGATAGC
GATTCTTGAAAAGGAATTGTTAGCTGCTGGTATTACAGGCCCTGAAGGGCATTATTTATC
TCGACCTGAAAAGGTTGAAGAAGAAGGTATATTTCGAGTGTTAAGTATAGCAGATATGGT
AAAATGTCCAATCCATTTTGGAAAGGTGTATGGTAAGTCATCTCTTGATAGAATAATAGA
ATGGCAACGAAATAGTTCTAATACAATATTATGCGGAACAAGCCTTATTGCTGTAGTTCA
TAATGCAGAGAAATATAGTGATCCTAATTGGAAAGTGGCTGCATCATTTGTAACAATGCC
AGCCCTTAAGATGAACAAAACTAATGACTTCCAATGCACTGAGTTACTAGAATCAGTTGC
TAGCAAAAAAATAAATCATGTTGCTTCTTCGCATTTAGTTTTTGATATGAAGCATAAAGC
CGGTGGTAAAAACGATTTTCGATGTATTGTTCCAGGAACTAATGGCGTTCAATATCGATT
GAAAATTCTCTGGGATAAAGCTGTGAAAAATGGAAGGATGGAGCCAACAACTTTTGTCAA
TTGCGTTAGTACAACTCCTGCCAAGTTAGCTAATCTCTATCCACAAAAAGGAACGATCTC
GATAGGATCTGATGCAGATATTGTAATTTGGAGTGGCCACGGAATATCGCATGCGCATCA
GGAAGCTGGCGATATTAATGTTTTTGAAAACATAACCTGCTATAGTGGTCCAAAATACGT
TATTATAAACGGTTTAGTAGTTTTAGATCACGAAGGCAACTTTTCAGCTGCTAAAGGATC
CGGCGAAATTATTTCTCCGTCATCTGAATCTATGAATGTATTTTTTCAAAAGCAATTAAT
TAATCACAAATTATCTCATGAAATGGAAAGGAAAATTCCACGAGAACCGTATGAGGGTCC
TGTCATCGATCCAAGTAGTGACAATAAAGAAGAGAAACATGTTCTCTATTCACATATGGT
GAATACCGATGTTTATAAAAGAGGTGGGCCTGTTGAAGATAAAAAAGAAGCTTTACCTCC
CGGTCAAAGAGTAATAAGAACTTCAGTGAAAACTGCGCAACCTCCTGGAGGTTCAGCACA
AGCAAGCTGGTGGGGATAAtctctaataacattatctggacgccattttcagtttcaatg
tttacttttatgtttatttcattttattatataggattacaaactattctaataggaata
ttttgtgtgtttgaaaatatatttttatttgttatcaa
>DjScaffold4233:78221-78651 strand:+
ttttttggccgttttcaacttagcagaaagtgcagcaccagaatgtagtttaccaaaagc
tgttggattgtgcaaagcacatATGCCTAGATTTTATTTCAATACTGAATCAGGTAAATG
CGAATCATTTATCTACGGTGGTTGCCGAGGAAACGCAAACAATTTTAATGATCTTTCCGA
TTGTCAAGCACGTTGTGAAAATTAAgaatcaatttgaaaattaataaaataatctattct
tta
>DjScaffold4233:19903-21921 strand:+

ATGCATCTCATCTTACGAGAAGAAAATTTTGAAAAATGTGTAATATACTTAGATGACATT
CTCATCTTTGGAAAAGATTTTGACGAACATAACGAGAGACTAAGAACAGTATTAGAGAAA
ATACGACAAGCAGGAGTAAAATTATCACCAACAAAGTGCACTTTTTTACGAAATTCAGTT
AAATATTTGGGTCATATAATATCTGAAAGAGGAATCGAAACAGATCCAGATAAAATTATA
GCCGTTCAAAATTGGCAGCCACCACAAACAAATGAGGATCTGCAATGTTTCTTAGGATTC
TGTAACTATTACAGAAAATTTGTCGAGAACTATGCCATCATAGTTAGACCTTTAGAAGAA
ATTCTTAGAGAAGCTAATATCATTGGAAACAGGAAAAAGATCGTAAATCTAAAATGGAAA
GAGGAGCACTCAACATCATTTTCAAAGCTAAAAGACGCTTTGACAAATTCACCAGTTCTA
GTTTTCCCGAATCGAACAGATACTTTCGTATTGGATACTGATGCAAGCCATCATGCTATG
GGAGCAGTTCTGAGCCAGTTACAAAATAATGAAGAAAAAGTTATCTGCTATGGATCAAAA
AAATTTACAAAGACAGAGCAGATGTATTGTGTAACTAGGAAAGAACTCCTGGCAGTATAT
TATTTTGCAACACGATTTAAACATTACCTTTTAGGCAAAAAATTTATCATACGGACGGAC
CATAAAAGTTTAACATGGATGCTGAATTGGAAAAAGCCAAATACTTCCCAATATTGCTCA
TGGATAGCAGAATTAGAAGCTTATGATTTTGAAATTCAACATAGACCTGGAAAATTTCAT
GTGAATGCGGACGCTTTATCTAGAGAGAAACAATGCGAACAATGCGAAATAATACATAAA
GACCCGAAAAAGAAACGGAATATAAAGGTGGTAAAATCAATAGATGAAAACCAGAAAATA
AATTATCGTGAAGTCAAAGAGATTGAGACAGAACGAGAAGCCTACGATATGAATAGAGTA
AATAAATTATTAAATTATTTTCACAATAGATTGGGACACCCAAGTGGAAGACAAACTCTG
CATAATGTACAAAGATACCACCAGTGGAATAATATGAAAATCGACATAGATACATTCATT
AACAATTGTATTCCATGTGCAGAAAGAAAAGAAGGAAATAAACTAGGGTGTAAGATCAAA
TTAGCATTCACCGCTTCAGAGCCATTTGAAACAATATGTGTAGATATCACAGGACCATTA
ACAACAGTAGCCGGTTATTCCTACATCTTAGGCATAATAGACGTATACTCCAGGTATGTA
TCATTAATCCCGATGAAAAATATTACCGCAGAGGGGATCACAAAATCTATACTAGAAAGA
TGGATAGCATATTTTGGAATTCCTAAAATCATTCATTCAGATAATGGAACACAATTTAAG
TCATCATCGATGAACAGATTATGCAAGGAGCTACAGATAAGTCAATCATATTCGTCTCCA
TATTACCATGAGGGAAATGGACTAATAGAACGGGCATTCAGGACGGCAAAAGATAAAATA
TTTACGATGAGCCAATCAAATAAACAAAATTGGGTGGAAACTTTACCATTTGCAGAAATG
AGCATGCGATCAGCTAAATCAAAGAAACAAAATATTTCACCATTCGAAATAATATTTGGT
ACGGAAATGAGATTAAGTACTAGGATTGCTTCAGGAGAAATAAAGAATCAGAAAACAAAT
GAGCAGGCAAAGAAAGAGTTTGCAGAAAACAATATTAAGGAGAAAAGGAACCAGCTAAAA
ATACCGGAAGTACCAAGAACAAAACAAAACAAAGGCAAAGTAGGAGATTTGGTTATGGTA
AAAACCAACGAGAGGGGTATGCTTACGAGGAGATTCTCAGGACCATGGAAAGTTAATAAT
ATTTTACATAATAATAATTTAGAGCTTTTACTGAACAAAAAAAGAATAACAAGGAATATT
CACCAAGTAAAACGGTTAAAGGGAGATTCGTCAAACTAA
>DjScaffold4389:8237-8829 strand:+
gtttattattctcctaatttcaaattagtcatttggtttaacaaaatttgtctttatttt
ccttaatataactatataatttaattaagttctatatgattcaacttaataatataagca
aagtcaatagaatttctaatttcttatttaggttattaattgcaaatcttagattttata
cATGTTTAGAAATAGAAATTTCTCAAATTCAGTTATTCCAAATAAAAACTGGAAATATTT
AATTGACATAGATTCCCCTGAGATAAATTTAAATGATGTGAAAAATGTAATCCAAAATTC
GGGAATACCATTTACTGATTTCCGTATAAAGGAATGTATAAATGCACTGGTAGAAGAATC
TAATGATTTAATTGTGCGTCGGGATAAATTTCTCCAATTACTGGAAGGAAAAGATTTCCA
CATTCTTAATAAGATACTCACAGACAATCTGGCGATTCCTAATTTCAAGAATTTCTCAGA
ACATATCAAAGATATTTAC
>DjScaffold4389:19106-26509 strand:+
AACAGATTTAATATTGGCGATTCAACGGTCAATTTCTGTCTGCAATCGACCAGCAAACCT
ATAAATTATGCACTAGCTTTGACAGAGATAGGTTCATACAATCTTCATAAATTTGTAGGT
TATGAACCATCAGGAACTTCATTCAATCATCTCACATTAAATCATGAAAATAAACCTCAT
AATCCAATGATCAATTCCGGCGCAATATTGATATGCAGTCTCATTAAACAGCATCTTAAT
TTACCAGAAAGATTTGATTATATATTTCGTCAATTCAAAAAGATTTGTGGAAATGAAGTA
CTTGGATTTAATAATGCTGTATTTATGTCGGAAAAATCATCAGCTGACCGAAATTATGCT
TTAGCTTATTACATGCGTGAATATAGATGTTTCCCTAAAAATTCCAATTTGCAAGAAATA
TTAGATTTGTACTTTCAATTATGTTCTTTGGAGGTTAACTGCGAATCAATGAGTGTTATT
GCATCTACTTTCGCTAATGGTGGTCTGTGTCCCACGACAGAAGAGAAAGTTCTCAGCTCC
GAATCTGTAAGAGATACTCTCAGTCTAATGCATTCATGTGGAATGTATGATTATTCGGGG
CAATTTGCATTCAAGGTATCATTATTATTGTTAGGGAGCGAGCTTATATACCCATTATTA
AGAAGGATTTCTTTTTCTTCGAACATAAAACCTATAACCGTTAATTGA

>DjScaffold4389:49881-50087 strand:+
ATGGTTTATCTTGATGACATTCTGGTATTTACGAACAATAAATATTCATACTATGAAGTA
ATTGAAAAAGTATTGACGGAAATAATGAAGGCTGGATTAATAATAATTCTAGAAAAGTTT
CAGTTTCATCAAAGCGAAATTAAATTTCATGGACACATAATTAACAGGGATGGTGTCAAA
ACTAATCCATCTAAAAAATGGCAATAA
>DjScaffold2957:9265-9609 strand:+
ATGCATGAACTCTTAGATGGATTAATTTTTAATGGTGTTATTGTTTATCTCGATGATATC
TTAGTATATGGAAAATCTAAGCAAGAACATGACAAAATATTAGAGGAAGTGTTTATACGG
ATTTTTAATTCCGGATTAAAAATCAACCCAGAAAAGTGCAAATTTCATCAAAAAGAACTC
ATTTTTTTGGGCCACACTATTAATTCCGATGGAATTCGAACAAATAACTTGAAAGTTGCA
GAAATACTTAAAGCAGAAATTCCAAAATGCTCATCAAAATTAAAATCATTTCTCGGGTTA
ACAAATTATTATCTCCAATTTATAAAGAATTATGCTGAAATTGCG
>DjScaffold4323:8747-9723 strand:+
AGTTTGTCACTAAAAAACTTGTCTGTTGGTTTGTTGAAACAGGATAATCTTCACTATTTA
ACCAGTCCTGGAAAATTGTATCATTGGAAATTGTATAAGATGAATAGAGAACTGTCTGAT
CAAAACTTTGATTATATGTTTAAATTACTGTTAATTGGAAATTCTTCCGTAGGTAAAACT
TCATTTCTTTTTCGGTATGCCGACAACAGTTTCTCATCAGCTTTTGTGGCCACTGTTGGA
ATTGATTTCAAAGTAAAGACGGTTTTCAGACATGACAAAAGAGTCAAACTACAAATTTGG
GATACAGCAGGTCAAGAAAGATATAGAACTATAACTACTGCTTATTATCGAGGTGCAATG
GGCTTCATACTTATGTATGATGTGACAAACGAAGAATCATTTAATGCTGTACATGACTGG
ATTATGCAAATAAAAACTTATTCATGGGACAATGCACAAGTAGTATTAGTTGGCAATAAA
TGCGATTTAGAAGATGATAAAATTATTTCAACAAGTAGAGGTCAACAACTAGCAGATCAG
TTAGGTAAATCACATTTTATAATGAGTTACTTGAATCTCTAAtttataaggcttaattgc
tattgaaataatctcaacaatccggttatactacactaatattggggattatggaaatta
ttaagatattgacctagtattgacaaatactgatcaatatcaaattgaaaattatataat
ttctgttaaatattaaatattagagtttatttcagttctatttatttgttgtattttaga
taaaattttattggttgactattcattcatcaataattttacgggataaatttgcgtgga
ataaattaatagcattttgaaattttttcagtaaattaagcaagaacaaa
>DjScaffold81:9294-14890 strand:+
acttgtaaaagtttattttaactactcacgtaattttaaaagaattcaataactcgATGC
AATTCTTAGTTAAAAATGTTATTTCCAAAGCGTTTGGTGATCAAAAACCGGGAACAAGTG
GTTTAAGAAAAAAAACCAAGCACTTTATGCAAGAACATTATGTGGAAAATTTTGTTCAAG
CTATTTTTAATGTTGTGGTTGAAAAAACCGATTATACTTTGGTGCTTGGTGGAGATGGAA
GATTTTATAATAAAGACGTAATTCTAAATGTGATTTTACCGGTTGCTGCAGCAAATGGCA
TCAACAAAGTTATTATTGGACAAAACGGGATTCTTTCAACACCAGCAGTATCACATGTTA
TTCGAAAATATTCCGCGGATGGTGGTATTGTTTTAACTGCAAGTCATAATCCAGGAGGAA
TCAATGAAGATTTCGGTATAAAATACAATGTTAAAAATGGTGGTCCAGCACCGGAAAATA
TCACTGATAAAATTTTTGAGCAATCTAAATTGATAAATTCGTTTAAAACAACAGATGTTT
TCCAAATTGATCTATCGAAAATTCACGAAATAACCTTTGAGAATAAAAACAACGGGAAAA
AATTTATTGTCAGTATTATTGATTCGGTTTCTGATTATTTGGAATATATGAAAGAAATAT
TTGATTTCCAATCACTGAAAGCAATGATAACTGGCTCAGACGGGATGGGTCCAGTCAATA
TTCTCGCTAATGCTCTCAACGGAGTTATGGGTGTTTATGTAACAAAAGTGTTTTGTAATG
AGCTTGGGGTGTCAACTGCAAATGCAGAAAATTGCGTCCCACTTGAAGATTTCGGTGGAC
GTCATCCAGATCCTAATTTGACATATGCTAAAGACTTGGTAACACGAATGGAAAGCGGAG
AATTTGATCTAGGATGTGCTTTCGATGGTGATGGTGATCGTAATATGATATTGGGGAAAA
ATGGATTTTTCGTATCACCATGTGATTCCTTAGCTGTTGTGGCTGACAATGTGAAAGCAA
TAAAATATTTTCAACAAAACGGTGTGCATGGATTCGCTAGAAGTATGCCAACTAGTTCAG
CAGTTGACAAAGTTGCTGAACATCACAATCTTCCGGTTTATGAAGTGCCTACTGGATGGA
AATTTTTCGGAAATCTTATGGATGCAAATAAATTATCATTGTGTGGAGAAGAAAGTTTTG
GTATGGGTTCTGATCATATTCGCGAAAAAGACGGCATGTGGGCAGTTCTTGCATGGTTGT
CGATTTTGTGTAAATGTCGTCATGAACAAAAGAAATACGTATCAGTTCAAGAGATAATGG
AGCTTCACTGGAATAGATTTGGGCGAAATTTCTTTACTCGGTATGATTTTGAGAACTGTA
CCAGTGAACAAGGAGAACAAATTATGAAAGTGTTGGAAACTTTTATTTCCGGTTCTGATG
TGATTGGTAAGAAATTTGAGGCCGATGGACACCAGTTTACCGTGAAGGTTGCCGATAATT
TCCAATACACTGATCCAATCGATCATTCACATACCAAGAATCAAGGAATCCGGATATTGT
TCACTGACGGTTCGAGACTGATTTATAGACTCAGTGGCACCGGTAGCAGTGGTGCTACTA
TTCGAATTTACATTGATAGTTACCAGAAAAATGCTGAAGTTGCCCATGTTTCATCAGAGG

file:///C|/Users/czh/Desktop/Data_S1.txt[2019/7/4 17:05:10]

TGGCATTGAAACCGTTAGTAGAGATCGGATTGAGTATTTGCCAAATCAAACAAATAACCG
GAAGAAACGCACCAACAGTTATCACCTAAatatgttttatatcctcctcggaatacagctc
tatatttacttcagtattttctatcctttcctgtcaattagaataaaatttgaagtttt
>DjScaffold541:8170-8644 strand:+
GAATTTAAAAATATATTTAGTAAAGTTAATTATTATACCAAACATTGTCTAATTTTAAAA
CATGGAATAGATGTTACTGATTTTAAACCTATTTTTCAACCATTAATTGATGAGATGATT
AAAAGACTAGAACAAAACGGAATAATACGCAAGACTTTAAGTCAGTGGTCTTTATCTATT
GCGGTTGATAAAAAAGTAATCTAGagacattgggtgatgtatagactatggaaaattaaa
ctcagttattaacagacctatacattctattccagataactagaagttgtttgatttact
tgcagggacaagattcttaagtgcacttgatttatctagtggtttatacaatgtagaaat
aagtgacaaaaacagaagtaaaacagcttttgctacacgaaaaggtcagtaagaatttaa
tcgcatgtcacttgaaatgtgtcga
>DjScaffold541:47383-57976 strand:+
tttttccacagtaaacaaATGTTTGACTATAAAGATATTTATGAAACAGAAGATTTACCT
GAAGCTGAGCAAGATTATGAAGATGAGAATAAAGATTGTGTTGAAAAGTTGAACATAACT
GCATCAGCTGCTTTCAATCGTTTCAAAGGCAAAGAAATTTCAATCAAGGAAGCAAATAAA
TCTAAATCCGTCAGAAATGATAATCCGTATGAACGGGCTAGAAAAATTCTTTCTGATTTG
GAACATTTAAGTAAAGAAATTCAAGAAAATTACGATTCAAATGCTAATAATAAAAGTGAA
CCTGTTACAAAGGAGCATTTAATGTTGCTGATCGAAGGGCTCAGTATACAAGTGAAAGAA
TTAGATTTAAATAAATATTTCGGTCCGGAAATTAGTTTCGAAGTAAAAGATCCGAAAAAA
GTTCTCTATAAGAAAGTGATTAAAAATATTGAGGAGTTGTCTACAGACAATACTATTAGT
AGTAATAAATCAGTGGAGCAAGCTGATAATCAGTCTTATGCAATTACTAATAATACTGAA
GTGAATGAACACATAAATCAATTAAAGATTGCAAATTTCCATAAACGGTTAGATGTTTTG
GAAAAGGTTCTTGGGGATGATAACATCAAGCTATCAAATGTTGTTAATGAATGTCCTAAT
AAAGGATTAATTGAGGCTGTCGAAAGACTGTTTGCAAAGACTAGTTTATTAAATAATCCT
CAGTTGAATGCAATCGAAAGTCGATTGACAGCTGTCATCAATAAGTTACAACATCTCACG
ACTGTTGCATCGAATGCTGACAAATTAGATCAGGAGTCACAAATGAAAATTGCCGAATTA
TATGAGATAGTTAAAAAGTGGGATCATGTGATGGATGCCGTGCCAAACATCATTGATCAA
TGTATTTCAATGCAAGAGTTTCATGCCAAAAGTATCAAGTTAGGTCAGTGTATTTCTCTT
CTTGACACTAAGCATCAACAAATGCATTTCAAGCAGCAAAACAGTCAAATGGCATTGAAA
AATATTCAGAACAATTTAGCAAATAATTTTCAAATAATGGAGAAAAATATAGAAATAATT
AATGAAAAATTGAAAAGTTTAAGTTGAaattgattattttacaaatggatttttgtcgga
ttgggcgggaattttgttattatagatttatatatcctttgatgactttggggttgtcgt
tttattgaatatatataatggttctctttcgtta
>DjScaffold5008:103-492 strand:+
ATGAAGACTGGTCTAAGAAATAATCCAGAGAAGTGTGAGTTTCTTCAAATCGAAATCAAG
TTTCTTGGACACATAATTAGCAGGGATGGTGTTAGAACTGATCTGTCTAAAATAGAAGCT
ATAAGAACATTTGTCAAACCTAACTGTGTGAAGAAGCTACGAAGTTTCTTGAGAATTTGT
AATTATTACAGGATGTTTATAGAAAACTACGCAAGAAAATCAAAGATATTAGAGCAGTTG
TGTAGCGGACGAAGCAACAAGAAATTAGAATGGACAGATGAAATAAACGATACTTTTGAG
AGTCTGAAAACATCTCTAATGAAACGCACTAATATTGTGTTTTCCAGACATAAACAAAGA
ATTTATATTAGATACGGATGCCAGTTTTGA
>DjScaffold2696:24621-35281 strand:+
attgacaatttcaaaatcaaattcaaacaatttatcacttgagctttttattcttagtga
taATGTCATATAATCCAACAACCACTACAGAACATGTTACTACAACGACAACGACTACTA
GTGTTTCTTTAAATAAAGAGTTTCTTACCACAATTCCTGGGATTCTAAAAATAATTGAAT
TGGTTGTCGATATAATCATATGGATTTGCGTAGGTGCAACTCCGTTTTTTGGCTTAGTTG
GGAATGGAGGCTACATTAATTTTTCAGCAACAATAACGTTTATTTGTACAATGATTTTTT
TCATTTTGTATTTGTTTAGTATTATATATCGGTTACCTGGACCATGGATTTTGATTGAAA
CCGTTTCAATTATTATTTCAATATTTTTTCTTACAATTGCGTTAATAGTAGGTGCAGTTT
CACATCATTTTGGACCTGGAGCAATTGCAGCAGCGGTATTTTGTGCATTGAATGCTGTTG
TTTATAGCGTGGAAATGTTCTTGAGATTTAGAATTACTCGCTCTAATGGATGGACATTTA
CTGCAACTGCAATGGATGGTGATATTGCCACTAGAAAGACCACTGTAATGGCAACCGAAG
TTACAACAAATGAGAAAAGTACTGGACAAAATGCATACGGTGACGTGTACAGTGTACCAT
AAaaaaccgattttgtcagtattcaatatatctagaaatattgcaaaattagtttattat
ctgtgttgtctctccaattatggattgaagtagatctttgattatcaatttttgtttta
tgaatatatgtttaattatata

>DjScaffold2696:47872-48690 strand:+
ATGATAACTTTGACAGTGATTCTTTCCTTAATTTTCTTGATATTTTCAGTTTCAATTAAT
TGCGACTGTTTAGGAGAATATAATGAAGAAAAAGTTTACATTAGAATACCACAGAAAATA
AAGAATCGTATCTCTATGAAAGTTCTTGGTCAGCAAATAGCTCCTTTAGAAAATGATTTT
ATGCCTCCATTATACAGTCTTAATGAAATAAAATTATTTAAAATCCTCGGAGACAATTTA
GATCCTTTTTGGTCATCAGTTAACAAACCGGTTGTCCAAAACGATAATAGATCATTATCG
TATGATCCGAGGCTAGAGCAATTTCTAGATAAATTGAATATTACTCATGTAAAGGAAGAG
AAAATATCTGAAAATTTAAAACAACAAATCGAAAACTGGTTGTTGGATAAATCAACTTGT
CAAATAGAATATTTCTGGGAGGATCTGGGAAACTTATTTTGGCCTCAATATATTAGACGA
GCAAAATGTGTGAACTTCAATTTCAGGTCTTGTTCTTGGCCACCAGGAATGAGATGTAGG
CTCGGAGGAAAAAAACTAATTCATTATCTTCGATGGAGTTGTTTTGAAACAGATCAGTCA
GAAATTCCAAACAGACGAAAACGTATTAATTTTAAACGTAATAGAAACCGAATAAATAAT
AAACAATCCAATTTGCGTTCATTTTGGAAACCGAATAAAAGGAAAAGGCGATTGAAAAAT
TTGATTAAAAGTACAGGAATTTGGAAAAATGGATACCGATGTGAATGGAGAATCAAAAGA
ATAAATATTTCAAATAGATGTGAATGTGGTTGTGAATAA
>DjScaffold2696:40031-40825 strand:+
ATGTTGTCAATGATAATGCTTTGTCATTGTAAATACGTTGGTGAGTTTACTGAAAGTAAA
ATTTATCGGCTCGTAAAAAACAGTGAATCAAATGGTATATTGGTTAAAAATTCAGCGGAA
AATATTAATCCAATTTTATTTAATGATCTGATGCCGGAACCTCATAGTTTAAATGAAAAT
CGATTGAAAAGGATTTTGGGAGACAGACTGAATACGATTTGGACGTCTATTAAAGAACCA
GTTAATATGGACCAAAGAATTGAACAATCCTATGATTCTATGCTAGAAATACTGGCTGAT
TCGTTAAATTTCACAGAGATTCGAGGTGAAAAACTTTCCCTTTATGCTAAACGACTGCTG
CAAAACTGGTTGGTGGATAAGTCAACATGTAAAATTAGATATCACTGGGATGATCTTGGT
AAATTGTTTTGGCCACGATATCTCAGAAAAGCGATTTGTGTTAATTCCATATCTAATTCA
TGTTCTTGGCCGCCAGGCATGAAATGCACACTTTCTGTTCCTGTTCCTGTGCACCACTTG
AGATGGACATGTTATAAATCCGAAGAACCACTATATCACAAAAGGCAAAAACGTCAAAAT
CGTTTGTATAGAAATGTGAAGAAATCCCATAGAAAAAGACAAATCAATCCGTTATGGAGT
CCAGAAAAAAGAGTAAAGCAGTTACGAAAATTATTAAGAAAAACCAGCTTATGGAAAAAG
GGTTACAATTGCCAATGGAGAATAAATGAAATCAGCCAACTGAGCACTTGTAAATGTGGA
TGCGAGAAATCGTAA
>DjScaffold2696:74928-75308 strand:+
ATGCCGTTCGGATTATGTACCGCTTCTGCAACATTTCAAGAATTAATGCATAAAACTTTC
AAGAAGATGTTATTTACTGGAGTGCTCGTTTATTTTGATGACATTTTAATTTTTGGTCAG
ACGCAAGAAATCCATAAGAAACATTTACAAAATGTGTTGGAATTAATTGAACCATCTAGT
CTCAAAATTAATCCTGAAAAATATATATTTAGTGTATCAAAACTCAAATTTCTTGGTCAT
AAAGGTGTTGATGATGACATTATTAATAAATTAAACGAGACTGTAATTCCAAAATGTGCA
CCCCAAATTCAAACCTTCTTGGGATTGCCAAATTACTACCCTAGATTTATTAAAGATTAC
GCAACAATTGCCAGTTCATGA
>DjScaffold849:80497-80981 strand:+
tattaagaattaaaaaagacaatcttaaaattaatgtaatttataataattaaataatta
aattttaaaaaaattaaaattaacccattaaaatttagtaaaaataaatgaattcaaata
aaattacttttgtaaaaataaatcttgaatgaatcaaaacgtcaatttctgaaaaagaca
aagtcacaatatagaattattataaatcccaaaaaaacaaattttgatagaatctttatt
ttaatgaattctgaaatacgatcaatgcttacgtcaaacaattattctcacaataattgt
aattgtttccgtgtttataaaataaattgaatattttttaatttttgaataatataaa
agttaatttaaatatttcagactttcaaacaatcctaaatgaaagtaatttattagaaat
ttttaattattagatatacaaagattctgcgtcgtttttccatgaaatctaattttttat
tatgcaggtaataaacagttactcaaagaaataaaacaaataaacagcgaaaaagaatat
tttacgatatttatagttgtctgtcacaaattgagatttcaattagtcaatcaatttgat
gaaacagaattatttgtttctaaatttgacaacgttataaaattaaagaaatcaatttgt
gatcgaaatATGAACATGAATGATTATAAAAGAATGATAATGAAATACAAAGGTGCCACA
CTTGATAATAATCAATATTTACTATCTTGTATTGATTCTACAAATATTCCAATTTTCTGT
GAATTTGATAATAATGTTAATGTCAATGCAATGATTGTTATAAAATTAAAATGTGACAAT
ATTTACTCCGAAATGATACAAATTTGGAAACACCAACAAATCAAAGATCTAAAAATTATC
ATTTCCAAATTGAATAAAATTAATCATAAAGATTTAATGATTTTATATAAATCTACACGA
TTAGATGATGATGATAAAAGAATTGATGATTACAAAATAGAGGATAACGATTGGATAAAA
GTTGTGCGGAAATTTGAAAAAATAGAAAATATTACAATGAAATCAATTTTCACTGTAAAA

GAAACAATATCAATTGAATTCTCAAAATCTGAAACTGTATCAGACATTATCAACGAATCT
ATAAAGAAAATACAACACAAAATGACAACCAATGATTTTGCTGTATTTCTAAATAAAAAA
TTACTGTCAAATGATCAATTAATTTATGAATATAATATTAACAGTAAATCATTGGTGGAA
TTTTGTTATTTCAAAAATTTGAATGAATTTGTAGATAAATCAAAATGGAGTGATTTTGGT
CATGTTTGTCTGTACAAAGCAAAACATAAAAGTTGCGATAAAACAATATTGTTAAAGGCC
TACAAAAAATCTTTCATGGAAACTTCACATGAAAATAATGATTTATCAATGATTTTGAGA
GAAGACTTACAAATGTTTCAAATAACAAAATCTCATCCAAGATTTATCCAATTATTTGGT
ATTTATATGGCTGATGATTGGATCTATGTAGGGTTTGAGAATTCGACAAAGAATCCAATG
AAAACATTTTTGTTTGCAAATAATGAAATATCAACTTATGAAATGTCTAAATGGGCCCAT
CAGATGCTTGAAGGACTAAAATTCTTGCACAATCATAATATTATTATGAAAGACATGAAT
CCAACTGATATTTATATTGATGGCAATCGTGATGTGAAGTTACTGAGTTTCAATATTAAT
CAATATTTACAACATATTAATGATGATGCCGATAAATATAAACATAAATATGGTATCTTG
GCATATGTTGCACCAGAGATATTGAGAACTAAACCTTATGAAGCGAATGTTGATGTGTGG
TCACTTGGTGTTATTATGATATATGTTGTCACACGTGGTCAACTATTTCATGGATTAAGT
GAATCTGAAATAGTTCAAAAAATAAAATATTCTCACCCAATTGAATATGATAAAAAAAAC
CAACCAGCTGGTTTAATCGATTTTATTGATTGTTGTGTGAAAGGAGACCCAAAAGAGAGG
GCCACAATAGATCAATTAATGAAACATGAATTCATTGAGAAATATATATTATAAttaaga
aattttgtttgaaataaatattctattttaaattttgtgttaaaaaaatatttagttaaa
gcacatttttagtgttttattttatatttttactttaaataaattataatttttttatta
taatattccccgggaaaaatatattctgtaatatagtaacttatcattttgattttatca
tatcacataatatgataacgtcattgaatttcataacataaaaattatattcaattattg
aaattaaagatgatcccaataattaataataagcatatggataaataacacaatggccta
tttctgttttaattacaattatataaattcatatagtttaacaatattttacgatattta
tagttgtctgtcacaaattgagatttcaattagtcaatcaatttgatgaaacagaattat
ttgtttctaaatttgacaacgttataaaattaaagaaatcaatttgtgatcgaaatATGA
ACATGAATGATTATAAAAGAATGATAATGAAATACAAAGGTGCCACACTTGATAATAATC
AATATTTACTATCTTGTATTGATTCTACAAATATTCCAATTTTCTGTGAATTTGATAATA
ATGTTAATGTCAATGCAATGATTGTTATAAAATTAAAATGTGACAATATTTACTCCGAAA
TGATACAAATTTGGAAACACCAACAAATCAAAGATCTAAAAATTATCATTTCCAAATTGA
ATAAAATTAATCATAAAGATTTAATGATTTTATATAAATCTACACGATTAGATGATGATG
ATAAAAGAATTGATGATTACAAAATAGAGGATAACGATTGGATAAAAGTTGTGCGGAAAT
TTGAAAAAATAGAAAATATTACAATGAAATCAATTTTCACTGTAAAAGAAACAATATCAA
TTGAATTCTCAAAATCTGAAACTGTATCAGACATTATCAACGAATCTATAAAGAAAATAC
AACACAAAATGACAACCAATGATTTTGCTGTATTTCTAAATAAAAAATTACTGTCAAATG
ATCAATTAATTTATGAATATAATATTAACAGTAAATCATTGGTGGAATTTTGTTATTTCA
AAAATTTGAATGAATTTGTAGATAAATCAAAATGGAGTGATTTTGGTCATGTTTGTCTGT
ACAAAGCAAAACATAAAAGTTGCGATAAAACAATATTGTTAAAGGCCTACAAAAAATCTT
TCATGGAAACTTCACATGAAAATAATGATTTATCAATGATTTTGAGAGAAGACTTACAAA
TGTTTCAAATAACAAAATCTCATCCAAGATTTATCCAATTATTTGGTATTTATATGGCTG
ATGATTGGATCTATGTAGGGTTTGAGAATTCGACAAAGAATCCAATGAAAACATTTTTGT
TTGCAAATAATGAAATATCAACTTATGAAATGTCTAAATGGGCCCATCAGATGCTTGAAG
GACTAAAATTCTTGCACAATCATAATATTATTATGAAAGACATGAATCCAACTGATATTT
ATATTGATGGCAATCGTGATGTGAAGTTACTGAGTTTCAATATTAATCAATATTTACAAC
ATATTAATGATGATGCCGATAAATATAAACATAAATATGGTATCTTGGCATATGTTGCAC
CAGAGATATTGAGAACTAAACCTTATGAAGCGAATGTTGATGTGTGGTCACTTGGTGTTA
TTATGATATATGTTGTCACACGTGACTATTATATTGATTATTATTAAtattattatttaa
ttaaaggtggtcaactatttcatggattaagtgaatctgaaatagttcaaaaaataaaat
attctcacccaattgaatatgataaaaaaaaccaaccagctggtttaatcgattttattg
attgttgtgtgaaaggagacccaaaagagagggccacaatagatcaattaatgaaacatg
aatattaagaattaaaaaagacaatcttaaaattaatgtaatttataataattaaataat
taaattttaaaaaaattaaaattaacccattaaaatttagtaaaaataaatgaattcaaa
taaaattacttttgtaaaaataaatcttgaatgaatcaaaacgtcaatttctgaaaaaga
caaagtcacaatatagaattattataaatcccaaaaaaacaaattttgatagaatcttta
ttttaatgaattctgaaatacgatcaatgcttacgtcaaacaattattctcacaataatt
gtaattgtttccgtgtttataaaataaattgaatattttttaatttttgaataatata
aaagttaatttaaatatttcagactttcaaacaatcctaaatgaaagtaatttattagaa
atttttaattattagatatacaaagattctgcgtcgtttttccatgaaatctaatttttt

attatgcaggtaataaacagttactcaaagaaataaaacaaataaacagcgaaaaagaat
attttacgatatttatagttgtctgtcacaaattgagatttcaattagtcaatcaatttg
atgaaacagaattatttgtttctaaatttgacaacgttataaaattaaagaaatcaattt
gtgatcgaaatATGAACATGAATGATTATAAAAGAATGATAATGAAATACAAAGGTGCCA
CACTTGATAATAATCAATATTTACTATCTTGTATTGATTCTACAAATATTCCAATTTTCT
GTGAATTTGATAATAATGTTAATGTCAATGCAATGATTGTTATAAAATTAAAATGTGACA
ATATTTACTCCGAAATGATACAAATTTGGAAACACCAACAAATCAAAGATCTAAAAATTA
TCATTTCCAAATTGAATAAAATTAATCATAAAGATTTAATGATTTTATATAAATCTACAC
GATTAGATGATGATGATAAAAGAATTGATGATTACAAAATAGAGGATAACGATTGGATAA
AAGTTGTGCGGAAATTTGAAAAAATAGAAAATATTACAATGAAATCAATTTTCACTGTAA
AAGAAACAATATCAATTGAATTCTCAAAATCTGAAACTGTATCAGACATTATCAACGAAT
CTATAAAGAAAATACAACACAAAATGACAACCAATGATTTTGCTGTATTTCTAAATAAAA
AATTACTGTCAAATGATCAATTAATTTATGAATATAATATTAACAGTAAATCATTGGTGG
AATTTTGTTATTTCAAAAATTTGAATGAATTTGTAGATAAATCAAAATGGAGTGATTTTG
GTCATGTTTGTCTGTACAAAGCAAAACATAAAAGTTGCGATAAAACAATATTGTTAAAGG
CCTACAAAAAATCTTTCATGGAAACTTCACATGAAAATAATGATTTATCAATGATTTTGA
GAGAAGACTTACAAATGTTTCAAATAACAAAATCTCATCCAAGATTTATCCAATTATTTG
GTATTTATATGGCTGATGATTGGATCTATGTAGGGTTTGAGAATTCGACAAAGGTTGAAT
TTACATGAacatgtcatatttaataataattatttttattgaagaatccaagcttatgat
aaactgctatccagtgttctccaccaagatttgatgtttgagtattgacgatttaggctt
tgttaactggaaagtatggtagtttgtctaatggatatactcccaaccaagatgatccca
ataattaataataagcatatggataaagtgattatttcctgattatattaacacaatggc
ctatttctgttttaattacaattatataaattcATGATATTTATAGTTGTCTGTCACAAA
TTGAGATTTCAATTAGTCAATCAATTTGATGAAACAGAATTATTTGTTTCTAAATTTGAC
AACGTTATAAAATTAAAGAAATCAATTTGTGATCGAAATATGAACATGAATGATTATAAA
AGAATGATAATGAAATACAAAGGTGCCACACTTGATAATAATCAATATTTACTATCTTGT
ATTGATTCTACAAATATTCCAATTTTCTGTGAATTT
>DjScaffold536:72014-72385 strand:+
ATGCCATTTGTTATCGCAGCAGCACCAGGGACATTTCAAGAGATGATGACAAAAGTGGTA
GGCGGAATCAGAGGAGCCATGGTTTATCTTGATGACATTCTGATATTTACGAACAATGTA
GATCAACACTATGAATTAATTGAAAGAGTATTGATGGAAATAATGAAGGCTGGTCTAAGA
ATTAATCCGGAAAAGTGTCAGTTTCTTCAAAGCGAAATCAAGTTTCTTGGACACATAATT
TGCAAGGATGGTGTCAGAACTGATCCGTCTAAAATAGAAGCAATAGGAACCTTTCGCAAA
CCTAACTTTGCAAAGAAGTTACGAAGTTTTTTAGGAATATGTAATTATTACAGAAAGTTT
ATAGAAATCTAA
>DjScaffold5038:19301-36685 strand:+
taatattactttggtattagtattattattaagatattacttatttagtattgATGAAAT
TTATATTGGTTTTACTTGTCTTGAAATTAATTACACAATCAAATGCTAATGGCCCCGGTA
AAGTTTTTGTTCAACTAATATCATATTACAATCCTTCCGGGAAAACGTATAAAGATGAAC
CCTGTGATGCTTGGTTCCAACAGTTTGTCCAGGATTCATGTGATTTAGTATTTGGTATAT
GTTTAAAAGATGTGGAAATAAAAAATTCAAAATTTCCTTGCAACATTGGCTCGATTTCGA
CATTAAATAATGTATTTTATAATACGTTAAGGCTTCGTTTCGAATCAAATATTGGATTTA
TTCCAAATCCGTTTAATTTCACTCTTTCATCAATGCCAGAGAATCTACAAATACTGATAA
ATGTCACTGATGATGACATATCTGGCCAACAACTAATCAGTAAAATGCAGCACGTAATTC
AGTTACCAATTATTGGGCATGATGAAGACCCTAGTAATTTGAACAAAATTGTTCTCAGTT
CCTCTAAATCAATACTTGAATTGAAAGTTTATGGCCGTTGTCCAGAGTTTCATTATGGGC
CTGGTTGCAATCAATATTGCAAACCATCATTCTCATCGAAAACTGGATTTTATGCTTGCG
ATAAAATTACCGGTCATAAACATTGTTTAATTGGTTGGACAGGTGTAGATTGCCAGCAAA
GAGATTTATGTATATTGGACAATCCTTGTCATGGGAGTTCTGAATGTTCTATGAATGAAA
CTGTTGTGAATTGTGACTGTCCAGAATCATTAATGGGTAAATATTGTCAAACTCCAAAAC
GACCATGTGATTCAAATCCGTGCCAAAATGGTGGTCTTTGTGTTCGAGATTCAACGAACT
CGTTAATGGGAACATCTGAATCTAATTGTATTTGTCCTCGAAATTTCAGTGGTAAACATT
GTGAAAATGATGTTGATGAATGCAAAATAGATCCTTGTAAAAATGATGCTAAATGTAACA
ACGTGATGGGATCGTATAATTGCATTTGTATAAACAATCATTGGGGTGTTAATTGTGAGA
AACCACCTGTGTACAGTGTTGAAGGAATTGATTCCGTTTATTTTCTCAAGCAGACCTCAA
CTGCAAGGTTTATGCAAAATACAATTATAATACTACTGATTGCTGTAATCTGTTTAATAT
TCATTATTGGAATATTGTCGTTGTTGTACTATAAGAATCGTCTACAATTTACTCGATCTC

AAAGTATTGGATCTGTTTATAGCGAAATTCACCATGATGATAAATTTACTAATGTAACTG
ATGCTCAAACCGATCCAAACGATCAGTATATTAATTACTATGAGTTGGAGAATGAAGATT
CGGGAAAAAAAGAAAATAATCTCAAAGAGGTTTTAAAATTTTGTGAAAAAGAATCTGTGA
TCTAGaaatcgtactatttttgtatgtgtaatcgaggcatttgtattactttcctcattg
tattcgttgtccctctcatttgtattaatacatctatattttatttcattccattaaata
aatatttacatttttatccaacgagttcttcatttacaagttgttacttttatgaaaatt
tatgtattatttagtgtttagatcgtattgctttattatgattgtaaattttatattttt
gtgtaa
>DjScaffold3353:15823-17080 strand:+
atcaataattgttgaataagcattagtaattcatgcggttatgaatcgttataccattta
acaaagagtatttatggtaaaaattattttaaatacggcgaaagttatgccgaagttcgc
agaaaatttaagtctgaatatggaagagaaagttgtccgtaccatgtgacaattcaaaaa
gttatcaaacaatttataataagtgagacgataaaagatgaacgacgcaaagaatatcat
aaaagtgatcgctcggaaacaaatattgcagcagtaaactgtagtgtgaatgagaaacca
aaaacgtcaatccgtcgtcgttcacaacaactatccattccaagaacgacgttaataaga
attttgcatgaggatcttcattttaagaaaatcaagattcaaataactcaacaaataaag
ccaatagactttgaaatacgccgcaaattcagtaaatgggccattgaaaatATGCATGAA
AATGAAACATTTTTCCAAAAAATGATCTTTAGTGACGAAGCATATTTTGAACTAGAAGGA
TTTGTTAACAGACAGAATTCTAGAATTTGGGGCAATGAAAACTCACATGAAATACTACAA
AAACAATTCCATTCTCCAAAGGTAAATGTTTGGTGTAATTTTTGGGCGGGTCCTTATTTT
TTTGAGAATGAAGCTGGGAATTCTGTAACAGTTACCGGAGCTCGATATCGAAATAGGATT
GGAAAATTTTTGTTTCATAAATTGAAAGGTAGGGATACCCAGAACATGTGTTTTCAGCAG
GATGGTGCCACATCTCATACGACAAAAGAAACAATTACCCTTTTAAGAGAACGATTTGGT
GAAAGATTAATTTCTCGAAATGCAAACATCGATTGGCCGCCCAGATCCTGCGATATAACG
CCTCTAGATTTTTTCTTTTGGGGTTATTTAAAGTAAaatatgtatattagcgatcctcaa
acaattgttgatcttaaggagaacatcatctatgaaattggtgaaattaagccccagttg
tgccaaaatgtcatagaaaatttttgtaaaagaatgggtgcttgctatg
>DjScaffold3353:44967-45896 strand:+
ATGGAAATCGCTAATTTATTTCAACTTTATCCAAACATTAGCAAAAATCGATTATGTGTT
ATTGCGTACAGGAAATGGAATGTGAGCTTATCAAAACTTCCAGAATTATGTTTCCAAGTA
TTGTCAAATGAAGCTGTCATTATTGTAACTGTTCTTTTATCATTTCTAATTACTGTAACT
TTCATGTGTAATTTTCTCATTCTTTTTTCATATTTTTTTGTCAAGAAATTGAGAATTGCT
TCAAATTTATTAGTTGTTAATTTGGCTATGACCGATCTCTTTTTAAGTGTTTCTGTTCTC
CCTTTAGCTGCAGTCTATCAGGTAAAAGGCACGTGGATTTTTAGCGAAACGATGTGTATG
GTTTTTATCATGTTCGATATGCTGCTTTGCACAGGCTCAATAATAAATATCTGCGTAATA
GCCGTCGACCGATATTTAACTGTTACCAAGCCACTCACTTATAATCTCAATCGCAGTATG
CTCAAAATGATCAGCATGGCTTCCATAGGCTGGTTCGGATCAGCAATAATAACGTTTCCT
TCTTTATTTGGATGGGAACGAAGTCAAGTAGAACGACAATGTGGTTACAGTAAAGATTTG
GCATATCAAATTTATGCTACGATTATTGCTTTTTATCTGCCTTTAGGTATAATGTTTACT
TTGTACTGGAAAATATATAAAGTGACTCGATCGGCTCTCAATACAAGATTTCAACAATCT
TTAAGACTTTGTGATATTGGTAAAACATCGAGAATCCGGCGGAACCTTTCAGTAAATCAA
CTGAGTAATGAAAATTTTGAAAAAAACATTCAAGCCATGAGAATTAGATCGGAAAGGAAA
GCGATAAAAACTCTTGGAATTATAATGGGCTGCTTTACGATTTGTTGGTTACCTTTTTTT
GTAATGCAGGTAATACAAATCTTCTTCTAA
>DjScaffold1169:4034-4476 strand:+
AAATCTACACATTCAACTCCAAATAAAACTCCAGTCGAAAAAGTTTATCACGACAGATTT
ATTCCAGTTCGCAATAGTGCTTTATGGAAAGAAAATAAACAAGAACATTATCAAATGTTT
GACATTGAAAATATTCAAAACCAACCGCTAAATAATAATGATTGTGATAGTAAATCCATT
ACAAATTTAATGGCGGATTTAATTGATAGTGAACTCGCTAGTGATACTAATAATTCACAT
AATTTCGACGCAGAATATAATCCAAATAGTGTTTTAGAAAATCGACGAAACAATAATATG
TTTTCTTTCAAACAAATCAGAGAATCTCCA
>DjScaffold1169:76970-77803 strand:+
caatattttttttacaggaATGGCGAGAATTGAAGAAAATCAGGAAAAGCTAATGGAACT
AAATGAACTTTCTAGTAGAAATCCAGCTCCAGAACTACCTTTTCAGCGACATTTATATAC
ACATTTATCTAAAATTATTCCTACTTATAAGCTAAAACCAATCGCTAGATATGATGAAGG
AGTTGCCTCGATTGGTGGGTCTGTTAAACCATTTGGAAATTTTGTTGTTCAAGGTCATTA
TAGTGCAAATTTACGAAGTTTAGGTGCATCATATCATACGATGCAATCTTCCAGATGGCC

GACTCATTGTCAAATTATGGAAAGGGATTATTACAGATGTTTAGCAAGAGTTGGATTCCA
AAATGAACAAAAGTATTGTAGAATTTACCGTGAAGATGTTTATGAATGTAGTTTTCAAGA
GAAATCAAAACGTCGGAAATTGTATATGTTTCGAATGGCAATCGAGAAGAAAGCCAAGGG
CATAGAAGAACCTCCGAAACATCTAATGTACTGTGAAATATAGaaatataatgttagtca
gttaggagtgtttgtgttgctgttaatgtttacttagctgaatatatcgatcgaattatt
acaatatttttttacaggaATGGCGAGAATTGAAGAAAATCAGGAAAAGCTAATGGAAC
TAAATGAACTTTCTAGTAGAAATCCAGCTCCAGAACTACCTTTTCAGCGACATTTATATA
CACATTTATCTAAAATTATTCCTACTTATAAGCTAAAACCAATCGCTAGATATGATGAAG
GAGTTGCCTCGATTGGTGGGTCTGTTAAACCATTTGGAAATTTTGTTGTTCAAGGTCATT
ATAGTGCAAATTTACGAAGTTTAGGTGCATCATATCATACGATGCAATCTTCCAGATGGC
CGACTCATTGTCAAATTATGGAAAGGGATTATTACAGGTTGAATTATTGAcgaattctag
ttaaaataaattaattgctatgctgatttgatttggtattttaaattttaactaatctca
cgaattaatcccattgaatacatacaaatacatttattttcagatgtttagcaagagttg
gattccaaaatgaacaaaagtattgtagaatttaccgtgaagatgtttatgaatgtagtt
ttcaagagaaatcaaaacgtcggaaattgtatatgtttcgaatggcaatcgagaagaaag
ccaagggcatagaagaacctccgaaacatctaatgtactgtgaaatatagaaatataatg
ttagtcagttaggagtgtttgtgttgctgttaatgtttacttagctgaatatatcgatcg
aattatta
>DjScaffold1169:103581-104500 strand:+
aaacaaagatattattcaaataatacgaataaatacacaaatagaaatggaagtcacgtt
aattcaattaaggaaaatgagaaagattgggcatgtagagaagacaaaggtcactattgt
aatgacgggtcaaatcattatcaagatgaatacccaaatggccaagattcgatcgaagtc
gagattgtccntttcttgacactttaattcaaaagaaacctggtgaaatattaactaccc
aattatatatgaaaccatttcacagtggacatatattaccttggagtaaaaattggtata
attaaaggtgaaagaattcgagctgagagactatcttcaaatgaagaaATGAAATTGATT
TCTCTCAAGTTGATAAAAAATAAATTTTTAAATAATGGCTATCCAAATAAAATTATATCA
AAATATTTATCTTTAAATTCAAAACAAAATAAAATCAAACCAAAAACAAATACAGTTATT
AAATTACCGTTTATTAATGAGAAATGTAGTCAAATCATGCAGAATATAATAAAAAATGCT
AAACTAAAACACAATCCAAGAATTATTTTCAAAACGCAAGCACCTTTGGGCATCCAATTA
CAGAAACCAAGACAAACTTTTTGTGGTCCCAAATGCATTTGTGATGGTAGAAATATATGC
AATAAGAAAAATGTCACTTATAAAATAACATGCACCATTTGTCAAGAAACATATATAGGT
GAAACATGCAGAACCATCCGAACAAGAGTTAAGGAACATTTGAAACAAAAAGAAAGTGAG
GTATTCAAACACTTTCAACACAAACATAAAATTTCACCAGAACAACATCACATTACATGG
AAAACACTAGGAACAGGCTACATTTCTACACTACATAGAAAAAAACATGAAGAAATACTA
>DjScaffold1169:160325-160788 strand:+
AAAGTCGAAAATTACATTTGTAATATTGAAGCGAAATTCGGAAGACAAATTAAAAAAAGA
GAGCGATTGACAAAAGCCTTAAATACTTTGAAGATTAAAAATGATCAGTGGTTATTGAAA
ATAATTGGAAGTAAAGAAGATGTTTTTATGACAGATAATTGGGATGTAGGACGCACTTAT
CTGATAAATCACAAAATAATCACAAGTGGTGAACCAATCAATGTAAAACCGTACGGGCAA
CCAATGAACTTAGGATCTAAAATTGAAGAAGCTANAAAAAGGAAAAGAAAGACATAAgaa
tatgtttggatttcaggcaattgaattaatcggatggagttacgaaaagacaagcctttg
tgtctatgctaatgtcgatgaaattctgaacaagttaaataaaacatttgggaaatgcat
actatcaagtagagcttgtagaag
>DjScaffold1169:182805-183765 strand:+
aaataaatgtaatttacattagtaatataaagtataagttaaactttttatatattactc
aattttttatgaagtaaattttcgtaattttttaattaacgtaattgtcaattttgaatta
tttaaaagttattaattaattgaaatttttaaaggataaaattagaaaactaacttataA
TGACTCAACTGACATTTCCTATTTTATTTTTAAACATGAGTGGCGAAATGCTTTATATTA
TCGAACAAAGATTAAATGCACAAAATGTGCCGGCTGAAAGATCTAATAAAGTAATGCATG
AAATTATAAGCACAATGTTTCGAACTGACTTTATTGATACACTGTTTACACCTCAGAATC
TCTATTCAAAGGTATCAATAAAGGAATTATTTGAGAAACTAGCTCATTCATCAATAATGA
AGTTGAATGCGGCATCAATGTCTAAATTGTACGACCTCATGACAATGGCTGTCAAATACC
AACTGTGTTTAGCACGGTCGGGAAGGGAACTACTCTTCATAACGCTGAATCATTTGTACT
CCATGTACAACTACATTAAGATGTCGCCCAACATCAAGAAATTGTGGGAATCAGCAGTTA
GTAAATTCTTGAAGGTAACAACGAAATATCTCATTATTTTACTATTTGGGGAATTTTTAC
GTAATATTTACTAAtaggattaagaggctcagaatttctcctgttttcgttgataaaaa
tattttccagctttttaggtggggaaaaatttttattggatttttatggaaattcgat

gaaaaagttcattttgtttaaataatatgaaaatttaaaattttgtaatcaaattttt
atgggaatccagatcaacagattctc
>DjScaffold4305:1939-2328 strand:+
ATGCCATTTAGCATAGCAGCCCCAGGAACATACCAAGAACTAATGATAAAAGTATTGGAC
GGTCTAGATGGAGCAATCGTGTATATGGATGACATAATCATATTCTCAGAGGGACTAAAG
GAACATTCTACAACTCTGGGTCAGGTATTAGAACGGATCCAAACGGCAAGGTTGCTAGTG
AATCCAGAAAAATGTTACTTATTACGCAAAGAATTGAAATTTCTTGGTCACATAATAGAT
AAAGAAGGAATTAGAACAGATCCAATGAAAATCAAAGCTATCAAAAATTTTGAGAAACCC
AAATACGTAAAGAACCTGAGAAGCTTCCTCGGAATATGCAATTATTACAGAAGATTCATC
AAGAATTATTCAAAAAATCAAGAGCCCTAG
>DjScaffold4686:12603-50683 strand:+
aagttgATGATTTTATGCCATTCTGAAAACATTTCGAATCAAGATCTGGGTTCTAATCGA
TCTTTATTCCCAGATAATTATGAATTAGTATTTCAAAAACCTGTTTATGATATTGCATTC
GTGTCAAATCACACTGAACTCTCATCTAAAGTTAATAGTCTACAGAATTATTTTCAATCA
AAGCACAAGTTTACAACGCACCATCTTTTTCTTGATGGTATTGAAGAAACTAATGCTTAT
TTGGGAGAATTAAAAAAACACGGCATATTATATGCTATCGTTCTTAACGAAAATAGCGAA
ATTACGAATTTATATGATCTTTATGTTTTGCACAGAGATGAAGTTTTGACAACAGTTACT
ATTGAAAAAGTTGAGGAATTAATAGAGAAAGATTTTAGTAATCTTATGAATAAAGTGAAA
ATATTAAAAACTATTCCGTCGAAGTCTCCACATCAGGAAGATTTAGTGCGTATAAGTGAA
CAGTTAAGACATTATCTTTGTCTGCTGTTTGATTATCGAACGTTGAGCATGAAAGAATTG
AAATATGTTAAAGATTATTTAATTAATCGAAAAGATCAATTGGAAAAAGAGGAAAGAGAT
AAATCTTTAAAATCTCAGAATAATGAATTACAAAGTTATTCTGTTTATGAACCGGCACCA
CTTAAAGATCATAGAAATTCATATGAATTTATCATTGAGGAGAAAAGTAATTGTATACAA
CCATCGAGATTCGTATTCAAAGGAAATAATCGAAATAGTAGTAGTACTGTTATTCAGTTC
CCACATTCTGAATTTAAACCAAAACCTTCAGCAAATTACACGATATTTGATGCTGAATCG
GACAGTATATTACCTAAAAATGTTTCTCAGTATCGGGTATCTAATCCGCATTTAGATTTC
GAAAATTCATTTATGAACAGTACCGATACCTTTTTTAATTCTAACGAAAGCTTCTCATCA
GATCCTATACATATATCAAATAATAGTGACAGTGAATTAGATCGTTCTTTTAAATATCTT
TTTAAAAGAACATCTGATTCTACAAATATTCTATTAAAGAAAATGCCATTGTGTAAAGCA
GAGATTATGTCAAAAAAACTCGCTTCCACTTCAGGCATTTTTGGAAAAGTAATTATGAAA
ATGGGTCGAGTTTCAAAGTTGCAAACAGAATACCAGAAAGCCAAGTTATCAAATAGTTAT
TCCAAGAAAAAAGATAATTCAGTTAAAGTTAAATATATTAACTCAACATATTTTCGAAAT
AAAATAAAGGAAAATCAAAGTAAATCATACAATCACAAGTCATCATCTGTCTCCAACCGA
AATAAAGAAGTTATAGGAAATTTTAATGATTCTAAAGAAAAGCGTCTTGATTTCGATAAA
CATGCATCTAAACGGAAACTTGAAATAACGAACTATACGCCAATTAAATTAAATAAATTG
GATATTGTTAGCTCAAACGCCAAGCCTCAAGTAGTAAATAATCCACTTTTTATTCCTCCT
AAAAGTTCTCCTGACAGCCTAATAAATTTGTTTGTGATGGTTTTAAATTTGATTCCTATA
GAATTGCCGAATAACAATGCCATCTACAATATAATGTCAAATTCGTGCGTTGAGATTCAA
GAAGGAATAATGGAACCTGCTTTGAAAAACGTAAAAATCAACTATGAAAATTGTAAAGCA
AATGGTGTGAATGAAATAATTGCTAACATATATTCCATTTATAATGAATCTGAAAAGGAA
GTAAAAAATGCATCATTTATGATTTTCATTTGGAATAAATACTTTCCTAGCCAGTTGTCG
AATTTCAATCTTATGATTCTGTTTTATAAATTTGTGAAGTTGTTGATGTTATATACAATG
TTCAACAAATCATTAGTGACCAAAAAATTGGTGTTGGATGTTATTGATAAAATTGATGTG
ATTGTTAATTTTGATTTTAAATCTTCTGTTTCAACTTTTAAAAGAAAACAACTATCTTGT
TTTTCTAATAAATTCGAAAAATATTTCAAAGAGCATGACCAATTACATGTTTTATACGCA
AAGCTGACCAATTCTAAATCGATAATTGTTGATAAAAAAAAATTGGATGCTACGATTAAA
AAGAAGAAAATTATTTTCACTGTTGGTCCATTGTTGCAATATTTTATCACTTCAATTAAT
TCCATACATAATCAAATGGAACCTATTCAAATTGAAATGATTTATGGTGCGATTAGAACT
CTTCGGTTTTGTCTTAGAAAAATTAATTTAAAATTACTTCAAACATCCTTCACAAAGCCA
CATTTATTTAATTTTAGAGTTAAAAAAATGGATAGTCATTTGGCTGAAATTTTGAAAGAA
ATAAAACTGTTATGTAAAGAACTCAAATATTATCAATGTGTTCTCTTTACAGAAAAGCAT
TATGATTTTATAAATTGCTTGTTAGATTTCTCATGGATGCTGGAAGGTACTATAGATTGC
AATTCAGAAGCATATAAATCTAAACCTTTAATTGAACCTTTAATGGTTTTTAATCCTAAG
GATTTCATTAATAATTTCGCATCATTTGAAAGTCCAATATTTCTCCAATATCATCCAGAA
TCAGAATTGGAATATTTGAAAGGGTATCTCAAAGAACGGTGGACAGCTATTCTCAATGAA
GCCTTTATTGGTTCAATTCCAGAGCCGTGTGGTTCTCGCATTATCAATGAAATTAATGTA
CTCTTACCACCATCGTGGGGGTTGCTTACATTTGACGAAGCCATTACCGAGGCATCCAAA

TTATATTACGAGGGCTTTCATAAATTGGTGGAGACCGATCCGAACGGATGGTACGCAAAA
TTTACCAAGTCTAAGTCGTGTGCAAAGGATTGGTTAACGAAATATCACCTACCAATTACC
AGTCTGGAAGTGTTGAACGACTACCAGTACATGTTAGAGAGCAAATATAAATATGCCACG
TAAtttcatgtcgagtttaatt
>DjScaffold4686:58572-86482 strand:+
aaaatttgaaaattcaaggttttattttaaaaatcagataATGTCTACAATTGATAAATC
AAAGAGTGTGTATGTTATAGGAATCGGAATGACTAAATTTCTGAAGCCGTCTGAAGACGT
TGATTATCCTGACTTAGTTAAAGAAGCCGTTCAAAATTGTCTCAAGGATGGTTGTGTAAC
ATTTAGTGATGTTGATCACGGAGTATGTAGTTATGTTTATGGAGATAGTACTTGTGGCCA
AAATGCTTTGTATAATATTGGTATGACCGGAATTCCTATTATAAATGTTAATAATAATTG
TGCCAGTGGATCGTCTGGAATATATATGGCATATAGCTTGGTGAAATCCGGTATGTCCAC
ATGTACAATTGTAGTTGGATTTGAAAAAATGCAAAAAGGATCTCTAACTAGTGTGTTCAA
TGATAGGTCAAACCCAATGGAGAAGCATGTGGATATTTTAGTTAATAATTTTGGTTTTGA
AAGTTCACCAGTAGTTGCCCAATTGTTTGGTAATGCTGGTCGGGAGCACATGAAAAAATA
CGGTACGAAACTTGAACATTTCGCTAAAATTGCATGGAAAAACCATAAACATTCTGTTAA
CAATCCTAATTCCCAGTTTCAAATCGAGTATAGCCTGGAAGAAATTATGAAATCTCCAAT
GATTCACGAACCATTAACCAAGTTGCAGTGCTGTCCGACATCTGATGGTGCGGCTGCTGT
TCTTATTGCTAACAGTGATTTTGTTAGGAAACATAATCTTTTCGACCAAGCTGTGGAAAT
TATTGGTATTTCTTTAGTTACCGATACACCGAAAGCATTTTCTGGAAATTCAGCTATGCA
ATTGGTGGGATATGATATGACTGTTAAGGCAGCGCAATCAGTTTATAATCAGTGTGAATT
AAAACCCAAGGATTTCCAAGTTATTGAATTACATGATTGTTTCTCTGCAAATGAATTGAT
TACTTACGAAGCTTTAGGTCTCTGTCCCCAGGGTGAAGCCGGTAAGTTTATTGATAACGG
GGATAATACTTATGGCGGTAAGCATGTTATAAATCCCAGCGGAGGATTGATATCTAAGGG
TCATCCATTAGGAGCCACTGGAGTTGCTCAGTGTGTTGAATTGTGTTTACATTTACGAAA
TAAAGCAGGAAAACGACAAGTCCCAAGTGCTACGTATGCTTTGCAGCATAATCTTGGATT
AGGCGGAGCAGCTTTTGTTGCAGCCTATAAAAAAGGGTTTTCTGTTGTCCCGGTTGCTCG
TCTGTAAttgatagaaacatttacttttaatttacactgattcggatggattaatttctg
aattttaataaaatttttcgtgattttttaa
>DjScaffold776:27627-31957 strand:+
attttttATGGGTACTGAATTTTTGCCTAAAGACTATTTAATTTTACTCAAAGAAGATAT
ACCGAATGGTACTGAAGATCTAATTCAAACCAGTAAAACTCTTGAGGATGTAGCTGACCA
TTGTTTGTTAACGTATGACAGATCTTTAGAACAAACAGTTAATTATACTGCTCAAGCATT
AGGTAGTGTAGCTTACAAAATCAATTTATTAGCTACTAATTTTCTTGAAGTATTGGATAA
GCAAAATATAAAATTAAATGAATTACAAACGAAAGTTAATCATTTAAATATGCAAGTACA
TATCCATCAAGAAAAAACTGCACGACTTTCAATAGGCAAATATACAATGCCTCGGGATCC
TAATGCTATGCGTTCATCAAGAAATAATGAGCCAATTTCGGAAATTCAGGGAAAATATAA
ACGAAAAGAAATAGATTTTACAATATTGGATAATGTTGGTCATGGTATCAAAGAACCTCA
ATTAGGAACCTCTGGAGTGTTCAGTAATACTAGCCGTCGCAGTAGCACAAGTGGTTCAGA
TGCTCATTCATCCACCCATGCATACACACAATCGACTATTGGGGGCTCTCTTGGACCAAA
AGCTGGATCGGATTACATAAATAAATTCGGGAAAATACAAACAATCGGTCGCACGACGGG
AATAAATATCAACAATCCTTCAACAGCTATATACCGTGCACCTGGAACTCTTTCACAGAA
GAGTCAAATTCCTATGGCTTCACCGAATTTGAATAGTGGAAATGGAATGCCACCTCCACA
GATTCCAAATTATGGCAATACGGTTCAATACCAACAACAAATGTCTCAAGGAGGGCCGAG
ACGAAGTAGTAGTACTAGCACTTCGACTAGTGGTTCTAGTCAAATGCAACAGCAAAAACA
ATATCAACCATCTGTCAATGTTGTTTCACCACATAAAATGGGCTCAATTCAAGGTAACAC
ACCACCATATGGCTATTCAGGAATGAATCATCCGATGGCGTGTATTCCCCCTCCGCCTCC
AGTTGTTGCAAGCCAGCCCACCGAACAGAATATTTCTCCCATAATGCGATACAATGAGTT
GCCACCGCCACCACTTGATCCACCAAGTCCTTACCAACCACTACCACAACAACTACAGCA
ACAGAATACAGCTAATCTACCACCTCCACCAAATGTTGAACAGTTCCAACGCCCTCCAAG
TAATGAACAAATCAATATTGACAAAAATCCAAATCGCTACATCTCCGACTCAGTAGGCAT
TGCTCATCTACAACCGACTCCTTACGATGTGCTTCACATAAAAAATCAAACTGACGAAGT
TTGGGCACCTACTCAATATATTTGCAAAGGTAATTATCGATCTATTCCAGCTTTCCAAAT
ATTCTAAttatttgtttttcgtcgtcttttcaagaaatttgtggaaatctttagtattaa
gagagatggaaattaattgttgtatattttaaattttatgttaaaattacaatttaatca
ctacaatcaataattttgaatgattttctgcaaaaaatacataatttggtttgtaagaaa
attttttATGGGTACTGAATTTTTGCCTAAAGACTATTTAATTTTACTCAAAGAAGATAT
ACCGAATGGTACTGAAGATCTAATTCAAACCAGTAAAACTCTTGAGGATGTAGCTGACCA

TTGTTTGTTAACGTATGACAGATCTTTAGAACAAACAGTTAATTATACTGCTCAAGCATT
AGGTAGTGTAGCTTACAAAATCAATTTATTAGCTACTAATTTTCTTGAAGTATTGGATAA
GCAAAATATAAAATTAAATGAATTACAAACGAAAGTTAATCATTTAAATATGCAAGTACA
TATCCATCAAGAAAAAACTGCACGACTTTCAATAGGCAAATATACAATGCCTCGGGATCC
TAATGCTATGCGTTCATCAAGAAATAATGAGCCAATTTCGGAAATTCAGGGAAAATATAA
ACGAAAAGAAATAGATTTTACAATATTGGATAATGTTGGTCATGGTATCAAAGAACCTCA
ATTAGGAACCTCTGGAGTGTTCAGTAATACTAGCCGTCGCAGTAGCACAAGTGGTTCAGA
TGCTCATTCATCCACCCATGCATACACACAATCGACTATTGGGGGCTCTCTTGGACCAAA
AGCTGGATCGGATTACATAAATAAATTCGGTAGGAGAAAATAAaaATAAATACAGGGAAA
ATACAAACAATCGGTCGCACGACGGGAATAAATATCAACAATCCTTCAACAGCTATATAC
CGTGCACCTGGAACTCTTTCACAGAAGAGTCAAATTCCTATGGCTTCACCGAATTTGAAT
AGTGGAAATGGAATGCCACCTCCACAGATTCCAAATTATGGCAATACGGTTCAATACCAA
CAACAAATGTCTCAAGGAGGGCCGAGACGAAGTAGTAGTACTAGCACTTCGACTAGTGGT
TCTAGTCAAATGCAACAGCAAAAACAATATCAACCATCTGTCAATGTTGTTTCACCACAT
AAAATGGGCTCAATTCAAGGTAACACACCACCATATGGCTATTCAGGAATGAATCATCCG
ATGGCGTGTATTCCCCCTCCGCCTCCAGTTGTTGCAAGCCAGCCCACCGAACAGAATATT
TCTCCCATAATGCGATACAATGAGTTGCCACCGCCACCACTTGATCCACCAAGTCCTTAC
CAACCACTACCACAACAACTACAGCAACAGAATACAGCTAATCTACCACCTCCACCAAAT
GTTGAACAGTTCCAACGCCCTCCAAGTAATGAACAAATCAATATTGACAAAAATCCAAAT
CGCTACATCTCCGACTCAGTAGGCATTGCTCATCTACAACCGACTCCTTACGATGTGCTT
CACATAAAAAAATCAAACTGACGAAGTTTGGGCACCTACTCAATATATTTGCAAAGGTAAT
TATCGATCTATTCCAGCTTTCCAAATATTCTAAttatttgtttttcgtcgtcttttcaag
aaatttgtggaaatctttagtattaagagagatggaaattaattgttgtatattttaaat
tttatgttaaaattacaatttaatcactacaatcaataattttgaatgattttctgcaaa
aaatacataatttggtttgtaagaaa
>DjScaffold2513:67309-68889 strand:+
ATGATAGAATTAATTCAAAATAGTACGATACACGGAGTCGGGCATATTTACAGCGCTAAA
AGTAAATTGTCTAAATTATTTTGGAGTGTTCTATTATTATTTGGTGTTATTGGGATAATC
GTCAATATAGTGTTTGTCTCAATGAAGTTCTTTTCCAATCCAGTAATAATTAGTTATACA
TTTGAAAATGAACCCTTTATTTGGCCAGATATAACATTTTGCAATCCAACCGCACCATAT
CATTTTAAAAATAATTCTGTATCATATAAACATTGGATGAAAATTATCAATGAAACGAAA
ACTACTGCAAAATTGATTGCAGCTCAAACAAAACTATCGAGCTATGTTCATAATCAAAGT
GATAAAGATTATGAGCTTGAAGAAAGAACAATAGCAATGTCTAGCGCTGATCCACGTAAA
TTGGGTGCACATATTTATCGAAATCATTTAACGAATATTGGCTACATAAAATATCAAAGC
GGTATTTATTTTGCTAAAGAAATCCCAAGAAATCCAACTAGATTTAAGGAAAATTTAAAA
CCACTATTTACATCTCAAATTGTTCAAATTCGAAATAATATTCCTTGTCATACATTCCAT
ATTGACAAATCAAAATTCATTAAACAAAGTTATGTTCGATCATTAATTTTTCGTTTTAGA
TTCAACGATAACAGCTACAAAATGTTTAATTCAAGTTTCATTTTCCAATCTATATTTCTA
TATATTAATAATAAAAATGAAATTCCCCAAGAAAATCAGATAATTCTAACACCGGGTTAC
AATAATATTGTTAGTCTTTCTCAAAATCGCTACATTTATACGGGAATTCGAAGAAAATGT
CGGCATAACGATTTTAAAATCCAACTTTACGATGCATTACTTCAAACAACCGATGAATAT
ATCGGAAATTACGAGGACTGTAAGAAAATAATCAGCCAACAAATGTATGTTGAAAGATGC
AATTGTTACAGTCCATTTCTGCCGATTTATAAAAACGAAAGTGGGTTTCCAAGGTTATGT
TTAAATGCTAGTTTATTTTCGAAATCAGATGTTTTGAAGAACACACAATGTATGATTAAC
ACTTATAATATTGTGGAAAATTCTACATATTTGAAAACTAAATTTATTTCTCAGTGTAAA
GACTATAGACCGATTGCATGTGAAAAACTGTCGTATAAAGTAGACCACCGAAAAGAATTA
CTGGCTGATTTATGGCTGAATTCAGCTAATGATAGACGAATGAATATGTTGAAAAAAATT
TTTATTAATATTTTTGAAACTAATCCAACCGGAGATAAATTTGTGGAAAAATTGAAAGGT
AATATAATGCAACTGATTATAACAAGAGAGTCGCCATTAGGTGATCAATTTAAAGAAGAA
AGAGCATATCCTTTTTCTCAACTCCTTTCGGATTTCGGAGGATTGATGGGCTTATGGTTA
GGAATGAGTATTGTTGGTCTGTTTGAAATTGCTGAATTTTTAAATGTATTTCTGAAAGGT
ATTTGCATTTTGAAACAAAAAAAGAATCACTCAAATAATGAAACGAATAATAATGAACAA
GAAATGATTGATATTTCCTAA
>DjScaffold4928:16069-16832 strand:+
tcattgaaaatgaaataattcttaaatcaaacaagaaaattcatctatggtttagataca
tagatgatattttctttgtaactaattctcctttagagaccattctagaattagccaaca
gtattaataataacattaaattcactttagagaaatcaattgactttcagattccttttc

ttgacactttaattcaaaagaaacctggtgaaatattaactacccaattatatatgaaac
catttcacagtggacatatattaccttggagtaaaaattggtataattaaaggtgaaaga
attcgagctgagagactatcttcaaatgaagaaATGAAATTGATTTCTCTCAAGTTGATA
AAAAATAAATTTTTAAATAATGGCTATCCAAATAAAATTATATCAAAATATTTATCTTTA
AATTCAAAACAAAATAAAATCAAACCAAAAACAAATACAGTTATTAAATTACCGTTTATT
AATGAGAAATGTAGTCAAATCATGCAGAATATAATAAAAAATGCTAAACTAAAACACAAT
CCAAGAATTATTTTCAAAACGCAAGCACCTTTGGGCATCCAATTACAGAAACCAAGACAA
ACTTTTTGTGGTCCCAAATGCATTTGTGATGGTAGAAATATATGCAATAAGAAAAATGTC
ACTTATAAAATAACATGCACCATTTGTCAAGAAACATATATAGGTGAAACATGCAGAACC
ATCCGAACAAGAGTTAAGGAACAT
>DjScaffold4928:29989-30539 strand:+
ttttctgtaattatcATGAGTGCTAAACATACATTTTTGAAATTAAGAAAATTTTCAAAT
ATTTTATTTACACCAAGATATTTGATTTATACCAATACCGTAATTGGATCTGGGTCTTTG
GTAGTAGGATATGGTATTGCTGAAAAACTTCGTAAGCATTTTACTAAAGATGCTGATTTT
AAAATGTTAAATTTTTCTAAAACAATTATGATTTCGTCCGTTGGAGCTATTTTTGGAATA
TATGAAACTATAGGCTATCATTTTATCGATAAAATAAAAATTAAAAAAGCTGTGTATTTA
AAATTACTTCTCGATCAAATGTTTGTGGCACCTCCTGCTGTTGCATTGTTTCTTTTTGCC
AATGAAGCCTATGATAAAAAATTACGATTTTAAAGCTGCCGTGAAATATACCAAAGACTCG
TTTCTACCAATATTTTTTCTAGATATGATATTTTGGATACCT
>DjScaffold2524:96296-97423 strand:+
cttaattttgtttaaattattttattataattcatatgaaaacaatgttttatttaattc
tttgtaaATGCAGGGGGTGGTGACAGTGGTTGGAATTACATTAGGAGTTGCTTTTTATAA
TGGGATATCATCTCGATTATCTCTACCCTCTTTATTATCTCTGCCATCAGAACTACTCGC
ACTAATACAACGATCAGAAACACTTGAATCACAAATATATACTGAAACTTTGTTACCTTC
CGAGGTTGTTAAAATTTCCGAGAATGTAACTATATCTATCCCCGATGAGTCATCAAAAGC
TGTTCAAAGGAATTCATGGAAACGTTGTGTGATTATCGTAATAATCTCTGTAGCTTATGT
TGTAATATGTTCTAAAATATCAAAATCAAAACCACCAAAACCAGAACCAGATGAAATTTT
TCATGGCTTAAGAAATAATAGTAATTTTTGTTATTTGAATTCCATGCTTCAAGCTTTGGC
GTCGAGCGAAGAATTTATTAGAAATCTTGAACGAATAAAAAGTGATATTGAAATATCTAA
ATCTAGTGCTAATCAACTGAATTTATTTATCGATCTTCTAAGAACCATAAATATGTCTGA
ATCAAATGTCAATGAAAATACAAGAAAACTTGATTTATTAATGGCTCAAATTAATTGGAC
AAAACCTGATCAACAAGATACATATGAACTTCTTGCATTCTTAATCAATACTATAGAGAA
AAACATAAATTTTCGAGGAAAGATTTTGATGCCTTTGAAACTGGAAATGGTTCATACATT
TTCTGGAATTAATATCCCCAGTAAAACACAGTTTCATCTTGGTTTATCAATCGGTTTATG
TGGAGAATATAATAAATATCCTGGAGTTACATTAAATTATCTAATTGATCAACATTTTGC
TCCGACAATAATTGATGATGATCCTAATAAATCCTCGAATTTAATGTTTTTGGGGCAGCC
ACCAAATTTATTGCTTTTACATATACAAGCTAATAAATGGAGCAGTGAGAAAGAAGAAAT
GATCAAACCAGAATATGATATTCAATATAACGAAGATCTCCGATTTGCTGCCAAATGTTA
TATTAATGAGAATTCTAAAATAGAAGTTGTGAATGTTCAATATAAATTGACAGCAATTAT
CCATCACATGGGAAATATTTCCGGAGGTCATTATGTGACTTACAGGAAACGGAAGAATAA
TTGGTATTTAACAAATGATGAACAAGTGTCCTTCTTTGATTTCAAATGTAATATTTGCCC
ATATATTTTATTATATGAAAAATCTTAAtttctgaactcattttaaacataaattttagg
aaatgtttttgtttatttatttattatatcaatttatgaaatataaaatatgaaaata
atgagaatgaatattatcagagcattcaagtgttgaaacagcactgaattttgtaacttc
cattgctttcgttatcgcttttgaaatatttccaaaggagtctcaaattacttgaagtac
ttgcactattatccattgttgtgcggtgcaaattttaaatgggttctatagtaaaattat
aataaaaaatgtatcctgaaaaactattgattttcatattcattattcggtaattaaatt
taagttcattattttcaaatatgtcattttatcctaatacctaccttcaaaagttagata
aaatagcgaggcacagttattaaaacaattaaattaaaataacttgctgttgaccttgcc
gatacattttttcgttcttttcttttgacaatggtcgagtttatctccgaacgtcaaac
acaaatgaatataatgaacataaagcgtaattcacaatagtttaacaaccaaagaaacct
ttgtctaataccggtattgggataagcaaataccgataattccgattgcatattttgatc
tgaaaaccggtattaccaatattgtaatctatatgtctactaataactaagaccctaaga
cgaaattatttctgcaccacgttattttgtacaatttacatggaaaattattttcttta
ttgacataataattgatctctgtattttataaattttagattatcgtaataatctctgta
gcttatgttgtaatatgttctaaaatatcaaaatcaaaaccaccaaaaccagaaccagat
gaaatttttcatggcttaagaaataatagtaattttgttatttgaattccATGCTTCAA

GCTTTGGCGTCGAGCGAAGAATTTATTAGAAATCTTGAACGAATAAAAAGTGATATTGAA
ATATCTAAATCTAGTGCTAATCAACTGAATTTATTTATCGATCTTCTAAGAACCATAAAT
ATGTCTGAATCAAATGTCAATGAAAATACAAGAAAACTTGATTTATTAATGGCTCAAATT
AATTGGACAAAACCTGATCAACAAGATACATATGAACTTCTTGCATTCTTAATCAATACT
ATAGAGAAAAACATAAATTTTCGAGGAAAGATTTTGATGCCTTTGAAACTGGAAATGGTT
CATACATTTTCTGGAATTAATATCCCCAGTAAAACACAGTTTCATCTTGGTTTATCAATC
GGTTTATGTGGAGAATATAATAAATATCCTGGAGTTACATTAAATTATCTAATTGATCAA
CATTTTGCTCCGACAATAATTGATGATGATCCTAATAAATCCTCGAATTTAATGTTTTTG
GGGCAGCCACCAAATTTATTGCTTTTACATATACAAGCTAATAAATGGAGCAGTGAGAAA
GAAGAAATGATCAAACCAGAATATGATATTCAATATAACGAAGATCTCCGATTTGCTGCC
AAATGTTATATTAATGAGAATTCTAAAATAGAAGTTGTGAATGTTCAATATAAATTGACA
GCAATTATCCATCACATGGGAAATATTTCCGGAGGTCATTATGTGACTTACAGGAAACGG
AAGAATAATTGGTATTTAACAAATGATGAACAAGTGTCCTTCTTTGATTTCAAATGTAAT
ATTTGCCCATATATTTTATTATATGAAAAATCTTAAtttctgaactcattttaaacataa
attttaggaaatgtttttgttttatttatttattatatcaatttatgaaatataaaata
tgaaaataatgagaatgaatattatcagagcattcaagtgttgaaggaattgatttttat
ttatttgaaatttatatcaaaataattaaaaatcatttttttaaaatatttttccagtat
agaaatttcacgcgctccaaaccaccaaaaagattaatatcaaaattgttcagggtgaaa
taaatgaaatctggtggagtattaggtagaatacggtattttttttatacagcaatatca
tttgttgtatcatttcgttatgtcggatacttatagtagttgtgattcataatatttttc
aggctaagatttacaaaatataaataaagttattgattgaatttataatcaagtcacggg
atcttttatttaataatctaacgtttatttagattttgATGCCTTTGAAACTGGAAATGG
TTCATACATTTTCTGGAATTAATATCCCCAGTAAAACACAGTTTCATCTTGGTTTATCAA
TCGGTTTATGTGGAGAATATAATAAATATCCTGGAGTTACATTAAATTATCTAATTGATC
AACATTTTGCTCCGACAATAATTGATGATGATCCTAATAAATCCTCGAATTTAATGTTTT
TGGGGCAGCCACCAAATTTATTGCTTTTACATATACAAGCTAATAAATGGAGCAGTGAGA
AAGAAGAAATGATCAAACCAGAATATGATATTCAATATAACGAAGATCTCCGATTTGCTG
CCAAATGTTATATTAATGAGAATTCTAAAATAGAAGTTGTGAATGTTCAATATAAATTGA
CAGCAATTATCCATCACATGGGAAATATTTCCGGAGGTCATTATGTGACTTACAGGAAAC
GGAAGAATAATTGGTATTTAACAAATGATGAACAAGTGTCCTTCTTTGATTTCAAATGTA
ATATTTGCCCATATATTTTATTATATGAAAAATCTTAAtttctgaactcattttaaacat
aaattttaggaaatgtttttgttttatttatttattatatcaatttatgaaatataaaa
tatgaaaataatgagaatgaatattatcagagcattcaagtgttgaa
>DjScaffold5045:289-7443 strand:+
aaaattataaatcgacaaaaatttATGACAATTACTGAAGAAACAAATGAAAAATGTACC
GAAACCATCGATATAAAGAAAATTAATATTTTAACTGTCGTTGATATGGATTGTGAGAAA
AATCAACTAATCAATCAAAAAAATATTGATAACTTTCCAATTTGTCAAGATGTCAAAGGA
ATAGTGTTTGATTCTGATCGTTTAACAAGAAAATTAAAAGAAATGAAGGAATTTCCAAAA
AAGCCAAATGAATCAAAATTGGAAAATATTGCAATGCAGTCTAATTTCCAAAAACTTTCA
AATGATAATTCATTCACATTAAATGACATGCTTATTAAAACAGACTATTTAAAAACAAAT
AGTTCAAATATAGCGAATTATATTCTTTCAAACACTTCAAATTATCCTCCTAAAGTTCTT
TCAAGTTCACCCACACAATCGTCTTATAATCAAAATTACAATTTGAGTACAGTTTCAAAG
ATAAATGAGGATTTGTCTGCAACTGAACCAGATTGGAATAGAGAAAAATTTAAGATTTAT
GATTATTATGAAAGTACAACATTACTACCAATCGGACAAGGAATCACATCATTAAATAGT
CCAAAATTTGGTTTGTAGtttattaattatataatttatggttttcaagtagaaatttga
aatttaactc
>DjScaffold5045:31800-32444 strand:+
TCAAGAAAATCAAAGATATTAGATCAGTTGTGTAGTGGACCAAGCAACAAGAAATTAGAA
TGGACAGATGAAATAAACGATACTTTTGGGGGTCTGAAAAAATCTCTAATGACTGCATTA
ATATTGTGTTTTTCGGAAAAAAACAAAGAATTTATGTTAGACACGGATGCCAGTTTGATA
CAATTGGATCAGTATTGTCACAGAGAGATAAAGAAGGAAGAGAATGAGTTGTTGCCTAAG
GATCACACTCAATGAcaagtcatgaaaagggatactgcattacacgaaaggagttattgg
ctgtgtattatttctgtatgcatttcaaacattacttgtacggtaaaatatttaccctta
gaacagctcacaaagctcttactttcatgatgatgacaaagaaaccaattacctaacaat
tccagacatggattaattatctgagcagtttagacatagttttggaatatagaaaaagaa
ttcctataataataatgcaaacggacaaattgaaagacagttcaggacaattcgagattt
gattaatgcaacacttcaggaaagaaagacaacatattgggcagatattctataggaaat

agaatttacaatgaacgt
>DjScaffold4345:94878-101954 strand:+
aaaattagatttgcgtagattttttttatgtttaaatacacttaaatatataaaaaatcg
aataaatattagttatcattatgaatactaattttttaagtcaatcaaaattagaaatat
taatggaattatatccaaatttcctatcatattcttttgaaaatgttgtttaggtggaca
ttccaagatcatcaaattcttcgacaacattcaaatatattgcgagtcaaggcccaATGA
GTCATACTTCTAGTGATTTTGTAACTATGATATGGGAAAATAAAGCTGAATTAGTAGTAA
TGTTGACTGATTTACAAGAAAAAGGTCGTACAAAATGTTATAAATATTGGCCTGATGTTG
GTGAAACTTTGAAATTACAACATGGTTTACAAATAACTTGTGAATCGGAAAATAGTCTTG
ATACATTTGTCATCAGAGAACTGAGATTGAAAAAATCAAATGAATCTCGTAAATTTACTC
ATTTACAATACATCAATTGGCCCGACCATGATGTTCCAATAAGAATGGAAGATTTTCTTT
CTTTTATGAAAATGTTGCGACAACTTCGATCTTCAAAACCAGAACCGATTGTTGTACATT
GTTCAGCTGGTATCGGACGAACTGGTGTTGTTATTACAATGGATATTGCAATTTCCTTTA
TGGAAATGAAACAGTCTTTTTCGGTTTTAGCTATTGTTAAGGACATTCGAAATCAACGAG
CAATGCTCATCCAATCTCAAAAACAATACGAATTTGTCTGCCGAGCAATTATTTATTATT
ACAACAATCAATCAAAGTAAtaaattcatccactattttcattgttttaatcagttggtg
aaatttgcagcatgtaccttttcatatctctttttatttgctgtgtgaatgtttgtttat
taatttaataaatagtattatgtaaataacaatcaatcaacgtttcctgttttaatggat
taaaagtattttgaaattgacagtctaaaagtgatgtgtattttcccagaatttctgtga
tagtaatctggttggtaaattcatgagatattgattaacgaattatatttgacaattcat
catttgagtaatgtaaatttttcattgtttttaatttgtaattctttcgccatattgata
agccttttttaaatcagctatagatttatttcttattatttcggataaatatttatctt
taattatcagttttaattaa
>DjScaffold4345:127328-128297 strand:+
ataaaaatataaattATGGTGATGTTAATAATTAACGTGTTAATAGTATTACTTAATTTA
TTCAATATTATTAATTGTGAGGTTTGTGAAAGATGTCAGCAAATATCTGGTGGAGCTATT
TATCCACAAGAATTGAGATCTCAAGGAACACACAATTTACATATTAGTAAAGCTAGAATT
TCCAAACATGCACCCGATTTTGAAGGAACAGCAGTTATTAATGGTACTTTTCGCGATATA
AAGCTAAAAGATTTTTATGGAAAATATTTAGTGTTTTTCTTTTATCCTCTTGATTTTACA
TTTGTTTGTCCAACAGAAATAATTGCATTTAGTGATAGAATAAAAGAATTTCAAGCTATC
AATACGAATGTAGTCGGTTGTTCAGTTGATTCCCAATTTACTCATTTAGCTTGGTTGAAT
ACACCAAGAAATCAAGGTGGTTTAGGTAACATAGAATTTCCTTTACTTTCTGATCTTTCC
CATCAAATTTCAAAAGACTACGGAGTTTATTTAGAGGAAGCTGGCCATACTTTAAGAGGA
TTGTTTATTATTGATTCTAAGGGAGTATTACGTCAAATTACTATGAATGATTTGCCAGTT
GGAAGATCAGTAGATGAAACATTAAGATTGGTACAAGCATTTCAATATACAGATAAACAT
GGAGAAGTTTGTCCTGCTGGATGGAAACCTGGAGCTGATACGGTTTGCTAAaacaaataa
ttttaatgttaatttttcatatccgtcgtaaatttaattttattattcggttattcctaa
aataatgtcattttaaaacaagtttattgatactt
>DjScaffold4725:76585-77340 strand:+
acgataATGGAAAATAGGCCTAACCATACATTATATGTTAATAATTTGAATGACAAGATA
AAGAAGCCAGACCTTAAAAAAGCGCTTTATTATTTATTTGGGCAATATGGTGGGATTGTT
GACATAATAGCTAGTAGAGAACCTAAAATGCGAGGCCAGTCATTTATTATTTATAAAGAC
ATAGTTTCATCAACTACTGCTTATAGAGCCTTACAAAATTATCAATTATTTGATAAGCCA
ATGAAAATTCATTATGCTAAAAAAGATTCTACTCAAATAACCGAAATGAAAGGTCTTCAA
AATGAAGCCAGTAAGCGTAGAGATGAGGAAAAAGAGAAGAGGAAGCGTAAGAAGGTCGTT
GGGGTGGTTAAAACAACTCCAACCATTCCAATTTACGTTGAATCAACAGTTATTATACAA
AATGATCAAATATCCCAAATGCAACAACAACCATCAATTCAACCAGCTCTCGACGAGACT
CCTGCTAGCAATACCTTATTTGTTTCATGTTTACCTCAAGAAACAACGAAAGCTATGTTA
ACTATGTTATTCAGTCAATTCAAAGGATTTCGTGAAGTGAGATATTCTGCTGAACGATAT
GATGTTGCATTTGTGGAATATGAATTAGAAAAACAGGCCAAGGATGCTAAAGAAAGTTAT
GATGGATTTCAGATTTTCTTAAATCACCGAATGAAAATTGAATATGCTAAT
>DjScaffold2616:58452-76029 strand:+
AATAAAAGAAAAAGAGTGGAAGAAGAATTTGAATTTGAAGGTTGCAAAGTAGGTAGAGGA
ACATATGGCAGTGTTTATAAAGCTAGGCGAAAAGATCGATTAGATAATCGTGAATATGCA
CTAAAACAAATTGAAGGAGCTGGATTGTCAATGTCTGCTTGTAGAGAGTTATCACTATTA
AGAGAATTACGACATCCTAACGTAATCAGTTTACAAAAAGTTTATTTATCTTTAAGTGGT
CGTCGAGTGTGGCTTTTGTTTGATTATGCTGAACACGATTTATGGCATATTATTAAACAT

CATCGAAGTGCAAAAGCTAATAAAAATGCAGTACCTGCTCCGCCTAATTTAGCTAAAAGT
CTTATGTATCAATTGCTTAATGGAATTCATTATTTACATCAGAATTGGGTTTTACATCGT
GATTTGAAGCCAGCAAATGTTTTAGTAATGGGTAGTGGGTTGGAACGAGGTAGAGTTAAA
ATCGCTGACATGGGATTTGCGAGAATTTTTTATCAACCATTGAAAGCATTAGCTGATTTG
GATCCTGTTGTAGTAACATTTTGGTACCGAGCTCCTGAATTATTGTTAGGAGCGCGGCAT
TATACTAAAGCAATCGACATTTGGGCTATCGGTTGTATATTTGCTGAATTGTTAACATTT
GAACCGTTGTTCCACTGTCGACAAGAAGATATCAAAACTAGTACTCCATATCACAAAGAA
CAATTGGAAAGAATATTCAGAGTAATGGGATTTCCTTCGGATGAGGCATGGCCTGACATG
AAGAAAATGCCAGAATATCAACAATTGATGAGAGATGCTATTTCTCGTAAAACTTATGGG
AGATTCAGTTTAGAAAATTTTTTAGTGGAAAAGGGTTTCTCAATCGATTCATTGGAATTA
TCATTATTAAAACGACTTCTTCATATGGATCCACAACATCGATTGAGTGCTGCAAACGCT
TTAAATGATCCATACTTTCAGAAAGAACCATTACCATCAGACGATGCTTTTGGTAACATG
CAAAACCCATATGCTAAGCGTGATTTTATTGTGGAAGAAGACAATGAAGCAGTGGAAAAG
CAAGTAAACAATGCCGATAAACAAGCACTGCAAACTGGTAACGCTCAACAGAAAAACTTA
ATTTATATCCAGAATCTCAATGGTGCAAATATAATGGATACATCGTCTGCACGACAAGCT
GCTAGTCATCAGCAAGGTGGGCCTCCAATGAAACGCAGCAGATTTGTTGCTCCACAAGCT
CCTACTGTTGGAACAACTAATGTCCCTAGTCATTTGATGACGTCGTCGGGATCAGGTGTT
TTGATGTCTAATTTTCATCACAACCAATCCAGTACTCATCATTCCAATAATCCCAGCGAT
>DjScaffold2616:96873-114883 strand:+
tttattagtaaaaatgtttttcttacttttatttactaaATGAGCTCTGATTTACCAAAT
TCCTTTAAGTTCTCTAGTTCAGAGACAAAAATACTTCGTGAAATTACTAGTTTTGATTTC
GGTTTCATTAATTTCAGTACTCCCAAAGGTCAAGCCAAAAAACAAATTTTAGAAAAAGCC
ATAAACTGCTTAGATTGTACTCTTTCTAAATTAGTAGAAGTATTTAGAGAGAAAAAGGAT
CAAGAACAATCCGAATTAAAAAATAGTGGTTTATTATCTGTTGAAAAACAGCTTGAAGTG
TATGATAAGACCATTAAATCACTTAATAACAGCATCGATTCACACATTTACTTATTTCTT
GGTCATTACCAACTATTATTGTACTTATACGAAGACGCTTTATCAGCATATTTAGAATTT
GAAACTCTAAATAAAGACAGTTGGAAAAATATACAATTTATATACGGATTAGCTATTTGT
TATTTTCATTTCAACGCATTTGATAAAGCGATTGAATTATTTCAGAAAATTCTATATCTT
CAACCGAATTGCACTAGATCTAAAGAAATTCATATTCGATTGGGATACATTTACAAAATT
AATATAAATTTTGATTGCAGTTTGAAGCATTTTCGCCAGGCACTCAACGATTCTTCTAAA
GGATCATTTACTGAAACTGAAATACGTTTTCAAATTGCACATCTTTTGGAAGTTTGTGGA
AAATATAAACAAGCTCAGAAAGCATACGAGCAATTAATTGGATTTAGTAAAGAAGACGCC
CCAGATCACATCAAACATTTGTGTTATAGACAATTAGGCTGGTTGAATTTCTCTGGATAT
ATTGGCAAATCTAATCCTAATACTCCTTCAAAGAAAGATTCTGTAATTAATCTTTTGCAA
CAAGCAATTAATTTCGACGATACAAGCGGTAAAACCTGGTATTTATTTGGAAGAGCTCAA
GCTAGAGAAAACAAAGTTCAAGACGCTTTTATGGCATATAGAAATTCTATTGATAAAACT
GAAGCTAATGCTGACACCTGGTGTTCCATCGGCGTTCTTTATCAAGAACAAAATCAGCCA
ATGGACGCACTTCAAGCCTATTTCTGTGCGTGCCAGCTAGATAGAAAACATGTAGCCGCA
TGGACAAATCTTGGTGTGCTTTATGAAAGCATGGGGCAATACAAAGAAGCATTAAAATGC
TATCAAAATGCTGTGAGTTCTGACAAAAGCAATGAAGTTCGAATTGAAATTAAAGAGCGA
ATGATGATTATACAAAAGTTATTGGCACAGTTACCGGAAAAAAATATGCCAAGTCAGGTG
ATATCTAATCAACAGAAAATTTCCCAAATACGTCCACCACAGCATCAGAACGCTAATTCG
ATACCTATCGGTGGGCCTGCTGGTTCATTATCACCATCGATGATCTCTCAGTCGATGATG
AAATTGCCGACTGTAGATGAAGCTTGGAATTTACCAATACCCACAGAATTAACGCAAAGA
CAAATGCAATTGGAAATGCAAGAAGCAGTTGCATCAGCATCTAGTGCAGGTGGAATTTCA
CGGATCTTCGCTCATCATTCTCCAAATTATTTGGCTAATAACGGCATTAATTCAGATATT
ACAATCGATCCAAACGGCAGCAAACGGAGAAGAATAGTAGAACCTGAAGAATCGGTGGTT
CCATTAACCCAACAACAAACACAATTTCTACTTTCTATGGAGCAACAAGGTTCTCAACTA
AATAGTCAACAGCGTCAGATGATGATTTCACTACAAAATCAATTACAAAGATATCAACAG
CAGCAGCAGCAACAACAATCGAAATTGTTTGTAAACACAAATAATCCAGCTCCGTCAACA
TGTACAAATATTAGTACAGATTCATCAGGTTTGAGTACAGACCAACAAAATAACAGAAAG
GATGCTAAAGGTTCCATAAATCAAGATCAATCGAGTAATAATCAAGATAGTAATAGCGCA
CATAACGAAGATCTTCATTTGATGCAAGAGCTTGATGAAATTCCTGAAGATTATGGTGAT
GATATAATGGTGAATTTAGATGCATTGCTCAATGATCCTATGGGAAGTTTTGATGAACAT
TTCGAAGATTCTAATGCAATTACAAGCAATAGCAACCAAATCGGATCAGTTGACAACATT
AAAGAAGAAACAGAATATCCAGTCACTGATCTGTTGATGTATTTGCGAATAGTTCATGAT
GAAAAATTACCAAAAACTGATATTTTGGATGAAATGGATATACTGATGACCGGTGCTCAA

ATAATTGATAAAGTTACTAAATTGAGTTGCACTAATCCCAACATTTGGAATATCACGTAT
CTTCCTGGAAATAGAGTTCCTCAACCACCTAATCCTCCACCTCTCGTTTGTACTGATAGA
TCATCTGATTCTAACAGTTTTAGCAACAGCAATAATAATAATAATAATAATAATTCGATT
TCAAGACAAAATTCCGAATATTTCCAGTTACTTCCACCCACACCTAGTGTCTACTTAGAC
TGTAAAAAAGATGTTCATTCACCAGAACTGAGACAATTTTGTTTATCGCAGCCAGTTGTT
GTTATTCGTAATTTGGCATCAACCTTACGGATGGATCTCGGCATGTTTTCAACTAAAATG
TTGGTTGAAACTAATCCCAATCATAAAATTGAGCAACGAATTCAAGATTTGCAACCTCCC
GATGAAAACAAAGACGCAAATGGAAAAACCATTTTACGTTGTTTGTCATCAAAGAGTACT
ACAACAATACAAGAATATGGAAATTACCAAGCTCGAATGTTTGTTGAAGCTAAAAAAGAA
GAAAAAATCAGTAATAATAATTCGTCACTTCTCTCAGGTGCAATTGGAAGTCAAACAATT
TCTAATGGTCCATCAGATGATCCAAAATATTCCAATAATTCATCAGCGGATGAAAAGAAA
AAACGACGCAAAATATACTTTGGTACTAATTGTGATCTCTCTGATGAATCAAAGTGGTTT
GCTCAACTTCACGAACTAAATAAACTTCCGAATTTTCTGAAAGTTGTGAGTGCCTGTAAC
ATGTTAAGTCATTGTGGGTTCACAATTATGGGAATGAATTCTGTTCAATTGTATATGAAA
GTACCTGGTTGCCGTACTCCAGGTCATCAGGAAAATAATAGTTTTTGTTCGATAAATATT
AATGTTGGACCTGGAGACTGTGAGTGGTTTTCTGTACCGGAACAATATTGGGGAGCTATT
AATACTCTTTGTGAAGAAAATAAAATTGACTATTTAACTGGATCGTGGTGGCCTAATCTC
GATGAGCTAAAGCAAAGACAAATTCCTGTATATCGATTTATCCAACGACCGGGAGATCTA
GTATGGATAAATGCAGGCACTGTTCATTGGGTACAAGCTATTGGATGGTGTAATAATGTT
GCGTGGAATGTTGGCCCATTAATCAGTCGGCAATATAGCCTTAGTATGGAACGATATGAA
TATAACAAATTAAAAATGTACAAATCCATTGTCTCCATGGTTCATCTCAGTTGGCAATTA
GCAAAAAATATAAAAATGACAGACAGTCATCTGTATCAATTGATAAAATATACGTTAATG
GTTAGTTTAATACATTGTACTATGACTTGGAATTTCCTGGAGAAAGCTCGTGTTCAAATG
GTTCTGCAGACTAGAGAAGAAAATGAACACACACACCATTGTGTCGATTGTGAAGTAATC
ATTTCTAATATTTTCAAAATTCATCTTTACAATATTTCTCTTTACAATTGAcatgttctg
aaacattagaaaaaataattaaattacacatgcagctattcctcgttacgttccagatga
ttttcgcgttatgtaagactcagctttcatataaagtagagggtgttgttctcgcaatta
gttattcctagagaaaatactaaattatacatttcagaaaataaaattaaccaagatttt
ccacaatttttaaatttctttgtaccagagtacagctttatacgatggatacctgcattt
ctaaattttaatttgattagtgcagtggtcggcaaagtgcgtgtggctctttggttcctt
aagtgcagctctgaaacaaatattcacg
>DjScaffold2616:55468-56061 strand:+
AATTTTAAGCTATTAAGAGAATTACGACATCCTAACGTAATCAGTTTACAAAAAGTTTAT
TTATCTTTAAGTGGTCGTCGAGTGTGGCTTTTGTTTGATTATGCTGAACACGATTTATGG
CATATTATTAAACATCATCGAAGTGCAAAAGCTAATAAAAATGCAGTACCTGCTCCGCCT
AATTTAGCTAAAAGTCTTATGTATCAATTGCTTAATGGAATTCATTATTTACATCAGAAT
TGGGTTTTACATCGTGATTTGAAGCCAGCAAATGTTTTAGTAATGGGTAGTGGGTTGGAA
CGAGGTAGAGTTAAAATCGCTGACATGGGATTTGCGAGAATTTTTTATCAACCATTGAAA
GCATTAGCTGATTTGGATCCTGTTGTAGTAACATTTTGGTACCGAGCTCCTGAATTATTG
TTAGGAGCGCGGCATTATACTAAAGCAATCGACATTTGGGCTATCGGGTTTGTTTTTCTT
TTTAAAAGCTCATATAATGAAAATTTCCTTGTTAAAATTGGTTTCATTAATTCTCAATAT
TTTAATTTGTAA
>DjScaffold2256:19632-26997 strand:+
CCAAATAATGATGATTTAGCAACTAATTCAAATTTTTCTTATACTCGAAGACCGAATACT
AATTGTAATTATGACCAACATAATAATATTCGAGAAAATCATTTTAATCACCAAACTTAT
TTTAGAGGCAATAGGAATAAATTTAGTGATATAAATTCATCAAGAAATAAAAATGATACG
AGTTCTTTTGATTATTCTTATTATGAAAATAAGCATGATTGCAAGCATAAAAGAAATTCA
AATAGTCTCAGAGGAGCATATAAAATGCAAAGGAATCAACAATATTTAAATGATCATAGA
AATAGTTATACAGATAGTTATAAAAATAACCACCAAATAAATGGATTTTCAAATAATAAG
TATTATGATAAAAAAGATGATGTGGACATGAACAAAATATATAGAAGTTCTTGGTTGTCT
AATGAGAGAAATTTTAATCCATATAATAACAATAAGGAAAAATCTTATATAAGAAAAAAG
CTAGATTTAAAGAAAAATTCCAGTGAAATAGAATTAGATGGTATGAATCAACGAGACTCG
TTAATCTGGCAATTGAATCATAATAAATACGAATGCATGATTTGCGTGACAAATATTCGT
CCAGAAACGCGAACCTGGTCGTGTGATAACTGTTATCATATATTTCATATCCATTGTATA
AAGAAATGGGCAACGACATGTGGCGGTAGTAGTGGTGGTGGTGGTAACCAAGACGATAGA
GATGGTTCGAAAATGAATTTACGATGTCCTGCATGTCAGAAAGAATATAGTATGAATGTG
AATAAAACCGTTGATGAATCAAAAGGAAAAGAATGGATAGATTATCGGTGTTTTTGTGGT

AAACAAATAAATCCAACATTCAAAAGAGGAGGCGATCTAACTCCTCACAGTTGTGACGAT
GCATGCGGTAAATCCAACAGAACCAATGATAAATGTCCGCATTTATGTGTGTTGATGTGT
CATCCAGGTCCGTGTCCGCCATGTGAAGCGATTGTAACGAGAAAATGTCAATGTGAACGA
ACGAGAATCACTGCGAAATGTAACGCTGATGAACGAGCAATGGTTTGTGGAGAGAAATGC
GGGAAATATCTGAATTGTGGAGTGCATCAATGTGAGCAGTTGTGTCATCCGGGTGAATGC
AATCGATGTGAAGTGGAAATTGAGCAAAAATGCTGGTGTGAGAGGAAATCAATGAGAATA
GTGATTTGTGGTGGAGTTGAAGCAGCAGCAGCAGCAGTACAAGTGGGAGGAGAAGGAGGG
AGAGGAGAGTACAGTTGCGATAATAAATGCTCGAGAACACTGGAGTGTGGTAACCATCAA
TGTGAAGAGATTTGTCACTCTGGGGATTGCACACCGTGCTCATTAACTGTGGATAAAATC
AGTCGATGTTGGTGCAAGAAATCGAGAATTGACGAGTTGGATGCGAGTTGTCATCGTGAA
TCTTGTGTAGATCCGGTTCCGAGTTGCGGCGAGGTTTGTGAGAGACTAATCGATAGTTGT
GGACATGTTTGTTCGAGAATCTGTCACGAGGGCCAATGTGGTCCTTGTCCATTGACACGG
GATGTTGTTTGTCGATGTGGCAAGACAACACATGTCGGGCTGGCTTGTAGCAGTCTGAAT
GATGGTGATGATGATGGAACCAATCATCAGCATCGATCTGCGGTGTTGTGCAACAAAGTG
TGCAATAAAAAGCTATCTTGTGGTCGTCATCGATGTAGTGAGAAATGCTGCACGTCAGAG
CTGCATATGTGTTTCCGACCGTGTGCTAGAACTCTGTCTTGCAAATCTCATCAATGCGAA
CGTGAATGTGGTCATCTTGGACTGTGTCCTTCCTGTCCGAATGTGAGTTTCGATGAGTTG
CATTGTGAATGTGGAGCTGTGACATTGCTGCCTCCTGTTGCTTGTGGTCAATCTCCCCCG
GTTTGCAATCGAGTCTGCAGTAGACAGCACGAATGCTCGCATCCTGTTCGTCACAACTGT
CACAATGAACCGACTTGTCCACCGTGCACTGAACTAACAAAGAAATCTTGTCCCGGGAAT
CACAAGATCTTGGAAAATGTTTACTGTTTCCGTGCAGTAGCTACTTGTGGATTACCTTGT
GGACGGCCGCTGGAGTTTTGTGATCATTCCTGCAATCGGATCTGTCACCAAAGTGACTGT
ATAATCGATAGTGCCAAATGTTGTCAGCCCTGTCCGCGTTTACGAGCCGACTGTGGTCAT
CCTTGCTCCGCACCGTGTCACAACAATGATTGCCAGGAAATGATGACAAGTTGCATGTTC
GAAGTCAGTCTGATATGCGCTTGTAAAACACGATCGATATCTATGGCATGTTGTAAGTTG
CAAAATACGATTTCCATTATAAAATTCAACACTAATAACGATTCGAAACGGGATAATTTG
GATCTCATTCTGTCGTCTCGAGACAATAGTGTGTCTTTACCATGTGGCAATGAATGCAAG
AAGAACGCTAGGGATTTAAGGCTTGCTGAAGCATTCGATATCAGCATTACCACGTCTAGT
TTGAACACTCATTACTCAGATTCCATCAAAAACTTCTTCAAAAAAAATATCGAATTTCTT
ATTCAGACGGAAAAGGCTTTGTTAGATTTGACTAAAAATGGGAAAAATAATATTGAATTT
AAAAATTATTCTGGTGAGAAAGCTGATTTCATTAATCGATATGCAGCCTGTTTCAAATTT
ACTGTGAAATGTACACTAAGCCAAAATCAAAAGCATAATGTATTTGTTGAATTGAATCGA
TTAAATTCCTGCATACCAAAACCACTGCTTAGTGAATTATTGAAAATCGAATGTCCTGAT
TTATTTCCATCAAAAACTGAAATGGAAAAAAACAAAGTTTCAACTTCAAATCTTGCTATG
TCTTTTGCTGACAAAATTCGATTGTCAAAAAGGGAATAAttttcaatttctttctcttaa
tacatttattttccctttatattataattaattgttcatgtaaatagcaaataatccagt
atttgtaacttgtaagttgctggaacctgttggattcattaaattttaattaaatataaa
tcattgtgcgccccaagtaaatatccttagttcgcgttggaacaattagtatgcttttgt
tgtgtaatgagaaatgcatgttaagt
>DjScaffold5088:73688-74456 strand:+
ataacgatttagttaagtcaatatcacaacagattcattcttttgaagatcttataactg
ctgaagtgttgaaaatttactatttaaactgattgaaaatataataaatcaattttgtta
cgaaaatgatattccaaatatttttctttgcgtttttatcacaacttatttacggtcaaa
atcaagaagatggataccatttcacaagagaacgaatgaattatgaggctgctgtgaaat
attgatctgaattagagATGAAATTGCCAGTAATCGATGATGAAGCAGCTAGTAAACTAG
TAAATGATATTGCTATCAAAAATAGTTTGGGAACATATTGGATTAATGCAAACGATATTA
ACTATGAAACAGATTGGGTGAATAGTGATGGAGAATTTCTAAAATACAAACCATTCCAAC
AAGTAGAACCAAATGGTGGTCGAGGTGAAAATTGTGTTCATGGATTATATTATCCAAATG
GTTTATGGAATGATATTTCATGTATGGGTACAAATGCAGTGATTTGTGATCCTGGAAATA
AAGAAAAACGTAGAAGATAAaaacaatttt
>DjScaffold3594:3265-20159 strand:+
agttaatattatttataaatattcgATGTGGATTTATTTCGATAAATATCAATGCTATTT
TGCCATTTTGGGTGCTTTTCTAATTCAATTAACACTTGGTGGTTTCTATAACTCTGGCAA
TATTCAGTCAATTATTGTGCAATACATGCAAAATTTCAGTCTTTCTAAAACTTCGGTTTC
AATGGAAAATACCGCTTGGATATTTGGAGTACTTTTATTGGGAATGGGAGCTGCAATTTC
TTTTGGAGGATTTATAGAAATTAAATTCGGTTCTTCAAAAACTGTTTTACTAGGTGGATG
GTTAATGAGTTTCAACATTTGTTTGATGTATGCAACAATTACAACATATCTACCAATTGT

TATAATCTCATACGGACTTCTTGGAGGAATCGGACTGGGAGTTGCTTACGGTGTATCACT
GGGTACACCATTAAAATGGTTTGAAAACAATAAAGGATTAATATTTGGATTGTGGTTTC
TGGAATAAGTTTCTCTTCTATAATTTTTGATGCAGTTCAAGTTGCCCTAATATCAGATAA
TGGAGAAATAAATATTCTACGTTTGCCTGTATCTTTCATTATATTGAGTTGTATTTATTC
CGTAATTCAACTGTTTGGTTCCATTTTATTGAGACATGATCTACAAAATAAAAAAGAAAA
GACACCAATTAATTCCAGTTTAAATAAAGTTGAATTAACTGAAAATCAATCTTTTCCATT
GATAAACATTTTGAATAAAACTGTGGAAATGCAGCCACATCACATTCTATTTGTACTTGA
TTTTTGGATATTGTGGATGATCATGTTTTTAAATTCATTTTGTATCTCTCTAATAACTTC
ATTATTCATTAGATATTCAGCAGTGCTTAGAATGGAAAGTAACAATATCAACCATAGTTT
AAAAATGGTAGCTCTCATTGGTAGATGTTGCAATAGTTTTGGAAGAATATTGTGGGGCTT
TTTAATGGATAAAGTTTCTTTCAAAAAATTAATGCTGATAATTACCTTATCAGTGACAAC
ATTAATTGGAATATTACCAGTTATACCGAAACTATATATAAGTTGGCATATTCCAGTTTT
TGGTTTATGGTTTGCAATTATTCATTTTTTCATCTCTGGAACTTTTCCAATTTTTCCAAT
AATGTGCTCAAGATTATTCGGAATAAAATATTTTGTTTGTAACTATGGATTATTATTTAC
ATCTTGCGTGAGTATAATTATAACTATAATTTCTTGAtttatcacactataaaaattcat
cttcacattgcgttgtatttctaattttaatgatgat
>DjScaffold27:46164-47162 strand:+
ATGCCATTTGGTATTGCTGCTGCTCCAGGAACGTTTCAAGAACTAATGAATAAAGTATTA
ATCGGGATAAATGGTGTCGTAGCCTTGATTTATTTGTATGATATCTTAGTTTTTTCTAAT
TCCTTGGAAGAGCACATGATTCGTTTAAATAATGTGTTAAGTAGAATAAAAACTGCAGAT
TTAAGAATTAATCCAGACAAATGTTCGTTATTAAAAAAAGAAGTTAAATTTCTCGGACAT
ATTATTACAAATGAGAGAATACGCACAGATCAAAGTAAAATTGAAGCAATAAGTAAATTT
GAAAGACCGAAATGTATTAAGAGTTTGAGAAGTTTCTTAGGATTATGTAATTATTATCGA
AAATTTATAAACAAATACGCAGAAAAAGCTAGAATTCTAGAGGCATTGTGCTGTAAGGGT
AATGAAAAGTGGATTTGGTCTGATCAGTGTGATGAGTCGCTTAATCAACTAAAAATTGCT
TTACAGAAAACTTCAGTGTTAGCATATCCAGATATCAACAAAGTTTTTATTTTGGACACG
GACGCTAGCTTTGATGCAATTGGAGCAATACTCTCACAATTAGATGAAAATGGAAACGAA
AGAGTTATTGCTTATGGATCACATGCGATGAGTTCACACGAAAGAGGTTATTGTATTACA
AGAAAGGAATTGTTAGCTATTTATTATTTCACTCAACACTTCAATCATTATTTGTATGGT
AAACAATTTATTCTTCGAACGGATCATAAGGCTATTACTTTTATGTTATCTACAAAAAAG
CCGATTACTTCACAATTCCAGACATGGATAAACTTTTTAAGCAGTTTAGATATTAAGATG
GANTGTATTACAAGAAATGAATTGTTAGCTATTTATTACTTTAACACTTCAATCATTATT
TGTATGGTAAACAATTTATTCTTCGAACGGATCATAAGGCTATTACTTTTATGTTATCAA
CAAAAAAGCCGATTACTTCACAATTCCAGATATGGATAA
>DjScaffold380:45575-47539 strand:+
ATGAATATTAATAGAAAAAGACACGGACTAGAAAATGAATTTTACAGTTCGAAGTGTAAA
TATAAATGTAATGATTATGATAATTCGACTGAAGAAATTCAAAGAAATTTGCCTGTCTAT
AAATGTAAAAAGGAGCTTTTAAAAGCTATTGATAATAATCAGATAGTTATCGTTACTGGT
GATACTGGTTGTGGAAAGTCAACCCAAATACCTCAGTATTTACTGAAAGCCGGATATACC
GCAAATGGAAATATCGCCTGTACACAACCCAGACGGGTGGGTGCCATTTCACTAGCTAAT
CGTGTTTGTGAAGAATTAGATTGTTACTTAGGCAGTGAAGTCGGTTATTCCATTCGTTTT
GAAAATGTGACTAGTTCAATCACGAAACTTCATTATTTGACCGATGGAGTCTTAATAAGA
GAATTGCAAACAGATCCCTTACTAAAAGCATATTCAGTTATTATAATTGATGAAGCTCAT
GAAAGAACAATTCAAACTGATGCTTTATTTGGAATTTTAAAATGCTTAACAAAAAAACGG
TCTGATTTGAAACTGATAATTTCATCAGCAACACTTCAAGCTGATGATTTTTCTAAATAT
TTTTACAATGCAGAAATATTTAAAATTCCCGGTAGATTATATGAGGTAAAAATTAAATTC
AGTAAAAAATCTGAAAATGAATATATCGATGAAGTAGTTAATAAAGTATTAAAAATTCAC
AAAAAAAATCGACATGGCGATATTTTAGTGTTTCTACCGGGACAAGAAGAAATTGAAGCT
TGTTGTAATATGATTAGAAAACGAAATCGATATTCTGATAAATCCTTACCGAACTTATTG
ATTCTTCCTGCATATTCGGCTTTAGTAAAGAGAGAATTATCGAAAATTTTTATCCCAACT
CCGAAAAATTACCGTAAAGTAGTTGTGGCAACTAATATTGCTGAAACGAGTTTAACGATT
GAAGGAATAGTTTATGTTATTGATCCGGGTTTAGTGAAACAAAAAGTTTATAATCACATG
TTAAGTATTGATCAACTGATAACAAGTGATATATCAAAAGCTCAAGCTGATCAAAGAGCA
GGAAGAGCTGGTAGAGTCGGAAGAGGGGTTTGTTATCGGTTATTCAGTGAAAAGAAGTAT
AGAAAAAATGCTGGAATATTCAATTCCTGAAATACAAAGAGCGAATCTCGCAAACACAGTG
TTGCAATTGAAATCAATTGGTATTCGAGACATTGAAAATTTTGATTTAATTGATCATCCC
GGACGTGAAAGATTACAAGAAGCTATAAAAGAGTTGATCGATATTGCAGCATTGGATAAA

ACCGGAGATCTCACAGATCTCGGCAAATACATGGCTGCATTCCCTCTCGATCCAATCCTA
TCGAAAATACTGATAATGTCCTCATATAAGAACTGTTCAGAGGAACTTCTGACAATTGTC
GCTATGCTTTGTGTTCAGAATCTTTTCGAGAAACCCACCAATCAAATGTTCGAAGCCAAT
CGAAAGAAACAAGAATTGAATCGACCCCAAGGAGATCACATCACTTTGCTTTACATTTAC
ATAATGTGGAGAGATAGCGATTACAGTAATCATTGGTGTGAGGATAATTTCATTCGTTAT
TCCACACTGAAACAAGCTCAGAATATCCGCAATCAATTGATTGAAATCATGAATCGATGC
CGACTAAAAATCGTTTCATGTGGCAGCAAAATCGTTGAAGTTCAAAATACACTTTCGTTG
TATTATTTTCGACGAAATGCTGCTCGCAAAGAATCCGGGATCGGTTACAGAACACTGGCA
GACAATCAGATCGTTCATATCCACCCGTCCAGTGCTCTGTTTGAGAGGCAACCCGGTTGG
GTTGTGTTTCATGACATCGTGATTACTAAAGAGAAATATATACGAGAGGTCACAAAAATC
AATGCTTTATTTTTACAAGAATTAGTTCCGAGTACTTTTCAATAA
>DjScaffold2640:28323-28938 strand:+
gaaacaaatattgcagctgtaatctgtagtgtgagtgagaaaccaaaaacgtcaatccgt
cgtcgttcacaaaaactatccattcctagaacgaacttattcagaattttgcatgaggat
cttcattttacggcattcaagattcaaataattcaacaaataaggccgaatactccgtaa
attcagtaaatgggccattaaaaatATGCATGAAAATGAAACATTTTTCCAAAAAGTGAT
CTTTAGCGACGAAGCATATTTTGAACTAGGAGGATTTGTGAACACACAGAATTTTAGAAT
TTGGGGCAATGAAAACCTACATGAAATACTACAAAAACAATTCCATTCACCAAAGAATGA
AGCTGGGAATTCTGTAACAGTTACCGGAGCTCGATATCGAAATATGATTGGAAATTTTTT
GTTTCATAAATTGAAAGGTATGGATACCCAGAGCACGTGGTTNACGTGGTTTCAGCAGGA
TGGTGTCACATCTCATACGGCAAAAGAAACAATTACCATTTTAACTGAACGATTTGGTAA
AATATTAATTTCTCGAAAT
>DjScaffold2640:29490-30084 strand:+
cggaATGAAGCCAGCGTTGGATTTTGCTCTGTGTCTAGAAAATTTGGCATTGGGTTTGTC
AAATGATAAAAAGCATCGTTCAGCTCTACGATTGTCGTGTGATAACGTTAAACAAGATCT
TTTATTTGAAGTTCAAGATAAAGTCTTAATGGGAGACTGGATAAAGTCATTCATAGAAGC
TCTTGGAATAAGTAATCAAAAACCTATTCCTCTTGCATCTAGTAGTCCAACCCCAAGCTT
GCCATCTCCTGAATTACCGGATGATATAATGCATAGATCATTAACAACCATCAAAAGTAT
GGGATCGGAATCTATATACGATGAAGTATGTTCATCTGATATAAAAGAATCAGATTTATA
TGAAGAAGCTCCGCCAACTCCAATAACTAGCAATCACTTTCAGCGCCGCTGGAGTAGTCC
GGCTGTCGAATTACCAAGTCGCAAGCTATTGAATCCGATGCAACCACCAAGTGCTCCTTA
TGTGGGTCGAAGATGTCTTCGAGAACATCCTTTACCATCATTACCTGCATCATCTCCTGA
TTCAGAA
>DjScaffold1938:47970-51564 strand:+
tgtgtttcaactgcagaagttgttggtttagaaataaaagacgttaatcaaagaatacaa
cattatcaattaATGAAGACTCAGTTTATTGAAATTGTTGATCAGTCTTCTGAATGGGTT
GAATCTCTCAATGATGATTACAGAACAAGGGAAGAAATTATGGAGATTGACACTAAAGAA
AAATTGGAACTTTATTCAGAATTTAAGGAAATATTTCAAGAATACCACCCAAAAAAGATT
GCAGCTTTATATGGAGCTCAAGATACACGAATTAAGATGAATCGATTTAAAAAAGAAATC
ACAGATTGGATTGAATCTGCTTCTAATTTACGAAACCGAATTGAAATTCGTTCAGCAAAA
CAACAACAAGACGATTTATCGAATCTGGATTTGAGTGATTTAAATGAAGAAATTAATCAA
TTGCAGCTGATTTTTGGAAATGAAGAAATGTCTTGTCCATGTGATGATGCATTTGAGAAA
TATAAATCACTCTGTGAAACATTGATTCCTTACATAGCTGACACTGATGCTATTGCACTG
AAAAGTGATTTAGACAGTTTGATAAATTCGATAGATGAATTAAAATCTTTCAACGAAAAA
TATACAGAGAAAATCAATATTTTGAAAAGTAACCTGGAAATATTCAAAACTGATTGTGAC
CATTTGGATACACAAATGGATTCAATTGAAATATTTTTGATGAATATTAGCAAAGGTTTA
AATGCGATCGACAATGAAGGTCATTTAGTCAATCAGAATCTAACTGATCTTAATAGAACA
ACGAACTATATCAACGATAATTTAACTCCGAGATTAGACAGAATGCTTGCAAGTTGTCAA
AGAATGTATACAATTTGTAATCCTGCTAGTTTACAAGTAATTATTGACGATTGTCAACAT
CGAAGTATACGATTGAAAGAAATCGAAGCTACTTGTAGTCAACTATCATCTTTTATTAAT
AATCAAATAAAATTGAAAGCTACCGAAGAATTGTTAAATAATAGAGTAATAATTGAATTG
ACAAATGCTGCCCAATCTATTCTGTCTGATGAAGAAGTACAAAAAGTTTCAAAAAATACT
TTGCTTAGCAATATTGAAAACATCAAA
>DjScaffold4707:17592-27648 strand:+
TTTTTATTTCGATTATGTGCAATTGTTGGTATATGCAAAGTAGATAATTTTAAGGAATCC
ATTTTGCATTCATCTGACTTAAATAGCTTTAGTTCTAAAACAGCTTATAGAAATTCCAAA
CTAACTGCGCTTAAAGAATCTACCAATTATTTAAATAAATGTAATCTTGTTCAAATAAAT

ACTGTGTTTCGTCATGGTACACGTTTTCCATCAGTCAAAGATATGAATAAAATAAGTAGT
GTAATTAAGAAGATAAACAAAAGTCACGGCAAGTCTATCTTGCCTTTGCCACTTCAGAAT
TGGAAAATGCCATTTTGTAAATCAAAGGACAAAGAATTAGCTTCAGTTGGTTATGAAGAG
ATGATGATTCTTGGAAATAAATTCAGAACTAAATTAAATGATGTGATACATTCTAAAGAT
ATTATGAAACAAATTAAAGTTTTCTCTAGTTCAATGGCACGTTCAAAATATAGTGCTGCG
ACATTTTTGCGTTCTTTTCTCAATGAGACGAAATCTCTCAAAGATATTGCCAAAGGAATC
ACCGAAAATGACCTTTATTTGCGTTTCTTTGATAATTGTCCTCATTATACGAACACTGTT
CTCAAAAATAAGGCGACCAAAATGGAATATATGCGATATAAAAATGGTCCTGAAATCAAA
CGTGTTATTAAAGATATTCAACAGAAATGGAAATTAACCGATATTGTATTGACTCCGGAC
GATGTGCGGGCACTTTTCGTTATGTGTGGTTATGAAATAGCTACTAATCTTTCAAGTTTT
ATGCGTCCCCACGAATGCCCAATATCACCAGCTGGTGATGGATTGGTTGTTTGTCCCTTG
AATCACAAATTTGCTTCTAAAGAAACTTCTCCGCTGTGCATGTTGTTGGAGCGTGATCAT
TTCCCAATTCTCGAATATCTTTATGATCTTAAGCATTATTGGGTTAAAGCTTATGGCAAA
GAAATCAATTGGTTACAAGCATGTGGTCTTATGTCTCCTCTTCTTGATCAGATCATGACA
GCTGCACGTGGTCATCAAGCAGCATTGCGATCTTCTGGTTCATCAAATAATCCATCATTT
CAGCAGGTTCTTAATAAACAGGTTTATAATCGTGCCACATTCTGGTTTGGGCACGCTGAG
ACTGTACTTCCGATAGTTTCACTACTAGGTCTTTTCAACCAATCAACTCCAATGTCAGCT
AAGGCATTTCCCAAACGGTTATCACTTATCAATGACAGTGAGCAACGATTCTCAACATTC
CGTACTTCCAATATAATACCATTCTCAGGCAATATCGGTCTAGAACTATATCATTGTTCA
CATTTGTCACAAGATGATCTCAGTGGCTATCGGGTCCAAGTAAAAGTAAATGAGAAAGCA
GTGGCTTGGCCGATGGCCAATGGTTATAGTCACATATCGATTGAGTTATTAATGAAATAT
TTGAAAATTTGCCAAGCAGATAAATATGACCATAATGCGATATGTGTGCGTGGTCTCTCA
TCAAATCAACTGCATGAAGTGCACAAACGCAGTGATGAGTTGTAGcgagtaattcttttc
ttgactttctgtatttttcc
>DjScaffold1070:24314-27709 strand:+
tttatatatgaaataattaattttacttttagataaaaatgatagaaaaactagttttcc
taataatttttataaatcgaaattgtgttggttttctccgacaacattgtaattttcgga
gaaagcacaaatccacatttatttagtgctgccaaaATGGGATTCAAGGATGTTATTACC
GAAATTTACACTTTTTCTGACGTATTTAATGATCAAGATAGTTTTCTCGATAAAGTTTGT
GAATTAACACCCAGTACTATTGATGGATTCATTGATCTGAGTCTTAATGTCATTGGAAGC
GATGATATAGCCAGTCATATGGATGCCCCCGTCATTTCCATGAAAGAGTGGACTCAATAT
TCTAGCTCAGAAACTTCAACCATACCTTCTGTAAGACCAACCTCCAGTGATTACATTGAA
TCTGTTGTCAGTTTCATTAACAGTCTCAACTCGACCAGAGATTCTATTTTAATTCTTTAC
TCAGCCAATTTT
>DjScaffold1070:43832-44289 strand:+
ataattcgccaggtttcttttggattaagatgtccagaaaatgaatctgaaagtcgattg
acttatttaaagtgaatttaATGTTATCATTAATATTATTGGCTAGTTCTAAAATATTTT
CTAAAGATGAATTAGTAACAAAGAAAATATCGTCTATATATCTGAACTTAAGATTTATCT
TCTTATCAGCTTTGGGAATAATTTTATTTTCAATGAAGTTCATAAATATTATCGCTAAAA
AAGGAGACAAATTGTTACCCATTGCGAGACCACTTTTTTGTTTAAAAAATTGTTCCGATA
AAAATGCCGATCTTAATTTTAAAGATAAATAAttaaatattgaaatttgtttttgcaaat
ctaaatgttacttgtttttaaattaacataaaaacaaatttgaagtttagataaagttg
t
>DjScaffold1059:9212-9696 strand:+
tgaagctgtaattagtattattgagaaagaatcaattttacaaatacctagctttatcga
aATGTTAATTAATTTAGCATACGATCCACAGGTAGAGAAAGGTATTATAAACGAAGAAGC
CGAACGTATACTAATTGCGATGGTTAATTCTCAAGCTAATGTTATTATGTTAAAACACTT
GTTTGAAATGCAAGGACCTTTGACAGATGAAAAGATAAAAACAATTAGAGGTAATAAAAT
TATTAAATCTTTACCGCAATGGAAACAAAATAAATACGATTATTTAATTCGTCTCATAAT
GACAAATTGCAATGGAATATTTTCCGTCATCAAAACTCTTGTTGACCTCACAAGTAGCAA
TAGAACTATAGAATTAGCTTTCACAATAGCAAAATGTATTTGTAAACCGCCTTCAACAAA
CCAACAAGAA
>DjScaffold1059:38937-43443 strand:+
actattttcccggttttttcaattaaatgctaaattctaaaatgaattcaataaattta
gaaatcagctaatcaaatttaattaactttcaacagtatattgatcaattgaattgattt
catttagataaaagttcagattttgataagccagaaattgttgttgaatctgaatgcttg
ATGGATTGTTTGAATTTGCATAATTCATTACTGAATACTCACCAACCTTCTCTTTATGTT

CAGTGGTGGGTGAGGAGGTTTTCACAAATTTATATCGCTATTTCATTGTCTGTGGTCGAT
AATGACAACAAATACAAAAAGATTGCCAGAGAAATTCTTGTCCGACTTATTGAATTAAGC
TACAATAATTCAAATCACGACACAAATAAATTCATCAGAGCTCTCTGCCAACTTGAAACA
TTAGAAGATGGAGAAGATCTCAACGATAAATTACAAATTAGATTGGCACTCGATAGAACC
AATCTAGAAATAGCAGTTATAATCGATACATTTGGAACAATATCGAGTTGTGTTGATAAA
GAAATGTTGGTTCTCGAGGAAATAATCGATATTCTTAATCAGCTGCCGATATCAGTGAAG
AACGATGTAGTTAGCAGTTTGTGCATCTGGCTGTTAGAGCAACGCTTGAGCATCACCACC
AGCAGCAGTAGCAGCAATACAGCAACCAGTTTCCAACCAGACTTGTTAATGGGCCTTAAA
ATCGATGAAATGGAACATTCTATCAAGTTCCGTAACGTGTTGCTCTATTTGTTATGCACC
AAGATGGAATCAAATTGCTTCACAAAAACCGGTGATATCTTGAAATTCGCCAAAATCGCA
ATTGAACAATACAAATCGATGCAGACTAATTCAACGACAATCGAGGGGGTTACCAAGGCC
ACCATTAAAGAGAATGTGGATTATGCATTGAATATGATAAAATTGGTGCTGGAAAATGGA
GAGCTAACTGACAATGACAAACAAATTCTCAACCAATTGTTCCCAGTATTGACCAGCCTG
ATTGCAAATTATCCGAATGATGGCTGTACAGACCTAATCACCGATATTTGCACAGTAATC
GCTACAATGGGACTTGTTAATCCTCTTCACGAAAAGAAAGCGTCTGTTCTCATTGAAGAA
GTAGAAGAAATCACTGATCCACAATTGAAAGAAGCTTTCGATGCTTTAGAAGATCCAGAA
ATCTGTTATAAAGGATTTGGTTTAGCGAGACTGCACAGTTTGCTCCAACAGAAAAACGAA
TCTGCTCTTCGTCAACGAGGGAAAATTTACAATATTTTCAGTAATTATCTGTTCCATTCT
GACTCATTTCTATATGTGCGGGCGANATAAagaaaagtaaaaattaatactagatttaat
taattactagaaaaattatttaataattatgacaaaccagtataatagttcaatctcgtg
ttactcttgtaggaaattaaaatttgcttgttcaactctctttatgaactaatttccagg
tcaattggctcctgcttatcgagatcagattctcaatagtatcatgcctcatttaaacga
tgaagaagatttgattcgatcgtcagctgtgacgaatcttggagaactgtgttgccaatt
ggaatatgcattcagtggagtaatgcatgagatagtgacacgagtgtgcaaccttctgga
aaatgatatcagttccagtgtgaaagttgcctgttcaatcatgattcgtaaactgattct
aggaaagttggatgatattcacgatccccgcccggatattgttagtctttcacctgtgtt
gagtattattcgcgactcattaattcgtgtatcgagaaacgaaaccgatgaaacagttct
cgatcatgtgtccgtggctaacaaggcattgattgaatctatcaaatctgttccattttt
cacagattctgcacaaatcgaaaggaaactcagaataggtaa
>DjScaffold3405:121145-122375 strand:+
TATTATACCGAAAACATATTTTTTCGACCTCTTGTCTCCGGCAGTGTTCCACACACATCT
ATAAATAATAGTTCAATATTTCGTCTGGTCTGCGTCTTTTCCGTTTCTTTCTTCGTTTTT
GTTGTCAACACTTTAGTTTTCTGGCATGTTATACAGCTTCGAACCTGTTATCGAAAAAGC
TTATTAGAAATTATTTGTTGCGATAGTAATGGCAAATTCCTAAAAACCTTCTCATCTTCT
TAAcgcattttggtttaccaaagttcttaattgcttctattttagttggatctgttttga
caccttctttactcataaagtttcctaaagatttgatctcctttcgcatgaattgacatt
tctccggattaattcttagaccagacttcttaatttctgttaatactttattgattattt
catagtgttgctcctttatttcggtaaaaatgagaatatcatctagataaaccatcgctc
cttttttctgattactttatttatcatttcctggaacgtacctagtgctgcttcaatt
>DjScaffold3405:128875-137732 strand:+
taATGAGCAATTTTGACTGGAATGATGTTGACTGTTTTTGCTTTGATGTAGATTCTACTG
TTACTATTGATGAAGGCATTGACGAATTAGCTAAATATTTTCATAAAGAAGATGAAGTAA
AGAACATTACCAATCGAGCCATGGGAGGATCAATGGATTTTAGAGCTGCTCTTGAATATA
GGTTAAATATAATCGAGCCTACATATCAAATGTTCACTAAATTTATTGAAGGTAGAAAAT
CGAAACGATTATTAACACCTGGAATTCAAGAGTTAATTGATTTGTTACGGAATTCTGGAA
AAGATATTTATCTGGTTAGTGGTGGTATGAAAAAAATTATTGAACCGACCGCTAATGAAC
TCGGAATTCCTCGTGAAAATATATTTGCAAACACTTTATTATTTCATAAAGATGGAAGTT
ATCGCGGATTTGATATTAATCAACCAACAAGTACTCAAACGGGTAAAAGTCGAGTAATCA
ATTGGTTGAAAGCTCATAGAGGCTACAAAAAAGTAATAATGATTGGCGATGGTGTTACCG
ATTTAGAATCTAGCCCACCGGCTGATTTGTTCATCGGTTTCGGTGGCAATGTTATACGAG
AAGAAGTGAAACGTCGAGCTCCTTTGTATTTTACAGATTTCTATGATTTGATTGAATATT
TGCAACAGAAATCCGTTATTATTTAAagagaaagatttataaaaaataatttttacttta
gaagatttttagaattttgtaaatattgatttgaatttacagtgattttgtatcaccata
tcctcttttgtgtgattagttccgaggtgggaattttctttttattaattattgattta
tttattatgttataaattatctaatacatgacgtaataataacaataatcgattgatttc
ctcgcggtaatttcagatgatactgataacaaattgttcaaatatttcttatttggtgat
caaatagcaacttatttctttcta

>DjScaffold14:160499-164563 strand:+
ATATATATTTTTTCTAAGAATCAGGATAAATCTGAATCAACTGATGAAAATGAAGAAAGG
TCTGAAACAGAAACGGAATGCTTTGATAAAACGGATTCCGATATTATATCACAAAGTGAA
AGTACTCTTGAGTTACGATGTATAGAATGCAACATCAATTATCAAAATTATGATGATTTT
CAAAAACATAAAATCCACAGACATCCAATAATTATGTACAAATGTGGAGTAGATCATTGT
CAAAGATGGTTTTCTTCGCGGAAAAGTCGGGATAAGCATTCAGAAAATTTAAAATCTCAA
CTTCATAAGTCATTAGTTAAATCTCCTCCTGTTAATGCCATATTTATTGAAAATGCTTCA
CAAGAAGAACTCGAAATACTAAAAGCGTTTTCAAATGAAAATATACGAAGCAAAAATAAA
CGTGGTCCAAGACCGAGAGTTTCTAACGAAACCGATTTTTTAATTGGATTGCCCAGAGAT
CAATTAAAGAGAAAATTAATGGAAAGTGTCGTGGAGAAAATGGAATCCAATCCAAATAAA
AAAATTCAAGAAACTTCGTCTTCAACAAAATATATTAAAAAAAATTCATTGTCGGGTCAT
TTATCATTGCCTTCGGATTCTAAATTTTCGTCTAATAAAGTACCTATAAAAAGTATGGAA
TGCAAAAATACTTTTTACATGGAGTCAAGCCAAAAGTTAAGATCTATACCCGATTTAAGT
CATATTAAAGTTCCAGTTTTATCCCTTGATTCTTCGTACTCATTGGAAAAATTTTCTGAA
GAATTAATAAATTTATTGGTACTTTGTGATCCAGCAGAAATTTTTAAGTATCCGCTAATA
GATTATTTCGCTCCATTATCCTCTCGATTAACAAATCAAATGGATATGAAGAGTATACAG
AAAAGATTAAAAGAAGGTATGTATTCCAAAAATCTCGAAATTCTAGAGAATGATCTTTTG
CTGCAATATTCCAATGGTATTCAAAGTTTTGATGAAAAATCTACAGGATGGTATGAAGCT
GCTAAACTTTATCAAACTGCTTTAGTAAACATACGAATTGCGAAAAAAATTTTTGATTCT
TCAATTAACATAAATGATTTAACTTCAAAAAATGATGCATTAAACAATTCACAAGTTAAT
GCTGATAATATATCTACTGTTCTCAATTTAATAAATCCATATAATAATGGGAAAATTAGT
GATAATGAACGAATTATGACAATTGGAGATCAAATAGGTAAATTAAAATCTGGCAGTTAC
CATTTGTCAGGTTATAAAGAAGATAGGCGCAATAAAGTTACAGTTTTATCTTACTTGGAC
TATGGCACAAATTTTTCATTTGGTCCTTCTTATGATTCTGGAAATTCCTATTGTTCTAAA
GAAACTAGTGATTTATTAATTCGAGCTTATGGCGATATAAATTATGCTGATGAATGTCAA
TGTGTGGAATTTAATGAAATTATTCCTGAAATAACATTAGGAGATGATTTTAGCATCTAT
TTAATGAATATTTTTACTAAAGACGTTATTAAATCATTTTCTTTCAACGAAAATGATGAT
AAATTAATTTCAGTTAAGCAATGTAAAGAATTAAAGGGGATATTAAATACAAACAGAGAA
GAAATGCCTAGTTTACAAACACTTTTGGACGAAAGCTGGAAAGATTTAGCATGTTTAAAA
TCTTTCAATTATCAAAGATTATCAAATTATCAAATACAACCTTCTTGTGATGAACAAAAC
GTATCGGAAAAGGTTACCTCAAATTTGATTAATTTAATTATTAAAACTAATCCTGAGTCA
ATTACCAATTTAAAAATGATTCGTAAAGCTTTAAATTGTAAATAAtgttataatattaat
aaatagaatgttaca
>DjScaffold14:208354-211867 strand:+
ttaattattatcattaattattatcattaattattatcattaattattatcattaattat
tatcattaattattatcattaattattatcattaattattatcattaattattatcatta
attattatcattactttagtcgaatgcgaaaaaatgtattgattcggttattaaattggc
aaatgacaaaaaaacaatcaatgaaccggagaagtggaaagaagctgaagataaatataa
agaatggaaaaaatgcctttctaagtttacaatagattcatattttaaaaatgagttatt
tatagctgagtatgttttaaagctatatgagattagatttacaagcttttataagctttt
caaatatttaggttcaataattgatttcaaagaatataaaacaattATGAGTCTTGGTTG
TGGTGCTTGTCCAGAAATTGTTGCGTTTCTTCATTATTATGAAAATGATTTGGGAAATCC
AAATTGCGAAAACTTAACAAACATTATTGGAATCGATGAGATTGATTATTGGCAAATTTA
TCATCCGTGTATAAATGAACATTTCAACAAGAGTGTTGAATTCAAGAATGACAAAATAGA
TAAAGATTACGTATCAAAGCTCGAGGAAGTCGATTTAATAATGATGTCTTATTCCTGTGG
ACGAGAATGTGAAGATATTTGGGAAGAACTGAAAACGAAATGCAAAACAGTAATAGTTTT
ACACAATGAAAGTGAATTATCTGGCCCTAAAAAAAAGGTTATACAATCAAGAAATTCCAT
GATATGCAAAAAGTCGAAAAATGTTATGCTTATT
>DjScaffold14:220948-221803 strand:+
gaaaatgaatgataaaatttcaacaatcaaatcaataatagaaataattcattcggaaat
aatcaatattgggaatgcagaggggaaacaaatgaaaaatgatcaaattgtcaccgagat
tgacacaaatgagaattacaaattgattattagatatgatgatggagaagggaataagat
caatattacatttgatcaatgttgctatttgcatgataaaaaatttggtagatcaatattt
gtgacgttctgctttgtgggatttttccaatattagacaacaaaacaacaataaaacaaa
taaatcagtttgcgatatcgtaatcgatgctttggattatttaATGAAATATTTGAAAGA
TTCCAACAAAGATCGTCAATATTGGGATAGATTATGGATGAATCGATATTTATTGACATC
TTTCAATGATTCTGATTTTAATGAGAGGATCAATAAATGTACAATCAAAGATTTCACAAT

TAAAATGAGAATGAATGGGAGGGATATAAACAATTTCATCCCTTCAAAACCAGAAACAAT
TATCGAAGCAAAACGAACCATTTCAGCAGTATACAAAGTTGACATGAAACATTTGTATTG
TATCAAGCCAGGAGGAGATCACAAACCAAATGATAAAGAGAAATTTGATGACGAATTTAT
CATTGAAATTGTGATGAGTATGGAAGATATCAAGATATTGAGATCACTAGGACGAGGAGC
ATTTGGTGCAACTTATCTTTATCTAGATAAAACATCCAACAAACAATTTGTTTTAAAGAA
AGCTATTCGAAATGATGGAGATCTTTGTCGAGATATAAATATTTATTCTAAACTCTCTTC
ATCAAGAAATATCATGAATTATTCTGGATTATTCAGAGATGGAGAACATATTTGCATTGG
CATGGAGTATTTGTCGGGAGGATCTCTCAAATCTTATTTGGATAAAGGCAAACATTTATC
GGAACAAGAAATAGTAAGATTTTCATATGAGATCTTGACACAAATAAATGAATTTCATAA
ATTGGAAATTATTCATCGAGATATAAAATCTGATAATATTGTTGTCGATGAACACAATCA
TGCAAAATTAATTGATTTCGGATTATCAAAATATACAATTGGATTAACAAGTAGAAATCT
AATAGATAAATTGAAAGCATATAAACCTGGAACAATTTGTTACATGGCTCCAGAATTGAT
TAGAGAAGAACCGTATTCAGAGAAAATCGATATTTGGTCATTTGGAATGTTGATTGTTGA
AATGGTGACAGGTAAAAGACCATTTCATTGGATGACTCCATATCAAGCAATTCTGGTAAT
AAGAAATTTTACAAAATACAAATTTGAACCAATTTTTCCAATTTCAAATGAATTGAATCA
AGTTATCAATTCTTGTTTGAATACGAATGCAGATGAGAGACCAAGTGCAGAACAATTGAT
CAAATCAGAATTATTTTTTCATCATTTAAATAAAaacaatttattgcagacatattatct
ccacatttggtacatttgatgaagaattgaagcaaattctaacagatttgccctcctcat
ttctcaatttgacaaattttttttgctttgatatgacagttcgaatttttgatatttgtg
gtggttattgggtatttgtgatggatcaatttgaaaatgatgttgtgacgatatttctta
aaaatttctgccctgcaatgtaagtttgattgaatgaacgatgaatggcattgtggcaca
aatcatttgaacatgcatatttgttatcaataatgacaataatcattacaaaatatgatt
tatcaaaatatatttgcagccatgattgatctttataatttgatattgattattacattt
atcgattaaataaaaaatgatttattcaaattgaatattaattttatgatttgatttgatc
tattcagacgttctgctttgtgggatttttccaatattagacaacaaaacaacaataaaa
caaataaatcagtttgcgatatcgtaatcgatgctttggattatttaATGAAATATTTGA
AAGATTCCAACAAAGATCGTCAATATTGGGATAGATTATGGATGAATCGATATTTATTGA
CATCTTTCAATGATTCTGATTTTAATGAGAGGATCAATAAATGTACAATCAAAGATTTCA
CAATTAAAATGAGAATGAATGGGAGGGATATAAACAATTTCATCCCTTCAAAACCAGAAA
CAATTATCGAAGCAAAACGAACCATTTCAGCAGTATACAAAGTTGACATGAAACATTTGT
ATTGTATCAAGCCAGGAGGAGATCACAAACCAAATGATAAAGAGAAATTTGATGACGAAT
TTATCATTGAAATTGTGATGAGTATGGAAGATATCAAGATATTGAGATCACTAGGACGAG
GAGCATTTGGTGCAACTTATCTTTATCTAGATAAAACATCCAACAAACAATTTGTTTTAA
AGAAAGCTATTCGAAATGATGGAGATCTTTGTCGAGATATAAATATTTATTCTAAACTCT
CTTCATCAAGAAATATCATGAATTATTCTGGATTATTCAGAGATGGAGAACATATTTGCA
TTGGCATGGAGTATTTGTCGGGAGGATCTCTCAAATCTTATTTGGATAAAGGCAAACATT
TATCGGAACAAGAAATAGTAAGATTTTCATATGAGATCTTGACACAAATAAATGAATTTC
ATAAATTGGAAATTATTCATCGAGATATAAAATCTGATAATATTGTTGTCGATGAACACA
ATCATGCAAAATTAATTGATTTCGGATTATCAAAATATACAATTGGATTAACAAGTAGAA
ATCTAATAGATAAATTGAAAGCATATAAACCTGGAACAATTTGTTACATGGCTCCAGAAT
TGATTAGAGAAGAACCGTATTCAGAGAAAATCGATATTTGGTCATTTGGAATGTTGATTG
TTGAAATGGTGACAGGTAAAAGACCATTTCATTGGATGACTCCATATCAAGCAATTCTGG
TAATAAGAAATTTTACAAAATACAAATTTGAACCAATTTTTCCAATTTCAAATGAATTGA
ATCAAGTTATCAATTCTTGTTTGAATACGAATGCAGATGAGAGACCAAGTGCAGAACAAT
TGATCAAATCAGAATTATTTTTTCATCATTTAAATAAAagataaatgtgattgacaaatg
caatttatacattcagtATGGAAGATATCAAGATATTGAGATCACTAGGACGAGGAGCAT
TTGGTGCAACTTATCTTTATCTAGATAAAACATCCAACAAACAATTTGTTTTAAAGAAAG
CTATTCGAAATGATGGAGATCTTTGTCGAGATATAAATATTTATTCTAAACTCTCTTCAT
CAAGAAATATCATGAATTATTCTGGATTATTCAGAGATGGAGAACATATTTGCATTGGCA
TGGAGTATTTGTCGGGAGGATCTCTCAAATCTTATTTGGATAAAGGCAAACATTTATCGG
AACAAGAAATAGTAAGATTTTCATATGAGATCTTGACACAAATAAATGAATTTCATAAAT
TGGAAATTATTCATCGAGATATAAAATCTGATAATATTGTTGTCGATGAACACAATCATG
CAAAATTAATTGATTTCGGATTATCAAAATATACAATTGGATTAACAAGTAGAAATCTAA
TAGATAAATTGAAAGCATATAAACCTGGAACAATTTGTTACATGGCTCCAGAATTGATTA
GAGAAGAACCGTATTCAGAGAAAATCGATATTTGGTCATTTGGAATGTTGATTGTTGAAA
TGGTGACAGGTAAGATATCAATTATGAATATGATTCTGTTTTTACGCGGGAATTTTAAGA
ACGTATCTATCTTGTAAaaatagaattttgtgtacattttattttattacattattatt

atattattaa
>DjScaffold14:116133-128904 strand:+
gataaaattttacaaaatattatgtagttaacactcattatttaaactcgatcatctaaa
ccttttacatttgtcttgataactgagtttcaatctatttatttagtctgtttcataaaa
tttaaatgtgtaatttttaaaattcttcaatgtaattataaaagttgaccaagatttaaa
aattcatcaacatagtttattttttcatataaattttaaattatccatttttttcaaacct
tttttcgaattctttagatttacatatttgaaatatttaaaaaaaaaccaatatattttttt
tatattagccattatcccgttggattactttacgattgtgcatcgaatcaatcattgccc
tggtccatcaccgtgcatttcagtaattttccatctgataaattgattgatttatctaaa
gaggaaaatttggaatctcattttatttatacagtgaaagaagccgattatttaaaaagt
cgtggattactgatacaatctttccaacaaaaagaaATGGAATCTATTTGGGCGGGGTTG
AAAACGGATAATTTTGAACAGTTTTGGTCCATAAATCGAAAGTTAATGGATTATGAAAAG
ATTGCACACGAATCAAATAAAGCTGGTGGTGAAAATCCGGTCGTCCATAATGGATTCAAA
TGTATTCCATGTAGAGTTTATTTACCTGATAAAATTGGTTTCGTACAAAAATTGATTAAA
TCTATGCACGATAATGGTGACCAATGCGTTTTATCAGATTTAATAGGAATTTTAGAGTTG
CCCCTTGATAATGTAACGAACTATAAATTCATTATTCACGGTATTGATGTTCCAAGCAAT
TCTCCTTTACAGTGGCTCAGCGAAAATTTCAGTTATCCAGATAATTTTTTGCATATATTG
GTAAAAACAATTTTTGAACAAATGTAGaattgagaatagaattgatgtagaattgatgat
tactaataattgatcaagtattttgtgattaacaataatctgaattaaagtggtcaattt
cgtgaataaattataatttggattttcaagtgagggtttgtaacccactttcttatcgga
gtgcttgtatgcgtctcatttaggtactgatattatctgaccctcgcttaaatcgtaata
attaacctttgataatattttcatttttgataaaaaattatttatcttcctaaagttgccg
tgaaaaaaatgtattactaaattgttactaattgtttctaacaactgaaaattttgaaatt
tgtaactacgtatgttggatatttaagaataaattagaaatgtg
>DjScaffold2144:3864-4142 strand:+
tcagaactctgtgcacaagtgttcagATGTCAGAGCTCTGCAAACTCTGTGCTCAGACCT
CAGAGCTCTACATTCTATACACAAGATTTAAGTTAACAGTTCTCTGCTTACAGTATACTG
ATTTTTAGttatcaatgattttctcactctttgctagtctttattcatcactaatggtaa
atttactgtttaattagtatttaattatttatcctgttctaaattttcacttataatttt
gaataatttaat
>DjScaffold2144:16527-20649 strand:+
actagcaattttaaaattctcaacggatggtaatcaggaaacaagatattatggtaaaaa
gATGTTGTTAGAGCTAAGTAAGCATCCAGATTTCGATAAGTTAATTAGCAAACATTTGCC
AGCAAAAGATTTGCGCGAATTGAAAGATATATTGGAAAATATTAAAGTAAAGGGAATCGG
AGATCCTTCCCAATTTTCCCATTCTGCTGGTATGCAACGGTCTGGATCTTTATCTCGAGG
TGGTAGTGCTGGAAATCTTGTGTCATTAGGTTCAAAATCTTCTACACTTTCACGAGGAAA
TAGCCGTTCAATGATACGTCTTCCAGGATCAGATCTGTCTGATGACGACAAAGATTGGTT
GACTAAAACTTGTTCAGAATTATCCTCTTCAGATTTTCGTAATCGTCAAAGTGGTTTAGA
GTCAATTTTAAATAAAGAAGATTTACATATTTTACCTCTGAAAAGTATTTCAAAATTATT
AAAAGACTTGATTCCACGCCTTAATGATAACAATACAAAAGTTACTGTTTGTGCCCTCGA
TGTTTGCCAGAGGATTATTCCAGTGATTTGCTCAAATTTCCCAACAGAATTTTATCCTGA
ATTTATAAATACTATAGCTCTGAATTTTCAATCAAAATCAATTGAACTCCGTAATTCTGC
TTCAAATCTTATGGACACATGTTTCAAAAATTTCGATCAGAGTCTGTTGATCCAACCGTG
CATAGAAGCTATTAATAAATCAAATCCAAAAACAAAACCCATCCTCATTGAAAACATGAA
TAAATTACTAACAATTATTGAATCAACAAAAACATCTTCTCTAAAAACTTCAATCAGAAT
GATAATTAATCATGTAATTCCAGAAATAAGTCTTTTATTAGAGAAAAATCGCTCATCGCC
ACAACAAATAAAAGAACAAATTCAAAATCTAGTCGTTTTATCTTAC
>DjScaffold2144:30241-51242 strand:+
taattcaaattttcttttaatttcacataaaattATGTCATTTAGGTTTTTCAAAGATAT
ATATTATTTAAAAAATTTTGATGCGTATTCTAAACCTATAGAAGATTTTAGAATTAAAAC
ATTATCTGGTGCAATAGTGTCATTAATTAGTGGGATCATTATTATAATTCTCGCTGTTTC
TGAATTAAATGAATATTTAAAGCCACAAGTTACTCAGGAATTATTTGTTGATGTTACACG
GGGTCAGAAGTTGCCAATTAATATTGATATAATTTTTCCTCACATAGCATGTGATTACCT
TAGTCTTGATGCAATGGATATATCTGGTGATCAGCAATTAGATGTTGATCATGAAATTTA
CAAAGTTAAATTAAATGAAAAAATTACAGCCGATTTCACCCGCCGAAAAACACAATGTAAC
GACCACTATAAAGCAGTCAAAAGTTTCTTTGAATAATTTAAATGGGACAGATTCTTCATC
GATTTGTGGAAGTTGTTATGGAGCTGAGCGCGAAGATTTAAAGTGTTGTAATACCTGCGA

ATCGGTACGACAAGCCTATAGAGTTAGAGGCTGGGCACTGAATAGTCTAGATAATATCAA
ACAGTGCCAAGAAGAAGGATGGCAACTAAAAGTTAATAGCATGAGTAAAGAGGGTTGTCG
AATGCACGGAAAATAACTGTGAACAAAGTTGCTGGAAATTTCCATTTTGCACCTGGAAG
AAGTTTTACCCAATCACATGTTCATGTGCATGATATTCAGTCTTATGGAAAAACACAGTT
CAATATATCACACTATATCAACTTTTTATCATTTGGTGATCATTATCCTGGATTCACAAA
TCCGTTGGATGATTTAAATGTCAAAGTCGATGAATTAAGCCACATGTTCACGTATTATGT
GAAAATAGTTCCGACTACTTATGTCAAATTAAATGGATCAGTACTTCACACGAATCAATT
CTCTGTGACCAAATATATTAAACCAACTGCTTTGTCCGGTGGTCATGAAGGACTACCGGG
TATTTTTTTTAATTACGACATTAACCCTATTATGGTTTGGATAAAAGAAAATGATAAATC
CTTTGTTCAATTTATTACAAATCTGTGTGCTATTGTAGGTGGAATTTTTACAGTATCGAG
TTTGATCGATTCATTTATTTACCATTCTTCAAAGGTTGTGCGAGAAAAAATTGGAAGCTGG
AAAATTATCATAAtttcaaacgaatattttgtttttatagtacatttaaatttattcaat
ctctaaaatattaagtgatgtattgaca
>DjScaffold2144:113174-113314 strand:+
ATGAAGTTTTATCTGAAATCAAAAAAGCTGATAACACATCTCCAGGCAATGACGGTATTT
ACTATAGAGATCTCGCACTTATTGATCCAGAAGGAAAATTGCTATCGTGTCTCTACAATA
ATATCATCACATCTGGCGTAA
>DjScaffold2601:26807-34670 strand:+
CCATTGATAGCTTTATTAACATATTCATTTAATATAAAAGCTTTGATCAAAATTTTGGTT
>DjScaffold3507:12356-13034 strand:+
caaaatatgtggcaaattagaacataccgagaaacaatgtaaaaataaatcggatatcga
acctattttaagaggagaaggttgcacaagatgtgggaaaagagaccgcacgttagcttc
atgtgggttaaaatgcaaaaaatgtgctgatttaaagcaagtagcgaagacttaggtata
ataatagaaagatcgaaccagttgtgtATGTTACAAGTAACTAAGGAAGAGGAAATAGTA
GAAACGCCTGATATTTTAAATGGAAACCAGTTAATGACACATGTGGAACAAGATAAGTAT
CGGTCAGTACCAGAAGACTGGCAGAAAATGCTTAATGTATATGATGACCGGATATCCAGA
TTAGAAGAAGCTTTACGATTGAGTCAAGTACAATGCCAAAATTTCAAGTTCTTAGCAGAT
AAAGCAATGGGAGAAAAAAGAGATGCAATAATATTAAGAGATACAGAATTGGCCCACTTA
CAAAGAAAATACAATTCAGCCGAAACCATATATCCAGATTAGaagaggccttacgttcaa
gccaaatacaatgtcaaa
>DjScaffold3507:128222-131006 strand:+
ATAAATGCAACTAGTTCCGAAATTTTATTCGAAGAGGATTTAGAAGAACAAATTTACCCA
GAAATTATCAAATTAGAAGATATTACAGACAATGAACCAGAAGATGACTTAGAAAGTTAT
GATATTTCCGGTGACTACTCAGGCTTCTATGCATCTGATATTGATTCATCTATTTATTCA
AGATTTATTAATTTCATTTGTTCTAAAGAAAAGCAATATTTTTCCGAAACATTTTGTGAT
AAATATTTCTCACATTTACGATTTTCCGAAAACCCAGAAGAAATCATAAACTTAAATAGA
GTAGATATTTGGAATATTGACTGGGCTTATGGCTTATTATTCCAAGCCTTTTTAGCTAGA
GAATACAACTTAGAATTACCATGCCTTAAAAAACGAAATAGAAAAGTCCCTATTATAGGT
GAAAAGTTAAAAAGGCCTTACAGATCAAAATCATATTATGACAGAGATCCAATTTATGAA
GAACTCCTGAATAAT
>DjScaffold726:14023-23575 strand:+
attccatttttactattgatatttattgtgacgagttgttggtttaggtgtggaagATGC
ATCGACAGAAGTTCTCATATTCTCTCAATGAACATAAAAACTGGGTTCGATGCGTAAAGT
TCTCATCGGATGGTCAATTACTTGCCTCTAGCAGTGATGACAAAACAATAAAATTATGGG
ATCGACATAATCAAGAATGTATCCATACATTTCAAGAACACGGAGGATTTGTACCGACAC
TCGCATTTCATCCAAGCGGATCATACATTGCGTCAGGTGGTACTGACGCGCATGTTAATA
TCTGGGATATTCGAATGAACAAATTACTGCAACATTATACAAGTCACTCAAGCCCTGTAA
ACAGTGTCAGTTTTCATCCAACTGGGAATTTCCTTATTTCTGGCAGTGATGATGCAAGTC
TGATAATTTACGATTTGCTGGAAGGACGAATGTTATATACAGTTAGGGGTCATGAAGGTC
CAGTCTTAGGAACTTCATATTCTCATTGCGGTGAAATGTTTGCATCATCTGGTGCCGATC
ATCAAGTATTTCTTTGGCAAGCGGGTTTGGCACCATCGAATGAAAACAACGTTTCACTTT
TAAACACAAAAAGCCTTAACCATTCTAATAAATCTCAACCAACAATAAGACGCACACAAG
AATTACGTAGACACGATTTAAATGGGGATAAATGTGCTCAGGAATCAGAATGTAATACTA
AACTGTTGACTCTCTCAAATAATAATACTGCAAATCACAATAAAGGCAATATTTCTGATC
ACGTTCCAATGAATCCACCTATGAAGGCATTTAATGAACGGCGGAAAGGTCTCAATGTTC
CTCCAACAGATGTGTTACGACATGCACCAGAAACTCCTCGTGATATTGATGTCACTACTA
TTGGTCCATCAATGTTTACAAGGGAAGACTATAAGGCAAACAATTTCGTTGAACAGCTCA

CTCAGCGCGAGGAAATGTCTCCATCAATGAGCTCAACTCTTGAATACATTGTCGGCCAAT
TGGACATTTTAACCCAGACTGTATCGATTCTAGAACAAAGATTAACGTTTACAGAGAATC
GATTGAATGACATATCAGCATCAGATCGTCAATATCAACCTCCTCCTCATCCTCCTCTCA
ATCGTAATCAATATGAATAAcaaatttgcctgcttgtgtttgtgtgtgtgtttaaatttt
gtaatgatttattgatcgatcgagatttttgctttttagtattattattattattatcat
agacatgtttcatcaatatattgattgattgattgattgatttttagcagtaattttata
taatcaatggataatttttattgattttccgtgcagataatcaatatcgataaataaata
tttttaatt
>DjScaffold3029:41558-42295 strand:+
ttttttttaccaaaacaccactgtatataaaggaaaatataatattaataatgcaataca
tcattctaagataaatatatttattttataggaaacctatgagcaaaaaATGAAATATTT
TTTATCAAAATATGGAATTGGAGAAACTAAAAAACCAGATGCGAATTTAGAAAATCCAGA
AATAGATGATGTGACTGAAACACACGAATTTTTAGAATTCAATGAAATTAAATCAGAAAT
AAATCTTTCTGTCAAAATTTATCATTTCTTGGAATACAAAACAACTCTGGCAGAGTATAA
TAGTTATGTAACTGAAGGATTCATGATTTTTTTTACAAATGATGGAATATATTCTTTTGA
AATGTGTGAAGGTAGAATTTTTTTTAAGAAGTTGAATAGTGTTACTGACAGGGTAAAGCC
AAATGATCAATTAATAAAGGATGGTGCTTTGGAAAATATCACACTGAATGAAGTCAAGAA
ATTTATTGAAAAATGCAAAAAACAATATTCAGATGTTCCAAAAATTAACCAATTTCAATT
TCTCCTCAAGTCATTATTTGGGTGTTTTACCAAAGGGAATAAAGAAAAGAAAGAAAAATA
Attaagaactttgttctttaaaagttaactttataaaggttaatcgaagtattttacaaa
acaattgttattgatatatgcggctt
>DjScaffold4739:1205-4703 strand:+
cgttgaaataaacttaatatacaaattgaaggtcaatcaaataccaataaaattacaata
accactgaaaattaatgttaaaattatcaagatacgagaaacaagggcttaattttattg
atttaaaaagaattggaaATGACTGAAAATATTGTACCTATTATGCAACAGAAATTGTCG
TTTAAAAAGGAAGTCTTTAATCATGTAGATGTATTAGCTGGTCTAGATAGAGGCTCTTCA
TTGACGAAAATTATTTATGTTGACAATGAAGATTCTACAAATCTGGATGAAAATTCGACA
AGTAACGTAATTATTCTAACTGTCGCTTTGTTTCCAAATTCGAAATTCGAAGATGCACTT
ATATGGTTAAAGGAGAAAGCAGTGTTTCCTGAAAATAAAGAACTCCCATTTCATGTATCT
GGAGCCGGAGGAGTTAATTATAGTGATAAAATCAAAGAAATACTAAAAAGAGATGTAGTT
TTCATTGATGAATTTTTGTCACAATCGAGAGGTGCATATTATTTATTCATGAACATACCA
ATAGCCGAAATAATTTATCCAGAAATTGAAAGTGATTGCGAGGAATGGTTTAATATACCT
TTTATCACAGACATCAAAATTCAATTTGAAAAGATGGTCGGTAAACGTAAAGAAGTAAAA
ACAAACGAAGATTTATTTCCATTCACTTTAGAAGTTCTTGGAAGTGGAGGAGGCTTGACT
TCAATTTATTCTGATGGGACATATCGCATTGTTAGTGATATACCAGTAGCTGGTAAAACA
TTTATTGGACTTTGTCGATGTATTCTTGGAACAAATGATTACAATGAAATTATGGCAATG
GCAGCAAGGGGTGATAGACGAAATGTTGATACGCAAATAAAAGATGTCTTGGGAGAGGAC
CGTTTTTCCAAGATTTCCCTGGATTTACCAATCATACATATGGGCAAATTGTCAGAAACG
AAGAAAAGTATAACAGAATTCAATAAAGATGATATAGCTCATTCTTTGGTGTCAATGTTC
GCTTATAATGCAATAAACGAAACTATTCATTTTTGCATGTCTGAAAAAAATTCATAATGTA
AATTTGTTTTTAATTATTTTGAATAAATTTTAAttaaatatttaaatattcattaatcac
tatgagaaattatttattggcttaataaataaataaataaatgttaattattaatacttg
aaaatacatagtcaactattaaattattagagaatttctcaaaagaaaattctctatttt
aattt
>DjScaffold3765:9533-18726 strand:+
aaaattttacataattaatataactccaatttcaaaaaggtttgatttaaagatctgata
aacgcactaagtgaaattgagatatagaggaattattttcggtacttcgcagaacaacca
attttttaaattcagaaaggtttagaaatgaaagtattttaaatattgaaaaaaattccca
attgaaaattattccacaaATGTCTACTGATGAAATTGAAACTTCACTAGAAAATATGAA
GCAAGATATTATAAAGTCCATTAAAGTTTTTCAGGATCAAATGAGGGCTACGAATGCAAA
ACTAATTAAAAAAAATTCCAAAACATTTACTGTTGAAGATTTCAAAGGTGTTAACCAAGA
TTTAATTTCACCAGTGAATAAATTAAATCAAAATAAAATCAATATGATGAGAAACGAAAT
AAAAGCTTTAGAAACTGAAAATGCAGGTTTATTTATTAATTTTATAATAAAATTATAAat
aacaaaataata
>DjScaffold4863:88185-92867 strand:+
atatatgatttaataatgtattaatattactttaataataactgattatttgtttaatat
aaatattttgtggaaaatacattataccagttcattattaatttttcatctataactct

file:///C|/Users/czh/Desktop/Data_S1.txt[2019/7/4 17:05:10]

cttgacatttccaatgaaacacataaattgcagcaactttcttcatccaatatgtatatt
ttaattattttaaatgttttttcaaggttatgggatttttttattgtgattttatccaa
ttgaaatactttagtgtacttaatttgtgtgtacttaataaacagaggaaaattttttta
cacaaaatcagtcaataacaaatacttaaaaatacaaattttgtatcttatacattattt
aattatatttaaaataaatctttattggaatctattgtaggaattattggacaggtgtgt
tacagaaaatacgactaataaaATGGATCCTAACTATGAGAAAAAGTACTGGTTTGGGTT
GATTGATGCTGGACCATACGACCCTGCAAGTATCAATCAAAGATATTATGCATTAAAGAG
CATGACACTTTACAAACGATTGAATTTACTTCAACATCCTTTAATCGAAAATTTAATACA
ACAAAAATGGCGAAAATTCGGATTGGGTTTTGTGATATCGAATGCAATTATTTATGCAAT
TTTTCTCGTTTCTATTATGTGCTACATATATATTTTGAGTCCAATACAGAATAATGAAGA
GGTGAATATAACCACACAGTGTCGAGACAACTCACCAGACAATGAACATATTTCCAAAGA
AAATGTGATCATTTATCAGATTGGTATTTATTTAATAATGAGTCTTATATTCTGTATTCA
ATTGGTCAAG
>DjScaffold4863:6472-7110 strand:+
ATGCTGTTTGGAATTGCAGCAGCACCAGGTACGTTCCAAGAAATTATGAATAAAGTAATC
GAAAAAATAAAAGGAGCGATGGTTTATCTAGATGATATTCTCATATTTACCGAAACAAAA
GTGCAACTCTATGAAATAATCAATGAAATATTAACAGAAATTAAGAAGGCTGATTTTATA
GTTAATCCGGAGAAATGCCAAATCATGCAAAAGAAGATCAAATTTTTGGGACACGTTATC
AGTAAAAAAGGTATCAAAACATATCCAACTAAAATAGAATCAATTAAGAACTTTGGTAAA
CAAAAATACGTTAAGAAAATGAAAAGCCATTTTAGAATTTGCAATTACTATCGAAGGTTT
ATTAAAGATTATTCAAATAAAGCTATATTGCTGAAACAGCTGTGTAGTATGCCAAGTAAC
AAGAAAATTGAATGGGCGATTGAATTAGAAGAAGTTTTTAATGGAATGATAGACGCACTA
ATGATGTCTCCAATATTATGTTTTCCAGATATAAATAGAGGATTTATTTGGACACGAAT
GCGAGTTTCGATACGATTGGGGCAGTACTATTTCAAAAAGAAAACCAATGGGCAAGAAAA
AGTAATTGCATATGGGTCACACGCACTGACGAGTCATGA
>DjScaffold349:115441-154399 strand:+
atttttatgtgtgataatgtaactttattgcagatatacgggtcgaactcaaaagcaact
acaagaatgtgttagagaaacaagttatagacgtccccaatttgaaaggtcattatccAT
GAGTCGTGTTAGTAACATACCGAAATCACCGGCTTCAAGTGTTCTTCCCACTCGTCATAC
TCGATTGGCGAGCAATTCATATGCACAGTACAAAAGTGAAAGCAACTCAGCTGGAGAATG
TCGATCACAACCAACTGTGAATCTAAGTGGTCCTATGGGAATGTTATTGATAACAGGAGG
GACTGCTAGTTCAGCATCTTACACTATGAATAGTTCCGCTGCTTCAGCAAGGTCTAGAAC
AGTTAGCACAAGCTCTGACAGTAGTCAGCATCAGAAAACACGTTCAAATAAATCAGCATC
TGCACTACTTCAAAATTTTGAACGCGTCCCAAGACGATCTATATCTAGGCCATCGTTGGG
ATCTAGAAATCGTAGATCACTCAGTCATTCACCTCCAGCCGGATCTACCAATCGATCAAA
CTTGCCCCATCAAAACAATGTTAATAGTAATCAACGAGATAACTATGTTGAATCTCCACT
TAATACTCGCACACTTCCCGCTGGCTTTGGTCGTGGATATTCTCTTATGTCAGATCAATC
TTTAGATTGTAATTCAAGAATAATTACAAAATCTCAAGAAGAGAGTTCAATTCCAAATTA
TACTGAATTAGCCCGTTTAATTTCTTCGGGTAGCAACTCTGGTCCAAAACCCCAGAATAT
TGGAAATACTCCTTTGTCACCAAATGGAATGACTGTATCCCATCCACAAGTAATAAGCAA
TGAAGAGTTGAAATCTTCTCTAACCTATCTTCGAAAATCTCAACAACGTCTTGACGATTT
GCTTATAGCAACTATGGCAGCTGTTGGTGTTGAAGACCCTGCTTTATCTGGTATGTGGTG
TCAAAATAGTTTGTCAAATTCTAAAAAAGGTGAAATAGATGTTGGATTATCAAATACAAA
TTCATATATATTTCTTTCCGGTCAAAAAACTTGTGTTAGTCCTATTTCTCAAAATGTTAA
CCATAAAGAGCCTTTTTCAACTTTGTTGCCTGGAAATCAAGTTGACAGTGATGTTATTAT
TCAGGATAGTTGCTCATTCGATATTTACTCAAATCCTCCCAGAAAAGACCAACAAACAGT
TAATTTGTCAACACGGTCAATTGAAAATTCCAGTGTATTTAATACAGTAACATCATCTTC
CCAGATGACCCAATTTCCTGGTTATATGGCATCAAGCAGTGATATGAATAGATATATGGA
TGCCAAAAATTTAAAAGCTGCTCTTTTAAATGAACTAAAACGTGACCGTGGACCACCACT
CCCTCCACTAAGATCAGGAAGCTCTTTAGGGAAATTAAATAGTCCAGTTCAAAAATATCA
GATTCAGAATCAGCCTGATGTGTTGCCAACCGATTTAAAAAATTCAAAACTAACTGAACA
GATTTCTGATGATGACCTCAGTAGACCAACGAGTGCTGATGGTTCTCGAGCCTCTATTAC
AAGAGATCCAAGTCTAAATCTCAGTAGAGGCGGATCTCTCAGTAGGCGTAGTTCAACATC
ATCAAGTAGAAGATCTCATCACCAGTCTAGCAAAAAAAGACCACCAATGGATAAATCATT
TCATATAATAAAAGAAGTTTTAATGACAGAACGAACTTATAAACGGAGTCTTGATATTAT
ATGCATATGGTTTAGAAAAGCTGTCGCCAGTGGTAATGTTGTAGATCTTAATAGCCGACT
ACCGGAGCAGTTATCAAAACGGATATTTAGTCTCCTTGATCCAATTCATGAGCAACATAC

TCAAATTTTGAAAGAATTAGAAAATCGAGTTATGGTTGAATTTAATTCATTTCAGTCTCC
TTATACTTCACGAGCTAATTCACGGTCTAATGTGGGATTGGATACGACTGAATCTGAATC
AAATACTGAAAAAAAATTTCAAATAGACTCAAATCCTGTTTATCGTGTTAGCGATATTTT
CCTGCAAAACCTTTGCATAATACCATTATACAAACAATATCTAGAATCAGCTGAGTCGAT
TTTATTATCCCTTGATCAAAATGTTAGAAGTAATTCTGTCTTGAAGGATAATATCAGAAA
ATTTGAAATTCAAAAGGTGTGTTTTATGCCACTTTATGCTTATTTTCTTACTCCTGCTCA
TCGAGTTCTTCAATACCGACAAACATTTGAACGGTTATTGCGTCATATAGGAGAAAATCA
TCCGGATAGCAAAGATTGTAAATTAATCCTAGTTCAACTTCAAGATTTGATACAAGGTTT
ATGGGACGTTTATAAAAAAACGGAAAACACATGTAAATTGTTAGAATTACAGAGAGATAT
TGTCGGATTTCAAAGTTTATCAAATGAAACCAATGAAAAATTAAATAACAGTGCTATATA
TGAGCAACACCGGAATTTTATCAGGGAAGGTTGGCTACAAAAACTGGGCAAAAAGGGCTA
TCAGCAACGCATGTTTTTTATGTTTTCTGACCAACTAGTTTATACTAACAAATTAAATGG
GAATATTTTACAATTTAAAGTACACGGACAATTTCCACTTCATGACTTAATGATTGAGGA
ATCTGAGCCGACTCACAGTTTTACTGTCTATTCTGGTAATCGATGCTTTTTGGTTGCTGC
CAATAGTCAGTGGCAGCGTGACCGATGGCTTGAAGATATTAGTAGAGCTATTCTCGCAGC
AAAGTCATCTTCCAGTCTCACGGGAAATGGAACAGTTGATGGCAAAAAATTTCTAATCGA
TGACAATGCAAGTTCTGAACAGCAGAAAACCGAGAAATCAAAAATTATCATCGATGAGCG
ATATCAAATACAGCAGAGATCTAACACCAGTGTTCATGTATGTTGGCATCGTAACTGTAG
TTTGGCATTTACTGAGATTCTCAGAGCAAATGAAAACGAACTATCGGGTTATTTGCTAAG
AAAGTTTAAAAATAGTAACGGCTGGCAAAAACTGTGGGTTGTATTCACTCAGTTGTGCCT
CTTTTTTTTCAAGAATTATCAAGACGACAGCCCTTTAGCTACTCTACCATTAATTGGATA
CTTGGTTTGTCAACCTTCGCCTGAAGATCAAATTAGAAAAGACTTTGTATTTAAATTACA
ATTCAAAAATCATGTTTACTTTTTCAGAGGCGACAGTCAATATACATATGATAGATGGTT
TGAATGTTTGAATGTTGCAACTGGATCTGCCAACAAATTGAAATCTACTCCTTCTGTCCA
AACTCATAATCGATAAtcatttgaaaatctactaagcatttagtaatattgccaaataaa
tgctgttggatttttttaaaatttcattgacctctttctaatgtttttcttatcactttt
ttttatctattcgatgaaaacataacattgaaatgtaatttttcattcaagttattcaaa
ttggaaaattatacttattcataaagaattataagacaaaccttttcgtataataatgat
gcattttcaaatttgtttgcaattttcctagtgatctcattgaaactattgctttttta
tgattttttagagaatattgacattgggctaatttaatgtaatttaatacgtattgaaa
caagtgataatattgttattattatgattttatgtttattcaattttgaaaaagacacat
tgatttaccatatgaaaattcttagtggctagatcaatgttattgttgtttattgcattt
tattaacagaattatttgatgaaaaataaattagtttatacttttaataatttttatgtg
tgataatgtaactttattgcagatatacgggtcgaactcaaaagcaactacaagaatgtg
ttagagaaacaagttatagacgtccccaatttgaaaggtcattatccATGAGTCGTGTTA
GTAACATACCGAAATCACCGGCTTCAAGTGTTCTTCCCACTCGTCATACTCGATTGGCGA
GCAATTCATATGCACAGTACAAAAGTGAAAGCAACTCAGCTGGAGAATGTCGATCACAAC
CAACTGTGAATCTAAGTGGTCCTATGGGAATGTTATTGATAACAGGAGGGACTGCTAGTT
CAGCATCTTACACTATGAATAGTTCCGCTGCTTCAGCAAGGTCTAGAACAGTTAGCACAA
GCTCTGACAGTAGTCAGCATCAGAAAACACGTTCAAATAAATCAGCATCTGCACTACTTC
AAAATTTTGAACGCGTCCCAAGACGATCTATATCTAGGCCATCGTTGGGATCTAGAAATC
GTAGATCACTCAGTCATTCACCTCCAGCCGGATCTACCAATCGATCAAACTTGCCCCATC
AAAACAATGTTAATAGTAATCAACGAGATAACTATGTTGAATCTCCACTTAATACTCGCA
CACTTCCCGCTGGCTTTGGTCGTGGATATTCTCTTATGTCAGATCAATCTTTAGATTGTA
ATTCAAGAATAATTACAAAATCTCAAGAAGAGAGTTCAATTCCAAATTATACTGAATTAG
CCCGTTTAATTTCTTCGGGTAGCAACTCTGGTCCAAAACCCCAGAATATTGGAAATACTC
CTTTGTCACCAAATGGAATGACTGTATCCCATCCACAAGTAATAAGCAATGAAGAGTTGA
AATCTTCTCTAACCTATCTTCGAAAATCTCAACAACGTCTTGACGATTTGCTTATAGCAA
CTATGGCAGCTGTTGGTGTTGAAGACCCTGCTTTATCTGGTATGTGGTGTCAAAATAGTT
TGTCAAATTCTAAAAAAGGTGAAATAGATGTTGGATTATCAAATACAAATTCATATATAT
TTCTTTCCGGTCAAAAAACTTGTGTTAGTCCTATTTCTCAAAATGTTAACCATAAAGAGC
CTTTTTCAACTTTGTTGCCTGGAAATCAAGTTGACAGTGATGTTATTATTCAGGATAGTT
GCTCATTCGATATTTACTCAAATCCTCCCAGAAAAGACCAACAAACAGTTAATTTGTCAA
CACGGTCAATTGAAAATTCCAGTGTATTTAATACAGTAACATCATCTTCCCAGATGACCC
AATTTCCTGGTTATATGGCATCAAGCAGTGATATGAATAGATATATGGATGCCAAAAATT
TAAAAGCTGCTCTTTTAAATGAACTAAAACGTGACCGTGGACCACCACTCCCTCCACTAA
GATCAGGAAGCTCTTTAGGGAAATTAAATAGTCCAGTTCAAAAATATCAGATTCAGAATC

AGCCTGATGTGTTGCCAACCGATTTAAAAAATTCAAAACTAACTGAACAGATTTCTGATG
ATGACCTCAGTAGACCAACGAGTGCTGATGGTTCTCGAGCCTCTATTACAAGAGATCCAA
GTCTAAATCTCAGTAGAGGCGGATCTCTCAGTAGGCGTAGTTCAACATCATCAAGTAGAA
GATCTCATCACCAGTCTAGCAAAAAAAGACCACCAATGGATAAATCATTTCATATAATAA
AAGAAGTTTTAATGACAGAACGAACTTATAAACGGAGTCTTGATATTATATGCATATGGT
TTAGAAAAGCTGTCGCCAGTGGTAATGTTGTAGATCTTAATAGCCGACTACCGGAGCAGT
TATCAAAACGGATATTTAGTCTCCTTGATCCAATTCATGAGCAACATACTCAAATTTTGA
AAGAATTAGAAAATCGAGTTATGGTTGAATTTAATTCATTTCAGTCTCCTTATACTTCAC
GAGCTAATTCACGGTCTAATGTGGGATTGGATACGACTGAATCTGAATCAAATACTGAAA
AAAAATTTCAAATAGACTCAAATCCTGTTTATCGTGTTAGCGATATTTTCCTGCAAAACC
TTTGCATAATACCATTATACAAACAATATCTAGAATCAGCTGAGTCGATTTTATTATCCC
TTGATCAAAATGTTAGAAGTAATTCTGTCTTGAAGGATAATATCAGAAAATTTGAAATTC
AAAAGGTGTGTTTTATGCCACTTTATGCTTATTTTCTTACTCCTGCTCATCGAGTTCTTC
AATACCGACAAACATTTGAACGGTTATTGCGTCATATAGGAGAAAATCATCCGGATAGCA
AAGATTGTAAATTAATCCTAGTTCAACTTCAAGATTTGATACAAGGTTTATGGGACGTTT
ATAAAAAAACGGAAAACACATGTAAATTGTTAGAATTACAGAGAGATATTGTCGGATTTC
AAAGTTTATCAAATGAAACCAATGAAAAATTAAATAACAGTGCTATATATGAGCAACACC
GGAATTTTATCAGGGAAGGTTGGCTACAAAAACTGGGCAAAAAGGGCTATCAGCAACGCA
TGTTTTTTATGTTTTCTGACCAACTAGTTTATACTAACAAATTAAATGGGAATATTTTAC
AATTTAAAGTACACGGACAATTTCCACTTCATGACTTAATGATTGAGGAATCTGAGCCGA
CTCACAGTTTTACTGTCTATTCTGGTAATCGATGCTTTTTGGTTGCTGCCAATAGTCAGT
GGCAGCGTGACCGATGGCTTGAAGATATTAGTAGAGCTATTCTCGCAGCAAAGTCATCTT
CCAGTCTCACGGGAAATGGAACAGTTGATGGCAAAAAATTTCTAATCGATGACAATGCAA
GTTCTGAACAGCAGAAAACCGGTAAGATAATATTGATTATCTAAtataaacagaaagaaa
accgtaataaatttgaaaataattaattttaaatatataaaatttctcattttataaaat
cacgacaatgaaatcaactaaatgcaattcgtctgattgacaatttcattcatatcaaaa
ttttgaggaaattattgtatgcttataataaatgtgtatctttattagaatgaagaaatt
atttcttttttcttatttgttttcgattctttatggaaatttctatataaatcatgtta
aaatggttttgtgttatcctattatttgttttacttctaattataaaaacggttttatat
atttaaataatatatatttgttgtaatttaacagataatttaatttttcagtcagcatat
tgtattcccatgagatatgaaattctaaaagaaacattgaatgcatttcatttggtgaac
taattcatttaattatattttc
>DjScaffold349:98329-98733 strand:+
ATGAGAAAAGATTTAGGTACAACAAAGGTAACAACGGTGTATTTAGACGATATCCTAGTA
TATTCAGAAAACTTGGAAGAGCATTACAAAACACTGGATGAAGTATTTAGTAAAATACAT
CAAGTAGGACTACGTCTGAGCCCGTCAAAATGTCAATCGCTAAGAGAAGAGATCAATTTT
TTGGTACACATCATAAATGATAAAGGGGTTCAGACTGATCCAGCAAAATTGGTTGCAATA
CAGACATACAAAAGGCCAAAATGCGTGAAAAGATTGAGGAATCTTTTGGAGATATTTTCC
TATTACAGAAGATTCATAAAGGATTACGCAAAGAAGGCTAGAGAACTAGAAAAACTGTGC
AGTGTAAATAATAAGAAATTAATATGGACAGAGAGTTGCGAATAA
>DjScaffold349:24711-24764 strand:+
ATGGACAAAAGTATTAGAATCAATAAATTGGAAGGAAGCAATTGTTTTTCTTGA
>DjScaffold4332:4057-4893 strand:+
AATGAAATAGATGTGCTCTATACTGATGTTCCACATCAACTTTTTCGCATTTCAGATTAT
GCAATTTTAAGCAAACTTGGTAGAGGAAGCTATGGTAGAGTTTATCTTGCAAAAAATAAA
CAAAACAAATATCTGTATGCCATCAAAAAATTGAAAATAGATCAAGGTATTCCTACTACA
ACTCTAAGGGAAATTGGGTTACTGAAAATTCTTCAGCATCCTAACATTATCAGCCTTCAT
CAAGTATTATTATCTGAAAAAAGAATCTATATGGTTTTGGAATACATGAAAATGAGTTTA
GATTTATTTATCAAGGATCATGAAATTTCTCCAGATTTGTTACAGGTAACATTTTTATAA
aacaatttaattgaaaaatttaattttactttattacttcattaatttaaaattatttat
gattttgattgtgttaaatgttttgatttattgaatcatgctaatctctgaatttattgt
ctttgctaacgaatctgttttcaaaataataagcgctgaaatcgtgtttgatgaattaca
ttaaattacatgaatggatttaattactgcaattactgaagatttattgagaaataaaat
tggaaattcgtaaagttggttgaagaaaatctgaaaacaaagagcgaatttacgtaaatt
ttcttcctcacgataaaacttggttaatcaaagaattttaattgattttctttcagaaat
attgtcttccaagaaaagaagt
>DjScaffold4332:29189-29718 strand:+

ATGTTCCAACTTTTACAAGCTACTTTATTCTGCCACAATCGTCGAGTTTTACATCGCGAC
ATTAAATCTCAAAATATATTGGTTCATCATTCATTGAATATCGTCAAGCTTAGTGATTTT
GGTTTAGCTAGGAAAGTTGATATATCTTCAAAACCTCTCACACCTACTGTTGTTACACTC
TATTATCGCAGTCCAGAATTGATTTTGAATGCGGGATATTATTTCTCTGGAATAGATATT
TGGTCTCTGGGGTGTGTTTTTGTTGAAATGGTATTAAAACAAGCTCCATTTCGAGTTGAC
GGGGAAATCGAACTATTTTACAGTTGGTTTAGTCAACTTGGGGTCCCACGTGACGAAGAT
ATGCCTGGAGCTTTGAATAAATTTAACTCTATGAAATTTAGAGAAGATAAGAATTCTGTA
AACGCAATAAAACTATTAAAAAATCAGGTTGATCCGAAATGTTTTGATTTGATCATGGTA
TAG
>DjScaffold2292:26937-27475 strand:+
ggttataataattatATGGGGAAAAAAACTTTTAGTTGTGTTAAATGGCTGTTGACATTT
TTTAATTTTTTATTTTTGATTTGTGGGTTAGCAATATGTGGCCTGGGAATTTGGATGTTT
GTATCTCCAGAGACATCTACTGCATTAATTGGGAAAAATTTAGCCCCTATTATTGGATAT
ATTGTATTAAGTTGTGGTGGGACTATTATATTAATATCTTTCTGTGGTTGTGCTGGAGCT
GGATTAGAAAATCGCTGCTTGTTATGCTTGTTTTTTACTGTATTATGTGGAATATTTGTT
GCTTGCTTAGTAGCATCAATTCTTGTTTCTATTTTCCGATTAGATGTAAGTTATTTATTA
AAATAAatagataaattgtaatttaaaatcgtaaatattttattttataagttatttttc
ctataatgagtttatttaatttggttatt
>DjScaffold2292:29539-38602 strand:+
ggttataataattatATGGGGAAAAAAACTTTTAGTTGTGTTAAATGGCTGTTGACATTT
TTTAATTTTTTATTTTTGATTTGTGGGTTAGCAATATGTGGCCTGGGAATTTGGATGTTT
GTATCTCCAGAGACATCTACTGCATTAATTGGGAAAAATTTAGCCCCTATTATTGGATAT
ATTGTATTAAGTTGTGGTGGGACTATTATATTAATATCTTTCTGTGGTTGTGCTGGAGCT
GGATTAGAAAATCGCTGCTTGTTATGCTTGTTTTTTACTGTATTATGTGGAATATTTGTT
GCTTGCTTAGTAGCATCAATTCTTGTTTCTATTTTCCGATTAGATTTAAGCAAATTGACC
AACGAGCAGCTCACAGAAAAACTGAAAAAACAATATGGTAGTAATCTAGCGTGGACAGAA
TTTTGGGATATTGTACAGTCCAATGTAAAT
>DjScaffold2292:56854-57299 strand:+
AAAGTCGAAAATTACAATTGTAATATTGAAGCAAAATTCGGAAGACAAATTATAAATGGA
GAGCGATTGACAAGAGCTTTAAATATTTTGAAGATTAAAGAGGATCAACGGTTATTGAAA
ATAATTGAAAGTAAAGAAGATGCTTTTATGGCAGATAAATGGGATGTAGAACGCACACAT
CTAACAAGACACAAAATAATCACAAGTGGTGAACCAATCGATATAAACCGCACATGGAAC
CAATGAacatagaatctaaaattgacgaagctatccgaaaccttttcgagaataatatta
tccgaaaatgcaattctccattgaataccctactgatatacaattgaatgcagtaacgaa
aagacaagcctttccgatgcctaatgtcgatgaaatg
>DjScaffold1484:64034-64495 strand:+
GAACTAAAATTCAATAAAGTATCACGAATCATTGATATAAAATCTGTTGTATGCAAAATA
TTTAATCATTCAATTAATTTATGCAAAGGAGATGTTGTAAAATTGAATACAAATGGAAAT
GATATTTTCTATAGTATTATTGATTTGTGGAAATTAAATGGAGAACAATGTGTAGAAGTT
GTTGAGTCAATTCTTTCAAATGGTGACAAATTTTATTTGCGTGAAATTGATATCTGTAAA
ATTGACAAATCAGATTGGGAATTGAAATATTTTCAACACACAATCATTGATCCATCATCA
ATAATTACATTAGAAATATATAGAAGTTCTGATTTAGAAATTGGAAATAAAATAAAATTT
AATTTGAATGAAACTAACGAGTTATTTGAAGGTGTAATCAAATTTATTGGGAAAGCAAAT
AATATTTTGTCAATTGCTGTTCAGTTGGTCGATGATAAAACAACTAGTAAAAATAATAAA
TTGTGCAAATATTCAATCAATCAAAAATATTCAATCAATCAAAATAAAACATTTCATTCC
ATTTGGAGAAATTCAAATTTACCAATCATTTTTCTACCTTTGAATATTCTGAAATTACAA
GATAATTGTGAATATGCTAACGGAATACCGATTGTTGATCCTAGATTTGTGAAGAGAATT
GAAGGAATGAAAGTTGAAACAGATCGGAAAATCATTTTCTTGTGTGAAGGAAAAAAATAT
TTCTCTCATATAAAATGGAAAGGAATAAGTAGATAAaaaatattgtttaggatgcgagat
tcattatccctgtgaattcgggaccaatggtaaatttggtgacaaaatacttttcacatg
tccggataaacatggaatattgattcgtgcagattcaat
>DjScaffold5359:7417-8018 strand:+
gtgatgatgtgaatgataagcaattaacgaaaATGGAAGTTCTGAAAGGTGCCTATATAT
TTTGGACTCCAGAAAAATTCGGCGCTGCAGGAGTATACTTTACTTTAATTGCAAATAAAT
TAATGGGGCAACCTTTATACAACAGATTGTATTTAACTATTTTAATTGGAGCTTTCGGTT
ATTTCGGCAGAAAAATAGATCTTCATTATAAATTCAAAAGAGCGGAAATTAGGGTATCCA
AATTAATGGATCACATGAAAAAGTATCCACATTACTATGATGTTGATTGTATTGAAATGA

TTGGGAACAAATTTTTACCTTGGTATCCTTCTCGTGTTTAGattgaatatcctaggattt
aaggaataatatcatatagtatattgtcctaattaaatagaaattgttaattaaa
>DjScaffold3919:9155-21538 strand:+
caagaaattgattcctgattttaatgaaaacATGAATAGGATTATTGAATGCATTGAAAC
GAATGCTAAAGTAATAGAACTTTTCGCAAAATATAAAGATGGATCTGAAATATTTTCCAA
AGATGACCAAGATGAACAAGAATTTAAACCCGTTAATGGAAATCGAAACTCTCAGAAAAA
TTTTTCCAATAATATTAAAAAATTAATTAATTCAAACGATATGGATAAAGTTAAGTCTAC
ATTGAAACAGTTTGTAAGAGATTGGTCGTCTGAAGGAGCTTTAGAACGACAGTTATGTTA
TCAACCAGTAATTGATGAAATTTTGAAATTATACAATACATCAAATTGTAAAGCAAAAAG
CGATTTTCGAGTATTGATTCCAGGTGCAGGTTTGGGTAGATTAGCATGGGAAATTGCCCG
ATTAGGATTCACATGTCAAGGTAACGAATGGAGTTTGTACATGTTGTTGCCAGCGTATTA
TATTCTCAATAATTGTTCAGAAGCAAATATTCTTAAAATTCATCCATGGGTGTCGAGTTA
TTGTAATAATTCATCAAGAGAACATCAGTTGGCTGCTGTAACATTTCCTGATGTTAATCC
AACTGATATTCCTGTCAATTCGAATTTTTCAATGGCTGCTGGGGATTTTGTGGAAATTTA
TACCGAACCTGATAACTGGGATTGTGTAGCCACTGTCTTTTTTATTGATACAGCACATAA
CATTTTGTCGTACATTGATACAATATGGAATATTCTCAAACCTGGTGGATTTTGGATCAA
TCTTGATGAACGGCTACATTTTTCTGCTTGGATGTTTGATTCTGTCCCTAGAAAAATGAA
AGAGTATTTTTAAagacattagaatttttgtaaaatatataatttacaacaaattttga
aaaaagttggcacttgtttttttttcaaaattgtaaaatttgaaactattgagaatatt
tgtgatttatttatttacattaggaccattattatatcactattctgacattgctaatga
agattcaattgaattaagttacgaagaagtgaaaaaagcaatcgaattaaagggttttaa
attccttgttaaaaaataccctgttgaagtgagctacacacaagaccgtaattcgatgct
cagttatcaatataaatgtgcctatttcgtggtccaaaaacctgagaaatcaattaataa
ttctatgtcaaatactaacggagatgttttgatttgaaataatgacgataaatgttgtaa
tagagcctatttaatgtgtcattctataatatacaagaaattgattcctgattttaatga
aaacATGAATAGGATTATTGAATGCATTGAAACGAATGCTAAAGTAATAGAACTTTTCGC
AAAATATAAAGATGGATCTGAAATATTTTCCAAAGATGACCAAGATGAACAAGAATTTAA
ACCCGTTAATGGAAATCGAAACTCTCAGAAAAATTTTTCCAATAATATTAAAAAATTAAT
TAATTCAAACGATATGGATAAAGTTAAGTCTACATTGAAACAGTTTGTAAGAGATTGGTC
GTCTGAAGGAGCTTTAGAACGACAGTTATGTTATCAACCAGTAATTGATGAAATTTTGAA
ATTATACAATACATCAAATTGTAAAGCAAAAAGCGATTTTCGAGTATTGATTCCAGGTGC
AGGTTTGGGTAGATTAGCATGGGAAATTGCCCGATTAGGATTCACATGTCAAGGTAACGA
ATGGAGTTTGTACATGTTGTTGCCAGCGTATTATATTCTCAATAATTGTTCAGAAGCAAA
TATTCTTAAAATTCATCCATGGGTGTCGAGTTATTGTAATAATTCATCAAGAGAACATCA
GTTGGCTGCTGTAACATTTCCTGATGTTAATCCAACTGATATTCCTGTCAATTCGAATTT
TTCAATGGCTGCTGGGGATTTTGTGGAAATTTATACCGAACCTGATAACTGGGATTGTGT
AGCCACTGTCTTTTTTATTGATACAGCACATAACATTTTGTCGTACATTGATACAATATG
GAATATTCTCAAACCTGGTGGATTTTGGATCAATCTTGGACCATTATTATATCACTATTC
TGACATTGCTAATGAAGATTCAATTGAATTAAGTTACGAAGAAGTGAAAAAAGCAATCGA
ATTAAAGGGTTTTAAATTCCTTGTTAAAAAATACCCTGTTGAAGTGAGCTACACACAAGA
CCGTAATTCGATGCTCAGTTATCAATATAAATGTGCCTATTTCGTGGTCCAAAAACCTGA
GAAATCAATTAATAATTCTATGTCAAATACTAACGGAGATGTTTTGATTTGAaataatga
cgataaatgttgtaatagagcctatttaatgtgtcattctataatata
>DjScaffold3919:66213-67406 strand:+
CAATGGACAGACGATGATGATAATGATGATGATGAATCCGATTCTTCTGGTTCAATAGAT
TTGGGTTCTGATGATTTATTAAATCGACACCGGCGTGGATTATCTCAATCAGGAAAGAAA
CCAAGAAAAGCTCGAACTGCATTTACAGATCACCAATTAAATGAATTGGAAAAGAATTTC
GAACAACAGAAATATCTTAGTGTTCAAGACCGAATGGAACTGGCAGCAAAACTCAGATTA
ACTGATACCCAAGTAAAAACTTGGTATCAAAATCGAAGAACAAAATGGAAACGTCAAACA
ACAGTTGGTCTGGAATTATTAGCAGAGGCTGGCAATTTTGCAGCAGTTCAGAGAATTATC
CAAACTAATCCATATTGGACATATCATCCAGAAGCTCGAGCTGTTATTTCAAATATGGAT
GCAATACTCAAACAACAACAAGCACTGAATTTTTGTCGAAACAATCCATTCCTCCAGCAG
ATTCTCGCTACCAATCCTGGAATTGACAAAATGAATTTATTAAATAGTACTTCGTTTAAT
TCCTCAAATTTTCCCACTCCATCTATCGGCTCATCAGATATCGGTAAATTGTCGTCTCCG
CCAACGTCTGATTTGACAAATGAAATAATCTCCTCACCAAATAGTCAAAAATTACCACAA
TTTTCACCTTCAAATAACCCAACAGATTCAAAATTGTTTTCAGCTTTTCATAGATTACTA
AATGTTTCAAAACCT

>DjScaffold3718:67310-76980 strand:+
AATTGCACTTTCTATAAATTAATAGAACAACTTACCCAGGAATCTCTTCTTAATGCATGG
CAGTTGATTGATGATGGAAAAGTACCACTAAATGTCATTTCTAAAGATGGTGACTCTTTA
TTACATTTTGTTGTATATAAATATTGTCATTGCGATCAATTAATCATCAGGCGATATCTT
GTCAGAATAATTTACAAACTAGTTCTTGCTGGCATTAATGTTAACCACCGAAATGGAAAT
GGTGAAACAGTTTTATTAGCTGCTTTTAAATTAAATGCCGACCGACGATTTTTGGACCAC
TTGATTAGTTTAGGTGTCGATGCGTCTTACCGATGTCGCGATGGTAAATTGATTTCAATA
AATACATATGCTACAAGAGGAGAACTGATTCTTACCCAACATTGTCAAAGAATATTTCTT
CCAGGATTATGGCCTGCTGTTGAAGACAATGATGTAAAACGAGTGAAGTTCTGGTTGTCA
TCATTGTGTAGAGTTCATAGCAAAAAATCTGGCAGATATTTATTGCAATATGCAAAAGAT
AATAATTACACTGAAATTGCAAATTTATTAGAGATATCACATTATTTAACAGAATTTGTA
GTGGCATCTCTTGCTGGAGATGTAAAACGAATGAAACACTGTCTCGCTATGGGACAAGGA
AACAGTAAAGTTAATGCAATTTACCATCATTATTATGCAGGATTAACAGACGATTGGAAG
GTTGAATTTATTGCGAGACCAATT
>DjScaffold3718:96022-96964 strand:+
GAAGAAGCACCGTGCGCACCAATAGCATTTTGGGCATTCAAAGATGATATAGATGAATCG
ATTACCGCTGTCATTGCTCAATATGCTGATTTAAATCTAAGAGATGAATTAGGTTCAACT
TTACTTCATAAAGCGATTACAAGAAAGGGAGACATTGAAAAGAAAAAGGAAATTCTTTCA
ATTTTATTAGAACGTGGTGCAAATGTAGCAGCAAGAGATATTAACAAATATACCCCAAGA
GACTATTGTGAAAGTGAGGAAGTTGAAAATGGTTCAGTATTATTGAAAATGATTGATGAT
CACGTGTTAAATATGGTTTATGAAGAAAATTATGATGCCATTGAAGAGCTTATTATTCAA
GGCTATGATCACATTACAGATATATGTGGAACTGTTCGAAATAAGTCGGCAATTGAACTA
GCCCAATTAAAGTATTATAAAGAACTTATTGATTTGCTACAAAATGTTGAAAGATATCAG
TACGAAGTTCGTCAGTTGCATGCTGCTGTTATTACAAACAATACACCGATTTATACAAAA
TTCCTATCAGAAAAAAAATTAGCTTGGGGTATGTGTTTGTTTATAAGTTTGCGGTTAACG
TCTAATCACGTGATTATTAAACCACAAATTCTGCATAAAATTTTTAGTATAATCAAATGC
ACTTCAGACCAATTGTTATTTTAGgattttgaatatagaatatattatcattaaatttat
taataatagaattattttaataacaagccctggatttataaccttcaattatttccgata
aattgattgtttacaaataattttacaacttaattaatctatgaaatttaaatctaattt
taaattaatggaa
>DjScaffold3718:121093-125531 strand:+
TTAGGTGTTGATAGTCGAAATAGAAATATTCTTCATATTGCTGTGCTTTATCGACATTCG
GAATTAGTAGCGTTAATATGTGATCAGTTTCCACAACTCATTAATCAGGGAGATTGTTTC
CAACGAACTCCAATTCATTATGCAGTCAGTTTGAAAGATCAACGGAAAATTCTATCCATT
ATTTCTACAAGTAATTCTGTGAATTTACAAGTGAAAGATGCGAATAATTTAACAGCTCTG
GATTACATTGAAGAACTGGAATTTCCAACTTCAATAAAAATGAAAACTCGTGGACGCCGA
GTCTATAATTATGATGTGAAAAATCTTGTAGCCATAGAAAAAGAGTGCGCAGAATCAGAA
ATGATATCTCTCGACCCAGATACCGGTATCCAATCGCGTAGCGATACATTTGTGACAGAT
TTCATTGAACGACCGAAAAGTATAGCTTGAtcacgtgatatcagtacaaataaaaacaat
ttcaatcattcagtaaacaataataataataataactttatgaaaatttataatttgtaa
aacaaaagaaaatacaatagatcttgattggtttatatttttaataaaaatcagtattt
a
>DjScaffold3718:179670-181187 strand:+
gttctagtATGAATTTTAATAAATCTTTGAATTCCATTTTTGAAGAAGTTAAAGATATTT
ATAATAAAGTTAATAATTCAAATTTATCCTCCTCAACTAATGAATACCAAGAACTAGTTA
TAAAAGGCATTGAGTTATCTCAAATTTTATGGAACAGAATACAAGAATTAGATTTATTTT
CTCATAATGAAAGTGTAGATGATATGTCTACTGAATCTCTTAAATACATTATTGTTCCTG
GAATGATTGGATTTTTCTTGTATAATTTAATGAGTAGTAATAGATTAGAAATCCTAAATG
AAGTCAAGAAATATTTTTCAATTTTTATGGAGTTAATAAACGATTATGGATTAGTACATC
CTGACAATACTGTAATCGCATCAAGAGACGATAAAATTAAACAATTTAGAGAAGACAAAT
TACTCAGCGAAAAATTAAATTATTTAGAGAAAAACATAAATGGAAGACATATTGATGATG
AAATGAAGAGAGAATATTTTATGATTCTTATCAATCATTGGATATCTAAAGTTGGAAAGG
ATATCAAATTTATTGATGATGAGATATTTATTCTTGAAAATCGAAATACCGTCGAGGTAT
CTAAAGATGAACCTAAACCATCAAATTTAAAATCATTCGTAATAACTAAAGACATGGTTA
AAAATACAGTTTTTGGAATGGGATACAAGAATCTTCCATCTTATTCTTTAGAAGAATTTT
ATATTATGCAAGTTGAACAAGGACAAGTTCCGTATCCCGAAGAAAATCATTTATCAAAAA
CTCCCGGAGGTGTTAATCGAATCATACCAGATGAAAACTTGGAGGACGAATTATTAGCGA

AAAAAAATGACTTCCTAGAAGATATTTGTGACGAAAATAAAAGATTAAAGGAAATTAATT
TCGACAATTATAAAGACGAAAATCGGCGTGGTTGTGGAAATACTATCAATCGTGGTTAAa
tcgaataaataaagctatgaatttattattttattattttctaaaggtatattttgat
taagtaatttggactgtatctgacttgtaaaggattttcatttaaattatttcgtacttt
tcttaaaatttcattttaattctgaataaaaattactgagaaatatatgaagaataaatt
attgacaattcatggatgtatttttggagtataatagattttattttattcgatcataga
ttattggaaataaaattgtagccatcaactgcttttaaacattattggtgctctattgta
aattgatactgatttcatagtaaccatgtttcgttaatgttaaacatttaaataattttt
tcttgatattaattttaaatttggttaatatacagttctagtATGAATTTTAATAAATCT
TTGAATTCCATTTTTGAAGAAGTTAAAGATATTTATAATAAAGTTAATAATTCAAATTTA
TCCTCCTCAACTAATGAATACCAAGAACTAGTTATAAAAGGCATTGAGTTATCTCAAATT
TTATGGAACAGAATACAAGAATTAGATTTATTTTCTCATAATGAAAGTGTAGATGATATG
TCTACTGAATCTCTTAAATACATTATTGTTCCTGGAATGATTGGATTTTTCTTGTATAAT
TTAATGAGTAGTAATAGATTAGAAATCCTAAATGAAGTCAAGAAATATTTTTCAATTTTT
ATGGAGTTAATAAACGATTATGGATTAGTACATCCTGACAATACTGTAATCGCATCAAGA
GACGATAAAATTAAACAATTTAGAGAAGACAAATTACTCAGCGAAAAATTAAATTATTTA
GAGAAAAACATAAATGGAAGACATATTGATGATGAAATGAAGAGAGAATATTTTATGATT
CTTATCAATCATTGGATATCTAAAGTTGGAAAGGATATCAAATTTATTGATGATGAGATA
TTTATTCTTGAAAATCGAAATACCGTCGAGGTATCTAAAGATGAACCTAAACCATCAGTA
AGATGTTTTAATCTTAGTTATAATATTACACATTCAGAATTTAAAATCATTCGTAATAAC
TAAagacatggttaaaaatacagtttttggaatgggatacaagaatcttccatcttattc
tttagaagaattttatattatgcaagttgaacaaggacaagttccgtatcccgaagaaaa
tcatttatcaaaaactcccggaggtgttaatcgaatcataccagatgaaaacttggagga
cgaattattagcgaaaaaaaatgacttcctagaagatatttgtgacgaaaataaaagatt
aaaggaaattaatttcgacaattataaagacgaaaatcggcgtggttgtggaaatactat
caatcgtggttaaatcgaataaataaagctatgaatttattattttattattttctaaa
ggtatattttgattaagtaatttggactgtatctgacttgtaaaggattttcatttaaa
ttatttcgtacttttcttaaaatttcattttaattctgaataaaaattactgagaaatat
atgaagaataaattattgacaattcatggatgtatttttggagtataatagattttattt
tattcgatcatagattattggaaataaaattgtagccatcaactgcttttaaacattatt
ggtgctctattgtaaattgatactgatttcatagtaaccatgtttcgttaatgttaaaca
tttaaataatttttcttgatattaattttaaatttggttaatatacaaaggtaatatat
ttagttatttactgctaaattttaattttagaaatatttttcaatttttatggagttaat
aaacgattatggattagtacatcctgacaatacgtattaacctattttattttagtgttt
tactaatttttgttcaaagtgtaatcgcatcaagagacgataaaattaaacaatttaga
gaagacaaattactcagcgaaaaattaaattatttagagaaaaacataaatggaagacat
attgatgatgaaATGAAGAGAGAATATTTTATGATTCTTATCAATCATTGGATATCTAAA
GTTGGAAAGGATATCAAATTTATTGATGATGAGATATTTATTCTTGAAAATCGAAATACC
GTCGAGGTATCTAAAGATGAACCTAAACCATCAAATTTAAAATCATTCGTAATAACTAAA
GACATGGTTAAAAATACAGTTTTTGGAATGGGATACAAGAATCTTCCATCTTATTCTTTA
GAAGAATTTTATATTATGCAAGTTGAACAAGGACAAGTTCCGTATCCCGAAGAAAATCAT
TTATCAAAAACTCCCGGAGGTGTTAATCGAATCATACCAGATGAAAACTTGGAGGACGAA
TTATTAGCGAAAAAAAATGACTTCCTAGAAGATATTTGTGACGAAAATAAAAGATTAAAG
GAAATTAATTTCGACAATTATAAAGACGAAAATCGGCGTGGTTGTGGAAATACTATCAAT
CGTGGTTAAatcgaataaataaagctatgaatttattattttattattttctaaaggta
tatttttgattaagtaatttggactgtatctgacttgtaaaggattttcatttaaattat
ttcgtacttttcttaaaatttcattttaattctgaataaaaattactgagaaatatatga
agaataaattattgacaattcatggatgtatttttggagtataatagattttattttatt
cgatcatagattattggaaataaaattgtagccatcaactgcttttaaacattattggtg
ctctattgtaaattgatactgatttcatagtaaccatgtttcgttaatgttaaacattta
aataatttttcttgatattaattttaaatttggttaatatacaa
>DjScaffold4569:64225-90894 strand:+
agataatATGAAATCTATACTGAAAAAATCTGACGGAAAGCCTTTAAAAAAAAATAGTTTC
TATATATCATGATGTTTCAATTATTGAAAATACAATGTCTAATGACTCAGAATTTTTTGA
AGGTGAAATTTTTCAACATAACGAAGAGAAATCAAAATATAGCGATCGATGTACTTCTCC
AGATAGTAAATTTTCAATGGAAGATTTTTTAAATAATTTGGATAGTAATATTGATATTAT
TAAAATTAACGAATATGAAATTTATGATGAAATTTTTAACAACGGTAAGCTTAGACATGT

TAATTGCAATACATTATATACGAATGATATTTACTCGTTGCAAAATGTGATTATCAGTAG
TCAGGATAAAAAGAAAACCAAAGAGGAAAGAAAAAAGGAGAGGGAGAAAAAAAAGGAAGA
AGAAATGATGAAAAAATATCAAATAGAACAAGAAGCTAGACACAAAGCGATAGCTAAATC
TAAATGTGGTCGATGGGTTAAGTTTAATATCAAAATTGGTGAAGGGGGATACAAATTTGT
ATATCGAGGGTATGATGTTGAAGAAGCAAAAAATGTAGCATGGTGTGAATTTAAAAGGGA
ACATGTAGATACTAAAGAAAAGCGCCAGTCAATGTTTAGAGAAACGGAAATTATGCTAAA
AATGAATCATTCACATATTGTTCGTTGTTTTGATGTCTTAAGGGAATGGCACGTTTATGA
GGATCCTCAAACTATTATAGATGAAAAAGGAATTGTAATAATACAAGAATTAATGGGGGA
AGGTACTTTAAAAAATATAATCAAGAAAAATTTTTTAGAAGGTCAATGTATATTAAAATT
TCCACTGATTACACGCTGGTGGCATCAAATATTGGATGCATTACGTTACATGCATATGAA
ATGTGACCCGCCTATAATTCACAGAGATTTAAAAGCTGATAATTGCTTTTTATATGGTCA
AACCGATGATGAATGTCTTAATGTGAAAGTCGGAGATTTTGGTCTCGCTACCCATGTACA
TACAAGTGGAAGAAAAACAATGCTAGGAACTCTCGGATTTATGGCTCCTGAAATTTTCGA
TGAAAAATATGATGAAAGTGTTGACATATATGCATTTGGTATGTTGATGTTAGAAGTAAT
GACTAACAGAACTCCATTTGATGAATGTGATACCATTGTTCAAGTAGCTGCAAAAACATT
AAGTGGTAAAGGTCCTAAAGTGATAGAAATGGTTTCTGATAAATGGTTGCGAGAAATAAT
TAATTCATGTATACATCCACTATCATGTTTTAGACCAACTGCTGAAGAATTGTTTTTCCA
TCCAATTTTTCAACCGAAAGGTCTCCCAGTTGAAGTAGAATTAAATTATGACAATATTTC
TGATCGAACTGAAGTGATCGATCGTTTTATTACTAACATTCCTAATTCCACTAAAATTAA
TTTGAGATTGAGATTTCGAGATAAAAGAATGCTTCAAGAGTTAGGGTTGGATGATGGCGA
GTCGTTGGAATTCGATTTGGATATTTATAAAGCTGAAGATCAAGATATCCCTGATCTCAT
TCAGAATTTAAGATTAGAATACGAAGATAAACTTTCGAGAGTTTATGATAATCCAAAAAT
AGTGGATAAAAGCACAATTAATGCTCATCTCGACAAATTATTTTTGAGCATTAAATTGCA
AATGCAATGCTTGGTTAAACATCTTCTTGGGAGACGATGGAGGGACATATTGGATTCATT
GGTTGCAAATAAAACGGCAAAAGTATCAGATGGGGAAATGGAATCAGGTGATGATCAAGA
TGAAGGGATAATGAATAGTTTCACTATAATTAGTCAGTATAAAGGAATGTGGACTAAAGC
TAAACGAGCTTTGGACAAAGAAATCGTAGCGTATAGAGATAATTCAATTAAAGAAAAGAA
TATTGTTCCTAATATTGTAACCAGTACTTTTCACGATGATGGGAAAACATTTAACAATTT
AAATGAAACCTCTACGATTTTAGAAACAGATTCTATAGAAACCGATAAACTTCCATTGGA
GAATTCAAATATTGAAAAAACAATTTCACAATCACCAATTGAAGGATCCATGACTTATTT
AAATTCTTTTACTGATATTGAACAAATGGATAATCCTAATATTATACAAGGAAATCTATT
CAATAAAGACTCTGTCCAGATTGTATCAGTTCATGGTCTTACTAATACTTCAACTATCAT
TTCAAATTCAATTACTGCTCTTGACACTCGCGAAGTAATCAAAGATACAAATCCCATGGA
AATTACAATGAATAAAGACATTCCCAATTCTTACTGTGAAATAGATACAACAAAAGAAAT
TTTAAATGGATCTTATATTTTGAAAGTTCACAATATAGAGTGCAATGCTCAATTAATCTG
TGTAACTTTCAATGTAGAAATTCCAGATTTTTCCAAGTCAGAAGGCGGAAAATGGGTTTT
GGGTTTCAAATGCGTTATCAATGAAAATACATCAATAATAGATAATTTAGAATTAAATAC
GTCAGATGTTCTAGCAGACAAAATTGATGCTGATATTTATTTTGCTTTACGAGAAACTCT
TCTGTGTATTTTGAAATTAATTCGAAATGATAAGAAAAGTGTTCAGATTAATCAAAATTA
TATATTTCATCCTAGAAAAGAAAATCTGTTTTTATCTGAAGGAATCGTTGATAAGCCTAA
AGCAAATATGAGAAATAATGTTACTTCTCATGAAGTTGTGATTCCAGAAGACCCAGTTCC
AGAATCTGTATCTGTACGACAGATTGATAATAAACTAATTAGCGCGGATAATCCAAATAT
TGACGGTCACCTAAATGTAGATTCATTTTTTGAACAAGAAAATACTGTTTTGCTTACTAA
GCGCATGGAAGAGGATAAATATATCGTAAAGGCATACGGGATTCCAAGTGGAAGTGCAGT
TGTGATAAATAGCAATGGTTCTATTCAAAACATAAAGAAAACGACAATAATATCTCCTAA
ATTGGATTCTTATGAAAATATATTTGTCCGCGAGTTAGATAAATCAGATTTATCCATGGA
TAAGGCTATCCATATTTTGAGTTTATTACAAGAAAAAATTGAAGAATTATCTTCTAAGAA
TCTTAATTTTAGTGAACTAACTTCAAAAATTATTTGTGAAATCAAAGCAATACATGACGT
CAATAAAAAATCACAAGTAGTTGATCCTGTAGATAAACAAAGGATAATAGAACAGGTTTT
AATGCAAAAAACTGAAATTGAAAAATTACAAAAGCAGCAAATTGAATTAATTTCGTTACT
ATCAAATCAAAAAATGATATTTCCTCCCGAAATGGATCAAAATGTCATCATAAATAATCC
TAACAAACAAATATCAAATTCTGAGCATGATATATCGAATACGCATAAAATAAACAATAA
AATATATTCCAATGCATTAGGCCAACAATTTATTAATCAAAACCAAATGGTAATGAATAA
CGACCCGAGATTGTTTATGCAAAATAATGTATATATTTCTCCAGAAGATCTAGAATATCA
GAATTCTAATAATTACTTATTAGATAATGTGATGCGTAATCAGATGATGCACATGAACGA
AAGTATGATTTATGGTCCAAATAAAGATTTCGTGACTATTGATAACAACATGCGATTAAA
ACAAAAAGAAATAGCAGATGTTAATAGAGTTTGGAATGATCGGATGGAATATGTTGAAAG

AAATATGTTTCAAATGCAGTTTCACGGAAATGATCAAATTTCTGAAAATGATCCGCATTG
TAAAATTAACCCGGTATCGAGTGTTCCTAATATTCAAGAACGTATAGCAGATTTCAAAAC
TCCTGATCCTAATTTAAGTCAGCAGAATTTTCAACCAAAATATCCATATTTTTATCAATT
TCCTTATCCAAATATTAGAAATGATCATAACTACATGACCGTAGGAGATAAGCCGGTAAT
AAATTATAGAGTTCAACCAGGTTTTTATGATACAATGTACGATCGCAGTGCTGTTGATGA
CTTCAAACAAATACAATCAGAAAACTTATTCAATGCGAATAACAATTTTCCTATTCCCGA
AAATCCAAACATCAATTATGATACTAATTTCAGTATTAAT
>DjScaffold2605:86622-90547 strand:+
aataataataataacaataaataataataaaaatataatccaatttttatttaagtctt
ttcatttaattcagatcgtaaatggacggaatatgaaaaatttcatataaaaacgtttat
ctatcgattagaaggttattatgatttagctgctgaaagttggcttgaccagatagtcaa
gaatcctctagatattcttgctttgaaaatattaaataatcattatataacaacaggttt
tacagagaaaatatcaaatccttttaatctatgtcgtagtgcaATGTCTATACAAGATGT
ATATTATCCATTTATTTTGTCTTGTGAGGCTTTTGGTCTCGAAGAAAAAGGGCACTTAAA
TCAGGCCCTTAATATGGCAACTGAATCTTTGAATATATATCCCTGGAATTCGTGGGCAAC
CCACACTGTAGCTCATATATTTGATTCCAATGGAAACTCTAAAAATGGAATCGAATTTAT
GAATCGTACGAAATCTGATTGGATTGGTGGTTCTTTACTAGCTTGCCATAATTTATGGCA
TGTCGGTCTTTTTCATTTAAATCAAGGAGAAATAGTTTCAGCTCTTGATATCTATGATCA
GGAAATTCTACCTCGATTAAATAGTCAATTTTCACCTTTTTCTATGAGTGATGCAGTGGC
TCTTTTAACAAGGTATTCATTTGTATTTCTTTATATATTTTTAAATAAAACAATAATAAA
ATAAttttgaagtaatgcatattaaaatctacaaattatttctttgttcatttgaaatac
tacatttacatatttcatgatataagatttgcatttgattaccgtcatga
>DjScaffold3982:132370-132762 strand:+
ATGCTCCTTGGAATCGCAGTTGCTCCTGTTACTTTTCAGAAATTGACGAATAAGGTTTTA
GGTCCATTAAATTGGAAGAAAGCAGCGGTTTACTTAGATGATACTTTATTTTTTTCTGAA
ACTATTAAAGAACACTATAAGAGAATTGACAACGTTTTGAAAACTATAAAAGAAGCTGGT
TTAAAAATAAGTCAATCTAAATGTCATTTCATGAAAAACGAGATAAAATTTCTGGGTCAT
TTTATTAATTCAAAAGGAATTCATACTGATAGTGCAAAAATTGAAGCAGTTTTTAATTTT
GAAACCCCAAAATTAGGCCTCACAAATTATTATCGGAAATTTTTTAAAGGATTACACAAA
GTATTCAAAATGTTTAGAAAATCTATGTGGTAG
>DjScaffold1982:22363-23466 strand:+
TTAGTTTTCTTTTTATTCACTCGTAGAAAATTAAAAGATGGAGTCTTACTCTTAGGCATT
AATGATTCAGGAAAGACATGCTTATTTTACAAATTAACAAATAGTGTAGACATTCAGTCA
GTTAGTTCAATTAATGAAAATATTGGATTTGTAAAGGTTTCAAATAAGATGTCTAAAATA
GTTGATGTACCAGGTCATGAAAAAAATAAGAAATTCAATAATTTCAAAATACAAAGATAAT
ACTAAAGCTTTAGTGTTTGTTATTGATAGCCAAACTATTTTGGCTAATATTCGGGATATT
TCAGAATATTTATTTGAAATTTTGGCCGATATGAAATTCCATAAATCTAAAACTCCGCTT
CTCATTCTTTGTAACAAACAAGATCTTGAGTTGAGTAAAAGTGCAAATGTGATAAAAACT
CGTTTGGAAAATGAAATATCTATAGTTAAACAAACTAAAGCAGGAGCTCTTGATTCAATT
GATTTGAAATCGAAAATCAACCGGTCATTGGGAGATACAGGAGAAACCTTCAAATTTGAT
AAATTACCTAATAAAGTGGATTTCAATGAATGTTCTGTTAAAAAAAATATATCGTGCATT
GAAAATTGGTTGAAAACTGTGTAAtataa
>DjScaffold1982:76495-95670 strand:+
cttatttgcttcccattaaaaagtaatatagaattaatttttgtacgaatttgtggaaat
atataatttattatagtattagtagtggtcactatgtacaataaatattaaattatgaat
ggaattgtagatatcgatatcatgtggactggcccaaaagtaatttcaaaatcgattcct
ttagaatctattgaggaattatcgcaagtcttgcgtcgatctccagtaatttgggataac
attcatgctaatgattatgatcaaaaacgattatttcttggtccttattgtggacgatct
aacaaattattagaaaaactaagaggagttttaacaaatcctaattgtgaattcgaagca
aattatattgctgtttttactttagctcaatggtttaagtatgtttttatcatattaaa
tgaatagttctaacaaaaatttcgaaagcagttttcattataataatacaaacaccaatt
attgttccttgtcactctaggtaactggtccagctcaagaatctgacataATGATGCAGA
GCAGAAAATCTGTAGCTAAGGCACTTATGGCTACTGCTGTGGGCTCTTTACCTCCTTCCA
CAGCAAGTATTGCTGTAGCTGCTACGCAAGTGTTGAGTCATGAAGTATGTACTGCAGCTG
TTGTAGCTGTTGCTCAACAAGATCCTTCGGCAGCTGTTGAAGTTGTATCAGAATCCATTT
TAGAAGCTCATAATATGCCTTCAGTCTCAAATACTGCTGACGCTTTGAGTACAATTGAAA
ATGCTCTTGTAGATGACGATAAAGATGTAACTGCTACTGCCTTTGCAGTTGCTTCTGTTG

TTACTAGGGCAGCTGAAAAAGAAGCTGCTGAGATTAGCAAATCAGAAACATTACAGGAAG
TGCTTCATCAGGAGATGATGAATACTTTACAAGATCATGAAAAAATGGAAAATGCAGAAA
TGCGCGAGGTTCTTTTTTCTCAGAATAATTTTCACAGAAATGAATCTGTAGATAATGGAG
AAAGCTCAAAAATGCAGACTGATATTTCTTTACATGAATCTATGGAAGGATCTTATCATC
TTGATAATGACGTTTTAAGCAATCAGAATACAGAAGTTTTAACGCTAGACAATTTACGCC
TTCTAGTAGACTTATATTATCTTCCATATGAACACGGTACAGAAGCCATTAAAATGCTCG
AGTTAGTCCATTGGTTGAGATCACATGCTCATTTAGTGGATAGAGATGACTTCGATGAAA
AAGATATGAATCTCAATCAAACCAAACCGAATGAAACAGCCGATTCTCTTGATCCAATCC
AATGCAAATTGACTCACTGGAGATTAAAAGCTGCTCAATTTCATCAACGAATGGAAATGG
TTCGTGAATTAACCAATAGATTTTGTCATATTCCAAACAAATCAGTTCTTGGAGATCTCT
ATTCATATCTACAGGACATTCGGCATGTGTTGGAAACTCTAGATTCATATATACAGTGGC
TTGAACGAGCGAAAATAACATATGCCAGTGTTTGTGCAATGAGACGATTATTATCATGGT
GTACCCCTGGGTACAAAGAAATGTTTACTGGAGATGATTATGAATCATGGATGTTTCGTG
GAGGTCTAATAACTGAATTTCAGAGAATGTTCCCCGTTGACAATGCCTATGATCTTTTCC
CAATCAGAGCTTCATTCATTCAATATTCTCCAGATTTCACAGTCAGTGGCTTATTATCAG
ATCCAATAGATGGTGAAAAACCATCTCCTTCAATATCACAAAATGGTTGCCATTTCATTA
AAATTCCTCGACCTATTTATTCAAAGCCTTCAATGTTCAATGTCCGCCCTTTTCGAGATG
AAGATCGCATATCTGTTTATTCACTGTGCTTTACACTTGTTAATAATCGTGCTTCCTTAT
CAGCAAATGATTATAAATGTTATTCAGATGATATGTTTAATCTTCCAGGAGACCGATATG
TTGGAGTTTTTCTTGATAATTTCCCTGAATATTGTCTAGTTTTGGAAAACGATGATTCTA
TGATTGTTGGATATGCCGTAGCTTGCATGAATGTATTGGAGTTTCAGAAATATCGTAATC
AGGAATATTTACCAAAAATGAGAAATAAATATTCTAAATATTTGACTAATTATTCTAATA
CAGACATCGAAATGGCAGACTCAACTTATTCAAACACAGCACGATCAGCCATTCAAAATA
TGATTAATCAGTTTCAGCGTGAACATACCTCAATGCCTCAATCTGAAAATTCTGTTGTTT
TTTATTTCGACATGAAAGAATGCTACAAGCCTAATGATCTTGATACCATTGACAGTTTGA
CTAGAAGATTATTGATATGTTTGTTCTCAGCTTTAAAGACTGCTGCTGCAGAAAATTCTG
ATCAGACTCCGACAGCAAATTACCCCACCTCTTTAATTGGTACTGGTAGTGCTCTTACGA
CTTCATCAATAGCTATCGTTTCTCAGGGTAGTCTTATTACTACATCATCAGTACCCTTAG
TGGCTATTGGCCCAGTTCTGCAAGTTACTAACAAAGATAGAGCTCCTGTGCCATCGCTGA
CGGTTTATGCAGAAATAGATTCGTTGAATAAATCTCATAAAGAAGTTTACAAACGTTACG
GATTCACAGAAATCTTGAGCACCGATTCAGGCAGCGATGATATAATTTTGACTAGAATTA
TTTAAagttttaatagtgtatgctgactttgaaaatatatattatatttactactatttt
taattgttgatttccagatgaaatttttaatattaatttaggtgaactcgaacattgata
taaacaatgattttatttatttttctattgtaaattttcacataaccatctcaattatat
ttccaaagaaagaatattcatcgaatcatgtatacaactgttttgaacttatttgcttcc
cattaaaaagtaatatagaattaatttttgtacgaatttgtggaaatatataatttatta
tagtattagtagtggtcactatgtacaataaatattaaattatgaatggaattgtagata
tcgatatcatgtggactggcccaaaagtaatttcaaaatcgattcctttagaatctattg
aggaattatcgcaagtcttgcgtcgatctccagtaatttgggataacattcatgctaatg
attatgatcaaaaacgattatttcttggtccttattgtggacgatctaacaaattattag
aaaaactaagaggagttttaacaaatcctaattgtgaattcgaagcaaattatattgctg
tttttactttagctcaatggtttaagtatgttttttatcatattaaatgaatagttctaa
caaaaatttcgaaagcagttttcattatataataatacaaacaccaattattgttccttgtc
actctaggtaactggtccagctcaagaatctgacataATGATGCAGAGCAGAAAATCTGT
AGCTAAGGCACTTATGGCTACTGCTGTGGGCTCTTTACCTCCTTCCACAGCAAGTATTGC
TGTAGCTGCTACGCAAGTGTTGAGTCATGAAGTATGTACTGCAGCTGTTGTAGCTGTTGC
TCAACAAGATCCTTCGGCAGCTGTTGAAGTTGTATCAGAATCCATTTTAGAAGCTCATAA
TATGCCTTCAGTCTCAAATACTGCTGACGCTTTGAGTACAATTGAAAATGCTCTTGTAGA
TGACGATAAAGATGTAACTGCTACTGCCTTTGCAGTTGCTTCTGTTGTTACTAGGGCAGC
TGAAAAAGAAGCTGCTGAGATTAGCAAATCAGAAACATTACAGGAAGTGCTTCATCAGGA
GATGATGAATACTTTACAAGATCATGAAAAAATGGAAAATGCAGAAATGCGCGAGGTTCT
TTTTTCTCAGAATAATTTTCACAGAAATGAATCTGTAGATAATGGAGAAAGCTCAAAAAT
GCAGACTGATATTTCTTTACATGAATCTATGGAAGGATCTTATCATCTTGATAATGACGT
TTTAAGCAATCAGAATACAGAAGTTTTAACGCTAGACAATTTACGCCTTCTAGTAGACTT
ATATTATCTTCCATATGAACACGGTACAGAAGCCATTAAAATGCTCGAGTTAGTCCATTG
GTTGAGATCACATGCTCATTTAGTGGATAGAGATGACTTCGATGAAAAAGATATGAATCT
CAATCAAACCAAACCGAATGAAACAGCCGATTCTCTTGATCCAATCCAATGCAAATTGAC

TCACTGGAGATTAAAAGCTGCTCAATTTCATCAACGAATGGAAATGGTTCGTGAATTAAC
CAATAGATTTTGTCATATTCCAAACAAATCAGTTCTTGGAGATCTCTATTCATATCTACA
GGACATTCGGCATGTGTTGGAAACTCTAGATTCATATATACAGTGGCTTGAACGAGCGAA
AATAACATATGCCAGTGTTTGTGCAATGAGACGATTATTATCATGGTGTACCCCTGGGTA
CAAAGAAATGTTTACTGGAGATGATTATGAATCATGGATGTTTCGTGGAGGTCTAATAAC
TGAATTTCAGGTATGT
>DjScaffold1982:235-612 strand:+
ATGAATATAGTATTAACTGGTGTCGAACATGTAGTTGTGTATATGGATGACATACTAATA
GCATCTGAAACATTTGAACAACATTTGGTTAATATAGAAAATGTATTGTCCAGAATTAGA
AAAGCGAATATTAAGTTAAAGCCTACAAAATGTAAATGGGCAGAGGAAAGTGTCCTATTT
CTTGGACATATTGTATCATCAAAAGGACTAGAACCCAATCCAGAAAAAGTGAAGGCAGTA
CAAGAATTTCCGGTACCAAAAAGTGTACAACAGTTACAAGGGTTTAATGGTTTAGTTGGA
TATTATCGAAGATTCATTAAAGATTTTGCAAAAATAGCAGCACCACTAGTTGAGTTAACA
AAAGGTATGAAAACAAAA
>DjScaffold1982:1689-2066 strand:+
ATGAATACAGTATTAACAGGTGTCGAACATGTAGTTGTATATATGGATGATATACTTATA
GCATCTGAAACATTCGAACAACATTTGGTTAATATAGAAAACGTACTATCCAGAATTAGA
AAAGCCAATATTAAGTTGAAACCTACAAAATGTAAATGGCGGAGGAAAGTGTTTTATTT
CTTGGACATATTGTATCATCAAGAGGACTAGAACCAAATCCAGAAAAAGTAAAAGCAGTA
CAAGAATTCCCAATACCAAAAAGTGTACAGCAATTACAAGGGTTTAATGGTTTAGTCGGA
TATTATCGGAGATTCATAAAGGATTTTGCAAAATTAGCAGCACCATTAGTTGAACTAACG
AAGGGTATGAAAACAAAG
>DjScaffold197:8658-9855 strand:+
cagctttagttgtcgacaatggatctggtATGTGCAAAGCCGGTTTCGCTGGAGATGATG
CTCCAAGAGCTGTATTTCCATCAATTGTTGGTAGACCTCGTCATCAAGGTGTGATGGTTG
GCATGGGACAAAAAGATAGTTATGTTGGTGATGAAGCTCAATCGAAAAGAGGTATTCTTA
CTTTGAAATATCCAATTGAACATGGTATTGTTACAAATTGGGATGATATGGAAAAAATCT
GGCATCACACTTTCTATAATGAATTGAGAGTAGCTCCAGAAGAACACCCAGTTTTATTAA
CTGAAGCTCCATTGAATCCAAAAGCTAACAGAGAAAAAATGACACAAATCATGTTTGAAA
CTTTCAATACACCAGCTATGTATGTTGCCATTCAAGCTGTGTTATCTTTGTATGCTTCTG
GAAGAACAACTGGTATCGTTTTAGATTCAGGAGATGGTGTCACTCACACTGTACCAATCT
ATGAAGGATATGCTTTACCACACGCCATCTTACGTTTGGATTTAGCTGGTAGAGATTTGA
CTGATTATTTGATGAAGATTCTCACTGAAAGAGGTTACAGTTTTACTACAACTGCTGAAA
GAGAAATCGTTAGAGATATCAAAGAAAAATTATGCTATGTTGCTCTCGATTTCGAACAAG
AAATGAGCACTGCTGCTTCAAGTAGCAGTTTGGAAAAGAGTTATGAGTTACCTGATGGTC
AAGTAATTACTATTGGTAATGAAAGATTCCGTTGTCCAGAATCTTTGTTCCAACCATCAT
TCTTGGGTATGGAATCTGCTGGTATTCATGAAACTACATTCAACAGTATCATGAAATGTG
ATGTTGACATTAGAAAAGATCTGTATGCCAACACTGTTCTATCTGGTGGAAGCACAATGT
TCCCAGGTATTGCAGATCGTATGCAAAAGGAAATCACTTCTCTTGCTCCAAGTACCATGA
AAATCAAGATTGTTGCTCCTCCAGAAAGAAAATACTCTGTATGGATTGGTGGTTCAATTT
TGGCTTCATTGTCAACTTTCCAACAAATGTGGATTTCTAAACAAGAATATGACGAATCTG
GTCCAGGAATCGTTCACAGAAAATGCTTCTAAgtttttatagctagtattagaaaattt
>DjScaffold197:18333-19042 strand:+
AAACTGCAAAAACAAGATTATGTATTGAATGAAAACAAGAACTACGAAACTCTTGCAGAG
TCTCTTGGTCTTGTAGGGTCTCCTAATATCATTATAAATGATGCTGTTTGGCAGAAGCAA
AAAAACTTTAGCATAAATAGAGGTGATTTTTCTTTGCCTTGTTCTATATGCCAAGAAGAT
TTCAAACTGATTTCCACTGTCCTATTATCATGTAGTCATATTTTTCATCGAAGTTGTATA
GAATCTTTTGAAAAATACTCAAATAAAAAATCCTGCCCGATCTGCCGAACTATCAATTAT
CAGAAAATGGTAGTATTCCAACAGGAAACTATTTATAAATCTAAATGTGCTACCAAGTAT
AATTTTATAATATTTTTAAAACTATTTTAAtactgtgagatgtgactgcgaattatgtaa
tatttctatgttgagatttgtatatttgaaggagtaaatcgagatctgaatttgataata
ggtgaatgcatgattgaatgtgtgtgtaatttgaagcactcaaaattattacataaccga
ttcctcaaaagctatatatatattaaaaaattacaaggtttctaactaaattttacaata
ataaatttacagcaaatcaataaatttacattttataattacaattattgtgagaatagt
>DjScaffold197:38328-39632 strand:+
ttcattaaataaattattctaaaaATGTCTGACGAAGAAATCTCAGCTCTAGTTGTCGAC
AATGGATCTGGTATGTGCAAAGCCGGTTTCGCTGGAGATGATGCTCCAAGAGCTGTATTT

CCATCAATTGTAGGTAGACCTCGTCATCAAGGTGTGATGGTTGGCATGGGACAAAAAGAT
AGTTATGTTGGTGATGAAGCTCAATCGAAAAGAGGTATTCTTACTTTGAAATATCCAATT
GAACATGGTATTGTTACAAATTGGGATGATATGGAAAAAATCTGGCATCACACTTTCTAT
AATGAATTGAGAGTAGCTCCAGAAGAACACCCAGTTTTATTAACTGAAGCTCCATTGAAT
CCAAAAGCTAACAGAGAAAAAATGACACAAATCATGTTTGAAACTTTCAATACACCAGCT
ATGTATGTTGCCATTCAAGCTGTGTTATCTTTGTATGCTTCTGGAAGAACAACTGGTATA
GTTTTAGATTCAGGAGACGGTGTCACTCACACTGTACCAATCTATGAAGGATATGCTTTA
CCACACGCCATCTTACGTTTGGATTTAGCTGGTAGAGATTTGACTGATTATTTGATGAAG
ATTCTCACTGAAAGAGGTTACAGTTTTACTACAACTGCTGAAAGAGAAATCGTTAGAGAT
ATCAAAGAAAAATTATGCTATGTTGCTCTCGATTTCGAACAAGAAATGAGCACTGCTGCT
TCAAGTAGCAGTTTGGAAAAGAGTTATGAGTTACCTGATGGTCAAGTAATTACTATTGGT
AATGAAAGATTCCGTTGTCCAGAATCTTTGTTCCAACCATCATTCTTGGGTATGGAATCT
GCTGGTATTCATGAAACTACATTCAACAGTATCATGAAATGTGATGTTGACATTAGAAAA
GATCTGTATGCCAACACTGTTCTATCTGGTGGAAGCACAATGTTCCCAGGTATTGCAGAT
CGTATGCAAAAGGAAATCACTTCTCTTGCTCCAAGTACCATGAAAATCAAGATTGTTGCT
CCTCCAGAAAGAAAATACTCTGTATGGATTGGTGGTTCAATTTTGGCTTCATTGTCAACT
TTCCAACAAATGTGGATTTCTAAACAAGAATATGACGAATCTGGTCCAGGAATCGTTCAC
AGAAAATGCTTCTAAatttttcaaattaatttgattaattatatatttatttaatgtcgg
tgttttataaataaaaaaaattaattttaaattcattaaataaattattctaaaaATGT
CTGACGAAGAAATCTCAGCTCTAGTTGTCGACAATGGATCTGGTATGTGCAAAGCCGGTT
TCGCTGGAGATGATGCTCCAAGAGCTGTATTTCCATCAATTGTAGGTAGACCTCGTCATC
AAGGTGTGATGGTTGGCATGGGACAAAAAGATAGTTATGTTGGTGATGAAGCTCAATCGA
AAAGAGGTATTCTTACTTTGAAATATCCAATTGAACATGGTATTGTTACAAATTGGGATG
ATATGGAAAAAATCTGGCATCACACTTTCTATAATGAATTGAGAGTAGCTCCAGAAGAAC
ACCCAGTTTTATTAACTGAAGCTCCATTGAATCCAAAAGCTAACAGAGAAAAAATGACAC
AAATCATGTTTGAAACTTTCAATACACCAGCTATGTATGTTGCCATTCAAGCTGTGTTAT
CTTTGTATGCTTCTGGAAGAACAACTGGTATAGTTTTAGATTCAGGAGACGGTGTCACTC
ACACTGTACCAATCTATGAAGGATATGCTTTACCACACGCCATCTTACGTTTGGATTTAG
CTGGTAGAGATTTGACTGATTATTTGATGAAGATTCTCACTGAAAGAGGTTACAGTTTTA
CTACAACTGCTGAAAGAGAAATCGTTAGAGATATCAAAGAAAAATTATGCTATGTTGCTC
TCGATTTCGAACAAGAAATGAGCACTGCTGCTTCAAGTAGCAGTTTGGAAAAGAGTTATG
AGTTACCTGATGGTCAAGTAATTACTATTGGTAATGAAAGATTCCGTTGTCCAGAATCTT
TGTTCCAACCATCATTCTTGGGTATGGAATCTGCTGGTATTCATGAAACTACATTCAACA
GTATCATGAAATGTGATGTTGACATTAGAAAAGATCTGTATGCCAACACTGTTCTATCTG
GTGGAAGCACAATGTTCCCAGGTATTGCAGATCGTATGCAAAAGGAAATCACTTCTCTTG
CTCCAAGTACCATGAAAATCAAGATTGTTGCTCCTCCAGAAAGAAAATACTCTGTATGGA
TTGGTGGTTCAATTTTGGCTTCATTGTCAACTTTCCAACAAATGTGGATTTCTAAACAAG
AATATGACGAATCTGGTCCAGGAATCGTTCACAGAAAATGCTTCTAAatttttcaaatta
atttgattaattatatatttgtgcttaacaaatacacgtttaattaattatatttattta
tttttttaattgaaataaa
>DjScaffold197:55204-56517 strand:+
ttcattaaataaattattctaaaaATGTCTGACGAAGAAATCTCAGCTTTAGTTGTCGAC
AATGGATCTGGTATGTGCAAAGCCGGTTTCGCTGGAGATGATGCTCCAAGAGCTGTATTT
CCATCAATTGTTGGTAGACCTCGTCATCAAGGTGTGATGGTTGGCATGGGACAAAAAGAT
AGTTATGTTGGTGATGAAGCTCAATCGAAAAGAGGTATTCTTACTTTGAAATATCCAATT
GAACATGGTATTGTTACAAATTGGGATGATATGGAAAAAATCTGGCATCACACTTTCTAT
AATGAATTGAGAGTAGCTCCAGAAGAACACCCAGTTTTATTAACTGAAGCTCCATTGAAT
CCAAAAGCTAACAGAGAAAAAATGACACAAATCATGTTTGAAACTTTCAATACACCAGCT
ATGTATGTTGCCATTCAAGCTGTGTTATCTTTGTATGCTTCTGGAAGAACAACTGGTATC
GTTTTAGATTCAGGAGATGGTGTCACTCACACTGTACCAATCTATGAAGGATATGCTTTA
CCACACGCCATCTTACGTTTGGATTTAGCTGGTAGAGATTTGACTGATTATTTGATGAAG
ATTCTCACTGAAAGAGGTTACAGTTTTACTACAACTGCTGAAAGAGAAATCGTTAGAGAT
ATCAAAGAAAAATTATGCTATGTTGCTCTCGATTTCGAACAAGAAATGAGCACTGCTGCT
TCAAGTAGCAGTTTGGAAAAGAGTTATGAATTACCTGATGGTCAAGTAATTACTATTGGT
AATGAAAGATTCCGTTGTCCAGAATCTTTGTTCCAACCATCATTCTTGGGTATGGAATCT
GCTGGTATTCATGAAACTACATTCAACAGTATCATGAAATGTGATGTTGACATTAGAAAA
GATCTGTATGCCAACACTGTTCTATCTGGTGGAAGCACAATGTTCCCAGGTATTGCTGAT

CGTATGCAAAAGGAAATAACTTCTCTTGCTCCAAGTACCATGAAAATCAAGATTGTTGCT
CCACCAGAAAGAAAATACTCTGTATGGATTGGTGGTTCAATTTTAGCATCGTTATCAACT
TTCCAACAAATGTGGATTTCTAAACAAGAATATGACGAATCTGGTCCAGGAATCGTTCAC
AGAAAATGTTTTTAAatgtattcttttagatctgttaattttagtttaataaagtttcac
atgatccaatgaaattttttaataatatttgtttgattaaaattttattagataaa
>DjScaffold2882:147326-148015 strand:+
cgttgagacaacgaatagctaattattatgaacatagatatcagggtaagATGTTTAATG
AAGATCAGATATTAACTGAGCTCAATGAGTGCTTGAGAGAACAAGTAATAAACTACAACT
GTAGAGCCTTAGTGGCGGCGGTACCATTTTTTACATATGCTGATCAGTCTTTTGTTTCTG
AAGTTGTTTCTAAACTTATGTATGAAGTTTTTCAGCCTGGTGATTTGATTATTAAAGAAG
GTACAATAGGAACAAAAAATGTATTTTATACAAGAAGGCATAGTTGATATCATTACTAAAG
ATGGAGAAGTTGTAACAAGTCTTTCTGATGGAAGTTATTTTGGAGGTAATTTTATTAACA
TGTTATGTATGAATAAATATAAGGAAAAAATATGAAATTGATTATCGTTATGATTATTACA
CATTGTCAATATTTCGAATAATACTGTAAataaattttatttttaaaagtatatttaaa
gcacatacattttaatgtaataactaaaaatataattactaaaatataagtattaaattt
taattaaaaaatatttattaaaatgtaagaatcaaaatgaaattattaaaatataatcat
taaaatatattaaaataaaagcttcgatcaaaaatatttttttgtt
>DjScaffold474:16900-24445 strand:+
tttttttagaaaaagatgttttagaggatcaagattgtctaggtcgaactcctttaATGT
ATGCTGTGTATTACGATCAAATTGATGTTGTTCGATGGCTTCTGAAAAGAGGGTCTGATC
CAAATTCATGTGATCTAAGTGAAATAACATCTCTTCATTGGGCTTGTTATAAAGGGAATA
TTCCTTTAATTGAGTTGCTACTCGATGCTGGGGCTAATATAAACTATCAAGATTCGTTTG
GCCGATCAGCATCCCATTGGGCGTGTACGTGCGAAAGTACTGAACCATTACAACTTTTAA
TAGAATTTGAATCCAAAGTAAACCAACATGACCGTAATGGATTGACCCCATCAATGTGGG
CTTGTCGATTGGATCATTTACCACATTTAATTGTACTATCAAATGTCAAAAACCAAGAAA
TTGATCATATTGACGGATTTGAAAGAGATAGAAATGGGCGCACAATTGTTCATTGGGCAG
TTAGAAGACAGCAGCCACTTGAATGTTTAAAATATCTTCTCAATGAACAAACAATTCAGT
TGAAAGATGATGAAGGGAAAACAGTTTTGTTGTTGGCTTCAGAACAAGGTTTAAATATTT
TTTTAATAAATTAGataaaatttatcaatgctcagttaaaaaataatttaattaattagt
ttctcgattattattgatatcgcattaaaacagttaatttaaataaatttgatttcaata
ttttgaaccgacttgtaaattgaaagttcgtttatttaatattgtttaataccaatgatt
tcgtaaattttattagtaaaattaagaatttc
>DjScaffold150:2334-16537 strand:+
ATCGGAGCTGGAATCAATGGATTATCTGTAGGAGTGAAGCTTTTAGAAATTCATAGAAAT
CTTTCAGTTACTATTATTAGTGATAAAGTAACTCCGGATACTGCTGGTGATGTTTCTCCT
GGATTGTGGATGCCATATGCCGTATGTGAAGACGATCCTCGTTTCGAGAATTGGTGCCGA
TCAACATATTCATATTTAAGTGAAATTGTACAAACTGATCCAAATCATCATGAATATGGA
ATTTATTATCAAGAAATTTATGATTTGAGTGACAATGAACCTCTTCAGATGAAATTATTC
AACGAAGTTGTTGACGTGAGCCGATTTATGAATGAACGTGAATTAAAACGATTTCCAGTT
GCTAAATATGGTATAGCTTCAACAACCATTCTATCCGAAGGAGAAAAGATATTATCGTAT
TTAAACAATAAGTTTATTGCTGGTGGAGGAATAATTAAGCATCATCTTGCTGAAAGTTTT
GACGAGGTTCAAGAAAAGGAAGATTTTTCTATTGTTATTAATTGTTCTGGTCTTGGATCT
CGAGAATTATGTAATGATCTTGATGTTAATCCAGTAAGAACACAAATTCTTCGAGTTTAT
GCACCTTGGGTTAAATCAGCCGTTGTCATGGATCATCGATTTTATATTTTGCCACAAAGT
AATGGAGTGGTAGTTCTCGGTGGAACTACACAAAACGATAATTGGGATTCTTCTCCAAAT
GATAAAGATACCGAAATTATTTTAGAGGGTGCTATGAAACTTTTACCAGGATTGAAAAAT
AGTAAAATTATATCAGTAAAAGTTGGATTACGACCCAGAAGATCCATTGTTCGACTTGAG
AGCGAAAATAGAACCAATAGTAACACAGGAAAATCATATAAATTAATTCATAATTATGGA
CAAGGAGAATCAGGATTTACATTATTTTGGGGCTGTGCTGAAGATGTTGTTAAAATAGTA
TCTGATGCAATTAGTAAATAAataaaagaaaccaacaATCGGAGCTGGAATCAATGGATT
ATCTGTAGGAGTGAAGCTTTTAGAAATTCATAGAAATCTTTCAGTTACTATTATTAGTGA
TAAAGTAACTCCGGATACTGCTGGTGATGTTTCTCCTGGATTGTGGATGCCATATGCCGT
ATGTGAAGACGATCCTCGTTTCGAGAATTGGTGCCGATCAACATATTCATATTTAAGTGA
AATTGTACAAACTGATCCAAATCATCATGAATATGGAATTTATTATCAAGAAATTTATGA
TTTGAGTGACAATGAACCTCTTCAGATGAAATTATTCAACGAAGTTGTTGACGTGAGCCG
ATTTATGAATGAACGTGAATTAAAACGATTTCCAGTTGCTAAATATGGTATAGCTTCAAC
AACCATTCTATCCGAAGGAGAAAAGATATTATCGTATTTAAACAATAAGTTTATTGCTGG

TGGAGGAATAATTAAGCATCATCTTGCTGAAAGTTTTGACGAGGTTCAAGAAAAGGAAGA
TTTTTCTATTGTTATTAATTGTTCTGGTCTTGGATCTCGAGAATTATGTAATGATCTTGA
TGTTAATCCAGTAAGAACACAAATTCTTCGAGTTTATGCACCTTGGGTTAAATCAGCCGT
TGTCATGGATCATCGATTTTATATTTTGCCACAAAGTAATGGAGTGGTAGTTCTCGGTGG
AACTACACAAAACGATAATTGGGATTCTTCTCCAAATGATAAAGATACCGAAATTATTTT
AGAGGGTGCTATGAAACTTTTACCAGGATTGAAAGTAAAATATATTTTTTAAtttt
>DjScaffold3767:353-6133 strand:+
GGAGTATCGAAGAATATTGATAGAGAAAAGGTCGAAAATATTATTTTATGGACTACGGTG
AATTGTGAGGGTTCTGAAAAGAGAGGATACAAAAAAGTAATAATAAATGTAGATGATGTA
GATGATAATAAACCTGTGTTCTCCCAAAATAATTATATTGCATTTATTAACGAAGGCCAA
CCAGCCAAAACATCAGTCATTCGATTAAACGTTTATGACCCAGATCTTGGTTTAAACAAT
GAAGTAGATTATTTTATTGAACTTTATTCATTGCTAGAGGAATTTATTATTGAAAATAAT
GGAGAAGTAAAAACAAAAGTACCACTTAATTTTCATACGAAATCAATTTATGATCTTGTT
ATCAAAGCCAATTCTAAGGCATCCCCACTTGTTTTTGCTGAATGCAGACTCACTGTTTAC
ATCATTAACATTAATGATCAGCAGCCAAAGATTTCAACGCCTATTCAAACAATAAAACTC
GATTCAAACACCGTTGTTGGGTCACTTGTTACAAAGATCACTGCAAAAGATTCCGATGGA
TCTGATGGGATTTCGTACAGAATTCGGTACCGTAACCTAACTTATCAAACTTTATTTCTT
AACATTGCATATGATTCTGGAGAAATATTCTTGTGCCGAAAATTAACGATATCCGATGTG
GGGAAACATAAACTAAATATTGTAGTCGATGATGGCAAATTTTCAACTTCAACTATAATC
AATGTTGAAGTTATTGATAATAATAACCATATTCCCATTTTACGAGAAAATGGTAAAACT
GTGGTTTTAACAAACCCGTTGAATCGAAACTCTGTTATTTTATATTTACATGCTGACGAC
GTTGATCAAGGTGATAATGGAAAAGTGACGTTTGACATGATCACTGCATCACCATGGTTC
GGATTACGAGCATTTGATAATAATACTGCTATTATCTTTCTTATTGATGAACCAATTGAA
AAAAATACTCCTCATTTTCTTGGTATTCAACTTACAGATAATGCAATAGATCCTAGAAAA
CGATTAATGTCAATTTCAGTTTTAACTATTCTTACAAAGTCTACAAGTCCTATCGCACCG
CGGATAATGAGCAATAGTAGCTCTGTAATAAGAGTACCAGAATCTTCTTCAATTGGATGT
AAACTAACTAATGTAAAATGCAAGGATGAAGATTCTAATCAACCAATACTCTATAAGATC
AAAAATTCTAATCAAGTGTCAAATTATTTTTACATGGATTTCGATACTGGAGATTTGTTT
CTAACTAAATCACTAAATTATAGCGTCCAACCGAGCTTCCTGGTGATTATAACATGTGAA
GTTGTTGGTGCACCTGAAAGAATAACAACTTTTAATATTTCTTTA
>DjScaffold3698:9924-10729 strand:+
ttcttgtttcattttttttcagtaataaaATGTGCCAATTAAAACCTAGTATTTTGTGGG
ACGTTCCCAAAAATTTTTATGTTGATGGAAAACTTAATAGGAATTCAGCATGTCGAGAAA
TAGAATTTCCTATTTTTAAAGTTCCAACTAAAAATTTTAAGGAAATAATGAAAATATTTA
AAAGATTTTGTCCAGATCTAATTAAATTTGATAAATCACTGAAATATTCAGATTCTATGG
AATTCAAAGGTATAATCTTGGACCCGTCTAAATTTGAAAATATTCCAACTGATCTTAGTG
AAAAAGAAGTGAAAATTTTAAAGCAATTTAATTGTGAATTCAAATTAAATAATATAGAAT
TTTCAAAAATTAATGTTTCATATGATAATTATAAATTGGCAACATTATTCAATTGGATTT
TACCAAATGACAATAATTTAACCGGATTTACTGTTGTTGGCCATATCGTACATTTAAATC
TCAAGGAGGAATTTTATCCTTTTAAATATGCCATTGGTGATTTGATATTAGATAAAGTAG
CAAACGTAACAACTGTTGTTTCGAAAGTTAATAAAATTGATACTGAGTTCCGAACATTTG
AATTAGATTTGTTAGCGGGGAAAAGTCTATTCATTACTACAATAAAGGAAAATGGTCTTC
AGTTTCAGTTTGATTTCTCTAAAGTCTATTGGAATTCAAGATTGAGTACAGAGCATTGTA
GAATT
>DjScaffold3698:27994-31270 strand:+
gATGTTTAGTAAAGCTATATTGCACCGAATTTATTACTTTTATTCACATAGATTAAATTT
CAGTTTATTGTGGGAAGCTCCGGAAAATTTTAGTAATCGAAAATTTAATAAAAATGATGT
TTGTCGTCAGGTGGAATTCCCTGTCATTAAAGTGCCAGCTGAACATTGCGAATCATTTAT
AAATATTTTTAATAGATTTTGTCCAAAACTTGTTAAATTTGACAAAATAAAATTGTTTTC
AAACGCTGAAGGTTACACGGGTATAATTTTAGATCCTTCAAAAGTTACAGAAATTCCAGA
AGAGCTTACAGAGAACGAAATTAATTTTTTTAAAGAAATCAACTATGAATTCAAAAAAGA
GAATTTAGAATATTCAAAAATCGAAGTTCTTTATAAAAATTATCCACTGGAAACTTTACT
AAAATGGGTTGTGGGGAAAGAGTTTACCTTGAGAGCATTTACTGTTGTTGGTCATATTAT
GCATTTGAATCTGAAAGATGAGGTTTTACCATTTAAAAATGCTATTGGTGAAATGCTGCT
AAATCATTATTATCCACGAATAAGGACTGTGGTTGCAAAAGCTAAACAAATTGAATCTAA
ATTTCGAACATTTGAACTGGATTTATTAGCTGGGGAAGAATTATATGTGACCACAGTCAA
TTCTAATGGTTTCAAATTCCAATTGGATTTTTCCAAGGTTTACTGGAATTCAAGATTGAG

TACAGAGCATTGTAGAATTGTTGATTCCCTACAACCCGGAGAAATTGTTTATGATGCATG
TGCTGGTGTGGGACCATTTGCTATTCCAGCCGCAAAGAAAGGTTGTCAAGTCATCGCGAA
TGATTTGAATGAAGAAAGTTGTAAATGGTTCAGATCAAATATTGAATTAAATTCAAAGAA
AAGTTGTAAATTAAATATCGATCTACACAATTTAAATGCAGAAATTTTCATTGAAAAGAT
TGTCATTCCTCATATAATATCCATTGCAAATAGAAAGGAAAACTTGACAAAATTTCATTT
AATTATGAATCTTCCTTCGTTTGCCATTGATTTTCTTTCATTTGTTGTTAATACTCAACT
TGAAGCGATTACTAGACCTGATGTTATTCTCTATGTGAAATGTTATTGCTTTATTGGTAG
AGAAATTACTCAACAAACACTTGAAGAAAAAATAATTCATCAGTTGGGCAAGAGAATTAA
AAAACTCAATTTTCGTTTCGTGAGAAATGTTTCTCCACCGAAAGATTTTTATTGTGCCGA
AATCGCGGTTTATTTGTCTCCGATGTCTAGTGAAAACGAAATCTAAaattttcaagATGT
TTAGTAAAGCTATATTGCACCGAATTTATTACTTTTATTCACATAGATTAAATTTCAGTT
TATTGTGGGAAGCTCCGGAAAATTTTAGTAATCGAAAATTTAATAAAAATGATGTTTGTC
GTCAGGTGGAATTCCCTGTCATTAAAGTGCCAGCTGAACATTGCGAATCATTTATAAATA
TTTTTAATAGATTTTGTCCAAAACTTGTTAAATTTGACAAAATAAAATTGTTTTCAAACG
CTGAAGGTTACACGGGTATAATTTTAGATCCTTCAAAAGTTACAGAAATTCCAGAAGAGC
TTACAGAGAACGAAATTAATTTTTTTAAAGAAATCAACTATGAATTCAAAAAAGAGAATT
TAGAATATTCAAAAATCGAAGTTCTTTATAAAAATTATCCACTGGAAACTTTACTAAAAT
GGGTTGTGGGGAAAGAGTTTACCTTGAGAGCATTTACTGTTGTTGGTCATATTATGCATT
TGAATCTGAAAGATGAGGTTTTACCATTTAAAAATGCTATTGGTGAAATGCTGCTAAATC
ATTATTATCCACGAATAAGGACTGTGGTTGCAAAAGCTAAACAAATTGAATCTAAATTTC
GAACATTTGAACTGGATTTATTAGCTGGGGAAGAATTATATGTGACCACAGTCAATTCTA
ATGGTTTCAAATTCCAATTGGATTTTTCCAAGGTTTACTGGAATTCAAGATTGAGTACAG
AGCATTGTAGAATTGTTGATTCCCTACAACCCGGAGAAATTGTTTATGATGCATGTGCTG
GTGTGGGACCATTTGCTATTCCAGCCGCAAAGAAAGGTTGTCAAGTCATCGCGAATGATT
TGAATGAAGAAAGTTGTAAATGGTTCAGATCAAATATTGAATTAAATTCAAAGAAAAGTT
GTAAATTAAATATCGATCTACACAATTTAAATGCAGAAATTTTCATTGAAAAGGTGAATC
AATTTACAATTTCTTTAATTCAACTGAAATGTTTTATTGTAATGTCAAAACAAGTGGTAC
AATTCCATGAATTAGTGTAGgttgaggttttccatttaaatttatttcgttgttcgtaaa
attcatgtcgatttttaatgaaaattcaaaagtaacacatgtactaggtcaaggagaaa
>DjScaffold1980:82966-83829 strand:+
atgtgttagaaATGAATCCAAATATCGAGCATCAACTGTTATCTGCTGCTAAAATTATTG
AAGAACAAGTTGATTCAGAAATACAAGCTTTGGAAAGGTTAGATGATGATGATCTGGAGA
AATTACGTCAGAAACGCCTTGATGCAATGAAATCAGCACAAAAAGAGAAACAAGAATGGT
TAAATAACGGACATGGAAACTTTTATGAAATTTCGGATGAGAAACAATTCTTCGAAGAAA
CTAAGAAAAGCAAGAATTTAGTGTGTCATTTTTATCGTGATGCAACTTTTCGATGCAAGA
TTGTTGATAAGCATTTGGGATTACTTGCCCCTAAGCATATCGAATGTAAATTTATTAAAC
TTAACGTCGAGAAAGCTCCGTTTTTAACTGAGAGATTGAAAATTCGAGTAATTCCTACAA
TTATGCTTGTCAAAGATGAGAAAACAAGAGATTTTGTCGTTGGGTTTGATGATCTAGGTG
GACATGATGAATTTACTACTAAAATGATGGAATGGAGAATTGCTCAAGCTGATGTAATTA
ATTATTCCGGTGATTTGTTAACTCCTCCAGATATTAAACCAAAAAGTGGAATATTGGGTG
TTAATAAAAAGAAAGCAATTAGAGGTAATGGGGATGATTCATCAGATGATAATGATTGGT
AGtgggattaaaataatatcagtttatttctactttgatttttaatttttttgcaatttca
ttgaaaaaaataataaaaaattgttattcaggtatgtaatgaattgttggaatattgatt
ttctgtaaatttttaaataatttgagttgtctttacag
>DjScaffold1980:171640-177240 strand:+
gatggtcaatccactgaccaacctccactttattattctcaaagtctggaaATGTTGGGT
GGTCTAAAGAATGTAAGAATACAACAACAAATGCCCAGAGATTTGAAAGAGTGTTGTCAT
GGAAACTCATATAAAATTTTTGACGAAAGTGGTAACGTCTTTCTAAGGCTTCTTGAAAAA
CCAGATTGCAATAATTGCTGTAGTCGATGGTGGTGTGGTCCTTCAAGAGGATTTATTATG
GTTGCATATGACAATAATCGTCGTCCTATAATTTACATGAGACGACATCCTTTCAGGTGC
GATGCCTGGTGCTGTTTGGATTGTGTATTTTGTAATCACAAAATAACTATTTCTGATGGT
GACAAAAACTTTTTGGGATATGCTTTACAAAAATGTTCTTGTTGCTCCCCGAAAATATTA
TTAATAGATTCAGCAAACGAAGCAGTAGGAAATATTAGGGGACCCTGTTGCCCCTGCAGA
TGTATTTGTGACATGACATTTAATATCTTCACTCCTGAGGGTAAAGGTCCTTCAATTGTA
AAAGACTTTACTATAACCGATCAGCCAGGTAGTATGGCATCATTGAAATCAGCAGAGAGT
TTTGTGGAAAAATTTCTATTATTCGGGTAGAATTCAAAAGAAATGGACAAAATCTGTATAT
CCTAGAGTAAATATGGATCACGAA

>DjScaffold1980:192209-193080 strand:+
ataaagtagaatgtctattagatacaggggctcgaattaacgtgatgagtttgagttgtt
ttaataaactacgagaagtaaagttagaaatgacttATGCGAAAGGTCGATAGCAAAACG
AATGAAAAAGTCGAAAATTACATTTGCAATATTGAAGCAAATTTTGGAAGACAAATTAAA
AAAGGAGAGCGATTGACAAGAGCTTTAAATATTTTTAAGATTAAAGAGGATCAACGGTTA
TTGAAAATAATTGAAAGTAAAGAAGATGCTTTTATGGCAGATAAATGGGATGTAGGACGT
ACACATCTGACCAGACACAAAATAATCACAAGTGGTGAACCAATCAATGTAAAACCATAT
AGACAACCAATGAACTTAGAATCTAAAATTGAAGAAGCTATCCAAAACCTTTTCGAGAAT
AATATTATCAGGAAATGCAACTCTCCATGGAATACCCCATTGATATGTGTTTGGAAAAAG
GAAAAGAAAGACATAAGAATATGTTTGGATTTCAGACAATTGAATGCAGTAACGAAAAGA
CAAGCCTTTCCGATGCCTAATGTCGATGAAATGCTGGACAAGTTAGATGGAGCCAAATAT
TTCAGTTCAATAGATTTGGGAAATGCATACTATCAAGTAGAGCTCGAAGAAGAATCTCAA
GAAAAAACGGCGTTTTCAACGAAACACGGGCAATATTGTTTT
>DjScaffold3690:88197-100075 strand:+
tgttattattattattatATGATAATTATTCATCAATCAAATTTAGGATACTTTTGGGAT
ATTTGTGATTTGCATTTGAATATTACTAATGAATTATACAGTAATTCTAGTTATGGAAAT
TACAAATCTGATTCAACTTTACTTTTGATAAGATCAGCTTTAACCGAAATGAAACGTATT
CATGGAAATCCTGATTTTATTCTTGTTAATGGAGACAACGTTTATCATGGTGATAATTTA
TTTAATGAAACCATAATTAATGTTAGAAATGAATTAATTCTTCAATTTCCAAATGCAAAT
ATTTTGCCAAGTTTAGGCAATCATGATGTAAGCCCTCGCAATGTTTTCGATGTTAATTCT
AAACGGTGTTATACTTGGTTTCAAATTTGGGAGAAATGGTTTAAACCTCTTGAGAGATCC
GAAATTGAAAATGTGAAGAAAGGTTGTTTCTATTCTCGAGAGATTTCTTCAAAAAATGGA
ACTGTAATTGTAATCGTTTTAAATACTCTTCTTTGGTATAATCGTAATAATTTTACGACT
TCTGTTAATGATCCCGCTGATCAATTTAAGTGGTTGATTGAAAAATTAAAAAATGTTAGA
AAAAATAATAAAAAGGCTTTTCTTGTTGGGCATGTACCTCCTGGCACATCAGAATACAGT
TCCAAAATTATTGAACATTTACAGATGAATTTCAATCGAAAACTTCTAACAATAATGAAA
TCATACTCTGATGTTATTGATGCCGGTTTTTTTGCTCATCAACATACAGATGCTTTCAAA
ATTGTATATGATAAAATGAATAAACCAGTAATTCCGATATTTCTACCCCCATCAGTCACC
CCATTTTTTTCTATCAACCATAGGATCAGTCAATCCATCATTTAGACTGTACAAATGGAAT
ATTTCTAGTGAAACTAGTCGTTATTATCAGTTTCATACAGAAATACCAATTAATTCAAAA
AATAAAATATTAAATTGGACTAAAGAATATTCTTCGTTGGATTTCATGGAAGGGTTGGGA
GCAAATCATTTGGATAGGTTGTTAAATGATTTTTCACAAAAGAACTCAAATTACTTTGAC
ATTTTTTTTCGTCATCATTTATCTGGTCAACGTCATTTAAATGATGATTGTTCTTACCAT
AGTTTATGTCGTCATAAACTGATTTGTTCTATGAGGCACATTGATTATTTTGAATTTTAT
AGTTGCATGAAGAATCGGCAGTCAAATACAGTTTGGATAATAATGGCAACATTTGGATTT
TTTCTTTTAATAGCAATTGTATTACTGTTCTATAAATATTTTTATTTGAGATATCAATAA
tcagagttaaattaaatataattaaccgatga
>DjScaffold974:50635-51693 strand:+
aaaagcatttttattaatattctaaaaagttttcattaattttaaaaaaatgcaattctc
aatttctttttctttaaaaaatattcaaaatgaaaggttaaattttagaaatcaATGCCT
TTTTTAAAATCTACAGTGGTTGATTTGCAACAAGATTTCGATGCGAATCCTATTGACCAA
CTTTCGAGTGTGTCAGAACATGACAATCCGATTTATGCGCAATGTAGAAACCAATTGGAA
ATTAATGAGTTATGCCAAGCTTTTGGATTAAAAACTAAAGTTGATAGTACATTATTTAAT
ACAATTGAGTCTATTAAACAAGAACAACAACAAATTCAATCGGAACAGTAACAAATATT
GATCAAGTAGAGGATCAGACAAATGAGTTGTTGATTGTAGCCACCTCATATTTATTGCCC
CGTATTGTTGGATCGAATTTTTACCATGTCGAATATGAGGCTCTTGATGAGAGTGCACAT
TGTGTTGCATATGCTATAAAAGAATTGATGTATGTCTTCTTTTTCAAGTATGGTGAAGTT
ACACTCGATGATCAAATTCGAAAATTTATCGCATTGACATCATCAAATTTATTAAGATTA
GGCATGGAAATAAACACTTCAAATCAAATTAATAAAGATGAGGCAGTGTTAATTAACAAT
CGAGATTCTATTTACATTTTCTTTGATTTCGTTATTCAAACATTGGAGAACATTTCAGAC
GATTTCTATGAGGAATGTTTTCCTTATGCACTTGTCAGGCAAGCCTATCACAATGTCGTG
AAACAATCCCGCCATGTCATGGCAAAAGCTGCTCATCCAACTGAATTGTCTTCAAAAGCT
GCAATTGAAGCTTGCAAAGTGGATAATAAATAAtaataataatatcttaacttatttttt
gaaatattttaatatgtaaaattgattaaaaaaactctagaaatatatcttgaattttac
aaa
>DjScaffold1182:104667-106518 strand:+
CTTTTTGTTGGAAATATACCTAATGGTACTTCTAAAGATGAATTATCCCGCCTATTTTCT

AAATATGGAGAAATTTTGGAAGTTACAATTGTTAAAAATTTTGGATTTATACATATGAGT
TCAGAAGAAGAAGCTAATCATGCAATTGAAGTTTTAGATGGTACAGAATTTGCAGGTAAT
ATAATAAATGTGGAAATAAGTCATGGGGGAAGGACAAACGGACTAGAGATGACAGGAGA
ATTCTTCCCCGTAAATCTTACGGGCGTAATGACGAACGTGATTCTCGTGATGGTTATCGA
AGTAGCAGATATGAAGGTCACTACTCTAATAATGATCGAACCCGACATAGAGATGCTCCA
CACAATCCTTTAGAAATGCTCAGTGGTCGACCAGATTTGCCTATCGCAGCTTCAATGCTT
GCCGCAACTCTTTTAAATCCCAACACTCTCAGTCTTTTACAAAACATGGCGAACATAAGA
AACATGGGAAATCGTGAAGAGAATGGCAATCGTGAAGAACGTCGATATTCACGACGATCT
CCGCCAAGAACCGATCGTTACAGTTCGTCAAGGTATCAAACACACGAAAGAAGACATCGT
TCAAGAAGTGATAGGTCGCCAGATCGCAGTTACTCAAGACGTCATTAGtctcctgttttt
gtaaatattctctattgtctaagattgtggaacatcagcttttattattcatcagtggtc
accgtcttcaattcaacataacagatggttcgtttgttcagcaatataatatatttttca
atgtagtatcgaacatgttttagtaaaatgtttCTTTTTGTTGGAAATATACCTAATGGT
ACTTCTAAAGATGAATTATCCCGCCTATTTTCTAAATATGGAGAAATTTTGGAAGTTACA
ATTGTTAAAAATTTTGGATTTATACATATGAGTTCAGAAGAAGAAGCTAATCATGCAATT
GAAGTTTTAGATGGTACAGAATTTGCAGGTAATATAATAAATGTGGAAATAAGTCATGGG
GGGAAGGACAAACGGACTAGAGATGACAGGAGAATTCTTCCCCGTAAATCTTACGGGCGT
AATGACGAACGTGATTCTCGTGATGGTTATCGAAGTAGCAGATATGAAGGTCACTACTCT
AATAATGATCGAACCCGACATAGAGATGCTCCACACAATCCTTTAGAAATGCTCAGTGGT
CGACCAGATTTGCCTATCGCAGCTTCAATGCTTGCCGCAACTCTTTTAAATCCCAACACT
CTCAGTCTTTTACAAAACATGGCGAACATAAGAAACATGGGAAATCGTGAAGAGAATGGC
AATCGTGAAGAACGTCGATATTCACGACGATCTCCGCCAAGAACCGATCGTTACAGTTCG
TCAAGGTATCAAACACACGAAAGAAGGTATGTAATTCATTCTTGCTCTTGAgctatgtga
atctcaaaaatccaatctaatgaattctcgttcaatcaaattataagtctaattataaat
taaattcgaatgttccctggaattaagtcttctattattccggtcaaaattttctgaaaa
aaaattctgaaattatctctaacaagtcgttttctgcttatattttctcatgtctaaaaa
atattattaacgagaaattataacgaaatacggacataaaatttattctcaataattaaa
ttcgtctatcttagttaattaataaagttggatcacctttatttctcaaaattttataca
atcttcacgtttcgatcgacaattcattctagaatgaaatatatctttattccatgatca
ttattatgtctactagtctaaatatttctacattatttcagacatcgttcaagaagtgat
aggtcgccagatcgcagttactcaagacgtcattagtctcctgtttttgtaaatattctc
tattgtctaagattgtggaacatcagcttttattattcatcagtggtcaccgtcttcaat
tcaacataacagatggttcgtttgttcagcaatataatatatttttcaatgtagtatcga
acatgttttagtaaaatgttt
>DjScaffold1182:140566-145869 strand:+
CATCCAAGTAAAATTGATGATGCAGAAGATAGATCTAAATTTTCAGCTATTCTCGACGAA
ATTCATGTCAAACAAGCTCCGTGGCGATCATTGTCAAGTTTAGACGATGCTTTAGAATTT
GCTTCAACCGTAGGTTATCCATGTTTAGTAAGACCGTCTTACATATTATCTGGATCTGCC
ATGAATGTCGCTTATACACCAATAGAGTTAATTGGATTCTTAAAGTCAGCAAAGCAGATA
TCACAAGATTATCCAGTAGTTATCACGAAATTTTTGGAAGGAGCTAAGGAGATTGAATGT
GACGCTATTGCTCAAGATGGTATTATTGTATGTCATGCACTGTCAGAACATATTGAAAAT
GCTGGAGTACATAGTGGTGATGCCACTCTAGTTCTACCTCCACATACAATTTCCAAAGAA
ATATCAAGTCAAATCAGAGAAATCTCTGCTAAAATAGCTAAAAAAATTTGAAATTTCAGGA
CCATTTAATGTTCAATTT
>DjScaffold1182:158439-161010 strand:+
AGCAATTTTCGTTTACCGAGAAAAAATTCAAATATTTTAATTGGAGTCCAGCATGAGTTT
CAACCCAGATTTTTACCAACAGCTAAGAAGCTTCATGATTACGGCTATAATTTATATGCC
ACTCAGGCAACTGCTGAATATTTGAAACTTCATAAAATTCCAGTTAATTCGGTTTCCTGG
TCAACATCGACTGAAAACGTCCATAATTCTGAAAGTCCAGTGGCTACAAAATTAATTTCC
GAAAAATTTTTTGATCTAGTAATCAATCTTCCAAATTATAAAACAAAATATGTGACAGAA
AATTATCTGATTCGAAGAGCATCGATTGATTCAGCCACTCCTTTGATGATCAATTTTGAA
TTGGTCAAAATGTTAGTGGAATCACTGGAGCACATCGATCTTGTAACAACAGATTCTCTA
TTCAACTATCAAACTGAAAAACCAGTCCATTAAaattttgtaatttttaattaattagtt
cagcactaaactttttgatttttattattcattaattattttattgattaattaggttgt
ttttatcggaatttcaattttttattaatttaaataaaagtttcattttagaa
>DjScaffold1395:11111-20193 strand:+
attattataattcATGTCTATTAATATTAAAGAGTTAGCTTACAGTGAAATAGATAATCA

TACTGAAGATTTATTTGATTTATCCAGTTTCTTGTTTACTAATCCTGAAACAGCATACAA
AGAAATAAAAGCTCACGATTACATTTGTAAATTTTTAAGGCTAAAAGGTTTTAAAATTGA
AAGTAATTTTTTGCATTCTACTGGTTTTCGAGCGACATATGAAACGAAATCCTATCCTCA
TTTTGCAATTCTTGTTGAATATGATGCACTTCCAATAATAGGTCATGCATGTGGACACAA
CATTATCGCTCAAGCTAGTATAGCCACTGCTCTTGGTATTAAAGCTGCCATGGAAAAAGG
ATTACAATGCAAGTTAAGTGTACTTGGAACTCCAGCAGAAGAAGGTGGAGGTGGTAAAAT
AAAGTTCGTGTCGGAAGGTATATTCAAAGATGTGGATGTATCGATTATGAGTCATTCTTT
TAATAAAAATATTCTTGATGGTCCTAGTTTGGCGATAATTGGTTTAATCGTCACTTTTAC
TGGAAAAGAAGCTCATGCTTCTATTTGTCCTTGGGAGGGAATAAATGCTCTCGACGCAGC
AGTTCTAGCATACCAATCGATTTCATGTTTACGACAACAAATGCCTGTCGATAATCGAAT
TCATGGAATAATAACTAAGGGTGGAGATGCTCCAAATGTAATTCCTCATTTAACTGAAAT
GGAATACTACGTTCGCGCACCAAATGCAAAACTTCTAAAAGAGTTTTTGCTTCATGTTTA
TGAATGTTTTTATGGAGCTGCTAAAATAACGAAATGTGAAGTTACCATTCAGGAATCTTG
TCCACTTTATGAAGAAATATTAAATAACAAAACCTTGTTGAATATTTTTGAGCAGAATGC
ATCTGCTTTGGGTATTTCATTTGAAACGATTTCAAATAATTTTCGTGGTTCATCGGATGT
AGGAAATGTTAGCCATTCTTGTCCGACTATTCAACCTTTGTTTTCTCTAGGTTGTTCATC
TGATTTACATACCAAAGAGTTTGCAACTGATGCAAGTAATTTTAAATTTTGTTTTATTTC
ACATGATAGAACTGACAACTAAtaataataataataata
>DjScaffold3685:119-28184 strand:+
tagtcatgagtaatgaaaaaattactaataatgataattttttaaaaaaaccagtaaat
tttaacaaaacaataggaattatttttataatttagttaaaataaatattatttaaaaat
tttttatttaaatttattacagtgtttgaatgtattaattattttataataataaacatt
ctaatttaaagtatttttcaaattaattttttttataaatttttaaatttaaatttga
atgttaattaaattatgtctttgaaaaacagtgtttgaatgtattaattattttataata
ataaacattctaatttaaagtatttttcaaattaattttttttataaatttttaaattt
aaatttgaatgttaattaaattATGTCTTTGAAAAATAATGAATTCAGAATAGTTGACAT
AGACTTGGATAATGACTTAAGGGTTCAATCTCAAAATAGTGATCATAGTGATGAAATACT
TAGTCACTATTCCTATACAGACTCAGATTTACATGATTTTCAGAAACTTGAAAAAGATAT
TTATGGTTCTCATGGTGACGATGAATCATCTATAGAGCAAGATGAATTTCTTGAAGATGC
TACAGATGAACAAACTGAATCTTTGGAATTTATTCGTCCTCATGTACCAGTTAGACCTTC
ACTGGGTGACCCAATAGATACTTACGATCAATTGCAGAGCAATTTTGAACCCGTTATTCG
GGGTGCGCTCGAAAATGACAAAGTCTTAATTAGTAATCAAACAAGTAACAAAGCAACTTT
CAATTCAAATCGCAATACCATGTCACCAATCCATAAATCACGAGGTAGCTCACGAGCAAG
TGTTGGTTCTGCTGAAACTGTTTTGTCTCAAAGTCAAAAAAATAGATGATATTCCATTAAT
TCCTAAATTTCTGGACAAAAGTCCTAAAACTCGTAATAAGCTTAGCTCAATAAGTTCCAT
AGAAAGTAGTGAAAAGCCATCTAGAAAAAGAGTAACTAATAGAGCTGCTTTAAAATCTTT
ACTTGATCAAGAAAAAGCCACGCGTAAGTCTCAAGAAACTTATATAGAACATCTTCATCA
ATCACTTAATCAACTTTTATCTCAACATGCACTTGCGAAAGAAACAATTGATCAACTACG
ACTTGGAATCGCTTTGAATGTAACTGAAAATGATAACCATCCCAGATTGCGACATCCACG
TTATCATAAACGTCCGTCAGCAACCAGTAATAGTCAAATTTTATCTCGTCGATATAGTAG
CATTATTGGTAATTCAGACATGCCTGATTTTGATAAATCAAAAATGTCTAGAGCTAGATC
GGTGGATATGAATATGTTTGACGAGGAGCCAATCAACGAAATTGCAGAACAGCACACAAC
AACGAATGAATATGGTGGATCTTTGAAATTTGTGAAAAATAATATGTCAGTAAATCAGGG
TCTATCTAGTGAAATAGCTCATGATTTTCAATTAAATTTTGATAAATTTCAAACTCGTGT
GGAAATCATTGAACAAGCCATAGAAGAATTATCAAGTTATATAGACGGTGGAGCATCAGG
TTCTTCTGGATCCCAATATGGAGATTTAGATGGATCAATTGATCGACATCAAACTGATTC
AGAAAATCGGCTACAAAATATTCTTTTAGCTTATGAAAATCTTCAGAAAGATTTCAATCA
AGTTTGTGACGTTTGGAAAGATACTGCTAAAAATTATATACCAGCAACAAAAGTGATGCA
TAACATGGATTCAAAAGAACAAATTGAAGCAAGTTTATTTCAACTACGTATGCAACTTGA
CGATTTGGAGCGACATGTCAAACAAGGTCATCAGCCTACGTTTATAAATCAAAGTATAGA
TACAACCCCAATTGGAAATTCAAACCCGAACAATAATTTAGTTAAACGTACAAAGGATCT
AGAAGAAAAATATCGCCAATTAATGGGACGATATAATGAACTGAAACGATTTTCATCGCC
AAAACAACAAAAAGATAAAGAAGAAGTAATAAAATTATTAAAGGATGTAATTGCTGAAGC
AAACGGCCGTTCATCGAATATTGCTACTCCTGAAGCTTTTGATAATATAATACATCAAGA
TGTAATAGAATCTATGAAGCTACATCTTTCATCAGATAGTCCACCATATTTCCTAACTGA
AGATGGTTTGTCCAGTGGTAACAGCTCGTTTGGAAAAGGTCGTTCAAAGCGTAAAAAAAT
AAGTAGACCAGAAGAAAACACTGTTTGGAAGCGTTCTTCTGATGAACGACTTTCCCCAAC

AAAGTCTCATGTTTCTTCTCATTCTGTTGATTTAAATAATTACGGTTCTTCAAATCAATT
TATTAAGTATGATAGTCAACCTGTGTCGCTAGTGAGTAGCGCATCAGGTTGGCAAGGTGC
AACTAGTGGTGAAACACTTTTAGTTGATGAACGAGGAATATTAACAGATGGATTACGAAA
TGGTTCAGTAAATAAAAATGATATATTTTCACATAAGTCTCGACCAACCGAAAATTCAAT
TAAAAAAGCTCGAAGTTATACTAACAGTGCTGATATAGAAAGTATAAAAAGTCGACCAAA
TAGTTCTGGATTACAAAATCTTGATGATGACGTAAAAAGACTTGATGAGCATCTAGCTCG
AATAGAGTTAGAAGAGAAGAACAGAATATCGGAAACTAATGCAAATCCTGATTCTGATGT
GGCCTTATCAAGAAATACACGATCATTATTAGGAACATCTCCTAGTAATAGTACTTATTC
TCGTCAAAGTAAACCGGCTAGTTCTGTGGGTTCCAGTCATAGATCTAATAAATCCCAGCA
ATCAAAACATAACAGTAACAGTCACAGTGATTTGCGAAAGAAACCGAAAGGATATAAATT
TAATATAGAATCTACAGATAGCGGTGAAGATAGTGCTAGTTATGAAGATGATTTAGACGA
GTATGTTCGGGCTGCAAGTTGTGGAATTGGTCCTCGATCAAGATATATTCGCAAGAAAAA
TAACAATAGCCAGGATAGATGGTCCCAGACTCCAACTAGGCCTTTACACAAGTCTCACAG
ATCATCAGCTTATGATATGAATTTATCTCCACAGTACGATGATTCCTACGTTGATGACGT
AGCATATTGGGATGGTGTAGCTTTTCATCCTGCCAGAGAAGGTTTACCTGGTAAATCCAT
TCTACGACGATCTTCTCCAAATTCCAAATATCATAGAAAGCATTTACGACCAGCTACAGT
TCCAATTAGACCACTCAGTGATTCATATTCCAGACCACCTTTTCCGTTGCCAAGAATGGT
AAATAACTATAATGAACCTCTGTCACTCAGAGAGTACTCAACAATCGGTCCTCATCGTTA
TTTTGTTCGAGAGTATGGTCACTTAAATAGAATTCCTTCTAATCAGCCATTAATGCTTGA
AGATTTCGAATGTGAGGATGATGAAGACGATAATGATTACTCGTATAGAAAACATGTTGA
AGAACGAAGACTTTGTAATTATTTAGATGAAGCAATAAAAGCTGCTCGTGATATGAAAGA
ATATACTCGTCGATTAGGCGATATTTGCCAACAAAGACAGCTGGCTAGAGAACCGGTTGG
TTCCAACCAATGTAGAAATTTTCGTCCA
>DjScaffold3685:34320-46855 strand:+
atatttttagagtactctaattgatttaatcATGAAAGGCGATGAAATTCCCCTAGTTAT
TAATGATTCAAATAAAATTCCAGATATCAGAAAACCTATTAAAAACAGAATATCAGTTTG
TAGTGATGATATGTCCCATGAAGAATCACCTTTATTAAATCGTTCGGATTATAGTAGTGA
TAATGATGCATTATCAAAGTACTGTAATTCATGTCCCGAAGATTCATTGTTTTCTAAAGT
AATTAAAGAATCAGAAACTGCAATTAGAAAAGGAATTGCCCCAGAAAGGATTTATCAAGG
TTCCAGTGGAAGTTATTTCATTAAAGATCTCAGTGGAAATATATGTGGTGTTTTTAAACC
GAAAGACGAAGAACCCTATGGAAAATTGAATCCGAAATGGACAAAATGGATTCATAAGCA
CTGTTGTCCATGTTGTTTTGGTCGTTCGTGTTTAGTTCCAAATCAAGGATATCTTTCAGA
AGCCGGTGCAAGTCTAGTGGATTATGCTTTAAATTTGAATATCGTTCCAAAAACCAAGGT
CATCCGTCTTGCGTCACATGTGTTTAATTACAACGCTATTGACAGAGCTAAATCACACAC
TAAAAAACAAATCGCACAGACCTTTCCAGGAATGGCCCGAAAGTTTCATCGTATAGGCCT
TCCTCCAAAACTCGGTTCATTCCAATTATTTGTTAGTAATTATAAAGATGCAGAATACTG
GTTATCGAGATTTGATTCGGAAACGTTACCGGAACAAACATTCCGGAAATTTAGATTTGA
ATTTGAAAAATTAGTCGTTCTCGATTACATAATTCGTAATACTGATCGTGGCAATGATAA
CTGGCTGATAAAGTATGAGAAATCAGAAGTCAACTCCAATACTGTAAATGGAGATACTGG
TGAATGGGATTTTGCAAACATTCCAGAAATTAAAATAGCTGCCATAGATAATGGTCTCGC
ATTTCCCTATAAACATCCCGATGAATGGAGAGCTTATCCGTTTCAGTGGGCATGGTTGCC
TATGGCTAAGAATCAGTTTTCTAATGAAATCAAAGAATTAGTTTTACCAAAACTGCAAGA
TTCCTCTTTCGTTCAATCGTTATGTAATTCTTTGTTTGATCTATTCAAAAGTGACAAAGG
CTTTGACGCTAACACATTTCGTAAACAAATGGCTGTAATGCGTGGCCAGGTAAAATTCTT
CATGTGTAAAATAATTTTATATACCTGGGAGCGCAATTCGTGTCCATGTCCATTTCCGTG
TTCCAATAATATAATCTAAtttgccgacgcgtcgcgacttaaaactagaatataaatat
ttaattaaattcattaaattgttgatttaataagtaaacaatagttactatagttttatt
ggtgacccgacaaaatagcccaggacaaaatttattgtcaaaaattattgttattattat
ttttaatatattattttatattattatattattaataatatattatataataataatatt
attttttaagctattttgtcttggtattattattattattacttcgtgtaattattcact
tttttaaaatttttaaccattgatctgtgaaataaaaaattaaatattttca
>DjScaffold5206:5006-6548 strand:+
gattctagggtttcaattagtttctaccctaATGGGTGCTAGTATTTTGTCTAAAGTCAT
TAATTATACGTCATTTGCCAGATTTCTTCTTTGCTTTAATTTATTTAGATACGTTTATCC
TTCTGCAGAGGAAATACTGAAAGCTGCAAATGTTAATAGGATAGATAAAAATCGAAAAAA
TAAACGAAACTATTCAGATCAAAATGCTCCAGTTAATATTTCTAAATCAATTTCCATTGA
TTTGAGACGAGTTCCTGTCGTATTTAAGGATTTAGTTCAATTGCATTATTACTCTGATTA

TCAATGGCTGATCGACTTTTCGCTTTGCTCTACCTTAGTCTTTTTTTCAAATGAATTTGT
TTATGGGTGTTTATTAGGTACATATGCTAAAGTTAATTTAAGTATTCTATGGTGCATATT
GGGAATCGCTTTCACAATAAAATCTTTGATTAATATAACAAAGATTTATTTTACTATGAC
TGATACTGCTGATTTAATGATTGTTATAATGTCTGCGTTTTCATTTCTTGTTTTTTCTAT
GATGATTTTGATTTTACCAGAAAAACATTTGACTTTTCAAATTGAAGAAGGTTATAGAAA
TTTCACAAATTCTATAACTAAAAATTTCCCAGATAATTCTGGTCCAATTAGTTTGATTTC
TATAAAAGGTGTGCTAGCAGTGCTTAGTGCATTTATTGGTGGAATTTTCACTCATTCTGG
GTTACGATATGGTAAAATGTATATTGATGCAATTAACGATTCTAAATCAAATGCCCAATT
ATTTGTTCTACATTTTTCTTTTCTATTTCCTTTAGCAATTTTAAGTCTTTGGTTAGTCCC
TGTTCAAAAGTTTTTAATTACACTTAAATTCGGTACTGCTACTTTGAAATACGATGATGT
TTTAGCATTGCAGATTTATTTACCTTTAGCATTTTGTATTTTGAGATTTTTTCTGTCCAC
TATGCATTTACAGTCTTATTTAAACCTCGCTGATAATCGAATTAAAGCTTTAAGAAAAGA
AGCTGGTAAAATTTCTATTGTTGAATTTCAAAAAATTGTTGCTCGAATTTTTCACTGCCT
GTGTCTTGTTGCCTTACAGTATTTTGCACCCATTATTTTATGTTTTAATGTCACACTACT
CTACAAATCTCTCAGTGGTTTAAGCTGGTTAGGATCTAACCGTGTATTATTTCACAATTC
TGAAGTTAACTCAAGCAACTTATCTACAAATTTTAGTGATACTTTCTTACCGTCTATTTT
CGGCGATGAAATTAATCATTCTGTTGAAGTATGGTCCATATCATTATCCAGTCTCAGAGC
AGTGTTTACTCCTATGTTCTACCAAGGAGTAATCGGTTACTTATTGTGGTTTACAGTCGC
GGCCATCCAACTAACTAGTTTTTTAGGACTGGTTTATCATAAAAATTTAAATAATTAAag
catgatctttgttaatattttttatcttattttagaatgtttatttagttttcagatta
>DjScaffold5206:20455-40785 strand:+
GAGAAAAAAGAAATATGTATAATTGGATGTGGTATCGCGGGTCTTAGTGCACTCCGAGTA
TTTTCAGAAGAAATTATTGATTTTCCTCGTAATTATTCTCTTTATGCCTACGAAAAAACA
AATAATTTAGGTGGCTTATGGAATTATAATGAAAATACAAATACTGAATTTATTACTCCA
GTTTATAGAGATTTAAAAACTAATTTGCCTAAAGAATTAATGGCATATCCAGATTTTCAA
TTTTCAACAAATATATCTGAATCATTTATTGGTCATAAAGATGTCTTAGATTACATTCAA
AATTATGCAAAGTATTACGATATAGAAAAGTATATTGAGTTTAATTCTGAAATACTTAAC
GTGGAGCCTGATAATTTTAATTCTTCAACTAAATGGAAAATAGTGATTAAATGTTTAAAA
TCTGGGACTACGTTTGAAAAGTTGTTTGATGTTGTCTTGGTTTGTAACGGACACTTTGAA
AAGCCTAAAATTCCTAAAATTAAAGATATTGAAAATTTTAAAGGAGAATTACTTCATAGT
AAATATTATCGTGTTCCTGAGAAATATGCGAACAAAAACGTTTTTGTTGTCGGGGCGAAG
TCTTCTGGTATGGATATCGCTCTTCAATTGTTAGATGTGTCTAATAAGGTGTATTTATCA
CATAGTGGACCAAAACTAATGTGGAAAACTGAAAAAAATAATTTCATTGAAACTGTCAGA
ATATCACATATAGATCTTCAAGGTTCAATTTGTATAACAGATGGATCCAAATATGATGTT
GATGTGATAATATTTTGTACCGGTTATGAAATCGATCTCAGTTTTCTTAGTGATAAAATT
GGATTAAAATATGACAATACTTATGTTCGACCTCTTTATAAACATTTAATTAATCCCTAT
TTTCCCAATAGTTTTTTTATGATTGGACTAGGTTATGAAATCACTCCTTTTAATCATAGT
TATATGCAATCTAAAGTGGCCCGGTCAATAGTTCAAAATTGTTTTAATGATTTGGATTTA
TCATTTGATAATTTAATGAAAAGTATTGATAAAGACGAAGAAGATTTTATAAATAATAAT
ATTGAAATGAGACATAGACATTATTTTAATAGTAAAGAATGGCCATATTTTAGGTGGTGT
ACAAATGTTGCAAAAAGCGTTATGACTTATAAAATACCAAATTATTTAGAAAATCTTTTT
GAAATAGTTAAAAAATGGAGATTCAAAAATACTAGTCAATACAGGGACATCAAGATACAA
TTGGTCAATAATAAATTAGGATTCGATATTGAAGAT
>DjScaffold4055:72354-72686 strand:+
ATGCCATTCGGGTTGTCAACAGCCCCAGCGACTTTTCAAAGTCTCGTGAATTTATTATTC
GAGGATATCCTTTATAACGGAGTCGCGGCGTATTTAGACGATGTCTTGGTTTACGGAACA
ACATTTGCAGAACACAATAGAAACCTAGATGAGGTTTTTAAACGAATACAAAAAGCTGGA
TTAAGACTCAATCCAAAGAAGTGTAGTCTGTATAAAACTGAAATTAGTTGTTTAGGTCAG
AAAATATCAAAACAGGGAATAATTACTAATAATGGTTTGATTAAGAAAATTGCTGAAGCG
GAAATTCCAAAGTGCACCAAACAGTTACGGTAA
>DjScaffold2833:2496-3021 strand:+
ttatcttggaaaagatagattctatattttaagatttaatcaatttttacaataaatttt
aaatagatacgattgcttcagtgctgacatttggtctATGGGTATAACACTATATACGAT
CATGTTTGGTGAAAACCCATTTTATGACTTCGAAGAAACTATAAATGCCAAATTGAAACT
TCCGAAAATTATTTCTTATGATTTAAAAGAAATGTTGTCCATGCTTTTGGATCCTAATCC
ATTTAAAAGAGTTAGTGCTAACAATTTATTGCAACATCCTTGGATTAATCAAAATGTCGA
TATAACAATATATTCTTTCGAAACTATTGTAAAAAATCCAGGTACAATTTTTTCACATTT

TTTACCTTTTAAATTTACTATTTTAAATTGAaataattaaaatgaattgaatgacataaa
attttaattatgcattttattgactttaaaatttgaagtcaatttctgacgaagaataag
tcgaaa
>DjScaffold2833:13034-13204 strand:+
ATGATTGCTCATCGAGATATCAAGGATGAAAATATTATATTAGATAACCAATTTAACATT
AAACTTATCGATTTTGGTGCTGCGGTTAAAGTAGAATCAAATGAAAAATTTAAAACGTTC
TGCGGAACAATGGAATTCTGTAGTCCTGAAATATTACTAGGAAAGGAGTAA
>DjScaffold4207:20608-20895 strand:+
TTTACAATTGCAATTATTGCGAGAATAATTGTTGAACATAACCATTTATCGTTGTTAGAA
TTCATTTAAaataaaaacttcgatcaaaatt
>DjScaffold317:81189-81704 strand:+
ATGGCATTCGGCACAGCAGCAGCCCCAGGAACATTCCAAGAACTAATGACAAAAGTATTG
GACGGTCTAGATGGAGCAATGGTGTATATGGATGACATAATGATATTCTCAGAAGGACTA
AATGAACATTATATAACTCTGAGTCAGGTATTAGAGCGGATCCAAACGGCAGTGTTGCGA
GTGAATTCAGACAAATGTCACCTATTATTCAAAGAAGTGAAATTTCTTGGCCACATAATA
GATAAAGAAAGAATTCGAACAGATCCAATGAAAATCGAAGCTATCAAAAAGACATTTGAA
TGGAAAAACTTCAAGAATCTGATGGAATCGATGAATATAGAAATACACTTTTCAAGTCCG
TACCATCACAATACAAACGGAATTATAGAGAGACAATTCCGCACAATTAGAGACTGTATT
AACGCAGCCGCAGATGAAGATAGAAGAAAGGATTGGAGTAACAGGATATTGAACATTGAA
TACATGATGAATGCGACAAGGCAAAAGACATTGTAG
>DjScaffold317:51667-52011 strand:+
ATGTCTTTTGGAATTGCGGCAGCACCAGGTACATTCCAAGAAATGATTAATAAAGTAATT
GAGAGAATAAAAGAAGCGATGGTTTATCTAGATGATAATCTCATATTTACCGAAACAAAG
GAGCAGCATTATGAAATAGTCAATAAAGTATTAACAGACGTTAAGAAGGCTGGTCTAAGA
GTTAATCCGGAGAAATGTCAATTCCTGCGAGACGAGATCAAATTTTCGGGACACGTTATC
AGTATAGAAGGTGTCAAAACGAATCCAACGAAAATAAAAGCAATTAAGAATTTTGGCACA
CCAGCTCAGCGTACCATGCAAAGATTATTCCAGGTGTTTCAATGA
>DjScaffold3454:32674-44552 strand:+
aATGGAAAATCCTGATATTAAACAAGAAGAAACAATTTCTGTAAACAATGTTGCAGATTT
CGATGGAAATGTCATTAATGCAACTACGTGTCAACTTATCGGGCAAATGATTGGCATTTC
TAATTTAAATATAAATAATCTAAAACCTTGTCTTGATGCTGTAAATAATTTAGCGAAACG
TTTAGTTGGTGAAACGGTATTTGCTGCTAGAAGAGGAAAGCGCTGTATTCCTATTCCTTT
TGATGCAAATACTGCTCTGAAATATCGAAAGATATCAACTTCGTTTACTGATATTCCATT
TAAAATGATACCCTTAAAACAAAATAAACAAAGTATTAATGGTGAAAGCTCACTGTTCTA
TCCAGACGATACAGAAATCGATTTGAAGGAATTAATAACAAAGGAACCTAGTAAAATTTC
CTATGAATCGTCGTTAACTGAGCATTGGCTCAGTATTTATTCAGTGCAACCAGCAACACC
GGAGAATCCTACTTCTTCATTTATCCATGCACAAAAGAAAATTCTCAGAGATAAAATGTA
TTCCAAATCTCAATTAATCATTTCTCATAATAATACGAAAGAAGGCACTGTTCAGAGAGT
TTTAGATGGAAATAAACCAAATATCATTAAGGGTAATCTCAACGGAATTGCTCTGGGATT
CAATGGCGATCGTATGCCGGCAACAGTGATCGACGTGAGACCACATTTCGTCGGAACTGA
ACATCAGGTTTACTTCAAAACTCTTACTGAAGCTTGTGTAGGAAATGATGAGAACGCAAG
AAACAATGCACTAGTTACAATCAAAAATGACAAAGCCCTCGATCCATTATTACCGCATTT
AATGCATTTCATTGCAGAGGGCATTCGATTAAATATTATGTATCAAAACCTGGCGGTTCT
TATTTATCTGGTAAGAATCGTACGATCTCTTGTTGATAATCCTCACGTCAATTTGGACCC
ATATTTACATCGAATTTTACCGGCTCTGTTGTCGGTAGTGCTTTGTGGAAACATTTGTCT
CAATCCTATTACTGATAATCATTTTGCATTAAGAAAGTTTGCGGGGAAAGTTGTAAACAG
TATTTGCGAACGACAACATACCGCTCGAAATGAACTAATCAATAGATTATGTGCTATTCT
TGTAACAAGCATCAAGCAAACCATCAACCCAAATTCGGAAACTATTGACAGTTCTAATAA
TAAATCAGCTGATTTTACGATGCGATTGAATACATTGTGTGGTGCAGTTTTATGTTTGTC
TGAACTAAGTGCGGAAGCCATTCGAAAATGCCTAGTTCCGAAAATTGGTGTCATATGTCA
ATTTCTCACAAACATTATTAAATTAAAGGGATCTACGTTACCAGGAGCAGAAGATAAAGC
TATAAGTTCTTTGAAGGATATATTATTGGTTGGAACCTTAATTTTATTTTGAttatttaa
a
>DjScaffold759:33515-44188 strand:+
aaataaatagaaacgtttttaattaaaaactaaattctttaatatgaaagggtttaaaaa
aacatttagaaactcattatgaattttagaataattaaaaatgttaatcgattttctagt
ttttaatgaatacataaatgtaaaataaaaaagacatctaaataaattaatctaaaaatc

attaaaatatccttcaacttctttataactaatttcagtatttgtcactttgataaatac
aatttctattttattgaaatttctctttaattaattttatataggtttaattgtaatttt
ttttaaaaatatagtacaaataaataattctatatattatctttgaagattaccgatttt
ggctttgctaaaagagttcaagggcgaacatggacactatgtggaactccagaatattta
gcgcctgaaattattctttgtaaaggatataataaatcagttgattggtgggctctagga
attttcatatacgaaATGACATCGGGCTATCCTCCATTTATTGCAGACCAACCGATTCAA
ATATATGAAAAAATTGTTTTGGGAAAAATGAAGCTGAATCAATATTTTTCTTCAGAGCTG
AAAGATATTTTACGTAATCTATTACAGTCAGATTTGACTAGACGATTTGGAAATCTTCGA
AATGGTGTTAATGATATTAAAAATCATAAATGGTTTAATGGAAAATGTGATTGGATTGAA
CTATATCATCAGCAAACTACTCCGCCTTTTATTCCGAAAATGAAAAGTCGGGACGACACA
TCACATTTCGACAAATATGAAGAAGAGCAATTTCCAATTTCAAATTCAGATAAATATTTA
ACTGAATTTATTGAATTCTAAaggaaa
>DjScaffold1871:1280-1889 strand:+
tttttattcttaataaattttatattaatctaaatatttttaatttgcttgagaaatgac
tgttaacgttcacgtaacttactgtaaagggtgaggatatcgccgaaaatttcaaagcct
taaacgggatttggaattagagttttctgataagttgattattacatgtactattgctcc
aagtaaaagtggattatttgaagttgaagtaaATGGCGAGCTTGTTCATTCAAAAAAGAA
TGGCGATTCTTTTGTTGATGACGAGAAGCTGCAAAATATATTTTATACTATTAAAAATTA
TCTTCGGAAAATCTAGTATTGTAAAAGATTATATTTTTGTATTCTTATATTTATTAGCTA
AAATAATGATGTTTATTGATCAAAGTCTTCATTGAtattcaataaattgatgttgatagt
tttaataa
>DjScaffold1622:5281-18085 strand:+
atatccgaataataaATGAAGCCGTCGATTCAAGAACCAAGTAAAATGAGTATTATAAGA
AAGTATTATAAATCTGCAAAGAGGAAATTTATATACATATCATACAATTACAAATTATTA
CTGATTCTAAGTTTTGGATTCTTCATATCGGTATACATGTTTTACACTATTTTCCATTAT
GCTTTCTTGAAAAATCATTTTGATCCCATTCACGGAACTTATGGTAAAAGTTGTCCGGCC
TGCTTTGGAAATAGAGCATGCCCGTATTTTTTCCGGGGTGAAATCAACATAAAAGGGATG
TATCGTTTACCATTTCTGCAAAAATTGAAAACAAGACAAATTTTTGAATCTTATCATGGA
TTATATCATATGAAAACGGATGTGATCATTAAAAAGTTGGCAACAAATAAGGAACATGAA
GAAAAAGATTTAAAGATTTGTAATGTAAATCGATATAGGAATGCGGAAGGGAAATGCTTT
CCAAAACATGCTCTATGGTATTATCTTGACTATCATCCATCAAAGGAAATGAAACTTTCA
CATTTGGCACAAACTCTAGAAACAGCTCCAATGATGCATTGTGTAACTGGTAGATTATTT
GACAGAATAAAGAAAAAATTCCGTGAAAGAGATTCTTCAAACACTGAACTACCTTTAAGT
CGAAGAGAGGAAATTCTTTTATTATACATATTGAATTTAAACCAACAATCATTGATAATG
CAACTTTTTCCGGCTGAGGAAGATTGGCCATTTCCGAAATATCTCGGTGCTTGTGGTCGT
TGGATAGTAGAAGAATATGTTGGAATAGAAATGGATAATTTTTGCCATGCGAAATTTTGG
CAGAAACTTACGATTCTCATTAATTTCTTGAAATTACCAAAACAACTTGATAAAAGCAGC
AAAACATTAGGAGAATGGGTGTTGTATATTGGTGATCCGGATCCGGAAAATTTTATGATT
AATCCTATCACATTCGCTGTGAAAATAGTCGATTTGGAACATATGATGGTTGTCGATATC
AATCAGTTAAGAAAAATACGTATGGAAAAGTCTGACGAGCCATATCACGTTAAATCTGAA
CCTTGTGGAAATCAATTAACTGATAGTTATTTCAATTGTAGCCAAGCAATAGATAAAGGC
ACCCTTTGTGCAAGAGAAAGCTCAGATCACAATATTCATTCCATTTGTGCCATGCTCTTG
AGTTCCACTGACCACACAGTTGGACCGGCATGTCTTCTTGACGATGCTCGCTTAGTGAAT
GTGAGAGAATTGTTGCAGAAATGCGTTTATGAAGAAAATAAGAGAGTAGAGCATCATAAT
GCCATATTAACAGAATTAAGTGAATTACTTCATTCCATTGAAAAGCAAGAATTTTAAtca
atattttctatcaaatattgct
>DjScaffold3501:20538-28239 strand:+
aaatttaaagaattaaaaataaaaattcataagagtaaaaatataaacgaataaaagtaa
taaattttaaaataagatttttaaattctttgaattaacgtcgaatctcatattgcttca
gagttcttaaatactgcgtggattcggcgagcacttgccaaacaaacatataaatagatc
atggaaatgtgttatttttgtcaggcaattcgtggggtaatttagaaaaaaaaacaattg
tttttatatcaaaaaaaattttgttttattaatgaagtctgtttcatattgatactgcaga
gatttctaattatcaactctttattattttacacattcaggattttgaagaagttcgcaa
aagaagaaatgaaATGCTTGTTTTTCGTAAGAATCAAATATTATTTCCATTCAGTTATTG
GCCTGAAATTAAACCAAGGCAATCAGAAGATTCGATATTTGAATTACGATCGTATACGCT
TAAACCGGGCACAATGATTGAATGGGGGAACTATTGGGCACGCGGAATCAGAGTACGTAA
AGAGCATTCTGAACCGATAGCTGGATTGTTTACCCATGTTGGAGATATTCATATGGTCCA

TCACATGTGGGCTTACAAGGACCTTGAGCATCGAAAGAAAATGAGAAATGAAGCGTGGAG
AAATCCTGGCTGGGATGAATGCGTTCTTCATACTGTTCCTCTAATTCGGCATATGAACAG
TAACATTCTTCGAGCTACGCCATTTAGTCTTATCTATTGAcgaattcaagttataatctt
gaaaatcaatttcaaaaattaaattagaaacattaatgaaataaattaaaataatttctc
atctttatagatagattgtgattacaatcaatgctgttaataaattaccgatttgtcatg
taacttgtcttctcattgtttttagtaaactgata
>DjScaffold3501:60700-68058 strand:+
aatttctttggggattttgatatcccagaataatcaagatatgtaccaaggaatcagtta
ttgattatatcaattttaatgttgaattgataaaagtttcaactaataaataaattttct
ctattgattacgtaaaaatagttatttaaatataacttgcttcctttaatttttagacta
aaattctagttgagatagttgagttgaaaaatttgagcgaaattttagctaggccattag
ttttaaccaacgattcaaaatcagaattagctgatattctaggagcagtgaatgaaaatg
tctactctttagtggatgttagaattcctgaaattaatggatactatttagcgaaatggg
aagaagacggaaacttttatcgggctaaattgttagagaagtcaggtaatagtcaatata
aagtctgttttgttgatgaagggacatctggtgacgttgtaaaagaattaaaagaaattc
ccagagagatttccggttttccttgccaatttttaccttgcgatATGCTATGTGAAACCT
CTGAAGAGTTGATCGATCTTATAATAAAGAGCGGTAGTGATTCCAAAGTCAGTATTTATG
TTACAAATCCTGAATTGAAAAACGGGAAATATCGAATAAATCTTTTCATTAATGATGTGG
ATGTAGTTGATTTATTTAAGAGAAAATATAAACAAGATCCACGTATTACTAGATTGCCAA
ATGAAGTAAATCTGAAAACTTATCAAATAGCGTCTAAATTTTCTAGTTTCCATATTATTT
CATTTCCAAAAGAACCCAGAGATATCTTTAAAATTGTTCTTACCGACACTTTTAATATGA
ACCCCAAAGTAGAACCTTTGAGTTGTTCACCGATTTCCGGTGACTTGGTAATCTATCAAC
AGTCTGAAAACTATCTACATCGTTGTGTAGTAGTATCTACGCGGTTTATTAATAGCAGTA
TTGGTTACAATATAAGATTAGTTGATGAAGACCGTTCGTTAGAGACATCGAGTGAATCAT
TACTGGAAACCAATGATAAATGCTTAGAAACACCGCCTAGAGTTGTACATTGCGTCCTTA
GTTTAGACGATAGTGTTTTGCGTCCCGGAGACGAAGTGGAAGAAATCATTATAGATGATA
TAGATAAAAACCAACCGTTATATTCGATTTCTTTAAAGAAAGCACAGCGAAATGAAGATT
TAACAGGAAAAAATCTCACTGTTAATGTTTGTTTTGTGGAAAGTTGTGATGAATTCTATA
TTCGATCTGCTGATACGAAAACTTTCCACAGTCTTAATGAAATAGAAACGATTCTTAATT
CCAATTGTAATCTTTTTGAATTCACATCGACCAGGGATGTTGTAATTAACCAAATTGTCG
CTGCGCCATTTGAGGGAGTTAATTATCGAGCACAAATTAAAGAAATCGATAGTGAAAAAC
GTTTGATTCGTGTGTTTTTCATTGATTTTGGAAATTCTGATTGGGTGGAAATCAGAGATA
TTCGTAAAGTAAAAGGTAACATAATGAATTTGCCGGGTTTCGTTAAGCGATGCAGATTTC
CCGTTAAAATAAAATCGAATTTCTCTTCACAAGTGAAAAATCTCTTGACGGAATATTCGG
AAAATGGAGAAATTAGTTTATGTGCTAATGTTGTCGCCGATGATGTTCATAATGGTGTTC
TTAGTGTGAATCTATCTGCAGAAAACAAAAGCCATTTCATTGATATATTGAAATTCAAAA
ATTTTGTCGATGAAGAAAATACAAAGAACATCACCGGTAGTGACGAACGAAGTCGTTTAA
TTGCTAAGATTATTGATTTAGAGAGACGATTAAACATTTTAGAAAAATTTCTCCCAAGAA
AATAAaaatatccttttatccatttgtaattcaaatttttaaaataaaaaaaagttgggtt
ttttttgtattaactaactagacaacattggaaatatcaatttgagtgttttatctattt
gtaatagtgtttttatatatatgtgattgattgtatcgaactagtcaatttgtgtttctg
ctgttaattatttctgtggagatctattagttattattatgacatgcattta
>DjScaffold4840:70556-80122 strand:+
tgatcttaaatcgatttgttaaatttaaacaatgaaaaaaattgaaaatttaaaacaaaac
tttattttctatatattatttctatttcaaattaacagttattattgttatcaaatttta
gtttactctatcaattattcgtcaaaaatgttgtttgaatgaagtcttggtggcgtttat
ttgcaccttgaacgaaagaatagatcaaatacacaatgaagaattttccacgagacttgt
tATGATGCTAGAAAGGATTATTTTTAACAAAGCAATTCCTGTTTCTGAAAATCCTTTGAG
AGATCTCTGCATCAGTCTTTCATTTAAATTGTTGTGGTCAGTGAGCAATTTTAATCTGAA
ATTACGAGTTACCCATATTCTCCAAGCTATAATTAGTCGACAATATTTACTGCAAAATAC
TATCAAAAAAATTATGTATTGAAAAACTTCTAAATGAATTTAATCAACCTTTTTTACCTGG
TTTTACGTATAGTGGTTGTTTAATTGGTTTAGTAAGTCTAGATTATTGCTTATACATGTT
ATCTGATGATTTCATTTCCAATCTGTGGTCATTACCAGATGACATTAAAGAACATTATTC
TGTTAAAGAAGCTGTATATTACCTATTTAATAAACTGGTAAGATATTAAttattattggt
aactgactgaattaaatatatcaaattaaccactacaaatcattaatgaccgaagcaaaa
taaatcatggtattaattttatattatattataaaattaatctgggaaataatctccttg
acgggctatgcattgtaaccatttttcgggtaattttcgaattatggtcaagaaaaatg

>DjScaffold3589:37466-45240 strand:+
ttcatttttatattgcaacttatattcaggagtgaattctaatcctgaaaatacggtatt
cgtgttttaaaataatttttaaaaatcaaatttaagtcgaaattaagtaatttaattgat
ttaactgttgatcttttaacacaaaaggatttcgagattgataataaaattgaatcttag
tcctgcttttattagtaaatattaatcaaaattttttatgattgattaatattttatcta
gcatgctgtagcacttgaacaaattttgcccaaattgaattctggtgcaaatattcttga
tgtcggttcaggtagtggatatttaactgcatgctttATGAAAAAAGTTGGTGCACAAGG
AACTGTTGTAGGTATAGACCATATTGAAGAATTGGTGCAAAAGTCAATATCGAATATCAA
TAAAATTCCAGAAATCAAAGAGCGTCTTTATACTGGAAATTTGCATTTGAAAAGTGGAGA
TGGTAGAGAAGGATTTAAAGATTTTGCACCTTATGATGCTATTCATGTAGGTGCTGCATC
TGAAACTATCCCAGAAAATTTGGTGAGTCAACTAAAAATTGATGGCATTTTAATTTGTCC
CGTTGGTAAGCCAGGTAACCAATATTTAATGAAAATTACAAAATTACCCAATGGTACTCT
TCTTGAAGAAAAACTAATGGCTGTTCGATATGTTTTGTTGACTGATAGATCGGCCCAACT
TCGTTCTGATTTGTAGctttttaaaaataattctaatgtacgatttgttcgtcgaaata
cag
>DjScaffold2339:35950-40970 strand:+
tacagtgaaatcgtagagaaatgagtttcactatcatataattattatttatttctttat
tttaacttttatttatgattgacagacgaaagaaaattatttgttgggATGTTGAGCAAA
AATCAAACAGATGAGAATGTTCAAAATATGTTTACGAAATTTGGAAAAATTGAAGAGTGT
ACTGTTTTAAAAGATCAGAATGGCAACAGCAAAGGGTGTGCATTTGTCAAGTTTCTGAAC
CATACCGATGCCCGTGCCGCAATAAATGCCCTTCATGCCAGTCAGAAAATGGAGGGTGCT
TCATCTAGCCTAGTGGTGAAATTTGCTGACACAGATAAACAAAAGCAAATTCGTAAACTG
CAGCAAAACTTACCAGACTTAAACATTTTAAACTCTCACATTCCAATACATATTCCATAT
TATACATCACAATGTGGTCCAGTTATTAATCCTGAAATGGTAAATGTTCAAATTACTGAT
AAATTAGGCAAAATTTAA
>DjScaffold3588:12067-12506 strand:+
AAAGTCGAAAATTACATTTTTAATATTAAAGCGAAATTTGCGAGACAAATTAAAAAAGGA
AAGCGATTAACAAGAGCTTTAAATATTTTGAAGATTAAAGAGGATCAACAGTTATTGAAA
ATAATTGAAAGTAAAGAAGATGCTTTTATGACAGATAATTGGGATGTACGACGCATACAT
CTAACAAGACACAAAACAATCACAAACCAATCAATGTAAaaccgtataggcaaccaatga
acttagaatctaaaattgaagaagctatccaaaaccttttcgagaataatattatccgaa
aatgcaactctcattggaataactcattgatttaaaattgaatgttgtaacgaaacaaca
agccttttcgaggcctaatgtcgatgaaatg
>DjScaffold3588:104275-116098 strand:+
ttagtgtaataatttcctacaatgatttatcagccttttttgaaatataacttttgttctt
catcttgacttgcaaattgacacaatttgaaaaagaaagaatatttcaataaaaataaat
agtacaagtaatttttttaattacttaatattttttaacaataaattcagaaaaataact
aatatttttgaatgtttatttaaatattcatttagtttagctaaacaacattgcgacgga
agtcattattttgttttacttattcttacaaatggttcagtagaagattggatcgaaacc
aaacgagctgtcattgaagcttcttcgttgcctgtttccataattgtggttggaattgga
tctggagcatttacagatATGAAAACTTTGGATCAGGATTTTGGTTTATTAAAAGTGGGT
AATGAAAAAGCTTTGAGAGATAATGTTCAATTTGTACCGATGCGGAAATTCCTTAGGATG
GCCGCCGATGGATCGAAAACTATACGATGGAACAAAGTGGCACTTGCTAAAGAGGTTTTA
GCAGAATTGCCGGATCAATTATTGGCCTACATGAAGCAAAATGATGTTCGCTCTAAATTT
GGTGATAATTTATCAGTACAAAAGCCGCTTGATATTCAAAAATCACCGAAAGAAATACAA
CATCAAAAATTACGTCGTTGCGTAACCGCTCACGTAAATATCAGACCGAACTCGAGTTCT
GATGTTGAAGGGAGAAAAGACAAACAAGATTCTACATTACATTCTGATTTCTTTTCAAAT
CGAAGTCTGCGAAAGAAATCTACTGATATGATTTATAAAAATGATGATAGCAATCAAATA
AGTAAAAGAAAATAActaaaagaaacgttttataaaaattattaactgatattgttattt
gttgaatttttatttttatc
>DjScaffold3588:127356-128011 strand:+
atttacacttaagctATGTTCATGAAATTCAATCGAATTCTTAAATCTACAATCTTAACA
TTTGATCAAAGAAAAGGATATGCACCAAACATATTTGGTTCAATTTTCAAGAATTTAATA
AGTAATCCTAAAATTAATAAGGTTGGTAGTGATATGGCTTGTGCTGAATATTTATTATCT
AATGGTGCTGCAGTAAAATGGTGTGGTTTTGACAGTTTTCATAGATATTTAGAATCTTTG
CCGACAAATTTAGAAGAAAGTAAATACTTGAAAATATCTCACATTATCGCAGATCGTTCC
AGTATTGACAGATATGGATTGCGTTATTTTGAAGACCTAAAATATCTCGAATATATCTAT

TTTAAGGAGTGTGCTCATGTGACAGATCATTTCCTCGAGGAACTGTGCCAAATATTGGCT
AATCAGTTGAAAATATTGAAAATAATTAATTGCAAACATGTCACAGAGAAAGGAATCAAT
CAAATAGCAAAACTTCAGTAAatttcattattaattaaattttattaattaatttatatt
tct
>DjScaffold3423:18402-22301 strand:+
caactttttattttttcatttactaacaaaagtaaatttgttaatataaaaaagATGCAA
CGAAGCAAGGAAGAAACTGTAAATGGTGTTATCAATAAAGAAAATGTTGTAACTCCGAAA
ATTTCAAAGCCACCAGTTGAAAGGCCTTATGAAAGAAAAAGTAGCAATGCAAATACAAAT
GCAAATGCAGAGCCTATTGCTGTTAAAACAGAAAATGAGCAACTAGTTGATTCAAAATCA
GTGAAACCAAAATTTCCTTCGGGAAATACAGATAATTCAAAGATAAGACTTGTTAGAAAT
ATTGGCTTCGAAAATCTACCCGATCAAATTTATCGTCGATCGCTTTTTAACGGATTCGAA
TTCAATTTGATGGTAGTTGGTTGCTCGGGATTGGGAAAATCGACTTTTGTCAACACTTTA
TTTCTTTCGGAATTGTACAACAATGAAAATCTGCCGCCTTCAAAAAGAACACAAAAAACC
GTCCAAATCAATACAACTACAGTTAATATCACGGAAAATTCAGTTTCTCTAAAATTGACA
TTGATTGATACCCCTGGATTTGGTGATTCAGTCGATAACTCTGAATGCTGGAATCCAGTT
CTTAAATATATCGAATCTAAATACACAGATTATATGCATGCACAAAATCGAATTAATCGT
AACCGAGTTGTTGATAATCGTGTTCATGCTTGTCTTTACTTTATCGAACCAACTGGGCAT
GGTTTGAAATCGCTTGATTTGGAATTCATGAAGCGTATCCATACTAAAGTTAATATTATT
CCAATAATTGCCAAAGCTGATACTCTAGTACCAGAAGAGTGTGCTGAATTCAAAAAAAAT
ATTTGCTGTCAAATAAAAGCTAATGGTATCAAAACCTATGATTTTCCAGAAGAAGCAGCT
GCTATTTCATTAGGGGTCTCAGTTGTTAAAAGTCGAGAAACGAGCTGGAAAGATATAACA
CTGAAACGGAAACGTCAACCATTTGCCGTTGTTTGCAGCAATATTATTATTGAAAAAGGT
GGTAATAAAGTCCGAGGAAGAACTTATCCTTGGGGATTAGTTGAAGTTGACAATTTGGAA
CATAATGATTTTGCAGCATTGAGAACTCTTTTGTTGAGTTCACATTTACAAGATATCAAA
GACACAACTGATCAAATTCTTTTCGAAAATTACCGATGTGAACAAATGAGCAATTTCGCT
AAAGATAGTCAATTCGGAGTTACTGACGGTAAAGATCCAATGAGTCAAATGGATGAAGAA
AAACGAGAGTTCGAAGCAAAAATTCGAAAAATGGAAGAAGATATGCACAATCTATTTCAG
CAAAAGGTTACAGAGAAAACTAGAAAACTCGAAGAATTCCAACAGAACATTGAAAGAACT
GAAAAAGAAATGTTATTACAGTTGGAGAGTGAAGTTAAAGATATCGAATATAAACGAAAC
AATTTCGAAGCACAAAGATTAACTTGGGAGGAAAACAATCGTGATTTGAATTTCCGAGCT
TCAATGGATGCATTGGACATAAAAGATAAAAATAAGAATAAAAGTAAATGGCTGTTGAAA
TAGtcgaatatcgtcgtgcatgaagattgtgcagattcttatcatttttctgtcaacagc
atgttttatattgatcattattttgtaataaatgtcagttcaatt
>DjScaffold488:22392-23159 strand:+
tatttaaatttcataattatagtgtcttatcaattattgataattagttttatttacgAT
GGATAATGATGATTTAAAAGCGGAAATAAGTAAATATGATCCACCAGAGTCAGAAAAACG
AAAAGACGCCCTTATATCACTATATTATGTAGTGATACTTCTTTTAATTGGCATTCCTAT
GTGGTATTTAACAACTACAACATATCGAGCTTCTTTACCCGTTTCAGATATAAATAAACT
TTTAAATTCTAAAATATCTTTAATAATAAAAGTAAATATAATTAGTTTCGATGGAAGAAT
TGAAATGGATGATCTGTTCAGCTTACAAGACAAATTGCTACAGGATTTTCAGAAAACTGC
AAATGGTAAATTTGTGAAATTTGATTTTACTGGATATGTTCGTCAAGCAACAAATGAAGA
AATGAAAACTTATCAATATGCCAAGATTTTGTCAGATTTAGATAAAATCACTTCTAAATT
GCCTTCAAATGATATTTACGTGCTAATTTTACCGAATGCATCTAATTTATTGGCTGATTA
TAAAAATGGAGTCATTCGAAGTAGAACTGATAGAAACGTTCTTGTGCTTCCGTTTGACTC
TCTTGATCGAATTGAGAAAGTTATTTACAAATGCATAACTGGAATACTCGCTAGAAACGA
TGATTTAAATGTAAGTTTTGTTATTGCAAATGAAATCTGAcgagaaacaattcgaatttg
ta
>DjScaffold488:40676-41346 strand:+
AGAAAGAAGGAAATTGTTGCGTTCGCAATTAGTAAAGATAGTACATCGTTTTATAAGGAG
CAAATCTTTGATCAATATAATGGTGGTTATAAATCATCTACTTCAACATTTCAACATTTA
AATGCTTCTTCCATATCTTTATCGAAAAACATTGATTATCGAGAGACATATAATCAAGAT
TTTGAAAACGATGATTATAGTACAACATATATGCAAAGTGTTGTTCGAAATATGGGATAT
TTGGATAAGAATATGAATTGTGGAATAACACTTGAAGAATTTAAAAGCAAATATCTGTTT
TTTACATTTGTATTTGCTCCAGATTTTGATATTAATCAGAGTCAATTACCACAACAAGGA
AATTTGAGATTAGATATAAAGTTTAAAACAGCTTTGACTGCGCCAGCATATGTTGTAATA
TATGGAGTATTTGAAAGCGAAATCCAAATAAATGCTAATAGAACAGTATTAGTGTAAaca
atatctaatataatcaaataatcaaaaattatgaagtaagttttttatttaatttcaata

ttttactttattagttcttttcagattaatcaaattggataaaaaatgttgggaacatca
tggttgggagtatacccattaga
>DjScaffold4001:39250-51148 strand:+
atttttagaatagcagttaacATGTATAGTGCCAGCTGTCCCAGTGAATCTGGCTCAGTT
AAGTTTTCGAATAATGAATCTGCATTTGTTCACGATTATGAAGATTGGCTGAAAAAAGAA
CCAAAGAAAAGTGCCAGTGAAGATTGTTTGTCAATAGAAAGTTATGAATATGATCAATGT
CCTGAGTTTGATGAGGGTGAACAATCAGATCATACTGATAATGAATTTGGTAGTTTAACA
AGTTTTGAAATGATCGGAAGCCAAGTCTGTAAGGATGGAATATCGACTTCACCCACTGCA
AAATATCGGAGGCGTGGATCAGATGCCATGAAAGAACTAAGTATGGTGCTAATGAATAAG
TTGAATCGAAAATATTATTCAATTCGAATTGGCGATTGGTTTAAATTAAAGCCTCAAATC
AATCCGAATAAACCAGTCTATCAGCTCGGAAAGAAATATTTGTTTAATTCTTATTACGCA
AATTTAATGGGTAAAAGTGAATTCATGCTGGATTTTGAAAGTCGATTGTGGTTCACATAT
CGGGAAAATTTTCGATTTTTACCTTGCCCAAAAATCGTTGACAGTATTGTACCTGATGTT
TTACGTACATCAGATTCCGGTTGGGGGTGCATGATACGATCTGGTCAAATGCTATTGGCC
CAAGCATTTTGTATGCATTTTCTTGGTCGATCATGGGAATATAACAGTAAAAATCCGAAA
GAACAGGCATTTCATTTGCAAATAATTAAGTGGTTTGGCGATTTCGATAAAGTAACAAAT
CCATTTTCAATTCATAGAATAATGCGATATAGTGGGCTAAATCCCGGTCAATGGTTTGGA
CCTGTCTCAATTTGTAGTGTCCTCAGGGATTCGTTTTATAAATCATGTGATTTAGAACCA
AAACTGCAATCTTTAGAAATTTATTTTTCATTCGAACAAACTATTTATCGAGATGAAATA
TTCGATTTGATGAAAATTAAACAAACGGGTGATGAAGTGGTATCTACAAAAAGATCAAAG
TCGGTAATCATTTTTCTACCATTATTTTTGGGGTCAAAAAGCCACAGTTCTTCATTTTAT
ACCGGTGTTTTGAATAAGTGGTTACGTGATCAATGTTGTATAGGTATTATTGGTGGTAAA
CCACATCACGCCATTTATATGATTGGTTATCAAGATAAAAACGTCATTTACTTAGATCCA
CACGTTTTACAGGACACTGTGGATGTCAATCGTGATCAGTTCTCTTCAGATACATTTCAC
TGCAAAAGACCTTTAATCATGCCGTGTTCTCAGTTGGATCCTAATTGTGCAGTTGGCTTT
TATTGTAGAGATATGAATGACCTAAATATGTTTTTATTACGTTTGCCAGATATGTGTAAA
ATTTATGTTAAAACTAATAAAGGCCCGGTCTTTCGTAATATTATTGAAATTCTTCCTGGA
TACCCACCGAATTATGACAGCCTAAATAGCATTATTTGAaagagttaattaaaccagaat
gagatgttttccaaccatattttatgtaaattttaatttacaagattattcttattatat
ttcatttttcttcggtttatttaatgaagaatttattacattttttagaattgtgattt
attcatgtcagttttgttcatatttactcagtaaaaaattaatgtatatttgtaaatatc
taattttgatggaatatttatattgattttcattctagacaaaattatgatgaaaaattt
gttttttattagctgattttaaattttaatgactctgtttatttgtgtcgctgatatta
aatgttttgtgtagaataactttaaggattataattaaattggaatttgttaattttcaa
ttagttgttactataattatcacatttttagaatagcagttaacATGTATAGTGCCAGCT
GTCCCAGTGAATCTGGCTCAGTTAAGTTTTCGAATAATGAATCTGCATTTGTTCACGATT
ATGAAGATTGGCTGAAAAAAGAACCAAAGAAAAGTGCCAGTGAAGATTGTTTGTCAATAG
AAAGTTATGAATATGATCAATGTCCTGAGTTTGATGAGGGTGAACAATCAGATCATACTG
ATAATGAATTTGGTAGTTTAACAAGTTTTGAAATGATCGGAAGCCAAGTCTGTAAGGATG
GAATATCGACTTCACCCACTGCAAAATATCGGAGGCGTGGATCAGATGCCATGAAAGAAC
TAAGTATGGTGCTAATGAATAAGTTGAATCGAAAATATTATTCAATTCGAATTGGCGATT
GGTTTAAATTAAAGCCTCAAATCAATCCGAATAAACCAGTCTATCAGCTCGGAAAGAAAT
ATTTGTTTAATTCTTATTACGCAAATTTAATGGGTAAAAGTGAATTCATGCTGGATTTTG
AAAGTCGATTGTGGTTCACATATCGGGAAAATTTTCGATTTTTACCTTGCCCAAAAATCG
TTGACAGTATTGTACCTGATGTTTTACGTACATCAGATTCCGGTTGGGGGTGCATGATAC
GATCTGGTCAAATGCTATTGGCCCAAGCATTTTGTATGCATTTTCTTGGTCGATCATGGG
AATATAACAGTAAAAATCCGAAAGAACAGGCATTTCATTTGCAAATAATTAAGTGGTTTG
GCGATTTCGATAAAGTAACAAATCCATTTTCAATTCATAGAATAATGCGATATAGTGGGC
TAAATCCCGGTCAATGGTTTGGACCTGTCTCAATTTGTAGTGTCCTCAGGGATTCGTTTT
ATAAATCATGTGATTTAGAACCAAAACTGCAATCTTTAGAAATTTATTTTTCATTCGAAC
AAACTATTTATCGAGATGTA
>DjScaffold4001:74194-83090 strand:+
GGTGGTGTACCGAAAATTGGAACGTTATTAGAGGATGCTTGGAAAATTGAAGATATTTGT
CGATTTAGTGTTAGACCAGTTCAGAGTTTAGTTAATGAAAGAAGAAATTGTCTTCCAAAT
TCCGTTCAGTCTATCAATCCAGCTTCTTTTGATCCATTAACTCATGCTCATATGTGTCCT
CAATTGAATACATCATTTTTATACGGTCTTCTTGGCAGTAGCAACAAAACGATAAATATT
AATGAGAATAATTCAATAATAGTGATAGTATAAATGGTCAAGGATCAAGTAGTAACTAC

AAAATGCCGTCTACGTGGGCGGAAATGCCTCCGCCTCCATCACCAGCCAGTTCTACATGT
AGCGATCAAAGTGGACCTGTGAAAATTAGTCCTGGAACATTCAAAAGGAAAAAGCAATCC
GTCAGTAATTCTGGAACTGGAGATTTAGTTGCAGAAGTTAATACTGAAAGTCGCCCTGTA
ACTAACAATAATAGATCAAGTAAACTACGTTCCTGTGATAATGACAAATCTACTACCACT
TCTACAAACTCTGAATATGAACCCATTGCTGGTCATAAGCGACCTTTGACCCCTGATGAT
AAATCAAATGGTTCTAATGGTATTCCACAAATGCAATTTCAGGAAGAAGAAGAAGATTGG
TGTCTGAAGGATGTTATTTTTCTAGAAGACGGAAGAACTCAACCTGTTGGTGTAGTTCTA
AAGGTCGATGGTTCAATTGCAGCTGTCAAATTTTTAAAGGACTCAGAAAGAGCATGTGTT
GCTGTAAATTGTTTATATTCACCTCTAGCTGCTGTATTCGGAAATAACAATTCCTCATGT
ACAGAATCTTCTTTGAATTCTAATACACCCAATTCTCCGGATCCTATTTCATGGCTCAAT
GATTGTAGATTGTTACGTAAAGAAGAATTGGTAGTTGTAAAACAACCGTTAGTATCTAGA
GTTCCCGATATTGTTCAGAAACATCCTAAATGCATACCAACCGAAACATTTCTTGAAATT
AAACAATCATCGTTGTCACCGAACTTTGGAATTCTAGGAATGGCTTTAGAAAATGAATTA
ATACATTTATTGATTAGGAAATCCACTAGTTCCAAATGTATTGAATATAAACTACTAAGT
TTGGGTAAAAAGGTTATTTCGTCTCATAGATTAAATACATCTGCGAATGGGTTTCTTGGA
CGCTCTCCTCAACCTTGTTTAATTGCTAGTCCCTTAGAAAGTGAATATTCCGAATTGAAA
AATGGAATGATATTCAACGCGGAAAGACATCGCCCGATATTCTTAAGAGATGGAAACGGA
ACAGTTTATCCATTGTTATTTAATGAATCACACAGTAGTAGCAATGTTTCATCGAAATTT
GAACCAATACCCAATCCACATTGGTTGGATTTGCCACCTGTCAGATGTGTCGATTTTGGA
TTTGTGAATGTCAATAATTATAATATCAAATCTCCAATTATTCCCGGATCGAATAATTCA
GTCAATGAAACCAATAAAAATTCAAAGAACAAAACCAATAGACTTCTTATCGGGGTTTTA
GTTTTAGAATCACAAACATTGATGCAACATATTTTACGAGCCGATGATAAAAAAGTTTCC
GGTGTTATGGATAGTTTATCAGCTGGTTCAGAATTTGATAGTCTGATACAAAAAATTATG
AGTGAAAAATTAGATGGTCAAAGAAACATAATTCATATAGCAGTTAATATGTGTCGACCG
CTATCAAATATAGAAACTGAATGGAAATTATCAAATATACTGAATTATAGATCTAAAAAA
TCATCAAATACTAATGTTGAAGATTTGTCTCAGCGATCAAATTGGTCATTAGAACATTCA
ATGAATATTTCTTCGTTTCCATATCGACGATGGATTGATATGGGTCGGCCGATAAATGAT
AATCAGTTTAATTATTCTCGTTCCAACGATAGACAAGCAAGTAGTGTTAGGTTTACGGTA
ATGAGTTCATCAAATACTTCAAGAACTTCTATTTTAAGTGGAGATGTAAATTCAGGGTGG
TTGTTACCACCAATTCATTTAGATGAAAAAGAAAAACGATCAAACAGTTATCGAATAATA
AATATTATCATTAATCATCCGTGTATGGAAAACAATTTGATATCTTTACTATCTCAAAGA
AATGCTGATGGCCAAACTCCTTTCATGCAATCAGTATCTCAACGAATTTATCCATTGTCA
TTGTTTCTACTTGACTGTATCTTAGATTTATGTGATATTCAATGGGTCTCAGTATTCAGT
TCTAAAAGTCAGTTGGCTTTAAGCATGATTTTTCCTCCTGCTATCAGTATAGCTACAAAT
AAAAATTTGGTATCCACTTTATTTGAAGATAATCCGTTACAAGTTCTTTGTCAAAACGAT
GGATGTAGTTTTACCTGGACAGGACCTGATCATATTCGCCAAGATATTTTTGAATGTCGA
ACATGTGGACTAACAGAATCACTTTGTTGCTGCACAGAATGCGCCAGGGTATGTCACAAA
GGACATGATTGTCATTTGAAAAAAACTTCTCCGACAGCTTATTGTGATTGTTGGGAAAAA
TGTCGCTGTAAAGCACTCATTGTTGGAGATAATGCAGCTAGACAAGAACTTTTGAAAAAA
CTTCTACTGCACACAGATTTAGCTACGCTTCCCAATCGAAGAAATGAACATCTCTTATTG
TTCCTTGTGAAAACAGTCGACAGACAATTTAGAGAGCACAAGCAAATTCGAACATCTGGA
CGACGTCATTCAACATCTGTTTTAAATGCTCGTCATAGTAACATAAACAACAGTAATTTC
ACAGCGTCACCACATGTATCATTATTGGAACCTCATCATCCGGCATATGAGCCACCGATG
CCGGAATATGGATTAGAACCATCGAATTTCGCATTAAATGCATTAGACGCGGTCTTATCA
AATTGGCCGTCCGTTGAAACTCTTCTCTTAACCGGTGATCGATCTGATTGGGTAAATAAT
CTTGTGAAAAATATGGATTCATCTTCAAGAAATAACGGAATTAATCCTCTAATAGACGAC
GAAGTATTTTTGAATTATCAAAGCTTTTCTGAACAACTCGATCAATTCGTTTTCACATTA
ATTGCGAAAACTGCATCATCATCTGTTGATATATTATTAAATACTCTAAATAAGGAATTG
CAAAATGAAAAGTCTCATCCTTTGACTTTAAAACCTCCGACTCTTACCAAAGTAAAAACT
GCCGCTGCTAGATTAGTAAGATCTGTTGCTCGAGTTTTCGTTGCTCTTCAATTACAAATG
GCTCCTGATTTTAATCGTAAACGGCAGTCGAATCGTCTAAGTAATAGTGTTCATGTTTAT
TCAGTTCAACCGAAACCATTAGATGTTTGTAAACGGATTTTTGAAAATTTGTTATTTATA
TCGCTTTCTGAATTACCGAGAATCGCGGCAGATATAATAGCACCAGTTCGTTGTGGCTTA
CTGAAACCGACAGCAAGTTATGCACATTTCTATCAGATTTCTGATGCTGTCAGAAATTTG
GATCAGATATTTTACTCCAATAATTCTGATATCGATAATCGTCACGAGTCCTCTGGAAAT
TCGGATAAAAACATCAAACAAATTCCTCAATCAAATCGTCCTGCTGATGGTATTTCAAGA
AATACCTCGAATGTATCTCGCAGAGCTAGTATTAATAATAGAAGTAATATGGCGTCTGAT

TCAAATTCATCAAATGGTATAAACATGCCAGATTTCCATAGCAATGATCGACAAAGTAAT
GCTAATGGTCGACAAAGTAGACGGCGACCGTATGATGATGATATTGATGGTTCGGGAGCT
GTTATTTCAATATCAGATGGCAACGGTACAGAAACAAGTAATCGAAATTTAGATTCCGTA
CCACCAGTTAGACGACGAAGGCAATCCACACCAATTAGCTTAAGTAGTAGAATGAATGCT
CTTATAGACGAGGAAGATGATGACGATGGTCATTCATCTGGGGATGACAATTGGAATTCA
AATAATGATTCCCGGGGTCTTTCTGAAGATAGGCAGCATTCAAATCAATTAAACAGCATA
TTTTTCGAAGAATCTGATGGAGATGGCAATACTATTGAAGGTGAAAATGATTTAGATGGA
GATGTGGACATGGAAGTGTATGACGAAGACCATGATACAGATATTGTGCCAGAAACCGAA
TCGGATAGTGATGATTCTATAAGCAGTAGCATTTGTGTAACAGAATACAATCCCGAGCCT
ATTAATATGACAGAATTATCTAATGCCAGATCTAATGAACAAAACCAAAACTCATCATTA
TCAGTTATTAATATGGTAGAGTCAAGTATAAGAATGAAAGTCGATAGGCCTGATGCAATG
GATACCAATATCGATAATTCTGAAACTCCATCTTCTATGAACAACACTACAGATAATAAT
TCTATAAGGATGGCAATAAGAAAATCAATGAATGTCAATGAGAACAAAAATGATCCGGAT
TCTGCTGTTATGGCAAATGATTCTTCTATTCGAATGTCATCGGATGATAATATTAGATAT
TCAGTCGTAACTAGTTCAGATTTAACTAGGTCTAATGTGGAAACTACACAATCAGTGTTA
GTTGAACTTGTCGCTCCATTAGTTTCGGTTTCAAATCTGATTTCACAACCCAGTCTTAAC
AATGCCACTGAATGGTTTCGTAATCAAGTTGATTTAGACGCTATTAGTGATGATGAATCC
GACGATGATGAATCATCTCAAAATACAGAGGATGATGAACAAGACGATATGAATGAATCT
GCTACTGATTTATCACAACCTTTGGCTCAACAAACTCAAAGGAACATTTCCAATTCTTCT
ATTCCTGATAATTCAACTAATTCTGAAAATCAAACCAATGCATCCACTAACAACCAATCA
ACAACTACTAATACATTTTGTGGACTTCAAACAACACAAGTTCAATTAGCTAGAGCATTT
TGTTGTTTAATCAAACTTCTTGCCGGCCTCCTTAATCAGATGTATTATTCTCGAGAAAAA
ATTGCTACTGCAAATCAAAAAGAATACGATCGATTTGGTTTAAATTTAGGCCAAGACAAT
GATTATTCAAATTCTTGTTCTCCTGGTGCTACTTTGAGTCAATCTGAAGAAGAACATGTA
ATGATCGAATCTTCTCAACAATTGGGACCTATTTGGATTTGGATTTGTCAATCGATGGAT
TTTCTCGAATCGCAAGTATGTTTTGCAAATGCCTTTCAGAGAAAACATGGCACTTATAAA
GCTCGTCCTGTTCCTACGTTTGAACAAGTAAATGCACCAAATAATTCTAATAATACTACA
TCATCTGCTGATTCTAATCGAGCTGATTTTCTGAAATATGTCGTTAGTATGATGCGAGCT
AGTTCCGATGAACATGGAGATCAAACACCAACTAGTCTTAATGCGGTTAACTATTGTCAT
TTGGCATATTTGGTCGATGCTTTGTTGTATTTAATGCGTATTTGTTTTGGTATGGTTACG
GATAAAGTTTGTTGTGATGGTGTAGATCCATCTGGACCCACTATAGATGATTGTGTTACA
TCAGTTACCGATCATTTAAAACGTGAGCCTACTTTGGTTCAAGAAGAAGTTGATGATGAA
CAAGAAGATTTAAATAGTTTAAGACCCACCATCGGATCAGAATGTGGTGATAGTATTTTA
GATGAAGATACGAATTGTCCTACACTTACTTGTAATTATCAATCTTCAAATAGTTCTAGT
TTGCTTAAACCTCAAATAAATCATAGAAGAGATTGTCAACAAAAAATTGATACATCAGAA
GACAGTTTTTTCTGTCGTACTGATTACCCACAAGGTTGGGTTGGTTTAGGTGCAGATCCA
TTCCACACTCCTGTTTCTGAATCATTGCCATTAGCTTATATGCCTCAGCTGTTGCAACCT
CCTGGCCCATCTGATGAAGTTTTTGGAGTTCTAAGAAGATTTGAAATAGATCGAAATACA
GTCAATAATCAAAATTTTTGCGGTAGAAGTAATGAAGGTCAATTACCTCTTCATATTGGA
TTCTCAAGTCGTTGGGCAGATCGTCTACTGGATAATAATATAATCTTCAGTGGAGTGCCT
GTTGGAAACCTTAGAACTAGATGGCGTTATTCATTGGAATTATTATCAAATTCATTCGAA
CAAAATGTTGGTTTAGAACCTGGAAGTGTACTAGTGGATTTCACTCATTTTAGTTTGAAA
GAAACTCGATTTCGAAAAGAAATGGAGAAATTACGAAATCAATGTCGCCGTGATCTTTCC
CTTATTGTTAACAGAGAACGAAATATGATTATTTGTGATACAGTAACTCAATTAAATACT
GAATATTCTCGTCGAACTTCAACAAATACCAGTACTCCATTAATGTGTCATAGATTCAAA
GTCAAATTTAATGATGAACCTGGAGAAGGAAATGGAGTGGCCAGAAGCTTTTTAACAGTT
TTTGCCGAAGCTGTTTTATCAGAAGAATTGTTAATCATGAATACATCAATTAATAATCAA
AAGAAACAAACTTCTACAATATCATCGCGTGGATCACTTTATAATCGGAATGAACATAAT
TATATCCGTATGTCAGCAATTTCGAGAATTAGTACAATTGCAGCAAATACTTCGGCTAAT
ATTTCTCAGACAACTAATTCGATTCCATCAAATAGCTGGTACAGTGACGCTTTATCGATA
ATTGGAATGAATCACAACAATTCTAATAATTCTTCAACGAGGAATTTTCGTCCAGCTATG
AGAATTATATCTAATCCTCAATCAGTTCAGTCTAATTCATCAAATGTACCTAGTACTAGA
TCAATTATTCGATCAGATCTTTCATATACAAACACAGCCCATAGACTAATTTCAGCAATT
GCAATATTTGCATCATCTGCTCCACTTGTATCAAATACCTCAACATCTCATGTTTGGGGT
ATCACAAGTAATTCATCCAATCTTAATAACACGGTTCCTCAATCCACCAGTATTAATTAC
TCGCAGTATAATGTTGATAATTTACCATTAGTTTCCACTCCTCATAGACTCGACTTTTCT
GATAGGAATAACTCTAGAAGGTCAATAAATATGACTTCTTCTAGACCACCTACGTCGACG

ATGAATGTTTTATCTGCCACTCCATTTAGTTCTGTCTCTCTTATTGGAAATAATGTCAGT
TCTATGTCATCACATCTTCACACATTATCGAGTGTTTCTAGTCGTTCAGTGAATAATAAT
ACAGATATGATTTATAGTTCTTCTCAGATTCCGATAACTGTAACTTCAGAATCAAATATT
TCTTCAAGTGCTCATTTGGGAAATTTAGATCCAATAAATCTGGCTACCGGCTTTACTGCC
AGCCTAGGAAATCAAGCCCCTATTTGGGTAAGTTCCAATTGGCTGCCGATGTATTCCAGT
TCGACATCCATATCCAATTCATTAACTTCTCTGACTAATCTTAGTCGCATTCAAGGTTTC
TCTCCATTACATAATCAAAATCCAACCAATCGATATTTAGAACCTCCAAGCATTCCTACT
TCATCAATATCTAATATTCAATCCACCCAAAACTTAAATATATATGATATGTTGCGAGGC
AAACTTCCAGTAAACGATCCAATATATACAATGCCAACATCTATCAGATTGCCAGATTTA
CCTCCTAGCACTGTTTCAAACTTGTCTAACACATCTGGACTGACATCATTACAAAATATT
AGTATATTTGACATTTTGCGTAGTAGAATACCCGGAGATGATTCATTAATTCAACAAATG
GCATACAACCTAACTGCGATTTCAGGTCGATCGGATCTCAACGGTCTATTGCATCAACTA
TCCTCCAAAAATATAAATATGAATGATGTTATCAATAATTTAATGAATTCTATGGCTCAA
GTTCCAAGATTTTCAGATTTACATCAGCCAGAATCAGCAATTTCGTATAATAATGCTGAA
GGCTCTTCCGAAACATCTCAGTCTATGGAAAGTAACATGGAAGTATCAATTATCACATTT
GCAATATACCTCGACAGCTTATTAAATACAAATAGCAACAATAATAATTTCAATGCAGTA
AAAACACAGTCTGACGACTCTAATTCTTTACCTGAAACAAATGCACCCAGTTCCGTACTG
AAAAGTCCTACCATAGACCCAGAGCAAACACCTTTATTTTGGCAACCTGGCCATCAAGGA
TTTTATTGTCCTCGTCGAGTAAATCTTAATGACAGTAATCAAGAATATACTCGCAATAAT
ATTTATCGATCGGTTGGTAGAGTAATGGGTATTTCGATGTTAATGAATGAGACCATGCCT
TTGAAATTATGCAGACATGTCCTTAAGTATATTCTCGGTTTACCAATAGGGTGGCATGAT
TTTGCATTTTTCAACTCTCAACTTTATGAAAGCTTAAGACAATTAATGAAAGTTGCTCGT
GAAACCCAATTAATGGGATTAGCAGGAACTCCCGACGATCCATTATTGGAATATAACCTC
AATTTTACGATAGTGCCGAGAGAAGAAGAAGGTGGTAATGGTAATTCTGGTGATAGAAAG
ATAACTGGAACTAACAGTATAAACCTAATTCCTAACGGTGAAGATGTTGATGTAGATTCA
AACAATGTTTTCCAATATGTGAAAAAGTATTGTATCACAAGAATGATAACCATTGTAAAA
CCTCAATTGGATCAAATAAAAAATGGGGTATATGATGTTTTACCAAAACATGCACTTAAT
GGACTATCGCCAGAAGATTTGCGGCTTTTGCTCAATGGTACTGATGAGATTAATGTGGAA
GTACTAATGAGTTATACTTCCTTTCTTGATGAATCATCTTGTACAATTTCTAATTCTCAA
GAGAAAGTAGCAAAATTCAAAAAGTGGTTTTGGCAAGTTGTGATGTCATTCAATAGTGTA
CAGCGACAAAATCTGGTTGCCTAAtataaacgagtcctgcattacctgcaagtaaccaag
gattccaacttatatatagcaattattgaaatcaatttacattatcaaaaatatttttaa
aatctgtaacgtactttgttttactattgttagatgggtttaaagtactcataggattat
taatatcaaaactcgattccatagcaaaatttattcaattgaacaatgccagactgtttt
gcaaggaaaatcacgccaattaataattcgagaattacagagaacaaatttagatgtcaa
tATGGCAGTTAATAATTTATTATCTCGTGATGATGAAGCAGAAGAAAATGGAGGAACCTC
CGCTCCTGATGATTGGGAAGAAGCTGATGATTTATTGTCATTGCTAGATCATGCTGATAA
TCCGTTATTGGAAAATGCAATTACTGACGGAACTCTAGGAATAACGGAAGATATTTTCCC
CAGCATTCGATTGCGTCGAGAATCACTTTACTCTTCAATGGCTAGTGATATTGAATCATT
TGCTGATCGCCGCAAACGACGCAGATTGGACTGTCAATTACGCAATTCTGTTGATGCATT
ACCACGTTCATTTCCAGACGCATCATGTATTTCTAGAAATGGAAATGAATCTCCAACATT
TTCCGCTGATGAATGCACGAAAAATGATTCAACATCTCTGAAAAAAAAATTGTTTGGTGCT
TGGTCCAACTTTAGAATTTTGGAAAGAAAAATTGAAAGATAATGGAAAAGTAAAAGATTT
AAAGTTCATCAAAATAGCTGGTATGTACTCGGAGTTAATCGGAATCACAGTAGAGAATGA
ATTGCATCAATGGAGATGGTGTGATATGGAACCATGGAGTAGTGTAAATGATTCTAATAA
TTCGACAGTTTTGCAAAATTCAAATATTTGGAATAATTCTATACCGAATTCCTCTGGTTA
TATAGGAGCTTATTTTCATACAAGAATTCCGAGTCTTAATTTGTTTGGAGAGAAAATTAT
TAAGTTGAGTGCATGTTCAAGTCGCGCAACTGTATTAACAGCAACTGGGAAATTAGCAAC
TTGGGTTGATGAAACGATTGCACCGATTTTCCAAGTATTAAGAAATTCCGATCAAAATTT
TACCACTCAAAATTTGCGAATAGAACATCCAGTTACACAATTTCCAGAGTTAAAAGATGA
AACGATATTTGAATTGTTTACATCATCTCTTGTTTCAGCGGTTCGATGTAATTCTGGCAA
TATTTACTGGTGGGGTGTTATTCCTTTTAAATATCGTAAAAGTGCTATGGAAAAAAATCG
AGATCGAACTCCGAAACCTGATTTAAAATATGCTGGTCAACGCAGTAAACGTAATAATAA
TAGTAATAATAATATAAATAACAAAGATATGACGAATATTATTCCTGGATCATTTGTTTG
TATGAGAAACGCTCCAATTTTTCATGCTGGAGCTATTGGG
>DjScaffold3233:241-1157 strand:+
aaatatttgaatttcttgaattatttttgaaaatatttcctaaaaaaATGGCAAGATTAA

CTGAAATGGAACTTAATGATGCATTAGAAACCTTTGCCTTATTTGACACTAAAGGTGATA
ATAAAATTGAAGCTAGACAAATTGGTGAAGTTGTTAGAGCTCTTGGCCTGAATCCAACTG
AAGCTGAAATTAAGAAATGTGGTTACAATAATCCTGAAGAAAGAATTTCATTCGAAATTT
TTATTCCAATTTACCAAAGTTTAATGAAGCAAAAGGTCAATTCAGATCCACAAGAATTTG
TCGAAGGTTTCAAAGTGTTTGACAAAGATGGTAATGGTATGATTAGTGCCGCTGAATTGA
GACATTTATTAACGGCTATTGGAGCTGGTATGGAAAATGCTCAAGGACAGATTAGTTATG
AAGGTTTGTTAACTTACAAATTTTGTAAAATTAAAGATTTTCACATATTAATTAAATTGA
AATCATTTCTAATGTCAAACGCTAGTTATTGGTTATTTTAAtgtatctaattcacatttc
gatactcttgatgttgtttggtgttgtgtgtaggttgattattcattattttgtttttaa
tctgtgccgattaacatttcaaatttgtgaaacattagaattaattaatatgaaatttat
tggtaagttaatattaattgaattttaaaagcttatttttaaaatatgagttcaattccg
gtattcaataatgtatttct
>DjScaffold3967:17563-34001 strand:+
aaatactaataaattttatggaatcgaagaaaaatacaaaATGAATTTTTTAACTACTGA
AATTATTGTACAGATTTACTATTTTTTAAATATTTTTCACATCATATATTGTTCTGGAGA
AATACATTCACCATTATTTCGAATAATTCCTCCTTTGAATATTTATCCTGAAGAATCAGA
ATTAAGTGTTAAAGGATTTAATCCCGAAGAGAAAACTGTAGATAATAGAATAATCACATT
ATTTGAAACTGATTTAAACGGTGTATCAAACTATGGATATTATATGCAAGTAGGCATCGG
TACTCCGCCACAAAAATTCAATCTCGTCATTGATACTGGCAGCAGCAATACTGCTGTAGC
AGGGACTCCTCAAAAATTTTTGTCTAATTATTTTAAATCTGAACAATCATCCACGTATAT
TGATCACAATTTACCAGTTGGAGTGCCTTATGATTCAGGCAGTTGGAAAGGAATTCTTAG
TTCTGACATTTTTACTTTTAATGGAATAGCCTACATTGATGCCAACAAAACTCTAAAATA
TGGCCAAGCAATAAATGTTAGATCTAACTTCGGACTTATTAAAGAATCAACAACTTTCTT
TCCAAGAAACGGAAATTTCCAAGGAATATTAGGTTTGGCTTATCCAATTTTGGAACAGCC
AAGTAATCTTATTCATAAGTCTACTTTATTTGACGATGTGGTAATGAAATATGGTCTGCC
AAATATGTTTTCAGTTTTGCTATGTGGGCACAATAAATCTAATTCGAAACCGACAACCAA
TGGATTCTTTATTGCGGGTGGTCAAAATGATTCTTTATATAATCCACCTTTAATTGATAT
TCTTATTCATCGTAAATGGTTCTATGAAGTTATTATAAGTCGTATTTCAGTTTCTGGAAA
GCCAATAATACTGACAAGTTGTAAAGAATACAATCGCGATAAAACAATTTTGGATACGGG
TACAGCAGACTCATATTTCCCTGGCAGAGTATTTCATTCTTTGATTTCTTCGATAATTTT
CAATATGCAAAGTACTCCATTTTTTGTTGGAAAAAATTCTCCTTTTTGGCAAAGACGTGC
TGTCATTTGTATCTTATCCACCAGTCCTAATCATCCAGCATCGTATTTCCCAGAAATTCA
TTTTGAATTGGTTATCAATGATACCTCATCTTTAGTGGTCTCTTTATCAGCTCAAGTAAA
TTATTTTCATTTGATTCAAATAGTTTTATGTACTTAAtacatttattaccagttcatata
ggttcgttaaaatggaatatttactttttattcatttattactgacttttgtattattgg
ctttgtgagatatttttagcgtttaaatttttaagataaagaataatattatattggtaa
gaaaataatgttagtttttgacttgaaaatttttgatctgaaataataagtcaaagacaa
taactaaactttattaaattataatttcaattattaattatttaaaagatgtgattataa
aggtggtctgtatcaatttcgtgtttgttttaataattcaaaaaattccgtcatttcaat
tgaagatttcaaatatggaacgaa
>DjScaffold3967:115559-115918 strand:+
ATGCAAAAGAAGATCAAACTTTTGGGACACGTTATCAGTGAAAGTGTCAAAATAGATCCA
ACTAAAATAGAAGGAATTAAGAACTTTGGTAAACCGAATTGCGTTAAGAAAATGAGAAGC
TTTTGGGGAATTTGCAATTACTATCGAAGGTTTATTAAAGATTATTCAAAGAATGCTAGA
TTGCTGGAACAGCTGTGTAGTCGCCCAAGTAACAAAAAAATTGAATGTGTGAATGAATTA
AAAGACGTTTTTAATGGAATGAAAGAAACTTTAATGAATTTATTTTGGACACGGATGAGA
GTTTCGATACAATTTGGGCAGTATTATCTCAAAAGGACAACAATGAGCAAGAAAGAGTAA
>DjScaffold3255:48295-57988 strand:+
aaaatatttcagaaattcaattttaatacattgaaaatttgagtcaattcttatttatag
ggaaacgaataatttaaataaacaaattaaaatcattgatatttttataggttcattgga
aacagctttggcaacatgtggtggatttagtgtcggatcaaaatatgtcgttgatcatca
acgtttaagtgggcaaggttattgtttttcagcatcattgcctcctttactcgcctgtgg
tgcgagtgttgttttagaaATGATTAAAACTGACGAAGGTGTTTCAAAACGAATTAGGTT
AACTGAAATTTCTAACAAGATATCAGAACTATTTTCCGGTTCTTTGGGTAAATATTGGAT
TTTACAAAGTGAAAAAATATCTCCGATAAAACACATTCGATTACGACTTAACAATACTGA
AGAACGTTTAGAGGAAGCTGTAAATAGTGCATATCGGCAGGGATTATTAATTACTGAAGC
CAGATACCTTAAAGAAGCAGAGTATAAGTGCCCTCAGCCAAGTATCAGACTTTCAATAAA

TTGTGCCTTAACTGATGAAGAAATCAGTTTCTTCCGTGATGTAATGGAGAAAATTGGTGA
AGCCTTGTCAAAATAAataatttggcatttggtttcagaaataatattgtgataatgatt
tgataacgaatttattctcttgtgaaatgttttataaaaggatttaattgttttagctgc
tcaaaagtaaataattggattatttttctcttttttaaaaagaaacaaattatattttt
gttcaaa
>DjScaffold1213:97311-98417 strand:+
attaattaattatttctataattagcATGGATGACATTGGGACATTGACACTACTTAAAC
AAGGAGCATTTGGTGAGGTGTTCCTCTGTAGTAATAAAATGTCAGAAAATCAATTTGTAT
TGAAGAAACAACTTTTTTCACAAAATGATCTTTATAGAGATATTAGCATTCAATGTAGAA
TAAGAAATTGTAAAAACATCATGAATTATTTGGGATTATACAACATGAATGGCAATATTT
GTATTGCAATGCAATATATGTCAGAGGGATCTATCCGATCATATTTAGATGAAGGGAAAC
GTTTTTCTGAAAATGATTTATTAAAGATTTGTTATCAAATATTATTGCAAATCAGAGATA
TTCACAAAGAAGGAATCATTCATAGAGATATTAAATGTGACAATATTGTTCTCGACAAAC
ACAATAATGCAAAGTTGATTGATTTTGGATTTTCTAAATTCATTGAAGAATTTTCTAATG
AAGAATTACTGAAAAATCTAAGAGCTTACAAACCTGGAACAGTGCGCTTCATGGCACCAG
AAATTCTAAAATGTGAGGAATACACAAATAAAATCGATATTTGGTCATTTGGAGCTGCAA
TGATAGAGATGATAACAGGAAAATATCCATATTATCATTTGGATGATTTACAAGTTTTCC
AGAAAATAAAAATGAGTGGTAAAATTGAATTTGAACCACCCGAATCAATTTCACAGGAAA
TTATTGAATTCGTCAATTTATGTTTGAATAGTGATCCTGCTAAGAGACTAAGTGCAGAAC
AATTAATTAGAGAGAATATTTTTAGAAAATTCCAAAATTAAaatatcctagttttaatga
tagtttttcatcgtgacatttttgtaaaatgtttgtaaataaaatcttaattttttttat
tttatcaataatagaaaaatatctcaaattaagataacctaatagtaatgagattgtacc
a
>DjScaffold1213:170004-173880 strand:+
tgatggttcacacggtttgtaatattcattatttaagaactatcacagttcatccggtct
atcaagttcgataataattaaaatttaaatattattaatcaactaaactttgtgtaatgt
ttgaaaagattggcaggaaatataaataatcaatttctaaataattataattgaatatta
tatacaacgaacaagttttggcttcaaactatgaatatcccagaaagtgttttaattgtc
tatacaaattattaattgataatgttctaataatttcgataaacaatttgttatttttgc
atttagggttctacttatttaatcattgaatatttgtttcaaatatatcgaATGATTGTT
CCAATGACACCGTATGTTCTTTTTCTCACTTCAACTGATCCACTACTCGACAGCCATTGG
TTCAATGCTGTCCTTGTAATATTGTATATCTTAATTAAAGCCTATTATTTGTATGATAAA
GTTTCAGAATTCATAATTGATATTAAAAATATCGATTTGCGATTGGACATGTGCAGACCG
TACATTGGCAATGAAGAATTTTGCTCAATTTGTCACAACAAATTTTCAGATCCTATGATT
CTACCTTGTGGGATATATTTTGTAAAAATTGTTTAATCGGTTCGATAATGCGATATTCT
TTA
>DjScaffold1213:196369-197075 strand:+
taaataataattcacataattagtcgaatggatgtaaattgtacattcccttggttaccc
caaggtcaaaataaaaaaatttaattgtgataaagccgtgttggtgtgccgaaatgaatt
cctcactatcgctagagattatgcgtgtctcagtcgcaacaaggaaaaacagattacatg
ctgtctctgatttaattttggaatcgtgtatgatcgattaaatttcgtgaATGATGCGTT
GGATTATAGCAGTTTGCTCAAAACTATCTCACGCACTTTCAAGAAGATGGTTTGGTGGAG
TTCCCATTGCTAAATTTATCATTGTTCCTTTGGAAGTTATTTACTCTGGTGGGGATGCGG
TGTGGTTGTACTTATGGCGAAAACAAAACGACGAGTATACAGTGATATTTCATCGGCTAT
TGGAAATTCTCTCTATGATGGATTTAATATTGGATTTACAGGGATACAATCACTATTTGC
CAACAATCTCATTTAATCAATGGACTTATTTTATAATGTGTTGTACAGATTTGGGTTTAT
CACCCTTTTAAtgctgatgctgttgtgattcttaatttatttctttcggatataatgatg
cagattcatgatcaa
>DjScaffold9:4822-10603 strand:+
ctatgttatgtttcacatttataaattctaaacATGAAAAATTTATTTAAGAATTCGAAA
GAAAGTTCAAACTATCAATCTCCAAAACGAATTCCAGATACTTGTTTTCAAGATAATACT
CATTGTGCTATACCAATCTCATCGAGATTGTCTCCATCCTTTGAAGAGTTGCTCAAATTG
TCTCCAGCGAGGCAAATGTTTTTGAGATATCTACGTTTACAGCGTTTACAGGGAATATTT
TATTGCCTTCGTGATTTACTAGTTTCAAATCATTGCAATATGAATTGTAATTTTCCTGAA
AAAAATAATTTAAATTTCTCCATAAAAACTGCATGCAGCACAATTTTGGATAAAATAAAT
TCTGAAAATGATGAAAATAATGAAATATTGATTTATCGAGAGCAACTAGAGAATAATTTG
GAACAGAATTCCTTTCAATGTCGCGTTGTAATGATTTATATGAAATATCTCTCGATCAAA

GCAAGCGAATCGTCTTTTGATAATTGCAATTCTCTCGATTGCATCAAGGATACAAATATT
ATTTACCAAATGCTCGTGGAAATATTCACTGAGTGTCATCAGAAACCGATACCATCATTC
CAAATTTTAGAGAAATGCATTTACAATTATTTATGTGAAAAGTGAgttattttagatgat
gataaattttattatcgcgattcaattactgttgtagattatttgtaataaaaatagggc
aaatcaatgttttcatctattagtttgacttaaaataaaacttaagacaacgaataaaat
ataaagagaatataaataaacgagtttattctataataaaattattaacaaaatgatcta
taccgttttattgtcaaaatgagaacagtaaaattcaatatgagaatttttattttgatt
taaaataattaatttaatactaaatatttttcattcaat
>DjScaffold9:27724-35585 strand:+
aatgatgtatctatgattctaaatacacaaatttgatattcaggtagcctatctctgata
attttgtttggtatcaatttggcaaatgaaaatttaataatttattatttaaaatctatt
taaaaatcatATGAAACGAAATGAAGTCAATTTAAATTTCTGCAATCTTAAATTACACTA
TTTAAAAGTACGCCCCCCCAAATTTATACTATTTAAAATTACTCATCGGGGCGCAGTTTT
TAGTAAATCCAAAAATTATATAAACAAACTAACGATCAGTGTATTTTCTGAGATTAAATT
TATAAAAATATCTAATAATAATGTTTTTAAATTATTCAGGATATTTCCGAATTATTTGAA
ATCCAGTTATCACAGTCAATATCAATTCCAAATGCTTAGTTCAGGGTTAAACTCTCTTCA
ACTCTCGGATTTCATTTATAGTGACGAATATATAATGATAATTACCGAATTTTTAGAATT
GAAAGGCCTTAATAAATATTTGGCTTTTTTATTAGCTGTAATAAATTTGGATAGGACGAG
CGACAAGTGTTCTTTTGAGGATATTCTTAATGTATATTCCAGATATCTTGTCATTGACTC
AGCTGATTACATTGAAATGCCCAATTTTATCAATAATATCGTGGTCGATAAAATGTGCAG
CATTGACGGATCCGGTATCGCAAATAAAATGCAACCGGCAGTCATTTTTGAGTGTGCTTT
TCGACCAGTTTATTTGTTTCTGGAACAATATTGGCTACCAGAATTTTTGACATGTTCTCC
CTATCAAAGAAATCTGAAGCAATTAGGTCATGTGATGGAAACAAGCCAGTTACGACGTAG
GAATGTATCAGAAAGCATTTGCAACCCACCTCATTCATTAACGAATGACATCCATAATTC
TGATGATGTTACGGCTGACAACACAAGTGTGCAATCGGAACCTGTTATGTCGTGTTCAAC
TCATACAGATAACTCATTAGAAATGAGTGGTATCAATCAAATATGGTGGCCAGATGAACG
TATTGAGGATCGGTTGTCTAGATTTCCGAGGCCCATGTCTCTTGGTAGTTTTGATGATTT
GGGTCGATATCAGCCTTTAATGACGACCAGTAAACGTCAATCCGGTGTTCCTAATTCTAC
ATCTTCTGAGATTCTAACAACAATCAGTGAAATTGGATCAGATTTCATAAATAAGCTTTA
TATCAATTCAACCCAGGAGGTAATCTTATCAATT
>DjScaffold3060:1000-8709 strand:+
attttatatttgattacataatatcgatttactatcaagtatgaggcgtaATGAAAAAT
TCATTTAAAGGAATCAGATCTGAAGATCTTCCAGATGGATCAGTTATTTTACCAACCAAA
CTTCAACCGTCGCCATTAAACATGGAAAATAACTCGGGATTCAAAACCGTTATTAAGCCT
GCTGACTATATTGGATCATTTCTTGTATTTGCTCCAACTCAAAAAGATCGAACTGAAATA
GTCTACAAGAAATTAGAATCAATGAGGAATACAACTCATCGAAAAAGCGTATTATTGGTT
GTATCGTTAAGTGGAATAAAAGTTTGCAACGAAACTGGCGAGGTATGTAATTTTGTTATT
TCACAAATGCATAGTTTTCTTTTTAAAAATTTGTATTTGATT
>DjScaffold3060:22799-35671 strand:+
gaaattattaatgtcatttttaaataattattcgaataatcagatttataaatattgta
taattaaaattttgtttaaaacttacagagtgtaagaATGGCTCATGCACTAAGGCGAAT
TTCTTATGCAACAAGTGATCCTGACTTTTGTCAATTTTCATTTCTTGCAAGAGAGCCAAA
AGGCCAAGCTAATGCTCAATATTGCCATACATTTATTACAAAAACCCCTGCATTGGCAGA
AGAACTAAATGATATAGTTGGTGAAGCTTTCCGTTTAGCTTATGCTAGACAAAAACAACA
GAATCGTGGTTTATTAACTAAAACACCGTCTTTCAATGACATAATTGAGCGTCAATTGCG
TCGTCAGCGTCCTCATGGCATGGAAATTGCGATTGAAGAAGCATCAAATGAAAGTGTTAC
CTCCAAATCAGATTTGGACAATCAATTAAAAGAAGGGGATGACTATAATTTACTGACAAG
TGATAAAAGTAAAAAGTCAGTACAACCGAATGTCAGTAAGAAGTTAACATCAAAGCATAA
TGACAATAGTCCTTTGACTGTTATAAAAGATAAGATTGATCGATCTCCTACATCAACAAA
CGCTACAAAACTATCTGATAGTAATAGTGATAGAGCTGTGCCAAATGCATTATGCAACCC
TAAAATCACTCCAAATAAACCTTTGCCTGTTCCTCCGACCAGTATTTCTCATTTATCAAG
CTCACGACAGACCAAAAACAATTATGATGAAACAGATGATCTTGTAGCGGAAGTATTACT
TGAACATAAGAACATTAATGACAACACTCCACCATGGATGTATGCTAACAGACGTGTTCA
ATCAGCCTTTGCGCCACAATCACAAAAGAAACCAATTGTTGGACCACATCAAATTCCACA
TCAGGACAAAATCATACCTTTTCCTGATCGACCATGTTCTGGGCCTCATCCAAATATAAA
ACTCAATAGTTCTGATCAGTATAAAATGAAAGAAATAGCTCCACTTCCTCCTCAAAGAAT
GGACTCTTTGCCTAGTTATGATTTGGCTGAAAGGGGCTGTGCTGAGGGAGAATCCGGTGT

AGGTACTGGTGCTACAAGTGTACCAGGAAAATATCACGCAACAGTTGATGTATCAACAAT
TTCTATAGATTCCACTAGTGGTAGTAGTGAAAAATCAGCGAGTCACGATGTGCTCATAGA
AGCGATGAAATGGCCTGAAAAATGGTATCAACCAGAAATACCTCGTGAAGTTGCTTTAGA
TTTGTTAACACAAGGCGAGATTGGAAGTTTTGTGGTTCGAGATAGTTCAAGTCATTCTGG
ATGTTACGCTTTATCAGTAAAAGTTTCTAAAACTGATAATCCAACTGGAGTTATACATTA
TTTAATTCAAAGAACATCTACTGGATTTAAATTGAAAGGATTAGAAAAAGAATGGCCAAC
AATGAAATCATTAGTGACACATCTTACAGTAATGCCAGAGATGTTACCATGTACATTGGT
ATTAGTCACTCAAAAACGGCACACTACAACTACTTCGAACCCTGCATTTTGTCCAATAGA
TGCACCAAAACTTTCGGGTTTAGGAGCAAATATTTGTGACACTCAAGTAGACGACGAAGA
AGACATTGATTATCAACGACTTTCTGATTTTTCTTCAATAATGGCAGATTTGTGTTTATC
GTAAtgtttatcaatttgtgcaatagcatccctgtgaaatttctggactttttttcaata
gtttccggttttcattaatatgaaacatttgattttagtttttataatctcgaacaatgt
tgtcaaatcagttctctatttttctttgattatgttattatataattcaatatttttatatc
tattagattgaaactacctgttgataattatacagaacttcatacttacatttatataaa
tatatattcaattcatttacaatccaaacaatgaattgaacctttccaattatgatgtta
ttattgtttattatagttatattctgaaattgttgataaggatgttgattttgataataa
tgaagattaataatgataattataattataattatgattattttgatgttaattaattta
tatttctatgttgtgattcaagttttagctatctctctcgatgtatttttaattaactat
cgattttgtttttcttttgaatttgagatgattatatgtagattcaaaagagctagtgat
ggaataatttgtgcgcttaattttgatgccttcgaagaatattgtttacaaaactgtaga
ttgttgaaattagtttgcttattatctctacctatctatctctgtcaccctttatacata
tatatttatggtgtagactgaacaataatgattaggatgtgtttttgtacagaattatta
ttaatgatattgtacagatcaaagattaagttgttccaatacgaaatatttagtttgaaa
gcattttattacattattattactattaaccagatatgccatttcgtaaatatgtgaaat
atatttaaatatatatatttgcagttatagactatttacaatgttggtcggcgttatatt
ttctttccaaaatatgtcaatgcaatgttttcaataaatgttatgtgattggttactctt
ttcagttgtaattcttgagtaaattataaaaaaatatatgaaggatgtatggtaaaattat
gttggcgttgtggaattttatatttttgattgttattctaattggtttgaaatggtgta
ttttttgtataacaatgcatgattaaatatttctgccagattgtttgtatgcccaattta
ctattttatgaattgcaataatatctacgttccatttgtttataaaaaaattttcattaat
ttgctgtttgtaactattgattactctgtacactttttaaattcatccacagagt
>DjScaffold4616:8832-12016 strand:+
cttaaaaattaatattttttcagATGGATTCAACAATTTGTTTATTTGACGTTGATGGA
ACTCTTACTAAACCTAGAAATGTAATCAACAATGAAATGCTTAATTTACTTGAAAGATTG
CAAAAGAAAGTTAAAATCGGAGTTCTAGGAGGTTCAGATTTTAGTAAAATATTAGAACAA
CTTGGTGGAATGAACAATTTAAAAAAAATTGAATATGTGTTTTCAGAAAATGGTTTATTA
TATCATAGATTTGGTGAACTTGTTAGTCAAACAAGTATCATCGAAGTTTTAGGAGAAGAA
ACTTTGCAAAATTTCATTAATTATTCACTAAAATATTTATCAAATATTCGACTTCCGATC
AAACGAGGAACATTTGTTGAATATCGAACGGGAATGGTAAATATTTCTCCTATTGGCAGG
AACTGTTCGCAGAGCGAGAGAGATGAATTTGAGAGATACGATAAAATTCATCAAGTTCGA
AATAAAATGATTGAGGATCTGAAGCAAAATTTTGGTGATTTAGGCCTAACTTTTGCAATA
GGCGGACAAATCAGCTTTGACGTGTTTCCTAATGGTTGGGATAAACGTTATTCATTACGT
TTCGTCAAAGATGATTTTAAAACAATTCACTATTTTGGCGATAAAACATTTGAGGGCGGA
AATGACTATGGAATATTCAATGATGAATTAGTAACTGGGCACACGGTAACTGATCCCGAT
GATACAAAGCGTCAATTATCGATTTTGTTTGATATCTAAtttttatcaattcttttagg
gtttctttatagattattccttttggtaaactagtgtctcctatttaattttatataata
tataataataataatcattaaaatcataatcaaa
>DjScaffold517:52644-53165 strand:+
CAAACCATAAATAAAAAGGCTGTGTGTGAAATAACAAGTAAAAATACAAATTCCACGATA
ACAGGTTTTATTGAAATTCATGAAACAAATAACATGATAAATATATCTGGCACAATAAGT
GGTGTAAAACCAAATGGTGTACACGCATTTCATATTCATGAGTATGGAGATTTAAGAGAT
TCATGTAATAAAGCCGGGGCACATTATAATCCATTTAATAAAAAGCATGGTGGACCTATG
GATGTTGATAGACATTATGGAGATTTGGGAAATGTACAAGCCGATCTGACTGGAACAATT
TTTATCAATATTCAAGATGCAATTGCTAAATTAAATGGAGTAAATTCTATAATTGGAAGA
TCAATAGTAATACATGAAAACAAAGATACTTTTGAATCTCAACCTAGTGGTAATGCTGGA
AAAAGAATTGCCTGTTGTGTAATTGGACTAGTTTAAtttcacactatttattatgt
>DjScaffold517:112876-127767 strand:+

ATGTCTGGTGTTGTTCTCGAAACTTTAAGTTGGAGAAAACTGGGAATTTTTATAACTATT
GTATTTACTTGTCAGTTTATTTGCTTTCTTATTGGAGGATTAATTGCTCCACATCCAAAT
GGTCATACGGATGTTCTTACTGAAAAATGTATTGATAAAGATCCTATACCTAATCCAGAT
AAATGGTTCTATTCTCGAATGGAAGGTCAAGATGGCACCTGCCAGGATGTAATGCCAGAT
GATATTTTAACAGACAAGGAAACTCCAACTAATTTAACTGCGAAACAAATCATTTTTGCG
GCTCAATTTCCTCTTCCAAGAAATGGAATTAGTCTAACAATGAGCAAATGGTTTCAGCAA
CTTATTGGAATATTAAATACTGATATGGATAAAGAGATTGTGTTAACACTGGATGTCAGA
CTAGGATATAAAAAATAAAGGACAATTCAAATGGACTGAAATTGCTAGATCAAAAGAAAAT
CGACCTCTCAGGTGTGTATTAGATGCAGAAACAATGAAAAAGAAAGCAAACGGCGAAAAG
ATCGATGGATATTATTATGATTGTGAAATTCTTCCATTATTTTCTCTTGGGAGTTGTTTT
TACGATGAATATCTTTTGAATATTCGTATCCCATATGATAGAAAAAAAGAAATCAATACA
GGATTAGGAAAATTACAAGATTTATGGGTAACTGAAATTCATCAAAATGGAGGTTTTACA
ATGGTATGGTTTGCTTTGAAAACTGTGATGTTTCCATTGACATTATCAGCCTTGGTGTTT
TTCTGGTATAGAATTACATTATTGGAACGGAAACCAAATTTGCTCGAACAAACTATTATT
TCATTGGGAATTATGATGTCAATTTTAAATTTTCCAATCGAATGGGTTTCTATATATATT
TTATTTAGATATAATTTATCTTTCTGGTTGGTTTTAAGTGACATTCGACAAGGAGGATTT
TATGCAATGCTTCTATGTTTCTGGGTAGTTTTCACTGGTGAACACATTCTCGATGACAAT
GGAAATGCACAAGCTATAGCTTCTTATAAAAGTGCAAAAGAACGTCTCTATTATTACTGG
CCCCAATTAGTCCCAGTTGGAGCCGCATCGTGTTTCTTATTTTTATTTGAATTGGCTGAA
AGAGGAGTTCAACTTAGTAATCCATTTTACAGTGTTTGGTCGGAAAAGTCTACAGCTTAT
ATTGCAATTGCATTTGTAGCAATGTCGGTCATTTGTGGACCACTGTATTTTATATTTCTC
TTGTATAAAATTGTTAGAGCAATTAAACAAATGCTAAATAAACGGAGTATTCTTCCTAGT
TTGCCTGAAGTTCGTAGAAGATTTTATTCCGGTGTTATCTATAGATTCACTTTATTCATA
ATATATACATTATTGTGCGCAACGCTAACAATAATATTCTTTTTAGTTAGCCAAGTAAGG
GTTTTTAATACTGGGAATCATTAA
>DjScaffold868:4361-47227 strand:+
acaccagataatgtatctatttctccattaggaaaattggtctttgctataaagaatcaa
tataaaccattttattatactccaaatattttatatttgcatcaaacttgggcgcaatta
aattatgatgtaaaactcaaggctattctttattctcttgggaaaatgttggaattgtta
atggaacacaggaatgaaagtcaatcaccaaaaagtaatgatctaagtgctataataaac
gagttaattgattcaaaagacggttctataacgatttcaaaaattcaatcgatttgtggt
aaaatcgctaaaagtttctcatttaaggataaactaatttcagtttatcatgaaagtgca
aataaaaataatgtgatcaaatcattaagtacttcaaatttaaatatcaatactcctgac
aagatctatgaaagaaacaattctattagagttttagaaaaacaaaaatcactcaatgct
gattctacttccttgtcttcaactatgagtggaaatactggtaattctaattccttacat
aattcaagaaatggcaatatttatcttggtcgtctggataattatgtgaagccaaatatt
cccgcagcactagaacgtttgaaagctcaaaggaaagtatcgaaaaccaatggacctatt
aaatcaccaagagttccagttgaaggaataagaaattcaaatgatctagaacaaatcaat
cttagaaattctataagtagtcaaaatatctccttcacggaaatgtctaacattgaaagg
ttaaatggggatcgaaaaattctcggtcctgagtttattattagaaaacctgaaacattt
aatgtttcctgtcatttgctggtagtcatttttagtattactcattaattaacaattaaa
ttgaatattttgagtctaaaccagagatttctaaataatgttcgtattattattattatt
attattattattattattattattattattattgttattattgttatcatcatcatt
tataatattaatattattttatattttccatatgtttagacgaagaattcttctttttga
aagaagatacaaatatacaaaaacttgctgattatttatttcgtaaaaataattcaATGA
AAGTTACAAATGGAATCGAAAACAAAAAATTTTTACATCTTTATTACCGAATTAAATTCT
ATCCAACCGAAGAATTAATTTATGAAATGAATTCTTTCAATCATAAACTCTTCTATCTCC
AACTTAGGCACGATTTGTTGTATTTAAACAATCCTCCAATCAATTTATCGGAATCGTTAA
CATTTGCCGGCATTGCTATCCAAGCAGAATATGGTTTTAATATCAATAATCTCGTAGAAA
ATTCAAAATATCTTAGAATAGAATATTATTTCTATAAATATTTTTGTCAATTTCTTAAAA
TTAATGACAATGAAATAGAATTTTTAAAATTACAACAGATGCCATGGATAAATATGAATT
GGGAAACTTGTGAAGCTTTATTTATTGAAAAAATTTGTCAGAATAAATTCTATGGAATGC
ATTGGTATCGTGTAAATAAAAAATCAATATGGTATTATAGATGCAATTGGAAAAAACACAT
ATTTAGTAATTGGACGTGATTCAATTCAGTTACTAGATTCAAGTCGATCAGACTCTGAAA
TACAAATACATAAGCATTTTTCATATAAATGGAGTAAAATTGACTCTATTTCAATTAAAC
AAAGAAAAATATTAATCAAACCAAAAATGCAATTAAAAATCGAAGGTTTGAATAAAACAA
CAAATTCATTTGCTTGCTACGTTGACAGTAAAACCAAAGCTGAACATATTATCAGATTAT

GTAATGAGCATCATTTACTGGCTTCACAAAACGATGAAACAGAAAGTAATTACAACAACA
ATCCTAGCGTCTTTGGATCTCATCATAGATCGTCAATTATTGATTATCCTTCCTCACTAA
CAAGAACATCATTAACATTCAATGATGATGAGAATGATAATGGTGATGATAATAATAGTG
AAAGTCTAGATGACAATCCAGTAAGTGATGATGAAATTTGTCAAAATTCTAAATGTTTCG
ATGAAAGGTCCAATTTGTCCAAACAAAAAAGATTTTCTGCGCATTCAATGATTAATATCA
GTGATCGAAAAATAAGGCAAACAAATAATACAAATGGTAACAGATTTAGCAACCTTGACC
ATGAAAATTACCGAGCCAATCTTGGAAGAACTCCAATAAAATCCCAATCAAAGAAAATCC
AAAATGAAAAATCCAATAATGAATTTTTTCAAGTTGATATAATTAGAGAAAATGTTTCCA
ATGAAAATTTGGGAATTGGAATCATTAAAATCCAAACTCAAACTTATGAAGGAATGTTTG
TCAAACGTATTGTGAAAGACAGTTCAATTGATAAATGTGGTCAACTGAAAATAGGTGACG
AAATCGTGTACATAAATGATCAGTGTGTGTCAGATTTAACGCAGTCTGAAGTTGGAAATG
TAATTAAAAACTGCGGTGAATCTATAAGATTTTTAATACGAAAACATCCAAGTTTTAACG
TTGATTGCAAGCAAAAAACAGTTAAGAAGGCTTTGAGCTTTTCTCAGCCAACTCATAATT
CAATG
>DjScaffold868:55724-60703 strand:+
tttattattaatcaataatttcagtcgcttacacaaatccttcattcaaataaaccattt
gttttctttgaaaaacttATGTCGGAAAGAATATTTCCAAATCTCAAATTATTTAATGAA
CAAATTCGATCAACAGTTTCTTCTTCAAAATTTGATGAAATTGTGAATCAAGAAATAAAT
TCTTTACAAAACAAAAAATTATTGATTTCAAATCCAGCTTGCAATAAAGAAAACAATGAA
TTTGGAAAAATTGTAAATATTCCAAATGGAAATAATGTAGAAAAGTATGCCAATTCTATT
AATAGAAATAACTATAAAATCAAAATAGAAATCGATAAAATATGTGCAAAAGAGAATCTC
GAAGTGAAAAATTTTAGCTTTACTTTGGTTCCAATAAAACAAAAAATCGATCAGTCTTTT
TGGAACTGTTGTATTGAAATGAAATATAAATTAATTATTTTATTCATGGATTGTCATCAA
AATGGTTTCAATTACACTCAACAAATCATCAATACGCTAAATATTAAAATTCAGAATGGG
AAATTTTCAGTTATCGCTGAGGAAATTCACGAATTTTCTGAGACAATAAAATTTCTCAGA
CTTTCTGTCAGAATTGACAATGATAAATTTGATAATATTACAAATCATCGATTATTTGTG
ATCGGATTTATTAATATTAGTGAAGATTTGAACAAATATAAATCAAATGAATCAATTTTA
CAAGCCATCAATATTGCATGTGGTATAAATGAAACGTTTTTGAATTCTGAACCAATCATC
ATTTATTCCGATCAAATTCATCAGAAAATTGGAATGTTTATTACAATTTATTCATTTATT
TATTGTGCTTTACATAGAAAACTGCCAATAAATCTTTTAATTCAATCTCAGACAACTATC
TTTGCAATATACACACAAATTATTAAT
>DjScaffold3459:913-6228 strand:+
ttttttcaatttttttaggagtgaattttttttattaaagtagatatagattatATGACGA
AAGAAAGTGAAGAAATATTAGAAAATGCTCGTTTAGCTCGGGAATATGCATTATTGGGTA
ATTATGAGTCTTCAGTAACATATTATACGTATGCAATTATTCAAATAAATAAATTGGTGT
CCACTATAGATAAAATCAGTTTGAGAGACAAATGGGTTAATGTGAAAATGGATTTAACTC
AAGAATCTGAAGACGTTAATGGAATAATTAATATAATTTCTAGTTTTAAAATTGAAGAAA
AATCGAGATATGAAGAACCAACACGAGATCCAGATAATCGCGCATTTGGTGGAGTTGATA
AAAAACCATTTAATACTCCTAAACTGGTTCCGCACGTTCCTGCTTCTGCTAAAATTGCTA
CGAAAAATGTAACTCGAAATGTAAAGCCCATCAAAGATGTAAAGAAACCAACTGCTAAAC
CTGAAACTCCAAATCGTGTTGCTAATCCAGTGGTTCAGAAAAAAGAAATTAAGAAATATG
TTCCATCATTTCCTGCTGAACAAGGCCTTATCGATATTTTAGAAAAAGACATAGTTCAAA
GAAATCTCAATGTCAACTGGGATGATATCGCTGATTTAGATGAAGCCAAGCGGCTTTTGA
AAGAAGCAATAGTTTTACCTCTTCTATTACCAAACTATTTCACAGGGTTACGAAAGCCAT
GGAAAGGTGTTTTGATGTTTGGTCCACCGGGTACTGGGAAAACATTGCTTGCTAAAGCAG
TGGCCACTGAGTGTCAAACTACGTTTTTTAGTGTAACTAGTTCTAGTCTTACGAGTAAAT
GGCGAGGAGAATCGGAGAAATTGATTCGAATTCTTTTTGACATGGCGAGATTTTACGCAC
CAAGCACTATTTTCATTGACGAGATTGACGCTATTGGATCGAAAAGAGACGATGGATCAG
GAAATGATAGCAATCGTCGAGTAATGTCGGAATTACTCATCCAAATAGATGGAATTTCTA
GCAGTAAGGACCAGGAATATGACGCGACGAAAACTGTTACGATTTTAGGTGCCACCAATT
TGCCTTGGGATCTCGATGAAGCTTTACGACGTAGATTGGAAAAGAGAATTTACATTCCAC
TTCCAGGAGGTGTTGGAAGAAGAGTTTTGTTAGATATCAATTTGAAATCTATTGACCTTT
CGGACGATGTCGATTTGGACGCGATAGCAGAGAAATTAGAAGGTTACTCCGGATCGGATA
TTACACAAGTTTGTCGGTAAattct
>DjScaffold973:42595-43589 strand:+
gtttgtactattgggaatatttattttatctattATGGTGAATATTGCCTTTATTATTCG
ATGTATTATAGTTCGCAGCTATCTTGATAAATATAGTGAGATATCTCGTAGAAAGAAAAG

AATGCCTATATGGTCGTGGGTGACTTGTTTTTGTTTGAAGGAAGAAATCAAAGCCACAGA
TGCATATTATAAAACCAGAGAGGATCCTTATGATAAAATTAGAGATCATCCAAGCTCTGA
AACTAGTAAAAGTAAATCATCAATTACAAATGATTATTTAAAACGTCCTACCAGTCAAGA
CACTGAAAGACTATGTTTTTCATCAAATACTAGTCAAATAAGCAGATCATCTACATTTCC
TGCTAGACAGTCTATAATTGTGCATACACCAACTGACCAATTCTCTATTTCAGCCCTTCC
TGGAAACACAATTGATAATTTCCAGCCTTTTATGATCCAATATGCAATGGACCCAAATTC
AAAAATTATGATGCCTATTATTAATACAAATCGTCGGATGAGCTCACATTGTATATATGA
TGATGTCCCCGGGGAACATATTATGATGAATCCGTCTTCTACAATGCCTATTCGACATTT
TGCTAGCACAGGCTCATTGCAAAATCGTTCCAGACAGGGCACTAACACGAATTTGAAAAC
GTCCCCTTTACCTGAAGAAACACAATTAAGTTTCACCAAATTTAATAAAGAACCTGTAAA
ACCAGATGAAGAGATTGGATTGATGATGAATGATTGTTCGTCTGTGTTACAAGATTATAG
TGGTTCCGAGGAAACATATTTATCAGGGAAAGACAGTGACTCAATGAAAGATCCAGTAAG
AGTCGCAAAATTAGAAATGAGTAGAGAAATGATGGCCAAGGCCGACACATTGCTACGAGA
AGCACATAATTTACTGAAAATGTCATAAccactcatttactgaataatgaag
>DjScaffold1636:32005-32738 strand:+
attgaagtttttcgtatttttgaatctcaacaaaacatttgccatcgtttttacttat
tcaataataaaaataatattaaaaccaaattttattgattcaatatcaacaattttatat
ttatttgttaaaatattttctagattgaatcgattgtgaaatcattgaatATGCATTGCA
CAATCAACTGTGATCACAAATTTTGTCCTAAAATTCTGAACAAAGATAAATTGCCAAAAG
GTGAACGGATTAATTCAGGATCGTTTGGCAATGTATATAAATATAAAATATCAAATACAA
ATCGAGAAATTGTTATCAAGAAAACAAAGCAAAGTGAAGACAAGAAAAATATTGAAAGA
>DjScaffold4659:27518-28090 strand:+
ttttctatttaactttttatctataaagattggtgattttatatttacatatttgaaatc
agtgaaaaatttcctacctaatagatacctctttagtcaatacaaatgcaattccgttgc
attaaaaagcaagtacatattgatgattggttgattttaattagttaggttgtgttaaaa
caaatttgtatataaaactatttattttaccgtttacatataatttcaaaATGATTTTAA
GAAATGCAGAAGAAATATCTGAACGAGATAAAGTTTTATTATTTGTTATTTATTTATTCT
TTTTTTCAGATCTTTCAAGAAGAATAATAAATGAAATGCTTCCAGAATGCGAAAGAAAAT
CCAAACTGTTGGAAGAAGAATATTGGGACTATGTTCTGGAAAAAGATTTGATTGATATCA
AGAGAGTTTTAGACCTTTTCGAAAGCGAATTTGAAATAGTATCAATGTGTGGAACCAATC
CATGTCCATACTGGATAATGCGGGACGTTATTTTAGAAAGATTTCCATGAatttgtttgt
tattaaat
>DjScaffold3891:81593-82134 strand:+
ATTAAGGAACATTTGAAACAAAAAGAAAGTGAAGTCTTCAAACACTTTCAACACAAACAT
AAAATTTCACCAGAACAACATCACATTTCATGGAAAACACTAGGAACAGACTACATTTCT
ACACTACATAGAAAGAAACATGAAAAATTACTAATTAAAGAAGACAATTCTATTTACAAT
TGCAATTATTGCGAGAATAATTGTTGGACATAAccattgatcgtttttcagaattcattt
aaaataaaagcttcgagcaaaattttttttgataataattctagaattgactaattcct
gacaaagacaaagtcgaaaactagaattattatcaaaaaccaaaaaaacaaatttccata
gaagcttttattttaatgaacaataataataataatattaataataataatgataatagt
aataataataaaaataacaacaattataatattaatatttataattatattaataataaa
atcagtccaattataatattattatcaataattatatta
>DjScaffold1975:11941-12470 strand:+
ttcttccaacaaatcttcatctaacaaacaagagactaatctctcatgttgtttcacatg
agtgATGTTTTTAGCTTCAAAATACAATTCACACCTTTTGATAAATTTCTCAATATCATT
TTCTCTGCAGAATATCTTTGGTGATAACCTCTTAGTAATAGAATTGGTATTTACCGCTGC
CACCATGTTAGGTAAATCTTCAATTGTTAATTCGTTTTGCAAATCAGAATTCATATTGTT
AGACATTTTTTTATTTTACTACTACAAACTATATCTTAATATTAGAAAGGATTATAATAG
TAAATAAtaataattataatatccagaaattactcaatttctattggctcaaaattatta
gataataaatatttcctcataatgattttttttcttcaatccacttgaaaggatcttcttg
gatatctttgaattctaaattaaaaaacagtaatatttcaaaaataataatattaaattt
accatatctaataatctatta
>DjScaffold1975:36152-38155 strand:+
ATGCAAAGTTGTGAAAATAAGTATTACCAAGATGATAATAACAGATGTGAAAACGATGAT
AAAATAGAATGGCAGTCGTTACAAAGCTATAATATTTGTGATGTAGGCGATATCGATGAA
CAAATTGAAGAATGTCCTAATGACAAAGAAACTGATTACAAGTATAAAGGTGAGTCTGAA
CCAGAAGATATCGGCTGTACTGATCAGTGGTTGCATTTACATTTTCAACAGATGGCAGCT

TTATTAATGCAAGGTACGGAAGATTTCATCAGAAAGGTTTGGAGGCGTGGTTGGGGTCTT
GTGCAACATCAGCATTTACCTGCGTGGTTGAAAGATAACAATTTTCTCGTCAGAAGTCAC
AGGCCAGAATTAAAGTCTTATAAGAAATGTTTTCAATCGATATTTCGTGTCCACACTGAA
ACTGTTAATATTTGGACTCATTTATTGGGTTGGTTGGGATTCCTTGCAATCGCTGTCGTG
ATTCTCTGTCAGCCATTCGACCATTTGCCGTGGCAGGACAAAGCTGTTATTATGTGCTTC
TTTCTGGGCGCAGTTTTGTGTTTGGGATTCTCGTGGTTGTTCCACACGGTTTATTGTCAT
TCTCAACGAGTTGGCAAAATATTCAACAAGTTGGATTACTGCGGCATCGCGTTTCTCACA
GTCGGATCATTTATTCCCTGGCTCTACTACTCGTTCTACTGCAGATTCATACCCAAAATT
GTTTACGTCTCACTGATTGTTGTATTGGGTGGCTTCGCGCTGATAGTATCGATGTTCGAT
AAGTTTGCAACACCGCCATATCGACCGTTACGCGCTGGCGTGTTTATCGGATTGGGATTG
TGCGGAGTTGTTCCATGCTTGCATTGCATAATTGTTGATGGATTCTGGAATGCAGTTTAT
CAGTCCTCTGTTGGTTGGTTGTCACTGATGGCGCTGCTCTACATCACTGGTGCGGTGATC
TATGCAGTGAGGATCCCTGAGCGCATTCTGCCTGGCAAATTCGATATTATTTTCCAGAGC
CATCAGATTTTCCACGTGTTTGTTGTTGTAGCTGCACTTGTTCACTACTATGGTATCATT
CTCATGTCTGGATATCGCCTCAGCAACGGTGATTGTGCTGATCCAGTCGCGATCACGCCG
CTGTTGTTGTGAgcagcactagagcatggttcattcttgtaaatattgaaatattgattg
attggttgattggttgaacatgtgattggatgatgggcttattgatgtgtgtctgtgtgt
gagtgtgtgttgagaacgattggttgaattattgatttattctgtgatcagatcagatca
gatttatcctcatttgattattattattatt
>DjScaffold4506:3534-4907 strand:+
taaatttacaagaagttgaaacaaatcaaatatatagctcagaacaagtaaactatataa
caatgacaaactcaaatcaagagtcagaattatcatcaaataacgatcttccagatcaga
tggaagagctgctataggtattaacgttacaactaaaggaattggaaaaggtaaccaatt
cagtacggaaagtattagtggaagcaatagtagaaaatgtcctaaaggcaaggctatcga
aattcagagaacaaggaaaaagaacgaaattcgcaatgaaattgttgttaaaaacctgga
acgaaacagatgaagtaacagtgaaacccacgatacctaaacaaaaagaaaactacaagt
gtaaagaatgtggtaaactcgtgcatttggcaaccttgtgctggaaaaataggctatgcg
aatattgtagggaatcagatcacccaaagaaactctgttggaaaaaaccatgcaaagtat
gtggcaaattagaacataccgagaaacaatgtaaaaaggaatcggatatcgaacctatct
ttagagagagggttgcacaagatgtggagaccacacgatagcttaatgtggggtaaaatg
caaaaaatgtggtcgcaagggacatactgaaatagtatgccaaccggacaaacaatgtac
taagtgtagaggaagaggtcatctggcagaacaatgtggtattcctcgatgtttcaactg
tggtagaaccggacacaccATGAGAAGGTGCCGATTTAAAGCAAGTAGTGAAGACTTAGC
TATAATAATGGAAAGATCAAACAAGTTGTGTATGTTACAAATAACTAAGGAGGAGGAAAC
AGTGGAAATGCCTGATATTTTAAATGAAAACCAAACAATGTCACATGTGGAACAAGATAA
TTATTGGACAGAACCAGAAGACTGGCAGAAAAGGCTTAATTCATATGATGACCGGATATC
CAGATTAGAAGAAACTTTACAATTGAGTCAAGTACAATGCCAAAATTTCAAGTTCTTAGC
AGATAAAGCAATGGGAGAAAAAAGAGATGCTATATTTTTAAGGAATATAGAGTTAGTCCA
CTTACAAAGAAGAATATCATTCAGCCGAAACCATATATCCCAATTGGAAGAGGCATTACG
TTCAAGCCAAATACAATGTCAAAACTTTAAACTATTATCTAAGAAGACGATAGAAGAACA
GAGAACAACCCAGGAAAAATTATTGGAA
>DjScaffold4506:84674-88751 strand:+
actgttttaatgatctccaatgatcataataaatcaaaatcacctacatattattaaatt
cagtattttaattgtctttgtaaatacattgaggtttttggtaggttttctcaatttac
tatatttgcaattattatgagtatcattttattaatgctttatagctccattgtctgcat
acttatcagaaagatttactacgtggaaaagtggattatttgcatcatttcttgcagtat
tatcaattataATGATGGCATTTTCATATAATATTTATACTCAAATCACATCAGCTGCTT
TTCTTGGTATTTCATTTTCAATAATTTACATACCAACCATGAGTATTCCTGGAAAATGTC
ATATAATCAGGCAAAATCTATCACAGGGTATCGTACTCGCTGGTGCATCTATTGGTGGAT
TAATTATGCCACTTGGTTTGTCTGAATTAATTGACACTTACTCAATTCGTGGAGTATTGC
TAATAATGGCAGGAATAATTTTCAATTTTGCAGTGGCTGCAGTTTTATTTCCAACGCATA
TATTTAAGATAACAAACGATAATAAACTGGCCATTGTAGCAATCCAAGATAGAAAAAAGA
GACCAGAAGGAACTATTTTACAGTTTGGTAAATGGAATTTTCCAATTAACGATTTTCCTC
AAAAGTCTAAATTAAAATGGCCACCTCGGAAATTATGGTTAACAGTTCTATTATATTCGG
TTGGCATGACAAATGTTATGAATGGTTATATGCTTGGTAGTGGCCTATATGGAGCTTTTA
CAAGATCATTATCCATCAATATACAAACGTCATCTTATATTCTTATTGTTCAATCATCAA
TAGATTGTGCTACAAGAATTTTTGTTGGTTATTTGATTGATTTCAAATTTGTGCCTAGAT

GGATTTTATACTGTTTAAGTGCAATTTTGCTGGGTATTATAACAATTATAACTGGGTGTT
TTCCGTCAATACCTTTATTTTATTTTTATATGTTTTCCAATGCATTACTTGGTGGAATTT
TGATGGTTTTGCATGTAATGATTTTGATGGACGTAGTTGGAGTTGATTATATTTCATCAG
CATTTTCAATTTATATTTTTATCCAAGGATTTATTGCAATAATTTTTTATTATATAATT
>DjScaffold5143:10579-33247 strand:+
TTTTATCCTTTAACATTGAACATTGTCAAGAATTTATTGGATAAACTTTATGAAAAACGT
AAATCTGCAGCAATTGATATAGAAAAAATAGTTAAGCAATGTCAAAGCCAAGGTAGAAAT
GATGAAATTCTATTAATAATTAAGCATTGTGGCGAAACATTTTGTACCTCCAGTAATTGT
CATAATAGAAAAGGTGGTACTATAGGGCTTGCTGCTGTTGCAATTGCTTTAGGAAAACAA
GCAGATGAATATTGCAGCTCTATTATATGTCCTTCGATTAAACTGCTCCAAGATTCTGAT
GCAAGAGTACGCTATTATGCTTGTGAAAGTTTATATAACTGCATCAAAGTGTTGGGAGGG
AATGTATTGCCACATTTTCAGTCGATATTTGATGCTCTAGCCAAAGGTTATGAAGATACA
GATAAAGATGTCAGATCAGCGATTATTCAATTGGATAAATCTATGCGTGAAATGGTAATG
GATTCTGACAATCTGGATGTTGCCAGTATCGTAACATATCTCAGAGACAAGATTTATCCC
AATTCTACGCAAGCAAGATTATTCTTAATTAATTGGATTACTTCTCTCGATCGTACAAAT
GGAGTTAATATGGTTCTTTATTTGCCTGAAATTCTCGATGGACTTCTTATCATGCTGGAT
GACCAAGATGAAACTCTAGTTAACAGATTGAAAACATTGTTGAGTCAATTTCTGAGTAAA
CTTATTTCGATTGAAACTGATTTGGGTCGCCGTAGATTGATAAATATTCTTGTCTTTCAG
ATAAATAATCCAAAATCTAGTAATATAGTTAATCATACCGCTTTAGTGTGGCTGGCAAAA
TTTATCGAATCTTTCAAACACAAAATGCTTCCATTTACATCATTGATTTTGAAAGCTATT
TTACCGTTTTTTGATGTCGAAAGGAAAGACGAAAATATTCGTAATCAAGCCAAAAACGTG
AGCAATCTATTATTAGGTCTTGTTCAAACTATTGATAATTTGGAAAATAAAGAAACTAGT
GAGGATGAAGACAAATCAAACACTCCTTGTTCAGGCAGATTATTAGATGCCAATCAAAAC
CAATTTACTTCATTAAAAATAAACGATTCTAAAACTGAAGAATCATTAGAAGATCCTAAT
CAAATTAAGCAAACAATAAATCAGAAATTGAGTCCAATTCAAAATTGCGGCTTGGCTTTA
TCAGAAACTTTGATTATTCTCTGTGAATTTTCTGGCTGTTACAGTCAACAGACAAAATTG
GCTGCTCTTAAATGGTTACATACATTAATGGATGTTGCTCCTTACCAAACACTTCGTCTA
TCAGAAACTGTCTTTCCTTCATTATTCAAATTTGTTATTGATCCGTCTCCAGAGGTAATA
AAAGCTGGATTGCAATTGGTTGCTAAATTCTCTAAGGAAGATGTACCTCTTGATCTGGAA
ATTGAAAACAGTGATTTTCTTTATGTTCCAGAACAAATCAAAAGTGTTTTGCCGCCTACT
GATAATCCATATTTTCTGCGATTTATTTTCGATCTCATTCATCATCTTGATACTGCTAAT
GAAATGCTAGAAGAGAAAGGTGATTTAATTATCGGTGATCTCTGTTACCAACTTAATCCA
AATCTTGTGTTTAACACCATTGCAGAAATCATTCAAACGTCAGACTCATTACATGAAAAG
TTTCGTCTTGTTCATAAATTGAATCGAATATTATTATCAAATCAGAATTTGGTTAATTTT
AGGTCAATGATCAAATCGATAAATTCTGAAGAATCGTGTGATATTTTCTGTCGACTTTAT
CGAGCTTGGTCATATAATCGAGTCGCTCTCCTCAGTCTTTGTTTACTAAGTCAGAACTAT
GAGCATTGTCGAATACTTATTAAGACTTTTGGGGATCAAGAAATGAATGTTGATACATTA
AATGAAATAGATTTGCTAATACAGTTTCTTGAAGCTCCAGTATTTGCATTTCTTCGTATG
CATTTAATGGATCCTCGTTATTCTGCTGCTTTGAGTGAAACTCTTTACGGTCTTCTTATG
TTAATTCCACAATCCGAAGCGTTCAAAATTTTACAGAATCGACTCAATTGTATTCCAAGA
CATAATATAACTAGTCTTTTACCTTATAATGATGTTTCTGATCCTAATCACGAATTGATT
AGGAAATTCGTTAATTTTGATAATTTATTGATCAACTTGAAAAACGTTCAAAATCGAGTC
CACAGTCAAATGTTGAAATCACAACATCAACAACAAAGTATTCATAACGATTTATCAAAA
GCATCATTTATGGATACCCATTTGAGTTTGTGAcagattttatgtgcataaaacattttt
atttacattttcattagttcaaatttttatccaataattattaatgatatttaaaacaaa
gttttctcttcatcataagaagatttttgattattaatcgttgttttttacagaaaatg
aaatttgattcttgtttttaa
>DjScaffold5143:46312-47763 strand:+
taatatttcatttattagATGGAATTAGCTCCGAACAATACTATTTATGTTAATAATTTA
AATGAAAAAATTAAGAAAGAAGAACTAAGGAAAGCTTTATTTGCTGTATTTTCGCAATTC
GGCAGAATTTTGGAAATTATGGTCAGTAAAAGGTTAAAATTGAAAGGTCAAGCATTTGTT
ATATTTGAGAACATAAAAAGTGCAACTGAAGCAATTAATTCAATGCAGAGTTTTCCTTTT
TATGATAAGCCAATGCGAATACAGTATTCGAAAACTGATTCTGATATTATTGCAAAGAAG
AAGGGTACTTACATTGAAAGGCCTAAGAAAAATCCTGAAGAATTAGTTAAAAAGAAAAAG
AAACGCATTACTATTAAATCAGAAGCTAGGTTGATGAAATCAGGTAAAAGTAAAGGAAGC
AATCCTATTGATCCTAGTCAAATGCCTGATCAACCACCGAATAAAATTTTATTCCTGACT
AATTTACCTGAAGATTCAAATGAATTAATGATAAGTGTATTATTTAATAAGTTTAATGGG

TATAAAGAAGTTAGAATGGTTCCAAGCCGTCCTGATATTGCTTTTGTGGAATTCGACAAC
GAAAATAATTCTGGTAGGGCTAAAACTCAACTGCAAAACTTCAAGATTAAACCCAATCAC
CATATAAGAATAACATTTGCAAAGAAATAAatgttatcggtgtgattttgtaaagtctct
tgtgtatgatggagttatagttaatgaatgcaataaatattaaagctctcttttctatcc
attataaagtcccagtgcctaacaaatgcagttggcttcggtagtagactttaaatttca
tttttctgttactattgaaactaatagtattttagtggatataattgatgcaagttgtca
gttttcacaaaatgctggctacagtttattgatctaatcagatgataattcagatgcttt
ttctgatcgattttaaaataaaatattctaaaacctcaagtgtatctgctgttgcagtag
accatattccaaaagctgtaatttacaaagccaaaggatatcgaatatcaaattaataag
tttgttaaactttacagagttgtttttttagattaaattcatattaataaatattaca
aaattcttgaatttactttaatgtctgccttaatatttcatttattagATGGAATTAGCT
CCGAACAATACTATTTATGTTAATAATTTAAATGAAAAAATTAAGAAAGAAGAACTAAGG
AAAGCTTTATTTGCTGTATTTTCGCAATTCGGCAGAATTTTGGAAATTATGGTCAGTAAA
AGGTTAAAATTGAAAGGTCAAGCATTTGTTATATTTGAGAACATAAAAAGTGCAACTGAA
GCAATTAATTCAATGCAGAGTTTTCCTTTTTATGATAAGCCAATGCGAATACAGTATTCG
AAAACTGATTCTGATATTATTGCAAAGAAGAAGGGTACTTACATTGAAAGGCCTAAGAAA
AATCCTGAAGAATTAGTTAAAAAGAAAAAGAAACGCATTACTATTAAATCAGAAGCTAGG
TTGATGAAATCAGGTAAAAGTAAAGGAAGCAATCCTATTGATCCTAGTCAAATGCCTGAT
CAACCACCGAATAAAATTTTATTCCTGACTAATTTACCTGAAGATTCAAATGAATTAATG
ATAAGTGTATTATTTAATAAGTTTAATGGGTATAAAGAAGTTAGAATGGTTCCAAGCCGT
CCTGATATTGCTTTTGTGGAATTCGACAACGAAAATAATTCTGGTAGGGCTAAAACTCAA
CTGCAAAACTTCAAGATTAAACCCAATCACCATATAAGAATAACATTTGCAAAGAAATAA
atgttatcggtgtgattttgtaaagtctcttgtgtatgatggagttatagttaatgaatg
caataaatattaaagctctcttttctatccattataaagtcccagtgcctaacaaatgca
gttggcttcggtagtagactttaaatttcatttttctgttactattgaaactaatagtat
tttagtggatataattgatgcaagttgtcagttttcagtaatattttatatctatattaa
cataatattcatagaaaaataatatttgcattgttctatgtataaatatatgtataaaat
agatttaaaatattatttataataaaattcattattaaaaataaattatatttttctaaa
ttaaaagtaagacattaagtaaattttagcaaaatgctggctacagtttattgatctaat
cagatgataattcagatgctttttctgatcgattttaaaataaaatattctaaaacctca
agtgtatctgctgttgcagtagaccatattccaaaagctgtaatttacaaagccaaagga
tatcgaatatcaaattaataagtttgttaaactttacagagttgtttttttttagattaa
attcatattaataaatattacaaaattcttgaatttactttaatgtctgcct
>DjScaffold4403:38040-46306 strand:+
tctatttaagttgataaataataaaaaagaggtagcacacatgtatagaatgaaattata
gaatagaatttaaattctcatgaaaatctgtgtcagtaatttttaaaagattttcattg
gtcaaaaaaaaagaaatttccaaaactctagagatgtttcagtgcgctactaactaccta
ctgtaaataattaaaattattttgttaaatgtttgacatttatatagcgtttatttattt
cattattaatcttagttatttttaaatataatttaaaaatttatgtaaaaaaaattataa
ttttcacttggaaatctgatgaattttttataattttgtctattaggatgtcgcaaaaat
tttttaacagtaaatacggaaataattacaaaatttacaatttatgcgaagagaaaggat
acgatacaagaaaatttaatggtcaagttgaaagatttATGATTACAGATCACAATGTTC
CAAGATTAGAAGAAATGATAAAATTTGCCGAGAGCTCTAGTAAATGGTTATCAAAATCCG
ATAAAAATATCATTGCTGTTCATTGTAAAGGCGGTAAAGGACGCACTGGCACGATGATAT
GTGTTCATTTAATTGACAATGAAGTATTCGATAATGCGGAATTGTGTCTTCAATATTTCG
GTCATCGAAGAACTGATTTATTGCAAGGTAAAAAGTTTCAAGGAGTTGAAACTCCAAGCC
AAAGTCGATATGTTGGCTATTTTGAAAAAATTAGAAAATTGCCTGAATTGAAAGTTCCCG
AAACAGTTCATTTGAAGATAGAAAAAATTATAATTCATGAAGCTCAACATTTATTTGTCC
AAGATGAATCCGAAATTTGGTTCAAAATTATTCTAGGAAATTTGGAAAATACTCGAGAAA
CTAATATTTTAGAAACGACTCTTGGTGAAATCAGTAAATGTAAAGTGGAAAAGGAAAAAA
CTGAAACAGGAATCTCATTGAATATTATCATATTAGATTGTCCAAATCTTCACGATGATG
TCAAAATTAATTTTTACACTTTAAATAATAAAGTACCTCTGAAATATGATAAATGTGCAT
TTTTCTTCTGGTTTCATACAAGTTTCATTGAAAACAATCAGCTTTCACTGTCTAGAGAAC
AACTGGATAATCCACACAAGAAAAATACCTGGAATATTTACGGTTCGAATTTCAGAGTTG
ATGTACAATTTTCAAATTTTCCAACAAAAAACCAAATTTTATTATAAtttctaatgaata
ttgcttataattttacatctattaaatatttgaatttcattattcattttacaaaatttt
ttaaaagattttcattggtcaaaaaaaaagaaatttccaaaactctagagatgtttcagt

gcgctactaactacctactgtaaataattaaaattattttgttaaatgtttgacatttat
atagcgtttatttatttcattattaatcttagttatttttaaatataatttaaaaattta
tgtaaaaaaaattataattttcacttggaaatctgatgaattttttttataattttgtcta
ttaggatgtcgcaaaattttttaacagtaaatacggaaataattacaaaatttacaattt
atgcgaagagaaaggatacgatacaagaaaatttaatggtcaagttgaaagatttATGAT
TACAGATCACAATGTTCCAAGATTAGAAGAAATGATAAAATTTGCCGAGAGCTCTAGTAA
ATGGTTATCAAAATCCGATAAAAATATCATTGCTGTTCATTGTAAAGGCGGTAAAGGACG
CACTGGCACGATGATATGTGTTCATTTAATTGACAATGAAGTATTCGATAATGCGGAATT
GTGTCTTCAATATTTCGGTCATCGAAGAACTGATTTATTGCAAGGTAAAAAGTTTCAAGG
AGTTGAAACTCCAAGCCAAAGTCGATATGTTGGCTATTTTGAAAAAAATTAGAAAATTGCC
TGAATTGAAAGTTCCCGAAACAGTTCATTTGAAGATAGAAAAAATTATAATTCATGAAGC
TCAACATTTATTTGTCCAAGATGAATCCGAAATTTGGTTCAAAATTATTCTAGGAAATTT
GGAAAATACTCGAGAAACTAATATTTTAGAAACGACTCTTGGTGAAATCAGTAAATGTAA
AGTGGAAAAGGAAAAAACTGAAACAGGAATCTCATTGAATATTATCATATTAGATTGTCC
AAATCTTCACGATGATGTCAAAATTAATTTTTACACTTTAAATAATAAAGTACCTCTGAA
ATATGATAAATGTGCATTTTTCTTCTGGTTTCATACAAGTTTCATTGAAAACAATCAGCT
TTCACTGTCTAGAGAACAACTGGATAATCCACACAAGAAAAATACCTGGAATATTTACGG
TTCGAATTTCAGAGTTGATGTACAATTTTCAAATTTTCCAACAAAAAACCAAATTTTATT
ATAAtttctaatgaatattgcttataattttacatctat
>DjScaffold4403:47512-48862 strand:+
tgaaatctggatacagaattcactaactaatttaattattattaatcattttaattagtg
aaaaatttatgttacaaatataaataattaccaatagaaaacaattcagaacaaattggt
attgtcagctattatttaatcaattgttttgtatagaattatcaattattattaaacaAT
GAGCAGTAACAAATTAGAACATCTTTATAAATTATTAACTGAACACAATTTTGATGCTAA
TAAAAATATTCCAGATAAATTCAAGTGTGCAAATTGTAAGAAAATATTGATTGAAGCTTG
TCAGACTCGATGTGGATGTAGATTTTGTCGGAGTTGTGTTGATGAAATAATGAATAATGG
ATGCCAAGTTTGTCCTGGAATCACAAGTGATTGTAAAATTGAATCATTGTCTAATGTTGT
GATTGATTACAGAGCAAATAAACAAATATCGAAGTTAATAATGAAATGTCCAGAAAAAAC
GTGCTCATATGAATGTGAACTATTAGAAATGAGTGAACATTTGAGAATTTGTAGAAAGAA
AAGTGTGGAATATTCATTTTACAAGTTAAGTTGTGATGTGAGGAAAATGACAAATGAAAA
TTTGATTGAACATTTCCAATTAGAGAATTATAATCACAACAAAATATTGATGGACATGAT
TGAGAATATAAAAAAGGAGATTGAAGAATTGAAAACTAATGAAGAAATCAATAAATTAAA
ATCCAATTTACAATTGAAAGATGAAAAACTAGAATTGATAAATGTAGAAATGGAAATGAT
GAAAGTAAAACAATTAGAAATGGAAATTCGTTTTAAAAATGTCGAAGAAATGTTTGATGT
TAAATTTACAAAATTGAAATCAAAACACAAGAATCAAATGCAATCGATAAAGACAGAGAA
AAACGAACTTGATAACGAAATGCAAATTAGGTTAAGAAATATGTGGTATAAAATAAATGA
AATCGAATTGGAAATGAATAAACGATGCCAAAATGGTAAAATTGAATCGATAGAATCGGA
GTTGATTGAGAAAATTGAAATTTTACAAGGAAATTTTGATAATAAAATCGATGAGGTTGA
ATCGACAAATAAGAAGGAAATCAAATCGATAAATGATGAAATTCGATTGGAAATGATGGA
AGTATGCAAACAAACAATCGATAAATCA
>DjScaffold388:17812-18569 strand:+
agcaaacataaaaaataATGGGCCAAAAGGTGTCTTCCATTAAATGCAAGAAACAATCGA
AAAAGAAGCAAACTCGAATTGTTAAAACGATGGGAAAAGCATATGAGGGACCTCCTGGTA
GTTATATGGTAAAAGCTAAATACAAATATGTTGCAAGTGGAGATACAGATATTTCATTTG
AAGAAAAAGACATAATGTATGTCATTAAAGAATATGACTCGTTTTGGTTGGAAGTTGAAA
AAACTGATAAATCAAGAGGTTTGATTCCTTCAAATTATGTTTCTAAGCAAGATGGAAGTC
CACAGAGTGTTGAGGCATGGCGAGAAATTCAAAGATGGGAAGCCGAAAAACTATTGATGA
GAGTTGGTTTACAGAAGGGTACTTATGTAATTAGGCCATCCAGAAGTAAATATATGATGT
ATTGAtaataataataataataataataataataataataataataataataataataat
aaaattaataataataataataatattaaatattttcaaaattacgaaaataaactaagg
cttatatcaaaaatgtttttgatttttaaaatttttattaaagttataaaaatttcaaa
aaattttatttttctaaaatttctattttattttctttagttgttcgataaaaacgaa
aatttaggaaaaaatgaaatttttgaaattttagtagcttt
>DjScaffold388:36243-43007 strand:+
aaaagcataatgaatcatactcttttcaaaataagaaattaacataagaataaatcctat
accacaaaaaatatttattttaagcaattcccgaatttgggctgtatagggagggttgac
tatattattaagtatatttttattagaggaataatgtaatttggtaatattttgaataat

taaaactaaataaatttcaaatttatttgattacccataaatatacgttataaataaaaa
tttagtttcatatttcaacaaaaattaatttgttaacttaaatgttctttttttaattt
aagaagaaaattgtttcgcattatcagttagagattttgatgaaaagaaaaagctttgca
ttgttaaacattttcaaatcaaagtaacttatgaagataaaggaacactttattcgatta
acatcagaggatttcctgatattttaacactgattcaattttatgaaaaaaatggaattg
gaaatactcatattccattaagtgatccacttccggataattatcaaccacccgttcatt
ttcaggatattgaaattaatcgtgaaaatattgaaatattaagtgaaataggtagaggat
gctttggctcagtacataaagcaaaatggggacgagcatatgaagtagcagcaaaaATGA
TGCAGTCAACAAAAGCTGAACGAGAAAAATTCGTTCAAGAAGCTAAAATCATGCATAAAT
TAAGACATAGAAAAATCGTAGAACTTCTCGGAGTATGCACAGAACCACAAGACATGCCAA
TGATTATTATTGTAGAATTTATGAAAAATGGATCCTTAAAAGAATATTTAAAATCCGAAG
ACGGGAAACAAACCAATCTTTCTCAAATGATTCATATGATGGCTGAAATTTCCGAAGGAA
TGGCGTATTTGGAAGGTGAAAAAGTTGTACATCGTGATTTAAGAGCAGATAATATTTTGG
TTGCTGATGATTTAACGGTAAAAGTAGCTGATTTTGGATTAACAGAATTAACAGAAGGAA
ATAATGACAGTCAAGAGAAAAAGAATTTAAGATTTCCTTATAAATGGACAGCTCCGGAAG
CAGCAAAAACGAAGATTTTTACTTCAAAATCTGACGTTTGGTCATTTGGAATAGTAATGT
ATGAAATTTTGACTTGGGCAAGCCCGCCATATCCAGATACACCCGCAAAAGAAGTTATTG
ATAAAGTTAGTTCAGGATATCGAATGCCAAATCCAGAAAAATTTATACCAGGCGCTCGTT
GTCCTGAAGAAATTTACAAAATCATGCTCAGTTGCTGGAATAACCTTCCCGAAGAAAGAC
CAACATTTTCATCTCTGCAAGAACAAATGGATACTTTAATTGTTGACAATACAGGAAGTG
GTACCTATTATAGTCACTCAAAATAAttattaattttcatggatatttgtggaattttat
atttttccaacaaatataataaaacttctagtttatttacatttttattgtgataatttt
aatattaaaaaatggaaattttatttactaaa
>DjScaffold388:27535-29637 strand:+
ATGCATCTCATCTTACGAGAAGAAAATTTTGAAAAATGTGTAATATACTTAGATGACATT
CTCATCTTTGGAAAAGATTTTGACGAACATAACGAGAGACTAAGAACAGTATTAGAGAAA
ATACGACAAGCAGGAGTAAAATTATCACCAACAAAGTGCACTTTTTTACGAAATTCAGTT
AAATATTTGGGTCATATAATATCTGAAAGAGGAATCGAAACAGATCCAGATAAAATTATA
GCCGTTCAAAATTGGCAGCCACCACAAACAAATGAGGATCTGCAATGTTTCTTAGGATTC
TGTAACTATTACAGAAAATTTGTCGAGAACTATGCCATCATAGTTAGACCTTTAGAAGAA
ATTCTTAGAGAAGCTAATATCATTGGAAACAGGAAAAAGATCGTAAATCTAAAATGGAAA
GAGGAGCACTCAACATCATTTTCAAAGCTAAAAGACGCTTTGACAAATTCACCAGTTCTA
GTTTTCCCGAATCGAACAGATACTTTCGTATTGGATACTGATGCAAGCCATCATGCTATG
GGAGCAGTTCTGAGCCAGTTACAAAATAATGAAGAAAAAGTTATCTGCTATGGATCAAAA
AAATTTACAAAGACAGAGCAGATGTATTGTGTAACTAGGAAAGAACTCCTGGCAGTATAT
TATTTTGCAACACGATTTAAACATTACCTTTTAGGCAAAAAATTTATCATACGGACGGAC
CATAAAAGTTTAACATGGATGCTGAATTGGAAAAAGCCAAATACTTCCCAATATTGCTCA
TGGATAGCAGAATTAGAAGCTTATGATTTTGAAATTCAACATAGACCTGGAAAATTTCAT
GTGAATGCGGACGCTTTATCTAGAGAGAAACAATGCGAACAATGCGAAATAATACATAAA
GACCCGAAAAAGAAACGGAATATAAAGGTGGTAAAATCAATAGATGAAAACCAGAAAATA
AATTATCGTGAAGTCAAAGAGATTGAGACAGAACGAGAAGCCTACGATATGAATAGAGTA
AATAAATTATTAAATTATTTTCACAATAGATTGGGACACCCAAGTGGAAGACAAACTCTG
CATAATGTACAAAGATACCACCAGTGGAATAATATGAAAATCGACATAGATACATTCATT
AACAATTGTATTCCATGTGCAGAAAGAAAAGAAGGAAATAAACTAGGGTGTAAGATCAAA
TTAGCATTCACCGCTTCAGAGCCATTTGAAACAATATGTGTAGATATCACAGGACCATTA
ACAACAGTAGCCGGTTATTCCTACATCTTAGGCATAATAGACGTATACTCCAGGTATGTA
TCATTAATCCCGATGAAAAATATTACCGCAGAGGGGATCACAAAATCTATACTAGAAAGA
TGGATAGCATATTTTGGAATTCCTAAAATCATTCATTCAGATAATGGAACACAATTTAAG
TCATCATCGATGAACAGATTATGCAAGGAGCTACAGATAAGTCAATCATATTCGTCTCCA
TATTACCATGAGGGAAATGGACTAATAGAACGGGCATTCAGGACGGCAAAAGATAAAATA
TTTACGATGAGCCAATCAAATAAACAAAATTGGGTGGAAACTTTACCATTTGCAGAAATG
AGCATGCGATCATCTAAATCAAAGAAACACAATATTTTACCATTCGAAATAATATTTGGT
ACTGAAATGAGATTAAGCACTAGGATCGCTTCAGGAGAAATAATGAATCAGAAAACCAAT
AAACAGGCAAAGAAAGAGTTTGCAGAGAACAATATTAAGGAGAAAAAAAACCAGCTAAAA
ATATCGGAAGAACCACGAACAGAACAAAACAAAGCCAAAGTAAGGGATTTGGTTATGGTA
AAATCGAACGAGAGGGGTATGCTTACGAAGAGATTCTCAGGACCATGGAAAGTTAATAAT
ATTTTACCTAAGAATAATTTAGAGCTTTTACTGAACAATAAAAGAATAACAAGGAATATT

CACCAAGTAAAACGGTTCAAGGGAAAGATTCGTCAAACTAATTACAATTCTGAATCTATC
ATCAGTATAACAACGAGACCTAATACATTAGAGTTACAGAAAACCAAAAGTAAAGACAGG
TAG
>DjScaffold1337:22007-22849 strand:+
NNNNNTGGATTACGAATTAATCCAGAGAAGTGCCATTTGTTGAGACAGCAGGTGAAGTTT
TTGGGACACGTAGTAGATAGAGAAGGAATCAGACCTGACATGACAAAGATAGAAGCTATA
AAAATGTACGAAAGACCGAAATGCATAAAGAAGCTACGAGGATTTCTAGGAATGTGCAAC
TATTACAGACGATTCATCAACAATTATTCACAGAAATCAAGGTGTTTAGAAGAACTCATT
GGTATGAACAACGTAAAGTTAATTTGGACTGACAAGTGCGATAAAGCATTTACGGAGATG
AAAGAAGCATTAATGAAAACGCCGGTATTGGGATTCCCGGACATCACGAAAACATTCATA
CTAGATACGGATGCTAGTTTCGATACGATCGGAGCAGTTCTGGCACAACGAGACGAAAAT
GGGAAAGAAAGGGTAATAGCGTACGGATCGCACGCAATGAACAACCACGAAAGAGGGTAT
TGCATAACGAGGAAGGAATTGTTAGCAATATACTATTTCTGTAACCACTTCAAACACTAT
CTCTACGGAAAAAGATTCGAATTAAGAACGGATCACAAAGCAATAACATTCATGCTGAAG
ACAACAAAGCCGGTAACACCACAATTCCAGACTTGGATCAATTATCTAAGCGGACTAGAT
ATATACTTACAATTCAGACCAGGAATAAAACACACGAATGCAGATATGTTGTCGAGACCA
CAGTGTGGGACCTGTACACAATTTCAGATGGAACACGAAGAACCAAAGACGGGTAAGAGA
AAGACAAGACAGTTAGATATGATTAGCGACAACAAAGAAGACAAGCAAAGGGATAAACAA
TGA
>DjScaffold2999:47619-57098 strand:+
catgtttttgtcttttagctttagtttaaaattttaaattttgagcatcacgcacaacca
aacatacacaactgggtttttaatatatttattatttatgaaaattgaatacttaaaaat
tcttgtttttttagataaattattgaatagagtaatttgatttatataatctataatagg
ttgaaaattgatatgaaaaattgattaaaataaacaatatcgaattagatttttataact
aacaaaacgatagttgaattaaaatcgtatttttcaaatttgtacacaaataaaataatt
gagagaaaatatatgaaatttattgatatttatttcctttagatttttggtgctttaac
aATGGATGTTATTGCAAGAACTATGTTCGGAATGCAAATAGATTCACAAACTAATCCTAA
TAATATGTTTATAGTTCATGCAAAAAAACTCTTTAGTGTATCAGCTTTTAATCCTTTCAT
TTTTCTTTTTTTAATATGTCCTAATTTAGCAAAATTTATAAGAAAACTCGTTCCTAAATA
TGAGATTTTTGGACGAAGCACCATGAAATTTTTCACTAATAATTTACAAAAAGCAATTGA
TGAACGAGCTAAATTAACAGATGAACCGAGAGAATATAATGATTTCTTGGCATTAATGTT
AAAAGCTCGTAAAGAAATCGATGGAAATGATACTGAAAGTGTTCATAATCTCGATTCTAA
ATCTTTAACAAATCAAGAAATAATGGCACAATCATTTATTTTCTTATTAGCTGGATACGA
AACTACTGCAACTACATTAACTTATTTGGCTTATTTTTTGGCTAAAAATCCCAGTGCTCA
ACAAGAAGTTTATAAAGAAATTGTCGACTCTGTCGGAACTAATGGTGATATAACTTATGA
AAGTTTAATAGGAATGCATGCTCTTGATGCTGCTGTATGTGAAACACTAAGAATTTGTCC
GCCAGCGGGTAGAATTGACAGATGTTGCACTCAAGATATAGTGTTAGAAGCTTCTGATGG
TAGTAAAATAGTTGCTAGAAAAGGTGATATTTTCGCCATACCAATATATGCTATTCATAC
GGATCCTTCAATTTGGGTGAACCCGGATAAGTTTGATCTTCATCGATTCGATGCTGACAA
GAAGAACATAAAACCTTATTCATACATGCCATTTGGTATGGGTCCACGAAATTGTGTTGG
AATGAGATTTGCTTTATTAGAATCGAAATGGGCAATTGTGAATATATTACAAAAATTTGA
AATTGTTCCATGTGCTGAAACTTTGAATGAAATTAAATTTAAACGAACTGGATTATCTAC
TCCAACCAAACCAATTTTATTAAAAGTTGTTAAGCGACAGTGAaagaaatgaattctgga
ttttgtttatttttattgattaataaattattattattatt
>DjScaffold1742:6921-7349 strand:+
ctaggtggtaattcgaggtttaagcttaagtttcgtaatggaactgatttcgatcaaact
tttgagaataatATGCCAGAAAATAAAGTGCGTTGTTTAAAAAGAGCTAAAATAATGGAT
GAAGATAAGCCAGCACTGGGCCAAATTTACTGCATTGTTTGTGATAGATATTTCGTTTCA
CAAGAAGTTTTAGACTCACATGAAAAATCCAAGCCACATAAGAAAGCCGCAAGAAGCTTA
AAGAGAGACAGAGTAATAACACAGGCTGATATCGAATATTTTGGTGGAGTTTCTAGACAG
GAATTACCAAGTAAACGACAAAAAATGGAATAAaatattgtcgtgttatgtttttgaaaa
ctcttaatttgtaatttgcgtac
>DjScaffold1742:13422-14277 strand:+
TTACGTTATATTAAAAGCAATAATTTAGATCTTTTAGATAATGAATTATTTTGTGACTTT
TCAATACGAGTAAAGGAAGATGTTTTTCGTTGCCATAAGTGTATTTTGTATTGTGGTTCT
GAGTTTTTTAAAGGATTTCTTAATTTTAATACCAATGCTACTGAAATAAATCTTAGTGAC
GAAATAATTACTACTACTGGAATCAAAAACTGTCTAATTTTTCTTTATAAATTAGGCTTG

GATTCATTTGTTTCTGATGAACAGTGTGAGGAAACCTATATTGCAGCTGATTATTTGCAA
ATTAATGCTTTGAAATCTGTAGCTAGTGAAAAGATTTCCGGTAACATTAATTGCCAAAAC
GCATTTAGCATTGTTCAATTCTCAATAATTTACAACGATACAAGTTTACGTTTGAAAGCT
CAAAATTTTATTAATAATAATTTCAATAAGCTTTGTTTCGATAGCAAAGACTTTCAAACT
CTACAATTCGATGTCGTTTCAGAAATAGTTTCAGATAGAAATTTATCCTCTACAGATTCT
TTGATTATGAAATCGCTTTTGAAATGGGCAACTTATGAATTGGCTAATCGTCAAATATTA
TTGGAGAAAAATGTGGAAAATCTTGTGCAACTGCATTTGGTATGTTTAATTAGTATTAAT
TATTAActaattaatttttatggagttttaattgtttgaaatttaattaatttacgatc
atttaataaaataataattaaataaaaagagtagcttcttctttcgatccgatgttccta
gataaagaatactaactggagatcaca
>DjScaffold1742:53023-64525 strand:+
gtgtgataataataataataattataataattataataattataattataatatact
tatataacataaataatttcatataatattaagttgattattatattaaataaattaagt
tcataatatataataaaatgatatttttatttaatatcaaaattaatataaaaaataat
aaatattcaatatactaagtatttttagataataactatagttaagaatcgatatttat
cttaaatattgatttttactaaaaaatgaaaagcaacgataattgtgtatacattgatat
tttttgctggaaaataatataaatttaatttttggtttatttttctagttaaaatatta
aaaattaatactgagattttattcagatttcaaatgaacaatggaataattttgcaaaag
aagcttgttacttaggtATGGTAGAGAATATTAGACAGAAATCCAAGACTTTAAATTTTT
ATAGTGATAAACCGCATAGAATTGGAACTCCAATTGTTTTATACTGCGTCGGTTCTAGTA
GGTCAGATAGACTTGACTCGAATAACAATTTTTTCTATCTAGATAAATCGACTAGGCAAT
TGTCACCAATGCAGGAAGATAGACTAAATCATAAAATGTGTTCATCAAGCACATTTATTT
TTGTAATTGGAGGGGAAAGTCCTGATGGGTATATGCTTAAAAGTTGTGAAAGATACAATT
TAGTGGAACGAACCTGGTCCTCAATGGCGGAATTAGACTGTTACAGAAGTAATTTTGCTG
TTGCCTGTCTAGAAGGATATGTTTATCTTATTGGTGGTTTCATTTTAACGGATAGTGGCC
AGAACTGCAAAGAATCTGATAACATTTTGATTTATGATAGTGGCAGCGACACGTGGCAGA
TAACAATTTTTAAAACTCCTTGTGTATTGATTGATGCCGTTGCTGTCGGGCATTGTGGAT
TAATTTATGTAGCTGGAGGAGTTAATGATTCCAATCGACCAACAAATAAAATAATGGAGT
TTGATCCTGCCACTGGTGATTGGACAAAATTAAACGTTTCTGGAATTCCTGAGGATATAT
TTAGTGAAAACAATATAAAAAACTTGTCAATCGCTGCAGGTGGTTCAACAATTGAAAATG
AGGGATTTCTACTATTATCTGGCAGATTGAATCATTCCCATATGGTTTATGGATATCATT
TCTCGTTGAATAGATGGCGACTGTCTACTACATTAGACGAACTAGACAACAGCATGATTG
AAATGACACATCGAGTAACGATATATTGTCAGCGCGAATTCATTGGTATTCTCAGTACGT
GTACTACACATACTCTCGAACAATTACGGTTACCTATTCCAAATACTGATGATATTCACG
ATGATTTATTGATCTTTCACAAATTCAAAATCACAAATGATTGGAGTGGCGAAATTGTAC
TAATGAACCATTGTTATTTGAATTCATTTTCAGCAAAATCAGGATTTCAATTTTTATTGG
CCGCTTTAGTTAACAATTAActatttctcagatctgtaattattatatatcgaatgcaat
tttgtgaattaaaattt
>DjScaffold1742:76799-79030 strand:+
aaccaattgaatatttaaaaaaatgaaattcaaattgattattttatataaggttattga
ttgcaattaaaagtttacttatttaatgaaagaattttataacaatttagtcaattaacg
agtgaaattagcaaataagcaaattctcttacatcaaATGAGCGATTTTCAATTATTTAT
TGATGCGATTAAATATGGTTCTAATGAAGAAATTGCCAGTCAATTCAAGATTGAATATCT
TGAGATGACAGATAAAGATTTATGGCGTCCGTTGCATTATGCATGTTATTATGGTCGAGG
TGGATTGGTTAAATTATTATTATCCAGTCATTGTGAAGTTGATTCACACACAAATAATGG
TTATCAACCAATATATTTTGCCTGTATGAATGGTTATATTGATATTGTTAATTTATTGAT
TGCAAATAAATGTGATATCAATGTTTCTAATGAAATTAATGGATATCAACCGATACATAT
AGCCTGTAAGAATGGTCACATTAATATTGTAAAAATATTGATAACAAGTAAATGTGATAT
CAATGCATCTGAAAGAATTAATGGATACCAACCAATACATTTTGCCTGTAAGAGTGGTCA
TATTGATATTTTGAAAATATTGATTACAAACAAATGTGATATCAATGCATGTAGTGGAGA
TGGATATCAACCGATACATTTGGCTTTCATAAATGGCCATATTGATATGGTGAAAATATT
GATTGATAATAAATGTAATATTAATGTATCCGATAAATGGGGATATCAACCTATACATTT
TGCCTGCGAGAATGGCCATATTGATATTGCGAAAGTATTGATAGAAAAGAAATGTAATAT
TAATGCACGGAGTGAAAATGGATATCAACCTATACATTTGGCATGTAGGAATGGTCATAT
TGAAATTGTGAAAATATTGATTGATAAAAAAATGTAATATCAATATATCTGATAAAAATAG
AGGATATCAACCGATACATTTGGCCATCATGAATGGCTATATTGATATTGTGAAAATATT
GATTGATAATAAATGCGATATCAATGCACCTGACTACTGGAAATATCAACCGATACATTT

TGCCTGTGAAAATAATTATATTAATATTGTGAACATATTGATTGATAATGAATGTGACAT
TAATGCATCTGATAAATATTGGGGATATCAACCGATACATTGGGCAAGTAAGAATGGTCA
TATTGATATTGTGAAAATATTGATTGCTAATAAATGTGATATCAATGCATCTAATTATAA
CGGATTTCAACCGATACATTTGGCCTGTTGGAATGGTCATGGGGATATTTTGAAAATATT
GATAGAAAATAACTGCGATATCAATGCTTTGACAAAAGATGGAAAATCACCGATCGAACT
TGCCAAACATAGAAAACATCAAAAAATAATCGATATTATTTCTACTTATCCAGTAAATTT
TATTTTATTAAAACTATTTTAAtaatattatttttgaattaattaatagttaataaaata
aatttatcgattgaaaacctgaatttatacacttattttgaaaaattatttctttgacat
aaattttatatattaatataatctaacctttcatcaaccagtttaactattcaaaatgat
ataacatctttcataaaccaattgaatatttaaaaaaatgaaattcaaattgattatttt
atataaggttattgattgcaattaaaagtttacttatttaatgaaagaattttataacaa
tttagtcaattaacgagtgaaattagcaaataagcaaattctcttacatcaaATGAGCGA
TTTTCAATTATTTATTGATGCGATTAAATATGGTTCTAATGAAGAAATTGCCAGTCAATT
CAAGATTGAATATCTTGAGATGACAGATAAAGATTTATGGCGTCCGTTGCATTATGCATG
TTATTATGGTCGAGGTGGATTGGTTAAATTATTATTATCCAGTCATTGTGAAGTTGATTC
ACACACAAATAATGGTTATCAACCAATATATTTTGCCTGTATGAATGGTTATATTGATAT
TGTTAATTTATTGATTGCAAATAAATGTGATATCAATGTTTCTAATGAAATTAATGGATA
TCAACCGATACATATAGCCTGTAAGAATGGTCACATTAATATTGTAAAAATATTGATAAC
AAGTAAATGTGATATCAATGCATCTGAAAGAATTAATGGATACCAACCAATACATTTTGC
CTGTAAGAGTGGTCATATTGATATTTTGAAAATATTGATTACAAACAAATGTGATATCAA
TGCATGTAGTGGAGATGGATATCAACCGATACATTTGGCTTTCATAAATGGCCATATTGA
TATGGTGAAAATATTGATTGATAATAAATGTAATATTAATGTATCCGATAAATGGGGATA
TCAACCTATACATTTTGCCTGCGAGAATGGCCATATTGATATTGCGAAAGTATTGATAGA
AAAGAAATGTAATATTAATGCACGGAGTGAAAATGGATATCAACCTATACATTTGGCATG
TAGGAATGGTCATATTGAAATTGTGAAAATATTGATTGATAAAAAATGTAATATCAATAT
ATCTGATAAAAATAGAGGATATCAACCGATACATTTGGCCATCATGAATGGCTATATTGA
TATTGTGAAAATATTGATTGATAATAAATGCGATATCAATGCACCTGACTACTGGAAATA
TCAACCGATACATTTTGCCTGTGAAAATAATTATATTAATATTGTGAACATATTGATTGA
TAATGAATGTGACATTAATGCATCTGATAAATATTGGGGATATCAACCGATACATTGGGC
AAGTAAGAATGGTCATATTGATATTGTGAAAATATTGATTGCTAATAAATGTGATATCAA
TGCATCTAATTATAACGGATTTCAACCGATACATTTGGCCTGTTGGAATGGTCATGGGGA
TATTTTGAAAATATTGATAGAAAATAACTGCGATATCAATGCTTTGACAAAAGTAATTTT
TATCAATATAAATTATTGGAAATGTAATCAATTTATGCAGGATGGAAAATCACCGATCGA
ACTTGCCAAACATAGAAAACATCAAAAAATAATCGATATTATTTCTACTTATCCAGTAAA
TTTTATTTTATTAAAACTATTTTAAtaatattatttttgaattaattaatagttaataaa
ataaatttatcgattgaaaacctgaatttatacacttattgaatttgtgttcatttaaat
>DjScaffold2914:25790-32458 strand:+
aataaattagagtggacaaactaaaaatcaaaaatctcagattaatttaccccaatcaaa
atcctatgaaaatcttgttgatgaaaattctggtgaagagaatttcttttttttactcg
aacttgtcgagaaaaagaagaaATGTTCCGACGTTTAATGGCAGCCAGTTTAGGAATGCC
CATTAATCTAACCATAACCGATATAATAGAAAAATATCTTAGTCAAGAAGTGTTTTCTAG
TTATCCAATACCTCTTTTAAATTCCAAAGATGATGAAGTAAATAAATCAGAATATTCCAA
AGAACATTTCAAAGAAGACTCTGTTTTTAGTTCTAAACCTGGTCCATCTTCTAAATCTAA
AATAAGTAAAGAGCCTCAAATAGTTTCCTATGTTCGATATATGGCGAAGTTTATGCCATC
AGATATGCTATTATTAAAGGCTAAAAGTCAACATATCGATTTAAATTATATTCAATGTGA
TCCAGAACTTCTTTGGCTCAATGCACTATTTGGTAGGGTAGCTTGGGATGTTTTCCGTGG
AAGTTATTGGAGTAAAGTAATCCGTGAAAAAATCCAGAAAAAATTAAAGAAATTGCAT
>DjScaffold2914:32585-44450 strand:+
cagccgaccatatttagatttcagaggtttatggatagaatttgaaattgtctatgccgg
tggtttttcaATGGGTCTTGAAACGAAAATCAATTTAATGAAATTGAAAAACGTTGCTGC
TCAAAAATCTGCGCATAATAAAGGTAAAAAGGGTGATTTTGCAGTCAAATATAATACAGA
AACAAACACAAATCAACCCGATTCAATTATTGAAGCTAATGAAACAGAACTCACATTTGA
TAGCTTAGACACCAAGTTACAAATGCACATGGTTACTGGCGGTGGAGCTGTTGGATCAGA
TGATGCAGAAGAAGACAGAATTGCATCAAATTTATCGAGTGACGACAGTTATGAGAGCAG
CACTGAATCTGATGCATCTGTAGATAAGTCGTTAAATCCATCTCAAGGTTCATCTAGCAA
GAGTTTGATGCATTGGGTGGATAAAATTGCACGTTCAACTTATTTTCAGAAAGCTGCTGA
TTATAAATGTGTACAAAAAACAATGGAATATGTTTCAAATACGAGTCTTGCCCTGCAGGT

AGACGCTACTTATTTAAATGGGACTCTTGTCTTAAATATTCCACCTCCTCCAAGTGATAG
ATTGTGGTGGGGATTTAAAAAGAATCCTGAATTGCAAATAAAAGCTAGACCAAAAGTAGG
GCAAAAAGAAATCCAAATGACAAAAATAGTTGAATTTATTGAGAAAAAG
>DjScaffold4775:29534-30521 strand:+
taccataaattgcaaggtagaacaagtgttacagtatcaattcaaggaaataaagtcgaa
tgtttattagatacaggggcttgaattaacgttATGAGTTCGAGTTGTTTTAATAAATTA
CGAGAAGTACATTTAGAAATGAGTGATGGAAGATGGTGTTGTGCAAATAATAGTACACTT
GAAATTGTTGGGAAAGTTAGAACCGGAGTAATAATTGATGACGTTTGCAAAGAGATTACT
TTTTTAGTAGCTGAAAGAGTGACACCAGATCTTATGGAAGGAATAGATATGCAGAAGATT
TTTGGAAAGAAGATTTTTGGAAAGGTTGATAACAAAGCGGATGAAAATGTTGAAAATTAC
ATTTGTAATATTGAAGCGAACTTTGGGAGACAAATTAAAAAAGAAGAGCGATTGACAAGA
GCCTTAAATAATTTAAAGATTAAAGATGATCAGCGATTATTGAAAATAATTGAAAGTAAA
GAAGATGCTTTTATCGCAGATAAATGGGATGTAGGACGCGCACATCTGACAAGCGACAAA
ATAATCACAAGTGGTGAACCAATCAATGTATAAccatacaggcaaacaatgaacttagaa
tctaaaattgaagaagaaaaaatgaaaaatcagaagtcgttaaattttaatggattttct
gataaacaatctcctccat
>DjScaffold4775:115734-115958 strand:+
ATGGCCTATCTTGGTGAATTTTTAATATTTATGAATAATAAAAATAAACAATATAACGTG
ATTAACAAAGTACTGGCTGAAATAATGAAGACTGATATAAGAATTAGTCCAGAAAAGAGT
CAGTTTCTTGAAAGTGAAATCAAATTCCTTAGACACATAATTTGCAGAGAATTGATCCGT
CTAAAAAGGAAGCAATACAAACATTTGGCAAGCCTAACTGCGTGA
>DjScaffold848:24271-24978 strand:+
AGGTTATTAGATAATTTACAAAACCCGTCTTGTGGATATAGATATAATGTGTTGTTCGTG
AAACAAAATAACTCTTTTACAAATTTTTTCAAGAATCACGAAGACGATTTACGAGATCGA
AATATGCTTTCAACTTACTACAATTGTTCAAGATATGGTGGAGTTCCACCGGAACATATA
ATTCTTGCAAATAAAGTCACTGGATTTTTAGCAATGAATGATCCGAATATGGATATTCGA
AGTCAAGATGTATTGGAAAACTGGAGACAATTTCATCAAGCAGCATTAGCTAACAATCTT
AATCCAATTACATTGGCTCATCTTCATACAATTAATAATAGCGATATAAATTCGATTCAA
GTGAAAAAAAATGCCACCAGAGAAAATTCCAGTACACTAAAAGCTTGGTTACAAGAGCAT
AAAGACAATCCGTATCCATCGAAAGGCGAAAAGATTTTATTAGCAATTTTAACACAAATG
ACTTTGACCCAAGTGAGCACTTGGTTTGCTAATGCTCGTCGACGATTGAAAAAAGAAAAT
AAAATCAGTTGGATGTTAAATGAAAAATCAGAAATTGTCGATTCTGATTCAGATTCAGAA
TTA
>DjScaffold848:121199-122096 strand:+
caataatATGAATACTAATAAACGTACGTATAATTCATCAGTTTTAATTGGAAACTGGTT
TGAAGATGTTGCTCTTAAAGAAGAATCAACTAAAGACTTTTTAGAACGCAAAGAAAAAGG
ATTATTACTTACCCAGCAATTAAAAAGAATTTTAGGAGATTCTGATACAAATGTTGGTCT
TTCTCCTTGTTCCAGTGGATTTTTGAAATTCGGAGATCGAATACAACTTTTAAATGCTGG
TTCTGATCCTATAGCTATTCAACTTAATAAAGCAGTTCCACGTAAACCAACTGTAATAAA
TATTAATGTTCCTCCAAATATATTTCAAAAGAAACAGGATTTCAACAACATTATTAATGT
TTCTGCTTCTGCAAATGTCAAACCTAATTCAAGAAATATATTTCAGATATTGAGGTTTGA
GAAATTGTTTATAATGAAAATATCTTATCATTAAttttattattattattatttttatta
ttattattaatattattattattattttttattattattattattattaattttattatta
ttanaacaatccaggagtttttgttgggaattaatagtaattttcaataatattaacttt
atttattaaaatataatttattttttctgggattatccattttattttaacgtttattga
tgaaagatacagttgggttctgtcccaacacatgtaaaagaaaaattataaaaatttcca
ggcgaaaaaaaatacctagacaatattattcaaattgagaaattcatcagtggatcaagg
aaatcaaatcacataaa
>DjScaffold4997:62795-63158 strand:+
taataataataattagatgatcaaacaagagtaattttgaatcgttcaccaccaaaaaat
gattatataaatgcaaattatattccatttattcaagacattaacaagcatgaatatatt
gcttctcaaggaccaattaatgatacaatttctgatttttggcaaATGATTTGGGAAAAC
AATGTTTCACTAATTATTATGTTAACGAGAACTTACGAAGAAGAGAAATTTAAATGTTTA
CAATATTGGCCTGAACATCTTGATCAGAAAATAGAATTTGGTGAAAACAAAGAATTTCAA
GTTAGAATTGCTGAAGAA
>DjScaffold3333:6488-9533 strand:+
aatttgcaaaattattgcaaaATGAAATGTAGAGTTTTAATTCATCCCAATAATATAATT

TCGACCGAGGGATTTGTTGTTAAAGGATTTGGACGTGGTAGTAAAGAATTAGGTATTCGT
ACGGCTAATTATGACCAAAAATTAGTGGAGCAATTGCCTCCTGGTTTTCAAACTGGAGTT
TATTATGGATGGGCTCAAGTAAATGACGGCCAGGTATTCAAATGTGTTATCAATGTTGGC
TGGTGTCCATATTACCAGAACAAACTAAAATCATTTGAGACACATATCATGAATGTTTTC
GAGAACGATTTCTATGGAGCAATTTTACGAGTTGTTATTGCAGGCTATATTCGAGAAGAG
ATGAACTTTGATAATCTCGGTAGGTTTGTTTGTTGAtgtaattggttaattacatgtaga
acatacgtgccgacagtgttgtcggaataattgatgatataaatacagttgtaaacaatt
gtgactgagttctgtgttgtggttggataaaactggatttattgatgattgattgattga
tgaaatctttatctaaatattattattat
>DjScaffold1340:1738-2306 strand:+
gtaaatttaaaatctaaattATGGGCGAAGAAGACTGTGATGATACAGGTCATCATAATT
CTCATGAAAATTCAAGTCATTTTAAGCGATCATACCTGGATGCACCGTCTACGCAATATC
AATCTTTATTTGGAAAAATCTTTTTTGGTTTTTTTGAAACAACGTCTAATGGACTTCATA
ATTTCCTGTTAAAGTTTCGCGGCGAGCCTTATCCATATTATCATCGCGTTTATAATCGAG
TTCCTCTAATAGATGAATGTATGTCAGATGATTATGCTTGTCAATATGAAGCAAATCATC
AATTCATTAGAGACAGGCTAGTAGAAGCTCAAATTATCCGAATTTTGCGAGCGAGAAGAG
ACGACTGCTTTATGTGGTATAAAACACCGTATAGTGCAGAAGAAAGAAAAACTATTTGTA
CGAAGGAATTTGAGGATTATGATACTGCAGTAATTAATTACTTTACAAAATACGGAGAAA
TCGGAAAATTTAATTCATCACTTGATGTTTATATGAAACAAAAACATCGTTTGGTTTATG
AAAGAAGAATCGGTAAGAAACTCGAAGTAGATATGAAAGAAGCCAAGAGAGTAGTAAAGA
ATGAGGACAACATGTTAAATGTATTAAGAGACAGTTTTACAACAACTAACTTACCGAAGA
ATATGTGAaattcttagttcttatctaaatttaaagtttattaataaataaatacctagc
gagtaaatttaaaatctaaattATGGGCGAAGAAGACTGTGATGATACAGGTCATCATAA
TTCTCATGAAAATTCAAGTCATTTTAAGCGATCATACCTGGATGCACCGTCTACGCAATA
TCAATCTTTATTTGGAAAAATCTTTTTTGGTTTTTTTGAAACAACGTCTAATGGACTTCA
TAATTTCCTGTTAAAGTTTCGCGGCGAGCCTTATCCATATTATCATCGCGTTTATAATCG
AGTTCCTCTAATAGATGAATGTATGTCAGATGATTATGCTTGTCAATATGAAGCAAATCA
TCAATTCATTAGAGACAGGCTAGTAGAAGCTCAAATTATCCGAATTTTGCGAGCGAGAAG
AGACGACTGCTTTATGTGGTATAAAACACCGTATAGTGCAGAAGAAAGAAAAACTATTTG
TACGAAGGAATTTGAGGATTATGATACTGCAGTAATTAATTACTTTACAAAATGT
>DjScaffold3560:121429-146355 strand:+
AATTTTAGGAAAAAATTAGCAGGTCCTAATAATGTAATATCACGTGATTATATTGGTAGC
CTTAGCATTTTTGCTGAATTAAATCAAAACGATGAAGATTTTTATTGTAATGAAATTGTA
AATTGTAATGAAAATAATGCATTGTGTAAGCGCGAAAAATCTCGTATTGCAGCTAAGAAG
CGTAGGAATCAAGAAAACGAAAACCTTAGCGACTTGTATATATCTTTACCTGTTGCAACA
CAATCTAAACTTTTATCTTGTTACGATAATCAATTTATTGGGAATTTCGAAAAAGCAACA
ATTATTAAAATATCAGCATTTGTACTTAAATTATATAATTCTTTATCAGATGTTCTATTT
AACAATTCTTTTCAATTTGATAAACATCCATTGGTTTCATTATTTAGTCAATCTATATTT
GGTTTATTTTGGTGCGAAAAAACGACAAAGTTAATATTTTCATCGCCTGGTTTAGCCAGA
CTAACAGGCTTGTATTGGTGCAACATGATCGGAATGCCCCGAAGTAGTATCATTGTTGAA
TGTGGAAATATAGATCCTTCAAATATGATATTTCTACGTTTCATGATGGGAGCATTAAAA
GATAAGGGCATTTTTACCGTGAATCCTCAAAGTTCTGGTAATAATAAGCAAACAGAAGAA
ACAAAAATCGAGAAACCACAAACTGTATTCAATCAAACAAAACGAGATTTTATTGGAAAA
CTACCTTGTACTGTATGGTGTTCTCAAACTTTGCCAAACGGTATAACCTTCGACCTCATT
CAGCCTTTAATTGTCAAATCGGATAATTTGTTCCATGAAAACAAATTCCCTCAATCATAT
CAACTGTGTCCTTATCGCTTGCCTATTTTCACTGATATTCGAAGTACAGAAACCAAACGC
ACAGGTCATAAATTCACCAGTTATCATTATATCGATTCATTAAACACAATGTCAATCACT
GGGGACGTATACAATTCTCTTGGATATTTTATGGAAGATTTTTCAAAGTCAAGTCTTCTA
AGCTTTTTTCATTTTGATGACATTGTTCAAGTTTCTAAACATGTAACTGAAGGTAAATCA
ATATTAATATTTTAAaatcatttaacaattctataactaaaacatttatttttaacattt
tcaatttatatatatatattaataaagccttaatcttaattaattaatcttaattaatcg
aagccttatcttattttaatgaaagtttttatattgattttattatttgttgggaaaata
tgaatagaatttcagtttctgaaaatttgatatgcaatcaactttaaaattttatccttt
aattagttttcatgacaatttctaactcaattgcttattaattatccgcattgattgtaa
tttttaatatataatattattatttgtaacaaaccgaaataaaacagaatacaatatgta
ttaataatcataataataatgtactaagattt
>DjScaffold4407:11198-11473 strand:+

ATGTCTTTTGGAATTGCAGCAGCACCTGGTACGTTCCAGGAAATGATGAATAAAGTAATC
GGAAAAGTAAAAGGAGCAATTTTATATCTAGATGATATTCTCATATTTACCGAAAAAAAG
AAGCAACATTATGAAATAATCAATAATGTATTAATAGAAATTAAGAAGGCTGGTCTTAGA
GTTTATACGGGAAAATGTCAATTCATGCAAAAGGAGATCAAACTTTTGGACACGTTATCA
GTAAAGAAGGCGTCAAATCAGATCCAACTAAAATAG
>DjScaffold5101:74159-92862 strand:+
aatttttagacATGCATGATGAAAGTTATGAATTTTCATTTAATAATGAAGATAATACAA
ATGTAGAGATTCCTCCATTGGAAGACACAGATTTATATTTAATTTTGAATATATCACAAA
ATGCTGATCAAATCGAAATACAAAATTCGTATAAAAAATTATCAAGAATTTATCATCCAG
ATAAACATTCAATAAATAAAGATGATTATCAAGCTGCTCAGATTCATTTCACTAAAATTA
AAGAAGCATACGATATATTAAGTTGTCCAACAAAAAGAGCGGTTTATGATTCCTATGGAA
TGAGTGGAATTAAAATGGAAAATTGGGAAATTGTAAGTCGAACCAGACATCCAAATGAGT
TGCAAATGATTTATGAAAGTCATAAACGATTAGCTGAAGACGATTTACTTAATAGAATTA
CTCGACCAGAAACTACATCAACATTTGGAATTGATTTAAGAGATATTTTCGATAGATATT
TACAAGAGGATCCAAGTGAACGATATGCAGGACCATCTGCAAATTTTTATAAAGCTGATT
TGAATCATTCTGTATGTTTGGGAATGACAACCAAAGATACAGTAAATTTATCTGGAATAT
GTTCAGCAAAACACGGTGTTGGAAAAGGAGAAATTGTAGCCAGTTGGAGAAGATTTCTTT
ATCCACAGTTTCACGATACATGGTTGGATTTTCATTCATCAATAAATACTGATATGAATG
TGTCTGGTGGTTTAGTTATTTCCCGGAAATTTATAGACAAATTCACATGTACACTCGGTG
GTTTCGTAATAATGCCAAAATATGGGTATCTCGCTCCAGGGCTGCAAGCGTCTATCAGTT
GGCAAATAACCAATCAATTATTTGGTAAAGTAGAATATAAGACACAGCCATTTTCAAAGA
TGCATTCTCAAATTGCATGGCAATCTTTAAGTGGTAATCACGGTATATATTTGGCAGCAA
ATGTTAGCATGCACAATTCATATTTATCGGCATATTATGATTATATTCTGAATTTTTCTA
AAACCAAATCTAATAATGATCAAGGAATACATGAAACCGACGATTGGACGATTGACGAGA
AAAAGGAACCAATTCATTTAAAGATTGGTGGGCAATATGGATTTAACCATTTTTATGCTC
GTTTTGGAATTACCAGAAGAATCACAAAATTTAGCACCTTTGGCATTTCATTTAGTGTAG
GATTACCTCTTGGTATCCTAGTTAAATTTAAGATTATCCGTGGCAATCAGACCTATATAT
TTCCTATTATTATGTCAGAAAATATCGATCCTGCTGCATCTTTTTATGGATCTGTAGTAC
CTATGGCAGTATTTCTTGCTGTTAAAAGTCTTCTTTATGATCCATGGAAGAAAAAAGAAC
TTGAAAAAGAAATTATTAAGCGTCGACAAATTTACCATGGTGAACTAAAACGTCGACGAG
ATGAAGCGTATTCAGCTCAGAATCTTATGACTGCAGCTTCGAAACGGTCATATCAACAAG
AGAAAGACAAAAATGGTCTTGTTATTGAGAGAGCAATTTATGGAAGACTGGTAAATTATT
ATTAGcttataactgaataaatagaaataaagaactttaaaaaaattcgatactattaga
atcttgcaatatttataaaaaatacttatatgattggaattaaaaatagattttttagat
atc
>DjScaffold5101:171551-171679 strand:+
ATGAGAAGCATTTTGGGAATTTGCAATTACTATCGAAAGTTTATTAAATATTATTTAAAA
AAGCTAGATTGCGGCAAAAACTCTGTCGTGGGCCGAGTAACAAGAAGATTGAATGAGCAA
ACGAATTAG
>DjScaffold2785:58991-59515 strand:+
CTGGATGTCTTACTGGATAAACGAAATTCAAAAATTAATGCGGTTGTTGAATTTAATGTT
GACGATACAATTCTTGAACGAAGAATTACTGGGCGATTATTTCATCTTCCAAGCGGTAGA
TCTTATCATGAAGAATTTAATCCTCCTAAGCAACCAATGAAAGATGATGTAACTGGTGAA
CAACTGGTTCGAAGAAAAGACGATAATGTTTTAACATTGAAATCTCGATTGCAATCATAT
CGCACTGAAACTGCACCACTGATTGGATATTATCAAAAGCGTCATGTGCATTACCAGGTA
AAGGCATCTCAGAAACCCGATAAAGTTTTTGAAGATATTTTAACTTTCTTTGAGAAAGTC
AATAAATAAaattccatttatatagtttttccaggaagatattaaagtaatcatttagta
gttagtttttataataaaattttttgaaca
>DjScaffold1378:28674-29682 strand:+
tattaaattatcgaatatacatactttggaaaattatgaaattatttgtgaaaaaaatttc
agtgctcttctggaagatgccaatgttaatcttacaccacatcgaccaccaccacaaagc
cttaaagtgactaccagagacagcaatagaATGAAGCGTTTCAATCCATATGGGGTGAGA
GAATCTAATGATAAGGGATCAGTAACTAATTTTAGTTCTGATAACGAAGAATTGAGAAAT
TCTCCCGAGGTAACTAGAGAAATCAATGATGACTATGAATATGCACCTAGTCCAAAACTT
AGTAAATCTACAAGTATTTCTCCAACAAAGAGTTCTTTGCGTACAAACAGCGATAATAGT
GATTTCAATAGTGAAGCTTCTTTCATTAATGAGTTGAAACGTAATCAAATCGAAAGAGAA
AAGTCTCATAAATCATCATTACAAAGATCATCTGGTGTTATTGTCACTCTTCCGACAAGT

TCTATATTCAAACCCAACCCTGCCTGTAAAGGAATTTCTATAAATGGAAAAACCCCGGAT
TACGATCCTTTGCATTTCATTCGATGTCACGCTCGAGATAACAATCCATTAGATTGCACT
ACTAAAGTTTGGCGATGCGCTTTCGAACCAAACCCGAATAATCCGTATCTAGAAACAACT
TCTGTTGTGGCAACTTGTGGTGGTGAGTGTGTTTGTTTAATCGATTGTCGAACAGGAAAA
GTCATGAAACGATTCAAGAGTCGTGAAGAGGAATTCTATACGGTTGCTTGGACAACTGTG
GAAATGGCAGGAGGACATTATATTAACATTCTTGCCGCTGCTGGTAAAATGAAAGACATT
CGATTGTTACATCCAGAACAATTGGTTTGTTATGCGGAAATGAAAGGCCATGAACAGGAA
ATTGCATGTATGACTTTTCATCCACGTTATCCGACAGTTTTGTTTTCCGGA
>DjScaffold4116:74337-78426 strand:+
aattttacggaaaATGTCGGTAGAGATTTGTTACAGAAATAAACAATTTACCCATGAAAT
AGACAAGAATATGATTGTCAAAGATTTGATTACCGAAATAGAGACAAAGCTGAATATTTC
GGAGTTTTCTTTGAAATTGGACAGCATTTTATTGTTGGAAGATAAACCGGTGAAAAAGTA
TCTCATCACAAAATTCACACTATCTGATAATAATAAGAGAAATATAGAAATTGAAATCAA
CGATTTAACATTCGATTTGAAAATAAATTACTCAAATTCTAAAGATATTTCCGAGAAAGC
TGCAGCAACGATTTCTGTTAAGGAATTAAAAACCAAAGTGTTGTCTAAATTAGCTTTATC
AGGTGACAATTGGGAGCTGAAGAAGAATGGAGAAGTGATGGAAAATGGAACATTATTGTC
TGCTTGCATTACTAAGAAAGTGTTTGAGGTTTCTTTTCAATTATTTGCAATTTTCGTGAA
ATACATGGACCGCAACTATGCAGTTAATATTACCAAGGATACAACCGTGAAAGAATTAAG
AAAAATTTGTTTTGACAAATTTGGTGAAGCAGTAAATGAATTTCTAATATTTTTCAATGG
GGAATCATTGGAATTCGAACATCCGATCACATTTGCTGACTTCTTTTTGCAAATAATTGG
TGTCAATATTATTAGTGTTTTAGATCAAGAAAGACAACATAAAGAAAATCTAACATTGAA
TTCATATGGAGTTGAAGCGGGTTGCACTCTATCTTTGATTGTACGTACAATTGGTGGTCA
GTACTTTCTTTCATTTACATTAGATGAAAGAATCAGAGCCTGAaaatttttatttacaat
gattatccaattttaatattgtgctactagtaattcctgatctttcagtatctgccaagt
gattgttgtcattactattattattatta
>DjScaffold4142:59311-71963 strand:+
aaaagacgatcagcaaaaatgccttattgcacacatggcagatagaaagggtcaagagaa
tgtttaatagctcagctcaaatattgtttcgttggtaaattattgcttcaaaacccttta
caaactttgctaaatggaatttttattcaatttaaaataaacgatcacaatcaaatcaac
gATGAATTATTATTGTGAATTATGCTCATTAAATTTCCAATTTGAAGATGAATATGATAA
TCATATGAAAAATGAAAATCATTTATTTGAAGAGAAAAAATTTGAAACAGACGATTTAAA
TGAAATGAAAGATTCTGATATTTACTGTGTGTTTTGTTCACAATTTTTCGATACAATTGA
AAAAGTAGAACAACATATTGCAGAAGAACATACAGGAAATGAACAGGTTCCAGAGAAGCA
GGAAACAAATGATTTTCTAATAAATAATTCGTCCAGTGAAATCAAATCGGAAAATCTTAT
TCCATTTGAAAAATCTCAACAAATTTTCTCGAAATTTCCTGAAAACAAAATCATTCCTAA
CAATAATTTAATGTTGTCGAATAAATTAACGGTTTATGATGTGGATTTGAATGTAGTTAA
TGTAGATGGAACTAAATTGGACCAAATAAAGTTCTGTGTCAGATGTAATAGACATTTTGA
TGGAGAGGATAATTTTCGACAACATCAATTGAAAGATCATGCAATCCGTAAACGTAAAAG
ATCCAGTCATCCAAATGGTGTTGAAGGTATTGTTGTAACTGGAGATGGTCGCTGGAGTTG
TGATGTTTGTGGAAGAGAATTTCCGACTTTGAAACAAGCAAAAGTTCATAAATTCAAATC
GCATATTGAGCCAGTTCAAAAACAAGAAATGCTTTCGCATGATTTTATGATTAGAAATAA
TCCCCCAAAAGAAACAGGAAATGTTACAGAATCAATGACAACCGGAAATTATTATTATAA
TAACAATAACAATCATATTCAATTTATTAACAACAATGAAAACGTTCATATTTTGCCACG
TCAAATATCTCCTGTACAAGAAAGTAATATTAATCAAATCAGTCCTAAATCCGATGTAAA
TAATCAACAAATAATCAATGAATCCGAGAGTGAAACACAGTCGCATGTCATTGATTTAAG
TCAATCATCAAGCCCTACTAATGTCAATAATCCTGATTGTGAAACGGTTTGGAAAATCAA
AAGTTTAGATCCAGAAGATACAAAAATCTCACTATCAAAAATTCAATTGAAGATCGAAAA
CAAAAAACATGTAACAAAATCAACTGAGCCATTCAATTTATCAGCAAGAAACTATATGTG
CTCACATTGTTCTATGTATTTTGGTAGTCGAGCAGTATATAGCAGGCATATATTGGTTCA
CAGACGAGGTAAAGCATTTGGCTGTGAAAATTGCGGAATGGCATTTACCCGGAATCATAC
TCTGAAACAGCATAAAGACATGAAATGCGTTGTCAGTGATCCAGTTGTTGGAGTAAATTG
TGAAGCTAATAATCAATGTGTTAAATGCGGCGTTTATTTTGCTAGTTCTAATAGTCATTT
ATCTCACTGTAAAAAGATTTGTAAAATTACTGTTGCAAATACCGATGAAGAATCATCGAA
AGATTCTGTAAAATCAGAAGAAACCAATGCTCAATGTGAAAGCCAATTGGAAATTGATCT
TGGGGAAAATGATAATTTTTCACCAATCATTATTAATGATTTTATAAACAATCCAGCTTT
TGTTACAAAACGAATTAATGAATTTAATTGCAATTTATGCTGCTGTTATTTTGCTTCATA
TAAAAGTATTGGGCATCATTTAGAGAATTATCATCGTATAGAAATCCGATCAGGATCCAA

TAAAAAAATCAATGAAACAGAAAATTCGAATGTGATTCAAGATTTTATTCATAAATGCGA
TGAGTGTGAAAGTAAATACGGTTCAAAGGAAGCTTTATTAAAACACAAACAACAATCTCA
TGCACCGAAACCGACTGAATTTACTCCGAATAAAATGAATAATATTATTGAATCATACAA
TAGGTTTTCTTTGGACAAACCAATTAAAATCGAACCTCATAGTAGTCCACCAAGAAAAAT
ATTAAATCCCAATAATTTCTCTTTACCTAATTTGGTACCAATGTTCAGTCCAGAAGCACT
CAATCTATCCACAACTCAATCTGTTACGTTAAATACAAATTCAGTATCTCAGATGAGTTC
AGTGATTTATAAGTCGCCGTCTGTAAAAAAATCTAAAGTACCACCAAATGCAAATGGAGA
ATTCGAATGCACTGAATGTCATAAAGCTTTTGGTTGTCGAAGTTCACTAAGTAATCATAT
AAAATCACATTTTAATCTGAGAAATAAGCCTTTTCCTTGTGAAGACTGTAAACAAGGATT
TATGTCTCAAATAAGTTATCAGGAACATCGTAAAAGTCAATGCCCGAAGCTGTATGGGAA
TAATATTTCTGAAAACGGTACAGCTACGAATTTTTCCAGTCCAAATATGTCGTACACTTT
GACAGGATCCAGTCCATCATCTGCTTCTAGTAGGGCTACTTGTTGTATGCATTGTAATGC
AGCATTTCCAGACAGATCGCTACTAGAAGATCACGCCGCAAAAGAACATTCTAATGAAAG
TGGAGAAATAATGTGCATGAAATGCACTCGGTCTTTCACAACTTATATGGCATTCAGGGT
TCATACAACAAAAACTCATGGAGGCCCCAATGGTTCCCGTTTGAAAAGTTCTCAAATAAT
CAAAACTCCCCCTCAATCTCCAATAAGTTCATTTACAGAATCACCATTTTCTAGTTTTCA
TCTTAGCACATTTTCATCTCTCAGTCGTCATACTTGTCAGTCGTGTAAAAAAGAATTTGG
TACCGCACAAGCTTTAGGAAACCACAAAAGGGTTTGTGATTCACCGATTTTCACTGAAAC
CAATGTAAGCAAAGACCAGTTGATAATTTAGagaatatcaaatattttcaatcacataat
tgtactttcttgcatttatttaatggtctcgattgtcatttttcattctatcaacatctg
atgatgatatttacctatttgacatgcatgg
>DjScaffold4142:1263-4183 strand:+
ATGTTGAATATTAGATTATTTTCGGGCTCTTCGAATGTTTTATTAACAGAAAAAATAGGA
GAGAGGATTGGAATTAAACTATCTGAAGCTATTTTAAACCAATTTAGCAATAATGAAACA
AGTGTTCAAATAAAAGAATCAGTAAGAGGTAAAGACGTTTTTATTTTACAGTCCGGATAT
ATAGATGTCAATAATCATTTAATGGAGCTACTTATTATGATAAATGCGTGTAAGAATGCG
TCATCTGCTCGCATTACAGCAGTTTTACCATATATGCCATATTCTCGTCAAAGCAAGAAA
GATAATAACCGTGTGCCGATTACAGCAAAATTGGTTGCTGATTTATTGCACATTGCTGGA
GCCAATCATATTATAACAATGGATTTACATGCTAGTCAAATTCAAGGATTTTTTAATGTT
CCAGTAGATAATCTTTTGTCTGAGCCATGTCATATCTCATGGATCAAGAAAAATATTTCC
AAATGGGAAGAGATCACCATATTCAGTTTAAGTGTGAGCAGTGCAGAACGTGTTGCCAAT
ATGGCTAATACCATCAAAGTAAGATTTGGATTAATATATGTGGAAAGAAAAGAAGCTTCA
GAACAGATCATAGTAATTGGAGACGTAGTTTCTCGAACTGCTGTAATATTATTGGACATA
ATTGATAATTGTGATCTAGTAATTAGTTCTGTAAAAAGGTAA
>DjScaffold1162:17477-17989 strand:+
atgaagaggcaaaaaaagtttcaaaaaaaacattttatttactataattatatttattaa
taacaaaattatattaatttactaaaataaactataacaaacttttataattatttacta
caatatgctataaactacaacATGATAAAATATGTAACAAACATGTATTTTATTACAAAC
AAATCAAATTTTACATTTAAATTACAACAATATATACTTAAAATTAACATCACGTTTCAC
AGGGCCAATGCGACTATAGTCGTTTTACGCGTCCAAAGGGTTAAA
>DjScaffold4784:447-995 strand:+
ATGAGCTGTGATTCGAATAATGATGGTCGATTGGATAAAAAAGATTTGATAACTATGTGT
CAGAAGATGAATATGTTGGATTTAGCAGAGGAAATAATTTCAGTATTGGGCGCAAGTGCT
ATCGGCAGTTCTATCTCTTTTCAGGAATTTCTGAAATGTCGTTCAAAAGTATTAGGATTT
AATGATAATAATAGTCCACGGAAAAAAAGTGCGGAATATCTAGTGCACTCTAATTATTAT
AGTCAACCGGTGAACTGTCAATATGATCAATTGGGAGGGTCATGTAGTGATGTCAGTCAA
ATATATTCAAGTGTAGACTACGATTCTGGCACTCAAGAAACACAAGAAGAACCTCTGAGT
TTATTTGCAATGTTGCATCAATGTTATGGAGAGCTTTATTGTCAATGGATGGCTGCTGAA
AGAGATAATACTTCTCCCAGCCTATCCCAACAATTGATGCAATGTGCCAATCAGATTCAT
CTTGCTCGTATCAATCGTCTT
>DjScaffold4784:29847-51291 strand:+
CAGGCTAGTCAGGATACCTATCAAAAGGAAATTACACGTCATCAAAAAGATCGGGAAAGA
TTGAAACAAGAATTACAAAATCAAACTTCGCGATACGAAGATCGGCTGACAGAATTACAC
AGTGTGATTGCAGAGTTACAGAAAAAGATTGAAAAAAAAGATAGAAATACGATAATCGAA
GAAGATGAGGATATTTTAAACGATGAAAACGAGGCTGTTGATAATACAGATTATGAAAAA
TTGAATTTAAACAATGTTGGAGAACTAATTAAAGATTCCGTAAATGCAGTCCATCAATTA
AGCTGTTCAACATCGTTTGTCGACAGCAGTATTTTCGGAGTTGATAAGAGCACAGCGCCA

TCTGAAAAACCAATGTCAAATATCAATTTTGTGGAAGTGCAAACTGATATGGTATGTGCG
ATGGACACTCGTACTGTCAATATACAAAAAAATGATAAATCAGGGGATAAACAACAGGAG
GCTACGGAGAAACAGCTTAAAGCGTTTCATCACAATCATATTTATCTGCAAGAAAAGTTA
TCCGGTTATGAAAACGCGGTAAGCAGCTTGCGTACTCAACTGGGCAGCGTTCTTGAGGAA
CGAGAATCTTTGAGAAAACAATTGAAAGATTTACAAGATTGTGTTGAATTAAATGCCCAA
TTATATTCTCAGCAGTCATCCTGTTCAGTATCCAAAGTTTCCCTGGATTCCAAATCAACA
AACAGTTCATCATCTTCACCTGCGCGACCTATTATGGGAGTACGGCAGCAGCAGTCTAAA
CAGAATCAAGTCAAGTCACAAAACCCCAATGAAATCGATTGGGTAGTAAAAGTTGTTACC
AAATATCAGAAAAGCCTCAGCAATCCCAACCAAAACATACTTCTTAATCACATAATCGAT
GATTCGCATTCTATTCTAACAAGCGGTCTTATTTTTCTACAGGAATTATCAGAAAATATT
CCTTACCCAGCGTTAGTGGAACCATGTAAAAGGATCAGTTTCGAATGGCAGCGACTAGTT
GCCAGCTCAGACAGAGAATCAGCTGATAAATTATTATTACAAACTACTCTAGCAGAAAGT
AAATCAATATCTGACCAATTACAGTGTTCCATCGTACAACTGCAGTCCGATTGTGCGGCT
CTTGCAAGGTCCAACTTGCTAGCAGATCAGATCATCGAATTGGCAGACATATTGATACAA
CTGCAAGAAACAGATCCCCTTGGTAGCGGTCTTGATATCACTCATCAATCACTTTGTCAT
GAGCCGGCCTACCAAAATAATTCAATGATAATGGCCAATGAAAACCACCGTTCCACCAAT
TCTTGTGGATATCGTGAACAAATAGAATCAGCGGCCAAATTACTTATCTATCGACTTGAG
TGTTACAGCTCAATGGGCAGACAAACTCCAGACAGTGGAGCAGGGGTGAGTAGTACACTG
AGTAGTGGAGGGGGCAGTGTTGATGTAGAAAGTGTGCTCCGACAGGCCTCAAGCACTGGA
GATTGGAGCAAGGCAGATGAATTAAAATTAAGACGATTATTTGATCAGTTAAAAGCAGAG
AGACATAGAGCTCGTTTATCTGTAGTCGGAACTCATCTAGGTCAATCTTTCTATGGCCAG
TGTTTAAGTTCATCAAATCAGTCATCTCTTCATCGGCATGACCCTAGTAAATTTTCGTCA
TCCCACGATTTTGAGAGCCAAATGTCGGTGTCTAATCAACAGTTGATAGAAAATGCAATT
CTTTTACAGGAACTTTGCAGTCTTAAAGAAGAGCGAGCTAATTTAAAGACTCGTATTTAT
CTATTGGAAAAAGAAGTGAAATCGATGCAATTAACATTGGACAGTAAAACAGCTTGTGAA
AAGTCATTGAAAGTACAGCTCAATCAGCTTATGCAACATCGCACAGATTTCATATCAGGA
ACTGATGAAAGCCGCCGATTGAAAAGTCAAATTGATGATTTGCTTCAAGCACTCGAGGAA
TTACGAGAACGGTGTCAGCTCCAACAAAGGCAAAATGAAGAATTTGTCAATGATCTCAAA
AAAGCAAACGCAGCATTAATATCAGCATTTGATAAAGCAAAACGGAAGTATCAAAACAAG
ATTTCCAGATTAGAAAGCCAGTTGTCAAACAGTAATTCTTGTTGTCATCAACAAATTATT
GCAACAATAGCAGCAACAACAACAACAACAAACACTGCATCTGATGAAACAGCATTATAA
attagtcggtgtctcgaaaagttttgtaaatatttctcagtactagatcagacataattt
cgccactagattatcaataatactgcagctgactaaccgcttattatacaaatttatgtc
tcatctttttcgaattataaaaatgaatttttttccttccaccaaatgatattgagcaagt
attgaccgaaaaaatgccgacaccccaactgatttaatttattttgacagcaaacaaca
aaccattaatgataatgatacaaccgaatcgtatagtataaatgagaacgatatcccatc
attacactgcctcatgggaattgcctattaatcttggtttcctttgttgccttggtaatt
aatagtttttctaattttttctaatttcttacacaaataaaacaccattgaatcgaat
atatattctcttcattgcaattcctctttttcttttatcaatatcgcatttcttgatc
ctttatttgacagataatggtcatcgatcagtgtggatcgcctatgtcaagatctatttt
ttctttaatcaattctcagttgaaatatatctctctaaatcatttatgatattcaatatt
ttcttaaataatacttattgatttcttattgatatcacttgatgatgatactgatcatga
catcacctcatttaatcagattttcttttttttgttttataatcgtttttaattttc
gtggtttactattatactgatcaatagtaatatatcagaatcggtgatgattattcattg
aatttccctgatatt
>DjScaffold5000:38012-38215 strand:+
AAAGTTAGCAAAACATACGAAAGGACAAAAAATATAAAAATAAAATCTTATTGTTCTTTG
TTTTACAATTGCAATTATTGCGAGAATAATTAAttcatttaatataaaagctttgatcat
aatttaggttttaattattgatattggataacaattcgatatttcgaccttgtct
>DjScaffold3331:4680-15732 strand:+
AATAAAAATGTTGATTCAGATGATGAAAGTAGTAATTCTAAATCAAATTTATCAGTTAAC
GATAAAATAATTTTTGAAAATCGATCAGTTTTAAGTTTTGGTGATGAACTAGATGAAGAT
ATAGATTCTTTTAAAGAAAAGAAATCTTCTCAAAGTAAAAAGTTGTCTATTAAAACGAAA
GAGCCTAGAAAACTATATAATAGAATTATCTCTGATTCTACACCATTGCCAAACGATTTA
GATATTCACGTTCAACATAATTTAAAATTAATTGTGAAGGAGCCTTCACCACCGGTTATA
GTGATTGATTCAAAAGAATTTGATGAATGTGGTCCTTCAAATATTGTTATAAATATAATG
CAAAAAGGAGACATCCCAGATGCAGCAGCTATAGATCACGCCAAAAAAACAAAGAGAATTG

GCTAAAAGGCAAATTATAGTTCACTCATCTTCATCTGAAGAAGATGACGTAACGCGTATA
AACTACATAAAATGTGGAACGGCTATCTCTGAACATGGACTTAATTTAGACGATTCCGAT
AGAATTGCAATGATTGGTCAAAATAACACGTTTGAGAAAGACAGACAAATGATTAGAAAT
AATTTTTTATCAGTAGAACACGGTAGTGACAGAGATAGTGACCAGGAGAACGAGTGGGAG
TCTCGTCAACTGCAAAAAGCTATTAATAAAAATATTTATCAACCAAGGGAACCGAGAAAA
ATTGATTGGCAATTATATACTAATGAAAATCTTGAAAACGATAAGAAATCTGCAGTTTTG
ATTGAATTGAATTCAATAAGTTTGGATGATTTGTCTGTAATCAAATTGTCGGAATTACTT
ACGAGGAGAAAGGAAGAAATTAACGAATCAAAGTATAAAAACAATCAAGATGTTTATCGG
AAACAACAAACATTATGCTCCAATGAAGATAAAGCAATTTTAGCTAAAGCTGAAATTCCT
GTTTTAGAAAAGAAATTTAATTGCTTCAATAATTTCCGAAAATACATTGCTATTCTTGTG
GAATGTTTCGATGAAAAAATGGAAAAAATGGTTCACTTGGAGAAAAAGTTTTTAAAAATC
CACAAAGAACGATCTGAAAAACTAATTAAACGTAATCGTCAAGATGTACAAGATCAGTTC
GATGAAATCATTTTGAGCACTAAATCGTCAGCTATTCAAAGTACTTTTGAAATGTCAAAT
AGAACCTCTAGGATGTCTGATAGAGAATCTCGTCGATTGAGAAGAATACGAACAAGAGAA
ACAATGAACATTATAAATCATTATGAAGGTTTGAGTTCTGATGACGAGGAACCGAAATCA
GCTATCAGTAGCAGGCAAAACGAAATAGAACAAATAAAAACTGATGCCAATCAATTATTC
AGTGATACTGATCCTGAATATTGCTCACTTGAAAAAATATTAAATGAATTTCTTGATGTA
AAGCGTGAATTTGAAAATGAGTACAAAGATGCTTACATAAATGGCTTTCTCGGTCATGTT
GTTAGTCCGTTAATTCGATTGGAAATTATTACATGGAATCCTTTGCAAACTCCTCATTTC
CACAAATGGAATTGGTTTAAAACTCTAATTCAATTCACCAAGAATTCAGTTGGTACAGAA
ATTCTAACCGACACCACATCGATTGAATATGGAGATTTGAAAATAATTCCATCGATATTC
GATAAAGTTATTATTCCGAAATTAACTGAATGGGTTTCTATATGGAATCCTCTTTCTAAA
TCCGAAACGCAGAACCTCGTCAAATTCATCAGTAAACTCGTATCGCAAATTCCCACATTT
AACGATAAAAACAACAATGTCAAGGAACTAATCAAAGCGATTGTTTGCAGATGTAATGAG
TGCATAGTTGAAGATATTGCAATTCCTTTGTATATAGAACAGTATAAAGAAAATCCGACT
GGCCAACCATTTCAATTTTTCCAACATCAAATGTGGGCAGCAATTAAAACCTTCACCAAT
ATTATGATGTTCCATGAGATTATCGAACCAAATTTACTTTGTAAATTAGCATTAGAAGGC
ATTCTGAATAATTACATAATAACTGGGCTTGTTATCCTTTCCGAGTTGAATTACGTTAAT
GACATAATCACAAAGGTTTCCATGATTAAATATTGTTTCCCTGAAGATTGGGTATCGAAA
TCCGAAGAACTGAAAATCGATATCGTTGTTGAGTTGAATAATCTGATAAGATTTATCAAT
AAATTCGGTGCTACTCATAAAAAGGACAAAGCTGTAATAAGTGTCGCTTTCTCAATATTG
GATCATTTTAATGCTAGTCGCGGAGATTTGTTGGCTTTGATGAAAGATTAGgtctg
>DjScaffold5235:80744-81707 strand:+
TTAATATTGATATTATGTCAATTTATTTTTAAAGCTAGAGGATATTATATTACTGTCGAT
GCAAATTCAGAAGATTGCTTCTTTGACAGATTAGTTTCTGGTGCTAAAATGATGCTAATG
TTTGAAGTTGTCGAAGGTGGATTTTTGGACATAGATGTTAAGGTTTATGGACCTGATAGC
AAGTCAATTTATGCTGGGGATAGAGAATCTAATGGTAAATACGCATTCTCAGCTCATGTT
GACGGCATTTATAAGTATTGCTTTAGTAACAAAATGAGCACAATGACACCTAAAGTAATA
ATGTTTTCTATGGATATTAGTCAAAAGCAAAGAGAAACTAAAGAAATGAACACAGATGCT
CATCAAAATAAATTAGAAGATATGGTTTATGAATTAAGCAATTCTCTTTTAAACGTAAAG
CACCAGCAAGAATATCTTGAAGTTCGAGAGAAAGTTCATCGATCAATTAACGAGAACACA
AATTCCAGAGTTGTTTTATGGTCGGCTTTTGAGGCAGTTTTGCTTTTATCCATGACAATT
GGTCAAGTTTATTATTTGAGAAGGTTTTTTGAGGTTCGACGCGTTGTTTAGtataaattg
taatacatggatcattttcaaatctaatgtcacaaagtttatgactaacttcaaattaat
taaatttaattaattaatgtaaatctaacttatttgaaatgcttttgtacaattattatt
caagtcaaatatcataatgccacctaaacgtgttaaatctaaatattttctatttgaatt
ggtttctggtgagttgtgattttatttttattaaagaaatttgttttgatgttt
>DjScaffold5235:109555-109764 strand:+
ATGCCATTTGGTATTGCTGCTGCTCCAGGAACGTTTCAAGAACTTATGAGTAAAATATTA
ATCGAAATAAATAGTGCAAAAGCCTTGGTTTATTTGGATGATATCTTAGTTTTCTATAAT
TTTTTGGAAGAACACAAGATTTGTTTAAATAATGTGTTAAGTAGAATAAAAACTGCCAGT
TTAAGAATTAATCCAGACAAATGTTCGTAA
>DjScaffold5303:70811-71297 strand:+
gaaactttaatttgaaatATGCGATACATTACTTCATATATCCTCTCAGTTCTTGGGGGC
AATTCAGAACCCGAAGCAGAAGATATTGGCGCTATTTTGTCATCAGTTGGAATTGAATGT
GATACTAACAAAGCCAAAGAAGTTGTTCAAGCTATCGATTGCCGGGACATCAATGAATTA
ATTGCTGATGGTTTGAAAAAAATATCATCCGTACCAACTGGTGGATCAGCATCTGCTGCT

GCTACTACTACATCAGCTCCAGCTCCATCTGCCGCTAAAGAAGAAGAAAAAGCAGCAGCT
AAGGAATCTAGTTCTGAATCTGAAGCTGATATGGGCTTTGGATTGTTCGATTAAttttaa
atgaataaaaatggaattattagttttggttataattgtttccatttcttaaaggaatag
ttattaatgtatattgtaaatat
>DjScaffold3656:17496-17708 strand:+
ATGATAAAGGTTTTAGGTGAAACAAATGGGACAATGGTGTATTTAGACGATATTCTAATA
TGTTCAGAAAACTCGGAACAGCATTACAAAATACTGGATGAAGTATTTAGAAAAATACAT
CAAGTAGAATTATGTTTGAGCCCGTCAAAATGTCAGTTGCTAAGAAAAGAGATCAAATTT
TTGGGACACGTCATAAGTGATAAAGGAGTTTAG
>DjScaffold4876:13530-14566 strand:+
ttttagtttcgtaatgatgacgaaataattttatatgccaatttttgctgttagtgcgaa
agcgctcaaatactatttaaacatggaacttttattatacataacctcaattacaaATGC
ACCACAACAACGATATTTTACTTTTGGATATACATCCAAAATTCCCCGATTATGGATCAC
TTAAATATTCGCCAGCGTCAAGTGACAATTATCTTGAATATAGTGATCCATTACTTTTTG
ATCAAAATAGTTTATCATCTCAATCCCCTTTATCCTTTGATATATCCGATGATTTCAATG
AATATAATGAAGCCCTTTTTTCAAAGCTTATCGAAGAATTTAATCAAGATCCCTTACTTA
ATCCTAATGAATTTGACTATCTTATAGCTGACAATCTATATTTTGATAATTGCTCTTTAG
ATTATACTCTTGCTTCTGTAGAAAATTGCACTTCAAATTCAAGAAAACGCAAACATAAAT
CGAATGACTGTGATATATTCGAAGAAACTTTTGCAAAGAAGCCTATAAAAGAAAAAATCA
ATAAAAATAACAACAATGATGCTGCTTTTAGATATAGACAAAAACAGAAAGTTAATAAAT
CTAAACTTGAAGAAGATTTAAATCAATGTGCCGTAGAAATGGTGAATGCTAAAAACGATA
TGCTTGAAATGCGTAAAAAATTGAATACTGTTAAAAATATTGTAAAAGATTTGTTGATAG
ATAAAGGCATCATTAATTTTTAAtcccttaaataaattttcattatttttaagattaatt
tttattttgtaaatattttcgtttgtctattagtttgtgggcggattttgtttgcttgta
tgatttattactaaaaataaatttattttaattagttattgttatcgcgatatctctgta
taatttgatttgtaaataattaaatatggttagttcctcctattttacaaaaatgtggct
actgtttattaaatatattttggattttgtg
>DjScaffold4452:64155-64883 strand:+
tttcttgacactttaattcaaaagaaacctggtgaaatattaactacccaattatatatg
aaaccatttcacagtggacatatattaccttggagtaaaaattggtataattaaaggtga
aagaattcgagctgagagactatcttcaaatgaagaaATGAAATTGATTTCTCTCAAGTT
GATAAAAAAATAAATTTTTAAATAATGGCTATCCAAATAAAATTATATCAAAATATTTATC
TTTAAATTCAAAACAAAATAAAATCAAACCAAAAACAAATACAGTTATTAAATTACCGTT
TATTAATGAGAAATGTAGTCAAATCATGCAGAATATAATAAAAAATGCTAAACTAAAACA
CAATCCAAGAATTATTTTCAAAACGCAAGCACCTTTGGGCATCCAATTACAGAAACCAAG
ACAAACTTTTTGTGGTCCCAAATGCATTTGTGATGGTAGAAATATATGCAATAAGAAAAA
TGTCACTTATAAAATAACATGCACCATTTGTCAAGAAACATATATAGGTGAAACATGCAG
AACCATCCGAACAAGAGTTAAGGAACATTTGAAACAAAAAGAAAGTGAGGTATTCAAACA
CTTTCAACACAAACATAAAATTTCACCAGAACAACATCACATTACATGGAAAACACTAGG
AACAGGCTACATTTCTACACTACATAGAAAAAAACATGAAGAAATACTA
>DjScaffold2438:86709-87383 strand:+
tacattttagcttttcagcatcaatacatggcgactcgtcaattaaatttaatggaaaat
ttttagtctgattatttttaacATGTTAAATTTGTTATATTTGATAGCATGCTCGATATT
TCTTAAATGTTCGAATAATTATTTGAAAGAATATTCTTATTTGAGTCACTTTAATTCGAA
AACAGCTTATCGTAATTGTAATCTTACTGAATATAACAAAGGATCAGATCAACTGAAACA
ATGTAATTTAATTCATGTAAATACTGTATTCCGTCACGGAACTAGATATCCTTCTGTTAA
GAATATTCAAAGTATGGAGAAATTATTTGATAAATTGAAGAAAAGTGAAAATAGAGAAAA
TCTTCCCAGGGCTTTTCTCAATTGGAATATTAATTTTGAAATAAATAAAGCGAATGAACT
AACCGGAGTTGGAATAGAAGAGATGATGGGACTTGGAAAACGATTTCGGAAACTATTTAA
TGCCATACCAAATAATGATGAACTGAAAAAAATTGTGAAAGTTTTTTCTACGTCAATTTC
TCGTAGCAAATTGAGTGGACAAACTTTCCTTTATTCGGTATAAttataaatatgataaag
aacgaaatttgattagctagaaattaaa
>DjScaffold2049:3884-4192 strand:+
ATGATATTTTCTATTGCAACGGCACCAGGTACATTTCAGAAGATGATGACAAAAGTGGTA
GGCGGAATTAATGTAGAAATGGTTTATCTTGATGAAATTCTAATATTTACGAACAATAAA
GATCAACGATATAAAGTAATTGACAGATTATTGACAGAAATATTGAAGGCTGGTTTAAGA
ATTAATCCAGAAAATTGTCAGTTTCTTCAAAGCGAAATTAGATTTCTTGGACACATAATT

AGCAGGGATAGGGTTAGAACTGATCCATCTAAAAAAGAAGCAATAAGAACATTAGAAAAA
TCTAATTGA
>DjScaffold4649:35290-38747 strand:+
aataaaatataactttacaattggaattattgcgataataattgttggacataaccattg
atcgtttttagaattcatttaaaataaaactgacgaatcaattattcctgatcaaatca
attcctgaagaagacaaagacgaaatatagaatcaatctcaaaaaacaaaaataaaacaa
caatttttgatcgaagctcttattttaaatgaataatagaacatagttaattattttaac
caataaaaatttattgaaaataaatatatttgaaaatttaaacttatttccataataaat
atgcaagaatcttttaaaaagatttccttggagtattaacctcactcaacagtttctgtg
atcATGATCAATGTGAAAGATGGAATGAAACTTATGAATATGATTAGTCAAAGATTTTGT
TACGCGCAATTAATGTCATATGATCTGCCTCCAGCAATTCTAATGATATACATTTTTCTA
ATGGGTGTGGTGATATTTATCACTTATTCAATTTACAAACTGGGACGCTACCTCAATATT
CTATGGATGCGAATCAAACATACTATAAACATGAAGATTTATATGTCGAATTTGAAAATT
CAGAAATATAAAGCTGGTGATGAAGATTCTTGTGCAATTTGCTTGATGGAGTTTTCTACT
AACTCCAAAGTCAGGAATCTGCCATGTGGTCATGGATTTCATCCAAGATGTATTGATCAT
TGGATACGAAGTCATAAACCTACATGTCCATTGTGCAATCAACAATTGCCTATTCCAACA
GGTGTGTTACATGTAAAATGTGCCATTTGTTCATCACCCGTTACTGGTGGTAACAATTCG
GGTTTAACAATTCATGATGATAGACCTAGTCAGAACACGATTTTGACAGCTGATGATTTT
GTAAACTCAAATGAAGAGCAAGTTGATAATAGATTAATAGATAATATTTTATCTGAAATG
GATTCGAATAATGATCCACATCAAAATGTCACAACTAGCCCAAATCATAATGACAAGAAT
GGAAATGATAAATTGCCGCTATATGAGCTCATACCTAGGATAGATGAAATAACAGAACAT
ACTGCATCAATAACGCTAGCCAATGTGACAATCAATGATGAAACGGTTTAAatatcaaaa
aatgttgctcaccgctttttcacaaaattttctcatattcatcaacaatgcaccgaaaaa
tattacattacaatcttggtgatctagagtagacattttgacaattacgattgtgtgatg
acatttcatgagactaaatataggaagctcttattttaaatgaataatagaacatagtta
attattttaaccaataaaaatttattgaaaataaatatatttgaaaatttaaacttattt
ccataataaatatgcaagaatcttttaaaaagatttccttggagtattataattacaata
atgtaaaaacgatatttctagttcaattgcctttcatttgcagaacctcactcaacagtt
tctgtgatcATGATCAATGTGAAAGATGGAATGAAACTTATGAATATGATTAGTCAAAGA
TTTTGTTACGCGCAATTAATGTCATATGATCTGCCTCCAGCAATTCTAATGATATACATT
TTTCTAATGGGTGTGGTGATATTTATCACTTATTCAATTTACAAACTGGGACGCTACCTC
AATATTCTATGGATGCGAATCAAACATACTATAAACATGAAGATTTATATGTCGAATTTG
AAAATTCAGAAATATAAAGCTGGTGATGAAGATTCTTGTGCAATTTGCTTGATGGAGTTT
TCTACTAACTCCAAAGTCAGGAATCTGCCATGTGGTCATGGATTTCATCCAAGATGTATT
GATCATTGGATACGAAGTCATAAACCTACATGTCCATTGTGCAATCAACAATTGCCTATT
CCAACAGGTGTGTTACATGTAAAATGTGCCATTTGTTCATCACCCGTTACTGGTGGTAAC
AATTCGGGTTTAACAATTCATGATGATAGACCTAGTCAGAACACGATTTTGACAGCTGAT
GATTTTGTAAACTCAAATGAAGAGCAAGTTGATAATAGATTAATAGATAATATTTTATCT
GAAATGGATTCGAATAATGATCCACATCAAAATGTCACAACTAGCCCAAATCATAATGAC
AAGAATGGAAATGATAAATTGCCGCTATATGAGCTCATACCTAGGATAGATGAAATAACA
GAACATACTGCATCAATAACGCTAGCCAATGTGACAATCAATGATGAAACGGTTTAAata
tcaaaaaatgttgctcaccgctttttcacaaaattttctcatattcatcaacaatgcacc
gaaaaatattacattacaatcttggtgatctagagtagacattttgacaattacgattgt
gtgatgacatttcatgagactaaatatag
>DjScaffold4649:7569-8942 strand:+
ATGATAGATGAAGCACACGAAAGATCAGTCGATTCTGATGTTTTATTAGGATTATTGAAA
CTAATAGCATCAAAAAGAAAAGATCTGAAAATTTTGATAACTTCAGCAACAATTAATGAG
GAAAAGTTTTCCAAATATTTTTCTCGAGCTCCTGTTATTTCATTTGAAGGTAAAATGTAT
GATGTTGATGTAATTTATGAGCCTATGTATACTTGGTCAATATATTTCATAAAGACTGTG
ATTGAGAAAATACATAATAAATCTGTTGATGGAGATATTTTAGTTTTCTTAACTGGTCAA
GAAGATATTGAGGAATGTTGTGAACTTTTAGAAAATGTAGCAAATCTTGTAGTTCTACCG
TTATATGGTGCAATGGATTTCGATAATCAACAAAAAGTCTTTAAACCAGTCGCGAATGGC
AAAAGAAAAGTAATTATAGCAACAAATATTGCTGAAACTAGTATAACTATTGATGGTGTT
CGATTTGTTGTCGACTGTGGTTATGTCAAGCAGAATCGATTTGATGCTAGATCTGGAATT
GACACCTTACAAAATGTTCGAATTTCAAATGATCAAGCTACTCAACGGAAGGGTAGAGCT
GGTAGAACAAATACAGGATGGTGTTATCGATTATATAGTAAAGATACTTTTGAATCTATG
AAAGTAGAAACTACTCCAGAAATTCATCGTAAAAATCTATGTGGAACATATTTGAAATTG

GTTTCATTTGGACTTGATCCTATGAAGTTCGATTTCATTGATTCACCAGATCCAGTTACA
CTTAAGAAATGTCTAGAAGAACTTCAAGATCTCGATTTACTTGATAGTTATAAAAAAATT
ACAGATTTGGGAAGAATGATGCAAGATTTTCCAGTAGAACCGAAAATAGGCAAAGTGTTA
TTAAAGTCAGCTTCGTATTCTTGTTCATTAGATATTATTACAATACTGGCAATGTTGACT
GAACGAAATGTGTTTGTTCGACCAAGGAACTCTAAAGGTAAATCAGATTTGACTAAGAAA
CAATTTTTCAGTGAAGATGGTGATCAATTTATGTTGTTGAATGTGTTTAACAAATGGACG
GAAAACAATAAATCAAAACAGTGGTGCTATGATAATTTTGTTAACTCTAAAACAATAGAA
GAAATTGATTTGGTTAGAACACAATTATTGGATTTAATGAAACGGAAATTTCCTAACCAA
GAAATGAAATCAAATAATATTTCCATAGATTCTATTAAAAAGTCCTTAATTTCTGGTTTT
TATAAAAATATTTGTTACCAAAGATCAAAAGGAGTTTACATAAACTATAGTAATCAACAA
GTTCTTTATATGCATCCGAGCAGTTGTTTAATGAATAAGCATCCACCAATGTAA
>DjScaffold5152:14449-15280 strand:+
tgttactaattcatctttagaaaatattttagaactagccaatagtatcaatgataacat
taaattcactttagagaagtcaatcgactttcagattccttttctggacaccttaatcca
aaagaaacctggcgaattattacttacccagttgtatatgaaaccatttcacagtgggca
tatattaccctggagtaaaaattggaataatcaagggtgaaaagataagagctgatagac
tatcttcaaacgaagaaATGAGACTGACTTCCCTCAGGTTGATAAAAAATAAATTTCTAA
ATAATGGTTATCCAAATAGAATTGTATATAAACATCTATACTCAAATACAGAATGTAATA
AAATCAAACAAAAGACAAATACAGTAATTAAATTACCTTTTATTAATGAGAAATGTAGCC
AAATCATGCAAAATATAATTAAAAAGTCAAAACTAAAACACAATCCAAGAATTATATTTA
AAACGCAAGCACCTTTGGGCATCCAATTACAGAAACCAAGACAAAATTTTTGTGGTCCCA
AATGCATTTGTGATGGTAGAAATATATGCAATAAAAAAAATATCACTTACAAAATAACAT
GCACCTTATGTAAGGATACATATATAGGAGAATCATTCAGAACCATCCGTACAAGAATTA
AAGAACATCTTAAACAGAAAGAAAGTGAAGTCTTCAAACACTTTCAAAACAAACATAATG
TTTTACCAGAAAATCATCATATTACATGGAAAACATTAGGAACAGGCTACATTTCTACAC
TTCACAGAAAGAAACATGAACAAATATTAATT
>DjScaffold11:272-1167 strand:+
ggaattgacaaatgtagattaatctgaactttagaactacaagtgtttatatctatcata
ttattataaatattacaattaataattttcagtaactaaaATGAAGTGGATAATACTGTT
CTGCAGCGTTTGTATAGTTTCAGTATTCAGCAAAGAAACTACCGGGAATATATGGAGTGA
ATTTAAAGAACAAATGGATAGTTTAATGAAGTTTGAAAAGTTCGATGGTAAATGGCCTCC
AGAATTCTGTAATACACTCAAGTGTCCAAAGTTTACAATTATTAAATCTACAAAGAACTA
TGAACTTCGAGAATATGAAAGTTCAACTTGGGTGCACACATCGACAACAGGTATACCTTT
CAGGAAAGCAATACGGAAAATGTTTTGGAGGTTATTCAAGTACATTAATTTTGATAATGT
TGAACGAAAGCACATGGCTATGACAGTCCCCGTATTGGTCAAATTTACTCCAGGAAAAGG
AGCAACTTGTTCAAATAATTTTACTATGTCTTTTTACATAGATACTCCTAATCCACCGGC
TCCAAGTAGTCCTACTGTTAAACTCTCAACGTATCCTAAAGGACGGGTATATGTGCGGTA
Aatatcgatgttttcgaatataattaacaagacttataactttgaaaataaattaattaa
taaaaatatttaaacaatgggataacgtattaattataaatattttaataaacagcttac
caccttcaacaaactaattaattgacgttattattattataattattattattattatta
ttattattattattattattctatta
>DjScaffold5207:20854-24303 strand:+
ttttataatttcgatttttatcttattggaaaATGTCCATTTCGTCTCACAATGCAACAT
TAGCATGGTCAATATTAAAAAAGTCACAAAAGAAAAAGAAAATAAATTTACTCAACTGTG
ATACACCTATTTCTAACGATTTTATTCGATTTGTGGCAATTTCAGATACACATAATAAAC
ATGATAAAATTTCAATTCCTAATTGTGACGTGTTACTTCATTGTGGTGATTTCACATTTA
ATGGTTTCAAACGATCGCAAATTGAAAGCTTTGATAAATACTTGGGAACTTTGCCGAATT
ATAAGAAAATCGTAATTTCTGGAAATCATGATTGGGATATGTATAAAGTAAACAAAAATT
CTTACCAATTTCAAAATTGTGAATATTTGCAAGATTCTGGTACAACAGTTTTTGGATATA
AGATTTACGGCGCACCTTGGATTCCTCATTATCACAGAGGAGCGTTTATGCTAAATCGAG
GTGATGAATTAAAAGAAAAATGGCAATTAATTCCCAGTGACACTGACATCTTACTAAGCC
ACGGACCACCTGTCGGATATGGCGATAAAACTAGGGGGAATAATAATGCTGGTGATGTTG
ATCTCCTTCTTGAAATACAGAATCGGGTCAAACCAATTATGCATATATTTGGGCACATCC
ATGAAGGATACGGTACATGGACAGATGAGACAACCACATATGTGAATGCTTCAATTTGTG
ATATTCATTATAGACCCATTAATAAACCTATTGTTTTTGATCTTCCCATATTGGAAAATC
ATTTTAAGAATTCTTAActtaaatttatgaaaagaaatt
>DjScaffold986:8234-15371 strand:+

TCTAATTCTAATAATTTAGCCAGCAATGATTTATTTTATTTAATTACTACAAGTAAATTC
AGCGACACGGAAATTTATAATCAATGCATTGAATTATTTATACAGTTAAATTTTATGGGT
CCCACTAATGCAACTATTGAAAAGTTGTTAGGTAGCAATTCTGAAATGATTCGATTAGTT
AATGAAGAATTAACATGTGAAGGAGTAGTCCCGTTTCTTATGACAAAGGGTATAATACTG
TTGTGGATAATAAAGGTGTTAGCTATAAAAATAAGTGAAAATAGAGAGAATTTGTATGCA
AACTGTTTGATAAATTTGAAGTATATTTATTTGCATCATTCTGTATTACAAGAAGAAAGA
TCAGAATTTCTAAAAAGAGAATTGGATGAATGTTACAATAAAGCAATAAATCTTAAATTT
GAAAATACAGAAGGCATAGAATCGATTGATGAATTTCAATTCTTAATGTTATCCAGTTAT
CTTTATGCGTTCTATTATGACGATAGAAAAGCTCTGAAAATCTTTAATGAAATCGAAGAA
TTGTTGGGTATTAAAACAGAGTTTATTGGAGCATTAGGCAGGAAAACAAAGCTGTACAAT
CGTAACATCTCACAATTATGTCTAAATATATCGAGGAAGCCGTCTAGAGCCGCGGAGGAG
ATCGATTCGGTATTAGAGAAAGTGAGAACGAAGAATCTACCGAATGATCAATTGCAAGAA
GACGACACGTTTTTCAATCAAATCGAGTTTGCAGAAAACGTAGAAGAAGAAGATTTTCCG
TTGAAGATTTCCCCGAGAACTTTAGCGGAACAAGCTGTCGTCGTATTGAAATGTCGAATT
CACAAAATAGCGAGAGGCAGATGCGAATCAACAATGGAGGAGTGCGCTGCGATTCTCGAC
TGTGCTCTTGGCAACAGCGCCACCAGATCGTCGATATGGGCGCTCGCAACCGAGGCGCTG
TTGCAGCGCTGTCAGCTGAGAACGAACAATCAATCAGCAAGACGAATTGAGCATTCTCTC
AAACAATTCGAGGAAATCTCCAATCAATATTTCCATTCCATCCCGACAGCAGAATCTCGA
GTTGTTTGGTTCTTCTTGTCGCGATTACCTCCAATTTGGGAAACTCAATTGAGATTGGCT
AATCTTTACCTGGAAATCGGACTGCTCAAGGCAGCACTCGATGTTATGTTGCCATGGCAA
CTGTGGAAAGGAATAATCGATTGTTTGATGAGATTGGGCGAGCGAGGTCGTGCGGAACAG
ATGATTCGTGAACGAATGCAACAATCTGGGGACACTGTGGAACTGCTGTGTTGCCTTGGT
GATGTGACGAAGGAGCGCAAGCATTACGAGAGCGCCTGGGAACTGTCGAAACATCGATCG
GCGCGTGCGATGCTCAGCCTTGCCCAGCATTTCATGTTTATCGATTCCGACCATTCGAAT
GCTGCTCGATGTTTCGAGTTGTCCCTCGCTTGCAACTCGATCCAACCGACAACTTGGTTC
ACTTACGGTTGCTGTTGCCTGCAGTTGCGGGATTATCGCAAGGCGGCTGACGCATTCATG
TTCTCAACACGATTGGAATCAGATAATTACGAAGCTTGGAACAACTGCGCAAGCGCTTTG
ATCGAGCTAGGAGATAAGAAAAGTGCTCTGAAATTATTAAATGACGCAACGAAATACAAT
TACGATAATTGGAGAATCTGGGATAACATTTGCCTGGTGTCAGCGCAAATTGATGATTTC
CCAACGACAATAAAAGCATACCATCGAGTAATGGATTTGAAGGAACAGTACGGAGATCCT
GCAATTCTGAATTCTATGACGAAAGCAGTTGTCAGCAACAAAATGGAAAGTATTCGTCCA
AAATTAAAGGAATTATTTGCACGTATTGTTTCTCATGACAGTCGTGATCCGAGAATCTGG
AGAATTTACGGTGAATTACTGGCGAATGATGAAGACAAATCAGATGAGATGAAACAGAGA
GTATTGCAATGTTATTCTCGAGCACATCACGGAATATGCATGAGTGGCTGGCAGAATGAC
AATGAGCAATGCAAACGTGCTCTGATTTCAGCGTTGGAGTTGGTCGATAAATCATTCGAA
TACGACATCAAATACACGTCTGCCAAACTTGCAGTCAAATCTGTGAGGGCGGTTTTTCAC
AATTTGCGGAGCTCTCGCACACAAGAATTCAGTGATGAGGTGAATAATTTGTTGAGCTTG
ATAGAGGAAGCAGATAATCGATTAGCTAGCAGAGAGAATGTAATGTAAtcaataaattgt
tctaattttcaaTCTAATTCTAATAATTTAGCCAGCAATGATTTATTTTATTTAATTACT
ACAAGTAAATTCAGCGACACGGAAATTTATAATCAATGCATTGAATTATTTATACAGTTA
AATTTTATGGGTCCCACTAATGCAACTATTGAAAAGTTGTTAGGTAGCAATTCTGAAATG
ATTCGATTAGTTAATGAAGAATTAACATGTGAAGGAGTAGTCCCGTTTCTTATGACAAAG
GGTATAATACTGTTGTGGATAATAAAGGTGTTAGCTATAAAAATAAGTGAAAATAGAGAG
AATTTGTATGCAAACTGTTTGATAAATTTGAAGTATATTTATTTGCATCATTCTGTATTA
CAAGAAGAAAGATCAGAATTTCTAAAAAGAGAATTGGATGAATGTTACAATAAAGCAATA
AATCTTAAATTTGAAAATACAGAAGGCATAGAATCGATTGATGAATTTCAATTCTTAATG
TTATCCAGTTATCTTTATGCGTTCTATTATGACGATAGAAAAGCTCTGAAAATCTTTAAT
GAAATCGAAGAATTGTTGGGTATTAAAACAGAGTTTATTGGAGCATTAGGCAGGAAAACA
AAGCTGTACAATCGTAACATCTCACAATTATGTCTAAATATATCGAGGAAGCCGTCTAGA
GCCGCGGAGGAGATCGATTCGGTATTAGAGAAAGTGAGAACGAAGAATCTACCGAATGAT
CAATTGCAAGAAGACGACACGTTTTTCAATCAAATCGAGTTTGCAGAAAACGTAGAAGAA
GAAGATTTTCCGTTGAAGATTTCCCCGAGAACTTTAGCGGAACAAGCTGTCGTCGTATTG
AAATGTCGAATTCACAAAATAGCGAGAGGCAGATGCGAATCAACAATGGAGGAGTGCGCT
GCGATTCTCGACTGTGCTCTTGGCAACAGCGCCACCAGATCGTCGATATGGGCGCTCGCA
ACCGAGGCGCTGTTGCAGCGCTGTCAGCTGAGAACGAACAATCAATCAGCAAGACGAATT
GAGCATTCTCTCAAACAATTCGAGGAAATCTCCAATCAATATTTCCATTCCATCCCGACA
GCAGAATCTCGAGTTGTTTGGTTCTTCTTGTCGCGATTACCTCCAATTTGGGAAACTCAA

TTGAGATTGGCTAATCTTTACCTGGAAATCGGACTGCTCAAGGCAGCACTCGATGTTATG
TTGCCATGGCAACTGTGGAAAGGAATAATCGATTGTTTGATGAGATTGGGCGAGCGAGGT
CGTGCGGAACAGATGATTCGTGAACGAATGCAACAATCTGGGGACACTGTGGAACTGCTG
TGTTGCCTTGGTGATGTGACGAAGGAGCGCAAGCATTACGAGAGCGCCTGGGAACTGTCG
AAACATCGATCGGCGCGTGCGATGCTCAGCCTTGCCCAGCATTTCATGTTTATCGATTCC
GACCATTCGAATGCTGCTCGATGTTTCGAGTTGTCCCTCGCTTGCAACTCGATCCAACCG
ACAACTTGGTTCACTTACGGTTGCTGTTGCCTGCAGTTGCGGGATTATCGCAAGGCGGCT
GACGCATTCATGTTCTCAACACGATTGGAATCAGATGTAGGTCAATTCGATAATCAATAG
gataataattcattcattcaatcatttctcttacagaattacgaagcttggaacaactgc
gcaagcgctttgatcgagctaggagataagaaaagtgctctgaaattattaaatgacgca
acgaaatacaattacgataattggagaatctgggataacatttgcctggtgtcagcgcaa
attgatgatttcccaacgacaataaaagcataccatcgagtaatggatttgaaggaacag
tacggagatcctgcaattctgaattctatgacgaaagcagttgtcagcaacaaaatggaa
agtattcgtccaaaattaaaggaattatttgcacgtattgtttctcatgacagtcgtgat
ccgagaatctggagaatttacggtgaattactggcgaatgatgaagacaaatcagatgag
atgaaacagagagtattgcaatgttattctcgagcacatcacggaatatgcatgagtggc
tggcagaatgacaatgagcaatgcaaacgtgctctgatttcagcgttggagttggtcgat
aaatcattcgaatacgacatcaaatacacgtctgccaaacttgcagtcaaatctgtgagg
gcggtttttcacaatttgcggagctctcgcacacaagaattcagtgatgaggtgaataat
ttgttgagcttgatagaggaagcagataatcgattagctagcagagagaatgtaatgtaa
tcaataaattgttctaattttcaa
>DjScaffold986:43083-44251 strand:+
aaaacttttaaagttattaattcctttttaatttattttaaaagatagtttacgtaaaa
ATGATGAAACCTGTTGATGAAAAATATGTAGAAGATGGGGATTTATTTCAATCATGTATA
CCTGGTAATATTACAACTAATAATCCCAACTGTGATGCAAATGGATCGAATAATAAATCA
GACAAAGTTTTAAATGTAGAAATATCAGATGCTATGAGTGAAAGAGAGAAAGTGGTATTT
ACAGTTCATACTAAAACAACATTACAACAATTTAAAAGCAAGGAATTTTCAGTTAAAAGA
CAGCATGAAGAATTTGTATGGCTCCATGATCAATTAGAAGAAACCGAGGATTATGCTGGT
GTAATTTTTCCTCCTGCTCCTCCGAAACCAGATTTTGATGATTCGCGAGATAAATTAATG
AAATTATCCGAAGCTGAAAATAATATGCCAAAAGAACAATTTGATAAAATGAAAGCTGAA
TTAGAAGCTGAATATCTAGCCACATTCAAAAAAACTGTTGCAATGCATGAAGTGTTCCTT
CAGAGATTAGCTGGTCATCCGATTTTACAAAGCGACGTTAATTTCTCGATTTTCCTCGAA
TACGATCAAAATTTATCGGTTCGCGGAAAAGCCGAAAAGAAAAATTTACTGGATTCCTG
AAACAAGTGACGGCAACAATGGACGAGACTTTGTTACGCTCAAATCAGAAAGAAGAAGAC
AGTTTTTTTCTCAATGAGAAAGTTTATTTGGTGGATTATTATTTGGCTGTGCGGGAAGCC
ACGGTTAAAGCTGATAAAATGACGAGGCAACATAAAACTATGTCAGATGGTTACATTGCC
GTGTCTACTGCATTGGAGGATGCAGTGCAGCCTCACACAGGAGACTTGGGTCTTTCAGGG
TAAatttattaattaattagctttatttgtaaattaattaattaaatttatttgttaatt
aataaattttttaatta
>DjScaffold3766:20591-22718 strand:+
tcgaattgttaaccaatatcaataaataaaacctaaattttgatcaaagcttttatatta
aatgaattctataaaacgatcaatagttaactccaacaattattctcgcaataattgcaa
ttgtaaagaataataaatattaacatagtcggcttacttttcaaataaataacttcatta
aattatgcatgtattttaaatagcttaatttattattagatttatATGTCCTGTCATGTA
ACCATGACATATTCGTGTTTAGTCTGTTTGTTAATTTTGGGTGACGATCTTAGTCGAATC
GATAAAGCTGGTATTGTAAAAAGTTTTAAAAGCTTACAAAATTCCGAAGACGGTTGTTTC
TATGCAGCTCTAGACTGTCCTGAAGCGGATGTTAGATTTATATTTTCTGCTTTAGCATCT
TGTGCAATTATCAACGATTTTAGTGCAGTCGATATCGAGTCGGTTGTGTCGTTTATTTTA
AAATGTCAAAATTACGATGGTGGATTTGGTCAGAGACCCCGTGAAGAAAGTCACGGAGGC
TCAACATATTGCGCTATTGCTAGTCTTAAGATTTTATCAAAATTAGATGTGTTATTTCCT
GAGAATTCCAATAAAAAAAATTTGATGTTACGATGGTTGATAAATCGTCAGTATGAAGGA
TTTCACGGTAGACCCCATAAAGATGATGACAGTTGTTATACATTCTGGATAGGAGCTTCT
TTAAAGATGCTTGGCTATGGTCATTTGATTGATTTTCCATCATTGTTTAATTTTATTGAA
TCGACTTATGATGGATTAGTTGGTGGATTTTGTAAATTAAAGAATGCTGGTCATTCTTTT
GCTGATCCAATGCACACTTATTTGTGTTTAGCAGGAATATCTTCATTAATTAATGATTGC
AAAGCTAACTCAACAAAGGACAACTCTCAAACTATTTCAATGGCAGCAAGTCAATTAATT
GAAATTCTTCAACCTTTGGAACCGTCTTTAAATATCTGTATTAAAAGAATTAAAAATAGG

TTGACTGATGATGACGAATTTTTATTGAAAAACATGGAA
>DjScaffold359:82632-93561 strand:+
caaacaaagcATGGACAAAGCTCGTTCCCGTATTCAAAATCTCCCAAATGATGCATTCGT
TGATCTTTGTATCGATGTGTGCGACGAAGTTGATCGTCGTGATACAAATGATATATTATC
TCCATCAAATAAAGATCTCACTCTTTATTTTCTACCTCCTAATCCAAATTATAGCCCTAC
CCGCAATCAAGGACGTCAAAAATTAGCTCGTCTATCAACACCTGAATTCCGTACACTTTG
CCAAGACGTTCTCGTTGAAGCTTCTTTACGCCTCAGTCCCCAGAGTCACGTTTATCAGGT
TTATAGCAATCAACCGAGACCCCAATCTAGGAAGCACAGATCTTCATTACCACTTGGTTC
GTCTCTCGATCCTTCTACCGCATTGGAGGAACATGACCCAGTCTACGATCAAGTTGCTGC
CGATGGTGATTATGCCTATGTAATGGATCTCCATCGCTCTCAGGGAAGTCCTTTACCGGG
ATCTGGTTTGTCGAATATTCAGAAAACCAATGGTTTATTAAAATTGAGTCTAAATGGAGT
ACCACCGTCGAACAGAACTAGTATGCCAGTAAAAGTATCATCATCGACTGATATTCAAAC
AGAAAACGCATCAGAAACAAAAGATAATTCTGTAATAAATTTATACAATCAAATAGACGA
ATTAACCGGTCAAAATAAACTATTGCAAGCGAAAAATACGGAATTGGAAAAAAAAATGAA
AGTCTTGGAATCACAGGTCTCTCAATTGAAAGATGAAAAAACAGCTCTCATAGCGGCCCT
ATCGAATATGGGTAATAAACATGGATTACCTAGTTCGAAAATTCGTTCAAGAAATCATAG
TAATCATACTGAGTCCAGTGATTCTTGTGATTACCAAGAAAATCTCGAGGATGTTACAAA
GTTACTAAAATCATCTCAGTTCGTGCCAGTATCTACTTCTACTCCTACCGCCAGTGTTAA
TAATAATGAAAGTCCTTATGATACAGGGTCCGATAGATTTAGTTCCGGTCCTTGGCTCCG
ACAAGAAATACTCCATAAAGGAAGTCAATTATCTTCTAATACTGGATCTCCAATTTCAAT
TGGACACAAGAAAAATCCAAATGCAGAGTCG
>DjScaffold3428:17361-41211 strand:+
atgatattcagttaacaactctttcatggaatactaataaaggttatatagcatgtggtg
gagttaatggactattaaaagtactaaagttggatcctcaagttgatggaaatactaggg
gtatagctgcacctacaaatttgacaatgaatgaaactttagaagaacataaaagtaatt
taacgaaaataacttggaatgaaaaatttgataaaatgacaagtgctgatgactctggtc
ttataatcgtttggatattatttaaaaatagctggcaccaagaaatgattaataattcca
attcaagtagagtcagtggaataaaagtaagtcttgaattgaatttaatgcacttaataa
tatttataagtagtagtaatttaattattaattatatttaataaatagtaattaatttta
attaatttgatttgttatcatattttttgattatgcagttaaactcatctaaaatcactt
tcgttataattccaaaattataatttggtttgctaaagaatttttgtagtaccgaagaag
aggtttctaaaattgtaggtattgaatggtataaaggcgaaaaaggatatATGTGCAAAA
ATTGTCCCTGTCTGGCAGTGTGCTATAATAATGGTTGTTGTCAAATAATGCGTAATGAAA
ATGATGAATATCCGGTTCTCATTAATACCGGTATTGAAGCGAAATGTTTAGCATGGAATG
GAAATGGGACGGTATTAGCTATAGGTGGTTTGCCCGTTGGTCAAATAACAACTAAAGACT
TCAATCTTATTCAATTTTACAGTCCTTTTGGATATCATTTACGAACACTAAAAGTTCTTT
CTACAAAACTCCATTCATGTAGTTGGGATAGTGAATCTCTACGTTTAGCCATCGCTGTGG
ATTCATCAATATATTTTGCCAATATTCGTCCATCTTATAAATGGGCATATTGCAACGATA
CAATAATTTATGCGTTTACAAAACCAGATCAATGTCATTATTTTGTTGTGTTTTGGAATT
CTAAGCTCGATTCGTATCAAACAAAATACTTTTTCAATTTAAAAGCCATAATTTCTAATG
GTATTCTTTGTACGATTATCTATCAACCGGATAATGACAATAAACAAAAGTATACTTTGC
TTGTCTGTAATTCAATTGGAACTCCAATTGATAGTAAAGAGTTACCATTTGAACCTAGAT
TCCAAGTGATGACAGAAACACATGTTATTGTTGCATCACAAAATATCATTTATGCATGGC
AATTCAAAAAACCGGAAAATCTTTTATCGATAGAAAGTCAGACGAAAAGAAAGTCAACTA
TGGATAATTTCAGACATGTCGATGATACAACTCAACAAATTGATAGTTCTACAATCACTG
ATAGTCACTTAGTTAATATGATAAGAAAAGTATCTGATGATCCAATTTGTTCTATATGTT
CATCAAGACTTACTCTTATAGTAGCAACTGAATCAGGCAAAATGAAACAATATTCCATGC
CGTTAATGGAATATGAGACTACTCATAAACTTCGAAGCAAACCGTTTAAGCTAGAAATTA
ATTGTACTGGAACTCGAGTCGCTAGCATTGATTTTGGAGGAATTGTTTCTTTTTTTGATT
TGGAAAAGAGTTATACTGATGAAAATGGAGTGACGAAACAAGGTATTATTGTGGAAAATT
TTGAATACAAAGATGTATGGGATATGAAATTTTCTGAGGATAATCCTCATCTTTTTGTTA
TGATGGAAAAGACTAAATTAAGAATTATCCAAGATATTCATTCAGAAGAGCCTGTAGTTT
TTTCTGGTTACATATGCGAATTTAAGGAATTAGAAGTAAAAGCAGTTCTATTGGATGAAA
TCATGAGTGATTTAGACAAACCAAACAAAAACAATGTCATCACATTGGAAACTAAAATTC
TTAGAGATTGTCGAAAACTTCTCGAAGAAGGACAGATAGAAAAAGCTAAAGAATTTATTG
AGGATCATCCAAATAATAAAATGTGGAAAATGCTTGCTGAATATGCCTTAGAAAAATTGG
ATTTAGAGACAGCTGATCAAGCTTTTGTACGATATACATATTATCCTGGTATAAAATTTG

TAAAAAAATTACAAAAACTTCAGAATAATAATTTGAAATTGGCTGAAGTTTCAATTTATT
TCGGACGTTTTGAAGAAGCTGAACGTCGTTTTATGTCAATTGATCGTCCGGATCTAGTTA
TTGCAATGTTAAAATCGATCGGAGATTGGTCTAGAGTATTACAAATGATGAAATCAGGAA
ATAGTGCTTCAGATTCTGAAATGAATGAAGCATGGAATGCAATTGGTCAACAATATTTCG
ATAAGCATAATTGGACACAAGCTGTAAACTATTTTACACAAGGAAGATGTTTTGAAAAAT
TAATTGAGTGCTATTACTATCTTGAAGATTATTCTGGCTTGGAAAGACTTGCTGAATTGC
TTCCTGAAAATTCTCCTTTATTAACTGAAATAGCTGAAATATTTGTCAATGCCGGTATGT
GTGAACAAGCAGTGTTTACTTATGTAAAGAGTGATCAATTGAAAAATGCAATTGACTGTT
GTATTCAGCTAAACCAATGGCAAACTGCCATCGATCTAGCAAAAAATCGAGATATGACCA
ATATTAATGATATGTTGACTCGATACGCAAAACAGTTGCTTGATAATAAATGTTACGTAC
AAGCAGTTGAATTATACAGAAGAGCAGGATGTTTTCTTGAAGCAGCAAAAATTGTACAAA
AAGTAATTTCTTAAaaacttaatgttcaatcttttaaaaaacgcttttaattttcatact
tgtta
>DjScaffold2244:43851-44165 strand:+
ATGGATGATATACTCATAGCGTCTGAAACATTTGAACAGCATTTAATCAATATAGAAAAT
GTACTGTCTAGAATTAGAAAAGCAAATATTAAATTAAAACCCACAAAATGTAAATGGGCA
GAGGAAAGTGTTTTATTTCTCGGACAATTTGTATCATCAAAGGGACTAGAGCCAAATCCA
GAAAAGGTAAAAGCGGTCCAAGATTTTCCAGTGCCAAAAAGCGTACAACAACTACAAGGA
TTTAATGAGTTAGTGGGATATTATCGGAGATTCATACAAGATTTTGCAAAGATATCGGCA
CTATTGGTAGAATTA
>DjScaffold3762:16469-17225 strand:+
tttttgaaatggcttaaatttaaattttaatagggttttattttaatcatgttttcaagt
tacatgatataATGATTACATTTCTATCAATTTTCATATTGATTTCCCTAAATAACGCAA
ATTTAAATGGCAATGAATGTTCAAATTGTGACAAAACCTGTAAAATGGAAAATCCTACTT
GTTTAAAAGTAATTAAGAACTTTACATGTGTTAATCATGATGAGAAGTGCGAGTGTTCTA
TTTATAAAGATAGACTTGGTCAACTTGTGAACTGTAAATATGAACCTATTTCTTTGAATT
TGACTGTTACGAGTCCCAAGCCAATTGAATTATATTGTAAACAGAGAAATATGGATGTGA
TTGTTATATCTGCTATTTATAGAAAAAATTATGGTAAATTTTATTTTATTAAACACAAAT
AAtacatattctttaaaatcaaaagttataaaacggctgaaatatttgaaaaaatgaaaa
aatttaattagcttatattatgaatatttaaactttaaaaaattttaataattattaata
tatattagtttttaattactgattatattcttattattattggcttttatttattcttca
tatttttcgaacattacttatgcggtagacgaattactcttaggctctaactcatgatga
cgacgaagaaaccaattacttcacaattcctgacatggattaattataaaa
>DjScaffold3762:35642-54212 strand:+
tggaatgaataattttaaacgcttcaattatttcgtagactgtttcccagcattaccatc
atcaaccattcagcatagtctccagattcaaatgctaatgacctcggctttttcaatacg
ctcaataagcgatttcttaggaaagaatttcaaaatttgaaaatccaattatcattacaa
gagaatttttgatcaactaaatcggaaacttcgacttctctttgtgaaagcaaatcttga
aaggaataaacgtcaatgtaccacatttgcgattataaactttgttaattgtctttgttt
cattacagttataattatttttaacaaattgggttttttatggaatggaatatcaatttt
atcactaaaatttatttcagggtcagagttaattcacgatgttaccagtgaaattcgtca
aatttgtgctctaaaacgattttgtgcctatccatttcatttacagaggaaatgtaattc
aacaaaaccatgttatgatgaatttgttattgattatttctgtaaaggtttatattaaag
ATGTTGGAATCAGATGATTGCATTGATGCATTTGTAGCGTTGCCCACTCTTATTGATATG
GATTTATTTTCCAATTGGTTAAAGGGTTTAAATGAAACAGAATCAGCTTGTAAATTACAA
GACAATTGTCAATTTAAACTCCCTAAAGGTTTATTGATTTCCGAGGTGCGATCAAATTAT
ACTCAGTTTATGTTACTAGAACCAGAATTATATGCTCCAGATATATTTCTAATGAAGATT
TTTGGTGGACAATTTCAAATGGATAAAATAATTAAAATGAAGCTAATCGATATGTATTAC
AATATTGATTCCATTGTACTAAGAGAATTTCTCGGTCGTAAGTTAAATAGTCGTGATCGT
CGAGATTTGGAGGATATTAGTGAGCGTACTGGTGTTTCATTAATCAGCTGTAAACGCCAA
TTTCAAAATTTAAGACGAATTATAAAATGTTGTGAAGATTTAATTGGCAATGTTAAACTA
TTTGATGCTATTTATCAAAAATTTTCTTTGAAAAATTCTCTTTCAAAAAAATATGTAGCA
GCAGTTTTTATAAATCACCATAGATTTGAAACCAATAAACGAAAACTGGGTCAAATAACA
TATGGAGATTTCGAAAAATGTGTGGATGATATAATGATAAATTGGTCTTACAACAATAGA
AATGATCAAATACCTGATTCAGAGATTTTTCATCCTGCTGATTTAGAAAATCAATTCCTT
CAAGATTTGAAAGTAGAATTAAAATACCTTTCAGAAAAAAAAGAAATTATGGATGAAATC
AAACAATTGACACTTTCAAAATTGAAAGATATTATTCCAGATAAAAGTCAAATGGATCTT

GATGTTTCTTTTAAAAACATTTACAAAGCCGTGTTAAGCATAGGTACTAATTTATCACAT
TCCAAAGCAATTCGAGATTTTTTATTGATCTAATGGAAAAAATCGTTGAACCATGTCGA
TTGTGTGGATTTTCAATTAAAGAAATTGATAATTTCCAAGAAGCGTTTTGTCAAAGTTTA
CTCGAATACAGTCTTGTGAAAAGGACACAGACTATTCGGTTTTTACAAACACTATTTAAT
TGCGTTTTAATATTCTATCAACGGATTTTATTAACTGAATAAaatttacaaactccttaa
tatcacaattttttatcattttaggtttatattaaagATGTTGGAATCAGATGATTGCA
TTGATGCATTTGTAGCGTTGCCCACTCTTATTGATATGGATTTATTTTCCAATTGGTTAA
AGGGTTTAAATGAAACAGAATCAGCTTGTAAATTACAAGACAATTGTCAATTTAAACTCC
CTAAAGGTTTATTGATTTCCGAGGTGCGATCAAATTATACTCAGTTTATGTTACTAGAAC
CAGAATTATATGCTCCAGATATATTTCTAATGAAGATTTTTGGTGGACAATTTCAAATGG
ATAAAATAATTAAAATGAAGCTAATCGATATGTATTACAATATTGATTCCATTGTACTAA
GAGAATTTCTCGGTCGTAAGTTAAATAGTCGTGATCGTCGAGATTTGGAGGATATTAGTG
AGCGTACTGGTGTTTCATTAATCAGCTGTAAACGCCAATTTCAAAATTTAAGACGAATTA
TAAAATGTTGTGAAGATTTAATTGGCAATGTTAAACTATTTGATGCTATTTATCAAAAAT
TTTCTTTGAAAAATTCTCTTTCAAAAAAATATGTAGCAGCAGTTTTTATAAATCACCATA
GATTTGAAACCAATAAACGAAAACTGGGTCAAATAACATATGGAGATTTCGAAAAATGTG
TGGATGATATAATGATAAATTGGTCTTACAACAATAGAAATGATCAAATACCTGATTCAG
AGATTTTTCATCCTGCTGATTTAGAAAATCAATTCCTTCAAGATTTGAAAGTAGAATTAA
AATACCTTTCAGAAAAAAAAGAAATTATGGATGAAATCAAACAATTGACACTTTCAAAAT
TGAAAGATATTATTCCAGATAAAAGTCAAATGGATCTTGATGTTTCTTTTAAAAACATTT
ACAAAGCCGTGTTAAGCATAGGTACTAATTTATCACATTCCAAAGCAATTCGAGATTTTT
TTATTGATCTAATGGAAAAAATCGTTGAACCATGTCGATTGTGTGGATTTTCAATTAAAG
AAATTGATAATTTCCAAGAAGCGTTTTGTCAAAGTTTACTCGAATACAGTCTTGTGAAAA
GGACACAGACTATTCGGTTTTTACAAACACTATTTAATTGCGTTTTAATATTCTATCAAC
GGATTTTATTAACTGAATAAaatttacaaactccttaatatcacaa
>DjScaffold747:87247-109202 strand:+
cagagctcgatattggggtcaaATGATGGATACATTGAAACGTACAATTGACGAAATATT
TTCAGCTTGTGAAACCGATGAAAGCGAAGTGGAATGCAAGGAAGTTATAATGATACTTGA
ACATAGCAAGAAAGACTTTTATTCTCTTATCAAAAAAATGAAAACATTTAAGGAAATCGA
GAATTCTACAATTGAAAATAGTTCAACAAATACATCTATTAATAAGCCAGTTTTTATTGC
ATGGGACGAAAGAAAAATTTCACCCGGTAAACCAATTATGAGACAAATTCTCGAGAAAAC
TAAACGTGCTCATTCTCATTCTGGTCAATCATCTTCGACCATGTCTCAAACTGATTTGCA
AACTCCAACTAAAGAAATTCGAGTTGTAACCGATAATGGAGATAATAATGACGTAGCAAA
TAATAATTTTTGTGTTAAATTGAATAGTCCATCTCAAACAATAACTGAATCAGTTATTTG
TACCTGGGCTGATAAAGTTAAATCACAATTAAACAATCAAAAGATCGATAGTAAAGATAA
TTCCGTTTTGAAAATATCAGAAAATAATTCGTCAATTAAATATCGGCGACTTACAAAACA
TTTGCAAAGATCAAACCAATTAAAACCAAATAAAACTCAAATTACTCAATCATTTAAACC
GAAATCCAATCGAAACGACAATAAAAAATCTGTTAATATTGATAATAGCAGTGATGGAGA
ATGGGAAACGGTTTGTAGAACGAAATTCAGTCAGCGTGGGAAACAGATACACAAAATAAA
TTCTAATCAAACTTTAAGTGATCGTCCGAAACGCAAATCTCTTCTAACTCGAACAAATGA
TAGAAATAATCAATACTTACGGCGAAATAAAAGTAAATCAGAAACTAATTTACGACTTTC
AACATTTAGTGGTGCAAGTCAAGTTGATTCAAATGATTTGTCCAATAGATTTTCAAATGT
TATTAAATGTTGCTCTTCAATTTTAATGCCAAATGAATCTGACCTGATAGATAACGATTC
ATTAAATAGTCAGTTACCGAATAATTATCAATTTAATTTCATAGATTATAATTTTATTAA
AAATAATGTTTCAAATTCTGAAAATCTTTCAATTGATAATAAATGTATTATTTCTCCTGA
AAACTCAAAATTATTTGTCGATTCAAGTCAAAATATGAATGATATATTTCCAGAATTATC
TTTAAAATCAAATAATTTTGGTAATCTCGAAACTAAAGCATTTCTGATGAATACTAATAC
TAAAGATTCTCCTAATACGTCTATAGAAAATTCAACAGATGAATTTGGTGATGATGATGA
AACTATTCAAAATATTAGAAAATTGAATCAAAAATTAGCTGATGTGGAAAATACTGAAAC
TCAATTACAAAATGAATTAGGGCGTGCACAAGAAGAAGAAAAGGCTTATAGAGAGCAAAT
AGAACAAGAGGAAGCTGCTTTGATAAATAATGAAGAAAAAGATAATGTTCACTGTATTGA
AATTTCAAATATTTTCTCGGAAAACCTAACAGCTGATGATAGTGATGATGGATATGATTT
CGTATTGAATTTGGAATTCGACAAAAGTAATTCTAATTCAAGCAATAATTCATCAGAAAA
TCCTTCCAATAAATTTTTGCTACCTCTTGAAGATATTAAAAATATATTTTCTCTTGATTG
GAGTCAAATTAATGATGAACCTGATGAGGATTTTCAAGATAACCCATCAAAATTGAGAAC
ACCAGGTAGATGTGTTGAAATGCATCAGAAATTATCAGCTCCATCTAGGAAAAGGTCAAT
AAATTCCATTCAACAATTAGAAAAGAAACAGGCTAAAGCGAAGGAACTTCGCGAGAAGCT

file:///C|/Users/czh/Desktop/Data_S1.txt[2019/7/4 17:05:10]

TCTATTCGACCGGACTGAGAAAGTTAGAGAATTAACAAAAAAAGTTGAGGAGGTAAGATT
GCATAAATCTGGATTATTAGACGAGAAAAAACTTTGGATGGAACAAAGGTTAAATAAAGC
TGAAACCAAGCGGCAACAACAACTTGATGAGCGAGTTAAAAAAGCACACGATGAAGAAAT
TAAGGGAAAAGAAATCGCATTTATTCAGTCATTGGAAGCTGCTCAGAAGCAACATACAAT
TTTAACTAAACATCAGGAGAGTGAAGCTAGACTTCTGGAAAGACAAGAGGAAAGAAAAAA
GCGAATGGAAGAAAAAGCAGAAAGAGAAGTTGCTGCAGAAATCAGACGAAAAACAATCGA
ATCCAATAGACAAGCATGGATAAATGATTTGCAATTACGATGGAAAACACGTGAAGAAGA
ATTAGCTTTTAAGGCCGAAAATCGGGAAAAGCAAAGATTACTAGCTGTGCGGGCAAAAGA
AGCTGAAAGAGAACAGAAAAAGCTGGTTTTAGAACAACAACAGAAAACACAAATCCAACA
ATTGAAAGAAAAAATTTTCAAAAAGCACGAAGATTCTGAACGACGTCACCAAGAACAGTT
AGCTGAAATAAGTCGGAAAGCGTTTGAAATGAGTATTGCTAAATACTCATTTGAAGAAAG
CTCAGATGCACCTCCCATTGAACCATATATTAATAAGAAATGGTGTAATGTCTGTAACAT
AATGATTGATTCTGATGTATTACTGCAAAGTCATTTGAGTGGGAAAAAACATAAAAATAC
CGTTATTGAACAACTTTCCGGAAAGAATATCGAAAAAGATCAATTTAGAATTTTCAATTT
GGAGCATATTGTGGATGCGCCTGATGATAAAACTGATCCTAAATTGCAAGCGAAGAAAGA
TCGCCGTAAAGCTGTCAAGAAACGTAATAAGAAAATTAAACAAAGAATTATTTCCAACAC
TACAGATTTTGAGAAATATTGTGTTCAATTTCATAACGACGGTAGTTTTGTACAAACACA
AAATAAGCAAAAAATTGCGAAATTTATCAAAGATATTAAAAGATTCGTTTCCATTCAAGA
TACTGGACCTTGGGTTGCTGTTCGAGTTTCATCATTGGAGAAATCTTTGGGAGATTTACT
TCGGTGTTTGAATATTAAGAAACCAGAAAATATGAATTTAAATGAAAAGCAACGAGCTAT
TTTCAATAATCAGGTTCAGTTGGAACAAAATACATTTCTCTATCTTGGTGGGATGGATGT
ACTTGGAAATATAATACAATTAATACAAGCCCAAAAGTCTGAAAATTCACCAATTCTTCC
AGAAAGGGCATTAAAATTGGTATTCAAATGTATTAGAAAAATTTGTAAATCTAATAATAA
TGTATGTCATTACGTTTTATTCTCTAAATTTATTGTCTTCTTATTGGATGGACTACAAAC
TAAGTTGAATTATTTGATCCCTGACAATTTCACAACTTATTTAAAAAATCTTAATGAAAA
TCAGTCTGGTGTACTTCCTGAAAATCGCGAATTACCTACTAACTCCATTGTGGAACAAAT
TTTATTAACATTAACTGTGGTTTTCGATAATTTCGTTTCCAATATTGGTTCTGATTCAAT
TTACACAACATATCATAAATCTCCTGAACTGAATAATGGAAGATCATCATCTTTATCCAG
TACCTCAAATCGATTAGATTTTCGGACAGATAGTTTAAAAGTACAAGATATAATCAGTTA
TGTTGTTTCAAGTGGTATAGTGGATATATTATCTGATTATCTTTGTCAAGTTCGAGGTCC
ATTGAGAAATAATAGTCCTACCGCAAATTTTGTTTTGATTATTCTATCTTTCCTCTCAAC
ATTGTTGAAAATGTTGGCATTAAACAGTGGAAAATCTTATGTCTTACCATTCAAAATTCA
ACCTGGCACTCTAAAATCTCCCTCTATTGATAGTAATTGCCTTGGAAACGGTGATGGAGA
TGGTTTCAAGCCTTTGATTGATCATACTCAATTGCTTCAATCTATAGAAGGTACAGAAAT
GTGTGGCTTAGTATGTCTTCTGTATGGAATATTACTTTATCAAGGAAATGTAGTGTCTAA
ATCTAATCGTAAGTTACTTATAAATTTGAATACCGATcagagctcgatattggggtcaaA
TGATGGATACATTGAAACGTACAATTGACGAAATATTTTCAGCTTGTGAAACCGATGAAA
GCGAAGTGGAATGCAAGGAAGTTATAATGATACTTGAACATAGCAAGAAAGACTTTTATT
CTCTTATCAAAAAAATGAAAACATTTAAGGAAATCGAGAATTCTACAATTGAAAATAGTT
CAACAAATACATCTATTAATAAGCCAGTTTTTATTGCATGGGACGAAAGAAAAATTTCAC
CCGGTAAACCAATTATGAGACAAATTCTCGAGAAAACTAAACGTGCTCATTCTCATTCTG
GTCAATCATCTTCGACCATGTCTCAAACTGATTTGCAAACTCCAACTAAAGAAATTCGAG
TTGTAACCGATAATGGAGATAATAATGACGTAGCAAATAATAATTTTTGTGTTAAATTGA
ATAGTCCATCTCAAACAATAACTGAATCAGTTATTTGTACCTGGGCTGATAAAGTTAAAT
CACAATTAAACAATCAAAAGATCGATAGTAAAGATAATTCCGTTTTGAAAATATCAGAAA
ATAATTCGTCAATTAAATATCGGCGACTTACAAAACATTTGCAAAGATCAAACCAATTAA
AACCAAATAAAACTCAAATTACTCAATCATTTAAACCGAAATCCAATCGAAACGACAATA
AAAAATCTGTTAATATTGATAATAGCAGTGATGGAGAATGGGAAACGGTTTGTAGAACGA
AATTCAGTCAGCGTGGGAAACAGATACACAAAATAAATTCTAATCAAACTTTAAGTGATC
GTCCGAAACGCAAATCTCTTCTAACTCGAACAAATGATAGAAATAATCAATACTTACGGC
GAAATAAAAGTAAATCAGAAACTAATTTACGACTTTCAACATTTAGTGGTGCAAGTCAAG
TTGATTCAAATGATTTGTCCAATAGATTTTCAAATGTTATTAAATGTTGCTCTTCAATTT
TAATGCCAAATGAATCTGACCTGATAGATAACGATTCATTAAATAGTCAGTTACCGAATA
ATTATCAATTTAATTTCATAGATTATAATTTTATTAAAAATAATGTTTCAAATTCTGAAA
ATCTTTCAATTGATAATAAATGTATTATTTCTCCTGAAAACTCAAAATTATTTGTCGATT
CAAGTCAAAATATGAATGATATATTTCCAGAATTATCTTTAAAATCAAATAATTTTGGTA
ATCTCGAAACTAAAGCATTTCTGATGAATACTAATACTAAAGATTCTCCTAATACGTCTA

TAGAAAATTCAACAGATGAATTTGGTGATGATGATGAAACTATTCAAAATATTAGAAAAT
TGAATCAAAAATTAGCTGATGTGGAAAATACTGAAACTCAATTACAAAATGAATTAGGGC
GTGCACAAGAAGAAGAAAAGGCTTATAGAGAGCAAATAGAACAAGAGGAAGCTGCTTTGA
TAAATAATGAAGAAAAAGATAATGTTCACTGTATTGAAATTTCAAATATTTTCTCGGAAA
ACCTAACAGCTGATGATAGTGATGATGGATATGATTTCGTATTGAATTTGGAATTCGACA
AAAGTAATTCTAATTCAAGCAATAATTCATCAGAAAATCCTTCCAATAAATTTTTGCTAC
CTCTTGAAGATATTAAAAATATATTTTCTCTTGATTGGAGTCAAATTAATGATGAACCTG
ATGAGGATTTTCAAGATAACCCATCAAAATTGAGAACACCAGGTAGATGTGTTGAAATGC
ATCAGAAATTATCAGCTCCATCTAGGAAAAGGTCAATAAATTCCATTCAACAATTAGAAA
AGAAACAGGCTAAAGCGAAGGAACTTCGCGAGAAGCTTCTATTCGACCGGACTGAGAAAG
TTAGAGAATTAACAAAAAAAAGTTGAGGAGGTAAGATTGCATAAATCTGGATTATTAGACG
AGAAAAAAACTTTGGATGGAACAAAGGTTAAATAAAGCTGAAACCAAGCGGCAACAACAAC
TTGATGAGCGAGTTAAAAAAGCACACGATGAAGAAATTAAGGGAAAAGAAATCGCATTTA
TTCAGTCATTGGAAGCTGCTCAGAAGCAACATACAATTTTAACTAAACATCAGGAGAGTG
AAGCTAGACTTCTGGAAAGACAAGAGGAAAGAAAAAAGCGAATGGAAGAAAAAGCAGAAA
GAGAAGTTGCTGCAGAAATCAGACGAAAAACAATCGAATCCAATAGACAAGCATGGATAA
ATGATTTGCAATTACGATGGAAAACACGTGAAGAAGAATTAGCTTTTAAGGCCGAAAATC
GGGAAAAGCAAAGATTACTAGCTGTGCGGGCAAAAGAAGCTGAAAGAGAACAGAAAAAGC
TGGTTTTAGAACAACAACAGAAAACACAAATCCAACAATTGAAAGAAAAAATTTTCAAAA
AGCACGAAGATTCTGAACGACGTCACCAAGAACAGTTAGCTGAAATAAGTCGGAAAGCGT
TTGAAATGAGTATTGCTAAATACTCATTTGAAGAAAGCTCAGATGCACCTCCCATTGAAC
CATATATTAATAAGAAATGGTGTAATGTCTGTAACATAATGATTGATTCTGATGTATTAC
TGCAAAGTCATTTGAGTGGGAAAAAAACATAAAAATACCGTTATTGAACAACTTTCCGGAA
AGAATATCGAAAAAGATCAATTTAGAATTTTCAATTTGGAGCATATTGTGGATGCGCCTG
ATGATAAAACTGATCCTAAATTGCAAGCGAAGAAAGATCGCCGTAAAGCTGTCAAGAAAC
GTAATAAGAAAATTAAACAAAGAATTATTTCCAACACTACAGATTTTGAGAAATATTGTG
TTCAATTTCATAACGACGGTAGTTTTGTACAAACACAAAATAAGCAAAAGTTAAAACAAC
AAAATTTTCTGTTATAActaattattttagaattgcgaaatttatcaaagatattaaaag
attcgtttccattcaagatactggaccttgggttgctgttcgagtttcatcattggagaa
atctttgggagatttacttcggtgtttgaatattaagaaaccagaaaatatgaatttaaa
tgaaaagcaacgagctattttcaataatcaggttcagttggaacaaaatacatttctcta
tcttggtgggatggatgtacttggaaatataatacaattaatacaagcccaaaagtctga
aaattcaccaattcttccagaaagggcattaaaattggtattcaaatgtattagaaaaat
ttgtaaatctaataataatgtatgtcattacgttttattctctaaatttattgtcttctt
attggatggactacaaactaagttgaattatttgatccctgacaatttcacaacttattt
aaaaaatcttaatgaaaatcagtctggtgtacttcctgaaaatcgcgaattacctactaa
ctccattgtggaacaaattttattaacattaactgtggttttcgataatttcgtttccaa
tattggttctgattcaatttacacaacatatcataaatctcctgaactgaataatggaag
atcatcatctttatccagtacctcaaatcgattagattttcggacagatagtttaaaagt
acaagatataatcagttatgttgtttcaagtggtatagtggatatattatctgattatct
ttgtcaagttcgaggtccattgagaaataatagtcctaccgcaaattttgttttgattat
tctatctttcctctcaacattgttgaaaatgttggcattaaacagtggaaaatcttatgt
cttaccattcaaaattcaacctggcactctaaaatctccctctattgatagtaattgcct
tggaaacggtgatggagatggtttcaagcctttgattgatcatactcaattgcttcaatc
tatagaaggtacagaaatgtgtggcttagtatgtcttctgtatggaatattactttatca
aggaaatgtagtgtctaaatctaatcgtaagttacttataaatttgaataccgatatcag
agctcgatattggggtcaaATGATGGATACATTGAAACGTACAATTGACGAAATATTTTC
AGCTTGTGAAACCGATGAAAGCGAAGTGGAATGCAAGGAAGTTATAATGATACTTGAACA
TAGCAAGAAAGACTTTTATTCTCTTATCAAAAAAATGAAAACATTTAAGGAAATCGAGAA
TTCTACAATTGAAAATAGTTCAACAAATACATCTATTAATAAGCCAGTTTTTATTGCATG
GGACGAAAGAAAAATTTCACCCGGTAAACCAATTATGAGACAAATTCTCGAGAAAACTAA
ACGTGCTCATTCTCATTCTGGTCAATCATCTTCGACCATGTCTCAAACTGATTTGCAAAC
TCCAACTAAAGAAATTCGAGTTGTAACCGATAATGGAGATAATAATGACGTAGCAAATAA
TAATTTTTGTGTTAAATTGAATAGTCCATCTCAAACAATAACTGAATCAGTTATTTGTAC
CTGGGCTGATAAAGTTAAATCACAATTAAACAATCAAAAGATCGATAGTAAAGATAATTC
CGTTTTGAAAATATCAGAAAATAATTCGTCAATTAAATATCGGCGACTTACAAAACATTT
GCAAAGATCAAACCAATTAAAACCAAATAAAACTCAAATTACTCAATCATTTAAACCGAA

ATCCAATCGAAACGACAATAAAAAATCTGTTAATATTGATAATAGCAGTGATGGAGAATG
GGAAACGGTTTGTAGAACGAAATTCAGTCAGCGTGGGAAACAGATACACAAAATAAATTC
TAATCAAACTTTAAGTGATCGTCCGAAACGCAAATCTCTTCTAACTCGAACAAATGATAG
AAATAATCAATACTTACGGCGAAATAAAAGTAAATCAGAAACTAATTTACGACTTTCAAC
ATTTAGTGGTGCAAGTCAAGTTGATTCAAATGATTTGTCCAATAGATTTTCAAATGTTAT
TAAATGTTGCTCTTCAATTTTAATGCCAAATGAATCTGACCTGATAGATAACGATTCATT
AAATAGTCAGTTACCGAATAATTATCAATTTAATTTCATAGATTATAATTTTATTAAAAA
TAATGTTTCAAATTCTGAAAATCTTTCAATTGATAATAAATGTATTATTTCTCCTGAAAA
CTCAAAATTATTTGTCGATTCAAGTCAAAATATGAATGATATATTTCCAGAATTATCTTT
AAAATCAAATAATTTTGGTAATCTCGAAACTAAAGCATTTCTGATGAATACTAATACTAA
AGATTCTCCTAATACGTCTATAGAAAATTCAACAGATGAATTTGGTGATGATGATGAAAC
TATTCAAAATATTAGAAAATTGAATCAAAAATTAGCTGATGTGGAAAATACTGAAACTCA
ATTACAAAATGAATTAGGGCGTGCACAAGAAGAAGAAAAGGCTTATAGAGAGCAAATAGA
ACAAGAGGAAGCTGCTTTGATAAATAATGAAGAAAAAGATAATGTTCACTGTATTGAAAT
TTCAAATATTTTCTCGGAAAACCTAACAGCTGATGATAGTGATGATGGATATGATTTCGT
ATTGAATTTGGAATTCGACAAAAGTAATTCTAATTCAAGCAATAATTCATCAGAAAATCC
TTCCAATAAATTTTTGCTACCTCTTGAAGATATTAAAAATATATTTTCTCTTGATTGGAG
TCAAATTAATGATGAACCTGATGAGGATTTTCAAGATAACCCATCAAAATTGAGAACACC
AGGTAGATGTGTTGAAATGCATCAGAAATTATCAGCTCCATCTAGGAAAAGGTCAATAAA
TTCCATTCAACAATTAGAAAAGAAACAGGCTAAAGCGAAGGAACTTCGCGAGAAGCTTCT
ATTCGACCGGACTGAGAAAGTTAGAGAATTAACAAAAAAAGTTGAGGAGGTAAGATTGCA
TAAATCTGGATTATTAGACGAGAAAAAACTTTGGATGGAACAAAGGTTAAATAAAGCTGA
AACCAAGCGGCAACAACAACTTGATGAGCGAGTTAAAAAAGCACACGATGAAGAAATTAA
GGGAAAAGAAATCGCATTTATTCAGTCATTGGAAGCTGCTCAGAAGCAACATACAATTTT
AACTAAACATCAGGAGAGTGAAGCTAGACTTCTGGAAAGACAAGAGGAAAGAAAAAAGCG
AATGGAAGAAAAAGCAGAAAGAGAAGTTGCTGCAGAAATCAGACGAAAAACAATCGAATC
CAATAGACAAGCATGGATAAATGATTTGCAATTACGATGGAAAACACGTGAAGAAGAATT
AGCTTTTAAGGCCGAAAATCGGGAAAAGCAAAGATTACTAGCTGTGCGGGCAAAAGAAGC
TGAAAGAGAACAGAAAAAGCTGGTTTTAGAACAACAACAGAAAACACAAATCCAACAATT
GAAAGAAAAAATTTTCAAAAAGCACGAAGATTCTGAACGACGTCACCAAGAACAGTTAGC
TGAAATAAGTCGGAAAGCGTTTGAAATGAGTATTGCTAAATACTCATTTGAAGAAAGCTC
AGATGCACCTCCCATTGAACCATATATTAATAAGAAATGGTGTAATGTCTGTAACATAAT
GATTGATTCTGATGTATTACTGCAAAGTCATTTGAGTGGGAAAAAACATAAAAATACCGT
TATTGAACAACTTTCCGGAAAGAATATCGAAAAAGATCAATTTAGAATTTTCAATTTGGA
GCATATTGTGGATGCGCCTGATGATAAAACTGATCCTAAATTGCAAGCGAAGAAAGATCG
CCGTAAAGCTGTCAAGAAACGTAATAAGAAAATTAAACAAAGAATTATTTCCAACACTAC
AGATTTTGAGAAATATTGTGTTCAATTTCATAACGACGGTAGTTTTGTACAAACACAAAA
TAAGCAAAAAATTGCGAAATTTATCAAAGATATTAAAAGATTCGTTTCCATTCAAGATAC
TGGACCTTGGGTTGCTGTTCGAGTTTCATCATTGGAGAAATCTTTGGGAGATTTACTTCG
GTGTTTGAATATTAAGAAACCAGAAAATATGAATTTAAATGAAAAGCAACGAGCTATTTT
CAATAATCAGGTATGCTAAactgattactaattcatatatataatctgg
>DjScaffold5068:1203-5929 strand:+
gttaaattcttaATGTCTAGGATTTTATTATATTTTATTCATGTTATTGTTCTTACTAAT
TTTAAAATTTGTGTTTGCAGTTTGACCATAAATGGTTCGATCTCAATACACTCTTTGATT
TTTGAAAAGGTTATTAATGCATTTGATTATGTTTTGGTAAAGTTTGATGAGCCTTTTCCT
TATGGAGATAAAGAAGATATTTTTAAAGAAATCGCTTCAGTGACAGTTAATATTCCAAAT
CTAATAACAGCTACTGTTGGTGTTGCGGATTATGGAGACAAAGAGAATTTCGATTTAGCA
AGCAGATATGAAATAGTTAAATCTGATTTCCCTACATATCTACTATTCAATAGAAGTATA
GATAATCCTATAATTTATAAAGGAGACATCAAAGTTGACTCAATTAAAACCTTCATAATT
AAACATACAGGAGCCTACATTGGTCTGAGTGGCTGTATCGAGGAATTCGATCAAATCGCT
AAAGAGTTCATCTCTGCTGATACTGAAGTTCAAACTAAAATTATTTCTCTTATGACTGAA
AAAGTTAAGGATTTGAAAAATGATGAAACAAAACATTCTTCGGCTGAGAAATATTTGAAA
ATCGCTACAAAGATTCGGGATAATGGAATCGGGTTTGTCAAAACTGAGCAGGAACGATTG
AAAAAATTAAGCGATGGAAATGTATCTGATAACAAAAAGATCGAGATCAAATTGCGATTA
AATATTTTGAATAGTTTCCAACCACCTCTTTCCCCCAATAAATCAGAATTATAAttataa
tctgaattgttcccaccaaataataattttattatttgtttatctgtttatcttgttgc
cactgattgataatttggataattctttgttctttctctgttcatttgttacgtccatgt

taataaataatcaattgtgttccttttcataaca
>DjScaffold2563:50-3415 strand:+
agaaaatatattttaagataatttagttaattgcggagtgaaattagcaaattcttgtac
atcaaatgagttttcaattatttattgaatcaattaaatctggtgttgatgcagaaattg
taaataaattcaaatttgaatatattgaggtgagacATGAAGATTCGTGGAATCTTTTGC
ATCATGCTTGTCGTTGGGGTCGAGTTGGATTGTGTGAAAATATTGATAGAGAACAAATGT
GATATCAATACAACTAGTGATTATAGATATCAACCGATACATTTGGCATGTGAGAATGGT
CATATTGATATTGTGAAAATATTGATAGCAAACAAATGTGATATCAATACATCTAATAAT
AGTGGATCTCAACCGATACATTTGGCATGTAAGAATGGTCATATTGATATTGTGAAAATA
TTGATAGCAAACAAATGTGATATCAATACATCTAATAATAGAGGATATCATCTGATACAT
TTGACATGCAAGAATGGTCATATTGATATTGTGAAAATATTGATAGAAAACAAATGTGAT
ATCAATGTATCTAATTATTATGGATATCAACCGATACATTTGGCATGCAAGAATGGTCAT
ATTGATATTGTGAAGATATTGATAGCAAACAAATGTTATATCAATGCATCTGATAAATAT
GGATATCAACCGATTCATTGGGCATGCAAGAATGGTCATATTGATATTGTGAAAATATTG
ATTGAAAACAAATGTGATATCAATGCATCTGATAAGAGTGGATATCAACCGATACATTTG
GCATGTCAGAATGGTCATATTGATATTGTGAAAATATTGATAGAGAACAAATGTGATATC
ACTGCAACTTATGAAAGAGGATATCAACTGATAGGTAATGCCAAAAAGCGAGGTCATAAA
AAAATAATCGATATTATTAAATCGTTTGATCCGATTGGATCGATTATTACAGCAATGAAT
TTGAAGTGTGCAATCAACTGTGATCACAAATTTTGTCGAGAAACTCTAAACAAAGAAAAA
TTGCCAGATGGTGAATTGATTGGATCAGGATCATTTGGAGAAGTTTATAAACACAGAATA
agaaaatatattttaagataatttagttaattgcggagtgaaattagcaaattcttgtac
atcaaATGAGTGATTTTCAATTATTTATTGAATCAATTAAATCTGGTGTTGATGCAGAAA
TTGTAAATAAATTCAAATTTGAATATATTGAGGTGAGACATGAAGATTCGTGGAATCTTT
TGCATCATGCTTGTCGTTGGGGTCGAGTTGGATTGGTGAAATTATTACTGTCCAATCAGT
ATGAAATTGATTCACGCACAAAATATGGATATCAACCGATACATTTGGCATGTGCGAATG
GTCATATTGATATTGTGAAAATATTGATAGAGAACAAATGTGATATCAATACAACTAGTG
ATTATAGATATCAACCGATACATTTGGCATGTGAGAATGGTCATATTGATATTGTGAAAA
TATTGATAGCAAACAAATGTGATATCAATACATCTAATAATAGTGGATCTCAACCGATAC
ATTTGGCATGTAAGAATGGTCATATTGATATTGTGAAAATATTGATAGCAAACAAATGTG
ATATCAATACATCTAATAATTATGGATATCAACCGATTCATTGGGCATGCAAGAATGGTC
ATATTGATATTGTGAAAATATTGATAGAGAACAAATATGATATCAATGCATCTGATTTAA
GAGGATATCATCTGATACATTTGACATGCAAGAATGGTCATATTGATATTGTGAAAATAT
TGATAGAAAACAAATGTGATATCAATGTATCTAATTATTATGGATATCAACCGATACATT
TGGCATGCAAGAATGGTCATATTGATATTGTGAAGATATTGATAGCAAACAAATGTTATA
TCAATGCATCTGATAAAGTAGTTTATTTCTTATTTACTTAAtaagttattaataaatgat
aatttagtatggatatcaaccgattcattgggcatgcaagaatggtcatattgatattgt
gaaaatattgattgaaaacaaatgtgatatcaatgcatctgataagagtggatatcaacc
gatacatttggcatgtcagaatggtcatattgatattgtgaaaatattgatagagaacaa
atgtgatatcactgcaacttatgaaagaggatatcaactgataggtaatgccaaaaagcg
aggtcataaaaaaataatcgatattattaaatcgtttgatccgattggatcgattattac
agcaatgaatttgaagtgtgcaatcaactgtgatcacaaattttgtcgagaaagttcgtt
ttttttattaattttaatactaaatgttttcaaaactaaatgtttaaataaattttcaaa
atattaattaattgttaatttaataattcaaataatatctgaattcattttatttattta
cttgatgacaactgtaatcagaatatttctatgagaatactaacaataggaaaatgttta
ttgtttggagatttaatatataaaacatttcatgattagaaaatatattttaagataatt
tagttaattgcggagtgaaattagcaaattcttgtacatcaaatgagttttcaattattt
attgaatcaattaaatctggtgttgatgcagaaattgtaaataaattcaaatttgaatat
attgaggtgagacATGAAGATTCGTGGAATCTTTTGCATCATGCTTGTCGTTGGGGTCGA
GTTGGATTGTGTGAAAATATTGATAGAGAACAAATGTGATATCAATACAACTAGTGATTA
TAGATATCAACCGATACATTTGGCATGTGAGAATGGTCATATTGATATTGTGAAAATATT
GATAGCAAACAAATGTGATATCAATACATCTAATAATAGTGGATCTCAACCGATACATTT
GGCATGTAAGAATGGTCATATTGATATTGTGAAAATATTGATAGCAAACAAATGTGATAT
CAATACATCTAATAATAGAGGATATCATCTGATACATTTGACATGCAAGAATGGTCATAT
TGATATTGTGAAAATATTGATAGAAAACAAATGTGATATCAATGTATCTAATTATTATGG
ATATCAACCGATACATTTGGCATGCAAGAATGGTCATATTGATATTGTGAAGATATTGAT
AGCAAACAAATGTTATATCAATGCATCTGATAAATATGGATATCAACCGATTCATTGGGC
ATGCAAGAATGGTCATATTGATATTGTGAAAATATTGATTGAAAACAAATGTGATATCAA

TGCATCTGATAAGAGTGGATATCAACCGATACATTTGGCATGTCAGAATGGTCATATTGA
TATTGTGAAAATATTGATAGAGAACAAATGTGATATCACTGCAACTTATGAAAGGATATC
AACTGATAGGTAAtgccaaaaagcgaggtcataaaaaaataatcgatattattaaatcgt
ttgatccggtaataaaaatatagcttccaaaaaatttcaaacaggggagtctttgtgata
tttacatgatacaatattcaaattaaaacttttctttacaagatgataaaaacattgaaa
atttttcattacta
>DjScaffold1160:29000-35967 strand:+
aattttgcattgaaatgtttagaaatccatttaatgaattcgtgaaagctataaaatcaa
aaaatctggctgatgttgaaaaaaatttcaaaccagattttctaaataaaccagataaag
atggattttatgcaatccatttggtatgtataaataattctgtggatattttcaaatatt
tcctgaaacaaaattgtgatattgaattacttgattcgtataataatcgacctatatatc
atgcgataaagaatggatctgtggaaattgtgaaattattgcttggttgtcgatgttctt
ttgatgatggaaatgaattgctggaactagctaaaacnatattgaaattattttaaacat
ctttaaacaaaagttgcttttttataaaatctaaatttactcagattggaattgaaATGG
ATCTGAAATGCAGTTTTGATTGTGATCACAGATTTTGTTTGATTTCATTCATGATGATCG
AGTTCGATAAGAGTGATATTATAGCTTGTGGAAGTTTTGGGAAAGTGTTTAAAGTAGTTG
AATCTGATGGGAAACGTGTGTTTGCTGTCAAAAAGGTTTCATGGAAACAATGTGGAGTGA
GCAATTTGCCAGGGAAAGAAAACGGTATAAGTGAAATTAAAAAAGAATCGATATATTTGG
AAAACAATATTAATGATTACAAAACAATCTTATCTCATCCACATATAATCAAATATTTTG
GTGCAATAAAGGAGAAAGATAAATTGCTCATTTTTATGGAATTTGCAGAAAATGGATCAA
TATATGATTTAATTGAGAAACAGTGTCAACAGGGCGGTCTACCATTCGATAAAATTATTA
AATATAGCAGGCATATTCTCCTGGCTCTAGATTACCTTCATAAAAAGAGTATTCTTCACA
GAGATATTAAACCTGCAAACATTCTATTGGACAAATATGATAATTGTAAATTGGCAGATT
TGGGCTGTAGTCGATTATTGGAAACGATTCACTTACACAAAACAAATTCCCAAATTCAAA
AATCTGCAAAAGTGTAAaaactaaacagcaagcaaaaacatattgtggaactgcacattt
tatggcccccgaagttatccacggagacccttattcatttccttgtgatttatggtcgtt
aggtggaacagttattgaaatgctgacaggttgattcattcataaagctcaaaattctgt
aaagtattaacatgtttgtaggtcaaattatttattagatgcgtttataccacaatatga
tggaatttacttaataattactagtgacttggactgagtgttggttaatctgagatctga
ctgaaattttgcattgaaatgtttagaaatccatttaatgaattcgtgaaagctataaaa
tcaaaaaatctggctgatgttgaaaaaaatttcaaaccagattttctaaataaaccagat
aaagatggattttatgcaatccatttggtatgtataaataattctgtggatattttcaaa
tatttcctgaaacaaaattgtgatattgaattacttgattcgtataataatcgacctata
tatcatgcgataaagaatggatctgtggaaattgtgaaattattgcttggttgtcgatgt
tcttttgatgatggaaatgaattgctggaactagctaaaacnatattgaaattattttaa
acatctttaaacaaaagttgcttttttataaaatctaaatttactcagattggaattgaa
ATGGATCTGAAATGCAGTTTTGATTGTGATCACAGATTTTGTTTGATTTCATTCATGATG
ATCGAGTTCGATAAGAGTGATATTATAGCTTGTGGAAGTTTTGGGAAAGTGTTTAAAGTA
GTTGAATCTGATGGGAAACGTGTGTTTGCTGTCAAAAAGGTTTCATGGAAACAATGTGGA
GTGAGCAATTTGCCAGGGAAAGAAAACGGTATAAGTGAAATTAAAAAAGAATCGATATAT
TTGGAAAACAATATTAATGATTACAAAACAATCTTATCTCATCCACATATAATCAAATAT
TTTGGTGCAATAAAGGAGAAAGATAAATTGCTCATTTTTATGGAATTTGCAGAAAATGGA
TCAATATATGATTTAATTGAGAAACAGTGTCAACAGGGCGGTCTACCATTCGATAAAATT
ATTAAATATAGCAGGCATATTCTCCTGGCTCTAGATTACCTTCATAAAAAGAGTATTCTT
CACAGAGATATTAAACCTGCAAACATTCTATTGGACAAATATGATAATTGTAAATTGGCA
GATTTGGGCTGTAGTCGATTATTGGAAACGATTCACTTACACAAAACAAATTCCCAAATT
CAAAAATCTGCAAGTCAAAAGCTGAAAGCCAAAAAAGTGTAAaaactaaacagcaagcaa
aaacatattgtggaactgcacattttatggcccccgaagttatccacggagacccttatt
catttccttgtgatttatggtcgttaggtggaacagttattgaaatgctgacaggtaaac
ctccattttacggatcaacaccgaacggcaagatcttcaagattggacggaatgaaaaat
atgattatgaaaaattaagacctacgccagaaaatttaaaaatatttatcgatcagcttc
ttagttttgaacctgaaaatcgtaaaactgcagcacacctattaacattaaacatctttc
aaaatgtgttataatataaatatgtttaatgtca
>DjScaffold3883:59073-60254 strand:+
agttATGCCTAGTGATAAAACGCATATTAATATTGTAGTTATTGGTCATGTAGATAGCGG
GAAATCTACTTCAACAGGTCATCTGATTTATAAATGTGGTGGTATTGATAAGAGAACTAT
AGAAAAATTTGAAAAGGAAGCTCAAGAGATGGGTAAAGGTTCTTTCAAATATGCATGGGT

TCTTGACAAATTAAAAGCTGAACGTGAAAGAGGCATAACAATTGATATTGCACTGTGGAA
ATTTGAAACAACAAAGTTCTATGTTACAGTTATAGATGCTCCTGGCCATAGGGATTTTAT
AAAGAATATGATTACCGGAACTAGTCAAGCTGATTGTGCTGTTTTAGTAGTAGCAGCTGG
AACTGGCGAATTTGAGGCTGGTATTTCAAAAAATGGTCAAACTCGAGAGCATGCGTTATT
GGCATATACTCTTGGTGTGAAGCAAATGATTGTTGGGGTTAACAAAATGGACTCTACTGA
GCCACCATTTAATGAAAAAAGATTCGACGAAATTAAGAAAGAAGTCTCGGGCTATTTAAA
AAAGGTAGGTTATCAACCCGAAGGTGTTGCCTTTGTCCCTATTTCCGGTTGGAATGGAGA
TAACATGATTGAAGAAAGCTCTAATATGTCGTGGTACAAAGGTTGGGATATTACACGTAA
AAATGCTAAAAAAGAAGAAACGAAAATCTCAGGTAAAACTTTACTTGATGCATTGGATTC
ATTAGAACCTCCTGCAAGACCGACAAATAAGCCACTTAGGCTTCCATTGCAGGATGTTTA
TAAAATTGGTGGAATTGGTACAGTTCCTGTTGGACGTGTTGAAACTGGTATTCTTAAACC
TGGAATGGTGGTCACATTTGCTCCACAAGGTCTTACTACTGAAGTTAAATCGGTTGAAAT
GCATCATGAAGCATTAACTGAGGCTCTTCCTGGTGATAACGTTGGGTTTAATATTAAAAA
TGTCTCTGTTAAAGAAATTCGCAGAGGAAATGTCTGTAGTGATTCTAAAAATGACCCTGC
GCGACAAGCTACTGATTTCATAGCTCAAGTTATTATAATGAATCATCCAGGTGAAATTCA
TTCAGGTTACTCTCCGGTT
>DjScaffold3495:8817-9613 strand:+
aattaatatattttaagggtgctgatcaaagttctattaaatcctcgaagatttctaaag
atgacATGTCTCCATATATGCATCCAATTGGAGAAACATCGTCAAATAAAGAAGGATTGT
TATCATTCAGATCAAAATTACCTTCTGCTGGTAATGTTAGATCTGGATCTGTATCATCTG
TTTACTCTCCAAATACGAATTATCCATCTAATAAGGAAAGTAAAATTAATCGAGCATTTT
CTACATCTTTCACAACACTTCCAACTTCTCAATCTCGTGAAATTGTTGTTGTCAATGAAG
CTAAACAATCAGATGACAGTAGTATAAAAGAATTAGATAAGCTGAATCGAATACCTCGAG
TGACTCCATTATACATGAATAAATCGTTGTCGTCGAGTTTTGATCATAAATCAGCTCTGC
ATTTCTGTGCACGCTACCAAGAGCACATTCATCAGTGTGCTGAAGCTGTTGCGTTTGATC
AAAATTCTTTGGCACTTCGCATTAAAGAGGTAAAATTAATATTTTGAttctaacttaaaa
cgtttttcattaaattaattaattaaataaaatgtttaatggtaaaattgaatagatttt
ccatctaatttttaattattgcgtaattgtttaatcataaaatgagacagattttcagc
taataaatctctattgaatgatcctgttagcctttt
>DjScaffold3495:34581-39452 strand:+
ACTAGTCTTCATGAATTAGCATTAATATCACAAAGTAATTTGTCCAATCATAATTTTTCA
ATTTGCAATGAAATCCAGTTTAAAAAGTCAATCTATGCAAAATTGTGTAAAGAAAAACCC
GAAGTCATCAGTTTAATGGCATTCGCAACTTCAGCAGCTCTAAATGAGTGTAATCAACAA
ATGAAATTTAACCGCTGGGACTGTGGATGCTCAAAAAAATTATTAAACAAAATCGTAGAA
AAATATTCAAAGGAAGCAGCATTTTTGAATGCTATAACCACAGCAGGAGTAACGTATTAT
GTAACAAAAGCTTGTAGTCGGGGTGATTTGAAATTCTGTCAGTGTAACAACAACCTACAG
GACTCATCTGGTATAGATAAAGATCACCAGACTCATTGCAATGAAAATGTTGATTATGGA
AAGTCATTTAGTGAAATTTTTCTCGATGGACCAGCGAAAATCTCATTCAAACCTTTGCTT
AATGGAAATCATAAATCAAAATTAGAACGGAAAAAAAATTACAAATCTGCGTTCAAAATT
TTTCTCGAGTTACATAATAATAATGTAGCTAGAAATGTTATTATTAATTCATTGGAAACA
AAATGCAAATGCCAAGGAGCGTCTGGATCTTGTACAGCAAAGTATTGTTGGAAAACATTA
CCGGAATTTTCTAAAATTGGAGAAGCATTGAAAAATAAGTATTTAAATGCTAAAAAAATG
CCAAAATTTTTGAAAAAACGCATTCGTCAAAATATGATTTCCGTTGATAATATCGATTCA
TCTGATGATGAATCATCTCCAAATACCGATCATCTAATGTTTTTACGAGACTCGTTTGAT
TACTGTTTACCAAATATTCGATTGGGAATAAAAGGTATTGATGGTAAGGAGTGTGAAGAT
GTTCCAAGTGGACCGACAAGTTGTTACAGTCTCTGTTGTAATTATGGTTTTATCAAAGTA
AAAAAGCTAATTGTGGAAAAATGCAATTGTAAATTTAAATGGTGCTGTGATGTAATATGC
GAAAAATGTGAAAAATTCGTTACAAAATTTTATTGCAATATACCAAAAACATCGAAAAAA
TTCCAGAAATATTATCTCAACGGAATGAAGATGTAA
>DjScaffold3430:95943-97247 strand:+
aaattaaagattttattttattatggcgtttatgtgATGGTTCATTCATATTTTGACGCC
AAATTAACACAGCCTGATTTAGATTCATCAAATTCATCTGATTGTGAAAATACCGACGAT
AGATTAGATAATATAATATCAAGTTTACTCGCAAATTTATCTGTATATAATTCTGAAGAC
GAAGACGATATGACTATTAAAAAATATAAAAAGGAACGTACAAAGCATGAGAGGTCATCA
GAAAGTGATAGAAATGATAAAAGCTCTAGATACCCCCATAGTCACTTTAATTATGGTGCT
ACGGCCAGCGATTCCAATTCTCATTTTCCGGTTTCAAGAAGGCAAGTTTCTGTTGATAAT
CATGTTCTTTCGTTCACCTCTGATCTGCGTTTATATAATTATGATTCTCAGCCCAGCTTG

CCTAAGAGACCAAGGTCATTGTTTCAAGGAGACAGTATAAATCCACAGAAGCTGACTGAT
AGAAGAAATAAAGTAACAGATATATTTAATGAAAATAGTGGAAATAATAGTGAAGATGAG
TATTATATGGCTACTGGGAGTCGATCACAAGTACCTTTTCAGGTCGAGGAAAGTCAAATG
AGTACCTTATTGAAAGAGAAAAAAGGATGGAAAATAATTGTTGTAGGTTCAGATGGGGCG
TGTTTATTTAGAGCAGTGGCACATCAGATTTTTGGTGATGAAGAGAAACACATGATTATT
AGGGAACAAGTTATAAATTATATGAAACAAAATCGAGAACATTTTTCACAATTTATTTCC
GAAGATTTTGAACATTATTTAATTAGAAAACTCGATCCACATACTCACGGGAATCACGTG
GAAATTCAAGCGATTTCCGAGCTGTATAATCGTCCGGTCGAAATCTATCACAATTCCTTG
GAACCTATAAATGTCTTTCATCGGGAATATAATGATTTGCCGCCACTCCGATTAAGTTAC
CATGGTGGAATTCATTATAATTCAATCGTCGACCCTCAAAATCCAACTTTTGGAGAAGGT
CTCGGAATGCCTAATTATCAACCAAGGCTGTCGGAGCCAGAATTAGTAGAAAAGGCAAAA
AACGAGTCCGAGAATATAGAGATTGAAGAAACGATCCTGCAAGATCGCATCTTGCAGTCG
GAAAAT
>DjScaffold4793:55814-56740 strand:+
gaagacaatcaagaaaatggaactgtatttcatgctttcccatcttgttcgcctcggttc
agcaggtttgtggagactgagctttgcagaATGAGTGAATCCAGCTCATTAGTATTTCCA
CAAGATAATGAAGACGCCGGAAAAATGGCCAGAAGTGTTAGTGCTGCACCAAGTTTGTTG
TCGAGTGAATCCAGCAGTGAGGATCCTGTGCAGCCAAGTAATACGCAAGCCAGTAGTACA
AGTAGTTTAGAGCTAAGTGCTCATGGAAGTTTAGCGCATCATTCAAGTGCTAGTAGTGAA
ACAGAATTACCTTGTTCACGAGATATTGGCAATCAAACAATATCTACACCTATTTGCACA
AGGGAACGCAGTGAATCATCTCCAGTTCATTTGTCTTCTGCTATTCCTCGGTCTTTAAAA
CCAGTTCGTGAACCAGTTTCTGCTGTATCTCCTCTTGCTCGTGTTGATAGTTCCACAAAT
ACTACCACTATTACTACTAGTGTTTCACGCTCAGCGTCAACACATCAACTGACTCCTCCT
CGTCGATCCAGTGCACCTCGAGCTCAGTCGCCTCTTAGTGTTCCATCTGGAGCTTCTGGT
GGACCAGCTACTTTAATTACACCTCCAATTGTCATTCATCGTAGATCTCGCAATTTCGGA
TTTGTAATTCGTGCTATTCGAGTATTTTATGGAGAAAGTGATGTTTATACACTTCATCAT
CTAATTGTTGGAGTGGATCCAGAAGGTAGTGCTTTCCAAGCTGGTTTGAGAGAAGGTGAT
TTGATTACTCACATAAACGGAGTTTCTATCGTTGGTGCTTATCATACAGAAGTGGTTTCA
ATGATAATTCAGTCCGGCGCTAGTATTACTTTACGCGTGGTTCCTTTGAACAAGAGTTTC
ATTAGAACGGATGGGCCGTGGCGTCCTGCTGGGAAAATGGCCACTAAACATCATCCCAGA
AAAGCTCAACATTTAAGAGCTGGTAAAAAGTGCTGTAAACCATCGAAACCTGTTTCACAG
GCACCTCCAGTTCCTCCACATGCGCAACATCAGATGAAACATATGTCAACAACTGGAGGA
ACGTATTTTGGGTATAGTTCTCCTAGTGCGGCTCCTCAGATAATCCGGAGGTCAACCGAG
AAACCCTTAATTCGTCAATTGAGTGAGAAACAACATTGGAGGCAATCCAATCAACCTCGC
CAATCAATCGGAGATCATGTTCGACCACGTCATCAGAATTCCACATCTTCATCGTCCCCA
ACTTGCGGCGCGAATATCATTAGTGGTGAAATAGAATGGCCTCAAGATCCTGATCCTTGT
GTACCCGAGAATGCAGTAAAACTTATTACTGAACTTCTTAAAAGAGACCCATTAGTGAGA
ATTGGGTCGGAAGGAACTCATGAAGTTAAAGAACACGAATTTTTTTCGGATTTAGATTGG
CAGGGCCTTTTACGCCAGAAAGCTCTGTTCATACCAGTACTTGATCATGAAGAAGATACG
AGTTATTTCGATCCAAGAACAAGTCGATATCAGCATGAATCTGAATCTGATGAGGTTGAA
GGAGGAGAGGACAGTGAGGCAGTTGAACATTGGTCCAGTGAAGACAATCAAGAAAATGGA
ACTGTATTTCATGCTTTCCCATCTTGTTCGCCTCGGTTCAGCAGGTTTGTGGAGACTGAG
CTTTGCAGAATGAGTGAATCCAGCTCATTAGTATTTCCACAAGATAATGAAGACGCCGGA
AAAATGGCCAGAAGTGTTAGTGCTGCACCAAGTTTGTTGTCGAGTGAATCCAGCAGTGAG
GATCCTGTGCAGCCAAGTAATACGCAAGCCAGTAGTACAAGTAGTTTAGAGCTAAGTGCT
CATGGAAGTTTAGCGCATCATTCAAGTGCTAGTAGTGAAACAGAATTACCTTGTTCACGA
GATATTGGCAATCAAACAATATCTACACCTATTTGCACAAGGGAACGCAGTGAATCATCT
CCAGTTCATTTGTCTTCTGCTATTCCTCGGTCTTTAAAACCAGTTCGTGAACCAGTTTCT
GCTGTATCTCCTCTTGCTCGTGTTGATAGTTCCACAAATACTACCACTATTACTACTAGT
GTTTCACGCTCAGCGTCAACACATCAACTGACTCCTCCTCGT
>DjScaffold4793:53384-53890 strand:+
ATGAAAAAAATTAGGAAAGACAATCTTTTATTACGCAACCAGGTTGACCAGATTTTCGCA
GAACGAGATATAATGACATTTGCTGACAGTCCATTTGTTGTAGGACTTTATTGTACATTT
GAAACACGCAAATATCTCTGTATGATTATGGAGTATGTTGAAGGAGGTGATGTTGCCACA
CTTATCAAGAGTATAGGACCATTACCTGATGAACTTGCTCGTATGTACTTTGCTGAAACA
GTCCTTGCATTAGAATATCTTCATAATTTCGGAATTGTCCATCGGGATTTGAAGCCTGAT
AATCTGTTAATAACTCTTGAAGGTCATATTAAGTTGACAGACTTCGGTTTATCTAAGGTT

GGATTGATGAATCTAACGACAACATTGCTGGAATCAGTGTTGAATAAAGATTGCACCATG
TTCCAAGATAAACAAGTATTCGGTACTCCAGATTATATCGCTCCCGAAGTGATATTGCGA
CAAGGATATGGTTGGTATAATTATTGA
>DjScaffold850:32811-35753 strand:+
agttttattttaatgagccaattttaagccattttaaaaatttatttttttattaagaA
TGGCCTCGTGTTTTGAAAATGTATCTTTGTTGCGATATAATGATATAGCAAAATGTTTTG
ATACTCTCATTGAAAATGTTGATCTCAAAATTGAAATTTCTCAACGTGAATTAGTTCTGT
ATATATCCAACAAAACTGGTGATACTTTAAATGTTCAATTAAGTTTAAATAAACTTAATA
CATGGAAATTATGTCCTAATCAAGTGATGATAGAAACTGAAACTCGAAGGATTGGAAATT
GTGATATGAATAGCTTTTTATTAGAATTTCACACAGAAAATGATTTGGTTTCTGCGTTAA
AAAGAATAGATCATTATAAAAATGGAAATAAACTTATTTGTAAGGATAAAAAGGATGGTA
TAAATCAATTTGATAATAGAACAGAAAAGTGGTCTGCAACTCAATATTTTCAATTTTACA
GTTATTTGAGTCAACAACAAAATATGATGCAAGATTACGTACGTACTGCTACTTACCAGA
GAGCAGTACTCAGTAATTGTGTAGATTTTAGAAATAAAGTGGTTTTGGATGTTGGTGCTG
GTTCCGGGATTTTAAGTTTCTTTGCAGCGCAAGCTGGTGCGAAAAAAGTATATGCTGTTG
AAGCGAGCAATATGGCGAAATTTTGCGAGCAGTTGGTACAAAATAACAAATTAGCTGGAA
AAATAATTGTAGTTAGTGGAAAGATTGAAGAAATTTCCATTCCCGAACCTGTGGATATTT
TAATTTCCGAGCCTATGGGCTATATGTTATTTAATGAACGGATGCTGGAAACATATGTTC
ATGCTCGTAAATTTCTCAAACAGAATTCACCTTGCATGATGTATCCAACCGTAGCAAATC
TTTATATTGCACCATTTTCTGACGAATCTCTTTATATGGAACAAGCTAGTAAAGCGAATT
TTTGGTATCAGCAAGCATTTCATGGGGTTGATTTATCAAGTGTAAGAGAAGCAGCTAATA
AAGAAAATTTCAGTCAACCTATTGTGGATACTTTTGACATTGGAATTTGTATGTCTCAAC
CTTTGGCTCACCGAGTTGACTTTCGAACTGTTGATGAAGAAAGTTTACATTCAATTGATA
TTCCATTGAAATTTACCTTCCATCAAACCGGTTGTTGTCATGGTTTAGCAATTTGGTTTG
ATGTTGGATTTCTTGGATCTCTTAAAGAAGTTTGGTTGTCTACAGCACCAACAGAACCAT
TAACTCATTGGTATCAAGTCAGATGTTTATTTGTAACACCTCTTTTTGTATACGAGGGTC
AAAACGTTCTTGGTCGAATGATAATGAACGCAAACGCTAGACAATCATATGATGTGGATA
TTGAACTTTCTCTTCCAGGAACTTCTTGCAAGGTATCTAATACCCTCGATTTAAAGAATC
CCTATTTTAGATATACCGGTCAACCTGTTCAACAGCCCCCTGGTAATCAAACGAAACCTC
CAACAGAAAATTATTTTAATTTATATGCCAACAATAATAGCAGCCAATCAGACAACGCTA
TGGATTATAATCAACAGCCAACCTCAGTTACAAATGATATTAATTCAAGTCCCGTTAAAG
GTATCGTATTATCTGGTGGTCAGCAACCATGCTACACATTTCAATTAAATAATAATAATA
ACGGTTCAGTTAATGATTATGCACCGATTTCTCCGTTTCCAAAAAATGATGAAGAAGAAA
ATTGGGGTTTATCGAAACATATAGTCTAAaaataattgaagattattctgtctgatgggc
gcgcaagtaatcgctggcatttatacaaatataatttatgattaaaaaatgttctatcca
aattattattataattattatttattgactatttttctaaatttatattgatcgtttgta
atggataataatttgtataattgaagattgagtgtttttttctttagcaatatgtaata
ataatattgatttttatttcaagttttgcattattgacttggtgtttttattatttttt
tcattaagtggaaaaaaatattaatgttggttgtacattattgaaggggtttatttaaatt
tttatatgcaatttgaattaatggatgttcaatttttattggattttcttgagattcgtt
atttgtatcaattaatttatatttatttatttcggttgtttaatattaaaaaaaaatactg
atagaaattggatatttttgtaaatatttagtatttcgagtatattttggaaatttaatg
ttattttaacacaatgatgtcttaatttgttcgaatgtcaaaattaattcgaattttctg
ggcttttatagtacagcaaaattttatttatctgttaatttttagaaattatgctatctt
tgatt
>DjScaffold2952:37622-38554 strand:+
ATGGATGCTTTATCAGTGATATTTGGTATAGGAATTGTATTAATATTTTTATTTTTATAT
ATTCTACTTTCAAATCGGCGTTTGACTGATAAAAAAATTAATAAATCTATATTGTTTAAT
ATTGGTATTTCATTATGTGATTGTTTGATTTGTTTAGATTACATTATAAACCAACAAAAC
AAACCAGATTTCAAAAATGGAATTATTGGAAAGATTATGTGTTTTCTATTTTACAATGAT
GTGATATTTTATTTCCTGATATCTTGTCGAAGTGGTTTAACTTTACTAATGTGTCTATAT
CATTTCATGAAATTATTATATCCATTTCTCAATCATTCGGTGTATTTCTCATTTGTGATA
GTTTCTGGATTTATGTTGTTATTAATTAATTCGTTAACAATACCGGTGATTTTAATTAAT
CATACAAACGTTTACGATAATTTGACAAGTTACTGTAGATTTACACCGTATGTTAATGAA
GGAACACAATTTCTAATTAATATATTTTACTTCACTTATTCATATTTGTTACCAGCTATT
GGAACTGTGTTCTTCTTTGCTCATATTTTGAATTCTATTAAAACTAATGAGATCAAATCA
AGACAAATAGCTTATAATGAAATAAGAAAAAGTTTCATTATCGATTCTTTATTATTTATT

GTCGTTTATTTCGGTTTCAGTTTCAGACTTTTGAGTTTTTGTTTAAATGTTACCTATGAT
GAAGAAAACCGAACTGGAATTAAAAGTTCATTTTCAATTCCAATTGTGGTTTATTCTTTG
ACTTATGCACCAGTTGTGATGATTTTCAAAGCGTCACATAGGAAATGGATTAAAAGATTA
TTTGTAATTAAGAAAACTATTGGACAAACTGATTCGAAAAATTTCACTACAGACTATGAG
AGTAAATGGAGTAGTAGATCGAGAAGGAAATGA
>DjScaffold4090:37358-64841 strand:+
CTTCTATTAGAACACGGAAGTAGTATTGAATCTAGTAACAAATATGGATCAAGACCGATC
CATTATGGCTGCACTAAAGGGCATGCCAATATTGTTAGAGTATTATTAGATAATCAATGT
AACATGGAGTGTGAAGATAGAATCAAATTAAGACCAATCCATATCGCTTGTTCTAATGGG
TTTTTACATATAGTTATATTACTACTCTCTCGGAATTGTAATATCGAATGTGAAGATAAT
TATGGATGGAGACCGATTCACCATGGGTGTGCAAAAGGGCAAATCGATATTGTTAAGTTG
TTAGCTAAACAAAATTGTGAAATTGAATGTAAATCGCATAATGGCGAAACTCCATATACT
TTAGCAAAATCGAATAATCATCAAGATTGTTTGGAATATATATGTAAAACACTGTATTCC
AAAAAAATTTTCAGTTGCATTAAAGAAAATAATCCCAACAAATTCATCAAAATTTACAAA
TCTTTCACTCTTGTTGTCCGTCAAGTTGATACTGGTTTATCAGTGCTGGAAGTTGCATTG
CATGACAAAGCTGATGAGATTATTCAATGTATCATGGATAAGCCAGTCACGGATGCAGAA
GTAGGATACCTTATTAATTCTCTTTTGAAAATAACTAGAACGGAAATGCTTAATTTAATA
AAAGAATGCCTTCTTTATCGATCTATAATTCACAATCATTCTGAAGGTTTCAAGGCTTTT
ATCTCAGCTGAGACAATTAATCGAAATCTTAAATTGTTTGAATCATCATTTCTGGAAATT
GCTGTTCACTACTCAAGCTATGCATTTATTGGAGACATTTTAAAGAAAATGATTTTTGAT
AGATATATCTGGGATAAAGCAAAGCGTAGTGCGACGACAACTAATCAAATGAAAATACTT
GATGAAATAGTAAAAAGAGAATCCCTTCTTCAATTGAGGCATCAATCGAATTTTACTATT
TTACATACAGCAGTCTGCGATAACGATGCTGATATGGTAGAATATATATTGCGTAATCAC
TCCGATAATGTTGATATCAATTCAAATAATCAAAGAGTTGGCACGGCTCTTGCTCTAGCT
CATATCCTACATTATAAATCATGCATAGATATTCTAGAACAATATAAATTTGATTCGAAT
ACGCAAAATCAATATTTGGAAACACCATTACATGAAGCAATATGTAGTGGTATGCGTGAA
AATTGTCAGTATTTGATTGATCAAACTCATGACATTAATAAATTTAATAATATGGGATTC
ACTAGTTTACATTGTGCAGTGATTAGAGGATTTGAAGATGTTTGTGAGAAATTAATCATT
AAAGGAGCTGACTGCACAGAATACATTAAGAATTCCACCGACTATTCTCACAGCCAAATA
TTAATACCAACTATAAATACATTTTTACCTTTTACATCTAAAGAGAATGCCTTGACATTG
TGTTTGAAAATTCATAAAACTATTTTTCCTTGTTTTATCAAAATTCTTGATGCGGATAAA
GCAAATATTTGTGATGGTGAGGACAATTCCTTGCTCCACTTGGCAGTGAAATGTTTGCAA
CCCGATATTTGTAGATCGTTACTTAATTTAGGAGCCATTGTAGACAAATTCAATAAAAAA
TCAAATAGTGCTCTTTTGGAAGTATTGTCTAGAACAGATGAAGAATTACTAGAAAATAAG
ATGGATCTAGAAAATTTGATCTTAGTTGTGAAAATATTGATTGAATTTGGA
>DjScaffold4090:66541-67341 strand:+
agattgATGATTAATTTATACAGAACAATTAATGAATTATCGATTTTTAATGAAGATGAT
TTTGTTCAAGTTTTTACAAACCATTGGTTTGATTTTGTCGAAGTTCTTCTTAATCGTCTT
ACGGATTATTTTCTATTAGAAAATATATCAATTGAAATTATTCAATTAATGATGACCGAA
AACAGATTACCACTTCTTCTATTATTATTTAATAAAGCAAATTCTAATGTACAACAATCA
GTGTTAGAAGACCTAATTTATCATTCTGCTCAATTAGACGATGAGAATTTATTCGATTAT
TTAATTAATAAAACATTTTTCTTTCCAAAACTATTCGAAAATGGTGAGACTTTGTTAACA
AATTTGATGAAAATGAATAAAATACATTTGTGTGAAAAACTACTACAATCTGAAATAAGT
GGGCAATATATTAATTTTAAAAATAAAAATTTACAAACACCCTTGCAAATAGCAATCAGT
TTAGAATCAATGAATTTAATCGATGAATTGATCAACAGAACTGATGATTTCAATTATGAA
GATATTGATGGTCAAACTCCATTAACTCAGTTATTGGAAAAATTATCGATTTGTGAAGAA
GAATTGCATGAATCGAAATTAATGTCAATCATCAAATCAATAATTATAAAGGTGGAAAT
ATTTATAGGTCTACCTCTCTTTATCAAACTGTTATTGATGCATGTAATCAACCTTTGAAA
AAATCTGCAATAGAACGTATTTATTGTTGCGGCTGA
>DjScaffold292:114298-115217 strand:+
tttttatgaacttcattgaaaataaaatcattctcaaaggtgagcagaagatacatcttt
ggttcagatatatagacgatatttctttgttactaattcatctttagaaaatattttag
aactagctaatagtatcaatgataacattaaattcattttagagaagttaatcgactttc
agattccttttctggacaccttaatccaaaagaaacctggtgaattattaattacccagt
tgtatatgaaaccatttcacagtggacatatattaccctggagtaaaaattggaataatc
aagagtgaaaagataagagctgaaagattatcttcaaacgaagaaATGAGACTGATTTCC
CTCAGGTTGATAAAAAAATAAATTTCTAAATAATGGTTATCCAAATAGAATTGTATATAAA

CATCTATACTCAAATACAGAATGTAATAAAATCAAACAAAAGACAAATACAGTAATTAAA
TTACCTTTTATTAATGAGAAATGTAGCCAAATCATGCAAAAAATAATAAAAAAGTCAAAA
CTAAAACACAATCCAAGAATTATATTTAAAACGCAAGCACCTTTGGGCATCCAATTACAG
AAACCAAGACAAAATTTTTGTGGTCCCAAATGCATTTGTGATGGTAGAAATATATGCAAT
AAAAAAAATATCACTTACAAAATAACATGCACCTTATGTAAGGATACATATATAGGAGAA
TCATTCAGAACCATCCGTACAAGAATTAAAGAACATCTTAAACAGAAAGAAAGTGAAGTC
TTCAAACACTTTCAAAACAAACATAATGTTTTACCAGAAAATCATCATATTACATGGAAA
ACATTAGGAACAGGCTACATTTCTACACTTCACAGAAAGAAACATGAACAAATATTAATT
>DjScaffold292:176350-183809 strand:+
agatcaacataccaaattgcggattatggttatgaagatATGGATGAAATTGAATGTGAT
CCAGGAGTTGATGTAGATGATCTCTTTAATCAGTCATTGGGTTTTGTTTGTGAGGAAGTA
ATCTTACAAAAAAATATTGATCAAAAATACGGATTGACTTTGTTTTATTGTCAGAACAGT
TCAGATTCTAATGTTTCAGATATATTTGTAGGAGAGATTGAGCCCCTTTCACCAGCTAGT
CTAGTGAAAGGAATGCGCATTGGAGATCAAGTTCTTCAGATAAATCATCAAGATGTATTC
ACCCGTGATCAAGTCATGCATTTATTTGCGTTATCAGTTAATCACATTCATCTTCTTCTC
GCCAGATCAGCATCTCAGCAGTTACAATTGATGGCTCAATCTCAGTTACAACCGCAATCA
AATGATCCCTACACGATGGCTGATCTACAGAATGCTCTTGAACTATATGAAACACTGGAG
ACTCCATCATCCATGGATGCTCTTGATGCTGATTCTGGAATGGGTCGACTAACTGATGAA
AATGTCAGAGCTGAAACCAGTAGTGATTGTAGTTCTTTTCATTCTAGTCGCCATGGCGCC
ATTCCATTCAATGCAAACACACCTTCATTACCAGATGACTTTATAGATTTAGACATGTCT
AAAATCAATGATGAATTAAACAAATTGAGATGGCAGTGCATTTATTTAGAACAGATTAAA
GCTCAAAAGTCTATGGTAGAATCTTCAGTTAAAGCTGGTCCTTCAACTGAACCCAATACA
AAACCAATTCCTAGAATGGGTACAAAACTTCCTCCAAGAATTAATCAAAATCAAATTGTG
CCACCATTGCCCAAAGAAAATCCCTTGCCTATCAAATCAGATCAACCTAATCGTCCTACA
CTAACTTTTATGCTTCCTCCAAATGACAGTAGCAAATGTTTTCCACCGGTTCTCAGATTC
TCAAGCTTTTCTTCCGAACCCGAAACTCCAGTTCAAGTTAAACCAGATCCTTCTCCAATC
ACAATTAATGATTCAAGCTCTTCACAAGATCCAACAGTTCAACCTCAAAACACCGTCCCA
TGTCAAAATAATGAAGGTGAACGACAAATGCAGTGGGTCATGAAAAAGCGTTCAGATGGA
TCAAAATATTTAGTAAAAAGACCAATCAGAAATCAGCTTTTGAAAGATAGAGCAAAGCAA
TTAACAGAAGACCGTCAAGCAATGAGTACAGATGATGATACAGCAAGTGAAATCAAAACT
GGGCGCTATTGGCCAAGGGCAGAGCGCAGAAGACAATTTGAAAAATACAGAGAATATAAA
AAACAAAAAGAAGCA
>DjScaffold1103:14302-21591 strand:+
aaggttttcagtcattataaagaaaaaggaatccaaataactgatagaaaatattttgaa
aatttggccATGATTTTTCAATCAATTTTGAAAAAACGAACAGGTTTTGAGCGTCAAAAA
ACATTTGACTTCTTTAACAGTGAACTTATCAATGCACTTATTGAACCTGTGGATTATGAA
GTTTGTGTGCAAAATAAAAAAGAAATGCTCATGAGATTAATTTATATTTATTTATCGAAA
AATGAGCATATTAATGCATTACATATATTTTTATACACGATACATTACTATAATGTCTGT
AGTAGCTTTATTTGGCGAACAGGGTTTCAGATTTTAATGAAAATCGGCGATGAACTATCC
ATGTATAATTTTTTTAATGCTATGCTTGACAACAATGTATGGGATCCGTCGAACATGATT
TTCGAATATTTTCTCTATTTATTGGATAAAAAAGACTATAACAAAATTCGGGAAATTTAT
CTAAATGAATACCGTGATATTTGTAAGCATAAAAGAGTTATGAGAGAACCTGAAATGGAG
GATTTACATCATTATATTAAATTAACTGTAGCTGTATCATTTTATTGTAGTTGGAAGCAC
AGCTTGATGTTGGGTAAAGAGGATGATGATTCTCTGTTTCAAGCTGAACTTGAATTCAAA
GAAATAATTCCAACAATCAAGAAAAACTTAAACGTTGATGTATACATCTTGAATTATCTG
GAAATATTGGAATATCAACAGAAATTCGAAGTTGCATTTGATGTGTTGAAACTTTACAAT
GAAAAAAGCCCGAAAAATATACACTCAACGGAATATCTTCTCGACTTTATGATACGCAGT
AAAGAATATTGTTCATTCATAACAAAGGAAAATATTGCAGAGATTCGTGCTCAACTGATC
ACGAAAGAAGTTGGCATCAGGCCGGAAGATTCCTCACAGTTTGTTTTGGAAGCATTCAAG
AAACATATTGAAGATCTCAATGAAGAAGGAAATCATGAAGAACTGGTGAAATTATTGTTC
AAGCACCTCGGAGATTCTCGTTGGGCAACGAGTGAATGGCCTTGGAGATGTCTGCTTGAT
GGAGTTAGACATGTCGGAGTTGATAGCTGCTGCGTTCGGAATACATGGTCATTCGTTGAA
GATTTGTGGACAGATCGGTTTTTCAGTTCTCCTCGCAACAGTTACATTTCTCAACTTTCA
CAGTTACTCTCATCATCTCCTGTTCCTAAATAAatcaatt
>DjScaffold1103:22895-23761 strand:+
aatgtaatataatgttttattttagatatttctataaaattatcttttaaaaatgtatat
cttttaaaattagatATGAAGTACCACAATGGTACCGAAATTGCATTAAATTCTTTCAAA

AAACAAAGTCTTTCAATTGATGACAAAATAATGCTAAATAAAAATATTAACTCTGAATTT
ATTTTTAAAAAGAATCCAGTAGCCGTTGGATTTATGAATGACATCAACCTGTATTACAGT
GATAACGATTTTCACCCGAATCCTGAATTAATCGGTTCAGGTTTCTTTGCTAAAGTGTAT
ATTGTGCACAATATTGTATTAAATATGAAAATGGCATTCAAAGTTCCTAAACAAACTCAG
TCGATTAATAAATTTATCAAAGAAATCAAATTATTAAAAAGTCTTAATAATGAAAGCATA
CTAAAGTAAttaattaaattttatttatttagtaatttaatctaaattcaaattaaaaat
aaacgttattatttgaattaagaattgataaattttaatttgaaaagataaatggttttt
taatatttaattttttagattattttaattaatttttgccatactttattattaaaatt
ttaaaaaatttttataattgacatataaagtacattacgttcaaatacaacacgcttaaca
cgttcgctacggtagcaccgtttcccattaaaatctatttttgtacggttacacgcaaaa
ctgtgaagaaaaaactgctgatattccatcatgacacatagttcctccttgttcaccactg
atgctccatcatgacacacagttctatagagtcattt
>DjScaffold1103:80862-81532 strand:+
AGAAAGAAGGAAATTGTTGCATTTGCAATTAGTAAAGGTAGTGCATCATTTTATAAGGAG
CAAATATTTTATCAATATAATGGTAATTATAAAACCTCAACTTCAACATTTCAACATTTA
AATGCTAATTCCATAACTTTATCGAAAAACATTGATTATCGAGAGACATATAATCAAGAT
TTTGAAAACAATGATTATAGTACAACATATATGCAAAGTGTTGTTCGAAATATGGGATAT
TTGGATAAGAATATGAATTGTGGAATAACACTTGAGTAAtttaaaagcaaatatacgttt
tttacatttgtacttgctccagattttgatataaatcagagtcaattgccacaacaagga
aatttgagattggatataaaatttaaaacagctttgacggcgtcagcatatgttataata
tatggagtatttgaaagcgaaatcgaaataactgctaatagaacagtactagtgtaaaca
atatgtaatataataaaataaacaaaaattatgaagtaagttttttatttaatttcaata
ttttactatattatttcttttcagattaatcaaattggatcaaaattgttgggatcgtcg
tggctgggagtatatccattaga
>DjScaffold3040:49449-52688 strand:+
CAACTTGTACAAATTCTTGAAAATACAGTATCTCCTAATCAACATGAACTTACTTCAGCA
AGAGATTTTTTGGAAAATGCCATCACTACACAATATCAACCTTTTATAATAAGCTTAAGT
CAGATTCTTAGTGATGTAACAAATAAGCCTGTTGTACGAATGCAGGCGGGATTGCAGTTG
AAAAACGTAATATATTCCAATGAAACCGAACAGAAAGCTATTAATCAGAGTAGATGGTTA
AGTACACCACCAGAAATTCGTCATCAAATTAAAGTCAATTGTTTTAATGCACTTGGAACA
CAAGAACAAGGATTCCATTCAGCTGCACAATGTGTAGCGTACATTGCTTGTGCGGAAATT
CCATCGAACCAGTGGCCAGATGTGATGCAATTGTTGGTACAGAACATCACAGGACCGAAT
AGCACGGAAACATTAAAGCAGGCATCATTGGAAGCGATAGGATATATTTGCAATGACATA
GATCCAAGCATTCTTGCCACACAAGCTGATCAAATTCTCACTGGTATTATATATGGTATG
AAGAAAGAGGAACCGAGTTATCGTGTAAAGTTGGCAGCTACGCAAGCATTATTGAATTCA
TTGGAGTTTACCCGGCACAATTTTGATAACGAGAATGAACGAAACTACATAATGCAAGTG
GTTTGTGAGAGTACTCAGTGCAGCGAGGCGAGTATTTCTGTGGTAGCGCTACAATGTCTC
GTGAAGATCATGAGCCAATACTACATGTACATGGAGCTCTATATGCAGCGAGCTCTGTTT
GCCATCACGGTGGAGGCGATGAGGAGCACTGTTGATGCGATGGTTCTGCAGGGTATCGAG
TTCTGGTCGACTGTGTGCGAGGAAGAAATCGATATTGCGCTGGAATTGGCGGAATCTCAA
GAACACGGTATTTCCGCAATACCTGGTTCAAATATCAGCAAGTATTACGCACGAGGAGCG
GTGAAATTTCTCGTTCCATTGCTACTGGATATTCTCGAGAAACAAGATGAGGGTGATGAC
GAGGACGAGTGGAACCCACACAAAGCAGCTGGAGTGTGCATGGCTCTGTTATCTCAGGCA
TGTGAGGGAGATATTGTCGAATTGGTGCTGCCATATGTCCAGGCTAACATCCGACATGAA
GATTGGAAGAAAAGAGATGCTGCTGTGATGGCGTTCGGGAGTATCTTGGAGGGTCCCTCG
CCGAGGAGTTTGGAGGGATTAGTGACGCAGGCAATGCCGGTAATCACTGAGTTGTTGGCT
GATCAGATATCAATCGTGAGGGACACAGCAGCGTGGACAGTTGGGAGAGTGTGTGAGGTG
CTGCCAGAGGTTCCCCTGGGGGACGCGTGCTTGCCAGGATTGTTGGCAGCGATGATGGCA
GCACTCAGCAGCGAGCCGAAAGTGGCAACAAATGTTTGCTGGGCATTCAGCAGCTTGGCA
GAAGGTGCATACACGGTGGAGATGCGCGCGTGCTCGGCAAATCCTGACGGCGGAGACGGG
TCATACAACAACGAACCTCTCAACTACAAGCTCAGTCCGTATTTCAGCAACATCGTTGAG
CAGCTGCTGCGCACAACAAATCGTGCTGATTGCGGCCAATCAAATCTGCGGAACGCTGCT
TACAGCGCACTGATGGAGATGGTGAAGAGTGCGCCCAAGGATTGTTACCCGTGTATCCAA
CAGACCACTCTGATCGTACTGGAGAAGTTGAACGGTATTCTCCAGATGGACCAAGCATCC
ATGTCGTCCCAGGACCGCATGCAACTGGCCGAGTACCAGAGCTTGCTGTGTGGCACGTTG
CAGAGTGTGTTGCGCAAGATCGAGCGCTCCGATGCACAGAAGATCGCTGATCAGATCATG
GAGGTGCTCATGCTCATGTTCGAGCGCACTCAAGTCAACGCATTTGCCAACAACAATGAT

GGGAGCACGCTGAGTCGCTACACTGGAGTGCAGGAGGACGCGCTAATCGCGGTGAACGCT
CTGATTGACGCGATTGGGCCTGCATTTAGCAAATATCTCACAGTTTTCATGAACATCTTG
ATCGTTTGCCTCCAGAACGTCACGGAACAACAAATCTGCTCGAGCGCTGTCGGAATTTTG
GGTGATTTGGCGCGTTCCGTGACTACCGAGCTCGTTCCGTATTTGGGTGACATTCTGATG
ATTTTGTTCAACACGTTACGACATCCGTCCACTCAGCGCTCGCTGCTCCCACAAATCATC
TCGGCATTCGGAGATTTCGCTCTGTTGCTAGGAGAAGCATTCGCTCAATATCGACTGCAA
GTGCTCGACACTCTCAAACAGGCCTGCCAAGCAGATGTAGACCACACCGATCCTGACATG
ATCGATTATTTGAACGGGTTACGGCAGAGTTGTATCGAGGCCTTCGTCGGACTGATCCAA
GGCGCCAAGTCGCAGTTCCCCCAGCACAACATCGAGAAGCTGCAGTTCATCATTCCTCAC
ATGACCTTCATCATCAACTTCATGGAACACATCGGTGCTCAGACCATCTCTTCCGAGGAG
CTCATTTGTGCGTGTTGCGGTCTCGTTGGGGACATTGCCATCGGATTCGGTAAGGACTCG
CTGCAGATTCTCGACACAGAGGCCATTCGAGTTCTCCTTGCTCGCGGTCGCAATTACAAG
AATGCCAACACCAAGAAATATGCCGTTTGGGCTCTGAAGGAGCTGACAAAACTGAGAAAT
ATGTAAtttgttgagatgcacttgcacccgcgctgctgtctcgcggtgtgattattcaat
tgagataatcgatggatcgttatgtaatgtttgcctgatgatgatgatgatttgagtgat
gttttcaatgtcgcgaatgactgctagattaataattactttatcatgttaataaagttt
ctatattttgcc
>DjScaffold2689:76686-80576 strand:+
gataatgctcatcgtcttacagttcgaATGTCTCCAAAGGAATCATTTGATAAGGATAAA
TATGCTGAAGAACAGAAACTCATCGAAAAATTAAACAATGCATTATCAACTGCTGATAAA
GCACAAATATCTGAAAAATGTACTAAACTCCTTGAGGCTCAGCAAAGTGCAGAAGATATA
AACTGTTTACCATGTTTACAAATCAATGATATACCAGTTACATGTGAAAAATTTCCTTTT
AAAATGTCGACAGATCAGCGTTGTCAGATCAATGAATATGATACCAATGGAATATTATAT
TTGAATATGTTATTTGATATCAATGGGATAGAACCAGATTTATTGCCATATATACCATTA
TTTTCATCAGTTTTCACTAATATGAGCACATCAAGTTTTCATTATCGTGAAATTGATCAA
GCTGCTGAATCATGTTCGGGTGGTTTGAATTCTAATTTGCATTTATCTGGGAACGTAAAG
ACTCTTGATAATGACGACGTCAATACTGGAATACTGCTTTCCAGTTATTGTTTATATGAC
AACACT
>DjScaffold2689:83720-86236 strand:+
atgcagtcgaatctactttgtctttattagataaatttgaaaaggatctcaatttccata
attttgaaaagataccaATGACCCTAGACTGTTCAATGGAGAAAAAATGTCAGTTCGTGT
TACCATATACTGTAAATTATGTTTCAACAGCAGTTAAATCGGTTCCATATACTCATCCGG
ATTATGCTAAAACAAGAGTACTCACAAAACTTTTGTCTTCTAAATTTCTTCATAAACAAA
TCAGAGAAATCGGTGGTGCATATGGTGGAGGAGTAGTTTTGAGCCCAGGACAATTACATT
TCTATTCTTATAGAGATCCACATTCCATCGCAACTCTGGAAACTTTTGAGAAAAGTTTTG
ATTGGGTAATAAATGAAGAAATAAATGAGCAACTGTTAAATGAAGCCAAATTGTCAGTAT
TTCAAGAAGTAGATAAACCAATCAGCGCAGGCTCTAAAGGTCTTGTTCATTTTTTCGATG
GAATATCCGATGAAACAAGACAAGAACAAAGGGAAAAGTTATTTATGGTCAAAGTTGAAG
ATTTGAAAGCAGTTGCAGAACAATTAAAGGCATGTTCGATAAAGAATAGCGTAATAATTG
GTCCAGAACAAGACGCTGGTTTTGATTGGCAGAAAATTTTGTTTAGTTGAtcgatttttt
ttattttgtgtagttgatcgattttgttattttgtacattaataaataaagttttaattg
aa
>DjScaffold2581:136512-152279 strand:+
gtttaaaaattctaacttttacgtgatgtttaaaaatttggtaatttgaaaactgttaaa
ctgttttgttacgttaaaatcaaaatttaactttgaattagtatttgtataattggtttg
aatattatttgataatcaaattgttatattttataataaattgtgataaaaattgaatttc
agaatgaaattaggccgatatgccattgtcatcacttatagtgcagttagatcattttct
acaggattaaaaatgaattacaatgctagtaattctgctaaaatatacgacttggttgta
atcggcggtggttccggtggtttagcgtgtgccaaagaagctgcaacatttggaaaagaa
gttgctgtgctggattatgtggttccaacattgaaaggcacatcttggggattaggaggt
acctgtgttaatgtgggatgtattccaaaaaaattaatgcatcacatcagtcaaatgcgt
gacataatagaaaatgctcacgaagcaggatggaatcaagtttcagtgaaaccagaatct
ttacagatagattggccaacaatggccacacgagttcaaaattacgttaaaagtctcaac
tggggtcatcgagttgcattacgagagaagtagcatttacttagatgtgtatattgatgt
tataaaggtgtatagcaaaatagaatacatcaatgccaaggcaaaattcaaggatggaga
aacgattgttgctactgacatcaaaggtggagaagtatgacatacataattcctctttct
gattgattgttATGATTAATCTTCTTCAGAAAGAAATCAAAGCCAAAAATTTCGTTATTG

CTGTTGGTGATGTGGCATTAGAGTGCGCAGGATTTCTGCATGCATTTGGGTGTGATACTA
CCGTTATGATTCGTTCCATACCTTTGAGAGGCTTTGACAGACAAATGGCAGAAATGGTGA
CTGAACATATGGAAAAAGAAAAGAAAATAAAATTTATCTATGGAGCAGAACCGTTTGAAG
TTATTCAAGGGGATTCTGGAAAGTATCGAGTCAAATGGAAAAAAGAAAAAAATCCCGGAG
AAATGTCAGATCTGTTTGATACGGTGTTATTTGCGATAGGAAGAGATCCATGCACTTTAG
AGCTTAATTTATCAGCAGCAAATGTTTCTGTTGATCCAAACACCAAGAAAATCCTAAGTG
GTTCCAATTCTAAAACTGTTGATATGGAAAAGACGAGCGCTCCGAATATTTATGCTATCG
GAGATGTCTTACAAGGTGGTGTGGAACTGACACCAGTTGCAATAAAGGCGGGTAAATTAC
TCGCAAGAAGATTGTTTCACAAAGAAACAGTTCAAATGAATTATGATTTGATAGCTACTA
CGGTTTTCACTCCTCTGGAATACGCCTGTGTCGGGATGACAGAAGAGGAGGCTTCTTGTA
AGTTAGGTGAAAACAACGTAGAAGTATATCATTGCTACTTCAAACCGTTAGAAATCGTCG
CGATGGGTCAGCAGCAATCATGTTATATGAAGATGATTTGTGATATTAACAAATCTGAGA
AAGTTGTTGGATTGCACATACTTGGTCTGAATGCAGGAGAAATCCTGCAGGGTTTCGCTG
CAGCAATCAAACACGGTATTACACACAGTCAGATATGCTTGAATACTATAGGAATTCACC
CTACTTCTGCAGAGGAAGTCGTCAAATTACACATCACTAAGCGGTCCAACATAGATCCAC
ATTTAACTGGTTGCTGAggttaatttcgatttcaagtcttgaaaaagttcttgaaatcgt
cgttgaaaaaaaatctttgaaattttggtcccagtttttcaatgatggagaatttggaaa
cgttggcaacaacgtcagattctcttgatatcgggcctgaatttcattttgataacgcct
atttatatcagaaataaatttcatttcgctt
>DjScaffold235:53488-54003 strand:+
tcaATGAAAAAACTTTTATTCGTAATTTTATTGGCGGGGTTTATAATATTTGCAATTAAT
GCATTTCAGCAATTCGGCAGAGAACTTGAATGTTATCAATGTAATTCATTAACGCAACCA
CATTGTTCTGATTTTTTTGACAATCAGACAGTGATTTTAGAAAGATGTCCAAATGACGGT
AAAAATTATTCCATGTGCAGAAAAATTATCCAAGAACTATATTATGATGGAGTTTGGGAA
CGAAGATATTACAGAGATTGTGCAGTTACTGGTTATATCGGTGCAGAAGAAGGTCGTTGG
TGCACAGATCGAATGGGAACTCTTCGTGTAAAAGTGAGATATTGCCACTGCAACAATAAA
CAAGGTTGTAACAGTGGATCATCGATTTCAATTGGAATTATTTTGATGATATTTTCATCA
TTTCTTGCAATTTTTACAGTGACTCCTACATAAtattttgatatatcactc
>DjScaffold235:60529-70264 strand:+
tttgaataattttaaaattagttattgttaaatttatctttagtctaagtaatctattat
ttaaataaaaaataaaattttatcctaATGAAATTGATAAACATTGTAACTAATCGTTCA
TTTTCGTTGTTATCATTTCTTATTGGTGTTATTTGGTTTCTTATGCTATCTCAATTTGAA
TTTAGCCAAAAAACGTATGTGTCAGAAAATGCATTGTTAGTAGGATTAATTAAACAACAA
TTTAATAATCGTTCTGCTATTGATCATTTTTATAATGAAATTTTAACTCTTTCTCGTAAT
GGGGATAGTGAAAAAACTAAACAATGGTTAAAAGAAACTATGGAAAATATTGAACTTGAT
GTTGGTGTTCATAATTTTACGTTTAAATATGATAAGTTCTTAAATTTCCAGCAATTAGAT
GGTCAAAATGTCTATGGAATCTTGAGATCACCAGGTGCAGCAAGAATGCAAGCGATCTTA
GTGACTGCCCCATTGAGATATGGTGATAGTAATGATGAAACTGAAAATCAACCGCTTTTA
TCAAGTGTTAGTATGATGTTGGCATTGGCTCAGCATGCAAGAAATAAACTATACTGGGCA
ACAGACATTATATTTCTATTTACCGAGATGGAATATGTTGGATTGAAGGCGTGGTTAAAT
GCATATCATGGGCTGAGTGACTCGAATGAATATATTCAACATCAACCACTGGTTGTTCGC
AGTGGATCCATTCTAGCTGGATTTAATGTAGAATTAGCTTCAATGAGAATTCCATATTTA
GAATTTCGTGTTGAAAGCTTAAATGGTCAATTACCAAATTTGGATTTATACAATACTTTT
GTACGATTGGCTCAATCTATGGAAATTCCTATCAGGATACACGGAGTGCCACCACCTATT
TCAAGTTCGTTAAAATCTCGACAAAATGGTCTCAAATCGTTTTTGTCAATGATGTGGTAT
CAGGCGTCTGGTAGTCCCAATGGGTTGCATGGATTATTACCAGATTTTCAAATACCTTCA
TTGACAATTTATTCTCCACCCGATTCTCGAGTGAATTCGAAAACCAATTTATTTGCAATT
ACTAAATTAATGGAAAGCACTCTTCGTTCACTTAATGGACTCCAAGAAAGAATGCATCAA
TCCTTCTTTTTCTATATTTTGCCTAGTCCAATGAATTACGTGTCAATTGGGATGTATATG
ATTCCATTTGGCTTGATGTATGGCTGTGGACTTGTTCTACAAGTTATCGGAGCTATTTAC
TATTTCCAATTGAAATTTAAAGAAGCTGACGAAAGCAATGACAATAGCAAACAAGTAATT
TATATATTGATTCTATTATTATATTTCCGTAGTATTTTTCAATCAATTAAATAAtatttg
caatcaataaatattatttgcaatcaattaaaataatatttgcaatcaataaatattatt
tgcaatcaattaaataatatttgcaatcaataaatattatttgcaatcaattaaaataat
atttgcaatcaataaatattatttgcaatcaattagataatatttgctatcaattaaata
atatttgcaatcaataaatattatttgcaatcaattaaatattatttgcaattaattaat
aaatacttgctatttcattaaaataagataaagcttcgatcaaaacaaaatttatttgta

aaaaagttcaaataaattttgttttgatcgaagc
>DjScaffold1973:51714-64193 strand:+
tatagaagtttcgatATGATTCGCCGAAAAATAAAAAATGTCGTTAAAAATTATTCGGAA
ACTGAAATTCTTGTTCGTGAAGCCACCTCAAACGAATCTTGGGGACCTAGTAATAGTGTT
TTAGTGAAAATAGCAGTATTATCAGCCGTTGATACCAACATTATCCAAATAATGCCAGTA
ATTTGGAAAAGATTAAAAGATAATGGGAAAAAGTGGCGACATGTATATAAATCATTAATT
TTAATGGAATTTCTTATCACTCGGGGAAATCCTGCAGTCGCAGAAAATTGTCGTCAAAAT
ATTCATTTCTTGCAGTCGCTGGAAAATTTTCAATATGAAGATAATGGTGTAGATTTTGGT
TGTCGAATTAGAAAGCGGGCAGAAACCGTTGTACAATTATTACAAGATAATGATTTACTG
AAAAGTGAACGAAACAAAGCAGCAATTACTGATATGAGGTTCTCAAAAGCAATAGTTTCA
CAAGGTTCTAATGTTCCTATTAAATATGGTAATGGAGTGAGTTCGTTAAGTCAAGGGCCT
TCTACTGGAAGCTTTGGTAGTAGTCTTAATGAGATTAATAAAGACTTTATTAAACCGGAT
TATGTTGTGCCGTCGAGTTTGTTGGAAGAAGATATTCAATTACAAATTGCATTGGCTGAA
AGTCAAGAAGACGCTAATCAGAAAAAAAAACTTGAAGAAATAGAACACTTGACTGATGAA
TATAAATTATCTTTAGCTGTTCAGAAAAACTTTAAAGAAAATGAATTTATAAAAGATGCC
GATTCCGAATTTAAATTAGACACATGGAGAAGCAATAAAGAAATTGCAAGTTGTCCCAAT
GATCCCTGGTCTCCGGTGGATTCAGAGAAAAAATTTGATTTCCCACCAGTGAATAATCCC
TGGCCTACTGTCGATGCTTCTAATGATAGTTTATGGTCGGATTCTCTTATACCTGATCAA
ACTGATCCCTGGACTGCACCTCAACCGCAAACTGAAGATGTTTGGCTAGTAAGTAACAAT
GAAAATGAAGTTAATCCATGGCCTGCTGTATCACCAGAGTCATTCCCATACGTAGATCCA
TGGAAAGAAAATAAAATGTCAACCTCCATTTCTCAGAGTGCAATAACCAGTGAATTAGAA
TATAATCCGTGGGCTAATAATGTGGCCAGCACAAATCCTATTCCCAGAAAAGTTATCAAT
CTCAATATAAGTGATTCAGTAACAAATTTCCTTGCTAATCACAGTCATCTAGTCGATTTC
AATAATTTGATTTCTTCAGAATCTCTTAAGTCTACGAAAGATCAAAATTATAATCCATTT
CTAAATCGGAACACTGAATCGTGCAATCCTTTTTTGTCTTCCAACAATGCTATTAACAAT
TTAACTCAGATTGATTGGGCATCATTAGAAGCTGAAAAAAATCAGAACCCTCAATCCAGT
CCAACTAATCAATTTCAACCATACATCGTAAACAATTCAAAAAATCCGAGTAGTTTGAAT
CCGTTTCTTACGTGAttatatttttgtgatgaaaaagatactgttattattgccttgat
taaaataatttattaaaaatattttctatctgttcgtaatttttatttttatttcataa
tttttatttattttcctagtgaaatagaacgaaaaatataa
>DjScaffold2649:76852-86595 strand:+
GTGGTGCTCTATTTGTGGTCATTGAAACTATTATATCCTAATACATTGCATTTATTGCGA
GGTAATCACGAATGCAGGCATTTAACGGAGTATTTTACATTTAAACAAGAATGCAAAATT
AAATACAGTGAAAAGGTTTATGAAGCATGTATGGAAGCATTTGATTGTTTACCATTGGCC
GCACTTATCAATGGTCAGTTTTTGTGTGTTCATGGTGGTATATCTCCAGAATTTCATTCT
CTCGACGACATCAGACGGCTTGATCGTTTCAAAGAACCTCCAGCATTTGGACCTATGTGT
GACTTGTTATGGTCAGATCCAATTGAAGATTTTGGTCAAGAAAGAACTTCAGAAAATTTT
TTACACAATTCTGTTAGAGGCTGTTCATATTTTTACACCTATCAAGCAGTGTGTAAATTT
TTGCAAACTAACAATTTGCTATGTGTCATCAGAGCTCATGAAGCCCAA
>DjScaffold2649:116674-127504 strand:+
tattaattacaattttgctaaaaaagcagtaaaataaaaaaaattccatttaaatattttg
gaattttttttatttcattattacatatttgtaaatatttttatattaattttcaggaa
attataaattttgactttaattatcaaacacttgtttatatatttgggaataATGATTTT
TTTTCATTTAGCTGCAGCCGTTGCGGCTAGAAGGGATGTCATCAAAAACAAAATCAGAGC
TATTGGAAAGATGGCTCGTGTGTTCACAGTGCTAAGAGAAGAGAGTGAATCATTACTTGA
ATTAAAAGGACTCACTCCAAATGGAATGCTACCAATGGGACTGTTGACAGGTGGTAAAGA
TTCTATTCACGGAGCAATGACTGGAGTTTCCCCACATCATCCAATTCAGTCATTTGAGGA
AGCAAAGGTTCTTGACCAAGTTAACGAACGAATGCCACCGCGTAGACCTAGCTATCCCGT
AAACAGTAAATTAATAGCAGCTTCACCTTCAATCGCTCAATCGAGTGCTGGTGCAGACAG
CATTTACCAAGCGTCTCCAATTAATGCTGTTGAATCATCTGCATCATCATCATCGCATGT
GGCATAGtgatttaatgtgcttattatcgatataattattattggtatctgaaccaagat
acgatagtatcatcactattcttattcttattcttcttattcttcttcttattattatta
taattattatttcttaaccttaatttaacaaaaacgaaactatcacacatttg
>DjScaffold1032:44276-49891 strand:+
AATATTGGTGCTTATTCTTCTTGGATTGAACTTATTTCAAAAATAATCCATCGACCAAAA
GAAAATTTATTGGTGTTTAATATTGACAAATCTTTTATAAGCACTGTTTTCTTAGTTTGT
TTAAATGCAGCTACAAAACTTTTTTATTCCAAATTTGTTTCTGTATATTACACAATCAGA

TCATTGCAATCCTCTGGACATCCAATTATCAATGAATTTGAGGAATTTAAATTCATTTTT
GAATTCTTGGAAAAGTGGATTAATAAAAAAAATAGTCAAGATGGGTTTGATCCTGATTTT
TCATTCCATTTAGAATTAAATCAAGAAAAAGCATTAGAAAAAGGTGCTTATAATCGTTTA
TATCCAAATTTAGATGATATGACATTTTCTGCCAAC
>DjScaffold4438:24344-25455 strand:+
GGCCAAAAAATAGGTATTGGTAGTGGATCAACAATTACATTTGCTGTTGAAAGGCTAGCC
CAAAAAGTAAAATCGGAAAATCTTAGTGTATCATGCACACCTACTTCATATCAAGCAAAA
CTATTAATTATAGATTATAAATTACCTCTAAAAAGCTTAGAAGAAAATATAGAACTTGAT
ATAGCCTTTGATGGTGCTGATGAAGTAGATAAAAATTTATGTTTAATAAAAGGAGGTGGA
GGTTGTCATCTACAAGAAAAAATCGTTGCTTCTTGTGCAAAAGAACTCATTATTATTGCT
GATGATAGCAAAGACTCTTTAAAATTGGGCGAAAATTGGAAAAATGGAATTCCAATTGAA
GTTATTCCTTTAGCAAATACAATTGTTAAGCAAAAAATTGAATCAAAATTCGGAGGTAAA
GCCGTTTTACGTCAAGCTATCAACAAAATGGGTCCATTAGTGACTGATAATGGAAACTTT
ATCATTGATTGGAAATTTGATATTAATGAAATTTACAATTGGTTGCAAGTCGATATTGAT
TTACATCTGATTCCTGGAGTGGTTGAAACTGGTCTTTTTATCAAAATGGCGAAAGAAGCG
TTTTTTGGTAAACCAGATGGAAGTGTTTACAGTAGAACACTGGATAATTGAaaaaaatat
tttattaaagaatcttgattagattttgtatgaaatgcctatgtaaatatacttattgtt
tatttatttgctgttgtttactttgtacattgatttactgtcaatttcatgttgtaatct
ttaatcttatattaattgttttgat
>DjScaffold1714:2961-4251 strand:+
ATGTTGGGTAAAAAATTGACTCAATTAATTGATTGGTCTAACCGAAAAGCTGTTATACGA
TTAGATACTGAAAAGTTTAAGACTTATGTTAAAGGTTCACCGAAAAACTATTCAATGATT
ATAATGCTCACAGCATTATCTCCACAACGGCAATGTCAAGTGTGCAAAGCTGCAAGCGAT
GAATTTTCCATAGTTGCAAATTCTTGGCGTTACACGCATTTAAGAAACTCTAAATTGTTT
TTCGCAATGGTTGATTTTGATGACGGTTTCGATGTATTTAGAATGTTAAATCAAAACACT
GCACCGGTTTTCATTCATTTTCCTGCTAAAGGCAACCCGAAAAAAGCTGACTTTATGGAC
GTGAGTCGCAGCGGATTCGCAGCAGAAGTGATAGCTAAATGGATCCAAGATCGATCGGAT
ATTCAGATTAGAGTGTTCCGCCCACCGAATTACACCGGAACAATGTTGCTGGCTCTGTTC
ATGTCCCTGGGCGCCACGGTTCTCTATTTCAAGCGGAACAGTTTGGATTTCTTGAAGAAC
AGGACATCCTGGGGCACAGTTTCTCTGACCCTGATATTCTCGATGATGTCCGGACAGATG
TGGAACCACATTCGAGGCCCACCAATGATGCACGCCAATCCGCAAACCGGACAACTCGTG
TTCATTTATCCAAGTGGAGATTTCCAATTTGTCGCTGAGACATTCATCGTGTTGCTGTTG
TGTGAGTTTTGTTTTGTTATTACTATTATTATTACTGTTTCTTGTTTGATTGACCAATCA
ATGAGCACCGCGGGGGCACAGAGGGGACAGAGGGGACAGAGGGACTCCTAG
>DjScaffold1714:23106-23489 strand:+
ATGTTCCAGGAAATGATGAATAAAATAATCAGAAAAATAAAAGGAGCGATGGTTTATCTA
GACGATATTCTCATATTTACCGAAACAAAGGAGCAACATTACGAAATAATCAATAAAGTA
TTAACAGAAATTAAGAAGGCTGATTTAAGAGTTAATCCGGAGAAATGCCAATTCATGCGA
AAGGAGATCAAATTTTTGGGACACTTTATCAGTAAAGAAGGTGTCAAAACAGATATAACT
AAAATAGAAGCAATTAAATACTTTGGCAAACCAAAATGCGGTAACAAAATGAGAAGCTTT
TTGGAAATTTGCAATTACTATCGCAAGTTTATTAAAGATTATTCGAAAAAAGTCAGTTTG
CTGGAACCACTGTGTGACAAGTAA
>DjScaffold3623:56573-60295 strand:+
aatttgaaaattgtttacttttagagaaaaatatggtacaaataatgtatttATGCTGTG
CTGTAAATTTATTACTGATAAGACAAATGAACCAACAATTAAGGTTCGTCTTTGTCTTCT
TGAATACATGAAATCCATTTTGCAAACGGCACAACCTAGCCTTCTAGATACAAATAATTC
GGATCTTAGTCCAACATTGGGTAGTATTCTTGTATGGACTGCAGAAACAAAATCAACTGA
TCTTCGTAAAATTTCTAGCAATATAGTGATTAAATTGTTTGACTTAAATTCGGCTCAGTT
CTCACGATTGTTAAAATTATTACCTACAACTTTTCAAGATCGAGCTACTGCGATATTAAA
TGCTTATATTTCATCTCAACAGTCGACAAGGAACTGTGAAACTGCTTCAGATGTTGGAGA
TTGCAGAGTCTCTGTGAAACCAACAAATAGTTCCATCAAATTTAATAGATCAAATTCTTT
TCATACGAAAACCACAAAAGTTTATAGTGCAAAGAAAACAACTCCTAATGTTTCATACTC
TTCTCACAACTCCAATAACTCATCGGTATCGTTTCATAAACGAACACCAAAACAGAAAAT
CGATATTGATAAAACAGATTTTATTAAAATTAATACAGAAGCCCTTCAAAATTTATCATT
AAATTATGATAATTTTGACAAAAGTTCTGTTTTATCAAATAGTTCCAATAAATCGGTCAC
AACTCCCGGAAGTCAATGGGGGTCGTATCAAAAAGGAGCGAGTCAAAATTCATTTTATCA
GTCAAAAAAATAGTCAAATTCCGACACCAAGACAAAACCTTAATAATTTTTCATCAGGAAA

CAACTACTTGTACGATAATACTAATATTCAGAAATTCTTGTCCTCCCAAAATTCCCACCC
GGAGCTTTCAATCGACATGGGAGGTTCAAATGTATCTTCAAGTCTTTATCCATCTACTAG
TAATGAA
>DjScaffold1237:15776-17286 strand:+
GTTATTTTGTCAATTTTTGTTTTTCTGCTTACTGTCGGTTTTGTTATTCCAGAAACAATA
GATAATGAGCTTATTCTGTATAGAGGTCTACCACAAATTAAGACCAGTTTAACTGAGAAA
GAAAAGAGTCAAATTATGAATGGAGTTCAAGGGGCAATCCAGTTTTTCAAATTTCTTGAA
GAAAAAAATTTCTTTTCAACATTGACGAAAATCTCGTCAGTAGCCGGACCGATTTTCAGC
GCGATATCAGCGATATTAACTGTGGTGTTTATGTTCATTGAAATAGAATCTCCTGAAATG
GCATTGATGAAAAAAGAATTCGCTGTCCTCAATAACAGAATCGATAAATGGAGTGTTGAA
TTTAAAATAATTAAACGAAAAATTGATTGGAGTGCTGTTCAGATCAATTTTGCTGAAATT
GAAAATAATATTATCGGTCTTCATAAGATTCTTTCAACTTTACCCGATGTACCGATTCAA
TCTCGTGAAACACAAGCGGCAATGTTCTTAAATAGTTATGAAATTAACTACAAAGCCGGA
GGAGATATTCTTTATAAAGCCATAATAAATGTCGAACAAATTTACTCTGTGAATTTGTTG
CGGGCAGCCCAACTTTACATGGAATATGATAGAAATCAAGTTCAAGAGTTCATGATTGGT
TTGTTGAAATTGATTACTGAAGCTATGCAACTTGAAGCAGCTTATGTTGGATTAAAATAC
AATAATGAAGAGGCTACTAAATTTACTCAATCACTTTGGGAAAATAAATTAACGGTTTTA
AAAGAGAAAATAGAAAAAGTCGATTTAGAAACGAAAGATAGATATTCTCAGCAATATCAA
ATTGATCTTGATAATTTATTGGCAACACATTATTCACAATCAAATGAGAAATTTTCCTCT
ATTGCTTATGATTTTTTTACAACAAAATACAACTGGAGAGAATGGTTTGTTGTAGTTTAT
AATCCAATATCGGGATTCAAAGATCACACAGTGGTCACATGTCAAGGAACACATAGATTT
AGAACTCATGGCAGAAATATAATTGTATCTAGTGTTGAACAAGTTCAGGGCGATAATGCC
ATATCACATATTGAAAGAAATCGACAAATAATTGGAAGTTACAACTCAAATTGGAAAGGT
GGTGATTATTCAAAAACCTGGGTTGAAAATATGGGAAATCAGCTACCATATTGCTCCGAT
GCTGACACAAGACTTTCAATTGCTTGGGGAAGTAATGTATGGTTTATGCAAACTAGACTC
GTTGTTTATCAAAAGAATTGTGTCAGGAATAAGTCTCCATGTTATAACACTTATCTTTTT
GCTTAAtaaagttataaatagaaaataaaattttcaaagtattt
>DjScaffold1237:55755-56060 strand:+
ATGCCTTTTGGAATTGCAGCGACATCGGGAATATTTCAGAAGATAATGACAAAAGTGGTA
GATGAAATTAAGAGAGCTATGGTAAATCTAGATGACATTCTGAAATTTACAAACAATAAA
GATCAACACTATAAAGTAATCGATATAGTATTGAAGGAAATAATGAAAGCTGATTTAAGA
ATTAATCCAGAAAAGTGTCAATTTCCTAAATTCGAAATCCAATTTCTTGGACACATAATC
AATGTTAGAACTGATCCATCTAAATTAGAAGCAATAAGAACATTTTACAAACTTAACTTT
TGTTAA
>DjScaffold876:52002-60374 strand:+
AAATTTAATCAAGCAATTATCGAAGGAAATTTACAAAAGGTAAAATTATTCTATCGAGAT
GAGTATTTAGAAAAAACAAATAATGATGGAATACGACCTCTTAGTTTGGCTTGTCAAAAT
GAAAATCTGGAAATTATGAAATTTTTATTAACAAAAAATTGTCAAACAGATTTTATCGAG
AATGGATCTTCTCCGTTCCAAATTCTATATAAAAAGGGCAATTTAAAAATTGTTGAATAT
TTATTGGAAAATAATTTGGGCGTTGAATCTTCTGATGATTTTGGTATGAGACCTATACAT
TGGGCATGTCTCAATGGTAATAAAGACATTATAAAACTTTTAATTGGACACGGATGCCAA
TTAGATTGCAAGACTTATACTGGTGACAGCCCTTACGACATTGCCATCAATAACTACCAT
GTAGACTGCGGCGATATGATAAGAATAGCAATTGAAATCTTACCAATAATCAACAGAAAA
GCACAAATAGAAATCGAGAAGGCAAATTTGAAATTAGACAAACTTAACAGAAGCAACAAA
AAAATTTGTACAATATGCTTAGATAATGAAATCGATTGTGTAATAGAATGCGGTCATTGT
TTTTGCATGGAGTGTGCAGGCAAATGTACTCAATGCCCTTAT
>DjScaffold2009:3907-19997 strand:+
ttttatccgtttgtatatcaatcagaagttatttatgatccaatctaatattactcttgt
tgaaatacctatattattgagaaacgtgactgcagtacgagtattaagttgtatatttt
aaatgaagatgaatcaaatgaatttgtatagaatcgttattattttacatttatatacaa
tctgattgttgtgatagaaacatgcatcacatnaaaagttgttgtaaatattcaatttaa
ttttattttaattagcttaagcctttataatttcaaaaaaaatcagattaatcctttgtt
aaaaaaatattttttaaatttaaatttatgtttttaacattgaaATGTTCAGAAATATT
GCGTTGCCGTTATTCTTGTTGTTCGCTCGAATGTCCTATTTCTTTGGTTCGATTCATTGG
ATCAAAGTAAAGGGTCGTCGATCAACGAGTAAAGAAGCTCCCATTCTTGTTTTAGCTCCT
CACTCTGCGTTTATGGATTCACTTTTCGTTGTTTTTCTAGGAATGAACTCAGTTATTGGC
AAAACCGAGACTGCCAATTCAACTGTTGGATATTTGTTAAAAATGACTCAGCCGATATTA

GTAACAAGAGAGGACCCCAATTCTAGGCAAACTACAATCGATGAGATTAAGCGACGATGT
TATTGTAAGGAAACTTGGCCTCAAATGTTAATCTTTCCTGAGGGTACTTGTACTAATAGA
TCTTGTCTTATCAATTTCAAAACAGGTGCTTTCATTCCCGGATGTCCTATTCAACCTGTC
GTATTGCGTTGGCCAAACAAAGTGGATTCAATCACATGGACTTGGGACGGTCCTGGAGTA
TGGACTCTATTATGGATGTTATTTGCTCAATTGCACACCTTTGTGGAAATTGAGTTTCTG
CCCATTTATCATCCCACTGAAGAAGAGAAACGAAATCCGAAATTATTTGCGGATAATGTG
AGGGCCGTTATGGCAAAAGCCCTTAATGTCCCAACGACCAATCATTCGTATGAGGATTGT
CGTCATATGACTCTCAGTAAGAAACATGGACTCCCGTTTACGGAAATAATTGTTGAATTC
TCACGACTTCAGAAACGACTATTATATCTTCGTCATGATTCGGTGTCGATGTTAGATTGG
TTGTTAGAAGCATTGCATCGCACAGGCGATGGTATGGTGTCGATTCAGGAATTGTGTCAC
CATATTTATATGACAGATAACGAGGCTCTCGTAGAACGAATTTTCAAATTTTATGACAAG
AACAATTCGGGATTTATGGACATCAAACATTTGTTTTGTCACCTTTCCTTGTATTCTCTG
CCTTTTAATCGAAAATTTGCGATAGAGTATATAATGAAAGCATATAAACTATCGGATCAA
ATAGATTATTCTATCATCAGTGAAATATTACAGAAATCATTTAGTTTGTCTTCGGAGGAA
GCCTATGAAATATTGTCATTCAGCAAATCTCACCAAACACCAAATAAGTCTTTCTTAAAT
ATTTTGGATGCCATTATGAACTCTGTGGCTGGCCCTTTCAATATGTATTCTGATTACCAT
ACTGAATATAATAAATCTATAAAATCTGGGACCACCACGTCGTGTGCCTCTGCCCAGCAG
CATTCAGTCAATAAAATCAGTAATAATACCACCGCTACAAAGACTGCCTCCTCCGTTACA
TATGATTCCTATTCCAAATTCCAGATTGATATT
>DjScaffold4515:20763-20993 strand:+
ATGCCTTTTGGTATTGTGACAGCACCAGGTACGTTCCAGGAAATGATGAATAAAGTGATG
GGAAAAATAAAAAGAGCGATCATTTATCTAGACGATATTCTCATATTTACCGAAACAAAG
AAGCAACACTATGAAATAGTTAATAAAGTATTGACAGAAATTAAGAATGCTGGCCCAAAA
GTTAATTCGGAGAAATGTCAATTCATGCAAAAAGAAATCCAATTTTTGTAA
>DjScaffold985:3736-13446 strand:+
ATTGATATAATAAATTATTTAGATGTTAAGACAAAACTCAGATTTTCAAGAGTTTGTAAA
AAATATAAAAATTTGGTGAACAATGCATTATCATGGAAATTTGTTTCTTTCGATAATGTA
AGAATTAAAATTTCAACAAATTCTAAAAAGCAACTAAAACTTATTATCGGTTCTCATACA
TTGTCGATTAAATTTAAATTCAAAACTCCGACCTCCTATAGAGACAGTTGGAAGAAAATA
AGTGACGAAATAATTTTTATTCTCAGCCTTTGTAGGAATTTGGTTGATTTATCCTTGTAC
CATGAAGTAACCGATATCGGATATTTGGGTGCCAACAATTTTCTCACAATCGAACTAAAA
GATTGGCCGAAATCTATTGAATTCTTTAAAATCGATTCAATTCCAAAAATTTCATGTTTA
CGATGCATGAGGCATAAAGATAAATTCTTATTAAATCTTAAAAAGCTAACGATTACAAAT
TGCAGTTGGTTTACAAATGACGATTTTCAATTCATCACTCCGAATCTGAATTTGAATCAA
TTGAAAATCATCAATTGTTTTCGAATATTTGAAATAGCAAATGATGATACAAATATTTTC
TCAAAATTTCCCAATCTATATTCGTTGAAAATACATTTGATTGCTGTTCCAGAAAATTTC
ACAGAATTTCTACTGAATTCTCCGGCAAAAAATATTGAAATAAAAATAGAGAAAAAACTC
GATTTCTCTGAAAGACTACTGAATgagttgatttttatttacaatatactgttgatatag
agtttgtaaaaaatataaaaatttggtgaacaatgcattatcatggaaatttgtttcttt
cgataatgtaagaattaaaatttcaacaaattctaaaaagcaactaaaacttattatcgg
ttctcatacattgtcgattaaatttaaattcaaaactccgacctcctatagagacagttg
gaagaaaataagtgacgaaataatttttattctcagcctttgtaggaatttggttgattt
atccttgtaccatgaagtaaccgatatcggatatttgggtgccaacaattttctcacaat
cgaactaaaagattggccgaaatctattgaattctttaaaatcgattcaattccaaaaat
ttcatgtttacgatgcATGAGGCATAAAGATAAATTCTTATTAAATCTTAAAAAGCTAAC
GATTACAAATTGCAGTTGGTTTACAAATGACGATTTTCAATTCATCACTCCGAATCTGAA
TTTGAATCAATTGAAAATCATCAATTGTTTTCGAATATTTGAAATAGCAAATGATGATAC
AAATATTTTCTCAAAATTTCCCAATCTATATTCGTTGAAAATACATTTGATTGCTGTTCC
AGAAAATTTCACAGAATTTCTACTGAATTCTCCGGCAAAAAATATTGAAATAAAAATAGA
GAAAAAACTCGATTTCTCTGAAAGACTACTGAAT
>DjScaffold1538:17252-18984 strand:+
atcATGCATGAATGGACTACTGTAGTAGAAGATCCTACAGTTGTAAACAACACTGAAACA
GTTAAAATTGATGTATCTAAACCCGGGGTGGAAAGAAATGCATGGAAAATTCATAAGGAG
TATTTATTGAAAGATGATTCCATGGCAGGAGAAATGATGCGAATTCTACGGGCTGGTAAT
GAAAATTTGTCTAAACCAAATCCTGAAGATGGTTACAAAGAAAGTATTGCTCGATTACAA
CAAGAACGATCTGATAGAATGCATCACGAGCAAATGATGTCCAGAAGAAGAATGAATAAT
AAAATAAAGTCGAAAGGTTTTCATCGTCATGCGAAAAGGAAAGATTTGAAAGAGTTTCTG

AAGAATGAAGAGAACAAGACTGATAATGACGAAAACTTGAAGAATAAATTATTAGAATTC
GAGAAAATACGGGCGCAAGAACGAATTTCATTGAAGCACAAAGTTGGAGGAAAATTTGCA
AAGATTCAAAAGTTGAGAGCGAAATACGATCCGAATGCAAGAGAGGCGCTTACTGGCATG
CGGGAGAAGGGCAAAGAGTTGACAGTGAAAATGAATGACGATGACGACAGTGATGAAAGT
AGAGAAGAAGAAGAGGAGGGAGAAAGCGAAATTTCTGACGAAAACGAGGAAGAAGATTCA
GAGGAAGAATCTCAAGACGAGGAAATGGTGGCCGCCGGAAGAAGTGGCGAAATGGAGAAC
TCGAAAAGTCACAAAGCGAAAATATTTGTCGAGAAAAATATGAAAAATGGAGAATCGGAG
GAGAATCCCAAGGTGAAAGAACAAAAAGTGAGCTCAACAATCAGCACAAACCAGTCGAAT
TTCCTCAATTTCCAAGATGGAATAGATCTTGACATGGAGGAGTCCGGAGAAGTTGTTGGC
AGTACGGACGAAGCGCGATTCAAGGACGCGATTTACGAGATTTATCAGCACGATTCTGAA
TTGGCTGCGGAATTCGAGAAGCAGAAATCGGAATTAGTGGAGATGGAGAATCCAAAAGAT
TTGCAAATGTTCCATCCAGGCTGGGGCACGTGGACAGGAGAAGGATTCGAGGCTGCAGAT
GAGCGCAGAAAGAAGCGAGGATTGATCAAGGCGCCAGATGTGAATCGTAAAGACAAGAAG
AAGACTCGTGTTTGGATTAAGCAAGGAGTCCATGACGGGCTGAAGAAGCACTTGATCAAA
TCGATACCGTTTCCGTACAACACAGCTGAGCAGTTCAATTTGTCACGCAAGGAACTCATC
AGCAGCACATTCAACTTCCAAACTGTCCACCGGGAACTCGTTAAGCCGAAATTCGTCAAC
AAAGCAGGACAACAT
>DjScaffold1538:32773-35701 strand:+
ctgagatttgtctcattagtattggtagaattttttttaATGTCTATTCTAGGTATTGCT
CTATTTTTTTCGTGTGTTTTGTTTAGTAGTGCCCTTAAATCATCATATTTAACTGCATTT
GACAAAGATTTGTTGAGATCGGTTTTTAATACAGGATATTCCAAAGGTTTCTTAGATGTG
CAATCAGTAGCTCTGTCAGCAAAGGGTTATTCGTTACTAGAAAATACGTTTGATCGTGAA
AAGGAAGCATGCAATTTATTTAAAATTACTTATCTAAATTCTGAAAATATTAATGAATTG
TATGCATCTGCTTATGGCGCTTCAGTTTTAAAATCAGAAAATTGTAATTTGGATGTAACC
AAACATAAAAAAGCACTTGTAGAAATAGAAAATATTAGTAATGTGGAAAATTTATTCAAA
GTAGTTAAAACACTAAATGTTTTAAATTTGAAAATCGACGTAGAATCTGTAAAGAAGTCT
TTAGAAAAATTTTTAAAAAAAGATTCCTCAGCATTAAGTCAAGCTTATAGTTATTATATT
GCGGGAATTATTTTTAAAGATAAAATGGATTTAAAAAAGTATTTTGTGGAAATAGAAGAT
ACAATTCAGCAAGCTGATGAATCTGATAATATTAACATGTATTTTGAGGAAGGTATTTAT
ACAACTGCTTTTATTATCAATAGTATTTTTGAATTATGTGATAAATATGAAGAAGCCCCA
CCCATTCCTATGGATAAAATAAATAAATTTTCAAATTTCTTTTTATCACGTCTTCGCACA
AATAATTTGAAAGCAGCAGTAGAATTGACTGGAGCTATTAAATTTTTATCAAAAAATAAA
TTCAAAGTTCCTTTAGTAATTTCTCTAGTTGAAAGTTCAATTAATAAAAATCAACCGAAA
GTGAAAATCAGTGTTAATAATTTGTGGGGTGACAAAATTAATGATGTAAAATTGATTGCA
GAAACTGTGAAGAACGTTCAATTAGCAAACACAATTTTATCAAGAAAATCACTTGTATAT
GATAGTACAACTGGAATTTACGAATTGGATTTGATTTCAGTTGAAGAACAATTACATAAA
GGATTTTATTCAATAAAATTGGATATAAAAACCAATGAAACTAGATATAATGAAGTTGTC
GGTGGGACAGTAGTTGTTAAATTGATATCTCAAATTTCTATTGATAATGTTGAATTAACA
ATCAAAGAACGCGATCAGATCACTGGAATTCTTAATTATAAATTACAGATGGGATCATTG
CTCAGTGACAAATTGAGTATGAATTGGCATCATCGACTCGATATGAAATTTGTTATTCGA
GACAATCAGCAATCATTTAAACCTCACCAAGTTTTTGTCAAATTAACTCATTATAACACG
AAACAAGAAATAATATTTGTTGTGGAAGCCGATCGCAGTGATTTTTATAAATTTAATTTG
GATTTGGATAAATCTAATAAAGATTTTGATAATTTATCTGGTGATTACAAAATGGAATTA
GTTGTTGGAGATTCAATGATTGAAACACCCGGCAGTTGGATTTTTGCAAACATCAAGCTT
GATATGCCGAGTAGTCCGTCAACTGAAAATACAGGAGAAAGTAGACGCACAGTTGCCGGA
TCAAAACGGTCAGTTGTAAATCCAAGAATTGGTTCTGGTCCGTCCTTGGTGAAACCACAA
ATATTTCACATGTTTAAACCAGCTGAGAAACGTGGAGGTCAATTTATATCAACAGTGTTC
ACAATTCTGTGCATTCTGCCACCTTTTATTCTGATGCTGGTGCTGTGGCTGGTGGTTGGT
GTTAATGTGTCGAATTTCAGTTTTGGGTTGAGTCCAATCATGTTTCATGTTGGCATAATT
GCAATTTTCTGTCTCTACTACTGTTATTGGGGATATTTGAACATGTTCACTACATTGCAA
TGCTTATTGCCTCTGGGAATGTTCACCGCTTTCTTTGGCAATCGAGTTTTGAGGCAAATC
GCATCGAAAAGGAAGGAGCTGTGAgtgattgatgtgagcaacgagcgagcaaggaaaatt
gatgattatatgtagttgctttttaattgatgttggaaataaactaataattttcttgt
aatttttgttaaatattttctatttttattatgggtttcgtttcttgtttactgggagaa
ttgtaattgttcgattagtagaaagatttaacatcg
>DjScaffold1538:53180-53772 strand:+
ATGGTTGTTGAAATTCCTCGCTGGACAAATGCGAAATTTGAAATTTCTAAAGGATTAAAA

TTAAATCCAATTAAACAAGATATCAAGAAAGGAAAACTTAGATATGTTGATAATGTGTAT
CCTTATAAAGGTTATATTTGGAATTATGGAGCAATTCCCCAAACATGGGAAGATCCCAAT
CATATTGATCCTGATACAAATGCAAAAGGTGACAATGATCCTTTAGATATATGTGAAATC
GGACAGAAGATTTATCCGAGAGGATCTGTGATACCTGTCAAACCATTAGGTATTTTGGCT
ATGATTGATGAAGGTGAAACAGATTGGAAAGTAATTGCTATTGCTAAGGGTGATCCATTA
GAATCTAAATTAAATGACATATCTGATGTTGAAAAACTAATGCCTGGTTTATTAAATGAC
ACAAGACGGTGGTTTAAGCTTTACAAAATCCCTCAAGATAAACCTGCTAACAAATTTGCT
TTTAATGGTGAATTTAAAAATAAG
>DjScaffold4672:37325-41148 strand:+
attgttaatttatattcaatttttacaagacctatcgacagttttgtcgttaaaatatgc
atctaattgactagaaattatgaaatttaatcaattaattttttttatcattattcttag
ataaattagtagattaaaacttttaaatttaactatttagatacaacaacaagttctatt
tttaagcatacaattgacaatgttattccattgtattgttcaatacttggtctagttatc
atttgtctaattggttatgtcatttataaactatggaaacagaaaaacgattctttactt
tgtacaggaattccttcaaaaggcagcagcggaaatagtttgagaataccaaaatattca
tctgcaaaatcagataataaagaaATGGCAGCATTAGTAACTTCTAATGAAGATGTTAAA
TTACCGAGTTTTGAACAATTTTTATTGAGTCAACCATTATATTGCCTTCCGGAAGATCAA
ATCGAAGCTATTGTATTCAGCCTTTCTGATGAAAGCTCTGGAGTTGGATGGTTACAATTA
GCTCTATATTACAATTTCAAACAAGACGAAATAGAAGCGATTGTGCAAAACAATACGAAA
ATTGAGGCCGTGCGAAAATTTCTAAAAAAATGGTCAGAAAATCCCAATCATAATTTAAAG
AAACTTATTAAAAGTTTACACGAAATTTCTCGACCGGATACAGCTTTTCTGATAAAATCT
GTTATCAGAAAGTTCAGCAATAAAGAACGTAATGAAACTTTTAATAAAGAAACAAACAAT
AATCTTCTACTCGGTGAGAAACAAAGTAACGGCACAAAAACTATTTCAACTTGAaatgaa
agagaaagtaacaattcagcaaaagaaacatatatttaattttccttattgatctcacgt
tggaaatgttttgtaaattaaaacaaaccattattttatattaaaatcgttttacaaaca
tttaaacaactgaaagccattatccat
>DjScaffold4924:16992-17516 strand:+
ttaattaaatctgcaatagtttgataattcaacaaatatactttattttaattaattttt
ataaataaaaaattttctaagtgttatctttaataaATGTCCTTAAAATTATATTATGATT
TTATGTCTCAACCTGCAAGAGCTTGTTATATAATGCTCAAATGCCTGGATATACCATTTG
AGTCTAAATTGATTGATTTAAAAAAAGGTCAACATTTAACTGATGAATATCAAAAAATTA
ATTACTTTCGAAAAGTTCCATCTATTGATAATAATGGATTTAAATTATCCGAAAGCATTG
CAATAATGAAATACATCATTGCAACATATAAACCGGATAGTTCATGGCTTCCTAAAGATA
AATGTGTTCAAGCAAAAGTCGAAGAATTTCTCCATTGGTATCATCTAAATCTGAGATTGA
ATTTCGGAATGTATTTTCGAACTATG
>DjScaffold3676:14340-15374 strand:+
caagagagagaaaataaagaagaaaaagatttgATGAGAAATGAAATAATCGATGAAATG
AGAAAGAAACAAGCTGAGATGAGGACAATGTCGAATAGAATTGATGAAACAATGAGAAAT
GTTGAGAATAAATTATTGAAAATAGAAATAATCAATCAAAATAAAATGAATTATGGAGAA
TTTGTGTGGAGAATCGATAAAATCAATGATAAAATGAAAGATGCAAAAAATGGAATCAAC
AGAGAAATTTACAGTGATTCATTTTATACACACAAACACGGATATAAAATGTGTTTGAGT
GTGTATCCTGATGGAGTTGTTTTTGGCAAGAGCACTCATTTATCTGTTGGATTTTGCTTG
ATGAAAGGAGAATTTGATGATATTTTGCAATGGCCATTCAAATATGATGTGACAATCTCA
TTGGTCAATCAACAAACTAGACTCGATCATGTCAGTGAGACAGTGAAATATGATAATTTG
CTAAGTTTATTTTACAAAAAACCAATTAATGAGAGAAATGATTTAATTGGAATTCCTAAA
TTGATAGAATTGCAAGAATTATCAACCAATGCAAATCTGACTCGCAACAATCAAATATTT
ATCAAATGTTCTGTAAATATTTCATGAtaatatcacaaagactgaattctatggttcttt
ttcatatattttatcatgataaaatcacaaagactcctctctttgaatttttttcatata
tttaatcatgatattatcacaaagactcccttctttggttatttattaaatatctatcat
gataatatcacaaagaataccctctatgaacattttaaatatattttatcgtgaaaatat
cacatagactcccctctgtgaactttttgacatattttatcatgataatatcacaaaga
cttaattctatggttctttttcacatattttatcatgaaaatatcacaaagactgccctc
tttaaactttttca
>DjScaffold3900:32801-34070 strand:+
aattaacacataaatatattcgaatatcaagtattcgaatattcagtttggaatctctag
caagcaaattatttcataaaagcaagtttatacttgttttcactcttttataaaacttaa
attaatttgaaaatataaaagcatctatttatgaagttttataaagtacaaaaatgttat

tgtgaaagataatgcaatattacattattgtaaaataaaattaccttttaaataatacat
catcaatattattacattttgtttaaaatttcgtatacaaattacctaatttaagatatt
tgaacaaATGAGTCAACGGAGGTTGGAGTGTTATAATTGTGGTCAAAGCGGTCATTTTTC
AAGAGAGTGCACTGAGAATAAGCGTTATGGATCTGGAGCGAGGTCTGGAGGATGTTATAA
TTGTGGGGAAAATGGACATATTTCGAGGGATTGTCCGCAAAATGATAGAAGAAGTGATCG
TAGAGGAGACGATCGTAGAGGAGATCGTAGAGATGATCGTAGAGATGATCGTCGAAGTGA
TGACCGTAGAGATGGCAGAAGAGGAGGATCACAGCGAAGCAATAATTGTTACAATTGTGG
CCAATCTGGTCATATTTCAAGTTTTTGTACACAGGGCTCGAGAAATAATGGTCCACCAAG
TCGTGGTTGTTATAAATGTGGTGACAGTGGGCATATGGCTAGAGATTGTCAGGATGGCAA
TGATAAACAAGTCAAGTGCTACAATTGCAACCAAAGTGGCCACATGTCAAGAGAATGTCC
AAATGGACCATCGGAAAAAACGTGTTACAAGTGCCATAATACCGGACATATAGCAAGAGA
TTGCAATGATGATTAAttctgatccaatcatgttttaattgttgaataaatggaatgtta
tttatttttcattaatatcgagttatgattaattggatgataatttgactaaatgattat
cggattacataatattgtcaattcgaagtaaaattttgtttataatgattatatataagc
aggattagtattgaattgaacgtcaaagtgttgacaaaattttttagtaatggattcaa
tcgagctatttcaacggtagtattagtgattaatttaaaatttccaataacaacgatctg
tggctatatcaaagcagattgaacttcaatcttcaagtaataaccgagg
>DjScaffold3900:37249-45921 strand:+
ctacatgtttcggattcaagaggcaatgtaattctttcttcagtcttaggttctttcgta
aaattaaaggatcgatcttagctgaaatgcaaagaaattagcacagttggtgactttagc
gactacgcaattttcaggagcagctaacactaatatagacttctggtttttacacagaat
aaaatATGAAAAATTCTTATTTTGATGATGAAGAAATGGACAGTTCTGACGATTCTATTG
AAAATGAGGAAGAGCTTATAAAAGAAGAAGGTCGTGGTTTTATTGTGGATGAGGATGAAG
ATGACGAAGATAATGATATTCCAAAAAATTCATCAAAAAAGAGATCACATGAAGATGATG
ATGATAATGACGATGACGATGATTATGGAGAAGAAGAAGACGAAGAAGATTTACTTGATG
AAGTTGAAGATTTAATAGGGAATAGTAGGAAAAGAAGATTGGTGAGAGATAGTGATGACG
ATATTGACGATGACTTCAATTATTCCAAAGAAAAACGTTCAAAATGTTTGTCTAAAGGAA
TTCCTAAAGCTGCTCCAAAAGTCTCACATAAAGAGTTTAACAATTTACCCAAAAAAATAA
AATCCAGACCCCAGCATCCATTGAAATCAAAACAAATATCTAAACCTAATCTTTCAAGTC
CTGATTTAGATTCCGATGAAGATATGGAAAATAACGATGAATTAGAAAATGAAGAGTTCG
AAAATGAAGATGAAGACGAATTTATTCAAAAAATGCCATCTGAAAACATTTCCAATTTGA
AATCTAAACCCCATCATTTAACGAAATCAGGTAAAATCTCTAAACCAAATGTTCCTAGTC
CTGACTTAGGATCCGAAGATGAAATAGAAAATGAGGAGGTTTTTGAAAATGAAGAGCAAG
AAAATGTGAATGAAAATGAAGAAAACGAGTATGATGAAGATGACTTAGATGATTTCATCG
TGGATAGTCGTAAAAAGAAAAAAACGTTGTGATGTATTGGAAGATAATGATAATTTATTAA
ATACTCGCCAAGAAAGAGAAGCTATGGATCTTTTTGGGTGTGATACTGCTGATTTAGAAA
ATTTAGATGCGGGCAGTGATTATAGTGATGTTGAGAATGAAGATATCGACCACACTGAAG
TTTTCAATTTAGAAGATTTGGAACGTGGGTTTCATACAGAACAAGATGAAATAATTCGTA
ATACAGATTTGCCAGAACGGTTTCAGTTGAAAAGTTTAGTTGTTGAGGACATAAAAGAAA
GTGACGAGTCTCTGCAAAATGAAGCTGAATTAGTTTACGAAGAACTATTCAACGAACGAC
CTTGGAATAAAAATACAACTTGTCATTTACTTGTAAAAATCTTACATATGATCAGATCAA
AATGTTACGAAATACCGTATATTTATTTATACGAACGTGACGAAATCACAGAAAAGGAAA
TTCAATTTTCCAATGACGATTTGTGGAATATCTTTGATTATCATTTGAAATGGAAGGTTG
TTTCAACAAGAAAAAGTAAATTGTTACAAATCGTTGACAGTTTAATTGAACATATTCCGG
AAAATGATAAAACCAAGTCTGACCATATCTGTGCTATTAGAGAATCAATAGAAAATATTA
AAAACGTCAATGAGGCTCGTTTAATTGACGAATTACTGAGTTTTACCTTTACCAATGAAT
ACAAATCTATCGTACCAGATTCAGCTAACATGAGACTATTAAACAAATTTCGATTGGAAA
ACGCTGCAAATCTTGATAAAATGATACGTCTTTTTGGAATGGAATCGTATCGATACTCTT
TGAATGTAAAAGAAAACTATATGCTGCATAAAATTCAAGACAATGACATTTCACCAGAAG
AAACTGCAAAATCGTTTATTTGCTCAAAATTTCCAACTACAGAAAAAGTTTTACAAAGGG
CTAGAAATATGTTTGCTTATCAGATCTCTAAAGAGATCTCAATTTATAATTATGTGAATG
ATTTCTTTAAGAAGAACGCTAAAATT
>DjScaffold3900:46177-49034 strand:+
AATCAGAGGAAATTGATCATTGAAGAAGCAATCGAAAAAAATTTGATACCAGAAGCTGTA
AAAAAAACTCGTCAATATCTTTCGGAGACAGCACAACAGAAACTAGCGAGAGAAATAGGC
GGTGTATTATATATAAAAATATAAACATCAGACCGTATTTAAATAGCACAGGGAATGATCAA
ACGATCATCCTTGGTATTAGTTATCAAAATGAAAATTCAGCTGCTGTTGTTATAAACGAA

ATTGGTGCAGTTATTCGATCAGTAAATCTGAAAAATCTTACGAATACCTCTAGATCTCGT
AATAACAAAGATTTGAAAAGATCTGATCTTGATAACATAATAAATCTAATTGAAACTCAT
CGGATATGTGCTATAGCAATCACTGGAAATTGTAAATTGTCTTTGGATGTGAAGAATGAC
CTTAATAATGCAATCAAGCATGCAGATAAAGAATTAGATATTCCTCATGTGGAAATAGAA
ATCTTGAATAGCGACTTCTCAAAAGTTTATGAAGCATCAACTGTCTCAAAATCTCTTTAT
CCACAACTGAACGCACTTCAAAGACAAGCTGTTGGAGTGGCTCTCAATTTTTCAAATCCT
TTAGTTGCGTTTTCCCATTTGATGAATTACGATAAAGACATTAAGAATATCAAATTTCAT
TTGCACATCTCTCTAGTCAATGAAGATATTTTGTTGACTCAGTATGAAAGACAATTCATT
GAAGTCGTTTCAAAGTCCGGTGTTGATATAAACCATTGTCTAAAACACAAAAACTTTCAA
TTTACACTTCAATTTGTCGCAGGTTTAGGTCCAAGAAAATCTAAAGAATTAATTGAGAGA
ATTAATAAAAAATGTCGGAAATATTACGAAAACCATATAAGAACGCATGTAGAAGATGCA
GAGTTGTTATTAACCAATCGAATCGAATTAATTTCTAAAAATTATCTCGATTGTATAGTA
GCTAAAAATTGCGCTCCATTTCTCAAAATTGTTAATGCACCCGATTATGATGGTGGTAAC
ATTCTTGATGAAACTCGAATCAATCCTATGGATTATGATTTCGCTATCAAAATTGCACAG
AATATTTTAGACATGGAAGAAAATCCAGAAAACGCTCAATTAGCTCTTAAAACTGTGATG
AATGACACCGATTTGTCTGATCTTGACTTTAAATCATTTGCAGATTCGATATACGAAAGA
AATAATATTAAAAAACATTTGATAATTCGGCAAATAAACGATGAATTAATGAATCCATTT
TGTGACAATCGACCGACATTTATTCCTTTAGCTGATTCAGATCGATTCTATTTGTTGACA
AATGAACACGAAGAAACATTGAAAATTGGAACAATCACGGAATTTAGAGTGCTCAATTTA
GTTTATAGAAGAAACCAAAAGGATTTTGATAATGCCAAACCGAAAAGAAATGAGAAAACC
GGTTATTTTTCTTGTCCACTTTGTAATAAGTCCGATTTTGTTGAACTGAATTCAATTTGG
GTACATTTTGATAATTCTTGTCTCGGGAAAATTGTCGGAGTAAATCTGATGGGTGATAAT
GAAATTTCAGCATTTGTATCGATAAGAAATATTGCCGATAGATTTGTGAAAAATATTTTC
GATTTCATAAAAATTGATCAATTGGTCATAGGTGTAGTTACGAAAATTGATTTTTTGAAA
ATGCAACTCGAAGTTTCATTGAAGGAATCAGAAATTACAAAAGCGAAAAATTCAATGGAA
AATATTCGAGATTATTATTATGATGAATTTGCAGAAAATGAGGAGAAACGGAAAGAAAAA
CGAGATTTTACAAAAACCAAAAATGCTACTACTAATTCACGAGTATCGTTCCATCAAAAT
TTTCAAAATATATCCTATCAAGAATTCCATAATAAATCCAATTCTTTACAAAATGGTGAT
TTCATCATAAGACCTAGTAGTAAAGGTAACAATCGGCTTACGATTAGTTTGAAAATTTCA
ACTGATTTATTTCGACATATCGATGTTTTGGAAGAAAACAAAGAAACCGATTATACTATT
GGTAAAGTGTTAATTGTTGAATCTGAAAAATATGATGACCTTGATGATATAATCGCCAAC
TATCTCCAACCAATGATTAATTACACTGATGATGTATTTAATTACAAATATTTCAAAAAT
ATAAAGGCAGAGGATAAACATTTATTGGAAGCGATGATAATCAAAGAAAAACAAAACAAT
CCCAATAGGATTCCATATTATATTGTACCTATGTACAAATATCCTGGTGGATTTATGTTT
GTACTTTTACCAAGCAATGAAGTGCTTTATGATTCATTTTTGGTGACTCATCAAGGTTAC
CGATTCAGAAATTCCGTTTTTCAAAATATCAATCGATTAGTGGACAATTTTAAATCAAAC
TGCCAACCTAGCACTAGA
>DjScaffold3900:67658-68659 strand:+
ttagattttcggtgaaataattttttcttagcctaattttcgacaATGTCTTTCACACCA
GCTTCAAATAATGTTGTTTTAAAAACTAGTGATGCGACGAATAATAGTTTGGACGAACTT
TTCGAAGTTGTCATTAATAAATCTTCTTGTCGACAAAAACCATTAACCGAGCGTCAATTA
CCAAGGTCGTTTTTTGTTCCACCAAGTGGAAATAGTAATTCTCCGTCGGTTTCTCATTTT
AAAGCTTTTTCGTCACCAGCTACACTTGAAGAAACATATAGAGCTGGACCTGGCCAACAG
AATAATTATCATTACAAACAGCGTAGCTTAGATAGTCAAAATGGAAATCAACACTTTTCT
GATTTGAATGGTGTTCCTAATATTGATTTGAAAGCCCATTCTCCTTCAATGGAATCAAAA
CCACATTTAACTATCGAAATAAAAGAGCTACCGTCAGGTTATGAAATGGCGATTAATGAA
AATTATCAAGTGTATTTTCTCAATCACAACACCAAAAAAAACGACATGGTTAGCATTTGAA
ATTAAATCAAATTTGAAATCTTATTGTTTAAACTTTAATTTATTATTGTAAataataatt
aataataacaataacaaataatgacaataatcattattaatattattgttttaatattgg
tattactatttttatcaactcaaattaaatcaactaaattagaattttattaaatctgaa
tttaattaaaaatgattttaattaaattttttattgctcttattattattattatctatt
tttgaatgataactttatttatgatgacgatgcaaataataaattttatattaacactgt
gtgcatttctacaacatgcatttctctataaaaattagtcagtattccatcacattaaat
tttgtg
>DjScaffold3900:112401-113331 strand:+
taatagtcattccaatgtactgctgcaggatttttatttttgtaaatgtgatagttcta
cattaaagtgtagaataaaatgcattgtcttcattatagttattaatgttttcctatttt

gtcttggtcttggtcttggtaagaaatttccattttatttttacttcctcgtggtgataa
tcgtgaattctgcaatattggccatcgaatattactacattgtttttatattgtaataat
taattagaattgtaaagatgtaATGAGAAAATGTCTAAAGACTTGTCCTCCACCTTGTCT
AGCAANNNNNNNNNNNNNNNNNNNNNTAAACGAATCAATCATAAAGAAAAATCCTGCTCAAT
ATTACAATCAAAATTATTTGCGAGAAAATTGAACATTGTATTTATCTGTACAAATTTAAT
TTTTGCAGCACTATTCTCTATATTTGCAATATTATCATTTATACAAGCCCCAACAGAATG
GAACAAACTTGCCAGTCTTTCTCGGGAAATAAATCAAGATTGTATTTGGTGGAACTTTTT
TGATATTCATGATAAATGGCATGTCCTCGCATCGATCGCAATCTTTCTCATTTATATGTT
CATTTTTCTAATGGACAATTGGATATACGAAAAGAAAGAGGAAAATCAAACAAAAATAGA
TAGTTTAGTAAATGAGGAAAAGAAAGAGGAAAATCAAACGGAAATGGATAGTTTAGTAAA
TGAGGAAAAGAAA
>DjScaffold3900:125423-127333 strand:+
gaatcatactacataattctagaaaacttgtttttcaaaattataacgtgATGTTTAGAC
TTGCAAATTCTTTGAGATTATTAAAACTTTCTAAAGTTAATACTCTGCGAACATGTACTT
ATGCAAAAGATGTTAAATTTGGAGCTGATGCTAGATCAGCCATGCTTGTTGGGGTTGACA
TTTTAGCTGATGCAGTTGCGGTTACGATGGGTCCTAAAGGTCGAAATGTTATTTTGGAGA
AATCATGGGGATCCCCTAAAATTACTAAAGATGGCGTCACGGTCGCAAAAGACATAGAAT
TGAAAGATAAATTTCAGAATATAGGGGCTAAACTAGTTCAAGATGTTGCAAATAATACAA
ATGAAGAAGCTGGTGATGGTACAACAGCAGCAACAGTTTTAGCTAGAGCTATTGCTAAAG
AAGGTTTTGAAAAAATAAGTAAAGGTGCAAATCCAATTGATTTTCGACGAGGTGTAATGA
TGGCAGTTGATAATATTGTCGCATCGTTGAAAGCTATGTCAAAGCCTGTCACGACACCAG
AAGAAATTGCTCAGGTAGCAACTATTTCCGCTAACGGAGATAGAAGTATTGGAGATTTGA
TTTCTGAAGCAATGAAAAAAGTTGGTAAAGATGGAACAATTACAGTTAAAGATGGAAAAA
CATTACATGATGAATTGGAAATAATCGAAGGAATGAAATTTGATCGCGGTTATATTTCAC
CATACTTTATAAATACTGACAAAGGAGCTAAATGCGAATTTCAAGACGCATTTATTCTGT
TCTCAGAAAAGAAAATTTCCAGCATTCAATCTTTAATTCCGGTTTTAGAGATGACACATC
AACAGAAGAAACCATTACTAATTGTTGCCGAGGATGTTGATGGAGAAGCATTAACAGCTT
TAGTGCTCAACAGACTGAAATTAGGATTGCAAGTTTGTGCTGTTAAGGCTCCTGGGTTTG
GAGATAATCGAAAAAATACTCTTAGAGATATGGCCACTTCCACCGGCGGTATTGTATTTG
GTGATGAAGCTGACACATACAAAATTGAGAACGTTCAACTACACGATTTGGGCCGTGCTC
AAGAAGTTTTAATTACAAAAGACGATACTCTAGTTATGAAAGGCAAAGGACTTCAAAAAG
ATATTCAAAAGCGAATTGATGAGATCAGAGATGAAATAGCTTTGTCAACAAGCGAATATG
AGAAAGAAAAAATGCAAGAAAGATTAGCAAAGTTATCAAATGGTGTTGCTGTTATCAAAG
TTGGTGGAAGTAGTGAAGTAGAAGTAAATGAAAAGAAAGATAGATATACTGACGCGCTTA
ATGCAACTAGAGCAGCTGTAGAAGAAGGTATTGTTCCAGGTGGTGGAACAGCTCTTATCA
GATGCATTCCAGGTCTAGAAAATTTGAAACCGAAAACAGAAGATCAGAAAATTGGAATAG
AGATTGTTAGACGAGCATTAACCACACCATGTTACACCATTGCCCATAATGCCGGTGTAA
ATGCATCAGTTGTTGTTGAAAAAGTGCTTAGTATGGAGAGTGCAATGGGTTACGACGCAA
TGAATGATGAATATGTAAATATGATCGAAAAGGGCATTATTGACCCTACAAAAGTAGTAC
GAACTGCTTTAGTTGACGCATCTGGAGTTGCCTCTCTTCTAACCACAGCAGAAACAGTTG
TAACAGAAATCATAAAAGATGACAAAGGAAGTGCTATGGGTGGAATGGGAGGCATGGGCG
GAATGGGAGGAATGATGTAAagaaatatgattgttaaattgcattttagttttataataa
atatttgcatttatca
>DjScaffold4451:82868-87636 strand:+
aaATGACTCATAGTTTTGTTGATTGGAACCATTTGGTTAGGTCAACTAATAATATCAGTA
CCATGCATAATTTTGATACTTCTCCAATTGTTTCATTAAAATTGGATATTATGGCTTATG
TGAAAAGATTAACACAAAAATATGAGTCTGATCGAGAGAAATTTTCAACTTTACAATGCA
TAATAAAGAGTGAACATAATTTTTCACAGTTAAATGTAGAATCAGCGACATATGTGATCA
GATCATTAAATAGATGCTTGATGTTTATAGAATATTTTCTTCAATATTTGTATCATTGGA
TATCAATTGACAGTTCTAACGATCAGATTGATGAAAATCTTCTGAAAACATTACTCACTC
GTGCATATGATGCCACCTTAGAAAACAAGCTAAATTGGCTTGATCAAGGTTTATTTACGA
TTATTATAAAGTCTTTGCCAAAGATTTCTGAGTTTTTCTGTCATTTATTATGTTTACATG
ATGAAATTGATAAACCGGTTAATATGATAATTAGCAACTTCAAACCAGAAATTGAACAGT
TGCTAGTTGATATGAATTCTTACCGTAATGGTTTAGTATCTATCAATTCATGTTTGACTC
AGTTTTTTTCTGATATGAAAATCGAATTGTGAtttctcttttggacacgatttttgttg
tctgtgatgtgttgatgagaataaattttttatattgaattaaaa
>DjScaffold4451:89609-93646 strand:+

tagaaattcaaaataacgttgtttgagtaatacgcattaacgttataatttatatttaat
aaatattctaagcacttaattattcttcaagtatgatagaggtcaaggatattgtaactg
ggaatcacgccagaattatgaatgcggtgacaagtcaacggaaatcaacggaaagtaatc
tttgatgcgtaaccaaagaatgcgcaaaaattaataaattttaacatcagaaaatatgat
ggaaaaggtgcagaagaatcttcaaaatggtttaagaatatcgaggaATGAATTTAAATT
TGTTAAATACCTTTGATTTACTTATGTCAAATGAACCAGGAATTCTTTGGAAAGATTTTA
AAAAGATTGAAATTACAACGGAGGAAGACAAAAGATCGTTTACAGACACATTTATGATTA
ATAAATTGTTTTCAGAGCTGATTTTACAATTACCAATGCAAAAAAAAGAGACTGATGAAA
GATTCGAAACATTCGAAATCAGAGTAAGAAATCTAATAGACAATGTCATGAATTCAGGAC
TAAGTAAAGATGAAATAATGATTGACGTACTATTAAAGAAGGTAAAAAATACAAAGTTGA
GAGAAACATCGACGATACATTCGGATACCAAGGTCGCAAAAACGAGATCCTTAGCAAAGG
TT
>DjScaffold4451:95762-96401 strand:+
tttgtcttcacagcaatccaatcttacttcaaccaatattgccaatttatctaatATGAC
ATATAATCAATTAGAAGAAATGATAAACAAATGGATCCGGGAATTAGATGATATGGAACG
TTGTTTTATTGATGAAGCTTACAAAATGAATGAGCGTGACCGTCAATTGAATATTAACGG
CGAAAAAATTGCTAATCTTCATGATAAAATAGAACAATGCCAACAAGATCAAAATCTATT
AGAACAAGAGTTACAACTTTTTGAAGATCAACAAGGTGAATTAGAAGCTGCTACAGATAC
TATTGAAAAGGAATTACTACAAAGTTCTATAATGAGTGTACGCTATCAATATTCAGATAG
AGAAAGAGAAAATATTTATCAGTCAGCTGAGAATTGTCAAAACGAATTGGATAATATTTT
GGGTGACGTCAAACAAATGGTTGATCGAATCAATTGTCAAAATAAAACATATGAAGGCGA
AAATAAAAATGAAATTTGTAATAATAATGATGTAATGACGCAAGTTACTCGTATTCTAAA
TGCGCATATGAAATCATTAAATTGGATTCACCAAAAAACGAGTAAGTTTCATTTAAAA
>DjScaffold4566:17862-21134 strand:+
aATGCCGCCTTTGGAATCCGGTGTAATCAGTAGATCTGCTATCAATTCGAATAGGAACTA
CTTTGGAGGACTTCCAATTGTTGGAAATATTAAAACTTGTTCTCACATTGAAAAATCGAT
TAAAGCAGAAGGATACGGAGGATCGATAGTTGTATTGTCGGAAGTCCATTTGAATCTGGA
AAAAGTTATCAGCAAATTAAAGATTTTATTTCAAGGATATTTGTCTTTGAAACCAACAGC
TTTTATTTTAAATGGAAACTTTCTTTCTCCAGATACGTTGTTACTTTCTAAACAAGAAAA
AATAAAATTACTGAAACTCGGCCTAAAAAAATGTCATAAAATGTTTCTCAATGTTTCCAGA
ATTGGCATTGAATAGTCATTTGGTATTCGTTCCAGGACCAAATGATCTATTTCAAGGACC
AACAAATGTTTTACCAAGACCATCTTTGTCAGCTGATTTACTCTTGACAGAGGCTTTCGA
TAATCTTCCTAATCTAACAAAATCACAGTGCGATGAAATTAAACAAAGAATTATTCTTGC
GAGCAATCCTTGTCGTATTACTTTTTATAATAAGGAAATTGTTGTGTTTCGCGATCGAAT
TCTGCAAAAGTTTCATAGAAATTGTGTGTACATACCAGATAAAGATGACACCTTTGAACA
AGCGGGGATATTGGACCAATCAACGACTTTGAATGATTCAATTAATGCAACGATGTTGGT
AAATTCTGAAACTGATGTAGGATCAGAAAACCAACACAAATTATCTCTTGAAAATCATAT
TGTTTGGACAATTTTGTCTCAAAATCATTTAATGCCTCTTCCTCTTCATATTTCGCCAGT
TTATTGGAAATACGATAACGCATTGCAACTGTTCCCCGTTCCAGATTTTGTCATCTTATC
CGATCCAACAGCGTCTCCATTTGACATTTCAATCAACGATTCAATGTTTGTTAATCCCCC
TGATTTCTCTGGAGGAGAAAACTTCCCATTTAAAATCATTTAT
>DjScaffold2715:86435-86836 strand:+
ATGCCATTTGGGCTTACCGATGCCCCAGCCTCATTTCAACGAATGATGAATTACGTGACA
CACGGCATATCTCAATGTATGGCCTATATAGACGATCTATTAATCTACTCACCAACATTC
GAAGGACATCTTAATGATTTACGAAGTGTGTTCGTGCGATTGCGTAAGTGGAATTTAAAA
GTGAAACCTACTAAATGCGAGTGGGCCAAGAATAAGGTGACATATTTAGGCCATTTAATA
TCAGATAAAGGCAAAGAACCAGACCCGCAAAACACAGAAAAAATACGAAATCTAGCACCA
CCAAATTGTGTGCTCAAAATACAAGAATTTCTCGGCCTCGCAAATTACTACCGTAAATTT
ATCAAGGACTTCGCGAAAATTGCAAAACCTATGCAAGAATAG
>DjScaffold967:48299-49792 strand:+
atttaaaatttatatttATGATTGTCAAAGGATTCTTATCAATTGTTGTCGCTTTAATGA
TGGGATTGTCGGCTTATTTTGGATCTCTAGCTTATCAATGCTTACAATTATGGCTGGCTT
TTGCTTCTCCTAAAGCATTTAGGTGGTATATTGATATAACAATGTTCATGTGGATTTGTT
CATCAATTTCTTTAATTGAATGGATTGTTGGAATAAAATTTCGATTATATGGTGACAAAA
TAATTTCAAACGAACGTGCTTTAATTATAATGAACCACAGATCACGGTTAGATTGGCTTT
ATTTCTATGGAATTTTAATGCGCTTTGGCACTTCTTTGAAAGATGAGAAAATCGTATTGA
AGAATGAATTAAAGCGAATTCCAGGCCCCGGTTGGGCTATGCAATGTGGCAGCTATATAT

file:///C|/Users/czh/Desktop/Data_S1.txt[2019/7/4 17:05:10]

TTCTCGAACGAAAATTAGAAGCGGATAAATATCATTTATCAAATATGATTGAATATCTCT
CGAGTGTCGGCCATAATACTAAAATTCTCATTTTTCCCGAGGGTACAGATCTTAGTAAAT
ATGCAAAAGATAAAAGCCATAAGTATGCAGACAAAATGGGATTATCGAAATACGAAAACG
TGTTGCATCCGAGAACTGCAGGATTCGTTCACATTACCGAGGAGATGACTCGTTATTTTC
GAGGTAAACTTTGAaacttgcaattcaatctctttgtaatttatcatcgattgtattttg
aattattatttgctattttaattattttaattaaataactaattttattaattaatcgat
tttattaaattttaactattatttaacattttaattaattattaataatatttcgatatt
attcttttttaattataagttagcattttaattattaattattttaattaattaactaat
tttattcattaactgattttttttatttttagatattattatttaccattttaaattatt
aattattttaattaattattattattaattaaccgattttattgatttttaattattatt
taccattttaattataattaattaatcgattttatttatttt
>DjScaffold4875:434-1148 strand:+
CATATTCTTTTTTCATATGTTTCAGTTGTAGTTACTAAATTTAACATCGTACTTATTACA
CCTGGATCGTTTATTGTTTCCACATCTGATGAACCTAATGTTAATCTCATAGATTTAAAT
AACCTTGGAAAGAAGTTATTCTCTAATGTTATTTTATCTTTATCTTTATCTGCAATTGTA
ATTTCAATTCTAAAGTATAATAATGAATTGCAAATGTCAAGTGGATTTGTAGATTGGAGA
GATTGAaatacaactcaccacttgcatttaaattatttattgatcctgatgatggtgaaa
ttttaagtgtatcatttttaattgatgaatgtgaaaattcaacattattatctaaatcta
acatttcttaaagcaattgtttcgtcattttcattgtttccatcattgcacgcactacta
ctgcatttttatttttatcattatctaccttttttctttataatcatagcttcaacttttt
catgatcatattctttcatactgcttatctcatcttctaatacgctatttttcatttgta
aagtataaatt
>DjScaffold4875:20172-21467 strand:+
ATGTTATGTGTTAATGTTCTAATTAATGCCACACAAACATCTTGTGGTTGTAAGGCTTGT
GCAAAATGTATTGAAACTTATTTCAACCAATCTATAAAACCATGTCCGAAATGCAATGAA
AATAATTTATCGTTTAATCGAGATGTTTATCAAGACAACGCTACAAATAAAGAAATTGCA
AATCTAGAAGTGTTCTGTCCATTTGAAAATTGTGAATATGTTGACATATTAAAAAATATT
GATGAACATATCAATAATTGTGAATTCAGTGATTACATTTGCATAAAGTGTAATAAAAAG
GTTCTATCGAAAAATAAACTAATACACGATGAAACGATTTGTGAAAAACGAACTGTGATT
TGTCATCTTTGTAATTTGTCATTTAATAATTCGGAGCTCAATGAAATTCATTACAGTGTG
AAATCGCAAGATTTTTGTGAGAAATTGACTGGATTTTGTCCTAATAAATGTGAAATCAAA
ACTAAAGTCAATTTAAAACAACATTTACAAACTTGTTCAAGAAAACTCTATGATTGTCCT
TATCAACCATATCATTGCAAAGCAAAACACATGTTTAAAACCACTTTATCAATGCATTTA
GAAAAAAAACGTTTAAATCACACTAAATCATTACTGGAAAACATTAAAACTATTGAAAAT
AAACTTGATAAATTAAATTCAAATCTATTACGATATCAACAGATCTCTGATGAACTAGGA
AACATGGAGAATAAAGCAGAAAATCAATGGGAAAAACTAAATGCCGATTTACCAGAAGCA
AAACATTTGGTACCGGAAAATTCGATTGGAACAACCGGAGATTCTATGTTTAGAAATGGA
AGTATGATTTTGAAAGTTGCCGATTTTGAAAATAAAAGAATGGAGACTTCATTAGACGAA
TTGAAACCGATAATTAGTAATCCATTTTACACCAATGAATTCGGTTATAAACTATGTTTA
AAAGTTTATCCTGGTGGTTTTGATGAAGGATTATCAAGCCACTTATCGGTCTATTCTGTC
ATAATGAAAGGAGATTATGATGATTTGTTACAATGGCCGTTTAGTAATCAAGTGAGTTTC
AGTATTCTCGATCAAGAAAATGGTGATCAGCATTTTTCGAAGATTTTAACGCCAGATATT
GAAAATTCCCATTTTCAGAAACCACTTTACGAATATAATGAAATATTCGGCTACGACAAA
TTTCTTCCTTTAACTGATATCATTCCGACATCTGTCTATTATAAGAATGATACGATTTAT
TTGAAGATTAGTGTCCAAGAGAAAACAAATTGCTAG
>DjScaffold2246:34147-36895 strand:+
tgctaatgttgaaattatattagcgcagaaacgacacgatttgattattgatccggaccg
cacaaactactgattattcattctttctacacgttcttgagatcaacacaacactactat
tttaaaaagacattacaagttacgaaaatcattaattttgcgatattctaaaattacaaA
TGCTAGCTCAAGAGTCAACAGGAGTCAACAACTATAACAATGTTACTGAAGATGGTTCAA
TTCCTAATGGCACAACAATGGCTCATCATGCATTAGAGGTTGTCAATCAAGTCAACAAGA
GATCATTAGCTTCCCCGCGTCGTGGAAGCGATAATATTTATGATCCTCCAGCTTTTTATC
AACAATTGGAGAATGAATTGACAGTTGAAGAAGGTGAACCGTTAAGATTGATGACTCGAG
TAGGACCAGCCGATGATAGTAGCTTGCAAATTAATTGGTTCAAAGACGGAAAATTGTTGA
GTGCAGGATCACGATTCAGCACAATAGTAGACCGAGGATTTATTGTTTTAGATATTCTGT
ACACAATTGAAGATGATATTGGAGATTATGTGTGTGTAGCAAAATCCCATTTTGGCACGG
CTGAATCGAATGTAGCTCATGTGAAAATCATACCAGAAAGTGGGATTGATTATCAAACTC

AATTACCAGAAGAAATGATGCAAGGGATCAATAATTTAAATAGAATCCTTGATGGAACTA
GTTACGATGCAATTGAAGAAGATAAACATGCAGCTAAAACTATTCCAAAATTTCTTTCTC
AACCTTCAAATAGTTTAACTTTGATTAATGGACAACCGTTTCATGTTGAACTATTTCTCG
AACATAATTCCGATTCTTCTCTACTAATAGAATGGTATAAAGATGGTCAAAATTTAAATA
TAGGCAGCAGAATCGATGTAATTTACGATCGGGGATATTGTGCTTTAGATATTGCTTATT
GTAACATTACCGATAGTGGAGAATACAAATGTGTAGCAAAGAACTCTCTCGGAAACACTG
AGAGTCAATCAATTAGACTCGTAGTTCAACCAGAAGAATCTATTGTAACAAAATCGAATT
TAACGGAATCTTCTATTTCTCAGTTGAAATCATTAGAGAATCCCGGAGAAGAATATTCTC
GGTATACTGATACTTCAACCAACCCAATACCTCCATCCTTTACTAGCTCTATTGATCCAA
ATGTTATTTCCGTTTCTGAAGGCACACCAGCATATTTTAAAGTATCAATCGATCCAGGAA
ACGGTCAGAAATTATCTCATGATTGGTATCACAATGGAGAATTAGTGCAAACCTGTTCTA
GACTGACAGGAAAACTAGAAATGAATCTGGTTTCACTAAATATATTATATACTTTACCAA
GTGATACAGGCTCTTATATATGTGTTTTATTCTCGGAATACGGTAGAGCAGAATCAGAAC
AGGTAACTTTAAATGTACAAACAGAAAGATCGATAATACTTGATCCGCAAAGCAAAGGGA
TTTCTGCTATTCAAGCTTTAGAAGCGGAATTGAACGCTCCAAAAGTAGAAATGAAAGTTG
ATGATGTAATCAAAGATGTACCATCATTTACAAAGCAACTGGATCATGTTGATCAACTAA
ATGAAGGCAATTCTGCTACATTGATAGTAAAAGTAGAGCCAGCTAATGACAATACCTTAT
TTGTAGAATGGTACAAAGACGAAAACCAATTAATAAATGGATCTAGATTACGAATTAGTT
ATGATAGAGGTGTTGCTATATTAAATATAACATCAGCGATAAGTGAAGATAGTGGAAACT
ACTGGCTGATAGCAAAAAACAGAAACGGTCAAACTGAAAGTGAAAGAATTTACTTAGTAT
GCATAGAAGGCGAAAAAATAGTAACTGCCAGTAGTTTACTAGAAGATTCTGATGGTTACC
GAAGGTTAAAGAATTTAGAAGAAGATTTATCAAACACAAATTATCAAGATTTTCAACATA
ATGAAGACATTCAAGAAGAATGTTCACCGAATTTTGATTTTAAACCAGTGAATATCACAG
CTGGAGAAGGAGGTCCAGCAAAATTCCTAGTTAAAGTCAGTGGATTTCCAACACCGATTT
TAGAATGGAAAATGGACGACATTACAGTTGTTCCAGATTCGATTTGCAAAACATATAACG
ATGGATCAATAAACTATTTAGAAATAAATCGATGCAATGAACTTGGTGATATACGTATAC
ATGTGACTGCAACTAATTCATTGGGAACAGCAGAAGCGGAAGCTACCCTTACTGTTATAC
CCATGGTTGATTTCAGACCAGATTTACGTCATATTACTCCTGAAAATCCATATAAAAAAC
TAGCCAGCTTAAAAAAAGTTGAGTGTACATCTGAACTTAATAATGCTTTCAAAAAATCGA
AACCAAGTGCTGAACAAATTGTTAAAATTGAAATGGGTTCAGAAAGCAAATCAAAATTTT
ATAAATCAGCGGATGTCATTGAAACGGAAAAACTTTATGATCAAATTGCAGCTGGACTGA
GAAAAAATATTGGTTACGGTGGCAATAATGGTTCAAGATCACCAAGAAACATGCATAGCG
GATCACCAATAACCAACGAAGATATGCCTTCACCTTCTCCATGAattacagaataaaaca
ttgccttttaaattcgtttttaaaaataattttataaatgcatgcaatttcgtttgtcaa
tatgtactgatatcaataaaattcatcgattgtaaaagaatttagaagaagatttatcaa
agtaaaattatcaagattttcaacataatgaagacattcaagaagaatgttcaccgaatt
ttgattttaaaccagtgaatatcacagctggagaaggaggtccagcaaaattcctagtta
aagtcagtggatttccaacaccgattttagaatggaaaATGGACGACATTACAGTTGTTC
CAGATTCGATTTGCAAAACATATAACGATGGATCAATAAACTATTTAGAAATAAATCGAT
GCAATGAACTTGGTGATATACGTATACATGTGACTGCAACTAATTCATTGGGAACAGCAG
AAGCGGAAGCTACCCTTACTGTTATACCCATGGTTGATTTCAGACCAGATTTACGTCATA
TTACTCCTGAAAATCCATATAAAAAACTAGCCAGCTTAAAAAAAGTTGAGTGTACATCTG
AACTTAATAATGCTTTCAAAAAATCGAAACCAAGTGCTGAACAAATTGTTAAAATTGAAA
TGGGTTCAGAAAGCAAATCAAAATTTTATAAATCAGCGGATGTCATTGAAACGGAAAAAC
TTTATGATCAAATTGCAGCTGGACTGAGAAAAAATATTGGTTACGGTGGCAATAATGGTT
CAAGATCACCAAGAAACATGCATAGCGGATCACCAATAACCAACGAAGATATGCCTTCAC
CTTCTCCATGAattacagaataaaacattgccttttaaattcgtttttaaaaataatttt
ataaatgcatgcaatttcgtttgtcaatatgtactgatatcaataaaattcatcgattgt
agttgtatttaggattcgatttgcaaaacatataacgatggatcaataaactatttagaa
ataaatcgatgcaatgaacttggtgatatacgtatacatgtgactgcaactaattcattg
ggaacagcagaagcggaagctacccttactgttatacccATGGTTGATTTCAGACCAGAT
TTACGTCATATTACTCCTGAAAATCCATATAAAAAACTAGCCAGCTTAAAAAAAGTTGAG
TGTACATCTGAACTTAATAATGCTTTCAAAAAATCGAAACCAAGTGCTGAACAAATTGTT
AAAATTGAAATGGGTTCAGAAAGCAAATCAAAATTTTATAAATCAGCGGATGTCATTGAA
ACGGAAAAACTTTATGATCAAATTGCAGCTGGACTGAGAAAAAATATTGGTTACGGTGGC
AATAATGGTTCAAGATCACCAAGAAACATGCATAGCGGATCACCAATAACCAACGAAGAT
ATGCCTTCACCTTCTCCATGAattacagaataaaacattgccttttaaattcgtttttaa

aaataattttataaatgcatgcaatttcgtttgtcaatatgtactgatatcaataaaatt
catcgattgta
>DjScaffold2246:74085-74796 strand:+
attttagccaactatttATGAATGATTACAAGATTGTTTTATTAGGCAGCGGTGGTGTTG
GAAAAAGTGCCCTTACTGTTCAGTATGTTTCAGGTGTATATGTTGAGTCTTATGATCCAA
CAATAGAGGACTTTTATGTTAAAAAAATTAAAGTTTCAGATGAAATGAAGACTGTGGAAA
TTCTGGACACAGCTGGAATTGAACAGTTTTCGTGCATGAGAGATCTTTATATTAAAAATG
GCGATGGCTTTGTCATAGTTTATAGTATTACAAATGATAAAAGTTTTAATGACATTAAAC
ATTTGAAAGAACATATAATGAAAATTAAAACCAATACAAAGGTAAATATTGAAATATGTA
AAATTATTTATTTATTAACTTAAtatagtattaatgaaatgtaatgtttgcattttaatg
ttgatttaatttcattttaaaaccaatttgattaaattatgaacttaaatttctctccga
aattagtttcattgttctatgtaaacaatgttaattaaattcatcgaattctaattcgat
aaatttgctttgatttatttttaccaatttgtgctgctgtgataaataaaattagataat
tgatgaaatgttttaaatttcgccaatattttattagtattttgtttttgttgtcacgtg
atttatgtat
>DjScaffold2246:75930-76469 strand:+
ATGCCATTTGGCATCGCAGCGGCACGAGGGACATTTCAAGAGATGATGACAAAAGGGTTA
AGCGGAATCAATGAAGCCATGGTTTATCTTGATGACATACTGATATTTACGAAGAATAAA
GATCAACACTATGAAGTAATTGAAAGAGTTTTGACGGAAATAATGAAGGTTTGTCTAAGA
ATTAATCCAGAAAAGTGTCAGTTTCTTCAAAGCAAAATCAAGTTTCTTAGACACATAATT
AGCAAGGATGGTCTTAGAACTGATCCGTTTAAAATAGAAGCAATAAGAACATTTGATAAA
CCTAACTGTGTGAAGAAGTTACGAAGTTTCTTAGGATTTTGTAATTATTATAGAAAGTTT
ATAGAAAACTACGCAAGAAAGTCAAAGATATTAGAGCAGTTGTGTACCGTACCAAGCAAC
AAGAAATTAGAATGGACAGATGAAATAAATTATACTTTTGAGTGTCTAAAGAAATCTCTA
ATCACTGCACCAAAATTGTTTTCCGGACATAAACAAAGACTTTATATTAGATACAGATGA
>DjScaffold250:7703-13868 strand:+
atgtatgtatagctaaaatttaagaaaagattcttttgttctgtatcgcgtgattttcca
caggaaaatatacttagtcacacaccagttttgttaaatgaagttcttgatgtttttct
tcaagtaataacaaaatagttttagatttaacttttggtaatggtggtcactctaaatca
ATGTTAATTGATAAAAATGTAATTAAACATGTTGGAATAGATAGAGATTATGTAGCTTAT
AAAACTGCCTTAGAATTAAGCAAATTACATTCCCAATTTATAGCAACCCAAGGCAAATTC
AGCGAATTAGACAAAATTTGTAAAGAAAACGATATCGTTGAAAACACTGTAGATTTTATT
TTGATGGATTTAGGATTTAGTTCAGTTCAAATGGATGACAGCAAACGCGGGTTTTCTTTA
AGTAGAAACGGTAAATTGGATATGAGAATGAACCAAAATTCCGATGAGTTAACAGCTGCT
GATGTGATTAACAATCTTGATGAATATCAGTTAGCCGATATTTTCAGAAAATACGGTGAA
GAAAAACATTGTACAAAAATAGCGAGAATGATTGTGGAATATCGAACTAGTGCTGGACCG
ATTTTGTCCACATTTCAATTGGCAGAATTAACTGCTAAATGTATTGGCTGGGGAAGAAAT
TATGATTCATTGAGGAGAAAAGTACATCCTGCTACACAAATTTTTCAAGCTTTACGCATT
TTTGTCAATGATGAATTAAATGAATTGTGTTCCGCTTTAAATCTTGCAAAACGCTATTTA
ACTCCCTTAACTGGTCGATGTGTTGTTATAAGTTTTCATTCGCTTGAAGATCGTCTTGTT
AAATGGACATTTCAAGGAAAAGATTTTAATGGTGTTGATAATTCCTTAAAGAATTTAGTG
TTATACAATCAAAAGACAAATCCAGAGGAATTGTATTCATGGAAAAAAGTGCATTATATT
>DjScaffold2580:47522-48553 strand:+
GGTTTTTCTGGTCCACAAGGTTCAAAAGGTGAAAGAGGAGATTCTGGATTGCCTGGTAAG
GAAGCGGATAAAATACGAGGTGAAAAAGGCATTAATGGAGATATTGGAGAGCCTGGAGAA
AAAGGATTAAAAGGAGAAAAGGGAAAAAATCAAAATATCAATCTTCTAATAACCAAATAT
AAAGGTGAAAAAGGACAGAAAGGTGAAAATGGAACCAGAGGTGTTGATGGATTTCCTGGG
AATAGAGGATCACCTGGCGAGACAGGCTTATCAGGAATACCCGGAAAACATGGAAAGAAA
GGAAATCCAGGTAAAGACGGAGAAAAAGGTGAAATGGGTAGTAAAGGAGAAAATGGTATT
GATGGAAGAAGAGGTAAAGCAGGAAGTAAAGGTGTAAAAGGTAAAAAAGGCTTGGAAGGA
ATAAAGGGCGATAAGGGTTTCATTGGATTCCCTGGAGAAAAAGGCTATATTGGTAGTACA
GGACTACCGGGTGATAATATTACTGGAGCAATTGGAGATAAAGGAGATAAAGGAGACAAT
GGTTTAAATGGTGAAAGAGGTTCCAAAGGTGACATTGGTTTAATAGGAGAAAAAGGAATA
ACTGGAGATAAAGGGGTTATTGGATTAAGAGGTGAAGAAGGCGATAAAGGTTTTGAAGGT
TTAGTTGGTGATCCTGGTAGTGTTTCCATAGGAGAAAAAGGACTTAAAGGATTTCCAGGC
GATATTGGTGAAATTGGTGATAAAGGAAATAAAGGTTTCAAAGGAAATCCAGGTTTGCCA
GACATCACTCTGAAAGGCTATAAAGGTATACCAGGAGAGAAAGGTCCGAAAGGATTATCA

GGTTTACCAGGAGAAAAAGGATTCAAAGGTGAAGGTGGTCTACCTGGTATATCC
>DjScaffold2580:48942-63046 strand:+
ttggtataaagggagacaagggtgacaaaggacaaATGGGAGAAATGGGAATAAAAGGCT
TTAAAGGAGATATCGGTCCTATTGGTTTACCGGGATCTAAGGGTTATCAAGGTCCACAGG
GTCAAAAAGGTAATCGAGGAAATAAAGGATTAACAGGCCGTCCAGGTTCTATTGGATTTT
CAGGAGATAAAGGTGACAAAGGTTTTAGAGGTGATAATGGACCACCGGGATTGCCGGGAG
ATAAAGGCAATATTGGTGAAACAGGTGAATCGCCTGATGTAGTTGAGAAAGGAGATAAAG
GAGAAAGTGGCGAAAAAGGAGAAATTGGTTTTAAAGGTGATAAAGGAAATACAGGACTTG
CTGGACAGGTAGGAGAAGAAGGACCTAAAGGCTATAAAGGATCAAAAGGTGAAGAAGGAC
CAAAAGGATTAACAGGACAAAGGGGAGATATTGGTCCTAAAGGATTACTTGGTGAAAAAG
GACAAGATGGACAAAAAGGAATTCTTGGTGAAAAGGGTATTAAAGGCAAAAAAGGAATAA
AAGGAATTATTGGAGTCAAAGGCGATTCTGGATTGCAAGGTGTAACGGGTAGAAGTTCAG
ATGGTGGTCGAGGTCAAAGAGGACCACAAGGAAATATTGGTGAAAAGGGGGATAAAGGAG
AATTTGGTGATGCAGGAAAAAGAGGTCCACCTGGTCCTGACGGTGATCAAGGAATTGGTT
TGTTAGGTGTGAAAGGAGAACGTGGAGATCCCGGACCAGAAGGATATGAAGGACGTCGAG
GTGTAAAAGGACCAAAAGGAGATAAAGGATTTTCGATTTTATATCCAGGACCCCAAGGGG
AAACTGGAGAAAAGGGGATAAAAGGAGAAATTGGTTCAGTTGGACCCACTGGAATTCCAG
GTCCGAATGGTCAAAAAGGTGAAATAGGAAATCAAGGTGATATCGGTGAAAAGGGTTTAC
GTGGACCACAAGGGTTAGCTGGTGAACGGGGACTAAAGGGATTTAAAGGAGACAATGGTT
TTAAAGGAGCACAAGGAGATAAAGGTGAAAGAGGATTTCTAGGTGGAAGGGGTTTAGTTG
GAGGAAAAGGAAGTATTGGAATTTCTATACCTGGAGACAAAGGTGAAAAGGGTATTCTTG
GTTCTGATGGAGATAAGGGAGAAAAGGGAGAACCTGGTATCGTGGGTCCTAGAGGGGATA
AAGGAAAACCTGGTGAACGAGGTGAAAAGGGTGAATCAGCATTTAAAGGTGAAAAAGGTT
TCATTGGGCCCCAAGGATTAGGAGGTAAACCTGGACCAAAAGGTGATCCAGGACCAGTTG
GTGACCCTGGTACAAGAGGTCCGCCAGGTAACCAGGGTGAAAGTAGAGGAAATACAGGAT
CCATTTTTGCAACACACAGTCAATCAACCACAATTCCTAATTGCCCATCAAAAACCAGAA
TGCTTTGGCAAGGTTACTCCTTTTTAGGAATGACAGGTTCAGATAGGGCACATATTAATG
ATTTATCTAGTCCTGGGTCTTGTTTACAAATGTTTAGTCCGATTCCCTTTGTATTTTGTG
AAAAACAAGAGCAGTGCTTTTATTCTGTTCGAAATGACAGAACATATTGGTTGAGCAGTT
CTGCTTTTATGGGTATGTCAATGCATATCAATGTATCTCAAGTTCAAATGTATCTCAGTC
GTTGTGTTGTTTGTGAAGCACCAAGCAAACCATATGCGTTCCATTCTCAAAGTACAATGT
TTCCAAAATGTCCTAATGGATGGACAAATTTATGGAGTGGAAACAGTTTTCTAATGAATA
CTGGTTATGGAGCTGCTGGAGGGGGTCAACAACTTTCTTCACCAGGAAGTTGTTTAATGC
ACTTTAGGAAGAGTTTATTCTTAGAATGCACCGGTAAAGGCCATTGTGGTTATTATGAAG
AACACAAACACTTTTGGTTAGTAGTGACAGACAGTATGAAATCGTTCCAAATGACAATGG
GTCATAATATAAAGACAATGCAGTCTGAAGATTTGATAGCTAAGTGCTTAGTTTGTATGA
GAAGCCAACCAATGGTCGGATATATTGCCTCTTAAcatctagaaaatgtttattttgttg
gattgttaattttgttcgatttttatatttattgatcttgaaattgacttgttaaattcc
gatttgaacaaataggaggccatgtgtgggtataaaattttatctagacttttgttacat
tggatgaatactttgagtataatattttgttattggtttttcattataactcaatttaat
tggttatcttttgaatgcttttctttgaaattgtaattagtatttcagataacataaaca
tttattgacattttggatttttattttcatttatagcaaaaatattgaatattataaaat
tggatcggacttattattataaaa
>DjScaffold2685:124037-124477 strand:+
GGTAAAAATTTCACATTTGATGGAATAGTGCAGCCGAAATCATCTCAGAAAGAGGCTTAT
GGAATTATAGGTAAACCCGTGGTAGATAATGTTTTAGGCGGATATAATGGAACGATTTTT
GCTTACGGGCAAACGTCGAGTGGTAAAACTCATACGATGACGGGAAATTTAAATGATGAA
AATCTAAAAGGGTTAGTACCTAGAATTATAGACGATATATTTGATCATATAGAAAAAATG
GATGAAAATCTATCGTTTTTGATTAGAATCTCTTATATGGAAATTTATTTGGAAAAAATT
AAAGATTTATTTGATCGTAAGAATTTATTAAATTCATATTTTATGTAA
>DjScaffold2685:3339-3626 strand:+
ATGAGAAAGGAGATCAAATATTTGGGACACGTTATCAGTAAAAAAGGTGTCAAAACAGAT
CCAACTAAAATAGAAGCAATTAAGAACTTCGGTAATCCAAATTGCGTTAAGAAAATGAGA
AGCTTTTTGGGAATTTGCAATTACTATCGAAAGTTTATTAAAGATTATTCAAAGAAAGCT
AGGTTGCTGGAACAGCTGTGTATTGGGTCAAGTAACAAAAAAATAGAATGGGCGAATGAA
TTAGAAGAAGTTTTTAATGGAATGAAAGAAAGACTAATGATTTCTTAA
>DjScaffold5331:17889-29608 strand:+

ACAACTACTACTACAACTACTGCAACAACTCCTTCATCGTCAAAGACACCAACACAATCT
ACAACAACACCAACTATTTCCACTACGACAATATCAATGACACCAACAAAATCTATAACA
TCACAAACAACAACACCATCATCAACTATTACAACAACCTTATCAAAGACAGCAACACCA
ACTGTAACAATCATAGAAACTAAACCTGTAACTTTACCACCTTGTCAAATTGTCGATGGT
TTTGTCGAATCATATTACATTAGTGATCGTCAAATCACTATCAATAATAAACCAGATAAT
CGAGTATCTCAATTACGACAGTCATCTACAACTGGTTTAGAAATTGTTTTGGAAGTTCCT
ACTGTAATTAAAGTCGATTTTACTGATAAAGGTGAACGTGAAATTCCATTTATTTATCAA
ATCAAAATTCACGGAGGAGTAGAAAAAGTATCTTATACAATTGTAGACAAAAATGGAATC
GCAACTTCAAAACAAGTAACGTTTGTTGAAGTTCCAGCAAAATTAGAAATAAATTCAAAT
ATAAAATCTATCGAATATGAAGTGATTAGTAAACTGAACAAAAAGGAGAGTTTATTTACA
AAACTAGAAGTTCAAGGATGCTTTGAAAAGGAAACAACGATAGCAATACCAACTACTACA
ATTCTAACTACCTCGCGTTTTACAAATATTTTTACTACTCCATGTTCCTTGGTGGATGGT
CTTATTTCTGAATATTTCATTCCTAGCCATCTGATTACTATTAATGGTAAATCTGATAGT
CGAGTGTCCAAATTACGGAAGTCTAATCTTTCGGGATTCATGTTTTCATTAAATAATTCC
CCTTCATTTGTTATTGATTTTAGTGATAAAGGCAATCGTGAAATTCCTTTCATTAATCAA
ATAATATTTGATGGTAATGTGAATGAAATTTCTTATAATTATGATCACGAGTCTTCCCGT
TTCTCTCTGTATCAGATGGGTGAGCTTCCCAGACGATTGGTTATGAATAAACAAATTAAA
TTCTTTCAATTCTTTGCTCGTTCTACAGTTAACAATTCTGATGATGCCATACTTAGTCTT
CAAATTATTGGATGTTTCAAAAATATTACTGGTTGCATTGTAAATGGTAGATATTTTGAA
AATCACCAATTAGTAAATAGCACTGCTAATCCTGATAAATGTTCATCAGAAATGTGTTAT
TGCAAGGATCATAGAGTTTATTGTCGAACAACCAATCCACAAACTATTTGCGCAGAAAAT
GAAGTTAAGTCTTGTTCAAAGGTTAACAAAAATCTAGAGTGTTGTACTTGTTTAAAAAAT
GTTAATATAACCTATTGTCAAATCGATTATCTTTGTAACCCTCATTGCGGAAATTGTCTT
GATGAGGGTGATACTTGGGGAAAGGAAACAATTGTTCAAAATGCTACTCAATGCAATTGT
AAAGATGGATACCAATCATTTATTGAATCATATGAAGATAAAGTTGAATCAACCACCAGC
ATTCATATTAATGCCAAGTGTATGTCTTATACCATTGATGAATCAACTTGTGGAACTACA
AGTACACCTTCTCATACTGTAACTAGACCATCACCAACTCAAACAATTTCATTTTCAATC
CCATCGATTTCATCTTCAACTCCAATTCAAAAAACGACAACAATGTCTATTTCAACACCA
ACAATCTCTATATCATCATCTGAGACTACAACAACTACAACCACAACAACTGCTACTCCG
CCAATCACACGGACAATACCAACTTCTTCAACAATAAAAGAAATATCCACTCTTTCTGAA
ACTGAATCAACAACTCCAGAAGTAACATTTATTACTACAACAAATAAACCTGTCTGTACC
TATATGTTCTGTTCCCAATACACTTGTGATAATGAATGTGTGATCGGTGAACCTCGGATA
CAGGATTGTTCCAAATTAGTTTGTACCAGTCGTTTGATCCAAGCCTTCAGAAATGAAACA
TATATTATTTGTACAAGACAAAAGCAAGAAAACTGTTCTTCAACATCAATACTTCCAACC
CAAAGCTTTATTACTCCAAATATAACCGTTTCATCTTTTTCCGTAACATTTCCAACATTT
ATTCCTCCTCTAACAACAGTTAAGCCTTGTGTTGTTGTATTCTGTTCTGTTTACACATGC
AGTAACTTATGCGTAAACCCAACTTATATTAATGAATATTGTTATAATTTGAACTGTACT
CGTGGATATAAAGAAGTAACTAGGAATGCCACTCATATACAATGTGCGCTATCTATTGAA
ATCCAATGTAAATCAACAACATTATTCATAAGTAGTACAACCAGTCTCCCGATTTCTACA
ACAGTTAAACCTAGTGAATGTGAACCAGAATTCAAATTGAATACTGAAAAGTTTATCGCT
GAAATTAAAAAGTCTACAAATCTTGCTGGTAACGAACTAGATTCATGGATAACATCTACA
GATGATTTTGTACCGTTACTTGAAATACGATTCAAAAATTCAATGACTGTTGTGAAACTA
GAGTTCAGTGAAATGACTAATGTCCAAGAAATGAATATTTTGACAAGTGAAGATGGAAAA
ACAAATAAAATTGCTGAAAGACTAGTTGATCAGTATCGTGGATATAAAACAAATCAAACT
GTAAATTTCCAGAAAACGACAATTTTAAGCTTGAAATTGATTATTTTAAAACGAGTTGAA
ATTTCACAACCAATTTCATTAAAACTGACAATCATAGGATGTGGAAAGGTATGTATCCCA
CCATTGATTCATGTTAACTGTTCGTGTCAACAAACATGTGATTTACTAGCAGAAAACAAA
GGAATTTCTCAATGCCCATCGAAATGTATTCCAGGTTGTCAATGTCCAGAAGGAATGGTA
AATAATAATGGTAAATGTGTCAAAGTTTCAGAATGCCCATGCTACTACAATGGTCAAACT
TATGCTTTCGGAGATAAAATACCAACTGAAAATCCCTGCAAAGTCCTGATTTGCACGAAA
GAAAGTTTACAAGAAATAACGGTTGGTAACTGTACAACAAAATGCAAAGAAACAATGATT
CCTTGTGCTTGTGAAGGCTGCCACAAAACGTGTACCAAAAACAATATGGATGCCAATTGT
GACTTGAAATCATGCAAGTCTGGTTGTTGTTGTCCACCCGGAACTGTTTATTCGGAAATT
CTTGAAAAATGTGTAAAACAATGCCCATGTATTTATAACAATCAAACCTACGCTGTCGGA
GAAAAATGGAAAAATCAATGTTTAGATTGCAGATGCGACGAACGCAAAGGAGCTTATTGC
ATAGAAAATGGATGTCATTTTGATCAATGTCCACCTCGTCATAAATTGGTCAATGAAAGT
CCAAATGATAATGTATGTTGTAAATGTGTGAAATTGGATCCTGTGTGTATTTTCAACGAA

AAAGAATATAAAGTAGGGGAAATATGGAGTGATGGGCCTTGTAGGAGTTTTAAATGTATC
ATAAAAGATGGTCAACCAATGGTTACTACTAGCAAAATAGAATGTCAACCAATTAAATGT
CCACCAACGCATACTCCAATTAAACTACCGGGCGAATGCTGCGAAAAATGCGTACTTAAA
AATATCACAACGACAACATTTATACCGACAACTACATCTCAAATTTCGACCAAATGCAAC
AAAATCAGTAATTTCACAACAATATCACTTGATGGTTGCGTTTCTAAAAATTTGGTGGAT
GTTGGAGCATGTAAAGGATTGTGTGACAGTGGATCTTATATAGACTACACCAAGGACCGT
AATTCAGTAACTAAAGATTGTCATTGCTGCAATGCAAATGTTTCTTCGATCACAGTGGAT
TATGTTTGTCCAGGAAATGTGTTTAAACAAGACTCGATTTTAGTTATAACGAATTGCGAA
TGTAAGAAATGCGGATAAtgtgataaaatacaactttgtttgaaaaataaatatttgtga
ctgtt
>DjScaffold2719:12388-13132 strand:+
gataaaattacgaatctttaccgtATGAGAAGCAGAGATGGACAGCAACCATTTTCAAAA
GAAATTGGCAGCGAATTTAATGTTCCGAAATTTCCAATTGAAAAACACGAATTAACAACA
CATATGTTGGATTTGAAGTTCCATAGTGAAATCGAATTTAGCGAACTTGAAGAGCCTTTA
AATTTTCTCAATGATTCACCAATAATGAATGACCCGCTTAATCAATTACATATGGTTGAT
AGAAAAATGCTTGGAGCAACAAATGAAAGTCTTTCTAGTACTCAATCAAAAAAAAATTGGA
ATTCCAAGTTATCAGAGAATTGCTCTTTGCGGAGAAGATGAAACTTCGGTTAGTTTGATA
ATGAAATAAttattaaaatttattttaagtagagatttttataattaaaaataattgat
tgatttgagcaaaaatatatttaattaaaaattttgatgaaaataaatattaaaaaagtt
tagtattttacaaaataaaacccatcaatatattttatcaaatagataatttgcttttta
ttgaaatattaaaataattattttgtttattgtgaaaattacactttgtgtgattattca
ctttttaaaatttttaaccattgatctgtgaaataaaaaaattaaatattttcaaaatt
acgaaaataaactaaggcgtagatcaaa
>DjScaffold2719:38014-42944 strand:+
atggagaaaaattaaattctcattaaataaacactaattgtttatactaatatttaatta
ttgattttaaaataaaaaactcaaaagcttttattttaaaatcaatctttagtggaaatt
tgaataaagacattatttcaaatatcaataatattatttcaataatattttaaatgtttc
aataattatgcaaattaaatgtagattaattcgaatattcttcctgttgatcaacaaatg
aatgaaattgttacttaaaatgaataacagtttaaattacttcaataaacaattgtattt
attaacaaatttaaaattaaattaatttagttggcattggaggaaatcgaggaaaattct
aaagcgatcATGACAGCAATTAGAATGCGTCGTAGATATATGAACGTATCAAAGCAACCA
TTCCATAAAACCACGGATCGATATTTGTCAATTATTGACAGTGGTGTTAATACTAATGAT
CACCACGTTGTCAATAACCAATTGACTATGCTGAAAGAGGATTCGCAAAGTGGTAATTCT
GTAAATCAAGCTAAAGATCATCCATTTAATGCTCCTGGAACTCAAGGAGATCCTTATAAA
GTTGATAAATTTCCAGACAAATTGAACGTTGAATTTCAGTTCAAAAATGGTGTCATGATC
GTCACAAATAAATCTGATGATACTCAAATTCAGTGCGGGATTGATTATCTAGAATTTGTC
AGAGATTATCATGCATTGTCGACTTTTGTAAGCGATGGCCCTTTAAAGTCATTTTGTTTT
AAACGATTGAATTTTTTAAATTATCGATTTAAAATGCATATTTTATTAAACGAATGGAAA
GAAACTGCTGAACAAAAATCGGTAGCTCATCGAGATTTTTATAACATAAGAAAGGTTGAC
ACTCATATCCACGCTTCGTCTTCCATGAATCAAAAACATTTGTTAAGATTCATCAAGAAA
TGCATTAAACAATTTCCAAACGAAAAGGTTATGGTTGATAAAGTAACTGGTAATATACTT
ACTCTAGTTGAAGTTTTCAAATTAATGGATTTGACTCCATATGATCTGACTATAGACAAA
CTAGATGTGCATTGCGACCGTGACACATTTCATAGATTTGATAAATTCAATGCCAAATAT
AATCCAGTTGGTCAAAGTGAATTGAGAGAAGTATTTCTAAAAACAGATAATTATAATAAA
GGCAGATTTTTTGCGCATGTTATTAATGAAGTAATGTCGGATCTTGAAGAATCCAAATAC
CAAAATGCTGAATTACGTTTATCTGTGTATGGGCGGAAAATAAATGAATGGGATTCATTA
GCAAAATGGGCTGTGGCAAATCGAGTTTACTCAGACAACGTTAAATGGATGATTCAATTT
CCACGAATCTATGATATTTTCAGAACGAAAAATTTAATTAAAAATTTCCAGGAAATTTTA
CACAATATTTTCACGCCTTTATTTGAAGCAACTCGGGATCCAAGCAGTCATCCTGAATTA
CACGCATTTTTGGATCATGTCAGTGGATTCGATTCTGTCGACGATGAATCAAAGCCTGAA
TCTTGCGTAATTGATAAAAATACTCCGTTTCCCGAAAATTGGGTTGGGGAAGAAAATCCC
CCATATTCATACTATTTGTATTACATGTATGCAAATATGTCCGTATTAAATGATTTAAGA
AGCCAGCAAGGATTAAATACGTTCACATTTCGTCCTCACTGTGGTGAAGCTGGTCCTGTA
CAGCATCTTGTTGCTGGATTTCTTTTGGGTTATAACATAAACCATGGGTTATTACTCAGA
AAAGCTCCAGTTCTTCAATATTTGTATTATCTTTGTCAGATTGGTATTGCTATGTCGCCT
TTGAGTAATAATTCTCTATTTCTTGATTACCAAAGAAATCCACTTTATGAATTTATGTGC
CGTGGACTGCATGTTAGTCTGAGTACTGACGATCCATTACAGTTCCATTTTACAAAAGAG

CCTTTAATGGAAGAATACAGTATTGCAGCACAGGTTTGGAAACTGACATCAACTGACATG
TGCGAATTAGCCAGAAATAGTGTTATGATGAGTGGATTTTCAGATACAGTGAAATCATTT
TGGCTCGGCCAACAATTCAAAGAAGAAGGCGTTCAGGGAAATGATGTTATTCGAACAAAC
GTTCCAAATATTCGTATTGCATATAGATTTGAAGCGTTAACTGCCGAACTTAATTTATTA
CTCAGAATGACTTTGAAAGGTCTTTCTAATCAAAATGAAGATTATTCCCAATAAaactta
acgtggtttggtggacagtgatatatagtgaagaagcatttgtgaatggtagtttccata
gataattaatctatgaatttttgtttatgtaattcatcgcttaaatgcaatatatatttt
tgatacttcattgaccgctctgactgtgttatagttgatttatattgcacttcatgtttg
atttttattgggttattattgtgatattttgtattgatttctagttattttgttaattac
agaagttaactattgaattaatgaattttttttaattatcacaaactatttgacatggag
aaaaattaaattctcattaaataaacactaattgtttatactaatatttaattattgatt
ttaaaataaaaaactcaaaagcttttattttaaaatcaatctttagtggaaatttgaata
aagacattatttcaaatatcaataatattatttcaataatattttaaatgtttcaataat
tatgcaaattaaatgtagattaattcgaatattcttcctgttgatcaacaaatgaatgaa
attgttacttaaaatgaataacagtttaaattacttcaataaacaattgtatttattaac
aaatttaaaattaaattaatttagttggcattggaggaaatcgaggaaaattctaaagcg
atcATGACAGCAATTAGAATGCGTCGTAGATATATGAACGTATCAAAGCAACCATTCCAT
AAAACCACGGATCGATATTTGTCAATTATTGACAGTGGTGTTAATACTAATGATCACCAC
GTTGTCAATAACCAATTGACTATGCTGAAAGAGGATTCGCAAAGTGATCATCCATTTAAT
GCTCCTGGAACTCAAGGAGATCCTTATAAAGTTGATAAATTTCCAGACAAATTGAACGTT
GAATTTCAGTTCAAAAAATGGTGTCATGATCGTCACAAATAAATCTGATGATACTCAAATT
CAGTGCGGGATTGATTATCTAGAATTTGTCAGAGATTATCATGCATTGTCGACTTTTGTA
AGCGATGGCCCTTTAAAGTCATTTTGTTTTAAACGATTGAATTTTTTAAATTATCGATTT
AAAATGCATATTTTATTAAACGAATGGAAAGAAACTGCTGAACAAAAATCGGTAGCTCAT
CGAGATTTTTATAACATAAGAAAGGTTGACACTCATATCCACGCTTCGTCTTCCATGAAT
CAAAAACATTTGTTAAGATTCATCAAGAAATGCATTAAACAATTTCCAAACGAAAAGGTT
ATGGTTGATAAAGTAACTGGTAATATACTTACTCTAGTTGAAGTTTTCAAATTAATGGAT
TTGACTCCATATGATCTGACTATAGACAAACTAGATGTGCATTGCGACCGTGACACATTT
CATAGATTTGATAAATTCAATGCCAAATATAATCCAGTTGGTCAAAGTGAATTGAGAGAA
GTATTTCTAAAAACAGATAATTATAATAAAGGCAGATTTTTTGCGCATGTTATTAATGAA
GTAATGTCGGATCTTGAAGAATCCAAATACCAAAATGCTGAATTACGTTTATCTGTGTAT
GGGCGGAAAATAAATGAATGGGATTCATTAGCAAAATGGGCTGTGGCAAATCGAGTTTAC
TCAGACAACGTTAAATGGATGATTCAATTTCCACGAATCTATGATATTTTCAGAACGAAA
AATTTAATTAAAAATTTCCAGGAAATTTTACACAATATTTTCACGCCTTTATTTGAAGCA
ACTCGGGATCCAAGCAGTCATCCTGAATTACACGCATTTTTGGATCATGTCAGTGGATTC
GATTCTGTCGACGATGAATCAAAGCCTGAATCTTGCGTAATTGATAAAAATACTCCGTTT
CCCGAAAATTGGGTTGGGGAAGAAAATCCCCCATATTCATACTATTTGTATTACATGTAT
GCAAATATGTCCGTATTAAATGATTTAAGAAGCCAGCAAGGATTAAATACGTTCACATTT
CGTCCTCACTGTGGTGAAGCTGGTCCTGTACAGCATCTTGTTGCTGGATTTCTTTTGGGT
TATAACATAAACCATGGGTTATTACTCAGAAAAGCTCCAGTTCTTCAATATTTGTATTAT
CTTTGTCAGATTGGTATTGCTATGTCGCCTTTGAGTAATAATTCTCTATTTCTTGATTAC
CAAAGAAATCCACTTTATGAATTTATGTGCCGTGGACTGCATGTTAGTCTGAGTACTGAC
GATCCATTACAGTTCCATTTTACAAAAGAGCCTTTAATGGAAGAATACAGTATTGCAGCA
CAGGTTTGGAAACTGACATCAACTGACATGTGCGAATTAGCCAGAAATAGTGTTATGATG
AGTGGATTTTCAGATACAGTGAAATCATTTTGGCTCGGCCAACAATTCAAAGAAGAAGGC
GTTCAGGGAAATGATGTTATTCGAACAAACGTTCCAAATATTCGTATTGCATATAGATTT
GAAGCGTTAACTGCCGAACTTAATTTATTACTCAGAATGACTTTGAAAGGTCTTTCTAAT
CAAAATGAAGATTATTCCCAATAAaacttaacgtggtttggtggacagtgatatatagtg
aagaagcatttgtgaatggtagtttccatagataattaatctatgaatttttgtttatgt
aattcatcgcttaaatgcaatatatattttgatacttcattgaccgctctgactgtgtt
atagttgatttatattgcacttcatgtttgatttttattgggttattattgtgatatttt
gtattgatttctagttattttgttaattacagaagttaactattgaattaatgaattttt
tttaattatcacaaactatttgacatttttgatttatttaggttttcaaattaATGGAT
TTGACTCCATATGATCTGACTATAGACAAACTAGATGTGCATTGCGACCGTGACACATTT
CATAGATTTGATAAATTCAATGCCAAATATAATCCAGTTGGTCAAAGTGAATTGAGAGAA
GTATTTCTAAAAACAGATAATTATAATAAAGGCAGATTTTTTGCGCATGTTATTAATGAA
GTAATGTCGGATCTTGAAGAATCCAAATACCAAAATGCTGAATTACGTTTATCTGTGTAT

GGGCGGAAAATAAATGAATGGGATTCATTAGCAAAATGGGCTGTGGCAAATCGAGTTTAC
TCAGACAACGTTAAATGGATGATTCAATTTCCACGAATCTATGATATTTTCAGAACGAAA
AATTTAATTAAAAATTTCCAGGAAATTTTACACAATATTTTCACGCCTTTATTTGAAGCA
ACTCGGGATCCAAGCAGTCATCCTGAATTACACGCATTTTTGGATCATGTCAGTGGATTC
GATTCTGTCGACGATGAATCAAAGCCTGAATCTTGCGTAATTGATAAAAATACTCCGTTT
CCCGAAAATTGGGTTGGGGAAGAAAATCCCCCATATTCATACTATTTGTATTACATGTAT
GCAAATATGTCCGTATTAAATGATTTAAGAAGCCAGCAAGGATTAAATACGTTCACATTT
CGTCCTCACTGTGGTGAAGCTGGTCCTGTACAGCATCTTGTTGCTGGATTTCTTTTGGGT
TATAACATAAACCATGGGTTATTACTCAGAAAAGCTCCAGTTCTTCAATATTTGTATTAT
CTTTGTCAGATTGGTATTGCTATGTCGCCTTTGAGTAATAATTCTCTATTTCTTGATTAC
CAAAGAAATCCACTTTATGAATTTATGTGCCGTGGACTGCATGTTAGTCTGAGTACTGAC
GATCCATTACAGTTCCATTTTACAAAAGAGCCTTTAATGGAAGAATACAGTATTGCAGCA
CAGGTTTGGAAACTGACATCAACTGACATGTGCGAATTAGCCAGAAATAGTGTTATGATG
AGTGGATTTTCAGATACAGTGAAATCATTTTGGCTCGGCCAACAATTCAAAGAAGAAGGC
GTTCAGGGAAATGATGTTATTCGAACAAACGTTCCAAATATTCGTATTGCATATAGATTT
GAAGCGTTAACTGCCGAACTTAATTTATTACTCAGAATGACTTTGAAAGGTCTTTCTAAT
CAAAATGAAGATTATTCCCAATAAaacttaacgtggtttggtggacagtgatatatagtg
aagaagcatttgtgaatggtagtttccatagataattaatctatgaatttttgtttatgt
aattcatcgcttaaatgcaatatatatttttgatacttcattgaccgctctgactgtgtt
atagttgatttatattgcacttcatgtttgatttttattgggttattattgtgatatttt
gtattgatttctagttattttgttaattacagaagttaactattgaattaatgaattttt
tttaattatcacaaactatttgac
>DjScaffold1998:11410-17074 strand:+
ctgatcaactggattcggctcttgatttaATGAGGCGATTGCCTCCACAAAAAATTGATA
AGAGTCTACAAGATCTTATTGAATTAGTACCGGATTTGTGTGAAGATCTCCTTTCTTCTG
TCGATCAACCTTTGAAAATTTCAAAAGACACAAAGACAGGAAAAGATTATTTATTGGGTG
ATTATAACCGAGATGGGGATTCTTATAGGTCGCCTTGGTCAAATTCTTATGATCCACCAC
TTGAAGATGGCTCATTACCTAGTGATAGAGTCAGATCTTTAGAAATTGAAGCAAATAAAG
CTTTTGAAGTTTATCGGGAAATGTATTATGATGGTGGTGTTTCATCGGTGTATTTGTGGG
ATTTAGACTCTGGTTTTGCTGGAGCAGTTCTTATAAAAAAAGCTGGAGATATAAAAGCAG
CCACAGGATGTTGGGATTCAATTCATGTAATTGAAGTAGAAGAAAAGGGGAGTGGCAGAA
ATGCTCGCTACAAACTGACGAGTACCATTATGCTTTGGTTGGAAACACAAAATTCTGAAA
CAGGTTGCTTTAATCTTGGTGGAAGTTTAACCCGAATGACAGAGAAAGAGGTTCCATTAT
CAGATGCTAACAATCACATTGTTAATATCGGTAGATTGGTTGAGGATATGGAAAATTCTA
TCCGGAAGCAATTAAACGACGTGTACTTTGGCACGTCAAAAAATTTGGTTGATTCTCTCA
GGTCTGGTCTTTCAAAGAGTGTGGAGGATCAGCAGCGTAAACTTTGCCAAGACGTCAAAA
TAGCAATTCAAAATAAAAACCAAGGGAATGCCAGTTAAataaaataaccctacttattat
ctattatttgattgacggcaacaaatagatttcatataatattccaaaatttgttggcgg
cttctatataatttttttctctctaaatttttgatgctttgcatcttctatagcgcagc
tgtgaatggaattaaggtgtttttgtgtttttgttttacgcaatcacgtattctttgtgc
atagtctactatttaaatgcagtatctgattggtgcagtcggtaaatacacaatatttag
ttattgaaataaaagtgattgttttcaggaaaaggggagtggcagaaatgctcgctacaa
actgacgagtaccattATGCTTTGGTTGGAAACACAAAATTCTGAAACAGGTTGCTTTAA
TCTTGGTGGAAGTTTAACCCGAATGACAGAGAAAGAGGTTCCATTATCAGATGCTAACAA
TCACATTGTTAATATCGGTAGATTGGTTGAGGATATGGAAAATTCTATCCGGAAGCAATT
AAACGACGTGTACTTTGGCACGTCAAAAAATTTGGTTGATTCTCTCAGGTCTGGTCTTTC
AAAGAGTGTGGAGGATCAGCAGCGTAAACTTTGCCAAGACGTCAAAATAGCAATTCAAAA
TAAAAACCAAGGGAATGCCAGTTAAataaaataaccctacttattatctattatttgatt
gacggcaacaaatagatttcatataatattccaaaatttgttggcggcttctatataatt
tttttctctctaaatttttgatgctttgcatcttctatagcgcagctgtgaatggaatt
aaggtgtttttgtgtttttgttttacgcaatcacgtattctttgtgcatagtctactatt
taaatgcagtatctgattggtgcagtcggtaaatacacaatatttagttattgaaataaa
a
>DjScaffold1998:41194-41670 strand:+
ttccttattaattaATGAATAACACCGGAACAGGCAATTATAACTCTAGATTTTATCAAT
CAACGAGTCATTTAAGCAATAATAACATTTCCAGTCAATTGGTAACTGATGTCAGTAATA
ATTTGACTCATACTAATAATGTCGCTAACTATCAAACACACCAGTGGGGGCCAGTGAAAG

TGTCTGGTGATAATATGCAATTTTATGGACAACATAATATCGAATCTATTATGCAACAAA
CTAATAATCAACAAATATCTCCGTTTGCATACTACAGCGCTTTATTAGGCCTGGCCGAAA
CTTTTCGAACAATGAGTCCTCCTAACATTCGTCTGTGTGTACACTGCTTGAAATCTATAT
TACATTACAAGCTTCCTGGTAATTTGGAAGCTAGAACATATTTGCATATCGGAAAAATTC
TTTTGATTCACAGTAAAAGTGATGATCAAATAAAATTCTATTTGGAAAAAGCTCGAGCAC
TTGGAGCTCATCTTAAACCACCTGACGATGTTATTAAATTTGAAGCAGCTGATTTATTAT
CGGATTTATTTGAGAAGAAAGGGAAGCGAAGTGAATCCATATGCGTTTTAATGGATGCCT
TGCGATTATCTGAACATAATCCATATTGGCATTGTAGATTATTATTAAAATTATCTAGTG
CTTATGTAGCTGATAAAAATATTTTGGGTGCTTGTGAAGTTCTTGCAATGGGATCGGAAT
TTGCTCAACAGCATCAATCTGATTATACCAAAGGATTGTTTTTGTTGAGTAAATGCATGT
TATTACTATCAATTCGACGTCCAGATGTAAATAACAGCCTTACTGTTGCCACACATCTGA
TAGATAATCTTAATGGAACTCCATATCAAAAAGAAGCTTTGAGAATATTTCAATTAGTCA
TACAGATCAGTCAGATTTTAATTGGTGGAGGAGCTAAATCGGCTAAACCTCAATTAAAAC
AATTACATACTAGCATACAGCAATTTGCAGCAATGGAAGAAGATGTGAGTAGTGGAGGAA
GTGATATTGATCAATTTTTATGGATGCCTCGCGAACATGTTGTGGTATTAGTTTATTTAG
TTACTGTACAACAAGCCATGCAAAGTGGACTTATGGCTAGGGCACTTCGATATGCTGAAA
AGGCCTTGACACAAATCGAGAAATTAGCGCTAATCGATTCGAGTCCATTACTCGCGATTC
TACAATTTTCTGTGCTTGAACATAGTGCCATGTGTCGGCTTGTAACTGGAAGTAAATTGC
AAGCCATTTCAGAAATAGGTATAGTCGTTCGACTGTGTCATAATGCACCACGGGGTATGC
GATTGCTTTATCGCAGGGGTCCCCAGCTGCACGCACTTATTGGTTTATATGCAATGGCGA
TGAACAAAATGGATGCTGGTGAAAGTCAATTCAAATTGGCATTAAAATTACTTGATCAAC
AAACTAGAGCACCTCATCGATGCAATCAACCTACAAGATCAGATGCTGGTCAAACTTCAC
AAACTGATAGCGCCAATAGTACTTTAGCTCAAACCACATCTGCAAATGATGACCCATCTA
ATCCACATCAGTTAACAAGTCTGCATGTTTTCATAATGTTGAATCTTGCACTGGTGCATA
TTCGTCAAAATCGTGTTGCTGATTGTAAAAGCATGCTACATCGAATCAGTAGTGAAGCGT
CAACAGTCCTTAATCAATGTTATTGCTTACAAGCTGCAAATTTCTATGTCCACAGTTTTC
AGGCTTTTTGTGAGCGTCGCTATCAGGACAGTAAATCTTATCTTAGAGCAGTTCTTCGTA
TGACAAATCTAGAAGATCTTAATCGACTAACTGCAGCTGGGCTTATGACATTGGGGCAAA
TTTTCATGAATCTCAGCAACAGCGATGTAAATTCGCCTGTTTTAATAGAAAGTAAAGAAA
TCATCCAACCCGTCATTCCATTAGCCAATCGTATTCCTGATATTGGCATCCAATTGTGGG
CAACAGCACTACTGAAGGATATGTATCAATTGTACCAGGACACGGAAGAAGAAACCAAAT
GGTTCCAACAACATAAATTGTTTTCCCAAGCGGTTATCAATGATCACACTGCAGCGGGAA
AACATGCCGATCATCAATTAGTCACTTGGTTGGAAGGCCCGCTGCCGGAGATGTGCATGT
CTAGTGACGCGCCCACAAGATTGTTGCCATCATAAtttcaattattgattttgtatatta
tagaaaaagaaacgtgttaatttgtacagattttgtatttattttcattcatctgtgatc
atttcgtgtaatataaaatatatttttgcatgtctcttcgaATGTGTTTTCAGTTACTTG
ATCAACAAACTAGAGCACCTCATCGATGCAATCAACCTACAAGATCAGATGCTGGTCAAA
CTTCACAAACTGATAGCGCCAATAGTACTTTAGCTCAAACCACATCTGCAAATGATGACC
CATCTAATCCACATCAGTTAACAAGTCTGCATGTTTTCATAATGTTGAATCTTGCACTGG
TGCATATTCGTCAAAATCGTGTTGCTGATTGTAAAAGCATGCTACATCGAATCAGTAGTG
AAGCGTCAACAGTCCTTAATCAATGTTATTGCTTACAAGCTGCAAATTTCTATGTCCACA
GTTTTCAGGCTTTTTGTGAGCGTCGCTATCAGGACAGTAAATCTTATCTTAGAGCAGTTC
TTCGTATGACAAATCTAGAAGATCTTAATCGACTAACTGCAGCTGGGCTTATGACATTGG
GGCAAATTTTCATGAATCTCAGCAACAGCGATGTAAATTCGCCTGTTTTAATAGAAAGTA
AAGAAATCATCCAACCCGTCATTCCATTAGCCAATCGTATTCCTGATATTGGCATCCAAT
TGTGGGCAACAGCACTACTGAAGGATATGTATCAATTGTACCAGGACACGGAAGAAGAAA
CCAAATGGTTCCAACAACATAAATTGTTTTCCCAAGCGGTTATCAATGATCACACTGCAG
CGGGAAAACATGCCGATCATCAATTAGTCACTTGGTTGGAAGGCCCGCTGCCGGAGATGT
GCATGTCTAGTGACGCGCCCACAAGATTGTTGCCATCATAAtttcaattattgattttgt
atattatagaaaaagaaacgtgttaatttgtacagattttgtatttattttcattcatct
gtgatcatttcgtgtaatataaaatatatttttgcatgtctcttcgttgattaggaaagt
aaagaaatcatccaacccgtcattccattagccaatcgtattcctgatattggcatccaa
ttgtgggcaacagcactactgaaggatATGTATCAATTGTACCAGGACACGGAAGAAGAA
ACCAAATGGTTCCAACAACATAAATTGTTTTCCCAAGCGGTTATCAATGATCACACTGCA
GCGGGAAAACATGCCGATCATCAATTAGTCACTTGGTTGGAAGGCCCGCTGCCGGAGATG
TGCATGTCTAGTGACGCGCCCACAAGATTGTTGCCATCATAAtttcaattattgattttg
tatattatagaaaaagaaacgtgttaatttgtacagattttgtatttattttcattcatc

tgtgatcatttcgtgtaatataaaatatattttgcatgtctcttcg
>DjScaffold1998:65453-66664 strand:+
attagtttataatacaaaaaaaaatattgaaatacaATGACTCGTCCATTGATTAATGTT
TATAAAGTTAATTTACCTACTGATGATTCAAAACCAAAGCTTGAATGCAGTATTGAAGGA
TCAGTTAAACTTCCATCAGTCTTCAATGCACCTTTAAGAAATGATATTTTGACAACTGTT
CATGATTTAATAAGAAAAAACAGCCGTCAACCATATGCTGTCAGTACTGGTGCTGGTCAT
AACAACTCTGCAGAATCATGGGGTACAGGTAGAGCTGTTGCTCGTATCCCAAGAGTTCGT
GGAGGAGGAACCCACAGATCAGGTCAGGGTGCTTTTGGAAATATGTGTAGAGGTGGTCAT
ATGTTTGCTCCAACTAAAGTATGGAGAAAATGGCATCATCGTGTTCCTATTCAACAAAGA
AGATATGCAATGGTAACTGCTATTGCTGCAACTGGATTACAACCACTTGTTAGTGCTAGA
GGACATAGAATTTCAAAAGTACCAGAGTTGCCATTAGTTATCAGTGAGGAATATGAAGGT
TTGAAAAAAACCAAAATGGCAGTTAAACTTTTGAAAGGATGTGGTGTTTGGGAAGACATA
GAAAAAGTTAAAGACTCAAGAAAAGTTAGAGCTGGAAGAGGAAAAATGCGCAACCGAAGA
TATGTTCAAAAAAGAGGCCCAATTGTTATTTACAAATCAGATAATGGAATTACCAGAGCT
TTCAGAAACATTCCAGGAGTTACTCTTATCAATGTCAAGAGCTTGAATATTTTGAAATTA
TCTCCTGGAGGTCACATGGGTCGTTTGGCCATTTGGACCAAACCAGCAATTGAAGAACTC
GAAAAAATATACGGTTCATTGAAAAGACCAAGTGAAACAAAAGTTGACTTTGTTTTACCA
AAAGCTAAAATGACAAATGCTGATTTGATGAGACTTTTGAAGTCTGATGAAATCCAATCT
GTCTTGAAACCAAGAAACATGAAAGCTCAAAAGAGCTCAAGAAAAGTCAAGAAAAACCCA
TTGAAGAATGATGGAGTTATGGCAAAATTGAATCCAAATGCTGCTGGCGAGAAAAAGATT
AATAAACGTAGATCAAGTGGTATTGACGCTGTTGTAGCAAAAAAAATCAAAGCTTAAttt
tggaaaaaaataaattttggatttaat
>DjScaffold1897:4789-9071 strand:+
tttcatcattattaagtttttaacattttaataattttaaatcaatttaaaaatagtaaa
tacttATGGAAAACGATAATCTAATTTTTGATGAAGAAGAAAGTGACGAATTAAAAATTA
CCCCGCTTGGAGCTGGTCAAGAAGTCGGTCGTTCATGTGTTTTATTAAAATTTAAAGGGA
AAAAAATTATCTTGGACTGTGGTATTCATCCTGGATTAAGGGGTAGCGAATGCTTACCGT
TTTTAGATATTATAGATGATATTGAAGAAACTGATTTGATTCTAATTAGCCACTTCCATC
TTGATCATTGTGGAGCGCTACCTCATTTTTTATTTAAAACCGGATTAAAGTCTCGTTGTT
ATATGACACACGCTACCAAAGCGATTTATAGATATATTTTAGCCGATTATATTCGTGTAA
GTAACAATGGATCAAGTGCACATTCTCTTTATAATGAACGAGATATTGTCAATAGCTTGG
ATAAAATCGATACTATAGATTTTCATCAACAAATTGAAATCAACGGTATAAAATTCACTG
CTTATCACGCAGGTCACGTTTTGGGAGCTGCGATGTTCATGATAGAAATTGCCGGCGTCA
AGATTTTATACACCGGAGATTTCAGTCGACAAGAAGATCGACATCTTATGAGTGCCGAGA
TTCCTCCTGCCAGGCCAGAAGTTCTCATCACCGAAGCCACTTATGGCACTCATATCCATG
ATAAACGCGAAGAAAGGGAAGCTCGTTTTACCACGTTAGTGCATAACATAGTCAGTCGAG
GAGGCCGCTGCTTGATTCCGGCATTCGCTCTCGGTCGCGCTCAAGAACTAATGTTGATTC
TTGACGAATACTGGTCGCAGCACTCTGAATTGCAATCAATCCCGATATATTATGCCAGCC
AACTTGCTCGCAAATGCATCAGCGTTTACCAAACCTACATTCACGCTATGAACGATCGCG
TTCGTCAACAACAAGCAGCCAATCAAAATCCATTTGTATTTAAACACATCAGCAATTTGC
GTGCAATCGATGATCTCGACGATAATCGTCCTTGTGTAGTCATGGCGTCTCCGGGAATGA
TGCAATCCGGGCTCAGTAGGGAGTTGTTTGAGAACTGGTGCTATGATGCTCGCAATGGAG
TTATTATTGCCGGGTACTGCGTGGAGGGAACATTGGCCAAACAGATATTATCCGCCCCAG
CTGAGGTGCCAACAATGAATGGACAGTCGCTTCCCCTCAAATGCTCAGTTGACTACATCA
GTTTCTCTGCGCACACTGATTACCAGCAGACGAGCGAGTTTATTCGGGAACTGAAACCAA
ACCACGTGATGTTGGTCCATGGTGAGAAAAATGAAATGTTGAAGCTGAAAGGAGCTCTGC
AGCGCGAGTATGAGGACGATGAAACCTACAGAATCGATATTAGTACTCCTTGCAATACTG
AAACAGTTCAATTCAAATTCCGCGGAGAGAAAATGGCCAAGATTGTTGGTCAATTGGCAG
GCCAAAAGCCAGTAAATGGTCAACGTGTTCAAGGAATCTTGGTGAAGAAGAATTTCTCCT
ATCACATCGTGACTCCCAACGATCTCCCGGTCTATACAGATTTATCCACCAGCGATGTTC
GCCAGAAATTATCTATGACATTCAAGAACCCTCCATCTCTTCTCCAGTTCCATTTGACTC
TTGTTGCCGGCAATATTGAAGTTCTCCTGTTGCGTGGAGACAAGCAAATCTTCCGTGTGT
TCGGTGTTATAGATGTCACATGGGAACCGAGAATCGTTGTTATAGAATGGAAAACTAATC
CAATCAACGACATGTTTGCCGACGCCGTAACTTGCATTGCAATGAAGGCAGAAATGGAAG
GATTGCCGACTCAA
>DjScaffold1662:46420-49637 strand:+
gattcagaaactgaaattggtcttgtggaaataaatgaacaaATGAAGGATCAAGAAAAG

AAAGACAAAGAACTAGAACAGAAAAGGAAAATGCTTACCGATATGGAAACTCACGCTGTA
AAACTGAAAGAACAATATCCTGATCGAGAAGAAGAAATTGAACAAATGCAACAAGAAGTT
CGAGTTAAATTAACTCAAGTTGAAGCTCCATTAGCAGGACGCCGTGAAAACCTCATGAAA
CGTAAACGTGTAAGGCAATTCTTGAGAGATATTGAAGATGAAAAACAATGGATTGAAGAA
AAAATTAATCTGGTTCAAAATAACGGCAAAATTGGAGATTCTTTGTTAGCTGTTCAACAG
TTTATTCGTCGGTTAAAAACTTTAAATAATGAAGTTGACAATCATCGTCCTCGAATTGAA
ACCGTTTGTGAAGACGGAGAAATAATGATTTCTGAAGGTCATCCACAAAGTGATAAATTT
AAAATCGCTATGAAAGAACTTTGGGAACTTTGGGATGAGTTACAAAGGAAATTATTTGAA
CGCTTTGCAGTTTTAGAAGATAATCTGGTAGCTCAGCAATATTTGTTTGATGCCAGTGAA
GCTGAAGCCTGGATGGGAGAACAGGAATTGTATTTAATGAGTCCAGAGAAAGCAAAGGAT
GAGCAAGGAGCAAATAATTTGAATAAAAAACATCAACACATTGAAAAAATGATTGAGAAT
TATGCTGATGAAATTAGAAATCTTGGGGAACGGAGCCAAGAAATGATGCATTCATTCCAT
CCGCAAAGCGATGGAGTGGCACAGAAACAGTCGAAAGTTGATAAAATGTACGCCGGTTTA
AGAGATCTGTGTTCAGAGCGACAATTTAAATTGGAAGAAAACGTTAAATTGTTTCACgag
actgctaatcatttgctaccgttatatgctggtcaaaaagaaagaATGATATGTGATCGT
CGAGATGAATTACTACATGCCTGGCGAGAATTACAAATGGCTTCAGAATTTCGCAAAGTG
CATTTATTGGATGCAGCCGATCTTCACCGATTTTTGGCAATGATTAGAGAGTTAATCAAT
TGGATGCACGAAATTCGTCAACAAATGGACACGAAAGATAAACCAAAAGATGTTGCTGGT
GTGGACCTTCTTGTAAACAATCATCGCAGTTTGAAAGCAGAAATTGATGCTAGAGAAGAG
AATTTTAGCATTTGCCTTAGTCTTGGAAGAACTCTTTTAAATCGTCGTCATCCTCGAGAA
AATGATATAAGGGAAAAATGTATCGAAGTTGTAACTACAAGGATTAATTTAATTGATCAA
TGGACTGAAAGATATGATTATTTGCATTTAATCTTGGAGGTTTATCAATTTGCTCGAGAC
GCAGCAGTTGCCGAGTCATGGTTGATGGCTCAATGTACTTATTTAAGCTGTCAAGATCTC
GGAGAAAATCTCGATGAAACTCTCGCTTTATTGAAGAAACATATTGCCTTTGAAAGAGCT
GTTCTGACACAAGAAGAAAGATTTGAAGCATTGAAAAAATTGACAACATTAGAAATGCGA
GCAAAGGAGGCCTCTCCAGAATTCCAAAAACAAAAGcaaataatttgaataaaaaacatc
aacacattgaaaaaATGATTGAGAATTATGCTGATGAAATTAGAAATCTTGGGGAACGGA
GCCAAGAAATGATGCATTCATTCCATCCGCAAAGCGATGGAGTGGCACAGAAACAGTCGA
AAGTTGATAAAATGTACGCCGGTTTAAGAGATCTGTGTTCAGAGCGACAATTTAAATTGG
AAGAAAACGTTAAATTGTTTCACTTGTTAAAGGAAATAGTAGATCTCGAAACCTGGATTG
CCGAAAGAATTGTATTGGCTAGCAGTCATGAGATGGGATCAGATTATGAACATTGTTGCT
TACTAAGAGATCGCTTTGCTGAATTTGCAAAAGAAACTAATATGATTGGAGGAGGAAGAA
TATGTGCTGCTAATGAATTATGTGACTCTTTGATAAATCAAGGACATGCGGAGTCAGCAG
AAATCGCTCAATGGAAAGATAGAATTAATGAATCTTGGGCTGATTTATTAGAATTGATAG
AAACCAGAATTCAACTCTTGAAAGCTGCTTGGGATTTACATAAATTCTTAAGTGATTGTC
AAGATTTGCTTGATAGAATTAGTGAAAAGAAATTAGTTATACCAGAAGAAGTTGGTCGAG
ATGCTAAGTCCGTAGCGACTTATCAGAGAAAACATCAAGCATTTGAACAAGAAGTGCATA
GATTAGGTCAACAAATTGAAACCGCAGTAGAGACTGCTAATCATTTGCTACCGTTATATG
CTGGTCAAAAAGAAAGA
>DjScaffold3742:44204-44652 strand:+
aaagaaatgaaaatgacatttgtaatattgaagcgaaattcggtagacaaatttaaaaag
gagagcgattgtcaagagctttaaatattttgaagattaaagaaaatcaacggttattga
aaataattgaaagtaaagaagatgctttcATGGCAGATACATGTGATGTAAGACGTACAC
ATCTGACAAGACCCAAAATAATCACAAGTGGTGAACCAATCAATCTAAAACCGTATAGGC
AACCAATGAACTTAGAATCAAAAATTGAAGAAGCAATCCAAAACCTTTTCGAGAATAATT
TTATCCGGAAATGCAACTTTCCATGGAATACCCCATTGATTTACAATTGAatgcagtaac
gaaaagacaagtcttttcgatgcctaatgtcgatgaaatg
>DjScaffold3742:49860-50597 strand:+
ATGCCATTTGGCATTGCAACGGCACCAGGGACATTTCAGGAGATGATGAAAAAAGTAGTT
GGCGGAATCAGTGGAGCCATGGTTTATCTTCATGACATCATGATGCTTACGAACAATAAA
CATCAACACTATGAAGTCATTGAAAAAGTATTGATGGAAATAATGAAGGCTTGTCTAAGA
ATTAATCCAAGGAGTTGTGAGTTTCTTCAAAGCGAAATCAAGTTTCTTGGACACATAATT
AGCAGGGATGGTGTTAGAACTGATCCATCTAAAATAGAAGCAATAAGACCATTTGGCAAA
CCCAACTGTATGAAGAAGTTACTAAGTTTCTTGGGAATTTGTAATTATTACAGGAAGTTT
ATAGAAAACTACACAAGAAAGTCAAAGATATTAGAGCAGTTGTGTAGCGGACCAAGTAAC
AAGAAATTAGAATGGACACATGAAATAAACGATACTTTTGAGGGTCTGAAAAAATCTCTA
ATGACTGCACCAATATTGTGTTTTTCAGACATAAACAAAGAATTTACATTAGATACAGAT

GCCGGTTTCGATACAATTGGAGAAGTATTGTCACAGACAGATAAAGAAGGAAGAGTTGTT
GCCAATGGATCACACTCAATGAAAAGGGATACTGCATTACACGAAAGGAGTTATTGGCTG
TTTATTATTTCTGTATGCATTTCAAACATTACTTGTACGGTAGAAGATTTACTCTTAGAA
CAGATCATAAAGCTCTAA
>DjScaffold3742:394-828 strand:+
ATGCCTTTTAGAATTGTAGCAGCACCAAGTACATTCCAGGAAATAGTGAATAAAGTAATA
GGGAACATGAAAAGATCGATGGTTTTTCTAGACGATATTCTCATATTTACCGAAACAAGG
AAGAAACACTATAAAATAGTCAATAAAGTATTGACAGAAATTAAGAATGTTGGTCTTAGA
GTTAATCCAAGAAATTGTAAATTCATGCAAAAGGAAATCAAATTTTTGGGACACCTTAAC
AGTAAAGAAGGTGTCAAAACAGATCCAACTAAAATAGAAGCAATTAAGAACTTTGGTAAA
CCAAAATGCGTTAAGAAAATGAGAAGCTTTTTGGGAATTTGCAATTGCTATCGCAAGTTT
ATTAAAGATTATTCGAAAAAAACTATATTGCTGGAACAACTGTGCAGTGGGCCGAGTAAC
CAGAAGATCAAATGA
>DjScaffold3742:44788-45018 strand:+
ATGCCCTTTGGCATCGCAGCGGCACCACGTACATTTCAAGAGATGATGACAAAAGTGGTA
GGCGGAATCAATGGAGCCATGGTTTATCTTGATGATATTCTGATATTTAAGAAAAATAAA
GATCAACACTATGAATTAATTGACCGAGTATTGATGGAAAAGGCTGGTTTAAGAATAAAT
CAGAAAAGTGTCAGTTTCTACAAAACGAAATCAAATTTCTTGAACACATAA
>DjScaffold4539:17581-21605 strand:+
aagttcagttcataatttactgaagaaaaatgaaagaaaccttttaacatttcaatttta
agaagtgttttaataaattaagactgttgaatttttatcagtgatttttaagaaaattca
taatttgctaatcgctgtcatatatctataataatttaattttgacttgaaatatttttc
atgttaatgattcacaaatgaaaaatgaaatattaattttgatacaatctagaaattttt
ttatatctcattttgcagtgatcttcaaggaattaaaactaaagctaaaaaagatgggga
tgactggattttatctggaagcaaaacgtttattactaatggatgtttatctgatttagt
aatagttgtatcattaactgatccagatgcaaaatctgccgcacatggaatcagtttatt
ccttgtcgaagctggcactcctggttttgtaaaaggtagcaagttgaaaaaaATGGGTTT
GAAAGCTCAAGATACGTCTGAATTATTTTTTGAAGATGTCAGATTACCAAAATCACAATT
ACTTGGCGAGGTTAATAAAGGATTTGTGTATTTAATGAAAGAATTGCCTCGGGAACGCCT
GATTATTGCTGATAGGTGTCAAGCTTTGATGGAATTTGTTTTTGAAGAGACACGTGCTCA
TGTACGTCAAAGAAAAGCTTTCAAGAGCACATTAAGTAATTTACAGACCGTTCAACATAA
ACTTGCCGAAATGAAAACTTCTATTTGTTCTTCTCGGTCATTTGTTGATGATTGTTTATT
ATTGCTAAACGAAAAAAAATTGGATACCCAAACTGCATCGATGGCTAAATATCACACAAG
TGAATTACTTAACAGAATTTGTTACGATGGAGTTCAGTTACATGGAGGTTGGGGGTATAT
GTGGGACTATCCAATTTGTAAGTCCTATGTTGATGCTAGAGTTCAATCAATATATGGTGG
CGCCAATGAAATAATGCAAGAATTAATCGCCAGATCTATTGTGAAGGATTAAtttactca
gtttatgtttaatttaaaataaattgtttttaatttatttttaaaattatttaacatcc
aataaattagttaatttttataa
>DjScaffold1015:18991-28973 strand:+
tacgactaattttaatatctaATGAAATCAATGATTGAATTATCGTCGAATAACAAAATC
AATGATTCCAATTCTTCATTTAAATTATATGCTGTATTGAATCCAATTAAATATGATAAT
AAATTTCTACCAAAAAAGAAAACTATTAAAATTCAAAAAATTGATGATTATGCATATTTA
ACGAGACAGCTTTTCCATGCTGTTCAACATGGCTATTTAAATTTTCTTGAATCAGTTCCA
CAGAAAAATTGGCAAAATCGATTGAACGTTTTGGATGATAAAAATGGCTATTCCCTACTT
CATTATGCATGTTTAAAGAATCAAAAGGATATTGTTTCGTTTCTATTATTGTTTAAACCA
GATGTCAATATTATCGGAGCAAAGCAAATACGTCCAATTCATTTAGCTGTACAACAGGGA
CATGATGAAATTGTTGAGTTATTATGCCGACAAAAATCAATCCAAATTGATGCTGCTGAT
GTAAAAGGACAAACCCCTTTGCACACAGCTTGTAGAAGAGGACATACAAAATCTGTTAAT
ATTTTGCTAAAACATGGAGCTTCTGTACATAAATTGGAGTTGGATAAAATTTCTATTATA
CATCATCCGGTAAGACGAAACGATATTGAAATGATTAAAATTTTGATTGCTTATGAAGCA
CCGTTAAATATTAGAAATAATAATGGTCAAACGCCATCTATGATCGCGGCTATTGAAGGA
CATTTTGAATTATTTGATTATTTGATGAAGGAAATGAAAAAGCAAGGTGAATACCTGCCT
AATATTCTAAATGAAATAGACAATGATGGATTATCTTGCTTGTCTCATGCAGTTCAGAAC
AAAAATGCAGAAATTATCAAGTTATGCATAGAAAATGGTGTAGACTTGAATGAAATACAA
ACAGATGGTAAAACAATATTGCACACAGCAGCAATAATAGGTTATGTTGATCTATTCAAT
ATTATTGGTGAAAAGTCCTGTAATATAAATGCAACAGATTACGGATTAAACACTCCATTA
CATCATGCTGTTATGAATAATAATATTGCTATGGTGAAAATACTGATAGAAAAAGGAGCT

AATTTAAATGTTTGTAATAGAAATTTTCAGACTCCGATAATGCTTGCTTGTATCGAAATT
GCTCAATCAAAAAATTCCGCAGAAATTATACAAGTTTTAAATGAATCCAATGCAGATTTG
AAAATAAAAGATTTTGAAATGAAAACTATTTTTCACTATCTTGTAGAAAAAGAAAATATA
GATATGATACAATATTTTACAAAAAACTTAAGACCAGATATTTTATCAGAATTGATCAAT
TCTCAAGATGATTCTCACAACAGCCCATTGCATTGTGCAGCAAAAAATGGAAATTCACAG
ATTTGCAAATTGTTTGTGGAAATTGGGGCAAATCCATTATTATTCTCTGAGAATGAATCG
TTGGCTCTTCATGTAGCTACAATTCATGGTCATTTGGAATGTGTGAAAATATTGCTGGAA
GCAAGTAAAGAAACTACTTATTACCGAGATGGCGACGAAAACTCTGCAATTAACTTAGCG
GCAGAAAATGGCAGATATGAAATATATAACCACCTTGTTGAAATTGGCAAAGATAAAAAT
TCCAAAGATGAATATGGGCGAAATACTTTAATGTATGCAGCAAAAAGTGGCTGCTTACCA
ATTTTAATGGATTTATTAAATGATGAGGTGAACGTTAATTTATGTGATCAAACGAAAACA
ACAGCGCTTCACCAAGCAGCAGAAAGTGGTCATTTGGAAATAGTAAATGTTCTGTGGAAA
CACGGAGCGAAATTATCTGAGGATATATTTGGTAACAGCCAAGTGGATGTTGCTATTATT
CATAATCAATCACATATTGTCAGATTCTTTTTAAACAATGAAAATTGGAAGAAAGTTATG
AAATTTCGACATAAAAATGGGCATAGTCATATTAAAATTCTAATCAAACATGATGAAGAA
ATCGCCTTAGAATTACTTAACAAATGTATCACTGAAAATATTTCTAATAGAATGGATCCT
AATTATGAGGTAAAATATTGGTTTGGTATTTTGGATTCTGGACCAGACGACACCGCAAGC
ATCAACAAAAGATTTCTAGCCCTGAATAGTATGTCCAAACATGGGAGACTAGAGCTTCTA
AAACATCCTCTTTCTGTAAAACTTCTTGAATTCAAATGGAAGAAATTTGGTTTAGGATTT
TATCTAATAGATTTTATTGTTTATTTGCTGTTCCTCATTTGCATCAATGTATACATGTCA
ATTTATAGTCCTTTGACTGATACAGATTCCTATACTAGTAGTACTGGCTGTAAAACAACA
AATAGCAGTGTTGTAAGTGTGTTTTTTAAAATTTAAaaataaatattcaaatttattaat
ataaatattaaaataattttataagtatgaaattagtaggcatgttgaattccgtttatt
aagctaaacaatttttttacggtacagctatacatttaattaacggttaaagaattaaatg
ttaaataaaattactgttaactgtttctagattttaaatatataaattatataaattata
taaattatatataaattataaattatcttttacgatatgttgatttaagttttagattct
taattaaaatgtatttaaaaaaatattcataacaaataaagagctgtcatcaatataaat
tatgattgtcaggattaattaacatttagtttacgactaattttaatatctaATGAAATC
AATGATTGAATTATCGTCGAATAACAAAATCAATGATTCCAATTCTTCATTTAAATTATA
TGCTGTATTGAATCCAATTAAATATGATAATAAATTTCTACCAAAAAAGAAAACTATTAA
AATTCAAAAAATTGATGATTATGCATATTTAACGAGACAGCTTTTCCATGCTGTTCAACA
TGGCTATTTAAATTTTCTTGAATCAGTTCCACAGAAAAATTGGCAAAATCGATTGAACGT
TTTGGATGATAAAAATGGCTATTCCCTACTTCATTATGCATGTTTAAAGAATCAAAAGGA
TATTGTTTCGTTTCTATTATTGTTTAAACCAGATGTCAATATTATCGGAGCAAAGCAAAT
ACGTCCAATTCATTTAGCTGTACAACAGGGACATGATGAAATTGTTGAGTTATTATGCCG
ACAAAAATCAATCCAAATTGATGCTGCTGATGTAAAAGGACAAACCCCTTTGCACACAGC
TTGTAGAAGAGGACATACAAAATCTGTTAATATTTTGCTAAAACATGGAGCTTCTGTACA
TAAATTGGAGTTGGATAAAATTTCTATTATACATCATCCGGTAAGACGAAACGATATTGA
AATGATTAAAATTTTGATTGCTTATGAAGCACCGTTAAATATTAGAAATAATAATGGTCA
AACGCCATCTATGATCGCGGCTATTGAAGGACATTTTGAATTATTTGATTATTTGATGAA
GGAAATGAAAAAGCAAGGTGAATACCTGCCTAATATTCTAAATGAAATAGACAATGATGG
ATTATCTTGCTTGTCTCATGCAGTTCAGAACAAAAATGCAGAAATTATCAAGTTATGCAT
AGAAAATGGTGTAGACTTGAATGAAATACAAGTAAGACTAGCTTCTAAAAAAAAC
>DjScaffold1015:53225-60747 strand:+
aatgtttcagaatatATGTATAGAAATTTCATTCAAAAATATTGCAAATTTAAAATACGT
AAAAGATTTTATTGTAAATCTGTTACTGAACAGCAAGCACCTAATGAAGAAATAATAGAT
TACGAAAAGAAAATTTTGAGGCCATTTACAAGGATATGTTAATAAATTATTTTAATATG
GATTTGTTTGTTTTCCCAGAAATAGATAATAATGCCGATTTGGATCGAGTGAATAATTTA
TGTAACAAATTGGAGAATTATTTGCAAGTTTTAAACGAAACATTGGATGATGATAAAAAG
AAAAGCATTATTGAACAGTTATCACATAGTGGGATATTTGGTCTATGTTTACCAGAGCTG
TATGGAGGTCATCGTTTTAATAATATGATGTTGGCTAAGGTTGTGGAGGTGATGGCTAAT
GATATGAGGTATCTATTCAATACCTTCATACCGCGCAATTTAGGAAGTCAGGTGGAACTT
TAAattaatattttattgttgacaaataagttatattatacacatgtgaaacttagatat
tttgaggctatataggattatttttatatatagaattaattaagatttatttttactaaa
tcaaataatttaattattaattaaccaat
>DjScaffold1991:41394-48276 strand:+
ccatgtaaaaattgaaatattatttttagaagaatcgacaggtgtcacaacatgaccact

taatataacgattccatgttatttagatattttgaataatttaaattatattttgatttc
taattaatggaaacaataaaaaatattttaaagtatgattttctattcagcgtactcttt
ttaattcaaattccacaATGATGAATAATAATGAAGGTGTAATATCTATGGGTGATTTAT
ACATGATCGCAGAGAATTATAATCCCATAGAGAAATTGACATTACCAGTTAATAATGGAA
TGTTGTGTAATCCGTTCATGCTTCCCAATAATCAGATTACTACAGAAGTTGAGTTTAATT
TAACTGAAGAATCTCTTAAAGAAATAGTTAATAGAGAAGATCTGGATATTGTTCTATTAT
CTCGATTAACTAAAGAGACCTCATTTAATCTTATAATATGGCCTTTATCGATTTCTATTT
CGATTAATAACTCCCCAGTAGTGTTGGACACGAAAATGTGCAACAAAATTCCTGTTCATC
TACCTCAATATATAAAAAAAGAATGTGTTATTGGAAGAAATGTTGTTAAATTTGATGTTA
TAGATATTTCAAAATTCAATGCGACAATGTTTCAATTTCAAATTTACATGGTTCACAGAC
CGACGATTGAACATGCAAAAAAACTTGCAATCTCACATGCTTTTGAATCAGAAGAATTAA
CATTTAAATTACTGCATGATATTTGTATGAATCTTAACAGAGAATTTGTCAATGTTTCTA
CTATTTGTCCGATTACCGGTAAAAGAATCGAAATTGCCGCTAGATTAAAATGTTGTACGC
ATTTACAACCTTTTGACTTAATTGCTTTCCTCAAAATTCAAGCCCGGTGGATGCTAACCT
CATGCCCACTTTGCTATAACTCCATGCCATTTAATTTTAATGGTTTGCAGCAGAATATTG
TTATTGATAAAATACTCCAGCAAATATTTGATCGAACGCCTACTCAGTATCAGTTGGTTC
AAGTTCATATGAATGGACAGTTTACTCCCGTAATTATGCATTCTGGAATGGAATTCAACG
CGGAAATGCAAAATGGCAACAATATTATGATGTCAAATCGTCATAATTCGAAACATTTTG
CAGAACTCAATAGCAACACAATTATGCATGCATCTCAACCGATGCCACCTGTTTATAGAC
CTCCAGATCAAACTTTAAATTTCCAAATGGTTAATGGAAATCCTCATCGAGTAATCAATA
CTCCATTTAACAATGTGTCAATTGATTCAAGATCTATGCTTGATCTACATCCCTCAGATG
ATCAATCCATCCTCATGTCTATGCAACCCAATATGAAATATCCACATCAAACTCTTCCAT
GCGTTGCAGACAAGCCTCTAACTAAAAAACCAGTCAAACGTAAAATACCCAGAAAAGCTA
CTCCAAAAACAAAAATCGATGTAAATCCAAATCTAATTAACAATGTTAACAACATGTCGA
ATGGTGCTATAACACCTCCCAGTACTAGAACTTCGTCAGTTGCTAGTACTAGCTCACCAG
ATTCTCTTAATGTTCCTCATTTAAATCATAAAAGACCGCCAAGCATTGATCTTTTGCCGA
GTACAGTTCTATCAGATTGTCATTTGTCTATAGGCGATGATCCTAATCTATTTTCAACAA
CTCCAATATCTCAAATGAATAACCTGTCTTTCAGTCATCCGACGCAAACACAACAGTTTC
AACATCAACAATTTCAACAATTCCAGCAGCAACAGCAAATTCAACAACAGCAAATTCAAC
AACAGCAAATCCAACAGCAACAACAAATGCAACAACAACAAATTCAACAACAACAAATCC
AACAGCAACAAATCCAACAACAGCAAATTCAGCAACAACAACAACTTCAACATCAACTCC
AACAACAGCAACTTCAGCAACAACATTATCATCATCTTTATCAACTTCCTCCTGAACAGA
AGCCTCTTCCAAATTGGCCTATTCCTGAACAAATATCTATGCAACAACGAAATTTACAAC
AGCAACAACAGCCACAGCATCATGCGCAAATACATCAGCCAATTCAACACATACCACATC
CACAAATTCCTCATATTCAGCAAGCTAAACTGCAACAATCTAAAGTTCAGGAATTCAACC
AGCAGCAGATTTTAACTCAACAACCATCTCAACCACAGCAACTTCATCATCTTCCACAAC
AACAAATACAGCATAAATCTCAGCAGTCAATACAAGATCAACAACAGCAGTTTAATGGAA
ATTTACCGTCACATCCACTATACCAAAATCAAGGTTCCTATCCAAATTCTGTTAAACAAG
AATCTATGCATTGTGTTCCTTCAAATAATCCAGAATCTTTAAATTATCCTCTCTCTGTCA
AACAAGATATTTTAAACTATTCTCCGTCTCTAAAA
>DjScaffold4008:43811-45555 strand:+
ttaaattcaatgggatgttgaaagaaaatttaaaaaATGAAATATACAGATTGTAATTTC
ACCTCAATTTTCAGTGCGAACCCTATCAATCTTCTTTGGTGTTTATTATCACTCATGGTA
GGCGGTATGGCTACATTTGCATTTCTTCAACCGTTTTGGTTAATTGAAGCCCAAAGTTCA
CTTTGTTTGGGAATTTATTCCCAATGTTTCAAATCTACTGAACCTCACCATGACAGCCAA
ATTTGTGGATTTTACCGTGGTAGATTTTCGTTAAAAAATTTACCAAGCCTTGAATGGAAA
ATCACTTGCATTTTGTTTGGAGGAGGTTGTTTACTACATTTAATCTGTGCGGTTTTTGGA
ATAAGCACATTATGTATTGGTAAAACCACCAGACGTAGATTGGCTTGCTTTTTGGGATTC
ATTCAAGCAATTGCAGTTATATTCTCAGTTGTTGGACTGCTTTTATTTCCAATGGGATTC
AATTCAACAATCGTTCAGACACATTGTTCTAGTGAATCGAAACGATTCAACGCAGGAAAA
TGTGAAGCTGGCTGGGGTTTATTATTAGCAACAATGGGGACCGCATTATCAATATTCTGT
CCGATTTTAGCACAATTTTTCGATGTACGAGTTTGTGATAAACCTTATTATAATAGTCAA
TCTATTTACAAACTTGAATTGAACAGTTTAGTGAAACATTTAGAGATTTTAGAGGAAAGA
GATCTTGAAATGTCAACAGATATGAATATGAATGAACAATTTCAAAAACCGATATTTACA
ACTAAATCATCAAACAGCAATATTAATTACAATAATAATTTCCCATTTTCCACGTATAGT
AGTTCAACTGAGCATTCTATTCCTTCATTTGCCTGAattgtcagtttctttgtacaattt
cgtttatctgtgatccatttaattgaaatttttcttaaaactacttatttttgtttgt

ttttattttttctctgtacagaatttcttttttttatttgtataaatttaacaaaa
>DjScaffold1750:11778-26004 strand:+
TCACAAAAAGAAGAAATTATCAAATTCGCAAATGTAGAAAATGAAGAAATACGATTTTCC
AAGCACAATGATATTAATCAATTGAAAATTGAAATGACAAACTTAATAAAAGAAAATAAA
AAAATAGAAGACTTACTACAGAAAGAAAAGAAACTGAATATTGATATGACATCGGAAATG
GATTCAATTGTTCAGAAACACGATAATATTTCTTTTATTAATAATGAATTGTTGAAGAAA
ATCAGTTCTTATGAAGTTGAAATATCTGCTCTAAAGAAACGACTCTCGGAAGCTGATGAA
AATCAAGAAATAAACAATAAAGATAGTAAAAAGCAAATATCCTCTCTACAAGAAGAAGTC
GTCAAATTGACTTTACAGTTGTCGTCAATTCAACAATACTCACAAGAAACTAAAACAAAG
ATGGAAACAACATTTAATCAAAAATGTTTACTTTATGAGCAAGAAATCGCAAATTACAGA
CTTAATGCTACCGAATCACAAGAAATAAAGGAAAAACTTAAATTAAAAGATTTGGAATTT
ACAAATTTGGAAAAATCTGTCAACCAATATTTGAATGAAAATAAAGAACTAAGGAAAGTA
ATAGATAATTTCGAAAATCAGAAA
>DjScaffold1467:7831-8673 strand:+
ATGATTTTAATTATCTCGTTAGTTATAGTAGATATTAAAATCAGGAAAAAGAAATTGAAT
TATAAAAAAGAAAAGAATGAAATAAATTCAAATTCTTTTTCAAACGATGTTTTAAACACA
AATTTACTTTTGATATCAAACAAAAATACTTTAAATTCTGATGATAATAATGAAACAATT
TCTCCAGCAATTGAAAATTTTATCGATTCTAAAGAAAAAATTAAAAAACAGAAAAGTAAA
AATAAATTAAAATCCATTTCGATTGTTGGTAAATTTGTGTTTGATATATATTTTATGGTA
TGTATGGGTGCTTTGGCAATTGTATATGTCAGTATATACATTCTCTATGCTACACTGCCT
AGATATATGAAAATTACATTTAATACATCAGAATCTCAACAAGGATTAATTTGGCTGTTG
AATTTACTAGGTTATATTGTCGGTTTCATTTTAGGAAATATTCTCAATGCAAAATTTTCT
AAATTTCGTCATCTTTTTTTAATAATGACAATATATATTCAATCGATTGCAACATTATCA
ATTGCTTTCTTACCAAATTGGTGGTCGATTTCAATTCCATTGTGTTTACTGGTAATGCAA
CACTTGATTTTTGAAATTATTATGACTCCAATTCTGACCAAAATTGTAGCAAAAAAATCAA
AATTCCAATTTTGGTATTATGACTGGTTTGGTAGTATTTACGCTTTCATTAGTTTCTTCT
GGAGGAAGTTTTCTAGGTCCAATCATTTATAGCAAAATTGGAATTTTATACTTGACTCTC
ATCAGTTTTCTACTAGGAATCATTTTCGGACCTTTTTTATACATTTTAAATAAATTTATT
TAA
>DjScaffold2686:48123-48557 strand:+
ATGAATATTATATTGCGAAATGAAAACTTGAAAAAAATGCGCAATATATTTAGACGATGTT
TTAATATTCGGAAAAAAATATAGAAGAGTGCAATGAAAGACTAAATCAAATATTAAAAAGA
ATAAGAAAAGCTGGGTTTAAATTACCACCAAATCAATGTACAGAAAAAAATACAATATTTT
GGACATATAATAACTAAAGATGGAATCGCAACATATCCTGATAAGATAATGGCAATAAAA
AAATGGAAGATTCCTCAATGTCGAAAAGAACTTCCCACATTTTTAAGGTTTTGTAATTAT
TATAAGAGATTCATTAAAAATTATGCAAATTCATCAGAACATTTCAGTTTGATGCTAAAG
AAAGAAAACAAATTTCAGTTGACAGATGAAAATAAAGAATATTTTTACAAGCTACGCAAA
AAGCTGACAGAATGA
>DjScaffold17:46042-46611 strand:+
agaaagagaaagatgttatcaaatattttcataatttgattaactacaggcgtggcaatg
aagagattcaagtttattttaaaaatgtaaaacatttagtaaaaatattatttacaaaca
aaaattttactgaagaaaaATGATAGCAAAGATGCTAGTATATTGTTCAGATAACAAAAA
AGTTGAAGAAGTGATTTTGCGGAAACGAGAGAATATAGAAGAAATTGAAGAAACGATGAC
ATTAGTATATCGACTAGAGAAAAACAGAGATCGAGTTGATGCAGTTAGAAGTTACGCGAG
AGTTACAAATCGAAATTATCAGAAACCGGTAAATCATACAGTAAATAGACATTTTGAGCA
AAGAAAGGAAGAACAAAATATCGAATGTTGGTCATGTCATAAGATTGGTCATACTAAAGC
AAATTATCCGACAAAAAAAGTGGTAGAGTTCTGGAGTTTTCATAAAACAGGACATATACA
GGACAAATAAaaaacgaataaaatgttttcgatgtggaaaggttggttatattagaaggt
tatgtcaa
>DjScaffold17:81438-87204 strand:+
ATTGAAGAATTTCAAAACCAGTACTACTGTGAGTCTGCAAACAAAATAAATGATACAGTT
GACTGTAAGCCATGGAAATTTACCTGTAGAAGCTGCCAGACTTATATTTGTACTTCGGAT
GATCTTAGATTATTAGATGGTTGTCATAGAATTTGCATGATTCCAGAACTGTTAAAAAAA
TTCACTGTTCGTGAAGAAGAAAACAAGTGTTACGGAGCGCAAAAGTCTTACAAAAATGCT
GGGAAAGTATTTTGTTTCGGCTGTAATAATCATCAGTTAGGAAGTTATATTTACTATCGA
GAGAACCCGTTTTTTCAACTAAAAATTGACTGTTGCAATACTACTAATTCAGACGGAGTG
ATGATACATTTTAAATCATGGAAATATTTTGACTTTTCACTTAAAAATTTGAGTTCTGCT

GAACTCGAGAATTATTGCAGTCAATTAAATGCATTAACAAATAATATTATTTGAgtgatt
attgattggtttagcataatttatcaggattactttgctaattattttatattctaatat
gtttataagcccgaattttgatagagtttccacaattcttctcttggattatcattatta
ttaaatttatttgtgtttata
>DjScaffold961:43996-60185 strand:+
taaaattgtgcaggaaATGTCTTGTAGAGCTGAAACTGAAATGGTGATGAATCTATTAAA
GAAATTATACAAGGAACTAGCTTCTGATTCTATTATATGGTTCATCAAATGCTGGTTAAG
ACTACCGGAAACTAGAGGGGAGAATTATTACAGCTGTGTACTCAGTCGAGGAGATGCAAG
AGGTCGTATGCGTCGTATTGATGGAGGCAAAGGTTCAGACAAGGTTTGGAGATTAGATAC
TATAGTGAGCATGTTATATCATGGTATGCCAATGTCCAGTACAGACACTAATATAATTGT
ACACACATGTGATCAGGAGCTTTGTGTAAGACCACAGCATCTCAGATTTCGAGCAAGTGC
TGTAGCATTAGCAGTAGTATTAAAAGCTCTGCAACAAGGTGGATATCAATTAATCCCACC
GAAAAAGAATTCAAAAGAAAAAGATAATGAAAAACCTGAATCTGCCGACATTGAAAACGG
ACAAGAACCTAACAGTAATCGCTCAGGCTCAGTTAGTTCAGTAATCAGTGGAACGCAAAA
CCAAGGCTTACAAGATGAAATGGAAAATTCAGTAGAGATATCTGGGCCATTTAATATTGA
ACAAATTCAACAATATTTAATACAAGATTTGAAGCCTTGTGTGCAATCTCTAGAAAAAAT
GGTTCTGAGTGCCACAGATAGATCAATATTATGCGCAAATCCTGACCTTGACAATATCAA
CAATAATACATCTATTTCAACGACATACGATTCACCAAGAACAATCGTTAACGATAACTC
ATTGCAGTATAAAGCGTTCCATTTTGATAAATCAAACAATTCCCGGCCAATGAAAAGAAT
GAGCAATCATTTAACGGCTGATTGTAAATCTCCAAACTCTATAAAACGAGGTCGAATGTT
GGATAGGAAAGCTGGAAGCAAAAGTCCTCCACTACTCATGACTGATATCACTCCTGTTAC
TAGTGGATATTTAAAGAATGCATCTGTACCAATTCCAACGATGAACAGTAAATCAAATTC
CAATAATATCAATCCTCATTTAAATGGATGCAATCTCAATGGTATTAACCGCTCTGATCC
TCATTCATTAACAAATTCCTTTCCTAATTTCTATCATAATCATCCGCCAAATCATAACCC
ATCCAATCATCATCCGTCAATGACCCTTTGTTCACCTAATCCCAATCCTAGATATATGTA
TCAATCACTTGGGAATCACAAATCAAACCCATTCCACCACAATCTTCATCATCCCTTTAT
ACCAGGAACAGGATCTCATACATTTCCGCCTCTTTTTCCGTCCTCTTCAAATATTCCTGA
TCCAGGAAATTCAATCAAACATCATCACAGATTTTCTCCTTTATCCTCACCTCGCCATGG
ATCAATGGATCATTTACCTTTATCACTTACGCCGACGAAAAATAATCGTCATGGTCATAA
CTCAAACATCATACATCCAGTTTCTGTGCCATATTATCCTTCATCTCAAACTCCATCGAT
CTTTCCATTCCGACACAATCCGTTTGAATCAATGCCTCCTCTTGGCTTTTCTCGTTTTCA
ATCTCCTAAAAAACCTCATTTTAACAATATTCATCGTCCGATTCCAGAACCCGCTACAAC
GTGTGCAATGCCAAAACTAGGCAATCCGAATATTCCACAAAACTTTGAAGAAGATACTCC
AATTATTGAAGAAACTCTACCTAATGAAACATTCAACAAACGATCAAATGAAGTTGAAAA
GAATCCATCTCCAATTGAAGAAGATAGTGCAAATGCATCATCATCGATC
>DjScaffold961:75527-81319 strand:+
cactgaatttcttttgataataaataattaatatattaaacattaattaataaatttcta
gatacaacaggcaatattgacaatgcaacattgagtaatataactgacagtattaaagtt
gacttgggcagtgacgatacggaattatcgaacattttaactgaaagatcaaaagttgat
aaagttttatcgaaaaaagttaatgagttatctggtcaactgttattacaaaaacatcaa
cgagataaggctaatgaaaataaatgttccggttcagcagcattattacatccgcgtaat
tctgacagtgcccaatcaggtcacaataaagaacaatcgtcgcattcatgtgtttgcttg
ggaaatgaagatgattgtccaagtgctaatggttatcctggtaattgctgctcttctgct
gcatcaagaacatcagtcaaatcatactcagcatctgcacctgatggagaaATGTTTGTT
CCATTCAATTTCGTATTCAGTGGTGTATCAAGATCAACACCAAACAATCAGACCACCCCT
TCAGAACCAAAATCTTGTGATAATGATAGTGTAAATGGAGTGTGTGCACGATTTATGGGC
AGCCTAAATCAAGATCGATTGAATTCTGGACAGGCTCACATGCTTTTGAATGCTATCCGG
AATTCACCAATATCTGCTAATAGTACTGATTTGTTGGACTTACTAAGTAGCATGGTTCAG
CAATACGGTATAGCCTCTGGATCAAATATTAATAATTCTGTTGATCATGGTTCATCAGAG
CAAGCAAGATCGGAAAGTCGAATTCTCGCGCATATCTATCAAAGATTATATAAAACTTCG
CCATCAGATAGACCAGCTAATCAACTACTTCAAAAAAATTATAGTTTCAGTCCTTTATCA
ACGGAAGTTGACTTGAACTATCTTCGTGCAGCTGCAGTAGCCCATCATAATGGCTCATTT
AACAAAAAATAAATCCAGACTTAGTACACGTTTCAATGATCATCATTTGAAAAATAATCTA
CCAACATCAAGAAGTGATAGTTGTACATCATTTGACACAAATCATTTGGATTCAGTTAAT
GGTAATCGTTCTCGTTATCATTCCAGTGATAATCCACCAACAACAGAAAGTACTCAGCCA
GTAACAGTATCAACAATATCGGCAACTCCTCCTGATAATGGACCAGTATCAAGTTGTTCG
GCATTTTCTGTTACCGATACTCATTTTGATTCGGATAATTCACAAAAAAAAATTCAATATG

GTTTAAcaaattgagatcattataagaagtcattattgttacattaaggtgagtagatat
ttcttctttggcaaagaataagattcatttcaaatagaaatcatttataatcccaattaa
acctataagaaatgcattattatgtc
>DjScaffold550:729-15738 strand:+
atATGAATTTCTCGAAATTGCTTATAACTAATCCATGTTTTGCTGTGATTGTTATGGCTT
TCTTTTCAATATTTTCAGGGGCCACTGTCACTTATATCAGTGCCGTTTTAACAACATTAG
AGAAACGATTCGAATTCAATTCTATGGAAATGGAGCTCTTTTAGGGGCGAATGATATCG
GATATTTATCGTCAGTTATTTTTGTAGCATATTTCGGTTCTAAGAAAAACCGACAACCAT
TTATGTTATTTTTAAGTGCTGTCGGATTGTGGTTGAGTTGTATGACATATTCACTACCGT
ACTTTATATTTGGTTCAAAATTTAATTCTGATTCTCTGATCAATACAAATGGTACAAATC
AAAAAAGTCTATGTCTAAATAATTCTCAAATAATAATCGGAATGAAAACTGTTGAAAAAT
TAAGTGGTGAAATTTCTCCGAAAGAAAAATCAGTGGGAATGCTGATTTTAATGATTGCTG
GCATTATTAATGGAGTTGCATCGACACCATTTTGGAGTTTAGGAATAAGTTTTCTAGATG
ATATGGCTGGTCCGACTAAAAGTGGAATTTATATTGGAACATTATATGGAATGCGAACTT
GTGCTCCGATTTTCGGGTTTTTACTAGGATCACATCTTTTAAAATATCATGAAAGTTTGA
AAATTACAGTCTCTCAGTATTCACCCGGACAGAAAGATATCAAATGGATTGGTGCATGGT
GGCTCGGATGGTTGATTTTGGGATTCGCTGTATTATTACCAGCACCTTTTATGCTACTAT
TAAAGGCTAAAAATCAACCATCAACCACTGAAAATGAAGAACAAAAAGTGGAATTCATAT
CGAAAACTGAAGCTATTGAAATCAACAATAAGGAATTAAAAGATTCATCGAAAGCTTACA
CTAATGAAGAAATAAAAACTGAAATAAACAATAGTTTCTTTATAAGTGAACTTAAAGATA
TGTTAAATGTCATCAAAGGTGCTATTAAAAATACCATATTTATGAGTTTGGTATTTGGAA
TGTTATTTGAAATTCTTAGTTTTATTTGTTTACTAACATTTTTTACCAAATATCTTGAGT
ATCACTTTCGAGTGTCTGCAAGTATGACAAATATTATGAGTGCAATCAGTTTGTCTGGAT
CCAGCGTTATTAGCGCTTTTACTAGTGGGCTCACTTCCCATTTTTACAAAACCAATATGA
GAAATATGAATAGATATCATGCAATTGTTAATTCAGTGGCAGCAATTTTGTTTCCAATTT
TATTTATATTCACTTGTCCTTCAACAACAGTTTCAGGTTTGGATGACCAGTCAAAGATAT
ACATGTGTTCGAATAATTGTAAATGTCAAATAACAAAGCTCGAACCAGTTTGTTACAATT
CCTCCACATATTTTTCACCATGTATTGCTGGTTGTACAAAACAAATAAATGAGACTTTTT
ATTCAAATTGCGGTTGTCAGGGTTCTCTATTTGCTACGAAAGGTTTTTGTGAAAAAATGT
GCCCATCTGTTTATTGGTTTTTGGTAGCCTTTGCTGTTATTAGAATTGTTGGTTCGGTGG
CATCAGTTCAGTTTACACAAGTTTTAATGAGGTTTTTATCTTTAATTATATTGCAATATT
AAtttcaaaattgtgtttttgctgaagtatgcaaagtataagaataatatattaaagtc
tgatatagtaaaatgacaaaaaggaaaatgagaaataattttagaaaaaatttaaatgta
agttacaatttttaaattttcttaatgaaat
>DjScaffold2770:30649-47128 strand:+
attactttagattttgaaaatatcaaATGGAAAGGAATACTACATATTTATCACCGAATA
ATTTATATATTATTTTACAGTCTATTAAATGCCATGTTACAAATGCACCATGCAATCATC
AAAATTGCCATAAATTTCTAAATTTTTTTGATCATATCCGTAACTGTGATAAAGCTGAAG
ATTGTAATATGGAAATGTGCAGGCAAACTGTTGCATATATGAACCATTATTTGAACTGTC
CAACTGTTAATAGTTGCGTTTGTAGGCAATTTGGTGAAAAATCTTTGGATAATATGACTT
TTGTAGCTGTAAAAAATATTCTACTGCGTAATCAATTCATGATATGGCGACGGAAGATAA
ATCCCTTTCGACCTCATTTACTTTGGAAAATATCCGATGAATTAGCAAGTTTCACGAAAG
AACCGAACAGTAGAAATATTATCTATAAATTTATCAGTGATTTCGATCAGGACAATTTCT
ATAAATCGAAAGGAATAATTAGTTATCTAACGAATTTTGCGACAAATGCCAACGATATTC
TTAATAAAATTCAATGGAAACATTTTCGTAGTAAAAATCCGAATCTCACCTATCAACATT
ACACAGAACTAGTCGATGGAAAAGCGAATATTCAGAAAATTTTGAATTATCTGAAGAGAT
ATTCTACCGATGTGTTGCATAATAGAATCAGATTGGAGCCTTCGTCGAATGTCGATGTAA
AACTCGAGGAGATAAAATCCGAAGAAATAACGAATCAAGAGAGTGTAGAAGGGATAAAAA
CGGAACCAACCGCTGTAGAAGTGATAAAAACTGAACCAACCGCTATAGAAGAAGATACGA
AATTGGAGTCGTCAGAATGTCATTCTCCAACGAATAGTAGCAGCCTGACTCCTCCTAAAT
CAATTTCATCCGTAAAAACGAGTCCAGAAGTTCCAATCGATGATAAATCATCGACGAGTG
TCTCCAGTGAGAAAGTTTGGATAGCCTCGGAGCTGGAATATTTGTTCAACGAATTACACA
ATAAAATCAGCCAGGAGAAAGAAGCGGAGATTTTCCTCCATCCGGTCGACTGGAGAGCTC
TCGAGATTCCAGATTATCCGAAAATAATCACTAATCCGATGGATTTGTCGACAATCAAAG
ATAAACTGGAGCACGGCAAGTATATCGATCCGTGGGAAGTGGTGGCCGACTACTGGCTCA
TGTTCAACAATGCGTTCACTTACAACAAGAAGAGCAGTCGCGTTTACAAAATGTGCTCAA
ATCTCGTCAAATTTTTCGAGATTGAAATCGATTCGGTAATGCAATCAGTTGGGTTGTGCT

GTGGGCGAGAATTCGTATTTCTGCCGCAAGTTTTGATCTGTTGCACGCCGCAAATGTGCA
TGGTGAATCGCGACGCAATTTATTACAGCTTCAATAATGAGGAACACGGAGAAAATGGCA
AACGACTCGTTCCAGATCTCGGATGTGATAAATATGTTTGCTGTGAGAAATGTTTCGATG
ATTCGGTTGAATCGATAGAACTGATGGTTTCGGAAAATTCTGCTGTGTTGGTACCTGTTG
AAAAATCGTTCTTTTCAAAATTGAAGAACAACGTGAAAGAAAAGGAAGAATTCGTTAAAT
GTAAATCTTGCTATAAGAAGTGGCACAAAGTTTGTGCACAACATATCGATGAAATTTGGG
GCAGTGGCTTTTATTGTCCTGAATGTGAAACAAACCGTCCAGTCACCAAGCAAAAGAATG
TTTTAATATCAGCCAATTTACCTCATTGTAATCTTAGTCAACACCTTGAGGCACGTATCG
ATGCTTGGAGACAGAAGGAAAGTCAAACAATTAGCAAAATTAATATTCGCGTTCTATCGA
TTTGTAATAAAAATGCAGAAATAAAACCGGAAATGAGGAGAATATTTGTGGAAAATGGAG
AAATGCCAGAAAATCTTCCGTACAAATTGAAAGCGATATTTGCATTTCAAGAACGACCTG
ATGGCAGTGAAATTTGTTTATTTGGAATGCATACTCAAGAATATGGCAGTGATTGTCCCG
CGCCGAATACGAGACGGGTTTATCTTGCATATTTGGATTCGGTTAATTTCTTCGAACCGT
CTAATCTCAGAACTGATTTTTATCATCGTTTATTGATATCGTATTTGGAATATTGTCGTC
AACTGGGATTCATATTTGCTCACATTTGGGCATGTCCACCAGGAGAAGGAGACGATTATA
TCTTTCATATGCATCCTCCAACTCAGAAACTACCAAAACCCAAGCGATTACAAGAATGGT
ATGTTGCAATGTTGGACAAGGCCATTAAAGAAGAGATTGTAGTAGAATATGCTGATATAT
TTACTTATGCATACAATTCGCAACTCACAAGTGCTTCAGAAATACCATATTTTGAAGGAG
ATTATTGGCCTAATATTATTGAAGATTTACTCAAAGAAGATATAAATCAAAATCTCGATT
CGCTGGCCGATGACGATTCAAAATTTCACGAACGTTTAGAATCTGTAGATGTTAGTCATT
CTAAGAAACGAAAATTAAAGAAAATACCAATCAGTAACAATTCTAAACGTCGTCGGCAAT
CTGATTTAACTGATGGATGTAATGATATTATGAGACGACTTCTCGACTTATTAGAACGCA
GGAAAGAAAATTTCTTCGTGATTCGTCTTCATAATCAATCAGACGCCTCAAAATTACCGG
AAATCGAAGACAAAGATGTTATCAAATTTAGTGATTTAATGGATGGTCGTGATAAATTTC
TATCGTTATCCCGCGAGAAGCATCTTGAATTTTCATCAGATCGTCGTGCAAAATTTGCGA
CTCTAGTGTTGTGTTACGAATTACATCTCGAATTACGCCATGGTGTAATTTATTCCTGTG
ATAATTGCGATTTGATGCTCGATGAAGTTAGATTCACATGCGAAGTATGTGAAGAATTTG
AATTGTGCGAAAGTTGTGCTTCCAAACAAAAACATCCACACAAATTAAATCGAATTCCAA
GTTTGGAAACTGAAAATAACAGCAACGAACAAAAATCTAATGAGAATGCCACCAAAGCTC
CTATTGATCAGATAGCAGCTGAATTGAAAAGACATATTGAAATCATCAAATTTTATGAAC
ACGCAAGTAAATGTCGTATTAGGTCTTGTAGTAACAAGAGCTGTCATAAATGGAGAAATG
TGCTCTTACACAACTCCAACTGCACGATGAAAAACGAGAATGCTTGTAATTATTGCAAGA
GAATATTTTCCATTATTAAACGACATAAGAGTACTTGTAAAAATCCCGAGTGTAGCCTGG
AAATATGTTTGGCGAAGAGAGAAAGATGCATTGATCAGTCTAGATTATTTAATCTGGCAT
CAAATCGAAGACGTATGCAGTCAGCAACCATAAATGTTCCAAATGTTCCGAATATTCCAA
ATGTTCAGCTGAAATCATCACCACAAATCAATGTAATGATACAAAGTTGTCCACAAATCG
ATCCAAACACATATAATTCTGATATATATACAATTCGAACTTTCATTGAACAATTAAAAC
AAGATTCAACTTTAAAAGAAGAAGAAATCCAAATAAGATTTGTACAATTTCTCAATTCTA
ATCCTCGTTATAAAGAAGCCTGGAAGCATTACAGCATTCAAAGGGGACAGATTACTTATC
AATCTCAAAATGGTCAGCGATTTTCTCAAACTTCACAAATGCGTCCTCCTCAATACTATC
AAACTCCTCAATCCAGGCAACAAAACCTCCAAGTTAATTCTTTATCACAACACCAAATTA
TACAACAACAAATAATGCAAAATCAAAACTCGCGACCACAATCAATTCTTTCAAACACAC
AGCCTTATTACGTAGTTCAAACAAGACAACCCTTCACTCAACCTAACCAATTACCATATT
ATAATCCTAGCAAT
>DjScaffold2770:85026-85556 strand:+
tctgatagcatttttatcggtgaacaactttttaaacaaaaaggtggtcttgcaATGGGT
AATAATTTATCTCCAATCCTAGCAATAATTTTCATGAACTTCATTGAAAATGAAATAATT
CTTAAATCAAACAAGAAAATTCATCTATGGTTTAGATACATAGATGATATTTTCTTTGTA
ACTAATTCTCCTTTAGAGACCATTCTAGAATTAGCCAACAGTATTAATAATAACATTAAA
TTCACTTTAGAGAAATCAATTGACTTTCAGATTCCTTTTCTGGACACTTTAATTCAAAAG
AAACCTGGTGAAATATTAACTACCCAATTATATATGAAACCATTTCACAGTGGACATATA
TTACCTTGGAGTAAAAATTGGTATAATTAAaggtgaaagaattcgagctgagagactatc
ttcaaatgaagaaatgaaattgatttctctcaagttgataaaaaataaatttttaaataa
tggctatccaaataaaattatatcgaaatat
>DjScaffold2770:88119-88750 strand:+
gaacaacatataatATGATGAGTGAATGGATCAACTATCCCTTGCATTTGAATATTTTCA
GTAGATACAAGCAAGAACCAGTTCCTTCTTTGCTATCTCTTCAAAATCAGATCAATTATA

ATCAGCCTTCTCATTTGATTAAAAGTCGAAAAATGAAGCACTTTGTCGGAGAAAATTTAA
ATTCTTTTCGTCGATATGATGATCTAGATAATTCTCTGTCTAGCAGAAATGGTAACACCT
CAACAATTGCATCGCCACATGTAGAAAATGACAAATCTTACTTGAAAACGAGACCGACTT
ACAACAATTTAACTCCAAGAAATTACTATCTAGGATATTTGAAGAGATTAAGAGAAGTTG
ACGATCCGGAATTAGTAGATAATTATAAATTTCGAAGTGGCAATGGCGGATCATCGTGGA
GACATGTAAATTGTTACTTAAAACCAGATGGTAAACGTTACTATTTCATTGACGGAATTG
TAAGCCAGAACGACCAATTTCATTACAACTAAtaatttatatttccaat
>DjScaffold2770:107426-117003 strand:+
tgaatgtatttattttagatatttttttaaATGGCTGGTGCATATATGAAAACATTTCCAA
GTATTGACGGTGATAATGATCAAAGTAGACGTCAATATTACGATCTTACTATCAGCGAAG
CGCTTACAGTAGCAGTTGATCAAACAAAACGAACATTAAAGACTTTTACTGGAGTGTTTT
GTCCTGTTGCATTGGCCCAGTTTTCATCTTTATTATTTTTACGATTAGGATGTGTATTGG
GACAAACTGGTCTTTTATTATCGATTTTGCAATTAATAATGGCGTATGTGATTCTTGTGT
TGACAGTTTTGTCAATTTGTGCTATCAGCACCAATGGTGCCATTAAAGGAGGCGGGGTTT
ATTTCATGATCAGCCGAACATTGGGACCAGAAGTCGGAGGGAGCATTGGAATCTTGTTTT
TCCTGGCAAACGTGTTTTCCTCAGCGCTGTATATGGCTGGGTTTGTCGAGGGATTAACTG
ATAATTTCGGTCCGGAAGGCCGCCTGAAACCCGGAATAATCCCTGAAGGAACTTGGTGGA
CCTACTTGTTTGCCACTTGTGTTTGCCTGTTCTGTCTCGGAGTCTGCTTGATCGGAGGAT
CGATGTTTGCTAAAACAAATCTCTTCATATTTTTGGTTGTGTTTGTCTGCTTCCTCTCGG
TGATCGTAACGTTCCTCTTTCATTATCCGATGCTCGTTCCAATTCCAAAAACAAACAAAA
TAGTTTATTTCAACTCATCTGTTGGTGTTGTTTATGGGAATTTCACAGCGCTCTCTGGAC
TCACCATGCGCAACAATCTCCATTACGAGAGTGGATACCAGATCGATTTCACCACTGGAT
CACAGATGAACTTCCTGTCGGTATTTGCAATCATGTTCTCGGGGGTCACTGGCATCATGA
ACGGTGCCAACATGTCAGGGGAGCTCAAGCAGCCAGCCGTATCGATTCCAAAAGGAACTT
TGGCGGCAGCATTGTTCACATTCATCTGCTATATCGTTTCATTCCTGCTATCGGCCGCCA
CGTGCGANAGGTATTTGCTACTTAATAATTATCTCTATATGCAAGCAGTTAATTATTGGA
GACCATTTGTAACTATTGGTATATTTGCAACATGTTTATCAGCAGGTCTTGGTAATTTAA
TCGGTGCATCAAGAATTCTTTATGCTTTATCTAAAGACGATATATTTGGTGTGCTGTTAC
GGCCTGCTAAATTAGCCACAAAGGGCGGTAATCCAATCGCTGCGGTAGTCATATCATTTG
TACTAGTTGAGTTAGTGCTTCTTATTGGCAAATTAAACCTCATTGCTCCAATCGTTTCTG
TATTTTTCCTATTAGCATACGCCTCAGTGAATTTAGCGTGTGGATTATTGGATTTGGCTT
CCGCCTCTAATTTTCGTCCAACATTTAAATTCTTTTCGTGGCACACATCAATGCTTGGTG
TCGTTTGTTGTCTGGGAATGTGTTTTCTTGTGAATTATATGTATGCAATCGGGATTTTTT
TCTTATTTATTTTGCTAATTGTGACACTCCATTTCCGACGTCTCGATTATGCATGGGGAA
GCATTGGACAAGCTTTGCTCTTCCATCAAGTAAGAAAATATCTATTGTTGATTGATATTC
GTAAAGAGCACATAAAATATTGGAGACCACAAGTACTCCTGCTCCTATCCAATCCGCGTA
GTCAAGCTAGTTTGGTTCAGTTTGTTAACATATTGAAAAAAGGTGGTTTATTCGTAATTG
GGCACGTAATAAAAGCAGATTTTGAGCAAATAGACGCGGATCCTGCTCAAGAAATGCAGA
AAAAATTCGTAATTATGAAAGACTTTCTAAAAATCAAAGCGTTTATTGATGTGACGACTG
CACCAACGGTGAGAGATGGATGTCGTCAATTATCTAGGTTATGTGGTCTTGGTGGACTGA
CGATCAATACAGTTTGTATTGGATTCAGAGATGATAGTGAGCCACAGGATGTCCTTCAAA
AAATCAATATTCAAATGAGAAATCTTGACAGAAATTGGCCATTTAAACCGAAAAATCTGC
CAGTATTTGCGGAATTGAGTTCTAATTTCAATAATATGCCTTCTACATCACATTATGGAT
ATCAAAATTTTTCAGCGGAAACATCAATGAATAGCCAAGCATTTCAAGTGAATGAAAACA
CTCATTTATTTAGCAAGAAAAGTAGGCGGATAACGATTGAAATAAACATACTTATTAATA
TTTAGgcgatttacctatgaatggtaaaaataataataataataataatgaatataacaa
taatattagtaagtgatctggtctgttttgcaactgcgctatgtttagtaattgcgtgtt
ttgttttacagtatcattatcatcatcatcataatattttaattttcagatttattttga
taatttccttttgaaaaattcaaaatttattaaactaattctatttttattattcatttt
attttattgtaactgttagagaatatttaattgaatgtatttattttagatatttttttaa
ATGGCTGGTGCATATATGAAAACATTTCCAAGTATTGACGGTGATAATGATCAAAGTAGA
CGTCAATATTACGATCTTACTATCAGCGAAGCGCTTACAGTAGCAGTTGATCAAACAAAA
CGAACATTAAAGACTTTTACTGGAGTGTTTTGTCCTGTTGCATTGGCCCAGTTTTCATCT
TTATTATTTTTACGATTAGGATGTGTATTGGGACAAACTGGTCTTTTATTATCGATTTTG
CAATTAATAATGGCGTATGTGATTCTTGTGTTGACAGTTTTGTCAATTTGTGCTATCAGC
ACCAATGGTGCCATTAAAGGAGGCGGGGTTTATTTCATGATCAGCCGAACATTGGGACCA
GAAGTCGGAGGGAGCATTGGAATCTTGTTTTTCCTGGCAAACGTGTTTTCCTCAGCGCTG

TATATGGCTGGGTTTGTCGAGGGATTAACTGATAATTTCGGTCCGGAAGGCCGCCTGAAA
CCCGGAATAATCCCTGAAGGAACTTGGTGGACCTACTTGTTTGCCACTTGTGTTTGCCTG
TTCTGTCTCGGAGTCTGCTTGATCGGAGGATCGATGTTTGCTAAAACAAATCTCTTCATA
TTTTTGGTTGTGTTTGTCTGCTTCCTCTCGGTGATCGTAACGTTCCTCTTTCATTATCCG
ATGCTCGTTCCAATTCCAAAAACAAACAAAATAGTTTATTTCAACTCATCTGTTGGTGTT
GTTTATGGGAATTTCACAGCGCTCTCTGGACTCACCATGCGCAACAATCTCCATTACGAG
AGTGGATACCAGATCGATTTCACCACTGGATCACAGATGAACTTCCTGTCGGTATTTGCA
ATCATGTTCTCGGGGGTCACTGGCATCATGAACGGTGCCAACATGTCAGGGGAGCTCAAG
CAGCCAGCCGTATCGATTCCAAAAGGAACTTTGGCGGCAGCATTGTTCACATTCATCTGC
TATATCGTTTCATTCCTGCTATCGGCCGCCACGTGCGANAGGTATTTGCTACTTAATAAT
TATCTCTATATGCAAGCAGTTAATTATTGGAGACCATTTGTAACTATTGGTATATTTGCA
ACATGTTTATCAGCAGGTCTTGGTAATTTAATCGGTGCATCAAGAATTCTTTATGCTTTA
TCTAAAGACGATATATTTGGTGTGCTGTTACGGCCTGCTAAATTAGCCACAAAGGGCGGT
AATCCAATCGCTGCGGTAGTCATATCATTTGTACTAGTTGAGTTAGTGCTTCTTATTGGC
AAATTAAACCTCATTGCTCCAATCGTTTCTGTATTTTTCCTATTAGCATACGCCTCAGTG
AATTTAGCGTGTGGATTATTGGATTTGGCTTCCGCCTCTAATTTTCGTCCAACATTTAAA
TTCTTTTCGTGGCACACATCAATGCTTGGTGTCGTTTGTTGTCTGGGAATGTGTTTTCTT
GTGAATTATATGTATGCAATCGGGATTTTTTTCTTATTTATTTTGCTAATTGTGACACTC
CATTTCCGACGTCTCGATTATGCATGGGGAAGCATTGGACAAGCTTTGCTCTTCCATCAA
GTAAGAAAATATCTATTGTTGATTGATATTCGTAAAGAGCACATAAAATATTGGAGACCA
CAAGTACTCCTGCTCCTATCCAATCCGCGTAGTCAAGCTAGTTTGGTTCAGTTTGTTAAC
ATATTGAAAAAAGGTGGTTTATTCGTAATTGGGCACGTAATAAAAGCAGATTTTGAGCAA
ATAGACGCGGATCCTGCTCAAGAAATGCAGAAGTATGTTAAACATTCATTGCATTTAAAT
ATACAATTATTAGAATTTTGCACGAAATCTGGAAATAGTTAAaaacgaattattaatata
ttttactttcaccatgaactgttgttacaattcaagtttccgaatgaaaatttatctgct
gactaagaatagcaatgtttttcgaaaacaaattatcgcatacgataccttcttgtctta
tttttgtaggaatttttatatttcaatgcagaattgtaaaagacttttcaaattctaatg
tgaaaaatgtgcggatggctacagatgactgcatttgacattattaaattacaaatggaa
cattttaaacattttagttgtttgagagccaagactggtcgttggcatgttacaaatcaa
taataagggtgatcattttggtaaaatggggtgaatatatcagtgacaattagagtggtt
gtcaaatattcaacattattaatattcatcttttttcttttatcatttatttttcatcat
ttacttttcgtaatttatctttcattattcatctttcttctttaattattcatcattcat
ctttaatcattcatctttaatcatttatctttaatcattcaactttctt
>DjScaffold2770:134170-137177 strand:+
gatgttgaatctgtagttaaagaaggtgatgaattttgtcaatatgaccaattatctgaa
ATGGAATATGTTGGAATCATTGCTGATTGCATGAAATTACAAAAAAATATTTGTTTGTGT
CGACATTTTCAGAAAATGGATTTGATGGATTTGACATCGAAGAGTTCATCGGAATATCAA
TATATTGATGTTTGGCCAATTAATTTTTTCCAACCAGGCAGTGGTAGCTATCTTGACATG
ACATGCATGTTTATGCTTCAGTTGAGTTGTATTCTTAATATGAGTTCTCAGTGGAGTTCG
AAATGCAAAATTCGAGTTTATATTTGTTTGGATTATTACACTGTCAGTAATAATATGAAG
AATATTCAAGAACTTTGGAATGAATTATTGGTGCAATGGAGAATAAAGGCAGAAATCAAA
ACTGTTGGATGGGATGACATCACAATGAATTTGGAGACAAATTTAGGAAGAGAAATTATG
TCAGGAAATCCTACTTCTGAAGAAATTAAAACCATTTCATTGTTTGACTGTACCTTGCCA
GACACATATATACAAGAAGTGAATAACTTGATAAAATTTAACACTTCTCCGAATACAGCT
GTTTGCTTCTTTTATCTTCCAGTACTACCTAACATTACGAAATCGCAATATTGGAAGAAA
TATATTAATCAATTAGCATTGCTTACTAACGATCTTCCTCCTTCAGTTTTTGTGAGAGGT
CAGCATGAAGTTATCACGTCAGCATTGTGAttttggaacttcaaagaatttatctgattg
ttatacccaattgctatttctatatttttacatgaaaataaatt
>DjScaffold2536:20660-24492 strand:+
tttaaatttcttaaattaagtcgtatttgatacatttcATGATGATTAAGAAAAATTTAT
TATTTTTAAATCCCAACTTTCAAATACTAAAATTAAATTTTAGTTCATTAAAGTTCTCAA
ATATCGAAATTAATAAAGTAGTAAACCGCAAAGAAATACCGAAAGATTGTGATAAAATTA
CTTTTGGTTCAGTATTTACTGATCATATGTTAAGAATTGACTGGAATATAAAGTCTGGTT
GGTCAAAACCGATAATAAAACCTGTTGAAAATTTTAGTTTGCATCCTGCCTGTATTGTAT
TTCATTACGCAACTGAAATATTTGAAGGTTTAAAAGCATTTTATGGAGTCGATGGTGTTG
TAAGACTTTTCAGACCTGATCAAAACTTAAATAGAATGCATAATTCTGCTAAAAGAGCTA
TGCTTCCAGAATTTGATAAAATAGAATTACTCAATTGTATTAAAGAATTAATTCATATTG

ATAAAGATTGGGTTCCCAAGAAACGTGGCCATTCTTTATATGTAAGACCAACGATGATAG
CAGTTGAACCGAAACTTGGTGTGGCGAAGTCTGATTCGGCAATACTTTATGTGTTATTGT
GTCCAGTTGGTGCATATTATCCAACTGGTTTCAAACCTGTTTCTCTTCTTGCCGATACAA
AATTTGTTCGTGCGTGGCCTGGTGGATGTGGTGACTCGAAAATGGGATCAAATTATGCTC
CAACAATTTACGTACAAAATGAAGCTAATAAACAAGGATGTCAACAAGTGCTTTGGTTAT
TTGGGAAAGACCATTATCTGTCTGAAGTGGGAACCATGAATATTTTCATTCATTTGGTTT
CTAAATCAGGCAAAAGAGAAGTAATAACTCCTCCTTTAAATGGTACCATTCTACCTGGTA
TTACAAGAATGTCTTTATTGGAGTTATTGAGACAAGAGAAAGATATTGAAGTATCAGAAA
GGGAAATTACCATGAAAGAATTAATAGAATCTCTAAAAGAAGGTAGACTGTTAGAAATGT
TTGGATCAGGAACGGCTTGTGTTGTTTCTCCGATTGAAAAGATTAGTTATAATGGAGAAA
TTTATAAAATTCCTACAATGGAAAAAGGTGCTCCACTTACAATGAGGGTTTTGCAATTAT
TGACTGATATTCAATATGGAGTCAATAAACACGAAGGATGGACAATTGAAGTTCGATAAg
cgttaggtcaatgttgtcttaatttaaattaattgtcagttattgagtgtaatagatttt
tggttttgagacttcaataaattatcttgtaataaacttttaataaattttttattta
catatgataataataattctcatacatcagaataactagagaagaaattaattagtaaaa
taagtcaaatcctaaaataaaatcctacacttattagattttacgattgttctggaattt
gttttattaaacgaaaagcagagatagaattattatttcagttttattaagtttcatctt
aattctcaataatgatcttcatcacaaataaactatttcatccaaggaagatctattaaa
atgaagagaatattttgatcggtacttcaacgatatattgattctaacttttcaataaaa
atctttcaatttcattttaaaattggatttgttgcagacatattaataaacattttattt
aaaattttatgtaaaatattttaatttttatctttcacccagcttacgctatttattaat
gaagataattattaaaatgtcctatttgattatgattgtttggttgattatagcttcaat
ttgatttgctaataaagattatggaaatagtaattctttttcgttgaaaattatttatta
ttattttacagcagcataaaatggttgaacaatttttcatgaaaaataaatgtttgcacg
tatccttgctggaattttttagtagattataaataaaattgaacatagtaaccagtagaa
tctgtcatcaatttaacaattttATGTTCTGTAAATTGTTGATGTATTCCTATTATTTTG
TAAAATTATACAGCATTTTTAATATTTTGCTGTTTTCTAAATTTCAGCCGAAACTTGGTG
TGGCGAAGTCTGATTCGGCAATACTTTATGTGTTATTGTGTCCAGTTGGTGCATATTATC
CAACTGGTTTCAAACCTGTTTCTCTTCTTGCCGATACAAAATTTGTTCGTGCGTGGCCTG
GTGGATGTGGTGACTCGAAAATGGGATCAAATTATGCTCCAACAATTTACGTACAAAATG
AAGCTAATAAACAAGGATGTCAACAAGTGCTTTGGTTATTTGGGAAAGACCATTATCTGT
CTGAAGTGGGAACCATGAATATTTTCATTCATTTGGTTTCTAAATCAGGCAAAAGAGAAG
TAATAACTCCTCCTTTAAATGGTACCATTCTACCTGGTATTACAAGAATGTCTTTATTGG
AGTTATTGAGACAAGAGAAAGATATTGAAGTATCAGAAAGGGAAATTACCATGAAAGAAT
TAATAGAATCTCTAAAAGAAGGTAGACTGTTAGAAATGTTTGGATCAGGAACGGCTTGTG
TTGTTTCTCCGATTGAAAAGATTAGTTATAATGGAGAAATTTATAAAATTCCTACAATGG
AAAAAGGTGCTCCACTTACAATGAGGGTTTTGCAATTATTGACTGATATTCAATATGGAG
TCAATAAACACGAAGGATGGACAATTGAAGTTCGATAAgcgttaggtcaatgttgtctta
atttaaattaattgtcagttattgagtgtaatagattttggttttgagacttcaataaa
ttatcttgtaataaacttttaataaattttttatttacatatgataataataattctc
atatttaaatttcttaaattaagtcgtatttgatacatttcATGATGATTAAGAAAAATT
TATTATTTTTAAATCCCAACTTTCAAATACTAAAATTAAATTTTAGTTCATTAAAGTTCT
CAAATATCGAAATTAATAAAGTAGTAAACCGCAAAGAAATACCGAAAGATTGTGATAAAA
TTACTTTTGGTTCAGTATTTACTGATCATATGTTAAGAATTGACTGGAATATAAAGTCTG
GTTGGTCAAAACCGATAATAAAACCTGTTGAAAATTTTAGTTTGCATCCTGCCTGTATTG
TATTTCATTACGCAACTGAAATATTTGAAGGTTTAAAAGCATTTTATGGAGTCGATGGTG
TTGTAAGACTTTTCAGACCTGATCAAAACTTAAATAGAATGCATAATTCTGCTAAAAGAG
CTATGCTTCCAGAATTTGATAAAATAGAATTACTCAATTGTATTAAAGAATTAATTCATA
TTGATAAAGATTGGGTTCCCAAGAAACGTGGCCATTCTTTATATGTAAGACCAACGATGA
TAGCAGTTGAAGTAAGCATTGATTATTTTCTGTCACATGATTTTCTTCATTATTTTATTA
TTTTAATGTTTAATTTTAGGAAATTGATTAAGATGAATGTTCAGGTATGCAAAGTATATG
TATATATATTTAGTAATCATTTGATTCATAATCTATCAAGCGCAATCTTCCCATACGTTT
CATTATATTGAttatagtcactaaggattatagtccaaaatcttg
>DjScaffold4926:3185-12389 strand:+
aaataggtttctatttttgattaggaagttaaactaaaatcaaaattctactgaattttt
tcgagtaatgtttgaatatctacttgcttattacacccaattaaatgaaaatgaaaatta
tatattttccaggataaaacggaatttttcgtattagttaataaattatccaactattcc

aaatttgatacggttaatcaagttctttcatttctgaatacaattccaaaatcgtttgat
gattgtgtttttgggcactcaatgaatggttttgtcattattcaaaaaatattcaacaa
gtcctacacagtttcccacctgattacaaaacagcagaaggagttccattttggagcggt
actaaacgtgttcctagatttgatgaattttgtgttgataattcaaatcatttggatttc
gtcaaatatgcaacgaaattacgggcgagattattcaacattgaaatcaaaagtgatgaa
tatataaaatcaatagcaaaatcatttcaatttgaaaaattcactccttcttccgatgta
aaaatcgcgtcacaagattcggaaattgagaaaacattggatctcgcattattaagtcgt
cgaattcaagaattacgtaatttagatttcaaagaagttcttagtttaaatccgcaagat
ttcgaaaaagacgacgattctaattttcatatcgactttattacagctgctagtaacATG
AGGGCTTATAATTACACGATAGAGCCAGTTGATCGATTTAAAACGAAAAAAATTGCTGGC
AACATAATCCCAGCTATAGCTACAACAACAAGTGTAATTTCTGGGCAAGTGTGCTTTGAA
TTATTCAAGTTGACACAAGGTCATCAAAACATTAGGAAATATCGAGATTCTTATTTGAAT
TTGGCTTCTCCCGTATTCACAATGACAATACCCGAACGACCAACAAGTTACGAAATATTT
GGTAAAACTTACACATTATGGGATAATTTTGAAATCCATGGAGACATTTCCTTGAAGGAA
TTTTTGCAATTCATTAAAAAGAATCATAATCTTGATGTCACTTGTGTAACATACAAAACC
GGTGTTTTATATGCTCTATGGTTTGATAAGGAATACTTGGAAGATAATATGAATCGATTA
ATTACTGACATTATACGTAACAAAATTAATCTAGTTATTTCTGAATCGGAATCTGTTATT
CCACTCTATATTTCTGTTGAAGATGAAAATGGAGAAGATATTGATGGAATTCCAGTGGTT
CGATATCATTTCTGAaattgattgatttctccaattaactggtttatgaaatttctaatc
ttaatgtttttaattgaaatatacgtaatactattca
>DjScaffold2749:121212-121668 strand:+
ttataactattttattattattANNNNNNNNNGAGGAGATGATATTGAGAAATTCATCAA
GCGATGTGAATTGTATTTTGAAGCTAAAAACATCACCCATGTGAAACAACAGGAGAGATT
AGTCCCTTATTTGTTAGATGAAGATTTGTTGGAAGAATATGTAAGGACGCAGAAAAAAGT
TGACGGTTTCCAGGCTCGTTTGCATATGGCATTTGAGAAAGAGAAAGATGTTTTTAAAGA
TTTTCAAAATTTGATAAACTACAGGCGTGGAAATGAAGAGATTCAAGTTTATTTTAAAAA
TGTAAAAGATTTAGTAAAAAGATTATTTATAAATAAAGATTTTACTGAGGAAAATCTGCT
AGCAAAGATTCTGGTATACTATTCAGATAACAAAAGAGTTGAAGAAGAAAATATAGAAGA
AATTGAAGAAACAGTG
>DjScaffold2749:122901-123966 strand:+
aataactacaagtgatgaaccaatcaacgttaaactgtatagacaaacaatgaattagaa
gctaaaaattgaagaagcaattcaaaaccttttcaagaataatattatccggaaatgcaac
tcgccgtggaatactccactgatttttgattggaagaatgaaaagATGGACATAAGAATA
TGTTTGAATTTCAGACAATTGAATGCAGTAATAAAAAGACAAGCCATTTCTATGCCTAAT
CTCAATGAAATGATGAACAAGTTAGATGGAGCCAAATATTTCAGTTCAATAGATTTGGGA
AATGCGTACCACCAGGTAGAACTCGAAGAAGAATCTCAAGAACAAACGGCGTTTTCAGCA
AAGCATGGACAGTATTGTTTTAATAGAATGCCTTTTGGGATCGCAGCGGCACCTGGAACA
TTTCAGGAGATTATGGCAAAAGGGGTAGGCGGAATAAATGGAGCCATGGTATATCTTGAT
GACATCCTGATATTTACAAACAATAAAGATCAACACTATGAAGTAATTGAAAGAGTATTG
ACTGAAATAATGAAGGCTGGTCTAAGAATTAATTCAGAAAAATGTTAGtttcttcaaagc
aaaatcaaatttcttagacacataattatcatggacggtgtcagaactaatccgtctaaa
atagaagtaataagaatatttggcaaacttaactgtgcgaagaagttacgaagtttctaa
ggaat
>DjScaffold2749:162222-162950 strand:+
tttcttgacactttaattcaaaagaaacctggtgaaatattaactacccaattatatatg
aaaccatttcacagtggacatatattaccttggagtaaaaattggtataattaaaggtga
aagaattcgagctgagagactatcttcaaatgaagaaATGAAATTGATTTCTCTCAAGTT
GATAAAAAATAAATTTTTAAATAATGGCTATCCAAATAAAATTATATCAAAATATTTATC
TTTAAATTCAAAACAAAATAAAATCAAACCAAAAACAAATACAGTTATTAAATTACCGTT
TATTAATGAGAAATGTAGTCAAATCATGCAGAATATAATAAAAAATGCTAAACTAAAACA
CAATCCAAGAATTATTTTCAAAACGCAAGCACCTTTGGGCATCCAATTACAGAAACCAAG
ACAAACTTTTTGTGGTCCCAAATGCATTTGTGATGGTAGAAATATATGCAATAAGAAAAA
TGTCACTTATAAAATAACATGCACCATTTGTCAAGAAACATATATAGGTGAAACATGCAG
AACCATCCGAACAAGAGTTAAGGAACATTTGAAACAAAAAGAAAGTGAGGTATTCAAACA
CTTTCAACACAAACATAAAATTTCACCAGAACAACATCACATTACATGGAAAACACTAGG
AACAGGCTACATTTCTACACTACATAGAAAAAAACATGAAGAAATACTA
>DjScaffold2473:17601-17681 strand:+

file:///C|/Users/czh/Desktop/Data_S1.txt[2019/7/4 17:05:10]

ATGAATATTGTAATGAGAAAGAAAAATTGGATCAAGTGTTATTTATTTGTATGTCATTCT
TGTTTTCTGCAGAACGATTGA
>DjScaffold4002:89808-104733 strand:+
ttgatgataaacatcctacttgcatcgtcaatacttgacgaaaattacacaagctcaacc
gtaacctttaatttttcattcagatcgatacagaattgtctatttgcaattactacacaa
atatttttatcacatattttttatccacaaatacgacattcaagtttactatgcattga
aaaatactcgttttattttattttattcattccagggttcacataggaataattatcaat
gtcttaacaaatttttaagacggattaatcaattgaatttttactttaaatacaaagagt
tacttttaaaatattaataaaactttgtatattaaataaaaactatttatagggtcgcaa
gaaaaccaaggaaaaaccattgaagaaatcagaaactgaagataataaaattaaagaacc
aaaacatggaattgaaatagctaatggattgtacgatgaaatcaatccgtctataaataa
taacttcaaaagaccagaaacaccaaactatgaatcagatagcccaatttattcaatccc
tATGCCAAGAAAACCCTCTAGATTAATACAATCAAATGAATACGAAAAAGAGCTATCAAT
TGAGGAATTTCCTGCGAAACAAACATTAGACCCGAAGGATTTGTCCAAGAAAATTGAAAT
GGAAGTTAAAGGTTTCTTTAATTCTGAAGAAGATTTTAAAAAGAGCAGTAGTAGTAAAGA
CGTATATGTTGATGAAATCGACAGTGATGAAAACATTGACTTTCAGATTAATGGAAGAAT
AAATAAGATCGGTTTTGAAGGTTCAAATTTAGAAAATCAAAATTCTATAGAAAGTTCTCT
TAAACAAATTCCAAATGGTTTACACAAACCCTCATCCAAGACAAATATTCCTAATTCTAG
AGTTTCAATAAACGCAGGATTTTACGATGAAATTGATAATGAATATTTTTATAATGATTC
TATGGGATTTAAAGAATCCATCCCGAATAAGAATACTGGCATGATAAATATTGAAAATCA
AAATTCTAAAGAGAATTTTCTTCAACAAATTCCCAACGGATTATATAAATCTTCACCCAA
GACACGAAATCCTAATTCCACAAATTCAAATAACATTGAATTTTATGCTGAAATTAATAA
TCAACCAAATAATGAACCTCTTAGTCACGACCATGCCGAATTTAAAGAATCTACAGCTAA
TAAGAACATTAATATGATAAATACAAAATTCTCGAACGGTCCTAATAACTCTTTGACTAC
TGATTATTATGGATTTAAAGAATCAATAATTGAAACAGACAATAAGCATAAAAATAATCC
AATTGTGGTTTTTCCAAGTGAAATGCAATACTCTTCAACTCAAAATGTTCCCAAACCACC
AATAAAATCTGATTTGAATAGTTCTGCTAGTCCTCCAGAATTAGGATTTATAAGAGAAGA
TATTGAAAATGTAAATGTAAAAAAAAAGATACCAAATGGATTAAATAAAACTCCGCCAAC
AACAAGAAATTATGAATTTTACGCTGAAGTTCACAATGAACCAAATAATAAACATCCTGA
TCACGATCCTATCGGATTTAAAGATTCGATTCCGAATAAAAATACTAATATGACAAAATC
GGAATATAATCAATCCAATAAGCAAGTAAAAGATGAAATTCGTTTACTTCAAGACACAAT
TTATCGTTTAAATATACAATTGAGTACACATATATCAAATAATGAGTCTGGTCGTCCAAT
TGGAAAAACGATTTTCCCTGGAAAAGGTCCTGCTCCAAGTTGGTTATTAAACACAAAATA
TTTGGCACCTCTATTAGTATTTTATGATAAAAGATATAATGACAAATCAGCCGAATTACA
AAAAATGCAGTTGGAATTGCAAAATTTTGAAAAAGAACTGTTACGTGTTATTAAAGAAAA
CGAAAGACTTCATTTAAAATTGCAAGAGAATCAAAAAGGATCACCGTTTTCAAATCAGGC
AGATTGGCAAGTAATAGTTCAACAAGCAAAACTCGTTATTGAAGAAAATCGTTTATTATT
AGAAGAAATTGAAATACAGCAACAAAAAATTGTTTTGCTGGAAAACAATAGTTTTTCCGA
AATGGCCAAACTTAAAAAATCTTTGTTGACTGTGACATCAGAAAGAGATGCTTTAGATGA
GGAAATTAAAACTTTAACACAGCGTCTTGCTGAACTAAAGGGTAACACATTACAATTATC
TTTTAATCAAGAAAGTCCTAAAAATTATCTTTTCGGTTCTTCAAATGAAAGTGTAAATTT
AATACATCAAAATCAGCTAAAATTTAATGATTCTGTTATTGTTGAACTGAAAAATGATAA
AAGAAAACTCGAACAAGAAATCGACAATTTAAAACAAAATGAAATAAAACAAGAAAAGAA
ATTGCAGAAATACAAAAGAAATATGGAATCAGAAAAGAAATTCAGAGATTATTTCAATGA
GGTTTTAGAAAAGAGTGATGAAGTCATAAGAGAACACAATTCTCTTTTAGAAATGTAGtg
aatttatttaattttaatttatttattattattattattattattattattattattatt
attattattattattattattattattattattacattaataaatggatgattatgt
aatcgtatataattaataaacaatgttgaatcgtatagatcagagacaaggaaccttttt
actcttagaggccacatttaaatcaggctgtaataaactaagttgatataaacgtctaaa
cactgttgaatggttaataccaaataattttatcaatatcttcatttcttctatgatgag
cactctcaataggcgccaatatgtcgtcgttatcggactcccggaacgtgatgcatggta
agattaaaatttttctggcgaaaatttctttgcctattcaaattaaatttgacgtattct
ctaatgcttttgtagtttggttgattaaacagtaaaaataatactaaaattgaaaaatgt
ttccctttagatttagaaaatcaaaattctatagaaagttctcttaaacaaattccaaat
ggtttacacaaaccctcatccaagacaaatattcctaattctagagtttcaataaacgca
ggattttacgatgaaattgataatgaatatttttataatgattctatgggatttaaagaa
tccatcccgaataagaatactggcatgataagtgtttttagatattgaaaatcaaaatt

ctaaagagaattttcttcaacaaattcccaacggattatataaatcttcacccaagacac
gaaatcctaattccacaaattcaaataacattgaattttatgctgaaattaataatcaac
caaataatgaacctcttagtcacgaccatgccgaatttaaagaatctacagctaataaga
acattaatATGATAAATACAAAATTCTCGAACGGTCCTAATAACTCTTTGACTACTGATT
ATTATGGATTTAAAGAATCAATAATTGAAACAGACAATAAGCATAAAAATAATCCAATTG
TGGTTTTTCCAAGTGAAATGCAATACTCTTCAACTCAAAATGTTCCCAAACCACCAATAA
AATCTGATTTGAATAGTTCTGCTAGTCCTCCAGAATTAGGATTTATAAGAGAAGATATTG
AAAATGTAAATGTAAAAAAAAAGATACCAAATGGATTAAATAAAACTCCGCCAACAACAA
GAAATTATGAATTTTACGCTGAAGTTCACAATGAACCAAATAATAAACATCCTGATCACG
ATCCTATCGGATTTAAAGATTCGATTCCGAATAAAAATACTAATATGACAAAATCGGAAT
ATAATCAATCCAATAAGCAAGTAAAAGATGAAATTCGTTTACTTCAAGACACAATTTATC
GTTTAAATATACAATTGAGTACACATATATCAAATAATGAGTCTGGTCGTCCAATTGGAA
AAACGATTTTCCCTGGAAAAGGTCCTGCTCCAAGTTGGTTATTAAACACAAAATATTTGG
CACCTCTATTAGTATTTTATGATAAAAGATATAATGACAAATCAGCCGAATTACAAAAAA
TGCAGTTGGAATTGCAAAATTTTGAAAAAGAACTGTTACGTGTTATTAAAGAAAACGAAA
GACTTCATTTAAAATTGCAAGAGAATCAAAAAGGATCACCGTTTTCAAATCAGGCAGATT
GGCAAGTAATAGTTCAACAAGCAAAACTCGTTATTGAAGAAAATCGTTTATTATTAGAAG
AAATTGAAATACAGCAACAAAAAATTGTTTTGCTGGAAAACAATAGTTTTTCCGAAATGG
CCAAACTTAAAAAATCTTTGTTGACTGTGACATCAGAAAGAGATGCTTTAGATGAGGAAA
TTAAAACTTTAACACAGCGTCTTGCTGAACTAAAGGGTAACACATTACAATTATCTTTTA
ATCAAGAAAGTCCTAAAAATTATCTTTTCGGTTCTTCAAATGAAAGTGTAAATTTAATAC
ATCAAAATCAGCTAAAATTTAATGATTCTGTTATTGTTGAACTGAAAAATGATAAAAGAA
AACTCGAACAAGAAATCGACAATTTAAAACAAAATGAAATAAAACAAGAAAAGAAATTGC
AGAAATACAAAAGAAATATGGAATCAGAAAAGAAATTCAGAGATTATTTCAATGAGGTTT
TAGAAAAGAGTGATGAAGTCATAAGAGAACACAATTCTCTTTTAGAAATGTAGtgaattt
atttaattttaatttatttattattattattattattattattattattattattat
tattattattattattattattattattattacattaataaatggatgattatgtaatcg
tatataattaataaacaatgttgaatcgtatagatcagagacaaggaaccttttactct
tagaggccacatttaaatcaggctgtaataaaATCGACAGTGATGAAAACATTGACTTTC
AGATTAATGGAAGAATAAATAAGATCTGTTTTGAAGATTTAGAAAATCAAAATTCTATAG
AAAGTTCTCTTAAACAAATTCCAAATGGTTTACACAAACCCTCATCCAAGACAAATATTC
CTAATTCTAGAGTTTCAATAAACGCAGGATTTTACGATGAAATTGATAATGAATATTTTT
ATAATGATTCTATGGGATTTAAAGAATCCATCCCGAATAAGAATACTGGCATGATAAATA
TTGAAAATCAAAATTCTAAAGAGAATTTTCTTCAACAAATTCCCAACGGATTATATAAAT
CTTCACCCAAGACACGAAATCCTAATTCCACAAATTCAAATAACATTGAATTTTATGCTG
AAATTAATAATCAACCAAATAATGAACCTCTTAGTCACGACCATGCCGAATTTAAAGAAT
CTACAGCTAATAAGAACATTAATATGATAAATACAAAATTCTCGAACGGTCCTAATAACT
CTTTGACTACTGATTATTATGGATTTAAAGAATCAATAATTGAAACAGACAATAAGCATA
AAAATAATCCAATTGTGGTTTTTCCAAGTGAAATGCAATACTCTTCAACTCAAAATGTTC
CCAATATTGAAAATGTAAATGTAAAAAAAAAGATACCAAATGGATTAAATAAAACTCCGC
CAACAACAAGAAATTATGAATTTTACGCTGAAGTTCACAATGAACCAAATAATAAACATC
CTGATCACGATCCTATCGGATTTAAAGATTCGATTCCGAATAAAAATACTAATATGACAA
AATCGGAATATAATCAATCCAATAAGCAAGTAAAAGATGAAATTCGTTTACTTCAAGACA
CAATTTATCGTTTAAATATACAATTGAGTACACATATATCAAATAATGAGTCTGGTCGTC
CAATTGGAAAAACGATTTTCCCTGGAAAAGGTCCTGCTCCAAGTTGGTTATTAAACACAA
AATATTTGGCACCTCTATTAGTATTTTATGATAAAAGATATAATGACAAATCAGCCGAAT
TACAAAAAATGCAGTTGGAATTGCAAAATTTTGAAAAAGAACTGTTACGTGTTATTAAAG
AAAACGAAAGACTTCATTTAAAATTGCAAGAGAATCAAAAAGGATCACCGTTTTCAAATC
AGGCAGATTGGCAAGTAATAGTTCAACAAGCAAAACTCGTTATTGAAGAAAATCGTTTAT
TATTAGAAGAAATTGAAATACAGCAACAAAAAATTGTTTTGCTGGAAAACAATAGTTTTT
CCGAAATGGCCAAACTTAAAAAATCTTTGTTGACTGTGACATCAGAAAGAGATGCTTTAG
ATGAGGAAATTAAAACTTTAACACAGCGTCTTGCTGAACTAAAGGGTAACACATTACAAT
TATCTTTTAATCAAGAAAGTCCTAAAAATTATCTTTTCGGTTCTTCAAATGAAAGTGTAA
ATTTAATACATCAAAATCAGCTAAAATTTAATGATTCTGTTATTGTTGAACTGAAAAATG
ATAAAAGAAAACTCGAACAAGAAATCGACAATTTAAAACAAAATGAAATAAAACAAGAAA
AGAAATTGCAGAAATACAAAAGAAATATGGAATCAGAAAAGAAATTCAGAGATTATTTCA
ATGAGGTTTTAGAAAAGAGTGATGAAGTCATAAGAGAACACAATTCTCTTTTAGAAATGT

AGtgaatttatttaattttaatttatttattattattattattattattattattattat
tattattattattattattattattattattattattattacattaataaatggatgatt
atgtaatcgtatataattaataaacaatgttgaatcgtatagatcagagacaaggaacct
ttttactcttagaggccacatttaaatcaggctgtaataaa
>DjScaffold867:2639-3011 strand:+
AACCACATCTGGAGCCCTGTTCAGCACCTGTCAGGATTTGTGGAGCACTATTATAAGATA
AGAGAACTACTGGTACAGCCTTGTAGATTCTATGGAGCGAAGGCTTTCCGCAATAATCGA
TGCAGCTGGAGGATATACCAAATATTAACGATATATTGTTTTCTGTATAAATATATATAA
tataaatgttgtaatattttgtactggacaatttaatgtaacagactgtatataaattga
tacattaataacttagataactctaaaattataaaacctaggaaattaaatttatcttaa
ctaaatatcttttcagatttaaaatttggtc
>DjScaffold867:6410-19404 strand:+
ATTGATCGATGTAAAATCCAATTTCAATTTCATCAAGAAACAATTAAAAACAATACAGTA
AAATGTATTTTTTTAGGCAGCACATCAGCTGGAAAATCAACTGTGATTAATGGTTTATTT
GGAAAAAATGTTATTTATAGTGGTGTATTTGATGGTACAGAATGTGTAACTCAATGCCAA
GCCTCTAGTAATAATAAAGACTATTTTACCATGGCAAACAAAATGGAAATTTTAAATAAA
GAATTCGAAATAACTGAATTGGATTACATAAATAGTCGAAATAAAAATTTAGAAATGTTT
CCCGATATTAGTTGCTTGATAAAAGTTTATTTAAATCCACTCATTTATCCAATTTTAGAA
AATCTAGTATTGATTGATTGTCCTGGTTATGATAATGAGGATATCGAATTGATTATGAAT
TACTGTGATAATGGAAATATTTTCGTTTTTTGTTTTGATGTAAAGAATGGAGTAACACCG
AATGATATAAAGATTATCAACACAGTACTGAATAAAGTTGGAAATTGTGAAATTATTATT
TTATTTACCAAATGTGACATCAATAAACCGATACAAAAAAATTTTACAGAAGAAAGTTTA
ATAAAAATATCAAATATGAAGAAGCTTTCAATTTTTTACATATCTGCGAAAGAAGAATTA
GAAGGGAAAATTAATAGTGAATTCCACAGCGATTTTAAAGATTTCAGAGACCTGCTTTTA
AAATACAGTATAATTATTAAACAAAGTATGAGAAATATATTCCTGGATGTAGTGGCATCA
GTGACAGCGATTATCCAATCTATGAGTACCATTATTCCAAGAGTGCCATTTAATAATATT
GAACATTTGCAAGACTTTATTAGAATTGTCAAGATATTCTGTCCAATTAAATTTAATACA
TTGGATTATAACATAAATTATCGAATTAAACTAATAAAAACACCTTATAATAGAAATCAA
TATAAAGAAGCGATTTCAGATTATTGCGAATTTGCCAATAAAGAGATTTCTAATTTTCTA
ACTGGAATCTTTCTAGAAAATACAACAGTTAATGATGCTCCTAATATATATTTTCTATTC
TATGAACGATTTATAAAGAGTGAAGAAATTCAGGTTACAAATTTATAAattactttttat
aaatataagttaaatttatttgttaaaaatatataagttgttcatttgacaaatcgatta
agtgaataagtaatatgtactgaactaatgaagcatgatgatttaggagtgttaatctaa
ttattgggaattaatataaaaggcattatgagctttgagactaactaatgcaattattgc
gagaataatatttggacgtaaccattgatcgtttttttgaattcatttaaaataaaagct
tcgactattgatcgatgtaaaatccaatttcaatttcatcaagaaacaattaaaaacaat
acagtaaaatgtatttttttaggcagcacatcagctggaaaatcaactgtgattaatggt
ttatttggaaaaaatgttatttatagtggtgtatttgatggtacagaatgtgtaactcaa
tgccaagcctctagtaataataaagactattttaccatggcaaacaaaatggaaatttta
aataaagtaaatagttaatattttcaaaaatagagataaatatttaggaattcgaaataa
ctgaattggattacataaatagtcgaaataaaaatttagaaATGTTTCCCGATATTAGTT
GCTTGATAAAAGTTTATTTAAATCCACTCATTTATCCAATTTTAGAAAATCTAGTATTGA
TTGATTGTCCTGGTTATGATAATGAGGATATCGAATTGATTATGAATTACTGTGATAATG
GAAATATTTTCGTTTTTTGTTTTGATGTAAAGAATGGAGTAACACCGAATGATATAAAGA
TTATCAACACAGTACTGAATAAAGTTGGAAATTGTGAAATTATTATTTTATTTACCAAAT
GTGACATCAATAAACCGATACAAAAAAATTTTACAGAAGAAAGTTTAATAAAAATATCAA
ATATGAAGAAGCTTTCAATTTTTTACATATCTGCGAAAGAAGAATTAGAAGGGAAAATTA
ATAGTGAATTCCACAGCGATTTTAAAGATTTCAGAGACCTGCTTTTAAAATACAGTATAA
TTATTAAACAAAGTATGAGAAATATATTCCTGGATGTAGTGGCATCAGTGACAGCGATTA
TCCAATCTATGAGTACCATTATTCCAAGAGTGCCATTTAATAATATTGAACATTTGCAAG
ACTTTATTAGAATTGTCAAGATATTCTGTCCAATTAAATTTAATACATTGGATTATAACA
TAAATTATCGAATTAAACTAATAAAAACACCTTATAATAGAAATCAATATAAAGAAGCGA
TTTCAGATTATTGCGAATTTGCCAATAAAGAGATTTCTAATTTTCTAACTGGAATCTTTC
TAGAAAATACAACAGTTAATGATGCTCCTAATATATATTTTCTATTCTATGAACGATTTA
TAAAGAGTGAAGAAATTCAGGTTACAAATTTATAAattactttttataaatataagttaa
atttatttgttaaaaatatataagttgttcatttgacaaatcgattaagtgaataagtaa
tatgtactgaactaatgaagcatgatgatttaggagtgttaatctaattattgggaatta

atataaaaggcattatgagctttgagactaactaatgcaattattgcgagaataatattt
ggacgtaaccattgatcgttttttgaattcatttaaaataaaagcttcga
>DjScaffold4376:20014-21151 strand:+
TTTTCCACCTGTGTTGAAGATTTTCCGAATTACGGTGAAGATTTAGTAGTATCTAAAAAT
GATTTGAAAGTTTTAGCTGACAAAATTATTAATTTTCTTTCTATTAATAATCAATTAAGT
TCTGAATCTATTTCTACGATTAGTGATTTCTTTGTCCAACAAAATCAGGATCTTCAATCG
ATTTACAGTGATAATGATAATAATATTAGAAAATATCCAACAATTACCGAATGCTTAGAA
AGTAAAGAGTTAATAATGAAAACCGAACATGATTTAACGAAATTGGATATAGATCATCGT
GGAAAGGAATTTAAACTAGCAAAAGTATTATTTGATGATATGTGTGTGCTAGATATGTAT
TATGTTAAAAGATTTACAACGGAATTCAAAAAATTTCGCCAATATATGGGTATGACCCAA
AAGGAAGTTAGTCTATCAATGTGTTCTAATTTAAAATGTGACTTCAGTCAAACAACCGTT
TGCAGATTTGAGTCAATGTCTCTTCCCCTTACAAACCTGTTTGACCTTATCCCGAATTTC
CGTAAATGGATTTCAAAAATTTACAATCCTAATAAAATTATCACTGTGTGTAATGTAAGT
AAAATTGAAAATGAATCCCATTTATCTTCTACTGAAAATGAATCTTTACAAGATAAAAGA
AAAATTAATATTAAAAGTGAAGATGATAAACCATTGATCGGGAAAAGAAAACGTCGGAAA
AAACGAACTTATTTTACTGAAAATCAGATAAAAACTATGGAAGAACTTTTTCAAAAAGTG
AAGCATACTCAAATTACGAATTTGGTATATAAGAAATATGCAAAAGAACTGAAAATTACT
AAAAAACAAATTCAAGCCTGGTTTACTAAACGACGAAGAAGAGATAATTTTGGAAATATC
AATAAAAGATGAgatttgatgttttttccaatataatgtttgtttcaaaatctaatctt
aattcttgttttctcttaattcttggtttgccttaaatcttggttaatcttaattccatg
ttgattttaattcttggtttatcttcattactggttggtcttaattcttggttgatctta
atgcttggttgatcttaatttctagttagtcttaattcctagtttatcttaattcttggt
ttgtcttaattcctagtttatctttattcttggtttgtcttaattcttggttaatcttaa
ttcctagtttatctttattcttggttaatcttatttcctagtttatctttattcttggtt
aatcttaattcctagtttatcttaattcttggtttgtcttaattcttggttggtcttaat
tcctagtttatcttaattcttggttaatcttaattcgtcactcttcatcctgagattata
caattaatccgttacatttaatctgttttagattgattataagttttaaaagaattgttc
agatttatagtgaaaaaaaattaactaagtcggtatttgttgtaaaattaattgatctag
gtATGACCCAAAAGGAAGTTAGTCTATCAATGTGTTCTAATTTAAAATGTGACTTCAGTC
AAACAACCGTTTGCAGATTTGAGTCAATGTCTCTTCCCCTTACAAACCTGTTTGACCTTA
TCCCGAATTTCCGTAAATGGATTTCAAAAATTTACAATCCTAATAAAATTATCACTGTGT
GTAATGTAAGTAAAATTGAAAATGAATCCCATTTATCTTCTACTGAAAATGAATCTTTAC
AAGATAAAAGAAAAATTAATATTAAAAGTGAAGATGATAAACCATTGATCGGGAAAAGAA
AACGTCGGAAAAAACGAACTTATTTTACTGAAAATCAGATAAAAACTATGGAAGAACTTT
TTCAAAAAGTGAAGCATACTCAAATTACGAATTTGGTATATAAGAAATATGCAAAAGAAC
TGAAAATTACTAAAAAACAAATTCAAGCCTGGTTTACTAAACGACGAAGAAGAGATAATT
TTGGAAATATCAATAAAAGATGAgatttgatgttttttccaatataatgtttgtttcaa
aatctaa
>DjScaffold1184:51898-58722 strand:+
ATATGCGTTGAGTTTACGCAAAATAAGAATTCTAATTCTAATAAGGCAAGTGCAAATAAT
GAAGAAAAACCTGTTTTGAATTTGCCTGACATTTTAAGAGAGAATCTAGATATGATTGTG
GTTGGTATTACTCCATCTGGAATGTTGGGCGTATCTAATCATCATTTTTCTGGTCCTGGA
AATCACTTTTGGAGATGTATTGCTGAAGTTGGTCTTGTTCCTCATCAGGTCACTTGTGAA
GATGATACTAAAATGTTGGACTATGGAATTGGTTTCATCAGTTTTTGTACACGACCCAAT
AAAGGCACCACAGAATTGAGCAGAAAAGAAATGAAAATTGGTGCAGCATTAATGGTGGAA
AAAATTAAGAAGTATAAACCGAGAATTGTTGTATTCAACGGGATTGGAATTTATGAGGCG
TTTATTGGTAATAAAAACTTTTCAATGGGTCTTCAACCAAATAAAATGGAAGGAACCGAT
GCTGTAATTTTTGTTATGCCGTCATCAAGTGCGAGATGTGCTCAACTTCCAAGAGCAGAG
GATAAAATTCCATTTTTTATTTCATTAAAAAAGCTCAGAGATCACATGAGAGGAGATTTA
CCGCAACTTGAAGAAAAAGAAGTCGTATTCGAGAATTATACTGAATTCCGAGTGACTCAA
CCCGATCCTAGAAGTCTATTGAAAGCTGAAAGACGTCGCAAACGAAAAGCAGAATTAAGT
GCAGAATCGGCATTGCAAGAGAGTTTGGTGGAGAATTCAAATCAAACACAATCGATAAAT
ATTGAAGAAAGTAAACCAAAAATTAAGACCGAAAGTGAAGTCGTTAAAGAGGAAAGAACT
TATGATTCATCACAAAATTCACTTGCATCCTCTATTCAACAGAATCAATCTGCTATTGTT
AATTCACCTTTGCTTTCTTGGCCGCAGAACGGAGGTTTGACAATAATTCACACGGACAA
CCAATTATAATGCCAACCAATGGTAATCAAACTAGTTCGTCTAACACGCTATTACTTCTT
GATCCGAGTGGTCTCTCGTTTTCATCTATTCCAGTACCGATGAATCCTGCTCAGCTACAA

GTTCGACCGATGATATTACCAACAGATACTGAACAACGAATTTCTCAGACATCTCACATG
TCTACAGTTCCATCTATAAAGCAAAATGATTGCTTGAATAACCTCTCAAATATACCGCAA
AATATAAAAGCTCGTCCGTCTATTGATGCTCAAAAATCAAACCCGATTAAAACTCCATTG
AAAACTATTTCAAACAATGAAAACACGAAGACCTCAACTACGTTAAATCCTGTATCAATC
ATAGCTAATCCTCCAATCAATAATAATAATAATAGTATCGTTCAAACAAACTCTGACAAT
AAATCGATGGTCGATTTACAAAGTCCAACAGGTAAACCAGTAACAATGGATTATGAAATC
GAAAAGGAGTCAACAAATAGTTCGCAACAAATCAATAAAGTAATAACGAGTGATTTAGAA
TTTAAATACAATCCTCTAAATGATCCAAATGATGAGTGGGAACTATTTATGAACGGAATC
TTTACAGGAGAATTCTCCTATCCCGCCCCACCTCGAATAATACCAGTTAAAACTATCGAT
GCTACAGAAGAATATGAACAATTATCTCAATTTAATGTGTCGCGAATATTAACTGTGTTA
TAAaataaaaataaaaa
>DjScaffold228:37463-48070 strand:+
tatttacttaagtttaggtcctcagcgtttttgaaatttcataatgtttcaaatggacat
ttaatatcaaccatatatatatatatgtcgtttctcaacatgtgtgataataaggtcaac
tctttcctcagaagattgaactaaaggacaagtgtgagtagtcctttgtagtcaggcgaa
attaactcatataacacgcctatatatgagtttggattttgcaatctaaagtttttgctt
tgttatcaatctaaaaatatttgacacaatatttcctatttaaattctctgaatttttat
tcattttataaaacattttataaagaatttcatacatttgaaatttttttaatatttaatt
ttagttaATGAGCAACGAAATTTCCTTAGAAAATATCGAATCAGAAGAAAAGACAGATGC
AACCATATCGGAAATCGATAAATTAAAACTTGAAAAAGATATGTTAAATCAAAAATGCCA
TGAATTGGAATCAAAAATATCACTGCTAAATGAGGAAAAACATTCACTAGAAGACGAAAT
AAAGCGTTATAAAAAGCAGTCCTTTTTTATTGATTCTAAAAATACAAAAACTTTCAATGA
ATCGTCATTTGAATCAGAAAATAACAGTCCTGAGTCAATGCGTGTTAGGACACTTAAGGA
CAAAATCAGTAGTCTTCAGTCAGAATTATATAAAGTTGAACAAAATCGTGATGAAGTCAG
CCAAAAATTAGAGCTCATGATCACTGAAAATTCAGATTTACAAGAAAAACTGAACGATTT
AAATGAACAAATTGGGGAACACCGTCACATCAAGGATGAATTGGACATTGCTAAAGAAAG
AATTAAAACATTAATTGGAATGGAAGCAACTCTTGATAAATATAAGAAACATGTAGATGA
ATCAAATGATTTAGCTCGACATAATAAAGAATTGGAGATTGAAAATTCCAAATATTTGGA
TAGAATTATGAAATTAGAAGAGGAAAGTAAGAAATCAGCATCTCTTAAAAGTCAAATTGA
AATGTATAATAACGAAATCAGAGATATTCAAGGTCTTTGTTCTCAAAAAGAAAAGGAATT
TTTGAAAGCAGATTTTGAATTGAAAAGAGCAATTGAGAAAGTCAAATCTTTGGAGCATGA
TAAAACAACGCTACATGAAGAAATAAAGAAATTAAAAGAAGAAAATGACGAACTGCATCT
GAACAGTTATACAGTGGGTAGCGAATCTAGCTCGGATCCTAACAGCGAAATTCACGACAA
TGTAAATCCTATTGATGATTCATTGGACATCATGGAGATTCCTGTTCATTTACGAGAACG
TGTCATTCGCTTACAAAAAGAAGTTAAACAACTGAGAAGAAATACTTTAATGAGCGTATG
TGGGTCTGAAAATTCTGCGGATATTAATTCTTTGAGTCCTGAACAACTGATTGTATCTTT
AGAGAGTTCTCAATCAAGATTACGACAAATTACTTTAGAAAACAAACAGTTACACCAAGA
AAATCTTAGCCTTAATGCTAGGATTGAAGATTTATCTTTATTATCTAATGTTATGGACGA
CAAGAATCCAGATTCTCAGAAAACAGAAAAATTATCTATTAGCATCAATGAAATAGAATT
AAAATACAAAGGTTATTTAGAAAAAGCTAAATATGTGATCTATCATCTTCATCAGAAGCT
GTTGCAGCATGAACAAAAAATGTCATCGAATATTAATGGATCTCAAGACATTGCTACACC
ACCTACACCTACTGGGGAACACGACGCATCATTTGCTCTTGTTGACAAAGAAAGTATCAC
TCGAAGTGATTATGACTCTCTGAAGTCGGCATTCATCGAGAAAGAAAAACAATTGGATGA
ATTACAAAAACAATATGAAAGATCACAATGTCTTCGAGAGGAAGAAGAGAAAATGGTTGT
TGCTGCTTGGTATAATTTAGGAGTTCGACTAAATCGTGAAGCAGCTCAAGATAGGCTTCG
AAACGACTCATCGGATACATCAACAATATCTTTAAAATCCACAGAGCCCAACTCATTCCT
TGCGAAACAACGTATGGCACATTTAATGTCTTCATCCCAGTCTCTGAAAAGCGATTCTGT
CGATCGGCGATAAataaaacccataattcctattttgtcattccattttatgatacatat
catacaagatcagaaatctttatcgtttcaacacatgcagttctcaagagatgccttacc
tagattaatgtatatcaacagctttagttattcattaaagtttcagtcattacagattga
ttctgatatttatcttatatttgaccgttatttttgacgatttctattctgtatgtattc
actacacaactttatcaattgagcaacttgtattattgtaagtaaaattttaattgtttt
ggctatttacttaagtttaggtcctcagcgtttttgaaatttcataatgtttcaaatgga
catttaatatcaaccatatatatatatatgtcgtttctcaacatgtgtgataataaggtc
aactctttcctcagaagattgaactaaaggacaagtgtgagtagtcctttgtagtcaggc
gaaattaactcatataacacgcctatatatgagtttggattttgcaatctaaagtttttg
ctttgttatcaatctaaaaatatttgacacaatatttcctatttaaattctctgaatttt

tattcattttataaaacatttataaagaatttcatacatttgaaatttttaatattta
attttagttaatgagcaacgaaatttccttagaaaatatcgaatcagaagaaaagacaga
tgcaaccatatcggaaatcgataaattaaaacttgaaaaagatatgttaaatcaaaaatg
ccatgaattggaatcaaaaatatcactgctaaatgaggaaaaacattcactagaagacga
aataaagcgttataaaaagcagtcctttttttattgattctaaaaatacaaaaactttcaa
tgaatcgtcatttgaatcagaaaataacagtcctgagtcaatgcgtgttaggacacttaa
ggacaaaatcagtagtcttcagtcagaattatataaagttgaacaaaatcgtgatgaagt
cagccaaaaattagagctcatgatcactgaaaattcagatttacaagaaaaagttattta
tatagaattgaaatgaacattgtcttaagacatttattattattattattattattatta
taactattattattattattattatataattttattagctgaacgatttaaatgaacaaatt
ggggaacaccgtcacatcaaggatgaattggacattgctaaagaaagaattaaaacatta
attggaATGGAAGCAACTCTTGATAAATATAAGAAACATGTAGATGAATCAAATGATTTA
GCTCGACATAATAAAGAATTGGAGATTGAAAATTCCAAATATTTGGATAGAATTATGAAA
TTAGAAGAGGAAAGTAAGAAATCAGCATCTCTTAAAAGTCAAATTGAAATGTATAATAAC
GAAATCAGAGATATTCAAGGTCTTTGTTCTCAAAAAGAAAAGGAATTTTTGAAAGCAGAT
TTTGAATTGAAAAGAGCAATTGAGAAAGTCAAATCTTTGGAGCATGATAAAACAACGCTA
CATGAAGAAATAAAGAAATTAAAAGAAGAAAATGACGAACTGCATCTGAACAGTTATACA
GTGGGTAGCGAATCTAGCTCGGATCCTAACAGCGAAATTCACGACAATGTAAATCCTATT
GATGATTCATTGGACATCATGGAGATTCCTGTTCATTTACGAGAACGTGTCATTCGCTTA
CAAAAAGAAGTTAAACAACTGAGAAGAAATACTTTAATGAGCGTATGTGGGTCTGAAAAT
TCTGCGGATATTAATTCTTTGAGTCCTGAACAACTGATTGTATCTTTAGAGAGTTCTCAA
TCAAGATTACGACAAATTACTTTAGAAAACAAACAGTTACACCAAGAAAATCTTAGCCTT
AATGCTAGGATTGAAGATTTATCTTTATTATCTAATGTTATGGACGACAAGAATCCAGAT
TCTCAGAAAACAGAAAAATTATCTATTAGCATCAATGAAATAGAATTAAAATACAAAGGT
TATTTAGAAAAAGCTAAATATGTGATCTATCATCTTCATCAGAAGCTGTTGCAGCATGAA
CAAAAAATGTCATCGAATATTAATGGATCTCAAGACATTGCTACACCACCTACACCTACT
GGGGAACACGACGCATCATTTGCTCTTGTTGACAAAGAAAGTATCACTCGAAGTGATTAT
GACTCTCTGAAGTCGGCATTCATCGAGAAAGAAAAACAATTGGATGAATTACAAAAACAA
TATGAAAGATCACAATGTCTTCGAGAGGAAGAAGAGAAAATGGTTGTTGCTGCTTGGTAT
AATTTAGGAGTTCGACTAAATCGTGAAGCAGCTCAAGATAGGCTTCGAAACGACTCATCG
GATACATCAACAATATCTTTAAAATCCACAGAGCCCAACTCATTCCTTGCGAAACAACGT
ATGGCACATTTAATGTCTTCATCCCAGTCTCTGAAAAGCGATTCTGTCGATCGGCGATAA
ataaaacccataattcctattttgtcattccattttatgatacatatcatacaagatcag
aaatctttatcgtttcaacacatgcagttctcaagagatgccttacctagattaatgtat
atcaacagctttagttattcattaaagtttcagtcattacagattgattctgatatttat
cttatatttgaccgttatttttgacgatttctattctgtatgtattcactacacaacttt
atcaattgagcaacttgtattattgtaagtaaaattttaattgttttggcTCAATTCAGA
TATCACTGCTAAATGAGGAAAAACATTCACTAGAAGACGAAATAAAGCGTTATAAAAAGC
AGTCCTTTTTTATTGATTCTAAAAATACAAAAACTTTCAATGAATCGTCATTTGAATCAG
AAAATAACAGTCCTGAGTCAATGCGTGTTAGGACACTTAAGGACAAAATCAGTAGTCTTC
AGTCAGAATTATATAAAGTTGAACAAAATCGTGATGAAGTCAGCCAAAAATTAGAGCTCA
TGATCACTGAAAATTCAGATTTACAAGAAAAACTGAACGATTTAAATGAACAAATTGGGG
AACACCGTCACATCAAGGATGAATTGGACATTGCTAAAGAAAGAATTAAAACATTAATTG
GAATGGAAGCAACTCTTGATAAATATAAGAAACATGTAGATGAATCAAATGATTTAGCTC
GACATAATAAAGAATTGGAGATTGAAAATTCCAAATATTTGGATAGAATTATGAAATTAG
AAGAGGAAAGTAAGAAATCAGCATCTCTTAAAAGTCAAATTGAAATGTATAATAACGAAA
TCAGAGATATTCAAGGTCTTTGTTCTCAAAAAGAAAAGGAATTTTTGAAAGCAGATTTTG
AATTGAAAAGAGCAATTGAGAAAGTCAAATCTTTGGAGCATGATAAAACAGTAATCTTTT
TTATTGATAAATAAttttaaattaaat
>DjScaffold228:83024-83641 strand:+
atactATGAATAATAGGAATTTCTTGTTATTTTCTATCTTATTTATTGGAATTTTATCTT
TACTTTTAACGCCTATTATTTGCGAAAATAAGGATGATAAGAGAGATAGTATATCTCAAA
TAAATCCAGATTGTCCTAAAAAAATTATGCAACACAAGTATATATATTGTCTATATTAGAT
CAAGCTCAAAGGAAGATGTTTTACACTTTTTATATTCTGCCGGCGAATATACACGGACTC
CTTCTGTAATTTTAATGAGGTCAACAGATTTGGAATCGAAAATCTCCATAAATTGGAACA
ATTTTACTTCAAATATTAGCGAATTTAGAATGGATTCGATTGAAAAGATTGGAAATCATT
TTACTTCATTATTTGGTTTAGTTTTTACTAAGTTATATCAATATGAGGACACCAATGACA

AATTAGCTATGACTGGCAAGATAACAATGTGGGCATTTAATCAATTTAAATGGGATTTTA
TTGTAGACGATAAAAAAATCATATTTCAAAGCAAAAGCGTCGAATCAAACAAAAATTTGT
TTAGCAAAAATGGCTCAATTAGTTTGATATTTTCCTATGAAGCGACTTCCAAACCAGGTC
CATATATGCCAAAATTAATGTTCAACACTAATAACACACAATTCGATTTCATTTTACAAG
AACCATCGTATTTTCAAAAAAATCCAAGATTTGCTGTCGAATTTGCTCTGATTAATATTA
ACAATACTGGTGAGATGAAGGTAGAAACTGTTCAATCAATAGATGATGAATCTTCTCCCG
GAGTCTTTGAAAGTGTAAACTATTATGGTCAAAGCCTCAACACAAGTGATTCAATCCAAA
CATTTGTTCAATGGAAACCTGTTTGTTATATTACCAAAAATGATTTTACCATTAATAGTA
TATCAGTGGCCAAAGATATGGACAGTCCGAAAGCACTTAGAAACAATTATGATATCATTA
AAACCAGTTTGGTTTATTATGCATATGGAGATCGAATTAATTTATCTACACGGGCGTCAT
CATTAAATGTTACATTTGGGATATCGAATGATAACTTTACTAAAAATTATATTTTATGGA
CAGGCAGTTTTGGCTATGGAAAACCTCCAACATATGAATTGTCGATAACAATTATCATGA
TTATTCTTTTTGGATTGGGAATCCCATTTCTGATAGTTGTGATTGGTTCGATTTATTTAG
TTGTTAAAAAAAAATTTATTCGTAAAAAGACTGGTTCATATGGGCAGATCGATGAAGCAA
TTAATTGAccttccacctctatgatttttactttatatttgatgcacactgatttgtat
attgattttataataaatgtattttattgactaatttgtttatcagttttcctatgaag
cgacttccaaaccaggtccatatATGCCAAAATTAATGTTCAACACTAATAACACACAAT
TCGATTTCATTTTACAAGAACCATCGTATTTTCAAAAAAATCCAAGATTTGCTGTCGAAT
TTGCTCTGATTAATATTAACAATACTGGTGAGATGAAGGTAGAAACTGTTCAATCAATAG
ATGATGAATCTTCTCCCGGAGTCTTTGAAAGTGTAAACTATTATGGTCAAAGCCTCAACA
CAAGTGATTCAATCCAAACATTTGTTCAATGGAAACCTGTTTGTTATATTACCAAAAATG
ATTTTACCATTAATAGTATATCAGTGGCCAAAGATATGGACAGTCCGAAAGCACTTAGAA
ACAATTATGATATCATTAAAACCAGTTTGGTTTATTATGCATATGGAGATCGAATTAATT
TATCTACACGGGCGTCATCATTAAATGTTACATTTGGGATATCGAATGATAACTTTACTA
AAAATTATATTTTATGGACAGGCAGTTTTGGCTATGGAAAACCTCCAACATATGAATTGT
CGATAACAATTATCATGATTATTCTTTTTGGATTGGGAATCCCATTTCTGATAGTTGTGA
TTGGTTCGATTTATTTAGTTGTTAAAAAAAAATTTATTCGTAAAAAGACTGGTTCATATG
GGCAGATCGATGAAGCAATTAATTGAccttccacctctatgatttttactttatatttg
atgcacactgatttgtatattgattttataataaatgtattttattgatattatagagtg
taaactattatggtcaaagcctcaacacaagtgattcaatccaaacatttgttcaatgga
aacctgtttgttatattaccaaaaatgattttaccattaatagtatatcagtggccaaag
atATGGACAGTCCGAAAGCACTTAGAAACAATTATGATATCATTAAAACCAGTTTGGTTT
ATTATGCATATGGAGATCGAATTAATTTATCTACACGGGCGTCATCATTAAATGTTACAT
TTGGGATATCGAATGATAACTTTACTAAAAATTATATTTTATGGACAGGCAGTTTTGGCT
ATGGAAAACCTCCAACATATGAATTGTCGATAACAATTATCATGATTATTCTTTTTGGAT
TGGGAATCCCATTTCTGATAGTTGTGATTGGTTCGATTTATTTAGTTGTTAAAAAAAAAT
TTATTCGTAAAAAGACTGGTTCATATGGGCAGATCGATGAAGCAATTAATTGAccttcca
cctctatgatttttactttatatttgatgcacactgatttgtatattgattttataata
aatgtattttattgac
>DjScaffold4663:13102-14033 strand:+
ttcttATGCGTATATTAACATCAGATAATGAATCTACATCCACAGAACTCACTGAACCTA
GGAATCTTAGTTCTTGGGTCAGTAATAACATGGTTTCGATTGCAACACAGGTTAAAATTC
AAGCTTTAAAAATGCTGAGTAAATGGATGGTGACATTAAAGGTGGAAGAAGATCGAAATC
TTATTCTAAGTGTCATGGTATGTCTTCGGAAGTATATCGGGTCCTATGGCAATCCGAAAT
ATAGTGGGAACATAATTGATGGGGATATGGCTCAAATGCGCTTGGCAGCAGGGAAATGTT
GGTTACGGCTTGCACAAGTTCAGTCTTTCTTATCGATTTTCGATGTGAAATGGCTGCATC
GCTTATCAGGACTCATTTATGATCCATGCAGTTATGTTCGTGAGGGATTTCTCAGGAAGT
TGCACAAAGGACTATTTAGATTGCAATTGCCATTGGATTATATGGCAATGTTTGCTTTCG
TTCCTTTGGTGCAAAATTCTGAATATCAAACTCTGGCCAAGAATTTCTTTGCAGCGAATG
TCACTCGTCGTCGCGAATTTCTGGAAAGAAATCCCCAATATCAAACAGATGCAAGTATGT
TATTGGTTTTTAATTAAtatcaattataatatatataatctttaatcaataatttatata
atttcaattatgcaattttaataaataatcaataatttatataatttcaattatgcaatc
tcaataaataatcaataatttatatttcaattatacaatctcaatcaataatttatatca
tttc
>DjScaffold3841:18163-20251 strand:+
ccgATGATGGAATACCCGTTCCCGCATGTCCAGTTGGAGGGAGAATATAATAATTTCATC
AATGAGTTGGGTATTGATGAGGCCACAGCCAAACCAGCATATTTGGGATTTATGCCATCA

CAATCGTCCGGTAACGCGAGTCAAGGCGCAAGTTCATTTATTTTCAGTGAAACAAAAAAA
AATTTTAAAAATACAAAAAATCTGTTAATTTAAcactaagattaataaccggcatattga
tttatttaataaaaaaactgacaatttttgggtttgatagaattttcaataaagatttca
tcaaattgattttacgaaatgagtgaaaatatcggaattttatttttaagatttatatg
cgcaaatatttaatgttatccttaattagattttttagtaatttcagtacctctttata
taactcaaatacgaaaaatactattgataaatttaaaaagATGACTAGAAAAGATAAATC
ACCTTCACGAAGAGATAAAAGACGTTCATCTACTGATACTGAATCATATCGTCGAAAGCA
TAAAGATCACAAACGAAGAGATAGAGATCGATCTTCACCAGATTCTAGAAAAGATAGATC
TCCTTCACGAACAGATCGTTCTTCACGAAGAAAGAAAGATAAAGATCGGCAATATAAGCA
CCGTGATCGTTCAAGTGATTCCAGAAGAGAAAAGTCTTTGAAACGAAAATATCAAGATTA
TTCTCCGGAATCATCTCATCGCAAGAAACACCATAAACGTGACCGTTCACAATCGAATTC
AAGTAAAAGAGAAAAACCTATCAAAGAAGAATCTATCTATACAGAAGTTTTTAGTTCAAA
ACGATTTTCTCCGAAAAAAGAAGAATTTGATTATCAACCTGAATCACAAAGTCTTTTTAA
ACAAGAAAGTTATGTTAAACAAGAATGCAGTGTTAAAACTGAAGATTCTGATGGAAATTC
TGAAAAAGCCGCTCGTAAAAGGAAACGCGAAAGTAAATGGAGTTCGGGTCAAGCTAAAGT
TCCTTTGTATTTAAAGCCGGATGATGCTGAAGTATATTCAATTCAGATACAGATAAACAG
TCTCACAACAAAACTTGCGCAACAGAATTATACAGAAGAAATTGAAACCATGTGTCGCTC
GGCGTCACCAGATCCTGTATATGGTCATGATGGTAAACGTATTAACACCAAAGAATACCG
AGCTCGTGAAAGAATGGAAGAACGTAAAAGAAATCTAATTCTTCGACTTTATGCTTTAAG
GCCAGATTTGGTCCCTCATGCCGATAAAATGATGGGTGCAAGAATTGAAGAAAAAGTTTT
TATTCCACAAGATGATTATCCGGATATAAATTTCGCTGGTTTGATTATTGGTCCCAGAGG
TATGACACAGAAAGATTTGGAAAAAGAAACCGGAACGAAAATTCTTTTACGTGGAAAAGG
TAGTGCTAAACCCGGCAAATTGAATGGCGGAAGTGCTCCGCTTCCGGGCGAAGATGAACC
ATTACATGCTTATATTACATCAATGTCGAGAGAAAGTGTGGATAGAGCTGTAGAAAAGAT
CAAAAAAATAATCCAAGACGGAATAGAATTTCCGGAATTACAAAACGATTTGCGCAAAAA
TCAAATGAAACAGTTGGCGATTCTCAATGGAACCTATCGAGAAACGGACGGTTTGAATAA
ATTAAAGATTCTCAGTGAAGCTCAGTCGATTGCAACCAATACGATAATTTGTAGTTTGTG
CGGAGGAACTGGGCATTTACCGAGAGACTGCAAAGTAAACTCCAATGAAATACCGATGAT
GGAATACCCGTTCCCGCATGTCCAGTTGGAGGGAGAATATAATAATTTCATCAATGAGTT
GGGTATTGATGAGGCCACAGCCAAACCAGCATATTTGGGATTTATGCCATCACAATCGTC
CGGTAACGCGAGTCAAGGCGCAAGTTCATTTGTTAGAAATTTTATATTTAGAAGTTGAac
atacgtttgtattattataattatcatcattattattattatctttgatacgaatatctg
gaaattgtaatattatttatcgtattaataataatatttaatttgactttttctttttca
ttaagattttcagtgaaacaaaaaaaaattttaaaaatacaaaaaatctgttaatttaac
actaagattaataaccggcatattgatttatttaataaaaaaactgacaatttttgggtt
tgatagaattttcaataaagatttcatcaaattgattttacgaaatgagtgaaaatatcg
gaattttatttttaagatttatatgcgcaaatatttaatgttatccttaattagatttt
tttagtaatttcagtacctc
>DjScaffold669:72176-74908 strand:+
attgctgctatacatgacagatcaacaattgcgaaatttttacttcgatgtggagcagat
ataaatactaaaacttataaaATGGAATTAACACCGATACATCTAGCCGCTATTTATGGC
AGTATAAAGGTATTAAACGTTTTGTTATTTCACAAAAATAATGCTGTTGATGTCGTCGAT
AAAAAATTGAATACGCCACTTATATATTCATGTAGTAAGAGTAATATTGACATTGTGGAT
TCATTACTAAAGGCGGGCGCTTCAATAAATTCTTCTAATCACCGAGGTTTTACTCCGTTA
CATGAAGCTGTTCGTCATAAGAAAGCAGATATTGTTGAATTATTGATTCGGAATGGTGCT
AATTTAACTAGTTTAAATTTAAATGGACTTTCGCCATTAGATCTTTCACAAGATTTGAAA
ATATATTATATACTAAAAAATGCTCTGCATTTAAAAGGGTCCAGAGAAATTGAAAATACG
GAGATAGAAAAAAGTGAATCAGGAATGGTTTCTCTCAATTCAAGTTTCTACGCAGATCAA
AATACATTTCATATTGATACAGATAATTTATCTCTTTGTAGTGGATTATCAGTTCCATCT
TCAGAAGATAAAGTAAAAAATATTATTGTTATTTTATTAATTTCGTTATATTTGACTGTA
ATATATTGTTGCCATTTTTGTCGTTATAATACAAGTCATTATTTCATGCAATATAACCGT
AAATGTTTATAAtttttattattattattattattatcattattattatcattattatt
attatt
>DjScaffold669:30452-32551 strand:+
ATGCCATTTGGTATTGCTGCTGCTCCAGGAACGTTTCAAGAACTTATGAATAAAGTATTA
ATCGGGATAAATGGTGTCGAAGCCTTGGTTTATTTGGATGATATCTTAGTTTTTTCTAAT
TCCTTGGAAGAACACATGATTCGTTTAAATAATGTGTTAAGTAGAATAAAAACTGCGGGT

TTAAGAATTAATCCAGACAAATGTTCGTTATTGAAAAAAGAAGTTAAATTTCTCGGACAT
ATTATTACAAGTGAGGGAATACGCACAGATCAAAGTAAAATTGAAGCAATAAGTAAATTT
GAAAGGCCGAAATGTATTAAGAGTTTGAGAAGTTTCTTAGGATTATGTAATTATTATCGA
AAATTTATAAATAAATACGCAGAAAAAGCTAGAATTCTAGAAGCATTGTGTGGTAAAGGT
AATGAAAAGTTGATTTGGTCTGATCAGTGTGATGAGTCGTTTAATCAATTAAAAATTGCT
TTACAGAAAACTCCAGTGTTAGCATATCCAGATTTCAACAAAGTTTTTATTTTGGACACG
GACGCTAGCTTTGATGCAATTGGAGCAATACTCTCACAATTAGATGAAAATGGAAACGAA
AGAGTTATTGCTTATGGATCACATGCGATGAGTTCACACGAAAGAGGTTATTGTATTACA
AGAAAGGAATTGTTAGCTATTTATTATTTCACTCAACACTTCAATCATTATTTGTATGGT
AAACAATTTATTCTTCGAACGGATCATAAGGCTATTACTTTTATGTTATCTACAAAAAAG
CCGATTACTTCACAATTCCAGACATGGATAAACTTTTTAAGCAGTTTAGATATTAAGATG
GAATATCGACAGGGAATTTTACATGCAAATGCTGATTCATTATCTCGACAATCATGTACA
ACTTGTTCCCAGTGTCAAATGAGTCATCCAGAAGCTAAATCAGGAAAGTTAAAAACTCGT
ATCTTGACATTAATGACTGATAATGTTGGAAAAGAATGGCAAGATGGAGTTCAAGAAATA
GAAGAAATAAAAAAGCAAATTGGAAAAACTAGAGGGGGTTATTTTCAGTCGGTGGAAGAT
ATTATAAGAACGAAAGATAATAAAATATGGATACCAGAAATTAAGAGAGCTGAATTTATT
AAAAAAACTCATTTTATTTTATGTCATGCTGGATGTGAAAAAGTAAAATCATATATTAAA
AATAATTTTGATATGAAAAATTTGGCAAGCTTTGTGAAAGAAATTATTGGTGGATGTGAA
GCATGCCAAAAACAAAAAATAATAACTACTAGAACGAAAGAGGACATAATTCCAGAGACA
TCTTCAAGACCATTTGAGAATATTTATATTGATATTTGTGGACCATTAAGGTCAACTTGG
CATCAGGAAAAATATATATTAGCAATAATAGATCATTTTAGTAGATATATTTCATTAACA
GCAATCGCTAAACAAGATGAAAATACTATAACTGATACTTTGGTTAAGAAATGGATTCTA
AAATATGGTGCCCCTCGTAAAATACATTTAGATTGCGGGAAAAGTTTTGAGTCAAAGACG
ATGAAAGAGTTGGCTGATAAATATAAAATACAAGTAGTTTATTCTAGTCCATATCACCAC
AGCGCTAATGGATTAATAGAACGACAATTTAGAACAATTAGAGATCGTATAAATACTTCC
GTAACGAATAATAAGAACAAAAATTGGGCAGATTTGATTCCGGAAATTGAATTTTCCATG
AATGCGTCAACTCAAAAAACAACTAAAAAGAGTCCAGCCGAAGTGGTTTTTGGCTTTAAA
ATAAACAGAACAGAATGGCATAAAACGGATAAAATGCAAGATAGAAATGCTATTCACGAA
GAAATAATAAATAATAATAAAAACACAGTTGATCATCATTTGAGTATTAGAACATTTGCA
GTTGGTGACACAGTATTAGTAAAAAAAGATAATCGACATAAAAATGAAGAAAGATATGAT
GGACCGTATAAAGTGACGAAAAGATTACATGAACGAAGTTATGAAATTACAGATATAAAG
GGAAGAAAATTTATAAGAAACGTTGAATGGCTCAAAGGTTTTAAAGAAGGGGGATATTAG
>DjScaffold669:58852-60003 strand:+
ATGCGAAAGGAGATCACGTTTTTGGGACACATTATCAGTAAAGAAGATGTCAAAATAGAT
CCAACTAAAATAGACGCAATTAAGAACTTTAGTGAACCAAACTGCGTTAAGAAAATGAGA
AGCTTTTTGGGAATTTGTAATGAATATCGCAAATTTATTAAAGATTATTCAAAGAAAGCT
AGATTACTGGAACAGCTGTGTAGTGGGCCGAGTAACAAGAAGATTGAATGGTCAAATGAA
TTAGAAGAAATTTTTAATAGAATGAAAGAAGCACTAATGATGTCTCCAATATTATGTTTT
CCGAATATAAATAGAGGATTTATTTTGGACACGGATGCGAGTTTCGATACGATTGTGGCA
GTACTATCTCAAAAGGACAACAATAGACAAGAAAGAGTAATTGCATATGGGTCACACGCA
ATGACGAGTCATGAGAAAGGATATTGTATTACGCGGAAAGAATTGCTAGCAATTTACTAC
TTTTGTATTCATTTCAAACATTATTTATATGGGAAAAGATTTATCCTCAGAACAGACCAT
AAAGCCATCACTTTTATGCTAAATACCAAGAAGCCGATTACATCACAGTTTCAAACCTGG
ATTAATTATTTGAGCATTTCAGATATAAAATTGGAATATAGAAAAGGATCGCTTCATACA
AATACGAATATGTTGTCTAGGAGTACATGCGTAACATGTGCACAATGTCTAATATCACAT
GAAGATTCCAAAAGTGGAAAATTAAAAACAAGGATGTTAAATACAGTAGATATTCAAGAA
AAACAAGGCAATTTTGGTGAGAATGATCAAGGAACATGGATACCTGATGATAAAGTAGAA
AAGGTCATAATGAGAATACACCATCTTTTGTGTCATGCTGGAGCAGAAAAGGTAGCAGCG
TATATGAAGCATAAATATAACGCGAAACATTTGTGGAGCAAAGTACAAGAGGTTGTCCGA
AGCTGTATAACATGCCAGGAAGCTAAATTGTTGACTACAAAAACGAAGAAAGAAACAAAA
AAGACGCAGCCAGTCAAAATAGTTGAACTATTATTTGTAGATGTTTGTGAACTACTGTCC
GAGACAAGAGTTCGGATAAAATATTTTCTCGGTATAATTGGAAAATGCATTAAGTATGTA
GCCTGGATGTAG
>DjScaffold325:7081-15500 strand:+
ATAACACTCAGAATTGAATCACCAGCTGAAACAGCTCCTAATTTAACATTAATTGATCTG
CCTGGAATTACACGTATTGGTCTTGATGATCAACCAAAAGATATTTGTGATCAAATTAGA
AACATTATTATGGATCATATAAGAAATGAAAACACGGTAATTTTATGCGTTATCTCAGCA

AATCTTGATGCAGCAGTGAATGAATCGATTAAATTTGCTAAAGAGGTTGATGAAGCAGGA
ACCAGAACATTAGGAGTTATAACGAAACCTGATCTTGCTGAAGACACTCACAAAGATGAT
GTTCTTGGATTATTGAATAATGAAAAAATTAAATTAGTAAAAGGATTTCATATGGTAAAA
TGCAGATCTCAAAAACAATTGAAAGAAGGATTGAGTCTCAAAGATTCTCTAGAGGATGAG
AAAACATTTTTCGAAAATGATTTATTTTATCGAGAAGCCGTACCAACCCAAATTGGAATA
ATGAATTTAGCAAAAAGAATTACTGATGAATTAGTTGGAGTAATTCATGATGCAGAAACA
AAGTTAATGAAATTTCCAAAACCTGTTCCACAGGGTGAACAGGAAATATTAATATATTTA
ATAGAGGTATTACATTTTGTATTTCATGTGTTTAAAGATTTATTTTCAAGAAAATTTCTG
ATATGAatcaagaaattaaatcaagtcttattcatggtaaaagtatttccaatttgaaga
atttgttttataattggcgtaatgtatttgatagaatgaggactcaaattgataaattaa
aggatgacgcatcaaaatttaaatttaatctcagaaatttgaaaaaggaaataatggaaa
aacgaggaagtgaaattagtggttttctaaattttcaactgtttgttgatcttatccaaa
accatgtaatgacttataaagaagaagcattaatacttctaaaagaaattaaagatgata
cttccaacattttaatacaaattatccatatttattttcaaaatttaccatcagtggaat
caagtttgaagtgtctcaattatctttgcctcagaactcgtttatactctcaaatgttac
tgaaagcatcggaacatatccgacaaagtgttctaagaatttggtgtttgaaaaatttct
gcaatttgaatggtcaattcatttcagaagaaagatttttcgATAACACTCAGAATTGAA
TCACCAGCTGAAACAGCTCCTAATTTAACATTAATTGATCTGCCTGGAATTACACGTATT
GGTCTTGATGATCAACCAAAAGATATTTGTGATCAAATTAGAAACATTATTATGGATCAT
ATAAGAAATGAAAACACGGTAATTTTATGCGTTATCTCAGCAAATCTTGATGCAGCAGTG
AATGAATCGATTAAATTTGCTAAAGAGGTTGATGAAGCAGGAACCAGAACATTAGGAGTT
ATAACGAAACCTGATCTTGCTGAAGACACTCACAAAGATGATGTTCTTGGATTATTGAAT
AATGAAAAAATTAAATTAGTAAAAGGATTTCATATGGTAAAATGCAGATCTCAAAAACAA
TTGAAAGAAGGATTGAGTCTCAAAGATTCTCTAGAGGATGAGAAAACATTTTTCGAAAAT
GATTTATTTTATCGAGAAGCCGTACCAACCCAAATTGGAATAATGAATTTAGCAAAAAGA
ATTACTGATGAATTAGTTGGAGTAATTCATGATGCAGAAACAAAGTTAATGAAATTTCCA
AAACCTGTTCCACAGGGTGAACAGGAAATATTAATATATTTAATAGAGGTATTACATTTT
GTATTTCATGTGTTTAAAGATTTATTTTCAAGAAAATTTCTGATATGAatcaagaaatta
aatcaagtcttattcatggtaaaagtatttccaatttgaagaatttgttttataattggc
gtaatgtatttgatagaatgaggactcaaattgataaattaaaggatgacgcatcaaaat
ttaaatttaatctcagaaatttgaaaaaggaaataatggaaaaacgaggaagtgaaatta
gtggttttctaaattttcaactgtttgttgatcttatccaaaaccatgtaatgacttata
aagaagaagcattaatacttctaaaagaaattaaagatgatacttccaacattttaatac
aaattatccatatttattttcaaaatttaccatcagtggaatcaagtttgaagcaaattg
ttcttaatcaactggaagaatacagtaaaataagtactgaaagaattaatattatattcg
aacaagaaatgcttgtattcacgatggattctacatttgagaaagaact
>DjScaffold325:47033-47507 strand:+
ATAACACTCCGAGTTGAATCACCAGCCGAAACAGCTCCTGATCTAACCTTAATTGATCTG
CCTGGAATTGCACGTATTGCTCTTGATGGTCAACCAAGAGATATTTGTGATCAAATTAGA
AACATTATTATGGATCACATCAGAAATGTAAACACGGTGATTTTATGCGTTATCTCTGCA
AATCTTGATGCAGCAGTGAATGAATCAATTAAATTTGCTAGACAGGTTGATGAAGCAGGA
ACCAGAACATTAGGAGTTATAACGAAACCAGATATTGCTGATGACGCTCACAAAGAAGAT
GTTCTTGGATTATTGAATAATGAAAAAATTAAATTAGTAAAAGGATTTCACATGGTGAAA
TGCAGATCTCAAAAACAATTGGAAGAAGGGTTGAGCCTCAAAGACTCTCTAGAAGATGAG
AAAATGTTTTTCGAA
>DjScaffold325:53637-59249 strand:+
cagagggtcaacctcaaatattgagatatttaattgaaatattttctgatATGAATCAAG
AAATTAAATCAAGTCTTATTCAAGGTAAAAGCAGTTCAAATTCAAAAAATTTGTTTTATA
ATTGGCGTAAAGCCTTTAGTAAAATGAAAACTCGGATTGACCAAATAAATAATGATGCAG
TGAGATTGAAATTTAGTGAAAAAAATTTAGAAAAGGAAATAATGGAAAAACGAGGAAGTG
AAATTAGCGGTTTTCTAAATTTTCAACTGTTTGTTGATCTTATCCAAAACCATGTAATGA
CATATAAAGAAGAAGCATTAATACTTTTAAAAGAAATCAAAGATGATACTTCCAACATTT
TAATAAAAATTATCCATATGCATTTTCAAAATTTACCATCAGTGGAATCAAGATTAAAGC
AAATTGTACTTAATCAACTGGAAGAATTCAGTAAAATAAGTAGTGAAAGAATAAATACAA
TATTCGAACAAGAAATGCTTGTATTCACGATGGATTCTACATTTGAGAAAGAAATGGATG
CCTTCCTCCCAGATGTAAAC
>DjScaffold325:76756-83855 strand:+

aacgtcacttggctactataattttaATGTCTTACTTACCTGACATAGAAGACATAGTTG
TAGAAATTGAAGATGCAAATGAAAGAGGCATTGCATCTGATGTTATTGTGACGCATATTA
AAAATCGAAAAGAAGTAGAAGATATTAAAGTTGAAACAGAAAGTTATGTTCCAGGTACTC
ATCATATTTATATTCAAACTTGGGGTTGTTCACATAATAGTAGTGACAGTGAATATATGG
CTGGAATTCTTTCGAAATATGGCTTTAAAGTCTCATTAAATGGTTCATGTCAGCAGACGC
ATGATTCTGACGAAATTGTTATTCATAAAAAAAAAGTTCCCGTAAATTCTTCAGATGAAG
AAAACAAAAGAAAAGCAGATTTATGGATATTAAACAGTTGTACAGTAAAAAATCCCGCTG
AAGATCATTTTCGCAATATGATCGCTGAAGCAATGAAGTACGATAAGAAAGTTATAGTAG
CAGGTTGTGTTCCACAAGGGCAACCAAAAGCGCCGTATTTGAAAGGTGTAAGTGTTATTG
GAGTTCAGCAAATCGATAGAGTTATCGAAGTAGTCGAAGAGACTCTCAAAGGAAATTCAG
TGCAACTTTATGGCAAGAAAAGAGAAGGTGGGAAAGCTCTTGGAGGCGCAAAATTGAATT
TGCCAAAAATTAGAAAGAATCCTTTTATCGAAATTTTACCGATAAACACGGGCTGTTTGA
ACGCGTGCACGTATTGCAAAACGAAACACGCTAGAGGACATTTAGGGAGCTATCCGATCT
CGGAAATTCTTGATAGGTTTGATCAAGCGTTTGAAGAAGGCTGCGTGGAGATTTGGCTGA
CTTCTGAAGATCTCGGAGCGTACGGCAGAGATATTGAGACAAATCTGCCGGAATTGTTGG
AAGCTGCGGTGGATCACGTTCCGCCTGGATGCATGTTACGATTAGGCATGACAAATCCTC
CGTATATTCTCGAACATCTCGACAGAATCGCTGCAGTGCTGAAACATCCGAGAGTTTATT
CATTCCTTCACGTTCCCGTCCAATCAGGTTCTGATAATGTGCTCGGAGAAATGAAGAGAG
AGTATACCAGAGAAGATTTCTGTAACGTTGTCGATACTCTTTATCAAGAAATCGGTGTTG
GCAAAGTCACTGTTGCTACTGATATTATTTGTGGTTTTCCAACTGAAACTGATGAAGACT
TTGAAGAAACGTTTAGTTTAATTGACAAATACCGATTTCCGGTATTGTTCAATAACCAAT
TTTACCCTAGACCGGGTACACAGGCAGCGAGAATGGAGAGAGTTGCTTCTTCAATGGAAG
TTAAGAAAAGAACGAAGAGAATTCACGAATTGTTCCATTCCTACGAGCCATACACCGAGA
GAGTTGGTGATGAAATGAATGTTCTCATCACAGAAGAAAGCACAGATGGCAACTATTTTG
TTGGTCATACCAAAGCCTATGAACAAGTTCTTATCCCGAGAATTCATAAATTGATGGGAA
AGGTTGCCCGAGTTAGAGTTATTGAAACAAGCAAGCATTCAATGAAAGCCGAGATAATTG
ATGACACAGTTACCAATGTAACTGATGAATTAGTAATTACTGAAATCAGAAATCAAACAA
AACATTTGCTAGAAACGGAATCCAAAGACATTCTCAAATCAACAATCCCAGCGGCGCTCT
TATCTCGTATTTTGGCAGTGTTTTTCCTATCTTTCGTATTGTTGAAAATTTACACTCTAT
GGTTACGGCTTGTGTGAtgtaataatacataccaataaatggttgcttcgtg
>DjScaffold325:26798-26893 strand:+
ATGCTTTACTTGCAGCACCATTGTATGCAGCAACGACAGGACAAGAAAAGAACATTGAAT
GGACAAAAGAATGTGAGAATAGTTTTCAATTTTTGA
>DjScaffold1800:11311-12474 strand:+
gtgatttaattttcaaattattttttttcttaaggcATGAATGCTATTGAACGTAAAGCT
TTAACAAAGAAAAATAAAAACAAAAATAATCAAATGAATAAAAATAGAAAAAGAAGCAAA
TCAATACAATCAAATTCTTCAAGTGAATCTGGAAGTCAATACGATTCCGATAATTACGAC
AGTGATTCTAATTATGATTGCAGTGACGGAGAACAAGAATCGAAATGCGATTATCGCAAA
GGTGGATATCATCCTATATTTATTGAAGAAGTATATAATAATAAATATAAAGTTATTAGA
AAAATAGGTTGGGGACATTTTTCCACCGTTTGGCTGTGTTGGGATATCAAATTGAAACGA
TTTGTAGCAATGAAAGTCGTAAAAAGCGCCCGGCATTACACTGAGGCAGCAATTGATGAG
ATTCAGTTGTTGAAATTCGTAAAAACTAAAGATCCGTTGAATATCGGGAAATTTGCTGTA
GTAGAGTTACTGGACGATTTCAAAACTTATGGACCGAATGGAACACATGTTTGCATGGTT
TTCGAGGTCCTCGGTTGCAATTTGCTCAAGATAATCAAGCAGTCCAACTACACCGGGATC
CCTCTTTCCCAGGTGAAAACTATCGTCAAACATACCTTGATGGGACTGTCCTACATGCAC
AGCGTTTGCCAAATGATTCACACTGACATAAAACTCGAGAACATCATCCTCACCGCTGAC
CAGGATTATATCAAAAGCTTGGCAGCTGACGCCATTCTTTCTTGGAAAACTGGGATTGAT
TTACCTTTAGCTTCAGGTAACAAT
>DjScaffold1800:2212-2685 strand:+
ATGCCATTTGGTATTGCTGCTGCTCCAGGAACGTTTCAAGAACTTATGAATAAAGTATTA
ATCGGGATAAATGGTGTCGAAGCCTTGGTTTATTTGGATGATATCTTAGTTTTTTCTAAT
TCCTTGGAAGAACACATGATTCGTTTAAATAATGTGTTAAGTAGAATAAAAACTGCAGGT
TTAAGAATTAATCCAGACAAATGTTCGTTATTGAAAAAAGAAGTTAAATTTCTCGGACAT
ATTATTACAAGTGAGGGAATACGCACAGATCAAAGTAAAATTGAAGCAATAAGTAAATTT
GAAAGGCCGAAATGTATTAAGAGTTTGAGAAGTTTCTTAGGATTATGTAATTATTATCGA
AAATTTATAAATAAATACGCAGAAAAAGCTAGAATTCTAGAAGCATTGTGTGGTAAAGGT
AATGAAAAGTTGATTTGGTCTGATCAGTGTGATGAGTCGTTTAATCAATTAAAA

>DjScaffold773:63117-64374 strand:+
aaattttgatcgaagcttttattttaaatgaagaaacggctgtgattaaggttgtaaaat
gaacaataaagctcatttaaatataagcttcgatcatatatttttcttctgataataa
ttcgaattttccgcttagtctaactaaattgaaaattcgaattattatcaaaaaccaaaa
acaatcttgatcaaagcttaaattatatgaattctgaaaaacaatcaatccaacaattt
aaaaaaaatgttttctatttttggattttatcaaaaaatttccctagtattcattaaac
actattttaagttttattcttggaatgtaaaatggaacttgtatttaaaatcaatcggat
gatatatttgtaaagaaaaataaatttttgtgtttctagaaatcaatatttatagaaaa
taatgaagaaatcgatttgaaattaatttacggtgaattgattgatatttttagaatatc
gtgcatacaggattgtgattatattgatttattgATGAATTTATTTGACATGATTTTGAT
TTTTCATCAAAATAAGATTCCCTGTTTATTTTCTCTTTCCGATTTCGTTTCCAATGGTTT
TCTTTCCATGAAAATTCAGAAAAACGATTTATTAGATCATTTCATGGATTGGAAAGAATT
ATTGGGAAATATTTTGAATTTTCTATGTGAAAATTCCGATGATAAATTCAAAGAGAAATC
TGCTGTAAAACTCTGGCAGAAAACATTCGAAATTCTCCCTCCAACTGAATTTGCTTCATC
CAATATTTGGATTCTATTTTTTTACACTGTCGAATTACTCAATAGTTTTCACATTTCTCT
TCAGTTCGATTCTAACACTTTTATAACGATTTTTTTGAAACAGTTGAAATTTTCATCTTT
GAAATTAATCGGTAAATTTTTAGGAAATGTTGCGAGATTTCTTATCAATAATTTTCAAAT
ACTCTCTCAATCTTTTCGACCAATTATCAGTTGTAATGAATTTCTCGATGATTTCGAGTG
TTTATTGAATTTGATTGAGCCTTTAAAAGATTCGAAAGATCTGTTTTTGTTTATTAAAAC
TATTAACCAATTGAAATCGTCTAATTATGAATGTACACGATTCGATAGTGTTTGTTTGAA
ATGGGTGTTCACGCGTATTAGTTTAGTTTATCCAATAGTTGATTTTACCTTTCAATTAAC
TGAACAAGAGGAG
>DjScaffold773:72048-77129 strand:+
tatttagaATGGCTGGTTATGATTATTGTGAATCTGATATGTTAAACTTTGTAAGTTATC
GAAATATCGAAGAAAAGTTCATAGGCGAAATATCTTGGGTGGAATCACCTTCTGATTTTT
ATGTCAATATTGTTCAGGGAAAAGAATGGAATATTTATTTAGATTTATTGGATGAAATGC
AAAAATTCTACAGGAATTATCAGTCTAAATGTTATATTAGTAAAGGATGTGTTGGACAGT
TAGTAGCTGTTAAAGTGGATGGAAAGTCACATTGGCTGCGGGGTTGTATTAAGAACGTTG
TATCGAGAGATCGTTATGATATATTTTATATTGATACGGGTTATATTCAATACGATGTAG
ACGAACGAGATATTCGTTTATTACGATACAAATATGCGTCATATTGTCCAGCTCAAGCGA
TTCCTGTTAAATTAGCTTTTGTTGAACCGACAAAGGGCCGCTGGTCTAAATCAGCAGTTT
CATTATTAAAGGACTACATGGATATGGAATTATCGGTGAAATGTTCGTCAACTAAGTTGA
CAAAAGATTGTTGTCGATTTTCGGTAGTGTTGCTCGATAAGAAAAAATACGACGAGAAGC
ACGTGGTTACTCCTGAATTGGTGAAAGGAAAGTACGCAGAGTTC
>DjScaffold773:85029-86162 strand:+
GCAGAATTATTTTTAGATTCAGCAATCAGATTTTGGGTATTTTTACCTTTAGCATTAATA
ACCTATCTTTTTGGTATAATTAGACATTATATAACAATTTTAATAACAAATGAAAAGAAA
CCTGATTTACAAGCTGTTGTTGATAGTCACGCTTTAATTAGAAGCAGGTTGTTACGGGAA
AATGGAAAATATCTTACAAAGCAGGCTTTTAATATGAGAAAACATTACTTTAATGACACA
GAAACTGGATTTTTCAAAGTACAGAAACGTGATTCCACAGCTAAAAATCCAATGACTGAT
CCATCTGCCATGTCAGAAATGGTTAAAGGAAATGCATTAAATGTAATTCCCATGTTAGTA
ATAGGAACATGGATAAATTCAGCATTTACTGGATTTCTAACAACAAAAGTTCCATTTCCT
CTTACTTATCGTTTTAAACCAATGCTACAAAAAGGATGCGAATCTCTAGTATCTCTTGAT
GCTTCTTGGGTGAGCTCAGCTTCTTGGTATTTTTTAAATATTTTCGGGCTGAGGAGCATG
TACGGGTTGGTTCTCGGTCAAGATAATTCCGCAGAACAACCGACAATGATGAGAGATCCA
ACCAGCACAATGGCTGCACAAACTGGACAAGATAGTCAGAAGGCATTTAAAACAGAATGG
GAAGCTCTAGAAATCGTTGATCACAATTGGGTGCTGAAGGATGTTCCGAATAAATTGATT
CTAAACGGAATGAACAATTATCAAAACTGTACTCATAACACGAATAATCGCATGATAACA
GCCGAATGAaaagaaaattttgatttaatttaataaaattgttttgtatttacaatagaa
attacgcgttga
>DjScaffold3520:27649-42428 strand:+
AATCGCGGACTTGTCGTTGTATTATTCTGTTTACCAGCGTCTTTCGTTTTTGGAATTTTC
ATAAATATTAGAGCATTTATTTTCCAATTAATTTCCAAATTTTCCGGCTCACATTTAGAT
AGAGTACGAAAAATCCAAAATCAGATTCGAAAATGGAATGAAATACCTAAAGAAAAAAGG
AAATTACTTTGTAATTCTCGACCAAATTGGTTGTCTTTATCAACAAAATTTTTCCAGAAA
GATGAATGTCACAAAGTTTCAATACCATTATTTCAGATTTTGGATTTCAATGAAAATAAA
CTAACTGTAACAGTTGAACCATATGTAACGGTTGGACAAATCACAGATTATTTAATTGAA

AAAGGCTTTGTATTAGCTTGCACTTTAGAAATAAAAGATGCGACTTGTGGTGGTCTTGCG
ATGGGAGTTGGGATGACAACTCATTCACATAAAGTTGGATTATTTCAAGAGACAATTGTT
TCATATGGTTTGGTTTTATCCGATGCCAGTTACATAAACGTAACAAAAGAAAATGAGCAT
TCCGATCTATTCTATTGTTTACCTTGGTCACATGGATCCTTGGGTTTCCTAGTGTCTTTG
GAACTGAAAATAATTCCTGCAACAAAGTATATACATTTAAGATACAGACCAGTTGTCGGA
CTTGAAAATTATTGTAATCAGATCAGACATTTTTCTGGAGCGGAAAATTCACATAATCCC
GATATTCCTGATTTTTTAGAAGCAACGATTTTTAGTCATGATGAGTCTGTAATTATTTTT
GGAAATTTTATTAATCAGAAAGAAGTTAGTAAAGTGAATAACATATCAAGATGGTACAAG
CCTTGGTTTTACAAACATGTAGAAAACTTTTTAAAGAAACCTGTTAATTCTGATGGATTT
TCTGGTGAAGAAATCGTTCCAGTTAAAGAGTATTTATTCAGACATAATCGAGCGATATTC
TGGGTACTTGAAGCAATTATCCCATTTTGCAATAATCCACTTTTTCGATTATTCTTTGGG
TGGATGTGTCCTCCTAAACCAGCATTTCTCAAATTTACTACGACTCCTGGAATTCGAGCT
TTTACTTTTGTTAAACAAGTATTTCAAGACATTGTTCTTCCAATGACAGTTCTTGAAGAA
TCCATCGAATTATCTGAAAAATTATTCAATATTTATCCTATTTTAATTTATCCCTGCAAA
ATATTTTATCGAAATCCTAAC
>DjScaffold4166:13140-16139 strand:+
gtaaacaatacaATGGATGATATTTTATCGCCCATCGCTAAATTAGAATTTGATGACTAT
GAGTCCTCAAATATCATTTGTAATGAATATAATTTAAGCGATGAAATAAATGGCAATTTT
CCTCTTGATTTATTTAAAAATAATCTCGGAACTTTTAAACACCAATCATTACAAGCTTCT
TTAGAAAATTTGGTCAACAACTTTGATTCAGTTATATCAGAATGTTTTAGTAGTGATGAA
AAAATGGATTCATTTTCCCCTATATCAGAGAAATATAATTTTTCATCAGATTTGGTTGAT
AAAAATCAATTGTGGTTGTCATTGACTGGATCTGTACAGAAGACACTTAATTTGGACTGG
AAAAGTTTTAGGTCATTTGAAGAATTTAAGAATGCTATTCATTTATGTGAGCATGAGGAT
ATGTTAAATCAAAATCTTGATTTGCCTGATGACGAAGAATTGACATTGGCAATGGATCAT
CACTCTTTGGTTACTTCTTGTTTACCATGTGATTATGAACCGCTTAAGACTGCTGATGAA
GTAATCCAAGAAATAGATAAAATTATGGGAATCAGCGGAATGGAAATTTCTATAACAGAT
TTGAGAACAGATCTTCAACACTCCGATTTCATAGGAAGTCAAGAAGCTATCTATTTT
>DjScaffold4598:19779-27139 strand:+
aaataatacattttaaagattattttaaattaagaattatttcttaaaatatATGGAAAT
TATATGCTTTCTATGCCGTGATTCTAAAGAATCGAGTGAACTAATTAAAATGAGATGTAA
TCACCAATTTTGCTGGATATGCCTAAATGATTATCTTAAAAAGAATCTTAATTGTAAAAT
AAAGTGTCCAATGAAAGATTGCCATGTGTTCATTCGTGATGCAGAAATCAGACGAGTGTT
ATCAGATTGTGATATTGAACAATATGAAAACAAATGTTTAGGAAAATTACAAAAATCTCA
CAATTGTTTGCTTCCACAATGTAACTTATCAGTGGTTTATACGGATCCAAATGTTCAATA
TTCTTGTAAAAATTGCACACTTTACAATTGCATTCAATGTCATGCATCTCATAGTTTTAC
ATCTTGTTCTGATTATCAATCGAAATCACCAAATACACAACGTAGAATTTGTCAGGAAGC
CATCAAAAATGCAGAAATTGTTTTCAATAACAAAAGAAGAACCGAATATCGAGAAATGTT
TTTAAAATTTGATGAAATGGATATTTTGTCGAATGATTCAGTATTTACGTGTAAATTGTG
CGATTCAGTGATTGCAAAGGGTGATGGTATAAAATTGCAAAATTGTCATGATATTTTATG
CAAAATTTGTTTGGTTACATCTATAGTAGAAACCAAGAAAATTCCAGCTGTTTGTTCACT
GTGTGAAACATCTCTAAACGATAGAGAAATCCAGAGTATCTTAACACCGGATCAATTTTT
GATTTATCAAGAAATGTGCAAAAATTATACTAAAAGATTTTTCAAACATTTCCAGTGCCA
AACAGTCGACTGTGATGGTTTCTATCAAATATCAGAGTCGATGACAGAATTCATCTGCAA
CAAGTGTGGAATCAAGAATTGTGTACCATGTCGAGCGGTTCATCCAAATGAAACATGTGA
TCAATATAAATTAAGAAAAAATAATTCAAATCTCAATGAAGAGCAAATAAAAAAATGATAA
CAAAATGGAATGCTTGATTTGTCACAATTATCTTACTAAAACAAAAGGTTGTAATTGGAT
ACAGTGTCCATTTTGTTCTACAGAATTATGCTGGATTAGGAAAATGCTTCGATGGGGTCC
TGGAGGTCGAGATGATATTTCTGGAGGTTGTCGATGTATGGTTGATGGTATTCCATGTGA
CCCTAAATGTACATCCTCCTGTACTCCCCATAATTCAAATAGTTCTATTATCAATCGTCG
TTTTCATAATCATTATTCAATACCTCCTCGCTTTCCTTATACAAATGACAGTGATAGTAT
TTCTACAAATTCTAGCAATTCAGAAGAATTTTATaaataatacattttaaagattatttt
aaattaagaattatttcttaaaatatATGGAAATTATATGCTTTCTATGCCGTGATTCTA
AAGAATCGAGTGAACTAATTAAAATGAGATGTAATCACCAATTTTGCTGGATATGCCTAA
ATGATTATCTTAAAAAGAATCTTAATTGTAAAATAAAGTGTCCAATGAAAGATTGCCATG
TGTTCATTCGTGATGCAGAAATCAGACGAGTGTTATCAGATTGTGATATTGAACAATATG
AAAACAAATGTTTAGGAAAATTACAAAAATCTCACAATTGTTTGCTTCCACAATGTAACT
TATCAGTGGTTTATACGGATCCAAATGTTCAATATTCTTGTAAAAATTGCACACTTTACA

ATTGCATTCAATGTCATGCATCTCATAGTTTTACATCTTGTTCTGATTATCAATCGAAAT
CACCAAATACACAACGTAGAATTTGTCAGGAAGCCATCAAAAATGCAGAAATTGTTTTCA
ATAACAAAAGAAGAACCGAATATCGAGAAATGTTTTTAAAATTTGATGAAATGGATATTT
TGTCGAATGATTCAGTATTTACGTGTAAATTGTGCGATTCAGTGATTGCAAAGGGTGATG
GTATAAAATTGCAAAATTGTCATGATATTTTATGCAAAATTTGTTTGGTTACATCTATAG
TAGAAACCAAGAAAATTCCAGCTGTTTGTTCACTGTGTGAAACATCTCTAAACGATAGAG
AAATCCAGAGTATCTTAACACCGGATCAATTTTTGATTTATCAAGAAATGTGCAAAAATT
ATACTAAAAGATTTTTCAAACATTTCCAGTGCCAAACAGTCGACTGTGATGGTTTCTATC
AAATATCAGAGTCGATGACAGAATTCATCTGCAACAAGTGTGGAATCAAGAATTGTGTAC
CATGTCGAGCGGTTCATCCAAATGAAACATGTGATCAATATAAATTAAGAAAAAATAATT
CAAATCTCAATGAAGAGCAAATAAAAAATGATAACAAAATGGAATGCTTGATTTGTCACA
ATTATCTTACTAAAACAAAAGGTTGTAATTGGATACAGTGTCCATTTTGTTCTACAGAAT
TATGCTGGATTAGGAAAATGCTTCGATGGGGTCCTGGAGGTCGAGATGATATTTCTGGAG
GTTGTCGATGTATGGTTGATGGTATTCCATGTGACCCTAAATGTACATCCTCCTGTACTC
CCCATAATTCAAATAGTTCTATTATCAATCGTCGTCATTCTAATCGTTCAGTTACCAATC
GTCGTCATTCTAATCGTTCAGTTACCAATCGTCGTaaataatacattttaaagattattt
taaattaagaattatttcttaaaatatATGGAAATTATATGCTTTCTATGCCGTGATTCT
AAAGAATCGAGTGAACTAATTAAAATGAGATGTAATCACCAATTTTGCTGGATATGCCTA
AATGATTATCTTAAAAAGAATCTTAATTGTAAAATAAAGTGTCCAATGAAAGATTGCCAT
GTGTTCATTCGTGATGCAGAAATCAGACGAGTGTTATCAGATTGTGATATTGAACAATAT
GAAAACAAATGTTTAGGAAAATTACAAAAATCTCACAATTGTTTGCTTCCACAATGTAAC
TTATCAGTGGTTTATACGGATCCAAATGTTCAATATTCTTGTAAAAATTGCACACTTTAC
AATTGCATTCAATGTCATGCATCTCATAGTTTTACATCTTGTTCTGATTATCAATCGAAA
TCACCAAATACACAACGTAGAATTTGTCAGGAAGCCATCAAAAATGCAGAAATTGTTTTC
AATAACAAAAGAAGAACCGAATATCGAGAAATGTTTTTAAAATTTGATGAAATGGATATT
TTGTCGAATGATTCAGTATTTACGTGTAAATTGTGCGATTCAGTGATTGCAAAGGGTGAT
GGTATAAAATTGCAAAATTGTCATGATATTTTATGCAAAATTTGTTTGGTTACATCTATA
GTAGAAACCAAGAAAATTCCAGCTGTTTGTTCACTGTGTGAAACATCTCTAAACGATAGA
GAAATCCAGAGTATCTTAACACCGGATCAATTTTTGATTTATCAAGAAATGTGCAAAAAT
TATACTAAAAGATTTTTCAAACATTTCCAGTGCCAAACAGTCGACTGTGATGGTTTCTAT
CAAATATCAGAGTCGATGACAGAATTCATCTGCAACAAGTGTGGAATCAAGAATTGTGTA
CCATGTCGAGCGGTTCATCCAAATGAAACATGTGATCAATATAAATTAAGAAAAAATAAT
TCAAATCTCAATGAAGAGCAAATAAAAAATGATAACAAAATGGAATGCTTGATTTGTCAC
AATTATCTTACTAAAACAAAAGGTTGTAATTGGATACAGTGTCCATTTTGTTCTACAGAA
TTATGCTGGATTAGGAAAATGCTTCGATGGGGTCCTGGAGTAATTAGTTAAtttatttat
tttcattgcttatttactgaaactagtagatattcaattatgtcgaatattaaagttttg
tcataatagtcgactttataaacaaaacctacaagtctgttactagtaaattatgagaat
actgagttaatggtggacaataatgatatagtttttgatagtgaaaaagtacattgttct
caaaactgttttaaaaaattttaggaagccatcaaaaatgcagaaattgttttcaataac
aaaagaagaaccgaatatcgagaaATGTTTTTAAAATTTGATGAAATGGATATTTTGTCG
AATGATTCAGTATTTACGTGTAAATTGTGCGATTCAGTGATTGCAAAGGGTGATGGTATA
AAATTGCAAAATTGTCATGATATTTTATGCAAAATTTGTTTGGTTACATCTATAGTAGAA
ACCAAGAAAATTCCAGCTGTTTGTTCACTGTGTGAAACATCTCTAAACGATAGAGAAATC
CAGAGTATCTTAACACCGGATCAATTTTTGATTTATCAAGAAATGTGCAAAAATTATACT
AAAAGATTTTTCAAACATTTCCAGTGCCAAACAGTCGACTGTGATGGTTTCTATCAAATA
TCAGAGTCGATGACAGAATTCATCTGCAACAAGTGTGGAATCAAGAATTGTGTACCATGT
CGAGCGGTTCATCCAAATGAAACATGTGATCAATATAAATTAAGAAAAAATAATTCAAAT
CTCAATGAAGAGCAAATAAAAAATGATAACAAAATGGAATGCTTGATTTGTCACAATTAT
CTTACTAAAACAAAAGGTTGTAATTGGATACAGTGTCCATTTTGTTCTACAGAATTATGC
TGGATTAGGAAAATGCTTCGATGGGGTCCTGGAGGTCGAGATGATATTTCTGGAGGTTGT
CGATGTATGGTTGATGGTATTCCATGTGACCCTAAATGTACATCCTCCTGTACTCCCCAT
AATTCAAATAGTTCTATTATCAATCGTCGTTTTCATAATCATTATTCAATACCTCCTCGC
TTTCCTTATACAAATGACAGTGATAGTATTTCTACAAATTCTAGCAATTCAGAAGAATTT
TAT
>DjScaffold4826:10363-21283 strand:+
tgtaatattcaaacaaggctattttaaatttagataacttctttaatagacaaatgagat
gtgaattgcaagtttactatttataaaaattaattccatacaagctctaataatctttta

ttactattacgtgatgtgttttattgaatgtaaatatgaacaataacattaagatgtatt
taatatcttatcttaataaattttccaagatttggaaacgtgaaatattgatattccaaa
aataattttattatattgattatttaccgaaATGAAATTATTTTCAGAGGATGATGAAG
AGATATCTCCAAAGGTAAATGTAGCAGTTACCATATATGGTATAATAAACGGTAAAAATG
AAAAACTGGTATATAATGGATCAAATCGTCATACTGAAAGAAAATTTAAGTGCATCAAGA
AAAATTGTGAAGAAATATTCATAGCTCATATAAATTTTATCTCGATGAAAATATATAAAT
TCTATGTAGAAATTTCTCATAAGAATTTTGTTATAAAAAATATAGAATTCACATTTTCTA
CATTCAGTCATTACTTTTCTCAATTGGAAATTTGGTTCAGATTTTCTTTTCTTGTTTTAG
GTTTTATTGCAACGGTGATTTATAACAATTCACTTAGAAAATTTTCCATTCGAGATTGGA
GCATGGAACAGAAATGGGTAGCTGTATTGTTACCGTCATTGGTGATTTATAATAATCCAT
TAGTTCCTCTAAAATTTATTATTAACACTCCGGTAACAAGTGTTTTGGATACTATATTTA
AGGCAACATTTGTTTGCTTATTATTATTATTTTGGGTAACTATATATCATGGAATGCAGC
AGTCTAAAAGAAGATGCCTCACATTCCATTTTCCAAAAATATTTATTGTTTTATTGATAT
GGATAATGATTGTTCTGTTGCCAACATATCAGGCGATTAAAGATTCCGACGATCCTACAT
TTTATTATAAATTACAGAATACATTTTTGTTTTATAAATTTGTTCTTTTGGGTAGCATAA
TTATTTATATTCTCTATGCATTGTTTCTCATAATAACTGCTTGTGGGGAATTGAGATCGG
TACCTTATTTTAATATTCGCATTAGATTTTTCGCTGGTTTCATGTCAATTGTTTTAATTT
TCAGTATCGTGATGATTGGGTTGCGTTACGGATCTGGATTGTTCAATAACAATTTTGTAT
TTGAAAGGGCCAATAGGTATGCGAATTCTGTGGAGTTCTGTGCTCAATACGGAATGATAA
ATTTGTATTTATTTGTTATGGCATATGTTTATTCTCCAGCGAAAAATGCTATTCTTGAAG
CTAACTTTCAAGATGATCCAAATCTTTCAATGGTTAATGAATCAGATGAAGATGTTCTTT
ACGGATCAGAAATTGAAGAAGCAAATTTATTAACACGCAAAATTTCCAAATCCAAATTAA
ATAATCGTTAAaattctatttcttaatgcatattctctttattttcgttatttttctggt
tgaggatttgttcgtatattgttatcgacagctatgaattttaatgaccgctacatttat
ttcatttgtgaaaatgttgttatttatgtaataaatatctcaatcatctacaattccata
caagctctaatattaagaatttacaattgacatagatcttttattactattacgtgatgt
gttttattgaatgtaaatatgaacaataacattaagatgtatttaatatcttatcttaat
aaattttccaagatttggaaacgtgaaatattgatattccaaaaataattttattatat
tgattatttaccgaaATGAAATTATTTTCAGAGGATGATGAAGAGATATCTCCAAAGGTA
AATGTAGCAGTTACCATATATGGTATAATAAACGGTAAAAATGAAAAACTGGTATATAAT
GGATCAAATCGTCATACTGAAAGAAAATTTAAGTGCATCAAGAAAAATTGTGAAGAAATA
TTCATAGCTCATATAAATTTTATCTCGATGAAAATATATAAATTCTATGTAGAAATTTCT
CATAAGAATTTTGTTATAAAAAATATAGAATTCACATTTTCTACATTCAGTCATTACTTT
TCTCAATTGGAAATTTGGTTCAGATTTTCTTTTCTTGTTTTAGGTTTTATTGCAACGGTG
ATTTATAACAATTCACTTAGAAAATTTTCCATTCGAGATTGGAGCATGGAACAGAAATGG
GTAGCTGTATTGTTACCGTCATTGGTGATTTATAATAATCCATTAGTTCCTCTAAAATTT
ATTATTAACACTCCGGTAACAAGTGTTTTGGATACTATATTTAAGGCAACATTTGTTTGC
TTATTATTATTATTTTGGGTAACTATATATCATGGAATGCAGCAGTCTAAAAGAAGATGC
CTCACATTCCATTTTCCAAAAATATTTATTGTTTTATTGATATGGATAATGATTGTTCTG
TTGCCAACATATCAGGCGATTAAAGATTCCGACGATCCTACATTTTATTATAAATTACAG
AATACATTTTTGTTTTATAAATTTGTTCTTTTGGGTAGCATAATTATTTATATTCTCTAT
GCATTGTTTCTCATAATAACTGCTTGTGGGGAATTGAGATCGGTACCTTATTTTAATATT
CGCATTAGATTTTTCGCTGGTTTCATGTCAATTGTTTTAATTTTCAGTATCGTGATGATT
GGGTTGCGTTACGGATCTGGATTGTTCAATAACAATTTTGTATTTGAAAGGGCCAATAGG
TATGCGAATTCTGTGGAGTTCTGTGCTCAATACGGAATGATAAATTTGTATTTATTTGTT
ATGGCATATGTTTATTCTCCAGCGAAAAATGCTATTCTTGAAGCTAACTTTCAAGATGAT
CCAAATCTTTCAATGGTTAATGAATCAGATGAAGATGTTCTTTACGGATCAGAAATTGAA
GAAGCAAATTTATTAACACGCAAAATTTCCAAATCCAAATTAAATAATCGTTAAaattct
atttcttaatgcatattctctttattttcgttatttttctggttgaggatttgttcgtat
attgttatcgacagctatgaattttaatgaccgctacatttatttcatttgtgaaaatgt
tgttatttatgtaataaatatctcaatcatctaca
>DjScaffold4826:42227-45111 strand:+
tttatgacaaaacatcccctATGGTTATAAATAGACAATCGTTTATTCAAAATTCTACCC
CAACTTGCGACCAAAAATCAAAAATTGAAGAAAAACAAACCAATTTACTAAATTTTTCCG
AAAAAGAATTACTATTAAAAGAGAAACAAACTCCAAAAATATATGATTATGATGAATATA
AAAGTTATGCCTATTTTACCATTTCAGTAGAAATTATTCATGATAGCACATTAAATACTT
TGGCAGTGAAAGTTATTCCTAATGAAAATATGCCTAAAAAGTTTGATGTAATTTTGAACA

ACGATGGTAATATACAAAAACATGTTAAATGTTATAAAAAAACTTCAAAATTTGTGAAAA
TTTCTGAAAATGATTTAGAAAAGGCAATACTAAGCATCAAATTTTATCAGAGTAAATCAA
TAAATATCGGAGATTTGAGTGAAATAAAATATGAATTAAATTCTTTGTTGAATGTTGCCA
TTAAAGATAAACAAAGATTTTGCAGAACTGATTTGAATTTATTTCAGAAAAATTCGAAAT
CATTAAAGGGATCAGTTGAAATTGGATTACAGTTTGATGAAAATAATAAAGAATTAATCG
TCTATGTTTATGATGGAAAGGATTTAGTAAATGATTCTGAAAAACAATTCAATCCATTTT
GTTTAATTCAACTATTTAAAGAAAAATCACAAATTATGAGTTTTAAAACAACTGTTCAAC
CTGATACAAAAGATCCGGTTTGGAATGAAATATTTAAATTTCCAATTCTGTATAAAGAAT
TGAGTAAAGATGTACAATTGGAAATAAGGGTTTTCAATAATACTGATTCTGTGAAATTAC
CAATTGGAAATTTAACATTCCCCACAATGGAATTGAGTTTTGAAACAAATCATTGGAATC
AAATCATAACGAAGCCCAATGAAGAAATTAAAATG
>DjScaffold4826:66871-67230 strand:+
ATGACAAAGGTTTTAGATACAACAAAGGGGACAATGGTGTATTTAGACGATATTCTAAAA
TATTCAGAAAACTTGGAAGAGCATAACAAAACACTGGATGAAATATTTAGTAAAATACAT
CAAGCAGGACAGCGTTTGAGTCCGTCAAAATGTCAATTGCTAAGAAAAGAGATCAAATTT
TTGGGACGCATCATAAATGATAAAGGAGTTCAAACTGATTCATCAAAATTGGTTTCAATA
CAGACATACAAAAGACCAAAATGCGTGAAAGGATTGAGGAGTTTCTTGGGGATATGTTGT
TATTACAGAAGATTCATAAAGGATTACGCAAAGAAAGCTAGAGAACTAGAAAAACTGTGA
>DjScaffold2718:23810-25150 strand:+
aatcattggtccatagcaattatttatgagtaataaaatgacaataattacacatttgct
gttagaattaatcaaaaaaaaataataataagaatacaaaacgaaaaatttacatgataa
aATGATTTTAGATGAAAACAATGGTAATGATACTATTAATTATATAAATAATTCGGTGGC
AATTTCTCCATATGAAATGATCAATTCGAAACAATTCGAAAATTTATTAAACAATCCAAA
TTACAACGAACATTACGATTTAATACAAAATTTAGATTTCAATGGTCTAGGATATCATAA
TGTTAATCAAAATAATAATAATCTTAACAATCAAAATTACAGTCTACTTCAACAACTGAA
TTCCGATCATTCCAATGATTTATTTAATCTTATCAATAACGATTCATTTGTTACTGCAGA
ATCAACTGGATATCCCCAGTCTCAATTTTTTTATCCTTACCAGCAATATATGTATCGTTT
TACTACTAATCCTACAAATAATAACAATCATTTCGAATTTCCCCATAATACACCAAATTT
GAGTAATTGTAATATTTATGAAATTCCCAAACCGAAGCTGATTGAAAACGAAACCCAAAT
TCCTGTTGAAATTAATAGACGACTTACAAATAATCAACTTCAAAGAAATTCAATTCCTAA
ACAAACAAATGTAAAATCTGAAAATCTTTTTCAGTTGAATGCTGACAAATTATTTTCGTG
TACAAATTGTCCTAGTCGATTTACTCGGAAAGATGAATTAAGGCGACATGAAAAAAATCCA
TAGCGGCATTAAAACGTTGACTTGTCCGTATTGTGATAAATCGTTTATTCGCTCGGATCA
CAGACGAACTCATATAAGAACACACACCGGTGAAAAACCATATTGCTGTGAAATTTGTTT
AAAATGTTTTGCACGCTCAGACGAACGAACACGCCATACAAGAATTCATAATAAAGACCC
AATTAGAAAACGAAGGAAGATAATAAAAGTCGAGCCTGTTAATAGTAATATTAATAATAA
TATTAGTGCAATAACTTTGAAAAATGTAAAAAATAATTTATCATCGAAAAATCAAAATGT
ATTCAAAGAGAAAAAATCTGCAATCCAATCTAATATTGATCAAATACACTCGTATATGAA
TGATGCAATAGTTGAATACAGTCCAGTATCGAACAATCATGTGCTGATACATTTTAAAGA
TTCAATTACTAATCCAAATACGAAAATTGAGCCGTATATTAATGATGCAATTTTAGAAGT
GAATAATTTGAAATTTAATCAATTTAGTGAT
>DjScaffold2718:83857-93384 strand:+
TATGCGGTTAAAAGCCCACTTAATCCAAAACAGGAGGATAATGCAAATTTTGAATGTACT
CTATCCAATGATTTTATTCTAAATGCTAAGGGATTTGTATTTATTAATATTTCTCTGGAA
GCAATTTCACCACAAACTCATCTGAAACCAATAATACTAAAAATCAAAATTAAAATGGTG
GCCAATATCGATTTGAAAGTATTAGTGACACCTGATAACTACCGATATCAAGAGAATCCA
TTGTTGGGATTTGATGGTATCACAAGTGAAGAAAAGATTGGTCCAAAAATGAAATTTACT
GTGACTTTGGAAAACAATGTTCAAAATAGTGTTGTATTAGCTTCTAAATTATTGATATAT
TTCCCTTGGGAAATTGCTGGAAATCCAAATGAGGTTGATGGGCAATATTTGTTATACTTA
CTGCGTACTCCTAAATTCAGTGTCACCACAGGAAATAAAAAAATCCAATGTAACGCTAAT
GTTCTTAATAATATTACAAATATCATTAAAACAAGAATTTATCAAAATACGATTGATAAT
CCCAAACTAGAGGATGTAATATTAAAAAAAGTATCTGTGCCAAAGAATGCACCGAAATTG
CCAAAGATTAGTGAAGAATTGAAATTTATTCCAAAGGAATCACATTCAGAAGAATCAAAG
TCTGAATGGGAAAATATTGTATCAATAGATTGTTTGGAAGGAAGTGCTAGATGTGTTCCA
ATTGTCTGTAGTCTTGGCCCTTTATCAAGACAAAGTGGAGAAATCACATTAGAGTTAGAT
ATTCGAATATGGGAACACACTTTAGTAGAGAATTTTCTCAAATATAAATTTGTCTATATT
GATATTCCAGTATTCTGGGTTCCATTGGTTGATTATGATATTCTATTAAAAGATCCAGAA

GGAAGTTTTAGAAAGGTTAATAATTTA
>DjScaffold2718:134512-140670 strand:+
tatttttgaaagtttattcgttttaaatttgttggaaATGACTAATAAAATGTTATATG
GCAAAGATTTAAGCAATTATAATGATGAAGATATTGACGATCTTTTAAGTAAACTAACTC
CAGAAGAAATAGAGGAATTGAATAATGATATTGATCCTGACAATCATTTGCTTCCAGCTT
CACTGAGATGTCGTGATCAAACAACCAAACAACCCACTGGTCCATATGATAGAGATGCGC
TTATCAAATTTATTGAAGAAAAGAGTAAGCAAGAAAAAGATTGGGAAAAAAATGTACCAT
TTGAAAAAAAAATTCGCGGTAAAATTTTTAAGCCAAAAATAGAAGAATCAGAAATATCTA
AAAAAGATCTTGAAGAAATGGGTTTTGATATGGAATTCGATACTGAATTTGATGAAGCAT
TAAAAAATGCAACTGAAGAAGAATTAGTTGACTTGGCTGCTGTGTTGGGTTTCAACGGAA
TGTTAAATCAAACCCAGTATCACGCTAGTGTTGAAAATAAAGAACAAATGTGTGGTGGAT
TTCAGGGTGTTGCCAGGCATGAACCCTGTAAACCAGTTCCTGATGAGCCAGATAACGACA
CAGATGTGGAAAGTGTTATTAAAAAACTGGAAGCTAATGATTCATCATTGAAAGAAGTGA
ATTTAAACAATTTAAAAAATATCAGCGAAGATGTTATTAAGCGTTTATGTGAAGCTGCAG
CCAATAATAAACATTTATTAACATTGAATTGTGCTAATATTCATTTGAATAACTCTGGTT
GTGATCACTTTGTGACAGTGTTGGAAAAAAATGATCACTTAGAGACTCTGAATTTGGAAT
CGAATTACTTGAATGGAGACAGTATTATCAAGTTAATTACGGCAATAAATCGCGAAAAAA
GTGGCCTTATTGAGCTTCGTATTGTTAATCAGCAACAGAAAGTTTTAGGTGTTCGTGTGG
AGCAAGAAATAATTCGGCTTATCAAAGAAAATGCAACATTGCAAGTACTTGGTATTGACT
TGGAGACATCGGAAGCTCGAATTAGAATTCGAGATCATTTAGCAATGAATGTTGACAGGA
AAACTCGCCAAAATCGATTAGTTAACAAGCATTAAggattatttttcacgtggaaattat
tataatgatcagatgaacaattcgtcgttatattctttcacttattatttctgattatta
ttattattattatcatcatcattattattactattactatttataggtttttcaattga
tttatccaacctggttaattttattatcatccgacaacacctttcgtgtaaatacatga
gttgcttctttatccagcttcgttgttgtttttgtccaatcagcgacgagtaataataga
ttgagtgctcggtgttgttattattacaaaaatatatatagaaatatttatttatttatt
tagtagttttattattagtttgaaattccaatttctacattttcgttttgttcttcaaac
ttgatgttacgaaatatgttgctcttatattttacagtgtttgatattaaattttggata
atagattttattaatttttgtgagaattgaatctgtttgaagtttctcggttgaattaat
actgcaattttcaatttattattcatatgggtctcacttcatgtagtcaatacgttacgc
tattgaaatttaattttttttatttattggaattactaaatcaattttaatgcgattttct
gggggtgtagcattaataaaatttcttgtcctgattttatctggaaaatatgttctttta
aaatagactgataactacataaaccctatttcacacttcattaatcaatgaacatgataa
aattaatatttcacgaataattgggattctaggtttgcatcggaagtacttttctttcca
aaaatatgatgacgcgtggtttgaatcattttaagacccttattgggtctttagtgctct
atgtctctaatagatgctacactgggatttgatgcatctgtatattggtgcactaatggc
tgccccggtgactcaatctgtaaataaattttgaaaaaatcacaagcaatgtcattaatc
atctttacagatatcaatgaaaatcttttgcgttttcttgagagggagctgtcgatgaaa
tagacggagaattgaactgtaatctccacagtcttgcactatcatcttttaaccatttaa
ctacgactggaagctctcatagcttcataccttgatctccatccttttgagaatactcaa
tatttgggccagttcaagtattgatatcttttactgcaaaatttttaccatcgaatgtta
catctttaaatcacccctttactgcaacaaaattatcgtaatgatctccttagaagctttt
aactgaggatgatattataatgaaattatgggctcaatttcaatgtattttgatatttag
aatttatcataaattcttttggagtatatctgtccaattcttattcgttttatcttgtaa
tgtatgaattgaatcttttatagnttatagttcgaaattgtcttttgatttgtccattat
tagatggtgattgagaattttaaccctattccatgtttttctgtcaattctctaatctaa
ctctcatgatcttcctctatcacatcctagttatttttgaaagtttattcgttttaaat
ttgttggaaATGACTAATAAAATGTTATATGGCAAAGATTTAAGCAATTATAATGATGAA
GATATTGACGATCTTTTAAGTAAACTAACTCCAGAAGAAATAGAGGAATTGAATAATGAT
ATTGATCCTGACAATCATTTGCTTCCAGCTTCACTGAGATGTCGTGATCAAACAACCAAA
CAACCCACTGGTCCATATGATAGAGATGCGCTTATCAAATTTATTGAAGAAAAGAGTAAG
CAAGAAAAAGATTGGGAAAAAAATGTACCATTTGAAAAAAAAATTCGCGGTAAAATTTTT
AAGCCAAAAATAGAAGAATCAGAAATATCTAAAAAAGATCTTGAAGAAATGGGTTTTGAT
ATGGAATTCGATACTGAATTTGATGAAGCATTAAAAAATGCAACTGAAGAAGAATTAGTT
GACTTGGCTGCTGTGTTGGGTTTCAACGGAATGTTAAATCAAACCCAGTATCACGCTAGT
GTTGAAAATAAAGAACAAATGTGTGGTGGATTTCAGGGTGTTGCCAGGCATGAACCCTGT
AAACCAGTTCCTGATGAGCCAGATAACGACACAGATGTGGAAAGTGTTATTAAAAAACTG

GAAGCTAATGATTCATCATTGAAAGAAGTGAATTTAAACAATTTAAAAAATATCAGCGAA
GATGTTATTAAGCGTTTATGTGAAGCTGCAGCCAATAATAAACATTTATTAACATTGAAT
TGTGCTAATATTCATTTGAATAACTCTGGTTGTGATCACTTTGTGACAGTGTTGGAAAAA
AATGATCACTTAGAGACTCTGAATTTGGAATCGAATTACTTGAATGGTAAATAAtttt
>DjScaffold2718:48948-49518 strand:+
ATGTACAGCATCGGCAAATTTTCAGAGTTTAATGAAGTTGATTTTAAAGGAATGCCGTAT
AAAAGTGTAATTGTTTATTTCGATGACATTTTAGTTTATGGAAGAATTGTCGATGCACAT
GATCAAAGATTAGAAAAGGCATTTGAATTAATTAAAGCATCAAAATTAAAAGTTAATCCA
TTAAAGTGTGTATTTGGAACTGATAAATTGAATTTTTTAGGGTACACAGTTAAGCAAAAT
GGAATTAAAACTAACGATGATAAACTTAAAAAAATTATAGAAGCACCAACTCCAGGTTGT
GTTACGCAGTTAAGATCCTTTTTGGGACTAACTAATTATTATCGTCGATTTATCAAGGAT
TATGCAATTATTACTGCGCCGCAGTATGCAATGATATCGGGAAATGAAAAACTGATCGAA
TGA
>DjScaffold2718:9350-9634 strand:+
ATGAAAGCTGGTTTAAGAATTAATCCAGAAAAGTGCCAGTTTTTTCAAAGCAAAATCAAA
TTTCTTGGGCACATAATTAGCAGGAATGGTGTTAGAACTGATCCGTTTAAAATTGAAGCA
AAAAGAACATTAGTCAAGCCTAACTACGTGAAGAAGTTGCGAAGCTTCTTAAGAATTTGT
AAATTCTACAGAAATTTTATAGAAAACTACGCAAAAAAAATCAAGGATCTTAGTACAGTTG
TGTACCGTACCAAGCAACAAGAAATTGAATGGACAGATGAAATAA
>DjScaffold2718:130051-130119 strand:+
ATGTTAAGAAGATTAAAAGCTTTTTGGGAATTTGCAATTACTATTGCAAGTTTATTAAAG
ATTATTTGA
>DjScaffold1876:27904-36533 strand:+
attgtattcagattgaaaattcttcatataatgatgaaacaATGAAAGAGTTAATAAATC
AAATAGTTAAATTTTTATTTCTGTTTGACAGTACCATGGCACAAAATTTATTAGACGCGC
TTGAAGGACGAAATAAAAGTGCATTATGTTCGGAATCGGAACCAAATAATGAAATGGCAA
ACACAATGAGTGGAATGATCACTGATGACGACACTACAATTCTATTTGCGAGTGCCGGAA
TTAAAAATTGTGACCTTGAAAATTATCAAGACATATTGTCTGGACAATTTTTATTAAACG
GTGAACATAAAACAGTCGCACCGTATAAAAATGATTACCAGTCAAATATGTCTAATGGGT
TTCGAAATCTTGTGCAGAAAACAGCTGAAGCTCTTGTCCATGACACACAGACGTTAGTTA
AAAACAGTGGTGATAAACAAAAACTTTTGGCCACCAACGCAGAAACTGTTATTAAAACTA
TTACCAATTTAGCAGATGAATGCGAAAATGGATTCGTACAAGAATCTAGAAAATCTTCCG
AAAGGAAAATGTTGAATACTATTCAAAGTGTTCAGTCAGCATTGGACAATACATACAATG
ATGGTAGCACTGAGATTTCAAGACTTATCGTATCAAACTCAGCGTCTCTGGTTAATCAAG
ATAGGAACAAAAATGAAAATTTGAAATTGGACGATATCTGTTCTATTGATGAAACTATTG
AAAATATTGAAATGAGGCTTGATAGATTTAATGATAACATAACTCGTGGAAACAACAATT
TACAATCAAAAAATAATGAAATGTTCAATATTAAAATTGACAGTTTAATCGAGAAGCTGG
TGAATCACGCTTCATCGGTTTTGGCGTTATCATCAGAATTGATGAGAATGCCATTTCAGA
AATTGTTTCAGAATTTAGATCAAAGCAAATCAATAATAACAAACATTAGCAATCAATGTC
AAGCAATATTATTTGATTATTTAGAATCGTTGGAGGAATTAGCTTTTAAATTGCCAAATA
ATAAAATCAATCAGAAAATCATTTCAATTGGCAGAAATCTTTCAATTCAATTTATCAATT
TCTTTCAGATCTTACAGAAGATTCAAAAAGATCCTGATAACAAATTTATAAAATCTTCAA
TGCTTGACGTGTTTTATGAAATACAAAATGGCATTAAAATATTTCTAGAATTTTCAGAAG
AAGTTCAAAAATCTTTGGAAACTCAAATTTCACCAACAAGAAAAGTTTCATTCAGGGATT
CTTTACCAGGAACAACTTATCAAAACTGTATGATTTTAATATTTTAGtattgtataaatt
ttttaaaaaattattatcaataataatattattaataataaaattattaataataataat
aaaattattaataataataataaaattattaataataataataaaattattaataataat
aataaaattattaataataataataaaattattaataataataataaaattattaataat
aataataaaattattaataataataataaaattattaataataataataaaattattaat
aataataataaaattattaataataataataaaattattaataataataataataatatt
aataataataataaaattattaataataataataaaatcattaataataattaattattt
aacacgtcatttaattcaacatttatttattacaactttcgacagtatatctgtcatctc
caggcaaactgtttaaaactaagaataaagtatatatacttaagttccgcattatgcaat
attatattcttaataaaattctaattaatcaaagacattcaattaagatttagaaacatc
agtttgaacaattatttttggatcatatgataaaaccatcaattttccgcacatgattct
ttcccaataatctttaataaatctttaatattactgctattatctaaccggctttgtttt
tgattctttttacagagatgatgtgaagtctctccaaagtatgtgacattacacccatta

cactcattaataagtaagattaaaatccaatccataaagaa
>DjScaffold1754:38180-46250 strand:+
gatgatgctaatattagtgctgatttagattttgcgttagttcagATGGAATTAATGACA
CATATGAAATCGGAGCTTGATCCTCAATTGCCCATTGGATATGAAATTACAACGTTTATT
GGAGAAGGAGGTATAGGAGCAGTATACACAGCTCGTCATAAAGAAAATGGAATGTTTCTT
GCAGTTAAAGTAATCCGAACTATGATAAACGAAATTCTACGAAAAAAAATCAGAGAAGAA
TATAGAATTCAAAAGAAATTAAAACATGAGAATATAGTAGAAGTTTATGGATTGATTAGT
ACAACTTGCTTTATTTCAATATTTATGGAATGTTTAACTGGGGGAGATTTGTTTGATATG
ATTCCAATAGGTCAAGGAATTGATGTTAGAGATGGATTTGTTATTTTTAAACAACTATTG
GATGCAGTAGATTATTGTCATGCACGAAAAATAGTTCATCTCGACATAAAACCGGAAAAT
GTCATGATTTCTTCATCTAAAATACCAAAATTAATAGATTTCGGTGTAGCTGTTGATCAT
ACAATTAAACCGTTTTTAAAAGATCATTACGGAACAACAGCTTATTCTGCACCAGAAACT
TTTTTAAAATGTGAATACACTGGGTTTAAAGCTGATTTATTTTCTCTAACGGTTACATTT
CTTACGGCTATTACTGGAGAACGTTGTTGGAACATAGCTTCGTTTAAGGACGATCATTAT
TCTTGTTGGCAAGATAAACGATGGGACTTGTCAACTTTTCAGCGGCTTCCAGCAGATTTA
TTAAATTTTTTAATAGGCGCATTAGCCCATAATCCATTAGAACGCTATTCAATTGAACAA
ATCAGGTCTCATATATGGTTTAAACGAAGTTTGAAAGGAGAGATGTCTGCAGTTAGAACC
CAAGTCAATTTCCAATGGAATAACATCATAGTTCCAAATTCACAACCAATGTTGTCCAAA
GATATTTTAAACACTTCCTTTGAGGCGGCCTATGCTGATTACGATTTTCCTCTTTCTCAA
CCAATTGATTTTGGCAAATTGTTAATACCACCGTCAATTAACGATTCTGATACTGATTTT
GCATCGATTGTGAAACGAATGACTCGATTTTTTTCAACTCTCGAATGTGATGTTACATTG
AGCAAAGTAAAAATGCTCGTCGAGCAACATCAACAATGCATGATAAAACATTGTCAAAAT
AATATCATCAATGTGCAATCGGTTTTCACTGATTTTGAAGTGTTTTTCAGAATTTTCATT
TTCCAATTTGGAGATTCGACTCTAGTGGATTTCCGACGAGTTCGAGGAGATGGGTTGTTG
TTTCACAATATATTTGCAGAAGTTCTTGATGTAGCCAAACAATTGGGATTTGTCAATGAG
AACCATCCAAATTTG
>DjScaffold3223:52957-53085 strand:+
ATGCCATTTGGCTTGAGTTCAGCCCCCAGCAACATTTCAACGACTTATGCATATGATACT
GCAAAATTAAAATTGGGAACAATGTCTTATTTATCTGGACGATGTATTGATATTTGGAAA
CTCTTATGA
>DjScaffold1115:26403-27182 strand:+
ATGTTTCCGATATTTGACAAATCAAATTTATTGTGCAAGTTGGTTATGATGTTCACAACC
AGATGGAAAATATTTGTGCAATCTCAAAATTATTTCAGAGAAGAAATCGATGAGCATTTA
AAAACAATTGATTTTAATTGTGAAGGTGAAGATTTGATGGATCGATTTTTAATTAAACAA
AATCAATTGAAACAACAAAATGGCGATGTTGAATCATTTACTGAATGGCAACTAACTAGA
TCATCTTTGGAGTTGTTCATTGCTGGATATGAGACAACATCAACAACTCTTTGTTGGTCG
TTTTTATTCATGTCAAAACATCAAGATATTCAACAGAAAGTACACAATGAAATCATTGAA
ATAATTGGTAAAGAAAGATTACCAACAATGAATGATAAAAAATCAATGAATTACACTCAA
GCAGTGATGGATGAAATATTTAGAATGAGTTCAGTGGGACCTCTAGCATTGATTCACAGA
ACTCTCAATGATGCCAATATTGATGGATATTTTATTCCCAAGAATACATTGATTTTTCCT
AATTTATATGCTTGTCATTTTGATCGTGATATTTGGGAGAAACCAATGATATTTTATCCT
GAACATTTTTTAACTGAAGATGATAATGGGAATTTAAAATACACATCGAGAGATGAATTG
ATGCCATTTGGTATTGGTAAACGACAATGTCTAGGAGAATCTCTAGCTAGAATGGAATTC
TTTATTTTCTTTACGAGTATTTTACAACGTTTTAAAGTTAAATTAGCAAACGAAGTAAAT
>DjScaffold1115:23094-23504 strand:+
ATGGCAAGAACAAAACAAACTGCCCGTAAAAGCACTGGGGGAAAAGCTCCAAGAAAGCAA
TTGGCAACTAAAGCAGCAAGAAAAAGTGCACCAGCAACTGGAGGTGTAAAGAAACCACAT
CGTTATCGTCCAGGAACTGTGGCTCTTAGAGAAATCAGACGTTATCAAAAGAGCACTGAA
TTGTTGATTAGAAAATTACCTTTTCAACGATTAGTTCGAGAAATCGCTCAAGATTTCAAA
ACTGATTTGAGATTCCAAAGTTCTGCTGTATCTGCTCTTCAAGAAGCAAGTGAAGCATAT
TTGGTTGGTTTGTTTGAAGATACAAACTTGTGTGCCATTCATGCTAAGAGAGTAACTATT
ATGCCAAAAGATATTCAATTGGCTCGTAGAATTCGTGGAGAACGTGCTTAA
>DjScaffold2393:17824-20575 strand:+
aattaattacaATGTCTATCATTAAAAGTGGCAAAACTTTTCGTACAATACCACAGTTTG
TTCGTCAGGATCCATTATTAACTATTAATTGTAAAAATCATGAACGAGATTATGTAAAAT
TATTTGATCAAAGTTACTATAATAATGTTTCCTATTCAATGAAAAAGAAAAACAGATGGA
TGCTTGCAACATTATTAACAAAAGATTTTTATGATCAAGATGATTTAATAGTTTATACTG

AACATCAGTCGTTCTCTGGAGACAATCCAATTAACACGATTTACCTCTATGATTCATTAT
CACAAAGAGAATTCGGTTGTCCAGATATTAATAATGATAAATTAATTGGTAAATCAATTA
ATAAAAAGAGTTTAATTCATTCTATTGAACATGAACATCAATATACAAAACAAGAATTGA
AAAATTTTGAAAATTTAAATAAAAGGAAAAATATCAAAAAGAAAAAGTCAAGAAAACATC
ATCGGTCATACATTCCAAAATCGGTCGATGCTTTAATTTCAAGAGAAGAATATTTATCAA
ATATAGAAAATCGAAAAGATTGCATTTATATCAATACTAATGTACATATTAATGATAAAT
CTAAAATGAATTATATAAAGAAAGCATGTCTAACAGATTATAAAAACGTTAACTGTAATG
TTAAGGAAAAAAAAGCTATGAGGAAACATGTATTGAAAGGTAAACTATTGATGAATGAAG
AAGAATTCGATGAAGAATTTGAAAACGAAGAGATAATTGAAGAAAGTGATGTCGACGAAA
ACGTTATAGAAATGTCTAAGAAAGAATTTATTTTAAGTGAAGATGATTTTCTTTGTAAAA
CTCTAACAAATAAAAAAAATGAAAGTTAAACATGAGAAAAAACAATTTCAAATTCCAACGA
ATATTACTTTGAAAAAAATAATTGTAAATGTAGATAAAAATGATGAGAAAATTGAATTAT
TTATCGATTTAAAAAGAGATGTTCTTCCATTCATTGAAATAGATATTCCAATTAAATCTT
TTATTGATGATCTATATATAATATTGGAAATTACACAATGTTGGTATAGTAATACATTGA
ATAAATCAATTTTAATTGATCTATCAAATCTACTTGGAGGAAATAATGAAGATTTTTTAT
TATTGAAACCAAAATTTTTTAAACAAGACATGATATCATTAAAAATAATCTTCAATTTAA
TAAATTTTAATTTCAATGATATATTAGATAAAAATTTCGAGAAAATAACAGAAATAATAC
AAACATTACAGATTAATGCTCAGCCAATCAAAAATGAATCAGCAATCAGTCGTAAAATAA
TGACTTTAGATAAACCGAATGAATGTTTGGATGGAATCTGGAAAACGAAAAAATTAGAAA
ATTATGAGAAAATTCATTTGTATCATGATGAATGGAAAACATATATATTAAATGATGAGA
CTATTGATAAAATTGTCAGTAGTATATCGAAAACTTCAACTTATTTACTAGAAACATGTG
AAATCTGTTTTAATGAAATTTGCGAAAGTGAAATGATTATTGTAAATGGATGTCAACATA
AAATATGTGAAGAATGTTTATCGAGTCATTTGATTTATCGTTTGGAAACTGGTATGAACA
CTTTAAATTGTCCAGGAACTGAATGTAAATCAGAAATCGATTGTGTTACTATATTACAAT
GTGTTAGTGGGAAACGGTACCGGAAAAGAATTGGAAAGTTAGAAGCAACAGTGACACAAA
AAATGGGAATATCCTGTGTTGAATGTGGAACTATTACAATAATGAATGATGGCGATTCAA
TTGAACCGCGAAGAAGATTGGATTACAAATGTAATTGCGGTAGAATTATTTGTAGTCTTT
GTGGTAGAAATCAACACTGGCCAAGCTCGTGTTACGAGTTTAGAAAACATAACCAAAAAC
TTAACTTACTTAAAGATACCGATTTATTCACTGAGCATCGTCCACCATTGATTTTTGAAG
GAATAAATTGTCCGAAGTGTTTGTATTTTGTTGTGAAAACTGTTGGATGCAACCATATGT
CCTGCAATTGTGGAGCACATTTTTGTTGGCTGTGTGGAGACTCTAGTCAAGAAGGTAAAC
ATTTGACATCTGGATTTCCTTGTTACAAACCTCCTGAAAACAATCATCTGATCTTTGATT
TAACGTATATTCGACCGGGTCCTAGTGAGTTTCGGCCAAAATTGTATGTTCTCAGTTTAG
AGCAAAAAGCTAACAATTCAGAATATTCCAGACGGAAATTGTTTTATCAACTGAATTTAG
CATTCAATTCAAAATCAGTAATCTTAATTTCAATTCTATTATTTCATTATCAAATTTAAt
tcaaatctacattaaaatcattttaaattaaattagaatgaatatgctgtaaatgttatt
ttaagccaataataataataataataattgtaagaataattcatagattttaaatatctg
gaagattttttaattttatatatattatattaaaatttatattttgaaacattcaaa
tattattgtaaaaagtaaaaacaaatttttaaaatataaaacattttttaaagataaatt
atttaagtaatttctatatattatattagtcaaaattataaaagtatgaaaactatttta
tgaaattatattt
>DjScaffold2901:88000-88228 strand:+
GACAAGGTCGAAATATCGAATTGTTATCCAATATCAATAAATAAAACTGAAATTTTGATC
AAAGCTTTTATATTAAATGAATTAAAAAAAATTATTTTAATAATTATTAAATATTTAGCA
ACTATTATTAGATTAAATGGAGAATCCTGTAATATCATTAAGCTTCCAATTAATTGTAGA
AAAAAAGAAGTTGTATTCAATACAAAG
>DjScaffold2023:28255-29159 strand:+
tcattgaaaatgaaataattcttaaatcaaacaagaaaattcatctatggtttagataca
tagatgatattttctttgtaactaattctcctttagagaccattctagaattagccaaca
gtattaataataacattaaattcactttagagaaatcaattgactttcagattccttttc
ttgacactttaattcaaaagaaacctggtgaaatattaactacccaattatatatgaaac
catttcacagtggacatatattaccttggagtaaaaattggtataattaaaggtgaaaga
attcgagctgagagactatcttcaaatgaagaaATGAAATTGATTTCTCTCAAGTTGATA
AAAAATAAATTTTTAAATAATGGCTATCCAAATAAAATTATATCAAAATATTTATCTTTA
AATTCAAAACAAAATAAAATCAAACCAAAAACAAATACAGTTATTAAATTACCGTTTATT
AATGAGAAATGTAGTCAAATCATGCAGAATATAATAAAAAATGCTAAACTAAAACACAAT
CCAAGAATTATTTTCAAAACGCAAGCACCTTTGGGCATCCAATTACAGAAACCAAGACAA

ACTTTTTGTGGTCCCAAATGCATTTGTGATGGTAGAAATATATGCAATAAGAAAAATGTC
ACTTATAAAATAACATGCACCATTTGTCAAGAAACATATATAGGTGAAACATGCAGAACC
ATCCGAACAAGAGTTAAGGAACATTTGAAACAAAAAGAAAGTGAGGTATTCAAACACTTT
CAACACAAACATAAAATTTCACCAGAACAACATCACATTACATGGAAAACACTAGGAACA
GGCTACATTTCTACACTACATAGAAAAAAACATGAAGAAATACTA
>DjScaffold2023:147743-148439 strand:+
tatcatgagaagaaattattgaagaaagtggattttaataattggcaatgcgataatcaa
ctacacgaatcaaaaataATGCAAAAATATTGTATTCAAAGGCGTGAAGATTATGTTGTT
TATAACGGACTTTCAAGGCAGATTAGAGAGTTGGCTAATACGTTGAAGGATATTGCAAAT
GAAGATAAAGAAAGAATAACATGTACAGAAACTCTACTTTCTAAACTATACAATCAAGGT
TTAATACCTGCTAAAACTAATCTAGCTTTAGCTAATTCAGTCACTGCTTCATCATTTTGC
AGAAGACGATTAACATATTTTATTCACAAAAGTCGCATGGCTGAATCTTTAGAACTCGCA
GCTAAGTTTATCGAACAAGGTCACATTAGAGTTGGTCCCAATATTATAAGAGATCCTGCT
TATTTAGTTCCACGATCATTAGAAGATTACATAACATGGGCCCCGAATTCTAAAATGAAG
AAAAAGGTTGAGGAGTTTAATGGTATTCGTGATGATTATGAATAAatgttaaataaattt
tgatcgactctaaattctttattccaaaaatcttataaacatttttgattctatgtctac
gccatcaaatat
>DjScaffold3926:5932-12001 strand:+
atcagtagtgATGGTCTACAAAAATGAAGTAAAACACTATTTTCAAGAACCTTTGCCTTT
CAATGTAACATGGAATAGTCGACGAGCTATTCTTCAATTTTTGACGTCAAATCCATTTAC
AATCAAAGAAAAACAAGAAATAAATCATAAAACGGAAAAGGATGTGCGTGAAGATCGTCA
CAGAGGAACAACTGGAAAATTGATGGATGCTGTTCCACAAGTTCCATTAATAAAGTCTAT
GGAATGTAATTCTGGGATGTCATCAGACTCAACTTTTATTCCTATTTGGGGTGTGAAAAA
TGAGATAATTGATGCAATTAAACAGAATCCAGTTGTTATCATCTTTAGTGCATCTGGTTG
TGGAAAATCTACGCAAATTGGGCAAATTATAATGCAAGAATGCGCGAAATCTGGTGAACC
GTGTCGAATGATTTGTACTCAACCATATCGGCTGACTGCTCATGGATATGCTCAATTTGT
TACTCAACAACGTAAAGAAAACATTGGTCAAACCGTAGGTTATCAAATCAGACTTGAATC
TAAAGTATCCCCGAGAACATTACTGACATTTTGTACAAATGAAGTTCTGCTGAGAACTCT
TTATGGCGATCGTGCATTTCTCAATGGTACCACCCACATAATTGTTGATGAAATGCAAGA
AAGATGTCGACATAGTGATTTATTGTTGTCGGTTCTCAATAAAATGCAAAAAATCCATCG
GCACATAAAAATAATTATATTAATTTCC
>DjScaffold3339:14944-25088 strand:+
agaacaaagtagcttaaatttctaaaactcttttgaaaatctattaacgacaaatgttta
acggtggtctttatcaaatagttccagtgatcacactactgatctcgagttgtactgttt
catgaaatattttcaaaattattgttccgtaaatgtccaatttatgtctttaaattatac
tgtattatgttatgtatgcatatttgacataacatcttttgaaggtggctcattaaatta
aaacaacgaaaaaatttattaatcgatgatacaagttttaatagtaatattaactataat
ttaaatgctttttacattcatctggtactaatctatttagcgttaattgaaatagttact
ctaaatttcaaaattttaattaaggtgtgggatgccagaacattagaatgtgtgcgcata
ttgacgggacatgttggcagtgtgttgtgtctacaatatgataataatgttatcattagt
ggttccagtgattcaactgtgcgcgtatggagtctttcgtcatggacatgtgtaaataca
atatatcatcattgtgaagctgtattgcatttgaggttcgctcacagcattctcgtaaca
tgttcaaaagatcgtagcattgcagtttgggatATGTTTGCACCCAGTGATATTCGTTTA
CGACGAGTTCTTGTTGGTCACAGAGCAGCAGTAAATGTTGTAGATTTTGATCAGAAAAAT
ATTGTTTCAGCTAGTGGAGATCGAACTATAAAGGTTTGGAATACTCAGTCATGCGAGTTT
TTGCATACAATGAATGGACACAGGCGTGGCATCGCGTGTTTGCAGTACAGAAACAATTTG
GTTGTTTCCGGTAGCTCGGACAACACGATTCGCTTGTGGCAAATTGAGACAGGAACATGC
TTACGCACCTTGGAAGGGCATGAAGAATTGGTTAGGTGTATCAGATTTGATTCAAAGTAT
ATTGTTTCTGGAGCTTACGATGGAAAAATCAAAGTATGGGATCTCGTTGCTGCTTTAAAT
CCTCAAAGTCCCGCCAACAGTCTTTGTTTACAAACACTAAATCAGCATACCGGCCGGGTA
TTTCGACTGCAATTTGACGAATTTCAAATTGTATCGAGTTCACACGACGATACAATTCTG
ATCTGGGATTTTCTCGATACCGGTGTTAATCAGGATTCTGGCATGAATATGGTACGCAGT
CCTGTTGTTAGTCTTGATAATGATGATGAGCCAATAAAACCTGTTGGAGAGCAGGGATCA
ATGAATAACGCGTCCACCAGTCAAAATCTTGTTTTCGAATACGATTTGAATAATCCACAT
GATAATTATTCAATGATGATGAGTAACACTAGTATTAGTGTTGATCCGTCTGGATTAAAT
ATGCTCGGTAACAGAAATTCTGCTAATAATAATAACAACAACAATAACAGATCCTCCAGT
AGCTTTAATATTAACAGTTACAGAAGACCCAGACAAACGCCTTCCAAACCTTCTACAGAT

CCTCGAGTTCGACAGAGCAATGATTCACTCAATGAATCCCGACTCAGACCAACTTTTGAC
AACTTTCTTTATGACAGTAGTCACACTAGTGTTACATCAGATCACGACGATATTCAAACA
CAAGATCACGATAATGATCATGATCATATGAGATCGTCTTCGTCGCGTCGATTTTCGTGA
tagaaatttcgtgaatttacaaatgttttgcaaatcaaattaataaataatcgatacgtc
agtttttcttttatctttttagcattaaaaatcaatcaatcaataatcactcaaaaaaa
aaaaatttttttttcttttcgatcgaaggtgttattaatcaataaataaacagaaacttt
cttgttgttactttttttattgctgttttgttgttttgttgatttttttttcattgtttt
ttggcagataaatttcgatatatatattgatatttataaataaaatttcgcgtgcgcgtg
tgtgtgatatcgatttatttactgtgttttctagtttttattattgtctagaataacaac
aagagttgaagtgtattttctttgttaattgtaatatgatacgaaacaaatcaataatta
cacacacacacacactatctttaattttgtgggctcgtgtagcccgatttttgatttggg
aatttgccgattatgatgatcattctattttaactggaa
>DjScaffold3339:37767-38504 strand:+
tcaaactgaatcaacaattgaagatttacctaacATGGTGGCAGCGGTAAATACCGATTC
TATTACTGAGAGGTTATCACCAAAAGTGTTCTGCAGAGGAGATGATATTGAGAAATTTAT
CAAGCGATGTAAATTGTATTTTGAAGCTAAAAACATCACTCATGGGAAACAACAGGAGAG
ATTAATCTCTTGTCTGCTAGATGAAGATTTGTTGGAAGAATATGCAAAGACGTTGAAAAA
AGCTGAAGGTTTTCAGGTTCGCTTACGCATGGTATTTGAGAAACAGAAAGATGTTATCAA
AGATTTTCAAAATTTGATTAACTACAGGCGTGGAAATGAAGAAAATCAAGTTTATTTTAG
AAATGTAAAAGAGTTAGTAAAACGATTATTTACAAATAAAGAGTTTACTGAAGAAAAAAT
GGTAGCAAAGATGCTAGTATATTGTTCAGATAGCAAAAAAGTTGAAGAAGAAGTGACTTT
GCGAAAAAGAGAAAATATAGAAGAAATCGAAGAAACAATGACGTTGGTAGATCGAGTAGA
GAAAAACAGAGATCGAGTTGATGCAGAGCGATTGACAAGAGCTTTAAATATTTTTAAGAC
ATCTGACCAGACTCAAAATAATCACAAGTGGTGAaccaatcaatgtaaaaccata
>DjScaffold5163:67085-67900 strand:+
ATAACTTATAGAGGTCGAATTAGAGATCTGGTACCTTATTTGTCTAGTCAGAATTTTATA
TGTCCGAGCTATCACAATCCCGCTGATTATTTTATGGAAGTAGCGTGCGGAGAACATGGT
GAAAATAGTGTAAAAAAATTAGTAAAATCATTAGAAAACGGAACTATGGAAACTGTACTT
AAACGACTTATGGAAGCAAACAATAAACACTTGTTGAACGAGAAAGATTCCAAAGATGAG
TTGTCTCATTCGCCGGCTCTAAGTATCAGATCTACTGACTCTTCAGAAAAAAAGCCCATC
AATCCAAATACAAACTTAATTATACCGATGGGTAATGATTCTATGGATACGTTAGAGAAA
AATATCAATGAAAAAGTCAAAATTTTAACAAAGAAAAATAATTCAGACGATATTTCAATC
AGTTCT
>DjScaffold5163:78263-79476 strand:+
gatttatcttttaattaaggaggaagcagacactcatacattctctaccagtctttttac
gcagttcaaagtgctttttgtcagaacatttATGTCCATTATTAGAGATACTACTTTGAC
AAGGCTGCGATTGGTTTCTCACTTAGCAGTAGGCTTCATGATTGGTCTACTCTATTTTAG
AATTGGAAATGAAGGAACTAAAGTAATAAACAATGCTGCCTTCTTATTTTTTTGTATGTT
ATTTATTATGTTTGCATCTCTAATGCCAACAGTACTTACATTTCCTCTTGAAATGTCAGT
TTTCCGTCGTGAACATCTCAATTACTGGTATAGCCTGAAAGCATATTATTTAGCAAAGAC
GTTCGCAGATCTCCCTTTTCAGATTATTTTC
>DjScaffold5163:79537-87852 strand:+
tttttcATGTTCCTTACTGTTTGTGTTCAAACAGCATTAGTTGGACAATCACTTGGCCTT
GTTATTGGTGCTGCAACATCTTTACAAGTGGCAGTATTTTTAGGTCCCGTGTCTGGAATT
CCAATTTTATTATTTTCGGGTTTCTTTGTTACCTTTGACACAATACCTAAATATCTTCAG
TGGATTTCATATGCCTCATATATTAGATATTCATTTGAAGGAGTATTACAAGCAATTTAT
GGATTCGAAAGAGAATCTCTAGCGTGCTCTGAGAAGAAAACTAGTATGTCAATGTGCGTT
AAGAAACCAGTTCAACTTTTAAGTTTATTGAGCGTAAATGAAGCTAAATTCTACGTAGAT
TTTATAGTACTATGTGTATTTTTCTTATTATTACGAATTCTTTGCTATTTCATTCTCAGG
TGGCGAGTGAAAGGATCGAAATAAtaacttcattaaaaagtattataaaaacagtcttag
atttttcctactggcatgtcagcaatgcatttgcaacagaatgtcaacatttttattaat
acactgtttgattgacagtagagaacaaacatgaaatttggtcataattcattattatat
tctgtcgactatttggtcaataaacataaatattttctaaccaccgaattgtttataagt
attatctcaatattttatttaataagtcgtaaaataattggataaaatatttatcctatt
aaaaattttaattttcggcggtttctttcctgttataatataagaaatattttcatgatt
taaatgtactgttgggcaacggatgcgattacgagatttacattgtaacttaatattttt
tgagttttattaaataaacattttatcgtta

>DjScaffold3894:75381-81478 strand:+
aaatttgctgttaattatttggtactcaataatttgtttagaattgcttaattatttaaa
taataaattgaaagtttattaactaatttttaatctaaaaaATGGTGAAACTAAATATGA
ATATTTTGATAAAAAGTACAAGTGGCCGAGTTATTAGAAAACCAGATGAATCTCATGAAC
AATATCTAATGAAAGTTACACATTTATATTTGGAAAATAAACAACTGGATTCTATTAGTA
GAGAAATCGGTTTATGTAAAAATTTAACAGTTTTATATTTGTACGACAACAATCTGACTG
AAATGCCAAATTTAAGTGCATGTCAATCTCTCACTCATATTTATGTTCAGAATAATAATA
TTACCAAAATTGAGCATTTGCAATATCTTTCAAATCTGACAAAATTATATATTGGACATA
ACAAAATAAATTTTGTTGAAGGATTGCATTTAAATACGATGTTACAAGAGTTGTATATTG
AATATCAAGATTTATTTCTCGGAGAAAAAATTGCTATTTGATCCGAGAACAATTTCAGGAA
TTTCTATGTCGTTAGAAATTATAAATATTAGCGGGAATAATATCGACAATCTTGAGCAAT
TGAAACAGTTGAAAAACTTGAAACAGTTATTAGCTACAGATAATAATGTTGACGATATTA
ATGAAACATTATCGATTATCTCAGAATTCAATTATCTTCATAGATTGGAACTACAACAAA
ATCCCATTTGTTCTAATAATAAAAAAATATCGAGATTTAATCATTATGGAAGTTTCTTCAT
TACAGTCTCTTGATGGCAAGGAAATAACCGATACTTCACGACAATTTCTCTTGAAATGGA
GAGCTAATAAGGATGCCATTGACAAGAAAAAAGAAGAAAATAAAACTAAAAATGTTCCAG
TTTATTTAACAGCAAATGAAATGAGATTTTCATCGTCAAATTCTCAGAATCGTAATTTCG
GCCCCGGTAAATATTCTGGGTAAgtcat
>DjScaffold230:78536-78919 strand:+
ATGTATTTTGGATTAAGAATAAACCCGGAAAAATTTAAGTATTATTATAATAAAATGTTG
TTCTTGGGACAAACAGTGAGTGCAGCTGGTATACGTACTAATGAAGATAAGATTGAGGAA
ATTAAAAATTCCATAGTTCCAGAGTGTTTTTCGAAACTTTGTTCGTTTCTCGGACTAATA
AAATACTGCATGAGTTTCATAAAAGATTATACAAAGATTGCAACTCTTTTATATGAAGCA
ATTTCATGTTGCAACAAACTATTAGTTTGGACAGAAGCATGCCAAAACAGTTCAAATGCG
TTAAAAGAGAAGTTATGTAGTGCTCTAGTATTAGAATATCCCAGGGGGGAAACGGATGTT
CATTTTACAATATTATGTATTTAG
>DjScaffold4961:1610-11693 strand:+
gcctattaacacATGGATTCTACTTTGAGATTCAAAAAAATCAAAAGAAGAAATAAAACG
AAATCAAACTGCACTGAAAATAGTATTTGTAGTGAAAATAAAGAAATTATTATCCAACAT
TCCGATACAGTTGGACCATTAGTTCTCAGGAATATTTTAAGTTCTAAGAAAAAGTTATTT
TTTAGTATATTGTCATTTCGAATAATCAATTCATTAATAAATCATACATCATACGTACCA
GATGAATATTGGCAGTCAGTTGAAGTTGCTCATAAATGGATATTTCAAAATGGTTATTTT
ACTTGGGAATGGTTACCGGAATTTGCAATAAGGAGTCCTTTATATCCTCTTTTATTTGCT
ATTCCGTTTAAAATTCTGTCTGTATTCAATTTGGACAACAGAATATTACTGATAAAGTTG
CCAATGTTAATTCACGCAGGTTTGGCAGCAATTGCAGATTTTCACTTGTATCTTCTTTCA
TTCAAACTAGCCGGACAAAAAGTAGCTCAGTGGACTCTCTTCAATCAATTGGTTAACTGG
TTCACGTTATATAGTTGTACCAGAACGCTCATTAACAATATAGAATGGCTGCTCGTCATT
TTGGCACTTTATGAATACAAATCATCGATCATCACGACAGATAATAATGCTCACCGTCCA
TTATCTCGCAGATTCATTCTGTTTGCCAGCTTGTGCCTTGTTTTGCGTCCGACTGGTGTT
ATTGTGTTGCTACCAGTTTGCATTTATCATATTCTCTCTCAAATACGCCAGAATCACAAT
CCTTGGCCAATCATATCTCACACATTTGTCAACTGTTTCACTGTTGGAACTTTCACACTT
TTGATATCTCTGTTAATCGACAAGCATTTCTATGGAAGATGGACTTGTTCTCAGTGGAAT
TTCCTCCATTTCAATCTGCTAACAGATGTCTCTCAGACCTACGGAGTTCATCCATGGCAT
TGGTATATTTCCCATGCAATCCCATCCATGTTTCTAACTCAACTTCCTATTCTCATATAT
TCCTTTGTGTCAGAATCGAGAAATAATCCAGATTCCAAGCGAATGATTAGTTTGATTTTG
TTGGTGTTGTATGCTTATAGCTGTCTCAGTCATAAGGAATTCCGATTTATTTTTCCAATT
ATGCCTATTGCTATGCATTTAATGGGTTGTGCTCTTGTCAGATTTCCTTCAAAGCGAAAG
GCTATCATCTTTCTTTTATTGATTTCTCAGATTCCCATGTCTTTCTACATGTCCCTTATC
CACCAAAGAGGAGCAAATGACATCATTATGACAATTGGTAGACGTATTGAACAGCAAGAA
TTAAAGGCAGACAATATAAGCGCTGTTTTTTTAATGCCTTGTCATTCCACTCCTTGGATG
AGTCACATCCATCACAACATATCAGCTAGATTCCTCACTTGTGATCCTCAAATTACCGGC
AGTATCAATATATTAAATAATCCCACTCAACCATCAATAAAGCTTAAACAATACGACGAA
GCCGATCTATTTTATGATAATCCTAAGAAATGGCTACTTGAGGAATATTATAATGTGTCT
TCTCGACCGATGATGTGGATTATGTTTGAA
>DjScaffold3971:5769-6019 strand:+
ggattaattgtttgttgaatttaatttcttctgtagttggtagtcttttgtaagctattg
tttcataattatttaaATGTTCAGAAAAAAATTGATTGTATGTTTGTTAGGTAATTTTAA

AAATTCGTTTTTATTAAATCAATATTTATTAGTTGAAATTTTTAAAATGTAAattcgaaa
tatttgtaaaatatattttaataaatataataaatttgtt
>DjScaffold3971:75351-75752 strand:+
ATGAAAAGAGAGATAAAATGTTTATGCCTTATAATTGGTGAGATAAAAATACAAACGGAC
CCATTTAAAATCGAGGCAATTAAGTCATTTGGCGAGCCAAAATGCGTTAGAAGGTTACGC
AGTTTATTGGGCATCTGTAATTATTACAGATTATTCATAAAAGATTATGCAATAAAAGCG
AGGAAACTAGAGAAGCTTTACAGTGGTCTGAGAAATATGAAGTTGGTATGGTCCTCAGAA
CTATCACAAATATTTGTAGGGTTTAAGGCAGCACCAGTCTTGGTACTTACATATATCCGG
AGAGAATTTATCCTGGATATGGATGCGAGTTTCAACAAAGTTGGCATAGTATTATCCCAG
AAAAATGAGCGCGGGGTGGAGCAGGTAATTGCTTACAAATAA
>DjScaffold108:2452-2937 strand:+
aagntgctcatatgaatgtgaattattagaaATGAGTGAACATTTGAGAATTTGTGGAAA
GAAAAGTGTGGAATGTCCATTTTACAAGTTGGGTTGTGATATGAGGAAAATGACAAATGA
AAAATTGATTGAACATTTCCAATTAGAGAATTATAATCACAACAAAACATTGATTGATAT
GATTGACAATATTAAAAATGAGGTTGAAGAATTGAGAGAAAAAGAATTGAAAAATCATGA
AGAAATCAATAAATTGAAATCAAATTTACAATTGAAAGATGAAAAATTGGAATCGATAAA
TGCAGAAATAGAAATAATGAAAAAGGAGAAATTGGAGATGGAAAATAGATTGAAAAATGT
CGAAGAAATGTTTAATGTTGAAATTAGAAAATTGAAATCAAAACACGAGAAAGAAATTCA
ATCGATAAAGACAGAGAAGGAAGATCTTGATGACCAAATGCAAATCGGGTTGAGAAATAT
TTGGTATAAAATCGATGAAATCGAATTGGAAATGAAGAAACAATGTGAAAATGGTAAAAT
TGAATCGATAAAATCGGAGTTGATTGAGAAAATTGAAATTTTACAAGGAAATTTTGATAA
TAAAATCGATGAGATTGAATTGACAAATAAGAAGGAAATCAAATCGATAAATGATGAAAT
TCGATTGGAAATGATGGAAGAGTGCAAACAAACAATCGATAAATCAATAATGCAAATGGG
AATCAAACAGAGAATAAATCAATTAGAAAGAAATGTTGAAAACAAAATGAAAGGAATATT
TGAAATGATTATTGATAAACTTGATGATTTGAAAGATAAATATCATCATGGAATCTACAG
TGAATATTTTTATACTGTAAATCGTCCGTATAAATTACAATTGTACATTTATTATTCATT
TGGTGAGGAATTGAGGATTTATTTATCTATTTATGATGATGTGATGAATCAAGAATTGCC
ATTCCCATTCTCAGCTGATGTAACAATAAAAATAATCAATAAAAACAATCGAGAACTGAA
CAAGAGCATCACAAAATATTGCCAAATAGAAAAACCAGAAAATAATTCATCCAAATTTAG
TGATAAATTTAGTTATTCACATTCAGAATTGAGAGAAAGTGGAATATTGCAATCAAATaa
gntgctcatatgaatgtgaattattagaaATGAGTGAACATTTGAGAATTTGTGGAAAGA
AAAGTGTGGAATGTCCATTTTACAAGTTGGGTTGTGATATGAGGAAAATGACAAATGAAA
AATTGATTGAACATTTCCAATTAGAGAATTATAATCACAACAAAACATTGATTGATATGA
TTGACAATATTAAAAATGAGGTTGAAGAATTGAGAGAAAAAGAATTGAAAAATCATGAAG
AAATCAAATTGAAAGATGAAAAATTGGAATCGATAAATGCAGAAATAGAAATAATGAAAA
AGGAGAAATTGGAGATGGAAAATAGATTGAAAAATGTCGAAGAAATGTTTAATGTTGAAA
TTAGAAAATTGAAATCAAAACACGAGAAAGAAATTCAATCGATAAATGATGAAATTCGAT
TGGAAATGATGGAAGAGTGCAAACAAACAATCGATAAATCAATAATGCAAATGGGAATCA
AACAGAGAATAAATCAATTAGAAAGAAATGTTGAAAACAAAATGAAAGGAATATTTGAAA
TGATTATTGATAAACTTGATGATTTGAAAGATAAATATCATCATGGAATCTACAGTGAAT
ATTTTTATACTGTAAATCGTCCGTATAAATTACAATTGTACATTTATTATTCATTTGGTG
AGGAATTGAGGATTTATTTATCTATTTATGATGATGTGATGAATCAAGAATTGCCATTCC
CATTCTCAGCTGATGTAACAATAAAAATAATCAATAAAAACAATCGAGAACTGAACAAGA
GCATCACAAAATATTGCCAAATAGAAAAACCAGAAAATAATTCATCCAAATTTAGTGATA
AATTTAGTTATTCACATTCAGAATTGAGAGAAAGTGGAATATTGCAATCAAATaagntgc
tcatatgaatgtgaattattagaaATGAGTGAACATTTGAGAATTTGTGGAAAGAAAAGT
GTGGAATGTCCATTTTACAAGTTGGGTTGTGATATGAGGAAAATGACAAATGAAAAATTG
ATTGAACATTTCCAATTAGAGAATTATAATCACAACAAAACATTGATTGATATGATTGAC
AATATTAAAAATGAGGTTGAAGAATTGAGAGAAAAAGAATTGAAAAATCATGAAGAAATC
AATAAATTGAAATCAAATTTACAATTGAAAGATGAAAAATTGGAATCGATAAATGCAGAA
ATAGAAATAATGAAAATGGAAAATAGATTGAAAAATGTCGAAGAAATGTTTAATGTTGAA
ATTAGAAAATTGAAATCAAAACACGAGAAAGAAATTCAATCGATAAAGGTGAAAATA
>DjScaffold3752:245-10933 strand:+
ATGATTTTGAGTTTTCTCGGCACTGCAACTACATCCTCGGTAATAGGTGTTTTCTACTAT
TACAAAAATACTCCCATTGTAAGAGCTTCTGGGCGAGAATTATCATTTTTAATTCTGTCG
GGCTGTTTAGTTTGTTATTTAGTATCATTTTGTTTCCTTCTTAAACCAACAATTATTTCG
TGTGCCACTCAACGTATTCTATTGGGACTCGGTTTTGCGGTAATGTATGCATCTCTATTA

ACTAAAACAAACAGAATTGCCAGAATATTTGAATCTGCTCAGAAAACTGCTAAACGACCT
CCATGCATTTCACCAAAATCCCAGTTAATGATTTGTGCTGCTTTGATTAGTGTTCAGCTA
CTCTCAAATGCAATCTGGCTATTTTTCTCACCTCCGGGAATTCGCTACGATCGTAAAAAA
CCAGAATACCCGGTGTTGAAATGTAAAATTGAAACAAATACGTTTCTAGTTTCTCTTGTG
TACAATATGCTGCTCATCTCAGTTTGCACGTATTATGCAATTAAGACACGAAAAATACCG
GAAAATTTCAATGAATCCAAATTTATTGGATTTGCCATGTATACAACTTGTGTTATATGG
TTGGCATTTATTACAATATACTTCGGAACTCTTCATTCATTTGAAGTGCAACTTTCAACT
CTTGGAATATCTGTTAGTCTCAGTGCTTGGGTAACGTTGGTTTGTCTATTTTTTCCGAAG
CTGCACATCATATTTCTGCAGCCAGAGAAAAATGTGCGAAAATTAACAATGAGCAGCACA
ACTTACAGAAAACCAACAAACTCATCAGGTTCATTTGCTAATCATCGTAAGAATTATTTA
AACAAATAAatttttcttctttaaaattggtataaatattcgaattatacagtgataatc
tgagtaaattaaaagttatatcaaagtttttgcatttagtacttaatactggataatata
atattaaaaaaaatactctaaaaatattcagatgaaaataaaaaataattatataaataat
gataatgacaatgaa
>DjScaffold429:4078-24805 strand:+
tttaattgtgatataaATGGCTAGTTCATCAGAAAATGTTAATTTACCTGGTCTTACTGC
TGTGGACATTGATAATTTGACATTAAAATTGTGTCGAATGACGGATGATGATTTATTTCA
AAATAATCTTATTACTACTTTAAAACCTATAATAATTGAAGTATCAAAAAATAATGAAAA
TCGAGTGAAGCTATTTGAATTATTAGTTCATATCAACAATCGTATTAGAGCAGTTAGGAA
TCTGAAAATGCCATTAGAACCATTTTTCGATCTATTAAATGATACATCTCTATTGGTTCA
CTCGACTAATTTGGCTCACATGTATATCAAGTTGTGTTTTCCTAAATACGATCAAGAATC
TCGAGTGCGATTGTTACCGTTATTGTTCAGTTCGCTTACTCCAGACAAAAACATGTCTCA
AAAAGATATAATGCTTCATGTGGCGTTGCCAACATTGCAACACGTGAAAATCACTGACAA
CAACAAGAGAGCATTCGAAATGTCGAATCTCGGCTACCAACGTCGATATATAATCGAATT
TCTCTCTCTGATTCTGCTTACTCCTTACAATTACAAATCTCTGATGAAAGAAAACAACAA
TTCGTTGGAAATCAGTCGAGTTCCTGACGGAATGAACGCGTCGGATTTCTCGAGAGTTGT
CAAGAACGATGCGACTTGTTTCCTCGCCAACGACGATTTAGAGAAAACTAAATGCGGAAT
TGTCAGTTTCTTGATCCAGCGAGCATTTCCCGACGATGAGATCGTGTTGGCGCTCTGCAT
TGCTCATTCCGACACTCGCCATTCTGTGATTTCTCTCGTCGACAAGGACGCCAAGAGTTT
GTATTTGCAGATCGATTGGGAGAAATTGGATGTTCTCAACTTGTTTCTCGATTGGTTTAT
TCCCCGCGCGAGACAGACAGATCAGACAAACAATCAGCAGCAGCAGGCCAACACGAGATT
GAGATTGCCCGTGGCTGCGCAGAACAAAATTGGAGCTTTGCTGATGAGATCGAAAATATC
TTTGCCGAATAAGTTGGCTGGAGCTCTCGCTGAGGCCGCCTCAGTTACGTTTTTGGATTA
TAACAACAGAAATATTCATCCGAAACAAATAGAAACTGGATTCGCCTTGGTGGCGCAGCT
TTGTGCATACTGCAGTGATATGACCAATGATATCTCAGAAAAATTGCTGAAAAACCTCAT
TATTTTCATGCAAAAACCATTAGACCAACACGAGTCTACGTTGAAAATTAAAGCGCTCAA
TCTAATACCTCAAATTCTTAAAAGAAATCACCAGTTTTTATTACAAGACACAAATTACTT
GTCTCAATTGTTTGATTCTCTATGTAATTCTAATATTGATACCGAAATTAAAGAGGCGGT
AAAGAATTGTTTAAAAAATATAGCTATTATATTCCAAAATAAAAAAGTCGGTGAGGATGC
TAACGCGTCCGTTAATTTAGAGAGCATTCTTTATCTCAACTTCAATAAGGAAGAAGAAGA
ATGTCGGACAGCTGTTGTTTATTTTGCAGGTCTTGTTTACTCATCAGTTCATATTCCAAC
CCGCTTTTTGCTCTTAGAAGCTCTTGGAGACCAAAGCAAACTTGTCCGGGAAGAGGCTGA
ACATGTATTTTCTGAATTGTTCACCGCATCCGCCCAACACGATATTGTCATGCAAAATGT
GAAATTGCCCTACTTTGTTGAATTAGTTAATTATCATGGGCATCTTTACAACAGGAACTT
ATTCAGTGATCCTGTGAAATTGATACCGGTGGTGGAGTATATTCGTATGTGCGTTCTGTC
GCTCGATGTGGGCCTCACGGATTTAATGAAGAAAGAATTGGTATATGACAATATGGATGA
TTATAGATTTCGCATTTACCGTAATATTCAATACTTCGTTAACATTCAAGAAAGTAGTGA
TGGTGACATCCCATCGGCCAAGGCATTGGAAATGTATTGTAAATTAGTGCAAGTTATTTA
TTACACCAAAAAATTATTTTATGAATTTTCTTGAAGAAATTATTGCTGCTTCACCAAAAAT
TTTGCCTACCAAAGAAGGAATTATACTCCATAGAACAAAAAAATTTGTTACTCAGTGATTG
CCAAGATGAATTGTTTAATAGAGAAAATTGTGCTAGTGCACATGCAATATTATCTTTAGA
AATGGAAGACGATAACGCTTTGGAAGCTAACCTTAAAAACTTGTTAGATTCCATGCAATT
ATTAAATGTCAAACAATTGACATCGCAAAAACTAATTTCTCATGTTTACGCATATCATGG
TAATGCTCTTGGATTTTCATATCTATTAGAACGCTTGGTTGTTCGGCAAAAGAAAATTGA
TGTTCGAGATTTTGCTTCTTCATTTTTACACAAGATTGATCCAATTTTCGTTGTTACGAG
CTCCTTTCAGGATTCCAATTATGCATATGGATCTTTATTTGAATCTCTGTGTGTCATGTT
TGATTGTTTGTCAGCGTTATGTCAATCTGGTGCCCTTGTATTTCTACCAGCTGGCAAATT

CACTGATGAACAGTCTTCATGGCCCAATAAAGCTCTTATTGCAAAGCGTTGTCTTTCGTA
CATACGCAGCGTCAATTCTCCAATAAAGGTAAAAGAAGCTGCTCTACATGCGCTGGCAGC
TCTCTGTATGGGTGAATCTAATTTTGTGCATTCTCAAGCTATTCTAGAGCAATTGTTGTT
AACACACACTATTAAGGATCCAGTTTTGCATAAAAATATTGCCCAAGCTATTGTCAGTGT
TATTATGGGGCCATATTCCCCGTATAAGGGTCGTTGGTATGAAAGGCAATTTTCATTACC
CACACAGAAAATGCCAGCAATAAAAAAAGCAATCAGTAAAGATTTGCAAGTAGTAATGAA
TAAGCTCCATGAAATTCTCTCACAAGGAAACAGTATTAGTCGACATAATGTCGCCGTATG
GAGTTTATATCTTGTTCAAAAATTGGGATGGCATTCTGCAATATCTTTGTCTTTATTGCA
TTCCCGAATATTGGTTGTACTTGGTGATAAAGATGAAATGGCACAAGATGCAGCAAGTTG
CGTATTAGGTATCATTTACGAGCAAAGTAATGCAACAGAAAGAAAATTGATGTCGGAAGA
ATTAATTCAAGTGATTTTGCATGGCCAAAGAGGTCGATTAGAAAGTAGTTTGGAAAATGA
AAAATTTACTGACGTTGAATTAGAATCGAGAGAAAATAACAAGGCTACTTATAAGGAATT
ATGCTCCCTCGCAAATGAATTAAATCAACCAGATCTTATTTATTCATTTATGCCTCTGGC
CAGTGGTCAGAGTAAATGGACAGCAAGGGAAGGAGCTTCAATTGGCATGAGCCATATGTT
GCTGAAACACAGTAGAGAACTTGAACCACATATTGCTGGTATGGTTCCTAAACTATTCCG
TTATCGGTTTGACCCACACGTGCGAACACAAAAAGCAATGGAACAGATCTGGAATAATTT
AAATGTGATAACGCCAAACCAAGAACTAATTAAACGTTGTTTCCCTGAGATAGTTGCTGA
GTTACGAACAGGGCTGATTAATAATCAACAGCGAGTCAGAGAAGCTAGTTGTTCTGCTTT
GCGCTCATTATTAAAACATGAATTGGCAGATCCACTTATATTATCAGAACTGGGAGAGTT
AGTTCGTTGCCTTATTCGAGTAATGGATGACACGAAAACTGAAATTAGAGAAGTGGCTAT
AGAAACTGCAAAGTCTATTGAAAAGTTTTGTACAATGGTTCTTGATGATCCAGCACGACG
GCAATCAATTGAACCTTTATTATCAAGCTTGGTTCCAGTGTTACTTGAACAAGGATTGAC
AAGCAGTTTAACACAGGTTCACCAATTCGCTCTCAGTACCTTAGGAGCCATCGTTCACAG
TGGTCGTGCTGCTATTAGGTGTGCTGTTCCCAGCATTGTTATTGCAGCCCTGCAAGCACT
CAGCAGTCATGAATCGAGTGAAAGCAGTTACATTAGTCAACAGTTGGCAGGAATGGGTAA
TCATGACTCTCGCGAACGTTGGGATATGCAGCGAGTAAACATGGCAAAACAGTCTCCCGT
TGGAGATATCTTATCGTCGTGCATTCGTTATATCGAGGATGACACTCAGGCTCAATTATT
GATTCCTTTAGCTGATATTGTGAGACATGGAGTGGGTCTCGCTACCAGATGTGGAACTGC
AGAATTCCTCATTGATCTCAGTCTCCATTGCGGATCTCGACTGATGGGAGCCACTGGAAA
ATTATTGGCAGCTTTGCTCTATGCCGTACTCGACAAAAATGAGGTGGTTCAACGTGAAAT
GGCTAAAGCTATCGGCTATCTTGTTAAATATTCCAAGAAATCTAGTGTTGAAAAATTATT
TGTCATAACTCGAGCTTGGTACTTTGAAAATCATGATTCAGAAGAACATCAGTTAGCATG
CTGTCATGTTTATCATTGCATTGAGAAATATGCTCCTGGAACGTTAGAAGCACATGCTAC
TACAACTTTACCACTTATTTTTCTGGCTAAACAGAATGAACCAGAAACAAGTGACAGTGG
AAGTAGTGGTCAAAAGAAGTTATCTCTACAAACCGAAAACTGTCAAAAACGTAGAACACT
TTGGAGTCAAACATGGGTTGAATGTGTTAGAGTGGAGGGATCAGTAATAATGGAAATGAT
GCCACACATTCTTGCTTGTGTTGGTGAAGCATTAAATAGTTCATTTTTCCATTTACGAAC
GCAAGCATGCTTATCGTTGATGGCTCTTGTTAGAGGAATCGGTAGACAATGGTCAAGTGA
AGATATCAGTCTATGGGGACAATTGCACACGGCCTTATTAAATGCTCTAGTCAATCAAAT
CAAATTCTGGCCCGGAAAAGTGTATTTATTGTATGCCACAATGAGTTTTGTTCGGCAACT
TGATGAAAACAACTTATTGAACATTATTCAGGACCGCGCCAGGCTAATTCAGATTCTTAT
GAAGGAAATCAATAAAAGCGATGTTTCCTATCGTTGTGTTGCATTCGAAGCTGTTGCCAA
GGTTTCCGATATTCTCGATAATGAATGCTTGAAATCAATGTTACCCATGATGGTTCCTTT
AATTGTGAAGGGGGAAACTCAAGTCGGTGCTCCGGAACGAGAAATGCTGCTCAGAGTAAT
TGGACACATGTGGCCCAAGAGTTGCGAGGATGTCCAAGAATCGTATTTTGTAACTGTGAT
TCAATTACTCACTCAGGCCATGGAGGAGTCGCAATGGAAACAACAAATTGCGGTACTCAA
AAGTTTTAAATCGCTGTTTAAAtttaattgtgatataaATGGCTAGTTCATCAGAAAATG
TTAATTTACCTGGTCTTACTGCTGTGGACATTGATAATTTGACATTAAAATTGTGTCGAA
TGACGGATGATGATTTATTTCAAAATAATCTTATTACTACTTTAAAACCTATAATAATTG
AAGTATCAAAAAATAATGAAAATCGAGTGAAGCTATTTGAATTATTAGTTCATATCAACA
ATCGTATTAGAGCAGTTAGGAATCTGAAAATGCCATTAGAACCATTTTTCGATCTATTAA
ATGATACATCTCTATTGGTTCACTCGACTAATTTGGCTCACATGTATATCAAGTTGTGTT
TTCCTAAATACGATCAAGAATCTCGAGTGCGATTGTTACCGTTATTGTTCAGTTCGCTTA
CTCCAGACAAAAACATGTCTCAAAAAGATATAATGCTTCATGTGGCGTTGCCAACATTGC
AACACGTGAAAATCACTGACAACAACAAGAGAGCATTCGAAATGTCGAATCTCGGCTACC
AACGTCGATATATAATCGAATTTCTCTCTCTGATTCTGCTTACTCCTTACAATTACAAAT
CTCTGATGAAAGAAAACAACAATTCGTTGGAAATCAGTCGAGTTCCTGACGGAATGAACG

CGTCGGATTTCTCGAGAGTTGTCAAGAACGATGCGACTTGTTTCCTCGCCAACGACGATT
TAGAGAAAACTAAATGCGGAATTGTCAGTTTCTTGATCCAGCGAGCATTTCCCGACGATG
AGATCGTGTTGGCGCTCTGCATTGCTCATTCCGACACTCGCCATTCTGTGATTTCTCTCG
TCGACAAGGACGCCAAGAGTTTGTATTTGCAGATCGATTGGGAGAAATTGGATGTTCTCA
ACTTGTTTCTCGATTGGTTTATTCCCCGCGCGAGACAGACAGATCAGACAAACAATCAGC
AGCAGCAGGCCAACACGAGATTGAGATTGCCCGTGGCTGCGCAGAACAAAATTGGAGCTT
TGCTGATGAGATCGAAAATATCTTTGCCGAATAAGTTGGCTGGAGCTCTCGCTGAGGCCG
CCTCAGTTACGTTTTTGGATTATAACAACAGAAATATTCATCCGAAACAAATAGAAACTG
GATTCGCCTTGGTGGCGCAGCTTTGTGCATACTGCAGTGATATGACCAATGATATCTCAG
AAAAATTGCTGAAAAACCTCATTATTTTCATGCAAAAACCATTAGACCAACACGAGTCTA
CGTTGAAAATTAAAGCGCTCAATCTAATACCTCAAATTCTTAAAAGAAATCACCAGTTTT
TATTACAAGACACAAATTACTTGTCTCAATTGTTTGATTCTCTATGTAATTCTAATATTG
ATACCGAAATTAAAGAGGCGGTAAAGAATTGTTTAAAAAATATAGCTATTATATTCCAAA
ATAAAAAAGTCGGTGAGGATGCTAACGCGTCCGTTAATTTAGAGAGCATTCTTTATCTCA
ACTTCAATAAGGAAGAAGAAGAATGTCGGACAGCTGTTGTTTATTTTGCAGGTCTTGTTT
ACTCATCAGTTCATATTCCAACCCGCTTTTTGCTCTTAGAAGCTCTTGGAGACCAAAGCA
AACTTGTCCGGGAAGAGGCTGAACATGTATTTTCTGAATTGTTCACCGCATCCGCCCAAC
ACGATATTGTCATGCAAAATGTGAAATTGCCCTACTTTGTTGAATTAGTTAATTATCATG
GGCATCTTTACAACAGGAACTTATTCAGTGATCCTGTGAAATTGATACCGGTGGTGGAGT
ATATTCGTATGTGCGTTCTGTCGCTCGATGTGGGCCTCACGGATTTAATGAAGAAAGAAT
TGGTATATGACAATATGGATGATTATAGATTTCGCATTTACCGTAATATTCAATACTTCG
TTAACATTCAAGAAAGTAGTGATGGTGACATCCCATCGGCCAAGGCATTGGAAATGTATT
GTAAATTAGTGCAAGTTATTTATTACACCAAAAATTATTTTATGAATTTTCTTGAAGAAA
TTATTGCTGCTTCACCAAAAATTTTGCCTACCAAAGAAGGAATTATACTCCATAGAACAA
AAAATTTGTTACTCAGTGATTGCCAAGATGAATTGTTTAATAGAGAAAATTGTGCTAGTG
CACATGCAATATTATCTTTAGAAATGGAAGACGATAACGCTTTGGAAGCTAACCTTAAAA
ACTTGTTAGATTCCATGCAATTATTAAATGTCAAACAATTGACATCGCAAAAACTAATTT
CTCATGTTTACGCATATCATGGTAATGCTCTTGGATTTTCATATCTATTAGAACGCTTGG
TTGTTCGGCAAAAGAAAATTGATGTTCGAGATTTTGCTTCTTCATTTTTACACAAGATTG
ATCCAATTTTCGTTGTTACGAGCTCCTTTCAGGATTCCAATTATGCATATGGATCTTTAT
TTGAATCTCTGTGTGTCATGTTTGATTGTTTGTCAGCGTTATGTCAATCTGGTGCCCTTG
TATTTCTACCAGCTGGCAAATTCACTGATGAACAGTCTTCATGGCCCAATAAAGCTCTTA
TTGCAAAGCGTTGTCTTTCGTACATACGCAGCGTCAATTCTCCAATAAAGGTAAAAGAAG
CTGCTCTACATGCGCTGGCAGCTCTCTGTATGGGTGAATCTAATTTTGTGCATTCTCAAG
CTATTCTAGAGCAATTGTTGTTAACACACACTATTAAGGATCCAGTTTTGCATAAAAATA
TTGCCCAAGCTATTGTCAGTGTTATTATGGGGCCATATTCCCCGTATAAGGGTCGTTGGT
ATGAAAGGCAATTTTCATTACCCACACAGAAAATGCCAGCAATAAAAAAAGCAATCAGTA
AAGATTTGCAAGTAGTAATGAATAAGCTCCATGAAATTCTCTCACAAGGAAACAGTATTA
GTCGACATAATGTCGCCGTATGGAGTTTATATCTTGTTCAAAAATTGGGATGGCATTCTG
CAATATCTTTGTCTTTATTGCATTCCCGAATATTGGTTGTACTTGGTGATAAAGATGAAA
TGGCACAAGATGCAGCAAGTTGCGTATTAGGTATCATTTACGAGCAAAGTAATGCAACAG
AAAGAAAATTGATGTCGGAAGAATTAATTCAAGTGATTTTGCATGGCCAAAGAGGTCGAT
TAGAAAGTAGTTTGGAAAATGAAAAATTTACTGACGTTGAATTAGAATCGAGAGAAAATA
ACAAGGCTACTTATAAGGAATTATGCTCCCTCGCAAATGAATTAAATCAACCAGATCTTA
TTTATTCATTTATGCCTCTGGCCAGTGGTCAGAGTAAATGGACAGCAAGGGAAGGAGCTT
CAATTGGCATGAGCCATATGTTGCTGAAACACAGTAGAGAACTTGAACCACATATTGCTG
GTATGGTTCCTAAACTATTCCGTTATCGGTTTGACCCACACGTGCGAACACAAAAAGCAA
TGGAACAGATCTGGAATAATTTAAATGTGATAACGCCAAACCAAGAACTAATTAAACGTT
GTTTCCCTGAGATAGTTGCTGAGTTACGAACAGGGCTGATTAATAATCAACAGCGAGTCA
GAGAAGCTAGTTGTTCTGCTTTGCGCTCATTATTAAAACATGAATTGGCAGATCCACTTA
TATTATCAGAACTGGGAGAGTTAGTTCGTTGCCTTATTCGAGTAATGGATGACACGAAAA
CTGAAATTAGAGAAGTGGCTATAGAAACTGCAAAGTCTATTGAAAAGTTTTGTACAATGG
TTCTTGATGATCCAGCACGACGGCAATCAATTGAACCTTTATTATCAAGCTTGGTTCCAG
TGTTACTTGAACAAGGATTGACAAGCAGTTTAACACAGGTTCACCAATTCGCTCTCAGTA
CCTTAGGAGCCATCGTTCACAGTGGTCGTGCTGCTATTAGGTGTGCTGTTCCCAGCATTG
TTATTGCAGCCCTGCAAGCACTCAGCAGTCATGAATCGAGTGAAAGCAGTTACATTAGTC
AACAGTTGGCAGGAATGGGTAATCATGACTCTCGCGAACGTTGGGATATGCAGCGAGTAA

ACATGGCAAAACAGTCTCCCGTTGGAGATATCTTATCGTCGTGCATTCGTTATATCGAGG
ATGACACTCAGGCTCAATTATTGATTCCTTTAGCTGATATTGTGAGACATGGAGTGGGTC
TCGCTACCAGATGTGGAACTGCAGAATTCCTCATTGATCTCAGTCTCCATTGCGGATCTC
GACTGATGGGAGCCACTGGAAAATTATTGGCAGCTTTGCTCTATGCCGTACTCGACAAAA
ATGAGGTGGTTCAACGTGAAATGGCTAAAGCTATCGGCTATCTTGTTAAATATTCCAAGA
AATCTAGTGTTGAAAAATTATTTGTCATAACTCGAGCTTGGTACTTTGAAAATCATGATT
CAGAAGAACATCAGTTAGCATGCTGTCATGTTTATCATTGCATTGAGAAATATGCTCCTG
GAACGTTAGAAGCACATGCTACTACAACTTTACCACTTATTTTTCTGGCTAAACAGAATG
AACCAGAAACAAGTGACAGTGGAAGTAGTGGTCAAAAGAAGTTATCTCTACAAACCGAAA
ACTGTCAAAAACGTAGAACACTTTGGAGTCAAACATGGGTTGAATGTGTTAGAGTGGAGG
GATCAGTAATAATGGAAATGATGCCACACATTCTTGCTTGTGTTGGTGAAGCATTAAATA
GTTCATTTTTCCATTTACGAACGCAAGCATGCTTATCGTTGATGGCTCTTGTTAGAGGAA
TCGGTAGACAATGGTCAAGTGAAGATATCAGTCTATGGGGACAATTGCACACGGCCTTAT
TAAATGCTCTAGTCAATCAAATCAAATTCTGGCCCGGAAAAGTGTATTTATTGTATGCCA
CAATGAGTTTTGTTCGGCAACTTGATGAAAACAACTTATTGAACATTATTCAGGACCGCG
CCAGGGTAAATCAATTTTGAaaataaatcgatttttccacaattttcatttaaatgacaa
tttttttattgtttttccagctaattcagattcttatgaaggaaatcaataaaagcgat
gtttcctatcgttgtgttgcattcgaagctgttgccaaggtttccgatattctcgataat
gaatgcttgaaatcaatgttacccatgatggttcctttaattgtgaagggggaaactcaa
gtcggtgctccggaacgagaaatgctgctcagagtaattggacacatgtggcccaagagt
tgcgaggatgtccaagaatcgtattttgtaactgtgattcaattactcactcaggccatg
gaggagtcgcaatggaaacaacaaattgcggtactcaaaagttttaaatcgctgtttaaa
aa
>DjScaffold429:42492-62261 strand:+
atttgagtaatattattttattaaatttcagttaacttttatattctgtaaaaattgtgt
atcttattactggaaaattttatcattagaaatagattatattattactaagaataaata
ataaatcgaatttttatattattgaacaATGAATTCTGAAGATTTTGGTAATACGCCCGA
AGATCGTGTTATTAATTATCACAAATTTCATTACAATTGGATTGATAAAGCAATAACTGC
CGATGAAGCCAATGATGTCCAAAGTGCGGTACATTGCTATATTAAAGGGCTAGAATTTAC
AAACATGTGTCTACAAGAAAATGTAAAAGGAAATAATCATACACAATTAAAAGCAAAAGA
TATCCAAAAAAAAATGAAACTGAACCGTCAATCAGCAATAGAAAGATTGGAGTTCTTATT
AAGCAGTAAGCTCCAATCTGACAAAGATTCTAGAACCTTAAAGAAGCCAGTGGCATGTGA
TTTGGATGAAATTGAATTCAAAGCTCCTTCAAAATTTTTAACCAAATCTATGCGAATAAA
AAAAAGCGGAAATGAAAAGGATAAGAAAGATAAACTGAGGAAAGATTCGTCAGGTGATGC
TGGTATAGGGCCTCGCAGTAATAGTGCAACTTGTATAAATAAAATTGGTACTGGTAATGA
AGGCAATCTTGCTAAAGCCCTTCAAACAATCAAATCGACAAATCCTCAACCTATAAATAT
TAATACTGTAAGGCCTAATATTAAACCATCTTCACGGCCTCGAGCAAAGTCTGGTCCTCC
TAAACCTACAATTGAAAATAAGGTTCCATCTAGTTCTGCTGCAAATATCGATAAAGAAGC
TATTTATAAAGTTTTAAAAAAATGTTGATAAAGAAGCTGTCGAAACTATTTTAAATGAGGT
TCTTGATAGTAAAGTGATAAAATTTGAGGATATAGCTGGAAATGACACAGCAAAACAATG
CCTTTTAGAAACTGTTATCTATCCAAAATTGAGACCCGAACTATTTACTGGTTTAAGAAG
TCCAGCCAAAGGTATATTATTGTTTGGACCACCTGGAAATGGAAAAACATTACTTGCAAA
AGCAGTAGCAAATGAATCTAAATGTGTATTTTTTAACATAAGTGCCGCCAGTTTAATGTC
AAAATGGGTAGGTGAAGGCGAGAAAATGGTTCGGGCTCTTTTTGCAGTGGCTCAGTACGC
TTCTCCTTCTGTTATATTTCTTGATGAAGTGGATTCATTATTATCTGCCCGAAATTCTGA
TGATAAAAGTGTTTCTCGAAGAATGTTAACTCAGTTTCTTCAAGAAATTGATGGAGCTGC
AACTAATGGAGAAGAGAAAGTTTTGGTAATGGCTGCAACTAATCGACCTTTCGAGCTGGA
CGATGCAGCTCTTAGGAGATTCCAAAAGAAAATATATATAAAAATGCCAGATGCTCCATC
AAGAAAGTTTTTATTAAAAAAGTTGTTGACTCAGCAAACTACAAAATTAAAAGAAGCAGA
AATAGACAAATTGATTGACCTTACGAAAGGATATTCATCGAGTGATTTAACTAATCTTGC
TAAATCAGCAGCAATGGTACCTATCAGGGAATTATCATTTGAACAAATATGCTCCGCTAC
AGTAGATAGTATGCGTCCGATAAAATACAATGATTTTGTGCACGCTCTTAAAGAAATTAA
ACCTAGTGTACCTCCACAGTCGTTGTTGAAATATGATAAATGGAACAGTGAGTTTGGAGA
TAATTCCTAAttttaaaaattctggtcatgtgttcggctgtgatgtaacgttttctatct
gttgcataattttttcaattgatctgtgtcattgtttattcttaataatgacttgaaat
cactttttagatttacagtataaactttgtatgtatatctacgatgaatttatgttctag
ttgtcgttgtatatttgttgttattgcaattgccattcgaaacagcgtagtaattaagct

tatgatataa
>DjScaffold652:137366-149212 strand:+
attattattattattattattattattagtagtattagtattagtattattaccatt
atttcattaaataattttactattctcctcaaatataattatttctaaaatgaaatatta
aattaaaaaatatttttaaagtctatttgttgaagcatttatgaaatcttaaattttctc
aacttgagtaccaataagattttttcgattaataaaaattatttgaaaaaaacaaatct
ttttctttataataatcatttttgaaaagttaattttttagattatATGCAACCAATTGG
AAATAGTGGTTTTACCTTACTTTATTTAATTTTAATTATTGGGACAGTGCTTGCAATATT
AATGGCAGTTACATCAAAAGCAGATACGCCACCACGATATTTTCCACTGTTTTCATTTTT
CGGATTTATAACATCAATTATTTGGATTTATGAAATTGCCAATGAAATTGTTAATATTTT
GGAAACATTTGGTGTTATTTTCTCGATACCACAAACAATACTTGGATTGAGTGTTTTAGC
TTTTGCTAATTCGATTGGAGATTTAGTATCAAATATAATGCTTGCTAAAAAGAATTTTCC
AAGAATTGCTTATGCAGCTTGTATAGGTGGTCCATTGTTTAATTTACTTATAGGAATCGG
CCTCCCATTTACTATAGCTACTACAAAATATGGAAATATAAAGATAAATATCCGTTCGGA
AACTGTAGTTTTGATATTCACAATTTTACTAATCACAACAATGAATATTGTTGCATTACC
TATAATGAAGTTCAATTTGAGACCTTGTTATGGAAAAAATTTTAATAATTATCTACATAAT
ATTTCTCATTATGGTTTTACTTTTTGCTGCACACGTTATTAAAGTTGTTTTATAAagata
taatataaatttgaatatttatttgattttaataaaataaaa
>DjScaffold1311:15129-21245 strand:+
AGTGATGTTATTACTGTTCCTATTATTTATGGATTGGAGTTTCAATCTCGGAGTTTGATA
GGTCTATATAGCGAAAAAGATGAAATTCGATTTGCCGCTGGAACTCAGTCTATTAAGTCC
ATGAACCAGCTCCATTTGATTACATATAATGATGAAATTCATGCAATAACAGCAAATATT
TTTAATCATTCTGATGGTGAAATATGGGGACTAACTGCAATGGCTTCAAATCCAGAGATT
TTAATCACAACATTTTCATCACTGCACAATAATTATTCCGAAATAAAAATGTTAGCAAAT
CTTTGGAAATTGCCATTAACTGACAATACTCATTTAAATGCTAATTTAGAATTTTTAACT
CAGCTTCCAACTGTTGAAATTGGCGATGTAAAACAAATTATAAGTTGTCCAAATGAGGAA
ACAAAAAATCTCATTGCATTAGTTGATGGGAATTTCGTTTTAATGGGATGTAAAGGATCA
GAATTTAGTATTATTTCTAAAGGTCGCTTAGATAGTCGTGGGCACTGCAAATTTAATGTT
GGTAGTTGGAGTCCACATCATGGTACTAAGCAAGTAGTTACTGCTTCTGAAACAAATATA
TATGGTTGGGATATAAATTCAATGAAAATGTCGTACAATATTGAAAATTCTCATTCCCAT
ATGGTACGAGATATAGATTATAATCCGAATCGCCAATATTGTATCATTTCTAGTGGTGAT
GATTGCTTTATAAAAATTTGGGATCTAAGAAACACAAAACCAGGACCAGTTAAAGAAATA
GATGAGCATACTCATTGGGTATGGAAAGCTAAATATAATCCTTTTCATGACGAATTAATT
CTTTCATGTGGTTCAGACAGTATGGTAGCTTTACATAGTGCACAATCTGTATCTTCAGAA
TCTCATTGCAAATCAATATCAGATGAAGAGGACGATTCATTTTTAAAAAAATAAtttca
>DjScaffold1543:22428-24051 strand:+
GAACTAGATACAGCTAACTTTAATCATGAAATTAACGACTTATTAACCGAATTGGAAGTT
TGTTGGCGTTGTAGACTTAGATTTATTCACGAAACTATTGATTTTCAACAATTCAATGAC
CAAGATATATCAGATGAAACTTTTTGCTGCTATACATGCAGTGGTATTTTGCAAGAGTCT
TTTGTTAGTGACAATGTAACAGATAAATTACAAAGTGACATTGTTAATTCTGGATTTAAA
TTTCTTTCTTTTCAAGTTGTTGTGAGTACTCCAATAAATTTATTTATTCGTGAATTCTCA
GTTTCTGTTGTATTGAAAGAGAAATTCCCTATTAAATATGCAAATCATTCTCTGGATAAT
ATGATTCAAATCAAGTCAGTTTGGCGTTTTCTTATTAAAGATAGATTGTCTAAATCACTC
GGAAAGGAATATATTAACGGTAGTCAAGTGCAATATGCTAGATTGTCAAAAGATTGTTTG
ATAGAGAATGATTTTCTTATTGTTAATGTTACATTCAATCATTTCCGAAATGAATTTGAA
TGCAAAGTTTTATCGGAATTAGAACCTGAATTATTTCAACTTAGGCGGAAAGGAGAATCG
AGATATAAATGGGATTTATTTACAAGGAAAGTTGCTGATAAAGTCTTACAAAAAACATCG
AAAGAAAAGTTTCAACGTTATTTCGGTGCTCCACCGAGTCAAAATCGAAACACTCGAGAA
ACCGATTCTCCGACAGTTCTCATGTCGACTGAAATCTCCCGACAAGATCCGGTAATGATC
GCCGGGAGATATTGTAAATTCAGTCGTCGTTTGCCGCAATCTCCATGGATATTCGACGGA
GTTCGGAAATTGGAGTCGTCCGTTGAAGAGTTGATCGCAGAAAAACTCGTTTCGACTTTC
GATTCGTCTTCGAATGGATTTATTTCGTCGGGAAGAGAAGATGTCGACGTGAGAATGTTG
GGTAGAGGCCGACCGTTTGTGATGGAATTGCGCAACTGTAAAAATATGGTGCGGAACGTT
ATTGATTTTACCGGAGAATTGGAGAACGAGATCAACACGTTCTGTCGACAGCTCGTTGTC
GTGAAGGATTTGAAAATCATTTCGAAGGAATTCGTCAATTTGATTAAACAAGGAGAAGAA
GAGAAATCGAAACAGTATCGAATGGTTGTTTGGTGCGAGAATGGGATCAGCGAGGATCAG
AACGAACTCGTTGCGATCAATAATACGAGAGATTTGCTCGTTCAGCAGTTGACACCGATC

AGAGTTCTCCATCGTCGAGCATTGATCGATCGACAACGCACGTTTTATTCGTTTTCCGCC
ACCCGATTAGATCGCGCTCTCTCGTCCGACGCAATCGATTCGGATCAATCGTTGATGGAT
TACAGGAGATTCCTTGTTGAGTACGATCGCGACCACTTGTTCTTGCTCCATTTGCGTTGT
CAGGCCGGTAGTTATGTCAAGGAATTTGTGCACGGCGATCTCGGCAGAACTCGACCGAAT
TTAGCTACAGTAATCAAGCAACAATGCGATATTTTGGCACTTGATGTCATG
>DjScaffold1023:7235-8602 strand:+
tatattttatttgtttaatttgcttacggaaaaatttaattcttttggtttccaagATGT
CGGAATACTGGGTGAAATTAAGAGGTATGCCGTATCGTACCACTATGGATGATATAATCG
AATTTTTACCAGGCATAACCATTAAAGGGGGCAAAAGAGGTATTCATTTTCCAATTAGTC
AAAGAAATATTGAGTGGGGAGAAGCTTTCATTGAACTAGATACAGAGGAAGATTTAAATA
AAGCTAAAGACCGACATAATCAACATATGGGACGGCGGTATATTGAAGTTTTTTCGTCTA
CAAAAAGTGATATGGTTAAGGCATGCAAAGAATTGCCTGCTGGTATTAATGAGAATGGTA
GAGATAGACCTAGGCGTGAACGGAGCAGAAGCCCTGTTCATGAAAGAATCAATGAAAATT
ATGTACTCAGGCTGAAAGGATTACCATATAATGCGTCAAAAGCCGACATTAAAGAATTCT
TTCAAGGACTGAAAATATTGCCGGATGGAATGAGTATTCTCGTTGATCATAATGGAAAGG
CTCTAGGAGATGCTTTTGTTCAATTTGCAACGTACCATGATGCAAAACTTGCCAAGGAAC
GAAATGGAAATAAGATTGCTCACAGGTACATAGAAGTGTTTGACAGTAACATAGAAGAAG
CAAACAGTGAGATTCGTCGACAAATGGAATTCAATGAAATACATATAAAGAGATATGAAA
AATCTCCACCACAATCAAGAGGACCAAGGTCCATGGACGATGGAATGGCTTATCAAGAGT
CACAAAGGTTTTATAGTAGAAATCCTGTGATAATCAACATGAAAGGAGTTTCAATAAGCA
CAAGACCCGATGATGTTTTCAGGTTTTTCAGACCTCTAGTTCCATTGAATATTACTATGA
GATTTATGAGTGGTGCAGCAGAAGGAGAAGTTGATGCGGAATTTGCGAATGAGGATGAAG
CCTATAATGCTATGTCGAAAAATAGAGAAAGACTTGGCAGTCATTCAGTGTTTCTGTCAC
TAATACCGATTAGGGACGATTTCGGATATAATAAACCACTTGGTGGTATACCAGAATACG
AACGCAGGGAACGAGAAATGAGTCGAGGAATGGGAGGTCCTGGTCGGAGTTTCGGTGAAC
GTGATAGTTTTTATCCGCAATCGAGATATTACTAAtactgattaatgaaatttcgatttt
gcttagttgtagttatttgttcatacttttaaataatactttgttgtattttatatattc
ctcagtgttgtcaattttatatatttattattataattaatatttaatgtgagtacaaat
cgataaaaaattccaaaatttatttaaatatgtaacctttgtattttgtaacgagagattg
gagaaattacataatcgctgattgttttattttttaattatgagttatattttatttgttt
aatttgcttacggaaaaatttaattcttttggtttccaagATGTCGGAATACTGGGTGAA
ATTAAGAGGTATGCCGTATCGTACCACTATGGATGATATAATCGAATTTTTACCAGGCAT
AACCATTAAAGGGGGCAAAAGAGGTATTCATTTTCCAATTAGTCAAAGAAATATTGAGTG
GGGAGAAGCTTTCATTGAACTAGATACAGAGGAAGATTTAAATAAAGCTAAAGACCGACA
TAATCAACATATGGGACGGCGGTATATTGAAGTTTTTTCGTCTACAAAAAGTGATATGGT
TAAGGCATGCAAAGAATTGCCTGCTGGTATTAATGAGAATGGTAGAGATAGACCTAGGCG
TGAACGGAGCAGAAGCCCTGTTCATGAAAGAATCAATGAAAATTATGTACTCAGGCTGAA
AGGATTACCATATAATGCGTCAAAAGCCGACATTAAAGAATTCTTTCAAGGACTGAAAAT
ATTGCCGGATGGAATGAGTATTCTCGTTGATCATAATGGAAAGGCTCTAGGAGATGCTTT
TGTTCAATTTGCAACGTACCATGATGCAAAACTTGCCAAGGAACGAAATGGAAATAAGAT
TGCTCACAGGTACATAGAAGTGTTTGACAGTAACATAGAAGAAGCAAACAGTGAGATTCG
TCGACAAATGGAATTCAATGAAATACATATAAAGAGATATGAAAAATCTCCACCACAATC
AAGAGGACCAAGGTCCATGGACGATGGAATGGCTTATCAAGAGTCACAAAGGTTTTATAG
TAGAAATCCTGTGATAATCAACATGAAAGGAGTTTCAATAAGCACAAGACCCGATGATGT
TTTCAGGTTTTTCAGACCTCTAGTTCCATTGAATATTACTATGAGATTTATGGTATAAat
tgttgcattgttgtacttcaataaaaatcaagctaatttgtattgccatttattaaatct
atttgaaaacagcagttttctgaatttaatgcaaaataaattagcttgatttgagttttg
gtactaaattcaatttggattctatttattggtcaatatgatctcattacagagtggtgc
agcagaaggagaagttgatgcggaatttgcgaatgaggatgaagcctataatgctatgtc
gaaaaatagagaaagacttggcagtcattcagtgtttctgtcactaataccgattaggga
cgatttcggatataataaaccacttggtggtataccagaatacgaacgcagggaacgaga
aatgagtcgaggaatgggaggtcctggtcggagtttcggtgaacgtgatagtttttatcc
gcaatcgagatattactaatactgattaatgaaatttcgattttgcttagttgtagttat
ttgttcatacttttaaataatactttgttgtattttatatattcctcagtgttgtcaatt
ttatatatttattattataattaatatttaatgtgagtacaaatcgataaaaaattccaaa
atttatttaaatatgtaacctttgtattttgtaacgagagattggagaaattacataatc
gctgattgttttattttttaattatgagttatattttatttgtttaatttgcttacggaaa

aatttaattcttttggtttccaagatgtcggaatactgggtgaaattaagaggtatgccg
tatcgtaccactatggatgatataatcgaatttttaccaggcataaccattaaagggggc
aaaagaggtattcattttccaattagtcaaagaaatattgagtggggagaagctttcatt
gaactagatacagaggaagatttaaataaagctaaagaccgacataatcaacatatggga
cggcggtatattgaaggtacatagaagtgtttgacagtaacatagaagaagcaaacagtg
agattcgtcgacaaATGGAATTCAATGAAATACATATAAAGAGATATGAAAAATCTCCAC
CACAATCAAGAGGACCAAGGTCCATGGACGATGGAATGGCTTATCAAGAGTCACAAAGGT
TTTATAGTAGAAATCCTGTGATAATCAACATGAAAGGAGTTTCAATAAGCACAAGACCCG
ATGATGTTTTCAGGTTTTTCAGACCTCTAGTTCCATTGAATATTACTATGAGATTTATGA
GTGGTGCAGCAGAAGGAGAAGTTGATGCGGAATTTGCGAATGAGGATGAAGCCTATAATG
CTATGTCGAAAAATAGAGAAAGACTTGGCAGTCATTCAGTGTTTCTGTCACTAATACCGA
TTAGGGACGATTTCGGATATAATAAACCACTTGGTGGTATACCAGAATACGAACGCAGGG
AACGAGAAATGAGTCGAGGAATGGGAGGTCCTGGTCGGAGTTTCGGTGAACGTGATAGTT
TTTATCCGCAATCGAGATATTACTAAtactgattaatgaaatttcgattttgcttagttg
tagttatttgttcatacttttaaataatactttgttgtattttatatattcctcagtgtt
gtcaattttatatatttattattataattaatatttaatgtgagtacaaatcgataaaaa
ttccaaaatttatttaaatatgtaacctttgtattttgtaacgagagattggagaaatta
cataatcgctgattgttttatttttaattatgagtaataagattgctcacaggttggaat
gggaatggataagtttatcaaattaaaaacatattaaatgggttaaaagaatattgaaaa
ttctggattattcgttaagtgacggataaaaataagaatcaggaaaagttaaggtttaaa
ataaaaaaatgaggaaattaggattggaggagagaatagcaaaacaaaattaatcaatat
acgttatagaattaaacctaattttatattaggtacatagaagtgtttgacagtaacata
gaagaagcaaacagtgagattcgtcgacaaATGGAATTCAATGAAATACATATAAAGAGA
TATGAAAAATCTCCACCACAATCAAGAGGACCAAGGTCCATGGACGATGGAATGGCTTAT
CAAGAGTCACAAAGGTTTTATAGTAGAAATCCTGTGATAATCAACATGAAAGGAGTTTCA
ATAAGCACAAGACCCGATGATGTTTTCAGGTTTTTCAGACCTCTAGTTCCATTGAATATT
ACTATGAGATTTATGAGTGGTGCAGCAGAAGGAGAAGTTGATGCGGAATTTGCGAATGAG
GATGAAGCCTATAATGCTATGTCGAAAAATAGAGAAAGACTTGGCAGTCATTCAGTGTTT
CTGTCACTAATACCGATTAGGGACGATTTCGGATATAATAAACCACTTGGTGGTATACCA
GAATACGAACGCAGGGAACGAGAAATGAGTCGAGGAATGGGAGGTCCTGGTCGGAGTTTC
GGTGAACGTGATAGTTTTTATCCGCAATCGAGATATTACTAAtactgattaatgaaattt
cgattttgcttagttgtagttatttgttcatacttttaaataatactttgttgtatttta
tatattcctcagtgttgtcaattttatatatttattattataattaatatttaatgtgag
tacaaatcgataaaaattccaaaatttatttaaatatgtaacctttgtattttgtaacga
gagattggagaaattacataatcgctgattgttttatttttaattatgagt
>DjScaffold1023:97588-125130 strand:+
aatttaaatatttaatattaaaaatatattacgaaataaatattaacatattttatatca
ttatccttatttattattataATGAAAATATTTACCTTTAATTCCTATTACAGTCAAGTT
ATCATTGCTGTTTCTCTTTACTGGTTCATATCAATATCATTGGTATTTCTAAATAAATAT
TTACTTAGTAGTGATGATCTTAAATTAAATGCTCCGTTATTTATTACTTGGTTTCAATGC
TGCGTTTCAGTTTTATTATGTTGGATTTTTTCAACATTATCTCAGAAATACTTATGGATT
AATTTTCCAAAAATTACTTTTGAATTTAATAAAACTATTAGAATTTTACCATTGTCCATA
ATGATGCTGTCAATGATAACTTGTAATAATTATTCTTTGAAGTTGATTAACGTTTCATTT
TATTTTATTGGAAGATCGCTAACAACAATTTTTAATGTGATATTTACATATTTTTTGTTG
AAAACCAAAACATCTTTAAAGGTTATTATTTGTTGTAGCTTCATCCTTTTTGGATTTTGG
CTCGGAGTAGATCAAGAAGATAAATTAGGTTCATTATCTTGGAGTGGAATGATATTTGGA
GTAGCATCCAGCGCATTTGTGGCTCTTAATTCTATTTATACTAGAAAAACAATTTCAGTT
GTTAATGATAATGTTTGGAGTTTATGTTTCTATAATAACGTTAATGCTGCGTTGATGTTT
ATCCCAATGATTTTCCTGACCGGTGAATTCGATGAATTGAAAAGATTTCCTTTCTTCTTC
AAAATACAGTTTTGGTTGTTGATGATTGCTGGAGGAATATTTGGTTTTGCTATCGGATAT
GTTACAGGACTTCAAATACAAGTTACTAGTCCATTAACTCATACGGTTTCTGGAACCGCT
AAAGCATGTGCACAAACTATTATTGCATTCTTTATTTTTAATGAAGCTAAAAGCTCATTG
TGGTGGTTCAGCAATATCATTGTAATGGGTGGTTCTTTGGTTTATGCACTTGTTCGAAAT
AGGGAATTAAAGAATAAGAATATTCAAATGCCTAAATGAtatttaattgaatcaattcct
ttcaatacc
>DjScaffold1023:89090-91683 strand:+
ATTAATACTGATCTAAAAGATATTTGGAATACACTTAATGACAAAGACAGACATCATGTT

TACGAGGAGACAAATTTTCCACAAAAGGATTCCATAGACGAAATTTTAGCTAACTACTCG
AATTTTAGAAATGATGTTAAATCCCCTAATTTAAATAATAGTAATCTGTTTTCAATTAAA
AGTATTTCCAGTCTGCAAGATGGAATTCAAGAAAATTTAAATTCAAAACTATTATGGCCA
TCTAATTTTGATAATATATCTTCTTCATCTTATAATACCAAGTCGGCTTCGTTAAATTCA
AATACGAATGATACAAATTCTCATTTTGATTTTCAAAATTTTTTAAATTTCTATAAATCA
GATAATGCTTTTGTGGAACCTCGACGATTTAAATCAGAAACCGATACTGCAGATTTGATG
AGCCATAGTTTACAAAATGGAGTAAATGATAAACTTTTAAATGCAATATTGGAAAAAATT
ATTGTATCATCAAATCAAAAGGACTTCTTTACGAAAAGTAATATAGTAAACAGTATTAAT
AGTTTGCTTCAAAAACCTTTAACAAAACAACAATATCAAAAAGTGCAAAATGCTGTTCTG
ACAATTTATAAAAAGAGCAATAGCTATTATCACGATCCATTGCAAGGATTTTACAATCAG
GAATTATTAATGTTACTGTTAAAAAACCAACCATTGTCAAATACTCAAAATGAGAGATTG
AGCGAACCGATTCATCGATCAGAACCACCGAAACCTATAAGTTCAGTAAATGACAGTAAT
ATTTCGTTAAAAGATGAAATTGCCATGCTTTCTATGTCATACACAAATATGATGGGAATT
TTAGAAAGTGATATAGAAAAAGCTGCTTCTCTTTATCGCACATCAGCTACTGCAATGGCA
CAGAAAAATGAGGCTGCTTATCAGTGGTCAGGGAAATTACCAATTACGTCTTATAAACTT
TGCCAATATTCAAGAAAAGTATTTCTTGGTGGAGTTCCTTGGGATAGTACTGAAAGCGAT
TTAATTTCTGCTTTTATCAAGTTTGGTTATTTGTCTGTTATGTTTCCACAAAAAGATCTT
AATAGTCCCATCAGGCATAATAGTTCTTCGAATAGTTTGAAAGGCTACTGTTATATTATT
TTTGAATGTGAAGACTCTGTACCTAGACTGTTAAATCAGTGCAACAAAAATGAATCTAAT
GGTGGAGAATTTTTTAAGATATCCAGTCCGAAATTTCGTTCTAAGGATGTTCAAGTGATT
CCATGGATTACTCAAGACAGTCAGTACTGTCGATTAGGATCACAAAAGCCTGATTCAAAG
AAAACTGTGTTTGTAGGGGCATTACACGGAATGATGACAGCATATGCATTAGCTAATGTT
ATGAATGAATTATTTGATAATGTTATTTTTACAGCACTTGACACAGACAAATACAAATAC
CCAATTGGATCTGGTCGAGTAACATTTTCCAGTCAAAAAAGTTATATGAGAGCAGTTACT
GCTAATTTTATTCATGTTAAAACTGGAAAATTTAGCAAGACAATTCAGATTGATCCCTAT
TTAGAAGATTCAATGTGCAACAATTGTTTTCAATCGCCTGGAATTTATTTTTGTCGATCC
TTTGAATGCTTTCGTTATTTTTGCTCTGGATGCTGGCAAGTGTGGCACAATAGATGCGAA
ACACTCTTTAATCACAAGCCTCTTAGACGAACATTTAAACCACTTTTTGATTTACAATAA
>DjScaffold1023:137816-138720 strand:+
AGAACATTATATAACTCTGGGTCAGGTATTAAAGCGGATTCATACGACATATTACGCAAA
GAAGTGAAATTTCTTGGTCACATAATAGATAAAGAAGGAATTTTAACAGATCCAATGAAA
ATCAAACCTATCAAAATCTTTGAGAAACCCAAATGCGTAAAAAACCTGAGAAGCTTCCTC
GGAATATGCAATTATTACAGAAGATTCATTAAGGATTATTCAAAGAAATCAAGAGCCCTA
GAAGAATTATGTGGAAGCAACAGCACGATGCTAATATGGTCAGATGGATGTGAGAAAGCT
TTCACAGGAATAAAA
>DjScaffold3174:52191-64686 strand:+
GTACGTTATGTTAGAGATTATACTAAAAGACTTGCGTTGCAAGAGTTTTATAACAGACGA
ATTGAAATCGGTCCAGAAAAACCAAGACATAGATCTGAATTCTCAAATTGGAATTATGAA
GCTGAAGTATTCGCTTTTGGGAAAAGACTAAAGGAGAATTTTGACTTGAGTTTATTACGT
CAGGCGTTCGTTGATAAATCTTATTATGAAACTGAAGATACCATAAAGTTGCAAGATAAT
ACTGAATTAAAAAATTATGGATACAATCTTACTAGTCAATACGTACGGGCGTTCCTTATT
CAAACATTTCAAGATTTACCAGCCGAATGGATAGATCAAATTTATAATCATCTTATGTCT
GTTAAAACTACAGGTCATGTTAGTGGTTATTTAGGAACAAAAGATTTAATTATGTGCAAT
TCGATCGAAGTTGATATTGAAACTGAATCCAACGTTTTATTTGCTATAATTGGAGCATTG
GCTAAAAGTCAAAATGAAAATGTGGCCTATTTGTTCGTTAAAGATTTTATATTGACTCAA
TTAACAACAATACATTTATGTGACATTATTCAAATCGATAAAGGGTTCGATTTACTAACA
GGTGTATTACTATATCACAAAAGAGGTCCTCCAGAATCCCGATTGTTGCGTCAATCTGGG
ATGAATTCAATTTATGCTTGTTATCGTGTTGGTATTTATAGTGATAAAGATATGATTGGT
GAAGCTGCTGGTGATTCATTACTTACTACAGAAGATATGGCGGTTAAACATTCATTGATT
AATCTCTTTGGAATATCAGAAAAT
>DjScaffold4325:43636-44094 strand:+
ATGAGAAAACTATCTTTTTCAGATTCATATCCTCCCGCTTTGACCGATGACGAAAAGGCT
GAATTAGATGTCAGATCGATTTACGTGGGTAATGTTGATTACGGCTCAACTGCCAAAGAG
CTCGAGGCCCATTTTAAAGGCTGTGGAGCAATTAATAGGATAACGATTTTAACGAATAAA
TACACCGGACACCCGAAAGGTTTCGCCTATATCGAATTTGCGATGCCGGATTCTGTTGAT
ACTGCAATGCTCCTCGACGAAAGTGAGTTTAGAAGTCGTCAGATTAAAGTGATGCCTAAG
CGGACGAATTTGCCAGGTTATTCAACAACCAATCGTCCTCCAAGACGGGTTCGTGGTGGA

CGTGGAGCAATCATTGGAAGGTTTCATACTCTTATTGGTGGCCGGCCAAGAACTTCACGA
TTCAGACGCAGAGGAAGTTTTCATTACGCTCCTTATTAG
>DjScaffold4589:72-7236 strand:+
aatattgattaATGAAATTATTTATTATAAATGGAGAAAATAATGATGAAAAAATAGAAA
TTAATATTAATGAAGATTCGATGGTGAGAGATATAAAATATAAATTAGATGAGATTCTCA
ATGGAAATGGATATTTTATGAAATATTATTTCAAATTAATTCATAAAAATATTATTTTAT
TAGATAATAATTCGACAAAATATTACAAAATATGTGAAAATGATGAAATCTGGTTTGGTA
AAATTGAGAATTATTTGAGAAAAGATGATGACGTCATTGTTTATGTGTTCATTTGGGGAT
TCACTTATGGTGGTGATTATTTGATTGTGAAAATGAATGATACAATTTCAATAATCAGAT
CGATAATAGAAAGAATTCATTCTGAAATAATCAATATTTGGAATACAGATGGCAAACAAA
TGAGAAATGATCAAATTGTCACCGAGATTGACACAAATGAGAATTACAAATTGATTATTA
TATGTGATGATGGAGAAGGAAATGAGGTTAATAATGCATTTGATGAATGCTGCTATTTGC
ATGATAGATCGATGGTGGATCGATATTTGTTTTATGATCAGACTCGTGATGATAACAAAG
ATGAATCAACAAGAACAATATTGATAAATCGAATGATGNNNNNCAGACAAATTATCTCAT
CAAATAACACAATTGATGACAGATTGAATCAGAATTTGTCAGATTTGCCCTCCTCATTTA
TCAATTCGACATATTTATTCTGTTGTGATATGGCAACTCGAATTGTGAATATTTGTAATG
ATTATTTGAGATTTGTGATGAATCAATATGAGAATGATGTTGTGAGGATATTCGATCAAT
ATTTCCTATTTGCAATACGTGCTGCATTGTGGGATTTTTCTATTATTAGACAACAAATCA
ACAATAAAACAAATAAATTAGTGAGCGACATCGTAATCGATGCTTTGGATTATTTTATGA
AATATTTGATTGATTCCAGCAATGATTGGCGATATTGGCTGaatattgattaATGAAATT
ATTTATTATAAATGGAGAAAATAATGATGAAAAAATAGAAATTAATATTAATGAAGATTC
GATGGTGAGAGATATAAAATATAAATTAGATGAGATTCTCAATGGAAATGGATATTTTAT
GAAATATTATTTCAAATTAATTCATAAAAATATTATTTTATTAGATAATAATTCGACAAA
ATATTACAAAATATGTGAAAATGATGAAATCTGGTTTGGTAAAATTGAGAATTATTTGAG
AAAAGATGATGACGTCATTGTTTATGTGTTCATTTGGGGATTCACTTATGGTGGTGATTA
TTTGATTGTGAAAATGAATGATACAATTTCAATAATCAGATCGATAATAGAAAGAATTCA
TTCTGAAATAATCAATATTTGGAATACAGATGGCAAACAAATGAGAAATGATCAAATTGT
CACCGAGATTGACACAAATGAGAATTACAAATTGATTATTATATGTGATGATGGAGAAGG
AAATGAGGTTAATAATGCATTTGATGAATGCTGCTATTTGCATGATAGATCGATGGTGGA
TCGATATTTGTTTTATGATCAGACTCGTGATGATAACAAAGATGAATCAACAAGAACAAT
ATTGATAAATCGAATGATGNNNNNCAGACAAATTATCTCATCAAATAACACAATTGATGA
CAGATTGAATCAGAATTTGTCAGATTTGCCCTCCTCATTTATCAATTCGACATATTTATT
CTGTTGTGATATGGCAACTCGAATTGTGAATATTTGTAATGATTATTTGAGATTTGTGAT
GAATCAATATGAGAATGATGTTGTGAGGATATTCGATCAATATTTCCTATTTGCAATGTA
Agtttgattgattgaatgatggatagaattgtggtggcacaaatcattggaacatgcata
tttgtgatcaatccagatcaatattgaccacatacaaacatgttgacatgatcgatcata
aatatcattgcaaaatataataataatttatcaaatgatatttgcagttatgattgtcct
ccctcatttgatattgattattgcatttatcgatttaataaaaatattatttaattgaaa
aatatttattttattaattgatttgtgttattcagacgtgctgcattgtgggatttttct
attattagacaacaaatcaacaataaaacaaataaattagtgagcgacatcgtaatcgat
gctttggattattttatgaaatatttgattgattccagcaatgattggcgatattggctg
attcaaataaattaaatttatattttattaatatgaaaatgtgattgtttctttcagatt
atttaatattgattaATGAAATTATTTATTATAAATGGAGAAAATAATGATGAAAAAATA
GAAATTAATATTAATGAAGATTCGATGGTGAGAGATATAAAATATAAATTAGATGAGATT
CTCAATGGAAATGGAAATTTTACTAAAGATTATTTCAGATTAATTCATAAAAATATTATT
TTATGGGAAGATACGATAAAACATTACAAAATATGTGAAAATGATGAAATGTGGTTTGGT
AAAATTGAGAATTATTTGAAAAAAGATGATGACGTCATTGTTTATGTGTTCATTTGGGGA
TTCACTTATGGTGGTGATTATTTGATTGTGAAAATGAATGATACAATTTCAATAATCAGA
TCGATAATAGAAAGAATTCATTCTGAAATAATCAATATTTGGAATACAGATGGCAAACAA
ATGAGAAATGATCAAATTGTCACCGAGATTGACACAAATGAGAATTACAAATTGATTATT
ATATGTGATGATGGAGAAGGAAATGAGGTTAATAATGCATTTGATGAATGCTGCTATTTG
CATGATAGATCGATGGTGGATCGATATTTGTTTTATGATCAGACTCGTGATGATAACAAA
GATGAATCAACAAGAACAATATTGATAAATCGAATGATGNNNNNCAGACAAATTATCTCA
TCAAATAACACAATTGATGACAGATTGAATCAGAATTTGTCAGATTTGCCCTCCTCATTT
ATCAATTCGACATATTTATTCTGTTGTGATATGGCAACTCGAATTGTGAATATTTGTAAT
GATTATTTGAGATTTGTGATGAATCAATATGAGAATGATGTTGTGAGGATATTCGATCAA
TATTTCCTATTTGCAATGTAAgtttgattgattgaatgatggatagaattgtggtggcac

aaatcattggaacatgcatatttgtgatcaatccagatcaatattgaccacatacaaaca
tgttgacatgatcgatcataaatatcattgcaaaatataataataatttatcaaatgata
tttgcagttatgattgtcctccctcatttgatattgattattgcatttatcgatttaata
aaaatattatttaattgaaaaatatttattttattaattgatttgtgttattcagacgtg
ctgcattgtgggatttttctattattagacaacaaatcaacaataaaacaaataaattag
tgagcgacatcgtaatcgatgctttggattattttatgaaatatttgattgattccagca
atgattggcgatattggctgattcaaataaattaaatttatattttattaatatgaaaat
gtgattgtttctttcagattatttaatattgattaATGAAATTATTTATTATAAATGGAG
AAAATAATGATGAAAAAATAGAAATTAATATTAATGAAGATTCGATGGTGAGAGATATAA
AATATAAATTAGATGAGATTCTCAATGGAAATGGAAATTTTACTAAAGATTATTTCAGAT
TAATTCATAAAAATATTATTTTATGGGAAGATACGATAAAACATTACAAAATATGTGAAA
ATGATGAAATGTGGTTTGGTAAAATTGAGAATTATTTGAAAAAAGATGACGTCATTTTTT
ATGTGTTTTATTGGGGATTCACTTGTGGTGTTGAATATTTGATTGTGAAAATGAATGATA
CAATTTCGGCAATCAGATCAATAATAAAAAGAATTCATTCTGAAATAAACAATATTTGGA
ATTTAGATGGCAAACAAATGAGAAATGATCAAATTGTCACTGAGATTAGCACAAATGGGA
ATTACAAATTGATTATTAGATGTGATGATGGAAAAGGAAACAAGATCGATAAAACATTTG
ATGAATGCTGCTATTTACATGATAGTCGGATGGTCGATTATTATTTTTTTATTGATCAGA
GTTGTGATGATAACGAAAATGAATCAACAAGAACAATATTGATAAATCTTATTGATCAAA
TCAAACAAATTATCTCATCAAATAACACAATCGATGATAGATTGAATCAGAATTTGTCAG
ATTTGCCCTCCTCATTTATCAATTTGACATATTTATTTTGCTATGATATGACAACTCGAA
TTGTGAATATTTGTAATGATTATTGGAGATTTGTGATGAATCAATATGAGAATGAGTGAg
attgatatttgtgtgtgacacaattgatttgttattgattgatcatttagtgttacaaat
tatctcatcaaataacacaattgatgacagattgaatcagaatttgtcagatttgccctc
ctcatttatcaattcgacatatttattctgttgtgatatggcaactcgaattgtgaatat
ttgtaatgattatttgagatttgtgatgaatcaatatgagaatgagtgagattgatattt
gtgtgtgacacaattgatttgttattgattgatcatttagtgttgtgaggatattcgatc
aatatttcctatttgcaatgtaagtttgattgattgaatgatggatagaattgtggtggc
acaaatcattggaacatgcatatttgtgatcaatccagatcaatattgaccacatacaaa
catgttgacatgatcgatcataaatatcattgttcaaataaattaaatttatattttatt
aatatgaaaatgtgattgtttctttcagattatttaatattgattaATGAAATTATTTAT
TATAAATGGAGAAAATAATGATGAAAAAATAGAAATTAATATTAATGAAGATTCGATGGT
GAGAGATATAAAATATAAATTAGATGAGATTCTCAATGGAAATGGAAATTTTACTAAAGA
TTATTTCAGATTAATTCATAAAAATATTATTTTATGGGAAGATACGATAAAACATTACAA
AATATGTGAAAATGATGAAATGTGGTTTGGTAAAATTGAGAATTATTTGAAAAAAGATGA
CGTCATTTTTTATGTGTTTTATTGGGGATTCACTTGTGGTGTTGAATATTTGATTGTGAA
AATGAATGATACAATTTCGGCAATCAGATCAATAATAAAAAGAATTCATTCTGAAATAAA
CAATATTTGGAATTTAGATGGCAAACAAATGAGAAATGATCAAATTGTCACTGAGATTAG
CACAAATGGGAATTACAAATTGATTATTAGATGTGATGATGGAAAAGGAAACAAGATCGA
TAAAACATTTGATGAATGCTGCTATTTACATGATAGTCGGATGGTCGATTATTATTTTTT
TATTGATCAGAGTTGTGATGATAACGAAAATGAATCAACAAGAACAATATTGATAAATCT
TATTGATCAAATCAAACAAATTATCTCATCAAATAACACAATCGATGATAGATTGAATCA
GAATTTGTCAGATTTGCCCTCCTCATTTATCAATTTGACATATTTATTTTGCTATGATAT
GACAACTCGAATTGTGAATATTTGTAATGATTATTGGAGATTTGTGATGAATCAATATGA
GAATGATGTTGTGAAGATATTTGATCAATATTTTCGTATTTGCAATGTAAGTTTGATTGA
TTGAatttcaaataaattaaatttatattttattaatatgaaaatgtgattgtttctttc
agattatttaatattgattaATGAAATTATTTATTATAAATGGAGAAAATAATGATGAAA
AAATAGAAATTAATATTAATGAAGATTCGATGGTGAGAGATATAAAATATAAATTAGATG
AGATTCTCAATGGAAATGGAAATTTTACTAAAGATTATTTCAGATTAATTCATAAAAATA
TTATTTTATGGGAAGATACGATAAAACATTACAAAATATGTGAAAATGATGAAATGTGGT
TTGGTAAAATTGAGAATTATTTGAAAAAAGATGACGTCATTTTTTATGTGTTTTATTGGG
GATTCACTTGTGGTGTTGAATATTTGATTGTGAAAATGAATGATACAATTTCGGCAATCA
GATCAATAATAAAAAGAATTCATTCTGAAATAAACAATATTTGGAATTTAGATGGCAAAC
AAATGAGAAATGATCAAATTGTCACTGAGATTAGCACAAATGGGAATTACAAATTGATTA
TTAGATGTGATGATGGAAAAGGAAACAAGATCGATAAAACATTTGATGAATGCTGCTATT
TACATGATAGTCGGATGGTCGATTATTATTTTTTTATTGATCAGAGTTGTGATGATAACG
AAAATGAATCAACAAGAACAATATTGATAAATCTTATTGATCAAATCAAGTTATTGATTG
ATGGAAATATTTCACAATTAATCAATAATAATAATTTATTGCAGACAAATTATCTCATCA

AATAAcacaatcgatgatagattgaatcagaatttgtcagatttgccctcctcatttatc
aatttgacatatttattttgctatgatatgacaactcgaattgtgaatatttgtaatgat
tattggagatttgtgatgaatcaatatgagaatgagtgagattgatatttgtgtgtgaca
caattgatttgttattgattgatcatttagtgttgtgaagatatttgatcaatattttcg
tatttgcaatgtaagtttgattgattgaatgatggatagaaatcaatattgaccacatac
aaacatgttgacatgatcgatcataaatatcattgcaaaatataataataatttatcaaa
tgatatttgcagttatgattgt
>DjScaffold2353:29975-40768 strand:+
tcgttgcatattattaattataataaattcattttaacacatgttataacatgagattca
attatatttctattttaatttttgttttacatttaccttatttttaattttattcgatag
aatattttgattgatttcaaagtccatgatgatatcagtaactcaattacaaaaactatt
gagaaagttattaaattagattcatttATGGAATATAGAGAAACTTTATCAAATTGTAAA
ACAACGTTGGATGAATTGATCTCTCAGATTCATGGACAATGGCTAAAAGCAATGGAATTA
AAATGTAAATTGGAATTTATGCCTAAAATTGTAATTAACTTTAATTCCATCACTAAATCC
GGAATTTCTTTGGTTGGACCAGAAAGTGGATTTGAATCAGAAATTAGTCCAATGAATCCT
ATTCGTGGTTTTCCAACAAATAATCTAAGTTTAATGAAATATATTTCGTCATCAGGTGAA
TGTTTTGCAGACGATAGCACAACTGATTTACCAATGGATTCGGAGTCTACTGATTTACCA
TTGGATTTTGCATATAATAGAAGCATCAGTTCGTCTTCCTCATTAGTTGAAATAATGTTG
GATTTAACACATCAATCCCAAATTTCTAAAAATGATTTTAATTCTCGAAAGAAGCAAAAG
CGATTTAAAAAACGGCGACAGTTTGTTTCAAAAACCACAGGAGTTCATTCTGAAAATTTA
ACTTTGGAAGAAAGGGAAAAGTATTTACAAATTCGTAATGCTTTTAGTAGGCCTCCATAT
TCTTTAAAACGACGAATCAGTGAAACTAGCATTATTCGAATTTTGGATCGAACTGATGTG
ACGACAAATAAGATATCAGATTTAGCTGCAGCCGCTGAGAAAATCGTTATGCAAAAAGAT
TTCAATCGCCACAATGTCTCTTTAAGCGAATCACCGAAGGAATCGGAAATTGAAGATCCA
TATGGTGTTGTAGCAAGTTCAAATAATGATATAAATGTAAAAGATGTTTGGGATAATTAT
CAGGCTTCGTTGTATTCCAATGATGATGGTGATTCTCAGATTCTAGAAGTCGATAAAATT
AAAGATTGGGAGCAAGATATAGAAACAGATGATCAAGAGTATGAGTCTTGTTATCGTGAT
AAAAAACTACAGTTTCTCACATCAGGTTATGAAACTTCTATGGAGTCAGATATACATTTT
TGCCACAAAAATTTATTAAGTATACTCAAATTCTCAGCCAATCAATTAGTAGATGTAAAT
CAACAAATTGATAATATAACCAAGCAATTGAATTCTGCTAATTTTTGCAAAATCGATAAA
ATTAATTCTATTATCAACGAACAATTATCTATTTCAAATATCCATGTCAAACTGCTTAAT
CAAATTTTGAATAATGATCCCTGTTGTGGAAATAATGAGAATTTAAAGAAGGATATACAT
TCTCTCCAAGACAAATGGGAAATCTTAATTAAGAAATTTATTTGTATTCAGTTCAAACAA
GAAAGATTAGCCAATAATCATACTTTAGATTCTATTATTGAAAGCATAGATAGAATTGAA
TGTAAAATCTCAAATCTTGACTTGGGGAATGATTTAAATAGTCTTAAATCTACTATTGAT
ATTTTACAAGAATATAAAATGGAGTTGTTAAATATGAATGATAAAATAATGAAAATTCGT
TCCGATGGGCGATATGTATTGGAAGAAGACTTAAAAAATTTGAGCAACAGAATAGTTATC
ATGACTGATAATATTTTTGTCATATTGAAATCATTGAACAAAGACTTTGAAATCATGAAA
AATTGTATGGAAAAGCTGATTGAATGTCAAAATGATAGAGTAAATTATGAAAGTGAAATG
AAGAAATTTTCGCAAAGTAACAGAACGCTTGATTGCGATAAACTTTCAAGTAAAATATTT
TTGGGACTTGAGAACAATAAAGGAATACTGACTAATTGCATGACGTATATATCTGACTTG
TCAAATGAAAAATATTTTGACAATATGAAATTTAATTTCGATCAAGAACTATGTTTATTA
GATGAGCTCAGTGTGAAAGTGAGATTGGGTTTACCTGTTATCACTAATTTTGGAGCTGAA
ATTGATTCATCGACATTCACAGCTAATAATCCAAATCGCAAAGTTCTTTGTTCTTTTGGT
GGATTATTAAATCGCTTACCAGTACAAGCTGCTATAGTTGTTTTCCTCGCAATATGTGGA
TTGTGGTATAAACAATCTTCATATTCTTGTCCAGGTTCCTCTGAGTGGTTGAAATTAATT
CGAAAATATACTAATAGCAATATGGAACCACATTGAttcaggacaaacaataattttttt
tataaaaatttgctgaattttataggtcgtcgtctagtcttctttgcctgcttttattcc
atctaatcatggaataatcatatatacgttattgtatttgtatggtgtcgagtccattta
gcttttattcaatatatttattaattaattatcgttgcatattattaattataataaatt
cattttaacacatgttataacatgagattcaattatatttctattttaatttttgtttta
catttaccttatttttaattttattcgatagaatattttgattgatttcaaagtccatga
tgatatcagtaactcaattacaaaaactattgagaaagttattaaattagattcatttAT
GGAATATAGAGAAACTTTATCAAATTGTAAAACAACGTTGGATGAATTGATCTCTCAGAT
TCATGGACAATGGCTAAAAGCAATGGAATTAAAATGTAAATTGGAATTTATGCCTAAAAT
TGTAATTAACTTTAATTCCATCACTAAATCCGGAATTTCTTTGGTTGGACCAGAAAGTGG
ATTTGAATCAGAAATTAGTCCAATGAATCCTATTCGTGGTTTTCCAACAAATAATCTAAG

TTTAATGAAATATATTTCGTCATCAGGTGAATGTTTTGCAGACGATAGCACAACTGATTT
ACCAATGGATTCGGAGTCTACTGATTTACCATTGGATTTTGCATATAATAGAAGCATCAG
TTCGTCTTCCTCATTAGTTGAAATAATGTTGGATTTAACACATCAATCCCAAATTTCTAA
AAATGATTTTAATTCTCGAAAGAAGCAAAAGCGATTTAAAAAACGGCGACAGTTTGTTTC
AAAAACCACAGGAGTTCATTCTGAAAATTTAACTTTGGAAGAAAGGGAAAAGTATTTACA
AATTCGTAATGCTTTTAGTAGGCCTCCATATTCTTTAAAACGACGAATCAGTGAAACTAG
CATTATTCGAATTTTGGATCGAACTGATGTGACGACAAATAAGATATCAGATTTAGCTGC
AGCCGCTGAGAAAATCGTTATGCAAAAAGATTTCAATCGCCACAATGTCTCTTTAAGCGA
ATCACCGAAGGAATCGGAAATTGAAGATCCATATGGTGTTGTAGCAAGTTCAAATAATGA
TATAAATGTAAAAGATGTTTGGGATAATTATCAGGCTTCGTTGTATTCCAATGATGATGG
TGATTCTCAGATTCTAGAAGTCGATAAAATTAAAGATTGGGAGCAAGATATAGAAACAGA
TGATCAAGAGTATGAGTCTTGTTATCGTGATAAAAAACTACAGTTTCTCACATCAGGTTA
TGAAACTTCTATGGAGTCAGATATACATTTTTGCCACAAAAATTTATTAAGTATACTCAA
ATTCTCAGCCAATCAATTAGTAGATGTAAATCAACAAATTGATAATATAACCAAGCAATT
GAATTCTGCTAATTTTTGCAAAATCGATAAAATTAATTCTATTATCAACGAACAATTATC
TATTTCAAATATCCATGTCAAACTGCTTAATCAAATTTTGAATAATGATCCCTGTTGTGG
AAATAATGAGAATTTAAAGAAGGATATACATTCTCTCCAAGACAAATGGGAAATCTTAAT
TAAGAAATTTATTTGTATTCAGTTCAAACAAGAAAGATTAGCCAATAATCATACTTTAGA
TTCTATTATTGAAAGCATAGATAGAATTGAATGTAAAATCTCAAATCTTGACTTGGGGAA
TGATTTAAATAGTCTTAAATCTACTATTGATATTTTACAAGAATATAAAATGGAGTTGTT
AAATATGAATGATAAAATAATGAAAATTCGTTCCGATGGGCGATATGTATTGGAAGAAGA
CTTAAAAAAATTTGAGCAACAGAATAGTTATCATGACTGATAATATTTTTGTCATATTGAA
ATCATTGAACAAAGACTTTGAAATCATGAAAAAATTGTATGGAAAAGCTGATTGAATGTCA
AAATGATAGAGTAAATTATGAAAGTGAAATGAAGAAATTTTCGCAAAGTAACAGAACGCT
TGATTGCGATAAACTTTCAAGTAAAATATTTTTGGGACTTGAGAACAATAAAGGAATACT
GACTAATTGCATGACGTATATATCTGACTTGTCAAATGAAAAATATTTTGACAATATGAA
ATTTAATTTCGATCAAGAACTATGTTTATTAGATGAGCTCAGTGTGAAAGTGAGATTGGG
TTTACCTGTTATCACTAATTTTGGAGCTGAAATTGATTCATCGACATTCACAGCTAATAA
TCCAAATCGCAAAGTTCTTTGTTCTTTTGGTGGATTATTAAATCGCTTACCAGTACAAGC
TGCTATAGTTGTTTTCCTCGCAATATGTGGATTGTGGTATAAACAATCTTCATATTCTTG
TCCAGGTTCCTCTGAGTGGTTGAAATTAATTCGAAAATATACTAATAGCAATATGGAACC
ACATTGAttcaggacaaacaataatttttttataaaaatttgctgaattttataggtcg
tcgtctagtcttctttgcctgcttttattccatctaatcatggaataatcatatatacgt
tattgtatttgtatggtgtcgagtccatttagcttttattcaatatatttattaattaat
tatcgttgcatattattaattataataaattcattttaacacatgttataacatgagatt
caattatatttctattttaatttttgttttacatttaccttatttttaattttattcgat
agaatattttgattgatttcaaagtccatgatgatatcagtaactcaattacaaaaacta
ttgagaaagttattaaattagattcatttATGGAATATAGAGAAACTTTATCAAATTGTA
AAACAACGTTGGATGAATTGATCTCTCAGATTCATGGACAATGGCTAAAAGCAATGGAAT
TAAAATGTAAATTGGAATTTATGCCTAAAATTGTAATTAACTTTAATTCCATCACTAAAT
CCGGAATTTCTTTGGTTGGACCAGAAAGTGGATTTGAATCAGAAATTAGTCCAATGAATC
CTATTCGTGGTTTTCCAACAAATAATCTAAGTTTAATGAAATATATTTCGTCATCAGGTG
AATGTTTTGCAGACGATAGCACAACTGATTTACCAATGGATTCGGAGTCTACTGATTTAC
CATTGGATTTTGCATATAATAGAAGCATCAGTTCGTCTTCCTCATTAGTTGAAATAATGT
TGGATTTAACACATCAATCCCAAATTTCTAAAAATGATTTTAATTCTCGAAAGAAGCAAA
AGCGATTTAAAAAACGGCGACAGTTTGTTTCAAAAACCACAGGAGTTCATTCTGAAAATT
TAACTTTGGAAGAAAGGGAAAAGTATTTACAAATTCGTAATGCTTTTAGTAGGCCTCCAT
ATTCTTTAAAACGACGAATCAGTGAAACTAGCATTATTCGAATTTTGGATCGAACTGATG
TGACGACAAATAAGATATCAGATTTAGCTGCAGCCGCTGAGAAAATCGTTATGCAAAAAG
ATTTCAATCGCCACAATGTCTCTTTAAGCGAATCACCGAAGGAATCGGAAATTGAAGATC
CATATGGTGTTGTAGCAAGTTCAAATAATGATATAAATGTAAAAGATGTTTGGGATAATT
ATCAGGCTTCGTTGTATTCCAATGATGATGGTGATTCTCAGATTCTAGAAGTCGATAAAA
TTAAAGATTGGGAGCAAGATATAGAAACAGATGATCAAGAGTATGAGTCTTGTTATCGTG
ATAAAAAACTACAGTTTCTCACATCAGGTTATGAAACTTCTATGGAGTCAGATATACATT
TTTGCCACAAAAATTTATTAAGTATACTCAAATTCTCAGCCAATCAATTAGTAGATGTAA
ATCAACAAATTGATAATATAACCAAGCAATTGAATTCTGCTAATTTTTGCAAAATCGATA
AAATTAATTCTATTATCAACGAACAATTATCTATTTCAAATATCCATGTCAAACTGCTTA

ATCAAATTTTGAATAATGATCCCTGTTGTGGAAATAATGAGAATTTAAAGAAGGATATAC
ATTCTCTCCAAGACAAATGGGAAATCTTAATTAAGAAATTTATTTGTATTCAGTTCAAAC
AAGAAAGATTAGCCAATAATCATACTTTAGATTCTATTATTGAAAGCATAGATAGAATTG
AATGTAAAATCTCAAATCTTGACTTGGGGAATGATTTAAATAGTCTTAAATCTACTATTG
ATATTTTACAAGAATATAAAATGGAGTTGTTAAATATGAATGATAAAATAATGAAAATTC
GTTCCGATGGGCGATATGTATTGGAAGAAGACTTAAAAAATTTGAGCAACAGAATAGTTA
TCATGACTGATAATATTTTTGTCATATTGAAATCATTGAACAAAGACTTTGAAATCATGA
AAAATTGTATGGAAAAGCTGATTGAATGTCAAAATGATAGAGTAAATTATGAAAGTGAAA
TGAAGAAATTTTCGCAAAGTAACAGAACGCTTGATTGCGATAAACTTTCAAGTAAAATAT
TTTTGGGACTTGAGAACAATAAAGGAATACTGACTAATTGCATGACGTATATATCTGACT
TGTCAAATGAAAAATATTTTGACAATATGAAATTTAATTTCGATCAAGAACTATGTTTAT
TAGATGAGCTCAGTGTGAAAGTGAGATTGGGTTTACCTGTTATCACTAATTTTGGAGCTG
AAATTGATTCATCGACATTCACAGCTAATAATCCAAATCGCAAAGTTCTTTGTTCTTTTG
GTGGATTATTAAATCGCTTACCAGTACAAGCTGCTATAGTTGTTTTCCTCGCAATATGTG
GATTGTGGTATAAACAATCTTCATATTCTTGTCCAGGTTCCTCTGAGTGGTTGAAATTAA
TTCGAAAATATACTAATAGCAATATGGAACCACATTGAttcaggacaaacaataattttt
tttataaaaatttgctgaattttataggtcgtcgtctagtcttctttgcctgcttttatt
ccatctaatcatggaataatcatatgttaatcatttgtttattggtcgctctgataattt
gtatgtttttaatttcagatacgttattgtatttgtatggtgtcgagtccatttagctt
ttattcaatatatttattaattaattattgttgctttgttcaattgttttatttcgttgc
atattattaattataataaattcattttaacacatgttataacatgagattcaattatat
ttctattttaatttttgttttacatttaccttatttttaattttattcgatagaatattt
tgattgatttcaaagtccatgatgatatcagtaactcaattacaaaaactattgagaaag
ttattaaattagattcatttATGGAATATAGAGAAACTTTATCAAATTGTAAAACAACGT
TGGATGAATTGATCTCTCAGATTCATGGACAATGGCTAAAAGCAATGGAATTAAAATGTA
AATTGGAATTTATGCCTAAAATTGTAATTAACTTTAATTCCATCACTAAATCCGGAATTT
CTTTGGTTGGACCAGAAAGTGGATTTGAATCAGAAATTAGTCCAATGAATCCTATTCGTG
GTTTTCCAACAAATAATCTAAGTTTAATGAAATATATTTCGTCATCAGGTGAATGTTTTG
CAGACGATAGCACAACTGATTTACCAATGGATTCGGAGTCTACTGATTTACCATTGGATT
TTGCATATAATAGAAGCATCAGTTCGTCTTCCTCATTAGTTGAAATAATGTTGGATTTAA
CACATCAATCCCAAATTTCTAAAAATGATTTTAATTCTCGAAAGAAGCAAAAGCGATTTA
AAAAACGGCGACAGTTTGTTTCAAAAACCACAGGAGTTCATTCTGAAAATTTAACTTTGG
AAGAAAGGGAAAAGTATTTACAAATTCGTAATGCTTTTAGTAGGCCTCCATATTCTTTAA
AACGACGAATCAGTGAAACTAGCATTATTCGAATTTTGGATCGAACTGATGTGACGACAA
ATAAGATATCAGATTTAGCTGCAGCCGCTGAGAAAATCGTTATGCAAAAAGATTTCAATC
GCCACAATGTCTCTTTAAGCGAATCACCGAAGGAATCGGAAATTGAAGATCCATATGGTG
TTGTAGCAAGTTCAAATAATGATATAAATGTAAAAGATGTTTGGGATAATTATCAGGCTT
CGTTGTATTCCAATGATGATGGTGATTCTCAGATTCTAGAAGTCGATAAAATTAAAGATT
GGGAGCAAGATATAGAAACAGATGATCAAGAGTATGAGTCTTGTTATCGTGATAAAAAAC
TACAGTTTCTCACATCAGGTTATGAAACTTCTATGGAGTCAGATATACATTTTTGCCACA
AAAATTTATTAAGTATACTCAAATTCTCAGCCAATCAATTAGTAGATGTAAATCAACAAA
TTGATAATATAACCAAGCAATTGAATTCTGCTAATTTTTGCAAAATCGATAAAATTAATT
CTATTATCAACGAACAATTATCTATTTCAAATATCCATGTCAAACTGCTTAATCAAATTT
TGAATAATGATCCCTGTTGTGGAAATAATGAGAATTTAAAGAAGGATATACATTCTCTCC
AAGACAAATGGGAAATCTTAATTAAGAAATTTATTTGTATTCAGTTCAAACAAGAAAGAT
TAGCCAATAATCATACTTTAGATTCTATTATTGAAAGCATAGATAGAATTGAATGTAAAA
TCTCAAATCTTGACTTGGGGAATGATTTAAATAGTCTTAAATCTACTATTGATATTTTAC
AAGAATATAAAATGGAGTTGTTAAATATGAATGATAAAATAATGAAAATTCGTTCCGATG
GGCGATATGTATTGGAAGAAGACTTAAAAAATTTGAGCAACAGAATAGTTATCATGACTG
ATAATATTTTTGTCATATTGAAATCATTGAACAAAGACTTTGAAATCATGAAAAATTGTA
TGGAAAAGCTGATTGAATGTCAAAATGATAGAGTAAATTATGAAAGTGAAATGAAGAAAT
TTTCGCAAAGTAACAGAACGCTTGATTGCGATAAACTTTCAAGTAAAATATTTTTGGGAC
TTGAGAACAATAAAGGAATACTGACTAATTGCATGACGTATATATCTGACTTGTCAAATG
AAAAATATTTTGACAATATGAAATTTAATTTCGATCAAGAACTATGTTTATTAGATGAGC
TCAGTGTGAAAGTGAGATTGGGTTTACCTGTTATCACTAATTTTGGAGCTGAAATTGATT
CATCGACATTCACAGCTAATAATCCAAATCGCAAAGTTCTTTGTTCTTTTGGTGGATTAT
TAAATCGCTTACCAGTACAAGCTGCTATAGTTGTTTTCCTCGCAATATGTGGATTGTGGT

ATAAACAATCTTCATATTCTTGTCCAGGTTCCTCTGAGTGGTTGAAATTAATTCGAAAAT
ATACTAATAGCAATATGGAACCACATTGAttcaggacaaacaataatttttttataaaa
atttgctgaattttataggtcgtcgtctagtcttctttgcctgcttttattccatctaat
catggaataatcatatgttaatcatttgtttattggtcgctctgataatttgtatgtttt
ttaatttcagatacgttattgtatttgtatggtgtcgagtccatttagcttttattcaat
atatttattaattaattattgttgctttgttcaattgttttatttcgttgcatattatta
attataataaattcattttaacacatgttataacatgagattcaattatatttctatttt
aatttttgttttacatttaccttatttttaattttattcgatagaatattttgattgatt
tcaaagtccatgatgatatcagtaactcaattacaaaaactattgagaaagttattaaat
tagattcatttATGGAATATAGAGAAACTTTATCAAATTGTAAAACAACGTTGGATGAAT
TGATCTCTCAGATTCATGGACAATGGCTAAAAGCAATGGAATTAAAATGTAAATTGGAAT
TTATGCCTAAAATTGTAATTAACTTTAATTCCATCACTAAATCCGGAATTTCTTTGGTTG
GACCAGAAAGTGGATTTGAATCAGAAATTAGTCCAATGAATCCTATTCGTGGTTTTCCAA
CAAATAATCTAAGTTTAATGAAATATATTTCGTCATCAGGTGAATGTTTTGCAGACGATA
GCACAACTGATTTACCAATGGATTCGGAGTCTACTGATTTACCATTGGATTTTGCATATA
ATAGAAGCATCAGTTCGTCTTCCTCATTAGTTGAAATAATGTTGGATTTAACACATCAAT
CCCAAATTTCTAAAAATGATTTTAATTCTCGAAAGAAGCAAAAGCGATTTAAAAAACGGC
GACAGTTTGTTTCAAAAACCACAGGAGTTCATTCTGAAAATTTAACTTTGGAAGAAAGGG
AAAAGTATTTACAAATTCGTAATGCTTTTAGTAGGCCTCCATATTCTTTAAAACGACGAA
TCAGTGAAACTAGCATTATTCGAATTTTGGATCGAACTGATGTGACGACAAATAAGATAT
CAGATTTAGCTGCAGCCGCTGAGAAAATCGTTATGCAAAAAGATTTCAATCGCCACAATG
TCTCTTTAAGCGAATCACCGAAGGAATCGGAAATTGAAGATCCATATGGTGTTGTAGCAA
GTTCAAATAATGATATAAATGTAAAAGATGTTTGGGATAATTATCAGGCTTCGTTGTATT
CCAATGATGATGGTGATTCTCAGATTCTAGAAGTCGATAAAATTAAAGATTGGGAGCAAG
ATATAGAAACAGATGATCAAGAGTATGAGTCTTGTTATCGTGATAAAAAACTACAGTTTC
TCACATCAGGTTATGAAACTTCTATGGAGTCAGATATACATTTTTGCCACAAAAATTTAT
TAAGTATACTCAAATTCTCAGCCAATCAATTAGTAGATGTAAATCAACAAATTGATAATA
TAACCAAGCAATTGAATTCTGCTAATTTTTGCAAAATCGATAAAATTAATTCTATTATCA
ACGAACAATTATCTATTTCAAATATCCATGTCAAACTGCTTAATCAAATTTTGAATAATG
GTAAGTTCTTTTGGATTATGATGATAAATTGAcattcattttcttcataatcaagttatc
aagatcttgagtaatgtagtattaaatttataaatgatttgcaaattttgttttagaaac
ttctgtatttagtcctttttgaaagtctctgaatcggacaatacatttctcaattgata
atatttgtataaatattagattgttcggaaactaatttcggttttttagtaaaattcaa
taataaatttttattgtaaaaattttgtgtcaaatatt
>DjScaffold2353:70395-88189 strand:+
ttatttgtcaacttctttacctctttttgataagtttaaaactttaattaataaaaatat
attgttgtaagaaattttagccacagatatttagaacttatttgaATGTCCAGTGAATTT
ATATCATCTATGCGTAAACAAAATGCGTATGGTTCTGCAGAATTTAATATGCCAAGCCGA
GCAATGACTAAAGAAGAAATTGAAAAGGGGAAAACTAGTGACTGGTTTCGTTTACAATTA
ACCGGTTATTTGAAAACTCTTCCAATTAAAATTTGTTCACATACATTTATCACTACATTA
GTTATAAAAAACAATTTATTTGATACTCTTCCACAAGAGATTTGCAAGCTTGAAAACTTG
AAAAATTTAGACGCCAGCTATAATAATATCATAGTTTTGCCTCCACAAATGGGCTTTATG
TTTGAATTGAGAAATATTTTGTTATCAAATAACAAAATTAAAGAAGTTCCATTAACATTT
GGACTTTTACATAATGTTAAAACGTTAGATTTACGTCAAAATCCATTGATCAATCTTACC
GCACAAACATATGAAATGGAAACGACCGGTAAATACCTTCTTCGACATTTGTTAGAACAA
TATTATCATAAAAATAAAGTTGTTGCTCCACCAAATCGTTCTTGGCAGCTTATTGAAAAG
CCTTCTGATGGAATGTCCTTTACGTTAATGTGCTACAATGTTCTCTGTCCAAAATATGCT
ACGACTCACATGTATCCATATTGTCCGGCATTCGCTTTAGATTGGGAATATCGAAAACGT
GGAATTCTCGAAGAAATTTGGAAATATAAAGCTGATATCATTTGTCTTCAAGAATTGGAA
ACATCTCAATACGAAAATGTATTTTCAGTGGAATTGCGAAAAAGCAACTATGATGGATTG
TTTGTTCCGAAAAGTAGGTCACGAACAATGCACGAATCCGATTCTAAGTGTGTTGATGGA
TGTGCAATATTCTGGAAAGTGGAATGTTTCGAGAAAGTTGACCATGTGACACATGAATTT
ATGTTGTCCTGTATCAGTGTTAGCAAAAACCCAGCCCCGGAATTGCTAAATCGAGTCATG
AATCGGGACAATGTCGCATTGAGTATTATTTTAGAAGCTAAAGATCCAAAAATGAATAAA
CGAAAGTTGTGTGTATCAACCGGCCATATTCATTGGGATCCAGAATATAGCGATGTCAAA
TTGATCCAAACACTGTTTTGGTCATCAGAGTTATGGAATTACATTCGGAATTATTACAAA
TCGGGAAATATGTCCCATCTTAATCCTAGTGATATGCCGGTTATTCTTTGTGGAGATTTC

AATTCATTACCACAATCGGGGGTTGTAGATTACTTAATCAAAGGAGAAGTCGAACGAAGT
CATGTGGATTTTAGAGATTTCGGTGATAAGTATCTGTTTAATGAGTGGAGTGCGATCCAA
GAAGAATTTGTGGAAAATGATGTGTTAAAGCACCGATTTAATTTCGATCGAGCCTACAAG
GATGAACATGAGAGTGGTTTAAAATGGACAAACTATACATACGACTTTCGAGGAATTATA
GATTACATATTTTTCTCAAAGGAGCATTTAAATTTGTTGGGTAATTTAAAACAAATTCCC
GAAAGTTGGTTCGAACAGCAGAAAATAGTTGGTGCCCCTCATGTTCATTTCAACAGTGAT
CATTTTTCATTACTTGTTGAGTTGGAATTGAAAAATCAAAGCTCACCTGGAGCAATTACA
CAAAACTTCAGCAGTATTTCACAGCAGCCCAATTCAGCAATCAATAGCAATTCAACCAAT
TCTTCTTCTAATATTAATACGAATAATAATCTACGACAGAATTGCAATCCACTCAACAAT
TCTCCGAATAATCTTTCAGCTGCCATCACTACTACTGCATATCCTTCTTATCGAGGCCAA
TCAACATGGTCGTCTTAGttttactatcaaaaaaataaaactctatcaattgttttgcgt
tatttctacttctactacctttacacacacacacatacacacacgattcgttttatattt
attgattgtgttttctctgtgtaaatgtgtaaatattgatttcttgcttgcttgtttcct
acttactcatcctaataatatttgaacacatttatctttgtataaatcaataataatcaa
tcaataatgcctaattatttatttattaataataataatatttcagcgtatgtgatgatt
ggttaattttatttataaattattgattaatcgatgattagttgcatgtgtgtatatgtg
tataaatgtgtgtatagttttctaatattattttattaattatatttttatatattatgt
ttatcaggactctaatatgtgtttcgcgcttcggcttatttgactgaaaattgtattctt
tgccgcatttattttttagtatttgtgtttgtaattaattttcttgataataatgctt
atgatgatgataatttcgctttaaaactcgactcgatattgatatattttatatatatat
atatatatattcagcagcaagttggcgtattttctgtacaaaatatcgattttaaagtga
aagttgtttgacgaaatacaaaagcgatgttaatatattttgctattttacaatcgtttc
gaatttcatttgactgaattattgaattggaaattaattggagttcaacacaaaacacaa
caaaagtagcaaggaagtaacatttttttctaaaacaATGAGGAATAATCGGAAATTTA
AAAACGCAACTTCTCATTACTCGACTGCAAATGAATTGGATATTTTTACGATTAAAAATT
CTAAAAATCGGAAAATTAAGCGCGTAAATTTTTCGCAACAATCATCAAAGTCTTATAAGA
AAAATTCGAAAACCAAATTGAAGTTCGACTCGAATACAGATAATACAGATTTAAGTAAAG
AATTTTCGAAAAATTGTAAATTAGAAGAGGAGCAATCTATGTCAGAAATTGTAAATCATT
TATCAAAAGTTCTTGATAAAGAATCGATTTCCAATGATGTACAGAATTCTAATTCCGGTG
TACATACTTTATTTCATTATACCCCCAATCAGCGTGTGGAGAAAATCAATTTCGAGAAAG
ATAAATGTCCTGTCTGTAATTATTTGAAACGACAAAGAATCCAATCTACGGATGCGGTAT
TAGAATCACGTTTTGGACAAAACAATGAAGTTCCAATTAAATCAGACCGATTTCGTGTCA
AATCAGCTAGTGAGGGATCTGAACCTAAAATTGCCGAAAGTGTTTCTTCATATTCATCAA
AAAAATTATCATTTACATTCGAATTTTCCTACCCCAATGATAACGACAGCTCTGATTCTG
CTTTCTGTAATGGAATCAGTGTTGATTTAACTAACTGTGAAAATGTTTCCTTTAGAATTG
AAAAGCTATCAGAAATTTCCTCCAGGTATTATTCCAATTCATGCTCTTCAATCGATTTCA
AAGATACCCAATCTGATTTAATAACTCAAAAACAAGATTTCATTAACAACAACTATAGCA
GCAGTAATTTCTCACTCAATAATTTCTCAAGCCTTTCTCTGAGTCCCTACGTATCAGTTT
TAATACCAAATAGCCTTAAAAAGTGTTTCTTTTGTCAGTCAAATTTGACATTTTGTTATG
AATGCCGCAAAACACCACCTTCTTCGTTTTCAAATTTCAATTACAGTTGTAGCTCTTGTA
AAGTTTGTAATTATAATGAGTATTCGGATCTTATTTCCAAATCTGCTTGCGTTATCGCTG
AGCCAATCCAATCTTCTCGGATAAATGATATATTTGATTCTTTGAAGACTTGTTTGATAC
CGCAAAGATTTCTCAATTATTATAAAGTTGTTGCTCCACCAAATCGTTCTTGGCAGCTTA
TTGAAAAGCCTTCTGATGGAATGTCCTTTACGTTAATGTGCTACAATGTTCTCTGTCCAA
AATATGCTACGACTCACATGTATCCATATTGTCCGGCATTCGCTTTAGATTGGGAATATC
GAAAACGTGGAATTCTCGAAGAAATTTGGAAATATAAAGCTGATATCATTTGTCTTCAAG
AATTGGAAACATCTCAATACGAAAATGTATTTTCAGTGGAATTGCGAAAAAGCAACTATG
ATGGATTGTTTGTTCCGAAAAGTAGGTCACGAACAATGCACGAATCCGATTCTAAGTGTG
TTGATGGATGTGCAATATTCTGGAAAGTGGAATGTTTCGAGAAAGTTGACCATGTGACAC
ATGAATTTATGTTGTCCTGTATCAGTGTTAGCAAAAACCCAGCCCCGGAATTGCTAAATC
GAGTCATGAATCGGGACAATGTCGCATTGAGTATTATTTTAGAAGCTAAAGATCCAAAAA
TGAATAAACGAAAGTTGTGTGTATCAACCGGCCATATTCATTGGGATCCAGAATATAGCG
ATGTCAAATTGATCCAAACACTGTTTTGGTCATCAGAGTTATGGAATTACATTCGGAATT
ATTACAAATCGGGAAATATGTCCCATCTTAATCCTAGTGATATGCCGGTTATTCTTTGTG
GAGATTTCAATTCATTACCACAATCGGGGGTTGTAGATTACTTAATCAAAGGAGAAGTCG
AACGAAGTCATGTGGATTTTAGAGATTTCGGTGATAAGTATCTGTTTAATGAGTGGAGTG
CGATCCAAGAAGAATTTGTGGAAAATGATGTGTTAAAGCACCGATTTAATTTCGATCGAG

CCTACAAGGATGAACATGAGAGTGGTTTAAAATGGACAAACTATACATACGACTTTCGAG
GAATTATAGATTACATATTTTTCTCAAAGGAGCATTTAAATTTGTTGGGTAATTTAAAAC
AAATTCCCGAAAGTTGGTTCGAACAGCAGAAAATAGTTGGTGCCCCTCATGTTCATTTCA
ACAGTGATCATTTTTCATTACTTGTTGAGTTGGAATTGAAAAATCAAAGCTCACCTGGAG
CAATTACACAAAACTTCAGCAGTATTTCACAGCAGCCCAATTCAGCAATCAATAGCAATT
CAACCAATTCTTCTTCTAATATTAATACGAATAATAATCTACGACAGAATTGCAATCCAC
TCAACAATTCTCCGAATAATCTTTCAGCTGCCATCACTACTACTGCATATCCTTCTTATC
GAGGCCAATCAACATGGTCGTCTTAGttttactatcaaaaaaataaaactctatcaattg
ttttgcgttatttctacttctactacctttacacacacacacatacacacacgattcgtt
ttatatttattgattgtgttttctctgtgtaaatgtgtaaatattgatttcttgcttgct
tgtttcctacttactcatcctaataatatttgaacacatttatctttgtataaatcaata
ataatcaatcaataatgcctaattatttatttattaataataataatatttcagcgtatg
tgatgattggttaattttatttataaattattgattaatcgatgattagttgcatgtgtg
tatatgtgtataaatgtgtgtatagttttctaatattattttattaattatatttttata
tattatgtttatcaggactctaatatgtgtttcgcgcttcggcttatttgactgaaaatt
gtattctttgccgcatttattttttagtatttgtgtttgtaattaattttctttgataa
taatgcttatgatgatgataatttcgctttaaaactcgactcgatattgatatattttat
atatatatatatatatattcagcagcaagttggcgtattttctgtacaaaatatcgattt
taaagtgaaagttgtttgacgaaatacaaaagcgatgtta
>DjScaffold2353:102092-102663 strand:+
GAAACAATTATTGCAGCTGTAAGCTGTAGTGTGAGTGAGAAACCAAAATCGTCAATCAGT
CGTCGTACACAACAACTATCCATTCCAAGAACGATGTTATTCAGAATTTTGCACGAAGAT
CTTTGTTTTAAGGCATTCAAGATTCAAATAACTCAACAAATAAAGCCGGCAGTAAATAGG
CCATTGAAAATATGCATGAAAATGAAACATATTTTCAAAAAGCGATCTTTAGCTAGAAGG
ATTTATGAACACAATATTGTAGAATTTGGGGCAATGAAAACCCACATGAAATACTACAAA
AACAATTCCATTCACTAAaggtaaaagtttgaatgaagctggtaattctgtaacagttac
cggagcttgatatcgaaatatgattggaaaatttttgtttcataaattgaaaggtatgga
tacccagattatgtggtttcagcaggatggtgccacatctcatacaacaaaagaaacaat
tatacttttacgagaatgatttggtataagattaatttctcgaaatg
>DjScaffold4322:3692-13536 strand:+
attaaatcgttaccaaaattctctATGTATCAAATCATTCCAGAAGATAAGCCGTTTAAG
AAACCTGAAGGTTATGTACAGTTTGCAGTACAAGAAAGGCTGCAGAGATTCGTTCTTTGG
GCAAATCAGAATTTCTTATTGGAAAAGGAATTTGAATTGAAATCAAAAGATCAAGATGAA
TTGAACCTTAGATTCATATCAATTCGTGAAAATGCAGAATTCGAAATCAATATCAATAAT
AAACTTAATCGGGTGACCATTGCAACAGATAATATGGATTTTGCTGGAAATATCGTTCAA
AGTTTAAGTGAATACCTTAATCTGGATAATTTAAGTAGCGTTTGCAATTTTCCCAAAGAT
TTTTCCCACTTAAACGACATTATATCAAAAATTGACGGTATACAGGCGAGTCGTCAAAGA
ATGACGGGAGATATCGCTGATAACTCAAATATCATCAAGAATTTTATTGTTAGAGCTGAG
GATTGTCGAATAATGCGTTATATGCCTGGAATGAATAAATGCTATTCGGAACTGCTTCAT
TTGAATAGAGATTTGATCAATCGATATCGTATCCGTTGCAATAATCATCAAGAACTCGTC
AATCATTTGAAATTTATAAATATCTTTATTCAGCAAGGAGGCAATTTACGAGTTGGGAAA
TATCGAACTCAGCTTGTAGCCAACTGTCGAGAAGCTATTAAAAATAGTCATCTTAATAAA
TTAGAAAAGCTGATTAAAACTGGAACTGATTAAcaaacacattacaaatagataattcaa
tgcaatttatatgattttatttatttgatcttggacagtgtatctattttggtgtaaata
tcgaattattattattattattatta
>DjScaffold572:38468-39375 strand:+
gcgatttgtgaatttgtgtattgagaaATGTCGTCTGATATTAAAATTGCTGTTGTTTGC
TCTAGTAATCAGAACAGAAGTATGGAAGCTCATCATTTTCTTAACAAAAAAGGTTACCTT
GTTAAATCATTTGGTATTGGATCTCAAGTCAAACTACCGGGACCTAGTCAAAGTGAACCA
AATATTTTCTCATTTGAAACAACATATGATCACATGTTTGATGAATTATCTAAACGAGAT
AAAGCATTATACACCCAAAATGGGGTTTTAAATATGCTTGAGCGTAATCGTCGGATTAAA
TCGAAACCTGAAAGATTTCAGGATGCAACCGAGCTTTTTGACATAATTGTAACTGTTGAG
GAGAGAGTTTATGACGTTGTTTTGGAGTGCTTGGAAAACAGGGATAACAAGTTAGGACAT
CGCCCGGTTCATATAATTAATATTGACATTCAAGATAATCATGAAGAAGCAACATTAGGA
GCATTTTTGATATGTGATTTAGTTTCCATGATTGTTCAAAGTGACGATATAGACAATGAT
ATAGACGAAGTTATACAGCATTTCTCAGAACATTCACATAGACATCTATTGCATACTGTG
TCATTTTACTGAatataataattattttaataacatttaactgttcgatcaaattaaata

agttactggttaatttaattaaatttagaaactgatttgtttaaagcgatttgtgaattt
gtgtattgagaaatgtcgtctgatattaaaattgctgttgtttgctctagtaatcagaac
agaagtatggaagctcatcattttcttaacaaaaaaggttaccttgttaaatcatttggt
attggatctcaagtcaaactaccgggacctagtcaaagtgaaccaaatattttctcattt
gaaacaacatatgatcacatgtttgatgaattatctaaacgagataaagcattgtacatt
ttaatatttattttcaaactggtattattagtattttcgtattagatacacccaaaatgg
ggttttaaatATGCTTGAGCGTAATCGTCGGATTAAATCGAAACCTGAAAGATTTCAGGA
TGCAACCGAGCTTTTTGACATAATTGTAACTGTTGAGGAGAGAGTTTATGACGTTGTTTT
GGAGTGCTTGGAAAACAGGGATAACAAGTTAGGACATCGCCCGGTTCATATAATTAATAT
TGACATTCAAGATAATCATGAAGAAGCAACATTAGGAGCATTTTTGATATGTGATTTAGT
TTCCATGATTGTTCAAAGTGACGATATAGACAATGATATAGACGAAGTTATACAGCATTT
CTCAGAACATTCACATAGACATCTATTGCATACTGTGTCATTTTACTGAatataataatt
attttaataacatttaactgttcgatcaaattaaataagttactggttaatttaattaaa
tttagaaactgatttgtttaaatgaatttgtgtattgagaaatgtcgtctgatattaaaa
ttgctgttgtttgctctagtaatcagaacagaagtatggaagctcatcattttcttaagt
attttttgtgatatatatttcgatcataatatatgtaaaacgaaatattttcagcaaaaa
aggttaccttgttaaatcatttggtattggatctcaagtcaaactaccgggacctagtca
aagtgaaccaaatattttctcatttgaaacaacatatgatcacatgtttgatgaattatc
taaacgagataaagcattgtacattttaatatttattttcaaactggtattattagtatt
ttcgtattagatacacccaaaatggggttttaaatATGCTTGAGCGTAATCGTCGGATTA
AATCGAAACCTGAAAGATTTCAGGATGCAACCGAGCTTTTTGACATAATTGTAACTGTTG
AGGAGAGAGTTTATGACGTTGTTTTGGAGTGCTTGGAAAACAGGGATAACAAGTTAGGAC
ATCGCCCGGTTCATATAATTAATATTGACATTCAAGATAATCATGAAGAAGCAACATTAG
GAGCATTTTTGATATGTGATTTAGTTTCCATGATTGTTCAAAGTGACGATATAGACAATG
ATATAGACGAAGTTATACAGCATTTCTCAGAACATTCACATAGACATCTATTGCATACTG
TGTCATTTTACTGAatataataattattttaataacatttaactgttcgatcaaattaaa
taagttactggttaatttaattaaatttagaaactgatttgtttaaa
>DjScaffold572:9642-11066 strand:+
ATGGCAGATATACAAACATCGCCAACTGAAAGATCCGTTCATGCCACTGAAAAGAAAATT
TTAGCGACAAAAATTAACGGCAAAGTGAAATGGTTCAATGTTAAAAGTGGTTATGGATTC
ATCAACAGAAATGATAATCAAGAAGACATTTTTGTTCATCAAACTGCCATTTTAAAAAAT
AATCCTCGAAAATGGCAAAGATCTGTTGGAGATGGAGAAGAAGTTGAATTTGATGTTGTA
GAAGGAGAAAAAGGGCTAGAAGCTGCAAATGTTACTGGTCCAGATGGAATTCCTGTACAA
GGAAGTAAATATGCCGCTGATAAGAGACATTATCGTTCAAGATCTTTTGGCCGTGCTGTG
GGTAGAGGATTTGGTGGTCGAGTTAGATCACTTAGAAATGGACCCAGAGGAAGATCATTA
TCAAGAGAAATGGATTCAACTAATGAGGACGCAGGACCAGAAGGAGATGCTGTATTTGGA
ACAGAAAGAAGACGAACTTTACCTAGTCGTAGAAGATCGTATTATCCTCGACCAATGCCT
CCTCCACCTCAAGGTGGTTATTATATGAATCGAAGACCCCTTCCTTCAAGAGATGATTAT
CGCGAACCTGACGAATATGAAAATCGTTCTATGATTATGGGTTATCGTGGTGGTGGAGTT
CCAAGAAGACGATTTATTGGTGGATTTGGCAGAGGTGGACATGCTAATGGTGGCTCAGGC
GCTAATAATTATATTAGAGGTTATTCTCCAGGACCCCCAAATACTCGTAGATACATGGAT
AGTAGACCTGGTCCAGTTATGAATAACTTTTCAGATTCAGGACCAATAATATCAAGAGGA
ACTAGTAGATTTAGACGAGGTCGAGGAGGACGTCCAATTATGTACCACAAACTTAACAGT
GGAGTAAATAGTAGATCAACCAGAGGCCGACAATTTGGAGGCGGTGAACGTAGAAATTGG
CGAGATTATAATTCTTATAATAACAGAAACAATGAAGACAATAACAATGAGGTCAGTATT
TATAAATTTAGAGCATCAGGTCGAGCCCCAAGTTTGGATGCCGAAAGCAACCAGAGTGAT
GACGATGCTATCCAGAAGACGTCTGAGTTAAATTTAGAAGAAATAAGAGCGTCTTAA
>DjScaffold572:47952-48383 strand:+
ATGACTTTTGGAATTGCAGCAGCACCAGGTACATTCCAGGAAATGATGAATAAAGTAATC
AGAAAAATAAAAGGAGCGATGGCTTATCTAGATAATATTCTTATATTCACCGAAACAAAG
GAGCAACATAATGAAATAATCAATAGAGTATTAACAGAAATTAAGAAGGCTGGTCTTAGA
GTTAATCCGGAGAAATGTCAATTTATGCGAAAGGAGATCAAATTTTTTGGACACGTTATC
AGTAAAGAAGGTGTCAAAACAGATCCAACTAAAATAGAAACAATTAAGAACTTTGGTAAA
CCAAAATGCGTTAAGAAAATGAGAAGCTTTTTAGGAATTTGCAATTACTATCGAAGGTTT
ATCAAAGATTATTCAAAGAAAGCTAGATTACTGGAACAGTTGTGTAGTGGGCCAAGTAAC
AAGAAAATTTAA
>DjScaffold4535:8460-16893 strand:+

TATGAAACTGGATTTATCATAAATCAAAAACATGAAATTGAACTTCCAAGTTGTTATGAA
CCTTGGATTTATTTGATTAAAAATTTAGGAGTTTTAGTGGAGACTTGTTCACTTGATGAA
TATATTAGCAAAATGCCAGTTCTTTCAATTGAATCTCTGAAATCATCCGAACAGTTTTTC
TATTGCCATTCAATCTTAGCATTTGCAACATCAGCTTTTGTATGGCATAAAAAATTACAA
GAGATTCCAAAAATTTTACCGAAATCTTTATCAATCCCGTTTCGTGCTTTATCGAATAAA
CTTGGAATGATGCCAATTTTATGTCATTCAAATATGGTTTTATGTAATACGATGATAGAT
ACCACCAAAGATTATTTGGAAGTTAAGATCAAAATCAATACCAACGATAACTGGAACATA
TTTCAGCGAGAGACTGCTAGAGTCGAATTTTGCTTTAGCCCAATAATACGAGCAATCTAT
GATTTAAATGCTTGTATTTTGGAAGAGAAATCAGAGGGTGTTATTACGAAATTGAGAGTA
ATAACAAAAACTTTACACTCCATGAAAGATGTTCTAAAAAATTTCAATGAAATGAATCCA
GATTATTTTCATAGACAAATGAGACCATTTCTAGTTGGATGGTCAGGAGATAGTTTTATT
CATGAAGGGGGTCTGTTATATGAAAACGAAAATGGTTCTACAACAGCAGTTGAATGTATC
GGAGCAAGTGCCGCCCAGAGCCCCACCTTTCAAGTACTGGATTCATTATTCAAAATTACT
CATTCACCTGGTATTTATTATTTTATGTTATTATTAAATAAACGTAGTGATATATTCAAA
AATAAATAAaatcaaagaactaatcactatattaaaagaaattaattttaaaacaaataa
taacaattttaattcaatatttattaaacacagatca
>DjScaffold4535:85734-95949 strand:+
aaacttagtattttaataatttatgaggattagcgattattcattccaaaacttatctta
attgtttaatatattaatttgtttaattaaaaattattaatgaatcgcttctccatttat
cacaatgattttattttgcatgtaaatataaaattcgttttatttaaaaaattgcattgt
atatgaaaaattttcacatttaagattttagagtgtaaatttgctgaagaaagaaagcag
cgcttgttggaatttgatagaacaagtgctcgtcgtacaaaagttattgatgacgaaagg
gattattatcaaactgaaggaaaagtgtggatcgacccgaaaaatagagaattattgaaa
cagaaagaacaaaaattgcgggacgagagacatgcttcaaagcattctcaaccaATGAAG
TATACATTAGATTTTAAAAGTCGTGAATTCATTGAGGAGAAACCGTCAAACGATGCTGCA
TATGAATTATTTGAAGAACCTGAACTTGAAATAATGAAGAACTATTCTACGGATGTCCAA
ACAGAATCCAATATTGATACAGGCTTTTTGGAATATATTGCTTCTAAAAAGAAGCAGCCT
ACTACACAAAAAGAAAATAATCAGACTACAAAAAATTACAAGTTGAAAAGAATTCAAGAT
AGAGAACTTCACGAAATTCGTGACGAAGGAATGTGTTTGAGTATGCATCAACCTTGGGCA
TCGCTGTTAGTTAAAGGAATTAAAAAACACGAAGGAAGATCTTGGTTTACGAATCATCGA
GGGCGATTGTGGATTGCTGCTACAGCTAAAGTGCCTTCGCCGGAATTAATAAATGAATGC
AAGCAATTTTATTTGTCCAGAGAAGTAGCGAGAAATGAATTTCCCACAAACTATCCTACC
GGTTGTATTTTAGGATGTGTTGATGTAATTGATTGTTTAACAGAAGAAGATTACACTGAG
ATGTATCCTGATGGTGAATCTGGTTCACCGTTCATTTTCATTTGTGAGAATCCTATTGAA
TTGAATTTAAAATTTCCCGTAAAAGGGAAACATAAAATTTATAAACTGGAAACACACGTA
CACAATGCTGCAAAATGTGGTATCCGTATAGAA
>DjScaffold819:9460-10057 strand:+
aATGAGCAGATATAAATTAATAGCTTTTGAACCAATTTCAGAAAATGAAATTGCCAGTTA
TAATTTTGAAGGAGTAGAAAACCAGGAAAAACCACTTAATCCAAATGAGGAATTTATACA
GTTAACAAGTAAAATAGATTTTAAAAAGCTAAATGGGATTTCTTTGGACTACACGTCTCC
TGATTTCCCATGTATGGATGAAGAGTCAGAACCTTTAGAAACTCTTCAGTTAAATTTAAC
AGAGAATTTTAAAAAGCACATTCAAACAACACTAAATGAAACGAATGCCTTAATTGATAT
TTTACAAGTATGTAAGGAAAATGTATATATGAATTTGAAAAAAGTTGTCAAAGAAAATCA
TGATGATAATGTTGCAAGTTATGTTGCTGCTAAAAAGATGGGTCTATCTGCAAGTTCAAT
AAATTTATTGAGTAGCACTGCAAGATTGATGAAAACTTTGTCATCTGTAAAAGATCAACA
TCAACAAAATTCGTTTTAT
>DjScaffold819:29105-30019 strand:+
TTTCTCCACGAAATCTTTAGTACTCTATGTTGGGAATCTCGATCACTAAATTGTCCGGTT
CTTGTCAAAGAAAATAGATTGATTATTAATATTAAAGGTTTAATGAACCTAGAAATGGAT
TTCATCATATTACCGGAGAAACATTGGTTTGGCTATAACGATAAACCTAGTAATGAGTTA
TTGGAAACACGACATCAAAATGAGCTTATTATTAATCAGCATAATCTAGTTTCAGATGCA
TTAATAATTTTCTTAAGAGATTTATTACGAGAAAAACATCGATCTAACTCCTGTGCTCGT
TTTTCTTTGGCGAATCCCATTGCGGGACCAGTACAAATTCCCAGTAATCAGCGATATAGA
GGAACCCAATTCTTCCGGGCAATTGATTTTACTCCAGCCGAGCAATCTCATTTTGTGTTT
CTGTTACCATCTCTCATAGATGCAATTGTATTTTATAAAGCTCGCGCAGATTTATTATTA
GTAATTGATAAAGTCTCAGAAATATCGAATTCCAGTATTTTTGTCAGGAATCACATTCAT
TTAACTCAATCAATGTTGAACTGTAGCATTTCGCTTGTAATCGGCTGTACAACTTACACT

AGTCTTCAACACTGTTTTTCATTGATTATGAGTGTTTCGAAAGAAACAATTAATCTTAAT
ATTATTTCTGATCAGATTAAACATAATCTCGTTTATGAATCAATTGATATCGACTTGTTA
ACCTTATTTTTTCAAACCCAGGTAATTATTGATTATTATTATTATTGGTTATTATTATTG
ATTATTATTATTATTGGTTATTATTATTGAtttttat
>DjScaffold4778:43021-47979 strand:+
tcagttgttattgtcattaattttaaccaaagagATGCTCTCAAAATTGCAATTATGCTT
TTGTCGTCACTTAAAACAGTCTTTTCCAATGAGAAATTATGCAAATAATTTCAGAGTGCG
TCAAAGCAGAAGTTCCAAGCAAGTTGCTCATGAAGCATTTAAGCCTACATCAACCGAAAA
AGCTGCAAATACTTTTAAAATTGTTGCTGCTGCTTCTTCAGCCACAGGAATCGGAATTAT
ATGCTACTATGCAATGCAAAAAACGAATGATCCATTTAATATTATAAATAAGTCTGCTAT
ATGGCCAGCTTATGTAAGAGAGCGAATTCATTCAACTTATACATATTTTGCTTTGTCTAA
TCTTGCTACGGCTGCTAGTGCTGTTGCCGTATTTCGAAATCCGACTTTATTGAAATATGC
AAGTCGAGCAAGCATGGCATCAATATTTTTAGGATTAGCTGGTGTTATCGGAACTGGAAT
AATGATGAGATCTATTCCCTATGATGAAACTAGTTTATTAAAACATGCAGCGTGGTTATT
ACATTGTGGGACTTTAGGTTTTGTTGTAGCTCCTGTATGCATGTTAGGCGGGCAGGTAAT
TCAAAAAGCTGCAGTGTACACACTTGGAATTGTTGGTGGACTTAGCGCGGTAGCAGTAAC
TGCACCTTCAGATAAATTTCTTTATATGGGTGTTCCATTGGCAATGGGATTCGGTCTTGT
CTTTGCTTCATCAATAGCTGGAATTTTTCTGAATCCTCTAAGTCAACTTGGAGCCGCAGC
TTTATCGTTTCAATTATATGGAGGACTAGCATTATTCGGTGGTTTTTTATTGTATGACAC
TCAACATGTTATAAAAAGAGCAGAAGAGCATCCTCCAGGTAGAACAATGCCTTCATACGA
CCCAATAAATAACTCAATGAATATATTCTTGGATACAGTGAATATATTTATTAGACTGGT
ATATATTTTCGGAATGGGAAATTCAAAAAAACGTTGAcattttaaattttcattgatata
aacaattgatttaaacagcttcaaaaattttatttcttgttaatatttactgtttagtta
gagttatccataaaaataatatctgattccattatttcagtaaataatagtagtagtcaa
ttaaaagtgtgtaaattatttattttcgattatagagATGCTCTCAAAATTGCAATTATG
CTTTTGTCGTCACTTAAAACAGTCTTTTCCAATGAGAAATTATGCAAATAATTTCAGAGT
GCGTCAAAGCAGAAGTTCCAAGCAAGTTGCTCATGAAGCATTTAAGCCTACATCAACCGA
AAAAGCTGCAAATACTTTTAAAATTGTTGCTGCTGCTTCTTCAGCCACAGGAATCGGAAT
TATATGCTACTATGCAATGCAAAAAACGAATGATCCATTTAATATTATAAATAAGTCTGC
TATATGGCCAGCTTATGTAAGAGAGCGAATTCATTCAACTTATACATATTTTGCTTTGTC
TAATCTTGCTACGGCTGCTAGTGCTGTTGCCGTATTTCGAAATCCGACTTTATTGAAATA
TGCAAGTCGAGCAAGCATGGCATCAATATTTTTAGGATTAGCTGGTGTTATCGGAACTGG
AATAATGATGAGATCTATTCCCTATGATGAAACTAGTTTATTAAAACATGCAGCGTGGTT
ATTACATTGTGGGACTTTAGGTTTTGTTGTAGCTCCTGTATGCATGTTAGGCGGGCAGGT
AATTCAAAAAGCTGCAGTGTACACACTTGGAATTGTTGGTGGACTTAGCGCGGTAGCAGT
AACTGCACCTTCAGATAAATTTCTTTATATGGGTGTTCCATTGGCAATGGGATTCGGTCT
TGTCTTTGCTTCATCAATAGCTGGAATTTTTCTGAATCCTCTAAGTCAACTTGGAGCCGC
AGCTTTATCGTTTCAATTATATGGAGGACTAGCATTATTCGGTGGTTTTTTATTGTATGA
CACTCAACATGTTATAAAAAGAGCAGAAGAGCATCCTCCAGGTAGAACAATGCCTTCATA
CGACCCAATAAATAACTCAATGAATATATTCTTGGATACAGTGAATATATTTATTAGACT
GGTATATATTTTCGGAATGGGAAATTCAAAAAAACGTTGAcattttaaattttcattgat
ataaacaattgatttaaacagcttcaaaaattttatttcttgttaatatttactgtttag
ttagagttatccataaaaataatatctgattccattatttcagtaaataatagtagtagt
caattaaaagtgtgtaaatta
>DjScaffold4778:91038-95304 strand:+
attgtaataattaagtaaatagtaattacaatatttatttttataaaaatttattttaaa
tctaaattttaaaatattatatttcaaaaaaaataattcaaatttaaaataatattggtg
ctgaacataaatgaaaatttttagaaatagctttcagtaaattgttatcaataatATGAG
TGAAATTTGTAATAATTCTGATTTATCTAATGAAAGTGAAATCGGCGAATTATTTAAACT
AACATTCCCAAATGGTTATAATTTTGAGGATTCTTATAAAAGACCAACCCGAGATTCTAG
CTCACAAACTGAACAAACTGATATAATAAAAATAATAGAATTAACAGAAGCTCTTTCAAA
AATTACAGTGAATTATGCTAATTTACATCGAGAACAAAATTTAATTAGATCAGCTTTACA
ATCAGAATTTAACAAACGACTGAACGACGCATGCAGTCAGTTATACTCCAGAATAAATCA
TAAATTAGAAGAAATTGAACGCTCTCAAAAAGATAGAGTTGAAGTTGTTCGTAGGGCTTT
TAAAACTCAATTATCTGACGCATTGAGTCATATGAGGTCGTTATACAACGAAAAATTACG
AAAAAAACTGACGAATGATCGAACATTTATGGAATCTGAAGAATCTATGAAATACATGAA
AAAAATCGCAGATTTGACAGCAAAATTTAAAAAATCAGAAGCCAAAATAAAAGAGTTAAC

AATGCAAATTAACGAATATCAAGAGTTTGATTCACAGCCAACATTAATGTTAGCAACTGA
AAGTGAAGTCAGTGAAAAGCCGATAATTGAAATAGAAGATGATAATTTAGAAGATGAAAT
TATGGAAGAAAATATTTCGCAAAAAAATATGATAAATGAATTAAATCAAAAAATTGAAGA
CCTGGAGAAACAGAATAACTCTTATGAAAAAGTTATAGAGAAATTGAATATGAATATTAA
AAGTATAAATGATGAACTTCTATCAACAATTAGAGTTTCTGAGCAGTTGAAGTCAGAAAA
AAATAATATGGAATTAACTTTGCAAAATTATGAAGAACAAAATAAGAAATATAAATCAGA
AGTTGAAACTTTGGAGGAACTACAAAAAATTCAAATACCACCACCAAAAGAAGAAATAAT
TGTTAAAAAACCTATAACTAAATCAGTAGATATTCAAAAAACATTTAAAGAATTTCCCGA
TTTGAGTAAAGAGGTGAAAAATCTAAACGATGTTATTCGAAAATTTCAAAGTAAGGAATC
AGAATCGAAAAATGAATTCGATGTTGTGAAAAACAATCTCATATCAGAAAACAGAAGATA
CATCGAAGAAATCAAGGTATTACAATCAATCAATATCAAATTAAAAGAAGAAATGTTCAA
TAAAATAGAATTACAAAACAATTTCAAGACTTTGCATAGTGCTTCAGTTGCTTATGCCAG
AAACAATCAAGCAATAGGGATTCTTCCAAATCAATTAACTGGAGATTTCAAACCAAATAA
TGCCAATATAAATAGCATTCCTAATTTCAAGAACAAATCCCAATTAGCAAATAATGACAA
GGAATTACTGAATAAATTTGTTGAATTTACAATATAAaacaaatttctgaaataaactga
atATGAGTTTACAGGACCTGGAGAAACAGAATAACTCTTATGAAAAAGTTATAGAGAAAT
TGAATATGAATATTAAAAGTATAAATGATGAACTTCTATCAACAATTAGAGTTTCTGAGC
AGTTGAAGTCAGAAAAAAATAATATGGAATTAACTTTGCAAAATTATGAAGAACAAAATA
AGAAATATAAATCAGAAGTTGAAACTTTGGAGGAACTACAAAAAATTCAAATACCACCAC
CAAAAGAAGAAATAATTGTTAAAAAACCTATAACTAAATCAGTAGATATTCAAAAAACAT
TTAAAGAATTTCCCGATTTGAGTAAAGAGGTGAAAAATCTAAACGATGTTATTCGAAAAT
TTCAAAGTAAGGAATCAGAATCGAAAAATGAATTCGATGTTGTGAAAAACAATCTCATAT
CAGAAAACAGAAGATACATCGAAGAAATCAAGGTATTACAATCAATCAATATCAAATTAA
AAGAAGAAATGTTCAATAAAATAGAATTACAAAACAATTTCAAGACTTTGCATAGTGCTT
CAGTTGCTTATGCCAGAAACAATCAAGCAATAGGGATTCTTCCAAATCAATTAACTGGAG
ATTTCAAACCAAATAATGCCAATATAAATAGCATTCCTAATTTCAAGAACAAATCCCAAT
TAGCAAATAATGACAAGGAATTACTGAATAAATTTGTTGAATTTACAATATAAaacaaat
ttctga
>DjScaffold4186:27965-58754 strand:+
ataataaatagagttcttattaaacatctatgcaaatatttaaatcttcagcgtccagat
acagttggattattgtacaaagattcgagaggttctttacagtggttagatgattccaag
acaattttcgatcatgatattgatttggataatgaatttgttcaactgatttttagaatt
aggttaattattttatgtgaaagaatatctattaattaaaattttcaaatgaaattttta
catcaattattattatttttattgcagtaattaattaatttacgaaatttatttgaaatt
acaaataatcagattatttatattattgaaatatagttctatttatttaataacctttac
atttacagtgctgtagaaattgaagatgccattattgaatcaaatttattaattaatgat
ccaatatccaaagacaatttattaaatataattattgatgcgtttgatgagtataaattg
aaaattgatactttagttggtttttattaaaatttcttcatttggccttaagctttcca
atcgtaaattccaagctatatgttgtttctgattccaaaagcaaaaattttgttttagga
ataagttctttagaattaaccttttttaatttggatcaatatcacagaccgttcATGCGG
CATAAATTTAGTCATATAGAAAAGGTTATGCGTCGCCGCATAAAGATTGTTCTTGATCTC
AAGTATAATAAAAAGCGATCAGTAACCCCAACAAATTGTTTTTCTGAAAATGAAATGGTA
ACGTTTTATGCTCTTTCTCCTTATCAAAGTGAATGTATTTATAATGAAATCAAAGCCTGT
GAAAAACAGTTTCACGAATCATCATCAATTTATAAAATAAGTGATCTGATGAAAAATTCA
CAATTCAGCACGACTTTTGATAAGTGGCTATTAAAACAATACAATTGTAAATTTGACGAA
AACTACAGTAATGACGGTATAAAAATGTTTGTTGAAATAGATGCTGACAAAGAAGATGTT
GAGGCAAAATTCGGTGAAATGGATTTGTTTGAAAGTGATATTTATCTAAGGCTATACCAA
AAGAAGGAAATCACTACAGATATTTATAAGAAACCCGATTATCAACAGTTGGTTTCTGAA
GAGCGTCGATTGAAAGAATTATTACATCAGAAATTAATACAATTACAGGAATCGTTTATG
GAAGAAGAAAAAATTACTGGATCTATGCCTGAAGAGGTGCTTCTTTATTTTCCAAATGGA
ATACAAAAATTTCAACGAAATACAAAACATTCAAATTCTAACTATTATCAAGGAAGAGGA
TGTGCAGCTAGTGTTCGTAATAAGTTGATGTTTATGGCAACAGAAAATTTGAAATCCAAT
GGGAAAACATTACCATCTAGCCGTTCTGTAACTGATCTCACATCTTTGAAAGACAATTCT
TTTTCAAAAACCAAGTCAAATTCAAGTTCATTTCGATCTTTCGAAGACAGCAAATCCAAT
GTTGTTCATAAATCTTCAAATAATTTATTTTTCAATGGATCGCCAAGTATTAGCAGCAGT
CAAAAATATAAACCTAAATCCATATCTCACAATAATAACTTTTCAAGTAATCACCAGTTA
AAATCAAGATTCCACGGTAGTACTGGTGATAACAAATACTCTCCTTCTTCGCCAAAATCA

GATGCGTTTTTGAGCCATACTCCTAATACAATACCTTCGACTCATTCGAGTCCACTTATA
CATCAATTTAAACAACAAAATTCA
>DjScaffold3466:19300-28232 strand:+
actaatatcatcatcgttaaaaaataataattttttaattaATGAGTAAAGATTATTCA
AATCATAACTCTTTCTATGCCTTTCAAAATAGTACTGCAGATGCATGGAATGTTGATGAT
GATGAGTTTTTAAATGGCCATATCTTCCAGATGTTCATTCAACCGCTGAAGCTGTTATA
AATCAGCATAAAAATGGTCAAATACCAAATAAAACTATTTTCTTGCCAAACAGATCTCAG
AAAAATCAAATTGAAACAAAATCAAATATATTTAATTCTAATTTTTCATGCTCTGATGTA
GAAGTTACTATTAAATCAACCATGAAAACTAAGAAAGCTAAAGTTGAAATTCAATCTAAT
TCTGTTGATACGTTTCCCAAAAAGGTTTGTCCTGGAATGGGTATTCGCCTAACAGATTGT
TGTCATTCAGTTTATCCCCAATTACCTCAAGGCGAAGTTGAAAATCAAAGATTAGAAAAG
TTTCATTCTCTTATTAAAAGCAGTGTAACAGATCTCGAACAATTAAGAAAGTTGAGTTGG
TTTGGAATTCCAGCTATGTTAAGACCAATTACATGGAAGTTACTTTCAGATTACGTTCCT
CCAAATGTTGATAAACGAGACCAAAAATTATCTTCAAAACGTGATCAATATTTCAGATTT
GTCGATCAGTACTATTTACACAGAGATAATCCTCAAAATGTTGAAATGTTCCATCAAATA
CAGAAAGATTTATTAAGAATGAACACTTTACAAAATAAAACTAAAATTCAGCAATTGTTT
GAAAGAGTATTATTTATATGGTCAATGAGACATCCTGCCAGTGGTTACGTACAAGGCATT
AATGATTTACTAACTCCATATTTCATTGTATTTTTATCAGAATTTGTCCAAGTTGATTTG
AATACTGCAGGGGAGCTTACGCTAAGTACTGATCCAACAGAAGATGAATTGAAAAAGGTT
GAGGCGGATTGTTATTGGTGCGTAAGTCGATTATTAGACAGTATCCAAGATCATTATACA
TTTGCTCAACCGGGTATTCAAACCAGAGTTCAAGCATTATCTTCTTTAATCAGTAGATTA
GATTCTGAACTACATCAACATTTAAATGATCAGAAAGTGGAATATCTACAATTTTCTTTC
CGTTGGATGAATAACCTTTTAATGAGGGAACTTCCTCTGCGATGTGTAATACGTTTGTGG
GATACATATCATGTAAGTGTTTTCAATATTTTTATTTTATTTATTTAAtttttactaata
tcatcatcgttaaaaaataataattttttaattaATGAGTAAAGATTATTCAAATCATA
ACTCTTTCTATGCCTTTCAAAATAGTACTGCAGATGCATGGAATGTTGATGATGATGAGT
TTTTTAAATGGCCATATCTTCCAGATGTTCATTCAACCGCTGAAGCTGTTATAAATCAGC
ATAAAAATGGTCAAATACCAAATAAAACTATTTTCTTGCCAAACAGATCTCAGAAAAATC
AAATTGAAACAAAATCAAATATATTTAATTCTAATTTTTCATGCTCTGATGTAGAAGTTA
CTATTAAATCAACCATGAAAACTAAGAAAGCTAAAGTTGAAATTCAATCTAATTCTGTTG
ATACGTTTCCCAAAAAGGTTTGTCCTGGAATGGGTATTCGCCTAACAGATTGTTGTCATT
CAGTTTATCCCCAATTACCTCAAGGCGAAGTTGAAAATCAAAGATTAGAAAAGTTTCATT
CTCTTATTAAAAGCAGTGTAACAGATCTCGAACAATTAAGAAAGTTGAGTTGGTTTGGAA
TTCCAGCTATGTTAAGACCAATTACATGGAAGTTACTTTCAGATTACGTTCCTCCAAATG
TTGATAAACGAGACCAAAAATTATCTTCAAAACGTGATCAATATTTCAGATTTGTCGATC
AGTACTATTTACACAGAGATAATCCTCAAAATGTTGAAATGTTCCATCAAATACAGAAAG
ATTTATTAAGAATGAACACTTTACAAAATAAAACTAAAATTCAGCAAGTTAGTTTTTTTG
TGATGATTTTT
>DjScaffold3466:159371-173805 strand:+
catatctccgatatcgaagttttggaaatcttatcaatttccgaatcgccagtcaattgA
TGGCTTCTCTGAGCAGTTATCAACACACGAGACGATCGTGGCGAAAAGACATATTCGATT
TACTTTTTGAATCTTACTTTTTTCAATTTGACGTGACTTCTTTGAAAGCATGGAAAGTTA
TTGTAGACAATTTGATGACCCAAGATAAAAGCACTTTTAAAGAAGCTTACACCAAACTAA
CTGTGAATCAAGGCGGAGGTCTAAATATCTTCACTACTAAAGAAGCTGAATACGACCAAA
CTGCTATGTGCATGAAAAAAATTGGGATTTATTATTTACAGTAGTGAACGTGATCAGTATG
TGAAATCAATGCCTGAAATTCAAGAAAAAATTGAGTGACTCAATACGACTGGGAATGATTC
CTCAAATTGAAAATCAAATATTTTTATGCTTCAGAGTTTTATTAATGAGAATTTCTCATC
AACATTTAACATCATTATGGCATATATTCATTCCAACATTGACTGCTGTTCTTCAGAGTC
TAATTGAAGATCTCAAATGTGATGCCAAATCCACAAGAAATAGTGATATCAATAAACGAT
TACCGATGTATCTTTCAGCATGTAAACTATTGGATATTATTTTATTAATGAAAGAAGACA
GTGTACCACAGTTTTTATATTATAAATGGGCATTCGTTGGAAATTTAGATGAGCAAAGTA
ATTATTATAGAACAAATGAAGTTCATCATTTGAATTCTCAACCAACATTCCGACCATTTT
TAACATTGGTTATCGATGCTTACATTCAATACTGTTTGGCCTCAAGCATTACTCTTACTA
CTATTGAAGTTGTTTATAAAAAATTTATTCAATGAAAAACCTTTACTTTATCGTCAATCAA
TTCACTCATTAATGGATCTAGCATCGTTCTTTTTCACAGCATCGTTGAGAAACGATCTCA
ACCAAGAGGCCAGATATAATCAATATGGAATTAAAGTTCTTTATGAAAAAGATATCGATT
CTGATTTTAACAATAACATTAGTGGTAGATTTTTCAATTTTGAAAATGAAGTCGAAATTA

TTCTTGACAATGAATTTGTTGAATCTTTAAATAAAACGTAActtcggtgcttacattgtg
aattaatattttctttcttggtttacgtatttatgttactaatcatgcattcaattatat
ttttaacaatcacttttacatcagttttgttgatatttaccaagtctttcagaaatggtt
ttcaataaaaatcggtttaacattgtgtcatggatactaaaattgttctagttaatgtct
aataaaggttat
>DjScaffold3446:32213-32930 strand:+
ctgattttaATGTCTGTTATTGCTTATTTTCTCGGAAAAGTAAAATTTCAACATGCCTA
CGAAATGCAAAAGCAATTAGTTCAATTATCAAATGATTTTAGATCGTATCAAAAATTTAA
TTCTATATTGTTATTGGAACATTATCCAGTTTATACTATAGGTATAAGAAATAAGGATCT
TGTTACAGAATATTCGGAAAATTATGTAAATAATTTGTTATCAAAAGGAGCTGATTTTGT
CAAAACAGATCGTGGAGGACTTGTGACTTTCCATGGACCCGGACAACTAGTTGTATATCC
AATTTTAAACCTTAAAACACCAGGCTTCAAAAATAGTATAAAATGGTATGTAGAAACACT
TGAAGATACAATCATTGATACATGTTATTCATTCGGGTTAGAAACAAAACGAACAAGCGA
TGTTGGAATTTGGGTGTCTGATTTTCGTAAAATTGCATCTATCGGCATCCATAATCGTAA
TTCCATAACTTATCATGGATTAGCACTAAATTGTGATATAGATTTGAGTTGGTTTGATAA
TATCATATCATGTGGTTTAGTTGGTAAATCAATAACATCTCTATCTATGGAATTAGGAAA
ACCAATTTCCGTATATGATGCTGCAATTAATTTCTTAAAATCTTTCGAAGCGAAATTTCT
TAAATCTGTTCAATTA
>DjScaffold3446:19340-25747 strand:+
ATGATGAATAACGTGATAAAGAAAATAAAAGGAGCAATGGTTTATCTAGACTATATTCTC
ATATTTACCGAAACAAATGGGCAACACTATGAAATAATCAATAAAGTATTGACAGAAATT
AAGAATGCTGGTCTAAANNNNNNNNNNNNNNNNNNNNNNNNNNNNNNNNNNNNNNNNNNN
NNNNNNNNNNNNGTTATCAGTAAAGAAGGTGTCAAAATAAATCCGACTAAAATAGAAGCA
ATAAAGAACTTTGGTAAACCAAAGTGTGTCAATAAGATGAGAAGCTCTTTGGGAATTTGC
AATTACTATAGCAAGTTTATTAAAGATCATTTGAAAAAAACAAGATTGCCGAACAATTGT
GTAGTGGGTCGAGTAACAAGAACATCGAATGGGCGAACGAATTAG
>DjScaffold1682:5428-5745 strand:+
ATGCCGTTTGGATTATGCAACGCTCCTGTAACATATCAAGAATTAATGCATAAAATTTTC
GACAAGATGTTATTTACTGTAGTACTCGTTTATCTTGATGAGATTTTAATTTTTGGTCAG
ACACAAGAAATTCATGATAAACATTTACAAAATGTGTTGGAATTAATTGAAAAATCTAAT
CCTGAAAAATGTATATTTAGTGTATCAAGACTCAAATTTTTTGGTCATACAATCAGTTCT
AAAGGTGTTGAAACAAATGATGACATTTTTAATAAAATAAAAGAAGCTGTTATTCCAAAA
TGTGCAACCCAAATTTGA
>DjScaffold1682:7867-8184 strand:+
ATGCCGTTTGGATTATGCAACGCTCCTGTAACATATCAAGAATTAATGCATAAAATTTTC
GACAAGATGTTATTTACTGTAGTACTCGTTTATCTTGATGAGATTTTAATTTTTGGTCAG
ACACAAGAAATTCATGATAAACATTTACAAAATGTGTTGGAATTAATTGAAAAATCTAAT
CCTGAAAAATGTATATTTAGTGTATCAAGACTCAAATTTTTTGGTCATACAATCAGTTCT
AAAGGTGTTGAAACAAATGATGACATTTTTAATAAAATAAAAGAAGCTGTTATTCCAAAA
TGTGCAACCCAAATTTGA
>DjScaffold4567:56824-57548 strand:+
cattaacatgtaatgatagagaatgtaaggatacttttgaattcgtaaattgcttaaaaa
ataaacaactgATGTCAGATAAGGAAATTATGGTTTCATTTGATATAGTTAGTTTGTTCA
CAAACGTTCCTATAAATGAGACTATCAATATTTGTGTTGATCTCTGGGAAAATCATTACG
CAAATTTAAACCATAAATTTGATAAAACAGCTATGAAAGAACTGTTGACTTTTTGCTGTA
AAGATGTTAATTTTCTATTTAATAATGATTGGTTCACTCAGAAAGATGGTGTTGCAATGG
GTTCTCCGTTGGCACCGACATTAGCATCATTATTTTTGTCTAAAATAGAGTCACGAATTA
CTAATTTTCCCGGTAATCAACCCATAGATACCTTTCCTTGAatatatgaacagtcttcat
caaaatatgaaattcacgatagaagttgaaaaagaaggtaaactaccattcttagatgtt
cttgtttgtagacagggttgcaactataatacatcactttataaaaaatcaacagacaca
ggactttataccactcctaaaagtttttgtgatgatcgatataagaatggattaataaaa
ggtttggttcatcgagtgtggtgtatatcatcaagctttgagattgctgatgagggcctt
aacaaatta
>DjScaffold4567:96322-100374 strand:+
atcgtgaatttttaatgagtaacttattcagtgtttaaattaataagaaaatttcaattt
attgaaagtaaaaaatttaatttcaataatttatcaaaaataaaatcttttttaaagat
aacgttttcaagtattaatttgaatttattttatttcttgtattcaaaataaaaaacacg

ttatttgtgcatcttttaaccaaaattcgtatttaggatttactaatagcactttctaag
ataacacgaggctcaattgaagataggcttagttggATGTTTTCAGTTTATGATAGAAAT
GCAGACGGATATATCACTTTCAATGAGATTCTAGAGGTTGATAAAGCAGTTCATGCAATG
TTGGGGAAACACAGTCAACCTGTTGAACCCGGACTTACCATTGCTAGTCACGCATCCGAA
GCCTTTGAAAGACTCGACATCGACAAAGATGGTCAAATATCTGAAACAGATTTCTATCAA
GCAATGTATAATAATGAATTTCTTTTTGATTCCCTGAAACAATTGGACACTATTTTATAA
taacagaatttttcatttgttaacataaaattccaattcatgttattttgttttaataaa
cgtttagttttgattataaat
>DjScaffold4567:177896-178141 strand:+
ATGCGAGCCGATGTTGTGTTAGATCCGCAAATTGAAATTCAAGAAACTTTTAAGGTTTTT
GATAAAGATGGAAACGGATTTATAACTGCTGATGAGTTGAGGTCTGTTTTGATAAAAATG
GGATACAATTTGTCTGATGATGATATTAATCAAATGATAAGTGAAGCTGATATTGATGGT
GATGGAAAAATTACCTATGAAGGTTTAATTTCATTATTTTGTTTTAATGATAAAATAATA
AACTAA
>DjScaffold3692:23008-59708 strand:+
GTTATCATCGAAACTCCTCATTATTTAACAGAATATGTTGAATATCCTATTAAGATAAGT
TATAGTTTAAACGAAGCTATTCCAGGTGAATCATATCTTATTGTGAAAAGATATTCTGAC
TTTGATGCATTGAAAAAGTCATTAAAGTTAGTACAATTTTCTACACAATTACCTCCGAAA
AAAATGTTTGGTAAATTTGATATGAATCTAGTAAATGAACGACAAATACTTTTGGAAAAG
TATTTAAAAAATATTTTAAATACATATATAATTGGAAAATCCGTTCCAGTAACTAATTTT
TTCCAGAAATCAAATGATGTCATTGTAGATTATACAAAATTAGGCTTTCAACAAATATCG
TTAGACTTTCGTTGTGAAACTAATTGGCAAGTTCACAGAGTTTTACCCGAAATCGGATGT
AGAATTAAAAAAGTATTTGCCAATGTAACCAGTTTAGGAGATCAAAGGAAACATTTGCTT
TCATGGTGCGAATTTGGACCAGACTATTCACCTTTAAACAATAAAGTCTGTCAAAAGTTT
TTGAAAACATTGACTTCTTTGAAACACGAATATATTTATCCCATAGCTCATGTAGCGGCC
AGTGATACTGGTGTGTCAATTATAAGATTATTCAGTGAACTAGGTTCACTTCATGATTTC
ATGTGCAAGGTGAAACCGATTAGCCCAGTCATTCAAAAATATGGTGTGCAAAATGGTAAA
TTCACTCTGATAAATCATTTATCTCTCTTCGGTCGACAAATACTACTGGGTCTCAAATAT
TTACACGATAAAGGAATTCCATATGGTCATCTTAATTTAAGAAATGTCCTGCTGTTTACT
GAAGATAGGTGTGGTCTGACCGATATTGAAAATTCTGTACTTGGTTTGATCCCTCAAGAT
CGTCATTTTTTTGTTGGTATATTAAAAAAATTGACTTTTGAAGAAATTGATATTTATTGT
TTTGGAAGAATTATGTACGAAATGATTTTTGGAGAACCGCTTAATTCCACATTTATTCTC
AGTGATGATAAATTGAAGTGGCCAACTCCTGCAATCAGAGAGTTTATGTTTTCTATACTA
ACAGATGAAGTATTAAAATCAGGTTTTCCAACAATCGATAATTTGTTAGAATTAACTTTG
TTCAAATCATGTTCAACAAAACTTGTTGATAAATCCCCATTCAAAATTTCTCCTTCATGT
AAAGAACTTCTACTATCTACACAACAATACTCATTGAAACGTTTGATTGCGGATCAGAAA
CTTGTAAAATCGCAAAGACGAAAATTAGAAGTAGATGCGATTGGAATGAAAAAAAAGCAG
CAAAGAAAAGAAGAATCACGACGTACTAAATATGCAGGTGATTATGAAGAAGAGCCAAAC
GTTGTGAGACAGAAAATTTCAATCGGTCAATCAACAAAATCCATTGATACAAATAATAAC
AATAATAATCCACCAGTGCAATCACCAATGGTCTGTCCAGCATCACCCGCACCCCTATCA
CCCCCACCTCCGCCTCCACCACCTCCACCAGGTCCTTCGACAACATCCTCTAAAGCTTCT
GATTCCAAAGTTGGCCCATTACTAAATGCTATTACCGGATTTAATAAAGGAAATTTGAAG
AAAGCCGTGACAGTCGATAAAAGTGTACCAAAATTATGAattcaattttgaaactgcttt
ccacttaaaaacatttatcattcaccagttgttgtaaaaatcaaatcaagttgattatat
gttaaatttatttacgcatacttttgatttatccactaatgaattgattatccgttgaaa
ttatttttcaaataaataattgattaatc
>DjScaffold2646:15949-16347 strand:+
ATGCCATTTGGCATCCCAACAACACCAGATAGATTTCAAGAGAGGATGACAAATGTGGCA
GGCGGAATCAATGGAGCTATGGTTTATCTTGATGACATTCTGATATTTACGAACAATAAA
GACCAACGCTATAAAGTAATTGAAAGAGTATTGACGGAAATAATGAAGGCTGGTCTAAGA
ATTAATCCGGAAAAGTGTCAGTTTCTTCAAAGGGAAATCAAATTTCTTGGACACATAATT
AGCAGAGATGGTATTAGAACTGACCAGTCTAAAATAGAAGCAAATAAAACATTTCGCAAA
CCTAACTGTGTGAAAAAGTTACGAAGTTTTTTAATAATATGTAATTATTACAAAAGTTTT
ATAGAAAACTACGCAAGAAAATCAAAAATATTAGAGTAG
>DjScaffold2077:131610-150308 strand:+
TTCTATGGATCATTAGCGTTGGCATTTAATTCTAATGAACATGCAAAAACATACAAAAAG
TATGAAAATGAAAAAGTTACAGGTTTGTTTGGTATATCTGAGTTAAAAAATCCTGCAGAA

TTAGTCTCTTTGAGATACAAAGCTGAAAATGATTGTCACCTACTCATTAAAGAAGCACTT
TCTCCTATGAGATTCCGAAAAATCGTTGAAATTTTAGATGATATGTCTGATTCTGTATGT
CGTGTTGCTGATCTTTCTGAATGCATGCGTCTACTTCATCCGAGTGATGAAGTCCGTCAA
ATATCCATGTCGGTATCTCAAACGATGTCGAAATTAGTAGAAGAATTAAACATAAATCAA
GAATTATTTTTAGTACTCGATAATGTAGTTAAAAATAATGTTGATGTTAAACCGCTTGAT
GATGTCGATATTCGAGTGGGTTCTCTACTACATACTGATTTTCAAGCTTCTGGTATTAAT
CTCAGTTTACATAAGCGTCAGAAATATGTAGAAGAAACTCAGAAATCTATTCAATTCGGT
TCATTATTTTATGAGGGAACAGAATTATCTGTAAAAATTAAAGCTTCCCTGTTGCCCAAG
AGATTACAAGATAATATTGGATTAAAACCTGATCAAAAAATTGAAATAGATCATCCGTAT
TCGTTAGAAACTAATTCATTTTTGCGTGAAATGGGATATAGAGTATTTTACTGTAATAAT
CCGGAACAAGAGAATTTACTGAGGAATCTTATGGAATCACGTTACAATGTTGCCAGTTTT
TCAGGATTTTCCTCATTTTCAGAACGAGCTAATATTCATTTAATTTGTGAAACACCGACT
AATATTAAAAATTTTCTGACTCGTCTTTCTTCTCGATTACTCCCTTTTGCCGAAATAGAT
CGTCTTAAAATTCTTCAGGCTAAATATGGTAAAAAACTGATGAAAAGTGATTTATATTCA
GAAAACTATAAAATGTTTCCCTGGGATTTTCCATATTATATGAACTCTATTAAAGAAGCT
ACTGTTCATGGAAGTTATGAACCTTATTACCCATATTTGTCACTTGGTAACTGTATGGAA
GGAATCAACATGCTTTTTAAATCTCAATTTGGAATTGAACTGGAAGTTGAAGAAACAATT
CCCGGTGAACTTTGGCACTCTGATGTCTTTAAAGTTTCAGTTCGACATGAAACAACGGGT
CATCTTGGAACTGTTTATTGCGATATGTTTTCTAGACCAGATAAACCTTCAACAGACTGT
CATTACACAATTCGTGCCGGAAAGCAATTATCGGATGATCTATATCAAAGTCCTATAGTA
GTTTTGCATGGTAACCTAAAACCAGTCTCACCTGATAGAAGTCTTGCTTTTCTTTCTCCA
AATATCGTAGAAACTCTTTTTCATGAATGGGGTCATGCACTTCATTCATTGTTAGCTCGT
ACTCGTTATCAACATGTTGCAGGTACACGTTGTAGTACAGATTTGGCTGAAGTTCCCTCT
ACACTTATGGAACATTTTGCGCTTGATAATAGAGTTATAAAAGAATATTCACGCCACTAT
CAAACTGGTGAAAATTTACCCGATAAAATGATTACTTCATTATCTCAGCAGCGTTATTTA
TGTTCTGCGATAGATCATCAAATGACGATTGTTGATTCCATGTTCGATCAAATAATTCAT
GGTGATGATCTACCATTATTTAAACGAGAGAGTTGTGAAATATACGCAGATATCTTTTCA
GATAATTCTATTGTGACTGGTAGTAAAGTTTTCGATCGGGCTGATGATTGTAAACCATAC
CGGCCACAAAGATTTGGGCATTTACTTAATTATGGAGCTAAATACTATTCATATCTTATG
TCTAATTGTATTGTTGGTTTTCTATGGAAAAATGGATTTCAATTAGATCCATGGTCGAAT
TCTGTTGGAGAACGTTATAAAAATACAATTTTACAACATGGCGGTGAAAAGAAAGCAATT
GAAATGATTGCTGATTTTGCTAATTCGAAAGAGATTACAATT
>DjScaffold3511:34865-37728 strand:+
ATATTAATTTTACTAGATGTCTCCTCATCAGTTGATGAAGAATCCGAAGTGTCGTCATGC
ATTCAATGTATTTGTTTTAATTCTAAAAATAGATCATTGAAAGAATTTCTTTCTAAATCG
GACAATTCCTCAAATCAGCAGCGTAAGCAAGGCATTTCACAGCCCTACAACAAACAGCAA
AGACTAGAGAAAAATGAAGAAGGCAATGAACAACAAAATACAAATTTACCCCTTATATGG
AAGTATGAAAACAAATTTCATGATTTTTATCCATATCCCGAATCTCCAGATTCAAATTTC
TTTATCAAATCTAGCTCAAACAAGCAAACCAATTTTTATGAGCCTCCTTTGAAGCAACTT
AAACCAAAATCCCCAATGATTAACATGCACAGTTATTCTACCGTGCAAATACCACCTAAT
AATGATACGGCTTCAGCAGCTCTTATTTATAATCAATCGAAATATGAAACAACTATTTCA
CAGACCGATCGATCAAATCCGGTACTAACCCAAACCACTTACCCGGTTTCTTGTTTCGCC
ATTCCAAATCCCAAAATAAATAATAGTTACGTTTGAaaaggaattatctttgatttcttt
ttttagaaattttgatgtgtacaaagaatttgttaattttattttctgtcgttttgtaat
aatatcattattattatta
>DjScaffold5424:3017-32625 strand:+
ttagcgatgaaaatctcaaggaaagatgtggttcagatgcagtaatctattgtcggtttc
aacgtaaattgatatttatcgtatcagtaatgatggtactatctctcactattattgtgc
ctttgaatttacatgggaatttgttgtcatatataactatataattaaatatgtgactat
atattattttcagatcttcatttttatgggcgcattttgcagtttctcttggatttatgc
tttttgtctttttcatatcaactcgatttctcactaatattgatgtcaaaactaaagaag
aacccagtttcacaataatgatcaataatgtttctacagaatactgtgatgaaatgacaa
tagcacaacattttcgggaagcatatcctgcaatcaatccaattaaagttcaagtcacat
acgaagtagccaccatgatctctctctacacagaaaagtaagtttgtacattattttcca
aaataataaaatcgataagaaatcacttgtgattaattgtaaaaattaaaatttagattt
tgtatacaggtgatttcaaaattaactaaattttagccattgatatggaataaatatgat
atgtaatgtaatgtattgttattataattacatttatttagacatttgtagacataacca

aatacaaaaaaatgatgaaatatttgacttaataatgaaacatttagaaaaaccctgta
aatattttcatttttcaatctacgtttattataggatttttaatgtttatcggcctgaat
ttatttttaattatatattttcaatagtttactctaaaatttaggcaaaatttgcataaa
cgtggttggaaatggtggattccaaatattataATGAACATGGTGGTTGGAGTAGTGATT
ATTGTTTTGTCAACTCCAGCTGTAGCTTTGAATATTTGGAATAATCTCAAATTCACTGAA
AAATTGGAAAAATTAAATAATCCGCTAGTTGCTCAATTTGCTCCATCTTTGGTGATGGTG
CTAGTTAGTGCTATAATGCCGTTGATTGTTTATTGGTCAGATCAGTTAAGTGGTCATTGG
ACTAAAACCACTCAAAACATTGCTGTCATGAATAAGTTATTTACAGTTCTTGTAATTACA
GTTTTAATCTTGCCATCTTTGGGTCTTAACAGTATTCCACAATTGGTCAAATGGTTATTT
GTTACTAATAATTCCTCCACTCCGAATACAATTGTATGGGAATGTATTTTTATGGCGGAT
GGAGGAGCTTTATTTTGTAATTATATTATAACAGCTTCATTCATTGGTACCGCCTTAGAG
TTGCTTCGCCTCGGCGAGCTCTTTATGTACACAATGAAACGCAGTTGTTCAAGATCTCGA
GCTGAAGATACTGCAATAATTAAAAGCGTTTTGTGGGATTTTCAATTTGGAGTTGGATAT
GCATGGACATTGGCACTATTTACTGTGGTGTCAGCATATTCTGTAATTGTACCAATAATT
ACTCCATTTGGTCTTACATACTTGCTTTTAAAACATCTGGTAGATAGATATAACATGATT
CATGTCAATGTACCATCCATAGTTCATAATTCAGTTCATTCGAATGCTGCTCAATTAATG
CAAGTTGGATTAGTTTTAACTCAAGCCACTGTAACTGTTTATGTTTCTTTGCGTCGTGGA
ACCACTGATTATTTATCTATTTGTTGTTTTTTGGCTTTTGTGTTGGGATTGATGTTAACG
TTTGGTAGAACTCTTAAACGTTGGATTTTTAGTATGTCTTGGGTCAAATACAGCAATATA
TCCAAATTTCCATCAAATATTGTAAGAACAAAATCAATGGATTTAGAAAATATGGATGAA
GATGGTTTTATTATGGTGGATGAAATCAAAAACAGTGTTTTTGTACCACCTCAAATGTCG
AATGGGGTTGATTTGTCATTATTAGAGAAAATCAAAACTGCCAGATCATCAGTTGCATCT
CTTAATGATGATAATGACACTATTCCATTTCATACTCGTAATTACGGAGCAACAGTACCA
AATTTATATTGAtcttttattttaccatgattgagagctattaatcgcaaattgatattt
gcg
>DjScaffold5293:5695-6176 strand:+
aactactgaatatcaaatagtcaactaatgcgaatttttatctacaATGTATTCTGGAAA
GTTTCAATTAAAATCTACGTTATCAGGTGTTGAAGAGAATAATTATTATAATCGTGGTAA
TAATTATTCTAAATCAGTTCGGTGTGACAATTGGTATTTAATACCAGATGACATTAAAAC
GATTTATTTAAATCAGAATTATAAGCCACCTTTACCACGACCTCTGCCAACTATTCACGA
AAAGTCTAGTTTTAAAAACCATATATGTAAAATGGTAGTTAAACTAAATGAATACCTTTC
AAAACGAAAGAAGAATATGAAATCAGAATTTAGATACTTAGATTTTAAACGAAATTATCA
TGAACAAAGTATTACAAATTTAAGAGAATTTCCAAATGAAAATTATCGGTACAAGTTTAA
TTGCCGCCTCAATAAA
>DjScaffold5293:66226-66620 strand:+
gtaaactaattctaagatattttctttttaataatttttggaaaattgtattcacaAT
GGCTACCAGCTTTGAAGAAAGTATTGGCGTTTCTCCAGATTATGATGAAGACTATCCTGC
TATGTTTAATATAATACTTTGGACGATGGTTGCGTTAATATTGGCAGTATATGCTATTGC
ATGGGGTATATGGAATATGAATCCAGGAAGAGATAGTATAATATATCGTATGACAACTCA
ACGTCAAAAGAAAGATTAAaagaaactgataatgtaatggcataatagtagattgtagtg
attgataaaatgtgattattgaatataatttgattacatttgcttca
>DjScaffold5293:69519-75582 strand:+
tatttagattaatgtgtttttgaaatttttgatttaatcgtcattcaATGGCAGTTGCT
AGTGTTTCATCAATCGGTGGTTTAACTGAATTTAAAAATCTTCAAATTAAATGTGATTTA
CACGATGAGACCAATTTAAATGGCTCCGAACAAATAAATTCAAACGATTTTGAATCGGTT
TCTGAATCCGTTGATATTTGTGAAGAAACTGTTAATCCTCCCGGGGAAAAAGTTACTTCG
CATGATTTTGATTTATTGAAAAAACTCGGGAAAGGTGGTTATGGTCAAGTATATTTAGCG
AAAAAGGTGAATGGAGCGATGGCTGAGGAATATTTTGCTATGAAAGTTGTGAAAAAAGCA
CGCTTGGTTACAAATAAGAAAAATGACACAAAATATGCAATGACCGAAAGAAATATTCTT
GAAGCTGTAAATCATCCATTTCTTGTCAAATTGTATTTTGCTTTTCAAACTAATCATAGT
TTATTTATGGTTTTGGAATATTTGGCTGGCGGGGAATTGTTTATGCATCTCGAAAGACAA
CATACATTAACAGAAGAAGAAGCTAGATTTTATTTAGCAGAAACCACTCTTGCTCTCGGA
CATTTACATTCGATCGGAGTAATTTATAGGGATCTTAAATCCGAGAATGTGATGTTGGAT
AGATATGGTCATGTAAAATTAACCGATTTTGGGTTGTCTAAAGAACGAAATGATGATGAA
TTGACACATTCATTTTGCGGAACTGTCGAATATATG
>DjScaffold5293:91659-96151 strand:+
aagaaattttaaatgggattttgatttttttttgaattttccaaattattttaaatgaa

aatttaatctaaaattttaaatttattgaaaaatgattcccaattaaagcataattgaat
aaacaaatagtgatggacatagttttgttttacgaataaatcttttatggaaacaagttt
tgacattaattacccaatgagtacttcaaactcatcgaatactcattatcagagttaggt
cagattggtagcagttaccaatctaatatattaatggaaactccaatgcattcacataat
attgattatattttaaaatatttaattgattaaacaggtcgtatttggctccagaaattc
ttcaacgtcgtggccatggcccgagtgttgattggtggagtATGGGAATCTTATTATATG
ATATGCTTATTGGCAATTCACCATTTATTGGAGGAAATCGTAGCCAAGTTATTCAGAAAG
TTATAAATGCCAAATTGAAAATACCGAGATTCCTTAGTGATTCCGGGAAACATTTACTAA
AAGGTTTATTAAATAAAAATCCAAAAGCTAGATTAGGTGGAAGTGAAAAGGATGTCGAAG
AAATAAAAAAGCACTTTTTCTTCCGATCTATCAATTGGAATAAAGCTTTTGCACGAGAGT
TAAAACCACCATTCATTCCTGAATTATCGAGTCGTGATGACGTCAGTTTGTTTGACAAAA
ACATAGTTTCAGAACCATGTGTAATCAGTCCAAGCACGGCTGATGATTCTAATTGCTATT
GGTTTGAAAATTTCAGTTATGTAAGTCCACAAGTATACCTCGAGTTATCGGAACAAAGCA
GTTTTTCACGACAACGAACACGAAGCGGAGTGTCGCATTCGAGCGGGTGAaaataatttt
tttattgattatcaatttccaaaaatattaaccaataacacaaaaacatattaatcaata
acaattaaatagcaactattactccaatttaaatgaaaatttaatctaaaattttaaatt
tattgaaaaatgattcccaattaaagcataattgaataaacaaatagtgatggacatagt
tttgttttacgaataaatcttttatggaaacaagttttgacattaattacccaatgagta
cttcaaactcatcgaatactcattatcagagttaggtcagattggtagcagttaccaatc
taatatattaatggaaactccaatgcattcacataatattgattatattttaaaatattt
aattgattaaacaggtcgtatttgtatatataatatttatacagggctccagaaattctt
caacgtcgtggccatggcccgagtgttgattggtggagtATGGGAATCTTATTATATGAT
ATGCTTATTGGCAATTCACCATTTATTGGAGGAAATCGTAGCCAAGTTATTCAGAAAGTT
ATAAATGCCAAATTGAAAATACCGAGATTCCTTAGTGATTCCGGGAAACATTTACTAAAA
GGTTTATTAAATAAAAATCCAAAAGCTAGATTAGGTGGAAGTGAAAAGGATGTCGAAGAA
ATAAAAAAGCACTTTTTCTTCCGATCTATCAATTGGAATAAAGCTTTTGCACGAGAGTTA
AAACCACCATTCATTCCTGAATTATCGAGTCGTGATGACGTCAGTTTGTTTGACAAAAAC
ATAGTTTCAGAACCATGTGTAATCAGTCCAAGCACGGCTGATGATTCTAATTGCTATTGG
TTTGAAAATTTCAGTTATGTAAGTCCACAAGTATACCTCGAGTTATCGGAACAAAGCAGT
TTTTCACGACAACGAACACGAAGCGGAGTGTCGCATTCGAGCGGGTGAaaataatttttt
tattgattatcaatttccaaaaatattaaccaataacacaaaaacatattaatcaataac
aattaaatagcaactattactccaa
>DjScaffold916:27395-27858 strand:+
gtATGAAATCTCGATCTCACAAAAAAATTGAAAAATCTCAAATGAAAAATGATATTATAA
TATCTGATGATATAGAGCCGACTGAATCATATGCGGGTGTTGGTGACCATAATATTGTGA
ATTTTAAAAATAATGAAAATATTATAAATTCACATACGTACGTCAAGAAATTAAAAATTC
AATAGatgtgaaattgaaatatatttctgatgatttaattaataataattcaaatactaa
tagtaatcaaatcagtaaaaaggtaataaatatattttttgtaaactttatatattctaa
atattattgaataaaactgttcctctagtctaattatacagatatattttctatgtttt
>DjScaffold916:131033-131904 strand:+
ataaagtagaatgtctattagatacaggggctcgaattaacgtgatgagtttgagttgtt
ttaataaactacgagaagtaaagttagaaatgacttATGCGAAAGGTCGATAGCAAAACG
AATGAAAAAGTCGAAAATTACATTTGCAATATTGAAGCAAATTTTGGAAGACAAATTAAA
AAAGGAGAGCGATTGACAAGAGCTTTAAATATTTTTAAGATTAAAGAGGATCAACGGTTA
TTGAAAATAATTGAAAGTAAAGAAGATGCTTTTATGGCAGATAAATGGGATGTAGGACGT
ACACATCTGACCAGACACAAAATAATCACAAGTGGTGAACCAATCAATGTAAAACCATAT
AGACAACCAATGAACTTAGAATCTAAAATTGAAGAAGCTATCCAAAACCTTTTCGAGAAT
AATATTATCAGGAAATGCAACTCTCCATGGAATACCCCATTGATATGTGTTTGGAAAAAG
GAAAAGAAAGACATAAGAATATGTTTGGATTTCAGACAATTGAATGCAGTAACGAAAAGA
CAAGCCTTTCCGATGCCTAATGTCGATGAAATGCTGGACAAGTTAGATGGAGCCAAATAT
TTCAGTTCAATAGATTTGGGAAATGCATACTATCAAGTAGAGCTCGAAGAAGAATCTCAA
GAAAAAACGGCGTTTTCAACGAAACACGGGCAATATTGTTTT
>DjScaffold916:183404-189476 strand:+
gtgctattttcttcttattttaaatcatATGCTATCTGCAAATTTGTTTTCATTACCTTA
TCCCTTAATGGTATTTCTTTGGGCCATGCTTTCGGTTCCTCGTCCATCTAAGAATTTTTG
GATTGCGTTAATCACATATACTGAAATCGTTATTGTAATCAAATATATATTTCAATTCAA
TTTCATTAATTTTAACTATTCAAGTGATATAACTAGAGAAGATCCATTATGGGTTCCACT

TATAATCGGAATTGAGAAGTCTAAAAATTACGCTGTTCTTGATTTAATTCAATTACTTTT
TATATTTTTGCATCGTGGTTATTTGAAAAACAATGGGTTGTGGAGAGATCATACTGAATT
CGCACAAGATCTCGAATTTGTGGATAAAGAACAAGATGGAAAAAATAAAATCAAATCTTC
ATATGACGAATCATCTCCTGCATCTAGCAATTGGTCATGCAATCCAATTTACAAGATGCG
AAGATTTTATTCAAAAATGACCAATCCGAAATTCAATAAAAAAGTTGATGTTTATGTTTA
TATGTTTCTTTGTGAATTTATTGCGTTTCTTATTATTATATTTGGTTATGTTTCATTTGG
TCCAGGGGTTAGTAATCTAACAAATTGGTAActttcatttatttaatatagaccgattct
gctgataatgcttttgattttattaaaacaaataaaattcctgtgcctttcataacaatg
attattactcagttcgtgtttattattattgacagagcactttttcttcgaaaacaagtc
aaagggaaatttatatttcaaattttccatgttcttttaatacatgcttggttatttttt
gcgttaccacatattacgaagaa
>DjScaffold916:191503-195486 strand:+
caaATGAAATCTGGATATCCACTGAGAATTTTGGGAAATTTTTTGACAAGAGATTTTAAT
TATATCAATCTATTTTTATTTAAGGGTTACATGATTGTTCCATTTCTTTATGAGCTGAGA
AACGTTATGGATTGGATGTGGACTGACACATCCGTTGCTTTGTTTCATTGGATGGAAGTC
GAAGATGTTTTCTGCAAAATATTTGTGCTTAAATGCTGGCGGCGATCTGAACTGGAATAT
CCGACTGTGCGAGGAAACACTCGACGTTTGGCTGCTAAGTACAGCGTTGGTGGTTTATTA
CTTGCATTTTTTTTCATTTGCTTTTGGGGTCCTCTTACCCTTAGTAGTTTTATTAGTACT
ACATTCATCAAAAATCCTCCAATTGAGTGTACATTTTCAATTAGTGTTGGTGGATACAGT
CCACTCTTTGTGTTTGTTTCTCGAGAAAATAATTTGAAATCCCTACCTAAAGAATTGTTT
GATAAAATGAAAGAATGCTACAAAGACAACACGGTTTCGACTGATTATTTAATGAGTTTT
GAAGCTGGTGATGTTACACAAATATCTATCGATGGATATTCGGATGATGTTTGGGTTATT
ACTCCACCTAGTCTTGTCAAACTAAAGAATAAGTTATCTTCACCCAATGAATCCCTAAAA
ATGAATATGCAAGCTATTTGTGAAAGGCGAAGTTCTGATTCCAAAACAACTGAGAAAATG
GTTCGGCATACAAATACCAAACAGCTAAGTGATGTTGACAAAAGGAATCTATTAACGGTT
ATTGAAAACAATAATGCTTCACATATAAATTTAAATATAAATAAAATATTTCCGCGATTT
TTAACGGCTTATAAACGCACACTTTCAGAAGCAATTCCACTTGTTAAAAATTTTAATAAT
ACCTTTTTAAATCTTACTTTATCAATGAAAAAAGATTCAGAAATTAAATGGTGGGAAATA
AAAGAAAGTATCCAAGGTGAAACTTTTaaaatattctttaatttaaatatgcgtttgtta
aaattataatttttttagttacATGATTGTTCCATTTCTTTATGAGCTGAGAAACGTTATG
GATTGGATGTGGACTGACACATCCGTTGCTTTGTTTCATTGGATGGAAGTCGAAGATGTT
TTCTGCAAAATATTTGTGCTTAAATGCTGGCGGCGATCTGAACTGGAATATCCGACTGTG
CGAGGAAACACTCGACGTTTGGCTGCTAAGTACAGCGTTGGTGGTTTATTACTTGCATTT
TTTTTCATTTGCTTTTGGGGTCCTCTTACCCTTAGTAGTTTTATTAGTACTACATTCATC
AAAAATCCTCCAATTGAGTGTACATTTTCAATTAGTGTTGGTGGATACAGTCCACTCTTT
GTGTTTGTTTCTCGAGAAAATAATTTGAAATCCCTACCTAAAGAATTGTTTGATAAAATG
AAAGAATGCTACAAAGACAACACGGTTTCGACTGATTATTTAATGAGTTTTGAAGCTGGT
GATGTTACACAAATATCTATCGATGGATATTCGGATGATGTTTGGGTTATTACTCCACCT
AGTCTTGTCAAACTAAAGAATAAGTTATCTTCACCCAATGAATCCCTAAAAATGAATATG
CAAGCTATTTGTGAAAGGCGAAGTTCTGATTCCAAAACAACTGAGAAAATGGTTCGGCAT
ACAAATACCAAACAGCTAAGTGATGTTGACAAAAGGAATCTATTAACGGTTATTGAAAAC
AATAATGCTTCACATATAAATTTAAATATAAATAAAATATTTCCGCGATTTTTAACGGCT
TATAAACGCACACTTTCAGAAGCAATTCCACTTGTTAAAAATTTTAATAATACCTTTTTA
AATCTTACTTTATCAATGAAAAAAGATTCAGAAATTAAATGGTGGGAAATAAAAGAAAGT
ATCCAAGGTGAAACTTTT
>DjScaffold916:131911-133902 strand:+
ATGCCATTTGGCATCGCAGCGGCACCAGGGACATTTCAAGAGATGATGACAAAAGTGGTA
GGCGGAATCAATGGAGCCATGGTTTATCTTGATGACATACTGATATTTACGAACAATAAA
GACCAACACTATGAAGTAATTGAAAGAGTATTGACGGAAATAATGAAGGCTGGTCTAAGA
ATTAATCCGGAAAAATGTCAGTTTCTTCAAAGTGAAATCAAGTTTCTTGGACACATAATT
AGCAGGGATGGTGTTAGAACTGATCCGTCTAAAATAGAAGCTATAAGAACATTTGGCAAA
CCTAACTGCGTGAAGAAGTTACGAAGTTTCTTGGGAATTTGTAATTATTACAGGAAATTT
ATAGAAAACTACGCAAGAAAATCAAAAATATTAGAGCAGTTGTGTAGCGGACCAAGCAAC
AAGAAATTAGAATGGACAGATGAAATAAACGATACTTTTGAGGGTCTAAAAAAATCTTTA
ATGACTGCACCAATATTGTGTTTTCCGGACATAAACAAAGAATTTATATTAGATACGGAT
GCCAGTTTTGATACGATTGGAGCAGTATTGTCACAGAGAGATAAAGAAGGAAGAGAAAGA
GTTGTTGCCTATGGATCACACTCAATGACAAGTCATGAAAAGGGATACTGCATTACACGA

AAGGAGTTATTGGCTGTTTATTATTTCTGTATCCATTTTAAACATTATTTGTATGGTAGA
AGATTTACTCTAAGAACAGATCACAAAGCTCTAACTTTCATGATGACGACGAAGAAACCA
ATTACGTCACAATTCCAGACATGGATTAATTATCTGAGCAGTTTAGATATGATTTTGGAA
TATAGAAAAGGAATCCAACATGCTAATGCAGATACGTTATCTAGACCAAGATGTGAGGTA
TGTGTACAATGTCAAATGTCACATGAAGATGCCAAGAAAGGGAAGATTAAAACGAGAATA
CTGAATATAATTGAAAAAGAAAAAGAAGATCATTTAAACATTTCTGAGGAAACAGATTTA
GTCAAGAAAATGAAAGTTCCGGATGACAAACTACGTGAAAAAATAGAAGAAATACATAAA
TTATTATGTCATGCAGGAGTTGAAAAGGTGTACGAATATATGAGAAACAAATATATTGCA
AAGAATTTGTGTAGCCAAATACAGAAGATAGTTCGAAGTTGTGAAATTTGTCAGAAGACC
AAAGTAGTGACAACACCAACTAAAGAGAAGACAAAGAGTTTAGAAGCTACCAATTTATTT
GAAGTGATATTCACAGATATATGTGGGCCAATGCGGGAAACAAGTGGACGGAAAAAATAC
ATTCTTGGAATAATCGACCAATACAGTAAATATCTGGTTCTTATACCATTGCCTAGACAA
GACGAGGAAACGATACGAAAAGCTATTTTTGAAAAGTGGATATTGAGATTTGGATGTCCA
AGAGAAATAAGATTAGATTGTGGAAAAGCGTTTGAATCCAGAGTTATGAGAGAGTTTGCA
CAGAAGCTAGACATTAAACTTTGCTTTTCGAGTCCATATCATCATAATGCTAACGGACAA
ATAGAAAGACAGTTCAGGACCATTCGAGATTTGATTAATGCGACCCTTCAAGAAAGAAAA
TCAACAGATTGGGCAGATCTTTTACCGGAAATAGAATTTACAATGAACGCAACAAAGCAG
AAGACTACTGGAAAGAGCCCGGCTGAGATCGTTTTTGGAAAGAAAATTAGCAGAGAAAAG
TGGTACGAGTCAGACGTTTCAGATATAATTAAAGAAAACATTTCAGACGAAATTACTAAA
AGAGAATTCAAAGTGGGCGATTATGTACTCATCAACGTAGAAACAAGAAATAAAAGTCAA
GACAGATTTGAGGGACCTTATCGAATAATTGAAAAAATTCATGAAAGGCGGTATGCAGTA
CAAGGAGAAAATGGACAGTGTTTAGAAAGAAATGTGGAAAAACTTAGAAAGTTTCTTAAG
AAGGGGATATGA
>DjScaffold5421:44324-54164 strand:+
ATGCATAAAATTTTTAGCAAGATGTTATTTACTGGGGTTCTCGTTTATTTTTATGACATT
TTAAGTTTTGGTTACACAAAAGAAATTCAATGCAAACATTTACAAAATGTGTTGGAACTA
ATTGAGCAATCTGGTCTCAAAATTAATCGTGAAAAATGTATATTTAGTGTATCAAAACTC
AAATTTCTGGGCCATACAATCAGTTCTAAAGGTGTTAAAACAAATAATGATATTATTAAC
AAAGTAAAAGAGTCTGTTATTCCAAAATGTGCAATCCTACTTCGAATCTTCTTGGGACTG
ACAAATTATTATCGTAGGTTTAGTAAAGATTACGCAACAATTGCCAGCCCATTATATGGA
GCAATAACTGGACAAAGCAACAAGAAATTGGAATGTACAGATGAAATAAACAATAATTTT
GAGGGTATGGAGAAGTCTCTAATGCCTGTACCAATATTGTGTTTTCCAGACATAAACAAA
GAATTTATATTAGATACAGATGCCAGTTTCGATAAAATTGGAGCAGTATTGTCACAAATA
GATAAAGAAGGCAGAGAAAGAGTTGTTGCCTATGGATCACACTCAATGAAAAGTCATGAA
AAGGGATACTGCATCGCACAAAAGGAATTATTGCCTGTTTATCATTGCGGCATGCATTTC
AAACATTACTTGTACGATAGAAGATTTACTCTTAAAACTGATCATAAATCATTAAATTTC
ATTACGACGACGAAGAAACCAATTACGTCACATTTCCAATCATGGATTAATTATCTGAGT
AGTCTAGGCATGGTTTTGGAATATAGAAAGGTAATAAAATATTAA
>DjScaffold2303:66-10657 strand:+
AGATTGGTATTTTTCAATAACATTATCGGTGAATTCTGGTATAAAGTGAATCTAATCGCT
GAATCACCGATTGAGAAATTTATTCCGGAGGTTAATTGTGAGCTTGGAAAATGGTGCTTA
ATTGAAATTCCTATGAAAAATATTGATCGAGATAATAAATTAATGATGTTTCCAGAGTTT
TCAAATCCAGAGTGTTTTAGTGTTCATATTCTTGATTTCGAATCATTTGATCAAGAGCAA
CAATGCCTGATTCTTCCTCCAGCTTCTGAGCGCATTTTTATAGTCAGATTTACACCTTGT
GAAATTGGAACCAATGGTCATCATTGCTTGTTAACTCTCAAATCTAAAGAGCTTGGAAAT
TATAAATTTAATTTAAGTGGATGTGGGATGCCACCATTGCCTAGTGATATTATTGGAGTT
TCATCTGAAGCTGGGTCTAACACGACATTTCTTATACCATTTCACAATCCATTATTGGAA
ACAGTACTGATTGACATTAAAATAACTGAGGAACATCAGTGCATCATAGATAAATATAGA
TTGGCATCTACTGATATTGCCAAAGAATTCAGTACAATGGGAACTGGAGTTTTTAAGCTT
TTACTGAAAAATAATCGAAAATTAAAGCTGGAATCTAAACAATCCATTGAAATTCCAGTT
TCGTTTGCACCCCAAGAAATGAAAAAGTATGATGCAATTTGTAGTGTAGTGATGAGACGG
AAAAATGGAGAACCTTTGGTTTCTGAAGAAGACTTTGAAAAATTGGTTTTTCCAAATTCC
GAAATAATCTCGACGTATGACAAAAACGTCTTGCGTTGGCTTTTTCCAATAAAAGGAGTT
CCTGAGGCGTCCGTAAAAATGAACGTATTTACTATTCCTTTGATTCAAGGTTGTTTACGA
GTTGAAACTAAGGAAGTTTTGGAATTTAGTTTATGGGGCTTACCACCCCCAGTTACTAGA
GAAGATTTAGTTACTAAATCAGTAAAAAGTGCCCCAGTATTTGGTGAAAACAATGAACTA
AAACCCATAGAAAGAATATTGCAAGAATTTACTACTGAATTAACGGTGATCAATGTTGAT

TACGGAGATTCTATGGATAGTATTACTTTCAATGAGTATGTGAAAATATTTCTCCTTAAA
CAAAACAAACAGTTGGAAACTGGTGTTACTCTTTTATCTTATGAGATTATAATGCGACCA
CTAAGAAAGTTCAGACTGAATTCAGAATTAATTATCACAGCAGCTTCTGGAGGTGTTTGG
AAATTTCCTATTCGTTTTGTAGCCCATGATTGTGAACCAGACGATGAAATCTTAATTGAA
GCCATTGGTCTCAATAAGCCAGTCAGTACCAGATTCTATCTGAATAGTAAAACATTTGAT
GCATTACCATTCAAAGCCTACTTTCTTAAAGGTAGTGATCCAGACTTTTCTGTTTCTCCT
GAAAACGGAGTTTTACCGTCAATCCAGTCTTGTTCTGAAACTCAGATACCAGGAACTGAA
TTTGTTGTTACGTGGATACCTAAAACATACAGTCGTTCAAAAGGTGCTAGATTAGTGATT
GAGACCGACATGATGGAATGGGTGTATAAATTTGTGGGTGTCAGTCCTCAATATGTGATT
CCTGATACCACTTCAATTGTTCAGTCAAACTGGCGTAGTGATAATGACAAAGGCATAAAA
CATAGAAGTGGCACAAAGAAAAACTTTCTCAAACAAAACATTGATTTAATATCCACAGCC
GTTTCATCCCCAATTAAAGGAGCTCCGTTGTTTCCAAAAATCGCCTAAatatacatatca
aacgatgttgattatcaaatagatttttaaaacaataaaataaattttttattattga
>DjScaffold2092:386-4111 strand:+
tcctcaaaaaataaattaattccaaatctgccattctaaattccaaaattcaaattaaat
ctaaaaaaacaattagtaattaaaaacttactaaatcatagtagtctcttatgaaattaa
atataattataacctattataaaatattacaagttgaaattcaattttctattgatttaa
ttaatataaatttagtttggttaataataataacaaacgcgagaaaaacaatgatttaaa
agttgaacattcaccggaagaccaaaaattatcattttctctctcaATGATTGCTGGAAT
ATTGAAATCGAAAATATTCAACAATGATTGCATTGTGTTGTATCTAGAGAAATTGATTCA
GTGTTTATTATCTCTACAAATCGTTCCAATCCAATGTCAAATATTGAATATTCTTTCGGA
TTTGTTTATTGGAAGAGTCCTCTTTCCAGAACAAATAGCTCGTTGTCTTACTTCATTATT
TAAATATGTCAATATTTGTTCTGTGAAATCTAATAATAGAAATTCATCGACTCCGGTTCC
ATCGCATTTTATCGGTGGAATTTGGTTAAATACGGTTTTAATGGGTTTCGAAAGTCTGAT
TTTGAATGATACTGAAACATTTGTCGATTCAGAAACAAATAACGTAATGAATTGTCGAAT
TATAGGTTTAAGTCATGCATCAAATCTAATTAAATTCTGTTCTGATTGGTTGTTATGCGA
CGATGAAGATTTCAGGAAAACCTCTTGTGGAGTTTTGAATAAGCTTTGTAGTGGATCTTT
AAATACTAGCGAATCGAAAATTGATCTTTATCTTGATCCATTACAATTTAACGATGCTGA
TAATTGGTTGTGTAAATGGGCAGAATCGATTAGTAGATTATTATTCGTGGAGAATGTTGA
ATTATCAACAAAATTATTGGATAATCTAATTTATCTAATTGTAAGATCAAAGGGAAAATG
TGATAATAACTTCATTAAATTAATTCTACAATGTGTACCGAGAATTTGTGAATTAAGAGA
TTCCTATGAAGATACTGAATCGGAAATTATTACTTCATTAACGAAAAATAAATTGAGAGA
TGTAAAATCGACATTGAGTTCAGAAGAGAAATTGTTCAGAAATTCTGCAGAATCATTTAT
TGGAAATATTATCAAATTCTACGGATTGAATAGATTATTTGAAGTGATATCTTTGATGGA
TATAATGGATTCTGATGATTCTTCAATGGATTTGTTGTCGACTCGTACATGGATTGTACC
TTTGATGAAAGCATATGTACAGAATACATCGTTGGATCAATTTAGAATTTATATTCTTCC
TTGTATTCAATTGATAACGAAAAAGATTGACAGTTTATCAATTGATAATAATAAGCAAAT
GGAAACAGATAATTTCACTAGCGCATCATCTGTTGCTACACTCAGTCTGGGAACATTGCG
ATATCAATTATGGTCATGTTTACCGTCATTTGGGAAACATGTTATTCCAGGTGGTATTGC
TCAAGTAACTAACAATCTCATTGAACCACATCAATATCCTCACCCATTCCCAGTTGGAAT
AGAATATCCCACTGTTACATTATGGGGTCTACTTGTCGATTTGTTGAATCCAAATCAAAC
GAGTAATTTAGAGATAATCGTTCTTACAACGATTAGAAATTTGGTGTTGACTGCACAAGA
TAAAGAAGAAATGAAAACCGTGTTGACATCTAATGTTTTATTTCCGAAAATTCTTCAGAA
TATGTTTAGTTTATATATGAAAAAAGAGAATGGAATCCAAATGAATTTAAAATCAACAAT
GGGTGCTTATTTGCAGTTGTTTGAACCGGAAACATTTGATCCGAATTGTCAATTTTTGAT
CAATAAGAAACTCTCCGATCTGAATATCAGCGAACCAACTGAAGATCAATTGTGTTGTAT
TGAATTACTGATCATTATCGGTCCTCATTTGTCTGAGTCTACAATTACGCAAGTTACAGA
ATTTCTAATTAAATATCTCGACAATAATAACAACAAAGTAAACCATCAAATACAGAAACG
AGTTTATCGATATTTAGAGATCATAACCGCATCAAATACTGTCACCAGTAATGAATATAC
GAAAAATAATTTGAATTTCATCATAGAAAAATTGAAATCCTGGAAAAACGTTAAAACCGA
CTCTTGGAAATGTCGAATGAAAACGTTGTATAATCTCTTCAAATTACTGAATACAACCGA
AGAAATATCGAATTTATTGAATGGATTCCTATCGGAAATTATCGGATGTTTAAATATTCA
AAATAAGAAAACGAGAGCAGTTGCCGGATTGATGTTGGTGGAAGGAATTCTTGCGTGTTA
TAATCAAACTGATGATGAAGTCGGATCGATCAGTAGTTCAATCGATAGAATGTTAAAACA
AATCGTCGCGTGTTTGAAAGAGAAAATCAATGATAACACTCCAGCTGGTATTCAATATAA
AAGTGCATTAGCTAAAGCACTGTATTTGATTCTTAGTGAAGATCGATTGGCTCGTTCTGT
TACTCGTAATCATATCACTGGGATTATGTCGGTGATTGTGGAGATTCTGAAAAATCACGA

AAACACTGAAAATACCGATCGACATTTGATCTATTGTGCATTGGATATTCTCGGAGGTCT
GTTGAAAAAGAAAACGAGTAATTTCCATAATATCTACATGAACGATATCGGTCAATGTTT
ACATGCTGCTGATCATGAGTTGAGAATGAAAATTCGTTTGCCAATAAAGAAAATATTGAA
GAAAATGTTGAAATTGTGGGATTACGAAATCATTAAAAAGTGTATTCCCGAAGAGTTGAT
TGCAGTGTTGAGAAATATTCATAAATCAATGAACCGATCGAAACGAAATAAAACTCAACA
ACAGCAACAGCAAGCGGAAGATCATGAATCAGTTGCGACTAGTTGGGCTGGTTTCAGTGA
TACTGCTAGTCAAAATACAATGAAAAATCCTCACATTAAAAATATTGCAGAACTTCTCCA
TGATTCCGATTCCGACGATGATGAGGACTTGAAAACGAGAATGTCAGCAACAACAATTGG
AAATCGATCAGTGAGATCTACACAATCAACTGCGAATGAGTTGAGAAAAGCTAGAAAAAG
AAAAATGGATGAAGCTAATTCTACTTTGCAATTGGTGGAGAACGCTGACGATCCAGTTGA
TTTATCTGATCCGATAAAATTGGTTCAGAATATGAGAATTGCCGATAGAAAGGATAAAAA
GGTGCGTATAAATGAAGAGATTGTTGTTGTCAAGACAACAGATAAAATCGTCGAAAAGCG
TGGATTTCATTTTCAAGATGGTCGTTTGGTTATTGGAAATGTCAATAAAAATAGCGAATC
GGAAAAGAAAACAGAAGATAATTCTGAGGATGAAAATTATCAGTCGAAGAAGGAAAGATT
CCAGCCACCTAAAAAGAGAGTACGAAAAGAGAAAATTCCTGGAAATGAATACAGATCACA
TAAAAAAGGTGCATTTGGTGATAAAATGGCTAAAAATAATGTTCAACCGTTTGCGTATGT
GCCACTGAAAAGCACCGATCAGTGTCACAGTGTCATGACGAAATATGAAAGAGAAATTAA
GAAAGCTCAAAGTGCTGCAAGAAAAATGTCTAAAGTTCGTAAACAACAAAAACGATAAac
atctaaaattttattga
>DjScaffold4350:5692-9950 strand:+
tgaaacaattacatcatgttgaagatgaagttcaaggaccaagtattgattctgaaaatA
TGGAAGAAAGAATTGAATGTCGCAGGTTAAGAATTAAAGCTAGAATAGAAAATCAAAAGC
GATTAGAACGTGGTGAATTAGATACTGACAAAGACAAACATTTGGAATTAAGTAAGAGTA
GAAAACAGATAGAACTAAGTCGTACTAGAGTAAATAAGCTTAATGATGATGGAATGGAAT
TTTTGACTAATGTTCGCGTCGCAAGTGATGCAAGAGAAAATGCTCGTCGAACGGAAGATG
AAGAATTAGCTAGAGTAAGACGTGAGAAACTAGAAAGTGAAGCCAAAACAGGATTAGATC
GATTTGAAGAGATCACAAAAAAAATGGGAAAGCGCGTTACAAAAAGAAGTGCCTCAAGACT
TGAATGAGATGTTGTTGCAACAGACAGATGCGTGTAATATCATGATTGATGAGAAGAACA
AATTAATTACGGACTTGCAATTGGAATGTAAAGCGAAAGATGATAGTTATGTTAAAGAAT
TAAAGAAACAATCTGATGATATAGATCTTATGATTTTACGGATGAATGAACAAATTGATT
GTATGAGAAAGGCATACAAAGAAGAGTTGGGTGAAATCGAAAAAGCTTTTCTAGTACAAA
GAGATGATTTAATAATTTTGCAAAATAAAGAGTGGAGTGAATTGTTGGATGCAATTAGAA
ATAAACAAGTAGATTATTTAGAAGCTCGTGCAAAACGAGCAGAGGAAAATGATTTGCAAA
TCAGTACATTGAGAGCATCGCATGCTGAAGAGTACAATGCGTTGAAGATCAAATTAGAGA
ATGAAGTGCAAGTGCTTGAACAAAACATACAGAATGCCAAAGCTACATTTCAATTAAACC
TTGAAAAACTCGAGTATAATTTCCAAGTGTTAAAGAAACGAGATGAAGAAAATACAATAA
CGAAATCTCAGCAGAAAAGAAAAATTACAAGAATGCAAGATAATTTGAATAATTTGAAAT
TAAAATTGGTCAAAGTAGAAAAACAATGC
>DjScaffold4350:37408-41151 strand:+
ccattgatttctgaggcggaaaaagtgaaggctaaatctgccactgaggtaatacgtgaa
gttttgaaaaaggaagaaacaatcctgaacATGAATGATGATGAGGGATGTGAGGAAGAT
ATTAATGAACCAATTAAATCACAAACGATTCAATCTCTTAATCCTTCAACCGTTCGTACA
TTGTTGGATTTGATATGTGATGAAGGGTCTTTCCTGATTGAAGAAAAATTAAAGAAATTG
TTGGAGCCTATTGGAGAAAATGAGGCAAGTTTGATGAAGTTGGATGCGATATTTACTGCA
TTAGGAATTGAGGATGAAGAAGACATTAATAGAATGCTACCATATTTTCTAATTCAGTTC
TCCAATGAAGACTCAAAAGTTTCAGAAGATCAAATTAATGATGAGATAGATCATTTAATA
AAACATGAAACTGAAGCCAGGAATATCATCCATCCAATAAAAGTCGTGCAAGCATTGCAC
GATTTTGTAACAGATTTTTATCAAGATGCTGCTGAAATGGAAAAGGCTATTGCTGCTCAA
CAAGCTGATGATTTCCATTTGGATGCAGTCGATACTAGAGACGATAGTCGAGATACAGAG
TATTGGTTGAAATATGAGCATAATCTAATCGACAGCGATAAAGAAATTGTGTGGGATGCA
CTCACAGAGGCCCTGGAGAAATATTTAAATATTTTAACATTTCGGAAAAAGTTGCTGGTA
GATGTTGATGCGTTCAAAACTCAGAATAATGAATTGAAATTCTTATTGCAGCAGTATATG
AATTCATCTGTCAATCGAGATCTGCAGGTTCCTCCATCAAAAGTGATCCAATACAATTGA
taattttctaccattgatttctgaggcggaaaaagtgaaggctaaatctgccactgaggt
aatacgtgaagttttgaaaaaggaagaaacaatcctgaacATGAATGATGATGAGGGATG
TGAGGAAGATATTAATGAACCAATTAAATCACAAACGATTCAATCTCTTAATCCTTCAAC
CGTTCGTACATTGTTGGATTTGATATGTGATGAAGGGTCTTTCCTGATTGAAGAAAAATT

AAAGAAATTGTTGGAGCCTATTGGAGAAAATGAGGCAAGTTTGATGAAGTTGGATGCGAT
ATTTACTGCATTAGGAATTGAGGATGAAGAAGACATTAATAGAATGCTACCATATTTTCT
AATTCAGTTCTCCAATGAAGACTCAAAAGTTTCAGAAGATCAAATTAATGATGAGATAGA
TCATTTAATAAAACATGAAACTGAAGCCAGGAATATCATCCATCCAATAAAAGTCGTGCA
AGCATTGCACGATTTTGTAACAGATTTTTATCAAGATGCTGCTGAAATGGAAAAGGCTAT
TGCTGCTCAACAAGCTGATGATTTCCATTTGGATGCAGTCGATACTAGAGACGATAGTCG
AGATACAGAGTATTGGTTGAAATATGAGTAAgaaggcattgtattttctttttatttgg
ttttgtattgtaattattaaatttatattacatccattagtaatatcatgcatcgatgat
attgttatattttcatgtaaccatcaccatttattctatttagttagagatcaatgcgga
taaatgtatttttgatcgcaccaagtagaggttttaggattcaagatatcagagaatgg
aatccttcctgaaactgaaacaatcgacagattgagagaaatgaaccttcca
>DjScaffold3336:22518-28007 strand:+
ataataatcgtaatcttaaacaattttcacgactggcattagtaagttttaatatgaatt
ttgattgaacaatatttatgttggaaaataaagattcgcaaaaatatttagaaccaaaac
agcatttactaattaacaattactaacaacacaataaaaacaagattttcatatattttc
tagtattctcggtttatacatactcattcttaccttaattcaaaaatcaatatttctact
aggaattagttttgcattcggcaacattttgtcgtacactcccatcgagataataataat
agtggaaatagtccaggaaattataaattgtttgttataaatattaaaatataactttca
ttttggcacgtatgaaattaacgataatttgtaattagaccgtcaataatgaaaacaaat
tggatattctgatccaatttctcaaagataaaagagataagaaagtgctgatcttctttg
ctgcttgtgcatgtgttgattattacaaatcgattttgtctctaatattaaagaaacatg
ttttcttagcattccacaagaaaattaaaaacaaaagagcaagtatttttgatagattca
aaaaaATGAAATCTGGAGTAATGTTGTGTACTGATGTGATGGCAAGAGGTATCGATATCC
CTGATATTGATTGGGTAGTTCAATTTGACCCTCCAAAGTCTGCAACTAATTTCGTACATC
GTTGTGGTAGAACTGCAAGATGTGATCAGTTTGGTCAGACGTTAATGTTTCTGAGATCTA
CTGAAGATGCTTATCTTCATTTTCTTCGTGAAAATCAAAATGTTGAAATGCAAGAATACA
CTCCTGATTTCAACCCAGACGAAGTCGAATCTTATGAAGTTCGTATGCAAGATTTTTGTA
TTTCAGATCGTCTGTTATATGAAAAGAGTGTTAATGCGTATATTGCATATGTAATGTTCT
ATAAGAAGCATGAATTGAAATATATCTTCAAATTAACAGATGTGAATATGGGCAAACTCG
GTAATAGTTTCGGTTTATTGAGAATACCTCAAATGTGCGAATTGAAAGATATCGATTTAT
CAGATTTCCATAAAAGAGATGTCGATTTGAAATCACTAATTTACAAAGATAAAAGTGTGA
GAAATATCCGAGAAAAAACTAGAGTTATAAAACAACAAAGACGAGAAACTGAAGCTATGC
AAGTTGATAATAATGAGGATGAAGTCGAAGTTGAAAAACCTGTTAAAAAAGTTAATAAAA
AAAATAAGAAAATTGAGAATGTCAAGTCTGATAGCAAAGAGAACAATCGTAAAACGATGA
AACGAAACAAACCGATGCAAGACACAAAGAAATCACAATCAAAAAAGGCAAAATTGAATG
CAGACCCAGTCGAACAACAAGAGAAAATGGAAGTTGATGAAGAGATATCGAAACCAGTTG
AGAAAACTAAGAAAAAACCAATAGTAGTTGAGGGAAAAGGAATTTTAGCAAGCTTCAATA
AAAAAAATAAATTGTGAataaatttata
>DjScaffold54:27244-27676 strand:+
tagattcctgaaatcgagacaattagtcattctgatgacctcagcatcgattcaacattg
gaaattgatcccttagaaaaagattcaatagaagcatccaaaATGCTTTTGCGACTGGGA
ATAACAGATAAACATATCGAAGTAGCCCAAGCTCAAACAGTTAGAAACTATGTGACAGGT
ACATATCGAATAATGTTACATCGGGTTCATCGAAATCGTCGGATGAGTAATATTCCTCAT
AGTATTAGTAATCCGGAATTGGGTCGGAAGTTTTTCAATAATCGTAAATTTCAAAACAAA
GGAATTACAAGAAAAGTGAATAGTGCAGCACCAAAATCTAAAACTTCAAGTTGTCCTGAT
AAAGGAACAGAACCCAATCAAAAA
>DjScaffold1196:49536-50162 strand:+
AGTTCTTTGGATAAAGTCATTCCTTCTAAATTCCAACCATTTTGGCAACACCCTGCAGGA
CCGAAAACCATTTTCTTTTGGGCTCCGACTTTTAAATGGTCACTAGTTATTGCTGGCTTA
GCAGATATTGCTAGACCAGCGGATAAATTGAGTTTATCACAAAGCAGTGCTTTAGCTGCT
ACTGGTCTAATATGGTCCCGATACTCAATGGTCATTATTCCAAAAAACTATAATTTGTTT
AGTGTGAATATTTTCGTTGCATCTACCGGGTTATTTCAAATTGCAAGAATTTTTAAATAC
CGCCAATCATTGGAAAAAGTAGAGTAAaccaataattagaaaccaataattcttttgcct
atttaaattgaatttttatgttttgtagtattttcaatgattatcaaaagtttaattgat
cgattatttctatccaaatttgattggctagattctatgcttgctgtgagaaatgtctat
tta
>DjScaffold1196:98875-106810 strand:+

ATCGCATTTAATGAAATACTGAAATTGGTAAAGGTTTATCAGCCTCTATTAACAGATATT
AAACAAGAATATGAAAGCCTTACAAATCATGTGATAAAAGCACAAAAATATACAGAGCAA
ATGGAAGAAGAAATCAAAGTGGCCGCTTCTTACCCAACTACTATTGAAAATCATGAAAGA
TATTGTAATGAATTAAGAGAAAAGCTCCAAACGCTAAGGAAAGCGAATAATCATCGTTTA
GAAAAGATAAGAGATGTTCAAATGAAATGTGACACGATTTCTAAATCGATTAATAAAGAA
GAAAATAAACCCAAAGACGAGAAACTTAAAATACGTCGCGATATCCGTCGATTACCAAAC
ATGAATTTGACAGATTTGACTGACTTAGATACCATGCAAAATTTCTATACACAATTAGAA
AATCAATATAAACAATTGAAACATACTCGCGATATCAAATATGTTCCAAAGGAAGAAGAG
ACCAAATTAAACTATCAACTGATGGAACAAATAAATAAACACGATCAATTGAGAATCGAT
GTTAAGAATTTTTTACAAAAGAAAATTTTGTTACAATCTGCACTGAAACAATCTGAACTA
TTTCAAATGAATCATCATATACATGAGAATCTTGGTGAAGTTCTTGTTAGAGCACTCGTT
AACAGTTCTAAAATTGTTGAAAAAGAAATTTCTAACTCTGGTAATATGTCTATTGAAAAT
GAGATAGACAAATTTTTCCCGGATGACAAACGACAAATAAGTTCAATAAAAGAGTCTGAT
TATATAGTTTTGAGTGTTCGAAAATTCGAGCAATTGTTTAATGAAGGTAAATATAAAGAA
TCGATTGATTTGGGTGTAGTGGTTCCCAGAGGAGTGCTGAGAAACTATGCTACGCTTTGT
AAATTT
>DjScaffold1196:117292-118123 strand:+
atcgctgacaatctgacaagtcatgctgatttaATGCCCGATAATAAGATCGCTTGTCTA
GAGCTTGCGACGTCAATTTATTTGGATCTAATGAACAATTTAACCAATAATAATCAACCA
GACAGTTCTAGACTTCATTCTTATCGACAATCAAATCCCAAATGGGTTTCTACTCGTGGC
AGATATTTTGATAGCGTTTTAGAATGTCAAATTAAATCTGGTAAAATCGAAGAAGTTATC
GATATGTTGACCAATGAAAATTCAACTCCAATCTCAGCAAGGTCACGTAAACAAATGTAT
AAAATCGAAGATTTGGTTTTGTTGATTCAAATTTACCCGAGTTTTGAGTTAGTACGCAGT
TTAATTGGCAAAAATCTTATGTCGTTTCCATTGGCAATGAAATCATTATTAGAGACAGAA
TCTTATCAATCGGCTGTTCAACTCATGATTCATAGCTCATCCTCTATCAGTATTTCCGAT
ATGACAGAACAATTAATTGAATCTATTCCATCGGGAATCAGTACAGATGTTTGGGAGGAA
ATGTTAAACTTCGTTAATGATTCAGACGAAAAGATTATTATTGAATTGGCGGCTTCTTAT
CTAGTTCAAATTTCAATAGAAAAATCATTTCAAAGTTTATTAGAATATGGAGTAACAGAT
TGAttttattttaacctttttttaaaaaaaaaaattatgacgatatttttgtatttcaaaa
tttattgtataaatacaaatttttcatctttatttaatttattaaaagtacacataaa
>DjScaffold1196:129905-130744 strand:+
ATGTTAGCTCGTATGATTTTTACTTCAAAATTCTTTTCATTCATGATGAATCCTTATACA
ATTATTGATTTTCTCTCACTAATACCATTTTATTATAATTTAATCGATACTGTTTCACAT
AGTAAACGATCGATTAATGTTGCATTTTTCAGCCAATCAAATGATTTAATTAGATTTTTG
AGTTTGAGTATTGGTTTACGTTGTTTTAGAATTTTACGCCATTACCGAGGACTTCAAGTA
CTAATCTATACAATCAAAGTAAGCTCGTTTGATCTTCTTTTAATGATGGTATTCATTTCT
TTGGCTACGATAATATTTTCTACGCTCATGTTTTACGCGGAAAATAAAACAAACCCAAAA
TTTACAAATATTCCCCGAGCATTTTGGTGGTGTATTGTGACGATTACAACCACTGGATAT
GGTGATATGGTACCAATAACTGGATACGGTTATCTTATTGCTGGGTTTTGTACTCTCTGC
GGGGTTCTTGTTATTGCTTTTACCGTACCAATTCTAGTTAATAATTTTATATTGTATTAC
ACTCATGCATCACAAAATCGAAAGAGTGGGTTATACGAATACGCACGGAAGCAAATCGAA
GAACATTTAAAGGATGCAGTTTATCAGGTGAAAAAGTTGAAAGAAATCACGAAAGCGTAT
CCAAAAAAGTTTCTAAAAATTTATGATGGCTATCAAAATAGCCAAGATGAAAATACTTGT
TCTTATACTTTGGAAAAACTTGATACTGCATCGTCTCTTAGTGGATGTTCACCTTCAATT
TACTGTAGTGGTGCTAGTTTTCATTATGCAAATAACGGATTTGAAATTTCGAGATTTTAA
>DjScaffold1318:138995-154677 strand:+
ATGATGTTTGCAGGAATGATGGGGATAATTATTAAAGGAAGCGTTGATGCTGGTGGATTC
GGGAATGTGATGAATACCGCTTGGGAACATGAGAGGATGAAAATAAATTTTTCGTTTGAT
CCTCGAATGCGTCATTCAATGTGGGGCGTGATTATTGGTGGAGGATTTCTTTGGATGGGA
ATTTACGCCATTAATCAAACGATGATTCAGAGAACAATTTGTGTGAAATCACTGAAACAG
GCTTATTTGGCATATTTACTCAATCTTCCTGGAATGAACTTATTATTGATTGTAGTAGTT
CTTTGTGGATTTGTTATTTTTCACACATTCTATAGATGTGATCCGATTATTGTTGGTGAC
ATTACGAAATCTGATCAACTTGTTCCCTATTATGTATCGAAATTTCTATTGAAATATCCA
GGCGTCGGTGGTTTATTTGTTGCTGGAATTTTCAGCGGTGGATTAAGTACAATGTCATCA
GGTCTGAATTCATTAGCTGCTGTTACTTTAAATGATGTTATAATGGTAAAGTTTCCAAAA
ATTAACGACAGAACGGCTTCTATTATATCCAAAGTTCTAGTAATAGTTTACGGAATGTTA
ACTTTGCTGTTTGCATTGTTGAGTTCGCTGACTTCTAAAATTCTGGAGGCTGCACTTGGA

TTATTTGGAATGTTATCTGGGCCTACTCTCGGAGTGTTTAGTTTGGGTTTATTTTTTCCT
TTTACCAACGCTAAAGGAGCCATTTCTGGACATATAGTAGCTCAGATAATTATTTTTGG
ATTGGATTCGGGGATATGCCCGAGGAATTAAGCATCCCCGATTAACCTTGAGTAATTTT
AATTGCTCCGGGAAAGGAAATATTAGCATTCCAACTAGTTATGATATCATATCATTTAAT
GGACGAATTCAAGGAATCGAGAATCTCTATGCGGTTTCCTATATATGGTATACTTTTATT
AGCTGGGTAGTCGTTTTATTGATCGGTATACCAGTCAGTCTTTTAACAAGATCGTCAACA
GATCGCAAAGTTGAATTTCAATATATTTCTCCGCCATTTCAAAAACTATGGAAAAACTAC
CTAGTTGAAAATACTCAAATGGAAAGCATGTTTGAATCAACACATAAATCAACAATAGTA
ATTTTTAAAAGCTACTTGTTAAACTACTAA
>DjScaffold2386:16321-16992 strand:+
GATAAATATATAACAAAAATTATACTTATTGGAAATGTTTCAACAGGGAAAAGCACATTA
ATTAATGCATTACTTGGCAGCGTTTATCTTGGTGCACAAGAAGGACATGGAACTACGTCT
TTCTATTACTGTTCTTTATCAGAAAAGGACTATGAATATTTTACTATGAATACTAGTGAT
TTTGAAAATAAAGAGATATCTGCTTTGAAAAATGCATCATTTTTCAGTGAGAATCGAAGT
GATGATATTAATCAGAACACTGTTATCAAGCTTTACATAAAAAATGATGAAATTAAAATA
TTGCAAAGAAAAACAGTTATTGTAGATTGTCCTGGATTTAAACAAGAAAAAGAATATGAC
GAAGAAATTGAGAAACATTGCTCTAATGCAAATTTATTTATGTTTATGTTTCAAATAAAT
AAACCAGCTACAGAAAAGCAAATAGAATATATCAAGGAAATTAGACAGAAATATAACAAC
CCAACATGTTTCTTTGTTTATAACAAATGCGATCTTCTTGGATTTGATGTA
>DjScaffold1455:14470-14991 strand:+
aataagtaaatttatttgcttgtcgaacagttgtataaaagctttgattaggatttgttt
tgattttctctatatttttagatttttcattaagtagaatacttattttcttttgtttat
tgagataacttatttcattatttccttctttttaactaATGAATCAGTTCCTTGGCCATT
GTTTATTATTTTCGTTGTTCGGATTACCGCTTTTTGACTTTCTTGGCTTTTTTATTTCCT
CGCTATCTCCTGCAGTAGTTCTAGTTCTTTCGTCCTTCGCACGAATTTTGGCCTTCTTAT
CCAATTTTGGCTTTCGTTATTCTTCTCTTCAACTAGATTATGCCCTTCTTGGGTCTGATT
TCTTCATTTTCTTTCTTAGATAGatttttcgaatttcgtgtttacttaggccattttatt
tgccctaatctggtgtttcaccccaacacatatatttcttcattaatacacttaggatca
cataaat
>DjScaffold3291:4065-4648 strand:+
TTTTTGGCAAGTGTGTTAAGCTTTGTAATAGGATTCGGAAAACCACCGAAAGCTAATGTT
AGAGAAAAGGTTGTACCACAACCGGATATAACTAAAGTTAACATTATTTTTATGAATCAT
TTAGATGTTGGTTACAGTGATCATTTAGTTGGATTAACAGGATTTTCAGCTAATGTTTTG
AATACATATTTTAATGTTCATTTTCCTAAAGCAATATCGTTGGCTAAGCAATTGCAATCT
CAAGGCTTTGTTGAAACATTTCGATATACTACTCATCCATGGTTGCTATCTTTTTATCTT
AATTGTCCTCAAAATATTGTATTAGCAAATATTAAAATTCAAGTAAGAATTTTTATAATT
ATTAAAACTAAATTTTTTTAGaaataaaggatattaactaatattaggttttataataaa
taaattaaattataagtattgtttcataatatttctaagttaacatgtttactttacgtt
atttataaactgttt
>DjScaffold3291:17288-17692 strand:+
ctaatcttgatcaacaatttggaATGAATAAACCTGTTAGAGTAGTCTCCTTGCGAGATG
TTCCAGGTCTAACTCGTGGAGTTATTCGAACCTTTCTAAAAAACAATGTTGTTGGTATTA
CTAGTGGTGTAAATTCAGGATCTGCGCCTCCAAATGTAACTAAATTATGCAGATGGCGAG
CGGCACGAAAATCATTTAATGAGAAAAACGTTCCAGAAATCATTTATATGCAAAATCCAT
GGGGTTACCCTGACAACGCTGGTCCTAATCCCAATAAT
>DjScaffold3786:23672-26678 strand:+
attttaaataaatattatattttctaaaattaaaaatatggtacagttgaaatatttact
ccaaggaaatatttcgacaagaaattattcaataaaataatttcttttaaaaaccgcaat
aatcggttttgagttcaactatactttttgcaatcactagtgcatatgctggattttaat
tagtaaaaaaagattaatacaaatcgccaacatgttctaccttcgttttccaaatttgatt
gtaaaattcctcgtttcatctaattgacagtaaattctggaaatcgttaggttgaaaaca
tatattttgagatgtgacaattataatacgatataatttggtgaaattttcaATGGAATT
ACTTGAAATTGTTTTTATTAGAATAATTGTGCCTCAAGATATGATAAATAAGGATTATTC
GATATGTTTCGATAATTATCAATCAAGTAGAGATACGAAAAGAGTTTATATGATGATTGA
TGTGTCTAATTCTAATATTATAAGTACTCCGGAATATGCTCAATTGAATAAATTAGATCT
AATGCATACATCACTGACAAACATTTATACCGCATTAAATCAAATGGAACATTTTCAAGA
GAAACAGCGGGCAATAGAAGCGGTTGATCGAAGTATTATGGAGAGAAATTATGAATATGT

GACAATGTGGTGTATTATACAAATAACAGTTATGATTCTCGTATCTGGTATTCAGTTAAT
ATTGATACGAAGTCTTTTTGATTCCAAAGGTGGTATTGGAGCTACTATTTGGAGGAAACT
CCATGATTTAACTGGTGGAAGATTTTAAaatcgaaaccaagccaaattatcaaatgtgca
gttacgtcattaatgaatttaatcaccataaccttttcc
>DjScaffold4991:63860-64147 strand:+
ATGCCATTTGGGCTTACCAATGCCCCAGCTTCATTTCAACGAATGATGAATTACGTGACA
CACGGCATATCTCAATGTATGGCCTATATCGATGATCTATTAATCTACTCACCAACATTC
GAAGGACATCTTAATGACTTACGAAGTGTGTTCGTGAGATTGCGTAAGTGGAATTTAAAA
GTGAAACCTACCAAATGCGAATGGGCCAAGAATAAGGTGACATATTTAGGCCATCTAATA
TCAGATAAAGGCAAGGAACCAGANTTCATAAGGAATCCGGTTATGTAG
>DjScaffold4263:25356-31233 strand:+
gaataattataaaaatgatttcattagaattatgttacagttaaattttcacttaaatta
cttatatttattttcttgttttaaagtagtaaaaccactgaaaattgaaatgtgtgatga
tgaaaaattgttagttaatattttattatctaagtctttttcatttataaatactgtta
tttatttaaagaaatgttttttatagtgatttactattaaaaattgtgattattggggat
agtggagttggtaaatcatcattactgATGAGATTTGTGGACGACACATTTCAAGAGAGT
TATACTGCAACATTAGGTGTGGACTTTAAATTGAGAACTGTCAAAGTAAATAATTTAATA
GTAAAAGTGCAAATTTGGGACACAGCAGGAACTGAAAAATATGCATCATTAACTTCAGCA
TATTATCGCGGGTGTCATGGAGTAATAATAGTATATAATTTAGTAGATGAATTAACATTT
GAAAATATTTCGCAATGGATTAAAGAAATCGATAGATCTGCTGGAATTCGAAATAAACGA
TTACTTGTAGGTAACAAATTGGATCTTGTTAATAAACGTAAAGTAACCACATCAATAGCT
CAGGAATTTGCTGTATCTAAAGGTATGCAATATATTGAAACCAGTGCTAAAACTTCAGAA
AATGTTGAAGAAGCTTTTCTTTCATTAGTTGATAATATAATTACATTTTCTCCGCACAGT
ATTCAACAGAATCGCGATATAATTAATTTGGAAAATCAGTTGACAAGAAATTCATCAAAA
AATTTTTGCTGTTGAtcaaaaatttttgaaaatttattttatttaaatcgaacaaattta
cgtctttaaattattttgagcaaattttaaattaattttagatttaa
>DjScaffold2637:3709-4771 strand:+
tagagaaaaggttattgatATGTCAGATCCATTTTATACCAGTGATATTGAAATATTAAG
CATGATAAAAGATCGAGATAGTTTGTTAGGTGCTGTTTATAAGCCTTTTACATTAGAATT
ATGGTTGTTCATATGCGTAATAGCGACTACATGCGCCGTTGCAATGGGCATATTTGAATG
GAATAGTCCATTTGGATTGAGTCCTTGGGGTCGGCAGCGACATAAAAATTATACATTAGC
TTCAGCTTTAAATGCAGTTTATGCAGTTATGTTTGGCCATACAATAAAAACAAAAGCTCC
AAAATCGTGGCCAGTAAAATGGGTTCAAAATTTTTGGGCATTTGCTTGTATTGTAATTGT
TGCTATGTATACAGCTAATTTAACAGTATTCATCGCTGGTGTAGCTCATCTAACAAACAT
TAACAGTGAATTAAAAGTAGGGAATACTCAAATTGGTATTATCAAAGACTCCAATATAAT
ACAAACATTAATAGATTTGGATGATAAAAGATATCCTGTTGTCAAATGGAACGTTCGTTA
TTTAGATAATTCAAAAGATGCTGAAGAATTGTTTCTGAACAAAACATTAGGTATAATTTT
GGATGAAAGTTTAGTTGTCAGAAATCTTAAAGCAAAAGTATCTCTATGTGGATTAAATAC
TCGAACAATAAATATAGGTCCTATCGGTTATGGATTTGGATTCTCAAAAGACTCTTGGAT
AAAAGTTATTATTTTAATTGTTAAATGTTTTTCTCGTATGTTTTGAaatttaaaaatctt
aaaaggcaaattaatttaaatttaagt
>DjScaffold2637:57280-58437 strand:+
aattttagaaATGTCGTGGATTAATGAAAACTCAAATTACACGTATTTACAAAAATTATC
CATGTTCTGTCTTAAAAAAGGATCTATTCCTAAGCATGTTGCAATTATTATGGATGGCAA
TAGGCGGTTTGCCCAAAAAAAATTAATAAATAAAATTGAGGGTCACAGATTTGGATTTGA
AAAATTGGCAGAAACTCTTACATGGTGTCGTGATTTGAATATACACGAAGTATCTGTTTT
TGCTTTTTCCATTCATAATTTCAATAGAGAAAATGAAGAAGTCACTTATTTAATGAACAT
GGTAAGAGAAAAAATTTCAACGATTAATTGATGAAATGAGCCTTTTAGAGAAATATGATGT
TTGTGTGAGAATTATTGGTAATTTAAGCCTTCTTCCAGAAGATATTCAGAGCATTGCGAA
AAATGTTATCAAGTCAACAACTTCACACAAAAAATTTTATTTAAATATTTGCATGGCATA
TACGAGCCATGATGAAATTAAACATTCTCTCAATCATATAAAATGGGGAATTACTAATAA
ATTAATTACTGAAGAAGATATAACTGAAGACTTAATCAATAAGTGCATGTATATGCGAAA
TTCAAGTAAAGTAGACTTGATGATTAGAACGTCTGGAGAAGCTCGTTTAAGTGACTTTTT
GGTATGGCAATCAGATAGGGCTTGTTTTCGATTTTTCAGTACTTACTGGCCTGAATTTAG
TATTTGGAATCTTTTTTCAGCTGTTTTAAAGTACCAAACTAATTATCTATTAAACGGAAA
TTTAAAACATATCAAAAAATTCAGTAAGTTCTGATGATTTCATTACAGATGCCAATAATAA
ACGGGTCGAGCATTTTTTACTTCAATTAGAAAAAAAATTCAATGATTGGCTTTATTGTGA

CGGAAATGATTTTTTCATAAAATAAaatttttacttgtttaaattttagaaATGTCGTGG
ATTAATGAAAACTCAAATTACACGTATTTACAAAAATTATCCATGTTCTGTCTTAAAAAA
GGATCTATTCCTAAGCATGTTGCAATTATTATGGATGGCAATAGGCGGTTTGCCCAAAAA
AAATTAATAAATAAAATTGAGGGTCACAGATTTGGATTTGAAAAATTGGCAGAAACTCTT
ACATGGTGTCGTGATTTGAATATACACGAAGTATCTGTTTTTGCTTTTTCCATTCATAAT
TTCAATAGAGAAAATGAAGAAGTCACTTATTTAATGAACATGGTAAGAGAAAAATTTCAA
CGATTAATTGATGAAATGAGCCTTTTAGAGAAATATGATGTTTGTGTGAGAATTATTGGT
AATTTAAGCCTTCTTCCAGAAGATATTCAGAGCATTGCGAAAAATGTTATCAAGTCAACA
ACTTCACACAAAAAGATTTTATTTAAATATTTGCATGGCATATACGAGCCATGAtgaaat
taaacattctctcaatcatataaaatggggaattactaataaattaattactgaagaaga
tataactgaagacttaatcaataagtgcatgtatatgcgaaattcaagtaaagtagactt
gatgattagaacgtctggagaagctcgtttaagtgactttttggtatggcaatcagatag
ggcttgttttcgatttttcagtacttactggcctgaatttagtatttggaatctttttc
agctgttttaaagtaccaaactaattatctattagtaagtagtatcgtaatttggttaaa
tttaaattttctgaaattaaattgtattggtttatttttaaaacaagaggttataataga
aagggacctcgactttcttgcataaaaaattcttgaaaatatgttagacaatattatatag
acaatattcattcatattcaatatatatgcgtacaatattcactcatagaaccaataaat
gcccatttaaattttagaaATGTCGTGGATTAATGAAAACTCAAATTACACGTATTTACA
AAAATTATCCATGTTCTGTCTTAAAAAAGGATCTATTCCTAAGCATGTTGCAATTATTAT
GGATGGCAATAGGCGGTTTGCCCAAAAAAAATTAATAAATAAAATTGAGGGTCACAGATT
TGGATTTGAAAAATTGGCAGAAACTCTTACATGGTGTCGTGATTTGAATATACACGAAGT
ATCTGTTTTTGCTTTTTCCATTCATAATTTCAATAGAGAAAATGAAGAAGTCACTTATTT
AATGAACATGGTAAGAGAAAAATTTCAACGATTAATTGATGAAATGAGCCTTTTAGAGAA
ATATGATGTTTGTGTGAGAATTATTGGTAATTTAAGCCTTCTTCCAGAAGATATTCAGAG
CATTGCGAAAAATGTTATCAAGTCAACAACTTCACACAAAAAGCAATATTTAATTTAAtt
atctgattaattttgaatttcagattttatttaaatatttgcatggcatatacgagccat
gatgaaattaaacattctctcaatcatataaaatggggaattactaataaattaattact
gaagaagatataactgaagacttaatcaataagtgcatgtatatgcgaaattcaagtaaa
gtagacttgatgattagaacgtctggagaagctcgtttaagtgactttttggtatggcaa
tcagatagggcttgttttcgatttttcagtacttactggcctgaatttagtatttggaat
ctttttcagctgttttaaagtaccaaactaattatctattagtaagtagtatcgtaatt
tggttaaatttaaattttctgaaattaaattgtattggtttatttttaaaacaagaggtt
ataatagaaagggacctcgactttcttgcataaaaaattcttgaaaatatgttagacaata
ttatatagacaatattcattcatattcaatatatatgcgtacaatattcactcatagaac
caataaatgcccattta
>DjScaffold2637:68217-70884 strand:+
ttgataacttcaaatctaattgacactcatgtaaatatcccattcaaatttgtattcttt
ttcgtaagatcaatgatgttagagagatcaatatcatcagttttagacatgcgccatgct
ataaagaataaatttattaaattcataatttttaaataaatcttatttcaggaagaaaa
ttctatataATGGACATAGTTTCACCAAAATCCAATACAAGTACTCTTAATACGTTGATC
ACGTATTCAAATTGTCCAAATATTGATATACAATCTAATGATATATCAGTTAATTTGACC
AATTCTCAAAACTTTCATGAGTTAGACCATAAAAACAACTATGGCTACAAAGTTTCAAGT
AATGATAACAAGTTAAATCATCTACCATATCTAGCTCAAACAAATTTGACAGAATGTATG
GGTGACATAACAAATGATATTAAGAATCAAAATTCACATCTTAACTATGGAAACGGAATA
TCATTCACCGAATTTTCTAAAATGTCTGAAAGTAATATTCCTGGTTATTCCCCGTATTGT
TTTTCAACTAAATTATTTCCTATTCCAAATCTACAAGAAAAACCACTAATAAATGACAAC
TATGCTGTAAAATCTACGGGAGCTTTTATTGCAGAATCTAATGAGCATCAAAACACTGTA
AATACCGGTACTTCTTATAATTCAAATTTAACCTCATACAGAACCGTTATACCAGCTCAT
TTTCATCTATTTAATCAGAATTCACCCGAATCAACGTCATTTTATAATACCTCTTGGAAT
AATTATCCATTCCAAATATCCCCAACTTATCCTATACAAAATTTACCAGATTCTACAATA
AACTGGACGCAAAACAGTCAAATTACTAACAACAATTTGGTTTTTAATCCCTGGAGCTGC
AAATCCAATCTTAACCTTCCTCCTATTAATGAATTTGGTTCAAATTGCGATGTCAGCAGA
GAGTGTGTTAATTGTGGAGCTAGTAATACTCAGTTGTGGTCTCGAGACAATACTGGTTAT
TATTTATGCGACGAATGCGATAGGTTCTCCCAAAATAATAGTAGAAACATTGAAAAAATA
CCCGCAAATCAAGTTACATCAAGTACCGAAAATGAATTCATGAAAAAGTCTAATGCAAGT
TTTCAGCAATATGGAAAACGATCAGATCTCGAATGCAGTAACTGTAAAATTACTAAAACA
TCTTTATGGAGAAGAAATAATGAAGGAGAACCAGTTTGTAACGCTTGCGGCTTATATTAC

AAATTACATAAGTCTCTGAGACCTCTTTCAATGAGAAAAGAAGGAATCCAAACTAGAAAA
AGGAAACGGAAAACACAATTGAAACCGGTCTCAGGTCATATTGCTAATAATCAGAAAGTC
CCTTCTTCAGAATCATCATCAGAACAAAATCAGTTTATCAATGCTGTAAAATCTTCCGAA
AATTGTTATAACATGATTTCTGAAAATTCTCCAACATATATGCCAAATATATCAATCCAA
CCTTACAATCGAATTTTAAATTCAAATCAATATCAATTTCAATCAAGTGAAAATCCAATA
AATCCATCGTATTTAAATTCTTCACTCAAGAATAACAATCCTTTCTATGATTCTGAAAGT
ATACTTTATTCGTACAACTCTTCAAATAAAATTAAAGACGAACCCTTATCCATGGATAAT
GAGCAAAAGCCATATTCAACTATTCCCGATAAAATCGATGTTAATTCTATAGTGAGAATT
AATGAAATCTCAAACAATTCGAATTCGATTTTGCCAGGTAAAGAAATAACGAATAATAAT
GTGGAAGTATGGCATTCTAAACTTCCAAGAATTACTTATACTAAATATCCATCAATTTGG
AAGTTCTTAGAATATATTCAAAGGGATGATAACGATAATGACAATTTAATAAATTAGtta
tctagtggtcatcgtacgataaagtatccaagaaaaagtatccaatttgtgataaaagag
ttgaaacaatagttgaaatggataatgagtataaaactaataacgagattaaaagatatt
cgttaaacattggtaggctgttgaattataatttttcatttaatcaataaagttgtactt
ttaattaattttgtgcatttttcttgttttttaccaaatattttataccttttgtttgt
gggttggaatattatgatacccaggcccggtcctagagccatggctcggggcacacggta
aattggggcgccaaatatatacaaaaaaatttaatcaatccttcacaagatattatggca
ccatgagacttaaaccttttttatagtaaattaaagtatttttggacggtgcataaacatt
gtaagatagtaagaggtttaaggatctaaaatatggtatgtacatttgataacttcaaat
ctaattgacactcatgtaaatatcccattcaaatttgtattctttttcgtaagatcaatg
atgttagagagatcaatatcatcagttttagacatgcgccatgctataaagaataaattt
attaaattcataattttttaaataaatcttatttcaggaagaaaattctatataATGGAC
ATAGTTTCACCAAAATCCAATACAAGTACTCTTAATACGTTGATCACGTATTCAAATTGT
CCAAATATTGATATACAATCTAATGATATATCAGTTAATTTGACCAATTCTCAAAACTTT
CATGAGTTAGACCATAAAAACAACTATGGCTACAAAGTTTCAAGTAATGATAACAAGTTA
AATCATCTACCATATCTAGCTCAAACAAATTTGACAGAATGTATGGGTGACATAACAAAT
GATATTAAGAATCAAAATTCACATCTTAACTATGGAAACGGAATATCATTCACCGAATTT
TCTAAAATGTCTGAAAGTAATATTCCTGGTTATTCCCCGTATTGTTTTTCAACTAAATTA
TTTCCTATTCCAAATCTACAAGAAAAACCACTAATAAATGACAACTATGCTGTAAAATCT
ACGGGAGCTTTTATTGCAGAATCTAATGAGCATCAAAACACTGTAAATACCGGTACTTCT
TATAATTCAAATTTAACCTCATACAGAACCGTTATACCAGCTCATTTTCATCTATTTAAT
CAGAATTCACCCGAATCAACGTCATTTTATAATACCTCTTGGAATAATTATCCATTCCAA
ATATCCCCAACTTATCCTATACAAAATTTACCAGATTCTACAATAAACTGGACGCAAAAC
AGTCAAATTACTAACAACAATTTGGTTTTTAATCCCTGGAGCTGCAAATCCAATCTTAAC
CTTCCTCCTATTAATGAATTTGGTTCAAATTGCGATGTCAGCAGAGAGTGTGTTAATTGT
GGAGCTAGTAATACTCAGTTGTGGTCTCGAGACAATACTGGTTATTATTTATGCGACGAA
TGCGATAGGTTCTCCCAAAATAATAGTAGAAACATTGAAAAAATACCCGCAAATCAAGTT
ACATCAAGTACCGAAAATGAATTCATGAAAAAGTCTAATGCAAGTTTTCAGCAATATGGA
AAACGATCAGATCTCGAATGCAGTAACTGTAAAATTACTAAAACATCTTTATGGAGAAGA
AATAATGAAGGAGAACCAGTTTGTAACGCTTGCGGCTTATATTACAAATTACATAAGTCT
CTGAGACCTCTTTCAATGAGAAAAGAAGGAATCCAAACTAGAAAAAGGAAACGGAAAACA
CAATTGAAACCGGTCTCAGGTCATATTGCTAATAATCAGAAAGTCCCTTCTTCAGAATCA
TCATCAGAACAAAATCAGTTTATCAATGCTGTAAAATCTTCCGAAAATTGTTATAACATG
ATTTCTGAAAATTCTCCAACATATATGCCAAATATATCAATCCAACCTTACAATCGAATT
TTAAATTCAAATCAATATCAATTTCAATCAAGTGAAAATCCAATAAATCCATCGTATTTA
AATTCTTCACTCAAGAATAACAATCCTTTCTATGATTCTGAAAGTATACTTTATTCGTAC
AACTCTTCAAATAAAATTAAAGACGAACCCTTATCCATGGATAATGAGCAAAAGCCATAT
TCAACTATTCCCGATAAAATCGATGTTAATTCTATAGTGAGAATTAATGAAATCTCAAAC
AATTCGAATTCGAGTTAAttattctgatctactttttaattctattaaaaaaacacttct
aaatataattttaatgataaaattttatatctaaaaaatgtaatttttaagtgcgaatta
ttaaagtatgcatttaaatagtgcaattattgtggtagttatttttattatttaataaat
gttgtttgtaaatttcaattgtacaaaataaaataaa
>DjScaffold3601:10017-10830 strand:+
tatgaattatatttattcagtgcaatcaacaaattcacaattaaaataaaaATGAGAGGA
AAGGATATGAATAATTTCACCACATTCAATCCAGCAACAATACTCCAAGCAAAACAATCA
ATTTCAGCAGTATATAATGTGGACATGAAACATTTGTATTGCATCAAGCCAGGTGGAGAT
CACAAACCAAATGATGAAGACAAATTTGATGATGAATTTATCATTGAAATTGTATTAAAC

ATGGAAGATATTGAGATATTAAAATTGCTAGGACGAGGAGGATTTGGTGAAATTCATCTT
TGTTATGATAAAATATCCAACAAACAATTTGTTTTGAAGAAAGCAATTGAAAGTTATGGA
GATCTCCATCGAGATATAAATATTTATTCTAAATTCAATTCATCAATAAATGTCATGAAT
TATTGTGGATTATTTATAAATGGAGAAAACATTTGCATTGGCATGGAATACATGTGTGGG
GGATCTCTCAGATCTTATTTGGACAAAGGAAAATGTTTATCTGAACTTGAAGTAGTGAAA
ATTTCATATGAAATCTTGGTACAAATATATGTGTTACATAAATTAGGAATTATCCATCGA
GATATAAAATTTGATAATATTGTTCTCGATGAATTCAAACATCCAAAGTTAATTGATTTT
GGATTATCAAAATATACAATCGGATTAACAAGTAAAGATCTATTAGATAAATTAGAAGCG
TATAAGCCTGGGACAATTTGTTACATGGCACCAGAATTGATAAGAGGAACACCATATTCA
GAAAAAATCGATATTTGGTCATTTGGAATGGTGATGGTTGAGATGGTGACAGGTAAAAGA
CCATTTCATTGGCTGGGACCATTGCAAGTAGTTATTAAAATTCGAAATTGTATAAAATAC
GACTTTGAACCAATTTTTCCAATTTCAAATGATTTTAATCGATTTATCAGTTCTTGTTTG
AATACGAATCCAGATGAGAGACCAAGTGCAGAACAGTTGATGAAAATAAATCAATTCAAA
ATTATTAATTATAAATAAgttaaataaaattgaaacttgcctaattaaaaatttatatat
tcagcATGGAAGATATTGAGATATTAAAATTGCTAGGACGAGGAGGATTTGGTGAAATTC
ATCTTTGTTATGATAAAATATCCAACAAACAATTTGTTTTGAAGAAAGCAATTGAAAGTT
ATGGAGATCTCCATCGAGATATAAATATTTATTCTAAATTCAATTCATCAATAAATGTCA
TGAATTATTGTGGATTATTTATAAATGGAGAAAACATTTGCATTGGCATGGAATACATGT
GTGGGGGATCTCTCAGATCTTATTTGGACAAAGGAAAATGTTTATCTGAACTTGAAGTAG
TGAAAATTTCATATGAAATCTTGGTACAAATATATGTGTTACATAAATTAGGAATTATCC
ATCGAGATATAAAATTTGATAATATTGTTCTCGATGAATTCAAACATCCAAAGTTAATTG
ATTTTGGATTATCAAAATATACAATCGGATTAACAAGTAAAGATCTATTAGATAAATTAG
AAGCGTATAAGCCTGGGACAATTTGTTACATGGCACCAGAATTGATAAGAGGAACACCAT
ATTCAGAAAAAATCGATATTTGGTCATTTGGAATGGTGATGGTTGAGATGGTGACAGGTA
AAAGACCATTTCATTGGCTGGGACCATTGCAAGTAGTTATTAAAATTCGAAATTGTATAA
AATACGACTTTGAACCAATTTTTCCAATTTCAAATGATTTTAATCGATTTATCAGTTCTT
GTTTGAATACGAATCCAGATGAGAGACCAAGTGCAGAACAGTTGATGAAAATAAATCAAT
TCAAAATTATTAATTATAAATAAgttaaataaaattgaaacttgcctaattaaaaaatgt
catgaattattgtggattatttataaatggagaaaacatttgcattggcatggaatacat
gtgtggggttgattctatttcactattgaaattattttgataacatttgtatttgacctt
attcagggatctctcagatcttatttggacaaaggaaaatgtttatctgaacttgaagta
gtgaaaatttcatatgaaatcttggtacaaatatatgtgttacataaattaggaattatc
catcgagatataaaatttgataatattgttctcgatgaattcaaacatccaaagttaatt
gattttggattatcaaaatatacaatcggattaacaagtaaagatctattagataaatta
gaagcgtataagcctgggacaatttgttacATGGCACCAGAATTGATAAGAGGAACACCA
TATTCAGAAAAAATCGATATTTGGTCATTTGGAATGGTGATGGTTGAGATGGTGACAGGT
AAAAGACCATTTCATTGGCTGGGACCATTGCAAGTAGTTATTAAAATTCGAAATTGTATA
AAATACGACTTTGAACCAATTTTTCCAATTTCAAATGATTTTAATCGATTTATCAGTTCT
TGTTTGAATACGAATCCAGATGAGAGACCAAGTGCAGAACAGTTGATGAAAATAAATCAA
TTCAAAATTATTAATTATAAATAAgttaaataaaattgaaacttgcctaattaaa
>DjScaffold3601:61086-64251 strand:+
ttactttttattcgtagattttataaatttgattgtatttaATGTCTGAAATTGAATTAA
TTTTAAAATCACCAACAGATCGTATAGCTCCGTTTACTATTAAATGCAATATTTCTTGGA
CAATAAAGCAATTAAAAGAACATATTAAAAATATTTATCCTTTAAAGCCAACTGTTCAAT
CACAGAAAGTTATATACGTAGGGAAATTATTAAAAGATGATCAAGTTTTAAAAAATCTCT
TTCAACCGGAATCAAGTACAATTGTTCATTTTATTTGTCAATCTTCAATTAATGAAACTG
AGAAAAAAGCGGATGATGTTAGTAAGAAACCAGAAATCACACCAGAGAAATGTCCAGATT
TGTATAAAGAGTATCAGAATTATGTTAGTAATTATTATGCTGCGTCACAATCGCAACCTT
TTGCTGCAATGAACCATTATCAACATCAAATGATGTGGATGATGAATCAGGCCTATATGC
AGCAATACGCCAATTATTACGCTCAATTCAGCAGCACTGGGTCGGATAGCAGTGTTCCGC
AAGTGCCATCTCCAATGGTAACGAGGGAATCATCTGTGCAGCAGCAGCAGCAGAATCAAG
ATCAGAATAATGAGAGAAGAGTTGATGCTCCAGCAGCAGCAGCAGCAGGGGAGAATGGCG
GAATGCAAAACATGATGGCTGCAGCGTTTGGAGGTTTGATGGGAGACGGCGGAGAAGACG
AGAATGATGGAGACGTCGAACCGAGAGATTTGTTGCATTATTTCTATATTCTGGTGCGAT
TCGCGCTGCTCTTGTTGATTTGCTATTATTACAGCAGCGTAGCGAGCGTCGTGACGACTT
TGATGCTGTTTTTCCTGTTTTTCGGAGTGAATTTGCTGAGGCGCAATCACAACAATCGCG
CTGTTCCTGCTGTTCCTGCAGTTCCCGTTGGTCAAATCAATCCAGCAGTTCCACCAGCAC

CAACAACAGTTGCCAATGGTGATCGTGGCGATCCAGATCGAGCAGCAGCAGCAACTGGCA
ATCTTCACGATCAGAATGAAGAAGGAGGAGACAACAACATCATCAATGTATCAACTACAA
CAACAGCAGCAGCAGCAGGAATATCAGCAAATGAGAATTCTCCCTCAGCACCACANTCAG
TTGTTGTTGTAACAGTAGAAACATTGCCAACAATCACAGAGCGACTGAAAAGAGCTGCTA
CAACATCTCTCCAGTTCGTGTTGACATTCTTCGTAACATTGTTCCCAGAACCGATTGCTC
AGATTGACATCAATTAAttgattgattgattgatttattgatcaactgtggtggaagaca
tcaaatatttgattggtcgagtacgtgtgtgtgtttgtgtgtgttgctgagacaatattt
ctcttcatttcattatataatttgattgggcacgatcaatcgatcgattaatttattgat
ttatgatgagtttgtgtgtctttgtgtgtgcaatatttgattggtggaacaaaataagga
actccgttgctatgcagtcaatatcgttggttgctatgtgataatcataaattagattag
atttttactttttattcgtagattttataaatttgattgtatttaATGTCTGAAATTGAA
TTAATTTTAAAATCACCAACAGATCGTATAGCTCCGTTTACTATTAAATGCAATATTTCT
TGGACAATAAAGCAATTAAAAGAACATATTAAAAATATTTATCCTTTAAAGCCAACTGTT
CAATCACAGAAAGTTATATACGTAGGGAAATTATTAAAAGATGATCAAGTTTTAAAAAAT
CTCTTTCAACCGGAATCAAGTACAATTGTTCATTTTATTTGTCAATCTTCAATTAATGAA
ACTGAGAAAAAAGCGGATGATGTTAGTAAGAAACCAGAAATCACACCAGAGAAATGTCCA
GATTTGTATAAAGAGTATCAGAATTATGTTAGTAATTATTATGCTGCGTCACAATCGCAA
CCTTTTGCTGCAATGAACCATTATCAACATCAAATGATGTGGATGATGAATCAGGCCTAT
ATGCAGCAATACGCCAATTATTACGCTCAATTCAGCAGCACTGGGTCGGATAGCAGTGTT
CCGCAAGTGCCATCTCCAATGGTAACGAGGGAATCATCTGTGCAGCAGCAGCAGCAGAAT
CAAGATCAGAATAATGAGAGAAGAGTTGATGCTCCAGCAGCAGCAGCAGCAGGGGAGAAT
GGCGGAATGCAAAACATGATGGCTGCAGCGTTTGGAGGTTTGATGGGAGACGGCGGAGAA
GACGAGAATGATGGAGACGTCGAACCGAGAGATTTGTTGCATTATTTCTATATTCTGGTG
CGATTCGCGCTGCTCTTGTTGATTTGCTATTATTACAGCAGCGTAGCGAGCGTCGTGACG
ACTTTGATGCTGTTTTTCCTGTTTTTCGGAGTGAATTTGCTGAGGCGCAATCACAACAAT
CGCGCTGTTCCTGCTGTTCCTGCAGTTCCCGTTGGTCAAATCAATCCAGCAGTTCCACCA
GCACCAACAACAGTTGCCAATGGTGATCGTGGCGATCCAGATCGAGCAGCAGCAGCAACT
GGCAATCTTCACGATCAGAATGAAGAAGGAGGAGACAACAACATCATCAATGTATCAACT
ACAACAGCAGCAGCAGCAGGAATATCAGCAAATGAGAATTCTCCATCAGCACCACAATCAGTT
GTTGTTGTAACAGTAGAAACATTGCCAACAATCACAGAGCGACTGAAAAGAGCTGCTACA
ACATCTCTCCAGTTCGTGTTGACATTCTTCGTAACATTGTTCCCAGAACCGATTGCTCAG
ATTGACATCAATTAAttgattgattgattgatttattgatcaactgtggtggaagacatc
aaatatttgattggtcgagtacgtgtgtgtgtttgtgtgtgttgctgagacaatatttct
cttcatttcattatataatttgattgggcacgatcaatcgatcgattaatttattgattt
atgatgagtttgtgtgtctttgtgtgtgcaatatttgattggtggaacaaaataaggaac
tccgttgctatgcagt
>DjScaffold2061:19632-29788 strand:+
aagtataaaATGGAAAAAGTAAATATTACATTAAGTGATTGTCTTGCATGCAGTGGTTGT
GTCACAAGTGCCGAAACAGTATTAATTAACGAACATTCTAACGAAGAATTTTTAAAAAAA
ATTAATTTAATGAAGACAAATAATAATAATGACACATTAATTGTGTTATCGATATCTCCT
CAATCTAAATCAAGTTTATCAGTAAAATACAATTTAGGAAATATTTATGAAAGTAATCGT
AAATTAAAGCAATTCTTTAAAACGCTTTCAATTGATGTTGTTCTCGATACGACTTTCGGT
AGAGAGTTTAGTCTCGTTGAAACAGCTAACGAATTAAAAGATAGAATTGAATTGAAAAAG
AAAAACGATCCATTGATGTCGAGTGCTTGTCCTGGATGGGTTTGTTATGCAGAAAAACAT
AAAGAAAATGGAATTTTAGTGCCGTTAATTAGTAAAGCAAAATCACCTCAACAAGTGATG
GGATATTTAGTGAAACATTATCTACCGAATTATCATGACAAATATCAGACGACACAGATT
TTTCACGTAACTGTTATGCCGTGTTTCGATAAGAAACTAGAAGCAACTCGAACCGAGTTT
TCGTTCAAGCAAACAAATGGTTCGATAGAAAAAGACGTGGATATCGTTTTGTCGACATTA
GAAATCGAACAGATTATCACAGAACAAGGATATGATTTTCATAGTTGCGATGATATTCTA
TTGGAAAATAATCACGAAGTAGAAGAAATGTTTTCCTGTGTAAATAGTGAAAATGAAAAG
GTAATTTACTCACACGAAGGAAGTGGTTCTGGTGGATATTTAGATTTTGCTATGAGATTT
TGTGCCAAGGAATTGTTTAATATCGAATTACAATCTAATTTGATGGAAGATCCACGGATC
AAATTCCAAACGATCAGAAATGTTGATTTTAAAGAAGTTTCTTTGATTTCTGAAAGTACT
GAAAACCCGGAACCTCTTTTAACATTTGCTATTGCGTATGGTTTTCGTAATATTCAATCG
ATTGTTCAAAAGATCCGACTGAAGAAATGTAATTATCAGTTTGTTGAAATTATGGCTTGT
CCTTCAGGATGTTTGAATGGCGGCGGTCAAATCAGATCTGAACCTAATGTTAAACAGAAT
GTTTTGTTGGAGCAAGTTACCGATTTTTATCAGTCTTCGAGCAAGCAGCCGCCTCTTGTA

AATAAAAATGTCCAGCGCATTTATGATGAGATTTGTCACACAGATTCTTTACACACAACT
TACAATGTTATA
>DjScaffold2071:85113-85990 strand:+
ATGAGCGCACCATCTGCGTGTAATTCGGATTCATTATGCATCGACGATAGAAGTCGTAAA
GAAAGAAAAATTATTTCAATTATGGTATTTTACACAAAATGTACACGACGTTCAAATGTG
ATTATAATTAATTTTCATCGGAAAGATCCACAAAAATCTTCCGGATCTTTCTTGGACGAG
AAGTTGAAGAGGACGTTGATGGTTGAGAATGTACTCTCTCGCTTCATTGCTTATTTTCTG
TGCGGAGCCATTCTACCTCGACTTCCATCTCCCATATCTTCATACCCTGGCTGTGTCCAT
CCGGGTATACTCAATATTTTGGCGACGGAGTTGGTTCATCTCGAATATTAAcattcctat
cttttcctgaagagcttgacaggctcttgaccgatttttgtggtggctaactgtccacc
aaagctagctgcgatttgcaactctgctccaaaggtccggcttggtctgagttggaacag
gtgctgattagccacatttggggggcattctcgtccatatctaaacatttcagattaaa
tcgtgcttaatctgtatctcattatgcgttaatgaaacgtttagagatgatctattttag
tgttaatattacatacgagctataacgatagtaatggtatccttgatacacccaagaaac
acaacataacgctaatttttctgagcttcctattaatagactaattggtagttgtttatt
gattgtaaaaacactattaaattgaggtaaagtatactaaaataaagggcaagtttaata
atttaataaatggaataattttgttattttaatttatgtattcaataataataataatga
taata
>DjScaffold1312:1241-11568 strand:+
tacactattttaatgtcaaaattgactatctatttcatcatggacaatatatagcggaaa
caatgtacagagaaatgatactcaatgatttttaactgaatacaaaaagtatagctcaca
aattttgtaagaaacatatcttaaaaattaggtggtcttggggggattaatggcaactaat
tgagagtcattgttaatatttacaggataatagatttagtttaaatttagatatttattt
aaattaagaagtgaaagtattttaattttattaagaatttagagcttattcctactgatg
tcaaattgaagaacgcaaatgttgaggatagacaagagctatgtttgatccgtagagcga
ttATGTATTGTAGATCATTTGAGGTTATTGAACCTGAACAACTATGTGACATATTAGGGG
TTGAAGGAAATGTTGGTAAAGGAATATTTCATACTTTGATTAAAGAAGGAGTTTTGGTTA
ATCCCAAAAGAAAAACTAAAAATAAGGGACTTTTAAAAGTAATCGATAAAGGGGCTTTGG
AATCTCATATTTTGAAAAAATTTTTCAATTCAATTAATAAAGTAAATTCTACGAAAGATT
CAAGAAAACGAAAAATTGAAACTACTGGTTTATCTCAGTTAACGGTGAACACTTGTACTG
ATCTGACTAATTTGAATATCTTGAATGCATCTCAAGATTCCATTATTGGCGAGATCACTA
AGAAAAGACGAAGAGCAGGTATTGCAAAAACTCCAGTTTTAGCGTCACGACGCTAAagta
gttaaatgttttattttgttcatttttggtttgttttaataaaatgttttctacgctc
>DjScaffold3790:54281-54716 strand:+
AAGGATACATATATAGTAGAATCATTCAGAACCATCCGTACAAGAGTTAAAGAACATCTC
AAACAGAAAGAAAGTGAAGTCTTCAAACATTTTCAAAACAAACATATTGTTTTACCAGAA
AATCATCATATTACATGGAAAACATTAGGAACAGGCTACATTTCTACACTTCACAGAAAT
AAACATGAACAAATATTAATTAAAGAGGAGAAACCTGAGATAAACGTGATTTTTTAAaaa
ttacagtaattaaaaaagaaaaaagaacattttgatctaagcattttaatttattgaaat
tatataaataatattttt
>DjScaffold935:80908-92171 strand:+
attgaattaaaattaaattaattttagatatattcaatagaaattaattATGGATGATCA
GAATGTGTTTTTAACTCTAAATGATAATCAAAGTATGCAAGATGGAGAGGAAATGGTTGT
CATTGCTGACGAAGGCTTAGATATGAATACATTACAATCTTTACTTGCTTCTGGAGCTGT
ACAAACATTATCGAATGATGATGAGATTATCCAAGTCTCAGATTCTGGTCTTATGAGTGA
TACTATTGTGCAACTTGAAGGTGAAAGTTATCATGTAATCAGTGATGAAAATGGTCAGAC
TTATTTGGCACCAACCAATCAAACGGGCTCATCTGGAAATATAGCTGTTGCACAAACGAT
TGATAATAATGGAATAACATATCGATGTCCTTCCTGTCCACAATCCTATCAGAATGCAAA
TGCATTCACTAAGCATATGCGGGAGAAACATTTCGTTAAAGTTTATACTTGTCGGCAATG
TGAACAGTTTTATGAAACTCTAAGTCAATTATTTCTACACGCCAGACGTACACATGTCGA
CCCGAAGAAACCGAAGAAGAATCCCGGCAATTATAAATTCCATTGTCCAGATTGTAATTA
TATGACGAATAACGGAGGAACAATGGAGCATCATCGTCGAGCTCACACTGGAGTCAAACC
GTTTTCCTGTGATATCTGTGGAAAACATTTTGCCCAGAAAGCCAATATGAAAACCCATAA
AAATCGTCATATTCATAAAGATCGGATTTTTCGATGTGAAACTTGTGGTAAAGCTTTCAA
TTCATATGATATCTATTTAGCTCATTTGAGAACGCGAGAGCATGAATTGCGTGAACATTT
TCAAATGATAGCTTCTTCAAAATTAATTCTTGGTTGTATGGGATGCAACAATACTTTCGA
TAGTATGTTCAATTTAAACAAACATACTCGTAGTTGTGGAATTCTAAAACAGATGGACAC

GAAATTCTGTTGTAAATCATGTGATTCCTATTTCAGTGATATAATGACATTGAAATCGCA
CGTTAAATCTCATGAAGGTAATCAAATAATCTCATGCCCGGTTTGTGGTGAGCGGGGCTT
TCCTGGTTTCAACCAACTTAATCATCATATTACTCGTGCTCATGCATTAAAAGTTAACCG
ACGACACGTTTGTCGTTATTGTGAATCGACCTTTGAAAGTAAAAAAGATCTTGACGGTCA
CATTTCCGATATGCATTCCGAAGAGCGAAATGCACGTCAATCTCGTGGAGCTGTCGGACG
ATTACGTTGCCGATACACGAATTGTGATTTCACATCGAACTCTGTGATGTTGATGGATCG
TCACCATCGACAACACACTCTTCAGGGCACCAATGGAAATTACGCTCTTACTCTTCCACA
GTCTCGTTATTCGAAATACAATAAACGCCGTAAAATTAGAGAAGCTAAAAAACAAGAAGA
TTTTGATGATGCAGAATTACTCGACGAAGAAATCGAACGAGTTAATGCCAGTTTGGAAGC
TAATGAAATCTTACGATATCCGACTTTGTTAGATGATGATGACGAGGAAGATGATTTAGA
AGATGAGGAAGATGAAGAGGAAGAACCGAAAGATGATGAAGATGATGAGGATTATCCCCA
TAACCTCCATCATAATCATAATAGTAATAGCAATCATCACCACAATCACCATCACCATAG
GGATGAAGTGAACGACATGCAACAGTTAGCAGATTCATTAAATTTGTCAGCAGAGAATTT
ATTGCCGATAAGAACGACCAAACATGAACTATTAAACAAGAAACATCAATCATTGGTTCG
GGTGAAATCTGAGCAGATTGTGCAAAATCTACCGAAAACAACAAATTTGATAACAAAACA
GAAACAGCGCAAAATGGAATCATCAATATTTTCCAATAATAGTAACAATAACAATAACAA
CAATGTTAGCAGCAACAGTAATATTGATGGAAATCTCTCATCTGGAAGGTCTAGAAAGAA
ACCTTGGTGGTATAAAGATTACGATACGACAGATATCATGTTGGACGAAGAAACTGTTCG
TCACTCTGCAGCAGGTCGCAACAAATCTTCATTTGTTGATAATAACACTGATGCCTTGAT
CAATAATGACACAGAAGAATGGAATATAGATATGTAGaattgattatttgtatatacgcc
tataaataatgattaattaatatttaattaataattaattaattaattaataattaatat
ttatatataaatataatagagaaattgaaaataaaaatgataaactgcatcaataataat
aataataataataataatggttgagttttttgtgtgatgatgttactttttaatgtttg
aaaattttattggaaatcataaaaatcgatttatttattttattggagccaaatgatcgt
aaaataatcgatttttaaaaaaaaaacggaattaacaatcaattgattcttcttgactct
tgttaaaattattattaattatgatcattagacattgaaat
>DjScaffold2973:14464-14823 strand:+
ATGCTATTTGGGTTATCAGCAGCTTCAACTACATTTCAGAAATTGATGCATGCAATTTTG
AATAGGATTTTATTTAAAGGTGTTGTTGTATATATTAATGATATTTTAATATTTGCAAAA
AGTCGATCAGAACATGATAAAATATTTGAAAATTTATTTCAAAGAATTCAGAATTCTGGT
CTCAAAATCAATCCAAATAAATTTAAACTATATCAAAGTGAATTGTTATTTTTGGGCCAT
ACAGTTAGCAAAGACGGATTGAGGCCTAATTCAAAGAAAGTCTTAGAAATTGTAAAAGCA
GAAATACTAAAATTTACGTTCCTTTTTAGGCATAGCAAACTATTACAGGAAGTTTGTTAA
>DjScaffold3830:3-7775 strand:+
AAAGTTTGGTCTAAATTTCCAAAAGAATTAGAAAATTCAAGTCTTTATGATGATGCAAAC
AAATTGAAAATTACATTTGAAGATAAGAAATATTTCTTTGAGGATTTTTTAACAAAAATT
GTAATTATTGGAAATGTTTCAACAGGCAAGAGCACATTAATTAATACTTTAATTGGTAAA
GATTATTTTGGATCAATAACAGCAAATGGAACAGTTTCTTTCTTTTATTGTTCTTTATCT
GAGAAGAATTATGAATATTTCACTCTGAATAATTGTCAAAATGAATATAAAGATATTTCA
ATATTACAATGTGCATCAAAGTTGAATGTTAATTTGACAGATTTTATTGATCACAATTCT
GTTTTAAAGGTTTACATAAAACATGATAAAAATAAATTATTAGAAAGAAAAACAGTTATT
GTGGATTGTCCTGGATTTGGATCTGATGAAAGATGCGACAAAGAAATTAAAAATCATTGT
TCAAGTGCAGATCTGTTTATATTTATGTTTCAATTTAATAAACCAGCCACTGAGAAACAA
AGAAATTATATCAAAACCATCAGAGAAAATTATAGAAATCCAACATTTTTCTTTGTTTAT
AACAAATGTGATTTCCCTGAATTTAATGATGAAAAGAAAGCTGAATTAAAAAAGGACTTC
TCAGAATTATTCGGCGAGCAATATTTTAGAAGTCTTAAGTTAAATAACGATCCATTGTAT
TTCATTTCTGCTTTGAAACCTAATGGATTTGAAGACTTTTCTCGACTGATAACAGATATA
TTGATTCACAAAAATGTGAATTTTATTCGATTTATAAATGTTCAATCAATATTATTAGAT
GAAGTAGATAAATTTTGCAAAACTGTCGGTAACTTTGTTACTAATGAAAGAAAAAAACGA
ATATTAATTGTAAAATTATTCATTAATCAATAAaatttaacaaaagtgaaattattgtca
catttttcaaatcatttgtgttgctcaattttgaaaaagtttgaaaatatttttaaatt
aaacggtatacaaaataagcaaatactgacttattaattatattatttgttagcataagt
taataaatcaaaaaagtaataggctgacttatttttctaaaatgtttttagttaatttga
cttttaaaatgaatttcattaataaacacacaataaatttttcaatttcaaaactacatt
ttattcatataataacatttgaataaaattaaattgttattttatggat
>DjScaffold3830:21552-32198 strand:+
atttagtaaataattaaagcatatattgatattattttaaaattttaATGGAATTAGAAG

TTTGCGTTGATTCGTTAATATCTGTCAAAGCTGCTGAATTGGGTGGGGCAGATAGGTTGG
AGTTATGCTATGGATTGTGTTGTGATGGGTTGACTCCTTCAGTTGGGTTTTGTCGAAAAG
TAAAAGAAATCACAAATTGTCCATGTTATCTCATGATCAGGCCTCGTTCTGGGGATTTTA
TATATAGTAACGACGAATTAGATATAATGGTAGATGATATCAATAATTTGAAGGAATACG
CAGATGGGTTTGTTGTGGGAACTTTGTTAGATAATGGAGAACTTTCTATTGATCAGATGA
GTGCATTAATGGATAATATTGGCAACCATCCCATCACATTTCACAGAGCCATCGATAGTA
GCGACAATCCCGTAAAACTATTAGATAAATTAATCGAGCTAACACGAGACAAGTGTTTTC
ATTCACCATTTCGCGTATTGACAAGTGGGGGTAAAGCTAACGCGCTAGAAGGAGCACACG
TGATTCACGAAATGGTGGAAAAAGTA
>DjScaffold4702:3691-4234 strand:+
GGGAAAAGTAGTCTCATTAACGCATTAAAGGGGGAAAAAGTTTGTAAAGTAGCTCCGATT
GCTGGAGAAACGAAGGTTTGGCAGTATATTTCCTTAATGAAATCAATTGATTTGATCGAT
TGTCCTGGTACAGTCACATCGGAAAAAGATGAGAAATTGAGTGAAACCGATTTGATTTTA
AGAAATTCGGTGCGTGTGGAAAAGGTAGAGGACGCAGATCGTCACGTTCCCGCGGTGTTG
AGCATTGTTAATAGAGAGGTGATCAATAAATTGTACAAGATAAATCAGCCATGGACCGAG
TGTGAACAATTTCTTGAAATGGTTGCTAAATCTCGAGGTCGATTATTGAAAGGAGGCCAG
CCGGATATCATGACCGTTGCCAAGGAGATATTAAAAGATTTCAACAGCGGAAAACTCATT
TGGTATGTAAAACCGGAAAGTTATGACAATTCTAAGGAGACAATCAAAGATTTTGACAAT
AAAAAT
>DjScaffold5093:29325-29735 strand:+
ATGGCAAGAACGAAGCAAACTGCCCGTAAAAGCACTGGAGGAAAAGCACCAAGAAAGCAA
TTGGCAACAAAAGCAGCAAGAAAAAGTGCACCAGCAACTGGAGGTGTAAAGAAACCTCAT
CGTTATCGTCCAGGAACTGTTGCTCTTAGAGAAATCAGACGTTATCAAAAGAGCACTGAA
TTATTGATTAGAAAATTACCTTTTCAACGATTGGTTCGAGAAATCGCTCAAGATTTCAAA
ACTGATTTGAGATTCCAAAGTTCTGCTGTATCTGCTCTTCAAGAAGCAAGTGAAGCATAT
TTGGTTGGTTTGTTTGAAGATACAAACTTGTGTGCCATTCATGCTAAGAGAGTAACTATT
ATGCCAAAAGATATTCAATTGGCTCGTAGAATTCGTGGAGAACGTGCTTAA
>DjScaffold5420:4943-17356 strand:+
atattcaggaaatgttgagtctttaatatttcattatATGAATAATAATAGCTATTCAAA
TCAAAATATTTCAAAAGAATCTTCATGGCTACTCGGATCAACATTAATCGGATTAGGAGC
CGCGGTTTCTTTTGGAGGTTTTCTAGAAATGAAAATTGGTTCGAAACTCACCGTTTTACT
TGGTGGATGGTTTGCAAGTTTCAATACTTGTATGGTTTATTTTACAATAAAAACTTATTC
GTCTATAGTAATAATAACATATGGACTTTTTGTTGGATTTGGAACTGGTATTGCATACGG
TGTTGCCTTAAGCACACCAGTTAAGTGGTTTTCGGTAAGAAGAGGACTTATTTTTGGAAT
CATATCTTCTGGATTTGGTCTATCTTCCGGAATTTTTTTTCCAATCCAGTTGGGAATATT
AAAGGACAAAGAAAAGTCAAGAAATTTACCATCATCATTTCTAATTATTGGTGCAATAAT
GGCAGTTCTTCAACTACTTGGAGCATGGTTGTTAAAAAACAGAAGAAAATCCATCAGAAC
GTGAgtgtatcgtttcgattgaagaaaacatatttcctaataaaacgtaagtttctataa
attattttattttaacaaatattttcattaacgatgtaaaatctgatgtgctattaaaca
tattttattgcttttactgttataatgatgaatgccccaaggatttcttatgatatcttc
taaattgggttgttgtgatgtacgctcacaagtgactttataaatggtaaacgtactaga
aataaaactagaaaaaaattcacgcaaccgaatttggttcaacatttttcaaacattgttc
aacattacagttatggatacaataatgtgaacaaattacgtatttat
>DjScaffold1078:22323-26694 strand:+
tatattgattctaacttcagcaaaatatcaaaatttctccgacaaatcctcttggaaaaa
tcatatacaacacatataatgaaagtgatacggatttctttcttaaatattataaaacct
attggaataccggatatggaaaatataattctacaatcaatgctcatcctgaaagtaaag
aatggaaaataattATGAAAAGTATTTATCGACAAAGCAGCAGTCCGTGCGCGTTTTTAT
GTTTAGTTGAACCTGAAGATTCCAGAGCCACTACGTATTATGGTTTTCCTGATTTAGTTT
TCGTTTCGTGGAATATAGACATAGGAACTGGAGAAATATCAACTGAAGTATTTTGGGATA
ATAAGCAGCCTACTCGTCTTATGGAAGCTATTTCATTTAAATTTCAACCAGAAATTGTAG
GTGAACGATATCAATGGTATTTCAGTAAGATGGGTGAACCTATTTCTCCGTATTCTGTTA
TTAAAAATGGTAGTCAATGGCAACATAATGTAGATGATTCTGGAGTTTTCATTTCTTCAG
CTCCTGATGCATATCCAATTTTTCGAGTTATTCCTTGGGATACTGGAATCACATGCCCAA
TAATCAATGGATATGGACCAACTGTTTGGCCAGCTCCACTTTTTCCAATAACTAGTAATT
TGACCGGTGTTGCTTTCAATTTTTTTAACAATGTATGGAACACCAATTACATTTATTGGT
ATCCATTTCGGGGTACAGATACTCAATTAGATCATCAGTTTAAAGCACGCTTTACAACCC

ATGTTACACTAATAACTCACAAAGCTGATGATACTCTTCGTTATTGAtcctcatttattt
gattgaaaaatattgtttggaaaacttgcataaattcgtctttatataaattacttgata
ttttatgtattaataaaattctttgatgattgtttataataattgtaataa
>DjScaffold1457:22169-24476 strand:+
acaagtcccaatatcaccttatgtaATGGGCAATCAATCATTACTGGTTGCTGAAGATGT
TTATGTATCAGATGTTATGACAAATTCTACTGTTCCAACTAATAGTTATTCAAGGAATTC
AAACTTGCCACAAAAATCTAAAGAAATGGATCTATTTTCTTTAAATTTTAGCTCAACTCA
GCCAAACTCAAATTATCACCCTCGTGCTACATATAGTGTTGCGAGTCTTGATACCGCTCT
TTTACTACAAGATACAGAAGCATTATTAAATGATATGGAAAAAAGAGTTTATTCCAGAAG
AGAAATTAAAACAGAAGAATTTACGAGAATTCAGCCATTTTCAACGTCTTTTCCTCAATC
AGATAAGATACCCTATGTTGATGACGGAGAAGAAAGAGAAGAAGATACAAGTTTATTTAA
TTATTCCGCTGATATAGACGAAAGTGTTAGATTATCTAATCATAAACAGCAAACATCTTT
ATCAAATCATAATGACTCCATGAGTACAGAAGATAAAAATCGATCTTACACTGTAAGATC
TCCGATTTCCTCAGGAATTATTCGAAGAAACAATTCGTGCAAAGTTGGTGTGTCAGAATC
CATTGTCACACAATCGAATATTGCTGCTAGAAAATTAAATTCAACAATTCGAAACCAGCG
GTCTTGTTCGTTAATCGAGGAGAGAAAATTGAATCATAATAATAATAACAATAATACTAA
TAGTAACAATAATATTGAAACATTCGCTGATCATCGAAATAAACCAAAATTGTCCCTT
>DjScaffold3413:39042-44690 strand:+
tttgtatacattgttattaaaattattgaaattaccaattatgaatgtttctcgtatgaa
gaaacctgagttaatggctgaattagagaagcgttcattaccaactaatggtctgaaaga
tgaattaaaatttagattaattgaagcgattgagaaggaaaaagggaacgatgtaattaa
tgttgatgaatctgacgaaagccgcgatgaactacaaagcatgcatctagttggaaacat
tgacaacgatacaattgttgaagatgttcgtgatgatattgttaacattggcgaaggtaa
atttattttaaatacagcttttgatattcgaaattcataaataaatttaaagtggaaatt
tattaattcaattccaatgaaatgtctcaatattttaccaagaaagaaaatatttaatac
tgatattcaatttattttcaatacaacaatgattgaaatgttaaaattattaatatttgc
actcattatttagaaacattaaaagaagatgaaatcattggtactgatcagtcagcagta
ttaacagtagaagacgcaattaaaATGAAAACTCCTCATGAAATTGAGAGTGAATCAATG
ATAGTCAATATTAATGAAGACGATGATAATGATTCCATGTCAAATTCAGCTAACGAGGAT
AATGTAAATTCTCTACAAACAATCAATAACAATAATCGTGAATTGGACAACGGTGATCAT
GAAATTATTGAAATCAGTGAAGAAGTTGTTATTGATGACGAAGACAATCAAGAAAATGAC
AACAATTATAATGACTTATCTTCTGACAGTAATCAACCGCAGTCAAGAGATATGGAAAAT
GAAATGGAAAAAATCGATTCAAGGAAAGAGGATCAAATGAAAAATCAATCAAAAACAGAA
ATATGGGTTACTAATATAGCTGAGGATGTTAAAGCTGTTGAATTAAAGGAATTGTTTGGT
AAATATGGAAAGGTTGTATCTGCTAAGGTTGTAATGAATGCAAAAAACCCCACAGGCAAC
TGTTTTGGTTGTATACAATTAGCGAGTGCAAATGATGTCAAAGCGTGTGTAATGAATCTT
GACCAAAAAGAGTTTCACGGCAATATTATCTCAATCACTAACGATATGAATGATAAAGCT
GCTAGTAAAGAAAGTATCATTAGAAATATTTCACCAGTTAGTTCGAGTGCATCATGGGGA
TCTAAAAATAAATCTGGCGACAAGAAATCATCGAAAAGTCCTTTGGATTCCAATGAACCG
TCATCTTATAGTAAAACGAGAAACGATCAAATTGATAAAGACAGAAGACCTCCTTATCGA
GAGAAACCTAGTTCAGGAGGTGGATTCTACAAGTCGAAAGATTTCTCAGACAGAGGCTCA
ATAAATAGAAGAGGCAATCGACCGCCGTATAATGATGATAGACGTCAAAATTATTCAGAG
AGATCAGGTGGAAATTCCTATAGTGACAGCAGAAATAGAGATGTATATCAGAAATCATCA
CGCGACACTCGAGATAATTACAATACTCACTCATACGAATCTCGATATGTTCCAAGTAGT
AGTGGTAGAGACAGACCTCCTGGAAGATCCGATTACGGAGGTGACAGAATGAATAAAACA
TCCAGCAGCAATTATAATGAATATCGCGGTCGATCGAACAAATATGAAAACTACGACGAT
TATGTAAAAAGAGACAAAAGATCATTGTATGATCGACCGATTTCCAGGGAAATTGATAAT
TATTCTAGTTCATCTAAACGAGATTCTTATAAAAGACCAAGGCCGGAAGACAGTGATGAT
TATCTTCATTCCAAAAAACGCAGATCCGATTTAATTGACGAAAGACCACATCATTATAGA
ACTGTTGAATCACCAAAGAAATATTACTCAGAAAAGGAATATAGGAAGCCTGACTATAAA
TCGAGAAGGGATGATTATCCTAGCGATTATCCTTCGAAAACAGATAGAGATTACTTTCCA
GCTCGTCGGAACATGGACTTCCCAGTTCGTCGTGATGCATATGACGCATATTCTCCTGAA
AGGCATGCTTCTCGTTCAAATAATTACAGCAAAGATTCATATGTGCAACATTCAACAGAT
TTCGGCGGTCGATCGAGACGCTATTAAtcttaatgttaaataataatactaataatatat
cgaaatatatatcaaagcagttattttcatgagtgaatatacattgtaacaaattgttaa
atcatgttggtgaaaataattacataataattcttttaaacttccttatgtaatttatat
acaagtcgagagaagaagctcgcatgcctaaattgtgaacataatgaattttgaatatag

ttaaatattttgtatacattgttattaaaattattgaaattaccaattatgaatgtttct
cgtatgaagaaacctgagttaatggctgaattagagaagcgttcattaccaactaatggt
ctgaaagatgaattaaaatttagattaattgaagcgattgagaaggaaaaagggaacgat
gtaattaatgttgatgaatctgacgaaagccgcgatgaactacaaagcatgcatctagtt
ggaaacattgacaacgatacaattgttgaagatgttcgtgatgatattgttaacattggc
gaaggtaaatttattttaaatacagcttttgatattcgaaattcataaataaatttaaag
tggaaatttattaattcaattccaatgaaatgtctcaatattttaccaagaaagaaaata
tttaatactgatattcaatttattttcaatacaacaatgattgaaatgttaaaattatta
atatttgcactcattatttagaaacattaaaagaagatgaaatcattggtactgatcagt
cagcagtattaacagtagaagacgcaattaaaatgaaaactcctcatgaaattgagagtg
aatcaatgatagtcaatattaatgaagacgatgataatgattccatgtcaaattcagcta
acgaggataatgtaaattctctacaaacaatcaataacaataatcgtgaattggacaacg
gtgatcatgaaattattgaaatcagtgaagaagttgttattgatgacgaagacaatcaag
aaaatgacaacaattataatgacttatcttctgacagtaatcaaccgcagtcaagagata
tggaaaatgaaatggaaaaaatcgattcaaggttcgttattacagatgaattttttaaaa
atctggaactgcaaattttcattaattttacataaatgttacacgaaaaatatttatttc
ttactgaaaaattttacaatggtcaaatgaaaccaaatcctcaactttagttctcagtaa
tagagcattacaaatttttgcaatttgaatttattaaATGTTTATGTCAAAATATTTCAA
TGTATTTAATGTTTTTTATTTTAGGAAAGAGGATCAAATGAAAAATCAATCAAAAACAGA
AATATGGGTTACTAATATAGCTGAGGATGTTAAAGCTGTTGAATTAAAGGAATTGTTTGG
TAAATATGGAAAGGTTGTATCTGCTAAGGTTGTAATGAATGCAAAAAACCCCACAGGCAA
CTGTTTTGGTTGTATACAATTAGCGAGTGCAAATGATGTCAAAGCGTGTGTAATGAATCT
TGACCAAAAAGAGTTTCACGGCAATATTATCTCAATCACTAACGATATGAATGATAAAGC
TGCTAGTAAAGAAAGTATCATTAGAAATATTTCACCAGTTAGTTCGAGTGCATCATGGGG
ATCTAAAAATAAATCTGGCGACAAGAAATCATCGAAAAGTCCTTTGGATTCCAATGAACC
GTCATCTTATAGTAAAACGAGAAACGATCAAATTGATAAAGACAGAAGACCTCCTTATCG
AGAGAAACCTAGTTCAGGAGGTGGATTCTACAAGTCGAAAGATTTCTCAGACAGAGGCTC
AATAAATAGAAGAGGCAATCGACCGCCGTATAATGATGATAGACGTCAAAATTATTCAGA
GAGATCAGGTGGAAATTCCTATAGTGACAGCAGAAATAGAGATGTATATCAGAAATCATC
ACGCGACACTCGAGATAATTACAATACTCACTCATACGAATCTCGATATGTTCCAAGTAG
TAGTGGTAGAGACAGACCTCCTGGAAGATCCGATTACGGAGGTGACAGAATGAATAAAAC
ATCCAGCAGCAATTATAATGAATATCGCGGTCGATCGAACAAATATGAAAACTACGACGA
TTATGTAAAAAGAGACAAAAGATCATTGTATGATCGACCGATTTCCAGGGAAATTGATAA
TTATTCTAGTTCATCTAAACGAGATTCTTATAAAAGACCAAGGCCGGAAGACAGTGATGA
TTATCTTCATTCCAAAAAACGCAGATCCGATTTAATTGACGAAAGACCACATCATTATAG
AACTGTTGAATCACCAAAGAAATATTACTCAGAAAAGGAATATAGGAAGCCTGACTATAA
ATCGAGAAGGGATGATTATCCTAGCGATTATCCTTCGAAAACAGATAGAGATTACTTTCC
AGCTCGTCGGAACATGGACTTCCCAGTTCGTCGTGATGCATATGACGCATATTCTCCTGA
AAGGCATGCTTCTCGTTCAAATAATTACAGCAAAGATTCATATGTGCAACATTCAACAGA
TTTCGGCGGTCGATCGAGACGCTATTAAtcttaatgttaaataataatactaataatata
tcgaaatatatatcaaagcagttattttcatgagtgaatatacattgtaacaaattgtta
aatcatgttggtgaaaataattacataataattcttttaaacttccttatgtaatttata
tacaagtcgagagaagaagctcgcatgcctaaattgtgaacataatgaattttgaatata
gttaaatattttgtatacattgttattaaaattattgaaattaccaattatgaatgtttc
tcgtatgaagaaacctgagttaatggctgaattagagaagcgttcattaccaactaatgg
tctgaaagatgaattaaaatttagattaattgaagcgattgagaaggaaaaagggaacga
tgtaattaatgttgatgaatctgacgaaagccgcgatgaactacaaagcatgcatctagt
tggaaacattgacaacgatacaattgttgaagatgttcgtgatgatattgttaacattgg
cgaaggtaaatttattttaaatacagcttttgatattcgaaattcataaataaatttaaa
gtggaaatttattaattcaattccaatgaaatgtctcaatattttaccaagaaagaaaat
atttaatactgatattcaatttattttcaatacaacaatgattgaaatgttaaaattatt
aatatttgcactcattatttagaaacattaaaagaagatgaaatcattggtactgatcag
tcagcagtattaacagtagaagacgcaattaaaatgaaaactcctcatgaaattgagagt
gaatcaatgatagtcaatattaatgaagacgatgataatgattccatgtcaaattcagct
aacgaggataatgtaaattctctacaaacaatcaataacaataatcgtgaattggacaac
ggtgatcatgaaattattgaaatcagtgaagaagttgttattgatgacgaagacaatcaa
gaaaatgacaacaattataatgacttatcttctgacagtaatcaaccgcagtcaagagat

atggaaaatgaaatggaaaaaatcgattcaaggttcgttattacagatgaattttttaaa
aatctggaactgcaaattttcattaattttacataaatgttacacgaaaaatatttattt
cttactgaaaaattttacaatggtcaaatgaaaccaaatcctcaactttagttctcagta
atagagcattacaaatttttgcaatttgaatttattaaATGTTTATGTCAAAATATTTCA
ATGTATTTAATGTTTTTTATTTTAGGAAAGAGGATCAAATGAAAAATCAATCAAAAACAG
AAATATGGGTTACTAATATAGCTGAGGATGTTAAAGCTGTTGAATTAAAGGAATTGTTTG
GTAAATATGGAAAGGTTGTATCTGCTAAGGTTGTAATGAATGCAAAAAACCCCACAGGCA
ACTGTTTTGGTTGTATACAATTAGCGAGTGCAAATGATGTCAAAGCGTGTGTAATGAATC
TTGACCAAAAAGAGTTTCACGGCAATATTATCTCAATCACTAACGATATGAATGATAAAG
CTGCTAGTAAAGAAAGTATCATTAGAAATATTTCACCAGTTAGTTCGAGTGCATCATGGG
GATCTAAAAATAAATCTGGCGACAAGAAATCATCGAAAAGTCCTTTGGATTCCAATGAAC
CGTCATCTTATAGTAAAACGAGAAACGATCAAATTGATAAAGACAGAAGACCTCCTTATC
GAGAGAAACCTAGTTCAGGAGGTGGATTCTACAAGTCGAAAGATTTCTCAGACAGAGGCT
CAATAAATAGAAGAGGCAATCGACCGCCGTATAATGATGATAGACGTCAAAATTATTCAG
AGAGATCAGGTGGAAAGCAATATTATAAAGGCAGTAATAACATAAGCTACAATTCTACAG
GTGGAAATTCCTATAGTGACAGCAGAAATAGAGATGTATATCAGAAATCATCACGCGACA
CTCGAGATAATTACAATACTCACTCATACGAATCTCGATATGTTCCAAGTAGTAGTGGTA
GAGACAGACCTCCTGGAAGATCCGATTACGGAGGTGACAGAATGAATAAAACATCCAGCA
GCAATTATAATGAATATCGCGGTCGATCGAACAAATATGAAAACTACGACGATTATGTAA
AAAGAGACAAAAGATCATTGTATGATCGACCGATTTCCAGGGAAATTGATAATTATTCTA
GTTCATCTAAACGAGATTCTTATAAAAGACCAAGGCCGGAAGACAGTGATGATTATCTTC
ATTCCAAAAAACGCAGATCCGATTTAATTGACGAAAGACCACATCATTATAGAACTGTTG
AATCACCAAAGAAATATTACTCAGAAAAGGAATATAGGAAGCCTGACTATAAATCGAGAA
GGGATGATTATCCTAGCGATTATCCTTCGAAAACAGATAGAGATTACTTTCCAGCTCGTC
GGAACATGGACTTCCCAGTTCGTCGTGATGCATATGACGCATATTCTCCTGAAAGGCATG
CTTCTCGTTCAAATAATTACAGCAAAGATTCATATGTGCAACATTCAACAGATTTCGGCG
GTCGATCGAGACGCTATTAAtcttaatgttaaataataatactaataatatatcgaaata
tatatcaaagcagttattttcatgagtgaatatacattgtaacaaattgttaaatcatgt
tggtgaaaataattacataataattcttttaaacttccttatgtaatttatatacaagtc
gagagaagaagctcgcatgcctaaattgtgaacataatgaattttgaatatagttaaata
tcaactttgatgatgatttacgattgggattgtcctcttacaccacaaactgtgagaaat
tagcaaatgagatgtcgaagattgtacataataaatatgtaggatgttatattaaatgtt
acaaaatgtatactataataaacgacatgtattattttttgcgccacaaaagtttgagaa
ttcttgcggagtaattgacgtgtaccatgtaaaataattccaaattcctagtttctaaaa
ttttgaatttattgagttctttttgataaatatttgtcaaagtaaaattaatataacctt
taattattccgtataatattattattattataattattttccagcaaaaacagaaatatg
ggttactaatatagctgaggatgttaaagctgttgaattaaaggaattgtttggtaaata
tggaaaggttgtatctgctaaggttgtaATGAATGCAAAAAACCCCACAGGCAACTGTTT
TGGTTGTATACAATTAGCGAGTGCAAATGATGTCAAAGCGTGTGTAATGAATCTTGACCA
AAAAGAGTTTCACGGCAATATTATCTCAATCACTAACGATATGAATGATAAAGCTGCTAG
TAAAGAAAGTATCATTAGAAATATTTCACCAGTTAGTTCGAGTGCATCATGGGGATCTAA
AAATAAATCTGGCGACAAGAAATCATCGAAAAGTCCTTTGGATTCCAATGAACCGTCATC
TTATAGTAAAACGAGAAACGATCAAATTGATAAAGACAGAAGACCTCCTTATCGAGAGAA
ACCTAGTTCAGGAGGTGGATTCTACAAGTCGAAAGATTTCTCAGACAGAGGCTCAATAAA
TAGAAGAGGCAATCGACCGCCGTATAATGATGATAGACGTCAAAATTATTCAGAGAGATC
AGGTGGAAAGCAATATTATAAAGGCAGTAATAACATAAGCTACAATTCTACAGGTGGAAA
TTCCTATAGTGACAGCAGAAATAGAGATGTATATCAGAAATCATCACGCGACACTCGAGA
TAATTACAATACTCACTCATACGAATCTCGATATGTTCCAAGTAGTAGTGGTAGAGACAG
ACCTCCTGGAAGATCCGATTACGGAGGTGACAGAATGAATAAAACATCCAGCAGCAATTA
TAATGAATATCGCGGTCGATCGAACAAATATGAAAACTACGACGATTATGTAAAAAGAGA
CAAAAGATCATTGTATGATCGACCGATTTCCAGGGAAATTGATAATTATTCTAGTTCATC
TAAACGAGATTCTTATAAAAGACCAAGGCCGGAAGACAGTGATGATTATCTTCATTCCAA
AAAACGCAGATCCGATTTAATTGACGAAAGACCACATCATTATAGAACTGTTGAATCACC
AAAGAAATATTACTCAGAAAAGGAATATAGGAAGCCTGACTATAAATCGAGAAGGGATGA
TTATCCTAGCGATTATCCTTCGAAAACAGATAGAGATTACTTTCCAGCTCGTCGGAACAT
GGACTTCCCAGTTCGTCGTGATGCATATGACGCATATTCTCCTGAAAGGCATGCTTCTCG
TTCAAATAATTACAGCAAAGATTCATATGTGCAACATTCAACAGATTTCGGCGGTCGATC

GAGACGCTATTAAtcttaatgttaaataataatactaataatatatcgaaatatatatca
aagcagttattttcatgagtgaatatacattgtaacaaattgttaaatcatgttggtgaa
aataattacataataattcttttaaacttccttatgtaatttatatacaagtcgagagaa
gaagctcgcatgcctaaattgtgaacataatgaattttgaatatagttaaatat
>DjScaffold291:12319-33455 strand:+
ttctgctgtatttctcaaaagcaataacattattctatacaagatcattatttattgaat
ttatgaaatcaataattacaattatcaattcttcaattattgtttactgatatttctgat
aaagtgttgattaaaaactgtatttttcacattggaatattacaaataaaaatataaaaa
tattggggtgcatatatgaagacttgtagatttattaaagatttaatggaataaatttct
gactcagtattaacttaacaagttaaataccctttgataaacttttcagcctgatcaagc
agtcaagaattaaattttgATGAATAATAATAATCAACAAGACGAAAATGAATCATTACA
ATATTATACCATAAAAGATAAACGAGACAATTTATGTCTCATTTTCAATGCTAGTTTCAA
TAGGCGTGGAAGTGAGCAAGATGTTAATGATATTAAATCAACATTTGAAAATATCAATGG
ATTTAAAGTTCTATTTCAAGAAATTACTACTAAAGAAAAATTTTTAAATGATATAAAGGA
ATCTTTAAAATTCGTTACGGAAGATAGTGATTTGATTGTTACGTTTATTATGAGCCATGG
AGAACAAGGAATAGTTTTTGATTGCTTTGACGAGTCAGTTATTATAAATGATATTCTTGA
ACTATATTCGTCTAAAATGGTTCCAAAATTATCTTTTAAACCGAAAATATTTGTATTTCA
AACATGTCGTGGAAATAAAAAGCAAGAAGTAATTCATTTCGATTCGCCAAGTGTAGAAAA
TATTGCATCAGACGCTCCAAGAAAATATTTGAATGATGACGTTATAAATGGTAGTGATAT
ATTTATCGTTTATTCTACTATACCGGGACATTCTTCAATGAGAGACACATCCAAAGGCGC
GTATTTTATTAGAAAATTTTGTGAAGTGATTAAAGAGAATTCTAAAAAACGGAATTTAGA
TGAAATCTGTACTCTTGTCAACACAAGTTATGAAAAATATGCGTTTGGGGAACAGGAAAT
TACCCAATTATGTGAAGTGAGAAAGGTTGTTATGAAATTTAACATTTATTTGGAAAAACC
AGATAAACTGAAAACTAAAGCATAAatgtttttattgtgataacttgttataatttcatt
tcagttttgATGAATAATAATAATCAACAAGACGAAAATGAATCATTACAATATTATACC
ATAAAAGATAAACGAGACAATTTATGTCTCATTTTCAATGCTAGTTTCAATAGGCGTGGA
AGTGAGCAAGATGTTAATGATATTAAATCAACATTTGAAAATATCAATGGATTTAAAGTT
CTATTTCAAGAAATTACTACTAAAGAAAAATTTTTAAATGATATAAAGGAATCTTTAAAA
TTCGTTACGGAAGATAGTGATTTGATTGTTACGTTTATTATGAGCCATGGAGAACAAGGA
ATAGTTTTTGATTGCTTTGACGAGTCAGTTATTATAAATGATATTCTTGAACTATATTCG
TCTAAAATGGTTCCAAAATTATCTTTTAAACCGAAAATATTTGTATTTCAAACATGTCGT
GGAAATAAAAAGCAAGAAGTAATTCATTTCGATTCGCCAAGTGTAGAAAATATTGCATCA
GACGCTCCAAGAAAATATTTGAATGATGACGTTATAAATGGTAGTGATATATTTATCGTT
TATTCTACTATACCGGGACATTCTTCAATGAGAGACACATCCAAAGGCGCGTATTTTATT
AGAAAATTTTGTGAAGTGATTAAAGAGAATTCTAAAAAACGGAATTTAGATGAAATCTGT
ACTCTTGTCAACACAAGTTATGAAAAATATGCGTTTGGGGAACAGGAAATTACCCAATTA
TGTGAAGTGAGAAAGGTTGTTATGAAATTTAACATTTATTTGGAAAAACCAGATAAACTG
AAAACTAAAGCATAAatgtttttattgtgataacttgttataatttcat
>DjScaffold291:51078-95658 strand:+
ctataccactggtaattcaaagtatttttcagatttatgaaaacttattttaattcact
ttttattttgaaaatagtgatttaaaattgaatttaaaaataaaaattattagctcttat
ttgtaacaaacaacgtttcctcgatatatacatatttactattagataatccaaaagctg
attattgtattccaattccaaaaaatgataatataatgtgcccatcattatttcagcata
aaaatttcttcaaaggaaatatttcaaaatattatcgattttgttgcttgaaaaaccgga
tggattccaattatttaccaattataaatagttgtctgttaatattcatatcaaaagaaa
atatgctgccaaattattccttatgcccaccaggatttatctataaaccaatattcaatg
aaattttagaagaaacttctatttataagtgttgcagattcagtcaggcgaATGTTTCAT
CTGGTCATGAAATATTTAAGATGGTTATTACTGGAAATACCAATACTTGAATTTTATTCT
GAAACTGTAGAATATTTTGATTCCCTGTCAGAAAAAAGTTACTTTCCTTATTCAGAAAAT
AAAATTTGCTTTTATTACTTAGACGTTTCAATAAACAAGATGACGAGTAATTCAGAACTA
GGTTTGTTAACTATGAATCTATGCCCATCGGATTTAATCCAATCAAATGAATTTGAAGTT
GATTTATTAATAAATAATAAGCCAATCAAAAGCTGCTGGCCAAATTCTGAGAATTACTTA
TATCCGCCAGGACAGTATTGTTTATTATTAAAAAGGAACCTAAAATTAGTAAGTAAATGT
CCACAAGATTTTCATGCGTACATTATTCGATCTATTAATGGAGTAGAATTGGTAAGGTTT
TGTTGCAAAATGGAAAATCTGACAAGTATTATATGGTTGGAAGCAGAAAACAAAACTGTT
TTAACAAATGTGATATTCATTTCGATTAGAAATGAATGCCCGTTTATGATTTCCGATATT
ACAATGTCTAGTTACACTGTGAATACTACTGCTGATAACTTAAGCCCCACCTTAAATAAA

TCGAACAATGAAAACAAATTTCCTGGAATAAATCTATGCATTTATGAAGTGAATAGAGAC
GAATTTGCATACATAAAAATCGGAATTTTTTGTCCAACAGGAATGAATGAAAGCCAAATA
ATTTTTACTTCGGTTTTACATTGGAACCAGATTCTACAAACAAAATTTTTCAAATGGAT
TTCTGTGTTGGAAAATCAAATTTGAAATATGAATCTTTTCCAAAAGGAAATTATTGTCTT
TACAAGATTGGTAGAATATGTCCACTTGGTTTTAAGTCTAAATCCATTCCCAATACTAAA
AAAATAATCAGTAAAAGTGGGTTTGTGCCGAATTACACTGAAGATGCATTTTGCTGTCGT
GATGATGGAAATATTCCACTGAATAAATTTAAACATCACCAGTTTCCATTTTTAAACAGA
AACGATATCCGATCTTTTGGATTGCCCATGTTTAATCGGCAAAATATTTGCCCTCAAATT
GAAGAATTGATTGATCACCATGATGCAAATATAGATGGGTTCTATTGTGCCTATAGAACA
TTTGAAGATAATTATAAATGGTCAAATGAAATTATATTTCCAAGTTTATCTGGAAATATT
AATGAAACATATCAATCTAGAGGATACTGGAAATCTCTTACAAAATTTCAGTACACACAA
TCAGTGGTGGTTGTTTATCCAAATGATAAATTTTGCATTGTTTCTGGAGAAAAATGTTCT
GCCATGAAAAATATCGATTTTGCAATGTTTTATCCATTAATGGGGAAATATTTGTGTTGT
TCATTAATAAAGTATAATTTATCAAATAGTATACTCCCGGGTAAACACAAAGAATTCAAT
TCTGTTGGGTTTGTCGCAATTAATGGAAAATGTCCTGTGATTAATAAAGATATTTACCGA
ATTGAGAATACGATTGAAGAAATATTTGAAATTGAAAAGATTCTATTCAATTCCAGTAAT
TTATTTGATATGATTAAATTAGGAATCAGTGTTGATCTTGATAGTAAACCTCAATTTGTA
CGAGTGAAATTTTTGTTTTGTTTACTTACATTAATTAAAGATCAAATATTACCAATTAAT
TCATATGGATTTATGAAACCTTTTCAATTTAAATGTCCAACTGGTTTCGTTGAAAAAACG
CAGACCTTCTCTGTGGATATTAAGGAAAAGTTTAATATAATTTCTTCCCACTTATTGAAA
AGTGCAGTTACTTCAAATATGGTTTATTCAAGTTTATGCTTTCCAACATCGATTACTAAG
AATTACCAAGCCTTATTTAACTCGTCATCATTGTGTTCTTTTCATTACAATACTTGTCCA
CCAGAATTTGCAACATTTCCAAAACATTTACTATTCAGAGATAATAATTCCATAAATAAT
TTGTCATTAATAGTCTGCTGCAAAAAAATATATAAACCAGAAAATCAGTATGATTTATCA
TCAATTGATATCCCATTTTCATTACCAATACATCCGGAGTCATCAGATTGTCCTATACTC
AGAAATTTTAATTTTAAAAAATGGCGATTGAATATAAAATATTCAGATAATTCTGAAATG
AACTCTTTTTGTTTCTATTATCGAATTTTACTCCCTATTTCCTGGCCATTGGGCAGTTAT
TTGACCATTTCATTTAATAAGAAATGTCCTCCATATTTTGTATTAATTGAACATAAACAT
TCATTTATGTGTATTAGAAATGAATCACACACTAATCAAATGATCTTTCCAAGAGGCAGT
TATTGTTTACTTAGTTATCAGGAGTTACATTGTCCTTTGTCAGGTATCAAATCTTGGACT
GAAATTATTTCTGTTGATGAAATACCATATGTGATCTGTTGTGCTCAAGACAAAGAAATA
ATATTAAACATTTATATTCCTGATGAAAGAGTTTTCGGTTTTCTTTCATATGAAAAATGT
TCAGTTATTCCTGGAACAACAGTGGTTGTTGAACCTTTTTCAAATTCTAATCACATTGTC
CGTTGTATATATTTATCAAATTGCTCAAATAAATTGGCGAAAAACTGCCTAAAATCGTTA
GTAAAAGATTCGCAAATAAACGTCCAGTTTTACACATCAGTGGCGTTGTTGACTTTGTCA
CTTATTTTAATTTTTCTGTTGTCGGGATTTATTGTTTATTTGAAAATTAAAAAAGATAAT
TCCCATTCATCTCAAACTAACCATTTCCGTTTGGAACCAATGTACAGTATGACAGATTCC
TCATCAAACAGATTGTATAGTGAATTAGAAGATAAGGCAATAAGTGTCACTTACAATACT
TTATCGTGAtttattagtttcttttgaatttgtctaaaggacactataccactggtaa
ttcaaagtatttttcagatttatgaaaacttattttaattcactttttattttgaaaat
agtgatttaaaattgaatttaaaaataaaaattattagctcttatttgtaacaaacaacg
tttcctcgatatatacatatttactattagataatccaaaagctgattattgtattccaa
ttccaaaaaatgataatataATGTGCCCATCATTATTTCAGCATAAAAATTTCTTCAAAG
GAAATATTTCAAAATATTATCGATTTTGTTGCTTGAAAAACCGGATGGATTCCAATTATT
TACCAATTATAAATAGTTGTCTGTTAATATTCATATCAAAAGAAAATATGCTGCCAAATT
ATTCCTTATGCCCACCAGGATTTATCTATAAACCAATATTCAATGAAATTTTAGAAGAAA
CTTCTATTTATAAGTGTTGCAGATTCAGTCAGGCGAATGTTTCATCTGGTCATGAAATAT
TTAAGATGGTTATTACTGGAAATGTAAATAAATCATTACCAATATTTGGTTCAATCACTA
ACGAATCGTGTAGACCAATACTTGAATTTTATTCTGAAACTGTAGAATATTTTGATTCCC
TGTCAGAAAAAAGTTACTTTCCTTATTCAGAAAATAAAATTTGCTTTTATTACTTAGACG
TTTCAATAAACAAGATGACGAGTAATTCAGAACTAGGTTTGTTAACTATGAATCTATGCC
CATCGGATTTAATCCAATCAAATGAATTTGAAGTTGATTTATTAATAAATAATAAGCCAA
TCAAAAGCTGCTGGCCAAATTCTGAGAATTACTTATATCCGCCAGGACAGTATTGTTTAT
TATTAAAAAGGAACCTAAAATTAGTAAGTAAATGTCCACAAGATTTTCATGCGTACATTA
TTCGATCTATTAATGGAGTAGAATTGGTAAGGTTTTGTTGCAAAATGGAAAATCTGACAA
GTATTATATGGTTGGAAGCAGAAAACAAAACTGTTTTAACAAATGTGATATTCATTTCGA
TTAGAAATGAATGCCCGTTTATGATTTCCGATATTACAATGTCTAGTTACACTGTGAATA

CTACTGCTGATAACTTAAGCCCCACCTTAAATAAATCGAACAATGAAAACAAATTTCCTG
GAATAAATCTATGCATTTATGAAGTGAATAGAGACGAATTTGCATACATAAAAATCGGAA
TTTTTTGTCCAACAGGAATGAATGAAAGCCAAATAATTTTTACTTCGGTTTTACATTGGG
AACCAGATTCTACAAACAAAATTTTTCAAATGGATTTCTGTGTTGGAAAATCAAATTTGA
AATATGAATCTTTTCCAAAAGGAAATTATTGTCTTTACAAGATTGGTAGAATATGTCCAC
TTGGTTTTAAGTCTAAATCCATTCCCAATACTAAAAAAATAATCAGTAAAAGTGGGTTTG
TGCCGAATTACACTGAAGATGCATTTTGCTGTCGTGATGATGGAAATATTCCACTGAATA
AATTTAAACATCACCAGTTTCCATTTTTAAACAGAAACGATATCCGATCTTTTGGATTGC
CCATGTTTAATCGGCAAAATATTTGCCCTCAAATTGAAGAATTGATTGATCACCATGATG
CAAATATAGATGGGTTCTATTGTGCCTATAGAACATTTGAAGATAATTATAAATGGTCAA
ATGAAATTATATTTCCAAGTTTATCTGGAAATATTAATGAAACATATCAATCTAGAGGAT
ACTGGAAATCTCTTACAAAATTTCAGTACACACAATCAGTGGTGGTTGTTTATCCAAATG
ATAAATTTTGCATTGTTTCTGGAGAAAAATGTTCTGCCATGAAAAATATCGATTTTGCAA
TGTTTTATCCATTAATGGGGAAATATTTGTGTTGTTCATTAATAAAGTATAATTTATCAA
ATAGTATACTCCCGGGTAAACACAAAGAATTCAATTCTGTTGGGTTTGTCGCAATTAATG
GAAAATGTCCTGTGATTAATAAAGATATTTACCGAATTGAGAATACGATTGAAGAAATAT
TTGAAATTGAAAAGATTCTATTCAATTCCAGTAATTTATTTGATATGATTAAATTAGGAA
TCAGTGTTGATCTTGATAGTAAACCTCAATTTGTACGAGTGAAATTTTTGTTTTGTTTAC
TTACATTAATTAAAGATCAAATATTACCAATTAATTCATATGGATTTATGAAACCTTTTC
AATTTAAATGTCCAACTGGTTTCGTTGAAAAAACGCAGACCTTCTCTGTGGATATTAAGG
AAAAGTTTAATATAATTTCTTCCCACTTATTGAAAAGTGCAGTTACTTCAAATATGGTTT
ATTCAAGTTTATGCTTTCCAACATCGATTACTAAGAATTACCAAGCCTTATTTAACTCGT
CATCATTGTGTTCTTTTCATTACAATACTTGTCCACCAGAATTTGCAACATTTCCAAAAC
ATTTACTATTCAGAGATAATAATTCCATAAATAATTTGTCATTAATAGTCTGCTGCAAAA
AAATATATAAACCAGAAAATCAGTATGATTTATCATCAATTGATATCCCATTTTCATTAC
CAATACATCCGGAGTCATCAGATTGTCCTATACTCAGAAATTTTAATTTTAAAAAATGGC
GATTGAATATAAAATATTCAGATAATTCTGAAATGAACTCTTTTTGTTTCTATTATCGAA
TTTTACTCCCTATTTCCTGGCCATTGGGCAGTTATTTGACCATTTCATTTAATAAGAAAT
GTCCTCCATATTTTGTATTAATTGAACATAAACATTCATTTATGTGTATTAGAAATGAAT
CACACACTAATCAAATGATCTTTCCAAGAGGCAGTTATTGTTTACTTAGTTATCAGGAGT
TACATTGTCCTTTGTCAGGTATCAAATCTTGGACTGAAATTATTTCTGTTGATGAAATAC
CATATGTGATCTGTTGTGCTCAAGACAAAGAAATAATATTAAACATTTATATTCCTGATG
AAAGAGTTTTCGGTTTTCTTTCATATGAAAAATGTTCAGTTATTCCTGGAACAACAGTGG
TTGTTGAACCTTTTTCAAATTCTAATCACATTGTCCGTTGTATATATTTATCAAATTGCT
CAAATAAATTGGCGAAAAACTGCCTAAAATCGTTAGTAAAAGATTCGCAAATAAACGTCC
AGTTTTACACATCAGTGGCGTTGTTGACTTTGTCACTTATTTTAATTTTTCTGTTGTCGG
GATTTATTGTTTATTTGAAAATTAAAAAAGATAATTCCCATTCATCTCAAACTAACCATT
TCCGTTTGGAACCAATGTACAGTATGACAGATTCCTCATCAAACAGATTGTATAGTGAAT
TAGAAGATAAGGCAATAAGTGTCACTTACAATACTTTATCGTGAttttattagtttcttt
ttgaatttgtctaaaggaca
>DjScaffold4850:3986-4312 strand:+
ataacttttcagtgatttccaattatttatcgatttgattaaatctggttctgatgaaga
aattgaaaataaattcaaaattgaatatcttgagATGAGAGATGAGGATTCGTGGAGAAT
CTTGCATCACGCATGTCGTTATGGTCGAGTTGGATTGGTGAAATTGTTATTAACCAATCA
TTGTGAAATTAATTTACGAACTGATAGTGGATATCAGCCGATACATTTGGCATGTATGAA
TGGTCATATTGATATTGTGAAAATATTGATAGAGAACAAATGTGATATCAATGCATCTGA
TAAATATTGGGGATATCAACCGATACATTTGGCATGTGAGAATGGTCATATTGATATTGT
GAAAATATTGATAGCAAACAAATGTGATATCAATtgaaattgttattaaccaatcattgt
gaaattaatttacgaactgatgtatttttattttacttattatgattaaatatttatat
agagtggatatcagccgatacatttggcatgtATGAATGGTCATATTGATATTGTGAAAA
TATTGATAGAGAACAAATGTGATATCAATGCATCTGATAAATATTGGGGATATCAACCGA
TACATTTGGCATGTGAGAATGGTCATATTGATATTGTGAAAATATTGATAGCAAACAAAT
GTGATATCAAT
>DjScaffold4850:27834-37404 strand:+
aataattgtataaatgctgtatcaaaaccaaatagaagtgattattgtATGGAAATTGGC
TTAATTCGATTTCATTTTACTTTTAATGTTTTCATTGTTGGATTGATATTATTTTTTGGT
TTAGTTGGTAATGTGATTTCTATATTAGTGCTGAAAAAAGATTGGAAACGATCATCAATG

AATGTGCTGTTGTGTGCATTATCTGTGTATGATCTACTGTTTTTATTGATGTCTATTGGA
TACATAGTCATTGCTAGGGGAATATCTCTTTATTTTCCACAAGTTATGTCATCTTCATTA
GGAGGTGATCGATATATTATTCCATTGGCTTGGTTTTTCGGTAATGTAACTCGTTATGGT
CGAAACTGGGTAGCAGTAGCTGTTGCAATCGACAGATTTGTTATGGTCCAAAATCCATTT
CTTGTTCACCGTCGAAACACATCAGTTAGAGCGAGAATGATAAGTATAATGATTGCAATT
GCTACAATTGTATACAATATTCCTCATTTATGGGATTATGCAGAGGTGTGGGATCAAAAT
TCATGTACTAAACGAATAAAAAGCAATCCGAATTTAACGAAATTTCATAGATTTGGATAC
AATATCGTTTTATTTTCAATAATTGTGGTAATCGGTCCTATGATTTTGCTGATAATTTTC
AATAGCCTTTTAACTATCAGTTTGGTAAAAGCAAAACATTTGCGTCAAAGTTTAATGGCA
AAACAACAACAAACAAGAAATTCCGGTCAAATGGAAACGAAAATGCTTGTTGTTGTTATT
GGTGTTTTTATTATTACCGAATTGCCAAGTTGCATTCTACAAATAGCGATTTTTCAAAGA
AATTATAGAAATTGTATATTTCACAGAATTATTCTACCAATTGTTGATGGTTTAGCAATT
TTAAATTCATCGATTAACTTTGTAATATACAGTTTGTGTGGCCAACGATTTCGGGATAAT
TTGAAAAAAATGTTTTGTAGTGGAAATTACAATCACGATCAACAAAATCGGCAAAAAAAT
ATTCAAACAAGTACAACAATTCTATCAATAAAAAAGCAAAATAATATTAACAATAAAGAA
CAAATGAATTTTTACACAATGGACAAAGTTGAATAAtttattttcagttcatcaataaaa
cgatttattattatta
>DjScaffold2706:1921-14799 strand:+
AAAGATGAAGAGAGAACTGCTGAGTTTAAACCCGCTAATAATTTACTAGAATTAGCGGAG
TGTATTGAACACGAAAGTACTGAATTTTGGGGTCGTGATCCAGATCCATTTGATGAAAGA
ATGCCGAAGAAAATACCTGTTGCCGGAACTTATGCTAATATATGTACAGATTTGATCGAT
CATTCTGATTTTATATACAGTTTGATTGATGATTATATCAAATCTCGACCCGGGGATGAA
ATTTGTACGAAATTGACTTTAAGATTTTTGTGCAGTGTACTAAATTGCTGCAGTTTAAAT
ATGGTTTTCAGTGAAAGTCAAAATGAAACGAAAGAACAATTCTATAATAAAATATTTGAT
TGGGCTTTATCAGAACGAGTTCCCGATAATATTTACGCAACATACATTGTTGGACATATC
ACAAAGGAAGATGATCAAGTAATTCCCCAAAGAAAGTCTACTTACATCAATTTGATATCA
CACACGATGTCGAGGCTGAAATCGTTAAATAAACAGTCAAAGGACAGCATGAGATCGAAT
TGGAACATAATTCGTGATAATAATTTTCCATTGGATGAAGGATCGAATGCTAGCGTGAGA
GATATGTCGATGTCACAGATCTGTGGAGTTTGGAATCTCTCATTTCCCATGAAGGAAGCC
ACTCAAGAACGTTTATTGATTTTCTTTTTGAGACATATTGCTGAATTCGAAACGTTGATG
CCATCGATTTATACGTGTGGCGTTTTACCTTTAATGTCATCTTGGCTTCGCGGGGAACGA
CATGATGATATTAGATTGACGTTTGAAGCTTTACGTTTATTGATGAAATTATTATGCCAT
CGAGTTATTTATATTGAGTTCTATGAGAAAATGGATGGTATAAATCTGTTACTCAGTATT
CCTAGGCCATCTGTGGCTGCAAACGCTATCAGTTTTTGCCTGTATAATCTGTTGTATTAT
GTTGAAATAGTTGATAAGGTCATCCAGTTACCATCTCAGAAACTGGAAAATCTCGTTAAA
GTCTTACTCTATATCATTGAAACGTGTCACCCATCAGCACGTTGCTATGGGATGCTGTGT
ATTCAATGTTTGATTCGACAACCATTAACATTGAATATCTTTGAAAAGTTAAATGGACCC
AGATATATTCTTAATTCACTCGCAGTTTTACCAACAATGCTTGGCGAAAATCAAAATAAA
AACGCTAGAAAAGATTTCACATATTTTGGTTTGTTAAAAACTATATTAAATACAATGAAA
GTTTGTTTGGAACAATCTCTTATTAATTGGAGTCTCAACACTGAAAATGCTGATAATACA
GGGCAACTCGCATTTCCCAGCGAAACAGAAACTCCATTCTCAAAAACCACAGTCAATTGC
ATTCCATTCGCTATGAAATTACAAACATGGAAGCATCCATACATTTCAGATCGTAGCAAC
ATCATAAAAGTTATCTATTCAAAATCAGGAATTTTCGATGACGCATTTTCAAATTTCCAT
GACATTATTTATTCCAGTCTCGATATTCTCAGTTTATTGTGTGTTTCAAACAAATTCGTG
GTGAAGTTTCTCGATTTTGCCGAAAGCGATACAAAAGCAACTATCAAATTAATTTTCGGA
TTTCTATTACGCCGTCCAAATAGCATGTCTGCAAATCACGTTAAAGCTTTGAAATCAATA
TTATTTTTCTTGGGAATTTTTTGTTGTCGTCCATTACAGGGCAGCCGTGTTGCAGCCCAA
TTAAATAAGTCAACAAGAAAACGGAAATTTGAAGAAATCGACAATTCATTAAATGTTGAT
GTTATTGAAAAAATACACATCACCGATCAAAGAGTTCCATCAAAAGTTAACATGGTAAAT
ACCAGTATTATAAATAAACCTCGACAAAATCTTCATCAAGAAATACAAACAAAATTATGG
AATCTAATACGTTGTTATAATGCCATTCCAAAATTGTTGAAAATATTAGAAATTCCATTA
TCACCATTGGATACTGTGTCAGTACGAACATGGATTTGCCATGTTTTATCTGGAATGTCG
AGATGCAATGATATTTTATTAATGCTGAGAAAGTTACCAACATTTAATAAAGGGATTCTT
GAAAATTTGATGAAAGAAACTGTAACTCCAGAAAGAATGAGTGAGCATGCAGAATTCTGC
ACAATTGCCAGGCGTTTGATCAGAGATTTACGTGGTCAAACTGGTAGCACTATTGACAAA
GTCGGTGATTTAAGACCAGATCAAGTTAGAAAAATGGAAATAGTTGGTCGGACTCAAATT
CAATGGGACAACCAACAATTGTTAAATCTAATTTATCATCATTTACAACAAACTGGTTAC

AACGATGTTGCTGATCTACTCAGACAGCAGGCTAATCTTCCATTTGATCGTGAAAATTTG
TCATTGTATGAAGATAAAGTCGATGATAAAATCTCATTCAGTCGATTACCACAAACACCC
AAACGTGCCATTGAAAACCGATTTAATATGCTTACATCGTCAACTCCGAAAGTGATCTCA
AATGATAACAGTAATTCTTTAAGGCCTAAATTATATCGAGATAACATCAATCAAACTACT
ATTCTTACCAAATCAGTGCCTGCAAATCCCGGCAAAACAACCACTTTAAATGACATTGTA
GAAACATTTTTACGTACACAACATGCTCAATGTCCAAATCCAATCAGTATTTGTCCTAAA
TTTGATCTACATACCAAACATATCTGTCCATCGGTTGGTGAATTGAACAGACCAACCAAC
ATTCTCAGCTATATTACAAAACGTGGATACGATTCAACTCATCACACTGTCAATAATCGT
ATGCTACATCGATATGTGCATCGATCATTTAAGCCTCGAGTGATTCTCAATGAAACTGAT
GTATTTGCCGATGATGTTTTCTCTGATGTGTTTTGTTTCGCCAAAGGTCTGGAAGATGGA
TTATTTTTCGCTAGTGAGAATTCTGGAATCGTCTTCACAGATGTTGATCTTGCTCAAGCT
ACAGTTGAAATTTTGTGTGTTCTGCAGTTTTTTAAGAAAGCTTATTATCTACGCTTTTTA
GATTATTTTAGATTTATTTTTTTGGTTAAAAAAAATTATTTTTGTATTGAATTATTTTTT
AATTACTTAAATTTTTCAAAAGATTTAAATTCAAAAAATAAATTTAAAACTGATAAAACT
CATTTAGAAAAAATATATTTTGTCTTATTAATAGAAACAATATTGTCGTAAatcaattaa
aaatcaattaatcaatgaaatgaaatgtttaaataaaagcttcatcactagtattaataa
atgaaaaataaaatgaattataaaaatatattgactttataatttattgactttca
>DjScaffold2381:41455-55324 strand:+
aaagaaaATGACTAGAAAAAGAAGTAAAATAAGGTCAAAAACCCGGTCAGATGATTCGCA
AAAATCAGAAGATGAAATTCTTTATTGTATTTGCCGGAGTTCAGATGGAACACGATTTAT
GATAGCCTGTGATAATTGTGAAGAATGGTATCATGGAGATTGTATAAACGTCACTCCGAA
GCAAGCCAACAATATTAAACGGTTTTTTTGTCATTTATGCATTGGTCGTAATCCGAGTTT
GGAAATCGAATACAAAACCATCAAGGAAAATCGACCTCGTTTACCTAATTTACGACAACA
AATCAAGAAAATCAATTTCTCTTCTGAAGATGAAAACGATGCAGACTTGAGTTCTTCGAG
TACTCCGGAAAAATCTTTCGTTCTCGGAAAAAATAAACGAACAATTCGCAAGCGATCCCG
TCCTTCAATATCTGAGTCTGTTAAATCAGCGACTATCAAAAAACGTCGCGGTCGGCCAAA
AGAAAAACCCGACGATTATGATGATGAAGCAACTGAAGATGAAATCGAAACTTCAGGATT
CGTCCCAACAATGAACGAGTGTCCATCGATAAATCTTGACGATCAATCCGAAAATCTCTA
TGTGAAAAAAACGTTTCGTAATCAAGTAATGGGTAACAACACGAATAGTAGTGGATATTC
TCGCACAAACAGCTACGGTCACTCAATATCAAATGATACTGGGCAGAAAATCTGTGGAAC
TTGTGATGGATGTAAAACTACGAACAACTGTGGAAAATGCGATCCATGTAAAAAACAGGG
TGGAAATATCAGTTCGAGATTTACTTGCTTGAAACGGGTTTGTAATCGAGAACAGAAAAC
TCAACTTCAATTCTCTAAACATTATTCAACATTGTTAGACGAAGATTACGAAGGTGTTTT
AGGTGAAGGAGGATTCACTCGACAAGACAATATTCGCCCCAGAAGTGGTGAATATAATAT
TACTGAAGTTCGTCAACGTTTAAATAATCAATCTAATTCTAACAAAGAATTAACTGCTGT
TGGTAGTAAAATGCGACGAGGAGATCATTTACCAGGAGGTGGTAATTCAACTTCTCATAG
ACGTGGTTTGAGTACCACAACATATTTTGGTGGCGAATCACTCGAAGTAACAAATGGAAC
TTCATACCGTTACAGACCACCGCCACCAAATCGTCTTTCAAGAAATCAGAATACCCCACA
AAATATACGCAGGCCTAGACCACAATCAGATACCGCTCAAATGTCGAGCAATACATCTTC
GGTGTCCGGTCAGTTATCACCCCATCATTATCAGTACGCAGAAGACTCTGAAGAGGATGT
TATGATTCCTGGAGAATTAATGTCACCTACAAATGGACAAAGACGATCTATAGTAGAATC
GGATCATAGAAATTACGCGAGCTACAATCAACAACAGCAGCAACGAATTGGCAAAAGTGT
AAATAATGATACGAGAAAATACCAGTATGGCCTTAGCTATCCCGATCATCTATATTGTAA
ACACCACCGATATTATAACTGGCACAGTAGTGTGTCGGGTGACTCAATCTCTCCTGATAT
GACAAACTGTAAAGTCGAACCAAATCTCAAATCAGACAAAGAAGGTGAAATTCTTTCTCA
TTCAACACTTCGGTCTCCTTCCCTTGAACGACAACTATCTCCTGGAAATTATATGGTGCT
TGATCGAACCGTAAAGCAAAGTCAAATTTCTTCCACAGGAATACGTCATAAATCTCAAAT
AAAAAATAGCAGCTCTATTACAAACGATCGTCACGACGTATCCGAATCTTTTGTAACTCT
ATATACAGAATCTAATCAAGTCTATCCTATTATTGATCTCAATGAACAGATGACATCAGG
CATACCATTAAATTCTTCGGCCCAAATAGCTATTGAATCCAATCCTCATTCCGCCTCAGT
ATTTTCAAAATTATCCATGGCTAATGTTCATCGCCAAGAATTCTACACTATAAACGATGA
AGATGATTTAGAAATTAACTGGCCTAAAGAAACTGTTAATGTCTAAtttataatcttcaa
tctttgtaactgcacttttattgtaatagctttattaatgttatgtcattgttaccattc
ttgagatgctagttgttttgtacataataagtatgtatgagtagattatcagtttatttg
ctataat
>DjScaffold2742:9733-10053 strand:+
ATGCCATCTAAAATCGCAGCGGCATCAGAGACATTTCAAGAGATGATGACAAAAGTGGTA

AGGGGAATCAATGGAGCCATCGTTTATCTTGATAACATCCTGATATTTACGAACAATAAA
GATCAACACTATGAAGTAATTGAAAGAGTATTGATGGAAATAATAAAGACTGGTCTAAGA
TATTATCCAGAAATATGTCAGTTTCCTCCAAGCGAAATTAAGTTTCTTGGACACATAATT
AGCAGGGATAGTGTTAGAACTGATCCGTCTAAAATAAAAGCTATAAGAACATTTGGCAAA
CCAAACTGTGAGAAAAAGTAA
>DjScaffold4189:54603-69771 strand:+
tagttatcggaaaatataatctgcttgaaaaataattttatatattatttcatgtaattt
attttactttttacaaatcctttaaaatttaatgtataaatgcttttatgaattaattaa
gaattttgttgaatgaaATGAAATCTAAAGGAATTAAATGCTTGAATAAAGATAAATATT
TTTTCGTCAGTGGTTACATCATAGCTGGGCAACCAGAACAAGCAGAAAACTTCATCAAAG
AAAATAGATTATATGTAGATAGTCACGATATTTTATACAATCGATGTTTCGGTTATGCTA
GCATCGGAGATGTTGCTAGCTTGAAAAATGTTCTTGCGGAAATGAATGAAAAAAATATAA
AATTATCAGATAAACGTTATTGTAATTTATACAAACGATTAATATATGCAAATGTAGAGA
ACTCAAATTTGAATGAGATAATCGATATGACAAAAAATGATTTTGTAAATTTAAAAGGGA
AATTGTACTCTCTTGTGGTTTCTTTAAATTCCGATGACAAATTCAAACACTCATTATGCT
TAATTGAAAAATTCATTGACTATATCGATGAAGGTTGTGGATTCATGTTCACATTCGGAA
CATATAGATGTCCTACAAACATTTTTACACAAGCTTTGACCATTTTGATTAGTCGATGTC
GTACTTTCTCTTTTAATGACAATATATTTGGTGATATAAAATTCGAACTAGCATTGTTAA
TTTCACATAGTTTCACTGCAAATGAAAATTGGAAAGTTATTCATTCTTCTCTCATTAATA
AAGCGATAAATGAAAAATCTCATTTTCTCATTGAGAACACATTAAAATTCTCAAAAAAAT
ATCTGAATTGGATTGAAAGTAGTTTGTTGTTTTATAAAACTTTGCCTGCAATGAAAATCA
ATGGAATGGTGTCGGATTATGATGAGATAGTTGATATGTTTCCAAGATTCAGAAATGATC
TTTTACCAACCATTCTTCTGAAATATCTTTGTTCTGGTGACTTTGACGGTTACAATAAAT
TAAGGTTAAAACATGCAAAGTATATAAATGCTTCACCACAATTCTTTCGGAATTTTTTGG
CGGTTGATTTGTGTAAAAAATTTATCCCTGAGTGAAATCAAGCAATGGTCAATGGAAGCTA
GCAAATGTTTTCAAACGAAATTTATAGAAGATATTTTGTCGAATATAGTAATTAATGAAA
TGGTGAAAATATTGAAGAATGAGTCTTTTGGAGAAATTGAACAATTAAATACTTATATGA
AGGAATGGAATGTAGAATTTTCAAAATATGTTATAGATAATTTGATAAATGCTGTACCGG
AACAATCTGAGTTAATCAAAACATTGATTGTTGCTAATGTTGATGATATTACAAAAGAAA
AGGACTGTGCTAATTAAaattttcaatta
>DjScaffold2228:14393-25974 strand:+
ttctattagtaaaagcacttcatcggtaATGTTGCTTAATGGATTATTTAAACATGAGGG
AAATGTATTTGTTTACAACAACAACAAATGGGGCCCAATATGTGATGATAACTGGGGTTT
TAATGAGGCAAATGTTATTTGTAAAGAGCTGGGATATTCTTATGCTTTGAAAGCAACTAC
AGGAGATTATTTTCGATCTAATGAACAAGATGATTATGTGATGGATGACGTAGCGTGCAA
TGGAAATGAAAAATCAATATTTAACTGCACATACTGGTCTGATCATGATTGTGGATTTAG
CAAGGCAGCTGGAGCTATTTGCTACAAAGAACCCGAAGTTAAACGTAGAAAAACTGATTG
GAATCCTCTCGAATTACCATTCAATAAAAAACAATTAAGAAAAATGTCCAAAGATTCATT
TCAAGTTCTTTTTAAGCATTATACAAATGAAAACGGCACCAAATTTGGTATTCCGGTAAT
AATTTATAAAAATAAAACTGGCGGAATTTGCCCATTGAATTTTGGTTTATTAGAAGCAAC
GGTAATTTGTAGGCAAATGAATTTAGGTTTTGGTAAAATGGTAGTTTTGATAGACCTTCC
ATCAAATTTTATTCCTAATACACATAATTTTCTTCGATTACACAGATGCTTTGGAAATGA
AACGTTAGTAAATAGTTGCCGATATTTTGTAGGATTAGAAAATAAATTATGTTCTCAGCA
CAATCAATCAATTATGGTTCAATGTATTGAAGCGCTTCCAGATTTGGCGCCAGATGTTCC
AGAAATTCAGAATTCGTTGTACATGGATATTGTAACAATAAGAAATGTTGCTTGTGCTGT
TGAAGAAGGTTGTTTTCCGCCTATAGTTTACAGAATGAAGGAAAGAGATCCTTATACATA
TCATGTTGTACAGAGACGATTGTTGAGATTTTCATCTATTATTAGCAATTTGGGAACTGC
AGATTTCCATCCTTACCAAGCTAGAGAAACCTGGGAGTGGCATGCATGTCACCATTCAAA
ATAGaatacatgaggcaattaaagtacatatgttatggcaataaatttaactttattaaa
ttaaccaatagcatttttgtatttatatcatcatataaacaataatggtctttgtagcaa
acttaatgagtattatcattaactaacaagta
>DjScaffold2228:1910-6525 strand:+
ATGGGACTATTTAGATGTACAACTGCTATTATGAATATGGGTGAAATGAAATTCAAACAA
CGACCTCGTGAAGAACAAGCCGAAGCTGATGGTACTTCAGAAGCTGAAAAAGTCGCCTTT
TTATTGGGAGTAAATGCGAAAGATTTACTACAAGCATTTTTGAAACCTAAAGTAAAAGTT
GGAAATGAGTTTGTTACAAAAGGCCAAACAAAAGAGCAGGTTCTATTTGCTCAAGGTGCT
TTGGCTAAATCCATCTATAGTCGGATGTTCACTTGGTTGGTAATGAGAGTTAATAAAACT

CTTGATACAAAGGTAAAGAGACAATGTTTTATTGGTGTTTTGGATATTGCTGGTTTTGAA
ATTTTTGAATATAATGGATTTGAACAATTGTGTATTAATTATACTAACGAAAGACTACAA
CAATTTTTCAATCATCATATGTTCGTTTTGGAACAAGAAGAATACAAAAAAGAAGCAATT
GATTGGGAATTTATCGATTTTGGATTGGATTTACAAGCATGTATTGAATTAATAGAAAAG
CCAATGGGTATTCTTTCCATTCTCGAAGAAGAATGTATGTTTCCCAAAGCTTCTGATGAA
ACGTTTGTTGCAAAATTATATGAAAATCATCTTGGTAAAGCCCAAATTTTGGAAAACCC
AAACCATCTGGTCAATCAACTGCTCATTTTGAATTACATCATTATGCTGGAAGTGTTGGT
TATTCCATAACAGGATGGTTAATTAAAAATAAAGATCCGGTCAATGATAGTGTTATTCAA
ACATTAGGTGCTTCTAAAGAACCACTTGTTTCCCAACTTTTTGCTCCAGCTGATGATGAT
ACATCTGGAAAAAAGAAAAAAGGTGGAAGTTTCTTAACAGTATCATTTGTTCACAGAGAG
TCTTTAAATAAATTAATGAAAAATTTGCAAAGCACTAGCCCCCATTTTATTAGATGTATC
ATTCCTAATGAATTAAAGCAAGCAGGATTAATTGATTCCCATTTGGTTCTTCACCAGCTA
CACTGTAACGGTGTGTTGGAAGGTATTAGAATTTGTAGAAAAGGATTTCCAAACAGACTT
ATCTATTCTGAATTTAAACAAAGATATTCGATTTTAGCGGCTGATGTACTTGGACAGGGA
TTCGTTGATGGAAAACAATGTACTGCAAAAATTATGGAAGCAATTCAATTAGACAAAAAC
AATTATCGGCTTGGATCGACGAAAATCTTTTTCAAAGCTGGAGTTCTTGCTCAATTGGAA
GATTTGAGAGATGAGAAACTATCATCGATTTTATCATTATTCCAAGCTCAAATCCGAGGA
TATTTGATGAGAAAAGCATACAAAACTTTACAAGATCAAAGAATTGCGCTTTCGCTTATT
CAAAGAAATATCAGAAAATATTTAGTTCTGAAAACTTGGCCATGGTGGAGACTTTATACT
AAAGTTAAGCCTCTTCTTAATATTGCACGACAAGAAGAAGAAATGAAAAAGGCAGCCGAG
GAACTCATTAAATTGAAAGAGGAATTTGAGAAAATTGAAAAATATAAAAAGGAATTAGAA
GCTCAAAATGTTGTTTTACTTGAACAGAAAAACGATTTGTTTTTGCAATTACAAACCGAA
CAAGATAATTTAACTGATGCCGAAGAGAAAATTCAGAAATTTATCAATCAAAGAGTAGAA
ATGGAAAATCAATTGAAAGATCTTGAAGAAAAAATGATGGACTCTGAAGATATTCAGGAT
GAATTAGAATCAGATAAAAAGAAGCTCGTTATTGAAATTGATGAATTGAAAACCGATATT
GATGAGCTTGAAGTGTCTTTAGAGAAATCAGAACAAGAAAAACAAGCTCGTGATCAACAA
ATCAATAACTTGAAAGATGAAATTCAAAAACAAGAAGATTCTATTGCTAAAGTTTCCAAA
GAGAAAAAAGGTCTCGAAGACAATTTAAATCAAACTAAAGAAGCCCTTAATGCTGAAGAA
GACAAAGTCAATCAATTGACTAAAATAAAATCGAAATTAGAATCGGAAATCGATGAGATG
GAAGAAAATTTAGAACGAGAGAAAAAAGTTCGAGGAGACGTAGAAAAACTTAAAAGAAAA
TTGGAGACTGAACTTAAATCAAGCCAGGAAAATTATAGCGAATTAGAAAACTCTAAACTT
GAATTAGAAGAAAACATTAAAAAGAGAGATATAGAAATATCGAAATTGAATGTTAGATTG
GAAGATGAGTCGGGAATAGTTGCTCAACTCCAAAAGAAAATTAAAGATTTACAAATACAA
ATACAAGATGTCGAGGAAGATTTGGAAGGTGAAAGACAAGGTAGAACAAAATCTGAAAAA
GCTCGCCAATTAGCTGAACAAGAACTAGATGAATTGGCCGATCGATTAGAAGAACAAGGC
GGTGCAACAATGGCTCAAGTGGAATTAGCAAAGAAACGAGAAGCGGAATTATTGAAATTG
AAAAAAGAATTACAAGATTCAAATTTGCAACACGAACAATTGATTTCTGCTTTGAAAAAG
AAGAATCAAGATATTACAAATGATTTTACCGAACAAATCGATCAAGTGAATAAAACAAAA
TCAAAAATTGAAAAGGAAAAGGTTGCTCTTAAACATGAATTTGATGAAATAAGTAATCAA
TTGGAAACAATTTCTAAACAAAAGACCAACACTGAAAAACAATGCAAACATTTAGAGAGT
CAAATAGTGGAACTTCAAAATAAATTAGATGAACAATCTCATCATATTTCGGAATTGGTT
AATCAATCTAATAAAGACGCTCAATCATTATCGGTTTTGGAACAACAATTAGACCAAATA
GAAAATGAAAACAAACAACTTAATAAAACAAAACAACAATTACAACAGCAACTCAATGAA
TCTAGAACGGCTTTTGAAGACGATGAAAAAGTTAAATCAAAACTTCTCAATGAAATTCGC
AATCTGAATGAAGAAATGGATAATTTGAGAACCAGTTTGGAAGACGAACAAGAATCAAAA
GCAGACATTCAAAGATTACTTAATAAAGCTCAAGCTGAACTCGTTCAAGTTAAAAGCAAA
TATGATAGTGAAGGCTCAGGTCGTATTGAAGAAGTTGAAGAAGCTAAAAGAAAACTACAG
ACAAAACTTCAAGAAATGGAACAAGAATTAGACACTTATAAAAATAAATGTTCACAACTA
GAAAAAATCAAAATTCGTCTTTCAGGAGAAATTGAGGATATGAGTGTCGATGTGGACAGA
GCTAATGCAGTGGCAAATCAATTAGAAAAGAAACAAAAGAATTTTGATAAAACAATCAAT
GAATGGCAATCTAAATGTAATTCTCTTCAAGCAGAAATTGATACTGCTAATCAGGAATCA
AGATTTTTATCCACGGAAGTATTTAAATGCAAAGGTCAAAATGAAGAATATTTGTCAGAA
ATCGAAAATCTCAAGAGAGAAAATAAGAATCTTAATGATGAAATTCATGATTTAACCGAA
CAATTGTCTGACGGTGGAAAATCAGTTCATGAAATCGAAAAAGCTAAAAGACGCTTGGAA
ATGGAAAAAGAAGAATTAGAACAAGCTTTAGAGGAAGCTGAATCTACATTAGAAAATGAA
GAAGCTAAATTACAAAGAGCTCAATTAGAATTGAGTCAAATGAAATCAGATGTTGAAAGA
CGTTTGGTTGAGAAGGAAGACGATTTTGAAGGAACTCGTCGTAACCATCAAAGAGCTATG

GAATCAATGCAAGCATCTTTAGAAGCTGAAATCAAAAGCAAACAAGATTCTATTAAAGCT
AAAAAGAAGTTAGAACAAGAAATAAGTGAACTTGAAGCCAACCTAGATCAAACTCAAAGA
TTGAAATCGGATGCTGATAAAAACATTACAAGAATGCAACAGCAAATTCATGATTTGCAT
TCAGAGGTTGAATATTAA
>DjScaffold3450:34948-35813 strand:+
aacaattttaATGACATTCACTTATAATCGACAGTTTGCAGTAAAGTTTGAAGGAGTATA
CGACGTAGTGGAAGCCTATGGGGCTGAAACAGTTAAGATGGCATTCAAAAACATGAAGGA
TGAAAAAAGAAAAATGAGAAAATCAGGATATAGAAATCCGAAATGTGTGATGATGATGAC
AGACGAAGAAATAATATTAACAAATAACAGAAATAATATAAACATTTTCAAGTGTCAAAT
CGCTAATATTTCGTATGTGATGAGCAGTTTATTCGATAGCCTCTACATAGCTATTATTGT
AACAAACAAATCTGAACAAGAGATCTTAAGTAAATGCCTTATTATTAAGGCAAAATATGG
AAAAACAAAATATATCATTTCGCATATTCGGGATTGTTTAATGGTTATTATTATTTATGA
TTATTAAatattatttattaataattactgttgttagacttggaaatttaattacgcata
gaaaatttgtataataatacaaaaatgatattgtaaccaatatatacagctttttatgaa
tcggttatgtaataatgcatgaccacttcaaataatacacacactcatatattcaatcat
gcatataccttttatcggatgaatatgttaatttactcttgcaaatatacaaattatcca
cagacattcgaaattaattttttttttgagtttatttattaaagtttaaattcttatata
tttttaatttaatcttgaaaaacagttaaattatctttaatggctttgttttgttaaatc
tttgcaatattctttttataaaaaaattttgctttaagtttgaaggagtatacgacgtag
tggaagcctatggggctgaaacagttaagATGGCATTCAAAAACATGAAGGATGAAAAAA
GAAAAATGAGAAAATCAGGATATAGAAATCCGAAATGTGTGATGATGATGACAGACGAAG
AAATAATATTAACAAATAACAGAAATAATATAAACATTTTCAAGTGTCAAATCGCTAATA
TTTCGTATGTGATGAGCAGTTTATTCGATAGCCTCTACATAGCTATTATTGTAACAAACA
AATCTGAACAAGAGATCTTAAGTAAATGCCTTATTATTAAGGCAAAATATGGAAAAACAA
AATATATCATTTCGCATATTCGGGATTGTTTAATGGTTATTATTATTTATGATTATTAAa
tattatttattaataattactgttgttagacttggaaatttaattacgcatagaaaattt
gtataataatacaaaaatgatattgtaaccaatatatacagctttttatgaatcggttat
gtaataatgcatgaccacttcaaataatacacacactcatatattcaatcatgcatatac
cttttatcggatgaatatgttaatttactcttgcaaatatacaaattatccacagacatt
cgaaattaattttttttttgagtttatttattaaagtttaaattcttatatatttttaat
ttaatcttgaaaaacagttaaattatctttaatggctttgttttgttaaatctttgcaat
attctttttataaaa
>DjScaffold3450:121995-123108 strand:+
tacactcctgaactttttaaaccattttttgatagcgatttactatggtattgtttgcag
tatgaaagtgttagctaacctttatagattaattgcgactaacgaagatttatcagattt
gtcagagatataaagttgtgatataactggaaaatatttcaaaaatcagaacaatgtgca
ttttgaaaattgtcaatttcagggtgccaatcatttaacagtgagttggaaagttgatga
aaacgtagtgcgtcacattgatgttattgaagaggataaagacaatgatttttcactcgg
taacacactggttatcgaaggtgaaaaatttgaagatctcgacgagataattgccagcta
cgtacaaccgATGGCGAATTTCGCCCGAGAAGTTCAGAGTTATAAATATTATAAGAGAAT
CGATGGAGATAACAAAAACCTTCTTGTCGATATGATTCGTGCGGAAAAAACGACGAATCC
CAACAAAATTCCTTACTTCATTGCTCCTTTACGTAAATATCCCGGCAGATTTATCATTGT
ATATTTACCAGAATCCAAAGAAAGATTTGATCAATTTAAAATCACACCGAAAGGCTTTAT
GTTCAGAAATATTTGTTTTAACTTTCTCATTAACCTGATCGATTATTTCAAAGCTCATTT
CAACGATCGCCCCTCGACCAGAACCACTCCTGGAACGTATTCACGCACACCCATGATCGG
TTCCACTCCCACATTTGGAACGCCCGCTAATAGAACTCCGAAAGCCGGAGGAGGGAAATG
GTATAATTCCAGTAACGGTCCAGAAACTCCGAGTTATTGAttgttttattgaataaatat
tatatatttaatgtgtataaatattcgagtcgcccgatgtgttttgattagttgattttt
cctcggccagtctggattacacagaaaaatcaataataataataataacattaacatttt
tccaagatttctttgtttggtaataaaattggaattattttgtaaa
>DjScaffold4078:74223-74354 strand:+
ATGGGCAGAAGAAAGTGTTTTATTTCTCGGACACATTGTGTCATCAAAGGGACTAGAGCC
AAATCTAGAAAAGGTAAAAGTGGTCCAAGATTTTCCAGTGCCAAAAAACGTACAACAATT
ACAAGGATTTAA
>DjScaffold2704:65353-82775 strand:+
aaattattaatcggaattttatagctaaaatatttagaacttcaattaatttgattttca
atatatttaattatgtattgtgccttataaaaATGATTGCTCCATTTTATTATGACCAAA

AAGCTGTGCAAACTGAAAAAGTTCCTAATTTTTATTTACCCAACAAAAAACTGTTTAATA
AAAGTGTGAGATTAGAACTTGATAACAGATCATGCAAATCTAATTTTTTAAACGAAGAGG
TAATTATTTTGGCTAATTTCTGTGAAATAGTTGGACCCACACATTTGATTGTTTATCCCA
GTAAACCTGGAATATCCGGTGAAATTATAGATATGGTTGTGTTGAAAGCCATGGCAGTTG
ATTATCAAGCTTGTGAGAGAGGAGATGAAACAAAAGAATCTTCAACTCAAACGCTGATAT
TAGATTGCTCTATTCCATTTTTTGTTCATTACTTCACTTTATGGGATATTTATGCTCGTG
GATACGTTAGACCATGTTGCATTATTGTATGCTGCCGAGATAAAATTCGAGCTAGAGAAC
TTTTTATCACCAAACATTCGGAAATTAAAAAATTGCTCAAACCACTTTTACGGAAGAATA
TTGGAAATTTTAACCAAGAATTACAAAGTAGTAAAGACATGATTGATAAAATATTTAGTC
TGAATCAAAAGGATGAATCGTTTCAAAAGTTGCTATTCGACAATAGAGAATTTGATGTTA
ATAAATTGAAACATTTATATGGCGAATATACCAATCTAATTTCAAGCATTAATGAACAAA
ATAGAGACTTTGAAACGCCTAAAAGATCAATGTCTTGTGATATTCTTCTTGATGTCAATA
TTGTTTTAAGTTCTGAAAGAAAAGAACTTCGTTCTATTTATTCAATTATGCAAATAAATG
AAAGTGATATGAATACTTGGATTAATAATCTCGGAAAGATTTTCACGAGTTCTACAGTTC
CTGAAACATTTTCACCAATAAATATAGATAATCATAAAGAATTGAATAACTTGCAAATGC
CAAATCATTTTTCTAAACTAAAAATCGATTATTTCAGCACTGCTTATACTGTCGATAAAA
ATTATTTCAGTAAAATATCTGGTGATATATTTTATATCGAAAAGTCCAGTCCGTTATGTT
TGTGTAGAACAATATTGGATAACAATAATATCACAGAGAATTATTACAATTTCGGAGATT
TAACAGAGCTCTTGTTTCCGAATTTATTAAATATTAAAGTGAATCAATGTCATTTTGTTA
GTAACTTATGTAAAGTTCTTGGAACACCCATTGTTATCAAATTAATTGCTTGTTTGTTCA
ATCAAATTTACTTAATAATTATTGTTAGCAGTCAACAATCTTATTTTTCATTAATGAGCC
TGTTGATGCTTTTCCTTCCAAAAACGCATAATTCAAATTATTTTATATGTAAATCGGACT
GGCCAAATCTCGAACAATTGAAGGAGTCACCAGTCATTTTCTTGAATAAGGAAGCAAAGA
ATGCCGAGGTTCCTTCACAGATACAAGGATTAGTGGCAATTTATGATTTTTCTTTAAACG
AGTTTGTAACCGGTCCGAAATATTGCAAAGGTCATATAAATACGTTTTTTGAATCCTTTA
AGCATCTAGAGTACAATCATATGCATCTTGAACCAATCGTTGGCATATTGGTGCTTAATT
TTGCCGATATGATTTATCTATGGATAAGTTTAATGGGACGTCGAGACAGGAGGTATGTAT
AAcaaagttgaagattaataatttaaaaattaaattttatatttcattgtaatcctatat
ttattgcaattatagttttcttatgttt
>DjScaffold2198:1366-5848 strand:+
ctatataaggtcatttctaatcctgagattttattattatattttagtaattaaATGACT
ACCGACATTGGTATTAAAGCAATCGAATTATATTTTCCAAATACATGTGTAGATCAAGTA
GATCTGGAAAAGTTGGATAATTGTTCTGGAAAGTACACTAAAGGACTCGGTCAGTTAAAA
ATGGGAGTTTGTAGTGACTTAGAAGATATAAATTCAATCGCTCTCACTGTCACTAAAGAA
TTAATGGAGAAGAATTGTATTGATCCGAAAACTATTGGATTTTTGGAAGTTGGAACAGAA
ACTTTAATTGATAAGTCGAAGTCCACAAAAACAATTTTAATGGATCTATTTGAAAAGTAT
GGCAATACAGATATTCAAGGAGTCGACTGTAAGAATGCATGCTTCGGGGGTACAGCTGCT
CTGTTTGCAGCATTAGACTGGAAGATTAGCAATAGTTGTGGCAGCAGATATTGCTGTGTA
TGCGGAAAAATCAGCCCGGTGTACTGGTGGAGCAGGAGCTGCAGCAATGCTTCTTGGTCC
TGAtgctccactggttttcgataagggagttagatcccatcatatgtcaaatcaatggga
cttttacaaaccaaaactggattcagaatatccagtttacgatggtcaattaagtgttat
atcatatcaaaagtccattgacaaatgttaccaattatacaagaaaaaatgtcttaataa
aagtaaaatatatgtttctttatttttatttgattggttttaatttaaatacaatcatt
caatatacaactacacaattttaaaacgtttttaagctttatgacaatacattgcacta
tataaggtcatttctaatcctgagattttattattatattttagtaattaaATGACTACC
GACATTGGTATTAAAGCAATCGAATTATATTTTCCAAATACATGTGTAGATCAAGTAGAT
CTGGAAAAGTTGGATAATTGTTCTGGAAAGTACACTAAAGGACTCGGTCAGTTAAAAATG
GGAGTTTGTAGTGACTTAGAAGATATAAATTCAATCGCTCTCACTGTCACTAAAGAATTA
ATGGAGAAGAATTGTATTGATCCGAAAACTATTGGATTTTTGGAAGTTGGAACAGAAACT
TTAATTGATAAGTCGAAGTCCACAAAAACAATTTTAATGGATCTATTTGAAAAGTATGGC
AATACAGATATTCAAGGAGTCGACTGTAAGAATGCATGCTTCGGGGGTACAGCTGCTCTG
TTTGCAGCATTAGACTGGTTAGAGAGTAGTAATTGTGATGGAAGATTAGCAATAGTTGTG
GCAGCAGATATTGCTGTGTATGCGGAAAAATCAGCCCGGTGTACTGGTGGAGCAGGAGCT
GCAGCAATGCTTCTTGGTCCTGATGCTCCACTGGTTTTCGATAAGGGTTAGagattaaat
ttgagattttaaacaataaataaatatatttagtaatgaaaatgcctaaattatacaaaa
taattataattattaattatatttaataactataaattatcattattatgattatt
>DjScaffold2198:18253-27888 strand:+

AAATACTATTTCTGTTTTCATTCTCCCTTTACTAAATTGGTTGAAAAATCATTTGCTCGT
TTGGTTTACAACGATTTAAAGGATAATCCTAATATTATTGAACAATACAATGCGGAACTT
GAATGTGTTAAACCATTTTTGAACATCAATGTTGAGAATACGTATGGAAACAGGGATTTG
GAATATTCACTTCTTAAATTTAGCGATTATTTGATAAAAGATAAACTTCATCCTGGAGTT
GTATTGGCACGAAATAATGGAAATATGTACACTGCATCATTATACGGAAGCCTTGTTTCA
GTTTTGCTTCATACATCAATTGATAACCTTTTGGGTACCAGAATTGTTTTGTTTTCATAT
GGGTCAGGTTTAGCATCTTGTATGTTTTCTTTGACAGTTACAGAAAATAAAGCTCAATTA
TCCAGTCTGGTTGGAAACTTAGAGAAAATTATTGTTAATATGAAAAATCGCATTGCTTTA
AAAATTGAAGAATTTGAAGAATTTATGGAAAGAAGAAATTTTCTCAATAAAAAATGTAAT
ATTGAACCTTCTACTAAACTTGGCTACCTAAAATCAGGAACATTCTATCTCACTAATATC
GATGATAAATATCGTCGCCACTATCGGCTATATAATACTGATATTTCAAATGGTCATGCA
CATTTTTGAattagatcatcactaattatcggatatcaattcatatttatatcgatctta
ttgttaaaaaaacgaatctggttattaaacattattatttaaattagtaaattcgttgaa
ttataatt
>DjScaffold3219:830-23946 strand:+
taacaataatttaaatcaaaagttagaaaataattcgaaatttaataaagaaATGATTTT
GGAACATTGTAAAACTATTGATCCTGCTAAAATCGCTGTTATTAATGTCAGTTTTCAAGA
TCTTGAATCATCGGAAGATGAATTTTATGAATTTTCATCAGTTTATAAAATAGACGCTTC
TAAAAATAGATTGAATTTAGATGATTTTATAACTTTTGTGTCACTTGTTGAATTGAATTT
ATCTTCAAACGATCTTAAAAATATTAATATAAATCCACAAGACTTTGAGAATTTGCAAAT
ACTTGATGTATCATTCAACAATCTCACTTGTGAAGATATTCTTATGTTAGGATTGTTGAG
GAAATTGAAAACTTTGATTTTAATTGGAAATAATTTACGAACATTACCTGCAGATATGGC
ACAACCTTATATGGGAGAAGATGGAAAAAGCCATACTAGATTTAATAAACTTGAAGAATT
GAATATGGAATATAATCAATTACATTCAATCAATTCATTTTCGGCTCTTGGTGGTTTGCC
AAAGCTTCGTATTTTAAAATTGGCACACAATGATTTTGATTCAATACCTTTACTGAAATG
CATTAAATTAGTGAATCAAATTGAAAATAATCATAGAAAACCTTATGAATTCGAATTAGA
TGATGATGAAGAAGAAATGGAAGATTATCCTAATACGTTTTTTCTTAATAAAAACATGGA
AATAAGAATACCAAGCACTTTACATGAAGTGGAAGAAGAAACAACAAATCGAACAACAAT
TGATACCAAAACTTCTGACGAAGAAGAACAATTCAAGATTTCAGAGGAACATAGACTAAT
AAATGAAATAATAAATGAAATTATCGACACGATAAATTTTCATGAAGTTACTAATGAAAA
TTTTGACACAGAAATGATTGATAATATTGTCATAAACCAATTTAACGAAGTAGCAGACAT
TTCTAATTCAAATGAATTGGACGAAATTATTGAAAATGATTATGAAGATGAAAGTGTTCG
TGAAGTGGAGAATATAATTAACTTTAATAATCAAAATAGTGAAGAAACTGATGATATTGA
AAATGGGGATTTAATTACTAACGAAAACAGTGATAATTTCAATGGGAATATAAATGAAAA
TAGCAATGAACGCCTGATGGATATTAATGAAAATTCCGAAAATTTACAGTCAAATGAAGA
GAAAGATTTTGACGAAAATATGGAAAAAGATAATGAAGATGATAGTGTGCATGAAGAGGA
AACTGTAATTAATCCTAGTAATCAAAATGATGAAGAAACTGATAGCATTGTAAATGAAAA
AAGTGATAATTTCCATGAAAATTTAGATGAAAATATGCTTATTAACAAAGAATCAAACCG
ACCAATCGAAGAAAACGAAATTCATTTTGATAAATCTAATGAAGAAAACTTTAATAGATT
AGACAACGATGAAATGGATATTTGCAATACAAGAGATATAATTGATTTTGAATATGATGA
AATTGAAATAGATAAAAATTATAAAAAAATTTCTGACGATAAAAATGTAGAATTTTCAAG
TGATTTACAGAATGAAGATAGAGATGAAAGTGATATCAAAATAATAATCATTAGACAGCA
TTCCAAAATCAGTGATGAAATCATAGAATATGTTGAAGAAGATAAATCTAATGATGTATG
TGTTGAAGAATATTTACCTCAAATAGAAATCAATGAAGGAAATCAATTATATTTATTAGA
ACAACATAATATAATGAATAGAGAATTGAATAATAATAGTCCTGACTTAGAACCTTTACA
GCAATATTGCAGCAAAAAAATTTCGGTAGAGAATCAAGAAAATCTATGTCATTCTTTGAT
AATTGTGAATGATTTTTGGAATTTTTCTTCAATATCAGATATGATAAGACAAATATCTGC
CAGTTCTTTTGGAGAAAATCTGCCGAAACCATTTCCAGTTTTAGAATTTTTGGACTTATC
TTTCAACAAGGTCAAATCAGAAAGGGCTCTCATTCCAATTATTGCATGGTTGAAATTGAG
AAATCTATCAATTCACAATAATCCTATTTCTATGAGAAAATCTCAGAAATCATCGTTTAT
CAAGGCATTATTAGAAAATACTTTCAAAATTATTGTTGAGTGGTTACCACCAATTTCAAA
AGAAAGGCCCAATCCTACTATCAAAAAATCAAAACTGAAACTTTCCGATACAATACCGAA
TATTCCAATTAATTGTCGAAAATCAAATAATTTTCCAGCTTTATCAGTTAAAGAAGAAGA
AAACAATCTGAATTTACCAAAATTGGTGCCTTTACCAAATATTGTAAATCAAACATATTC
AGTGAAAACACAAGAAACAGAAAATAATATGGTATTAGAAATAAAAAAGTCACTTGAAGA
AATGGATAATGAAAATGACAATAATCAATCAATAAAATTTATCGGAAGAAGTTAGcgata
ttttattatgaatttttctaaattaatgaagatttaactttttctattaaatattat

taatttattgaaaatatcaaataattatcttaattatttaataagtaatatatgcaaata
atgttatttccaaacattagtatcagataagtaatccaaattttcggcgaacttcaaaa
tttattttaaaaaagtgacacaaattattcaattaaagtaatcgtaacaataatttaaat
caaaagttagaaaataattcgaaatttaataaagaaATGATTTTGGAACATTGTAAAACT
ATTGATCCTGCTAAAATCGCTGTTATTAATGTCAGTTTTCAAGATCTTGAATCATCGGAA
GATGAATTTTATGAATTTTCATCAGTTTATAAAATAGACGCTTCTAAAAATAGATTGAAT
TTAGATGATTTTATAACTTTTGTGTCACTTGTTGAATTGAATTTATCTTCAAACGATCTT
AAAAATATTAATATAAATCCACAAGACTTTGAGAATTTGCAAATACTTGATGTATCATTC
AACAATCTCACTTGTGAAGATATTCTTATGTTAGGATTGTTGAGGAAATTGAAAACTTTG
ATTTTAATTGGAAATAATTTACGAACATTACCTGCAGATATGGCACAACCTTATATGGGA
GAAGATGGAAAAAGCCATACTAGATTTAATAAACTTGAAGAATTGAATATGGAATATAAT
CAATTACATTCAATCAATTCATTTTCGGCTCTTGGTGGTTTGCCAAAGCTTCGTATTTTA
AAATTGGCACACAATGATTTTGATTCAATACCTTTACTGAAATGCATTAAATTAGTGAAT
CAAATTGAAAATAATCATAGAAAACCTTATGAATTCGAATTAGATGATGATGAAGAAGAA
ATGGAAGATTATCCTAATACGTTTTTTCTTAATAAAAACATGGAAATAAGAATACCAAGC
ACTTTACATGAAGTGGAAGAAGAAACAACAAATCGAACAACAATTGATACCAAAACTTCT
GACGAAGAAGAACAATTCAAGATTTCAGAGGAACATAGACTAATAAATGAAATAATAAAT
GAAATTATCGACACGATAAATTTTCATGAAGTTACTAATGAAAATTTTGACACAGAAATG
ATTGATAATATTGTCATAAACCAATTTAACGAAGTAGCAGACATTTCTAATTCAAATGAA
TTGGACGAAATTATTGAAAATGATTATGAAGATGAAAGTGTTCGTGAAGTGGAGAATATA
ATTAACTTTAATAATCAAAATAGTGAAGAAACTGATGATATTGAAAATGGGGATTTAATT
ACTAACGAAAACAGTGATAATTTCAATGGGAATATAAATGAAAATAGCAATGAACGCCTG
ATGGATATTAATGAAAATTCCGAAAATTTACAGTCAAATGAAGAGAAAGATTTTGACGAA
AATATGGAAAAAGATAATGAAGATGATAGTGTGCATGAAGAGGAAACTGTAATTAATCCT
AGTAATCAAAATGATGAAGAAACTGATAGCATTGTAAATGAAAAAAGTGATAATTTCCAT
GAAAATTTAGATGAAAATATGCTTATTAACAAAGAATCAAACCGACCAATCGAAGAAAAC
GAAATTCATTTTGATAAATCTAATGAAGAAAACTTTAATAGATTAGACAACGATGAAATG
GATATTTGCAATACAAGAGATATAATTGATTTTGAATATGATGAAAATGAAAATGAAATC
GAAACAAAAGATGAAATTGAAATAGATAAAAATTATAAAAAAATTTCTGACGATAAAAAT
GTAGAATTTTCAAGTGATTTACAGAATGAAGATAGAGATGAAAGTGATATCAAAATAATA
ATCATTAGACAGCATTCCAAAATCAGTGATGAAATCATAGAATATGTTGAAGAAGATAAA
TCTAATGATGTATGTGTTGAAGAATATTTACCTCAAATAGAAATCAATGAAGGAAATCAA
TTATATTTATTAGAACAACATAATATAATGAATAGAGAATTGAATAATAATAGTCCTGAC
TTAGAACCTTTACAGCAATATTGCAGCAAAAAAATTTCGGTAGAGAATCAAGAAAATCTA
TGTCATTCTTTGATAATTGTGAATGATTTTTGGAATTTTTCTTCAATATCAGATATGATA
AGACAAATATCTGCCAGTTCTTTTGGAGAAAATCTGCCGAAACCATTTCCAGTTTTAGAA
TTTTTGGACTTATCTTTCAACAAGGTCAAATCAGAAAGGGCTCTCATTCCAATTATTGCA
TGGTTGAAATTGAGAAATCTATCAATTCACAATAATCCTATTTCTATGAGAAAATCTCAG
AAATCATCGTTTATCAAGGCATTATTAGAAAATACTTTCAAAATTATTGTTGAGTGGTTA
CCACCAATTTCAAAAGAAAGGCCCAATCCTACTATCAAAAAATCAAAACTGAAACTTTCC
GATACAATACCGAATATTCCAATTAATTGTCGAAAATCAAATAATTTTCCAGCTTTATCA
GTTAAAGAAGAAGAAAACAATCTGAATTTACCAAAATTGGTGCCTTTACCAAATATTGTA
AATCAAACATATTCAGTGAAAACACAAGAAACAGAAAATAATATGGTATTAGAAATAAAA
AAGTCACTTGAAGAAATGGATAATGAAAATGACAATAATCAATCAATAAAATTTATCGGA
AGAAGTTAGcgatatttttattatgaatttttctaaattaatgaagatttaactttttt
ctattaaatattattaatttattgaaaatatcaaataattatcttaattatttaataagt
aatatatgcaaataatgttatttccaaacattagtatcagataagtaatccaaatttttc
ggcgaacttcaaaatttattttaaaaaagtgacacaaattattcaattaaagtaatcg
>DjScaffold3219:41644-41976 strand:+
GCGTTGAGACATAGAGTTGAACATCCAATAACATATTTTGAAAGAAATGTGGATTTATTT
GAAAGAAGTAATTCGTTAAAACAATCAAAAAAATACCAAGGGGAAAATAAATTATGAAAAG
ATAAAAGTAGATAAACGATCAGAACTTATTTCATCAGTTATTGAAAATCAAAAACACAGA
TCAGTTCACTCAGAAGTATCGTTAGATAAGGTGAATTTTATGAATTTATTAGTAAATTTA
CCAAGTTTATCTTAAtcaatatttttaacttgtaataatat
>DjScaffold2823:74554-76273 strand:+
atatcgttgtagaataccaaattttatttacagaatttttccaataattatagactctct
ctgttttccgtttttcagtcgactttcaatatttttggaaaactttagattaaattttta

aaATGTCAGACGACGAAAGAAGAGAAAGCATCAGTGATGAAGGAGGAAAATCAGAAGCTC
AAATAAAAATGGAAGCTGATGCAAAAAGAAGAAAGGAAAAAGCGGAGGAAGAATGGAAGG
AATATGAAGAAATGAGAAAGTCTGAAAAAGAGAAGGAAGCCGAAGAAATTCGTCAATTAC
GTGAAAGACGAGAACAACGAAAAAAGGAACGAGCTGCTGAAGAACAAAGATTAGCAGAAT
TAAGAGTTGTTGAAGAACAAAAACGTCGAGCTGAAGATGAAGAAAGACAACGTCGCAAAA
GAGAAGAAGAACAAAAAAGACGTGAAGAGAGGGAATTGAAAAATAAAGAAGCTGAAGAAC
GATCAAAAACCTCCAAAAGACCAAATTTTATTATTAATAAACGTGCTAGCAATGAATTGG
AAAAAGATGAACCAACAAAAGAAGAAGTTGCAAAATCGAAAGAACAATTAGAAGCCGAAA
GGAAAGCTATTCTTGAACAAAGAATTCAAGCATTAGTTATAGATGGATTTGATTTACAAA
AGCTTAAAGAAAAAGCCAAAGAACTTCATCAAAATATTTATAAATTAGAAAGTGAAAAAT
ATGATCTGGAACAACAATTCCAAAGACAAACTCTTGACTTGATGGAATTGGCTGAGAGAG
CTCGTCAAATGAACAAAGGTGGTAAAGGGCAAAAGGTTAATGTGGCTCAAACAGATGCAT
TGCCCTCTAAGATGTCAAGAGCTCCACCAAAAGTAGTAATGTATAGCCAATATGAAAGAG
TCAAAGATCCAAGAAGTTTTAGAGACAGATCTACAGTATTTGATGGACCTCTGTACTCAG
AATACTTTACAAAAATTGAGCCTCGGTTTAAAGTTCAGATGGGAGAACAAGGTGGAGAAG
TTGTCATGGACAATGATACACATAATGCGAACGAAGCACCAGCTGCAAATGAAATAACGG
CTGAAGCTTAAtttatcccttatttcaaatattgatatgatgtcgaatatctgtgtacga
tagatagtaataactttcccaatctttcgataatgcatgtttatgataaaaaaaagtttt
tttcgaatggctttgaaaatataaggttattgaaattcaaaagctttattattgttttac
aataaattttaatttaaattg
>DjScaffold2823:76378-89301 strand:+
aaaaattattttcaaaaaactaatttttgttactaaataaaattttaagaatattaaatt
ttaattaaaatttaaagttataaaaatattagtattcgaaattgtgtttttcaagtttca
aataattttaaaatATGGGTGATTCTGAAGATGAATTCGAAGAAAAATGTATAAAATTAG
TTGTGGTTGGTGATGGTTCAGCTGGAAAAACTTCGATTGTCAATCGTTTTGTTAATTCAT
GTTTTAATAAAGACTATAGGCAGACCATTGGCATCGATTTTTTTAGCAATATAATAAATT
TGCCAGGTTTGAGTGTTAAAGTTAACTTATGGGATATTGGAGGTCAAACACTTGGTGGTC
CATTACTTAATGGCTACATTTATAACGCAGATGGCATTTTATTTGTTTATGATATAACGA
ACAACAATAGTTTTGAAAACATTGTTGATTGGCTAGATACTGTCAAGAAAATATTTGAGA
TTGAAAATAAGAAAATGCCATATATGGCATTAGTTGGAAATAAAGTTGATCTCGAACATT
CTCGAGCTGTTCGTATAGATAAACACGAAAAATTTGCTCATGATTCTGGCATGTCTAGTC
ATTTTCTTTCGGCTCGCACTGATGATTCGGTAACTCTTTGTTTGTACAGCATAACTGCTG
AAATTTGTGGGATTCGATTGACTCAAGCAGAATTGAATCGTCAAAAAACAGTTGTTAAAG
CTGGAATACAAAGTTTTACAAATTCTCGTTCATCCGGACTGAAAGGTCCAGATAATCAAA
GTGTTTTACCAAAAGTATCAGCTAATTCCAATGTTGAAGATGAAAATAATAGCAATTCTC
TTAACGATCAACATAATAGTCAAGCTTGTTCTATATTATAGtctatttgaagaatttcta
tttaaccactaaattgtcattttacagagtttttgaaattcatgtattctaaattactat
tatttatttttctaaacgataatggcctttgttgaaaaattttgttgtaaattaaatttg
atgttgtgtaattttgatataactatcgatttt
>DjScaffold2823:145158-149488 strand:+
TTAAACTTAAAGGATTTAGCTCAACGAATCAGTGCAGAATTGAAACGCTACAGTATCCCA
CAAGCAGTTTTTGCCCAAAGAGTATTATGTAGGAGCCAAGGAACTTTATCAGACTTATTA
AGAAATCCGAAGCCTTGGAGTAAGTTAAAATCGGGTAGAGAAACTTTTCGACGAATGTGT
AAATGGTTAAATGAGCCAGAAGCAAATCGAATGGCAAGTTTAAGAACTGCAAGTTGCAAA
CGAAAAGATGATTTACCAAAGACTCCCGAAGACCAAAAACAATCAAAAAAGCCTAGATTG
GTGTTTACAGATATTCAAAGAAGAACATTACACGCTATTTTCAAGGAAACAAAGCGACCA
AGTAAAGAAATGCAGACTACAATTGCACAACAATTAAATCTTGATGTGTCGACTGTTGCC
AATTTTTTTATGAATGCTCGTCGAAGATCCGTTGATAAATGGCAAGAAGATATTACAGGT
GATGGTACAGATTCTATACTTGATAATAACAATTCAGAATCTATTTATGATGTAAGTGGT
AGTCCACAAACAGATCATTTGTTGAGTAACTATTCTATTTTAAATAGCAAACATGAATTG
TTAGATCCTGTTGATAAATCTATTTTAAATGACAATTGTTTTTTGAATTCACCAACAAAT
TTTTATGATCACAAATCTCTTCAAGATCTCAGTGGAAATGAATCTATCCAATCAGTTGCT
AAACGTCAGAAGATAAAGGGTACAGACAACACATTACTACTGTCAGCAGTTAATGAAACT
AAATCCATTGTGGATTCTGTCATCTCAGATGCCTTGTCGGATTCTGTTACCAATTCATCG
ATGATTAATACTTTGCCTTTT
>DjScaffold254:97166-98143 strand:+
ttaaattaATGTCTCACAATAACATTGAATTAGCAACTAAAATGTCCAATATTAGTATTG

GACCTTTAAACGAGGATTACAAAAGTATATATCAATTAATAAATATAGATAGAACTGATT
TAATTAAAGGACGTTTAAAAGAAATATTGTCAACACTGAAAACAGACGATAAGATATATA
GATTTATAAACAGTTTAGTAGACATTTTGAAAAGCGATTTGAACAACTGGAAATCCAATG
AAACTATTAAAACCATATTTAATGAATTGGAAGATGCATATCTAAGTATTTCTTCTGGAA
AAAGTGATAAAGCAGCAATGGAGTCATTGAACACGATACTAGAAAATATTTTTATGAAAT
ATACGAAAGAAGTTGAAATGCAAATAAAGAATTGGATCAATAATCCAGAATTTGAATTAT
ATCCATTAAATACAATTTTAATGAACTTTACTCAACTTTTAGATATAAAAAATTATGAAC
CAATTCTTTACCCAATGGCTTACTTAGTAGACGTACTTCTTAAGCTAAACCACAAATCAT
TGCAGCATATTATCATTGAATTTATTCTTCATATTAATCATCGATTGGTGAATTATCTTG
CCGTTATTCCTGGCAATCCGGTATCAAAAGGTTTGAAAGAGTCGATTGCAATTTACAATG
CCGTAAGAACAAATATTGTTCAACTAAATTATACTAGTCCGGAAATTATCACAAAAGCGT
TAAATATTCTTGAACACCGCTCCAGATTCTTCGGACTTACAGCTGATGCTATTCCAGAAA
ATGAAAATCATGGAGATTATATTGTAGTCGACCCTCTTAATACAGCATTTGAGGATGATG
ATGTATATGATGATTATGAAAATTTTCTAGCTAATCTACCCTCATAAttaaagttttctg
tgtataatatttgttatggaattataaa
>DjScaffold254:116682-119025 strand:+
cggagtttataattttatatgaagaATGAGTACTGTCATGGAACACGATGAATATGAAGT
TGAAGATTCCTTAAATGAAGTAGAATTTTACAATGGTGAAGAAGTATTTCTATCTCATTA
TCAGCGTCATGAAGAAATTGGCCAAGGTGGATTTGGTACCGTTTTCAAAGCAACTCGATT
ATCAGATGGTTTAGCTGTTATTGTCAAATTTATTTCTAATAAAAAAGTCACAGACACATA
CAAATGGAAGGCTCAAAAACTTGTTATACCTCTTGAAGTAGCTCTTTTAGCAAAATGTAG
ACACATCGATGGTGTCATTGATATGTTAAGCTTTTACAAATCAGACGAATCAAAGGGATG
GTATTTAATCATGAATCGAGTTGAACGATACCTCGATTTATTTGATTATATCAGTCAAGC
AAAGTATTTATCAGAAGAAAAAGCTGCTTTCTTTTTCAGACAACTTATAGAAATTCTTAA
AAAATGTCATCAGCAAGATGTACTACATCGAGACATAAAAGATGAAAATATTCTTGTTGA
TGAAAGCACAGACATGATTTACCTCATTGATTTCGGTTCAGGTGCATTTTTAAAGAGTGA
TGATTATCGAGATTTTGATGGTACACGAGTATACAGTCCGCCAGAATGGATCAAAAATCA
TCGCTATAACGGATTCAAACTGGAAGTTTGGTCCCTGGGTGTATTGCTATTTGACATGGT
GTGTGGTGATATTCCTTTCAACACTGATGAGCAAATATTAAATGGGAAATTGAAATATCG
TCATTGTGTCAGTTCAGATTGTCGTGATCTTATTGAAAAATGTCTGAAACATGATCCATC
TCTACGTATAATGTTGGATGAAATTTCTTCTCATCCCTGGATGCAGAAACATCGGCTTAC
AACAAAAAATAATTTGCCAAATTTTATTATCAATTCAAATTCGATTGCTCAACGTTGCTA
TCATGCGACATCAGGATCTTCCTCTCTTTATTCACCAGCTGAAACCATATCTTCTTCATC
GGGAGATGAATCAATCGATGAGAAATACAGTCAAACCATTCCTACTTATTTTTCCAAACC
CAGAGATCCTCCAAAATTTTATATCTCCGAAAATGAAGATGAAGATTCCAATGAATTCTC
AGAAAATAATGTTATCAAATTTTCACAAATAACTCGATCTCAATCAGAAAAACCAAACAA
AGAATGTTTGTGTTTTGTTCAAAATTGTACAGTAAATCCTCAATAAtttttttttctttt
ttttgtacaaaattgatttataaatatgctgagtgatatttataaatttattattaattt
aaatataatttatacatcactaata
>DjScaffold254:26439-26498 strand:+
ATGCGTAAAGAAAAAAAGAAGCTTTTGGGGAATTTGCAATTACTATCACAAATTCATTAA
>DjScaffold4805:44186-45930 strand:+
TCGTGGAAGTCGGATTCCATTCTGTGGGACAGCTTAGCCAGAAGTGGTAAAGCTCCTTCC
ATAGACGAAGTTACGCCACCTGAACGAATTGAGCAGTCATTCAAACAAGGAAATAAAAAA
CTCATTGTGGAATCAAATCGTGAACGAACTGTTACTAAATCATCTATGATTACCGGTAGA
CGTTTGGGTGGATCAACACCACTCAGCCAAAACACTCAGAATAATCAGGGCTCATTTCGT
CATGAAGATGACGAATCAGCAAGAGCCGCTGCACAATTATTATCAGCAACTGATGACGAG
AAACAAGAAAATGTTGAGATAGATACTTCAAACAAATTGGTCTATTCCATTCGACATGAT
TCGTTAGCATTGTTTTTTAGGCATATGTACTTCCTGAGTAGTTTGCGATTAAGAGATATT
TGTGATAGGTTACATATTAGTAGAGATCTGATGTGCAAAATATGGACGACATTTGAGCAC
ATCATAATAGAGGAGACCCACTTACTCAAAGATCGCTGTATCGATCAAATAATACTTTGT
AGTATATTTGCTGTTACACGAGTGGCCTCCAGTCGGAATTTAACGTTTCACGAAATTTTA
CAAATGTATAGAATGCAACCGCAGTCCACAAAGGATATATATCGATCGGTACTAATTGAA
GTTGTTAAAACCAATACAGGAATTACTACCGGGGGTTTACCTACCGATTGTAGAACTAAT
GTTCACACGTCACCCGGTGGAACTCAGAGATTTATCCGAACCAAAGAAGTAAGGGATGAC
ATTACGAGATTCTACAATCAAGTGTTCTTGCCAACGGTCCATAACTACGTTAAGCGTTTC
CTAATCAATTATCTGATGACTGGAGGATCGAATGTATCGGTTAGTTTGACAGGGCCCAAT

TCAAATATATCGAAATCGTCCAATCAGCCTCAACTGAACCAGCCGCATTTATCACCAATG
CCAATTAGAAACAATTTCGATCAGATTCTCGGATCTACATCTAGGCGGATATCGAACAAT
AAGAACATATTTGTTAGTCCAGCCAAGACGCAAATCACAATGTCACCGAAACGAGTCACA
TTCACAGTGAACAGCAGTCCCAGTAAAGAACTCCGAGATATTAACGCCATGATCGCTAAT
GCTGCCAACAAAATCAAAACTGGATTGTCATTCAATAGTTCCAAGAGATCAGAGCCGACA
TTTTTCAGTTCACAATCGAATATATCAGTTTTAGATAGATCATCGAGCATACAGTCCGCC
AAACGATTGCGATTCGATCAATAAatatatttgttttaatttttccaataatcttatgag
atatttgtgtttttggtgtattgtattataattaattaaattattagagttcttttttac
tttaaaatcaatcaaattattccagcttttaggcgtgattcgattcaatttctcgaatct
cctagagcttcatgtaatcgatttctaattttctcaaacaactgtaaattcaatatcaga
gttatgctaagccgtgttcagtttcgatggagtaaatgaattggtaaatttttgaaatac
atttaaattatggacatcattggttgattagtccttaaattagatccaataaatcctttc
tttattcgatgatcacagttctttcatagagattcttctattcgttttgtcttcctcgat
tgcatgactgttcttgattactttctctatagctttgtgatcttttgtgtttaattccac
ctgttacagagcttcctggtttctactatacaaataactgcgaatagaattcggcgtctt
tataatgcttatcttcctctttacattttttctatttcttttggttgttgaagcctatt
tttgaatcatctggaactttaacatctaggaggtctcagtttgaagtgaagttccatatg
gtacacgtaatccctatttcacagtattgtattgtatattcatattattaatgtcattat
taatgtgattatcaacaatttcatttatccagatgtacttcctgagtagtttgcgattaa
gagatatttgtgataggttacatattagtagagatctgatgtgcaaaatatggacgacat
ttgagcacatcataatagaggagacccacttactcaaagatcgctgtatcgatcaaataa
tactttgtagtatatttgctgttacacgagtggcctccagtcggaatttaacgtttcacg
aaattttacaagtagaaatgtctggtgttgatagattggtaatttgatgtgtaaatttgc
ttgtccagATGTATAGAATGCAACCGCAGTCCACAAAGGATATATATCGATCGGTACTAA
TTGAAGTTGTTAAAACCAATACAGGAATTACTACCGGGGGTTTACCTACCGATTGTAGAA
CTAATGTTCACACGTCACCCGGTGGAACTCAGAGATTTATCCGAACCAAAGAAGTAAGGG
ATGACATTACGAGATTCTACAATCAAGTGTTCTTGCCAACGGTCCATAACTACGTTAAGC
GTTTCCTAATCAATTATCTGATGACTGGAGGATCGAATGTATCGGTTAGTTTGACAGGGC
CCAATTCAAATATATCGAAATCGTCCAATCAGCCTCAACTGAACCAGCCGCATTTATCAC
CAATGCCAATTAGAAACAATTTCGATCAGATTCTCGGATCTACATCTAGGCGGATATCGA
ACAATAAGAACATATTTGTTAGTCCAGCCAAGACGCAAATCACAATGTCACCGAAACGAG
TCACATTCACAGTGAACAGCAGTCCCAGTAAAGAACTCCGAGATATTAACGCCATGATCG
CTAATGCTGCCAACAAAATCAAAACTGGATTGTCATTCAATAGTTCCAAGAGATCAGAGC
CGACATTTTTCAGTTCACAATCGAATATATCAGTTTTAGATAGATCATCGAGCATACAGT
CCGCCAAACGATTGCGATTCGATCAATAAatatatttgttttaatttttccaataatctt
atgagatatttgtgtttttggtgtattgtattataattaattaaattattagagttcttt
tttactttaaaatcaatcaaattattccagcttttaggcgtgattcgattc
>DjScaffold4805:12161-15602 strand:+
ATGACAAATTTAAATGATGATTATGAAGATCAGGAAGAACAATTGGGTAAACGCTTTGAA
GATCTATGTGTAGATCTATGTATAGAAAGTACTGTTAAAGAAGAAGCATGGGATAAATAC
AAAGAAGTGTCAACTAATTATTGTATTGATGGGGATCAATTGCAGTGGCTTGTATGCGCA
TTATATGAATGTGTTCGTAAAAGTACTACCGATAATATAAATAGTTTAGATAATACTTAT
GTTTCTCTGGCAAGATTGCTTTCTGTTTCGAAAATGAGTTTTGTTCAGTTTTTTCATCGA
ATTGGTAAATGGGCTGACATGGTTGATATGAACGAAGACTTACGTGACCGTATTGAACGA
ATAGAGAGTCAATTTTCAGTTTCTGCAGTTATATTTGATAAATATATAATTGCATTTTAC
GATATATTTCAAAAAACCGATGATTCTCATGTTACACAATCAATGGAAGAAAAATTCAAA
GTTTTGTGTACAGATGAAGAATTGTTCCGATTTACATGGTTATTATATATAAAAATCAAA
ACGAATTTCCCTGCAATTTGTGGGGATATTATACTTTCGTTTCAATTATTACTGTGCACT
CTTGATTGGATTTCAAGCAATGCTTTAATTTCGAAAAGTAAACAACTGTTTAATGTCAAT
TTCAAAGGCCTGCCAAATGATTTTCACAATAGAAATTGGAAATCACCTGAAAGATTTCCT
ACAGTTTTAAAATACATTTGTGAAAGTAATGATATGAATTATATTGATTGCAAATCAACG
AGAGATAGTTTTTTTCGAAATTATATTGAGAAATTATTGGAAAAAGAAGTAATTAAAGGC
CGAGAAACAAAAGATGATAACGTGTTGAGTAGTGTGAATTTTGAATTCAATTCTCGAGGA
ATAATGAATTTATATGGAGAATATATGATGGCATCGGGAGATTTTGACGAGCGGATTTAT
TTGAATCCAAACGCTTTAGAGGAAATTGGTTCTGTAAATTCTCGAAATAGTGAAATGATG
CTGAGTAGTGATTTGACCCCGTCTGCAATGCGTTATATGCAAGAGGTAATCAATTGTGGT
GCTGTTCCTGGCAGTGTCGATACTAGAAAAGCTGCACCGAAAGCATGTCCATCTAGACAT

AATGCTACTCCGAGTGGAATTGTCAGTCGGTTGAATGGTGGAGTAAATTTCAATAATTAC
GATTCCAGAGTGATCAATTCGAATTATCTCATTACTCTACTATCGAAAAGACAAGCATAT
GCTAGCGAGGAATTGACAGACATGCTACAAACTCACTGTTTCATAGTCCTATGGAAGAC
ATCATTTTGCGATTAGAAAAGTTGAGTCTTTATTTTCTCAGCGCATATAACGGTAAAGTT
TCCGAAAACAAAACAGATTTGCGTATACTCTTTCAGAAAAAGCAATCTGATCCGTCTCTG
CAACGTTGTCAAACAACGCATAAAATCTACTATTATGCCTTAGAGATGATACTCAAATCG
GAAATTAGTAAAATTGAATTACGCTATAAAATGCGCTCCAGAAATTCAGAAGGTGGAGAA
ATAAAGCTGAATAACATCGATATTAGCGCGCTTTTAAAGAAAGATGAATTTCACAGAGCA
CTCTATGCAATTTGCGTGGAAATTGTGTTGGTGTGCTCGGATTCCAGTAAACATTTTCCT
TGGGTTCTAGAAGTTCTTGAATTGCAAGCTATTCATTTTTATAAGGTAAAAATTTCCTTG
ATTTTCAATTGTGAATTTTAA
>DjScaffold928:886-8860 strand:+
gtcgcgttattgaggacctacaatcagaattaacgtttttaaaatcagaacaaatagata
aagtgccaactctaaattttggtgcacaagaaATGTTGATTGAGCAATTGAAAAAAGAAA
TAAGCCAATTAAGAGAAAAATTAGTTTTTCAAACCGAACTTGATACATGTTCAATAAAAG
GTGAAGATAAAGAAAATTTAATTCTTCGTAGGAAACTAAAAGCTGCTGCAAATCAAATAA
GAAATCTGGCCAGTGAAAAACAGGTTTTAATTCAAATCAGTAATCGGTTGCGGTCTGAAC
TGAAACTTGCTGGAATAAAATTACCAATTGGTGGTAATAATATCAAATGCGTCACTGAAA
ATCCTATTGAAAATGATGGTAACAAAGATATTAAACGAACAATCAATGATTTAGAAGACA
AATTGTCGAAATTAGAACAATTACAAAAGAATTTAAAAATCAATTGTATAGATGAACAAT
ACATTAAAACAAAATCCAATTTAAAATCTTCAAGAGACATTATTAGTTCAGTTGGTCGAC
AGTCTGTTGATGAAGTTTTTAAATTATTGGATGATTCAGATTCTCTAGGCCAGTCGTCTG
TGATAGACAATGACAGAAATATGCCCATCAGAACGTTTATCAATGATAAATCCAATGATA
ATAATTTTCTATCTTCAAAATCCGTAAAATTGAACACATCTAAATTAAATACAAAACGAA
ATATGGTTACAAAAAGATAAtgataattattattattattatt
>DjScaffold137:3976-21678 strand:+
AAATATGGATGCAAAAATGATTATTCAACATTATATTGCAAAGATTGTAAACTATCATTT
TGCTCCAAATGTCAATCAAAGCATAATTCGCATAAATACCTGAAATTGGTTATAGAGGAT
GGAGATATATGTGAAAGTATTTGCAAAGAAACATTATCAAATTACGCAGATGTTGTATGC
AGTGTTTGTAATAAACGATATTGCTGGAATTGCTTTAAAGACGTTGATAAATCGCATTTT
AGTAAAAAAAAGTATAGTATATATGAGAAAGATGCGGATGACGAAAATACATATCAAACA
ATTCATTTACAGTCTATTCCTCCTAGAATTATGATTTTTGATGAAGATGAACACTTTAAG
TTTCAGTCAGCAGAAGAGTTAACAAAATTGCTCAATGTAGAACTGTCAGAAAAATTTTTG
GTAATATCAATAATGGGACGTACAGGAGATGGAAAATCTCATTGCTTGAACCATACTTTT
TTTAATGGTATGGAAGTTTTTCAAACTGATTCAGGACAAACACGTTGCACTGTGGGAGCT
TGGGCAGCAGTTGCTAAAAAACAAGGTTACATGCTTATTGATACTGAAGGATTACAAGGA
GAATCAAAAAATCCAAGTCAACAAAAACGACTTTTAATGAAAATTCTTGCTGTTTCTGAC
CTCGTTATTTATAGAACTAAAGATAGTCGACTCAACCAGGAAATATTTAACTTCATTGGT
GATGCATTTGCGGCTTTTGACAAATATTTTCGACAGCATCTTGAAAATAAAGCAGAAGAG
TTCCAATGTAAACCAGAATTATTAGCACCGGCATTTTTATTAGTTCATACTGTGGATCAC
ACTGAATTGGTACAACATGATGACTTTATTCTCGATAGAATCAGAAGTACTCGACCTAAT
AAAGTTTTAATGTTTTCCAATATATTTTATGAAGGCCGTAAAATAAACGATAAGGCCTCA
GAAGATGCTGTAGATTACAAAAAATTGGAAACTCGTATAGGCGAAATATTTAAAACTGGC
GGTAATATCCGTACCCCGCGAACATGTGATTTCATTTTCTCATGTATGCAAATGCTCAAT
CAAATTTTTAATAAAAATATAACTGATAAACCTCAATCTATTTTTCCCGATGAATTCTTT
TCATGTGGTAGTCAATGTGAATCTTGTAGCAAGAAATGTGACCTTTCAATGAATCACGCT
GGTAGACATAAAAATAAATCGAGTTGTGTTCTTACAAACAAATTACAAAATTTTGTTTAT
TTATGTAAAGCTTGTAATCATGGCAATGTAATCCCTGAACGTTTTTTAGTTCCAAAACCT
ATGTCGAGTAATGAGTCAACATTTACAGGTGCTCTTAATTACCTTTGGAGAGGTGTTGTA
ATAGAATGCGAGAAATGTGGTATTTTATTCAAATCGAGAGAAAACTGGTTTTATAACGAT
TCTCCTAATGATCACCCCAAATTAATTCAAAAAATTATTCACTATTGGCCTGGAGATCAA
TCTCTTATTTGCTCTGTTCAGCCCATTGGCCAAAGATGCATCGATTCTGTTTCAGAAATA
TCATCAGAACTGGCAAAATGGAGCAATCCGGCCCTTAAAGTTGTCAAGGATACAATTGTT
GATGCGTATCTTCCCGAAGGCATGCAACCTAATTCTGAAATAACTAATTGTGTTGTTTGC
AAGCGTGGTATAGATACTTTAACTAATCCAGTGCACCATTGTCGACAGTGCAGGAAAGGT
GTTTGTAACAAGTGTTCGGATTACCATATAGATTTTCCTAGGCATGGTAAACAACGTGTT
TGTTTCAACTGTTTTGAAGAATCCAAGAATACAGATTTCAAAAGAAAAAATTTACCTGAA

GAAACCAGCGCATTTCAAGTGATTAGAATTGCGAATAAAACAACTAGTATACTTGGAACT
TTAGCTAAGGCACCATTTTCTGTAGCAAAGTTGGTAGTACAGCCGTCGTATTGGCAGTCG
GATAGTGAATGTAAAAAGTGCCCAATATGCAGCAGTGATTTCGATGCTGATTGGCTTCCA
GTTCATCATTGCCGTGCTTGTGGTAAAGGAGTCTGTGATCTTTGTTCCCTTAAAAGAGTT
CCCGTTCCTCTCCGTGGATTAGATAGTCCACATCGAGTCTGTGATAAATGTTATAAAGAA
AAACGATATGAATTACCCTATGAATAGtgcctattttcagatttaaattatataagtatt
gattttattatcaattttacaaaaggctttccacactatcaagatcacaaattggataat
gtaccaattgtctaattgttatgctt
>DjScaffold137:45164-46035 strand:+
ataaagtagaatgtctattagatacaggggctcgaattaacgtgatgagtttgagttgtt
ttaataaactacgagaagtaaagttagaaatgacttATGCGAAAGGTCGATAGCAAAACG
AATGAAAAAGTCGAAAATTACATTTGCAATATTGAAGCAAATTTTGGAAGACAAATTAAA
AAAGGAGAGCGATTGACAAGAGCTTTAAATATTTTTAAGATTAAAGAGGATCAACGGTTA
TTGAAAATAATTGAAAGTAAAGAAGATGCTTTTATGGCAGATAAATGGGATGTAGGACGT
ACACATCTGACCAGACACAAAATAATCACAAGTGGTGAACCAATCAATGTAAAACCATAT
AGACAACCAATGAACTTAGAATCTAAAATTGAAGAAGCTATCCAAAACCTTTTCGAGAAT
AATATTATCAGGAAATGCAACTCTCCATGGAATACCCCATTGATATGTGTTTGGAAAAAG
GAAAAGAAAGACATAAGAATATGTTTGGATTTCAGACAATTGAATGCAGTAACGAAAAGA
CAAGCCTTTCCGATGCCTAATGTCGATGAAATGCTGGACAAGTTAGATGGAGCCAAATAT
TTCAGTTCAATAGATTTGGGAAATGCATACTATCAAGTAGAGCTCGAAGAAGAATCTCAA
GAAAAAAACGGCGTTTTCAACGAAACACGGGCAATATTGTTTT
>DjScaffold137:67243-67892 strand:+
AGAAAGAAGGAAATTGTTGAGTTTGCAATTAGTAAAGATATTACATCATTTTATAAGGAG
CAAATATTTAATCAATATAATGGTAGTTATAAAGCATCTACTTCAACATTTTTACATTTA
AATGCTACTTCCATATCTTTATCGAAAAACATTGATTATCGTGAGACATATAATCAAGAT
TTTGAAAACGATGATTATAGTACAACCTATATGCAAAGTGTTGTTCAAAAAAATGGGAATT
TTGGAATAAcacttgaagaatctaaaagcaaatatccgtttttacatttgtgcttgctc
cggattttgatattaatcaaagtcaattgccttaacaaggaaatttgacgttagatataa
agtttgctaaagctttggttgtgccagcatatgttgtaatatatagagtatttgaaagcg
aaatacaaataaatgctaatagaacagtactagtgtaaacaaaatctaatataatcaaat
aatcaattatgaagtaagtttttattttatttcaatatttttactttattagttctttt
cagattaatcaaattggatcaaaattattgggatcgtcatggttgggagtatatccatta
ga
>DjScaffold137:110912-111687 strand:+
actaaaatttgttaaaaattgttactaactcatctttagaaaatattttagaactagcca
atagtatcaatgataacattaaattcactttagagaagtcaatcgactttcagattcctt
ttctggacatcttaatcaaaaagacacctggcgaattattacttacccatttgtatatga
aaccatttcacagtgggcatatattaccctggagtaaaaattggaataatcaagggtgaa
aagataagagctgatagactatcttcaaacgaagaaATGAGACTGACTTCCCTCAGGTTG
ATAAAAAATAAATTTCTAAATAATGGTTATCCAAATAGAATTGTATATAAACATCTATAC
TCAAATACAGAATGTAATAAAATCAAACAAAAGACAAATACAGTAATTAAATTACCTTTT
ATTAATGAGAAATGTAGCCAAATCATGCAAAATATAATTAAAAAGTCGAAACTAAAACAC
AATCCAAGAATTATATTTAAAACGCAAGCACCTTTGGGCATCCAATTACAGAAACCAAGA
CAAAATTTTTGTGGTCCCAAATGCATTTGTGATGGTAGAAATATATGCAATAAAAAAAT
ATCACTTACAAAATAACATGCACCTTATGTAAGGATACATATATAGGAGAATCATTCAGA
ACCATCCGTACAAGAATTAAAGAACATCTTAAACAGAAAGAAAGTGAAGTCTTCAAACAC
TTTCAAAACAAACATAATGTTTTACCAGAAAATCAT
>DjScaffold137:46042-48042 strand:+
ATGCCATTTGGCATCGCAGCGGCACCAGGGACATTTCAAGAGATGATGACAAAAGTGGTA
GGCGGAATCAATGGAGCCATGGTTTATCTTGATGACATACTGATATTTACGAACAATAAA
GACCAACACTATGAAGTAATTGAAAGAGTATTGACGGAAATAATGAAGGCTGGTCTAAGA
ATTAATCCGGAAAAATGTCAGTTTCTTCAAAGTGAAATCAAGTTTCTTGGACACATAATT
AGCAGGGATGGTGTTAGAACTGATCCGTCTAAAATAGAAGCTATAAGAACATTTGGCAAA
CCTAACTGCGTGAAGAAGTTACGAAGTTTCTTGGGAATTTGTAATTATTACAGGAAATTT
ATAGAAAACTACGCAAGAAAATCAAAAATATTAGAGCAGTTGTGTAGCGGACCAAGCAAC
AAGAAATTAGAATGGACAGATGAAATAAACGATACTTTTGAGGGTCTAAAAAAATCTTTA
ATGACTGCACCAATATTGTGTTTTCCGGACATAAACAAAGAATTTATATTAGATACGGAT

GCCAGTTTTGATACGATTGGAGCAGTATTGTCACAGAGAGATAAAGAAGGAAGAGAAAGA
GTTGTTGCCTATGGATCACACTCAATGACAAGTCATGAAAAGGGATACTGCATTACACGA
AAGGAGTTATTGGCTGTTTATTATTTCTGTATCCATTTTAAACATTATTTGTATGGTAGA
AGATTTACTCTAAGAACAGATCACAAAGCTCTAACTTTCATGATGACGACGAAGAAACCA
ATTACGTCACAATTCCAGACATGGATTAATTATCTGAGCAGTTTAGATATGATTTTGGAA
TATAGAAAAGGAATCCAACATGCTAATGCAGATACGTTATCTAGACCAAGATGTGAGGTA
TGTGTACAATGTCAAATGTCACATGAAGATGCCAAGAAAGGGAAGATTAAAACGAGAATA
CTGAATATAATTGAAAAAGAAAAAGAAGATCATTTAAACATTTCTGAGGAAACAGATTTA
GTCAAGAAAATGAAAGTTCCGGATGACAAACTACGTGAAAAAATAGAAGAAATACATAAA
TTATTATGTCATGCAGGAGTTGAAAAGGTGTACGAATATATGAGAAACAAATATATTGCA
AAGAATTTGTGTAGCCAAATACAGAAGATAGTTCGAAGTTGTGAAATTTGTCAGAAGACC
AAAGTAGTGACAACACCAACTAAAGAGAAGACAAAGAGTTTAGAAGCTACCAATTTATTT
GAAGTGATATTCACAGATATATGTGGGCCAATGCGGGAAACAAGTGGACGGAAAAAAATAC
ATTCTTGGAATAATCGACCAATACAGTAAATATCTGGTTCTTATACCATTGCCTAGACAA
GACGAGGAAACGATACGAAAAGCTATTTTTGAAAAGTGGATATTGAGATTTGGATGTCCA
AGAGAAATAAGATTAGATTGTGGAAAAGCGTTTGAATCCAGAGTTATGAGAGAGTTTGCA
CAGAAGCTAGACATTAAACTTTGCTTTTCGAGTCCATATCATCATAATGCTAACGGACAA
ATAGAAAGACAGTTCAGGACCATTCGAGATTTGATTAATGCGACCCTTCAAGAAAGAAAA
TCAACAGATTGGGCAGATCTTTTACCGGAAATAGAATTTACAATGAACGCAACAAAGCAG
AAGACTACTGGAAAGAGCCCGGCTGAGATCGTTTTTGGAAAGAAAATTAGCAGAGAAAAG
TGGTACGAGTCAGACGTTTCAGATATAATTAAAGAAAACATTTCAGACGAAATTACTAAA
AGAGAATTCAAAGTGGGCGATTATGTACTCATCAAGGTAGAAACAAGAAATAAAAGTCAA
GACAGATTTGAGGGACCTTATCGAATAATTGAGAAAATTCATGAAAGGCGGTATGCAGTA
CAAGGAGAAAATGGACAGTGTTTAGAAAGAAATGTGGAAAAACTTAGAAAGTTTCTTAAG
AAGGGGGATATGAGGAAATAA
>DjScaffold3042:60709-67139 strand:+
GATAAAGAGCCTATTTACTCTAGTATTCCACCTGATAGAAAACGAAGTTCTATTCAGAAA
ATACAAACCGCTGCTCCCATTACTAAAAAAGTGTCTGTAAATGAACAACCAGTACAAGAA
GTTCCTCCACCAAAAACATTAACACAAAAAGAACACCCGACAAAAGCTCAGATTATACCC
GGTGTAGAAACGGTTATTGAATTAACAAAGGACCGAGGTCTAGGTTTATCAATTATTGGT
GGAAGTGACACCATTCTGGGAACTGTAATGATTCATGAAGTTCATGAGAAAGAAGCAGCT
GCTTTAGACGGGCGATTATGGGCTGGAGATAGAATTCTCGCTGTTAATGGAAAAGATTTA
CGTAATGCTACTCACGATCAAGCAATACAAACATTGAGATCAGTTGGAAATCGTGTTAAA
TTGAAAATATTTCGTGATAAACAGCCGTTATGTGAAGATTCTCTTTATTTCAAATTGAAA
GTAGATCTTTTGAAAAAAAGTAGAAAAGGTCTCGGTATTACTATCGTATCAAAAAGTGAT
GTCGGTGTTTGCATATCTGACTTGGTGAAAGGATCTATTGCAGAACAAGATGGTCGGTTG
AAAAAAGGTGATTTACTATTAGAAATAAATGATACAAATGTTCGTTCGTATACTCAAGAT
CAAGTAGCAGCTCTTTTAAAAAGATGTTCTGGAAATGTCTTTCTAATGGTGGGTAGACTC
CGATCGAATATTTATTCTTCTCAAACTTCATCAGATAAACCAACGAAAAAAAAAAAATGA
tcagaaaattcgagaaaagagaaactttcttattattaattaattatttccttgttttt
tttattgtaaaaaatcacaaaatcaatgagaaactgttaaagtttatgtttttaaattat
tattgttgatattccattatgactggctaactctgaaatatttttagtaatttttctat
caacagttcattattagatttaatatagattattcattaa
>DjScaffold3042:747-1031 strand:+
ATGCCATTTGGCTTCGCAGTAGCACCAGGGACATTTCAAGATATGATGGCAAAAGTAGTA
GGCGAAATCAATGGAGTCATGGTTTATCTTGATGACATACTGATATTTACGAACAATAAA
GACCAACACTATGAAGTAATTGAAAGAGTATTGATGGAAATAATGAAGGCTGGTCTTAGA
ATTAATCCAGAAAAGTGTCAGTTTCTTCAAAGTGAAAGCAAGTTTCTTGGACACATAATT
AGCAGAGATGGTGTTAGAACTGATCCATCTAAAATAGAAGCATAA
>DjScaffold3802:53841-55032 strand:+
cgtttgcctcctagacaaactaatagaggatatagagcagcagactggaagcttgatagt
cctgattggactggcagaATGCGTCTTATTGATATTAACAATGAATTAATACTTAAATTA
GAAGATAAAAATACCGGTGATTTATTTGCTAAAAGCCCAATCGATGCATATCCTGGTTTA
GCTATAGAATCTGTTTTAGATTCTAGTAGATATTTTGTTATTAGATTGAAAGACGAACTA
AACAGCAAAACAGCTTTTGTTGGTATTGGTTTTGCTGATCGTAGTGATTCTTTTGACATG
AATGTTTGCATTCAAGATTATTTCAAGCAGCTTAAAAATGAAGATTTGGGTAATAGTCCT
GACGAATTAGTTGAACAATCAAAACTTGATTTACGATTTAAAGAAGGTGAAACAATAAAA

TTAAATCTAGCAACTAAAAAATCAGGTGACACTAAATCTAAAGCCAGTATACCTAAATCT
TATCCTGTTGGAGGACCGATAGGATTACTACCACCCCCACCCTCCAATAAACGTAATACC
AATAATGTCGTACCAAATAATTCAAGCAAAAGTTCAAACATGGATAATTATGTTGATGAT
TGGTTAATCAAATAAagaggataataaagatgctatcttgattgtcaatataaaaaatta
attgatcattcctcaaatcattaataaatcaataaataaatataaaattattatgttcag
tgatattattatttcaattgttttagaatcaaatttattaaagtagatatatgcatgcat
gatgattttattttcaatctgtttgtcgtcaatgatgagtaattaacttgaaatttatta
ttattaatgttattattttatattagattattattatgtaattatggtgagctgttttt
tcgaaactcaaatatttttcgtatatttataatttttagttaatttgaattaacatggtg
tgagtgagtcttaaatttattgtgattatttgtaaagtctagttgatcggcatttctggc
tgattttgaagattttccgtatttgcgattgcatttgttgaaatgaaatgatttgccata
ataatattgttgagcaatagatattaaattctaaatttttgttataaaaatacaccagt
ttatggattcct
>DjScaffold3802:38774-39532 strand:+
ATGATGACAAAAGTGGTAGGCGGACTCAATGAAGCCAGGGTTTATCGCGATGACATTTTG
ATATTTACGAATAATAAAGATCAACACTCTGAAGTAATCAACAAAGTATTGACGGAAATA
ATGAAGGCTGATTTAAGAATTAATCCGGAAAAATGTCAGTTTCTTCAAAGTGAAATCAAG
TTTCTTGGACACATAATTAGCAGGGATGGTGTTAGAACTGATCCGTCTAAAATAGTTGCC
ATAAGAACATTTGGCAAACCTAACTGTGTGAAAAAGTTACGAAGTTTCTTGGGAATTTGT
AATTATTACAGGAAGTTTATAAAAAACTACGCCAGAAAATCAAAAATATTAGAGCAGTTG
TGTAGCGGACCAAGCAACAAGAAATTAGAATGGACAGGTGAAATAAACGATAATTTTGAG
GGTCTAAAGAAATCTCTAATGACTGCACTAATATTGTGTTTTCCAGACATAAACAAAGAA
TTTATATTAGATACGGATGCCAGTTTTGATAAAATTGGAGCAGTATTGTCACAGAGAGAT
AAAGAAGGAAGAGAAAGAGTTGTTGCCTATGGATCACACTCAATGACAAGTCATGAAAAG
GGATACTACATTACACGAAAGGAGTTATTGGCTGTGTATTATTTCTGTATGCATTTCAAA
CATTACTTGTACGGTAGAAGATTTACTCTTAAAACAGATCACAAAGAAAGATTAACATTA
ATGATTACGACAAAGAAACCAATTACGTCGCAATTCTAG
>DjScaffold3802:46827-47108 strand:+
ATGAATAAGATATTAAAAGAGATGTGGAAGGACGGAGTAATGGTATATTTAGATGAAATT
TTGATTTTTACCGATTCTATGGAAACACACTATATTCTATTGGAAAAGGTTTTGAAGACA
ATTAAAGACGCAGGATTAAGAATTAATCCAGTAAAATGCCAACTGTTAAAAAGAGAAGTT
AAGTTTTTAGGTCATATTACAAACGACGAAGGTATTCAAACAAATCCGAGTAAAATAGAA
ATTATCCAGTCATTTCAAGGACCAAAATGTGTAAAAACTTGA
>DjScaffold2402:5744-8568 strand:+
ttttatttttattattagcaataattttgtttatttaattttttattcaagaaacaat
aaaataaaaATGAAAATCGGAAAAAGTCTCTTTAGAAATTCATTGGCGTTTTTAAAATCA
AATTCGACTAGAAATCAAATCAGACGTTGTTGTACATGGAATCTTTCAAATAATCCATTT
ACTAATCCTGCAGCTTTAGCATCTCGTCAAAAGAAGAGCCCTGTTTGGTCATGGAATGAA
TGGGATCATCTGGAAGAATGCATGGTTGGAATACCTGATAATGCTGTTGTTCCCACCATG
ACACCAGAAGTAAAAGCATGCATGAGTCCAGATTTATGGGAATTTCTTGAGACCCACGGT
GGTAAATATTGGTCAGATATTTTACCAGATGGACATTGGAAAGAAATTCAAAAAGAAATA
GAAGAGTTTTCTAGAGTTCTCGAAGGAGAAGGTGTAAAAGTTATTAGACCAGAAGCTCAG
GATTTCGGAAAAAATATTCACTATTCCAGGATTTTCATCACATGGAATTTATGCTGCTATG
CCAAGAGATTTGGTGCTTGTTGTTGGAGATGAGTTGATAGAATCGACTATGACTTGGAAA
AGTCGATATTTTGAATTTATATCCTATCGAAAACTATTGAAAGGTTATTACGATAATGGA
GCTAAGTGGACAGTTGCACCGAAACCTTTGATGCTGGACGAATTAATTGAGAAGAATTAC
GATTTTCACGGGAAAAATAAATTAAAGCGAGTAACAACCAATGCAGAAATATGTTTTGAT
GCTGCTGACTTTTTCCGATGTGGTCAAGACATATTTGGCCAACGTAGTCAAGTTACAAAT
TCTGCTGCAATAAATTGGCTAAGACGACATTTAGCTCCTCAAGGAATACGAGTTCATGAG
CTGAATTTCTATGATGCTTTCTGTATGCATGTAGATGCAACATTGGTTCCAGTAAAACCG
GGAATTTTTCTTAAAAATCCAAAATATCCATTGGTTGAAATGCATACATTTACCAATTCT
GGTTGGGAATTTGTGGATGTTCCTGCTCCACTTTTAAGTGAAGGTAAAAATACTAAAGGA
ATTTTAAAATTCAATTTAGAAATTATTATTTATATAGAATTTTTAAATTATGAGTGTTAG
gtaaatcatgaa
>DjScaffold2075:29881-30326 strand:+
ctgatggagtggaaaacttttggATGTATGCAGTAATACTTGCTTTAGTTGTATTTATAG
TCTTTGTAATACTTGGAATTATAGCAATATGTCGCGAACCAATCAACAACCCCAAATCTG

GTCACATGATGGCTCAAGCCTCGTCCATTCAAATGCATACATCTTCAATTTATGTACCAC
ATACAGGAGGAGCTAACATTCCAATTATAAATAGAAATCATAGTATAAAGTACCAAAGGC
ATGGTAGTTTTACAAAACAAAATGACTTCCTGAATCATAGCCCAGCTTCTTCTGTCAACG
AATATGATGTATCAATAATGCAAAATGATTAAttgacttaattaatttttattgtcgtac
ctcatttaattttaatgaatttctatgtgtacataccaaa
>DjScaffold3326:10382-11576 strand:+
tttttgtaaATGGAGAGTAATACAGATAAGGGTTATAAATTATACATTGGAGGGTTATTG
GATTCTGTTACTAAGGAGGATATAGATGAAAATTTTAAAATTTATGGCGAAATAAACGAT
ATATGGATTGCTAGAAATCCTCCTGGCTTTGCGTTTGTTTTTTATAAAAAGTTGGCTGAT
GCAGAAAAGGCAATTAAATGTCTTGACGGTGGAAATACATTTGGTTCTCAAATTAAAGTT
GAATTTGCTAAGAATACTGGTAAACGTAACAAAAGTTATCGGCAAAATAGAGGGCCTACT
GATATGAACAGAAACCAGAGAGATTCTTCAAGATACTTTGATGATAGTTCAATGTTGCCG
CGAGGGAATAATCCCAGATCAAATACTGGCATAATTGAAAATTTGATGAATAGAAATACA
TATGCATATAAAAATGAATCAAATCCAGTGAATATTGACCTCTTGAAAAATATTCTTAAA
TTGGTTAACAATAGCAATGTTTATGGTCCTTTAACTAATCCTAATCGTCCCAATCCCTAT
GATGATCTTCGATTCGGCGAATCTTCATACAATCGACGAATAAGAGATCGATCTCCTATA
AACGCTAGGGATTCTTTCGACAGACGATATCCAAGAGATAATAACAATTCACCATATTTC
AACAACAGAACTTACGGACGAAATTTCAACAATATGTAAagaaaatgtgtgggtcttgca
ttctgatgcgattttgaattgtttctagttgttattcgcgaagaatgagttcaaatttgt
actatttaaaatacaattttaaagtatttataataattattcaagtgttatttaataatg
agtacattttgaaaatctagccttattatatttgtaaattaacgcgtttgtaattgaatt
gttgagtacgacaattaaaatttccgggaaaaatgaagttacattaatttttatgtatg
ccgtgacagttgaaattatatttctatttttgtaaATGGAGAGTAATACAGATAAGGGTT
ATAAATTATACATTGGAGGGTTATTGGATTCTGTTACTAAGGAGGATATAGATGAAAATT
TTAAAATTTATGGCGAAATAAACGATATATGGATTGCTAGAAATCCTCCTGGCTTTGCGT
TTGTTTTTTATAAAAAGTTGGCTGATGCAGAAAAGGCAATTAAATGTCTTGACGGTGGAA
ATACATTTGGTTCTCAAATTAAAGTTGAATTTGCTAAGAATACTGGTAAACGTAACAAAA
GTTATCGGCAAAATAGAGGGCCTACTGATATGAACAGAAACCAGAGAGATTCTTCAAGAT
ACTTTGATGATAGTTCAATGTTGCCGCGAGGGAATAATCCCAGATCAAATACTGGCATAA
TTGAAAATTTGATGAATAGAAATACATATGCATATAAAAATGAATCAAATCCAGTGAATA
TTGACCTCTTGAAAAATATTCTTAAATTGGTTAACAATAGCAATGTTTATGGTCCTTTAA
CTAATCCTAATCGTCCCAATCCCTATGATGATCTTCGATTCGGCGAATCTTCATACAATC
GACGAATAAGGTAAtcgtctaaagttaatttttatgtctctttttaaagaagtcttcgtc
gattttaattaaaggtgaaagtcaacaaatgttaataatttagtttttttttcgaatgtt
atttttggatttattacttgaacctttcagagatcgatctcctataaacgctagggattc
tttcgacagacgatatccaagagataataacaattcaccatatttcaacaacagaactta
cggacgaaatttcaacaatatgtaaagaaaatgtgtgggtcttgcattctgatgcgattt
tgaattgtttctagttgttattcgcgaagaatgagttcaaatttgtactatttaaaatac
aattttaaagtatttataataattattcaagtgttatttaataatgagtacattttgaaa
atctagccttattatatttgtaaattaacgcgtttgtaattgaattgttgagtacgacaa
ttaaaatttccgggaaaaatgaagttacattaatttttatgtatgccgtgacagttgaa
attatatttcta
>DjScaffold3326:63228-73099 strand:+
AGACTCGAACAAAAAATCATTGAACTTGAACAAACACTTTCCAAAAAATCAGATCATTAT
TTAAATGAGGTTCAGAATTTAGAATACAAATTATCAAATACTCAACAAAATCTTGACATG
GAGAAACAAAACTCAAATAATCTTTCTGCAAAATTAACAAATATGCAATTAATGATTGAC
AACAAACAAGAAAAGAAAGTGTATAGAAGCAGAACTAACAGTGGTACTAGTGTTGCTGAA
ATCGAACCAGATGATTCTGTCAGTATAATCTCTAATAAATCTTTGGAAAATGCTGATAAG
AAAATTAATGGAGCATATTCAGTTTATGAACAAATACGACAACAAGGAGTGGCTGTGATG
ATCGAATATCTAGAGGCTTCATTAAAGCAAAAAGAGGGGCAAATAAGTAAACTTCACAGG
GAATTGCAAAATATGAGTAGATCGAGAGACAGTATGATTGAAGAAATGACAAAACTTACC
CAGACCATAGAGAAATTAGAAAATAAATTAGAAGAGCAGCAAAGTGCTATCGATAATGTT
TTGGAATTAACCGATCGATACAATGCACTATTGCAAATGTATGGAGAGAAATTAGAAGAA
TGTCAAGAGCTGAAAATGGATATTTCAGAAATGAAAGAAGTTTATCGATCACATATTGAT
ATGCTTTCCAAATCAAATGAAGCGCTGTGAtgtatttgtagtatatgactgtgatatgta
tatataaataacgatgttgggtgcttattgggattttctgaattattgttaatatatgaa
taggctttcaattttcatttttgaaatttattttacagacaatgactatgagttgacatc

tcactaataatttctttgtaattcatttacagacactatttaaaaatatgaaatttgatt
tttaa
>DjScaffold3326:100640-101016 strand:+
ttgtgcattgcatcagcaactttccaaagtttgATGCATCAATTATTCAACGAAATGTTG
TACAAAGGAGAACTAGTATACTTGGATGATATTTTGATATATGGACAAACTCAACAAGAG
CATGATGAACGGATTGGTAAATTTTATTTTTACTCAAACAAGCAGGTTTAAagataaata
cagataaatgtgtattcaattctgagaaattacgattattgggtcaaaaaattagtgcta
aaagtacaccaaaatgcaccacacaaatacgggcatttctaggtcttaccaattgttaca
ggcggtttataaaagactacgctctcgttgca
>DjScaffold5100:107934-108539 strand:+
ATGCCTTTTGGATTAGCTACAGCACAGGTCACTTTTCAAAAATTAATGCACGAGTTATTG
GAGGTATTAATTTTCAAAGGAGTTATTGTATATCTAGACGATATTCTGGTGTACGGAAAA
ACTTTAGAGGACCACAACAAAAGGTTAGAGACAGTCTTTTACAGGATCAGAGACTCGGGA
TTAAGAATAAACCCTGATAAATGTAAGTTTTATCAGAAAGAAGTGTTATTCTTGGGACAT
ACAGTTAGTGCAACTCGTATACACACTAATGAAGTTAAGATTCAGGTGATTAAGAATGAT
ATAGTTCCAGAATGTTCTTCAAAACTCCGTTCGTTTCTTGGACTAACTAACTACTACAGG
TGTTTCATAAAAAATTATGCAAAGATAGCAGCTTCTGTATATGAAGCAACGTCAGGTTGC
AACAAACCGTTAGTTGGGATAGAAGCCTGTCAGAACAGTTTCAATGCGTTAAAAGACACG
TTATGTAGTACTCCAGTATTAGAATATTGCAGAGAGGATCGTATGTTCATTTTAGACACG
GACGCTAGTTTGAAGCTATCGGTGCAGTCCTTAGTCAATGCGAAGAGAAAAAGGAAGTGG
TTATAG
>DjScaffold4032:7666-8519 strand:+
gtgggcatttagcaaagatttatgctATGCAATGGTGTAAAGATAGTAGAAATCTGGTAT
CGGCTTCTCAAGACGGAAAATTAATTGTGTGGGATGGTTACACAGGATATAAGATTCACG
CCATACCATTGAGAAGTAGTTGGGTAATGACATGCACCTATTCTCCATCAGGTGGAATGG
TTGCCAGTGGAGGCTTGGATAATACTTGTTCTATTTATGATTTACGAGGTAGAGAAGGAC
AAGTTCGTATTTGCCGGGAATTACGAGGTCATACTGGATTTTTGTCTAGTTGTAAATTTG
TTGATAATGCTCGAATGCTGACAGCGTCAGAAGATGGAACATGTGCATTGTGGAATGTTG
AAACTGGAACATTATTATCAAGATTTTCTGAGCACACAAGTGATATAATGGGTATATCAG
TGTCGGATGATGGATCGATATTTGCTTCGGGATCCTGTGATCACTCTGTTAAGGTTCATT
TTATTGAAAATTTTAATTAAttttgtaataatttcatattgataatttaatgggtaaaa
tttgatgaccttgaccttgggtttcaattttgattagttttaacaagaattgtgactaat
aatcgttttgattgaataaaaggaatatttctcgcacctcattttatataaataacctag
tgtgatttttttttgacaaaataataattttgttctgtattgattaaattataataatt
ttttttgtttagtattctagaaactaaaagaattg
>DjScaffold3579:11364-39960 strand:+
atttagtgtttttttcgtcgaATGGTAACCAATTCAATTTTGGTTCAAGTGGTATTGCAG
TGTACTGGATCTAATAAATACAAATGTGAAATAGCTCCAGATACTCGTAATAATTGTCAG
CGTTGCCGATTACAAAAATGTTTTCAAATGGGAATGACGAGATATAAAACTCGTCTTGGT
CGCCATACAAAAGCTTTTGCATTAAAATTGGAACGAAGTACTTCTCGATCAAGGGTTTGT
TTGTTTGTTAATGATGACGAAATGGTGAAATTAATTAAACGAGAAAATAAGATTTTCAGC
AATAATGATACCGAAGACATTATTCAACCAATAGAAGAAATACCGCAAGCGAAATGTGTT
AAATATAAAGCTAAACTTTATAGTGAAAGGAATTTGGGTATCCATCAAATGGCCACTGTT
TCAAGCATCGGTTCGAGCCAATCGGAAAATAATTTTGAATTGGTTTCAGGAGGACATCAA
TCAGTCAACAATACTCAAATGAATTCAATACATCATACTAATAACATTAATAGAAACAAT
TGTTTCTTTGACACTGTTAAAGCCAATTCAGAAAACTCTCAAATTAAACAAATATGTATC
AAGATTCGTTCATTTTTTAATTTAGACTCAAATTCTGAAGACTTTCAACATTTTATTTAT
TATAAAACAACAATATCTGCATTAACGGATTTTTTTTACCATCCGAATCTTGGGGAAATG
GAAGAATATTATAGGACACGTATTGAACAGGTGACATTTATTTACAATCCGCTGTTGGTT
TTCTTTGAAATGATAAAAGAATTATCAAACATGTCTAGAAAAATATTTGCATTTTTCAAA
AGACTTCCAGATTTTGAATTATTTGGATTAGTCAGTCTAGAAAGCAAATATATTAAAAAA
ACGATAGTTCAAACATTGTATGTTTGGTTGATCAGATTTGTAAACGTTTCAGAAAATGTT
ATATATAATACTAAATTGACGATGAAAATTCCTCGTTCAGTACTTTGGCAGACTCCATTG
AATCGTTTCTTTGAAACACTTTTTCAATATACTGGTATTTGTAATTCTACTTTAACAATG
GAAATCGATGATTACGAAATTTCACATATTAGTGCTCTTATAATAACATTTCCAGAATAC
GATTTGAATAATAACTTGAAATTCATGTTACAATCTGATGATTTTTTGAAAGTTCGATTT
TTATATTGTGCTTCATTGTATGATCATATATTTACTCAACGATGTAATCGAAAACACGAT

CTCAGTAAGTACATTTCGGCCGCTAATTGTTTTCCGTGTTTTGTGAAAGTATTTTCTATT
CTTTCAAATAATAATAATAATAATAATAATAAATTTTATTCTTTATAAttggcttttcac
aagtcatttctaataattaattattaataatttatgttttgtttatttttaaaataaa
ttctgattaaaaggcaaattatagtaagaaacttgatgaaatgttatcgaccatactctt
catacatcaga
>DjScaffold3583:21393-24241 strand:+
tttataaATGTATGATTATGACGATTCTGATGGAACTGATTACTCTTATGAAGATTCAGA
TTATGAATATAATAGTTTAGAACCATATGCTTATGAACTTAATAATTCTAAAATAAATAT
TCAAGAAACACCAGAAGATAAATTATTTAAATTTATTCAAACAAATGACTTGACCGAAAT
TCAATCACTTATTCCGAGTTTGTTAAAAGAAGATGATTACACTGTATGTCAGCTTAATGG
TAAAAGCAATACCAATAATAATGTTGATGAAAGTGGAGATGTTGCTAGTGATAATTCTAA
TGATGAGTACAGTGATACCGAAGAAGATGAAGATAGTAAATGTGATATTTCATCGCGCAG
TAAATTCAAAGAAATAATATTGAAAAATGATGATACGCCTCTTATTCTAGCTGCTAACGA
AGGCAATGTTGAATTGGTTAAGTATTTCTTATCTCTTGGATTTAACGTTAATTATAAAGT
ACCCAATACTAATAAAACCGCACTTTTGGCGCTATTTTGTAAAAGCAATTCGGGTATTTG
GGGAGTAGATAGAGATTCTGAAACAATAGAAAATCTAATAAAATGTCTGAAAGTTATAAT
TGATGCTGGTGGAGATGTAAGAGCAAAATGTTTCATAACAACACAAGATTATGGAAGCAC
TGCTCTACATCTTGTTGTTGAGAAGGATTATTGGGAAATGATAGAAATATTGATCAATGC
TGGTGCAGATATTGATGCTGTGAACAACGATAAATTGACTCCCTTGCATATTGCTGCTAA
AAGAGGTCATGTTGCCTGTTGTAATATTTTGTTACTAGCTGGTGCTGATGCCAATTTAAC
AGATAAATTCGGATATTCAGCTGCACAGTTTGCAGAACATGAGGGTTATACTCATTTATC
AGATGCAATCAATGTTTTTTGTGGTAATGCTCAGCATAATGTTAGACTATACAATATTTG
GAAACAATATGCTCCACAATTCAAACCTCTTGATCACGATACCAGTTTTCTAACGGATAT
TAAAGATAATATGTTAGTTTTTTGTTTATTTATTATCAATAAAAAATTGAATTTATTCGG
ATTAAGAAGGAATCTAAAATATATAAATAAAATCTATAAATAGaatagaaaagataatta
ggaataaaaaataattaataataaagacaattaataaaaaaaagacaattaataataaaaa
taattaataataaaaataattaataaaaaagataattaggaataaaaataattaataata
aagacaattaataaaaaaagacaattaataaaaaaaagacaattaataataaaaataatt
aataataaaagataattaataataaagataattaataataaagataattaataataaagc
aattaataatacagaaaattaattaaaaaaaaagataattaataataaagata
>DjScaffold3583:56956-57462 strand:+
ATGCCATTTGGCATCGCAGCGGCACCAGGGACATTTCAAGAGATGATGACAAAAGTGGTA
GGCGGAATCAATAGAGCCATGGTTTATCTTGATGACATATTGATATTTACGAACAATAAA
GACCAACACTATGAAGTAATTGAAAGAGTATTGACGGAAATAATGAAGGCTGGTCTAAGA
ATTAATCCGGAAAAATGTCAGTTTTTTCAAAGTGAAATCAAGTTTTTTGGACACATAATT
AGCAGGGATGGTGTTAGAACTGACACGTCTAAAATAGAATCTATAAGAACATTTGGCAAA
GCTAACTGCGTAAAGAAGTTACGAAGTTTCTTGGGAATTTGTAATTATTACAGGAAATTT
ATAGAAAACTACGCAAGAAAATCAAAAATATTAGAGCAGTTGTGTAGCGGACCAAGCAAC
AAGAAATTAGAATTGACAGCTGAGATAAACGATACTTTTGAGGGTCTAATGACTGCACCA
ATATTGTGCTTTCCGGACATAAACTAA
>DjScaffold3583:46743-46985 strand:+
ATGAATTATATACTGATGGATCTGGATCATGCATTAGTATATATTGATGACATTATAGTG
TTCTCAACAAATTTTGAGGAACACTTAAAAGATCTAGAAATGGTTTTGAAACGATTAAAA
CTGGCAAATTTTAAAATAAAATCCATCAAAAGTGCGAATGGGCGAGGATTCAGTAGTATA
TGTGGGGCATGTGATATCACAAAATGGTATACAGCCAGATCCTGGAAATGTGAGCAAAGT
AAA
>DjScaffold3583:31034-31928 strand:+
ATGGAAATAATGAAGGCTGGTCTAAGAATTAATCCAGAGAAGTGTGAGTTTCTTCAAAGC
GAAATCAGGTTTCTTGGACACATAATTAGCAGGGATAGTGTTAGAACTGATCCATCTAAA
ATAGAAGCAATAAGAACATTTGGCGAAGCTAACTGTGTGAAGAAGTTACGAAGTTTCTTG
GGAATTTGTAATTATTATAGTAGTTTTATAGAAAACTACACAAGAAAATCA
>DjScaffold3605:4425-6737 strand:+
ATGCCGTTTGGACTCACTGGAGCACCAGCAACATTCCAAAGGTTAATGAACAAAGTGTTG
GAAGATGTACCATTTGCGATGGTATACATAGATGATATCTTAGTTTATTCAGAAACGTTT
AATGAGCATCTCAGCAATCTGGAGGAAGTATTTAAAAGATTAGAAAAGGCTAATATCAAA
TTAAAACCAACTAAATGCAAATGGGCAGAAGATGAAGTAACTTATGTTGGTCATGTAATA
TCTCGTAAAGGGATAAGTCCTGATCCTGCTAATATCACCAAAGTTAAAAATTTTCCAGCA

CCAAAGAACCCAAAACAAATTAAAGAATTTTTGGGTATGGCAGGGTATTACCGCAAGTTT
ATTAGAGATTTTTCAGCATTAGCTTTTCCATTAACTCAATTAGAAAAGCAAGGAGTAAAA
TTTGTTTGGGGGCCAGAGCAACAGAAAGCTTTTGAGGAGCTTAAGAAGCTGTTGACAACA
GCACCAATTTTGAGATACCCTGATTTCGAAAAACAATTTATAGTCATGACTGACGCCAGT
AATAAAGGTATTGGTGCAGTACTAGGACAATTAGATGAGAACAAAAACGAATATGCTATA
GCATACGCAAGTAGAGGATTGAAACCTCACGAGAAAAACTACGCGGTAGTTGAATTGGAA
ACTTTAGCAGTAATATTTGCTATTGACAAGTTTCGGCATTACCTATGGGGTAGAAAAATT
GTTATATTTACTGATCACAAACCATTACAATGGTTAATGAAGCACAAAAACGACTCATCA
AGATTGATTAGATGGGCACTTAAACTGCAACCATACGATATTGAGATAATTTACCGAAAG
GGAAAAGAGAATTCAAATGCAGATGCACTATCTCGTATGGAGCAAGATGATGAACCAGAA
GATTTAAAAGAAGAGAGACTTGAAGGAACTATATTCGCAATCTTAAAACAGGTCAATAAT
CAATCCGAAGTAATTCAAGCTCAATTAGAGGATGAGGAGATCAATCAGATCAAACAAGTA
TTGCAGACTGGGATAATACCAGCTGAATTTCAAAAATTTTGGTCAAAAAATGGAGGAAAG
TTCATAATGGAGGATGGACTTGTCAAATATCAGGAAACTATGGACAACGTCGTCATGGTT
CCAAAAATGCTTAGAGAAGCAGTAATGTTGCAGTATCATGATGGTCAACTTGGAGGACAT
CTATCTGCAAAGAAAACCATATCAAGAATTAGACAGAAATATTTTTGGCCAGGTCTTAGG
AAAGATGTAAAAGAATGGTGTCAAACGTGCAAAGTCTGTGCTACAAGGAGAAATACTGGG
GTTAAGCCTCGAGTACCACTAAAACCTATGCCAGTTCCAACCTCGCCAATGGAAATCTTA
GCGATGGATGTGTTGGGACCATTTAAGCAAAGTAAAGATGGAAATAAATTTATCTTGGTA
GCATGTGACTACTTAACCAAATGGCCAGAAGCATTTCCTATGCCAGATCATAAAGCAGAT
ACAATGGCTAGGATATTTGTTGAGCAAATTGTATTTAGGTATGGAGTGCCTAAGAAATTA
TTAACAGATAGGGGTACTGATTTCATGAGTAACCTGTTAAAAGGTATAAATGAATATTTC
GGAATATTGAAGTTAAATACATCTCCATATCACCCACAGACTGATGGATTAGTCGAACGA
TTTAACGGGACATTAGCACAAATGTTGGCATCGTATGTCAACGCAGATCAAACGGATTGG
GATGTTCATATTCCAACTTGTTTGTTTGCATATCGAAATGCAGTGCACTCAGGTACTGGT
TATTCACCATTTTATTTAATGTACCTGCGAAATAGTAATATGCCAATAGATCTTATTTTC
CAAACTCCATTGTCACAATATCTTGATGAAAAGGATTATTTGTTGGAGTTGAAAGATCGA
ATGCAAGTAGCATGGCCCAAGGCCAATCTCAGCATTAAATATGCTCAAGAGAGTATGAAA
GAGAATTATGATAAGAAAACAAAACCTCATGAATTTAAGGTTCAAGATTTAGTTATGTTG
GAAGGTCATAACACGACAAAAGGGTTGTCACCTAAATTAATAAGAAATTTCAAAGGACCT
TATGAAATCATCAAAGCAACAGAGACAAATTTAGTATTAAAATTATGTTCAAATAAAAAG
GCAGAACCTATATTAGTTCATGCTAATAGATGTAAAAAGTATTATCCTGATGAGAAAGAA
AAGAAAAAGATACAAAAATCTGATAAAAGAAAAGATCTAACCAAACAAGATTTACAAGAA
ATAGGTATCCAATTCGAAATAGGATGTCGATGA
>DjScaffold5295:75125-79918 strand:+
ATTTTAGAGACAAATAATGGATTGTCTGATCATTTTCGAAATCATGTTGAGTGGGAAATT
AGTAAAATTGAAAGTATAGTAGAAAACCACACTGAGAAAGAAACTGGTGTCATCATGTCC
GCCTATCAAACTACGATCAATATTAGCCGACGAAGCGCTAGCTATCTGATATCAATTTTA
CTTCCAGCGATCGGATTAATGATAGTTTCGATCGTTATTTTTCTCCTCCCCCCTGAGTCT
GGTGAGAAAATCGGCCTAAGTATTACTGTCCTGCTATCCTATACAGTTTTCAATTTAACT
TTGTATACAGCGTTACCAAAATCAAAGACTATTTGTTTAGTTGGTTGGCTTATGGCATAT
CTTGAACTGCTTTCTGTTGTAGCTATTCTCTTGAGTGGAATTGTATTACGATGCTATTAT
CGATCAAGGAATGAACAACTAGAGAAATGGGCCCAAAGAGTGATTGCCTCTAGAGATTTT
CTGAAGAGAATCTGTTCCAGACGAATGATGAATTGTAGAAAGTATGAAAAGAATGATCAA
CGTATCACTACCTGGCAAGAATTTGCTGTGTTTTTAGACACTTTATTCATCTATTTGTAC
TTAATTATATATTCCGTAATGATTATTATTTCAACATATTTTTTCATGTATTAAttagag
tgaagtaaaacaaactttgaatcatc
>DjScaffold4796:46018-62687 strand:+
TTAATATCACTTTTGCACGAAAGTCGAGAAGCAAATAAAGTGTTGCAATTTGAATTGGAG
GAGAAAAAACGCAAATTATTTGAATATCAAGGAGATATAAAATTATTACGGGAACAATTG
ACCAGAAAAAATCTTGGTGTTGTCGAAGATAGTGGAATCTCAATCAGAAATTTTCCACAG
CATGAAAGGGCTGCTTTAGTGGCACGCATTGAAGAAATAACGGATAAATACAAACAATTA
CGTCGTGATCTAGAGCAATGTTTAGACGAGAAAGAAGAATTAGTCACAGAACGAGATGTT
TACAAAACCAAATATGAAAAACTAAATACTGAAATAAATTATATATTGAAAGGAGAATCG
AAAGAAATGATTGACGTTGACGCATTAATTTTAGAAAATAAACTCCTTAAAGATCAAGTG
ACATCTATGGAAATTGAGCGGTCAGAAGCTGTTGCCACCATGTCTAAATACAGGAGCATG
ATTGATAAGAAAAAGTCAAGGTCATCTTTGTCTGGAATGGATACCAGCTCTATAACAAGT

TCTCCAAGTCATGATTTAGGAGTTCAGTCTTTCATTTCTGAGTCTACAAATATTTCAATG
ACACCACAAGCAACCTCAGATTTACAACTATTAACTAGAGGCCTGTTGGATACCATAAAT
GATAAGAATTTGGCTTTATCTCATCAAAGGAAAACCAATAAGTTGGTATTTTAActttaa
tattatatttaacataatcaaatagcgagattttgaaattttcatcgaactttttttaat
agatgttaatatatttgttgtgttctggtaataaacttttaattaatatttaccttaaaa
atgaa
>DjScaffold4010:5662-6649 strand:+
atATGAATTTTCAATCTTCATGCAAGGAAAATCAAGAAAATAATTCATCAAATGGATTCT
CGAGGAGAGAAATAAATTCTCTAGTTCTCGAATATCTTATTATAGAGGGATTTGAAGAAG
CTGCATGTAAATTCAGTCAAGATACAGCAATTCCATATTATGGAGATTGTTTTCACATAT
CGGAAAGATTGAAAGTTATTGATATGGTAATGAAAAATGATATTGAAACGTGTATCATGG
AAATTAATACTGCGTGGCCTGAGTTTTTTGATAGAAATCCTGATATATATTTTCAATTAC
GTCAATTGAAAATGTTAGAGTATATTCGATCCAAAGAAATTATGGAAGCTTTAAAATTTG
CCCAGATATATTTTGCTGATGCTGTTTGCAAAATATCAGATAAGAATTCCAAAATGTTAA
CCGAATTACAAAAAACTATGACTTTATTAGCTTTTGATAATCCTATAGATTCCCCTAATG
GGTATTTATTAAAGAAAGATTTCACAAAAATCGTTGCTGGAAACCTTAATGCAGCCATTT
TAAAATATATATCCGGTGACACATGTGATGTAGTTGAAGAGGAATCATTTTCTTCTGATT
CAATCCCAAAATTGACTCAAATGATGGCTCTAATGATGCACTTCAATTCACAATCTAATT
GTAAAATTGATTTCCCCGAAGAGTTTTACGATAATAAGTCATAAtatccggcgtttactt
tattttacgtttttcgtgtaaatttttccattcatgaattattgcaacatcaaatatctt
ttgtagaaaaaatttcattaattatttatttcacaagaaatttgatacatttaatgttaaa
tttgttttcttggaatttgaagttttatgatttataattcgaaataaaatatttaaattta
atattcgtatggtaaaatttatctaattagtggtttcaaaatatat
>DjScaffold4010:40111-41553 strand:+
acatttatatatttatttgcagtgtttgttaatatagtttatttaatcaataaacattga
tcatctttatcgatattaattattacttaattaattataatatttatgatggtaaaatga
cgaacttatatttataaataatttttattgaaatttcaaatgtagccattgttacaagga
gatgaaaataaaacaATGCCAATGGTAGTATTTGCTTCACCAGGAATGCTTGATGGAGGT
CATTCATTAAACATATTCAAAAAATGGGCTAATAATGAAAAAAACATGGTAATCATTCCA
GGATATTGTGTTGCCGGTACTGTCGGTCATAAGATCCAATCTAATGTAAAGAAAATAGAG
TTTGATAAGCAAACTATTGATGTTCGATTGAAAGTTGAATATCTTTCATTTTCTGCACAC
GCAGATGCTAAAGGCATAATGCAACTTATCGATCAATGCAGACCAAGAAATGTTTTGTTA
GTTCATGGTGAACCGAAACAAATGGATTTTCTCAAGGATCGTATTGAGCACGAGTTCAAA
CTTAAATGCTTTAAGCCTGCTAATGGAGAAATGGTCACTGTAAAATGTGATGATCGGATA
TACTGTCAGGCATCTTTGTTTTTCTTAAGGAAATGTTTCGAGCATCAAAGATCAGTTGAA
GATGAAAATGAATTATTTATACGTCAAGATAAATCTTCAAAATGCGGAATTCTCATAAAG
AAAAGCACAAACGAATCCAAATTACTTGATAGACAAAGTGCTATTGTTGCTGCAGGATTA
GTTGAACATCATCTTCAATTCACATCCACCGTTAAGTTTCTGTCCAATCACACCCTTACA
GATATAATGAATCAATTAGTTCATTTTTTGAAAAGCATTTTCAATACTGAATCTGGACAT
GAAATTCATTGCCATGAAGAAAATTTCACGATTGGACCACTCGACGATCCATTAGTCAGC
ATATTAGTGAAAGAAAGCAACTTAATAACGAATAATTCCGATGAATCCAACACCTTGGCA
AATAGTCCGAATGATTTGCAAGTTTTCGATGTTCTCAGAGTCACTGATAAAAATAATGGA
TCATTTGAATCGCTACAAAATACTCGTCAAGTCAGTCTGCATGTAACTTATTCTTTCCAA
GAAGAACCAATGGGATCACGGCTTTATGAAGCGATTCAAATAGCTTGTACAGAACGAAGA
TTAGAATTTGTAAATTAAattatgaacaatttgtattgttgatttttctctattaatatg
tgtcattgtaaatataattgaaacatttttctaata
>DjScaffold232:12730-15718 strand:+
tttatcaaagcaagcatttgaagaagcgATGGCAGATTATCCAGCAGCATATGAATTACT
GAAGGATAAGGCTAATAAAGTTCTTAATCGACACAAAGTTATAGAAAAATCTGCTAAAGT
AGAATCTGCACCAGAAGTCATTATTCCAAACAAAAAAGTAATTCCTGATTTTCGAAACAC
AGTCTTAAAGATTTTAGAAAATAAAAGAAAATCGAGTTTGGCTAAAGATAATGAAAGTTA
TTGTTCCACTCCTTCGGAAATATCTCTTCATTACAGAGATAGCATAAGTGTTACATATGA
TAGTAAAACACCATCTCCAACATTTGATAGACGGTTTGATTATGATTATGACAACTTTCA
ACCGATATTGGTACATACAACAGGTTCTCCCTCGATGAATAATTCATCGTTTCATCCTAT
TAAAACGGTTTTAAAGAAACAGTCAACCTTAAACAGTTATGAAAATGAGATTGGAGATGA
TGAATTATAAaaataaatca
>DjScaffold232:19678-39950 strand:+

taatatctaaatcttaaacatgaaaaatttcttggattctaaaacttaatactcgtttaa
ttaattcatttatatagtaatctataaatcttgaaatgaattacgtagatgaaaaatatt
taaATGAAAAGCAAAAAATTTAATTCAATTCTATGTGGTAAAAACATTGAACTATCTGAA
TGTGGAAAAAGTGCTAAGCGATTAGAAAGTTATTGTCATGGCATAGTTTTATCAGATCAG
TACATTAAACCTGGTGAATTGTTTGCTGTTGAAATAGAGGAATTAGAAAATGGGTGGAAT
GGTAATTTGCGCATTGGATTTTCTATGTTATCCGATGACAAATTAAATCCGTTGCCAAGT
GACTCTATTCCTGGCTTAGTTGATAGAGGATTTTCTTGGATTTGCGGAGTTGGACAATGT
AAAAGCAATTTAGAATCATTTGGAAATTTTAACCAACAATTGCCAAATAGAGGCAATTTA
AACGTTTCTGATAATCATATCTATAATCGACAGCCGTTTAAAATTGATTCTAATTTTAAT
AATCAATCCAAATCAGGAATTTCATGTCCGCCATATTGTAATTGTTGTGTGAGAACAAGG
TTTGGTACAGTTCCATTTGGTAAACTTGTTAGTTCTGCTAAAATGAATAACTCTTCTTAT
CCTTTACCATTTGAATCTGGAAGTGTTGTAGCTATTTTCTATGAAATTTATCCTCTTAAT
TCCATTAGTAATAATGCCGAAAATAATTCATTGTTCGATTCTCTTCATTTTCTGCAAATA
TCTCAAAATTATAATGAAAGATTTTACACAAAATCAAATGGTTATCGTAACTCTTATAAA
TTGAGATTTCATTTATTGGTCAATGGTTTCCAACAACTTGTTTCAGAAGAAATTATTAGT
ATTCAAAACCAGTATGGGCAGATTTTAGAAGATAATTCTTCCCAATCTAGGTTGCGATCC
TTATCTTGTCCAGAGTCTTTATTTCCTATAAATATTAGAGCTATTGTTGATGTTTATGGA
ATGACAAAAAAAGTTCGAATCAAGTCTTTAAAACAGGCACGAAAATCAATGGCTGCCTCG
GTTTTGATAGAACATGTTGACAGCTTGGAAAGTCTGTGTTACCAATTTATTTTACATACT
CTTTTACTGTATATTGATCGAAAAGTTGAGAGATCACTTATAGTTTATTCTCTTCATTTA
CCTCCCCAATTGACCGAATCCCTTTACCAGCATTGCGTTGCACTCTGTGCTAAGTGAttg
tattatatgccaatatctaaatttattttagatttttatttgtagatttgtttttttat
aaatttccatttattaataaagttatttgagatttttataataataatatctaaatctta
aacatgaaaaatttcttggattctaaaacttaatactcgtttaattaattcatttatata
gtaatctataaatcttgaaatgaattacgtagatgaaaaatatttaaATGAAAAGCAAAA
AATTTAATTCAATTCTATGTGGTAAAAACATTGAACTATCTGAATGTGGAAAAAGTGCTA
AGCGATTAGAAAGTTATTGTCATGGCATAGTTTTATCAGATCAGTACATTAAACCTGGTG
AATTGTTTGCTGTTGAAATAGAGGAATTAGAAAATGGGTGGAATGGTAATTTGCGCATTG
GATTTTCTATGTTATCCGATGACAAATTAAATCCGTTGCCAAGTGACTCTATTCCTGGCT
TAGTTGATAGAGGATTTTCTTGGATTTGCGGAGTTGGACAATGTAAAAGCAATTTAGAAT
CATTTGGAAATTTTAACCAACAATTGCCAAATAGAGGCAATTTAAACGTTTCTGATAATC
ATATCTATAATCGACAGCCGTTTAAAATTGATTCTAATTTTAATAATCAATCCAAATCAG
GAATTTCATGTCCGCCATATTGTAATTGTTGTGTGAGAACAAGGTTTGGTACAGTTCCAT
TTGGTAAACTTGTTAGTTCTGCTAAAATGAATAACTCTTCTTATCCTTTACCATTTGAAT
CTGGAAGTGTTGTAGCTATTTTCTATGAAATTTATCCTCTTAATTCCATTAGTAATAATG
CCGAAAATAATTCATTGTTCGATTCTCTTCATTTTCTGCAAATATCTCAAAATTATAATG
AAAGATTTTACACAAAATCAAATGGTTATCGTAACTCTTATAAATTGAGATTTCATTTAT
TGGTCAATGGTTTCCAACAACTTGTTTCAGAAGAAATTATTAGTATTCAAAACCAGTATG
GGCAGATTTTAGAAGATAATTCTTCCCAATCTAGGTTGCGATCCTTATCTTGTCCAGAGT
CTTTATTTCCTATAAATATTAGAGCTATTGTTGATGTTTATGGAATGACAAAAAAAGTTC
GAATCAAGTCTTTAAAACAGGAACATGTTGACAGCTTGGAAAGTCTGTGTTACCAATTTA
TTTTACATACTCTTTTACTGTATATTGATCGAAAAGTTGAGAGATCACTTATAGTTTATT
CTCTTCATTTACCTCCCCAATTGACCGAATCCCTTTACCAGCATTGCGTTGCACTCTGTG
CTAAGTGAttgtattatatgccaatatctaaatttattttagatttttatttgtagattt
gtttttttataaatttccatttattaataaagttatttgagatttttataataa
>DjScaffold4834:32214-45882 strand:+
AACGATCGCGTATTCGCGAAGTTGTCCAACTGTCCATACTGGCCAGCTAGGATCAACAAA
TTACCAGATTATATTTCTGTACCTGATGGAAATTATCCAATATTCTTTTATGGTACTCAT
GAAGTGGAATTTTTACCTTCCAAATCACTTGTTCCATTTATGGAGAATAGAAATAAGTAC
GTCCGAAACAGTCGATATCAGCATTTTACCGAAGCCATGTGGGAAATCGAAAACAATCCC
GATATTTTGGCTATAGACAAGGATCCCGATGCAGAAGATTTCCTTTGTGATATGTATGAG
GTAGAAAAACCGCCTGTAAAGATTTCTGAAACAAGCACTGATGGAATTGTAGAGAAGTCG
ATAGATGCTGAAATCCCAGAAATAAATGATGTTGAAGGAATTATGAATCTTGGGGAAAGT
GAATGGAATGAATTATTTACAATTGAAGAACAAGAAATTCATAATGCTTCCACAACAAAT
TCTTCATTAGTTGTAATCAATATGTCTCCTGTTCGAAACAATACACCTGATTTTCGAAAA
ACCGCGATTATCCGTGAAGCAATTTCTATTTCACCTACCAAAATCAAAATATGCCAAACT
TCTGTTCCTGTTATTTCTGAATACACAGACGATTTATATTTATCTGCACTGAATAGTAAT

TGTAAAGTGAATTTAGAGAAATTATCACTTATCCAAATAAATCGTTTGATTCCTAGTAAT
TTTTCTGTAGGCCTACTTGAAGATTCGTATTCTAAAAAAATTAAAAAATCTTGTGTAGTT
CCAAATAGTGTAAAATCAAATAAAGAATCAACTGTTGAAATTAAACCGAAAAAAATTGTC
AACACATCTTCTGAAATAGAGAAAATTGTTCCAAAAGTACAAGTTGTACCTGAGCAAAGA
AAACCTATTAATTCTTTGAAAGGTGGTTTATCAACAATTAATAAAAATTCTGTTTCTAGA
ATTATCGATGCGATATCTATAGATTCTGACGATAACCAAAGTTGTGTTATTGTAAGTTCT
CCGAAAACTAAAGTGGATTTGAAAAGAAAACAAGAGAAAATAGAGAATGAACTAAAGGTG
CTACCGAACATTAAATTGAAATTAGTAAGACCAAATGAAGAAACAGATAAAAATGATAAG
CTGGTTAATTCAGACGGTAAAAAAGTTTCATGTAAACCAAAATTTAAAGATAGAAAAATA
ATCAAAAGTGATTGGTCTAATAAAAATACCAATGGAAAGCCTATTACATTTGAAAATTTC
TTCAATAAAAAGTTAAGTATTCTCGGAAGTGGTTTAAAAATTGGTAAAAAAGTTAGAACT
CATGAAAGTGGTAATTTTACCAAAATGGAAGATGACGACAAAGACCAATGGTCATCAATA
AAAGATTCGAGTGATAATCAGAATAATAAATGTACAGAGAATAATAATGACGATGATGAT
GAAGATAACCTTTGGCCAAAAATGAAGGACCAAAATGAATCTGGTAATTATGGAAAATCT
GATGATTACGGAGATTTTGCTGGTGATGATGACGATGATGACTGCCAGTGGCCTGTTGCA
AAGGATGAAGGTGGAACTCTAAAAAATGAAAAACAAAATAATGTTCAAAATGAATTGGAT
GATGAAGAAATTGATTCGGCTCAAAAATGGTATTCAAATAATGATGAGGAAGTGGATGAC
GATTCTTTGAAGAAAAACCACAAGGATCCTTGGAGCAATTATTTATCAAAAAATAAGAAT
ATCAATCAAGCAGAAAATCAAACCGGTGATGATGATATTACTAAATCTGGTGATTTAGCT
GAATTTGCCGAGATCGATGATGACATAATCGATGTAATTGTGAAAGGAGAACCCGTCAAT
TCGACGAAGAAGTCCGTTGGTAACGTATGGAGTCACATTCAAGAATATCAAATCCAATTT
GAGAACAGTGGCGGTGCTGGTGGTGAAGGGGTTGGGGATATTGAAGAACTGGAAGATATC
GGAATTTTAAATGACGTGGACGAAACCATTAAAACCATGAGTTATATAAAAGATGCTAAA
TGGTTAAATTCTAAAAATGAAGATATTCTCGATTTATCTCCTGGAGATTATTGTAAAAAT
ATAGATTCTATTGATTCAGCATCAAATGACAAATGGTATGGACAAGGAAAAAGTATATCT
CCAACCGATCGGATTCCGAATTTAATGGAAATAGATACGAGTCGAGGTTATAAGAAATTT
ACGAATATCGAAAAAACGAAAGCTCCAGAATCTTGGGTTGATAGTGGACCACCATTCAAA
ACGTTCAGAGGATCTGTACGTAACCGTCAAAATCATTTACCAAATAATTCCCGGTCGAAT
TATATCTCCCCGAATTCAAAGCCACAACCTTATCCAAATATACCACTAGACCAATTATTC
CCACCGTTCAAAGAAGAATATTTTAAAAATAATAAATCTGCAAACAACAATTTAAATAAA
TCTCCAGAAAACTCAGAAAAGCCTAATTGGACAGATAAGGTGACAAAACCAAATCATCCC
TTTGTGAATCCCAATTTTCATCAAATGGGTCAACGTTTTATTAATAAACCACCGCCGTTA
TCTATTGATCCATATTGCAATGGTGCTGCTCCAAACAAAACTAAGGTAATGCCTTATGGT
CCTCCTTTTCCATATTATGGTGACCCGTATAATCCATATCAGAAAGAATCAGTTATTAGG
GGATTTCCTGTAAGTAGTTCTTACGATTATTATGGAAATCCCTTCTCAAAAATTAATACC
TATCCTTACTATCCGCCTAAAATGACAGCTGTGCCAATTACACCCGAATCTCCTGATCCG
TATACAGACGAAACTGACTCATTATCCAATTCCAAACTTGCATCAAATATCAACCAAACA
AAATCTGTACCTAAAATCAATAATGATAGTTCTGTGATTAATAAGAAATTATCAATTAAT
CCATTACACGCTCCTAATTATCAGCATCATCCGCATCATAATCCATACATGACAGAAAAT
GGTCAATATCTATCATATGACGACATGTATGCATACTACTATCAATTTTATGGGTCTTCA
TTTAATCCAATGAATCCATATTCGAATCCTAATAATTTTTATCCGTACACACAGCATGAT
CCTATCTCGTACACTCCAGCTTCCCCAGATCCAGAAGAAATGTGTCCAACAGAG
>DjScaffold4834:82738-91523 strand:+
gatagtcgagagattatcctcctggagtaactagagagtatacaaaagaaATGACAACGG
AGACCACAACTACAGAAGCGCCAAAGATTATTGCTCCACAAGAGCCTCGAAGGCCAAAAA
AACCTGGTCGTTCAGGATTACCAGGACCGTCTGGTCCCCCTGGACCACCTGGGCCTCCAG
GAGAGCCAGGAACAGATGGACAAAATGGAGAACCAGGAGATCCTGGAGCACCTGGAAAGC
CTCCGCAAAGTCAAGATGTAAAAAAATTTGCTGCCAGATCAAGGTCTGTAAAAGGTCCTG
GTGGTAGAAGTGTTTCTACTCATGAGGAAGTGTTAACTATGGGAAGGTCTGGTGAGCCTG
GTCAGCCTGGATTACCTGGACAAATTGGAATTAATGGTCCTGTAGGACCAGTGGGTGGAC
CAGGTAATCAAGGTCCACCTGGATTGCCTGGCTCGAGAGGACCACCTGGTATTTTGGGAG
AAGATGGTAGACCTGGTCCACCTGGTCCGGATGGATTTAAAGGAGCGGTTGGCAGACTAG
GACCAACCGGATCGGATGGAGAACCTGGCTACAAGGGTCCAATAGGTGCTCCTGGAATTC
CAGGTAAACCTGGTGACATTGGTATGAAAGGAATTGCAGGTAATTCGGGGCGCAAAGGTC
CACCTGGGAAACCAGGTACGAAAGGTCCAATAGGTTTAAGAGGATTAAGAGGAGAAACTG
GCCCAAGAGGTCCACCGGGATCTATTGGAAAAATTGGAATACCAGGAAATGACGGAAGTG
GAGGAACTCCTGGTGATCCGGGACCTCAAGGATTACCTGGACCACAAGGCTTTCAAGGAA

TTGTAGGACCAAAAGGCAATGCTGGTCCTCCAGGTTCTAAAGGTGAACCAGGATTCCAAG
GAAACAGGGGAGGTAGAGGTATTCCTGGAGCCACTGGAACTACAGGAAAAAGAGGAACTG
TTGGAGTGCAAGGAGAATCAGGTTATAAAGGAGAACCTGGAATATCGGTAGATCTGGTC
GACCAGGGCAAGGTGGCATGAGAGGACCTCCTGGTGTGGAAGGAGAAATTGGTAACATTG
GACCAAATGGGCCAGAAGGTGATTCTGGATTACAAGGATCTCAAGGAATGCCAGGATTGG
AAGGAACAAAAGGAAAAAGAGGAGAAAGAGGACCGTTGGGAATACCTGGAGAACCGGGAA
AACGCGGTGTCAGAGGTTCAACTGGAATTGACGGACCTCCAGGGTATGCTGGTGGACGAG
GTTTGGCAGGAATGAGAGGTTCTCCAGGTCCAGAAGGGCCACCAGGATCATCAGGAGTAA
TTGGGGACCCAGGCGCTCCTGGTACTATGGGAATTAAAGGAGCTCCAGGAGATTTTGGAC
CATCAGGTGTTCGTGGGAATCCGGGACCAAGAGGTCCTAGAGGAAAACGAGGTCCACGTG
GACCTGTTGGTGAACAAGGTTCAAAAGGACCTAATGGGGCTATCGGAAATCCAGGTAATA
TGGGGCCAGTAGGTATGGTTGGACCCATGGGCCCTCAGGGTCCACAAGGAATGCGGGGTT
CTAATGGAGCTCCTGGAAAAACAGGGGAATCTGGACAAATAGGACCAAGAGGTCAAAGAG
GTGACAGAGGTCCAAGAGGTAATCCAGGTTCTCCGGGACCACCTGGTATACCAGGACAAA
AAGGTCAACGTGGTCAACCAGGAAAACGAGGTCCGATTGGTTCAGAAGGAGAGTTGGTC
CAGAAGGTGCACCAGGATCTCAAGGTAAAGAGGGTGATCCTGGGCCAGTAGGTCTTCCAG
GTGATATAGGTCCAAAAGGTCCCTTAGGTGAAAGAGGTCCACCAGGAGATATGGAAAAG
TTGGAAATCCTGGTAAAAAGGGACAAAAGGGAGCAACAGGACCTGTTGGTTTTGATGGCC
TTAAAGGTTTAACTGGCCAAAAAGGAAATATTGGAATAGTAGGTGGTCCAGGTCCGATGG
GTCCAATGGGAAATTCAGGAATAAATGGTCCAAGAGGTCAAAAAGGAGAGAGAGGTGATG
ATGGTCCAAGAGGAATGCCTGGTCCAAGAGGAAGGCTTGGTCCACGGGGTGAAGATGGTC
CAGTTGGGGCGCCTGGAGATACTGGAGTTCCCGGAATCAAAGGTCTACCAGGAGATACTG
GTCCTATGGGGGATAAAGGCGCTATTGGTGATACCGGTGACCAAGGTCCGACAGGAGATA
TTGGTGGTCCAGGTCAGCCTGGTCCTCAAGGTGAAATTGGACTAAAAGGAATGAAAGGCG
AGATAGGTATGAGAGGTGGAAGAGGAGATCCTGGAGATGCAGGAATTAAAGGAGTACCAG
GATTAGTTGGGCCTAAAGGTAAACAAGGCCACCCTGGTCGTGCGGGAGACAGAGGACAGC
CAGGTGAACAAGGTGGTCAAGGGCCACCTGGTCTTATCGGTGAACCAGGAATTATTGGTT
CACCCGGACAACAAGGTAGGCGAGGGCCTGTTGGACCACAAGGTGTACATGGAAGTAAAG
GTGAACCCGGCGATCCAGGAGCTCAAGGTCCTCCTGGTTCTGATGGTAAGCCTGGAGACA
TGGGGCCTGCCGGTGAAAAAGGTGAACGAGGCGAAAGAGGTGTCGTGGGTATCCAAGGAC
CACCGGGTCCAATCGGCATTCCGGGTCCAAGAGGACCTTCAGGAAAAATTGGACCACAAG
GTCCAGTTGGAGATATTGGAAATCCTGGAACTACTGGGTATGCTGGAAATCAAGGTCCAC
CTGGAAGACAAGGTATTAAAGGTCCACCAGGAACCGATGGGCCTCCAGGCAAAGAAGGAT
TACCAGGTGAAGAAGGTCCACCAGGGAAAGATGGAGAACCAGGCTCTCCTGGAGAAGTTG
GTAAACCCGGTATACGCGGCCAACCTGGTGAAGTTGGACCTCCAGGCCCATCAGGCCTTG
CTGGTCCACCAGGATTGAGAGGTGAACCGGGTCGTATGGGCAAAGATGGTCCTCCAGGAC
CTCCAGGTAAATTAGGAGATCCTGGATTTGCTGGAAGAGCAGGAGATACAGGAGATAAAG
GTCCTAAAGGACCACCTGGCCCACCGGGTTCTATCGGCGATGCTGGAGAACCAGGGGATC
CCGGTGTAGCTGGAAATGCTGGATTAATTGGACCTCCTGGTGAAGCAGGATCAGCTGGAA
ATGATGGTCCTAAAGGGTCACAAGGTCCTCCTGGACCCACAGGTAACATTGGACCTAAAG
GTGAAAGAGGACCAAAGGGAGTAGGAGGACCTGAGGGCATTCCAGGCCCAGTGGGTCCCA
CTGGTCCGCCTGGTCCTCAAGGAGAAAATGGACCTAAAGGACCTTCTGGTCCAGTTGGTC
CTAAAGGTCCTCCTGGAAAACCCGGAGAAATTAATACAGACTTTCAAAATATATTTAAAC
GTCAAAGAACGACAGATGAGGAGGAATCATTCAATTTTCAACGGGATGAACCTCAAGCTG
AGCAGAAGTCTAGTATTGATCCTAACGATGAAGATGAAGATATATTTGAAGCTCTTTTGA
GACTTTCAACGATTGTTGATAATTTATTTAAACCTCCTGGAAGTAAGACTTATCCAGCAA
GAAGTTGTGCAGATATTCAAAGAGATTACAATAATAAATCTAATGGCATGTATTATATCG
ATCCAAATGGTGGTCACTGGAAAGACGCTATTTATGTTTTTTGTAATTTTGAAAAATTGG
AAACATGCATTGAACCAGAAGTGCCTAATTATAAGAAGACATCTTACACAAAGTCATATT
CATCATGGTTCAGTATTTTGGCAAGTCTCAATAAACAAGTATCGTATAAGATTCCCAAAG
AACAATTGGTATTTTTACAGTTATCCAGCGAAAGCACTTCGCAGAATTTCACCTTATCAT
GTGATAACATAGGTCTTGTTACAGATAGTTCTGCCAATCAAGAGAACAAATACAATAATT
CTCTCCAATTATTGGGCGATGACAATCAGATTTTGACAGTTAATAATGAAGATGATTTAT
TTCAATATCAAATAATAGAAGATAATTGCAAGGTGAAATCTAGTTCCGGACAAGTTATAA
TAAAAGTTGAATTAGATCGACCCAGACGTCTTCCGATCAGGGATTTCAATTTTAAATCTC
TCGAGAGTTCACCTCAAGCAAAGATTGGAGTGGAAATAGGCCCGGTTTGTTTCAGTGGAT
TGAGTGAAGAAACAAAAAATTCAGCTAAAGTTCAACGTCAAAAAAGAGATACTCATGCCG

ATATTAGTTTAAAGTCGTTATTGTCAGTTCTATCACAATGGTAGactaaatgtaaccaat
gattgtaattatgtgattttcaatgatattattcagtttgtttgttgagtttacgttggt
tcagtcatatgttaagacgagtaatgaatatgtataatttgtttatttctgatcgaacat
tgtggtgaaatttgttttgtaaaatattatttataattaaacttggtttcatttattttt
tggacataattcttcttgtgaagatatgtaaaaaatttcacaaaggtttagatgtcaatt
ctaattaagtgttttgttttagtttttcaatcatattgttatatatgaagttattaa
>DjScaffold4193:19389-20405 strand:+
ATGCAAAAAACGCTCCGATCTATTAAATTCAACAAACAAATATTTCTTTCTATCGTATTT
GGCCAAATTCTATCATTATTCATTTGCGGAACAGCAATTTCTAGTGCTTATCTAAGTAAT
TTTGGCGTAAATATTCCTCTTACACAGAGTTTATTGAATTATCTAATTTTGGCATTTACT
TATGGATTATATTTTGCTTATAACTCATCATTGGATTTGTGGAAAATAATTAAGGATCGC
GGATATTTATATATTATTGCAGCTGTAGTTGATGTTGAAGCTAATTATTTGATAGTGAAA
GCATATCAGTTCACTAATGTAACTAGTATTCAGTTACTTGATTGTCTAACTATTCCAACA
GTTTTTATCCTAAGTATATTTATTTTCAAGATCAGATACTTATGTACTCATATTTCTGGA
GTTATTTTATGTCTACTTGGTTCAACCGGTATGGTTTTGATAGATTATTTCTCAAAAGAA
TCTGTTTCCTTCACAAGTAGTAGTAATCCATTATTAGGTGATGTTCTTGTAATTTTTGGT
GCTATATTATACGGAATATCAAACATTTTTCAAGATTACTTAGTCAAAAAGCACAATCGA
ATTGAGTATTTAGGTTTTATAGGAATATTGTGCTCATTAGTTTGTGGTTGTCAAGTAATT
ATAATTGAACGTCACGAAGTGTTCACTGCATTCAGTACCAATCTATTTAATCCATTGTTT
TGGGCGTGTTTTGCTGGGTTTACATTATCGATGTATTTATTGTATTCTCTTATGCCTGTT
ATGATGAAAATTAGCAGCGCTGTGATTACGAATCTTTCTTTATTAACCTCAGATATGTAC
GCTTTAATCGTGGGTATTTTTCTGTTTAATTATACTTTTCATCCTCTTTATTTTGTCGCA
TACGTTGTAATTATGATTGGAGTTGGAGTGTATGTGGCAAAGAATCCAGTTTCTCGAGAT
GATAACGAATCTATAGGCAGCATATTTTGTTGTAAAAACTCCAAAGTAGATGTTTAA
>DjScaffold4193:77206-77676 strand:+
ATGCCATTTGGCATCGCAGCGGCACCAGGGACATTTCAAGAGATGATGACAAAAGTGGTA
GGCGGAATCAATGGAGCCATGGTTTATATTTATGACATACTGATATGTACGAACAATAAA
GATCAACACTATGAAGTAATTGAAAGAGTATTGACGGAAATAATGAAGGCTGGTTTAAGA
ATTAATCCAGAAAAATGTCAGTTTCTTCAAAGTGAAATCAAGTTTCTTGGACACATAATT
AGCAGAGATGGTATTAGAACTGATCCGTCTAAAATAGAAGCTATAAAAACATTTGGCAAA
CCTAACTGTGTGAATAAGTTACGAAGTTTCTTGGGAATTTGTAATTATTACAGGAAATTT
ATAGAAAACTACGCAAGAAAATCAAAGATATTAGAGCAGTTGTGTAGCGGACCAAGCAAC
AAAAAATTAGAATTGACAGATGAAATAAACGATACTTTTGAAGGTCTGAAA
>DjScaffold1385:8197-9068 strand:+
ataaagtagaatgtctattagatacaggggctcgaattaacgtgatgagtttgagttgtt
ttaataaactacgagaagtaaagttagaaatgacttATGCGAAAGGTCGATAGCAAAACG
AATGAAAAAGTCGAAAATTACATTTGCAATATTGAAGCAAATTTTGGAAGACAAATTAAA
AAAGGAGAGCGATTGACAAGAGCTTTAAATATTTTTAAGATTAAAGAGGATCAACGGTTA
TTGAAAATAATTGAAAGTAAAGAAGATGCTTTTATGGCAGATAAATGGGATGTAGGACGT
ACACATCTGACCAGACACAAAATAATCACAAGTGGTGAACCAATCAATGTAAAACCATAT
AGACAACCAATGAACTTAGAATCTAAAATTGAAGAAGCTATCCAAAACCTTTTCGAGAAT
AATATTATCAGGAAATGCAACTCTCCATGGAATACCCCATTGATATGTGTTTGGAAAAAG
GAAAAGAAAGACATAAGAATATGTTTGGATTTCAGACAATTGAATGCAGTAACGAAAAGA
CAAGCCTTTCCGATGCCTAATGTCGATGAAATGCTGGACAAGTTAGATGGAGCCAAATAT
TTCAGTTCAATAGATTTGGGAAATGCATACTATCAAGTAGAGCTCGAAGAAGAATCTCAA
GAAAAAACGGCGTTTTCAACGAAACACGGGCAATATTGTTTT
>DjScaffold1385:100291-109869 strand:+
CAACTTAGAGTACCTTCTAATAATAGTAACTTTCACTTCATATCACAACTGCCTAATACT
ATTACTGTTAATCAACCTTTACAAGTTAATCAAAGTAATCAACGCTTTGAATCAAAGCCA
AATGAAGGATATATTATTCAAAATCGACCAATGATAATAAATACTAATTCTCTTGGGTTT
AACCAAAGATTTATATCTCCAGCTAATATTGTCAATCAAGGTCAGCTTAATCAAATTCAA
AAATTCTCTAGTGTTCAGCCACAAATTCAAATTCAGCAACCTCAACAACAGCAGCAGCAA
TCAAGACCAGTTAATGCTCAAATTCAAAGAATTATTACTAATCCAATTTCTGATAATCAA
GTTATCGAAATTAAAGATAATACCATTACGCCTGTAGTTGCAAATACCACTTCAGTTAAT
TTGGCTTGTAAACAACAACAACAACAACAGCAAATAAAAATTAAAGAAGATGTCGTTGAA
GAACGAGAAGAGCCAGTGAATACAAAAGATATGGTAAACAGCAACAGAATCAATCAGATA
GTTCGTCAAATTGATGAACATTTGCAATTAGACGAAGATATGAAAGGAATTTTTAGTCAG

TATTTAGACGATTTTTTGCAACATGTAACCGAGAATGCTTGTACTTTGGCTAAGTTGAGA
AACTCTAATACTTTAGAAATGAAAGATTTCCAATATGTTATGGAGAAAGATTGGAATATC
TATGTGCCAGGTTATCCATCTATTAATGAGAAACCTAAACCG
>DjScaffold1385:125857-126632 strand:+
CAACTTCGAGTACCTTCTAATAATAGTAACATTCATTACATATCACAAGGGCCTAATACT
TTTACTGTTAATCAACCTTTACAAGTTAATCAAAGTAATCAGCCAAATGAAGGATATATT
ATTCAAAATCGACCAATGATAATACATGCTAATTCTCTTGGGGTTAACCAAAGATTTATA
TCTCCAGTTAATATTGTCAATCAAGGTCAGCTTAATCAAATTCAAAAATTCTCTAGTGTT
CAGCCACAAATTCAAATTCAGCAACCTCAACAACAGCAGCAGCAATCAAGACCAGTTAAT
GCTCAAATACAAAGAATTATTACTAATCCAATTTCTGATAATCAAGTTATCGAAATTAAA
GATAATACCATTACGCCTGAAGTTGCAAATACTACTTCAGTTAATGTGACTTTTGAACAA
CAGCAACAACAATATCAAGTAAAAATTAAAGAAGATGTCGTTGAAGAACGAGAGCAGCCA
GTAAATACAAAAGATATGGTGAACAGCAACAAAATCAATCAGATACTTCGTCAAATCGAT
GAAAATTTGCTATTAGACGAAGATATGAAAGGAGTAAGTTATTAAtattaatgatcctga
aattttatttaatattaatcattaaaatgataaattatgtaacttttaataataaattaa
cgcaatttcgtttaaaatttaacaaaaatgaataacaatagtttatatatattataaaat
cgttggggcagtgtt
>DjScaffold1385:9075-11075 strand:+
ATGCCATTTGGCATCGCAGCGGCACCAGGGACATTTCAAGAGATGATGACAAAAGTGGTA
GGCGGAATCAATGGAGCCATGGTTTATCTTGATGACATACTGATATTTACGAACAATAAA
GACCAACACTATGAAGTAATTGAAAGAGTATTGACGGAAATAATGAAGGCTGGTCTAAGA
ATTAATCCGGAAAAATGTCAGTTTCTTCAAAGTGAAATCAAGTTTCTTGGACACATAATT
AGCAGGGATGGTGTTAGAACTGATCCGTCTAAAATAGAAGCTATAAGAACATTTGGCAAA
CCTAACTGCGTGAAGAAGTTACGAAGTTTCTTGGGAATTTGTAATTATTACAGGAAATTT
ATAGAAAACTACGCAAGAAAATCAAAAATATTAGAGCAGTTGTGTAGCGGACCAAGCAAC
AAGAAATTAGAATGGACAGATGAAATAAACGATACTTTTGAGGGTCTAAAAAAATCTTTA
ATGACTGCACCAATATTGTGTTTTCCGGACATAAACAAAGAATTTATATTAGATACGGAT
GCCAGTTTTGATACGATTGGAGCAGTATTGTCACAGAGAGATAAAGAAGGAAGAGAAAGA
GTTGTTGCCTATGGATCACACTCAATGACAAGTCATGAAAAGGGATACTGCATTACACGA
AAGGAGTTATTGGCTGTTTATTATTTCTGTATCCATTTTAAACATTATTTGTATGGTAGA
AGATTTACTCTAAGAACAGATCACAAAGCTCTAACTTTCATGATGACGACGAAGAAACCA
ATTACGTCACAATTCCAGACATGGATTAATTATCTGAGCAGTTTAGATATGATTTTGGAA
TATAGAAAAGGAATCCAACATGCTAATGCAGATACGTTATCTAGACCAAGATGTGAGGTA
TGTGTACAATGTCAAATGTCACATGAAGATGCCAAGAAAGGGAAGATTAAAACGAGAATA
CTGAATATAATTGAAAAAGAAAAAGAAGATCATTTAAACATTTCTGAGGAAACAGATTTA
GTCAAGAAAATGAAAGTTCCGGATGACAAACTACGTGAAAAAATAGAAGAAATACATAAA
TTATTATGTCATGCAGGAGTTGAAAAGGTGTACGAATATATGAGAAACAAATATATTGCA
AAGAATTTGTGTAGCCAAATACAGAAGATAGTTCGAAGTTGTGAAATTTGTCAGAAGACC
AAAGTAGTGACAACACCAACTAAAGAGAAGACAAAGAGTTTAGAAGCTACCAATTTATTT
GAAGTGATATTCACAGATATATGTGGGCCAATGCGGGAAACAAGTGGACGGAAAAAATAC
ATTCTTGGAATAATCGACCAATACAGTAAATATCTGGTTCTTATACCATTGCCTAGACAA
GACGAGGAAACGATACGAAAAGCTATTTTTGAAAAGTGGATATTGAGATTTGGATGTCCA
AGAGAAATAAGATTAGATTGTGGAAAAGCGTTTGAATCCAGAGTTATGAGAGAGTTTGCA
CAGAAGCTAGACATTAAACTTTGCTTTTCGAGTCCATATCATCATAATGCTAACGGACAA
ATAGAAAGACAGTTCAGGACCATTCGAGATTTGATTAATGCGACCCTTCAAGAAAGAAAA
TCAACAGATTGGGCAGATCTTTTACCGGAAATAGAATTTACAATGAACGCAACAAAGCAA
AAGACTACTGGAAAGAGCCAGGCTGAGATCGTTTTTGGAAAGAAAATTAGCAGAGAAAAG
TGGTACGAGTCAGACGTTTCAGATATAATTAAAGAAAACATTACAGACGAAATTACTAAA
AGAGAATTCAAAGTGGGCGATTATGTACTCATCAAGGTAGAAACAAGAAATAAAAGTCAA
GACAGATTTGAGGGACCTTATCGAATAATTGAGAAATGTCACGAAAGGCGGTATGCAGTA
CAAGGAGAAAATAGACAGTGTTTAGAAAGAAATCTGGAAAAACTTAGAAAGTTTCTTAAG
AAGGAAGATATGAGGAAATAA
>DjScaffold5386:2673-17017 strand:+
gaataagatgcagttggtaaagaagttcaaacttatatactgattattatttaaattgtt
ttatttgaaaaatttactagtaaaattttgaattagatttcaaaaagctttgaataaaat
ttttttgaATGAGTGATATTGATTATATCGAAGAAAGTGATACAGAAAATTCAACTGATT
TTTGCCGGGTTTGTAGGTGTGGAGCTACCCATAACAGGGCGTTATTTTACCCATGTTTAT

GTACAGGAAGTATTAAATATGTTCATCAAGACTGTCTCATGCAGTGGTTAAAACACAGCA
AACGAGAATTTTGCGAATTATGCAAATATAAGTTTGCATTTGCTCCTATTTATTCAGCAG
ATATGCCTACTAGACTTCCAGTTGCTATTTTAATTCAAGGTCTTTTATCTTCAATCAAAC
GAGCTGTTTTATCTTGGCTACATTATAGTCTTGTAGCTATAGCGTGGCTATTAGTTGTGC
CATTGACTGCATGCAGAATTTGTCGATCTCTCTTTACCGGTTCAGTAAGCAGCTTGTTAA
CTTTACCATTAGATATGTTGTCTACCGAGCATATATTTATCGATTGTTTATATGGTATAG
TAGTTGTTGGTCTTACTCTTGGTACTTTTGTTTGCTTTATGTGGGTCAGAGAACAAATAA
TGCTAAATGGTGGTCCAGAATGGCTTGAAATTAGAAATTTAGACAATCTAAACAACAATA
ACGATGCTGGTGCAGGAAATGTTATTCCGAATAATATAAATTTACTAGATGGTGCTAGAA
ATTTATTGCCAAATGTACTTAATGCTTTAGGCGCAGGGGCCGAAAATGATGATAATCAAG
ATGAAGGAAATTGGAATAATGATGAGCAAGTGAATCAAAATTTTGGGAACGAGAATGCGG
TTGTAGTTGAAGCCAACAATCAAGAAAATGATCCGTTATTAGGAAATCATCCTCAAATTC
TCGGGAATCAAGGAGTTGGAAATTTCAATTTAGATTGGGGAAGAGGTGGTGAAGAGTTGA
CATGGGAGAAATTAATAGGATTAGACGGTTCTCTTTTCTTTTTGGAACACGTATTCTGGA
TTGTTTCATTAAATACGTTATTTATTCTTGTTTTTGCCTTTTGTCCTTATCACATCGGTC
ATTTCACAATTGTGGGTTTTAAATTAACCAAACTTATTTCCTGGACCAAAATTGATGGAG
TTGTGACTACTATTGTTGGTTATGTGATTATAGCATTTGGGCTACTTTTTCTGCATAGAA
TTTTGAAGTATATCAAAGTCAAATGCCTGGAAAAACCTTGTCATATTGCTGGACTGGCAT
TTATTATTATCAAAGTTGCTTTATTCACTATTCTGGAGATAGGAATTGTTCCGTTGCTAT
GTGGAGTTTGGATTGATGTTTGCTCCTTAAGCATGTTTAATTCAACACTTTACCAGCGTC
ATAATTTGTTTCAATATGCTCCTGCTACTTTTATTTTTATGCACTGGACTGTTGGAGTGA
TGTATTTGTTCTATTTTGCATCTTTTCTTTTATTACTGAAAGAAATGCTTCGACCTGGAA
TATTTTGGTTTCTGAAAAATTTTAACGATCCGAATTTCAATCCTATTCAAGATATGATCA
CTCAGTCCGTATTTCGCCTTTTAAAACGATTCTGTATCAATTGTTCTCTATTTGGTATAA
TCATATTTATTATGGTTTGGATTCCAAATAAACTTATTCGAATTACCATTCCTGGATTTC
TTCCATATAAATTTCAGAGTACAAAAGATTCACCAATGGATTATTCTTTGGAAATACTTT
TTTTGCAAGTTGTTTTACCAATGCTATTTGATGGACACACAGTTTTTTGGCTTAAAACAA
TAATTCGATTTTGGTGTATCACTGTTTCAAATTTGCTGGATCTGAGAAGTTTCCTATTGG
GGGATGTGCCGTTTCTTGAAGGAGATATTATTATGAATTACGAAGGAAATATTCAAAGAG
TTATTCAAAATCGAACGAACGATAACACTGATTCATCTTTTCGTCCAGTTAACAATAATC
ATCTTGCAACTCAAGGACCGTCAGGAAATTATAGACCATATAAACAGCCTATTAAAGGAT
GTTTTGGTCTTCGCTTTCTAACTCTAATATTTTGTGTATTAATTAGTACTGTTGCTTTCA
GCACTGTTATTTTAATTGCACCAGTTTATATTGGTCGAAATGTTCTTTACATCGGTGGGT
TTGATGTATTTTGCGATTTAGTGAGTTTACTGTGTGGTTTGTGCAGTATCGCATTTCTGA
CAAAAGCATCGTATTCATTATATCAAAGGTTTCTTAATGGTGATGGAGATATTCAGAGAC
TTAGAACTCGATTGATAAATGGAATTTTGATACTTTGGAAATTAGTTGTGTTAGGAATCG
TAACCTTTATTTGGATACCATTAACTTTAGGACTGCTTATAAATCTATGTGTGTTATCTC
CGATACGTGTTCCTTTAGACAAATCAATTATTCTATCTTTTTGGGAGAATTGGATGTTTG
GAATGATGCATCTAAAAATTTGGGTTATGGTTCTACTTTTTGGTCCTCGTTGGTGGTTCC
GAGAGAGACTCGATATAATAGAACAAGAACTAGCAAGAAGAGAAAATTTACGTTTGTTTA
ATCTAACTGAGCAAGCTGGGCCATTGGTGGCTTTTGTAACGCTAGCAATAACTGGACCTT
TTGTTTTTTCAAAATCTATAGCTCCAATGTTTTTTTTAGATGATCATTATATATACAGAA
GAATATATCCAACTATGTTCTTTTTTGGATTATTAATTCTTATAATCTGGATTCAGTTTC
GTCAAATGTATCGATTGTATGAACATATAAAAAATGAAAGGTATTTGGTTGGTCAGCAAT
TGGTAAACTATGAACGGTGAcattttgaaatttgctattgtaaattttactaataaat
>DjScaffold1717:128314-130188 strand:+
ATGCCATTTGGCATTGCAGCAGCTCCTGCTACATTTCAAAATTTAATGACAAAAGTATTG
GGACCATTAAATTGGAAAGAAGCAATAGTGTATCTTGATGATATTCTTATTTTCGCTAAA
ACTGAAAATGAACATAATAAAAGATTAGAAAGAGTTTTTAAAAGAATTAAAGATGCCAAT
TTAAAGTTAAGTCCTGATAAATGTGAGTATCTAAAAAATGAGATTAAGTTTTTAGGTTAC
ATTATAAATAAAAACGGAATTTGTACAGATAAGAATAAAATCCAAGCAGTAATGGATTTT
CAACCACCTAAGTGCATAAAGAAATTACGAAGCTTTTTAGGACTAACGAATTATTATAGA
AAATTTGTTAAAAATTATGCACAACATGCCAGAGTACTCGAAAAACTTTGTGGATCTAAC
AAAAGTAAATTGATTTGGACTGAGGAATGTCAAAATAGTTTTGATAATTTAAAAAAACAA
TTAACAACAACTCCAATTTTGAGTTTTGCTGACATCACTAAGGATTTCATTTTAGATACT
GATGCAAGTTTTGACACAATAGGTGCAGTTTTATCACAACGCGATGATCATGGATTCGAA
CGTGTTATTGCTTATGGATCTAAATCGATGACCAAACACGAGTTAGGGTATTGCATAACT

AGGAAAGAATTGTTGGCTATTTATTATTTTACTCAACATTTCAAGCACTATTTATATGGA
AAGAAATTTTTAATCAGAACGGATCATAAAGCAATTACTTTTATGATAAATACCAAAAAA
CCTGTAACGCCACAGTTTCAAACATGGCTGAACTTTTTGAGCAGTTTGGACATGAAAATG
GAATATCGAAAAGGAGAATTACATGGCAATGCAGATGCAATGTCTAGGAAAGAATGTCAG
TCTTGCAATCAGTGCCAAATGATTCACGAAGATCCTAAAAAAGGCAAAATTAAAACAAAG
GTTTTGGTAATAGAATCTTCAGAAAACGGATTAAAATGGCAAAACAACTCTGAAGAAATT
GAAAATATTAAAAAAGACATTTTTTCTAATAAAGTAGACTTTATTTTAGTCGATAACGTT
GTCAGAACAAAAGAAAACAAAATCTGGATTCCATTTGATAGAAAAGTTGAATTTATAAAA
GAAATTCATCATAGTTTATGTCATGCAGGGATTAAGAAAGTATATGATTTTGTTCGAGAA
AACTTTGACATGAAAAATACTTTAAAAGCAATTATGGATGTTATAGGGAGTTGCAAATTA
TGCCAAAAGCGAAAAACTCTTACAGGAAAGTCAAAAGAAGATAATAAGATATTATGTGCT
CAAGATACATTTGAGAATATTTTTATCGATTTCTGTGGCCCACTGAAAAGAACATTATCA
GGAAAAGTTTATATCATGGCTATTATTGATCAATTTAGTAGATATATTTTGTTAAATGCT
GTAAACCATCAAGATGAAAGAACGGCATCAAAAGTTATTGTTGAAAAATGGATATTGAAA
TATGGGGCTCCGAAAACTATAAATTGTGATCGGGGTAAGGTGTTTGAATCAAATGTAATA
CTTGAACTAGCTAAGAAATATGGAATAGAAATGCGATACTCTAGCCCATATCATCATCAG
ACTAACGGTATTATTGAGAGGCAATTTAGAACCATCCGAGATGCAATTTCAATAAAAATG
AGAGAGAATCAATTTAAAGATTGGACAGAGACACTACCTGATATTGAATTCATGATGAAT
TGTACAATACAACAAACTACTGGATATTCACCAGCAGAAATAGTATTTGGTAAAAGAATA
TTCAAAGAATGGTTTGATAAATATAAATTTTCGTCCAATGAAAAGACAATTATGAAAACC
GATTTCAAATATTAG
>DjScaffold1717:219858-220331 strand:+
ATGCCGTTTGGAATAGCCGCCGCTCCATCTACTTTTCAGAAGTTAATGACAATTATTATA
GGAGACATGAATTGGAAAGAAAGTTTGGTCTATCTGGATGATATTTTAATTTTTTCGAAA
ACAATTGATGACCATATTTTAATTTTGGACAAGCTTTTTACAAGGATATCTAAATCTGGT
TTACGAATTAATCCTGATAAATGCAAATTTCTCGTATCGGAAATAAAATTTTTAGGTCAC
GTAATTAATACTGATGGAATATTGACTGATAATGATAAAGTGGAATCTATAAAGAAATTT
AAAAAACCATGTTGCGTTAAACACGTCCGAAGCTTTTTGGGTCTAACTAATTACTATAGG
AAATTCATTGAAGGATATTCTCAGCTAGCAAAACCACTAGAATCTTTAATTAGTACGAAA
GAAAAGAAAATCATTTGGACATCTGAATGTGAAATTGCATTTGAGAAACTGAAA
>DjScaffold1717:123685-124065 strand:+
ATGACAAAAGTATTGGACGGTCTAGATGGAGCAATGGTGTATATGGATGACATAATGATA
TTCTCAGAAGGACTAAAAGAACATTATATAACTCTGGGTCAGGTATTAGAGCGGATCCAA
ACGGCAGGGTTGCTAGTGAATCCAGAAAAATGTCATCTATTACGCAAGAAAGTGAAATTT
CTTGGCCACATAATAGATAAAGAAGGAATTCGAACAGATCCAATGAAAATCGAAGGTATC
AAAAATTTTGAGAAACCCAAATGCGTAAAGAACCTTAGAAGCTTTCTCGGAATATGCAAT
TATTACAGAAGATTCATCAAGAATTATTCAAAGAAATCAAGAGCCCTAGAGAATTATGTG
GAAGTAACAGCATTAAGCTAA
>DjScaffold3673:51883-52086 strand:+
TTAATCGATTATAAACCAAATCCGCATTATTTATCTGTACCCATAACATTAATTAAATTG
TTTTACAATTGCAATTATTACGAGAATAATTAAttcatttaatataaaagctttaatcaa
aatttaggttttaattattgatattggataacgattcgatatttcgaccttgtct
>DjScaffold1521:12061-21487 strand:+
aagtttgttgtataattaatatgcaacttgtttcacatggaatatttggaaaaatatgtt
tacattaaacttattaaccatcaacctttaacatttatttATGCGATTATTTCCTTCAGT
ACCATGGAGTCTGAAAGCCGTACAAATAAAAAAATTACCATTTAAAAAATCCGATTCTCA
TACCCGTAATAATTCTATTGAAACTGGTGAGAAAAGGTGGCCTTATGATTTACGGAAAAA
TCACAGCTTGCTTGTCATATGCGAAACTAATTGCGGTTTGCCAGGTCCAAAATCGATACT
GGTGAAATGTGGTAATTCTACGAAATCTGGGGAATTATTTGGTGGTACACAGAAAAGTGA
AATCTGTCCAGAAAACAGTGAGCTACAGAAAATGACACACACGATCATTATTAATTGTCA
ATCACAAAGATTAACTGGTCGTAACGAAATACGTTGTGAATCTCAAGATTCAAATGGAAA
TTTAATTTCAAACAAACCAATTTTTTTTCTTTGCCTAGCATTTTCTGGAATGGACATTTT
AACTCTTGGTGAAAAAGTGGCCATCGTAGTTTCAGTTTTGTTATTTATAATTGATGCTAT
TATAATTGTATTTTTGCTCAGAAAAAAATTGCCGAGAATTATCAGATAGTAAACTTTCACA
TTATAAAATAGACAAATCACATTGTTCTGAAGTGATGCTTTGAtttaatgtacacattat
acatattataatattattgtattgaagttagatttgtaataaaacagtttcttcaatttt
ta

>DjScaffold5221:24964-41883 strand:+
aaacaaatttattaaagtaaattcgatgaaaaatgaaaaattatgacaactaagttaaaa
ttgaaatttttaaaacgttaaaatgttattttaattgaaatgaaggaataagacgaatca
aatgcgttaataaagtcataaatattaATGAATAATAAATATATATATATATATATATAT
CATTTAGGTGATTATTTTAATCTTCTAAAATATCTGACAAAACTTTTCATTTTAGTTAAT
GAGGACGCTGACAAATTCGTATCAGTATTTTTTGATGATCAAGAATGGAATATTTGGCTG
CATGACACTGAGAATAAAAAAGGCGCAGATTCAAATGAAGTTACAGTTGAGTTACTAAGT
GGCATGAACGGCTATATGGTTGTGTACGCAATAGACAATATGTCCTCATTTATCTATACC
AAACATATTCTTACTGACCTCGTAGAAAAGGCAAGTAGAGATAGTGTTATTGCGCTAGTT
GGCAACAAAATCGATTTAGAGAGATGTAGAGAGGTATCAAGAGAATCTGGAAAACAATTG
GCTAAAATCCATCGTTGTCTGTTTTATGAAGTTTCAACAGCCATAAATCATAAAATAGAT
GAGCTGCTCATTCAAACTCTAATGAAAATCAGGAATAATCTTAAAGCCAAAGAAAAACGA
TTTTTAAAATACTCCAACTTGCAATCTACAAATCGTCGAGTTTCCAGTTTTCGA
>DjScaffold5419:2741-3344 strand:+
tcgatATGGAAAATTTGAAATTAAGAATGTGGTCGATTCTGAAAGATCGTATTGGCGCGG
TTGATATAAATATTTTGGATGAAAATAAATATCCAGATCTGCATAGATCTACGAAAACAT
GTTATTTTTCTGATCTATTGCTCGACTTGAAAGATCAACTGTCTGCTCAACAATTGAAAG
ATGTGAGTGTACCACTCGTGTTTAACTATCTTTTGCATTTGGCCAGTGAAAAAGGCTTGG
ATATTGGGAACACAGATGACTTCAATGATCTGGTTATTGGGCAAGGATACTCAGATTACG
ATATTAAACAAATCAATAAATTTGGTGTTGGAAATGTTTTGCTCAAAGAATTTGGTGTGA
AAGATAACCAAGATCATCTCGAGAAATGAtattccggattgattaattttctattgtttt
gttaaaagttatcctcgagtttttattttatagtttttactaaacgaggaagattgat
ttattgttttcatttttttgttaaagataatcatgtttattctgcatatctgaatacat
ttttgttagtgt
>DjScaffold1052:23136-29296 strand:+
taataaataagtgtgcattttatgttaattgatgagattattagatcatattgattttca
gcatttgtcaaagatgtgttATGAAAATGGATCACCATTGTCCATGGGTGAACAATTGTA
TCGGTGAAAACAATCAGAAATATTTTGTTCTTTTCACGTTCTATATATGTGTGATATCGA
TCTATTCCATTTATATTAGTATACATTTTTTTGTCATGTGTGTTGGGTCCGAATGGGAAG
AATGCAAAAACGGATTCAGTCCGGCAGCGTCTACAATTTTGATAGTTTTTCTTTTGGTTG
AGGGTGTTCTGTTTGGATTATTTACCGCAATTATGTGTGGTGAACAGCTTCAATCTATTT
GGATGGATGAAACAGCTATCGAAACTTTAAGGAAAGAACGTGCTACTTGGGAACGTAGAT
CCAAGTGGGTTAATATGCAAGCGTTATTCGGTTATCCACCCTCATGGAAATGGCTTTGTC
CATTAATTGAACCAAATTTTCAGTCCTTAATATCAGATTCAAATACGAATTCTTTGCATA
TTGTGTGAaaatgtatgcagaattcgatttttatctagaagccttcatttaacacgattt
attgatttgtttgggattttaaatttttaatcaaagttacacaatgattttaatgttgtt
ttatgctgaattttctgtatttattagtgaatattgtaatgaaattgttgatttatggaa
tcataatggaacttttaattgtcacatataattaagtattttgtaataaaatattaattt
gtta
>DjScaffold1052:33781-33987 strand:+
ATGAAATGCCATTTTTTGAGAGAAAAGGTAAGATTTTTGGGTAACATTATCAGCAAAAAT
AGAATGTGCACGGATCATGGGAAAATAGATGCTATCAAAAATTATATTAAACCAAAATGT
GTCAAAATTCATAGAAAGATACGCCAAAAAAACAGGAATTCTGGAAACGATGTGTTGGAA
GATCAACAAGAAACTAGACTGGAATAA
>DjScaffold222:113684-123619 strand:+
aatctccaatttatactccattctttggtgttATGGGTGTGACATCTGCAATGGTTTTTT
GTGCTTTAGGTGCTGCTTATGGTACTGCAAAATCTGGTACTGGTATATCGTCAATGGCTG
TAATGCGCCCAGAATTGATAATGAAATCCATCATTCCTGTTGTAATGGCGGGTATTATTG
CCATATATGGTTTAGTAGTTGCAGTTCTCATTGCCCAACAGATTAATGTAACTTATTCTA
TGGAAAAATCTTTGCGACATCTTGGTGCAGGCTTGAGTGTTGGCCTTAGTGGTTTAGCTG
CTGGTTTTGCAATAGGAATAGTGGGGGATGCCGGTGTTCGTGGAACAGCGCAGCAACCAC
GTTTGTTTGTAGGAATGATTTTAATTCTTATTTTTGCAGAAGTATTGGGTCTTTACGGAC
TGATTGTGGCATTGATCTTGTCGACCAAATAAacattaactcttattgaattgattacag
catatttctattctttcttcagttgatttagttattgttttcagaggattgttattatta
ggtaattgagtagtagtatcgatctagaggtgtgttggtcagatgtatttacttgtttat
tgtttatcagtcatttgtaactggattagatgattattatatttgttaatttatttatgt
ttatattaagaatctctaattgtttattaaattaaaatcggtt

>DjScaffold222:131687-132620 strand:+
ttttcacctcaatttatttataataaattattatatttcaaattcgagtcATGTCTACAG
ATCCATTTTTGATTGTAAAAGAAGAAGTAATTAAATCACTAGCAATTGCTGAGAAGCAAT
TGAAAAAAGTTAAAACTTCTAATGGAGACAATAAAAATGTGACAGAATTATCAACTACCC
TTAAAAGCATTTCATGGGATTTGGAAGATTTAGAGGAAACAGTTTCTATTGTTGAAAAAG
ATCCCCATAAATTCCCTGCTGCTTTTGAAGAGTTAACAAGTAGAAAATTGTTTGTTGAAG
AAACAAAGAATAAATTAAATTTTCTTCGATCCAAGTTAAATAATGTTTCCTCTAGCGATC
ATGTCCGCGTTAAAATCGGAAAATCTAATACAAGATTAACTACAATTAATATTGATGATA
TGAAAGATTCTAATAATGAACATTTCGATCAAATCAGAAATCAACAAGAACAGTTATTAG
AGAATCAAGATAAACAAATAGATCAGTTGGGAAGCAGTGTATCTGTATTGAAAAATATTT
CTCAGAAAATGAATGATGAACTCGACAGTCAAGCATTACTATTAGACGATGTTGGCCAGG
AAATGGATAAAACTGAGTCAAGAATGGATGCCAGCATGAAGAAAATGGCTCGATTATTAC
ATTTGACATCAGACCGTCGTCAATGGTGTGCAATAATTGGTCTTGTGGTGATTATTATAT
TATTGATTGTTCTTCTTACCGCTTTGTGAaagattgatattttttataatattcattctt
tggtttttttgtgaatgacgattaaatattttcccttgatgaatttgt
>DjScaffold2220:24771-29061 strand:+
TTAAATACCAACTCTAAACTTATTTCTTGTCCACCAACGAAAATAAATAAAGTTTGTAAT
TTTAACATTGGCAGAGGCAAAGAAATAACTACAAAATACAAATTGGTCAATTGCAAATGT
ATTGAAAATAAAATTGTTAGACGAATACCTTGTATATGCGATACAAGACTGGTTCTTGAA
CATCGTGGTGAATGTCGCAGAAATAATATTAGAATTGATGTTTATTCACATAAAGAAGCA
AGAGGAAAACATTGTGTTACCGTTAAAGTAAGAAAATATGTAATTTGTCGTTGTTCCTTA
GAAGAATCTATCAAAAATTGCATTAACAATAGAGTTGTGCATATTCGTACATATTTCACA
TTAGTTAATAATATTTGTGAAAAAAGGAACATTGTAAGATATGAATCAGTTTTCTGCACT
CAACAAGTATGGAAAGTGGAAACAAAATGTAATGCACGTGGCTTTAAAACTATAAGGTAT
TACAGATACATTGTCCACAATTGTAAATGTGTACCTAGAAGAATAAGAGTACAGTTTTGT
AGATGTCGATGTCCTCGAAACAGAAAATTTCGAAAATGTCATCGATCAGGAAAAGCAATA
ATCAATATTCATATTTCCTATAAACTTGTCGGTTGTTCTTGTATAGGAAAATTAACAAGA
AATATTCAAGCAATTCAATGTCCTTTAAGACATGTATATATATCCAGATGTAACGAAGTG
CGATGTAGAGGCTACTGGTTTCGAACAACCACAGTTACCAATTATCATCTCCGTCATTGT
ATTTGTGTACGAAATGTTAAGAGAATCAAATCTATTTGTAAAATTAAAATATTTAAACAA
GTCAGTGTAACATGTGATAGAAAGAGAAGACTTGAAATTAGGAAAGAATCAAAACTGATT
TTCAAAAATGGGAAATGTGTCAAAATAACAAAAGAAAAACGAATACCAATTACATGTAAT
AATAGATGGGTCATTATTAACAGACCGATTTGTCATTTGGATGGCACTCAAATTCGTATT
TACCAAAGATTGTATTTTAAAGATTGTCGATGTCGTCCGGAAAGAAAAGTCAAAAGATGT
AATTGTAGATGTAAGAGACCATTTGTGGTAAGAAAATGTGTAGGAAATATTATTCGTTTA
TACTATTATCGATATGAATTAAGACAATGCAAATGCAATAGAAAACTAATAAGAGTGCAA
ACAAAGAGAATTTATTGTAAAAGCAAAATATTAAAAGTTGGGCCTTGTTTTGAAAAGAAT
AATCATTGTGTAAGAAATGTAGAGTATCAAGTGTCTATTTTGGTAAATTGTCAATGCATT
AAAAGAAGAAGAATTATTCAAAAGATTTGTCATAGTTGTTGCCAACATTCGACTAAAACC
AAAAACCATTGCGAAGAGTGTCCACCTAGAATCATTACTAAGATTACCAGTTGTCGGGTT
AATCATTATGGTCGTATTGTAAGAGTTTCACGTGTTACATCAAAAGGCATTTCTTGTAAA
GAAGGAACTACTTGTGTATCGCAGAAAGATTGTAATCGTTTGACATCTACCGAAACATTT
AAATGCAAAACAAAAGCCATAAAACGTTGTATGTGTGTTTTAAAAAAAACATTTATAAAG
AAAGATCTTTGCAAATGTGGTCCAACTAAACATAAAACTGGTGCTTGCAATTTGAAATCG
TGTAAACGAGCAGTTTATCAAATTGAAAGAAAACTTGTTATTTTAAGAAATAACAAGAAA
ACTTGCCAAAAAGTTGTAAGGAAACTAAGAGATGAAATATGTTGTTGTCCGAAATCAAAA
GTTACAAAGAAATGTTTAAATAATAGATTTATTCTTAAGACAAAGAAATATACATTTAAT
AAAACAAAGAAAATATGTAAATTAATAATTAGTGTAAAAGATATAACAAGAAAATGTGGA
CCAAAGAGAGAAATTAAAGAAACAATTTATTCGAAAATTTTCAAAAAGAAAATCATAATT
ATAACGAGACGATTGGTAAATTGTAAATGTATAACAAGAAGAAATATTAAATGGGAATTG
TATCGTTGTCCTGGAAGTAGAACCGAAACCAAATGTGTTAGAAATAAATTGGAAACAATT
CGTCATAAATATTCTATGAATAATAAAAAGAACAAATGTATTCTGACAGTTAATAAAAGA
ATTAATAAAATTAGATGTCCACCTTTACGTAGAGAATCTTTCAGATACAATATAAATAAT
CTCAGACAATGTGGAAAAAGAATTTATAAAGTGCAGAATTGTCGGTGTATTCTTGTGATT
ATTCCTCGGCCTTGCCCTTGTTTTCTCAGTAATTTTATTTTATTAAATTGTTTATTATTT
CAATTAATATCGATTCTATTATTTGTAATTAATAATATAATTAGATTTATTTGAaaatat
gattgtgtagaaatatttaatattttctttaaatgtagtttttgtttatgaaattaattg

gatattaaatatttaaaatgctaattaaaatgaaaaaaatgaaaatttgttatgtaaatt
ttgaagttaaaatattttaaatctcaaattattaatttaataaataattattaataactt
tttagttaacaattcagaattttataaaattataaaatttcattgaaatatttataataa
tttctaaattaattagaatgatagctccaaagtaaaaaacttgagatgaaaaaaTATGCA
TTAAAACTCAATTACAGAACAAAGCAATGTAGACCAGTTGTTATTAAACAACAATACAAA
ATATGTCGTTGTCGTAAATCATTTGTTAAAAAATCATGTTTCAAGAACATCTATATTAAA
ACAAGAAAAGTTACTTTTAAAATCGATAAACCAAAAAATGTCTGTCATAAAAGAATCGAT
GAATCCATTTCAATTGTTTCTGGATGTCACAAAACTTTAAATATTGTAATGAATGAAAAA
GAAACAAAACCTGATGGATGGTGTATAAGAACGAGCAAAATAACCACTCCAGTAAACTGC
AAATGTTTAGAAAGAATAGAAAAAACTAGATTCAGGTGTCCATGCTACAAACCAGTACCT
GTAAAGATCAAAAAATATTGTAGAAACAATTTTATAATTGTTGTTGAAAAACATATTCCA
AAATGTAGATTAAATAAAATTTATTGGAGAATTGAGAGAACCTTTACACCAATAGTCAAA
TGTTCACATAAACAAATTTTATATGGTCCTACTTTTCCTATTTCAAGGTACAGAGGTCAT
GTTTCAATAAGATTCTGTACTAAAATCATATTTGCAACTCATATACAATGGTATGTGGAC
AATTGCGTATGTAAAAGTAAAATAATTAAGGAACAACCTATTTGCACAGAATGCTTGCCA
CCACGTCAGAAAGAGCAATGTTTAGGATCGAAGATAAATGTTGTAAGTTATTCTCACTCA
ATCGTTAATGGAAATTGCAGAAGAACTGTTACTAGAAAATTAGTTCATATTCCATGTCCA
ACACAACCAAAAATCCACCAATCAAAATGCATCAATAATAAATATACAATAACAACAACA
AATTATTTCTACGATAATTGTCGATGTAAATCTAGAGTTGAAAAGAGAACTTGTGATTGT
CAATGTGGTAAAGATTCTAAAGTAATAAAATGTTCAAGAAATTATTTCTTGAATGAAATA
ACAACGAAATATTATAAAAAAGATTGTAGATGCTTCAAAACAGTAAATGTTAAACAAATT
TCACCAATTTGTCCTCGGTTTATTCATCTAAGTGTCGGAAAATGCGAAATACGAAAGCAT
CATTCAGTTTATCAAAAAATAACTCGTCGATATATTAAAAAATATAACAAAAAGTGCAAG
TGCAAAATAGTAGGCAAGACCAAATATAAAGTTTGTGACTGTAAGAAACAAAGCATAAAA
CGTAAAGTTTGTACTAATAATAACAATTTTGTTATTCAAACATGGGGATGGAGAATTAAT
GTAAAGAAAAACAAATGTGACAAAGTAATATTAAATACCAACTCTAAACTTATTTCTTGT
CCACCAACGAAAATAAATAAAGTTTGTAATTTTAACATTGGCAGAGGCAAAGAAATAACT
ACAAAATACAAATTGGTCAATTGCAAATGTATTGAAAATAAAATTGTTAGACGAATACCT
TGTATATGCGATACAAGACTGGTTCTTGAACATCGTGGTGAATGTCGCAGAAATAATATT
AGAATTGATGTTTATTCACATAAAGAAGCAAGAGGAAAACATTGTGTTACCGTTAAAGTA
AGAAAATATGTAATTTGTCGTTGTTCCTTAGAAGAATCTATCAAAAATTGCATTAACAAT
AGAGTTGTGCATATTCGTACATATTTCACATTAGTTAATAATATTTGTGAAAAAAGGAAC
ATTGTAAGATATGAATCAGTTTTCTGCACTCAACAAGTATGGAAAGTGGAAACAAAATGT
AATGCACGTGGCTTTAAAACTATAAGGTATTACAGATACATTGTCCACAATTGTAAATGT
GTACCTAGAAGAATAAGAGTACAGTTTTGTAGATGTCGATGTCCTCGAAACAGAAAATTT
CGAAAATGTCATCGATCAGGAAAAGCAATAATCAATATTCATATTTCCTATAAACTTGTC
GGTTGTTCTTGTATAGGAAAATTAACAAGAAATATTCAAGCAATTCAATGTCCTTTAAGA
CATGTATATATATCCAGATGTAACGAAGTGCGATGTAGAGGCTACTGGTTTCGAACAACC
ACAGTTACCAATTAT
>DjScaffold2220:41750-45634 strand:+
tgtattttgataaatcaaagatttttcttgagaattttttctagaacttattttgcgtaa
attctattagcaatcaaattgttgtatcgcagtgacaaattttctagatatttcaataaa
tcttcaatttattaatctattattaaaaactgtttttgaatatttaggttgtccaaaaga
tacgattactcaatcatgtattgctcgatacggagtttttagaattgttcgcagaagcta
caaatttaatagagaagggtgcaaatgtattgcaaatatcagaacacaagataaacaaat
aagatgtgcgagatcaccaagaaatgtaattagaatggctgcaaaagtctatcagaATGG
TATTTATGATTCTGTCACTACAATCTGTTGAACGACGACAAAATTGTGCATGCATAAAAC
AATCATCAACGAGATTCGTGTTAAATAGATGTTATTATGGTTATATAAGAACTGGAAGTC
AATGCAGAAAGCGTTATGTACCTCAAACAAACGTAAATATCAAAAAACCCTATCCGTGGA
GAAAACCACGGTGGACAATATTAATCAAAGGAAACCTTCTAGATTGGAGGAAAAATTGGA
GATATAACCATAGAAATTGGAGATATAACCATAGAAAT
>DjScaffold1675:34763-35743 strand:+
ATGAATTTTACTCTTTGTCATAAAAATATTTCTCATGTACACTATCAAATTCAGTGGTAT
TTTTACCCATATTATTTTGCATTATTTTTTTGCAGCTTATTACTGAATATTGTTTTCATC
AATGCATTTGTGAAATACAAAAAACGATCAGTCTACAAATTCAATATTTTCAACTACCTC
ACTTTTATTTTAATACTTATTGATTTTAGTCAATCGATTATTTTACTTTTCGATGTACTT
TTTTTAATGATATTTGAAAACAATCCAAATCAATATTAATGGAAGTATTTTTGTCTCTA

CGAAATTGTCTCATTTGTTGTCATGCAATGACTTTGCTTTTCATGGCTACAAATCGATAT
CGAGCTGTCAAGCATCCACTAATGGTTAAAGATCCAAATTTAATATTGATAAAACTGTAT
AGAGGAATATACATTTTTATGAGCATTCCATTTATTATAAGACTAATATTGTTATCAGTG
TTAGAAAATTGCCAATTGCTAGAAAGTATTGACATAGTTCTGGAAATAATGATTGGAATA
GCAATAGCCGTGGTAATGGCAGTAATTATTTGCATTTATTCAGTTCTGTTTGTATTGTTG
TGTTCTCGAAATTTCAAATCAGATCCAAAACAAAGAAAAATTATCAGCAGAATCCATCCA
TATCCAAATGAATTAAGCTCAATAACGAATTCAATAACTGTCAAAAAACATTTCAGTAAA
CCTGCTAAATTTGCTCAAGTTCTATTTTCAATTACTATGTTAATGGTGATTTCTTACATG
CCAGTTTTTTTGGCAAATGCAAAAATAATTGATTATCCCGGACATGCGATATATTATCTG
TTTTATCTTCCGGTTCTTGGTGATCCTTTCATATTGATGTTATCTAGAGAAGACATTCGT
CAAGAAATGATTCGATCATTCAACCAAACAGGAACACAAAGTGTAACAAATACTCTGATT
ATTCAACAAGTAAATTCTTAA
>DjScaffold2029:2952-6252 strand:+
tgtaATGAAAAGAGATTGGGATTATGAATATGAATTAATCATTGCTACAACAGATGCTGG
TGAACGGGAAACATTAACCAAGATATTTAAAGAAAATGGCTTTACAGTTGAAACTACAGA
CAGTTTAATAAAAGGTTGTAGTGAAGATAAAGCGAAAGCTGAAGCTCTTTTGGCAACATT
GCAGAAGAAAGGATTCACTGTAAAGTTAGAAAAGGAAGAATCTTCGTATACATTTTAAgc
attgaaaactgtcgattcttt
>DjScaffold3947:16854-24020 strand:+
caatatttattggatcattaatatgatttggattgcagctaattaattttgttctaattt
acgatattaattaacattgcttgttacgatgtaaaggaagaaattaaatcagaatacaac
accattttcacgatataattaccattttaccatacaggtaatagcattcatatcaaaggt
tggcatattaaattgaaattaatagctaatgttaactatatgtatgacaaacatactgaa
aaagattttattctactctgattttcgtgaaagtttaaaattaaaaagtcgccctacttt
ttgaccacagtctataaataaactaataaataattctataattcataaatagaatatgac
ttggatattttttattagtcatcatggaaacattttcgaagcATGGTAAATGAAAATTT
TGCACACGACCAATGGGATTTGGCAGCTAAATGGAAAATACCTTTGAAAAATTCTATTGC
TTTGCTATATACTCGATTACCGGATGATCAAATTATGATAAATAAATCCCAGGAACTCAA
TTTAAGATGCTTACAGCTGCCGTACAAATGCAGATATTTGTTAATTGCTGGGTTTATTGC
TTCATATAATAGTAAATCAAATGACAAAGAATTATTTCTTACGAAAGGATTATCCAATAG
ACTGAGTAAGCGACAAAAGAAGTCGATAAATAAACGCGAAAAGGCAAATAGTTCTCTATC
TGGCCCAAAATCATTTTCTCTTGACAGGTTGTTTGCCATTTACTCTTGCATTATTGATGA
AAAGATCGAAGACAATTATTGGTTTGTCATGAAACTAGTTAGCAATCTCGTTGATACGAG
ATTATTCATGTCAATCCATTCGAAATCT
>DjScaffold3969:50155-50551 strand:+
CAGCGAAATATTTTTGCTTGTATTTCACTTAACATTATCCGATATACTCTTACTTATATT
AGTGTTTGGTTACTTGTTGCTGTTGCTATAGATCGTATGATTTGGGTTATATTTCCATTC
CGAGCAAAGATGATTTGTACAAGAAAAATTGCCAAAATAATTGTTACTATATTAATTGCC
ATTTTTAGTATTATTGATTTACATTTTATTTGGACTCTAAATTTCAGAATTTTTCCAAAA
TGTAATTTTATATATTGAtttgactttagtaatgggtttgccatttattattatgtctat
tgccaattcgttaattatttcaaagttgagagaaatggagaaaataagaaaaggaatga
>DjScaffold2186:4573-21603 strand:+
atttttttttatacttttaattttaaattttaatgatttttatattttaatcaactcaa
attttttaaaattttttgaagttttttgagttgagttgatttaaaaaataaaaatcatta
aaatttaaaattaaaagtataaaaaaaaaaccaataaaatttcgataaaatttgaagaatt
gagtaacaaagatctattgtattgaatatctcaagaagtcaacatgttcctgaagaagac
ttttgtcgaaaatttgaaccttttttacaaaatgaaggtaaaatttcttactaataaaaca
taattgaaatttaaaattagcgatattaattttcagagaatattaaaattgtaaatttaa
tgtaaaaaatatttcattttagatggtttcgcattcggtttaggccgacttgggaaactaa
tgaaaataattttcagttgacttttATGCATCGATTTTCAGAGATAAAAAATTCGACTAC
ATATTTTGCATTTTGTTATCCTTGGTCTTATAAAGATTGCCAGAAACAATTAGAAGATCT
GGACAATAGATTTGCTATAGTCCGATCAAGTAGCATAGATCAATTATCGAAAATGGATTC
AGGAACTATTTACTATAATCGAGAATTATTGACGAGATCACTTGATGGTCGAAGAATTGA
TCTTTTGACAATTTCAGATTTGAATGGTTTGACAAATGAATATGAAGATATTTTCGATGA
AAAACTTTTTCCTGAATCCAAATGTCGACCTTGTAAATTTTCTAACAAAAAGGTATTTGT
TGTTACTTCACGAGTTCATCCCGGCGAAACTCCAGCTAGTCATGTTTTTAACGGTTTTCT
TGAATTTATTCTTCGCGAAGACGATGCACGTTCTATTCAATTACGGAAACAGTTTATTTT

TAAACTGATTCCATTGCTCAATCCAGACGGTGTGTTCCATGGACATTACAGAACTGATAC
GAGAGGCGTTAACTTAAATCGCGTGTATTTATATCCTGATATTAAAACCCACCCTTCGAT
CTATGCAGCGAAATCACTTCTCGTTTATCATCATATCGTCAACAGTTCAGAATCAAAGAT
TTTCGTCAATACTGTGAAACAGAACGTTTTCGGAAACATAAATCCGTTAAGTTGCGAAAA
TGATAAAAATTCCATTGATGACAATGAATCAATTCCTGAAAATTGTGAATATCAAATAAA
TTCATACACACGAGATACAGGAGAAATTGAATTTGCATTATGTAAAGATCTCATTTCAAA
TCCTATTGAAACTCAATTAAAAGTTCAACCAGAATTAACATTGCCACCGTTACAAGTTGA
CTTGAATATTGTAAAACATCTAATTGATGCTGGATCAATCAATGATTTCCTGGAAGTGAC
AGTGGATGTTGAAAATTCATTATCTGATCCTTTATCAAATAAGAATCTAATTAATAATCT
TTCTGAATCTAAAACGATAAAAGAATTCCATTTATCGAATAATCAAAATGAGATTTCTAT
CTCAAAGAATGAACATGTAAACATTTCAAATGAAATACAAGATTCTTCTTCAAACAATTT
GAAATCTGTTTATAAGTTAACAAAATTAGCTGGAAAACGATCTGAAATCTTATCTAAATC
AAATGATTCCATTATGAAACCGACAGTTGAGCATCAATGGAATTTGAAAAATTTTAAAGT
ACCACCAGAAACAAATGTCTTGCAATTGTCGAAACCATACAAAAAACTGAGCGCGTCTAT
GATTCCTCTTCCTTTAAGACCAGCTATAGTTTCTATGAAATTTTCATCGAAGAAAATGAA
CAAAATTACACTAAGTTCAGCTCAATTATCAAAAATGAAAAAAGAATTACAGCTGAGAAA
CGGCTTTAAAACTGAACCGAAAATCGGAAATGAAGGATCAGACGATGAATCGGAAAGAGA
AATGGGTAATAACGAATGCCTTATCAAATATAGCTTACATTTATCTGATTCTTCTTTGTT
AAAAATATCCCCGGACAATAGCGGAATAGCATTTTATGTTGATTTACATGGACATTGCTC
GAAAAGAGGTTGTTTTATTTATGGAAATTGCTTAGAATCAGAAAACGAAATGATTGAAAA
TTTATTATTTCCAAAATTAATCTCGTTAAATAGTCCTCATTTCGATTTTTCGGGATGTAA
TTTTAGTTTCAAAAATATGTATACTCGTGATAAACGTGATGGTTGTTCTAAAGAAGGGGC
TGGCCGAGTAGCGATGTGGAAGCATTTGGGAATTGTTCACAGTTACACGTTAGAGTGTAA
TTACAATACTGGAAGGATAGTAAATTCAATTCCACAAGTGCTCAAAGATAATGGTAGAAC
GTCTCCACCAACAGCGGAAATATCCAGTTGTTTGGCTCCGAGATATACCCCGTATCATTT
TGAAGATGTTGGTAAAGCAATGGCAATTTCAGCATTAGACGTGTACAAAATTAATCCATT
ATCGCGTCTTATGAACTCTGAATACAATAATTTACAGGTATATAATTTCTTTTCAAATAT
CTGCTTTAATTTATCTTTAAAAATCTATTAAtttaagtttattacttta
>DjScaffold2186:58296-59108 strand:+
taagatctaatcattaatgactctctcccactaatctttttaaatatcaattaataaaa
gaaaaggtcgcacaagaaacacaaaaaaagctacatgtggcctcgaggctgcaggttccc
aatctctgatataatatattaccataaaacttcatatatttttataaatttagggaacaa
ttgtgagttatttgaattctaatgaaatattgttatatcaacttactctatggataaaac
aaattatttctggcctcaattattttcatgaaaacggaatatatatcggatacttgaatc
cattaaatattttaattgatggcaatcatcatgtaaaaattctctcttttaaaactgatt
cgaacgagtttgccaaccaaATGAATGAAAAGATTGACATTAAGGTTTTTGTTGAAAATG
ATATTTTCGAAAACAAAAGACAGCCTGATTCTCAATCTGACGTTTGGTCTCTTGGAGCTA
TATTGACATACATTACTACTGATAAATCCCCTGAATATATATTAGAAAATAGAGATACAG
GAAGAATAAGAAGAACTTTGCATAGAGATATGATTAATAGAAAAGACCCGTCAAAATGTT
TACATGAGTTTATTTTGTTTTGCATGAAAGAAAATCTGGAAGAGAGATCAACACTTGATC
AATTAATGAATCACATGCTTATGAGAACTCGGACTCTTCGTTATTAAaaatatgaatttt
atttgttt
>DjScaffold2186:67217-68032 strand:+
ttgatatttatttattctatgtttaaatttactctcattcattataaatcaattaacgct
tgaaaattattaaaaatttttgattgtcaattgtttattctcagaATGCTGGCTTATATA
ATTTTTTCCCACTCTCTCTTAACTTGTTTATCCTGGGCATCGGGAAAATCGATTTGTCCT
CCAGATTTTCCTTTTTCAACTAAAGATTTTTCATGTGGAAATTTGCATGATGTTTGTTTT
AAAATGGGTGATGAACATTGCGATCGACTTCAAAATTTATGCAAACAATGTCCACCTAGC
AATATTACAATGATAGTGATGGAAAATATTCTTCATCGTATCACAGAACTCGAATCAAGA
TCCAAAGAATGCCAATCAAATATTACACGTAATTATATTTGGATTTTCATAATTTAAtaa
aaatatttataattatttgctatgtgaacaaattaacatcgcagttaattacgaatattt
tttttacttttcacacactaaat
>DjScaffold2186:78439-82508 strand:+
GAAGAACATATTTTGTTAGATGATGAATCCAAACATGTTTCTGTTGGACCGGAAAAAGTT
TTTTCACAAAAATTTGCATCCACACAAGTTTCAGTTGATTCTCAACAAGAACGATTTTTG
TTACGTGAGCATGCATCTATACAAGGAAATATTAAAATGAAATTCAAAGTACCGAAATAT
AACAAAACAATTCAAGTTTATGTAAACAGTTCAGACAATATTGTTAAATTAAAAAAGAAA

ATCTGTAAGAGAATCGGTAATCTATTGCCAGATGCTTATAAGGCAATGATTTTCAAATAC
GATGGAAACCAACTGAATAATAACCATTATTTGTTATCATGTGTAAAAGGCAATACAGTC
CCAATTTATTGTGAATTTCCTAATAATATTAATGTCAAGGAAATCTGTGTTCTAAAAATA
GAAGGAAAAGAAAATTCCCCTGGGCTATTGCAAATAAATAGAAGTAATAAAATAAACGAT
TTGAAAAAAATAATTGCGAATCTTATTAACTGTGAATGTGATAAGTTGATCTTATACCAT
AAATCTGTTGAATTGGAAATAGATAAAACCATCGAAGAATGCAAATTAAAAAATTAT
>DjScaffold2948:52411-60629 strand:+
tgtataatttttttaatgagtaaagttataaactgaattaataagaattgtgagaatatt
ttttttgaagtattaatttgttaattacaattatattgatttataatctgttatcggaga
tATGTTGTTTAAAGATTCCAATGATATCACATTTCTAGAAACTAATTTTACAAATACATT
TGGTCATATTTTCCCTCGTCATTGTAAAATTGCCATTCATTTTACCCGAATTAGTGGGCT
GCTCTTATACAGCATAGATTACAATTCCAATCAATTGATCATAAATCTACAAGATACAAA
ATATCCGACAATGAAAATCGAGTTTAGAAATGATAAACATATTTTAAGCATAAACGATCT
ATGTAAATGCAATATACCTTCATGTCTTTATGAGACATTCTACTTTACATTTCAAATAAG
TGATACTTTGAATATTCACTGCTCTAATGGATCGACAGTAAAATTCAAGTTGGCCAACAA
ATGGATAAATCGTGATTATCCTATTCCAAGTTTTCATAACAGCAACATATCTGCCTATTT
ATGGTTAAATAATATTCCAATGTATCATAACAAAGATAAAACGGTTTATCCAAACGCAGA
GTATCTAACAGGGTGCTTACACAATGTCGAGTATGATGGTAAACCAGTTAATTTTACTCA
TCGTGGACCAATTGATATCTGCAAACGGCAATGTCCATGCAAAGAATGCAGAGAATATAA
TTGCTTGGAAACCTATCTAAAGCCGAAATGCGACTGTAAAGATTCATTTTACAATGGGAA
CTTTTGCAATATTTCTTTATCTGATATCGGATTTAAATATTTTGAATTTAAATTTAAAAT
AATGAAATTGTCTTGGAAATGGAAATACAAATCACCAGGGAATGGAGATATTTTAGATTT
ACAAAATAAACATCATCAAATCAAAATTTATTTGAAATCAAATGATCTAATATTTCAATA
TATCGATTCTGATCTGATCTTCACAGGTTAGtttttcgtgcaaaaataaattaaacaaat
aaataagataatatattaaattttgaaaccttttactaaatgaaataaattaaaaataaa
agttaatattgcaagatgagtcagaaacgttcgcagtagaattcataaaatttttatttt
cattaataattttatttaaaaagttgttttaattattttgtttattcaaagaaaataaat
ctttcatacaaagccaagtaattagaaagtcaactgcaattttcaaaatatacttacaaa
taaattttacattttgcaaatttatttagcttgatttattttataattttaaaattacaa
gaattattgagagaatatttgttgtacttctctattgatagttttttttagatatcattaa
>DjScaffold795:41679-57754 strand:+
GATGTAATTGATGAAACACACATTCCTGTAAAAGAAATTGAAGAACATCAAGATTCTGAA
GAAGAACGTGATCCTCAAACTTCAGAAGATATCGAAGATGTATATCACGAAAAAGATGTA
ATTGATGAAACACACATTCCTGAAAAAGAAATTGAAGAAAACCAAGATTTTGAAGAAGAA
TGTGATCTTCAAACTACAGAAGATATCGAAGATTCATATCACGACAAAGATGTAATTGAT
GAAACACATATTCCAGAAAAAGAAATTGAAGAAAATCAAGATTCTGAAGAAGAACGTGAT
CTTCAAACTACAGAAGATATCGAAGATGTACATCACGAAAAAGATGTAATTGATGAAACA
CATATTCCAGAAAAAGAAATTGAAGAAAATCAAGATTCTGAAGAAGAACGTGATCTTCAA
ACTACAGAAGATACCGAAGATGCATATCACGACAAAGATGTAGTTGATGAAACACACATT
CCTGAAAAAGAAATTGAAGATAATCAAGATTCTGAAGAAGAACGTGATCTTCAAACTACA
GAAGATATCGAAGATGTACATCACGAAAAAGATGTAATTGATGAAACACATATTCCAGAA
AAAGAAATTGAAGAAAATAAAGATTCTGAAGAAGAACGTGATCCTCAAGGTTCAGGAGAT
ACTGCAGATACTTATCATGAAAAAGACATAATTGTAGAAAATAGTGTTTCTGAACATATT
ACAGATTATACAGAAAAAGAAATGACCGAAGCACATGATATTGACCTTCATGATAATGTT
AAGTCAGATGACGAATCACACGAAAAGCTTGAAAATTATACAGAAGTTCAAGATCAAGGT
TTGAATACTGAAAATACATTCGAGACATTAAAAGTGGAACTTACAAAGTCTAATAATGCA
AATGTTTTAAATGATGATGCTTATATTCATAAAGAAATTGAAGAGCAAGAAAATAAACAA
AGTGAATATGAAGAGGAAAATCAGTATAAGATACCAAACATTGAGAATATGCATCAAGAT
ATGGATTTATACGATGAAACTCGTGTTCATGAAGATATAGATGAAATGAAAGAATTTCAA
AATGAAATGAAATTTAAACATAACCAAAATATTTCGAATAATTACGATTTGGAATTATCA
GAAAAGGAAATGATTGACCCATATCCTCAGCCTACCATCGAACGTAAAGAAGTTTTTGAA
GATATTTCAGAAACTAATATTAGGCAAGAAGAAAGTGAATTTCATCCTCATCAAGTTGAT
TCTATGAATATGGAAGTTGCAGATAAGTCAAAAAATTTTTGAAGATGATGAAGAAAATGAT
GATCAGGAAATGATTATTCATGATGTGGAATCTGTTGGTGAAATGGACCATATAAATGAA
TCTGACGAAAATGAAGATATATCCGAAAATCAACATCAATCAGAGACTACTAGTGAAGTT
GAAAATAATCGAAATCAAGAATATTTAGATGAACCTGAAAACGAGAATGAATATAAACAT
GAAATTAATGAAAAAACAGATAGTAAATTTGATCAAGATCAACAAATTGAAAATACTGTA

AATAACAAAATATTAGAACATAATTCAAATGAAGACATTATTGAAGACAGAAATAATGAT
CCACATAGCTTTGAAAATGATTTCGATGTTTCGAATGAAAATATAAAGTTATATAATAAT
GAAGAGGAAAATACTCAAGAAATGAATGATAATCAATTTAATCTACTAGAAAAATGTCAC
AGTGACAATGAAGAAAATGATCTTTTAAATGAGGCAAATCTTGGACATCATAATATTACT
GAACGGCCGGATTCTCTTTATATGGATAACTATCCCATCAATGAGAATTTAACGTATGTA
CAAGGTGATTCGGCGTCTTCAGAAAATCACCAAGAAACCGATCGAATTATGCCTGATTCG
GAAAGTAACGACAGTGATCTTAGCGTAGATATAGTTAAGGATCATACTGCTGATTCGTCT
AAAGATTCAGAACAAGAAACATATGTTCCATCAATTCCTGTTTTAGATGATATCATTGAA
GATGCATCTCCATCTTCACCTCTTTTCCACGAATCTTCTGAATTTGACAGACCTGAGTTA
GAACCAACTATGTGCAATATAAATGAAGTCGATAATTCTCAAGTGAATATGAGCAATGTA
AATATAACTCCTGATATTCTAAGAGGCCTGAAACATATGAATTTGTCCGAACCAACGGAG
ATTGAAGTGGAACAAATTGTTGAAGATGTGGAAATAATTAAATATCAAACAAGCATACAC
GATTCCGATCAAATTTCAAAATCAGACATTACTGATCAATTTTTAAAAGATTCAGTTGAA
ATCAGTGACAAAGAAAGTAAGGAATCAGATGAATCAGTTCATATTCCATCGCCTACGAAA
AATGATAATGATGATATAATTAATATGTCATGTAAACAAGATCATGATAATTTGAATGGT
TCTTTGAATGGTAATCATCACTATGAACATTTTCCCGAATTAGACTCCGATCACACTACC
TACTGGAACGAACCTTTAGGACTACCAATGCCAACACTATTAAGTTCAAAATCTAGTACT
AAGCCAGCATCAGAAAAAATTTCATCTCATCCATCAACTAAATTAAAATCTGCTACTGCA
ACTTCGGTAAAACTTAATGGTACATCTTCAAATGCTGCAGCCACTATAACGCACAAATCA
GCAGATTCGTCCAAAAGTGCGTCAAGTCCTCGGAATAGTATTCCTCCAAAACCATCAGCC
CCTAAAAGTGCTCCTATTTCAAAAGCACCACCATCGAAATCCGCTAAAAAGCCAGAAATT
GTTGATCCAAGTAAATCATCAGCTTTCTATATGGAGATAGCTTATATTCCTGTGGATCAC
GATGGCAACGTAAATCCAACTAGAGTTAGTGAAGCCTTTTTCACACGAGTCAGAGCTCGG
CAATATATACTTCCTGGATTAGAGCATAGTTCTGAGGTTCTTGATATGATATTGGCAGCT
AGAAGTAAATGGTCTGACGAAGATAACGAAATTGAAGTGAGACTCACTCCTATCAAAATG
ACAGAAACATTGAAAAAATGGGCTGGGCAACATGCTCAACAATTGTCCCAACTCAATATC
AAAATCGGTGCCGCTGTAGAACATTGTTCAGTTAGTCTAGGTGGTGACAACAATATGCCA
TGCATCGCTCACAGAATTGATTTTTAAccgcaacaatgaatttatttttttttcaatttt
gagttttttgttgtattaatttttttgtcatttacaatatttagttgatcgaacaattt
cttacttgaggaattatcgagttactgaattgtccgtggcattcgttggtaactcttatc
aaccattattatgttgtgtgttttatttgtattaaattgattttaaatcattaAATATAT
CGAATAAATATAATCTTTTCATTTTTACATTTGTCATTGTAGATAATTTGAATGGTTCTT
TGAATGGTAATCATCACTATGAACATTTTCCCGAATTAGACTCCGATCACACTACCTACT
GGAACGAACCTTTAGGACTACCAATGCCAACACTATTAAGTTCAAAATCTAGTACTAAGC
CAGCATCAGAAAAAATTTCATCTCATCCATCAACTAAATTAAAATCTGCTACTGCAACTT
CGGTAAAACTTAATGGTACATCTTCAAATGCTGCAGCCACTATAACGCACAAATCAGCAG
ATTCGTCCAAAAGTGCGTCAAGTCCTCGGAATAGTATTCCTCCAAAACCATCAGCCCCTA
AAAGTGCTCCTATTTCAAAAGCACCACCATCGAAATCCGCTAAAAAGCCAGAAATTGTTG
ATCCAAGTAAATCATCAGCTTTCTATATGGAGATAGCTTATATTCCTGTGGATCACGATG
GCAACGTAAATCCAACTAGAGTTAGTGAAGCCTTTTTCACACGAGTCAGAGCTCGGCAAT
ATATACTTCCTGGATTAGAGCATAGTTCTGAGGTTCTTGATATGATATTGGCAGCTAGAA
GTAAATGGTCTGACGAAGATAACGAAATTGAAGTGAGACTCACTCCTATCAAAATGACAG
AAACATTGAAAAAATGGGCTGGGCAACATGCTCAACAATTGTCCCAACTCAATATCAAAA
TCGGTGCCGCTGTAGAACATTGTTCAGTTAGTCTAGGTGGTGACAACAATATGCCATGCA
TCGCTCACAGAATTGATTTTTAAccgcaacaatgaatttatttttttttcaattttgagt
tttttgttgtattaatttttttgtcatttacaatatttagttgatcgaacaatttctta
cttgaggaattatcgagttactgaattgtccgtggcattcgttggtaactcttatcaacc
attattatgttgtgtgttttatttgtattaaattgattttaaatcattaAATACCAAAGA
AAGATATATTGTTTATATTGGAAGTCATGGTGCCAGTTATTCATCGAACTGGTTATTAGA
GGACGGTATTTTTTCAGCCAATAGCATGCTTACAACTCTCAGTGAAACTTCATGGATCGA
AAATAATTCAATGGAATCTTCATCAATAAATATTTTAACACCCGGTAATTCAGCTCATTG
GAATAAAATTCTGTCAACATTAAAAATCAAAGAAACAAAACAGGATCGAGTACGAGTATT
TTCATTTTCATCTGAAAATAAAGTTGGTGTAGAATCGGCATTTAACAAAGACATTGAAGA
ATTTTGTCAAACTCTGTATAAAAAATATCAACCAATTTTACTTAATGATGATATTATAAA
CTCATCGCAAAACTTCGGAACTCTTCAGTTAATGAATAAAAATGGTTCATTCTATGTCTT
TGATGAAGGCTGTGGCCAAAGTAGTTTACTCGCATTAAATGGATTCAATTTATTAATCGA
TGGAGGATATAAGAGAGCTCCTGAATTTTGGAATTTTTTACAAAAATTGGAAAAGATTGA

TACCTACATGATGACATCATGGAACGAAACCAATCTTTTCGGAATGGAAAGCTTGTTGGA
CTTCAAATCTAAATCTTCTGAAAATAGTGGGGATTTGTCCCGACCAAATCTCAATTATTG
TGTCTTGCCAATTGGACCTAACCCACTTAAGGAAACAGAAAAATTTGAAATTAAATCTCA
TCTAGAAATAAATATTGAAATGGAATCTCAAAAAATTATTCATCAAATAAAATCTTTGGA
AATACCATTGCTTCAAATGTCACGTGATGCAAAATCAATGCTGAGTCCTCATCAGCTGTA
TTTTAAATTAGGTCAAGGTTGCTTAGATGCATTTCCTCTTTGGCCTTGTAGTGCTGAAAC
ATCTGGAGATTCTAAGCGCTATATTGATGAATGGCAAAAAGAATCCAAACTTCAAACAAT
TTATTTTGATGAATTAGTAAAAGATAATGCTAGCGCTTTCATGCTTATCTGGAATGGATC
GAAAGTCACAGTAGAATCTCCAACTGAAGAAGCAAATATGCTTACGAGAATATTAATTAG
TAGCAGTTGTCACGAATCTAACTTATTTAAATCATTAGAAGAATTTTCTAAGGTAAATCC
AATTTGTAATTCTTTTTCCGGAAAACCCACTGTAAAGAAAACAGTTGAAAAGAATCCAAT
TTTTGCTGATCTAAATAAAAAGTCTACTGTTCCAGCTAAAGAAAGTTCCAAATCTATGAC
TGACAAGGCTAATGCAGCAAAGAAACTTATTAACAGTAATGATAAAAATAAAGAAATTAA
TAAAGGTGACGTTCAGAATAAATTAATTGAAAAGAAATTACAACTCAGTGCTGATAAATC
CGTAAAATTAAAATCTAATGACAAAAAAGATGAAAAGAAAGATGATATAGCTGGATCAAA
GTTAAGTGATGAAAAGATCAAGCATGCAGAAAAAAAAGTTCCATCGTCTAAAGCTTTGCC
TGCTTCACAACCTATCAAAAAACTGAATGATAAAGTTGTAGATGTATCTGATTCTAAGAA
GTCTGTTAAAAAGTCAACACCAACCGATAAAGTATTGGAAACTAATGAAGCTAAAAAGTC
TATTAAGAAATCCTTAACAAATGATAAAACGCACGAAATAGATGATAGTAAAAAAAATGT
GAAAAAATCATTAACGACTGAAAAAGTTACTGAAGCCGGCGATGGAAAGAAACCTTTAAA
AAAGTCTGTAGTAAGTGATAAACTTGCAGAAAGTTTAGACACTAAGAAATCAACAAAAAA
GACTATGGTTGGTGATAAGGGAGCAGATTCTGTGGAAGTTAAAAAGCTCATAAAGAAACC
TTTGTCAAATGAAAAACCTATTGAAACTCAGGATAATAAAAAATCTAAAAAAACAATGCT
TGCAGATAAGGGTTCTGAAGCAGATGCTAAAAAAGCAATTAAAAAGACTATTCCAAATGA
AAAAACTGTGGAAAAAGCGGATGGTAAAAAAGCAGAAAAGAAACTTACTAAGGATGAAAC
TGCAGATCAAAAGAAATTGCCTAAGCGACCAATATCAGAAAAACTATCAGAGAAACCTAT
TTTAAATAAAAAGTCTACTACGGGTAAATCAGAAGTGGAAAATAAGAAAGCTAAAGATTC
AAAATCCTCGTCTCAACATAAGATTACTAAAAAAGTAGATGCAAAATGTCCTGATATAAA
AAAAGATGATAAAAACATTGCTGATGTAACAGTTATTAAAAACGAGCTAGAAAGTGACTT
TGAAAAAATAGAAGCTGTTGGTCAGCCATCAATCAGCTTGAATACTGATGATACCCTTAA
TAATATTGATACATCTGAATTACTTGGTAAAATGGAGGTGATTGGTTCCAATAGTAATCA
TGTTGAAGATGAACAGTTACTAATAACTGCCGAAAGATATGATTTACAAAGTACTCCGTT
ATCTGATATGATCACAGATAGTCCTGAACATCCTAAAACTGAAGAAGATGTTGAAGCATT
GAAAACAGATGCATTATCCATCGATCAAGATTTGCTCATAAATAATGAAGAATTTCCTGA
GGATGAGAAAGTTACAGTGCCAGCTAATGATGAAGCTGAATGCGAAGTTAAAACGAAAGA
TGAAATAGAACATACAAATATTTCTGATGAAATTCCAATGGTTGGAGATAATGATGAGTC
TGGTTCAAAAGATTTGCATCAAGAATTCGTATTAGAAAGTCAAAATTTGAAAGACTATGG
TATCTATGAAGATGGTGAATATGATGATGAACATAAAATTGAAACTGGAGGAGATGTGCA
TGAAATTAATGAAGGTACATCAGAGAATTTTGAATTTGTAAATGAATTTGAAATAGAACC
ACATGGGTCAAAAGCTGATTATCAAGAACCTTTTGAATCTGAAAGTCAACAAGTCGAAGG
AATAGAGCTTAGTTTGGATTTGCATCATAAGGGATGCGATAAAGAAAAAGAAACTGAAAA
AATAAATAATTTCAATGATATACATGATTTAGATCGTAATATAGAAAATAATGTTATTAC
AAATAGTTTAATAGATGACAAAAAAGGTGAAAATCAGAAGAATTTAACTACTGAAATAGA
AAATGAAATTATTATAGATGAAAATCAAGAAATGAATTGTAACTTTGCTACAATTGACGA
TAAAAATCAAAATAATCCAATAAATGAAATTGAAAATGAAGTCAAATCTCATTTATATGA
ATATGCAATTGGAATTGAAAATAATTATGAAAGTAAACAAGAAATTGAAGAAATTCAAAA
AGAAATTTTATTTCAAAAAGAAAGCCACTTGGAAGATATTTTAAATATGGACAGTGATGG
CATTGATACAAATATAGAGGAAATTCCTGAAAACTATGGTAATGATGACCTACAGGAAGA
TAAAGAAATCTCAGAGACTGAAGAGTACGACAAAAAACAGACTGATATCGATGATAATTT
GAATATTGATAACCAAGATCTTGAAATTGATTATAATAAGTGTTATGTCGAGGAAAATGT
AGTAGATACATTGGATAAAAATGATTTTGAAAAAGAAAATTATCTTCAATTTGAAGATGT
TGTAAAGGAAAGACATATTGCAGACAAAACTTACACCACCGAAATTATTGAAGATATTAA
TCACGAGGAACAAGTACAGAAATGTGAAGATTTTTCAGAGAAAGAAATTCAAGATGAAAA
GTATGACATTAATAAGGATGATGATGTAAAATCTAATATTCAATCTGAAACCCGTGAAGT
TGCTGATGACTTTTCAGTAAAAAATGATGAAGATGAAATGTGTGACTCTGAAAAGGAACT
TGAAGAAAACCACAATTTTGAGACAGAATATAATCCACAAGCTTCAAATTATGTCGAAGA
GCCATACAATGAAAAAAGTTTAATTGATCACACAATTAATCCTGAACATGTAGATTATCC

TGAAAATGAAATTGAAGAAAAACAAGATTCTGAAGAACGCGATCTTCAAGCTACTGAAGA
TAAAGAAGATTCATATCACGAAAAAGATGTAATTGATGAAACACACATTCCTGTAAAAGA
AATTGAAGAACATCAAGATTCTGAAGAAGAACGTGATCCTCAAACTTCAGAAGATATCGA
AGATGTATATCACGAA
>DjScaffold4743:35439-47200 strand:+
ATTTCTGAGATCACATATTTAAATCGTCAACGACCAACTACAGATAATCTGGTACACTAC
CGAAACATTTTTGCAAGTCCTGATAATTTTATAAAACGAAAGGTGGATTATAGTAAGATG
AAAGTTTTCGACGAGAAATTTCGCAAAGAATTCGAACGCCTGAAAACTTTCGAAAATGCT
AAGTTCTCGGATAAAAATATGATCAGAAATATTGCAAAATCTGGATTTTACTATAAAAAG
ATGTCAAATGTGGAATATGAAGTGAGATGTATTTATTGCCAAGGAAAGGTTTCAGATTGG
AAAAAAAATGATGTACCAGTGAAGCGCCATAAGTTTAGTTATCCAACTTGTCCTTACATA
ATTTATCTAAATGTTGGAAATGAGGCGTTGGAATATGAAAAATTGAAAGAATCACACGAA
GATCCTGATGTTACTAGACCAACAATGAAAATTAAAATTTATATTCCAAAATTAAAACTA
AAAACCATAGATCAATTAAATGAAACTGATTATTTGGATGTTGATACCAGAGAACAAACA
TTTCAATCAAATCCTGAATTGAAATATTTAGCATTAAAAGGATTTTTTATTGTAAACAAA
ATATCGGAAAAGGAGTTGAAATGTTGTTCGTGTTTGAATGAAATCATTATTTTGAAAGGT
TTTAAAAATCTTAATGAATACATAATGCACAAGGATAATTGTTATTATAAAAACATTGAT
GAGATTTTGAATAATGAAATCCAAGTATCAACATATTCCAATGAAGAATTTTGTGATGTT
GAATTCTCATTAACAGCAGAAGAAATTGAGGCTCGTGTTGAAATGAAATCAAATTTAGAG
TTATTTAGACTCGAATTTAAACAAGAACAGTTAAAATCTGTTTTGAATCATAAACTAAGA
ACTACAGGAAAGGTATAAttgaaacgaagccgccaaatacaaaagaaaatgaattcactt
taacagattatcaatgtgtagtttgtttagataaatccgtagaagtcgtttttttaccat
gcggacatttaaaaacctgcgaaaactgttcgggaatgatgagtagttgtcctgtttgta
gagtgtacattgataaacgtgtcagacgaattcgttttaataagccatgatagctcattg
ttataattcttagattattatttattatttattattatattcaagtgataataataactg
aaacaagccaccatacaccaagatagatttttccccgacagaatattgattaccagcaca
cgttaccatcgatttgattggtcgaaataaatatgtttcttatatattatattcaagtga
ttaggttcgaaATTTCTGAGATCACATATTTAAATCGTCAACGACCAACTACAGATAATC
TGGTACACTACCGAAACATTTTTGCAAGTCCTGATAATTTTATAAAACGAAAGGTGGATT
ATAGTAAGATGAAAGTTTTCGACGAGAAATTTCGCAAAGAATTCGAACGCCTGAAAACTT
TCGAAAATGCTAAGTTCTCGGATAAAAATATGATCAGAAATATTGCAAAATCTGGATTTT
ACTATAAAAAGATGTCAAATGTGGAATATGAAGTGAGATGTATTTATTGCCAAGGAAAGG
TTTCAGATTGGAAAAAAAATGATGTACCAGTGAAGCGCCATAAGTTTAGTTATCCAACTT
GTCCTTACATAATTTATCTAAATGTTGGAAATGAGGCGTTGGAATATGAAAAATTGAAAG
AATCACACGAAGATCCTGATGTTACTAGACCAACAATGAAAATTAAAATTTATATTCCAA
AATTAAAACTAAAAACCATAGATCAATTAAATGAAACTGATTATTTGGATGTTGATACCA
GAGAACAAACATTTCAATCAAATCCTGAATTGAAATATTTAGCATTAAAAGGATTTTTTA
TTGTAAACAAAATATCGGAAAAGGAGTTGAAATGTTGTTCGTGTTTGAATGAAATCATTA
TTTTGAAAGGTTTTAAAAATCTTAATGAATACATAATGCACAAGGATAATTGTTATTATA
AAAACATTGATGAGATTTTGAATAATGAAATCCAAGTATCAACATATTCCAATGAAGAAT
TTTGTGATGTTGAATTCTCATTAACAGCAGAAGAAATTGAGGCTCGTGTTGAAATGAAAT
CAAATTTAGAGTTATTTAGACTCGAATTTAAACAAGAACAGTTAAAATCTGTTTTGAATC
ATAAACTAAGAACTACAGGCAAAGACTTTGATTCATTATTTGAATTAATTCAAGAATTAA
GGAAAGGTATAATTGAAACGAAGCCGCCAAATACAAAAGAAAATGAATTCACTTTAACAG
ATTATCAATGTGTAGTTTGTTTAGATAAATCCGTAGAAGTCGTTTTTTTACCATGCGGAC
ATTTAAAAACCTGCGAAAACTGTTCGGGAATGATGAGTAGTTGTCCTGTTTGTAGAGTGT
ACATTGATAAACGTGTCAGACGAATTCGTTTTAATAAGCCATGAtagctcattgttataa
ttcttagattattatttattatttattattatattcaagtgataataataactgaaacaa
gccaccatacaccaagatagatttttccccgacagaatattgattaccagcacacgttac
catcgatttgattggtcgaaataaatatgtttcttatatattatattcaagtgattaggt
tcgaa
>DjScaffold4743:24914-25354 strand:+
ATGCCATTTGGTATAACAAACGTACCAGCCTCATTTCAGAGATTGATGAATACAGTACTG
ATAGATGTGGAACATTTAATAGTATATATGAACGATATTTTGATTGCGTCAGAATCATTT
GAGCAACATTTAATAAACATAGAAAATATGATGTCCAGATTGCAAAAGGCAAATATCAAG
TTAAAGCCAACAAAACGCAAATGGTCAGAGAAACAAGTATTATTTTTAGGTTATATTGTC
TCATCAGAACATTTGCAACCAAATCCAGATAAAATAGAAGCAGTAAAGAATTTCCCAATA

CCAAAAACAGTACAACAACTACAAAGTTTTAACGGATTAGTTGGATATTATCGAAGATTC
ATAAAGGATTTTGCTAAAATATCTGCCCCTCTACTGGAATTAACAAAAGGTTTAAAACAA
AAGGAGAAAGAAAAAATGTGA
>DjScaffold3299:14753-14967 strand:+
CAATTACGAGAAATCAATAGAGAATTTCTACGAAGAAAATTAAATATATATTTTTTGTTG
AAAAGTTTTGCTATTATTATTAATACTATTATTATTATTAATATTATTATTAATTCATTG
AATATAAAAGCTTAGatcaaaatttaggttttaattattgatattggataacaaatcgat
atttcgaccttgtct
>DjScaffold3299:54085-54715 strand:+
ctgcattatgtttttcgtttgggaaatagaaaagaaattaactcatttttcgttggtatt
ttggggATGAAAGTTTTAAGGCATGAAGAGTTTAAGGAAGGTTGCGAAGCAACTTGTAAT
GGGCCGTATGATGGTAAATGGAGTAAAACTATGATTGGGTACGGAAATGAAGATGACCAT
TTTGTAATCGAGTTAACTTATAATTATGGAATTTCATCATATAAAATAAGCAATGATTTA
AAATACATTAAAATTTATTCTCGAAAAGCACTTTTAAATGTTAAACAATCAAACTATGAA
CATTCGGATATATCATCAAATCCAGAAACAGTTTCTTTAATCTCTCCAGATGGTTATAAA
TTCATAATTGTTGGAGAAGACAATCATGAGAATTCCGATCCACTTAGGGAAATTTGTTTA
TCTGTTAAAAATCTCGTGGAAACAACAAGTAAATTGTCGAATCTAATTAAATAGaaatat
tatttcataa
>DjScaffold3299:69521-71881 strand:+
aaaattctcctttccaggaaatggggtttgtaaattcttttttcatatgaatactatcc
agatacgtctgtgaatattctcaataatttattagtttcaattgattggaacatttgttg
atgagtatttgtgatgtaaattctaaatatttatattattatacaattaaaaaatgtgaa
tctgactgattggtacttaatttgacaagtaactgtaaatttagatttttaaaattgaag
aaaaaATGAAAAAGAATCACCAACCAATTAAAACTACTCTTGTAACGTTGGAAACTCCGG
GAAAAGCATCTGTTGATGTTGTTATACTTCTTGATCCGAATGACCATGAAATCTGTGTAG
TTGGCGATGAAGGATTCAGAGAGTTATCAAAATTTGATCCTTCTGCTCAACAATTATTAG
AAAATGCTATGAAAACTGATAAGAGCGATGCGTGGTTTGCTAAAAAAGGTGGAAAATTGA
TTTCGTCATAAgatattatttttagtcaattcaaaaaaccgaaaaatatctccaataaac
aaatcgtgtttttttctaattaaatgtgtcgtgtgaattta
>DjScaffold3299:78157-78662 strand:+
tcatttcttgacataaccttctaatATGACCAACCTTTCCACAGCCAAAACATTTTGTTG
GTTTTTTATTTGGACAATCTCGACTTATATGTCCTGTTTTATGACAAGTCCAACATTCTA
CCACTTTTTTCGTTGGACAATTTGCTTTATTTTTTCTTTCTATGATCCATTTGTCTATTC
ACGGTATAATTCACTGGTTTCTGATAATTTCGATTTGTAActcttgcgtaacttctaacg
gcattgatttgatctctgtttttctctactcgatctactaacgttaatgtttcttcaatt
tcttctatattctctcgttttcgcaaagttacttcttctttaacttttttaaaatcttt
atttgtaaataatctttttactaaatcttttacatttttaaaataaacttgaatctcttc
atttccacgcctgtagttaatcaaattttgaaaatcttt
>DjScaffold3299:137871-147879 strand:+
attaagttattttaaaatttaaatttcggaaaattttttattaatttctttttttacaaa
tttcattaacttaagcaaacttcacattATGGCTGATGATGATAATGATATTTTTGAAGA
AGCAATGGAAAATAATGAAGATTTATTTGTTAACACAGAAATCAAATTGGATGAGCCCGG
ACCTATTTTAAGTTCATCACCGCCAAAAACAAATGTAACAGAAATTTTGCCCAGTAAAGT
AACCAGTCAAAGGACAGCGAGAAATGGTAAATTTATCGAGATAACTGTCAGTTACCCAGA
GAAAATTGGCTCTGGGATGGGAGCTTATTTCATTTACAAAATTAACACAAAGACAAATCG
GCCTTCATTTCTTCATCAAGAAATTACTGTTCGCCGTAGATTTTCTGATTTTCTCGGACT
ACATGAACGCTTCAAACTGAAATATCTTTGTGCAGGTTGTGTTATTCCGCCTGCGCCTGA
AAAAAATGCTTTCGGAAATACCAAGTTGAAATTGTCGAAAGAAGATCAAACGAATAGTGA
ATTTCTCGATAGACGTCGTGCATGTCTAGAAAGATATCTACAACGTATCGCTCGTCATCC
GTTGCTCGTTATTGATGCCGATTTCAGGGAATTTCTCGAGGATGACAAAGAGCTACCAAA
AGCAGTTAATACTCAAGTATTATCAGGGGCTAATGTTAAAAAATTGTTTAAAAATGTTGG
CGACGCAGTAGGAAAGATTACAACGAAAATGGAAGATTGTGATCAGTGGTTCGAGGACAA
GCAAGTTGAGATGGACAATCTCGATAGGCATCTCAGTCAGATTCACGGTAGTTTTGAACA
TTTAATTAATCAAAGAAAAGAACTTGCCTCACAAATAAAGATTTTTGCACAAAATTGTTC
CCAGCTCTCTCATGTCGAAGAGCACAACGGTTTAGCTAGAGCATTAGCTCAATTATCTTC
AACCAAAGAAGAAATAAGCGCTATTGAAGAACGCCAAGCTGAAGTGGAATTTTACACTCT
TCTTGAACTGTTGAAAGATTACCTTGGTATGATTGGTGCTGTTAAAGACGTGTTGAATGA

AAGAATCCGTCAATATCGAATTTGGCAAGAAGCAGAATCGAATTTAAATAAAAAGCGAGA
ACAGAAAGTGCGTTGTGAATTATCAGGAAAAGCGGATAAAATTCCAGCACTCGATGCTGA
AATCAAAGAGTGTCTCAATAAAGTAGACAGAACAAAAGAGGATTTTGAGAAAATCTCTAA
AACCATTAAAACAGAAATGGAAGTTTTCGACACCACAAAAGTAATCGATTTCAAGGAAGG
AGTGATCAAGGTTCTCGAATCAATGATTACAAGTCAAGAAGAAATAATTCATCAGTGGGA
GAAATATATTCCGGACACCGCAGGAATTTCAACATGAatctttctgtctgtctgagaaac
aaacaattggaatttgttgatttttactactactactactactgactaatttatgaagta
gtatgatagctttatttttactaaatataaattgaaattgatttatttgtttatcgattt
gattaaatgtgattttgtgatgaaatcgttattgatttatcaatttataattttaataca
ttttctggtttctttttatcaata
>DjScaffold3299:159844-160727 strand:+
caaaaacggacacatgtccaaggattgttgatctaaaaagagctctggaaaaataataat
aataataatagaaaatattttagaactagacaatagtatcaatgataacattaaattcac
tttagagaagtcaatcgactttcagatttttattctggacaccttaatccaaaagaaacc
tggtgaattattacttacccagttgtatatgaaaccatttcacagtggacatatattacc
ctggagtaaatattggaataatcaagggttaaaaaaataagagctgaaagattatcttca
aatgaagaaATGAGACTGACTTGCCTCAGGTTGATAAAATATAAATTTCTAAATAATGGT
TATCCAAATAGAATTGTATATAAACATCTATACTCAAATACAGAATGTAATAAAATCAAA
CAAAAGACAAATACAGTAATTAAATTACCTTTTATTAATGAGAAATGTAGCCAAATCATG
CAAAATATAATTAAAAAGTCGAAACTAAAACACAATCCAAGAATTATATTTAAAACGCAA
GCACCTTTGGGCATCCAATTACAGAAACCAAGACAAAATTTTTGTGGTCCCAAATGCATT
TGTGATGGTAGAAATATATGCAATAAAAAAAATATCACTTACAAAATAACATGCACCTTA
TGTAAGGATACATATATAGGAGAATCATTCAGAACCATCCGTACAAGAATTAAAGAACAT
CTTAAACAGAAAGAAAGTGAAGTCTTCAAACACTTTCAAAACAAACATAATGTTTTACCA
GAAAATCATCATATTACATGGAAAACATTAGGAACAGGCTACATTTCTACACTTCACAGA
AAGAAACATGAACAAATATTAATT
>DjScaffold2656:9078-10145 strand:+
taatttcaaaATGTTAAAATTAATAATAATTTTTACTTCATGTATTCGAATTTATTTGTT
TACAGAGATTAATATAAATAATTGTATTAATGGAGCAAATATCACCACCGAATGTGGATA
TAATATAAATTTTAAAAATATTGGAAACAAAAATCTGTATTGCATATTAAACTTTACTTT
ATCAAATCCGACTGTAAGAATTAATAATAATTGGATAAAAATAAAATCTGAAGAGATTAA
CAAATGCCCATATGTTGAATTGAATACAATTACTGTTGTTAACGGTAAAGAAAAACATAA
TAGATTATCCAGCACTTGTGGAGGGCAAATAGAGAAATTAGAGAAAATATTTTCTAATTT
TTCATCACTTATTATTAAGTTAGATACGAAAGATTTCGATGATCAAGATTCCCAGGTTGA
TTTGCGTGTGGTAATTGATGGAGATTGCAAAAAAAGTTTGACAATTTGTAGTTGGAATAA
TCTCAGGTTAATTATAATAACTTCATCAATTATTGTTTGGATCATTTTTGTATCGATGTG
TTACTGCTGCTATCGAAAACGGAAATATGAAGCTTTTGGTAGACAAGAGAAGAGAAAATC
ATGTAAAATTTATATTGATTTGGTGAAATTAAATACTTAAtattaattttataaaataat
tcaaagtcaattgaaatactaatttttatttatcaaccaaattaagaaatattaataatt
atcgatttacaaaatggagaaatttaaataaatatagaataaaacaatcaaacaataatt
tattatctatgaaaaattattgtaaaattaac
>DjScaffold2656:25945-27037 strand:+
taatttaaaaATGTTAAAATTAATAATAATTTTTACTTCATGTATTCGAATTTATTTGTT
TACAGAGATTAATATAAATAATTGTATTAATGGAGCAAATATCACCACCGAATGTGGATA
TAATATAAATTTTAAAAATATTGGAAACAAAAATCTGTATTGCATATTAAACTTTACTTT
ATCAAATCCGACTGTAAGAATTAATAATAATTGGATAAAAATAAAATCTGAAGAGATTAA
CAAATGCCCATATGTTGAATTGAATACAATTACTGTTGTTAACGGTAAAGAAAAACATAA
TAGATTATCCAGCACTTGTGGAGGGCAAATAGAGAAATTAGAGAAAATATTTTCTAATTT
TTCATCACTTATTATTAAGTTAGATACGAAAGATTTCGATGATCAAGATTCCCAGGTTGA
TTTGCGTGTGGTAATTGATGGAGATTGCAAAAAAAGTTTGACAATTTGTAGTTGGAATAA
TCTCATGTTAATTATAATAACTCCATCAATTATTGTTTGGATCATTTTGGTATCGATGTG
TTACTGCTGCTATCGAAAACGGAAATATGAAGCTTTTGGTAGACAAGAGAAGAGAAAATC
ATGTAAAATTTATATTGATTTGGTGAAATTAAATACTTAAtattaattttataaaataat
tcaaagtcaattgaaatactaatttttatttatcaaccaaattaagaaatattaataatt
atcgatttacaaaatggagaaatttaaataaatatagaataaaacaatcaaacaataatt
tattatctatgaaaaattattgtaaaattaac
>DjScaffold2656:6028-6612 strand:+

ATGTCAAGTGATGGACAATTTGCTTTATCTGGATCTTGGGACGGAACCTTGAGATTGTGG
GACTTAACGTCAGGATTAACCACAAGACGATTTGTTGGTCATACTAAAGACGTTTTAAGT
GTAGCATTCAGTGCAGACAATAGACAAATTGTTTCTGGTTCAAGAGATGGAACAATTAAA
TTGTGGAATACTTTAGGTGTATGTAAATACACTATTCAAGAAGACGGTCACTCAGATTGG
GTTAGCTGTGTCAGATTTTCTCCAACTTCTGAAAATCCTAGCATTGTTAGCTGTGGTTGG
GACAAAGTCGTTAAAGTGTGGAATCTTTCAAATTGCAAATTAAGAACTAACCACTTTGGA
CACAATGGCTACCTAAACACAGTTGCTGTTTCACCTGATGGTTCCTTATGTGCAAGTGGA
GGTATGTATGTATATCCTAAAATATTTAAATTAAAAATGATAAAGACGTCAATAAATAGT
ATTGACAGTAAATTCAAATTGAAAATAAAATTTATAAAAAAAGAAACTTGGCAACGGCCA
AAAATGACAAAGAGAAATTTAAAGAAATTGGAATTAAAACTTTAA
>DjScaffold1745:64651-65016 strand:+
ATGTCTTTTGTGTGGAGCACCAGCAACTTTTCAAAGATTAATGCATTTAGTTCTCAGAGA
AGAAATTTCGATAAATGCTTAATATATCTCGATGATATTTTGATTTTCAGGAAAAGCATA
GAGGAACACAATGAAAGACTGAAAATGGTTTTAGAGAAGATACGACAGCCAGGAGTTAAG
TTATCTCCAAAGAAATGCATTTTTTTGAAAACTAAAGTAAAATATTTGGATCACATAATA
TCAAGCGAAGGAGTGAGAACTGATCCAGAAAAAATTAGTGCAGTAAAGAATTTGAGTCTC
CCTAAGACAAATGAAGAATTATCAAGTTTTCTAGGGTTTCGCAATTATTACAGAAGATTT
ATTTAG
>DjScaffold5392:37654-41237 strand:+
GATCAGCTGGGTTTAGCAGGAGAAACTTTATTTCGTTTATTTCAATCTGGCTCAGATGAG
CTTGTAGCCCAAGCAATTAAAGTAAAGTTGGTTGAATATGTTCTTAGTTTATTAGAATCA
GGGTTAGTTACTGTGAAAGAACCTGGTCAAGTGAGAGCTTATTTAGTTAAAGCCCTTAAA
GCAATGCAAAGGAGTCCCACGTATGGGACACAAATAACTACAATGCTTGAAAACAGTTCA
GTTTGGTCTCAATTCAAGGATCAAAGTCACGATCTCTTTTTGACTAACACTCCCCAATTT
GCTGGCTATTTGACAGGTCGATCTGTTGAAACCGCTGGTTACCTTACTTCATTTGCTCAA
GATACTCCAACAAGGCCGCTGTAAatctgccatgaggtttgagttataaatgattaaaca
atgaataaaaaccatttattttatgaagacattgattgaaatttgttgaattatttcttt
tcgaatatgtaaatgagtatattggtttattaccgatgtcaattctaatatatgtcaaat
taccgccgttataggaaattatttaatcgaataaataatattttttaa
>DjScaffold5392:92490-93208 strand:+
TATTATTTTTATTATTATTATTATTATTATTATTATTATTATTATTATTATTATTATTAT
TATTATTATTTTGAATGCGATCATGTTGGTATTGCAGATATTTTATTCATTTTGTTAGAT
TATTGTGTTATATTAAGGCGATTGAAAAACGAAAGCGATATGGCGAAAACTATTATTGGC
ACTCCTTATTATATGAGCCCAGAATTATTTCAGAATAAAGCATATAACTTCAAATCCGAT
ATTTGGTCTTTAGGTTGTGTAACTTATGAGATGGCCACTTTAAAGCATGCTTTCAACGCT
AAAAATTATACAGCTCTTTCATATAAAATAATTAATGGTCAAACTCCTTATATGCCATCG
AAATATAGTAAAGAATTAACTGTTCTCATACAGGATATGCTCAACAAATCACCAGAAAAA
CGTCCCAGCACAACAAGAATATTACATCATCCATTTATACAGCAACATATTCGAATGTTT
GTAGAAAACTCAAAAAGGTAAttaacataaaaataaattcactttagaagctgttaatta
tctttttaataaatttttaaatttaaatttaaaaaagattatggaagtgaatgaatagat
tgaataattacaaatataaaactcaaccagtatttttaatatttaaatgtacattgggct
cctacaatgtatgttacgtcattcgaattagtgttattcaaaagaaaaatggtttagaaa
taaggttaagtttcataagttttaaaaatataatatttcctttactaaattataaaaa
tTATTATTTTTATTATTATTATTATTATTATTATTATTATTATTATTATTATTATTATTA
TTATTATTATTTTGAATGCGATCATGTTGGTATTGCAGATATTTTATTCATTTTGTTAGA
TTATTGTGTTATATTAAGGCGATTGAAAAACGAAAGCGATATGGCGAAAACTATTATTGG
CACTCCTTATTATATGAGCCCAGAATTATTTCAGAATAAAGCATATAACTTCAAATCCGA
TATTTGGTCTTTAGGTTGTGTAACTTATGAGATGGCCACTTTAAAGCATGCTTTCAACGC
TAAAAATTATACAGCTCTTTCATATAAAATAATTAATGGTCAATCAAACAACAAAAAACA
ATATTTCTAAtttttatattttttttgaaaatcttcaattattaattaattcttttatta
gtttaaatttctctattcttctttttgttaatcattttcttaaaaattgttctcttgatg
aaattcatattatttgtggtattgaatttcttcatttttccatatctttcttaaattcaa
cagataaaacattttctaatgatattcacctttttttccattgaacaggaacaaataaatc
tctaatccaatttaaaaaggaaaaaaaaata
>DjScaffold2217:37594-41404 strand:+
tgatttataatttgatgatttttttatttctaagagaaaaatatgattctttcattgtcaa
aacaattgatggacattccactggtataatttctttcaaagactcaaatgtctttcatta

ttggaaagaagctatcaaatttaatattaatgctttacttattaattctattaataaaat
aaatcggacaATGAGAGGTGATGATCAAATTCATTATATGGGTTGGTTATTCTGTGTTGG
TCACACGAAAGTTAAAGGTTCATACTCATGTAAACGATATTATGAATCTGAATATGCGAA
TATTGACGAAATTAAAAAGGAAATTAAACTACCAATTATTCCAACTGAAAAGTCACAGTG
TATATCGAGAAAACAAAGGTCACTTGAAAACTCTCCTGGTAATAAATGGTGCTATTTATT
TAGTAACTTTTCACAAAAGAATTCTGTGGCAAATTCCCAACCGCAAAAAACAAATAATCA
TTCCCATTCCAATCTTTCAGAATTATGTGTTTCTTCACTTCATCAAACTCAGCTCATACA
AGAACCAAAATATTCCAAAATAGATAAGCCTTTGTTTCTTGTGTTGAGAGGAATCCATTT
GTATTTATTTGTGAAACCTCCGATATTTTTAGGCGACTGGTTTTCTCCATCTGTTAAAGC
TGTAATTTCCAGGTCAATGATTAACCAAATAAAAATCGTAATAATTATTTTTTGAaaaaa
aaattatttaatatatttttaatgattttaatttttcttaaagacattaatcaacgaaaa
tttatgcaaaatatcaaatttaagggaaagaattctggctttttgatctcgcaaactaaa
agtcaataattcat
>DjScaffold2217:57494-57841 strand:+
NNNNACAATAAAGACCAACACTATGAAGTAATTGAAAGAGTATTGACGGAAATAATGAAG
GCTGGTCTAAGAATTAATCCGGAAAAATGTCAGTTTCTTCAAAGTGAAATCAAGTTTCTT
GGACACATAATTAGCAAGGGTGGTGTTAGAACTGATCCGTCTAAGATAGAAGCTATAAGA
ACATTTAGCAAGTCTAACTGTGTGAAGAAGTTACGAAATTTCTTAGGAATATGTAATTAT
TACAGGAAGTTTATAGAAAACAACGCAAGAAAATCAAGGATCTTAGAGCAGTTGTGTAGC
GGGCCAAGCAACAAAAAGTTAGAATGGACAGATGAAATAAACTTTTGA
>DjScaffold5158:1058-3686 strand:+
GTTGAATTTGTGATTAGATGTGACGATGGAGAAGGAAATAAGATCAATAATACATTGGAA
GAATGTTGTCATTTGTGTGACATACGTGCAGTCGATCGATATTTGTTTCAAGACAAGATT
CGTGATGATAACAAAAATGAATCAACAAGAACAATATTGATAAAAATTATCCGTCAAATC
AAACAAATTATCTCATCAAACGACATAATCGATGAGAGATTGAATCAAAATTTATCAGAT
TTGCCATCATCATTTCTCGATTCGATAAATTTATTTTGTAGAAATATAACAGATTACATA
TGGGATATTTGTAATGATTATTGGAGATTTATAAAGAATCAGTTTTCCATTGATGTTGTC
AAGATATTCGATCAATATTTCGAATTTGCAATTCGCGGTGCATTGTGGGATTATTCCAAT
TTCAGGCAAAAAAATAATGATCAAAAAAATAAATCAATCAGCAACATTTTAATTGATTCT
TTGGATTATTTTATGAAATATTTAAATGATTCTAGCAAAGATTGGAAATATTGGCTGAAA
CTACAAATGAATCAATTTTTATTGGGACATCTCAGTGAATTTGATGTGAATGAAAGAATT
GATAAATGTGAAATCAATAAATTTTCTATCAAAACAAAAATCAATGGGAAGGATACAAAT
AATTTTATTTCATTCAAGCCAGACTCAATAAACAAAGCAAAACAATTAATTTCAAAACTG
CACAATGTGGACACGAAGCGTTTGTATTGCATCAAACCAGGTGGAGATAACAAACCTAAT
GATGAAGACGCATTTGAT
>DjScaffold738:28738-31451 strand:+
aatatttttcaacgaatattagttatttatatattaattgaaaatatattatagaattaa
aacgaATGTCTGATTCGAGTTTTCTAGAGTATCTTTTTGAAACTCATAAGTCACAATTGA
ATAAACCTTCTGATTATTTGGCGTTGTTATGTCATTCTATGCTTTTAGATAGAAAAGTGA
AGTTGAAAACTGGTAAAGCTATATTACCAGATGATTGGAATCAAACTGACCCAATTCGGC
TTGTTTATTTATGTGAAGATGGAACAGAATTAAATGTATCTGTGGTTGAGACTGCTGGTG
ATGCCTTCATATCGGTCACTGAATCGAAATCTGACCAATTGGGTCATCTAGAAGTTCCTA
TGGATGATTTTGTCAACACATCTTCTTCCAATTTGTCACTTGATCAATCATATCCGCAAT
TGAAGGAACTCAAGAAACGGTTCGAAGAGAATATTTGGTCGATAATATTTCCTGTCAGAA
AACAAACTACACCGAAAACGACATCAGAATTTGTTCCATACCAGCCAAATCCGTATAGAC
GTTCCAATCCATTGATTGATGATCGATTTTCCGGTCCCTCGATTCCATTCGATTACGGCA
GACGCGATTTGGATCCGTTCGGAATGAGTGATCCTCTGAGACATGGACCTAGTGTCGGCA
CTGGTGGTATCGGAGGTGGAGGAGGGATGCTGTTTGATCCTCGTCACCCTAATTCCGATA
TCAGACCTCTCTATCCTGGTGCTCCCCCTGGTGCCTCTCCACCTGGAGCCAGGATCGATC
CGATATATCCACTGCCGGGCAATCCCAGTCGTGGTGGCAGAGGTAGACAAGGACCTCCAG
ATCCAGACATGCCAACACCTCCAGGTTGGGAGGACATGTACTTGTGAgttgatcaattat
caataaataatctattccgttaggttacccgttatatcgagttcattattgattttcacc
ctgttttatccgataatttattttcatgccatgcagaataatctcgctattttgtcgacc
ctcattggctcctgccacgctaaatcaatattaattttgtttcgatttgatgtcagtaat
ttatttcaagtatttattg
>DjScaffold5259:7971-8366 strand:+
ATGGTAATTAAAGGAAAGGTTGCGGTTAAATCTGTCAAAAAGATTTCTAAAGATGTTAAA

CCCAAAACTGACAAAAAGAAGAGAATTCACAAACGTAAAGGAAGTTACGGAATTTACATT
TACAAAGTATTAAGACAAGTTCATCCTGATACTGGAATCTCTGGTACAGCTATGTCAATT
ATGAACAGTTTTGTCAACGATGTTTTCGAAAGAATTGCTTCTGAAGCTAGCAAATTAGCC
AGCTACAATAAGAAATCAACGATTTCAAGCAGAGATTCAACCTGCTGTCAGATTGATTTT
ACCTGGAGAATTAGCAAAAATAGGGATACTTTTTATAAGAAATTTCTTTTAACTAAATTT
AATTTAAATCTGAGTTATTTAAATGTAATTTATTAA
>DjScaffold2467:38277-38928 strand:+
aaaaagtttaaattattATGGGAATCGATATTTCACATAAAACAAGAATTGTCAAGAGAA
CAACTCCAAAATCTAATGACAGTTATACTCGTTTAAGTTTCAAATTGTTTTCTTATTTAC
ACCGTAAAACTGAAAAAAAAATTCAATGGTATTATTGCTAAAAGATTAGCTATGGCCAGAA
GACTTAGACAACCTCTTTCCATTGCTCGTTTAATGAAACAAATGAAGAAGCCAGGACGTG
AAGACAAGATTGCAGTTGTTGTTGGAACTGTTGCTGACGATCTTAGAATCAGAGAAGTAC
CAAAAATGACTTTATGTGCTTTACGTGTAACTGCTGGTGCTCGTGCTCGAATTTTAAAAG
CCGGTGGTGAAATCCTCACTTTCGATGAATTAGCTTTGAGAACACCTTTGGGAAAAGGAA
CTGTTTTATTACAAGGTCCAAGATCTCAAACTACTTCTCAAAAATACATGGGTTCAGCTC
CAGGTGTTCCACATAGCCATACTAGACCTCATGTAAGATCAAAGGGAAGAAAATTCGAAC
GCGCCAGAGGTAGAAGAAGCAGCAGAGGTTACAAAAAATAAactataataaatttttatg
gatta
>DjScaffold2467:50735-52299 strand:+
aattattgcagaaattatttcttcaaaaATGGATCAAATGTTCAAATTTATTTCACAATA
TAAATATGAAATCAATGAAAATGTTCGAGATAATCTCAAATGTGGAAAATGTAAAAGTAT
TCTAATTAATGCGTACTCTGTTCCATGCGGATGTTCGTATTGTTGTGATTGTGTAGATGA
AATGTTGAATAATGATATACAGAATTGTGCTGGGAAAAATGAGGATTGCAAATTTTCGAA
GATATCGAGTAAATATCTGGATGTTAGAATAAATAGAGAAATATCAAATTTTGTGATAAA
ATGTCCAGAAGAAATGTGTTTTTATGAGTGTGCGATAATTAATATGGAAGATCATGTGCG
TGTTTGTGAGAATAAGAGTGTAAAATGTCCATTTGCTAATGACGGTTGTGATGGAAAGAA
GATTTCACGAGAAATGCTGAATGATCATTGTCAATCAGCTAATTTCAATCATTGCAAAAT
ATTGATAGAAATTATTGATAATATGAAAAATGAGATCAATATTTTGAAAGTTAATGATAA
AAAGAGGCAGTTGAAAATTGAAAATTTAGAGAAAAATTTGAAATCAAAAGACGAAAAAAT
TGAGAAAATCAATGAAGAAATCAATTTGATAAAAGCATTAGAATATTATTTTAATAATGA
AAATTTTAATTGTTTTCAAATTTTCAGTTGGAGAAAGAAGCTGTCCAACAAGAATTTGAT
TCATTCAAAGAAAATTTCGAGATGAaaattgaagtaatcgatggaaaaatcaaaagaaaa
aatgcaaaaatgaagaaagaaaatgcaaatttgaagattgagaacgatgagaacaagcga
gaatgcaacaggataaatgagaatttagaacggaaaacaaatgaaatatttgcaggtatg
cgaaaagaaaaggaagatcgaatcaaatcgataaatgaagagatcaagatagagacaaag
aagaaatcagaaatgatggaaacaaggataaataaattgattggaattatcgaattgaat
tatccactgattaaatggagaatcgatagtttggatgcaaggaggaaggaaggtggaatc
agttggagtgaacattttaccggattgataacaccataccaattacgattaacattttat
gctaatggaacagacgctatcaaaggaaaaggttgtagtaattatttacgtatttattct
agtgagaatgatcaatttttgagctggccattctcagctgatgtcacaataaaaatgacg
agcaaaaaagatcctaatataaacaaaagcgtcacaaaatattgtaaaatagaaaaacca
tcgaataattcattcaatcgcagtgaatcattcaattatttatattgtgatttgacaaaa
gatggagaaaagcttgacgactgttttattattgagtgctttgtcgatttcaaatagtta
ttaaatattatcttgccgaccaaacaattcaaatattgcaatattatttctttaattatt
ttgctttttacaatcgcaatttggc
>DjScaffold2467:57748-59128 strand:+
aagttgaggatacatattatcggtataagATGCCTAAGCTTATGACGAAAATTGAGGGAA
AAGGAAATGGTATCAAAACGGTAATCACTAATATATCTGATATTGCTAAAGCAATTTATC
GTAAACCGTCATATCCTATTAAATATTTCGGTATGGAACTTGGTACTCAAACTATTTTGG
GTGAAAGCACTGATCGATATATTGTTAATGGTAATCACGATGCTTCGAAGCTTCAAGAAG
TTTTATACACATTTATTAAGAAGTTTGTTTTATGTTCTAAATGTGGAAATCCTGAGACTT
TTCTTACAATTAAGAAGAAAGTTGGAACCGTTGTAGCTAAGTGTAAAGCTTGTGGAAATT
CTGATATCCTTGATATCAAACATAAAATTACACAATATATTTTTAAAAATCCACCGGAAA
CTGAAGCTGAAGGTGCATCTGTTAATTTGAAAGCTACTGAAGAAAATGAAGAAAATGATG
AATTTGACGCTATTGAAGAATCCACTTTTAATGAGCAGAAAAACACAGTAACAGATGAAA
TGATGGAAGAAGCTGAAAATAATCTTAAAGAATTGAGTGTTGGAGATAATTGTAAAGTAG
CTCCAAGTCATGCAAGAAGTCAACAAGAAAGTATTGATGATTTTTTTCAAATGGTTACAA

AGAAATTCAAAGAAGGCAAGTTATTATCTGAGCGTTTTGAAATTCGGAAAGTAGTTAAAC
AATTAAAATTGAAGGAAGGCTGTATTATGGCTCTAATGGAAATTATTTTTGTTAGTCCAA
GAACGATTCTTACTGATATTGAAAAGTACAAAAAACTTTTTCATTTATTCACCAGGGATG
AGGAATCTAGTAAATATAAATTTCAGAATTATGTGATTGCTGGAATGATGAAATTAGTAG
AAAAATTTAATTTAGCATCAAAATCTTGTTACTTATTGAAGGCATTATATGATCATGATA
TTATTGAAGAGGATATTATCATTAATTGGCATGAAAAAGGACCATCTAAAAAATATGTAG
CTAAAGATTTAAGTAAAGAAATATTAAATGCATGTGATCCTATGATTCATTGGCTAAGGA
ATGCTGATGCCGAATCTGGTGATGAGAATGAAACTATGCAGGAAAGGGAAATTCCTGTCA
ATACAAATGTAACTGGTCATAAGTTTAGTCTCATTCAAGGAGATGATGATGAAATAGATA
TTGATGACTTA
>DjScaffold3394:34992-41235 strand:+
ccagattcttatactacgtgatttttatataaaATGAATTTGAGTAAGAAATTATCGGTT
GATAAACTTGATTTATCAAATAAGCGAGTGTTAATTAGAGTTGATTTCAATGTTCCCATG
AAAGAAGGAAAAATTACAAACAATCAAAGATTGGTTGCAGCTTTACCCACTGTAAAATAT
GCTTTGGAAAAAAACGCTAAATCTGTTGTGTTAATGAGTCACCTTGGTAGACCAGACGGA
ACTCCGAATAAGAAATATTCTCTAGAACCGGTGGCAGCTGAAGTATCCAAATTATTAGGA
AAAGAAGTTAAATTTTTGCATGATTGCGTTGGAGACGAAGTTGTAGCGGCTTGCAAAAAT
CCAGCTCACGGAAGTATATTTTTATTAGAAAATCTTCGTTTCCACGTCGAAGAAGAAGGT
AAAGGAGTTGATGCAAGTGGAAATAAAATTAAAGCTTCTGCTGATGCTGTTAAGAAATTT
AGCGAATGTCTTACATCTTTAGGTGATGTTTATATCAATGATGCCTTTGGAACCGCTCAT
CGAGCTCATGCTTCAATGGTTGGATGTTCATTGCCACAAAAGGCATCAGGATTTTTATTA
AAAACTGAGTTAACCTACTTTGCCAAAGCTCTTGAAAATCCAGCAAGGCCATTTTTAGCA
ATAATTGGCGGCGCGAAAGTTAGTGATAAAATCCTTCTGATAAAAAAACATGTTGAACATT
GTTGACGAACTCATAATCACTGGTGGTATGGCGTTTACCTTTTTAAAAGTTCTCAACCAT
ATGGAAATCGGAAACAGTTTATTTGATGAACCCGGATCGAAAATTGTCAATGAAATTATT
GAGAAAGCAAAAGAAAAGAATGTTAAAATTCATCTACCGACCGATTTTGTGACTGGAGAT
AAGTTCGATGAACATGCTAAAGTGGGACACGCAACTCTCGCTACAGGAGTACCAGTTGGA
TCAATGGGATTAGATTGCGGAACAGAAACAATGAAAGAGTTTTGCGCTGCTATCCATAGA
GCGAAAACTATCGTATGGAATGGACCGATGGGAGTGTTTGAATTCGAAAATTTCTGTCAT
GGAACCAAAGCAGCGATGGATGCAGTTGTGGCAGCAACTAAGTCTGGAGCTACAACTATT
ATCGGAGGCGGAGACACAGCCACTTGTTGTGCTAAATGGGGTACCGAAGACAAAGTGAGC
CATGTTAGCACTGGAGGAGGGGCCAGTTTGGAATTATTGGAAGGGAAAATTCTTCCTGGT
GTAGCTGCTCTTTCGGATTTGTAAtcgattatcatctctagcaactagaataggattctt
tccctatattttatgttttattgaataaattgtgaaggctgttaatttagttctgttgtt
atgtcgtcattagatatttttgtgtgctgtatttaaataaaatatttgaactttca
>DjScaffold1766:20478-51512 strand:+
tgatatagATGTCAAAAAAATCAAATTTGTCAACAGATGAAATTATATCTCATACAAAAT
CATCATTGATGGTGTTAGAATCACTGAAAAATGATCATTTTCAAAGTTTAAATAATTTTC
GTGAACAAATTGAAAACTTTTCTGACAATTCAGATTATGACATTAAAATTCCTTGGTTTG
AGCAATCTATTAATAAAATAGAACTTGGAATTGAAGAGGCTGAGACTATAATTCAATTGT
ACGAATTTCTGCAAATAAGTGAATCTGAGCGTAATAAATTAAAAGTCCAGATTGGTAGAT
TAAATCAAGAAAACAATTGGTTAAGAGATGAACTTACTACAACTCAATATCGTTTAGAAG
CTAGTGAGAAAAGTGTTTGTCAATTGGAAGAAGAAAATAAACAAATGGCTTTCATGAATG
AAATGAAAAAATACGATACTCCTGACAATGAAAGAGATCCTACAACAAATGGCACAGACA
AAGCCGATAATTTATCATCAGATCTCGGATTTACAGATGAAGAAAAAGATCAAGAACCAA
ATGATGTGATGATGCAAAGTCAATCGTCATCAGTATCTCATGGAAGTGGTTATGAAATTC
CTGCTCGTTTGAGGACTCTTCATAATCTTGTTATACAATATGCCAGTCAGGGCCGTTATG
AAGTTGCAGTACCACTTTGTAAACAAGCTTTAGAAGATTTAGAACGTAATAGTGGTCGGG
ATCATCCAGATGTCGCCACTATGCTCAATATTTTAGCATTGGTTTATCGCGATCAAGGCA
AATATAGAGAAGCTGCCAATCTTCTCAATGATGCTTTGGCGATCAGAGAAAAAACACTTG
GACCAAAACATTCAGCGGTTGCTGCTACTCTTAATAATTTGGCTGTGTTATATGGTAAAA
GAGGTCGATATAAAGAGGCTGAACCACTTTGTAAAAGGGCATTACTTATTCGAGAAGAGG
CACTTGGTTCTGATCATCCAGATGTAGCTAAACAATTGAATAATCTGGCTTTACTCTGTC
AAAATCAAGGGAAATATGAAGAAGTTGAAATGTATTATCTGCGTGCATTAGAAATATATT
TACAAAAATCCGGTCCTGATGATCCAAATGTAGCCAAAACGAAAACAAATTTAGCCACAG
CATATTTAAAACAAAATAAATTCAATAAAGCAGAATTATTGTACAAAGAAATATTGACCA
GATTTCATGAACAAGAATTCGGTAAAGATAATCCTCAACAAAAACCGATATGGGTTTTAG

CAGAAGAGCGTCAAGAATGTTTTTCCAAAAATAAAGAAATTCCATATTTTTCAAGTGATA
ATTGTTGGCATAAATCATCAAGAATTGACAATTCAACAGTTTATACAACTCTCAAGAATC
TCAGTGCACTTTATCGTCGTCAAAATAAAACTGAAGCTGCAGAGACTGTAGAAAATTGGA
TTAGTAAAAGCAAAACGGTGACGGATTTTTCTGATTCTAATGGAAGTTCCAATGATTTAC
AGTCATTGAAATATTTACCCTTTCATAAAGAGAAGAATACTTTAAATGGTGAACCTTCTA
ACATTCAACGACATCATGATACTGGTAATGATTTATTAATGAAAAAATCACCTAGTGATC
CTATTGGTAGTAAAGACATTCGATATGAAAAGAGCCAGTTCAATGTCAACTCTAGTTGGA
AACAACATTGAatctgaagatttaaagaacaatcagaagccaatacagtcgtcatttttc
aagaaaggcaacaatattagtatcgaaaattttaaaaaattgttatcgcgaattgtttga
tatctatcataaagttgtattgcaaatttttgttaactttattatctagtcactgataa
ttgatatttatattttggtctattcggtgattttcgaaatctctgttaaaagaaaatcta
aaatagttaattataatatatttacatttacagttgatatcatcatttttattatcatga
ttgatttttagtgctctttttttatatcagtacttgacgccttcttgctgcacattccaa
tttgattattttaccgaattatatttgttgtgcattgatgaagttctttaatggtgaata
ataatgataataaaaaaataataataattttgttacgttaataaattttagtggtcacgat
ggaaaatttttagtgttcatttttatttttctaaggtttgtaatcattttatacgtcagta
ttggcaatagtttgtatatatctttcagaattcaatttgttgtggaatcagtgttagaaa
tagatatttatgtttacattttgcactactattatgcaaatcagggaataataaacgcat
ttagatttgctacacataaacagtttgatgaatttaaatttattctccccaaataactgc
acattgttatcataatttgtggaatttgtatctattttgctatgtgttcacttgtatttt
ttgggattatgtctgtaatattacagctgctacctttctattacgtaatagattagaaac
acttttgtgcgtttgcttatttcaaataattattgattactatcgtgcatgtaattcaaa
ttttagtgATGATGCAAAGTCAATCGTCATCAGTATCTCATGGAAGTGGTTATGAAATTC
CTGCTCGTTTGAGGACTCTTCATAATCTTGTTATACAATATGCCAGTCAGGGCCGTTATG
AAGTTGCAGTACCACTTTGTAAACAAGCTTTAGAAGATTTAGAACGTAATAGTGGTCGGG
ATCATCCAGATGTCGCCACTATGCTCAATATTTTAGCATTGGTTTATCGCGATCAAGGCA
AATATAGAGAAGCTGCCAATCTTCTCAATGATGCTTTGGCGATCAGAGAAAAAACACTTG
GACCAAAACATTCAGCGGTTGCTGCTACTCTTAATAATTTGGCTGTGTTATATGGTAAAA
GAGGTCGATATAAAGAGGCTGAACCACTTTGTAAAAGGGCATTACTTATTCGAGAAGAGG
CACTTGGTTCTGATCATCCAGATGTAGCTAAACAATTGAATAATCTGGCTTTACTCTGTC
AAAATCAAGGGAAATATGAAGAAGTTGAAATGTATTATCTGCGTGCATTAGAAATATATT
TACAAAAATCCGGTCCTGATGATCCAAATGTAGCCAAAACGAAAACAAATTTAGCCACAG
CATATTTAAAACAAAATAAATTCAATAAAGCAGAATTATTGTACAAAGAAATATTGACCA
GATTTCATGAACAAGAATTCGGTAAAGATAATCCTCAACAAAAACCGATATGGGTTTTAG
CAGAAGAGCGTCAAGAATGTTTTTCCAAAAATAAAGAAATTCCATATTTTTCAAGTGATA
ATTGTTGGCATAAATCATCAAGAATTGACAATTCAACAGTTTATACAACTCTCAAGAATC
TCAGTGCACTTTATCGTCGTCAAAATAAAACTGAAGCTGCAGAGACTGTAGAAAATTGGA
TTAGTAAAAGCAAAACGGTGACGGATTTTTCTGATTCTAATGGAAGTTCCAATGATTTAC
AGTCATTGAAATATTTACCCTTTCATAAAGAGAAGAATACTTTAAATGGTGAACCTTCTA
ACATTCAACGACATCATGATACTGGTAATGATTTATTAATGAAAAAATCACCTAGTGATC
CTATTGGTAGTAAAGACATTCGATATGAAAAGAGCCAGTTCAATGTCAACTCTAGTTGGA
AACAACATTGAatctgaagatttaaagaacaatcagaagccaatacagtcgtcatttttc
aagaaaggcaacaatattagtatcgaaaattttaaaaaattgttatcgcgaattgtttga
tatctatcataaagttgtattgcaaatttttgttaactttattatctagtcactgataa
ttgatatttatattttggtctattcggtgattttcgaaatctctgttaaaagaaaatcta
aaatagttaattataatatatttacatttacagttgatatcatcatttttattatcatga
ttgatttttagtgctctttttttatatcagtacttgacgccttcttgctgcacattccaa
tttgattattttaccgaattatatttgttgtgcattgatgaagttctttaatggtgaata
ataatgataataaaaaaataataataattttgttacgttaataaattttagtggtcacgat
ggaaaatttttagtgttcatttttatttttctaaggtttgtaatcattttatacgtcagta
ttggcaatagtttgtatatatctttcagaattcaatttgttgtggaatcagtgttagaaa
tagatatttatgtttacattttgcac
>DjScaffold1:16403-20216 strand:+
tttggaaatcgatgaagaaaacgatgttctcactgatttaacgaaagctgcctgtggtga
aaacaatattgataattatctaacattagataatttgagttgtcgacgttgtaacgattt
gaattctgctattatgtttttcaacgagataaaacaatatcagaattccaatgaaaatca
aattaggaaacacgttctttattcgtttcaaatttaccaacaaacaagtttgaaatctga

atcttgttcggttttctccaaatgttcgaaaatctatcttctcaatcttgatttaacaaa
atctcgcaatcaacataatacaatgtcgttgccgtttttatcaaaattcgttacttctct
tattaataaagaatataaacgagttccttgtgagaatcagttccagaaaatattcgcgga
gatctttcataaaaatataaatttctactcttttataactcaaatttcaatgaaagaaaa
aaaatacaatgacacctatcaattgttacagttgtctggtagaattcaacgattgaaaat
caaattgaaaaagagttgggatttttccgaccgtgaatcgaatgaaattgaaaattatca
tcattcaaaattaaggaaatccagttttcataaatcgggcaaattttccgattcagaaat
cacatcaaacagtgagatttcttgtgctgaaactgtgatttgtttgagagaagaaaaacg
tagaaatagtgatgatgaatcaatgatttctaagagattgcggtcagtattcatttcgtc
cgattctgattctggttgcaaagatatcagaacttcgcattcaagctattctcgagacgg
gacaccagttggtaccatgaaaaaaaaatcccacccgagaacaaatgcacttgtttggcc
aaaatcagccataagaatggcaaaaacaacaattttaaatggacaagagttatgggtcga
cggccctatagtttttcaaaaaactgaaaatgctagttacaacaacataaatcaacaatc
agctagtacaatatcaactccggaaaccgtaaaacaagtactcggtacactaaataaatc
aaataaaantatcaaatattcaggaaacccaatatgagtatcaaaaactcaacacaaatg
attctaaacaagatttccatctactctctgatatcagtgaaataactgacgaagtcgata
ttATGAAGCATGCCAATGAATTAATGGGTTTGTCTGATGTCAGCTATAACAGCCTCAATT
CAAGAATTTCTCAAATTAAACTCAATTTACAAAACGAACAAGCCAATCCAATGATTTTAT
CACCTAAAAACGATGTCAAAAAAGTGTCCAGATGGATTGATGATGTCAATCAACTGACAT
CTTCTCAGGTTCCATTATCGAATTCATATCACGAACAAATTCACCCAAAACAATATCTTC
AACCGATATCGAATTCTTCATCAACTAATAGTCACAGTTATCAGTACTACGATCCTGACA
GTCAAGATGACTATAGCAGTTATCCCAGATTATATTCTCCTGTACAGACTAATTTACCCA
TCGTTAAAAATGACACCAAACAGATCAGCTATGTCAGTGAAGAAGAACAAAAAAATGTAC
TAATCAGCGATAAGAAATTATTTAATCGTTATAAGAAAAAAGATTTGAATAACAGCGATT
CCAAGGATTGTTCGAAATTGCGCTCGTTAATGTCTAAAAGTCCTCTGGGAATATTAAAAA
AAACCTTATTTCAAAGCAAATCCGGTAAAAAGAAGATCGATATTCCGGAATATTTAGCAG
AAACTAATTGCAACATATCAAACAGTCAGGAAATACGATATTTAATGCCAGCAATGCAAG
TCGAAGATAGTTCTGATTCAAGTAGTGACAGTGAATGCAATGAATGGTTCAGAAACATTA
AGACATTTAAAAATAAACTGGATGTTGCAACTGGTGGTAAATATTCTGGTTTGCGCCCAC
CTAGTGCACCTTTATTAGTGGGATCTGGATCTGTGGTGAATTCACAACACACAACAAGAC
AAAGATCAAGTAGCAGTGGATACGGAAGTGTAATTACGAAAGATAGTGAAATATCTTCCC
ATATTGATTCTAATAGTGATCAATCAATACACAGTTTTTCTAAGCCTTCAATTAAACAGT
CAACTTCATTCTCACATACCAGAACTATAGGATCAAATCAAAATGATTTAGATCCGGATA
TTATTGCGCAAAGATGTGAAAGTCTTGTAAAACAAGAAAAAGCAATACTTTTAATGCAGC
ATCAAGAACTAATGATTCAGGATTTTTATAACACTACTCGACCTTTGTTGACGAATGACG
AATCAAAATACACGTTGAGAATGTCCGCAGTCAACTATGATCGTCTTATTTATGACAATC
AATATCTTGATGAATTGGAAGAAGATACTAACATCATTAGAGATTCGATAGCGTTTTATA
AAAGTTTGGTTCATTTGGAATCGtaaaaatatttttatcataaaatattcaggaaaccca
atatgagtatcaaaaactcaacacaaatgattctaaacaagatttccatctactctctga
tatcagtgaaataactgacgaagtcgatattATGAAGCATGCCAATGAATTAATGGGTTT
GTCTGATGTCAGCTATAACAGCCTCAATTCAAGAATTTCTCAAATTAAACTCAATTTACA
AAACGAACAAGCCAATCCAATGATTTTATCACCTAAAAACGATGTCAAAAAAGTGTCCAG
ATGGATTGATGATGTCAATCAACTGACATCTTCTCAGGTTCCATTATCGAATTCATATCA
CGAACAAATTCACCCAAAACAATATCTTCAACCGATATCGAATTCTTCATCAACTAATAG
TCACAGTTATCAGTACTACGATCCTGACAGTCAAGATGACTATAGCAGTTATCCCAGATT
ATATTCTCCTGTACAGACTAATTTACCCATCGTTAAAAATGACACCAAACAGATCAGCTA
TGTCAGTGAAGAAGAACAAAAAAATGTACTAATCAGCGATAAGAAATTATTTAATCGTTA
TAAGAAAAAAGATTTGAATAACAGCGATTCCAAGGATTGTTCGAAATTGCGCTCGTTAAT
GTCTAAAAGTCCTCTGGGAATATTAAAAAAAACCTTATTTCAAAGCAAATCCGGTAAAAA
GAAGATCGATATTCCGGAATATTTAGCAGAAACTAATTGCAACATATCAAACAGTCAGGA
AATACGATATTTAATGCCAGCAATGCAAGTCGAAGATAGTTCTGATTCAAGTAGTGACAG
TGAATGCAATGAATGGTTCAGAAACATTAAGACATTTAAAAATAAACTGGATGTTGCAAC
TGGTGGTAAATATTCTGGTTTGCGCCCACCTAGTGCACCTTTATTAGTGGGATCTGGATC
TGTGGTGAATTCACAACACACAACAAGACAAAGATCAAGTAGCAGTGGATACGGAAGTGT
AATTACGAAAGATAGTGAAATATCTTCCCATATTGATTCTAATAGTGATCAATCAATACA
CAGTTTTTCTAAGCCTTCAATTAAACAGTCAACTTCATTCTCACATACCAGAACTATAGG
ATCAAATCAAAATGATTTAGATCCGGATATTATTGCGCAAAGATGTGAAAGTCTTGTAAA

ACAAGAAAAAGCAATACTTTTAATGCAGCATCAAGAACTAATGATTCAGGATTTTTATAA
CACTACTCGACCTTTGTTGACGAATGACGAATCAAAATACACGTTGAGAATGTCCGCAGT
CAACTATGATCGTCTTATTTATGACAATCAATATCTTGATGAATTGGAAGAAGATACTAA
CATCATTAGAGATTCGATAGCGTTTTATAAAAGTTTGGTTCATTTGGAATCG
>DjScaffold1102:40032-44125 strand:+
atttaaatctagacgaaacggattgctaATGAATCATTTATTTGTTATTCTCGGAGCAAT
TATCAATGGGTTGACAGTTAAAACTAAAATCGCTGCATTATTATTTGTTGGAAGATTTTT
TATTGGAATAAATTCCGGTGTTACAGTTGGATTAGCATCAATGTATCTTACTGAGATTGC
ACCAAGAGCTTATCGTGGAGCAATAGGAGCTTGTCATCAACTTGCTGTTACACTGGGAAT
TGTTTTCGGTTACTTATTAACAATGACACATACATTGAATACAGATTCACTTTGGCCAGT
AGCATGCGCTTGCACAGCTATTCCAGCAATCATATCATTATTTCTTTTACCTCTTTGTCC
AGAAAGTCCGAGATTTATATTTTTGAAACAATTCAAAGAAGATCTTGCTCGAAAAGAGTT
TATCAGAATTAATCAAATGGAAGATGTGGATGTATTTTTAGGAGAATTGAGAGAAGAAGT
TGAAACCGCAAAAAATCAGCCAAAATTCAAATTCTTTCAATTATTTACACAAAAAGATTT
AAGAATGCCATTGATAATTGCTTGTTTAATTCAAGTGATGCAACAACTATCCGGTATTAA
TGCTGTGATTTCATATTCAAGTTCCATGTTGAAAACTGCTGGAATACCAGAGGAATACAG
TCAATATTGTGTGATAACAATTGGTGTTTTAAATGTGATTTGTACTATGTGTTCTCTTCC
ATTACTCGAAAAATTCGGTAGAAGAACATTGTTATTGTGGCCATCAATTTTATTAGGTGT
TTCTCTCTTACTTCTTACTATTACTGTCAATATTGTCAATGCACCTGGTTTATTATCAGT
TAGTTCACAGAAAATCGTTGGTGGAATTTCAGTTTTCTTCATTATGACATATATTATTGG
ATTTGCCTTTGGTTTGGGTCCTGTTCCAGCATTAATTGTAGCAGAAATATTCCGCCAAGA
ACCTCGAGGAGCTGCTTATTCCGTTAGTCAAGCTATACAATGGGCAGCAAATCTTATCGT
TTTATTCAGCTACCCAAATATGAATAAAGCTATTGGTGGTTATTCTTTCCTACCATTCTT
AGTTGTAGTTATCGTGTGTTGGGTGTTTTTCTTCCTATTTATGCCAGAAACGAAAAATCG
TACATTTGATGATGTAGCACGTGATTTGGCATTCGGTACAACAGTAATCAGTAAAAAGAA
AACTCAAGAACTTAATCGTCCGATTTACAAAAAGGTTGCAACAGACGAAAACAAATCCGA
TGATATATTGTAAtattaaacaattttattaattcatccgatttcgtagttagaaatcca
ttatgagaaagataattttataaatttttaattattagtaaatgttttcaaatatcgtcg
tttatcttatcttttttttgatttaacctttcaaatgaggatatttaacatttacacatt
gtagtgattcaattgttattaataaatgtgatcatttttgctt
>DjScaffold4923:12542-12753 strand:+
aacaatactaattcgaagaataactgaaagATGCAACAAACTAATGAAGCCGTTCTACCC
AAACCAAAACCAAAACCTGTGGATTCGCTTAGTAAAGTTTATTCAAAAGAAATTCGACCA
CTTATAGATGTTATTGATAATTTACGAAGATTTGGCTTA
>DjScaffold4923:55471-58372 strand:+
taaaaatattttgtcctagctttgataatttatactttcaataacaatttaatttaaaat
ttttcatttataaattgttgccttcaatgaattcataaaaaataaacttttaattctgta
attattccagcaaatagcacatcttcaattggaacactttaataatATGTCGTCAGAAAG
AATTTCTGTATTATTTGAAGAAGAAAAACTCGTTTTCACTCTAGACGCCGCATTTGAAAG
AGAAATGGAATTGCTTAAAAGAAAAGAAGAAGAGGAAAGAGAAGAGCAGGAAAGAATATA
CAAGAGATCAACAATTAGCGGCATAATAATGAAAGAAGAAACAATGGAAGAAAATGCAAA
GCGCATGGTTGACATAATTGAATGCTATTTAAAGATTGTACATTCTCGCCTGGCAGACGT
GATTCCAATGTCGATTCGACAAAGTCTTCATGTTGATTTGAGTAATAATTTGAAAGACAA
ATTTTTCAAGTATGCGACAAGTTGTGATGTTGAAGAAAAAGTCAATGAAAACAAAACTAT
TAACGAAAACAGAAAGAAATTACAGGAAAAAATTGAAAGATTACAAAAAGCAAGAATTGA
ACTCGCAAAACTTTGAttttactttatcttgtcacatcataaatgatttgtgaaacattt
ataagaaatcaatcgtatcatcagaaataattatttttcaaatagttttgtttaaatag
ttatatattttagactcggatttttaatgtatttttacttggaatgttgctgtatttgta
gttcttattaaacaatacacaaaatggatttattaccattcttgacttgaaattatttca
aatattttgtttga
>DjScaffold3671:12269-18327 strand:+
agcatctggaaacgaaataaatattcttgattattccaataaaaatgtcaaaaaattaac
gttaaataaattaaaaataattcatggaattgaattaagcgaagatattgttatagtcta
tggcgggttttattttgtatttgctttgttattgataataagtaattattttgatttat
taataatcgataaattcattcaatatagtattttattttctactgtATGATTTATTTCAA
CCAGGGTGATGAAATAAGATTTTGTGTGTCGTTGGCACATGGATCGACGCATTTCTATAG
ATTTCGGAAAGATGAAAAGAAATTACTGAGTCTCGGAGAAGCGATGATTGCTGAGGAGAG

ATCGTTGCTATATTGTTGTCATCTTTTCCGAAATAAAGCCGTCAGTAATGTAACTAATGT
GTTCCTGGCTGGCGGCGGGGTTCAGAATGAAATATTCATATGGGACATAGCATCACCTGA
ACAGTCAATTGACAAACGGCCTCGAACATTGGCCAAATTGAATGGTCACAAAGGGGCTTT
ATTTGGTATTCACATGAACTGGGATGATAGTTTGTTGGTCACAATGGGTGACGATCGAAA
TGTTATTTTATGGGAATCATCTAGCAAATTGAGTTGTAATGAGAATACATTAGACAATAT
CAAATGGGAAATATCTCGAATTTTAATCGGTCATGATGCAAGAGTTTGGGGAGGAATGAG
CCTTGCTAATGATGATTCGGTGATTGCTTGGGGAGAGGATTGTTCTGTACGATTTTGGGA
TAGAAATGGAGTAAATCGTGGTTGTATTACACCTCATACCGGGCGCAGTGTGTGGGCAGG
AGATGTGAAGATCAACAATGACAAATATTTGATTGTTACTGGAGGCAACGATGGAGCTGT
AGTTGTTTCAGAATTTCAGAGAACTAAAGAAAAAGGAGAATTTAAAAATTTTGAATTGGA
CGACAAGGTTGATTTTCCTCGGAGATTGATTGTTCTGCAATCCGGAGATGTTCTGATTGT
TACAGATCTCGGTCGGCTCTACATGGCTGTTGGTGGTGGGAATGGTGGTGGACAGAGCTA
TATTGCACGAATCGAATGCAGCGGTATTGACGAGAATCTGATCAAACATTATTGTTTCAT
TTATGAGTATGATCTGTCTGGTATTATCTTGCTTGGCAATATGTTCGGGTGTCTCATGGT
GGCCAAATTGGAAGTGGTTGATGATTTATTCCACATTTCCTGTCTACAGACATTTCAATT
GGTCCACAAAATCATGGCCATCGATAGGGCTGGAACAGATGTTTTGATATCGTTGAATAA
TGGACAAATGATTTCTCTGAGAACTCAAGAATTGGGCAATTACAGTGTTCACAATCATGT
TTGTTTGACGAGTGGTTGGGCAACATGTTGGGCCCAATGGCAGCGGATTGTAAATGGAGG
AGAGGTTGTGACACATTTTGTTTGTGGTGATAGAGACGGACTATTGCATTCATTTGATAT
TAAATATAACACAGAAATGGATTTATCTGTTGTTTCTACAAATATGAGGAAACTGTGTAA
CAAATCTGGAATTACTTGTCTCATCTGGCACGAGAAATTTCAACGGATTTATGTAGGAAA
CAAATCTGGTGATGTGTGGTCATTGGATGGTTACACGTTAGATAAAGATATTGTGATGCT
GAAGAATCCTGATATATTAGGATGGACTTGTCGATTAGAATTACTGGATAATGAGGGAGA
TGGAGATGGAGATGGAGATGGAGATGGGTTGATAATTTCCGGATTTAAATCGGTTCAATT
T
>DjScaffold1238:19084-22495 strand:+
GATGATGAAAATATCAAACCGAAAATAAAAGCAAAAGAAATTTTCATAAATCATTTAGAT
ACTTATCACAGTTATAACATAGGAAAATTTCTGTCCACATGTGTTACTGGCGCGAGTTTA
CGAGAGGAAGAAGAAGAGGAAGCCGAAACTGAAACCAAAACCATTTTAAAAGAAGGTCTT
TTTGAGATTATTGGCACTATTTTGAATGAAGAATCTAGCCATAATAATTTTGACTTTGCC
AAACAAATAATCAAATTTGAAACGAGAGAAGAATTATTTAAATCTCTTCATGAAACCGAT
GTTATTATTTACAATGTATTGGATGACGAATACCAATTGGAAGAAGCTCATTGGTTGCTG
AAAAAGTTCTATGATGAATCGGCCAAAATTACGAATCAAAAGCAATTTATTCTGTTATCA
ACTGTGATGAGTTGGGCAAAAACCAAACAATTAGATCCAGAAGAAGAAATGCAAATTCCT
TTTACTGAGGAAGATTTCAGACGTCGCATCGCTCATCCGAACTTTAAAAATCATTTAACT
TTAGAGAAAACAGTTATTAAGCGAGGAAATGATAAAAAAGACAAGCTCATTACTTATGTT
GTGGCAACTGGATTGACATATGGTTGCGGAGAAAGTGTGTTTCACCCTTATTTCAAGGAA
GCTTGGCAAAATTCTCTGCAATTGGAAATAATTGGAGCCGGTCAAAACATTATTCCAACT
ATTCATGTGCATGATTTAGCTGGAGTTCTCCAGAATCTTATTGATGTTCATCCGAAAACC
AGATATGTTCTTGCTACTGATGATTCAAAGAATAATCTCGAGGAAATTATTCGAGCGATT
TCGAAAAGTTTAAGCACTGGTAAAATCAAATATGTCACCAAAGAAGAAGTGCTAACTAAT
GAAGATATTAAAGTAGATTTTTTTTAAtgaaaattgtacttaaaactt
>DjScaffold3481:25458-26261 strand:+
ccagaATGCAGCCTATGAATTACCAAAATCCCGATAATAAGCCTCCAATGACAATGGATA
ACACTAATATGCCAATACCCAATGTTGGTTATAAACCTGATGTAACACAAGGGGAACAGT
CACATCCATATTCCCAGCCCTATCCTCCTAGTGGAATACCGGTTTCGAACAACAATAATA
ATAATAATAATAATAACAATATGTATTGGAATAGACCGCCACAGGGTCAACCATATAATC
AGCAAGGACCACCTTATAACCAGCAAGGCCCACCTTATAACCAGCAAGGTCCACCTTATA
ATCAACAAGGTCCACCGTACAATCAACAAGGGCCCCCTTATAATCAACAAGGTCAACAGT
ACAATCAACAAGGGCCACCTTATAATCAACAAGGTCCACAGTTCAATCAGCAAGGACCAC
CATACAACCAACAAGGTGCGCAGTACGGACAATCAGGGATGCCAATGCAAATGCCTATGG
GTCAACAATGCAATACAGGAATGCCACAACAAGGGATGAATCAACAAATTTGGATGCAAA
GACCTCCACAAGTAGTTGGTTGTCCACCGGGCTTAGAATATTTAACGATGGTTGATCAAA
TTCTAGTACATCAGCAAATTGAATTACTTGAAGTGTTTACCAATTGGGAAACTAAAAACA
AATATCAAATTAAAAATTCTCTTGGTCAACAGGTCTATTATGCTGCCGAAGGTTATGTTT
TT
>DjScaffold3481:66191-67628 strand:+

ttttaagtatatgtaaagaaagatattcagaattgcagaatatgtcatgaaggaaaagtg
tttgcaatgaaaacgaaaaaagaatgcctaaaattagcatctaatgaaccatttaaatct
atttacatagatctttgtggaccatttaaattgacaaacacaggaaaatggtatatattt
gcagttatcgatcagtatagcaaatatattgaactaaaagcagtaaaaaattaagATGAT
AATCATAATTAATCAAGATTTAATTAAATGTTGGCTACTGAAGTTTGAATTACCAAAATC
AATTCATTTTGACCATGAAAAATGCTTTTTATCATCAAACTTCAAAGATTTGGCAAAAAA
ATTTAATTTTAAAATTGAATATTCTAGTCCATACCATCATCAAGCAAATGGTCAAATAAA
ACGGCAATTTCAGACAATAAGAGACTGGATATATTGCTGTGTCGAAGATAAATTATTCAT
AAATTGGGAAAAATGTTTACTTCAAATAAAATTTACTATAAATTCGACAATTTATAAAAG
TACTAAAGTAAGTTTTGCAAAAATAATTTTTTGAaaaaagctaagtatagataaatttga
aagctcagataaatttttaaccaatgagtatatagagtatagtctgttacattaaattgt
cctgaaaaaaaattcaatactttttcaattaaataaagttgtttatatatatcagattat
ttatagttgtcaattcttatttaaatttttgtatttcattttgtaaaaccatgtaagtat
tttatgctttataattcagctcgaatattagtttagtt
>DjScaffold3481:70710-86675 strand:+
tgtgATGCATATTACTGATAACATGGGCACGGAAGTTATTCGTGCAATGAGAGAATTCAA
GTGTTGTGCAGGTCCATGCTGTCATTGCTGTGCTGGTATAGATATGTGTGCCCAAGAATT
GACAGTGGAAGCTCCACCTGGACAAGCTATAGGCCGAGTTATTCAACAATCTAGTTGTTT
GGAGCCTAGTTATGCAATTCAAAATGCCGATGAAGAAACTATGCTTCAAATTAGAGGACC
GATTTGTATTTGTCAAGGACCTTGTTGTACTTGGGATCAAGAATTTAAAGTGTTTTCATC
AGACATGTCTACTGAACTTGGTAGAATCAGTAAACAATGGTCAGGATTTGTACATGAATA
TTTTACTGATGCTGACAACTTTGGTGTTTCTTTTCCGATTGATCTTGATGTGAAAATGAA
AGCAACAATGTTGGCTGCTGTGTTTTTAATTGATTTCATGTTTTTCGAAAATAATCAACA
AAATCAGAACAATTAAatatgaaaaattaaaatcttgttctacatgttactcgaatttcg
gtcactttgattttcaatgtcatttaatgatgtattataatgaaaacagtccatattttg
atcaagattgtcgattaactaaatttagttttaatgtcgagatttgatttatcgataatt
taaagtatttatattcctatttaactattaaattatttccttta
>DjScaffold447:98417-100573 strand:+
attaaacactcaaggttactttaggaattctgtgtcactgttcattgtatattttttaATG
AATTTTTATAATAATTTTCTCAAATCTTGGAGCGACAAATTCCCCAATATTGCTGAACCA
GATGATTTGAAATGTTTAAAAGCAGATGAATTCTTTTCTGTTAATGATCAAAAGGCTGCA
ATGGAAAAAATTAATGACAGCAGTTTCAACACAAATGTTTGAACTTAAATCTAGACAAGAA
CAATTATTATATTTTAATGATTTTCTAAAATCATCTGTTGCCAAGCTTAACACTATAGCT
CAGATGTTTGACGGTAAAGCGACATCTGTTTCTAATGGTCATAGCGACGATACTGCTTCA
AATAATACATTTCTATCTAGCTCTTCAAATTTAAACAACAATATAGACAATAATAATTCC
AACATGTTTCTCACTTCTTTCGATAGTGGCTCAACTGATCTCAATTGGAAACCTTTTGCG
TTCTCTTCACTACCTTTGAATCAAAATGATCGTATTTCAAACAATTTCAGCTTCCTCAAT
GGAGATATCAAATTGGAAACTCAATTACCGGAAATCAGTCATTTTTCCAGCACATTTATG
GCTCCAACAATAAAGCCAGACTCAGAAATTTGTAAAACGAACAATAATTATGAAACACAG
AATGATGCTAGTCGACGGCCATTCAAATGCAATCAATGTGATCGGGCATTTGCCCAGAAA
AGCGGTTTAGAAGCCCATTTAAAATTGCATACCGGTGAACGGCCGTACGAATGTCAGATC
TGTGGTCGAGCATTTAAATTGAAAGCACAATTACAAACTCACGAAAGAGTTCACAGCGGA
GAACGACCATTTGGATGCAAAATCTGCGGAAAGCTGTTTCGACAACAGTGTCATGTAGTC
CAACATTTACGCAGTCACACCGACGAAAAACCTCATCAGTGCGAAAAATGCGGCAAGGGC
TTCCGGCAGAAACACAGTTTATTGTCTCACGAAAAACGACAATGTCAAACTCGACAAAGT
CGTCGAATGTTGCCAGCCGGAACGACAATGTGGATCGGATTGTCTGATGTGCCGGGTGGA
TCTTTAACAAAAGAATTACGTCCTAGTTCGGATAATCCGATTTTATTGGCTACCACCAAT
CCCTCCTCTCAGACAAACGATTCGGCTTGGTCGCAATCGTGTTCTACCGGCGCAGATGAC
TCCAGTAGTGTTGACACTCACGACGAGTGAggctggcacattaaggggcacatcacaaca
gttttacctgcatttttacttgtggtattatttaaaaaaaaataataataataagaatcc
attttgtaaatagtttcgatcgtttttcgctgtcattatctctctctctctttctcgc
tttttatttgtaagtttaatgtttgtgaaacgataacaaagtatctctatcaaaacccgt
gttgatctactgtgttaataatattcaaatttatattaaatgattggtgtgtgtgtgt
tttagtgcgtgcgtgtacgtgtgtttttgcgtgtgtacgtgaatgttgtggaaaatcata
tctcaggatggaaaatgcttgattgtcttcctttatttagtttagaaatttaaaaccgat
atgaatatagatgataaattggcactttttgttattgatatattgttgaataatttttt
tttctgtataaaaaatacatttttttcaaaatttattttcatcgaaacatgatttgttat

gagtatactatcaattattgatcaattgaactatggattttacttgaatcatgcaatatt
tccattagaaaagacctgccaaattctatgaaaataatcaatactttccaaaatcaattg
tccaatcgtagaagatttagtaattttcttgaaaatagacattggattattgtttttta
tttgaatttacatgcaatttgatgaattaatcgatttttgagacatctatggaatctaat
tccggaaaatttttagtggtcaatgcatttccgaatttttactctaaatcaattttatt
cagatactgttatcgtgtgttttataattagaatgggaaatgattctgctaataaatatt
tatattttc
>DjScaffold3135:3817-4770 strand:+
TTCTTCTCTAAACATCTGAATAATTATGAGACCATCATTCATAAAAGACTACCAATTACT
GATGAGAGTAAATTTCATAAAGAATTCATTAAAATTGCAGGGAAATATGAAGACCAAATA
AAATCGTTATTTTTAGCAGTTAAGTACTGTGTTCCTAAAGATCATAAAACAACAATGAGA
GGAAGACCTATTATGAATGCTTCTGATACTCCATCTACATCATTATGTAAACTTATGGCT
GATTATTTGAGACTATTATTGAATTTTGTTCCAGCACATTTGAAAAATAGTTATGAGTTT
GTTAAACGAATTAAAAGTTATTACTGGAATAACGATGAGCAATTTGGTTCATTGGATGTA
AGAAATCTCTATGGATCAATTCCCATTGAAGGACCGGAAAATGTCATCGATATTGTAAGT
GATTTCTTTGAGAGATATAAAGAACATACAGATATTCCTGAAATCACTAAAAATGATTTC
AAGTCTCTTATTAATTTGTGTCTATTCTCTGACAGCATTTTCATTGGTGAAAAACTTTTT
AAACAAAAAAGTGGACTGGCAATGGGTAACAACTTGTCTCCTATTTTAGCAATAATTTTT
ATGAACTTCATTGAAAGTAAAATTATTCTCAAAGCTGAGAAGAAGATACATCTTTGGTTC
AGATATATAGACGATATTTTCTTTGTTACTAATTCATCTTTAGAAAATATTTTAGAACTA
GCCAATAGTATCAATGATAACATTAAATTTACTTTAGAGAAGTCAATCGACTTTCAGATT
CCTTTTCTGGACACCTTAATCCAAAAGAAACCTGGCGAATTATTACTTACCCAGTTGTAT
ATGAAACCATTTCACAGTGGGCATATATTACCCTGGAGTAGTAATACTACCATCAGGCAG
AAAATTGGAATAATCAAGGGTGAAAAG
>DjScaffold3135:28284-51200 strand:+
aataataataataataataataataataaattttcattctgtatataggataatgaagag
gtattgaaggaaatatcgacgaaatgtcaacatttcttgtggtcatacaaaaagaattta
accaccagtgattacttagacaagttaactaataagaaatgtagtactagtcttgataaa
atagatgagtgtaaaaatgcacaatttgggcatcaagtaccatgtttattgtatcataaa
gataaaatttctgatccagtttgtcataaatttctcaataaagttggaatcatagttttc
tctgattatcatttaatctacaaatttattgataaatgtaatgaagatattacaactttt
aaatgtggtcgccttcataaggcaagtgaagtagatcccgaggatgacaaagaatcaaaa
gaaagtctagtagattatgttactactcaaacggataccATGGATTGTTTGGTGAACTCG
ATTCTTAATATTAGCAAAATATGTCAACATGAAATATTGCGATTATCAGAATTACAATCC
GATGATTATCATTTAAATAGAAAGTTGTATATGTCTTGTAGAGACGATCGAGATACTCTT
TGTGCAAATGTCGAATCTGGAGATGGTCGAGTTTATTCTTGCTTAATGAAGCACAAATTT
GATGATAGAATGTCTAAGAAATGTCAAGATCAATTGACAAAAAAACAACAACTCGAATCA
ATCGATGCAAAAGCTGATTATCCTTTGTTCACTCATTGTAAAACTGATATCGAGAAGTAC
GAATGCACAATTCAAAATCCTGGAACTGCTGAAGAGGTATGGCAAATGTCAGCTATTCTG
CTTTGTCTTGAAGATGTAGTGCATCGTGGCAAAAATATCAATCCAAATTGTAAAGCAGAA
CTTACTCAAAGAAGGGTGGAAATGCTTGAAGACTTTAAAGTCTCTCCGGAAGTTGTTGTT
GCCTGCATTGATGACGTTAAAAATAATTGCGAAAGTGCTGGAAGACAACCAGGAGCTGTT
TTGCATTGTCTAATAGGTTTAAATATTGCCGATGAAGAAAACTCAAAAGGGAAACTTAGC
GGACATTGCCGAATTGCATTGACTCATCTATTGAAAGTTGCTAATCCAACAGAAGACATT
ATTTTATTAGACCCAACTATACGAAAGTCATGTAGTAGCTCGTTAAGAAGTCTGTGTGGA
GAAGTAGGGACGAAAGATCAAGCATTTCAATGTTTGATAGAAAACTTAGACCATCCAGAT
ATGACTCAAGAATGCCACAAAGATTTATCCAATCTTCTTAGTTTTGTGTCCTATGATTTC
AGAATTGATAGTGCAGTTGAGAGAGCTTGTCATCAAGATGCAATCAGGTTATGTAACGGT
CCGAGAACGTATAATGAAACATTGCAACGCAGCGAGAATGACAGAAAATTAGAATATTAT
TGCATGTACGAACATTACAAGAATGATAGAATTCAAATGGATTTATCCGCGGAAAAGGGA
AGTACACATTTCAATAGATCTTTGTCTGCAGAATGTTCGACAGAAATATTTAGTTTAATC
AAAGCCCAAGCTAGTCAAATTCAGCTTGATCCAATCGTGTTTGAATCATGTTTACCAGAT
TTGAGTAAGTTTTGCAAAAGTAAACAGCATAAAGGCCGTGAGCATGAATGCCTTCAGGAA
AAGTTTGAAGAGCTTCAGACACTTTGTCAGAAAGCAATCATAGATTTCAGTCAAATGGAA
GACGCAGATGTTCGACTTAATCGTCAACTGACAGAAGCTTGTATGCCGATGACGAAATTG
TTCTGTAAAAACAAAAGTCCAGAAGAATTACTAAATTGTTTGATTTTTCATAAGCGACAC
CCTGATATGATCACGTCATGTCAAGCTGCCATCGAGCATGTTCAGATAATAAGTCTTGAG

GAATACAAAATTGATCACAGGTTTGCAGGATTCTGTGAAACTGAAATCAAAAGATATTGT
CAAGATGAACTGAAAGGAAACATCAATAAAAAAGGCAGCAAAGTTATCAGCAAAATTTCC
ATAGTTTCTTGTCTCAGTAAAATTGTTGTTAGCAAATTAATTGAAGGTGAAGAACATCCA
TTAAGCAAAAATTGTCGCCGACAATTTTCATTCGAATTGCTGCAACGTTCTGATTCATTT
GTTCTTGATCACAATCTTTTAAAAGATTGTAAATCAGATATTGACAAGTTATGCAGTAAT
GCTGGTCCGGACAAGGTTTCTTCTTGTCTACGAAAACACCAAGACGAATTAAATGAACAA
TGTCATCGAAGAATATTTCAAAGAGAAAGAATGGAAGCATTAGATGCCAACTTTGATGGT
AGATTAAAACGAGTCTGTTCCCAAATGATAAATGAGTATTGTCGTGAAGTAAAAGGAACT
AATCTTTTCCAGTGTCTTCATAGACGAATTCAAGAAATTGAGTTTGATGAAGACTGTCGT
AAATTAATTCTTCTGCGCCTAGAAAGACGAATGCATGATATTCGACTAAATCCGGGTCTT
ATGAAGGATTGCGAGTAAttgttttctaaattatttattaatgtcagaaattgttaacat
ttattgattttggcaccttgaataatgtaaaaataagttgttgtacgtgtttgtacaata
tttatttgtagaataaatataatgataattgaggagaggcaaaatatttttatattttaa
atacggaaaccataacacttgatcgcgatcaaacaatgtttgaaaattgataatctaccg
atattttcaattttgggattttcttgatactaattgacgctgataaattacaaataatta
gtgtttctttttatactcaagacacctcattaatgttcaaactttaattaagcatacgcg
cgcgtgtatcattatggaatccgaaggcagagcagatgtgcatgttgatactcaattgtt
tgaggcgtgtgaagatgccgttttaaaattatgcagtagtgaagaatttggtagaggccg
aaaaatgagttgtttgttgcaatttgctgcggatgagaaaggttcaaaattacttgatcg
tagatgccttgacatgatccaatctcgacgagaaatgtggtcgtttgctgcaaatattga
accacaatcaatgcaagatttatatatacaagttaatcagagttcaatgagaacgtattt
atttgctatgattggtcttatgatttttgcattaatatttcttggtatttgttgtggtcg
agttaccaagcgcgttccagttgatttaaagaacaaataatcatcatcattgtcatattt
tctaattgttgtctaacatcactcctctctattgttagccttttccaaaattaatcaata
actgcaataatccaataaccgttctggtggcaatgttgtatcattgatttgttttacgta
atttagaaacatcgttctatgaatgtctcgtccgt
>DjScaffold2161:87652-88244 strand:+
ccccccttctttggtgttATGGGAGTTACTTCGGCTATGGTGTTTTGCGCTCTTGGGGCCG
CTTATGGAACCGCGAAATCAGGAACTGGCATAGCTTCCATGGCTGTAATGAGGCCAGAAT
TAATTATGAAATCTATTATTCCAGTTGTTATGGCTGGTATCATAGCCATTTACGGCCTTG
TTGTTGCTGTTCTGATTGCACAACAAATCGGAACAGGTTATACTATGGAAAAATCTTTAA
GACATCTTGGAGCTGGCTTGAGTGTTGGTCTTAGTGGTTTGGCAGCTGGATTTGCAATAG
GAATTGTAGGTGATGCTGGAGTCCGGGGTACTGCCCAACAGCCTCGTCTTTTTGTTGGAA
TGATTCTAATTTTAATTTTCGCTGAAGTCCTTGGATTGTATGGTTTAATTGTTGCATTAA
TATTGTCAACAAAATAAatgcaaacaattaaaatgtaattaacttaagcataattcgtta
ttaagtaacgccattatttgtaaaatgttagaataaattttgacaatttgtaaaatgtta
gaataaa
>DjScaffold2161:33617-33916 strand:+
ATGCGTGATACATTGGCAGAGGTCATTTTTAAAGGTATCGTCGTATATCTTGAAAATATA
TTAGTGTATGGAAAGACGGTCAAAGATCATTATAAAATTTTGAAAGAAGTTTTTAATAGA
ATCAGAGAATCAGGATTGAGGATAAATCCAGAAAAGGTTAATTTTCATCAAAAACAACTG
GTCTTTCTGGGACACACAATAAGAGCAGAAGAAATAAAAACAAACAAAACTAAGATTCAG
GAAATAAAAAATGCTGAAGTACCAAAATGTTCATCTAAACTCAGATTTTTTTTGGCTTAA
>DjScaffold640:14800-24641 strand:+
atttgaaaaacgaattgcaaaatctcaatcatcgttatttacaatcagaatctgataaac
aaaaatcagaaATGAAATTGGATAAAGTCTGTACAGAGAATGAGAAAGCAATAGCTCATT
ATAAAAATGAGTTGGAAATTGAAAAAACCGAACGTGAAAAATTAAAAGAAACACATAAAA
TTGAAGTCGGTGATTTGAAAAGAAAGATAAAATGGTATCTTGAAAACCAATCTATTCTTG
GTCAAAATGAAAAAAAACTTAAAATGAAAGATAATGAATTAGCAGAATTAAAAGAGCAAC
TCAACTCTGCTAGTACTATAAAGATAAGCTCAAATATTCGTGCTAAAGAACGATTGATGG
AATCAGAAAAGATCAAGAGATTGGAAGATGAAGTTATGTCTTTGAAAATGGATTTATATT
CTTTAGTTAAAGAAAAAAATATCGGAAATGTTGCCGAAAAACGAACTATTCAATTTTTAG
AAGAAAAAATAAAATCTCTGGAATCTGATAATGATATGAATAGTAAAAAGGAAAACAATG
CTCTTCGTTCTCTTCAACAGCAATTTGAAGCCATGAAATTTCAGTATGAAGAAAAAATTC
GTGATTTAGAATTTGATCTATTGAAAAAAATTGATAATAAATCTCACCCCGAAAAACTAA
TGAGAATATCTGATTTACGATCGAGAATAGATGATTTAAACAATGAACTGGAAAAGAAAT
CATCTATTATAAATAAATTGAAAGCTGAAATCAAAAAACTTAATAAAAATAACAACGATC

AGATTCAAAATGAAAACAAAACAAAAAATATTCGTAGAATTGAAGTGAATAATCGAATGG
AAATGTTAGAGAAAATGTTAAAAGAAAAAGAAAATGAAATTCAAAGATTAGCAATGCACA
ATACATACGTTAAAAATCATGAAGAAAAAGAACGGAATAATAATTTATCTTCTAGCCATC
TTGAATTGGAAGCAGAAAATGCTAGATATAAAAGAAAATAGgttattgatacatacaata
tacatatgtatatttggtgtcagtttatatcttaatgttaatacattactttattttgtc
taaagaaacgaatttcttgttttagttgaagcaactaatggtttttgtcattaataaaat
taatgtttttcaatatttttctattaaatatttatcttcttcaatattattcagtctcaa
atcgaagttcagaaaacctatataaatcatttgcaaaatcaaacagctgactataatatc
aaattgatgggcaatgaagctaaatcaaacgaattaattgaagctaaacaacagattcaa
aatcttaaaaccgaattagctgatcttagattaatttggtctaaaaagcctggtcttgaa
atgattgatagattacggacaaaaatagttcaattagaagaaaaagaaaatttcaaaggt
agacaattagatgaatatgtagagaaagtggttagaaatcatagtaaacaatatcaggat
gaagtggaaaggtggcggttaatcgttgctaaaaagaatcaaaatctaatccgtgttcag
catgaacttgatcatttacttgaagccttagaagagcttagacgccaaggaatagttatt
cctaaattttctataaagtccaaatcgttaaatgaataaatagttttaaataatgatttg
taattataaatgaaattaatattctattttatccaagggttatgtgaaaatatctttgaa
atgatgtttctaataattagaagtcagtaaatgagacaaatccacatacacatgtgagtt
aaactagttgcatagattcggaatttttatagtttccatttgcgcttaaattagaaacat
aaggaaactgaccacaaaacaaaaaattaattagttaaaatattggtttccaaaactttt
tggttttgctgtgctctagaatttcaattttttgacggtaccttgaataaaaatattggt
ctattgttgtaaa
>DjScaffold4494:9825-28656 strand:+
atttaaATGAATAAGTTATATTTTAGTGAAGTGAAAATGAAAATAGTAATTTGCGGTGGA
GGTATAATCGGTGTATCGACTGCTTATTTTCTTGCAAAAAAGAATATTTCGTGTGTAATT
ATAGAACAAGAAGCGATAGCATGTGGTGCTTCTGGTAAAGCTGGTGGATTTTTAGCTAAA
TCTTGGTGTAAATCAAACGAATGCGATGAATTAGCGAATTTTAGTTATGAGTTACATATG
CAGCTTGCTAAGGAGTTCAATGAAGTTGATTACAGAAACGTAACAACATATTCTGCTTTT
ATCGATAAAGTTCATTCACCCATATCAAAAAAAATGTTCTGAATATTTAAAATGGATCGAT
GGTCCAGCCTCAAATCGAACATTGCTTGAAAGTTCTGATTCGACATCACAAGTTCATCCT
AAAAAATTGTGCCAAAAATTACTTGAAGAAACACAGCGAATGACTAATAATGAAACACAA
ATTATATATGATACTATAGTTGGTATAGAATTTGATGATTCTGGTTCTATGCCAATAGTG
AAAGCTGTGAAATTACAATCAGGATCTAACATAGACGCTGATCACGTCTTGATTGCTATG
GGTCCCTGGTCTAAAAATGCCCTGAAATGGTTTCCTAAACAATCTTTTCCAAAAATTAGT
GGTCATCGGGCTCATAGTATTGTGTTGAAAGTTCCAAGAAATGTCTCTATTCCAGAGGAG
TGTGTATTTCTGCAATATAAGAATGATCGTGGAATTTCATCCCCAGAAATTTATCCAAGA
CCAGACGGTACAGTTTACCTCTGTGGATTTGGAGATGACCCCGAACTTCCAAAAACACCT
GCTGATGTAAGTATAGAACGTTGGAAATGTAGTCAGTTACGCGAGGTTGCCTATGCAGCT
TGTTCAAGTCTTGAAGCGTCAGAGGTTATTGCAGAACAAGCTTGCTTTTTACCTTTATCT
GCTGATGGAATTCCAGTAATTGGGGAAATCGATTCAGTTAAAGGAGCATATATTGCTACT
GGTCATAGTTGTTGGGGAATTTTAAATTCAACTGGTACTGGATTTGCTATGGCAGAACTC
ATAGCTGATGGGAAATGTCAATCAATTGATCTAACACCGTTTAGACCAGGAAGAATAAAA
TAGgttttgatttatgaacaatgttatgagtgatgagttttaataaattttgattttaac
attttatccttgaagtttaaatattgattttggttttcatgtcattttggagtatctgaa
atatataatacgatgttctataagttcttatgtaatcagtgaattcttttttctcgatt
tttttgttattctatcacatgtattattttgcaaataaatgtaagtttgaca
>DjScaffold2846:39983-40471 strand:+
agttaattgaagacgaagaagatttggagcaatgtaatgataagaaaagacgcacattca
gcaaagacttaaaaaccATGTTATATGGATTTGGTGATGAAGAAAATTCTTGCCCGGAAA
CTATTCAGTTATTAGAAGAATTAGTTGTTAACTACATTATTGATATGACCAAGCAAGCAC
TAGAAATAGGTAAAGTGGGTAAAATAACTGTGGACGATATTTTGTATTTAGTTCAAAATG
ATCCCAAAAGGTACTCTAGAGTTAAGGAATTAATAACTTTGAATGAAGAACTAAGGAAAG
CCAAGAAAATTTTCGATTCTGTCGATATTGTTAAATAAtttgttgtacaaccttattcat
tgatctgaaattttattatggaatttaatttaataattattttataccgg
>DjScaffold734:8038-21549 strand:+
ATGAATAAATCCAAAGAAGCTAAAGTTAAAAAACAAAAAGAAACACACAGTGAAATCGAG
AGAAAACGAAGAGAAAGAATGAAAAGAGAAACGGAATTTTTAAGCACACAGTTACTTGAT
AAAACTGTTAGAAAAGATAAGTTGTCAATTTATAAAGCTGGTGCAGATCAGTTATTAAAA

TATGCAGAAAAACTTGGAAAACGAGAATTTATTTTGAACGATTCAGAATATTCCGCTATT
ATAGTTAATAATTTTAGTGGAATTGCTTTGTTCATAAATTGTAGTGATGGAGAAATCTTT
TGGTGTAATGATAATATTAAAACAATTCTTGGATTGGATGAAAATTCTGTAATTGGAAAT
ACAATATACAGAATATGTGAACCATCGTTTGAAACATTGAATATTATTGAAACCAACTTG
TCTGTTGATTCGAACGATGAAGAAAAATTAAGAAATGGAGAATTAATTTCTAGAAATTTT
ATTATCGGTTTAAAAATATCAGATAAATTATCCAAAGAAAACTGTACCAGAGGTTTTGAT
GGTAAATGGTATAGATTTATTGAATTATGTGGTGATCTGTCTTTAAGTACTGATGAAAGA
GGTTCTCAAGAAACCTCGCCTATTGTATTTAAGGGTCTTGGTCGACTTCTTGATCTAGCT
AATTCAAATAATACTGAATCAGTATCATTTACAACTAAACTTCATCCAGATAATTATCGG
GTGATAGAAATACTAGGAAATGTTCAGTACTTGTTGGGATTAAAAGCTGCTGAAATGATT
GACCAAAACCTATCAGACTTTGTTGTTGAAGAAGAAAGGCAGTATTTTAAAAATTCACTG
CTCGAATGCATTTCAAAAGAATCTATCAATCTTCTTATTAATTTGAAATCTTCTGATAAA
GATAAATGTCAATTTTCTGTGTCTCTAAATGCAGTATCTGTAAACAAGAAACTTCTATTT
ATCATTGGGAAATTTGAACCTTACAACCCGGTGCAACTTCTAGATCATCAAAATTGGACT
AAAACGGAACCTATTTTAGTAAACAATTATTTTCTGAATTGAtattaatttaattaattt
attaacttaatttttaataattaattaatttttactaatgatattttcgtaaggatttat
gtgccaatcaaattttcattcaaattcactcaaattagtgcaattatttaattaaattta
ccaaaaaataatgagtgatttttaataaagagttttaaaccatatttcgagatgtttttt
tttattaaatttttccatagaaaatctttattgtcagatataactttttaatctattgaa
aagttcttatttattcaa
>DjScaffold2771:2915-19774 strand:+
taataataataataataataataataattaaatattataaatgatatcattttaaaaata
aaaatacattcagtgtcaaaaactggcatcgtagaattcgaatatttcgtggaagtcgac
tgactatttataacatatccagcatccaatcaggatattacgtatgtgatgtaaactccA
TGAGTAGTTCGGATCACATAACATCAATTTCTATTATAACCGTGAAAACACCACCAAAGT
CAATCGCAATTCATTTCAACAAATCAATAAATGTATTTCAAGAAAACGAAATTATCGCTA
TTCAATGTTCTGTAAGTGGAAGTATTCCAGCACCGAATATATTCATGTATATAGTAAAAG
AAAAAGAAATGAAACCTTTAAAGCTGATATCAAAGAGTTCTCAGCATGTAGCATCTTCAG
AATTTAGTGCAATATCTCTCTGGAGACCTCATTATCTTGATCATCGAGGATTTATTATGT
GTACAGCAAGTAATGATGCTGGTAATCTGACAAAGAATTCTTCTTTACTCAATATAAAAT
ATAAACCAAAAATCAAACCGTTTTCCCCAAATATAATTGATGCAATGATTAATCATACTA
TCGATCTAAGTTGCAATGTCATTGCTAATCCTTCCGCCATTGTTCAGTGGATTATTGAGA
AAGGTTTACAGCGAATAGATTCTTCCGAACTGATTACAAAGATTAAACCAGAAATGACTG
GGTCTTTGATTTGCCAAGCAAATAATTCAATTGGACTAGCAAGTGAATCACTACAGTTGA
ATGTCTTAAGAGACCCAATAATTCACATTGAGTCAATTTATCGAGTTAGAGAAAACACTG
ATTATTCAATCGAATGCATTGTTAAATCAAATCCACCACCAGAGTTTATAAAATGGTATA
AATTATCAAATACCACTGATTCTTCTGCTGTATTATTTTCGGACGATGTAAAACTAAACT
TTATACGAATTGACAGAAGCTCATCGGGAATGTATGCATGTGTAACTCAAAATATTCAAA
TTCCTACTATTGGTAAAAAGAAACTCGCTAATGCAGAAAAAACTATGCTTCTTATTGTTA
CATGTTAGtttctgtttagaatatttcgtataaatagaaaattattataagtattctttg
tagctaattctgtaggatttatatttatttataataaattataaattagttataattaa
ttatatttattgacaaataatattcactttgttttatttataaagaagtgtttctgaacc
aataaa
>DjScaffold1069:144667-144738 strand:+
ATGGACGAACTCAACAAGAGCATGATGAACAGGTTGGTAAAGTTTTTTCTTTACTTAAAC
AAGCATGGTTAA
>DjScaffold2672:4501-5070 strand:+
aatgttaaaaaggttacatcagaatttATGGTTTTAGAAATGGTGCAAACCAATAAAACT
AAGGATGGTAATGAAATCCGAACTTTTAAAATAGCGGATAAAACCGGATCAATGTTTTTA
ACTGTATGGGATACTTTAGGAGCCTTTATAAATCCTGCTGACATTTTAAGATTATCAGGA
GGGAATACAATTTTAAGAAGTGGTCAATTGATTTTAAATGTAGGTCGATATGGAACTATT
TCAAAAATTGGCGAGTATTGTTTCCTATTTAACGAGAATCCTAATCACAGTTTACAATAT
GATGACTCTCATTCAGAAGTAACCAGCGTTAATCCAAGTAAAAGATTT
>DjScaffold4841:8915-12293 strand:+
atttacttcattttttatcaactagttttaaattaatttgaaggaatacatacaaagac
actttgtaaATGCCCAAAATTTTAGATAATTTTCTGAAAAGAATCTATGATATGAGCAAC
AATCATATTTTTACTAGTCATAGTTCTTCACAAATAACTGAAACAGAAAAAAAGTCCCAT

GAGAAACGATACGTTGACTGGATTAGGAAAAGATTTATTTTTCATAAATTATCACTCATA
ACAGAAGTAAACCTCCGAAATTCGAATGAAACTGAAATATTTATATCTCACTGGATACCA
TTTATGTTTGACGAATCATATGTTGATATTTCCGAACAAGAGCAAAACTCGTGGTCTGCT
ATTCATAAATCAGTCAATATCAATTTGTTATCTTCGAACCCTTATGCTTTTCATAGAAAT
CCAGTCGCAAATTCCACAGATGCTATAAGAGGAAATCGAGGGTATTCGGAAGGGATACAT
ATCTGGGAAATTAATTGGCCTGTTACAATGAGAGGCACTCATGCAGTTATTGGAGTATCC
ACAGAAAAAAGCCAATTGCAGCAAGATCGCTACGTTTCTCTAGTTGGCATGGATAATGAA
TCATGGGGTTGGGATATAGTGAGTCGAATGAAAATTCATGGAAAAGAATCTGGCAATCCA
ATTTGCAATGAGAAATATCCAGATGCAAAGAAAATTCGTAAAATGTGCTCAATGGGAAAA
ACTTTCAAAATTCCTTCAAAGATTTATTTGATTTTAGATATGTATCAAGGTTCATTGTCA
TTTGTGATGGGTAATGACTTTCTGGGAATTTGTCATACAGGGCTTCAAGGAAAGACTCTT
TATCCAACAGTTAGTGCTGTATGGGGTCATTGTCAAGTAGAAATCAAATATTTTGGTTGT
CTATCAAGAAGTCCACCGTCCTTGCGTAATTTGTGTCTTTATCAAACCAGACGATTACTC
AGATATCATTGTCGATATAATGAATTGCGCGACCAACAAGGTCGTTTTCAAACCGTTACA
AAAATCAATTCATCGACAAACCTATACGATGCTGTCATGGATCAGAATGTACCAAATTAT
ATAAAAAACCTTCTATTAAATAACGATGCTGTGGTACTTCAATTTTGAttttatgttcgt
atttgagtaatattttaaatattttttatcattttttaagagaaaataaattttactttg
tta
>DjScaffold1744:7514-8331 strand:+
tatcattgtaattattgatattattgttttagtcATGATTTTGAGAAATTTGTCATTCAT
GAAAAGCTGTTTACCAACTGTAATAAACCGGGTTTTAAGAAATATCAGTTTCTCAATTCC
ATTATTAGATGATAAAAAGGAGAAACAGGAAGCATTTAAAAAAGAAATGAGTCTGAGATA
CCCTGGTTATCGAATAGTGTATGGGAAAATGCCATATGAACCAGTTGATGTTAACACTAG
TATATTATATTTAAAATCTCCTGAGTATCGAGAAGTTTACAGAGATAAACCGATTTGGTA
TTTTTATCGTAGGAATTCAAAAACCCATTATCCTACAGTTAAAACGAGAAAGAATTGTGT
GTATTCAGGAGTTTTAGGCTCGAAAAATGCGTGTCCAATTTGTAGAGATAATCACTTGGT
TCTTCACTATAGTGTTAGATAAtaatattttatgaatttatttttaatttaattttacta
aattctaatttaattcattaaatttttaaattaaggttttcgatttgggggtcaaatgta
aatctagttctaattttctgtgatcagtgtgatatttgacacttctttttacgacacaaa
tcggaaatatttgctttgatatttgataatagattgctagtaattctatccgtgtaacac
aatatcctttcttatgactcgtcattgcgtgcgatctgtgtgtaattactcgttctcgtt
cat
>DjScaffold2380:14472-47656 strand:+
gggaccggtaaaacatatactatgttgggagtcaacgaacagccaggcatcatgtttaaa
gctttggagagcttattcaaacatatgcgaatgatggaggatgagttcatctatcaaatg
tcgctatcgtatttagaagtatacaatgaaatgattcgggatttacttgggtcaaattcg
gtttatcttgatcttagggaagatcaaaatggagtacaggtagctgggctgacagagatc
gatgtcaacacaaccaatgaagttatggatttacttaatcgcggtaatgctatgagaaca
gttgaagctacaggagcaaataaacatagcagtagatctcatgcagtccttcaagtaaat
gtcgttagtcgaccgcgaatcagaaacactcaggaacaaatattaaaaggcaaattattt
ttaattgatttagcgggttctgaaagagcttctaatactttgaatcgtggaaaaagatta
accgagggagctcatatcaatcgatcattattagctcttggcaattgcattaatgcatta
tcggatccaaacgggaaaagatatgtcaattacagagatagtaaattaactcggttatta
aaagatgctctcggaggaaattgtagaactgtcatgatagctcacattggtccaagcgcc
ttgcattttgaggacagtagaaatacattggtatatgctgatcgagctaaacatatccga
actaaagtgaattgctaatacatttatttcatgattcttatattgatttcatttcaacta
tcaattatatatgtaatatttgagagaaattgattaaaaactcgtgttaaaaatattaat
atttatagtagttgtatctATGAATGAGCAAATTGAAGAAATGAAGATCAAAGATCAGTT
GTTAGTTGCATTTCAGAAACATGTGGATTGCAAAAAAATATTATCTGAAAATATAACAAA
TGATATTGATTTAAATTTGGAAGAAACTCGTTGTCAAGCGATTATTAAAGAATGGGAGAA
CAAAAAGGCTGAAAGAATTCCCGAAAGGGAAAACAAAAGTCGTGATAAAGAAACTGAAGT
TGAAGAAAAACGATCAGAAATCTCTGGATTATCACTTCTTGATGAACCGGAAAATATCAA
ATTAACAAAAAATGAATTAAGATTGACTCAAATGGAAAGATTGAGATTAAAAACACAACG
ACAAAAATTAGAACAAGATTTGGAAAATCATTCGAATAAAATAAAAAATTTAGAATCTTC
TATTTTGGAAAAAGTGAAAAATAACCATGCAAGACAAATGTTACAGTTGCTATGTAGGAT
GCATAAATTGGAGATTCAACAAACCGATTTACGCACAGAAAATATTGTATTACGACAACA
TGCTTCAAAACATGATCTCGTCCAGTCGGTATTAATGAAACACAATGAACTGTGTAAGAG

GATTATTACAACACAGTCCTCGCTTATTAAGGATTATGGAATCGATTTACCGAAAGAATT
AGAAATGCTTTATGAATTATACAGTGAAGACGATATTACTATGACAAATAATATCAAAAT
TGGTCTTCTTGATATTGATAAAGATCTGTATAATTACGACAGAAACTTACCTGTGGAAAT
CGATTCGTCAAGTGATATCCGCGATATGACATCCAATGTAAGTGTAAATGAAGTTCGTGA
GGAAGATGAATCACTAGCAAGTTTGCCTCTTATCAATAATCCTCATGCTACGCCCGAATC
ATGGGACAATGAAAGCTTACGCCAAGGTCACAAAAAATCATCTTTTAACAGTTATTTTCA
AACCAATTTTGACACAAAATATAATACCGGACCAAAACCAGCGTTTGAAAATATTTCAAA
CAGCAATCGAAATTTCATGATATCTAAACCGCCAATTAATCAATTACCATATCGTACGAC
GCATAGATCAATTCTTCCAGACCTTCGTGGAAGGCAATTAGTAGGTCCAATTGAGAAAAA
AAGTAATTCCAGATCTCGTTCATTTGAACCAGAACCTGATACATCAAGAAATAATGGAAA
TTCCACCACATTAAATAATCGTTATGCGAATTCAAATGTGCGAAAGTTGGTAGTTGGACC
GCGATTAGACCCATTTGGAAACACAATTTATGACCTTGATCTTCGAGGTAAAAAATATGT
ATCAAAATACGATGCCTACAAAAGTACTCCCATTTCTCATCGACCTGACAAAACGAAAAA
GCGATCAACTTCACAGAAAAAAATTCCTAACAGCACACAAATAACGAAACAAACTGATGA
AAGATATGCAAACAACCATCTTGTTTTGCGAGCCAATGAAATTAAGAAACTAAATGAACG
GAAAGCTCTTGATAATTTTGATGAAATGGATTCATTGAACAATTTTCGTTTGTGAtgaaa
ttattttttctatattttgtaaatgatttacgatttccaatgtatatttgagtgatatcc
ttatgttttcctcttatacatttcgactttctataaatcatgctttcaaaaccagatgaa
cgtattctttgattattgaaaattgtattagattttatgcatgttccgatgatgttggtt
gaacattgaattatttctattatat
>DjScaffold5184:2204-17395 strand:+
atagaaaattctaaatcaatatagagagctatcagtttgtgtgagtcaactgaaagcgtg
tgctttaacaatggtgcagacacacatgcacccatttaatgcattacaatagatttcgat
aaatcttttacgttaaatttgattttggatattttaaatacttaaatgacaagcaaattA
TGCGTTCTTTCGCGCGTTTAGATTATTTGTGTCTCGAAATAGATCGAGCGCGATTTTTGA
AATTTTCAATGGCAGACAAAGGCACTCAGTCTGATGAATGTCAATATAATGAAATCGCTA
CTGAACATTATAATTCGCTCTCATTGAAATTAAATACTGTCAAACAACCACCAAAACGGC
CGCCACCACCGAAATTGAAAAGCGTTGATGATAGTGTTCTGTTGAAATCTCAACGGAAAA
CTTCAAAAGTCGATTGGTCGAGAATTTCTAAAGATTTAAAAATCAAAAGAAATTTTAATA
CATTTAAAGAAAAAACGTCAACATTTACGAAAAACGTTTACAAGAAAATGGCTAAAAAAC
AAAAATCCCAAACGGTAAATGAATACACTCAAGCGAATGGGGATATTTTCAATGAAACAA
AACCAAACGTTTGTTTAGACGAAACTAACTCTGAAAAAATAATTCCTACAACAGCTGAAG
TAAATTATCAACCAAAACCTATCGATGAAATGGATTCATCGAAAGCAATCAATCAAAAAT
CAACCCCGCCAAAAATTTCAAAATCCGTAAGCAGTGTTATATCAATAGAAGATATTCTCA
ATGAAAATGAATCGATCTTTTCAAAATTCGATATTTTATCGATGATCAATGATTATCTCA
TCAATGATTTATCGGAAACGGAAATTCCAGATTTCGATCAATACGTTGACAGTTGCTTGA
CGGCATTTACCAGAACTTGTCATCTTCAAGTCAAAGACACAATAGCTGACCTCAATGATA
AATTGGCCTATTTTCTTGATCTGGATTCAATAAGTCTCACATCTAATCAACCGGATGCTT
ATATTGTTCATTGTATTCCTGGTTGGAAACAAATAAGAATTATTGAAAACACTAATACCA
AAGAAAAGTATCTTTCCTTGAAAAATTCTCGTTACCAAGCATGGCTTCAAATAAATTTGG
AAAAATCCAATCTAAATCAAAGATGGGTATGTTTTCCAATTCACTCAATAAATACTCTTG
AAAATGAAATCCAACTATCGGAAGAAAACGATTTTAAGAGAAATTTACTGAAATTAATTA
TGTTGATTTGCATAAATCATATTTGTAATGATGACAAATTTCCGAAAAATATTGATCCGA
ATACAATTTTGTTGGTCTCCTCAAAATGTGGAATTATCGTCATATTTAAAGAATGCCTTA
ATGAATGTGAATTATTCTCACTTGAATTTCTTGGATGCGACTCGATTGAAAATGTTCCTG
ATAATTTATTAGAATTAAAAATCAATATCATAAAAATTATTAAAAACTTCTTTCAACGAC
CAGAAGTTATTTCGATTCTGATTTCT
>DjScaffold4757:94992-96958 strand:+
taatcagtgttacaactagaagatatcttgttgatgataaatcaataaatagtggttcaA
TGAAAAAATGGCTCAGGGATAGACGACATGGTCCGGTAAGAAGAGGTTCAAAAGTGGAAT
CTCAATCTGTCAGACCCATTCCGACACCAATTATTCCATCTAATATTACTCAAATATCGA
CTGTGCCATCAGAATCAAGTGTGGAAAATAATTTGCTTGATAATGTTTCTGAAGAAAGTG
TTGACGTGGAATTACCACCACCAATGGCTTTGCAAGAATTTCCTGTGGTTTTGTCTCCAA
ATAATGCTGAAAACAATTCCAGCGCTGGTTCAAGTGCCCCGTCTAGTCAAACGATAGATT
CAAATACAATCGAGCCTATTTCAGAGTCGCAACTGGATTTACAACCGTATATTCAGGAAT
CACACGAATCCAATAATATTATTGCTTCTTGTAATACTCCAATTAAAGAAGAATCAAGGC
CTTTAGAATTGGTTTCAAATATACAAAATACCGATCAATATTCAGCATGTGATGATCAAG

ATAAACGAGCTTCCAGTTTGACCATAAAAGAGTTTACCATGTCACCATGTCAAAGTAACA
AAATATCCGCCTTTACATCTATATGTAATCAGTCCACTAGTCCAAGTTGTGTTTTACCTA
CAGAATTGGATGAATTGGGTATGGCACTCGAGAAAAATTTGCGCCTTAGTCGTCAGGCGA
GACAAGAGAATGAACGATATCAAGCTTGGCAGCAACATATTACTTCAGGGCAGCACACTG
GAACATTGGATTCTATCTCAAGTAAAGCGTCCAAGGCCAGTCAAGAAAGTGAAAGAGCTC
TTTCAACTTCTGAAATGAGCCAATCTTCTTGTTCTGCGGCTTTTCCAGGACTCGAAGAAG
ACGAATTTGATGATAGTAACCACTCACCAATTCTCATCGCTCCACAGATTCTAGATTTAC
CGGGCAATGGAGATGAGAAATTGCTCGGGGAACTTCGTCTTAGCGATGACATGAACGTTG
CCGGACGATTATGGTCTCCAAATGACGATGAAATGGTTGAA
>DjScaffold4757:124877-125287 strand:+
ATGCAATATTTCAAGAATTTATTGCATAACCTTTTCCACAAGATGTTATTTACTAAAGTG
CTCGTTTATCTTGATGACATTTTAATTTTTGGTCAGACACAAAATATTCATGATAAACAT
TTACAAAATGTGTTAGAATTAATAGAACAATCTGTTCTAAAAAATAATCTTGAAAAATGT
ATATTTAGTGTATCAAAACTCCAATTTCTTGATTATACAATCAGTTCTAAAGGTGTTGAA
ACAAATGATGACATTATTAATAAAGTAAAAGAAGCTGTTATTCCAACCCAAATTCGAACC
TTCTTGGGATTGAAAAATTATTATCGTAGATTTATTAAAGATTACGCAACAATTGCCAGT
CCATTATATGCAGCAATAACTGGATCAAAAAAAGTCGTAGAGTGGACTGAA
>DjScaffold4073:1316-47223 strand:+
tatacatgtataaactaagatttctacatactccacacaaaaagaaaaacatttatcaat
aagtataaacataaaattaatgtaattcaaaagaaaaataagtaattattaaaatttaat
taatttaataatttaataaaataatttcactttcaaatagtataaatcgaaaattggaaa
aatttaaaatgtaatgaaaatgaaattattttcatttttattgtaaattaaattacttta
tgaaatagagaaatttcatttATGCGTTTTTATCGATATAGAATAAATAGATTTTTTTAT
TTTAGAGTTGCTGCTGAATTTTACTTTTCAATTAGTAACCTCAGTTCTTTCAGTGCTCAA
CAAACAATTAACGGATTTGAAAAGATAAGAATTGTTGGAAAAGGTGCCTACGGAACAGCT
GTACTTTATAGGAAAAAAGACGATGATTCATTGGTAATTTTAAAAGAAATTAATATGCAT
GATTTAAGTGCTACAGAAAGACAATTAGCAATGAATGAAGTAAAAGTGTTATCCCAACTT
GATCACCCGAATATCGTGGCATATTTTGACAGTTTTGAGGAAGATGGAGTTTTAATGATT
GAAATGGAATATGCTGATAATGGAACATTGTCTCAGTTTTTGTCTGTTCAAGAAAAACCC
ATGGAAGAAAAAAAAATTCTTGAAATGTTTTATCAAATTGTGTCAGCTATACATTACATT
CACGAACATAAAATTCTTCATCGAGATTTGAAAACTGTTAATATTTTTCTCACAAAGGAA
GATATCATTAAAATTGGTGATTTTGGCATATCGAAAATGCTTTCGACGACAAACGATGGA
GCCAATAGTATCATAGGTACTCCGTATTATATCAGTCCAGAAATGTGTCAAGGGAAATTA
TACAATGAAAAATCAGATGTTTGGGCTCTTGGCTGCATTCTATATGAAATGACTTGTTTA
CATAAAACATTTGAAGGATCCAATTTACCAGCACTAGTCAATAAAATACTCAAGGGTCAA
TTTGAACCTGTTAAAGGAAATTATTCTGACGAATTAAAAGAATTAATATCTTCACTTCTT
CAGAAAAATCCAGACGAAAGATTATCCACTGAAGTTATACTTCATCAGAACTTACCAATG
ATGATGAAAAAATTTCAAGCAAATAAAATTGAAAGTGCTGATATCAATAAGCGACAAAGC
AGATCAGTTGTTTATGTTATGGATATCAATACATTGACTTTGTCTCCTATACGATTACCA
TCGAAGATTAGAATAAAACAAGTATGCAGTGGAGAAAATCATACTATAGTTTTGACAACA
GATCGAACTGTGTTTTCGTGGGGTACTAACGAATTTGGTCAATTAGGACATGGAGATTTA
ATAGATCGCAAAGTGCCGGAACTAGTCACTAATCTCACCCAGAAATCTTTGATCAGAATT
GCTTGTGGAAATAACTTTTCAATTTTTGGACCAGAGAATGGAATTATTATGACATGTGGC
AGTGGTATCAGTGGTTGTCTAGGCCATGGAGATTTTAAAGATGTTTTAAAGCCGAAATTG
ATTGAAAGTTTGTTAACTGTTGATGTTGTTTCTATTTGTGCTACTAATAGTCATGTAGTT
GTTGTAAATTCTGAAGGTGTTATATATATGTTTGGGGTAGTAATAAAAATGGGCAATTAGGA
TTAGATAATTTAAGTCTTTATTGTTCACCTCAAGAAATTTCATTTAAGGAACCAGTGACT
ATTCGTAATGCGTTCACTTCCGAAGATGCTACTATGTTATTAACTGACACAGGAAGTTTA
CTAGCTGCTGGCAATAATTATGGAAATAAGTTGGGTCTTAATGCTAGATTGGGATTTTTA
ATGACAATGAAAAGCATAAATCAACAACCCACTATTTATGGTGTTAATGAATTTACTGTT
GTTAAAGCTTTAATGCGTCATTGTGTGAGTCAAGTAAGTATGGGTAAAAATCACACTATT
GTATTGTGTGAATCCGGTAAAATGATTGCATTCGGAATGAATTCAAACGGGCAGCTAGGT
ATTGGAAATACAAAAAATATTCCTATTCCTAGTGAAATTAAAATAATGAAAAAATGCAAA
ATTACGAAAGTTACGTGTGGATCGAATTTCTCTGTTGGATGTGTTGATTTCAAGTCGCTA
TATTTTTGGGGTAAAAGACATTTGCAAGAATGTGATATGAAAAATTTCAATGGTGATGGA
TTGATAGAAAGCAAATCGTTAATAAAAGACGAAACCCTTACCGATCAATTTTTTCATGCT
ACAGAAAATTCCAATATTCTGATAGAATCTAAACAAATATCCAAGCAATCTAATGTTCTA

GATGATATCGCCAATGATTCAGATGACGTTATAAAAATCTATTCAAATCCTGTTCTTATT
TTACATTTAAAAAATCCTATAACAGATTGTGAAGATAAAGAACTCATTAGCTTGACTAAT
TGTATAACTTATCAGGATCACAGTTTATTGGTTCAAATAGAGACCACTGCACCACCTCCT
AAGAGAAGACGGTCTAAAACGATTCCACGGCTTAAAAAACATAATTCATCTCTATTTGAA
AATGGTTCTAATCCAACAAATTCACAAAATAATCCTAAAAGTCTTAAAAGTCTCTCCACT
GAAACACCTGCAGATTCAACATTTCCAACGGAAACATTTGAAAACGAAAAGATACCGACT
TGGATAAAAAATGAATTAATAGATGCTGGAGCTGAAATTCCAACTGACAATAAAATTAAT
AATGATTATCCGAATTTCAAATTCAGCTCAATTAAATCAAAAAGTTTCAACTCAAAAAAT
GGATCTTTTAAAAATATTTTAAGAATTTATTCCTCATCAGAACATGATAAAACTCCTGAT
TCTTTTGTAAAGAGATCATCTGATTTTTTTAAAAGTTTCTCTTCGTCTTTAATTAGCAAT
CAAAGTTTTCATAAAATGAACCCGGAGTTAACTATTACACCGAAAAGTGATCGAGAAAAA
GAATTAGAGAAGGAAATAATTATTTTACAAGAACAATTGCAAATTCAACAGAATAATCTT
GTACAGAGCAATTTGATAATTAATCATCTACAA
>DjScaffold1695:81057-99966 strand:+
GCACAAGTTGGCAAGACTACTCTTATTTTATCTTTAGTCAGTGAAAAATTTTCGGAAAAA
ATACCAGATAGGGCTGAAGAAATTTTAATACCGGCAGATGTAAATAATGAAGGAGTTCCC
TCTCAGATAGTTGATTATTCCTCCAAAGAACAGAATGAACAAGATTTAGTAGAACAAATT
CTTTTAGCCAATGTGATTTGTTTGGTTTACGCAGTAAACAATTCTAGTTCCATAGATAAA
TTAACATCATATTGGTTACCATTCATTAAAGACACATTTAAACAGCATGACCGTAATCTT
ATTCCGATTGTACTTGTTGGCAATAAATTAGATTTATATGAGAATGGGAAAATTAAGAAA
GTTTTGCCGATCATGAATAAATTTTTAGAAATTGAAACTTGTGTAGAGTGTTCCGCAAAA
ACAATGCAGAATCTAAGTGAAGCGTTTTGGTTTGCTCAAAAAGCAGTTTTGTATCCAACA
TCACCATTATATTGCCCTGAAAAACGCCAATTATCAGAAAAGTGCATTCAATGTCTTACA
CGAATTTTCCGAATATGCGACCAAGATAATGATGGATTCTTGTCTGATCGCGAACTGGAA
GCCTTTCAGCGTCGTTGTTTCAAAATTGCTTTAACTTTACAATCATTGGACGAAATCAAA
ATACTTATTCGACAAAACACTCCAGAAGGTCTCACTCACAATGGAGTTTCTTTGAGAGGA
TTTCTCTATCTGCATTTGCTATTCATTCATAAAGGCCGCCAAGAAACAATATGGGCGGTT
CTCAGGAGATTTGGATATGACAATACCTTACAGTTAAGCAAGGAATATCTCAGACCAAAG
CTTGAAATTCTCCGTGGTTGTAATACTGAAGTTTCACAACAAGGCAGAAAATATCTAGAT
ATTATATTTTCCAAATATGACGAAGATCGAGATGAATGTCTATCTCCCCAAGAAGTTACA
TCGATGTTTTTTACTTGTCCTTGGTTAACTTGGGGGTCTGATGTTAGTAATACGGTTCAA
ACAAATTCCTTAGGATGGATTACTAAAAAAGGTTTCTTTGCTTAT
>DjScaffold1695:112189-119227 strand:+
TATCTCACAGGTCTTAGTCGAAGCGAGTTACCCTGTTCTACAGCCGCGGTGATTCAAGGT
TACGGTGATACGGAAAGAGTTATAATATTACATGAAATAGAAACTGGAGTCGGGGAACAA
CAAAGTCTTGAAGAAGCCTGCATGGCAGATGTAGCTTGTCTCCTTTATGATACAACCGAC
GCTGATTCATTCAAATATATAGCTGATATATACTTGAATTATTACAGAGGTACCAGAATT
CCTTGTCTACTTGTTGCTGCAAAGGCTGATCAAATGGAAGTTCCACAATATTACGTACTA
GATCCAGATGAATTCAGTGGAAAATATCAATTGGAATCTCCTGAAAAATTTACATGTCTA
ACTGATCAAATCAATATGTTTGTTTTTACAAAATTGGTTGTTATGGCTAATTATCCGCAT
TTAAGAGGAATGCAATTAGCACAAAATGATTATGGATGGAAATTTACTTTAGCTGCTACT
GTTTTAGCAGGATTCGGTTTCGTTGCTTTTAGAATAGCTAAGACCCATTTGTGAtttaca
aatcttcattgctacaatcaattttaattaaatatttatttgc
>DjScaffold1695:78496-78750 strand:+
GAACAGCACTATAGAGTATTAGACCAAGTTTTTAAGAGATTTCAAGAATTAAGATTGAGA
ATTAATCCAGGCAAATGTCATTTATTGCGAGTAGAGGGCAAAGTTTTAGGACATTTCATA
TATATGAATGGAGTTAGGACGGATTCGACTAAAATGGAAGCAATAAAAAATTTCAGAAAA
CCTAAATTTGTAAAGAACCTGAGAAGCCTTTTAGTAATATGCAATTACTATAGATGGTTT
ATCAATAATTACTAG
>DjScaffold3061:32380-33290 strand:+
gtttataatttcaaaaaaaaattcaattgcaaactatattaatctagatatagaaaaATG
GGACGTGTTATTTTAACTCAAAGAAAAGGTCGTGGATCAGTTTTCAAGGCACATACTAAA
CATCGTAAAGGAGCTGCTAAATTGAGAGCAGAAGATTACGCTGAAAGAAATGGTTTTTTG
AAAGGAATTGTTAGAGATATCATCCATGATCCTGGACGTGGAGCCCCTTTGGCAAAGGTT
GAATTCAAAGATCCATACAAATATAAGAAAGTTATGCAAACTTTCATCGCTGCTGAAGGT
ATGTCAACTGGACAATTTGTTTACTGTGGAAAGAAAGCTAAAATCAATATTGGAAATGTT
CTTCCAATTAAATCAATCCCAGAAGGTACCGTTGTTTGCAATCTTGAAGAAAAATCTGGT

GATAGAGGACGTATTGCTCGTGCTAGCGGTAACTCAGCTATCATTGTTTCACACAATTCT
GAAACTAACAAAACACGTGTTAAATTGCCAAGTGGTCAAAAAAAATTATTGTCCTCTTCA
TGCCGTGCTATGATTGGTATCGTTGCTGGTGGTGGAAGAATTGAAAAACCTATGCTTAAA
GCAGGTCGCGCTTATCACAAATACAAAGCTAAGAGAAACTGCTGGCCACGTGTTAGAGGT
GTGTGCATGAATCCAGTCGACCATCCTCATGGTGGTGGTAACCATCAACATATTGGTACT
CCATCAACAGTTTCAAGACACACTGTTCCAGGTAGAAAGGTTGGTCTTGTTGCTGCCAGA
AGAACTGGTTTATTGCGTGGAACTAAAGTTATTAAAACTACTAAGCAATAGttgaataat
atatttttggattttataa
>DjScaffold5415:46988-53351 strand:+
GCTGGTCCGTCATTTGAATATTGGAAAAATAGATGGAGCAAAAATGATATTGCTTGGCAT
TTGGATGATGTTAACAAAAGTCTTGTTAAGTATTGGGAATTATTAAAACCTAAACCTAAA
TCTGTTGTGTTTTTCCCTTTATGCGGTAAAAGTTTGGATATGGTATGGGTATTATCAAAA
GGACATGTGGTTATCGGTGTAGAATATTGTGAAGAACCTGTGAAATTATTTTTCCAAGAG
AATAATATTGAAATTGTTGTAGAAACTGGCGAAAATTATAAATTATACAAATCTTCTGAT
GGTCATTTACAAATATTTGTACAGGACATATTTCTTTTTAATTTACGGAATGAAAAATTT
GACATAATCATTGATAGAGCATCTCTGGTAGTTTTAGAACCAAATTTAAGAGAAGATTAC
ATAAAAGTTCTCCACGATTATCTCGATGATGAAGGCCATATATTTTTTGAAACTATACAA
TATGATCAAAGTTTATCCAGTGGTTCTCCATTTAATGTTTCTCATGATGATTTCGATGCA
TTAATGAAGAAAAACCATTTGAAGCATGAAACGGTTTACAATGGTATTTATTGGAAAAAA
GTAAAAAACCTGGAAGTAAACGAAACAATTAGCATTGCTTCAAAATTTTAAtaagtcaat
agtggttttgtacaattttattcaagcaattttcaatcagaaattaggaatatattaatt
tgattttgacattttaatattttttttattattgacattgtaatatttttattaataaa
atattagaatctctttaggaataataatagaagtcttgttaagtattgggaattattaaa
acctaaacctaaatctgttgtgtttttccctttatgcggtaaaagtttggatATGGTATG
GGTATTATCAAAAGGACATGTGGTTATCGGTGTAGAATATTGTGAAGAACCTGTGAAATT
ATTTTTCCAAGAGAATAATATTGAAATTGTTGTAGAAACTGGCGAAAATTATAAATTATA
CAAATCTTCTGATGGTCATTTACAAATATTTGTACAGGACATATTTCTTTTTAATTTACG
GAATGAAAAATTTGACATAATCATTGATAGAGCATCTCTGGTAGTTTTAGAACCAAATTT
AAGAGAAGATTACATAAAAGTTCTCCACGATTATCTCGATGATGAAGGCCATATATTTTT
TGAAACTATACAATATGATCAAAGTTTATCCAGTGGTTCTCCATTTAATGTTTCTCATGA
TGATTTCGATGCATTAATGAAGAAAAACCATTTGAAGCATGAAACGGTTTACAATGGTAT
TTATTGGAAAAAAGTAAAAAACCTGGAAGTAAACGAAACAATTAGCATTGCTTCAAAATT
TTAAtaagtcaatagtggttttgtacaattttattcaagcaattttcaatcagaaattag
gaatatattaatttgattttgacattttaatattttttttattattgacattgtaatat
ttttattaataaaatattagaatctctttaggaataa
>DjScaffold4565:80734-83975 strand:+
gttggtttataatactttcaattcttATGCAGTGCACAAGAAATGTAAAATTCTGTGGAT
TTAATTTATCGAAAAAAAATAAGAAAAGTAAAGATAAAAGACATTCCAATGATAGTGCTA
ATATGCGTTCACAAGAAACAAATAGTAGTTCCGGTGTAATTAGTGATCTCAGTGATAAAA
GTCAATTTGGAAAGCAATACAGTATTTACAGTTGTCAATCAAGTGTACCCTGGTTAAATT
TTGGCACCAGTTCCGTTAATTCTTATGGAAATCTTTATGGAAATAAATCCACTTTCTATG
CTCTACCAAACGATATGATGGAGCCTGGCTGTTCAAAAGATCATTATAAGAGAAGATTAC
GAAGTTCAGCTCGTTCTGATGAAAGTGTTACACCGTTTTCAGTTCATTCAAATCATCCCT
TATCAACATTTTCTATTTCATCATCTGAACCATATGAATCACACCATTCATTTTCTAATT
TTGATCAAAGTGATCCTAGTCCTTTTACATTGCTAAAAGCAGAGATTTTGTCCAAAATTT
TACATTTCTTACCCGTTAACGATCTGTGTAATTGTGCCCGGGTTTGTAAACATTGGTATT
CAGTGGTTTGGAATCAGGACATGTGGTACAAATTAGATTTATCAAGATGTAAAAATGATT
CTCGAGTTGATATTCATAAAGGAATCGATACTATATTAAATATTCTGAGTTATAAATATC
ATCCAAATTGCTATTGTTTGCGAATTATAGACCTTTCTAAATGTCAAATTACTAATGATG
ACTTATTTAAAATAGCAAATAAATGTCCGGAGTTAGAAGTCATTAATTTATCCTCAAATT
TTCAATTACAAGATCGAGCAGTTTGTCATTTATCAGTCATGTGCAAGCGGATTTATTCTC
TAAATTTATCAAGTAAAACTTTATTTTATATATATAATTTGTTGAGAGTTTTCAAACACA
AAAGCGCGAAATAGaatatttgcatgcaaatttggtacactaatctgactctgttaatca
agtaaaggttcgtcctgcaaaaaaaagttaatgttttcttcaataaaattttattgtccc
tcaaactaaactttttgatagattcacaaaatacactgagatgtcttctgaaaaaatttt
gcaaagttttacaatgatttcattcatttaaaaactctttcatgcacatacacaacttaa
ctacacaaaaataaaaccaattggttgtatcaacgaataaaaagctttgaagataatttt

tacactcttatatatatcaataaaaa
>DjScaffold4565:110962-118055 strand:+
tgattttaagttaaaatttgaaatccatgaaaataaatctgaaATGCTTAATTATCTGGC
TAATGATTTGAGTAGACCATATGAAGAATTATTGAATAAATTTCTTGAAAATTGGAAAAT
TCCAAATATTCCAAAACATTTTTGTTTTCGTGAAGGATGGACCAAGTATGACAGTAGCAC
AAATTCATTTATCCAAGTACCATATCCTGATGAAAATGTCCTTATTTTTGATATAGAAGT
TGTTGTTAAAGAAGGTTATTTCCCATCATTAGCGGTTTGTCTTGGGAAAAATAACTGGTA
TAGTTGGGTTTCAAAGCGTTTGATGAATTTAGATAATTCCAAAGGAACAATTTATTCTGA
TGAATTTATTCCGTTATTTAATAATGATAGAAATATATATCGATGTATAATTGGACATTT
TGTTTCTTTTGATAGAGCTAGAGTGGCTAATGAATATTTAATTCAGGGATCGAATACAAG
ATATATTGATACTATGTCGATGCATATTGCTGTATCAGGCATGATAAATGATCAAATATT
GATGTATAAGAGTCTTAGAAACTTCAAATGGAAAAAAGTTACTAGTAACAATAATCTCTT
AGAAGTATTTTCACTTTATAATCCGAAAGATATTCTTCCTCAAAACAAAGACAATCGAAA
CATTTTTGTTACAGGGTCTTCTACTGATGTAGTTCAAAATTTCCAAAAATTAATGAGATA
TTGTGCTAATGATGTCTTTATGACTGCAAAAGTTTTCAAGGAAATATTACCAATATTCAA
GAGCAGATTTCCTCATAAATCTGTTTTTTATGGAATGTTAGAGTTGAATTCATTATATTT
ACCAACAAATGACAAATGGTTTGACTTTATCAAGCACAGTGAAAATAACTTTGAAAATTT
TGGTTTAAAGTTTCAAAAAGATATTCTTATGAAAGCTGCTAATTTGAGCGTAGAATTGGG
AAAAGATGAA
>DjScaffold4565:123339-133753 strand:+
TATCCACTTGCCCATAAAAATGGCCATAAATCTTATGTTAGTCGTCCTCTTGGTGGAACT
TTTTTTCCATCGTTTAATAATCGATTGACATCAGACCTCAACATTGGCCAAACGTTATTG
CATTCAATGGTTACTTGCCGATTTTGGCAGTCCAATGAAGACATTATTAAAAATCAGATA
ATTGTAAAATTAAACCGATTGCCTGAAAACAATCAAATGGCAATAATTCCAATGGTGGTA
CCAATAAGTGACCCATACCGTAAAATTGCAGAGAAATTGTGGTTTAATAACTTTTATTTG
TCGAATAAAATCGGTGCTGAATATAAATCTATGATTGTTGCACCTGAAAATTATGTATTT
GTCGGTTGTTCCCTTAATTACAGTGTGGATTGGTTAATTGGAATCATTGGAGATTCGGAA
CATGGTAAACTTGGAGGCTCTGTATTTGGAAAGCACGTTCTCAATGCTTGTTATCATTTT
TTGCCTAGATTTAAAACTGAACTTTATTCTAATTTATCTCGTATCACTAATATTGATCAG
AATATTATACGCGATTTAATAAAAATCATGGTCCTTGGCGGCACTGATGATGATGTTGCT
AAACATGTTTGGAAGAATTTATTTTTCAATTTTAATGAAGCAAACACACAAGCCAATATA
TACGTAAATACATTGAAAGGGAAAAAAACTGACAACATTTTTCACGGTGGAATTCTATCA
GATTATTTCAATATTATTAATAAAATAATAAATTCTGATATTAAGACTCCATTTTTAAAT
TCCAAAGTGACAAGACCGGTTGAATTACAATCTAGTATTCCTGCTTTACTAAAAGTTATC
TTAAAAGCATGCTTAGCAGATTACATTAGCACTCTAATAGTTTATTGCAAATCTTTAATA
ATTGATTCGGGATTAGAATCGAGATTAAGTATATGTAACAATCACGAAATAAGATTCTTG
TGTCACGAAAAAGATAAATACAAAATGGCGTGTACTTTACACTCAGCTGATTTGCTTACT
AAAAGTACATTTGCTTCAATTTTATCTCTTGGTGATTTACCTTCAGTTTTTTAAtaatgt
cgatattgataaaagttggagaatggatcctgttgaagatttcagttatgaattcaataa
tggtatttatcataattatggaattaaacccggagtttcgataaattttgacgaaatttt
cacagaaagtcggaaataatttttctgcccttttttttggcattccacctaataaattt
tgcatttgtttttattcgttcaattaatatgtaaaatgaacattgcaacttatgcttgg
taactcagcttggaatccggacaaattgttcttgatagcaaaactaggtcagagttgaat
atttttgatctttaacaatcaa
>DjScaffold636:50996-53714 strand:+
ATTTTAGACGATCGTTGTGCTATTCTTCTATGGATCATTATTGTTATTCTTGTCGTCCAT
TTTCTGGAAACACGATTCAAATTGTTCACTAAATGTCTCCAAATGTGTACAAGAAAAGGT
ATATTTCATTACGCATTAGTAAAAAGCAGAAAAAGAAAATATCAAAATGAATTAGAAGCA
AAAATTAATCAATGCTTTGGTAACATAAAAATTGAATTAGAATCAAAAATCAATCAATTA
TTTGGTAAATATGTATTTTATGAATTGTTTCAAACTAATATTAAG
>DjScaffold1970:43218-54068 strand:+
aatgaattaaataaatttttaataATGCTTCATTTCCTTCCTTACCGTTATAAAAGATT
TTATCTTTTTCAAGTTTTGCTTCTTGTGACATTTTTGTTAACGGGACTCATTGTCTATCC
AATTACATTAGTGACTTTCATTTTATTTTACAGAACTAATAGGCTACTATACAAAGCAAT
CATTAGTAGATTGTGCCATTTTTTATGGATTCATTTTACTTATATAGCTGAATGGTGGGG
TGGTTTACGAATTACGGTTTACGCTGATGATGAAATTTTGAATAAATTTGGCAAAGAAAA
TGCCATAGTGATTATGAATCATAAATATGATATCGATTGGATTACAACATGGATATTTGC

TGAAAAGTTTGGAGGATTAGGTGGTGCCCAATCAATTGCAAAGAGAAGTCTGAGGCTTGT
TCCTATTATTGGTTGGATATGGAATTTTAATGGTACAATATTTTTGAAACGCGATTGGAT
TAAAGATAAAGAAATTATTGCAAATAGTTTGAAAGACTTCAATAAAACATTACCTGGTTA
TATAAACTTGTTATTGATATATCCGGAGGGAACGAGATTTACTCAGAAAAAGTTAGAAGC
TAGTAATGAAGTTGCTCGATCTAAAGGAATGCCGGAATTAAAACATTGTCTGTTGCCACG
TACTAGGGGTTTTGTGGAAATTGTTAAAAATTTAAGATCCCAGGTAGACTGGGTATATGA
TTTCACAGTTGCGTTTCCAGACAGTGTTAATTCGTCAGTTAAGGAGGTTAACACTAGTCA
ACCACCATTAACCATGAAAAATATTCTTGATGCGAAAGTAAAAGAAGGGAATATGACTTA
TGAGCGTATTTCGATGAAGGATTTACCAGAAGATGATGACAGATTAGCACTGTGGCTGCA
AGATAGATTTAGGAAAAAGGTAATTTAAatacaaatgcgtttcgatttttgaaaatttat
tgattaaaatcaccatttttgaagtcagtatttaacgatttttccatcttcaaataacaa
taaattcataattataaattttgttttaagcaaatttagatacgacncatacgttttatg
gattagtagttggacggttcgagttgttattttagggattattttatcaatattgatcat
gattcaaatactaatgcgcatcactcaagcagcaaaaggatcatcatacggaacgaag
>DjScaffold1970:74996-75528 strand:+
cgagttttatatttatattcattaaataaagtatttatttatcgaccaattATGTTGTAT
CTCCTTCCAGTTCGTTTTAAAAAATTGTTCATTCTGCAAATGATACTCCTAATGACATTT
ATCATAGTTGGATTTGTAACTTTTCCATTTATTTTATTAACATATGTAATTTTGTTCAAA
ACACACAAATTATTATACAGGGCTATCGTAAATCGATTATGTTATTTTGTTTGGATTCAT
TTTACTTATATAGCACAGTGGTGGAATGGATTGAAACTGAGGCTATACATAGATGAAGAT
TCACTAAAACATATCGGTAAAGAACATGCACTGATCATTATGAATCATAAATATGATATC
GATTGGGTATCTACATGGATATTTGCTGAGAAATTCGGTGTTTTAGGAGTAAGTGTAAAT
ATTGATTATTATTATTACTTTCTCAAAATCATTATTTTATATGGATCGAATAATGTAAAA
GAAATT
>DjScaffold1970:81504-82562 strand:+
cattgaattaagtaaatattatattaaaatgagtcaccggctttttggtgcttgttttt
atttctaattttttttattaccaggaattaaatttaATGAATTCAAGATTTGCAATATTA
GGTTTGTTACTTCATGTCTATTTTTCTGTATCACAGATGGGAAGAAAACCAGTTATTACT
TTATTTATTATTGGAATTCTAACATTTTTTTATTTAAATAGCAGTATTTTGGGAATTTAT
GCTGATAGCTCAAGAGCATGTATATCATACGAATCGATTATTTATAGAAAACAGTTTGAT
AGATTATGGTGGCATTCATTTATCCACTTATCTGATTTGCATCTTTATTATAATATGATA
AATTTATTATTGAAAGGTGGTGATTTAGAATTTCTTTTGGGCAAATTTCATTTTGTTGTA
TTTATTATTGTGGCGATCCCAATTACCGGTGTTGTATATATATCTATCGCTTTGTTCTTA
AATTGTATTGATGGTGGATACTATGGAAATCTTTATTTAAACGAATGTGCAGCGGGATTC
TCAGGTGTTCTCTTTGCCCTGAGAGTTGTGGAATTATCAGTTTTACCTCCAAATACACCA
TTTTCATTATTAGGATTTCCTTCGCCAATACCTAATCATTTTATAGTTTGGATTGAACTG
TTATTAAATTATTTATTAGTGCCAAATAGTTCTTTTATCGGTCATCTTAGTGGTATTTTA
GTTGGATTAATGTACAGTTTTGGTCCATTGAAGACATTAATCGATATAATTTGTCAATCA
CATACCGAGACGAATATATATTGGATTCCTTTCATGTCAATCTTTCGACGCAGG
>DjScaffold1970:95113-98015 strand:+
taaaagttaataattcaaagataaatgtaaatctttcgattccataataaattgataaaa
agatttactatcaatgactaataacaatttgcttttaattgtcataatttatgttttaaa
tcaataaacattattgttcaaataagtacgaaatttaattcgattattacacagctccta
tcattctttaaatacgaacttaaattaattagctgcgtatctgcattgacattgtcaaga
aaattgattaccggataattatagtaaagtatgatcatattcagaaattttccaaaattt
aattttttaaattaaaatcATGCCCCAACACTATTATTATTATTATTATTATAAAATTA
TCATTATTAAGGGAGGTCAATCGTTTGCTAAACATAGTCTGAAATATGTACCAATTATTG
GCTGGATTTGGAATTTCAATGGAACTATATTTTTAAATCGTGATTATAAAAAAGATAAAG
AAATAATAGATGAAGGTCTTTTGGGATTCGGTGAAACTTTGCCGGGTTATTATCATGTGC
TGTTGATTTATCCAGAAGGAACCAGATTTACTCAAAAGAAATTGGAAGCAAGTAATGAAG
TTGCACGGTCTAAAGGAATGCCAGAATTAAAGCATTGTCTGTTGCCACGCTCAAGAGGAT
TTGTTGAAGTTGTCAAGAATTTAAAGCCACAAATTGATGTCATTTATAATTTGACTGTTG
CATTTCCAGATAGCGTTGATCCGTCAGTAACCAATGTTAACACCAAACAACCTGGATTTA
CTATGAGAAATATTTTAAACGCTGATGTTAAAGAAGGTGACTTAGCATGCGAACGACTTT
TGCTCAAAGATTTACCAGATACTGATGATGAATTATCGGCATATCTACAAGAACTTTTCC
GTAAAAAAGATGATTTGTTCGATCACTATGTAAAAACAGGTAAATTTCCAGGATCATATC
ATGTTATCAGTCCTTCATATGTCGGCCTCATAATCAATTCTATCACATTTGTAATTATAT

GGTCAGTTCTAGGATATTGGTTATGTTATACTCTCTGTATAACAAGTTGGATTATTCGAT
CAACAATTTTAGCAATTATTTTACTGGTGTTATTTTTGATCCAATTATTTATGAATGTTA
CTCAGGCAGAAAAGGGATCATCTTATGGAATGAAATCAAAAAAAATAGATTAAtagaact
cagaggaaacaatttaattttagaaatttttatttgtgacaaaacactgtattatttata
atatattagcaaaaattcagcctgtcaaacgtaggagatatccatggatgggcaagacat
tccgatgctgacattcgcttttctggttctaatataaaaattgtttttag
>DjScaffold1970:111280-128449 strand:+
atttagtaaagATGTCTGAAGGAATTTTATTCGTAGAAGAACTTCCGAATATCTTTAAA
TGTCCCAATTTAAATTGCCTCAAAAACATGATAGATGCAGTTACCGTAAAATGTGGTCAT
TCATTCTGTAAAAAATGTGCATTATGTATTGATAAATGCCCAATTGATAATTATAAGATT
GATAAGAAAAATTACTTTCCAAATTTGGTGTTGTCTAGTCAATTACAAGATATGCTTATT
CATTGCAGTAATGGAGTTCAATATAATCCAATGAACCAACAATTAGTAATAGATGAAACC
GGATGTCAAGAAAAAATAAGTCTAAAAAACAAAGATATTCATGAAAATCAGTGCCCATTC
AGAATCGTCCATTGTCCTTTCAATAATAATAAATGTAAAGAAAAAATGAGATTTAATGAA
ATAGAGTCACACAAACAAAATTGCCAATACTCTCAAAAAACAAATAATTTATTCATTTAT
TCTAAATCGGAAATTTCCAACAAATCTTTAGGTCAGAAAATGCAATTTAAAATTTCAGAA
GATAAACCAGATATGCAGCAAATTGCGGATAATTTAACATTTAGAATTAAACGTTTGGAA
ACTCAGATGAATGGTTCTTTAAATAGAATGGAAAATATGTTTTATGAGATTCATTCTGGA
ATGCAATTTATAATAAATCATTTAAATATGTTACCAGATTACGAAAAGAGTAAAAGAGAG
TCAACAGCAAGTAGTTCGCCTTCATCTTACATGGATAATAAATTAAATGAAATCAATCGG
CCTATACCACAATTAAATAAAAATAATCTACTGTCATTGAAAGAAATTGCCAACATTAGT
GGAATAATGTCAAATAGTGGTGGAAATTCCGATACATATAAAAGCTCACACTGGATTTGC
CCCTTTTCGTTTTTAAATGCTGGCATTTTGAAAGGGCATCACAAAATGATCACAAGCTTA
ACACAATTTGAAAATTTTATCATAACAAGCTCTCAAGATCAAACTATCCGTATTTGGGAT
ATATCATTTTTGATTCAAGGTGAATTCGGTAAAACGTTGACTAACGGTGGATGTGTTGCC
ATTCTTACCGGTCATAGCTTTTCGATCTCTTGTTTAGCCATTACTCGCTTAAGTCGTGAA
ATAATTTCTATTGATGCGGAATCGAATTTAAAAATTTGGGAATTTAATTCTGGATTAATG
GTTAGAGAAAAGGAAAAGCTCCACGAAAAGCCAACAACAAGTGTTGCTGTTAGCAAATCA
ATAATCTTCACTGGTTCGAATGCCGAAATTAGGATGTGGGATCTAATAAGTTTAGAGCAA
GTCGGCTGTGTTGTCAACCACATAAATCATTTTATTCGATGTCTCGTGTTGAACCAGCCT
CAGGAAAATCTTCTATATAGTTCAGGTCATAATCAAGTTCTTGTTTGGCAGGTAAAATTG
GCTAGAAAAGTTGATTCCAAATCGGTTCATAATGTAACTTTGGATCTAATGAAGTCGATT
GAACATTCTTTCTATGGTGTTTATAGCATGGTAATTTCTGCGCGATATATAATAATTGGT
TTGTTTTTATTATTTTATTAAtcatattctctcagtctgttgaaattatttaaaatggtt
gaaatattttggacaaactacaaaatgattgttaaaacgatatgattggttaaacttggt
tggtataagaaatgcactgtgacaacaaattatactaagtattaaggttatattggaata
ttctcaatcgaaataat
>DjScaffold1603:54552-55562 strand:+
ATGGGTGCCAGATGTGTAGCAATATGCTCAGTCTTTGTGATTATATTATCAATTGTTATT
TTTTGTATTGAAACCTTGCCTCATTTTAAACATTATCGTATTGTAAACACAACATATTCC
GGTACAAATCAGTCGTCTTACAAAATTGAAGAAGACGATATACCTCAATTTTCAGAACCG
TTTTTTATTATTGAAACAGTGTGCATTATTTGGTTTACCTTTGAACTGTTAGTTCGATTT
TCTTCTTCGCCAGAAAAATTAATGTTTTTTAAAACTATAATGAATTTAATCGATATAGTC
GCTATCATTCCATATTTTATAACTCTTGGTACCGTTATTGCTGATGATAGTAGAACACAA
AATCAAGCCATGTCTCTTGCGATATTACGAGTCATTAGGTTGGTAAGAGTTTTCAGAATA
TTTAAACTTTCACGTCATTGTAAAGGTTTACAAATTCTCGGCCAGACTTTAAAAGCCAGT
GTTAGAGAACTTGGATTATTAGTCTTCTTTTTATTAATCAGTGTAATTCTATTTTCAAGT
GCTGTATACTTTGCTGAAGCTGACGCAGATAAATCGTATTTTCGAAGTATTCCTGATGCT
TTCTGGTGGGCTGTAGTTACAATGACTACTGTAGGTTACGGTGACATGAGGCCGGTAACA
GTGTGGGGAAAACTTGTTGGAAGTCTTTGTGCCATTGCTGGAGTATTGACAATAGCTTTA
CCTGTTCCTGTTATAGTCTCAAATTTCAATTATTTCTATCACAGAGAATCAGAATCAGAA
GATAAACAAACATATATTCATATAGGAAGTAATCAGAAATTTAATTCTGAACATGAAAGT
AGTGAATCAAAAAAATTCATCATCTGGAAGTTTAGATAATTGTAATATGCTCAAATACTCA
ACCGCCATCCAGCCTCTTATTGAACCAAACAAACAAGCAAATCATATTCCAAGGCAAACA
TCAGTCGAATTGTCTAAACGTTATGACAGAACTATTGAGACCGATGTGTAA
>DjScaffold285:15632-29778 strand:+
ttaaacattgttaatttccttaatttatttttaaagattttattgaaaattttttcATG

TACGCCGATAAAATTTGTTATAAAAATAATATTACTGAAGATAGAATAAAATATGAGAGA
AAAATTCATCAAAGTAAAAACAGATATTCCACTGACGAAATTCATTCAAAAAGTCCTATA
ATTATTGATATAAGAGAAGAAAAAGTTATAAATTTCGGGACTGTGGATAATGTAGACGAA
AAATTAACATTTTTGCCAAATTATTTATCTGAAAAAGAAGCTAAGGATTTGTATGATAAT
CTCGTGAAAAAATATTAATTGGAAGCAAAGACAAGGTGTCAATAAAAATGGAGAGACATAT
TTAGAACCTAGGAAAACATGCTGGTTTGGTAAACCTTACAAATATTCAGCTGTAGCTTGG
GATGAAAATGAAGAATGGAATGATCAAATCGACAAGTTGAGAAATCATCTAAATGAAAAT
CATCCTATTAGTCAAGGAAAAAATTACAATAGTGTAATGTGCAACTACTATAGGGATAAA
AATGATTCAATTGGTTGGCATGCCGATGATGAAACATCACTTGGGAGATTCCCTAAGATT
GCTTCAATTTCTTTGGGATGTGAACGAGTATTTGAAGTTTGCAAGAGAAACGAATACTAT
AACCGAAATAACTATAATTCAAAACAATCTGTCTTTGAAGGTTGTTCGGTTCTATATCGA
TTTCATTTAAACTCAGGAAGTCTTTTATTAATGGAAGGAGCTATTCAAGAGGAGTGGCTT
>DjScaffold2126:43691-44128 strand:+
ATGGTTTATCTTGGCGACATTCTGATATTTATGAAGAATAAAGATCAACACTATGACGTA
ATTGAAAGAGTATTGTTGGAAATAATGATGCCTGGTCTAAGAATTAATCCAGAAAAGTGT
GAGTTTTGTCAAAGCAAAATCAAATTTCTTGGACACATAATTAGCTGGGACGGTGTCAGA
ACTAATGCGTCTAAAATAGAAGCAATAAGAACTTTTGGCAAATCTAAATCAAACGCAAAT
TCAGACATATCCTGCCCCTTCCTTCCAGGGGTAGGGAGGTGTGCAATAACTCATCTTCCT
ACTTTGATCAAGAAAATGAAAATTCCCGATGACAAAATATATGAGACAATAGAAGAACTA
CAAAAATTATTATGTCATGCAGGAGATGAAAAGGTGTACGAAAAAATAAGAAACAAATAT
ATTGCAAAGAATTTGTAG
>DjScaffold3197:22882-25989 strand:+
TTTTATAACTTTATTAAATTATTTTATTTTTTATATGACTATTTATTACATTTTGTTACC
GGTAAATGTTCATTGTTAAGAATTATTCATTCAAAGCATTCTAAATCTGAAAAATATATT
TATCTTGAAAATAATATAGTGAATTCTGGAAGTTTAGATAAATCGAAATTGTTGGTTGAT
TTACAAGAATCTAAACTTCGAGAAAATATTTTAGATGTTATTAAAATAAAAAAAGTGAAA
TACGAATTTCATGAAGAGTTTTCCCATTATTTATTGAATTTATCCAGATTATTTGTGAAA
TATAATTCTCTAATAAGAGAAATAACCGAATTGAAAGAGAAGAAAGTCGATACAACAAAT
CAAGATCATTTAGAAATATTAATTTCGGTGTGGAACAATTTAAAACCACAATTAGAAACA
GCTGACAAGAAACCACTCGAATCAATTAACTGGTCGGAGATTGGATTCCAAGGGAAAGAT
CCTCAAACTGATTTTCGTGGAATGGGAACGTTAGGACTCATTCAACTAGAGTATTTCACC
ATAAATTCTCTCAAATCTGCTAAGGAAATGCTTGTTCAATCTGATAATATCATGCAAGGG
TTTCCTTTCGCTATAGCAGCGATCAATTTCACTGCTCTTCTCTACTCCATGCTGACAGAT
AATCGATTAAAACTGTATTTCTATTCGTTACCAGCAGATTGTGTCAATATTGACACATTT
AATAAATTGTTCGGTACTAATTTCTTTCTTATTGATTATTAAtattg
>DjScaffold1861:880-11013 strand:+
cttttatgtaatattatttattaaaaataaaATGAGTAATTCTTCGCCAAATGAACATTT
AAAAGATAAGAAGAAATCAATATTTGAAAGAATTGTCAATTTTGCTAATCACACTGTGAA
AAGTAACAAAAAATCCAGTAATAAAAAAAAGATTAAAAAGTCTGCTGATTTTATTTTAAC
TTCGCCTCCATCTAAATTGGAAAATAATTATATTGAAACATGTACATTTTGTGATTATTG
TGACAAAGAAATTTTTGCTGAATTCACAGGACAACCCTTAATTTATCATGAATCATGTAA
GAAGAATAATTTGGATATTATGAAGCAAGATAATTTCAATGAATTCAATTATTCCATGGA
GTCATCTGATCTTACATTAGTTTCAACCAATGAGACTTGCTTAGGTAATGTGAATACAAT
TAACCGTAGATTGTCAGTCAGCTTGCAAAATTTACCGATACCTTTTGCTGATGATGCTAC
ACTTGACAACAGTTCAACTATAAGCACTTTTCGATTTAATAAAAATAGATCCAATTCGAC
AGTCAGTAGAAATTCGGTTCTGACATGCAATGTTGATTCATACACGTTCAGCACCGTTGA
CTTATCGACAATCAAGCAATTCGGCATTTCTATTAAGAAATTACCGTCTTTTCGTGAGAA
GAATATACCTCCCAAATTATCAGAACTTCAAGAAAAAGAAGTTATATCAAAACCTAATAA
CAATTTGGATTATACGACTTTACCAAAGGATCTAACAGATTTGATTAAAATTTTCAATGA
TAACGACCAGGGCCTCTTTATAGAAAAAATTGATGATCATATTATGGGAAAAGTTCGTGT
TTATATCAATCTAATCAGGCCAATAAAAATGTTACTTACAGCCCGACCACCTTCTATTTT
CGAACAATATGACGATACGGATAAGGATAAAAGTACAATTCAAAGAGCATATTATAATAA
GGAAGCCATATTTTGGatctaaaattttagATGAGTAATTCTTCGCCAAATGAACATTTA
AAAGATAAGAAGAAATCAATATTTGAAAGAATTGTCAATTTTGCTAATCACACTGTGAAA
AGTAACAAAAAATCCAGTAATAAAAAAAAGATTAAAAAGTCTGCTGATTTTATTTTAACT
TCGCCTCCATCTAAATTGGAAAATAATTATATTGAAACATGTACATTTTGTGATTATTGT
GACAAAGAAATTTTTGCTGAATTCACAGGACAACCCTTAATTTATCATGAATCATGTAAG

AAGAATAATTTGGATATTATGAAGCAAGATAATTTCAATGAATTCAATTATTCCATGGAG
TCATCTGATCTTACATTAGTTTCAACCAATGAGACTTGCTTAGGTAATGTGAATACAATT
AACCGTAGATTGTCAGTCAGCTTGCAAAATTTACCGATACCTTTTGCTGATGATGCTACA
CTTGACAACAGTTCAACTATAAGCACTTTTCGATTTAATAAAAATAGATCCAATTCGACA
GTCAGTAGAAATTCGGTTCTGACATGCAATGTTGATTCATACACGTTCAGCACCGTTGAC
TTATCGACAATCAAGCAATTCGGCATTTCTATTAAGAAATTACCGTCTTTTCGTGAGAAG
AATATACCTCCCAAATTATCAGAACTTCAAGAAAAAGAAGTTATATCAAAACCTAATAAC
AATTTGGATTATACGACTTTACCAAAGGATCTAACAGATTTGATTAAAATTTTCAATGAT
AACGACCAGGGCCTCTTTATAGAAAAAATTGATGATCATATTATGGAAAAGTTCGTGTT
TATATCAATCTAATCAGGCCAATAAAAATGTTACTTACAGCCCGACCACCTTCTATTTTC
GAACAATATGACGATACGGATAAGGATAAAAGTACAATTCAAAGAGCATATTATAATAAG
GAAGCCATATTTTGG
>DjScaffold3679:15186-16431 strand:+
atttcattttgaaatttatattattgtataattttgtataaattgtgatattttaatcat
tgcagtcaccagttgtgtgaaatctgttggaaattaatttgataattattcaaaattaat
taatattgataaatgaaattattcattataaatggagaaaatgatgatgaaaaagtagaa
atttacattaatgaagattcgaaggtgggagatatgaaatataaattagacgagattctt
catggaaatggaaattttacaaaagtttatttcatattaattcataaaaatgttattta
tgggagaatgtttcgatgaaacattacaaaatatgtgaaaatgatgaaatttggtttggt
aagattgagagttatttgagaaaagatgatgttattgtttatgtgttcatttgtggaggc
atttatggtggtggtttatatttgattgtgaaaatgaatgatagaatttcaataatcaga
tcgataatagagagagaatttattctgaaataatcaatatttggaatgcagatggcaaacaa
atgagaaatgatcaaattgtcaccgagattgacacaaatgagaattacaaattgattatt
agatgtgATGATGGAGAAGGAAATGAGATCGATAATACATTTGATGAGTTGTGCTATTTG
CATGATAGAGTTTTTGGTGGATCAATATTTGTTTTATGATCAGACTCGTGATGACAACAA
AAATGAATCAGCAAGAGCAATATTGATAAATCTTATCGATCAAATCAAACAAAATATCTC
ATCAAATAACAGAATAATTTATGACAAATTGGATCAAGATTTGTCAGATTTGCCCTCCTC
ATTTATCAATTCGACAGATTTATTTTGCTTTGATTTGACAAGTCGCATTTTGATTATTTG
TAATGATTTGTGGAGATTTGTGGAGAATCAATATGATAATGATGGTTTGTGGATATTCTG
TAATTATCTCAATCATGCTATATGTGCTGCATTGAGGGATTTTTCTATAATTAGACAACA
AGACAACACTAAAACAAATAAATCAATAAGAAATATCTTAATCGATAGTTTGGATTATTT
TGTTGAATATTTGAATGATTCCAGCAATGATTGGCGATATTGGCTGAGATTATGGACTGA
TCGATATTTATTGAGATCTTTCACTGATGATGATTTGAATGAGAGGATTGATGAAATTCC
ACTCAATAAATCAACAATCAAATTAAGAATGAAAGGAAAAGATAGAAATAATTTCTTTGA
TATCAAACCAGAAACAATTATCGAAGCAAAACAAATAATTTCCCAAAGAATGTATGTTGA
TATGAAACATTTGTATTGCATCAAGCCAGGAGGAGATCACAAACTAAATGATGAAGACAA
ATTTGATGATGAATTTATCATTGAAATTGTATTGAGAATGGATGATATCGAGATATTGAG
AATGCTAAAAAAAGGAGCATTTGGTAAAACTTATCTTTGTCGACATAAAATATCCGACAA
ACAATTTGTTATGAAAATACCAATCAGAATTGATGGAGATCTCTATCGAGACATCAATAT
TTATTCAAAACTCAATTCTGCAAGAAATATCATGAATTATTCTGGATTATTCAGACACAA
AGAAAGAATTTGCATTGGCATGGAATACATGTCTGGAGGATCTCTTCGATCTTATTTGGA
CAAAGTGGAACAATTATCCGAACAAGAAATATTGAGAATCTCATATGAAATCTTGATACA
AATAAATGAATTGCATAAATTAGGAATTATTCATCGAGATATAAAACCTGATAATATTAC
TCTCAATGAACGCAATCATGCAAAATTAATTGATTTCGGATTATCAAAATATATCATTGG
ATTGACAAGTAAAGATCTATTGGATAAATTGAAAGCATATGCTCCAGGAACAATTAATTA
CATGGCACCAGAATTGGTCAAAAATGAATGTTATTCAGAAAAAATTGATATTTGGTCATT
TGGGATGGTGATGGTTGAGATGGTGACAGGTAAAAGACCATTCCACTGGATTCTTGCTGA
AGTGATGATAAGTATTCATATTCATCGTTGTACGAAATATGAATTTGAACCAATTTTTCC
AATTTCAAATGAATTGAATCAATTTATCAATTCTTGTTTGAATATGAATCCAAATGAGAG
ACCGAGTGCAGAACAATTGATCAAATCAGAATTATTTATTGATTATTTAAATTAAattaa
aatttttggaatattgtttttgtttgtttattttgagttgttatttaatgtttttcaatt
aatattggaattataagttattgatctttctaatgaaattatcaaacacttattattcac
ataatgtacttacttaaatcaactattcgatttcattttgaaatttatattattgtataa
ttttgtataaattgtgatattttaatcattgcagtcaccagttgtgtgaaatctgttgga
aattaatttgataattattcaaaattaattaatattgataaatgaaattattcattataa
atggagaaaatgatgatgaaaaagtagaaatttacattaatgaagattcgaaggtgggag
atatgaaatataaattagacgagattcttcatggaaatggaaattttacaaaagtttatt

tcatattaattcataaaaatgttattttatgggagaatgtttcgatgaaacattacaaaa
tatgtgaaaatgatgaaatttggtttggtaagattgagagttatttgagaaaagatgatg
ttattgtttatgtgttcatttgtggaggcatttatggtggtggtttatatttgattgtga
aaatgaatgatagaatttcaataatcagatcgataatagagagaatttattctgaaataa
tcaatatttggaatgcagatggcaaacaaatgagaaatgatcaaattgtcaccgagattg
acacaaatgagaattacaaattgattattagatgtgatgatggagaaggaaatgagatcg
ataatacatttgatgagttgtgctatttgcatgatagagtttttggtggatcaatatttg
ttttatgatcagactcgtgatgacaacaaaaatgaatcagcaagagcaatattgataaat
cttatcgatcaaatcaagtgattgattgatggaaatatttcacaattaatcaataattat
aataataatttattattgcagacaaaatatctcatcaaataacagaataatttatgacaa
attggatcaagatttgtcagatttgccctcctcatttatcaattcgacagatttattttg
ctttgatttgacaagtcgcattttgattatttgtaatgatttgtggagatttgtggagaa
tcaatatgataatgatggtttgtggatattctgtaattatctcaatcatgctatatgtgc
tgcattgagggatttttctataattagacaacaagacaacactaaaacaaataaatcaat
aagaaatatcttaatcgatagtttggattattttgttgaatatttgaatgattccagcaa
tgattggcgatattggctgagattatggactgatcgatatttattgagatctttcactga
tgatgatttgaatgagaggattgatgaaattccactcaataaatcaacaatcaaattaag
aATGAAAGGAAAAGATAGAAATAATTTCTTTGATATCAAACCAGAAACAATTATCGAAGC
AAAACAAATAATTTCCCAAAGAATGTATGTTGATATGAAACATTTGTATTGCATCAAGCC
AGGAGGAGATCACAAACTAAATGATGAAGACAAATTTGATGATGAATTTATCATTGAAAT
TGTATTGAGAATGGATGATATCGAGATATTGAGAATGCTAAAAAAAGGAGCATTTGGTAA
AACTTATCTTTGTCGACATAAAATATCCGACAAACAATTTGTTATGAAAATACCAATCAG
AATTGATGGAGATCTCTATCGAGACATCAATATTTATTCAAAACTCAATTCTGCAAGAAA
TATCATGAATTATTCTGGATTATTCAGACACAAAGAAAGAATTTGCATTGGCATGGAATA
CATGTCTGGAGGATCTCTTCGATCTTATTTGGACAAAGTGGAACAATTATCCGAACAAGA
AATATTGAGAATCTCATATGAAATCTTGATACAAATAAATGAATTGCATAAATTAGGAAT
TATTCATCGAGATATAAAACCTGATAATATTACTCTCAATGAACGCAATCATGCAAAATT
AATTGATTTCGGATTATCAAAATATATCATTGGATTGACAAGTAAAGATCTATTGGATAA
ATTGAAAGCATATGCTCCAGGAACAATTAATTACATGGCACCAGAATTGGTCAAAAATGA
ATGTTATTCAGAAAAAATTGATATTTGGTCATTTGGGATGGTGATGGTTGAGATGGTGAC
AGGTAAAAGACCATTCCACTGGATTCTTGCTGAAGTGATGATAAGTATTCATATTCATCG
TTGTACGAAATATGAATTTGAACCAATTTTTCCAATTTCAAATGAATTGAATCAATTTAT
CAATTCTTGTTTGAATATGAATCCAAATGAGAGACCGAGTGCAGAACAATTGATCAAATC
AGAATTATTTATTGATTATTTAAATTAAattaaaatttttggaatattgtttttgtttgt
ttattttgagttgttatttaatgtttttcaattaatattggaattataagttattgatct
ttctaatgaaattatcaaacacttattattcacataatgtacttacttaaatcaactatt
cgtctaataatattttgatgtacaaaaattatatttcattttgaaatttatattattgta
taattttgtataaattgtgatattttaatcattgcagtcaccagttgtgtgaaatctgtt
ggaaattaatttgataattattcaaagtaattaaaattaattaatattgataaatgaaat
tattcattataaatggagaaaatgatgatgaaaaagtagaaatttacattaatgaagatt
cgaaggtgggagatatgaaatataaattagacgagattcttcatggaaatggaaatttta
caaaagtttatttcatattaattcataaaaatgttattttatgggagaatgtttcgatga
aacattacaaaatatgtgaaaatgatgaaatttggtttggtaagattgagagttatttga
gaaaagagatgatgttattgtttatgtgttcatttgtggaggcatttatggtggtggttt
atatttgattgtgaaaatgaatgatagaatttcaataatcagatcgataatagagagaat
ttattctgaaataatcaatatttggaatgcagatggcaaacaaatgagaaatgatcaaat
tgtcaccgagattgacacaaatgagaattacaaattgattattagatgtgatgatggaga
aggaaatgagatcgataatacatttgatgagttgtgctatttgcatgatagagtttttgg
tggatcaatatttgttttatgatcagactcgtgatgacaacaaaaatgaatcagcaagag
caatattgataaatcttatcgatcaaatcaaacaaaatatctcatcaaataacagaataa
tttatgacaaattggatcaagatttgtcagatttgccctcctcatttatcaattcgacag
atttattttgctttgatttgacaagtcgcattttgattatttgtaatgatttgtggagat
ttgtggagaatcaatatgataatgagtgagattgagaatattgattttattaattgattt
gttctattcagatgtgctgcattgagggatttttctataattagacaacaagacaacact
aaaacaaataaatcaataagaaatatcttaatcgatagtttggattattttgttgaatat
ttgaatgattccagcaatgattggcgatattggctgagattatggactgatcgatattta
ttgagatctttcactgatgatgatttgaatgagaggattgatgaaattccactcaataaa

tcaacaatcaaattaagaATGAAAGGAAAAGATAGAAATAATTTCTTTGATATCAAACCA
GAAACAATTATCGAAGCAAAACAAATAATTTCCCAAAGAATGTATGTTGATATGAAACAT
TTGTATTGCATCAAGCCAGGAGGAGATCACAAACTAAATGATGAAGACAAATTTGATGAT
GAATTTATCATTGAAATTGTATTGAGAATGGATGATATCGAGATATTGAGAATGCTAAAA
AAAGGAGCATTTGGTAAAACTTATCTTTGTCGACATAAAATATCCGACAAACAATTTGTT
ATGAAAATACCAATCAGAATTGATGGAGATCTCTATCGAGACATCAATATTTATTCAAAA
CTCAATTCTGCAAGAAATATCATGAATTATTCTGGATTATTCAGACACAAAGAAAGAATT
TGCATTGGCATGGAATACATGTCTGGAGGATCTCTTCGATCTTATTTGGACAAAGTGGAA
CAATTATCCGAACAAGAAATATTGAGAATCTCATATGAAATCTTGATACAAATAAATGAA
TTGCATAAATTAGGAATTATTCATCGAGATATAAAACCTGATAATATTACTCTCAATGAA
CGCAATCATGCAAAATTAATTGATTTCGGATTATCAAAATATATCATTGGATTGACAAGT
AAAGATCTATTGGATAAATTGAAAGCATATGCTCCAGGAACAATTAATTACATGGCACCA
GAATTGGTCAAAAATGAATGTTATTCAGAAAAAATTGATATTTGGTCATTTGGGATGGTG
ATGGTTGAGATGGTGACAGGTAAAAGACCATTCCACTGGATTCTTGCTGAAGTGATGATA
AGTATTCATATTCATCGTTGTACGAAATATGAATTTGAACCAATTTTTCCAATTTCAAAT
GAATTGAATCAATTTATCAATTCTTGTTTGAATATGAATCCAAATGAGAGACCGAGTGCA
GAACAATTGATCAAATCAGAATTATTTATTGATTATTTAAATTAAattaaaatttttgga
atattgtttttgtttgtttattttgagttgttatttaatgtttttcaattaatattggaa
ttataagttattgatctttctaatgaaattatcaaacacttattattcacataatgtact
tacttaaatcaactattcgatttcattttgaaatttatattattgtataattttgtataa
attgtgatattttaatcattgcagtcaccagttgtgtgaaatctgttggaaattaatttg
ataattattcaaaattaattaatattgataaatgaaattattcattataaatggagaaaa
tgatgatgaaaaagtagaaatttacattaatgaagattcgaaggtgggagatatgaaata
taaattagacgagattcttcatggaaatggaaattttacaaaagtttatttcatattaat
tcataaaaatgttattttatgggagaatgtttcgatgaaacattacaaaatatgtgaaaa
tgatgaaatttggtttggtaagattgagagttatttgagaaaagatgatgttattgttta
tgtgttcatttgtggaggcatttatggtggtggtttatatttgattgtgaaaatgaatga
tagaatttcaataatcagatcgataatagagagaatttattctgaaataatcaatatttg
gaatgcagatggcaaacaaatgagaaatgatcaaattgtcaccgagattgacacaaatga
gaattacaaattgattattagatgtgatgatggagaaggaaatgagatcgataatacatt
tgatgagttgtgctatttgcatgatagagtttttggtggatcaatatttgttttatgatc
agactcgtgatgacaacaaaaatgaatcagcaagagcaatattgataaatcttatcgatc
aaatcaagtgattgattgatggaaatatttcacaattaatcaataattataataataatt
tattattgcagacaaaatatctcatcaaataacagaataatttatgacaaattggatcaa
gatttgtcagatttgccctcctcatttatcaattcgacagatttattttgctttgatttg
acaagtcgcattttgattatttgtaatgatttgtggagatttgtggagaatcaatatgat
aatgaatgtgctgcattgagggatttttctataattagacaacaagacaacactaaaaca
aataaatcaataagaaatatcttaatcgatagtttggattattttgttgaatatttgaat
gattccagcaatgattggcgatattggctgagattatggactgatcgatatttattgaga
tctttcactgatgatgatttgaatgagaggattgatgaaattccactcaataaatcaaca
atcaaattaagaATGAAAGGAAAAGATAGAAATAATTTCTTTGATATCAAACCAGAAACA
ATTATCGAAGCAAAACAAATAATTTCCCAAAGAATGTATGTTGATATGAAACATTTGTAT
TGCATCAAGCCAGGAGGAGATCACAAACTAAATGATGAAGACAAATTTGATGATGAATTT
ATCATTGAAATTGTATTGAGAATGGATGATATCGAGATATTGAGAATGCTAAAAAAAGGA
GCATTTGGTAAAACTTATCTTTGTCGACATAAAATATCCGACAAACAATTTGTTATGAAA
ATACCAATCAGAATTGATGGAGATCTCTATCGAGACATCAATATTTATTCAAAACTCAAT
TCTGCAAGAAATATCATGAATTATTCTGGATTATTCAGACACAAAGAAAGAATTTGCATT
GGCATGGAATACATGTCTGGAGGATCTCTTCGATCTTATTTGGACAAAGTGGAACAATTA
TCCGAACAAGAAATATTGAGAATCTCATATGAAATCTTGATACAAATAAATGAATTGCAT
AAATTAGGAATTATTCATCGAGATATAAAACCTGATAATATTACTCTCAATGAACGCAAT
CATGCAAAATTAATTGATTTCGGATTATCAAAATATATCATTGGATTGACAAGTAAAGAT
CTATTGGATAAATTGAAAGCATATGCTCCAGGAACAATTAATTACATGGCACCAGAATTG
GTCAAAAATGAATGTTATTCAGAAAAAATTGATATTTGGTCATTTGGGATGGTGATGGTT
GAGATGGTGACAGGTAAAAGACCATTCCACTGGATTCTTGCTGAAGTGATGATAAGTATT
CATATTCATCGTTGTACGAAATATGAATTTGAACCAATTTTTCCAATTTCAAATGAATTG
AATCAATTTATCAATTCTTGTTTGAATATGAATCCAAATGAGAGACCGAGTGCAGAACAA
TTGATCAAATCAGAATTATTTATTGATTATTTAAATTAAattaaaatttttggaatattg

tttttgtttgtttattttgagttgttatttaatgtttttcaattaatattggaattataa
gttattgatctttctaatgaaattatcaaacacttattattcacataatgtacttactta
aatcaactattcgtctaataatattttgatgtacaaaaattatatttcattttgaaattt
atattattgtataattttgtataaattgtgatattttaatcattgcagtcaccagttgtg
tgaaatctgttggaaattaatttgataattattcaaagtaattaaaattaattaatattg
ataaatgaaattattcattataaatggagaaaatgatgatgaaaaagtagaaatttacat
taatgaagattcgaaggtgggagatatgaaatataaattagacgagattcttcatggaaa
tggaaattttacaaaagtttatttcatattaattcataaaaatgttattttatgggagaa
tgtttcgatgaaacattacaaaatatgtgaaaatgatgaaatttggtttggtaagattga
gagttatttgagaaaagatgatgttattgtttatgtgttcatttgtggaggcatttatgg
tggtggtttatatttgattgtgaaaatgaatgatagaatttcaataatcagatcgataat
agagagaatttattctgaaataatcaatatttggaatgcagatggcaaacaaatgagaaa
tgatcaaattgtcaccgagattgacacaaatgagaattacaaattgattattaggttaat
ttgaatattgattaattgattaattgaatttgtgaatagatgtgatgatggagaaggaaa
tgagatcgataatacatttgatgagttgtgctatttgcatgatagagtttttggtggatc
aatatttgttttatgatcagactcgtgatgacaacaaaaatgaatcagcaagagcaatat
tgataaatcttatcgatcaaatcaagtgattgattgatggaaatatttcacaattaatca
ataattataataataatttattattgcagacaaaatatctcatcaaataacagaataatt
tatgacaaattggatcaagatttatgtgctgcattgagggatttttctataattagacaa
caagacaacactaaaacaaataaatcaataagaaatatcttaatcgatagtttggattat
tttgttgaatatttgaatgattccagcaatgattggcgatattggctgagattatggact
gatcgatatttattgagatctttcactgatgatgatttgaATGAGAGGATTGATGAATCC
ACTCAATAAATCAACAATCAAATTAAGAATGAAAGGAAAAGATAGAAATAATTTCTTTGA
TATCAAACCAGAAACAATTATCGAAGCAAAACAAATAATTTCCCAAAGAATGTATGTTGA
TATGAAACATTTGTATTGCATCAAGCCAGGAGGAGATCACAAACTAAATGATGAAGACAA
ATTTGATGATGAATTTATCATTGAAATTGTATTGAGAATGGATGATATCGAGATATTGAG
AATGCTAAAAAAAGGAGCATTTGGTAAAACTTATCTTTGTCGACATAAAATATCCGACAA
ACAATTTGTTATGAAAATACCAATCAGAATTGATGGAGATCTCTATCGAGACATCAATAT
TTATTCAAAACTCAATTCTGCAAGAAATATCATGAATTATTCTGGATTATTCAGACACAA
AGAAAGAATTTGCATTGGCATGGAATACATGTCTGGAGGATCTCTTCGATCTTATTTGGA
CAAAGTGGAACAATTATCCGAACAAGAAATATTGAGAATCTCATATGAAATCTTGATACA
AATAAATGAATTGCATAAATTAGGAATTATTCATCGAGATATAAAACCTGATAATATTAC
TCTCAATGAACGCAATCATGCAAAATTAATTGATTTCGGATTATCAAAATATATCATTGG
ATTGACAAGTAAAGATCTATTGGATAAATTGAAAGCATATGCTCCAGGAACAATTAATTA
CATGGCACCAGAATTGGTCAAAAATGAATGTTATTCAGAAAAAATTGATATTTGGTCATT
TGGGATGGTGATGGTTGAGATGGTGACAGGTAAAAGACCATTCCACTGGATTCTTGCTGA
AGTGATGATAAGTATTCATATTCATCGTTGTACGAAATATGAATTTGAACCAATTTTTCC
AATTTCAAATGAATTGAATCAATTTATCAATTCTTGTTTGAATATGAATCCAAATGAGAG
ACCGAGTGCAGAACAATTGATCAAATCAGAATTATTTATTGATTATTTAAATTAAattaa
aatttttggaatattgtttttgtttgtttattttgagttgttatttaatgtttttcaatt
aatattggaattataagttattgatctttctaatgaaattatcaaacacttattattcac
ataatgtacttacttaaatcaactattcgtctaataatattttgatgtacaaaaattata
tttcattttgaaatttatattattgtataattttgtataaattgtgatattttaatcatt
gcagtcaccagttgtgtgaaatctgttggaaattaatttgataattattcaaagtaatta
aaattaattaatattgataaatgaaattattcattataaatggagaaaatgatgatgaaa
aagtagaaatttacattaatgaagattcgaaggtgggagatatgaaatataaattagacg
agattcttcatggaaatggaaattttacaaaagtttatttcatattaattcataaaaatg
ttattttatgggagaatgtttcgatgaaacattacaaaatatgtgaaaatgatgaaattt
ggtttggtaagattgagagttatttgagaaaagagatgatgttattgtttatgtgttcat
ttgtggaggcatttatggtggtggtttatatttgattgtgaaaatgaatgatagaatttc
aataatcagatcgataatagagagaatttattctgaaataatcaatatttggaatgcaga
tggcaaacaaatgagaaatgatcaaattgtcaccgagattgacacaaatgagaattacaa
attgattattagatgtgATGATGGAGAAGGAAATGAGATCGATAATACATTTGATGAGTT
GTGCTATTTGCATGATAGAGTTTTTGGTGGATCAATATTTGTTTTATGATCAGACTCGTG
ATGACAACAAAAATGAATCAGCAAGAGCAATATTGATAAATCTTATCGATCAAATCAAAC
AAAATATCTCATCAAATAACAGAATAATTTATGACAAATTGGATCAAGATTTGTCAGATT
TGCCCTCCTCATTTATCAATTCGACAGATTTATTTTGCTTTGATTTGACAAGTCGCATTT

TGATTATTTGTAATGATTTGTGGAGATTTGTGGAGAATCAATATGATAATGATGGTTTGT
GGATATTCTGTAATTATCTCAATCATGCTATATGTGCTGCATTGAGGGATTTTTCTATAA
TTAGACAACAAGACAACACTAAAACAAATAAATCAATAAGAAATATCTTAATCGATAGTT
TGGATTATTTTGTTGAATATTTGAATGATTCCAGCAATGATTGGCGATATTGGCTGAGAT
TATGGACTGATCGATATTTATTGAGATCTTTCACTGATGATGATTTGAATGAGAGGATTG
ATGAAATTCCACTCAATAAATCAACAATCAAATTAAGAATGAAAGGAAAAGATAGAAATA
ATTTCTTTGATATCAAACCAGAAACAATTATCGAAGCAAAACAAATAATTTCCCAAAGAA
TGTATGTTGATATGAAACATTTGTATTGCATCAAGCCAGGAGGAGATCACAAACTAAATG
ATGAAGACAAATTTGATGATGAATTTATCATTGAAATTGTATTGAGGTGAgtttgttatg
ttgcattgtgatatttctatggatttaatagagttctcgtgaaagatttataatatttat
cgataatttctactcaattaattcacatgtttgctacaaaatattgattatatttctctt
atattcagacatattttagttgttatcaaattatttttacacaatatgaatatcttcctc
agtttcaatgaaattatcttcatcaacaagatcgttttggtagataaactttgtcactgt
caaatgtaatgcatagtttaaccataataccgatttgacatttgatattgattattttca
attatcgattaattatattaatattatttaattgaaaaaatattgattttattaattgat
ttgttctattcagatgtgctgcattgagggatttttctataattagacaacaagacaaca
ctaaaacaaataaatcaataagaaatatcttaatcgatagtttggattattttgttgaat
atttgaatgattccagcaatgattggcgatattggctgagattatggactgatcgatatt
tattgagatctttcactgatgatgatttgaatgagaggattgatgaaattccactcaata
aatcaacaatcaaattaagaATGAAAGGAAAAGATAGAAATAATTTCTTTGATATCAAAC
CAGAAACAATTATCGAAGCAAAACAAATAATTTCCCAAAGAATGTATGTTGATATGAAAC
ATTTGTATTGCATCAAGCCAGGAGGAGATCACAAACTAAATGATGAAGACAAATTTGATG
ATGAATTTATCATTGAAATTGTATTGAGAATGGATGATATCGAGATATTGAGAATGCTAA
AAAAAGGAGCATTTGGTAAAACTTATCTTTGTCGACATAAAATATCCGACAAACAATTTG
TTATGAAAATACCAATCAGAATTGATGGAGATCTCTATCGAGACATCAATATTTATTCAA
AACTCAATTCTGCAAGAAATATCATGAATTATTCTGGATTATTCAGACACAAAGAAAGAA
TTTGCATTGGCATGGAATACATGTCTGGAGGATCTCTTCGATCTTATTTGGACAAAGTGG
AACAATTATCCGAACAAGAAATATTGAGAATCTCATATGAAATCTTGATACAAATAAATG
AATTGCATAAATTAGGAATTATTCATCGAGATATAAAACCTGATAATATTACTCTCAATG
AACGCAATCATGCAAAATTAATTGATTTCGGATTATCAAAATATATCATTGGATTGACAA
GTAAAGATCTATTGGATAAATTGAAAGCATATGCTCCAGGAACAATTAATTACATGGCAC
CAGAATTGGTCAAAAATGAATGTTATTCAGAAAAAATTGATATTTGGTCATTTGGGATGG
TGATGGTTGAGATGGTGACAGGTAAAAGACCATTCCACTGGATTCTTGCTGAAGTGATGA
TAAGTATTCATATTCATCGTTGTACGAAATATGAATTTGAACCAATTTTTCCAATTTCAA
ATGAATTGAATCAATTTATCAATTCTTGTTTGAATATGAATCCAAATGAGAGACCGAGTG
CAGAACAATTGATCAAATCAGAATTATTTATTGATTATTTAAATTAAattaaaatttttg
gaatattgtttttgtttgtttattttgagttgttatttaatgtttttcaattaatattgg
aattataagttattgatctttctaatgaaattatcaaacacttattattcacataatgta
cttacttaaatcaactattcgagattaatgtgataagcaaatgtaattttacattcagaA
TGGATGATATCGAGATATTGAGAATGCTAAAAAAAGGAGCATTTGGTAAAACTTATCTTT
GTCGACATAAAATATCCGACAAACAATTTGTTATGAAAATACCAATCAGAATTGATGGAG
ATCTCTATCGAGACATCAATATTTATTCAAAACTCAATTCTGCAAGAAATATCATGAATT
ATTCTGGATTATTCAGACACAAAGAAAGAATTTGCATTGGCATGGAATACATGTCTGGAG
GATCTCTTCGATCTTATTTGGACAAAGTGGAACAATTATCCGAACAAGAAATATTGAGAA
TCTCATATGAAATCTTGATACAAATAAATGAATTGCATAAATTAGGAATTATTCATCGAG
ATATAAAACCTGATAATATTACTCTCAATGAACGCAATCATGCAAAATTAATTGATTTCG
GATTATCAAAATATATCATTGGATTGACAAGTAAAGATCTATTGGATAAATTGAAAGCAT
ATGCTCCAGGAACAATTAATTACATGGCACCAGAATTGGTCAAAAATGAATGTTATTCAG
AAAAAATTGATATTTGGTCATTTGGGATGGTGATGGTTGAGATGGTGACAGGTAAAAGAC
CATTCCACTGGATTCTTGCTGAAGTGATGATAAGTATTCATATTCATCGTTGTACGAAAT
ATGAATTTGAACCAATTTTTCCAATTTCAAATGAATTGAATCAATTTATCAATTCTTGTT
TGAATATGAATCCAAATGAGAGACCGAGTGCAGAACAATTGATCAAATCAGAATTATTTA
TTGATTATTTAAATTAAattaaaatttttggaatattgtttttgtttgtttattttgagt
tgttatttaatgtttttcaattaatattggaattataagttattgatctttctaatgaaa
ttatcaaacacttattattcacataatgtacttacttaaatcaactattcg
>DjScaffold3077:6733-7731 strand:+
AACATATCAAATTCTACCCTAGAAATTTCACTGGATAGAAAACTGCTTAATTTAGATAAT

CTCAAAAAATTCAATTCTTACAATGTAGTTAAAGCAATAAAATATGAAAAAATTATTAAC
AGTGAATTAGAAAATATTTCACTACCGAATTCATCAAATACTGATACATCTTCAGATAAT
CATTTAACTGAGTCAACTGAATTAGAACAGGACTTGAATTATGAGGAAAAGAGTTGTGAT
TCGTTATCTGAATTTGAAAATGAGTCAGATTCAAATGATAAAAATAAAATTGTAAATAAT
TTAGAATCAATCGGAATTCCATGTACGTTTAATGGTTGCTCAAAAGTATTTAAAGATAAG
TCAAGAATGAGAAAGCATTTGCATACACATAAAACTAAAAGTCACATTTGTGAAGTTTGT
AAAAAGGGATTTGTTGAAAGTAGCAAACTCAAAAGGCATTATTTGGTTCATTCAGGCAGA
AAACCAATAAAATGCCAATTTGAGGGCTGTTCTAAAACGTTCTCATTAATTCACAATATG
AAGACTCATCTCAGAATACATACTGGCGAAAGACCATTTCATTGTCCGTATCCAAACTGC
AACAAATCATTTGCTCAATCAACG
>DjScaffold3077:8258-13745 strand:+
tatgataaaacttttagttttagattatagaaaattatttaattccaaattttacaagtt
ATGAATATATCAGACTTTTTTAATTTTAATGATCAATTTTTGGATGATGACAGTGAAGAT
ATTGTATTGGATCCTTTGGAAAATCCGTTAATGAATTTAGATACTTTTCAAATTCCAAAC
GATTCACAAGATGAAAATTGTCATGATTATGTTGATGAAGTTGTCGACGAAGATACCAAT
AACGTTTTCCTCTGTGAAGGTATCGATGTTTGTCGAGAGGAAATTATTGAGAATTCAGCC
ACTACTGGTAATGCAATAAACAATAATGATATGAATGATTACAATTTAAAACATGGGATT
GAGTATTTTTCTGAATGCAAAGATTTTCCTGTTTATAATTACAATGACGATTATTCGATA
AGCAACAATGTAAAATCACGGAAAAAAATCGGATCCGCTAATGACTCTCTGATCAAATCA
AGTGTAATCAAGTCAGAGTCTTCACTATCTAACTGTGGAGTGGATATAATTAATTTCCCC
TTTTCTAAAAACACACGTTATTTTCGTAATTATCCAAATGAAGTTTCGAAAATCATTTTT
AAGACCGAGCCAAATTCAGCTGTATTCAGACCTGTTATATCAGATCAAAGATCAAATGGA
TTGCTTAATTTAAAGAAAGAACGCAGATTAATGTCTTTTGATCATGGACATGAGTTCACT
AAAGAAGATGTTCTAGAAGATGATGATGATGAATTACCGGATGATCCAGTGGCGCCAGCT
GCACCACAACATCGTCGTCGACGAAGAACAGTCGACTCAATGCAATATGGAACAAGCAGC
TCTATGCGTGCGGTTCCTTGCCCTCACAAAGGCTGTAGTAAAGCATTCAGGGATAATTCA
TCAATGCGTAAACATCTACATACTCATGGTCCTCGAGTTCACGAATGTAGTGAATGTGGA
AAAGCATTTGTCGAAAGCAGTAAACTGAAACGCCACAGATTGGTCCATACCGGAGAAAAA
CCATTTCAATGCTCATTCGAAGGATGTGGCAAACGTTTTTCATTAGATTTCAATTTGCGC
ACGCATATCCGTATCCATACCGGTGACCGACCCTACACATGTCCCTATGCCGGTTGTGGC
AAGGGCTTTGCCCAATCAACTAACCTCAAGTCACACATTCAGACACATAACAAAAACAAA
TACAAAACTCTTAAAACGTTTTGTTCACCAAATAAAATCTCCAACAATTCCAGCAACAAC
AACAACAACAATTGTCGTAATTCTAATGTCGTAATTGATGAACACACTCCAGTCGGTAGC
GCCACTACTCAATTGCGTCGACTCAATTCATCTCAAGGCTCATCGTCGAGCTCCTCATCT
CGAGAATTACGACTGCAAAAACGCGCATCTGTCGATGTCGAAGGGAATTGTCTCGATTCG
TCTCCTGGAATTTTATTACATCACCAAAGTAGCACTGTAGGAGGAGGAGGAGGAGGGAAC
AGTGTCCGGCGGAGGAGGAGGAGTAAGTCTTCCAATCGACGAAGCCGAAGATAAttcatc
atttgaattttgataatttctcatcttcttcttctccttgttctcctagttctccttgtt
cgtgatctttgatatcaaaatattgctacttacaacaacaacaagctgttcagactgatt
tttactcattcaactgtcgttttagtgtttattattgcaagagtctgatgatggtgattc
cattgatattcattcattcgttcgttcgttcaatcaatcaatcaatcgatatgtttctct
ctgtctcatcatgcgcgccatgtaaatacccattctctctctctatctctctctgcagct
actactgctacctgaggagattcgtgtacatagatatatctatttattaattatcatcac
tgcgccccctccccccctccttctccccgtctgcctcttgttattccttactaatatttca
tttcgagtgcgcatttctcactgtaaatcatctacatttctcctatcggtacaatctcgt
aatatccaaaaatcgtaagttccctatgtgttctcctttttattattgttgttccccccgg
ccccgcaccgccccactcatgtttttgtatttgagagaaattttttgcatttagttatga
ttttgcgcaacattttttacatttaaatcgtttttttctgtttgtgtgtgttgattctgt
aaatttattggtcgatgatgatgattggatgattgaatgatgatgattggatgattttaa
aaattgtgttctagttatgatttttattacaattttaatgtgtttcgtttttgttttctt
ttttttctatcacttgatgtatttataattattattattatttattatcattatttataa
ttatcattatttataattattattatttattattattataattattattatcaatttaca
aattacaattaatgtattcgttttgttactatttgttattgtgatttattttcaaattca
attcaattaaatgataacctgtgtatgtgtgtgtgtggatcttcaacaattcggtcggta
actcagttctatgtgcgtctattaaattc
>DjScaffold3261:44852-69729 strand:+
ttaatctttatcgttattttcATGAAAAACGTTTCTGGAATTTATTCAAATTGTTTATCT

ATTAATACAACATCTCCAAAATTTTCAACACCAGTTGATACCGATGTAGAAATGATTGAA
GGAAACAGCCAAGGATTAACTTGTTGTATTTTTGGGGTTACTAGAATTTTGTGGTATTTC
AAACGTAGATACTCAAATGAAGCTTGGAAAATTAAAAGCGAAGTTATTGAAAATCAATTT
TACTGGTTTAAAGATTTGAATAGGACCTTGTATTTTTATCAGCCGAAATTTTATGATACT
GGAAATTACAAATGTATAGGAGAAGACAGCAAAAGTAATAATATTAGTGCAGAAATCGAC
TTATTCGTCGGAAATGGCTTAAAAGATTTCAAATTAATTGGTTATAGTACTTCTCCAAAG
GTGTACTTTAATACCAATACTGAGCACACAATTTATTGTTTAGTAAAAGCTCACAAGAAA
TTGTTCTGCATATTTTCATGGATTCGTGAAGAGAAAAATAAAAATAACTCCATAATAATG
GATTCTAATTTAACCACTTCTAGAACAAATAGTTCTCTTATTAGCGGTTATAATGGATAC
ACAACGTTGATTTGGAAGCCGATTGAAGATAACAACAGAGTTTATAATTTTTCTTTGAAT
ATACGAAATTTGAAAGAATCACAGGAAGGCTATTATAGTTGTAGAATCTATGAAAGTACG
AAGAAATCTGATATTACTTATTCATTTAATTTTTTGCAGATCAGAATAAATCCGGTATAT
TTGGCAATTGGATTAATAATCGGTTTATTAATTGTATTTCTAATCATTTACGTGTTTAAA
TATGACATAAAAATTATTTATCTCCAGATTTTTCGCAAGTTTCTTAAAGAAAAAAATGGA
AAATTCAAGTATTTCAAAAGTATTCGTAGATGTTTTAGATGAaataaaacatttaaaatc
aattaatgaagaagtaatgcttaagtaattttataaattactaaattttacaattcattt
tttacaaaggttaaattttctatttccaaataatctgaaagaagctttacatatagttga
ccaaaattttataaaattttctcgacaaacaacggtagaaatctttatagagtttatga
tgacaatactaatctttattcttctgttttattggacggaaatttctgtaattgtgcttc
gtataaaaatgaagtagttaaaactcaaactaatttcatggttgtttttattcattattt
cgataattaatttacaaagaaacatttcagtgttgtcatcagatttctgttaatattgcc
gttgcactctcagctgtttcttttagtgaaaatattagtgatgaaataatgactaacatg
attgaaatgagttttctttaatacaaatataattaataaatttatattaatctttatcgt
tattttcATGAAAAACGTTTCTGGAATTTATTCAAATTGTTTATCTATTAATACAACATC
TCCAAAATTTTCAACACCAGTTGATACCGATGTAGAAATGATTGAAGGAAACAGCCAAGG
ATTAACTTGTTGTATTTTTGGGGTTACTAGAATTTTGTGGTATTTCAAACGTAGATACTC
AAATGAAGCTTGGAAAATTAAAAGCGAAGTTATTGAAAATCAATTTTACTGGTTTAAAGA
TTTGAATAGGACCTTGTATTTTTATCAGCCGAAATTTTATGATACTGGAAATTACAAATG
TATAGGAGAAGACAGCAAAAGTAATAATATTAGTGCAGAAATCGACTTATTCGTCGGAAA
TGGCTTAAAAGATTTCAAATTAATTGGTTATAGTACTTCTCCAAAGGTGTACTTTAATAC
CAATACTGAGCACACAATTTATTGTTTAGTAAAAGCTCACAAGAAATTGTTCTGCATATT
TTCATGGATTCGTGAAGAGAAAAATAAAAATAACTCCATAATAATGGATTCTAATTTAAC
CACTTCTAGAACAAATAGTTCTCTTATTAGCGGTTATAATGGATACACAACGTTGATTTG
GAAGCCGATTGAAGATAACAACAGAGTTTATAATTTTTCTTTGAATATACGAAATTTGAA
AGAATCACAGGAAGGCTATTATAGTTGTAGAATCTATGAAAGTACGAAGAAATCTGATAT
TACTTATTCATTTAATTTTTTGCAGATCAGAATAAATCCGGTATATTTGGCAATTGGATT
AATAATCGGTTTATTAATTGTATTTCTAATCATTTACGTGTTTAAATATGACATAAAAAT
TATTTATCTCCAGATTTTTCGCAAGTTTCTTAAAGAAAGTAAGTTCTTTATTTTATAGaa
ctttattttagaattttaatgttaaaattttatgcatacaataataaaatttcaattaaa
aaaaacttttaacgtagtttattgttgtaaaaagctaataatatcaataaaacagtaact
acacttttctctgagatcgatttgtaaatcaaatagactttacacgaagatccaaaagta
aataacttaattttaaaataaaattaataaataactcttttaattaatgattgtaaccat
taatctgtaataattaaaaaaatatttttaatttctgcagcaaataacggagaatatatt
ttaaaaaattaatgttataattagcaaccaatggaaattaattttaatcggattaattta
aattgtgtttgtatttaa
>DjScaffold3696:57037-57687 strand:+
CTAAAACATGAAATAAATTTGCATCCAAAGTATTTGGGTCCTAATATTATTGAAACTATA
AAAGCTAAATTATTATCTGAAGTTGAAGGCATTTGTACTCCAAATTATGGTTTCATAATT
GCTGTAACATCAATTGATAAAATTGGAGATGGTGTTGTTCAACAAGGTCGTGGATTTACT
TTGATTCCAATTGAATATAAAGCAATTGTATTCCGACCATTTGTTGGTGAAGTTCTTGAT
GCCGTTGTATTAAGTGTTACTAAAATTGGGATTTTTGCCATGGCTGGTGCATTTCAGATA
TTTATTTCAGTTTATGCTGTTCCCCAAAATTTCCAATATGATGGTGACACAGTACCTCCT
TGTTATAAAGCTGACGAAGAGGAACAAGTTATTCAAGAAGACGATGAAATCCGGGTTCGA
ATAGTCGGTTTGCGACCGGATGCTTGCGGCATTCTGGGAGTTGGAACATTAGCTGAGGAT
TATCTTGGGCTGATTAATTAAt
>DjScaffold91:54958-55546 strand:+
atagaagccatgaaaaaatagaatacgtaattatcggaaaacgcatagtttacacatggg

attatagatcgtccgataggtcaatgactatttttctgagcttcttgggtggaggagctt
ctgatgatgttgaaggttgagggtggaggcgatcactgagttgtttgttttcggtgcgca
accactcaacttctgcttccaactcccagattctcataccttgggcagtgtcccgtcgat
tgaattcgacattctgccatcggagttgcgacatttcgaagaccaacATGCCAATCTTCT
CCTGTAAGGCTTGGCATGCTCTCGACCAATTCGTAGAAGTGGCTAGCAAACCATCAAAAC
TGGCGGCTAGTTGGACTTCTTGCCCGAAGGTCCTACTTGGACGGAGTTGCATTAAATTCT
GGTTGGCAACGTTCTGGGGAGAATTCTCATCCATTTCTAAATACTCCCAGATTAATTCAT
GCTTAATCTGTAGTTCATTATGCGTTAACGAACTGTTTAGCGACGGGGTGATTTAAtgct
tgagaagtta
>DjScaffold91:115210-125864 strand:+
GAAAAAACAATTATCTATGTCCCCTCAAGACATTTAGGCTATATAATTGGAAAAGGAGGA
TCACGAATTAAGGAACTTTCAGAAGAAACTGGTTGTCGAATAAATATAAAAAAGGATCAG
GTTGAAAATGATGAAGTGCCCATATCTTTAGAAGGTTCTACAAGAAATATCGATAAAGCT
AAATCTAAGATTGACGAAATTACTAAGCGACAAAATAAGCGCCACATTGATGATAATAAT
GACCGTCGTTATAATAACAATTCCAATGAAAAATCGTACTCATCAAATAATTTTTCAAAT
AAAAGGTCATCAAAGTATGGGAACGATGATGAAGATGATGATGGTGATAGTTTTTCGCGG
AAAAGAAAATCACCAAATGATTATTGGAACGACAAAGAACGAGATAATTCAAAAGATTAT
TATAGTAATTCGAACGATAATGGAAATAGTCGTAATTACAACAATAATAATAACAGCAGA
GATTATAATAATAGCTCTAAATATAATGGAAATTCTAGAGATTATGATAATTCTAGAGAC
AATGATTACTCACATGGATCTCATAATTCACGTAATAGTAATAATAATAATTCTAAATAC
AATGGAGACGAGTGGGGTGGATGGGGAAAGAAAAATGATGATACTTCAAATTTAGATTCA
TTAATATTCGACAGCAATGCACCTTTAAAAGGCGCTTCAAAATTTAATGACCCGCCATCT
GAAAGTTATGTTCCGGATGATGATTGGGGGATTCCAACATATTTGGAAGAAGAGAAACCA
ATAAGAAAGAAACCAGAAAATGCATATCTGAAAATGAAAAGTACGAAAGACAGTAATTTC
TATCCAGTTCATAGAAATTTCTACCGTGAACATCCAAACGTGTCAAAAATGTCTACTGAA
GAAAAAGATCAATTTTTATTAGAGAAAAATAATATCATGATAAAAGAAATTGAATCTGAA
ACTGGAGAATCGTTTTGCAATTTTGGAAATACAACTTTCGAAAAAGATCCCGAAGAGATT
ATTGAAGAAATTCCTATTCCAAAAATTATTCCGAATCCAGTTCGAAACTTTGAAGAGGCA
TTTTTTGATCATCCAGATATATTAAGTGAAATTAAAAAACAAAAGTTCCAGGAGCCATCT
CCGATTCAGTGCATATCATGGCCAATTTTAATGACAGGTCGTGATTTTATTGGGATTGCT
CAAACTGGAACTGGAAAAACCTTAGCGTTTTTATTGCCGGCTTTTATTCATATAGAAGGA
CAACCAAAACCAAGAGAAAAACGACAAGGTCCAACAGTTTTAATATTAAGTCCAACTAGA
GAACTGGCTATTCAAATTCAAAATGAAGTTCAAAAGTATACTTATCATGATATTGATTCT
GTTGTATGTTATGGTGGCGCTGATAAGCGCGATCAAATTTATCAATTGAAAGAAAATCCA
GAGATTGTTGTTGGGACACCTGGTCGTATCCAAGATTTGAAAGATTCTGGAGTATTAAAC
TTAAAAGATGTCAGTTATTTGGTTCTCGATGAAGCCGATAGAATGCTTGATATGGGTTTT
GAACTACAAGTTGTTAATCTGATTTATGCCGTCAGACGACAAAGACAAACAGTTATGACA
AGTGCAACATGGCCATTTGGAGTTCAACGAATTGCGCTAACTCACATGAAGAATCCTATT
CAAGTTAATGTGGGAACATTAGATCTAAATGCTTGTCACACTGTTAGACAGCACGTTGAA
TATGTCGAATATCACGATAAAACTCAGCGATTGGTTGAAATATTATCAGAAATTAAACCT
GAAGATAAAATTATCATTTTTGTTGCAAGAAAAGCACAGTGCAGCGATTTGGCGTTTGAG
TTGTTTTTGAAAAGATCAAAGGGTTTATTAGGACCAAAAAGTGCTGTAAGAGCTTGTGAA
ATTCACGGCGATCTTGATCAACAGGAAAGAGAAATGGCATTGAAAATGATGAGATCGGGA
GCTAGAAATATTATGATTGCAACAGATGTAGCTTCTCGAGGTCTCGATATTGTCGATCTG
ACTAAAGTCATAAATTATGATTTTCCAAAAAATATTGAGGAATATGTTCATCGTGTAGGT
CGAACTGGCAGAGCTGGTCGAACTGGTGAGGCTTTCACATTCATGAATAAAATCGATTGG
AGTAATGCAGCGGAATTGATCAGAATTCTCGAAGACGCAGATCAAGAAGTTCCAGATTTT
TTACCACAATTAATGAAACAACACCAGGAATTTCGAATCAGTGGTGGTAGTCGAGGTGGA
GGACGATTTGGTGGCCGAGGAGGCGGTCGAGGTAGAGGAGGAGGAGGAGGAGGCGGAGGA
TTTCGTGATGGCGATCGAGATCGCGATGGAGGTAATAGAAGAAATTCAAATTTCAACCGG
GGACACAGATCTGATGATACGGATGATGTTCAATCTGATTCCAGTTGGGCGGATTTCTCG
AATTCATCAAACACTTTAAGTATAAGTAAAAAATTCGGTGCATGTTATATACAAGATGAT
ATCAGTGATGAGGAAAATTGGGGAAATTGGAATAAAGTAGACGATACCGATAAAGAAAAA
TGTAAAGAAATAATCGAAGCTAAAATAATTGAATCTCCTTCAAAACCCATTGAAACACAA
TCTGTAGAAAAATCGATTATTCGCCATGCAACAAAACCATATATTTGGGAAACAAAGAC
CGAAATATATATCCAATTTATCGAAATTTTTACGAAGAACATCCCGATATTACGAATTTA
TCTTTTGATGAAGTCGAGTCGTTTTTGTTGCTAAGAAATAATATAATGATTCAAGAATTG

AAAACTTATCCTACACATTGGGATCAAAATGATGACGACATTAATGAAGATCAAGTTGAA
ATTTTGCCTGGAATTCCAGGTATTGCCAAACCCATAAGAACATTTGAAGAAATGTTTTGG
AAACATCCGGAAATTTTAGACATAATTAAATCCTTAAAATTTTCAAGTCCGTCGCCAATT
CAATGTATCGGGTGGCCTGTTATATTGAGTGGTCGTGATTTTATTGGCATTGCTCAGACG
GGTACTGGTAAAACACTGACTTTCGTTTTACCAGCTTTGATACATATCGAAGGACAAGTA
AAACCCAGGCAATATAGACGTGGTCCTACTATCCTTATTCTCAGCCCAACAAGAGAGTTA
GCTATGCAAATTGAAAATGAAATCTTGAAAATACATTATCATGAGATTAAATCTGTTTGT
TGTTACGGAGGAGCTGAAAAAACTTATCAGATTAATGCTCTTAGAGATAATCCAGAAATC
GTCGTTGGTACTCCTGGTCGCATAAATGATCTTTTATTGATGGGATGCTTGTGTTTAAAA
GATGTCAGCTATTTGGTACTTGATGAAGCTGATCGAATGTTAGATATGGGGTTTGAACCT
CAGGTAGTTAGTATTATTAATGAAACAAGAAGACAAAGGCAAAATATAATGACCAGTGCT
ACGTGGCCTGAAAGTGTTAGGAATATAGCTTTATTGTATATGGATAATCCGATACAAGTC
AATGTTGGCTCATTGGATCTTATTGCTTGTAATGACGTTGTCCAAGAAATTAAATTTGTT
AGTAATTTTGAAAAGACAAATGCATTGGTCGACATTATTTGTAACATGAATGAGGACGAT
AAAATAATTGTATTCGTTACTCGAAAGGTTCAATGTAGATCATTGACCAAATATTTGGAT
AAGCTAATACGATCAGATAAATTGGGCCCTTATGCAAATATTTGTGAAATTCATGGTGAT
CTGAGCCAAAGTGAACGAGAGCAATCATTGTCTTGGTTTCGTAATGGAGAATGTACAATT
TTAATTGCCACAAATGTAGCATCAAGGGGTCTCGATATTGCGGATATCACATGTGTTGTA
AATTACGATTTTCCTAACGATATTGAGGAATATGTACATAGAGTTGGTCGAACTGGAAGA
GCAGGAAAAAATTGGAAGATCAATAACGTTCATAAACGAGGCAGATTGCAAGCATACATAT
GAGTTAAGAAATATTTTAATTCAATCAAATCAAGATGTACCATCTTGGCTTTTGAATTTG
CGACAAATGAAATACACCGAGCAAGAGATTTCCAAGAAAAATAATCCAATAAATCCCGTT
TCTAAAAGTCATAAAAAAGAAAGGATCAATAATGCATTTTCATCTACCGATTATTTTGAT
GTGAATTACTCATCTGATGATTACGATGATTATTTCGATGAATATCAATACGAGCGTGAT
CCGAAAATGAATGATAAAGTAACTTGTGATGATGACGAAGAAGATGTTCCTTTTTCAGAA
CTGGAAACAGTAGAAAATAAGAAAAAAGAATCGATTAATAACAACGGAGTTGTAGAAAAA
GTTATTGAATTTCATAGTGATTGTTTGAACGAATTGTATGAAAATGAAGAAGAGGGTTTT
TTTGATGAATATCAAAGGAATCTCGATCTCGAATGTCCAATAAAATGTCCAATTACAGAA
TCTTTGCCTGTAGATAATATTTGTGAATATGATTATGAGTATGAATCTGACAATGATAAT
GAGGGTTGGTGGTGAtgatgatgAAAACAATTATCTATGTCCCCTCAAGACATTTAGGCT
ATATAATTGGAAAAGGAGGATCACGAATTAAGGAACTTTCAGAAGAAACTGGTTGTCGAA
TAAATATAAAAAAGGATCAGGTTGAAAATGATGAAGTGCCCATATCTTTAGAAGGTTCTA
CAAGAAATATCGATAAAGCTAAATCTAAGATTGACGAAATTACTAAGCGACAAAATAAGC
GCCACATTGATGATAATAATGACCGTCGTTATAATAACAATTCCAATGAAAAATCGTACT
CATCAAATAATTTTTCAAATAAAAGGTCATCAAAGTATGGGAACGATGATGAAGATGATG
ATGGTGATAGTTTTTCGCGGAAAAGAAAATCACCAAATGATTATTGGAACGACAAAGAAC
GAGATAATTCAAAAGATTATTATAGTAATTCGAACGATAATGGAAATAGTCGTAATTACA
ACAATAATAATAACAGCAGAGATTATAATAATAGCTCTAAATATAATGGAAATTCTAGAG
ATTATGATAATTCTAGAGACAATGATTACTCACATGGATCTCATAATTCACGTAATAGTA
ATAATAATAATTCTAAATACAATGGAGACGAGTGGGGTGGATGGGGAAAGAAAAATGATG
ATACTTCAAATTTAGATTCATTAATATTCGACAGCAATGCACCTTTAAAAGGCGCTTCAA
AATTTAATGACCCGCCATCTGAAAGTTATGTTCCGGATGATGATTGGGGGATTCCAACAT
ATTTGGAAGAAGAGAAACCAATAAGAAAGAAACCAGAAAATGCATATCTGAAAATGAAAA
GTACGAAAGACAGTAATTTCTATCCAGTTCATAGAAATTTCTACCGTGAACATCCAAACG
TGTCAAAAATGTCTACTGAAGAAAAAGATCAATTTTTATTAGAGAAAAATAATATCATGA
TAAAAGAAATTGAATCTGAAACTGGAGAATCGTTTTGCAATTTTGGAAATACAACTTTCG
AAAAAGATCCCGAAGAGATTATTGAAGAAATTCCTATTCCAAAAATTATTCCGAATCCAG
TTCGAAACTTTGAAGAGGCATTTTTTGATCATCCAGATATATTAAGTGAAATTAAAAAAC
AAAAGTTCCAGGAGCCATCTCCGATTCAGTGCATATCATGGCCAATTTTAATGACAGGTC
GTGATTTTATTGGGATTGCTCAAACTGGAACTGGAAAAACCTTAGCGTTTTTATTGCCGG
CTTTTATTCATATAGAAGGACAACCAAAACCAAGAGAAAAACGACAAGGTCCAACAGTTT
TAATATTAAGTCCAACTAGAGAACTGGCTATTCAAATTCAAAATGAAGTTCAAAAGTATA
CTTATCATGATATTGATTCTGTTGTATGTTATGGTGGCGCTGATAAGCGCGATCAAATTT
ATCAATTGAAAGAAAATCCAGAGATTGTTGTTGGGACACCTGGTCGTATCCAAGATTTGA
AAGATTCTGGAGTATTAAACTTAAAAGATGTCAGTTATTTGGTTCTCGATGAAGCCGATA
GAATGCTTGATATGGGTTTTGAACTACAAGTTGTTAATCTGATTTATGCCGTCAGACGAC
AAAGACAAACAGTTATGACAAGTGCAACATGGCCATTTGGAGTTCAACGAATTGCGCTAA

CTCACATGAAGAATCCTATTCAAGTTAATGTGGGAACATTAGATCTAAATGCTTGTCACA
CTGTTAGACAGCACGTTGAATATGTCGAATATCACGATAAAACTCAGCGATTGGTTGAAA
TATTATCAGAAATTAAACCTGAAGATAAAATTATCATTTTTGTTGCAAGAAAAGCACAGT
GCAGCGATTTGGCGTTTGAGTTGTTTTTGAAAAGATCAAAGGGTTTATTAGGACCAAAAA
GTGCTGTAAGAGCTTGTGAAATTCACGGCGATCTTGATCAACAGGAAAGAGAAATGGCAT
TGAAAATGATGAGATCGGGAGCTAGAAATATTATGATTGCAACAGATGTAGCTTCTCGAG
GTCTCGATATTGTCGATCTGACTAAAGTCATAAATTATGATTTTCCAAAAAATATTGAGG
AATATGTTCATCGTGTAGGTCGAACTGGCAGAGCTGGTCGAACTGGTGAGGCTTTCACAT
TCATGAATAAAATCGATTGGAGTAATGCAGCGGAATTGATCAGAATTCTCGAAGACGCAG
ATCAAGAAGTTCCAGATTTTTTACCACAATTAATGAAACAACACCAGGAATTTCGAATCA
GTGGTGGTAGTCGAGGTGGAGGACGATTTGGTGGCCGAGGAGGCGGTCGAGGTAGAGGAG
GAGGAGGAGGAGGCGGAGGATTTCGTGATGGCGATCGAGATCGCGATGGAGGTAATAGAA
GAAATTCAAATTTCAACCGGGGACACAGATCTGATGATACGGATGATGTTCAATCTGATT
CCAGTTGGGCGGATTTCTCGAATTCATCAAACACTTTAAGTATAAGTAAAAAATTCGGTG
CATGTTATATACAAGATGATATCAGTGATGAGGAAAATTGGGGAAATTGGAATAAAGTAG
ACGATACCGATAAAGAAAAATGTAAAGAAATAATCGAAGCTAAAATAATTGAATCTCCTT
CAAAACCCATTGAAACACAATCTGTAGAAAAATCGATTATTCGCCATGCAACAAAACCAT
ATATTTTGGGAAACAAAGACCGAAATATATATCCAATTTATCGAAATTTTTACGAAGAAC
ATCCCGATATTACGAATTTATCTTTTGATGAAGTCGAGTCGTTTTTGTTGCTAAGAAATA
ATATAATGATTCAAGAATTGAAAACTTATCCTACACATTGGGATCAAAATGATGACGACA
TTAATGAAGATCAAGTTGAAATTTTGCCTGGAATTCCAGGTATTGCCAAACCCATAAGAA
CATTTGAAGAAATGTTTTGGAAACATCCGGAAATTTTAGACATAATTAAATCCTTAAAAT
TTTCAAGTCCGTCGCCAATTCAATGTATCGGGTGGCCTGTTATATTGAGTGGTCGTGATT
TTATTGGCATTGCTCAGACGGGTACTGGTAAAACACTGACTTTCGTTTTACCAGCTTTGA
TACATATCGAAGGACAAGTAAAACCCAGGCAATATAGACGTGGTCCTACTATCCTTATTC
TCAGCCCAACAAGAGAGTTAGCTATGCAAATTGAAAATGAAATCTTGAAAATACATTATC
ATGAGATTAAATCTGTTTGTTGTTACGGAGGAGCTGAAAAAACTTATCAGATTAATGCTC
TTAGAGATAATCCAGAAATCGTCGTTGGTACTCCTGGTCGCATAAATGATCTTTTATTGA
TGGGATGCTTGTGTTTAAAAGATGTAATACAGTGAttcaatatgtgagataaaatttatt
gataaataaaggtcagctatttggtacttgatgaagctgatcgaatgttagatatggggt
ttgaacctcaggtagttagtattattaatgaaacaagaagacaaaggcaaaatataatga
ccagtgctacgtggcctgaaagtgttaggaatatagctttattgtatatggataatccga
tacaagtcaatgttggctcattggatcttattgcttgtaatgacgttgtccaagaaatta
aatttgttagtaattttgaaaagacaaatgcattggtcgacattatttgtaacatgaatg
aggacgataaaataattgtattcgttactcgaaaggttcaatgtagatcattgaccaaat
atttggataagctaatacgatcagataaattgggcccttatgcaaatatttgtgaaattc
atggtgatctgagccaaagtgaacgagagcaatcattgtcttggtttcgtaatggagaat
gtacaattttaattgccacaaatgtagcatcaaggggtctcgatattgcggatatcacat
gtgttgtaaattacgattttcctaacgatattgaggaatatgtacatagagttggtcgaa
ctggaagagcaggaaaaaattggaagatcaataacgttcataaacgaggcagattgcaagc
atacatatgagttaagaaatattttaattcaatcaaatcaagatgtaccatcttggcttt
tgaatttgcgacaaatgaaatacaccgagcaagagatttccaagaaaaaataatccaataa
atcccgtttctaaaagtcataaaaaaagaaaggatcaataatgcattttcatctaccgatt
attttgatgtgaattactcatctgatgattacgatgattatttcgatgaatatcaatacg
agcgtgatccgaaaatgaatgataaagtaacttgtgatgatgacgaagaagatgttcctt
tttcagaactggaaacagtagaaaataagaaaaaagaatcgattaataacaacggagttg
tagaaaaagttattgaatttcatagtgattgtttgaacgaattgtatgaaaatgaagaag
agggtttttttgatgaatatcaaaggaatctcgatctcgaatgtccaataaaaatgtccaa
ttacagaatctttgcctgtagataatatttgtgaatatgattatgagtatgaatctgaca
atgataatgagggttggtggtgatgatgatgaaaaaaaataattttgatttttccattttg
attgaaaatttaatatttaaaaatttatggggatcggtctatttcaaaatataatttgtc
atgtcattatttatttaataatttattcataatgtttaagaattacagtgaaacttccat
ataagatacaacttaatccattagcgattgttgggttgaaaatttgaggacgcatgaaaa
taaaaacgtttgtacttttctatgagctgtttatgattttgttctgttatgacctgatct
atataaacaaacgaaccgatttgtaaccttatttcatctcccttaaaatattttttttat
ttattaatttctccgtcgatgttaatgggttaaatcatcttaaaattttaatttatactt
tatatacctagttgttagattgtaacgaactggggtgaggtttcactgtattattaaaaa

aacagaatttggttatggatttgaaatatattcgaacaatttactaataataaaatttgt
aaattttaaaatcggttatggtttaaatgtatattttgaatggttacactgttatagaac
ggtgtaatatatgtttaaaaaaagtgaaatccggtttcagttatccctgctctgttgtag
ctttatattaaagctcattagaaattgaatctttattcagttgggcggatttctcgaatt
catcaaacactttaagtataagtaaaaaattcggtgcatgttatatacaagatgatatca
gtgatgaggaaaattggggaaattggaataaagtagacgataccgataaagaaaaatgta
aagaaataatcgaagctaaaataattgaatctccttcaaaacccattgaaacacaatctg
tagaaaaatcgattattcgccatgcaacaaaaccatatattttgggaaacaaagaccgaa
atatatatccaatttatcgaaatttttacgaagaacatcccgatattacgaatttatctt
ttgatgaagtcgagtcgtttttgttgctaagaaataatataATGATTCAAGAATTGAAAA
CTTATCCTACACATTGGGATCAAAATGATGACGACATTAATGAAGATCAAGTTGAAATTT
TGCCTGGAATTCCAGGTATTGCCAAACCCATAAGAACATTTGAAGAAATGTTTTGGAAAC
ATCCGGAAATTTTAGACATAATTAAATCCTTAAAATTTTCAAGTCCGTCGCCAATTCAAT
GTATCGGGTGGCCTGTTATATTGAGTGGTCGTGATTTTATTGGCATTGCTCAGACGGGTA
CTGGTAAAACACTGACTTTCGTTTTACCAGCTTTGATACATATCGAAGGACAAGTAAAAC
CCAGGCAATATAGACGTGGTCCTACTATCCTTATTCTCAGCCCAACAAGAGAGTTAGCTA
TGCAAATTGAAAATGAAATCTTGAAAATACATTATCATGAGATTAAATCTGTTTGTTGTT
ACGGAGGAGCTGAAAAAACTTATCAGATTAATGCTCTTAGAGATAATCCAGAAATCGTCG
TTGGTACTCCTGGTCGCATAAATGATCTTTTATTGATGGGATGCTTGTGTTTAAAAGATG
TCAGCTATTTGGTACTTGATGAAGCTGATCGAATGTTAGATATGGGGTTTGAACCTCAGG
TAGTTAGTATTATTAATGAAACAAGAAGACAAAGGCAAAATATAATGACCAGTGCTACGT
GGCCTGAAAGTGTTAGGAATATAGCTTTATTGTATATGGATAATCCGATACAAGTCAATG
TTGGCTCATTGGATCTTATTGCTTGTAATGACGTTGTCCAAGAAATTAAATTTGTTAGTA
ATTTTGAAAAGACAAATGCATTGGTCGACATTATTTGTAACATGAATGAGGACGATAAAA
TAATTGTATTCGTTACTCGAAAGGTTCAATGTAGATCATTGACCAAATATTTGGATAAGC
TAATACGATCAGATAAATTGGGCCCTTATGCAAATATTTGTGAAATTCATGGTGATCTGA
GCCAAAGTGAACGAGAGCAATCATTGTCTTGGTTTCGTAATGGAGAATGTACAATTTTAA
TTGCCACAAATGTAGCATCAAGGGGTCTCGATATTGCGGATATCACATGTGTTGTAAATT
ACGATTTTCCTAACGATATTGAGGAATATGTACATAGAGTTGGTCGAACTGGAAGAGCAG
GAAAAATTGGAAGATCAATAACGTTCATAAACGAGGCAGATTGCAAGCATACATATGAGT
TAAGAAATATTTTAATTCAATCAAATCAAGATGTACCATCTTGGCTTTTGAATTTGCGAC
AAATGAAATACACCGAGCAAGAGATTTCCAAGAAAAATAATCCAATAAATCCCGTTTCTA
AAAGTCATAAAAAAGAAAGGATCAATAATGCATTTTCATCTACCGATTATTTTGATGTGA
ATTACTCATCTGATGATTACGATGATTATTTCGATGAATATCAATACGAGCGTGATCCGA
AAATGAATGATAAAGTAACTTGTGATGATGACGAAGAAGATGTTCCTTTTTCAGAACTGG
AAACAGTAGAAAATAAGAAAAAAGAATCGATTAATAACAACGGAGTTGTAGAAAAAGTTA
TTGAATTTCATAGTGATTGTTTGAACGAATTGTATGAAAATGAAGAAGAGGGTTTTTTTG
ATGAATATCAAAGGAATCTCGATCTCGAATGTCCAATAAAATGTCCAATTACAGAATCTT
TGCCTGTAGATAATATTTGTGAATATGATTATGAGTATGAATCTGACAATGATAATGAGG
GTTGGTGGTGAtgatgatgaaaaaaataattttgatttttccattttgattgaaaattta
atatttaaaaatttatggggatcggtctatttcaaaatataatttgtcatgtcattattt
atttaataatttattcataatgtttaagaattacagtgaaacttccatataagatacaac
ttaatccattagcgattgttgggttgaaaatttgaggacgcatgaaaataaaaacgtttg
tacttttctatgagctgtttatgattttgttctgttatgacctgatctatataaacaaac
gaaccgatttgtaaccttatttcatctcccttaaaatattttttttatttattaatttct
ccgtcgatgttaatgggttaaatcatcttaaaattttaatttatactttatatacctagt
tgttagattgtaacgaactggggtgaggtttcactgtattattaaaaaaacagaatttgg
ttatggatttgaaatatattcgaacaatttactaataataaaatttgtaaattttaaaat
cggttatggtttaaatgtatattttgaatggttacactgttatagaacggtgtaatatat
gtttaaaaaaagtgaaatccggtttcagttatccctgctctgttgtagctttatattaaa
gctcattagaaattgaatctttattcagttgggcggatttctcgaattcatcaaacactt
taagtataagtaaaaaattcggtgcatgttatatacaagatgatatcagtgatgaggaaa
attggggaaattggaataaagtagacgataccgataaagaaaaatgtaaagaaataatcg
aagctaaaataattgaatctccttcaaaacccattgaaacacaatctgtagaaaaatcga
ttattcgccatgcaacaaaaccatatattttgggaaacaaagaccgaaatatatatccaa
tttatcgaaatttttacgaagaacatcccgatattacgaatttatcttttgatgaagtcg
agtcgtttttgttgctaagaaataatataatgattcaagaattgaaaacttatcctacac

attgggatcaaaatgatgacgacattaatgaagatcaagttgaaattttgcctggaattc
caggtattgccaaacccataagaacatttgaagaaatgttttggaaacatccggaaattt
tagacataattaaatccttaaaattttcaagtccgtcgccaattcaatgtatcgggtggc
ctgttatattgagtggtcgtgattttattggcattgctcagacgggtactggtaaaacac
tgactttcgttttaccagctttgatacatatcgaaggacaagtaaaacccaggcaatata
gacgtggtcctactatccttattctcagcccaacaagagagttagctatgcaaattgaaa
atgaaatcttgaaaatacattatcatgagattaaatctgtttgttgttacggaggagctg
aaaaaacttatcagattaatgctcttagagataatccagaaatcgtcgttggtactcctg
gtcgcataaatgatcttttattgatgggatgcttgtgtttaaaagatgtaatacagtgat
tcaatatgtgagataaaatttattgataaataaaggtcagctatttggtacttgatgaag
ctgatcgaATGTTAGATATGGGGTTTGAACCTCAGGTAGTTAGTATTATTAATGAAACAA
GAAGACAAAGGCAAAATATAATGACCAGTGCTACGTGGCCTGAAAGTGTTAGGAATATAG
CTTTATTGTATATGGATAATCCGATACAAGTCAATGTTGGCTCATTGGATCTTATTGCTT
GTAATGACGTTGTCCAAGAAATTAAATTTGTTAGTAATTTTGAAAAGACAAATGCATTGG
TCGACATTATTTGTAACATGAATGAGGACGATAAAATAATTGTATTCGTTACTCGAAAGG
TTCAATGTAGATCATTGACCAAATATTTGGATAAGCTAATACGATCAGATAAATTGGGCC
CTTATGCAAATATTTGTGAAATTCATGGTGATCTGAGCCAAAGTGAACGAGAGCAATCAT
TGTCTTGGTTTCGTAATGGAGAATGTACAATTTTAATTGCCACAAATGTAGCATCAAGGG
GTCTCGATATTGCGGATATCACATGTGTTGTAAATTACGATTTTCCTAACGATATTGAGG
AATATGTACATAGAGTTGGTCGAACTGGAAGAGCAGGAAAAATTGGAAGATCAATAACGT
TCATAAACGAGGCAGATTGCAAGCATACATATGAGTTAAGAAATATTTTAATTCAATCAA
ATCAAGATGTACCATCTTGGCTTTTGAATTTGCGACAAATGAAATACACCGAGCAAGAGA
TTTCCAAGAAAAATAATCCAATAAATCCCGTTTCTAAAAGTCATAAAAAAGAAAGGATCA
ATAATGCATTTTCATCTACCGATTATTTTGATGTGAATTACTCATCTGATGATTACGATG
ATTATTTCGATGAATATCAATACGAGCGTGATCCGAAAATGAATGATAAAGTAACTTGTG
ATGATGACGAAGAAGATGTTCCTTTTTCAGAACTGGAAACAGTAGAAAATAAGAAAAAAG
AATCGATTAATAACAACGGAGTTGTAGAAAAAGTTATTGAATTTCATAGTGATTGTTTGA
ACGAATTGTATGAAAATGAAGAAGAGGGTTTTTTTGATGAATATCAAAGGAATCTCGATC
TCGAATGTCCAATAAAATGTCCAATTACAGAATCTTTGCCTGTAGATAATATTTGTGAAT
ATGATTATGAGTATGAATCTGACAATGATAATGAGGGTTGGTGGTGAtgatgatgAAAAC
AATTATCTATGTCCCCTCAAGACATTTAGGCTATATAATTGGAAAAGGAGGATCACGAAT
TAAGGAACTTTCAGAAGAAACTGGTTGTCGAATAAATATAAAAAAGGATCAGGTTGAAAA
TGATGAAGTGCCCATATCTTTAGAAGGTTCTACAAGAAATATCGATAAAGCTAAATCTAA
GATTGACGAAATTACTAAGCGACAAAATAAGCGCCACATTGATGATAATAATGACCGTCG
TTATAATAACAATTCCAATGAAAAATCGTACTCATCAAATAATTTTTCAAATAAAAGGTC
ATCAAAGTATGGGAACGATGATGAAGATGATGATGGTGATAGTTTTTCGCGGAAAAGAAA
ATCACCAAATGATTATTGGAACGACAAAGAACGAGATAATTCAAAAGATTATTATAGTAA
TTCGAACGATAATGGAAATAGTCGTAATTACAACAATAATAATAACAGCAGAGATTATAA
TAATAGCTCTAAATATAATGGAAATTCTAGAGATTATGATAATTCTAGAGACAATGATTA
CTCACATGGATCTCATAATTCACGTAATAGTAATAATAATAATTCTAAATACAATGGAGA
CGAGTGGGGTGGATGGGGAAAGAAAAATGATGATACTTCAAATTTAGATTCATTAATATT
CGACAGCAATGCACCTTTAAAAGGCGCTTCAAAATTTAATGACCCGCCATCTGAAAGTTA
TGTTCCGGATGATGATTGGGGGATTCCAACATATTTGGAAGAAGAGAAACCAATAAGAAA
GAAACCAGAAAATGCATATCTGAAAATGAAAAGTACGAAAGACAGTAATTTCTATCCAGT
TCATAGAAATTTCTACCGTGAACATCCAAACGTGTCAAAAATGTCTACTGAAGAAAAAGA
TCAATTTTTATTAGAGAAAAATAATATCATGATAAAAGAAATTGAATCTGAAACTGGAGA
ATCGTTTTGCAATTTTGGAAATACAACTTTCGAAAAAGATCCCGAAGAGATTATTGAAGA
AATTCCTATTCCAAAAATTATTCCGAATCCAGTTCGAAACTTTGAAGAGGCATTTTTTGA
TCATCCAGATATATTAAGTGAAATTAAAAAACAAAAGTTCCAGGAGCCATCTCCGATTCA
GTGCATATCATGGCCAATTTTAATGACAGGTCGTGATTTTATTGGGATTGCTCAAACTGG
AACTGGAAAAACCTTAGCGTTTTTATTGCCGGCTTTTATTCATATAGAAGGACAACCAAA
ACCAAGAGAAAAACGACAAGGTCCAACAGTTTTAATATTAAGTCCAACTAGAGAACTGGC
TATTCAAATTCAAAATGAAGTTCAAAAGTATACTTATCATGATATTGATTCTGTTGTATG
TTATGGTGGCGCTGATAAGCGCGATCAAATTTATCAATTGAAAGAAAATCCAGAGATTGT
TGTTGGGACACCTGGTCGTATCCAAGATTTGAAAGATTCTGGAGTATTAAACTTAAAAGA
TGTCAGTTATTTGGTTCTCGATGAAGCCGATAGAATGCTTGATATGGGTTTTGAACTACA
AGTTGTTAATCTGATTTATGCCGTCAGACGACAAAGACAAACAGTTATGACAAGTGCAAC

ATGGCCATTTGGAGTTCAACGAATTGCGCTAACTCACATGAAGAATCCTATTCAAGTTAA
TGTGGGAACATTAGATCTAAATGCTTGTCACACTGTTAGACAGCACGTTGAATATGTCGA
ATATCACGATAAAACTCAGCGATTGGTTGAAATATTATCAGAAATTAAACCTGAAGATAA
AATTATCATTTTTGTTGCAAGAAAAGCACAGTGCAGCGATTTGGCGTTTGAGTTGTTTTT
GAAAAGATCAAAGGGTTTATTAGGACCAAAAAGTGCTGTAAGAGCTTGTGAAATTCACGG
CGATCTTGATCAACAGGAAAGAGAAATGGCATTGAAAATGATGAGATCGGGAGCTAGAAA
TATTATGATTGCAACAGATGTAGCTTCTCGAGGTCTCGATATTGTCGATCTGACTAAAGT
CATAAATTATGATTTTCCAAAAAATATTGAGGAATATGTTCATCGTGTAGGTCGAACTGG
CAGAGCTGGTCGAACTGGTGAGGCTTTCACATTCATGAATAAAATCGATTGGAGTAATGC
AGCGGAATTGATCAGAATTCTCGAAGACGCAGATCAAGAAGTTCCAGATTTTTTACCACA
ATTAATGAAACAACACCAGGAATTTCGAATCAGTGGTGGTAGTCGAGGTGGAGGACGATT
TGGTGGCCGAGGAGGCGGTCGAGGTAGAGGAGGAGGAGGAGGAGGCGGAGGATTTCGTGA
TGGCGATCGAGATCGCGATGGAGGTAATAGAAGAAATTCAAATTTCAACCGGGGACACAG
ATCTGATGATACGGATGATGTTCAATCTGATTCCAGGTACTTTAATAAGCCTAAACTATC
TGAAGCGTCGGTTAATGATGAATGGTGGTAAaaataaaacgaaagatttgtaaaacattc
atgtattgttagtgtttgtggctgcatttaatgcatttaatgcttaattaaaaatttatt
aataactggtttcttttttggtttgtttgacacattttatatatttatatatcaacagt
ggagtacggataactcgaaattggcattgatctacacttcgagttatcctaacgtatttt
atgaattattatactatactgtttcgtttttatcaatttcgaactatttagatgtatata
ttgattcgctgggaatattttgacacgata
>DjScaffold91:141787-142272 strand:+
ATGATGACAAAAGTGGTTGGCAGAATCAATGAAACCATGGTTTATTTTGATGACATCCTG
ATATTTACGAACAATAAAGATCAAAACTATGAAGTTATTGACAGAGAATTGATGGATCTA
ATGAAAGCTGGTATAAGAATTAATTCAGAAAAGTGTCAGTTTCTTCAAAGCGAAATAAAA
TTTCTTGGACACATAATTAGCAAGGACGGTGTCAGAACAGATCTGTCTAAAATAGAAGCA
GTAAAAACATTTGGCAAACCTAACTGTGTGAAGAAGTTGCGAAGTTTCTTAGGAATATGT
AATTATTACAGAAAGTTTATAAAAAAATACGCAAGGAAATATAGGATCTTAGAGCAGTTG
TGTAGCAGACCAAACAACAAAAAATTTGAATGGACAGATGAAATAAACGATACTTTTGAG
GGTCTGAAGATATCTTTAATGACTGTACCAATATTGTGTTTTNGTTTAGGTTGCGAAGTT
TCTTAG
>DjScaffold91:62193-62483 strand:+
ATGAGAATTAATCCGGAAAGATGTAATTTATTACGAGCAGAAGTCAAATGTTTAGGACAT
GTCATAGACAAGAATGGAGTTCGAACTGATCCAACAAAAATTGAAGCGATAAAAGATTTC
GGAAGACCTAAATGTGTGAAGAACTGGAGAAGCTTTTTTGGAACATGCAATTACTATAGA
CGGTTTATCAATAATTACTCAAAAAAGCTAGAAGCTTGGAAGAGTTATGTGAAAATAATA
ATAAAAAACTTATGTGGTCTGAAAATTATGATAAAGCTTTTAATAAGATGA
>DjScaffold3863:57694-58240 strand:+
ttccagttcagcttgatATGCTATGCACTTTTGAAAAACTGAAATATTTTAGACATTGTC
AAAGTTTTATTGGTTATGATTATGGAAATAGACTGTTTTCTTTAAATTTAAATTCGAACA
AAATCGAAAATTTTCTATTAAAATCAAATCTGTTAGAAAACCACAATTTTATAAATGGTA
AGAGAATTAATGGCTTAGGTTTGAAATTTGATAATCGTAATCCTGCAACTGGACAGATTC
TAGCTAAAATAAATTCATGTTCAAATGATCAAATAGATGAATGTATAAAAAGTAGTATTA
CAGCACAGAGTGAATGGATTAGTAAATATTCAAACATTGACAAGCATAAAATATTAATTA
ATGCTGCTAATATTGTTCGTTCTAAAGCTAAAATTTTAGCTTCTGTTGATGTTCTCGATA
CTGGTAAAGCCATGTGGGAAGCTGAAAGCGATATTTACACTTGTGCCGATGTTATAGAAA
TGTTTGCCGCTTTTCTT
>DjScaffold3863:116568-118315 strand:+
aacactatcATGGACTTTATTAATTCTGCAAAAGATGATGGTGCAAAAGTATTATACGGA
GGAACACCAATTATCTTTAGTGAAGATTCTCCACTCGCTGGTGGCAAATTTATCAAACCA
TGTGTCATAAGCAAATGTACTGATACAATGAAAGTTGTGAAAGAAGAAATATTTGGTCCT
GTGGCTTGTTTAATGCCATTTGAATCAGAAAACGAGGTTATTGCAAGAGCTAATGAGAGT
ACATTGGGTCTTGCTGGAGGTGTTTTCACAAATGATATAAATAGAGCTCATCGAATAGCT
TCTCGTCTAAAATGTGGGAATGTATATATAAATACTTACAATACTTATCCAATAGGAATG
CCATTCGGTGGTTACAAACAATCTGGGTTTGGTCGAGAAAATTGCAAGCAAACATTAGAA
TATTATTCACAAGTAAAATCCGTTTTTGTTGAGTCCAATCCAATTGATTATCCATTTTGG
CCAATGAAAAAATAAaatctagactgttattttcaattttaaatgattcaaattatttat
tttccattttaaatgttccaaatactagaaattattttaattatactatcataatttgt

atta
>DjScaffold5017:58342-65807 strand:+
taatttaaccgttttactatttaaatacaatcaggaatatttctaattttgcatacttaa
taattaactgtattatattatattaaaATGCCAAAGTCAGTAAATAATAAAGTGGAATAT
TTTTCTTGTCGATATAAATACATTTTACAAAAATCTAATGAAGCCTTAACTATAGAATCG
CAAGGAAATAAATTTGAAGTGTATTCTATGTACAAAAGTGTAATTGTGGATATAGATAAT
ATTATGAGTTTCGATATGAGCTTAGAAAATATGTCCATAAATAGTGATATCTTGAAGAAA
ATTAACAATTTGAAGTTACTGAGAAACAATATATGTGAATGTATTAATCGTATTGAAAAA
GAAAATCAAAATGATGAGTTGATAGTTGCTGAATTATTGTATTCGATTCAGTCAGAATGT
CAGTATACTAGAATTGATTCAAATGGAGTGTTTGATATTCCTAGTTATCCAGGATCTTTA
GAAATTTATAAATTTCCATCAAACTTAGAATGTTCAAAAGGATTTATAATAGTATCAGAT
TGGATTGGTGTATTAATTTCTAACGTAACTCCAATTCTTATTACTAGCGAAGGTGCTTTG
ATGTTCCTTGATATGGAAAGAAATGATTCTAAAATTGCTGTCACACTTCCTTTTAATGTT
TCAAAAGAAGAATTTCAACAATGCATTTCTGTATTATCAACTCTTTCAATCATTGTCGTT
GATCATATTCAAATAAATGAAAAATCAACAATTAATATTGATGAACAATTACCTAAAACA
AAAGGTGAAATGATTGCAGATGGATTAAATACAGTTTCTCGGAATATATCAAAAGGTGTT
GTAATTGGAGCCAATAAGTTAGGTGAATTAGTTTGTAAAGGTTCTGAAGAGCTGTGTCAA
CGAATAAAACCAGGAGAAGCAACACTTAATCCAAATGTTAAGACAGGATTTGAATATGCT
CGAACAGGAACACACGCTGTGATTGAAATTGGAGGAATGGCGGTTGGAGTTTTAACAAAA
GCTACAAAGTACGTAGCCTCAAAAGCAGCTCCTCATATTGTATCAGCTGCAAATAATTTA
ATACCAAATGAATGGAAAGAAAAGGGTGTAAATGGAGAAATATCTACAGTTGATCAGGTC
GCTTTAGTAACCGGGTCTGGCTTACAAGGATTAGTTTTATTATTTGATAGTTTACATAAT
GGAGCAGTAACATTTGGTCGTTGTGCAGCTCGAAGCAGTGGCAAAGTTATGCGTCATCGG
>DjScaffold1848:13119-25266 strand:+
tttatatttgaagttttaataaaataatcaattaaattaattataattaagctaattttaa
aatttaaatttaaatagtctgaaactattaagtattgttatgaatcaagtaaaccaagca
aaaacatatggctattctcgactggtaggatgtatttatgtgtttaatttaattgttggt
actggtgcattgacattacctaaagcatttcaagatgctggatggttattaagtggaatt
gttttatctgctttatgctttttgagttttatcacatctacatttgtaattgaagcgatg
agtatatctaatggcttacaaaaactctccaaattgaaatatatatttacaaacagttca
ccatttactgctgatgatatcgaagatgaaactacaaaaactctattcacctatactagg
aattatggtttgacaaatggtaaaataatgattttatacatttatatgatacatttcaac
tttgaactgttgctaatacgattaacctttgcatcaacagatttattaagttgttataga
taattattgaaatacatgtgtatttttgtgcaatacatttcattgatgtttcaatgtaat
aagatctaatctattgttcttatctattacaattatataattgattgtactataacttga
caacttcttcatcttacttcaattatctaactgaaaactcatcttttccatttattcttc
ctgtaagggattcagatccatgctggccaaattcctcaatttcccggattaatgcctaca
gattatttctgactgcttttgtattactgctgtgtccattcacatttttcaatttaacaa
aaacaaaactaattcaatacgctactctatcaATGAGATGGTTTGCGTTTGGATTAATGT
TTACTTTAATGAGCATCTATTTGGCTAAAGGATACAAATACGGTAATCCAACAATAGCAA
ATTTTTCTAAGTCACCAGATTTGCTTGGAATTTGTATATATTCTTTCATGTGTCATCATT
CTCTACCGTCACTTGTTACTCCAATCAGGAAAAAAAATAAAATGTTCCTGTTATTTTTTA
TTAATTTTCTTGTTATTCTCGGGTTTTATTTGCTGTTATCACTGTTATCTACTTTTACTT
TTTCAAATATTCAATCTTTAATTACACTCAATTTTATACCTAATATTGGTGCTTCTCAAC
CTCCAATCACCAAAATTGAATTCTTTCAATATTATTTATCATTGTTTCCAGTGTTTTCTA
TTAGTGCTAGTTTTCCAATTATCGGAATAACTCTAAGAAACAATTTAAGGAACCTCGTTA
CTTGTGGTACCGGCAAGAGATATAACAATTGGTTTATAGAGAAAATGTTTTATCCATTAT
TAGCAATCATTCCTCCGATTGCTGTAGTTTACGGAACGAATAATATTACGTTTCTTATTG
GATTTACTGGCTCGTATGCAGGCACTTTCATTCAATATGTCATCCCAATCGGCTTAGTTT
TATGTGCTCGACGAAGCGTAAATATTTTCATGTCCAAATTTAACGAACATTATGAAGGCA
ACATTGGTATAACGAAGGATCAATTATATCAACCATATCAGTCGCCATTCAAACACAAAG
CTTGGGCGATATTAATATTAATTTGGTCTGGCGTATCCATTGTTTTTGTAACAGTTAATC
ACTTTCTATCAAATTCAGGTCATTGAattttatttttttatatttgaagttttaataaat
aatcaattaaattaattataattaagctaattttaaaatttaaatttaaatagtctgaaa
ctattaagtattgttATGAATCAAGTAAACCAAGCAAAAACATATGGCTATTCTCGACTG
GTAGGATGTATTTATGTGTTTAATTTAATTGTTGGTACTGGTGCATTGACATTACCTAAA
GCATTTCAAGATGCTGGATGGTTATTAAGTGGAATTGTTTTATCTGCTTTATGCTTTTTG

AGTTTTATCACATCTACATTTGTAATTGAAGCGATGAGTATATCTAATGGCTTACAAAAA
CTCTCCAAATTGAAATATATATTTACAAACAGTTCACCATTTACTGCTGATGATATCGAA
GATGAAACTACAAAAACTCTATTCACCTATACTAGGAATTATGGTTTGACAAATGGTAAA
ATAATGATTTTATACATTTATATGATACATTTCAACTTTGAACTGTTGCTAATACGATTA
ACCTTTGCATCAACAGATTTATTAAGTTGTTATAGATAAttattgaaatacatgtgtatt
tttgtgcaatacatttcattgatgtttcaatgtaataagatctaatctattgttcttatc
tattacaattatataattgattgtactataacttgacaacttcttcatcttacttcaatt
atctaactgaaaactcatcttttccatttattcttcctgtaagggattcagatccatgct
ggccaaattcctcaatttcccggattaatgcctacagattatttctggttagacatattt
tcaaatttacgtttttcaaattcaaaatcgttaaaattgtattaccaaaatttacgtca
acaaaataatgaatattccaagccactagtttcttgcttcctgttgattcgaaacctttc
tgcatccttaaatttttctataggaaaatgtaataatttttgagaaattagttttagaaa
aatcaatttaaatgcgttttttc
>DjScaffold4748:65097-65565 strand:+
ttaaagttttcaaaatttttatttattgaaATGAGGTATATTTCTGCCTATATGTTATGT
CTTTTGACTGGTAAAACACCTAAACCAGCTGATATTGAAAATGTTTTAAGCAGTGTAGGT
GTTGAATGTGACAAAAAAGCTGTTACTGAACTTTTCAACTCAATCAAAGAACAATCGATT
GAAAGTTTAATTAATGCCGGTATTTCTAAATTAGCTTCAGTCCCCGCTGGAGGAGCAGCT
GCACCTTCATCTGCTGCACCAGCTGCTGCAACTTCAGCCCAAGCTACAAAAGCTGAAGAA
GCTAAAGCTGAAAAAGAATCTTCATCTGAAAGTGAAGCTGACATGGGCTTCGGTCTTTTT
GATTAAataaaaaatgttaatttggattatattttaatattaagttttaaagagacaataa
ttaata
>DjScaffold3230:128096-128879 strand:+
ATCATATATAGGAAGTATTTTTATCAGAAAATTTTAGATATAAAAAAATATCGTTTTTCT
AATTTGATTTATAATAAAATTAGACAGCTAATTAGTTTTAAGAATGACGTGGTTTTGTTA
CATCCTTATTATGAAAATAGAAAAAATTTTAATTGAAAACAATGAATTACAAACATCAGCA
AACGTATATATAACATCAGCTGTTACCTCTATTAAAAAATACAAATATGTAACTATAATT
CTTTCTAGTTGTTACATGATATATGGTTTTGTAAACTACTATAAAGCAAGTGCACAAAGA
GAGTTGTTAATAAGAAAAGTAAATTTATTCAAGTATTATAATAAATATTGAatttagtat
gaaaaatcttgcttgaatccgactttatttcaagatttatttgacagtcttcggtcatac
tttgtgaaaagtaactgtgaaatttattttgatgtgtactacagtttgccaattttcgaa
ttttctcctattaacattattgtcgatcttgtcgattatatattgcagaaattgtttgaa
atactaggcaaatctacaggaagtttactgaaaaatttatcgttgtcctcacaattatca
cttctctcgtttatttccgttatttattggatttatttgaaacggtaaaaatgaagatgt
tcaaaataccaatttgtctttctatta
>DjScaffold3205:67447-70710 strand:+
cgtttctaccaatgaatatttaataaaaatttttaaccaaatatgtttgaatattttttt
agaaaaattactgcatatataattgtgattttaagtttgatattcttttcattttctagg
aattcttaactttttaataaggcaacacttaacggataacttttagagacatgtgaaagc
tatcattaatactttttttttaatttgaaaattctaattcttgctctttattttaatttt
ctagaataataccaaattcttatttctttgaattttttttgtgtagaatattaaattttct
acgtattgattgctaatgaatttttcatagttgttattaataaataattattttgttttt
atttatttatgattttcagatcatcgcagtactgctgaaaatgtccgtgatttaATGAGA
CCTTTTCTTCATGAATTTTTAGGAAAAATTTATTCTAATTATGACATTGGTATCTGGTCT
GCCACTGGTCTTACTTGGATTAGAACAAAGTTGTATGAAATGGATCTTATATCGAATTCT
ACAAATCGATTATTAGCACATTCTGAAGGTCGAGAGCAACTGTTAAATCGACTAAGCGAT
GAAAACAGTAGTATTGAAAGCCCTTTTAAAATCTGTTTACTGTTGGATCAAGGAGGAATG
ATCAGTGTACATTTACCTAGTGAAGGAGTTAAAGAAGTAAAACCGTTAAAAGTTATATGG
GACAATTTTCCTCAATGGGGACCTCATAATACAATAATGTTTGATGATATTCGGAGGAAT
TTCATTATGAATCCGCAATCAGGACTAAAGATAAAACCATATAAAAATGCTACAACTTCT
CGAAATACAGATAGAGAATTATTGGATCTGGCTAAATATTTAAAATTGATTAGCAACATT
GATGACTTCACAAAATTAAATCATAAAAAGTGGCGGCGTTTTATTGAAAATGGTAACCCT
AATTCTTCAAAAAGAGCTAAAAAGGAATAAattatttgataaattatttgtgtgtcaaat
ttcatttatgaattttcttaatgttttgttgtattttactaatgatgatgtctaataatg
tatttgcgttttctta
>DjScaffold2598:29113-30269 strand:+
attgattaatttattttagatATGCCTCGTAAAAATAAGGATACAAATGTTAAAAAGGAA

GAAGCTCCTGAGTTAAGTAGACGTAGAAGTGCGAGATTAGCGGCTGTGGAACCCAAACCT
AAAGTTAAAGAAGAAAGTCCAGTTAAAACTCCTAAGAGACAGGCAGCTAAACGAGCTTCG
CATGAAGTGAAAAAGACAATGGCTGATGAGGAAAACGTAAATTCAAATATTGAATCAATT
CCTTCTGTAGAAAAAGTTGATCAAGATGGCGACAAAAGCTCTGTTGATAATAAAACTGGA
AGTGATACTAAAAAGCTAAAATTGGATGAAGAAGATAAGACTACAAAAATAGCTGATGAG
GAATCAAGTGAATTAGTTATTGATACACCTAATGAGAAGAAAGATGTAAATGAAGAGAAA
AAGGAAAAAAGTGAATCTGAAGAAAAGGATTCTAAGAGTGGTGAAGAATCTGGAAAAGGT
TCTGAACAGAATCATGTTAATAATATCGAATCAAATGATAAAGTAGAAACTCTTGGTAGT
TAAatgctaaatataaataattgtctggttataaatgtttgaaatattttatcatttatt
ttgtattgtcattgtgaatattcatatttaaatggtttttggtgcttgaaacaaattaat
gcatgcaataaaaaccgcaccatttatgcatttgaaatatttatttgtgaaaaatggttg
tttgttatgttggtttttaatgtaattaaaatccattgtacacaaagtggtgttataat
ttgtattataatttatattttatttgtgtagaaaataaataatatggatttaaatttttt
taatttgagcagttgattaagtttttttggtattgtgtttaaataatttaaactattaac
agaacttatttttattatataaaatgatgtgttataggtgtgaatgtttgaagttaggga
ttttattgtaatttcttttctgatctgcatatatatgcatactaattactc
>DjScaffold2598:25029-25604 strand:+
ATGGATATGATTAAAGCTGCTATTAAATCCAGTCAAGATCGTACAGGAATGTCATTAATA
TTGATTAAAAAATTCATCCAACAGAATTATAGTATCAATATAGAAAAATATGGTCCATTT
CTACGACGTGCTCTTAGTCATGGTGTTGATAAAGGAGTATTGATTCGCGTTGGCAATAAA
GGTAAAGGTGCTTCGGGAAGTTTCAAGCTACCATCTAAAAAGGACAGTGGTAAAGCAGAT
CTTGTTGAAAAAGTTTCTAAAAAATCGTCTGAAAAGAAAACTATTAAAAAGCCTAATAAA
AAAGGTGACAAGAAGTCAACTGTTAAAAAAGTTGTTAAGAAATCTGAGAAAAAAGTGGAA
GATAAGAAAGATGAGAAAATTGAAAAGTCTGATGAGAAAAAGTCTGTTAAAAAGTCTCCT
GTCAAAAGACCTGCTCAAAAGTCTAAAAGTCCAACTGCCAAGAAAATAGTTAAAGTCGAT
GCAACAAAGTCAAAAATAGTGAAAGCAAAAGCTACGCCTAAGAAAGTGGCACCTAAGAAG
ACTCCAAAGAAAGCACCAGTCAAAGCAAAGAAGTAA
>DjScaffold2598:135138-146556 strand:+
GATACTCTTATCCCTAAAACTGTTCTTCATCGATATGTTTCTTTGTTGTTGGAACATCGA
CTTATTATTTTATGTGGACCGTCAGGAACTGGGAAGACTTATTTGGCTAACAAATTAGCG
AAACATCTTATTACTCGACATGAACAAGAAATATCATCATCTACTCTAGCAATGTTTACA
GTGGATCACAAATCAAACAAGGAATTGAGAACCTATTTGGCGACTATTGCTGAACAATCT
CAAAATAATTTCGAGTCAAACAATTCTAGAAATATTGGTTGTCGTAACCCGCCAGTGGTT
GTGATTTTAGATAATCTACATCATGTTAATTCTCTTGCTGAAATTTTCGATAGTTTTCTC
AGCTCAAATATTCAAAATCGACCATATGTAATTGGAACAATGACTCAGGCATCTTTTTCT
AACACAAATCTTCAAGCTCACCACAATTTTAGATGGGTTTTGTGTGCTAATCATATGGAA
CCCGTAAAGGGATTTTTGGGAAGATATCTTCGAAGAAAACTAATTCAAGCAGAATGGCAA
GAGAAAAAACATGATGCAGCTTTATTGACAACAGTTGAATGGATTTCAAAAGTCTGGCAA
CATATAAATAAGTTTCTCGAAAGTCACAGTTCGTCAGATGTGACAATAGGACCGAGATTA
TTTCTTTCGTGTCCAATGAGTATGGAAGGTTCACAAATATGGTTTACTGATCTGTGGAAT
TATTCATTGGTTCCATATTTGTTAGAAGCTGTTCGAGAAGGGTTACAGACATTTGGACGT
CGTTCAAATTGGGAGGATCCAGTTGATTGGATATTACAGACATGGCCATGGACTTCATCT
AATGTCAAAACTAGAAATTCAGATCCAAGTTCTTTAATCAGATTACGACCAGAAGATGTT
GGATATGACAGCCAAGTACCTCTTAATTTAACAGTCGCATCACCATCATCTTTATCAAAT
TGTCAGTCTATTTCCTCTTCACAGTTAATACCACAAAGTCAGCATTCTAAATATCTACAA
GATTCAAAAATACTTTCTCTGTCAAATTCATCACCTCACAGACATGCCAATTCCAATAAC
ACGTTTCCACGTACCGATCCCTTACTGAATATGTTGATAAAATTACAAGAAGCTGCTAAT
TATTCTACTAGTCCTAGTAGTAATGACAGTAGTGATTTACCTTCCAGTAAATCGAATTCC
TTATCAAGAATCAACTCATCTATCAATTCAGTTGGCCAATCATCACAAATTCACAAGATT
TTTAAAACTGATATTCTATAAtacgaaaattatacatattagtattaatattataatcat
tcatatttatactgaaaatagtaattagaatgaaactttttatatttaaagattgtttga
gatacaatttgtacagttttgttctttccccaagatttagtagaaacgcagctctcatt
tttgctcatgatagtttattatctctgttgataattatgatgatgtcttaatattattat
tattaatatgat
>DjScaffold4126:21266-22137 strand:+
ataaagtagaatgtctattagatacaggggctcgaattaacgtgatgagtttgagttgtt
ttaataaactacgagaagtaaagttagaaatgacttATGCGAAAGGTCGATAGCAAAACG

AATGAAAAAGTCGAAAATTACATTTGCAATATTGAAGCAAATTTTGGAAGACAAATTAAA
AAAGGAGAGCGATTGACAAGAGCTTTAAATATTTTTAAGATTAAAGAGGATCAACGGTTA
TTGAAAATAATTGAAAGTAAAGAAGATGCTTTTATGGCAGATAAATGGGATGTAGGACGT
ACACATCTGACCAGACACAAAATAATCACAAGTGGTGAACCAATCAATGTAAAACCATAT
AGACAACCAATGAACTTAGAATCTAAAATTGAAGAAGCTATCCAAAACCTTTTCGAGAAT
AATATTATCAGGAAATGCAACTCTCCATGGAATACCCCATTGATATGTGTTTGGAAAAAG
GAAAAGAAAGACATAAGAATATGTTTGGATTTCAGACAATTGAATGCAGTAACGAAAAGA
CAAGCCTTTCCGATGCCTAATGTCGATGAAATGCTGGACAAGTTAGATGGAGCCAAATAT
TTCAGTTCAATAGATTTGGGAAATGCATACTATCAAGTAGAGCTCGAAGAAGAATCTCAA
GAAAAAAACGGCGTTTTCAACGAAACACGGGCAATATTGTTTT
>DjScaffold2005:32916-33858 strand:+
tttttattagtttaaatctatctagcccattagacaaatttttagtacacgttgcatttc
aattaataaatcaaATGTCTAATGAATGGGAAATTGTTACTAAAGGTGGTAAGAATAAGT
CGAAGGCACAATTATCGAAACAGGAATTGAAAAAAATTACTCAAAGTAATTTAAACAATA
ATAAATATCGTTTAGCAATAGATGAAGATACTGACAAATCGCTTTCACCTGAACAGAACA
AAGTGTTAAACAAAGTAAAACCGAAAAAAGTAAATGCAATAAAGGCCGAAGATGATATTA
ATCGACAAATTAATAAAATTAGCAGTGATTCAGTAAAGAAAATATACAATGAATTAACAG
AAAAATTTCCAAATCAAACAACTGTTTCACTATTAGATTTAGCAGTACATTTAAGCTTGA
TGGTAAAACCGTCAGATGATCATATGAAGACCATTAATTTCGATCAAACTGATTTCCCAT
ATGCTTTATTGTCTAGTGACACGCAAACTGTTTTACGAGGTATTTTCAGCGGTTTATCAA
ACATAAATATTAGCAACTTTTTAAAGTCAGCAATGATTCAACTGTTAGATGATTACGATA
AAGGTGTTCACTCGATTGGTTTGCGTATAATGATTCAAGTATGTGTCAGAATGAAGTCAG
TCGTCTTCATTACTAATAGAAAAAACTTTTTTGATCTTTTCAATCTTCACCGTGAAAACA
CCACAAAATTCAAATTACTAACGTGGGTGTTGAGACAAGCATGCTCAAATTATAATATGG
CTTGTCAAATATGGTTGATATATTTTCTTCCTTTTATCACTCAAAAATGCCATACAGAAT
TTGTAATTAGTTTTGGTGAAGAGATAAAAGATTATCCAATCGATACAGCCGGTAATGATA
CTAGCTTATTGGTTGCTGATTTTGTACCAATTATCGATCTTATTTTCACTCAGAACATTA
ATAATCAACACATAAATGATAAGAAATCAAAGAAAAAACAAACTAATTTCCAATTAACTA
GCACGCAAATCGATAGATTGAAAGTTATTTATCTTAAAATCAAAGATCAAGCTTTTCTTT
CAAAATCTCCTGGAGCTTTAAAAGATTATTTTGCATCGTTGTTTTTAAGATTATCACCAA
ACAACAAAAATGCACATCTTAACAAAGAACTGTTGAACTGTTTGGAGCATTGCGTGAAAA
CAAGTCCAGAATCGATCAGTCAACTTAACAATTTACATTCGGGATATCCCCAACAAACCA
AAGTATTTCTTGATTATTTATCGAATGAGAACAGCAGTGCAGCTCAATCGATTAAAAAAT
CTAAATTATTCAAATCGTTGATGGATAAAATACATTCCAACCATTTAGCAGCACCAATTG
GATCAGTACAAGATAATTTACGACAATTGACAAAAACATGTAAATCTTCTGATAACAACT
TCATGTCGTGCTTTGGAATCTTTTTCCGATTGTTGACTTATATCGTTTTGATGGGCTTCA
TTATCGGTGGCATCTTATGTTACCAATATCCACAACAGTACAAATTGCTACAGAAACATG
TCACTCCCTATCTCGATTACGTTGTCGAGAGATTGTCACCTTCTTACAAATATTGCGAGA
ATCAAGTTCTAGAGGCTTACAAATACGTACAACCAGCAATTATAGATGGTTACAATAAAA
CCTGGACGTATTTAAAACCGTTTTTAAACAATTTGGCTGCTAAGATTGATTTCTTATCGA
TTTATTTACGAACAAAAATAGATGAAGTGTTTCCACAATTGATAAATCAAATCGGGGAAA
TGTTGCAGTCATTGAAATATTATATGACACAATTGGCCAATAGTATTCAAGAAAAAAATCA
ATAGTAATGAATTCCAAAGCTGGATGAAATTTGTCAGTGAGAAGTCGTTTTATTTCTTGA
GACAAATCGAGTTTGGGTATCAGAGTATTGTCAGTTACACAATGAAAATGTTGGGCTATT
AAtaatcacaattgacttttagtgtacaaatcgtaataataataataataatgtctt
gtttcttggaatatttgtataatataatttattaaaaaaaatactagagtatttcttgatt
atttatcgaatgagaacagcagtgcagctcaatcgattaaaaaatctaaattattcaaat
cgttgATGGATAAAATACATTCCAACCATTTAGCAGCACCAATTGGATCAGTACAAGATA
ATTTACGACAATTGACAAAAACATGTAAATCTTCTGATAACAACTTCATGTCGTGCTTTG
GAATCTTTTTCCGATTGTTGACTTATATCGTTTTGATGGGCTTCATTATCGGTGGCATCT
TATGTTACCAATATCCACAACAGTACAAATTGCTACAGAAACATGTCACTCCCTATCTCG
ATTACGTTGTCGAGAGATTGTCACCTTCTTACAAATATTGCGAGAATCAAGTTCTAGAGG
CTTACAAATACGTACAACCAGCAATTATAGATGGTTACAATAAAACCTGGACGTATTTAA
AACCGTTTTTAAACAATTTGGCTGCTAAGATTGATTTCTTATCGATTTATTTACGAACAA
AAATAGATGAAGTGTTTCCACAATTGATAAATCAAATCGGGGAAATGTTGCAGTCATTGA
AATATTATATGACACAATTGGCCAATAGTATTCAAGAAAAAAATCAATAGTAATGAATTCC
AAAGCTGGATGAAATTTGTCAGTGAGAAGTCGTTTTATTTCTTGAGACAAATCGAGTTTG

GGTATCAGAGTATTGTCAGTTACACAATGAAAATGTTGGGCTATTAAtaatcacaattga
cttttagtgtacaaatcgtaataataataataataataatgtcttgtttcttggaatatt
tgtataatataatttattaaaaaaata
>DjScaffold2005:70011-74517 strand:+
actgcaaatatttaaaacattttcggatataaaatattcacatttgttgaATGGATAGTG
ATTTAAAAGAAAGTCTTAAAAGCAATTTTCTAGATTTGCTCAAGGATCCTACCTGTGTTT
GTGTTGAAAACTTTTATGAAAAGATGGAAGAAAAAAATAAAATATTAGTTTGGAGTGAAA
AGAAAATAGATTGGAATGATTTTAACAAGCATTTTAATGAATACAAGCCTGAAGAACTTG
AATTATATATTGAAGGTTATAGATGTCTGGCACAAATCCATATTAACCTTTTGAAAGATT
TTGAAATTTTTAAAATTTATCCTATTCGGAAATATTCCAGCGCATATCATTATATTTCCA
AAATTCCTGATTATGAAAAATTATTGATAGCAATCGATTATAAATTAATAGAGCAAGAAA
AAAGATATAAGTTAAAAGAGAATCTAAAGGAATTAAAGGATAGAGAAGTTGTTGAAGACA
CAGCATTGAAATTATTGATTTGTTTCATGGAGTTGTCTGAAATGATTAAATTACGATATG
ACTTGTGTGAGATGCAAGATTTGATAAGTCATAGAGAAAAAGAAATTAAGAAAAATCGTG
AAGAAATCGAATTTAATCAAATGTCAGATGAAGAAAAATTGAAAAAATTTGAAAAATGTG
AAATTTGTGAAAATAACATGAATATGGAAAGTGGAGAATGTGAAGTTTGTCAATTTTCTG
TTGCAGAGTTTAGTTTCTCTCATAAATTCAAAGAACTATTGGAAAAGACATTTAAAGTAA
CTGTAAAAATGCCTGAAAATCAAACTAATGATGAGAAAGAAACACTTGAAGAACCGCAGC
ATGCGAAGATACATCCAGCTTCATCAAAGAAAATTGAAACAAAAGAAAAAACATCAAATG
AAAATTGGCCATGTTCCAAATGCACTTTTTTTAATGAAGCTGCTAATTTGAATTGTATCA
TTTGTGAAGCTAAACGAGATAAGTGAatagtagaactataaaatttggttatcgattttt
actgaatatttatgaaataataataataattttaaacaaaatattttgaaaaatattttc
aatttatttcataataaattattagtattttgctagatttgctcaaggatcctacctgtg
ttgtaattaaaatttttattataaattaattttctatagaaattatttacttttaaaat
attattttattcattaagtgtgttgaaaacttttatgaaaagatggaagaaaaaaataaa
atattagtttggagtgaaaagaaaatagattggaatgattttaacaagcattttaatgaa
tacaagcctgaagaacttgaattatatattgaaggttatagatgtctggcacaaatccat
attaaccttttgaaagattttgaaatttttaaaatttatcctattcgggttataaatttt
ttttcaattcataaaaaatcgttttctagaaatattccagcgcatatcattatatttccaa
aattcctgattatgaaaaattattgatagcaatcgattataaattaatagagcaagaaaa
aagatataagttaaaagagaatctaaaggaattaaaggatagagaagttgttgaagacac
agcattgaaattattgatttgtttcATGGAGTTGTCTGAAATGATTAAATTACGATATGA
CTTGTGTGAGATGCAAGATTTGATAAGTCATAGAGAAAAAGAAATTAAGAAAAATCGTGA
AGAAATCGAATTTAATCAAATGTCAGATGAAGAAAAATTGAAAAAATTTGAAAAATGTGA
AATTTGTGAAAATAACATGAATATGGAAAGTGGAGAATGTGAAGTTTGTCAATTTTCTGT
TGCAGAGTTTAGTTTCTCTCATAAATTCAAAGAACTATTGGAAAAGACATTTAAAGTAAC
TGTAAAAATGCCTGAAAATCAAACTAATGATGAGAAAGAAACACTTGAAGAACCGCAGCA
TGCGAAGATACATCCAGCTTCATCAAAGAAAATTGAAACAAAAGAAAAAACATCAAATGA
AAATTGGCCATGTTCCAAATGCACTTTTTTTAATGAAGCTGCTAATTTGAATTGTATCAT
TTGTGAAGCTAAACGAGATAAGTGAatagtagaactataaaatttggttatcgattttta
ctgaatatttatgaaataataataataattttaaacaaaatattttgaaaaatattttca
atttatttcataataaattattagtattttg
>DjScaffold1824:24665-36170 strand:+
TCAGAAATACCATTCGAAACAACCAGTATTACTAATTTACCTTTTGTATTACCAATTAGA
CGACAAAATAATGGAATTTTAAACGTGAAGGTTTGCAATCATTTTACTATTAATGAAGTG
ATAAACAAAGTCTCAAAGAAAATATCTTGCGATTCAAAATCAATAGCATTATTTTATAAA
AATACTCGACTGGAGAGACATATTACACTGTCATGGTATAACATTACCATAAATTCTCAT
GTGAAAATGCAAATTTTGAAAAAAGTACAATATTATGGATCTTTTCAAAACCAAATTTAC
CCTATCTCAATATTTACGTCAGAAAAAGTATTGGAAATTAAACAATTATTTTCAAATGAA
ATTAACTATAATCCACATTTGTTAGAATTGAAATATAAAGATATTGATTTGAACAATAAT
AGACAGTTTTTTCTATATGATATTCACGAAGGTAGTAAAATTGATATTTTTACAAAGAAA
GATAGTGTCACTGACGGAAGTTCAGATAATATGGATTATGGAAAAAGTCCTGGATATGAG
TTTACTCAATGTTTTCTAGCAATGACAAGTAATTTACAGCAATGCCGATGTGCGGAATTG
CGATATGAATCTATGACTTGTGGCTTTTTGTCATCACATGTTGAAAGTTATAAAAAACAC
AAATGCTTGGAATATCTGGATATATTTTTAAAAAATGAGCATCTTCCATCTTGTTCCAAA
TCAAAAACTTCACCTTCCACAAATATAAAACAAGATCCTGAGTCAGAGGTTTTCTTTGAT
GAAAAAAGTTTAAATGAAAGTATTCATGCAGAACTAGATAACATAGATTTGAATCAAATT

CAAAAGACCGACTTAGAATATTTGTGTCAATGTAAAAATTTCAAAGCATTTAAACATTTT
CTACGGAATAAAATTGGAACAAAGTGTGATAAAACAAGTGTATCAATGCTTTACCATTAT
GCAGAGACTTTGGTGAAATTTTTAAATTCAATTAGTGAAAGTGATGAAATGATCAACAAA
ATGTTAGATTATATACGACGGTATTTTCGGAAATATTTTGAGAAACCGATAATAGCACGA
GGTGGTCTGGTA
>DjScaffold3635:96838-97906 strand:+
tggtaaccatgttagacttggaaatagatcaaaccataaaatatatctattcaacaaaaa
tgactgagtaagaaatatatatagcttttaagaaatcggttatttaataatgtttcaaca
tttcaaattataatcacattcatacattcaatcacgcattcacgtattattaaataaata
ggtcattatataaatattatacaattcttagttatatctcacaataataataataataat
aataataataataataataataataataataataataataataataataataataataat
aataataatcataataaacgttacttacagaaatcacatttgatgtaataATGAATGATC
ATAAATCAGGTGAAAAAGTCCATTTCGTAGGGGAATCAAGTATGACTGTTGGTGAATTAA
AGAAAGTTATTTCAGAAAAGCTTGAACTAAATCTTCCTTCTTTGATTTTCAAATCACCTG
ATAAAGTGTTCAAAAATAATTTAATATTGAAAGATATAATCGAAAAGAAAACAGTATATA
TAGTCAAAAATGAAGAATTCACGGTCCACGTCATCAACGACAAACGAAATTATAAAATAT
TTGCTAAGAGAAATCAAAAGATTTCTGAATTCAAACTGGCTATATCAGAGAAAATTGGAC
TTCCTGTGGATACATTTTATTTGACATTTAATGGTCAAATTATTGAAAATGTTTTACCTC
GAAAGTTTAGTCTCAGAAGCAGAAAGTTTTTCAAAAACCTGTTTCGATCAAATAGTGCTC
ATAGACCACCTAAAGATAATCCTGAAGGAGATCGTCAAATGCTTGAATCTTATGAAATTA
ATAAAAATTCTATTGTTCATGTTATTTTGAGAGCTAGAGGGGGAGCTAGATTATTATTCC
TTACACGTTAGactttggataaaaatattatttttgaagaatatgtacaatgaatatgag
tgttattttaataatgttttgtattttaataatttgtaattatttt
>DjScaffold668:3884-4854 strand:+
catttctagattcgaatcaattttcaATGACTTCAGAATATCGCGATGCAATCGCAATAT
TTGAAAAATTTGAGAAATATGAAAAACAAGAAAACCTTGGATATTATCGAACTAATTGGT
TATTATCTAATGATGACTTCAAAATATCTGTAGAGCCTCCTAGTGGCATATGCAAAACTA
TGCCAACATTGCCAAATAATCCTCTTATCGTTCTGTTTCCTTATGAAGATGAAATTGATC
ATTTGGATTATAGAGAAATTTATCAATTGATTAGGGAGTTGACTTTAGGTATTTATGGTA
GCAATCAAATTCCAGGTATCGAGTTGGAGGCTAATTACGATAAAAGTACAGAATGTCAAA
TTCCCCCTGCGTATATCAATACAAGAGTTGGACAGCATCTTATCAATGTAGATTACACTA
TGAAAAGTCTGTGGCATGGCATATATTTTCCACAAGAACTAAGAAAACCGTGGAATGATA
AATGGAAAAATATTCTAGGAGTAAATTATAACGGAATTGCGGAAACTAAGAAAGATATTT
TCGAGGAATTCATATTTGCTGGTTAAtattattataaactttataacttcgacaaagttt
ttctcgttttgtaataactgtaacgtttaaattagtctaaactaaccattttctaactta
aattgatagtttctttatattc
>DjScaffold668:9720-45053 strand:+
GATAATTTAGCCAATAAACCAAGTGTAAACCATGGAATATATTTATTAAAGTTTATCAGT
AGTTTAATTCCGTTTTTAATTGGTTTGAAGAAAAGAATGAAAATTCCCAATATTTTTAAA
TTGTTTTCACCAATAACAGGAGATGAAGTCAAAACGGAAAAGGAATTACCTCCGATAATA
ACTAATGAAAGTTTTCCTAATGTTCTTTTTAATAGTAAGAAAAAGGAGTATTTTCACCTC
CATGGAGGAATTCAATTTGATCTTGATACGCCGAACATGCAAGACTTTCCAAAACAAATC
CAGGATGAAGAGTTTATCGAACAAATTAGAAATGTGTCATCGGAAGCGTCGATAGTTGCT
CAATCGGAAACAACTTCTCAAAACAAAAATCCAGTTAGAACTGTTTGTGATCCAACTGGA
AAAAGATATTACCTCATACCAATATTTGTGGACACTTATTATAATCAAGCACCAAAGAAT
CCTAATTGGGTAAGAGCAGTTAGTGAAGAATCTATGAAATTGTATCACAAAAATCTTCCA
CTAAGTGACATTGCAATGCATGAACAATTCAAAAAGTTTTTTGGTTTAGACTCAGCAATT
AAAAACAAAAATATAATGGAAGCACTGAAAACATGCAGCAAAAGAGGATTAGTAGCTATG
TTACAAACATTATGCAGAAAATGCTCGGTATCTCGAGTCAGTAAACAAGATGAATTAGGG
TGGTCATTAATGCACCATGCAGCAATGAACAATAAAATTCACCTTATTGGTTCTTTGATT
TTACAATCGCAGGACGTTAATGTTAGACGATACAATACAGTTTCTGAAACAAGTCCAACT
CCTTTACATATGGCATCAATCTGTGGAAGTCTAAATGCAGTAAGCTGTTTGGTTGGATGT
GGAGCTAATTTAAAATCAGCTGACAAATCTGGTTGGATGCCCATTCATTGTGCTGCATTC
TATGATCAAGTAGTCATTATCAAATCATTTGTCAGAAAGGATCCTGATCTCAAAAACATT
TTCTCTAGAAATGTAACAAAATCGTCACCGATTTTGTTAGCATCGAAATCTGGAGCACTT
GCTTCAGTAAAATGTTTGATTGAACTTGGAGCCGATCTGAAATATCGCGATTCGGAGAAT
AGATGTGTAATTCATATGGCAGTTCTTGCAACAAGAACCGAAATTATTGAGTATTTTATC

AAAGAAAATATTGACTGTCTTCCAGTGTGGCAGATAATGTTAGAAATGCTGCGCCATTCT
GATATGTTGTATGTTCAACAAACCGTAAATTGTCTTGAGATCATGACGAAATATTCTTCT
AATCATTGGAATAGTATTTTAGAATCAGGCTTGGTGCTCGCATTAGTGGAGTTGTTGCAG
AAACAGGAATCATTACATTTACTGATCACCACTTCATCCGTGTTGTGTAATATTTGTACG
ACTAAGCAAGCACAAAATGAAATTGCTAAAACAGAGATTGTGAAAATATCCATTCAACTT
TTTGATTTGTCTGATATACTACTAAAATCCCAAATGGCTTTTATTCTCACACAAATGTGT
AAAATAGAATCCTGTCAGCAACTGTTTTCAGATTTAAAAGGCTATGAGTCTCTCGCAAAA
ATGATAGATGAAGCAATTATTTCCCAAGATATCACAGTTCTTGTCAATGGTGTTCAGGCC
ATCGATTCACTGTGTCGTGATAATCAACAAAATCAAAATCTGGTTGTTGAAAGCACCAAA
ATTGTCAGCTTTATTATTGAATTTTTGTCGTACAAAAACAGTGAAATAAATGATACTTTA
TTGATTGCATCGAGTCTAGCTATATCATCGTTGGTTAAGTTCAATGAAGTCAATCAAAAC
ATATTCATTGAAGCAAAATGCGTGGACTCAATATTAAAATTGCTAAAATACAGAACGAAT
GTTAACGTTCAAGTAGCTGCAGCCAATGCATTAGAAAATCTCTGCTTCAATAATAATTAT
TCTCGAGAATCTTTTCTTGTGGTTGAATCAGAGACCGCTTTGATTCATTTATTGAAGATT
TGGTCAGTTGAGGTAAAGGAGCAAGGCTTAAGAAGTTTATGGGCTCTCGCAGGAAATCAT
ATTAGTGGCCAACGTTTGATTGCTGGAAGGATTGGTCCGGGACTTCTCATTGAATTGATC
ATGACAAAATCAGAAACCCTTCAATCTATAGGCTGTTGTGCTATCTCGGCCTTAACAAAA
GAGATTAAAACAGCTCAAGACAGAATTTTGAGAGAAGGAGGAATAGAACCTTTAGTTAGA
CTGTTAAGATTGTCATCATCTACAAAAACAGTTCTGAAAAACACAGTTGAAACATTAGGA
ATATTATGCATAGGTGTTGCTCATTGTAACAATTTCGCTGTCCAGAGTCACATAGCTTTA
GAAAATGCTTTTAAAGATTTGGTTGATATTTTTAAACGGCCAACCACTGATACTAGTCTC
AAAATCGAAATAGGTTTCACTATGGCCGCTTTGATCTTGAATCATAAAGAAAATCAAAAG
AAAATGAATGATCTATTTAATTTCTGTGAATTATTAGATATAATGGCTAACACAACGGAA
AAGATTTATTTATAAaatctgataaatatttcattgttattgatttttgaaagattatca
acacaaaataaaactgtgtggattaatagcattcaatcaatgcattgcattaaattcatt
gttcactctaagtattcccattttagatggtaatactagcgaaggatatcataaatagta
accaagtgactattactgctgtctgtgttgaaactctcactaccaatcttagaaccgatg
aaaaccatgttataattctctcagctagtcttattgcaagtttatgtcatactcgagctg
gaattccagatgcatttgtggctctaggaactgtggaatatttattagaattattagatt
caaccaatgctgaagttagagctgcaagtgcagtagcattaggttatttatctttcaatg
gaactgcttctcgaataatgttaagagaatgtcgaaagaatttcacagtatttaataaat
ttatagacgaagttacaaaaatggattcaaaagtttctggtgaattggttgaagcttatc
agagagcagaaaaaattggattaccttataaatatttggagtgcaatggggggatatccaa
ttatcccgaaatcatcgcgtttaagaataaaatcatcagtatctcatccaggaggatcta
attcaaaatctaaaattaattcatctaaacgacctgtgacagcaaacgtaaccggtggat
acatggcactatgaaactagaagaattattttgtcacaaattata
>DjScaffold668:51788-53622 strand:+
TTGAAAAAAGTTGAAGAATTGGAAACTTTATATGATAACCTTTTAAATCCTAAAAAAGAT
ATTTCTAAAGAGGATCTTGAAGACGAAAATTTAAAATTAAAGTATCGGTTTACTTGTCTT
GAGAGATCTGTTAAGTTAGCAAAAGAAAATTTATCAAATCATTCAATTAGTGTGCTAGGA
TGTCTTACTGCATTGTTTGACAAAGCAATTAAAACCGCATATCCTACCATTAAAAATCCA
GAAGTATTACTTAATCCATGTGTGAATCCTAAATTTGGTGACTATCAATGTAATAGTGCC
ATGAGTTTATCAAAAAATTCTCGAAAAAGAAGGTACCAAGAAATCTCCTAAAGAAATAGCG
GAATTTATTGTTTCAAAATTAATCGAATGTAATGTGATTGAAAAATGTGAAGTTGCTCCT
GCTGGTTTTATTAATATTTTCCTGGAAAGAGAATTCATTGCTGGAGAGATATCAAAAATT
ATCAATTGTGGAGTTTTACCTCCAAAAGTCAACTCGACACGAGTTATAGTCGATTACTCA
TCTCCGAATATCGCAAAAGAAATGCACGTTGGACATTTACGATCGACAATTATCGGTGAT
TGTCTAGCGAGGTTGTTACGATTCCTGAATAACGACGTGCTCGCCGTTAATCATATTGGA
GATTGGGGAACCCAATTCGGTATGCTCATCGCTCATTTACAAGATAATTTCCCGAATTAT
CAAACCGAATCTCCGCCGATTTCCGATTTAGCAACCTTCTATAAAGCTGCTAAAGTGAGA
TTTGATTCCGACGAAGAATTTCAGAAAAGAGCGTACGAATCTGTAGTTCGTTTACAGAGC
CATAACGAAGATTCTATCAAAGCTTGGAAATTGATTTGTGATGTGTCACGTTTGGAATTC
GAGAAGATCTACATCAGACTCGGAGTTGAAGGATTGGTGGAGAAAGGAGAATCGTTCTAT
CAGAAACATATGGAGGAGATCGTTGCGGAACTCGACGAGAGAGGATTAATGGAATTCAGT
GAAGGCAGGAAAATTCTCAAATCAGAAGCGTGTGAAACTCATTTACTGATGGTGAAGAGC
GATGGAGGATTCACTTACGACACTTCGGATTTGGCCGCTTTAAAATATCGATTGGTTGAG
GAGAGAGCCGAATCGATTTTGTACGTGGTCGACAATGGGCAAGCCAAACATTTCGAGGGT

TTGTTCGCTGTCGGCGAGAAGGTCGGTATTGTTAATCCACTGAAACACAGATTGGAACAT
GTCGGGTTCGGAGTGGTTTTGGGAGCAGACAGAAAGAAATTCAAATCTCGAGCGAACGAA
ACTGTGAGACTTGCTGATTTATTGGATGAAGGAATGTCGAGATCCTTGGCTAAATTGAAG
GAAAAAGGAAGAGATCAAGAACTCTCGACTGTGGAACTTGAGAAGGCGCAAACTGCTGTA
GCTTACGGGTGCATAAAATACGCAGATTTATCTCACCATCGTACCAAGGATTACATTTTC
TCTTTCGATAAAGTGAGACTTCAGATTTGCTTGATTTCAATAATTATTTAATATTTTTG
TAAttattttaatatttttataattattttaatatttttgtaattattttaatattttta
taatttaatatttttataatttaatat
>DjScaffold2440:70908-71604 strand:+
ttaagttctatctatatctataattttatcagcttatagcaatatatttttATGACTTC
GCTTTCATCAGCAGTGGTTGTATTACCTAAACCTGAGCCAGTAGTTGAAACAAATCAATT
ACAATCAGTAGTTGAAGTAACTAATCCGGTTTTAACTGAAATTGATAGAAAATTGACATG
TCCAATTTTAATTAATTGTTTCATAAGTATGAGTGGACGGTGGAATGCTCTTACAACCTA
TAAAAGATCAAGAAATGAGTTACATAAAGTAGAAATAAACACATGGATTGATGCTTCTCT
TCGAGAATTATCGAGTGAAATTCGAGATACTTGTATCGAAGCAAGAAAACCTGGATTAAC
TCTATCTTTTGCCATTATCTATCCCGATAAACATTCAAGCGATTATAGAAGAAGAGAAAT
CGGCAAAGTTATTGTTGGAAGAAATAAGCGTAATGTAGATGGTTCCTTCCGATCTCCTGA
CGATTCATTTGTCACTTTACTATCAAAGAAATTCCAAATTGGTGATTTCGTCGAAGTTTC
TATTTTGGAAGAACCTAGACAAAATAATAATAATAATGCGAACAACAGAATCAATAACAC
GCGCAGATTTTAAttttcattaaatttgcttgtaaaatagtgattgtc
>DjScaffold2440:38983-39228 strand:+
ATGCCATTTGGCATCGCAGCGGCACCAGGGACATTTCAAGAGATGATGACAAAAGTGGTA
GGCGGAATCAATGAAGCCATGGTTTATCTTGATGATATACTGATATTTACGAACAATAAA
GATCAACACTATGAAGTAATTTACAGAGTATTGATGGAAATAATGAAGGCTGGTTTAAGA
ATTAATCCAGAAAAGTTTCAGTTTCTACAAAACGAAATCAAATTTTTTGGACAGAGATGG
TGTTAG
>DjScaffold3499:2180-5080 strand:+
aatttatattaaatggtaaataataaaatgttgtttgaagccaaattaggtaaagcagat
gcttttaaaaagttaattgattccgtaaaagatctggttcaagagggaatttttgaatgc
ttaataaatggtatagaaatgcaagctatggacagttcacacgtcagtttagtagttatt
ttaattaaaaattaagcttttgtaaattataaacattcgagagatatctaattaagggtt
aatcttgttagtctgtctaaaatccttaaatgtgctcaagtcaacgattccattacttta
aaagtggataatgattgtgaatcattggaatttgtttttgaatctccagatggtgaacgc
atatctgaatttagcctaaaggtaATGGAATTACTCATCGACAATTTAGGAATACCGGAT
ACTGATTATGATTGTGATGTTAGAATACCTTCTATTGAATTCAATAAAATCTGTCAACAG
TTAGCTGTAATTGGCAATTCTGTAACCATTACAGTAACTAAAGAGTCTATACAATTTAGT
TGTGCTGGAGATATGGGGAAAGGGAGAATTTTATTGACTCAAACCACAAATAAGCCTAGA
ATTGAGGATCAAGTTTCTATTGATGTCGTGAATCCAGTTACAATGGTTTTTGCTTTACGA
TTTCTTCATATGTTTTCAAAAGCTTCTTCTCTCAGCAGTAGAGTTCGATTATCGATTTCC
AAGGATGTACCAGCATGTATTTATTTTAATGGATTATTTCATTTCATTTAAattacacgt
ttatcacggaatttaag
>DjScaffold2007:29681-42136 strand:+
gagtttaatttttcgatagaatatttagttttcattacataattaaagttattttatgta
aaatttcggaaacattgttgtcaacagtaattgtagtaaaccaatgagaaaaatccgcga
ggtggagtctcgttttattgatatttaaatgaaatagttttattatctaaattgtgctta
ctcgtgagtaattgttggttaaataatttctaatatcacaaatgcttggaagagtgtttc
gagaagtattgatttatgtgtgtttcatgtagaaatatgtgcacaatgacgtgatataag
ttattatttgagcagtgaaatcaaatattgatatgttgtgtcattgattaatatgtacct
gtattcaggtcgagttcgagggaattaaacacgaattgaaacgcagtcaagaagaaaatg
atattctccacagtcagttggaggaaatcaataaacttttgcaaatcaatggaaaatcat
tggaaaatgctcttgaatcgcttcagttagagcgagagcagaaatacgcaataaaaaaag
aattagaaaatagaattaatgctgattcatattatcaattgaatagcattcataatgata
ttcggATGGCCGCTGAAAATTCTGCACGCAATGTAACAAGTCAACAGAAAAATCATAGTT
TGGAGGACTCCGCGAATCTCGATAATGTTGATTTTGAATTAACTAATCAAAAACACATAA
TGGAAAGTGAAGATGCTATGGATTTATTTTCTGAATTGCAACGGACTCATATGACTTCAG
AAATGAAAAAACTGGAAGAAGATAAATTAGAATCAATGCGCCAAATTAAAGAATTAAAAC
ATTTACTAGAACGACATCAAAGAGAAGCAACTCAAAGTCAGGATACAATTGGCGAACTGA

AATCTCAACTTGAAGCCATTACAACATTTGATAAAATGACAGTTGATGGATCTGGTGATA
TGAACGAATCTGTCACTCTAGGAGAATCTCTGGAAAACAGGACTGAAGAAGAATTACGAG
AAGAAATTTATAAAATGAGGAAAAACGTTAAAGTGTTAGAAACTCGTTATAAAGTTGGCT
TAAAACAAATGTCTAGCATGCAACACGAATTAAAACGGTATCACACTCGTGAAAATGTTG
ATCAAAGCTTCCAATCAGAAGAAGAATTAAAAGAAGAAATTGTACGACAACAAGTTGAAT
TGGAACAAATAAGAACAGAGCTTAAATTTACGAAAGATGAATTGGTTACAATGAGTTCAA
ATCACCAGCATTTAGTGTCAGAAGTTGGATTCTCGGCTCGAAGCATGAAATCTTCATTGG
CAACCCTAGCATTGCTGTATACATCTATCTGTGCAATTATCAAAGAAACACCTTCGAAAG
TAATGGCTGACATAATTGCAAATTTAGGTCTTGTTTCTCGTGATGATATTTCTCGTAAAG
ATTCACCAGATGATTCTATATCATCAGATATTGCTGATTCATCTCAATCCAGTGTTGAGT
CACACGAAGAATTGGCGAAAGAAGTAGAAAATCATCATAATATTGTGAATCATCTCAAGA
GTTCTTTATCGACACTAATAGAAGTGGTAAGTCAATTACAGAAGTTTAGAGAGAATAGCA
ATACCACAAATGACAGTACCAATTCTTTTAGCGGTGTAGAAGTAGAGGAGTTGCAATCTC
AAATTGTTAAACTCAAATCTATGCTAAGTACAAAACGTGATCAAATCAATACATTACGTA
ACGTTTTAAGAGCAAACAAAGCTACAGCAGAAACTGCACTTGCCAATTTGAAACAAAAGT
ACGAGAATGAAAAAGTTGTTGTTACTGATACTATGGTTAAATTACGAACTGAATTAAAAG
CCTTAAAAGAAGATGCAGCAACATTTGCTTCGTTACGAGCAATGTTTGCACAAAGATGTG
ATGAGTATGTTACTCAGCTTGATGAATTACAACAACAATTGCAGGCAGCTGAGGATGAAA
AAAGAACTGTGAATTCTTTACTTCGTATGGCCATCACACAAAAATTATCTTTGACACAAC
GTTTGGAAGATCTGGAAATGGATCGTGAAAGAAACAACATTCAGCACTTTCAACAAATGC
ACCAAGTTATGCGAAGTAACACTAATTTCCGTCCGACAACGCCAATGAACACCATACCCA
TGACTCCTGGTCAAAATCCTGCTAATGGTGTAATGATTAATCCTGTTCCTCCTGGAATAA
TTAATGCACATAGCCAACCTTTCTATGGGAATATGACATCAAATGCAAGACCGTTTAATA
GTATGCAACATCAGTATATGCCAATGACTCAACACACTTTATCAAATTTTCAACCACCTC
GTCAACCAATCAGTCATCCACTTTCTATACCGTCATCATCAAGTAACACAGCAACTCACT
CACATGCTCTTAATGTCGTCACTCCACCACAGTCAAATCCACTCACTCACAAAACTAATC
AGAGTAAAGTAAGTAAA
>DjScaffold3537:18492-18972 strand:+
TTTATTTTTCAAACGCCAAATTTTAGAAAGCATTTCGATTTAATCAAATCGGAATCCCGT
TTTAAAAGTCAATTAATTACTCAAATATCAGAAGCTAAAGAATTGCATGAAAATTCCTAT
GACAAATTAATTGATTTATGTAAAACAGACGAAGGAAATCAAGCTTTTTGGAGTGAATTA
GCAAAAAATAGAATCAGTTGGCATGAAAACTTCACAAAAGTTTCTAATATTGATTTTCAA
AATGGAATAAATCAATGGTTTATCAATGGAAAATTAAATGCATCGGTAAATTGTCTTGAT
AGACATGTGAAAACACATCCGGAAAAAACTGCTGTGGTGTGGGAAAAAGACGAGCCGGGA
AATGAAGAATTCGTTACTTATAAGTAAttttttttaattttaagttaattacatttctaa
ttaaataatactattt
>DjScaffold3537:79401-82648 strand:+
ttattattattattattATGAAAATAGCTAAAGCTAAAGTTATTATAACAACTGATGAAG
TGGTAAGAGGTGGTAAGGTAATCAATTTGAAGAAAAATATTGATGAAGCAATTAAAATTT
GTCCACCAAATACAATTCATTCGGTGCTGGTACAAAGAAGAAATAATACTTCCTTGGATC
AGTTGTCAAAAATTGATATTTGTATGGAAAAACATTTATCTTCCATGCCCATCGATTGTC
AACCTGAAATCATGGATGCTGATGATATGCTTTTTATTCTATACACATCTGGTAGTTCAG
GAGCCCCAAAAGGTATTGTTCATAACACTGCTGGATATTTACTTTATGCGGGTTTCACTT
TTCAACAAGTATTTGATTATCATCCTGAGCAAGATTTGTTTGGCTGTGTTGCTGATATTG
GGTGGATAACTGGTCATAGTTATATTGTTTATGGACCACTTTTCAATGGAGGAACTACAT
TAACATTTGAAAGCACTCCAACCTATCCAAATGCCAGCCGATACTGGGAAATGGTAGAAC
GGTTAAAACTAACTCATATATATTTAGCACCTACGGCTTTAAGATTGTTGATACAGTATG
GAGAAGAATATGTAAGGAAATATGATCGATCTTCCTTACGAGTATTAGGCTGTGTTGGTG
AACCATTAAACACGGAAGCTTGGAAATGGTACCATGAAGTTGTCGGAGAAAAGCGTTGTG
ACATTGTTGATACCTGGTGGCAAACAGAAACTGGCGGAATTATTATTTCTCCAAGACCAT
CTGGAAAAGAAGATATTATTAGACCTGGAATGCCAATGAGACCGCTACCTGGTATAGATC
TGGAATTAATTGATTTAGAAACCAATCAAGTTATTGTTGAAAGAGAAGGATACGGTGCAT
TAATTTTAAAGCGATCTTGGCCAGGTATTGCTAAAACTATATTCGGAGATCATGATAGAT
TTGTTAATTCATATTTTAAACGATATCCAGGATACTATTTTTCTGGGGATGGTTCACACA
GGCATTCAGATGGATGTTATCAAATTACTGGTCGAATCGATGATGTTATAAATACATCTG
GTCATAGAATTGGAACTGCTGAATTAGAAGATATTCTAGACATGCATGAATATGTATCTG
AATCCGCAGTAGTGGGATATCCACATTCTGTGAAAGGTGAAGGAATCTATGCTTTTATCA

CTCTAAAAAATTCGGCTACATATCTGCTTAAAAACGGAAATGAAAAATTAATCAGAAAAT
TAATCCAACAATTAAAACAACTTACTAAAGATAAACTAGCCCATTATGCTATTCCTGATT
TCATTTTAATCACACCTGGACTTCCAAAGACGAGATCCGGAAAAATAATGAGACGAATAC
TGAAAAAAGTTTCAAGTGATAATTTTGAAGATCTTGGAGACATTACAACACTTTCAAATC
CTGAAATAGTTGAATTTTTAACGTCAACCCATAAGAAAATCCAGGAAAATCCACAATGAt
taaattactaattaattttattaattgagcactttgtattatgatttcataaatttttta
cgaaaaaacttataattaatgacgaataacattgtaaatttccaacttagaaaaaattat
atttattattattattattattattaaaacaaaaaaccagcgatgttgctacatgccact
taggtgtttgtctaatttttaattcgcgtattttaatcaaaaaataatgataattatttt
tattttattttatcaaatatgaagaaactggcggaattattatttctccaagaccatctg
gaaaagaagatattattagacctggaATGCCAATGAGACCGCTACCTGGTATAGATCTGG
AATTAATTGATTTAGAAACCAATCAAGTTATTGTTGAAAGAGAAGGATACGGTGCATTAA
TTTTAAAGCGATCTTGGCCAGGTATTGCTAAAACTATATTCGGAGATCATGATAGATTTG
TTAATTCATATTTTAAACGATATCCAGGATACTATTTTTCTGGGGATGGTTCACACAGGC
ATTCAGATGGATGTTATCAAATTACTGGTCGAATCGATGATGTTATAAATACATCTGGTC
ATAGAATTGGAACTGCTGAATTAGAAGATATTCTAGACATGCATGAATATGTATCTGAAT
CCGCAGTAGTGGGATATCCACATTCTGTGAAAGGTGAAGGAATCTATGCTTTTATCACTC
TAAAAAATTCGGCTACATATCTGCTTAAAAACGGAAATGAAAAATTAATCAGAAAATTAA
TCCAACAATTAAAACAACTTACTAAAGATAAACTAGCCCATTATGCTATTCCTGATTTCA
TTTTAATCACACCTGGACTTCCAAAGACGAGATCCGGAAAAATAATGAGACGAATACTGA
AAAAAGTTTCAAGTGATAATTTTGAAGATCTTGGAGACATTACAACACTTTCAAATCCTG
AAATAGTTGAATTTTTAACGTCAACCCATAAGAAAATCCAGGAAAATCCACAATGAttaa
attactaattaattttattaattgagcactttgtattatgatttcataaatttttttacga
aaaaacttataattaatgacgaataacattgtaaatttccaacttagaaaaaattatatt
tattattattattattattattaaaacaaaa
>DjScaffold2788:40205-42453 strand:+
aaatgtaattatttttgattttatgtttaaaattataatttattttggaatttataaaat
tgaatacatttagaaattttgatattttacttattgttgtcatctgttggaaattaattt
gataatttttttaattattcaaattatttaatattgattaatgaaattatttattataaat
ggagaaaataatgatgaaaaaaaagaaattcttattcatgaagattcgaaggtgagagat
ataaaatacaatatagatgagaaactcaagggaaatggaaaatttataaaagattatttc
tcattaattcataaaaatattattttatcggattatgatacgataaaatattacaaaata
tgtgaaaatgataaaatttggtttggtaaaattgagaattatttgaaaaaagatgatgac
gtcattgtttatgtattcatttgtgaacagatttatggtggtgaacatttgattgtgaaa
atgaatgatacaatttcaataatcagatcaataatagaaagaattcattctgaaataatc
aatatttggaatgcagatggcaaacaaatgagaaatgatcaaattgtctccgagattgac
acaaataagaattacaaattgattattagatgtgatgatgaagaaggaaacgagatcgat
aatacatttgatgaatgctgctatttttacgatagcaattgtgtggatagatatttgtct
attgatcagattcgtgatgataacaaaaatgaattaacaagaacaatattgataaatcga
atgataaaaatcaaacaaattatctcatcaaataacacaattgatgacagattgaaccag
aatttgtcagatttgccctcctcatttatcaattcgacaaatttattttgcgacgaaatg
acaaatcgcatttttttatatttgtgaagatttttggaaattcgcgatgattcattatggg
aaagatgttgtgaggatttttttgtcaatatttctcatttgcaatgtaagtttgtttgaat
gaatgatggatagaattgtggtggcacaaatcattgaaacattcatatttgtgatcaatc
aaaatcaatattcaaacatgatcgatcataaatatcattgcaaaatataataatttatca
aatgatatttgcaaccattattgctctctctctctctctctcatttgatatttattat
tacttataacaatttaaaaaaatataatttcattgaaaaatattaattttatgaattaat
ttgtctatttcagtcgcgctgcattttggaatttttccaatattagaaaacaaaacaaca
gaacaaataaatcaATGAGCGATATCGTAATCGATGCTTTGGATTATTTTGTGAAATATT
TGATTGATTCCAGCAATGATTGGCGATATTGGCTGAGATTGTTCACGAAACAATATTTAT
TAAGATATTTCAGTGATTCTGATTTGAATGAATGGATTGATGAAAGAACCAATAAATTCA
CAATTAAAATACGAATGAATGGAAAGTATATAAATAATTTCATTCCATCCAAACCAGAAA
CAATTATCGAAGCAAAACAATCAATTTCAGTAGTACAGAAAGTAGACATGAAACATTTGT
ATTGCATCAAGCCAGGAGGAGATCACAAACTAAATGATGAAGACAAATTTGAAGATGAAT
TTATCATTGAAATCGTGTTGAGCGTGGAAGATATCGAGATATTAAGATCGGTAGGACAAG
GAACATTTGGTGCAACTCAGCTTTGTCTGGATAAAATATCAAACAAAAAAATTTCTTTTGA
AGAAACCAATCATAATTGATGGAGGTCTCTATCGAGATATAAATATTTATTCTAAACTCA

ATTCATCAAGAAATATCATGAATTATTCTGGATTATTCAGAGATGGAGAAAACATTTGCA
TTGGCATGGAATACATGTCCGGAGGATCTCTCAGATCTTATTTGGACAAAGGAAACATT
TATCGGAACAAGAAATAGTGAGAATCACATATGAAATCTTGACACAAATAAATGAATTAC
ATAAATTAGGAATTATTCATCGAGATATAAAATCTGATAATATTGTTTTCGATAAAGAAA
ATCATGCAAAATTAATTGATTTCGGAATGTCAAAATATACAATTGGAGTAGCAAGTGAAT
ATCTAAAAGACAAATTGAAAGCATATAAGCCTGGAACAGTTTGTTACATGGCACCAGAAT
TAATCAAAGAAGAACCGTATTCAGAGAAAATTGATATTTGGTCATTTGGACTGGTGATAG
TTGAGATGGTGACAGGTAAAAGACCATTTCATTGGCTGAAACCACCGCAAGCAGTTCTAA
AAATTCGAGTTTGTACAAAATACGAATTCGAACCAATTTTTCCAATTTCAAATGAATTGA
ATCAATTTATCAATTCTTGTTTGAATACAAAGCCAATTGTGAGACCGAGTGCAGAAAAAT
TAATCAAATCAAATTTATTTAATGATTATTTAAATAAAATTGAATTTATTTGAatatgat
atttgtttttgtagaatattagtgcatattagttttgtatattatatgtttgtttgtac
atgatttgtggttgatgaatttattggtagaaaaaattttaaactggaaatgtaattatt
tttgattttatgtttaaaattataatttattttggaatttataaaattgaatacatttag
aaattttgatattttacttattgttgtcatctgttggaaattaatttgataattttttaa
ttattcaaaattatttaatattgattaatgaaattatttattataaatggagaaaataat
gatgaaaaaaaagaaattcttattcatgaagattcgaaggtgagagatataaaatacaat
atagatgagaaactcaagggaaatggaaaatttataaaagattatttctcattaattcat
aaaaatattattttatcggattatgatacgataaaatattacaaaatatgtgaaaatgat
aaaatttggtttggtaaaattgagaattatttgaaaaaagatgatgacgtcattgtttat
gtattcatttgtgaacagatttatggtggtgaacatttgattgtgaaaatgaatgataca
atttcaataatcagatcaataatagaaagaattcattctgaaataatcaatatttggaat
gcagatggcaaacaaatgagaaatgatcaaattgtctccgagattgacacaaataagaat
tacaaattgattattagatgtgatgatgaagaaggaaacgagatcgataatacatttgat
gaatgctgctatttttacgatagcaattgtgtggatagatatttgtctattgatcagatt
cgtgatgataacaaaaatgaattaacaagaacaatattgataaatcgaatgataaaaatc
aagtgatttattgatggaaatatttcacaattaatcaataataatttattgcagacaaat
tatctcatcaaataacacaattgatgacagattgaaccagaatttgtcagatttgccctc
ctcatttatcaattcgacaaatttattttgcgacgaaATGACAAATCGCATTTTTTATAT
TTGTGAAGATTTTTGGAAATTCGCGATGATTCATTATGGGAAAGATGTTGTGAGGATTTT
TTGTCAATATTTCTCATTTGCAATTCGCGCTGCATTTTGGAATTTTTCCAATATTAGAAA
ACAAAACAACAGAACAAATAAATCAATGAGCGATATCGTAATCGATGCTTTGGATTATTT
TGTGAAATATTTGATTGATTCCAGCAATGATTGGCGATATTGGCTGAGATTGTTCACGAA
ACAATATTTATTAAGATATTTCAGTGATTCTGATTTGAATGAATGGATTGATGAAAGCAC
AACCAATAAATTCACAATTAAAATACGAATGAATGGAAAGTATATAAATAATTTCATTCC
ATCCAAACCAGAAACAATTATCGAAGCAAAACAATCAATTTCAGTAGTACAGAAAGTAGA
CATGAAACATTTGTATTGCATCAAGCCAGGAGGAGATCACAAACTAAATGATGAAGACAA
ATTTGAAGATGAATTTATCATTGAAATCGTGTTGAGCGTGGAAGATATCGAGATATTAAG
ATCGGTAGGACAAGGAACATTTGGTGCAACTCAGCTTTGTCTGGATAAAATATCAAACAA
AAAATTTCTTTTGAAGAAACCAATCATAATTGATGGAGGTCTCTATCGAGATATAAATAT
TTATTCTAAACTCAATTCATCAAGAAATATCATGAATTATTCTGGATTATTCAGAGATGG
AGAAAACATTTGCATTGGCATGGAATACATGTCCGGAGGATCTCTCAGATCTTATTTGGA
CAAAGGAAACATTTATCGGAACAAGAAATAGTGAGAATCACATATGAAATCTTGACACA
AATAAATGAATTACATAAATTAGGAATTATTCATCGAGATATAAAATCTGATAATATTGT
TTTCGATAAAGAAAATCATGCAAAATTAATTGATTTCGGAATGTCAAAATATACAATTGG
AGTAGCAAGTGAATATCTAAAAGACAAATTGAAAGCATATAAGCCTGGAACAGTTTGTTA
CATGGCACCAGAATTAATCAAAGAAGAACCGTATTCAGAGAAAATTGATATTTGGTCATT
TGGACTGGTGATAGTTGAGATGGTGACAGGTAAAAGACCATTTCATTGGCTGAAACCACC
GCAAGCAGTTCTAAAAATTCGAGTTTGTACAAAATACGAATTCGAACCAATTTTTCCAAT
TTCAAATGAATTGAATCAATTTATCAATTCTTGTTTGAATACAAAGCCAATTGTGAGACC
GAGTGCAGAAAAATTAATCAAATCAAATTTATTTAATGATTATTTAAATAAAATTGAATT
TATTTGAatatgatatttgtttttgtagaatattagtgcatattagttttgtatattat
atgtttgtttgtacatgatttgtggttgatgaatttattggtagaaaaaattttaaactg
gaaatgtaattattttttgattttatgtttaaaattataatttattttggaatttataaaa
ttgaatacatttagaaattttgatattttacttattgttgtcatctgttggaaattaatt
tgataattttttaattattcaaattatttaatattgattaatgaaattatttattataaa
tggagaaaataatgatgaaaaaaaagaaattcttattcatgaagattcgaaggtgagaga

tataaaatacaatatagatgagaaactcaagggaaatggaaaatttataaaagattattt
ctcattaattcataaaaatattattttatcggattatgatacgataaaatattacaaaat
atgtgaaaatgataaaatttggtttggtaaaattgagaattatttgaaaaaagatgatga
cgtcattgtttatgtattcatttgtgaacagatttatggtggtgaacatttgattgtgaa
aatgaatgatacaatttcaataatcagatcaataatagaaagaattcattctgaaataat
caatatttggaatgcagatggcaaacaaagattgacacaaataagaattacaaattgatt
attaggtgaatttgaatattaatagttggtagatagtggcgcactggaacattcgatgat
gtaattaatacgcactgatgacgtcatacttttagaataacacctatattgattgattat
tctcgatttagtattgatcagattcgtgatgataacaaaaatgaattaacaagaacaata
ttgataaatcgaATGATAAAAATCAAACAAATTATCTCATCAAATAACACAATTGATGAC
AGATTGAACCAGAATTTGTCAGATTTGCCCTCCTCATTTATCAATTCGACAAATTTATTT
TGCGACGAAATGACAAATCGCATTTTTTATATTTGTGAAGATTTTTGGAAATTCGCGATG
ATTCATTATGGGAAAGATGTTGTGAGGATTTTTTGTCAATATTTCTCATTTGCAATTCGC
GCTGCATTTTGGAATTTTTCCAATATTAGAAAACAAAACAACAGAACAAATAAATCAATG
AGCGATATCGTAATCGATGCTTTGGATTATTTTGTGAAATATTTGATTGATTCCAGCAAT
GATTGGCGATATTGGCTGAGATTGTTCACGAAACAATATTTATTAAGATATTTCAGTGAT
TCTGATTTGAATGAATGGATTGATGAAAGCACAACCAATAAATTCACAATTAAAATACGA
ATGAATGGAAAGTATATAAATAATTTCATTCCATCCAAACCAGAAACAATTATCGAAGCA
AAACAATCAATTTCAGTAGTACAGAAAGTAGACATGAAACATTTGTATTGCATCAAGCCA
GGAGGAGATCACAAACTAAATGATGAAGACAAATTTGAAGATGAATTTATCATTGAAATC
GTGTTGAGCGTGGAAGATATCGAGATATTAAGATCGGTAGGACAAGGAACATTTGGTGCA
ACTCAGCTTTGTCTGGATAAAATATCAAACAAAAAATTTCTTTTGAAGAAACCAATCATA
ATTGATGGAGGTCTCTATCGAGATATAAATATTTATTCTAAACTCAATTCATCAAGAAAT
ATCATGAATTATTCTGGATTATTCAGAGATGGAGAAAACATTTGCATTGGCATGGAATAC
ATGTCCGGAGGATCTCTCAGATCTTATTTGGACAAAGGGAAACATTTATCGGAACAAGAA
ATAGTGAGAATCACATATGAAATCTTGACACAAATAAATGAATTACATAAATTAGGAATT
ATTCATCGAGATATAAAATCTGATAATATTGTTTTCGATAAAGAAAATCATGCAAAATTA
ATTGATTTCGGAATGTCAAAATATACAATTGGAGTAGCAAGTGAATATCTAAAAGACAAA
TTGAAAGCATATAAGCCTGGAACAGTTTGTTACATGGCACCAGAATTAATCAAAGAAGAA
CCGTATTCAGAGAAAATTGATATTTGGTCATTTGGACTGGTGATAGTTGAGATGGTGACA
GGTAAAAGACCATTTCATTGGCTGAAACCACCGCAAGCAGTTCTAAAAATTCGAGTTTGT
ACAAAATACGAATTCGAACCAATTTTTCCAATTTCAAATGAATTGAATCAATTTATCAAT
TCTTGTTTGAATACAAAGCCAATTGTGAGACCGAGTGCAGAAAAATTAATCAAATCAAAT
TTATTTAATGATTATTTAAATAAAATTGAATTTATTTGAatatgatatttgtttttgta
gaatattagtgcatattagttttgtatattatatgtttgtttgtacatgatttgtggttg
atgaatttattggtagaaaaaattttaaactggaaatgtaattatttttgattttatgtt
taaaattataatttattttggaatttataaaattgaatacatttagaaattttgatattt
tacttattgttgtcatctgttggaaattaatttgataattttttaattattcaaaattat
ttaatattgattaatgaaattatttattataaatggagaaaataatgatgaaaaaaaaga
aattcttattcatgaagattcgaaggtgagagatataaaatacaatatagatgagaaact
caagggaaatggaaaatttataaaagattatttctcattaattcataaaaatattatttt
atcggattatgatacgataaaatattacaaaatatgtgaaaatgataaaatttggtttgg
taaaattgagaattatttgaaaaaagatgatgacgtcattgtttatgtattcatttgtga
acagatttatggtggtgaacatttgattgtgaaaatgaatgatacaatttcaataatcag
atcaataatagaaagaattcattctgaaataatcaatatttggaatgcagatggcaaaca
aatgagaaatgatcaaattgtctccgagattgacacaaataagaattacaaattgattat
tagatgtgatgatgaagaaggaaacgagatcgataatacatttgatgaatgctgctattt
ttacgatagcaattgtgtggatagatatttgtctattgatcagattcgtgatgataacaa
aaatgaattaacaagaacaatattgataaatcgaatgataaaaatcaaacaaattatctc
atcaaataacacaattgatgacagattgaaccagaatttgtcagatttgccctcctcatt
tatcaattcgacaaatttattttgcgacgaaatgacaaatcgcattttttatatttgtga
agatttttggaaattcgcgatgattcattatgggaaagagtgagattgatatttgtgtgt
gacacaattgattttcgcgctgcattttggaatttttccaatattagaaaacaaaacaac
agaacaaataaatcaATGAGCGATATCGTAATCGATGCTTTGGATTATTTTGTGAAATAT
TTGATTGATTCCAGCAATGATTGGCGATATTGGCTGAGATTGTTCACGAAACAATATTTA
TTAAGATATTTCAGTGATTCTGATTTGAATGAATGGATTGATGAAAGCACAACCAATAAA
TTCACAATTAAAATACGAATGAATGGAAAGTATATAAATAATTTCATTCCATCCAAACCA

GAAACAATTATCGAAGCAAAACAATCAATTTCAGTAGTACAGAAAGTAGACATGAAACAT
TTGTATTGCATCAAGCCAGGAGGAGATCACAAACTAAATGATGAAGACAAATTTGAAGAT
GAATTTATCATTGAAATCGTGTTGAGCGTGGAAGATATCGAGATATTAAGATCGGTAGGA
CAAGGAACATTTGGTGCAACTCAGCTTTGTCTGGATAAAATATCAAACAAAAAATTTCTT
TTGAAGAAACCAATCATAATTGATGGAGGTCTCTATCGAGATATAAATATTTATTCTAAA
CTCAATTCATCAAGAAATATCATGAATTATTCTGGATTATTCAGAGATGGAGAAAACATT
TGCATTGGCATGGAATACATGTCCGGAGGATCTCTCAGATCTTATTTGGACAAAGGGAAA
CATTTATCGGAACAAGAAATAGTGAGAATCACATATGAAATCTTGACACAAATAAATGAA
TTACATAAATTAGGAATTATTCATCGAGATATAAAATCTGATAATATTGTTTTCGATAAA
GAAAATCATGCAAAATTAATTGATTTCGGAATGTCAAAATATACAATTGGAGTAGCAAGT
GAATATCTAAAAGACAAATTGAAAGCATATAAGCCTGGAACAGTTTGTTACATGGCACCA
GAATTAATCAAAGAAGAACCGTATTCAGAGAAAATTGATATTTGGTCATTTGGACTGGTG
ATAGTTGAGATGGTGACAGGTAAAAGACCATTTCATTGGCTGAAACCACCGCAAGCAGTT
CTAAAAATTCGAGTTTGTACAAAATACGAATTCGAACCAATTTTTCCAATTTCAAATGAA
TTGAATCAATTTATCAATTCTTGTTTGAATACAAAGCCAATTGTGAGACCGAGTGCAGAA
AAATTAATCAAATCAAATTTATTTAATGATTATTTAAATAAAATTGAATTTATTTGAata
tgatatttgtttttgtagaatattagtgcatattagttttgtatattatatgtttgttt
gtacatgatttgtggttgatgaatttattggtagaaaaaattttaaactggaaatgtaat
tatttttgattttatgtttaaaattataatttattttggaatttataaaattgaatacat
ttagaaattttgatattttacttattgttgtcatctgttggaaattaatttgataatttt
ttaattattcaaattatttaatattgattaatgaaattatttattataaatggagaaaat
aatgatgaaaaaaaagaaattcttattcatgaagattcgaaggtgagagatataaaatac
aatatagatgagaaactcaagggaaatggaaaatttataaaagattatttctcattaatt
cataaaaatattattttatcggattatgatacgataaaatattacaaaatatgtgaaaat
gataaaatttggtttggtaaaattgagaattatttgaaaaaagatgatgacgtcattgtt
tatgtattcatttgtgaacagatttatggtggtgaacatttgattgtgaaaatgaatgat
acaatttcaataatcagatcaataatagaaagaattcattctgaaataatcaatatttgg
aatgcagatggcaaacaaatgagaaatgatcaaattgtctccgagattgacacaaataag
aattacaaattgattattagatgtgatgatgaagaaggaaacgagatcgataatacattt
gatgaatgctgctatttttacgatagcaattgtgtggatagatatttgtctattgatcag
attcgtgatgataacaaaaatgaattaacaagaacaatattgataaatcgaatgataaaa
atcaaacaaattatctcatcaaataacacaattgatgacagattgaaccagaatttgtca
gatttgccctcctcatttatcaattcgacaaatttattttgcgacgaaatgacaaatcgc
atttttatatttgtgaagatttttggaaattcgcgatgattcattatgggaaagatgtt
gtgaggatttttttgtcaatatttctcatttgcaatgtaagtttgtttgaatgaatgatgg
atagaattgtggtggcacaaatcattgaaacattcatatttgtgatcaatcaaaaatcga
tgctttggattattttgtgaaatatttgattgattccagcaatgattggcgatattggct
gagattgtgagatgcaatgatgattgtgagattctgatttgaatgaatggattgatgaaa
gcacaaccaataaattcacaattaaaatacgaATGAATGGAAAGTATATAAATAATTTCA
TTCCATCCAAACCAGAAACAATTATCGAAGCAAAACAATCAATTTCAGTAGTACAGAAAG
TAGACATGAAACATTTGTATTGCATCAAGCCAGGAGGAGATCACAAACTAAATGATGAAG
ACAAATTTGAAGATGAATTTATCATTGAAATCGTGTTGAGCGTGGAAGATATCGAGATAT
TAAGATCGGTAGGACAAGGAACATTTGGTGCAACTCAGCTTTGTCTGGATAAAATATCAA
ACAAAAAATTTCTTTTGAAGAAACCAATCATAATTGATGGAGGTCTCTATCGAGATATAA
ATATTTATTCTAAACTCAATTCATCAAGAAATATCATGAATTATTCTGGATTATTCAGAG
ATGGAGAAAACATTTGCATTGGCATGGAATACATGTCCGGAGGATCTCTCAGATCTTATT
TGGACAAAGGGAAACATTTATCGGAACAAGAAATAGTGAGAATCACATATGAAATCTTGA
CACAAATAAATGAATTACATAAATTAGGAATTATTCATCGAGATATAAAATCTGATAATA
TTGTTTTCGATAAAGAAAATCATGCAAAATTAATTGATTTCGGAATGTCAAAATATACAA
TTGGAGTAGCAAGTGAATATCTAAAAGACAAATTGAAAGCATATAAGCCTGGAACAGTTT
GTTACATGGCACCAGAATTAATCAAAGAAGAACCGTATTCAGAGAAAATTGATATTTGGT
CATTTGGACTGGTGATAGTTGAGATGGTGACAGGTAAAAGACCATTTCATTGGCTGAAAC
CACCGCAAGCAGTTCTAAAAATTCGAGTTTGTACAAAATACGAATTCGAACCAATTTTTC
CAATTTCAAATGAATTGAATCAATTTATCAATTCTTGTTTGAATACAAAGCCAATTGTGA
GACCGAGTGCAGAAAAATTAATCAAATCAAATTTATTTAATGATTATTTAAATAAAATTG
AATTTATTTGAatatgatatttgtttttgtagaatattagtgcatattagttttgtata
ttatatgtttgtttgtacatgatttgtggttgatgaatttattggtagaaaaaattttaa

actggaaatgtaattatttttgattttatgtttaaaattataatttattttggaatttat
aaaattgaatacatttagaaattttgatattttacttattgttgtcatctgttggaaatt
aatttgataatttttttaattattcaaattatttaatattgattaatgaaattatttatta
taaatggagaaaataatgatgaaaaaaaagaaattcttattcatgaagattcgaaggtga
gagatataaaatacaatatagatgagaaactcaagggaaatggaaaatttataaaagatt
atttctcattaattcataaaaatattattttatcggattatgatacgataaaatattaca
aaatatgtgaaaatgataaaatttggtttggtaaaattgagaattatttgaaaaaagatg
atgacgtcattgtttatgtattcatttgtgaacagatttatggtggtgaacatttgattg
tgaaaatgaatgatacaatttcaataatcagatcaataatagaaagaattcattctgaaa
taatcaatatttggaatgcagatggcaaacaaatgagaaatgatcaaattgtctccgaga
ttgacacaaataagaattacaaattgattattagatgtgatgatgaagaaggaaacgaga
tcgataatacatttgatgaatgctgctatttttacgatagcaattgtgtggatagatatt
tgtctattgatcagattcgtgatgataacaaaaatgaattaacaagaacaatattgataa
atcgaatgataaaaatcaaacaaattatctcatcaaataacacaattgatgacagattga
accagaatttgtcagatttgccctcctcatttatcaattcgacaaatttattttgcgacg
aaatgacaaatcgcatttttttatatttgtgaagatttttggaaattcgcgatgattcatt
atgggaaagagtgagattgatatttgtgtgtgacacaattgatttgttattgattgatca
tttagtgttgtgaggatttttttgtcaatatttctcatttgcaataaattattttgatatt
gatttatagaatcgatgctttggattattttgtgaaatatttgattgattccagcaatga
ttggcgatattggctgagattgttcacgaaacaatatttattaagatatttcagtgattc
tgatttgaatgaatggattgatgaaagcacaaccaataaattcacaattaaaatacgaAT
GAATGGAAAGTATATAAATAATTTCATTCCATCCAAACCAGAAACAATTATCGAAGCAAA
ACAATCAATTTCAGTAGTACAGAAAGTAGACATGAAACATTTGTATTGCATCAAGCCAGG
AGGAGATCACAAACTAAATGATGAAGACAAATTTGAAGATGAATTTATCATTGAAATCGT
GTTGAGCGTGGAAGATATCGAGATATTAAGATCGGTAGGACAAGGAACATTTGGTGCAAC
TCAGCTTTGTCTGGATAAAATATCAAACAAAAAATTTCTTTTGAAGAAACCAATCATAAT
TGATGGAGGTCTCTATCGAGATATAAATATTTATTCTAAACTCAATTCATCAAGAAATAT
CATGAATTATTCTGGATTATTCAGAGATGGAGAAAACATTTGCATTGGCATGGAATACAT
GTCCGGAGGATCTCTCAGATCTTATTTGGACAAAGGGAAACATTTATCGGAACAAGAAAT
AGTGAGAATCACATATGAAATCTTGACACAAATAAATGAATTACATAAATTAGGAATTAT
TCATCGAGATATAAAATCTGATAATATTGTTTTCGATAAAGAAAATCATGCAAAATTAAT
TGATTTCGGAATGTCAAAATATACAATTGGAGTAGCAAGTGAATATCTAAAAGACAAATT
GAAAGCATATAAGCCTGGAACAGTTTGTTACATGGCACCAGAATTAATCAAAGAAGAACC
GTATTCAGAGAAAATTGATATTTGGTCATTTGGACTGGTGATAGTTGAGATGGTGACAGG
TAAAAGACCATTTCATTGGCTGAAACCACCGCAAGCAGTTCTAAAAATTCGAGTTTGTAC
AAAATACGAATTCGAACCAATTTTTCCAATTTCAAATGAATTGAATCAATTTATCAATTC
TTGTTTGAATACAAAGCCAATTGTGAGACCGAGTGCAGAAAAATTAATCAAATCAAATTT
ATTTAATGATTATTTAAATAAAATTGAATTTATTTGAatatgatatttgtttttttgtaga
atattagtgcatattagttttgtatattatatgtttgtttgtacatgatttgtggttgat
gaatttattggtagaaaaaattttaaactggaaatgtaattatttttgattttatgttta
aaattataatttattttggaatttataaaattgaatacatttagaaattttgatatttta
cttattgttgtcatctgttggaaattaatttgataatttttttaattattcaaattattta
atattgattaatgaaattatttattataaatggagaaaataatgatgaaaaaaaagaaat
tcttattcatgaagattcgaaggtgagagatataaaatacaatatagatgagaaactcaa
gggaaatggaaaatttataaaagattatttctcattaattcataaaaatattattttatc
ggattatgatacgataaaatattacaaaatatgtgaaaatgataaaatttggtttggtaa
aattgagaattatttgaaaaaagatgatgacgtcattgtttatgtattcatttgtgaaca
gatttatggtggtgaacatttgattgtgaaaatgaatgatacaatttcaataatcagatc
aataatagaaagaattcattctgaaataatcaatatttggaatgcagatggcaaacaaag
attgacacaaataagaattacaaattgattattaggtgaatttgaatattaatagttggt
agatagtggcgcactggaacattcgatgatgtaattaatacgcactgatgacgtcatact
tttagaataacacctatattgattgattattctcgatttagtattgatcagattcgtgat
gataacaaaaatgaattaacaagaacaatattgataaatcgaATGATAAAAATCAAACAA
ATTATCTCATCAAATAACACAATTGATGACAGATTGAACCAGAATTTGTCAGATTTGCCC
TCCTCATTTATCAATTCGACAAATTTATTTTGCGACGAAATGACAAATCGCATTTTTTAT
ATTTGTGAAGATTTTTGGAAATTCGCGATGATTCATTATGGGAAAGATGTTGTGAGGATT
TTTTGTCAATATTTCTCATTTGCAATTCGCGCTGCATTTTGGAATTTTTCCAATATTAGA

AAACAAAACAACAGAACAAATAAATCAATGAGCGATATCGTAATCGATGCTTTGGATTAT
TTTGTGAAATATTTGATTGATTCCAGCAATGATTGGCGATATTGGCTGAGATTGTTCACG
AAACAATATTTATTAAGATATTTCAGTGATTCTGATTTGAATGAATGGATTGATGAAAGC
ACAACCAATAAATTCACAATTAAAATACGAATGAATGGAAAGTATATAAATAATTTCATT
CCATCCAAACCAGAAACAATTATCGAAGCAAAACAATCAATTTCAGTAGTACAGAAAGTA
GACATGAAACATTTGTATTGCATCAAGCCAGGAGGAGATCACAAACTAAATGATGAAGAC
AAATTTGAAGATGAATTTATCATTGAAATCGTGTTGAGCGTGGAAGATATCGAGATATTA
AGATCGGTAGGACAAGGAACATTTGGTGCAACTCAGCTTTGTCTGGATAAAATATCAAAC
AAAAAATTTCTTTTGAAGAAACCAATCATAATTGATGGAGGTCTCTATCGAGATATAAAT
ATTTATTCTAAACTCAATTCATCAAGAAATATCATGAATTATTCTGGATTATTCAGAGAT
GGAGAAAACATTTGCATTGGCATGGAATACATGTCCGGAGTTGATTTTATTTTACTATTG
AAATTATATTGAtaatatttgcatttgaaattattcagggatctctcagatcttatttgg
acaaagggaaacatttatcggaacaagaaatagtgagaatcacatatgaaatcttgacac
aaataaatgaattacataaattaggaattattcatcgagatataaaatctgataatattg
ttttcgataaagaaaatcatgcaaaattaattgatttcggaatgtcaaaatatacaattg
gagtagcaagtgaatatctaaaagacaaattgaaagcatataagcctggaacagtttgtt
acatggcaccagaattaatcaaagaagaaccgtattcagagaaaattgatatttggtcat
ttggactggtgatagttgagatggtgacaggtaaagctgaaaccaccgcaagcagttcta
aaaattcgagtttgtacaaaatacgaattcgaaccaatttttccaatttcaaatgaattg
aatcaatttatcaattcttgtttgaatacaaagccaattgtgagaccgagtgcagaaaaa
ttaatcaaatcaaatttatttaaaatgaatgatacaatttcaataatcagatcaataata
gaaagaattcattctgaaataatcaatatttggaatgcagatggcaaacaaatgagaaat
gatcaaattgtctccgagattgacacaaataagaattacaaattgattattagatgtgat
gatgaagaaggaaacgagatcgataatacatttgatgaatgctgctatttttacgatagc
aattgtgtggatagatatttgtctattgatcagattcgtgatgataacaaaaatgaatta
acaagaacaatattgataaatcgaatgataaaaatcaaacaaattatctcatcaaataac
acaattgatgacagattgaaccagaatttgtcagatttgccctcctcatttatcaattcg
acaaatttattttgcgacgaaatgacaaatcgcatttttttatatttgtgaagatttttgg
aaattcgcgatgattcattatgggaaagatgttgtgaggatttttttgtcaatatttctca
tttgcaatgtaagtttgtttgaatgaatgatggatagaattgtggtggcacaaatcattg
aaacattcatatttgtgatcaatcaaaatcaatattcaaacatgatcgatcataaatatc
attgcaaaatataataatttatcaaatgatatttgcaaccattattgctctctctctctc
tctctctcatttgatatttattattacttataacaatttaaaaaaatataatttcattga
aaaatattaattttatgaattaatttgtctatttcagtcgcgctgcattttggaattttt
ccaatattagaaaacaaaacaacagaacaaataaatcaATGAGCGATATCGTAATCGATG
CTTTGGATTATTTTGTGAAATATTTGATTGATTCCAGCAATGATTGGCGATATTGGCTGA
GATTGTTCACGAAACAATATTTATTAAGATATTTCAGTGATTCTGATTTGAATGAATGGA
TTGATGAAAGAACCAATAAATTCACAATTAAAATACGAATGAATGGAAAGTATATAAATA
ATTTCATTCCATCCAAACCAGAAACAATTATCGAAGCAAAACAATCAATTTCAGTAGTAC
AGAAAGTAGACATGAAACATTTGTATTGCATCAAGCCAGGAGGAGATCACAAACTAAATG
ATGAAGACAAATTTGAAGATGAATTTATCATTGAAATCGTGTTGAGCGTGGAAGATATCG
AGATATTAAGATCGGTAGGACAAGGAACATTTGGTGCAACTCAGCTTTGTCTGGATAAAA
TATCAAACAAAAAATTTCTTTTGAAGAAACCAATCATAATTGATGGAGGTCTCTATCGAG
ATATAAATATTTATTCTAAACTCAATTCATCAAGAAATATCATGAATTATTCTGGATTAT
TCAGAGATGGAGAAAACATTTGCATTGGCATGGAATACATGTCCGGAGGATCTCTCAGAT
CTTATTTGGACAAAGGGAAACATTTATCGGAACAAGAAATAGTGAGAATCACATATGAAA
TCTTGACACAAATAAATGAATTACATAAATTAGGAATTATTCATCGAGATATAAAATCTG
ATAATATTGTTTTCGATAAAGAAAATCATGCAAAATTAATTGATTTCGGAATGTCAAAAT
ATACAATTGGAGTAGCAAGTGAATATCTAAAAGACAAATTGAAAGCATATAAGCCTGGAA
CAGTTTGTTACATGGCACCAGAATTAATCAAAGAAGAACCGTATTCAGAGAAAATTGATA
TTTGGTCATTTGGACTGGTGATAGTTGAGATGGTGACAGGTAAAAGACCATTTCATTGGC
TGAAACCACCGCAAGCAGTTCTAAAAATTCGAGTTTGTACAAAATACGAATTCGAACCAA
TTTTTCCAATTTCAAATGAATTGAATCAATTTATCAATTCTTGTTTGAATACAAAGCCAA
TTGTGAGACCGAGTGCAGAAAAATTAATCAAATCAAATTTATTTAATGATTATTTAAATA
AAATTGAATTTATTTGAatatgatatttgtttttgtagaatattagtgcatattagttt
tgtatattatatgtttgtttgtacatgatttgtggttgatgaatttattggtagaaaaaa
ttttaaactggaaatgtaattatttttgattttatgtttaaaattataatttattttgga

atttataaaattgaatacatttagaaattttgatattttacttattgttgtcatctgttg
gaaattaatttgataatttttaattattcaaattatttaatattgattaATGAAATTAT
TTATTATAAATGGAGAAAATAATGATGAAAAAAAAGAAATTCTTATTCATGAAGATTCGA
AGGTGAGAGATATAAAATACAATATAGATGAGAAACTCAAGGGAAATGGAAAATTTATAA
AAGATTATTTCTCATTAATTCATAAAAATATTATTTTATCGGATTATGATACGATAAAAT
ATTACAAAATATGTGAAAATGATAAAATTTGGTTTGGTAAAATTGAGAATTATTTGAAAA
AAGATGATGACGTCATTGTTTATGTATTCATTTGTGAACAGATTTATGGTGGTGAACATT
TGATTGTGAAAATGAATGATACAATTTCAATAATCAGATCAATAATAGAAAGAATTCATT
CTGAAATAATCAATATTTGGAATGCAGATGGCAAACAAATGAGAAATGATCAAATTGTCT
CCGAGATTGACACAAATAAGAATTACAAATTGATTATTAGATGTGATGATGAAGAAGGAA
ACGAGATCGATAATACATTTGATGAATGCTGCTATTTTTACGATAGCAATTGTGTGGATA
GATATTTGTCTATTGATCAGATTCGTGATGATAACAAAAATGAATTAACAAGAACAATAT
TGATAAATCGAATGATAAAAATCAAACAAATTATCTCATCAAATAACACAATTGATGACA
GATTGAACCAGAATTTGTCAGATTTGCCCTCCTCATTTATCAATTCGACAAATTTATTTT
GCGACGAAATGACAAATCGCATTTTTTATATTTGTGAAGATTTTTGGAAATTCGCGATGA
TTCATTATGGGAAAGATGTTGTGAGGATTTTTTGTCAATATTTCTCATTTGCAATGTAAg
tttgtttgaatgaatgatggatagaattgtggtggcacaaatcattgaaacattcatatt
tgtgatcaatcaaaaatcgatgctttggattattttgtgaaatatttgattgattccagc
aatgattggcgatattggctgagattgtgagatgcaatgatgattgtgagattctgattt
gaatgaatggattgatgaaagcacaaccaataaattcacaattaaaatacgaatgaatgg
aaagtatataaataatttcattccatccaaaccagaaacaattatcgaagcaaaacaatc
aatttcagtagtacagaaagtagacatgaaacatttgtattgcatcaagccaggaggaga
tcacaaactaaatgatgaagacaaatttgaagatgaatttatcattgaaatcgtgttgag
cgtggaagatatcgagatattaagatcggtaggacaaggaacatttggtgcaactcagct
ttgtctggataaaatatcaaacaaaaaatttcttttgaagaaaccaatcataattgatgg
aggtctctatcgagatataaatatttattctaaactcaattcatcaagaaatatcatgaa
ttattctggattattcagagatggagaaaacatttgcattggcatggaatacatgtccgg
aggatctctcagatcttatttggacaaagggaaacatttatcggaacaagaaatagtgag
aatcacatatgaaatcttgacacaaataaatgaattacataaattaggaattattcatcg
agatataaaatgtttgattttactctgcacaatattttattatttgtgttgtctagctga
taatattgttttcgataaagaaaatcatgcaaaattaattgatttcggaatgtcaaaata
tacaattggagtagcaagtgaatatctaaaagacaaattgaaagaaaatgaatgatacaa
tttcaataatcagatcaataatagaaagaattcattctgaaataatcaatatttggaatg
cagatggcaaacaaatgagaaatgatcaaattgtctccgagattgacacaaataagaatt
acaaattgattattagatgtgatgatgaagaaggaaacgagatcgataatacatttgatg
aatgctgctatttttacgatagcaattgtgtggatagatatttgtctattgatcagattc
gtgatgataacaaaaatgaattaacaagaacaatattgataaatcgaatgataaaaatca
aacaaattatctcatcaaataacacaattgatgacagattgaaccagaatttgtcagatt
tgccctcctcatttatcaattcgacaaatttattttgcgacgaaatgacaaatcgcattt
tttatatttgtgaagatttttggaaattcgcgatgattcattatgggaaagatgttgtga
ggattttttgtcaatatttctcatttgcaatgtaagtttgtttgaatgaatgatggatag
aattgtggtggcacaaatcattgaaacattcatatttgtgatcaatcaaaaatcgatgct
ttggattattttgtgaaatatttgattgattccagcaatgattggcgatattggctgaga
ttgttcacgaaacaatatttattaagatatttcagtgattctgatttgaatgaatggatt
gatgaaagcacaaccaataaattcacaattaaaatacgaATGAATGGAAAGTATATAAAT
AATTTCATTCCATCCAAACCAGAAACAATTATCGAAGCAAAACAATCAATTTCAGTAGTA
CAGAAAGTAGACATGAAACATTTGTATTGCATCAAGCCAGGAGGAGATCACAAACTAAAT
GATGAAGACAAATTTGAAGATGAATTTATCATTGAAATCGTGTTGAGCGTGGAAGATATC
GAGATATTAAGATCGGTAGGACAAGGAACATTTGGTGCAACTCAGCTTTGTCTGGATAAA
ATATCAAACAAAAAATTTCTTTTGAAGAAACCAATCATAATTGATGGAGGTCTCTATCGA
GATATAAATATTTATTCTAAACTCAATTCATCAAGAAATATCATGAATTATTCTGGATTA
TTCAGAGATGGAGAAAACATTTGCATTGGCATGGAATACATGTCCGGAGGATCTCTCAGA
TCTTATTTGGACAAAGGGAAACATTTATCGGAACAAGAAATAGTGAGAATCACATATGAA
ATCTTGACACAAATAAATGAATTACATAAATTAGGAATTATTCATCGAGATATAAAATGT
TTGATTTTACTCTGCACAATATTTTATTATTTGTGTTGTCTAGCTGATAATATTGTTTTC
GATAAAGAAAATCATGCAAAATTAATTGATTTCGGAATGTCAAAATATACAATTGGAGTA
GCAAGTGAATATCTAAAAGACAAATTGAAAcaatgagcgatatcgtgtaaaatgaaatga

tttattaataaattattttgatattgatttatagaatcgatgctttggattattttgtga
aatatttgattgattccagcaatgattggcgatattggctgagattgttcacgaaacaat
atttattaagatatttcagtgattctgatttgaatgaatggattgatgaaagcacaacca
ataaattcacaattaaaatacgaATGAATGGAAAGTATATAAATAATTTCATTCCATCCA
AACCAGAAACAATTATCGAAGCAAAACAATCAATTTCAGTAGTACAGAAAGTAGACATGA
AACATTTGTATTGCATCAAGCCAGGAGGAGATCACAAACTAAATGATGAAGACAAATTTG
AAGATGAATTTATCATTGAAATCGTGTTGAGCGTGGAAGATATCGAGATATTAAGATCGG
TAGGACAAGGAACATTTGGTGCAACTCAGCTTTGTCTGGATAAAATATCAAACAAAAAAT
TTCTTTTGAAGAAACCAATCATAATTGATGGAGGTCTCTATCGAGATATAAATATTTATT
CTAAACTCAATTCATCAAGAAATATCATGAATTATTCTGGATTATTCAGAGATGGAGAAA
ACATTTGCATTGGCATGGAATACATGTCCGGAGGATCTCTCAGATCTTATTTGGACAAAG
GGAAACATTTATCGGAACAAGAAATAGTGAGAATCACATATGAAATCTTGACACAAATAA
ATGAATTACATAAATTAGGAATTATTCATCGAGATATAAAATCTGATAATATTGTTTTCG
ATAAAGAAAATCATGCAAAATTAATTGATTTCGGAATGTCAAAATATACAATTGGAGTAG
CAAGTGAATATCTAAAAGACAAATTGAAAGCATATAAGCCTGGAACAGTTTGTTACATGG
CACCAGAATTAATCAAAGAAGAACCGTATTCAGAGAAAATTGATATTTGGTCATTTGGAC
TGGTGATAGTTGAGATGGTGACAGGTAAAAGACCATTTCATTGGCTGAAACCACCGCAAG
CAGTTCTAAAAATTCGAGTTTGTACAAAATACGAATTCGAACCAATTTTTCCAATTTCAA
ATGAATTGAATCAATTTATCAATTCTTGTTTGAATACAAAGCCAATTGTGAGACCGAGTG
CAGAAAAATTAATCAAATCAAATTTATTTAATGATTATTTAAATAAAATTGAATTTATTT
GAatatgatatttgtttttgtagaatattagtgcatattagttttgtatattatatgtt
tgtttgtacatgatttgtggttgatgaatttattggtagaaaaaattttaaactgg
>DjScaffold1439:5432-5872 strand:+
TTGATTCTTGCTTTGACAATTTCGTTAGTGGCTTGCAATCCTTTGCAAAATGCAAATTTT
GAAACAGAATCTGGAAAGAATGTTATTTTACAAGAAAGATATTTGAAAGAAAAATCAACA
GAATTACTTCATAAACTAATTCAAAAATTAAGAGCTGCTTATGATAAAGGAGCAGATGCA
TTCTACAAAGTTATCAATGATTCAGACATCAAAGAAATCTTAGATCGTATNCCGTTGACA
AGGAAAATTGCTGATTCTTTAATAAAAGAATTTAGAGATGTAATTAAATTTATAAATTTA
TACGATAATGAAATACAAAATAATAAATTAAATAAAATTTAAtaaaacaaaaaatacaat
aaatatataaaaaataaaataataacatacaa
>DjScaffold1439:24274-24666 strand:+
TTGATTCTTGCTTTGACAATTTCGTTAGTGGCTTGCAATCCTTTGCAAAATGAAAATTTT
GAAACAGAATCTGGAAAGAATGTTATTTTACAAGAAAGATATTTGAAAGAAAAATCAACA
GAATTACTTCATAAACTAATTCAAAAATTAAGAGCTGCTTATGATAAAGGAGCAGATGCA
TTCTACAAAGTTATCAATGATTCAGACATCAAAGAAATCTTAGATCGTATGGATTCACTC
ACCAGACGAATTGCTGAATCGATTATCAACAAATTCAAAGAAATTATCAAAAAAATTGAT
GAAGCAACAAGAGAAAATCCAGAATTGTTGAAAAGAGATCTGAAAGGAGATTCGAGAAAA
TATTTTAATAAATTGGTTCAGATTTTATTGAATGCATACAAACAAGGTTTGGACTACTTC
AATAAAGTTGTCAATGATCCTGATGTGCAAGGAATTCTCGAACATCTTGGACCGTTGACA
AGGAAAATGGCTGATTCATTAATAAAAGAATTTAGAGATGTAATTAAAATTTTAAAACAA
TTAAAATAAatctctattattaatctataaataattcttttccatttctaatttaaatac
caaaataaatttttccagatatttgaatatgaattttcaatattattttatttaaaatca
atttatttttcaaaagttaacaagataatattattaataataattatttttacgaagaa
aaaacaaaattatttcataaataatttgtgaaaaatagaaaattaattaagttattaatt
aaatatataatatgaaatataattttagttaatcgacaaaattgatgaagttaccaaaga
aacaaaccatattgaaaaaagagatttcaatgaaagctcatttagtcatttaaagaaatt
aaaaagcattttaacgaaagcttttcaatctggtccaaaagccttcaatgaaactctaga
aggttcagaagttaaagaaattttaaccaaacttaacgatctttgtctaaaaactgcata
ctgcatgaatcacaaatttgaagaaatgtttatgaaattactcgttagtttgagtaaatt
aatgtggtctgcatcagtgaatttaattgaataaaactttatatctcgttggaactttgt
taagtaatctaacattagatttttagtcagagtaattaataaatttgtaTTGATTCTTG
CTTTGACAATTTCGTTAGTGGCTTGCAATCCTTTGCAAAATGAAAATTTTGAAACAGAAT
CTGGAAAGAATGTTATTTTACAAGAAAGATATTTGAAAGAAAAATCAACAGAATTACTTC
ATAAACTAATTCAAAAATTAAGAGCTGCTTATGATAAAGGAGCAGATGCATTCTACAAAG
TTATCAATGATTCAGACATCAAAGAAATCTTAGATCGTATGGATTCACTCACCAGACGAA
TTGCTGAATCGATTATCAACAAATTCAAAGAAGTTAGAAGA
>DjScaffold1439:78682-80521 strand:+

aataataataataataataataataatcataataataataataataataataataataat
aataatactatactttttaattaattttcagttggttgataaaatcgatgaagccacca
aagagaatacctctgttgcaaaacgagatttcaaagaaagttcaataaaccttttaaaga
aattaatcaatattcttgtaaaagcttttgaaaaaggctcagctgcatttaataaagctc
ttgaagatccggatgttaaagaaattctgagaagtctcgacaacatcagtaggaaaactg
caaattttctcatagaaaaatttaaaaatttgattataaaactcgatgagttaacaagtg
aaaaaccaaaatcagaaagaaatttatttgaaacttcaaagaataacATGAAAAAATTGA
TTCAAATTCTGATAAGTGCTTCGAAAAAAGGAGCAGAATATTTTATTAATATAATAAATG
ATCCAGACGTCATTGCTATTCGAGAACATCTCAACTCGGTTGAACAAAAAATTATCGATA
AAATTATTGAAAAATTATTGGAAATTTTCAACAAAATCGATAATGCTACCAAAGTTGAAT
CAGATGTTGCATTCGGAGTTTCTGATTGGAAAAACGATGCAACTGTTCAATTGAAAAAAT
TAACTCAAATTTTATTGAATGCTTATAAAAAAGGTGGAGAATATTTCGAAAAGGTTATCA
ATGATCCTGATGTACAAAATATCCTTGATCATCTCGGTCCATTGTCAAGAAAAGTAGCCA
ACTCAATAATCAATAATTTCAGAGAGTTGATAGATAAAATTGACAATGCTACTAAAGAAA
AGTGAttcattttgattggataataaatatgaaaactatattttgattgaaataaaaatt
ttaatcaatgaaataataataataataataataatcataataataataataataata
ataataataataatactatactttttaattaattttcagttggttgataaaatcga
tgaagccaccaaagagaatacctctgttgcaaaacgagatttcaaagaaagttcaataaa
ccttttaaagaaattaatcaatattcttgtaaaagcttttgaaaaaggctcagctgcatt
taataaagctcttgaagatccggatgttaaagaaattctgagaagtctcgacaacatcag
taggaaaactgcaaattttctcatagaaaaatttaaaaatttgattataaaactcgatga
gttaacaagtgaaaaaccaaaatcagaaagaaatttatttgaaacttcaaagaataacAT
GAAAAAATTGATTCAAATTCTGATAAGTGCTTCGAAAAAAGGAGCAGAATATTTTATTAA
TATAATAAATGATCCAGACGTCATTGCTATTCGAGAACATCTCAACTCGGTTGAACAAAA
AATTATCGATAAAATTATTGAAAAATTATTGGAAATTTTCAACAAAATCGATAATGCTAC
CAAAGTTGAATCAGATGTTGCATTCGGAGTTTCTGATTGGAAAAACGATGCAACTGTTCA
ATTGAAAAAATTAACTCAAATTTTATTGAATGCTTATAAAAAAGGTGGAGAATATTTCGA
AAAGGTTATCAATGATCCTGATGTACAAAATATCCTTGATCATCTCGGTCCATTGTCAAG
AAAAGTAGCCAACTCAATAATCAATAATTTCAGAGAGTTGATAGATAAAATTGACAATGC
TACTAAAGAAGTAATTAATTTATTT
>DjScaffold3337:11587-17581 strand:+
atttttagttgATGAATACTACTCCGGATACAAATAGTTTATCTTTTGTTAATGAAACT
AAAGTTTTACTCTTACATGTAACCATTATTAGATCCTTATTTTTGATTATTTGTATGTGT
GGTGGAATCATTATTAATGTTCTTTTCATTGTTATTATAGCCAAAAGTAAAAAATTTTCAT
CGGCGATCTCTATTTGGTTTATGTATCAATTTATCTTGTATATTTTTTCTTGATATTATC
ATAAATCAATTTTTAACTCTTATTTCGTCAATAAAGAATGAAAGGTTTATTGACGACGTT
TCATGTATTCTTCAATCCTTTTGTATTATTCTCGTTGATACTTGTTTATCATTAACTCTT
TTTATGTTAGGTATTGATAGATTAATTGCTATTCAAGCTAATGTCAATGATAAAATCAAA
CTGTCCAAAGGACTGAATGCTGATGCTATTGGTTTATCTATCTTTTGTTTATGGCTTGTT
AGTATTATCTTTTCTATTCCTCTTGCAACGAAAACGGTCAACAGTACTTATATAGACAAT
AAATACTCTTGTGGACCATCTATGAAAGACGATTATAAGTATACTTTATTTTGTTTAACG
ATTGCGTCGCCAGTATTGTGCGTTTTAATTATCTATATATACATTGCAAAAGTTTGGTGG
AATGCTAAACAAGAGATAAATACTGTTTCTTTATCTTATACTTTAACCGATAAATTTCAA
ATTGAAAAAGAAGTAAACAATTCTCTATGTACATTCGTATTTCATTTCTTTTGGGTTTTG
GCTGTTGGTCCGTATATTATTTGGCTATATAATCCCAATATTCCTACTGAAGCAACAGTT
GTGTTGGTTTGGTTAAGGAATTTCTATTGTTGGCTGCCTTTTTTAATTGCTTTAATTTTC
CACAAGTCTTTCAGAAAGAATTTAAAGTATTTGATATGTCGAGATAGAGCGGGCAATAAT
TCTTCAAATAACCCTCCTGATACCAATATAAATTGCGACTCAAATCGAAGTGGTACTGTT
ACAAATAATTCTCAGCAACAAACTTATTCGACAAATGCTGCATTTGATTGTGATGAAAAT
AATGAAAATCGATATTGTTTTAATGATAGTATTAATGGAAATTCATCTCCATCTTTATCA
TGCACCTCATCGGCTATTTCAATCAAAACAATCGAACGTATACCAAAAGTGCCAATTATG
TTTGCTACTGTAAAAGGAGTACATATAACTAATGGATATTCTTCTGAAATGTCAAACTCT
AATGATGAAGAATCGCATTGTCCAGCAACTCTTTGCGATGTGAACGAACCTGGATTTACT
GATACATCAGATGCAGCATCAATGACTACGAACAATTCTTTCAAAAAGAACAGATTATCA
AATCCTATTAATGACGTCAAACTACCCGCCGATATTATTGATACTATTAATCAAGATGGT
GTTATACACAAAAGACTTAAGAGACGACCCCAGTCAAATCTTAAAATTAAAGCTACT
>DjScaffold3337:8545-8916 strand:+

ATGTCATTAGGCATAGAAGCGGCACCAAAGAAATTTCAGGAGATGATGATGAAAGTGATA
GGTAAATTTAATGGAGCCATGATATATCTTAATGACATACTGATATTTACAAACAATAAA
GATCAACACTATAAAGTCATTGAAAGATTATTGACGGAAATAAGAACTATGGTTTATGAA
TTAATCCAGAAAAGTGTCAGTTTCTACAAAACGAAATCAAATGTGCACATAATCAGTAAA
TATGGTGTTAGAACTGATCTGTCTAAAATAGATACAATAAAAACATTTGATAGTCCTGAT
TGCATAAAGAAGTTACGAAGCTTCTTAGAAATGTTTCATTACTACAGGAAAATTATAGAA
AACCATGCATGA
>DjScaffold3097:14796-28063 strand:+
tgaaaatacaattgatATGAATATTGGGTACACTTGTAAATCGAGTTTAGTTGAAGCGGG
TAAAATTCTTAAAAGAGCATTCAGTGAATTAATTGATTTTGAAATTGAAAATAATGCACC
ACGTTTGATTTATCTTGAAGAACAAGATAAAATGAAAATGTTAACAATTTTAGCAAAGTT
TCTATCCAATCAATACGAAGATCAAGTTTGCGAGTCAAATCTCATTTTTACTAGTGGAGC
CAGTTCAGCTTTTGTTTACCTTTGGACTCATTTACTCCCAAGAGATCGGAAAGTAATTGT
TGCTTCACCAACATATCAGTTATTACTCAATTGTTTGGTTAATGATTTGAACTATGAAAT
AGAATCCATTCCTGAAGATGAAGATGGATTGAATCTCGATATAGTAGAAAATATATTAAA
GAAAGAATATGAATTACATGGATCCGAACAACAATTCTGTTGTGGTATTTATATTGTTCC
AACTTTCAGTAATCCAACTGGAATTCTTTATTCTGAAAAATTCTGTATTAGAATAGCACA
AATAATTGCAAAGTACAACGCCTTAGTAGTTTGTGATGATGTGTATAATTTATTGTATAC
CTCTATACATCCACCGAAACGTTTATTAGCCTATGAATCGAATGAAACGAAAGGCCATAT
TGTTTCAAGTGGATCATTTTCAAAATATTTTACTCCTTCAGTAGGTTTGGGTTGGATTGA
AGCTCATACACCTTTGATTGATAAAATTGAGAAATGGGGAATGTTACAATCGACTGCTTG
TTTATCGCAACTGTCAGCAATGCTTGTTACAACAGCTATTAATAATGGCGACATGTTAAA
ACATTTAACTGCCGAGCGTTCAAATATTAAGGTAATTTTTCATTGAatttttatttgtaa
ttaataattaaagcatcaattaattgctttctgtcagtgttacaaatttggtttatcttg
ttaactactacatcagaaattgtttaactaagaccataaggttagtataagttgttacaa
gaatatttgctcagttctaggttttctctcgtattaagaaattttaaaaaaaattaaaaat
ttgacaaattaataaatcaataggattttgaaattgaaaataatgcaccacgtttgattt
atcttgaagaacaagataaaATGAAAATGTTAACAATTTTAGCAAAGTTTCTATCCAATC
AATACGAAGATCAAGTTTGCGAGTCAAATCTCATTTTTACTAGTGGAGCCAGTTCAGCTT
TTGTTTACCTTTGGACTCATTTACTCCCAAGAGATCGGAAAGTAATTGTTGCTTCACCAA
CATATCAGTTATTACTCAATTGTTTGGTTAATGATTTGAACTATGAAATAGAATCCATTC
CTGAAGATGAAGATGGATTGAATCTCGATATAGTAGAAAATATATTAAAGAAAGAATATG
AATTACATGGATCCGAACAACAATTCTGTTGTGGTATTTATATTGTTCCAACTTTCAGTA
ATCCAACTGGAATTCTTTATTCTGAAAAATTCTGTATTAGAATAGCACAAATAATTGCAA
AGTACAACGCCTTAGTAGTTTGTGATGATGTGTATAATTTATTGTATACCTCTATACATC
CACCGAAACGTTTATTAGCCTATGAATCGAATGAAACGAAAGGCCATATTGTTTCAAGTG
GATCATTTTCAAAATATTTTACTCCTTCAGTAGGTTTGGGTTGGATTGAAGCTCATACAC
CTTTGATTGATAAAATTGAGAAATGGGGAATGTTACAATCGACTGCTTGTTTATCGCAAC
TGTCAGCAATGCTTGTTACAACAGCTATTAATAATGGCGACATGTTAAAACATTTAACTG
CCGAGCGTTCAAATATTAAGGTAATTTTTCATTGAatttttatttgtaattaataattaa
agcatcaattaattgctttctgtcagtgttacaaatttggtttatcttgttaactactac
atcagaaattgtttaactaagaccataaggttagtataagttgttacaagaatatttgct
cagttctaggttttctctcgtattaagaaattttaaaaaaaattaaaaatttgacaaatta
ataaa
>DjScaffold5036:21774-29932 strand:+
ttatttaattaaatggtttgattttgtagacttaatATGCTGCACGATTTGAATACTTGG
AAACTTTCCAAAAGGTGGGTTATTATATTTACGGTAGTTATGTTTGTTGGTGGCATTTCT
TTAAATGAATTTTGGAAGTCATATCAAAAGAAACTTCTTTTAAGTAAAAGACGTAAGGAT
AAGCAGAAAGCAAAAATCCGTCACAAAGGAATGGCTACTTCACCAAATGAGAAAATACAA
TATGCAAATCATGCTGGTGGCGATTTTTATAGTATAATCAATATGAACACACAGGAATTA
ATAAATTTCTTAAAAACTAACGAAAATGGTTACGAGACAGTGTTTAATGTTTATCAACGA
CAAGCTTTATTGCAAGACCAAAGATTAAATTGTGTAGTTGAGTTTGTTAAATTGGACATA
TCCTGTGTGAACTTGTCCGGTCCATTTTATGGTATACCAATAAGCTTAAAGGATAATTTG
ATACTCAAGGGATATGAAAGCTATGTTGGCTGTTCATCATTTCTTAATAAACCAAAGGCA
ACCGAAAGTTGTGTATTGGTTCGAGTTTTACAGAAATTAGGTGCAATTCCATTCGTAAAA
ACAACAGTTCCTCAAACAATGATGACAATACTTGCTTCAAATCCAATTGATGGAACTTTT
AAAAATCCGCAAAACAGAAATTTTATTTCCGGAGGAAGTAGTAGTGGAGAAGCGGCTCTT

ATCGGTGCCAAAGGATCAATATTAGGATTCGGGTCTGATATTGGGGGTAGTGTTAGAATT
CCAGCTGCATTTTGTGGAGTTGTGGGTTTAAAGCCCACAATAGGCAGAATCAGTTCAAGA
GATGTGTTTAAATTAGGAATACCTACTCTTGTGAAATCAGTGATAGGTCCAATTTGTCCA
AACGTGGAAACTTGTGCTTTTGCACTAAAATGTATTGTCGATTCTGAAGAAATGTCTAAA
TTAGATCCGGAAATTCCATATTTACCATTTAATGAGGAGAATTACAACAAAAAAAAGTTA
AAAATTGGTTATTTCTTTCATGATGATAGCTATTATGTGGTTCCGGCTATGCGCCGAGCA
GTAGAAGAATCAGCTAAAAAATTAAAAGAAAAAGGACACGAGGTAGTACAATGGAAACCG
CCTTTGTGTGGTAAAGAGTGGTGGAAATTGTATTTACGTTCTATATCAATCGATGGTGGT
CAAAAACTAAAAGCAATAATCAAAAACGATCAAATTGACAATTCAATCATTAATGGAATC
GTTCATATCGATAAAAATATTCTGAGCTATTTTTATAACTTTTTCAAATATAAGTTGCCG
GATTGGGTAGTTGATCCAGATATGAAATTGTTCCATCAATGCAGTATTGAAACGCTGAAT
AGTTCGGATTGTATGGCATTATTAATGGAAATAGAATCGTTTAGAATTAGTGTAATTAAT
GAATGGTCAAAATGCGGTTTAGACGTTGTCATCTGTCCAGCTGGGGGTTTTACTGCGCTT
CCACTGAAAGCCAATTGTTTATACAGCCCGTTGTTGAGCTACATATTCATTTGGAATCTT
GTGAACTTTCCTGCAGGTACTGTTCCAGTTACTAAAGTTAAACCTAATGATATTAAAAAC
TTGCAATCTGATCCTATTTATAAATCGACACCTACACAGAAAAACATTTCAATGTTGCAA
AAAGATACTGAAAAACTCCCTGTTAGTGTGCAATGTGTAAGTCTTCCATGGAAAGAAGAG
TTGTGTCTTAATTTAATGAGAGAATTGGAGAAGATTGCGGCAGAAAGTGGGGAGATTGTG
GAGAAAATTGTGGCAGTAAGTGGGGAAATTGTGGAGAAAACTGTTGGTGTAAGTGGAGAT
ATTGTGGCAGAGAGTGGACAAATGGTGTCGAACGATGTCTCTTAAttttaggagataatt
ggaggaaatctgttactatatgtatcttgtatttttttaatcagtcttacactttttg
agatttggtgatttttatttgattcataaagtttggattataaaaacactttgtaatacg
ttattttaaccctttgaacgcgttgaacgaaaattcaaattggagtccgatgtcgctttg
actctccagcaattcagaaatgactgtccaaattataaatgcgaggcgacaaaagtcgac
taccccgtcgtaaagtagcgttttttttacaagccatggacttaagtcgttaatcgaaa
agggttaaccgaaaacaattatcaatttgcacaaaattaaaatttattttagtatttaca
gccaaaggctgtctaaaatataatgcgaggcctttatgtactaacaaaattgtaattttt
gacaacttaaaatatatattttggaggctgtagtgaaatcagtgataggtccaatttgtc
caaacgtggaaacttgtgcttttgcactaaaatgtattgtcgattctgaagaaATGTCTA
AATTAGATCCGGAAATTCCATATTTACCATTTAATGAGGAGAATTACAACAAAAAAAAGT
TAAAAATTGGTTATTTCTTTCATGATGATAGCTATTATGTGGTTCCGGCTATGCGCCGAG
CAGTAGAAGAATCAGCTAAAAAATTAAAAGAAAAAGGACACGAGGTAGTACAATGGAAAC
CGCCTTTGTGTGGTAAAGAGTGGTGGAAATTGTATTTACGTTCTATATCAATCGATGGTG
GTCAAAAACTAAAAGCAATAATCAAAAACGATCAAATTGACAATTCAATCATTAATGGAA
TCGTTCATATCGATAAAAATATTCTGAGCTATTTTTATAACTTTTTCAAATATAAGTTGC
CGGATTGGGTAGTTGATCCAGATATGAAATTGTTCCATCAATGCAGTATTGAAACGCTGA
ATAGTTCGGATTGTATGGCATTATTAATGGAAATAGAATCGTTTAGAATTAGTGTAATTA
ATGAATGGTCAAAATGCGGTTTAGACGTTGTCATCTGTCCAGCTGGGGGTTTTACTGCGC
TTCCACTGAAAGCCAATTGTTTATACAGCCCGTTGTTGAGCTACATATTCATTTGGAATC
TTGTGAACTTTCCTGCAGGTACTGTTCCAGTTACTAAAGTTAAACCTAATGATATTAAAA
ACTTGCAATCTGATCCTATTTATAAATCGACACCTACACAGAAAAACATTTCAATGTTGC
AAAAAGATACTGAAAAACTCCCTGTTAGTGTGCAATGTGTAAGTCTTCCATGGAAAGAAG
AGTTGTGTCTTAATTTAATGAGAGAATTGGAGAAGATTGCGGCAGAAAGTGGGGAGATTG
TGGAGAAAATTGTGGCAGTAAGTGGGGAAATTGTGGAGAAAACTGTTGGTGTAAGTGGAG
ATATTGTGGCAGAGAGTGGACAAATGGTGTCGAACGATGTCTCTTAAttttaggagataa
ttggaggaaatctgttactatatgtatcttgtatttttttaatcagtcttacactttt
tgagatttggtgatttttatttgattcataaagtttggattataaaaacactttgtaata
cgttattttaaccctttgaacgcgttgaacgaaaattcaaattggagtccgatgtcgctt
tgactctccagcaattcagaaatgactgtccaaattataaatgcgaggcgacaaaagtcg
actaccccgtcgtaaagtagcgttttttttacaagccatggacttaagtcgttaatcga
aaagggttaaccgaaaacaattatcaatttgcacaaaattaaaatttattttagtattta
cagccaaaggctgtctaaaatataatgcgaggcctttatgtactaacaaaattgtaattt
ttgacaacttaaaatatatattttggaggc
>DjScaffold513:8971-17005 strand:+
aatgaatttcgaattttaacgtgctataatttttcataattaaaattaaataaatatttt
tactaaggttttattaataataggaaggccttgaaatttatcccagcgaggattctacct
atttttcttctgaacttcctcaaggtgatgctattttcgattattatgcgattgtcttac

atcaatcactttattttaccaagtgctttgttgtatcgaattctaatggagaattgataa
cttaccacgaagaaagcaataatttagtaattaaaagggatggttgtaaagagttatatc
cagaagagctgattaataatacgtataaatgcatcaaatgtgctgttgaaaatttcaaat
cgttaaattacgatgtgaaaaaaatcgtttgtATGAGTTTGTGCACTCATCGAAACGTTG
TTGTTGTATGGAGCAAAGAAACAGGTAAACCGTTGTACAATGCGCTGCCGGAAGATGATT
GCAGATGCGATGATGCCTGTCAACGATTGATTCACAAATCTTGGCGCAAATCCAGAACCA
ATTTCCAACATTTATGCGGTCTGCCGATCAGTGGCAAATGGGCTGGAGTTAAAGTGAAAT
GGCTGATTGATAACATCTCAACTGTACAAGATGCGTATAAAACTGGTGATTTGCGGGTTG
GCACTCTAGATTCCTGGTTGCTATGGAAACTGACAGGTGGGGAATGTTATGCGACAGATG
TAACCAATGCCAGCTACACGATGCTCATGAATCTCCACCGGTGCTCGTGGGACTCCTCTA
TTTGCAAATTTATTGATATCGACATCAATATGTTGCCGGAGATCAAGAGCTGCACTCAAC
TGTTTGGGAAAGTGGCTCAGAATTCCTCGACTGGAATAGACGGGTTACCGATAACAGGAT
CTGTGTGTGATGTCAAGGCGAGTGTTATTGGTTGTCAGAGCACTCAGCCGGGTTCTGTTA
ATCTACATATCGATCCAAAATTTGGTATTAGTCACGTCACAGTTACAGTTGGAGAGGAAT
TGCTTTTCTATCGATCTATGATGAAAGGTGTTGGATACATGTTGAGCACAGTTTCATATC
AATTCGGAACAAAATCTAATGTTGTCTACTCTCTTGAGGCAACTCTCTACAACAGTCAGA
ATCTAATGAATTGGTTCCGTGATTGCATGCGCCTTCTATCAAAATCCGATGATATCGACT
CTGTTGCAAATTCTGTTACTGATTCCAACGGCTGCTATTTCATTCCAACAACTGGTGCTT
CCCACACTCCTCTCGAACGAAGTCAGTATCGACATGTCAATCTATGGGGAATCAATTTAA
CCAATTCTCGTCCTGGACACATTGTACGCGCCGCATTGGAATCAGTAGCTGTAGAAGTCA
GTCAATTGCTGAGTTCCATTTCAGGTGTTGTTGGGAGACCATATAAAATACTCAATGTCA
GCGGTCGTGGTTTGGACCGCAGCGATGCCTTTTGCCAGACTTTAGCAGATCTTACCGGAC
TGAGAGTTGAACGATTGGCAATGTCCGAAGCTGTCATATTGGGAGGTGCTCTTGTTGCGG
TTCTCGGTGCAAATTTAGATCCATCAGGTTTGCAAGGACCAATCAAAGAAGGGTTTTCCT
GTTTGATGGTGCCAGGTGAACAACGCAGACGATGTCGACAGTGGGTTAGTCTGGCCAACG
AACAGGAATCCCTCGATCATCGATTTTGGATTGGCAGTGAAGATGAAATCAATATCAATA
TTGATTCCGGGAAACAAAATCGAAATAAAAAGTTTTTATCGACCAATCGAAATTGGGGCT
GGAAATTTTTGTCTGTCATTATGATTGGCACTCCGTTCACATTAGTAGGTTTTGGAGTTG
CTCTGCTCATTAGTaatgaatttcgaattttaacgtgctataatttttcataattaaaat
taaataaatatttttactaaggttttattaataataggaaggccttgaaatttatcccag
cgaggattctacctatttttcttctgaacttcctcaaggtgatgctattttcgattatta
tgcgattgtcttacatcaatcactttattttaccaagtgctttgttgtatcgaattctaa
tggagaattgataacttaccacgaagaaagcaataatttagtaattaaaagggatggttg
taaagagttatatccagaagagctgattaataatacgtataaatgcatcaaatgtgctgt
tgaaaatttcaaatcgttaaattacgatgtgaaagtagtgataatggctgataatgaatt
tatggtgaatctttattttaatgtttccagaaaatcgtttgtATGAGTTTGTGCACTCAT
CGAAACGTTGTTGTTGTATGGAGCAAAGAAACAGGTAAACCGTTGTACAATGCGCTGCCG
GAAGATGATTGCAGATGCGATGATGCCTGTCAACGATTGATTCACAAATCTTGGCGCAAA
TCCAGAACCAATTTCCAACATTTATGCGGTCTGCCGATCAGTGGCAAATGGGCTGGAGTT
AAAGTGAAATGGCTGATTGATAACATCTCAACTGTACAAGATGCGTATAAAACTGGTGAT
TTGCGGGTTGGCACTCTAGATTCCTGGTTGCTATGGAAACTGACAGGTGGGGAATGTTAT
GCGACAGATGTAACCAATGCCAGCTACACGATGCTCATGAATCTCCACCGGTGCTCGTGG
GACTCCTCTATTTGCAAATTTATTGATATCGACATCAATATGTTGCCGGAGATCAAGAGC
TGCACTCAACTGTTTGGGAAAGTGGCTCAGAATTCCTCGACTGGAATAGACGGGTTACCG
ATAACAGGATCTGTGTGTGATGTCAAGGCGAGTGTTATTGGTTGTCAGAGCACTCAGCCG
GGTTCTGTTAATCTACATATCGATCCAAAATTTGGTATTAGTCACGTCACAGTTACAGTT
GGAGAGGAATTGCTTTTCTATCGATCTATGATGAAAGGTGTTGGATACATGTTGAGCACA
GTTTCATATCAATTCGGAACAAAATCTAATGTTGTCTACTCTCTTGAGGCAACTCTCTAC
AACAGTCAGAATCTAATGAATTGGTTCCGTGATTGCATGCGCCTTCTATCAAAATCCGAT
GATATCGACTCTGTTGCAAATTCTGTTACTGATTCCAACGGCTGCTATTTCATTCCAACA
ACTGGTGCTTCCCACACTCCTCTCGAACGAAGTCAGTATCGACATGTCAATCTATGGGGA
ATCAATTTAACCAATTCTCGTCCTGGACACATTGTACGCGCCGCATTGGAATCAGTAGCT
GTAGAAGTCAGTCAATTGCTGAGTTCCATTTCAGGTGTTGTTGGGAGACCATATAAAATA
CTCAATGTCAGCGGTCGTGGTTTGGACCGCAGCGATGCCTTTTGCCAGACTTTAGCAGAT
CTTACCGGACTGAGAGTTGAACGATTGGCAATGTCCGAAGCTGTCATATTGGGAGGTGCT
CTTGTTGCGGTTCTCGGTGCAAATTTAGATCCATCAGGTTTGCAAGGACCAATCAAAGAA
GGGTTTTCCTGTTTGATGGTGCCAGGTGAACAACGCAGACGATGTCGACAGTGGGTTAGT

CTGGCCAACGAACAGGAATCCCTCGATCATCGATTTTGGATTGGCAGTGAAGATGAAATC
AATATCAATATTGATTCCGGGAAACAAAATCGAAATAAAAAGTTTTTATCGACCAATCGA
AATTGGGGCTGGAAATTTTTGTCTGTCATTATGATTGGCACTCCGTTCACATTAGTAGGT
TTTGGAGTTGCTCTGCTCATTAGT
>DjScaffold2758:48507-63802 strand:+
aatctgacagtttactgatatccataattgttagaattcgagattattgtttgataaacg
tctatgagttataatttatttgccaaagcaactattgttgattgtgttttatgatttact
atacaggtcatcaaacagtttattaatgaaacattagtggaacgagatagttcaatcagc
gaaagtaaaataaaattattgttagctacggttgttgccatatttgctgttgggggcctt
attggtgcattaattgcaggctggttatctgacagatttggaagacggaacggaATGCTT
GTTACTAATCTATTGATGGTTATTGCTGGGGCATTTATGTTAATTGCTCCTTATGCTCAT
TCGTTTGAAATGATAATTATTGGTCGCTTATTGTCTGGTCTTGGTTCTGGTATTTATACT
GGCATTGTTCCTGTTTATATAAATGAAATAACTCCTGTAAATCTTAAAGGAGCATTAGGT
ATTATGTCTCAACTTGGAGTTGTCTGTGGAATATTTCTCTCTGAAATTTTAGGATTAACA
GTTGTTATGGGTAATGAGAAATATTGGAATATTTTATTGGGCATTATTATGATTCCTGCA
ATTTTACAAACAATATTTCTACAATTTTGTCCCAGTTCTCCAAAATACTTGTTGTTTTTT
AGGAATGATGAAATAGAAGCTGAGAGAGCTCTTAAAACATTGAGACCTTTAATGTATGAT
ATCGATTCTGAAATGGAACAAATGAAGAAAAATAATGAACATAGCAAACAAATACACAAG
ATCGGCCTAAGAAGTATGTTAAAAAGTAAAGCTTTGTTAATGGCATTAGCAGTTGGTATA
GTTCTTCATTTATCTCAACAATTCTCAGGAATAAATAATATATTCTATTATTCGTCTACT
TTGTTTGAATCAGTTGGCGTTAGTAAGCAAAATAGTGAGTATTCAACCCTTGGAGTAGGT
GGAATTATGATTATAATGACTTTAATTTCTATTCCATTGGTTGAAAAATGTGGTCGACGT
GTTCTGCATATTGGTGGCTTATGTGGATGTCTTTTAACATCGATTTGCTTTACAGTATTT
TATTCTCTCAGTTCAAAAGTATCAAATTTCAATTATTTAAGTGTTATTTCTGCTTATTTG
TTTGTTGGAGCTTTCGGTATTGGACCGGGTTCCATACCCTGGATGATGATACCGGAAATG
TTTCCGGAAGATAGTAGATCGACTGCAACATCAGTTGGAGTATCTGTTAATTGGTTGTCA
AATATTGTGGTTGGATTAATATTCCCATCACTTCATGATGCACTTAAAAATTTAATTTTT
ATTCCATTTATTGTTTGTCAAGTACTTTCAATTTTGTTCCTATGGTTCAAAATGCCAGAA
ACCAAAGGTCGACCTGTTAATGAAACCTCTGACTTGTTTTCTCAGCATTCGGAAGATAAA
AATTCGCAATTATAAtttgtgatattaaaattatggaaaaggaaaatattttgtgcattc
taatcatattaatttattgtttata
>DjScaffold2758:94307-94980 strand:+
aattttttagcaaaaaacacagtccaattcttaattatcaaaaatATGATTCAAATTTGT
AATGATGAACATAGTGGCAAAATTAAAGAGGTTAGTAGTAAGGCAATGTGGTCGTTATCA
TCTTGCAAACCTGATCATGGTATAAGACAACTTCTTGACGAATCTCATGATACTTTCTGG
CAATCAGATGGTCAACAACCTCATTATGTCAATATTGAATTTAATCACAAAACAACTATA
TGTGACATTTGTTTATATGTCAACTTCAAGATTGATGAAAGCTATACACCTAGCAAAATC
ATTATAAAAGCTGGTAGCCATTTTGATGAATTGACAGAAATTGATCAAGTTGAAGTTCAA
GATCCAGAAGGTTGGATTGTTATTCATTTACTCGATAAAAATAAAAGACCTCTGCGAACC
AACATGCTACAATTGGTAATTGTATCGAATCATCAAAATGGTAGAGACACACATTTAAGG
CGAATCAAGATTCATAGTCCAAATGACGATTGGGATCGTCATGATACATCAAATTCAGTT
CTAAATATGGATATGTCCAGATTACTTCGTTAAttgattgttgtaatgttg
>DjScaffold1119:53724-62888 strand:+
GCTTCAAATTCCGAATTCTGTTTATGTGAAGTATCAGTTGAGCCTGGATCAATAACCAAA
CAAAAAATAATTAAAGATAATTCTTCAAATTTAATGGCCAATGTAAAACTCAACAATCGA
TTTTATTTAAAACGGCGAGATTTTTCGGGAAAGTTAATCGATAATATTGACATTGAAGAA
ATCAATTCAGAAATGAATGTTTCATTTTTCGATTTACCTGTTTGTGAATTGGCGACTCAA
GTCTTCGTAGAAAGTTACAATGTATTCAGATTGATCAATATATCAGAAGTAATTGATTTT
GTTACTGGAAAATCAAGAAAAGAACTATCAAATTGTAAATCGAAATCCGAATCAAACAAT
CGAAATTTTGCTATTGGAAATAATTGTGAAAATCATGAATCTAAAATGGATAATGTGAAT
AATTTCCAAGAATTTTTGAAACTCAGCGAAAAGTACATGTATTTTGTTATCAATGTGGTG
TGCAGTGAAACTAATAGTACTTCAAAACGTGGTAAAATATTGAAAAGACTAATTCGAATG
GCACGATTATGCAAAGAACTCGGAAACATTGAAGTTTTTTTTTCTTTAATGAATGGCTTA
GACCGAGTGGAAATTAGTCGGCTTCGCAATACATGGGATCGAGTGCCGAGTAAAGAAATG
AAAATGTATCGTCAAATGCAAGTATTACTAGATGTGTCAGGCAACATGCGATTATTACGT
AGTCTTCACAAATCTTGTAAACCGCCTGTCATTCCAATGGCCGCTTTAGTCACAAAAGAT
TTGACAACAATCAACGAAGTTCATCCAACAAAAGTTGACGCAGACGGCCCTGGAAATGAC

CAAAAGTCCGAGAATGAATGTTTGATAAATTTTGATAAACTACGTATTATGGCCAATCGA
GCCAGATCGTTCAAAAATCTCCTTTATGATTGTGATAACAATAATAATAGAATTACTCAG
TCACCTCCTATTTTACCTGATGATGAGCCAGCACAAATTGCATTTATCAGAGAATTACTA
TTAAAAATTGATCTCAAAGAAAGTTCTAAAGGTTTGTCTGGTAGTCAAAATCATTTAAAT
CGCAGAAGATCAACGTGCATCAATCCGAAGAAGATCTATGAAAATGAAATCAGTCGCCTT
AGAGTAAAACATTTTATCAGTAAAATTGAACAAATGGAGCGTGATGATGCAATTCTGTTG
GCAATGTCTTATAAATGTGAACCGCGATCAGAAATTAGTGGACTCACACCAAATGTATCT
CGGTCGACAATAGCAGGAATTTTAGCATCGCCTACT
>DjScaffold1119:88017-90856 strand:+
aataattaaatttcagaagaaATGATGAATATTCGCAATTCAACTGGTGGCTCAATATCA
TCAAAAGAATCTAGTCAAACGACCTATTCTAGTGGATCAGAACCATTTTCTGAACTAAAC
AATCAGAGCAGTTATCGATTGAATGCAAAATACAATCGAATGAATACCAGTACTCCTCTG
TCTGGTCAATTGCAATATTTAGAGCCGTCCGGATCTCAAAGAAGACATATATTGCCCCCA
CCATATTCATTGGTTAGCTCAGTTCCTAACCCACCTATTCCACCTCGCAGAAGATCATCT
GTAGTGTCTGCAGAGAAAACCAAACCATTTTCAAATCAACTATCAAATGACGAAGTAGCT
ACACATCTTTGAgatatcaaatattggtatcggttttaacaaatctgtgataattatcga
ttttgcagctgcctaattttattctaaaaatcattcattttattgagatttatatttgat
ttaattattcatatctttcaaatttgtcaaatgatttattctacacacacatacacacat
tatttctatccacgggatgttggttcataaagtactggtcaaacagtactagtcagcata
gaaaacactttttactcagtttgtttgaaaaatagaatgaaaatgttagtgcctaaataa
tttatatctattaatcgcattataatgataataatgaaaatcattatcattattatttat
attatttataatctctgttgatattttttgttaatagtattgtattaatttaaattttaa
taaaattatgaacttcaaataattaaatttcagaagaaATGATGAATATTCGCAATTCAA
CTGGTGGCTCAATATCATCAAAAGAATCTAGTCAAACGACCTATTCTAGTGGATCAGAAC
CATTTTCTGAACTAAACAATCAGAGCAGTTATCGATTGAATGCAAAATACAATCGAATGA
ATACCAGTACTCCTCTGTCTGGTCAATTGCAATATTTAGAGCCGTCCGGATCTCAAAGAA
GACATATATTGCCCCCACCATATTCATTGGTTAGCTCAGTTCCTAACCCACCTATTCCAC
CTCGCAGAAGATCATCTGTAGTGTCTGCAGAGAAAACCAAACCATTTTCAAATCAACTAT
CAAATGATATCAAATATTGGTATCGGTTTTAAcaaatctgtgataattatcgattttgca
gctgcctaattttattctaaaaatcattcattttattgagatttatatttgatttaatta
ttcatatctttcaaatttgtcaaatgatttattctacacacacatacacacattatttct
atccacgggatgttggttcataaagtactggtcaaacagtactagtcagcatagaaaaca
ctttttactcagtttgtttgaaaaatagaatgaaaatgttagtgcctaaataatttatat
ctattaatcgcattataatgataataatgaaaatcattatcattattatttatattattt
ataatctctgttgatattttttgttaatagtattgtattaatttaaattttaataaaatt
atgaacttca
>DjScaffold1420:8865-11849 strand:+
tttaagttcgatatcaaaatggtataattctgaattcactggaattaataattctatatg
taatATGGCTTTATTACCATTAAAAACAAAATTTAGAGGTCCTGCACCAACACAAATGGA
TCAGAATGCTCAAGATATAGTCGATGAGTCTATATATTTATTTAAATCTTTAGTATTTTT
CAAAAATTTTGAAATACAATCAGATTCTGATCGAGTGCTAATGTACTTGGTTCTTTATAT
TATTGAATGCTTAAAATTATTACAAAAATGTAATAATCGCTTACAAGGAGAAAAGGAGCT
AACTGCACTTGGAATTGCAAATTTCAATATGCCTGGTGATCCTGGATTTCCTCAAGAACT
TTCAAATTTTTATTTAAAACCAAAAGACAATAATGAACGCGAAAAATTGAGATCTTACAT
GCAGCAATTACGACAAGAAATGTCTGCTAGAATTATAGATAAAGTATTCACTGGCACTGC
TAGTGATCCACCTTCTAAGTGGTGGATGTGCTTCTCAAAAAAGAAATTTCTTGATAAATC
TCTTAGTACTCCTGGAAGATTTTGAatgagaaattaacatttgaattttctaatttctta
attaattccacaaaaagtataataactaaaatatattttcaattttataataaaatgt
>DjScaffold1420:22559-57093 strand:+
aattttatatttaaggaatatctgatacATGCAGGAAAATTCTGAAAATGCTGATTCACG
TTCATCAGTAAAAAGTCAATCGACTTCTATAAAAAGATCTAATGCAAGAGTTTGTAATCT
CAGTTTAAGATGTAGAACTAAAAGTGAAAACGGTATATTATGTGGTGATAGTGATTGTTC
AATTTTGGAAAGTAACTTAACTTCAAAATTGGATTCCCGTTTGATTTCCATTGATAAAGA
AGCTGAACAGTATATTCAGCTGCGAAATCGACCTCAGAAGCGACATATTAGTAATACTCA
AGAAGACCAAAATGATTTAATTGTTACCGCAATATACAGTTATACGCCTAATAATGAAGA
TGAATTAGAATTACAACCTGGAGATAAAGTTCGAGTATTATCTAATGATTTTAGTGATTC
TGGAACTGAAGGATGGTGGGTAGGTCAGAGAGAATCTCAAATTGGAGTTTTCCCCATAAG

TTTTGTCGAAAGTGAAACCAGTTGGTCTATTAAATCAGGAAACGAGCAAAATGTCCCCGA
AATAACACCAAGAACACCAAATGAAAGTTTTAAAATCAATTTATCTAATGAAAACAGTTT
AAAACGAGGAAAATTAGAAACTTCGTCTTGTAAAATAATTAGTAATAATTCCGTTGTAAA
TGGATCTGGTATTTCGAAAACAACTGGAGTTAGAATGATACCATGTGAAGAATTAGAAAA
ATTAGAGTGCTTGGGATCTGGTGCATTTGGTGTTGTTTATCATGGTAAATGGCAAAATAA
AGAAGTAGCTATGAAAACTTTTTCCATCAACGCTAACGAGGTTCATATTTATCAGGAAGC
TGGGCATTTGGGTTGTTTGTTACACAGGAATATTGTTCGTTTGTTTGGAATTAGCATTTT
CAATGAAATGAGTAATCAAATTTCTCTCATAATGGAATACGCCAAGGGCGGTGCATTGCA
TAATTTATTGCATTTCCGCCCAAATATTAAAATATCGCCGAGAACCCTCCTTATATTGTC
TAGACAAGTAGCTCTGGGAATGCGTTACTTACATCAAGATGCAGGTATCTGTCATCGTGA
TCTCAAATCTTCTAACGTACTTATTAAAGAAGCCATATCAGACGATGCAACAGATGCTCA
AATATTAAAATGCACATTACTTATTACTGATTTTGGAATGGCACGCCGCACTGTAGAATT
GGTATCTCAACAATCCAAATTAGGGACTTTGGCATATGCTTCGCCAGAAGTGTGCAAACA
GCAGGGTGTATTTTCCACTAAATCAGATGTTTGGTCATATGGTGTTCTTCTGTGGGAATT
ATTGACAATGGAAATTCCATATCGAGATATTGAACAACCACATCTTCTTTATAAAATTGC
ATTTAAAAATCAAACTCTTTATATTCCTGATGGAATTCCGAATTTATTAGTGAAATTATT
AACAAGCTGCTGGGCAGAAGATAAAAATCTAAGACCAGATTTTGAACAAATTGTTCAGGT
TCTTGATCGTGCCGAGAAATATAAATTTATGGATATATCCGCAGACGAATTTTTCGTAAT
CCAAGACAGTTGGCGTGAACAAATTCGGATTGCTGATAAAATAAGCGAAGAATCTATGAA
TTCTTCTGATAATGAAAAGGATGAATTTCTAGAACAGAGAAGAGAACTTGCTTATGCTCA
AGAGGAATTACAAAAAAGAGAAGAAGACATGCTGAGAAGGGAAGCGGCGTGTTCTCGTGC
TATGGGCACATTTAGTCAATGGATGCTATTTACTGTTTTAGCAGCAAATCAATTTCCTAG
GTTAGGCAGTGCAGAAGACTACTTAAGTCCTACTCCTCCAGTTCCACCGCCTCGGAAGCG
ACCGTTTGTTGGTCGTTTGAAATTCTGGGCTAACAATGGTAACTCTAGTCTTAATCATCA
AAGTCCAGATACTGGAACACCAATTACATGTGAAGATATTAGTGGTAATCCAAATATTAG
TGCGCCTAGTAACCTTCAACACGTAGTTCATATTTCACAAAAAGATTTCAATCTGACAGC
TCCTGCTCTTAGGTTCTTTCCAGATTCATTAAAAATGCACACATGGGGACCTAAAAGCAA
CGATAAGTTAATTTTCAGTAACAGATCTCGTCTGAAAAGTTCAGACGAGACCGATCAAGA
AACCATGTCTCCTCCTAATCAATTCAATTATGAAGAATCGCGTTTATTGACAGAATATGC
CGATAGTGTTGAATCTCCATCTCCTTGTGTAGAAACACCCAGTAGTACGTATGGGATAAC
TATTCAAGGAGATAGTAATCTATGTGCCAGTGCTCCTAATTTACGAAAAATATCTCTTGG
AAATCTACCTGTATTTGAGTTTTCTAGTTTTCGACGACCAATTTATGAAAATGTTAAAGT
TGAAGAAGATCCAATTGAGCCACCAGCAATATCAAATTGTCCTTCCTGTTACTTTAAATG
GCTGGATCATAGAAAACGAATACCATGTGAAAGATGTCGGTGGCGAACAACAAATAAAGC
GGCTACAATTGGTTCTACCACAAAAACAAACTTAAATTGTGATAATGACTTGTGGACCTG
TATTCCGAAACATCCTCCAAAATCAACTGAACTTAGATCTCAATCAGAATCTATTCAAAA
TCGAAAGTTTAGTTCTTCCCAACAATTTGATTCAAAAAGAAACAAAAAATCCAGAAATAG
AAATAACAAATCAGAATGGCGCCAAGTTGATCATGCCATGTGGTCAGTAATGAGAATGCT
TTCTGCCGTTGTTCTTCCTGATAGAAATAATGAAAATCAAAAGGAAACAACTACAAATGC
TTCAATGCCAACTTTTGATATTCTCGATAAAGCTCATGGGCATAGAGAATCTTGCAGTCC
AACTTTGTCTTTAGATATTACTACTGAAGATGAAGATCCTTTACCTAATGCTAAGAATGA
TTCAATATTTTATTTTCAAAGTAGCGATCAAATTTCATCTCCAGTCAAAACCAACTCAAC
AAAACCCAATTCTATCAGTAATTTTAAACCGATTACTACACCGAAACCAATCACATTTCA
GAACTCTACACGAGGTCGATCGAAACAAAACTACAATACTGCAGTTGATAAAAATTTTAA
ATTCAAACACACAGTTAAAAATGACCAATCAACAGGTCGTGGGTCATCAGCTCCTCCATC
CCGAAATCTCAATTTCACATTAAATCAGCCAGACGCAAGTAAAGACAGTTGTAAAAATTC
AAATTATTTCGCCTCTTGGTTGCCAGCTGTTAATTTTAATTCAGATCTCAAAAGCGGATC
TCAATCAACCGCTAATCAATTTAGAAATTGTGATAAAGTATTATAGtcgccattatgtat
gtctcttggatataattctctatttttatgttatatccattcccatttgcctttcatttg
aatttagtaagatattccattgtataaagaaattctttctctttgtccatgatgctttct
cattgaagtgcactagaaactgtatttcaaagaaatttgtttcaaattcttgctttgcag
ggacgttgcattttacaccgtaataaattagctaatggtaatgttgtcccaatcattgtt
ttgtatgtctttcattgtcttatttacaagcatgtttctcaaaaaatgatcatatattat
gtgtatttcaatccctctctttctcaatgatttggttttgaaaatttagaatttttattc
gtagataaatgtgtaaatattttgagaaatattataatatttagaaaacaaacattaatt
ttataatttcacagtttaatgaaaattgagtttatgttatgttatatgaatatgtttaca
gtgattttgtattttttataaaattatac

>DjScaffold1420:105648-107402 strand:+
ATGGTTGGATTTGGAAATGCTTGGAGATTCCCAATAATGGTTTATGAAAACGGAGGAGGC
TCTTTTCTTGTAACTTATACTATTTTATGTTTTATTATGGGTATTCCTCTTGTAATTATG
GAATTTGGGATTGGTCAATTTTCTGGACGTGGACCAGCTAAAGCATTTAAATTAGTTCCA
GTATTATATGGAATTGGTTGGTCAATTATTCTTATTTCAGTTTTCATCGGAATTTATTAT
GCTGCAATTATTTCTTGGTGTTTTTATTATTTTTTTGCATCATTCAAAACACCTATGCCA
TGGACAGTTTGCACTCATAAATTTAATACTCCAAACTGCCTTGGTTCACTTGACAATAAA
TTTAATTATAATTTATCCAAGTTTGTTTCATCAACAGCAGAATATTGGGATTATTATGTT
ACAGGTTTTATTAAATATCCTGGTCGACTACCATATACTATGAATGAAATTCCTGGTACA
CCAATGTGGCATTTGACGCTTTGCCTATTAATAACGTGGTTATTGGTAGGTTTTGCCATT
ATGAATGGAATAAAAATAAGTGGTAAGGTGATGTATTTTACAGTTGTGTACCCATATATT
GGTTTAGTTATTTTAATTTGTCTCGGCGCTACTCTTCCTGGCGCCATGGACGGTATTAAA
TTTTTTTTAACTCCAAATTGGTATAAAATTTATAATCCAAGAGTATTATATGTTGCCATT
GTGCAATTATTCTTTTCAAGCGGCATTGCAAGAGGTGGATTGATTACTTATGCTAGTTTT
AATGATTTTCGTGATAATTTTTATCGAGATTCATTTGTTGTTCTCTTTGTAGATTTTGCC
ACAAGTTTGTTGTTTGGGTTTGCTATTTTTCCGATTTTGGGCTACATGTCGCAAAGAACT
GGTGTAGAAATAGAAAAATTGATAACTGCAGAATCAAGTTTATTTTTTATTTCCTTTACG
GAAGCACTGGCAACTTTACCTGGATCACAGTTGTGGACAATGCTATTAATGTCTATGGCA
ATTACTTTGGGTATTGATTCGCAATTTGCATTATTTGAAACCCTAACTACAGGGATTTTT
GATGTTTTTCCTGATTTACAATTGACAAATGTATTTAAAGCTACAGTTATTTGGATAATA
TGCTTTGTGTATTTTCTATGTGGATTGTTATTGATTACGCCTGGTGGAAAGTACTGGTTT
GTTATTTTAGATAGAAGAGTTGTGGCATATGGACTACCATTGATGTTATTTTTGGAAACA
GTTTGTGTTGGGCATTTTTATGGATACCGAAACTTGAGAGAAGATTTTCGAATGATGACG
GGTTTTTATTTAAGCTGGTACTGGTTTATAATGATAGCATTTTTTTGTCCGGCAGTCGTT
TTAGCGATTCTTATAGCCAGTTTAATCAAATTAGAAAGGATTAAAGTATATGGATCATCA
ATACCATTATGGGGAGAAATAATCGGATTATTTATGTTGTTCATTTTAATATCCCCAATA
ATTTACGGAATTATTTATACAATATTTTCTTTCAAGAAAAAATATTCAAAACTTTTTAAG
AAAAATTTCTTAAAGAAGCTTTTTGATCCACCAATTTATTGGGGGCCCTTTTATAAACAC
GAATGGATGAAAAATCAGTTTTATAGAAATAAACTGATAAAAAATAAAGATGACCCCAGA
TTCATTTTATACGTAACACCTTATGAAGATGTACATTCCATTGCTGAAAAAACTAATATC
AAAGTTTTATTCTAG
>DjScaffold5197:52449-54792 strand:+
cttgatgacatattatttggtcgtacaactataaaaaccATGGCAGATATGGTGTTTTGG
GGAGAAGAATTTGAATTCAGGGGTTTGCAGAATATCACAACTGTTAGTATATGCCTGTAT
AAAATGATCGATAACAAGAAGAAGCATGAAAAGTCAGAATTATCTGCTAAAATTAATATA
CCATGTTCTGAATTTACTGATTGCACATTCTCCGAGAAGTGGTATTCACCTACTTCAGAA
ATATCTCAAAGTAATCGATTTTCCCAATGCAAATCTTTCAATTCAGAGAAATCAAATCAA
TTCGATTTTGAAGAAATGATGAAGAGTGCATTAGATTGTGCACCAATGATACGAATTAAA
GCTCGATATAGACCTTTAGAAATTTTCCCAACTCGATTCTATTCTCCTTTATTTGAATAT
CTGGTTAATCAATGCATTCCATTGGTTGAATCACTTGAACCCCATCTAAAAGTGAAAAGC
AAAGAAGATTTGGTCAAAATATTATTAGTGATTTTGGAGAATAAAGAATGTCATATTGAT
TTTTTAGCAGATCTGGTTACAATGGAGGTTTCAAAAGCAGATGATAATTTGATACTTCGC
GGCAACAGCATAGCTTCTAAAGCAATGGAATGTTACTTAAAAATGGTGGGAGAAAAGGTG
CTTTAGtttaataataataataataataataataataataataat
>DjScaffold5197:76335-86209 strand:+
TATCTTGTGTCACTCTTAAAAGATCTCATAAATGAAGTTATTTTCAAAGAAATCGACTGT
GAAGTTGATCCTTCGCGAATAGTTAATTGTAAGGAATCAATATTGATTTCTGAAAATCAA
ACAAAATTGGTACGGTTGGTTGAAAACATTTGGAGTAAAATTTACAACAGTTCCCATTTG
GTTCCAAACGATTTAAAATCAATTTTCACCAAGATCCGAGAAAAACTTTCAGAAACCAAG
CGTGTGTCATCATATACTTCTCCAATGATTGATCAAGTCATTTCATCGTGCCTGTTTCTC
AGATTTATATGCCCAGCTATTATGTCACCATTTCTTTTTGGCTTATCAAATGAATATCCA
GACAATATAGCCAAATCTATTCAATCGTTAGCCAATTTTTCCCAAAACCCTCAAAGCAAA
GAAACCTACATGGAGTTCATGCAACTATTTATTAATAAAAATTCTAGTAGTATGCGTTCA
TTTCTGAAACAAATATCAACAATCAAATCCAGTTTTAACAAATCAAATGAGACTCATGAT
ATTGTAAAGTTACTATCTCCCGAGGCTTCGATAAATTCATCACCAAGAGATGATCGATAT
GTCGATATTGGTTATCATCTAGCAGAGCTTATGTTTCATTTAAATGTTCATTTAAAAGAT
ATTCCTGAGAAAGAATATACTGAAGCACTTCATGATGTTCGATCAGTTATTGAAAATATC

TCTAAAGCCGCAGTACATTATGAGTCTGATACTGATAGTTGTTCAAGTAACATTTCAAAT
TTCAATGTTGCACAGAGTCGTATTCCAACATTAATTAGTAAAATTGATAAAAGAAGTAAT
TCATTAAACAAATCCGTTCTTCGAGTTAATTCCACAAATTCCAATCGACCTTTATCATTA
ATAAAACAAAAATCGGATGTAGAGGAAAATTTCAGTTACATTCCTAATCGTAGTAATGAA
ATCAAAATGCCTCTGGAAGTGAAAAAACCTTCGCTTAACAATGACAATTATGTGAACTTT
GAACATTGTGAAAGTAATATTTAT
>DjScaffold5320:63361-66244 strand:+
ttagtttggtgaagctccaattcatgtcgcggcgactaagggacaggagaaaattctcaa
attgctgttgcaaaataaagcgaatccaaatattaaagacaatagtggctggacaccaat
cagttgtgctgccATGCAGGGACATAGAAGTCTTGTGAAACTTTTATTACTGAATGGGGC
CGATATTGATATTCAAGATTATAATGGAAATACCTCCCTACATCATCTTTCGATAAACGA
TTCATACGAAGGTATCAAAATTCTCATTGAAAACGACGCCAATGTCACTATTAAAAATGA
AGAAGGTTTGACAGCAAGCGAATTTGCGGAAAATGTAGACGCCTCAATGCAATGAAAAT
CTTTACAAAGTACGGATTACTATCACAATCAAATTATAATCCAGTGGTTTGCAATTATTA
TCAATTATAAtccaatttttacatattcattcatctaggacttttgtcgaaatagtcaaa
ccgaattttgaatctgataatctttatctgcaaatatcttttctagatctttcattcgat
caatgctaaacattttttaaaaaagtcacagatttttattttgtgtgaatattcattat
cttgtaaaaataa
>DjScaffold5320:68370-86812 strand:+
tgttttagagatttaaATGCACGGTGAAAATTTTGTCTATGAAATAGCTTACGAATTGGA
GAACGATTTGAAATATTTGAGTGATTCATTAGATAAGATTGCAATCAGCCCGCTGATTCA
TAAAATTGTAAATGTTTTGGAAGAACTAGAACGGCATTCTCAGCTTTATGAAATGAAAAA
GAAAGAAGTGGAGAGCTACAGAAAATCAATAAAGAATTTAGAAAATGAAGCATCAAACAA
AGAACAAGAACGACAATTTTATGAAAAAGACCTTGAGAAACTTGAGGAGTCATGGCATGC
AGAATCGATGGTGTTGATGAAAACAATGGAAAAGTTGCAAGAAGAGAATCAAAACATAAA
AACTCTGCTGGAACAAGCACATAAAAAGCCTCAGATTGATAGAAACAAACGATTTAGTCA
ACAATTGGAAATCAAAAATGTTCATTTGAAACTTAAAGAAACAATTGATAGTCAAGTTGA
TGAGATTAAACAGTTGAAAAAAGAACTTGCTCAAAGATGTATTGATTTAGATGCGATGCA
ACAACAATGTCTGAATCTCAGCAATGTCAATGCGGAAATATGTGAAAATCACTGTAATAA
TAAAATGATGTCAAAACGATTGATTGAGGAAAAAGTGAACATTGAAGCACAAATCCTTAT
CAATAAGAAATCAATTCAGCAAACTAATGATGACGTATTTGTTGAAGACGGGGTGAAGAT
TGAAGGACTTCCTAAAAGTGAAACGAGCGATATCAATTTCAGCTGGGAAGAAGTACGAGA
GTATCTTCAAGAATGTCGCCGACTTAAATCAGAAGTCAATGAAAAAGAAGAAGAACTGCT
GACTTTTAGGCCTAGCATAATGGAAGAAGGCGATGTTCAAGGACCAATCAATAAAGAACC
TAATGATAAAATGAGTCCAACTTATCGTCAAGATAGTGGAATAAGAAAATGCTTTCAGAA
ATTATTGGTTGGGATCAAAATTTGAttcttgatcattggttgtatgttctggaattttca
aatattgagttttgaatgtttttcgttgttttagagatttaaATGCACGGTGAAAATTTT
GTCTATGAAATAGCTTACGAATTGGAGAACGATTTGAAATATTTGAGTGATTCATTAGAT
AAGATTGCAATCAGCCCGCTGATTCATAAAATTGTAAATGTTTTGGAAGAACTAGAACGG
CATTCTCAGCTTTATGAAATGAAAAAGAAAGAAGTGGAGAGCTACAGAAAATCAATAAAG
AATTTAGAAAATGAAGCATCAAACAAAGAACAAGAACGACAATTTTATGAAAAAGACCTT
GAGAAACTTGAGGAGTCATGGCATGCAGAATCGATGGTGTTGATGAAAACAATGGAAAAG
TTGCAAGAAGAGAATCAAAACATAAAAACTCTGCTGGAACAAGCACATAAAAAGCCTCAG
ATTGATAGAAACAAACGATTTAATGCAACAACAATGTCTGAATCTCAGCAATGTCAATGC
GGAAATATGTGAaaatcactgtaataataaaatgatgtcaaaacgattgattgaggaaaa
agtgaacattgaagcacaaatccttatcaataagaaatcaattcagcaaactaatgatga
cgtatttgttgaagacggggtgaagattgaaggacttcctaaaagtgaaacgagcgatat
caatttcagctgggaagaagtacgagagtatcttcaagaatgtcgccgacttaaatcaga
agtcaatgaaaaagaagaagaactgctgacttttaggcctagcataatggaagaaggcga
tgttcaaggaccaatcaataaagaacctaatgataaaatgagtccaacttatcgtcaaga
tagtggaataagaaaatgctttcagaaattattggttgggatcaaaatttgattcttgat
cattggttgtatgttctggaattttcaaatattgagttttgaatgtttttcgt
>DjScaffold5320:96467-97080 strand:+
gtaattatatcatcaaacagtcaaacatatcagatttttcaggcatacatctcacaagca
tcattatgcaaacttATGGGAGATTATTTAAGACCATTAATGATTTTTGTTCCAGCACAT
TTGAATAATAGTTATGATTTTGTTGAACGAGTTAAAGGTTATGACTGTAATAAAGATGAT
CAATTTGGTTCATTGGATGTAACAAATCATTATGGATCAATTCCCATTGATGAACCGGAA

AATATCATCGATATTGTAAGTGACTTCTTTGAGAGATATAAAGAACATACAGATATTCCT
GAAATGACCAAAAATGATTTCAAATTTCTTATTAATTTGTGTCTATTCTCTGACAGCATT
TTTATCGGTGAACAACTTTTTATGGTAATGGGTAACAATTTGTCTCCTATCTAAgcgata
atttttatgaacttcattgaaaataaaattattctcaaaggtgagcagaagatacatctt
tggttcagatatatagacgatattttctatgttactaattcttcttcagaaaatatatta
gaactagccaataat
>DjScaffold4911:4630-20298 strand:+
ATTAAAAGCTATTTTTCATTATTTCAAAATAATGTTATAAACGAGTTCAGTGCTATTGCG
CCATCTCCACAAAGAGATTTTCATCAATTTCTTATTACAACTAGCAAGTTTATTTGGAGA
TGGTGGAAAATAACTGCTAGAAACGATGGTGCCAAATTCCATCCTTGTGAAATAATAAAT
TCACATGAAGATTTTCTTCATGATGAAAGGCTGCATGGAGAAATTCGTCGTATTTTCGAT
CGAGAAACTTTACAATATGCATTGAAAATTGCTGAAGGTTGTTATGATGTTTTACCGCGA
TTGCCAAGAAATATTCTTACACACGTATTTTATTATTTAAATTTGGAAGATATTTTCAAT
TTGGGTTACACTAGCAAGTCGATGTATCGAATATGCAATGATGAACTTTTGTGGCAAGAA
CTTTATCACAGATATAATCATTCATCCTCACCAACATCAGTCGCAGATTTAGCAGAAACC
TTTGGCTGGAAAAGATTATTCTTCACTAATAAATTGCAATTACAAATGAAATTGAGACGC
CTCAAGAACAATCGCTCAGCTAATGGTAGTCAAAATGAAGAGATTAATGATGAAACATTC
GCTGAAACTGAATTGCCATTGGATAATAAACATGCATTACAAAACAAAACAGTTTCTCCA
GTCAATCACCTAATGCCTGATACATTGTTACCATGTGACCCTCTGCCCTACAATCGATAT
GCTTCATCAACCATCGCTGGAGCTGAAGATTGGAATGATTTAGAAAGTGAATTTTTAGAA
AGAAGTTTCTTTGAAGACACAGAAGAAGAAGAGCTAAAACATTTGAAAGATGATTAAaat
taccttttaatatttagtattaaagaaattatattatcaattttg
>DjScaffold3695:3590-4556 strand:+
catatttggtcgattttaatATGCAGAAAGGTGAAAATATATTAAATTTAATTCCACAAG
CTCCATTAACTGAAAAAGAAAGATTAACTTACAAGTCTCAATTTAAATCCACAGTTAAAG
ATGAATTCAATAAATCCAAATTCTCTCATAAAACAATGGGAGAACCTGAATATAAAGGTG
CTGATCCAAAAGAATTTTTAAAAAAGAAAGATGAAAAATCAATGTCTGAAAAAGTTCCAT
GTGATTTTCACAACGAAAAACGAAAACCTCCAGTTCCAAAACACAATGAAAATCCTCCTC
CAATGGGAATAAAGACAACGAAAAACTTTATTGCGCAAAATTCTGTGGATAATATCATGT
CAGTTAGTAAAAAACCACAGAAATATGTTGTGGATACACGAAATGGTGACAAATTTCAAT
TGGAACCATCGGGTCTTGATACAGTTTACGTGGGCAAGAAAGATTATGGAAAAACACCAG
AATATCTGGAAAAGAGAAAAGAAGAACTAAAAGAAATGCAACTGGAATACGATGCGTATG
TTAAAGAAATGCATCGTCGAGGAGAAATGCGTCGTTTGACAGAAGAAGAAATACAGGAAA
TTCTTTACGGTTTGAAAACGAATTGGGAGAACATTTATCGTCAATATCAAACATTATCCG
TTATGGTTGACAGCATTCCGAAAAAGAACAGAAAGGAATGGATGGAACATGAAATGAAAG
AATTGGAGAAAGATATTGAAATGCTGGAGCGATATCAAATCATTTATGTTGCTAACTAAt
ttacttgaatttagatgggagaataaaatttacttttatcttt
>DjScaffold808:118596-125051 strand:+
GCAGTACAATTCAGTTGGACTGGACCACCGTTATTTAAAGAGTTGTATAGGAAAGATAAC
GTTTTATCTAATAATAATAGTAATCATTCGAAGATAACTTTCAAATTAAAATCCAATAAA
AGACATATTGATGGAAATTCAGAAACTGTTGCTACGAAACGATCGTATTTATCAAATCAA
AATTCGAATGATGAACCATCAACATCGGGTTCAGCTTTGTATGAAACAGAAATGTTATCA
TCGACTTTAATTCTCGGGAAATCATTTATTTCAGATTCGAGATTCAATCTCAATGTCACA
TTAATATCCCGACTGAAAGAAAGGATTGTAATTTCTAACATTTTGAATCAGAATTGGAAT
CTCCTTGAATTACTACCAAATAAAATTTTCGAAAACTATTTAAGTTATAAAGAAAATAAT
GTTGAAAAACATTTGCATTTGTCGTCATCTGTTATAAATAATCTTCCCGGAAATATAAAT
GATCGGAAAAAACTTGGAGCTACGTTCCGTCCTAAATATGCTCTGCATTTCACTATGAAT
CCTGTTAGTTATCGTGACAAAAAGTTAATGCTAAAAGTGATTGAACAATTAAAATCAAAA
AATACGGTGGGAGTTGTCAGATGTAATACTCTTTGTAATATATTATTCTATGTTTTCCCT
AGTGGACCAATTTCAAAATTCACAGGTGTATCATCTCACGATGCAAATTGTAATTTACAT
GTAGTAATTTTCACTGAACAACATCCTCAGTTTTTACCATGTAATTATACACCTCAAGAT
AAGATCCTTAAACGAGAACAGAAATTATTCCAAGATATTTTATCAGATTATGAAGCGACG
AACAACCCTAAATTTGTCCAGAAATCCGTCATTTTAATAGATGTCTTAAGGAGTTCGATA
ATAGCTCACAAAAATAAACTCTACGATAAACTATCAGATAAAATATTGAATAAGTTTCAA
GTTGTCAGTAAAGTTCCCATAGAATCTGTAAATAATGATAGTGATATGGAAATTGATAGT
GATTTCGATACATCTTCATGTTCTAAATCAAAACATCAGATTGAATCACCATCGATATCT
AAAGAAACGAATAACCTATCAAATAAAACTCCAATAGTTGAGCAATCAACATCACAACCG

AATTATTTTAGCAATCAAACTTTACCACAACCTCATAATTTCATTGCTTCTAATACTAAT
TCTTTAGTGATTAACCAATTCTATAATTCCGTACAGTCATTACCAGTAACAACCCACCTA
CTAAATGGAACATCAATATCTCCAGTTTCA
>DjScaffold808:57630-66858 strand:+
TTATTAGCTGACCATAAAGGATCTATTTTTCTATTTAACTTTAAAGATAATAAATATCAA
TTTATTACTAATTGTAGTCAGGCTTGTACATGTTTAACGTTCAGTCTAAAACCAAAACCT
ATTAAGGAAGTATATGTTGGATTAGTAGATAATTCATTTAGATCTTTTGATCTTGGATTA
AATCAATGGATATCAATAATGAGAGGGCATACATCAACCATTCGTACAATCTCTATACAT
GACAGTGGAGAAATTGGGATAACAAGTACTGTGAATTCTGCTATTATATGGAACCTGCTC
AGTTGGACAAGATTGCGTCAACTTAACGTCAATCAGAATATTGGAATTACACAAGTATTT
TTTCAAAACAATTTCATTGTTTCTTGTTTTCGTGATGACTCAATATTCATGTGGGATTAT
GAAAGCCTGGATTGTAAACTGAAATTACCAGCTGCATCAGAAGATCGAGAACCTGTTTAC
CGCAGTTTAGCTTGTTCCTGTGATGGACGATTGTTAATGGCGGCTGGCAAATCTCGGTTT
ATCGACATTTGGAATTTAGAAAGTGGAACATTAAGACAAACTATTGAGCTTCCAAAACCT
ATTCTTGGTGTGAGAAAAGTATTATTTCTTGAACCAAACAAAATAGACAGTGATGGACAA
GAAACACTATTGGCCGTATTGGGTGAAGATCGACGATTGCGCTTGATACAGATGTCTACT
TGTCGGTTATTGATGGAATTCAACACTGATAATCCCTGTAAAGATATCACAGATCCGTTA
GTATTAACTTTGACTACCGGTTTAAGAAGAGCTGACAAATTAGCTATCATTTCAGCTTCA
AATGGTAGTTTGCATTTATATGATTTAGGAGCTATTTGGAGGGAAGCAAATAAACCTGAA
TTACCAATTCGTAAGATTTGGAGGAAACCAGACGGTAAAGTAGTTAACACCTCAGAGTCG
CCGTTGGTTGGAGCCACATCGCTTCAATTTAATGAAAACAATAAAGAAGATGATAACATT
ATTGCATTG
>DjScaffold698:31734-40697 strand:+
aaacaatttgcaattaaagatcttggagaaaatccagaacaagttaatgcccatattgag
agttttcgccgatggctaaaatcaATGCCCCATTTGAAATGCCCAACAGATTCAACTTTT
CTACTTAGGTTCTTAAGGGTTTCAAAATTTGATCATAAAAGAGCAGAAAGAAGATTAGAC
AATTTTTGTACTGTTAGAAAGTCACCAAGAGGTTCACCTGATTTTTTAAAAGTGTAGGA
ATCGATGATCCTATGATTGATTACTACTTAGAAACTGGAATGCACCTTTATCTAGGACAA
GTTAATAGTGGAGAAGTAGTTCTTTTAGTTAGAACAAAAATGTGGGATCCGGAAAAAATT
CCATTTCCAAAACTACTTATTTTCATAATGATGGCACTGGATATAGTAATGTCTGATGAA
AACAGTCAGATTTACGGATTTAAAATGATTCTCGATTTGTTGGATTCTTCTAGGAAGTCA
ATGGAATCTTGGAGTGATCCAAAAGCTATGAAACAAATGAATCGAGTTATGCAGGAATCC
TATCCGATTAGAATGAAAGGGACAATTTTTTACAATGAACCTGTGATTTTTGACATTGTT
TTTCAAATATTCTCGTTTTTCCTTAACGAAAAAATGAAAAGCAGAACTATACGAGTTAAA
AACGACATTAACAAAGCTATAGAGAAAATACCAGGATTAGAAAAGATTTTACCAGCAGAA
TATAATGGCCAAGGAGGTTCTATTAAAGAATGTTCTGAACAATGGAAAGCCAAATTTAAA
GCTTTCTATAAAGGCGGACATCCTTTGGATTGTATTTCTGTCGATGAAAGTAAACGTCCT
AAAGAAACTGCATCTTATTTAGTTGAATACGCTGATTATAAAGGCCAAACTTTAGGAACT
GAAGGAACTTTCACTAAATTCGAAAATGTTTTTTGAgtatattcaattggtataacctgt
ttttacaaagattgaatctatatggtagacaattaggcatctaataaatttatttatttt
aataaatattttttttaatgtgttaattagttattcaataatttattaataaa
>DjScaffold3484:8195-19850 strand:+
aggttaagttttactaatttgaaatttttaattaaatacttaataattcattgaaaatt
tattttatcgaaaatctcatcataaaaaaATGAAATCATTTTGTATATCATTCGTTCTTT
TATGGAATTTTATTAATTTTTCTAAAGAAAACTCTTGCAATATATTCAATCGTATCATGA
ACTGCACTGGATTAAAGTTAAAAGATTGTCCTCTTGTAAATGACACTATTTTAACTTTAA
TTCTCGACGATAATTTAATACATGATTTTAATACAGGAAATTGTCAAAATTGGACCCAAT
ACGAACGTCTTAGTATTCGACGAAATCAATTAGAGTCTTTCTTAATTCTTAATAAGCCTA
ACATGCTCGATACTATCGATTTGAGTCAAAATAATTTGAAAAGCATAACATTGGATTCCA
GTCAAATACGTTTTGTTAATATTTCCCATAATCATATCTCTAATAAAATAACTTTGACAT
TGCCTAGTTTGAATCAGTTGATTATCAGTAACAATCTAATCGATAATGCTGGTTTGGACA
CAATAATGCACGATTTGCATTGGTCCGCATCATTATCGTTAATAGATGTTAGCTACAATA
AATTTAATAAAACAAGTACTAATTTCATGGATTTATCTGTGAATAGAAGAATATTGATAA
ATTTAACGGGTAATCATTTATCGGAGTTTAGTTTTATATCTAAACTGAAATCTGGAGCCG
ATTATGATTTATCCTTGAATCAAATTGCAAGTATTCGGGCAAGAGTAGATAATGGTGTGC
AGAAATTGAATTTATCCCATAACAAATTGCCAAATATCGACACTACTGAACTGGTGAAAT
TAATAAATCTTCAACTACTTGATTTGTCTGGTAATCCCCTGAACTGCTTGTGCAGTGAAT

CTCGATCATTTATTCAATGGATGATTACAAACAAGAACAAAATTACTAATCAAGACAAAA
CGGTGTGTGACAAACCTGATAAGAAATCAATATTTGATGTCAAGTGCACCAATGGATCTT
CGTCGACGACTCCGTTCGTGATAACTTCTACAACATTCCATCCACCCCATTCAACTCATT
CAACTCATTCAACTCATCCACCTCATCCACCGATAAGCCGTACTGCAGTCTGGAAAATTC
TTGTACCGATTTTCATCATTCTTCTTCTAGGATTTGGATTATTTGTTTTCCTTAAGCGTC
GACGGAGAAATGAATATCAAAGAATAAGTGAATCTCGGAATTCTAGTCCTTTGTTTGGTT
GCGAATGTTTTCCAATGTTATCATTCCGACCGCGAAAGAATTCAGAATTGCTTATTAATC
AAGAAGATAGCAGTCAAGACGCATACAGAGGAGGACGTTTGAACTTTGAAGTAACGCTAA
ATGTAATCTGCTCTAGTAGAGACAGGAAATGGACTGCTAGTAATGTCACATGCCATTTAG
ATATTAAGAAGAATATTATTAGTTCAAAAATCAAAAATTTGCCATTTTGCGATTATACAT
TGATTGTTGTTAATGGTGACAATTGTTTGAAGGATAATACATTTTTGACTAATGCAGACA
AGAGTTTAAATACGAATAATCGAGACGATATAAAACTTCTAATTAATGAGATGCCAGATT
TTGATAACTGTAGGAGTAATTCGTTATTTATTAATTTTCTTAGAGAAAAAGTGAATAATA
ATATTTATTTCAAATATAATCAAATCGACAGTGACACTTGTTTATCTAATTTGAAAATCT
GGTTGAGATTGGATTTTGTTCCTCATAATGTTTGAtttttgaaatgcatgacaacaggac
taaatgtttacaccattcagttgttgaatttttaaatatgttaaataaaatatttgatt
ttgc
>DjScaffold3062:21061-36038 strand:+
tttgtcagagatattagaacagattaacaataatgtcaacaaaaatgatgagggattcAT
GGAAACACAGTATTTGATGAACATAATACTGCAAAATTCAAATTTAACAGATGATTGGAA
ACGTTTTCTAAACACTATTTTTGAATCAGTAAATATTATCAATGAAAAACAGTTGAAAAT
TGTTTTGGAAACCCAAACTGTTTGTCACCATTCTCAAATCAATTTTTCAAAATTTGAAAT
AGAAAATGCCAAATTAATGGTTCCTATGATATTTAATGAATTTCTGAAATCGGAAGATCG
TTTAAATATAGATCAGATTTTAAATTTCTTAACAAATTCAAATCAATTTGTGATATCTCA
AGACCAAATCAATAAATATTATGATGAATTTTTATCAATAATAACTCAGGTTGAAACTCA
CAGCCTAGACAACATTCCCCGATTTCATTGTTTGATATTTATACCATTTTTACTGAACAA
ACTGAAAAGTGAAATCTAAtatttgtaaaagtaaatgatatgaagtgttatttattattg
tacatccagatttctattattgtaatttttttagattcatagtaaatttgtgaatttta
>DjScaffold3062:92015-110464 strand:+
atcacaacaatagaacaaaattagaaaatagttttttatttttcgctttaATGTTGTGG
GGATTATCTAAAATATTGACCAGGCATTATTCTATAACTAAAGTTATAGATACCACTAAA
TTAAGACAAAGTTGGAAAACTTTAAGCGATAATAACATAAAATCACTGAGGAAAATTCTA
GACCATGATAATCTTGAATTACGCGATCAATTAAGATGTTTTTTAAAAAACGAAGCATTT
CAGCCAAAATATGATATCGGAATTAAAGAGGAACGAGAATTGGCACTTATACGATTAAAA
TCTTTATGTGATAATGGCTACATATCAGTATTGAATTTTAATGATAATCCTTGGAAAATT
TTTGCAACTCATGAGTTGGCTGCGATTGTCGATGCATCTATGACTACTAAAATGACCGTA
CAATTCAATCTATTCGGTGGTACTGTTTTAAAATTAGGAACAGAAAAGCACTTTGATCCT
CTTCTTAAAAATATTGATACTTTGAAAAGTGTAGGCTGTTTTGGATTAACTGAATTAGGA
TATGGAAATAATGCAGTTGAAATGGAAACAACTGCTTTATTTGAAAAAGAAACTGATCAA
TTTATTATTAATAGTAATACCATACAAGGTCAGAAATATTGGATTACAAATGGTGCAATT
CATGCGCATTTTTGTGTGGTTTTCGCACATCTTTACAGTTTAGGACAAAATCATGGAATT
CATGCTTTTTTAGTACCAATTCGTGACGAAAAAACAATGGAGGTCAAACCTGGAGTTATT
GTGGAAGATATGGGTTTCAAAATGGGTCTAAATGGAGTTGATAATGCCAAACTTAGTTTC
ACTAATGTTCGAGTACCTCGGAGTGCTCTGTTAGATCGTTTTTCAACATTAAGTCACGAT
GGTATATTGACTAGTTCAATCAAATCAACACGGGCTCGTTTTCTGACAGTTGCAGATCAG
CTGTTATCTGGAAGAATTTGTATCGCAGCAATGGCAATTGGCGCTGCTAAAGCTTGCCTT
GCAATAGCCGTTCGATATTCAGCTACTCGAGAAACTGTTGGTCCTACTGGAAAGTCTGAC
ACTCCAATTCTTAAATATCAACTTCAACAGAAAGCTTTATTACCATTGATAGCTGCAACA
TTTGCCATTAATATTGGCATCAACCATGTCAAAGATGAATGGGCAAATAAGAAAATTGGA
CATGAAAAAATCGTTATTATGTGTTGTGCTATTAAACCAATATCTAGTTGGAATCTTGAG
AGAGTTGCTTCTATTTGTCGGGAAAGATGTGGTGGTCAAGGATATTTGAGTTGTAATAAA
TTGGGTACCTACGTTGCTTTGTCTCACGCAACGATAACAGCAGAAGGAGATAATTCAGTT
TTGATGCAAAAGGTATCAAAAGAATATTTAGCAATCTTCAAACCATCTGGTAATCCTTTG
AAAGCAGAATTCAATTCTGATTTTTCTGATAGACATGTTCTTCATAAAATTTTACAAATG
AAAGAAGATTTTCAATTCATAACTCTGAACAAAAAACTTTCACCAGTTCTTTCTGATAGA
ATAAAACTTTTCAACACATGGATGCTTGAACAAAGTGATCTTATTCAAGATGCAGCAAAA
TCATACGCTCATAAAATGATCAGTGAAGAATTTCATAAAGTTATAGAAAAAATGTGACAAA

GCTGAAAAAGATATACTATTGAATCTTTACCGACTTTATGCAGTTTCTTTGATTTTAAAT
GATTCCGGATTTTATTTGAGACGCGAATTAATGGATTCGAGTACAATTAACAAATTGCAC
GAGTATCACGACAAATTGTGTAATGAATTATGTCCTCTTAGTCTTGATTTGGTTGATGCT
TTTGGATTAGATGGAGAATTACTCAATGTTCCTATTGGAATGAATTGGATTAAATACAAC
TCTGTCAATAATGTCGGTGAAGTTAATCCTAAATGGTTATGAatattgatgtgatgtttt
aatttacaataatatttacatttattgatttttagcatattgatgtttcttgaaatttta
tgaaatggtatcgttaatttgacaattcatgtattttattttggtatatttagtaccttg
atttaattacaaaatatgttaacgtggctgattaa
>DjScaffold3062:116543-117613 strand:+
gaaactgtgttgataaccgagttcaataaagtacaaacgaagcactctATGATACAAGTG
TGGCCTGTTCGTCAACCACGTCCTGTTGCAGATAAATTACCCGCTAATTATCCATTATTA
ACAGGCCAACGTGTGCTCGATGCTCTATTTCCATGTGTCCAAGGAGGAACTACTGCTATT
CCAGGCGCTTTTGGTTGTGGTAAAACTGTCATATCGCAATCATTATCAAAGTTCTCGAAT
TCCGATGTGATTATTTATGTTGGATGTGGAGAAAGAGGAAATGAAATGTCGGAAGTATTG
CAAGATTTCCCCCAATTGACGATTGAAATCGACGGCAAGAACGAATCTATCATGAAAAGA
ACCGCACTGGTTGCCAATACGTCCAACATGCCTGTTGCTGCTCGTGAAGCATCGATCTAC
ACCGGAATCACTTTATCTGAATATTTCCGCGACATGGGTTACAATGTTAGTATGATGGCA
GATTCGACTAGTAGATGGGCCGAGGCTCTCAGAGAAATCAGTGGTCGATTGGCTGAAATG
CCTGCTGAATCTGGATATCCTGCATATTTAGGAGCTCGATTGGCTAGTTTCTATGAGCGC
GCAGGACGAGTGCAGTGCATTGGTGGACCGGAAGAGCGACAAGGCTCAGTGACCATTGTC
GGTGCCGTGTCACCACCTGGAGGTGATTTCTCTGATCCCGTTACCTCGGCCACTCTCGGT
ATTGTTCAGGTGTTCTGGGGTCTCGATAAAAAGTTGGCACAAAGGAAACATTTCCCCAGC
ATAAATTGGTTGATCTCTCACAGTAAATACACGCGAGCTCTCGATGATTTCTACGATAAA
CACTACAATGATTTCGTCGCGCTGAGAACAAAGACATCAGAAATTCTCCAAGAAGAAGAG
GATCTGTCTGAGATTGTGCAATTGGTCGGCAAAGGATCGCTAGCTGAGAGTGACAAAATC
ACTCTAGAAGTGGCTAGATTGATCAAGGATGATTTCTTGCAACAAAACGGATATTCGTCC
TACGACAAG
>DjScaffold5384:14284-27101 strand:+
cttgataataaatgtccatcaattaggaatgtgaatgaaattttcaaatttttcttcttt
ttaaaaatttatctaaatttgtgttgaaacttaactactgtagttaacagattgaaATGC
TTCCACTTAAAGGAATAAAGGTTTTAGACTTATCTCGTGTTTTAGCTGGACCATTTTGTT
CCATGTTGCTTGGAGATTTAGGTGCAGAGATTTTAAAAATAGAGAATACAAAAATTGGTG
ATGAAACTCGAACATGGGGACCTCCATTTGCTGAAGAAGATTCTTTATATTTTTGGTCAG
TTAATCGTAATAAATCAAGTATTGCTGTAAACTTTAAAGACACTGATGGCCAGAAAATCA
TTGAAGAACTTGTTCCACAATGTGACGTTTTCATTGAAAATTACCTTCCAGGCACTTTAA
AAAAATACAATTTAGATTATAATTCTATTAAAAAATTGAATTCCAATATTGTCTATTGTT
CTATTACTGGGTTTGGACAAAAAGGTCCCATGGCACATAGACCTGGATATGACGTTGTTG
TTGCAGGCATTGGTGGATTATTAAACATAACTGGTCCTGAAAATGGTCCGCCTTGTCGTG
TTGGTGTTGCGGTCACAGATTTAATGACCGGTATGATGGGTGTCACATCCATTTTATCGG
GTTTAATCAGTAGAGTTAATAACGGAGGATGTCATATCGATTGTAATTTATTAAGTACTC
AAATAAGTATGCTGTTTCATATTGGAGCCAATTATTTAACTGAAAATATTGAATCATCTC
GTATTGGAACTGCTCACAGTACTCTGGTTCCTTATCAAGCATTCACTACATTTGACAATC
GATATTTTATTGTTGGAGCTGGAAATAATGACCAATATTTTAATCTATGTAAAATTCTTA
AAATTCCAGAAAACGATAAGAGGTTCTCAACAAATTCTCAAAGAGTGATTAATAAGGTAG
AACTTAACAAAATATTAGAAGAAACATTTTCTAAACACAATTTGGATTATTGGATCGATT
TATTTAAAGACAGTTCTTTGCCCTGTGGTCCAATTAATAATTTGAAAGATGCTTTCAACA
ATCCTCAAGTAATTAATTCATATATATTTTAAtttataaattgctaaaatacacttttca
ttgcgtatatatcaaccgcttataatattgtgaaat
>DjScaffold5384:51356-51799 strand:+
agaaaacctagaatcgatATGCGGAATGCTGGAAGGAGGAAAACAATCATCACAAAGAAT
TCTGATAAATATGTAGAACAATTAAAAAGAAATACTATGAAAATTCATAGCCATGTTATA
AAAAATATAGAAGAGCATAATGAAGTTACAGCATCTAAATATAATAACTATCGCTGTAGA
GATGAAATTGAAATTGATGATAAAGTGCTTATAAAAATCAATGCACATGAGGGTTTTAAA
TGGCGATATATTAGACCGTTTAAAGAAATAAAGAAAATTATTGAATGGACTTACACTTTG
CTCGATGAAAATAGAAACACATTAATTCAAAGAAATTACAACCAAATCAAAAGAATACCA
AGAGACGAACTTTCCTACAAGTCATGAagcacacaaatatcagtcttaccagtacaatat
gac

>DjScaffold5384:132731-133018 strand:+
ATGCCGTTTGGGATAGCGGCACCGCCGGGTACTTTCCAAAAGATCATGAGCCAGGTAGTT
GGCGATATCAAAGATGCGTTGGTCTACCTAGACGATATTCTGGTTTTTTCCGATAACGTG
GAAAATCATTACGAGGGACTCAGGCAGGTACTGGGTAGAATTAGAGAAGCAGAGCTGGGG
ATTAATCCAAAGAAATGCCATTTTTTGAGAGAAAAGGTAAGATTTTTGGGTCACATTATT
AACAATGTACACGGATCCAGGGAAAAAAGATGCCATCAAAAATTATAA
>DjScaffold4324:8457-9391 strand:+
aaaaATGGATATTCTCAGACTGAGAAGCAGTATCAAAGATATAAAATCGTTTAATCTTTG
GCAGTCCTTATTTGCAGAATTTCTTGGTACATTTCTATTAGCTTTTATAGGATTTGGTTC
TGTGTTCAGCAATATGAATCCAGTTAAAGTAGGATTAGGATTTGGATTAGCTGGAGTTGT
GATAATTTATTGTATTGGTCCGATTAGTGGTGGTAATATTAATCCAGCTGTGAGTTTTGG
ACTACTTATTGCTAGGCAAATAAGTTTAATTAGATGCATACTGTACATTTTCGTACAAAC
TATTGGCGCAACGTTTGGCTATAAATTCATTTTCATTTTAACAAATGGTAACACGTTGGA
GAAGTCTTTTTTAACGATGAATTTGAATAAGAATATGTCAAATAACATAATGAAATTTTT
TAATGAGCTCTTTGTAACGACGAATTTGAATAAGAATATGTCAAATATCATAAAGACATT
GATTAATAAGACATTGTATATAAATGACAATTTGATTATTACCGAGAAAATTGATAAAAT
AAATAGTTTATATTTGAAGATATTTTGTGAAATGGTGATCTCATTTGTTTTAGTATTTAC
AGTAATGAGACTTTGTGTGGAAAAATCAAGAAGTACAACAGAAAAAGGTTTAGCTTCTTT
GATTATTGGGATTAGTATCACAATTGGAGTTTACTGTGGTATTAATATAAGTGGAGGTTC
TATGAATCCAGCTGTTAGTTTTGGAAAATATATATTTACAGGTAGCAAAGACATCGTGAC
TCATATTGTTGGACCATTAGTCGGAGGTTTATTAGCTGGTGTTCTGCAACAACTTGTATT
CACATCGGATCTGTCAAGGAAGCATATTATATCGTATTTGATAAGCAATGATTTTGAGAA
CAAAGAGTTATCTAATCAGGAAAATTTAAACTGTATACAGTTAAATGATGTAGAGAAAAT
TGATATTGAAAAGGAAACAAAATCAAATCGTAATTCTCAAAACGGTGAACATGTTGGAAA
AAGTAGTGAAGAAGTCAATATTGAAAAGTTAAACTGCATACAGTTAAATGATGTAGAGAA
AACTGATATTGAAAAGGAAACAAAATCAAATCGTAATTCTCAAAACAGTGAAAATGTTGG
AAAAAGTAGTCAAGAAGTCATTATTGACAATGAACAAAAGATGAAAGAAAACCCTTAGtg
aattgatgtctcgataagaaatattttctcactttaattaatgaactcttattattttg
attattaacattattgataaaaATGGATATTCTCAGACTGAGAAGCAGTATCAAAGATAT
AAAATCGTTTAATCTTTGGCAGTCCTTATTTGCAGAATTTCTTGGTACATTTCTATTAGC
TTTTATAGGATTTGGTTCTGTGTTCAGCAATATGAATCCAGTTAAAGTAGGATTAGGATT
TGGATTAGCTGGAGTTGTGATAATTTATTGTATTGGTCCGATTAGTGGTGGTAATATTAA
TCCAGCTGTGAGTTTTGGACTACTTATTGCTAGGCAAATAAGTTTAATTAGATGCATACT
GTACATTTTCGTACAAACTATTGGCGCAACGTTTGGCTATAAATTCATTTTCATTTTAAC
AAATGGTAACACGTTGGAGAAGTCTTTTTTAACGATGAATTTGAATAAGAATATGTCAAA
TAACATAATGAAATTTTTTAATGAGCTCTTTGTAACGACGAATTTGAATAAGAATATGTC
AAATATCATAAAGACATTGATTAATAAGACATTGTATATAAATGACAATTTGATTATTAC
CGAGAAAATTGATAAAATAAATAGTTTATATTTGAAGATATTTTGTGAAATGGTGATCTC
ATTTGTTTTAGTATTTACAGTAATGAGACTTTGTGTGGAAAAATCAAGAAGTACAACAGA
AAAAGGTTTAGCTTCTTTGATTATTGGGATTAGTATCACAATTGGAGTTTACTGTGGTAT
TAATATAAGTGGAGGTTCTATGAATCCAGCTGTTAGTTTTGGAAAATATATATTTACAGG
TAGCAAAGACATCGTGGTAAGATTTTATTCATTTGTACATTTAGATAACTATAATAAACT
AATTAATAATTATAAAGTACTTGTATTTAATTACATTTTTCGCAATATAAATTTAAATAA
TAAT
>DjScaffold4324:41620-60121 strand:+
agataaactttcgtttaactATGAAAAAATTTTTCAAATTATCAGCTTTAACTAAACCAG
CTTCTTTTATTGCTTCGAAACTTGGCAAAAAGAAGCATTCGGTTAAAGATGATTTTGAAG
AAGTTTCGGAATTACCTGACTTAGACGTTAAGGGTGATAGTATTTTTGAGGAAAATGTTA
TACAACAAGCAAGTGCTCCTGTTGATGAATCTTTAGTTGACCCTCTGAAAAAGATGGACA
TATTAGAAAATCGTAATCCAGGTCTTACATTATTAATAAATCAGTCTGACGAAACATTTT
CTTCTCCTTCATTTGATCCTTACTTTGAAAGTTCACAAATTAAAATTCCAGAACCTGTAT
CATTAAACGTTTCTTCTGTTAGCACTGTAAAATATTACGAAAAACCTGAAAGAAATTTAA
CATTACTGGGCAAAAAAGCAAAAAAAATCGAAGAGGATGATATTATGCAGAAAAAAGTCG
ATGAATTGGAAAAGGTTTCAGAAAAGCAGGCAAATGAAATAAATGAACTAAAATCGATAT
TAACCGACTGCTTAGTTCGAATCACCAATCTCGAAGGTGGTTCAAAAACAACTAAGGAGA
AAAGTTCCCCAACTCCAAATTCATTGAAAATTACGTCACCAAAAGCAAAAGATGTTCAAT
TACGAAAATCAATGGTGAGTCCCAGAACTCCAGTAAAAGCTACAAGATTGTCAGGAACAC

CTACTCGTAGAAGCACAAGTGATTTGGCTAAAAGATCGAAAACACTGCCCATATTCAAAA
GACCCTGGAAACCACCAGGAGGTTTGATTATATGTCCAATTATTATTAAAAGGGGAAATT
TGAACAGATCGAAAAGTCTTGATCAACAAAAGATAACAAACCAAATTTGCAATCAACTGG
CTAATAGAAATTTTAAAATATCCAAAAAGTGTTGTTCTTGGAAACCCCCAGGAATTTCAA
TTTTATTTTGTAATTTCACATGCAAAGTGAAGATTAATCAATTCAGACCAATGTTAAAAC
TAAATTTGAATAAAAATCAATATCTCTATAAATCAAACAAAAATTATAATCACAAAGAAG
CCACTTTCATCAAAGGTGAAGGAGTTTTACGAATATTTATTAAAGGAAGACCAATTAATT
TATACGTTCCGAATTCATTGACCGACGGATTTGATATTAAGAGTCCAATACAAGAACCTC
AGGAAAATTTAAGAATGGAATGGGTATATGGATATCGAGGTAAAGATTGCAGGAAAAATT
TATTCTATTTACCAACGGGGGATCTTGTATATTTTGTTGCTGGTATTGTAGTATTGCACA
ATTTAAATACGCAAATTCAGAGACACTACGTGGAACACACGGATGATATCAAATGTATGG
CATTGCATCCTGATAAAGTTACTTTTGCTACTGGACAAGTAGCCGGTCATTCTAAAGTTG
AAGGAAAACCCCATGTTCGTATTTGGAGTTCGATTTCACTACAAACATTGCACACTATTG
GCTTAGGATTTTTTGAAAGAGCTGTCAGTGCTTTGGGCTTCTCTAAATTAGATGGAGGGA
AATTATTATGTACTGTTGATGAAGCTAATGATCACAATATAACGGTTTGGAATTGGGCAA
CTACTAAAAGTATATGTACATCCAAGAGTTCAACTGAAGCCGTTATAGAAGCTGAATTCA
ATCCCACTGATAACAACGTCATTGTAACTTGCGGTAAAAATCATCTAGCATTTTGGATTT
TAGAGAAAAGCACTCTGGTAAAGAAAATGGCTATTTTTGAAAAATATGATAAACCGAAGT
TCACAACCTGTGTAACATTTACTAATACTGGTGAACTCATTACAGGAGATTCTTCGGGAA
ATATTTACTTTTGGAGTCGTGAAAAACGATCAATTACTTCAGCAATTTTGCAAGCGCACA
ACGGTCCAATATTTTCGTTATTTATTACCGAAGCTGGATCACTTCTTACAGGTGGTGGAA
AGGATCATGCTATTTTTGAATGGGACTTATCAAAAAAAGAAAAATCTGGTAGAAATATAC
AATTGACCAATGAACATGGATTTTGCAGAACAATTTCGGAGGTGAATGATAAATTGGTGG
TTGGAACAACAAATAACGCTATTATAGAGGTAACTGGAAATGATGAATTCATTAATTTAC
TTAACGGCCATTCTGATGAGGTGTGGGCTATTGACAGCCATCCTTCAAAGGAGCAATTTA
TAACGGGTGGTCATGATCGTAAACTAATTTTGTGGGATGCTTTGACTCATACTGTTCTAT
TGTCGAAACCGTTTGAGAAAAATATCCAATCCGTTTCGTTAAACCCAACAGCTGATTTGG
TAGCAGTAGGATGTATTGAAGGTCACTGGTATGTGTGCGATATAAATGAATTCAAAGAGA
TTCATCAAGGTAAAGATGGCAACGAACCAATTCAAAGTATTGAATTTAGCCCAGATGGGT
CTCATCTTGCAGTAGGATCAAGAGACAATTTCATCTACATTTATAAAGTAAGTCATGAAA
CTAACGAAGTTTCCAAAGTAGGAAAATGTTCCGGTCACTCTAGTTTTATTACACACTTGG
ATTGGTCTACTGATGGATTGTATTTGAGAAGCAATTCAGGTGATTATGAACTTCTTTTTT
GGAACATACCAGAGTGCAAACAATTAACAGCTGCAAGTACACTGTCAGATGTTAAATGGC
ATTCTGCAAATTGCACATTAACTTTTGAAGTAGCTGGAATTTGGCCAGAAGGTTCTGATG
GAACTGATATCAATGCTGTAACTAAAGCACACAGTAAATTAATCCTCGCTACAGCAGATG
ACTTCGGAAAAGTTAATCTTTACCGTTATCCAGCATGTAATCCAAAAACAGCCCATAAGA
TATACAAAGGTCATAGCAGTCATGTAACTGGCGTGAAGTTTCTTGTTGAAGATCATCGAC
TTATGAGCGCTGGAGGTCAAGACACAGCAATAGTTCAATGGGCTCTTACTTGAaagataa
ttataggagaaatgaaaattggaatattgacatctgaattattagtttttattgttatta
attctatttagtttatatgatttaaaatttgcaagatttaatttttgtatcagtgtgcgt
gtgtgtgtgtgtgtgaaatcgatgttgcaattatttatggaattttaatttatctggctc
tcattagtgtatctttaacctttcaagggttatagtgcttaatttgtttagactatttat
ttcgaattttatctaaatcaaatataaaaataatattgttatgaataaaatattattttt
cgcaagataaactttcgtttaactATGAAAAAATTTTTCAAATTATCAGCTTTAACTAAA
CCAGCTTCTTTTATTGCTTCGAAACTTGGCAAAAAGAAGCATTCGGTTAAAGATGATTTT
GAAGAAGTTTCGGAATTACCTGACTTAGACGTTAAGGGTGATAGTATTTTTGAGGAAAAT
GTTATACAACAAGCAAGTGCTCCTGTTGATGAATCTTTAGTTGACCCTCTGAAAAAGATG
GACATATTAGAAAATCGTAATCCAGGTCTTACATTATTAATAAATCAGTCTGACGAAACA
TTTTCTTCTCCTTCATTTGATCCTTACTTTGAAAGTTCACAAATTAAAATTCCAGAACCT
GTATCATTAAACGTTTCTTCTGTTAGCACTGTAAAATATTACGAAAAACCTGAAAGAAAT
TTAACATTACTGGGCAAAAAAGCAAAAAAAAATCGAAGAGGATGATATTATGCAGAAAAAA
GTCGATGAATTGGAAAAGGTTTCAGAAAAGCAGGCAAATGAAATAAATGAACTAAAATCG
ATATTAACCGACTGCTTAGTTCGAATCACCAATCTCGAAGGTGGTTCAAAAACAACTAAG
GAGAAAAGTTCCCCAACTCCAAATTCATTGAAAATTACGTCACCAAAAGCAAAAGATGTT
CAATTACGAAAATCAATGGTGAGTCCCAGAACTCCAGTAAAAGCTACAAGATTGTCAGGA
ACACCTACTCGTAGAAGCACAAGTGATTTGGCTAAAAGATCGAAAACACTGCCCATATTC
AAAAGACCCTGGAAACCACCAGGAGGTTTGATTATATGTCCAATTATTATTAAAAGGGGA

AATTTGAACAGATCGAAAAGTCTTGATCAACAAAATCACAAAGAAGCCACTTTCATCAAA
GGTGAAGGAGTTTTACGAATATTTATTAAAGGAAGACCAATTAATTTATACGTTCCGAAT
TCATTGACCGACGGATTTGATATTAAGAGTCCAATACAAGAACCTCAGGAAAATTTAAGA
ATGGAATGGGTATATGGATATCGAGGTAAAGATTGCAGGAAAAATTTATTCTATTTACCA
ACGGGGGATCTTGTATATTTTGTTGCTGGTATTGTAGTATTGCACAATTTAAATACGCAA
ATTCAGAGACACTACGTGGAACACACGGATGATATCAAATGTATGGCATTGCATCCTGAT
AAAGTTACTTTTGCTACTGGACAAGTAGCCGGTCATTCTAAAGTTGAAGGAAAACCCCAT
GTTCGTATTTGGAGTTCGATTTCACTACAAACATTGCACACTATTGGCTTAGGATTTTTT
GAAAGAGCTGTCAGTGCTTTGGGCTTCTCTAAATTAGATGGAGGGAAATTATTATGTACT
GTTGATGAAGCTAATGATCACAATATAACGGTTTGGAATTGGGCAACTACTAAAAGTATA
TGTACATCCAAGAGTTCAACTGAAGCCGTTATAGAAGCTGAATTCAATCCCACTGATAAC
AACGTCATTGTAACTTGCGGTAAAAATCATCTAGCATTTTGGATTTTAGAGAAAAGCACT
CTGGTAAAGAAAATGGCTATTTTTGAAAAATATGATAAACCGAAGTTCACAACCTGTGTA
ACATTTACTAATACTGGTGAACTCATTACAGGAGATTCTTCGGGAAATATTTACTTTTGG
AGTCGTGAAAAACGATCAATTACTTCAGCAATTTTGCAAGCGCACAACGGTCCAATATTT
TCGTTATTTATTACCGAAGCTGGATCACTTCTTACAGGTGGTGGAAAGGATCATGCTATT
TTTGAATGGGACTTATCAAAAAAAGAAAAATCTGGTAGAAATATACAATTGACCAATGAA
CATGGATTTTGCAGAACAATTTCGGAGGTGAATGATAAATTGGTGGTTGGAACAACAAAT
AACGCTATTATAGAGGTAACTGGAAATGATGAATTCATTAATTTACTTAACGGCCATTCT
GATGAGGTGTGGGCTATTGACAGCCATCCTTCAAAGGAGCAATTTATAACGGGTGGTCAT
GATCGTAAACTAATTTTGTGGGATGCTTTGACTCATACTGTTCTATTGTCGAAACCGTTT
GAGAAAAATATCCAATCCGTTTCGTTAAACCCAACAGCTGATTTGGTAGCAGTAGGATGT
ATTGAAGGTCACTGGTATGTGTGCGATATAAATGAATTCAAAGAGATTCATCAAGGTAAA
GATGGCAACGAACCAATTCAAAGTATTGAATTTAGCCCAGATGGGTCTCATCTTGCAGTA
GGATCAAGAGACAATTTCATCTACATTTATAAAGTAAGTCATGAAACTAACGAAGTTTCC
AAAGTAGGAAAATGTTCCGGTCACTCTAGTTTTATTACACACTTGGATTGGTCTACTGAT
GGATTGTATTTGAGAAGCAATTCAGGTGATTATGAACTTCTTTTTTGGAACATACCAGAG
TGCAAACAATTAACAGCTGCAAGTACACTGTCAGATGTTAAATGGCATTCTGCAAATTGC
ACATTAACTTTTGAAGTAGCTGGAATTTGGCCAGAAGGTTCTGATGGAACTGATATCAAT
GCTGTAACTAAAGCACACAGTAAATTAATCCTCGCTACAGCAGATGACTTCGGAAAAGTT
AATCTTTACCGTTATCCAGCATGTAATCCAAAAACAGCCCATAAGATATACAAAGGTCAT
AGCAGTCATGTAACTGGCGTGAAGTTTCTTGTTGAAGATCATCGACTTATGAGCGCTGGA
GGTCAAGACACAGCAATAGTTCAATGGGCTCTTACTTGAaagataattataggagaaatg
aaaattggaatattgacatctgaattattagtttttattgttattaattctatttagttt
atatgatttaaaatttgcaagatttaatttttgtatcagtgtgcgtgtgtgtgtgtgtgt
gaaatcgatgttgcaattatttatggaattttaatttatctggctctcattagtgtatct
ttaacctttcaagggttatagtgcttaatttgtttagactatttatttcgaattttatct
aaatcaaatataaaaataatattgttatgaataaaatattatttttcgcaagataaactt
tcgtttaactATGAAAAAATTTTTCAAATTATCAGCTTTAACTAAACCAGCTTCTTTTAT
TGCTTCGAAACTTGGCAAAAAGAAGCATTCGGTTAAAGATGATTTTGAAGAAGTTTCGGA
ATTACCTGACTTAGACGTTAAGGGTGATAGTATTTTTGAGGAAAATGTTATACAACAAGC
AAGTGCTCCTGTTGATGAATCTTTAGTTGACCCTCTGAAAAAGATGGACATATTAGAAAA
TCGTAATCCAGGTCTTACATTATTAATAAATCAGTCTGACGAAACATTTTCTTCTCCTTC
ATTTGATCCTTACTTTGAAAGTTCACAAATTAAAATTCCAGAACCTGTATCATTAAACGT
TTCTTCTGTTAGCACTGTAAAATATTACGAAAAACCTGAAAGAAATTTAACATTACTGGG
CAAAAAAGCAAAAAAAAATCGAAGAGGATGATATTATGCAGAAAAAAGTCGATGAATTGGA
AAAGGTTTCAGAAAAGCAGGCAAATGAAATAAATGAACTAAAATCGATATTAACCGACTG
CTTAGTTCGAATCACCAATCTCGAAGGTGGTTCAAAAACAACTAAGGAGAAAAGTTCCCC
AACTCCAAATTCATTGAAAATTACGTCACCAAAAGCAAAAGATGTTCAATTACGAAAATC
AATGGTGAGTCCCAGAACTCCAGTAAAAGCTACAAGATTGTCAGGAACACCTACTCGTAG
AAGCACAAGTGATTTGGCTAATCACAAAGAAGCCACTTTCATCAAAGGTGAAGGAGTTTT
ACGAATATTTATTAAAGGAAGACCAATTAATTTATACGTTCCGAATTCATTGACCGACGG
ATTTGATATTAAGAGTCCAATACAAGAACCTCAGGAAAATTTAAGAATGGAATGGGTATA
TGGATATCGAGGTAAAGATTGCAGGAAAAATTTATTCTATTTACCAACGGGGGATCTTGT
ATATTTTGTTGCTGGTATTGTAGTATTGCACAATTTAAATACGCAAATTCAGAGACACTA
CGTGGAACACACGGATGATATCAAATGTATGGCATTGCATCCTGATAAAGTTACTTTTGC
TACTGGACAAGTAGCCGGTCATTCTAAAGTTGAAGGAAAACCCCATGTTCGTATTTGGAG

TTCGATTTCACTACAAACATTGCACACTATTGGCTTAGGATTTTTTGAAAGAGCTGTCAG
TGCTTTGGGCTTCTCTAAATTAGATGGAGGGAAATTATTATGTACTGTTGATGAAGCTAA
TGATCACAATATAACGGTTTGGAATTGGGCAACTACTAAAAGTATATGTACATCCAAGAG
TTCAACTGAAGCCGTTATAGAAGCTGAATTCAATCCCACTGATAACAACGTCATTGTAAC
TTGCGGTAAAAATCATCTAGCATTTTGGATTTTAGAGAAAAGCACTCTGGTAAAGAAAAT
GGCTATTTTTGAAAAATATGATAAACCGAAGTTCACAACCTGTGTAACATTTACTAATAC
TGGTGAACTCATTACAGGAGATTCTTCGGGAAATATTTACTTTTGGAGTCGTGAAAAACG
ATCAATTACTTCAGCAATTTTGCAAGCGCACAACGGTCCAATATTTTCGTTATTTATTAC
CGAAGCTGGATCACTTCTTACAGGTGGTGGAAAGGATCATGCTATTTTTGAATGGGACTT
ATCAAAAAAAGAAAAATCTGGTAGAAATATACAATTGACCAATGAACATGGATTTTGCAG
AACAATTTCGGAGGTGAATGATAAATTGGTGGTTGGAACAACAAATAACGCTATTATAGA
GGTAACTGGAAATGATGAATTCATTAATTTACTTAACGGCCATTCTGATGAGGTGTGGGC
TATTGACAGCCATCCTTCAAAGGAGCAATTTATAACGGGTGGTCATGATCGTAAACTAAT
TTTGTGGGATGCTTTGACTCATACTGTTCTATTGTCGAAACCGTTTGAGAAAAATATCCA
ATCCGTTTCGTTAAACCCAACAGCTGATTTGGTAGCAGTAGGATGTATTGAAGGTCACTG
GTATGTGTGCGATATAAATGAATTCAAAGAGATTCATCAAGGTAAAGATGGCAACGAACC
AATTCAAAGTATTGAATTTAGCCCAGATGGGTCTCATCTTGCAGTAGGATCAAGAGACAA
TTTCATCTACATTTATAAAGTAAGTCATGAAACTAACGAAGTTTCCAAAGTAGGAAAATG
TTCCGGTCACTCTAGTTTTATTACACACTTGGATTGGTCTACTGATGGATTGTATTTGAG
AAGCAATTCAGGTGATTATGAACTTCTTTTTTGGAACATACCAGAGTGCAAACAATTAAC
AGCTGCAAGTACACTGTCAGATGTTAAATGGCATTCTGCAAATTGCACATTAACTTTTGA
AGTAGCTGGAATTTGGCCAGAAGGTTCTGATGGAACTGATATCAATGCTGTAACTAAAGC
ACACAGTAAATTAATCCTCGCTACAGCAGATGACTTCGGAAAAGTTAATCTTTACCGTTA
TCCAGCATGTAATCCAAAAACAGCCCATAAGATATACAAAGGTCATAGCAGTCATGTAAC
TGGCGTGAAGTTTCTTGTTGAAGATCATCGACTTATGAGCGCTGGAGGTCAAGACACAGC
AATAGTTCAATGGGCTCTTACTTGAaagataattataggagaaatgaaaattggaatatt
gacatctgaattattagtttttattgttattaattctatttagtttatatgatttaaaat
ttgcaagatttaatttttgtatcagtgtgcgtgtgtgtgtgtgtgaaatcgatgttgc
aattatttatggaattttaatttatctggctctcattagtgtatctttaacctttcaagg
gttatagtgcttaatttgtttagactatttatttcgaattttatctaaatcaaatataaa
aataatattgttatgaataaaatattatttttcgcagcgaaataaagatttacatATGGA
AATCGAGAACGATATTATGCAGAAAAAAGTCGATGAATTGGAAAAGGTTTCAGAAAAGCA
GGCAAATGAAATAAATGAACTAAAATCGATATTAACCGACTGCTTAGTTCGAATCACCAA
TCTCGAAGGTGGTTCAAAAACAACTAAGGAGAAAAGTTCCCCAACTCCAAATTCATTGAA
AATTACGTCACCAAAAGCAAAAGATGTTCAATTACGAAAATCAATGGTGAGTCCCAGAAC
TCCAGTAAAAGCTACAAGATTGTCAGGAACACCTACTCGTAGAAGCACAAGTGATTTGGC
TAATCACAAAGAAGCCACTTTCATCAAAGGTGAAGGAGTTTTACGAATATTTATTAAAGG
AAGACCAATTAATTTATACGTTCCGAATTCATTGACCGACGGATTTGATATTAAGAGTCC
AATACAAGAACCTCAGGAAAATTTAAGAATGGAATGGGTATATGGATATCGAGGTAAAGA
TTGCAGGAAAAATTTATTCTATTTACCAACGGGGGATCTTGTATATTTTGTTGCTGGTAT
TGTAGTATTGCACAATTTAAATACGCAAATTCAGAGACACTACGTGGAACACACGGATGA
TATCAAATGTATGGCATTGCATCCTGATAAAGTTACTTTTGCTACTGGACAAGTAGCCGG
TCATTCTAAAGTTGAAGGAAAACCCCATGTTCGTATTTGGAGTTCGATTTCACTACAAAC
ATTGCACACTATTGGCTTAGGATTTTTTGAAAGAGCTGTCAGTGCTTTGGGCTTCTCTAA
ATTAGATGGAGGGAAATTATTATGTACTGTTGATGAAGCTAATGATCACAATATAACGGT
TTGGAATTGGGCAACTACTAAAAGTATATGTACATCCAAGAGTTCAACTGAAGCCGTTAT
AGAAGCTGAATTCAATCCCACTGATAACAACGTCATTGTAACTTGCGGTAAAAATCATCT
AGCATTTTGGATTTTAGAGAAAAGCACTCTGGTAAAGAAAATGGCTATTTTTGAAGTATT
T
>DjScaffold4324:91992-92489 strand:+
caacatagtaatgcaaatATGTTATCTAGACCAAGATGTGAGGTATGTGTACAATGTCAA
ATGTCACATGAAAATGCCAAGAAAGGGAAGATTAAAACGAGAATACTGAATATAATTGAA
AAGAAAAAAGAAGATCATTTAAACATTTCAGAGGAAACAGATTTGATCAAGAAAATGAAA
ATTCCGGATGACAAATTACATGAAACAATATAAgaaatacataaattattatgtcatgca
ggagttgaaaatgtgtacgaatatatgagaaaaaaataaaattgaaaagaatttgtgcag
ctaaatacagtacataggtcgaagttgtgaaatgtgtcagaagaacaaagtagtgacaac
>DjScaffold4324:34619-34957 strand:+

TTAATCGAATGCCCTTCGGAATATGTACTGCACCAGCAACATTTTAAAGCTCGATGCATA
AATTTATTTGAAGGAATATTGTACAAAGGAGTATTAGTATGTCTGGATGATATTTTAATA
TATGATAAAACTAAAGAGGAGCACGACAAACAAGTTGGTAAAGTTTTCCAGCTACTAAAA
GATACGAGTTTAAAAATAAATCCAAGTAAATGTGTGTTCAATTTCAAGAAACTTAAATTT
TTAGGACATGCAATTGGCGTTAAAGGTGTAAAGACAAATGATGAAACTATAAAAAAATTT
CGAGAACCCGTAATGCCAAAACGCGCCACGCAAATATGA
>DjScaffold4324:29419-29751 strand:+
GTGCTCGTTTATATTGATGAAATTTTAATTTTTGGTCAGACACAAGAAATTTATGATAAA
CATTTACAAAATGTTTTGGAATTAATTGAACAATCTGGTCTAAAAATGAATCCTGAAAAA
TTTAGTGTATCAAAACTCAAGTTTCTTGGTCATGCAATCAGTTCTAAAGATGTTGAAACA
AATTATGACATTATTAATAAAGTAAAAGAAGCTGTTATTCCAAAATGTGCAACCCAAATT
CGAACTGTCTTGGGATTGACACATTATTATCGTAGATTTATTAAAGATTATGCAACAATT
GCCAGTTCATTATATCGACCAATAACTGGATGA
>DjScaffold5349:9175-9693 strand:+
attgtacagtaattttaagttaaggaaaatATGACAATTCCTCCAGGTGATATTGAAAAG
GGTAAAAAAATATTTGTTCAAAAATGTGCTCAGTGTCATACACTTGAGAAGGGTGGAAAA
CATAAAACTGGACCAAATTTATATGGTCTTTTTGGACGAAAAACAGCTCAGGCACCGGGA
TTCGATTACACTGCAGCAAATAAGAAAAAAGGAATTATATGGGGAGTAGATACTCTTTTT
GAATACTTAGAGAATCCCAAAAAATATATACCAGGAACGAAAATGGTTTTTGCGGGAATA
AAAAAAGAAAACGAGCGAGCAGATTTAATTGCGTATTTAGAAAAATCAACGAAAGAATAA
atttaattagtgaccttcccatagtgtacttatttgtgaatgctggttttactagtgaat
tgtagagttatttcatacatttttagtaggaataaataattcagggcacttg
>DjScaffold1449:87475-88443 strand:+
agtgggcgtcgattttgtagtttgttacatattgcatatgaatatttgttaattacaaAT
GTCTTTTTTAATTATTATAAATATTATTACACTAAATATATATATATATATATTTAAATT
TAAAAATTTAGATCATCGATCTCAAAGAGCTTTGTACATTGGAGTACACATGCCTCCTGA
TCGAATACATCAACCAACTCGACAACGCCGTCGTCGTCATAAAAAATCCAAACAAGCTCA
ACATGATAAAAAAGATTATCTAACAGAAATTGCGGAAGATGATACAGAGAAGGCCACTGA
TCCAAGTCAACATGTTATGTTTCTTCTCGGTGAAGATGAGGAAGAAGAAACTCATAATTT
ATTTTGTGAAATGGACGAATTGAGATTAGATGACGAAGGAGAACCTGAGTGGAAGGAAAC
AGCTCGATGGATAAAATTTGAAGAAGATGTCGAAGAAGGAGGTGAAAGATGGTCCAAACC
TCATGTTGCTAGTTTATCTTTGTATTCGTTATTCGAAGCTCGAAATTGTATTAGTTCGGC
TGTTAACATGTTCAATGTTGAAGCTAATAATTTTGAAACTATAACAGAAATCACATTAAA
TAAAATCATCTCGGAAAAGAAAATCTCTCCAGATCAACGGGAAAAAGTAAAAGAAATTTT
ACAACGAAAGCATGTTCATTTACACGAAAGAAAGCACAAACTTAATACTAAAGGTAGTCA
ATTACCACTTATCAGATCGTTAGCAGATATTGGTAAAAAGTATTCTTCAAAAGAAGTCGA
TAAAGTCCAATTACAACGTCGCAATTCAGAATATCCGACAAAATGTATTAGATTTGATGC
AAACTGTAGCCAACCGAACATTTTGAAATCAAGTAGCTCAATCTATGTCGATAAGAATAA
AATCGATGTTGATCAATGTGGCTCAGACAATGCCTCATCACAAAAATTTGATTTACATTT
CTTTAAAAAAATACCTCCTGGTGCTGAAACATCAAATATTATGGTTGCAGAAACTGATTT
CCTTACTCAAATGGTCATTGTATTTATTCGACTAAAAGAAGCTGTGATTTTAGGAGAACT
GACTGAGGTTCCTGTACCTACAAGATTCATTTTTCTATTATTAGGTCCACAAGGCAGTGC
TATCAAAAACCATGAAATTGGTAGATCTATGGCAACGTTAATGTCGGACGATGTTTTTCA
TGACGTGGCCTACAAAGCTCGTAATCTCGATGATCTGTTAGCTGGAATCGATGAATTTTT
ATCAAACTGTACTGTTCTTCCACCAGCAGAATGGGATCCTTCAATACGAATTGAACCACC
AAAAAGTGGTCCTATAGCAAGAAAAGTTCCAAGCATGCCGAATGGGGCACAAGCATTACT
TACTGCAAGTGGGGATGGAAGGAGTGGAAATCCTAATGGTACTCCTAGCAGTGGTACAGG
ACCAGTTACCACGGCCAATAAATCGCAGCCCGTTGAGGAAGAAAGTCATGCTGATCCAAC
TCTTAGAAGAACTGGAAAACTTTTCGGTGGTCTAATTGAAGATGTTAAGCGAAAAGCTCC
ATTTTATCTTAGTGATTTTAAAGATGCTCTTCATGTTCAATGCTTTGCTTCTTTTTTCTT
TCTTTACTTTGCCTGTTTAACTCCAATTATCACATTTGGTGGTTTGCTAGTCCAAGCTAC
TGGAGGCTATTTGGGTGCAATAGAATCGATTTTGTCCGGCACAATAGTAGGAGTTGTTTT
TGCCTTGTTTTCTGGACAGCCTTTGACAATTATGGGTAGTACTGGTCCTGTTCTAGTATT
TGAATCAATCATTTATAATATGTGCGAAAAATTGCGATGGGATTATTTATCTTTTCGACT
TTGGATTGGAATATGGATAGCTGTTTTGCTATTTGTTATGGTGGCCTTCGATTTGAGTGC
ACTTGTTCGCTATATTACTCGTTTTACCGAAGAGAGTTTCGCCGCGCTTATTGCATTGAT
TTTTATAGTTGAAGCTTTCAAAAAATTGTTTTTGATTGTTGATTATTATCCCATAGACAC

TAATTGGAATATTGATATATTCAAAGATCGGAATTGTACTTGTCGACCGAGAAATCATAC
TGAAATGGTTAAATTCTTTCGAATAATAATCGTCCGAGTAATATTTACAAACCACCTAT
GCTAATAAATGGATCGTTTAATTCAACATTGAAGATTGCGGAAAATATTGATTGGGAAAA
TATATCTAAATATCACTTCTCTTGGGATTCAAAGAAATACAAACAAATTTGTACGAAATG
GGGAGGTATATGGACAGGATTAGGATGCGGAAATTACTATATTCCCGATGTGTTTTTCTT
CTCGTGCCTGTTATTTATTGGTACCTTCATTCTGTCATACGGACTGAAATTGATGAGAAA
TGCTCAATTTTTCCCTAATTGGGTTCGTAATTTAATATCAGATTTTGCTGTTATGATTGC
TATTTCTTCAATGACTCTACTTGATTGGCTACTGAATTTTCATACTCCAAAATTGAATGT
ACCTGCCAAATTTGAACCCACATTGGGCTATGAGATGCGTGGATGGTTAATACCTCCACT
TGGAAAAAATCCTTGGTGGACAATTATTGTATCTATAGGTCCTGCGTTACTTGCAGTTAT
TTTAATTTTTATGGATCAGCAAATCACGGCAGTTATTGTAAATAGAAAAGAAAATAAGTT
AAAGAAAGGCTGTGGTTATCATCTTGATTTGTTGATTCTATCTATTCTTATTCTTGTTAA
TTCAATCCTTGGTATACCGTGGTTTGTAGCTGCCACTGTTTTATCAATTAATCATGTTAT
GAGTTTGAAAAAAGAATCAGAAAATACAGCTCCCGGTGAACGCCCAGTATTTCTTGGATG
CAGGGAACAAAGAGTTACAGGAACTATAATTTTCCTTGTGATTGGATTGTCAGTATTTTT
AACTCCTTTATTGAAACAAATACCAATGGCAGTTCTATATGGTGTATTTTTGTTTATGGG
TGTGGCCTCTTTGAAAGGAGTACAATTAATGCAACGAGTATCACTATTTTTTATGCCTCC
AAAATATCAACCGGATTACATATTTTTAAGATATGTTCCTTTAAGAAGAGTCCATTTATT
TACATTTATACAAATAATTCTCCTAGTATTGTTGTGGGTTATTAAAACAATAAGCTTTAT
GTCGATCTGTTTTCCTATTATGGTGTTGGCCTTATGTTTCGTTCGCAAAGGTCTCGAGTA
CGTGTTTACATATAACGAATTGAAATGGCTTGATGACATTATGCCAAATCACAGAAAACC
ACCGCAGGATGACAATGACAATGATGTTGAAAAATGCGGATCATCAGAAGTAATAGATTA
Gtttttcttataatggttatttagaggtgtgtaaaagataaaataatttttaaaatgttt
caatttattaagttaaataagaaaattcgtttttgactttcaatttttaaattgaaataa
ctgaaaggcagcaactaagcctaattaattaaactaacaatcaccacaatcaacacgtgc
ttgccaacgataaacaggatttctcattctagtcgtatagacatcctgttctctggattc
aataaagttatgagtgggcgtcgattttgtagtttgttacatattgcatatgaatatttg
ttaattacaaATGTCTTTTTTAATTATTATAAATATTATTACACTAAATATATATATATA
TATATTTAAATTTAAAAATTTAGATCATCGATCTCAAAGAGCTTTGTACATTGGAGTACA
CATGCCTCCTGATCGAATACATCAACCAACTCGACAACGCCGTCGTCGTCATAAAAAATC
CAAACAAGCTCAACATGATAAAAAAGATTATCTAACAGAAATTGCGGAAGATGATACAGA
GAAGGCCACTGATCCAAGTCAACATGTTATGTTTCTTCTCGGTGAAGATGAGGAAGAAGA
AACTCATAATTTATTTTGTGAAATGGACGAATTGAGATTAGATGACGAAGGAGAACCTGA
GTGGAAGGAAACAGCTCGATGGATAAAATTTGAAGAAGATGTCGAAGAAGGAGGTGAAAG
ATGGTCCAAACCTCATGTTGCTAGTTTATCTTTGTATTCGTTATTCGAAGCTCGAAATTG
TATTAGTTCGGCTGTTAACATGTTCAATGTTGAAGCTAATAATTTTGAAACTATAACAGA
AATCACATTAAATAAAATCATCTCGGAAAAGAAAATCTCTCCAGATCAACGGGAAAAAGT
AAAAGAAATTTTACAACGAAAGCATGTTCATTTACACGAAAGAAAGCACAAACTTAATAC
TAAAGGTAGTCAATTACCACTTATCAGATCGTTAGCAGATATTGGTAAAAAGTATTCTTC
AAAAGAAGTCGATAAAGAATATCCGACAAAATGTATTAGATTTGATGCAAACTGTAGCCA
ACCGAACATTTTGAAATCAAGTAGCTCAATCTATGTCGATAAGAATAAAATCGATGTTGA
TCAATGTGGCTCAGACAATGCCTCATCACAAAAATTTGATTTACATTTCTTTAAAAAAAT
ACCTCCTGGTGCTGAAACATCAAATATTATGGTTGCAGAAACTGATTTCCTTACTCAAAT
GGTCATTGTATTTATTCGACTAAAAGAAGCTGTGATTTTAGGAGAACTGACTGAGGTTCC
TGTACCTACAAGATTCATTTTTCTATTATTAGGTCCACAAGGCAGTGCTATCAAAAACCA
TGAAATTGGTAGATCTATGGCAACGTTAATGTCGGACGATGTTTTTCATGACGTGGCCTA
CAAAGCTCGTAATCTCGATGATCTGTTAGCTGGAATCGATGAATTTTTATCAAACTGTAC
TGTTCTTCCACCAGCAGAATGGGATCCTTCAATACGAATTGAACCACCAAAAAGTGGTCC
TATAGCAAGAAAAGTTCCAAGCATGCCGAATGGGGCACAAGCATTACTTACTGCAAGTGG
GGATGGAAGGAGTGGAAATCCTAATGGTACTCCTAGCAGTGGTACAGGACCAGTTACCAC
GGCCAATAAATCGCAGCCCGTTGAGGAAGAAAGTCATGCTGATCCAACTCTTAGAAGAAC
TGGAAAACTTTTCGGTGGTCTAATTGAAGATGTTAAGCGAAAAGCTCCATTTTATCTTAG
TGATTTTAAAGATGCTCTTCATGTTCAATGCTTTGCTTCTTTTTTCTTTCTTTACTTTGC
CTGTTTAACTCCAATTATCACATTTGGTGGTTTGCTAGTCCAAGCTACTGGAGGCTATTT
GGGTGCAATAGAATCGATTTTGTCCGGCACAATAGTAGGAGTTGTTTTTGCCTTGTTTTC
TGGACAGCCTTTGACAATTATGGGTAGTACTGGTCCTGTTCTAGTATTTGAATCAATCAT
TTATAATATGTGCGAAAAATTGCGATGGGATTATTTATCTTTTCGACTTTGGATTGGAAT

ATGGATAGCTGTTTTGCTATTTGTTATGGTGGCCTTCGATTTGAGTGCACTTGTTCGCTA
TATTACTCGTTTTACCGAAGAGAGTTTCGCCGCGCTTATTGCATTGATTTTTATAGTTGA
AGCTTTCAAAAAATTGTTTTTGATTGTTGATTATTATCCCATAGACACTAATTGGAATAT
TGATATATTCAAAGATCGGAATTGTACTTGTCGACCGAGAAATCATACTGAAATGGTTAA
ATTCTTTTCGAATAATAATCGTCCGAGTAATATTTACAAACCACCTATGCTAATAAATGG
ATCGTTTAATTCAACATTGAAGATTGCGGAAAATATTGATTGGGAAAATATATCTAAATA
TCACTTCTCTTGGGATTCAAAGAAATACAAACAAATTTGTACGAAATGGGGAGGTATATG
GACAGGATTAGGATGCGGAAATTACTATATTCCCGATGTGTTTTTCTTCTCGTGCCTGTT
ATTTATTGGTACCTTCATTCTGTCATACGGACTGAAATTGATGAGAAATGCTCAATTTTT
CCCTAATTGGGTTCGTAATTTAATATCAGATTTTGCTGTTATGATTGCTATTTCTTCAAT
GACTCTACTTGATTGGCTACTGAATTTTCATACTCCAAAATTGAATGTACCTGCCAAATT
TGAACCCACATTGGGCTATGAGATGCGTGGATGGTTAATACCTCCACTTGGAAAAAATCC
TTGGTGGACAATTATTGTATCTATAGGTCCTGCGTTACTTGCAGTTATTTTAATTTTTAT
GGATCAGCAAATCACGGCAGTTATTGTAAATAGAAAAGAAAATAAGTTAAAGAAAGGCTG
TGGTTATCATCTTGATTTGTTGATTCTATCTATTCTTATTCTTGTTAATTCAATCCTTGG
TATACCGTGGTTTGTAGCTGCCACTGTTTTATCAATTAATCATGTTATGAGTTTGAAAAA
AGAATCAGAAAATACAGCTCCCGGTGAACGCCCAGTATTTCTTGGATGCAGGGAACAAAG
AGTTACAGGAACTATAATTTTCCTTGTGATTGGATTGTCAGTATTTTTAACTCCTTTATT
GAAACAAATACCAATGGCAGTTCTATATGGTGTATTTTTGTTTATGGGTGTGGCCTCTTT
GAAAGGAGTACAATTAATGCAACGAGTATCACTATTTTTTATGCCTCCAAAATATCAACC
GGATTACATATTTTTAAGATATGTTCCTTTAAGAAGAGTCCATTTATTTACATTTATACA
AATAATTCTCCTAGTATTGTTGTGGGTTATTAAAACAATAAGCTTTATGTCGATCTGTTT
TCCTATTATGGTGTTGGCCTTATGTTTCGTTCGCAAAGGTCTCGAGTACGTGTTTACATA
TAACGAATTGAAATGGCTTGATGACATTATGCCAAATCACAGAAAACCACCGCAGGATGA
CAATGACAATGATGTTGAAAAATGCGGATCATCAGAAGTAATAGATTAGtttttcttata
atggttatttagaggtgtgtaaaagataaaataatttttaaaatgtttcaatttattaag
ttaaataagaaaattcgtttttgactttcaatttttaaattgaaataactgaaaggcagc
aactaagcctaattaattaaactaacaatcaccacaatcaacacgtgcttgccaacgata
aacaggatttctcattctagtcgtatagacatcctgttctctggattcaataaagttatg
agtgggcgtcgattttgtagtttgttacatattgcatatgaatatttgttaattacaaAT
GTCTTTTTTAATTATTATAAATATTATTACACTAAATATATATATATATATATTTAAATT
TAAAAATTTAGATCATCGATCTCAAAGAGCTTTGTACATTGGAGTACACATGCCTCCTGA
TCGAATACATCAACCAACTCGACAACGCCGTCGTCGTCATAAAAAATCCAAACAAGCTCA
ACATGATAAAAAAGATTATCTAACAGAAATTGCGGAAGATGATACAGAGAAGGCCACTGA
TCCAAGTCAACATGTTATGTTTCTTCTCGGTGAAGATGAGGAAGAAGAAACTCATAATTT
ATTTTGTGAAATGGACGAATTGAGATTAGATGACGAAGGAGAACCTGAGTGGAAGGAAAC
AGCTCGATGGATAAAATTTGAAGAAGATGTCGAAGAAGGAGGTGAAAGATGGTCCAAACC
TCATGTTGCTAGTTTATCTTTGTATTCGTTATTCGAAGCTCGAAATTGTATTAGTTCGGC
TGTTAACATGTTCAATGTTGAAGCTAATAATTTTGAAACTATAACAGAAATCACATTAAA
TAAAATCATCTCGGAAAAGAAAATCTCTCCAGATCAACGGGAAAAAGTAAAAGAAATTTT
ACAACGAAAGCATGTTCATTTACACGAAAGAAAGCACAAACTTAATACTAAAGGTAGTCA
ATTACCACTTATCAGATCGTTAGCAGATATTGGTAAAAAGTATTCTTCAAAAGAAGTCGA
TAAAGATGCAAACTGTAGCCAACCGAACATTTTGAAATCAAGTAGCTCAATCTATGTCGA
TAAGAATAAAATCGATGTTGATCAATGTGGCTCAGACAATGCCTCATCACAAAAATTTGA
TTTACATTTCTTTAAAAAAATACCTCCTGGTGCTGAAACATCAAATATTATGGTTGCAGA
AACTGATTTCCTTACTCAAATGGTCATTGTATTTATTCGACTAAAAGAAGCTGTGATTTT
AGGAGAACTGACTGAGGTTCCTGTACCTACAAGATTCATTTTTCTATTATTAGGTCCACA
AGGCAGTGCTATCAAAAACCATGAAATTGGTAGATCTATGGCAACGTTAATGTCGGACGA
TGTTTTTCATGACGTGGCCTACAAAGCTCGTAATCTCGATGATCTGTTAGCTGGAATCGA
TGAATTTTTATCAAACTGTACTGTTCTTCCACCAGCAGAATGGGATCCTTCAATACGAAT
TGAACCACCAAAAAGTGGTCCTATAGCAAGAAAAGTTCCAAGCATGCCGAATGGGGCACA
AGCATTACTTACTGCAAGTGGGGATGGAAGGAGTGGAAATCCTAATGGTACTCCTAGCAG
TGGTACAGGACCAGTTACCACGGCCAATAAATCGCAGCCCGTTGAGGAAGAAAGTCATGC
TGATCCAACTCTTAGAAGAACTGGAAAACTTTTCGGTGGTCTAATTGAAGATGTTAAGCG
AAAAGCTCCATTTTATCTTAGTGATTTTAAAGATGCTCTTCATGTTCAATGCTTTGCTTC
TTTTTTCTTTCTTTACTTTGCCTGTTTAACTCCAATTATCACATTTGGTGGTTTGCTAGT
CCAAGCTACTGGAGGCTATTTGGGTGCAATAGAATCGATTTTGTCCGGCACAATAGTAGG

AGTTGTTTTTGCCTTGTTTTCTGGACAGCCTTTGACAATTATGGGTAGTACTGGTCCTGT
TCTAGTATTTGAATCAATCATTTATAATATGTGCGAAAAATTGCGATGGGATTATTTATC
TTTTCGACTTTGGATTGGAATATGGATAGCTGTTTTGCTATTTGTTATGGTGGCCTTCGA
TTTGAGTGCACTTGTTCGCTATATTACTCGTTTTACCGAAGAGAGTTTCGCCGCGCTTAT
TGCATTGATTTTTATAGTTGAAGCTTTCAAAAAATTGTTTTTGATTGTTGATTATTATCC
CATAGACACTAATTGGAATATTGATATATTCAAAGATCGGAATTGTACTTGTCGACCGAG
AAATCATACTGAAATGGTTAAATTCTTTTCGAATAATAATCGTCCGAGTAATATTTACAA
ACCACCTATGCTAATAAATGGATCGTTTAATTCAACATTGAAGATTGCGGAAAATATTGA
TTGGGAAAATATATCTAAATATCACTTCTCTTGGGATTCAAAGAAATACAAACAAATTTG
TACGAAATGGGGAGGTATATGGACAGGATTAGGATGCGGAAATTACTATATTCCCGATGT
GTTTTTCTTCTCGTGCCTGTTATTTATTGGTACCTTCATTCTGTCATACGGACTGAAATT
GATGAGAAATGCTCAATTTTTCCCTAATTGGGTTCGTAATTTAATATCAGATTTTGCTGT
TATGATTGCTATTTCTTCAATGACTCTACTTGATTGGCTACTGAATTTTCATACTCCAAA
ATTGAATGTACCTGCCAAATTTGAACCCACATTGGGCTATGAGATGCGTGGATGGTTAAT
ACCTCCACTTGGAAAAAATCCTTGGTGGACAATTATTGTATCTATAGGTCCTGCGTTACT
TGCAGTTATTTTAATTTTTATGGATCAGCAAATCACGGCAGTTATTGTAAATAGAAAAGA
AAATAAGTTAAAGAAAGGCTGTGGTTATCATCTTGATTTGTTGATTCTATCTATTCTTAT
TCTTGTTAATTCAATCCTTGGTATACCGTGGTTTGTAGCTGCCACTGTTTTATCAATTAA
TCATGTTATGAGTTTGAAAAAAGAATCAGAAAATACAGCTCCCGGTGAACGCCCAGTATT
TCTTGGATGCAGGGAACAAAGAGTTACAGGAACTATAATTTTCCTTGTGATTGGATTGTC
AGTATTTTTAACTCCTTTATTGAAACAAATACCAATGGCAGTTCTATATGGTGTATTTTT
GTTTATGGGTGTGGCCTCTTTGAAAGGAGTACAATTAATGCAACGAGTATCACTATTTTT
TATGCCTCCAAAATATCAACCGGATTACATATTTTTAAGATATGTTCCTTTAAGAAGAGT
CCATTTATTTACATTTATACAAATAATTCTCCTAGTATTGTTGTGGGTTATTAAAACAAT
AAGCTTTATGTCGATCTGTTTTCCTATTATGGTGTTGGCCTTATGTTTCGTTCGCAAAGG
TCTCGAGTACGTGTTTACATATAACGAATTGAAATGGCTTGATGACATTATGCCAAATCA
CAGAAAACCACCGCAGGATGACAATGACAATGATGTTGAAAAATGCGGATCATCAGAAGT
AATAGATTAGtttttcttataatggttatttagaggtgtgtaaaagataaaataattttt
aaaatgtttcaatttattaagttaaataagaaaattcgtttttgactttcaatttttaaa
ttgaaataactgaaaggcagcaactaagcctaattaattaaactaacaatcaccacaatc
aacacgtgcttgccaacgataaacaggatttctcattctagtcgtatagacatcctgttc
tctggattcaataaagttatgatttagggaacaaagagttacaggaactataattttcct
tgtgattggattgtcagtatttttaactcctttattgaaacaaataccaATGGCAGTTCT
ATATGGTGTATTTTTGTTTATGGGTGTGGCCTCTTTGAAAGGAGTACAATTAATGCAACG
AGTATCACTATTTTTTATGCCTCCAAAATATCAACCGGATTACATATTTTTAAGATATGT
TCCTTTAAGAAGAGTCCATTTATTTACATTTATACAAATAATTCTCCTAGTATTGTTGTG
GGTTATTAAAACAATAAGCTTTATGTCGATCTGTTTTCCTATTATGGTGTTGGCCTTATG
TTTCGTTCGCAAAGGTCTCGAGTACGTGTTTACATATAACGAATTGAAATGGCTTGATGA
CATTATGCCAAATCACAGAAAACCACCGCAGGATGACAATGACAATGATGTTGAAAAATG
CGGATCATCAGAAGTAATAGATTAGtttttcttataatggttatttagaggtgtgtaaaa
gataaaataatttttaaaatgtttcaatttattaagttaaataagaaaattcgtttttga
ctttcaatttttaaattgaaataactgaaaggcagcaactaagcctaattaattaaacta
acaatcaccacaatcaacacgtgcttgccaacgataaacaggatttctcattctagtcgt
atagacatcctgttctctggattcaataaagttatg
>DjScaffold1449:101599-102751 strand:+
tctcaaacaagtatcactcacttttgagataaccatgataatccaaataagttaaacaaa
ataatttatgatattattgatatttcttgacataaatcattgatataataaacttttaaa
aacataacatttgctgttgtgcaaaaaacattcatttaaagttagcaatccgataataat
tgatttatttccggtacccctgataatataaagaaataatgtgttcttaattaaaatcta
aataatgaataaatatattttagctcattaaaATGACCGATACAAACTTCGATTGCAATT
CTTCTACCAACAAGGTTATTCGCAGCAATGATTCAGGAATTAATATCAGTGAAGAAATGG
GTAAAACTGCAATTTGGAGACAACTTTCTGGAGGATCTAAATCCAGTCAATCAAAAAATA
AAAAACGCAAGAAACGGGAACATAACGGTGAAAAATCTGGTAACAAAGATTTAGAATCTG
GTGATCAGGAAAATTTAGATAATAGTCATGTTCAACCGTTACTCAATGACTCAGCTGACG
GTTCAAAAAGCAATAAATATCACTATATGACAACTCCCGAAATCGTTGTAAATCCTCCTT
CAGCAAGAGAATCACCAGAATTAAAACATCAAAATCCGAAAGTTTAAttcactgtttatc
ttagctttagatatgctctatgaacttgtgctcatatgtagttcttagtataatttcata

tgta
>DjScaffold715:5391-15159 strand:+
attatcataaatgacattaaattagtttcctttataaacgttttgaataaataagattat
atttgaaatttattatttatatattatctttttgaattttataattccatgtagttATG
TTGCGTAAATATGACAAATATTTAGTTTTATTTGGAGCATTTTTAGTTGAATTAGCTGTG
GGTAGTTATTATATTACCGCAACTACTATATATGATATTCTGAGCTATAAAAACAAAACA
CATGAAAGCAACTCGGCTACATCAAATGCAGACACAAACAGTGTAATTTGGTTAAGTAAT
TCAATTACCATTGGGATGGGAGTTACACTTTCATTTGGAGGATATTTAGAAAAGAAAATA
GGTTCATTAATTACCATTTTAATTGGTGGATGGTTTTTAAGTATAAATATTTGTTTAATG
TATGTAACTTTGAAATTGAGTTGGTCAATAATAATTATCACATACGGATTTCTTGGAGGG
TGCGGATCTGGAATTGCTTACGGTGTTGCATTGGGACTTCCCATTCATTGGTTTCACAGA
AACAGAGGTTTGATACTTGGAATAACGATATCTGGATTTGGTCTCTCTCCAACAATTTTT
GAAATTGTAAAGAAAAGTATATTAAATGTCCATAACGAAAAAAAAATCGAAACATTGCCA
TTATGTTTTCTAATTCTTGGATTGTCATTTGTATTGATTCAAGGAATTGCAGTATTTTTA
ATTTCGAGGCCACATTTTAATAAAGTAAGATTTTTATTTATCACAGTTGGAAACTTTTAT
TGAttgccagtatcacgtgttttcttaaaatggaaaataaactatgaaaaatctatttta
catattgtatattgaataaaaatatttctcagtattctgtcctt
>DjScaffold3674:28955-29737 strand:+
ATGACAAAATCATCAACAAAATCTACAACACCATTTTCAAGTTGTTTTAGTATTCGTCAA
ATGCTAGACAATACTGATGAAGAGAAGACTAGTAATTCCGGTGATCCACCAAATTCAATT
GCTGGTCACGATAAACCACCTTTCAGTTACAATGCGTTGATAATGATGGCAATTCGAAGT
AGTGATCAAAAACGTCTTACTCTCAATGGGATATATGAGTTTATAATGAGAAATTTTCCA
TATTATAAGAATAATAAACAAGGATGGCAAAATTCCATTCGACATAATCTTTCATTAAAT
AAATGTTTCATCAAAGTTCCCAGAGGATATGATGATCCAGGAAAAGGAAATTATTGGATG
GTAGATCCTGCTTGTGAAGATGTGTATATTGGTGGAACAACAGGCAAATTAAGAAGACGA
TCAAGTTCAGTTCAAAGGATGCAAAGACTTGGTTTAAGATGTGGTCCTTGGCCGACTTCT
TTAGATCACTGTTTACGATTGGGGCCTCCATCTCTCAACAATATATTTCCACATCCATCA
GATCCATTACAAAGCGACGCTTCTACTTGTTGTTCTCCAGTAAAAAACACTTTCCCAGAT
TTTCAAAACAAATCCCTACCTTGGAATTCGGTCAGTCAAAATTGTTTCTTTCCCAGTGTT
CTTTCTCATCATCCCATGCCTCCAAATTTGGATTTTTATCGACATTTACGATTACAGAAT
ATCTTTCCGAAAATGTGGTGTCCTCATCCAATTTCTTTAATAAAAGAAAAAAAGATTTTT
TAA
>DjScaffold3066:24995-26983 strand:+
gacaaaacaaccctaaacgtagaaataccacattattctgtttttttcaaagaaaatat
taaaaaaaaaaccaaatctatattgcttaaatatttatcaatATGACAGCTAGTTTGGAA
TTAGAAGATTGCGATGATGGAGTTCGATCGAATAGTAAACGATCCATCAAATCTTGTTCA
CTCTGTGTCGGATGTGGAGGAGCAATAACTGATAAGTTCATTTTACGGGTGCAACCTGAT
CTCGAGTGGCATGCTCGTTGTTTAAGATGTGTCAAATGCAATCGGGGATTGGATGAGAAA
AATACATGCTTTGTTAAAGATGGAAAAACATATTGCAAAGAAGACTATCAGAAATTATTT
TTACAAGTATGTGCAGCTTGTAATCGCGGTCTACACAAATCGGATTATGTGTTACATGTC
GGTTCACGAATTTATCATATTTCTTGTATGAAATGCGTTGCGTGTAATCGAATTTTACAA
ACCGGAGACGAGTTTGCTTTGAGACCGAATGGAGTTATTTGTTTACATGATCATCAAAGA
GATGGGCATTTATTTATTGAAAATTCTGGAAATCTATTAACTCGACCAGAACGATTAGCT
ACAAATCATCAACCGCCGAGCCCCATTAAACAACCGAAACAAGAACTCGAAACAGAATCT
TTATTAAAAATTGAAGATGAAACGAATTCAAGAAAAAGCGGATGTTGTCGGAATTATTTG
GATGTGGTATCAGATGAAAGACTATTAATGGATACAATTAATAATAACAATACTGGAAAT
ATCAGTCGGGCTAACTCTAGATTAAGTTATGTAAAAGGAATTCTCGACGATTCTTCAATG
AATAGCGATATCGATGATATTAGTCAAGATGGTAGCAGTGTTTGTGGGAATCAATCAATC
AATGGAGGTCATGGCGGTACGATTAGTGCTGCTGGAAGTTTACCTTCATCGGGAGGTGGA
GTGACATGTACGGGTAATGCAAAAATGAGTGGCACCAAACGGAGTAAAGAACAGAAAACA
ACGAGAGTTCGAACAGTTCTTAATGAAAAACAGTTACATACTTTACGAACTTGTTATGCT
GCAAATCCGAGGCCCGACGCATTAATGAGATTATTTTTACAAGTATGTGCAGCTTGTAAT
CGCGGTCTACACAAATCGGATTATGTGTTACATGTCGGTTCACGAATTTATCATATTTCT
TGTATGAAATGCGTTGCGTGTAATCGAATTTTACAAACCGGAGACGAGTTTGCTTTGAGA
CCGAATGGAGTTATTTGTTTACATGATCATCAAAGAGATGGGCATTTATTTATTGAAAAT
TCTGGAAATCTATTAACTCGACCAGAACGATTAGCTACAAATCATCAACCGCCGAGCCCC
ATTAAACAACCGAAACAAGAACTCGAAACAGAATCTTTATTAAAAATTGAAGATGAAACG

AATTCAAGAAAAAGCGGATGTTGTCGGAATTATTTGGATGTGGTATCAGATGAAAGACTA
TTAATGGATACAATTAATAATAACAATACTGGAAATATCAGTCGGGCTAACTCTAGATTA
AGTTATGTAAAAGGAATTCTCGACGATTCTTCAATGAATAGCGATATCGATGATATTAGT
CAAGATGGTAGCAGTGTTTGTGGGAATCAATCAATCAATGGAGGTCATGGCGGTACGATT
AGTGCTGCTGGAAGTTTACCTTCATCGGGAGGTGGAGTGACATGTACGGGTAATGCAAAA
ATGAGTGGCACCAAACGGAGTAAAGAACAGAAAACAACGAGAGTTCGAACAGTTCTTAAT
GAAAAACAGTTACATACTTTACGAACTTGTTATGCTGCAAATCCGAGGCCCGACGCATTA
ATG
>DjScaffold1710:83335-84306 strand:+
agttatgagtttgttgatcgaattaaaaattatgactggaagaacgatgatcaatttggt
tcattggatgtaacaaatctttatggatcaattgccatggaagaaccaaaaaatgtcatc
aatattataagtgattgtaaaattattctcaaagctgagaagaagatacatctttggttc
agatatatagacgatattttctttgatattaattctcctttagaaaatattttagaacta
gccaatagtatcaatgataacattaaattcactttagagaagtcaatcgactttcagatt
ccttttctggacaccttaatccaaaagaaacctgttgtatATGAAACCATTTTACAGTGG
ACATATATTACCCTGCAGTAGTAATACTANAGACGATATTTTCTTTGATATTAATTCTCC
TTTAGAAAATATTTTAGAACTAGCCAATAGTATTAATGAAAACATCAAATTAACTTTAGA
GAAGTCAATCGACTTTCAGATTCTTTTTCTGGACACTTTAATCCAAGAGAAACTTGGTGA
AGTATTAACTACCCAGTTATATATGAAACCATTTCACAGTGGACATATATTACCCTGCAG
TAGTAATACTACCATCAGACAGAAAATTGGAATAATCATGGGTAAAAGAATAAGAGCTAA
AAGACTATCTTCAAACGAAGAAATGATACTGACTTCCCTCAGGTTGATAAAAAAATAAATT
TCTAAATAATGGTTATCCAAATAGAATTGTATCTAAATATTTATTCTCGAGGGCAAAATT
TAATAAAATCAAACAAAATACAAACACAGTAATTAAATTACCTTTTATTAATGAGAAAAG
TAGCCAAATCATGCAAAATATAATAAAAAAATCAAAACTCAAACACAATCCAAGAATTAT
ATTTAAAACACAAGCACCTTTGGATATTCAGTTACAGAAACCTAGACAAAAT
>DjScaffold2815:87655-89088 strand:+
tcattattcaaaaattatttaaagaaatttgagcttttttgtataaacgaattataatt
agatttgtattaaacttataatttaaaaataatattaataataataataatcaattaat
cattacaataatattaacatttttaatttttatcataaatattgaaaattatatttatta
ttcagaaaaaATGACGAATGTTGTCATTGATCAACGGAACAAAATGTATAAATTGATGTC
ACAACAGAAATTTGTAATTAGTTGTGATGTTAATGATGATTATAAATGTGGAATTTGTAA
AAATATCTTAATAAATGCTCATCAAGCTATTTGTGGATGCAATTTTTGTTATGATTGCAT
CAATCAATATATAACTAATGGTGCCATCAATTGTCCTAACAACACTGAAGAATGCGAATT
GGAAAGTATATCAAATATTGTTCCAAATCATATAAAGAATAAACTAATATCGAAACTAAT
TTTGCGATGTTCTAACAAAATGTGCTCACATGAATGCCAATTAATAGAAATGGAGAATCA
TTTAAATTCATGTTCGAACAAAGCTGTAAAATTTCCATTTATTGATGTTGGTTGTGAACA
TGATGATACGATCAATGATAAATTAAATGATCACATGTCAGATATTCACAATCACAAAAT
ATTGTTTGATCTGATTGGAAATTTGAGAAATGAAATGCGAGAATTGAAAGAGAAAGTAGG
ATGGAATCGATTAGAAATTGAAATGTTGAAAGAGAATGATCGTATAAAGAGTGAAATAAT
TGAATCATTGAAAGAAGAGGTCAAATCATTAAAAGAACAGAATGAAGATCTGACACAACA
AATGGAAATTGGTGATCGGAATTTATGGTACAAATTTTGTGAGATCGATATTGAGATAAA
GAATAATTACAAGAAAGAAGAATTATTGAAAGAATTTGCTGATAAATTCAATATGGAAAT
GAATGCGAGATATTGTGATATGAAAGAAGAATGGAATGAGAAATTCGATGAGAAAATAGA
GAAAATGAGAGAAGATATTTTAAAATTAGATATTGAAAGATCAAATAAAATTGATAAATA
TTTGAATATGAGAGATTATGGAAGATTGGATTGGCAAATTGGTAAAATAAATGAGATAAA
ATGTATCCAAAGTGAATATTTTTATGACAAACAGAATCCATATAAAATGTATTTGGAATT
GCAAAATATAAATAATGAATTGTATATTTGGTTGAGCATTCGCTCTGGTGTATTTGATTA
TGATCTCGAATGGCCCTATCAAGCTGATGTTATAATTATTATATGTGCATTGAATGATTT
AAAGATATACAAAACAGTAACAAAATCATGCAAAATAGAAAAACCAGAAACAAATTTATC
CACATTTGGTGATCCAATCGTAATTTCTCATTCTGACTTGACAAAATCAAATCTAATCAA
TATAAATGGCTTATTGCGTTGCTCTGTAAAATTTAAATGAtaaatgttttgttaaataaa
tttgaaaattatatttattattcagaaaaaatgacgaatgttgtcattgatcaacggaac
aaaatgtataaattgatgtcacaacagaaatttgtaattagttgtgatgttaatgatgat
tataaatgtggaatttgtaaaaatatcttaataaatgctcatcaagctatttgtggatgc
aatttttgttatgattgcatcaatcaatatataactaatggtgccatcaattgtcctaac
aacactgaagaatgcgaattggaaagtatatcaaatattgttccaaatcatataaagaat
aaactaatatcgaaactaattttgcgatgttctaacaaaatgtgctcacatgaatgccaa

ttaatagaaatggagaatcatttaaattcatgttcgaacaaagctgtaaaatttccattt
attgatgttggttgtgaacatgatgatacgatcaatgataaattaaatgatcacatgtca
gatattcacaatcacaaaatattgtttgatctgattggaaatttgagaaatgaaatgcga
gaattgaaagagaaagtaggatggaatcgattagaaattgaaatgttgaaagagaatgat
cgtataaagagtgaaataattgaatcattgaaagaagaggtcaaatcattaaaagtaaat
taattttatgattgatttaataatattttgttATGATTCAGGAACAGAATGAAGATCTG
ACACAACAAATGGAAATTGGTGATCGGAATTTATGGTACAAATTTTGTGAGATCGATATT
GAGATAAAGAATAATTACAAGAAAGAAGAATTATTGAAAGAATTTGCTGATAAATTCAAT
ATGGAAATGAATGCGAGATATTGTGATATGAAAGAAGAATGGAATGAGAAATTCGATGAG
AAAATAGAGAAAATGAGAGAAGATATTTTAAAATTAGATATTGAAAGATCAAATAAAATT
GATAAATATTTGAATATGAGAGATTATGGAAGATTGGATTGGCAAATTGGTAAAATAAAT
GAGATAAAATGTATCCAAAGTGAATATTTTTATGACAAACAGAATCCATATAAAATGTAT
TTGGAATTGCAAAATATAAATAATGAATTGTATATTTGGTTGAGCATTCGCTCTGGTGTA
TTTGATTATGATCTCGAATGGCCCTATCAAGCTGATGTTATAATTATTATATGTGCATTG
AATGATTTAAAGATATACAAAACAGTAACAAAATCATGCAAAATAGAAAAACCAGAAACA
AATTTATCCACATTTGGTGATCCAATCGTAATTTCTCATTCTGACTTGACAAAATCAAAT
CTAATCAATATAAATGGCTTATTGCGTTGCTCTGTAAAATTTAAATGAtaaatgttttgt
taaataaattt
>DjScaffold1051:73910-87037 strand:+
tttaaggaattgactcgacgagatgctgaagctagaATGAATGTAGCTTTGGAAAATCTC
GTACTGACGGCTCCAGAACCAAAAAGGAATCATATCGAAAAAGAATTTGAAGGATTTAAA
ATTTTATTTAGACGTTATTTGGATGAATCGTCTTCTCAAATTAAATGGGATAATATAAAG
ATTTTAGATGGTGGAGAAGTTAAACCTTACGAAAATCTTTCAGAAATTGATCCATCATTA
ATAAAAAATTACTTGGATAAATTAATTGTTATTAAACTCAATGGTGGTTTGGGAACAAGT
ATGGGCTGTACTGGGCCAAAGTCATTAATTTCTGTACGATCGGACTTAACTTTTCTTGAT
TTAAATGTACAACAAATCGAAGCATTAAATACCAAATACAAATGTAATATTCCTCTTATT
CTAATGAATTCTTTCAATACTCAGGAGGATACATTACAAGTTTTAAGAAAATACGCTAAA
ATAAATGTTACTATCGAAACTTTTGAACAAAGCATGTATCCAAGAATTCATCGTGAATCC
TTAATTCCAATTAGTAAAAAAACGCGCCCAGGTTCTGCTGAATTTAGACATAATAAACAA
AAAACCGACCCAGAAGAAGAAGCTTGGTATCCACCAGGGCACGGTGACATTTATAGAAGT
CTGATGAATTCTGGGATTCTTGAAAAGTATTTGAAAGAAGGAAAAGAAATAGCATTTATA
TCAAATATTGATAATATGGGCGCCACAGTCGATTTAAAAATTCTTTATTTTCTGTTTGAA
TCCATGCAACATTCAAATTATCCTGAATTTGTTATGGAAGTTACTAATAAAACAAGAGCT
GATGTTAAAGGAGGAACATTAGTAAAATATGAAGATAAATTACGTTTGTTAGAAATGGCA
CAAGTTCCGAAAGACAAAACAGAAGAATTTACCTCGGTTCGAAAATTTCGAATATTCAAT
ACCAACAATTTATGGGTGAATCTAAATGCAGTGAAAAGAGTAATAGAAGAAGAAACCTTG
CAAATGGAAGTCATAGTAAATTTGAAAACTCTT
>DjScaffold736:15540-36212 strand:+
taggtggATGTTTAACATCAGTAATACCAAGCCAGGGTCGACTTCGTCCACCCCATTAGT
TATGTCAATGAAATTTCTTGATCTACAAACAAATACTTTACCAAAATTTAAAGGTCAACT
TTTACGCCGATTGGTCTTTGAATTGCTGCTAGATTTTTGTCAAATTTCTACTGAAATTTT
TCAAACGCTTTGCACGAAAATAGTCGATATCCACATTGAAAGATCTTTACATGATGATTT
TAAAATTTTTGCCCTTGAATATTGGCCACGAGATGATTTACGAGAAGAAACTGGATTTTG
TGGAATTATCAATTTAGGAGCTACATGTTATTTGGCAACGACAATACAACATCTCTTTAT
GATTCCAGAGTGCAGAAAACTCATTCTCGAATGCACCGAATCTAACTTGAAATGGTTTAA
TAAATTACAATCAATATTTTCTTTTTTGCAATTTTCAGAACGAAGTGGACTCAACCCGAA
AGCGTTTTGTGATGTTTATGAAATGAACAAGCAAGTAATTAATACCGGAGAACAAAAAGA
CATGTCAGAGTTCTTCAGTGATTTAATTGGAAAACTTGAAACAGTTTCATTAACCCTGAA
AAATGGAATAAAAAACCTATTTGGTGGAGAATCCAGTAGCTATATTGTTTCTCTTGATTG
TCCTCATCAAAGTCGTATTCCTGAAGAATTCTATTTTCTTAGTTTGAATGTTAACAATAT
GACAAGTCTTCAACAGTCGTTTGCTCATTTTGTTGAAAAAGATATTCTTGATGGTAATAA
CAAATATCGATGCAGTGATTGTGACTGTTTAGTTAGAGCTGAAAAGCGAATGTGTCTCAC
TAAATTACCGTCTATTCTTTGCCTAAATATAATGCGTTATTATTTCGATGTTAACACTTT
GAGACGAGAGAAATTGAATAATTTCTTTGAATTTCCAATGCAGCTTGACGTTGAGCCATT
TATGAAACATTCTTTAATGGCATTAGAACCGGATGCAGGGGTTGCTGGCAATTGTAAGTA
TAGATTGATTGGAATAACAGTTCATTCTGGAACTAGTGAGAGTGGCCATTATTACAGTTT
CATTAAAGATCGTAGTGATGGAAAGGTTTTATTTTGGAATGATCAAAACTCTTCATCTGA

CAAGTGGTTTTGCTTTAATGACTCAAAAATAAAATGCTTCGATCCAACAAATATTCCATC
AGAATGTTTCGGGGGATTAAATGTTAACAAAAGAAATATTGAAACATCACAGCTATTTCT
TGGAACCTCCGAATATAAAACAAACAATGCTTACATGCTATTCTATGAAAGAGTTTCCGT
TGGAGACATTCGAAAAGAATCTAAATCTATGGAGATTGTTATTCCACCAAATATCTATTC
AGCTATTGAAATGGAAAATCAAAGATTTCTTCATGATTGTTTATTATTTGATTTGAATTA
TTTTCAATTCCTTTTGCAATTTGGAACCAATGTTCCCAGATTGATTAGTAAACCTCATAC
TGAAATGCTAGTAACTACTGATGTAGGAAAAGTTTGCGCGTCTTTCATTTTAGAAGTTCT
ATTGCATTCAAGAGAAAAACCGGAACTTAGTCAATGGGTGGAATTATTGTTCCGTATAAT
TTCATCTTGCCGACCTTCTCAATTATGGCTCATTGATTTCATGACAACGAAGAGTAAATG
GATTACGCAATATTTATTTCTTTGTTCCGATAAAATTGTGAGAAATTTATTTATAAGATT
TGTTCAAGAATTAGCCAAACTTTTGTTGAATCAAAATGATGCCAACACAAATATTGAATT
TATTTCCAAAAAAATTAAACTATTAGTTAAGTCAATGTTTGATTCACTTTCATTAACCAC
TTCTGATATAAATAATATTACTTCCATTAATATGAAATTGAATCAATTTGATGAACCAAT
ATTGTCAGGTATGTCCCGTCCAACTTTGGTTCAATGTGGTCCTAATAATGCGACTTGTTC
ACGATTCAATTCTAGCAGTTATCATGATCAGTTGGAGAATTATTTCGATTTATTTTATTC
GCTTGTTAAATTGAATCCAGATATAATGTTACCTCTTCTTTTTAAATTTGGAGGAATTCA
CAATTTGATAACATTTTATCTCAATATTCGACTTTGTGATTCAGAAATCAAGGTATGCAA
TAAGCAAACAAGGGGGCACAAATATCTTGAAATGAAATCTTTACTTCAATCAACAATGAA
CGATTCTAAACAAATATTAATTATCGACAACAAACAAATATCTAATTTGATCATGCAGTC
TGTTGTACAATCTCAAAGTGAAATCGGAGCAAATTCAAGAAATAAAAGTTATTTAGAAAA
CCTTATTGTTGTTATTGTTGAAATTCTCAGTTATCTTTCTCACACTTCACTTATTCAAGA
TAATTCATCTTTATCCAAAGAAGATGCTGTTGCACTGTTATCAGAAGATTTTATCAATTC
AAATTTTTCCTTATCAACAGATCCTTGTCAATCCATGCCGTTTCTTTATCATCAGATTTG
TGATACAATCAATCCATCTTACACTATGAAATTGTTGGCTTTACTAATTTCATATAGAAA
AGAGATTTTACCACCATTAATTTGCTTTCTCATGTTATCAGCTACTGTGCAGGTGGATCC
AATTCGATGTCAAAACTTCTTGTATTATTTAAACGTTCTGCTGCAAATGGAAGAAAAGTG
TAAAATTTCCAATAATAGTATGGAAAATGACGAGGATATCACAAGACCAATAGAACATTT
AGTTTTATCTCGTTCTATGGATTTTGCTCTTCTTAGTCGTTGTTCATATCAATGGCTGAA
TTGGATGTCTACTCGATGTGCAGATACAGAATTTTTGAATTGTCTTCTAATGAAATTAGA
TGATTGGCTTTATAAATTTCTAGTTTTAGATCCGAATAATAGAGTGAGACAAAATTCTCG
AGACTTAATTAAAGCCTTATTGAGATCATCTCAGGAATCTGATTCTGATTCGTCTGATGA
ATGTGCCCAAGAAACCGATATACAACAAAAACTCTTTAATAAATTATTAAGTTTGCTTAA
TGAAATTAGAGAAAAGCGGTGTTCAGTAGCAAAATCATTAAATAATGAGTGGATCAACAC
ATCTGGTCCGGAAATACAATCTAATGAGCCATGGAGACAAGAAGACCATTTAACTGCATA
TTTTAATGCATTAAAAGATTGTATGTATTCACAGTCAGATAGATTAAAAGTCAATGAGTA
TCTTCCTGATATTTGGGCTATTTACCATCCGGGAATGAGTGTCGCCCTGTGTCCAATAAA
TTGGGAAAAGCAAGCTTTATTAGCTTTTCTCTATCGTGTTTGCCTGAACAATAATGCAGC
TGTCGATTTCATTACAAAAACGTCGTCTGTTTCTATGCCAATCAGTACAAATTATATTCT
CAGCGATTTGGAAGATCCAACTGTTGTGAATTATAACAGGAAAATGTTACCACATTTTTT
TGGAGTATTAACAGTGTGTTGTCAACGATCACCTGTGTTTCGCTTGAATCTTTGTCACAA
ATGTGATACTATAGATTGGTGTTACCGTCATATTTTACCTCACAGGCAGTACTATGGTTC
TGTTATTGATGACATAATTTCACTGACTCAAGCAATGATTGAACCAATCGGACAAACTAA
TTTAACGATTAAAGACGATGCTTTAGCATCACAAAGCAAGTTTCGGCGAAAGTTCCTCAG
AAAACATATATCAACCGAGCTCCGTTCGAAATTATTGTTAAAATTATGCACCATGCTTAT
TCAAGATAACGAAGATAGGCTTACGTTAGCTATGCATGATGGATTTTACGCTCTTTGCTT
GGCTGCTTTGGATTTAGTGAATAAATATTGCTCGACTAGTACTCCGGAATCTGAGAAACT
TGTTATCGAGATTTTATCCATAATAACTCAAATCATTCGTTATTGTTTATTGGTTATTGA
ACAAAGCACCGGTACTTCTTTACGCCGAGACATATTTTACAGAACACAAGGAAGATTTAA
AAGTCAGATGCTGGAATTAGCTACAAAATGTTTGCATTTGATTTGTTCAGAATCACCAAG
ACAAATGCGATTATCGTCAATTGAATTTGTTCGCCAAGTGCTTGATGTTTATCAGAAAGA
TTTTCTTACTATGATCACAAAAGAGTTATTAAGCTATCAAAATAAGCTCAATATTGAGAA
TATTGTTAATCGAGATACATCTGTTTTTCAACGATTAATGAACATTAATTCCATTGTTAA
ATCGTCTCCTTTGTCAAAGAAAGATTCAGATATTCATAAAGTGACAGATGACAAAGTTAT
GCCCTCATATTTCAGTCTGGTGGATCGTGTTTCAAGAATGGCTGTAAATCTACATAGATT
CAACTCTGACACAATGACATTGTGTTTAACAGCTGCCATTCAGTCATATCCAATAGCATG
TCCTCTATTTGCTCAATTTTGTTTGGAAATTCTACATTCCGATCTTATTGCGGAAGTCGA
ACATATGATGACTTCGTCATTGTTTCATGATTACATTTCATTAATTATTTCCGGTGATGT

TAGCCATGTTTTAAGAGAAAAGGCGATAATTCAACTACTCGAAGAAGTCTTTCCAAAAAT
AGCGAATTCTCTTTTTTCCCGTTCCGAATCTGAGCAAATAACTTTTAAAATTATTGAAAT
TGTAGGTGCTAGATTATTACAATTTAAGCCAGGAGATGTAACATTCGATCTCAGTGGTAT
CGACCATCTACTCTCTGTTTTGTACATATTTTTAAGATATCCTTTACTTGAACCAATTCT
CAAAAAAATCAGTAGTGCTTTAATGAGCAAGTCTTCAACCTCTGAACTCAGTTTATCAGT
CATATCTAATTTGGACTTTTTATTTGGTAACAATGCGCTGAATATTAAACAGATACTTCA
CAAATCATCTTTAAATAAAGAATCTACATCTGAAGATATCATCGATGTGCAGTCGCCATC
GAATCTTATTACCACCTCTGAATCTGTAAAA
>DjScaffold4026:26574-39722 strand:+
tgactactttaatattaaaattctcgcatttcttacaccaaaatttcaatgtagaaagtg
ttataattttcttcagatatttctaaattgtgttatttggaattatttttaatactgaat
tattattattattatcattattaatattattattaataatattattattaaattatatat
tttaggtttcagattttatcacagcatggaataggactaatccaaaatatttattagaga
ataattcaaccaatcagatcgaaaggaaaATGATAGTTTTATATCAATTACCTATTTCTG
ACCGACTAAATAACGTAGATATAATTTATGAAGATACCGAAGATGAATTAATAAATAGAT
TAACAATGGCTTTGAAAAAAAATCGTGATGAAAATTTTTTGGGTTTTGGTGAAATCGAGC
AAGTTGATCCTGATGAGTTTTGTACAGTTTGTAACGAACAATTTGAAACAGATGACATTT
ATTGTATCCGAATCACGCATTCTGCTACTTTTGCATCTCATATATCGTGTTTCAATTGTA
AAACATGTAAAGATAATTTGATTGATTTTCATTATTGCTGGCACAAAGAATCATCAAATG
TATATTGTATGAGACACTTTGGAGAACAACGTTACAGTCGATGTAAGGGATGTGATGAAT
TAATTTTAAGCAAAGAATTCACAAAAACTATGGACAATGATTATCATGTCGGACATTATG
TTTGTTATAATTGTGAAAATTCTCTTACCGGAGCGAGATATATTTTAAGAGAAGAAAACC
CATATTGCATAACATGTTATGAAATTAAATTTGGAAATACTTGTGCTAGATGTTCAGTGA
AAATCGACTGCGAAAGTAAAGATCTTGCATATAAAGACCAACATTGGCACGATAAATGTT
TCTTTTGTGATGTCTGTCATGATTCTCTAGTGGATAAATCATTTACTACGAAAGAAAAGG
ATTTATACTGTCCTAATTGTTTTGATTTAAAGTTTTCATCACCTTGTGATGGCTGTGGTG
AATTATTCAGGAGTGGGATGAAGAAATATGAATATGATGGCAAACAATGGCATGAGAATT
GCTTCATCTGTGATGAATGTAAAGAAAGCATGGGAGATAAAAAATTCATTCCCAATAATG
GGAAAATGATTTGTATTACATGCTATGAAAGTAAACATGCTCAAAGATGTCTTAAATGCA
ATGAGATAATTCGTAAAAATGGTGTAACTTATAAAGGCAATCCATGGCATAAAGAATGTT
TTCTTTGTACAAATTGTCAGACTATGCTAAGCGACACGAAATTTACTACTAAAGATGAGA
AACCATATTGCGGCGATTGTTTTGCTGAATTATTCAGTAAGAGATGTAAAAAATGTGGCA
AACCAATTTCGGGATTTGGAGGTTGTAAATTTATTTCCGTTGAGGATGAAAATTGGCATC
CTGAATGTTTTACATGCAGTAAATGTGATATCAGTATGGTTGGTAAAGGATTTCTTACGC
AAAATGAGGAGATGGTTTGTCAAACATGTGGAGGATATACTGGTGATGATTTTGCAGAAC
AGAAAGCTGAGACTGAGAAAACTGAATAGtcgacattgtttttatttaattttacaattg
tgtggttgttcaataattcctgttttatttaatttccaatggtagcggttaagaggaatt
cggttttcatatttcgtaataatattaaaatatatttttgctaaatagtatgaattta
taatcaacaatgggttgtacacatatttttcgt
>DjScaffold5187:31290-31770 strand:+
attaaatatctgtcgtagatcatacgATGGTACAATTCGTTAAACCTAATCTTCTTGGTA
CACAAAAGGAATTTTTAAATAGATTTGTTAATCCAATAAACAACGGGCAGCATATCAACT
CTACACCTTACGATGTCAGTTTAATGAAGAAACGGTCGCATATTTTATTCAAAATGCTTG
ACGGCTGCGTTCATCGTCGCGATTATTCTGCATTGGTGAAATACTTACCACCGAAATATG
AATACGTTGTAAAGATCCGTTTGTCTGATATACAAGTTCAACTTTATCGACAATACATTT
CAATTTGCAAAGATAATAAACATAGTTTATTCCAGGATCATTTGACTTTCTCTAGAATTT
GGACACACCCCTTTGTCATATGTATGCATCAGGAAATTATGGATAAAAAAGTTGGTTTTT
TATTTAAA
>DjScaffold912:16566-17052 strand:+
gacaaatttgagattttcataaaaagttttcctttgaagaatttttatctgttgagtaaa
aaaattaaaatataaacaATGGCGAGAGATGCTTATAGCAAATACAGTAAACAATCTGAT
AAAGTCATGATTCCGGAATACAATTATCCGATGGGCGAACGTAAATCATTATGTTATGAA
GGTGCTGCTTGGTACATACCTTCAGAAAGACGCTGGGAAAACTATCATGAACATCGATGC
TTACCAACACTAGCAAGAAGAGATGCGATTGATTTTCAAAGAGAAGAAGATTATATGAGT
TTCGTAAGAAAGCGAGACCGTCCAAAAGGTTTATTGATTACATTCAATTAGataattatg
ataaatatgtaaaacaatgataaaatcgagatg
>DjScaffold5312:109656-117001 strand:+

GGCGTGTGGAGTTATTTTAATAGCTTTTTTTGGGATACTAAGAGAAGAATTTTAATAATT
GGTTTAGATGGAGCCGGAAAAACAACGATTTTATATCGTTTAAAAGCCGGAAAAATCGTG
ACAACTGTACCAACAATTGGATTCAATGTTGAAACTTTGGAGCATGGTAAATTGAAATTA
CAAGTTTGGGATCTCGGTGGACAAACAAGTATTCGACCGTATTGGAGGTGCTACTACTCA
AATACCGATGCAATCGTTTACGTTGTTGACAGTATGGATCGTGAGCGTATAGCATTATCC
AAACAGGAGTTATTAATGATGCTTCAAGAAGAAGAATTAAACAATTGCATACTAGCAGTA
TTTGCCAATAAACAAGACCTGGAGGGATGTTTGACATTATCAGAAATAGCTACAGAATTG
GGTTTATCAACCATTCGAAACAGACGATATCAGATCTTTAAAACTTCCGCAACCCAAGGT
TTTGGATTAGATTTAGCATTGGATTGGTTGACTTCAAATCTCACGAAGTGAtaagtaaat
atctgagaagtggtttattattttgaaatttaggcattaattttcactgatcaagtttt
aatggtttctaatgtgcctttttagctttgaacttaactatgtacatttttaaataa
>DjScaffold5232:44169-56957 strand:+
ggaagattaaaagattatttatttaaatataaaggttataaccatttaataagacctgtt
gtcaatttatcagatgtcattgatgtcaaatttgatttagcattaatacagttaataaat
gtggtatatatttattttatttcttaacataatatttaagtaatgttgaaaattttctgg
aaaagattttcatgtgatgcattttaataattcaattttaactatttcagataattaaca
tttttaaaatttttctaaattattaatgctgatggcaattatgaagttttatggcaaccg
aatattttgattcaatatgatggagaaattctctggattcctccagctatttatgaaagc
tcttgtagtattttggttgaatattttccatttgatgaacaagtctgtgaaATGCGATTT
GGCAGCTGGACTTTTAATTCCAAGCAGGTATCTCTCAGTTGGTTAGGAAGTTCAAAAATT
GACTTATCTACTTATTGGCTAAGTGGAACTTGGGATATTATATCTTGTCCTGGTAGAATT
ACTAACGGCACTATGGACTCAAGTGGAATCGATACCCAAGAAATTATTTACCAACTGACA
ATTCGAAGAAAGACCTTATTTTACACAGTCATACTTATATTACCTTGTTTATTAATTTCA
TTGTTAACAGTTTGCGTTTTCTATTTACCAGCTGATGATCACCATAAAATGACTCTAACA
ATATCTATTCTTTTAGCATTAATTGTGTTCTTTTTGTTAATTAGTAAGTTGCTTCCTCCT
ACTAGTACATCTATTCCGCTTATTAGTAAATATCTGTTGTTTACCTTTATTGTTAATATT
ATAGTTATTGTGATTACTGTGATTATAATCAACTTGAATTATCGAACACCTCGAACTCAT
TGTATGCCATCTTGGGTTAAAAAAGTTTTTATTGAATTTTTTCCAATTGTCTTGGGTATG
AGTAGACCAAAGCACATTGAACAAGAATTGATTGATAGAAAGAAAATCAGTCCTATTTCT
CCGGGTATTATAAAACAGCAAAAGCAAGTTATTAATACAAATAAACCGGCTTCAAGCGCA
TTTCAAAAGTTAAAACCGAACTCAAAACGTTTAATAAGAATGGTTGATTCTCAAATTTCA
ATGGGGCTTATGAATGAAAAATATAAACCATATTCAGATGATACGGACAAAAGTATTTGG
AAATCTCAAAATAGCAGAATGATTCCATCAACTAGTTATAGTAAAATTACCAATAACACT
CATAGTTTTTCAAGAAATTGGGAAGATATTGAGCATGTTCCACAAAGATCTTTTACTGAT
AGCACATCTTCTAGAAGTGACAGTAGTACTCGGTTAACAGATGAATTAAATTCCAATTGG
CTGTTTCGTGGGTATGAAATCAATCAAGAAATGAGACAAGCTATCCTCAGTATTAATTTT
ATTTCATCTCACACTGAATCTCAGGGTGAATTTTCAATGGGAAAACAAGATTGGCAGTTT
GTAGCATCAGTTATAGATAGATTAATGCTACTTATATTTTCCACTGTTACACTTATTGGT
ACAGCAGCTTTATTTTTCAGAACCCCATATTTTTTCTCACTTTTCTACCCAACTGTAGAT
GTTAACAATTGTTAAgtagagataatttagattcaatgttttcctcataattaatctttt
gtcataataaatataacatttgagtttta
>DjScaffold5232:61149-71559 strand:+
ttttttagattccttcaatataataattagtctgaaaagtttaccATGCAAAACAACTTA
TCATCAGTTCCCCCCAATAGTCTTTACCATCAGAATAGTATCGCAAGTTTGGCAAGCGTA
TTTAATGCAATACAAACTCCTAATTCTCATATTTTGAAGAAAGATATAGTTAGTGAGACT
CCTTTGAATTTAATATCTCATTCAAATAATTTGTGTAATGATGCAACGGGTCAAATTAGT
ATTGGTAGTATTGTTGGCCGTACTCCAATAACTCCACATACCCCATCTACTCCTATGATT
GATAAGGATCATACTATGCCTACACCACAATTACAGAATATTGTCTGCACTGTAAATCTT
GGTTGTAAATTAGATTTAAAAGCCATTGCACTTCATGCAAGAAATGCTGAATATAACCCA
AAACGTTTTGCTGCAGTTATCATGCGTATTCGCACTCCTAGAACAACTGCATTGATATTT
TCTTCTGGAAAAATGGTTTGCACTGGTGCTAAAAGTGAAGATGAAGCTAAATTAGCTGCA
AGAAAGTTTGCTCGAATTATTCAAAAATTAGATTTTGATGCTAAATTTATGAATTTTAAA
ATTCAAAATATGGTTGGTTCTTGTGATGTTAGATTTCCAATTCGATTGGAAGGTATTTGT
CTTTCACATACTCAGTTCGCTACATATGAACCCGAACTGTTTCCCGGTCTAATCTATAGA
ATGGGTAAACCAAAAATAGTTTTATTAATTTTCGTTTCTGGAAAGATTGTTTTAACAGGA
GCTAAAGTCAGAGAAGAGATCTATGAAGCATTCAGAAATATCTATCCTATCCTCAAGACA
TTTATGAATACGTCTCAGAATCCCAAATAAtttgtttgattaactcatttttggataatg

tgaattttgcaaaacgacagaaataataatattatatttgaattgtgatattttaaaaat
cgcttattttcaatgtcgaacaataaccataatgtataaagatgactttttattgaaat
aaataattttctaatcactgattccaattcggttttct
>DjScaffold5232:73992-98409 strand:+
atctacagaacaaatcacatcatggagatagtATGAAATTTAATTTAATTTGTTTCAATA
GTGATGTTGCAAAATGGTGTGTGTCAATGGCACCTTGTACTAATGAATATATAACAGCAG
CAGCTGATTGGTTGCGTCATATATTACCGGAAGGTTCTTGTAATTTTCTGGGTGCTTTAA
TTAGTGCCTTGAAAGAACCGCTAAGTTGTAATATATGTTTCATTGTCACACAACAATCAG
ATCAAAATATCAATGAGATATTGGATTTATTCGCTTACGTTGGGCGTAAAATTCCTTGTC
ATATTAGGTTATGTTTACACGATAATGAAGTTTCTGAAGAATCGAAAAATGCTCAGACTA
TCTACAGCCAAGTAGCAAGAGAAACTCATGGAACGTTTGCATTTGTAAGATTGAATTCGA
AAAATGAATTAGTCATTATGCAAAGTGCGAAAAACATTTTGAATTCAACAGGTTTAATAA
CTGTCAACAGTTATGTTAATTCGAGTGAAGAAGAAAATGAAAACATTAAAAAACTTGAAC
TATTACGTGATGAAGAGAAAAGAGAACTTTTCAGTCCATTTCAGCCATTCATTCCAGTAA
CGGATTCTTTTTTTCTTGGTAAGCAATCCGATGATATGCATTATAAGCCATTCCACTTTT
GGAAACGAGTTTCAGCTTCTCAATCTTACTTGGATTGTATGCGGAATTTCAATGAGTCCA
ATATTTCCAGAATGGCTCAAGAAAATGTATCAAAACTCGCTGCATCTGTAATTGTTGGTC
GGGAAGTCTTGGCGCGAAAATATACAAATGGACAATATTATCTTGGTTACATTAAAGATC
AAGTTAACAATGAATTATTTAATGTTGTGTTTGGCCCGTATAATTGGGGTACCGGACAAT
TTGTAGCCACAGATTATCAACAAACAAAATTACTGGATTTAATAGACTTTAATGATGCTA
TGAAGCGTTCTATTCTACCAGGTGATAGAGTTTTAGCTCCAGATCCTACAAATCCTAATA
AAGATGCATATGCATGTCGATATTTAACTGGGACTGTTCTTCATGGTTCAGTAATTAGAG
AGTATACGACTGCAAAATCTCATTCGACAGAAGCACATGATGGGAAAAATATTGACAGTC
CTATTAAAACTTCGAATAGTCTTATTGTGGAATTTACTAATCCACTTAATAAAACAGAAA
AGGTTTTAAGTGATAAAGTTATATGCATTCCAGAGCCATTTTTCCAAAGATTGGTTTTGG
AAAGTGCAATGCCAAAATCAGCCCGTGAGAAACTTTACAAAGTGCCAAATTATCCATTGA
ATTCTTTGCCGGGTTATCCAACGTCAGGAAGTAAAAAAACACCTGATGACTACACTTTCA
CCAATTTGAATGTTATGAAGAGTAGAACATTACAATCAATCGAAAATAATGTTGTCAATT
GTTGGAATAAAATTGTAACACATAAAAAAGTGGATTACCTTATGCCTCCATATTGTTTGC
CTCTTTATCCTGTTGTTGAAATATTCGATAAAGATGAACGTACAGGAAATGTTGTAATGA
AAAGTGACTGGGCTGTCGGGAATGTCGTAAAAAAATCTTCAACTTTCAAGAAAGATGAAA
CTATGGTTCCTGAATCTATATATACCATAAGTGAACTACAATTAAAAGTTAAAGATCAAT
TAGAAACTTTTAAAAATCTACCTCAAGTGAGGATAAATTCAGAGAAGTCTTTCTATCGAA
GGGGACTTAGTAAAAAGTTTGATAATGATGCAAAATTAGCCAAGTCTGTACATATCAAAT
CCAATGAAAAATTATTAACATCACAGAAATCCAATTTACAAGACAAAAAAATTCGTTTTT
CACCAGAAACTGAATCAAAATTAGAAGCGAAAAGCAAAATTGACAACAAATCAACAGTTA
CACCTTATGCTATTCATGAAGAATATTTCGATGATATACAAAAAATTCGGGAAAATATGG
ATTTTGATCAAGTCGAGTCGGTTAGCAGTTCTGTTTTCGAAGTCTCAGATAACAAACAAA
AAAAAGTTATTTCTTTTGCTGATGAATCTTCTAAAGAAAAAATTGGAATTCAACCTGGAA
TTTTATTTCAAAACAAAATCTCTAATAAACCAATTGCACAAAATTCCAATGTTTTAAATA
TTACTGATCTCGGAGAAAAAGACAAAGAGAAAGAATTTAACGAAAAAATGAAATTAGAAA
GACATCAGCGACAAATTCAAAAACGCCTTGATGAAGAAAAGCATAAATTTTGTAAAGAAG
AAATAGCAAAAGATAAACAGTTTTATTATCACCAACAAAAAACTGCTGACAGACTTCATA
AAGAAAATATGATTTTTCAAAGAGAACAAGAAGCAATCGATAATGCTCAATCATTAAAAA
GAGCTATTTTAACTGAAGTAGAAAACAAAGTAAAACCAATCAACTCTGTTCCTGTTTCGT
ATGATGAAATTGATCAAAAAatctacagaacaaatcacatcatggagatagtATGAAATT
TAATTTAATTTGTTTCAATAGTGATGTTGCAAAATGGTGTGTGTCAATGGCACCTTGTAC
TAATGAATATATAACAGCAGCAGCTGATTGGTTGCGTCATATATTACCGGAAGGTTCTTG
TAATTTTCTGGGTGCTTTAATTAGTGCCTTGAAAGAACCGCTAAGTTGTAATATATGTTT
CATTGTCACACAACAATCAGATCAAAATATCAATGAGATATTGGATTTATTCGCTTACGT
TGGGCGTAAAATTCCTTGTCATATTAGGTTATGTTTACACGATAATGAAGTTTCTGAAGA
ATCGAAAAATGCTCAGACTATCTACAGCCAAGTAGCAAGAGAAACTCATGGAACGTTTGC
ATTTGTAAGATTGAATTCGAAAAATGAATTAGTCATTATGCAAAGTGCGAAAAACATTTT
GAATTCAACAGGTTTAATAACTGTCAACAGTTATGTTAATTCGAGTGAAGAAGAAAATGA
AAACATTAAAAAACTTGAACTATTACGTGATGAAGAGAAAAGAGAACTTTTCAGTCCATT
TCAGCCATTCATTCCAGTAACGGATTCTTTTTTTCTTGGTAAGCAATCCGATGATATGCA
TTATAAGCCATTCCACTTTTGGAAACGAGTTTCAGCTTCTCAATCTTACTTGGATTGTAT

GCGGAATTTCAATGAGTCCAATATTTCCAGAATGGCTCAAGAAAATGTATCAAAACTCGC
TGCATCTGTAATTGTTGGTCGGGAAGTCTTGGCGCGAAAATATACAAATGGACAATATTA
TCTTGGTTACATTAAAGATCAAGTTAACAATGAATTATTTAATGTTGTGTTTGGCCCGTA
TAATTGGGGTACCGGACAATTTGTAGCCACAGATTATCAACAAACAAAATTACTGGATTT
AATAGACTTTAATGATGCTATGAAGCGTTCTATTCTACCAGGTGATAGAGTTTTAGCTCC
AGATCCTACAAATCCTAATAAAGATGCATATGCATGTCGATATTTAACTGGGACTGTTCT
TCATGGTTCAGTAATTAGAGAGTATACGACTGCAAAATCTCATTCGACAGAAGCACATGA
TGGGAAAAATATTGACAGTCCTATTAAAACTTCGAATAGTCTTATTGTGGAATTTACTAA
TCCACTTAATAAAACAGAAAAGGTTTTAAGTGATAAAGTTATATGCATTCCAGAGCCATT
TTTCCAAAGATTGGTTTTGGAAAGTGCAATGCCAAAATCAGCCCGTGAGAAACTTTACAA
AGTGCCAAATTATCCATTGAATTCTTTGCCGGGTTATCCAACGTCAGGAAGTAAAAAAAC
ACCTGATGACTACACTTTCACCAATTTGAATGTTATGAAGAGTAGAACATTACAAGTGGA
TTACCTTATGCCTCCATATTGTTTGCCTCTTTATCCTGTTGTTGAAATATTCGATAAAGA
TGAACGTACAGGAAATGTTGTAATGAAAAGTGACTGGGCTGTCGGGAATGTCGTAAAAAA
ATCTTCAACTTTCAAGAAAGATGAAACTATGGTTCCTGAATCTATATATACCATAAGTGA
ACTACAATTAAAAGTTAAAGATCAATTAGAAACTTTTAAAAATCTACCTCAAGTGAGGAT
AAATTCAGAGAAGTCTTTCTATCGAAGGGGACTTAGTAAAAAGTTTGATAATGATGCAAA
ATTAGCCAAGTCTGTACATATCAAATCCAATGAAAAATTATTAACATCACAGAAATCCAA
TTTACAAGACAAAAAAATTCGTTTTTCACCAGAAACTGAATCAAAATTAGAAGCGAAAAG
CAAAATTGACAACAAATCAACAGTTACACCTTATGCTATTCATGAAGAATATTTCGATGA
TATACAAAAAATTCGGGAAAATATGGATTTTGATCAAGTCGAGTCGGTTAGCAGTTCTGT
TTTCGAAGTCTCAGATAACAAACAAAAAAAAGTTATTTCTTTTGCTGATGAATCTTCTAA
AGAAAAAATTGGAATTCAACCTGGAATTTTATTTCAAAACAAAATCTCTAATAAACCAAT
TGCACAAAATTCCAATGTTTTAAATATTACTGATCTCGGAGAAAAAGACAAAGAGAAAGA
ATTTAACGAAAAAATGAAATTAGAAAGACATCAGCGACAAATTCAAAAACGCCTTGATGA
AGAAAAGCATAAATTTTGTAAAGAAGAAATAGCAAAAGATAAACAGTTTTATTATCACCA
ACAAAAAACTGCTGACAGACTTCATAAAGAAAATATGATTTTTCAAAGAGAACAAGAAGC
AATCGATAATGCTCAATCATTAAAAAGAGCTATTTTAACTGAAGTAGAAAACAAAGTAAA
ACCAATCAACTCTGTTCCTGTTTCGTATGATGAAATTGATCAAAAA
>DjScaffold256:10755-19947 strand:+
aattttggtaaatatttttagaattaattattcaaaATGTCTATTTCTGATCCCTTACTG
AAGTCATTAAAAAAATTAATAATCGACTTATGTGTTGTTGGTTCAAGTGAAAATATTGTA
ATAACTAAAGAAGAAATTTTGGATGGAAATAAAGCATTAGGAAATTTATGTGAAACAATT
GAAGATATTCTTCGTAAAGGTCTTTTTAGAGTTGGAGGATTACTTAGCTTAGAGCATCGT
GATTATTGGTCTTGGTTTAATGACCTTCCAAAACTTTTAAAACAGCAAGAAAATCAAAAT
CCAAATATGAAATACAAGTTACATCACCGATATGGTGATTGTTTAGACATCGTTAAAAAA
TATTCTGATAAAGTCGATACAATTCAAGGTAAAGGAAGATTAATGATAAGAGCCTTAATT
CAAAATAAAATCCTACACTGGCCGTTTGAGTGTTTATGCTTTACTTTGAATAATGTTTCT
CGATATTATTCCGAATTTAACTCTCTTTTAAGCAATGAAATTCTTGCACAAATGCTTATT
TCACAATTGTCACAATTAGCAGTATTAAATTTTAAATTGAATTTGAAAAATGCAATGTTT
CTCGATGAAACCTGGACAATTCCTTGCTATGAAACTTATGAATTCGTTCCATGCAAACAG
CTGGGAGTAACTTTACTATCCGCTGATGCATATCAAATTGTTGAAGCAGTGAAAAAAGGT
AGTGTAGCAGAAGAAGAGAAGTACTTCCAGCCCGGTGATGTACTTGATGAATTATTTGGT
TGTTCATTGAAATGGTTCAAAACACCTCTGAAAGATCTCATGAAAGCTCATGAACACAAA
CCAGTTCGTTTGACTGTAGTAAAATTACGATTGAAAGATGGTTCTTTATTCCCACCGATT
TTAAATTTGTTAAGAAAATATGATTTAATTCATATGATTGAAGGACGTGAAATGAATCTG
AGCATTCCA
>DjScaffold931:21298-25531 strand:+
atgatATGCATATCATTCTGAAATGTGTTGAAGAACATGATATTTCCTATAGTGATAATT
TGCATTATTTAACTTGTGACAAAAATGATATTGTAATATGTGACTCCTTTGTTATTTTAA
AAGATCAAACTTTTGGATTCGGTAAAAATATCTCTAAAAACATGTGTGGTTTATTTTTGA
TAAATCATTTTGCAATGATTGATTCGACAAATGTTGTTAATAGAGAATTATTGTCAATCG
TACTAAAAAATCAATCAAATACTATTCAAAATCGAAGCAAAGATACTCCTGGAATATCCC
ATCGTATAGCAAAAAAAAGCGTTGGTGTTATTTCATTATGCTTTAATTGTGGTGAAAATC
TAATGGGGCCACGACAAGATTTTATTCACTGTACAGTTTGTAATCAGATTTTCCACGAAA
TGTGTTATGAAAATCACAAATCTATTAAAACGTGCCATAGAATTCTGAAAAATACAAATA
TTGAATGTGCTGCATCTTTCAAGGCAGCAAATGTGTTAGTGTCTGATTGGTCTAATAAAG

AAGTTGTACAGTGGTTGTGTTCCGTAAATTTTTTTCGACTCATCGATTTATTTGAAAATA
AAAATATAACTGGGAAAGATATAGTTGCACAAATGCCGAAATATTCGGATGAATTAAATA
ATATAGAAATTTCTGGTTTAACATTTTGGACACAAATAGCTGATTCATTCTATCGGCTAG
CTCTTTATGAAGCATTTAAAGTAAAGTTTTTAAACTTTATTATTTAT
>DjScaffold931:44102-45458 strand:+
AGTTCAATATTTGGTGTCAGCATTGGTGAACAGAAATTGTGTTCTAATGGTGTTCCATTG
TTTATTAAATGTTGTTTTGAAGAATTGGAACGTAGATTTATTTCAAACAACAGTGGCAAG
AAACTACTAGACACGTATAGTCAAACTGCAACTACTGATGACACAATTTTTTTGAAAAAT
AAATTTTCAGATCCAATTTTTAATGGAGAATCTGATATTGTTGGTTTAACTGAAATCAGT
GATATCCATCTTGCCAAATTGATAAAATTATATTTCAAAGAACTTCCAGAACCGTTACTT
ACTCATTGCTTTTATCATAGTCTCATAGAGAGTGTAAATGAAGAGGAAGATAAAATATGT
TGTTCACGAATTCATTCAATACTGTCTGAACTTCCATCTCCAAATAAACAAACTCTTCGA
TTTTTAATGAAACATTTTGAATTAATTTGCTCAAATATTTGGGACAATTGTAATAAATTA
AAAATTGTATCAAAAAAATGGTCATTAATATTTTCAAAAACACTTATTTGGCCTCCACAA
ATTATGGTGGATCAATTTGCTATTCTTAAATCAAGACATCAAAGAATTATTCAATTAATA
TTTGAA
>DjScaffold931:61447-77019 strand:+
aaaacaatgttatattttcaaaaaatatcaattctaataatttgattaaaattaattaat
tattgatatttttgtatttgtttatttcagtttcaacttgttactttctttgtttttatt
ttgttttaaatatcgaattgatttcgtgctgcattttatttttaattgacttcatttaa
cataaaattccagatttatttcaatttctaaaaatgttttattaatttttattatgaaaa
agatttctgaattttattaaagatattttctgtaggaaagaaggagaaaataaattggtg
cgaataatttgcagggatggtctttatggatttcaggaaccgtgtcagtttgaatctgtt
ccaaaactgatcaaatattatcaatcgcataaattatcagaatataatgatcaattaaat
atcATGCTACGATTTTCATGTTCAAAAGCAAAGTCCGAAGTGAAAGGCAATGTTAATGAA
TTGTATCAACTCTCTAATGACGATCTTATTCAATTACTTATCAAAACTATGAACGATATT
TTGAAATTAAATTCCAAAGTGGAAGATGACACTAATACTTATAATGAAAATCAAAAGCAA
CTTGATGAAGTGACAAAAGTTTTGAAAGGTATCGATCGAATTCGGTCGTGGTGTAAAAGT
CATGTCATACATATTATGAATATAAACAATTCTGTTGATAAATCTGCTTGGATTGATCAT
TCTAGAAAATGTTTGGCACAGTTGAACACAAAATTGAATAATTGTGATCAAGTCACAAAT
GAAATGAATGAACGTCGCGAGAAACTCATCACTGAATCCGATGAAAAACACAGTCAATTA
CAAATGGACAGACCAGAATTAAAACGACAAAACCGATTGCAATCTCAAATTATTAGATTA
TTTAAAGATCGAGGTGTTCCAGATGAAATTTTTAGTTCAGAAACATTAGATAGCAGTTTT
GACAATTTGTGCGACAAAGAGAAACTGGCTGAATCAACAAACATGGATCATAACTACTTA
ACTTTGAACATAAGACAAGCTCTCAATGTAAATAATCCCATAAATATTAGGTCAAATTGG
TTTGTTGGAAATATTGAAAGACAGGAATCTGTCGATTTGTTAAGTAGTAAACAACATGGT
ACTTTTTTAATCAGGAAAAGTCGAGAGCCTGGAAATTATGCTTTGGATATTAATGTTAAC
GGTACAGTTCAGCATTGCAGAATCTATTGTCAAAATGGTAAATATGGATTTATTAAAAAC
GATTGTTTATTTCAAAGTTTGGAAGAACTAGTAAAAGCTTTTCATATATGTAGTTTGTCA
AAGTACAATCCGAGTTTAACCACGAATTTAACTTATCCAGTTAATATGTGAtcgatttaa
caatttcctttgatttaaatatggtttgattgatatatttagatatttttgttaatctt
gaactggttacattttatctatgctcgtttgtatataaagaggcttattatttcggatgt
ttggcttatatatatattagaatgagtacagtcataataatgagttttacaattacaatt
ttgtttttatatcatattacaatatcttatttattaaaattcaatatgcctatattttgt
ttaatgtgctcatttatagtgtgaatcatttattctttaagcactaatttggaaatcaaa
gtattttaaagtgtgattaaataaaaaatgaatttttaaaaacaatgttatattttcaaaa
aatatcaattctaataatttgattaaaattaattaattattgatatttttgtatttgttt
atttcagtttcaacttgttactttctttgtttttattttgttttaaatatcgaattgatt
tcgtgctgcattttatttttaattgacttcatttaacataaaattccagatttatttca
atttctaaaaatgttttattaatttttattatgaaaaagatttctgaattttattaaaga
tattttctgtaggaaagaaggagaaaataaattggtgcgaataatttgcagggatggtct
ttatggatttcaggaaccgtgtcagtttgaatctgttccaaaactgatcaaatattatca
atcgcataaattatcagaatataatgatcaattaaatatcATGCTACGATTTTCATGTTC
AAAAGCAAAGTCCGAAGTGAAAGGCAATGTTAATGAATTGTATCAACTCTCTAATGACGA
TCTTATTCAATTACTTATCAAAACTATGAACGATATTTTGAAATTAAATTCCAAAGTGGA
AGATGACACTAATACTTATAATGAAAATCAAAAGCAACTTGATGAAGTGACAAAAGTTTT
GAAAGGTATCGATCGAATTCGGTCGTGGTGTAAAAGTCATGTCATACATATTATGAATAT

AAACAATTCTGTTGATAAATCTGCTTGGATTGATCATTCTAGAAAATGTTTGGCACAGTT
GAACACAAAATTGAATAATTGTGATCAAGTCACAAATGAAATGAATGAACGTCGCGAGAA
ACTCATCACTGAATCCGATGAAAAACACAGTCAATTACAAATGGACAGACCAGAATTAAA
ACGACAAAACCGATTGCAATCTCAAATTATTAGATTATTTAAAGATCGAGGTGTTCCAGA
TGAAATTTTAGTTCAGAAACATTAGATAGCAGTTTTGACAATTTGTGCGACAACATAAG
ACAAGCTCTCAATGTAAATAATCCCATAAATATTAGGTCAAATTGGTTTGTTGGAAATAT
TGAAAGACAGGAATCTGTCGATTTGTTAAGTAGTAAACAACATGGTACTTTTTTAATCAG
GAAAAGTCGAGAGCCTGGAAATTATGCTTTGGATATTAATGTTAACGGTACAGTTCAGCA
TTGCAGAATCTATTGTCAAAATGGTAAATATGGATTTATTAAAAACGATTGTTTATTTCA
AAGTTTGGAAGAACTAGTAAAAGCTTTTCATATATGTAGTTTGTCAAAGTACAATCCGAG
TTTAACCACGAATTTAACTTATCCAGTTAATATGTGAtcgatttaacaatttccttttga
tttaaatatggtttgattgatatatttagatatttttgttaatcttgaactggttacatt
ttatctatgctcgtttgtatataaagaggcttattatttcggatgtttggcttatatata
tattagaatgagtacagtcataataatgagttttacaattacaattttgtttttatatca
tattacaatatcttatttattaaaattcaatatgcctatattttgtttaatgtgctcatt
tatagtgtgaatcatttattctttaagcactaatttggaaatcaaagtattttaaagtgt
gattaaataaaaatgaattttta
>DjScaffold104:25462-26263 strand:+
taatttaaaattacttttgggttcttttatttttataatttatttaaaatatttagggt
ttattaggatggtatATGGTAAAAAGTGGGCTTAAAGAAACGAATAATCAAAGTTCTATA
CCGAGAGTTTCTCAGTATCGATTGGCAGCACATTTGGGATCTGCCATAGTTTTGTATTCT
CTATTTTTATGGAATGCATTTTCACATTTAATCGAGCCGATCAATACAGCAGAATTTCCC
AAATTGAAGATGATACGTGGAATGACACACGGGGTTAAATTTTTCATCTTTCTAACGGCA
ATATCTGGTGCTTTCGTAGCGGGATTAGATGCCGGTTTAACGTATAATAGCTGGCCGAAA
ATGGCAGATCGATGGATTCCATCAGATGTGCTTTCGATGCAGCCGAAATGGATTAATTTT
TTTGAAAACCCTACTACTGTCCAGTTCCAACACCGTTGTCTAGGTCAATTTACCGGCGCA
TTGGTTTTTGCTTTATGGTTCGTTTGCCAAAGAAATAGCGCTAATTTACGGTACCCTGTT
TTATTGGCAGCCAATGCGTTGGGATTCGCAGCACTTTGTCAAATCGGTCTCGGTATTTCG
ACTCTACTGAATTACGTCCCGACTAATCTTGCCGTTGCTCATCAATCTGGCTCATTAGTA
CTTTTATCCACCGCACTTTTCTTGTCGCATTCTTTGAGATATGTTCCCAAATAAtcttgt
aatttttgaataaatttttctattttaa
>DjScaffold104:124858-130807 strand:+
gttaataacagtaataatcgaagtgtgtattttgatgaattaaaaccaaattggATGTTA
TTATCAGAAACAGATAAAGCCGTCGCTGACAGTTCGAGTTTAGCATCGTCAGGTGAAATT
GAAGATCTTTTACGACTTCTTGAAGTTCGATATGAATCCGCAGATCTTTCTTTTCGTTCA
ACTGCAAATAAATATGGAGCTGAGTCAAGGCAACCACAAGAATTTACTGATTCAGTAGAT
AATCACACTGCATTTATTGAGCTGGGTAGACGTGTTCCATCTCTTTCTACAGATCCCTTG
AGAACCATTGGAAAGAGTTGTTCAAATAATCGTTCATCAAAACATCACGAAGAGTATGAT
ATTGATGATGAGACTATGAGTGTCGATTTTGATGTTATGGAAACAGTCTTAGATTCAGCA
ACTGGAACAAACACATCTAATTTGTTTAATCCAACAACAAAACCATTAATTGATAAAACA
AGTTGGTGGTGTAGTTCTCGGTGGCGTCGTCCATTTCTTGCATCTAATTCACCTAACAGA
CGACCAGTTATAGGCAGCGAAACTATCGTTAAGCAAAAATGGAATCCTAACTCTAATTCT
GAGACTTCTATTGATCAAACTCCTTTTTCAAGTTCTTCAACCAGAAATATTTCAGAACAA
ATGCAGTACTTTCAAGTAAAATTACCTTCACTAGAAAGTGATGGCCAAGGATCAAATGTA
ACAGATGCTGCTAAAAAATCAGCCAAGAATAATCTGAAGAGATTACAAGATGCTATCAAA
GAACTTACAGGAGAGAATTATGATCATAGAAGATCGTTAATTTCAGAATCAGGACTGGAT
CTATCTAATCGAACTAATTTGTCACTAGCTAGACATAATGTTCATGGAAACGACAGTCAA
CAAAATAATCAACAAGAAATGCGTGATATCAGAAAGCGGTTGATTAAATGTGTTGATAAA
TTAAGAAATTCATCTAGCACTAATCATGAGGTTTCTAATATACCGATCTTCGATTCTACT
ACCTTTTCGAATATAAGAGATTCAGATTCAACTACTGATGGAAATGAATCACCTGATACT
GTAGCCCGGAGATCAAGGTACCTTTAGtagaggactttgaattctaaataaatcaattat
tttcagatcaacttctccctgattagcgtttaaaagttgaatatattgcctcacattatc
caacattccgttaataacagtaataatcgaagtgtgtattttgatgaattaaaaccaaat
tggATGTTATTATCAGAAACAGATAAAGCCGTCGCTGACAGTTCGAGTTTAGCATCGTCA
GGTGAAATTGAAGATCTTTTACGACTTCTTGAAGTTCGATATGAATCCGCAGATCTTTCT
TTTCGTTCAACTGCAAATAAATATGGAGCTGAGTCAAGGCAACCACAAGAATTTACTGAT
TCAGTAGATAATCACACTGCATTTATTGAGCTGGGTAGACGTGTTCCATCTCTTTCTACA

GATCCCTTGAGAACCATTGGAAAGAGTTGTTCAAATAATCGTTCATCAAAACATCACGAA
GAGTATGATATTGATGATGAGACTATGAGTGTCGATTTTGATGTTATGGAAACAGTCTTA
GATTCAGCAACTGGAACAAACACATCTAATTTGTTTAATCCAACAACAAAACCATTAATT
GATAAAACAAGTTGGTGGTGTAGTTCTCGGTGGCGTCGTCCATTTCTTGCATCTAATTCA
CCTAACAGACGACCAGTTATAGGCAGCGAAACTATCGTTAAGCAAAAATGGAATCCTAAC
TCTAATTCTGAGACTTCTATTGATCAAACTCCTTTTTCAAGTTCTTCAACCAGAAATATT
TCAGAACAAATGCAGTACTTTCAAGTAAAATTACCTTCACTAGAAAGTGATGGCCAAGGT
AAATGAacgttattatactattaa
>DjScaffold104:271310-274305 strand:+
gaaacaagaaatcatataaaaaatcctgaatctttcgggtctgattacgatttttctcaa
ATGAATTTAAATAATAGAGATTCAAGCTCAGATTGGTATGGTGATAAAAATTCGAATCAA
TATTCTGATAATATTTACCAAGATAATAATCCTGTGAAATATTCCAATGATCGTAATGGT
TATTTGGATTACACAGAGCCGGATGATGATATGTACTCAAAATCCCGAGACAGATATTTA
GCTGATACAGATTATTACAATCCAAATGCATATTATCAAAATTCTTTTGAAAAATCGATG
TATCAGAATAAAGCAAGAAAAAATAAAAAAGAAAGAGTTAATCGAATTTTTGTCCAACCG
AAACCTCAATTGGGTCAAAACAAAATTCATTCCACCAGTACTTCTGGAAATCAAATGAAA
GTGAATTCTGATGATACAGATATTAGAAATTCTCAAGAATGGAGTACTGCGGTTATCATC
CTTAGTTTGGGATTGTTAATTACAGCAGGTTTGTTGATTACCATTGGAATTAAAATGCGT
TCTTGGCGTAATAGATTGAGAAAAGGTAAAAAAGGTAAATCCCTAACAACAGATGAAGGA
GATTATCTAATAAATGGAATGTATTTATGAtccatattttctttagatgaaacattgact
attcagtgactattttgtcgtcagatattgtttaaagttcttattgttcaattttatcgt
ttttggaatgatctcttagtttgtcgcataattattatcaaatgagatatttttatactt
tgcatttgtcctgttttttgtgttatccttacatttgtttttgtctgtttatttaataa
atgtatgttctatatcaaattaactcgaacaa
>DjScaffold104:160746-160817 strand:+
ATGGAATTCAAACAAATGATAAAATTAAAAAAATTAAAGAAGCACCAATTCCAGGTTGTG
TTACGCAATTAA
>DjScaffold3220:1899-21897 strand:+
ttatttatactatttgtaatattattttactttaattcttatttcttttagtcatggatg
aagtgaaatatgacattaatagtattaaagaaaaaatcactcaattttatttcaatggta
gccatcatcaagatttagtggattttatcaatcacgataaaaccactcgtatatgttttg
atctgtatctagataataatcaatgtataaaacattttggaattactggattaataaata
aactaaaaaaacagtctgttaatgaagatgattttgtttggcttcattcgcaaatcaatc
aaagattttatgaagaaaaaagcaatttattatattttattaaattgtcagcttatatta
agctttcatttatttatcaatgtgaaaataaatcatttattcaagttttaaaagaactac
aaaatgacaatgaaaattgcgtttgtcgtaatcaaacattgattagcaacatgatagaga
ttttcagtaatcgcgacatgccggtgtttgtattgagcgttctcagtcaggttttgtata
aagaagctaacgatcttttgtctttcattcttgagacaagtacgagatttgccggtaatc
tcaatttgcaccaattgacattgaaatcaattagttatctatacaactactgcactgtaa
atggtcgctctattcccaatttacagcaattcgtaatgcactgtgttcataaacctgaat
tatttgctgatgccgttgatttactaacaaacattctcgttggtactggtctatcaaatt
ctgtgatggaatatgttatcaaatttactttgatgtgcaatcctgtcatcgagaaaatta
tccaagatcataaagaagctgttagagacaatatcgaatggaaggaacgagataataatg
caaaacaaatggtctatgctctttgtgaatatgttggatcaataatcaatttgactatag
atccatgacttggatatcttaggatatccaataatattaattatattattattattatta
ttattattattattattattattattattattattatccaaataatattaatagagc
aataagtgatatatcaataaattatttaattattaattttaatATGACGAAAAAATGTAC
GTTTAATGCTTCCATGAATTTTATGTCCAGTTTTTGTTGTGTCTACAAACTGAAACATCC
ATTCTCATATGGAGACAAAGATGTAGACATGGATGAATTTCTTGATAACAGAGACAATGT
CGGATATGCATTTGCCAGCATGAATCATGAGATGCAAGTTGATTGGTTGAAACAAAAACT
GTATTTCTATTCGCAATCAGAAAATCTCAATGTGTCGGATATTGAGACAGTACTGTATTT
CTATAGTCAAATACCAGAATTCTCTGGCGAAATATTACCATTTGAGCAATTACACAATAT
TTTTAAAATGGCATTTTTGTCAGCTGGAGATTTCCAAGAAACTTTGATTGACACTTATGT
TGGATTATTAAAAGAATTATTTCAAATACTAAAGGAAAACGATCTTAATTTACTCATTGG
ATTTCTATGGGATGTATTGAGAAATTATGAACCAGCGATGGGATCAGCAGCTTGTCTTTT
GAGTAGTGTTTATCAATCTGAGCACATTACTCTATCTGTGATCACATCTGAAAGAGAAAC
ACAAACAATAATTTTAATAAAAGAAATTATTGATTCGTCGAATTGGAGAGAATCGAATGT

AATACTTTATTCTATTGTCGGTAGACTGGCTTGTATTTGTCGGGATAATGAAGAATTGAA
CAGTTTATCAAATGCATTCATCAGTTCAATATTTGAAAAATCGACTGAGTCTATTCAACG
TATAATTAACAAAAAAGTCGATATAAATCAAAATCAACAACCAATCGAATGCAAGGACGA
ATTGATTATTTCAAATCAATTATCTTTAATTTCGAGTTTTCTCACCCCGATTAGTAAATT
TCCAAACAATTGTACACAATTCATTCAGGAAATGATTGATGCTCTTTTACAATTGTTATC
CCAGTTAGCCGATACTGTTATTTTATCAGTAGAATTTTATTCAGCGCTGCTCAAATTAGT
TCACAGTGCATTGCTCACAAAAATTATCGAAAATATTGCAATTGTACAGAAATTTTCCGA
GATTCTCATGGTTTTCATGAACACAGTTTATCGACTTAATACAGGCCAATCAAGATGCGC
CTGTTTTGACTTAATGATTGCTACTCTCAATATTGCATTCCAATTGATCGTTCCTTATCA
TCCAGAACTTGCTACTTTGATTTATAAATATTTCTCCGAACTTTTACTTGATTACACTTT
CAACAGATACAGAAACAATTACTACATGCCAGCTTTTATATCTCGTTGTATAAAATTCGT
CCATTCCACCCTGATCGAACATGGTTTGTCGACTCGAATGCGTAACAGTGATTCACTTTG
TTTTGATCACACTCCGAACACTTCTCGTCCGAATTTATCCAGTCAAAAACTGGATTGGTT
TGAGAAAAATGCCACAGTGCTTAAACAAATTAATTGGTCGAGTATATTTTTCATTCCATC
ATTCTATCTCGGTGATCTAACTCCTGATGTTAATCGAGCCTCAATAAAATTGTTGATTTC
TGCTATTAAATGCCGGTCTCAATTGCCAGATTATATCTCTCAAGCTTTAATCAATAATTT
GCAACTCGTCATTTTCAATGTTGTCAAACTGTTCAGTTTCGGTGTAACAGAATTGGGTGA
TATGGTTTCTTTGGTTACTGCATTCAGGGAGAATATGTGTGGTGAATGCGTCAAAATGTT
AGCTTTGGTAAATAACGAACCAATTGTCCAAGCAAATCAGTATCTACAAACTGTTACTAA
ACTTGTTCATTATCTTTTGACGGGTTCTAAATTAAGCAGTTCCACTATGAGAATATACAT
CAGTAACTTCCAGGAAAATGTAAAATTATTACAGCCTCCCGCTCAGTGTTTACAGTCGTA
Acttgtccaatactatttaatcaattctattgtatcataatgtgtctgtgcaatgacaaa
tggcttgaaatctttgccgtgtctctttaatgttgtactcaaattctctgcaaatagtat
ttatatagaaaccgcacgtgctctctgcaatccatctaccttgtggccattttgccgggg
aaatgttgtatttttaataaatatttgttgat
>DjScaffold2463:19115-28073 strand:+
AGTGGCCTAGATAACAAATGTACAATCTATCCTTTGGTTACTGATGAAGACCCAGTTCTC
AAGAAGCGATTAGTCGCTACTCACACCTCATATTTATCATGTTGTCAATTTAATTTGAGT
GAATATCAACTGTTGACAGCTAGTGGAGACAGCACTTGTGTTTTATGGGATGTTGAATCA
GGACAAATGATACAGAGTTTCCATGGACACTCTGGTGATGTTATGAGTTTAGATTTGTCA
CCTTCGGAATCAGGGCGGGTTTTTATATCTGGGAGTTGTGATAGATGCGTCAATGTTTGG
GATATGCGGACTGGTCAATGTGTACAAGTATATGAAGGCCACGAATCTGATGTTAATAGT
GTTCGATTTTATCCTAGTGGGGATGCATTTGCAACGGGTTCTGATGATGCCACTTGTCGA
TTATTTGATTTGCGTGCGGATAGTGAAATTTGCGTTTATAAAAAAGACAGCGTTTTATTT
GGTTGCAATGCTGTGGATTTCTCTTTAAGTGGCCGGTTATTATTTGCGGGATACAATGAT
TACATCATGAATATTTGGGATGTGCTCAAGAGCAATCGTGTTGCAATATTAAGTGGGCAC
GAAAACAGAATATCTTGTTTGAAGACAAGTCCCGATGGAACAGCTGTATGTACCGGCAGC
TGGGATTCAACGTTGCGGGTATTTATTTTATTTATATAA
>DjScaffold1706:17241-18090 strand:+
gtaaggatacttttgaattcgtaaattgcttaaaaaataaacaactgATGTCAGATAAGG
AAATTATGGTTTCATTTGATATAGTTAGTTTGCTCACAAACGTTCTTAGAAATGAGACTA
TCAATATTTGTGTTGATCTCTGGGAAAATCATTATGCAAATTTAAACCATAAATTTGATA
AAACAGCTATGAAAGAACTGTTGACTTTTTGCTGTAAAGATGTTAATTGTTTATTTAATA
ATGATTGGTTCACTGAGAAAGATGGTGTTGCAATGGGTTCTCCGTTGGCACCGACATTAG
CATCATTATTTTTGTCTACAATAGAGTCACGAATTACTAATTTTTCCGGTAATCAACTCA
TAGATACCTTTCCTTGAatatatgaatagtcttcatcaaaatatgaaattcactatagaa
gttgaaaaagaaggtaaaccaccatttttagatgtttttatttgtagacagggttgcaac
tataatacatcactttataaaaaataaacagacacaggactttataccactcctaaaagt
tttgtgatgatcgatctaagaatggattaataaaaagtttggttcatcgagtgtggtgta
tatcatcaagctttgagattgctgatgatggtcttaacaaaatta
>DjScaffold1127:358-7249 strand:+
aaatctaatttatattctttatttatttcaacagcagtttgttattaatttatattgaaa
actaatacatagttgaaaaccaaATGAAAATATTATGTATATATGATGCTTCGGGAAATG
AAACCAGAATTGAAGTTGAAAGAAGTGATAACGTAGTTGCTGTTAAAAGAAAAATTCGCC
AGGCTATTTATCTTAAAGAAACTAAAATGGAGTTATATGATGAAGGTGTAAGATTAGACA
ATAATCAATATCTTCCTGACCTTATTACAAAATTAGAATATAAAGAAATTCCTGAAAAGG
CACAGGATATAATAATGTCTGTAGCGAGAAATATTCTCCAAATTCCTGAATGGATCCAGA

TCAACAAAGATGTTCAAATTTCTGACTTGAAAGACATTATCATTGATGTGTCAAATATTA
ATAAAGACATTTCGATTTTCGATGAAGATGGTAATGAACTTGATGATAATGAAATTATCA
AGAATTATGATTTATTCAATGATTCAGAAATTAAAATATGTTTAAAAGTAGACCGTTACT
TGAATCTTCAAGTGACAAATTTATTACCAATTCAATCTACATTCCCCTTAGAATGTAGAA
TTTCCGATACAATTTTAGAAGTAAAACAAAATATTAATCGTGTTTTTGGTTTTCAAATGG
AAATGACCTTGTTTTCAGAGATCACTATCTTGGAAGATGATAAACGATTGATAGATTATG
CTGTGCAAAATAATGATTTATTATTCTACATAGAATCTAACAAAGAAGATCAAATAAAAA
TTCTTGAGCTCATCAAAAATGGAAAATTTGCCAAAGTCTATAAAGGGAAAAATTTGATAA
CTGGCTCTAACATAGTTTTGAAAAAGTACAGTTTAAAATGGAAGAAAATTCGCAATTTTA
CACCATTGAAAGATGAATTAATTACTTTGTCGAAATTTCAAAATGACGATAAAGTTGAAT
TCGTCAACTTAGACTGCGTATTTCAGACCAAGACACATTTCTTCATTGGAATGGAATATA
TTGAAGGGGGATCTCTAGATGCACGTATCCCTCTAACAGAAAATAAGATTATCGATTATT
CCAAACAAATATTGGGAGCATTGGATTATTTACACAGATCAGACATAATTTATCGGAATA
TGAAATGTGAGAATTATCATTTTCCTATTTACGCCTGTATAATGATATGTAAATTATGGA
TTCTAACAACTTTGGCAAAAATGAAAAAATTAAAATATGAATTTTTCAATTTTAAATGAa
catttattg
>DjScaffold920:49004-49480 strand:+
aagattcagattggtttgaagcatcagaagtcgcagaaaaacttgttgacaaatattata
attctATGGATAAAAGCCGTAGTACTATTAATTTGTATTTTACTCCAAAGGGTACGTTGA
CTTGGAATGGTCATCAAATAGATGCTACAGAAATTCAGAAATTTTACGATAATTTACCGG
GTTCAAAGACTCAGATCTTATCGGTTTCTGCTCAACCTATTAGTCCTATCGTATCTCCTC
AATTTAAGTCTGTTGCTATTTTTACTGTTGGAAAAATAAATTATGACAATCAATCTAAAA
GCAAAATTTTCAACGAAAACCTTATTGTTATGAAGGATACAAATGATACTTTATGGAAAA
TTATTTCTTCAACTTTTCGTTTTGTGAGTGAATCTTGAatatatatttttaatgaatttt
gtttgcttat
>DjScaffold1242:20604-21239 strand:+
tattttcagggaaatacggtatagattctttatattatataatactaaatccgattcata
accttcaaactctttaaattgtaatgaactattttcatattttttaattataattttcaa
tgattatttgtttataatattattcttgtttgtagttgccacgatcattgtaatcattat
tctgttagtcattttattttctgggttaatttggttttactgcttttatcaacggcaaaa
gcctatattagacgagaaatcacacgaaataaacgattattcatttttactaaataaaaa
tgacaaacctgacaaaattaaaccaaaacttgatggaaaaccaagtggactaaaaaataa
ttcaacagaatgttacATGAACTCAATGATCCAAGCTGTAGCATCAAGTGATAAATTAAT
TAAAGAATTCAAAAAAATTCAATTGAAAAACTCAGAATTTGAATCTTTGAATTGTTTTGT
CGAACTCCTGCAAGGTGTAAATACATCTGATGATTCTTATAAACATTTATGTAAAAATAC
TGATCTACTTGGACAACTGATCAGATCTGTCAATTGGAAAAAAATATCTCAACAAGATGC
ATTTGAATTGTTTTCTGCACTTGTCAATGGCATTCATCAAGATTTCAAGTTACTCAATAT
AGAAAATTTTGAAATGCCGATGAAATTGAAAATGGTTTATCAATTTAAAGGTATAAACAT
TCCAAATAAACTTCAATATAACATTGGTCTATCACTGGGATTATTTGGAGAATATTCAAC
CAAACCTGGAGTTTCAATAGAAAATCTTATTCATCAATATTTTAAACCAACAATCATTGA
TGATGATTCAAATAAAACATCTCAAATAACTCTATATCGTACAGCTCCAGAGGTTTTACT
GCTTCACATTCAAGCAAATAAATGGGATAATGATAGAGGAGAAATGATCAAACCAAAATA
TCATATAAAATATGATCAAAAAAATGGAATTATGTTTAAACATTGTGAATCAAATAGATGA
TGATGAATTTAAACAAATCACAGTGAATTATGAAATCAAAGCTGTTGTACAGCACATTGG
AAACATTGGTGGAGGACATTATGTGACTTACAGAAACTGGAATGATAAATGGTTTTTAGT
AAATGATGAACATGTTGAATTGATAAAAATTTAATAATGATATTTGTCCATATATTCTGAT
CTATGAGAAATATTGAatttgttaattaaatttgttaaattctttttgtagtttatcga
tcttttcagtagttcctaatatatatttacaagcaatgtttcaatgttcatttttaatcg
cttctttCTCAATTCATATATCTTTATAATTGTTTATATAAATATAGCCAAACCTGGAGT
TTCAATAGAAAATCTTATTCATCAATATTTTAAACCAACAATCATTGATGATGATTCAAA
TAAAACATCTCAAATAACTCTATATCGTACAGCTCCAGAGGTTTTACTGCTTCACATTCA
AGCAAATAAATGGGATAATGATAGAGGAGAAATGATCAAACCAAAATATCATATAAAATA
TGATCAAAAAAATGGAATTATGTTTAAACATTGTGAATCAAATAGATGATGATGAATTTAA
ACAAATCACAGTGAATTATGAAATCAAAGCTGTTGTACAGCACATTGGAAACATTGGTGG
AGGACATTATGTGACTTACAGAAACTGGAATGATAAATGGTTTTTAGTAAATGATGAACA
TGTTGAATTGATAAAAATTTAATAATGATATTTGTCCATATATTCTGATCTATGAGAAATA
TTGAatttgttaattaaatttgttaaattctttttgtagtttatcgatcttttcagtag

ttcctaatatatatttacaagcaatgtttcaatgttcatttttaatcgcttcttt
>DjScaffold1242:25542-26012 strand:+
ATGAAAAAACGGAGATAAAATCTTTTCGACACATAATAAGTGAGAGAGGAATTCAAACA
GATCCATTAAAAGTAGAAGCAATCAAATCATTTGGAGAGCTAAAATGTGTGAAGAAACTA
CCAAGTTTTCTTGGCATAAGCAACTACTACAGGAAGTTTATTAACGATTATGCGATGAAG
GCTAGAAAACTGGAACAATTATGCAGTGGCCAGGGAAACACAAAATTGGTTTGGTCTGCA
GAGTTGTCGGAGATTTTCGAAGGATTGAAAACAGCACTAACCACAACACCAGTGCTGGGG
TTCGCAGATTTACGGAGAGAATTCATCTTAGACACGGATGCAAGCTTTAGCACGGTCGGG
GCAGTGTTGGCACAAAAAGACGAATATGGGAAAGAAAGAGTGATTGCGTACGGTTCACAC
GCGATGTCCAATCATGAGAAGGGTTATTGTACAACTAGGAAAAATTGCTAG
>DjScaffold1528:9546-10217 strand:+
taacctttattttttaaacaaaactcttgataaattttattataataattaataataat
acctaacatcatcttcaaagaaagtgcagggtagatatcaataatcatagagaaaaattt
ttttgcaacgttgaggcaaaatttggaaggcaaatacagataacgattgagacatgcaaa
ggagattctagaaattaacaaagatcatagattgactgcgattattcagaataatgagta
tgtatttATGGCGAATAAATGGGACATTGGATGTACACACTTAACAAAGCATCGTATATT
CACTGAAGAAGGTCCAATCAACATGAAACGGTATCACCAACCAATTAATCTAGAGGAGAA
GATTGAAGTGGCCATCAAGAATCTATATGATAATGGTATCATCTGGAAGTGTAATTTACA
ACATAATATGCCATTCATCTGTGTTAGGAAAAAGGAAAGAAAGGATATACAAATATGCTT
AGACTTTAGACTGCTCAATGCAATAACAGAAAGACAAATTTTTTCTATGCCAAATGTGAA
CTAAatgctaaatggtttggaatgtacgcagtattttagctcaatagacttaggaaatgc
atactaccaagtgaaattcagtgaagattctcaagaaaagacggccttttcg
>DjScaffold1528:136968-138803 strand:+
gaaagtgtttccgagtcacctttctgctgttcaaaggattgcagctgctATGCCAAAGCA
ACCATCAAACAGATTTATTTATGAAGATGCAGAAGAACTATTCCAGTCTCTACAGGCAAA
TGTTTCTATCGGTCATGATACTTTAACTGATTTGCAACCTACTATTCCTCTTAGTACTTT
TAAATTTCAATCATTTGACAACAATGAAAAATCTTCAACCAAAGATGTTTATCAAAATGT
TTTTAATATATCTGTTGACACACCAACAAAGTCTACTTCCAATAATGCTTCAAATGGCTT
TAATCGATTAAATTCCAATATTCCTACTAGACCTAATCAAAATCGATCAGTCATTTATAA
TGTGAACAATAACCAATTTGGAAGCTTTTCAGATCCAAGACAAACGTATTTAAATGTGCA
ATCGATACCAAAATAAtttttatttcatattttgtctcaaatgtcttcaacttttgtct
ctcgtaccgtgcattttcagtctttaataattatttactttggatagttttccttccgtt
gttttatttttgctaatgccgtatattttctgatttcctgagccttaaatccacatattc
agcgttaaacacaagttagacttataatcaatccgagtcaattttattattatcattatt
actattatgaacatcatcattggtatttttgtccttaaaacaatagtaataatacagatt
tatttaactaataagaaactcctatatttattattacatcatgagaggcattaaatgttg
aaatagaataacgcttattattattatgattaatattgttattgaatttattaattgaag
aaatcaatatttgaatattttcagattgtttttccattgttattatttgtaaataaaatt
ataatttattcattattttattaatgtgatatgtctactc
>DjScaffold1528:145208-160988 strand:+
ATGGCGTTAGGGTCTAGTGCTCCAGAAATTCTTCTATCAGTTATTGAAACAATTGGAAAA
AACTATGAATCAGGTGAATTAGGTCCAGGTACAATTGTTGGTTCAGCCGCTTATAATCTC
TTCGTAATTATCGGAATTTGTGTTCTTATTATTCCAGATGGAGAAGTGAAAAAAATAAAA
CATCTTAGTGTTTTCATTTTAACCGGTTTATGGTCAATTTTTGCTTATTTATGGTTGTAT
CTGATTCTAGCGAAAATCTCAGTTGGAGTAGTAGAAATCTGGGAAGCCGTTCTGACGTTT
GCATTCTTTCCACTTACTGTTTTTACTGCTTATATAGCTGATACAAAACTCTTTTTCAAT
AAATTTTTAAAGCGTAGAATTTTACAGAGGAATATCAAAACGTTTACTAAAGATGGAAAA
GATATCGAAAATAAACTAGCTTTTAGAAACAAAAATAATTTAGAAAATGGAGTAAGGTTT
TCAAATGATATCTATGAAGATGTTGACAATGATAACTATGGAGAGTTTGAAAAGCATAGA
TTGGAATATTTAGACATAATCAAAGAAATAAGACTACAGCAACCAAATATTGACAGTAGA
AAACTTGAAGAATTAGCTCAGTACGAAATTTTAAAACGAAGTTCGAAAAGTCGAGCATTT
TATCGAATTCAAGCGACTCGAGTACTGACTGGAAGTAAATGCATAATTCGAAAATCTAAT
ATTGAATCAGATTTCCAGAAAGATTTTGAGAAATTATTAGATAAACCAAAACAACAAGAA
AAAACTATTCAAGATTTATTTTTTGATCCGGGTCATTACACGGTTTTTGAAAATGTAGGC
ACCATGCAAGTTTCATTAAAACGAACTGGACCACTTATTGATTGTACATATGCTGAAGTC
GAATTTTATTCTGAAGATGGAAATGCTACCGCTAATGAAGATTATATTCCTGTAAAAGGA
AAATTAGTTTTTCTACCTGGCCAAAGTGATACTACAATAAATATTACAATTATTGACGAT

GACGTTTTTGAGGAAGACGAACACTTTTTTATTCACTTAAGAAACGTTCTTGTATACAAG
ATTTCAGAGGAAAATTTTGTAACACGGGAAGTATTTCCATCACAGCTATCTAATCCGTCT
ACGGCAACCATAATGATTCTCGACGATGATCATCCTGGAGTTTTCAATTTTGGATTTTCA
TCTATAACAGTTCCGGAAACCATTGGTCTTTTAGAAATAAAAGTTTACAGATCATCTGGA
GCTCGTGGTCACATAAAGGTACCCTATAAAACAATTGAAAACACTGCAACTGGAGGAGGC
CATGATTTTGAAGATGTATTTGGAGTTTTAGATTTTTATAATGATCAAACCGAAGCTACT
ATTTCAGTGAAAATTATCGATGATACAGAATATGAAAAAAATAAATCATTTTTTATTGAA
CTTGGTCAGCCAATTCTATTGGATAAAACATCTGATCGAAAAAGTTCTATTCCATTCAAT
TTGATTGAGGATTTTAGTAAACGGTTGTCATTTAGTCGTCGAAATTCGAAATCAAATCAG
ACTAGTTTGTTGGAGATTCCTAAAACCATTATTAATATTGAACAACCTGAAAATCAGTGT
AAACCACAAATTGGAGACAAAAATAAATTAGAAATTTGTATTCGTGAAAGTCAAGAATTG
AAAGTACATTTTCTCTTTTATTTTTATATTAGTATTACTAGGAAATGTTAA
>DjScaffold1528:78519-78842 strand:+
ATGGGTTTTAATCAAATAAGAAATTCAGGTTTGAAATTAAGTTCTGATAAATGTGTATAT
ATGAAAAGTGAGATTAAGTTTCTGGGCTATATCATTTCAAAAGATGGTTTAAAAATCAAT
CCAGAAAAGATAAAAGCAATTAAAGAATTTGAACAACCTAAGTGTTTGAAAGAACTTCGT
GGATTTTTAGGGCTTACAAGTTATTACAGAAAATTTATTAAAGATTATTCAAAGCACTCG
AGGATATTAGAAAGTTTATGTGGAAACAATAAAAATAAATTAATTTGGTCAGATCAATGT
AAACAAAGTTTTAAAGCTATAAAA
>DjScaffold3938:8645-21895 strand:+
agtgaagaATGCCAGGAGGAGCAGAATTTAGTGCACCACTGCAAAAGTTTAAATTAGTTT
TTCTTGGTGAACAAAGCGTGGGGAAAACCTCTTTAATTACTCGATTTATGTATGACAGTT
TTGATAGTACGTATCAAGCAACTATTGGTATAGATTTTCTTTCAAAAACAATGTATTTAG
AAGATCGAACGATACGATTACAATTATGGGATACTGCTGGACAAGAACGATTCCGTAGTT
TGATACCGAGTTATATTCGTGATTCATCCGTTGCAGTTGTTGTGTATGATATAACAAATG
ATAATTCTTTCAATCAAACCACGAAATGGATTGATGATGTTAGAACTGAACGGGGTACTG
ATGTGATAATAATGTTAGTTGGTAACAAAACAGATCTGGTTGACAAGCGGAAAGTGTCAA
CAGAAGAAGGAGAAAAAAAAGCTCGAGAATTGAGTGTGATGTTCATAGAAACAAGTGCTA
AAGCTGGGTACAATGTTAAACAATTATTTCGAAGAATTGCAACCGAATTACTAAGCAGAG
AAAAACCAGCAATGCCACACGAGGACTTGGTTGAAGTACGATTGAAAGATCATGCCAATG
AACCGGCAAACAATTCTAGTAATTGTTGGTGTTGAaacatcgaagaaattattaactgtc
ctaccattttattagtcgaataataatattgatgccggtttaacatgttttgttactttt
catttgaaaaatgtactttaagcattttattggttattccttgatattttatgatatatt
tgcaagaggtgatattgaaaagttattcgttcatttagggttcatttgtaaaatttaatt
tgaaaaactgaatgaatttgagttttaatttaaaattgaagttgtttatttctataattt
acattttttttataaataaatcgttattgaatgtttctccaggaaacattactaaactat
ctgctcaaataaatagtgatttgaagctattaattcattaactacgatacttttaattta
tgttaattatcgtggcacatgaattccaatcttgttaaactattgaaaatcggctaattt
tctttcctgatattaagtattgcttatttgtattgatagctgttaataatttttatatta
ccagtgatcgatgatgatgatgatgtgataccaatttaattttggtatgacagtaatgt
>DjScaffold1285:31575-31937 strand:+
ttgtgaactgcaccagcagctttccaaagtttgATGCATCAATTATTCGAAGGAATGTTG
TATAAAGGAGTACTAGTATACTTGGATGATATTTTGATACTTGGACGAACTCAACAAGAG
CATGGTGAAAGGATTGGTAAAGTTTTTTCTTTACTCAAACAAGCAGGTTTTAAGATAAAT
CCAGATAAAAGCGTATTCAATTCTGAGAATTGCGATTCTTTGGTCATAAAATTAGTGCTA
AAGGTACACCAAAATTCACCACACAAATACGTGCATTTTTAGgtctaaccaactcttaca
ggtggtttataaaagatt
>DjScaffold1285:32083-36645 strand:+
ttggatacagatgctagttttagggcaattggatgagttttaagccaagttacaaaggat
ggattagaacgagtaatagcttatggttctcgcacattaaATGGTCATGAAAATGATATT
GCGTAACTCGCAAGGAGTTGTTGGCAATCCACGAGTTCATGGAGTTGTTGGCAATCCACG
AGTTCATGGTGTAATTAGAGCAAAAACAGATCATAAAGCTCTGACATTTATGACGAAGAC
AAGTAAACCTATTTGTTCGCAGTTCCAAATATGGATAACAAATTTAGCAGAATTTATTTT
CGATATAGAATTCAGAAAACGAGCAGCACATGGGAATTCCGATGGACTTTCAAGAATAGG
CGAAAGCTGGTGTTTACAATGTCAGACGTCACATCAAAATGCCAAGGGAGAACGAGCTCG
GGTTAAAAGAATCAATGTATTAGATAAAACAAATTTTGATAAATGTCGTATCAAAGAAAT
ACAAAGAAATGATCTTGTTACAAGAGAAATTATTGAAAAAGAGAAAGGTGATAGTAAGCT

GAGCGCT
>DjScaffold4509:9771-29836 strand:+
aaataaaatattcgcctttagataatgatctagaaacaagaaagcggtttagttttttaa
cggatacttcaactctattttattattgtgcccttatttctgcttggattcatctcataa
tcttgattctgttaattctaagagtgaaaagtattttggaatttactgatctactttaag
tcaaattagatgatagaagctggtttttcttgATGGGTATTTATCTGATTGTACTGGATC
CTTCAAATCTCATTTTGACTGCTGTTTTTGGATTTTTATCGTCCGGAATAGTATTACTGT
TTGGATCAGTTATCGGTTCGTGGACTGACAGATCATCAAGATTGAAAGCGGCTGTAGTTG
TTCTAATCTGTCGAAACATTAGCACCTTGATTTGCTCAGTTTGTCTATGTGTAGTTTTAT
ACTATTATGATTGGATAATAATAACTGACAAATCACCGATTCTTCGTTACATTTCTCAAT
CTTTATTGGTTATATTCGGAGTTATCGCGCATACAGCTGGTTTAGTCAACAATAATTTAT
TAAGCAAGGATTGGCTGCCTGTTTTATGTTTAGATGATTCTTCATTGTTTTCAACCATGA
CAGCGAGATTTCGATTAATCGATTTAGTTTCGAAAATAATTGCTCCAGTAATCATTGGAC
AAATAATCTACTTTCAAGTTCTGTTGACTCTTCTGTTATTTAGTTTTTTGTTGAAAACTG
TTTATAACAGAATGCCTCGATTGCATTTCAGAGAAGGTTTATCCAATATAAAAGACAAAT
TGGAAGAAACTATACCAATTCTGTCTAATGAGGTATTATTTTTGTAAatatataattgta
aaatttatt
>DjScaffold996:200032-200202 strand:+
ATGCCGTTTGACATAGTTGCAGCGCCTGGGATATTCCAGGAACTCATGACAAAGGTTTTA
GGTACAACAAATGGAACAACGGTGTATTTAGACGATATTCTAATATATTCAGAAAACTTG
GAAGAGCATTACAAACAGTGGATGAAGTATTTAGTAAAATACATCAAGTAG
>DjScaffold3529:588-1267 strand:+
AGAAAAAAGGAAATTGTTGCGTTTGCAATTAGTAAAGATAGTTCATCATTTTATAAGGAG
CAAATATTTGATCAATATAATAGTAATTATAAAACCTCTACTTCAACATTTCAACATTTA
AATGCTACTTCCATATCTTTATCGAAAAACATTGATTATCGAGAGAAATATATTCAAGAT
TTTGAAAACGATGATTATAGAACAACATATATGCAAAGTGTTCTTCGAAATATGGGATAT
TCGGATAAGAATATGAATTGTGGAATAACACTTGAAGAATTTAAAAGTAAATATCCGTTT
TTTACATTTGTGCTTGCTCCAGATTTCGATATTAATCAGAGTCAATATCAGAGCCAACAA
TCACCAAGGAAATGTGAGATTAGATATAAAGTTTGCTAAagctttggctgcgccagcata
tgttataatatatggagtatttgaaagcgaaatacaaataaatgctaatagaacagtact
agtgtaaacaatatctaatataatcaaataatcaaaattatgaagtaagttttgtattta
atttcaatatttcactttattagttcttttcagattaatcaaatcggatcaaaattgatg
gaatcgtcatggttgggggtatatccattaga
>DjScaffold3529:70635-75188 strand:+
tgatttgataataatatcaatttaccatgaaaatatagcttttctggacgttgtatacat
taattcaaatattttcaaaatataaaaaagcatgaaaatgtatttttcatcatgttagat
tggctaatgctgtattaaaattacctgtaaatttaattgtaaatgtattattattttaat
aattaatttttgaaagattacgaaaatattATGAGTGTTGTAAATCATTTCGATTTGGTT
TCTCATCATGTTCGCAGCAAACTCGATGAAATCAAGCGAATTGAATTGAATAGATTAAAC
AAAATGGAAGCAAGACTAGAAAAACTTCAGAAAACAAGTAGTCTTTCACTGAATCCGATT
GATAAAGATCACATAAATATACAAAATGATAACAAATTTGAAGAAGAAGATCTTCGTCGT
TTAATAGCAAAGACTGTTGAAATTGCTAATAAATTGGATGCTGAAGCTGAAGAAGAATAT
CGCCGGTATCAAATGGAAAAAGAAATTAAACGAAGGTTAAGACTATCGAAAATTAAATCA
AAAGAAATGAGGGAAAAACAAAGATTATTGGATGAAGCTGTAAAACAAAGACACAAAAGT
CATCCAGCAATACATTATCCTGGCACTGAAGAACAATTAAAAGAAGTTTGGGAAAAAGAA
GACAAATTGGATCCCCGTGCTTTTAATCCAGTAACGTTTTTCAGGCTCCATGATACAAAT
GAAGATGGACATTTAGATATTGCCGAAATTGAAGCCATAATGCAAACTGAGCTTAATAAA
GTTTATGAAGTTAATAATACTGACGATGATGTTGTAGAAATGTACAACGAAATGCTGAGA
ATGCGTAAAGAAGTATTTAAAGAAGCTGATACTAATAATGATCGAATGATTTCTTTTCCT
GAATTTATAAGATGGACGAAGTCTGATGAATTTAAAAATGAAAAGGATTGGAAGGTAAGG
TTTCACATAAATCATTTACGTAAATTTCTTCAATTTTTGTTCATGATTCATATAATTATT
TAActatattgtatttattgtataaatcatgcgagacaatagtaatttataatatattat
tacatctgaccgatgtaataacagaaatacttttgaattttactgctcacgttttaaacg
taattctttactaatacagattattaaattctaaaacctgattttctatttattatggaa
ctcacaagattttcggaaatagttctttttaaatattcaataatttcgtagaataaatac
tgatttgataataatatcaatttaccatgaaaatatagcttttctggacgttgtatacat
taattcaaatattttcaaaatataaaaaagcatgaaaatgtatttttcatcatgttagat

tggctaatgctgtattaaaattacctgtaaatttaattgtaaatgtattattattttaat
aattaatttttgaaagattacgaaaattattgaaaaaacattttgacttagattATGAGT
GTTGTAAATCATTTCGATTTGGTTTCTCATCATGTTCGCAGCAAACTCGATGAAATCAAG
CGAATTGAATTGAATAGATTAAACAAAATGGAAGCAAGACTAGAAAAACTTCAGAAAACA
AGTAGTCTTTCACTGAATCCGATTGATAAAGATCACATAAATATACAAAATGATAACAAA
TTTGAAGAAGAAGATCTTCGTCGTTTAATAGCAAAGACTGTTGAAATTGCTAATAAATTG
GATGCTGAAGCTGAAGAAGAATATCGCCGGTATCAAATGGAAAAAGAAATTAAACGAAGG
TTAAGACTATCGAAAATTAAATCAAAAGAAATGAGGGAAAAACAAAGATTATTGGATGAA
GCTGTAAAACAAAGACACAAAAGTCATCCAGCAATACATTATCCTGGCACTGAAGAACAA
TTAAAAGAAGTTTGGGAAAAAGAAGACAAATTGGATCCCCGTGCTTTTAATCCAGTAACG
TTTTTCAGGCTCCATGATACAAATGAAGATGGACATTTAGATATTGCCGAAATTGAAGCC
ATAATGCAAACTGAGCTTAATAAAGTTTATGAAGTTAATAATACTGACGATGATGTTGTA
GAAATGTACAACGAAATGCTGAGAATGCGTAAAGAAGTATTTAAAGAAGCTGATACTAAT
AATGATCGAATGATTTCTTTTCCTGAATTTATAAGATGGACGAAGTCTGATGAATTTAAA
AATGAAAAGGATTGGAAGGTAAGGTTTCACATAAATCATTTACGTAAATTTCTTCAATTT
TTGTTCATGATTCATATAATTATTTAActatattgtatttattgtataaatcatgcgaga
caatagtaatttataatatattattacatctgaccgatgtaataacagaaatacttttga
attttactgctcacgttttaaacgtaattctttactaatacagattattaaattctaaaa
cctgattttctatttattatggaactcacaagattttcggaaatagttctttttaaatat
tcaataatttcgtagaataaata
>DjScaffold3529:32247-32297 strand:+
ATGACCTTCTTATCTTTGGAAAAGATTTTGAAGAACATAACGAGAGACTAA
>DjScaffold3058:26468-27415 strand:+
attagaattaattaaatattcaatttaatcaatttctattggctgctatatttaaataat
agttatttccttataataacaatttcaatttaaatttttgattaatttgcaagagaatta
tgaattagatttactttatttcaaatccttggtcacaatcttgattatgaattcttatgt
ttcaaggaatatccaaaataaaactattgttgaatgaatattcaaaaaccataataataa
cagtaaataaaattaaaaattttgatttacgatttaacgaaactattgttaaaagctca
gttaacttacaaattcattgacaattttacaattcgtttattattaaaatattttttta
attttgcaaataaaaaattttagaatttcgcagaaattATGGAAATGGCTTTCAAAGGAAA
TAGAGAAAATGTTGACACACAAGTTTTTGAAGTATTATCGGATGATAAAAACTCTCCATA
TGGAAAATTTCCACCAAATTTACCAATATTTTCCTTTGGTAAAAGTGCAGACTTTGATAA
AGGGAAAGAAGATTTTTCCAAAAAATGACCTAATGGAATCATTGAGTTGTCTTATATTTAA
TGAGATACTTACTACATCACATCTTTTATGTAAGATTCACAAAGTACAAACGATTTACTT
TTGCGGAGATATCTTTAATCAGCCAAGTTTTTCAAAGATAGTACAAATTTCTGAAAAAAT
TCTTCCAAGAAATGTTAATATGAAAATTCTTAACTGTGAATATATTTCTGCATTAGGAGC
TATTAGAAAATAAatttgaaaaaccaagagttttttagaatataaacaatttacattgt
ttttttgaaactattaattaaaatatgtttaatcttttgaaaaaaaataatgtttacgaa
aaatttta
>DjScaffold3058:35554-36213 strand:+
GCAATTGACAGTGGAATTTCGAGATCAAAAATCGTTTTCATTTTACGTGAAGAATCTCAA
CAAAAATCTTTACGTTCATTATCAATTATTAATGAAGAATTAGAAAATTATCTTCGACAA
CTAAATTCGATTTTAAAATTTGAATCGATTGATAAAATTTATTTAACTGGTTTCAAAGTA
ATGTGGGTTAAAGAAATTGTCAAATCGATATTTAACATTGATGCAATCGCTATTGATGAA
ACAATCGCTTTATCCAATGGAATCGATTTGATTTCTAATAATTTGAAATTATCTGATTTG
GAATTTGAGATCGATGAAAAATCTTTGGATTTAGATAAATATAAAATCGATATAAACAAT
CCAATGATTGCTCAAGTAAGTAAACGCTTTTTTTTTTATAAAATACTTAATAAATTTTTT
ATGTTTAATTATTTTACAATTATTATTATTATTATTATTCATTAAattaaaaagctaaaa
ttaaaatgaattccttgaaagttgaaaattcgaaatataaacaactaaaaaaaaatattga
aaaaaaccagattttgatggaaacttttaatttaacgaaatgtttaattatattcaaatc
aaaatgtt
>DjScaffold3058:60129-67878 strand:+
atttaattcaattatatcgtagttggcaattttaattccgatttcaatttaagaATGAAT
ATATTTATTGATTCATCAAGAAAATTGCAAAGGTATAGTAAAATTAGATTTCTTGGCGAA
GGTCAATTTGCTAACGTTTATCTGGCCAAAGATACTGAAAATAACAATAAATATGTCGCC
ATAAAGAAAATTAAAAGTGGATCAAAGTACGATGTACAAGACGGTATTAACAGATCGGCT
TTGCGAGAAATTAAATTGTTAAGAGAAATGGAGCATCCGAATATTATAAAATTACTAGAT

GTATTTGGTGAAGAAAATAATATCAATTTGGTTTTTGAATATATGCTGACAGATTTAGAA
GAAGTTATTAAGGGAACTCCGGTTTTTGAAGTGAAACACATAAAAAATTTATCCATTCAA
ATTCTACAAGGAATGGAATATTTGCATTACCATTTCATTTTGCATCGTGATTTGAAACCG
AATAATCTATTTCTCAGTGATAAAGGACATGTGAAAATCGGTGATTTCGGTTTAGCACGA
CATATCACAGATGATCCCGATCGTCCTATGACAACTCAAGTAGTTACTCGTTGGTATCGA
TCACCGGAATTATTACTCGCGGTTCCTCTCTATTGTTACGGAGTTGATATGTGGGCGATC
GGATGTATTATTGCGGAAATTTGTTTAAGACGACCATTTTTCCCAGGAGATAGTGATTTA
CAGCAAATATCATTGATCTTTGAGGTTATTGGTGTGCCAGATAACAACAATTGGCCGGAT
TATAAGTATTTTGAAAATAAAATGGCTTTTACTCAAACCACTCCAACTCCGTTTCAACAG
ATTTTCACAGCTATTCCCGACGATTTGATAAATCTTCTTTATAGACTTCTATCACTTGAT
CCTCTCAGGCGGGGTACTTGTACTGAAGCTTTGAAACATCCTTTCTTTGTCAATGAACCT
TATCCTTGTGACGATAGCGAACTGTTGACATTGAAGCAGCAACCAATTGAGAGTTTATCT
CCTCTCAATACTAAACGACGCAAAACTGATTTGATAAATTACTAAgattt
>DjScaffold3058:82281-84153 strand:+
accgctaactctatctttcaatgtctgtatttggcctgtaaattttcatttttagaaat
aaatgtttggggaaggggatgcatattttgtgactaagtgtaacaagggggtgtaaaga
gtcatccaaaatcgtgtgacgtaatttatggatgaccctttatccaataaaattatacct
aaaaaaatcatctattataatttgtttataataatttttaataattcttattttagttt
cgaaatgcaATGAAGATTAAATTTGATAAAAATGACATTACTGATGAACTAGCTATCAAT
ATTCCATGTATTCTTGTAATGGCAGGTGCAACTACGAAATTCTTTTTAGTCAATAAAGAT
AAATCGGTTAAACTATTGAACACTACATTTTATTCTGGTGAAACTTTAAGAGGCCTATCA
AAATGTTTAATTGATACAAATAATTATCAAAAGTTAATGGCAGCTGCCAGAATAGGAAAT
CGAGAAAATACTGACACCCAAGTTTGGGAAGTGATGTCAGAAAATGAAAGTTCACCTTAT
GGAAAATTGCCACCTCATCTTCCGATTTTGGCTTTCGGTAAATGTGCTGATTACAATAAA
GGAAAAGAAGAATTTTCTCCGAACGATTTATTGGAATCTTTGTGTCATTTAATATTTAGT
GAAATGAATATACAAACTATTCAGCTATGCAAACTTTTTAAATTAAAATCCATTTACTAT
TGTGGAGGAATATTTGCCGATCCGATATTCGCCAAGTTTTCAATACATCGTAATAGAGTG
TTTGCCGGCGACTTAACTGTGAAAATATTAAAATGCGATTATATTTTTGCTTTGGGCTCA
ATGAAAAAAAATTAAtacagatttcaatttttaaaaatcttttgaaatgtgacaaatttg
attaatattaaatagaatatttttaagtaaataaactaatagttatttaatcgaattgaa
tgaaa
>DjScaffold3058:129140-129934 strand:+
actttaaaatatacatttttcattaaaaaatataactcaaatagttagcttcaaaaatag
atttaaatattcagataacATGTCAAACATATTCGACTATGATCAACAGGTATTGGAATT
GAGATTGGAGTTATCTCAGATAAAAAAATCGTATGAAAAATATTATCATAAATATACTAA
GAAAACAAAGGAAATTGAGAAAGAAAAGGATGAATTGTGTAAAGAAAAAATCAATTTACT
TAAAGAAATAAATGAATTGCGTAGTGTACAAAAAAATTTTAATGATGAAAAAATTCATTA
TCAAGAGCAAATTAAAGAATTTGAGTTGAAACAAAATACTTGTTTAAATGAACGTACAGC
TTTTAATGAGCAAATAAAATTAATGAAAAAAGATTTTAATGATCAAATCAATGGTTATGT
TGAATCAGAAAGNACAACAATTATTAATTGAtaatgattcagcaaaacgcatgattattc
gattagaaaatgagttaggccgtttgagttctgaaataaaaattaaagagaaaacagttt
gtaaaatggaacaagttattaaagtttctgaaaagacaatcaatgaaaaaaatgaagtta
taaggtgtaaatacatttaatttttatatctttaaagtggttttaaagtattttatttat
gata
>DjScaffold3058:132806-154554 strand:+
aataaattacttattaaattggaaaataattactttttgttaaagttaatatcagtaata
caatgaaaactgatgaaagttgaatctcagaaatagtaaagctgcaaaaagaaaaaatta
caaatggcaataaaatagaaattatatcaaaagtgataataataatgtaagttaatataa
tttcatattttgtctcatatttttaatattataatatacattttattatttctaattaat
aaataaacttttataaataaaaataaattatccaataaagtttcaataaaatgaaaaata
ttttgtaaatttttataatattgattaaatcgatcgaaaagttaaactcatccgtaaaga
agatttggaagtgaatttaaaacaattatcaatattggaaaatgaaaaatccattttcag
tgaaaaaataaaaaccttagaatgtgttattgataaaaaatctgacgaaatagctaattt
gaaatcaaaattggaaaatacagaattaaacaataaggaattaattcaacagttaaataa
agaaaaagaagaacaagtgaataacaatgataatattacaaggaccagcaataaaaagaa
attcgaagatgaatgtctaATGCAATCGACAGAAAGCTTAATGAAAGAAAGAAATTATTT
ATTAAAATTTTGTGAAGATAAAGACTTAATTATTCATCGTTTACAAATGGAAAATCGAAT

CCTTCAAGATTTCATATCGAAATTGAATAATGGATTATCTGATGAAGTTTCGTTTTTCAA
TAATGATTTAATTGGAGACAATGGTTTGTCGATGTATGATGGCTACCATCAACTTAAAGA
AACTTTCACAGATTTTGAATCGTCAGTGATAAATCCAGATAAACCATTAGAAACTAATAA
TCAAAATATTCAAATTTCTTCAAAATCTTCATCCAGTAAATATGGACGAGACTTTGTTAC
AAATTCTTCAGATTCCTTTTCCAATATAAACCTTTACCATTCGCATGAGTCATTTTCAGA
TAATATTAACAAATCATCAGATGAATTCCTAACTCGTTTGATTCAATCAGATACTGGTGA
TCAGATGAAAACTAATGAAAATGAATCATTTATGCAGAATATCTATCCTACATTAGCAGC
TGCCAAGTATTTCACAGATGAACGTGAAAAAACTAGGGAAATTGAAAAGAAAATTGATTT
TCACATGAATCAATTG
>DjScaffold4006:35489-36154 strand:+
ATGTCATTTGGCATTGCAGCACCAGAGATATTTCAAGAGATGATGACAAAAGTAGTAGAC
GGAATCAATGAAGCCATGGTTTATCTTAATTACATACTGATATTTACGAACAATAAAGAC
CAACATTATGAAGTAATTGAAAGAGTATTGACGGAAATAATGAAGGCTGGTCTAAGAATT
AAACTAGAAAAGTGCCAGTTTCTTCATAGCAAAATCAAATTTCTTGTATACATAATTATC
AAGGCGGGTGTCAGAACTGATACGTTTAAAATTGAAGCTATAAGAACATTTGGCAAACCT
AACTGCGTAAAGAGGTTACGAAATTTCTTAGGAATTTGTAATTATTACAGAAAGTTTATA
GAAAACTACGCAAGAAAATCAAAGATATTAGAACAGTTGTGTAGCGGACCAAGCAACAAG
AAATTAGAATGGCCAGATAAAATAAACGATACTTTTGATTGTCTAAAGAAATCTCTAATG
ACTGCACCAATATTGTGTTTTCAAGGCATAAACAAAAGATTTATATTAGATACAGATGTC
AGTTTTGATACAATTGGAGCAGTAATGCCACAGAGACTTAACGAAGTAAGAGAGAGAGTT
ATTGCCTGTGGATCACTCTCAATGACGACTCATGAAAAGGTATATTACATCACACAAAAA
GAATAA
>DjScaffold372:6174-10157 strand:+
atcatcatataatatctcaataaaaacatgaattaatattccaaaaataaattttattag
aagtttgatgaaatctcaaaaacgatcaatggttatgcccaacaattattctcgcaataa
ttgcaattgtaaaattattattattaatataaatattattaaattattatattatattaa
atttataatattatattaaatttataaatttattctcacagaATGATTATGATTCACGAT
GTTTTATCAGAGAAAAAGAAATCGAAAAGTAAAGTTTTGCAAAATTATACTCTTCGTCAA
ATACTTCTTGATGCTTTACAACGTTTGGGAAATGAAGAAACTGATGGTTCATATATCATT
AATTTATTTGTGAAAAAATTCTTTTTGTCGAATTTCCAATCAGAGAATTTCAATAATTTC
AGAAGAAATTGCTTAGAAAATTATGCTGTCATCAAAATTGGTTGTTCAAAATTTACCGAA
CATTTACCGATTCCTCTAGGTCAACGAACCTTGAAGGAATTAAAGGAAATAACTTCATGG
CATTTTGGAATAACACCGAACGATTTACACTTTGTTGGGTATGAAACAGAATCTCTCAAT
CATGCTGACGAAATAACTCTTACAAAAAACATGACTATACATTTTCTGTTAATTCTCAAA
ATGGATGGAAGTGTTGTAGAATCTATTGGCGCAGGATCATTTGGGGAAATATTCTTATAT
AGGTCATCAGATAAACTG
>DjScaffold3644:77322-79111 strand:+
aaatgtcataactagtaaattgtttattataatttgtcaaaattgtctctaggttttaca
tttatatttatttaggatttttgttgaatataaacaattgtattattatttaaaataata
taaattccttattccttccattgttaaattttattatattgaatgaattttacagaatga
catttggaatttgatataaattttactcaagttactattccattatttttcactgtcttt
atcgataatcgacttaattaaagttatttctaatatatgtgtttattatttattcctaat
ataatctgttgttgaaacttgtcttttaagactaggtaaagtcgaatccaaactgccttc
gaaaaggtctaataaatgtttagaagatgaaaatgaagaaattattactcctgaagatcc
aaaggtattaattgcaatagaaaatttggtaaatcaggcttctcttttgcctcaaaattt
acaattggcctatattaaacaatttgaagatcaagaagaatattggtttctgacattagc
ggacagtgttgaatatagctactttcattggagactggaacaagcaaagttagaatactt
gaagaaaatagaagaaagtgagacaacctttaaatttgaatcaaatcttttaaactccaa
ttcctcgagttctgtgaggaaaagaaaatcaagatggagttctgaacttcccaaagtact
tttagttccagttgtttctcaaaactctcaaattttaacagaatcacaaaagcagcaaAT
GGAATATCAAAAGGAGCTTCAAGCAATGCATGAATTTATTACAGCTCAACAAAAAGCAAA
GGAACAAGAACTTATGAACAGCATCGTTGGAGTCACATATGGACCAGCCAATACAAAATT
GGGGCAGTTCAAATTCGAATACGATTCAGATGAAGAAACAAAAGAGGGCACCTGGGAGCA
CAAACTTCGTAAAGAAGAAATGGAAGCAACTCGAGACTGGGCCGATAAACTGACGGAAAT
GGGACAAGGCAAGCACCACATCGGGGATTTTCTACCACCTGAAGAATTAGATCGATTTAT
GGAGACGTTTCGGGCTTTAAAAGAAGGTCGAGAGCCAGATTATTCAGATTATACTACCTT
TAAGATAACATGTGAAAATGTTGGTTTTAAAATGCTGAAAAAGATGGGTTGGAAAGAAGG

AGATGGACTGGGTTCAGATGGTCAAGGGATCAAAGTTCCATTGTGCAATGGTCCAATAAA
AGCAGATAATGTCGGTATTGGAGTTACAAAAATGTCAAAATTGACTAAATTTGATGACGA
ATATGATGCATACAGAAAAAGGATGATGTTAGCTTACAGATTCAGACCTAATCCTTTAAA
TAATCCCCGTCGTGATTATTATTAAcgcagaatattcagtgtcgaaattttatgtgattt
gtttttattgtaaattttcaaagttctttttaactcacaataaaatattttcaaataaat
gttggacattcttttaggctttaaaagaaggtcgagagccagattattcagattatacta
cctttaagataacatgtgaaaatgttggttttaaaATGCTGAAAAAGATGGGTTGGAAAG
AAGGAGATGGACTGGGTTCAGATGGTCAAGGGATCAAAGTTCCATTGTGCAATGGTCCAA
TAAAAGCAGATAATGTCGGTATTGGAGTTACAAAAATGTCAAAATTGACTAAATTTGATG
ACGAATATGATGCATACAGAAAAAGGATGATGTTAGCTTACAGATTCAGACCTAATCCTT
TAAATAATCCCCGTCGTGATTATTATTAAcgcagaatattcagtgtcgaaattttatgtg
atttgtttttattgtaaattttcaaagttctttttaactcacaataaaatattttcaaat
aaatgttggacattc
>DjScaffold3644:79517-91527 strand:+
tattatttattgttaataatttcaggtataATGGACGGGATTCCAAAAGATATTCTAGTT
AATTGTAAAAAGTGTTCCTCGCCGTTAATTTTAGATTTGGATCTAAGTTTAATGAAACCG
CCTGTTGATGAAAAATTTTGTGAGGAAAATCCAGATATTATTATAATTCCAACGACACAG
CCAAATGAATCTCTTGAGTATCAGGAAGTTTTATCTGATTATAGTCATGACAATGTTGAC
TATGGAACAATAATGGGAGGTGCTTCTGATTTATCAAGGAAAATAAGGGTTGCTGAAGAA
ATATTCAATTATATTAGTGGTAAATCAACTTCAGATCATCCCATGTGTCAAGATTGTGCA
AATACTGTTATTCAGAATCAAGAAAAACTTTTAAATCAAACTAAATCGGAAATTGAGTCA
TTAGAAATCTATTTATCAACACTCGAAATTAAACCATTTACTCAGGATAACTCAGATGAG
TATTCTAAGTTGGAAGAAGAAGAACAGAGTTTACTGGCGGAATTGAAAAATTTACAATTG
GAAAATGATCAGTTAGATAGAGAAATAGCCGTAGAAAATGCTATATTGGAAAGCAAAAAC
ATTGAATACGATAAAATTGTGATGAATTATAATACCAAAAAACAAATACTGTGGGATCTT
GAAGATGATATTAGATCATTAAATCAACGTAAAAAATATCTTAGTTTCCTTGCGGATAGA
TTAAAGCGAACTAATTTATTAAACGTTCTTTTTCCAATTTGGCACGAGGATCACTTTGCA
ACGATTAATGGTTTTAGAGTAGGTAAATTACCTGAAACTCCAGTCGAATGGCCAGAGATT
AATGCTGGATTGGGGCAATGTGTCCTATTATTGCATAATATTCTTAGGAAGATGGATATG
ACACTAGAGAACTACAAATTAGTTCCTTGTGGAAATTCTTCATATATTGAATCATTGAAA
GACAAGAAAAAATATTTGCTTTTTAAGACTGGTGGATATAGAATTCTTGCAGATAAATTC
GATTACGCTCTTATTGCTTATTTGGATTGCTTAAATCAAGTTATAGTTAAAATAGAAAAT
CACGACCCCAATTTCAAGTTGCCCTATCGTATATTCGATAAACATAAAATAGATGAGCCT
GATGGACCGGGATATTCAATAAAGTTATCAGGTAATTCGGACGAAAATTGGACTAAGGCA
ATGAAATTTATGCTCATTAATCTTAAATGGATCACAACATGGATGGTTAAAGCATCATTT
AAACAGTATTGTGATTCAATTAAAGAACAATCATTATCATAAtgaatcttttatattaat
ctatttttcttatgaaattaattgtttttaataaataatttttgaaatattatttattg
ttaataatttcaggtataATGGACGGGATTCCAAAAGATATTCTAGTTAATTGTAAAAAG
TGTTCCTCGCCGTTAATTTTAGATTTGGATCTAAGTTTAATGAAACCGCCTGTTGATGAA
AAATTTTGTGAGGAAAATCCAGATATTATTATAATTCCAACGACACAGCCAAATGAATCT
CTTGAGTATCAGGAAGTTTTATCTGATTATAGTCATGACAATGTTGACTATGGAACAATA
ATGGGAGGTGCTTCTGATTTATCAAGGAAAATAAGGGTTGCTGAAGAAATATTCAATTAT
ATTAGTGGTAAATCAACTTCAGATCATCCCATGTGTCAAGATTGTGCAAATACTGTTATT
CAGAATCAAGAAAAACTTTTAAATCAAACTAAATCGGAAATTGAGTCATTAGAAATCTAT
TTATCAACACTCGAAATTAAACCATTTACTCAGGATAACTCAGATGAGTATTCTAAGTTG
GAAGAAGAAGAACAGAGTTTACTGGCGGAATTGAAAAATTTACAATTGGAAAATGATCAG
TTAGATAGAGAAATAGCCGTAGAAAATGCTATATTGGAAAGCAAAAACATTGAATACGAT
AAAATTGTGATGAATTATAATACCAAAAAACAAATACTGTGGGATCTTGAAGATGATATT
AGATCATTAAATCAACGTAAAAAATATCTTAGTTTCCTTGCGGATAGATTAAAGCGAACT
AATTTATTAAACGTTCTTTTTCCAATTTGGCACGAGGATCACTTTGCAACGATTAATGGT
TTTAGAGTAGGTAAATTACCTGAAACTCCAGTCGAATGGCCAGAGATTAATGCTGGATTG
GGGCAATGTGTCCTATTATTGCATAATATTCTTAGGAAGATGGATATGACACTAGAGAAG
>DjScaffold1348:85218-94697 strand:+
tatttttgtttttagtcgcatttaattcgtaaattaaataaattaagcaaaccgtttttc
agattaaacaaaagatattattcttatttggtccaaacatctgaatattcatggcttaaa
gatttgggtattgaagaatataataatggagtatttgatggcaataattggtctggtagt
ggttcagataatgattcaatttgtccatcaaataatagaaaaattgccaaagtaaaaATG

GGCTCAATAGAAGATTATAATCGATGTATTAAAGAAACAAGTAAAGCTAGTAAAATATGG
AAAAATTTGCCAGGACCTTATCGAGGAGAAATAGTTCGTCAAATTGGTGATCGATTACGA
GATAAAAAGGAATCGTTGGAAAATTATTATCACTTGAAGTGGGTAAAATATATTCAGAA
GGTAAAGGAGAAATACAGGAGATTATTGATATTTGTGATTTTTCCACCGGGCTTTCTCGC
ATGTTTAATGGATCAATATTTCCATCTGAACGGCCAAATCACCTTCTGATGGAACAGTGG
AACCCGCTTGGAGTGGTTGGAGTTATCACCGCGTTTAATTTCCCAGCTGCAGTGTTCGGG
TGGAACATGGCTCTCGCTCTGGTCTGTGGCAATTCGATAGTGTGGAAAGGAGCACCGACA
ACCAATTTGACAACGATCGCAGTGACGAGAGTTGTGACGGAGGTGCTTAAAGACAACAAT
CTGCCAGTTGGAATTGTAACTGCGCTTTGCGGTGGTGCAGAGGTTGGCAGAGCTCTTGCT
GGAGACAATCGAGTTAATTTGGTGTCTTTCACTGGCAGCTGCACTGCAGGAGCAAAGGTG
CAAAATGTTGTTACCAGTAGATTCGGTCGTTGTTTGTTGGAATTGGGCGGAAATAATGCC
ATAATTGTCAACAACGATGCGAATATCGACATGGTAACGAGATCAGTGTTATTTGCGGCA
GCAGGTACCGCTGGTCAGCGATGTACGACCACTCGAAGATTATTTATCCACGAGGATGTA
TATGAATCGGTTTTGGGCAAATTAGTTGCTGCATATAGCAAATTGAGAATCGGTGATCCA
TTAGATGAAAAAAGCATTATTGGTCCGTTACATTCCAAAGCCGCAGTTGAATTGTATAAG
TCTTGTATAAGTAAAATTAAATCCAGCGGAGGGAAAATAGAATACGGCGGGCAAGCAATT
GAATCCGAGGGAAATTTCGTACAACCAACCATTGTTTCAGGTTTGGCACACGATCACCCT
GTTGTTCACACGGAAACATTCGTCCCGATCATGTACGTAATGAAAATTAAAAATCTTGAA
GAAGGAATTGCCTGGAACAATGAAGTAGAACAAGGTTTGTCGAGTAGTTTATTTACAGGA
AATCTCAGCGCAATTCATCAATGGATTGGTCCAAACGGTTCCGATTGTGGAATCGTTAAT
GTGAATATTCCAACTAACGGTGCAGAAATTGGTGGTGCTTTTGGAGGCGAGAAAATGACT
GGAGGAGGACGTGAATCTGGAAGTGACAGCTGGAAACAATACATGCGCAGATCAACATGC
ACAATAAATTGTGGAAACGAGTTGCCATTAGCACAAGGAATCGTATTTGAATGAgactac
aatattacagaattgtgattttcatgaactgcaataaatatatttatctatttggg
>DjScaffold1348:39041-45090 strand:+
ATGCACGAGGTATTGGAGGGATTAACTTTCAAAGGAGTTATTGTATATCTGGACGATATT
CTGGTGTACGGAAAAACTTTGAAGGACCACAACAAAATTTTAGAGACAGTAGTTTACAGG
ATCAGAGACTTGAGGTTAAGAATAAACCCGAGAAATGTAAGTTTTATAAGAAATAAAGTG
TTATTCTTGGGACATACAATTAGTGCAACTGGTACAAGCTTTAATAAAGTTAAGATTCAG
GAGATTAAGAATCCTGTAGTTACAGAAGGTTTTTCAAAACTTCGTTCATTTCTTGGAATA
ACAAACTACTACAGGTGTTTCATAAAGGATTATGCAAAGATAGCAGCTCCTTTATATAAA
GGTATTTCAGGTTGCAACAAACCGTTAGTTGGGACAGAAGCATGTTAG
>DjScaffold3488:9492-9839 strand:+
ATGGAAACACATTGTAATATACTGGAAAGATTTTTAAACACAATTATGAAAGCAGGATTG
AGGATTAATTCAGAAAAATGCCAACTGCTAAAGGTAGAAGTTAAGTTTTTAGGTCATATT
ATAAACGACGAAGATATTCAAAAGGATCCGAGTAAAAGTACAATTTCAAAAACCAAAATG
TGCAAAAAATTTGAAAAGTTTCTTGGGTATATGTAATTATTATTGGAAGTTTATTAGAGAT
TATGAAACAAAATCAAAACCATCAGAAACTAGGTGTGGAAAAAATAATTCAAAGTTGATA
TGGTCTGATTTGTGGGAAAAAGCATTAAAATATATGAGATTAGCATAA
>DjScaffold1703:22651-23673 strand:+
atttttactacatgtATGCAGGTTTATTTGCTTGAAGAGATTAATAATAGTAATTGCATT
AATGGAACACATGTCACTACTGAATGTGAATATAATATAAATTTTAAAAAGATTGGAAAC
AAAAATCTGTATTGCATATTAAACTTTACTTCATCAAATCCCACTGTAAAAATTAAAAAT
AATTTTATAAAGTTAAAATCTGAAGAGTATAATAAATGCCCATTTATTGAATTGAATATA
ATTAATGCTAACGGAACAATTAAAGATAATAAATTATCCAGCACTTGTGGAGGGCAAATA
GATGAATTAGAGAAAAATTTTTCCTATTTTTCATCATTTGTTATCATATTAGACACGAAA
GGTCTCGATGATCAAGATTCCAAGGTTGATTTGAGTTTGGTGATTGCTGGGGATTGCAAA
GAAAGAAATTATTTAATGTGGATAATAATAATCATAATAATAATTATTATCAGCATTGCT
ATTGTTATTATTTATTATCAACGAAAGCAAAAATATGGAGCTATTGATAAAAATGGCAAG
GGAAAATCGAGTAAAATTTTGATTAATTTTAAAAATTTCAATTAGaaaatcaaatttaat
tttgttttaataaatccatgaaaatattaattataattgaaatttagataataataattt
tttaataacttaat
>DjScaffold5191:992-10077 strand:+
gaaatatttgaatctttaacaataactgtctaatttttaaagtttcattataattaaca
ttaacaaacacatcttATGTATGATCGAGCTGCAAGAAATTTCACATTTCAATCCTCTGT
TGGGACATCTTTAAATATCGGACCAGGATCTTATCAGCATTTTAGTACTATTAATACTAA
GAATGACGGATATGCACCTTTTCAGTCTTTAGCATTGCGGGATTTTAATTTAGCACACGA

TGATGCTTTGGTTCTTCCTGGTCCTGCTCAATACGATTCAAAATTGACGGCAAATAAAAT
ATTGGGAGGCTCATCAGTTGGCAATGGTGAAGTTCGATTTAAACCGATAAAGTCGGATAT
TCCTGGTCCAGGCCAGTATTGTGTCGAAGATAAAATTAAATGTGTCTCAGCTGACTCGAG
AAAAAAATTAGTAGACAAAAATAAAGTAACTTATAAAAGAGAAGAAATGCCACCAAGTAT
ACCGCATGCGAGAACCGCATTCGGTTACGAAGAAGGACCATATGGGAAATTAATCAAACA
AGGTCCACCAGTAAGAGATAATAGTATTGGTCCAGCGTTCTATATTCCAAAGATTGGTGG
TAACAAGATCACCGATAAATATAAAGGATGCTTTTGGAGTTATGATAGGGCCAAGAGAAC
TGATTTTGGTGGCTCCAAGGGTCCAGGCCCATGTGAGTACTCCCCATTTGGGGACAGATG
GACAGACATGGCGGAGTACTACCAGGATCGCGACAGTGGGAAATTGGCACAACTGCGCAT
TCCGAGATACATTGATGATTTGCAAATGAAGTCTATTAAAGATCACATCCCTGGACCAGC
ATCATACATGCATTCCGGATTAATTGAGTGTCCGAAACCAATTTATAACGAAGAAGGCGT
CAAAATAGAACATCCTCCATTTGGAGTTCAAGATAAAAGGTTCCGTAGTAATGTGGATAA
TATTCCTGGTCCAGGCAGTTATAATGATCCCAGAACTGCTCTCAACTCGTTGAAAAAAGT
AACTGGAATGAAGCGAAGTCCATTTGGTGTCACAGCTACTCGATTTACTGAAAAGATCGA
TGTT
>DjScaffold5191:53738-54583 strand:+
TGTATTTCAGGACCACAGGTCTATCATGTATCCGATGAGTTGTCAAGAGAAAAGCCTAGC
TATATATCAGTGACGAGAAAAGGCGGTTTCGGTTCTACTAGTATCAGAGATGTTGATCTG
GGAAATAGAAATGCCAACGAAATTCCTGGTCCTTCACACTATCAACCAAAACAAGGAGTG
AATGCGGCAAAGGCATATCAACCGATGTCATCCATGTTTGCCTCACTAACTAATCGAGAT
TATGATCCAATCGTTCAAGGGAAATCTGACTTGCCCGATGGTACTCAATATGACGTGGTC
AGTTCATTCAATAAATCCCAAGGCAAGAAACATTATGCAGAACCTAGAAACAAAGAAGCA
AAACGAAGGCATTGTAGTTTCCTTTCTAATGCTGGTCGATATGCAAAAGGTTCTCCATTG
GATTGTGCGGATCCCTCTTTACCGGGACCATCAACCTATGAGCACAAGACTTACTTGAAT
ACGAAAACTGGGAAATTCTATTACCGAGAAAAAAGATTCAATACTCACGTGGAGGAAACT
CCCGGCCCAGGACAATATGAATATTTATATCCAATAATCATGTATACTGATGGTCAAATT
CTTTGAcacacctaatatttttataatcgatgctcaatcaaaatacttttctaattttaa
tattctcatttaaaatatttaatcaaatatacttgtttatataatatagttatatttagt
tatattatattatatttagttatattatatcttatattatattatatttagttatattat
aTGTATTTCAGGACCACAGGTCTATCATGTATCCGATGAGTTGTCAAGAGAAAAGCCTAG
CTATATATCAGTGACGAGAAAAGGCGGTTTCGGTTCTACTAGTATCAGAGATGTTGATCT
GGGAAATAGAAATGCCAACGAAATTCCTGGTCCTTCACACTATCAACCAAAACAAGGAGT
GAATGCGGCAAAGGCATATCAACCGATGTCATCCATGTTTGCCTCACTAACTAATCGAGA
TTATGATCCAATCGTTCAAGGGAAATCTGACTTGCCCGATGGTACTCAATATGACGTGGT
CAGTTCATTCAATAAATCCCAAGGCAAGAAACATTATGCAGAACCTAGAAACAAAGAAGC
AAAACGAAGGCATTGTAGTTTCCTTTCTAATGCTGGTCGATATGCAAAAGGTTCTCCATT
GGATTGTGCGGATCCCTCTTTACCGGGACCATCAACCTATGAGCACAAGACTTACTTGAA
TACGAAAACTGGGAAATTCTATTACCGAGAAAAAAGATTCAATACTCACGTGGAGGAAAC
TCCCGGCCCAGGACAATATGAAGTAAATAAATGAattattcagtatttatatccaataat
catgtatactgatggtcaaattctttgacacacctaatatttttataatcgatgctcaat
caaaatacttttctaattttaatattctcatttaaaatatttaatcaaatatacttgttt
atataatatagttatatttagttatattatattatatttagttatattatatcttatatt
atattatatttagttata
>DjScaffold4100:116876-117361 strand:+
ATGCCGTTTGGAATAGCCACCGCTCAATCTACTTTTCAGACGTTAATGACAATTATTATA
GGAGATATGAATTGGAAAGAAAATTTGGTTTATCTGGCTGACATTTTAATTTTTTCGAAA
ACAATTGATGATCATATTTCAATTTTGGACAAGTTATTTACTAGAATATCTAAATCTGGT
TTACAAATGAATCTTGATAAATGTAAATTTCTAGTATCAGAAATAAAATTCTTAGGTCAT
GTTATTAATACCGATGGAATATTGACTGATAATGATAAAGTGGAATCTATAAAGACATTT
AAAAAACCGTGTTGCATTAAACACGTCAGAAGCTTTTTGGTTCTCACTAATTACTATAGA
AAATTCATTGAAGGATATTCTCAGCTACTAAAACCAGTAGAATCTTTAATTAGTACGAAA
GAAAAGAAAATCATTTGGACATCTGAATGTAAAATTGCATTTCAGAAACTGAAAGAGAAA
ATGTGA
>DjScaffold1707:6932-12822 strand:+
ttttggacacgtccctgcgtataattattcagcgtcaacaattctattcgctATGAATAT
TTTTAGATTTTTAGGTGATATGTCTCATTTGTTAGCAATAATAATTTTGCTGAATAAAAT
TTGGAAAAGCAGATCTTGTTCAGGATTATCTGGAAAATCTCAGATAATGTTTGCTATTGT

TTTCATAACAAGATATTTGGACTTGTTTTTCCTCTATATATCTTTATATAACGTTTTAAT
GAAAGCATTTTTCATTATTTCATCACTTCTTACAGTTTATTTTATTTATTTCAAATTCAA
AGCTACATATGATTCCAATCATGACACTTTTAGAATGGAATTTATTATGGTTCCACTTGG
AGGTTTAGCAGTTCTTATAAACCATAAATTTTCTTTTGTTGAAATTCTGTGGACATATTC
AATATATCTTGAATCACTTGCAATTTTACCACAGTTGTATTTAATAAGTAAAACGGGCGA
AGCTGAAACCATAACATCTCATTATTTATTCGCATTGGGTGTATATAGAGCACTTTATAT
ATTAAATTGGGTCTATCGTTTTTTTGTTGAAGATTTTTATGACCTGATTGCTATTTTTTC
TGGAATTGTTCAAACAATTCTTTACTGCGATTTCTTTTATCTCTACATTACCAAAGTATT
AAAAGGCAAAGCATTCACGCTCCCTGCATAGcaagccacgctttgtgtcgatgtgctgtc
tccaagctgaatcagtcattgttataatcaaaatcgttatcttatcaaaaaatttgttta
ccaagcatatttctattaaaaaaatatatttgcatgatgtttatttgtcttttgttaaat
ttttataatacactatcttttctttttaattttcaattattgaatgtgcagtgtttctga
gttgtgttcaaaatgtattattgtacttgtatctaataaatatggaatttttctgaacta
tgg
>DjScaffold1272:86281-94744 strand:+
caatattactttcactatcaactagacattatcttacaaacgattctaaccctagaaaac
atattgattattgtgtcttgacgtattactttgttgtattaaccctttaaacgctttgaa
tggaaaatataattgggtctgaaaaatgaaatttttacattaaaaataaagtattctatc
ctattgattctaaaaataataatttttttattaattagactgagattccatttctggagaa
aactgcaaagattATGATTAATGGAGGAGCATCTCCTTTTTTTACAGATTCCAACAACAT
CCCAGTGATAGATCACTGTGTTGATAGTAATGTCAAATCACGAATCATAGCTTATGCAGA
GAAGAAAAATATTGGTGCTAACTCGTCAAAAAGCGAAACGGAATGCAAAATTTGTTTGAC
TAGACCTGTAGAATATGTTCTTGATTGTGGCCATCTTTTCTGTGTAGTGTGTGCTGATAA
GTGCAAAAGGTGCCCAATATGCAGGATGCCATCTAAATCATCGAGAAAAATATTTTTTTA
Aacaaatcttattgatatttttatcatcattttcgattttattgaataatgattttcatg
tggtgcagcataattctgaactatttatttattgatgattgtatgtatttaatcaataaa
tattgtttgctaaatcaataacctgaaata
>DjScaffold4992:10941-11447 strand:+
ATGCCATTTGGCTTAACAAACGCACCGGCTTCTTTTCCGAGATTAATGAACACAGTCTTG
ATAGATGTGGAACATGTAATAGTCTACATGGATGATATTCTGATCGCATCAGAATCATTT
GAGCAACATTTAACAAACATAGAAAACATGATGTCCAGATTACAAAAGGCAAATATAAAA
TTGAAACCAACAAAATGTAAATGGGCGGAAGAACAGGTATTATTTTTAGACCATATCTAC
TCATCAAAGGGTTTGCAACCAAATCCAGACAAAATAGAAGCAGTGAAAAACTTTCCACTA
CCAAAAACAGTACAACAGTTACAAAGTTATAATAAATTAGTTGGTTACTATCGTCAATTT
ATAAAAGATTTTGCCAAAATATCTGCTCCATTAGTAGAATTAACAAAAGAATTAAAAACA
AAAGGAGAAAGTAAGAATGTAATGATCACCAAAGAAATAGATGAAAGTTCATCAAAATTA
CAAAAATTATGGGGAAATAATCAATAG
>DjScaffold3141:88990-89963 strand:+
TATTATTATTATTATTATTATTATTATTATTATTATTATTATTTCAAGTTGATTGTACTT
GCTCGTGTTATTCCTGATGAAGAATTAGCAATTAGTACTGCCTTTGGGATTAAAACACTA
ATTGAAACTCTGGCAGTTACCAAAAATTCTGATGTCCAGGAATTGGGTTGCGATTGTGTT
GCTAGATTATGTCATTCTAAGGCTGGTATTGCAAACGCAATGATTAGTTTTGGAATTATA
GATATTTTATGTTCACTTATGGTAAGCTATATTGATTCTGTAAAAGGATCAGCTGCTTTA
GCGGTGAATTATTTATCGCATTATCCTGAATCTAGACGACAGATCCTGCGAAAATGTCGA
CGAGATCCTTACTTGATGAAAGTGATTTTGTTTTATAATAAGGATGGCAAACTTGATTCA
GATTTTATCGACGGTTGGAAACATTATAAACGCGTTGGTCTTCCAAAAATTGAGTAAttt
aaattataaaaaattctatgaaaatattttaagtattcttttctaatttagtttattttga
tgaggtataataatttttttgctaagctcttcatatattttagttattaatatatataagata
tttttaaaagaattataattttatgaattcaattttatgaatttttttctacttaatatat
ccttaatttttaggttttatttttttatatatattatacattattaatccttatttcgtt
ataatccactgtcttaatttctaataaaaaatatctgtgagcagtgttgaaaagcaagttg
aaatttttgtgtgattggtgatattttgtttaaaagagagatctaaattatggacgaaaa
ttttaaataaacaaatatttggtttaa
>DjScaffold3141:99014-99962 strand:+
acttgttataaatgttatcgtcatttcagggattctaaaataatattttaaaatatatat
aaataaaatatatttttaaaatattccaaaatttcttatatttataaagtggtacaaatt
tttaaaaatcttttgaaattaatagaatattttgaaaattaacaaataaattaagtggct

gtttttatatgaaaaattcaaaccttttgtgttgggattttagaaattgagtttttgaaa
ttttagagaccttcaaaaattttgcaactaattggttcggaagctatttacgtaataagt
ggaggtcctacaagtcaaagagacgtcattggaaaaaagaatggaatttcacctttggtt
aatttattaaaatccgataacaaagaaatagttATGAGTTGCATTCGATCTTTGAGACAA
CTGTGTGTCAAAATCGGTTATATTGCCCATGTATCAAACCAATTTCTGGTAATATCAACA
AAAGGTCTTCAAATGTTGGTAGGATTGATGCGTTCCTCCGACGATGAGCTCATTCAAATT
GAAGCGGCTTATACAATTGCTTCAGTTACAATGAGAAATGAGGAAGCCCTAAATGAACTA
CATTTGAATTCGAATTTCAATTATGTCTATATATTAGAAAAAATGTATAGTTCCATAACA
AAAGTTCGATTATTAGCTGGAGCTTCTTTAGCTACATTTGCTTATAATAATTTGACAGAA
CAAAAGAAAATTGCTTTGTATGGTGGAGTTCGTTTTAAATGTTTGCATCCATTTTTATTT
GCCGAAGATGAATATGAAAGATGTATGAGTGCCTTTCAAGTGAGTTACGTTTGAtaaatg
gaaattattattagtattatttattattattattattattattattattattatt
>DjScaffold329:37639-45361 strand:+
gttttattaattaaattatgacctatcacaattgggttttgcgagatggttttgattgta
atgatacactattgaaaataaattttaataattaattatttcgaaagccaattaattaat
tttgctaataatttccagaaaattgctaaataatgccgaagaagcgtttatcaatgtgaa
atttaaaaatgtggtatagataaaaaaatatgtttataatgaaattttatgattattagt
gtatgtgtctgtgaaactccaacagtatgatatccaagcatgaattgaaataattttata
cgtgacttacaaaaataaaattaattcattttaactttttaattttacatataaattta
cttattaatttttaatttaagttttgtgaaaATGACAGTTGAACATGAATGTGAACTCAA
TTTCTATCCTCCAAAACAATGCACAAAATTCTGTTTTCCTATAGAAAATGAATACTCTTG
TGATGGTTTTGGTAATAAAATTTGCAAAGAAGGGTATTATTATAATGCTCAAGAAAGCAA
ATGTTTACTAAATAATTGTATGTCATCAAATATTCAATGTCAAAATAATGGATATTGTAT
CAACAAAGGATCAACGTTCGAATGCAAATGCTTAGAGAGCTATATTGGAAAATTATGTGA
AAGTTCTAACCCGTGTTATCCTAATCCATGTGCAAATAATGGAAAGTGTTTTGTTCAAAA
TCATGGGGAAAAACTTTATGCATGTTCTTGTTCAGAAGGATTCACAGGAGAAAAATGTGA
TTTAAGAATTTATCAACCATGTATATGTCCAAAAAATATGAAATGCAATTACTCAGAATC
TGGAGTATTATTTTCTTGTACTTCTGAAAATGAAGCTGGTTCATCGAATCCTAAATCCGA
TTATAGTAATAGAAAAATAAAACAAAATTGGAAAGATGTGAGTTTATCCACAACAATTGT
AGCAGCTGTCGGATGCATGATGATTCTTATTTCTGTTGGAGTATCAATATTTGTATGTCA
TCGTACAAGAAGCTATTCCTCACTTAAAAAATCTTCAGAAGCTTCGAAATCAAACTTTTT
CAATTCGTATCCTCCACAAATTATAGAAAGTTCTTTTAAACGATCCTTTTCTCTCGATGA
TAACTCAGCATATCTACCAATCAATCAAACCGTATTAACAAATGCACAAGGACTTGTAAC
TATGACACCTAGTCCAGGATTTTCGATCAATTCAAATTCTTACCAGGAAACCCCTGCCAT
CAGTTTTATGAATTATCCCTGTACATATAACGAGATATCTAAACAAGATTCCCGTTCTGG
TTATTTAATAATGAGTAGAAATTGTACTGAAAACAGCAACACTGATAGTAAATCCTTGTC
TAGTGGAAGTATACCGCCTCCTCCAGCTTTGCCCCCCAGACCAACTCGTGAAAATGAAAG
TCCAATTTTACCAACTCAGAACTTTGAATAAtttcttttattttaagtatttgtaatttc
acaaattttgtaacctctcaattttatagaatatatacatctttaaaatttggttttatt
aattaaattatgacctatcacaattgggttttgcgagatggttttgattgtaatgataca
ctattgaaaataaattttaataattaattatttcgaaagccaattaattaattttgctaa
taatttccagaaaattgctaaataatgccgaagaagcgtttatcaatgtgaaatttaaaa
atgtggtatagataaaaaaatatgtttataatgaaattttatgattattagtgtatgtgt
ctgtgaaactccaacagtatgatatccaagcatgaattgaaataattttatacgtgactt
acaaaaataaaattaattcattttaactttttaattttacatataaatttacttattaa
tttttaatttaagttttgtgaaaATGACAGTTGAACATGAATGTGAACTCAATTTCTATC
CTCCAAAACAATGCACAAAATTCTGTTTTCCTATAGAAAATGAATACTCTTGTGATGGTT
TTGGTAATAAAATTTGCAAAGAAGGGTATTATTATAATGCTCAAGAAAGCAAATGTTTAC
TAAATAATTGTATGTCATCAAATATTCAATGTCAAAATAATGGATATTGTATCAACAAAG
GATCAACGTTCGAATGCAAATGCTTAGAGAGCTATATTGGAAAATTATGTGAAAGTTCTA
ACCCGTGTTATCCTAATCCATGTGCAAATAATGGAAAGTGTTTTGTTCAAAATCATGGGG
AAAAACTTTATGCATGTTCTTGTTCAGAAGGATTCACAGGAGAAAAATGTGATTTAAGAA
TTTATCAACCATGTATATGTCCAAAAAATATGAAATGCAATTACTCAGAATCTGGAGTAT
TATTTTCTTGTACTTCTGAAAATGAAGCTGGTTCATCGAATCCTAAATCCGATTATAGTA
ATAGAAAAATAAAACAAAATTGGAAAGATGTGAGTTTATCCACAACAATTGTAGCAGCTG
TCGGATGCATGATGATTCTTATTTCTGTTGGAGTATCAATATTTGTATGTCATCGTACAA
GAAGCTATTCCTCACTTAAAAAGTATaaattagaattggaaagatgtgagtttatccaca

acaattgtagcagctgtcggatgcATGATGATTCTTATTTCTGTTGGAGTATCAATATTT
GTATGTCATCGTACAAGAAGCTATTCCTCACTTAAAAAATCTTCAGAAGCTTCGAAATCA
AACTTTTTCAATTCGTATCCTCCACAAATTATAGAAAGTTCTTTTAAACGATCCTTTTCT
CTCGATGATAACTCAGCATATCTACCAATCAATCAAACCGTATTAACAAATGCACAAGGA
CTTGTAACTATGACACCTAGTCCAGGATTTTCGATCAATTCAAATTCTTACCAGGAAACC
CCTGCCATCAGTTTTATGAATTATCCCTGTACATATAACGAGATATCTAAACAAGATTCC
CGTTCTGGTTATTTAATAATGAGTAGAAATTGTACTGAAAACAGCAACACTGATAGTAAA
TCCTTGTCTAGTGGAAGTATACCGCCTCCTCCAGCTTTGCCCCCCAGACCAACTCGTGAA
AATGAAAGTCCAATTTTACCAACTCAGAACTTTGAATAAtttcttttattttaagtattt
gtaatttcacaaattttgtaacctctcaattttatagaatatatacatctttaaaatttg
>DjScaffold3815:12631-15185 strand:+
tagaaattatttattcataattgtgatttaattctaacaaatttatttgtaaattatttg
gtcaaaatgtgtttatttaataaaaataaatgtttatgatcaaagaaaacgtttctattc
cagagagatttatgtctcaattgttattattattttcatatttaacaatccttttcagca
actagtaaaaccaattcaagtacatccttgtcagatgttaatgaagtcattaaagctaaa
agagttgctggtttatcattgtgtgttggaatttcacagtttataATGGGAATATTCAAT
TTGGGATTCTTAACTAAATATTTATCGGATCCATTGATATCAGGATTCACTGTTGGTTCG
GCAATAATCGTGTTTTCTACGCAATTAAAATATTGTTTTGGCGTTACGATTCCAAAATAC
AATGGGATATTCAGCGTTTGGAATACAGTTGCAGCAGTTTTTGGAAAACTGCCACAAACT
AATATTCCCACATTTGTTATGACAGTTATTTCTATTGTTCTGTTGGCTGTTACAAAAATT
GTTATCAATCCAATTCTAACAAAAAAAAATTCATGTTCCAATCCCAATAGAATTATTAGTT
>DjScaffold184:11880-18544 strand:+
taccaaatcagacccgcaattgttaacaaaacgaaattttattaatattcaaaatcttca
atatcggcacataaatgatgatATGTCGGGAGAAGTAATTAAAATCAATGGAGATGTTGG
ATTAATTAATCAGAATCCTAATTCATTCATTTGTAAGGAATCTGATTTGAAAAATATTTT
TGTTGAAACCACTGAAGATTTAAATTCGAGTTTTCCAAATATTGAAAGAAATTTATTAAC
AAAAATTAGAACAAAAAAATATGGTTGACAATAAACCATTAAAACAAAATCGGGGGTCTTC
AACGATTAGTATGACTGCTTCTTTTACAAAAGATTCTTTGGGAAACGTATCTCGAAGAAC
TGGGCCGTACGAAAAAAACCCAGTAAATCCGGAAACAGCAATGAAATTATATATGAATAA
ATTAACTTCATTTGAACATCACGAAATATTTAGTTATCCCGAAATATATTTTGTTGGTCA
AAATGCTAAAAAACGATATGGAATCATGGGCGGACAGCATAATTGCGGTTTTGATGATGA
ACAGAATTCGTATATTTATGTTGGTCATGATCATATTGCTTATCGATATGAAGTGGTGAA
AATTTTGGGTAAAGGAAGCTTTGGCCAAGTTGTTAAAGCGTATGATCATAAGTATAACAT
TTACGTAGCATTAAAAATGATCCGTAATGAAAAACGATTTACAAGACAAGCCTCCGAAGA
AATTCGAATTCTAGAAAATCTTAAAAAACAAGATCCCTCATCAAATCATAATATTGTTCA
TATTCTAGAAAACTTTACGTTCAGAAATCATGTGTGCATGACTTTTGAATTGCTGAATAT
AAATCTTTATGAACTCATCAAGAAAAACAAATTTCAAGGATTTTCTTTACAACTTGTTCG
AAAATTTGCCCATTCAATATTGTTATGTTTGGATTTATTGCATAGAAATAAAATCATTCA
TTGTGATTTAAAACCGGAAAATATTCTTTTAAAACAACAAGGAAGAAGTGGCATCAAAGT
AATTGATTTTGGATCTAGTTGTTATGAGCATCAAAGGATTTATACTTACATACAATCGAG
ATTTTATCGTGCCCCTGAAGTTATCCTTGGCAGTTCATATAATATGATGATAGATATGTG
GAGTTTTGGATGCATTTTAGCTGAATTATGGACAGGTACACCATTATTTCCTGGAGAAGA
TGAAGCCGATCAACTTACTTGCATAATTGAATTAATTGGAATTCCACCAAAAAGTTTGCT
TGAAGCGTCTAAACGCACAAAAATGTTCATAAATTCTAGAGGTTATCCAAGATATTGTAA
TATAGAATCAGATACTGATGGTCATATAATTTTAAAAGGAAATCGTTCTAAACGTGGAAA
ATATCGGGGACCACCTGCAAGTAAATCACTGACAGCTTTATTGAAAAACTGCAATGACCT
TGCGTTTATTTCATTTTTGAAACGTTGCCTTCAATGGCTACCTCAAGATCGAATAACACC
GAGAGAAGCTCTTAAACATGAATGGATAAGGCGAAAGCCTCAAATTCATTCTCAAGATAT
TTGTGTTTCTACTAATATAGAAGGAGAATTGATTTATAAAGAAACTGCAACTGGTGATCG
TATCGGTGTAAATGAAAATTTTCTCATGAATAACACTTGCCTTGTGCCAGTTTAAaaatg
aacagatcgtacgttatctgttacagattgagctggaaatttcatttgttatttagtcct
catgctatcatttatgtttaatgcgaactttgaaaataataaattaaaccgttcttattc
taacagattagaaaataacaattatgtaagcataataacatataatcattcgtattacgt
ttttttgatattaaagaaattatttccagtattttgatgattatttattttactttttta
attggaaaataacagaaaaaacttgtctaacgagattaattgtatttgatttgcagacag
attttttctttatcttatttaatatttttattcttgttttgtacttttgaaaaaatta
aacttatccatcgtttttccgaattttgacatt

>DjScaffold184:104727-107307 strand:+
aaatagactaaatttcatttaaataaattaaaatctctgtttttagtaaataccaataa
aagtggtggtgatattaaatattctcggatattagctagtggaaccattgaaaatcgtta
tagaattgagttgtcactggcaagagcatatcggatcaataaacaatatcgtgaaagcgt
aaaatattacaaaattagtggtatagacaaatttcttaacaatattaacgataccATGCA
AGAATATATTGAGACATTAATCTCTGCTCAGGACTACGAATCTGCACTGAATGTTTATTA
TCGTTTGCAAATTAATGAACAAAATCCATCCTTCGATGACGGATATATTTGTGGGAAAT
CGTTCGATTATTAATGAAACTTGAAAAGCATAAAGATAAAATGTTAATTAAATATGCGTC
TTTGTGGGCAAAAATATTTGGCTTTGGTATATTA
>DjScaffold3319:4333-4494 strand:+
ATGGAAGCAATAATAAAATTTGGCAAACCTAACTGTGTGAAGAAGTTACGAAATTTCTTA
GGAATATGTAATTATTACAGAAAAATTATAGAAAACTACGCAAGAAAATCAAGGATCTTA
CAGCAGTTGTGTAGCGGACCAAGTAACAAGAAATTAGAATAG
>DjScaffold3319:124821-140227 strand:+
atgcttttaaacaaatgtatgcaaaccaaatcaagtaatatgtttaagcaaacccaatta
atatcatttataaacttaaaatttaaatatatttatttatttaaatcaaggtcaaaggat
tccttcaaatataaatttagacgggattaaaaatcaaatagacatttttattacacgaat
aaacgatttagcgaaatattttccaggtcaaaggattcctttaaatatatatttagacag
gattaaaaatcaaatagacatttatgttacacgaataaacgatttagcaaaggatttccc
agaaatagaaagtctttttaattggttaacaaagaaaagtatttcgttaggcattgatgg
agaaaaatatgacttcgaaagaattgtatatgaaATGGGAAAACTAATTAATTTGTTAAC
AAGGGAAATCAATTTTGATATCGATGGAAAAAAATATGACTTTGAAACAATAGGAAAACA
AATCGAAAGACTATTTGATTCATTTGCAAAGAAAAATATTTCCTTATACATTGATGGAGA
AAAATATGAATTCGAAAGAATTAAAAATGATATGGAAAATCTACTTAATTGGTTGATTAC
AGTAGCTACTCTTGACGTGAGTGGGAGAAATTATTATTTAGAAAAAATTGGAAAAGAAAT
GATGAAACTATTTAAATTGTTAACAGAGACAGATAGTTACTTGGATATTGATGGAGATGA
ATATGACTTTGATAGAATTAGAAATGAAATAGAAAGACTATTAAATTGGTTAACGGAAAG
ATTCGCTCTTGAAATCTATGGAAAAAGATATAATTTCGAAAAAATGAAAAATGAAATGGA
AGGACTATTTTATTTACTAACAAGGAAAGTTATATCTTTATACATTGATGGAGAAGAATA
TAATTTGGACAGATTTGTAAATGATATTGTAAGACTACATAATTGGTTTACAAAAAAAAT
CGCCCTTTTAATGGATGGACAAAGATATGATTTAGAAAGAATTGGAAATGCAAAGAGAAA
TATTTTCTTAGTCGTTGATGGAGAAATATATGACTTTAAAAGAATTGAAAATGAAATCAA
AAGATTTCGTTTCTTGGTAAATATGCAAAACGCTCTGAATATCTTACAAAATAATTATGA
TCCAGAAAGAATGGAAAATAGAATAGTAAGATACTTGGAGTTGTTATTAGAGAAAGATAT
TTACTTAAACATTGATGGAGAAGAATATGACATAGAAACCATTCTAAATGAAATAGAAAG
ATACTTTGATTTGTTTATAGAGAAAGATATTTACTTAGACATTGATGGAGAAATATATGA
CTTCGAATCTGTTCTAAATGGTGAATATATTATACAGGGTATCTCATAAgtttccataca
tataaaaaagaaagggaaaccattaattatataacatgttttacatggccaccgttgtta
ttgaagcacaatttaatgtggattgtcccctgctaaagaacacgtgcaagcaaagctgta
gatatgcttgtaatggcgtcaatgattcgttcttcaagaggacttatattttgaatcttt
ctccaatacaccatagccttcaaatgggataaaagataaaaatttaat
>DjScaffold3319:253449-255676 strand:+
ATCCCAGTTGCTCTTACAGGCGTTGATATAATTGGGCTAGCCGAAACTGGTGGAGGTAAA
ACTGGCGCATTTTTGTTGCCCATTTTAAATGATTGGTTAAATAATGAAAAGCCTGTGATG
TTTGCTTTAATTTTAACACCGACTCGCGAATTAGCCTCACAAATTGAACAACAATTTATA
ACAATGGGCAGCGAGTTTGGTTTGAAAACTACATTGCTCATTGGTGGGGATGAAATGACC
GAACAAATCGTTGAATTAGGTAAATGGGAATCGCATATAATCGTTGCAACTCCCGGTAGA
ATTGTAGCTTTATTAGCAAAATCGAAACCTGCAAGAACAATGTTGGAAACTATTAGAGTT
CTTGTTATTGATGAAGCAGATCGAATGTTACATTTGGAATTCGAGTCAGACATGGAGGCT
ATTTCGAAGGTTCTTCCGAAGAAAAACAGTTTAAATAATAAAGAAAAACTGGAAAAAGGA
AAAATTAAATTTGTTCGTCAGACTCAATTGTATTCGGCTACGTTTAGTAAACATATAGAG
AAAGTAGCTGGATATTTACTTTATCATCCGGAGAAAGTTTTCGTTGATAACAGATTGCAA
ATTCCTAAGCAATTGAAACAATTTATTCTTGTTACGAAATATGTCGAAAGAATTCCGAAT
TTATTTGGTTTGTTACGTGAACATTTAGCAAAGAAAATCAAATCAAATAAAGAAGAAGAA
GAGGGAAAGGAGGAAAGAAGAGAACTGAATAGTGCTATCGTGTTTGTAACAACTTGCAAG
CAAACGAAACCATTGGAAAGTTATCTTAACAAAATGGGTGCCTCGGCAGTCGCGTTACAC
TCGCAAATGTCCCAGAATCAGCGCGAGCAAGCAATCGATAGTTTCAAATCAGGCGTGGCA

AACATAATGATTGCCACTGATATTGCAAGCAGAGGAATCGATATCGACCACGTCCGACTT
GTTGTCAACCTTGATTTGCCCCAGAATCCAGATTGGTACAAGCATCGAGTTGGGAGAACC
GCTCGAGCTGGAAAGGAAGGAGTTGCAGTCACATTCGTCAGTGAAGTGAATCAAGTCGAA
TATCTTGGAATGGAGAAACGATTAGCACTGCCGAAACTTCGTCCGCATCCACCGATTCAT
CTGAGTAAGAAAGATTTGATCAAATCAGAAGCGGCAATCAGAGAAGCGAGAAAAGAACAA
CGACAGAAAGAAGAGAAATTGAATGGAATCGACATGGATTTCTCGAATATCAACAACAAT
TTCAGTACGAAACGAATCAAATTAGCTGAGAAAGAAAATAGAAAGAAAAAATAAATGAttt
ttgtgcatttttgaaattttatatttctcttaattttcattgg
>DjScaffold3319:263062-264108 strand:+
TCAGTTGGTTGGCGACTTCCACAAAATAATTCAACAAACGATTCAGCTGAAAATGATTTC
ATATCCAATGCTAATCTTAAAATCTCTTCGTCGAATATACCAAATTTTTTCTATCAATGT
CCTGAGAGCAGTCAATCAACCAGCAATTCAAATGGATTTTACTCGGTTGATACAAATTGT
TCTGCTGGATTTCAAGATATGAATACTTACTATTCGAGAGTTGCAGCTGGTTTCAATCAG
TCTTTTGATCTGATTCCTCAACATCAGTCTTTTGTCAGGCTCAATTCAACTGGATTATCT
GAATCTGATCAAATGTCATATATGAATCCTAATCTTCATAGTTCATTTCATAGAAATTTA
TCCTATCCGAATTATTCTACCAACTGTCCACCAAATGTCAATATTGGTGGTGTATCAATG
ATGCAAGGATCATCATCATCGTTTTTAGGTTCCAATTATTCTGCTATACAACAACCGATT
CAAGTTGTAAATCAAGGAGATATTGAATATGAAACTAGAGAATTGGAAGCGTTTGCTGAA
TGGTTCAAACAAAGAAGAATCAAATTAAGAGTTACGCAAGCAGATGTTGGCCGAGCTCTT
GGCAATTTGAAATTAGCCGGAGTTGGATCACTGAGTCAGTCAACAATATGTCGTTTTGAA
TCACTAACTCTCAGCCATAACAATATGATAGCTTTAAAACCAGTCTTACAAACCTGGCTG
GAAGACGCAGAAACCGAAGCAAAAGAACGATTAAATGGTTCAGATGCATTCTCTGATATC
GATGAAAGAAAACGAAAGCGAACATCAATAACAGATTCAGAAAAACGTTCACTGGAAGCA
TTTTTCACAATTCAACCGCGACCTTCGAGCGAAAAAATTATACAAATTGCAGAGAAATTA
AATTTAAAGAAAAATGTTGTGCGGGTTTGGTTTTGTAATCAGCGACAAAAACAGAAACGA
ATGAAATTCTCCACTCTTGGTTTTCAGCATAAT
>DjScaffold4319:66260-67309 strand:+
aatggatatatagtggatattcgaactacagtgaaattgattgatgaattagacaaactg
agaggtcaaATGCAAAATTTACGATTGTTATGTAGTATAATGATACCTTTGGTTTATACA
CAGGTTGTAATAATCGCTGTTTACAGTTATTTTCTTTGTCAACTCTTTGCCTCTCAATTT
TCAGTTAAGTATCCCGAACATGATAGAATGAATTTCACAGCACATGAACCTCGGAGAACA
AGCAGCGATATAGATTTTATATTTTATCCTTTATTTACAATTTTCCAATTTTTATTCATT
ATGGGTTGGTTAAAGGTCGCGATTTGTTTATTGAATCCTTTTGGAAACGGAGAAGAGGAT
TTTCAAACCTCTGATATTCTTGATCAGAACTTAGATGTTTGTTGGCGATCTGGATGCGCT
CATGATGATAACTATCCAGATCTTTTGAGTCCACCACTTTTTAATTTTAAAGTAACAGCT
CCACCAAATGATTCATTAAACACCTTTATATCACTTACAGAAGAAGATGTTTCTGAAGTT
GAACCAATAAATGATGATAAAAATCTGGCATCATTTGAATGGAGTTTGAAAGGCTTTAAT
CGACTTAATAAATTCTTTTGCTGTCATCAAGAAGATGTGCAAAGTAATTGGGATAATAAT
ATTGGTAATACTTTATTTAAACAT
>DjScaffold2743:43620-54831 strand:+
TTAACTCAAAATCACCTGTTATTCGTTTTACTGATAATTACAATACCATTTATAAATGCC
AAGATTAGAAATAGAAGAGCGGTCATTTGGAGTCAAGCTAGACAATGGCCAAATCGAACA
ATTCCATACTTTATTGACCAAAGTTCTTTCAATATCGAAGAAATCACATTGTTAAATGAT
GCAATTGACGCATGGAACATGGAAACCTGTTTAAATTTTCGAAAGAAACTTGATGGAGAT
AAAAACTGGGTTAAGATCACTGGGGGAACAAATTGTCACTCTATTGTTGGTATGAATGAA
ATCGGTGAACAAGAACTGAACCTGGCATCGCCTGATTGTAGGTTTTATGGTACGTATTTA
CATGAAATTGGTCATGTTCTCGGACTTTATCATGAACATACTCGGCCTGGAAGAGATGAG
CACATACATGTTTATAAAGATAAAGTACAAGAGGGTCTTGAGGACTTTTATGATAAAATT
GAAACATCGGAGCTAAAAACATATGATTTACCGTATGACATTCAATCAATTATGCATTAT
GGACCAAAGTCATTCTCAGGATCAGACGGACAATCAACAATAAATGTGAAAAATGATTCA
ATTTCTCATCTTCTTGACGAAGTCCATGATAAAGATTTATCGTTTCTTGATGTGAAAATT
ATAAATTTATATTATAACTGCTCAAATTTGTGTAAGCAAACAGTTTCATGTCCATCATAT
GGATTTGTTGATCAATTTTGTCAGTGCCATATTCCTGAAACTTTCGAATCTCGAAGGTGC
CGAAATAAAAATGACAACGAATGGTGTGAAGAACAGAAAAAAACAGGCAAATGTGAATAC
AATCCGAAATATATGTCAGTTTATTGTCGGAAAACTTGCGACAAATGTTTCAAACACAAA
GCCAAATTTTTAAAGCGAAAAAAAATATGTAAAAATTTTGAAAAAGATAAAATATGTGAT
AATTGGGCTAAGCGCGGAGAATGTGAAAAAAATGCAGGTTTCATGAATATCCAATGTAAA

AAATCGTGTGGTATTTGCGTTGAAGAAACAACAACTCCAGTATCACTGAGAATTTGTCGA
AATAAACTGGATGATGAACACTGCGACAGATTGGAATCAATTGGAAATTGTGTGGAAAAT
AAAACATTTATGGAAAGCAATTGTCAAAAAGCTTGCGGCTTTTGCGGTTTGAAAGCAATA
TCGACAAAAATTTGGAAAATCAACGAATTTTGTTCTGATAAATTAAGTGTAGAAACTTGT
CAGAGCTGGGCAAAAAACAATGAATGTGGCACAAATTATATGTATAAAATGTGTAAGAAA
ACTTGTAAGTTTTGTAAACTTCGTAAACCTGATTCTTTGAATTGTAAAAATATTTTCAAA
GATGAGGTTTGTGAAAAATTTTCCATTGTTGGCAAATGTCAGTCGGATTCGGAATTTATG
AGAAAACACTGTATGAAAACTTGCGATGTTTGTTCGATGAATTCCGATGGGAAATTATCT
AAGAATATAACATGTAAAAATATTTACGGTGATAGAAAATGTGAAACTTTGAAAAAGAAA
GGTCAATGTGAGAAATATCCGTATTTTATGTTGGCAAAATGTGGTAAAACCTGCTGGAAT
TGTGTAGATGTAAAAAATAATAGTTGTAAAAATTTAGAAGCAGATCATATATGTAAGAAA
TTAGCGAGTGAAGGTAAATGTAAATCAGATATTATTAATATGTCAATTAATTGTGCAAAA
ACATGTAAGTTATGCTTGGTAGGAATAAATAATGTTGGTTGCAGAAATTTAAATCCCGAT
AAAGTTTGTGAAGTCTGGAGTAGAAATGATAATTGTTCGAAAAATTCGGTTTTTATGATG
GTTCAATGTGCTAAATCATGTGGTCGTTGTAATTCTAGTAGTTATACAGAATGTTCTGAT
TTGTATAAAAAAGAATTATGTAGATTATGGGAAAAAGTTGGACGATGTTATAAAAATCCT
AATTTTATGCTTCATCATTGTGCAAAATCTTGCGGAAGTTGTGATTTGAAAAGTAAAGGA
ATTTCAAATTTTGTTTGTCTAAATAAATTCGAAGAGAAATTTTGTGAGATTTGGAAAACC
GATGGAGAATGTAAAACCAATCGAAAGTTTATGTTGGAAAATTGCGGTAAAACTTGTGGT
ACATGCCGAAGAGAAAATGAAACGGTGTTCGAACAAATAATTTGTGAGAATATTTATAGT
GATTTACTTTGTAATAAATGGAAATCAAAAGGTCTTTGTCATGGAAATAATCGGAACGTT
CTTAATAAATGTGCTAAAGCATGCGGAAAATGTCGACAAACTGTAAATAACATAAATTAT
TTATTAGAAAAATGTGAAAATGTTTATAATAATATCCATTGTGACAAATGGAAACAATCG
GGCGAATGCCGGAAAAATCCGGAATTTATGAAATTACAATGTAGAAAATCTTGTGAGTAT
TGTGACTCAAAGATAAAAAAGTCAAATTGTAAAAATCTCCTTAAAGATGAACAATGTGAT
GATTTCGCGAAAAAGTTCTACTGTGAAGAAAATCCGATTTATATGCTTCAGATGTGCGGT
AAAACCTGTAATAATTGTACATACTACGGAAAATTGATGGAAATGAATAATTCTTGTTCG
AACAAAATCGACGAAGAATCATGTGAGTTGTGGGCTGGCACTGGAGAATGTTTGAAAAAT
CCAAAATACATGCTGAAAAGCTGTGCAAAATCCTGTGGAAATTGTCTATCAATGCCGAAA
AGATTTGAAATTTGTAAAAATAAAGTTGATGATAAATCCTGTGAAAATTGGAGTGAATTA
GGTGAATGTGAGAAAAATCCGAAATATATGCTACCGAATTGTGCTAAATCATGTAAAATT
TGCCAAACAACTCAACTCACATATCCAATCGACCTATCCACAGCCAGTCCAATATCGAGT
ATAAGTACGCCAGTTTTTTCAAAAACTCTTTTTCCTTTTGTCAGTTGTCAGAATTTATAC
AGAGATGAACTCTGTGAAGAATGGAAAAAGAACGGGGATTGTAAAACTAATTTTAAACTT
ATGTTCTCTCAATGTCGAAGATCGTGTGGTCGATGCAAAAAAACATTGGAATGTCAAAAT
GTTTACAATGATCAATCATGTGAGAGTTGGGCAAAAAAAGGGAAATGTTCAAAAAATATG
AAATTTATGTTAATGCTTTGTATGAAATCATGTGGAAAATGTTCTATATTAAATTTTGAA
ATTGAGGAATCAGACAAACGCAGTTCCAATAGATGTAAAAATAAGTATGGTGAAATGACA
TGTGAATCATGGAAAGAAATCGGTGAATGTGAAAAGAATTCGATATTTATGAAAGATAAT
TGCGCTAAATCTTGTGGTTATTGTAATACAAAAACGGAAAACTCCACGTCAATTCATAAA
TTCAAAAAGAAAATATGTAAAAATATTTACAATGATAAAATTTGTAATGAATGGGCAAAT
AATCGAAAATGTAAAAGTAATTTTGTTTTATTCGTTTGTGACAAGTCTTGCTCAAATTGT
TCTCGGCGTATTTCGGAAAATAAATTCTCTAATTGCAGAAATCAAGCGGGTAATAGAAAT
TGTACCAAATGGAAATCGTTAGGACACTGTGAACGATTTCCTTATACAATGAGTTTACAA
TGCGCTTTGATATGCGATAAATGTAACTTGAATTCATCAAGTTTACTTTTAACTTCACAA
AAGAAAGCGATTAAGTCACAATGTAAAAATATTGCTGGTGATTTACATTGTTCATATTGG
GCAAGACAAGATCAGTGTGAAACAAATCCTTCTTATATGAGAATTCATTGTCGAAAAGCA
TGCAACGCGTGTATAACGGGGAAAATAGCGGAACATAATTGTTTTGATAAATTTTCAAAT
ATAAGGTGCTCAACCTGGGCTAGAAGGGGAGAATGCAATCGAAATAAAATTTGGATGAGT
GAAAATTGTGCA
>DjScaffold1559:11587-12387 strand:+
ATGCCTGAACATGATGAATGTGAATTCTGTATGTCTTCCAAACGAATGCGAACGAGTTAT
CGGAGTAACTCGAATAACAATAATAATCTAACAACTGTACCTCGGCACTATTATGACAAG
TGTCCTCCTCGACTGTTACAATTAATGGATATACCTCACTTTACTTATAAAACGCAATTA
GCTTACGCTTGGAACCCTCTTGATTCTTCGATCAATATCGATGTTAAACTAATCGATCCG
TTGACACTCCACAGGCACCCTGTGGCTCAGAGCACAGATTCGATCCGTGGAAAAGTGGGA
TTTAGTAGAGGTTTTCATATGTGGCGAATAGATTGGAAATTACGCGAAAGAGGAACTCAT

GCAGTTGTCGGCGTTTGCACCAAAGATGCTCCTCTCCATTGCCATGGATACTGTTCTCTG
ATCGGATTGACAGAGGAATCCTGGGGCTGGGACATCATCCGAAAGAGATTGGTGCACAAG
CAGTCGGTAGATGTTGAAGATCAAACATATCCAGTTATGATCCCGTCGGAGGAACAGAAC
AGCAGTGCAGATGCGAACGGAGGCTCGAATATCGAGGTTCAGGAATCGATTTTTGTGTTA
CTCGATATGGAAGAGGGCACCTTGGCATTTATCAACGATGGTCAATATTTGGGAGTGGCA
TTCAGCGGACTGAAGGGTCGCACGCTCCATCCCGCCATCAGCGCGGTGTGGGGACATTGT
GAGGTTACCATGGCACACATGGCCTCTCTGAACCGTGAGTCTCGATTTTTCTGTCTAAAC
TTTTTGTCAATTAATTTTTAA
>DjScaffold2408:69324-70528 strand:+
gcttctaatgacATGCCTATGGACATTAGCCCTGTTACTAACGATTCTAAATCGAATGCT
TCAAATGAACCTACTCCGATAGATAGAAATTCAGTTATTATTCCATTAAAAAGAAAGAGA
ACTAACGATAGTAATATTGGCGGTCTTGTTTCATATGGTATGGATGAAGATGATATTATT
GACGACGATGAATACGATAGCGAACCATATCTACAGTTTGCTAATGACAAAGAATCTTCC
AACGAAAAAGATACTGTAGATTTGCGAGAATCTGTTCCGATTGACAACGAGGAATGTTTC
AATGCGGAAGGCCGAGAATCAGATGTAGAAAATGACGAAAGTGTCCGGCCACCGGGAGAT
GTAGATTTGAAGAGTAGAAGTGGTCAATCCTCGTCTCAAGTTAAAGCAATAGTTTTAACT
AAACGTCCGAAAGGCAGCGAATTAGCATATTTACTATCACGGCAGATACTGAACAATCGA
TCAGAAATCCATTTACCTCCTCCTCCGTCCGGCAAGCCGCCACCAGAGCTCGAGCAGCGG
ATAACTGAATTGCGTGCAAAAGCGAAAAGTGCTCGCTGGAACATGACGCAATCGATTCTC
AATAACAAGGAATACAGGAATCCCAGTATTTACGAGAAACTGATCGAGCACATGAAAATC
GATGAAAGAGGAACGAATTATCCGCCGGTGAGTTGTTGAgttgttttctgagttttcgta
atgaattaatcaattaatcactgcaaattatccaaataaaatgcgatatttgagagatcc
agcgggaaaataccgactaaattaacatagagcagagttacccaatgtgacccagatttt
tgctttgtatttgtaaatatcaatatcgataggttttattcactgttttacttaattagg
gccgatatttaccaagattcggtaggaaattccaaggaaaatgaactaatccaaagggga
acagttcggggttgttgtgaccccccgcgcaaaatattaaaaat
>DjScaffold4461:11759-16499 strand:+
ttattcggcgaATGTTGATAAAATGTAGTGATGTTAATAATCCGACTAGACCGTTTCATC
TTTGCAAAGAATGGGCTTATCGAATTAGTGAAGAATATTTCAGCCAAACTGATGAAGAAA
TCAGCAAAGGTTTACCTGTAGTTATGCCACAATTTAATCGCCAAACCTGTGATATACCAA
AATCTCAAATATACTTTATAGATATATTCCTTCAGGACATGTTTAAGGCTTGGGATCAAT
TTGCAGATGTTCAAGAACTTTTAACCAATTTACAAGAAAATAAATCAAAATGGACCTCAT
TATCAGAAAATAATACTGACAGTGCCAAAACGTTAAGCGATAATTCTAAATAAaatgatt
ttatttttatctctttgtaaagtaaattttattagacaatactcttttactaggatgaga
tgagattaaactatccattttaaatttttactttatttattggtattttatattttgta
ttatttttgattatgtgtatatcatttgtaaattgtcaatggtatttgacaatcaaagta
ctagtttgattgtgtgaatataatgctttggtttttatt
>DjScaffold3531:13933-14481 strand:+
ATGACAAAAGTATTGGACTGTCTAGATGGAGCAATGGTGTATATGGATGACATAATGATA
TTCTCAGAAGGACCAAAAGAACACCATATAACTCTGGGTCAGGTATTAGAGCAGATCCAA
ACGGCAGGGTTGCGAGTGAATCCAGAAAAATGTCACCTATTACGCAAAGAAGTAAAATTT
CTTGGCTATATAATAGATAAAGAAGGAATTCAAACAGATCTAATGAAAATCAAAGCTATC
AAAAATTTTGAGAAACCCAAATGCGTAAAGAACTTGAGAAGCTTCCTCGGAATATGCAAT
TATTACAGAAGATTCATCAAGAATTATTCAAAGAAATCAAGAGCCCTAGAAGAATTATGT
GGAAGTAACAGCATGAAGCTAATATGGTCAGATGGATGTGAGAAAGCTTTCGCAGAAATG
AAACAAGCACTGATAGAAGCACCTATTTTAGGATCTCCTGATATAAAGAAGACATTTATT
TTAGATACTGACGCCAGCTCCGACGCAATAGGAGTGGTTCTATCACAAAATTATGATGAT
GGAAGATAA
>DjScaffold3531:130939-131304 strand:+
ATGAAAATCAAAGCTATCGAGAATTTTGAGAAACCCAAATGCGTAAAGAGCCTGAGAAGC
TTCCTCGAAATATGCAATTATTACAGGAGATTCGTCAAGAATTATTCAAAGAAATCAAGA
GTCCTAGAAGAATTATGTGGAAGCAACAGCACCAAGCTAATATGGTCAGATGGATGTGAG
AAAGCTTTTGCAGAAATGAAACAAGCACTGATAGAAGCACCTATTCTAGGACTCCTTGAT
ATAAAGAAGGCACTTATTTTAGATACTGACACCAGCTTCAACCCAATAGGAGCAGTCTTA
TCACAAAATGATGATGATGGAAGAGAAAGAGTTATTTCTTACGATGTCGGTTTTAGTCCA
ATATGA
>DjScaffold1400:42793-62003 strand:+

acaattaaattgatagtgttgatgatctatcaaaatatattacaattaagttttatttta
aatggtgttatttatttttttaaataaattattagacaaatttttaaatttcttcaatt
caataataataataattcaggaaacagaaatgctggaaaaactacatttcttaaacaatt
tcgaattcactacggcgatggttatccttatttacaaagagcgatattagtgcctgacat
tctgagtaatttggctgatgcaattcatatcgttATGGATAACATGTCTCGATGGGATCT
TAGATTTGAAGATCCATATGTTCAAACATTGGCTAAAGATTTTCATAATAACTGTCCTAG
AAGTGCTACATTATCAAACAGTCAATCCTTCTTTTATAAATCAAATTTGAGTAATTCTCC
GAAACCATTTAATCGATTTTGTAAAGCGCCAAGTAGAATTAGTGATTTCTGTTTACATAT
TAAAAATATTAGTGATCAAGATGACTTTCAAGAATGCTTAGATTGTAAAGAATTTTTTAA
ACCCAATATTACATATTCTGAATTGTATTTTATTCAAAATATTGATCGGATTATTCAAAG
TGATTACATACCTACCCTACAGGATATTTTGATTATGAGGAAACCAACATATAGTGTCAA
AGATTTTATATTTAATTTGGGAGGAGTAACATTAAGGTTTATTGACTTGGGAGGATATAA
ATGCCAACGTAAAAAATTTATTCATTATTTTGAAGGTGTGTCTACTGTATTTTATTTTTG
TGCCATTTCTGAATACTCGGAAAATGTTGAAGAAACAACGAAAAATAAAGTCGAAAATAG
TCTGGAAAATTTCAAGGAAATCATTAATAATAAATATCTTTGGAGAAAAGATTTCATAAT
TTTTTTGAATAAATCAGATTTGTTTAAATCCAAAGTTGAAAATCTTTCTATCAACGTTTT
TTTCAGTGATTATGAAGGTAACCCGAGTGATGTGGAAGATGGAATTAACTTTTTCCGAAT
GAAATTCCTAGATCTTCGACCTTCCAATGCTCCAAAAACGAAAAAGATTTATACTCATGT
TACATGCTCATTGGATATTGATAAAATGAAAGTAATAATCAAACAAATGATACAAAATAT
GCTGGATAGTAATGTAAAAAGAATGACTTTGTTTTAAttagaattaatataaaatttttc
aagaaga
>DjScaffold2141:121187-125421 strand:+
ttatagaatgagcaatattattcaagtttctaacattgctgcaactgtaacagatgaaaa
tataaaaacattatttggttttgttggatcaattagggaaacaattatttattttgaaga
ctcacagcaggaagagactaaagtaataagaatttttagcatgttttattttttgcattt
gggactctattcatttttaacaaagaaaatgattgaaattttattataggttgcatacat
tgtttatgaaagcaatagtgacgcagaaacttctttgcatctgcacaacactgtatttca
tgatcgagctttaattgttttgcctttagcagattgctgcgctggccagattcccgatat
caattatgctaaagttattcgtgctccagataaaaccgtaactggaattttgccgaaatc
caaaagctggcctcgcgatgtaatcagcatggttgtcggtcgtactggggaacagtcgat
aataacaattgagccgaactttattaagcagggtttgccgacttatccgaaattaccata
cagtactgaagttgcaaaaatcgatgaaatccgcagaacggttttggtgactaaccttga
ttctgctacaaatgctcagcaaaaaaatttcattctaacaattttgaataattctcaggct
tcaataaaacacactcactccttgattactgtaatcaaattattcgtatttagctcattg
attttcttgataattcttattgatttaacactgatatgttatattttattatagataata
atatttgtggatcatcaccgattattctgtttcggaaattacctgttaataatttgagct
aaaccgaatgattatatacatatatatattctgttataaatcaaataaaattaattaatc
acattaagattgatgcaagatgtaaatagtaacatgaagtctcttagcggagggaggagg
ggaatatattattaaatacattatttcgattgttttatctacatttaacacaaatattgt
tcagtttaataatatttattttgttaaattcatcaaatattagtttcgagaattaaaatt
gtgctattactatggcacaaaatgtggatttaatatattttgatattcagtcaagatgtc
atgttatttgtgcgcgtgtgtcagtcttaatgtgagatttattaaaataatatatttttc
aattgttattttaaactgtcttttctatatttgcatttatgaattttgcatcaagatgga
ttcaagcttagagaactagacagttctgtatggattgattttagtcccctaattttctcc
agctctgcATGAAACTGCAGGTCCTGGATTTTTTTCAACCGGTTGCTGAAGTAAAATTTA
TTAGAATGGCTGGATCTGATAGTGACAATGCTGCCTATGTTGAATTTAGTGAACAATCGG
CAATTGTGAAGGCTTTCGGTATGATGGGAACGAATTTGAACACAAGACCGATTATGATTC
AACATTCTAATGTGGCGATTGTGAAACCTTCTAATTATTTGAGAGGGTTGGAAGAGACGA
GCAGACATGTTCGTGACTCTGGAAGTGTTAATGGTCGCAGTAAATCTCGAAGTCGCGATA
GAAAAGTTTCCAAGTCAAGGAAGCGTTCACGGTCTCGCAGAAGAAGCCGATCTTACAAAA
CCAACAAATCTCGTCGGAAATCCAAATCTCGATCAAATCGTCGACGTTCAAAATCGAGAT
CTCGTAAACGATCAGTTTCCAGATCTCGTCGTAGTCCAAGTAGATCAAAGAATAGAAAAA
TAAGAAAACGATCAAGAACTCGATCAAAAGATCGTAAAAGACGAAGTCGAAGCAGAGAAA
AACGAAAAGAACGATCGAGATCTCACCATAGAAAACCCAAGAGAAGATCAAGATCAAAAC
GTCGAGATGAATCAGTGGGTAATGGCAAGCGGAAAAATCGGGAGAAATCGATTGATGATG
CCAAAGAGAAAGAGAAATTAAAGGATAGAAAACGTAAAACCACAGAAAAACACAAAGAGG
AACGGAAGAAGAAACATTCCAGTTCCGATCATAAAGAAAAGCAAGATAAAATCGATCCGG

CGAGAAAAGAAATTCTTGTGAAAACTGAAAAGATGAATGGAGAAGAGAATGTGGGGGAGA
ACGCAGCCAAATCGAGTTCTCAGGAGCGAGAATCCAATCATAAGAAGAAGCCCAACCAAA
ATTGTGAACCTAGTGATACAGAGAATGTAGAGAAGAATTCTGAAGAAAATACTGCTGTCG
TCCATAGCAACAATAATATCGATATCGATATCattaattttggtcaaattaaatttttag
agtattttccttatttatagaATGAGCAATATTATTCAAGTTTCTAACATTGCTGCAACT
GTAACAGATGAAAATATAAAAACATTATTTGGTTTTGTTGGATCAATTAGGGAAACAATT
ATTTATTTTGAAGACTCACAGCAGGAAGAGACTAAAGTTGCATACATTGTTTATGAAAGC
AATAGTGACGCAGAAACTTCTTTGCATCTGCACAACACTGTATTTCATGATCGAGCTTTA
ATTGTTTTGCCTTTAGCAGATTGCTGCGCTGGCCAGATTCCCGATATCAATTATGCTAAA
GTTATTCGTGCTCCAGATAAAACCGTAACTGGAATTTTGCCGAAATCCAAAAGCTGGCCT
CGCGATGTAATCAGCATGGTTGTCGGTCGTACTGGGGAACAGTCGATAATAACAATTGAG
CCGAACTTTATTAAGCAGGGTTTGCCGACTTATCCGAAATTACCATACAGTACTGAAGTT
GCAAAAATCGATGAAATCCGCAGAACGGTTTTGGTGACTAACCTTGATTCTGCTACAAAT
GCTCAGCAAGTCCTGGATTTTTTTCAACCGGTTGCTGAAGTAAAATTTATTAGAATGGCT
GGATCTGATAGTGACAATGCTGCCTATGTTGAATTTAGTGAACAATCGGCAATTGTGAAG
GCTTTCGGTATGATGGGAACGAATTTGAACACAAGACCGATTATGATTCAACATTCTAAT
GTGGCGATTGTGAAACCTTCTAATTATTTGAGAGGGTTGGAAGAGACGAGCAGACATGTT
CGTGACTCTGGAAGTGTTAATGGTCGCAGTAAATCTCGAAGTCGCGATAGAAAAGTTTCC
AAGTCAAGGAAGCGTTCACGGTCTCGCAGAAGAAGCCGATCTTACAAAACCAACAAATCT
CGTCGGAAATCCAAATCTCGATCAAATCGTCGACGTTCAAAATCGAGATCTCGTAAACGA
TCAGTTTCCAGATCTCGTCGTAGTCCAAGTAGATCAAAGAATAGAAAAATAAGAAAACGA
TCAAGAACTCGATCAAAAGATCGTAAAAGACGAAGTCGAAGCAGAGAAAAACGAAAAGAA
CGATCGAGATCTCACCATAGAAAACCCAAGAGAAGATCAAGATCAAAACGTCGAGATGAA
TCAGTGGGTAATGGCAAGCGGAAAAATCGGGAGAAATCGATTGATGATGCCAAAGAGAAA
GAGAAATTAAAGGATAGAAAACGTAAAACCACAGAAAAACACAAAGAGGAACGGAAGAAG
AAACATTCCAGTTCCGATCATAAAGAAAAGCAAGATAAAATCGATCCGGCGAGAAAAGAA
ATTCTTGTGAAAACTGAAAAGATGAATGGAGAAGAGAATGTGGGGGAGAACGCAGCCAAA
TCGAGTTCTCAGGAGCGAGAATCCAATCATAAGAAGAAGCCCAACCAAAATTGTGAACCT
AGTGATACAGAGAATGTAGAGAAGAATTCTGAAGAAAATACTGCTGTCGTCCATAGCAAC
AATAATATCGATATCGATATC
>DjScaffold3170:48086-62265 strand:+
ATTGGTGGTGGAATAGGTGGAGTAACATGTGTTGAAACTCTTAATCAATTAGACGAAACT
TTAACTATTGGTCTGATTAATTGCTCTAATAACGTAAAAGCTGTTACAAATTTACAGAAA
GTTACTAATTTAATTGAAAACTTTAATGTTGTTGACAAAGATTTTATTGATATATCAAAT
AATAATTCGAGGTTATTTATGATCAAGGGTGAAGCTGCATCAATTAATTCAGTAGAAAAA
TTTGTCAAACTAAAAGACGGCAGAAAAATATTCTTTTCTAAATTGTGTGTTTCTACTGGA
GCTAAGCCAAAATTAATTTATGATAACAATGATTTTATTATGGGGATTAGAGATACAGAT
TCAGTGGAAATTTTTAAAGAAAAATTAACAGGATGCAAGCGTGTGGCGATTATAGGGAAT
GGAGGGATCGCCACAGAATTGATATACGAACTCGTTGATTGCGAGTTAGTTTGGATTATA
AAAGATAAAAATATTAGCGCAACTTTTTTCGATTCTGCCGCTGGAAAATTTTTATTAAAT
TCTTTTGATGATAATGAAAAAATAGAGAATAAAGGCGCTGAAAAAATTTCAATTCAACGA
CATCATTATGATATCGATGGAACAGTTTATACAACAGAGAGGAAAATAATTGAAACAGAG
AAATCTCAATTCGGTGGAGCACTTGGTCCTGACTGGGCACAAAAAATGACAATGAGAGGT
TGTGGAAATAAAAAATTGGAGGTAAGATATAATGCAGAAATCGCATTTATTGAAAGTGAA
CCGAATAAAGTTGCTGAAATGTGGAATAGTCTCAATTCAACCAATCAAAAATGTGATTAT
AAAGTTGCTCTAACTTTAAACAATGGTTCAATTGAATTTTGCGATCTTGTAATAAGTGCT
ACTGGTGTTACTCCAGAGTTTCCACCATTGATTGACCAAAAATGGTCCATTTCTGATACC
GATGGAGGCATTTTAGTGAAAGAAACTATGGAAACAAATATCGATGGAATCTATGCTGCA
GGAGATTGTTGTTCTGCAAATTGGGAGCACGCTGATAATTGGTTTCAAATGAGACTCTGG
AATCAGGCACGATTGATGGGATTTCAAGCTGCCAAGAGTATGTTCACGTCGCAACATAAT
AAACCTCCAATGCTCGATGCCGGTTTTGAATTGTTCACTCACGTTACTAAATTTTTCGGT
TTCAGTGTTATCTTATTGGGTCTTTACAATGGTCAGAAACTAAAATTGGAAAATAACGAA
AATGCAGAGCTGTTGATTCGTACATCACCCGGTTTAGAATATATTAAATGTGTTGTCGTT
AACGGTCGTATTCGAGGCGCTATATTGATCGGTGATACCGGAATGGCAGAAACATTAGAA
AATCTCATTCTTAATGAAATCGATATCTCACAATTTGGTGAAAATTTACTGGATCCCGAA
ATTGATATCGACGATTTTTTTGATTGAtctgcttataatattctagtttctagatgtttt
ggaatatttatttattattttcttt

>DjScaffold4447:215353-216020 strand:+
agcaatcactaaaaaagaaattcatcagaatattccaggaattttaatttaatttccatt
gttgaatattattgataatttATGGAAAGATCGTCAAGTCCTACTGTTCCTATCGTAGAT
GCAATCTATCCTAAAAATATTATACCATCGAGTAAAGTTTTGGAAACGTTCCCAAAATTG
ACTTTGAATGAAAAGGAATTTATTCACAAACCAGTACCTAGATCAAATCGAAAACGCCAC
ACACGATCAACCAGATTCAAAACTCAACCAATCACATTTTCTGAGATCAAAGAAGTTGAT
GAAGAAAGTGTAATTACTATTTCCAATCAAAATACAAAATCCCAAATAGATGAAATGAAA
TCCACTTCAGAAATCGATAATTCAAAAATATTTACTCAAGTAAACAAACCAGTTGTTGTA
TCTGATGAAAGAATGGATCAAAAATTTTGCAACAAAAGACCTTTTTTGCCTCCATAActc
taaataaaaatggcattgttaaatattaaataataataataattaaacaacttgaacaat
ggacaagatgtagtagaaatgttgtctagctatttattattgatgttaaacttaattttg
atagtcaga
>DjScaffold4447:128946-129161 strand:+
ATGTCGTTTTGCATAGCTGCGACGCCTCGAACATTTCAGGAACTCATGACAAAGGTTTTA
GGTACAACAAAGGGAATAACGGTGAATTTAGACGATATTCTAATATATTCAGAAGACTTG
GAAGAGCATTACAAAGCACTGGATGAAGTATTTAGTAAAATACATCAAGCAGGACTACGT
TTGAGCCCTTCAAAATGTCAATTGCTAAGAAAATAG
>DjScaffold936:11293-18376 strand:+
tataacatttaataatcgtatattctaaaaataatcaaattaatttaatagtaattgtga
taaataaaattaaaataattatatgaaaataaaaatatatcaaatatttcaatacaaatt
tttcctgatacaaatgaattattttgattaatttttagaaactttgatcagatcgcaagt
ttgaatATGGAATCTCAGTTATTTGCAGGTTTAAGTAAATTTAAATGGTATGCAGGACGG
TTTATGAGAGACGATGATTTTGCTGATCGGTTGAATCATACCTTTACTTCCTTTCTACTT
GTTATATTTACACTAATTATTTCTGCTCGACAATACATTGGAAAACCAATTGCTTGTTGG
GTACCAACTGAATTCTCTCGTGCTCAAGAGGAATATGCAGAATCTGTTTGTTGGGTAACT
AGTACGTATTTTGTTGAGCCGGGATTTCACTCAATTCCACAGAGTATTGATGAGCGAGAA
GACAGGAAATTACTTTATTATCAATGGGTACCATTTATTTTAATGGGGCAAGCTTTTTTA
TTCAATATTCCTTGTATAATATGGCGTCTTTTCAACTGGCAATCAGGTATTAATCTTGTC
AGTGTTATGCAAGTAGCGTGTGATGTCGAAATAATCAAAGATCGAGAATGCCGTAAAGTC
AATGTATCATATGTAGCTCATCATATTGATCAATGTTTGAACACCCGTCACGTTAATCGA
AACACACTTGGTGGTAGAATGCGAAACTTTATTGCAGAAAAATGTTGCCTTTGGTGCAGC
AAGATGTACGGCAGTTACCTCATCTGTCTTTATATAATTTGCAAGTTACTGTACATAATG
AATGTTATCAGTCAGTTTTTCATTATGAATCGAGTTCTCGGCACAAACTATACATTTTAT
GGATTTGATCTCGTGAAGCAACTGGCGGATGGGCGAATGTGGCATGAATCTGGTCATTTC
CCCCGAATAACAATGTGTGATTTTGAAATTCGCAAATTGGCTAATAAACATCGCTATACA
GTTCAATGTGTTTTACCAATCAATATGTTTAATGAGAAAATATTTATTTTTCTTTGGTTT
TGGTTTGTTTTGGTTTCAGCTGTCACTGTGAGCAGTTTCATATCATTGACTTACAAAATC
GGCTTAGTTTATAATAGAATAAATTTTGTTTGTAAATTTCTCAAATTACGACAAAAACCA
GACTCAATCTCAAAAGGTAAATGCCGGAATTTTGTCGAAAAACATTTACGTTATGATGGA
GTATTTTTATTACGTTTATTGGCTCAGAACGCCGGTGATTTGATAGCTTCGGAAATCACT
GACGCTTTATGGAAATTGTATATTGATCATAAATCAGATACCAAATCAGATACCAAATCA
GATTTGTTTCGTTCTAAAGAAAACCATCGACCGTTAGCATCTAGTCAACATATTGTATTA
CAAAAGCCTACAACAAATGAATCGTTTTTTCCAGGAAGAAAAGACACTACATCACATACT
AAAATCAGAGCACCCGAAAAGACCCGCCATAAGATGCCTTTAACTGAATTCGGAACAGTA
AATACAACTGAAGAAGATCCTGGTGAATTCGTTTGAggaaatattttcagtttaaagata
aactgaaataataatgattacttgtgataatattaataataataaaaaataataataa
taaaaataataataataacaacaacaataataataataataataat
>DjScaffold936:77959-86512 strand:+
cgattcgtgatgccggtggttcatttggtaaaagggaagctgcacaagaagaagaatatt
tcaaacgattagagcaagaacaagttaaaaatATGAAAAAGTTTTTACAAGATGAATTAA
AAAGTCACCAAGAAGAAATTGAACGTCATTCAGAAGCCATAGAACGACATAAATCAAGAA
TTAAGGAAATAAAAGATGCTGTTATAAAACATTAGatgaaacatttttatttaactttat
ttgcttttcataaaataatagtaatgcatgaagccaaggt
>DjScaffold936:36056-36349 strand:+
ATGTGGTTTGGGTTGTCACCAGCCCCAGCAACTTTTCAAAGTCTCATATATGTATTATTC
GAGAATATCCTTTATAACGGAGTCGCGGCGTATTTAAACGATGTCTTGATTTACGGAACA
ACAGTGGCAGAACACGAGAAAAACCTAGATGAGGTTTTTAAACGAATACAAAAAGCTGGA

TTAAGACTCAATCCAAAGAAGTGTAGTCTGTATAAAACTGAACTTAGTTTTATAGGTCAT
AAAATATCAAAAGAGGGAATAATTACTAATAATATTCAAAGAAATGTTTTGTAA
>DjScaffold4084:21011-21682 strand:+
CAACCTCGAAAAGTTATTATAGTTGGCCTAGATAATGCTGGTAAGACTACCATTTTATAT
CAATTAGTCACAAAGGAAGTATTGCATACTTCTCCGACAATTGGAAGCAATGTTGAAGAA
TTTCATTGGAAAAATATAAATTTTTTAATGTGGGATATTGGAGGTCAGGAATCTCTTAGG
CCCTCGTGGGAAACGTACTTTATGGGAACTGAATTTGTAATATTGGTTGTTGATGGAACT
GATAAAGAACGTTTATTAATAACTAAAAATGAAATGCTGAAAATGTTGCAATCAGAATTT
TTATCGAAATCTAAATTTCTCATTCTTGCTAACAAACAAGATGTTAAAGGTTGTATGTCT
GCTGCTGAAATTAGTCATCAGTTAAATTTAACCACTATTAGAAAGCACTCTTGGCACATA
CAGTCTTGTTGTGCGTTAACAGGAGAAGGATTGTACCAGGGTTTAGAGTGGATGATTAGT
CAAATTAAAAAGTCGTAAtctgcatttttataattttgtgattaaaatgacttgtctttc
caatcatgt
>DjScaffold2727:16502-29178 strand:+
ATGAGTGGTGAAGCTGTTAAAGTAATTGTCAGAGTTCGGCCATTAAATGAACGAGAAAAC
AATCTTAATTGTAAAAATGTCATTGATATTGATTCTAGTCGTGGCCAGTGTTCAATTAAA
AATCCTAATGATTCTAAAGCTCCATTAAAACCATTTTTCTTTGATGGAGCTTATAATGTT
GATTCAACTACTGAACAAATGTATTCAGATATAGTTTATCCACTTGTTGAAAATGTAACT
GAAGGTTATAATGGTACTATATTTGCATATGGTCAAACAGGATGTGGTAAATCATTTACA
ATGCAAGGAATTATCAATCCATCAACACAAAAAGGAGTTATTCCAAGAGCATTTGATCAC
GTATTCGAAACGATTTCATTGGCAGCAAATTCCAAATATTTAGTTCATGCTTCGTATCTT
GAAATTTACAACGAAGAAATTCGTGATTTATTAGCAAAAGATGTAAAAGCCAAATTAGAT
TTAAAAGAACATCCAGAAAAAGGTGTCTATGTGTCTGGATTATCAATGCATAAAGTCTCG
AATGTCAGCGATTGCCAGGTAGTGATGGAAAGAGGCTGGAAAAATCGAGCTACCGGATCT
ACATTAATGAATGTTGATAGTTCACGTTCACATTCTATTTTCACTATATACCTGGAAATG
TTTGACACTGATGATAAAGGTGAAAACCACATTAAAGCGGGAAAATTAAATTTAGTGGAT
CTTGCTGGATCTGAAAGGCAATCCAAAACCGGAGCGACTGGTGATCGATTAAAAGAAGCT
ACTAAAATCAATTTAAGTTTATCTGCATTAGGAAATGTTATTTCAGCATTGGTTGATGGG
AAGAGTAAACACATTCCATATCGAGACAGTAAACTTACAAGAATGTTGCAAGATTCTCTC
GGTGGTAACACCAAAACTCTCATGATAGCTTGTCTCAGTCCAGCTGATAATAATTATGAG
GAAACTCTTAGTACCTTACGATATGCTAATCGAGCAAAAAACATTCAGAATAAACCAAAA
ATTAATGAAGATCCCAAAGATGCTTTATTAAGAGAGTTTCAAAATGAAATAAATCGTTTG
AAACAAATGCTGGCCGGTCAAATTCCTATTAATATAGATTCAGTTAACACGGAAAAATCA
ATGAGCACCAAAGATAGAAACAAATTAACAGAAATGGAGCGAGAGAAAATTAAAGCGGTG
AGTGTTTATTCATAG
>DjScaffold301:1733-5227 strand:+
tatttaataaagttttgcaATGGAAAACTTGTTGCCTCATGTTATTAAAAAGATAGGACA
AGAAATTCGCCAACTAGTTTTAAATCCAATTGAAGGAATAACTTTATTAACTAATGAACG
AGAAATAACGGATATTCAAGCTATTATTGATGGTCCTACTGAAACTCCTTATGAAAATGG
TAAATTCAAAATACGAATGGCATTATCAAATTCCTATCCACAACAAGCTCCAAAAGGCTT
TTTTATTACTAAAATTTTTCATCCAAATATTTCACCTAAAAACGGTGAAATTTGCGTGAA
TACTTTGAAAAAGGATTGGTGTGCTGATTTTGGAATCAAGAAAATACTATTGACTGTAAA
GTGTCTAATGATTTATCCAAATGCCGAATCAGCATTGAACGAAGAAGCTGGTCATTTGTT
ATTAGAAGATTATACAGAATACGCTAAAAGAGCCCGATTATTTACAAGTATTTACGCTGC
TCAAAACAATCAGAACATTTCTAATTCCGGAAATTTCTCATCTGACCAGCCTCAAGTTTC
GAGTTCCATTAATAATCCCTCAATGAATGCAATTTTAAAAACGAATTCCGACAGTAACGA
TGCTGGTCCAGTCGCTAAGAAATTGAACTCAGATGAGCATTCTAGTAATATTTCAGATCA
AAATGCTCAAAAACGACCAGTTCCTCAACATAAAAAAGCTCTCAGACGCTTGTAAtacac
tcggaaatgatgttatcattattattattattattattatttattaaggagcgagta
taattattatttgttgaaaatatgaaattagtgttttatttataaatgaatctttaaatt
acaattatcatcacttttattaaactttgtaaacagttaattcgccttaaattcgcttta
catcagaattatctttattattattattattaatattataattattataataataattat
tattactattattacatcaaaattggattattattaaccatatagatatattgtgtttat
gtaatgatatgtatttatttatgtttggatgtgtgtgtgtgtgtttatgtgtccgtgtct
gatgcttgtaaatctaactcaactactcgtgcta
>DjScaffold3305:14931-24608 strand:+
ACATTAAACCGTTTGGAGAAAAGTATTGACCAAGATACTTTAACGGAGTCTACCGTTCCT

AGTTCATTTTCAACAATTGAAAATTCAATACCAATATTTACTGCTAAATTAACCGTAACA
AATCCAAATTTCAGAAATATTTGTCTCTTTGAATTGAAGTTAAAAGACGAAAATATCATT
CAGTTCCATAATAACCAATTGGACAATATTATTTTGTATTATTGGAAATCAAATCAAATA
GTTTTTCATCGAAATATTTTTCAAAATAAAGTTGTACTTTCACTAGGTTTTACAGTAATG
TTTTCTGTAAATAATACTGCAATAACTTTGAAGATTTGTCAAAAAAATCATGCTTATAAA
TATACAGAAAATTTAAATGTATCTATAAAATCACTCTATGTTGGAATGACTTCAGGAAAA
AGTAATTGCAGAAAATTCGATGGATCGTTAAGTTTGAAATATGATTCGAGGACTTTTTCT
CTAGACAATTATTCGTACGTAATTACAATAGTTTTGATTATATTCGGAGCAGTTCTTGTA
ATCACTGTGATGGGTGGACTTGCGATT
>DjScaffold3251:2837-3968 strand:+
aatgttgataatgttgaatataatttaaagttttgtaattgtttggtgagacatttattt
tttaaaattattatgtgctaaattattATGAAGTCAAAAATATGTGGTTGTGACAAAAAC
TCCCCTCTTACTAAAGGATTTACTGATAATCTATTGAAATATTTTCAAGTTAATGATATA
ACTGAAAGTATAGATTCTGGAAAACGGTTAAACTCTATTCATTTATGGGGTACTAGAGAT
AGATCTACAGAAGATTTAGAAGTATGGTTGTCAGATTACACACCATTCAAAATTGAATGG
ATCGATGATAACACATGCAATCTGGTGTTTGATTCAATCACAGATTGCTTCAAAGTTTTT
GAAAATTTCACTGACGAATACGATAATGAGGAGGATAGAGATATTAAAGTAAATGATCCA
GAATTTTGTTTGAGAACCGACATTTGGAGACAAGTTGTCAATTTACCAGAAGGAACAGAA
AAAATGTACATGAGATTGGCAACTAAAGATGATGTAAAATCTCGAGGAGCTGCAAAAAAA
AGCCAGTTTTACGTAACTCATGGAAGTTCTCGACACGATAATAACAGGAAAGGATTTACT
TCTAAAACTCATCGTGATAAACTGAAAAGAAGCCACGCAAAGTATCGGTCGTCTGCTGAA
GTTGTTGATGATTCCGGGCTGGTTGCGTATGACGATTTACCGGAAGTCGATAACTTGTCT
AAACGATCACCGCTTTCAGTTGAATCGATATCAGACAGCGAAGATGATGAAAATTCATTT
GGAAAGAAACGCAGAATGATTTCCAGTATGAAATTGTATAGTGACAGATTCGTTGAAAGA
GATTATGGAGAGCGAGTCAAACCAAAGAAGAAAAAAGGATCAGTTCACAGTCGGTTAGGC
TGAacggaaggaaataaatgtttcagaaagataagaaaatcgttttatctaa
>DjScaffold4068:64361-75668 strand:+
attttcctcaatatatttaatataaatttgttttacgttttcataactcttaatcataac
tgttattaatgaaataaaaataatacgaagaaattaaattgtttgatttttattaaatat
tctctagttatatattttcataaatgtagattttgtatttgttttcctttagttttgtga
tgataataataataataataataataataataataataataataataatcataataa
tagttgctaaattttattgatattaaaatattttattttttgtttattttcaattaattt
ttatgtttttgatataattaataaacgtttaatttgataaatttgtatcattttatcaga
ttctgaaagtacctctttattggaagcaaccaatttatttatgttgcggaggaaaggaaa
ataaacctATGAAAATTGACGATGTCATCAGTTTCTGGAGGAACATGTTAAATAACTGTG
GAACAGAAGCAGCAAAGTTTATGCACATTATGACTCGGGGTAGTACTCGAAATTTTCTAG
AACGTTCAGATTTTGAACCTTTAGTACAAGATGTTTTAGAAACACATCCTGGACTACAAT
TCTTACAAAGCTCAGTGGATTTCCACATTCATTACATCAACACAGTTATTGCGAGAATTT
TCTTTAATCTCGATAGATACTGGAATGATCGCATAACGCTTGCAGAAATATCTAAAAGTG
ATTTAATTTGGGTGATTAATTATCTTGAATCACACGATGATATTAACATGGTTACTCAAT
ATTTTTCATATGAACATTTCTATGTGATTTACTGTAAATTTTGGGAACTTGATAATGATC
ATAATCTTATCATTAATAAGAATGATTTAATCCGACATAACAATTTCGCCCTTTCTGATC
TTGTTATTGATAGAGTATTTTCTGGAACTGTTCTTAGAAAATCATCAAATGACAAAACCA
TGACTTATCCTGAATTTGTTTGGTTCCTATTAGCAGAAGAAGATAAAAGGCATCCAAGGA
GTATTGAATTCTGGTTCAGACTGATGGATCTGGATGGGGATGGTGTATTGTCACTGTATG
AATTAGAGTATTTCTATGCAGAACAACTAAAGAAAATGGAAGAATTGTGCATTGAACCAG
TCAGTTTCACCGATTGTATGTGTATGATGTTAGACATGGTCAAACCACGATATGAAAATA
AGATAACGCTGGCTGATTTGAAAGCTTGTCGACTTTGCCATATTTTCTTTGATACATTTC
TAAATTTGCCGAAGTATCTCGATTATGAACAAAGGGATCCTTTTAATAATTTACGAGATA
TTGAAGAAGGTCTCACGGAAATATCAGATTGGGATCGCTATGCCAGTGAAGCTTACGAAT
TGTTAGTGTCTGAAGACGGACAACAACAAATTGGAGCTAGCAGCGACGAATATAATTATG
AGGACGATTTTGAAACAGAAGAAGCTGCAGCGCAAATAAAGCCTGGGTATAATCACGACC
AATTTCATTTACATCAGAATAATATGACTTCAGAAACAAATTTTAATGTGGGAATTAATG
GCAGAATTGGTCGTAGCTGAatttttcatattatctttg
>DjScaffold101:44819-46167 strand:+
cctaataagagcacttatttaaaagttctgtattggacaATGACGGAGAAAATGAAATTT
AATCCCACATTGGATGGTGATACAATTTCCAAACTCGGTCTATTAGACTTCAATGTAAAA

GATCTTCATCCTATTGAGCTTTTTGGAATAGATCACAGTGATTATTGGCATGGACTATTA
TTGGATTATTGTCATTACAGTATTCATTTGTCAGTAATATATGTCATTTTAATTTATGTT
GGGAAAATGTATATGAGTTCGAGACCTGCATTCGATCTCCGTTTACCCATGACAATTTGG
AGTAGTTTGTTAGCAATCATCAGTATTATCGGAACATTACGTGCAGCTAATGTTCTCTTA
TCTACTGCAATAAATTATGGATTCATGTATTCTGTCTGTCATCCACACTTCTATTACAAC
TCTAACGCTTTGTGGGGATTCATTTTCGTTTTGTCCAAGCCAATAGAACTCGGCGATACA
GCGTTTATTGTGCTCCGCAAGCAACCTTTGATATTCTTGCACTGGTATCATCATATCACT
GTCATGATTTACGTATTTCTCAGTTTCTATGAACATTCTGCACCTGGTCATTACTTTTAC
GTAATAAATTACGGAGTTCACGCAATTATGTATACATATTACTCTTTGAAATCTCTCCGT
ATTCGTATGCCAAAATACATTCCTATAATGGTTACGTCAATACAATTATCCCAAATGGTA
ATTGGAAGTGTGATCTTATCATATGTTTTAGTGTTAAAATCGAGAGGGGACACCTGCCAA
ATAACTAATAGATTGCTTTGTTTAGGTCTTGTAATGTACTTGAGTTATTTTGTCCTGTTT
TCCCACTATTTCTATAACGCATATTTCAGAGGAAAGTATTGGTCTCGTGGTCGAGAAGAA
CAGACTACGAAATCGTTACATTCCAATGGTGTATCTAATGGGGTAACGAAAAGTATAGCT
AATGGATTTTCTAATAACACAAAACAATCTAATGGAACTTTGAATAATGGCAAAAAACAT
GTGTAAaccaatctcgataaattgctttctttatcgtattgttgtagctgatataatcgc
ggtttatattttaatattatttatcaaattgatgtcgaggtttgtgataattacaatgat
ttttcgctttggtatgtgatgaaataaactgcttttatttattattttgttggttattat
tatcgatcattttgttttctggttttcttttttttgaaatattgatttttgggatatta
gagagattttatgattggaaataaatttaatattttcgt
>DjScaffold4607:11734-21485 strand:+
gtatattgctgccaaataatttttgtaatttgaatttatttatatctctATGGTTTGGAA
AAACTCCAAAAAAACACTTTATTTAACTGCTCAGCATTTGAACAACTTAAAAGATCATAA
ATACAGTTGTAATGGAAAATCCATTATAGAACCATTATTTCAGTTGTATTGGAACTGGAT
TATTAAATATGTTCCAATGTCAATTGCACCAAATACTTTAACCTTAATTGGGTTAATTGT
AAATGTTTTTAGTGTTTTAATTATAGCATATTACAGCCCTGATGGTACAAATTCGATTCC
TGCTTGGTCATTGGTTTTATGCGCATTTGGTTTGTTTTTCTATCAAACACTTGATGCTCT
TGATGGAAAACAAGCAAGACGTACTAATACTTCTTCACAACTTGGTGAATTATTTGATCA
TGGCTGTGATGCAATATCGTCGAATTTCGTTGTATTGGGATCGATTTTGAGTATAGGGAT
GGGAACATATCCCTGGCTAATGTTTTTGCAGTATTTCATTACAGTTGTTCTTTTTTACAT
GGCACATTGGCAGGTTTATGTGACAGGAACATTACAATTTGGGAAAGTTGATGTTACAGA
AACTCAAGCCTGTGTTGTTGTAGTTTATATGGTTTCAGCGGTACTTGGCGTAAAATTCTG
GGAATGCAATGTACCAATATTAAACTTGGAAATGAAGATGCTTCATTTCTTTATAATGCT
CGTTGGTGCCATTTATATTTGCTGGCGATTTGTTAGAATAATTTTAGCAGGTGGCTGCGG
TCGTCATGGAGCAACCATTGCAAATACTTCTATTCTCTTTCCTTTTTGCCCAATTGCTAT
CGTTATCGGATTAGCAATAACTGTTGCTATCAAATCTCCAAATCAGATCTATCAACAGTA
TCCATGCCTTTTTCTTATAAGCTTCGGTATGGTTGCTTGCAAAGTAACACATAAATTAGT
GGTAGCACACATGACTAAAAGTGAATTGTGCTTATTTGACAATGAGTTAATTGGTCCTGG
ATTACTTATTATGAATCAGTATTTGTTCAATTCTTACTTCAATGAACACACGGTTCTTTG
GCTTTGTTGTGTGAGTTTATCATTTTTTAAACATTTTTTTTTGATAATTTTCTATTTTTA
TTGAaatatctatttttctaaatttctctctagtttaaaattgcgaaatctgtttctcac
aacaatgttaaatttatatatttattttttttacaaatattgaaaaattttacaattaaa
attattgcgcggataattgttgggcttatctattgatcgcatttgtgattttattggata
tatatattagtttttgagaatctctttacaagtatacatttttttaaaatgcttatatata
aaacattctgattacgcaatcaattatgcaagctctccaatatatcaaatttattaaaaa
cattcaataaagatttgaagagaccagtttgaacattaacttatgga
>DjScaffold1020:7618-14074 strand:+
GATTCAAATAATGATATACATCCTAAAATCAGAATAGATGAAGACGGAATAGAAGTAAAA
TGGGATGAAGATAAGAGAGCTTGGTTTCCAAATATTGATGATGAATTTATTTATAAACAT
AATTTAAGTTATGGATTTGTAACAAAAGAAGAAAATAAAATTGAAGTGAATGAACCTCCA
GTTCAACCAGAATGGTTTGTTCGGTTATCGAAAGATTTTCTTGAAATATGCAAATTGGAA
AATATAGATTTAACTACTTCTTCTGAAGAATATAACAATAAGTGGTATAAATTTTATTCC
GAAAATGCTGAAAAATATGAGAAAAATAAGATCAAACCGAAAAAGCGACCTGCTAAATCT
AATGAAATGACTTGGTTTGAAGTAGATGAAAATAAAAACAAAAACATTTATATAGAAAAT
CTCCCACTTGATATTACAATAGAGGAATTTGTTGAATTAGTTTCTAAATATGGATTATTA
ATGTATGAACCTTTCACAAACATTCCAAAAGTCAAATTATATACAGACCAAGATGGAAAT
AAAAAAGGCGACGCTAAATGCTGCTATTTAAAAATCGAATCTGTGGATTTAGCACTGAAT

CTATTGGATGGGTATAATCTTCGTGGAAATATTTTAAAAGTATCAAAAGCTAAATTTGAA
AAAAAAGCTGATTTCGTTCCGAAAAAAATGTAGAAGAATTACAAATAAAGAGAAACGAAAA
TTAAAGAAGAAAGAAGATAGCTTATTTCGATGGGGTCCAGAAACTAAAACAATTCGACCC
AAGAAAGATAATACTGTTATTTTGAAGAATGTTTTTGACACTGAATTAATGAAAATAAAT
CCAGAATTGGCAATTGACATTAAAGAGAAAATAACCAAGGAAACAAAGAATATTGGTGCT
GTTAAATCGATTCAAGTGCATGATTTACACCCAGAAGCTGTTGTTCAAATAAACTTTAAA
GATGCAAATCTGTCTGATATTGCAGTGGAATATTTTAACAATAAAATATGGAATAGTAGA
AATATCTCAGCGGAAATATGGGACGGTAAAACGAAATATAAAGTTAAAGAAACTGCAGAA
GAGGAAAAAGAAAGATTGGAAAAATGGAACAAAGGCTTGAAAATGCGAAACCTAGTAAT
AACGATGATGAGGATATCGATGAAAAGGCAAGCAGTTCAGATCTCGGAAGAGATAGAAAT
ATTTCCGTTAATGATCAAATAGAATGTAATCCGGAAAATGAA
>DjScaffold3795:8880-12667 strand:+
ttatttcaatgtccgggaATGTTCCAATGTCATAAAATAAAGTATTGCATTGATATGTAT
CATTTATGTGATGGAATAAAAGACTGTCCTGATGGAGAAGATGAAGATGAAGAAAATTGT
AAATCATTAAGATGTCCATTCAATTTTAGATGTTGGTCATTAGATCGTACTAAATTACTT
TGCATAAATCAGAAAGATGTGTGTAATGGAATAAAATATTGTCCATTTGGTGAAGATGAA
CAATACTGCGATATTTCCCATTGTCCTGATCGCTGTACTTGTTTAGGATATTCAATAAAT
TGTGATTCGTCTTTGTCAGTAAACATTTCTAATTTGAATTCAAAAACACGAACATTGACC
ATTGTTAAAACACGATTTCTTGAGCTAAAATCAGGAATTTTTTCATCATTTATTTATCTT
GGTTACCTAAATCTAAGTGAAAATTCAATTTTAGAACTGTATACAAATTTATTCGATGGA
CTCTTTAATCTAAAATATTTAATCATCAGTAAAAATTTTATTCGAACAATACCTTCTTAT
ATTTTTCAATTTCTTAATCAACTTTTGATTTTGGATTTAACTGATAATCCAATTAAAACA
ATTGAATCTTACGCTTTTCATAATTTACTATCGTTATCTAAAATTCAACTTTCCTCGATG
CAGTTTCAGGATATCAGAGATTACACATTTCTTGGTATGTCTGATAACCTTGTCAATATA
GACATGTCAAACAATGATATCAAATCTATCAGTCAATTTGCATTCATTGGAATTAATAAT
TTATCTACGTTGGCCATTAGTGTCAATAAAAACGATATTGCAATGTATGGGGAACCGTTA
AAGCATTTAACTGTGATAAATGTGTCCACAAATGTTTATAAAATATGCTGTAGTCTAGCT
AGTTATAAAAGATGCTCTGCAAAATCTGATGTTTCCAGCAATTGTGAAGATCTTTTAGCA
AATAAGTCTATTCAGGTTATTGTTTGGTCAATGGGAATCATTGGATTGATTGGTAATATT
ACGGTAATATTGTGGAGGATATTAGACGCTAGAAAAGGCAAATTTTCTACTGAGGATCTA
TTAATTGTAAATTTGGCTATTTCGGATATGTTCATGGGAATTTATTTAATTATTATTGCC
TCAGCAGACAAATATTATAGGTAAgagaatttatatttaatcatatctatatttcaatat
ttatcctattaaatattttaaattcaaaaaatctcaataatacagataagcaattattgt
tgataaaataaatttcactgctaatttcattaaaataagagttttgatcaaattaatttg
atcgaagcttttattttcacgaaatctgaaaaaagatcaatggttacgtccaacaattat
tctcgcaattattgcaattgtaaaattcactactaatatttgggttttacttttctccaa
attaagaactttagttaattgctaatttaacctaccttttttaaagtcttaataatataat
aaaatatttataaatcaaatctataattttgattttaactaaaaattactgg
>DjScaffold3795:50973-51170 strand:+
attttagATGTTTCCTATTGGATTCCAAGGAGATGAGCCACAAGAAGAACCATCGAATCT
GCCTTATCTTTTTAAAGATAAATTCCATCGAAATTTAGCTAAAGGAATTGGAATGTTTGC
AGTTACAATTGTCTTATGTTTTATGATCGATGCCAGT
>DjScaffold3649:14113-14959 strand:+
tgttactaattcatctttagaaaatattttagaactagccaatagtatcaatgataacat
taaattcactttagagaagtcaatcgactttcagattccttttctggacaccttaatcca
aaagaaacctggcgaattattacttacccagttgtatatgaaaccatttcacagtgggca
tatattaccctggagtaaaaattggaataatcaagggtgaaaagataagagctgatagac
tatcttcaaacgaagaaATGAGACTGACTTCCCTCAGGTTGATAAAAAAATAAATTTCTAA
ATAATGGTTATCCAAATAGAATTGTATATAAACATCTATACTCAAATACAGAATGTAATA
AAATCAAACAAAAGACAAATACAGTAATTAAATTACCTTTTATTAATGAGAAATGTAGCC
AAATCATGCAAAATATAATTAAAAAGTCAAAACTAAAACACAATCCAAGAATTATATTTA
AAATGCAAGCACCTTTCGGTATCCAATTACAGAAACCAAGACAAAATTTTTGCGGTCCCA
AATGCATTTGTGATAGTAGAAATATATGCAATAAAAAAATATCACTTACAAAATAAcatg
caccttatgtaaggatacatatataggacttctacagaaccatttcttttcagaaccatc
cgtacaagaattaaagaacatctcaaacagtaagaaagtgaacttttcaaacacttatga
aacaaacataatgttttaccagaaaatcatcatattacatggaaaacattaaaaacaggc
tacatttctacacttcacggaaaaaaacatgaacaaataattattaa

>DjScaffold3649:16080-27442 strand:+
agtcgatatttgaaATGTTTTTACCAAAAACTAATGTGTTCTATTCACGTATTCTAAATC
AATTTACATTTAATATAACTGCTAAAGCATGTTATTCTGATTCATCAAGAAGTACTACTT
CTTTAAAACGTGGAACAGGTGGGAGATCATCATTTAGTGGAGTAGTCGCTACAGTTTTTG
GTGCTACCGGATTTCTTGGTAAATATGTTGTAAATGAATTAGGTAAAATCGGATCGCAAA
TTATAATTCCGTATCGAGGGACAGCATATGCTGTTAAAGAATTACGAGTGATGGGTGATT
TGGGTCAAGTTCTGTTTATGCCTCTGCAAGTCGATGATGACGAAAGTATCAGAAAAGCCA
TCAAATATAGCAATGTTGTGATTAATTTAATTGGCAAAGACAATGAAACCAAGCATTTCT
CTCATCAACATGTTCATGTTGATATTGCAGCTCGAATTGCACGAATCAGCAAGGAAATGG
GAGTAGAAAAATTAATTCACATGTCAGCTCTAAATGCCAGTCCCACTGTTCCCGAATTTA
TCAAAAAAGGTGGTTCAACGTTTTTAAAAACAAAGGGGATTGGTGAATTGGAAGTGTTAC
GAGAATTTCCAGAAGCTATTATATTCCGACCTTCAGATATGTATGGTCATCACGATCGAT
TTTTCTTTTATTTAATGAGATGGGAACGCAGAAGATTCATGATTGTAAAGTCATATATTC
CTTTATGGAAATATGGCAAAAGGACTTTTAAACAGCCGGTTGCTGGACAAGATGTAGCAA
AAGGAATTATCAATGCTGTAACTGATCCGACAATAAGTGAAACCATAATAGAAGCTGTTG
GACCACATCGATATCGTTTTGATCATCTTATTTGGTGGCTCTATATGGTATTGAGGTTTA
AACCAGAAAAATTAGCAATTAGTAACATGGACCCGCTTATTAAATTACAGATCAAAATTT
TGGAAACAATTAAACCGATTTATCCGATAGTTTGCATGGATAAATTGGAAAGGGAATGTT
TCACTGATACATTAAATGATTTGCCTACTTTAGAAGATATTGGAGTTGATAATTTGACAA
ATTTGGAAGATAGACTGTTCTATGAGGTTCGGTTTCGCAGGAGAGATAGTCACTATTGGG
ATTCTATTGGAGAAATTACCACTATTCCGCCCCCTCCTAGAGCAGTGATTTAGgttttat
attaaataaatatcaatatacttgg
>DjScaffold1469:10514-11473 strand:+
ACTTTCAGACTTTTCAAATATTATAGTTTTAATAAAAGATGTTTTAAACTAACTGCTTTT
ATTTCACAAAGCGAATATGAAGCTTTAAATTATCCGGAATGTAAATTGAGTCAAAAATCT
ATTGATGATATTATTTCTGCTAATGAAACTTGTGAAAAAATCAGCAGTGATAGAATTTCC
CATTTTCATTCTAATCAAGTAGGAGTTAATCGGCCTGTAGAAGACCGATGGAATGCAGGC
TCGTTTAATTTATCAAATTTAAAGCCTTCAAATTTAAATTCCTATAATCTTGGTCATATA
TTTTCAGTTGTTGATGGACATAATGGTCATGCATGTTCACATACAATTAATTATTGCCAT
ATGGATTATATCGCTGTAGCAATGATTAAAGATGTAGAAGTTTTAAAAGAATTTTTGAAC
AGTTTAAATGATAATGCATATTTGACTTTAACGAAATCTTTACTTCCTCATAATACTTGG
GGAACAATGGCTACATCAGTTAGAGAATCACATTTTCGAAATTTAAGGTCGTATATTAGT
GATATTATTAAAAACTTCGATGATGTAGATAAAAGTCGATCATCAGCTTTACGAAGGAGT
CTTTGTCGGTTAGATCAGGACATGATTTCGGCAGCATCCCCAGATTCAAATTTTATTAAT
AGAGCTTTGTTAAGAACTGTTACAAGTGGATGTGTTGGAACTTTTATATATGTTCCTGAA
ACTGACACAGACGTTTATATTGCACAAGTATGCTCCGATAAATTTAATTAAatttttta
aactgaattttggtattacgcaatacactctacaaatttattaagtgattacaaaaaata
agcaaaaaccttataataaatttaagcaatcctattggt
>DjScaffold1469:39301-47456 strand:+
GATTCTGAAACAAATCGATGGAGTGCTGAATTATTAATTAAGCCTCATAATTCTAGTAAT
CCATCTGAAATCGAACGAATTGAAAGTGAACATCCAATTAGTGAATCTAATTCTGTTATC
AGAGAAGACCGATTACTTGGAGAATTAATGCCGCTTCGAGCTTTCGGTGATGTGAAATTC
AAATGGTCTACAAATACTCTTAAAACAACGGCGGCTCTGTTAGGCCTGTCTCCATCATAT
CCGGTTACTCCACAATTTTATTTCTCACCTCCTTATTTGACTTCAGAACCTGAAATCAAA
AAGTTTCATGTTGATAAAAATTCAGATCAATTTCTCATAATTGCTTCAGACGGATTATGG
GATAAACTGACACCGAAAGAAGCAGTCGATACTGTTGCTAAATGTTGGAAAGAACAAAGA
TTAAAAATACAAAACACTAACGAAGATTATTGTGTCGGTGAAAATATTGCGTCTGTTCTT
ATTAAAACCGCTCTTTCGGGGCGTGATACAGATTATGAAAAATTATCTTTATTACTATCT
ATGCCAGGCAACGTAGGGAGGTATTACAGAGATGATATTACTGTGATTGTTTTATTTTTC
AAATGAatatacctattttattga
>DjScaffold64:45838-54634 strand:+
TCATGTGGACGTAACCATTCTCTTATATATGGTATAATTGAAAACTTCACTCACCCATCT
ATTATTTCAATCGGTAATAATACTTTCGGTCAGTGTAGTAATCAGAATAATACGATGAAT
ATTTCTGATAACATCATTGAATTTCCCGATCTAATTTCCCAGATTCAATGTGGACTGGAT
CACTCATTAGTTCTTACGGATAAAGGACGAGTATATTCCTGTGGTCTTGGTACTGATGGT
CAAACAGGCTTACAAACATATGAAAATGTTTTCAGATTTAATGAAATTTCATTTCCTGAG
TCCTCAATCGTAATCAAAAAAATTTCCTGTTGTGCTGACACTAATTTGGCCCTAACAAAT

GATAATCGAATTTTCGGATGGGGAAACAACGAATATGGCCAATTGTTTGAACATATAAAT
GAAATTCAAATTAATCAACCAACTGAATTAATATTTGACAATAGTGAAGAAGTTCAAGAT
GTTTTAGCATTAGGCTCATTTTGTTTAGTTGTTACCAACACCGATAAAATAATGGCTTGG
GGATTTGGAAAATTCAATGACCACGCAACAGCATATAGATATCCAACAGAAGTACAATTA
ATTGGAAATAATGGGAAAATTGTAAAAGAAAAGATAATTAAAATTCGAGGATCTGTTCAT
TATGCTGTGTTGATGACTATATCGGGTAGAATGTTTGTTGTTGGCAATTATCCAAGAGAT
AAGCGGAGCATAAATTCAGATAGATTTGTATCAAATGAAGTTATATTTCCTAGACCTGTT
ATTGATTTCTGCAGTGGTTCTGATAGTATTTTCTACTCTATGTGAataaattggcaatag
ttgtattaaatatcacttgaaaaagcaaatgtcaacatttgatatgcattttagagataa
actgtaaaagaatactat
>DjScaffold2567:13181-17701 strand:+
GATGATCAAACAAGAGTTGTCTTACAAATTCGAAATGAAAATAACGATTATATAAATGCC
AGTCGTATTCCATTTGTTGATAATAGTGAGCTTACAGAATATATTGCTACACAAGGGCCT
CTACCAAATACTATTTCCGATTTTTGGTATATGGTTTGGGAACAAAATATTTCAATAATT
GTTATGCTAACAAAACTTTATGAAGGAGTACAACTTAAATGTGACCAATATTGGCCGTTA
TTATTAAATACGGAGTTTGCATTTGATGAAAATTTAGGACTTTCAGTAATTATGGAAGAG
GAAATTCAAAACAATTCATTTTTCGAAAGAAAATTTAAAATTCTAAATCGAAATACAGAT
GAGGAAAGAAGAATTACTCAAATTCATGTACAGAAATGGAATGATTTTTCGAGACCTGAA
AACCACGATTTTGAGGAATGTTTGACTCGATTTCATGAAATAACAACAGGAAACAATTTA
TCTCCTTATTTGATACACTGCAGTGCTGGAGTGGGACGAACCGGAACATTCATCGCATTA
GATCGTCTAATAAAAAAACTAGTGAAAGAAAATTTAAATCTAGAAATCTACGATGTTGTT
TTAGAATTAAGAAAATTCCGTCAAAATATGGTCCAACAAGAATCTCAATACATTTTCATT
CATGAATTTATGGATTATTTCCTGTTCAAGTTCAAAAGACAAAGCGTTGAATGTCAATAT
GATAACGACTTCACGGAAATACCCCTAAATTAGatttattggttattaaaattgaacaga
ggttttcaaaaaaacagaaactgacagaaagttttatttttatttataacaaatacacta
tctaaagtcTTATTATTATTAACAAAGAATACAGATGAGGAAAGAAGAATTACTCAAATT
CATGTACAGAAATGGAATGATTTTTCGAGACCTGAAAACCACGATTTTGAGGAATGTTTG
ACTCGATTTCATGAAATAACAACAGGAAACAATTTATCTCCTTATTTGATACACTGCAGT
GCTGGAGTGGGACGAACCGGAACATTCATCGCATTAGATCGTCTAATAAAAAAACTAGTG
AAAGAAAATTTAAATCTAGAAATCTACGATGTTGTTTTAGAATTAAGAAAATTCCGTCAA
AATATGGTCCAACAAGAATCTCAATACATTTTCATTCATGAATTTATGGATTATTTCCTG
TTCAAGTTCAAAAGACAAAGCGTTGAATGTCAATATGGTAAAATTGAAAGTTTTGATTTT
TTTCATTGGAGATTTTTTTTTTCATAGataacgacttcacggaaatacccctaaattaga
tttattggttattaaaattgaacagaggttttcaaaaaaacagaaactgacagaaagttt
tatttttatttataacaaatacactatctaaagtcttctta
>DjScaffold2933:55650-59662 strand:+
ATGCCCTTTGGTATAGGAGCAGCGTCGGGTACGTTTCAGGAGCTGATCGTAAAGGTGACG
AAAGACATCAAGGGATCAATGGTATATTTAGATGATTTATTAATTTTTCTAAGGACACGA
GAGGAAAATTATGGAATTGTAAAAAGAGTTTTGGACAGCATTAGTGAAGCAGGTCTCCAT
ATTAACCGAGAAAAGTGCATTTTCTTTCAGAAAGAAATTAAATTCTTAGGACACATTATC
AGTTCACAAGGAATTTGTACAGATCCGGTAAAAATAGAAGCTATTCAAGCTTTTGGGGAA
CCATCATGTGTGAAGAAATTTCGTAGCTTCCTGGGCATCTGTAATTATTATAGAAAGTTT
ATTTGTGGGTATGTAAATAAAGCTAGAAAATTGGAGCAGCTATGTAGCGGTCAAGGTAAT
ACGAAGATAGTTTGGTCTTTAGAGTTATCAAATGTGTTTAAAGAAATGAAGAAAGCTTTA
ATTTCTACACCAATTTTAGGATATCCAAATCTGCATGAGGAGTTTATTCTAGACACCGAT
GCTAGATTCGGAACGATTGGTGCGGTGCTGTCGCAGAGGGACGAAGACGGAAGCGTACGG
GTCTTACGCAATGTCTACCCACGAACAAGGATACTGCATAACTCGTAA
>DjScaffold2562:20528-21099 strand:+
GTAACTTCACCAGATACAACCATAACCCAAATATTGAATTCCTACATTTTGCATACGGCT
TCATTGCCTAATAATAGTCAAGAAAAAATCAAACATTTACAGTATCTTAATGACTTTCGT
ACGCTTCAATGCCCATTGTTTCTTGATCATAAGTGCCAAGTGCATCGGCCATTTGCATGT
TTTTACTGGCATTTTCCAAATCAACGCAGACGTCGTCCAATAAAAAAGAGTGACGGAACG
TTTAATTACAACCCAGATGTATATTGCACAGTTTACGATGAAAATACTGGAATTTGTCCA
AACGGCGAGGAATGTTTGTATGTTCATCGGAATGCTGGAGATACGGAGCGCAGATATCAT
CCTCGCTATTTCAAAACTGGCCAGTGTATTTATGAAACATCAGATAACGGTACGTGCGTG
AAGAACGGACAGCACTGTGCATTTGCACATGGCTTGGAGGATTTGCGG
>DjScaffold4470:11008-12995 strand:+

TATGACAGTGATAATTATAAAAGCAGGCGTTCTCCTATAAAATATGAATCATCACGAACT
CATAAAAGCAAAGTAACACATTCCCATTATTCTTCTGAAAGTCGTAAACATGAGTCAAGT
AAACTAAAAAAAATAAGTGACAAATCTCATTATGATGTTCACAAAGATTCTGATTCTGAT
GATAAATATTTTAAAAAGATTGAAGGTAATTATAGTCGTGAATGTAAGAAATCTAAATAT
AATCGCGGAGACAGTCCTGCCATGAAAAATTCGCGAAAATATGACGCATACACTCATTCG
TATCGGAGAAAAAGTATTTCTAGCGATACTAGTCATCATACTTATGAGGATAAGTATAAA
AAAATTGAAGAAAAGTCTGATAAAAGGGCTTACAAATCAAACAGAGAACAAAAAAGGAAA
GTTTCTAGAAGTGTTTCATCAGATCAGTTCAGTGACGTTAAAAATAAAATTGAACGCGTT
AAAGAAAAAACAAAAGATAAGAAACTTAAAAAGAAGACAAAGAAATCTGAGAAAGATAAA
GAAAAAAAGAAAAGAAAACGACAGAGACATTCTTCTTCAGATGATGTTAAAATTAATAAT
AAGAGACATGGTGAAGATAAACGTAGTCCCACAAAATTAACAATAGTTGCGAATGAAACA
GAAGTTAATAAAGAAAAAATAAAATTAGAACCTTTTAAACTTAAAGAAAGTTCTAATTTA
AAAAGTCAAATCAACTTTTTAAATTCTTCAAATATTTTTACTAAAGATGAGTGGCAGAAA
GAGTCATCAGATGATGATTCACATAGAAAGATTGAAAAAGATGGAATCTTTGATAGAGAA
ATCAACTCAAATGTAGAAATAACAGAAGAAATTATTAAAGAGTCGAATATAAAAGTCCTT
AAGACTGAAGATGGCAAAGATTTTGATAGAGAACGTATGAAAAAAAAACGTCACAAATCT
CCTCAATCAAAATCACGTGAAAGTAACATCAAGATTTCTAAACACATCTATGTAGAGGAA
GATAAACCACATTTAAAATTTAGTGAATGTTCTCGTAAAGAAAGATCTTATTCACATAAT
AAATCTGGTGAAACACTAAAATTGATTGATTCTAAAGAAAAGATAAAACCGAAAGACCAC
AGAACGTCTATGAAAAATATTGAAGATAGGAAAAGAGTAGAAGATCTATTTGAAGATGAA
CGAATCGAATTGCCTAGAACTCCAACAGATGCGCAGTATACTGGTTTGTTTGCTCGTCTT
CAAGAAGAACCGGTATCTGATACTGAAATTGAATCAATTTTACCACCTATTTTGCTTGAT
GATAGAATGAAAGCATTGTTAGTAAATGATCCAAATGTTACTCATGAAACATGGGATTCT
GAAGATGATGTATTGTTGAATAAACGTGAAAATATAATTGCACCTAAACATCGTGGTGTT
CTAGAAACACTTTCTGTTCCTTGGAGTACTCTGATTGAAGAAGCTTCATTAAATAAAAAT
AAACACGATAAGGATTCTGGATTAATAAAATTTAATGTATCAAAATTGCTTGATAACATT
GGGATTAGTAAACAACTAAGTGGTGATTTACTATGGGATGATGTTAAAAAACATTGTGTA
GATGGTTTCCTACATCCAATGGCTCCTGTTCATCAGACTTTATTATCACAAAAAATTCGA
AGAAATTCTATATTTTGTGAAAGAGGATTGTTCGCAGGAGCTTTATCTGCTAGAAAAGAT
CTTTTTATTAGAAGGGTTTTGTATGAAAAAATAATTTAAtgttaatttttgttagtgttt
atttattattttcaggaatgaaaacgttttctacggtaaaaatattaaataacaaaaagg
ca
>DjScaffold4470:92558-93445 strand:+
GTTTTGGTTATTGATTGTGCTGGTCATCTTTTGGGAAGATTAGCCACTGCTGTAGCAAAA
GCATCTCTTCAAGGACAAAGAGTTGTTCTTGTTAGATCCGAAGATATTAACATTTCTGGT
TCATTTTACAGAAATAAACTTAAATATCAAGAATTTTTGAGAAAGAGAATGAACACCAAC
CCATCTAGAGGACCTTTCCACTTGAGGTCTCCATCAAAAATTGTTTGGAGAACTATTAGA
GGTATGATTCCACATAAAACAAAGAGGGGTGCTGCTGCATTGGATAAAATTAAAGTTTTT
GAAGGAGTTCCATCTCCTTACGACAAAATGAAGAAATCATGTATTCCATCTGCTCTTCGT
GTCATAAGACTCAAGCCTAATCGCAAATTTTGCCAACTAGGTCGTTTATCTCACGAAGTT
GGATGGAAATACCAATCAGTCGTTTCGACTCTTGAAGAAAAGCGAAAAGCTAAGGCCGCT
CTCTGGTACAGTAAACAAAAAGAAGCTAAGCAATTGAAATCTAAGGCACTCGAAGCATCA
TCAGCCAAAATCGCAAAATACAATGACATTTTGGCTAAATTCGGTTATTAG
>DjScaffold4470:25913-26350 strand:+
ATGAACACAGTTTTAATAGATGTTGAACACGTAAAAGTATATATGGATGATATCTTGATC
GCATCAGAATCATATGAGCAACATTTAATAAACATAGAAAATATAAGATCCAGATTACAG
AAAGCAAATATAAAATTAAAGCCAACAAAGTGTAAATGGGCGGAAGAACAGGTGTTATTT
CTAGGTCATATTGTCTCATCAAAGGGTTTACAACCAAATCCAGATAAAATAGAAGCAGTA
AAAAACTTTCCAATACCAAAAACAGTACAACAGCTCCAAAACTATAACAGATTAGTTGGA
TATTATCGAAGATTCATAAAAGATTTTGCTAAAGGATCTGCTCCGCTAGTTGAATTAACA
AAAAGTTTAAAAACAAAAGGAGAGAGCAAAAACGTGATGATCACTGAAAAAATCGATCAA
AACTCATCAAAATTATAA
>DjScaffold93:31072-38095 strand:+
aaatttattaatatattcgaaagagttatctgaacatttattctcggattattctactaa
tattttttaaagacaaacattttacacaccatttttatttagaaaaattctttttcaat
tatacattgcgttttaggaagttatttcttggcaaacaaatcaattttgaaaatcaaatt
aaaactttcgttcgtttagcaaaagatggagacttcaattcttccatcaagattcagaaa

ggtttagatttactgcttaaagaacgaaatagaaagatttctttatttaagcatcgatta
aaggacttagaaaaaataatttataagaaaaaatctttttcgataacaataagtgatgaa
gaggaagagtgcATGAAAGAATTTCAATATATCCTTGATAGTGATGACCCTTTATCTGAC
ATTGTTGATCAACGCTTTAATAAGATTATGGAAAAGATTACAAGCGATCCAGATGAATTC
AAGAAATCTGAGTTATATGCTGAATATCAGTCGAATACATCTCTGGAATTCAAATCTTTA
TCGTTGTGTGATGTGATAAACCGCTGCCAGAATGAAAATGAAACCATATCTGAGGAACCA
TTTGTCAACAACAACAATAATAATAATAATGGCAATGGTCCCATGACTCGCAAACGACGA
CAATTACAAAATGGTTTCAGTAACAGCCTTATTAAATATGCCAAATTCAATGAATCTAGT
GATAGCCAATCAGATTCATCAGATGATTGTCGTAGTAACGAAGATAATAGTGATGATTCT
GTTAGCAGTGACACCGATTCTGTTGGAAGTGGAAATGCTGCAGTAAATGAAAATCACAAA
CGTGTTCGTTTAAATCGTCATGAACGACGTGAATTTCGTTGTCCTCAAGATGATTTCACG
GATTATTTCTCTTTGTTACAAAGATTATTTGCTGGAGCTCATCAACAAAAATTAACAACC
CATCAAATCGAAGATCGCATTAGACATTGGCATATTGTTCATAAGCGTAACATTGGTGAT
AATTTCAATCGGTGGGCAGGTATGAACACTGATTGGGTTTGTCTGGCGGTGCCAGCATTG
ACATATCTTAGTTTGAATGAGTTCTCTATTGTCGATCATCGCAAGAAATCCCAAACGTGG
AGATGGAAAAATGAAATAGATAATCATGTTCTCCGGAAATTATTCAACGAATGGCATCAA
ATTTCCTACAATACCGGTGGAGGAGGTGGTGGTGGTAGTGTTGGTGGGGGACCAACTAGT
CGCGGAAGTGGTATTCAAGTAGAATGTGAACAAATACCTCCACCGTTGTTCCAAACAGAA
TGGCGAGTTCGAACATCGTCTGCAGAAGAACGAATACGGTTTCAAATGCAAGAACGAAAA
AGATTTATGCGTCCTTGGAGAGGATTTATCTACAATGTTCACGGATATAATTGTGCAGTA
GCTCCAATCAGAGGTTGTCTTCCAGGTCGCGACTACAGTAATGTTAAAGCGCGTGGACAT
CCTCTACTTGTCAATGATCGTCCGCCTCATGTGAATATCGTCGAGATAGTACGCGATGCA
GTCGCAAGGTTGCCAAACGGAGAAGGATCTCGTGCCGATATTGTCTCTTTGGCAATGGAT
AGTGCATTCTGGAGTTGTAGCAGCACGACTGGGGCAGCAGCCAATGAGAAGAGTATTAGC
CAATGCATCAGTTCTGCCTTGGATCGGTTGCAAGGTGAAGGCTGTGATCCGTCAGTCAAA
TACAATGCTCGACTGAAACTCTGGTTTTATCGGCATCGCAACAAAAGCTTAGAAGAACTC
GAACAAATTCATTTACAACAACATAATCAACAAGACGATGTCAAACGGTATCGTAATAAC
ACAAATCGACCCTCTTCTTCTTCTTTATCTCAGCAAACTGTTCCGACAAACTCCTCTTTG
CATTATGGCCAAATTGTTCTTAGCAACACACCTAACAACAACAACAATATTAATTATCCG
CAGCAGCAACAGCAACAACACGTATCTGCATCTGCTCCAACTAGATACGGACAAGTGCAA
ACCTATCAACAGTTCTCACAGGCGGTAAATCACGCACGAGACCACCTCTCATTACAGCAG
TCAATTTACATTTCTAGACCTCCTTCTGTTGCTGCTTCTGTTCCTGCTGGTTCCATTTAT
CAGAACAATCACCATCACCAGCAGATTCAGCAGAACCATCCTTCCTCAGCACCAACGAGA
ATAATCACTCCTACTCGACACGATAACATACCTTCTCACCATCCACAAGATCACTATTCT
AATCATAACAATCAGCAACATTTCTATTATTGAacatttctattcaacaatattactatt
atgatgatgatgatgatgatgatgatgattattattattaataataataactgtttgtta
ccttatatactgaattgatttcttcgttttaatgtacttgtatttattattattatt
>DjScaffold4092:15363-18442 strand:+
aaaATGTCTAATTTAGCTTTATCTCGTATTCAAAAGGAATTTAAAGAATTGATGAAATCA
CCAGAAAGTGTTTCAAGTCAATTAAAAGTTAAACCAATAAATGAAAACTTGATGCACATG
ACCGGTACAATTGTTGGACCTCCAGAAACTCCTTATGAAGGTGCGGAATTTCATTTAGAT
ATTGTTATTCCAGAAAATTATCCATTCGTCCCTCCAAAAGTAAAATTTCTAACGAGAATT
TGGCATCCAAATATTAGTTCGGCAACTGGAGTTATATGCTTAGATATACTCAAAGATCAA
TGGGCCGCTGCTATGTCGTTACGAACAGTATTGTTATCTATCCAAGCTTTATTAGCTTCT
CCTGAACCAGATGATCCACAAGACGCCATGGTAGCTACTCAATATGTTAATGACAGAGAA
TTGTTCTCTAAAACGGCGAAACACTGGTCTTGTGTTTACGCTAATGGGCCACATAAAAAC
AAAATTTTTGAGCAAGATGTGGAAAAATTGAAACAGATGGGATTCAATGAGGATGAAGCT
CGAAGTGCTCTTTCAATGAAGAATTGGAATATCGAGGAAGCCACAGAACATTTGTTAAAA
TAAtcaattacagatgaaatttggaaaatattaatttgtgatttgttctattttaacttt
aagccgtttcatgtaatcccccagcttattatttcagctggactttaagttgtttattaa
aatttaataatgttc
>DjScaffold2270:10801-16558 strand:+
ttcgcaaATGAATAGTAATTGGAATACAAAGACTTTGCCGAATTTCGGTGGACATCATAG
AAGTGCAAGTGTAGTTGGGTATCAAGACACATCATATTCAGACTATGAATCCTCAAAATA
CAATCACAGTTGGCTCAATAACAATCAAGGAACGTATTATAACAGCTCGATGGATATAAG
TAAAACAAAGCCAGATTCTAATGATTTTCAAAAATTGTTATGGAATTCCCCATCACGTTA
TCATCCATCAAAATCAGTATCGACATATTCCGAATCAGCAGACCATTCTCAGAAACAGAG

GCCATTTGAAACAGCTTTCAAATCGGCTATAATTAAATTGAAAGAATCTAGTTTATCCAG
ATTAGGAACAGTAGAACAGAATATTCCTACAGTTAAACTAATGAAAGACAACATTGCAAA
TGAAACTGACGAATTTTATCAAAGAAAATGTCAAACTCTCCAACCATCTAGAAAGTATTC
TACAAATTCTACGAAAACCGATTCAAAACGAAACATATTTCGATGTGAAATTCCAACAAA
TAAAAATCAATCCCACGCTTATTCTGTTTCCAAAGTTTCGAAAGAAATCACTTCTGATAC
AGAATCTGACACTAACACAAAGTACAATCAATATAAATCCAATAATGGCATAAGTGAAGA
TTCTAACAAAGTAATTAAATTCATTTATTTCAGAAATAAAGGAATCATAAATTAAaattt
taaaaaacgaaccggaataaataaatgacatatttcatattgtgatgtactttaatgttt
aatcatgtgtagtaatgttttactatgcatcataatgtattacgttaattattttgttca
gtgctccgcaacctatacaggtgtaccgaggtaaaataaaaaatattctaactaaataat
acgtgtaattaagagaatacctataataaaagaacacttaataaaatatattgaagaatt
cgaatatcaaacttttg
>DjScaffold3981:7909-8517 strand:+
cagattgtttaatattcagtgtatattgtagtgcgattttcactttatttgtaaattata
aattagttttATGATCAAAAAATTATTTCAATCACTTTCTATTAATTTTACTCCTTTGTT
TCTGAGACGTTTTACAGTTCCACCCGGGGAAAAGAAATTGCACTGTATACTTAATAAAGA
ATTTCCATCTGCAACACAAATAGTTGTTGAAGATATTAGCGGTGGTTGTGGAGCTATGTA
TCAAGTGTTTATTGAAACTCCAGAATTTAAAGGATTAAAAATTTTAGAACAACATAGACT
TGTTAAAGAAGCTTTAAAAGATGAAATAAAAGAAATGCATGGTATAACTTTAAAAACGAA
AGTATCTAATTAAtgttataattatgaattaataaataaaagatttaaactaattacact
taatgattatcctacatattttaattttgaattgtttaattagagtaatctcaccaactt
gagttccaaacttataaatgtgtgcaaatatttttcacaaatggaat
>DjScaffold3981:17093-27760 strand:+
agaaacagtttagttcataaaattgaattttctattttatggacatgtccaaatgaatgt
aaatacgtttgtATGTGGAAAACAGTGGAAGCTTTTAAAAATGATGGTTTAAAAATTCCA
CAGTTTTATGGAAAATGGCCTTTAATACGAATATTTGGAATTCAGGAAATAGCTTCTGCT
ATAGCTGCTGTATTGAATTTTATTGTTACTAGTTTAATGTTTAAACGTTTTTGGTCAGCT
GTGCCAAAAGATAGTCCCTGTTACAATTTATGGTGGCTTCATTACATTACTGCATGCATA
ACTTGGGCTTGTGCTACTCTTTTCCACATTTCCGATGTTTGGCTAACTGAAGTTGATTAT
TTCAGTGCTCTTCTTTTTGTAATTTCCGCTTCATATGTAACCGTTTTAAGAATACTCGAA
TTATCTAATGGTTTCTTAAAAGTGCTGATTGGAATATTGTTTTTCGCATTTTTTGTATTC
CATATTTCTTATTTATTGCTGGTCAGATTTGATTATACCTACAATATGATGGTATTTATT
ATAATTGGCACTATTAATATAATTTCTTGGGGTTTATGGGGATTCAAAAATCGCAAATTG
CCGCAATCAAGATATATAATATTATGGGCCTTCTGTTTGTCGATTTTATCTATATTAGAA
ATAAAAGAATTTACACCAATTTTATGGTTATTTGATTCTCATTCAATATGGCATTTTGGT
ACAATATTCATAGCAATTCCTTTATATAAGTAAgtttatt
>DjScaffold4645:19902-20684 strand:+
gttgagcatacattttatttttctgtagaaatctagatttattctaatcttctgaattt
tatttttatctctctaattatttgttacttggcttacaaagatttttaattcatttcaaa
ttcccaataaaaaatcaaatttaacaataaaaattaatattaaattgtaaaactgtgaat
tactttgaataataaaaatattgtatttcagatgcaaattatgattatgatctggctttg
attaaaattgagagaaacgatggagttggaattaaatttaataattacgtgcaacctgta
tgtttaccaaaactaggcATGATGCCTGATCCTAATACAAAGTGTTACGTTTCTGGTTGG
GGAGATACTTTGGCAACTTATCCAGAAAAGTTACTAGCAGCCGAAGTTCCTTTGATTAAA
CCTGAAAGATGCTATGATTTTTACAAAGGGCGTTTGACAGAAAGAATGCAGTGTGCTGGA
TACGAAGCCGGTGGTATTGATACATGCCAAGGTGATAGTGGAGGACCTTTAGTTTGCAAA
GTCAATGGTGTTTATACAATATTTGGAGCTACTTCATGGGGAAATGGCTGCGCGCAACCA
AGAAATCCGGGTGTTTACGCCAAAATTCAAATTATGTTAGAATGGGTGAAGGAACAAACT
GGATTATGAttttatcatagttactacaacttgaaaatatattagtaccattttattaaa
tataatattgtttttca
>DjScaffold1592:21158-22009 strand:+
ATGAGTAATGGAAAATCTAAAACACCTGAAATTAAATGGTATTTTGGTGGAATATCAGGT
ACATTAGCTATAAGCGCTACTCATCCACTGGATTTGGCCAAAGTCCATTTGCAGACGTCT
AAAAATCAATCGAAAAGTTTAATTTCTGTATTATCGAAAGTTGTAAAAACAGATGGTATT
CTCGGATTATATAATGGACTCAGTGCTAGTATTTTACGTCAGATAACATATTCTTTTCCA
AGATTTGGACTTCATGATTGGTATAAAAATACAGTTTCGAAATCTAGAAAAGTATCCGTA
TACGAGAATATTTTAGCAGCAGGTTTTGGCGGATTTGTTGGGGGTATTACTGGAAATCCG

GCCGATATGATTAATGTTCGAATGCAAAATGATATGAAATTAGCAGAGGCAGCCAGACGT
AATTATAAAAGCGGATTTCACGGATTGTATCAAGTTGTTAAGCATGATGGATTTCCAGCT
TTATTTAGTGGTGTAACAATGACAACTAATCGAGCTGTGTTTATGACCATTGGCCAAGCT
GCTGGATACGATCAAATGAAAATAATTTTGCTCTCGACAAATCTATTTAATGATAACCCA
TCAACCCATATATTGAGTGCGTTCGGAGCAGCAGGAATCGCAACAATTCTTACACAACCG
TTTGATGTAATGAAAACAAGATTAATGAATGCTAAAGCTGGACAATATACGAGACTGGTT
TATTGTGCAAAGGATATTTTATTAAGTGGACCATTTGGTTTCTTTAAAGGATTAGTACCA
GCATTTGTCAGAATTGGACCACACACAATTATTTTGTTCATTATCAAAGAACAGTTAATT
AAATACTTTTGA
>DjScaffold3100:6084-11108 strand:+
ttacttaacaagtgcattttcaaataatgagttagttgagcattctattgaaATGGATAT
TGAATCACTACAAAATTTTTCGACAGAATATTCAAATATATCAATGAAGGAAAGCTACTT
TTATATTCAATATTATTTTCCTCTGCAAAGTTCTATATCAGAAAATAATGGCAAAAATTG
GGAGAGCGCTGTGTTTCGTTCAATTAATAATCCTGTTGCAAAAAATGTTGATATTTCTGA
TACAAATCGACATCGGTTTCGATTACCAGTTGACTGTTCTTTAAAGGCAATGCTTGGTAA
ACATTTTGATGCATTAGGTAAGAAGCACATTTTATTTGAGGTTTATTTGAAACAATATCA
ACCATTTCCAATTTCGAAATCTATTGCTAAAGGTCGATTGCTCGTAACATCATTGTTTCA
GTTAATAAACCAGCATTCGGTTGATGTGGATAGTTTAGCAGACACTATAACTCAGCAACA
AATCCCCATTCGATTAGTAGTTGATGATAATGGTGATTCAAGCAATCCTATCGGGATTTT
GAATCTTAGATTGTGCTATCGAACTAATGTTGTTCAGAATATGATTCGACAACAATGTTT
GAATAACAGTTCACCTTATGATCAACCTATCGTTCGTATCGAAATATTATCAGCAACTGG
ATTGCAATGTGCGGCAGAAGCTCTAGTCAGTACTCATCCAGAATTACAATATCCTTCTGT
TACTGGATTAAATAGCCATGTAAAATTCTGGTTGACAGATGAAAGTAATAAGCAGTCTCG
TATTTATGTGAATTCCAAA
>DjScaffold3100:11383-27879 strand:+
caagttctaaaatactcattggtaaaggaagaatttccattgctgatcttctactgagat
ccagtggatttATGGGTCCGATATGGTTGCCAATTTTCTCCCCAATAAACTCAGATGCTG
GAATTGGTGGATTTAAACTCAACTGGCCACCGAAAAAGAGACATGAATCATTTTACTCTC
TTGATAAGCGAAAATCTGTCGGTGCTGTAGAGATTCGATTAGGATTTCTTGATAAATCTG
ATCGGAAAACAATTCTTCAATTGGCTATGGATGCTGGGTATGTTGTACCCTGGACAAATA
GATTAGAAACTGAGGACCTTATCTGGCAGCAAATGGATATTCACGGATGTACATTCAAAT
ATATTACTATTAATATTGAAGAAGTGGAACTTTCAGAAAAGCTTCTTAATTCAATTTTCA
CCCCTGAAGCTGGTTTCAAAAAATCTTCTATAAAGATTCTAACTAGATATCGCATGTTTA
AGCAATCAGTCTCAAGTTCTCCTAACTTTCTCATCAATGAAGATGAAGTATCTGATAAGA
TATTTATTCCGGTTAATCATGAGCAACAATACTGTTATCGAATCTCAAGTGATTTGTTCA
GATATCTTCGAGAGTCAAGACTTGAAATCCAAGTTTGGGGAATTTATGGACCTGTAACAA
TGTGCAATATGGATTATAATTATGATCCAAATGATAATCCCAATGATGTTCGGTTATTGG
GCAGTGCTTTGTTTGATTTAAGTAGTCTGATTTTACCGAGTGACCAGCTGAATAAATCTG
TCAGTCATATTCTACCTATTTTGAAACGAGGTCTTAATAATTGTGGAAATTCGAATATCA
CTGTTACTATCAATATTTGTGGAGCATCATCCCCGATTGCAGGATATAATTCTGAGAACG
ATGAAGATCAAGATGTAAATCATTCAAATAATCCCAATAATCATTCAACAAGAAATGAAA
ATGTCTATCGTATACGATCTGATAATAAATCATTTCCAGTTCATGTCATCATTGAAAAGG
CCGTTAATTTGAAACCAATCTCATTAGGAAACGAAAATCTCGGCAGTACTGACAATTCTT
ACAAACCTTTGAATTATTTTGTATTATTTCCATGTGCCGATGAAAATAAATCCATCAATT
CTTCAATTGTTAAAGGTTACAAAGACGTTGTTTGGAATTTCGAATGTGATACTCGAATGT
CGTATGATTTATTGACGAATGAGAAATCGACATTGGATTTTAAAGTTTATTATTCTATTG
CTGATTCTGAATTGACAGGAGAAATTCTCGATGAGAAAATTTTGATTGGAATCGTTAAAC
TTGATCTTTCACTTCTTTTAAAAGGATTCGAACGCGTAATTGGATGGTATAATATTATTG
ATATGCAAGGAGATTGTCAAGGTCAGATCAAAGTTGGGTTGACTCCAACAATACCTTTGG
TAGCCAGTACAGCTCCACTGTTTAAACGGAATCATTCAAATGCCCATCAATTACCATTTA
ATAAACCGTTTACAATTGAAGACCATCAAAGTTATTTATCAAGTGTTCGAATGCGTTTAT
TTGGTCAAAGCGAACCAATTAACGATTACCCTGCTCCAATTTCAATGGAAAATCATTCAA
TGCTTGATAATGATTCTCAAGATTTGATGTCGAAATCTTTTCTATTTGATACTTTGAAGG
AAAAATTGAAGGAGTTAGACAATTTAAATGAAACATTTAAAAAGAAAATGGAACCAAATG
AAGATAATAATAAAATCGAAATTAATCTTCAGCAAAGGGTTTCCAGCGTTCCTAATTTCC
TTTTGCCGTCATCATCATCATCCGTAATGCCAGTAAAAGTTGATTATGGTACAAATACAG
AAGTAGAAAATGATCAATATGAACACAATGATTGCAATAATAACAATTGTGATCTAGAAT

TCAAATATAATGAAGATGACTTTGATAGTGATGAGAAGGTTGATGGTGTATCAGACAATG
AGGATTTGTCAAATGATAAATATAATAATTACATGGCTTGTATAAATGAATTGGAGCCAT
GTCGTCAAGCAGCCAATATTGAAAATATGAAAATGCCAGTTGGCACATCCGAATATCAGC
ATAAAGATGATCATGATAATTTATCTAATGATCAATCAGAATCTGATCGTTATAGTACAT
CGTCATGTGAGATTGAGCACCCTCAGTCAATCACCACATCCAGATCAAGTTCACCATGCA
GTAATTACAGAAAATCAATAGAAGAAATACTTGAACTTAATCAGCCTATCAACAAATCGG
ACATTAACAAGGATAAATATGAAACGCAAAATGATTCATGGGATGAAGCTCTATCTCAAG
TTTCAACCCCTTTTGATAAAAACCAGCCAAGCAATCAACCT
>DjScaffold3615:53165-54562 strand:+
ttgaaaatgattatttttattatttagaataaaATGAGGAATTTAGTGAGTGATCATTTT
AACAGAATGTACAAATTGATGTCGCAACACAAATTTGTAATTAGTTGTGATGTTAATGAT
GATTACAAATGTGGAATTTGTAAAAATATCTTAATAAATGCTCACCAAGCTATTTGTGGA
TGCAATTTCTGTTATGATTGCATCAATCAATATATGACTAATGGTGCCATCGATTGTCCT
AACAACACAGAAGAATGCGAATTGGAAAGTTTATCAAATATTGCACCAAATCATATAAAG
AATAAACTAATATCGAAACTAATTGTGCGATGTTCTAACAAAATGTGCTCACATGAATGC
CAATTAATAGAAATGGAGAATCATTTAAATTCATGTTCGAACAAAGCTGTAAAATGTCCA
TTTATTGATGTTGGCTGTGAACATGATGATACGATCAATGATAAATTAAATGATCACATG
TTGTCAGATATTCACAATCACAACAAAATATTGATTGATCTGATTGGAAATTTGAGAAAT
GAAATGCGAGAATTGAAAGAGAAAGTAGAAGGAAATCGATTAGAAATAGAAATGTTGAAA
GAGAATGATCGTATAAAGAGTGAAATAATTGAATCATTGAAGGAAGAGATCAAATCAATA
AAACAAGAGAATGAAGATCTGACACAACAAATGGAGATTGGTGATCGCAATTTGTGGTAC
AAATTTGATGAGATCGATATTGAGATGAAGAATAAATGCAAGAAAGAAGAATTATTGAAA
GAGTTTGATGATAAATTCAATATGGAAATGAATGCGAGATATTGTGATATGAGAGAAGAA
TGGAATGAGAAATTCGATGAGAAAATAGAGAAAATGAGAGAAGAGAATGACAAATTAGAT
ATTGAAAGATCAAATAAAATTGATAAATATTTGAATATGAGAGATTGTGGAATATTTTAT
TGGCAAATTGGTAAAATAAATGAGATAAAATTGATGGTTAGTGAATGGTTTTATGACAAA
CAGAATCCATATAAAATGTGTTTGTTATTGCGAAATATAAATAATGAATTGAAAATTTGG
TTGAACATTTGTTCTGGTGCATTCGATGATGATCTCAAATGGCCCTGCAAGCTGATGTTA
CAATTGGTATTTATGCATTGAatgattggaatatatgcaaaacaataaagaaagcgtgca
aaatagaaaaaccagaagaaatatactccaaatttgatgaataaataaagatttctcatt
attctttgacaaattcaaatcttatcaatgtaaattgttgaaaatgattatttttattat
ttagaataaaATGAGGAATTTAGTGAGTGATCATTTTAACAGAATGTACAAATTGATGTC
GCAACACAAATTTGTAATTAGTTGTGATGTTAATGATGATTACAAATGTGGAATTTGTAA
AAATATCTTAATAAATGCTCACCAAGCTATTTGTGGATGCAATTTCTGTTATGATTGCAT
CAATCAATATATGACTAATGGTGCCATCGATTGTCCTAACAACACAGAAGAATGCGAATT
GGAAAGTTTATCAAATATTGCACCAAATCATATAAAGAATAAACTAATATCGAAACTAAT
TGTGCGATGTTCTAACAAAATGTGCTCACATGAATGCCAATTAATAGAAATGGAGAATCA
TTTAAATTCATGTTCGAACAAAGCTGTAAAATGTCCATTTATTGATGTTGGCTGTGAACA
TGATGATACGATCAATGATAAATTAAATGATCACATGTTGTCAGATATTCACAATCACAA
CAAAATATTGATTGATCTGATTGGAAATTTGAGAAATGAAATGCGAGAATTGAAAGAGAA
AGTAGAAGGAAATCGATTAGAAATAGAAATGTTGAAAGAGAATGATCGTATAAAGAGTGA
AATAATTGAATCATTGAAGGAAGAGATCAAATCAATAAAACAAGAGAATGAAGATCTGAC
ACAACAAATGGAGATTGGTGATCGCAATTTGTGGTACAAATTTGATGAGATCGATATTGA
GATGAAGAATAAATGCAAGAAAGAAGAATTATTGAAAGAGTTTGATGATAAATTCAATAT
GGAAATGAATGCGAGATATTGTGATATGAGAGAAGTGATTATTGATTACAATTTGATGAT
TGATATTGAGATTATTGATTCAGGAATGGAATGAgaaattcgatgagaaaatagagaaaa
tgagagaagagaatgacaaattagatattgaaagatcaaataaaattgataaatatttga
atatgagagattgtggaatattttattggcaaattggtaaaataaatgagataaaattga
tggttagtgaatggttttatgacaaacagaatccatataaaatgtgtttgttattgcgaa
atataaataatgaattgaaaatttggttgaacatttgttctggtgcattcgatgatgatc
tcaaatggccctgcaagctgatgttacaattggtatttatgcattgaatgattggaatat
atgcaaaacaataaagaaagcgtgcaaaatagaaaaaccagaagaaatatactccaaatt
tgatgaataaataaagatttctcattattctttgacaaattcaaatcttatcaatgtaaa
ttg
>DjScaffold3165:51716-52118 strand:+
agtctttcataaataaaaaATGGTTGATTTAAAAAATCCAAAAGGTCCTTTTCAAGAAGG
GATTAATAATGCAATTTTTATTGTTCCATGTATTGTGATAGGATTTATACTGTCATATAT

TGTTTATAAATTATATAGAAATCAGAAAGATAAAGTTGTGGCTAAAGAAAATAAAAAACG
AATTAAACAAATGCGCAAAGAAAAAGATATAAAGAAGGCAAAATAGttaatacgacaaaa
tgtgcaattgtcccatctattatatttattttctttatgttattattattcctattgtgt
gtttttgtttggaatataagatttgttaatgagttgtggcttgtgtaaataataataaa
a
>DjScaffold2499:10914-17815 strand:+
atagttaattatggatatcaatacaataaaatttgaatatattaaatggcccctattatg
aagttatttaggaaatgttttctctgaaatatgctctgtaaacattttgttacatctatt
caaaataATGATGATAAATCTTATTGTACTGTCAAAAACCATCATATTATATGCTCAATG
GGTTCTTGTTTCCAGCACATATTACTCCAATATTGATTATAGTAATGATAACAAGAAACC
TATTTGTCCTCCAGTTTGCAACCAAATGGCATGTAATATGGGCATTCTTACATGCTCAGA
AACCGGTTTATCTGAAATACCAAAGATAATTCCACCAAAAACTACGAAAATCTTATTAAT
AAACCAGGATTTTTCCGAAGCTGGAACTTTATCACGTTCCAATCTCAGCGAATACAGTTC
AAATAAACATAATATAACAAAAATTACAATTCAAAATTGTTCTTTACAAGCGATTGAATC
CAGAGCCTTTTCCAAATTGATTTATCTGGAAACATTAGATCTTTCGTTTAATAAATTAAC
TGAAATCGAATCATTTACATTTGCAGATTTAGATCTGAAATTTCTTCGACTTGACGGAAA
TTTGAAATTAAAATTGAATTCCAATTCATTTCAAGAACTAACTGTCAATTCTCTATCACT
TATTGGCTGTCAACTAACACATTTGCCTTTTTCAATTTTAGAGCCTATCTCATCAGGTCT
CACTCAATTGATGCTGACAAAAAACCAGTTCACCACTCTAGATTCAAAATATGAAACATT
ATTCCAGCGTCTTGCGGTTTTCGATTTAGCTGATAATAGATTCGACTGTAGTCAATGTTC
GTTGATCTGGATGACAAATAGTATGAAATCCAAATTTTATAAAAATCAAAACAAAGACCA
ACAAACGATAATTTATCCGAAATGCTATCAACCCCAAAAATTAAAAGACAAATTCATTCA
CACTTTATCAAGTGCTGATTTCCAATGTCCGAAACCTTCAATAGAGAAAATTAGTATTTT
CATTTTACCAAATAATACAGCACAGTTGATTTGTACTGCATCAGATATTCAATCGAATGT
TATTTGGAGTAAAATCAGTGAACCAGTTGAAAATCAATTTGAATTTTTCGAACAAAAAAC
AATGATATCAGAATCAGCTCACACAAATCGGTTGGTAATTAATCAAAATCATGATGTGGA
TATATTTTTGTGTACTGTAACTAGTCTTATTGGAAATGAGTCAAGTACGAGATTGCAAAT
CTCCTGGCCGAAATACAAAACACAGTTTAGACTTTTAAATAATACCCATAGAAATATTTG
GAATGATCCGTTTACTTCGATTCCAGTAGAAAAGTACTCGAAAAACTATTTTTTCCAACC
GCAATTTTCATTGATCGAAATGATTTCTGGAGTGGTCGGAACTTTTATAGCTACTTTAAT
TGTGTTCATAACAATGTTACGATGTATATTTTATCGAATTAGACTTTTTTCAAAGAATTC
CCAGTTGAGAGATCATCAATTGAGTTTAACCAGTTCAAAGAAAGAGAAAAGAAAACAATA
TATGACATATGAGCCACCCATTTATTCTGACAGTCAAACCTATGACATTCCAACCCTGCC
TGCAAATCAGTCTACTCAAGGTCATTTTATCGATTTTAAAACTCAACAAACATTATATAG
TGAAAAAAATCAATAAtaaccataattctggaaaatctataaataatttataaaaaaaca
cacttactaaaaatatgttcatcatctgtagttttgaaacatttatcagtaaaaaaatat
ttttttgagttttaaataataatgaaattatcattttttttaataggttatttaggaa
atgttttctctgaaatatgctctgtaaacattttgttacatctattcaaaataATGATGA
TAAATCTTATTGTACTGTCAAAAACCATCATATTATATGCTCAATGGGTTCTTGTTTCCA
GCACATATTACTCCAATATTGATTATAGTAATGATAACAAGAAACCTATTTGTCCTCCAG
TTTGCAACCAAATGGCATGTAATATGGGCATTCTTACATGCTCAGAAACCGGTTTATCTG
AAATACCAAAGATAATTCCACCAAAAACTACGAAAATCTTATTAATAAACCAGGATTTTT
CCGAAGCTGGAACTTTATCACGTTCCAATCTCAGCGAATACAGTTCAAATAAACATAATA
TAACAAAAATTACAATTCAAAATTGTTCTTTACAAGCGATTGAATCCAGAGCCTTTTCCA
AATTGATTTATCTGGAAACATTAGATCTTTCGTTTAATAAATTAACTGAAATCGAATCAT
TTACATTTGCAGATTTAGATCTGAAATTTCTTCGACTTGACGGAAATTTGAAATTAAAAT
TGAATTCCAATTCATTTCAAGAACTAACTGTCAATTCTCTATCACTTATTGGCTGTCAAC
TAACACATTTGCCTTTTTCAATTTTAGAGCCTATCTCATCAGGTCTCACTCAATTGATGC
TGACAAAAAACCAGTTCACCACTCTAGATTCAAAATATGAAACATTATTCCAGCGTCTTG
CGGTTTTCGATTTAGCTGATAATAGATTCGACTGTAGTCAATGTTCGTTGATCTGGATGA
CAAATAGTATGAAATCCAAATTTTATAAAAATCAAAACAAAGACCAACAAACGATAATTT
ATCCGAAATGCTATCAACCCCAAAAATTAAAAGACAAATTCATTCACACTTTATCAAGTG
CTGATTTCCAATGTCCGAAACCTTCAATAGAGAAAATTAGTATTTTCATTTTACCAAATA
ATACAGCACAGTTGATTTGTACTGCATCAGATATTCAATCGAATGTTATTTGGAGTAAAA
TCAGTGAACCAGTTGAAAATCAATTTGAATTTTTCGAACAAAAAACAATGATATCAGAAT
CAGCTCACACAAATCGGTTGGTAATTAATCAAAATCATGATGTGGATATATTTTTGTGTA
CTGTAACTAGTCTTATTGGAAATGAGTCAAGTACGAGATTGCAAATCTCCTGGCCGAAAT

ACAAAACACAGTTTAGACTTTTAAATAATACCCATAGAAATATTTGGAATGATCCGTTTA
CTTCGATTCCAGTAGAAAAGTACTCGAAAAACTATTTTTTCCAACCGCAATTTTCATTGA
TCGAAATGATTTCTGGAGTGGTCGGAACTTTTATAGCTACTTTAATTGTGTTCATAACAA
TGTTACGATGTATATTTTATCGAATTAGACTTTTTTCAAAGAATTCCCAGTTGAGAGATC
ATCAATTGAGTTTAACCAGTTCAAAGAAAGAGAAAAGAAAACAATATATGACATATGAGC
CACCCATTTATTCTGACAGTCAAACCTATGACATTCCAACCCTGCCTGCAAATCAGTCTA
CTCAAGGTCATTTTATCGATTTTAAAACTCAACAAACATTATATAGTGAAAAAAATCAAT
AAtaaccataattctggaaaatctataaataatttataaaaaaacacacttactaaaaat
atgttcatcatctgtagttttgaaacatttatcagtaaaaaaatattttttttgagtttt
aaataataatgaaattatcattttt
>DjScaffold2499:32461-59542 strand:+
ATATTATTTCTGTATATATCTGTATTGTCATTTAATTATAATTATCCAACGGCAGTTGAT
AGATGCTTTTGTAAATTGAGTGGAACTGTAGATGACTGTGATTGCCCTGTTGTAGATCTT
GAAGAATACAACAATCATAGAATTTATCCTATTTTAAAAAGCCTCCTTATTAAAGATTAT
TTTCGCTATTTTAAAATAAACTTAAATCGTGAATGCCCATTTTGGGTTGATGACAGTCGT
TGTGCGTTGAAAGATTGTCATGTTAAAGGTTGTGGGCCTAAAGAAATTCCTAAAGGTCTT
CGAGAAGATGACCCTGTTTATATTGAGCATCAAAAGTCAAAGTATTTTGGAACTCCAAAC
TTAGTTCATCACCCAGAATGTTTGGATGAAAATCATGGAGGTCTAGGAAATCTCGACACA
TCAATTAGTCCAATACAAAAAGAAGTTATGCACAACTGGACAATATATGATGACCAGCAA
GATAATTTTTGTGAAGTTGATGATGATAATGTTAATGGGGCATCTTTTGTGGATTTGACG
AAAAATCCAGAAAGATTTACAGGATACATGGGTAAATCTGCACATAGAATTTGGAATTCA
ATTTACAAAGAAAATTGTTTTAACGAACATCGAAACAAGTATGGTATTAGTTTACACGAC
GCAGAATATCTGGGGTTTCTTAATGTTGTTGATAGAGAACAATGTTTAGAAAAACGAATT
TTTTATCGTATAATTTCCGGCTTACATACCAGTATAAGTATACATCTTTGTCGAGACTAT
TTACTAAAAGATAAAACGGGATTTGGTACACCACAATGGGGACCTAATGTTACAGAATTT
GCTCGAAGATTTGATCCAAAATTTGAACCGGATGGTCCGCAAAGATTGAAAAATCTGTTT
TTTACATATTTAGTTGAATTGAGAGCTATCGAAAAAGTCGCAGATTATCTCCGAGAAGAT
GAATTTTATACCGGGGATGAAGTTGATGATAAAGAAACGAGAGTAGCTGTTAGACAATTT
CTTAAGCAAGTAAAGAGCTTTCCTAATCATTTTGATGAGGAAAAACTATTCAAAGGTGAT
CCTAAAGAAGCTAAAAAATTGAAACAACAATTCATACAACATTTCCGCAATATAACTCGG
ATAATTGATTGTGTCGGTTGCGACAAGTGTAAATTATGGGGAACTCTACAAGTCAAAGGA
ATGGGTACAGCTCTCAAAATATTATTCACAAATCCTTCAAAAATGACTAAATTATATCCC
GATAAAGATTCTTTTTCGAATATTGGCGCAAAATTTCAGCTCACAAGAACAGAAATTGTT
GCTTTATTTAATGCTTTTGGCCGCCTTTCAACCAGCATTAATTCACTAACATTGTTCAAG
GATCTAATGAAAGATTGGGTTTGAagaaaaatatatttcttataatattaatattccagc
aatattttattatttttgtaacattcaaacacatacatataacgagaaatctgacaccg
attcttaattttaatgaaataagaatattttttctttgtattttggagtaatcaattgac
tttatttttccattgatgaagttttatttgaatgataacaagtattattatt
>DjScaffold5410:8765-19205 strand:+
agcgaaaacatcaacaccgattagaaatgattttaccgggagaaaatgtcagctttgtAT
GAGCCATTTTGTGAATATCGATAATCAGCTGCATTTTCATCAGGAACTCGGTAGGGATGT
TATTGAGAAATGTCATGATGTGGACGAATCTGTTGTTGATAAACAATATAAGAGAATTCT
GTTGCAGAACCGAGAATCGGTACTCACTTGTAAACCATGTAATAAGAAGACTTATTTGTC
GGATATTAGTGCTTCCTATCAGAATTTAATTCTGTATAAGCTTAATGTTGGCAACTCAGC
GCATGTTTTACTGGAGTATGATGAAATTCACGGTGATGTGCTAGGCATATCGAATGTGGA
CGGTAGTGGTAGTTATCAGTGGGCTCCTAATCACACTGAATCAACCAATGTGGCTTTAAT
AAATGAAGAACTTGTAATCAATAGTAGTTCATGGTTTCCGGCATTGGATGGACAAGTTAC
TATCCATTGTCTCGGTATGGCAACAGGTAAGTTATCACTTGAGAAAGTTATTCTCATTCA
TATTACCGACGAGGTTAATGCTATGTCATACTTGGTTGAATTGGATGTTAATACAGCATC
AGTGAAATTTATTCTTCAGCAGCCTACATCTATAGAATTTTGTACATTTGTCAAGAATGC
TCAGATTTGTAAAAAATCCATTAATACTTGATTTGAATAATGACCTTCAAGTATTAAACGG
AACTTCTCTTGAATCAATTACAGATTACAGCGGTTTAATTGAATTGAAGCATTAAttaat
ttatctcaggaggaa
>DjScaffold3813:62095-62933 strand:+
gtattttaggaatttgattcattaattgctgtacatcgattatgttatgaaaatttatca
aaatatctggagttagatgattttgattgcgtgttatccgaaataactcaaagtacaaca
tctacttttggaaatatatctgagtttattattaaagaaataattgaaatttatttgtcct

aatttctcttataacagcggaacaagtcgatttgtacctATGATAGAAACTTATCGAGTG
CGACCTGCAGAAACTACAACTCATGATCGAATTGATCAATTATGGGGAAGCAAAACCTTA
AACTCTTGTTATGAAATTATAATGAAACTATTTAATGGATATCTCGGAAAGGAACACATA
TTAGTTATATGCAAATACGTTGGTTATGATGGTATTTATTGGTTATTGATAAGACTAAAA
GAACTTGCCGAATTACTATTGAATGGAACTTTGAAACACTATGTATCACTTTTAATGGAA
CAAATGCCTTTAAATTTATCATCGCGTTTTGATCGA
>DjScaffold3813:64386-65062 strand:+
gatttaatatgttaacgtttatttgtttctttttactcaattatttttacatgaaact
attacgacattatattcaagtttacattaaacaaaaacttttgtataaattgaaatttga
atgacattcgacgtattaaaataaactcagatttttgatggagcttttaaagttaaataa
tttttgaacactttaagtgtgacatttttaaataaagaaaattataaatttagcgaaAT
GTTCGGTGAAGGTCTATTTTGGGCTGGATGTACTATATTGGCTCTTTTAAACCAACAGCG
CTTTTTTGAATATTATGACATTTGTGGAAAAATTATGAGAGCTCACAAAATTTCCAATAG
AGATAAAGTAGAAACAGATTTAAATTTGATGTATAAACGTATTCGGTGTTTATTACAAAC
CAACAGTCGTATATTCGATACATTAAATCGATTTTTGTTTACAATTGATGATTATAATTA
TATTAAACCAATAATTTTTCATATGAATCCAAATATTGTCACAAATGCAACTTCCGATAA
TGTTGAAGAATTTGATGATTGAaaattacataccacaattcatttaatcaaatattataa
aaataaatttaacaatattaaatcaaagt
>DjScaffold3813:83841-89597 strand:+
attggatgttggaatagccttgacaATGCATGTTATTTGTAATGAGTCTATAGTACAATA
TCAAATTATTTATATAGATTTAATTCCAAAAAGTTTTGACTTTGTTTATTATTACATTAA
TACTATGCAGGACCAATTTGAATTAGCCAATATTTTGGTATCAATGGTAAATAGTGAATA
TGCTGACCAATATCGGTCTTTAAAATTGAAACTTAATCGAACTCGAACAGCTAAACCGTC
AGTACGAAATTACATTGCTGAAAAAAATCCAAATTTGACATCACTGCTTCAAGCGATTGA
TTTCTCTCATTCTAACCATTTGACAACTGACAATTACGAGCATAATAAATCGGAAACAAT
GTTAAATTCTATTAATACAAAATTTTCAAATCATTCTAAGACTCTTATTACTAACAATTG
CCAATCAAATGTCAGACAAAAATTATTTAAATGTCACTATAGCAGCTGTTTGAAAGCGTA
CTACAAAAGCTCTCATCTCAAAGCTCATATTAGAGTTCATACTGGCGATAAACCTTACAT
TTGTAATTGGGAAAATTGTCAGCGACGATTTACCAGATCTGATGAATTATCTCGACATAA
ACGAACACATACCGGTGAGAAAATGTTTTCATGTTCAATATGCCAACGGAAATTCAATCG
TAGTGATCATTTATCAAAACATTTCAAAACTCACAAGAAACAATCGAAATTCAATTGTGT
TATATTACCAAATCAAGTTATTGTTTTATCTACA
>DjScaffold3813:17411-18538 strand:+
tcaaaagtttggaattcctatcacctcattaagtaagattggagaattcagaaggtttgt
atagagtggctaaaaaagtgttgaaaatctggatgagacggagaaggtggaaattgttgt
gaccttgaagttttgtttaaatattaaataataaatatatttttgaatttaaagatgtca
atcattactgatgtaactaaagttttaaccagaacttttaaaaatcattaaacattttgt
agagaattaaaaagaaaaccaaatcttttgaaaaaacaattaaatattttgaattctcta
ttattaaaacttagttttgtaatatttagcaacacctgagtctgcatttattttgtcaat
tatttctgctggtgtatcatatcaagtaactaaagcttgtagtcaaggagtccatagtaa
ttgtggatgtgacagaacagtttatgatgctcctaaagattctaaatttacatggtctgg
atgctcacataatatacattttggagctgccttttctagaagatttttagatgcaagaga
gaaaaaaagattaaaaggacaaactgctgcagctttaaccaacATGCATAACAATCATGT
TGGCAGGGAAAGTGTCATTAATAAAATGAGAATTAAATGTAAGTGCCATGGAGTCAGCGG
ATCTTGTGAAATGAAAACCTGCAGTAGAAATTTACCCTCATTTCGAGTCGTTGGAGATCA
ATTAAAAGAAAGTTTTCAAAAAATAATTCAAGTTGATTTCGTTGATAATAAATTAACACC
GAAATATCCATCTAAGGAAATGTTTGCTGAAGTCGATATGCTTTATTTAAATGATTCCCC
AGACCACTGTAATCATGATTTCCAAAGGGGAACTTTAGGCACAAATGGGAGAATATGTAA
TGCTACTTATAAAAATACAAATCAAGAACCAGAAAACAGTTGTTATCGTTTATGTTGTCA
ACGTGGGTATCATATTAAAACATATATTAAAGACGAACAATGCCGATGTAAATTTAAATG
GTGTTGTAAAGTGATTTGTGAAATTTGTCAAAGAAACGTTATTGATGCTATATGTAATTG
A
>DjScaffold3813:52903-52971 strand:+
ATGTCATTTATTGCGAGCAGAAGCCAAATTTTTACGATATGTCACAGACAAGAATGGAGT
TCGAACTGA
>DjScaffold4820:32129-42311 strand:+
CTTAAAGCAAATTACTGTTTTGCTGAAGGTCATTCAGTGTCTAGACAATCCTTGTATGAA

AATTATCAGTCATTCTGTAAAATTTATAACTATATGCCAATAAGTACTGCTACATTTGGT
AAGGTGATAAGACAACAATTTCCCCAAATCACTACACGCAGACTGGGAACAAGAGGCAAC
TCAAAATATCACTATCATGGCTTGACGGTAAAAGATAACTCAATTTATTCAACTGGAGAA
AATTATAAAAGTTTCCTATCTTCATCAATTAAACCGTCTCCAGCAATCAATGTTCAACAT
CCGAAAAATAGATTTGATTCAGTTCCCAGAATTCAGATAACTTTAGAAGCATTTAAAGGG
TCGACCAGTGAAGATCAATTTAGTTCAGAGATCGGAAATTTCTTTATCATGTACCAAACA
CATTCTCAACAGATTTTGGATTCGATTTTTCATGGATCATTTGACGAAATCTCAAAATTT
CTATATTCATTTTGGAAAAGTTGTCCTGGTCATGTTAAGCCTTTATTTGATGAACCGTTA
TTAGTTGATGGAGTGATAATAATTGATTTACAAGTTTATATCTCAGTTTATAAATATGTC
CTTCATTCAATGAACCAGTTATTACCAGATCAATATTTAAACAACTTTGTCAGTTTTCTC
AATGAGTACTCATTGGTTTTATCCGATTCGATTCCAGAACCAAACGTATTACTACGAAGC
AGAAAATTATTTTATGCGTCAATGTTCAAACAAATGAGTTTTAGTTGGTTAAGAGGATCA
CATTTATATCAACTGATCTTTCCAACTCTCAGTAAAACTGATATTGTTTCTCAAATTCTT
GACGATTTTCAATCGATTTGCGTAGAAAATGTAATTGAAGATACAGTTAAAATTGTGCAA
TATCCAGCTATGTCTCATGTTCAACTGCATCTTTATAATTATTTTGAGCTATTTGAAAAT
CTATTAGAAGTGGACTTTTCGTTTGACAGTATAATTAAATGGACCAAAGAAATATTCCAA
TGTGACTGGGTTACAATGGGTTTAACTTATAAAGATTGCTTTCTGACGTGGTCAATTTTC
TTTTCAAAATTTCTCCAGGAAATGTTTCTTGAAATGTCAGAGAGTTTTGCTCAAGTAAAT
CTGATAGTTATTTGGTTAGAACACATATTCTATACCGAATCAATGATCCGAGAATTTGAA
ATATTAATCAAGACGCTAAATGATATCATTCAACCACACACTAATTGTTATTCTCAATTT
CATAAAATGGCACCTCAAGTTTCACAGGAAATACGTGACGTTCTGGAAACAACTTATTGA
attaataacagcatcgaaataggaacacttt
>DjScaffold1659:72191-72373 strand:+
ATGATCAATAGTTTTCTTATAATCAAAATAGGAGATTTCGGAATGGCTAGAGACATTTAT
TATGATTGCTATTATAGAAAAGAAGGTAAAGGGAGGTTGCCGGTTCGATGGATGGCACCA
GAATCATTAGTATCAGCTTATTTCAGTTCAAGTTCTGATGTTTGGTTAGTTTTACAAATT
TAA
>DjScaffold3019:66563-87637 strand:+
ttttttgatatttttacttcattacatatatccattaaatatcttatttttattactttt
gtaaaatgataatccgttgttttaaaatttaaagtgtttaaactaatatttatattacta
attataatttaataaATGAATAAAAAACATGAAGGAATAAGTTGTGATTTTTGTCACAAA
AGTGGATTTTCATTACGTCGTTATAAGTGCCTCTTGTGCTTTGATTATGATTTATGTGGG
CCTTGTTTCGATTTTAAAAGGGAGTCAAACAATCATCACTGTGATCATCCAATGCAATGC
TTATTAACTAAATATGATTATGGTACATTTTACGCTGGGGAGGTTCAAACAGATTTAGCA
TTTAGTTACACATGTCCTATTTGTGGCCGTTTAGGACACTCAGTTATAACTCTTAAAGAA
CATTTAAGCCAAGAGCACAGTAATTCTAATCTTGAAGTTATTTGTCCTATTTGTGCAAGC
TTGTCTGATGGAAATGCTAATCAACAAACCAAAAATCTTATTTATCATATGTCTACGAAG
CATAATACTTTGATAAATGGAGACACTCAAGAATATTCTCCAAAGGTTACCAGACGATTT
TCGAAACAAACTCGTAGCACAGGTGAAACTTCTAATTCTTTGCAACCAACAAATAATAGA
AGTCGGAAAACTATACAACCTAATATCAATTCAGATACAATTAATCCAAATAGTTCAACA
GTTCACACATTTTCCACCGGGAATTTCTTTACCGGTCATGATCAAAATCCACTGGGAAGT
TCGGAAGAACCATTATTACATACACTTTTAAATCATTTGTACACCTGGCGAACAAGACGA
GAAAATGTCTCTATGCCGAAAAAAAATATTGGCTCTGAAATTAAAACACTTCCAAATGTA
TCAAGTGAAAACAATTCGATTATAAATGAAACAATTATTAACCAAGCTAATTCACTTCGA
CGAGGACCATCATTATTAAATGAAACCTCTACTTCTGGACAGAGTAGTAATAGTTTGTCC
AGGTCGTCTACAATTAGATCTACATTTCATGAGAAAAATAAAAATATTAGACGGAAGCTT
GGATCTAGACATATCATAGATTTAACTGGTGACAATTGGCGACTTTTAGATCCCGATAAT
GATTTGTTTAGTTTCGACGATGATGATCTAGAAAGTATATCTTTAAGCCTTAATCAAGCA
TCTCTTCAAGCTGGGGATATTGATGACTCTGAATATATGGATGATGACGAAGACATTGCA
TCCTTAATTCCATGGGGACAGGATCTTGGACCAGAGTCAATCTTTAATCAATTAAAAGCA
ATTACAGATCCAAAATCAACGGTTAGCTCTTCAAATAACAGTTTTGTGCGACCAAATGGA
TCAATTTCCTCAAATTCTAATTTAAATGCTAACAACCTTTTGCATATGATTAGTATGATG
AGTCCAAATCTAATTCCTGGAAGTGATAGTTTGGATTTTAAATTACCACATAATATTCAA
TTACCCACAACATGTGAATTTATTATTGACAATGAAAAGAGCCCAATTTCAGTTAATCTT
GAGGAGCATCTTGGATCACAGGGACTTTTTTCGAGGGATATTCATGTGATGAATCAACAA
ATTATCGAGGAGCAAAATTCTATTTTGGCACAAAGATCTCAGTTTGTTCGGGATTTAATA
TTATCAACTTTCGTTATTGATTGTGATTCTACCGGTGATTACGTGTGTAATGAAAATCTT

GAACAACCCCATCTTAATTCAAATGGTGTAGTTGTAAAAAGTTCATTGGAAAATGATAAA
AAGGAGTGAtcaaattctatttttattcatttaggttttatttacaattccatacatgcc
tgctgctactgttg
>DjScaffold4608:2101-2142 strand:+
ATGCAAAAAAGAGATCAAATTTTTGGGACACGTGATCAGTAA
>DjScaffold4432:65037-65793 strand:+
gaagagagaattactatatttagtaaatattttgacgacaacttgatgaaggaaataaaa
tgtgatccaaagtttgagagtacgatggtagatatcgattacttggaaaaacatcttatc
aatttactggaagctaaaccatcaaaaattaaccaagctatgtcgatacttgaattaatg
cagtatcgaggagaaaaaaataacggattttgctaagcgtattcgtatggagagcagtgtt
ttatctgacaagaaagaggaaatcatggtaaaagcattattaatgaattggggcatagaa
tattggcaaatgcactaaaaatacgaaccaaaatcattggaagaggcagtataaaagatg
aaaagATGCCAGATGACTACGAAACGAATGAAAATGTATTATGCAATATGCGCCAAGAAA
ATGAAACTATAATGAATGTGAAAATTGAAATAGAAGAGCTAAAGAATGAAATTGCCGAAT
TAAAGAGACACAATTACAAATCTCTTGCACCAAATGTGTTTAATAAAAATAGGAATAATA
ATGCCCAAAGTTCTGAAAAAACCCAAAAATGGATAACTGTGGGTAGACAAAAAACAAGAA
CATGCTTTACATGTGGTAAAAAAGGCAATATAGCTAGGGATTGCAATATCAAAAAATGTT
GCAAACAAACAAGCAATCTAAGATACTATTATGAAGAAGAAGAGCAAACAGAATCCGTAA
TTAATGAGGAA
>DjScaffold1633:11662-20059 strand:+
gactacttaaaattttaatcataaagttataacttaaaatatttgttcaatagtaaaatt
ttagaATGGGTGAAAAAATTTTACGACCACAAGATAAATTGGGGTCTTGGTATTTTGGTG
GTATAGCCAGTGCAATGGCTGCTGCTTGTACACATCCTCTAGATTTATTGAAAGTTCATT
TACAGACTCAACAGAAAAAAGAATTTGGTATGGCTGGGATGGCAATAAAAGTAGTTAAAA
CTGATGGAATTTTGGCTCTTTATAATGGCCTCAGTGCATCATTGTTGCGTCAATTAACTT
ATTCCATGACTCGGTTTGGTCTGTACGAAACTTACAAAGCAAGGAGAAAAGTTGCTACAC
CATTCTACGAGAAAGCTGGTGTATCCATGGTATCAGGGTTTATTGGTGGAATTGTTGGTA
ATCCTGCAGATATGGTAAATGTCAGAATGCAAAATGACATGAAGGTTGCTAAAGAACTCA
GAAGAAATTATAAAAATGCTGTTGATGGAATGTTCCGAGTTGCAAGAGAAGAGGGTGTTG
TAGTGTTATTTAGTGGTGTCTCAATGACTGCTACCCGGGCAGCTTTTATGACATTTGGTC
AGTTAGCATTCTATGATTTATTTAAACAAATGATGATACAAAGTGGTTTATTTTCCGATA
ATATCGTCACGCATTTTACAGCTAGTACTGGCGCGGCTGGAATTGCAACATTGATGACAC
AGCCATTCGATGTAATGAAAACACGATTAATGAATGCTAAACCTGGCACGTACTCGGGAT
TGCTTGCTTGTGGAGCAGATATAATGAAAACAGGCCCAATGGGTTTTTTCAAGGGATTAG
TACCGGCTTTTATTAGACTTGGTCCGCAAACTATATTAACATTCGTATTTTTGGAACAAT
TGAGAATGAATTTTGGACACTATCCAGAGAAATCTAAGTAAcaaacagcatttgttcaaa
aacatagtcatagttatttttccaatttgatctaatcaatttggtatatttcgatagttt
ctttttaaaatgtatttcgcaattagatctattgactttttatgtttaaatcaatacttt
aattggtaactttatcaaatcaataataaaatgtgaaatttcagttcaattgattcaata
tttcaaaaaatatatctggcaaaattagactacttaaaattttaatcataaagttataact
taaaatatttgttcaatagtaaaattttagaATGGGTGAAAAAATTTTACGACCACAAGA
TAAATTGGGGTCTTGGTATTTTGGTGGTATAGCCAGTGCAATGGCTGCTGCTTGTACACA
TCCTCTAGATTTATTGAAAGTTCATTTACAGACTCAACAGAAAAAAGAATTTGGTATGGC
TGGGATGGCAATAAAAGTAGTTAAAACTGATGGAATTTTGGCTCTTTATAATGGCCTCAG
TGCATCATTGTTGCGTCAATTAACTTATTCCATGACTCGGTTTGGTCTGTACGAAACTTA
CAAAGCAAGGAGAAAAGTTGCTACACCATTCTACGAGAAAGCTGGTGTATCCATGGTATC
AGGGTTTATTGGTGGAATTGTTGGTAATCCTGCAGATATGGTAAATGTCAGAATGCAAAA
TGACATGAAGGTTGCTAAAGAACTCAGAAGAAAGTAAgttccatagtaatggaaaataat
ttttataatgtctagttataaaaatgctgttgatggaatgttccgagttgcaagagaaga
gggtgttgtagtgttatttagtggtgtctcaatgactgctacccgggcagcttttatgac
atttggtcagttagcattctatgatttatttaaacaaatgatgatacaaagtggtttatt
ttccgataatatcgtcacgcattttacagctagtactggcgcggtaaatttttggatttt
ctactaataattattagttgatttttcaataaatttagacctatatatttttctaggctg
gaattgcaacattgatgacacagccattcgatgtaatgaaaacacgattaatgaatgcta
aacctggcacgtactc
>DjScaffold2399:12126-22442 strand:+
CACGAGCCGTTTAATAATCAAACAGTATTCATTAGTTTATCTACTGGTATAACTTCAGAA

AAGAGGCCATTAAATGTCGATATAAAAGATGCTTATGATTTTGATTGGTGGGAGTTACTA
GATTCTAAATCTAATAGATTTTATTATTTCAACACAACAAATAAACAAACTACGTGGATT
AAGCCTCAATCAAATTCGATTATCCCATTTGGCAACGTGCAGGACAAATTCCAGTCGCCA
ATCTCATCAATGCTTCAGTCCGATTTAAACGCAGAGGTTATGAAACCTCAAAAAATTCTT
CATTATGGTACTGCAAAACGTAGTACTGATGTCAAATCAATTCCAAAACGACCTCGATCT
CCTATTGTTCATTCAATGAGTACTGATGCAACACCAGTTTCAACAGCATCAGAGTCACCG
TCTCTTGGCAACAGAGAA
>DjScaffold2399:34749-54777 strand:+
gcaccgatatgattaataatcacatattgataaaaattataattcagaacttgctgttga
aacaggtttgaatATGAATAATGGTGGAATTCATTCAGGTCTAAGTCCTCCGAATCCAAT
TAATGGATATCCAGTATATGATTATCCACCAAATTCAAATAATTATTATATGACACATCA
AAATTTCATGCTGATGCAACAACAAGGTGGAATGACAGGTCGAATCACATCTCAAAATAT
GGGTTCTCAACCCGAAACATTTTGTTTGAGAAGACCCCAATCCGAACAAGCAATTAGTGC
TGATATGAATAAACAAAGAATGACTATGGTCAGTCCTCGTAGTGATATCCTTCCTGGAAT
TGTCCAACCAGGTAGGTATTATCCTGCAACTACTGGATGGGTTGAAAGGTCTTTATCTCC
TACTCCTATTGGAGCAATTATACCTCCATATTCTGGTCCCCTTCCTGGGTGGAATTCTGA
ACTCTTTCGTAAAGATCAACCACCAGCAAAGTTATTAAATTGGTGCAAGGATAGTATTCA
GAAGCCATTGCTTGTTTCAACAGATAAAATTCTCAAATCTGATGCTTGCCAGTCTTTTAA
ACAAATCCAAGGCTATATGGGTGATAGAAAAAGCAAGCATTCTCCATCCGAATTAGTCAA
TTCCATAATGCAAAAGGGCATTAATTACTATCCATTGCGTGATGAGATATTCATGCAAAT
TTGTAAGCAGACCACAAATAATCCGGACGACTTGAGTACTCTGCGGGGCTGGGAATTGCT
TCGTACTTGTTTGAATTATTTTGCTCCATCCCCAATGTTCCGACCTTATTTGGAAGGTTA
TGTTTTACGCCATCTTGATAATTCACATAGCAATGAATGCCCAACGCCACAAGCCATTCA
ACAAGCTGCAGAACAAACACTTAATATTCTTTCTCGTCAAACTAATTCTACTCTTCGTAT
GGATTCAAATATTCAATATCAATATCAACAACAACAACAAATGATTATGTTGGCTAATAT
GCAAACGATGTCAGGTCAGAAAAACCATTTGCCAATGCATTTATTTGAATCGGGAGATAA
TTCCGATGATTCTGCATCTCAACGATGTTTGCCACTCAGTTCCCATTTCGGATACACATT
GGATGAAATAATGCGAGCTCAGTATCTCAGGTTTCCTGAACGAGAATTGCCTTGGATTTT
AGTTACTTTATGTGAGGAAATTTTAACTCGTGGTGGTGAACAAACAGAAGGCATTTTCCG
CGTTGCTGCAGATAGTTCGGCTGTGCAAGCAATGTGTACTGTACTTGATCTTTGTGAAAA
TTTAGCTGATCATCCACTTACAAATGGAGAACGCAGTCCTCATACTCCAGCGTCTTGTAT
AAAACGGTGGCTTGATTCATTGTTGGAACCTCTTGTTCCACCGTCATTATGTAGCAACAG
TCAAGCTGGGCGTGCTCGTAGTCCTTTAGAAGTTTATCAGTGCTTAACAAGGTTACATCA
AGCTACC
>DjScaffold2399:88513-89514 strand:+
ATGTGCTTGAGTTTAAATAATCTAAGAGTTTTTGATTTATATTTCATGTTGATTTATCCA
CCGATATTTTTCATTCTTGGAATTTTTAATAATATCATAATACTTATTTTAATGACAAAT
AAAATGAAAAAAGGAAAAACACGTCAAGATCACATAACAGTATATTATTTTGCATTATCA
TTGGGAGACACTTTAACATGTATTTTTCCAATATTCCCGAAATGGCTGAAAAGTGCTTTT
AATATCGATTTGTATTCTTTATCGGAAATTACTTGTAGAATATTTCCAAGATTTATTCCT
TATATATTTAGTGATTTTAGTGTATGGCTAGTAGTTGTCATGTCAACTGATATTTTCATT
CGAATTTATTTTCCAATTCATTTCAAAAGAATGGTTAAAGTAAAACAAGTTTGCTTCACG
ATTTTTCTATTGATAATTTTGGCATTATTGAAAAACGTCTGTTACGTTTATGAGAATACA
ATTATTAGTAGTTGTGTATTTAATAACAAATCGATAATTGCTAATTATTTCACATCGGGT
TTGAGTTCAATAATTTCATTGGTGTTCGTTTCAATTATTCCAGTAGGAACCAATATTATT
CTAAATATTTTGATCTTTATTAAATGGTGTAAATTATTCCCGAAAATTCCAGTCGATAAT
AATAGCTCAGTTGTAAATGAAAATTCTCAAAGAAAGATAATTAAATTGTTTTTAGCAATA
TCAATTTCTTTTACAGTTTTTGTCATTCCAAATTTTATAATACTGCCATTTTCTCGTTTT
CCATTTACAGATGATTGCATCGTTCTTCTGTTTTCAGTTCTAGTTCATATAACAACATTT
ATATTTAATTTGAATTTCATTACAAAACTGTATTTTTATATACTACTTTCAAAATCTTAT
CGAAATGAACTAAATAAAATTTTTTATTTATTTAGAGTAACGTGTATTGAAAGAAAGGTT
TTCAAAAATATTGTTAAGCCAATTAGTAGTGAATTTAATTAG
>DjScaffold2399:97172-98152 strand:+
ATGTGTGACAATTTAAATGATTTATTAAATCTTGATAAATATTACCTTATTATTTATCCG
CCAATATTTATCGTATTCGGTGTTCTAAATAATATTATAATTTGTTTTGTAATGACATCA
AAATTAAGAAAAGGAAAACCGCGCCCAGATCACATAACTATTTATTATTTGGCATTATCA
GTTGGTGATGCTTTATCCTGTTTATTTTTATTATTACCAAAATGGCTAAGAAATGCATTC

GGTATTGATCCATATTCTATTTCAGAAGTTGCTTGTCGAATATTACCAAAATTTCTTCCA
TTTACAATTAGTGATTTTAGTATTTGGCTGGTAGTTGTCATGACAATCGATGTATTCATC
AGAATATATTTTCCAATTCAATTTAGATTTCTTGTTAATGCTAAAAGGGTATATTTGACA
ATTTTTTTTCTGATTATTTTAGCATTCTTCAAAAATTGTTGGTACACAATGGAAAACACA
ATTGAAAGAATTTGTCAAGTCAATAACAGTTCATTAGTTTCTAATTATTTCTCATCGGGA
ACAAGTTTCATTATTTCAATGGTAACTGTATCAATTTTACCAGTAACAATTATTGTCATT
TTGAATATTTTCATTTTTATCAAATGGAATAAGCTTTTTCCTAGAAATCGAACCAGTAGT
AATTCCATATCAGCTTATAATTCTCAAAAATCTACAGTTAAAGTGTTTTTAGCAGTATCA
TTTTCGTTTTCGTTATTTCTTTTTCCGAATTTCATAGTATTACCATTTTCCAGTTTTCCG
TTCAAATCCAAATGTGATAAAATGATTTACAATTTTGTATTTCATTTCACTGAAAGTATT
TTCTATCTTAATCATATTACAAATTTATATTTTTATATTATACTCTCTAAATCTTATCGA
GCAGAATTGTTAAAGATAATCAACAAGTTCAAATGTAAAAAGGTTAATTTGATTAAAAAT
TGTAAACAACTTTTCTTTTGA
>DjScaffold836:51805-52970 strand:+
AAAAAAACTCCTGTTCAAAAAGGCAAATCCAACATTAAATGGAATTCGTTATTGCAATTT
ACAGATGTTACTTTAGAAGAACTAAAAGATAGATCTTTGGAATTATCTATTTATCATTAT
AAAAGACTGAAAACCAATGAATTCCTTGGAGGTGTACGTCTTAACCATGGACTTGGATTA
CATTATGCAGACCATGTTTCATGGATGGATGCACAAGGCACAGAAAAGACAACTTGGGAA
CGATTTCTGTCTAATCCAAATGAAGAAACAGAAGCTATATTAAATTTACGACCAAATCTT
GAGTATTAAatgtttttttaaaaaaaaaagaggaaaaaatgaattttagaaatattacga
atttttattgttattgtttacgctattctaagcagatgcttatttctattttttgaaat
tatttggtatgtttttgagatcatttaataaaatctctgtttactaaatatttagtagtg
tttaaattgattataaaatcattttatttataaaaactattttattaaaatggtgattat
caaagtcattgtaaatgcttttgttaaataccgattttcattttataaataaatgtcatt
cagcta
>DjScaffold995:46823-55538 strand:+
GAATTCATTCCTAATTTTCAAATACAGTCTCAAATAATCTATGAATTGAGCGATCAATAT
TCTAAACTAATCGAATTTATAAATTCATGTATACCAATGCATAGATCTGAACTTAGAGTT
TTATTATATCCAGTATTTGTGCATATTTTTTTACATTATATATCAGTGAAACAGTATTCT
CAAGCAAATGAATTTTTCGAATTATTTGTTCGATTACAAGAAGAAATTTATAAACAAGAT
TTAATTGATTTACGACTAAATTTATTTGAACACAATTCATTCAAGAATTCAATTTTTGAA
GTTTTTAACATTAATAAATTTGGTGTTCGAATTTGTCATAGTAGCTTCGATTTTGTTAAG
CGTGTTTTTTTGGAAAATCAGAAATCTTCTGATTTAGTTAAAATTATAAATGAACGTTTG
AAAATTAATTTTTACGATGGTGAGCCAAGAACAGAAAAAGAACTTTATTTAAACAGGGGT
GCTTATTTTGGTGAAACAGTATCGGATGTTAATTCAATTCCTATTTTATATGGAAACTTG
AAAGATCCACAACTCGATGGTATAGTTAAAAGAATTGATAATAATATTAAAGGTTATGGA
GATGATATTGTAAATAAAAGAAAATCTAAAAGAGAAAGTCCTACAATATTGACTAGTTAT
TCTAAAGAAACTGAATCCATATCGCCTGATATGAGTAATATTCCAATACCGACTTTGAAT
TTGCGCACAATGGAATATATAAGAAAAATGCACACGGAAATTAATTCAGCTGTTCATCTT
AATAAAAAAATACGTTTATTTGAAGAAGACTTAAGTTTAACGGATAACATTTTACTTGTC
AAAAATGCTGGAATTATTACACATAAATTTGTTAAAGAAGGATATCCTCTCAATAGCATC
ATGTGTACGGAGAACAATTTAAATGGTCACGCAACATCAATTAAGCTAACTGAAGATTGT
TTGATGTTAGCTGTCGGCTTAAGTTCAGGAATTATTAATTTCCGAACGATAAACGGAGAA
AAGTTAACTAAATTAAAACGAGTCGAGAATTTACCAGAATTAGATACATATGTTCCAGAT
ATTGAACGCTTTATATTAGAAACTATTCCCGAAACAAATTGTATAGAAATGAAAGGTCAT
TCTGGTGGTGTTTATGACATATCCTTTTCAACTACTAAAGAACACGTACTTTCTGCTTCT
AGTGATGGTACAGCTCGATTATGGTGTACATATACATGGGCAAATATAGCTAAATATCAA
ATCAACAGTGATCCTTTGTGGAGCGTAGATGTAGCTCCATTATATGGACATTATTTTGTT
ACTGGTAGCAATGATCGTTGTGTACATCTTTTTGGTTTGAATAAATTGAAACCTTTACGC
AGATTTATTGGTGACTGCTTTGGTCCTGTGACAAGTGTGAAATTTCATAACAATATTAAT
TATGTGGCCTCAGGAAGTACAGATAGAGTAGTAAGACTATTTGATGTCAGATCTGGTGAA
ATGGTTAGATCATTTACTGGACACAAGGGAACTATTAATTGTTTGCAATTCTCTGATGAC
GGTAAGTATTTATTAAGCGGTTGTGATAACGGTTCAGTAAATATTTGGGATTTGGCAATA
CCTAGTGGATTGATGGATACTTTAAACATAACCGGTGGTAATGATAAAACAGGTGGTTCA
GTAACAAGCTTATCGTTTTGTCGAGATGGAGCC
>DjScaffold2305:60025-71635 strand:+
tactttccattttttagatatgaacagaagaggtagaggaggtatgatgcaaagggacaa

caaaccgtttaatattcctggtaatcgaattgaatttcgtttttaatacctagcaaagc
agccagtattataattggaaagggaggagaaagaattcagagacttcgacacgagtgtaa
tgcacgattgtctattcctgataaccatggccccgaacgagtatttattcttgaaagcac
tatggataattgtataaaagtatggcatgattgtttgccagatttgaaagattgtatgtt
tcctaatacagatcaaaggaaatcatcgggatcttttagagatgataatcctgaaatgga
cttgagatttctggttcatgacagtcaagtcggagcgattatagggaagggtgggattaa
tgtaaagaatttacgaatgcaatatcaagttaaatgtgattaaagttttccccgttacag
ctcctaattcttcagatagattagttcaaATGGTGGGGAAATTTGATAAAGTTTTTGAGT
GTTTTCGAGTCATTTACGACACATTAGAAAGTCAATCGATAAAAGGAAAAATAGAGAATT
ATAATTCTGAAAATTTTGATGAATCTTTAACCGCATCATATGGAGGCTGGAGTACTCAAT
ATGGTGGCGGCAGTGGTGGCAGTGGTGGTGCTGGCAGTGGTAGTAGTTCAGGTGCATCTG
GTGATAACATGAGGAAATCTGGTCCACCGTTTTTTGCATCTAACGATCGCAATCCACCGC
AAAATCCTCGGTTTACTAATCGATCGAGTGAACCATTTTTCCAGAAACCTGATATAAATC
GGAACAATAATAATAATTTACCGAAGCAACCACCTATGAATCGATTCGATAATTTTAATG
ATAACTACAACGATAGTTCTCCTAGTTTTGAAATGGAAATGGATAATTTTCAAATGCCGT
CATTTACCAATAGATTACCTCCAATGTCTCAGGAACAAAGTGCTCCTGGATTGCGCAAAA
GAAATTTTATGGATAATGAACCAGACAACAACAGGAGAGGAGGAGGAGTAGGAGGAGGAC
GAATGAAAATGAGTAATCCGAATACTTTTGAAAATAGGATTGAGAAATCGAGTTTTGACA
ACAGAAATCAAAAGCCATATAATTTTGATAATAGAATAGAAAAACCAAATTTCTATGATA
ATCGTATTGATAAAATCAATCGAATGGATAATAATAATGCTGTTGGTGTGTCACCCAAAA
ATTCATCGATTGAAAAATTGCTAGTTTCTATTCCTACATCTTTAATTCATTCTGTCAAAG
GCAAGAATGGCGAAGTAATGTCGAATATTATAAGTGATTCTGGAGCTGAAATTTCAATCG
ATGGTGATGATGAACAGAATATGGAAACGATTTTAAGTTTAACAGGAACAAGAGATCAAA
TAGATGGGGCGCAATATCTTTTACAAATTTGCATTAAAAAGTCGGTTCTCAATTTGTGAt
ttgatcattttgttgtttttgtgtgttaaataaataatttgttgtacacgttgcataaat
tactaattcatattcataagatatgttttcatggaaaattaataaataatttttttggt
ttgcatcgtatatttatatatttacagtaaaaatgtcttcaatttttttctgtatatag
aagtagtaaatactttccatttttagatatgaacagaagaggtagaggaggtatgatgc
aaagggacaacaaaccgtttaatattcctggtaatcgaattgaatttcgtttttaatac
ctagcaaagcagccagtattataattggaaagggaggagaaagaattcagagacttcgac
acgaggttaatatacttattattttcagttaatttatattaatttaattattttctaaat
tatctgtttttaatgaatttttatttcgattattcaaaccattactttggacattcaaat
gaaaattggtctttttttaaatatgatcaaatttgacaaaacattaatcttgatactcac
ttttatagtgtaatgcacgattgtctattcctgataaccatggccccgaacgagtattta
ttcttgaaagcactatggataattgtataaaagtatggcatgattgtttgccagatttga
aagattgtatgtttcctaatacagatcaaaggaaatcatcgggatcttttagagatgata
atcctgaaatggacttgagatttctggttcatgacagtcaagtcggagcgattataggga
agggtgggattaatgtaaagaatttacgaatgcaatatcaagttaaatgtgattaaagtt
ttccccgttacagctcctaattcttcagatagattagttcaaATGGTGGGGAAATTTGAT
AAAGTTTTTGAGTGTTTTCGAGTCATTTACGACACATTAGAAAGTCAATCGATAAAAGGA
AAAATAGAGAATTATAATTCTGAAAATTTTGATGAATCTTTAACCGCATCATATGGAGGC
TGGAGTACTCAATATGGTGGCGGCAGTGGTGGCAGTGGTGGTGCTGGCAGTGGTAGTAGT
TCAGGTGCATCTGGTGATAACATGAGGAAATCTGGTCCACCGTTTTTTGCATCTAACGAT
CGCAATCCACCGCAAAATCCTCGGTTTACTAATCGATCGAGTGAACCATTTTTCCAGAAA
CCTGATATAAATCGGAACAATAATAATAATTTACCGAAGCAACCACCTATGAATCGATTC
GATAATTTTAATGATAACTACAACGATAGTTCTCCTAGTTTTGAAATGGAAATGGATAAT
TTTCAAATGCCGTCATTTACCAATAGATTACCTCCAATGTCTCAGGAACAAAGTGCTCCT
GGATTGCGCAAAAGAAATTTTATGGATAATGAACCAGACAACAACAGGAGAGGAGGAGGA
GTAGGAGGAGGACGAATGAAAATGAGTAATCCGAATACTTTTGAAAATAGGATTGAGAAA
TCGAGTTTTGACAACAGAAATCAAAAGCCATATAATTTTGATAATAGAATAGAAAAACCA
AATTTCTATGATAATCGTATTGATAAAATCAATCGAATGGATAATAATAATGCTGTTGGT
GTGTCACCCAAAAATTCATCGATTGAAAAATTGCTAGTTTCTATTCCTACATCTTTAATT
CATTCTGTCAAAGGCAAGAATGGCGAAGTAATGTCGAATATTATAAGTGATTCTGGAGCT
GAAATTTCAATCGATGGTGATGATGAACAGAATATGGAAACGATTTTAAGTTTAACAGGA
ACAAGAGATCAAATAGATGGGGCGCAATATCTTTTACAAATTTGCATTAAAAAGTCGGTT
CTCAATTTGTGAtttgatcattttgttgtttttgtgtgttaaataaataatttgttgtac
acgttgcataaattactaattcatattcataagatatgttttcatggaaaattaataaat

aattttttttggtttgcatcgtatatttatatatttacagtaaaaatgtcttcaattttt
tttctgtatatagaagtagtaaatactttccatttttttagatATGAACAGAAGAGGTAGA
GGAGGTATGATGCAAAGGACAACAAACCGTTTAATATTCCTGGTAATCGAATTGAATTT
CGTTTTTTAATACCTAGCAAAGCAGCCAGTATTATAATTGGAAAGGGAGGAGAAAGAATT
CAGAGACTTCGACACGAGTGTAATGCACGATTGTCTATTCCTGATAACCATGGCCCCGAA
CGAGTATTTATTCTTGAAAGCACTATGGATAATTGTATAAAAGTATGGCATGATTGTTTG
CCAGATTTGAAAGATTGTATGTTTCCTAATACAGATCAAAGGAAATCATCGGGATCTTTT
AGAGATGATAATCCTGAAATGGACTTGAGATTTCTGGTTCATGACAGTCAAGTCGGAGCG
ATTATAGGGAAGGGTGGGATTAATGTAAAGAATTTACGAATGCAATATCAAGTTAAATGT
GATTAAagttttccccgttacagctcctaattcttcagatagattagttcaaatggtggg
gaaatttgataaagtttttgagtgttttcgagtcatttacgacacattagaaagtcaatc
gataaaaggaaaaatagagaattataattctgaaaattttgatgaatctttaaccgcatc
atatggaggctggagtactcaatatggtggcggcagtggtggcagtggtggtgctggcag
tggtagtagttcaggtgcatctggtgataacatgaggaaatctggtccaccgtttttttgc
atctaacgatcgcaatccaccgcaaaatcctcggtttactaatcgatcgagtgaaccatt
tttccagaaacctgatataaatcggaacaataataataatttaccgaagcaaccacctat
gaatcgattcgataattttaatgataactacaacgatagttctcctagttttgaaatgta
at
>DjScaffold288:2121-17857 strand:+
atttatttatttctctcaattcaccaattctttctattaaaaaatttatttactttttttg
ttcgaattattattattattaatattagaataattttaaaaattcaaagaatcaaaataa
aattagagtatatttttaatttaaaatttcctattaattagcctcatcaagtaactgctc
aagaagtcgtaataaaaattgcaacaatattagaaaattctcaaaaggaaaatgaaattc
aaaacaggatattaaATGATCAGATTTCGACAGACAGAAAGTACTAGTAATCATGATCAT
GATCAACTGTCTCATAGGGAAAACGAAGATAATAAAAGTTTATATAGTATATCTTCTGAA
TTTATTAATATTTGGTATGGCAAAATGGATTCAATTCACACAATACAACCAAATGCTTTA
TCGTCTTTAGCGAGTGTTCATGAAGAATTACATACGATAAATCAGAATTTAAAAGACATA
AGATATTTTATGAATAAGCTTTTCAATGCAATTCATTGTGCCGTTCAGGAAAGAAAGCGA
AAACTCCATGAAACATTGCGCAGTAAAAATGAAATTGATGCCCAAATTGTTCGACTTCCA
TCAAAGGAAGAATTCATAGAAGTTATAAAAAAAATCAAAACATGTCTGAATTTAACAGCA
GCACACAAGGAAATCATCATTAATCCATCATCAGAGGAAATAATAAATCAACTTTTCATT
TTTTTAAAAATTATTGGTGATGCAACTATAAACAATAACTCCGGTAAACCCGATTTATTA
TGTGGAGTGAAAAGTCCACAAATCAATTCTACAACTGTCAAATTTCTATCATCGAAATTA
AATATTGAAAATTTAAAATATTGGATGGTTCTCGGTTATAATTTTGGTTATCTTAAAGGA
AGTTTACCAAATCAAATAAATAGAACTCCAATTGATGAATTTCAAATAAAAGAAATATCA
AATGTTTATGAATATGATAGGAAAAACAACCCATTAACGCTTCAACAACAATTTTCACCA
AATCGTTACACTAGCGTTGATTCATTGCATGAAAATGAGCCTATGGAACTCAATACTCCA
ATGAGATCTTACAGAAACAATATTCGATCAAGTCCAGTTCCTCTAATTAATAATGATTTG
GAATTCAATACTCCAATTAAATCTCATAGAAACAACAATATTCGATCAAGTCCTGTTCCT
TTAGTTAATAACGATTTGATTTACCGCAGTTATCCACGACTGTTTGAATCAGAGAGAAAA
GCACCGCTTTTTCATGAATCTGAGACTTTGCAATATCAGAATTTGTCCTCCAACTCACCC
CATGTCAATTGCTATAACCGTTCTAATAACTCAACATGGGATGATAGACAATGCCGATTC
ATAGATGCTATTCAACTGAGAAATGGAAGACTCGGAAAAGCACTTTATGATAGATCTGTT
CAAAATTATAGAGACCTAGGTGTAAGCAGCAACGAAATAGTGGAAATTTTAAGAGATTAC
GGTGATGTGCTCTCAATTAAAAATGCTCTCGGTGAAATTGGAGAATGCCCGAAATCTATT
ATTACCGAATTACATAACAATGCTTCTCGATGAttgagatttatctatttaattccatat
caatcttttctagaaacaaatgatagttattaaaaagcaaaaaaaattgtttaaagaaaac
agtttgatatctttgattttagtttcgatttttataagtttcaatttcagtgtttttctg
aaactttggttttatgtttttttgattttacgtataataaagatttataatatatt
>DjScaffold288:22054-22299 strand:+
NNNAAAGGATTAAGAAAGGGAAGAACTAAAATATCCAGAAAAGGAATTACAAAACCAGCA
ATCAGAAGATTGTCTCGTCGTGGAGGTGTCAAGCTTATTTTGGGATTAATTTATGAAGAA
ACACGAGGGGTTATAAAATTGTTTTTAGAAAGTGTAATAAGAGATGCAGTTACATATACT
GAGCACGCTAAAAGAAAAACTGTCACAGCTATGGATGTTGTTTATACTTTAAAACTCCAA
GGCTGA
>DjScaffold1250:58876-59817 strand:+
ATGAATGATTATGACACTGATGACGAACTAAAAATCGTTGAGCATAATGATAACCTGATA

CCAAGTTTAGATGGAAATTCTGTAATCAGCTATATCAGTCTCAGTAGTTTAAATTCTACT
CCAGTTAATAACACCGATGTTCAAAAAACTCACAGACACTGTCCGAGATGTTATACAGCG
GAAGTTTTCGTAAAAGGAATAAGTACTGTTCTTAGAAAAAGAATTAAAAACTCTTCTCCT
AGTAATATTCCTGCAGAACGAAAATATTTGATTCAATGTTCCGTTGATGATGGATATCAA
GAAGATAATCCATCTCCTTTAATGTCTTCTGTTTCTAGTGATAGAGATCAAAGTACATTA
TCCGACGCGTCATCCGAAGGATTTTATTGTCAAAATGATTACAGTCATGTATGTAACGAA
GGTCTCTGTATGCATGTTACACCATTTATTAATAAGATGAAAAGAAATTTCATCCTTCCT
TTTAATGATCAATGTCCATCACTTCCTAAATTTGATGACAAATTCTATCTTTCTCTCGAA
CATATGATATTTTATTTTGAAGAAAAATTCAACTCCGATTCATCGGATTGGTTGCGTACA
AATGCTTTACAAATGAATCAAATGGACAAAGTTCAAGCTAGACAAGAATTAAATAAAATC
TTTGAATCTATTTTCAGTGAGAAAATTAATTGGGGTCGAATCGTTGCAATGATTGGATTT
GTATGCGAATTGTCGTCTTCAGCATTTATGATAAATCGTCAAGATCTTATAGAAGAATAC
ATATATACAAGCGTAGCATTTGTCGACGAATTTTTATGGAATTGGATATTTCAACATGGT
GGCTGGGTGAGTCGCTTTCATTCAAATTTAAAGGAAAATAAATCTATCAATCATAAATTA
CATGTTATAAATTCCATTAATAATAATTCTTATTTAATTTAG
>DjScaffold134:58138-59038 strand:+
gaactttcgaATGGCTTATTATCAGCATCAAAACCCAATACCAGATCCAAATTGGTTATA
TCAAATGTTTCAAAAAGTGGATAAAGATGGAAGTGGAGCTATTAACCAATTGGAACTTCA
ACAGGCTTTATCCAATGGCACATGGACACCATTTAATCCTGAAACAGTAAGACTTATGAT
AGGGATGTTTGATCGAGATAACAACGGCCAAATAACTTACAATGAGTTCCAACAATTATG
GAAATATATTACTGATTGGCAGAATACATTCCGCACTTATGATCGTGATGGCTCTGGTGC
AATTGATAGAAATGAGTTAAAAATGGCTCTTACTTCTTTTGGTTATCGTTTGTCTGATAA
TTTTTTCAGCCTAATGATCAGAAAATTTGATAGACAAGGACGTGGTACAATTGCTTTTGA
TGATTTTATTCAGGCGTGTGTGTCATTACAGACTTTAACCAGTGCTTTTCGTGGATTTGA
CACTGATCTTGATGGGTGGATTCACATAAATTATGAGCAATTCCTTATGCTTGTTTACAA
TCTCAATTTATAActgatttattcagaaagccaatcaatttcaaatttgattttttgtct
tttaatattaaatttttgatattgtaaatattgattgttatattttgctttaattatata
tttattaattctgtaata
>DjScaffold134:105104-105863 strand:+
atatatatcaattaaaatttaaaataaatttgaattactgaattttgaaaaagcATGGAT
TCTGACTATATTGAAATTTTACCTACGATAACGTGTCCTACTTGTAATGGGAAAAAATAT
GTTTTAAAAAGAAATCAACCTGTATATGTGGCACTCATACCTGCTACTGATGAACGGCTC
AAACCTCCAAGAATTTGTGTAAAGGTATCTTTAGCTGTATTGATTACTCTATGTATTTCG
GGATGTATAATTTACTTTCTAATAACTCGCAATATTTCTTTATCAAGCAATAGTGACAAT
ATTATCCCATCATCAGTAAAAATTAATACGGCTTCAAACACTTTGCAGCTCTTTTTTAAA
AAAGTGTTCAATGTTACTAATAAAAATTTTTTCGCACTTTATATATCGAATATTAATGCG
TATGCAACAATTGATGGAAACACAAAACAAATATTTAATGAATCAGTTTATCATGTTATT
AATATGAGGTCAATCAAAGAAATTGGTTTCAATGTTACAATTAGTTTTATTGATGAAGAC
ATGTGTTTCAGATGCACCACAGAATTTATGATTCATAAATTGTATTTTTTCTTCAAGTTT
GTAATAATTCATTAAtaaattaatga
>DjScaffold4811:41738-42792 strand:+
ttcagaagctattgacaaccacgctttgtctgaaacatggtgtcaagaatggtttcggca
atgtaaaATGGGTGACTTTGATTTGCAGGATAAACTACGTCCAGATGAAGATTTAGAAAA
GGTGTTGATCAAAGATCGATCCAAAACAATGTCAGAAATCATTAATGTAAATAAATATAC
CATAAGCAGACGATTACAAGCCATAGGATACAAACAGAAAGTCGATAAATCGTTGCCTCA
TCAATTGAAAGAAGCAGATATCACCAAAAGATTAGACATTTGTCAGACGCTGCTCAGCAG
GCAAAGGAAAAAGTCATTTTTACATCACATTGTTACTTGTGATAAAAAATGGATTTCTTA
TGAAAATTCGAAACTAAGAAAATCATGGGTGAAGCCTGATCAACCCGGAAAATCAACACC
AAAACGAGACTTGCATGGAAACAAGATGATGTTATCCATTTTTAGGGATCAAAGGGGGAT
TATTTATTTTGAGCTTCTGAACAAACGAGAAACGATTGATGGTCGGCGTTATCAACAACA
ATTAATAAATTGAggcaagaattggacgaaaaacgaccagactattccaataaaaatgag
acggtgttgctacaacatgataatgctcgaccccacacttcgagtttagtgaggaatacg
atagtagaattaggatggaatgtttcatctcaccggccatactcaccagacatcacatca
agtgattacaatccttttcgctcaatgcaatatagcctttgtgaacagcacttcaacagt
cattatgatatccagaaatggcttgactattgggtaaattccaaggaagaaaattttttt
tatcatggaattcatttattgccagaaaggtgggaaaatgttgttagcaataatggacgc
tactttaaatgagatgttatgtaaaaataaatgtatttagagtttttatttacatttctt

cttattattaaaaaacagcgataattaatgcaactacctaataatattacgttatacctc
ccatgagatattat
>DjScaffold4781:13721-31193 strand:+
ttggagaattttgataattttgataaattcataggtaaaagaagcagtagtagttgttgt
agacaatagttcattatgtgaaacatgtggagaagcatttattaagaaaactgatgaaaa
aattgacaatcaatgttctccatgtaaaaaagctgcaaaagaattatccaatgtatataa
tgaagaaaagaataaaatttgtgaaaattgcaaagtaaacctattaaatttctgttttaa
ttgctcgtttaaaatttccaaaggaaacttccttgtctttagataatagaATGATAGGAT
GTATGATATGTATGAAATACTACCACACGAATTGTCTTCTTCCATCTGATTTACCTAGCG
ATTTGAAATTCGATGCATCAAATTTAGTCCGTCGAAAATTGGGTTGCATAAGATGTGCTG
TTGTTCATTTGTGTTACAAAGTAAATAGTATAATCACCTGGAAATGGCGTAAGGAAGCGA
TGGACGTGGCAAACGCTGATAAATATTACATGCCTCGTTTGTTCCTGATCCGTTATTCGA
ACATGTCATTCTGGGATTGCGAATGGGTGTGTGAGGCTCAGTTAGAAATATCAGAAGCGA
ATTTATATCGCGCATATAAACGGAAATGTATAGATTTCATCACTCCTCCCGCCGTAGATG
TTCTAAGTGACATTCAAAAGCGGAAACTGAGTGCTGGAGAAGTGAAATTACACGAGAAAT
ATTTCCAGTGGGGTATTGAGCCTGAGTGGTTGCAACCGCACAAGGTGCTTCAAATGGACG
ATGTCGGCTGCTTTGTGAAATGGCGCGGTCTCGGTTACGAATTCGTGTCGACAGAACCGT
TGAGTCGAGTGAAGGAAATTGCCGGATTGAAGGAGGCTATAGCTCGACATAAGAAGGTAA
CAAAGGAGCTGACTGCTGCTGGTGGTGCTGGTGGGGGCGAATCGTTGTTCTCTCGTGACA
AAACAAAACTGACGAAATTGAGCGAATCTCCCGAGTATCTCGGACCTGCATCGACACACA
GCAAGAACAAGGCAACTCTTCATCCGTACCAGCTGGAAGGAGTGAATTGGTTGAGGAGCT
GCTACTCTAACGATATCAATACGATCCTCGCCGATGAGATGGGATTAGGCAAAACTATTC
AAGCGATCGCCTTCCTTTATTCTCTTTACAAAGATAATGTTTCCCCAGGACCTTTCCTCA
TTACCGTTCCGCTGTCAACCATGAAGAATTGGGAACGAGAATTTGAATTTTGGGCTCCGG
ATTTCTGTATTGTAACTTATTATGGAGACAAAGATACTCGAGTAGTGATCAGAGAGAAAG
ATATGTTCTACGAAAATAAGAAAAAGAGCGCAGTCACTTTCCATGTCTTACTGACAACGT
ATGAATTGATTTTTATGGATGAAAGCTTTCTAAGTCGTATAAATTGGGAAATATTGATTG
TTGATGAAGCTCATAGATTGAAAAATAACGAAACAAAGCTGTTCAAAAGTCTGAGAAAAT
ATAGTATCAATTATCGAAATTTGATGACCGGAACTCCGCTGCAAAATAATTTAGAAGAAT
TGTTTAATTTATTAAATTTCATGGAACCTAATTCATTTGGAGATATGTCGGCATTTCTTT
CTCAATTCAATGATATTTCTAAAGAAGAACAAGTTAGAACTTTACAGGATATGCTGAAAA
AACGTATCTTACGTCGATTAAAATGTGACGTTTTTGATGACATGCCAACAAAAGAAGAAC
AAATAGTCCGAGTTGAACTTACTCCTCTTCAAAAGAAGTTTTACAAGATCGTTTTATCGA
AAAACTATGATGCTCTTAGTGGAAAGTTCAGAGGAGTAAAAATGATAAATATAATGATGG
AGTTGCGAAAGTGTTGCAATCATCCATACCTATTGTCATCTGAAATAGCAGAAGAAAATA
TTTTGGAAGATGGTTCATTTGATCCTCCAGCTTTAATTGCAGCTTCAGGGAAAATGCAAA
TTCTGAAAATAATGCTTCTGAAACTGCAACAGACAGGTCATAAAGTGCTTATATTTTCCG
GTATGACAAAACTTCTCGATCTTCTCGAATATTTACTGGAGCATATTAACCTTCAATATG
AACGTATTGACGGCAGAGTATCTTCAACTGCTCGACAAGAAGCTATTGATAACTTCAATG
ATCCTAATTCTACCAGCTTGGTTTTTCTTTTATCTACTAAGGCTGGGGGTTTGGGTATTA
ATCTATGTTCCGCCGATACAGTTTTCATTTATGATGCTGATTGGAACCCTCAAAACGATA
TTCAAGCATTGTCACGAGCCCATAGAATAGGACAAAAACGGAAAGTTATGATTTATAGGT
TTGTGACACGAAATTCAGTAGAAGAGAAAATTATGGAAAAAATCGAAACGTAAATTGATGG
TAACCAATCTCGTCACAAAATCTTTCACAAATGATGAATTTCATCAAATTATCAGTTTTG
GAACAAAGGAATTGTTTGATGAAACCAATGAAAATAATGATCAAAAAATTATTTATGATG
ATGAAGCTATTGAACATCTTCTTGATAGAAATAGAACTGAAGCTGTTAATCCTGATAGTA
GCTTTTTGGATTCTTGTTTCAGTGGATTTAAAATGGCACAATATCAGTTTGCAAGTGAAA
AAAGTGAAAATGAAGAAAGTTCAAAGAAAATTGATAAAATTGAGGAAGATGCAATTATTG
AAAATCCTACCGACTATTGGGCATCAATTCTTGAACCTTTTATTAAAACTATCAGTCAAG
AAGACAATTCAAAAGAACCAGAATTAGGGAAAGGGTTTAGACATAAACGGAAGATTAATT
ACAACACACACATAAACCGATAATAATCGATAAGAACTCATATGCTGGAACTGAAATCATTA
ATGAAAAAGATCCAGATGATACTGATTATGATATTTCTGAAGGTGAAAATGTTGATCATG
TATCATCATATTCACATAGTGATGATAGTTTCAAATTTGCTGAGACTGATAAAAAAACCCA
TTGGTGAAAATAGTAGGCAACCTAGTAAATTTAATATGGATCGAGATTATAAAGACTTGG
CGAATTATGGAATTTCTCCGGAATTGACAAATCAAATTAAATGTGACGTGATTAATGAAA
ATACTATTTTGCATTTTAAACATTTTTCCATCATAAATGGATTCATGCCAAAAGAAAGAA
TGGAAATCTACGAAGCATTAATGAATTATGGGTTGCCTCCGAAAAGTCTCAAAAAAGGTG

ACATTTGGTGGCCAAGCGCTCTAGAACATAAAACAATTGACGAAATGGATAAATTTATCA
CGACTCTCATGGTAAATTTAGCTGAATTTCACGAAACACCATCTACCAACAGCAAAAATA
ATCTTATTAAAGACGAAATTGATTATGATTTGTTATTGGCTAGAATCGGTATAAATGGAC
TTATCAATTTCAAGGTCAATCAATATGAAGGAATAAATCGAACGATTTTGGAAATTGTAA
CTTACAAGACGTGGAATGTTCGTACAATTCCTATTGACGAATTACTGAAACTATTCAATG
AATTAAGTCATTTCGAAGTGGATTTCAATATTTACGAAGGTGGATTTTCTGAAATCGAAA
ACGATTGGAATGATTACAATGTAAAATACAAACAGAATTCAGAATCGAACCCCACATTAC
ACTGGAATAAACTGCATGATTATTATATGTTATCTGCAATAGCTACTCACGGTTATGATA
AATATATCGACCAAATATTCATAGATCCCCAATACCAGATTTTGTTCATTCCGTTACGAA
TAGCCAACATGGACTTTGTTGCAAATAACGCTGACATTAGGCAATCTTTTCGTAATCGCC
AACGATTGCTGGAAAATGCATTATTGATTGAGGCTCAAATAATGAAAATTGGAGCCACAT
TTGACGGTTACTCGATCGAATCTTTTTCCGTAGAACGATTGAAATACTATCACGAATTCT
TTACGAAACAAGGCGATAATCTTTCTATCACTCCTACATTAGCCAATAATAAATTTAAGC
AGAATGCAATGGCTCAATTAGAACTGCTAATTAATGATATCGAATCGAGTTCTGATTCCT
TTAGACCGATCGTTGTTAAATTTGGGAACCATGAATCTTCTAATACCAATACTAATACCA
AAACTAATGCCAATACCAATACTAATGCCAATACCAATACTAATACCAATACTAATttgg
agaattttgataattttgataaattcataggtaaaagaagcagtagtagttgttgtagac
aatagttcattatgtgaaacatgtggagaagcatttattaagaaaactgatgaaaaaatt
gacaatcaatgttctccatgtaaaaaagctgcaaaagaattatccaatgtatataatgaa
gaaaagaataaaatttgtgaaaattgcaaagtaaacctattaaatttctgttttaattgc
tcgtttaaaatttccaaaggaaacttccttgtctttagataatagaatgataggatgtat
gatatgtatgaaatactaccacacgaattgtcttcttccatctgatttacctagcgattt
gaaattcgatgcatcaaatttagtccgtcgaaaattgggttgcataagatgtgctgttgt
tcatttgtgttacaaagtaaatagtataatcacctggaaatggcgtaaggaagcgatgga
cgtggcaaacgctgataaatattacatgcctcgtttgttcctgatccgttattcgaacat
gtcattctgggattgcgaatgggtgtgtgaggctcagttagaaatatcagaagcgaattt
atatcgcgcatataaacggaaatgtatagatttcatcactcctcccgccgtagatgttct
aagtgacattcaaaagcggaaactgagtgctggagaagtgaaattacacgagaaatattt
ccagtggggtattgagcctgagtggttgcaaccgcacaaggtgcttcaaatggacgatgt
cggctgctttgtgaaatggcgcggtctcggttacgaattcgtgtcgacagaaccgttgag
tcgagtgaaggaaattgccggattgaaggaggctatagctcgacataagaaggtaacaaa
ggagctgactgctgctggtggtgctggtgggggcgaatcgttgttctctcgtgacaaaac
aaaactgacgaaattgagcgaatctcccgagtatctcggacctgcatcgacacacagcaa
gaacaaggcaactcttcatccgtaccagctggaaggagtgaattggttgaggagctgcta
ctctaacgatatcaatacgatcctcgccgatgagatgggattaggcaaaactattcaagc
gatcgccttcctttattctctttacaaagATGTTCGTATCGATCTCGCATATTATACTAT
ACTATCATGCTTATTTAATACGATTTTCTATGCAGAATGTTTCCCCAGGACCTTTCCTCA
TTACCGTTCCGCTGTCAACCATGAAGAATTGGGAACGAGAATTTGAATTTTGGGCTCCGG
ATTTCTGTATTGTAACTTATTATGGAGACAAAGATACTCGAGTAGTGATCAGAGAGAAAG
ATATGTTCTACGAAAATAAGAAAAAGAGCGCAGTCACTTTCCATGTCTTACTGACAACGT
ATGAATTGATTTTTATGGATGAAAGCTTTCTAAGTCGTATAAATTGGGAAATATTGATTG
TTGATGAAGCTCATAGATTGAAAAATAACGAAACAAAGCTGTTCAAAAGTCTGAGAAAAT
ATAGTATCAATTATCGAAATTTGATGACCGGAACTCCGCTGCAAAATAATTTAGAAGAAT
TGTTTAATTTATTAAATTTCATGGAACCTAATTCATTTGGAGATATGTCGGCATTTCTTT
CTCAATTCAATGATATTTCTAAAGAAGAACAAGTTAGAACTTTACAGGATATGCTGAAAA
AACGTATCTTACGTCGATTAAAATGTGACGTTTTTGATGACATGCCAACAAAAGAAGAAC
AAATAGTCCGAGTTGAACTTACTCCTCTTCAAAAGAAGTTTTACAAGATCGTTTTATCGA
AAAACTATGATGCTCTTAGTGGAAAGTTCAGAGGAGTAAAAATGATAAATATAATGATGG
AGTTGCGAAAGTGTTGCAATCATCCATACCTATTGTCATCTGAAATAGCAGAAGAAAATA
TTTTGGAAGATGGTTCATTTGATCCTCCAGCTTTAATTGCAGCTTCAGGGAAAATGCAAA
TTCTGAAAATAATGCTTCTGAAACTGCAACAGACAGGTCATAAAGTGCTTATATTTTCCG
GTATGACAAAACTTCTCGATCTTCTCGAATATTTACTGGAGCATATTAACCTTCAATATG
AACGTATTGACGGCAGAGTATCTTCAACTGCTCGACAAGAAGCTATTGATAACTTCAATG
ATCCTAATTCTACCAGCTTGGTTTTTCTTTTATCTACTAAGGCTGGGGGTTTGGGTATTA
ATCTATGTTCCGCCGATACAGTTTTCATTTATGATGCTGATTGGAACCCTCAAAACGATA
TTCAAGCATTGTCACGAGCCCATAGAATAGGACAAAAACGGAAAGTTATGATTTATAGGT
TTGTGACACGAAATTCAGTAGAAGAGAAAATTATGGAAAAATCGAAACGTAAATTGATGG

TAACCAATCTCGTCACAAAATCTTTCACAAATGATGAATTTCATCAAATTATCAGTTTTG
GAACAAAGGAATTGTTTGATGAAACCAATGAAAATAATGATCAAAAAATTATTTATGATG
ATGAAGCTATTGAACATCTTCTTGATAGAAATAGAACTGAAGCTGTTAATCCTGATAGTA
GCTTTTTGGATTCTTGTTTCAGTGGATTTAAAATGGCACAATATCAGTTTGCAAGTGAAA
AAAGTGAAAATGAAGAAAGTTCAAAGAAAATTGATAAAATTGAGGAAGATGCAATTATTG
AAAATCCTACCGACTATTGGGCATCAATTCTTGAACCTTTTATTAAAACTATCAGTCAAG
AAGACAATTCAAAAGAACCAGAATTAGGGAAAGGGTTTAGACATAAACGGAAGATTAATT
ACAACACACATAAACCGATAATAATCGATAAGAACTCATATGCTGGAACTGAAATCATTA
ATGAAAAAGATCCAGATGATACTGATTATGATATTTCTGAAGGTGAAAATGTTGATCATG
TATCATCATATTCACATAGTGATGATAGTTTCAAATTTGCTGAGACTGATAAAAAAACCCA
TTGGTGAAAATAGTAGGCAACCTAGTAAATTTAATATGGATCGAGATTATAAAGACTTGG
CGAATTATGGAATTTCTCCGGAATTGACAAATCAAATTAAATGTGACGTGATTAATGAAA
ATACTATTTTGCATTTTAAACATTTTTCCATCATAAATGGATTCATGCCAAAAGAAAGAA
TGGAAATCTACGAAGCATTAATGAATTATGGGTTGCCTCCGAAAAGTCTCAAAAAAGGTG
ACATTTGGTGGCCAAGCGCTCTAGAACATAAAACAATTGACGAAATGGATAAATTTATCA
CGACTCTCATGGTAAATTTAGCTGAATTTCACGAAACACCATCTACCAACAGCAAAAATA
ATCTTATTAAAGACGAAATTGATTATGATTTGTTATTGGCTAGAATCGGTATAAATGGAC
TTATCAATTTCAAGGTCAATCAATATGAAGGAATAAATCGAACGATTTTGGAAATTGTAA
CTTACAAGACGTGGAATGTTCGTACAATTCCTATTGACGAATTACTGAAACTATTCAATG
AATTAAGTCATTTCGAAGTGGATTTCAATATTTACGAAGGTGGATTTTCTGAAATCGAAA
ACGATTGGAATGATTACAATGTAAAATACAAACAGAATTCAGAATCGAACCCCACATTAC
ACTGGAATAAACTGCATGATTATTATATGTTATCTGCAATAGCTACTCACGGTTATGATA
AATATATCGACCAAATATTCATAGATCCCCAATACCAGATTTTGTTCATTCCGTTACGAA
TAGCCAACATGGACTTTGTTGCAAATAACGCTGACATTAGGCAATCTTTTCGTAATCGCC
AACGATTGCTGGAAAATGCATTATTGATTGAGGCTCAAATAATGAAAATTGGAGCCACAT
TTGACGGTTACTCGATCGAATCTTTTTCCGTAGAACGATTGAAATACTATCACGAATTCT
TTACGAAACAAGGCGATAATCTTTCTATCACTCCTACATTAGCCAATAATAAATTTAAGC
AGAATGCAATGGCTCAATTAGAACTGCTAATTAATGATATCGAATCGAGTTCTGATTCCT
TTAGACCGATCGTTGTTAAATTTGGGAACCATGAATCTTCTAATACCAATACTAATACCA
AAACTAATGCCAATACCAATACTAATGCCAATACCAATACTAATACCAATACTAAT
>DjScaffold2790:93547-94541 strand:+
atttaatgttaattcaattaaattaaggttaacgatttgtttgataaaatttatttatcg
aattttattctttgatttacatgtattattaaggtcatataatttagttcATGCAAAAAA
TATTCGTTGAAAACCACGATCCGACTATTGAATCGACTTATTATAAAAATTATGAAGTTG
ATGGCGAATGGAGTAATTTGGAAGTTCAAGATACCGCTGGGCAAGAAGAGTTTGCTTCTA
TGCGAGAATCATATTATCGAACCGCGAATGGGTTCCTTATTGTTTTTTCCTTAACAACTA
GGGAAAGTTTTGACATGGTTCGTAGATTCCATAAGAGTGCTCTTCGTTCCAGAGATCGTG
ATCATTTCCCAATGCTATTAGTAGCCAATAAAACCGATTTGGAAACAAGTCGTAAAGTTT
CTCCTTCAGAAATAGAAGATTTGGCGAAAGAACTACAAATAGATTATACAGAAACCAGTG
CTAAAAATCCACCAGTAAATATCGATGATGCATTTTCTCAGATTGTTCGCAAAATCAGAT
CCCATCAACTTATTAATGATGTGAAAACAAAATCAAGGCCAAGGCAAAAACAGAAATGTT
GCTTAATGTGAtagtccaatattatttttctcgcgcgataaacaaaatcgaaccttgtc
tccttttctgcttcacaaaaataacaagtttaattgatctataccaattataatgaactg
aaatccaatttattcagttgtatatatagtttgaaaattatcccttattaccatttttca
cacatgtatacacagatccctcacacctatctatgatagcctttcaa
>DjScaffold2790:54533-60480 strand:+
ATGGGCAATGCAATTTGGGAGTTATTATGCACAGAACATGGAATCGATGAAAATGGAATG
GCCAATGGGAATTTATTACTTGAAAATAATAATGAAACTTTTTTCAAAAGAACTGTAGAA
GAAAAATATGTTCCAAGGAGTTTGTTTGTAGATCTTGAACCGACAGTTATTGATGAAATT
AGAATTGGGTCATACAGAACACTGTTTCATCCTGAAAAATTAATTACTGGTCCAGAAGAT
GCTGCCAATAATTTTGCAAGAGGATTTTTTACTGTTGGAAAATTGATTTTGTTTAATGTT
GACGCTCAAATCCGAAAACTCTTTGAATCTTGTGATTTTCCTCAAGCGATTATACTCTAC
CGATCATTGGGTGGGGGTACTGGAGCCGGTCTTACATCGGCGATGTTTGAGGGTTTAGCC
GAGTATCCCAAAACTCTTAAAATAGAAATGCCAATTTTCCCATCTTCCAGAATTTCAGTG
TCGACTGTCGAACCTTATAATTGTCTGTTAGGGGAACACTTCTGCATGGAAGATGTAGGA
TTAGGAATAATCGTCGATAACGAATCTTTATTTGAAATTGTGATCAAATTCCTTCATAAC
AGCAGTCCTACTTATACTTCAATAAACCGTTTGATAGGGCAAGTGATTAGCAATATTACC

GCATCGTTACGATTCCAGAGTGTTTGCACAAGCGACGTGACAGTTCTGCAGAACAATCTC
GTGCCGTATCCACGAATCCATTATCCAGTTTGTAATCTTTCTCCTCTGATGGCCCACGAG
ACGGGACCCTTTGAATCGCTTACGGTAAAGGATTTGACACAAATGCTGTTTCGACGAGAT
AATCAATTAGTGAAAATCGATGCGACTGTTGGACGGTACATGTCGTGTGTATTATTTTAT
AGGGGAAATATAAACATTTGTGACGTTTATGACTCTCTGTTTGACATAAAAAAGAATAAA
ACTATAAATTTTGTCGATTGGTGTCCAACTGGTTTCAAAGTCGGTATTAATTTGAATCCG
CCAGCATTTATTCCTGGCGGATTTCTAGCACCAACTGATAAGAATGTGTTGATGTTGACG
AATACTTCGTCGATAGGTCAAGCATGGCAGAGATTAACGCATCACTTTTATCTGCTTTAT
TCGAAACGATCGTTTGTGCATTGGTTTGTAGGTGAAGGAATGGAGGAATGCGAGTTTACA
GAGTCACTCTACAATATCAGTTCTTTAGTAAAGGATTACGAAGAGATTGTAACACCGACT
GATTTGTCACAAATAAGTACTTCGAAAAGTAAAGAACAACACATGGCTAACAATACAGAA
TCAAATGCTCCGCAAATACCACACGAAAACGTATCGTTGTATGTTACTATGGCAAATGGT
TTGCCTGTAGCAGCTCCCGATAGGGAATCTTTGACAGTTAAATCCAGTTTAGTCAAATCG
GGGTACCAGAAACCATCGGTTTACCCAGATCAAAACGATAAAAGTTCAGAAAATAAACTA
GGGAATTTAGAAGCAGTCCCAGAGGAACCACAAAACAATGAAAATGAAGTCCAAGGAGAA
TACAATGAACACATGTTCAGTAGTAGCGAATTGGAAGCAAATGTAAAGGTTAATGTTGAT
GAAGAACAAATTATTGCCGAAACAAATGAAGATGAACCCGTAAATGCCGAAGAACAATAT
GAAATCGATGAAAACAATGGAGGAGGGTCAAACGAGAATGCGTTCGAGGATGATTTGACA
AACAGTGAAGAAAGCACGGGATATTTATTTGATGAAATGAATAAGATAACTTACAGGAGA
TATTAA
>DjScaffold2790:47875-48213 strand:+
ATGATTCGTTTAAATAATGTGTTAAGAAGAATAAAAACTGCAGGTTTAAGAATTAATCCA
GACAAATGTTCGTTATTGAAAAAAGAAGTTAAATTTCTCGGACATATTATTACAAGTGAG
GGAATACGCACAGATCAAAGTAAAATTGAAGCAATTAGTAAATTTGAAAGGCCGAAATGT
ATTAAGAGTTTGCGAAGTTTCTTAGGATTATGTAATTATTATCAAAAATTTATAAATAAA
TACGCAGAAAAAGCTAGAATTCTAGAAGCATTGTGTGGTAAAGGTAATGAAATGTTGATT
TGGTCTGATCAGTGCGATGAGTCGTTTAATCAATTAAAA
>DjScaffold2790:50969-51310 strand:+
ATGCCGTTTAGTATTGCTGCTGCTCCGGGAACGTTTCAAGAACTTATGAATAAAGTATTA
ATCGGGATAAATGGTGTCGAAGCCTTGGTTTATTTGGACGATATCTTAGTTTTTTCTAAT
TCCTTAGAAGAGCACATGATTCGTTTAAATAATGTGTTAAGTAGAATAAAAACTGCAGAT
TTAAGAATTAATCCAGACAAATGTTCTTTATTGAAAAAAGAAGTTAAATTTCTTGGACAT
ATTATTACAAGTGAGGGAATACGCATAGATCAAAGTAAAATTGAAGCAATAAGTAAATTT
GACAAATGTATTAAGACATGTATTAAGGCCGAAATGTATTAA
>DjScaffold4132:3958-10224 strand:+
tataaatgtaggtgtgtttaatacaaacaaagctcattgataaaatcataaagtatcgat
tttttgtacgattaaatttttcgagATGAGTATACCTATGTTTAAGAACTTTGTTGGAT
TAAAATTAAGCGATGAAGAAATGCAAAACATTGATAATCCTAGTGCAGAATTAAATATCC
ATGTTACTTATAAAACAATTCAGGCTTGTACAACATTGGGATTCGCAGTTTTAGCTCCTA
TTGTAAATTCAATCAAAGGTCCTCGAACTTTGCCTGGTTACAGTAAAAGGATGCTAAAAT
ATGGAAGCCGGGGATTTATTATTGGACTTCCTTTAGGATCTCTGTTGACATTTATTGTAA
CAAGAAATGATACTAATGAAAGAAATTATGATAGATGTTATCGGCTTAGGCATAATAAAC
GTCAATTAAATATTGATCGGTTGACAACCATTGGTGGAATAATTGGCCTAATTAGTTATC
GATTTATCGGTGTTACTGTTCTTCAAGGAATTTCATTGGGAATTTTTTCTGGAGTTATTT
GTGGCACTGTTGCGGGTATAACGATTTAAataggaattgaatttaaacagcaaatagaaa
tttgtaatcggtgtcttatgaaattaaatttttaaatgagtctataatgcatttttgatt
ctgtaaagataattaagatttatgcagctatggttttgattttaatttttatagttaata
aattttatatttgctttgttaaa
>DjScaffold4132:39338-41977 strand:+
aattaaatagttatttgatatcattATGACTGAAGATAAAGTCGAGTATAAAAGAATTTT
CATAAACCATGTTGATAGATATACCGGTTTAAATTTAGCGAATTATTTAAGTGCTTGTGT
AGTTGGTTCCACTCTGGATGATGATGAACCTGAAGAAGAAGAAGCTGCAGCTGAAATTGA
GGGATTTATTCCAAAGAAGAATTCTGAAAGTTTTTTTGAAATTCATGGCACTCTAAAGCA
TAAATTTTCAGAAATACCTAGCTCAGTTAAAGCCATTTTAAAGTATGATACACGGGAAGA
ATTTTTCGAACACTTGGTCGAATCAGATGTCATTGTGTACGATATTACGGAAGATATTAA
TCAAATAGAAGAGGCATTATGGGCAGCAAACACTTTACATGAGAATTTTCATTGGTTGGA
AAAAGAAAAAGTATTCATACTTCTTAGCTCAATAATGACATGGGCTAAAACCAAACCAAC

TGATTTAGATGATACCGAAGCTAGTTTTAATGAAGAAGACTATCGACGCCGAAAACCTCA
TCCAAATTTTAAAGAGCATTTTAATGCTGAAAAGGAAATCTTAAAATTAAGTAAAAAACA
TAAATCGAAGATAAAAATGTATGTTTTAGCAGCTGGTATTCTTTATGGAGAAGGGGAAAA
TATATTTCATGAGTTTTTCAAGAAATCGTGGAACAATATTTTTGAATTAATTATATATGA
CAAAGGAATGAATATTATTCCAACAATACATGTCAAAGATTTGGCTGGAATTATTCAAAA
TATCGCTGATAAGAAACCAGTAAATAAATATATTTTGGCTATCGATGATTCGAAAAACAC
ATTGGAGGAAATAGTCAAAGTTATTTCTTCAAAATTATCATCTGGGAAAGTGAAGAAAAT
ATCA
>DjScaffold4132:58215-64139 strand:+
GAAGAAAATATTGATACGGAATTTGGAATTGAAGAACTTGATGAAGCAGAAACAAAAGCT
TCTATTGCCACTGAAACCCAAACAAAATTTTTAAATAATATGACTGAAAATCAAGGAAGA
CTTTATGACGAAGATTTAGCTATGATTTATAAGGAAACATTAGAAAGAAAAGAATGCCAA
AATCATGGATTTATTTTGGATGGTTTTCCGAAAACTAAAGAACAAGCCAAACTGTTATTT
TCAATGGAAACTGACGAAGAAGAAAATATCAAGAAACCTGGATTTAATCCACTGATTACC
CCTCAACATGTTTTTGTTTTAAAGGTATCTGATAAATATTTGAGTATAAGAAACTCAATT
TTAAAATCTTCTAACGACGATACAATTGATGAAGAGTTCAAACGCAAATTAAAGGAATAC
AGAAGCCTCAACGATGATTTAGAGGAAATCGACGAAGAAACTGTTTTTACATTTTTTGAT
TTACGCGAAATTCATCCAATTGTTTTGCATTTACAAGAAGATTTGTCTAAAGATGGTGAT
GAAAATCACATTAATCTTCTTAATGAAATGTTTTATGAACTCGGTCAGCCCCATACTTAC
GGTCCAAATGAAGAGGAAAAAAATTCTTATTAATTTAAAAGAACAAGAAGCTGCGAAAAGA
CAGAAATTAATAGAAAGTGAAAACAAAAAAAATTAAGGAATCTGAAATGTTGACTATTCGA
AAACATAATCAATCAGAATGGAATGAAAATCTTGAATTAGTTAGGCAACAGCAATATGAG
TATCTTGAGACTCATAATCTTCCATTAAGAAATTATTTAATGCACTTAGTTATGCCGGTT
CTTACAGATGGTTTGATGGATTGTAGCAAAGTTAGAACTGAAGATCCGATTGATTATTTA
TCCGAATTTATATTGAGATGTGGTAATCGGAAGTCGAATGACACCAAGAAATGAc
>DjScaffold4132:88727-93075 strand:+
tatgctaatgtctttcttaatttttatacattaaatactttaaaatatatggttattttt
gtttttttaattaaagttaatgaaattaaattgtttagttggcctaatggaaaaattat
agttaatttaaaaaacccgttaatgaaattaaacccattatttaatataaaagcaattaa
gtaattatttacattttaggtagtggaaaaacattagcatatttgtcgccgattcttaat
aatttaatttcaaatgataaattgattgaaacttctatttgtcctagatctataattATG
GCACCAACTTGGGAATTATGTCATCAAATATATAAAGTTACTCTGACACTAGCAACTTAT
TTAAATTTAAATTGCTGCTTGTTTAGAAGTTTTAATGATATAAATGTTGATTCCGATATT
ATTGTTACTAATCCACATTGTTTAACTATTGCCATGAAAAATGGAGATATTAGTACCAAA
AAACTTAAATCAGTCATTATAGATGAAGCTGATATGCTTTTAGATAAATCATTTGCTGAT
GTTACGTGTGAAATATTGTCATCATTATCCATTCAAACTTCGAAACCTTTAGATGGAAAT
TCAATTGGAAGTCAGTTAATTTTATCAAATGCGACATGTCCTAATTCATTGGAAGAATTC
GGAAATTATATACCAATTGATAGTTTCGCTGTATTAATTGGAAATGCCATGCATAGATTG
ATTCCTAATATATCGCATAAATTTATCAGGACAACTAAATTACAAAAATCTGGTATGTTT
AATTTTATAAGCTTCAGTTACCGGTTATCGGCAAAGTAAaaaaattctaagtacgactaa
atttggaagatatgtaaagattatttgatacaatgctttatgtttcttctgtgtaatact
tatcacattaaaatttaattcaccactttctataattacatcggtatttgtcgaaaacaa
attattttcaataagaatacaattatattatcacattatttattcaatgaaattctgagt
tagatttaacaaaactaaaaaataaataattttccttattattatttaaataattaaatg
gaggttatttgtaatttttttgtagatttttaaaaaaaaatttgttttatttataggaaa
gatgaacaaaaacagattaggataattatgctatatgactttaaaaaagataactaagtt
ttgaaaccgctcaatcaattgcacaatgttatgaatacacaataaaaatcataaaaaatt
aacatcaaaataattcatattctattgaagc
>DjScaffold4132:95660-101990 strand:+
CCTTTAGATGGAAATTCAATTGGAAGTCAGTTAATTTTATCAAATGCGACATGTCCTAAT
TCATTGGAAGAATTCGGAAATTATATACCAATTGATAGTTTCGCTGTATTAATTGGAAAT
GCCATGCATAGATTGATTCCTAATATATCGCATAAATTTATCAGGACAACTAAATTACAA
AAATCTGTTTTGTTAATTACATTACTTAAACGAAAAGGATTGAATAAATCACCATATACA
ATTATTTTTTGTAATAATAACGAATTTGTACAAAATGTTTCATCAATATTACAAGAAAAT
GGAATTGAACATGTTTGTCTTCATCAAAGGATATCATTACAAATGAGACAACAAAATTTT
AATAAATTTCAAAATGGAGAGGTAAGAGTACTTGTTTCTACAGATTTGGGGTCACGAGGC
CTAGATACTATTAAAGCAACTCATATAATAAATTATGATTTTCCAAGATTCATTGCTGAT

TATTTGCATCGAGCGGGTCGCACTGGCCGTGTTGGAAGCAATGGAGTAGGAAAAGTTATT
AATTTAATATCTTCCAAGAAAGAAATCAATTCTGTTCAAATAATAGAAAAATCAATTAGG
TGTGGAATCCCTTTACCCATTATTGATACTAACGAAAGAACAAAAGACAAATAAatctcg
cattatttaaataaaattattttgaatcgc
>DjScaffold3236:15960-22520 strand:+
ttaATGTTAGTTGGGTTAGGCGGTGCTGGCAAAACAAGTCTCATGAGAGCCTTGACTAAT
GATAAATACTGTGCTCCTCAAATCGCTGAAGAAGCTATAACCGATGGTATAGATATCAAT
AGTTGGTCAACTAAAACGGATAATAAAAGTTTGAAGTTTTCAATTTGGGACTTTGCGGGC
CAAACGCTTTATTACAACACTCATCAATTTTTTCTTACTGGTCGTGCTATTTATTTATTA
ATTTGGAATGTACGACAAGGATTTGAACACGCTGGACTTGATTTTTGGCTCAATAGTATT
ACTTGTCATGCACCAACAGCGCCTATCTTTATTGTTGGAACACATTCAGATAAAGTTGAT
AAAAGTGAAATATCAACGAATGAATTTCAATTTCGTTACCCATCGGTTGTTGGTTTCTTT
TTTATTAGTTCTTTAACAGGAAAAGGAGTTAAAGAACTCCACGACAAAATTGTTGATGAG
ACTAAAAAGCAACCATACTTTGGTGAGATAGTTCCCAATGCTTGGCTATTATTTGAAAAA
GATTTGATCAGCCAGAGAAGTACTGCTGATTATTTGAAATGGGATGCAGTAAAAAAACTG
GCCAATAATAATGCAATATATGATGGGGAAATTCATCAAGCTGTAAGATTCTTGCATGAT
TTGGGCAGCATTCAATATTTTGAATTTCCTTTGTTGAAAGATTTTGTAATAATCAATTCA
CAATGGCTTGTCGATGTTATGGCGTGTGTAGTAAGTGTAAAAAATTCCATTATAGAGAAC
GGTAAATTAGAACATTCAAAAATTAAATTTGTTTGGTCAAAATATCCAGAATCTATTCAC
AGTTGGTTGTTGAGATTAACAGAAATATTTGATTTGACTTTCTCTGTTCCTGGACAAAAT
TTCAATTTTGTTCCTTGCCTTTTACCAAATGACCAACCAGACTTTTATTGGGCAGATTTC
GTAGATTTTTCTCAAACAAGAGAAGCTAATATGTTTTATCAATTTGAATATCTTCCACCA
GGTCTTTTCAATAGAGCTCAGGTTCGTTTGTCTCAATTTTCTGATAGTTTCGTAATATGG
CAAAACGGATCTTTGTTAAATAAAAATGATGATAAAGCATTATTGACAAAGTTAAACGAC
ACTACTTTGAAAGTTTGCGCTAGAGGATTTTGGCCAGAAAATATATTATTCATCATTCAT
GAAATGTTTGAAACTCTTATTTCCGAAGCATTTAGTGGTGTGAAATATGAATATCAATTA
TCATGCAAGAAATGTATAGCAGAAAGAGAGCCATTTCCCTATGTTTTCAATAAATCTATT
TTACAAAGAGCAATTCAATCAAAAACAATGTTCATTCAGTGCCACAGAAACTTTCATATT
ATAACAATGAGTGAAATTCAAGAAAGTACTCCACCAGATAGTATTGAAGATTATGATTTA
CAATTCAAAAAATCTGTTCATTTTATATCAGCTAATCGAGAACAAACCAATCACTCATTA
ATGATACTTGTATCTGACCTTGATTATCAAAACGAAACAGAATTGTTGAATACACCAAAA
CGATTAGCTAAAGATTTGAATAAAAATCAATACACAGTATGGTGTGAACCATTAGTAGAA
GGAACTCCAGAAGATAAATATATTGATATGCTAACCCGTTCAGAAGCTGTCATCAGCGTA
GTTTCTGGAAATTTTACAAATAATAAATTATGTATGGATTTATTAAAATTGGCTAAAAAT
ACTTTAAGAAAGAATCTGATTCTTGCATTAATTGATAAAAATTATACAGAAAGTACTTTG
GGCTTTGAAATCGCTGGAGACAAATATATCAATTTTCAACGTGTCAATCTTTATGATAAT
AAGTTTAGTGAACTTCTTCATGACCTCGGAAATCTTTTGGAAGTAAAAGATACTCAAACT
GGGAATGTGCAAAAACACAGAGTTTTCATTTCATATTGTTGGGATAATTCCCAACGAGCA
TTTGATAAAGGAAATTCGAAAGTAATTCACAAAGACTCAATTGGGTGGGGTGACCCTAGA
GACATCAAAGATTATATCGAACAACAAGGTTTAAGTTGTTGGATAGATGTGGAAATGATA
GGATCGACATCGGTTTATTCAGAAATAACCACGGGTTTACGGCACAGTGAAATTATGGTT
GCATGTCTTTCGAAAGAGTATGCTCAATCAGATGCTTGTAAAAAAGAATTTTTGTACGCC
AGACAAAATCTTAATTTACCAATAGTAATTGCAGAGGTTGGTTGTAATCATGATTGGATG
CAAACTGAAATAGGTTTACTCAGCATTGAATATTCAAAAATACAATTTCAAACTGAAAAT
CCAACCGCGTTTTCGGATCTTTATAAACTTGTGAGTAATGCAATTCCTGCCGAAGCGCCA
GAAGCTGAATCCGAGTCTTATGTTGCAAAAATGAATAAAATTGCTTCAGAAGAAGACTTT
GAATTAATTCAAAGGAAAATATTCCGTCTTCTGTCTTCTGGATTTGGAAGTGAAGAATCA
AGACTATACCCTAGATTGTTTTTCATTGATATTTCCGATGAAATTAATGAAACCACAATA
ATTAATGATTTATTTTCAAATAATAACTCAATTTTGGCAAATAGTCTCTCCACAAATATG
CCAAAAATATCACTTTGTTTCAAAGCTTTATGTGAAAGTGAAACCGGTTGGCATACTGTT
GATAGCGGTATTCCAATAAATCTTGGTTATTCTGAATTGGAGCGATTATTGATTCTTGGA
GCTCCGTATTTATTTCGAATAACTCTTCTATTAAAATTTTCAGGTGGTGTTAATATACGT
TTATTTAATCCCGACACAAATCCAGAGGTGACAAAATATATCATGGAATTACTTGAAAAA
AACTCTAAATCTTGGAGTTATATGAAATCTATACACAACGCCATAAGATCTCATATTATC
TCTACGGATATCAATCTTAAATACGGTGGTCTTCAATGTTGTTGCCTTAATAATGGTAAA
GTTGTTTGGTTATGTCCAGAACACTGCAGCTTACCTTATGTTTCTCTGATTGGCTCAGAA
GAATCTGGTTCATTGATTAAATTAGAGAGACAAAGAACTCAAAATGAAATTGATGTGACG

AAAGAATCTGGAAATTATAATGAAATTGTATCATCGAAAGATAATGTTTTGGTAGATAAA
TTGGAAGAAATTATTAAAGATCAATTTTCTGATGCAGATTCTTCAACTGATAATCGTAAA
GGTTCACCAGGAACTACTGAAACCAACAAATTTCTTCAAATGGTAACTTCAACTCCAAAA
ACAAACAAAGTGTCAACTGAAATCAAATCAAAAACTGGAATACTTCACAGAAAATTGATA
CCTCCTCTTGAAGTTGTTAAATATCCATTAGCCTTTCAAAGCAAAACATGTTTAATTATG
TGAattcttttactttttatgaatacttgctttgttagattattagacaattttattaag
aaaataaattttaATGTTAGTTGGGTTAGGCGGTGCTGGCAAAACAAGTCTCATGAGAGC
CTTGACTAATGATAAATACTGTGCTCCTCAAATCGCTGAAGAAGCTATAACCGATGGTAT
AGATATCAATAGTTGGTCAACTAAAACGGATAATAAAAGTTTGAAGTTTTCAATTTGGGA
CTTTGCGGGCCAAACGCTTTATTACAACACTCATCAATTTTTTCTTACTGGTCGTGCTAT
TTATTTATTAATTTGGAATGTACGACAAGGATTTGAACACGCTGGACTTGATTTTTGGCT
CAATAGTATTACTTGTCATGCACCAACAGCGCCTATCTTTATTGTTGGAACACATTCAGA
TAAAGTTGATAAAAGTGAAATATCAACGAATGAATTTCAATTTCGTTACCCATCGGTTGT
TGGTTTCTTTTTTATTAGTTCTTTAACAGGAAAAGGAGTTAAAGAACTCCACGACAAAAT
TGTTGATGAGACTAAAAAGCAACCATACTTTGGTGAGATAGTTCCCAATGCTTGGCTATT
ATTTGAAAAAGATTTGATCAGCCAGAGAAGTACTGCTGATTATTTGAAATGGGATGCAGT
AAAAAAACTGGCCAATAATAATGCAATATATGATGGGGAAATTCATCAAGCTGTAAGATT
CTTGCATGATTTGGGCAGCATTCAATATTTTGAATTTCCTTTGTTGAAAGATTTTGTAAT
AATCAATTCACAATGGCTTGTCGATGTTATGGCGTGTGTAGTAAGTGTAAAAAATTCCAT
TATAGAGAACGGTAAATTAGAACATTCAAAAATTAAATTTGTTTGGTCAAAATATCCAGA
ATCTATTCACAGTTGGTTGTTGAGATTAACAGAAATATTTGATTTGACTTTCTCTGTTCC
TGGACAAAATTTCAATTTTGTTCCTTGCCTTTTACCAAATGACCAACCAGACTTTTATTG
GGCAGATTTCGTAGATTTTTCTCAAACAAGAGAAGCTAATATGTTTTATCAATTTGAATA
TCTTCCACCAGGTCTTTTCAATAGAGCTCAGGTTCGTTTGTCTCAATTTTCTGATAGTTT
CGTAATATGGCAAAACGGATCTTTGTTAAATAAAAATGATGATAAAGCATTATTGACAAA
GTTAAACGACACTACTTTGAAAGTTTGCGCTAGAGGATTTTGGCCAGAAAATATATTATT
CATCATTCATGAAATGTTTGAAACTCTTATTTCCGAAGCATTTAGTGGTGTGAAATATGA
ATATCAATTATCATGCAAGAAATGTATAGCAGAAAGAGAGCCATTTCCCTATGTTTTCAA
TAAATCTATTTTACAAAGAGCAATTCAATCAAAAACAATGTTCATTCAGTGCCACAGAAA
CTTTCATATTATAACAATGAGTGAAATTCAAGAAAGTACTCCACCAGATAGTATTGAAGA
TTATGATTTACAATTCAAAAAATCTGTTCATTTTATATCAGCTAATCGAGAACAAACCAA
TCACTCATTAATGATACTTGTATCTGACCTTGATTATCAAAACGAAACAGAATTGTTGAA
TACACCAAAACGATTAGCTAAAGATTTGAATAAAAATCAATACACAGTATGGTGTGAACC
ATTAGTAGAAGGAACTCCAGAAGATAAATATATTGATATGCTAACCCGTTCAGAAGCTGT
CATCAGCGTAGTTTCTGGAAATTTTACAAATAATAAATTATGTATGGATTTATTAAAATT
GGCTAAAAATACTTTAAGAAAGAATCTGATTCTTGCATTAATTGATAAAAATTATACAGA
AAGTACTTTGGGCTTTGAAATCGCTGGAGACAAATATATCAATTTTCAACGTGTCAATCT
TTATGATAATAAGTTTAGTGAACTTCTTCATGACCTCGGAAATCTTTTGGAAGTAAAAGA
TACTCAAACTGGGAATGTGCAAAAACACAGAGTTTTCATTTCATATTGTTGGGATAATTC
CCAACGAGCATTTGATAAAGGAAATTCGAAAGTAATTCACAAAGACTCAATTGGGTGGGG
TGACCCTAGAGACATCAAAGATTATATCGAACAACAAGGTTTAAGTTGTTGGATAGATGT
GGAAATGATAGGATCGACATCGGTTTATTCAGAAATAACCACGGGTTTACGGCACAGTGA
AATTATGGTTGCATGTCTTTCGAAAGAGTATGCTCAATCAGATGCTTGTAAAAAAGAATT
TTTGTACGCCAGACAAAATCTTAATTTACCAATAGTAATTGCAGAGGTTGGTTGTAATCA
TGATTGGATGCAAACTGAAATAGGTTTACTCAGCATTGAATATTCAAAAATACAATTTCA
AACTGAAAATCCAACCGCGTTTTCGGATCTTTATAAACTTGTGAGTAATGCAATTCCTGC
CGAAGCGCCAGAAGCTGAATCCGAGTCTTATGTTGCAAAAATGAATAAAATTGCTTCAGA
AGAAGACTTTGAATTAATTCAAAGGAAAATATTCCGTCTTCTGTCTTCTGGATTTGGAAG
TGAAGAATCAAGACTATACCCTAGATTGTTTTTCATTGATATTTCCGATGTTAGTAGTAA
ATTATTTAATTACATCAATATTCCCGATGCTATTACTAATATCAGCAAAGTTTATAGAGA
TATTATTTTGTTTGATATTGTAGCTAAGATTAGCATATATAGTAATAATATTCTGTCTTT
TGATAGAGATAATGAAGGTGGTCTCACTCCAAGAAAACAAAAAAATAAAAAAAATAAAAA
AGTTATAATTAAAAAAGCTACATTGGCTAAATTATATGAAGAAAACGATGAATTAATGCA
GTTACCCTGGACTGATGATGACATTTTAAAAATCCAAGACATGCTTGAACAAAAATTTAT
CGGATGTGAAAGCCATAAGTCGGCATCAAATGTTATGGGAAGAACTAATCTAGGTGCATA
TTGTCGATGCATTGCAAATATCGTAATCAAACGAAGTGAAATGGATAATGTGGCAGAAAC
CGCATTAGTAACTTGCAAAAAACTAAGGAAACTTGAAGTTGATTTTGGAACTATGATGAA

TATATCATGGATTGTTAATTTGCCAACATTATCTTCTCTTACTCTGTCAAATTGTGAACT
GGAAGAAATCGGTCCAGAAATTATTCATTTAAAAGAAAGCTTGAAACAACTTTGCTTTGA
TGGCAATCCAATTAAAGAACTTCCTGAAATAATATGTTCATTTAAGCAGCTCAATAGCCT
TGAATTAAACAATACATTGATAACGGCATTACCAGATAAAATAGGAAATTTGCATTTATT
GAAATATTTATCCATTGAACATACAAAATTGTCTACTTTGCCAGAATCGTTTTCCCGACT
AAATAATTTAGTAACTCTTAAATTGAGCGGAGTTACAATACAAAGTCTTTTTACCAAAAA
ATCCTCAATCTCTTTAAGACAATGGACAGAATATTGCAATAAAACTAACATCAATGGATA
TGTGTTAAAAGAAACGAAAATTTTCGATTTATTTCAACATTTAAGTGGCAATGACAGCAC
TTTAATTGGATCAAAATTAGATGAATTTAAAGCATTGATATTTGAAAATATTCCGAGATT
TCAGGAATTTCCACCTCAAATATTTAACTGTAACTTACTCCAAACAATAATTTTCTCCAA
TCAAGCTTTTCCGGATGTACCTGATCAAATTCAAAATTTAAAACATCTGAAACGTTTAGT
ATTCGATGGTTGTTTGAAATTACTAGCAATAAGTCCATATGTTGGAACTTTACCATTAGA
GGTTCTAAGTTGTCAAAACTGTGATATTCTCAAAACACCGCCATTTGAAATTCGTAAACA
AGGAATGAGAACAACAATTTCGTATTTATTAAGATTAGTCAGTGGAAGTGAAGCTTGTTA
TCGAACG
>DjScaffold1601:12829-25783 strand:+
agtaatggaagttgtttatttgaacttgctcaacaattggctattataATGGTCGGGAAA
CAAATAATAAATAATTTTCAAGAAATATTTGTACCAAAAATTCTTACATGGTTTTACCAG
TGCTCTAAGGGCCTTGATAAAGTATCTAATTTAGACGAAAACAAAGAATTAATCAAATTC
TGGGCTGAAGATTATAAACTTCTTCCATATGAAGGATTATTTGAAGAATATCTTGAAATG
GTGTTACAATTTGGATTCATTACGATATTTGTGGCAGCCTTTCCACTCGCTCCTTTATTT
GCACTGCTGAATAATTGGGTTGAAATTAGATTAGATGCAAAAAAATTGGTTTGTCAAACT
CGTCGGCCAATTTCTGAAAGAGCTCAAGACATTGGAAAATGGTCAGTCATATTAGATGCA
ATAGTGCATTTATCGGTTATATCAAATGCATTCTTGATTGCATTCACATCAGAATTTTTG
CCAAGATTAATTTACAAACATCGATATAACGAAAGTCTTCAAGGTTATGTAAATTTTACT
CTGTCATGGGCACCAAATGGCTCACTTTCAGAACCTTGTCGGTAAattaattaatgaaat
tgttttaatttaataataaagatgcaaattaatattttctgtcaaaagttatgtttctta
tacgattaattcctaaagcatttattatgaattttattaaatttttctattgaaactaat
gtttaacaatttcaatgaaattatcagaataaattatattaatttaagttataaa
>DjScaffold2279:105923-111671 strand:+
aaattagaaaaattttattttacaattctagaatatttttaatttggtttaaaattttt
caataaaaaaacatcctataataaactcaaatttaatctgaaattgaatattaatgtttaa
tctaatcattattcatcaatcaactaaaagaatatattatactttgacaaactggattt
tctgtaaattgaagcttttggattaataattttggtgcttctttgaattatatacagaag
aacaataaaaatatttttaaatttgtgaggatattttatatttattatataaatcattatta
aaattgcatgtttcataatttatatttgactatgagttcaaatattttgtatgttgcata
acaggtcaatgtttgtttgctgattgaggaaatcaaaggtggcaaatcatatttaaaaAT
GGGATATGTTCTTGTCAACATTGCTTGCTTTGCTAATCTAGGTGATACAGCGTGCCGGAG
ACGATATATTCTTGAGGGATACAATCAAGATAAACGACAAGATAATTCTATGCTTCAAAT
GCATTTCACGGTTTGCCAACTATTCGGTGATCCCTGTTTTCGAGTTCCCGATGAAGATAC
AAGTAAATGCGTCCCATATGAAGTGTTTTCTCCAACGAAACTTATTATGCTGACTCCTAA
TAGTAGTCTTGAAGGAATCGACGGACTTCTGGATTCAGAACCAATTGATTCTGAAGGAAA
TGTAGCATCTTTTCTGTTACAGCATGATCAAGAATATGGACAAGCAGGTTCCTCTGTAAA
AGTAGGAACTAAAGATACAGTTACAGATATTTACAATGAAATCTCCCGTCAGACCTTAAA
CAGATCTCAAGAAAATTCCTCACCTGATTTCTTTAAAACAACCTCAAAAGATCATTGTTG
GGATGCATCTTTTGGGGAGCCTTTAATTGCAACAATTCCCAAATTTTCCAAACAAACTGC
TTTAACACAAAATCCTGGATATTCAACTTGCCCACATCCAATGTCTCATAATAGACTGTC
AAACGTATCAACATCACAGTCATCGAAATTTCAGCAAATTCAAATAGATTACAGTACTAA
ATCAGATATCGATCATAAAAAGCATCGAAGATTCGGTAGTAGTACTCAGGGAAGTGGTAG
TACTGCTGCGAAAACCACAAGTTCAGGTTATGGCAGTCACTCTAGGCAGTCTAGCAATGA
ATCAACATCATGTACTCACTCCAGACTACTATCTAATTTTTTAAGACCATCATCAGCAAT
CTCAAAGCTTGATTCCTCCATGAACACCGTCATCACTAGAAGATATTATATTAATGGATA
Agaattttaaaattatcatcaattttctatttccatattttgtttattttataattaaaa
ttgttttgcgtctgattttagatggtgcttcgttgctgaaattttctcagttttaattat
tcttcattcttgaataaatgatgtatacaattattatcagagtcaattatttaaatacat
aattagtgagcattattttgattggttgcaaaatttttaacaatacgttatcatttaatt
ttaatttttctaaacttattctatattacattaattaaaaaatttagtttttactttcaa

aaattttttcttagtatattttaatcgtaaaaggcctttatatgctctttattgttgttc
tatctggttatagtaatgatgtattcaaatataaacacattttttatgtaggaaatcaaa
ggtggcaaatcatatttaaaaATGGGATATGTTCTTGTCAACATTGCTTGCTTTGCTAAT
CTAGGTGATACAGCGTGCCGGAGACGATATATTCTTGAGGGATACAATCAAGATAAACGA
CAAGATAATTCTATGCTTCAAATGCATTTCACGGTTTGCCAACTATTCGGTGATCCCTGT
TTTCGAGTTAATGAATGTCTCTTGTACATCTAAtttgaagtgttttctaattaataagat
tagataagttgatttatttgtcaaataattcaaatattttactgtctatatgttaaactg
attttaatgattttaggaaatcaaaggtggcaaatcatatttaaaaatgggatatgttct
tgtcaacattgcttgctttgctaatctaggtgatacagcgtgccggagacgatatattct
tgagggatacaatcaagataaacgacaagataattc
>DjScaffold2279:232423-247019 strand:+
ttattcaagattttaccttcatttttagATGTCTGATGAAGATGGTGTTGATAAATTACC
CAAGGCTTACTATGATGAAATTATTGATGATTTCGTTAAACTAACCAATACCGATATAGC
CTTGGCAATGTATTATTTAGGAGAGTGTAACTATGACTTAAAAATTGCTTTTGAAAAATA
TGAATTAGACGATCATATTGTTTTGAACGGTTTTCATGTACCATGTTCTCAATTAAAATC
TAACGAACTGGAACAGTTTTCAGTTCTATCATGGAATCTTGAAGGTCTTTTGGAGAAAGA
TTTGACTAGTAGAACACAGGCAGTTATAAATACAATTATCAAGCGAAAAGCAAATATTGT
GTTTTTACAAGAATTAGTTAAACAAAATGAAGTTGAAATACGTAAACAGTTAGATAAAGA
CTACAATATTTTCACAGCTGATGGAGATTATTATTGTGGAATTCTTGTGAAAAAGCATCC
GGATATTAGAACACGAAAGAAATCATTGCAAATTAGACACTTCGATAATACAAGAATGGA
TCGTCATTTGGTTATTTTAACAGTGGAAATAAATGATATTCCAGTGAAATTAATCACATC
TCACTTGGAAAGTACGAAAGAATTTTCAAACATTAGAAAAGCTCAATTGAAAGAAGCGTT
TGATGAAATGAAAATGGATACTAATTTTTCTATATTTGCTGGGGATTTGAATTTACGCGA
TAATGAAGTTACAGCAATTGGAGGTTTACCCATAGATATCGTTGATGTTTGGGAAGCAAC
TGGGAAACGATATGAAATTCGATTTACATGGGATCCACAAAGAAACTTTAATGCCGCTCA
AAATTGCCCCGGAGCATATGGTGCACGATTTGATCGAATGTATTGTTATGCAAATAGTAA
AGCAACTTCCAAGGTCATTAAACCTAAATTTCTTGGGTTAGAAGGCTTGCAAAAAATTGA
ACGTGGAGTTCGATATCCTAGTGATCACTGGGCACTTTTAGCGTATTTTGATGTGGAATA
Aatgatttatagttgtct
>DjScaffold800:21786-22097 strand:+
AACAAATGGAACACCGGTTTATTTAAAAGTATTATTGAAATAAATCAAGCTGGACTACGT
TTTAGCCCATCAAAATGTCAATTACTAAGAAAAGAGATCAAACCTTTGGAGTACATTATA
CATTATAACAGAGTTCAGACCAATCCAGCAAAGTTGGTTGCAATACAAACATATAAAAGA
CCAAAATGCGTGAAAGGATTGAGACGTTTCTTGGGAATATGTTGCTATTACAGAAGATTC
ATAAAGGATTACGCAAAACAGGCTAGAGAACTAGAAAAACTATACAGTGTAAATAATAAA
AAGTTAATATGA
>DjScaffold2665:5626-12062 strand:+
tgcaacttttcaaactagatcatttttaattgcatаatacgattaaaacaatttgttgaa
catttacataggaaaatacgcaatacttctcggacagtgtgatacttaaatcattttatt
ttactatatttgtgttggtttttacaatgtgatttatatattatttaaacaaagattttc
gtttattgtttttatcatttagctagctggtgctacgtatttagatattcacaacgccgg
tggaggtatacacttttctgttgatgcaactaccaaggcttccgagaaagtattgtttga
atctttaaagaaaagattgacgagcATGATGCGTCATGGCACAACTACAGCAGAATGTAA
AACTGGCTATGGATTATTATGGGAAACTGAACGTAAAATGTTGCATGTTTTGACAGAAAT
GAAGAAAAGTATTTCTATGGATATTTCAATAACGTATTTACCAGCACACGCAATTCCAAA
AGAATCGAATGCTTTGCAACACATGAAGGATATCATTAATTATCAAATACCGAAACTGAA
ACAATGCATCGATAATAATGAATTACATTGTGACAATATTGATGTATTTTGTGAAAAAGG
TATTTTTGAACTTGAAGAAACTAAATTAATTCTAGAAGCTGGAATGAAAATTGGTTTAAA
TATCAATGCTCATGCAGATGAGTTGTTTCCACTTGGTGGTACAGAAATGATTTCAAAACT
TAAATCAAAGGCTGTCAGCCATCTTGAAGAAATATCAACTGATGGAATTAAAGCATTGAA
AGAAAACGAAACAACGGCTGTTTTACTTCCAACAACAGCGTATTTGTTGTCGCTTAAATC
TCCTCCTGTAAGACGTATGATTGAAGCTGGTGTTCGTTGTGCTTTAGGATCTGATTTCAA
TCCAAACGCTTTTTGTTTGTCTATGGTGAATTTTTAAtaattttagaacaaaatttattg
taaatttatttgtaaattttatatgcatatataatcaataaaatttattttgattaata
aaaacaacgaatttttaatcaacaacaatcctagtatttattattaatttattccgcaca
cacgcattataataaagttttttgaaatacgtgtaccgataaataatgaagatttctcc
tttatatacaaggaaaaagtattcattatccgttcaagattttcttatttattgttttaa

ttaattattggattatatctccgattctatgattttcgttaat
>DjScaffold4455:6461-14361 strand:+
tattattattttcaatttttatttacaatccttttattgATGACTGAAGAAAATGCTAGT
GATTTAAGACCTCAAGATAATTCTAATGAACCCATAGATCCTAAAAAGTTACTCGCATTT
GGTTCAAAACAAATAATAAGTTTATTTGTACCAGTTTCTTTATGTATGTTAATGGTTGGA
ATTGTGGCCAAAACTGTCAGTTACTATGCATCAACAGACATATATTTAATATATACACCA
TTCCATACTAAAGATGCTGACATTGGAACAACAGTGTGGCAGTCTTTGGCCAATGCGTTA
ATAATGATAGTATTAGTTGTCGTAATGAGTGTTTTTCTCGTTCTTCTTTATAAATACAAA
TGCTATAAGGTAATTAATGGTTACTTGATCGTATCAACAGTTATGATTCTTTTTGTGTTG
TTCACCATGTTCTTCGTGGAAGTTGTAATTGCCATGAATATTTACATGGATTTTCTTACT
TTGGCGTTATTGATCTGGAATTTTGGCGTTGTTGGATTTCTTATGATTCATTGGAAAGGA
CCCCTCGTTCTTCAACAAGCCTATCTGATCATCATATCAGCCAAAGTTGCTCTTATTTTA
TTGAAATTTATGCCTCTTTGGACAACGTGGGTTGTCCTTGGAGTACTAGCAATTTGGGAT
TTGATCGCTGTGTTGTGTCCATTTGGACCTCTAAGAATCCTAGTTGAAATAGCATCAGAA
AGGAATGAACCGATATTCCCAGCATTGATTTACTCAACCACAGCTACGATGATGGTAAAA
GTTCCTGGATCTGAGGTTGATAATGCCAACAACAGTCACAGTGTTACTCCACCAGAAGAT
GACAATAATTCGCATTTATTCCCAGCTGATATCGAGGAGAATAACACAATTAACAACACT
GTACCTCTGGAAATGCATCAGCAGCATTATCGCACTTCACTCACAAATTCACATGACAAC
ACTAATAGGGACAACAAGATCGTACAATCGACAAACAAAGAAAAGTTACCAACCGATTGT
AATGAGGACCAGAATGAAGTAAATATTATTTGAttgataacatttctcacaggaggatct
gcagttttgtcgtgatggttacatcttagatctcttttcttattattctgaggatgtgat
ggatcgtggagatcgcggtgtcaaattgggtctcggtgattttgtattttacagtttatt
gatgggcagagcaacattggacagcgatttcaacacaataatggcatgctatgtggctat
tctcgttggaatgtttctcacaattcttctactaacaattgctggccgagctctgcctgc
tttgccgatctcaatggcttgtggaatcttgttctattttctcacttctttgataatctc
tccattcacaagagctctctggctgcagagtgtattcatttgatactcatctgtctagag
tcttatgatttcgattttgttcaataataataatcaatttccacc
>DjScaffold4455:49377-49915 strand:+
CTATCAACAGATGAAAACATTCCATTAGTGAAATACAAGCTATGGTTTGTTACTGGTACT
GATTTAGGATCTGATTGTAGTGCTCCAATCTCATTTGCATTATATGGTGAATATGGTGAC
AGTGGTGACAGAGAATTCTATTCACCATTTATGGATATGGAACCCAGCTTTAGAAGAGGA
ATGACAGATCAAATGAATATCGAATGCATTTATCTCGGTGAACTCAAAAGATGTAAAGTA
ACTGTTCACACAACACAACCTAATTCGGGATGGTTTTGCCATAAAATAATGGTTGAAGAT
CCACTTACACAAACTCAAGTGGTATTTCCCGCTAATGTGTGGTTTGATCCTAAGAAAGGT
GATAGATTAACTTCAAAGTTTTTAGACGTTGAAGGTAAAAGTTTTTAAtta
>DjScaffold4455:46975-47652 strand:+
ATGCATGATATATTGGCAGGGATAATTTTTAAAGGTGTCATTGTATATCTAGATGATATA
TTAGTGCATGAAAGAACGGACGAAGATTATAATAAAATTTTGAAAGAAGTTTTTAACAGA
ATAAGAGAATCAGAATTGAGAATAAATTCAGAAAAATGTAAATTTTACCAAAAACAACTA
GTCTTTTTGGGACACACAATAAGTGCAGAAGAAATAAAAACAAACGAAAATAAGATTCAG
GAAATAAAAAGTGCCGAAGAACAAAAATTTGCATCTAAACTCAGGTCTTTTCTTGGATTA
ACGAATTACTATAGACGTTTTATCAAGGATTACGCCAGAATTGCAACACCTTTATATGAA
GCTATTTCAGGATGTGACAAAACCTTGACGTGGACAGAAGCATGCAGAAAAAGTTTTGAA
AACTTAAAAGACGAGTTATGTAATGCACCAATTCTATATTACCCAAGAGAAAATAGAATG
TTTATCCTAGATACTGATGCAAGTTTCAGAGCTAAGCTTGCAGTGTTTAGCCAAAGAACG
GAAGATAGTGAAGAGGTGGTAATATCATATGGAATTAGATACTTCATTGCTCATGAAAAA
GGCTACTGCGTTACAGGAAAGGAATTGCTGGCATTACATGAATATGTGGTACATTTCAAG
CAATATCTATATGGATAG
>DjScaffold1386:33895-51466 strand:+
gttaataataaattaataagaatattctaatggaaactaatgattctgaagtaagtagtg
aaggtagtgattttatagctacagatgagatgctgacactagcttatgacgacgaaagca
ctattaatgaggaagaagatattgctgatgaagacgaagtagataaactcaaacttgaaa
gtgatatgcctattgaagaattacttaaattatatggatcaaaactggagtctagtgatc
aagatgaggaagaatctgacgatcttaatgttgaatcgaatctagaaaataagaattcag
acaaatctgaagaatgtcggccaagagattccatcaacgaatccagtaataatttagagt
tgtctgacaatgaggtagaaaatgaggttccaaggggatcaaacagattgtggtgcaaag
cgttgataggtgaattatgattcgattatcagtagttctgtttgaattgtcctttaagaa

aataattccaattataattcggaagatgatggtgattatattccaattattacatcgaaa
ctgttctccaatgcaaaaatacgaattggtgataattatcaagctcttattcctggaATG
TTCAATAAAATCAATCAGAATGATTTGCGCTTGCAATTAAAAAGAAATGAAATCTTGTTA
TGGAAATGTCTCGACGACAGTGAAAATTTCCAGTCAGACTTGAAACGTTATTTAAAAAGA
TATGGAGAAATTAGTGGTCAAACTGGATTAATAAATGGAAAAATCGATGATCCTACGGCA
TTATACACATTAATGGAACAAGATTACGATTTAGAAGCAGCCGTAACATATCTGAAAAAT
AAGCCAATCAAATCTTCAGATTTACCGACTATCATGCAATCATGGTCTGAACAGGAATGT
ATTGAATTTGAAAAAGGATTCGAAATACATAATAAAAATTTTCGAATTATCAAAGAGCAA
CGATTACGAACGCGAAGTGTTGCGGAAATTGTAGAGTTTTATTATTTTTGGAAAAAAACC
GCTCGATATGATGAATTTATTCGATGGTGCAAACGAGATAAGAAGAAACCACTTAATCAA
ATTACAGATTTTATTGATTGTTTAGCACCTGAATCTAATGGTCGAAAGGAACATTGTCTA
CTTGGAAATTGGCCTGGAGTAAAGGTAGACAATGAAATACTCCAAGTCCAAGGTATGTGA
ttataaaattattcaacgaaatatattatattacagtttatattccactaatataaatta
aaagattaagaaatatatat
>DjScaffold1386:31950-33522 strand:+
AAAGAGATCATTTCAATAAATATTGGTCAAGCTGGAGTTCAACTTTCTAATGCCGCGTGG
GAGTTATTTTGTATAGAGCACGGTATTAGGTCAGATGGAACAATGCATAACATGGTTGAA
AATGGAGAATCTGGACAAGATACATTTTTTAAACTTACAGAAACCACGTATGTACCACGT
GCTATTTCAGTGGATTTTGAACCCACAGTTATAGACGAAGTTCGTGTCGGAGCTTATAGA
CATTTGTTTAATCCAGAACAATTAATATCTGGATTTGAAGATTGTGCAAGTAATTTTGCC
AGAGGAAATTATACGGTTGGAAAATTGTTTCTTAAACCTGTTATGAAACAAATTGTAAAA
CAAATTGAAAATTGTGATAGTCTACAAGGATTTTGTATATTAAATAGTTTTGATGGAGGC
ACTGGTGCAGGTTTTACTTCAACTCTTCAAGAAGAATTATCAATGGAATTTGGGAAAAAA
CCAAAATTTCAAATTGGAATTTATCCATCTCGACGAATGGCTACCAGCACAGTTTATCCA
TATAATGCAATTCTTCATACTCATACAACGATGACTCATACAAATCTAAATACATTGATG
GATAATGAAGCTATTTATGATATATGCACAAATAAACTCGGTGTAGAAAGACCAACATAT
AGTAATATTAATCGGATAATTGTGCAACTGTTTAGTAGTTTAACTATTTCTGTAAGATCT
AGAAATCTTTTAAATAGCGATTTAGACCAATTATGCACAAATCTAGTTCCATATCCTAGA
ATACATTTCCCATTATCTCATTTGTCTCCATTATTAAATGCAAATAAATCTGTCAAAGAA
TCAACAGGTATTGGTGATATGACACGAGATATATTCGATCCTGAAAATCAATTAGTACAA
TGTAATACGGCCAATGGGAGGTTTATGGCATGTGTGCTTCAATTTAGAGGTCCAATACCT
CCAACATCAATTAATAAAACAATTTTTGAACTAAAAAGAAGAACGGATATAAACTTTGTA
GACTGGTGTCCCACTGGATTCAAAATAGGTATTAGTAGTATGCCTTCTGTAAGTCCTCCT
GGAAGTGCATTGGGGGATATGAAAAAAACACTAGCAATGCTATCAAATTCGACTGCAATC
GTTGAAACATGGTCGGGACTACTTAAAAAGTTTGATTTACTGTTTTCTAAAAGATCATTT
GTGCATTGGTTTGTAAGTGAAGGTTTAGAAGAAGGAGAATTTTTAGAAGCTTACCAAAAT
ATAAGATCTCTCGAAAATGACTACGTTGAAATTGCTATGTCAACACAAGAAATAGAAAGT
CCTGGTCAAAAACCTCAATCCCAACCTGTACCATTGAAAAGAATTGGATCTAGTGAACAA
AGTAGTAGAATTGATGACTCAAAAAGCACAATTAAAAGGGACAAAACAAAAAGCCTGAAA
TCTACGACATCAATTTCCAAAAGCATTAAAAGTGTAAATAAGTCAGGAAATAATCCATCG
TCCTTTCACACTGAAATTATGAATTATTAA
>DjScaffold4633:2620-3559 strand:+
GCTTTAACCCACAAAATCACTGGAGAAACTAAATATATAACTGGTTCTAAACCTATCATA
TTTACACCTGTTCTTGAAAAGTTGAAACCAAATGAATCTGGCAGCAATTTAGGTACTTCA
TTTGCTGATTTAACAAGAGAATTCGGCTCAGCTAAAGCTCAGAAAGTTTTACGTGAAAAA
GAAAAATTAAATATTAACAAAGAAGCACAAAAAACTTTAAAAGATGCCCAAAGAAGTGCA
ATAAAAGAAGAAAAATCTGAAGCTGCAAAAGGAAATAAACTCGATAAAAGTTATATGGAT
AGCTCCATCGATGGAAGTTTCTCGTGTGGAACAATTACAAATATCATAGATTCAGATAAT
CGTATTAAACTTTCGACTATCGCTCCAGAAGAAGTATTAGAAAGTTTGAAAACTGAATCC
GAATCATTCTTGCATATGTCATATGAGGAAGTGGAAAAACTTGAAGGAATATTATCATTC
CATCGATGCATTTTGGATGTGATTAGAAAATCCGGTACGAATGATTTGAGAAAAATGCAA
CTTTGTGTTTTATTGGAATATATGATAAGATTCCATGGATTACCAATCAAACAAATTCGT
TCGAAAACTCCATTAAATGGATGTCCAGATGTAACGATGAAATTTCTTTTAAATAACTTT
TCCAAAGAGGTTCTAGTTGATGGGGGTAAATCAACAATGTAAgtaaattatattattcat
attttcattattaatatgcatttcttcctaataattaattaataattaattgatttttta
ttgatttttaattttcaactaggtaaagaagaaaaagataaattgacattaatgattat
ggttttatatgga

>DjScaffold3258:2354-5944 strand:+
ctatttatctctgtgctggataagatagatcacaATGAGCCAATACGATGACCAAAATGA
TGATATTGAGTTCATAAGACATGTCTATAGAGCCAGCTCTCCCACAGTGACTAGACAAGA
GAATAGAATAAAAGAACTTGAAGAATTATTGGATTTAGAAAGAGATATTAAAGTTCGGTT
GGAGAAAACTAATGCAGATTTATGTGAACAAATTGGTCAATTGAATGATCAAATAGAGGA
ATTATCTCTCACTTCCAGTCAGTATAATGATGTCATTCGCCGAAGAGATACCGATCTACA
GAGATTGAGGAAGGAATATGAAAAAGTAACTTTACAAATGGAAATGTCTGAACAAGATAA
TCGAAAGAGAAATCATAATTCCATGATGGAGTTGTCTGCAGAAATTGATAATTTGCAGAA
ACAGAAAATCAAATTTGAGAAAGAAAAAATGATGTTGTTACAAGAATTGGAAGCATCTCT
AACACAAATAGAATCTCTTTCGAAAACCAAAATCAATATTGAATCAAAACTAGAAAATTC
AGAAATCCACAATTTAAGATTGAAAACAATGCTAGATGACCAAGAAAAACAATTGAAAGA
ATTTTCTGTGATAAAGACAAAACTTTCCGAAGACAATGTTAATTTGAAAATGCAAATAAA
TGAGATTAATCATCAAACAGTTTCTTTGAAGAAATCTAAATCGAATTTAGAATCTTCATT
AGATGAAATAAAAAAACAATTAGAAGAAGAAACAAAGATGAAAGAAGTTCTACAAAGTCA
ATTTCATCAAATTCAAGGTGATAACGACCAACTGCGAATTCAAATCGAAGATGAAACTGA
TAACTATCATCGTCTACAAAATCAGGTTTCAAAACTCAATTCCGATCTAATGTTTATGAA
AAGTAAGTACGATAAAGAAATTACTCAGAAGACTGAAGAATTTGAAGAAATGAAACGAAG
AAACACTGCTAGAATCAATGAATTACAAGACGAGCTGTCAAGAGAACATTTGCGACTATC
GTCATCAGAAAAGGCAAAGCAGCAATTATCGATGGAACTCCGAGAAATTTATACCGATAT
TGATAGAGTTTCTACTGAAAATGAAAGGTTGATGCATGAACTGAAATCATCAGAAAATAT
AATAAAGGAACTCAATAATCGTCTTCATGATTTCGATATGGAATTTACACAATTAAAAAC
CTATTTAAACAAGTCAAATGCAGAAAATTCAAAACTTAAAATGAATCTCGAAGAAGAGGA
ACAAAAAGTTTTATCAATGACGAAAGAGATTGGATCATTATCGAATAGTCTAAAGGAAGA
AAAAGACAACAATCGTATAATACAAAAGGAGTTGTCTGATCTTCAAATGATCAGAAATCA
GATGGAAGCCGATCGGAGCAATTTATTGGCTGCATTGGAAGAAACCAAAGAAAGATTGAT
TGATTCGGAGCATAAGATGCAGCTGTCTCACAATACATTATTACAATTAAAAGGAGATAT
GGAACAACGTTTGCATGAGAAAGATGAGGAGTTGATGTTGATAAAGAAAACCACTCAGAA
AACCATTGAAGAATTAACAAATACGATAGCCTCTATTGAAAACAAATATAAGACAGAAAT
AGGTAAACTAAAAAAGAAGTACGAAACTGATATCAAAGAAATCCAAAATGAAGTGACTGA
GCTGCAAAAAGAAAATACTCAACTCAATGATGATTTGAAAGAGACAACATTAAAATCACA
CGAACTAGAACAGATCTTGGAAGAAGAAAGACGAGAATGTAATGTTTTGCAAGAGAAATT
AATTTCTTCAGATAAACGTAAAAGTCAAATACAAGGAGATCTCGATGACTCCAACCGCCT
TCTGGAAGCGTCAGATAAAACTAGGAAAAAAATCCAAATAGAAATTGAAGAGATCTCGTT
GAAATTAGCAGATTCACAATCCCATATCAATCTGTTAAACAATGAAAAAAGAAAATTGGA
AAGTGAAATTGTTGTCCTACACAATGATATGGATGACGCAATAACAAGCAAATTGGAAAC
TGAGGACAGAGTCATCAAGCTGGGGAGTGAAATTAACAGATTAAATGATGACTTCAAACA
AGAGCAAGAAAATCATAAGAAATGTGAGAGTGCAAGGAAAGGCTTGTTCAGTGAATTAAG
AGAAATAAAAACACGATTGGAAAGCAGAGAATCTAGCACTTCAAAGGAAATACAGAAAAA
TACTCAAATATTCCAGGAAAGAATAAGAGATTTAGAGATAGAATTAGAAAATGAAAGAGT
TAGAAGTAAGGATGCACAAAATGCCATCAGAAAACAAGAAAGATCAATTAGGGAAGTACA
GCAGCAGGCAGATGATGACAGGCGAACAGTTATTGATTTGCAACAGCTGCTGGACGCAGC
AAATGGCAAAATAAAATCTTATAAACGGCAATTAGATGAATTGGAATCAGCAAGCACAGA
AAATCTCAATAAGTACAAGCGGGCCCAAAAACTGATTGATGAAGCGGAACATAGAGCATC
AGTTGCAGAAAGAAGGGTTGGAACAGCTTTAAATTATATGAATCAACCTATTAGTTCTAT
TAGTCTATCTAGCTACACCAAGCAATCTGGCACACGATAAatccaccttgaacaccaata
gagatagattagataactttcgatgccaatttcgacaactgtagactgtttatgtatta
>DjScaffold3258:38320-40165 strand:+
aaatttaaataattaaaATGAGTGTTCCAAGAGCAATTACTGGTATTAGTAAAGCAGATT
CTGTTTTATCTCATTTATCTCAGCCACCTTTGACTCTTTCCGTTCCTAATGATGTTCCAA
CTGCGAGTCCAAATGCTTTGAGTTTTATTGAAAAAATTGATATGACGGACTTGAAATATA
CATTTTTTGAAGACGATTTAATGACATTTATTGATGAAGACATTGAAATAGGATTTTACA
AAGCTACAGTGAAAGAAGGTTGTTATAAAGGAGAGAATTGTATCGTTGTAGAAGCGCGTA
GCGAAGGTGTGGTGGAAGGAGTGCCTTGTTTCACCGATGTGGAGACCTATTTGAATCCGT
CGTTGCAGACGATTTTCCAGCGATTGCACGAAGTCGTCAGACTGCCGCAAGGAGGACAAC
TCGACCGAGTAACAACGATGGAAATGGACGACTCGATCGATGGTTACCGAGTGCGCAAGG
AGATCAAACAGGGAATCGACAAGCGCGACGTGCTGGAGCGAGTTTTTGGACGTGCGGTGA
TGGAGGGATACATTTCTGAGGGTGCCAACATCGTGCTGCAGCGCGTCCTCATGTCGCGAG

GCTTGCCCGACTATTTCGATGTGGTCAGCTTCGATGCAGACACAAATTTGTGCTTGGCGA
CTTACAAAGATCTCGCTGCTACCAAAATCATTGTGGACTCCTCAGAGGCAACAGAAGTGA
ACTGTTATGGAATCGAGCGACGGTTGCGAAGCGTGACGGACGTGCCTGTGACGTGGCACA
CGTATTTCCTGTCAGATGGGCATCTGATCAGTCGAGTGCAAGTTGGCAGTCCAGTGCTGA
TGAAGGTGCGAACGATTCCTAAACTGATGGAGCATGACGAGTTCCTGCCTGCTCCTACAT
TTCCTCGACCGCAGCTCATTTGGCAAGATGATCTCCAACTAACAAGAATGTTCCAGGAAC
GCAAAGAGGAACTCGCTAGTGGAACTCGTCAATATATTCTCACTCATCCGGAACTGCAAC
ATTTGATTGCGGATTTCCTGCAAGCTCTGCTCGCTCACAAACCGACTGATACGATTAAAT
TTGCTGGCGATTTCTTTGCTACTTTTTCAAACAGATTTATCTCTGGTCCAGCAGCATCTC
AGCTACACTCGGAGCGCCCTGGCATTCTTTTATCAGATAGAGTTCCTTTGTGAtcctgat
gataattatcaaataaaataaatttatatttttctatttaaataattaaaatgagtgtt
ccaagagcaattactggtattagtaaagcagattctgttttatctcatttatctcagcca
cctttgactctttccgttcctaatgatgttccaactgcgagtccaaatgctttgagtttt
attgaaaaaattgatatgacggacttgaaatatacattttttgaagacgatttaatgaca
tttattgatgaagacattgaaataggattttacaaagctacagtgaaagaaggttgttat
aaaggagagaattgtatcgttgtagaagcgcgtagcgaaggtgtggtggaaggagtgcct
tgtttcaccgatgtggagacctatttgaatccgtcgttgcagacgattttccagcgattg
cacgaagtcgtcagagtaatttacaatcgattttctatgtgaaataattgataattctcg
ctcgttattgtgttgttattagctgccgcaaggaggacaactcgaccgagtaacaacgAT
GGAAATGGACGACTCGATCGATGGTTACCGAGTGCGCAAGGAGATCAAACAGGGAATCGA
CAAGCGCGACGTGCTGGAGCGAGTTTTTGGACGTGCGGTGATGGAGGGATACATTTCTGA
GGGTGCCAACATCGTGCTGCAGCGCGTCCTCATGTCGCGAGGCTTGCCCGACTATTTCGA
TGTGGTCAGCTTCGATGCAGACACAAATTTGTGCTTGGCGACTTACAAAGATCTCGCTGC
TACCAAAATCATTGTGGACTCCTCAGAGGCAACAGAAGTGAACTGTTATGGAATCGAGCG
ACGGTTGCGAAGCGTGACGGACGTGCCTGTGACGTGGCACACGTATTTCCTGTCAGATGG
GCATCTGATCAGTCGAGTGCAAGTTGGCAGTCCAGTGCTGATGAAGGTGCGAACGATTCC
TAAACTGATGGAGCATGACGAGTTCCTGCCTGCTCCTACATTTCCTCGACCGCAGCTCAT
TTGGCAAGATGATCTCCAACTAACAAGAATGTTCCAGGAACGCAAAGAGGAACTCGCTAG
TGGAACTCGTCAATATATTCTCACTCATCCGGAACTGCAACATTTGATTGCGGATTTCCT
GCAAGCTCTGCTCGCTCACAAACCGACTGATACGATTAAATTTGCTGGCGATTTCTTTGC
TACTTTTTCAAACAGATTTATCTCTGGTCCAGCAGCATCTCAGCTACACTCGGAGCGCCC
TGGCATTCTTTTATCAGATAGAGTTCCTTTGTGAtcctgatgataattatcaaataaaat
aaatttatattttttc
>DjScaffold3258:24351-24932 strand:+
ATGACATTTGGTATTGCTGCTGCTCCAGGAACGTTTCAAGAACTTATGAATAAAGTATTA
ATCAAGATAAATGGTGTCGAAGCCTTGGTTTATTTGGATGATATCTTTGTTTTTTCTAAT
TCCTTGGAAGAGCACATGATTCGTTTAAATAATATGTTAAGTAGAATAAAATCTGAAGGT
TTAAGAATTTATCCAGATAAATGTTCGTTATTGAAAAAAGAAGTTAAATTTCTCGGGTAT
ATTATTACAAGCGAGGGAATACGCACAGATCAAAGTAAAATTGAAGCAATAAGTACATTT
GAAAGGCCGAAATGTATTAAGAGTTTGAGAAGTTTCTTAGGATTATGTAATTATTTTCGA
AAATTTATAAATAAATATGTAGAAAAGGCCATAATTCCAGGAGCATTGTGTGGTAAGGGT
AATGAAAGGTTGATTTGGTCTGATCAGTGTGATGAGTCTTTCAATCAATTAAAAATTGCT
TTACAGAAAAATCCAGTGTTAGCATATCCAGATTTCAACAAAGTTTTATTTTGGACACGG
ACGCTAGCTTTGATGCAATTCGAGCCATACTCTCAAAATTAG
>DjScaffold4651:22252-38499 strand:+
atattttagtattattatcacttattaatttccaATGATTCAAAACGGCCCCCCTGCTGC
ATTAGTTATTAAAAATCGAATGAAGACACTTAATAATGAAATAGAAAATTTAAAGGACGA
AATAGTGAATCGGGATAAATTATTAAAAATTGAAATCGAAAAGAAACACGAAGTTGAAGC
TGAAGTGAAAAATCTTTCAAAAAAATTAGAATTAATTGAAGAGGAAAAGCTAGATTATGA
AGCAAAATATAAAGAAGCTTGTCAAAATTTAGAACTTATCAGTAAAGATGCCCATAAAAG
TGAAAGAAGCCGAAAAGTCATGGAAAATCGCCAAAATGCTGATAGTGATCGAATGGATTT
TTTAGAAGAATCCATTAAAATATCAGTTGTAACTACTGAAGAGGCTAACAAAAAATCTGA
AGAGTTATTAAAGAAGCTTACTCAATCTGAAAATGCATTAGTAGAAGCTACAGATCGTTC
TACCACAGCTGAAAATCATATTAAAGAACTGGAGGCAGATTTAAAATCGATTGCTTCAAA
ACTGAAATCCTTAGAAATTCAAAAGGATAAAGCCGCAGAAAGAGAAGTAAATTACGATGC
TCGCATTCGATGCTTAACATTACAAGTAAAGGAGGCTGAAGAACGTGCATTGATAGCGGA
ACAAGATGTTAGAAAACGAGAAAGTGAATTAGACAAAGTAAAAGAGGAAATCGATCGTGA

AAAAGCCAATTATTATCACGTTAAAAAGGAACTGGATAAAATGCATGCAGAATATTTTTC
AGTTTGAacacaaaaattataggaatattggattttctgattttaatttagaaatctat
tactattgtaaattaaggcttttaagtatatttgttttattatttatattgcatagtggt
actccagaatccaggcttatgttggttaatatatctggagtattgttgtaattaatttta
ttgctttacttttctttcgaattttcaattgattttattaaaaattataattataatcga
tacaatttttattgaatatcttgtgttttatccaataaataatcgatcaatgttatgttt
aattagtgttatatgtgttccattggatttgttgaaaatctggagaaattttaatttcag
tttttttttatttgtaatgtttatttgttcgatgtttaatattgtgcaatgaaa
>DjScaffold4227:60031-70656 strand:+
taatttaATGGATAATTTGAATAAAATTAAATCAACGTTAAAATTATTTAAAGATTTTCC
TAAAAAAGACATCGAATTTATTGATATTTTAAATGTTTTTGCTGAACCTGAAATTTTAGA
TATGACAATTGATCTTTTTGTTGACACACTTAAAAATGAACACATTAATATTATTGCTGG
TGTTGAAGCCAGAGGATTTCTAATTGGACCTTTAATAGCTTATAAATTAAAATGTAAATT
TATACCTATTAGAAAAAAGGGTAAATTACCTGGAAAATGTATTCAGCAAAGTTATTCGTT
GGAGTATGGTGAAGACTGCATTGAGATTCAAGAAACAGCTGTGAAACAAAACGAAAATGT
TGTAATTATAGATGATCTACTTGCAACTGGTGGTACATTAAGTGCTGCTATTCAATTGAT
TAAAAAAACTGGAGCTAAAGTAGTCAAAGCTATCACATTAATTGAAATTGATTCATTAAG
AGGTCGAGAGATATTAAGTGCTCCATTCCAGTCATTTATCCATTATTAGtttaaaattta
agtgacaatatcgggttcttattttcatttataaaagtca
>DjScaffold4227:81902-88314 strand:+
TTCTATAGCGGAGGTAAAAACAGCAATAAAACTTTTAAACCGCGAAAAAATATTCCTGAA
AACACAAATCAACAAGATTTATTGGAGAAGGCTTCTGTTACATTGGGCAGTGGTAATTTA
AGATTAGCTGTTAAATTACCTGAAGGAGAAGATTTAAATGAATGGATAGCGGTTAATACA
GTAGATTTCTTTAACCAAATCAATATGTTGTATGGGACACTTACTGAATTTTGCACCGAA
GAAACGTGTCCAGTGATGTGTGCAAGTCCTAAATATGAATATCACTGGGCTGATGGTCAG
TCAATCAAAAAACCCATAAAATGTTCAGCACCTCGATATATAGACTATTTAATGGTTTGG
GTTCAAGATCAACTCGATAATGAATCAACTTTTCCTTCAAAAATAGGTGTTCCTTTTCCA
AAAAACTTTTTATCAATAACTAAAACCATTTTAAAACGTCTTTTTCGTGTTTATGCTCAC
ATTTACCACCAACATTTTTCTGAAGTTTGCAAATTACAAGAAGAACCTCATCTAAATACA
AGCTTCAAACATTTTATATTTTTTGTTCAGGTTTGTAATATTTTTCTTGTTTTTACTTCA
GTAAAATATTTTGTTTTATTTTCTAACTGTTTAAACTTTTTTCACTTTTTTTTCACTTTG
TTTTACTTTTAA
>DjScaffold4682:20678-20998 strand:+
ATGATGATCAAAGATCATAATAAAATTTGGAAAGAAGGTTTTCATAGAATCAAAGAATCA
AGATTGAGGATAAATCCAGAAAAATGTAAATTTTACCAAAACCAACTGATCTTTTTGGGA
CACACAATAAGTTTAGAAGGAATAAAAACAAACAAAACTAAGATTTGGGATATAAAAAAT
GCCGAAGAACTAAAATTTTCATCTAAACTCGGGTCTTTTCTTGGCCTAAGGAATTACTAC
ATGCGCTTTATGAAGGATTATGCCAGAATTACAACATCTTTATATGAAGATATTTCAGAA
TGTGACAAAACTTTGATGTAG
>DjScaffold2103:27165-27904 strand:+
AGAATATCAGAAATTGTTGTGAAATGTCGAAATAAATATTGTGAATTCACAGACGAGTTG
AGTTTGATAGAAAATCACATGAGAACATGTGACAAGAATCCAACAAGTTGTCCATATTTC
GAAATGGGATGTAAGAATAATGAAGTGATTAATGATGAAATGAAAGATCATCTTTGTACA
GAGATTTACTGTCACACTAAATTATTATTAGATTTGATCAATAATTTACGAAATGAGATG
GAATCAATGAAGAATGACATTGTAGAATTAAAATTAGAAAATAAAAATTTGAATAAAGAA
TTAGATAAAATAAAAATCGAATCAAAAAATAATCAAGTAAATTTATTTATTGATTATTTN
NNNNNNNNAAATAAAGAATAAtttatcagaaattgaagaattaaatgaagaaatggttgt
aataaagaatgaaaatagatcattgatgaataaattagagaaaattgagaaatcgcgaat
tgaaatgaggagaataattgatgaaaattattcagatttgatgaagaaatcgaataatta
tgatgagaaaatcaataattatgatgagaaaatcaataattatgatgagaaaatcaataa
ttatgatgagaaaatcaataattatgatgagaaaatcaataatttacaaataaaaataat
caatattgaaagaatcacagaaaaa
>DjScaffold1101:30586-32472 strand:+
tttctgcaaatagaaaaaaatcgaaaatgccatttacgtaaagatgatgaatcatgtatat
tgcttagaATGATAAAAGTTATTGACGGAAATACTGTTGACGTTCATTACACGCAAGTAG
ACAAGTTCCATAATCAAACAATACATTTGTTTGCAATATCAACTCCACTTTTAAATGAAA
CATTTGGAATCAAAGCAAAAGAGCATTTGCAGAAACTATTGAAGAAAAGAAAAAGTATTA

TTGCTAAAATAAAAAAAGAAAGAAGTATTAATGGTCAATTTATTGGAATTTTATATTCAG
GAAAAACGGATATTAATAAAAAAATGATCATGGATGGATATGCTTGGTGTAAAAAACCAA
TGAAATGTCCACAAAGTTATTACAGAGCAACTAAACGCGCAATTAAATTTCAAAAAGGAA
TTTGGAAAAAAAATAAAGTTGTTAAGTCGAAAAATTCTGGAAAAAGTAAATCTTCAAATG
GGAAGAATCCGGGAAAAGGAGAGTATAATATAATAATAAATTCAGTGATCGATGGGGATA
CTGTTACGGCAAAATTGATTAGTTCATCAATGAATTCAATCATAAAAATAAGAATGGGAG
GAATCGATGCACCAGAAATGAAACAATCATTTGGTGTTGAAGCTAAAAATAAATTACAGG
AAATAATAGGAAATTTACCAAACAAACAAGTTAAATTAATTGTAGAAAATATTGATATTT
ATGGAAGAAGTATCGGTTATTTAATCGCTGGAAATATTGACATTAATAAATCAATGGTAG
AAAAAGGATATGCGTGGTGTTATAAGTATAGAATATGTCCTTTGTCGTACCAAATCGCAA
TGAATGATGCTAAAACTCAAAGAAAAGGATTATTTATTCAAACTAATCCAACTACTCCTT
GGGATTGGAGGAGAGAGAACAAGAAAAGAGATTAAttaat
>DjScaffold3672:5079-6677 strand:+
aattaataagctagatttcacaggctcattcacatctgaaaatatttactgaaaATGAGA
GAAATTGTTCACTTGCAAGCTGGTCAATGCGGTAACCAAATTGGTGCCAAATTCTGGGAA
GTTATTTCTGATGAACATGGAATCGATCCAACTGGTACCTATCACGGAGATTCAGATTTA
CAATTAGAAAGAATTAATGTTTATTACAATGAAGCTACCGGAGGCAAATACGTTCCTCGT
GCAGTTTTAATTGATTTAGAACCTGGCACTATGGATTCAGTCAGAGCTGGACCTTTCGGT
CAATTATTCAGACCAGATAATTTTGTTTTCGGACAAAGTGGAGCTGGTAACAACTGGGCC
AAAGGTCATTATACTGAAGGAGCTGAATTAGTCGACTCAGTTCTAGATATTGTAAGAAAA
GAAGCTGAATCTTGCGATTGTTTGCAAGGTTTTCAACTTACTCACTCATTGGGAGGAGGA
ACTGGATCTGGTATGGGAACTTTACTTATTTCAAAGATCAGAGAAGAATATCCTGATAGA
ATCATGAACACATTTTCGGTTGTGCCATCTCCAAAGGTATCAGATACAGTTGTCGAACCA
TACAATGCTACATTATCCGTTCATCAATTAGTTGAAAATACAGATGAAACATTTTGTATT
GATAACGAAGCTTTATACGACATTTGCTTCAGAACATTGAAATTGACCACTCCTACATAT
GGAGATTTGAATCATTTAGTGAGTGCAACAATGAGTGGTGTGACAACATGTTTGAGATTC
CCTGGTCAATTGAATGCCGATTTGAGAAAATTAGCAGTAAACATGGTTCCATTCCCACGT
TTACATTTCTTCATGCCAGGATTTGCACCATTAACTAGTAGAGGTAGTCAACAATACCGT
GCTTTAACTGTTCCAGAATTGACTCAACAAATGTTTGATGCCAAAAATATGATGGCTGCA
TGTGATCCAAGACACGGTCGTTACTTAACAGTAGCTGCTTTGTTCAGAGGTCGTATGTCA
ATGAAAGAAGTTGATGAACAAATGTTGAATGTACAAAACAAGAACAGCAGCTATTTCGTC
GAATGGATTCCAAACAATGTTAAGACCGCAGTTTGCGATATTCCTCCACGAGGATTAAAA
ATGTCTGCAACATTCATTGGTAACAGCACTGCTATTCAAGAATTATTCAAACGTGTAAGT
GAACAATTTACTGCTATGTTCAGGAGAAAGGCTTTCTTGCATTGGTATACTGGCGAAGGT
ATGGATGAAATGGAATTCACTGAAGCTGAATCGAATATGAATGACTTGGTTAGTGAATAT
CAACAATACCAAGATGCCACAGCTGAAGAAGAAGGTGAA
>DjScaffold3672:19834-20359 strand:+
gatgcatctagtaggcaagattgatgagaagattaaaacacttgtcaaaagcggcaatat
aaaagaatgaagtagcccgtggaatttacctATGGTAATTATTAACAAAAATAATGGAGC
TATTCGTATGTGTCAGCATTTCAGATTATTAAATGAAGCAACTGATAAATTTTTGTTTCC
TATTCCAGATCCACAAATGTTATTCGATCATCTTAGTGAGTGTACCATATTCTCAACACT
AGATACTTGTCAAAAGCGGTGAtaagaaaaagctagactgcgctgaaattttacaa
>DjScaffold1140:18706-23128 strand:+
taataattaattattttaacaatttttctttaaatttattaatttgtttaaaacataatc
tttccatttacaataacaaacttttaaaatcgtaatatattaaaATGAACTACAGAAATA
GAAGAGATCGATCTCGTTCTCCAGTTAACAGATATGGAAACAATTGGCCAACTATCCGCT
TAATGCCTTTTAAGAAAAACTTTTACGTAGAGCATCCAATTGTTACCTCTAGAAGTTCAT
ATGAAATCGAACGATTTCGCAGTGAGAAAGAAATGACAGTTGTAGGAAAGAATGTCCCTC
GACCAATATTTAATTTTAATGAAATGAATTTTACTAATTTTATTATGTCAACAATTGTAA
AGAATAAGTGGGTCAAGCCAACGCCGATTCAATGCCAAGGCTGGCCAATGGCAATGAGTG
GACGCAATGTTGTAGGAATTGCTCAAACAGGTTCTGGAAAAACAGCTGCATTTTTATTAC
CTGCTTTGATTCATATACGAGCTCAACCTCCGTTAGCAGCTGGAGATGGTCCTATTTGCC
TTGTTCTTGTACCTACAAGGGAGTTAGCTCAACAAGTTGAATCTGTTGTTGAAGAATTTG
GGATTTCGGGTGGTTTTAACATGGTCTGTGTATATGGAGGATCTGATCGACTTAATCAGA
TAAAAAGCTTGCAGTCAAAACGTCCAGAAATATTAGTTGCTACTCCTGGACGCTTATTGG
ATCTTTTGAAACAAAACGAAACTAACCTGAGGCGAACAACGTATTTGGTCTTAGATGAAG
CTGATCGAATGTTAGATATGGGATTTGAACCGCAAATTCGTGCAGTTTTAAATCAAGTCC

GACCTGATCGACAAATGCTTCTATGGTCGGCAACATGGCCAATAGAAGTCAGACGATTGG
CTGAAGATTTTTTAAATGAATATATCCAAGTTAATATTGGTGCTATGTCTTTACACGCCA
ACCATAACATTTGTCAATATGTTGAGGTGATTGACGAACTCAGCAAAGAACGGAGATTAA
TTGATTTATTAAATCAATTTCGTGGTCTAAAAACACTTGTGTTTGTGGCTTTGAAGAAAA
GATGTGATAGAATGTCAGATATGTTAAATTCTAAAAGTTTTAAAGCTATTGCCATGCACG
GAGATAAGCCACAAAGAGAAAGAGAACGTGCACTTGAAAATTTCAGAAATGGTAGATCAA
ATATTATGGTTGCAACAGATGTCGCTTCAAGAGGATTAGATATTGATGATATAATGTTGG
TGATTAATTACGATTTTCCTACCCAAATAGAAGATTATGTACACAGAATTGGAAGAACTG
CAAGACAAGAAAAGAAAGGAACTTCTTACACTTTTTTCACAAACAATGAAAAGAAACATG
CACAGAAACTTGTTGCTATTCTAGAAGAAGCTAATCAGAATATTTCAGAGAAATTATACG
ATTTAGCAGAAGTACCATTTTCAAGTCGAAAGAAACCGACTTCTAATGTACCTAAAGGAT
TAGTCAGTAATTCTGCAACTAATAACTGGGGAGAAGCTAATAAACGTGTTCCAAAAAGTG
ACTATAGGAAGTCAGATTATTACGAATACAATGGACGTCAAACTGAAACAGAGTCTTTTA
CTCACAGTAGTAATTCATATCGAGAAGCTGGGAACTGGAGAAATAATGGATCCTATAATA
ATTACAATCAACCTCAACCAGCATTTAATGGTAGTAATAATAATACTAATAACTTCAATT
CTAACAAAGCACCTAATTATAATGAGGATTTCAATTCAATGGGAATTATTGCTAACAGAT
GGTAAatgaaatctcctgtttgttgtatgtcttagttaaactattaatttgtaacggaaa
ttgttgtaattattttaataaatagtatatttattaataattaattattttaacaatttt
tctttaaatttattaatttgtttaaaacataatctttccatttacaataacaaactttta
aaatcgtaatatattaaaATGAACTACAGAAATAGAAGAGATCGATCTCGTTCTCCAGTT
AACAGATATGGAAACAGTAAATTTCAAAAAAGGCAGTTTGACTGTGAAATTACAAAAATA
GATTGGCCAACTATCCGCTTAATGCCTTTTAAGAAAAACTTTTACGTAGAGCATCCAATT
GTTACCTCTAGAAGTTCATATGAAATCGAACGATTTCGCAGTGAGAAAGAAATGACAGTT
GTAGGAAAGAATGTCCCTCGACCAATATTTAATTTTAATGAAATGAATTTTACTAATTTT
ATTATGTCAACAATTGTAAAGAATAAGTGGGTCAAGCCAACGCCGATTCAATGCCAAGGC
TGGCCAATGGCAATGAGTGGACGCAATGTTGTAGGAATTGCTCAAACAGGTTCTGGAAAA
ACAGCTGCATTTTTATTACCTGCTTTGATTCATATACGAGCTCAACCTCCGTTAGCAGCT
GGAGATGGTCCTATTTGCCTTGTTCTTGTACCTACAAGGGAGTTAGCTCAACAAGTTGAA
TCTGTTGTTGAAGAATTTGGGATTTCGGGTGGTTTTAACATGGTCTGTGTATATGGAGGA
TCTGATCGACTTAATCAGATAAAAAGCTTGCAGTCAAAACGTCCAGAAATATTAGTTGCT
ACTCCTGGACGCTTATTGGATCTTTTGAAACAAAACGAAACTAACCTGAGGCGAACAACG
TATTTGGTCTTAGATGAAGCTGATCGAATGTTAGATATGGGATTTGAACCGCAAATTCGT
GCAGTTTTAAATCAAGTCCGACCTGATCGACAAATGCTTCTATGGTCGGCAACATGGCCA
ATAGAAGTCAGACGATTGGCTGAAGATTTTTTAAATGAATATATCCAAGTTAATATTGGT
GCTATGTCTTTACACGCCAACCATAACATTTGTCAATATGTTGAGGTGATTGACGAACTC
AGCAAAGAACGGAGATTAATTGATTTATTAAATCAATTTCGTGGTCTAAAAACACTTGTG
TTTGTGGCTTTGAAGAAAAGATGTGATAGAATGTCAGATATGTTAAATTCTAAAAGTTTT
AAAGCTATTGCCATGCACGGAGATAAGCCACAAAGAGAAAGAGAACGTGCACTTGAAAAT
TTCAGAAATGGTAGATCAAATATTATGGTTGCAACAGATGTCGCTTCAAGAGGATTAGAT
ATTGATGATATAATGTTGGTGATTAATTACGATTTTCCTACCCAAATAGAAGATTATGTA
CACAGAATTGGAAGAACTGCAAGACAAGAAAAGAAAGGAACTTCTTACACTTTTTTCACA
AACAATGAAAAGAAACATGCACAGAAACTTGTTGCTATTCTAGAAGAAGCTAATCAGAAT
ATTTCAGAGAAATTATACGATTTAGCAGAAGTACCATTTTCAAGTCGAAAGAAACCGACT
TCTAATGTACCTAAAGGATTAGTCAGTAATTCTGCAACTAATAACTGGGGAGAAGCTAAT
AAACGTGTTCCAAAAAGTGACTATAGGAAGTCAGATTATTACGAATACAATGGACGTCAA
ACTGAAACAGAGTCTTTTACTCACAGTAGTAATTCATATCGAGAAGCTGGGAACTGGAGA
AATAATGGATCCTATAATAATTACAATCAACCTCAACCAGCATTTAATGGTAGTAATAAT
AATACTAATAACTTCAATTCTAACAAAGCACCTAATTATAATGAGGATTTCAATTCAATG
GGAATTATTGCTAACAGATGGTAAatgaaatctcctgtttgttgtatgtcttagttaaac
tattaatttgtaacggaaattgttgtaattattttaataaatagtatatttattaataat
taattattttaacaattttttctttaaatttattaatttgtttaaaacataatctttccat
ttacaataacaaacttttaaaatcgtaatatattaaaATGAACTACAGAAATAGAAGAGA
TCGATCTCGTTCTCCAGTTAACAGATATGGAAACAGTAAATTTCAAAAAAGGCAGTTTGA
CTGTGAAATTACAAAAATAGATTGGCCAACTATCCGCTTAATGCCTTTTAAGAAAAACTT
TTACGTAGAGCATCCAATTGTTACCTCTAGAAGTTCATATGAAATCGAACGATTTCGCAG
TGAGAAAGAAATGACAGTTGTAGGAAAGAATGTCCCTCGACCAATATTTAATTTTAATGA
AATGAATTTTACTAATTTTATTATGTCAACAATTGTAAAGAATAAGTGGGTCAAGCCAAC

GCCGATTCAATGCCAAGGCTGGCCAATGGCAATGAGTGGACGCAATGTTGTAGGAATTGC
TCAAACAGGTTCTGGAAAAACAGCTGCATTTTTATTACCTGCTTTGATTCATATACGAGC
TCAACCTCCGTTAGCAGCTGGAGATGGTCCTATTTGCCTTGTTCTTGTACCTACAAGGGA
GTTAGCTCAACAAGTTGAATCTGTTGTTGAAGAATTTGGGATTTCGGGTGGTTTTAACAT
GGTCTGTGTATATGGAGGATCTGATCGACTTAATCAGATAAAAAGCTTGCAGTCAAAACG
TCCAGAAATATTAGTTGCTACTCCTGGACGCTTATTGGATCTTTTGAAACAAAACGAAAC
TAACCTGAGGCGAACAACGTATTTGGTCTTAGATGAAGCTGATCGAATGTTAGATATGGG
ATTTGAACCGCAAATTCGTGCAGTTTTAAATCAAGTCCGACCTGATCGACAAATGCTTCT
ATGGTCGGCAACATGGCCAATAGAAGTCAGACGATTGGCTGAAGATTTTTTAAATGAATA
TATCCAAGTTAATATTGGTGCTATGTCTTTACACGCCAACCATAACATTTGTCAATATGT
TGAGGTGATTGACGAACTCAGCAAAGAACGGAGATTAATTGATTTATTAAATCAATTTCG
TGGTCTAAAAACACTTGTGTTTGTGGCTTTGAAGAAAAGATGTGATAGAATGTCAGATAT
GTTAAATTCTAAAAGTTTTAAAGCTATTGCCATGCACGGAGATAAGCCACAAAGAGAAAG
AGAACGTGCACTTGAAAGTATGATGAATGGCATTATTAATCTTAATATCTGCGAAGCTTC
TCTATTCAACATAGTTACTGGAATCATAGAAATTGATTTGAGGAGGCCAGCCCATATCTC
GTGAgccacagatatctttgctgacacagatattgatgatataatgttggtgattaatta
cgattttcctacccaaatagaagattatgtacacagaattggaagaactgcaagacaaga
aaagaaaggaacttcttacactttttcacaaacaatgaaaagaaacatgcacagaaact
tgttgctattctagaagaagctaatcagaatatttcagagaaattatacgatttagcaga
agtaccattttcaagtcgaaagaaaccgacttctaatgtacctaaaggattagtcagtaa
ttctgcaactaataactggggagaagctaataaacgtgttccaaaaagtgactataggaa
gtcagattattacgaatacaatggacgtcaaactgaaacagagtcttttactcacagtag
taattcatatcgagaagctgggaactggagaaataatggatcctataataattacaatca
acctcaaccagcatttaatggtagtaataataatactaataacttcaattctaacaaagc
acctaattataatgaggatttcaattcaatgggaattattgctaacagatggtaaatgaa
atctcctgtttgttgtatgtcttagttaaactattaatttgtaacggaaattgttgtaat
tattttaataaatagtatatttattaataattaattattttaacaatttttctttaaatt
tattaatttgtttaaaacataatctttccatttacaataacaaacttttaaaatcgtaat
atattaaaATGAACTACAGAAATAGAAGAGATCGATCTCGTTCTCCAGTTAACAGATATG
GAAACAGTAAATTTCAAAAAAGGCAGTTTGACTGTGAAATTACAAAAATAGATTGGCCAA
CTATCCGCTTAATGCCTTTTAAGAAAAACTTTTACGTAGAGCATCCAATTGTTACCTCTA
GAAGTTCATATGAAATCGAACGATTTCGCAGTGAGAAAGAAATGACAGTTGTAGGAAAGA
ATGTCCCTCGACCAATATTTAATTTTAATGAAATGAATTTTACTAATTTTATTATGTCAA
CAATTGTAAAGAATAAGTGGGTCAAGCCAACGCCGATTCAATGCCAAGGCTGGCCAATGG
CAATGAGTGGACGCAATGTTGTAGGAATTGCTCAAACAGGTTCTGGAAAAACAGCTGCAT
TTTTATTACCTGCTTTGATTCATATACGAGCTCAACCTCCGTTAGCAGCTGGAGATGGTC
CTATTTGCCTTGTTCTTGTACCTACAAGGGAGTTAGCTCAACAAGTTGAATCTGTTGTTG
AAGAATTTGGGATTTCGGGTGGTTTTAACATGGTCTGTGTATATGGAGGATCTGATCGAC
TTAATCAGATAAAAAGCTTGCAGTCAAAACGTCCAGAAATATTAGTTGCTACTCCTGGAC
GCTTATTGGATCTTTTGAAACAAAACGAAACTAACCTGAGGCGAACAACGTATTTGGTCT
TAGATGAAGCTGATCGAATGTTAGATATGGGATTTGAACCGCAAATTCGTGCAGTTTTAA
ATCAAGTCCGACCTGATCGACAAATGCTTCTATGGTCGGCAACATGGCCAATAGAAGTCA
GACGATTGGCTGAAGATTTTTTAAATGAATATATCCAAGTTAATATTGGTGCTATGTCTT
TACACGCCAACCATAACATTTGTCAATATGTTGAGGTGATTGACGAACTCAGCAAAGAAC
GGAGATTAATTGATTTATTAAATCAATTTCGTGGTCTAAAAACACTTGTGTTTGTGGCTT
TGAAGAAAAGATGTGATAGAATGTCAGATATGTTAAATTCTAAAAGTTTTAAAGCTATTG
CCATGCACGGAGATAAGCCACAAAGAGAAAGAGAACGTGCACTTGAAAATTTCAGAAATG
GTAGATCAAATATTATGGTTGCAACAGATGTCGCTTCAAGAGGATTAGATATTGATGATA
TAATGTTGGTGATTAATTACGATTTTCCTACCCAAATAGAAGATTATGTACACAGAATTG
GAAGAACTGCAAGACAAGAAAAGAAAGGAACTTCTTACACTTTTTTCACAAACAATGAAA
AGAAACATGCACAGAAACTTGTTGCTATTCTAGAAGAAGCTAATCAGAATATTTCAGAGA
AATTATACGATTTAGCAGAAGTACCATTTTCAAGTCGAAAGAAACCGACTTCTAATGTAC
CTAAAGGATTAGTCAGTAATTCTGCAACTAATAACTGGGGAGAAGCTAATAAACGTGTTC
CAAGTAAATATAATGGAACGATTTCGAATCGTGATTCTTATTATCAACCTGTAGAAAGTG
ACTATAGGAAGTCAGATTATTACGAATACAATGGACGTCAAACTGAAACAGAGTCTTTTA
CTCACAGTAGTAATTCATATCGAGAAGCTGGGAACTGGAGAAATAATGGATCCTATAATA
ATTACAATCAACCTCAACCAGCATTTAATGGTAGTAATAATAATACTAATAACTTCAATT

CTAACAAAGCACCTAATTATAATGAGGATTTCAATTCAATGGGAATTATTGCTAACAGAT
GGTAAatgaaatctcctgtttgttgtatgtcttagttaaactattaatttgtaacggaaa
ttgttgtaattattttaataaatagtatatttattaataattaattattttaacaatttt
tctttaaatttattaatttgtttaaaacataatctttccatttacaataacaaactttta
aaatcgtaatatattaaaATGAACTACAGAAATAGAAGAGATCGATCTCGTTCTCCAGTT
AACAGATATGGAAACAGTAAATTTCAAAAAAGGCAGTTTGACTGTGAAATTACAAAAATA
GATTGGCCAACTATCCGCTTAATGCCTTTTAAGAAAAACTTTTACGTAGAGCATCCAATT
GTTACCTCTAGAAGTTCATATGAAATCGAACGATTTCGCAGTGAGAAAGAAATGACAGTT
GTAGGAAAGAATGTCCCTCGACCAATATTTAATTTTAATGAAATGAATTTTACTAATTTT
ATTATGTCAACAATTGTAAAGAATAAGTGGGTCAAGCCAACGCCGATTCAATGCCAAGGC
TGGCCAATGGCAATGAGTGGACGCAATGTTGTAGGAATTGCTCAAACAGGTTCTGGAAAA
ACAGCTGCATTTTTATTACCTGCTTTGATTCATATACGAGCTCAACCTCCGTTAGCAGCT
GGAGATGGTCCTATTTGCCTTGTTCTTGTACCTACAAGGGAGTTAGCTCAACAAGTTGAA
TCTGTTGTTGAAGAATTTGGGATTTCGGGTGGTTTTAACATGGTCTGTGTATATGGAGGA
TCTGATCGACTTAATCAGATAAAAAGCTTGCAGTCAAAACGTCCAGAAATATTAGTTGCT
ACTCCTGGACGCTTATTGGATCTTTTGAAACAAAACGAAACTAACCTGAGGCGAACAACG
TATTTGGTCTTAGATGAAGCTGATCGAATGTTAGATATGGGATTTGAACCGCAAATTCGT
GCAGTTTTAAATCAAGTCCGACCTGATCGACAAATGCTTCTATGGTCGGCAACATGGCCA
ATAGAAGTCAGACGATTGGCTGAAGATTTTTTAAATGAATATATCCAAGTTAATATTGGT
GCTATGTCTTTACACGCCAACCATAACATTTGTCAATATGTTGAGGTGATTGACGAACTC
AGCAAAGAACGGAGATTAATTGATTTATTAAATCAATTTCGTGGTCTAAAAACACTTGTG
TTTGTGGCTTTGAAGAAAAGATGTGATAGAATGTCAGATATGTTAAATTCTAAAAGTTTT
AAAGCTATTGCCATGCACGGAGATAAGCCACAAAGAGAAAGAGAACGTGCACTTGAAAGT
ATGATGAATGGCATTATTAATCTTAATATCTGCGAAGCTTCTCTATTCAACATAGTTACT
GGAATCATAGAAATTGATTTGAGGAGGCCAGCCCATATCTCGTGAgccacagatatcttt
gctgacacagatattgatgatataatgttggtgattaattacgattttcctacccaaata
gaagattatgtacacagaattggaagaactgcaagacaagaaaagaaaggaacttcttac
actttttcacaaacaatgaaaagaaacatgcacagaaacttgttgctattctagaagaa
gctaatcagaatatttcagagaaattatacgatttagcagaagtaccattttcaagtcga
aagaaaccgacttctaatgtacctaaaggattagtcagtaattctgcaactaataactgg
ggagaagctaataaacgtgttccaagtaaatataatggaacgatttcgaatcgtgattct
tattatcaacctgtagaaagtgactataggaagtcagattattacgaatacaatggacgt
caaactgaaacagagtcttttactcacagtagtaattcatatcgagaagctgggaactgg
agaaataatggatcctataataattacaatcaacctcaaccagcatttaatggtagtaat
aataatactaataacttcaattctaacaaagcacctaattataatgaggatttcaattca
atgggaattattgctaacagatggtaaatgaaatctcctgtttgttgtatgtcttagtta
aactattaatttgtaacggaaattgttgtaattattttaataaatagtatatttattaat
aattaattattttaacaattttctttaaatttattaatttgtttaaaacataatctttc
catttacaataacaaacttttaaaatcgtaatatattaaaATGAACTACAGAAATAGAAG
AGATCGATCTCGTTCTCCAGTTAACAGATATGGAAACAGTAAATTTCAAAAAAGGCAGTT
TGACTGTGAAATTACAAAAATAGATTGGCCAACTATCCGCTTAATGCCTTTTAAGAAAAA
CTTTTACGTAGAGCATCCAATTGTTACCTCTAGAAGTTCATATGAAATCGAACGATTTCG
CAGTGAGAAAGAAATGACAGTTGTAGGAAAGAATGTCCCTCGACCAATATTTAATTTTAA
TGAAATGAATTTTACTAATTTTATTATGTCAACAATTGTAAAGAATAAGTGGGTCAAGCC
AACGCCGATTCAATGCCAAGGCTGGCCAATGGCAATGAGTGGACGCAATGTTGTAGGAAT
TGCTCAAACAGGTTCTGGAAAAACAGCTGCATTTTTATTACCTGCTTTGATTCATATACG
AGCTCAACCTCCGTTAGCAGCTGGAGATGGTCCTATTTGCCTTGTTCTTGTACCTACAAG
GGAGTTAGCTCAACAAGTTGAATCTGTTGTTGAAGAATTTGGGATTTCGGGTGGTTTTAA
CATGGTCTGTGTATATGGAGGATCTGATCGACTTAATCAGATAAAAAGCTTGCAGTCAAA
ACGTCCAGAAATATTAGTTGCTACTCCTGGACGCTTATTGGATCTTTTGAAACAAAACGA
AACTAACCTGAGGCGAACAACGTATTTGGTCTTAGATGAAGCTGATCGAATGTTAGATAT
GGGATTTGAACCGCAAATTCGTGCAGTTTTAAATCAAGTCCGACCTGATCGACAAATGCT
TCTATGGTCGGCAACATGGCCAATAGAAGTCAGACGATTGGCTGAAGATTTTTTAAATGA
ATATATCCAAGTTAATATTGGTGCTATGTCTTTACACGCCAACCATAACATTTGTCAATA
TGTTGAGGTGATTGACGAACTCAGCAAAGAACGGAGATTAATTGATTTATTAAATCAATT
TCGTGGTCTAAAAACACTTGTGTTTGTGGCTTTGAAGAAAAGATGTGATAGAATGTCAGA
TATGTTAAATTCTAAAAGTTTTAAAGCTATTGCCATGCACGGAGATAAGCCACAAAGAGA

AAGAGAACGTGCACTTGAAAGTATGATGAATGGCATTATTAATCTTAATATCTGCGAAGC
TTCTCTATTCAACATAGTTACTGGAATCATAGAAATTGATTTGAGGAGGCCAGCCCATAT
CTCGTGAgccacagatatctttgctgacacaggttaatcttgtgaatttgcattgatagt
aaaatcgatcgtttatataataaatttgttgatacttaggtatcagccctttctcatgg
gagggttggccacttcggaactactaaatatcaaaattttcataaacaattgtgttaaat
tctttactggaatgagtaaattattttattttagaaaccatgttcatttactagttgtcc
acaagtttattccaacaaaagaaatcaacttaattaaccacatttctagtatattattt
atctaaagttcaatttaaaatcacattaaattttagatttatatcaatataacatttaga
taatgaagtttttctaatatgtttcataggtttacttgattgaaacttaattttcatatt
attgagtttatttgaacagttaatccggtaaaataaatataaaaaatttcaaataattaa
atatttgaatccaagttttaataaattgtgattttaaaaatcatgtgtcgggtttaatgc
taaattcacgtgggtttatgacgtaaaataattaaataaaaatttcaaataaaattgtg
ttctaattgatttttgatttgttttaggtttcgtaattgtattaatttcattgtttaaa
aaaatttaatatcttgaaatttttgcttagttttggttccaggattgattgaatttagt
ctaaaatcagctccaagttcagatctgacagaattatacaatttcgctggaaaataatga
tttaaaaccaatttaataattatttaatcgaatgaatacacttaagtaggctacattttt
tcactcctatttcgttctattacaggaaagctgaatttttataaaatttcggaaaattg
aacatgttagttaagtgggggtaataatcgattggacaaatgtaaacttgagttcattac
atgtgaataataataagaaaataatttattaaaatttgtttaaaagcatttaatgttttg
gttattatatttttaataaatcttattatcaaagcagaaattacttaagaaaaatgatag
aaatgaagttattcagtgtttttaatcgattattataaaaaaaatttcaaatcaaaacag
aaaatttaaattgctttcactttaacgaccgtggttaaactttcaaaacacaaatatttt
gaatatatttttataaatttaacaatttagttacaaaagtacggctattgtagaattga
taatttaaatgtattaaatctttgaaaaaactttaaacatttcttctgaaatcctatggt
gtcactagattaagcattagaattttgtttcgttatttttaattagaaagattccagta
atttttaagagaagctagcaaaagtgaagataaaattattataaaggttacttgacttta
tgtagatttcagaaatggtagatcaaatattatggttgcaacagatgtcgcttcaagagg
attaggtatttaatatctatcttgaaaatgtttttaattcgtctttgctctcttaatatg
atgctgttcttaagagttttaaagaaagtttttacaaataaattcgcaattttattggaa
ctttaaccataaataattgtggatattatttgattaagtttaatatttattaatagattt
gcccaaatcattttttatttttagtttgaattttcaatgaacagtgttatttaagagag
ctataattgaaacttttatttattttcttataaattttatagatattgatgatataATGT
TGGTGATTAATTACGATTTTCCTACCCAAATAGAAGATTATGTACACAGAATTGGAAGAA
CTGCAAGACAAGAAAAGAAAGGAACTTCTTACACTTTTTTCACAAACAATGAAAAGAAAC
ATGCACAGAAACTTGTTGCTATTCTAGAAGAAGCTAATCAGAATATTTCAGAGAAATTAT
ACGATTTAGCAGAAGTACCATTTTCAAGTCGAAAGAAACCGACTTCTAATGTACCTAAAG
GATTAGTCAGTAATTCTGCAACTAATAACTGGGGAGAAGCTAATAAACGTGTTCCAAGTA
AATATAATGGAACGATTTCGAATCGTGATTCTTATTATCAACCTGTAGAAAGTGACTATA
GGAAGTCAGATTATTACGAATACAATGGACGTCAAACTGAAACAGAGTCTTTTACTCACA
GTAGTAATTCATATCGAGAAGCTGGGAACTGGAGAAATAATGGATCCTATAATAATTACA
ATCAACCTCAACCAGCATTTAATGGTAGTAATAATAATACTAATAACTTCAATTCTAACA
AAGCACCTAATTATAATGAGGATTTCAATTCAATGGGAATTATTGCTAACAGATGGTAAa
tgaaatctcctgtttgttgtatgtcttagttaaactattaatttgtaacggaaattgttg
taattattttaataaatagtatatttat
>DjScaffold3916:1254-5441 strand:+
TTATTTGTTGTTTTTTCTTTGGCTGTCAGCTTTGAATCACTAGAAGATTTTGAAGACGAA
ATTAGCCACGACGAAAAACGTGAAACTAAAGAAGAAGAAAGAAAAGTTGATGTTGAAGAA
ATAATCGACGATGCAGTTGAAAGTGATACTAAAGAATACGAAAGTGAATTAGAATCCAGA
AACGAAGAAACAGTTAAAGCAAATGATGATGATATTGACGTGCCATTCGAATAGaaaatg
atcatcactttataggaatgattggacacaaatatatgcaattatataaaaaatattattt
attaacaactatatttttttaatacattgtgctagtgaaaattatttgaattgtttacttt
gttgatttaaatataat
>DjScaffold529:17880-19582 strand:+
atgaataagaaaatgttgagaatattcaaagagaaaaataaagcaaatttattttataga
ttttaaaaatatgatcaattgatattcatttgtgaaaaaaaatataaaacacaatataat
gatATGAAAAATTTAAGAAAGAATCTTGATGATTTTATTGCACAAATGAAAAATATGACA
TTGACAATTGATAATCTTCACACGTGCTTCGGAACTCTATATGAACCCAACTGGCTGGGA

CGAATGGAATTTCAACAGTCTTGTCAGAATTCAATAAATGCATGGAGAACATTTATTGAA
GAATTCAAAAATAACATTCAAAAGGACATTGATAGTTACGCTGAACGTTTTCCTCATATT
CAAAAGTCCATTGACAAATGCAGTAAAAAGGCCAGTGATTTACAAAAGCATAAAACAATA
TATGAAACATGTAGAAGTAGTAAAAAGTCTGATAGTTCCAAAATTAAAAAGGCTGAAACA
GATTTTTCCAATGCTCAGGTCCAATACAAAGAAATTAATGATTCATTGAAAAAAGACTTG
CCTGAATTTTGTGATAGTCGAATCCAGTTATATCATCATGTTTGGTCATTTATGTTTCAA
TTAGAAGGTAGACTTTATCAAACTCTTCAAGAAGAAAGAAAGAATCAGTTGCTTATACTT
GATCAGTTTAATGAAGCTGTCAGTTCTGGTAAGTACCAAATTTCAAATCAGACGAATGCA
GCTGAAAATCAAACAAATTTGATATATGACAAGAAAAAAGCCGTATCTGTAATGAATTTA
TTTTCATCGGAGAGGCGAAATGTAGAAGTAAATTTAGTAAAAGGATCGAGTGAATGGAAT
ATAAACAATCAAGATTCAATTTATGATCAAATTCCATATAATCCCATTTCAAGAGAAGAT
GCCTCACTTTCTCGAACTCAAAGTTATCAAGTTGGTCAGAAAAATTCACGTGAAAATTTA
AATAAAATCTTTACAAATGCGGATACTATTAATTCGAAAACATACAAAGTCAAGGATTCA
TATCAGGAGGTTTTTTAAaaatatttttaataataatttaatcacttattcaaatgccga
tccaatgaaattatcagtggcagatttggatgatattgatttatcgccgaatctgaaaga
tttcaaggtcgatataaagtcatccgtggagactctgaaccaaacacttaccagtgatag
cgccagttcgatgtattctattgatagaaaagaaccgattacaaaaaagctttcgtttga
tacacgatccagttcaccaaaaagaacatcgatacctggagtaataatgaggccgtcacc
gtcaattcagatggatagaaataatgatgttgaattaaacaatggaggctatgtctatgc
tcaattttctgataagaagccgccggtttttaataggaattcttcatctcaatcacaaaa
ttcagaccttaaaaaaaccctcagtaaaaaagtttcaatctcactcaattccggtgattt
ttataatacaagttttgataatcagcattactatcacgttattgccaaattcaattacaa
tcggcaagatgatgacgaattagacttgaaaaaagatgatattattatagtattaccatg
ggaatgtgaagatcaaaagactaaacgggtcgatcatagaaacattaaaaatgcggagga
cggttggttatttggagaaaatgtagaaacgaaagcaaaaggacttttcccggaaaatca
atgaataagaaaatgttgagaatattcaaagagaaaaataaagcaaatttattttataga
ttttaaaaatatgatcaattgatattcatttgtgaaaaaaaaatataaaacacaatataat
gatATGAAAAATTTAAGAAAGAATCTTGATGATTTTATTGCACAAATGAAAAATATGACA
TTGACAATTGATAATCTTCACACGTGCTTCGGAACTCTATATGAACCCAACTGGCTGGGA
CGAATGGAATTTCAACAGTCTTGTCAGAATTCAATAAATGCATGGAGAACATTTATTGAA
GAATTCAAAAATAACATTCAAAAGGACATTGATAGTTACGCTGAACGTTTTCCTCATATT
CAAAAGTCCATTGACAAATGCAGTAAAAAGGCCAGTGATTTACAAAAGCATAAAACAATA
TATGAAACATGTAGAAGTAGTAAAAAGTCTGATAGTTCCAAAATTAAAAAGGCTGAAACA
GATTTTTCCAATGCTCAGGTCCAATACAAAGAAATTAATGATTCATTGAAAAAAGACTTG
CCTGAATTTTGTGATAGTCGAATCCAGTTATATCATCATGTTTGGTCATTTATGTTTCAA
TTAGAAGGTAGACTTTATCAAACTCTTCAAGAAGAAAGAAAGAATCAGTTGCTTATACTT
GATCAGTTTAATGAAGCTGTCAGTTCTGGTAAGTACCAAATTTCAAATCAGACGAATGCA
GCTGAAAATCAAACAAATTTGATATATGACAAGAAAAAAGCCGTATCTGTAATGAATTTA
TTTTCATCGGAGAGGCGAAATGTAGAAGTAAATTTAGTAAAAGGATCGAGTGAATGGAAT
ATAAACAATCAAGATTCAATTTATGATCAAATTCCATATAATCCCATTTCAAGAGAAGAT
GCCTCACTTTCTCGAACTCAAAGTTATCAAGTTGGTCAGAAAAATTCACGTGAAGATTTG
GATGATATTGATTTATCGCCGAATCTGAAAGATTTCAAGGTCGATATAAAGTCATCCGTG
GAGACTCTGAACCAAACACTTACCAGTGATAGCGCCAGTTCGATGTATTCTATTGATAGA
AAAGAACCGATTACAAAAAAGCTTTCGTTTGATACACGATCCAGTTCACCAAAAAGAACA
TCGATACCTGGAGTAATAATGAGGCCGTCACCGTCAATTCAGATGGATAGAAATAATGAT
GTTGAATTAAACAATGGAGGCTATGTCTATGCTCAATTTTCTGATAAGAAGCCGCCGGTT
TTTAATAGGAATTCTTCATCTCAATCACAAAATTCAGACCTTAAAAAAACCCTCAGTAAA
AAAGTTTCAATCTCACTCAATTCCGGTGATTTTTATAATACAAGTTTTGATAATCAGCAT
TACTATCACGTTATTGCCAAATTCAATTACAATCGGCAAGATGATGACGAATTAGACTTG
AAAAAAGATGATATTATTATAGTATTACCATGGGAATGTGAAGATCAAAAGACTAAACGG
GTCGATCATAGAAACATTAAAAATGCGGAGGACGGTTGGTTATTTGGAGAAAATGTAGAA
ACGAAAGCAAAAGGACTTTTCCCGGAAAATatgaataagaaaatgttgagaatattcaaa
gagaaaaataaagcaaatttattttatagattttaaaaatatgatcaattgatattcatt
tgtgaaaaaaaaatataaaacacaatataatgatATGAAAAATTTAAGAAAGAATCTTGAT
GATTTTATTGCACAAATGAAAAATATGACATTGACAATTGATAATCTTCACACGTGCTTC
GGAACTCTATATGAACCCAACTGGCTGGGACGAATGGAATTTCAACAGTCTTGTCAGAAT
TCAATAAATGCATGGAGAACATTTATTGAAGAATTCAAAAATAACATTCAAAAGGACATT

GATAGTTACGCTGAACGTTTTCCTCATATTCAAAAGTCCATTGACAAATGCAGTAAAAAG
GCCAGTGATTTACAAAAGCATAAAACAATATATGAAACATGTAGAAGTAGTAAAAAGTCT
GATAGTTCCAAAATTAAAAAGGCTGAAACAGATTTTTCCAATGCTCAGGTCCAATACAAA
GAAATTAATGATTCATTGAAAAAAGACTTGCCTGAATTTTGTGATAGTCGAATCCAGTTA
TATCATCATGTTTGGTCATTTATGTTTCAATTAGAAGGTAGACTTTATCAAACTCTTCAA
GAAGAAAGAAAGAATCAGTTGCTTATACTTGATCAGTTTAATGAAGCTGTCAGTTCTGGT
AAGTACCAAATTTCAAATCAGACGAATGCAGCTGAAAATCAAACAAATTTGATATATGAC
AAGAAAAAAGCCGTATCTGTAATGAATTTATTTTCATCGGAGAGGCGAAATGTAGAAGTA
AATTTAGTAAAAGGATCGAGTGAATGGAATATAAACAATCAAGATTCAATTTATGATCAA
ATTCCATATAATCCCATTTCAAGAGAAGATGCCTCACTTTCTCGAACTCAAAGTTATCAA
GTTGGTCAGAAAAATTCACGTGAAAATTTAAATAAAATCTTTACAAATGCGGATACTATT
AATTCGAAAACATACAAAGTCAAGGATTCATATCAGGAGAATCCAAACAAAGTTTTCAAG
AATAATTTAATCACTTATTCAAATGCCGATCCAATGAAATTATCAGTGGCAgttttatct
atatttagaagtccattgacaaatgcagtaaaaaggccagtgatttacaaaagcataaaa
caatatatgaaacatgtagaagtagtaaaaagtctgatagttccaaaattaaaaaggctg
aaacagatttttccaatgctcaggtccaatacaaagaaattaatgattcattgaaaaaag
acttgcctgaattttgtgatagtcgaatccagttatatcatcatgtttggtcatttATGT
TTCAATTAGAAGGTAGACTTTATCAAACTCTTCAAGAAGAAAGAAAGAATCAGTTGCTTA
TACTTGATCAGTTTAATGAAGCTGTCAGTTCTGGTAAGTACCAAATTTCAAATCAGACGA
ATGCAGCTGAAAATCAAACAAATTTGATATATGACAAGAAAAAAGCCGTATCTGTAATGA
ATTTATTTTCATCGGAGAGGCGAAATGTAGAAGTAAATTTAGTAAAAGGATCGAGTGAAT
GGAATATAAACAATCAAGATTCAATTTATGATCAAATTCCATATAATCCCATTTCAAGAG
AAGATGCCTCACTTTCTCGAACTCAAAGTTATCAAGTTGGTCAGAAAAATTCACGTGAAA
ATTTAAATAAAATCTTTACAAATGCGGATACTATTAATTCGAAAACATACAAAGTCAAGG
ATTCATATCAGGAGAATCCAAACAAAGTTTTCAAGAATAATTTAATCACTTATTCAAATG
CCGATCCAATGAAATTATCAGTGGCA
>DjScaffold1506:733-1170 strand:+
aactgaagaagacaatcacagaagtgataagacagggtgaagcgtgccaaaagagacaaa
agagacaacagtaaaattaagcgcaaattgtccatttgaaaagatatttatatatatgaa
aatatatatgtagaataatagaccaatacagtcgtttcgtaagcctaATGGCAATCAGCA
AGCAAGATGATGGCACAATTAAAAGAGTGATAATGGTAAAATGGATCCTGAGATTTATAG
CACCAAGAGAAGTTTATGTAGACAGTGGTAAGACATTTGAATCGAAAAACTTCAAGAATT
TGATGGAATCGATGAATATAGAAATATAAgtcattaccatcacaatacaaacggaattat
agagagacaattctgcacaattatagactacattaacg
>DjScaffold202:65493-72984 strand:+
aaatgtgtgttgtcatttacattaataataataatttttattaagaatgatttatgtcat
ctatttttaacaggaagaaATGAATCGAGACAGCACAGATGATTTATTCAAATACAATCA
TGGAGATGATGAAACACTTAATATGGATGCAATTCTTCCTGAGCAATTAAAAATTTTGTT
ACAAATGAATTATGAAATAGAAGAAAATTTTTTTGCATTTAAAAGATTGGCTGCTACTCA
ACAAATGCAAAATGTGAAAGATATGTGCGAAAAATTGAGAAAAAAGAAAAGTTCATTGAT
AGGCTCATTGCGCATTGCACATTCAGGAAATCTTGATGATATGGACAAAGAGTTACACGA
AGCCAAGATTGCTGTGGAAGAAGTTAGGAAAGATTTAGTTGAAAGATCACAAAGATGGGA
TTATATTGAAAAATTAACAAAATTCAAAATATTTCCTACACCCGGTTTATCAGCACTGCG
ATCAGCATTCGCAGCAACTATGAATGGAGAAAATGACTTCAACAGATGTCCAAATTGTTT
TCCATCGTTATGCATCCGACATTAAtgtaccataatctgaacaataaagatatatccact
ataaatccggtactactcccttttcaaaaaattcaatgatcatttcaccatcttcctctg
cactaagcacgctggtaactgcta
>DjScaffold202:157049-158167 strand:+
AAATGTAAAGATACATTCAAGTTGATAACGATAATTTTCAATGTAGCAATAAGCTTTATC
GGATTCGGTATAGTTATCGCTTCTGGAATCGGAATTTGCAGTAGTACAACGTATAATATG
ATTAATAGTTTTGACCAATTTTTGAATATCCGTCAATCTTACGGTATATTAATTATTATC
GGAGTTTTTATGATTTCTGTTGGAGTATATTCTCTTTATTTAATTTCAAACAAAAATTTA
AAACAATTATTCATATCGGTAATATTTTATTTTTTCATGATTATAACACTGGGTGCCATG
CTGTTTCATACTTGGTATGACAAAGAGATGCATATGAAAAAACGAATGGCAACAAATATT
CGATCTTACAATTGGCAAAATATTCATTCTTATCATTTGGATAATGTGCAGAAAACCCTT
GGTTGTTGTGGATATGGAGGTAATTTAATTGAAATTTCTGATTTGAAAAAAAGATTTCTT
TCAGAAAAAAAATCTTTAAATTAAcaatgaatagaagtttaacataaaagttttaaaaag

tagatttaaaatgtttacttttaaataggatacaaaataacacgatttacacgaatattt
tcaatagttaattacagtttttttaaaaataactacgagatttttgtgtgtaagataa
aagatctccggaaaaaagtcatggagttgattatttgtatatttctatgacagagttga
aatatttctttttgaactttatcaccaaaatttgattagcccttgtgtgagcgggtagga
aattattcattaattttaaaacactattagtatttttaagattgaaaataattcacatat
t
>DjScaffold1877:9031-9585 strand:+
cacactcgtaatggttctataaaatacgatgaattcgtaaatctattcttaccaagaaat
tgtcttcaaactttagacaaaccaaaattattctactttgcttgttgtctaggaggctta
ataaataaaatagataaatcttctaatcctgagaatattactgaaactaagtttgtaaag
ttgtgaaaacgttaaaaaaatcaaacatcttctggaatactcaaatgtatttATGGGATT
TTCTACAGTTCCAGATTATTACAGTTTTCGAGTAAATCACGTTAATCCATTTATACAAAC
TCTTTTGGGTGTTATTAGAAGAAATCCTGCATTAGAAGTTACAGAAATTCAAAAAATTAC
TCAGCGAGAATTGAGAAAAAAATGGGAAGATTACTTTTCAATTGCAAGTGTATTTCACTG
TATCACTGAAGACGTTTTTTTCATACGGAATAAAAATTAGtaatctgaaactttttatcc
tcggtttggcagaattcaaataaaatattcttt
>DjScaffold680:14694-19557 strand:+
caatttttcaaaaagcgaaataaaaactaagttattaattATGAAAAAACTAATTTTTAG
TATACTGGTCAGTATTTCGTTTTCACAAGTTCAAAAAGGTTTTATTTATGAAAGCTGCAC
AGGAAATAATCTCTTAGATTTTCCAGAATCACCAGGAGGAACTATATATTTTTCTCAAAA
GGATAAAAATATCTCAGCTAATGAATATGGAAATATGAACTACTCTTCTATTTTTAATTG
TATGGTTGTATTAAGAGGAAAGTCACAAGGATATTTTCCATATAAGAGATTTTTTGGAAT
ACACTTTAGATTATGGAATTTTGACACAAATTCTGGTTCAAGATATAATGAAACCGTAGC
ACAATTATATTATCAAAATGTATCGGCAGATATCAAAAGCATATGTGGAGATTCTTGTAA
AACTGGTTGTATGCAATTAATACATCCAGAATCTAAAACAAATGTTAATTTCTGTGGATC
GAATCAGCTCACTGGAACAGATTATAGAGAATAC
>DjScaffold680:23633-24164 strand:+
gcgaaataaaaactaagttattaattATGAAAAAACTAATTTTTAGTATACTGGTCAGTA
TTTCGTTTTCACAAGTTCAAAAAGGTTTTATTTATGAAAGCTGCACAGGAAATAATCTCT
TAGATTTTCCAGAATCACCAGGAGGAACTATATATTTTTCTCAAAAGGATAAAAATATCT
CAGCTAATGAATATGGAAATATGAACTACTCTTCTATTTTTAATTGTATGGTTGTATTAA
GAGGAAAGTCACAAGGATATTTTCCATATAAGAGATTTTTTGGAATACACTTTAGATTAT
GGAATTTTGACACAAATTCTGGTTCAAGATATAATGAAACCGTAGCACAATTATATTATC
AAAATGTATCGGCAGATATCAAAAGCATATGTGGAGATTCTTGTAAAACTGGTTGTATGC
AAGTACTTAAGTTTTTAATTAAATTTTGAaattagtaattttgagaggtcaataattaat
tgatcaaa
>DjScaffold680:65671-69469 strand:+
caagtaatattttaaaagagggtactgatATGGGATATTCAGAAACTGAAGTTAGAGAAA
TTTGGAAATTATTTGGAGTCTTTGACCAAGAAAATACAGGATCTGTTCAACAAACTGAAG
TTATGGCCATTTTACAAAGTTTTGGGCAAAATCCTACTCCAGATGAGGTCGAAAAAATTA
TCCAGAAAACTCAAAAAAAAGGAAATCAAAGGATAACATTTCTGGAATTTCTTCGACATA
CCAAGGATTTTATTAAAAATTCAGCAACTCTTAAAGATGAACTGAAAAATGCATTTTTGG
CGTTTGATGCTGAAAAGACAGGGTATATTTCTCAAAAGGAATTAAAGGATATTTTAACGA
GTCTTGGTGATAGATTGGATGATGAAGAAATAAATGAAATGTTTATGTTAGTTGATGTTG
ACAATGAAGGCAGAATAAACTACGAAGGTTTTATTAAGCAATTTGTGATTGAAAAATAAc
agcaatacattggacatttaatttgaacttcgaattgttattttacatttattgtaataa
ttattattgcttcatttatttctcacagttaattattaataaatataatataaattgt
>DjScaffold680:30282-30839 strand:+
ATGCCATTCAGCATAGCAGCAGCCCCAGGAACATTCCAAGAACTAATGACAAAAGTATTG
GACGGTCTAGATGGAGTAATGTTGTATATGGATGACATAATGATATTCTCAGAAGGACTA
AAAGTACACTATATAACTCTGGGTCAGGAATTAGAGCGGATCCAAACGGCAGGGTTGCGA
GTGAATCCAGAAAAAATGTCACCTATTACGTAAAGAAGTGAAATTTCTTGGCTACAAAATA
GATAAAGAAGGAATTCGAACAGATCCAATGAAAATCGAAGTTATCAAAAAATTTCGAGAAA
CCCAAATGTGTAAAGAACCTGAGAAGCTTCCTCAGAATATGCAATTATTACAGAAGATTC
ATCAAGAATTATTCAAAGAAATCAAGAGCCCTAGAAAAACTATGTGGAAGTAACAGCATG
AAGCTAATATGGTCAGATGGATGTGAGAAAGCTTTCGCAGAAATGAAACAAGCACTGATA
GAAGCTCCTATTTTAGGATTTCCTGATATAAAGAAGACATTTATTTTAGATACTGATGCC

AGCTTCGACACAATATGA
>DjScaffold680:76501-76839 strand:+
ATGCCGTTCGGAATTGCCGCAGCTCCAGGTACATTCCATGAATTCATGACAAACTTACTG
GGAAGCACACCTGGAGCTTTGGTGTATATGGATAATATTTTAATTTATTTCGATAATGCC
ACGGACCATTTCAAGACACTGGACACTGTGCTGAAAGAAGTAGCCAAAGCTGGAATACGT
ATCAACCCCAAGAAATGCAGTCTCCTGCTAAAAGAGGTAAGGTTTCTAGGACATATCTTG
GACGAAAATGGCATCCGAACAGATCTTACAAAAATAGAAAGTATACAGAACTTCAAGAGC
CCCAAAATAATCAAAACAAGTCAAAAGCTTTCTAGGTAG
>DjScaffold680:86403-134589 strand:+
AAAAATCAAGTAATCAATTTCTCTGAAAAATACAAAGAGATTGACAGTTGTAATAATTGT
TGTAATCAAGATGTGCCTATTTTTACTTCAGATAAAAGTGTTGTAACAACTAAAACTATT
CGTAGAACAAAACGAATAATAACGACTAAATTTATTGCAAAAAGTAGAAGTATGGACTCT
GAAACTTTGAAGGATTGCTTAGAAAAAGGAAATTTCCTAAATATTGAAACTGGGAATCCC
GAAATAACAATGGAAACTATTACAAGTGAAATTACAACATTAACTAAACCTGATCCCCAA
GATGAAAGTAAAGTGATCACTCATGTAATTTATGGATCTAGTGAAAATAGTAAATCTTTG
TTGACTGATTCTAATTTATCATTAAATGCCAGTTTAAATTTACCTTTGAATGAAAACTCT
CCAAGTAAAGTAAATATTCTAATTCCAACTGAAAATGAATCAATATTATTAGGAGTACAT
GGAAGTTCTGATAATTGTACGTCTTGCGATGAAGTATTATCAACTACCAATGAAGGCAAA
ATTAATAAGATATTTTCAAATGATAATTTAGATCTGCCGTCTAATCAATCTTTAATGGAT
TGTGAAACTTCTTATGATTGTCAGAACGCTAGTACACCGACAGAAACTCTAAATGATAAA
GGTGAAATAAAGACTTCAAATAGTATCATAGATGAATCGGATCATATGACTGAGAATATA
ACTAAATCAGCATCTTCAAATATTTTAAAATCAATGCAAAGTTCTCCATCTATTGCTGCC
CAATTTAACATCATTGCAAAGTCTAGAAAAACTACAGTACCCAGATGCAGAAGAGCGGAT
CCACCTAACAAATCAACTATTAGATATATGACTCAACCTGTAATTTCAGATAAACCGGGA
AGCGCTGAATGCATTAGAGATGGGGGGTATATAAATTTAACAGATTCTAGCTGCACAAAT
AGCGTTCAAGATATCACAATGATTCATGGAGATGATAAGTCTAATAACTCTTACGATAAT
TCAACGGCATCTTCTCATAACTTAAATAAATATTCCCGTTCTTTATCAGGTACTGCGAGA
ATAAGCAAAGAATCTATTGATTGTTTAGAGGGCAACACATCTCGGGCATATAATATTACA
AAGAAGGAAAATATTTCAGAGATTGATAATAAATCAGAAAAGAGTTACGGTAGTGAAGAA
CTGTCAGCAAGTCCAGAAAATAATGTTGAATCAGTATTTTATGATGAATCAAAAGGCACT
TCTGCCAAACCCATACGTCGGAGTGAGCGTCATTTATCTGGTGGATCTGGTAGCTTCAGT
GGTAACAGAACTTTTCAAAGTAGTTTACCTGGAACTCTTAAAAGACCTACTCCACAACAT
ATTCGTATTCATAGTGATGAAATTAAAGCTCCTTCGGTAGCTATTTCGGATTTAACTTCA
GAATATTTAAATAGAAAAATTTCACAGACTGACAGAAAAGATATTGATAATTCCTTATCA
ACAGGTCCGATGAGTTCTAGATATTCACCTTTTAGACACGGAGTTGATTCAGGTGGTCTT
GATAATGTGGTTCTAAAGTCGCCAAGTGCCCAAGCATTGATGCGAAACAATATTAGTAGA
AATAGATCATCAACAGTAAATGCTCCAAATAATTCGAGACAAAGTCACCGATCAATAAAT
AGCAGTGAAAGAACTTTGGAAAAATCAAAACAGGATGAAGATAAAGATTCAAATTTTAAT
TCAACAAATTTAACAGATTTTTGTGAAGAAAATAAAATAGAATCCAATCAAACAATTCCA
GACGAAACCATTTTAGAGGAAGTCGACGATAATTTAACCCATGAAAAGCAAAAATTGGAT
AAAAGATTCTTAATAATTCAAGAGCTATTAAGTGTGGAATCTGAATATGTCAAAGTTCTA
GAAATTTTAGTGAATCATTTCATGATACCTTTGAAATTAGAAAAAATATTAGATGAAGCT
TCAATTGATACTATTTTTAATAAAATTCATGAATTAATGATTTATCATGCTGAATTTTTA
TCAATTTTGCATATATGGGAAGTGAAGGATACTATTGGGGATAAAATAGCTGAAATGTTT
TCCAGAACTACTGTCCAAGACTGCTATATTTCATTTATAGAAAATTTTTCAATGTCAGAA
AAGCTTCTTGAAAATGCAATTAATTCAAAATCATCATTGCTAAAATTTGTCGAACAAAAA
CAAAAGGAAAGTAAAAATAAATTACCTCTTAAAGCTTTAATGGTTCAACCTGTTCAAAGA
ATACCTAGATATGAACTGCTACTTAAGAGATTAATTGAATACACACCAACAGATTCTATT
GATTATAAATATTTGTTAGATGCACAAACAGCTGTTCACCAATTAGCTATTAAAATAAAT
GCAGTAAAGGAAGCCAAACAAGATGAAACTTTTATTGAGGGGCAAAAATTACTTGATCGT
TTACTCTCACCAATGACAACTAATCAAAACCGGCAGTTTATACGCCATGATTCAGTAACT
ATTGAGGATCGAAAAGAGGCATATTGCTTGTTTTTATTTTCCGATCAGATTATTGTGACA
ACGTTAAAACGGAGAGGAACTCAGACAACAAAAAAGCCACCTGTTTTGAGACTATTGACT
CCAGCTGGTCTAGACTTTATTGATAATGTAAAATACAAATTTATATCTCGAATAAATATA
GAAAATATTGAATTTGAACAAAATGAAGACACTGATCAAAAGGTTTTCTTAAGAAATCGG
GAAATGAAAGACAAAAAAGATCTATCAATATTGCAAGAGATCGAATCTTTAGTGTCAAAG
CTTGATCGCAAACATCAGGATATAGATATATGCATCAAGCGATTGTATGCAAATGTGCTG

CAAGATATGGAAGAATCGAGAAATTCTAGGGCTGCCCCGACTGTGAATAATTCCCTAGCA
TCTCAATTTCTAGTTACAACTAAAGAAGGTATTGAAAGAATGGAATTCACTTTTACGTCA
ATGGAAAAAAAGCAAAGCTGGGAGAAAATTGTGAAAGAATTAAAAACAAAATTAGTTGAT
GTACCGAGAACACCACGTTTCAACCAGGCTCTTTCAATAACTAGAACACAATCTGGAATG
CAATTTTGTTGCGCTTGTCCAATTGAAGGATATAATAATTTTGGTTTAAGAGATGTTTGG
ATATGTCAATCAGATGGTTTTACGGGACATTTATGTTTATTAAGTATGCAAATTGATCCT
ATTGTCACAATGAATAGTCCATTACCCGGTTGTAATGCTAAAGTTATTTGTATTACAGCT
GTTCCGGGATATCTAGGTTCTACCAGAAGATCATCATTGTCTATGGATGGAAGATATAGT
AGACCGATTGAACGTAAAATAAGACAAGTTGGGGATGCTTATCAATTGGATACGCAAATT
GAAACTCAGGAAGACGATGGCGAAGCTGAACAAGATCCATCAGCAAACGCAGACGATTCA
AATACGTCACAATCTTGTTCAGAAAGTGGAGAATCAGAAACAGACGAATCAAATAATAAA
TTAATAAGAAAAATAAGTTCAGAAGAAAGTCCCAAGAATAAAAAACTTAGTGATCCTTTC
AAAGCAACAATGTGGATTGGCTTGGAAGATGGAATAATTTTAGTATTCTACAGTGCTGAT
AACGTGAGAATGACAAAATCTAAGAAAAAAATTGATTTGGGATCAGCTGTGATCAGTATG
CTCCATTTTGACAATAAAGTGTACGTATCTCTTCAAAATGGGGAATTGGTGACATTTAAA
AGACATATAGAAGGTTATTGGGACTTAGATTATTCTGTAATTACCAAATTAACACCGCAA
ACTTCTCCAATAAAAAATCTTATTGCTGTTACTGGATTCATTTGGGGAACTTCACAAAGA
AATATAGTTATTATTAATCCAACATCAGTTAAGATTGAAAATACATTTACGTTATCGGAA
AGTAATGATCAGCGTTCAATATCACATCTTGCATGTTCTGGTCAGTGCGTTTGGATTACA
CAAGAAAATTCATCGAAGATTCTGTTATATCATGCAACCTCCTATGAGTATCTCTGTGAA
GTCAATATTCGTCCAGCAGTTGTTCAGAGATTACAAGTGTGGGATGATATTATCATTCGT
CACAAAGAAGCTTGTTTGAGAATAACTTGTCTTCTGGTTTGTAAAGATGTTCTTTGGGCT
GGTACAAGTGCCGGTGTTATTCTAACTATTCCAATTCCTAACGTCACAACATCTACGACA
AAGGCAACTATTATTCAACCAAAAGTCGAATCTCTCGACAGAGGTCATACTGGAAATGTA
AGGTTTTTAGTAAGCTTAGAAAGTTCGATAAGGCCATCTCATCAGTCATCAAGAAGGCAA
TCATTAAGTCCATGCACAACAATGGCAACTCAAATGAAAATTATTTGCGGAGGTGATGGA
TATGAAGACTTTTCGAGTAAATCAACTCGTGAAACTAATCAAGACGTTGGTAAAGATGAC
GCGATAAATCATATTCAAATATGGGACATTCAATGAttataagagcatatgaaagacatt
ttatgaaatcttatatattcttaaatctttagattatcagaaaacattaatttgttactt
taagtattgagtataaatattttacaagcattttcattattaaataaaatttattcgttg
tca
>DjScaffold680:215733-216244 strand:+
agtcaagaagttgaaaatacaatattgtaatgaatgcaataatATGCTTTATCCAAAGGA
AGATAAAGAAACTAGGTGCTTATTGTACGCATGTAGGAATTGTAACTATAGTCAAATTGC
CGAGGAATACTGTATATATGTTAATAAGATTGAGATGAGTGTTGATGAGTTGTCCCAAAT
ATGTACGGATGTCATTCATGATCCAACTCTTTCAAGAACTGAAGACCATATTTGTCCTAA
ATGTAAAAGAAAGGAGGCTGTATTTTTTCAATCTCAATCTGCCCGAGATGAAGATTCCAT
GAGACTTTATTATGTATGTGTTAATGATAGTTGTCTTCATCGGTGGTCTGAATAAaatct
tttgttaattgaatagtaaatcagatttaataatttagaaatattgaattttataggttg
aatttataatttttaaataaatactaagattaattgaattatatatact
>DjScaffold910:31915-51527 strand:+
ttatgagtttattttaatatctgaattaacgaggcagatttgcttttgaaattgaataaa
taaattttggttacagtcgatttttacttattgttctcaacatattcataataaatgaat
taattaaattttattatttaattaataatttatttttaattctcacacaaattaattctt
agaagttgattttttttctaaagaaaaatatttactgaaactttttatattcagaattg
gaaattctgaacatatcagaagagatgtgtcgtatgattctATGATTCAAATGAAAGATA
TTCAACCGGAAATACAAACTGGTAAGAAATTTAGAAAATTTTCTGCAATTGCGATCACTC
CTAGAGCGAGTTATGCTGGAAATTCTTTTGATATTTCAGAAGATCAACTAAGTATAAAAG
TATTAGAGGATTCAACAATACCTCAATTATTTGTTGAGTTTGATTGTTTGCAAATCAATC
CACTAACAAATACGTATTGTTGGATTGAAAAATCCCGTTGGTTACGTTATGAAGAAGATT
ATGATCTTGATTTAAAGCGTTTCAATAATCCCCATTTGTCTGCAATGTCGTTAAGAGGTT
TAATTGGATTCAAAAAGTTTTTCAAACAAGGGGATGCAATTCTCAATTGTCAAAGTGAAA
GATATACAGATATTTTACAAACCCTATCTGATAGAATAACGAAATCTGGAAATTTTACCA
TGCAAGAAATTGCCGATATCAGAAATGCTCTGGTTTTAAGACATTATCATGCAGGCGAAC
AACTCGCAGCAACAGCTAATGAGGCTTCTATGTTTAAATATGATTATGAAAAGCAACGCT
TTGAAGTAAATGATGCTGCGCAACAAGTAACAAATCGCAAATGGAGTATGGCTTCAATGT
ACGAAGGTGGCCAAAGAAAATTAAGTCGCATTCGACTAAACGAACGACTTGATCCTAATA

CGGAAGCTCTTTCTGTTCAAGTCGGGGCTTTAGACAGCTTCAAACATATTTTTTCGTTAT
TTGTTCGATTGGATCCGCCAATTCGAGCTGACGGACTTAACGAAGTCAATTGCCCACTGC
GTTTTATATATATTTTATTTGGACCGAAATCAATTGAAGAAGAACTGATGGAAGTCGGTA
GAGTGTTTTCAATTATGATGTCAAATCCTACATTTCGTGAAATCGGTTATTCTGCTGAAT
CTAAAGAAGAAATTTTGGAAGCTACAAAAGTATTTCTTCAGCATTCAGTTATTGTTCCAC
TGACAAAAGTGATCACCAATGATGCTTTATCAGCTATGTTACCCCAGCTAAAACATTACT
CAAATTTTTTTCTGAATGAACAAAACAAAAAAAACATTTGAAGAATTCAAGCAAGAAATGT
ATAATAAAAATCAAGATTTGTTTATAAATCAATGCAGTAACGGTGTAAATCGTAAGATAT
CTGTTAATCAAGCTATTAATCAAGCAAATTTTACTGAAGTTGATGGTAAAATAAATTCAG
CTATAACTTCATCGGAAAAAATAGAAAAATCGTCAGTAACATTTAAGCAATGTTGTTTTC
AATTTTTTCCACCTTTCAAGCGTGTAATACTTGATCTGAAAGGATTGAAAAAGAGATACG
TTTCCGATTATAAAGATGCATTTTTACCGGAAAATGTTTCTACTGTATTTATCGGAATGA
TATTTATCTACTTTGTTGTACTTTCACCTGCTATTACTTTCGGTGCTCTTTTCAATCAAC
TAATTACAAAAAAAATGTCTGTCAGTATGTGCCTTGTGTCTAGTGGATTTTTCAATTGTT
TTATTCTCTATTCAATGGTCAGCCACTAGaaatcgttggtataacgggtcctgtttatgt
agttgaaaaagttcttggatcactatcaaaaagtctcaatattgaattggaagttatgcg
gttctggatcggtatttacgtttcaattctgggatttctgttcactatgtttaacggagt
cttaattataagacattttcgaagatctgttgaagaagtatataatttcttcgtcggaat
aatttttattttatcggctttgtttactatgttcaaagcgcttccctacagttcaactcc
aatcaatggaatttatacaaatgtcacagttattgatttggagagattaaaattcggtcg
tggtggtactacacttttcttggcttttctaatgctaaatttctgtcttctcatgataca
aatcaaacggggctcatttttcgacgtcaaatacgaaacttgttgggatcattcaatgt
tccactcggaataattcttgtggtcgttattgaaagaattttttccaagcgtttaatat
gccgactttaagtgtgcccccaagtaatcaaatcaaaccgacgacgtggattaacccacc
aaacttttcatcagttgctaatgcaaaaaaagaagctcatctcttttgttttgctattag
tttgagtttatttttcattctaatcgttgaagtagcaacaaattcaatttccgttactaa
agcagaaagaaaattggcaaaggaaagcatgttttcattgaacttttttatattcttgt
catatttcctgttatatcggcttttctcggatggcctttcgtatctggtgctatggttag
atcgaatgctcactgtattgctatggctaaatggagtataaataatcttcccggagtttc
tccaaaagttattggagcagctgagcaaagggtttctggactaatagttggtgttcttgt
attactttcagtatttttggggtcagcattaaaatacattcctatggctgctatgtacgg
aatgtttctatatatgggtgttatgggtgttagagacttggttgtaaccaggcgtgttaa
aaatcttttccaacgaagaaagcattggcatcagcatgatttcttaaaggattttccttc
atttcatttgttaatactatgttcaatacaagcatttttcattgggatactgatagtttt
aaattgtttatcagaatttacaatttattcgtggacaccacttattttcccatttttcat
aggtttgtatgcatttatcagaggatacattctgcccctctggcctagacttaataccca
tttatccaaagtttgtttaaatac
>DjScaffold3400:36332-40935 strand:+
taactcaactcaacacaacatatttaatgagaacattaactatttacaattacaatgatc
aaataactggttaagtaaaatggcaaatcaaaattctagaaaagtttaattgttctctaa
attacgaaaattgctgtgaaaaatcatgaaagaaacactgataaaaagaaagaatgaaat
atttatattttgtcattacaagtcatattaatcaatttcatctctaataacttcatcatt
tatattttcttgattattaattaataaatcttctatccaaccaattactgtgaatataat
tatgaatattattatcgatacacaaaATGATCCTCACACTCAAATATATTTCTCTAGTTC
CATCGATTATTGACGATGTAAAGAAATTGGGACTAAGAAAATTAGGTACAATCGTTTATT
ATGGAGAAAATCGCAAAGTTATTTTTGATCGAACTGGATTGAAAACGATTCTGGATGATT
TTGATTTGATCAATAATGATTTGATTCAACAATTTAATCCTCATTGCATTGTTATTGGTC
GATTTCCTTTTGTACGCGATGATTTAATTGATCGAATTTCTGATGCAGTTGGATATTTCT
CTCAATCGCTATCATCCATTCCTGCAGGAATCATTATTCATCATCAATGGGGATTGATGG
ATAATGTTGAAGTCGGAAAATTCCTTGTCAAACATTTTATTCCACATTTGGATTCCATTG
ATCTTGGCCTTCAAGAACTTCATCAGTTGCATTCTCTAGTCCGAAAAGGTGATTTTAACA
TATCTGCATATACGAGTGATATCGAAACGAGTTTGGATATTCTTCAAGATCTTTGGAATA
TCTTGAATTCTGGCTCTTTCTCAGTAAGAACTGGAACACGCAAGATATCACGAATTCATC
TTCGTGCTCATGACTATCATATTTTACTCACAAATCCAGATACTCAAATGTTTTATTTGA
ATCTGAAATATGCTTTAGCTAAGGGTTTGATGGCACAATTACATGCGCGATGCAATCGGA
AATTTGCTGTTTTGAAGGAAATTGTCCATTTCTATTTGGAGGATGAATTTTTCATCTCAA
ACAATTTAACAGCTTCTCCTATTCAATGCTGGAAAAATACGAGAATGTATGAGATTTGTG

TATCACCAGTGCCGACATGTTTCGAGTTGAAATATCCATTTGAAAACGGAGAATCGACAG
CAGCTGCAGCTCTTGCTGCACAAATCAAAATTCAAACTGAATGAttttttattttaacc
tttattttttgttaatttttcgaaaatattatttattgtttgtttttaataataactcaa
ctcaacacaacatatttaatgagaacattaactatttacaattacaatgatcaaataact
gctgaactcgatcatcgatctcatttttcaagttaagtaaaatggcaaatcaaaattcta
gaaaagtttaattgttctctaaattacgaaaattgctgtgaaaaatcatgaaagaaacac
tgataaaaagaaagaatgaaatatttatattttgtcattacaagtcatattaatcaattt
catctctaataacttcatcatttatattttcttgattattaattaataaatcttctatcc
aaccaattactgtgaatataattatgaatattattatcgatacacaaaATGATCCTCACA
CTCAAATATATTTCTCTAGTTCCATCGATTATTGACGATGTAAAGAAATTGGGACTAAGA
AAATTAGGTACAATCGTTTATTATGGAGAAAATCGCAAAGTTATTTTTGATCGAACTGGA
TTGAAAACGATTCTGGATGATTTTGATTTGATCAATAATGATTTGATTCAACAATTTAAT
CCTCATTGCATTGTTATTGGTCGATTTCCTTTTGTACGCGATGATTTAATTGATCGAATT
TCTGATGCAGTTGGATATTTCTCTCAATCGCTATCATCCATTCCTGCAGGAATCATTATT
CATCATCAATGGGGATTGATGGATAATGTTGAAGTCGGAAAATTCCTTGTCAAACATTTT
ATTCCACATTTGGATTCCATTGATCTTGGCCTTCAAGAACTTCATCAGTTGCATTCTCTA
GTCCGAAAAGGTGATTTTAACATATCTGCATATACGAGTGATATCGAAACGAGTTTGGAT
ATTCTTCAAGATCTTTGGAATATCTTGAATTCTGGCTCTTTCTCAGTAAGAACTGGAACA
CGCAAGATATCACGAATTCATCTTCGTGCTCATGACTATCATATTTTACTCACAAATCCA
GATACTCAAATGTTTTATTTGAATCTGAAATATGCTTTAGCTAAGGGTTTGATGGCACAA
TTACATGCGCGATGCAATCGGAAATTTGCTGTTTTGAAGGAAATTGTCCATTTCTATTTG
GAGGATGAATTTTTCATCTCAAACAATTTAACAGCTTCTCCTATTCAATGCTGGAAAAAT
ACGAGAATGTATGAGATTTGTGTATCACCAGTGCCGACATGTTTCGAGTTGAAATATCCA
TTTGAAAACGGAGAATCGACAGCAGCTGCAGCTCTTGCTGCACAAATCAAAATTCAAACT
GAATGAttttttattttaacctttattttttgttaatttttcgaaaatattatttattg
tttgtttttta
>DjScaffold3737:19712-26614 strand:+
TTAACTGTATCTTCAAAAATCTTAAAGCAATTTAAAAGATTTATTACAGTTAATAATAAT
GCCGTAGATGTTTCTAAAATTCGAAATATTGGTATTTCGGCTCATATTGACAGTGGCAAA
ACTACGGTTACAGAGAGAATATTGTTTTATACTGGAAAAGTGAACACTATGCACGAAGTG
AAAGGTAAAGATGGAGTTGGTGCAGTGATGGACTTTATGGAACTAGAACGACAACGAGGC
ATAACAATACAATCTGCCGCTACCTACACAGAATGGAAAGACCATGTAATTAATATTATT
GATACACCGGGACACGTAGATTTTACTGTTGAAGTTGAAAGAGCTCTCAGAGTATTAGAC
GGTGCTATTTTAGTTCTATGTGCAGTTGGAGGTGTTCAAAGTCAAACAATTACTGTAAAT
CGACAAATGAATAGATATAAAGTTCCCAGAATTGCTTTCATTAATAAATTAGATAGAATT
GGAGCTAATCCTTACCGTGTTTTGACTCAAATTCGATCGAAATTAAAGCATAATGCTGCA
TTTATTCAATTACCAATCAAACTAGAGTCTCAAAATGAAGGAATTATCGATATTCTTAAA
GAGAAAGCAATTTATTTTGATGAACCTTTTGGATTGCAGATTCGAGAAGAATGCGTTCCG
GATTTTATGATTGAAACTGTTAAAAAGAAACGATATGAATTGATTGAATGTCTAGCTAAT
TCTGACGAAGAAGTTGGAGAGTTATTTTTAACGGAGAAACCAGTTTCAAATGAACTCCTA
ATGAAAGCAATCAGACGAGCAACATTAGCACAAACATTTACTCCGGTTTTGATGGGAACC
GCTTTGAAAAATCGAGGAATTCAACCGTTATTGGATGCAGTGGTTGATTATTTACCGAAT
CCAACTGAAGTGGAGAACTATGCGATGCCAGCAGCAACAAACGAATCGAAAAATGGAGAA
AAAGTTGTTCTCAGTTCCGAGCGATCAGATAAACATGAATTATTATCACTGGCATTCAAG
TTGGAGCTCGGTAAGTACGGCCAATTGACATTTCTGCGCATCTACCAAGGCAAACTGACC
AAGGGCGACATGATTGTCAACGTGAGAACAGGTAAAAAGTTCAAAGTATCGAGGCTTGTG
AGGATGAATGCAAATTTTATGGACGATCTCAATGAAACTTACGCTGGAGACATTACAGCT
CTTTTGGGGATCGATTGTTTCTCTGGGGATACTTTTATGGGCGCCAATTCGAAATCTCTC
CTTTCAATGGAATCCATGTTCATTCCCGATCCAGTTGTGTCGATGAGCATCACGTTGAAG
GACAACAAGAACGCAGATAATTTCATGAAAGCTATGAGGAGATTTCTTAAAGAGGATCCC
ACTTTTAAAATGAGTTACGATGAGGAGAATAAAGAAACAATAATTCATGGAATGGGAGAA
TTGCATTTGGAAATATATTCGCAGAGAATCGAGAGGGAATACAACGCACCGTGCACTCTT
GGGAAACCTAAGGTTTCATTTAGAGAAACATTGTTTGAACCAATCAAATTTAATTATCTA
CACAAGAAGCAGTCCGGTGGAGCTGGACAGTTCGGTAGAGTAATTGGTGAAGTTATACCA
TTAACAGGAGATCAATTAACTACAGTCGATTTCGTTGACAAAACCATGGGTTTACGTTTG
CCGAAAAATTATATACCGTCAGTTGAGAAAGGTTTTCGACTAGCATGTGAAAAAGGTGGA
TATATTGGCCAGAAAATCACTGGTATTAAAATGATTCTGAGGGATGGTGACTCCCATTGT

GTGGACAGTAGTGATTGGTCTTTCCAACTTGCTGCCCAAGGAGCAATGCGTCAAGGTCAT
ATTTTATTTGAAATTCATAATTATATAAATTTCATTGCAAAATTAAAAATCCTAATGTAT
TTATAGaattgggaaaaaattattttaatacctaaagttactgagatataaatcaatatg
ttaataggttaataataatgccgtagatgtttctaaaattcgaaatattggtatttcggc
tcatattgacagtggcaaaactacggttacagagagaatattgttttatactggaaaagt
gaacactATGCACGAAGTGAAAGGTAAAGATGGAGTTGGTGCAGTGATGGACTTTATGGA
ACTAGAACGACAACGAGGCATAACAATACAATCTGCCGCTACCTACACAGAATGGAAAGA
CCATGTAATTAATATTATTGATACACCGGGACACGTAGATTTTACTGTTGAAGTTGAAAG
AGCTCTCAGAGTATTAGACGGTGCTATTTTAGTTCTATGTGCAGTTGGAGGTGTTCAAAG
TCAAACAATTACTGTAAATCGACAAATGAATAGATATAAAGTTCCCAGAATTGCTTTCAT
TAATAAATTAGATAGAATTGGAGCTAATCCTTACCGTGTTTTGACTCAAATTCGATCGAA
ATTAAAGCATAATGCTGCATTTATTCAATTACCAATCAAACTAGAGTCTCAAAATGAAGG
AATTATCGATATTCTTAAAGAGAAAGCAATTTATTTTGATGAACCTTTTGGATTGCAGAT
TCGAGAAGAATGCGTTCCGGATTTTATGATTGAAACTGTTAAAAAGAAACGATATGAATT
GATTGAATGTCTAGCTAATTCTGACGAAGAAGTTGGAGAGTTATTTTTAACGGAGAAACC
AGTTTCAAATGAACTCCTAATGAAAGCAATCAGACGAGCAACATTAGCACAAACATTTAC
TCCGGTTTTGATGGGAACCGCTTTGAAAAATCGAGGAATTCAACCGTTATTGGATGCAGT
GGTTGATTATTTACCGAATCCAACTGAAGTGGAGAACTATGCGATGCCAGCAGCAACAAA
CGAATCGAAAAATGGAGAAAAAGTTGTTCTCAGTTCCGAGCGATCAGATAAACATGAATT
ATTATCACTGGCATTCAAGTTGGAGCTCGGTAAGTACGGCCAATTGACATTTCTGCGCAT
CTACCAAGGCAAACTGACCAAGGGCGACATGATTGTCAACGTGAGAACAGGTAAAAAGTT
CAAAGTATCGAGGCTTGTGAGGATGAATGCAAATTTTATGGACGATCTCAATGAAACTTA
CGCTGGAGACATTACAGCTCTTTTGGGGATCGATTGTTTCTCTGGGGATACTTTTATGGG
CGCCAATTCGAAATCTCTCCTTTCAATGGAATCCATGTTCATTCCCGATCCAGTTGTGTC
GATGAGCATCACGTTGAAGGACAACAAGAACGCAGATAATTTCATGAAAGCTATGAGGAG
ATTTCTTAAAGAGGATCCCACTTTTAAAATGAGTTACGATGAGGAGAATAAAGAAACAAT
AATTCATGGAATGGGAGAATTGCATTTGGAAATATATTCGCAGAGAATCGAGAGGGAATA
CAACGCACCGTGCACTCTTGGGAAACCTAAGGTTTCATTTAGAGAAACATTGTTTGAACC
AATCAAATTTAATTATCTACACAAGAAGCAGTCCGGTGGAGCTGGACAGTTCGGTAGAGT
AATTGGTGAAGTTATACCATTAACAGGAGATCAATTAACTACAGTCGATTTCGTTGACAA
AACCATGGGTTTACGTTTGCCGAAAAATTATATACCGTCAGTTGAGAAAGGTTTTCGACT
AGCATGTGAAAAAGGTGGATATATTGGCCAGAAAATCACTGGTATTAAAATGATTCTGAG
GGATGGTGACTCCCATTGTGTGGACAGTAGTGATTGGTCTTTCCAACTTGCTGCCCAAGG
AGCAATGCGTCAAGGTCATATTTTATTTGAAATTCATAATTATATAAATTTCATTGCAAA
ATTAAAAATCCTAATGTATTTATAGaattgggaaaaaattattttaatacctaaagttac
tgagatataaatcaatat
>DjScaffold3144:37593-38042 strand:+
AAAAAAGTCGAAAATTACATTTATAATATTGAAGCGAAATTCGGAAGGCAAATTAAAAAT
GGAGAGCAATTGACAAGAGCTTTAAATATCTTGAAGATTAAAGAGGATCAACGGTTATTG
AAAATAATTGAAAATAATGAAGATGCTTTTATGGCAGATAAATGGGATGTAGGACGCACA
CATCTGACAAGACACAAAATAATCAAAAGTAGTGAACCACTCAATTTAAAACCGTATAGA
CAACCAATGAACTTAGAATCTAAAATTGAAGAAGCTATCCAAAACCTTTTTCAGAATAAT
ATTATCCGAAAATGCAACTCTCCGTGGAATACCCTACTAATATACAGTTGAatgcagtaa
caaaaagacaagcctttccgaggcttaatttcgatgaaatg
>DjScaffold919:186-3992 strand:+
agtaacaggatctaacatttatgttataaattgttgtgataatactaaATGATAATACAA
ATAGAATGCGGGCTTTTAAATAAAAATATCGATGTTTCTCAGAACATCAGTGTTTCAGAA
TTGCGACAACAAATTGCAAATTTGTTCGCATTGAAACTAGAAAATTTTTGCTTACAATAT
GCACAAGTTTATCTGCTAGATAGCAAATATTTATCAGATTACGGAATTATGGATAACTCT
GTTATTAGAGTAGCAAATCAGAAAATAGATTTAAAGATTATCAATAAAGATTCCGAAGTA
ATATTAAAACAATTGGATATCAACATGAAGGTTGGCGATTTAAAAAAACAATATTCTCCT
GCTAATTGGGAAAATATCAAATTTTGTTGTTATTATGAATATGATGTCGACAATCCAAAC
AAAAATAAAGTTGATATGAAAGTTATTGATGCAAAACTCAATATAAATGATTTGACAACT
GAAGAACAAAAAGAAGAAATAAGGCGGAAATATTTACGACGATATCATGGAGATAGACGA
TATTATTTTAAATATGTAAATTTGGACAAAAATGAGCAAACACTTGCATATCATCAAATG
AATGATAATTATAAAATTTACATTACATTTCAAGTTTTTATCAAACATCTTGGAATGAAT
TATTCTTTGGAGGTGTTTGACAAGCTTTCGATTCATGTTTTTAAAGAACGTATCAAGGGT

AAATTAAATGTATCAAATGTGGAAATTGGACTGATTTTCTGTGGTGAAGCTCTCCAGGAT
TCAAATGTAATAGGTGATTACGATATCCAACCAAATGATTATATACTAACAACAGCGACG
ATAATTGGTGGAGCTTAAaaaattatatcatttattatttgttatcttacaatttgttt
cacttactttagttttcaattaatatattaatttattatttattaattttagtgtaatgt
gttacaattctttatgatggattgtaatttaagtgtgattgattataatttttatgtgct
ggcttagaaattgtatgtgttgacttaaaaatttacttgttcagtgttaatttccatgaa
tcaaataagactttgtgagatggattataattttaattttttactgctgacctagaaatt
>DjScaffold4972:48529-49079 strand:+
cgaattagaattaaacatatttaatgaaaagaattctgaatattttgggagtattaaatt
agctgaaaacttatttATGAATATAATTAATTCAAAACAAATGATAATTTTGCATAAATT
AATTTCTACATCATTAATTATCAAAAGCCACCCGAAAAGAATTATTAAAAAAGGTACTAA
AAGTCAAACTGGCACACAATTTGAATCCTCAATAATGTGTTTACTTAATTCGGATTATAT
AACATTTACCGGAGTTGAAATTTCTCCAGTTTTACTTATGTCTGGTGAAGAAAGTTTTCT
TGAACACAAGCTTCAATTTTTTGCAGAAAATCCCAATTTATGGATATTAAAAAACATGAG
ATGGTGTTCAAGATCTGTTGGTCGATCATCTAGAAAAAGTACATGTGATAAATATGTCAT
AAGATTTAAATTTTCTGCCAAATATCTTTGTAAACATATTACAGGAAACGTCGAGAAATC
TCTTTATTCCGATACAATAGTA
>DjScaffold942:9039-17899 strand:+
caattcaatttcgcttcaatagggattatttttcaATGCATGTTTTATTGTATTGTAC
GCTCAGTATTTTTCAATTATTTATTTTATTCTCGCGATTGAATGCAATAGGAGAGATTAT
TTGGGCGGTCAATTGTGGTGGTGATGCACACGTAGACTCTCATGGTATACGTTATCAACG
AGATACTCTTAATATTGGGATTAATTCTGATTATGGAAAAAGTATGGTAATCAATAGGGT
TCATACAGATGATCAAATATTATATCAAACTGAAAGATATCACACTGCTAATTTCGGGTA
TGAGATACCAATACCTCATGACGGAGAATATATTTTGGTCTTAAAATTTTCAGAAGTTTG
GTTTACTGCTCCTAATCAAAAAGTGTTTGATGTCATGGTGAATGCGGAACACATTGTTGT
GGAAAGATTAGATATATTTGGAAAGGTGGGTCGAGGAGCTGGCCATGATGAAATTATTCC
ATTGAGAGTGAAAGATGGCAAATTATATGTGAAAGGGGAGTCATCTGATCTTTATTACAA
CAAAATATCAGTGGAATTCATCAAGGGAGACAGAGACAATCCGAAAGTCAATGCAATTTA
TTTGATGAAAGGAAGCATTGAAGATGTGCCGAAATTACCACCGTTATCATTTGCCAGTCA
CGGTCACGATGATGGCCTTGAAGACTCACATCATCAGAATTCCGATGAAATGGAAGAAGA
TTACTCCAGTAACAGTCGAATTCCTCAACCGGAACAACCAAAACGTAAAAGATTAACAAG
CGGGCCAAAGTCTCCTGATCCGTATTCCACGGATGATACATCTTCGATGTTTTTACCAAT
TGCGATGGCCATTGGTGCATTTTTGCCAATTCTTTTCTGTTTGTGCAAGCTCTAGaatca
tgtagatcgatcgattgagtgatgcgctgaattaatccccgtcgcggtgttcagtaatcc
ttagtgctgtgtttttatttaatgatgatgctggtgatgatgattgattgtgttgttgca
gtttggaggaacgtgttataatttttgctaataaatgtgtcttat
>DjScaffold1044:15705-49911 strand:+
aataatttagctggatgtggagaatatgcttttcaagatgattttcaatcccatcgagaa
ttcaaacgaaattttaagaatctcttctccgagcaaacgttcggataatattaatttaac
gaataatttaaaaagaatgtgttttgctaaagaaggaatttacatggctggtgacgacgg
aattcttcgactggccaaacaatctgaaaatgatatcacagactttgtagaagaaattca
taaaattaaaggcccagtatcaaatttaacctttaattataattacacccagctagcagt
tgtttcttcacatggttcaattgatatactttctgccaatgaaaatgttgagccaccgaa
atcattgattgacagtggacatggttcgttagtaggtatcggtgttctaccgggtgataa
atattttgcaacaatgcgaaattttggaaaaattgcaaatttgggatgttgcgaatcaaaa
tgaaatttcgtttctttatttaaatgaaaacgcttcatcaatgaatgtatcaccattatg
gaatattattggtgttggattagagaatggtaatttagaaatttttgatgttaatgatcc
tctacatcctcgacgaattcatcaaatcaaactacatataatacccctatcacaaaaattca
atttggaaaacgaggtcatgtcctaataacagctgctaatgatggatatgtttcaattct
ctctggattagtctcagactgcttcaaagttttaggcattacttcctacgaaggtttctt
attggatttatgcttatttcaaaatcctacagctgatgaaattgcccaagttgctattag
ttcacaaagttctccagatattaatggagctaacaaactagtttatttcgaaatgtcgcc
aacagttattaatgatccaactatttttttatgataatattaatggttcttttgatgaaga
tgcaattcggaaaatgaatctgatatttgaatcaccaacttttggttgtactttaggcgt
tcaatctcatgaagagaagtcacctgtattgtatgcagtagagaaagaatcaagatgttt
atgtaaataccaaataccttcaactccacctaaaaaaactaaatcgaaatatcgaattgt
tatcaaatataaatttaaaaaacaaaaattttgatcgaagcttttattttaaatgaattc

tgtaaaacgatcaatggttatgtccaacaattattctcgcaataattgcaattgtaaagt
aaatatcaatatcaatattaatgcaatattaatacaatatcaatatcaatcttaatttag
gaatatgaaaacaaatatggataataacgattttattatgattaaataattaacaaatgt
ttctattaatttcagtgtttctcattctactcaaatctcaaatgaacaagattatttgaa
cattgtttcaaaatttgatagtaaaatcttacaacaaaatagtgaattacaatcacttgc
aactattgaaattcaaggacgatcagaatcgcccaatgaatcagtatcaaacctaagctt
aaaagcttcaactggtttaagtaccaaagaaagtaaagaatttaaaacgtggagtgagaa
aagagtaattgaacatcaggaagaagaagatcagcagtatcgtgaaataaaagcatctat
taaaaaaggaataaaagcaatacgaacaactatacaagaaATGTTAGATGAAAATGAAAC
ATTGAGTGAACTTGAGCAAATTGATCGACAAGAATTTGAATTAGATATGGACATGAAGAA
GAAAATTCACGCTGATATCGAAAATGAAATTAAAAAGGTTGTTGATGAAATCGAATACAA
TAATTTAGCAAAACAATACATATTGGATGTAATCAAAAAAGAATGTTGGGATCAAATGAA
TGTAAAAGGCAGATCTTTATGTGCATTTAGTTCTGTAATTGAAGTAACAAATTTTCCCAT
GAAAGATAGATTACAAGTTACTCTCGATCAAATTGAAATGGTTGTCAAACGTCGTAAAAT
AGAAATAGAAGACGCAAAGGATCGTAAACTATATAGTGAACATTTACAAAAGAATACTTT
GAATTTAGAGGAATTGGAAGAAATCGAAGATGAAGAAAGCAAAGACTCTTCAAATTTAAC
TCCTCATGATTTGAAAGATTATGAAAAATTTGATAAAATTGCTCCAAATATCAAATATCT
CTACCCACAAATGGAATTGGAAACACGCGAACAAAAAATTTATCAAATAGTATTATTAAA
AGATATAATTTATCAAATGAAAGTACAATTTAATAAGGAATTTACTGAAGTTTACCAGAA
GAAAGAACAAGAAATAAATCGTATTAAAGAAAAGAATGCCAGAATTGTAAAAATTTTGAA
TGATATTGATATGCCACATTTAATTCAACCGGATCCTGAACTTTCTAATTCTGAGAAACC
GGAAAAACTTTTGATCGTTGAAGATCACGAAATCAAAGTTGAAAAATATCTTACAGATGA
ACAGAAAAGAAAATTCGAAGATGATAGATTGGCAGAAGAAGAGAGAAAACGTAAAGAAAA
ACTTGATAATTGGAGAGAAAGGGGATTGGAAGATATGATGGGAGGAGTTTTAGAAGTGAA
ACGAGAAGATGAATTAAAAAAAGACATTCCAAAACCAGCGTTTTTAATTGCTGGCAAACC
TCAAGAAGATTGGACTGATGAAGAAAAGAAACTTTTTGAAGAGTATGAAAAAAAAGTAAA
AGAACTTAATGATGAAAGAGAAAAATATAAAAAGCAATTAGAAGCTGAATTTAAAAAATT
GTCCAGTATTATTGATGATGCTAAAAATGCTTTTGATGAAATGTTATTAAATTTATTCAA
CAGACAAATTACAATACAAATGGCCATTTATCAAGAGGACGTGAAAATTCTTAGACTTCA
GTGGTCTTTATTAGTTGATGAAGAATTGAATGCCCGTCGAAAAGAATTGTTGGAATTAAA
AACAGAAAATAAAATCGAAATTCAAACATTGAAAAACATAATCCAAACGTCTAAATCTGT
TCTCGATGCTAGTCAAGATACTTATGACAATTTTGTTGCTGAAGAAAAGGTTATGGACAA
AAATTTCCGTAAAGAATTTGTTGATGTACATGGTTCACTTTTCGATCATATTACCAAACT
TTATAAGAGACGACCACGAAATCGATTGCTTGGTACTCAAATTATTCCAAGTAGTCAGAT
GCCAAATTATGCAAAACATAATCCTTATACTGGAAGACCAGCATCTAGTAAACAAAAACA
ATTTCAATTGGAAACATTGAAAAATGCCATTATTGAGTTGGATAAACAAGACGCTAATGA
TACTGTAGAATCAGCACTTTACGAACGTCTTTGTAAATCGAGAAGAAAACGAATAATGAA
AGAATTTGAAATCAAACAAATGGCAATTGAAATGGCTGATATGCAAGCATTTTACCAAAA
AAGAATAGAAGAAGAAAAGAGTCTGGAAAATAATGATGTTCACCTAGATACTTTGTTGGA
GACTTTAGAAAATTATCGATATCAGTTCCATGTGAATAAAGAAATCCAGTTAGTTGTAAA
ACAAGGTCAAGTAGAAGTTGAAGCCACTTCTGGTTTTATTCATGATTTCACTGAATCTGT
ACTTCTCCATCGTTCAAATATAGAAGATTTAAATAAGGAAATCAAAGTTCTTGGTCATAA
TAAAGTGAAGCATATGTTAAAAGCCAAAGATTCCAAAAAGAAATTTATTCATTTGGAATG
GGAATTGAGACGAATGCTAATGGAATATGAAGATTTAGAAAGAAAGAAACGTGAAATCCA
ATGTTTCAAAATAACTAAAGAAGTTCAAAGGTACTTGGCTACCAATGATTATGACGCACT
AATAAATGCTGAAATTCAACAAATGGAGCAAACTATTAATCATTTAAAAATGCATCATTC
AAAGAAGTTAGATCAGAAAAAGAATCGATTGAGATTTATCAATGATAATAAACTCATAAA
AATCAAAAATGAAAATGAAGATATCAGTCATCAATTGACAGAATTGAATGTTCAAGTTAA
TGAAAGAAAACATATTAATGCAGGATTCCCAAGTGACAGTGTCGGACAAAGAAATGTAGA
ATTACGATATCAGAAAATTCTTCAAAGGCAACATTTGGTTGATTTGGCAAAATCACAAGC
TCGAGAAGTGGCAGTGCTTCGTTCAGAAGTAGAACGACTTAGAATGAGAACATTCCCTGC
ACTTATACAG
>DjScaffold1044:55217-56015 strand:+
caccgtaataagcaatttttttttaaatagtcattatcaaaacaaaaataaagttttaaa
attgaaattgtgtaaattgaaaactaaaaatctacttttgtaatcgagttttatgatttt
aattattttgctttttgacttctcgattaccaatcatcggccATGATTTGTAAATCGGGC
ACTCTCATAGGCCGTTCCATTCAATTTTGCAATTTGAACTGCTCCATGTGCACACGAACT

TTCTTCCTCTCCGGTGTATGTGTAAGTTTCGCTTTCCTGCCGATGATTGACACCGAATTT
GGTTTTCCAAATTCGTGTCGTCAGGTAGATAATTTCTCATTTTCCTTTGTCTGGTTTACT
GAATTTTTGCTGATTCAGCATTTGGAAAACCAAATAAATTGGACGATTTTGATGACTGGA
CCAGCAAATTAAtttataagaataatatcaaacgattaattccgttattataataaataa
aacgggtcgtagagacccttctaatataatttatattatagtatattatttttattatt
taattttttatttcatctttttacacataattttcattttggatctcgaatttagagtc
tcccacgcccgattttcatttttcgatctttttataataattccataataattataataa
ttataattata
>DjScaffold412:87387-96029 strand:+
tagttcaaatttgaattgttatttataaacgcttaaaATGGCGAGTAAAGTTGTAAAGCA
ACTAGCTGGAAATGGCACTGGACAAAGTCTAGTTGATCGAATGAGTGCTGCTAAATACAG
TTTAGCTGGCTCAGGATTGGGAAGAGGAGTTGTGAAAGCTACAACTGAAGAAATTACCCC
TCCTAAAAAGAAACATTTAGACTATTTAACTAATTGTACTAATGAAGTTAATGTTTCTAT
TCCTGAGTTGGCTGAATATCTAATTGATCGTACTCAACAAACATCATGGGTTGTTGTTAT
GAAGACTTTTATTACAACTCATCATTTAATGAATTTTGGAAATGAAAAATTCTCACAATA
TTTAGCTGCCAAAAGTACATCATTATCCTTGCCTAATTTTGTCGACAAAACTAGTTCTAT
TGGTTATGAAATGTCGCAATATGTAAAACCGTACTCCAAATATATCAATGAAAAATCGAC
AACTTACCGATTGATGGCATTTGATTTTTGTAAAATAAAAAGAGGACGTGATGATGGTAT
TTTAAGGAGAATGCCATCTGAGAAGTTAATGAAAACACTTCCAGTTTTACAAGGCCAGAT
AGATATTTTATTGGAATTCGATGCCAATCAAAAAGATTTATTAAATCCTGTTCTGAATGC
TTCTTTCACATTGCTATATAAAGATTTAATTCGTCTGTATGCATCCTATAATGAAGGAAT
GATTAATTTGATTGAATTATATTTTTCAATGAGCTACAAATCTTGTCGAGAAAGTCTCGA
TATGTATAAAGCGTTCTTAGAAAGAACCGAAAAGGTTACCAAATTTGTTACAGTTGCTGA
GGGTGCTGGAGTTGGAGATAAAGCATCTTTGGGATTACAAGCTGTGCCTCCAAAAGTTCT
TGAAACTTTAGAAAATCATTTAGCAAATTTAGAAAAAAATAAATCCTCGATAGATTCTGA
TGATGGAAAGCAACCACCAGTCACTACAAATGTATTGCCGCTTAAAATTAATAAACCCGA
ATCTTCACATGGTGCTAAAAAAATCAAATCAACATACTCCTTCTCACCCGCCTAAACCAGA
AACAGCAAAATGTCAGGAATCTAATAAGCCACATAATGTACAAGAAAATTTGCTTGGTGA
TACATCTTCCACTGAAAAGCCATCTGAAGTAGCAGTTCGAACTCAATTAAGAAGGCCTGA
ACAGCAACATTCAGTTGCTGTTACTGTCAAAAGACCTGGTAGATCTCCATCTCCAGCTAG
ACCACCCAGACCAATTCCTAATAGATCTAGAGAAGCTTCACCAGTAAAATCTGAACAGCC
TTCTAAAGAAAATCACGACGAAGAAGATTTCTTTAGTCATCTTCGTGACGAATCAAGTGC
TAATTTAGAGGTATCTCCCACCACACCTATCAACGGTAGGGCTAGTCCTTTTTCAGCAGA
TGAGAAACCCCTGGAACCTCCTTCATCATTCTGGAATTCCGATGTAAAAAATAATGAAGC
AACAAAACACCTTGGAGATATTGATTTTGATTCTGCTTTCAAATCTGACACTGAAACAAC
TAATCCCAACACACAACAAACTGAAATGAACCTTTTTGAAACTGATTTACTCGGTCTTGA
TCTTGGTAATGAACCATCAAATAAACCAATTACACCTGTTGAAACGAAAAGTTCAGTATT
TGCAGATCTCGACGATCTTTTTGCATTGGGGGCTTGTGAGGCCAATACTCTAACTGATGC
TCCATTGGCACCATCAGCAATTAGTCCGATTGCTTCTAAAAATCTGAATGATGACTTATT
GTCATCATTATCCTCAATCAGTCCAAGCTATTGTAGTAACAATTCTATAGTTCAGTCTAC
AACTTCTTACGCAACGTCTGCTACAAAACCGTCAATGAATACACCTGTTAATAAAAGCAA
TAAACCAGTCGACGCAGCATTTAATGATACATTGGCTGATTTAGCCCAAAGTCTGGGTAA
AAGTGAAACAAAGTGGTCTTCAAAAGTAAGAAAATTTAAAGAAAAATTTTCAAAAGCAAA
TAATTATAACCGATTTAAAATAAAACTAAAATTTACGATTTCCTTTATGAATCCGAAATT
TGGTAACATTTATATATAAatttccgcaagataaagttttaatggcacatcggaataccc
gcgttgacgtaatactgtttttgagtttgtctacgtaattattatgtaaacatgtgcac
atttttagactaattacgtaatgggtgttttgatacgcaatcaaaatgacctttaacgca
atccttcgaatcacgcactcaaaaagtatatatgacacaataattgcataaatatattta
ttacataatcgttacatgaccagatttttgaagcattgttatatttgtagttcaaatttg
aattgttatttataaacgcttaaaATGGCGAGTAAAGTTGTAAAGCAACTAGCTGGAAAT
GGCACTGGACAAAGTCTAGTTGATCGAATGAGTGCTGCTAAATACAGTTTAGCTGGCTCA
GGATTGGGAAGAGGAGTTGTGAAAGCTACAACTGAAGAAATTACCCCTCCTAAAAAGAAA
CATTTAGACTATTTAACTAATTGTACTAATGAAGTTAATGTTTCTATTCCTGAGTTGGCT
GAATATCTAATTGATCGTACTCAACAAACATCATGGGTTGTTGTTATGAAGACTTTTATT
ACAACTCATCATTTAATGAATTTTGGAAATGAAAAATTCTCACAATATTTAGCTGCCAAA
AGTACATCATTATCCTTGCCTAATTTTGTCGACAAAACTAGTTCTATTGGTTATGAAATG
TCGCAATATGTAAAACCGTACTCCAAATATATCAATGAAAAATCGACAACTTACCGATTG

ATGGCATTTGATTTTTGTAAAATAAAAAGAGGACGTGATGATGGTATTTTAAGGAGAATG
CCATCTGAGAAGGTGAAT
>DjScaffold412:23179-23703 strand:+
ATGCCATTTGGACTTACTAATGCACCAGCTTCTTTCCAACGAATGATGAATTATGTGACT
CATGACATATCTCAATGTATGGCTTATATCGATGATCTTTTAATCTACTCGCCAACCTTT
GAGGGACATCTCAATGACCTGCGTAGTGTGTTTGTACGATTACGTAAATGGAACCTAAAA
GTAAAACCTACCAAATGCGAATGGGTCAAGAATAAAGTAACATATTTAGGCCATTTAATA
TCACATAAAGGTAAAGAACCGGATCCGCGAAATACAGAGAAGATACGGAATCTAGCACCA
CCAAACTGTGTGCTCAAAATACAAGAATTTCTCGGTCTTGCAAACTATTATCGCAAATTC
ATTAAGGATTTCGCGAAAATTGCAAAACCTATGCAGGAATTGATCAAACAAAATACTGCA
TTCATATGGGGACCAGGACAACAACAATCATTCGAAACTTTACGAGATAAATTACTAACG
GCACCAATTCTGGTACACCCAGATTTCAAGAAGCCATTTATATAA
>DjScaffold789:24868-32632 strand:+
atattactttttcagaATGAATTGTAAAAAAAGTTTAGAACGAGCAATTGTTTTTGGGAA
AATGATACATCATGGACTAGAACCATTATTTATGTTTTCATCAGTTGAATCTTACAAAAA
CATTCCTCCAATTGATGATGCTGGAGAAGATGATGTAGACATTTTTAGTGTTCAATTTAA
TGAAGATGGTACGGAAATAGTTGCAGGAGGAATGAATAGTACAATTAGAGTTATAAATAC
CTCTCAACCAAATCGAAAATTAACTATTAAACCATCAAAATTTATTTTGGGAATGCCAAT
TACCTGTGTTCGTTATATGCCATTGAAATACGATAGAATTATTGCTTGTTCACCAAGTGG
AAATCTTTATATGTCAAAGGCTAATAAAGAAGGCTTTGAAACAATAATCACAGAAAATAA
TGAAACCTATGCCTTTGATATATCTACCGATGGAATGGAATTAGTGACTGGTGGTAGAGA
TAGAAATGTACGAATATATCGCTTTGCGAATGCTGGTTATTGTGAA
>DjScaffold789:80485-81388 strand:+
taaattttttataatagtaatagaATGTCGCCATTTCCAATTATAAAATTGGCTACATTG
GCTATGAAACAAATGTCAAAACCACTTGCAAATTTTTTTAAAGTCAAAGCCAAATCAAAT
AATTTTTTTCGGAAATACATATGCATGCCTCCTGCTCAAATGTATCACTATTATGACACC
TCTCTTAAAATGAAATTACTTGGCTTGGGCAAATCCAAAACTGTAGCTAAATTAAATGAC
GAAGCAGCTGTTGAACTTGGAGCAGATATGTTAGGAGAAATGATAGTTTTTTCAGTAGGT
GCAATTTGTCTATCATTGGAATATATTCGACAACAGAACATTTCCAAAAGAAAAGAGGAA
GATGTTGTTGACAAGTTAGACGATCTTCGGAAATCTGTCAATTCATTAACTCTTCAAATT
GAAAATCAAGACGCTAAAATTAGAGAAATCTCTCGAGAATTAGCAACTCATAGAAAGTGA
ccttttcaaagattattacaataaatattggcgctggaatcttggtttaatattaattt
tcacatttgataaataaatatgcagattctttgagactctttgttgaaaaattcagtgat
tacccaatgatttgagctgattgaagattggagattcggtttctatttcgaatgaaatac
ctttttgagtcagtaatggtaattttagatgtttgttgcaattagaataatttatataat
tataatattcagttttgtttttctataaattggtcatttccaattggttttttagtgttt
gtaatattgattt
>DjScaffold789:85895-86798 strand:+
taaattttttataatagtaatagaATGTCGCCATTTCCAATTATAAAATTGGCTACATTG
GCTATGAAACAAATGTCAAAACCACTTGCAAATTTTTTTAAAGTCAAAGCCAAATCAAAT
AATTTTTTTCGGAAATACATATGCATGCCTCCTGCTCAAATGTATCACTATTATGACACC
TCTCTTAAAATGAAATTACTTGGCTTGGGCAAATCCAAAACTGTAGCTAAATTAAATGAC
GAAGCAGCTGTTGAACTTGGAGCAGATATGTTAGGAGAAATGATAGTTTTTTCAGTAGGT
GCAATTTGTCTATCATTGGAATATATTCGACAACAGAACATTTCTAAAAGAAAAGAGGAA
GATGTTGTTGACAAGTTAGACGATCTTCGGAAATCTGTCAATTCATTAACTCTTCAAATT
GAAAATCAAGACGCTAAAATTAGAGAAATCTCTCGAGAATTAGCAACTCATAGAAAGTGA
ccttttcaaaaattattacaataaatattggcgctggaatcttgggttaatattaattt
tcacatttgataaataaatatgcagattctttgagactctttgttgaaaaattcagtgat
tacccaatgatttgagctgattgaagattggagattcggtttctatttcgaatgaaatac
ctttttgagtcagtaatggtaattttagatgtttgttgcaattagaataatttatataat
tataatattcagttttgtttttctataaattggtcatttccaattggttttttagtgttt
gtaatattgattt
>DjScaffold394:41396-42226 strand:+
gatccgaaaatatttgctgctagatacattcagaatttcATGATTGTTATAATTCTGTGT
GTGTTTTATTTTCAACAGAAATACAATCAAATCGGAGTTAACAATATCAGTGGAGTTTTG
TTTAATCTAATAACAACTATAACATTTTTAGCCGTTGTTCAATTGAATTCTTTTGTTTCA
AGTATGAAGTTTTATTTTCGGGACCATCAAAACGGTATGTGTCGATCAGGAACGTTTTTT

ATTGCTCAAAACCTTTCCGAAATGCCATTTTTTCTTGCTACCACTTTATCATCTGTAATA
CTTATTTATTGGTGTGTCGGTCTTTATAATGGAATAGGAGAATTTCTTATTTTTAGTTTG
GTTATTTTACTAATTTATCAATATTCTTATAGTTTAAATATTATGTTTATCGGAACACTA
TTTAAGAGTCTTGATTTACTTTTAAATATAGCTCCATTGATAAGAATTGTAATGATGTAT
TTTGGCGGTTATCTAATCAACGATGCTTCTTTGCCGGGTTATTTAAGTTGGTTGAAATAT
TTATCTTGGCATCGATATGGCTTTGAGGTTTTATTGGTCAATCAATGGCAAAATGTCAAT
GAGATCATCTGTGAAAATCAAGACTCGTGCTACAAAACTGGAAACGATGTCATTAAATTC
TATTATTTTTCAGCTTCAAATAATTTGTGGATTATTTTTTATAATAATAAGAAGCAGAGA
GAGAAAATTAAATTAAATTTTTGTTTTAAATTAATTTTATTCAAATAAaaaatttgtgaa
attt
>DjScaffold394:65250-72469 strand:+
aaacaatcaaggagaacacaaataccacccaagccaaatattaatATGAATTTATGGAGT
GTTGTAAAAAATTGTATCGGGAAAGATTTAAGCAAAATACCAATGCCAGTTAACTTTAGT
GAGCCAATTTCCATGTTACAAAGATTAGCAGAAGATTTTGAGTACTCTGCAATTTTAGAT
AAAGCTGCTACTTGTCAAAGTCCAATACAACAAATCGCTTACGTGGCAGCTTTCACTGTA
TCATCATATGCTGCTACGGCGTATCGAATTAATAAACCTTTTAATCCGTTGTTAGGTGAA
ACATATGAATTAGATCGCACAGATGATTATGGATGGAGATTGTTTGCCGAACAGGTAAGT
CATCATCCTCCAAGTTGCGCAGTTCATTGTGAAAGCAATGAATGGGTTTGCTGGTTTGAC
TTCTCAATGGGTAATAAATTCAGAGGGAAATATATTCAAATTAACCCAATCGGAAAATGT
CACTTATTTTTCAAGCGTTCAAAATATCATTATGTCTGGGAAAGAATTCCAATGACAGTA
AGATCTTATTTAATTTCCTATAATTACATTACTTCAATTCATGTTGAAACTAATTTATTA
TTTTGAtaaattcttctgaattttttccataacgaattcaagcatagaaaaatgattag
aattgaaagtaaacctataaaaaatcttaactgagaaattttttttatatttctgaaata
tttatttaatacagttgtttcctgagagtataataaagtatttacaatgttggctagtct
aaataataccgtaatttgttttataattacaa
>DjScaffold2825:35706-36789 strand:+
atattttgaaaataatagttcacaatttatttaaacttgtcaATGTTTTATGCTTTCCTA
AATAATAAAATGAATTTTAATGCATTTATTTATATATTTTTTATTCTAAAATTTTCATAT
TCCACATCATCAGAGATATGCTCTAAATCAAAAACACTATGGCATTCTTTGAAATATCCT
TATTTAAATTTGTTGGAAAATTTCAAACTGCAAACCACACCATTCACGTGTGCACTATCA
ACAACCACAAACTGTTGTAATTCTGATAAATTAAAAGAAATGTCAAGCATCAGTGAAATA
AAATTGAAAGGCTTAGCTGAAGATAAAATAGAATTTCTTCTGATGATGTTTAGTAACGAC
AGTTATTTTCTCATGAATATGATTACTAATGATTTGGAAAACACTAAAAACGATCTCAAT
ACATTATTCAGTACAACATACGGCTATAACTACAAAGTGGAAAAAGATTTCTTTTTCAAT
TGGTTTGAGAAATTAAAATTATACATGGAAGGAAAAAAACATAATATCGGCCAGTTATTA
GACGATTTTTTTCTTGAAATGGCCCAAAAAATCGTCAAAATTCTTATTCCTCCAGAATCG
GGTCCAGAAGTCACCAAATGCCAGATAGCTCGTTTAATCGAAGCAAAACCTTATCAGGGA
ATCGATGCTGATTTGAAAAAGAAAATAATCAGCTCATATTCACCAGCAAGATTACTTGTC
AATGCGATAGCTGTTGGACGAGACGCTTTCAAGAAATTAATGCAAAAGGTGAAAATATTG
CTTGTATTACTCATCAAATCTCAATTGAAAATTGCATTCAGTAATTAAttacctaattta
atagtaaaaaaaaattcaaaaatcccgaaaatttttcatttttaaaaatcaataaaaaaa
ccgctacataaattgtg
>DjScaffold2825:74809-84479 strand:+
AAAGCATTGGAAGGATATTTCTTAAAAATTGTTCCTGATTATTGCACTGTTAACTCTGAT
TCGCTAGATTCCGCACAATGTTGGAATGTTACTAGTGGCCAAAGTTCTGGAATTTCTTTG
GAAAAAAATAATAAAAAGAATAATAAATCAACTGGTTCAATATTCAAATCAGTAACCAAT
CAGATCAAAGAAATCACTACTAGGATTAATGACTTTTTAAAAGATAAAACACTAGATCCA
GAAACTATTCAAGTTGAAGAATTTATTTTGTATAAATCAAAACAAGAAAATAAATTTGTG
GACAAAAATTTTAAAACATCGCAAAGTCCAATAGAAGGAAGTGGTGATTTGGATAAGTTT
CCTATTCAACCATCTATATTACCACCAAACAAAAAACCAGAAATTGTAGATCCGCCATCT
ATTTTAAAACCAGTTCCCGATGAAAATGAACAAGAAAATAATAAAAAAATTGTTGAAACT
CCAGTTAAGAATCCAAAATTTCCTCCTTGGGAAGGAAGTGGTGATGCAGAAGAAGGAAAA
TCTGATCCAGATAAAGAGAAATCATCTAAATCACTGAAAAAATCATCGACATCAAGACCT
AAAACTATGTCGACAGCTTCAGTTCAGAAAATTGTGACTCAATCATCGACAGAATCTAAT
CTCAATACTGACGATGAAGATATGATTTCAAAACAGCCCAAAGGCGTACCTGATAAACCT
GCTACAACACCATCTTTAAAACCAGACATTTTACCTGAGGGCAGTGGGTCGGATCCTGAC
CTTACTCAGTCGAAGCAACCACCTGCGATAGTTGAGCAGCCTACTACTACAACAAAGTCT

ACAGTACCCGAGAAGACTGTTGAGGGCTCCGGATCGGAAGTTGAACCTGGACCAGATACA
GAGAAACAGCGTCCAGTATTTCCTAAAGTACCAGAAAATACCGGAGTTTCAACTAAATCC
CCTAAAGAGAAAGATTCAAAATTGAATCCTGATGATGAAAAGAATATTGAAGGTAGTGGT
AGAGCAGCTGTCAACCCATTTCAAACGTCACAATCACCTACAATTTCCGACTCTACTTTG
AGTCCCACAAAACCTTTACCACCTGATCAGTATCCTGGTACAATTCCATCAGAGGTTTGG
ATTCCAGGTATAGATGGTGCAAAAAATGCCGATAAAGACAATCAGAAAACCACACCTGGA
AATCGGGCAAACAAAAATAGAAATCAGAGATCTACTATTACAAAGATAATTATAACGCTC
AGTCTTTTATTGATTTTTACTGCCTAAcataatttgcatcaaataaatcgatatag
>DjScaffold49:47385-62383 strand:+
TCAGAATTAGCTACAGGATACGAAAATGAAAGCTCGGATGATGAAGAATCTTCGAAGCCC
GAAGTTAATTCTCAAGGAGATAATTTATCTGAAAAAAATAAGGCATCTTATTTTGAAATA
GCTGGTATTTTGGTTCATGTTTCAGGTGTTGTTGGACAAATTCTTCTATCTCATCCATGG
ATTGTTATACGTAGACAATGCCAGATTCGTCATGATAGTTTGAAATTTCATTGGACACCA
TTCACAGTTATTCCAGTAGCATTTAAACTTGTTAAGAGTCAAGGACTTCCAACTTTATTG
AAAGGAATTAGTAGCACGTTATCTCTTTGGTTGATGGGGATAGGAAGTGAAACGGTTTTA
AGTGAAATAACTAATTTACCAAAAGATATTTCCAAACATAGTTCTCTTAAAAAAAATCGGG
CAGCATTTTTTATTAAAAGGAATTTCTTTGATAATTTTATCACCATTCTATGCGGCAAGC
CTTGTTGAGACTGTTCAATCAGATTTATCTACTGAGCCGACTAGTATTTTAACATGTCTG
CTTGAAGGTTTTCGAAGAATGTTTATTTTTTATCGTGAACCAGTTTCCAGATTGTTGCCA
ATATGGAAAATCGTTCCAATTGTGGCGTCATTTGGTTTACTTCATTACGCAATTAAAACA
TTCACTGGATTCATGACAAATTCTTATCTTAGTCGTAATTCTGTCGAGATTATGAACACT
CAATTAGACGATGTATATTATAAACATTATTGCGAGTTATTCAGTGCCATCATGGGAGCT
TTTACAGCTGATGTCATCTTATTTCCGATGGAGGTTATCGTTAATCGATTATGTATTCAA
GGTACAAGAGTCTTAATTGATAATATGGACCATCCATATGAAATGGTTCCGATCACAACA
GCCTATTATGGGACAAGACACGCACTTAATACAATTTTGAATGAAGCTGAGAGAGGAGAA
GGCTCCGGTGGTTTATATAAAGGTTTTGGTGCTCTAATTATACAGTACTCTATACAATTT
TTATTAATTTTTTGTATTCGATTGGGTTATGAAAATTTATTGATTAAGTGTTCTAAATCC
AAACCAGATTCGACTACTTATTCTCCCAAAAAATCTTATAAAAATTTGTAGtttttctca
aaaaaaaaggattttataagatttcaattattatcaaaataaagaatttcattttgt
>DjScaffold4194:53246-65061 strand:+
gtttaaactacaattaatatataaagtaatcgttataaaatacatttttcacttgatcca
attgtaaacataaatATGCTAAAAGTTGCTCTGATTCAAATGATTGTTACCGCTAGCAAG
TCGGATAACTTAATTAAAGCATGTTCTAAAATAAAATTAGCAGCAGAAAGAGGGGCTAAA
TTAATTTGCTTGCCTGAATGTTTTAATTGTCCGTATGGAGTAAAACATTTTCAAGAATAC
AGTGAAAATATTTCAAATGGCAAAACATCTGAAATGTTATCAACAGTTGCCAGAGAGCAC
GGTGTTTGGTTAGTTGGCGGTTCTATTCCTGAAAATAGGAATGGAAAAATTTATAATTCA
TGTTTAATTTATAATCCAAATGGAGAACTTATTGGAAATTATTCCAAAATACACTTGTTT
GATATTAATATACCAGGAAAAATTGTTTTCAAAGAATCTGAAACTTTAACTGCTGGAAAT
AGATTGGTTTCATTTGACATTCCAATTAGTGACGATAAAAATGTTACTGTTGGAATAGGA
ATTTGTTATGATGTTCGATTTGCTGAATTAGCTCATATATATACACGTAAACGTAACTGT
AATTTGTTACTGTATCCCGGTGCATTTAACATGACGACTGGTCCGTTGCATTGGGAACTT
CTTGGGAAGTTAAGAGCTATGGATAATCAATGCTATATTGGTTTGTGTAGTACGGCTCGT
GACGAAAATGCTGAATATATCAGTTATGGTCATTCATTAGCTACTGATCCATGGGGAAAA
ATTATTGATTCTCTTAATGAAAAGGAGGGTATATTGGAATTTTCTGTGGACCTAACTGAA
GTAGAACATGTTCGTTCAATGATACCAATTAGTAAACAAAGACGAGAAGATATTTATACT
GTAGCAACAGAAAATTAAttagttttattatgattactgaattctta
>DjScaffold4194:100282-104419 strand:+
tgttttagtatgtttaaaaattgattattttattcagtgacatttttgatgcaATGTTTC
CACTTCACAAAAAGGCTAATGAAACAATAATTCAACAAGGGGATGATGGAGATAATTTTT
ATATAATCGATCAAGGAGAAGTCGAGATATATGTCAATGACGAATTGAATTGTAATGTTA
GTGAAGGCGGAAGTTTCGGTGAATTGGCCCTAATTTATGGCACTCCTCGAGCTGCTACTG
TAAAAGCTTCCACAGATGTCAAATTATGGGGTATCGATAGAGATTCATATCGACGAATTT
TAATGGGTAGCACTATAAAGAAAAGAAAACTTTATCAAGAGTTTCTTTCTCGTGTTCCAA
TTTTGGAGGATCTTGACAAATGGGAACAACTTACTGTTGCGGATGCTTTGGAATCAATTA
ATTTTGAAGATGGTGAGTACGTAGTAAGACAAGGTGATCTCGGTGAAGATTTTTATATGA
TAATAGAGGGTGTAGCGAAAGTCTTTCAGAAACCATCAGACTCAGAAAATGAAATTGAGG
TTGGACAATTATTAACGTCAGACTATTTTGGTGAAATTGCTCTTTTATTAAACAGACCGC

GTGCTGCTACAGTTCAGGCGTCTGGGCCTCTGAAATGCGTTAAACTTGACAGAAAACGAT
TCGAGAGAGTAATGGGAAGTTGTGCTGAAATTCTAAAACGTAACATTAAAAAGTACAATA
GTTACATTTCGATAACGGTTTAGtg
>DjScaffold1790:70590-71559 strand:+
ggaataattgcacaacttgcggacgggtaggacaccaggcagagaggtgtttccaaaatc
atagagcaagcaaactacagaacgacaagactggaaagaacacaaggacacgaagatagt
gtcaatagatacaaccagtacgaggggaagcagaacgtatataaaagcaaccaacaaaga
aatggatacggatatgacaattatcagaataagggatatggtgtacgcaacgatagaaac
agaaacctgaggcatgtgaatattatagaagcagatgcgtaggaacaatattattacgct
ggtggagacaacagagaagagtatggtagaggacgacagagtgaaagaaaagaggacaaa
cacgtccaaagacgagttgatcggagctgttcattaaaagaaaaatcagaatcgcacata
cgccgagaagaggaaggagaagcagagatgacggagaaaatatacagatttctgaagaga
gaagtacaacgaaaagacgatattgagatagtaacgattggtgaattagacagagATGAG
ATTAGATATGATCGCATGCAAGGAAGATCCTAGTATTATAATATGTTTGCAAGGGAGAGA
GTACGAATGCTTGTTAGATACAGGTGCCAGAATTAACGTAATGAGTGACAAGATATACCA
AAGATTGGAAGATATAGAGATACAACAAAGAGACGAGCGGTTGAGATGTGCAAATAATAG
TCAAATGAACATTAAAGGTAAGATAAATATAGAAACGTCGGTGGGAAATCAAAAAGAACT
AACGGAATTTACAGTAGTACAAGAAATGACTCCAGATATTATTGGAGGAGTCGAATTGCA
AAGGAAATTCGGATTCAGTCTAAGACGAGAAATAAAAGAAACAGAAGACCATCAAATATG
CAGCATAGAAACAAAATTCGGG
>DjScaffold4410:54134-56531 strand:+
attttagttaatacgtaaagatcgccaactATGGGGTTCAAGTTTCTTGATTTGGTGAAA
CCGTTTTGTCAAGTTTTGCCAGAAATATCAAAACCAGAGCGAAAAATTCAATTTCGTGAA
AAAGTGTTATGGACAGCTATCACTCTTTTTATCTTTCTTGTATGCTGCCAAGTACCTTTA
TTTGGTATCATGTCATCAGAATCTGCAGATCCATTTTATTGGATGAGAGTAATTCTTGCA
TCTAACAGAGGTTCTCTTATGGAATTGGGTATCAGTCCAATTGTAACATCAGGCCTTATC
ATGCAATTATTGGCTGGTATTAAGATTATTGAAGTCGGTGATACTCCAAAAGATCGAGCA
CTATTTAATGGAGCACAAAAGTTATTTGGAATGGTTATTACTGTTGGTCAAGCAATGTTG
TATGTTATGTCCGGAATGTACGGTACTCCCGGTGAACTCGGTGCTGGAGTTTGTTTACTT
ATCGTTTTTCAACTCACATTTGCTGGTTTGTTGGTCTTGATGTTGGACGAATTACTGCAA
AAAGGTTATGGTCTTGGATCTGGAATTTCTCTTTTTATTGCAACGAATATTTGTGAAACT
ATCGTATGGAAAGCCTTCAGCCCAACCACAATCAACACTGGTAGAGGAACAGAATTCGAG
GGAGCAATCATTTCGTTGTTCCATTTAATGGCTACCCGAACTGATAAAGTCCGTGCACTA
CGTGAAGCATTTTATCGAACAAATTTACCAAATCTAATGAATCTGTTAGCAACAGTACTC
GTGTTTGCTATTGTTATCTATTTCCAAGGATTCCGTGTTGATTTACCAATCAAATCCGCA
CGTTATCGAGGTCAACACAGTTCTTATCCAATTAAATTGTTCTACACCAGTAATATACCG
ATTATCTTGCAAAGCGCGCTTGTATCGAATCTCTATGTGATTTCTCAAATGTTATCCAAT
AAATTCCGAGGAAATCTGATTATCAATTTGCTGGGAACTTGGTCGGAGGGTGAGGCCGGT
TCCCGTTCGGTGCCGATCGGTGGAATTTGCTATTACATGTCTCCTCCTGACAGTCTAACT
GCCATGTTCCACGATCCAGTTCACGGATTTATCTATATCGCTTTCATGCTCGGAAGCTGC
GCATTTTTCAGCAAAACGTGGATCGATGTGTCCGGATCAAGTGCCAAGGACGTTGCTAAA
CAACTGAAAGAACAACAAATGGTTATGCGAGGACATCGTGAGAAATCAATGATCCATGAA
TTGAATCGATATATTCCAACTGCTGCAGCATTTGGAGGATTGTGCATCGGTGCGCTGTCG
GTGCTCGCTGATTTCCTCGGAGCAATCGGCAGTGGAACTGGAATATTGCTGGCTGTCACA
ATCATTTATCAGTATTTCGAGATATTCGTGAAGGAGCAAAACGAAATGGGTGGAAGCATG
AGCACTTTGTTGTTTTAAtattaataataatacatttgtttacttttggaataatcataa
ttattattataatattgaccggcgcgtgtgtctgtggtgtgctttattgattttgttttc
atgtaatttatgaactccgtgtcgaattgtagatatatcgtgtcgttattattattattt
tatcgatcggttcaatcaaatacagaattctggatttcatttttgattttactttaggga
tattaatgtgtgtgtgtgtgtgcagctgatctaattcgaatgcgtcactacagatcgttt
cagttatgtaattcagttgaagtgttttcggattataaatagcgttgactaacttttacg
aacaatatttagaaatcaaataaaatttaatcaatatttgtacacgtgttgtcacatgtt
tgttcatgttagcattacattaattgaatttgtttgtatagcaataatgcatgcatttta
attcactttaggcaataactggagtttttcttattgtaatgtgtggttagatttgtgca
ttttattaatttcgttttgacaatagaggctatcatttggtgttgtgcataacaatgagt
catatccaataaaacttgatttgttaaattcaattgatatttatagattatatttgaatc
gttttaatatagataatcaatggtggaatattgatttgatttggcataatattggcagaa

ttgaataaaaattgttatgaatttgtaaacttgataatcagag
>DjScaffold4326:32797-33668 strand:+
ataaagtagaatgtctattagatacaggggctcgaattaacgtgatgagtttgagttgtt
ttaataaactacgagaagtaaagttagaaatgacttATGCAAAAGGTCGATAGCAAAACG
AATGAAAAAGTCGAAAATTACATTTGCAATATTGAAGCAAATTTTGGAAGACAAATTAAA
AAAGGAGAGCGATTGACAAGAGCTTTAAATATTTTTAAGATTAAAGAGGATCAACGGTTA
TTGAAAATAATTGAAAGTAAAGAAGATGCTTTTATGGCAGATAAATGGGATGTAGGACGT
ACACATCTGACCAGACACAAAATAATCACAAGTGGTGAACCAATCAATGTAAAACCATAT
AGACAACCAATGAACTTAGAATCTAAAATTGAAGAAGCTATCCAAAACCTTTTCGAGAAT
AATATTATCAGGAAATGCAACTCTCCATGGAATACCCCATTGATATGTGTTTGGAAAAAG
GAAAAGAAAGACATAAGAATATGTTTGGATTTCAGACAATTGAATGCAGTAACGAAAAGA
CAAGCCTTTCCGATGCCTAATGTCGATGAAATGCTGGACAAGTTAGATGGAGCCAAATAT
TTCAGTTCAATAGATTTGGGAAATGCATACTATCAAGTAGAGCTCGAAGAAGAATCTCAA
GAAAAAACGGCGTTTTCAACGAAACACGGGCAATATTGTTTT
>DjScaffold4326:33675-35666 strand:+
ATGCCATTTGGCATCGCAGCGGCACCAGGGACATTTCAAGAGATGATGACAAAAGTGGTA
GGCGGAATCAATGGAGCCATGGTTTATCTTGATGACATACTGATATTTACGAACAATAAA
GACCAACACTATGAAGTAATTGAAAGAGTATTGACGGAAATAATGAAGGCTGGTCTAAGA
ATTAATCCGGAAAAATGTCAGTTTCTTCAAAGTGAAATCAAGTTTCTTGGACACATAATT
AGCAGGGATGGTGTTAGAACTGATCCGTCTAAAATAGAAGCTATAAGAACATTTGGCAAA
CCTAACTGCGTGAAGAAGTTACGAAGTTTCTTGGGAATTTGTAATTATTACAGGAAATTT
ATAGAAAACTACGCAAGAAAATCAAAAATATTAGAGCAGTTGTGTAGCGGACCAAGCAAC
AAGAAATTAGAATGGACAGATGAAATAAACGATACTTTTGAGGGTCTAAAAAAATCTTTA
ATGACTGCACCAATATTGTGTTTTCCGGACATAAACAAAGAATTTATATTAGATACGGAT
GCCAGTTTTGATACGATTGGAGCAGTATTGTCACAGAGAGATAAAGAAGGAAGAGAAAGA
GTTGTTGCCTATGGATCACACTCAATGACAAGTCATGAAAAGGGATACTGCATTACACGA
AAGGAGTTATTGGCTGTTTATTATTTCTGTATCCATTTTAAACATTATTTGTATGGTAGA
AGATTTACTCTAAGAACAGATCACAAAGCTCTAACTTTCATGATGACGACGAAGAAACCA
ATTACGTCACAATTCCAGACATGGATTAATTATCTGAGCAGTTTAGATATGATTTTGGAA
TATAGAAAAGGAATCCAACATGCTAATGCAGATACGTTATCTAGACCAAGATGTGAGGTA
TGTGTACAATGTCAAATGTCACATGAAGATGCCAAGAAAGGGAAGATTAAAACGAGAATA
CTGAATATAATTGAAAAAGAAAAAGAAGATCATTTAAACATTTCTGAGGAAACAGATTTA
GTCAAGAAAATGAAAGTTCCGGATGACAAACTACGTGAAAAAATAGAAGAAATACATAAA
TTATTATGTCATGCAGGAGTTGAAAAGGTGTACGAATATATGAGAAACAAATATATTGCA
AAGAATTTGTGTAGCCAAATACAGAAGATAGTTCGAAGTTGTGAAATTTGTCAGAAGACC
AAAGTAGTGACAACACCAACTAAAGAGAAGACAAAGAGTTTAGAAGCTACCAATTTATTT
GAAGTGATATTCACAGATATATGTGGGCCAATGCGGGAAACAAGTGGACGGAAAAAATAC
ATTCTTGGAATAATCGACCAATACAGTAAATATCTGGTTCTTATACCATTGCCTAGACAA
GACGAGGAAACGATACGAAAAGCTATTTTTGAAAAGTGGATATTGAGATTTGGATGTCCA
AGAGAAATAAGATTAGATTGTGGAAAAGCGTTTGAATCCAGAGTTATGAGAGAGTTTGCA
CAGAAGCTAGACATTAAACTTTGCTTTTCGAGTCCATATCATCATAATGCTAACGGACAA
ATAGAAAGACAGTTCAGGACCATTCGAGATTTGATTAATGCGACCCTTCAAGAAAGAAAA
TCAACAGATTGGGCAGATCTTTTACCGGAAATAGAATTTACAATGAACGCAACAAAGCAG
AAGACTACTGGAAAGAGCCCGGCTGAGATCGTTTTTGGAAAGAAAATTAGCAGAGAAAAG
TGGTACGAGTCAGACGTTTCAGATATAATTAAAGAAAACATTTCAGACGAAATTACTAAA
AGAGAATTCAAAGTGGGCGATTATGTACTCATCAAGGTAGAAACAAGAAATAAAAGTCAA
GACAGATTTGAGGGACCTTATCGAATAATTGAGAAAATTCATGAAAGGCGGTATGCAGTA
CAAGGAGAAAATGGACAGTGTTTAGAAAGAAATGTGAAAAACTTAGAAAGTTTCTTAAGA
AGGGGGATATGA
>DjScaffold2569:5147-7361 strand:+
aagcgtgtctggcagaacaataatttcaattatgacaacatcgtaaactctATGATTACA
CTATTTACCGTGACCACAGGAGAGGGCTGGCCCGATGTGCTGAAGAATTCAATTTACGCT
ACGCATGCAAATGAGGGCCCAATCGATAATTACAAACAGGAGATTGCCATATTTTATATT
GTGTTTTTCATTGTGTTCCCATTTTTCTTTGTCAACATCTTCGTGGCTTTGATTATTATA
ACCTTTCAGGAACAAGGAGAAAGTGACAAAACAGATTTAGATCTAGATAAAAATGAAACA
AAATGTATAGACTTTGCTATTAATGCAAAACCTACCTCTCGATACATGCCAGCAAATAAG
AAATCTATTTACTATAAAGTTTGGCTACTTGTTGTGTCCCCACCATTCGAATACCTCATC

ATGATAATTATAGCTCTGAACAGTGTTACTCTTGTAATTAAGAGCCACACAAGATTCCCA
GATAAATATTCCAAGAATAATTCATTTCTCTGGAAAGAACAAACCACACAAAATGATTTT
TGTTCAAGTTTGAAATATTTAAACATCGCCTTCACAATAATTTTCACATCCGAGTGTGTG
CTGAAGATGATTGCGTATCAATTCAAATATTATTTCAAAGATCCGTGGAACATTTTTGAT
TTTGTAACAGTGCTCGGCAGT
>DjScaffold2851:19753-29235 strand:+
aaaatcgtgtaATGAGGTCTAACGCCGCTCAATTAAATAATTTTAATGGAATAATCAATC
AATTATTAGAGATAAGTTATGAAAATGAAATTTTAGTACTTTTACTTTTGGTAAATGAAA
GTATTATAAAAATAGAATGTTTTGTAAGTTGGAAAGTGGATAGTGATTCAGAATTGTTCA
CAATATTCTTGCAAATTAATCGAACAATCAGTATTTTCCGAGCGGAATATAGAATTAATC
ATTTTGAAGTGATAAATAATAAAATAATAATTTCCACTGAAACAGGTAAATATGGTATTT
TTAATTTGATCGAAACACTTTTGGAAAATAACATTGAATTGAATTGCAACATTCTTGAAT
TTCCATATGGCCCATTTCCATGCAAACCAATCAATAAATGTTTGGAGACAACTTTCTCTG
GGTATTGAaatttatattatttttgaattttattaaattagaattgttagctgtctctaa
attattgggttcgttttcaatatctataaaataatttcaataacttcaagatttatttaa
caataaactttttttttattcagaatatttaaaatatcaatgagattgtggttctaacaa
aaacagaatacatatcaattacctatcagaatggaatgtggaattcatgttataaaaatc
aaattagaccgatttcacaaaatcaaattacaggaatgagtaatgcaatcgaaattaaaa
attttttaatcgatgaaaactgtaacgattcatccgacaaaagatttgaacaaatcaaaa
gaagcaaatctgtaaaaatgtttgccttatcaaatgatgacaatactgtttcaacatgga
ttataactccagataattttttctacacgaaactgttaatttaaatcggttttacaaag
aatcagaaattcaaaaattagagaaaatagcaccatggccgaaatttgtgaaatgtgga
>DjScaffold2851:65510-74631 strand:+
gtcgaattaaaaaggttggaatacactgtattaattgtaattaatcaaataatagtttgt
tttttctataaaaagttttaataaaaaaacgcgtgaacttatttaccaaccttatactaa
attgaatatttaattaaataaaatcattttcaatctatataatcgttctttattaataaa
tactcgtgtaatgctgtctttattaggcagaatatcagactcgtcaaaatataatgtctt
atattttcctttgcatttttacatatatttaaaacaaaaacatttgttgaccgtttttat
tgtgacacgctctatatttctattatatatatttatattatattgtatattcctattata
ttattaggtaatatttctatttttaaataggtATGGCGCGTCTACTTATAACAGTTAATA
AGTGTGATATTAGATTTCCTGATAACGTTGTCAGTCTTAATATGACACAGTATGTAATGA
CACGATGGTATAGAGCTCCAGAATTAATAATGGGCCAAATATCTTATAATACCAAGATTG
ATTTATGGTCCTGTGGATGCATTCTTGCTGAGCTTTACTTAAAAGAACCTCTTTTTCGTG
GGGATAATATGATTAATCAATTAAAAGAAATTATTCAAAAAATGGGAACTCCTGAAAGTC
AGTTCAGAGCGAAGGTTTTACCAATTGTAAATTCTCTTATTAATGCCGAAGGGCACTTAA
AACCCATTCCATTAAATCATTTGATCAAAAATGCACCAAAAAAGGTAATAGATTTACTGT
CTCGTTTATTGGTGTATGATCCTGCTCAACGATGGTCCGCCAGTCAAACTTTGAAACATA
TATTCTTTTTAAAATATAAAAATCCTTCAAAGGAAGAAATATGTAAATCTATGTTAGATT
TATCATTTGAAACAAAGTTGAATTCTATTGATTTGCTTAAAGACGCTTTATTGGTTGATA
TTTCATCAATAAATTCAAAACAAATCAATTTTAATTTAATGACTCATACAGACTTTTCAA
AGGAAATTGATGACTATTCAAGAAAACCAATTTTAAGTAGTTGTGAAATTCAATCATTGA
ATCGATCTGAATCTCCTATTTCTCAAAGAACTTTATCAGCCGACAGTGGCAAAGAATTAG
GATATCCAATCATGTTTTTTGAACTAGAAAATTCATCTTCAAGTTCACTTTCCACAAATA
ATATTTATAAGAATAATCGCTTGAAAACCGTTTCTCAATTCACAAAAAATAAATTAATCA
ATTCAATTCAAAAACCTAAAAATAAATCAAAAAAAATTATTACTGCAGCTCAAAGGCAAA
AAGAACGGCAAGATCGAAAATTAAAATTAAAACATAAAAGAGATGAAAATTTCCAAATTA
ATAATGTAATTTCCAAATGTCAGGAACCACTTTCTGATTTGGATAAATCTTATTTAAAAA
AATGGGAGCATTTAACAAAAAGTACTGTGAATATTAATGAAAAAAATCTAGATCAAGAAA
CCTATAAAAACTGCTATGAAAATACATCCACTATTAAATCTGCAAAGTTTCAAGCAAATG
AAGTTGATGAAATGTCTGCCGGATTATCCGCATTGTTATTGTTGGATGATATTATTGAAA
ATGATCAAAATATAACCACAAAAACTTCGAATTTTAATTCAAATAAAAATGAAAATATTT
CAACTGACAATCTTGATTTACTGTTCGATGTAGATATCGATTGTATTGATTTAGACCTAA
ATATTTTAGAATCTTATTCTGTTGATTTATTAAAAGAATGAttataaaaattaacttaaa
tacattcacttttaaagaaatttttaatgtatatttttaatcgaatagattta
>DjScaffold2851:88737-89108 strand:+
CAAATAGCAGAGTTGTACATTTATATTGATGACATCCGGATATTTACGAACAATAAAGAT
CAACACTATGAAGTAATTGAAAGAATATTGACGGAAATAATGAAGACTGATCTAAGAATT

TATCCAGAAAAGTGTCAGTTTCTTCAAAGCAAAATCGAATTTCTTGGAGACATAATTAGT
AGGGACGGTATAAGAACTGGTCCGTCTAAAATAGAAGCAATAAGAAAATTTGAAAAACCT
AACTGTGTGAAGAAATTACGAAGTTTCTTAGAAATATGTAATTATTACAAAAAGTTTATA
GAAAACTACGCAAGAAAATCAAGAATCTTAGAGCAGTTGTGTAGCGGACCAAGCAACAAA
AAATTAGAATGA
>DjScaffold5210:98116-98795 strand:+
AGATTGAAAACAGGTGATGTGATTGTTGTTGATGATTTGTCTCAAGAAGCTAATAAGGAA
AAGAACTTTAATAATTTATTCACAAAAATATCAAGATATTGTGGTGTTACTGTAATATTC
ATTACACAAAATCTATTCCATCAAGGTAGTGATCATAAAACTCGTAATTTAAATGTACAG
TACTTGGCTATATTTAAAAATCCAAGAGATCAAGCGGTTGTTGATTTTCTAGCAAGACAA
GCGTATCGAAATAATCGGCAATTTCTATTGGATTCATTTAAAGATGCAACAACAATGCCT
CGTGGATATATATTTCTGGATTTTACTCAGCAATATCCTGATGATCTTAGAGTTAGCAAT
CGCATGACAAATCAATATGAATATAAGAATAATATTGTTTCAAATGATATCAAATCGATA
AGGAAGGCATTAAACAAGTCGATAAAGAGAACAATAAAGAAATCCATAAAGAAATCGATA
AAACGACAATCAAGGAAGCAATCAAATCTTTCGAACAAGGAACAAACAAAAAAAATTAGC
AAGAAAAATTCAAAG
>DjScaffold5210:192102-193006 strand:+
tcattgaaaatgaaataattcttaaatcaaacaagaaaattcatctatggtttagataca
tagatgatattttctttgtaactaattctcctttagagaccattctagaattagccaaca
gtattaataataacattaaattcactttagagaaatcaattgactttcagattccttttc
ttgacactttaattcaaaagaaacctggtgaaatattaactacccaattatatatgaaac
catttcacagtggacatatattaccttggagtaaaaattggtataattaaaggtgaaaga
attcgagctgagagactatcttcaaatgaagaaATGAAATTGATTTCTCTCAAGTTGATA
AAAAATAAATTTTTAAATAATGGCTATCCAAATAAAATTATATCAAAATATTTATCTTTA
AATTCAAAACAAAATAAAATCAAACCAAAAACAAATACAGTTATTAAATTACCGTTTATT
AATGAGAAATGTAGTCAAATCATGCAGAATATAATAAAAAATGCTAAACTAAAACACAAT
CCAAGAATTATTTTCAAAACGCAAGCACCTTTGGGCATCCAATTACAGAAACCAAGACAA
ACTTTTTGTGGTCCCAAATGCATTTGTGATGGTAGAAATATATGCAATAAGAAAAATGTC
ACTTATAAAATAACATGCACCATTTGTCAAGAAACATATATAGGTGAAACATGCAGAACC
ATCCGAACAAGAGTTAAGGAACATTTGAAACAAAAAGAAAGTGAAGTATTCAAACACTTT
CAACACAAACATAAAATTTCACCAGAACAACATCACATTACATGGAAAACACTAGGAACA
GGCTACATTTCTACACTACATAGAAAAAAACATGAAGAAATACTA
>DjScaffold5210:89347-89922 strand:+
ATGTCAGATGAAGATGTTGCCGCATTGGTAGTTGATAATGGATCTGGTATGTGTAAAGCC
GGATTTGCTGGAGATGATGCTCCACGAGCAGTTTTTCCATCAATTGTCGGTCGTCCACGA
CATCAAGGTGTGATGGTTGGTATGGGCCAGAAAGACAGTTATGTTGGTGATGAAGCTCAA
TCAAAAAGAGGTATTTTGACTTTGAAATACCCAATCGAACATGGTATTGTTACAAATTGG
GACGATATGGAGAAAATTTGGCATCACACTTTCTACAATGAATTAAGAGTTGCTCCTGAA
GAACATCCAGTCTTATTGACTGAAGCTCCTCTTAATCCTAAAGCCAACAGAGAAAAGATG
ACACAGATCATGTTTGAAACTTTCAATGCTCCAGCTATGTATGTAGCAATCCAAGCAGTT
TTGTCCTTATATGCATCTGGTCGAACAACTGGTATTGTATTGGATTCTGGTGATGGAGTA
ACTCACACCGTACCAATCTATGAAGGTTATGCTCTTCCACATGCTATTTTGAGATTAGAT
CTTGCTGGAAGAGATCTTACCGATTATTTAATGAAA
>DjScaffold3934:1441-8250 strand:+
tgggaatctattttcacagtgaattatttttcatgcaaatatttttatttgaatagaaaAT
GACAATTTCATATGTTTCAAGAATTGCTACATCGAATAGTTTTCGGGTTTTCAAACTTTT
GCTGACATGGAAAGGAAGTATCTATAAAACTATTCGAATTGAATTATTCATATACATTGT
TTTTTACGCACTCATATCTCTATTGCACCATTTTGTTCTCAATGGTACCTATAAATTAGT
TTTTGAGTCGTTTTGTTCATTTTGTGCAAAAGGAACAGAATTCATTCCCGTTGGTCTTGT
TTTAACGTTTTATGTGACACTTGTCGTTCAACGATGGAATGATCAATTTATTACATTACC
ATGGCCGGATAGTTTGGCAGTTTATGTCAATACATTTATTCTTGGAACGACTGATGTGCC
TCGACTTATTCGTCGAAGCATAATGAGATATGTGATAGTGAGTCAAATTTTGTGTTATTC
TAGTATTTCTAGTAAAGCCAGACGCGTATTTCCCACTAATAAAACGCTTGTTGAACAAGG
ATTATTAACCACAGAGGAACTTGATAGACTAGAAAATTTTGAAACCACAACTAACAAACA
TTTTGTTCCAATTGCTTGGGCACAAGAATTAGTGGTAAAGGCTCAGAAAGAAAAATATTT
ATATGATAATAAACGAGTCTGGTTGTTAGTAAACGAATTAAATGAATTCAGAGATCGACT
TCGAATATTGTTTGCATATGATTGGATAAATCCTCCTCTTATTTACACTCAGGTATTTTT

AGATTTATTGTTTAATGGAATTTTTACATCATCATTTTCATTTATTGGTTTTCATTAAtt
aatcatcgctgtcaatatctgtgacatttgtaaatatttatttcatttgtttgactaatt
ttataacaaagtttttaattaattcatctgctttattattattatt
>DjScaffold1550:22495-22983 strand:+
ATGCCGTTTGGGTTTGCAGCAGCGCCGAGTACGTATCAGGAGCTGATGGTAAATGTGACG
AAAGACATCAAGGGATCAATGGTATATTTAGATGATATATTAATTTTTTCTACGCCACGA
GAGGAACATTATGGAATTGTAAAAAGAGGGTTGGACAGCATTAGTGAAGCAAGTTTCCGT
ATTAATCCAGAAAAGTGCATTTTCCATCAGAAAGAAATTAAATTCTTAGGACCCATCAGT
TCAGAAAGAATTCGTACCGATCCGGTAAAAATAGAAGCTATTCAATCTTTTGGGGAACCA
TCATGTGTGAAGAAATTGCGTAGCTTTCTGAACATCTGTAATTATTATAGAAAGTTTATT
CGGGGGTATGCAAATAAAGTTAGAAAATGGGAGCAGCTATGTAGCGGTCAAGGTAATACC
AAGCTAGTTTGGTCTTTAGAGTTATCAGATGTGTTTAAAGAAATGAAGAAAGCACAAGAC
GCGAAATAA
>DjScaffold1550:25168-25656 strand:+
ATGCCGTTTGGGTTTGCAGCAGCGCCGAGTACGTATCAGGAGCTGATGGTAAATGTGACG
AAAGACATCAAGGGATCAATGGTATATTTAGATGATATATTAATTTTTTCTACGCCACGA
GAGGAACATTATGGAATTGTAAAAAGAGGGTTGGACAGCATTAGTGAAGCAAGTTTCCGT
ATTAATCCAGAAAAGTGCATTTTCCATCAGAAAGAAATTAAATTCTTAGGACCCATCAGT
TCAGAAAGAATTCGTACCGATCCGGTAAAAATAGAAGCTATTCAATCTTTTGGGGAACCA
TCATGTGTGAAGAAATTGCGTAGCTTTCTGAACATCTGTAATTATAATAGAAAGTTTATT
CGGGGGTATGCAAATAAAGTTAGAAAATGGGAGCAGCTATGTAGCGGTCAAGGTAATACC
AAGCTAGTTTGGTCTTTAGAGTTATCAGATGTGTTTAAAGAAATGAAGAAAGCACAAGAC
GCGAAATAA
>DjScaffold3890:8506-10358 strand:+
ttttatttttttattatactaaaatatcaaaATGTCATCAAATGTTACAAGCAATCTAGC
TCTTAGTGGTGAAAGAACAAGTGGCGATAATGTTAGACGACAAAATGTTTTGGCTGCTCA
AACCATTGCTAATATTGTTAAAACAAGTTTGGGTCCAGTTGGATTAGACAAAATGCTTGT
TGATGATGTTGGTGAAGTTACAATTAGCAATGATGGAGCTACTATATTAAAACTTTTGGA
AGTTGAACATCCTGCAGCAAAAAATTCTAGTTGAATTAGCTCAACTACAAGACGAAGAAGT
TGGTGATGGAACCACTTCTGTGGTTATAATTGCTGCTGAGTTATTAAAATTAGCTGAAGA
ACAAATCAAACAAAAAAATTCACCCTACAACTATTATTAGTGGTTACAGAATTGCATGTCG
TGAAGCTTGTAAATATTTACAGAATAATTTAACAATCGATGTTGATAAACTCGGTAAAGA
ATGTATTACAAGTGCTGCTAGAACATCGATGTCTTCAAAGTTAATCGGTTGTGATGGTGA
TTTTTTCGCCAATATGGCTGTTGATGCAACAATGGCCATAAAAGTAGCTGATGGTAAAGG
AGGTTTCAGATATCCAATTAAAGCCATCAATATCTTGAAAGCTCATGGAAAATCAATGAA
AGAAAGCATGTTGATACAAGGTTACGCTTTGAATTGCACAATTGCATGTCAACAAATGCC
TAGAACTGTGAAAGGTGCAAAAATTGCTCTATTGGATTTCAGTTTACAAAAAACGAAATT
AAAACTTGGCGTTCAAATCCTGATTAATGATCCGGAAAAATTAGATGAAATTCGTAAGCG
TGAATCTGAAATTACCAAAGAAAAAATCGAAAAGATTTTGAAAACCGGAGCAAATGTTAT
TTTAACTACCGGTGGAATTGATGATATGTGTATGAAATATTTCGTCGAAGCCAAAGCAAT
GGCTATTAGAAGATGTAAAAAAGTGGATTTAAAAAGAATTGCCAAAGCTACAGGAGGTAT
GTTGTGTGTCAGTCTTGCGACAATGGACGGCGAAGAAGCATTTGATCCATCTTATCTCGG
TTTTGCTGAAGATGTCAGCCAAGAAAGAATATGTGATGAAGAATTGGTTCTGATTCGTGG
TTGCAAAGCTCGAACAGCTGCCAGTGTTATATTACGAGGAGCTAACGATTTTTATGTTGA
TGAAATGGAAAGATCATTGCACGATGCTCTGTGTGTTGTTAAGCGCGTTCTTGAAAGCAA
ATCTGTTGTACCAGGAGGTGGCGCATGCGAAGCTGCTTTGCATGTTTATTTAGAAAAATA
TGCAATGACTATAAGCTCCAGAGAGCAAATTGCTATTGCTGAATTTGCTAAAGCTTTATT
AGTTATACCAAAACAATTAGCAGTTAATGCTGCTCTTGATTCAACCGAAATGGTGTCTAA
ATTGCGCGCTTTACATCATTCGAGCCAACAATTTCCCGATTTAGTTCATTTCAAATGGTA
TGGATTGGATCTATCTAATTTCCAAGTCGCTAATAATAAAGAAGCTGGAATTTTTGAGCC
ATCAATGTCGAAAATCAAATCATTGAAATTCGCAACAGAAGCTGCCATCACAATTTTACG
TATTGACGATATGGTGAAATTAGATGAAGAAAAGAAACCGAGAAAGCCGGGATGTGGTGA
TGAATAAtttcagtgtttcattttgttgttaaataaaaccggattagacgtgaatcgatt
ttgtaattgagttgtattgttatgttttatttttttattgtaaggtatcttagaaattat
agtaaaatataacaatttgtcctagatactaaaatatcaaaATGTCATCAAATGTTACAA
GCAATCTAGCTCTTAGTGGTGAAAGAACAAGTGGCGATAATGTTAGACGACAAAATGTTT
TGGCTGCTCAAACCATTGCTAATATTGTTAAAACAAGTTTGGGTCCAGTTGGATTAGACA

AAATGCTTGTTGATGATGTTGGTGAAGTTACAATTAGCAATGATGGAGCTACTATATTAA
AACTTTTGGAAGTTGAACATCCTGCAGCAAAAATTCTAGTTGAATTAGCTCAACTACAAG
ACGAAGAAGTTGGTGATGGAACCACTTCTGTGGTTATAATTGCTGCTGAGTTATTAAAAT
TAGCTGAAGAACAAATCAAACAAAAAATTCACCCTACAACTATTATTAGTGGTTACAGAA
TTGCATGTCGTGAAGCTTGTAAATATTTACAGAATAATTTAACAATCGATGTTGATAAAC
TCGGTAAAGAATGTATTACAAGTGCTGCTAGAACATCGATGTCTTCAAAGTTAATCGGTT
GTGATGGTGATTTTTTCGCCAATATGGCTGTTGATGCAACAATGGCCATAAAAGTAGCTG
ATGGTAAAGGAGGTTTCAGATATCCAATTAAAGCCATCAATATCTTGAAAGCTCATGGAA
AATCAATGAAAGAAAGCATGTTGATACAAGGTTACGCTTTGAATTGCACAATTGCATGTC
AACAAATGCCTAGAACTGTGAAAGGTGCAAAAATTGCTCTATTGGATTTCAGTTTACAAA
AAACGAAATTAAAACTTGGCGTTCAAATCCTGATTAATGATCCGGAAAAATTAGATGAAA
TTCGTAAGCGTGAATCTGAAATTACCAAAGAAAAAATCGAAAAGATTTTGAAAACCGGAG
CAAATGTTATTTTAACTACCGGTGGAATTGATGATATGTGTATGAAATATTTCGTCGAAG
CCAAAGCAATGGCTATTAGAAGATGTAAAAAAGTGGATTTAAAAAGAATTGCCAAAGCTA
CAGGAGGTATGTTGTGTGTCAGTCTTGCGACAATGGACGGCGAAGAAGCATTTGATCCAT
CTTATCTCGGTTTTGCTGAAGATGTCAGCCAAGAAAGAATATGTGATGAAGAATTGGTTC
TGATTCGTGGTTGCAAAGCTCGAACAGCTGCCAGTGTTATATTACGAGGAGCTAACGATT
TTTATGTTGATGAAATGGAAAGATCATTGCACGATGCTCTGTGTGTTGTTAAGCGCGTTC
TTGAAAGCAAATCTGTTGTACCAGGAGGTGGCGCATGCGAAGCTGCTTTGCATGTTTATT
TAGAAAAATATGCAATGACTATAAGCTCCAGAGAGCAAATTGCTATTGCTGAATTTGCTA
AAGCTTTATTAGTTATACCAAAACAATTAGCAGTTAATGCTGCTCTTGATTCAACCGAAA
TGGTGTCTAAATTGCGCGCTTTACATCATTCGAGCCAACAATTTCCCGATTTAGTTCATT
TCAAATGGTATGGATTGGATCTATCTAATTTCCAAGTCGCTAATAATAAAGAAGCTGGAA
TTTTTGAGCCATCAATGTCGAAAATCAAATCATTGAAATTCGCAACAGAAGCTGCCATCA
CAATTTTACGTATTGACGATATGGTGAAATTAGATGAAGAAAAGAAACCGAGAAAGCCGG
GATGTGGTGATGAATAAtttcagtgtttcattttgttgttaaataaaaccggattagacg
tgaatcgattttgtaattgagttgtattgttat
>DjScaffold3890:32925-34125 strand:+
aaacagtctgatataagtcgagtatattgttcaatATGACCAAACCAAGAGTTGGAATCA
ATGGATTCGGAAGAATCGGAAGATTGGTTTTGAGAGCTGCCCTGGAAAAGGGAACGGTTG
ATGTTGTTGCTTTGAACGATCCATTTATTGAACTAGATTACATGGTTTATATGTTCAAAT
ATGATTCAACTCATGGAAAATTTCATGGAAATGTTGAAGCTAAGGATGGAAAATTAGTAG
TTAATGGTCATGCCATGTCGGTTTATAACAAGAGAGCTCCCGAAGAAATTCCTTGGGATC
ACGACCAATGCTACTATGTTGTGGAATCAACCGGTGTTTTCACTACAATAGAAAAGGCTT
CGTTGCATACAAAAAACAATAGAGCTAAAAAGGTTATTATTTCAGCACCATCAGCTGATG
CCCCAATGTTTGTTATGGGAGTAAATCATGAAAAATATGAACCAAGTTTATCTATTGTAT
CAAATGCTAGCTGCACCACCAACTGTTTAGCTCCCTTAGCAAAAGTCGTGAATGATCATT
TTGGAATTGAAGAAGGTTTGATGACTACTATTCATGCTTATACAGCAACGCAAAAGACTG
TTGATGGACCATCATCTAAATTATGGCGAGATGGCAGAGGGGCAGCTCAGAATATTATTC
CAGCTTCCACTGGAGCTGCAAAAGCAGTAGGAAAAGTGATTCCAGAGTTGAACGGCAAAA
TAACTGGAATGGCTTTCCGTGTTCCAGTTCCAGATGTATCTGTTGTCGACTTGACCTGCA
AATTAAAAAAACCAGCAAAGTACGATGAAATTAAAGCAGCTATTAAGCATGCATCTGAAA
CTACAATGAAAGGAATTCTTGCATATACTGACGAAGAAGTTGTCAGTCAAGATTTTAAAG
GTGACCACAATTCATCAATTTTCGATGCAAAGGCTGGTATTCAATTGACCGATACATTTG
TAAAATTGGTGGCTTGGTATGATAACGAATATGGCTATAGCTGCCGGGTTATTGATCTGA
TACACCATATGTCTACCAAAGATCATTAAatgtttacaagagctttagtttgaaatagtt
tagatgtttctggacctgacgctattataaagtttaatgtggtttagttgaataaatttc
tttatcctataatttttgcactttaattataatttttagtatattgttcaatATGACCAA
ACCAAGAGTTGGAATCAATGGATTCGGAAGAATCGGAAGATTGGTTTTGAGAGCTGCCCT
GGAAAAGGGAACGGTTGATGTTGTTGCTTTGAACGATCCATTTATTGAACTAGATTACAT
GGTTTATATGTTCAAATATGATTCAACTCATGGAAAATTTCATGGAAATGTTGAAGCTAA
GGATGGAAAATTAGTAGTTAATGGTCATGCCATGTCGGTTTATAACAAGAGAGCTCCCGA
AGAAATTCCTTGGGATCACGACCAATGCTACTATGTTGTGGAATCAACCGGTGTTTTCAC
TACAATAGAAAAGGCTTCGTTGCATACAAAAAACAATAGAGCTAAAAAGGTTATTATTTC
AGCACCATCAGCTGATGCCCCAATGTTTGTTATGGGAGTAAATCATGAAAAATATGAACC
AAGTTTATCTATTGTATCAAATGCTAGCTGCACCACCAACTGTTTAGCTCCCTTAGCAAA
AGTCGTGAATGATCATTTTGGAATTGAAGAAGGTTTGATGACTACTATTCATGCTTATAC

AGCAACGCAAAAGACTGTTGATGGACCATCATCTAAATTATGGCGAGATGGCAGAGGGGC
AGCTCAGAATATTATTCCAGCTTCCACTGGAGCTGCAAAAGCAGTAGGAAAAGTGATTCC
AGAGTTGAACGGCAAAATAACTGGAATGGCTTTCCGTGTTCCAGTTCCAGATGTATCTGT
TGTCGACTTGACCTGCAAATTAAAAAAACCAGCAAAGTACGATGAAATTAAAGCAGCTAT
TAAGCATGCATCTGAAACTACAATGAAAGGAATTCTTGCATATACTGACGAAGAAGTTGT
CAGTCAAGATTTTAAAGGTGACCACAATTCATCAATTTTCGATGCAAAGGCTGGTATTCA
ATTGACCGATACATTTGTAAAATTGGTGGCTTGGTATGATAACGAATATGGCTATAGCTG
CCGGGTTATTGATCTGATACACCATATGTCTACCAAAGATCATTAAatgtttacaagagc
tttagtttgaaatagtttagatgtttctggacctgacgctattataaagtttaatgtggt
ttagttgaataaatttctttatcctata
>DjScaffold3890:61694-62599 strand:+
attgaaaataaaattattcttaaagctgagaagaagatacatctttggctcagatatata
gacgatattttctttgttactaattcatctttagaaaatattttagaactagccaatagt
atcaatgataacattaaattcactttagagaagtcaatcgactttcagattccttttctg
gacaccttaatccaaaagaaacctggcgaattattacttacccagttgtatatgaaacca
tttcacagtggacatatattaccctggagtaaaaattggaataatcaagggtgaaaagat
aagagctgatagactatcttcaaacgaagaaATGAGACTGACTTCCCTCAGGTTGATAAA
AAATAAATTTCTAAATAATGGTTATCCAAATAGAATTGTATATAAACATCTATACTCAAA
TACAGAATGTAATAAAATCAAACAAAAGACAAATACAGTAATTAAATTACCTTTTATTAA
TGAGAAATGTAGCCAAATCATGCAAAATATAATTAAAAAGTCAAAACTAAAACACAATCC
AAGAATTATATTTAAAACGCAAGCACCTTTGGGCATCCAATTACAGAAACCAAGACAAAA
TTTTTGTGGTCCCAAATGCATTTGTGATGGTAGAAATATATGCAATAAAAAAAATATCAC
TTACAAAATAACATGCACCTTATGTAAGGATACATATATAGGAGAATCATTCAGAACCAT
CCGTACAAGAATCAAAGAACATCTCAAACAGAAAGAAAGTGAAGTCTTCAAACACTTTCA
AAACAAACATAATGTTTTACCAGAAAATCATCATATTACATGGAAAACATTAGGAACAAG
CTACATTTCTACACTTCACAGAAAGAAGCAGGAACAAATATTAATT
>DjScaffold3127:98367-105262 strand:+
GCTTCTGAGGTACCTGTATCTAACCTTCAACCTCCAGAACCTATATCCCAAGAAAATATA
ATTGATAAATTATCTAAAGATTTACAAAAGGTCCAGTCTAATCTTGATGTAATGTGTGAA
TTAATGAATGAATATTTACCAACAGAACACATTCCGGATGATGTATTAAGCCTTATGAAA
AATCTTTTTGAAATCAATAGTGAAATGCAAAAGAGACTTCTGCAATTAATTAATCACTGG
GAAGAAACGAAACTTAAAGAATCATCAATCTTGTTCGATATGTTGCGAATTAATGATGAA
ATGAATAGTGCACTAGCACAATATGAAAGATATGAACGCACAGTTTCGGGAGGAATAAAT
AAAATCGACGAAACCAGTGATTTAAAAAACCGGATATCTAAATCTAACGTCGAAGATATT
TTTTCTGCTCAACAAGAAATGAGTGGTTTCTATGATGGATTTTCAAAAGCAATAAAGGCT
AATAAAAATGACCAAAAAAATGTACCTCATTTCCCTGAAGAATTACAACCCTTAGTTTCT
ACAGAAAAAAATACTAACACTTCCAAATCATCGATTGGTGCTTTATCAAGTCAAGAATTC
GACCAATTTCTTTCTGAGCAATCCAATACTGCTTTTTCAATTAATAATGATAACAATCAA
TCTAATTCTTCCCGAAATGATCGACAAAACCCGTTTGGTGCAAAGCCAAAAAGTGAAAAA
AGCGAATTATTTGAACTGTAAtacatgataatgcccaccagataatgttttgtgatattc
agtgttgaagatatttcatttttattgcataatttatatacgaaataaatcacgtttgtt
attattaataata
>DjScaffold4208:30040-33744 strand:+
AGTGGTATCTTGACAATTGCAGATTTAGAGAAGTTAAATGGTCACATTGCTTGTGAAGTT
CCAAATAGACATCTTTATGAATTCTGTGGGAATATTGCACTAAAAGATGAACCAAAAATA
TCTTTGGGAAACGACCAGTTACTTTTACGTGGAGCTACTTTGAAAAATACAAAATGGGTG
TTTGGTATTGTTGTCTATAGTGGTCATGAAACAAAGGTGATGCTTAATAGCACCACTCCC
CCATTAAAACGATCATCTGTCGAACGTATGACAAACAGATATATTCTAGGTTTATGTGGA
CTGCTGTTAATTCTAACTTTGGCTACATCGATTGCCAATGTTATCTGGACTAATGCAAAT
AAGAATACCGCATGGTATTTAGGGTTTGACAGATACCCACCAAACAATTTTGGACTTATG
CTTCTAACTGCCTTTATCATGTTTCACACTATCATTCCTATTAGTCTACAAGTTACTCTG
GAAATGGTTCGTTTTATTCAAGCACATTTTATCAACGTGGATATAGAAATGTATGATGAA
GCTTCAAATACA
>DjScaffold4208:65500-66331 strand:+
aaagctgaagtagtgactttagtgaaaacgaatgttaaagatgctattacacttgcaata
ggagatggagctaatgatgttggtATGATACAGGCTGCTCATGTTGGAATAGGAATAAGC
GGATTAGAAGGTCAGCAGGCAGCATGTAATTCAGACTACGCTATTGCACAATTTAGATAT

CTTAATCGTCTCTTATTGGTTCATGGTGCATGGAATTATAACAGATTAACAAAATTGATT
CTTTATTCCTTTTATAAAAATGTTTGTTTCTTTGTAATACAATTCTGGTTTGCAATCGTA
AGTGGATTCTCTGGTCAAATTATCTTCGAACGCTGGTCTATAGGATTCTATAATGTGATT
TTCACTGCAGCTCCTCCTTTAGCTCTTGGATTATTTGATCGTAGTTGCAGTGCAGAAAAT
TCTCTGAAATACCCCCAGCTTTATAAAGAGATTCAACATAGTTCAACATTTGATCTCAAA
GTTTTCATCACATGGATTATGAATTCTTTATATCATTCTCTCATCCTTTTCTGGTTCACA
GTTTTGGCTAATAATCACGGTATGAATCCTTTATTGATTGCATTGTGAtaattattagga
cttttcatttatcgttagttcggtaaattgaaatttgaataccgataattaacataggta
ataaatttatagtttatgtttcgcaatt
>DjScaffold4208:77122-78185 strand:+
gattcatacaagttcataattattcagttggaatagatttgcactttatctgcatttaaa
tatttacagttacattgaaaatatcaaagatattccattcaaaaatggtcagactggaag
tttattatttttgggaaattgtatttatacatatgtggttgtgactgtatcagtgaaggc
ggggttggaacatattgcttggacatggctttcacatttagcaatttggggaagtgttgc
agcgtggtttatctatttcattatatactctcatctgtttcaattaattgggttatcagc
cagcATGGCTCTTATGTATCAAGCTGTATTTAGTAGTGGGACATTTTGGTTAGGTCTGAT
ATTAATACCTTCAGCTGCTCTCACTCGTGATATCGCTTGGAAAGCCGTAAAAAGAACTTT
ATTTGAATCCCTTCGCGAACGTGTAATGCAAATGGAAAAAAGAAATCAAGATCCTGGTAA
AATTATACCGGGTGTTGTTGAAACTAGAAAAAGAGGTCAAAACAGATTTTGTAATCCTTT
CCGCCAGAAAGATTCCAAATCCGGAGGAGCAGCTACTATTGTTGACGATGGAAATCGTAA
GTGGTTTAATGTTTTCATTATGGAATATCTATTTCTATATTGTTATTTATATATATAAaa
ttatatatttatatatttctccacgaatcagttatctaaattgagatttctatgcaaata
atttccatttcacagtgattggtttcggaagattcgtgaagaaatatttggagaatttat
aacttataatatacacatttatcggattttatattt
>DjScaffold3043:1132-7256 strand:+
gcctaaaagaaaatccacagataatgaatttgatttacctgcgaaatttagtcggatcga
tgaagtaATGTCTAGAGGAAATAATTCATTAATGCAACTAAATGAAATCGGAATAAAAGG
ATTAAAATGGACAATGGTGCAAATTTTTAACACTTTTCCACAAAGATTTCGAGGTTCATG
TGCATTTGGAGAACCTTTTACTGGTGTTTATGAAGTTATTCCTTCGGGGACCGTGTTTTA
TCACGAAGGGCTTAGCAAATCAGTTTGTAAACATGAAATCGCTAATCAGATTATGGATCG
ATTGTCAGGAAAAAAATATACCTGTAGATTTTATACGTGTTCCATTACCAACACCTAAACC
AACAGAGAATGAAACATTGAATACAGGTATGACAGGCAGTAATGGTGATACAATACCATC
AGATTCAGCTGTGAGAATTCTCAATGAAAAAAATGCGAGACATTGAATATAAATTTGTTTT
GCAAGAAGAGGGATATTGTTGTTATGTTTCATTTCGAGGGTTTGTTATTAAGGGGGTTGG
AACTAATAAGAAAACAGCTAAGCGTGTTGTAGCAAGTAAAATCTTAGAGAAATATTATAA
AATAAAAGTTGCAGATGAAAATAAATTATTTGTTCCACAATCTAGTTCTGCAACAGATCT
TCCTATGGAAACTGATAGTTCTATTTCAAAAATTCCTAAAAGTCGTCAGAAAAATTTTTC
AAATCTCATTCAGTCGATGGTGAAAAATGGATATGAAAAATTAGTGAGCACACTTGATGT
TCAATATTCAAAAAGGAAAGTGCTTTCAGGGATTATTATTAGTAGGAAAATGAAAGTGGT
TTTCCCGGATTATTATCGGGAAGACGATGAAATCGGATCTAATCCAATGGTGATTGATCC
GATATTGTTTTATTCGAATACTGGTTGGCATATAAATCCAGAGGATCAGGGAGAAATATT
GAAATTAATTACGATTAGTTCCGGCACAAAATGCTTATCTGGTAATGAAATCAATGATAA
TGGACTCGTAATCAATGATTGCCATGCTGAAGTTGTTTCCAAACGTGCCTTTCAATTATT
CATTTGTCAAACAATATCTCGACTGCAGAACAATGTGCAGTGTGATTGTTTCGATCAATT
ATTTGGATGTGATATTCTTGAACCTCTCGATAAAAGTAGGAAATCTATCTATgcctaaaa
gaaaatccacagataatgaatttgatttacctgcgaaatttagtcggatcgatgaagtaA
TGTCTAGAGGAAATAATTCATTAATGCAACTAAATGAAATCGGAATAAAAGGATTAAAAT
GGACAATGGTGCAAATTTTTAACACTTTTCCACAAAGATTTCGAGGTTCATGTGCATTTG
GAGAACCTTTTACTGGTGTTTATGAAGTTATTCCTTCGGGGACCGTGTTTTATCACGAAG
GGCTTAGCAAATCAGTTTGTAAACATGAAATCGCTAATCAGATTATGGATCGATTGTCAG
GAAAAAAATATACCTGTAGATTTTATACGTGTTCCATTACCAACACCTAAACCAACAGAGA
ATGAAACATTGAATACAGGTATGACAGGCAGTAATGGTGATACAATACCATCAGATTCAG
CTGTGAGAATTCTCAATGAAAAAAATGCGAGACATTGAATATAAATTTGTTTTGCAAGAAG
AGGGATATTGTTGTTATGTTTCATTTCGAGGGTTTGTTATTAAGGGCGTTGGAACTAATA
AGAAAACAGCTAAGCGTGTTGTAGCAAGTAAAATCTTACAGAAATATTATAAAATAAAAG
TTGCAGATGAAAATAAATTATTTGTTCCACAATCTAGTTCTGCAACAGATCTTCCTATGG
AAACTGATAGTTCTATTTCAAAAATTCCTAAAAGTCGTCAGAAAAATTTTTCAAATCTCA

TTCAGTCGATGGTGAAAAATGGATATGAAAAATTAGTGAGCACACTTGATGTTCAATATT
CAAAAAGGAAAGTGCTTTCAGGGATTATTATTAGTAGGAAAATGAAAGTGGTTTTCCCGG
ATTATTATCGG
>DjScaffold3043:18033-23460 strand:+
gcattttgggaggcgagcgattactaaccATGTCATGTTCTGATAAAGTCTGTACATGGA
ATGTTCTTGGTATTCAGGGTGCACTTTTGAGTAATATTCTGGCTCATCCTATTTATCTCA
ATAGTATAACTATTGGTTCTTTATTTAATCAGGAACACTTGGAAAGAGCTCTTTCTGAAA
GAGCTCAAGGCGTAATTGAAGAGTTAGATTGGCGTAATGGAAATGCTCCACCAAATGAAG
AAATCGAACAAGGCGAATTGAAAATTTCGTCAGATAATATTGATTTGTTCAATGTTAATA
AACCCAATATCCTCGCAGTTACTGATCAGGATGATCGATGTCCAGGAAAAGCATCAAATT
TCAGTGGGAATTGGATATTAGGCAAAGAAAGCTTTGAAATAATATCTACCGCGAAAGGTG
TCAAAATTGACAATACTCCATCAAGGTTATGTAAACGGAATTTATTAAACTCTTTCCTTT
CCATATTCAAATGGCTCGAGTTTATAAGTTCTGATGATGGATATTATCGTTACAATGAAA
TGAAATGCTTGGAAATGTTTCCGGACAATGCAGTTGGAGCTGTAAATTATCAGAAGAAAA
AGATTTCTTTGTTTAAATTTTTCGAAAAACAAAGTTTTGGTCTTTGGATTGGAAAACCAA
TCGAACAAGATTCGTTTTCTATTAAATTCCCAGCTAAATAAaaagtggtttttatctttg
atgacatcttcaggccaataaatctattcttttgtagattttctaggtgtaatatcaatt
gttttatttaaaataatc
>DjScaffold3043:29491-30274 strand:+
tttgcctgcgaaatttagtcggattgatgaagtattgtctagaggaaataattcattaAT
GCAACTAAATGAAATCGGAATAAAAGGATTAAAATGGACAATGGAACAAATTTGTAATGC
TTTTCCGCAAAGATTTCGAGGTTCATGTGTATTTGGAGAACCTTTTTCTGGTGTTTATGA
AGTTATTCCTTCGGGGACCGTGTTTTATCACGAAGGTCTTAGCAAATCAGTTTGTAAACA
TGAAATCGCTAATCAGATTATGGATCGATTATCAGGAAAAAAATATTCCTAAACAGGATTT
TATCTGTGCTCATTTGCCAACAACTAAATCAATCGTGAATGAAATATTAAATACAAGTAC
GACAAGCAGTAATGGTGATACAATACCATCAGATTCAGCTGTGAAAATTCTCAATGAAAA
AGTACGAGACATTGAATATCAATTTGTTTTGCAAGAAGATGGATATTGTTGTTATGTTTC
ATTTCGAGGGGCAGTTATCAAGGTTAGTTGGAAATCATTAATTTTTTTAGTGTAAattaa
caattttttaataattatttcatgatatttttctctaaatttttaaagttaattaatta
attaaaataattatattttatgaatgatatttagcatattctgttaatcaaacaggtttc
tttctgtttgttaacaaaaatttctgatttctatacaaaagcggaattttaaatttata
tggaaaataaaataaaaaa
>DjScaffold484:384-7876 strand:+
TTTATATTTATTAATAATAATAATAATAATATTTCAGTCAAATGTATTTCTGAAGAAACA
TATAAATCTCTGAGTGAAACTTATTATAACATAAATGAATTAAGCGAATCACAAATTAAT
AAATGTCTACAAATTGTAAACGAGTTCATTGAGAATTATCAATCAATAATTATTGATTGT
TTAAAAGAAAAGAAACTACTTGATGAAAACTTGAAAGTTTCTCTAAATTGTTTGTATGTA
TCAATCAATATTATATCAAAATATATTGAAAATCATCAACTTTCTACTACTATAAATGAT
TCCAGTTTGATTCGAGTCTTTGATGGATTCTTTGAGATGATTTTTACTCATAATTTGGCT
GAAAGAATTTGTGAATCAATTTTGTATGCATTGTGCAGTCTAATGAAACTAGAAAATAAG
AAGATTATTATGGAATTTTGGAAAGAGAAATTAGAAATATGCTTTCAAACTGATAAAATA
TCTGAAGAAAATAAGTTGGCATCTTCTGTCATTCTTGTGCAGATGCAGATTTCCAACAGC
AAATTATTGTTTGAAATTACTAATTATTTGTTTAACAAAATAGATTTGGATTTTTCTGAT
AATTATTTTGGATTCTCGAGATCAGAAATAAATTCTGCCAGGAGAAACGCATTGAATCAT
TTAAAAATTCTCTTGAAAACCACATTGGATAATGATAAAGATCATGATCTATTTATCATA
TATTTAAAGAATATTATCAATTTACAATTTAACAACAAATCAAACCAATCCTATGTAATT
GAAATGAACAATCTTTCTGATGTTTCTGTCAAATCATTTGAAACTAACTATGAGAATTTG
GTAAATGAATTTGAGCAATTTGAAACGCTAAATGTTTTCATTGAGAAATATTTTAATTTT
TTTGTTCACGAATTTCCCACTATATTAATAATTGTTGAAAATATACTTTCACACTTGGAT
AATGAGTTGGATAACCAATTAATTGGAAGAAATGAGAATATATCAAAATATTTTCAGAAT
TATCTCAGAAATTGTGGTCCAATAAAAGATTTGTCTTCTACATTGGCAATTTGTTTTATA
ATTGATTGTGACAAAACTACAATTATTGAAATTCGAAAATATTTAAATGAAAGTAATCAA
CATTTCCTTGATAAACTTTCTAAAGAAAATATAAAATTGGCACTAAATATTCTGGATAAA
ATTGCAAAACGATATTTTCATGTGAAATAAtggcatcatgttttcagcatataaaatgac
atgaatattttaaagacttttcga
>DjScaffold484:17920-18598 strand:+
gacaattgaatgcagtagcaaaaagacaagcttttctgatgcctaatgtcgataaaatgc

tggacaagttaaatgccatttggcatcgcagtaccagggacatttcaatagATGATGACA
AAAGTGGTAAGCGGAATCAATGGAGCCATAGTTTATCTTGATGACATACTGATATTTATT
AACAATGAAGATCAACAATATGAAGTAATTGAAAGATTATTGATGGACATAATGACGGCT
GGACTAAGAATTAATCTGGAAAAGTGTCGGTTTCTTCCAATTGAAAATAAGTTTCTTGGA
CACATAATTAGCAGGGATGGTGTTTTAACTGATCCGCCTAAAATAAAAGCTGTAAGAACA
TTTGACAAACTTAACTGTGTAAAGAAGTTACGAAGTTTCTTGAAAATTCGTAATTATTAC
AGGAAGATTATAGAAAACTACGCAAGAAAATCAAAGATATTAGAGCAGTTGTGTAGCGGA
CCAATCAACAAAAAATTAGAATGGACAGATGAAATAAACGATACTTTTGAGGGTCTGAAA
AGATCGTTATGActgca
>DjScaffold635:81013-82275 strand:+
ttattatcaattctaacgatatattgatttattaataatcaattataatttatgattttc
ttggcgacattttcaaaatcaatatagaattttatccatatttaattgatatttattttt
tctcaacgtattcttatctttagatttttacgtttttatcagcttccatttatttcatat
ttattatttagaaataacaacgtatgtgtttgtttttgtataagtgggctttattaatga
agactgtatatttttgattgatgctttcttattgatatttacgactttttactcagaatt
tatttatatatttaaacgtttaatccgattctatttgattccataattaatagaaatgaa
aagtatATGCATGACTATTCCGTAATTATTGCGCATTTCAAATGGTCAGATTTGGACAAG
AAGAAATCACCTACTTCCATGACAATGAAATTAAATTTACCTCCAAGAACAGCACTGATT
CGAATTAATAATCCAGTTTTGACAGATTCTAAGATCAAATTCAAAATCAAAATAAAAGCC
AATACAGGATTCTTTGTGATAGATCAAGACCATCTGACTATTGATGGGAAAGATTTTGAA
GTCATTGATAGAAATGATGCTGATGAAAAATCTGATAATTGTGATTTAAACGAATCGTTC
TTATCCAGCACATTGTTTTTCCAGGGAGTAGAACGGTTTATGAAGCATATTGAAAACTGT
CGTCAACATGGGGCTCCACGACTAGTTGACGTTGGTACTCACAACGAAGAAGATTGTACA
GCCGCAAAAACCAATCTTATTGAATTAGCACAATTTATTCAATTGTGCAAAGTCGTCAGT
GCAATCAATAAAGCAGTTTCCTCTTGTCAGACTGTGGTTGTTGATTGAttgccacgaaac
ttgcgcgcgccaacaatatttttataacatcattttagagtctatttctaattagttttt
attgtgttttattgataaattaatattattattgattattaattttattattattgatta
ttaatattattatgattgattattaatattattattattgattattaatattattattat
tattgattattaatattattattatttgttattaatattattattattgattattataat
attattattgattattatttaagaattgttttt
>DjScaffold635:91122-100302 strand:+
gaatgtttttattaatttaaattaatttatttttattttatttccttttaaatcgaaaat
cttaaatagcagcttatttattaaatacatATGACTTCTCATGCAAAACGATTACAATTT
GTAAAAAAATTACACGAACCAGATAATGATTATACACCTAGTATTATAAGTGGAACCGGC
TCAGTAAGCAGTAATAGTAGTCGAGGAACTGTTAGTAAATTGAAGAAAAATGCATATCAA
CCTCAGACAATACCTGAAAATCATGAATTAGACACATTTAACGAGCAGCAGGCAATATTG
CTTCCCAAGACTCCTATCAAATATAGTGCTGGTCCTTGCAGCAGCATTATATCAGGGAAA
TCTGGCCAAAGTTCTGTTAAAACCGGATCACATTTATCTATCGCGTCTGGCAGTCGACCA
CTTTCTGCTTCTAGAAGTCTTGGTGGTTCTAAGGCTTCGTTAACCAGTGCTAAAAGTTAT
AAATCAAGCATCCAACAAGAATTCCATGACATTAAAGTGCAAAGAAAAGTAACATCTTTA
TCATATGGTCGTGCCAAAAAACCTTTTAGATACAGTTTGATTTGTTTTGTAATTGGTGGA
GTTCTTTTCTTTATCGGAATTACTGTGATAATGTTTCGATTGTATGGACAATTGTTTCTC
AGTGTAGACAAGGGGGAAATTATTGGGCCGATTTTTATAGTTAGTGGTTTGATTTTGGTG
AGTTTCGGTATAAAATTTATGGTCGATGCTTATCGATATGGAGTTATTGAACGGAAAAGG
CTTGGATTTAGAGCAGCTGGACCTAATATGGTAGCATCTAATGATAATGGAAAACCCAAA
GCGAAGTAAgttcaaacactaatatattatcaattatattgggtctgctggcaaagaagc
tgttcgtcaagttttgcatacggtatctttgggcgaatttcactccacgtcgatgcttcg
atgaaacatatcttttaattttatatatttttcattttataaatgctcaattttcgaaag
gaatccaataatcaattttgaatgtttttattaatttaaattaatttatttttattttat
ttccttttaaatcgaaaatcttaaatagcagcttatttattaaatacatATGACTTCTCA
TGCAAAACGATTACAATTTGTAAAAAAATTACACGAACCAGATAATGATTATACACCTAG
TATTATAAGTGGAACCGGCTCAGTAAGCAGTAATAGTAGTCGAGGAACTGTTAGTAAATT
GAAGAAAAATGCATATCAACCTCAGACAATACCTGAAAATCATGAATTAGACACATTTAA
CGAGCAGCAGGCAATATTGCTTCCCAAGACTCCTATCAAATATAGTGCTGGTCCTTGCAG
CAGCATTATATCAGGGAAATCTGGCCAAAGTTCTGTTAAAACCGGATCACATTTATCTAT
CGCGTCTGGCAGTCGACCACTTTCTGCTTCTAGAAGTCTTGGTGGTTCTAAGGCTTCGTT
AACCAGTGCTAAAAGTTATAAATCAAGCATCCAACAAGAATTCCATGACATTAAAGTGCA

AAGAAAAGTAACATCTTTATCATATGGTCGTGCCAAAAAACCTTTTAGATACAGTTTGAT
TTGTTTTGTAATTGGTGGAGTTCTTTTCTTTATCGGAATTACTGTGATAATGTTTCGATT
GTATGGACAATTGTTTCTCAGTGTAGACAAGGGGGAAATTATTGGGCCGATTTTTATAGT
TAGTGGTTTGATTTTGGTGAGTTTCGGTATAAAATTTATGGTCGATGCTTATCGATATGG
AGTTATTGAACGGAAAAGGCTTGGATTTAGAGCAGCTGGACCTAATATGGTAACATTTAA
AGTAGCATCTAATGATAATGGAAAACCCAAAGCG
>DjScaffold635:117912-118942 strand:+
AAAGTGCCCGTTACTATATTAACTGGATGTCTTGGATCTGGAAAAACAACATTATTAAAT
TATATCTTAAATGAAAATCATGGGAAAAAAATTGCTGTCATTTTGAATGAATTTGGATCT
GGATCAGCTATCGAATTAAATGAAAATATAAAAGATAATGGTGTTTTGTATGAAGAATGG
TTAGAATTGTTGAATGGTTGCCTTTGCTGTTCTATTAAAGATGTTGGAGTTAAAGCTTTG
GAAAGTTTAATGAAAAAGAAAGGAAAATTTGACTATATCTTATTGGAAACAACTGGATTA
GCAGATCCAGGACCTATTGCATCTATTTTTTGGTTAGATGATAGTTTATGTAGCGATTTA
TACTTGGATGGAATTGTTACAGTAGTTGATGGATTTTACATAGATAAACAGATGTCTGAA
AATAATGATAATGAAGGAATAAATGAATGCCTTAGACAAATATCTCTTGCTGATGTTATA
GTATTTAATAAAATCGATCTAATTAGTCAATTGGAAATGGAGCAGAGAAAATTGTCGATT
CGGTGAgttaattttgataaatgctttttaaataactattttcatttttaaatttttata
aataattaacttaataattaaattaattaataattaaattaattaatttaaataattcat
ttataaattactgaaaaatgtttttgtaatgtggaagtatagagtttngttaattttgaa
atgtgctttttatataactattttcatttttaaatttttataaataattaacttaataat
taaattaattaatttaaataattcattaatttaaataattcatttataaattactgaaaa
atgtttttgtaatgtgaaagtagagtttttataactaaatatactgttcacctggtaga
aatta
>DjScaffold1358:104164-105238 strand:+
ACTGAAGATGAAGAAATATTTACGAAATCTATTTATCACTTTTTACATGAGCGTGATCTT
AATTCTGGAAAAAAAGAGTCAATACATCGCATAACAATTGTTTCCAAACTACCTCCAAAT
AAGTCATTAGATTCAAGACAATTAGTGGCCGAATTTTTTGAAAAACTTATGGATGGCTTT
GTTGATACCAACAAAGTAACAGGGTTACTTCTTTTATACCCTAATCACGCAATTCATATT
TTGGAATCTACTGATCATACAATCCTAGAAATTGTTAGATTATTTAACTTAATGGAAATG
AATGAAATTGATGATTCTATGTTGATGTTTTCGAGAAGTAGAATATTAGTTGTATCGAGA
AATATTAATCAGAGATTATATGTTCAATACAATACCAAAATATTAGACATGGAACCAGCG
AATGTCAATTTGTTTGAATCTAACGATTCTGATTATAAAGTTGTTATTGATGTTATGAAT
CAATTAACAAAGTTAGGAGTATTTCTTTTTAAAGAGCCATTAAAAGATCAAAGAGAATTC
GTTCCAGAAATTTTTTCACTGAAATTGTCGGAAGTGTTGCAAAATATTCGTGATCGTGTA
CCTGATCTTATTCCTCAACAAATAATTGTTGGGTATTTGGCAGAATTATCTCAGGATTCA
TGTTTAATACGCTTAAGTGAATTCATTCAATACTATGAAAAACCGTTTTCTGTTCTTTTG
GAATCTGGTATTGAA
>DjScaffold1771:6958-9648 strand:+
CAGCAAATAGATGAAACATTTTTCGAAAGTCATTTTGATTTGATTTATAAAGAATTTATT
CTAAATGATAACAAAATATTACCATTTTATAAAATAAAATCAGGAGACGAAATACGGATT
GGAAAAATTGAAAATTATTTGCAAAAAGATGAGATTATTTTAGAAGTTTTTCTAATTCAA
TCGATTCCTGAATGGATGTATTTAGTTGTAAACATGCATTGTACCATAGCTGAAATAATT
GATATTATTGAGAGAGTTATTATTGATTTTCATAATCTTTTCATTTGGGACAAAAAATGGA
AATTACATTTTCAAGAATAAAGTTGTTAGTGAAATTGATAAAATTGAAGGATATCACAAG
ATTTTGATTAATTGTGAAAGCGAAAACAACACATTTTTCGAATGTTGTGAAATTCATGAT
AGAACTATTTTGGAGAATTATTCTGCGAATTATCAAGATGATTTCTTACTGTTCGAGAAT
TGTTATTACATGCCCAAACCAATGGTAAATCCCACTAAAGATCTACTAAATTTCGATACA
AGTGATTATATAAATCCGTCAAAATTTCTCGAATTGAAAGATTTGGTGTCGAATATAAAT
ATTTCTTTTCAAAATATGAAATTTCTATTTTGTACGAGAATCGCTGATCGTATCTTTTAT
ATATCTAAAATGTTTTGGAACGATCTTAGAGAGGAAGTAAAGAAACAATCTAACACCGAT
TATTTATCAGATTCACTTAATGTTTTTGAAAGCTTTGACAGATTTGACCACTATTTTCGA
ATTGCTTTGTTAGTAAAGATTTGAtgatttcattatattatctaaatacctttatttata
attgatttttaattgatatttaatgaagtttattaataataattatttctataataaata
ttgaacttttccatttttgataaatctcatcaatataattataaacgtttaattctctt
tgaaaacattctatgattgtcgtattctcttttggtatgattcaagaaatcagataaaa
aatattctaatggacatactataccttgaggataataaactattttcaattaaagaaacg
aaattgatttctctaatgttattaaaacaaatttcttagtaat

>DjScaffold4985:12185-22032 strand:+
AGTCCCAGAACCGCTACAATTGATTGGTGTGAAGGAAACTATGAAATTTCACATTATATA
GCTGAGTTTTGGAATACGATTAGCAATGCTTTAATGATTGTATTTCCAATTTGCTTAATG
TGGGTTTCAAGGAATTGGAAAATGGATTTAGAATTCAGGTTATGTTATTTAAGTCTTATT
GTAGTGGGAATTGGATCGTGGGCATTTCATATGACTCTTCTTTATCCTATGCAACTATTA
GATGAATTACCAATGATATGGTGTTCCTGTTTGTTATTGCATACTGTATTTAAAGTTGAA
CGGACGTCTTCGAATTTAAAAACGATTTTGTTAATAATTTATTCAACAATTGTTACTTTG
GTAAGTTTATAGcaattaaatataattatatatatatatatatatttatacatatttata
tatatttatacatatttatataaattatttatgtatcaaatgtaaactgactcaatttaa
cttattgaatttttaattgtttatgaaattttcacaaattattggcataaatttatcaat
aattagaaaatatttacatcatttaaataaaatttgtgttcaattttcattattaatattg
atttaattagtttttgttaaataaaaatatattccaacgaaagtagtcattatcgattaa
tatgtgttgcctgttgtatgttaattaattaaaacaaatatgaatccaatatttatataa
atcttttgtataggaattagtgatttttaatgtattttagtt
>DjScaffold4985:34831-47235 strand:+
taggtgcatacaagaactttaaaaattctatttgaagattacagattttctgtcaaaac
gtaataaaattatatattttgaaatggcgattgacactttttaattgatttgcttgttta
taatttacgatttaatataattttactatttataattttatcataataaattataattgt
ataagtttatttatattttagatttattcctttaattcaaaatctaaactaaaatttata
ttcgaaaatgtacacttaaattttaatcaattttaaaacttgttttcaaaacattttaat
attaaattatcttataaatttaataaaactattttATGTATTATTTTATGAATTTTAAT
TTTGTTTATCTCTGCTTACCAAATCCATTATTCCATCATTCCAGTTACGCTGTATTGGTG
ATTTATACTATATATAGAACATCAAAAATAATAAAAATAGAAAAATGCTCAAAACAGATT
GCTTTAGTTTCAGCGTCCACTTATACAATGGGATTTGTATTTTGGAATATTGACAATATG
TATTGTAAATATTTAACAAATTTCCGGTCTTTTGATATTTCTTTAATAAATCCATTCACT
CAAATGCATGCTTGGTGGCATATCTTCAGTGGATTTGGCAGTTATTTTTATTTAATTTTT
TTAACACATATTTCTTTGAATCATCTGAGTGAAAATCCTCAACTAAGTTTTATTGGATAT
ATTGTTCCTATTGTTAGACCAACTTCCTCTTCAAACAGCAGAATTGGCTTTCGTTGCTAG
tttttttttaaattgaaaagatatgcgtgatatctcaagaattttttatgaaattataa
aaattatattattgtaaaaattgatcatgttag
>DjScaffold1992:1198-1989 strand:+
caaagcaatatattaatttcctccaacATGGTGGTAAAAACATACTTTGATATTGTTGCT
AACAAAAAGAAACTAGGAAGAATTGTCTTCGTTCTATATGAAGGCGATGTCCCTAAAACC
GTCGAAAATTTTAGATGTCTTTGTACCGGTGAGAAAGGATTTGGTTATAAAGGAAGCAAG
TTCCATCGAGTAATTCCGAATTTTATGCTTCAAGGTGGAGACTTTACCAAAGGAAACGGA
ACAGGTGGAAAGAGTATATATGGAGAAAAGTTTGGTGATGAAAACTTTAAATATAAACAT
ACCAAACCAGGACTTTTATCAATGGCGAATGCTGGTCCCAACACAAATGGATCACAGTTT
TTTATAACCACTGTTGTAACCAATTGGTTAGACGGTAAACACGTTGTTTTTGGAGAAGTT
GAGGAAGGTATGGACATCGTAAGACAAATTGAAAAGATGGGAAGTGAAAGTGGAAAGCCC
TCACAAGAAGTATTTATTGAGGATTGTGGTCAACTCAAATAAttccttgtattatgctaa
tagtgaaaacttggtaaagcatttccatgtttgacagttattaacttgaatgtgttgttt
tttcgctattctgttagtcaataatagatggtttttaaatttgattttgataaaaaaagtt
taatttatattcccgtcttgtaagccaacatagtattaaaagttaattccgtattgttat
tgatttgtgtcagtagtaaataag
>DjScaffold1992:16653-28035 strand:+
gattATGTCAAAAGCATTTGATAAAATATTGGAAGTGTATAAAAAATTTCCAAATGAATT
TAAAAATTCAAGCCTTTATGATGATGTAAATAATTTGAAAATGACATTTGAAAATAAGAA
ACATTTCTTCGATAATTTTCTAGCAAAAATCGTAATCATTGGAAATGTTTCAACTGGCAA
GAGCACATTAATCAATGCTTTACTTGGTAAAGATTATTTTGGATCAATAAGTGGAAATGG
AACAGTTACATTTTTTTATTGTTCTTTATCTAATAAGAATTATGAATATTTCACTGTGAA
TAATTGTCAAATTGAATATAAAGAAATTGAAACTTTTAAAAATACAACAAACTTGAATAT
TAATCTGCCAGATTATTTTGATGAGAAATCTGCTATAAAGATTTACGTAAAATATGATGA
AAATAAAATATTAGAAAGGAAAACAGTTATTGTGGATTGTCCTGGATTTGGTCATAGTGA
AGAATATGATGAAGCAATCGAAAATCATTGTTCAAATGCAGATATGTTCATATTTATGTT
TCAAATTATTGAACCAATCACTGAGGATCAAATGAAGTTTATAAAAAAATTAAGAGGTAA
CAATGAAAGGCAAACGTGTTTTTTCGTTTATAACAAATGTGATTTACCTAATTTCAATGA
AAATGTGAAAATTTCTTTACGTACAGATTTTTTTGCATTATTTGGTCAGCAGTATGCAAC

GAATCTTATGTTAAATTTCGATCCATTATATTTCATGTCAGTTAAGTTCCCCATTCAATT
TGAAGATTTTGCTCGACTAAAATGTGATATTATGATTCAAAAAGATGTTAAATTTCATCT
ATATGCAAATGTTCAATCGAAATTATCCATTGAAATTGATAAATTTTGTGAAGTATTTAG
TCATTTTGGATCCAAATATACTGAAAAAATATTATCATTAAAATTGATTAATGAAAAGAA
AAATCAGATTCTTGAATTTGTAAAAAGAAGCATTGATATTTGCAGAAACAATCTTCTTCT
AGACAAAATAATTGAAATAATGCTTACAGAAATTTATGATTGTGGTGACAAATGTTATAA
ACAAACTGCACCAATATTAATTTTAAAAATTGAAGAAAATATTAAAAAGAAGGTGAATGA
ATTTGTAGAAAATCAATTAAACAAATTGTGCATTGAACCAATAAATAAAAAATTACAGGA
AATTAGAAATGATACAAATGGAAACATAATTATCAATGATATTGACTTGAGAATTTCAAC
GGATCATTTGATAATACCAATTTATAGAAACATAGAAGATATATTTTTTTGGTTCGAA
>DjScaffold826:81825-92680 strand:+
atattttttaatctattttaattattataattttagaaatcataaaagatataaaatttt
taattaaatagtgaaatgtttttttgttgtatattaaaatttgacattaataaaatttta
aattggaatattttacataatgttgatatttgatatttttcgctttgaatatgaattttg
aaatttgtttgcgaaaagcaaattattatacatgtatatagtttttgtgaataaatttt
ctatgaaattcagttttgatattaatgatccgatgagtacttcaaactcatctaacagta
ttattgagattagttccttaataaatctagaataaattcatgacatcaatttcagaattt
gtgataaattatctgaattaatgtattacattttttaggaagcgtctaattttggagcc
aaagtggcagttttagattttgttcaaccaacaccgATGGGAACAACTTGGGGATTAGGA
GGAACTTGTGTTAATGTAGGTTGTATTCCAAAGAAACTAATGCACCAATCAGCTCTTCTT
AAAAGTGCTTGTGAAGACGCTAAATCGTATGGATGGAATATTCCCGACAAATTGGAACAC
AATTGGGAAAATATGGTCACTGAAATACAGAATCATATTCAATCACTTAATTGGGGATAT
CGTGTTTCATTGAGAAATAAAAGTGTAGATTACATCAACGCTTATGCGGAATTTATTGAT
GAGCACACAATAAAAGCAACGGATAAGTACAATTCATCAAGAGAAATTACATCTGATAAA
TTTCTAATAGCAACAGGCGAACGTCCCAAATATCCTGATATTCCTGGAGACAGGGAATAT
TGCATAACAAGTGACGATTTGTTTTCCTTGCAATATTTCCCTGGTAAAACACTCGTTGTT
GGTGCTAGTTATGTAGCTTTGGAATGTGCTGGTTTTATTGCTGCTCTTGGTGGTAAAGTC
ACGGTAATGGTTAGATCAATTTTCCTTCGTGGATTTGATCAACAAATGGCAAATAAAGTT
GCTGAATATATGAAAACACATGGTATAGAATTCATAAATGAATGTGTACCAACAAAGATC
ATTCCAATGGAAGAAGGCAGTCCCGGAAAGAAAGAAGTATTTGGAAGTTATGTAAAGAAC
AATAATGAATACAAAGATTGGTTTAATACAGTTTTATTTGCGATCGGTAGGGATCCATGT
ACAAGCAATATTGGGCTTGGAAATATAAATTTGGATTTGGATAAAAATGGTCGAGTTAAA
GTTACTAATGAAGAACAAACAAATGTTGAAAATGTTTACGCAATTGGAGATATTATTTCT
GGAGGACTGCAGCTTACACCGGTTGCAATTCAAGCCGGTAAAAATCTGGCTAGGAGATTG
TACGGTGGTGAGCGAGTATTAACAGATTACGTCAATGTTCCAACTACAGTTTTCACTCCG
TTAGAATATGGCTGCATCGGGTTGCCTGAAGAAGATGCTATTTCTAAATTTGGCGCAGAA
AACATTGAAGTATTTCATCAAAATTTCAAGCCACTTGAATGGACAGTGGCACATAGAGGG
GATAATGATTGTTACGGAAAACTTATTTGTAATAAGTCGGATAACATGCGAGTAATTGGT
CTGCATGTTTTGGGTCCGAATGCTGGTGAAATCACTCAAGGATATGCTGTAGCCATGAGA
ATGGGTGCAACGAAACGAGATTTTGATGACACCATTGGTATTCATCCAACTTGTTCAGAG
ACATTTACGACATTGAATATTACCAAGTCATCAGGTGCCGATCCTGCTCAATCGGGTTGC
TGAggttaatttccgattatatcgattcctcaattatttccagaaatctggatgttgagt
tgagtctataatcggaatagcatattaacattttacttatggctgtttataactacgttt
ttcgatgtgcttttggacaacttgctgtatacattctttcattgatgtcgttttacgatc
tatagaaattatcttttcaatgataactaatgcagataaattcctttggaggtctgttta
gtttgatgcgatttcttagattatgacacattcattgttgtgtaatatatttttctataa
attttaaactctttattgtgctgtgtgtgtgaagataagttattcaaatgtttatgatct
tacaaaccaatgttttaaatgtagagataattaaataattttcattttccataaaatgaa
tgagacgagtaaacaaataaaatatagcaaaagtttaaactttgtttacttcattaagga
aactattttgtgacttatcaaacaaatcaggtaagaattgattaactgttagatgtagac
aatttattcttttcattaaatctgcaaacaaatactacatttttacagtgaaaataaat
tgttttttggcttgttttatctcgttttccagactatcgctgatttgtattcatcgaaaa
aattattaaaaaatattaattttttttgttattatattttattggattatttttatact
tgggaatttgtatatatatatatatatcaatgtttcaagatcattccaATGGAAGAAGGC
AGTCCCGGAAAGAAAGAAGTATTTGGAAGTTATGTAAAGAACAATAATGAATACAAAGAT
TGGTTTAATACAGTTTTATTTGCGATCGGTAGGGATCCATGTACAAGCAATATTGGGCTT
GGAAATATAAATTTGGATTTGGATAAAAATGGTCGAGTTAAAGTTACTAATGAAGAACAA

ACAAATGTTGAAAATGTTTACGCAATTGGAGATATTATTTCTGGAGGACTGCAGCTTACA
CCGGTTGCAATTCAAGCCGGTAAAAATCTGGCTAGGAGATTGTACGGTGGTGAGCGAGTA
TTAACAGATTACGTCAATGTTCCAACTACAGTTTTCACTCCGTTAGAATATGGCTGCATC
GGGTTGCCTGAAGAAGATGCTATTTCTAAATTTGGCGCAGAAAACATTGAAGTATTTCAT
CAAAATTTCAAGCCACTTGAATGGACAGTGGCACATAGAGGGGATAATGATTGTTACGGA
AAACTTATTTGTAATAAGTCGGATAACATGCGAGTAATTGGTCTGCATGTTTTGGGTCCG
AATGCTGGTGAAATCACTCAAGGATATGCTGTAGCCATGAGAATGGGTGCAACGAAACGA
GATTTTGATGACACCATTGGTATTCATCCAACTTGTTCAGAGACATTTACGACATTGAAT
ATTACCAAGTCATCAGGTGCCGATCCTGCTCAATCGGGTTGCTGAggttaatttccgatt
atatcgattcctcaattatttccagaaatctggatgttgagttgagtctataatcggaat
agcatattaacattttacttatggctgtttataactacgtttttcgatgtgcttttggac
aacttgctgtatacattctttcattgatgtcgttttacgatctatagaaattatcttttc
aatgataactaatgcagataaattcctttggaggtctgtttagtttgatgcgatttctta
gattatgacacattcattgttgtgtaatatatttttctataaattta
>DjScaffold2479:15811-22744 strand:+
TCAAAATCTGACCAAAAATTTAATGACGATGAAAGAGCTGAATGGCAACATGATGATTTA
GCATCGGTGAAAATTAGAGTTTATCACGGTCATAAAGATAAGGTTTCCAGTATAAATTAT
TGTTCTAATAACAATAAAATTATATCTTCAAGTTTTGATAAAACCTGTAAATTATGGAAT
ATTTCTACTGGAAATATTTTACATACTTATCAAGGTCATGATTCATATGTAAATTATTGC
GAACCAAATAAAAACGAGTCACAATTTCTTAGTTGTGGCTGGGATAGTAATTTGATGGTT
TGGGATACTGAAACTGGACAAATACCACTAAAAATAAAATTTGGAGGAATAATTTATTGC
AGTCGATTTTCCAGTAATGATAATTTAATAATTACCGGAACTGATTTAGATAATCTCATC
ACGATTGTCGATATCAAATCAGGCAAAAAAATCCGATCAATTAGAGGTGTAAATTTCTTT
CAGATCTTCAATTATAACTATTAAtataaataaattttaaaaaattattagttcaaaaaa
ttaattttactcccaaataaacttaattattgacaaatttcacattgattaataaataaa
taattatgaagaatttcaaccaatagaaatcaattgcaattattgcgagaataattgttg
gacaaaagattttaaaaatatgatcaccaatcacacaaaaattgcaacttgcttttcaaa
agccattcacaattattattagaggcctagttaacaaattttaaacttaactgattttta
ttatctttggggttgagaaacactgatatgtagtttctttaatatttcaatattaacatt
ttcgatctcctcaaaaatagggtcatatagattgggttgaagacgttaaaattagcagtg
atcaaaactggattctttcttgctctaaggatcatacaattcggttatggaatattgaac
aaaccgataagctatcaacaccttccgtaacgaatacaaaagccagtagaggagttcaaa
tttttgagtgtcctaaatgtggcaaaaaaatacaaaggaactgatgatgccatgtgtgtat
tttgtcgattggaatctgattcaaataccagtatgaatattgttaaacaaagattctcaa
tgatatcattataaatttTCAAAATCTGACCAAAAATTTAATGACGATGAAAGAGCTGAA
TGGCAACATGATGATTTAGCATCGGTGAAAATTAGAGTTTATCACGGTCATAAAGATAAG
GTTTCCAGTATAAATTATTGTTCTAATAACAATAAAATTATATCTTCAAGTTTTGATAAA
ACCTGTAAATTATGGAATATTTCTACTGGAAATATTTTACATACTTATCAAGGTCATGAT
TCATATGTAAATTATTGCGAACCAAATAAAAACGAGTCACAATTTCTTAGTTGTGGCTGG
GATAGTAATTTGATGGTTTGGGATACTGAAACTGGACAAATACCACTAAAAATAAAATTT
GGAGGAATAATTTATTGCAGTCGATTTTCCAGTAATGATAATTTAATAATTACCGGAACT
GATTTAGATAATCTCATCACGATTGTCGATATCAAATCAGGCAAAAAAATCCGATCAATT
AGAGGTGTAAATTTCTTTCAGATCTTCAATTATAACTATTAAtataaataaattttaaaa
aattattagttcaaaaaaattaattttactcccaaataaacttaattattgacaaatttca
cattgattaataaataaataattatgaagaatttcaaccaatagaaatcaattgcaatta
ttgcgagaataattgttggacaaacactgatatgtagtatctttaatatttcaatattaa
cattttcgatctcctcaaaaatagggtcatatagattgggttgaagacgttaaaattagc
agtgatcaaaactggattctttcttgctctaaggttttatataatatttctcttgttttc
aatcagtgaaaaattactcaggatcatacaattcggttatggaatattgaacaaaccgat
aagctatcaacaccttccgtaacgaatacaaaagccagtagaggagttcaaatttttgag
gttagtgtcctattatcttaaaattgaaaagattaactaggaaatattgctttgttttat
tatttagaataatgaagaaccatcggcattgaatggtatacaggttgttggtcgaattat
tgctcggcttatt
>DjScaffold1993:48742-49065 strand:+
ATGCCGTTTGGATTATGCTCAGCACCAGCGACATTTCAGCGGTTTATGAATATTGTATTG
CGAAATGAAAATTGGAAAAAGTGCGTAATATATTTAGACGATGTTTTAATATTCGGTAAA
ACTGTAAAAGAGCATAATGAAAGACTAGATCAAATATTAAGAAGAATAAGAGAAGCTGGA

CTTAAATTAGCACCAAATAAATGTAAATTTTTACAGCAAAAAATACAATACCTTGGACAA
ATAATAACTAAAAATGGAATAGCAACAGACCCTGATAAGATAATGCCAATCCTCAATATC
GAAAAGAGCTTCAAACAGTTTTAG
>DjScaffold3542:7486-8369 strand:+
taggatattgctcatcattggcacatgcgtgtgttgaaaaagcaggaATGATTGCTTTTG
TGAAAATCCGTCAACTTGATGTCGATGATGATTTTTCAATGCGTGCTGCGACATTGGAAG
AAGCAATTGAAAATGATATTAAAGCAGGATTGATACCATTTATTGTTATTGCAACTCTCG
GAACTACTGCTTGTTGTTCATTTGATAGTTTAGAAGAAATAGGTCTTGTTTGTAAGAGAT
ATAATCTCTGGCTTCATGTTGATGCTGCTTATGCCGGATCAAGTTTTATCTGTCCAGAAT
TTCAGCATTATTTAAAAGGAATTGATAATGTTGATTCAATAAATTTCAATCCAAACAAAT
TTATGCTAATTGCTTTCGATTGTAGTGTTCTGTGGGTACGTCGGAGTGCTCAAATTACTG
ATTCGATGATTGTTGATCCAGTGTATCTGCAACACAAACATTCAAGTATGGAAACGATGA
ATATTTTCTTTTTTATATTTTATTTGTTATTCATATTTTACTATCAATATTTATATTTAT
ATTTTGAT
>DjScaffold4555:6482-7941 strand:+
ttttagaatatattgatgttatctcATGAATATTAAAATGATTTCTCCTCGAACTATTTT
TCATCGAAAACCTTTTGGTAAGAATATATTAGAAGTAAAGAATGCTTCCAAAGTTAATTA
TAAACAATCTAAAAGTACTGATGAATCAAATGAAAATGAATTTTGGATTGTTGAACCAGT
TTTAAATCAGCAGCTGGAATTGTATATTAAAGGATGTGATGCAGTACTTCATAATTTTAC
ATCTCAAGATCACAGTCATTTGTTTACTGGGTTTACGGAAACACCAATTAAATGCGCAGT
GTTTACGTGTTTATCAGATAAAGTTTCAACAAATGCCACAATCGATTCAATTTTTCAAGA
TTTGAACAACTCATATTCGAAAGGAGTTGTATTATTCACTGATACCATTGCAAAATATTA
TGGATTTAATGCAGAAATGCACACTTGTAAATTACCTTGCCAAGTGAGTGGTGCTTGGAA
AATTAGAAACTGTTTGTTTGTTGAACGAGAAGAAACAGAAACAGAGTTTTCTGATGAGAT
TTCTCCACATTCTTTGTTTTTAATGAGTCACACGCTCGCTGAATTCACCCCAGTCGTATT
TAGAGATGAATTCGGTTACTGCGGATTCATAAATGATACTCGTTTTCGTATTGTTTCTAT
ATGCGAGGCTTCGTTCCTGCTGTTGGCCTATGATAAATTTGATGAATCGTTTTCGTTGTG
GAGAATCAGAGATGCAGTTCAACAAGATTTTGCAGATCTTTATGATGTGCATTGGGACGG
ATCTATCGCTGTGAAGAAGGAGAAATTGTTGCATTGGTGGAATAATAATGACAATTCTGT
TAGTTTCCGATCTTCTCGTGCTGCTGCTGGTGGCAGTATTGGTGGCGGCACTGCTGGGGA
ATACTCGATAAAAATGAGAACTCATCATCATCATCATCATCAAATGAACCACTCTCATGT
CTCATCTGAGTATTTCGAGCTCACTACTAACGAGTTGACAGTAGCAACAACAACAACAAC
AGTGAGTAACGCAGCAGCAGCGCACGGTAATTATCTATTTATATTTCTCTCTCTATCAAT
TATTCAATAAtttgtttgtttgttccattcattcattatgaatacattattgattgcact
cctctcttctccttgcacaaacaattctctcttagtgcagttcctccttcacatttctca
atgtatctcatctcctctatcttccttgttctcctacaatcgatcctcctcctc
>DjScaffold4555:35683-44806 strand:+
gagcgtacagttgacgatctcgacattcgcagtccaATGGCAAGATCATTAAAAATAACA
AAAGATGCCCAAATACCGGAAAAAATGACATTGCTCCAAGAAATCAATAAAATAAATCAA
ATTTATCGACAAATGGTCGAACCACTCTATCCAAAAGTTTGTCTGGATTTCTTATTTCAG
TGTAAATCGAGCATTCCTGAATCAGTATTTGATTGTAAAGATGTCGGTCACCATAGCACT
CCCATGGGAAATGATAATAGAAAAATGAAAACCGTTATAACGGAAATTGCTACCAAAGGA
TTCTTTGTCGAAGATTTCTATCGACGAAAATATTTATGCTGCTTAAATAAATATGGGAAA
GAAAATATGCTGAAAATTGTCAAATTCAACTATGAAAATAATTGTTTCTCTCATCTAGAT
GAGCATTGTTGGGCTGTTTTAGATGCAGTTTATCTAGATGGACTGAAATTGATCATCATT
CTAGAAATAGGCAGCACTCTGGCACTTTATACTGGGATGACGTTTGTCTGCAAGATTTCT
TTATTGAACGCACTAATTTACGAACCGAATATGTTTGATGAAAGTGGTAGAGAAATGTCA
ACGAACGACATCAATAATATGGTTTCATTGTCAGAACCAACGAATAATGGGTTCACAATA
CACACTCAATCGAATTTATCAGTACGATTATATATGCCGGAAATGGTTCGATTTCATCTC
GTAAAGAAATGCTTGTTGGTATTAAAGAAATTGTTACCGATCGATGTTGGAATGGTTTTG
CTTTGTAGATGGTATTCTTTGAATCGAGCAGTTGACAACAATGAGGCTACATCAACAGAG
TGGCGTAGATTTGTTGACTTTCTACTGGAAAATATAGGATTTGTGAAGAATCTGCACCAT
GAGCACAGTTCGATACTGAGAGATGTGAAGGAATTATGTTTGAACAATATTGCTTCAAAT
AAAATGGAACAAGACCTGGAAAGCAGTAATGATATAAGTATGGAAGAGATTTGCACAAAG
AAATTGCGGACGAATTCTGGTTTATATTCATTAGAAAACAAAAACATCAACAGGGATAAA
TTGGAGGATTATTCTGGTTTGTCTCACAACGTTGCCGATCGGTTGCTGTCTCGATACGCT
ACAACGGTTTTGTTTTCTCTTCATCTAGTGTATGAATGTGAAAAAATCAACTATTCATCA

TGTCTATATTTGGATAATTTGGCAGCTCTCAATTGTTCCTTGGCTTGCAGTGTCGAGGCA
AATCAATATGTGGATTATTATGTGAGAGATAATCCGGATTTGTGGTCGAGAGTAAATCAA
TTGCCATCTCTAATGAAATATGTGTCTCCTGTGGATAATGAAATGATCTGGAATCCTCAG
CCTCCAGATTGGAATAAATGGTTCACCGACATTCTTCTAAATAGACCATGTGATCGATAT
CCGATTATTGAAGATCTCAGTGAAATGATTTTGCACCTCATTAAGATATGGCTTGTGATG
TTTCGACACGACAAATTAATAGAGAATCCGGAAATGGAGTTGAACAATTATTTGTTTCGA
ATAAACTTTAATTTGAACGGTAAACCCTGCCAATTTCCAATTCATGTGTGCCAAGATATT
GTTGATAGCATTAGCAAAGGTATTTTGGATATTGTTGACATTTTCGAGAGAGTTGCTGCC
TATTTGGGCCAAATCAGCTTTCATACTGCAGATCTTGATTATATGAACATCGGGCTGGCA
ACGATTTTCAAACTAGTGTTCTTCCATTGCAATAAATATCCGCATTTGTACAACATTCTA
CATAGATCCGATCTCGATAAACAACGCCAACTCATAATGCCAATCATTAAAGAGAACAGT
GTTGTTGAAGTAATGCCGAATCCTCTTCACAAATTTAGATCATTGAGCGAGGCTCGACGT
AATTGTTTCTCGTATTGGACAAACAATGTCAAGCCATCAATTAATATCAAGAAATTTGTC
GATTTTAACAAGAATGATAATCATTACTTATTGGCAGTACTTGACTCAGACCCGATGGTT
CAATTATCCTATAGTGACTTGAGAATCCACGAGGCATATAAAATGTTGCAATCTTGTTGT
CCAATCGAATTGGATGCTAATATTTGCAGCAATTTCACTGTGGATGACGGAGATATTCTT
TCACCAGCGTTAAAAGATGAGTTGGACGGACATTTGAAAATTATTAATTGCCAAAGAACA
TTTTCATTACCGATTGGAAGAGGAATGCTGGGATTACAAACAGTGCAATCGAACTGGTTG
GGGGAGAAATTACCAGTTCCTCAGTTATGTCTGACTGGAAAATTCAGATTATCGGAAAGT
GGTAAACCGCAAATCATTGAAAGTAGCGCCTCAACAAAAGTTTGGCCTAATTTTCATAAT
GGAGTTGCTGCCGGCCTGATGATTTCTCCAATGAATGACGGAGTGGATTTTGTTTGGATT
CTCAAACAATGTCCGTCGATACATCTTGACAAGATTGAATTAAATCCGGAACAAGCTGGC
ATTCTGTTTGGATTGGGTCTCAATGGTCATTTGAACAAGTTGAGTGCAGATACTATTGGG
CAAACAATTAAATCAATGAACGAAATGGCAACCATGGCAATTCTAATAGCAATGAGTGCA
TTTAATCTGGGATCAAAGAATTCAGTCTTATCAAAACACATCTCATTCCATTATCGAAGA
TTGATACCAGGAGAGAAAGATCATATTGATCCGATTCCAACACTATCTCAAGTGTCAGCT
ATTATTTCAGCTGGATTATTGAATTTCAAATCTGGTGATCGGAAAATGGCGAGAATATTT
ATTGAAGAAATGGGTCGCTCTCGAGAACACTTGTCAGTAGAAGGAACTCCGCCATTAGAT
GTGGAATTGACTTCTGTGACCGCAGGATTCAGTGTAGGATTGATAATGTTTCAACAAGGC
GACATTGTTTTCGGTCGAGAATACAATCCAGAAGAGTTGACTCGTCTCATGAGCTACCTA
AAAGGAACATATCGATTCCAATCAAAAGAAAATTTATGCGCGGATGTTCCTACAGATTTG
CAAATTAGAGCAATTCTGAAAAACAATGACGTGAAAAAACCGCAACCGATTTCAGAAGTT
GATAGTTGGAATTCGTTTCAAACGAAAAATCCGAGGGTACAAGAAGGTGAATATTACAAT
ATGGACATCACTGGATCAGGTGCTGCTGTTGCATTGGGATTGATATTCATGGATTCGGGA
AATGATATCATTGCCAGGCAGTTGGAATGTCCTGAAACATTCTATTTACTAGAGCAAATT
AGGTCTCCTGTTTTGTGTTTCCGACAAATAGCACACGGATTGATAATGTGGAGAAAAATC
AAATCGAGTTCTGAATGGGTTGACAGTACAATTCCAGAATTTGTCGCATCGAAAGCACTG
ATGGTCAAACCAACTGAAATACAGGAGAAATACTTTGAAACTGTCAACGACTCTTATTTG
CAAATGCTTGTTGGATCGTGCTTCGTGATGGGCTTGAGATACGCTGGTACTGATAACGAC
TGTGCTAGGAAAACTATAATCAATTATGTGGAGAAACTGATGTCAATCAAGTCCAATGCA
GAATTGATGCAAAATGTGAACCGTAACCAATATGATTGGGCACTATCGACATTGTTCCTG
TCTGCGTGCATGATATCAGCAGGCAGTGGAAATCTCCAACTGTTGCAACTATCTCGACAT
TTGCGGTCGATTCACCATCCAAAGGATTGTGTGAATTACGGAGTTTATATGACATACTCG
ATGTCGATCGGACTACTATTTCTTGGTGGTGGAAAATTAACTTTATCCAACTCTCCAAGA
TGTGCTGCAATATTACTTATTGCATTTTTCCCAAGATTCCCACCAACGTATTCTGGTAGA
AGATATCATCTCCAATCATTACGTCATCTTTACGTTATCGCTACCAGGCCTCGTGTGATG
TGCGCCGTTGATCTCGATACACAAATTGCAGTTCCGATCGAATCTAAAATCAAACTGAGA
AATGAAACTAGAGAATATCACGGTTGCTTCGTTTATCCTCCCGATAATCCAGCATTAATA
GAACAAATTGAATTTTATGACAAAGCTGGAAAATATTGGCCCCAGATTTACAAACCTAAA
ACAGAACCATGGAACATCTTGCTAGAATCACTACAGAGTGGAGGATATTTCTTTGTGAAA
AAGAAATCAATAGATCTCGAAATAAAGGAATTAGTGGAGGAATTCCGTGTTGAAAAAGCA
TCGATCGGTTATCAGTTGTCTGATTATCGATTGTTTGAACAATTCGTGTACGAAACTCAT
GATCTAGATTGCAAATTAATGTTTGACCCATTTGCAATTCACGATAAAGTTCTGGGATTC
GTGTTTGATCATATTTCTGAACTGAAACATTATTTTCTGAAAATCGTTTCAACCGACATT
AAAGATTTCCCTCAATTGATTATGCTGATGAATTGGTGTAACCAGAGCAATCTTATGAAG
ATTTGGTCGGTTTTCAGTTCATATTTCGGATTGAGTTTGCATTTAATCAAAGAAATTCAA
TGCGGTGAGGAGATCGGAGGAGATTATTCACTCTGTGATGAGGACACTAGATTTATTCTT

AGAACAATTTTGCAATAAataattaatttga
>DjScaffold1649:23530-24620 strand:+
attttagATGAGTATGATCGCCAATATTGATTGTCCCGCTGGTATCGGTAATGTGAAGAG
GATTATTTTCGTTTCCTCTGGGAAAGGTGGAGTTGGTAAATCAACTGTGGCAACTCAGTT
AGCATTATCACTGTGGAGATCGAAACACTCTGTTGGATTGCTCGATATCGATTTGTGTGG
TCCCTCGATACCGCGCATGTTGGGAGTTGAGAACAATAAGGTGAATGAATGCGACGGGAA
ACTGCTGCCTGTTTACGCTGATGCTGGCAGTGCTGAAAAGAGCCCTCGATTTGCTGTGAT
GTCGATTGGATTCCTGTTAGCTCATCCAACTGACAGTGCCACTTGGAGAGGACCCAAGAA
AGATTCCACGATCAAGATTCTTCTTAATGAGGTTCATTGGGGCCCTCTCGATTTCCTGAT
AATCGATACTCCACCAGGATCATCCGATGAGAAGATCACTATTGCTAATGCTATTAGAAA
TGATCATTTATCGCAATTCTCTGGTGCAATTATCGTGAGCACTCCTCAAATGGTCTCGCT
CTCTGACGTGAGGAAGGAAATCACTTTCTATCAGAAACTCAATTTGCCAATGCTGGGATT
GATCGAGAACATGAGTGGATTGTGTTGTCCTCGTTGTGGAAATATCGATCGAGTGTTCTC
GATGGGAGGCGGAGAGGCGCTAGCTGCTGCTCGACAAATTCCATTTCTCGGTTTCTTGCC
GATTCTGCCGGATATTGCCGCATCTGCCGACTCTGATGATGGATTTGTTGTGAAATCGGG
TTTCCATTCCGCTAGCAACAATGCTTTCTCAGATTTGATGaaacaattatcgaatattct
gtttactaacaacaaacacaacattttgatcgaaattttttatttattgaaacagaaat
ctagaaattctacatcattttaaattggctaaatatttgttggaattggctcaacaattt
aaacatttaatcattacatcacatcacgcttgatatttatacagttttctgttcatcgcg
ctgataatgcgatattccgccgagctgtttgcttattattcatttctctatatattattg
agtgcagtattcacaagaagaagaacgtacgcataggggaacacaaaaggtaccaaatg
taacatgtgatatgatacaatcgtatacggATGCCCCCAAGCTTGGTTATCGATATTTCT
CAAGATCCACCCGTTCATTGTTATTGTTATTCACCCGTTGATCTAATTTCTGCGACTCAG
GTGAATGAATGCGACGGGAAACTGCTGCCTGTTTACGCTGATGCTGGCAGTGCTGAAAAG
AGCCCTCGATTTGCTGTGATGTCGATTGGATTCCTGTTAGCTCATCCAACTGACAGTGCC
ACTTGGAGAGGACCCAAGAAAGATTCCACGATCAAGATTCTTCTTAATGAGGTTCATTGG
GGCCCTCTCGATTTCCTGATAATCGATACTCCACCAGGATCATCCGATGAGAAGATCACT
ATTGCTAATGCTATTAGAAATGATCATTTATCGCAATTCTCTGGTGCAATTATCGTGAGC
ACTCCTCAAATGGTCTCGCTCTCTGACGTGAGGAAGGAAATCACTTTCTATCAGAAACTC
AATTTGCCAATGCTGGGATTGATCGAGAACATGAGTGGATTGTGTTGTCCTCGTTGTGGA
AATATCGATCGAGTGTTCTCGATGGGAGGCGGAGAGGCGCTAGCTGCTGCTCGACAAATT
CCATTTCTCGGTTTCTTGCCGATTCTGCCGGATATTGCCGCATCTGCCGACTCTGATGAT
GGATTTGTTGTGAAATCGGGTTTCCATTCCGCTAGCAACAATGCTTTCTCAGATTTGATG
>DjScaffold2249:4383-16072 strand:+
aaaatggcgcatggagcatctttttctttgccgctactgccacttgtattatttgtatta
ttaaaccagaacctcgtttaATGGGAATTATTGGAATTTGTGCGATTTATCTCGTATTTA
ATGAATCATATATGGAATCTAAAGTTTGGCACTCATTAAGAGGACCGTTTATGATTCTAC
TTATGAAGGTCATTTCCGTTGCTATCGATATCGATAATGGCGGATTAAAATCTGTGTCCA
TATTGAAATTAGTTGGGTATCTTATGAGTCCCTGTACTATTTTATACGGACCATGGTTTG
ATTATCGTTCATATGAAAAATGGGCAAATCACTCTGCATTTAAATTTAGCTCATTTCTCA
ACTTCGTCAAAGCATATTGTTTAGGTTCAGCTTTTCTTCTTTATTCGACTTGTTTTAGTC
AAATGATATTTGCCAGTTTGCCAGATAACAAATGGATCCGCTCATATCAGGATGCTCAAT
CATTCAGATTTAGTCATTACTTCATCAGTTATTTGTCATTGAGTTACCTACATGTCATCA
ATATCGATCATGTTGTTGACACCAGTGCCAATCACGATGCTAGAAAAATTGTTGATCCAT
TATCTATCGAACTTCCTAGATCACTGATCGATGTTGTCACTTTATGGAATTTACCTATGC
ATTATTGGTTAAAAAATTACATATTCTTTCCTTGTCGACGATTTGGTAGATTTATG
>DjScaffold2296:24158-25279 strand:+
aaaatttaaaATGAGTTTTGCATCTTTTTTTGGTAAAAAAAAGAAGGAAAGTGAATCACC
CCAAGATGCCATTCAGAAGTTAAAAGGCATACAGGATATGCTTTTAAAAAAGCAGGACTT
TTTAGAAAAGAAAATTGAACAAGAATTATCAACCGCTAAAAAACATGGAACTAAAAATAA
AAGAGCGGCTTTACAAGCATTAGCTCGTAAAAAACGATATGAAAAGCAATTAACTCAAAT
TGATGGCACTTTAACAACCGTTGAATTTCAAGTTGAAGCTTTAGAAAATGCTAGTACAAA
TACCGAAGTGCTTAAGGCTATGCAAGTTGCCTCTCAGTCTCTAAAGAGTGCTCATCAGAA
TATGGATATTGACAAAGTTCATGATGTTATGGATGAAATACAAGAACAACAGGAATTAGC
TAAGGAAATCGCTGATGCTGTTTCCAACCCTGTGGGATTTGGTCATGACTATGATGAAGA
TGAATTATTAAAGGAATTAGAATCTTTACAGGAAGAAGATCTAGAAAATGCTTTATTGGA
TGTAGGTGGTCGTAACGCAAACACAGAACCAACTTTGGGACTTCCTAGTGCACCTCAGCA

TGAATTGACACGTGCTAGAAAAGATCGCAAAGAAGAAGATGAATTAGCTGATCTTGAAGC
ATGGGCATCATAAaaatttgacagataactgattttttacgtatcttggttgaataaat
cgaggttgatatattaattgtgttttgccgtttttatgttaaatactgtcggtagtacg
catgttttcgtggaaaagagactcttaatggctttcattcaatatttgtaatttaattct
ttacattaaatatataaatgttaaatttgtttatcatcaccaaaata
>DjScaffold2296:39427-40714 strand:+
tatatttttcaaattttgttaggaatattctttttaaATGAGAAGAAATACAAATATTC
GTGATCCTTGTGCTCGGGTAAAAGCACTTATAACAGAAGCTGAAAAGCATGATGTAGAAA
TTGGAATTTCACTTAAAGTTTATTTTAGAGCTGCTCAGAACCTTTTAAATAGTATGTACA
GTTATTTAGATGATGAAGATGTGGAAAGTGCATATATTTTGGCTACGAAATTCATTATTT
TATTTATTGAAAAGTTACCTAAACATCCAGAATATAAATCAGTTTCATCGGAAATGATAT
CAAAATGGAATCATGAAGTTAGCAAAACTCTTAAAGGAGCAGAAAAAATGAAAACGATAA
TTTTGGAAAATTATGTTAAACAAGAAAATGAGCGATGTAAAGAAGAACAAATTCAAGCTG
AAATTGATAAAAGAAAAGAAGAAGAAAACAAGAAACGTATCGAGAAAAAATGGTTGGAAG
AGCAAGAAAATAAGTTACATTTAAAAGAAAAAGAAAATTTGGATTTGTTGGAGGATTTTC
TTTCTGGAAATAAGACAAATTTACCTTCAGTTGATCGATCATTAAAACCTCTGTCTTCAT
TGGGAGTTGAAGATAACAATTCCAAAAATGCTTATAATTGGAGATCTTTACTGGTGCCGA
AATTTTTGTTTCAAAATTTTTTGAATCATGCTCAATCTAATACGATCAATAATATCGAAA
CTTGTGGGATTTTATGCGGTAAAATAACCAGCGGAAAATTAATCATTACAACTGTCGCCA
TTCCGAAGCAGAAAGGAACGAGTGATTCTTGTGCAACTGAGTATGAAGAAGAATTGTTTG
ATTATGTCGATCAGAAATCTCTAATCACTCTCGGATGGATTCATACTCATCCGACACAGA
CCGCATTCTTGTCGTCGGTGGATCTTCATACGCAATTATCATATCAGATCATGTTGCCAG
AAGCTATCGCAATAGTTTGTTCTCCCAAATATGATCAAAATTTGTTTTTTTCTTTAACTC
CAGAATACGGATTAAACTATATTAAAAACTGTAAAAAGAATGGATTTCATCCTCATCCGT
CGGAATCTAAATTATTTGAAGATAGTCCTCATGTTTGTTTGGTTGATTCTCCACTGGAAT
TTCACGATTTACGTACTAACAAAACGATTTACTTTAAATAGatttttcaatgaattttct
gggtttctggagaggacacattgagggcgacacatcacatttta
>DjScaffold2296:34148-34762 strand:+
ATGAATACAGTATTAACAGGTGTCGAACATGTAGTTGTATACATGGATGATACACTCATA
GTATCTGAAACATTCGAACAACATTTGGTCAGTATAGAAAATGTGTTATCCAGAATTAGA
AAAGCAAACATCAAATTAAAGCCTACAAAATGTAAATGGGCGGAAGAAAGTGTGTTATTT
CTTGGACATATTGTATCATCAAAAGGACTAGAACCAAATCCAGAAAAAGTGAAAACAGTA
CAAGAATTTCCAGTACCAAAAAGTGTACAACAACTACAAGGATTTAATGGTTTAGTTGGA
TATTATCGAAGATTCATAAAAGATTTTGCAAAAATAGCAGCACCATTAGTTGAATTAACA
AAAGGTATGAAAACAAAAGGGGAAAGCAAGAACGTTATGATTGTGGAACAGTTGGAAGAG
AATTCAAAGAAACCTCAGAAGAAATGGGGTCCAAAACAAGAAGAAGCTTTTGATACGCTA
AAACAACGATTACTAACAGCTCCAATATTAAGGTATCCTGATTTTACAAAGCAATTCATA
GTAATGACAGACGCAAGTGGATATGCCATGGGGGCAGTACTTAGACAAGAGGAAAAAAAA
ATAAAGATTATGTAA
>DjScaffold4295:10723-11721 strand:+
ATGATAAATAAAACTTCACATACTGATTCAAATCAACAAATTGAATTTGAAGAAGACGAT
ATTCCAAAATTCACAGAACCATTTTTTGTCATAGAGACTTGTTGCATAATTTGGTTCACT
TTTGAATTACTAGTCCGTTTTGCCTCTTCTCCAAGAAAATTGGAGTTTTTCAAAAATATT
ATGAATCTTATCGATATAGTTGCTATAATGCCCTATTTTATTACTCTTGGAACAGCTCTT
GTTGATGATACACGTCGGAGCAATCAAACAACCTCCCTTGCCATTCTTAGGGTAATTCGA
TTAGTTAGAGTTTTTCGTATATTTAAACTGTCTCGCCATTCGACTGGCCTTCAAATACTC
GGACAAACATTAAAAGCCAGTACCAGGGAATTGGGATTATTAATATTTTTCTTGTTAATA
TGTGTGATTCTATTCAGTAGTGCAGTTTATTTTGCCGAGGCTGATTGTGATCAAACATTG
TTTCGCAGCATTCCTGATGCATTTTGGTGGGCAGTTGTAACTATGACAACAGTTGGTTAT
GGAGACATGCACCCCGTAACAATTTGGGGTAAATTAGTGGGTAGTTTGTGTGCTATTGCA
GGAGTGCTAACTATTGCTTTACCAGTGCCCGTTATTGTTTCTAATTTTAATTACTTTTAT
CATCGTGAAAACGAAATCGATGAAAAACAAACTTATGTGCATGTTCGATCTAGTGATAAT
GTGGAAAATATTTCAAACAGTAATTCAAGTTTAACAAGAACGTCATTACATCATCGAAGT
TCGAATAGCTCTTCCTCATCTTTTAAGAATGGCTTATCGGACAACCTTACTTTGCCAAGT
ATTTCAATGAACAATAATAAAAGGATAATTTCTAAACAGAAGTCGTCTTCCATTCATTGC
AACTCAATTGATATTAATCCCAAAAAGCTTGGTGCTAACAATCCTAAACCCGACACGATC
GCTTATAAGTTTATTGGAAGTGTGGAAACGGATGTCTAG

>DjScaffold115:3444-7749 strand:+
TGGACAACTGCAGCAAGTTTAGGAATTCCTTTATTGATCGTATGTAAAGTCGAATATCCG
CCACCAAAATGTAAAGAGATCTGGAGTGACAAAGCCAGTACTCTCTATTCTTTAGTTTTA
CTGCTTACTCAGTACTTTGTCCCATTATTTTTCCTACTCAGTACTTACAGTGCAATTGCA
ACAAGAGTGTGGCTTCAACAGGCTCCTGGAGAACGTGATGAAAATCGTGATCGAAGAATT
GCCAAATCCAAAAGAAAAATGGTGAAAATGATGATAGTTGTATCGCTTGCTTACGCCATA
TCGCAACTTCCGCGGCATTTAACGTGGCTGTATATATTTTACTATGGAGGACCGCAGTGG
GCAAATATTATTTGGAATATAGTTGTGTTGTTACAAATAAGTTCAACATGTTACAATCCA
ATTATCTATTGTTGGATGAATCATTCTTACCGTCATAGATTCATTTATTACTTATGTGTT
TTAACAAAAATCGATGCGTTTCGGCAGTTAAATCCTCCAAAATATCGTAAAACTGTAAAC
ACTGAATTCTCAAGTGGTATGATTACCCAATATGTTAAACAAAATCCGAATATTGATAAA
TCGCGCTATTTAGCAAGTGGAATTTTAAAATCAAAGCCAGATAATTTAACAGATGGTATT
GAACTAGACCATCGG
>DjScaffold5246:19588-55468 strand:+
acaattaattaaattaattaattaaatgatataaatgaaatcatataaattaaatataaa
tttaaaaatttaaatttaaaattaaaaataaatataaattaaatttattaaatgatataa
tttaattaattattttaaaaaaactttataagcttaaaatattttgtttaaaagttatta
taaaaattattataaatacaaatttattgataatcacttttgtaaattatatctgagtaa
taattttcattttgaaggcatacgctgatatttatgttattcaccattggaaaaacagtc
gtaaatttcaaagtggcaaaaaaacaacaaaatttctttcaaatcttcttattaattgta
acggtagaccgttggaagatggaaaatttcagaaaatacatgaattctcacaatcacaat
tatttcacgatactagtcatttgagaatcgtttattcatatttattgttcaaattgcgtg
ttctagaacggcgcacagttgactattccaagattgaatcaaacttacaaatcaaccaaa
acaatggactttacgctgtccattatgaattgaaatttccattttataaatttcgttcga
atgataacgatttgaaattttatctcttaccaggcgaatacgagATGCGTCTAGAAGTCA
GTTTGGATCAACCTTCAGAAAGTCTTGCGGATTTCGCAAATAATGAAATGGTAATTGAAA
ACTTTTCTGATTCGCCAGAACGGATTTGGGTTAGAGAGGTTAGTCCAAGCAGCAGTGTTG
CCAAAGAATATGATAAATGGCCGAGAATTTTATTCAGACTAGAATCTTCCAATTCACAAA
CCAAATTGGATACTAACAGTGTTGAAATCAAGCCATATATTTCAAATGGATTAATTGACA
AAGTGTCAAAAAAAGCAAGAAAAGCTAAATTAGAAGTAAATATTGTTTATAAATTTTATC
ACAACAATCATATGTTACAATGGTCACGCAGCCAATCAGTAAAATGTCCTTGGTGTAATT
TTATTTGTGAAAAAACCAACGCTAAATTGAGTTTTAGTTGTTTGGTTAAACATTTACAAA
CATTTCATGCTAGATTTACATTTAAACATTGGGTGATATCTGAGACCAATATTTCATCGA
TGATTATTGAAGTCAGATTAAATGAGTGTTACGATGGATCCATTGAAATAGGACTTAGAA
GTTCTATGGAAAGTCGATCACTTGAGAAACCCGAGAGAAAGCTTCCATTTACCCATATAG
TGTATTGCTCACAAATGTGTAAAATAAACCCAATGGTTAATAATCCATCAATAGACTTAC
GGCCTTTAGTTTGGGGTCATAATAGAAAATATTTTTATTCAACAACCGGAATAATGAAAT
GCATCGGCGATCTCGATGTAGATTCTGAAGCAGAAGATAATTTAGAGTGGTTGCAACAGA
TGTATTTCAAAAGAATTGAAGAATTTACCGATGTTAATCCAGGAGAAAAGCGTATTATGC
AATTGTGGAATTCTCATTTATTAGATCATAAATATAATGGAGATTTCATGGTACCAATTC
TTACCAAATCATTCATTATTCAAAAAGGTGCAGAAATCAAAGAACATAATCTTCGAAATA
ATCTATTGCTCCATTTAGTCAATTTGGTGGAGTTTGGTGTTTTGAGTCAAAAACAATTGA
TTGAAGTTATTGGAATACTTGATCAAATGTAAatctgattcgatcagtttccattggcat
ttattttagttttattattttgataccgagaaccatt
>DjScaffold4203:8939-9743 strand:+
gtctcacactgatattttattcagagaattgcacgcagatgacctcccagaggtcATGGA
AATAGAAAGCTTGTGTGTGAATTGTTATGAAAATGGTATTACAAAATTGTTTTTAACTAA
AATTCCATTTTTCCGGGATGTTGTAATTTCATCATTTGAATGCACGCATTGCTATTATCA
AAATAATAATGTTGATGCAAGAGGTGTTATTCAAGATAAAGGTGTTAAATTTCTTTTGAA
AGTTTTAAATAAGAAAGATCTAAACCGTCGAATGGTTAAAAGCCAATCAGCAGTATTTAA
TATTCCTCATTTAGATGCCTCATTTCCTTTGACAGATGCTTCATTCACAACAATTGAGGG
TGCCCTAAATTCTATTGCAGAGAATCTAGAATCACTCCAAGAAGAACGAAGAAAAGTAAC
ACCGGATTTAGCAACACAAATTGATGAATTTATTTTTAAATTGAAAGATTTAATGGAGTT
GAAATCTGAATTTTCTGTGGAACTAGTTGATCCTACCGGTAACAGTATCATTGAAAACTT
TCTGGCACCCAAAGTAGATCCACAATTATCGGTGGAGCATTATAAAAGAAGTCGAGAAGA
AAATGAAGCACTTGCAATTAATGAAGAGAATTTCATTGAAAACGAAGGAAGTAATAATAA
GAATTATGTCGAAGAAGCTCAAGAGGAATTTAATCCAAAAAATGAAGTTTTGATTTTCAA

TTTGTTATGTCCGGAATGTAACTCTCCAATTGAAGGCAGAATGAAAATGTTGGACATTCC
TCATTTTAAAGAAGTGATCGTAATGGCGAGCAATTGTGATAATTGTGGATTTAAGGAAAA
TGAAGTGAAAGGCGGATGTGGTATTTCTGAGAAAGCACGTAGAATTACTTTGAAAATTGA
AACTGCGGAAGATTTGACGAGAGATTTTTTAAAATCTGATACAGCGGCTATGAAAATTCC
GGAATTGGAAATTGAAACTGAATCGGGTTCACTTGGAGGAAGATTTACCACAGTTGAAGG
AATTCTACATCAAATGGCTGATAAATTGGAAGAGAGCAATCCGTTTTTGAAAGGTGATAG
TCAAACAGCCACTTCAACAGAGATTATTCTTAGTAACTTGATTCAGTCGTTGCATGACGT
AGGATACGGTAAAAGATTCGGAGTGACTCTAATCATTGAAGATCCAGCAGGAGGAAGTTA
TATCCAAGATTTGTATTTACCTGAACAAGATCCGCGATTGACTATTGAAGATTATGAGCG
TTCGTTTGAGGAAAATGAGTTGTTGGGATTGAACGACATGATCACTGAGAAATATGTAAT
TTAAtgaataaattatcaattttcaaattaatgtttagaattatgtcgaagaagctcaag
aggaatttaatccaaaaaatgaagttttgattttcaatttgttatgtccggaatgtaact
ctccaattgaaggcagaATGAAAATGTTGGACATTCCTCATTTTAAAGAAGTGATCGTAA
TGGCGAGCAATTGTGATAATTGTGGATTTAAGGAAAATGAAGTGAAAGGCGGATGTGGTA
TTTCTGAGAAAGCACGTAGAATTACTTTGAAAATTGAAACTGCGGAAGATTTGACGAGAG
ATTTTTTAAAATCTGATACAGCGGCTATGAAAATTCCGGAATTGGAAATTGAAACTGAAT
CGGGTTCACTTGGAGGAAGATTTACCACAGTTGAAGGAATTCTACATCAAATGGCTGATA
AATTGGAAGAGAGCAATCCGTTTTTGAAAGGTGATAGTCAAACAGCCACTTCAACAGAGA
TTATTCTTAGTAACTTGATTCAGTCGTTGCATGACGTAGGATACGGTAAAAGATTCGGAG
TGACTCTAATCATTGAAGATCCAGCAGGAGGAAGTTATATCCAAGATTTGTATTTACCTG
AACAAGATCCGCGATTGACTATTGAAGATTATGAGCGTTCGTTTGAGGAAAATGAGTTGT
TGGGATTGAACGACATGATCACTGAGAAATATGTAATTTAAtgaataaattatcaatttt
ca
>DjScaffold4203:17614-17864 strand:+
AATAAAGATTGCGAATTTAGATCAGTTGATTTAGATCCATTAATTCAAGCGTTATTTAAA
TTTCGTGAAAAAGTTTTTCAATTGCGTAGTCGTTTAGAATTTCAATTCACAGATTATGAA
TGGTCCAAATATTTGAATGTTTTTGGATTATTAGCAACCGATCTAAATAACATTGTTAAA
GTTATGTTAACTGATAAAAATTTACAATTA
>DjScaffold4203:58830-68224 strand:+
ttttagtaattcgaactgaagtatttaatttaaatattagactttatttttaagtctttt
aagcATGTCATTTTTTGTCACTCCTCGACTTTTTGTGCAATCGCTTATAAAATCAGAAAG
TATTTTCAAGAACATTGTTAAATCACACAAATATCCATCTAGTGTTGTTGACAAATATAT
TAATGAAATTAAAGAAATTCAATTTCATCAAAGTGACACCAATGTATCGGAATATTTAGA
ATTCCCATTGAAAACATTTTATGGTCTTAATAAAGATTCTATGAAATATATTATTAGTAA
AACATTTGATTTTATAAAGGAGCAAGGATTGGGGTCGAATCCAATGATTTATAGTTGCGT
TTTGAAAGCGTATTTGCAATATGATATCGAAATTGATATTGAAAACGTGTTGCAAGATAT
TATCAAAAATGGAGTTGCTTTGAATCAAGAAATTTGTAATAAAATAATTGAATATTATTG
TAAGCACGGTGAAATGAGTACTGTTGAAATTCTGTTGAATGAAATGAAATCCCAAAGAAT
CTATCTTCAGGAAAGTAATTTATTTCTGATTGTCAGTGGTTACATCATAGCTGGACAACC
AGAAAAGGCTGAAAACTTCATTAATAATAGAGGTCTAAACTCCGAGTGTCAATTTATGTT
TTACAGTCGCTGTTTTGCCTTCGCGACTAGCGGAGATGTTGCTAGCTTGAAAAATATTCT
TGCCGAGATGAAGGAAAAACATATTAAATTTGTACCATTTCGATATTTCAATTTATACGA
GAGATTAGTTTGTGCAAATGTTGAAATATCAGTTTTGGAAGAGATAATCGATATAACCAA
AAATGAAATCGCATTTGCCAGCGGCTGCTTATTCGCCTTGGTCGCTACCTTGAATAGAAC
TGATCAATATTTCAATTATTCCGTATTTCTCATCTCCAAATTGATTACTACCAATGACGA
AATATCCAAATTCATTCAATTATTCTCTACATCAAGATGTCCTCCGACTATTTTCCAACA
ATTTATCACCATCTTGGAGCCTCAATATACAAATGTTTCATACAACATCAATATTTTCGG
TGACATGTGTTTCGATACTGCTTTGGGTATTTCACAGAAATTCACTAAATGCAAAAATTG
GAAACTTATTTTCTATTCTCTTATTAATAAAGCTGTTTGTGAAAATTCTATCACAAATAT
CGAAAATGCATTGCATTTTTCATTTAAAAATCTGAAAGTTCAGAAAATAAATATATTGCT
GTCGCAATCCCTGCTACCTTCGATGTTGAACAGCGGAATGGTGTCGAATTATGATGAGAT
CTTAGATCTATTTCCCACGTTCAGAAATCAGATTTTACAAGTGCTTCTTATGAAATATCT
TTGTCATGGTGATTTTGATCAGTATAATAACTTGAGATTAAAAGAAACACACATGACAAA
CCTTTTTATGAAATCTGAGTATAGCTATCTTGCCTCTGAGTTACGTAACAATTTACTGTT
GTTTAACATCGAACAATGGTCAATGGAAGCTCGGAAATGTTTTCATTCACAATTTATTGA
TAATGTTTTAGAAAATGTAGTGGTTTATGAAATAATCGCTCTGTTAAAGAATAAATCAAT
AAATGAATTTAAACAGTTAATTGATCATGCAAAGGTTGTTTTATTGAATTTTTATTATCA

TTATTATTATTGAttattattgaatattaatattattgattattattgattattattatc
attattattgaatattaatattatttattattattgattattattatcattattatagtt
attgattattatttattattattataattttagtaattcgaactgaagtatttaatttaa
atattagactttatttttaagtcttttaagcATGTCATTTTTTGTCACTCCTCGACTTTT
TGTGCAATCGCTTATAAAATCAGAAAGTATTTTCAAGAACATTGTTAAATCACACAAATA
TCCATCTAGTGTTGTTGACAAATATATTAATGAAATTAAAGAAATTCAATTTCATCAAAG
TGACACCAATGTATCGGAATATTTAGAATTCCCATTGAAAACATTTTATGGTCTTAATAA
AGATTCTATGAAATATATTATTAGTAAAACATTTGATTTTATAAAGGAGCAAGGATTGGG
GTCGAATCCAATGATTTATAGTTGCGTTTTGAAAGCGTATTTGCAATATGATATCGAAAT
TGATATTGAAAACGTGTTGCAAGATATTATCAAAAATGGAGTTGCTTTGAATCAAGAAAT
TTGTAATAAAATAATTGAATATTATTGTAAGCACGGTGAAATGAGTACTGTTGAAATTCT
GTTGAATGAAATGAAATCCCAAAGAATCTATCTTCAGGAAAGTAATTTATTTCTGATTGT
CAGTGGTTACATCATAGCTGGACAACCAGAAAAGGCTGAAAACTTCATTAATAATAGAGG
TCTAAACTCCGAGTGTCAATTTATGTTTTACAGTCGCTGTTTTGCCTTCGCGACTAGCGG
AGATGTTGCTAGCTTGAAAAATATTCTTGCCGAGATGAAGGAAAAACATATTAAATTTGT
ACCATTTCGATATTTCAATTTATACGAGAGATTAGTTTGTGCAAATGTTGAAATATCAGT
TTTGGAAGAGATAATCGATATAACCAAAAATGAAATCGCATTTGCCAGCGGCTGCTTATT
CGCCTTGGTCGCTACCTTGAATAGAACTGATCAATATTTCAATTATTCCGTATTTCTCAT
CTCCAAATTGATTACTACCAATGACGAAATATCCAAATTCATTCAATTATTCTCTACATC
AAGATGTGTATGTACTAACACTTGTTTTCTTTTCTTTTTCTTTTCCTCATTATTGTTATA
Ataatttctctttttcatctcagcctccgactattttccaacaatttatcaccatcttgg
agcctcaatatacaaatgtttcatacaacatcaatattttcggtgacatgtgtttcgata
ctgctttgggtatttcacagaaattcactaaatgcaaaaattggaaacttattttctatt
ctcttattaataaagctgtttgtgaaaattctatcacaaatatcgaaaatgcattgcatt
tttcatttaaaaatctgaaagttcagaaaataaatatattgctgtcgcaatccctgctac
cttcgatgttgaacagcggaatggtgtcgaattatgatgagatcttagatctatttccca
cgttcagaaatcagattttacaagtgcttcttatgaaatatctttgtcatggtgattttg
atcagtataataacttgagattaaaagaaacacacatgacaaacctttttatgaaatctg
agtatagctatcttgcctctgagttacgtaacaatttactgttgtttaacatcgaacaat
ggtcaatggaagctcggaaatgttttcattcacaatttattgataatgttttagaaaatg
tagtggtttatgaaataatcgctctgttaaagaataaatcaataaatgaatttaaacagt
taattgatcatgcaaaggttgttttattgaatttttattatcattattattattgattat
tattgaatattaatattattgattattattgattattattatcattattattgaatatta
atattatttattattattgattattattatcattattatagttattgattattatttatt
attattataagcaattaatcaccaatttattgattgaattaaatcaatcaccaatttatt
gattgaaagcaattaatcaccaatttattgattgaatgcaattaatcaccaatttatcga
ttgaattcaatctaataatgcaatcagtcaccaacgtgatgattatatgatcctagATGT
ATTTATCGAAAATATTCAGAATTCTGTTGAATGAAATGAAATCCCAAAGAATCTATCTTC
AGGAAAGTAATTTATTTCTGATTGTCAGTGGTTACATCATAGCTGGACAACCAGAAAAGG
CTGAAAACTTCATTAATAATAGAGGTCTAAACTCCGAGTGTCAATTTATGTTTTACAGTC
GCTGTTTTGCCTTCGCGACTAGCGGAGATGTTGCTAGCTTGAAAAATATTCTTGCCGAGA
TGAAGGAAAAACATATTAAATTTGTACCATTTCGATATTTCAATTTATACGAGAGATTAG
TTTGTGCAAATGTTGAAATATCAGTTTTGGAAGAGATAATCGATATAACCAAAAATGAAA
TCGCATTTGCCAGCGGCTGCTTATTCGCCTTGGTCGCTACCTTGAATAGAACTGATCAAT
ATTTCAATTATTCCGTATTTCTCATCTCCAAATTGATTACTACCAATGACGAAATATCCA
AATTCATTCAATTATTCTCTACATCAAGATGTCCTCCGACTATTTTCCAACAATTTATCA
CCATCTTGGAGCCTCAATATACAAATGTTTCATACAACATCAATATTTTCGGTGACATGT
GTTTCGATACTGCTTTGGGTATTTCACAGAAATTCACTAAATGCAAAAATTGGAAACTTA
TTTTCTATTCTCTTATTAATAAAGCTGTTTGTGAAAATTCTATCACAAATATCGAAAATG
CATTGCATTTTTCATTTAAAAATCTGAAAGTTCAGAAAATAAATATATTGCTGTCGCAAT
CCCTGCTACCTTCGATGTTGAACAGCGGAATGGTGTCGAATTATGATGAGATCTTAGATC
TATTTCCCACGTTCAGAAATCAGATTTTACAAGTGCTTCTTATGAAATATCTTTGTCATG
GTGATTTTGATCAGTATAATAACTTGAGATTAAAAGAAACACACATGACAAACCTTTTTA
TGAAATCTGAGTATAGCTATCTTGCCTCTGAGTTACGTAACAATTTACTGTTGTTTAACA
TCGAACAATGGTCAATGGAAGCTCGGAAATGTTTTCATTCACAATTTATTGATAATGTTT
TAGAAAATGTAGTGGTTTATGAAATAATCGCTCTGTTAAAGAATAAATCAATAAATGAAT
TTAAACAGTTAATTGATCATGCAAAGGTTGTTTTATTGAATTTTTATTATCATTATTATT

ATTGAttattattgaatattaatattattgattattattgattattattatcattattat
tgaatattaatattatttattattattgattattattatcattattatagttattgatta
ttatttattattattataa
>DjScaffold2307:12288-18585 strand:+
ATTGAGGCAAAAAATTTACAATATTTGCAAGTTGATAAAAGCTTGGTTTCTGATTCTGCT
AACCTATCCGATTGGGCAAATAAAAAATTGGTTTGGATACCCGATGAAATTGAAGGATTT
ATATCAGGTTCACTAATCGAGGAGAAAGGTGATGAAGCAACAGTTAAATTAGAAAATGGA
AAGTCTGTAAAGTTCCCCATAGAGAATATTCAAAAAATGAATCCACCAAAATTTCTTAAA
TCTGAAGATATGGCTGATTTAACTTACTTAAATGAAGCTTCAGTTTTATTCAATTTAAAG
GATAGATATTTTTCAGACTTAATTTACACTTATTCTGGATTATTTTGCGTTGTTGTGAAT
CCATACAAGCGTTTGCCTATATACACCGACAACGTTATTGAATGGTATAAAGGTAGAAAA
CGTCACGAAAGACCACCCCATATTTATGCCGTTACTGATGTAGCATATCGAAATATGCTT
CAGGATAAAGAAAATCAATCAATACTCTGCACTGGTGAATCTGGAGCTGGAAAAACTGAA
AATACTAAAAAAGTCATTCAATACCTTGCATCTGTTGCTACTTCATTAAAGAATCAAAAA
ACAACTGCTAATAATGCTTTATCACAGTTTTATGCACATGATGTAAATATTGGAGAATTA
GAAACACAATTATTGCAAGCTAATCCAATTCTGGAAGCTTTCGGAAATGCGAAAACCATT
AAAAATGACAATTCATCACGATTTGGTAAATTTATTCGAATTAATTTCGACAATTCTGGA
TTCATCTCTAGTGCAAATATTGAAACATATCTTTTAGAAAAAGCTAGAGTTATTAGACAA
GCAGCTAGCGAGAGGTGTTTTCATATTTTCTATCAATTACTTATTGGAGCTGATGATAAT
TTGAAAAAAGAATTAATTTTAGAGAATGTGTCCACATATAAACTGCTTTCAAATGGAATG
ATTACTGTCCCAGATTATGATGAAAGACAAATGTTTAAGGATACTGTTGAGTCGCTCGAT
ATTATAGGAATTTCAAAAGACGAGCAAGATTCAATTTTCCGTGTAATTTCTGCCGTATTA
CATATGGGTAATATTGAATTCAAACAAGAAAGAAGCTCAGATCAAGCTGCTTTACCAGAT
AATACAGTTGCTCAAAAGGTCGCACATCTTCTTGGTTTACCAGTTACTGAAATGACTAAA
GCTCTTTTGAAACCTAAGCTAAAAGTTGGAAGAGAAGTTGTAGCAAAAGCTCAAACTAAA
GAACAGGCTGAATTTTCAGTTGAAGCTATTAGTAAATCTACTTATGAAAGAATGTTTCGA
TGGCTGGTAATGAGAATCAATCGATCTATTGATAGAAATAGACAGAAAACTAATTTTATT
GGAATTCTTGATATTGCTGGTTTTGAAATATTTGAAATCAACTCTTTTGAACAATTATGC
ATTAATTACACTAATGAAAAATTACAACAACTTTTCAACCATACTATGTTCATACTTGAA
CAAGAAGAATATAGAAAAGAAAATATAAAATGGGAGTTTATAGATTTTGGTCTTGATTTA
CAGCCTACTATAGATTTGATTGAGAAACCAATGGGTATTTTATCTTTATTAGATGAAGAG
TGCTTCTTTCCAAAAGCTACTAGCAAAACATTTGTCGAGAAAATAATTAAAAATCAATCA
AGTCACTCTAAATTTAAAGCAACAGATTTCAGGGCTAAGGCAGATTTCGGCGTGATACAT
TACGCAGGTAAAGTAGATTATGTTGCTGATAACTGGTTAGTAAAAAATATGGATCCCCTC
AATGAAAATGTTGTGAGTTTGTTGCAAGAATCTAATGAATTATTTGTTCAAACTATTTGG
AAAGATACTGAAAATATCATTGGTTTATCAACAACCACTGCACAGGAATCAGCATTTGGT
GGTGCTAAAACTCGTAAAGGCATGTTTCGAACTGTTGGTCAACTTTATAAAGAATCTTTA
ACTAAGTTAATGGATGTTCTTAATAATACCAGTCCGAATTTTGTTAGATGCATTATTCCA
AATCACGAAAAAAAAGCGGGAAAAATTGATTCCAGATTAGTTATTGATCAACTGAAATGC
AATGGTGTATTGGAAGGTATTAGAATTTGCAGACAAGGGTTTCCAAATAGAATTCTATTC
CAGGAATTTAAACAACGTTACGGAATTCTTACTCCAAATGTCATACCAAAAGGATTTATG
GACGGTCGTAAAGCTACTGAACTCATGCTTGGAGCTTTGGACATTGATGTTTCAAATTAT
CGAATTGGGCAAAGTAAAATATTTTTTAAAGCGGGTGTTTTGGCTAGATTGGAAGAAGAC
CGTGACACGAAATTAACAGAAATTATTGTTAAATTTCAGTCTTTTGCACGTGGATACTTG
GCAAGAAAAAAATTTGCAAAACCGATCACAACATCTTAATGCTGTAAAAATTATACAAAGA
AATTGTACTGCTTATTTAAAATTGAGAAATTGGTCATGGTGGAAATTGTTTACTCGTGTA
AAACCTTTATTGAGTGTGACCAGACAAGAGGAAGTAGTTGCACTGAAAGAGGAAGAATTG
AAAAAAACCAAAGAATCTTTAGAAAAAATGACAAATGAATTCGAAGTGACTAAAAAGAAT
TATGAATCTCTAACGGAGGAAAAAAGTAAACTCCAAGAAGATTTGGAAAAAGAAAGATTC
GCATTACAAGATCTCGAAGGAGAAAGAGAAAATTTAATTAAACGATTAAATGATATTGGA
TTAGAAGCGCAGGAATGTGAATTGAGAGAGATAGAAACTAGAGATAAATGTAGCAAATTG
GAAATAGAAAAAAAGAAATTAAATGAAGAAATTGGAAATCTTAGTCAAAATTTAGAATTA
GAAGAACAACATTTACAAAAAATACAAACTGAAAAGCTTACTGCTGATAAACGAATAAAG
GAATTAGAAGAAAAAGTGGCTGAATTAGAGGATAAGCTTAACAAACTGATTAGAGAGAAA
AAGTCTCTTGAGGAAAAATTAGCTGATATTATGTCTACCTTAACAGAAGAAGAAAAAAAG
AGTAAACAATTGATGAATCTGAAATCTCGTCATGAAGCAAGTATTAGTGAATTAGAGGAA
AGATTGAGTCGTGAACAAGCTGCGCGACAAGATTTGGAAAAAACTAAAAGAAGATTGGAA

ACAGAATTAGCTGAAAAATCAGATAATTTATCCACACAATCTCATACTTATGAAGAAATA
AGAATTACTCTGGAAAAAGCTGAAATTCAAATTACAGAAATGCAGACTAAACTCGAAGAA
GAAACAATGGCAAAATCAGCTGCCCAAAGACAAATAAGAGATGTAGAGAATGCAATTTCT
GAAATAAAAGAAGACCTTGAAGCAGAAAAACGTTCGAAGGAAAGAGCAGAAAAAGCAAAA
CGAGATTTAGCTGAAGAGATTGAATCACTCAAAATGGAATTACTTGACACTGGTAATACT
TCAGAAGAACAACAAACTATTTTACGTAAAAAAGAAGTTGAGTTACAAAACATTAAAAAA
TCGTTGGATGATGAAACTAAGAGAGCTGAAGCTGAAATTCAAGAAATTAGAAAAAAAGCT
GCCGTTTCAATAGAAAATCTTAATAATCAATTAGAGACATCTAATAAGTCAAAAGTTGCA
ATTGAAAAGGCTAAATTGCAATTACAAGAAGAACTTAATGATGCAAATAATGAATTATCA
ATTTTGCGATCATCAAAAGCTGAATCTGAAAAACTTAAAAAGAATACCGAACAACAACTT
TCTGAATCATTGAATCGTTTAGCAGAAGTTGAGGAGTACAAATTAGATATTGAAACAAAA
CTCAAAAAACAAACAGAAGTATGTGATAAATTAGCCCTTAGTCTTGAATCTTCTGAGAGC
AAGCTATCACAAATTACTAAATCTGAAATTTCGTTAAAAAGTCAATTAGAAGATTTACAA
AAAAATTTTGAAGAAGAAACTCGATTAAAATTAGCAGCTCAAACATCATTAAGACAAGTA
ATAGGCGAAATCTCGCAAATTAAGGATCAGTTAGAAGAAGAACAACAAATGAAAGAAAAT
TTGGAAAAACATGTTCAAATCTTACAAACACAAATGCAAGAAGTGAAAAAGAAAGTAGAA
GATGATGCTAATCAATTGTTGGAATTAGATGATTCTCGTAAAAAAATAATGCGCGAACGA
GAAGAATTACAAATTAGGTTAGAAGAAGCTTTAAATCAAGTTGACAGATCTGAAAAGGCT
AAAAGAATTATTCAAGCTGAACTTCAAGATGCGTACCATGCCTTAGAAGGTCAGAAAAAT
GATCAAACTCAAAGTGATCGAAAGCTTAAAAAAATGGAATCTCAAGTCAATGAAATTATG
AATTTAAATAAAAAATATTTGTCTGAAAAAGAAATATTGGAAAAGGAAAATCGTGAAAAA
GAAACTCGAATTATGCAATTACAAAGAGAAATTACAGCAAACGAAGATAAACTAATTGAA
TTAGAAAATTCTAAGTCCTTATTAACTCGTCAGTTAAATGAATTGGTATCTAACAAAGAT
GACGTGGGTAAAAATATTGGAAGTTTAGAAAAAGTCAAAATTCAGTTAGAAACTGATTTG
GATAACTGTAAAAATACTATTGAAGAATTAGAAGAAGAAAACGCGACTCTTCAAATGAAT
AAAGAAAGAATTGAAATGCAATTAACTGCCACAAAAGCACAACTTGACAGAGAATTGAGT
TCTAAAGATGAAATGCATGATGAACAATTTAAAATCCTTAACAAAAAACTCCGTGATACA
GAAGCAACTTTAGAAGAAGAACAAAAACAAAGAACTTCTTTGGCTTCATTAAAAAAGAAA
TTAGAAATTGAAAATGTTGATTTGATGAATCAGTTGAGTGAATCGGAAAAGATTAAAGAA
GACTTGTCAAGGCAAATTAAAAAACACCAAGGAACATCTATCAATAGTCAAAGAGAAATG
CAAGAAGCTTTAAATGCGAAAAATGAAGCTTTGAAACAAATTAAGGAATTGGAGAAGCGC
TTACGAACAGCTGACAATAGCATTAATCAATTAACAGAGGATTTATCACAATTAGAACGT
GTTAAAAGAACAATAATAACTGAAAGAGATGATGCTTTAGAAGAAATCGCTACTTTAACC
GTGAATAAAGACACCGCTATTGCTGATAAAAAACGAATTGAAAGTAATTTATCAGCATTA
AATATGGATTATGAAGAATTGCAGTTACTTCTTGCTGAATTAGAAGACAAAAACAAAAAA
ATAACAAATCAATTAGAACAAGTTCAAAATGAATTAAATGTTGAACGCCAAAATGTCACA
CGCTTGGAAAATCAAAAATCTACTGCTGAAAGACTAAATAAAGAATTACACGAAAAAATC
GAAGAATTAGAAGTGGAAGCTGGTAAAAAGTACAAAACTACCGTTGCAGCTCAACAAAAT
AAAATCCAGAGTCTTGATGCTCAATTAGATGCCAAAAGTGAAGAAATTAATCAAGCAAAC
CGAAATAATGGCCCTTATGGTTGAtgaacaaaagcgggctgataatgcgattgagcaaat
tgaaaaagctactggaagatataaaaaagcagaaagaagtttgcaaatggaacaagaaaa
taattctgctttgactactcaaaatcgtagattgcaacgtgatttggaagaagtcaatga
aacgaaagaagcccgtgatcaagagataaaagttcttaaaactaaaattgaaagattgga
acgaagagttcgtactggtggtcgcccaaatagaggagatgccgtatctgatgatggttt
atctggtgatggagaatcgttaactgattctggtcttggagcagtccagcaggattaatt
tactatacatttaacaatttatatagataattagcttatacagcgaaatttgtcggttta
atttaaaaaatggacttatttggtttgctcaatatgttattgttttgaatttttaataaa
tttttcacgagATTGAGGCAAAAAATTTACAATATTTGCAAGTTGATAAAAGCTTGGTTT
CTGATTCTGCTAACCTATCCGATTGGGCAAATAAAAAATTGGTTTGGATACCCGATGAAA
TTGAAGGATTTATATCAGGTTCACTAATCGAGGAGAAAGGTGATGAAGCAACAGTTAAAT
TAGAAAATGGAAAGTCTGTAAAGTTCCCCATAGAGAATATTCAAAAAATGAATCCACCAA
AATTTCTTAAATCTGAAGATATGGCTGATTTAACTTACTTAAATGAAGCTTCAGTTTTAT
TCAATTTAAAGGATAGATATTTTTCAGACTTAATTTACACTTATTCTGGATTATTTTGCG
TTGTTGTGAATCCATACAAGCGTTTGCCTATATACACCGACAACGTTATTGAATGGTATA
AAGGTAGAAAACGTCACGAAAGACCACCCCATATTTATGCCGTTACTGATGTAGCATATC
GAAATATGCTTCAGGATAAAGAAAATCAATCAATACTCTGCACTGGTGAATCTGGAGCTG
GAAAAACTGAAAATACTAAAAAAGTCATTCAATACCTTGCATCTGTTGCTACTTCATTAA

AGAATCAAAAAACAACTGCTAATAATGCTTTATCACAGTTTTATGCACATGATGTAAATA
TTGGAGAATTAGAAACACAATTATTGCAAGCTAATCCAATTCTGGAAGCTTTCGGAAATG
CGAAAACCATTAAAAATGACAATTCATCACGATTTGGTAAATTTATTCGAATTAATTTCG
ACAATTCTGGATTCATCTCTAGTGCAAATATTGAAACATATCTTTTAGAAAAAGCTAGAG
TTATTAGACAAGCAGCTAGCGAGAGGTGTTTCATATTTTCTATCAATTACTTATTGGAG
CTGATGATAATTTGAAAAAAGAATTAATTTTAGAGAATGTGTCCACATATAAACTGCTTT
CAAATGGAATGATTACTGTCCCAGATTATGATGAAAGACAAATGTTTAAGGATACTGTTG
AGTCGCTCGATATTATAGGAATTTCAAAAGACGAGCAAGATTCAATTTTCCGTGTAATTT
CTGCCGTATTACATATGGGTAATATTGAATTCAAACAAGAAAGAAGCTCAGATCAAGCTG
CTTTACCAGATAATACAGTTGCTCAAAAGGTCGCACATCTTCTTGGTTTACCAGTTACTG
AAATGACTAAAGCTCTTTTGAAACCTAAGCTAAAAGTTGGAAGAGAAGTTGTAGCAAAAG
CTCAAACTAAAGAACAGGCTGAATTTTCAGTTGAAGCTATTAGTAAATCTACTTATGAAA
GAATGTTTCGATGGCTGGTAATGAGAATCAATCGATCTATTGATAGAAATAGACAGAAAA
CTAATTTTATTGGAATTCTTGATATTGCTGGTTTTGAAATATTTGAAATCAACTCTTTTG
AACAATTATGCATTAATTACACTAATGAAAAATTACAACAACTTTTCAACCATACTATGT
TCATACTTGAACAAGAAGAATATAGAAAAGAAAATATAAAATGGGAGTTTATAGATTTTG
GTCTTGATTTACAGCCTACTATAGATTTGATTGAGAAACCAATGGGTATTTTATCTTTAT
TAGATGAAGAGTGCTTCTTTCCAAAAGCTACTAGCAAAACATTTGTCGAGAAAATAATTA
AAAATCAATCAAGTCACTCTAAATTTAAAGCAACAGATTTCAGGGCTAAGGCAGATTTCG
GCGTGATACATTACGCAGGTAAAGTAGATTATGTTGCTGATAACTGGTTAGTAAAAAATA
TGGATCCCCTCAATGAAAATGTTGTGAGTTTGTTGCAAGAATCTAATGAATTATTTGTTC
AAACTATTTGGAAAGATACTGAAAATATCATTGGTTTATCAACAACCACTGCACAGGAAT
CAGCATTTGGTGGTGCTAAAACTCGTAAAGGCATGTTTCGAACTGTTGGTCAACTTTATA
AAGAATCTTTAACTAAGTTAATGGATGTTCTTAATAATACCAGTCCGAATTTTGTTAGAT
GCATTATTCCAAATCACGAAAAAAAAGCGGGAAAAATTGATTCCAGATTAGTTATTGATC
AACTGAAATGCAATGGTGTATTGGAAGGTATTAGAATTTGCAGACAAGGGTTTCCAAATA
GAATTCTATTCCAGGAATTTAAACAACGTTACGGAATTCTTACTCCAAATGTCATACCAA
AAGGATTTATGGACGGTCGTAAAGCTACTGAACTCATGCTTGGAGCTTTGGACATTGATG
TTTCAAATTATCGAATTGGGCAAAGTAAAATATTTTTTAAAGCGGGTGTTTTGGCTAGAT
TGGAAGAAGACCGTGACACGAAATTAACAGAAATTATTGTTAAATTTCAGTCTTTTGCAC
GTGGATACTTGGCAAGAAAAAATTTGCAAAACCGATCACAACATCTTAATGCTGTAAAAA
TTATACAAAGAAATTGTACTGCTTATTTAAAATTGAGAAATTGGTCATGGTGGAAATTGT
TTACTCGTGTAAAACCTTTATTGAGTGTGACCAGACAAGAGGAAGTAGTTGCACTGAAAG
AGGAAGAATTGAAAAAAACCAAAGAATCTTTAGAAAAAATGACAAATGAATTCGAAGTGA
CTAAAAAGAATTATGAATCTCTAACGGAGGAAAAAAGTAAACTCCAAGAAGATTTGGAAA
AAGAAAGATTCGCATTACAAGATCTCGAAGGAGAAAGAGAAAATTTAATTAAACGATTAA
ATGATATTGGATTAGAAGCGCAGGAATGTGAATTGAGAGAGATAGAAACTAGAGATAAAT
GTAGCAAATTGGAAATAGAAAAAAAGAAATTAAATGAAGAAATTGGAAATCTTAGTCAAA
ATTTAGAATTAGAAGAACAACATTTACAAAAAATACAAACTGAAAAGCTTACTGCTGATA
AACGAATAAAGGAATTAGAAGAAAAAGTGGCTGAATTAGAGGATAAGCTTAACAAACTGA
TTAGAGAGAAAAAGTCTCTTGAGGAAAAAATTAGCTGATATTATGTCTACCTTAACAGAAG
AAGAAAAAAAGAGTAAACAATTGATGAATCTGAAATCTCGTCATGAAGCAAGTATTAGTG
AATTAGAGGAAAGATTGAGTCGTGAACAAGCTGCGCGACAAGATTTGGAAAAAACTAAAA
GAAGATTGGAAACAGAATTAGCTGAAAAATCAGATAATTTATCCACACAATCTCATACTT
ATGAAGAAATAAGAATTACTCTGGAAAAAGCTGAAATTCAAATTACAGAAATGCAGACTA
AACTCGAAGAAGAAACAATGGCAAAATCAGCTGCCCAAAGACAAATAAGAGATGTAGAGA
ATGCAATTTCTGAAATAAAAGAAGACCTTGAAGCAGAAAAACGTTCGAAGGAAAGAGCAG
AAAAAGCAAAACGAGATTTAGCTGAAGAGATTGAATCACTCAAAATGGAATTACTTGACA
CTGGTAATACTTCAGAAGAACAACAAACTATTTTACGTAAAAAAGAAGTTGAGTTACAAA
ACATTAAAAAATCGTTGGATGATGAAACTAAGAGAGCTGAAGCTGAAATTCAAGAAATTA
GAAAAAAAGCTGCCGTTTCAATAGAAAATCTTAATAATCAATTAGAGACATCTAATAAGT
CAAAAGTTGCAATTGAAAAGGCTAAATTGCAATTACAAGAAGAACTTAATGATGCAAATA
ATGAATTATCAATTTTGCGATCATCAAAAGCTGAATCTGAAAAACTTAAAAAGAATACCG
AACAACAACTTTCTGAATCATTGAATCGTTTAGCAGAAGTTGAGGAGTACAAATTAGATA
TTGAAACAAAACTCAAAAAACAAACAGAAGTATGTGATAAATTAGCCCTTAGTCTTGAAT
CTTCTGAGAGCAAGCTATCACAAATTACTAAATCTGAAATTTCGTTAAAAAGTCAATTAG
AAGATTTACAAAAAAATTTTGAAGAAGAAACTCGATTAAAATTAGCAGCTCAAACATCAT

TAAGACAAGTAATAGGCGAAATCTCGCAAATTAAGGATCAGTTAGAAGAAGAACAACAAA
TGAAAGAAAATTTGGAAAAACATGTTCAAATCTTACAAACACAAATGCAAGAAGTGAAAA
AGAAAGTAGAAGATGATGCTAATCAATTGTTGGAATTAGATGATTCTCGTAAAAAAATAA
TGCGCGAACGAGAAGAATTACAAATTAGGTTAGAAGAAGCTTTAAATCAAGTTGACAGAT
CTGAAAAGGCTAAAAGAATTATTCAAGCTGAACTTCAAGATGCGTACCATGCCTTAGAAG
GTCAGAAAAATGATCAAACTCAAAGTGATCGAAAGCTTAAAAAAATGGAATCTCAAGTCA
ATGAAATTATGAATTTAAATAAAAAATATTTGTCTGAAAAAGAAATATTGGAAAAGGAAA
ATCGTGAAAAAGAAACTCGAATTATGCAATTACAAAGAGAAATTACAGCAAACGAAGATA
AACTAATTGAATTAGAAAATTCTAAGTCCTTATTAACTCGTCAGTTAAATGAATTGGTAT
CTAACAAAGATGACGTGGGTAAAAATATTGGAAGTTTAGAAAAAGTCAAAATTCAGTTAG
AAACTGATTTGGATAACTGTAAAAATACTATTGAAGAATTAGAAGAAGAAAACGCGACTC
TTCAAATGAATAAAGAAAGAATTGAAATGCAATTAACTGCCACAAAAGCACAACTTGACA
GAGAATTGAGTTCTAAAGATGAAATGCATGATGAACAATTTAAAATCCTTAACAAAAAAC
TCCGTGATACAGAAGCAACTTTAGAAGAAGAACAAAAACAAAGAACTTCTTTGGCTTCAT
TAAAAAAGAAATTAGAAATTGAAAATGTTGATTTGATGAATCAGTTGAGTGAATCGGAAA
AGATTAAAGAAGACTTGTCAAGGCAAATTAAAAAACACCAAGGAACATCTATCAATAGTC
AAAGAGAAATGCAAGAAGCTTTAAATGCGAAAAATGAAGCTTTGAAACAAATTAAGGAAT
TGGAGAAGCGCTTACGAACAGCTGACAATAGCATTAATCAATTAACAGAGGATTTATCAC
AATTAGAACGTGTTAAAAGAACAATAATAACTGAAAGAGATGATGCTTTAGAAGAAATCG
CTACTTTAACCGTGAATAAAGACACCGCTATTGCTGATAAAAAACGAATTGAAAGTAATT
TATCAGCATTAAATATGGATTATGAAGAATTGCAGTTACTTCTTGCTGAATTAGAAGACA
AAAACAAAAAAATAACAAATCAATTAGAACAAGTTCAAAATGAATTAAATGTTGAACGCC
AAAATGTCACACGCTTGGAAAATCAAAAATCTACTGCTGAAAGACTAAATAAAGAATTAC
ACGAAAAAATCGAAGAATTAGAAGTGGAAGCTGGTAAAAAGTACAAAACTACCGTTGCAG
CTCAACAAAATAAAATCCAGAGTCTTGATGCTCAATTAGATGCCAAAAGTGAAGAAATTA
ATCAAGCAAACCGAAATAATAAGAAATTAGAAAGAAAATTAAAGGAAATAATGGCCCTTA
TGGTTGATGAACAAAAGCGGGCTGATAATGCGATTGAGCAAATTGAAAAAGCTACTGGAA
GATATAAAAAAGCAGAAAGAAGTTTGCAAATGGAACAAGAAAATAATTCTGCTTTGACTA
CTCAAAATCGTAGATTGCAACGTGATTTGGAAGAAGTCAATGAAACGAAAGAAGCCCGTG
ATCAAGAGATAAAAGTTCTTAAAACTAAAATTGAAAGATTGGAACGAAGAGTTCGTACTG
GTGGTCGCCCAAATAGAGGAGATGCCGTATCTGATGATGGTTTATCTGGTGATGGAGAAT
CGTTAACTGATTCTGGTCTTGGAGCAGTCCAGCAGGATTAAtttactatacatttaacaa
tttatatagataattagcttatacagcgaaatttgtcggtttaatttaaaaaatggactt
atttggtttgctcaatatgttattgttttgaatttttaataaatttttcacgagATTGAG
GCAAAAAAATTTACAATATTTGCAAGTTGATAAAAGCTTGGTTTCTGATTCTGCTAACCTA
TCCGATTGGGCAAATAAAAAAATTGGTTTGGATACCCGATGAAATTGAAGGATTTATATCA
GGTTCACTAATCGAGGAGAAAGGTGATGAAGCAACAGTTAAATTAGAAAATGGAAAGTCT
GTAAAGTTCCCCATAGAGAATATTCAAAAAATGAATCCACCAAAATTTCTTAAATCTGAA
GATATGGCTGATTTAACTTACTTAAATGAAGCTTCAGTTTTATTCAATTTAAAGGATAGA
TATTTTTCAGACTTAATTTACACTTATTCTGGATTATTTTGCGTTGTTGTGAATCCATAC
AAGCGTTTGCCTATATACACCGACAACGTTATTGAATGGTATAAAGGTAGAAAACGTCAC
GAAAGACCACCCCATATTTATGCCGTTACTGATGTAGCATATCGAAATATGCTTCAGGAT
AAAGAAAATCAATCAATACTCTGCACTGGTGAATCTGGAGCTGGAAAAACTGAAAATACT
AAAAAAGTCATTCAATACCTTGCATCTGTTGCTACTTCATTAAAGAATCAAAAAACAACT
GCTAATAATGCTTTATCACAGTTTTATGCACATGATGTAAATATTGGAGAATTAGAAACA
CAATTATTGCAAGCTAATCCAATTCTGGAAGCTTTCGGAAATGCGAAAACCATTAAAAAT
GACAATTCATCACGATTTGGTAAATTTATTCGAATTAATTTCGACAATTCTGGATTCATC
TCTAGTGCAAATATTGAAACATATCTTTTAGAAAAAGCTAGAGTTATTAGACAAGCAGCT
AGCGAGAGGTGTTTTCATATTTTCTATCAATTACTTATTGGAGCTGATGATAATTTGAAA
AAAGAATTAATTTTAGAGAATGTGTCCACATATAAACTGCTTTCAAATGGAATGATTACT
GTCCCAGATTATGATGAAAGACAAATGTTTAAGGATACTGTTGAGTCGCTCGATATTATA
GGAATTTCAAAAGACGAGCAAGATTCAATTTTCCGTGTAATTTCTGCCGTATTACATATG
GGTAATATTGAATTCAAACAAGAAAGAAGCTCAGATCAAGCTGCTTTACCAGATAATACA
GTTGCTCAAAAGGTCGCACATCTTCTTGGTTTACCAGTTACTGAAATGACTAAAGCTCTT
TTGAAACCTAAGCTAAAAGTTGGAAGAGAAGTTGTAGCAAAAGCTCAAACTAAAGAACAG
GCTGAATTTTCAGTTGAAGCTATTAGTAAATCTACTTATGAAAGAATGTTTCGATGGCTG
GTAATGAGAATCAATCGATCTATTGATAGAAATAGACAGAAAACTAATTTTATTGGAATT

CTTGATATTGCTGGTTTTGAAATATTTGAAATCAACTCTTTTGAACAATTATGCATTAAT
TACACTAATGAAAAATTACAACAACTTTTCAACCATACTATGTTCATACTTGAACAAGAA
GAATATAGAAAAGAAAATATAAAATGGGAGTTTATAGATTTTGGTCTTGATTTACAGCCT
ACTATAGATTTGATTGAGAAACCAATGGGTATTTTATCTTTATTAGATGAAGAGTGCTTC
TTTCCAAAAGCTACTAGCAAAACATTTGTCGAGAAAATAATTAAAAATCAATCAAGTCAC
TCTAAATTTAAAGCAACAGATTTCAGGGCTAAGGCAGATTTCGGCGTGATACATTACGCA
GGTAAAGTAGATTATGTTGCTGATAACTGGTTAGTAAAAAATATGGATCCCCTCAATGAA
AATGTTGTGAGTTTGTTGCAAGAATCTAATGAATTATTTGTTCAAACTATTTGGAAAGAT
ACTGAAAATATCATTGGTTTATCAACAACCACTGCACAGGAATCAGCATTTGGTGGTGCT
AAAACTCGTAAAGGCATGTTTCGAACTGTTGGTCAACTTTATAAAGAATCTTTAACTAAG
TTAATGGATGTTCTTAATAATACCAGTCCGAATTTTGTTAGATGCATTATTCCAAATCAC
GAAAAAAAAGCGGGAAAAATTGATTCCAGATTAGTTATTGATCAACTGAAATGCAATGGT
GTATTGGAAGGTATTAGAATTTGCAGACAAGGGTTTCCAAATAGAATTCTATTCCAGGAA
TTTAAACAACGTTACGGAATTCTTACTCCAAATGTCATACCAAAAGGATTTATGGACGGT
CGTAAAGCTACTGAACTCATGCTTGGAGCTTTGGACATTGATGTTTCAAATTATCGAATT
GGGCAAAGTAAAATATTTTTTAAAGCGGGTGTTTTGGCTAGATTGGAAGAAGACCGTGAC
ACGAAATTAACAGAAATTATTGTTAAATTTCAGTCTTTTGCACGTGGATACTTGGCAAGA
AAAAATTTGCAAAACCGATCACAACATCTTAATGCTGTAAAAATTATACAAAGAAATTGT
ACTGCTTATTTAAAATTGAGAAATTGGTCATGGTGGAAATTGTTTACTCGTGTAAAACCT
TTATTGAGTGTGACCAGACAAGAGGAAGTAGTTGCACTGAAAGAGGAAGAATTGAAAAAA
ACCAAAGAATCTTTAGAAAAAATGACAAATGAATTCGAAGTGACTAAAAAGAATTATGAA
TCTCTAACGGAGGAAAAAAGTAAACTCCAAGAAGATTTGGAAAAAGAAAGATTCGCATTA
CAAGATCTCGAAGGAGAAAGAGAAAATTTAATTAAACGATTAAATGATATTGGATTAGAA
GCGCAGGAATGTGAATTGAGAGAGATAGAAACTAGAGATAAATGTAGCAAATTGGAAATA
GAAAAAAAGAAATTAAATGAAGAAATTGGAAATCTTAGTCAAAATTTAGAATTAGAAGAA
CAACATTTACAAAAAATACAAACTGAAAAGCTTACTGCTGATAAACGAATAAAGGAATTA
GAAGAAAAAGTGGCTGAATTAGAGGATAAGCTTAACAAACTGATTAGAGAGAAAAAGTCT
CTTGAGGAAAAATTAGCTGATATTATGTCTACCTTAACAGAAGAAGAAAAAAAGAGTAAA
CAATTGATGAATCTGAAATCTCGTCATGAAGCAAGTATTAGTGAATTAGAGGAAAGATTG
AGTCGTGAACAAGCTGCGCGACAAGATTTGGAAAAAACTAAAAGAAGATTGGAAACAGAA
TTAGCTGAAAAATCAGATAATTTATCCACACAATCTCATACTTATGAAGAAATAAGAATT
ACTCTGGAAAAAGCTGAAATTCAAATTACAGAAATGCAGACTAAACTCGAAGAAGAAACA
ATGGCAAAATCAGCTGCCCAAAGACAAATAAGAGATGTAGAGAATGCAATTTCTGAAATA
AAAGAAGACCTTGAAGCAGAAAAACGTTCGAAGGAAAGAGCAGAAAAAGCAAAACGAGAT
TTAGCTGAAGAGATTGAATCACTCAAAATGGAATTACTTGACACTGGTAATACTTCAGAA
GAACAACAAACTATTTTACGTAAAAAAGAAGTTGAGTTACAAAACATTAAAAAATCGTTG
GATGATGAAACTAAGAGAGCTGAAGCTGAAATTCAAGAAATTAGAAAAAAAGCTGCCGTT
TCAATAGAAAATCTTAATAATCAATTAGAGACATCTAATAAGTCAAAAGTTGCAATTGAA
AAGGCTAAATTGCAATTACAAGAAGAACTTAATGATGCAAATAATGAATTATCAATTTTG
CGATCATCAAAAGCTGAATCTGAAAAACTTAAAAAGAATACCGAACAACAACTTTCTGAA
TCATTGAATCGTTTAGCAGAAGTTGAGGAGTACAAATTAGATATTGAAACAAAACTCAAA
AAACAAACAGAAGTATGTGATAAATTAGCCCTTAGTCTTGAATCTTCTGAGAGCAAGCTA
TCACAAATTACTAAATCTGAAATTTCGTTAAAAAGTCAATTAGAAGATTTACAAAAAAAT
TTTGAAGAAGAAACTCGATTAAAATTAGCAGCTCAAACATCATTAAGACAAGTAATAGGC
GAAATCTCGCAAATTAAGGATCAGTTAGAAGAAGAACAACAAATGAAAGAAAATTTGGAA
AAACATGTTCAAATCTTACAAACACAAATGCAAGAAGTGAAAAAGAAAGTAGAAGATGAT
GCTAATCAATTGTTGGAATTAGATGATTCTCGTAAAAAAAATAATGCGCGAACGAGAAGAA
TTACAAATTAGGTTAGAAGAAGCTTTAAATCAAGTTGACAGATCTGAAAAGGCTAAAAGA
ATTATTCAAGCTGAACTTCAAGATGCGTACCATGCCTTAGAAGGTCAGAAAAATGATCAA
ACTCAAAGTGATCGAAAGCTTAAAAAAATGGAATCTCAAGTCAATGAAATTATGAATTTA
AATAAAAAATATTTGTCTGAAAAAGAAATATTGGAAAAGGAAAATCGTGAAAAAGAAACT
CGAATTATGCAATTACAAAGAGAAATTACAGCAAACGAAGATAAACTAATTGAATTAGAA
AATTCTAAGTCCTTATTAACTCGTCAGTTAAATGAATTGGTATCTAACAAAGATGACGTG
GGTAAAAAATATTGGAAGTTTAGAAAAAGTCAAAATTCAGTTAGAAACTGATTTGGATAAC
TGTAAAAAATACTATTGAAGAATTAGAAGAAGAAAACGCGACTCTTCAAATGAATAAAGAA
AGAATTGAAATGCAATTAACTGCCACAAAAGCACAACTTGACAGAGAATTGAGTTCTAAA
GATGAAATGCATGATGAACAATTTAAAATCCTTAACAAAAAACTCCGTGATACAGAAGCA

ACTTTAGAAGAAGAACAAAAACAAAGAACTTCTTTGGCTTCATTAAAAAAGAAATTAGAA
ATTGAAAATGTTGATTTGATGAATCAGTTGAGTGAATCGGAAAAGATTAAAGAAGACTTG
TCAAGGCAAATTAAAAAACACCAAGGAACATCTATCAATAGTCAAAGAGAAATGCAAGAA
GCTTTAAATGCGAAAAATGAAGCTTTGAAACAAATTAAGGAATTGGAGAAGCGCTTACGA
ACAGCTGACAATAGCATTAATCAATTAACAGAGGATTTATCACAATTAGAACGTGTTAAA
AGAACAATAATAACTGAAAGAGATGATGCTTTAGAAGAAATCGCTACTTTAACCGTGAAT
AAAGACACCGCTATTGCTGATAAAAAACGAATTGAAAGTAATTTATCAGCATTAAATATG
GATTATGAAGAATTGCAGTTACTTCTTGCTGAATTAGAAGACAAAAACAAAAAAATAACA
AATCAATTAGAACAAGTTCAAAATGAATTAAATGTTGAACGCCAAAATGTCACACGCTTG
GAAAATCAAAAATCTACTGCTGAAAGACTAAATAAAGAATTACACGAAAAAATCGAAGAA
TTAGAAGTGGAAGCTGGTAAAAAGTACAAAACTACCGTTGCAGCTCAACAAAATAAAATC
CAGAGTCTTGATGCTCAATTAGATGCCAAAAGTGAAGAAATTAATCAAGCAAACCGAAAT
AATAAGAAATTAATGGTTGATGAACAAAAGCGGGCTGATAATGCGATTGAGCAAATTGAA
AAAGCTACTGGAAGATATAAAAAAGCAGAAAGAAGTTTGCAAATGGAACAAGAAAATAAT
TCTGCTTTGACTACTCAAAATCGTAGATTGCAACGTGATTTGGAAGAAGTCAATGAAACG
AAAGAAGCCCGTGATCAAGAGATAAAAGTTCTTAAAACTAAAATTGAAAGATTGGAACGA
AGAGTTCGTACTGGTGGTCGCCCAAATAGAGGAGATGCCGTATCTGATGATGGTTTATCT
GGTGATGGAGAATCGTTAACTGATTCTGGTCTTGGAGCAGTCCAGCAGGATTAAtttact
atacatttaacaatttatatagataattagcttatacagcgaaatttgtcggtttaattt
aaaaaatggacttatttggtttgctcaatatgttattgttttgaatttttaataaatttt
tcacgagATTGAGGCAAAAAATTTACAATATTTGCAAGTTGATAAAAGCTTGGTTTCTGA
TTCTGCTAACCTATCCGATTGGGCAAATAAAAAATTGGTTTGGATACCCGATGAAATTGA
AGGATTTATATCAGGTTCACTAATCGAGGAGAAAGGTGATGAAGCAACAGTTAAATTAGA
AAATGGAAAGTCTGTAAAGTTCCCCATAGAGAATATTCAAAAAATGAATCCACCAAAATT
TCTTAAATCTGAAGATATGGCTGATTTAACTTACTTAAATGAAGCTTCAGTTTTATTCAA
TTTAAAGGATAGATATTTTTCAGACTTAATTTACACTTATTCTGGATTATTTTGCGTTGT
TGTGAATCCATACAAGCGTTTGCCTATATACACCGACAACGTTATTGAATGGTATAAAGG
TAGAAAACGTCACGAAAGACCACCCCATATTTATGCCGTTACTGATGTAGCATATCGAAA
TATGCTTCAGGATAAAGAAAATCAATCAATACTCTGCACTGGTGAATCTGGAGCTGGAAA
AACTGAAAATACTAAAAAAGTCATTCAATACCTTGCATCTGTTGCTACTTCATTAAAGAA
TCAAAAAACAACTGCTAATAATGCTTTATCACAGTTTTATGCACATGATGTAAATATTGG
AGAATTAGAAACACAATTATTGCAAGCTAATCCAATTCTGGAAGCTTTCGGAAATGCGAA
AACCATTAAAAATGACAATTCATCACGATTTGGTAAATTTATTCGAATTAATTTCGACAA
TTCTGGATTCATCTCTAGTGCAAATATTGAAACATATCTTTTAGAAAAAGCTAGAGTTAT
TAGACAAGCAGCTAGCGAGAGGTGTTTTCATATTTTCTATCAATTACTTATTGGAGCTGA
TGATAATTTGAAAAAAGAATTAATTTTAGAGAATGTGTCCACATATAAACTGCTTTCAAA
TGGAATGATTACTGTCCCAGATTATGATGAAAGACAAATGTTTAAGGATACTGTTGAGTC
GCTCGATATTATAGGAATTTCAAAAGACGAGCAAGATTCAATTTTCCGTGTAATTTCTGC
CGTATTACATATGGGTAATATTGAATTCAAACAAGAAAGAAGCTCAGATCAAGCTGCTTT
ACCAGATAATACAGTTGCTCAAAAGGTCGCACATCTTCTTGGTTTACCAGTTACTGAAAT
GACTAAAGCTCTTTTGAAACCTAAGCTAAAAGTTGGAAGAGAAGTTGTAGCAAAAGCTCA
AACTAAAGAACAGGCTGAATTTTCAGTTGAAGCTATTAGTAAATCTACTTATGAAAGAAT
GTTTCGATGGCTGGTAATGAGAATCAATCGATCTATTGATAGAAATAGACAGAAAACTAA
TTTTATTGGAATTCTTGATATTGCTGGTTTTGAAATATTTGAAATCAACTCTTTTGAACA
ATTATGCATTAATTACACTAATGAAAAATTACAACAACTTTTCAACCATACTATGTTCAT
ACTTGAACAAGAAGAATATAGAAAAGAAAATATAAAATGGGAGTTTATAGATTTTGGTCT
TGATTTACAGCCTACTATAGATTTGATTGAGAAACCAATGGGTATTTTATCTTTATTAGA
TGAAGAGTGCTTCTTTCCAAAAGCTACTAGCAAAACATTTGTCGAGAAAATAATTAAAAA
TCAATCAAGTCACTCTAAATTTAAAGCAACAGATTTCAGGGCTAAGGCAGATTTCGGCGT
GATACATTACGCAGGTAAAGTAGATTATGTTGCTGATAACTGGTTAGTAAAAAATATGGA
TCCCCTCAATGAAAATGTTGTGAGTTTGTTGCAAGAATCTAATGAATTATTTGTTCAAAC
TATTTGGAAAGATACTGAAAATATCATTGGTTTATCAACAACCACTGCACAGGAATCAGC
ATTTGGTGGTGCTAAAACTCGTAAAGGCATGTTTCGAACTGTTGGTCAACTTTATAAAGA
ATCTTTAACTAAGTTAATGGATGTTCTTAATAATACCAGTCCGAATTTTGTTAGATGCAT
TATTCCAAATCACGAAAAAAAAAGCGGGAAAAATTGATTCCAGATTAGTTATTGATCAACT
GAAATGCAATGGTGTATTGGAAGGTATTAGAATTTGCAGACAAGGGTTTCCAAAAAAAAA
GAAATTAAATGAAGAAATTGGAAATCTTAGTCAAAATTTAGAATTAGAAGAACAACATTT

ACAAAAAATACAAACTGAAAAGCTTACTGCTGATAAACGAATAAAGGAATTAGAAGAAAA
AGTGGCTGAATTAGAGGATAAGCTTAACAAACTGATTAGAGAGAAAAAGTCTCTTGAGGA
AAAATTAGCTGATATTATGTCTACCTTAACAGAAGAAGAAAAAAAGAGTAAACAATTGAT
GAATCTGAAATCTCGTCATGAAGCAAGTATTAGTGAATTAGAGGAAAGATTGAGTCGTGA
ACAAGCTGCGCGACAAGATTTGGAAAAAACTAAAAGAAGATTGGAAACAGAATTAGCTGA
AAAATCAGATAATTTATCCACACAATCTCATACTTATGAAGAAATAAGAATTACTCTGGA
AAAAGCTGAAATTCAAATTACAGAAATGCAGACTAAACTCGAAGAAGAAACAATGGCAAA
ATCAGCTGCCCAAAGACAAATAAGAGATGTAGAGAATGCAATTTCTGAAATAAAAGAAGA
CCTTGAAGCAGAAAAACGTTCGAAGGAAAGAGCAGAAAAAGCAAAACGAGATTTAGCTGA
AGAGATTGAATCACTCAAAATGGAATTACTTGACACTGGTAATACTTCAGAAGAACAACA
AACTATTTTACGTAAAAAAGAAGTTGAGTTACAAAACATTAAAAAATCGTTGGATGATGA
AACTAAGAGAGCTGAAGCTGAAATTCAAGAAATTAGAAAAAAAGCTGCCGTTTCAATAGA
AAATCTTAATAATCAATTAGAGACATCTAATAAGTCAAAAGTTGCAATTGAAAAGGCTAA
ATTGCAATTACAAGAAGAACTTAATGATGCAAATAATGAATTATCAATTTTGCGATCATC
AAAAGCTGAATCTGAAAAACTTAAAAAGAATACCGAACAACAACTTTCTGAATCATTGAA
TCGTTTAGCAGAAGTTGAGGAGTACAAATTAGATATTGAAACAAAACTCAAAAAACAAAC
AGAAGTATGTGATAAATTAGCCCTTAGTCTTGAATCTTCTGAGAGCAAGCTATCACAAAT
TACTAAATCTGAAATTTCGTTAAAAAGTCAATTAGAAGATTTACAAAAAAATTTTGAAGA
AGAAACTCGATTAAAATTAGCAGCTCAAACATCATTAAGACAAGTAATAGGCGAAATCTC
GCAAATTAAGGATCAGTTAGAAGAAGAACAACAAATGAAAGAAAATTTGGAAAAACATGT
TCAAATCTTACAAACACAAATGCAAGAAGTGAAAAAGAAAGTAGAAGATGATGCTAATCA
ATTGTTGGAATTAGATGATTCTCGTAAAAAAATAATGCGCGAACGAGAAGAATTACAAAT
TAGGTTAGAAGAAGCTTTAAATCAAGTTGACAGATCTGAAAAGGCTAAAAGAATTATTCA
AGCTGAACTTCAAGATGCGTACCATGCCTTAGAAGGTCAGAAAAATGATCAAACTCAAAG
TGATCGAAAGCTTAAAAAAATGGAATCTCAAGTCAATGAAATTATGAATTTAAATAAAAA
ATATTTGTCTGAAAAAGAAATATTGGAAAAGGAAAATCGTGAAAAAGAAACTCGAATTAT
GCAATTACAAAGAGAAATTACAGCAAACGAAGATAAACTAATTGAATTAGAAAATTCTAA
GTCCTTATTAACTCGTCAGTTAAATGAATTGGTATCTAACAAAGATGACGTGGGTAAAAA
TATTGGAAGTTTAGAAAAAGTCAAAATTCAGTTAGAAACTGATTTGGATAACTGTAAAAA
TACTATTGAAGAATTAGAAGAAGAAAACGCGACTCTTCAAATGAATAAAGAAAGAATTGA
AATGCAATTAACTGCCACAAAAGCACAACTTGACAGAGAATTGAGTTCTAAAGATGAAAT
GCATGATGAACAATTTAAAATCCTTAACAAAAAACTCCGTGATACAGAAGCAACTTTAGA
AGAAGAACAAAAACAAAGAACTTCTTTGGCTTCATTAAAAAAGAAATTAGAAATTGAAAA
TGTTGATTTGATGAATCAGTTGAGTGAATCGGAAAAGATTAAAGAAGACTTGTCAAGGCA
AATTAAAAAACACCAAGGAACATCTATCAATAGTCAAAGAGAAATGCAAGAAGCTTTAAA
TGCGAAAAATGAAGCTTTGAAACAAATTAAGGAATTGGAGAAGCGCTTACGAACAGCTGA
CAATAGCATTAATCAATTAACAGAGGATTTATCACAATTAGAACGTGTTAAAAGAACAAT
AATAACTGAAAGAGATGATGCTTTAGAAGAAATCGCTACTTTAACCGTGAATAAAGACAC
CGCTATTGCTGATAAAAAACGAATTGAAAGTAATTTATCAGCATTAAATATGGATTATGA
AGAATTGCAGTTACTTCTTGCTGAATTAGAAGACAAAAACAAAAAAATAACAAATCAATT
AGAACAAGTTCAAAATGAATTAAATGTTGAACGCCAAAATGTCACACGCTTGGAAAATCA
AAAATCTACTGCTGAAAGACTAAATAAAGAATTACACGAAAAAATCGAAGAATTAGAAGT
GGAAGCTGGTAAAAAGTACAAAACTACCGTTGCAGCTCAACAAAATAAAATCCAGAGTCT
TGATGCTCAATTAGATGCCAAAAGTGAAGAAATTAATCAAGCAAACCGAAATAATAAGAA
ATTAGAAAGAAAATTAAAGGAAATAATGGCCCTTATGGTTGATGAACAAAAGCGGGCTGA
TAATGCGATTGAGCAAATTGAAAAAGCTACTGGAAGATATAAAAAAGCAGAAAGAAGTTT
GCAAATGGAACAAGAAAATAATTCTGCTTTGACTACTCAAAATCGTAGATTGCAACGTGA
TTTGGAAGAAGTCAATGAAACGAAAGAAGCCCGTGATCAAGAGATAAAAGTTCTTAAAAC
TAAAATTGAAAGATTGGAACGAAGAGTTCGTACTGGTGGTCGCCCAAATAGAGGAGATGC
CGTATCTGATGATGGTTTATCTGGTGATGGAGAATCGTTAACTGATTCTGGTCTTGGAGC
AGTCCAGCAGGATTAAtttactatacatttaacaatttatatagataattagcttataca
gcgaaatttgtcggtttaatttaaaaaatggacttatttggtttgctcaatatgttattg
ttttgaatttttaataaatttttcacgagtatttaatatttcataaactaggcgggaaaa
attgattccagattagttattgatcaactgaaatgcaatggtgtattggaaggtattaga
atttgcagacaagggtttccaaatagaattctattccaggaatttaaacaacgttacgga
attcttactccaaatgtcataccaaaaggatttATGGACGGTCGTAAAGCTACTGAACTC
ATGCTTGGAGCTTTGGACATTGATGTTTCAAATTATCGAATTGGGCAAAGTAAAATATTT

TTTAAAGCGGGTGTTTTGGCTAGATTGGAAGAAGACCGTGACACGAAATTAACAGAAATT
ATTGTTAAATTTCAGTCTTTTGCACGTGGATACTTGGCAAGAAAAAATTTGCAAAACCGA
TCACAACATCTTAATGCTGTAAAAATTATACAAAGAAATTGTACTGCTTATTTAAAATTG
AGAAATTGGTCATGGTGGAAATTGTTTACTCGTGTAAAACCTTTATTGAGTGTGACCAGA
CAAGAGGAAGTAGTTGCACTGAAAGAGGAAGAATTGAAAAAAACCAAAGAATCTTTAGAA
AAAATGACAAATGAATTCGAAGTGACTAAAAAGAATTATGAATCTCTAACGGAGGAAAAA
AGTAAACTCCAAGAAGATTTGGAAAAAGAAAGATTCGCATTACAAGATCTCGAAGGAGAA
AGAGAAAATTTAATTAAACGATTAAATGATATTGGATTAGAAGCGCAGGAATGTGAATTG
AGAGAGATAGAAACTAGAGATAAATGTAGCAAATTGGAAATAGAAAAAAAGAAATTAAAT
GAAGAAATTGGAAATCTTAGTCAAAATTTAGAATTAGAAGAACAACATTTACAAAAAATA
CAAACTGAAAAGCTTACTGCTGATAAACGAATAAAGGAATTAGAAGAAAAAGTGGCTGAA
TTAGAGGATAAGCTTAACAAACTGATTAGAGAGAAAAAGTCTCTTGAGGAAAAATTAGCT
GATATTATGTCTACCTTAACAGAAGAAGAAAAAAAGAGTAAACAATTGATGAATCTGAAA
TCTCGTCATGAAGCAAGTATTAGTGAATTAGAGGAAAGATTGAGTCGTGAACAAGCTGCG
CGACAAGATTTGGAAAAAACTAAAAGAAGATTGGAAACAGAATTAGCTGAAAAATCAGAT
AATTTATCCACACAATCTCATACTTATGAAGAAATAAGAATTACTCTGGAAAAAGCTGAA
ATTCAAATTACAGAAATGCAGACTAAACTCGAAGAAGAAACAATGGCAAAATCAGCTGCC
CAAAGACAAATAAGAGATGTAGAGAATGCAATTTCTGAAATAAAAGAAGACCTTGAAGCA
GAAAAACGTTCGAAGGAAAGAGCAGAAAAAGCAAAACGAGATTTAGCTGAAGAGATTGAA
TCACTCAAAATGGAATTACTTGACACTGGTAATACTTCAGAAGAACAACAAACTATTTTA
CGTAAAAAAGAAGTTGAGTTACAAAACATTAAAAAATCGTTGGATGATGAAACTAAGAGA
GCTGAAGCTGAAATTCAAGAAATTAGAAAAAAAGCTGCCGTTTCAATAGAAAATCTTAAT
AATCAATTAGAGACATCTAATAAGTCAAAAGTTGCAATTGAAAAGGCTAAATTGCAATTA
CAAGAAGAACTTAATGATGCAAATAATGAATTATCAATTTTGCGATCATCAAAAGCTGAA
TCTGAAAAACTTAAAAAGAATACCGAACAACAACTTTCTGAATCATTGAATCGTTTAGCA
GAAGTTGAGGAGTACAAATTAGATATTGAAACAAAACTCAAAAAACAAACAGAAGTATGT
GATAAATTAGCCCTTAGTCTTGAATCTTCTGAGAGCAAGCTATCACAAATTACTAAATCT
GAAATTTCGTTAAAAAGTCAATTAGAAGATTTACAAAAAAATTTTGAAGAAGAAACTCGA
TTAAAATTAGCAGCTCAAACATCATTAAGACAAGTAATAGGCGAAATCTCGCAAATTAAG
GATCAGTTAGAAGAAGAACAACAAATGAAAGAAAATTTGGAAAAACATGTTCAAATCTTA
CAAACACAAATGCAAGAAGTGAAAAAGAAAGTAGAAGATGATGCTAATCAATTGTTGGAA
TTAGATGATTCTCGTAAAAAAATAATGCGCGAACGAGAAGAATTACAAATTAGGTTAGAA
GAAGCTTTAAATCAAGTTGACAGATCTGAAAAGGCTAAAAGAATTATTCAAGCTGAACTT
CAAGATGCGTACCATGCCTTAGAAGGTCAGAAAAATGATCAAACTCAAAGTGATCGAAAG
CTTAAAAAAATGGAATCTCAAGTCAATGAAATTATGAATTTAAATAAAAAATATTTGTCT
GAAAAAGAAATATTGGAAAAGGAAAATCGTGAAAAAGAAACTCGAATTATGCAATTACAA
AGAGAAATTACAGCAAACGAAGATAAACTAATTGAATTAGAAAATTCTAAGTCCTTATTA
ACTCGTCAGTTAAATGAATTGGTATCTAACAAAGATGACGTGGGTAAAAATATTGGAAGT
TTAGAAAAAGTCAAAATTCAGTTAGAAACTGATTTGGATAACTGTAAAAATACTATTGAA
GAATTAGAAGAAGAAAACGCGACTCTTCAAATGAATAAAGAAAGAATTGAAATGCAATTA
ACTGCCACAAAAGCACAACTTGACAGAGAATTGAGTTCTAAAGATGAAATGCATGATGAA
CAATTTAAAATCCTTAACAAAAAACTCCGTGATACAGAAGCAACTTTAGAAGAAGAACAA
AAACAAAGAACTTCTTTGGCTTCATTAAAAAAGAAATTAGAAATTGAAAATGTTGATTTG
ATGAATCAGTTGAGTGAATCGGAAAAGATTAAAGAAGACTTGTCAAGGCAAATTAAAAAA
CACCAAGGAACATCTATCAATAGTCAAAGAGAAATGCAAGAAGCTTTAAATGCGAAAAAT
GAAGCTTTGAAACAAATTAAGGAATTGGAGAAGCGCTTACGAACAGCTGACAATAGCATT
AATCAATTAACAGAGGATTTATCACAATTAGAACGTGTTAAAAGAACAATAATAACTGAA
AGAGATGATGCTTTAGAAGAAATCGCTACTTTAACCGTGAATAAAGACACCGCTATTGCT
GATAAAAAACGAATTGAAAGTAATTTATCAGCATTAAATATGGATTATGAAGAATTGCAG
TTACTTCTTGCTGAATTAGAAGACAAAAACAAAAAAATAACAAATCAATTAGAACAAGTT
CAAAATGAATTAAATGTTGAACGCCAAAATGTCACACGCTTGGAAAATCAAAAATCTACT
GCTGAAAGACTAAATAAAGAATTACACGAAAAAATCGAAGAATTAGAAGTGGAAGCTGGT
AAAAAGTACAAAACTACCGTTGCAGCTCAACAAAATAAAATCCAGAGTCTTGATGCTCAA
TTAGATGCCAAAAGTGAAGAAATTAATCAAGCAAACCGAAATAATAAGAAATTAGAAAGA
AAATTAAAGGAAATAATGGCCCTTATGGTTGATGAACAAAAGCGGGCTGATAATGCGATT
GAGCAAATTGAAAAAGCTACTGGAAGATATAAAAAAGCAGAAAGAAGTTTGCAAATGGAA
CAAGAAAATAATTCTGCTTTGACTACTCAAAATCGTAGATTGCAACGTGATTTGGAAGAA

GTCAATGAAACGAAAGAAGCCCGTGATCAAGAGATAAAAGTTCTTAAAACTAAAATTGAA
AGATTGGAACGAAGAGTTCGTACTGGTGGTCGCCCAAATAGAGGAGATGCCGTATCTGAT
GATGGTTTATCTGGTGATGGAGAATCGTTAACTGATTCTGGTCTTGGAGCAGTCCAGCAG
GATTAAtttactatacatttaacaatttatatagataattagcttatacagcgaaatttg
tcggtttaatttaaaaaatggacttatttggtttgctcaatatgttattgttttgaattt
ttaataaatttttcacgag
>DjScaffold2307:47616-50564 strand:+
GGACCAAATCAGCAGTATCCCCACGCTCAAATTCACAATCATGGATTGACGAACTTTGAT
AAACAATTGGCAGTAAAAAATGCATTGAGATATTTTCCTAAATATTTGCATGGACTTCTG
AATAGTGAATTTGAATTAGAATTAGAAAAATATGGTCACATTTATATGTACCGTTTTAAG
CCACCAAGAGAAATTTTGAAAGCGTATCATATAAGCCTTTATCCAGCAAAAATACCAGAA
GCAGCATCAATAATGTTAATGATTTCCAATAATCTAGATCCGGATATCGCTCAGTATCCT
GATGAATTAGTAACGTATGGTGGAAACGGTCAAGTATTTTCTAATTGGGCTCAGTATTGG
ACAGTTATGAAATTTTTATCTAAAATGAATAATGAGCAATGTTTAGTAATGTATTCCGGT
CACCCTTTAGGATTATTTCCAACTAATTCATTGTTTCGAAGTCCAAAAATGGTTATTACA
AATGGAATGGTAATACCTAATTATTCGTCTCCAACAGATTATGAAAAATTATTTGCTTTG
GGGGTTACACAATATGGACAAATGACCGCTGGTAGTTATTGTTATATTGGACCTCAAGGA
ATAGTTCATGGAACAACACTAACACTTATCAATGCAATCAGAAATCAAACAAAAATTCAT
AAGAAAAATGAAGTTGATGGAAAAATAAAGGTTTTTGTAACATCTGGTCTTGGTGGAATG
AGTGGTGCTCAACCAAAAGCTTCTGTTATTGCGAATTGTATTGGAGTAATTGCCGAAGTT
TCAAAAGCAGCATTAAATAAAAGATTTAAACAAGGCTGGGTCGAACAAATTTGCAATTCG
ATAACAGAGGTCATTGCAAAAATTAAGGAACATATTCAATTAAACCAATCGACAAGCATT
GGATTTCTTGGAAATGTTGTTGATCTCTGGGAAGCATTAGTTGAACACCATAAACTCACT
GGAGAATTATTAGTTGACATAGGATCAGATCAAACTTCTTGTCATATCTTGCTAGAAGGA
GGTTATTATCCTGCAGGACTGTCCTACGAAGAATCCCGAGCTTTAATGAACGAAGATAAA
GATAAATTTAAATCTATGGTTTATCAAAGCCTACAGAGACAGATTCAAGCAATAAATTAT
TTATCAACTAAAGGAATGTATTTCTTTGATTATGGAAATGCTTTTCTTTTACAAGCTGGT
AGAGCTGGTGCCGATGTTTTTATAAAAAAATTCAATTCAAGAATCTAGCACAGTTAAAAAT
AAATTCAAATATCCATCATATGTACAGGATATAATGGGTCAAATATTTTCAATGGGTTTT
GGTCCATTCAGATGGGTTTGTACTTCATGTGATGACAATGATTTGGTAAAAACTGATGAA
ATTGCCGCAAAAGAAATAGAGAAAATACTGAATGATAAGTATTTTGAAAACTGTGAAAGT
GATTGCTATAAAACGAATGTAAAACAACAATATGAAGATAATTTGAATTGGATTAAAAAT
GCAGGAAAATATGAATTAGTAGTTGGTTCAAAAGCCAGAATTCTTTATACGGATGTTGAA
GGAAGAGTTAATATTGCAAAAGCATTTAATGATGCTGTTTCTCAAAAGATCATTAGCGCG
CCAATTATATTAAGTAGAGATCATCATGATGTCAGTGGAACAGACAGTCCTTATAGAGAA
ACTTCAAACATTTATGATGGATCAGCTGTTTGTGCTGATATGGCAATACAAAATGTTATT
GGTGATGCATTCAGAGGAGCAAGTTGGGTAGCAATCCACAATGGGGGTGGTACTGGTTGG
GGGGAAGCTATTAACGGTGGATTTGGTCTTTTACTTGATGGATCAAAAGAAACTGAAGAA
AGGGCATTGTCAATGTTAGAATGGGATGTGATTAATGGTTTATCAAGAAGAATGTGGTCT
GGAAACGAAACCGCTAGAAGTACCATGGAAAAATGGGATTTGAAATGCAAAAATTTAGAA
TTATTTTATCCAAATTAAttaactaggaaattttaatttataatcattatttgtgatgta
tttgaattagaattttataaaagtttcttatcaaattttaaatttcttaccgcttgtatg
gaaatacatttaagatttgaattgaaatgctttaactcctgatcgatattttcacaggca
ataattattatacttactactttcagaatttagtggatagctttaatccacttaaacttt
aatttcctactacgtttatcgtatatttcttattcatgtaatatgagatattttaagaga
tttatttattctggattttctggtcctatctgccgcggatcagattttccgagatttaat
gtttttcacataaaat
>DjScaffold118:6828-15656 strand:+
GATCTAGATTTATCTACCAATGGGGCGTCTATTAAAGAAAGTATTTCAAAATTAAACAGT
GGAAAAATCTCTTGGTTAATTTTGGGATATTCAGGAAATTGTACACTCAATGTTATTGAA
ACCGGAGATAATTCTTTGGAGGATATGTTTGACGAATTTCAAGGTAGCGAAATAATGTAT
GGGTTTGCATCAGTTATCGATCCAAATACACAATTAAAAAAGAATGTCTATATTCATTGG
CAAGGCGCTGGATCGAAACAAGTAATGAAAACAAAATCTGTACAACATTCTAATATTATT
CGTGATCTTTGTAAGCCTATTCACGTAACTATCAACGCCAGAGATTCTCAAGACCTTGAA
CTTAAAGACGTTATGCAAAAAGTAGCTTCCGCATCAGGAGCAAATTATAGTTTTCACAAT
GAAACAAAACAAATATTCGAAACCACGCCTAATATAAAAAGTCAAAGCAATACAGCCAAT
ACTGCACGAGATGAACGCCGAAGAGAACTCGCCACAATAGGCGATATGAAAAAGCAAATT

TCTACAAATCAGAAAGTCAAATTACAATTTAAAAATTAAtttttttccaaatctttatta
aaagtggtttattattaaagttcttattgaattaataaattaaatcatgatttttaatat
ataaacttttcatttttaaaattttaccaagaagtaacattctatcgtaacatgaatttg
tttttataatgaagtagttgttaataatttaaaaattaaatttcaggaactttcgaatct
agtcaatcggacttttgttgacgaaccatttcaacaagtgggtacgaattatactaaaat
taaccctttggctgaaattcagcaattgaaaaggcaaagttctatcaattcagaagaatc
taaaagttcaccagaatattcaaaatccactaagaatacttactctgaagaacaaataca
gaaagtgggtacgaactacactaaaattaatccgtctgttgaaattgaacgattgagaag
acaaagttcttctaatgattctgaaaaacctataaatacaccaagtagtagctacacaaa
atgcattcctagagatgaaataataaaattaagaaaagaaagtattactgacaacgatga
cactccagttctctcctgtgtaagctgataaataGATCTAGATTTATCTACCAATGGGGC
GTCTATTAAAGAAAGTATTTCAAAATTAAACAGTGGAAAAATCTCTTGGTTAATTTTGGG
ATATTCAGGAAATTGTACACTCAATGTTATTGAAACCGGAGATAATTCTTTGGAGGATAT
GTTTGACGAATTTCAAGGTAGCGAAATAATGTATGGGTTTGCATCAGTTATCGATCCAAA
TACACAATTAAAAAAGAATGTCTATATTCATTGGCAAGGCGCTGGATCGAAACAAGTAAT
GAAAACAAAATCTGTACAACATTCTAATATTATTCGTGATCTTTGTAAGCCTATTCACGT
AACTATCAACGCCAGAGATTCTCAAGACCTTGAACTTAAAGACGTTATGCAAAAAGTAGC
TTCCGCATCAGGAGCAAATTATAGTTTTCACAATGAAACAAAACAAATATTCGAAACCAC
GCCTAATATAAAAAGTCAAAGCAATACAGCCAATACTGCACGAGATGAACGCCGAAGAGA
ACTCGCCACAATAGGCGATATGAAAAAGCAAATTTCTACAAATCAGAAAGTCGTAAGTTA
TAAAATATTATTTAATGATTTGTATTCGATGAAACTTTAGtatttatgtgatgggatttt
atgtaaataaactttaaatataaacaaacatttaatagtttaaactaatttattaaagtc
atatgatactc
>DjScaffold130:34551-35659 strand:+
atttgaaataatatttaatattcacttgttagattaataatttttttattaataaataatg
aattctctttctgtaaaatattttcgcattataacaaatcacttcaaaaacgttgaattt
attagaaaatacgtaagagatattctgatttatatttgagattgttgtcataaatagtca
tttcatactaaaccagctcatcaaagtccaaccgtaactgtcttacctggtgatggtgtg
tggccggatcttattgaacatttgaaatatatatttcggataatcgatgttaaattgaat
ttggaaacaaaatatctaagtgaattaccgggagttaagtctgattcagtaaatgatgtt
gttgatagtttcctaagaaataaattaatatgaaatttcggtaaatcatttcgatttgaa
aagttatcagaataaattcgtctcatcagtagccagttggatttatttgcaaatgttATG
AACGTTCGTAGTTTCGATGGGGTTCCAACGAGACATAAAAACGTTGATTTTGTTGTTATC
CGCGAACAAACCGAAGGTGAATATTCAGCTCTGGAACACGAATCAGTTCCAGGAGTTGTG
GAGAGTTTGAAAATTATAACGAAAAACAAATCGGAAAGAATTGCAAAATTTGCATTTGAT
TTCGCAAAAAAACACGACAGAAAGAAGGTAACAGCGGTTCACAAGGCAAATATTATGAAA
TTGGGAGATGGATTATTCCTGGAGAGCTGCAGAAACGTTTCCAAATTCTATCCCGATATT
GAATTGGACAACATCATCGTGGACAACTGCTGCATGCAGTTGGTGTCGAATCCTCAGCAA
TTCGATGTGCTCGTCACTCCGAATTTGTACGGCGCCATCGTGGACAATGTAGCTGCTGGC
CTCATTGGCGGTGCCGGACTCGTTCCAGGTGCCAGCTTTGGATACGATGCTAGTTTATTT
>DjScaffold130:46257-46371 strand:+
tacaacttttcaactcctgcatgacataataatttATGTATTTCATCTATTGTTTCATCT
TATATCGTTACAAATTTCTCAGTTGTTTTTTACAT
>DjScaffold3278:6639-8100 strand:+
ttttgtaatataatatcattttattaaaatgagattacttattaattatttaatttaaac
taatgttattattacatttttaaaagtagacctacataagttcccATGAATGATTGCGAA
GTTTTTTTTAATTGTGTCAAATCTGGAAAGATCGATGAAGTTCGACAGAACTACAAACCA
GATTATTTAGAATTATCTTTTGAATTTTGGAAGCGACCAATACATTGGGCTTGTCATTTT
GGGCATTTATCTGTAGCAGAATTCCTTATTGAAAATCATTGTAATATTGAAGCTGTTATG
AAAAGAGGTGTACGGCCGATACATTTAGCTTGTGAATATGGACACCTAAATGTAGTAAAG
TTATTAGTAAAAAATAATTGCAATATCGAATCGTGTTCAGAAGATGATTATCGACCTATT
CATTTAGCATGTGTTAATGGCCATTTGGAAATAGTTAAATTTTTAATTGATAATAGATGT
GATATCGAATCTTCTGCCAAATTTGATTGGCGTCCAATTCATTTTGCTAGCGCCAATGGT
CATATTGAAATTGTGAAATTGTTATTTGAAAACAAGTGCAATATTGAAGCATTTACTGAT
GTATGTGCAGGAAATGTTATTTTTATAAATAATACAGTAAACCAACTAATATCCGCCAAA
AAATTGGAATTCTGTAAAATGTAGatattaa
>DjScaffold3278:72344-80172 strand:+

aatatcaaaatttgATGCTACACAAAAGAATTGTTCAATATTTCGGAGCAGTTGAATTTT
CTAATAAACTTTATATATTTATGGAATATGCGAATGGAGGTTCGTTGAAAAGTCATTTGA
CAAGCTTACAAATGAAATCTCATGATATTGTAACACAGTATACTAAACAAATATTAGAAG
GACTATCTTTTCTTCATAGCAATAGTATTATTCATCGTGATATTAAACCGGAGAATATTT
TGCTTGATCAGAAAAATAATATAAAACTAACAGATTTCGGTTTGAGTCGTTTGTTGGAAA
GCATATCCAAAACAGGAATTGATAAAATTGAAGACGCATCAACATTTTGTGGAACACCGA
ATTACATGGCTCCAGAAATTATAATGGGTAAGCGTTATAGCTTCCCAGCAGATATCTGGT
CTGTGGGCGCAACAGTTGTAGCAATGCTATCAGGTAAACCACCGTATTGCGCATGGTCTG
CTGAGGCTGTAATATTCAAAATTGGAAGCACTAAAAAAATAGAGTTTCAACCATCAAAAC
CTATCTCGAAAGACATGAAATCACTCCTCAGTGATATGATTAATCCAAATCCATCTAAAC
GATTAACAGCTGATTTGCTATTGAAGTCCAAAATCTTTCTTGATGAGAAATAGgtttaat
tttacaaattttacgatgtattgattttcgagattttgtcaaaatgtatctccagtatta
attacgatgctatattgaatttcattcacagattatcttaaattcgtgttgttattatat
tattactattatatccaattttttaattaaattagttaatttattatttata
>DjScaffold4302:52271-60985 strand:+
AACTTAATTTACGGAATTTCATATGGGAAATTGAGATATCACTCTCTTCATCATGCAATT
CACGACAGAAGATTAACACAAATCAGATTATTATTAAAACTTCAACCGGATTTAATAAAT
TTTCGAACTGATCTTGGACTAACTCCTTTAATGATTGCATGTCTTCTGCCAGAAAGTGAA
AGAAAAATTAATATTATCAAAATATTGATAAAAGCACCTTTACTGAATCCATACTGCAGA
GACCATGGAAAAAGATCAGTTGTTTTTTATTTCATTAAATATTTAAAACTTAACGATTTG
AAAGAATTTCTGCAAGTCAGTAACTTTAATTATTTCACTCAAACGGATATTGACGGATTT
ACTATTTTACATTATGCAGTAGTTAGAGAAAATCTGGAAATTCTGGAATTTGTTTATAAT
TTATTTTTACGATATAAAATAAATATTGATACACCAAGCAAAGTTAATTTAACACCTTTA
TTACTTGCATGTAAATTATCTAAATATCGCTCAATAGCATTAATTATAAGAATGGGAGCC
AATTACTTTAATGTTCGAGATAATGAATTTTTTAGAAATTGCTTAGAGTGGCTGGAATGC
AACAAAGAAAGTAAATTAGTGTTAAAGGAAGATGAAGTGTTTTATTTTGATAAATTTGAA
ATGGAGCAGGATTTAGTATCAGGAATTCCCAGTGACACTGGAACTCCAAGATTTCGCGAA
ATCAAACCGCTAAGTGCCAGAGTTCAAAATGGGGTTGCTAATTGTCTGAATAGAGAATAT
TCATTTTCTGGTAGCACTAAATTCAGACTTAATACGAGTCATATTTCAAAATTAGATAAC
GAAGATAATGTTGAAAGCAAATCGTTATTAGTGTTGGAAATTGAAAATATGTCATTAGAA
GAACTTGAAGAATTTGTAATGGATTCTTTAAAAAACAACAAAAGAAAATTAAATACAAAG
AAAAGAGTTTCAATAGTGATAAATGACAGAATATTTTTACCGAAAAGAAATTCAATTTCA
ATTGACAACTTACCTCTATGTCGAGAAACGTCGGCAAGCATAGCT
>DjScaffold4435:3373-5843 strand:+
gaatgttatttgaacattagtaaacctagactagaagagagaagatacaattttagaagc
gaacaacaaacaaaagattggagtcggaccaaaccaattcatgccattcgcgaagaatat
gatgacgagaatgaagagagattttctgagagcataatagaaaatgatgattcaagaaga
gaatacccaaaagacagagctccatcagtagaggagttggtcggagctatgcatttaacg
agataccatttgaatatcctcaagaggccgagaaatgggttgagacgaataaagagagag
aaaagatttacagattcctaaaaatagaaagtttcaatgatgcgaaagttaataaaacag
aagtcctagaaataaaaacagaatgcagaaatttcgtatgataaaatgcaagggagaccc
agtatttcagtgatgatcaagggtgaacagtttgattgtttattggataaatgagtaaga
ataaatgtgATGAGCATGAAAAGATTTAATAGACTCGGAGAAACAATGTATGACAAAAGC
GAGGAACGATTGCATTGCGCGAATAACAGCTCATTGGAGACTATTGGAAAGATACGAGTC
AAAGTTGTGGTTTCAGAGGTGTGCAAGGAAGTAAAATTTATATTAGTAGAGAAATTAATA
CCAGATCTAATTGGTGGAGTGGAATTTCAGAATAATTTTGGGTATCATCTTCAGAAAAAG
TGCAAGGTAGACATCAATAATCATCGAGAAAAATTTGTTTGCAACATCGAGGCAAAATTT
GGAAGTCAAATACAGACAAGTGAAAGATTGAGACATGTAATGGAGATTCTGAAAATTAAC
AAAGATCATAGATTGACTGCGATTATTCAGAATAATGAGGATGTATTTATGGCGGATAAA
TGGGATATTGGATGTACACGCTTAACGAAGCATCGAATAATCACTAAAGAAGGTCCAATC
AACACAAAACCGTATTGCCAACCAATGAATCTAGATGAAAAGCTTGAAGAGGCCATCAAG
AATCTATATGATAATGGTATCATCCGGAAGTGTAATTCGCAATGGAATACGCCATTAATC
TGTGTTTCGAAAAAGGAAAAAAAGGATATACAATTATGCTTAGACTTTAGACAGCTCAAT
GAAATAGCAGAAAGACAAGCTTTTCCTATGCGAAATGTGGACGACATACTAGATGCTTTG
GAACGTACGCCGTATTTGAGCTCAATAGACTTGGGAAATGCATATTACCAAGTGGAACGC
AGTGAACATTCTCAAAAAAACACAGCGTTTTCGACCAAACAAGGGTAAttttgtttcaat
agaatgccttttgaaattgcagcagcaccaagtacgttccaggaaattatgaataaagta

atcggaaaaattaaaggagcaatggtttatctagatgatattctcatatttaccgaaaca
aaagagcaacactatgaaataatcaatagagtattaacagaaattaagaaggctggtctt
agagttaatccaaagaaataatgatgaaggaacatgggtacctgatgataaagtagaaga
atttataatgaggatacaccatcttttgtgtcatgctggagcagaaaaggtagcagcgta
tatgaagcataaatataacgtgaacaatttgtggagcaaacaatgacaagacaagatgaa
gatacc
>DjScaffold4435:174478-177339 strand:+
ATGAAGTTGTTAAGTGGTGCTAAAATCAATTCATTTTTTTATAATAAAATTTGTCGAATT
TTAATTAAATTATGGATTGATTATCTATTATTTATTCTTAGTTCTACGTCTCACAAAAGG
TTTGAATCCATAACTTTGGAACAACGACGAGCTAGTCAAAATCCTGTTGTGATAAAAAAT
CTTCAAAGGTTTTCCTTCATTTTCCCATTCCATTCATATCTCCATTCGGCTGCATTATAT
GTCAATCGTAGAATACTTGCTTCTCATTCTTGGCAGTTTAGTTATGATGGCATAAATCCA
TCTCCAAAGATCTTTGAAGAAATCAACAATTCTTACGATCAATGGAATCAGAAATTCCAA
TTTTCTCACCATGTTAAAGAATCGAAAGAAACTATTACAGAGCCTTATATTCCAGTTGAA
TCAAATCAGAACAATTATACGGAGTCTTCAACTGATGATCAAGTTGTAAATTACCCAGTT
AAAGATGTTGGTTATTATTCCTCCAATAATGGCTCTGGAAGTACAATATCAAACCAGCAG
TCTTATTTTGTTTTACCAGATAACAATGAGTCGATTAATGATTATTCAAAGTCAAAAATA
GAATTTCTTTGTCAGCCTGTAGATACCGCTAAAATATACAAATTGAACTTTTTTAATTTT
GATTATCAAATTAAAGAAGCTCAAGATTCAGAATTTATTAATGAAATCTCAGATCAAAGT
GAAATAGAAAATTTATCTTTTATCGAGACGTCTAACAAGAATGAAGATATCAATGAGCAT
AATGTTTTACTAACAACACATGAAGACTGCAATTTTTCTGATTTTCTTGTTAGTTTAGAT
TTGTTAGAAATTCAACAGAAAATAGATTGGAATATAAATTGGGATAATGTTACTTTGTTG
CTCATTGATCATGGAATTGTTGAAAATCGAGAGTACGGAAGATCAAGTTTGACCAGCGTT
TCACCTGTTGAAAGCTCTGAAGAAAATTATGAATCACCCAGTCGATCTACTCCAACTAAT
GAGGTAATTTTCAAACAGCGTCTCCAGTCTTTTGATCAATCGATTCTATGTTCTCCGAAC
ATTTTCAATTGTCCTATTAATTTAGGACCATTTCTCAGTGCCATATTTCAACGCCTAAGT
GCTCTTCCCTCAAATACATTTTTTATGAATTTGCTCATTAGTGGCATTATTACAGATCTG
GCAGCATATCCGCATCCACTTATCAAGAGTCTCCTTTTAGATATTGATATAATTTTGCAA
CCTTATATCAGTAAACTTTTTGAAACATTGTCTGAAGTAAAAGTAAATATATTAGAGAAG
TCGCAAAATATTTCTGATTGGGAAAGTATTCTTTCAAATGCATATGAATATCTTTATTCG
CGAGTTGATGTGAATTTGGAGGGAATGGAAATAAATTCAAATTTCGATGAAGAATTTGAA
TTAAGATTAACTAATAATCCATCTGGTTCGATGAACAACTCATTTTTTGATTTTGATGCC
TTTTCATTGTCAAGAAAATTGTCACAGTCTTCTCCGACAAAGGTTAAAGATAAAAGTAAC
GCTGGTGTTAAAATAGCAAAATTTTTATCAATTTATTGAatgtttttttccagaaaata
gaagaactcctttcggtatactgaagcgtaaaaagaaggaatattctccacaatataagt
aagaattattttatatttagattgatttcgtaatgatgaatatataaaaataaaatacat
ataatgcgcgtagaaaaaaatcatttacagaatttttttgaattcaattttatttatgta
tcattatattt
>DjScaffold4435:94753-103344 strand:+
TTAGCTTATTTCAAGGAATCAACTTTACCAGAAGAAACGAAATCTGATTGGAGTAAATCA
TGTGAAGAAGCAATAGAACGTTGTGAAACAAGCCCTCATGTGATGAGAAAAAGACCGCGA
ACAACGTTTTCACAATCTCAATTACTGGAATTAGAAAAAGAATTCATTCACACTGCCTAT
CTTGACCGAGCCAACAGAATATCTCTGGCTGGAAAATTGAACTTGAAAGAAAAACAAATC
AAAGTATGGTTTCAAAATAGACGAATGAAACGGAAACGTGACGAAAGCGAACAAAAACGA
CGGAAATCATTAACCGAGAACTTAGTTGAATCAACAAATCCTATTGTGGATCATAGTAAA
TATCATTTGCCATTTCAAAGTTCCATTAATCGTAATTCAACTCACTGTTCGGATTCAATA
CCAGGAAATATTCCTTGGAATTTGGAAAATGGGTTCTCAAATTACAATTTCGATACAGCA
CCACGCAGTCCATTGATGAAAAACGAATACATTGGTCGTTCAGGAATTAATTTCGAATCT
TTTATTAATCATTATAATCATCTGTATGACTTACCAACAACATTGGAATCACAATATTCG
TGTCATAACGATCATTCTTCATTTAGTTCATATGAATCAATGCCGCCTAATGTTACGACG
AATTACCATCAAACATTACCAATCCATAATTATTGTCCAGAACTGTTTTATGATGACTAC
CAGAATTGTCAAAATTATTATCAGAACAATTATTTCGATTGA
>DjScaffold4224:1798-7126 strand:+
atctaatttcatgaagtgtaacatataatcccataaattattgaattgattaaagtcttg
ttcaaattaattattttcttgatcagttattttcttttctcttaatgtattgaagaaatc
ttcttttgatggtttatggtttctttttaataattctattacttcattaatattattacc
ttttacattatcataaggaaatgatattttctttggagtaagttttaatgttcttaaatg

attaaattttcctgattcatttaatgttgaacccattttatcaactgagtacacattctt
gctgaatctaaaaaataattcttcttctgaggaggtggctatgtgcgttactatttacct
actgtaatactattatatgatatttgataattttcctttagagtaaaaaagaaatatcta
acgacttggtcttatttttcaaagataaacttcatttacctgaaagcgaggcaaaaaaaa
gaatatttttcgtgtctgcagaagacatttatataaaaaaaaaacatcaaaaaaagaaatt
ctgtagaaATGCATGGTTATGACGAACGATGTCGTATGTTCGAGGAATTTGAAAATACAT
TATCGCAATTTATAAACACATTAAACGAAAATAAATTAAATCATTATAAAGAGGAACTCA
ATAAAACTAAAAAAACCATAAATGATTATTTGGAAAAATTTAAAACTGAATTCATAAAAA
CAAGCTATTTTAAAAATATTCTTGTTGAATGGCCAAATTATAAATACGAACTCCAAAAAT
CATTAAATGAAAAGATAAAGTACATTGAAGATAAACATGATAATTTAAAAGTGGAAATGG
GAAAATTTATTGATGAATGTAAAACTAATTTACACCGAGATTCAATTAGTTCATTTGAAA
TAAAGTTGGACAAATTAATTAAGCAAGGACAGATCGATTTTCGAGATTCAAAAATTGATA
TAAGTGATTATATCGACAAGGAACATAAAAAATACAGAAGTTCAATTTCAATAGAAAAAG
AAAAAGATGAATTCAGT
>DjScaffold5121:50440-53716 strand:+
ATGCAATCTGTGACTTATCAAGAACCGAAAAATTGGTGTTCAATCGTTTATTACGAGTTG
AATAATCGAGTTGGAGAAGCATTTTTTGCTAGTAAATTAAGTATCATTGTCGATGGATTT
ACCGACCCTTCAAATAATTCTGATCGTTTTTGTCTAGGATTGTTGTCAAATGTGAATCGA
AACTCAACAATTGAAAATACAAGACGTCATATAGGAAAAGGTGTTCATTTATATTATGTT
GGGGGTGATGTGTATGCTGAATGTTTATCGGACAGCAGTATTTTCGTCCAATCCAGAAAC
TGCAATTATCACCACAATTTCCATCCAACCACTGTTTGCAAAATTCCCCCAGGATGTTCA
TTGAAAATATTCTCTAATCAAGAATTTGCTTTTCTACTTAGTCGAACAGTGCATCATGGG
TTTGAAGCAGTTTATGAATTGACAAAAATGTGTACGATTCGAATGAGTTTTGTCAAAGGT
TGGGGAGCTGAATATCATCGTCAAGATGTCACCTCTACTCCTTGTTGGATTGAAATTCAT
TTAAATGGTCCTCTTCATTGGTTAGACAAAGTTCTAAGTCAAATGGGTACTCCAAGAAAT
CCCATTTCATCAGTTTCTTGA
>DjScaffold3509:6178-32267 strand:+
aaaaaatagttttaaaaattatttttaacgttaatttgaaatttaaaaacacatttttat
aaatagaggataaccaataagtttttgtcgagattttataatatattcttgcaatttttt
cgggattgattttttttatccaaaacttgaatttcttagttttgaatatATGACTATGAA
AATGTTCAAAAAATTATTTACTATCATTTTAATATTTTTAATTTTGATTTTGATGCTATA
TTTTTTTATGGATTCCACTATTTTTGTTCGACAAATCAAGAACTACACAGTTACACATGA
TAAATTTGATAATTCCAAATTAATGAATCATTATGGAGTTATTATTGATGCAGGAAGTAG
TGGTAGTAGATTGTTTATTTATACTTGGCCTCCTCATACTGGAAATCCGGGCGAATTATT
GCAAATCCAACCTTTATTAGATGAATCCGGAAAACCAATTGTGAAAAAAGTCAATCCGGG
ATTATCGTCGTTGGCAGATCAGCCAGATAAAGCCATGTCGTATATTAAGCCATTATTGGA
TTTAGCTGCGAACAGTGTTCCCAAATCTCTTCATAATGAAACTCCATTTTATTTGATGGC
AACAGCGGGAATGCGAATGATAACAATGGAGGCTCAAACTGCCATATTGAAAAATATCCG
TGAATTCGTTAAGAATGAATATAAATTCCTGTTTTCTGATTCTCACGCCAAAGTGATCAC
TGGACAAGAAGAAGGCATATTTGGTTGGATTACAACAAATTATCTACTTGGTCGATTCAA
TCACAACCATTCTGATGGAGTGCCGATCATCCCCGTGCATTTCATTGATTCATCATTCAG
ATCTGAACAAGTGCATCATCGGAAACGCACTGTTGGTGTGATTGATATGGGTGGTGGCAG
TATGCAAATTGCATATGAATTAGAACCGAATTCTGGAAAGGATATCGATTCTGCAGTTTC
TTTTAATCTCGGATGTCGGGAGAACGATGCAGATCACACTTATAGAGTCTTCGTAACAAC
CTTTCTGGGTTTTGGAGCAAATTCAGCGCGAACAAGGTATGAAAGAATGCTTGTTCGTGA
GTATGTGGGGTCGGAATTTTACAGAAATTTTACAGAAATTCAAAATAATACAACGAAACC
TATGGAGATAGTAATCGAGGATCCGTGCCTTCACAAAGGATCTAAAATAATACAAAAAGT
GAAGTTGGTAAATGATACACAAGGTGGTTTGGGGTTGTCATCAAAGGAAATCAAGAAATT
GCCATCTATAAAAGCCACATTTCAAGGAAACGGGAAATACCATTCTTGTGAAGCTCGCTT
GTTATCAGTCTTGAACAAAATTCCTTGCCCTACAGAGTCCAATTTGAAGTCAACCTGCAC
CATGTCCGGTCGATTACATCCCAAAGTAAATCTTGCTAACACTCAGTTTTACGGTTTCTC
GGAATTTTGGTATTCGACACAAGACGTGTTAGGCCTTGGTGGACAGTACAATTATTTCAA
GCTTCACTCTGAATCTGTTGACCACTGTAAGAAGGATTGGACGTCTCTTTCCATGGATTA
TAAGAAGGGACTCTTTAAAAACGCGGATTTGCCTCGAATGAAATATCAATGTTTCAAAGC
GGCCTGGATTACCGCTGTTCTCCACAACGGATTGGGTTTTCCGAATGCTTTCAACAAGTT
TACATCTGTACAAGAAATTAACGGAGCCGAGGTTCAGTGGAGCCTCGGAGCTCTTCTTTA
TCAAACAAGATATTTCCCTTTACGAGTTATTGAACAGAAAGAACAAGTTCGGAAGAAACA

AGCACGAGAAATACGAAACAAACCAAAGAATATTTACATGTACTTGTTCTTGTTGATAGC
AGCATCTGTCGTTATGGTAACTGTTTATTGGATGCACAATCGCGATAAATGGAAACATTT
TCCGTCAGTCACATCATACAGAAAGGATTCGTATAAGAACAGTTTGAATTTCGATACTCA
CTCATCAGATGACCTATTTAACAAGATCGAATTGGCCCATAGCTCAGGATCAATAAAGGA
TTTTAGGAGTTCTCCAAATTTCAAGAATTGGATCCAATAAtctttccctttaatcaattt
tcttaataatcgatatgtcaagtctttctgtataaacttccatcacaattccgcgatgtt
ttcgtttgaaatatatttttaatgataa
>DjScaffold2293:17149-17917 strand:+
tggatgaagatattaatatcgaaaatttcaatgaaacattaaacaacccaattatcgaat
cttttaaaaaaATGATGGCTGACGAAAGATTAAATGAATATGGAAAGCTTTTTAAAAACG
ATACTAATGAAGACGATTTCTATGATGGAGAAGCAGATTTTATAGATGAAGAAGAAAGAG
TAAAGGAATTCAATTCGCATTTCATTGATACACCTATTCCAAGTGATGCGATTCTGAATT
GCCCAGGATGTATGTCACTGTTAACCATTGATTGTCAGCAACACGAAATATATAAGAATC
AATTTAGAGCAATGTTCGTAGAAAATTGCAGATCTGATGAAACTGAATCCGTTATTTGTC
CAATTGAAAAGAAAGATAAGAAAAAAACGCGCAATGCAGAAATTAAAACTTGTGACATAT
TTCATCCAGTTTATTGTCAGATTTGCGATACAAAAGTAGGAATGTTAGACGAGAAAGAAG
TTTATCACTTTTTTAATGTTCTCCCGAGCGATTAAgatttaataaactttaaaatgtatt
tttaattaatattatgatattgaaattttaaaatattcaaaaattatttacacttctttt
tggaat
>DjScaffold824:33716-33937 strand:+
ATGATATTGAGAATAATAATTAGCAGAGATGGTGTTAGAACTGATCCGTCTAAAATAGAA
GCAATAAGAACATTTGGCAAACCTAACTGTGTGAAGACGTTACGAAGTTTCTTGAGAATT
TATAATTATTACAGGAAGTTTATAGAAAACTACGCAAGAAAATCAAAGATATTAAAGCAG
TTGTGTAGCGTAACAAGCAACAAGAAATTAGAATGGACAGAT
>DjScaffold3617:70593-76874 strand:+
gatgctggaattactgctttgATGGAAGTTGGATTAGATCCAGGAATAGATCATATGATT
GCTAAGGATATTTTTGATGATATGTCTGATAGTGGATTAAATATTGTGTCCTATAAATCT
TATACTGGTGGTTTACCGGCTCCAGAATTTTCAGAAAATCCACTTAGATATAAATTTAGT
TGGAGTCCAGAATCAGCACTAATGACAGCTCTTAATCCAGCTAAATATATTGAAAATGGA
AAAGTTAAAGAAATTAAAGCGGGTGGAGATTTAATGTTAGCCTCAAAGAAAATGTCAATT
TTGCCAGGATTTAATTTACAAGGTTATCCAAACAGAGATTCTACAGCTTATATAAAAAAG
TACGGTTTAACAAACGTGCACACACTCATTAGAGGCACACTTCGTTACAAAGGATTCACA
GATGCCATGTGGTCGTTGATACAACTAGGTCTTCTTTCACCTAAACCTGATCCCCATCTC
CATCCGAATGGGCCGAATATCACTTGGAAACAATTTTTGTGTAAACAAATGGAAATGAAT
GTAGATGTTTTTACGGGAACATTGAAAAATGCTATTTTTGAAAAATTAAGTGAAGACAAA
TTCAAATTTTCTTGCCTAGAAGACTTTGGTTTATTTAAAGATGTAGAAATTGAAAAGAAA
GGCACGCCACTTGCTACTATTTCACATTATCTTTCTCAAAAACTAGCATATGAAAAGAAT
GAAAGAGATGTCGTTATTATGGCACACGAAGTTGTTGTTAAATGGCCAGAAAATAAATTT
CAAAAGTTGGACGTTCGATTGGTTGTGTATGGTGATCCAGATGGCTTCATGGCTATGGCT
AAGACTGTGGGTTATCCAACAGCAATCGGTGTTAAAATGATACTCGATGGAGAAATTCAG
AAGAAGGGTATTGTTTTACCATTATCAGTTGATATATATCGACCAATGTTGAAACGTTTG
AAAAAAGAAGGAATTGATATAATTGAAACTACAAACATAATTGACAGTTTTTAAggaatt
cttagtttctttaacttcccctttgaatcatttttcttattcacgattattatggatatt
taatatttaacgaaaataaattcgttgttttggtt
>DjScaffold4050:78761-90040 strand:+
gaataatttattttaaaattcatttacatttaatgccataaatactacataatttttaat
ttattcatttaaatattaaaactatctgattaatattgactttaaaaaatactactttat
ttttaaaatttagtaaaccaaataaattttctataaatatgtgagtcttgactATGGCAA
GTTCAAAATGTTCTCAAAAACCGTTAACTAATAAATTGGAATTTATGTTGAAAAAAATAA
TGCCTTTTCTCATGAGGCATGAAGCCGCAAAGCCTTTTCTCAAACCAGTTGATTTCATAG
CATTTAAAATACCAGAATATTCAAATATAATTAAATGTCCAATCGATTTAAGTACGATAA
AAGAAAGATTAATTATGAAATTCTATGATAATTTTGAGTATTTCTTAAAAGATTTTAATT
TAATGTTAGAAAATTGTTACGTATACAATTCACCAGATGATCCAGTAATATCAATGGGAT
TGCGATTGAAAAATTATTTTGATACTAAAATAAACGCAATGCCCGCTGAAGAATTTAAGC
TGAAAAATTCCATTAAACGAAAATTTGTGCCTTTGAAAAAAAATATCAAAATAGTTTGTA
AAGAAAATTTAAAAAAATCCGAATCAAAACTAACCAATCTAAATCAGTCTATAATGAAAG
AAAATTGCGAAAATCAAATTATTCCTATTAGTAATTCAATGACGAATAATGAATACGAAA

AATGTTCATTGCTTATTAGCGAATTGATGTCTGTAAAGTATAAATCGATAAATTATTTGT
TTCTCCAACCTGTTGATGCTAAAGCGATGGGACTGATCGATTATCACGAATATGTCAAAT
ATCCTATGGATTTGAGTACAATTCAATCGTATCTGTTCGATAGGCTTTACGATACGAGAA
TGGCATTTATCAATGATATCTATTTGATGCTCAATAACTGTATTATTTATAACGGTCGCG
AAACTGATATTGGAGTTATTTGTCGACGATTTCAACACGATTTTGAAAGTCTTTATTATG
AAATTTTCAAAGAAAAATGCCGAGAAAATATTAATGATGAAACATTGTATTTTCTAATGA
AAATCAAACGAAAACACAAAGAAATCGAAGAAATGATCCGTTTGAATACTGAGAAGATAT
CAGCAGCACGCTCCAGAATCGATATGATTGTGAATTTATTTGACAAAAATAATAAAGCGC
AGTTCAAACATGAATTGAAACGAAAAACGTTTAAAAAATTTCCTAATTTTTCATCACCAT
CACCATCACGA
>DjScaffold477:44682-52146 strand:+
caattacggaATGTTAAATGAAGAATCAAATGTCTCGTTTATTAGACCCGAATATCATAT
TTGGATGAAATCCGAATGGAGTTCTTGTTCTGTTACTTGTGAAGCTAATTCAAACAAAAT
AGGTTATCAGAAACGAATTGTGACTTGCGAGAAAATAGAAGCCCAATATTATAAAATTGT
GGAAGATAAGTTTTGTTCACACACCAAACCCATTTCAATTAAGAATTGCAAATCGTCGGC
TTTATGTCCCTCATGGAAAGTGGAGAAAACTGATAATTTACAGTGTTCAGATGTTTGTGT
TAATGTTGGTGTAGCGAAAACAAATACCAATATTATCTGTCAACACAACAACAAAACTGT
AGATCCATCGTTTTGCGATAATGATAAACAACCAATCATTACTTGCCCAAATACAATTTG
TCAAATCATTTATGATTATGGAAATTGGTCTCAGTGTTCGATGAGTTGCAGCAACACAAC
CAATGTCAGTATACGAAGTCGTACCATGAAATGTGTTTGGATACACACAAAAAAACCAGC
GTACAATCTTTGCCAACTAAATAAACTCCCCATTCGGCGTTTAACGAAGTCCTGTAGAGT
GCCAAAATGTATTCTGGGTTGCAAAGATTTGACTCCAGCCTGTGTGAAACAATCAAAATT
TCGATGTCGGAGCAGTTTATTTTCTCATTTTTGTTGTGCATTTTGCAACAAACAATTTTC
ATAAtttctatcgcttcattgtgaacattattagattattattattattattattat
tattattattattactattattattattgctgctgttgttattatttacaggagttcaaa
tttcataaatcctattttttgttaattatttcaaatattttcgttaataatttttacaaa
aaaatttgtaaaaaattgtctaattttatattggaaataattcaaaataaaaaagaaaat
atacatgtgcatttttttattattcttttttttgtttatattcattgtgaaaataataaa
attagtgtttgagataaactgttttgcattttcaattacggaATGTTAAATGAAGAATCA
AATGTCTCGTTTATTAGACCCGAATATCATATTTGGATGAAATCCGAATGGAGTTCTTGT
TCTGTTACTTGTGAAGCTAATTCAAACAAAATAGGTTATCAGAAACGAATTGTGACTTGC
GAGAAAATAGAAGCCCAATATTATAAAATTGTGGAAGATAAGTTTTGTTCACACACCAAA
CCCATTTCAATTAAGAATTGCAAATCGTCGGCTTTATGTCCCTCATGGAAAGTGGAGAAA
ACTGATAATTTACAGTGTTCAGATGTTTGTGTTAATGTTGGTGTAGCGAAAACAAATACC
AATATTATCTGTCAACACAACAACAAAACTGTAGATCCATCGTTTTGCGATAATGATAAA
CAACCAATCATTACTTGCCCAAATACAATTTGTCAAATCATTTATGATTATGGAAATTGG
TCTCAGTGTTCGATGAGTTGCAGCAACACAACCAATGTCAGTATACGAAGTCGTACCATG
AAATGTGTTTGGATACACACAAAAAAACCAGCGTACAATCTTTGCCAACTAAATAAACTC
CCCATTCGGCGTTTAACGAAGTCCTGTAGAGTGCCAAAATGTATTCTGGGTAAGTTAATT
AGACATTTGTAAaaatatgaaatcatctgttaatcggatgattcttaacagttttcgaga
taaactgacaatttcaattttagattagtagtggaattttatttaatttcataaaacttt
acattgcttatattaattaaccgatgttactggaaaatattcaattcattaattattatc
gaaaatttctaatttataaaaattttattgtgaatttaaattgatttttttcaggttgat
aaaaaataaattcttaaataa
>DjScaffold3471:15287-15870 strand:+
tttcaatgatatttgaaggaaacaattatcaatattttaaagaaagatcaacctatcaaa
aatgatttagtttccaaatggacaaagcagattcttgaaggcttcgtcttccttcataat
aaaaaaaataatctttcgggcttttagtccgtcctgcattctagtaaacaaagataatgat
ataaaactgcacggattaaatctttctaaatatacgaatttcaacaaaataagttcttta
ATGTTAAATTATGGAAAGGAAGAACTACCAATTAGATATTTGTCACCAGAAATACTATCA
AATAAACCATATAATGAACTCACTGATGTTTGGTCTTTAGGAGCTGTTTTGATATTTATA
ACTACTGGTAAAGATCCCTTTCCAAATATTGGAAATGATGAAGTAATTGACAAAATATGC
AAAAATTCAAAAATTGAATATCAATTACCCGATGAATATAAAACCGGAAAATTAAGAGAA
TTTATAGATTGTTGT
>DjScaffold3534:43295-45459 strand:+
tcaattattgttATGTTAACTAGAACTTATGAGGAAGATAAATTTAAATGTTTACAATAT
TGGCCTAATAATAATTTTCAACAAGAAGAATTTGGACAAAAGAAGGAATTTATTGTTTCA

ATTATGGAAGAAAATGTGAAAAATGCTTTTGTTGAAAGAAAATTAAAGATTGTAAATAAA
AAAGAAAATGTGGAGAGAAGTCTAATTCACTTACATATGGTTGAATGGTTTGATTATTCT
GCTCCAAAACTAGAAGACTTTGAACAGTTGCTTTTTCGATTTCAAGAAATTGTTTTACAA
AAAGATTACCATCCATATTTAATACATTGCAGTGCTGGTGTCGGTCGAACTGGAACGTTT
ATTGTGCTGGACAGAATAATTCGAGAATTAATCGATGGCAACAAAAATATTGATATTTCA
CATCAAATATTAGAGCTACGAAAGTATCGAAAATCTATGGTCCAACGAGCAGCTCAATTC
ATCTTCATTCATGCTTTTTATCAGTATTTCCTTAATAAATACACCTGTCCAGGTGATATT
AATGAAGATAATATATACTTCAATGCAACATAGaactgcaacgttttctgttaacttcag
atttggaaatgttctattgatgtagattttatgaatttgttttgataaaaattagattat
aaattcgtc
>DjScaffold4355:117211-118296 strand:+
aatttatatagtaacaaagaaagttaaaatatttctaaaatATGGGAGTTCAAGGACTAT
GGCACATTTTGCACAGTACTGGACGAGATATTGATTTATCATCTCTTGAAGGAAAAATTC
TTGCAATTGATTTAAGTATTTGGTTAAATCAGGCTCTTAAATCTGGAATTGATTCACCAA
ATGCACATTTAGAAATTTTGTTTAGAAGAATATGTAAATTATTATTTTATAAAATTAAGC
CAGTGTTTGTGTTTGATGGTCCTTCGCCATCTCTAAAGAAAGAAACAATTTTAGCTAGGC
GTAAATCGCGTCAATTAAAACTAGAAAAATCAGAACAAATTAAATCGACTATTTTAAGTT
TATTAATGAAGAAAAAGATAGTCCAAAAGCGAAAGCATTCTGATGATAATTCTTCAGAAG
TGCACGATTTATTTAAACTGCCAGAAAATGAGTATTATGATTTAATTTTGAATAATATTA
ATAATGATAATAATTTGGAATCAGAATTTGGATTTTCTGATATTGCTCATGATTTTTTGT
GTAATTTACCATCTATGAATGAGATAAACTTTGAATCAGAATCTTTTCGATCTATGCCTC
TTGAAGTTCAGCATGAAGTGATCAGAAATGTCGCTAAAGAAAAACGCTACGGTTATGATA
CAAATCAGAATAGTTTTTCAGATAAAAGCGGAGAAGAGTTTTCTTCTCTTCAAATTCAAC
GTCTATTAAGTCGTCATACATTACAACAAAAAGTAGAAGATTTGAGAACATCTATGAATT
TTCGAAACAATCAAACCGTTATTGGTTCTCTAAATATGAAACAATTTCATAATTTTCCCG
TAGCAAATATTGATACCAAAAAAATTATGTCACGAAATGATGGTTATTTAATGATGATTG
ATCCGTCAGAAACCGACATTGTTGAAACACTTACAAAATTAAAAGCTGAATATTTATTTA
AGAATCGAAATATAAATGATCAACCAAATGTATCAATGTCAACAGATGAAATTGGAACTT
CGATGGTTCTTAGCAATAGTTTAAATTGTGAATCAAATAGAGTAATAAATTCATCTGATA
ATCTGGAAGATATGACGAAAAATGAAGATTATGAAAAAAGTATTAATTATCATAATAAAC
AGAATGATAGTTCTCATTGTGACATACATAACAAACTGGACTCTGTTTATTTCGAAAGTG
ATCTCGACAAAAATGATTCTCCTGTAAATTTAAATAATGTTTGTGAATCTTCATTAGTAA
TAAATAATTGTACCGGAAATGAAAATAGAGAATCTTCTGAAATTGAATCGAAATTCTGCA
ATATAGGCCATGGCGTTAGTTTGGAACATAACAAGGAAATTAATATACCAAAGGATGACC
AAGAAATGGAATTAACAAAAAACCTTTCAAAAATCTCTGACTGTGAGTCCAAATCAAAAT
CTTCGGACTTAGTCAATGTAAACAACGAGACTACGAATATATCAGCAAGTATAGATCAAT
CAGAGTTTAATAATACAGATTATCAATCGATATCACCAAATTATGAAGAAAAAGTAATGA
GAAAAGAAGAACATTCAAAGGATAACAAATCATTTGATGCAAATATACATGAAAATAAAG
AATCAGATGACGACATTGACAATGATGATGATGACGATTTTATTGATGTACCAGAAATTA
TTCCCGATGATGTTAAAGCGAAAAGTGATATTGGTTTTTCTGAAGAGGAAATCGAAATGA
TTATTAGTGATCCATTACAGTGGGATAAAAATATTGAAGAAACTATTGATAATCTTTACA
AAGAAGAACAAAAAATTGAGTGTCAAGCAGCAAGTATGAGTGAAAGATATTATCAAGAAG
CCAAAGAATTGCTGAAATTATTTGGTATTCCTTATGTCGAAAGTCCTGCTGAAGCGGAAG
CTCAATGTGCATATCTCGAAAAACATAATTTTACAGATGGAACTATCACTGAAGATAGTG
ATATATGGTTATTTGGCGGGAATTTCGTCTATAAACATGTATTTTCACACGATTTCTCAG
TTCGTGCTTACAACAATAAACACATTCAGAAAGTTCTTGGTTTATCACAGGATCTTATGA
TTTGTATAGCTTTATTGAGTGGTAGTGATTATACACCTGGAGTTTACGGAATCGGACCTG
TAATTTCTATGGAAATTTTAGCTGAATTTTCAGATCCAGAAATTGACCAGTCTCATTTCC
AAGTTTTAAAAGATTTTAAATCGTGGCATGAAAAATGGTGTAAATTTCCCGAGCAGTTTA
AGGGGAATCCCGACAGAAAACGATTCTTAAAATTTCAATTAGACAAGAATTTCCCTGACC
TTAGAGTTATTGATGCTTATAATAAACCCTTGGTAGATGAGTCCATGGAAGCATTTATTT
GGTCGAAGCCTTCAATAGAAAAATTATTGGAATATCCTTTTAAAatttatatagtaaca
aagaaagttaaaatatttctaaaatATGGGAGTTCAAGGACTATGGCACATTTTGCACAG
TACTGGACGAGATATTGATTTATCATCTCTTGAAGGAAAAATTCTTGCAATTGATTTAAG
TATTTGGTTAAATCAGGCTCTTAAATCTGGAATTGATTCACCAAATGCACATTTAGAAAT
TTTGTTTAGAAGAATATGTAAATTATTATTTTATAAAATTAAGCCAGTGTTTGTGTTTGA
TGGTCCTTCGCCATCTCTAAAGAAAGAAACAATTTTAGCTAGGCGTAAATCGCGTCAATT

AAAACTAGAAAAATCAGAACAAATTAAATCGACTATTTTAAGTTTATTAATGAAGAAAAA
GATAGTCCAAAAGCGAAAGCATTCTGATGATAATTCTTCAGAAGTGCACGATTTATTTAA
ACTGCCAGAAAATGAGTATTATGATTTAATTTTGAATAATATTAATAATGATAATAATTT
GGAATCAGAATTTGGATTTTCTGATATTGCTCATGATTTTTTGTGTAATTTACCATCTAT
GAATGAGATAAACTTTGAATCAGAATCTTTTCGATCTATGCCTCTTGAAGTTCAGCATGA
AGTGATCAGAAATGTCGCTAAAGAAAAACGCTACGGTTATGATACAAATCAGAATAGTTT
TTCAGATAAAGTTTGCAATTTTATTTTATTATTTTATTTTCTTTTA
>DjScaffold1477:1831-20031 strand:+
gattgcagctatccatatcaatgaatttataaaatatttagctttatatgcatttccttg
cATGTTGAACGATAATGATATTGAATTTAGAAAAAAATATGAATTTTTTTATTAGAACC
ACAAAATGAAGAATATTTGATAAGATTTATAAATGATCCACAAACATCAACATTATCGAT
TCACGAAACCAAAAAAGGAGACGATGATGAAACAGAATCCGAGTTTATTTATAAAATATC
AAATGATGTTAATTTTGAATCAAACAAATCTCAAACATCAATTTTTATAAAGAACAGTCA
AATAATAGAAGCTGACAAACCTATCACTTCTCAATTACAAGTTTTAAGTTATTGTGATGG
TTCACCTTTCGAATCAATGCATGCATTAGTTTGCCATGGACTTCTTCCATTTTTTAAATC
GTATATCAAGAAGATTGGTAAAGATAGCAGCTCCTCCAGTGACAAAATGGTACCAAGTGT
TGAAAAGAAAATTGTTGAATTAGAACTTGGGTTATTGCATTTACAACAAAACATAGAAAT
TCCAGAAATCATCTTAACACCTCATCCATATATTGCTCATATAATCCAAAAGAAAGCTTC
AGAAAACATAACTGCAAAACTTTCAGACTTTGAAGATAAATATGAAGACTCGCAATTTTT
AAACCAATTACAAGCCGGTGTTACAAGGTGGATTAAAGAAATTCAAAAGGTTACAAAATT
AAATCGTGATCCCAGCAATGGAACAGCATCGCAGGAGATATCTTTTTGGTTAAATTTAGA
ACATAGTTTAATAAAAATTCAAGAGAAGCGCGATTCTTTAGAGGTGGTGTTGACACTTGC
AATTCTTAGACATGCAAAACGATTTGTAGTGACGACCAGTTTTGATACTGACACAGCTCT
TAATGCAACCAAGAATCTTGTTAAAGATTATAATCAACTTATGAAAGATTTTCCCATCAA
TAATTTACTGTCAGCTAGCCGTTTAGATAAAATAAGTGAAGCTTTGCAACAAATTTTCGC
ACATCTTAAAAAAATTAAAAATACCAAATATCCAGTCAGCAGAGCTATAAAGTTAATGGA
AGCTTTATCAAGAGATTTGTCTAATCAAATGATGAAAGTAATCAGCACAATGCATTTAAT
GAGCATGCCATATGACGAATTTGATGTTATAATGAGGGATTGTGAACAAGTCTTTAGAAC
CTGGGAAGATGAGTATGATAAAATACAGAATCATCTGAGAGAAATTGCCAAAAAGAAACG
AGAAGAGTTTACTAAATTTTTTCAATTTGCCAAATATGCTTGGAAAATGAATGCGATGCA
TAAGAAATTACAAACTCGTTTTGAAAACATAAAAAAATTTCGGTATCAGCATGAACAATT
ACGTCAAGTGATTATACGAGTACTAAAACCACTGGTTAAGGTTGATTCCGGAAACGAAGA
ACTATCTCAAAATATCTCACATAAAAAAGGAGATAGGCCTTATGGTTTACTATCAGAAAC
ATTTGATACATCTTCCATCGATGACGTAAATTCTGCCTATGAAATTGTAAAAAACATTGA
TTACCTCGATTTTACGAAAGAAGAAAACAATGAATGGGATGCTGCTATTAAACGATATGA
CGAGAAAATCGATCGAGTAGAATCACGAATTGCATCACATCTTAGAGATTTGTTAGGACA
ATCAAAAAATGCAAACGAAATGTTTCGATATTTCTCTCGGTTTAATGCACTTTTCGTCAG
GCCTCATATTCGCGGTGCTATTGCAGAATATCAAATTCAATTAATTCAACGCGTTAAAGA
CGACATTGAGGCTCTTCAAAACAAATTCAAAATCCAATACACATCAAGCACTGCTTACAA
AATGAGTATTATTAGAGACTTGCCTCCAGTTTCTGGTTCAATCATTTGGATGCGTCAAAT
TTTAAGACAATTAAATGCCTACATGAAACGTGTTGAAGACGTTCTAGGTAAAGATTGGGC
ACAACATATTGAAGGGCAAAAGTTAAAAGCAGATGGAGAAAGTTTTAAATTCAAATTAAA
TACGACTGAATTATTCAATGAATGGTCTAAAAAAATATCTGAAAAATCATTGTCAGTTAG
TGGATTTATATTTAACATTGAATCAATAAAAACATTAGTACCAGTTCGTGATAAAGAACT
TCAAAAAAAGTCTATCTTGAAATTGAAAGTAAATTTTCTACCTGAAGTTATTACATTAGC
AAAAGAAGTGCGAAATATGAAAGCATTAAAATTTCACATACCATCA
>DjScaffold1477:20227-20680 strand:+
GATTACAAGTTGGAAGCTTACGTTCAGGAATTCGCTGAACTAGTATTTAATTTCCAAGAA
AAAGTTGATGATTTAGTAATTGTGGTGAACGAAATTTATTTCAACTTGAATACTCTTGAA
ACCTGCTCATTTAATCAAAATTCATTTGAAGAAATATTATTAGCCATTCAAAAAGATCTC
GATAAGTTGAATTTCCAACAGTTTTCAAATCTAGCTCAATGGATATCACATTTAGATGAA
TTAATTGAAAATAGGCTAGTAATGCGATTAGATGCGGCATTGATAGAATGGACAAAAGTT
TTCAAAAAGCAAAATCTAACAGATGAAAATGATCAAGACACTGATATTGATCCAAATTCT
ACGGGAAAACTTTGGATTGGTGGAAAGCCTACCATTAAAAAATTG
>DjScaffold1477:20711-30534 strand:+
ATGCATTTGAATCCAAGTATTGAATTTGCAACCCAAGAACTACTTCATGAAATATCATCT
TGGCAAAATATAATTCTAAATCTACCAAGAATTGTTCACTTTAGATACCAGGTTGATCTT

GAAACTGAAATAGAAAATCAAAAAACATACAAAAATTTGTTATCAAATTTACCAAATTCT
GGAGAGAGTTTGGATAATTGTTATACAGCAATCTATAATAGTTTGCATGGCGCTAATGAA
TACATAAATACCTGGACCAGTTATCAGTCTTTATGGGATCTTCAAGGGGATCAATTATAT
TCTAAAATTGGTGACAATTTTACAATCTGGATGAATGTTTTGGAAGAGATAAAAAATATG
AGAAAGCACTTTGATGTTGCAGAAACACAGAAAGAATTCGGACCTTTAATAATAGCTTTT
GGAAAAGTACAGTCTAAAGTATCTATGAAATATGATAGTTGGCACAAGGAAGTTCTTACA
AAATTTGGTTCAAATCTTGGCAAAACTATGAATGCATTATACAGTGATTTACAAACTGCT
CGAAAAGAACTTGAGTTACAATCAATTGATGCTGGGAAAACAAATGAATGTATTAATTTC
ATTATTTACAATCAAAATATAAAAAGAAAACTAAATACATGGGAAAAACAAGTTGAAATC
TGTAGAGCTGGTCAAAATATTTTATTTAGAGATCGATTCCAATTTCCCAATGATTGGCTA
GATAGTGAAAATATTCAGGGAGAGTGGAGTGCTTTCAAAGAGATCATGTCTCGTAAACAT
AATATCATTTCTAATCGAATAGCCAGTTTACAAATGAAAGTGACCGAAGAAGATAAAATA
GTCGAAAATAAAACTTTAGATGTTTTGAGTTATTGGGAAAACGAAAAACCAATTGCTGGG
GATTTAACACCAGAAGAAGCAACGAAAATAATTCTTGATTTGGAAGAGAAATTTTTGCAA
ATTCGAGAAGAACGTAGCAATGTACAAAAAGCTAAAGAAGCATTAGAGTTAACTGAACCA
>DjScaffold1477:47621-48418 strand:+
tatttgttagcaatttgaagaagaagcaaactcttgggaagagaaattaaataaaattaa
tgcactttttgatgtatggattgatgttcaaagacgatggatttatttggatggaatttt
cactggttcagctgacattaaagttcttttgccaattgaatctcaaaaatttcaaaacgt
cagcgcagaatttttagcattgATGAAAAAAGTTCAGAAATCACCCTTGGTAACAGACAT
TCTCAATATTCCTAATATTCAAAGACAATTAGAACGGTTGACTGATTTGTTGGGAAAAAT
TCAAAAGTCATTAGGAGAATATTTGGAGAGACAAAGAGCTTCATTTCCAAGGTTTTATTT
TATTGGAGATGAGGATTTATTAGAAATTATTGGAAATAGCAAAGACCTGAAAAAATTACA
GAAACATTTTAAGAAAATGTTTGCTGGGGTGCATTCAGTTATATTAAGTGAAGATGAGAA
ATTGATTTTAGGCATGATGTCTAAAGAAGGAGAGGAATTAAGATTCTTATCAAAGATTTC
AATTACTGAGAATGTTAGAATTAATGACTGGCTAACTGCTATGGAAAAAGAAATGCGAAT
ATCTTTAGCAAAATATTTAAACATTGCCGTAAAAGAAATATCTAGCTGGAGAACTCAGTC
ATTTATTGACAAAGATAAATTTATGATTTGGTTAGATAAA
>DjScaffold1477:48545-73433 strand:+
GCTGATATCGTCCTTTATGAGCAACCACCAGTAAGAAGAAGAAAGTTAGAGCATTTAATT
ATTGAGTTTGTTCATCAAAGAGATGTTCTGAGAGAATTACATAAAGATGGGGTGAATAGC
AATAAGAATTTTAGTTGGATCAGTCAAATGCGTTTCTACTTTGATTCCAAACATAAAAAT
CCTTTACATCAATTGAGTATTCACGTTGCTAATGCCAAATTCAATTATGGATTTGAATAT
TTGGGTGTATGTGATAGATTAGTTCAAACTCCATTAACTGATAGATGTTATTTAACCATG
ACTCAGGCTTTAGAAGCAAGATTAGGTGGATCTCCGTTTGGTCCAGCGGGTACTGGAAAA
ACTGAATCTGTAAAATCCCTTGGTAATCAGCTGGGAAGATTTGTTTTAGTATTCAATTGT
GATGAAACCTTTGATGTACAAGCAATGGGTAGAATTTTTGTTGGTCTTTGTCAAGTTGGT
GCTTGGGGCTGCTTTGATGAATTTAACAGATTAGAAGAAAGAATGTTATCTGCTGTTTCT
CAGCAAATTCAAGGAATCCAGGAAGCACTAAGAGAAATGGTGATATCAAAGAAAGACTCC
ATTACTGTTGAATTACTCGACAAACAAGTTCACGTAAATTCTGATATGGCAATTTTTATT
ACAATGAATCCTGGATATGCTGGTAGATCAAATTTACCAGATAATTTAAAGAAACTTTTT
AGATCTCTTGCTATGACTCAACCGGATAGACAGCTTATAGCTCAAGTAATGTTATATTCC
CAAGGATTTAGAACTGCTGAAACACTAGCAGGAAAGGTTGTTCCGTTTTTTAAGTTATGT
GATGAACAACTTTCTGCACAATCACATTATGATTTTGGATTAAGAGCACTGAAGAGTGTA
CTTGTATCAGCAGGAAATGTTAAGCGTGAAAAAAATTAAAGAAATAAAAGCAGAAGTAGTA
GCCCGAGGGGAAGAAATCAATGAAACTACTATATCAGAACAGTTACCTGAACAAGAAATT
TTAATCCAATCAGTTATGGAAACTATGGTTCCTAAACTAGTAGCTGATGATATTCCATTG
TTAAATTCACTATTAACCGATGTTTTCCCAGGGGTTGATTACAAACAAGCAGAAATGAAG
CTGTTACGAAATGAAATAAGAAAAATCTCAGAGGCTCGACTTTTATCTTATAGTGATGAA
CCGAATAAACCTGGCTATTTATGGGTAGAAAAGGTTTTACAGCTTTATCAAATTATTACT
ATTCATCATGGTTTGATGATGGTTGGGCCATCAGGAAGTGGAAAAACGATGTCATGGCGA
GTACTTCTACAAGCATTAGAAAATCTTGATGGGATAAAAGGTGTTGCTCATATTATTGAT
CCAAAATCAATAAGCAAAGACTCATTATATGGTTCATTGGATCCAAATACAAGAGAATGG
ACTGATGGATTATTTACTTATATACTTCGAAAAATAATTGACAATGTCCGGGGCGAAAGC
AACAAAAGACAATGGATAATATTTGATGGTGATGTTGATCCAGAATGGGTTGAAAATTTG
AATTCCGTTTTAGATGATAACAAACTATTAACATTACCGAATGGTGAACGACTTGGAATT
CCTTCAAATGTAAGAATAATGTTTGAGGTGCAAGATTTGAGATATGCTACCTTGGCAACA

GTTTCTAGATGTGGTATGGTTTGGTTCAGTGAAGAAGTATTATCAACTGAAATGGTTTTG
ACTAATTTCTTAAATACCTTGAAAGCAGTCCAACTTTCTGATGGAGAGGATTCACCAAAT
AACATAAAAGATAATGAAACAGAAAATTCAAAGTTGACCAATTCACCGACCATGCAATTA
CAGAACCATGTGGCATCAATTCTTGCTCCATTTTTTGCTGAAGATGGTTTGATTGTAAAA
TGCTTGGAATACAGTTCAGCCTTTGAACACGTAATGGATTTTACTCGATTGCGAGCTTTA
ACAAGTTTATTTTCAATGATTAAACAAAGTATTCGTAATCTGATAGAATACAATCAAACA
CACTTGGATTTTCCTTTAACCGCAGAACAAATGGAAAGTTTCATTACGAAATCTTTGATT
TCTTCAATTATATGGAGTATGAGTGGTGATAGTAAGTTAAAAGTTCGTGAATTATTAAGT
GGATACATTAAAGATATAAGTATGATTAATATTTCAAATTCTATTGGAGGAAATAGAAAT
ATTATTGATTTCGAAGTAAATCTTCAGGGATCATGGGTACCTTGGTCAGTTAAAGTACCA
CAAATTGAGATTGAATCCCATAAAATAACAGCAACAGATATTGTTGTACCAACAATAGAT
ACAGTTCGGCACGAAAACCTATTATATACTTGGCTAGCTGATCACAAGCCTGTTCTTCTA
TGTGGGCCACCAGGATCTGGGAAAACTATGACACTTTTCAGTGCATTGAGAAGTTTACCT
GATATGGAAGTAGTTGGATTAAATTTTAGTAGTGCTACTACTCCAGAATTAATGTTAAAG
ACTTTTGATCAATATTGTGAATATCGAAAAACTCCAAATGGAATTGTATTGGCACCACAA
CAAATAAATAAATGGTTAATTTTCTTTTGTGATGAAATTAATTTACCTGATATTGATAAG
TATGGAACTCAAAGGATAATTAGTTTTTTAAGACAAATGGTTGAACATCATGGATTTTAT
CGAATAAGTGACATGCAATGGGTGAGCTTTGAACGTATTCAATTTGTTGGAGCATGTAAT
CCACCTACTGATCCTGGAAGAAAACCATTATCACATAGATTTCTTCGTCATGTACCAGTA
ATCTATGTAGACTATCCTGGAGAAGAATCCTTAAAACAAATTTATTCCACATTTAATAGA
GCAATGCTCAGAATGCTACCATCACTGAGAGGCTATGCAGAAACATTGACAGATGCAATG
GTAGAATTGTTTTTAAAAACTCAGGAAAGATTTACTCATGATATGCAACCTCACTATATT
TACAGTCCTCGTGAAATGACTAGATGGGTTAGAGGTATCTACGAGGCACTTAAACATTTA
GAAACACTATCTATCGAAGGTTTAGTCCGTATTTGGGCTCATGAAGCTTTAAGATTATTC
CATGATCGGCTAGTGGAAGAAGAAGAAAAAGATTGGACTGATGAAAATATAAATGAAGTA
GCATTAAAACATTTCCCCAACATTAATAAAGAAGAATCTTTACAACGACCAATTTTGTAT
AGCAACTGGTTAAATAAGGATTATACTCCTATTGATCAAGAAGAACTAAGAAAGTATGTG
AAAGCCAGATTAAAAGTGTTTTACGAGGAAGAATTGGATGTTCCTTTAGTATTATTTAAT
CAAGTATTAGATCATGTGTTGAGAATAGATCGAGTATTTCGTCAATCACAAGGTCATTTA
TTATTAATAGGAGTCAGTGGTGCTGGTAAAACTACTTTGTCACGATTTGTTTCGTGGATA
AATGGTCTGAGTGTTTTTCAAGTAAAAGTACATAACAAATACACTGCTGAAAATTTTGAT
GAGGATTTAAGAAGTGTTTTAAAGAGATCAGGTTGCAAAGGTGAAAAAATAGCATTTATA
ATGGATGAATCTAATGTTATGGACTCCAGTTTTCTTGAAAGAATGAATACACTTTTAGCT
AATGGTGAAGTTCCAGGATTGTTTGAAGGAGATGAACTTATTGCACTTATGACACAGTGT
AAAGAAGGAGCCATTCGTGATGGATTAATGATTGATTCTCAAGAAGAATTATATAAATGG
TTCACCCAACAAATTTGCCGAAATTTACATGTAGTATTCACAATGAACCCTTCTACGGAA
GGATTAAAGAACAAAGCTAGTACATCTCCTGCACTTTTTAATCGATGCGTGTTAAATTGG
TTTGGCGATTGGTCTCTAGAAGCTTATTTTCAGGTTGGAAAGGAATTCACAAATCGAATG
GATCTTGAAAAAACTGATTACAGTGCGCCAAATACTCTTCCAATTGTTGTTGAGACAATG
TTACCTCTTAATCCAGTTTATCGAGATGTTGTAGTAAATGCATTAGTATACGTTCATCAA
ACTATGCATCAAGCTAATGTCCGTATTCAAAAAAGAGAAGGACGTGTAGCTGCTATAACT
CCAAGGCATTACTTAGATTTTATTGCTCAATATATAAAATTATCAGCTGAAAAGCGCGCA
GATTTAGAAGACCAGCAATTACATTTAAATAATGGATTAAAAAAGATCGAAGAAACAGTT
AAACAAATTGAAGTGATGCAAATGTCTTTGACTGAGAAACGACAAGAATTAGAAATTATG
AATGAAGCTGCAAATGCAAAATTAAAGCAAATGATTCAAGATCAAAAAGAAGCTGAGCAG
AAAAAAACCTCCGGACAAATTCTAAAAGAAGAACTTATTAAACAAGAATCCTTTGTGAAT
GAAAAACAAAAAGCAGTGAGATTAGAATTAGACACAGTTGAGCCAATTGTCAGTGAAGCA
AAACAAGCGGTACAGAATATTCGTAAAAAAGATATTCAAGAAATAAGAGCTATGAGAAAC
CCACCAAATGCTGTAAAAATGGCTGTGGAATCAATTTGTTTATTGTTAGGTGAGAAAATC
TCAGATTGGCAATCAATTCTTCGAGTTATTATCAAGGACAATTTTGTTCAAAGTATACTT
AATTTCAATACGGACGATATCACTGATAATATAAGAGAAGTTTTAAAGAGATATGTTAGC
AATCCTGATTTTACTTTTGAAAAGGTCAACAAGGCTAGTGTTGCATGTGGACCTATGGTT
AAATGGGTTTTGGCACAAATTAGTTATGCTGAAATTTTGAGAAGAGTTGAACCACTGAGA
AATGAATTAGAAGAATTAAGTAAGGCAGCTGAAATCAACAGAGAAAAATCACAAGAAACT
GAAGAATTGATACTGCAATTAGAAAAAAGTATTCGAAAATACACTGAAGAATATGCGACT
TTGATTAGTCAAGCACAAACGATCAAATCTGATCTATTACATGTTGAAGGAAAAGTAAAC
AGATCGGTAGCTCTTCTTCAATCTTTAAGCGCTGAAAGGTCACGTTGGGATAACGGATCT

GAAACTTTCAAATCACAAATGGCAACAATCAATGGAGACTGTTTATTATCATGTGCATTC
ATTACGTACGGTGGTTATTTTGATCAGCATATGAGAACCAGTTTGTTTAACACTTGGTGT
TTTCATTTGACTCAAGCTGACATTCGTTTTCGATCAGATTTGGCTTATGTTGAATATTTA
TCTAACCCAGATGATCGGTTAAGATGGCAAGCAAATTCGCTTCCAGCAGACGACCTTTGT
GTGGAAAATGCAATAATTTTATGTCGCTATAATAGATTTCCTCTCATTATTGACCCAAGT
GGACAAGCTACTGCATTTTTAATGAATGAATTAAATGACAAAAAAATAACTAAAACTTCT
TTCTTAGATGATAGTTTCAGAAAAACTCTTGAATCAGCTTTAAGATTTGGAACTCCACTT
CTCGTACAAGATGTTGAATCATATGATCCAATTTTAAATCCAGTTTTGAATAGAGAAGTT
CGAAGATCAGGAGGAAGAACATTAATAACTTTAGGGGACCAAGATGTTGATTTATCTCCT
ACCTTCAAAATATTTTTATCTACTAGAGACCCATCTGTTGAATTTCCACCAGACATTTGT
TCTCGTGTTACTTTTGTCAATTTCACTGTTACTCGCAGTAGCCTTCAAAATCAATGTTTG
AATGCTGTGCTAAAAATTGAGCGTCCTGATATCGATCAGAAAAGAACAGATCTATTAAAA
GCACAAGGAGAGTTTCAATTAAAATTGAGACATCTGGAAAAAGATTTACTTCAATCATTA
AATGAATCTCAAGGAAATCTTTTAGAAAATGATATAATAATTTCACGATTAGAAAAATTG
AAACTTGAGGCTGCTGATGTAGCTAAAAAAATTCGAAGAAACAGATATTATTATGCAAGAA
GTTGAGAACGTATCTAGAGAATATATTCCGCTAGCACAGTCTTGTTCAAACATTTACTTT
ACATTGGAATCCATGAATCAAATTCATTTTCTTTACCAGTACTCTTTGCAGTTGATGTTA
AACATATTTATGACAGTATTGACAAATAATCCCGAATTAGTTAATGTCAAATTGGCATCA
GACCGATTAAATATTATCACAAAAAATCTTTTCCAAGTCACGTATAACCGTATAGCAAGA
GGAATGCTACACAAAGATCAAATCATCTTTGCTACTATTTTGGCTAGAATCTATCTTAAA
GGAAGTACTCCAGCTGGATTTTTGGAAAATGAATTCAAACATTTCCTTCGAAATCAAGAA
GCAAGCATTATTTCATCTGATGAAAATAAAGATAAAAGTTACGATTTCTTGCATCAAGAC
CAAATTTATTCCGTAAAGCGCTTAAAAAAATTGAAAGCATTTTTACAAATTGAAGAAAAG
CTTAAGTCATCAAACTATATAAAAACTTGGATAGCATCAATTAGTCCAGAGAATAACGTT
CCAGAAATATTGTTTGAAAATAATTTTACTAGTAATGATAAACTATGTAAAATTGCAACA
AGTATGCTCAATCTCCTAGTAATACAAGCTATTAGACCAGATAGATTATTAGCTAGTACA
CAAAAGTTTATTTCTTCTGTTTTCGGTGACAATTTCACTGAATCCGCCAACGGTCCATTA
GATCTGGTTCGTCTAGTTCAATTTGACGTGACTGCATCAATGCCAATATTGCTATGTGCA
GTTCAAGGTTATGATCCAAGTAGTCGAATTGAGGATTTAGCTTCAGAACAGGGTAAACAA
TTAGTCTCAATTGCTATTGGCTCAGCAGAAGGTTTTAATCAAGCAGAGAAGGCAATAAAT
TCAGCGGCCAAATCTGGAAAGTGGGTTCTTCTAAAAAATGTTCATTTAGCAACAGTTTGG
TTGACATCTTTAGAGAAAAAACTGCATATTCTACAAGCACATCAAAATTTCAGATTGTTT
TTAACCATGGAAATTACTCCTAAAATACCCGTTAATCTTCTTAGAGCTGGCAGAGTTTTA
GTATTTGAACCTCCTCCTGGAATCAAGGCAAATATGTTACGAACGATATCATCAATATCT
AGTAAACGAATGTCAAAGGCCCCCGTTGAAAGATCGCACTTATATTTTCTTTTAGCTTGG
TTTAATGCAATTGTTCAAGAAAGACTGAGATATGTTCCGTTAGGATATTCAAAAAAATAT
GAATTTAGTGAAAGTGATTTTAAATGCGGTCTTGATACGATTGACACGTGGGTCGATATA
TCTGCACTTGGCAGAACTAACTTACCACCAGACAAAATTCCATGGATAGCTCTTCAAACA
TTATTAGGGTCATGTATTTATGGAGGGAAAATAGACAATTTATTTGATCAAAGATTATTA
AATTCTTTTCTTGAATATTTATTCAATGAAAAATGTTTTAGTAATAATTTTGCTTTGACA
TTTGAAAAAGATGATAAAACAGAGATAGTTTTTCCTGAATTCAAAAATCACGATGATATT
TTACAGTGGATCTATAAATTACCAGATTCCCAAACTCCTTCATGGCTTGGATTACCTAAT
TCAGCTGAAAAGGTTTTATTGACAAATTTAGGTCAATCGTTAATTGGAAATATTTTGAAA
ATGCAACAAACATTTGAAGATGATGAAGAAATTGTTGTTGATCAACGTAAATCAAGAAGA
ACTATTGAGTTAGTTGACAGCCGACCTGTATGGATGCAACAACTTTACCAGAGTACACAG
ACTTGGTTTCAACTATTACCAAAAGTCGTACTACCTCTTCAACGAACTACAGAAAATATT
AAGGATCCCATATATCGATGTTTTGAACGAGAAGTGAATTCAGGATCAAACTTGCTAATT
ATCGTTAGAAAAGATCTAGATGATATTTCGGAAGTATGTAGTGGAACCAAAAAACCAACC
AATCACCATAGATCAATGATGGCTGGTCTAGCAAAAGGAATTATACCTATTTCTTGGATA
AAATATCCTATTCCTCCAGGTCTTACTGTAATTCAATGGATTCATGATTTTGCGAACCGT
ATCAAACAGTTAATACATATCAGCAATACTGCAATTAGTAGTGGTGCAGCTGGACTTCGT
AGTGTTAAAATTTGGCTTGGAGGGTTGTTTATGGCTGAAGCTTATACAACTGCAATCCGA
CAAGCTGTTGCTCAATCAAATAAATGGGCTTTGGAAGAACTTTTTCTTGATGTTGAATTC
GGTGAAGGGAAAATTGATAAAAACACATGTGTAGTTACTGGTCTAAGACTGATGGGAAGT
GAAATTGTCGATAGTCAAACTCTGAAATTAAGCACATCAATTTATAAAGATATAGATATT
TGTCGTTTGAAATGGATTAGAATTGAGAAATATGAAAATTTTGAAGAAACTCTATTCAGA
GATAAAGTTTGTTTGCCAGTCTATCTGAACGCTGCGAGAACATCTCTTTTGTTTACTTTA

TATTTTAATACGACTGATTTGAATTATGATTTCTATAAAAGAGGAGTAGCTATATTGGCT
TCGTCATTAGGATAAtagattttaacaatatttctttttgaatctttttatattccaat
ttttcatgataacatttgaagttttgtgattacatctaaagaaaataa
>DjScaffold1477:88268-88751 strand:+
aaaaactcaaggccaacaacatcattaagcacgctttcgaattgattaatcttATGACTG
GTGAGAATCCTCTTCAAGTTATGGTTAATGCTATTTCTAACAGCGGTGTCAGAGAAGATT
CTACTCGTATTGGTAGAGGAGGTGCTGTTAAACGTCAAGCCGTCGATGTTTCCCCATTAA
GACGTGTAAACAAAGCCATTTCACTTCTTTGCCAAGGAACCAGAGAAGCTGCATTGAGAA
ACTCAAAATCCATGTCAGAATGTCTCAGTGATGAGTTGATCAATGCTTCAAAGGGTTCAT
CAAACTCATACGCAATCAAAAAGAAGGACGAACTT
>DjScaffold1354:62910-63390 strand:+
atatttgcctccaaaacacttccgtgacttcaatttagtaaaaatctacaaaaatagaaa
cattattttaaaaggtttaataATGAAAAACTCATTATTTTTATATTTTGGGGATGACCG
AAACAGTTTTTCGGGGTTTTCATTGGGTTTTGAACATTCAAACAAGTTAATAGCAACTAA
CTGCCCACTAAATCCAAATGTAGATTTTAACCTCAATGAACTTATAACAAAAAAATTAGT
GAAATATTTCCAATGTCCTATTTATTTGAGTTACAATTTACCATATGAAGAATGGGATCA
AATTGATGAAATCATACAAATAATTAAGCCAGATTTAGCTGATATCATATTGGGTAATAA
AGAACCTAATTGAaagtttcagtgatgctgatgtttatatatatttttaaaatattttca
tttgtttttta
>DjScaffold1354:131807-139627 strand:+
ATTGAATTTTGGAGAAATAAAGCCCAAATTTTACAAGACGAACTTAGAGAAACTCAAGAG
GAATTTCAGAATTTTGAAATTAGCAGCAGGGATTTAGAAAATGAATTAGAGCTGCAATTA
AACGATTATGAATCAAGAAATAAAAACTTAATGGTAGCGAATGAAAAACTAAGTCTTGAA
TTAGAGAACTTATCTATTCGTTACTCAGAAACTGTAACTAATATGAATCATCAAATGTCA
GACTGCCAAGATGAAGTTACTAGATTAAAAAACGAAAAATCTACTTTGCAGAAATATGTC
AGGGAATTAGAACAGACAAATGATGATCTGGAAAGAGCAAAAAGAGTTACAGTTATGTCA
TTGGAAGATTTTGAAGAGAAACTAAATTTAGCAATTGAGAAAAATGCTATTTTGGAAAAT
GAATTGGAGGAAAAAGAAACTCTTGTTGAAACTGTTCAACGATTAAAAGAAGAAGCTAGA
GATTTACGAAACGATCTTCAAGTAAAAGAAATGTCATTAAATCAGCAAGGAAAAAAATCT
GAACAAGATATTGAAGCTTTGGCCACTAGTATGATAGAAGAGCGTGATAATTCACAAATG
TGCCACAAATACTGCCAAACCGATTTAACAATACCTCAAGATTCAAATTTATCAAATGGT
TTCACCGATTCAAATTATTCTCATTCCAGATCATCTGGACATTTAAATTATAATCCCAGA
TCCAGTCACATTGTCAATAATCCATCATACACACCTCTAACACCAGCAGCTAGAATTTCT
GCGCTTAATCTAGTCAGTGATATGTTGAGAAAAGTTGGAGTAAGTACTCATTTTCTGTTT
CGATTACTCAAAATTATATGAatagtgtagtgtgattggatgatatcagtattac
>DjScaffold1354:177039-188491 strand:+
acagaaataaatggaattaaatttaaaatgcaaccttaaattggagcactctgtattaat
tataatcggattaaactgctagaaaattttcacacaaaacatttacatttattagtttgt
gtacaattgtttgaaataagttttaaattgcagaataatttttattaggactagttaaaa
tacgatctgattttacttttcattatgatttggatttaatctctctaaacaattaatcga
cgcaataaattttATGCATTCCTATTGTGTCATTCATCGTGATTTGAAACCAGCCAATAT
TCTAATTAACAAAAATGGGACCGATTTAAAAATCACAGATTTTGGTTTGGCAAGAATTCT
CGGATTCGATTCAAGTTTAACTCCTGTAGTAGTAACATTATGGTATAGATCACCTGAAAT
ATTATTACAAAGAAGTTACTTGACTGCGTGCGATGTATGGTCAGCTGGATGTATTATTGC
GGAAATGGTTTATGGAAAACCAATATTTACGGGTGATAACGAATTATCTCAAATCGAGAA
AATTGTCGCACGCCTGGGATTTGAAGCAATTGACAATTGGCCTGAAGATAATGGGATTGA
TGCCCCGTCGTTGAGAAAATTGATCGATAAATCTCGTAGTCTGGAGAATATTCTAAAACG
AGTTGATCAGAAATTTTTGAAACTGATCAAAGACCTGTTACGATTTGATCCAGATATGCG
TATTTTATCGAGTGAAGCATCGAGTAAACTGGAAAAATTGAACCAATCGTATTCAAGAGT
AAAGGAAATAAAGAAAACTGTAGATAATTGTATTTCACCGAGTGTGTCCGACTATACGAG
AATAGATTGTAGCTTGAAAGAATGGGAAATTGAAACTCAAAATGAAATGATTGTGAATTC
AGTGGTAAGAAATGTGCTCGACGGAGATGATGATGATGATCTAGTAATTCCTGGAATCAT
CGACAATGAACCAATCAATTGTTATCCTCACAAAGAAATAGAAATTGAGGACGACATCGA
TGAAGCTGAAGAATCAAGTGTCAGTGATGACGATTATAAAGGTTCCGATGAAAGTGATTC
GTGTTCTGAAACTGAATCTTGTGAAGAAAGTCACATTTATCAAGATGGGGCTTTTATTCC
TGAAAAGGATGAAGTGGGTGACAGTTTCAGTAGCTATAGGAGCTGCCAAAATAATTGTTC
TTTCAGTTCACAAGCGCCTTCTTCCCGAACTGGAGCAGATACGACAACCGATGATGAGCA

TACCGAGTGTAGCAACAATAAAGAAATAGCAGATTTCAGTATATTTAAAGAAAAATTTGA
TGACACAGTGATTCGCGATGATGGCTTGCCGGATTTAGTGGTTCCAGTGCTAATCACGAG
TTCCGATGATGAGAATGCCGACGAAACGAATGAAGAAACCTGCAGTGCTACGGAGCCATC
TCCGATATATGTGAGAACTTTATCGAAACCATCAAATGCTTCACCTTTGTCATTCTCATT
GAGTCGACAAAATTCAGACAGATCTTATTCAACTCCATTCAAAGAGGTCAATGATTATAA
TCGATTGCTACGGACCTCTTCTAATCAGTCCAAAGAACAGTTCGCTAGAAAGCGCAATTT
TAGTACTGATGACAGCAGCGATACAGAAAACGAAACAGGCGAACCTTCTCCAATCCCAAG
TGTAGAAATCAAGCCAAACCTACTCAGACAGAAATCCTTGCGTAGAAGAGTGTCCATGCT
GGGTCCCCCTCATGTCAGTAGTTCCGACGTGAAGCAGAAGAGCACTTTGTCGCGCAGTGA
AAGCGTTCGAATTCAAACGATTTCTGAAATCGAAGAAAACCGCGATATCAGCTTTGGTGA
GCGAAGTTTATCGCGAGCCCTGGACTCAATACAAAAATCAGACTCTCAAAAACGCAACTC
GCAATTATCTGACAGGCCAAAAATTCTCGTTCGACGAAGCACAATAGCAAAGAAACAAAA
CTGAtgatttcgtgaacgattt
>DjScaffold1354:196164-196340 strand:+
GTTGGAAAATCATCTTTATTGATTCGGTTTGCTGATAATACATTTGATTCAACTTTTATT
GCTACGATTGGAGTAGATTTTAAAGATAAAATTATCAATATTGATAATGAAGTTATTAAA
TTACAAATTTGGGATACAGCTGGTCAAGAGAGGTTTCGTACAATCACTTCAACGTAA
>DjScaffold1011:74074-81880 strand:+
NNNGGTATTGCACCACAAATATATTCTTTACATGCTTTTCTCAGATTCATAAAATTATTT
GGAATTGGTAGTTCGATAATTGGTTTTGTTTTAGTTTCTGAATTGACTCCACAGAAATAT
CGAAATTTCATCATTATTAATTATTGGTCAATTTTCAGTGTTGGATTGGGCGTTACACCT
GTTTATGTAAAATATATTTCTCATTGGCAATGGTCATGGTTGATTTCAGCTTGGCCAATG
TGTTTCTATTGTTTAGCACCTTTTGTTTTACTGGAATCTCCAAAATGGTTGTTAGTGAAT
AATCGATATGATGAAGCAGTTGAAAATTTGAAGAAATTGGATCAATATTCTAGTGAAAAT
AGTTTAGCAGAGATCAATGATGAAACTTTAGAGAATTTCGTAGATACTAAAAGCTTTTTT
CGTTCAAAAGTATTAATGATAAGAGGGGTAATTTTGTTGTTGACACAATTTATTTTTTTT
CTCGTTTATCAAGGAACATCAACTAATTTAGAGTTTGCAATTTCTGATATTTATATAAAT
GTTATGATTGCCGGTTTAATTGAAATACCAAATGGATTTGTGAATTATATTTTACTCAAA
TATATTGGTGATGTTACATCAGTGGTTCTGTGGACATTTGTGGGAATGTTTTTATATGTC
ATGATTCCAGACCACTGGGAACCGACATTAGCATTGAATAAATATGGACAAATTATTCAT
GTTATATTAGCGAGTTTAGCAAAGTTTTGCTTAGTTTTGGCTTGGAATGTTTGTGATATT
TTCAGTGTTGGTCTTTTCCCAACAAATCAAAGAAATTTTGTAGCAAATGTAATTATGATG
ATTGGTTATATCGGATCAGCTGTTGGAGGATTTCTTGGATATTATTTCAAGAGCAACAGA
TTTACAGTTGATATTCGTATTTATGGAATGTTATTAACTGTTTCTATGTTATTGTTCGGA
TTGACAATTCCTTCGGTTAGCAAAAGAGATTTGCCGAACACAGTTAAAGAAGCCGAACAA
ATCGAAAAGTCTATTTCATGTTGTCAATCAAGATCTTTTGAAGAATATGAAAATAAATAA
>DjScaffold965:94929-99186 strand:+
tgatcgagcgtatgattacgccagtaaattactattggatatcATGTTTAATAAAATGAA
ATTGAAGGACCATTTCAAATTTATGAAACATTATTTTTTACTATGTGAAGCAGACTGGCT
CAGTTTCTTTATGGAACTTGCATGTGATGAATTGAATGAAAAAATAGCAGAATGCAAGTT
GGGACGCTTAGAATCTCTTCTTGATGTTGCCATTCAAACTAGAAGTTCTGGTATTGATCC
ATTACGCGAAAATCTCCGAGTTGAATTATCCAATCAAGAATTGATTCCATTCATTTTCCA
CGTTCTTTCCTTTAAGACAGACACTAAAAGAATTCATACTGAAGTCATTGGTAAAAATCT
CAGAGTATTGGAAGCGTTCACTTTGAACGCTGTCACTCAATGGCCGATATGTCTAGTATT
TGATAGTTCAATAATGCAACGCTATCAAATTCTGTTTCGTCATTTGTTTTACTGTCATCA
TGTAGCACGAGTTCTATCGAGTGTTTGGTTGATGAGGAAGAGCAGTCATAATCATCACTT
CTACAATCCGAGATTAATAAATAAAGCTAATGCTTTAGGTGGTCGGATGCACAATTTTAT
TCGGGATCTCCTTTATTATATGACAACTGAAGTAATTGAGCCGTATTGGATTGATTTTCA
GAAGAAACTTGAACAAGCACAAAATATCGATGTAGTGTTTGAAGCACATCATTCTTTTAT
TGAATCTTGTATTGCTAATTGTATGCTTAATTCTAAGGAAATGTTAAAGAGTCTGCAGAA
AATGATGGTGCTTTGCGTCACATATACCAATAGATTCAATCGACTCTTGAGTGGTAGCTC
TTTTGATCAAAATATAGCGATGGGAGATGTAGAAGGCTTATTTGAAACCCAACCATTTGA
AGATATGATCAATCAAGATGATGCAACATTTACAGGAATAATGACGACATTAATCAGTGA
TATCAATGAATACGAAAAGGGTAATAAGAAATTACTCAATTTAGCCCAAAGGTTATTATT
ATTATTATTATTATTATTATTATTATTATTATTATTATTATTATTATTATTATTATTATT
ATTATTATTATTA
>DjScaffold1381:34099-35197 strand:+

agtagttgtataATGGTTATTCAAAAGAAAGTTAATTACGCCGAAAAATTGAAGACTAAA
TTTTCGCAATATGAAAAGATATTCATTGTTAATGCTGATAACGTATCTAGTCAGCAAATG
CAAAATGTTCGGAAAGAGTTAAGAAACACAGCTGAAATTGTTTTGGGGAAAAATACAATG
ATGAAATTCGTTATTACGAAAAACAAAGCAACTAATCCGGATTTGTTAAAATTGTTTCCT
CATCTTAAAGGAAATGTTGGTTTTGTTTTTACTAATGGCGATCTCAATGAAGTTAAAGAC
ATTATTTTGTCTCAAAAAATTCAAGCTCCAGCTAAAGCAGATGCTGTAGCTCCTGTTAGT
GTAATGGTGCCTAAGCAACTTACTACCTTAGGCCCTGAAAAAACTTCTTTCTTTCAAGCT
TTGCAAATTCCAACAAAAATCACCAGAGGAACAATTGAAATAATCAACGATGTGGATTTA
ATTAAAGCTGGTGATATTGTTAAGCAATCTGAAGCCCAATTGTTACAAATGTTGAAAATA
TTTCCGTTTCAATACGGGCTAAAAATACAGCAAATTTATGAAAATGGTTCATGTTATAGT
CCTGATGCTTTAGATATCACTGAGGATGATATATTGAGAGGATTTATGGAATCAACGCAA
AAAGTTGCTGCAGTTTCATTAGCTCTTAATAAACCAACTCTTGCGTCAGTTCCACATATT
ATTGCCAATACAATCAAAGCAGTGATTGGTTTATCTGTTGTTACAGATTATAATTTGAAA
GAAGCTGAAGGCATTAAAGAATATCTTAAAGATCCAACTAAATTTGCATCGACAACGGTT
ACAGCAGCAGCGCCAGCTGCAGAGAAACCTGTTGTTGTTGAAGAAGCAGCCAAATCTGAG
TCAGAAAGTGATGGCAGCATTGGAATGGGATTGTTTGATTGAattaaataaatattcttg
gattttgtc
>DjScaffold1381:101440-106927 strand:+
tgtaaattttactccagtcaactctttatttcgggcgatcaaatgtttacgatacaattt
caaaATGAATAGTGATAAAAAGAATTTTGAACACCTTTGTCGACAGCTTAATGCTAGTAT
TCATTCACCAAAAAGCACACATTCCTATATCAGCATGGTATTAAAAAAAGATTCTATCGT
GGATTGGATCCGGATGACAAATTGGATATTAAAACAATCCTGTGAAGCTCTGGGTGCTAT
CGATCCTGCCTTACAACACGATTTTCGATTAATGAATATCGTTCTCACTTTAGCAATCAA
TTTCTTGACAGTTAATCGATGGAAACTTGTTACTGCTATGAATAATTCCAATCTCAATCA
GCAATTGGGTAAAATCACGGACACACTCTACATTCGAATGTTAAAATCAAATTTGTTCAA
AACATTGAAGGTATAAtcataaagttttaaatttatttaaatttagctctcacgcactta
tcgatgaataaattaatttattcatatttatttgtactaaattaatatatctctatctcg
gttataacatgggtttaggggaaatattctgaccttcactgatgcataaccgaggatcat
ggttattaatgtttactgaagttgaatacaaatgataatttatttatgaataaaat
>DjScaffold1381:113137-128445 strand:+
actttctttagctaatattattgatcttggattactggagATGGATGTCTTAGCTAATCA
CGGCTCAGAATTTTGTACGGTCTTGTCGAGTCTTTTAGTGAAATTAAATCAGTCAATTAA
TGAAACAATATCAACATCCTCGCATTACCATCACATATTTGGATGGTCTGGAGTATCGAT
GGATGAAAATATTCAATGCTCCATGCTTCATTTACGAAATCAATTGAAACTTCTTTGGAG
TAGTAAAATGATACGATCAATGTTTGATGAATTATATGATCAACCAATTCTCGATAAAGA
TAAAACCAGATCCTCATTCAGCAATAGTAAAGACAACAAAAAGTTGTCGAGTCCTTTGTT
TAAGTTACCACGGTTATCAACAAGTAGTAATTTTTGGAAGAAATTACATATTTCAACCAA
AGCGTCATCAAATAACGAACAAAATGCAATTTCTCAGATTCCAATCTCCATACAATCTGT
TTGCATGCTTTATTTTTTCACCACAAATTCATTATCACAAATACGCGATGATATACTTGC
AGGTGTTTCTCTGACCGATCTTCCCGCACGAATGTGGCGACTTATTCAAAGTAGTGGATC
AGTAAAACAATGGGCAAATATAGTTAAAGATCCTGATTCTGGCTGGTATTCCCAGCCTCA
TGTCAGTCATTTACTTCACATATTTGTGCTGATAGCAAGAAATTTATTCATAATTCTTGA
TGATCACGAAGTCTTTGAAAAAGAGAAAGTTTTTACAGTTGTCGAACTTGTTGAAATGGC
TGATTTCTTAAATCATTTATTCATTGAAGTAGCAAACAGTGCTACTTCTGAAATTGAATT
GAACAAATTAATGTCAAGAGATTTTGAATCGGAAAGAGCTAAGAAAACTGGAAAAGATTT
AAAAGGAACTGTAGAAATGTCAACATTATTCAAATGTTGTCATAACTTGTTAATGGTGCT
TTTTGAAAGAGATATTCGACATCAGTTCACCAAAGAAGGATTTTGGATATCGAGTTCACA
CAAACCATCTCAATTTATGGCTGATTTGAAGAAAGAGAAATTTGTTGCAAAATTTCTATT
GAAATATGTACCTCATATTATTGCACATAAAGAGCGAGTTGTATTATTTCACGAGAAAAT
AAAAACGGATAAAGCGTCTTTAGGATTGCTGTCTCGTAATTGGAACCACGATGATAGTCC
TATTGGA
>DjScaffold1381:128712-131013 strand:+
ACTATAAAAACATTACTTAATCCTAGTCTTAATCTGTTTTGTACGACTTCAGATAATCGC
TTATATCCATCTCCATTGTCTGGATTATACGAACAGAATCATTTACTTCTTTTCCAATTT
CTTGGCCGAATGATGGCAAAAGCAATTTATGAAGAAATTCTCATTGAAGCACCATTTGCT
AATTTCTTCTTGACTCAAGTTTTACGCAAAGAGCAATCCAATATGTATAGCTTTATTGAT
GAACTAAACACTCTAGATAAAGATTTATATAAAAGTTTGACATATGTTAAACATTATGAT

GGTGATGTTAGCGATTTAGAGCTAACATTTTCTTATGATGAAGATCGTTTGGGTAAAATC
GAAACACATGATTTACGCCCGGGTGGTAGATATATAACTGTTACAAATGACACCAAAATC
AGTTATGTGCATATTGTTGCTCGATTTCGCATGTTTCAACAAATTCGTGAACAAACACGA
GCTTTCATTTCAGGGTTTTATTCAATTATTCAAAGAGATTGGTTTGATATGTTTTCTGTT
CCTGAAGTGCTGAAACTGATCTGTGGGGATACTGGTTCAATCGATATCGATGACTTGAAA
CAAAATGTTCGATATTCTGGAGGATTTCACGCAGGGCACCGAGTAATTGTTTGGCTGTGG
GATATCCTGAAAAATGAATTCTCTGAAAGTGAAAAGAGTTTATTTCTCAAATTTGTCACT
AGTTGTTCCAAGCCTCCTTTATTGGGGTTTTCTTATTTAGAACCATCTTTCTCTATTCGT
TGTGTTCACTCTTCCGATGAAGATCAAGAAGCCGAGGACACAGTTGGTTCGTTACTGAAA
AACATATTTACAATGTCAAAGAAAAAGAACGATATCAAAAGGTTGCCATCTGCTGCAACT
TGTTTCAACTTGTTGAAATTACCAAATTACGAATCCAAATCAACTCTCAGAGAAAAATTA
CGATACGTTATTAACTCAGGTGCCGGATTTGAGTTATCGTAAtaccgataatgtttatat
attctggtgtttattgcaacacaatgctgattccatttgattggttcagtcacgtcataa
agaggtttgattgatcttttaggtcgtcatctacggattttctacttcttttttcattc
aatatctgaacaacctcattttctttcacagtatcgattgttatttaattttcattattt
atccttgtaaatttat
>DjScaffold1063:165934-166404 strand:+
ATGGCCTTTAGAATCGCAGCTGCTCCTGCTACTTTTCAGAAATTGATGAATAAGGTTTTA
TGTCCATTAAATTGGAAGGAAGCAGTGGTTTACTTAGATGATATTTTAATTTTTTCCGAA
ACTATTAAAGAATACTATAAGAAAATTGACAATGTTTTGAAACTTATAAAAGAAGCTGGT
ATAAAAATAAGTCCATCTAAATGCCATTTCATGAAAATCGAGATAAAATTTCTGGGTCAT
ATTATTAATTCAAAAGGAATTCATACTGATAGACCAAAAATTGAAGCAATTTTTATTTTT
GAAACCCCAAAATGTGTTAAAAAGTTGGGAAGGTTTTTAGGCCTCGTAAATTATTATAGT
AAATTTATAAAGGATTACACACAGTATTCAAAATGTTTAGAAAATCTATGTGGTAGTAAC
CAGAAAAAATATACTGGACATATGAGTGTGAAATTTCTTTTAAAAATTTAA
>DjScaffold4456:57838-66988 strand:+
taacttaaaagttaaatcctgaagaagacaaaattctaaaattctaaatactaacaacaa
tttaaacacattttgatcgactcttttaatttaatgaaatatattcttaatttaatttac
aataaatatttacaatttaaaatatatttccaaataattttactaataaatatttacaat
ttaaaatatatttttccaaataattttactaataaatttatttattaaaaattttatttt
atttttcaaaagatgaaaatgaaattatgtatttacactcgtcttacactcttgacttca
gtaataaattttctcaattgtatgtataatatcaataattttctatattatttaattcta
aactttcccgttagaattataataaacctaattcctatcaaccttatcaaaagaaatcag
ataattataacgatgatcggagaagaactcataatccaATGAAAAAAGAATACTTTGAAA
ATCCAAGAGAGCAACAATATTATCGAAAACAAGATAAATCACCACATTATGAAGAAAATT
ATATGCAAAACAAACAGAGAAATGAAGATACGAGAAATAAAACAAACAGAGAAAACAATA
AATTTGACAACGTGCAAATAGCACGGAAACACTATGACAATCAAGCTAATTTTGATAATT
ATCAACCAAATACCCAAAACGTAGAAAGACCACCTAAGAAAAATGATGGTACTGATAAGT
GGAAATGTAAAGTTTGTGAAATGATGAATTCGTTTCAACGTCTATGTTGCGAAGCCTGTG
ATAATAAGGGACCCAGTTTTCAAGAATGGTCTTGTCCTCAGTGTACTTTTCGCAATAAAC
CAGGTTCTGTTAATTGTGAAGTATGTGGGACAACTAATCCCATGAGACCGATATAAcccg
aatccaacgaaagagtataattaattaaggttttaattcaaagttttgatttatttcgtg
taaaatttgtaaaaaaaaatatgttttaatgagataaatatcaacaaatcatttacataaa
ttctacttgcatta
>DjScaffold3804:12823-23839 strand:+
tttattatttattaattaacatttttcaaattgtgatctataatttctagctatttgat
atatataattattatcaatcgtaataatatctggactgttaaaccaattcttactgtgaa
ctaacaacaatatttgaaaattccgtcaaatttttttcatcccatttttgaATGAACAT
GAAAAAGTTCCATTCTTTATTTTCCTTATTATATTTATTAGAATTGTCTAATGCATGGAT
ATTTTCACCATCAACAAATAACTTTATTGATTATTCTCAAAATATTCCAGTTCCACTAAC
TGGATTTATGTCTGCATTTGGGGATTTCAATGCTGATCGAACTGTTGATATTTTTTTACT
GGACAAAAATAAAAACAACCAAGTTTTGGATGTAATTTTAATGGGTAAAGAAAATCAAAA
AAGTTCAGTGCGAAGAATTACATTACTTAATGATAATAATAATGAAATAAGAAATGTTGT
TGTTGGTGATTTTGATGGTGACAGCTTACTAGATGTAGTAACATTCAACTCAGTTAACAT
TACAGGCCCATATACAGTTAATATATCTTATGGTTTTCCTACAAGAGACAAAGTTAGCAG
TCCTATTTCACTTAGTGAATACCAGTTTAAATCCCAACCGTTACACTTAGATTGGAATAC
TGACATGATTGGAGATTTATTTGGACAAACTAAAAATGGAGATTTCATTGTTTTACTCGG

AAACATTAATCGAACTTTTATATTGTATAAGTTACCAGATTTATCAGTAGTACAATCATA
TTTCGATGAATTGTTATTTTCATATTTTGTTGATATTGATCAAAGTGGAGAAACAGATAT
TTTATTGGTTGGACGAAAAAGTAGTAAACTTGTTATAGTCAGTGTATTGAGGGACAGTTC
GACATCTGTTAAACCGATTAAAATGTCGGATCAATATGCAGTGACTCTTCTCGGACAACC
AGTTATTGCTGATTTTTTATTCAATGGGAAACTACAAATGATCATTCCATTTTGTAGAGA
TAATGCTTGTGAAACTAGTCAAATAATTGCATGTGAATTTCTTGATATGAAATGCAATCC
TGCAAATTTTACACGGACATTTAACGGAAATATAATGAACTTCAAGATAACAACTCCGTC
AAGCAGTAAATTATCTTTGTTGGTGCCTGAAATCGTTCCGATGTCTTGTGGTGACGTGAA
AATGACTGGCAGAATTGGATGTCTTGTAATTGTCCAACATCCAACGAAACCTCAATTTTC
CACTTTAGCTCGATTGAACAATAATGGAGATTTCAAATTCGATTTGGTGGCGATTGAAGG
GCAGTCGGATTTATTTATGAATGTTACACAAGCTGCAGTATTCGATCTGGAAGAAAACGG
AATAGTAGATATTATTACAGCAAGCCAACAAGGAGAATCTGGCATTATTAAACCACTTCT
TCAAGATGCATCTGGGGATTCATATTTTATTAAAGTTATGGTGCTAAATGGATTGTGTGA
TGGCGATTCTTGTCCTGAAAAGAAATTGCCATATGGTATACCTCTTCCTGGATCAGTTGT
CAGTTATTCAACTGAGGGCACTGATGGCGGCACACAATCGTCTGTTGTATCAATGGTGTC
GACCACTTCTTATTCATCTTTAATGCTACCCTATGCCTTTTTCGGATTGAATACGTTTTC
CAATTATATTGAAAAACTACTGATTCAGCTGCATTATAAATCTGTAAGATTTTATTAGct
tataacaaaactctttggtgatttaatattttactaatgctattgaatgctacaataatt
ttatattctcattaaattgttataaaa
>DjScaffold3804:47092-53418 strand:+
tattaataatttttagattagtttgtcttctattttttgttatctattaagcttttcaag
attttttaaatgataattttaatttagattctcgaaaaacacATGTTGACTTTCATTGT
TCCAAAAACTCAGATTGTTATTATTCCAATACCATTAGATTCTCCAGAAAAATGGAAAAA
CAGATTATTTCTGGAACCGTTTGCTGACAAAAAAGTGATATATGTGGCTGCTTCATTGTT
AGGAACAATGTTTATTGTTTCAATCGTTCTATGTATTTTACAGTACTTAGAAGTGAGAGA
AGACAGATTAGAAAAAATGCAGGAAGCGCAAAAATTCAATTTTGACGCTATGTGAttctc
atcagattgaacccataattaactttgattagcttcttatatgatatttttgggctttgt
aatattttagcctttcatgaaatggttgcattccaaaatatttatgatataataaacgtt
ttatttta
>DjScaffold990:41938-71547 strand:+
caaatggttatgtagttgctcaaataattaaagagggtcctgaagaatatggtgtgcaaa
ttcctgaaaccggtaaacaaaaagccattcacagagacctcgttcaaaaaatgaatccgc
caaaattttcgaaatcagaagatatggcaaatttaacatatctcaatgaagcttcagttt
taagtaacattaaagaaagatatttcagtggattaatatatacatattctggattgtttt
gtgttgtattaaatccttatagaaaactaccaatttatagtgatgagataattgaatggt
ataaaggaagaaaacgccacgaaagaccacctcatatatacgctattactgatacagcat
atagaaatatgttacaagacagagaagatcagtctattttatgcacaggagaatcaggtg
ctggaaaaacagaaaacacgaaaaaagtgattcagtatttagcatcagtcgcagcaagta
tgaaaaaccaaaaactcaccgcatcaaatgcgatggcacaatttcacgcaagttcagtaa
atattggtgagttggaaacgcaattattacaagcgaatccgattcttgaagcattcggaa
atgcaaaaactattcgtaacgacaattcttctagatttggtaaatttatcagaatcaatt
ttgatagttctggattcatcgctggagctaatattgaaacatatttgcttgaaaaggctc
gagtaattcgccaagcttcccaagaaagatcatttcatattttttatcaattactttcaa
cgaaatccgaagaaatgagaaattatttgcttcttgatcaaatgtcaacgtataaatttc
tctgtaatggtgcttcctccatatcaggtgttgatgaagttctggctttcaatgaaactc
gtgaatctttaaaaattatgggaataacagatgaagaacaagaatgtaagttactaaatt
aatattggttaacattgaaatagatgctgttaaaatttaaaatatgatttaaatttaatt
attttacaattgcgattattgcgagaataattgttggacataaccattgatcgtttttta
gaattcatttaaaataaaagcttcgatcaaaaattttttttataattttttgagattga
ttgaatcaagaaagcaataaaaangatgctgttaaaatttaaaataggatttaaatttaa
ttattttacaattgcgattattgcgagaataattgttagacataaccattgatcgttttt
taaaattcatttaaaataaaagcttcgatcaaaaattttttttcgtaataaagtaattat
ttttcggaaatatattaaatttcatcattttaaaatattaaatatcaacacaactactta
taggttgcatttaaataaaaatatcaaacactatgtttaatttgataaacaatcatgcta
catgggtaagccacattatcggtaattattaatcgggttatttatttatattggagattg
aatttgattttatttgatatatgccttaatattattattattattatttatttattatta
ttattattattattatataatctcatcttgttaatcgaaaaatctactgaaagaata

ttatgtcttgagttataaataaataatgaataacgataattgatatattttgttgtgcaa
atagtgtaaggaatatttataatggtagatttatttggtttatgtgacgtatgatattgt
attaaaagtttgtacactcaattctgtttttacacgatagttccaggagaaaagatacct
caagtaagtgtaaaaatggatactgcatatgaaaaataaaggatccagacttgaaaaaat
aatgcaataaagaaaattaaaattcattttcacattaaatctattataattatcgttaat
atattattactatttatttatattaacttataattaatcaaaataatttgttaatacagc
ctgctaaaattgattcaaaactggttctcgaccaattgaagtgtaatggtgtcttggaag
gaattagaatatgcagacagggatatccaaatagaattcttttccaagaatttaaacaac
gttatcaaatattgtgcccacattctattaatcagggttttATGGATGGTAAAGCTGCAG
CTGAACAACTTCTTAATGCTTTGGAAGTTGATAAAAGCTTATACAAAATTGGTCAAAGTA
AAATATTCTTTAAAGCTGGTGTCGTTGCACAATTGGAAGAGGATAGGGATTTACAAATTT
CGTCTCTTATTATCAAATTCCAAGCATATGGTCGTGGCTTTTTAGCCAGAAAAAATTGCC
AGGCCCGTATTCAACAAATTAATGCTATCAGAATAATACAAAGAAACAGTGTAGCTTATT
TAAAATTGAGAAATTGGCCATGGTGGCGACTTTTCACAAAAGTTAAACCACTGTTAAGTG
TTACTCGACAAGAAGAAATTGTTGCTATGAAAGAAGAAGAATTAAAGAAGGTTAATGATG
TATTAGAAAAGACAACATCTGAGTATAATGAAACCAAGAAAATCCTTGAACAGATGAAAG
AAGATAAAACTAGACTTTCAAATGAATTACAACAGGAGAAATTTAATTTTCAAGATGCCG
AAGACGAACGCCAGAAACTCGCACAAAAATTGAAAGAAAGTGAAAATTTAATAAGTGATC
TTGAAGCCAAAGAAAGTGAAGATAAAGATAAAATCAATAAATTAGAAGAAGATCAAGAGA
GATTGAAGCAAGAAGCTTCTGATTTATCAGAACAATTAGAATCTGAAGAACAAACTCGCC
AGAAATTGCAGTTGGAAAAAGAATTCTCTTGAACAAAAGAATTCACAATTACAAGATGAAA
ATGCAGAATTAGATGACAAATTGTTGAAATTGAATAAAGACAAGAAAATTGTAGATGATC
GTCTGACGGAAACAGTCAATACTTTATCTGAAGAAGAACAAAAGAATAAACAACTCAGCA
AATTAAAAACCAGACACGAAACATTAATCACCGAATTAGAAGATAAATTAAGCCGGGAAA
ATTCTGCTCGACAAGATTTGGAACGTACACGTCGTCGATTGGAAAACGAACTTAAAGAGA
CTGTCGAACAGCTTACTGATAGAAATTCAACACTTGACGATTTGAAATTGAGATTGGAAA
CTGCCGAAGCTGAATTGATAAAATTACAAACTAGACTCGATGAAGAGTTGGTAGCTAAGG
CTATAGTTACGAAACAGCTGCGAGATAGCGAAGGTTTAATTCAAGATCTCCGAGAAGATG
TTGAAACTGAACGTGCTGCTCGTGATAGAGCTGAGAAAATCAAACGAGACCAAGCTGAAG
AAATCGAAGCATTGAAATCTGAATTATCTGATACTGGATCTACCTCAGAGGAACATTTGG
AGGCTCTTAGAAAGCGCGAAGAAGAAATCACAATGCTCCGTAAAACTGCTGAGGTGGAAA
ATCAGAATTTCGAAGCTAAAATCCAAGAATTAAGGAAGAAACATACGATAAAAGTTGATC
AATTACAAGAACAACTGGAGAAAGTGAACAAGGAAAAAATCAATATTTGAGAATTCTAAGA
ATCAATTGGACAAAGAATTAAATGAAATGACTGTAGAATGTAGAAATTCTCAAAGTGCAA
GAGCCGATGTGGATAAAAAGAAAAAAGCTCTCGAACAACAAATTTTGGAATTGTCCAATA
AGTTTGTTGAATCTGAGAAAATCAGAAAAGATCAAATTGAAAAAATTTCTAAACTTTCTT
CAATTGCCGAACAGAATACTTTGTCTCTAGATACATTGGAAAACAATTACAATCAGGCTG
TTCGTCAAATTCAATCGTTACAAGTGGAAAATAAAGATCTTAAATCTTGTCTTAGTGATG
AAACTGAGGCTAAATTGAAGATGCAAGCAACTTTAAGAGCAGTTGAAGAAGAAAGAGCAA
ATATTCAAGATCAAGCCGATGAAGAAGAAAGAAATAGAATTATTTTAGAAAAGCATTGTC
AGACTCTTGAAATTCAGATGCAATTATTTAAAAAAAAAGCAGAGCAAGATTCTGAACAAA
TTCAACAGCAAGATGAGAACAATAAGAAGCTAAGTAGAGATGTATATAATCTCAATCAGC
AATTGGAAGAATATGCAGCGAATATTGAAAAATTAGAGAAATCAAAAAGGAAATTACAGG
CAGAACTGGAAGATGCCAGCCACGCTAATGAAAGTCAAAGAAATGAATTCCTCAATAATG
ATCGTAAATACAAGAAAATTGAGAGTAATCTCAATGAATTATCGAAGGAAAGAGCAGCTT
TATTACAAGCGAAAGAAATCGTTGACAAAGATTGTAGAGAGAAAGAAACGAAAATTTTGA
CACTTACTGGGAAAATTGAAGAAATGGAGGATAACTTCGCGGAATTAGAAAGACAGAAAA
ATAATCTCGAAAAGGAACTCAACGATTTATCCTTTGCACAAGATGATGCTGGTAAAAATA
TGATGTCGCTTGAAAGAGAGAAAAACAAATTAGAAAACGAAGTTCAAGAATTGCAGTTAA
CAATGGAAGATTTGGAAGAAGAAAATGCTACATTATGTATGATGAAAGAAAGATTAGATG
TCGAGATCGCTAATATTAAAAATAGTTTTCAAAGAGAATTAATTCAAAAAGATGAAAACT
TTGAAGAACAACGGAAAATGATGATTAAAAAACATAAAGATATTGAATCAGAGTTAGAAG
AAGACAGAAATCTAAGAGCATCAATTCAAGCTTCTAAAAGAAAGTTAGAGATTGAGATAG
AAGAATTGAATTCAAGATTGGATGCAGCCGAAAAAAATCGAGATGATTATTTACGCCAAT
TAAAGAAGTTCCAAAGTGCTGGAGGAGAAAAACAAAGGGATCTACAAGAAATTACTGCGA
AAAGAGACGAATTAGCAGCTCAATTGAAGGAGGTTGAAAAGAGATGGAAAGCTGGAGAAA
ATGAGATTATGAAACTTACAGAAGATTTAGCTCAGTCAGAGAAGCAAAAGAAACTAGCAA

TTGTAGAGCGAGATGAATTTGTCAGTGAATATCAAGCTTTGGTAGTATCTAACAACAAAG
CTCAAGAAGAACGTAAGAGGTTTGAAATGAGATGCCAAAACTTGGAAGAGGAATTAGAAG
AACTTCAGAGTGCTTTCAATGAAACAGACGACAACCGTAAAAAACTCAGTTTACAATTGG
AGGCTGCTCAATCTGAACTCGGAAATGAGCGGGAACGCTTGGTTACAATTGATGGACAAA
GAGCAAATTCAGAAAAAATGCTGAAAGATTTACAAATTCAATTCCAAGAATTAGAAGCAA
ATGTTGCCAAGAAATACAAAGCTCAAATCAACAATCTTCAAACCAGAGTCAACCAAAGTG
AATCTCAACTCGAAAGTGAAGTCGCAGATAAAAATCAATTAGCGAGAGTGATGAGAAAGA
ATGATAAGAAAACAAAGGAATTCCTTTTCCAATTAGAAGAATCAAGAAAAAAACGAAGAAC
AAATGAAACTTCAACTCGATAAATCCTTTAAACAAGTCAATAGAATGAAAGTTGAGCTCA
GAGATTTAGAAGATGAAAACACAAGTTTGAAAACAGCAAAACGTAAATTAACACGAGACT
TGGAAGACGCTACAGAATCGAGGGACAGTTTAGAAAATCAGCTACAAAGCATGAAAAATC
GCAGAAATACG
>DjScaffold3812:46463-52104 strand:+
tttctaacctttaatatatattgttggcttttaaagaaaactgtatttatctagttactg
tttattttagtccgttcggtgaatataatcctttagttgaaaaggcattgtggatcaatt
ctgacagtcagcatgttATGGTTTCTCACGAATACAGACAAGCTTCATCAGAACGAAATT
TGGAAGAACTTTGTAGTAGTTTTGATCAACTGTTTCGAGAAATGCTTAATTTTTCAGAAT
CTCTCATCAGAAGAGACATTTTAACTGTAAGTGATTTTTCAAGGGTTAATTATTTTACAA
ATGGTTCTTTCTTGAGATCTGAACATGGAACTGACTGGGATTTGCTAACGGACATTCCAT
GGATTAGTGAAGTTTGTTGTAGTATGTTGCCAAGTGATTTAACCAGAACACAACAAGCCG
ATCAGCTTTATGAATTATTGTCAGAAATACAATCTCATTTGGGTCACATGAAATTGCGCC
GTCGAGGTATTCACTTCCATGTACAATTTAGAGATGTTCATAAACCGACTATTATTGATG
AGAGCAGCTTAATTAACGAAGTACTTCCGAATGATAACCAATTTTCTTACTTGCCTTGCT
CCTATACAGATTCATATCTTGAAACAACACATCCTAGAGATGACATAAATGCCAACTTTT
GTGAACCAAGCATTCATGATCTCTTTTCCGAACTTAATCAAGATCAATGTGATTTGGATT
TTATCAGTGATTTATCGATGTTTCCGTCTCATTTACGTGATCTTTTGGATGATAGTTCAC
ATGATGATATCACATTTGGTGCTGACATTAATGAAAAAGTG
>DjScaffold4578:4870-14043 strand:+
aatttataaaatATGATATTTTGCTTAATTTTTTGTGTAATAATAAAGACGGTATTTACT
AACCTATCGAAAAACTGGATACCCACATATCAAAATGAAAGTACATTCGATGAACCTTAT
GATTTTGTGAATGTCAATTCATTACCAAATTTTGAAAAATTTGGGAAAGATTATGATTGG
ATTAGTATTTCTGGAACATCTCTAACAACTTCTTCTTATATAGCCTTTACTGGCACCAGA
AACAACAATGCTTCCAATTATGAATCAGTTTTCAGGAAACAATCGGGAATTATTATTTTA
TGTTCTAGTATTTCAAATTCTTCAAATGAAAAAGGATTTGATATTAACTGTCAATCTTTA
CAAGAAAAATATTTAGAGTCTAGTGACGGTAAATATTTTGGAGCTGTTGTTAAATCGACT
AAATTTAAAAATGATACTATTGCTTTTATATACTGTGATCCATTATGGTTTAATCCAAAT
TTTACATTTAAAATATCAAATGGAAAATGTCGACTTACTCTAGTCAAAGATTTCTCGGAT
ATGAAACATATAGAATATCAAGTGTGTGATAAATTTAAAAAATGTCTGGGTGGATTTTCT
GTTGATCTTATTGCTATTGATAATGATCTAAAAATACTTTTTGGATTACCTTTCGAAAAT
TCTACAAAATTATCGAATCTTGTTGGATCTCAAGAAATTAAAGATGGTCCTGAAAACAAA
GGAACATATTTCAATTATGGGTATTCGGTGGCCTTTTCTGGAGATGGCTCTCAATTTGCC
TCATCCCCAGTTAATGATGGTCATTCGCCATTCGTCAAAATGATAAATATTGGAAATTTC
TATCCTACTGATATATCAATGCAACGTTATGGTGGATTTGGTACCAGTATTGACACTGTA
AATGTTGGAAATAATTTATATGGTGTTATAATAGGAGCCCCATATCAAAGTAAGCCTAAT
TTAGTAAATGTCGGCGCGGTTTTTTTAGCTTGTAAAGGAAAAAATTCAACTGATGTAGTT
TTTGGTGAAAATGCTAATGATTATTTTGGATATTCAGTTGCACGTTTAGGTGATATAGAT
TCTAATGGTCCTGAAGAGTTCGTTGTTAGTGCCCCAAGTCTCAACTTTGAAAAACCTGGG
AAAATATATATTTTTTCGACTACCAGACATTGTCAAATTTCAAAAATCCCATTGCAAGTA
TATCAGAGCAACATTAACGGATTCGGTATAGCAATTTCAAGAGGATTTGATATTAATTTC
AATGGAAAACCAGACTTCTTTGTTACTGCATTAGGTTATCAAAAACCATTTTTATTTGTT
ATGCCAAATCGAGAAAAAGTAACATGTTCTTGGATTGAGCAGAATAAGGGTGATTTTATT
ACAATAGAAAACTTTAAAATAAAAGTTATATTTCAGTCTTCTAACATAAAATATGAGAAT
TATAATTATCAAGTTGCAATTGATTTTTTAAATAAAGGAGAACCTCGTTTTAAACTCGTT
GATAATGATACAGTATTAAATGATGAGGGCTATACAATGACTTTACAACCATTACTAGAT
GTCCAAGATATGGATTTTAAAACAGTTCCATTAAAAGTTGCTATTAAAGGTATTCGAAAG
TGTAAAATTTCTGGAGTTCAAGATTGCTTTCGTTCTAAAACTGCTCAAATTGAATGGTTG
AAATGCGAAAAAATTATTAAACTTTCACCAATGTCATGTTCTAAAATTAATTGTCAATCA

GAATTAAGTATAAAATCTGTTAATTACACCAAAGGACTGGTTATTTACCATGGAGAAAAA
TCAGATTATCAACGCAAAATCGAATTTTTAATTAGTAATAATGGTCCGGATTTCACGCAA
AGAGTGTTTGTTTCCATTAATTCAGAACCCGATATTCGCATTGATAATGTCAGATGTGAT
GGTTATTGCAAATCTGAACATATAACAACACAACATACAAATATTGTTATAAAAGATTCT
ATGGAAAAGAAAGATGATTTCAAAATTTCTGTTGATATTCTTTATAATGAGTTAGAAAAA
GTTAATGAAAAAACTTCAATTAATTTCAAAATTTGGAGTATTAATGATGATGATAATATA
AATAATAACAACTATACGTTATTGTATACTGTCTTATTTAAACCGAAATTAATCATTTCA
TCAAAAAATACTAAAGAAAATAATATAGATTCTAGAATAAAACCCCCATATTTAGAACGA
TATAATCGTATTTCAGTTCAAGATTTAGGGACTGAATTAAGTTTTTCATATTTAATACAA
AATAAAGGCCCTGAATTGAAAAATGTGGAACTAAAATTTCTTTTTCCTTACAAAATAACA
TCGGACAAATATATTTTATATTTAATGGATAAGATTCGTTTGTTAAGTGATGAAAGTTGG
GAAAATGTCAAGCCTTCCATTAATAATAAAAATGGATTAGTTAAACACTGTGATGTTGGT
AAGGATATGTTGAATCCTTTAAACGTAATTGGACATAAATATAACAAGAAATCAGACAAT
CTTAATAGATTAAAAAGAAGTCTTGAAGTAAACATTCTTAATAATTTAACAGTTAGGGAT
AAGCAATCGAATTCGGACTTGAATAATAATATTTTTTCCTCCAGCAGAACTTCAAATATA
AAATGTAACGAATCGATTCGAAATTTGGGATATCCAATATGCGGAGAAGTTACGTGTAGT
CTAAATCTAATTCCGAAAAATAGTGAATTTACTTTTGATTTTCAAGCATGGCTTTTCACA
AAAACATTCTTTGATGCTCGTATTCCCCATTTAACTATTTTAACTACTTTGGAAGCTTCT
TTAAGAGATTTGCCAAAAGGATTAAAAATTTTAAAAGAGGACCAGTTTATTGGGCCAATT
GTTGCCTCTCAAAATATATTGTTTAATAAAATGAAAGTAGATGAAGATAAACCATTACCA
TTATGGCCAATTATTGTAGCAATAATAATTGGAATATTTCTTCTTGTTATTATTATCGTC
ATCGCATATTATTGTGGATTCTTTAAAAGAAAAAGATTAAGAAAAGAAAGACAAAAAATT
AGAGAAAGA
>DjScaffold2355:38858-48172 strand:+
attttccaaaaaacaaaggattgataattggaatcacagtttctggttttggactctccg
catcgatatttgagccgatacaaggtgcattgATGGATAAAATCGGTATTCCTCAATCTT
TTCTTTGGATGGGCATAATTTTTTGTGGAATTCAACTTATAGGTTCTATTTTGATGTTT
>DjScaffold2355:112585-113135 strand:+
ttctttacaaatcaaaaatttaataaattgtgagaaATGTATAATTTCAAATTACAACCA
TATTTTATAATATTTGGAGCATTCATAATTAATATAACTCTTGGTGGTTTCTTTACAGCA
GGAGGTGTTAAAAGCATGATCTTTGAATACATGAGAAATAATAGTCATTCACAATCTGAG
GACACTTTAGAAAATATTTCATGGTTATTTGGAACAATTTTATTAGGAATTGGAGCAACG
ATTTCACTTGGAGGATTTATTGAAATGAAAATTGGTTCTTCGTTAACAGCAATTTTAGGA
GGGTGGTTTATGAGTTTGAATGTTCTATTAATGTATGCAACTCTTAAGTCGTATTTATGG
GTTGTTATTTTATCATTTGGTTTACTCGCCGGATTTGGTATAGGAATAGCATATGGTATA
ACACTTGGAGCTACATTAAAGTGGTTCAAAAAGAGAAAAGGATTAGCTATAGGAATTTCA
ATTTCGGGATATGGATTTTCACCAGTAATATTTCTCGCTATTCAAACCTCATTAATCTAC
AAAAATAAGATATTATGTTTACCTGATTTTCCCTACTCATTCATTATTATAGCATCAATT
TTCGCGTGTCTACAATTTATTGGAGCATTATTAATACGTTTTGAAAATATTAATTTGTTG
AATTCACTTTTATCTGAACCCACTTCAATCAATTCGGATCCAGGTCAAAGATTATTTTCA
CAAAGTTCAAAATATCAAAATAAAATGCACAATAGTGATATACAATCTCATCAACTTATC
TGGATTCCGGATTTTTGGATTCTTTGGATAATTTTGTTACTGAATTCAATTTGTGTTACT
TTTATAAATGCTGTTTATGCGTCTTATTTAGAATTTCAAGAAATAAATATGAAAACTATG
TCGATTATTTTTGCTTGTTTTAATGGATTAGGACGAATATTTTGGGGTTATTTTATCGAC
ATTAAACCATTTAAAGTAGTTATGCTTTGTTTGACTTTAACAAATTCATTTCTTTTTTTT
ACTTTACCAACAATATCATATTTACCCTATTCAAAATCGCAATACATTTATATAACTTTG
GTTTCAATTATCAACTTATGCACTGCTGGAAATTACTCAATATTTCCAACAATATGTGCA
AATTTGTTTGGAGTCAAATTTTTTGTTTGTAATTATGGAATGTTATTCACCGGATGCGTA
AGTtaaaattctaaatggtttttaaaagaaatttttcttaaattttattggataaataa
cttcaatttaatttctaatgaaaaatgtaaagaaaatagttttgattattcaatttcatt
attaatataattattttataaacataaatatttaacattgttaagtcttatttagaattt
caagaaataaatATGAAAACTATGTCGATTATTTTTGCTTGTTTTAATGGATTAGGACGA
ATATTTTGGGGTTATTTTATCGACATTAAACCATTTAAAGTAGTTATGCTTTGTTTGACT
TTAACAAATTCATTTCTTTTTTTTACTTTACCAACAATATCATATTTACCCTATTCAAAA
TCGCAATACATTTATATAACTTTGGTTTCAATTATCAACTTATGCACTGCTGGAAATTAC
TCAATATTTCCAACAATATGTGCAAATTTGTTTGGAGTCAAATTTTTTGTTTGTAATTAT
GGAATGTTATTCACCGGATGCTCTGTAGGTGGAATTATCAGTAGTTTATTGATGGCTTAT

AATAATCAAGACAACAAGATTGTCTTTATTATCGGAGGAGTAGTTTCATTCATTGGTAAG
TTTTAAatttcaaatttttattattatatcaagcagagtgaatatagctatagaaaacgt
taacagttgagttgacatcattttgataattttattataaaattctaaatggtttttta
aaagaaatttttcttaaattttattggataaataacttcaatttaatttctaatgaaaaa
tgtaaagaaaatagttttgattattcaatttcattattaatataataattattttataaacat
aaatatttaacattgttaagtcttatttagaatttcaagaaataaatATGAAAACTATGT
CGATTATTTTTGCTTGTTTTAATGGATTAGGACGAATATTTTGGGGTTATTTTATCGACA
TTAAACCATTTAAAGTAGTTATGCTTTGTTTGACTTTAACAAATTCATTTCTTTTTTTTA
CTTTACCAACAATATCATATTTACCCTATTCAAAATCGCAATACATTTATATAACTTTGG
TTTCAATTATCAACTTATGCACTGCTGGAAATTACTCAATATTTCCAACAATATGTGCAA
ATTTGTTTGGAGTCAAATTTTTTGTTTGTAATTATGGAATGTTATTCACCGGATGCGTAA
GT
>DjScaffold189:37736-38347 strand:+
ATGGAATTAGTTTTGAGTAATGATAATCAAACAAATGAGAATTCTGAAGTTAGGTTCAAC
TTTCAAATTGCCAATATTGTTTGTTCTTTTGAAGTTGGACATGAAATAAAATTAGAAGTT
ATTTCAAATATTTTACGATTTTCAGAATTTAATCCTAATAACTTTAATGCATGTGTTTTC
CGGCTTTTGAATCCTAAAGTAACATTCAGATTATATTCAAATGGTAAAGTTACTTCTGTT
GGTTCTAAAACTGAAAGTCAAAGTTATTTAGCTGCTAGAAAAAATGGTTAGAATACTAAAT
TTACTTGGTCATCCATCAAAATTACTAAACTTTAGAATTCAGAATATTGTAGCAATCGCT
AATTTTAAGTTTCCTATTAATTTGCCTTTATTATCAGAATGCAGTCCTCAAAACACTAAT
TATGAACCAGAAATTTTTCCAGCTGTACTCTATCGAACGTTTCAACCAAGGTTGGGAATT
TTGGTGTTTGTGAATGGTAAAATAACAATTATGGGTGCTAAATCTAAAAGCCAAGCTTAT
GAGGCATGTAATTTAATCTATAATGAGTTACTTAAATTTAAGAAATCCAAATTTAAATCA
CTAGAGGAATAA
>DjScaffold4714:70503-75503 strand:+
ataaataattttcataataaaattaattcattgattatatttcgacggagttgtagtcga
aactttgagaagaaatctaaaaaaaaatttaaagtacattttcaagtatgctttatacttt
tattgaaatattgaaatcgatgtgtaaaatatttcattactttaaaatttattccaacca
tgagataaaaaataaatttataagtttaaaaaaatttattaagaaaccatgctcatatttaa
tatttattaccacagATGATAAAACCTCTTTCATTGGCAACATTTCGAAAGCTTTTACAA
ATAGAAGACGATGATGATCTAGAAACTGAAGATGACGATGAAAACGATACCAATGCAAGT
GATAACACGGAAGAAGTAGATGAAGATAATTCAAATGAAACAGAAGAGGACGAAGAGGAA
AGTGAACAGGAAGCTGAAGAGGAATCTGTCGATGTAAGTGAGGAAGAAGACAAATCTGAA
GATATAAATTCTGACGAAAATGAAGAGGAGGATAGTAATGAATATGATGAGGAAAACGCA
GAAGACGAAACAGAGATGGAAGAAGAAGATAGTGAAGAAGAACAAGAAATTCCAAAAACC
ACAAAAAACGTCCATAGAAGTCGAAAACTACATTCAAAGAATCACGTTTTTGAGCCTACT
GAGAATGATCACGACTGTGATGGAATACCAGACGATGAGGATGATGACATTGATAACGAT
GGATTGTTAGATCATACACAAGACTCAGATTGGGATGGAGAACTAAATCATATTGATGAA
GATGACGATAATGATGGTATTCCGGACAAGGATGATCCAGATTCAAATGGTGATGGCATT
CCAGATTGTCGAGAAGATACGTCGGATCATAAATTAAAACTTAGATTTAAAAGTAAACAA
AAGCAATTGGATAGTGATATGGATGGAGTTCCTGATGATGCGGATATTGACGATGATGAG
GATGGTATTCCAGATATTCTAGAGGATATGGATGGGAATGATATTCCAGATTTCTTAGAT
AAAAACAGGGATATTAACAACAAAGCAGATTTTGCAGCAATATTAGCAGATGCAGCTCGC
CAAGACCATGATTGTGATGGAATTCCAGATCAATATGATCCTGACGATGACAACGATGGA
TTTTTTGATAAGAAACAAGACAGTGATCGTGATGGTATATTGAATGAATATGACGATGAT
GATGACAATGACGGTATCCCTGATTCTGAAGATCCAGATTCAAATGGAGATGGAATACCT
GATTGTGTTATGAAAGATAGTGATGGAGATGGAATTCCTGATCATATTGATACAGATGAT
GATAATGATGGTCTACCAGATTTGAAAGATCCTAATCATCCGTTGTTTAATTTTTTCATG
GACAGTGATAATGATGGGATTCCGGATAGTATGGACAATGATGACGACAATGACGGGATT
CCTGATCACAGGGATTACGATGCTGATGGAGATGGTGAAAGTGATTTATTTCCAGACAAA
GACAAAGATGGAGTTCCAGACAATATTGACAATGATATGAATAATGATGGAATTCCAGAT
CATCTACAAGATCGAGATTGTGATGGTATTCCAGATTTAATTGATCCTGATGATGACAAT
GATGGATTTTTTGACAACAAACAAGATAGTGACAATGATGGTACTCTTAATGAATGGGAT
GATGATGATGACAATGACGGTATTCCAGATATTAATGATCCTGATGCTAATGGGGATGGC
ATAATCGATTGTAGACCTCGAGATTCTGATGGCGATGGTATTCCTGATCATTTAGATCCT
GATGATGATAATGATGGAATACCGGATTATCTTGATCCAGATTCTATGGCATTCCTTCTT
TATAAACGAACCCAACAGATGGATGCAATGCCAGCTTCTCAGGCTAGACATGAGGCAAAC

ATTGATGTAGATCTTGGGGATCCCGGAGACAAAGATTGCGATGGAATTCCAGATGATATT
GATCTCGATATTGATAATGATGGAAAAATAGATAGAACTCAGGATTCAGATTTAGATGGA
GATCTAAATCATATTGATGAAGATGATGATAATGATGGTATTCCAGACGCTGAGGATCCA
GATGCCAATGGTGACGGAATTCCTGATTGTAGACGAGATGATTTCATTAATTACAAACTT
ATTAAATCTAGAACTGGCAAAATTAAAAGAGTTCTAAGAATAAGAAAACAAACAGAAGAA
GAAGATGAAATTGATAATCAAAAGAAGTCCGTCAATATAAGATTAATACATCAGCAAGCT
CTAGAAATTGGGAAATCTGACAAACCAATCAAAAAGATTGTTAAAAAAGTCAATCCTAAA
GAAGCTAAAAAAGCTGCAGAAGAATTGAAAAAGAAAACATTGTTAACAACTCGTGCCCCA
CCATCACCATCAACCACTAAAATGATGAAACCTAATAAAATTATAAAGACCACTAAATCT
CCAAAACCTGTTGAAGTAACTAAAGAAAAAGTAGAAACCAAAATTAAAAAAACTACTAAA
GTCAAAGCAACAACTGAAAAAAAAACGGTCACAGTTGCAAAAGTAACAAAGGCTCCAGTG
ACAACTACTAAGGCCCCTAAAAAAGAAAAGAAAGTTGTGCATTCCAAAAAGAGTAAAAAA
TCTACGAAGAAAAAATGTTCCAAAGAAAAAAACTAAAAAAATTGTTAAAGAAAGTCCAAAG
AAAAAGTCGTCAAAGAAATCTAGAAAAATATATGATCATGATTGCGACGGAATTCCAGAT
AGTAAAGACAAAGATTCAGATAATGATGGTATAGATGATGATAACCAGGATTCAGATAAT
GATGGAATTGTAAATAGTTTAGATCCTGATGATGATAACGATGGTATTAAAGATGAATTA
GATGATGATGCTAATGGCGATGGTGTACTTGACTGCAAAGCTAAAACAAAAAATATTAAA
AAAATTATTCCTACAAAGGCTCCCATAAAGAAAAAAAGTAAAGTTAAAAGCAGAAAATTA
CTATCAATAAATGACGAAGAAGAAGAAGAAGCTCAATCGGAAGAAGACAGCGAGGAAGTT
GAAAATGAAGAAGATGCAGAACCGGAAGAAGAGGAAGATGAATCAAATGAAGATGAAAAT
GAAACTCCAATTATTGTAAATAAATTACCTGTTTCGCAAAATAAGTTTGAAAGAAAATCT
AATGAACAGTCAATTCCACCTAAAAGTAAAAATGCATTTTCAAAAACAAATGTCAACCAA
GCTTCATCAACAGATTCTTCTAAAAAGACTATTAAAAAAGAAGAAACCGTTGGGAATTCA
AATAAAGACTCTGATGGAGATGGAATTCCAGATTATCTTGAAAAAGATTCCGATGGAGAT
GGTATTCCCGATTTCAAAGAGGATGAAGATGGTGATGGAATTCCTGATTATTTAGAAAAT
GATTCAGATGGTGATGGTATTCCTGATTATTTGGAAGATGAAAATGGGGATGGAATTCCA
GATTATCTGGAAAAAGATTCTGATGGAGATGGTATACCAGATTATAAAGAAGATTCTGAT
GGCGATGGAATACCAGATTATTTAGACAAAGACAATAAACAAATAAATCCAAAAAATGAC
GCAAAAAAAGATGTTATTTCAGAAAACATCAAAAAAGATAGTGATGGAGATGGAATTCCA
GATTATTTAGAGAAAGATTCAGATGGTGATGGAATACCGGATTATAAAGAAGATGAAGAT
GGAGACGGAATTCCAGACTATTTAGAAAAAGACTCTGATGGTGATGGAATCCCAGATTAT
CAAGAGGATGAAAATGGTGATGGAATCCCAGATTACTTGGAAAAAGATTCAGATGGTGAT
GGAATACCGGATTACAAAGAAGATGAAGATGGAGATGGAATTCCAGACTATTTAGAGAAG
GACTCTGATGGAGATGGAATTCCAGATTATAAAGAAGATGAAAATGGTGATGGAATACCA
GATTATTTGGAAAAAGATTCAGATGGCGATGGAATACCTGATTTTAAAGAAGATTCAGAC
GGTGATGGAATTCCTGATTATTTAGACAAAGACTCGGATGGAGACGGAATACCAGATTAT
AAAGAGGATGCAGATGGTGATGGAATCCCTGACTATTTAGATAAGGATTCAGATGGCGAT
GGGATACCTGATTACAAAGAAGATGAAGACGGAGATGGAATTCCTGATTATCTCGATAAA
GACTTTAAAGGTAAGCCTTTTAAACAAATTAAAGAGTCAAAACATTCAGAAAAAATGTCT
GGTAGCAGTGGTATACCCAATAATAAAGAAGATTCCGATGGTGATGGAATTCCAGATTAT
TTGGAAAAGGACTCTGATGGTGATGGTATACCTGATTACAAAGAAGATGAAGATGGCGAT
GGGATACCTGATTACTTGGAAAAGGACTCCGATGGTGATGGTATACCTGATTACAAAGAA
GACGAAGATGGCGATGGAATTCCAGATTACCTAGAAAAGGATTCCGATGGTGATGGTATA
CCTGATTACAAAGAAGATGAAGATGGCGATGGGATTCCAGATTATCTAGAAAAAGATTCC
GATGGTGATGGTATTCCTGATTATTTGGAAGACGAAGATGGGGATGGAATCCCAGATTAT
TTAGCAAACGATTCAGACGGGGATGGTATTCCTGATTATTTGGAAGACGAAAACGGAGAT
GGAATCCCAGATTATTTAGAAAAAGATTCAGATGGCGATGGAATCCCAGATTATCAAGAA
GATGAAGATGGGGATGGAATCCCAGATTATCATAAGATTAAAAGTCTACATCAAAACTTT
ACAAAGAAAAATGTTCCAGTTGATTCAAATCGAAAAGTTGTGAAACCAGGTAAAGAAAAG
ATAAAAATAGATAAAACTAAAGACTCCGATGGTGATGGTATTCCAGATTATCTTGACGAT
GATAACGACAGAGCCGATAAAAAATCGTGCAAATCAAAAGAATCATGTGAAAAATTTATT
AATTTAAACAAAGAAGAAATACAGAACGAAATTAATGACAATAAATTAACAATGCCTCGA
ATTATTATTCGGCAAGTTCTACAGACTTTGGAAAACTTTTTCGGTACAGACGAATAGgat
atttaatgaagaaataatttagaggcactttccgtagataatatccattattagtgtttg
tgatagtttttctctttttgattttctgctaaaaatgctctgttaataggttcttaaaat
ttattattagttttgaaataattttaaaattgttggtttgccgatttatttatttgattt
attttataaacgctaccattgattattcaattttcggtacatttaaacaagattttcgtt

agttttcgattttggaaaaaatcttgtagagaatatgattttatttatcgaacaaatttt
tgctgaataatcttgtttttcataacaaaattcattttatttaattgaaaattcgattta
acaaaacaaatccaattttcaaattatattttattttaaaaaaaaaaattggtataaatg
ttactcttttgcagcaaattaattatttaaaattttgagagaaaattaaatttgatttct
taaatcaaattcaattttattttcgtgaaatttagtttctcttgaaaaaaaattgttattt
tacatttgtttatttgtaataTATTTAGATAAGGATTCAGATGGCGATGGGATACCTGAT
TACAAAGAAGATGAAGACGGAGATGGAATTCCTGATTATCTCGATAAAGACTTTAAAGGT
AAGCCTTTTAAACAAATTAAAGAGTCAAAACATTCAGAAAAAATGTCTGGTAGCAGTGGT
ATACCCAATAATAAAGAAGATTCCGATGGTGATGGAATTCCAGATTATTTGGAAAAGGAC
TCTGATGGTGATGGTATACCTGATTACAAAGAAGATGAAGATGGCGATGGGATACCTGAT
TACTTGGAAAAGGACTCCGATGGTGATGGTATACCTGATTACAAAGAAGACGAAGATGGC
GATGGAATTCCAGATTACCTAGAAAAGGACTCCGATGGTGATGGTATACCTGATTACAAA
GAAGATGAAGATGGCGATGGGATTCCAGATTATCTAGAAAAAGATTCCGATGGTGATGGT
ATTCCTGATTATTTGGAAGACGAAGATGGGGATGGAATCCCAGATTATTTAGCAAACGAT
TCAGACGGGGATGGTATTCCTGATTATTTGGAAGACGAAAACGGAGATGGAATCCCAGAT
TATTTAGAAAAAGATTCAGATGGCGATGGAATCCCAGATTATCAAGAAGATGAAGATGGG
GATGGAATCCCAGATTATCATAAGATTAAAAGTCTACATCAAAACTTTACAAAGAAAAAT
GTTCCAGTTGATTCAAATCGAAAAGTTGTGAAACCAGGTAAAGAAAAGATAAAAATAGAT
AAAACTAAAGACTCCGATGGTGATGGTATTCCAGATTATCTTGACGATGATAACGACAGA
GCCGATAAAAAATCGTGCAAATCAAAAGAATCATGTGAAAAATTTATTAATTTAAACAAA
GAAGAAATACAGAACGAAATTAATGACAATAAATTAACAATGCCTCGAATTATTATTCGG
CAAGTTCTACAGACTTTGGAAAACTTTTTCGGTACAGACGAATAGgatatttaatgaaga
aataatttagaggcactttccgtagataatatccattattagtgtttgtgatagtttttc
tctttttgattttctgctaaaaatgctctgttaataggttcttaaaatttattattagtt
ttgaaataattttaaaattgttggtttgccgatttatttatttgatttattttataaacg
ctaccattgattattcaattttcggtacatttaaacaagattttcgttagttttcgattt
tggaaaaaatcttgtagagaatatgattttatttatcgaacaaatttttgctgaataatc
ttgtttttcataacaaaattcattttatttaattgaaaattcgatttaacaaaacaaatc
caattttcaaattatattttattttaaaaaaaaaaattggtataaatgttactcttttgc
agcaaattaattatttaaaattttgagagaaaattaaatttgatttcttaaatcaaattc
aattttattttcgtgaaatttagtttctcttgaaaaaaaattgttattttacatttgttta
tttgtaata
>DjScaffold4714:32509-33969 strand:+
GTTGAAAGCGAAACCGATAAACGGTCAGTTGAAAAAACCGTTTGGGACTGGGAATTAGTA
AATAAAAACAAACCTATTTGGACTCGAAAGACTGGAGAAACTTCAGATGATGAATATAAT
GAATTTTACAAATCTTTTTCTAAAGATACGGAAAATCCACTTTCAAAAACACATTTTACT
GCCGAAGGTGAAGTTTCGTTCAAAGCCATTCTTTATGTTCCTAAAAAATCAAATATTGAA
ATTAATAATATGGTAGAGAAAAAAAATGATAATATTAAGCTTTATGTTAAACGAGTTTTT
ATAACTGACGATTTCGATGATATAATGCCAAAGTATTTATCTTTTATAAAAGGAATTATA
GACAGTGATGATTTACCATTAAATGTATCGAGAGAAACTTTACAGCAACATAAATTACTT
AAATTAATTAAGAAAAAACTTGTAAGAAAAACTCTAGATATGCTTAAGAAAATGCCTGAA
GATGTATTTAAAGAATTTTGGAAAGAATATGCAACAAACATTAAATTAGGTGCTATTGAA
GATGTTTCAAATAGGAACAGATTGGCAAAACTCCTTAAATTTTGGTCATCACACTCAGAA
GAAAATCTAACAAGTCTTTCCGATTATGTTTCAAGAATGAAGCCAAACCAAGATAAAATA
TTTTTCATTGCAGGAGTATCATTAGCAGATGTACAATTATCGCCAATGGTAGAAACACTA
TTGAAAAATCAATATGAAGTACTCTATTTAATTGAACCAGTTGACGAATATACTCTTCAA
TCAATACCAGAATTTGAAGGAAAAAAATTCCAAAATGTGGCGAAAGAAGGATTAAAATTG
GATGATTCTGATAAGAGAATAAAAAGTAAAGAAAAACTAGAGAAAGAATATGAAAGCTTA
AATAACTGGTTAAAAACTGATGCATTAAAAGATTATGTAGAGAAAGTAGAAATCTCTGAA
AGATTAACAAGTAGTCCATGTGTATTAATCGCAACTACATATGGGTGGTCAGGTAACATG
GAAAGAATTATGAAAGCTCAAGCTTATCAAAAATCAGATGAAGTAATGCAAAGCTATCAA
GTTAATCAAAAGAAAATTTTAGAAATAAACCCCAGACATCCATTAATAAGACAATTGAAG
TATTCTATCAATAAAAAATCAAAACATTAATGAAACAAGAACTCTTGCCAAATTATTATAC
GACACTGCTTTATTAAGATCAGGATACATTTTGAGTAATGTAGCCAGTTTTTCCAAACAA
GTTGACACAATTATTAGAACATCAATGAAAATTCCACACGATGAAAATATTATAGATGAC
GAAGAAATAATGGAAGATACTTTTAAAGAACCGGTAACAGAAAAATCTGAAGATGAAAAT
AAAAAGAATGAGAATAAAGTTTGTTTTAAAAATTTATTGTTTAAGTTTTCGTTATACAGA

TTTTCTATAAAATTAAAATGA
>DjScaffold3018:43188-56356 strand:+
atatatatatcatatataacagatgatttatatgtgtgtaaaagtgtgtttatgtgtgta
tgttataaattattcaaaaattattttatttcaaaattttagataatcgaaaaggttga
tgcaattctcattgaaaatccaataaaagaattattttataatgaagacaagaatgaaat
aatttcttcacatggtacttatttggtcatttggcaatttcgatatgctagcaagcatct
aataccaagatcgacaataaatttaaattataaaagtcagtgtgctaaaatattcacaat
cgaacgaactcaaagaaaatttcaacggttatttgttgttaacgattgcggaataacagt
agtcgagttgtcaaaaggtgtggtcattgctgatcacttatcacttcatgagcgaccaat
tactacattgacatttttcaatcctttaaaatttctagtaactggatgtaaagatgggtc
gataaaggtttggtattttatagacaatgatattaaattaaagATGGTATTCGTTGGCCA
TACAAGTTATGTAAATAATTTGGCATTTTTTCCTGGAGGTTCTCAAGCTACGATTGCTTC
ATGTGGTGATGATAAAACTTTGCGCGTTTGGAGTCTAGAATTGGATGATGAGATTGAGTG
CTTAGATTTGGAATATACCTCTAAGTATATAGGATCTGAAATCACTAAAACGTCGTTTTT
TACTCTCAGCAATAATTTTATAGATATATGGCACCAGAATCATCTGTCGGATGATCTAAT
GAGTCTCACTGCACCAGTTTCAAGAATGTTAAATACTAGTGAAAATAATGGAAAAATTCT
TATTTGTTGTCGAAATAATCTCGTATTTTATATCACACCTGGAAAAACTGCCAGCATTAT
TACAAAATCTGTTATTGGAAAAATTGATAAATTTTATTCAAAAACTCCAAAAATATTTTC
CACTGTACATTTGGAAAATAGAAATTCTCTTTTAGTGCTAGTACAAGATAACCATATTTT
ACAATATAACACCAAATCTAATCCAATGTTATTGAATTCGATCTCTCAATACGAATCTGA
AGAAAGTTTAGTCATTCTTACCGCTTATAAACCGTCATCAGAAATCAATGGAAAACCCGA
AAATAAGAGTCTGATTTTATCTGGTTCTAATCTAGGTATAATCAAAGTGCATGATCTTAA
TAAATTGGATATAATAACTTTTCAAATTGAAGCCCATCCCAATTCAGAAATCGTTTGTAT
AATCCCAGATCCAGTTAATTGTAAATTGTACACTTCTGGATTGGATAACAGTATCAAAGT
TTGGTATATCTATCCTTTTGCTACTGAACCTTTATCAAAAATTCTTTCTTTCTTTTGTGG
TCTTTTACCATTTCATTTAATTAGCATTAATAATTATCTCTTTACCGTTTTTCATGATTC
CGATGGAGCCCATTATCCTTGTGTTGTTTATAATATTCAAACTCGTGAAAGATTTGACCA
TTCCCCGTCTGATGATCATATAGAACCAGTAAATTCTGTCAGTGCATGCAACCGAATGAA
ACTTTTTCTTTCTTGTTCTGCAGGAACTGAAAATATGATAAAAATTTGGACACAGAAAAA
TATTCTTCTAAGACAAATAGAAATGCATTTTCCCGTTTACAGTGCTATTTTTACTTCGTT
TAAAGGAGACATAATGATGGGATTGGGTCATCAAATCTGCTTTATTGATCATACAAAATA
CCTACCGAAAGCGTACATTAAAAAACAGGTTTTAATGTATTTCAAAAGTGATTCCGATTC
AGTAGAGAATTCTTTGGAGTTTGACAATGATAATTTCAAAAGTAAATTTGATTCTGAAGA
TCGAGATAAAATGAATGGAATTAAATCCGTTTTCCGGTATGATACGTATATTGATATTTT
AACAGAAAACGAAAAACAAGAACTGTTTAATTTAAATGAAATTAAAAATCAAAATCTCGA
CCGATTAAAGAAAATTGAAAACGATATTAGAAATATTCAAAATGGTGATCATGAAAAACC
GAAAAGAAAATATGAACATCTCATTGAAACACCTTTGAAATCGGATATTTTTAATAATTA
TTTCTCACAAATTGTGAAATTGTCGAAAATATTACTACCGGATGATGATGATAAATTACA
AACTGATTATAGAAATCGATATTCAGAGAAATACATAAAATCGGAAAAAGAAGGATTTTT
CCCATCTGTAGATACAATCAGTAAAATAATGGCTCCAAATAATCTGAAAACAGGAGGTTA
TATACCTAATTCTACGGTTTTACAGAAGCTTTGGCCTCCAAAACTACTCAAAGAAAAAGC
TGATACTTGGAAACCATTGATGCTATCAGCACTTGATTTACAACAAATGGAATTATCAAA
AATCGCAACGATTGAAAACGATGAAGATGAAACAAAAATTTTTGATTGGGAATCCGACGA
AGAACCGGAACAATCTATCATATCTCCCACACCTACTGAAATTATTTCTGAAACTTTATC
TGACGACATTCTTAGCAAATTTGGATCTTTATTAGAAAGTAAACCATCAGTCAATGACGA
TATTAAAGATGATTTGGCCGATACTAAATCATTGGAACCAGAAACTCCAAAAGAAAATAA
AAGACCAACGACCACGAAAATAACAAGAGAACATTTGAAATTACCACCTATATCCAGAAA
TCCAGCGTCGATGCCGGAAATTAAACGAGTTAGATTTATTTCTTCAGCTGAAAATATTCC
TGAAGAAAATAAAACTCTTGATATTTCGAATACTAAAGAACAATACCATTCTGATTCATC
TCTATTTATTACCCAGGTATCGGATCAAGATGCAGAATTAAAGGTAATTACAAAACTGAA
GGAAAAACAATCAGTTTTACCTGAATTTCTTGTCAGATTTCAGGGTCAAAATTGGTTCGA
AAGAATTTGTCCACCTCCTTATCAAGATGAAAAGTGCCTTGGAAATTTACCTTCACCACT
CAATTCAAATAGTTTTACCATGTATATAGTACAATTTCTAAATAATTCAAAAGACAACAA
TGAAAAATACGGAATCGTAAACGCAATAATTTATCTTTACTCCAATGAAAATATCGATAA
GCAAAATTTCCCTCAAATATATAAAACAATTCAAAATATTGTTAAAGAAATTAAATTAGA
CAATGAACCAAACGTTCTATTGTTCAAATCATGCCTACATCTAATGGCATCATTGGAAGG
TGTTTCTGATAAAGTACTTGTCACAATTTGCTTGTGTAGACATTTTGATGCAAACGAAGA

ATTGAAAGCATTTTTGAAATCTATATTATATCATATTGGATTGGAAGATAATACAGACAC
AATAGAACAAGAATTAAAAAACTTTAAATTATTTTCTATTGGATCAGGAGATGAAAGAAC
TGGAAAGTTGTATGATATGGTTTCTGATTGGTTGGAAAAATGGTTTCAGTCAATGACCAC
CTTTGCTGTTGAATGTAGAGAGATTATTCGAAAAGAATTGCATTTAATTGGCAGCTCATA
TAGTAAATTATCAAAAAGTACCATAACTTGTTTAGATAACGTTAAAAACGTTTTCCATTG
TCATATTATGTCATCTGCAGATTTAGTATTGTTATATCCAGTTGATAAAAATCCAAAATC
AATTTTAAATTACATAAACTGCCTTAATTACTTTAGATTTAAAAAACTTCAGAGGGAAAC
AAAAGAAAAATCTATTTTGGTTGAAAATACTGGTCCTAGAAAAACAATTGTGTCGTTACC
AAAATTAAATCCAAACAAGCAATGTCTAGTAAGATTAGGAGAATCAAATGCGATGGGAAT
AGAAACGAAATCTAAAAAAGACTTTATTACTTTGAATTCCGTTGGTAATAAATTTAGCAT
ATCTGATTATACTCAACATCCTTCAATGCAACCGTTTCATCATTATCAAGTTGCTCTTGG
CGGTGGATTTGTTCCATTTCTACATTTGCATCTC
>DjScaffold386:26916-34398 strand:+
agtgtcaatatacagaaaaggtatccactttatcaaccgcagtttccatacatccatccg
atatgccaaataccaaacgagaaagcagtggtattgttgctgatgccagtagtattgaat
gcatttcatgtcaaaaagaaggaaacaataaacctgaaaatataataacagatcaattta
atgatctgaagtctccgttattggaagttgagtctgatgaattaggagaaattgaaagat
taaacagttgtcgattttctcacaagacaattttatccaaactatagaaaagatgcaaca
ataaatccgcactgatatctttttctggactttacacaaaaatgtgagaactgatatttt
caaatttaccaagcttgttatagttttagattactatattatcgatcattttagtttaat
aaaatattttattactgtaataaatagggcttctggcgtggtaATGGGTGGACGAGATTT
GGAAACAAAGAAAAAGGTTGCCATTAAACAGATGAATTTGAAACAACAACCAAAGAAAGA
TTTAATTCTAAATGAGATTCTTGTTATAAAAGCCAATAAACATCCAAATATTGTTAATTA
CCTTGATAGTTTTATTATGAATAATGAACTATGGGTGGTTATGGAATATCTCGATGGCGG
TTCATTGACAGACGTCGTAACAGAAGCATGTATGGACGAATATCAGATTGCCACCATTTG
TCGAGAAGTTACTATGGCATTAGGATTTTTACATTCCAATCGTGTTATACATCGTGATAT
CAAATCGGATAACATTCTTCTTGGTTTAGATGGATCTGTTAAAATTATTGATTTTGGATT
TTGTGCTCAATTAATTGGGGGTCGCAACAATCGATCAACAATGGTTGGGACACCATATTG
GATGGCTCCTGAAGTTGTTACCAGAAAGCAGTAC
>DjScaffold386:86034-89943 strand:+
aattaaaattctcgaggatggaattgaaagaATGGAATCTAAATTAAATAATAATCTTCA
TCTTATCAATGAAATTAACTTAACCACTGAAGAATACAACAATGAATATAGTAATTTAGC
AGACAAAGATGATTGTAACATTGATAAAGTTGAAACACTAATTTCACAACTGAAAATAAT
GGTAGAAGAGTCAACTGTTGCCGAAATGAAATGTATTGAGGCATCACAAAAATTGATCAG
TCAAGAAGAGAAAGTTAAATTTACAATAGAAAAGGCTATTCAATTTGAATTTGCACTCGA
AAATCTTAGTAAAGAAATCACTAATGTTTCGTCTAATCTTCAATGCTTGGAACAAATGGG
TCATGAAGTA
>DjScaffold804:14112-32591 strand:+
atgaagtgttttgtgattttctgttgttgttgagtgtgccacatacaacatacatcgtta
aaatagccctccaggagcaagaatgggaggttctttcacatccataaaatgatgcggtac
ccatttatccgatttcttaacttttccgattagttgattgtgaaacttgctttttttgctg
aaatatctgatatatctcccttgactattggttttaaaacatcgccatcaacagcataag
gttaccaagttcttttatctgtaagagttattccctttttcttacatccagcctaaggaag
caatcattggacatcatactgtgcaaaaacagccgactttcgtccttcacaacaacgcac
gtctccatatttcacgaatggtcgtacaaaaactacataattcagagtaggaaactcttg
ctcacacacaataatcactggaccttgcttccacagatttccattttttgaaactgtgat
atcgaaacagcattaaaaaatttctttgactccagaaatccagaatttttccaaatcgg
aatcataatatttacaattacaattattgcgagaataattgtcgatcttaactattgatc
gcatttgagatttcattaaaataaagtaatggtttcgatcaaaataaattgttgttttat
agatattcaagtttacgacaattgccttcttcaggatataatatatgaagacaattgtcg
aaaatttgaatatctataaaacaaaaatttatttcgatcgaagctgctattactttattt
taatgaaatctaacgtttaaataaatttttaataaatgattcaaatgatctattttgatt
tattctgatatccaaatcgtaactaatttctctattaacaaactgtcataaatatctgca
tttttccgttaaaattttatctattcaatttttaatatttgtcgctagaaaatttgaa
tttcaacataattttgcatttgtattatgtaaaaaatatttacagaaataatattctttg
tgatgttgttttaaaagctggagaattagagattaatgctcataaagttatcctagcaag
ttgttctccttattttcatgctATGTTTAGCGGGAATATGTGTGAGTCAAAAGCTCCATG

CGTTTATTTAAATGATGTTAATGGTGAAGCGCTTCAACAATTAATTAATTTCATTTATAC
ATGTGAAATCCACGTTAGTGAAGAAAATGTTCAAACATTATTGCCGACAGCTAATTTACT
ACAGCTTAATGAAGTGAAAGATGCGTGCTGTGAATTTCTGCAATATCAATTACATCCTAG
TAATTGTTTAGGTATTCAGAGATTTGCTGATTTACACAGTTGTAAAGAGTTACTGTTGTA
TTCTAAACAGTATTCTGAAACTCATTTCTGTGAAATATTGAATCAAGATGAATTTGCCAA
CCTTCCAATTGATCATTTGGAATCACTAATAAGTAGTAATAATTTGAGTGCTAAAGAAGA
GGAAGTTTTTGAAGCTGTAAGAAAGTGGATAAAACATAATTACATAAGTCGTCAGCAATA
TACGCAAAGATTATTTTCTCATGTTAGATTTCCGTTATTATCAAAAGAATTTCTTGTTAA
ACTTTCAGAAGATGACGAATTTATTAAAACAAATGAAGCTTGTAAAGATTTTTTAATATA
TGGTTTACGATTCCATTTATTATCGAGTGAAGAGAAAATCGAAAATGAGTCATTACAAAC
AACTCCTAGAACTCCTTCAACAAACCCAAAAATACTAATGGTTATTGGAGGACAAGCTCC
AAAAGCAGTGCGATCTGTGGAAGAATATGATTTTAAAACAGAACAGTGGCGTCAAGTAAC
AGAATTACCAAGCAGGCGATGTAGATGTGGAGCGGTTCTTAATAAAGGATTGGTTTATGT
AATTGGGGGATTTAATGGCTCATTGAGAGTGAGAACTGTTGAAACTTATGATCCCGTTAA
AGATAAATGGGAATCAGTTTCTCAAATGGAAGCCAGAAGATCAACACTTGGAGTTGCTGT
TCTTGATGGATTAATATATGCCGTTGGAGGGTTTGACGGAGCACAAGGATTGTCTTCTGC
TGAAGTTTATGATCCTAATAATGATACATGGAAAAATATTGCCAATATGTCGTTGAGAAG
ATCTTCTGTCGGTTTAGCTGTATTAAATGGTTTGCTCTATGTTGTTGGTGGATACGATGG
TTTAGATAGGGAATGCTTATCAAGCGTTGAATGTTATTCACCGAAAAAGGATGTTTGGAC
TACAGTCGCTAATATGAGCTGTAAAAGAAGTGGTGCAGCCGTAGGGACATTAGATGGTTA
TTTATATGCAGTAGGAGGTCATGACGGACCTAGTATTCGAAATACTGTCGAGAGATATTC
TCCAGAAACAGACAGATGGACTTTGGTAGCCAAAATGACTTACAATAGACGCAACGCAGG
AGTAGTTACACATAATGGAATGTTATTTGTTGTCGGAGGAGACGATGGATCATCAAATTT
GAGTTCAATCGAATATTATAATCCCAAAACAAACACTTGGACATTACTTAATTCTAATAT
GATGACCGGGCGGAGTTACGCAGCTGTTGTTTTGATACCATCTGATAAAAGAAATTGAtt
tctttttgtgtttttgttatttactattgatttattgatttccaattttacatacacaca
aaaattattatcatgttttcttatttcaacgttttgcattattgatttaaatgatttttt
aaaagcaaacaaacaaaaagttgtaagataagtgataaaaaaaaattgtaaattattatct
ttattcttagaatcctttattgtaagtcgttttattgtaaagatttttttagaaactta
ttattgatttatattatttgcaaaatgttacgtgataaatttagtgagttacaaatttat
taatttattcataatttggcagaaatcgaagatagtttgacaaatttgtg
>DjScaffold462:101062-109794 strand:+
tttatcaatATGGGACAAAATTTATTACCAATGTCCAGCCAACCGCCGATGACTCAATCA
AATTTTATTCAATATCCTGGTATTTCTATACCAAAAACTACCTATATTCAAATGAAAACA
CAATTACTCGGACAAAGACAATTGTTTTGGGATTTCGAATATCCAGTAAATAGACTTGAA
TTGATATCTTCAAATCAATCTATTGAATGGAAAAGACCATTTGAAATATGTCAAAATCCA
CAAATGTTTACTTATGGAATCAGTAGATTTGATATAAATCAAGGAAATCTTGGTGATTGT
TGGTTATTAGCTGCTATTGCCTCTTTAGTCATATATCCTAATCTGTTAACGCGAGTTGTT
CCATTAGATCAAACGTTTAATTCAGGAGATTATTGTGGAATTTTTCGGTTTTATATCTGG
TATTACGGACAATGGCTGGAAGTGATCATTGATGATTTATTACCTACTGTCAATGGGAAA
TTAATTTATCTGGCTTCAAAAGATTCAAATGAATTTTGGTCACCTCTTTTAGAAAAAGCG
TATTCAAAGGTTTTTGGATCATATAAAAATTTAAAAGGAGGAATTACAAGTGAGGCAATG
GTTGATTTTACCGGTGGAATAACAGAAAAAATATATTTGAGGAAAAACAAAACTTTATCA
AATAATTTAAATTCAATTCAACAGTTATTCTTTTCAATTCTACAAGCGTATACCAATCAA
GCATTGATGAGTTGTTCCAAAGATGGGGGTCTCGAATTCAAGGATAATTCTGGTTTGGTT
TCACGACATGCATACAGTATTACCGCAGTTTGTTTAGTAAATACAAATGGTTATGGTCAA
GCAAATTTGATTCGTTTAAGAAATCCATGGGGAAACGAAACAGAATGGAGAGGACCCTGG
TCCGATGGTTCTCATGAATGGCAATATGTTCCTCCTCATGAATTACAAAGATTACAAATT
GTTAAGAAAGAAGATGGTGAATTTTGGATGGCTTTTCATGATTTTGTAAATAACTTCGAT
AAAATTGAAATTTGTCATATTGAACCTCAATCCTTAGAAGATTTGTCACAATTGAGAAAT
CATGAAAATCTAATAAAAAGAACTTGGGAATTAAAATCATTTTATGGAGATTGGAAAAAG
GGATTGAATGCTGGTGGATGTCAAAATCACAATACGTATTTCACGAATCCACAATTCCCG
TTAACGATTTTAGAACCAGACGATGGCTCTAGTAATGGTACTGGAACTATAATTGTCGGA
TTAATGCAAAGAAATCTTAGAATGTTTAATAAAGAACTTTTCGCAATAAGTTATTCAATA
TATCAAAACCCCCATGGAACAATTTATGGTCAAAATTTCCAGAATCTTACTGAAGTAGCT
TCTTCAGGAACTTACAATGATATGAGAGAAGTAACAGGAAGACACAAACTCTATtttatc
aatATGGGACAAAATTTATTACCAATGTCCAGCCAACCGCCGATGACTCAATCAAATTTT

ATTCAATATCCTGGTATTTCTATACCAAAAACTACCTATATTCAAATGAAAACACAATTA
CTCGGACAAAGACAATTGTTTTGGGATTTCGAATATCCAGTAAATAGACTTGAATTGATA
TCTTCAAATCAATCTATTGAATGGAAAAGACCATTTGAAATATGTCAAAATCCACAAATG
TTTACTTATGGAATCAGTAGATTTGATATAAATCAAGGAAATCTTGGTGATTGTTGGTTA
TTAGCTGCTATTGCCTCTTTAGTCATATATCCTAATCTGTTAACGCGAGTTGTTCCATTA
GATCAAACGTTTAATTCAGGAGATTATTGTGGAATTTTTCGGTTTTATATCTGGTATTAC
GGACAATGGCTGGAAGTGATCATTGATGATTTATTACCTACTGTCAATGGGAAATTAATT
TATCTGGCTTCAAAAGATTCAAATGAATTTTGGTCACCTCTTTTAGAAAAAGCGTATTCA
AAGGTTTTTGGATCATATAAAAATTTAAAAGGAGGAATTACAAGTGAGGCAATGGTTGAT
TTTACCGGTGGAATAACAGAAAAAATATATTTGAGGAAAAACAAAACTTTATCAAATAAT
TTAAATTCAATTCAACAGTTATTCTTTTCAATTCTACAAGCGTATACCAATCAAGCATTG
ATGAGTTGTTCCAAAGATGGGGGTCTCGAATTCAAGGATAATTCTGGTTTGGTTTCACGA
CATGCATACAGTATTACCGCAGTTTGTTTAGTATGTAATTTCACAATTGGGTTCTGGTTG
TTTTCTTTATACAATTAAggtaaatacaaatggttatggtcaagcaaatttgattcgttt
aagaaatccatggggaaacgaaacagaatggagaggaccctggtccgatggttctcatga
atggcaatatgttcctcctcatgaattacaaagattacaaattgttaagaaagaagatgg
tgaattttggatggcttttcatgattttgtaaataacttcgataaaattgaaatttgtca
tattgaacctcaatccttagaagatttgtcacaattgagaaatcatgaaaatctaataaa
aagaacttgggaattaaaatcattttatggagattggaaaaagggattgaatgctggtgg
atgtcaaaatcacaatacgtatttcacgaatccacaattcccgttaacgattttagaacc
agacgatggctctagtaatggtactggaactataattgtcggattaatgcaaagaaatct
tagaatgtttaataaagaacttttcgcaataagttattcaatatatcaaaacccccatgg
aacaatttatggtcaaaatttccagaatcttactgaagtagcttcttcaggaacttacaa
tgatatgagagaagtaacaggaagacacaaactctat
>DjScaffold462:60150-60503 strand:+
ATGGTTTATCTTGATGACATCCTGATATTTATTAACAATAAAGACCAACACTATGAAGAA
ATTGAAAGAGTATTGACGGAAATAATGAAGACTGGTTTAAGAATTAATCCAGAAAAGCGT
CAGTTTTTACAAAGCGAAATCAAATTTCTTGGGCACATAATTAGCAGAGGTGGTGTTAGA
ACTGATCCGTCTAAAATAGAAGCAATAAAAACATTTGGCAGGCCTAACTGCATAAAGAAG
TTGCGAAACTTCTTAGGAATTTGTAATTACTACAGGAAGTTTATAGAAAACTACGCAAGA
AAATCAAGGATCTTAGAACAGTTGTGTAGCTTGGAGAGAAATACGAAAAACTAA
>DjScaffold3398:2194-7580 strand:+
aatagtttggaggaactaattacacgttcaaATGGCTTTCATACATAAATTTGGACAAGT
ATCTATCATTTGTCATGCCTTTAATGGCAATAATACTGAAATGGCTGTGTCGATTAATTC
AAACGATGTTTTCATTTTTAAAGTACCCGATGTGGGAAGGGGAAAATTCCAATTTTTACA
TAGGTTACAAGAACATACAGCTACTGTTCGTTCTATAGATTGGGGTAAAAAAGAAGATAA
AATTGTCAGTTGTTCTACTGATAGAAATGCTTATGTATGGGCACGCTCGGATAAATCATG
GAAGCCACTATTGGTTATATTACAGTTAAATCGAGCTGCCACATTTGTCAGATGGTCACC
ATTAGAGAACAAATTTGCTGTCGGAAGCAGCGCTAAACTTATATCTATTTGTTCTTTTGA
TACAAACAACGATTTCTGGGTCAGTAAGCACTTGAAAAAAAATATTAATTCTACTATTAC
ATGCTTGGACTGGCACCCAGATAACAATGTTTTAGCGTGCGGATCAACAGATTTTCACGT
CCGAGTATATTGCGCTTTATTAAAATCCCATGACGCTGCATCTAAAGACAGTAACTGGGG
CACTAAGATGACTTTTAACACTCTTCTTTGGGAAAGTGAAAGCGGTCTTGGTTGGATCCA
TTCTATTGCATTTTCGAATGACGGAAATAAACTAGTATGGAGCACTCATTCTAGTGCGAT
CTGTTTGGCCACAGTAAATGGGTCTCATCAACCACCTACAGTGAGAATGATTCTAATGGA
GAATTTGCCATTCCTAACATTAGTTTGGGCGGCTCCAAATACTGTTATTGGTGCTGGCCA
TGATTGTGTTCCCTACACGATCTCAACAGACAGAGATCAGCTGAAATTAGGCACGAGCTT
GGATAGTAAAGAAGAAAATAACACAAATACAACTATGTCAGCTAAAGCTAGATTCCAGCA
AATAGACAGAGTTGCAACAGTCGACTCCAATGATTCAACTATAAATTCCAAACACAAAAA
TGCAATCAATGAAATTCGCATTATGAAAGGAAGTAATGGGTCCGCTCAAGTTATTTCTAC
TATTGGTCGAGATGGACAGCTTATTTTGTGGGACCTCAAAACCTTAAGCCAAAAAATGAG
TGATTTAAAAATTCAATGAgattgattttgcaatttattcattgctttgttgaattttta
tgtgaaaacaacacttttctagtgttaatttcaatagtgctaattatataacgaactttt
ctattgattttcagtcggaatttattattttaccttgcatcattgtgtgcatgtttgtgt
gtattcgtcggtttaattattttcgaattgttatttttggtttacgttggtccataattt
agaaatagtttggaggaactaattacacgttcaaATGGCTTTCATACATAAATTTGGACA
AGTATCTATCATTTGTCATGCCTTTAATGGCAATAATACTGAAATGGCTGTGTCGATTAA

TTCAAACGATGTTTTCATTTTTAAAGTACCCGATGTGGGAAGGGGAAAATTCCAATTTTT
ACATAGGTTACAAGAACATACAGCTACTGTTCGTTCTATAGATTGGGGTAAAAAAGAAGA
TAAAATTGTCAGTTGTTCTACTGATAGAAATGCTTATGTATGGGCACGCTCGGATAAATC
ATGGAAGCCACTATTGGTTATATTACAGTTAAATCGAGCTGCCACATTTGTCAGATGGTC
ACCATTAGAGAACAAATTTGCTGTCGGAAGCAGCGCTAAACTTATATCTATTTGTTCTTT
TGATACAAACAACGATTTCTGGGTCAGTAAGCACTTGAAAAAAAATATTAATTCTACTAT
TACATGCTTGGACTGGCACCCAGATAACAATGTTTTAGCGTGCGGATCAACAGATTTTCA
CGTCCGAGTATATTGCGCTTTATTAAAATCCCATGACGCTGCATCTAAAGACAGTAACTG
GGGCACTAAGATGACTTTTAACACTCTTCTTTGGGAAAGTGAAAGCGGTCTTGGTTGGAT
CCATTCTATTGCATTTTCGAATGACGGAAATAAACTAGTATGGAGCACTCATTCTAGTGC
GATCTGTTTGGCCACAGTAAATGGGTCTCATCAACCACCTACAGTGAGAATGATTCTAAT
GGAGAATTTGCCATTCCTAACATTAGTTTGGGCGGCTCCAAATACTGTTATTGGTGCTGG
CCATGATTGTGTTCCCTACACGATCTCAACAGACAGAGATCAGCTGAAATTAGGCACGAG
CTTGGATAGTAAAGAAGAAAATAACACAAATACAACTATGTCAGCTAAAGCTAGATTCCA
GCAAATAGACAGAGTTGCAACAGTCGACTCCAATGATTCAACTATAAATTCCAAACACAA
AAATGCAATCAAGTAAatt
>DjScaffold3398:18137-27030 strand:+
AAATATGAAAGAAAATCAGTTAAAAAACGAAGAAATGAAAAATATAATTTTTTCCACTTA
AATGATTCGGAAAATAGTAGTAGTTATGATGAAGAAAATGGAATACGAGTAGCATCGAAA
CAGCAATATTTGAGATTGAAAAAAAAGAATTTCATTGAAACGCATTGTCAGAAGAAAAAA
ACAAGTAAACAAAGAGGTTTTAATCAAATAAAAATTCCATCGCAAAATTCAATCTTAAAA
GAAATTCATAATATAACTCCAAAATCAACAGAAACTGTAGTAAATGATTTCAGTGATACT
GCATCGTTATTTAACTTTCATTTTCAACCAAATTGGTCAGACTTTGAAGACAATGATGAC
ATAAAATTTGTTTCAGAAGATGAATTCGATTTGAATAATGTACTTCAATTATTGGATTAT
CATCAGACTTTTGAATATGATGAATCGAATTATCCATTCGGTTATCCTAATAAATGTTTT
AATAATTGGACACCCGAACTTTGCGATGAGAGATATGATCAAACCGCTATTTTAGCTAAC
AATTTAGAAAAGGTTATTCGAAATAATTATCAATCTGTCTTCGAATTGGATCATGATGTG
GTTTTTTGGGCTGATATTCACAAAGAAATTAATAATTTATTGATTATTAGCGAATCAAAC
AAATTCCTGTTACAAGATATTTTATTAAAAGGTTAGtttttatacagaagtttttttcca
attggtaatcttattactttaatacaagggtcttcaactcacggcctggccaatatatct
gcgcacaataatatataataaagtaacaaaccgacgttgaataaaaataagataggtaga
atccgcctaacatttttttaattatactcttttttgcaattattaaaaaaatgcgttca
tttattttgaatataccaccaccattagataactacggtagctaaacatgtataatatct
aaaatatgtcaaatttggattactgattttttgacagatgaaaaatgttgaaataatttt
aaaaaatacaaacaaaatgctttaggaaaagttgattaaattttcaactgctaaataa
>DjScaffold3921:42100-42820 strand:+
aaaaggatttgaatttattttgattttgattttgtcttcaagttttgatttttcattaaa
tagaatactaattttcttttgtttattgcgaaagcttatttctttatattttgtaatttc
tttatttcttaataatttagtaatttctatgtatttctttatatttgatattagatcacc
aaaaatgatttcagatgtcttatcaatttgtgtcttttctctgatagcatttttatcggt
gaacaactttttaaacaaaaaggtggtcttgcaATGGGTAATAATTTATCTCCAATCCTA
GCAATAATTTTCATGAACTTCATTGAAAATGAAATAATTCTTAAATCAAACAAGAAAATT
CATCTATGGTTTAGATACATAGATGATATTTTCTTTGTAACTAATTCTCCTTTAGAGACC
ATTCTAGAATTAGCCAACAGTATTAATAATAACATTAAATTCACTTTAGAGAAATCAATT
GACTTTCAGATTCCTTTTCTTGACACTTTAATTCAAAAGAAACCTGGTGAAATATTAACT
ACCCAATTATATATGAAACCATTTCACAGTGGACATATATTACCTTGGAGTAGTAATACT
ACCATCGGGCAGAAAATTGGTATAATTAAAGGTGAAAGAATTCGAGCTGAGAGACTATCT
TCAAATGAAGAAATG
>DjScaffold3921:22122-22388 strand:+
ATGCCTTTCGGTATAGCTGCAGCACCTGAAATGTTTCAAGAACTAATGACAAAAGTCTTA
GGAAGATTGAGAGGAACGATGGTATACATGGATGATATATTGATATTTTCAGAAAATTTA
GAGCAGCACTATAGAATTTTAGACCAAGTTTTTAAAAGAATTCAAGAATCAGGATTGAGA
ATCAATCCGGAAAAATGTCATTTATTGCGAGCAGAAGTCAAATTTTTAGGGCATATCATA
GACAAGAATGGAGTTCAAACTGATCCA
>DjScaffold2642:10823-13185 strand:+
gctactgaagcttttaaaATGATCGGTAAATCATTTGCAGTATTGTCAGATCCTGATAAA
CGGAAACGGTACGATGAAACAGGAAGCATAGAAGATTCACAACCAAGACGTCGGAATGGT

TTTACTCACGAGTTTTATGATTGTTCCCGAGGTTTCGATTCTGATTTCACCGCAGAAGAG
TTGTTTAATGTCTTCTTTGGCAGTGGTAATTTATCAGGTTTCACAACTCGCACTACTCGT
CATAGAACTGCTCATCGCCCAGAACAAGAGAACTCAAATTATGCGTGGATTCAATTTTTG
CCTCTCCTCTTTCTATTTTGTATGTCCTTGTTTAGTGGATTCTTTGTCAAAGATCCATAT
TATCAACTGCACAAATCCGAGAAATACAACAATGAGAGGATCACAAGCAACCTCAATATT
CCTTATTTTGTCAAGAACACATTCGAGCAAGATTTTGGTCGTCAATCGATAAATCAGCTA
GACAGAGAAGTTGAAGCT
>DjScaffold893:36224-41914 strand:+
tagccgtttttatataatcgaaatcaccaaggttttccaccgttttcaaattttacatac
tcgaataattcaaacaaccttactgggttcaatacccctggaatcactaaattcgataac
aattcctcgagaaccttcgataattttcaaatccaagttcacgtaaatcaaaataatgac
gctttcaacgatcctaatgtttatgagatcagtagtgatagtgaaagcagtgatgatact
agtattgaatgtaacacttcagtagtttcaatacaaactacagaaatagaatacacgcga
ataaccacgtcgatatcatctttaaaatctctagaattcgtcggatttgttgtcgatata
attgtggaatttaaacctctcaatcttgacaatgttgtgtttttcaaaatcagaagcgat
ttatattattatttcaatttatttaccggactgaatctcgatccttgtttatttaatgac
atcgttttgaatgttctttcatctctgaaatgcgaggaattgtatgaagatctgatttac
ccggatacttctggtaaatattgttgcttgaatttatcaacaactaaatacaccttgact
catctaccatatcttgaatttaatATGAATACAAGTTTTGAAGTCGAAGTCATGGTTGTC
AGTTCCGACGGGATTTGTGCTGTAAGATTTTCTGAAGATTCTTCTGATTTATTGAGGTGG
AGTAAAAGTCGTCCTTTATCTGATCAAGCTAAAGTTGTTATAGAGAATCAACAATTTTGG
TCGTGGATTCCTGGTAGTTTGTGTCTAGTTCAATTACCAGATAATTTAGTTAAATCTCTT
CGATTATCGTCTGTTGAACGAGCAATAATCGTATCAACTGAAATCGCATCGCCTACTGAT
GCTCAGAAACATAAATGTTGGCGTCAAATTCTTCATAAATCGTCGTTTTATCTCATAGAT
CATAAGCGTTATATTAGTAGTATGCCATCATTGAGCAGCAGCTTTCAGGTCGTTAAGCGC
AGCGCTTATAGAACTAATGACACAAATATTGCTTATGTTTGTAATGTATCAATTCGTGCG
CGTTGTATGCCATCAAAAATACAATACATTAAAATTAATAATTTCAAAGATGTTTCTCAA
ATATTGTATAATAAGCGTGGAGATTTCCTTCCAAATAAACATTTAATTATTTATCAAGTT
CATCGAACAAGTCTAATGATGAATTTCCAACCGGTTTTTGAATGTTATTCACGAATTAAC
AAAAATGACTTGGATACAAATCGTGTTCTTGAAAAATATGACGACTATGTTGTTGAATGT
CCTGGCAAAAAAATTAATAACAAAGTTGAGAAAAAAGTAAATAGTGAAGAAATTGAGGTG
ATCGTAATTTCAGATAGTGAAGAAGATGAAGTTGAATTGAAATCAAAAACGAAGCATGAT
TCCGATATGTCCATCAGTTCCACGGAAGATGAGCCTATTCTTGTTCGAAATCACTTAAAA
ACCTTAACGAATGGAAATTCGTCTAAAATTTCCACAAGTGAAGTTAAAACTGCAATTGAT
ATTAACCAATTAATGAATTCCATTTCAAAAAAAGAAAGTGAAGATGATAAAATTTCAGTT
ATTTCCTTTACAGACGATGAACTTCAAGAAAAAATATCGATAAGTTCCAGTAATTCTGAC
ATTGAAGAAACTGATAATGAAAATATCGAAAACATACAAAATATTGATAATATTACACTT
GAATCTGAAATTGTACCTGAAACAAAAAAACCAGAAAAATGTATCATACCAATCTCTACT
GATTTATGCCATTATTTCCATGAAAAAATTGTTAAAATCATGTACATACAATCAGGATCG
GCCGTGATTGGTCTTCTTGAAACAACTGCTTTAATGAAACGTGAATTGAAAATGATGTCT
CTAATACACCAGGATTTATTTGGTGTCATCAATCAGATTTCTATAGGAGATTATTATTTA
TGGTGTTGTAATGTTGAAATTGAATATATTAAAGATAAAAAGATTGTTCTTGTAGAAGTA
TTACAAGTAATCCAAAAGGATATGGCATTAGTTTCTTTAGTGCATTATGGATTGGTAACT
ACTGTTAAACGCGACGATTTGCGTCACATTCCAAATTTTAATAATAACTCTGGTAGTGGT
GGTTCTAATAGTAGTGATCTTCAAGATTTAATCGGCCCAATAATTGGTGCATTTGATCTA
CATATGGTACCGAATTGGTATAGTACAGAATTAGACACTTCACTTACAGATCAATTATTC
ACTGCATATTTTATCAACGAAAATGAACTTGTGTTGGAATACTTTGATGATCCTTGTAAA
TTTTTCAATTTACAGTTGATCGAAACTATTGTTAATGATTTAAATAATAATCACTGTAAT
ATCCGCTCTAAATCTTTTATTACTACTTTATCAGAAGCGTTGCTAAACTGTAGATTATTT
GTGGGTGACTTACTAGTATTGATGACGACATACAATGACTCTTCATCTGTTGGTTATTCT
CTACCGATAACAAATACATTGGTTCAAATAACTGAAGATGATGAACCTGGTGTTTGGTTT
GCTTCAATCGATGTGGAAAATGAATTTGGTGACCTAATCAAGAAGCTGTCTCCAAACTGT
TTCGCTGAAGGAAAGTTCTGCATTGATCGTGTTCAAATCAACAAATCAAAAAATCAACTG
CAATTATTGGACTATCAAAATATCCATGTCGACATTCCAGAAAATTTCCTAGGATTATAT
AAGTTTGACAAGAGTTTACTGAATGTATTCAACAAGTCCAATGCAGAATTCCTATCGCGT
TACATGCTTATTGGCGCAAGTGAAATCCCAGATTTAACTTACTTGCTTAACAAATTAATA
TTTTGAacagtttttgcatttccatagattttgtgtttagactttttgtaatttgtttgt

atagattcggtttgtgtttgtcaaataatctttgtgcaataaatttttataataataaa
ttctgtaatttttatattttacaggtgttgtaatgttgaaattgaatatattaaagata
aaaagattgttcttgtagaagtattacaagtaatccaaaaggatATGGCATTAGTTTCTT
TAGTGCATTATGGATTGGTAACTACTGTTAAACGCGACGATTTGCGTCACATTCCAAATT
TTAATAATAACTCTGGTAGTGGTGGTTCTAATAGTAGTGATCTTCAAGATTTAATCGGCC
CAATAATTGGTGCATTTGATCTACATATGGTACCGAATTGGTATAGTACAGAATTAGACA
CTTCACTTACAGATCAATTATTCACTGCATATTTTATCAACGAAAATGAACTTGTGTTGG
AATACTTTGATGATCCTTGTAAATTTTTCAATTTACAGTTGATCGAAACTATTGTTAATG
ATTTAAATAATAATCACTGTAATATCCGCTCTAAATCTTTTATTACTACTTTATCAGAAG
CGTTGCTAAACTGTAGATTATTTGTGGGTGACTTACTAGTATTGATGACGACATACAATG
ACTCTTCATCTGTTGGTTATTCTCTACCGATAACAAATACATTGGTTCAAATAACTGAAG
ATGATGAACCTGGTGTTTGGTTTGCTTCAATCGATGTGGAAAATGAATTTGGTGACCTAA
TCAAGAAGCTGTCTCCAAACTGTTTCGCTGAAGGAAAGTTCTGCATTGATCGTGTTCAAA
TCAACAAATCAAAAAATCAACTGCAATTATTGGACTATCAAAATATCCATGTCGACATTC
CAGAAAATTTCCTAGGATTATATAAGTTTGACAAGAGTTTACTGAATGTATTCAACAAGT
CCAATGCAGAATTCCTATCGCGTTACATGCTTATTGGCGCAAGTGAAATCCCAGATTTAA
CTTACTTGCTTAACAAATTAATATTTTGAacagtttttgcatttccatagattttgtgtt
tagactttttgtaatttgtttgtatagattcggtttgtgtttgtcaaataatctttgtgc
aataaatttttataataataaattctgtaatttt
>DjScaffold2132:1639-9812 strand:+
attttatttagtattatataatactcattggtccataaattgattgctgctattcagaATGA
TTATTAAACCGAGATTCATTTTTAATCTGTCCAAATTTAGAAACAGTTCTGTTAGAAATC
TCATTTTAGGCATAGAAACTAGTTGTGACGATACAGGCGTAGCTGTTGTAGATTTAGATG
GAAATTTGATTAGCGAACACGTTCATTCTCAAATTAAAATAAGTTCAAATATCGGTGGAA
TTATTCCAACAGTTGCCAGACAGATGCAATTATTCCATCTCGACCCCACTGTCAGAACAG
TTATGAGCAATAGAAACTTGTCTTTCAAAGATTTGTCAGCCATTGCGGTAACAGTGACAC
CTGGTCTCTCATTATCTTTGGAAATCGGCAGGAATTATGCCGTTAATAAAGCAAAAACAA
ATTGTATTCCGATAATCCCAATTCATCACATGGAAGCTCACGCATTAACTGCAAGAATGT
TGGATAAAAATGTCGATTTTCCATTCATGGTTTTCTTGATTAGTGGTGGAAATTGTTTAT
TGGGATTAGCAAATGATATAGAAGATTTTTCATTATTTGGCAAAAAATTGGATTTATCTC
CCGGTGATTGTCTTGAAAAAATTGCTCGTCGTTTAAACTTTGTTGGCAATTATGGAACAC
ACACCAATATCTCCGGTGGCAGAGCAATCGAGTTATTTGCAGAGGAAAATATTGACAACC
TTTTAGATTTTCCATGTTTTCGTACATCTAAAGCTGATTGCAATTTCACTTTCTCAGGAC
TGGAGGGAACAGTTTGTACATATTTGAAAAACTGCGAACAAAACGGAGTGAAATTGAATT
TGCCTCAATTGTGCGCATCAATACTGTATTCAATGGCATTTCAAATGTGCAGAAAATTGC
AAAGAGCTATGATGTATTGTTATAGATATGTAGATTGTCCACCGAAAACATTGGTAATTC
GGAAATCGTTACATTAAtaataattaaaa
>DjScaffold2687:9525-18402 strand:+
gtgattgattgtttacactacataaactaaagaattacaacagaaattcaaaacattaac
aATGTCTGAAACCTATTCTTATGAAGGATTGACCGAAAGAGATAAAAAAGTGGTAATGGC
TGATTTAAATGAGGATCCGAAAACTGTGAATGCACACATTGAAAGTTTTCGAAGATGGGT
GAAATCGATGCCTCATTTAAAATGTCCGACAGATTCAAAATTTTTATTGATGTTCCTTCG
AGTAGCAAAATTTGATCACTCAAAAGCTCAAAAACGATTGGATAATTTTTGTACAATTAG
ATCATCAACTAGAGGACATCCGGAATTTTTTGAATATTTTAAATATGATGATCCGATTCT
GGAAACATTTTTTCGCACGAAACAACAAATACCTTTAGGAAGAATGGAAAATGGAGAAAT
TTTAAATTTTGGAATTCTTGGTAACTGGAATCCAAAAGAAATACACATTAAAAAACTAAT
GACATTAATGCTTATGGGATTAGACGTGCAATTACAAAGTGAATACGCTCAAATATATGG
ATTGAAATCGTTCATGGACATGCATGGTTGTGGTAAAAATATGATGGAAGCTATGAGTGA
TCCGAAATTTTTAAAATCAGTTACGAGAATTTGGCAGGTAATTTAAttcttttctgaaaa
attatttgtgaagcaaatttctgagttcaagggttaatggtctgacaattttccgcgaca
caaccgtaaaataaaacctgataattgtaaaaaaaagagaaacaatttattaataacatg
gaaaaaatatttttgattattgaaaacgctgagattgtttatcagccgaaatgagactta
tgcgaggtataatattgttgaaaattctcccaaaaaatgggcaaatgaatttcgagaatt
ttataaaaaaggttccccgtgggcatctatttctgttgatgagagtaaaaggccaatgga
aacgttaaagtatatggaacaatatgctgattatccaggtgaaaacgttggaacgaaagg
aacatatactaaatttgatgcgttctgagcattttccatttaaaattttagtttgactca
taaataaatttaattaaaattgaatgataatgataattgttaaaatatatttgatcatga

tga
>DjScaffold229:45340-55636 strand:+
ctggattgatcccaaaaattgatcaatgctggcctttgtttgccaaaatgatgaatattt
ttttacttattaaactttgttttaggaaataaaattttaattgaaatttcttatagcaac
aacgctcattcatacccaaatttttgaaaatttatgttcatacaattgaattcttttatc
aacatttatccagactttttttaattataatttaaagatttatatattattctagtttta
aatttttcaaattaattaataaaaacaaataataaaattatttcagataaaaccaatagt
cattcacgtgaagaaacaccaacgacaaatacactaccaattccaatctcaagttctggt
agaaaatctccatcaaaactaaaaacattttttcatagtttaccagctaaattcgagtct
gacaaaagattatcattatttttacacaaaacacataaagaaagtgtagaaaatacagca
agttctaatcaagatgttagattaccaaatattaacatttcggaaattgaaaatgaaaaa
aatgatgaattcaaacgtcctgttgatcttcgacagaaagctgattcgtcaaagttatct
ttaaattcgcttttcaaaaagcgaccaaagaaaattcgtgattctgggctcagtctgaat
agttcagctagtactggaagtagtagtcgtaaaagtgatacaATGACAAATCGAGAAAGC
ATGTCAACTGACGGGAAAGAAATAGTAATTTCATCTAGTGATCATTCTCCAGTTATTCAT
ATTGCACAAGTACAAAAAACAGAAAAACATAAAGCTAAAATTGAAGTTTTTAAAGCTAAA
GCAAATAAAGTTATACGTTCAACAAGCTGTCTTCATAGTCCGAAAGTAAAACGATCAAAA
CACAATGTTGCAAAAGTAATGCATAACGTTGCCAATTTTTCTAGTATCATTATAGCTGCT
TCATCTGACATCGGAAATCAAAGTGAAAATGTTACAAGAAAAGTTCTTCCGTATCTGCTG
AATAACAGAGGTAAAAGTGAACCTCCAATGGATGGGCTAATTAACTCAAAGAATGATATA
GTGTCAAATGGAATAATCAAATATTATTCATGGAATGACATTAACAATGATGAAAAAGAT
CATTTAAAATTTAATGATTTAGAATTAACTACTTCTATTGTCCACCCAATTTTAAATGAT
AGCATGAATGAAAATATCGATTTTGACAATAGTAATCATGCAAATGAAAATGAAAATATA
AATAATATTCAATTTACCAGCATTGATTTAATTAATGATGGCATAAATGATGAAGAGGTT
TTCTGTACAACTTTTAAACCTTCTAGCAGTGTTGAAATATTTGATGAAAATCAAAATGTT
ATGAACAGCAATTATACTGAATCGTCATATTCTACTTTTCTACCATCACAAAATAAAGCA
GCAACAAACAATAAAATAGATATAAAAATTGATAATGATTTTCTACCAGAGTCCAAATCA
CAAAAGTTATCATCACCAACCACTAAACTTAAAGACGATCCAATGGAATCTCTTGATAAG
TTGATTAAAAGTATTCAGATAAGTTGTGAAGAAATGAGAGCTGCCAGAAAATCTCAAGAA
TTTTGTACAAACGATGAAATCGATAAAACATCTGTTGAAAATGATTTAATGAATATCGAA
AATAAAATGTGTCCATTAGAAACACATCAGCTACTTTTAAATAATCAAGATTCTATGGAA
CTGATTTTAGATGACGAAAAAACTATGGTCGAGTCTGAGGAAAAAATCGTAGAAAATTAT
AATTTTAAAGAAGAAATTTTACCTACATTAAAATTTTCTGTTGTGGAAATCAATTCATAT
CGAGAAATGTCTCCTGAATTGATAGAAGTTGCAGATTCAATTGCGAATTTATATGTTTCT
CCAATCAACTTGGTACAACAAACGTATCAAAGAATGATTAGAAATCCGATAATAGTATCT
GGAATTTTTGCAGCAACTACCTACACTGCTCCAATTTGGTGCTCTTATCTACCACGCAAT
GTGTGCCTGTCTAAACTATTCTATGGGACTTTAGATTATTTGTTTTACAGTGAAGTTGTG
CAATCAATTCCAAACCAAATAATATCGACTGGTCGCAATACAGCCTCGGCTGCTTCCAAA
ACGATAAAGCGTTTCTTATTTTGAtagtctacattataactcagttaattcaaatctatc
gtagcttgacagtcatcaaacttattttgtaaaatagcatttttggtgaagatttcagat
caattatttggatcatatttttgagtgcctcctatttctttaaacatttatacccaaaat
tatagataaatctgatatttcatacaaagatctttctgatttcttttgttgtttaaatat
ttcgatattttgcatgattaaatatggttttgttatttcgatttttcgaatatgaaaaag
attcattataaaataaaaattataatcatttaatttttgaaaaatataaataa
>DjScaffold229:140299-141070 strand:+
atttctcagcatagcgaaataagatgtttgtttttatttatacattttttaaaatgctac
tgtagttatttaattattaaccgttaatatataattggaaattttaagtaatttaatgtc
aaagaatttttggaattaaatctataaatataataactattacttaaaataaataaaaat
atttctagtatcaATGCAATCAAGAATAATTGATAGTGATATAATGATTATTGCGGAGGA
CATTCCTACTACTCGATCGCATTGCTTACTTTCTAAATACAAGATAAAAAATGAGTCCAT
AATTAATGAGACTGTTGATCTAACCCAAATGGCTTCTCCACCTATGAGTGAAGTAATTCC
CAGAAATCCGGTACAATATCAAGTGCGTCCACAGTATCCATTAAACCCATCACGTTTTAG
AAATGAGTTGTCAAATCAGACCTTTGTGAATATATGTCCACAAGTTAATTCATTGCAAAA
CAATATACAAAGAAATCCAATTTCAGATATGCATGTCAATCGAAACCGTGGTCCACAAAA
TTTTATTAATGGAAATGTTGTTCCTAATGTTGGAATTGTAAGTAATGGTGGGAGTTCATC
GTCTGTGTTAACAAGCAGTGTTTCAATTAATTCAAAATCTAACTTGCATAATATTGAATC
AGATTTAAATGATTGTTGTAAAGAATTAGCAATGACAAGTCTACCTGATTTGTTAGTTTC

file:///C|/Users/czh/Desktop/Data_S1.txt[2019/7/4 17:05:10]

CATCCAAAGCCGAAAAGGCTGTACAAACATTTCCGAACTTCAAAATATAGGCAGTGTTAA
TATGCACGAATTTCTGAAATCAACTCCAGCTGAATCATTTTCTCGAAAAGGCTTTTATTC
TTGTGCTGGTTGTTCTTCAAAATTTACCTCATCAGACATGTATAATTTTCATTTGCGTCG
TCAAATTGGAGATTTTAACTATGACTGTGTCCATTGTTTAAAAGTAATAAAAACGACCAA
CATATGCCAAATTGTTATACATTTACTTACTAATCATAAAGTTTCCACTCCTGCAATGGA
TAAATTATCTTTTAAATCTTTTCTAAACATCAATGAATCAGTTGAAGAAATAAATGAAGA
TTTCGAAGAAACTGCAAAAACTAGTATAAAGAAATGTATCGAGAATTTTCTAAATGATTG
GAATGTCGATATGTTTAATTCTTACCAAAGAGCTCCTAAAGATCAAAGATATTTTTTATT
ACCTATGGCCAGAAAAGATGTTGTTCAATCTCATAAATTTTATTCTGATCATTATCGGGC
AGCCAATCAGAAGTTGAAAGATTACCCGGTTGAACTTATGCATTGTTTTATGTGTGATTT
CGTCTGTGAAAGTCATAATGATCTCATCAATCATCTCCAAGGCGATTGGCCAAACAGTGT
AACAAAATGTGCCTTTTGTACTTTATCAGTTCCACTTAACAAAGAATTTCTCTGTGCGGC
TAAGGCACACGTTTTGTTACACATGGGAATTTTTATTATGTGCCCTCGATGTGGATTTGT
GCCACCACCAGATTTAAATCCCGAAGAGTTTGAAATAACTCTCAGATTGCATTTAAGATA
TGTGTGTTTTCATTTTAATTTGATCCAGATTTTTATTTGTCCTCATTGCGGAGACTCGAA
TAGCAAAGCATTCAATACAGCCAGTCAGGTTACTATGCACATTTTTGATGAACATTTAGG
AGATTATAGAACTTGTTCACTTTGCTCAACAAATAATAACGTTCCCAATGAAGGTCCGTT
TAGCTATGCAGAAATGACTCAACACCTTTACACAGTACATAATAGATCTGATATTGCAGT
TATGTGGAATGATTGTATTTTTACTGGGAAGAAATGTGCTGAATGTACAGAAATGATTGC
TCAACGAGACGCTTATTTCTCCCATATGATGAATAATCACAAAATTCAAATTATCAAAGG
AAAATCAGCGTTCCGATGTTTTGGTACTTGCAAACGTATTCATCAATCAATTGATGAATT
CCAAAGTCACAGATCTGAATGCTCAGAATGCATTAATGTTAAGAACAAATTATATCAACC
ACATCTACCTAATGCTTCACTGGAATCTACTTCACTATGTTTTTGTCTTTATTGTGGGGT
TGGTGTTCGTCGAAAGAGGCTAGAAAATGGTGACATAGAAACACAAAGTGAACCACAAAT
GTTCGCAAATCTTAGTATTACCCACGAACATGAACAGGATCATATTCTATGTGAAACTAA
TGTAACGGCTTGTCCTTGGTGTCCTAATCGAATGTCCGCTAAGATCAATGAAATGACTCA
ACATATGGAGACTCATTTACCTGAAAATTGGTTTCTCTCATGGTATACAAAACGAGCTGA
TAAATACAATAACAATAGAAATGATATTGTCTATTGTTATTGTTACGCTGCTATTGACAA
TCCATTGTCTTTACATTGTCACGCTATTCCCCAGATGCTTGGGAGTAGCAATTCTAATGC
ATCAATTACTAAGCATCTTGCATTGACCGATGCCATCCATAGAGAATTTCTTACTGCTCC
TAGACATCGAATACCCGGAACTTGCCCTATGTGTTCAGAATCAGATATAATAGCTAACTC
AATCGAAATGTCATGGCACATTTTTTTTAAACATCGGAATGTGCTTTGTTATGTGTGCAA
AAATTTTCGTCTGAGCAATGCTGATAACAGTCCAAAATGTCGTATAACTATGTGGAATCA
TGTTTACTCATGCTTAAGGCGGCATACTCCAAAATTTAATATGAAATCAGGTTTAAGTTT
GTTGCAGTCAATTGCTAATTGTGAATCAGTGTTATCAGATTGTACCGTATTTGATTTGAA
AAAGTCTGGAGAGATCCATGTTGGATTATTTCCAGATATACCAGAACAAGATGATACCGC
TTTAAGTAGTGGAAACACAGAACTTGCTAAAAGCATATTTTCTGATCTCAAATTTGAATG
CGGTTTTTGTAAGGAAACTGCAATACATTCGAATTTTTTCTCAACAAGAATCATGCTCAA
TGATCATTTAAAAGCATGCCATGGAATTACAGGGGATTCACAAACTGCTAATGATTTCTT
GTGTTACATTTGCGGATTAAAATTTGATTCTCGTGATAAATGGGAAGATCATACATTAGC
AGAGCATTCTAAAGGTTTCAGAAAAATAGGTCTCAGCTTAATCAATCCATCAGCTGTAGT
ACTAGACACGACTTGTACTTCATATAGATGTTATCTTTGTAATCACAGATTCGACAAAAA
AATATCTTGTGCCAATCATATGAAAACTAAGCATTCCATAGATGATGGCGAGCTTAACTG
GGGTATGCTTACAGACGCATCAGTTATTTCAGATACTAATAAAAGAGCTAAACAAAGTAT
CAATAGTTCTAAAATGTGTCTTTCAACATATCCTTCCAAACGTTTGAAAGTAGAGAGTAG
TTCCATCTCAGAAGAGAGTTTATTTATTTGCGGAATTTGCCCGTTCCAGTGTAATATACG
AAAAGAAATGATTGACCATGCCATTTCTTTTCATAGCAAATCTTCAGATAAAAGTCTCGA
CAATCCAAGTTATTTCGTGTGTCCAGATTGTGCACTATCGTTTATATCAAACAAGTCTCG
AGCAATACACCTGAAACTGATCCACAAAATTTCCAATCCAACAGTTTTCATGGAATCTAT
TCTGGATTGAtgaagaattttagttttattcaattctgtatattttgtaaattaataat
tacatattgctcattattgatgttttcgtggaatgacctttgtgtgtgtgcgtgtttgtg
tgtgtgtgtagctataattataattattattgatttgtatgtataatattttgacaatg
gaatttcgttcttcttcgttgatttctcagcatagcgaaataagatgtttgtttttattt
atacatttttaaaatgctactgtagttatttaattattaaccgttaatatataattgga
aattttaagtaatttaatgtcaaagaatttttggaattaaatctataaatataataact
attacttaaaataaataaaatatttctagtatcaATGCAATCAAGAATAATTGATAGTGA
TATAATGATTATTGCGGAGGACATTCCTACTACTCGATCGCATTGCTTACTTTCTAAATA

CAAGATAAAAAATGAGTCCATAATTAATGAGACTGTTGATCTAACCCAAATGGCTTCTCC
ACCTATGAGTGAAGTAATTCCCAGAAATCCGGTACAATATCAAGTGCGTCCACAGTATCC
ATTAAACCCATCACGTTTTAGAAATGAGTTGTCAAATCAGACCTTTGTGAATATATGTCC
ACAAGTTAATTCATTGCAAAACAATATACAAAGAAATCCAATTTCAGATATGCATGTCAA
TCGAAACCGTGGTCCACAAAATTTTATTAATGGAAATGTTGTTCCTAATGTTGGAATTGT
AAGTAATGGTGGGAGTTCATCGTCTGTGTTAACAAGCAGTGTTTCAATTAATTCAAAATC
TAACTTGCATAATATTGAATCAGATTTAAATGATTGTTGTAAAGAATTAGCAATGACAAG
TCTACCTGATTTGTTAGTTTCCATCCAAAGCCGAAAAGGCTGTACAAACATTTCCGAACT
TCAAAATATAGGCAGTGTTAATATGCACGAATTTCTGAAATCAACTCCAGCTGAATCATT
TTCTCGAAAAGGCTTTTATTCTTGTGCTGGTTGTTCTTCAAAATTTACCTCATCAGACAT
GTATAATTTTCATTTGCGTCGTCAAATTGGAGATTTTAACTATGACTGTGTCCATTGTTT
AAAAGTAATAAAAACGACCAACATATGCCAAATTGTTATACATTTACTTACTAATCATAA
AGTTTCCACTCCTGCAATGGATAAATTATCTTTTAAATCTTTTCTAAACATCAATGAATC
AGTTGAAGAAATAAATGAAGATTTCGAAGAAACTGCAAAAACTAGTATAAAGAAATGTAT
CGAGAATTTTCTAAATGATTGGAATGTCGATATGTTTAATTCTTACCAAAGAGCTCCTAA
AGATCAAAGATATTTTTTATTACCTATGGCCAGAAAAGATGTTGTTCAATCTCATAAATT
TTATTCTGATCATTATCGGGCAGCCAATCAGAAGTTGAAAGATTACCCGGTTGAACTTAT
GCATTGTTTTATGTGTGATTTCGTCTGTGAAAGTCATAATGATCTCATCAATCATCTCCA
AGGCGATTGGCCAAACAGTGTAACAAAATGTGCCTTTTGTACTTTATCAGTTCCACTTAA
CAAAGAATTTCTCTGTGCGGCTAAGGCACACGTTTTGTTACACATGGGAATTTTTATTAT
GTGCCCTCGATGTGGATTTGTGCCACCACCAGATTTAAATCCCGAAGAGTTTGAAATAAC
TCTCAGATTGCATTTAAGATATGTGTGTTTTCATTTTAATTTGATCCAGATTTTTATTTG
TCCTCATTGCGGAGACTCGAATAGCAAAGCATTCAATACAGCCAGTCAGGTTACTATGCA
CATTTTTGATGAACATTTAGGAGATTATAGAACTTGTTCACTTTGCTCAACAAATAATAA
CGTTCCCAATGAAGGTCCGTTTAGCTATGCAGAAATGACTCAACACCTTTACACAGTACA
TAATAGATCTGATATTGCAGTTATGTGGAATGATTGTATTTTTACTGGGAAGAAATGTGC
TGAATGTACAGAAATGATTGCTCAACGAGACGCTTATTTCTCCCATATGATGAATAATCA
CAAAATTCAAATTATCAAAGGAAAATCAGCGTTCCGATGTTTTGGTACTTGCAAACGTAT
TCATCAATCAATTGATGAATTCCAAAGTCACAGATCTGAATGCTCAGAATGCATTAATGT
TAAGAACAAATTATATCAACCACATCTACCTAATGCTTCACTGGAATCTACTTCACTATG
TTTTTGTCTTTATTGTGGGGTTGGTGTTCGTCGAAAGAGGCTAGAAAATGGTGACATAGA
AACACAAAGTGAACCACAAATGTTCGCAAATCTTAGTATTACCCACGAACATGAACAGGA
TCATATTCTATGTGAAACTAATGTAACGGCTTGTCCTTGGTGTCCTAATCGAATGTCCGC
TAAGATCAATGAAATGACTCAACATATGGAGACTCATTTACCTGAAAATTGGTAAttttt
cttattctttaatttctcagcatagcgaaataagatgtttgtttttatttatacattttt
taaaatgctactgtagttatttaattattaaccgttaatatataattggaaattttaagt
aatttaatgtcaaagaattttttggaattaaatctataaatataataactattacttaaa
ataaataaaatatttctagtatcaATGCAATCAAGAATAATTGATAGTGATATAATGATT
ATTGCGGAGGACATTCCTACTACTCGATCGCATTGCTTACTTTCTAAATACAAGATAAAA
AATGAGTCCATAATTAATGAGACTGTTGATCTAACCCAAATGGCTTCTCCACCTATGAGT
GAAGTAATTCCCAGAAATCCGGTACAATATCAAGTGCGTCCACAGTATCCATTAAACCCA
TCACGTTTTAGAAATGAGTTGTCAAATCAGACCTTTGTGAATATATGTCCACAAGTTAAT
TCATTGCAAAACAATATACAAAGAAATCCAATTTCAGATATGCATGTCAATCGAAACCGT
GGTCCACAAAATTTTATTAATGGAAATGTTGTTCCTAATGTTGGAATTGTAAGTAATGGT
GGGAGTTCATCGTCTGTGTTAACAAGCAGTGTTTCAATTAATGTACGTTTGATCAAATTA
TTAATAAAATATTTAATAAATGTAAACCAAAAGTTTTAAagttttaatcattttataatc
acaaaatttaacttataa
>DjScaffold1046:34975-53034 strand:+
TTAGAAAATAACAAAATCTTATCAATTCAAAGTCATGTCGTATCTGGTTTTGTTGGAAAT
AAATGTTGCATTTTACCTTTACAAATTTTAGGCAATGATGTTGATTTTATCAATTCAGTA
CAATTTTCAAATCATACAAAATATGAATTTTGTAAAGGTCAAGTTTTGAATTCTGACGAT
TTAAAAGCTCTTTACGATGGATTGAAATTAAATGGATTATCAAATTATACGAATATTATT
ACTGGATATATTGGATCTAAAACATTTTTAGAAGAGGTTTGTAATATAGTCAAAGATATA
AAATCATCAAATCCATCTGCTTTGTTTTATTGTGATCCTGTGATGGGTGATAACGGTAAA
ATGTACGTACCCAATAATTTACTTGACGTTTATCGGACACAAGTGCTTCCATTAGCAGAT
GTTCTTTTTCCAAATCAATTTGAGGCCGAATTATTGACAGGCATTGAAATAAACAATGAA
GATGATATGATAAAAGCCATGGACAGTTTTCATTTGAAAGGTGTGAAAATTGCAGTAATA

ACGAGTTCGAATTTGGGATCATCAGATTTTTTGTTTGGTTTTGTTAGTAAAGTTAATCCA
ATAGATGGATCAACGCAACGTATTCGAATGAAAATTCCGAAATTTCCTTATGATTTTGTG
GGTACTGGTGATTTGTTTGCTTCTTTATTTTTGGGGTGGTCTTTAAAACATCCGGATGAT
TTAGTGGCTGTATGTTCTAAAGTGATCGGATCAATGCAAATCGTGTTACAAAGGACAGTC
GATAATTTTCAATATTTGAAATCGAATAGTAATGTCTCGGAAAGCCGCGGTTTGGAATTA
CGAATAGTTGATTGTATATTAGATTTGATGAATCCACAGGTTTTCGATGGAATAATGAAT
ATTATCTAGcttagtttctatattgtgtgtcacaattatgtatttaattaattatttttt
aattttcataacaatatttctttttgtttttcatttcaaaatattctattaacttct
>DjScaffold1046:32961-33754 strand:+
ATGCACGAGTTATTGGAGAGATTAATTTTCAAAGGACTTATTGTATATCTAGACACTATT
CTGATGTACGGAAAAACTTTTGAGAATCACGACAAAATATTAGAGATAGTCTTTTACAGG
ATCAGAGAATCGGGATTAAGAATAAACCCAGAGAAATGTAAGTTTTATCAGAAAGAAGTG
TTATTCTTGGGACATACAGTTAGTGCAACTGGTATACACACTATTGAAGTTAAGATTCAG
GAGATTAAGAATGCTGTAGTTCCAGAATGTTCTTCAAAACTCCGTTCGTTTCGTGAACTA
ACAAACTACTACAGGCGTTTCATAAAAGATTATGCAAAGATAGCAACTCCTCTATATGAA
GAAACCTCAGGTTGCAACAAACTAATAGTTTGGACAGGAGCATGTCAAAACAGTTTCAAT
ACGTTAAAAGATAAGTTATGTAGGGCATTATGTATTAGAATATCCCAGGAAAGATTGGAT
GTTCATTTTAGAAAAAGGATACTGTGTGACAAGGAAGAAACTATTAGCTATAACTAA
>DjScaffold2926:22149-22919 strand:+
ATGAAAGATTGCCAAGTGGTTTTGATTTTTTCGATTCTAATTGGTTTCACACTGATTTTA
CCACAATCTTGTTCTGATTTAATATCGGAAAATCTTTGGAAAACATTCCAGTTGAATAAT
GAAGATTCAAAACCAAAACAACAAATAAAAGAAAATCAAACGAATTCTTTTAATATTTCA
AAGGATATCTCAATTGTACAATCGTCGATTTTGGGAGTGGAATTTCCGCATTTGGATGAG
CAGCCATTTGATGCAAATGAAAAACCACCGAATGAAGAAATGCTACGATTGTTTCGATTA
GAAAATTACAATTCGGACTTTCTGAGAATTTTCAAACCGAGAGAATCTCTTTTGTTTCCC
AACGGTCGAATACAAACAATTCCCTACAGTTTTATGATGAAAAACTCCGATAAAAAATGG
ATTTTCAGTAGATTTCTGTCTCTTCCTTATGGCAATAATTTAAAACCGCAATTAAATCAA
AAGAAACGCAGACAAATTCGTAAATTCCTAACGAATTTCAATTTCTGCCCGGTATTTTAC
AAATGGATTGATCGGGGTATCAAATTCTGGCCTCGATGGATAAAAGAAGGTAACTGTGTA
AATATTCATGGTTCATGTAGTATACCTGCTGGTATGTTTTGCCATGAAGACAGCCATAAA
ACGATAATGATTTTCCGATATATTTGTTTGAAAACCTGGTCGAAAGAAAACTGCAAATGG
TACCGAATGCATTTACCAATAGTGACGTCATGCCGATGCTCGTGTGGGTAA
>DjScaffold741:111508-112318 strand:+
aattATGCTGATAAATACAATTTGTAATTGGTTCACAGTATTAACAGGAATTTCTTCACT
TTATTGTGATTTTGATTTTGTGTCCAGAGAACAATGGGGAGCTCGAAATTCGAAAAGCAT
AGAATTATTAAACTCTACACCAAAATACGTATTTATTCATCACACTAATACACCTGGTGC
ATGTTATGATTTAGATCAATGTATAAACGCGATGCGCGATATACAAAACTTTCATATGGA
CATAAGAAAATGGGATGATATCGGATACAGTTTTGCAGTTGGCAGTGATGGACGTGTTTA
TGAAGGACGTGGATGGGATCGAGTTGGAGCTCACACTTTGGGATATAATGCTGTTGGATT
AGGTTTTGTGGTGATTGGAAATTATGAAACAAATCCACCAAATGAATTGTCAATCAATAC
ATTGATTAAATTAATTGATTATGCAGTTGAAAATGAAAAAATTGATAAAAATTACATTCT
TCGTGGCCACAGAGATGTTCGACCAACTTCTTGTCCCGGAAAAACTCTTTATGATATCAT
TCAAAAATGGCCGCATTATTGAattgtttgtattaatatttaaattttaaaacctgaact
tgctaattcatttccgatcaaatcactggtcacaatattttcatacataatatgtactaa
ttttataattacttagaatttttatttgcaaatatagttgtgtatggggggttttagg
>DjScaffold1260:8253-20141 strand:+
tggtagagcaatagacgtgccggtttatatagaagatcagacaatttctATGGATATTTG
CATGATTGATAGACTCTATCAAGACACTGTCGTTTTCAATAGTAGAGCGGCAACTGCTCT
TAAACTGCACTTTCATGTTCCATCTGAATTGAAAGAATATCTCGAGATTCTACCAAATTC
TGGTTTCATTCAAGCTGGGCAAAGCTTCAAAGCACAAATTAAACTGCTAGCTAGATTGGG
TTTACTAAATGGAAATTGTCCGTATTTTGATTTAGTCACAGGAATATTAGATGTTCCAAT
AAAGACATCAATAGCCAATCAGGTGAAAACTGTAGACTTTAATCTACAGGCTATATTAAC
AACAACAGATTTGGAAATCGTTGTTCCAGATTCAAATGAAAATATTGTCGAATTTGGACC
ATGTACAATTAAAGAAACCAAAATTAGAAGTGTTAAATTAATAAACAAATCTTTATTGGC
TCAAGAATTTGGATTTACCGGTTATCCAGACTTTATTGAAATTCAACCTAATTTTGGTTA
TGGGACAATTCTACCGAGTGAGACCTTAGAATTTCAAATTCTTTTTAGTCCAACAAAAGC
AGAAAATTTTCAATTCCAACTTCAATGTCAAACATTAATTGACAGAACATTTACGGTTTC

ATGTTCCGGAGTTGGTGTACATCCGGCTTTATATCTATCTCAACAGGTCATTAGTTTTCC
TCATACTGCAATTAATGATCGTTCGATTATTGAGATTTTCCTTCAAAATAATCATTTAAG
TACGAATATTTTTACTCACGAACCGCCGAAAATTGGATCTGGTCCAGTTGTACCAGTTGG
ACCACAAACATTCCAGTTTATTATTCCTGATAATTCACCGATATCTATTCATCCACAA
>DjScaffold1260:28472-30011 strand:+
AAACCGGATTTAATAATTCTTTCCAATTCTGGCAAATCAATTATCAATTTCGGTGAAAAT
ATATGCAAGAAAAAAGTGAAAAGAACCATTTCAATTCAGAATATTTCCAATGAACGATTC
CAGATAAAATCATCATTTTTGAATCCAAATGGAGTTTTTCAAGTTTGTAAGGCTGTGAGA
TCAATAGAGCCAAATGAAGTTATAAATATTTTGGTGTCTTTTCTTTCAAATGAAGAAGGC
ATTTTTCAAAGTACTTGGTCAATTTTCGGAAATCAGAAAAATAAAATTCTCAATTTAACT
TTTATAGCTAAAGCAATATTGCCAAAATTAGAACTCAAAGAGCTAATAAATGGCGAGTTC
AATTCTGGAGCTGTGCTCATTAACGAATTTAGTGAAAAACCACTAACTATTTTCAATAAT
ACTCCATTAAATATTGATTATCGAATTGAATTGTTCTCAGATTTCCAGCAGAAATCTAAT
AACTTGATTATTAAAAATAAAAATGCCCTATGCATCTTTAATGTTACACCACAAAGAGGA
GTTATAGCAGCAGGTAAATCACAAACATTAACGGTTAGCTTTTCACCTGACCATATCAGT
GATAATTATATTGAATGCACAAAACTTTTTATTTTCGATGATGTTGAATTTACCACATTT
GTTTTACGCGGGGAAGGAAAAAACAATACTTTATATTTAAAATATAATTTACAATCGGCA
ATTAAGTCTTCGGAGTATTTTGGAGAAAGTTGGAAATCAGAATCTGTTAAATCTGACGAA
CATATTGCTGATGAAAAGCAAACTATAGATTCGCCAATAATTTTGCAATCGACCTATTCT
GATGAGGGCAATTTCATTGAGGCTAAATACAGTTTTCTAATCGGATGTGTGCGGACTTTA
GCTGTGAGTCAAAAGAAGGTACACTTGCCAATGTTATATATATATATATATATACTGCTG
GATATATAAatttagatAAACCGGATTTAATAATTCTTTCCAATTCTGGCAAATCAATTA
TCAATTTCGGTGAAAATATATGCAAGAAAAAAGTGAAAAGAACCATTTCAATTCAGAATA
TTTCCAATGAACGATTCCAGATAAAATCATCATTTTTGAATCCAAATGGAGTTTTTCAAG
TTTGTAAGGCTGTGAGATCAATAGAGCCAAATGAAGTTATAAATATTTTGGTGTCTTTTC
TTTCAAATGAAGAAGGCATTTTTCAAAGTACTTGGTCAATTTTCGGAAATCAGAAAAATA
AAATTCTCAATTTAACTTTTATAGCTAAAGCAATATTGCCAAAATTAGAACTCAAAGAGC
TAATAAATGGCGAGTTCAATTCTGGAGCTGTGCTCATTAACGAATTTAGTGAAAAACCAC
TAACTATTTTCAATAATACTCCATTAAATATTGATTATCGAATTGAATTGTTCTCAGATT
TCCAGCAGAAATCTAATAACTTGATTATTAAAAATAAAAATGCCCTATGCATCTTTAATG
TTACACCACAAAGAGGAGTTATAGCAGCAGGTAAATCACAAACATTAACGGTTAGCTTTT
CACCTGACCATATCAGTGATAATTATATTGAATGCACAAAACTTTTTATTTTCGATGATG
TAATA
>DjScaffold1399:4206-13134 strand:+
gaatttttaaaaaaatcagctttggtgcgattattaaacattgaagtattgttATGCAAT
TTAATACAGTAGGTAGGTCGAGTACTATAAGGAAATCATTTACACCATCTAAATCAAATA
CTATTGGAAGATCTCACAAAGAAAATCTTTTTGAAACTTTTACAAAGCGAAAGAAATTAA
ATAGACAAGAAGTTGAAAATCTTACTCTTGAAGGAAAAAATGCAATTGAAAGTCCTTTTA
GGAGAGTATTAAATGAATTCGATGATTACGACATTAAAGATAAAACTCTTATTGTCGATA
AAACCGAGATGAATCGAGAAGAAGTTAAAATGCTTGTTGACATTTTAATGAATTGGATGA
ATAAAATTCTCGAACCAAAGCGGATAATTGTTAAAAATTTAACAGATGATTTATGTGATG
GTCAAATTCTTCATGAATTAGCTCAACATGTTTGTGGAACTAAAATTGAATTTCCTGAAG
TAACTATTTCAGAAGTTTCACAAAAACACAAACTCAAAATGGTTCTCGATGTATTGCATT
ACAAAATGGGTATTCCAGATGATGAAATTAAAGATTTGAAATGGAATGTTAACGAAATTT
ACGAAGGAAATGTAATTGCGATTCTTTGTTTATTAGTTAGAGTGGTGCAATTTTTCGATA
AAGATGTCAGATTACCGTTGAAAGTCGTTATCGATGCGACAAAAGTAATAAAATGTAAAG
ATAAATATGAACATTTAAAAGAATCATATGAAATTACGGGAATCATTGAGGGCGGCGACC
AAACTAAGATTGGAAAAGATTCATTTGACACATTATTCGCTTCCGCTCCTGAAAAAATTG
AAGATGTTAAAAAGTCACTTCTGAATTTTGTTAACGGCCATTTTAAAAAAATGAATCTTG
AAGTGACAGATATTCAAACCGAATTCAGAGATGGAATTTATTTAATTTATTTGCTGGGTT
TATTGGGCTATTATTTTGTTCCAACATATGCATATAATTTGCCTGCACAAGAGTTCAATG
TTAGATTGGACAATGTTAACTTTGTTTATGAATTAATGGAAGATTCTGGTATTCCCAGAC
CAAATTCTGTACCGGAGGACATCGTTCTCGGTGATTTGAAAGCTACCATGCGAATTTTAT
ATTCATTATTTTCTCATTTCAAGGACGTTTAAaatttaaattttccaaatattataaaag
ctcatcaaattgctttttctataattttgtataatttattatattataattttctaaaa
atatatgtcttttttaa
>DjScaffold61:15158-22069 strand:+

taataaactattattttaaatgaattatttcatgtactttattccttttagaaatatata
attaatttcatttttatcgtaattataaaaagaaaaaATGTTTGGAAATTACGGATCTAT
TGATAGTATTGATACCAAAGAAAATGTCCAGAATTTAGTTTCAAGAATAACAAATAATTT
TCAGCAAATTCGTCAAATAACAAAGGATCTCAACGATTTATATCAGATAATGGGTGGACC
TCATGATTTACCATCAAGACGAAATAAAATACTACAATTGCAAACAAGAGCTAAAGATCT
TTTGTCTTTGACTAAATCATTATTTGAAAGTATTTCACACTTAAAAATTGCTGATGAATC
ATCCCGATTAACTGTAACGAGAGTGAAAGAAGAATACAACATAATCATTCCCAAATACAA
TGAAATTCACGAGAAAATTATAACGAAAATGACTCAAATGGCATATGAAGCTCGACAATC
AGTATCTTTGATTGAGCCTTTAAAAGATGACGAACTTTTTATGAACTCACAAATTGTTAA
AGAT
>DjScaffold2865:33809-34566 strand:+
ttgaaaatcaaataattcttaaatcaaacaagaaaattcatctatggtttagatacatag
atgatattttctttgtaactaattctcctttagagaccattctagaattagccaacagta
ttaataataacattaaattcactttagagaaatcaattgactttcagattccttttctgg
acactttaattcaaaagaaacctggtgaaatattaactacccaattatatatgaaaccat
ttcacagtggacatatattaccttggagtaaaaattggtataattaaaggtgaaagaatt
cgagctgagagactatcttcaaatgaagaaATGAAATTGATTTCTCTCAAGTTGATAAAA
AATAAATTTTTAAATAATGGCTATCCAAATAAAATTATATCAAAATATTTATCTTTAAAT
TCAAAACAAAATAAAATCAAACCAAAAACAAATACAGTTATTAAATTACCGTTTATTAAT
GAGAAATGTAGTCAAATCATGCAGAATATAATAAAAAATGCTAAACTAAAACACAATCCA
AGAATTATTTTCAAAACGCAAGCACCTTTGGGCATCCAATTACAGAAACCAAGACAAACT
TTTTGTGGTCCCAAATGCATTTGTGATGGTAGAAATATATGCAATAAGAAAAATGTCACT
TATAAAATAACATGCACCATTTGTCAAGAAACATATATAGGTGAAACATGCAGAACCATC
CGAACAAGAGTTAAGGAA
>DjScaffold1367:2165-11636 strand:+
tttacttacagatacatgtgtcgttcaatgtcatttcttttattttgATGTTTATTGGAT
ACATTTATATTCTTTACTATGAAAAATTCAAATTGCAAGTTCATGGAAGTTTTGGAACAA
TTGTTATGTTATTGACTTTCATAAATATACTAATGGGATTTCTAAGACCAGATTTGAGTA
GTAAAAATAGAATTTATTTTTATTGGGCTCATTTAACAAATGGAATATTAATCAGAATAT
TCGGATACATTACATTTTGGCTGGTATTTTCAACAATAAATTTAAGAAATAGTATCCAAT
TGTGGTTGAAAATTGTTGTAATATCATGGACAATTGGAGAACTAATCTTTATCGGAATAT
TTGGAATTTTACGCTTCAAAATATCTCGAAAAAATGATGATAAAACTCTAGTAAATGCAG
CTTTCAAATATCATATCAGTAATTGGGCATTAATAATTTATATATTATTTTTGTGCACG
>DjScaffold1121:63840-65246 strand:+
ttattaattattaaatattctattactttcaagtATGAACCATTTTACTAAAATTTCTGA
GGAAATGATTATCGGTGGTTCAGCTGCAAGTTTCGCATGTATTTTTACTAACCCTTTGGA
AGTTGTAAAAACAAGACTCCAGTTACAAGGTGAATTAAAATCAAGAGGATCCTATTCAGT
TCATTATAAAAATTTTCTTCAAGCCTTTTTCGCAATCGCCAAATCAGATGGTATACTTGC
TCTACAAAGTGGATTAGTATCTGGTTTATTATATCAGTTTATGATGAATGGAACTCGACT
AGGATCTTATCAGTTTTTAGCAGGATACAAGTTACATATGAATAAATCAGGCGAAGTTAA
CTACGTTAAAGCATTAATAATGGGTGCAATATGTGGTGCTTTAGGATCATATATTGGTAG
CCCACTTTATTTAGTAAAAACACATCACCAAGCATTCTCCAAATCCGAGATTGCAGTTGG
ATTTCAACGAGAACGCCGAGGAATGATATCATCATTGAAATTAATTTATAAAGAAAAAGG
AATTACTGGTTTATGGCGAGGTTGGCAAGCACAAGTGATTCGCGTAATGGTGGGATCCAG
TGCGCAATTGTCAACATTTTCCAAATCTAAACAATTACTTCATAATTATCAAGTAGATTC
TTTTACAATGCTTTTTCTTGTTATAAATTATTTACTTATTAATAATAGCTTTATAAATTG
TCGAAATGTAATAAATATTTAGtctaaaaaatttcattaagtaaaagaatttataataaa
tcggaacaattcaataatataataaatttataaaacaatttggtaagtgacaagtggata
atttcgcttgatcggtataatataaggggtagaaaatatttgtgtgcacacacacattat
agacacacacatacacacatttatttattattaatttttaggaagtcccttttttaaattat
taattattaaatattctattactttcaagtatgaaccattttactaaaatttctgaggaa
atgattatcggtggttcagctgcaagtttcgcatgtatttttactaaccctttggaagtt
gtaaaaacaagactccagttacaaggtgaattaaaatcaagaggatcctattcagttcat
tataaaaatttcttcaagcctttttcgcaatcgccaaatcagtagctattatttcaatt
aattcacattagcaatcttacattaaaaggatggtatacttgctctacaaagtggattag
tatctggtttattatatcagtttATGATGAATGGAACTCGACTAGGATCTTATCAGTTTT
TAGCAGGATACAAGTTACATATGAATAAATCAGGCGAAGTTAACTACGTTAAAGCATTAA

TAATGGGTGCAATATGTGGTGCTTTAGGATCATATATTGGTAGCCCACTTTATTTAGTAA
AAACACATCACCAAGCATTCTCCAAATCCGAGATTGCAGTTGGATTTCAACGAGAACGCC
GAGGAATGATATCATCATTGAAATTAATTTATAAAGAAAAAGGAATTACTGGTTTATGGC
GAGGTTGGCAAGCACAAGTGATTCGCGTAATGGTGGGATCCAGTGCGCAATTGTCAACAT
TTTCCAAATCTAAACAATTACTTCATAATTATCAAGTAGATTCTTTTACAATGCTTTTTC
TTGTTATAAATTATTTACTTATTAATAATAGCTTTATAAATTGTCGAAATGTAATAAATA
TTTAGtctaaaaaatttcattaagtaaaagaatttataataaatcggaacaattcaataa
tataataaatttataaaacaatttggtaagtgacaagtggataatttcgcttgatcggta
taatataaggggtagaaaatatttgtgtgcacacacacattatagacacacacatacaca
catttatttattattaattttaggaagtcccttttttaaa
>DjScaffold1121:68921-69160 strand:+
ATGCCGTTTGGCATAGCTACATCGCCTGGGACATTCCAGGAACTCATGACAAAGGTTTTA
GGTACAACAAAAAGAACAACGGTGTATTTAGAAGATATTCTAACATATACAGAAAACTTG
GAAGAGCATTATAAAACATTGGATGAAGTATTTAGTAAAATACACCAAGCAGAACTACGT
TTGAGCCCGTCAAAATGTCAATTGCTTAGAAAAGAGATCAAATTTTGGGACACATCATAA
>DjScaffold4011:7295-18613 strand:+
atttacgaaaatgtcccattaaacgtttcggtactgaatttgaaaagtgaaatagaaaaa
tttcaaagtgattttaaatatgatttattaATGGGTTTGTCTCAAAATAAATACTTTTTC
ATAAATAAAAACTGGGATTTAATTGTATTAACTAATATTGATAGAGATAAAAATCCGGAT
TTGTGTAAAGTAATGACAGCAGATTATTTATGCATCTGGACCGGAGTAATCATGTTTCAA
GGGGATAATGGGATCATCAGTTTAAGAGTGGAAATAAAGGATTTGAACGATAATATTCCG
TTTTGGAATCAACCGATTCTAATTGAAATTTTGGAGAATAATCCACCCAATAAAATATTC
GATTTAACTCAAGCTCAAGATGATGATTCTCCGCCAAATAATGTGCAAAAATACCATCTG
GTTTCTAATAAATACTCATCAATATTCCAATTATCACAAGAAACAGGACCAAATGGCATC
ATACCAAAATTAACTATTCTTTCATCGATTGATCATGAACAATTTAAAGAATTTACACTA
ACATTAAGTGCATCGGACGGTGCTTCTCCATATCACGAAGGACGTGTAAATATTACGATA
AAAATTCTTGATCAAAACGATAATTCACCGAAATTTCCTCAATCGCATTTCTTGTTAACA
ATTCCTGAAAATTATCCTCTTCATAAAGCTTTGCCAATAAATGTGACAGCAGTTGATAAC
GATTCCGGTTCATTTGGTTTTATTAACTATCAAATATCTGATTCGTCACCACCGATTTCA
TTGAAAAAATTTGCCATTGATAATCACGGTATTATAACAATAATAAAAAAAATTGATTAT
GATAAAGGAGATAAATATTTTAGTTTTATTATAATTGCTAAAGATCAAGGAAATCCACCT
TTATCTACCTCTGGTACAATTGATGTAACTATACAGGATGTCAACGATCAGCCTCCAACA
ATTCTTATTAATTCATATTCAACATCCGGAAATAAGAATAACCCATCAATAATATCGATT
GAAGAAAACAATCGCATCGGTCAATCAGTGGCGAGAATTTCTGCTACAGACTCTGATAGT
TTTGAAAATGACTACTGTAACTGTACATTAAAATCTTTGATAGATTTTCAGTTGATTTTC
GAACGAAATATTCAGAAAATGACAATTTACAGATTAATAGCTAATAAAATATTCGATAGA
GAAACAAGAAATTTCATTAAAGTAACTGTAATATGCTCTGACACTGGGTCACCACCAATG
ACTAGTAGTGAGGCATTAGAAATCAGTGTAGAAGATGTAAATGATAATAATCCAGTGTTT
GAGAAAAATATTTACAATTATGTGGTTCCAGAAGGAGATATTCGTAATAAAATAATTTCC
AAATTAAACGCCACAGATGATGATTCTGGTTTAAATGGAAAAATCAGCTACAGTCTTTCA
CCTTGTTGCAATTACAGCGATCCAAGACCTGTTATAAAGGTCAACGAAAATGGAGAATTA
ATAGCTGTCTACCCACTTGACAGGGAATCAAATTTACAGGGTTACAAATTTCAGGTGATT
TTGATATTTAAAACAAAATTAATAATTTTACAATTGCAATTATTGCAAGAA
>DjScaffold4011:72492-83026 strand:+
attgttattattattattattattattatattatcacatttattagtattcttattatat
ttatccataattaacatgacaaagtcaaagaatattatttgataaaatcgaaaattgtga
aacgttaaattatttcaaattaatatttaaagcaaaataattatgttgttctttttgtgt
attttcttagagatattaaaataagttgaggattaattattttcttattttataataaaa
tttaggcatcgattgctctgacaatactagctcgagatcacgggaatcccagtttctcat
cgacaataattcttcatttacagctcacagacagccaagcaattggagctgaaaatagta
aactaaacaatcgaccgacaaatagcactcagactaatcagatactaataATGATAATTG
TCACTATTACTTGCATTATTTGTATTGGTCTCATAACGATTATTTGTTTAATGGCAAGAG
GAATTGTGTTCAGGCAAAGATATTGTAAAAATCTAACGGAGAAACAAATAGCACTTGATG
AGAGATCATCAGCAATGGCCGGACATGGTTATTACCCAGCTATGATATCAATGGGTGATA
ATGAATTTGATACAATGAGTCATTACAGACATTCTCTGCTTGATAACCCATACTGCACAA
TGCAAATGGCCACGATCAACTACAATAATAATATTAATCCACTGTGTTATTCTGAAAAGA
TGTCAAAAGATAGATATCTCACAACAACAAAACTAATAAATGATCAACAAAACGGTTCAG

ACAGTGACAGTGGCCATGGAAACAGTATGAATAATAATTCTATACCATGTGACAGCAAAG
ATAATCTAGTATATACATCATTCAGTACATTTTCATCTCAACCGATGTATTCTCCCATTT
CATCATTCCCAAAATCATGCAATTCACCTTCAATAGCATTTGCAAAAGTCCATGAAAATA
TCCCTTCAACCTTTGTGAAATATTCCACCTAGattctatatttaaacaataaaaaattac
atttcatttataataattactaatattattttattactattatta
>DjScaffold2418:106497-106859 strand:+
ATGAATTCATATTTGCTGTTTATCCTATTTCTTTGTTGTTTATTTTCAACTTTTACAATT
TCAGTTACTCAGCCAACCAAACCAGGATTGATTATCAGACATGAATCCTTAAGACATGTG
ACTCTCAATGATCCAGTTCATACAGAGAAATTAAGTTTACAGACTCGTCAAAAAAATAAA
CGCACAAGAAACAAATGCAAGAAAAAGAAATGTAATTTAAAGTGTCGTCAAAAAAGAAAG
GAACGTCGAAAAGCTTGCAAAAAGTTAAATAATTGTCCTAAGAAAGTTAAAGGAAACAAG
AAAAAATTATGCATTAAAAAATTTAGAAGTTCTATTAAAAGTCCATCTATTATTAAGAAA
TGA
>DjScaffold2418:15683-15769 strand:+
ATGGGATACAAACAGACCAAATCAAAGTTGATGCGATTAAATCCTTTGGTGAGACAAAAT
GTATCAAGAAATTACGTAGTTTCTTGA
>DjScaffold1247:4-14194 strand:+
TATTATTATTATTCAGTATTTGGCTTTGTGTCAGTAATTATTGCACAAAATTATGCATCA
TTAGTTGTTAAAGAAATCTCAAGATTTCCTCACCCAATTAATAAACCATTTTATCTTCAA
GCTCTACCGAAGACTGATACTGAACCACTTATTAATTTTCCCGGTGATATAATAATCATT
CAAATAGCTGATTCACATGGTTTAGTAAAAGAACGGGTGATGTTTAATTATGGTTTGTAC
GATCAGTTCGTTAAGCAATTTGATAAAAATTATATCAAAACTGTACAATTAAAAATCAAC
TCAATTTCCCCTTCAATAGATATTTCTGTCGTCTTTAGAAACAGCAGTAATCTGTATTTG
TTTGAAATTTATGTCATCAGCACTACATCAAAGGATTTAAATAGAAAAATGTTGAACGAT
TTAAAATTGGCGAGAAGAATTTATTGGAACAGAATAAAATTATTAAAACCATCTTCATTA
GATTTATCTTTCAATCCGATTGGAGAATACGACAATAGTTGGGATATCGGAGACGTTGTT
GTTGCATCTTGTCGTTACTACAATGGAATGGTTAAAAGAATACCACTTTTTAATAATTAT
CAAAATTTGACGATTATTGAAAGCTCATTTCATCCGGATCGAAATGAACGAACCATAAAG
TTTTCTGTCAAATTATCAAATCATTTAACAGTAATCCAATGTTTTGCAGGAAACTATTCA
CAACACCATATTATTAAAGTTAAACATCATCCCACCGAGGTTTATATCAGACCGGACCAT
AAGAAATCACTGGAACCCAATTCAACATTTGAATGCTATTCCAAAGATGGTTATCCTATT
TCTATAATTGATTGGATTCTTATTGATGGACCGATAAATGCATCTATATTTTACGATATC
AGTAGAAATGGGTCTCAATTACATTTCAATGGAAATATACCGCATAAATCAAAATGGACA
TTCATATGTGTAATAATTCACAATTTACCGAAATCTGTAAGAAAAATTAAAAAGAAAATC
TCATTTACTGCTAATAATTGTGATATGAATTGTATGAATCGGAAAATGTCTAAAACTATT
GACAAATCACTTAAAGAAACACAATATCTTAATAATACAGAAAAAACTAAAAATGAAAGA
CTTTTGGAGAAATATGGAAAAGAGATAGAAGATATACAAAAATTTTTAAATAAAACAATC
AGATTATTCAAACAAGAAAATAACCAATCAAAATTTAATCAAAAATTAGAAATTCAATTT
ATAAAAGAAGAATTGTTATCGATTAAATCCCAAATTAAAAATCTTTTTAAATCCGAAACA
AATAAATTATCAAAATTATCAGAAATTGAAAACAATTTTAACAAAAATTTGTCAAATCAT
ATTATTAAAGTTGAAAATCAGTTTAATACTGAAATCAATTTATTAAAACAAGAAAATCAA
AAATTGAAAAATAGTATTAAATCAATTGAACAAATTAAAAATGATAAACGAGTTCAAAAC
TATTTGAAAGAAAATCTTCAAAAGGAAAACAAAGAAATAAAAAAATTACAAAGCAAATTT
GAACAAATGAAAAAATTTATGGAAATGAAATTAATTAAGAAAAAAAACAATTTACTTTGT
GAATACTTTACAAATGTTGGTGAAATTGTTAACAAAGGACCAAATAGTGAAATCAATGAA
TGGGTTATAAAAAACTATCAGAATTTACAAGTGGACAATGAAGATATAAATAGTACATCG
TTTTATTCTCATGATTGGGGTTATAATTTACAACTCAATCTACATCCAAATGGATTTGGT
AAGGGCAAAGATTCATATCTCTCTTTATTTTTACGAATATTGCCTGGGGAATATGATAAT
CAATTGGTTTGGCCAATGAAACATTCGATAAAATTATCTATCAGAAATCAAGTAAATGGA
AAAGACTATTTTCAAAAAACTATTAAATTTGATAATAAAAAAGTCCAGTATTTTCAAAAG
CCATCTTGTAAAGGCAATAGATTTATTGGATATCAAAAATTTCTAATGTTGGAAAATTTG
GAAGATGAAGAATATTTGATCAATGATTCTCTTATTATAACTTGTGAATTAATTAAGTAA
atatttaatt
>DjScaffold3338:3657-4554 strand:+
tataatttttagaatttgagtattaataatttccaacttgttgaagtctgccttgataga
agtgttactgaaagaccaataagtgatgaggaatttccttgggatattattcagaggtta
cgcaaagattctatcagaagtttgagattaacaaggttttatctcaaagctattgaagat

file:///C|/Users/czh/Desktop/Data_S1.txt[2019/7/4 17:05:10]

ccacatggacctggtgttgctatatttgtaggaaatttggtggagaatctcaatcagaac
gtttatgaaaagATGTTACTAGAAAAATTAGGAATAAATAATAAATGGAACGCTATTGAA
GTCATTTATTATGAAAGTGGTTGTTTAGTTCTTTCTTACACTTGTGGAGAAAAGGCCGAG
ATTTGTTACGAAATACTCAAAGACACAGAAATAAAAGATCGACCATTAATGGTGATGATT
CTTCCAAGAATTCTACCAAGAATGATTCCAGATGACATTACTCCATTATTAGTTTTTGTT
AACTTAAAATCTGGTGGATGTCAGGGCACGCAACTCATTACTGCATTTAGAAAACTACTT
AATCCTCATCAGGTCTTCAATCTTGAATATGGTGGTCCACTTCCAGGATTGTATTGTTTT
AGGCAGGTTAAAAACTATAAAATTTTAGTATGCGGTGGAGATGGGACAGTTGGGTGGACT
TTGTCGTGTTTAGACAATACTGGTCAAGATGCTGCGTGCAACTCGCCTCCAATAGCTGTC
TTGCCACTTGGAACAGGTAATGATCTGGCCAGAGTATTGAGGTGGGGTGGAGGATACACC
TCTGGAGAAACACCACTGTCAATTCTCAAAGATGTGGCCATGGCTGAAGAATTAAAATTA
GACAGG
>DjScaffold3338:34012-35139 strand:+
tcattattgattattaataatttaaacgacgatttattattaacttcaaatcctaatcgA
TGATTTATTATGCTTATTCTTTGCTGTTTCGCATTGTAAGTTTTATAATTCTTTATTGGT
TATTTCGTTACATAATTGTTCCGATAGTAATATCGATAATTAAATTTTACTGGATTACGG
GTTTTCATAATAATCATAAATTCATAAAAGACAGTGGGGAATGGGCAGTTGTAACTGGCG
CAACTGATGGAATAGGACTTGAATATTGTCGACAATTTGCTAAAATTGGATTGAATGTAT
TTCTTATTAGCAGAGATAAGGAAAAGTTGAATTTAACTTGTAAAGAATTGAAACAATATT
ATAAAGTTGAGGTCGATTATTTCCAAGCTGATTTTACATCAAATGAATTTTATGGAGATT
TAAAAATTATCTTGAAAGATTTAGATATTGGAGTTTTGGTAAATAATGTTGGTATGATTG
AACAATTTCCGTCATATTTTCACAATCTTGATGATAATTTCTCACTTCCCAATATAATAA
ATTGTAACATTTTATCAGCTGTTTCTATGTCAAAAATAGTCTCAATCGGAATGTTAAAGC
GAAAACGTGGAATTATTGTTAATATTTCATCAGCGTCGTCTATTTTTCCAATGCCAATGA
GTGCTCTTTATAGTTCAACAAAATCGTTTTTATCTCAATTTTCTCGTACAATTAGTGAAG
AAATGTCAAACTCGGGAATTTATGTTCAAACTGTTCGCCCATTTTTTGTTTTGACAAAAT
TAATCGACAAGTTTCGTCCTCCGCACTGTCTTACTCCAACAGCAGAAGAATATGTAAAAA
CAGCTATAAAAACTATTGGTTGTGAAAAAGACACATTTGGAACTCTAAGTCATTCAATTC
AAGGAGTAATAGCTTCTAGCCTTTCTTTAACTATTGGAATCAAATTAGCTAAAATTGTTA
ATCTAATACGCCGGAATGCCTTATTAAAAGAAAATTAAattttattaatttgttggaat
cgaatattaaattttctatttt
>DjScaffold4437:13756-14587 strand:+
tgttactaattcatctttagaaaatattttagaactagccaatagtatcaatgataacat
taaattcactttagagaagtcaatcgactttcagattccttttctggacaccttaatcca
aaagaaacctggcgaattattacttacccagttgtatatgaaaccatttcacagtgggca
tatattaccctggagtaaaaattggaataatcaagggtgaaaagataagagctgatagac
tatcttcaaacgaagaaATGAGACTGACTTCCCTCAGGTTGATAAAAAAATAAATTTCTAA
ATAATGGTTATCCAAATAGAATTGTATATAAACATCTATACTCAAATACAGAATGTAATA
AAATCAAACAAAAGACAAATACAGTAATTAAATTACCTTTTATTAATGAGAAATGTAGCC
AAATCATGCAAAATATAATTAAAAAGTCAAAACTAAAACACAATCCAAGAATTATATTTA
AAACGCAAGCACCTTTGGGCATCCAATTACAGAAACCAAGACAAAATTTTTGTGGTCCCA
AATGCATTTGTGATGGTAGAAATATATGCAATAAAAAAAATATCACTTACAAAATAACAT
GCACCTTATGTAAGGATACATATATAGGAGAATCATTCAGAACCATCCGTACAAGAATTA
AAGAACATCTTAAACAGAAAGAAAGTGAAGTCTTCAAACACTTTCAAAACAAACATAATG
TTTTACCAGAAAATCATCATATTACATGGAAAACATTAGGAACAGGCTACATTTCTACAC
TTCACAGAAAGAAACATGAACAAATATTAATT
>DjScaffold5237:40897-48027 strand:+
atggaactttgtctaactttataaaatcaagatttttatgtatactctttagtatttatt
ttttgtagaatagtaaattgttttctgtttctgctgatggcactgttaaatgctggctaa
ttgatctttgtgttttggaagaaatcaagggaaatgctcagcataaatgtcttctatata
cttatacattcagtgatttggaaattccgacgtgtattgatttttaccaactactgacg
atctgtttgttgttgggtttaccaatggatcatgtagtgttattaatATGACTACCAAAC
AATTAAAGATACGATTTCAAGTTCCTTCATCATTATCTGAATGTGATGATAGCACATTTG
GAATTAGTAGAGTTAACTGTCTGGTAGTTCATCCCACTAAGCCAATGACAATTGTTGGAT
ATGATGATGCACATATTCGGTGTTTCGATAACAACACTGGTGAACTCATCTCTGCACTCT
TAGTACATCAGAAAGCAGTATCCTGCATAGCACTTGATTGTAGTGGAGGATGTGTTTTCA
CAGGAAGTCATGATACTGCCGTACGAATATGGAATATGGCTACTTGGACGTGTAATCAAG

AGATAATTTGTCATCGAAAGAAATTCGATGAGTCAATCCATCAAATTGCATTTCATCCAT
CGAAAAATTTGTTTGCAACAGCTGGAGCTGATGGCTTGTCTAAAGTGTTCATCTGAttta
atctgacagttaattgtaaatttcaacttcgttattttcatttatttgctattgaaaatt
tttgctatcagaattttttctgttattttcattaaccaattatttatattggttatcat
tgtagaatttgtaatagattattttgctttcaaatacaaatgatggtattcgt
>DjScaffold4756:49350-50098 strand:+
agacataattaattatctgagcagtttagatatggttttggaatatagaaaaggaatata
acatatttngttatctagaccaagatgtgaggtatgtgtacaatgtcaaATGTCACATGA
AGATGCCAAGAAAGGGAAGATTAAAACGAGAATACTGAATATAATTGAAAAAGAAAAAGA
AGATCATTTAAACATTTCTGAGGAAACAGATTTGATCAAGAAAATGAAAATTCCGGATGA
CAAACTACATGAAACAATAGAAGAAATACATAAATTATTATGTCATGCAGGAGTTGAAAA
GGTGTACGAATATATGAGAAACAAATATATTGCAAAGAATTTGTGGAGCCAAATACAGAA
GATAGTTCGTAGTTGTGAAATTTATCAGAAAACCAAAGTAGTGACAACAGTAACTAAAGA
GAAGACAAAGAGTTTAGAAGCCACCAATTTATTTTAAgtgatattcacagatatatgtgg
gccaatgcgggaaacaagaggacgaaaaaaatacattcttggaataatcgaccagtagac
taaatatctggttcttataccattgcctagacaagacgaggaaacaatacgaaaa
>DjScaffold4756:13834-15777 strand:+
ATGCCATTTGGTATTGCTGCTGCTCCAGGAACGTTTCAAGAACTTATGAATAAAGTATTA
ATCGGGATAAATGGTGTCGAAGCCTTGGTTTATTTGGATGATATCTTAGTTTTTTCTAAT
TCCTTGGAAGAACACATGATTCGTTTAAATAATGTGTTAAGTAGAATAAAAACTGCGGGT
TTAAGAATTAATCCAGACAAATGTTCGTTATTGAAAAAAGAAGTTAAATTTCTCGGACAT
ATTATTACAAGTGAGGGAATACGCACAGATCAAAGTAAAATTGAAGCAATAAGTAAATTT
GAAAGGCCGAAATGTATTAAGAGTTTGAGAAGTTTCTTAGGATTATGTAATTATTATCGA
AAATTTATAAATAAATACGCAGAAAAAGCTAGAATTCTAGAAGCATTGTGTGGTAAAGGT
AATGAAAAGTTGATTTGGTCTGATCAGTGTGATGAGTCGTTTAATCAATTAAAAATTGCT
TTACAGAAAACTCCAGTGTTAGCATATCCAGATTTCAACAAAGTTTTTATTTTGGACACG
GACGCTAGCTTTGATGCAATTGGAGCAATACTCTCACAATTAGATGAAAATGGAAACGAA
AGAGTTATTGCTTATGGATCACATGCGATGAGTTCACACGAAAGAGGTTATTGTATTACA
AGAAAGGAATTGTTAGCTATTTATTATTTCACTCAACACTTCAATCATTATTTGTATGGT
AAACAATTTATTCTTCGAACGGATCATAAGGCTATTACTTTTATGTTATCTACAAAAAAG
CCGATTACTTCACAATTCCAGACATGGATAAACTTTTTAAGCAGTTTAGATATTAAGATG
GAATATCGACAGGGAATTTTACATGCAAATGCTGATTCATTATCTCGACAATCATGTACA
ACTTGTTCCCAGTGTCAAATGAGTCATCCAGAAGCTAAATCAGGAAAGTTAAAAACTCGT
ATCTTGACATTAATGACTGATAATGTTGGAAAAGAATGGCAAGATGGAGTTCAAGAAATA
GAAGAAATAAAAAAGCAAATTGGAAAAACTAGAGGGGGTTATTTTCAGTCGGTGGAAGAT
ATTATAAGAACGAAAGATAATAAAATATGGATACCAGAAATTAAGAGAGCTGAATTTATT
AAAAAAACTCATTTTATTTTATGTCATGCTGGATGTGAAAAAGTAAAATCATATATTAAA
AATAATTTTGATATGAAAAATTTGGCAAGCTTTGTGAAAGAAATTATTGATGGATGTGAA
GCATGCCAAAAACAAAAAATAATAACTACTAGAACGAAAGAGGACATAATTCCAGAGACA
TCTTCAAGACCATTTGAGAATATTTATATTGATATTTGTGGACCATTAAGGTCAACTTGG
CATCAGGAAAAATATATATTAGCAATAATAGATCATTTTAGTAGGTATATTTCATTAACA
GCAATCGCTAAACAAGATGAAAATACTATAACTGATACTTTGGTTAAGAAATGGATTCTA
AAATATGGTGCACCTCGTAAAATACATTTAGATTGCGGGAAAAGTTTTGAGTCAAAGATG
ATGAAAGAGTTGGCTGATAAATATAAAATACAAGTAGTTTATTCTAGTCCATATCACCAC
AGCGCTAATGGATTAATAGAACGACAATTTAGAACAATTAGAGATCGTATAAATACTTCC
GTAACGAATAATAAGAACAAAAATTGGGCACATTTGATTCCTGAAATTCAATTTTCCACG
AATGCTTCAACTCAAAAAACGACTAAAAAGAGTCCAGTCGAAGTGGTTTTTGGTTTTAAA
ATAAACAGAACAGAAACGGATAAAATGCAAGATAGAAATGCTATTCACGAAGAAATAACA
GATAATAATAAAAACACAGTTGATCATCATTTGAGTATTAGAACATTTGCAGTTGGTTAC
ACAGTATTAGTAAAAAAAAGATAA
>DjScaffold4038:15448-25684 strand:+
tttaataaagtaaaaatcattgactctgaagcaaaaagtggaataaatattcagatttat
ataagtcgaatttcctattcaattattgtttgatattacatattgattttaatccgatca
agtaaaacttcaaagtcattaagtATGATAAACAATAATTTAATAAAAAGATTGATATTA
TATTCCTGCCTTTGTATTGAAATAATTTTTATTATATTTTCATTCTATGTTGGGATAACG
CAAAAATGCATTCTAGTTTTCATTGATCTTCTAAATGTATTTAATGTATTGGTTGGAAAT
ATTATCGCAATAAAGATCTTCAGGAATTTACAAATGAAGAAACCAAAAATACCTCGTTTG

TACACATTATTAGAAATTATAAACTGTGTGGTTTTTTATACTTTTCTTATTACTTGTATA
GTACACTCAGTAGAAAGTATTTTTCATCATAATACAGTAAGAGAACCTATTAAACTAATT
ATTCTTGGATTTGTCGGGATTTTTTGTCATTTCATTGAAATTATTACAGATATACTTTAC
TCAAAGGAGAACAGTGTAATTATGTTGAAATTTCAACCAAATCAAAAGTGTTCTTCAGGC
AATATTAATTTCAGTACATTAAAACTCGAGACAGAATATCTGATTGATAATGACAAAAGA
AAACTGACTAATTCATCGTTACTGAACTTTTACAAGATTTTTCAGAAAATTTATTTAAAA
TATCCAAGAATTGTGAGAATTTTTGTCATGTGGCACATTGCATTTTCTTTGCTCAGTTATC
ATTACTATTTACGGGTATCTTCAATTTAATAAAAGGATTCACGATATAGTTGTCATCTAC
ATCGATCAGGTAGCCAGTATTACAATGGCATTGGTTCTGATTTTGTCAAAATTATTTCCA
CTTATAAACAACGGTATAACAGTTATGGAAGCAGCTCCATCGCATTTATGGAATGATTCT
ACAATCATCCATCAAATCAATTCGGTTATTTATTAAcctatttgtttattagaaattgaa
attttttaaaattaagttgatcattttttattcattaaatatctattttaagtaaagaaa
ttagttaaaacaagaatttctttcataattgctagacaaattccatagttagtaaacaca
agaattttgtcaaaatacttattgctttgttaatttatagttttcaataaaatttgaggt
ttatactaataatactaataatacttataataataataataattatgataattattatta
ttataataataat
>DjScaffold438:50874-51466 strand:+
AATCAATCAAATCAATTCATCAATATTAAACATGAAAATGAAGAGAATATTTTAGTAGAT
CGAGATGAATATTTGATTGGATATCAGATATGTGGTGTGCATGTTATCAAGGATGATGAT
CAATCGAGGTTAAATGAAATGCAAATATTTATCAAAACAAGTGATGGTAAAATATTAACA
TTTCAAATGGAACATTCTAATAAAATCAGAGATGTAAGAATGAAAATTGAAGAGAAAACA
AAATTTTCTGCAGATCAACAAATTTTATTTTTTCAAGGTGAAATATTGGACGATGATAAC
AAAACAATTGATGAATATCAAATATGTGAGAAATCTGTGATCGATATTGTTGGTAGATTA
AGAGGAGGAATACAAATATTTGCTAACACCGTTGATAAAAAACAACTCACATTAGAAATA
TTCAAATCAGATAAAATAATCGATATAAAAAATCAAATACAACAGAGAATTGGATTGCAT
CCAGATGAGCAAAAATTGGTTTTTCTTGGAATAGAATTGAGTGATGACAAATCTATTGAT
GAATATCAAATATGTGAGAAATCTGTAATCAATATTGTACCCAGATTGAAGTTAATAAAT
AATGAATTTAATAATTATAATAATTAGtgcagaggtagaatgcaaatatttgccaaaaca
acagatgggaaacattgttagaaatgaaATGCAAATATTTATCAAAACAAGTGATGGTAA
AATATTAACATTTCAAATGGAACATTCTAATAAAATCAGAGATGTAAGAATGAAAATTGA
AGAGAAAACAAAATTTTCTGCAGATCAACAAATTTTATTTTTTCAAGGTGAAATATTGGA
CGATGATAACAAAACAATTGATGAATATCAAATATGTGAGAAATCTGTGATCGATATTGT
TGGTAGATTAAGAGGAGGAATACAAATATTTGCTAACACCGTTGATAAAAAACAACTCAC
ATTAGAAATATTCAAATCAGATAAAATAATCGATATAAAAAATCAAATACAACAGAGAAT
TGGATTGCATCCAGATGAGCAAAAATTGGTTTTTCTTGGAATAGAATTGAGTGATGACAA
ATCTATTGATGAATATCAAATATGTGAGAAATCTGTAATCAATATTGTACCCAGATTGAA
GTTAATAAATAATGAATTTAATAATTATAATAATTAGtgcagaggtagaatgcaaatatt
tgccaaaacaacagatgggaaaca
>DjScaffold4357:96652-96936 strand:+
ATGCCATTTGCCATCTCAGCGGCACCAGGGACATTTCAAGAGATGATGACAAAAGTGGTA
GGCAGAATCAATGGAGCCTTGGTTTATCTTGATGACATACTGATATTTACGAACAATAAA
GTTCAACACTATGAAATAATTGAAAAGGTATTGATGGAAATAATGAGGGTTGGTCTAAGA
ATATATCCAGAAAAGTTTCAGTTTCTACAACGCGAAATTAAATTTCTTGGACACATAATT
AACAGAGATGATGACGATACTTTTGAGGGTCTGAAAAATCTTTAA
>DjScaffold3701:13242-14129 strand:+
ttatatattttcatttgatatttaacattaaacatttttgttaagtatatttttgaaa
tattttccattgagttttttgtgattatcaATGAATTATTTGCGTATTTTAATTTCAAT
ACCGGTTATATTTTTATTATGCTTCGTTTGTGGCGTATATTCAATTACCTTTTTATTATT
GTATCCGATTAAGCTATTTTTCCCAAAAATATATTTTGCTATGGACTCGTTAGCAGTAAA
ATCATTGGTTAATGCTATTAGTTCGGCATTTAATATAGGCGGTTGTGCAGTTTATGAATA
TGGTGAAGATTTAAGTGATTTCGAGAACGAGAATTGTTTGCTTTTAATTAATCATCAATC
AACCGTGGACGTTCCTTTGATAATGCATCTTCTTAATTTTCGAAAATATTTAATTTTTCA
TTCTTCTTGGATGCTTGATGAAATGTTTAAATATGCACCATTTGGCTGGGTGTCTTTAAT
TCGTAATGATATTTTTGTGAAACAAGTTTGTTTATTTTTAAATTATTTAAGTTTCATATT
TAGTAAAAAGTAAttaaattttaaatgttaaatttacataagaaacgacaaagtaaatag
attttcgttaattctttttgttattagtgtatttaaaaatatgaatattggttaaataaa
tttaatgaaacgtatataaaaccactcaaattttactgttgagtcatttattgtaacacc

file:///C|/Users/czh/Desktop/Data_S1.txt[2019/7/4 17:05:10]

tggttggtttttactcaaattttattttaatatacgctacgtgaataatgctttatttgc
aagcaataaccgatatttaccgagaaaaataaaatttttgttaaccttaaatcacgataa
gaat
>DjScaffold3701:32297-38472 strand:+
gctaaacacaaaattaaatatattattgatatcacagttgcttatgacaatatagagtgt
ccgccatcgtttgtaagcATGCTTTTGGGTTCTCATAGTACTCGACCTGTGTATGTTTAT
TATCGAGTTTATGATATCGGAAAAATTCCAGTTGCATCGTCTTATGAGTTGCAGTCATGG
ATGTATAAGATTTATAAGGAAAAAAACGATAGATTGTCATTATATTATTCAACTCGGAAA
TTTCAGCATTTACCATCTGATGAAAAAGAAATTCCTGCAAACAGGCTAATGCATCGTCAT
AAATATGTTTACATTGGATGGGATTCGATTTTATTTTCAATATTGGTAATGTCATTATGG
ACCTATTTGTGGTTGTATATTCTTCCTGTGAAGTTTATGATGAATTTTCTAAATTTTTGA
acattatttctttccgataatcattattactcgtgataacgacatattcaagctatatta
gaaaaataagttcatttaaaaatatactttattaagtcgtttgtttagttttaaattgaa
caagttgaatgtttttattaaaaattcatttttgaataatattatgtatttatgttagca
aatggaaccgatttccaattcagttgcctttgcatttgcgagagtaagtagttttagcac
tacgtgacactcgatcggctttgcaaattcgttactgactgtcggtgatttcccttcttt
tctggaattatttttgtcagattttttctcacctgaattaaaaatataaatttttgatg
>DjScaffold5034:18151-18561 strand:+
ATTTATCTACTTCTGCCATTTTTGTCCAAATCAGTTTTCTTAAATTTCGACAAAGTCTGT
GACAATAACTCTTTTGATACAAGAACTGGATTCTTTTTGTGTAATGTTATATCAGCAAGA
CATTGTTTGAATCTTTGTGCTTCTGCTGAATGTTATGAATGGTCATTCTACAATTATGTT
GATCCATTTGACGAAGCATTGCGATCAAACCATGTTTGCCGGTGCTTAAGATATTTAAAT
ACATGCTACTATGCTTTAATGACTCGGAAATTACGAAATTTAAAATTTGTTTAAaaataa
attcggtcttattgaatgaaata
>DjScaffold5034:20677-21090 strand:+
ATTTATCTACTTCTGCCATTTTTGTCCAAATCAGTTTTCTTAAATTTCGACAAAGTCTGT
GACAATAACTCTTTTGATACAAGAACTGGATTCTTTTTGTGTAATGTTATATCAGCAAGA
CATTGTTTGAATCTTTGTGCTTCTGCTGAATGTTATGAATGGTCATTCTACAATTATGTT
GATCCATTTGACGAAGCATTGCGATCAAACCATGTTTGCCGGTGCTTAAGATATTTAAAT
ACATGCTACTATGCTTTAATGACTCGGAAATTACGAAATTTAAAATTTGTTTAAaaataa
attcggtcttattgaatgaaata
>DjScaffold148:7750-8327 strand:+
TTTAAATTACGACAATGCATTGATGCTCATGATAATGATGTGAAAAGTTTATTCGTTTTA
TGTGATGGTTCTAGTTTTCTTTCGGCTTCCAGAGATTTATCTGTAAAATTATGGCAAGAA
CAAAACGATCTTTTCGAAAAACGTCAACATTATTTATTTCATAAAAGCTTTATATCAGTT
GTTCATTTCATGGAACCATGTTTGGGATATCCCAATGGTTTAATATTCTCTGCCGGATAT
GACAAAATAATTAATGGGTATTTTATAAATAGTTCGGAAGTTCAATTCTCACTTATTGGA
CATACAAATGCAGTTACTTCCATCACTACTACCAACAATTGTGAATTGATTTCCGGAAGT
ATGGATGGGTCGGCGATATNCTAAaacgataaaaatgaatttcaaaggccacaaacatgg
aatatggtcaatcatacttttgaatagttt
>DjScaffold5142:85177-92603 strand:+
CTTTCTGATTTGGGATCTGCAAAATTTGATAAAACTACAAAAACATTGAAATTGGATCTT
CCAATAGAACAAATACCAGAAAATTCAGAAAATATGAATGAAAATTTGACAAACATATCG
AACAATTTAATTGAAGAACATCAATCAGAAATATTGGAGGTGTATAAAGAGACTAGTTCA
CAAATCTCTGAAGAAATAAATGTCCAAAATCCTCCATCAGATATTCCCGACAATATAGAA
GGGAATGAAAGTGAATTTCATGAGGAATCTCTCTCATATAATATTGATCAATATCGTCAA
GAATTTAATGAAATTGAAAGAATTGTTCCGGATATCGATTTGAAATTTCGCAACAATTCC
AAATTGGCTTTCTTTATTTGTAATATAAAAAATGTTGAAAAATCGAGTTTGAAATTTTCA
TGGAGCTCAGTTTCAACAACTAAACAAGAAGTGATTGTTTCATTTTCTAATTGCGGTCAA
GGAGGATGGAAAAATTTGTATAAATTACCTTTAATTATTGATTTCAAAGGGGAAATAATT
TCCATAAATAATGACCTTCAGTGCTTTGTATCGAATGACAACATGGTTATACAGTTACCT
TTACTAATCAACTCACAACTTATTGAATCTATAACATTCCGTGATCAGACTTTTCAATGT
AAATATGAAATACCGGTCTATTTCACAGATGAATTGTGCAACATAGTGACATCACATGGA
ATCGAAAATAACGAGGAAACTTTTCAATCTGCTTATGGTATGATAAATAAGGAAACACTT
AAAATAATATGTGCTCCGATTGATTTTACAATCGATTTAGAAAAAGAATTTCCTAAGATA
GATGAAAATATTATCAGTGATAATTGTGATGATAATGATGATGAACTTACTCATTCGGAA
CAGAAAATAATGGAAATAAAAATCGAAAATGAAGAAATTATAGAAATGAAAGAAGTCCAA

AATGAAAATACTAGTAAA
>DjScaffold5142:75811-85158 strand:+
agaactacaggatccagaaaacagacgaaaatatgaagaagaattaacagctATGGAAAA
TGAGAGAGGTATGGATGTTGAATTTATTAATCCACTACCGGGACACGTTTTAAAAACAAT
CCAGAATTCAGAGAAAAAAATTTTTATTAATATTTGTCAATCAGACAAAATTGAAAAACC
AAGTGTAACTAAATCAATTAAAGGAGATATTCGAGGAGCAAATTGGTCTATTCCTCATAG
TTTTTCGCCACCCAGAGAAGATGTCGATAAGAAAAACGATAGTTGCCTGGTATATGATGT
AGTTTACAATGTAAATGCTTATCAAATGGGAACTAAAAATCCTCGATTTCGTAAAATGTT
ACATGATACAGCATTAGAAGGAATTGAAAAAATGTTTAAAGTTAATTTAGATAAAATCAA
TTACAAAATACTGAAATTAAAAATGAAAGGTGTTCCTGTTGCAACTGTCATCAGAAAGAA
GAGATCTGATTTTGAAGAGAAACAAACAGAGCTTAAAACTCGAGATGATGATCCATTAAA
TTTTCCTTATCCTGAAATAAAAGAAACTGCAACAACAGAATATAAAGTTAATGAAGAAAA
AAAACATGATAAATCTGATGAATTCACAAAACCAAATTTTAAAATAAAATATTCAAATGA
TTTTGAAATGATGAATTACGTTGGAGATGAAATAAGGCATTCAATCAGACCGAAACGAAT
GATAATTGAAATAAAATTACCAATGATTTCTTCAGCTAATTCTGTTGATTTGGACATTGA
TAATCGTACGCTTAAATTGACGACTGTTAAACCCGTGAAATATTTATTGGAACTG
>DjScaffold930:46984-62021 strand:+
tataataaaataaattaattaattaaaatttttttattaaggacaatcttggtaaatttt
tacgttgttgcgaagatgattttggattaagtccacttattttgtttgaaatatcagatt
ttgaagatttgagcgaaaaatcgagctatcaacttgaatcagaaaaaagattagaaagag
tattgatatgtttattccacttggctaaatctgtagaaaatcatctaacatcctttacag
ttgattcaactccgtttcaattactcttgaaaagtcaagatattcgagaaatgaaaaaaa
ataaaactgctgccaacactgatgattcggtttttgaatctactgttccagaattgatac
aaaaacaaactccaaattatttagcacataaagccgaacaaacactgaagcagacaaaaa
tgaatcatgaaattcttcaaaaatccttggaaaatgatgattggcaggaggttatttaaa
cattgtagcgaacgttctaacaaattattcttttatttaaattaaattttcaaaaaacac
gtttcaataaattattttaatttaaatattaataaataaaaaacattaaaatccagaaaa
atttaaacattttaaaaaaaataaaatattctaaacatattgcagataaatgatgaagga
gattgtaaaagtagttcatcgatcccagatattgatgaagaaATGAAAGCTATGATTGAA
CGAGTTAAAATGAAAAAACTTGCCGAAGAACAGAAAAGACAATTCACTCTTTCTTCACCT
ATAAAATCATCATTTGCATCAGATAGTGAATTAAGTACTTCAACTGGATTAGACAAGTTT
GAAGAAGACTCATTGAGTATATCAGAAAAAAATGAAAACGATCGTTCATTATCACTTTGT
GATAATCAGTTAATTCCAAATGTTACTGACAAAATTATTTTATCAAATAACGAAATAAAG
AAACCCGATAACATTCCCATGGAATCTGACACTCGTATTGTACAAATAAAATTAATTCAG
CGATCAAAACAAGATAGTGATCAGTTCTGGGGGATGGATTTAGTAGATAAATCTGGCATA
ATTCTGGTTTCTAAAGTTAAAATTGGAAGCACTTCTGATGTTTGTGATGTAAGAAAAGGT
GATGAGATTATTTCCATAAACTCGACAAATCTGACAAATCAATCACTCGAAAGTGTAAAA
TCAATATTCACTAAATATGCTTCTGCATTATCTCCAACTGGATTTATTACATTATCTTTG
AAGCGAAACGTAAATGAAGATGAAGACGAAGACTTTCTTTATAGTGATATTTCAGAAAAA
ATTGAAGATGATCAATATCATCCAGTAAAAAGTTCAGAACTTGAATGCAATTCTAAAATA
CATTTAAATCACAATGATGTTAAATCTATCGCTAAAAATTTTGAACAAATAGATAATTTA
AGCATTGAATCTATAAACACAGAAATGTATCCAATTATAACAAAAGAAATCGCAAATAAA
TCATATGAAAATGCAGATAAAAATTTAGTCATTTCAGAAACTGTTGAACGAAAGCGAAAT
TATGATGAGAAATCATCTAATTCATTAAACAATATTTCTATTATAAAACCAGAAATTCCT
CGTCTCATTGCTAAAAATACAAATGAAGCTGATAACGATTCTCAGCTTTCTGTTCAAGCC
AAAATCGCCAAATTTGAATGTCAAAAAATATCAAATCCTTTAAATTTAAATTCCTCTTTG
AATATATCTAGAATCCCACAGAATCCAGAGTTTTCATTAATCAATACAAAATTCCGGCAA
CAGTCAAGTTGTGATGGTCTATCATTGAATCAAGATGATACAATTTCAAAATTATCAGAA
GAAAATCATTCCAGTATTTCTAATTTGTATAATTATTGGGAAACGGAAGCACCAAAGTCC
ATTGACTCATTATCTAAAAATTTAATAAATAAAAATGCTCCATATATTCAAACTCCAGTT
TATTCTCTTGTAAACGAAAAAGAAAAGAACGATAAATTTATTTTAGAAGCAGCTGAACAA
CATTTGGATACTTCAAATGTAAACACTGTTAAAACTTGGAGAAATGGTCGTTCCTCTTGC
AATTCTAGTACTTCTGACGATGTATCTGCATTAAACGATTCCATTAATATGCCTCAATAT
GGATCATGGGGGAAATCAGTTGAGAAAACTCGGGAAAATCCTGCAAGTACAAATGCAAAA
AGCAATTCAAGTGTCAGCATAACGAGGGAATTCTCGCCAAATGCTCCGGTGTCCACATCA
TCTGGAAATAAATTTGCAAACACTGATATCAATTATAATCCAAAAAGCAATATATCCACT
GGTATTCAAACAAATATTCAAAGTCGACTTCAACCGGTTTACACAACAATAACTACAGCT

TTCGAAAGTCTATCACAAAAACCTGGGACCAATTCAATATCTAAAATTTCAGACGAAGAA
AGTTATTCTGTTTCTTCCACAAAATTCGTTGGAAATATTTGCAATTTTACTGGAACCTTA
GTGCCATCATCGGAAATCGGACAACCAATAGTAAATCCACAAATGTCATGGCTTCCAAAT
AATAATAACGATTTGAGCAAAGATTTTACATTGATTAGTTCATCAATTACTGGAAAAGAA
AATCAAAAAAATCTCTCAGAAACTTATTCATCACGAACAATTGGATCTATTATTTCTGGT
AGTTTAGATGAAAATGAGAAATCAATTGATGTTGAAGTTTCCGGTCCACCGAAATTGTTC
CCTATGAGAAATAAAACATTATTACCTAAATTAAGCAATGATAAAATCCTTAGTCATAGA
ACCAGTGATCCGAATTTAAAAAATCAATCATTGGAAGAAGATGAAACAACATTAAAATCG
AATTGTATCAAAATCACTGACCTCGATGATGTAATAACACCAGTTAATCAAAAATCTAAT
GCATCCATTAATATTGATATTAAAACCTCTCAGTCGAAAAAATATGATGGTGATTATTTG
GATATTACAAAAATGAAAAATTCTCCTAATCAGTGTGTTGCCAATTGCATTAATAGTGAT
TCCTTTGATAAGCACGAAATTAATGATTGCTCTGATGTTTTGGAATCAAATATCGATATT
GAACCAACTGTTTCGGCAATTTCAGCTCCAACTTTATTTAAAAGTAATAAAAATGCAGAC
AATACAATCTCTGTTGGTATGACTCTTTCGAAATCGGAAAATTACGTTCCTATCAAGAAT
GAATCTAATTCATCCGATAAAAAATCTAGAAAAaattttttaaggtcgacttcaaccggt
ttacacaacaataactacagctttcgaaagtctatcacaaaaacctgggaccaattcaat
atctaaaatttcagacgaagaaagttattctgtttcttccacaaaattcgttggaaatat
ttgcaattttactggaaccttagtgccatcatcggaaatcggacaaccaatagtaaatcc
acaaATGTCATGGCTTCCAAATAATAATAACGATTTGAGCAAAGATTTTACATTGATTAG
TTCATCAATTACTGGAAAAGAAAATCAAAAAAATCTCTCAGAAACTTATTCATCACGAAC
AATTGGATCTATTATTTCTGGTAGTTTAGATGAAAATGAGAAATCAATTGATGTTGAAGT
TTCCGGTCCACCGAAATTGTTCCCTATGAGAAATAAAACATTATTACCTAAATTAAGCAA
TGATAAAATCCTTAGTCATAGAACCAGTGATCCGAATTTAAAAAATCAATCATTGGAAGA
AGATGAAACAACATTAAAATCGAATTGTATCAAAATCACTGACCTCGATGATGTAATAAC
ACCAGTTAATCAAAAATCTAATGCATCCATTAATATTGATATTAAAACCTCTCAGTCGAA
AAAATATGATGGTGATTATTTGGATATTACAAAAATGAAAAATTCTCCTAATCAGTGTGT
TGCCAATTGCATTAATAGTGATTCCTTTGATAAGCACGAAATTAATGATTGCTCTGATGT
TTTGGAATCAAATATCGATATTGAACCAACTGTTTCGGCAATTTCAGCTCCAACTTTATT
TAAAAGTAATAAAAATGCAGACAATACAATCTCTGTTGGTATGACTCTTTCGAAATCGGA
AAATTACGTTCCTATCAAGAATGAATCTAATTCATCCGATAAAAAATCTAGAAAA
>DjScaffold4752:29120-29830 strand:+
tgtcaaaacagctccaactaaaatagaaaccattgaaaaatttggtaaaccaaaatgcga
ttaagaagatgagaagctttctatgaatttgcaattactattgcaagtctattaaagatt
attcgaaaaaatctagattgctggaacaactgtgtagtggaccgatttttaatggaATGA
AAGAAGCACTAATTATGTCTCCAATATTATGTTTTCCAGATATAAATAGAGGACTTATTT
TGGACACGGATGCGAGTTTCGACACGATATTTCAAAGGGACAACAATGGACAAAAAATAG
TAATTGCATATGGGTCACATGCAATGACGAGTCATGAGAAAGGATATTGTACTATGCGGA
AAGAAAATCTCGCAATTTACTACTTTTGTATTCATTTCAAACATTACTCATATGGGAAGA
TATTTATCTTTAGAACAGACCATAAAGCTATGACTTTTATGCTAAATACAAAGAAACCGA
TCACATCACAGTTTCAAACTTGGATGAATTATTCGAGCAGTTTAGATAAAAAAAATTGGA
ATATAGAAAATGATCGCTCCATACAAATGCGGATATGTTGTCAAAGAGTAAATGCAATAC
ATATGCACAATGTCTAAtgtcacatgaagatcttaagagtagaaaattaaaaacaaggat
gctaattacagt
>DjScaffold1610:30760-31609 strand:+
tacatgcgaggtgcagttactgtaagtgagataggttaagtaagagaagagatgaaattt
agcggattttaagagcaggaacgtcgagtagttttgattagtttgaaaaataaatagtac
aatataggttcacaatcaaccaaatttaacatattcttatttaaaaattttttacttaat
ttcaattttctatattgtttttgtcaaaaaaatttgaataaatcttaaaacttccagct
ctcttcatacttcaattgattaatttggattttaattgattaatttttatagattttaat
tttagagagaatcaagtccaggaattaatgaaattATGCAAATAATGTTTGATTTATCCT
CGATATTAACTGAACTTGATAACGTTTGCAAAGAGTTTGACAGCATGAAACAAACTCAAC
TTGAAGAAAAAGAAAATCTGATTGAATACTTTAAAGATTCAGATAAAAAACAAAAATCTA
TATTTTCCAAATTAAATGAAACAAATCAAAGAATTGCATTTCTCACTTCAAAATCAGTAA
AATTCGATAAATCAATTGAGAATATAGAGAATCGAACCAGTGATTTCGAAGACAAAATGG
ATTATATTGTAACAAGAGTAGATACACAGAATTATGGTTATTTTATGTGGAATATTGAGA
ATTTTTCGTTAAAAATTCAAAATGCCAAAAACTGGTCAAACCCTGTCGTTTACAGTGATC
CATTTTATACACACAAATATGGATATAAAATGAGATTATGTATTTATCCAGATGGAAATG

GAGATGCC
>DjScaffold1610:17321-17461 strand:+
ATGCTAGAGAAAGAAAACGAGTTGAACAAGTTAATAATGCTTTTGCCGAATTACGAAAAT
GTATACCGATTGAAAATCGTAAAAAGCGAGTATCAAAAGTTCGAACTTTACGAGTGGCAA
TTGATTATATTAATTCTTTGA
>DjScaffold2734:2596-6150 strand:+
aaattttagttATGGAATTTTTTACTTCATTATTTTCAAGAATGGAAACAATTACTCAGG
AAACAGCGATTCCAGGTCGTACAGAACCGTTATTTAAACCAAATGAAATTCATGCTGTTT
TGAAAACTTCAACAGTTCCGCCTTTTCCTAAAAATACTAAATGGATAATTTTTGGTATTG
GTTGTTTTTGGGGCGCTGAACGTTTATTTTGGAAATTAAATGGAGTATATTCTACACAAG
TCGGTTATTGTGGTGGATTTACGAAAAATCCTACTTACGAAGAAACCTGTTCAGGAAGAA
CTGGTCATAATGAAGTTGTTAGAGTAATATATGATACCGAGAAAATAAAATTATCAGAAT
TATTAAAAGTATTTTGGGAGAGTCATGATCCTACATTACTCAATAAACAAGGAAACGATG
TTGGTACAATGTATAGATCTGCTATTTACTGCGCAGATAAATTCGATTTGGATGAAATTG
AAAAATCGAAAATTTATTTTCAAAAACAATTAGATAAAAAAGGTTTCGGTAAAATTGTTA
CAGAGATAAAGTTGGTTAAGGAATTTTATTACGCAGAAGATTATCATCAACAATATTTAC
ATAAGCATCCAGGTGGTTATTGTAGTATTCGAGGTACTGGAGTCGAATGTGTTTAGagat
ttattgattttatttcactttgttacatgatctagatttttctataattaacataaaata
ttaatataataaacaattaatgtaatatgatacaatcaaaactttaaataagcgtcagtt
acattgttttcaagcacttcgttccaaccaatatattctgatttattcttttcgtcgttt
attttccatttattaattactcccagagttagacaccttataaaccgcaagtatttataa
ttcgccatcactaattcgtttttgaattcagcgaccttcagtgttgcatgtcttctggt
tcataattaatttatcagtagtcgcaaatatctattttcataacatctcctcattcttct
aactgacttataattctttctcttactgtgtttcatcttgaatttttctcaggcaatcgg
acgttgtgtagtcttttatatcttattgtacagtttcagttcgcagtttcttgcaacact
gtggttgctttcgatagtatcattccaatttcgtaacttaaattcactcttcggtgtcga
aatttctgcctgatatttcttcatgttatcgaagaaaagtgccagttgcactgtattttg
ctctgtgtaacacatgtattcgacattttttttataatattgttggggatttctacccaa
caagttatttatggttcgattattttataactattagttgataatatgtttgcgaatttt
acgggtgttgatttttcccttgtatgttcgtacagtattatat
>DjScaffold4184:34524-35856 strand:+
AAAGCTAAAATATCTAAATTAATTGATTTTACAAATTTGACAAAAGATATTTCAATCCTA
CAGACGTTTTATGATTTTATTGATGATACGGAATATGATGTGATTGATGATTATTTAAGT
AATCATGATTTCAATGACTTTTTGGACTATTTTATCTCTCATCATCTTTACAAGCCAGAT
GATTTTTGCCTTATTTTTAAAGTCTGTTCTGAAATACTAATGCGGATTGTCGGTGATATT
AATAAAAAACACTCATTATCCGGCCAATCATTTGCCGAAGGATTTCTAAATTCTCATAAA
CCGATAATTTATCTCTATTTAAAAAATAATAAATCCAATGAACGAATAATGTCTTGTTTG
AAGTTTTTGACATCTGTAGTTTTACAAAGTAAAGATATCGCTATAAAACTGGCAACCGGT
TTGAATTTTTCCAAGGCAAATTTCAATCACTTGCTAACTATCCGAAAATTAAAACACGAG
GAAGATGTGCACAGTTGTGCTTTGAATTGTTTTGCATCGTTTTTCGTTATGGGCAGTAAT
TATTTGATCAGAAAAGCTTGTGAAGAAAGCATGCTTGTTGGAAACATTTTGAAGGATTTG
CGATTTGAGAGATTTCATAATGTTCAGTTGATAATTGAGATATTGCACGACAAGATTATT
AAAAATCAGTTATTCAGCAAAACAGCGAAAGTTAAATTTTTTAGTGTTACAAATTTACGA
TTTCTACTTGCAACATATGAATGGAAAGGTGAAGCAGCCTCATATGATGAATTCCAAAAC
CTATCCAATGTCGATGTAAATTGCAACACAATCCACGATCAACTGCATCGCATGTTGATA
GATTTGTGTTCCAATCATCACCTTGGAATAGCTTTTGTCGATAATCTTCTATGCCAATCA
ACATTAGGAAATCTTAACAAGAAATTATATTATTTCATCACATCAGACGGAATGCAGGAT
TTCTTTTCTCATCCATTCAAACACGAATTACTCATTTCGATTTTGACAGCTTGTCCTGAT
CTTATTAGTTCGTTTGTCGAATATCTTGTCGGTTTGATGCTTGTTCGTAAACCGGAATTT
TGGTCGAATATTCTCAAATATATCGTCGAATGTTATCGATTAGTTAGTTGTGAAAAAATC
AATGACATTCTTACAGCGGCATGGAATTATAACAAAGGTGAAAAATTCATAGAAATTGCT
ATGATTCTTTGTTTACCGTCAAAATCGATAATTGAGTTACTACAAACGGATGAGAATATT
CGCGTGTATTGTGATGAAAATATCAGATTACTGAGAAGTGCATTGATTGACACATGTGAA
AAAGTGATTACATGGATCAATATTTCCGATGTGTTTCGATCACAACAACAAAAGTCGGTG
TTTAAAACGAATTTCGAAGAAATATTATTGGAGAACTTCACTAAATGCAGCTGGGATTAT
GAGTTGAAACTTTTGTTGTCTGAGGAATTACAAGATGAGAGCATGTTTCGGAAATTGAGA
AATAATAATAATAATAATGAAATTGAGTCAGTGAGAGAAGAGATTGAAGAATTTGATTTG

GAAATATCAGAATACGCCAATAATTTGAGAAAGCTAAACACAAGAAATTCCATTTGTGAT
TTAAATGGAATAGAATCTCTGACAAAATGCTATCAACACTTTATCTCTACTCCTTTCAAT
AAAATTTATCTATTAAAATTGATTGGAGAAACGCTAAATATCGGTGAAATTTCTCTTAAG
AAAAAATATAAACGTTGTTTGATTGAATCATTGACTAGCAATGAATTCATATCAGATCAT
TGGACGCTTATGTTACCTCAGGACGAAACATATACCGATAGTGAATTGAAGCGAAATAGT
ATTTTCGACGTTATCATCGCTTGCATCTCTGACAGTAAAAACCTGAAAATAACATTTTAC
GAACGTGTTCATCCATTAAGATTGTTGAGTGTTTATGGCGCAACTCTTTCCGCCAGTGAT
CAAAAGCTTTTCTATCTTTTAACGTTACAATCTTTTTCCGGAGTTGTTTGTTCGGATTAT
AATTTCATTTTGTTCGGTAGTGCTGTCACTGAGCATTATTCGTTGGATAGTAGTAAAACT
CCGGTTTTGCGGAGACCGATAATGAATTCCCAGTGTTTCAATTTAATAGAAGATGAAAAG
CTAGCTGTTACCATTTCGCATTATCCTATTTCCCGACCGCCACCGACTAATAACATGATT
TTGATTAAGTCTCAACCCAATCTTTACGATCCATTCTTTCTGTTAGAATGGATTTCTCAA
TTGCTCAACATCCATTTTGAATCTGAATTACAATTGGAGTATGGAATAGATCTGAAACAG
TTGTACTCTAAACAGGTGTTATCATTCCTTATCACATGTCAAACCAGCGAGTCTCGACAT
GTGAGACTGGTTAGTAAGCTACATTTGGAGCATTTATTGGCAATATTGGAAGCATCGGAA
AATGTGATTGATTTTCCAATGAAATTCCAAGTACAAATGGTGTTGTATCATTTGTTGCAT
TCATTATCCAATCACCAAGACTCTGTTCAATTGTCGTCAATAATATCAAATTACTACGTA
AAAATGCTGAAACTCATCTCTTCTCCCGGTAAAAAGTTGTTCTCTCCTATTATGGATATC
TTCATTATGAACCCTAAATTCGATTTGCTTTACGTTCCAAACTTTCTGAAACTTATGTAT
TCACCGGATATCGACGCAAAACTTCGTCAAATATGGTGTTTGAAATTGCTTTCGGAAAGT
GTTAAATGTGCTGAAGATTTTCGGATAATTTTGAACACAAAAGTGCTCACTCATGTTTTA
CCGATTTACGCTAATAGCGAACAAAGTTTAAAGAAAGTGATTTTATATTTGATAAAAAGC
TGTGTATCGATTCCGAAAGTTGCAGTTATGCTCTATAATTTCCCGCAATTCGTTATGTGG
TTGAGTTTCATAGCGTATATTAAAAAGGACAAAATAAATCACTATTTAATATTGGAAATT
ATTGAGCAATTTATCGGTTCATTGAAATGCGGAGAAAACACGAAAAAAATCAATATTGAA
GATTTGGAAAGTACTTTCAAACATCATATTCCAGATTTAAGGATTTAAttgaataaattt
cgagttgaatcgtattttgtttaaatattttttcccccaatgttttcttgtaaattaat
cacttgattgaaaacagtagaaaacagacgatttcgatttaccaatatggagttctcgca
tttatttatcaggagctaggaAAAGCTAAAATATCTAAATTAATTGATTTTACAAATTTG
ACAAAAGATATTTCAATCCTACAGACGTTTTATGATTTTATTGATGATACGGAATATGAT
GTGATTGATGATTATTTAAGTAATCATGATTTCAATGACTTTTTGGACTATTTTATCTCT
CATCATCTTTACAAGCCAGATGATTTTTGCCTTATTTTTAAAGTCTGTTCTGAAATACTA
ATGCGGATTGTCGGTGATATTAATAAAAAACACTCATTATCCGGCCAATCATTTGCCGAA
GGATTTCTAAATTCTCATAAACCGATAATTTATCTCTATTTAAAAAATAATAAATCCAAT
GAACGAATAATGTCTTGTTTGAAGTTTTTGACATCTGTAGTTTTACAAAGTAAAGATATC
GCTATAAAACTGGCAACCGGTTTGAATTTTTCCAAGGCAAATTTCAATCACTTGCTAACT
ATCCGAAAATTAAAACACGAGGAAGATGTGCACAGTTGTGCTTTGAATTGTTTTGCATCG
TTTTTCGTTATGGGCAGTAATTATTTGATCAGAAAAGCTTGTGAAGAAAGCATGCTTGTT
GGAAACATTTTGAAGGATTTGCGATTTGAGAGATTTCATAATGTTCAGTTGATAATTGAG
ATATTGCACGACAAGATTATTAAAAATCAGTTATTCAGCAAAACAGCGAAAGTTAAATTT
TTTAGTGTTACAAATTTACGATTTCTACTTGCAACATATGAATGGAAAGGTGAAGCAGCC
TCATATGATGAATTCCAAAACCTATCCAATGTCGATGTAAATTGCAACACAATCCACGAT
CAACTGCATCGCATGTTGATAGATTTGTGTTCCAATCATCACCTTGGAATAGCTTTTGTC
GATAATCTTCTATGCCAATCAACATTAGGAAATCTTAACAAGAAATTATATTATTTCATC
ACATCAGACGGAATGCAGGATTTCTTTTCTCATCCATTCAAACACGAATTACTCATTTCG
ATTTTGACAGCTTGTCCTGATCTTATTAGTTCGTTTGTCGAATATCTTGTCGGTTTGATG
CTTGTTCGTAAACCGGAATTTTGGTCGAATATTCTCAAATATATCGTCGAATGTTATCGA
TTAGTTAGTTGTGAAAAAATCAATGACATTCTTACAGCGGCATGGAATTATAACAAAGGT
GAAAAATTCATAGAAATTGCTATGATTCTTTGTTTACCGTCAAAATCGATAATTGAGTTA
CTACAAACGGATGAGAATATTCGCGTGTATTGTGATGAAAATATCAGATTACTGAGAAGT
GCATTGATTGACACATGTGAAAAAGTGATTACATGGATCAATATTTCCGATGTGTTTCGA
TCACAACAACAAAAGTCGGTGTTTAAAACGAATTTCGAAGAAATATTATTGGAGAACTTC
ACTAAATGCAGCTGGGATTATGAGTTGAAACTTTTGTTGTCTGAGGAATTACAAGATGAG
AGCATGTTTCGGAAATTGAGAAATAATAATAATAATAATGAAATTGGTTGGTAAattatt
ctgttatctctttatttgtatcatttgattcttggacactttttttggatttcaaaatta
tacttcttacacgtctttcaaactttgcaagttttattgatgatttcgctgtattctcaa
cttccaataagagtaggaatgtttgcacacagtcttttgctctatcaaaacattgcatat

atcgcattcatgacagagataggaaagaagagcatacaaacatcattgcattgattacaa
cgtgatatttcttttagataaaaataccttttgttaattatattacacgtattaaaaattc
gtaaaaagaaaatagtaatcgttagaaatgctgatttttaccttatccgattgagaaagg
caactatataatctggtataaataggtccgtagttacagatgtcgacagtattaacaaat
cttatcgaacatacatttgatatcttccagtttctcacatgaaaatgagtctttgtgtct
cattaattagtgtattttctattattatttgaagtctcaacccaatctttacgatccatt
ctttctgttagaatggatttctcaattgctcaacatccgtgagtttacttatcgacgatc
aataatgataattcgcctcgagttttagattttgaatctgaattacaattggagtatgga
atagatctgaaacagttgtactctaaacaggtgttatcattccttatcacatgtcaaacc
agcgagtctcgacatgtgagactggttagtaagctacatttggagcatttattggcaata
ttggaagcatcggaaaatgtgattgattttccaATGAAATTCCAAGTACAAATGGTGTTG
TATCATTTGTTGCATTCATTATCCAATCACCAAGACTCTGTTCAATTGTCGTCAATAATA
TCAAATTACTACGTAAAAATGCTGAAACTCATCTCTTCTCCCGGTAAAAAGTTGTTCTCT
CCTATTATGGATATCTTCATTATGAACCCTAAATTCGATTTGCTTTACGTTCCAAACTTT
CTGAAACTTATGTATTCACCGGATATCGACGCAAAACTTCGTCAAATATGGTGTTTGAAA
TTGCTTTCGGAAAGTGTTAAATGTGCTGAAGATTTTCGGATAATTTTGAACACAAAAGTG
CTCACTCATGTTTTACCGATTTACGCTAATAGCGAACAAAGTTTAAAGAAAGTGATTTTA
TATTTGATAAAAAGCTGTGTATCGATTCCGAAAGTTGCAGTTATGCTCTATAATTTCCCG
CAATTCGTTATGTGGTTGAGTTTCATAGCGTATATTAAAAAGGACAAAATAAATCACTAT
TTAATATTGGAAATTATTGAGCAATTTATCGGTTCATTGAAATGCGGAGAAAACACGAAA
AAAATCAATATTGAAGATTTGGAAAGTACTTTCAAACATCATATTCCAGATTTAAGGATT
TAAttgaataaatttcgagttgaatcgtattttgtttaaatattttttccccaatgtt
ttcttgtaaattaatcacttgattgaaaacagtagaaaacagacgatttcgatttaccaa
tatggagttctcgcatttatttatcaggagctagga
>DjScaffold4184:42181-43447 strand:+
gtaaaatcgtaaggttaattatattttcgagaaactattaattttattgcgttATGATAA
ATGTTAAACTTGTCACTGATATTAATGACAACCGTTATGACTGTGTAGTTGTTGTTAATG
ATTCTTTATCACACTTCGGAGCTGCATTAATATCGATTGAAAATGCTTTGAATAATTTTA
AACAATATCATCCCAAATTAGAATCAACGTTGACACTTGTACCGTTTGATGGACATGCCT
CAAAGAAATTGATTTTCAGTCCGATAAAATCATTGAATGATGATTATGATGACAGTAGGC
GGATAACCGAAGCATCTAATTCCGGAATAAAAATGGCAATTGATTTGGGCCTAAAAAACC
CTCTTTTGGTTTTCGGTAACTTTCAAACTTTACCTAAAGAAACACCAGAATGGGGATCTC
AAAAATTAATTGAAATTAACGTCATTCTTGGAGCTTTATTTGCAGCCTATGTTCCAATTG
AAATCCGTGAAGACATTCCAGCTCGAGCTTCCAAATTAAATAAATTGGGAGTTTTCGGAG
TCAAGAGTAAATCTATTGATATTGCTGTAGCTCTAGAGGAAGGTAGACGTGTCGCTAGAG
ATATTGGTGGATCAGATCCAGAAAGAATGGCTGCTATTAAGATAGCTGAATTTATTAACA
AAGAATTTTGTGGATCTGATATTAAAGTTGAAATAGAATTAATTGATAGTAAGAAATATC
CATTAATGGCTGCAGTTAATCGTGCAGCATCAGTCATAGAACGACATGATGGAAGAGTGG
TGAAATTCGAATATGACTCAAAGGATGAAGTTGAAGAAACGATATTTTTAGTTGGTAAAG
GAATTACATATGATACGGGAGGAGCTGATGTAAAAGCTGGAGGCCATATGGCTGGAATGC
ACCGGGACAAATGTGGTGCAGCTGCAGTAACTGGGTTTTTTAAGGCGTTGAGTCTTTTGA
AACCAAAAAGATTTAAAGCCGTAGGGTTGTGTGCTTATGTTAGAAATAGTATTGGAGAGA
ATTGTTATGTTGCCGATGAAATAATCACTTCACGAGCTAGGAAACGTATCCGAGTTGGTA
ATACTGATGCAGAAGGTCGAATGGTAATGGCTGATCTGCTTTGTGAAGCTAAAGAACGAG
CTGCTACAGCTGTGAATCCGTTTTTGTTCACAGTTGCAACTCTAACAGGACACGTTATTC
GAGCATATGGGGAACATTATACCGGTGTTTTGGGTAATGGACTTTCATGTGAGAAAAATA
TTCCTCGCTTGCTGCAACAAGCAGGAGATCTGTTAGCTGAGCCGGTTGAGGTGTCAACAA
TACGCAGAGAAGATGTTGCATTCCACAAAGGATTCTCTGAATATGAAGATATTCTACAAT
GTAACAATATGCCCAGCACAATGACTAATCGTGGACATCAAAGCCCAGCTGCTTTTTTGA
TCTTAGCAAGTGGACTCGATGAGCACAATTTGACATCTAAACAACCTCTAGCTTACACTC
ATCTGGATTGTGCTGGCAGTGCAGGAAGTATCGGAAGTTTACCGACTGCTGCTCCTCTGC
TGGTGCTTGCAGCTAAATACATTCTACCAAAAATTCATTAGttaaatcatttactttagc
taaatatttttcaaattttgtgaaataatcaatagttttaacttctaagttaataattga
tttatataattattttaataaatatatttctgtgaaatcagtaaaatcgtaaggttaat
tatattttcgagaaactattaattttattgcgttATGATAAATGTTAAACTTGTCACTGA
TATTAATGACAACCGTTATGACTGTGTAGTTGTTGTTAATGATTCTTTATCACACTTCGG
AGCTGCATTAATATCGATTGAAAATGCTTTGAATAATTTTAAACAATATCATCCCAAATT

AGAATCAACGTTGACACTTGTACCGTTTGATGGACATGCCTCAAAGAAATTGATTTTCAG
TCCGATAAAATCATTGAATGATGATTATGATGACAGTAGGCGGATAACCGAAGCATCTAA
TTCCGGAATAAAAATGGCAATTGATTTGGGCCTAAAAAACCCTCTTTTGGTTTTCGGTAA
CTTTCAAACTTTACCTAAAGAAACACCAGAATGGGGATCTCAAAAATTAATTGAAATTAA
CGTCATTCTTGGAGCTTTATTTGCAGCCTATGTTCCAATTGAAATCCGTGAAGACATTCC
AGCTCGAGCTTCCAAATTAAATAAATTGGGAGTTTTCGGAGTCAAGAGTAAATCTATTGA
TATTGCTGTAGCTCTAGAGGAAGGTAGACGTGTCGCTAGAGATATTGGTGGATCAGATCC
AGAAAGAATGGCTGCTATTAAGATAGCTGAATTTATTAACAAAGAATTTTGTGGATCTGA
TATTAAAGTTGAAATAGAATTAATTGATAGTAAGAAATATCCATTAATGGCTGCAGTTAA
TCGTGCAGCATCAGTCATAGAACGACATGATGGAAGAGTGGTGAAATTCGAATATGACTC
AAAGGATGAAGTTGAAGAAACGATATTTTTAGTTGGTAAAGGAATTACATATGATACGGG
AGGAGCTGATGTAAAAGCTGGAGGCCATATGGCTGGAATGCACCGGGACAAATGTGGTGC
AGCTGCAGTAACTGGGTTTTTTAAGGCGTTGAGTCTTTTGAAACCAAAAAGATTTAAAGC
CGTAGGGTTGTGTGCTTATGTTAGAAATAGTATTGGAGAGAATTGTTATGTTGCCGATGA
AATAATCACTTCACGAGCTAGGAAACGTATCCGAGTTGGTAATACTGATGCAGAAGGTCG
AATGGTAATGGCTGATCTGCTTTGTGAAGCTAAAGAACGAGCTGCTACAGCTGTGAATCC
GTTTTTGTTCACAGTTGCAACTCTAACAGGACACGTTATTCGAGCATATGGGGAACATTA
TACCGGTGTTTTGGGTAATGGACTTTCATGTGAGAAAAATATTCCTCGCTTGCTGCAACA
AGCAGGAGATCTGTTAGCTGAGCCGGTTGAGGTGAATTAGccacattgaggttttatagg
aatgaaatttgatttactaaaataggtgtcaacaatacgcagagaagatgttgcattcca
caaaggattctctgaatatgaagatattctacaatgtaacaatatgcccagcacaatgac
taatcgtggacatcaaagcccagctgctttttgatcttagcaagtggactcgatgagca
caatttgacatctaaacaacctctagcttacactcatctggattgtgctggcagtgcagg
aagtatcggaagtttaccgactgctgctcctctgctggtgcttgcagctaaatacattct
accaaaaattcattagttaaatcatttactttagctaaatatttttcaaattttgtgaaa
taatcaatagttttaacttctaagttaataattgatttatataattatttttaataaata
tatttctgtgaaatcagtaaaatcgtaaggttaattatattttcgagaaactattaattt
tattgcgttATGATAAATGTTAAACTTGTCACTGATATTAATGACAACCGTTATGACTGT
GTAGTTGTTGTTAATGATTCTTTATCACACTTCGGAGCTGCATTAATATCGATTGAAAAT
GCTTTGAATAATTTTAAACAATATCATCCCAAATTAGAATCAACGTTGACACTTGTACCG
TTTGATGGACATGCCTCAAAGAAATTGATTTTCAGTCCGATAAAATCATTGAATGATGAT
TATGATGACAGTAGGCGGATAACCGAAGCATCTAATTCCGGAATAAAAATGGCAATTGAT
TTGGGCCTAAAAAACCCTCTTTTGGTTTTCGGTAACTTTCAAACTTTACCTAAAGAAACA
CCAGAATGGGGATCTCAAAAATTAATTGAAATTAACGTCATTCTTGGAGCTTTATTTGCA
GCCTATGTTCCAATTGAAATCCGTGAAGACATTCCAGCTCGAGCTTCCAAATTAAATAAA
TTGGGAGTTTTCGGAGTCAAGAGTAAATCTATTGATATTGCTGTAGCTCTAGAGGAAGGT
AGACGTGTCGCTAGAGATATTGGTGGATCAGATCCAGAAAGAATGGCTGCTATTAAGATA
GCTGAATTTATTAACAAAGAATTTTGTGGATCTGATATTAAAGTTGAAATAGAATTAATT
GATAGTAAGAAATATCCATTAATGGCTGCAGTTAATCGTGCAGCATCAGTCATAGAACGA
CATGATGGAAGAGTGGTGAAATTCGAATATGACTCAAAGGATGAAGTTGAAGAAACGATA
TTTTTAGTTGGTAAAGGAATTACATATGATACGGGAGGAGCTGATGTAAAAGCTGGAGGC
CATATGGCTGGAATGCACCGGGACAAATGTGGTGCAGCTGCAGTAACTGGGTTTTTTAAG
GTTTTCTTATTGTGTTTCTTAAAGTCTTTTTGAttttctggcaggcgttgagtcttttga
aaccaaaaagatttaaagccgtagggttgtgtgcttatgttagaaatagtattggagaga
attgttatgttgccgatgaaataatcacttcacgagctaggaaacgtatccgagttggta
atactgatgcagaaggtcgaatggtaatggctgatctgctttgtgaagctaaagaacgag
ctgctacagctgtgaatccgtttttgttcacagttgcaactctaacaggacacgttattc
gagcatatggggaacattataccggtgttttgggtaatggactttcatgtgagaaaaata
ttcctcgcttgctgcaacaagcaggagatctgttagctgagccggttgaggtgaattagc
cacattgaggttttataggaatgaaatttgatttactaaaataggtgtcaacaatacgca
gagaagatgttgcattccacaaaggattctctgaatatgaagatattctacaatgtaaca
atatgcccagcacaatgactaatcgtggacatcaaagcccagctgctttttgatcttag
caagtggactcgatgagcacaatttgacatctaaacaacctctagcttacactcatctgg
attgtgctggcagtgcaggaagtatcggaagtttaccgactgctgctcctctgctggtgc
ttgcagctaaatacattctaccaaaaattcattagttaaatcatttactttagctaaata
tttttcaaattttgtgaaataatcaatagttttaacttctaagttaataattgatttata
taattatttttaataaatatatttctgtgaaatcaatatgacgtgacaaataaacagata

gtaaaacattaagcatttgtaattatttggaattgttttagtcatagaacgacatgatgg
aagagtggtgaaattcgaatatgactcaaaggatgaagttgaagaaacgatatttttagt
tggtaaaggaattacatatgatacgggaggagctgatgtaaaagctggaggccatATGGC
TGGAATGCACCGGGACAAATGTGGTGCAGCTGCAGTAACTGGGTTTTTAAGGCGTTGAG
TCTTTTGAAACCAAAAAGATTTAAAGCCGTAGGGTTGTGTGCTTATGTTAGAAATAGTAT
TGGAGAGAATTGTTATGTTGCCGATGAAATAATCACTTCACGAGCTAGGAAACGTATCCG
AGTTGGTAATACTGATGCAGAAGGTCGAATGGTAATGGCTGATCTGCTTTGTGAAGCTAA
AGAACGAGCTGCTACAGCTGTGAATCCGTTTTTGTTCACAGTTGCAACTCTAACAGGACA
CGTTATTCGAGCATATGGGGAACATTATACCGGTGTTTTGGGTAATGGACTTTCATGTGA
GAAAAATATTCCTCGCTTGCTGCAACAAGCAGGAGATCTGTTAGCTGAGCCGGTTGAGGT
GTCAACAATACGCAGAGAAGATGTTGCATTCCACAAAGGATTCTCTGAATATGAAGATAT
TCTACAATGTAACAATATGCCCAGCACAATGACTAATCGTGGACATCAAAGCCCAGCTGC
TTTTTTGATCTTAGCAAGTGGACTCGATGAGCACAATTTGACATCTAAACAACCTCTAGC
TTACACTCATCTGGATTGTGCTGGCAGTGCAGGAAGTATCGGAAGTTTACCGACTGCTGC
TCCTCTGCTGGTGCTTGCAGCTAAATACATTCTACCAAAAATTCATTAGttaaatcattt
actttagctaaatatttttcaaattttgtgaaataatcaatagttttaacttctaagtta
ataattgatttatataattatttttaataaatatatttctgtgaaatca
>DjScaffold3308:20022-20987 strand:+
AGATTGAAAAAAGGTTCAGCCTACCATTGGGATTTACTAATTGTTGCTATCATTAATCTC
GTCTTGTCTTTATTTGGTCTCCCTTGGGTGCATGGTACATTACCCCACTCTCCACTTCAC
GTTAAAGCCTTAGCAGATTTAGAAGAACGCATAGATTCAGGACACAGAATTTATGAAACA
ATCGTTAAAGTTCGTGAAACCAGACTAACGGGATTAATATCGCATTTATTAATTGGTTTA
TCATTACTTTTATTACCAATACCATTAATTTATGTGCCAATTGCTGTTCTCGACGGTCTA
TTTGTGTATATGGCAATCACTGCGCTTTATGGAAATCAACTAGTTGAAAGAGTTATTTTG
TTATTCACAGAGCAGGCAGCTTATCCACCATCTCATTATTTAAGAAGGGTTCCACAAAGA
AAAGTCCACTTGTTTACAGTACTTCAATTAACTCAATTAGGACTGCTTTGTACATTGGGT
TTTGCTCCAACTCCATATGCGAAAATAGGCTTTCCAATTTTACTTTTACTACAAATGCCT
ATCAGGTTAATTTATTTTAATTAAatttatttaaataaaattgttttgttttattaaatt
ataagaacttaaaaaataattattaattttcatttttctatatcaatcatattacgttga
agatttatttaacagttttacgaatagaattcattaaaacaaacgaactatagaaaacag
ttttctattcaattaattaagaattgatatctgttttgtaagtgttgtgacacaaaaata
aattgtgattatgtaattattagttaataaaaaaaaa
>DjScaffold4330:41280-57426 strand:+
gttttgaaagtttttattttaaaatttactgcaagtgatttacaaattcattatttttt
aattttacaATGTTTTCTAATGAACCCTTATATAATGATATAGATACTTATAATAGTGGA
AATAGATTATTTAATCTAAATGATTCACAGAACATTTGGTCATCAGATAAAATGTGTTCT
ATTGATAACAACTATTTTATGAAAAATAGAAATGTATTTAATAATATACAAGTGTCAGAT
CGATTTAACAGAACTAATCGAAAACCTCCTGGAAACCGAACTGAAACTAATTTAATTGTT
AATTATCTTCCAGATGGATTTTATGAAAAGGATTTATATGAGTTATTTAGCAAGGCTGGA
CAAATTGTACATTATCACGTTGTTAGAAATAAAGACAATAGAAGTGAATGTTATGGTTTT
GTTGATTATGACAAACCATCTGACGCTGAAAAAGCGATTGAAATGTTTCATCAGTATCAA
ATTCAGAATAAAAAGTTGAAAGTTCAAAATGCTTCTGAGGGTTTACAAATTAAAGAAGAA
GAAAAATTGCGAGCTATTAAAGGATTTAATAATATAGGATATAATGTGGAAATACGAAAT
GCTCCAATCAGATGGACGGTTAACGAGGTTAAGAACATTTTCGGAAAATATGGGGAGATA
TTGAAAGTTTATGAAAGTACAACAGAAGAAATTACTACAGTCGATAATTCCAATGATATC
AAAAGCAAAACTTTCAATATTATATTTCGATATCACACAATAGCTGCAAAAGTAGTTAAA
AAGAAGCATAATCAAATTGCTGAAGCAATGTGGCCTCTGCAATTAGAATTATCGATCGTC
AGCCAGGAAATCGTCGATATCCCCACAGATAGTACTCGAGTAAACCGACCGAGACCAAAT
AAAATGCGATTTGATCAATTATCAGAGAGTTCTTGGGAAAACAAAGTGATTGAAAGTAGT
TACTATCAGAAAAGCTACGAAAGTCCCACTTTTAATTGGGCGGAAAGTATTGATCCGCAA
CGATCGGGTGTTCCGTTATCCGCCAATGGGAGACCAATTCTCAAACATAAAGACAATTTA
ATGCATCTAAACGGCGGCGGTGGATGTGGAGGAGTAGCAGGGATGTACGATGTCAACTCG
CGACTCGACAATGGCGATAAATGGAGCAATATGCTCATGAACGATCCCAACAGTTTCTTC
GACACCAAACTTCCTGGTTCCTGTAATGATATCAAAGCCATTTGGGAATCGAAAGAGTTA
CTGTTGAATGATATCACTCCGCAAGATGTGTTCGTGAAAACACCTCTCCTTCCGTCGAGA
TTCAGAGGAGACGATAAAAAAAAATTACAAAGCGAATATTTGGTTCAAAATGGAAAATATG
CAACCGACGGGCTCGTTCAAATTACGAGGGATGGCTTATTGGTGCTATAAAGCGAAACTT

GATGGTGTACAACTCGTTGTGTCGTCCAGTGGTGGTAATGCAGGCCTGGCAGCTGCTTAC
GCCTGTCGAATGCTAAAATTGAAATGCACAGTGGTTGTATCTCACGCTACACCCGTGAAT
ATAATCGGGGAATTGTTAAAAGAAAACGCAGAAGTCATCCAAGAAGGATCATGTTGGAAG
GAAGCTCATATGAAAGCTGAATCTATTGTAGCAACTTGTCCACGAATCAGTCGATTATGT
CATCCATTTGATAGTCCATTATTATGGAGTGGTTATGAATCAATAGTCGACGAAATAGTC
ACAGACATGAAGGGCGCCAGACTCGATATGATAGTGTGCTCAGTCGGAGGAGGAGGATTG
TTGAATGGAATCGCACAGGGAATAACAAAAAACAAATTAAACAAAACTACTGCTATATTA
GCAATGGAGACAGAAGGTGCTGCATGCTTTAATCAGTCTTTATCAGTCAATCACATAGTT
TCCATCGAACCTGACAGTATTGCGACCTCTCTTGGTGCTTCTAGTATCAGTCAGAGAACT
TGGGAAATTTACTGCAATAAAGAATTTCATCTTTTCTCAAAGATTTGTTCTGACGCTGAT
GCTATCGATGGATGTTGTCAATTTCTCAGTGAACAAAACTGCTTAGTAGAACCCGCTTGT
GGTGCTACTTTATCCGCGCTTTATAATGGAACTCTTGATCAAATGGCTGAGTGTCGCGAA
TGCGATCTGAATGTCGCTGTAGTAGTTTGCGGTGGTCGATGTATTGATGTGAACAGTCTC
ATAGAACTGAGGCAAGTATTCTCAGCACTGATAAATAATCCCATCCAAACCACAACTGGA
ACAACAGGACTGTACGGTGATAATTTATCGAATTACGATATCACCGACAGATTTCTCACA
ATGAATCTTATTAACAAAGATCACCAATCTTATCATCATCAAACTGCTACTACTCCTATC
GAATCCTCATCTTCCACCGCTACCGCAGTCACTACCGACATGTATTATGCATCATCATCT
GCATCAGCATCAGCCTCCACCAATCGGTTCGATCCTTCCATTCCCAGTTACAACAACATG
TTTCCATCTCTCACAAAGAATAATAATACAGTCGAGCCTCCAGCAACAATAAATACGATA
AATTCTATCAATAACTGTGGAGGTCAACATAAATCCAATATTCTCACAGAGAACAAGTAC
ATCGCAACCAACAGTGCTGGTGGTGGTAGTGGTTACCACCGACAACGGAATAATAACACT
AATTTGTTGTTGAACGAGAGTAATACGAATAATAATTCTCTCCTCCTCCACCACCACCAC
AATTCCCTCCATCATCATCATTACAACTACCAAATGTCCTCATCCTCCTCAATCAATACT
AACAACAATAACGATCTAATGCTGCTACAACAACAAAATAACAATATCATTAACAACAAC
AATAACAATAACAACAGCAGCAACAACAGCAATAGTTATTTATTATCCAATGATTTAATG
TCATCTCCAGTCGGTAGTCAGCATTCCGATAAAGAAAATCGAATGATAGATACCCAATGA
aaactaattaattgtgtaaatcctcctaaaagactccaaaacataacaaaaaaaatcgaa
aataactggcatagattagttttataatttctttattattattattttattattaatttg
aataacttgaataacgaactaactaactaactaactaactactcatttttaatgcggaa
tgtgctgtgttgtatttgcattgttgtgtcttgctattatttgataaatctctgttttg
attggtcgtcgtttgtcgcgagtgatttgtgcgatttgttcgccggtttcgattattttt
actattatcatttgtactataatctatcatttattattactataattaattacttttact
aattgaaatctgtcagtttaccaatcattcatttcgattggtttattaatgatcgtgttt
gttttattttaccggttattataagcatgtagtattaattattatcattattgattatta
ttattattgatgattatt
>DjScaffold4726:5162-5623 strand:+
tgtaatgtgatgttgttctggtgaaattttatgtttgtgttgaaattgtcttaagacttc
attttttttgtttcaaatgtgccttaattcttgttcacatgcttctgtatgtttcaccta
tatatgtttcttgacaaatggtgcatgttatttttaagtgacatttcttttattgcatat
atttctatcatcacaaATGCATGTCCAAAGGTGCTTGTGTTTTGAAAATAATTCTTGGAT
TGTGTTTGAGTTTAGCGTTTTTTATTATATTCTGCATGATTTGGCTACATTTTTTATTAA
TATAAGGTCATTTAATAATTGTGTTTGTTTTCGGTTTGAGATTATCATGTTTTGTATTCA
AAGATAAatattttgatataattttattttgatagccgttatttaagaatttatctttta
t
>DjScaffold4726:12418-26831 strand:+
aatttatttataccaccaattgcatttcattaatgccaatattttagattattttgaatt
aatgaaaaattgctttttattattatttaaagagggctttctgatttaaaacttacacaa
accaatatttatttttttattaacATGGATGCTGATCAATACTTTTTAATTCCTTTTTATG
AGTATATGAAAACCATAATTGGAATAACTCCACTCCAATTAAAAAATTCAAACTCTAATG
ACAAGCATTCTGAATACCACGGAAATATTACAACGGAATTTAGTTTGAATTTCGATAATA
ATCTCTTATTAACTGAATCACGAAAGCATAAATCGAACAGATTTGACATTCGCTTAGTAA
TGAGAATTTGTAAATTGCAAAATAAAACTTTTCAAAAAAATCAATATTCACTGGTAGATT
GTTTCCCTGAAAATTTGAAAATATGTATTAATGGCCAACCTGTACAGTTGTCTTCATATC
TTCCATATGATAGATATCGACGACCTGGTAGAATGAATCCACGACTTGTAGACATAACCG
ATTTGATTCTCGACAGCAATAATTCTAATCCGCATACATGTAATATTTATTGGGTTCAAG
ATGATCTCGAAAATCAATTTAATTTTGCTCTCTATCTTGTCCGGCGGAGAAGTTTCGATG
AATTATTATCTATTTTAATCAAATATCGCATTTTATCTGTACAAAATACCATAAATTTAC

TTTCCGAACGAATAATTATTCATCCAGTTAATAACAAAAGTAATATTGAATATATGTTTC
AAGATCGCAAAGTTTTAAATACATCTCTTGTTTGTCCGCTTTCTTTGAGTCGAATGAAAA
TACCTGTCAGAGGAGGAAATTGTACTCATTTAAAATGTTTTGAT
>DjScaffold4726:31586-31822 strand:+
ATGGTTATCATGTTAATTGGTAACAAAAGTGACCTGGAAGCAAGGAGAGATGTTAAACGC
GAAGAAGGTGAAGCGTTTGCAAGAGAACACGGTTTAGTTTTTATGGAAACATCGGCGAAG
ACTGCTGCAAATGTCGAAGAAGCATTTATTAACACAGCTAAAGCAATTTATGAAAAGATT
CAAGAAGGAGTTTTAGACATCAATAATGAAGTAATTTATTTTTTATTTAGTCAATAA
>DjScaffold3198:40854-50397 strand:+
atagtcaaatggcttatacacctcattcatgttatatgataataatttaaacttaaatat
ttatattttcaattattaatcaaatggcagatattattcgtcgatttaatctccagtcaa
taatattattacaactaatgcatcgcaaactggtggtcaaataaaagtattcagccaatc
aaatcaatttaagcataagaaatttttttaatagtagtagtaatattatcATGACTACCGG
GATTTATGAAGAAATATGGAAGTTGCTAAAAAGATACGATTTTGACATTGATTTTCAACT
AAACAATGATTTACTATGCTCACTCTGTAACAAAATATTGATTAATGCAGCGGTTGGTCC
ATGTGGTTGCAAATATTGTCATCAATGTATTGAAATTTATTTGAATGGTGATAAGAAATA
CTGTCCCACACAAAAGATTGCTTGTAAGAATAAAGAGATTACTTTAAAATGTGATTTGAA
AATCGATAATATTATCAATGGGAAAATATCATTACTCAAAGTTAAATGTCCGAAAACTTT
CTGTCAATTTACAGATGAATTGAAAAATATTCCATCGCATCTGAAATCTCATCAAAGTAA
ATGTGAATTCTCAGAAATTGGATGTGTAGAATCGAGAAATCATTTGGATAATTACAATTC
TCTACATCTGAATTTATTGATGAATACTTTACAAAATGTACAAAATGAAATGGAAATAAT
TAAAGAAAAATGTGGTAAAAATGCAAATTTAATTAATAATTTAAACATGAAATTAAAACA
AATGAAAGAAGAGAGCAATTCTTTGAAATCACAATTGATGACACTGGAAGCATACAAAGA
AAAATGCGATAACAATGAAGATTTAATTAATAATATAAATATGGAATTAATACAAATGAA
AGAAGAGAACAATTATTTGAAATCACAAGTGATAATACTGCAAACATCAAACGAAAATCA
AAATACAAACAATAACCAAATAAATACGAGATTTAACTTACTCGATTTAATCAAGATTCA
TATATCAAAGCAATCGCTCGATTTGGCCCCATTTCGATATGAAATTAAAAACTTTCGAAA
TAAAATTAATAGTAGAGGTAAATGCATTTATACAAATGAATTTCTTTCTAAATCATGTGG
ATATACTTTGCAATTGAGGATTCATCCTAAAGGTGATGATTCGGATTTTAACAAAAACTT
ATCAATTGATTTTGCTATTGTCGAAGGAAAATATGACGACATGTTATCATGGCCAATGAA
AGATAAGGTCAAAATTGCAGTGATGAATCAAGAAACTGACGAAATTTATGTTAGTCGCAT
ATTTTGTTTTGACGATGCACCTAAGTCGCTCAAATGTATTTTTTTCAAACCATCCGTGCG
ACGTGAACGTGATAATAATTATTTTTATGGAACTACCAATCTGATTAGTTTAAATGAATT
GATACATAATAAAGATCTATGCAAAAATGATTCAATTGTTGTGAAATGCTTCGTTGCTTG
Agttttcttaatttaagaaaattcgtttaaattaagattaaatgaacttacgcttggaac
atctttataactcttatattgcacgttactttgataaatatttattacact
>DjScaffold4511:26225-27055 strand:+
aataaaaatttaaaacATGGTCAAAGTAAAAAAAGGTATTTTCATTGGAGTTAAAGATAT
TCCACAACAAAAATATACTGTTGCCTTGGCTGAATTTTTGAAAAATTCTGGAAAAATTGA
ACAACCAGAATGGGTTGATTTGGTTAAATTGTCAAGAGCTAACGAATTAGCACCTCAAGA
TGCTGATTGGTATTTTGTTAGATGTGCCAGTATTCTTCGTCATTTGTACAACAGATCATC
TGGAATTAATGGTTTGACTAAAGTTTACAGTCGTAACAAAAGCAATGGCAGTAGACCAAG
CCATATGGTTAAAGGAAATACTGGAATTATCAGAAATTGTCTTAAAAGTTTAACTAATTT
AGGAATGGTTGAAATTTCAAATGAAAAAAGGACCTTGACCAGCCAAGGAAGACGCGATTT
GGACAGAATTGCTTTCCAATTGAAAAACAAAATATAAatcatttaataaaatttggatta
gttttatttttcattcaaatttagtgcattattttattgatattttgttattttattgcc
tgtttaaattaaatttattgtattattgcattcnnnnnnnggttaattaaattttaagctt
atcaatttatattttttaagtgtacttttctgtaattgtatcagtggattatgatttcgg
tacaatt
>DjScaffold4511:18077-18424 strand:+
ATGCCATTTGACATCGCAGTGGCACCAGAAACATTTCAAGAGATGATGACAAAAGTAGTT
GGCGGAATCAGTGGAGCCTTGGTTTATCTTGATGACATACTAATATTTACGAACAATAAA
GATCAACACTATGAAGTAATTGAAAATGTATTGACGGAAATAATGAAGACTGGTCTAAGA
ATTGATCCAGAAAAGTGTCAGTTTCTTCAAAACGAAATCATGTTTCTTGGACACATAATT
AACAAGGATGGTGTCAGAACTGATCCGCATAAAATAGAAGTAATAAGAACATTTGGCAAA
CCTAACTGTGTGAAGAAGCTGCGAAGTTTCTTAGGAATATGTAATTAT
>DjScaffold4800:36335-36568 strand:+

ttttgcatgggatgggcagtctaaaatacataggcatgtgattttcattgtatgtgtgtt
gcatctgagttgcatacATGTTGCATACATTTTTAAAAATTCTTAGACGCATGATTTTTA
GTGTATGTGTGTTGCATTCTCATGTCATCTGAgttgcatgcctattgcatttattaataa
aaa
>DjScaffold4337:5636-6507 strand:+
ataaagtagaatgtctattagatacaggggctcgaattaacgtgatgagtttgagttgtt
ttaataaactacgagaagtaaagttagaaatgacttATGCGAAAGGTCGATAGCAAAACG
AATGAAAAAGTCGAAAATTACATTTGCAATATTGAAGCAAATTTTGGAAGACAAATTAAA
AAAGGAGAGCGATTGACAAGAGCTTTAAATATTTTTAAGATTAAAGAGGATCAACGGTTA
TTGAAAATAATTGAAAGTAAAGAAGATGCTTTTATGGCAGATAAATGGGATGTAGGACGT
ACACATCTGACCAGACACAAAATAATCACAAGTGGTGAACCAATCAATGTAAAACCATAT
AGACAACCAATGAACTTAGAATCTAAAATTGAAGAAGCTATCCAAAACCTTTTCGAGAAT
AATATTATCAGGAAATGCAACTCTCCATGGAATACCCCATTGATATGTGTTTGGAAAAAG
GAAAAGAAAGACATAAGAATATGTTTGGATTTCAGACAATTGAATGCAGTAACGAAAAGA
CAAGCCTTTCCGATGCCTAATGTCGATGAAATGCTGGACAAGTTAGATGGAGCCAAATAT
TTCAGTTCAATAGATTTGGGAAATGCATACTATCAAGTAGAGCTCGAAGAAGAATCTCAA
GAAAAAACGGCGTTTTCAACGAAACACGGGCAATATTGTTTT
>DjScaffold4337:46910-47509 strand:+
ATGGTATTGAAATTATTTCATGATTTTATATGTGAATTTATTCCAAATGCTCTGAGAAAC
GTTCCCATTCCTGCAAAATTTGCTGATATAAAATCTTTTTCATTTGGAGATATTTTGAAG
CTAACGGTGTTTTATGGAGTAAGTGGAGGAATTGGATATTGTATTTATTACACTGTTCAA
AACCAATTATGTTTACGTAAACGTAAAGTCTGTGGTAATCTAGTAAATCTTTCTATTGAT
AAAGACTGTGGAAAAGTTGTAAATACAGTCGACATAGAAGAATCGGGTCAAAAAGCCGTT
TTATGTCGTTGTTGGAGATCTAAAAAATTTCCATATTGTGATGGAAGCCATACATCACAC
AATAATGAAACCGGAGACAATGTTGGACCAGTCATAGTTAATAGAAAATAAttttcatcg
gtgtttattagtttttaataaaaatttagatttttaaaagtaac
>DjScaffold4337:6514-8514 strand:+
ATGCCATTTGGCATCGCAGCGGCACCAGGGACATTTCAAGAGATGATGACAAAAGTGGTA
GGCGGAATCAATGGAGCCATGGTTTATCTTGATGACATACTGATATTTACGAACAATAAA
GACCAACACTATGAAGTAATTGAAAGAGTATTGACGGAAATAATGAAGGCTGGTCTAAGA
ATTAATCCGGAAAAATGTCAGTTTCTTCAAAGTGAAATCAAGTTTCTTGGACACATAATT
AGCAGGGATGGTGTTAGAACTGATCCGTCTAAAATAGAAGCTATAAGAACATTTGGCAAA
CCTAACTGCGTGAAGAAGTTACGAAGTTTCTTGGGAATTTGTAATTATTACAGGAAATTT
ATAGAAAACTACGCAAGAAAATCAAAAATATTAGAGCAGTTGTGTAGCGGACCAAGCAAC
AAGAAATTAGAATGGACAGATGAAATAAACGATACTTTTGAGGGTCTAAAAAAATCTTTA
ATGACTGCACCAATATTGTGTTTTCCGGACATAAACAAAGAATTTATATTAGATACGGAT
GCCAGTTTTGATACGATTGGAGCAGTATTGTCACAGAGAGATAAAGAAGGAAGAGAAAGA
GTTGTTGCCTATGGATCACACTCAATGACAAGTCATGAAAAGGGATACTGCATTACACGA
AAGGAGTTATTGGCTGTTTATTATTTCTGTATCCATTTTAAACATTATTTGTATGGTAGA
AGATTTACTCTAAGAACAGATCACAAAGCTCTAACTTTCATGATGACGACGAAGAAACCA
ATTACGTCACAATTCCAGACATGGATTAATTATCTGAGCAGTTTAGATATGATTTTGGAA
TATAGAAAAGGAATCCAACATGCTAATGCAGATATGTTATCTAGACCAAGATGTGAGGTA
TGTGTACAATGTCAAATGTCACATGAAGATGCCAAGAAAGGGAAGATTAAAACGAGAATA
CTGAATATAATTGAAAAAGAAAAAGAAGATCATTTAAACATTTCTGAGGAAACAGATTTA
GTCAAGAAAATGAAAGTTCCGGATGACAAACTACGTGAAAAAATAGAAGAAATACATAAA
TTATTATGTCATGCAGGAGTTGAAAAGGTGTACGAATATATGAGAAACAAATATATTGCA
AAGAATTTGTGTAGCCAAATACAGAAGATAGTTCGAAGTTGTGAAATTTGTCAGAAGACC
AAAGTAGTGACAACACCAACTAAAGAGAAGACAAAGAGTTTAGAAGCTACCAATTTATTT
GAAGTGATATTCACAGATATATGTGGGCCAATGCGGGAAACAAGTGGACGGAAAAAATAC
ATTCTTGGAATAATCGACCAATACAGTAAATATCTGGTTCTTATACCATTGCCTAGACAA
GACGAGGAAACGATACGAAAAGCTATTTTTGAAAAGTGGATATTGAGATTTGGATGTCCA
AGAGAAATAAGATTAGATTGTGGAAAAGCGTTTGAATCCAGAGTTATGAGAGAGTTTGCA
CAGAAGCTAGACATTAAACTTTGCTTTTCGAGTCCATATCATCATAATGCTAACGGACAA
ATAGAAAGACAGTTCAGGACCATTCGAGATTTGATTAATGCGACCCTTCAAGAAAGAAAA
TCAACAGATTGGGCAGATCTTTTACCGGAAATAGAATTTACAATGAACGCAACAAAGCAG
AAGACTACTGGAAAGAGCCCGGCTGAGATCGTTTTTGGAAAGAAAATTAGCAGAGAAAAG
TGGTACGAGTCAGACGTTTCAGATATAATTAAAGAAAACATTACAGACGAAATTACTAAA

AGAGAATTCAAAGTGGGCGATTATGTACTCATCAAGGTAGAAACAAGAAATAAAAGTCAA
GACAGATTTGAGGGACCTTATCGAATAATTGAGAAAATTCATGAAAGGCGGTATGCAGTA
CAAGGAGAAAATGGACAGTGTTTAGAAAGAAATGTGGAAAAACTTAGAAAGTTTCTTAAG
AAGGGGGATATGAGGAAATAA
>DjScaffold4337:52079-52423 strand:+
ATGGTTTATAAAGGTGTAATTGTTTATTTAGATGACATTTTAGTTTATGGAAGAACTGTC
AATGAACATAATCAAAGATTAAAAAAAGCATGTGAATTAATTAAAGTATCAAAATTAAAA
GTTAATTTAGGAAAGTTAGTATTTGGAACTGATAAATTGAATTTTTTGGGGCACACAATT
AGTCAAAAAGGAATTCAAACAAATGATGATAAAATTAAAAAAATTAAGGAAGCACCAATC
CCAGGTTGTGGTACGCAATTAAGATCATTTTTGGGACTAACTAATTATTATCAAAGATTA
TGCAACTATTACTGCGCCACTGTATGCAATGATATCAGGACATGA
>DjScaffold5170:100329-103605 strand:+
ttaattcgtggaaattagtttgtctctaaaatcatttagtttattcatgttttcttaata
tttatctttataaaataaaatttcatATGGTTCTTTTTGAAAATATTCAAGTTGGTCAGA
CAGTTGAAATTTTAAGATCCAATGGATCTATTAATTTAGGAAAAGTTCGTTTCAAAGGAA
GTATTAATGGACGTAAAGGAGACTGGGTTGGAATAGAATTGGTTTCGAAAGTTGGTAATT
CCGATGGCATGTTGTGTGGCCACCGTTATTTTATAGTTCGAAACGGTTATGGACTTTTCA
CAACTGCTGAGAAAATAAGATTTATATCTATTACTCGAAATTCCTTTAATACTTATAAAA
GTCGACCTGAATTAAAAACCGAAGATGATTTATTTAAATTCAAAACAGAAAATTCCTTAA
ATTTAGGAAATGAAAAGAAAGTACTTGTTACTAAAGATTTTGTTGAAAAATCGTTTTGCT
TAGATAAAACAATCAACAGTACTGATTATTTTACGGAAACTAGATATCCATTACGGCATC
TTATTGGTAAGGTTGAAGTTAAAAAAGATCCTGAAATAAAGCCCAAAAGATCTAAATCTT
ATCAAATACCTCTAGAAAGAAATTCATATTATTTACATCATGAAGATTTCATAACACCAT
CAGCAATTCCAGACTATTTCATTCCTAGAAACAAATTGAAAACGTTAACT
>DjScaffold3937:19200-20013 strand:+
tgttactaattcatctttagaaaatattttagaactagccaatagtatcaatgataacat
taaattcactttagagaagtcaatcgactttcagattccttttctggacaccttaatcca
aaagaaacctggcgaattattacttacccagttgtatatgaaaccatttcacagtgggca
tatattaccctggagtaaaaattggaataatcaagggtgaaaagataagagctgatagac
tatcttcaaacgaagaaATGAGACTGACTTCCCTCAGGTTGATAAAAAATAAATTTCTAA
ATAATGGTTATCCAAATAGAATTGTATATAAACATCTATACTCAAATACAGAATGTAATA
AAATCAAACAAAAGACAAATACAGTAATTAAATTACCTTTTATTAATGAGAAATGTAGCC
AAATCATGCAAAATATAATTAAAAAGTCAAAACTAAAACACAATCCAAGAATTATATTTA
AAACGCAAGCACCTTTGGGCATCCAATTACAGAAACCAAGACAAAATTTTTGTGGTCCCA
AATGCATTTGTGATGGTAGAAATATATGCAATAAAAAAAATATCACTTACAAAATAACAT
GCACCTTATGTAAGGATACATATATAGGAGAATCATTCAGAACCATCCGTACAAGAATTA
AAGAACATCTTAAACAGAAAGAAAGTGAAGTCTTCAAACACTTTCAAAACAAACATAATG
TTTTACCAGAAAATCATCATATTACATGGAAAACATTAGGAACAGGCTACATTTCTACAC
TTCACAGAAAGAAA
>DjScaffold2543:15556-16263 strand:+
taaaaattcttttagtcacatagtgttATGAATCTTAGAAGTAAGGCGTCTAATTCAACA
GAAATATCTATTCATCCGTCTATTGTAATAAATGAAAATTTTGATGACGAATATGATGAA
ATGGAATACATTTTGAATTGTAACGAAGAACTCTTAAATGAATGTGTTAAGCGTACAATT
GAAATTAGAAAACAATATAAAAAGCATGTTCCAAGAGATATTTTAATTAAAAACGCATTT
GAAGTATTATTTGGGTTATCTAATGAAATTGAAAAGCAACAAAAAGCAGAAGAAGAAGAT
ATTGAACGACGTTTTAAAACTCCTGCATCCGTTATGAAACCGAAACGTAAAATGAACGAT
ACTCTCGATTCGGTATCTAAAACAGCTAAAAAAGCAAGACTAAATAAAGACTACCAAGAT
ATGCAAACACCGAATAGGAAAGATATTGATAAAATATGGGAAAGATTAATTAGTGGAAAA
GATGATGTTTTATTCAGCAAAAACAATTCTCCTATTATCAATCCTTTTTTCAAAGAAACA
GTTACAAAGCGAAATCTCTTTTCTCGTGCTATCAAAACGATACGAAGTAAAAAATAAacg
gattccttgttatttttgtta
>DjScaffold2543:33774-48027 strand:+
ggaatcagaagtaatttaaaattatgaaattatttctattgctcttttgtcattaatcat
tgatatcgtattatacaattatcgagaagcttctcaATGGCTGAATCTTTCAAAAGTCAT
AACCCACATAAAGAAGTAGAAAATTACTCTCAACATCATTCCGATGCAGTAGATGCAAAT
TCAAAATTCAAAAACTTTTTGAAATATTTCAAGCTTAAAAATCCTAATTTTTTTTATTGT
GATGTTCTAATGGAAAATTATCACCAGAAAAAATATTATTTGGTCATTGAATTAACAGAT

TTGATTTTATTTGATAATAATCTCGCAAATAATTTGATTAAGAAACCTCATCAATTCATA
AAAATTTTTGAAACAGTAGTTAATGAAATTGCTATTGAAATGGACAGTTCTCTACTTGAA
TGTAATAATATTCAACTTGTTATTGAATGGAATATTCAGCCAATCAAAATTAAAGAAATA
ACATCAAATCAAATTTCCAAATTGGTTAAACTCAGTGGAATGGTTATTTCTGTTTCGGAA
ACTTTGTCAAGACCTGACAAAGAAAATGTATCTGTTTATTCATATCAAATGGTTAATTTA
CAAGAATCTCCGGAAAATATGTCCGATTTTGAAATGCCAATGAAAATGGAACTTTTGGTC
GATCGTTACTTGTGTGACATTGCAATACCGGGTAATCGCGTCGCAATTGTTGGAATATAT
ACGATAATATCCACACATACTTTAAATGAATTTACAAATGCCACCAGAAATTGTTACATC
AAAGTTGTCAGTATTGTCATTGAAAAGACAGGTTCCACAATAAATAGATTTAGTTCTCTT
GCAATAAATGATAATGAAGAGAATGAATTTCGTGAATTTTCTACAAAACCTGATCTTTAT
GACATAATTATTCGCAGTATTGCTCCTGGAATATATGGATATGATGACGTAAAGAAATCT
TTAGCGTGTATGTTGTTTGGAGGGTGTAGAAATAGAGTAGCAGATGATATGTATTGTCGG
GGAAATATTCATATATTGATTCTTGGGGATCCTGGTACTGGTAAAACGGAATTTCTGAAA
TTTATTAAGCAGTGTGCTCCGATTGCAGACTTGAGATCTGGATTAAACATTTCGTCGAGT
AGTTTAACAGCTTCGATCACCAGAAATAATGAATCAAGGGTTTTAATATTAGAAGGAGGA
ACTATGGTTCTTAGTGATGGTGGTGTTGTGTGTATCGATGATTTTGATAAAATAAATGAA
GATATTCGTGTGGAAATTTTCGATTCAATGACGCATCAATTCATTTCAATTTCCACAGAT
GGCATGAGACCAATAAAGATTGATAGCAGATGTTCGGTATTGGCAGTAGCAAATAGTTTA
TATGGTCGTTCAGATAACACAATTTGTCAGCAAAATATAGATTTTATTTCATCCGTTCTG
TCGAAATTCGATTTAATATTTTATCTACATGATAGTCATAACAAAGAAAAGGATCGACAA
ATCGCCAGACATATTATATCTTTATTCCAACAAGATTTTCAGGATTTCGATGAAAACATT
AGTTTGACAAGTTTAAATGGCGAGTCTTGTATAATACCATTACATAAGTTGAAGAAATAT
ATTGCATTTTGTCGAGATCGATGCGAACCCACATTGACAGATTCTGCTAGAAATATAATT
GTTGATCATTATGTTGTTATGAGATCCAATGCGCTTCGTTACAAAGAAGAAACTGGTCAA
TATTCGTCGAGTCCAGTCGATATCCGAACACTTGAAACTCTTATAAGAATATCAGAATCC
TTGGCAAAAATGCGTCTCTCTTCATATGTTGACCAATCAGATGTTCTCGAAGCGATCCGT
TTGTTAGAAATTGGGAAATGAaatgcatgaattcttttatatatatttaaaaacttcat
tttttcttatattttatcgttgataaatactttgtaaaattttgttattatactattta
aaataaattgtttctgacttga
>DjScaffold1829:38291-38765 strand:+
CAATTATTATTTCTAGTGATAATTACTCAATTTATTCAAGGACAAGAAGACAATGGTTAC
CATTTTACAAAAGAAAGAAAGAACCATGAAGATGCTGTGAAATATTGTGAATCGAAAAAT
ATGAAATTGCCAGTGATCGATAGTGAAGAAACAAATAAACTTGTGTACGATATTGCAGTA
AGAAACGGTTTAGGATCATATTGGATTAATGGAAATGATATTGGATACGAATCTGATTGG
AAAACTACAGATGGTGAATTCATTACGTTCAAACCTTGGCATCAAGTAGAACCAAATGGT
GGTCGTGGTGAAAATTGTATTCATGGACTGTTTTATCCAAATGGTATTTGGAATGATATT
TCATGTGGTTCTACACAAGGTGTAATCTGTGATCCTGGAGTATCGAAAGAAAACGCAGAA
CCTGGTAAAACTGAAGAAAAGAAAAAAATAGatctaaataaataatttaaaatgcgaTTTC
AATTATTATTTCTAGTGATAATTACTCAATTTATTCAAGGACAAGAAGACAATGGTTACC
ATTTTACAAAAGAAAGAAAGAACCATGAAGATGCTGTGAAATATTGTGAATCGAAAAATA
TGAAATTGCCAGTGATCGATANTGAAGAAACAAATAAACTTGTGTACGATATTGCAGTAA
GAAACGGTTTAGGATCATATTGGATTAATGGAAATGATATTGGATACGAATCTGATTGGA
AAACTACAGATGGTGAATTCATTACGTTCAAACCTTGGCATCAAGTAGAACCAAATGGTG
GTCGTGGTGAAAATTGTATTCATGGACTGTTTTATCCAAATGGTATTTGGAATGATATTT
CATGTGGTTCTACACAAGGTGTAATCTGTGATCCTGGAGTATCGAAAGAAAACGCAGAAC
CTGGTAAAACTGAAGAAAAGAAAAAAATAGatctaaataaataatttaaaatgcgacgaaa
aatATGAAATTGCCAGTGATCGATAGTGAAGAAACAAATAAACTTGTGTACGATATTGCA
GTAAGAAACGGTTTAGGATCATATTGGATTAATGGAAATGATATTGGATACGAATCTGAT
TGGAAAACTACAGATGGTGAATTCATTACGTTCAAACCTTGGCATCAAGTAGAACCAAAT
GGTGGTCGTGGTGAAAATTGTATTCATGGACTGTTTTATCCAAATGGTATTTGGAATGAT
ATTTCATGTGGTTCTACACAAGGTGTAATCTGTGATCCTGGAGTATCGAAAGAAAACGCA
GAACCTGGTAAAACTGAAGAAAAGAAAAAAATAGatctaaataaataatttaaaatgcga
>DjScaffold972:11809-30667 strand:+
tttatccgaaaacattaaacagtttttcaaactgaaaaatatttaaattttaaaaattgt
aatgattttttaattttatgaagtaaaattgaaattaagtaaagtttagttaaaaattt
ttgaaatttataacgataaaattttgaaatattatctctgataaacgttttggaaacatg
ttaaattcttttaataaatattttgttgtatttggagcgtttctaattgaattaactctt

ggatatttgtatagttccggaaatcttcaaaagttcattatcacttacatgaaaaataac
agttattcaagtagttctgtaaatgagggaaatacggcttggatatttggagctgaaaca
ttgggaacaggagcttcgatatctttgggggatatttagaattaaaatttggagcgtgg
atagctgttttgattggttcgtggtttatgagtttaaatttcgcattgatgtattttact
ctaaaatcatatttatcacttgttatcatatcatttgggcttttcggtggatttggagtt
ggaatcgcttacggagttgctttgagtgctgcagcaaaaagattttccaaacatagaggg
ttaatatttggaattacaatttccggaattgtcttatctacaatgctttctaaagctgtg
caaagactatttttccgagATGTTTCTAAAAAGTATCATTAATTATTACGTTAATAATAT
TAATAATGATGATAATAATAATAATAATAATAATAATAATAATAANNTATCACAGTTAAA
TGATATCCCAGCCAGTTATGAGTCTTTCAATTCAGGGACAAATGAAATTTATTTTTTTGG
CAATTCAAGTCATCCCATAGAAATTGCACCTCATAAATTAATATATGAGTATGATTTTTG
GAATTTATGGCTTCTCATGTTTTTAGTCAGTGGAAACAATCATTTCATGGTTTTATTAAA
GAAAAAATATTTTGAGATTGTTAATGATAAGTCAAGTTTTATTGATCTTTCTATTATTCT
CTCTATTTTCAACTGCTTTGGTAGAATATTTTGGGGATATTTATTTGATTATGTGCAATT
TAAGAAAATAATGATTTTTTTAAATCTGATGAATGCAATATTTATTGGAACCTTACCCGT
TTTTATTTTTCTGAAATATGAATACAAAATATATCCTGCTGTTGCATGGTTTTCAATACT
TCATTTTTTATTTGGCGGTCATTTTGCTGTTTTCCCATCTATTTGTGCGAAGCAATTTGG
AATGAAATATTTCACATGTAACTATGGAATGATTTATACATCATGTTCCGTATCGTCCAC
TATTATAAGTGTTATTTTAAATTCGATTGAAATGCGAAATACCCAAATGAGTATAATATT
TGGAACTGGAGGAATATTATCATTATTGGGTtttatccgaaaacattaaacagtttttca
aactgaaaaaatatttaaattttaaaaattgtaatgatttttaatttttatgaagtaaaa
ttgaaattaagtaaagtttagttaaaaatttttgaaatttataacgataaaatttttgaaa
tattatctctgataaacgttttggaaacatgttaaattcttttaataaatattttgttgt
atttggagcgtttctaattgaattaactcttggatatttgtatagttccggaaatcttca
aaagttcattatcacttacatgaaaaataacagttattcaagtagttctgtaaatgaggg
aaatacggcttggatatttggagctgaaacattgggaacaggagcttcgatatctttggg
ggatatttagaattaaaatttggagcgtggatagctgttttgattggttcgtggtttat
gagtttaaatttcgcattgatgtattttactctaaaatcatatttatcacttgttatcat
atcatttgggcttttcggtggatttggagttggaatcgcttacggagttgctttgagtgc
tgcagcaaaaagattttccaaacatagagggttaatatttggaattacaatttccggaat
tgtcttatctacaatgctttctaaagctgtgcaaagactatttttccgagATGTTTCTAA
AAAGTATCATTAATTATTACGTTAATAATATTAATAATGATGATAATAATAATAATAATA
ATAATAATAATAATAANNTATCACAGTTAAATGATATCCCAGCCAGTTATGAGTCTTTCA
ATTCAGGGACAAATGAAATTTATTTTTTTGGCAATTCAAGTCATCCCATAGAAATTGCAC
CTCATAAATTAATATATGAGTATGATTTTTGGAATTTATGGCTTCTCATGTTTTTAGTCA
GTGGAAACAATCATTTCATGGTTTTATTAAAGAAAAAATATTTTGAGATTGTTAATGATA
AGTCAAGTTTTATTGATCTTTCTATTATTCTCTCTATTTTCAACTGCTTTGGTAGAATAT
TTTGGGGATATTTATTTGATTATGTGCAATTTAAGAAAATAATGATTTTTTTAAATCTGA
TGAATGCAATATTTATTGGAACCTTACCCGTTTTTATTTTTCTGAAATATGAATACAAAA
TATATCCTGCTGTTGCATGGTTTTCAATACTTCATTTTTTATTTGGCGGTCATTTTGCTG
TTTTCCCATCTATTTGTGCGAAGCAATTTGGAATGAAATATTTCACATGTAACTATGGAA
TGATTTATACATCATGT
>DjScaffold972:63358-73246 strand:+
cttaacatttcaaaaaggacatcttaattgaactttatttaacttttaatatttatttaa
tattcttatattaaatacgtagatatttcttttcatgtaaattaattATGATTTTACATG
AATTAGTTTCTTTGAAATCTGACTTAAAGGACTATTCAATTTTTGTGTGTGTCATTTTGT
CTCATGGTGACGAAGGAATTATTTATGGGTTTGAGGATTCTATCGAAATAAATAAATTGT
TGGCTATTTTTAATGAGGAAAACTGTGAAGCTTTACGCAATAAACCCAAAATTTTTCTAA
TTCAAGCATGTAAAGGGGATAAATGTGACTCGGGAGCTTATGCTGATTCAATTCAATCTA
ATAATATCAATAAAATGTTCATTGAACCTAATTTTTTTATTGGATATTCAACTGTACCAG
GTTATTTTTCCTGGAGAAATCAAGTTGTAGGGAGTTGGTTTATCAGTTCGTTATGTGAGG
TTATAAATAGCAACGAAAACAGAGAAGATCTCGTTCAACTATTAGTTCGAGTAAACAATC
GAGTTTCAAAATATCGATCAAATGTCAATCGGAATGATATGAATAATAAGTTGCAAATAT
CTTCTTTTGTTTGTATGCTTATGAAAGATGTTTATCTTTAAatgctgaaatgtcttgttg
acatgttatgacgcgtaatataatattttactttgtaactatt
>DjScaffold972:143442-143663 strand:+
ATGTTCAGAGATCTGGCGGCTAGAAATGTTTTAATTAACGATAAAGATTTAATCCAAATT

ACTGATTTTGGTTTGGCTAGAATTGTTGATGAAACGGAAGACAGTATTATTATCAAATCT
GGTAAAGTTCCTATACGATGGCTTGCTTTGGAAACTTTACTTAATGCTACTTATTCTCAA
GCTTCAGATGTGTGGGCTTACGGTAAATATAATATTAAATGA
>DjScaffold3328:16581-22581 strand:+
ACAGCGGATAATGTCACACTTGTGTATAAAGGAGAAGTTCAATTTGAACAAAAAAAAGGA
ACTGCTGGAAGAGTGGTATATCGAGATTTAACTGGAGTAGCCTTGGAAAATTGTCTCTTT
TTCTTAATTGCAATAACAGAACCCGGAAAAAATATTTGCAAGTATCAATTATTTCAAGAC
AGGGATCGGGATAAATTTCCTGCCTTGCTAGAATGGGGTCATCTTTATGGATACTTCAGA
GAAGAAGACTCAAAAAATATGAATTCATTCAGCTTTTATGTAATAATTGAGAAACCAGAA
CCCTCTATGAAGCGTCTATTTGTTAGTACGACAACGGAACGTAACTGTTGGAAAAAATTGT
ATGATTCAAGGATTTTCTCGTTGGCAAAATAAAGATTCCGCTTTAAAAAATGCCGAGGAT
AAATTGAATGAAGAATTGAGAAATATAAATATGAAATGGAGTAATACTCAACATGGAATC
GACTTGATAAAGTCTCTCGATCACTCTTTAAAAGTTCAATTAGAGTATCGAACTGTTGGA
TTTCGTTGTATTTTGGAGGAAGAACTTAAACGATTAAGCGCTTCTGGTCGAAATAGTACC
ACTTCAACAGCAACAGTCACAGACAATGAATCGCGAAAAGATCAAAACGATGGATCTACT
GATGAAATTTTGGACTCATATATACCAGATTTAACCTCTCAGGAAGGAATCAATGACTGG
CTTGAAGTCTTTGTAGAATGGGGT
>DjScaffold2157:30298-44676 strand:+
caggtctgcctctccgagctcaagaaagcttgaaaagaatttaaagatactaattaggaa
aacctgacgataaaacaaaaaatgtcattcttacggcaacgattaagtataagtcgtcga
cgtgaactatctatgattaataataaATGAATGTTGGCCGAAATCGTCAGAAGTTATGCA
GACTGAGAAGCTACTGTTCTAGCCAACGTCAAAACTTTATGGAAAGAGTATGGCTTCAAA
CAGAATTAGAAACTTCAAACAAATCTATAGAAAAGAAATTCAAGAAGCAATTAAAATTAA
AAGATCAAGAAATGGAAGATTTACACGAAGAATGGAAATCATTGGAAACGCAGAAACCTC
GAGATAGTAAGGATAAAACCAAAATAAAAGAGCTGGAAGACAAAATTGAAGAATTAGAAA
ATGATATTGAATCGAAAAATAAGATTGAAGAATTAAAAAAAGAAATCGATGAATCGAAAA
AAACAATTATTAATCAAAATAAAGAAATGGTAAAAATAGTTGAATCTAAAGAGAAAGAAA
TAAAAGATATAAAATCTAAGTATAAAGAAAATGAAAATAATTTTGAGACTCGAGGTAAAA
TTTATGGAATTATTAGACAATATACACAATTGATTAAATTTAGAAAAAGAATTAGAAAGT
GAaatcgataaattgaaaaaaaataatatcgatttaacacaaacaattaataatcaaaaa
tttgaaaaagaaaagattgttaaagataaagatgaaaaaataacgaattctaattgaatg
tcgattattaaatttaaatgttctcaaataaaaagatattattaaaacaatttcataaca
atgatataacaaaaaatgcaattacttattcattaattcaatataatcatttcaatgttt
cttcatatttgtttgccaaacaattattcgtgcccatattatttccgtgattgtaaattc
ataatgttaagagttactttactttgataatctgacttcattaaattagaatatttatta
aaattcaaaattacaacaaaagttttgcaATGTCCACAGATGGTGATAAAGTAAAATCAC
TCGTGATAAAAATTGAAAGACTAAATGATAGAATATATGAATTAGAAACTTCAAACAAAT
CTATAGAAAAGAAATTCAAGAAGCAATTAAAATTAAAAGATCAAGAAATGGAAGATTTAC
ACGAAGAATGGAAATCATTGGAAACGCAGAAACCTCGAGATAGTAAGGATAAAACCAAAA
TAAAAGAGCTGGAAGACAAAATTGAAGAATTAGAAAATGATATTGAATCGAAAAATAAGA
TTGAAGAATTAAAAAAAGAAATCGATGAATCGAAAAAAACAATTATTAATCAAAATAAAG
AAATGGTAAAAATAGTTGAATCTAAAGAGAAAGAAATAAAAGATATAAAATCTAAGTATA
AAGAAAATGAAAATAATTTTGAGACTCGAGGTAAAATTTATGGAATTATTAGACAATATA
CACAATTGATTAAATTTAGAAAAAGAATTAGAAAGTGAaatcgataaattgaaaaaaaat
aatatcgatttaacacaaacaattaataatcaaaaatttgaaaaagaaaagattgttaaa
gataaagatgaaaaaataacgaattctaattgaatgtcgattattaaatttaaatgttct
caaataaaaagatattattaaaacaatttcataacaatgatataacaaaaaatgcaatta
cttattcattaattcaatataatcatttcaatgtttcttcatatttgtttgccaaacaat
tattcgtgcccatattatttccgtgattgtaaattcataatgttaagagttactttactt
tgataatctgacttcattaaattagaatatttattaaaattcaaaattacaacaa
>DjScaffold3262:65562-69960 strand:+
gtcgtagctttaacgtggtctattATGTTTTACAAAGCTCTGTTTTTTCTATTCTTGATT
AGTGCTCCATTTATTAAAGCTCAATCAAATGAGGATCCAATATCGGCATCGGCGTCAGAC
TCAGATGAAGAGGACATTGTCACGGATGACTCTGCTGCTGAAGAAGCTGAAGATAAATTA
AACGAAGATAAAATTGTTACAGAAATATTAGATAGTCAGCAAGATCAAACAGAACAGACT
TATATTGATGGAGTGCCAGTTGCATCTAAAAATGTTCAAACAGAATTATTGTTCACTGAA
CCTCCGTATAATAAGAGATCCAGTGGTGCTGGTATTGAATTACCTGCTAATAAACCTGTT

AAGATGTTAATAAGCTTCACTAATGCTGAAACTGAAACCAATAAAGACTTTAAATTAGAT
TATGTTGAAGGATTTTTACGGTATCCATCAGACTACTCGTATTACTTACAAAATTTTACT
GCTCGTCCTCTTGACATCACAGTTGAACCTAAAAGAGAAGCGACAGTTTATTATTCATTT
ACTCCAGCTCAACAATTAGCTGGAAGACCTTTTGGATTGACTTTAAGAATTGGATATCAC
AATAATGATGGTAAACTGTTCACGAGCACATTTTTCAATGATACTGTCCAGGTAATCGAA
ATTGAAGAAGGTTTTGATACTGAATCTCTATTTCTGTACGTTTTGGTTGCAGCTGTTTTG
GGATTAGTGTTATTTGGTGTTTACCATCTGTTTACTGTTTATGCATTGAAAAAATTCAAG
AAAAGTGGAGTCAGTAAACAAGTAATGGAACTGGGAACATCTAACAAAGCAGATCTTGAT
CTTCAGTGGATTCCTGCTGAACACTTGAAAGTTCGATTATCACCAACCATTTCTCCCAGA
AAACGAAAGAGTCGGCGACATGAAGATAATCACGTCACTGGACATTAAataaactttttt
tttgattggaatatttacttacgaaaatttgtatttagttttactgataattcctttga
atgattgattgattgattgattgactaattgattgtgcaataaaagtatggtgttgattc
atattccttgtttgtataatatatatattgattggtttgttaataatttgtgacgtgtgt
gtgtgtcaatagattttttgctgtgaataatcgattaattgatgagagaaaacgattttc
taatgcagcttacatctatttttgttttacgctttcattttttaattaacaattaactgt
ttaataatatttcgtaccttttttgtgtgcggtttataaattttatgtcaattggtggctt
tttgttctgtttaatcaaaatggatttcaatgttagacgcgttctttcattttacttgct
gcttagtttgtaattaaatagtattttgctgatgacatcaataatgagaaagattcttgt
ttcacaaagtgctggttaattgatttttacctgcgaatattgatccaaaattttaatagg
aaatcggtaaaaacttgacttcattttgtaccaaaattatttgtgattaatcttggctaa
ccgcagaagttgtatatttttcacaaaagaatcaatttgtggtcaatacatgtaggtttt
gtcaattttataatttttgcacgaatactttttttgagaaataattatttggattttgttt
aaatgaatttttacatttacga
>DjScaffold3262:86423-90415 strand:+
GAATTATTGAAAGCTTGCAAAGAAGGAAGTCATAAGAAAGTAAAAAAACTATTAGAAAAT
TGTACTGACATTTGCGATGTCCGTGATCTTACTGATAAAACTTTACTTCACTATTGTTTT
AAATATGAACCGTGTGCTGAATTAATTTTGCAAGCTGCTCCTGAACTCTTAGATATTGGA
GAAAAGAATGGGTATACTGTTCTACATTTGGCATCAATGATCAAACCTCCTACTTCTCTG
GAATTTCTTTTATCGAAAAACCCAGACATAAACAAACGAAATAACGATTCTCGAAGTTCT
CTACTTCTTGCGGTTATTTATGACAATGCGGAAGCTGTTAAAATTCTTTGTGAACATAAA
GCCGAAGTAAATATTACAGATATTAATGGAGTTTCTCCCTTGCATTATGCGGCCAAAGTT
AAATTTGACAATAATTATGAAAATGAAACGCATATAATCAGCATTCTATTAAAATATGGA
GCTAATATCGATGGTTTGGATTTGTATGAGAGATCTCCTTTGATGTGGGCTGTCTGTTCC
AATAATATTGTTAAAATGAAATATTTAATAGAAAAAGGAGCGAATATTTATTCAAAAGAT
TCAGGAGGATTGTCAATTTTACATTATGCTACATCCAACGGAAACAAAGATATCATAAAA
CTTCTACTCGAAGATTACAATTTCAATTTGGAAACTCTTGATTTCAATCATTGCACAGCG
TTAATGTACGGCTGTTCATATGACAGATTAGAAATTGTGGAATTACTATTGGAAAAAGGA
TCTAATGTGAACGCTGAAGATTGTAAACGAAGAAATGCAGCTCATTTTGCCAGTGGAAAA
GGTGACTTGAGAAATCTGGAGTGTCTCTATAGATTTAATGTCGATCTTTGGAAATTAAAC
AACGACAGTAATTATCCTCTTCATGAAGCTGCTTATAAAGGTCATTTGGAAATCGTCGAG
TTCTTGATGCAACATTTGGATTCTAATCATAAACTTTTAAATGTAATTAATCATAAAAAT
TCCAACGGCCAAACTCCTCTTCATTTGTGTGCTGTCAAAGATAATTTTCCGATTTGTAAA
ATTCTCCTCGAAAATGGAGCAGATCCGAATATTCCGTTACGTGTTAGTAATGGAAAATCA
TTGACAGCTTTGGATTTAGCTAATCGAGACGAAACGAAACGTTTGATTAGTGAATTTAAA
GGAAAAACTTCAGATAATTTATTGAAAACTTCGGTTATCAATGAAGAACCTACGAAATCT
TTTAGTGGAGAATCATCGGAAATCGATGAAAATGAGATTAGTAGTATTTTGTCGGATAGA
GAAATTGATGAAAGTGAAAATGAAGATGAAGAGAAACTAAGTGATGGTAATGTTTCAATT
GAAAGATCTAATTCAAAATCGAGTCTGTCGAAGAAACAAAATAATAATGAATCTACAGAA
AATAATACACAACAAACAATTAATGAAACATTTTACGAAACCGAAACATTGGAAATAAAT
AAAAATAATAATAACAATAATAATAATAATAATAATAACAATAATAATAATAACAATAAT
ATTAATATTAATAATAATAATAAAGAAAAAGAAAATAAAATTGAATCCGATAAAACAAAA
TCAAAAATTGAAATATCTAATTATCACAATACAAATACTGATACAGAAAACAATAAATTA
GAATTAACACAATATGAATCAAATACAAATGATAATTTGGAAAATAATAGAATTAATCAT
GCAACAACTTATGAAAGAAAACATTTTGAGATAAATGAAATTTATAAAAACCATTTATTG
AAATCATGTTTGGAGGGGAATCTCGAAGAAATATTATCTTTCATTAACAATGGAGGGAAT
CTGTTAAACATTACTGATAAATTAGGAAGAACTTTATTGCATTTTTCGTTGAAAAATGAA
GATTTATTTGAAATGATCATTGGAAATGCTCCGGAATTGGTAAATATGTGTGATGAGACT

AATGTTACTGTATTGCATTTAGTTATTATGTCAAAAGAAACCAAACGTTTACCGTCATTA
TTAGCCAATAAAGCAGATATTAATTTCCCCGATAACAAAGGAAGAACTGCGTTGCATTTA
GCTGTGATTTACAATAATTTTGAAGCGTTAGAGTTTATGAATGATTACATTAAAGATAAA
ACTGAATTTCATCATTTACCCGATCTTTGTGGAAATTATCCATTACATAATACTGTGGAA
TTTTGTAAAGCGAAAAAATCTGTTTGTCAGTGTAAAAAATTTATTGAATTATTGATGGAA
ATGAATGGTAATATCGAATGTCGTGATAAAATCAACAGAACACCTTTATTATTAGCTATT
AATATCAATCACACTGAAGCCGTTTTTACACTTCTTAAAAACGGTGCGGATTACATGGCA
AAAGACAGTCAAAATCTTTGCAGTATACATTATGCATGTTCTGAAGGAAATTTAGAAATA
CTGATTGAGTTGACTGGATTGGATAATTTTGATTCTAATGTTCAAGATCCTAATGGTTTA
ACTCCAATATTCTATGCGGTATCGAATTGTAATGAAAAAATTGTCAAGAAACTTATTGAA
TTCAATGCGAAAATCGATATTGTCGATCAATATGGGAGAAATCTTTGTCATAAAGCTGCA
GAAAAAGGAGCTTTTAAATGTTTAGAACTTTTATTGTCATACAATATAGATCCATGGTGT
AAATCAAAGGATGGAACAATGCCACTTCATGAAGCAGCGTATAGAGGATATAAGGAAGTA
GCTGAAATTTTATTGAATACGCCGTCAGCCGCAGAAAATATCAATTTGAAAAATAGTAGT
GGTCAAACAGCATTGCATTTAGCAGCAGCTAGAAATGATTGCCCAATGTGCTATTTGCTA
ATTTCTAAAGGTAGTAGTATTAATGAATTACTTCACGGTCACAATGATTGTGAAGATCTA
ACAGCCTACGATCTTGCTACAACACCAGAATGTAAAGGTACATTACGGGATTACGGAGGA
CGGTCTGCAAAAAGAATTATAGATTGTATTGAATCCGAAATAGAATGTAATAATCCGGAT
AATTTTGAAGAATTTCCAATAATTAAAGATATTGATTATGAAAATAATCTCCCAGTATCA
ATTAGAAGTACAGAATTGGCTTTAAGTACAATTCCAAGTGTTGTTTATACTGTTCGCACA
AATGAAGTGTTTAATTCAGCAAATACGACATCGATTCAACCAGCAAAAAGTTCAAAAACT
TCATCGAAAAAATCTGATAATTCTAGTAAAATGAAAATTTCCATTTCAAGCAATGAAAAA
TCACCAAAGAGTCTCTTATCTACCAGTGAATGCAAACCGGAAATTTGTGTTTCTCCGAAA
AATGATAATGTTTCTGCGATTAATAAATTTGATAGTGAAAGAGATATAAAAAACCATCAA
GAAAGATTATCAAGTAGAAAAAAAAACAAATTTAAGTTCGCATTCAGAAATTATAGAACCG
AAAAAACCTCCATCGAATCGTACTAACTCTTCTAGTGGATCTGAAATGAAAAAATCCAGT
TCCCGATCAAAGTTTAGTAGTTCATCCTATAAGAAACCGATAAAACAATATTCAACACAG
AAAGTCATGGAAATTAAAATACAAAATTCAGTTATAAATTACGAATATATCAGATTGAAT
TGTAAAATAGCGAATGATCTCGCCAGACTGAAAATTCGCGAGAAAAATATTGAAAAATTG
ATTAATCCGAAAATTAGTAAACTTCAGAAATCTTCAAAATCTTTAAAGCAAATATAAtaa
acaaaagatttgagtagcctttaattcgaaatatatgaagaaatgttttcagtttgggat
gattttctttaaatttctttattaatttcaacagttttgatatattttccaatattggat
atattttccaataataatctttcataaaaataaaaatgtgattttgg
>DjScaffold2255:31725-32196 strand:+
GGTTTCGGATTAGCTTGGGCTATTTCTGAACATATTGCGACAGAATTAAAAGCATTCAGT
GTGTTTGCAACACACTTTCATGAGTTAACAGAACTGGAAAAGATACATGGAAATGTGAAG
AATTTGCATGTATCTGCGGAGACTATTAGAAATGAATTGATATTTTTGTATAAAGTCGAA
GAAGGAGCCTCAATGAAAAGCTATGGTATTGAAGTTGCGAAATCATCGGGTTTACCTGAT
GAAACTATTGAATTGGCTAAAATCAAGTTAGGGGAATACGAAAATTTGATGGAATGGGAT
TGCAGCAGCTCAAATAAAGTTGAAGAAAATAGAAAGTGTCTCATTGAATTTTGTGACAGA
AAATTAGATGAAAAAATTGATTTATCGGATTTTCTTAAAGACTTACGTGAACATTTGAAT
GTTTAActg
>DjScaffold2119:182253-191739 strand:+
acaatgattgaaagcaccaaacgaactcacatacaaacaagtaaaatatacacaacgtta
ggaattagtttaactgtgttgttattttatttgattgttttcaattaaattaatataaag
ttggtcttatacatagaatattatgtggttgattatattgatctagtaattagtgatcat
tttcttttgatatcatcaggatatagaaaactggtaaataatattatgaatttaccagca
aattttgctgtaagattttatttatttttaattgactttattttaatattttaaaatat
atttttcttcagaatgaattagcgcttaaattgaaaaataaaaatccaccaaagccgaaa
aaagatgacgaaagtgattcgtcaaaaactcccgattcacctcctggaaattgtactgga
acttctcctacccgtcaagtattcaacggatctagaactccttattcaATGTCATCTTTA
TCTCACAGCTCAAACAGCCTGGTATCCGGAAATACTGCTAACAATTATGGAACTAGTGAT
GGTGAATCAATGTCTCGTTCTGTAATTGATTCTACCGATGACATTGGTATTGTTGTCGAT
TTAGCAACACCAATTAACAAAACCACAACTGCTAGTTTATTAGACTCAACTGCTGCTCGC
CAAAAAATCGCTGCATCTCGTCCAAGGCGTGCTCGTCCGAAGGCTAATGATCATACAAAA
TCTGTCTCAGGGGATAAATTAAAGGATAGTGGAAATTTTCTCCTGTTAGAGAAAAAAGA
GATATTTTAGAAGAAATCAGTGAAGAAGATTCTCAAGCACCTTCCAATTCACCTACAAGT

ACAGAAGATGATTTGCTTGGTTCTTCTTTGGTTACAGAAACAAAGTTAGAATGCACTTTA
TTATCTGAAACTACTAGTTCACCTATTCAATCAAGTCCTTTAGATCTTATTAGTCATAAT
GATAATAAAGATGTGCCTTTTCAGAATTTTCCTAAACCAGATTTAAATATGCTTCCTCGT
CCTGTATCTGATCCGTCCTCTGTTATTGATGTTCAACCTAGTGGATGTGGTGATTCAATT
TTAAAATCAATATCACATGATAACAGTTCTAATAAAAATAATAAAGTTGAAACTATAAAA
AGTTCACATTCTGAAAAAGAAGCAACACTTTCGAATTTTAAAACAAGCAAAATGAAATTT
GAAGAGCAAGAAAAACTTCCACTATCCTTTGTCAGTTTACGTCCAGTAAATACAAGAATT
AATGCTGGATTAGAAGTTCTGAATAAAGATGGTGTAGGATATCAAAATAAATGCACGTCT
TGGGCACCTGGTAATAAAATCAAGATTGAACCGGGAAATGAATTAAAACATCAAGCTTTT
GATCATATTGATAGCAAACCCAAAATAAATCGTTATACTCCGATAAGTACAACCAAAAAC
CAGCACATTGTTATTAATAGCCCTTCTTGTCAAGCAGTTCAGAAAGATGAAGTAAATGAT
AAACGAAATTCACGTGTATATGACATGATCAAAAATTTAGAAAATAGTGTCCATTGAtct
tgattcttattgagactggatttttggagttatcttcttaaatatgctcattatttatt
tactacattaaagttataactatattactttcgtgttgatttattaatcctgtcgtagtc
aaaatgaattgaaaagagttgttatgaagagtttttaaattttattttgatttctattat
tgtattattggtaataactatgattatttttatctcggttatttttgtgaaatatgtat
aaattttgacgctttgtcgggtgaatttcattttaatattatgtgaccaacttccgtatt
cagtttgtttaaattgtttcatttcaagtctataatatcgttttaatgtatgccttttcg
aaattgtcttagatttgtattacagcataattcaataaacatatatgattag
>DjScaffold3293:10560-11192 strand:+
ATGAAAATTGGAAAATGCTTAATATATTTAGACAATGTTTTAATATTCGGTAAAACATTA
GAAGACCACAATGAAAGACTTGATCAAATATTAAGAAAAATAAAAAAAGCTGGACTTAAA
TTAGCAACAAATAAATGTAAAGTTTTACAGGAAAAAATACAATATCTCGAACATATAGTA
ACTAAAAAATGGAATCGCAACAGATCCTGATAAGATAATGGCCATCCAAAAATGGGAGATT
TATCAATGTCGAAAAGAACTTCAGATATTTTTAGGGTTTTGCAATTATTATAGGCGATTC
ATAAAAAATTATGCAAATTTATCAGAACCTCTCAGTTCGAGGCTAAGGAAGGAAAACAAA
TTTCAGTGGACAGATGAAAATAAAGAATGTTTTTACAAGCTACGTAAAAGGTTGACAGAA
TCACCAGTACTAGCTTTGCCAACAAAGGATGGTAAATATATCTTGGATACAGATGCTTCT
CACAACGCGATTGGTGGTGTACTTTCTCAAGTACAAAATGGCAAGGAACGAGTATTATAT
TATGCATCGAAGACACTAACAAAAAGTAAAAAACAGTACTGCATCACAAGAAAAGAGCTT
CTGGCAATATATATATATTTGTCCTCAAGTTTTAG
>DjScaffold1915:90092-106542 strand:+
aatatttttaagtaaaaattttatattattagattattgaaaataaatctgaaacatatt
atggagacagtgaagaagataatgaagatgaagaagacATGGATATTAATAAAATTAATG
ATTATGTTGATTATTTACTCACTAAAAAAAAAATTTCAATCAGTGATGATAAAGCAAATG
ATATTATTAAAACTCCGTTAACAATGTTTCAAGAATCGTCTTCTTCAATTAGCAAATTTA
ATACAACAGAAGCCTATCAAAAATGTAATCTAGATTATATTCCGAATACTGAATCCACCG
ACATTTTAGCTGAGTGGCGATTGCGCAGAAAAATGAATGAAGCTGAAAATTCTGTTAGAA
TAGAATATCAAAGACATGACCTACACATTCATTCGCTAAATTCTCAAATAAATGAAAACT
GTCAACCTTTATCAATTAAACCATCTATTTCTTTATCAAATAACATAAGTACTATTAACA
AAACAAACCAATCCAATAAATTAGAAGACTGTGATCAAGTTCCAGTTCGTGATGTCATTA
TTAACGAAAAATCTTTTACTTCAACAATTGACAAGGATAAAATTAAAAAAAATCCTTTTA
CTACTGATGCAAGTTGTCAATGCCAATTAAATCAGTCTAATTTGACAAAAACTTCTCAAA
AAAGCCATGCTGTAAAAACTTGTAGAAAAACTCGAAATGTTGCAGTTATGACAAATATGA
GGCATAGTAAGGTTTCTGAACTTACTAATGATTCCACTCAAACTGAAAACACTTCAAATG
ATTCTATCAATTCAATAATTGTTAATAGGTCGACAGAATGGGTTGATAAAAATGTCGGTC
ATGATTATATTGACAGAGCAATTGAAAAAACTCTTGAATCGCAATTGTTTGATAGAAATT
ATTCACCTGCAATGTCTACAATTACTTCTATTAATGAATCACAAAATAATGATGTCACTG
TGCAAAAAAATCCACAGAATCTCTGTACAGACGATAATAATAGAAAAATTATTAATAATG
GTTAGcaattgaatgcattttatttttctattcaaatattctaatttatagggtttaca
tatatactaaataatttttaacatttcaattaatttttgattaatattttaaataatcat
aacctcgatactaaa
>DjScaffold2493:18010-20985 strand:+
ctattttaattttagagtcaatttcattctttacagtattctgagttcttaaataatagt
tgtccaaatggagttccaatatttttaaacgttttagatctctgtgttaataatacccga
acatggtttgctgtaatatgtggaatattatctattttagcttgggttgtatatggccta
cctcaaATGATAATCAATTGCAGAAAAGGAATTCCTGATGAAGCAATATCACCATGGCTG

TTAATATTCTGGACAATTGGTGATACAAGTAATATGGTTGGTGCAATTTTAACCAAACAA
TTGATATTACAAATTGCTATGGCTGCATATGCAATATTTTGCGACATTATTCTTGTTCTT
CAATTTTCTTATGCAATTCTAAAAAATCGATGGAAAAGAAAAAAATCATTTGGACCACAA
AATGCAATTTATAATGAAAATGAAAATTATATAGATTCTGTCAGTGTAAATACTCCAGGA
AATTCCAGTCCTAATAAAGCAAAAGTGACTGGAGCAGCATTATTTTGCATATCAATATTT
TGTATTAATTTTATCTCATTTCAAAATTATCCTGACACAAAATACGTTGGCAGAACTTTA
CTAAGTCAAAATGATTCACCAAACCAATTTTTTACAGGGTTAGTAGAGATTTTTGGATAT
GTACTTGGATGTCTTTCCAGTTTATTCTACAGTAGTGGCCGTATCCCACAGATTTGTATG
AATCTTCGTCGGAAATGTACTGATGGTTTAAGTGCTGGATTATTTATTTTCGCTATTCTC
GGTAATGCTACGTATGGTGCACAGATTTTATTAGTATCTGTGGAATATATGTATATTATA
AAGCATTTGCCTTGGATTTTAGGAAGCTGGGGTGTGATGATTCTTGACTGCATTATTCTT
CTTCAGTTTTATACTTATCGAAAGAATAAACTGCCGGCGTACACGATCATCAATGATTTG
GAGGACGATGATTCTGGATCAGATAGCGGATTATTATCTTGAatttttactgaattctgc
ttggcattatttattgttaactgatatatcggtttatcgtgagggttggagagagaatgg
attgatttttaacgagttataataataatatttttactaataaaattattctttttgga
tatttcagattgctATGGCTGCATATGCAATATTTTGCGACATTATTCTTGTTCTTCAAT
TTTCTTATGCAATTCTAAAAAATCGATGGAAAAGAAAAAAATCATTTGGACCACAAAATG
CAATTTATAATGAAAATGAAAATTATATAGATTCTGTCAGTGTAAATACTCCAGGAAATT
CCAGTCCTAATAAAGCAAAAGTGACTGGAGCAGCATTATTTTGCATATCAATATTTTGTA
TTAATTTTATCTCATTTCAAAATTATCCTGACACAAAATACGTTGGCAGAACTTTACTAA
GTCAAAATGATTCACCAAACCAATTTTTTACAGGGTTAGTAGAGATTTTTGGATATGTAC
TTGGATGTCTTTCCAGTTTATTCTACAGTAGTGGCCGTATCCCACAGATTTGTATGAATC
TTCGTCGGAAATGTACTGATGGTTTAAGTGCTGGATTATTTATTTTCGCTATTCTCGGTA
ATGCTACGTATGGTGCACAGATTTTATTAGTATCTGTGGAATATATGTATATTATAAAGC
ATTTGCCTTGGATTTTAGGAAGCTGGGGTGTGATGATTCTTGACTGCATTATTCTTCTTC
AGTTTTATACTTATCGAAAGAATAAACTGCCGGCGTACACGATCATCAATGATTTGGAGG
ACGATGATTCTGGATCAGATAGCGGATTATTATCTTGAatttttactgaattctgcttgg
cattatttattgttaactgatatatcggtttatcgtgagggttggagagagaatggattg
atttttaacgagttataataataatatttttactaataaaattattcttttaaAAAAAT
CTATTGCTTACAGAATCATTTGGACCACAAAATGCAATTTATAATGAAAATGAAAATTAT
ATAGATTCTGTCAGTGTAAATACTCCAGGAAATTCCAGTCCTAATAAAGCAAAAGTGACT
GGAGCAGCATTATTTTGCATATCAATATTTTGTATTAATTTTATCTCATTTCAAAATTAT
CCTGACACAAAATACGTTGGCAGAACTTTACTAAGTCAAAATGATTCACCAAACCAATTT
TTTACAGGGTTAGTAGAGATTTTTGGATATGTACTTGGATGTCTTTCCAGTTTATTCTAC
AGTAGTGGCCGTATCCCACAGATTTGTATGAATCTTCGTCGGAAATGTACTGATGGTTTA
AGTGCTGGATTATTTATTTTCGCTATTCTCGGTAATGCTACGTATGGTGCACAGATTTTA
TTAGTATCTGTGGAATATATGTATATTATAAAGCATTTGCCTTGGATTTTAGGAAGCTGG
GGTGTGATGATTCTTGACTGCATTATTCTTCTTCAGTTTTATACTTATCGAAAGAATAAA
CTGCCGGCGTACACGATCATCAATGATTTGGAGGACGATGATTCTGGATCAGATAGCGGA
TTATTATCTTGAatttttactgaattctgcttggcattatttattgttaactgatatatc
ggtttatcgtgagggttggagagagaatggattgatttttaacgagttataataataat
atttttactaataaaattattcttttaa
>DjScaffold3645:24929-44771 strand:+
atactacttgaattaatattcgtttcatgacaacagatcacgcaacaatcaaaatgaaga
aaacATGTATTTCATGGGAATAAAAACAGAAAATCTGTTTGTAGAAGCTATATCTAATAG
ATTATTGAGATCTTTGTTAGATAGTAAATATATTCAATGTGAAATAGTTTATCAATTTTG
TTTGGGGTTACTAGCACACCAAGTCATGAAACGCACAGTTGAATATTACTGTGATCCTAT
AAATCATAATCAATGGTTGTTCCAAAAACTCATTGAAAAAAAAATTACTCGTTCAAAACT
AAATAAACAATTACCACATTTCCAGTATGATATGAGTTTCGAGGATTTTTTCAAGATGGT
GGAGTCGATAAATGATCTTAATGAGGTGAAGCAAATAAAACAGAAACTGTTAAATGAATT
GAGAATGGCTGAAGTTTTAAAATTCACTCAGAATATTGATTCAAAAAATCCAAATAAGAA
GAGTTTGGATCGAAACAAAATCAGCAGAAAAACCTATAAAGGTCATTTGCTTACCCAGAG
GGATTTAAAATGTTATTCTAACCAATTATCGACTTTGTTGGACACAGTGAACACTAAATT
GAAAATGATGACCCGCGGAAAAGCAAGCATAAATATCAGTACAGTGTCGTTGACAAAATC
ATCGAAACGTCATAAAAGTATTCGTCGAAACTCTAAATCTGAAAAGCAAACCTTATCAAA
GAAAATTAAATCCAAAGTGACTGGAAATTTCAATAGAAAATTGAGTAGTGCGAGATCTAT
AAATGAGAAGAATGCCGCTTTAAATGAGTGCATCCCATATGAAACAATTATGGATAGCAA

TGAAGGCAGAAAATTTTTCAAAGAATTTTTAAATACGACTGCTTATAGTCGAACCGTAGA
GTTGATTGAAATGTTAAATCATCTCCAAAATCATCCGGAAGATCAATATGTTATTGCCCA
AGGAGTAGTCAAACGATATATTCATTCATCTCCTCCAGCCTTTGACTTTGATAAACAAAT
AGTTAAGGAAATGGAAGAATTTCTTAAAGGAAATTCGAGCGAAAAAGTATTTTTGAAAGC
AAAATCTATCTGTGATAAGGAATTGAAGCAACGATGGTATCCGTATTTCATTTTGAGTCC
TCATCACAGTAAATTTTCGAAACATCTGAGCAAAGAATTGAATTCCAGGGAATCTATGAA
AATGATGGATATTTCGCCTCCCGGTTTGTCTACTGATAGTGAAAATTCAGATATCGATTC
TGATGTATCTGGAGATGAATCACAAGATGTCCTTGTTTCAAGAAACAATAATGAAAAATT
ATTGACAGATAATGATGACGATAATCCACCACTGTTATTTGACTTCACACAAAGTCTTCC
TAAAGATAATCAAGAAGAAACACAATCAATACAAACAGATGATATCGAAAATAATAAACT
CGTGTCGGATACATTTATTTTACAAACAGATGAAGAACTTGAAGAAGAAGAAGATGGTTT
CCTTAAACCAATATTTAATAGCGCTCAAATAGTAAGTGGCAGTGAAACCATGGAGTATTA
CCATCAGAAAATTCAAAAATTAAATAAGAAAATTGAAGTTAAAGAAAAGGCATTTAATGC
TGGTGTTGCTGTATCTAACCAGATGCCATTAAGTAAAAAGGAAAGATCTAAGATGAAAAT
TGACTTAAATTCTGTGAAAGAAGATTTAAATCGATTGTATGAAAAACGAAACAAATGCCA
ATACAATTTAAGTAGAGTTGATAGATGGATAATTTCTATGGGAAATTGGAATGCAATAGT
TTATGATGTTATTTTGCCAGAACCTAAAAGAGGTACATCATTAGATTTAGGATATTTGCC
AACATTAGCTGAGTCCAAATCGGGAACCATGGTTATGGATAAAGATGATATATCAACTGA
TGATGATACGAAACAAGTCATTATTTTTATAATGGTTAGTCTTGCATCGTCCTCATTGGA
TCAATACAATGGTAAAGAAGATAATGGCTGGCTTGTTAAACACACTCTGAAAGATTTTCA
AGCTTTAAAGCGAGAGCTAATACCCGTTCATCCTGCAATTGATAGAATCAGTCTTCCGAA
AGTTCCTCAGCGTTTGTTCATGCGCAGAAAGTACACAGATAATGATCTCACTCATATTAA
AGATTCGCTTAATAAATTTATTCAATTTGTTATGAAAACAGAAATTTTGGCTAAATCGGA
ACCAGTTTTTAAATTTTTCTGTTCTTCAATGGAAAGTAGTCAAATGCCAAACGAAGATAC
TTTAATGGGTCCTTTATCGACCTTTGCCAATACTTTGTCAGATGCCAGTGCTAAAGTTAT
GTTAATGGCTAAAATACCCAGTAAATTTGTTCAGAAAGTTCTTGCCCCTCGAAAACATTC
CGGAAGTGTTACTAGTTTAGACAGTGAAAGCACAGACAGTAAATCTGACAAAAAGAATAC
TTCTAGACGAATTTCTGGTTCAAGTTATATTGGTGGTAGTCTGAAACACAAACTTAGAAA
ATTCAGTCACTCCAAAGATATTTCCATGTCTGCATTTGAATTGTTAGAAGAACTGATACA
ATTACGGAAATCATTGACGTTAACAAGGAGATCCATGATTACTTTCGTTCAAAGTTTTTT
CGGTAAAAATCTTACCCAAATATTAACTGAAAAAGTAGCCTGGATAAAGTCAGAGTCTAC
AGTTTTCGTTTGTTTGCAAAAATATCGTGATAGTTTTCGAGTAGACATGTCAACATCATT
ACAAAATAGTATGGAAGCACCAGCAGTAGAAGGAGAATCAAAATTACATCCGTCCGCATC
CGCATTTTTAACTCCACACCTGTCAGGTGCTACTGAAACTAATCTACAGCATGTTAATTC
TAATTCATCTCCAACAATGAACAAGGCGAGATTTTATCAGAAAAATGACACGAGCAGTTG
TGAGAATTTTGACAGTTATTATCCAGCGCAGCCAACTGATAAACAAGTAGAAAATGAAAC
TGGTTCCATGAAAGCTGGTATCAATTTTAATCAAACTTGGACATATTCTATTAAATGCGA
TGAAGCAGTCAAACGGTTATTATGGGAATTAGTTCCAGAATACTGGAAAACTTTATTTGG
AGCGATGAGTGTGGCCGACAGTGTGGGTTTCGTTTTTGAGTCCTTGCAAAGTTTAGAAGC
TAACAAAGCATTGGTTTACAGAATCCTACAATCATTTGTCGATGAGCTTTTGGAAGAAGA
TGATGAACCGCTTTATTTAACAGCGTAAaattgagattctattgattattctgttctttc
tgtcgtgatttttaaattttgttatcagagtgttattgtaacaaccgtatttttattaaa
aataaattgtttttattgttttgtgacaa
>DjScaffold2481:69790-70486 strand:+
attgcttcaaataatttttcaaatccagctATGAAATTAATTATTAATTTCAATAATATTG
ATAAATTTGAAATTGATTTCCCTATATTCACAATTGGAGATATATACAGGGAAATAAAAA
GAGAAATATTCTCTCTGGATTGTGAATTAATTTATAAAAATATTATAATTGATTCAAGCA
AATTATATAAATTCTATCAAATATCAGAAAGTGATGAAATTTGGTATGGGCCGATTGGTA
ACTATTTGAAAGAAGATGAAATTATTATGGAATTGTTTATACCAATATCGGATGGAGATA
TTATTTACATTATCATCAAGAAATCTGATTCGATATCGAAAATTAAATCAATAATTAATA
AAATAATTCCAAACATTTTTAATATTTGGAATATGAAGGGACAGCAACTAGACGATGATC
AAATTATCAGTGATATCGATTTTGAAAATGAATATTACAAATTAATTGTTGATGTGGAAC
CTGACGACGGAATATTTGAAGAATTTTGTGAAATTCATGATGACTATTTCATACATAATT
TACTAAAAGACTTTGTTATGATA
>DjScaffold2481:81013-89446 strand:+
tctaagtcttttaatttattgaaatattttaaacaataatttgttggcataaatatcaata
taagctatatcatattttaaatgttttattttgaaagaaagtatttatttagcaagaaat

atatttttttagttattatggtttatttgataaaggaaaatcgattattgatttattata
tattgagATGCACGAGTGGATTGGATGTTTCATCAGAGTCAAAAATACTTTACTAGATTC
AAAATATTTGAATTTACTTGAGATTTTTGATGAAAATAAATGTTCTAAAAAAATCTATAA
TTTTGGCATAAATGAATGGAAAGCAAGAATAAATAATGCAAAACAGGAAGTCATGTCAAA
ACCAATATATGATTACTATTTTCCATCTGGATATGCATATTCAGTATTTTGGTTTTTCAA
TGAAATTTTTGATATAATTGATGAAATTTTGTTAGAATTCACTGATTATCATTGGAAATT
CCAAACAATATTAAGGATAGCAATAAAATATTATATACAAGAACAAAAATATCATTTGAA
TATTGCTGATAAATCAATTAGAAACATTTTAATGGATATTTTGAATTATTGTGAAAACAT
TTTTTATCATTTAGATGATAAATTTGAATGTTTAGTCGAACAATTAGTCAATTCATTCAC
AGTGATAAGTTACAAATATGATGAAATTTGTGATATTTTTGATGATTATCCTGATGCAAT
GAATATTTCTTTCATGTTCAAAAGGCAAAAACGCTGGATCTCTTCAATCAAACCCAAAAC
TATTCTTGGTGTTAAAAAAGCTCTAGAGTATCAAACAAAATATCCAAAAGAACAATTTTA
TATAATAAATAGTGTCGGTGAGAAACCAAATGATAATGAAAAATTTCAAACAGATTATAG
TATTGAATTTAGCTTCCCTATGGACGATATTGATATATTACGAATGCTTGGTTCCGGAGC
GTTTGGAAATGTTTATTTGTGTGAGGATAAATTCTTCAGAAAACAATTTGCATTGAAAGT
CCCGCTTAATAATCAAAATGATTTGACTAGAGATATTGTCATTAATCGACATTTGAGACA
CTCAAAGACTTTATTAAGCTATTCAGAATTATTTAGATATAATGAATGCATTTGTATGGC
CATAGAATATTCAAAAAAGGGTTCTCTGTCATCATATTTGGAGAAAAACAAAATAATATC
AGAACAACAAATTGCAAAATGGTGTCATCAAATATTAATACAAATCAATGATTTACATAA
ACAACGTATAATTCATCGAGACATAAAAGGTGACAACATTACTTTGGATGAAAATCTGAA
AGCTCATTTAATTGATTTTGGAGTTTCAAAGTATGCGGATGACACACTTTTGGTAAAAAA
TTATTTTGTAGGAACAAGAAACTACATGGCACCAGAATATATTGATAAGGACACATATTC
AAATAAAATAGATATTTGGTCGTTTGGAGCAACTGTTATAGAAATGGTGTCAGGAGAAAT
GCCATTTTCCAAAATGAATCCAGTTCAAGCATCATTTCAAATTTCCAAAGCAAAAAAAAT
CAGATATAAACCTCGTCAACGAATATCGAATGAACTAAAGGATTTCATTGATTTATGTTT
AACACCAAACGAAAATGAGAGACCATCTGCTGAACAATTGATTCAGTCACAATTTATATT
GAACAATTCGAATGAAATTAATAAATAAaattttattaacacttgaaatttattgaataa
cctaatcaataatttcaataaagagtgactcgccaattttactctaattgtatttttat
gtcgagggtagtatgaaatgagattaaaataaagatactattttaagtattttctcgatg
aattgtttatatcttattggatgatatcaattgatctggaaatgaaattataaaatatgt
tatgtaataatgtatgatgtaattattgaaaaaaataatattatgacattgatcaattaa
atattgcattaaattattaataaatatctcggttttatgataaattgagtcaacgttaaa
tatatctgtttgaaagaaagtatttatttagcaagaaatatattttttagttattatgg
tttatttgataaaggaaaatcgattattgatttattatatattgagatgcacgagtggat
tggatgtttcatcagagtcaagtaagtggtatattgttgaaaatttatattttgcaaaag
aaatactttactagattcaaaatatttgaatttacttgagatttttgatgaaaataaatg
ttctaaaaaaatctataattttggcataaatgaatggaaagcaagaataaataatgcaaa
acaggaagtcATGTCAAAACCAATATATGATTACTATTTTCCATCTGGATATGCATATTC
AGTATTTTGGTTTTTCAATGAAATTTTTGATATAATTGATGAAATTTTGTTAGAATTCAC
TGATTATCATTGGAAATTCCAAACAATATTAAGGATAGCAATAAAATATTATATACAAGA
ACAAAAATATCATTTGAATATTGCTGATAAATCAATTAGAAACATTTTAATGGATATTTT
GAATTATTGTGAAAACATTTTTTATCATTTAGATGATAAATTTGAATGTTTAGTCGAACA
ATTAGTCAATTCATTCACAGTGATAAGTTACAAATATGATGAAATTTGTGATATTTTTGA
TGATTATCCTGATGCAATGAATATTTCTTTCATGTTCAAAAGGCAAAAACGCTGGATCTC
TTCAATCAAACCCAAAACTATTCTTGGTGTTAAAAAAGCTCTAGAGTATCAAACAAAATA
TCCAAAAGAACAATTTTATATAATAAATAGTGTCGGTGAGAAACCAAATGATAATGAAAA
ATTTCAAACAGATTATAGTATTGAATTTAGCTTCCCTATGGACGATATTGATATATTACG
AATGCTTGGTTCCGGAGCGTTTGGAAATGTTTATTTGTGTGAGGATAAATTCTTCAGAAA
ACAATTTGCATTGAAAGTCCCGCTTAATAATCAAAATGATTTGACTAGAGATATTGTCAT
TAATCGACATTTGAGACACTCAAAGACTTTATTAAGCTATTCAGAATTATTTAGATATAA
TGAATGCATTTGTATGGCCATAGAATATTCAAAAAAGGGTTCTCTGTCATCATATTTGGA
GAAAAACAAAATAATATCAGAACAACAAATTGCAAAATGGTGTCATCAAATATTAATACA
AATCAATGATTTACATAAACAACGTATAATTCATCGAGACATAAAAGGTGACAACATTAC
TTTGGATGAAAATCTGAAAGCTCATTTAATTGATTTTGGAGTTTCAAAGTATGCGGATGA
CACACTTTTGGTAAAAAATTATTTTGTAGGAACAAGAAACTACATGGCACCAGAATATAT
TGATAAGGACACATATTCAAATAAAATAGATATTTGGTCGTTTGGAGCAACTGTTATAGA
AATGGTGTCAGGAGAAATGCCATTTTCCAAAATGAATCCAGTTCAAGCATCATTTCAAAT

TTCCAAAGCAAAAAAAATCAGATATAAACCTCGTCAACGAATATCGAATGAACTAAAGGA
TTTCATTGATTTATGTTTAACACCAAACGAAAATGAGAGACCATCTGCTGAACAATTGAT
TCAGTCACAATTTATATTGAACAATTCGAATGAAATTAATAAATAAaattttattaacac
ttgaaatttattgaataacctaatcaataatttcaataaagagtgactcgccaattttac
tctaattgtatttttatgtcgagggtagtatgaaatgagattaaaataaagatactatt
ttaagtattttctcgatgaattgtttatatcttattggatgatatcaattgatctggaaa
tgaaattataaaatatgttatgtaataatgtatgatgtaattattgaaaaaaataatatt
atgacattgatcaattaaatattgcattaaattattaataaaatatctcggttttatgata
aattgagtcaacgttaaatatatctg
>DjScaffold4089:19228-39523 strand:+
caaattgggaaATGGGAACAAAGGCAGGAGTATATTTTTGTAATGTTACTGAAACATTTA
CACCTCAAAATGGACCAACGTACACCGGAACAAATAGTTTCTCAGAAGATCCCAGAAAAT
TACCAAGACCAGCATTTGGAATGCCTCTTGATGACAGTATTTATGACACAGGAAATGATA
ATTTCCATCCAAATGATACAGATAGCGACAGAGGACATGACGATGAAATTCAAAGTTTTT
TTGATGCTATTGTATCACAACATAATTTACATAGAAAAACTCATAATGTACAAACTTTGC
AATTAGATCCGCAACTTTCTAATTATGCTCAAGACTGGGCTGATGGACTGATGATGGAAG
ATAAATTATCATTCAGTGAATATTTGTATGAAAATAAACCGGTTGGCGAGAATTTAACAA
CCACAAAAAGTGAACTTAATAAAGATTCAGGTATTGCTCTGGTGAAAAGTTGGTATAATG
AAGGTAAAAATTATAATTATCGAAATGAGCCAGGCAATTCTTTCAAACATGTCGGACATT
TTACTCAATTAGTTTGGAAAGATTCAAAGAAAATAGGAATCGGAATAGGTCAACATAGAT
CAGGAAAATCATATATTGTTTGCTTTTATCATCCATCGGGCAATGTTAAAGATAAATTTA
CTGCTAATGTTCTGAAACCATAAattgaaaacatgaattgctttgatattaattatgctt
ttgctattgccatactttatttgatgaatttattttttagtaattctaaaatggaacata
ttttaaaaaaactgttaaagtagattgtcataatatttcaatttgttttcttattatttga
ataatttttaatttatttgtagttactataatcttgtcatctatcacaattgaacaataa
ctaaatacttcgaatatttattaattaaaaatttgaacatta
>DjScaffold1410:71904-72511 strand:+
tcaaatacgaaatataaaaccaccaaatctaatttcattttgttattctactaattttgt
ttattaaagATGAGTAAAGGTATTGAAGTAAAAGAAACGAAAATGGATCGTGACTTGACC
AATGACGTTCAGGATATTGTCAAGCGTGTATTTGATGATTGTGAATCTGCAAAGGAGCGA
GCCGAACAAATTAAGAAAGAATTGGATAATCGTTATGAGCAATATTGGCATGTAGTTATT
GGTAAACAATTCACTACATTCGTTACTTATGAGGAATCTAAATACTGCCATTTAGTATAC
GGTGGTAATACAGCTGTTCAAATTTTCAAATGTGGCTCATAAagatttccagtaaaactt
ctagaatattttatagtgttatctcttgctttgcctttgttaaattaactaattatttga
ttttaaattacatgatgctttattgatatgtttatgtgattaagatttgaattaaattta
aaattaaccattgatagaatttttaacaactgtacagaatattgatgattttttttcattt
aattttcaaata
>DjScaffold2871:12562-13187 strand:+
aatgtgcaattttaaattaatttaaaaaggaaatttttataaattttttgtaatttatcg
tttaaatttttaattgttgatattaATGGCTGCACCACCACCTCCTCCACCACCACCACC
TCCACCAGGTGCAATTAATTTAGGTAAACCAGAAAACAAAAAATTGCTCGATAGTATCTG
TAAAGGTACATCTCTTAAAAAAGTTCCTGATTCAATGAAAAATGATAGAAGCGAAGCCCT
ATCGAATAAGCCTCAAGGAACTGGTAATAGTAAAAATAATTCAGGACCCTTAGTTCCACC
ATCTAATAAAACTATGACATTAGCTGAAGAAATGGCTGCTAGAAAAAACCAAAAAGCTAC
TAGTAATAAAGCAATGTCATTGAACGAAGAATTAGCTGCTAGAACAAATCAAAAACCTGG
TATTGCGCAAATCGCACCGCAAGGTCCACCTGTTCCGCCTAGTAATAAAGCAATGTCATT
GAACGAAGAATTAGCTGCTAGAACAAATCAAAAACCTGGTATTGCGCAAATCGCACCGCA
AGGTCCACCCGTTCCGCCTAGTAATAAACGGATGATATTGAATGAAGAATTAGCTGCTAG
AAAAAACCAAAAATCAACTGATCTGCAACCTGCTCCTCAACCGTCACCAATTCTACCAAA
TCCTAAACAAAAAAGTGGTGCTCCTTTACCTAATTCTCTTCCACCACCAGTTGCTCCTCC
TCCACCTAGTGCGTTTAATAAGCCCCCAAGTATTGCTCAACCACAGGCTACTATAAAAAT
GCCCACGAATTTACCACCACCTCCCATGCAAACATATAATACTACTGGGCAACCACCACC
TCCTCTTCCTAATATCTTGAAGCCAAATTTTCGACAACCAATACCTTTATGCAGTAAACC
TACGACAAATGGTGATTCTCAAACTCGTGGTTTGCCCAATCAATTTTGTGGTCCAATAAT
TCCACCACCACCTCCTATGCTTCCTTCCTCAACTGACATTCGTCCACCTCGATTTGGACC
ACCACCACCGCCACCTCCATCAACAATACCAATTCAAGCTCTTAAGCCTAATACGCCATT
ACGTGGGGATTCTAGATGCGATTTTGATTCGAGATTTACTTTTATGGAAATGAAATACTT

CCCTGATCCTAACACAATGATTAAAAGTTAGctatcaatcgttttgaatacaattaattt
cagtaagacaaacataatgttgcgcttggataatttttgttttcattattcataaaatac
tcaactgattatttttgactcattggttgtgattatttcaatattaatgacttaaatata
ttattagaaattttagttaaatgtgcaattttaaattaatttaaaaaggaaatttttata
aattttttgtaatttatcgtttaaatttttaattgttgatattaATGGCTGCACCACCAC
CTCCTCCACCACCACCACCTCCACCAGGTGCAATTAATTTAGGTAAACCAGAAAACAAAA
AATTGCTCGATAGTATCTGTAAAGGTACATCTCTTAAAAAAGTTCCTGATTCAATGAAAA
ATGATAGAAGCGAAGCCCTATCGAATAAGCCTCAAGGAACTGGTAATAGTAAAAATAATT
CAGGACCCTTAGTTCCACCATCTAATAAAACTATGACATTAGCTGAAGAAATGGCTGCTA
GAAAAAACCAAAAAGCTACTAGTAATAAAGCAATGTCATTGAACGAAGAATTAGCTGCTA
GAACAAATCAAAAACCTGGTATTGCGCAAATCGCACCGCAAGGTCCACCTGTTCCGCCTA
GTAATAAAGCAATGTCATTGAACGAAGAATTAGCTGCTAGAACAAATCAAAAACCTGGTA
TTGCGCAAATCGCACCGCAAGGTCCACCCGTTCCGCCTAGTAATAAACGGATGATATTGA
ATGAAGAATTAGCTGCTAGAAAAAACCAAAAATCAACTGATCTGCAACCTGCTCCTCAAC
CGTCACCAATTCTACCAAATCCTAAACAAAAAAGTGGTGCTCCTTTACCTAATTCTCTTC
CACCACCAGTTGCTCCTCCTCCACCTAGTGCGTTTAATAAGCCCCCAAGTATTGCTCAAC
CACAGGCTACTATAAAAATGCCCACGAATTTACCACCACCTCCCATGCAAACATATAATA
CTACTGGGCAACCACCACCTCCTCTTCCTAATATCTTGAAGACAAATGGTGATTCTCAAA
CTCGTGGTTTGCCCAATCAATTTTGTGGTCCAATAATTCCACCACCACCTCCTATGCTTC
CTTCCTCAACTGACATTCGTCCACCTCGATTTGGACCACCACCACCGCCACCTCCATCAA
CAATACCAATTCAAGCTCTTAAGCCTAATACGCCATTACGTGGGGATTCTAGATGCGATT
TTGATTCGAGATTTACTTTTATGGAAATGAAATACTTCCCTGATCCTAACACAATGATTA
AAAGTTAGctatcaatcgttttgaatacaattaatttcagtaagacaaacataatgttgc
gcttggataatttttgttttcattattcataaaatactcaactgattatttttgactcat
tggttgtgattatttcaatattaatgacttaaatatattattagaaattttagttactcc
tccaccaccaccacctccaccaggtgcaattaatttaggtaaaccagaaaacaaaaaatt
gctcgatagtatctgtaaaggtacatctcttaaaaaagttcctgattcaatgaaaaatga
tagaagcgaagccctatgtaaaattatgtaagtataacatgtattattttattattagcg
aataagcctcaaggaactggtaatagtaaaaataattcaggacccttagttccaccatct
aataaaactATGACATTAGCTGAAGAAATGGCTGCTAGAAAAAACCAAAAAGCTACTAGT
AATAAAGCAATGTCATTGAACGAAGAATTAGCTGCTAGAACAAATCAAAAACCTGGTATT
GCGCAAATCGCACCGCAAGGTCCACCTGTTCCGCCTAGTAATAAAGCAATGTCATTGAAC
GAAGAATTAGCTGCTAGAACAAATCAAAAACCTGGTATTGCGCAAATCGCACCGCAAGGT
CCACCCGTTCCGCCTAGTAATAAACGGATGATATTGAATGAAGAATTAGCTGCTAGAAAA
AACCAAAAATCAACTGATCTGCAACCTGCTCCTCAACCGTCACCAATTCTACCAAATCCT
AAACAAAAAAGTGGTGCTCCTTTACCTAATTCTCTTCCACCACCAGTTGCTCCTCCTCCA
CCTAGTGCGTTTAATAAGCCCCCAAGTATTGCTCAACCACAGGCTACTATAAAAATGCCC
ACGAATTTACCACCACCTCCCATGCAAACATATAATACTACTGGGCAACCACCACCTCCT
CTTCCTAATATCTTGAAGCCAAATTTTCGACAACCAATACCTTTATGCAGTAAACCTACG
ACAAATGGTGATTCTCAAACTCGTGGTTTGCCCAATCAATTTTGTGGTCCAATAATTCCA
CCACCACCTCCTATGCTTCCTTCCTCAACTGACATTCGTCCACCTCGATTTGGACCACCA
CCACCGCCACCTCCATCAACAATACCAATTCAAGCTCTTAAGCCTAATACGCCATTACGT
GGGGATTCTAGATGCGATTTTGATTCGAGATTTACTTTTATGGAAATGAAATACTTCCCT
GATCCTAACACAATGATTAAAAGTTAGctatcaatcgttttgaatacaattaatttcagt
aagacaaacataatgttgcgcttggataatttttgttttcattattcataaaatactcaa
ctgattatttttgactcattggttgtgattatttcaatattaatgacttaaatatattat
tagaaattttagttaaatgtgcaattttaaattaatttaaaaaggaaatttttataaatt
ttttgtaatttatcgtttaaatttttaattgttgatattaATGGCTGCACCACCACCTCC
TCCACCACCACCACCTCCACCAGGTGCAATTAATTTAGGTAAACCAGAAAACAAAAAATT
GCTCGATAGTATCTGTAAAGGTACATCTCTTAAAAAAGTTCCTGATTCAATGAAAAATGA
TAGAAGCGAAGCCCTATCGAATAAGCCTCAAGGAACTGGTAATAGTAAAAATAATTCAGG
ACCCTTAGTTCCACCATCTAATAAAACTATGACATTAGCTGAAGAAATGGCTGCTAGAAA
AAACCAAAAAGCTACTAGTAATAAAGCAATGTCATTGAACGAAGAATTAGCTGCTAGAAC
AAATCAAAAACCTGGTATTGCGCAAATCGCACCGCAAGGTCCACCCGTTCCGCCTAGTAA
TAAACGGATGATATTGAATGAAGAATTAGCTGCTAGAAAAAACCAAAAATCAACTGATCT
GCAACCTGCTCCTCAACCGTCACCAATTCTACCAAATCCTAAACAAAAAAGTGGTGCTCC
TTTACCTAATTCTCTTCCACCACCAGTTGCTCCTCCTCCACCTAGTGCGTTTAATAAGCC

CCCAAGTATTGCTCAACCACAGGCTACTATAAAAATGCCCACGAATTTACCACCACCTCC
CATGCAAACATATAATACTACTGGGCAACCACCACCTCCTCTTCCTAATATCTTGAAGCC
AAATTTCGACAACCAATACCTTTATGCAGTAAACCTACGACAAATGGTGATTCTCAAAC
TCGTGGTTTGCCCAATCAATTTTGTGGTCCAATAATTCCACCACCACCTCCTATGCTTCC
TTCCTCAACTGACATTCGTCCACCTCGATTTGGACCACCACCACCGCCACCTCCATCAAC
AATACCAATTCAAGCTCTTAAGCCTAATACGCCATTACGTGGGGATTCTAGATGCGATTT
TGATTCGAGATTTACTTTTATGGAAATGAAATACTTCCCTGATCCTAACACAATGATTAA
AAGTTAGctatcaatcgttttgaatacaattaatttcagtaagacaaacataatgttgcg
cttggataattttgttttcattattcataaaatactcaactgattatttttgactcatt
ggttgtgattatttcaatattaatgacttaaatatattattagaaattttagttactcct
ccaccaccaccacctccaccaggtgcaattaatttaggtaaaccagaaaacaaaaaattg
ctcgatagtatctgtaaaggtacatctcttaaaaaagttcctgattcaatgaaaaatgat
agaagcgaagccctatgtaaaattatgtaagtataacatgtattattttattattagcga
ataagcctcaaggaactggtaatagtaaaaataattcaggacccttagttccaccatcta
ataaaactATGACATTAGCTGAAGAAATGGCTGCTAGAAAAAACCAAAAAGCTACTAGTA
ATAAAGCAATGTCATTGAACGAAGAATTAGCTGCTAGAACAAATCAAAAACCTGGTATTG
CGCAAATCGCACCGCAAGGTCCACCTGTTCCGCCTAGTAATAAAGCAATGTCATTGAACG
AAGAATTAGCTGCTAGAACAAATCAAAAACCTGGTATTGCGCAAATCGCACCGCAAGGTC
CACCCGTTCCGCCTAGTAATAAACGGATGATATTGAATGAAGAATTAGCTGCTAGAAAAA
ACCAAAAATCAACTGATCTGCAACCTGCTCCTCAACCGTCACCAATTCTACCAAATCCTA
AACAAAAAAGTGGTGCTCCTTTACCTAATTCTCTTCCACCACCAGTTGCTCCTCCTCCAC
CTAGTGCGTTTAATAAGCCCCCAAGTATTGCTCAACCACAGGCTACTATAAAAATGCCCA
CGAATTTACCACCACCTCCCATGCAAACATATAATACTACTGGGCAACCACCACCTCCTC
TTCCTAATATCTTGAAGACAAATGGTGATTCTCAAACTCGTGGTTTGCCCAATCAATTTT
GTGGTCCAATAATTCCACCACCACCTCCTATGCTTCCTTCCTCAACTGACATTCGTCCAC
CTCGATTTGGACCACCACCACCGCCACCTCCATCAACAATACCAATTCAAGCTCTTAAGC
CTAATACGCCATTACGTGGGGATTCTAGATGCGATTTTGATTCGAGATTTACTTTTATGG
AAATGAAATACTTCCCTGATCCTAACACAATGATTAAAAGTTAGctatcaatcgttttga
atacaattaatttcagtaagacaaacataatgttgcgcttggataattttgttttcatt
attcataaaatactcaactgattatttttgactcattggttgtgattatttcaatattaa
tgacttaaatatattattagaaattttagttaaatgtgcaattttaaattaatttaaaaa
ggaaatttttataaatttttgtaatttatcgtttaaatttttaattgttgatattaATG
GCTGCACCACCACCTCCTCCACCACCACCACCTCCACCAGGTGCAATTAATTTAGGTAAA
CCAGAAAACAAAAAATTGCTCGATAGTATCTGTAAAGGTACATCTCTTAAAAAAGTTCCT
GATTCAATGAAAAATGATAGAAGCGAAGCCCTATCGAATAAGCCTCAAGGAACTGGTAAT
AGTAAAAATAATTCAGGACCCTTAGTTCCACCATCTAATAAAACTATGACATTAGCTGAA
GAAATGGCTGCTAGAAAAAACCAAAAAGCTACTAGTAATAAAGCAATGTCATTGAACGAA
GAATTAGCTGCTAGAACAAATCAAAAACCTGGTATTGCGCAAATCGCACCGCAAGGTCCA
CCCGTTCCGCCTAGTAATAAACGGATGATATTGAATGAAGAATTAGCTGCTAGAAAAAAC
CAAAAATCAACTGATCTGCAACCTGCTCCTCAACCGTCACCAATTCTACCAAATCCTAAA
CAAAAAAGTGGTGCTCCTTTACCTAATTCTCTTCCACCACCAGTTGCTCCTCCTCCACCT
AGTGCGTTTAATAAGCCCCCAAGTATTGCTCAACCACAGGCTACTATAAAAATGCCCACG
AATTTACCACCACCTCCCATGCAAACATATAATACTACTGGGCAACCACCACCTCCTCTT
CCTAATATCTTGAAGACAAATGGTGATTCTCAAACTCGTGGTTTGCCCAATCAATTTTGT
GGTCCAATAATTCCACCACCACCTCCTATGCTTCCTTCCTCAACTGACATTCGTCCACCT
CGATTTGGACCACCACCACCGCCACCTCCATCAACAATACCAATTCAAGCTCTTAAGCCT
AATACGCCATTACGTGGGGATTCTAGATGCGATTTTGATTCGAGATTTACTTTTATGGAA
ATGAAATACTTCCCTGATCCTAACACAATGATTAAAAGTTAGctatcaatcgttttgaat
acaattaatttcagtaagacaaacataatgttgcgcttggataattttgttttcattat
tcataaaatactcaactgattatttttgactcattggttgtgattatttcaatattaatg
acttaaatatattattagaaattttagttaaatgtgcaattttaaattaatttaaaaagg
aaatttttataaatttttgtaatttatcgtttaaatttttaattgttgatattaATGGC
TGCACCACCACCTCCTCCACCACCACCACCTCCACCAGGTGCAATTAATTTAGGTAAACC
AGAAAACAAAAAATTGCTCGATAGTATCTGTAAAGGTACATCTCTTAAAAAAGTTCCTGA
TTCAATGAAAAATGATAGAAGCGAAGCCCTATCGAATAAGCCTCAAGGAACTGGTAATAG
TAAAAATAATTCAGGACCCTTAGTTCCACCATCTAATAAAACTATGACATTAGCTGAAGA
AATGGCTGCTAGAAAAAACCAAAAAGCTACTAGTAATAAAGCAATGTCATTGAACGAAGA

ATTAGCTGCTAGAACAAATCAAAAACCTGGTATTGCGCAAATCGCACCGCAAGGTCCACC
TGTTCCGCCTAGTAATAAAGCAATGTCATTGAACGAAGAATTAGCTGCTAGAACAAATCA
AAAACCTGGTATTGCGCAAATCGCACCGCAAGGTCCACCCGTTCCGCCTAGTAATAAACG
GATGATATTGAATGAAGAATTAGCTGCTAGAAAAAACCAAAAATCAACTGATCTGCAACC
TGCTCCTCAACCGTCACCAATTCTACCAAATCCTAAACAAAAAAGTGGTGCTCCTTTACC
TAATTCTCTTCCACCACCAGTTGCTCCTCCTCCACCTAGTGCGTTTAATAAGCCCCCAAG
TATTGCTCAACCACAGGCTACTATAAAAATGCCCACGAATTTACCACCACCTCCCATGCA
AACATATAATACTACTGGGCAACCACCACCTCCTCTTCCTAATATCTTGAAGGTAAATaa
tgtgcaattttaaattaatttaaaaaggaaatttttataaatttttgtaatttatcgtt
taaatttttaattgttgatattaATGGCTGCACCACCACCTCCTCCACCACCACCACCTC
CACCAGGTGCAATTAATTTAGGTAAACCAGAAAACAAAAAATTGCTCGATAGTATCTGTA
AAGGTACATCTCTTAAAAAAGTTCCTGATTCAATGAAAAATGATAGAAGCGAAGCCCTAT
CGAATAAGCCTCAAGGAACTGGTAATAGTAAAAATAATTCAGGACCCTTAGTTCCACCAT
CTAATAAAACTATGACATTAGCTGAAGAAATGGCTGCTAGAAAAAACCAAAAAGCTACTA
GTAATAAAGCAATGTCATTGAACGAAGAATTAGCTGCTAGAACAAATCAAAAACCTGGTA
TTGCGCAAATCGCACCGCAAGGTCCACCTGTTCCGCCTAGTAATAAAGCAATGTCATTGA
ACGAAGAATTAGCT
>DjScaffold5284:28902-31752 strand:+
tatgtttatatttatttttataaattaatcacttttattgcgtgtagcttttggctgttt
gtactacagaATGGTGAATTTTACTATTGATCAAATTAGATCTCTTATGGAGAAAAAGAG
AAATATTCGAAATATGAGTGTCATAGCTCATGTTGATCATGGGAAGTCAACTTTAACTGA
TTCCTTAGTAAGCAAAGCTGGAATTATTGCAGGTGCTAAGGCTGGTGATGCCCGTTTTAC
AGATACGAGGAAAGATGAACAAGAACGATGTATTACTATTAAGTCAACTGCAATTTCTTT
ATATAACGAAATGACTGAAGATCATCTAGCTTCTGTTAAAGTTGTTCAACCAGTTGCTCT
TAGTGATGATGGCAAAACTGATGAGAAAGGATTCTTAATAAACTTGATTGATTCACCTGG
CCACGTCGATTTCAGCAGTGAAGTTACAGCTGCTTTAAGAGTTACAGATGGAGCCCTTGT
TGTAGTAGATTGTGTTTCAGGGGTTTGTGTTCAAACAGAAACTGTGCTTAGACAAGCAAT
TGCTGAAAGAATAAAGCCAGTCTTGTTTATGAATAAAATGGATATGGCGGTACAAGGTTT
ACAGTGCGAACCAGAAGATTTATATCAAAAATTCCAAAGAGTTATAGAAAGTGTTAACGT
TATAATTGCACAATATGGTGAATTAGATGGACCAATGGGAGATATACAAGTTACTCCTGA
AAATGGAACAGTTGGCTTTGGTTCCGGTTTGCAAAGCTGGGCTTTTACAGTTAAAAATTT
CGCAGATATGTATTCCAAAAAGTTTCAATTGTCACCAGATAAGCTAATGAAAAGACTGTG
GGGAGATAACTTTTTCAACGAAAAAACAAAAAAATGGACTAAAAAGCCAGAAGAAGGTGG
AGTTAGAGGTTTCAATCAATTTGTTTTAAAACCCATTTACATGGTTTTCAATACTGTTAT
GAGCAAAACATTAGAAGACTCTGTAGAATTAGCAATCAAAATGGGTATTAAATTGGATAG
CGAAGAAAAAACACTTCAACCCAAAGGATTATTAAAATGCATAATGCGTAAATGGTTACC
AGCAGGTGACACTTTATTACAAATGATATGCATACATTTACCAAGTCCTGCTATATCACA
AAAGTATCGAACTGATTTGCTTTATGAAGGACCATTAGATGATGAAATTGCCGTGTCTAT
GAAAAATTGTGATCCTAATGGTCATTTAGTTATGTACATTAGTAAAATGGTTCCGACTTC
TGATAAAGGAAGGTTTTATGCTTTCGGCAGAGTGTTTTCAGGAAAAGTTGGTACTGGTCA
AAAAGTTCGAATAATGGGACCTAATTATGTCGTTGGTAAAAAGGAAGATTTATATGAAAA
GAGTATACAAAGGACAATCTTAATGATGGGAAGATATGTTGAAGCAATCGAAGACGTTCC
ATGTGGAAACATATGTGGACTTGTTGGAGTTGATCAGTATTTGGTAAAAACTGGGACTAT
AACAACTTTTCCTGGTGCTTATAATTTAAGGGTGATGAAATTCAGTGTTAGTCCTGTTGT
TCGTGTTGCAGTAGAGCCTAAAAACCCCGCTGATTTGCCTAAATTAGTTGAAGGTTTAAA
AAGATTGGCAAAATCAGATCCTATGGTACAAATTTCGATTGAAGAATCAGGAGAAAACAT
TATTGCAGGTGCTGGAGAATTACATTTAGAAATTTGTCTTAAAGATCTTGAAGAGGAACA
CGCGTGTATACCAATTAAAAAAAGCGATCCAGTTGTTTCCTACCGAGAATCAGTAAGTAA
GACCAGCAGTGAAGTTTGCTTGAGTAAATCGCCAAATAAACATAACAGACTCTACATGAC
CGCTACTCCAATGAGCGAAGACCTCTCGAGTGATATTGACGCAGGAAAGATCACTGCTCG
TTCTGAAATTAAAGAAAGAGGAAGATATTTAGTGGAACATTATGATTGGGATGTATCTGA
ATCACGTAAAATTTGGTGCTTTGGTCCTGACGGAAATGGGCCTAATTTGGTTGTCGACGT
TTCGAAAGGTGTTCAATATTTGAATGAAATTAAAGATAGTGTAGTCGCTGCTTTCAATTG
GGCTACTAAAGATGGAGTTTTATGTGAGGAAAACATGCGTGGTGTGCGGTTTAATATCCA
TGATGTGACGTTGCATACTGATGCTATTCATAGAGGTGGTGGACAGATTATTCCAACAGC
AAGAAGGTGTTTCTACGCAACAGTACTCACCGCTAGCCCTTGTTTATTAGAGCCAGTTTA
TCAATGCGAAATACAAGGTCCCGAGACGGCATTAGGTGGTATCTACAGTACTCTCAATAA

ACGCCGTGGAATTATTTTCTCTGAGATGCAAGTAGCTGGTACCCCTCAGTATGTCGTAAA
GGCTCATTTGCCTGTTAACGAATCATTTAATTTTACATCCGAGTTACGAGCTCAAACTGG
TGGACAAGCATTTCCCCAGTGTGTGTTTGATCATTGGCAACTGTTCCCCGGTAATCCATT
GGATCCATTAAGTAAGCCAGGAATTACAGTAAAAGATACAAGAAAACGAAAGGGTCTATC
TGAGGATGTTCCGTTGTTGGATAAATTTTTGGATAAACTTTAAattccaataaataaata
aatctggatcttttagttttgtgaattttgtttcgatattgtaattggataaaataattt
tcaaat
>DjScaffold591:4166-11033 strand:+
taattttacgaaatgaattttctcgaattcattaaattgttttagattcgaaagaaataa
aaaatttatacagtgaaacctttgtagaaattgatttattctataaggccacATGTTGAC
TCCTGATCTTAGTGTTGGCGTTATCATAGCAATAGTAATCGGAGCTATAGTAATCTGCGT
GTTATCCTTTATTGTATATTTCATTTTTAATCGAGATGATTTTTTTGATGATAAAGAAAC
ACATTCAATGGGATATAAATATACAGAATTGAATGATTATGAAACTAAAACTTCGAAACC
TGAAATTCAACCGATTCTAGGTAAACATAAATATAAATGTATAGAGTTTTGTGAATATTT
TTGAtttttttcttttgaaatatttctaagaaattatatgcatgataaaagaa
>DjScaffold591:23110-33418 strand:+
aattttattttaaatgtatttttaattttaaaaaagctatcgattagtttttatttatat
tttttacctataataattatttaattttatttaaaatgaaggtaattttgctggcctaga
aaatgtcgttcaggaatgttttATGAACTCGGTTGTTCAAGCTCTTGCAAGCAGCAAGAA
AATATATGATCAATTAAAAGAAATGCAGTCCTATGTTCCCACTTGTAAATCACTGAAAAT
ACTTCAAGAATTAATTAATGGTCTTAATGATGACAGTCCAGAGAGAAGTTCTGAAAAAAA
TACAACATTGTTGAGAGCACTTGTGAAAGCAATTAAATGGACGGTGAAACCAGAGCAGGA
TGCGTATGATTTCTTGTCTGAAATTATCAATGACATTGAAAAAGAACTAATTGATGCTAA
AGTGAAGATAGATGCCCCGTTGAAATTCAAGGTTAAATATAAATATTGTGGCGTGAATAT
ATCTGATAGTATCAAAACGCATCTGGGCTTGTCATTAGAACTTTGTGGAGAATACCAAAA
AAACCCCGGAGTACCTTTAGATTTTCTTATCACAAAATATTTTTCTAGAACACTTATTGA
TGAGGATCCAAAAAAATTCTTACAGTTGTTTATGCGCAAAAAAATGCCCTAACGTATTGGT
GCTTCATATTCAAGCTAATAAATGGAATAATGAACTGCAGAAAATGGTTAAGCAAAAATA
TTTCATAAAATATGAAGAATATTTGAATTTATACTTAGATGTGTTGGATTTCGATAATAA
TGAAGAATCAAAAGAAAATGTTCAATATAAATTAACAGCTGTTGTTCATCAAAATGAAAA
CTTTTCATCTGGTCATTACGTAACATACAGACAATTCGAGGAAGATTGGTTCCTGGCAGA
TGATACAAAAATCGAATACATCCCATTCAATTATAAAGTTTCTCCATATATATTAATTTA
TGAAAAACTTTGAtaaaaactataaattctaaaataccacagttcctcataaatttcaat
ttgtcatttaatttttaattataatgaagaatatatatatttatcaaatttctttttgta
aataaa
>DjScaffold3723:45420-58026 strand:+
ctcactgagaaaatcggtgatataaaaaatagtgaaaatgttaaaagcactttaacagaa
ATGAGTGATGCCATAAATTTGCAATTGATTGCTAATCAAGTTATTAAAACTGCATCAGAA
AATGTTAATGTTAAAAATCTAGTTGAAGCGTTAAACTGGCTTAGTGATGCAATAAAGGAT
TTTGGATTTAAAGTTCCAATAAATGATCTTGTACCGTTTATTCGATTGGCTGTTCAGCAT
ACCAACCCTGCTGTTCGGCAATCAGGAATCAAACTTGCTGGAACTATATCTCTTTATGTT
CGAGATGTAATATCTACTCTTTTAAAAGATGAAAAAGCTTCTGTTAAGCAATTAATCGAT
ACTGAAGTTGAATCATTGAAATCCGTTAATCCTCCAGTTCCAACGCGTGGTCTGAAAAAT
GGACCTCCAGCGGCCGAAACAGCCACTGATTCTGAAAAAGCCACTGGCCCAGTTGTTCAA
GAGTTAGAATTGGATGATATGATTCCACGAGAAAATATTAGCCATAAAATTACCGAAAAT
TTACTTACTCAGATAGCGGATAAAGCCAACAAAGTTAAAAGAGATGCACTTGAGGAAGTA
ACGAAAATTATAAGTGCTCCGGTTGGAATTTCGGGAGAAGGACTACAAAGTCTAATCACA
GCTATTTCGAAAAGTACAAACGATTCGAGCATGGTTGTTAGTAAAATGGCTATTGGTATT
CTGGTTACACTTGGTAATGCACTGAACAAAAAAGATGCAGAAAGATATATAAAACTTGTA
ATTCCAGCACTATTTGCAAATTGTAGTGATCCGAAACCAAAAAATAGAGAAGACGCTGTT
GCAGCTCTAAATGTGTGGACTGAAAAGGTGGGATTTCCCAATGTTGTCGATGGGGGGATG
CTTTCTGAAGGCCTTAAAGTCGATAATCCCTACCAAAAAGCCACAATATTAGCATGGATT
TCTCCACATCTCACAAAGTTATCGCAAAATAAATCAGCAAAAAAACTTCTTTATCCGGAT
TTTGCGTCTGAAATTCTTCCACATCTTTATTCAGCAATCGAAGAAAGAAGTCCAGATGTT
AGAAAAAGTTCGCAAGATGCTGTCCTTCCTTTTATGCTCGTTTTAGGTTATGATGTGATG
GTAAAAAACTATAAAAACAATTCCAAAACTGCCTCAAAGAATACTGTAATGGGATTTTTA
GATAAGGCAAAATTGGAATTGCCAGCCCCCGAGCCGTCTGCCAATAAGCAGACAACGAAA

CCTGCTTCTGCAAATGCCAAGCCACCTTCAGCCAAAGAAGCCTCAAATAAAAAGTCATCA
GATGCTCCTGGTAAACCGAAGAAAGAAGAAATGAAAGCTAATGAGCAATCTGACAAAGAT
GCAGTAATTGTTGCTCTTCTTTCGAAAACAAGTAATAAAAAGCAAAGAATTCAGGAAGAT
AAGAAAAAGAAAATAACCAAATGGGAAACGAATATTGCAAATTCTCAGCAATTAAATGCA
CAATTCTCTACAAGTTGTTCTCCAGAATTGTTGCAATTGTTGTTTACAAATGATCTTAAA
AAATCAATAAAAGCAATTGAATTGATGAATAGTTTTCTTTCCGAAGATGATCAAGATTCT
ATCGATGCAACAAGACAAAATTTAGATTTAATTCTTAAATGGATGGTTATTCGGTTTAAT
GAAACAAATCCATCAGTTTTAACAAAAGGACTTGATTATCTATGTAGAGTATTTACAATA
CTCTGTATGGAGAATTCTTTTCAATTATTGGAACAAGAGGCTGTAGGATTTATACCAGAG
TTAATTATTAAAATGGGCGATTCTAAGGATCAGATTAGAATCCCCTCGCAGAAAATTTTG
AAACTGATTTCGAACTGTTATGATAATTTAAAAATTGTATTGTTTTTGGAAGAAAATCTA
GCTAATAAAACTGCTCGAGTTCGACAGGAATGTGCTGATTTTTTGAAATTTTTGATAGAT
GAGAGAAAAGTTGATGTTTTAAATCAGAAAAACTTATTGAAATCTGTCGCTTTGTTAATT
TCTGAAAGAGATAATAGTGTTCGTACTGCAGCATTGAACTGCTTGGTTTCTGCCCATAAT
TTTATGGGAGAGGATTTATTGAAATTACTTGATTCACTTCATGATAAAGACCGTTCAATG
CTATTAGAACGAATAAAGCGTTCAGGTAAACCTGTTTCTTCAGAAACTGAATCTTCACAG
TCTACAATAGCAGTTGAAAAAAATCACAAACTTTCAAAACCTCGTGAATCATCAGCTTCA
AGAGAACAGATCAAACCTCAAATGCAATTACCTCCTCAAAAGTCAATTGATATCAATCCA
TTATTAAAGGAAGAAATTGGTTACAAATTAATCAATGTCGATAAAGAAATTGAAGAGCTT
CTACAACCTTTAGAAAAGCTTACTCTTCGTTTTTCTGGAAGAGCTTCTCAAGTCGTTAAT
TCAAATGATGCAATTACATCAGTTATACACGCTATTAATTCCACTGATCTTCAGACTAGT
ATACAAGCAATGGCTCAAATTGACGAAGTTATTAGAAATCAAGATGTTCGTTCACAATTA
ATCAATTACATGGATAATCTTGTTATGTCCATTACAATGCAGTTAAAAGCAACTTTAGGA
AAATTGTCAGAAGAGAAATTCAGTGAATCTGAAATAACGGATATGTTTAAAGGAATGCTA
GCTACTCTTGTTTCTATTTTCAATGCCAAAGAATTGGCTAGTGAACTTAATCGAGACAGA
CTAATGAATTTGATGCAATCTATTTTAAGAATTGTTATCAATGATCAATTAGTTTCTCTG
TCTGAAGGTGGTACCATTATTCGTGGTATTAATATACTCAGTGTTCGCATACTGGAATCT
TCTAATCAAACTAGAATGTTGAGTGCTCTTCTTAGAATGATGAGTGATTGCTTGAAAAAT
GGGGATACAAATAGCAAATATTTAAATCTGATATTCAAGTGCATTTGGTCAATGACTAAA
AATTTGATTAATACCGTTAACTCTATCGATTTGGTTGTGATCCTTAAAGATATTCATGCA
TTTTACACAGATTTTCCTCCTCATGTATGGGAGGCTAGAGAAAGTGGTAGAGATGTTCCA
TTAAGAACTGTGAAGACTTTAGTTTATTCCATTATTAATAACAGAAATGGGGGCGTTTTA
GAAGCTCTTTATCAAATTCCTAATGCGCCTCACAAAGATTTAGATACTTATATCAGAAAA
TCATATGCCAAACTCAATGTACCCATTCCTACTGTGATGCCTTCACCATCAGATAACTCA
AGTGCTACAGAAGCCCCAGCGCCTACTTCAAATAAAACTAAATTCGCATTTAAATCAAAT
TCTCGGCCATATCTAGATGAATTGAAGGAAATATTCGGTAAAATCGGCAATCCTGATCAA
AATAAACAAGGATTGAATGATTTATTTGACTTCAGACAGAAATATCCCTATGTTGATTTG
TCACGATATTTGTCACAAAGCACACCTTATTTCCAGAAAACTATTGAAACAGGATTGAAA
AACATTGCTGACGAGCGAGGACAAGCTACTATATCTGTTTATGGTTCCAATCAGTTTAAT
AATAATAGTGGTGCTACCGCGTCTTTAGAAAAAGACAATTTGGCTAATTGTAATATGGAG
CCAGAAGCATTATTGCTTCGTTTGAAACAGTTGCGGAAAAATGCCGGTCTAGATAAATCT
CATTCAGAAAAAATAGATCAGAATCCACCAACATCCCCAATAAGAGAAGAAATGGAAATC
TCAGTAATATCAAAGCTGGTTCCGTCCGGCGATGGGCCTGTCGAACCATCACCAAATAAT
CAGATGAATATGAAAAGAAAAATGTCAGCTGTTGATCTAGCAGCAATCAAACAACGTTTT
GAAGCGATCAAACGGCCTAATTGAgataattttatctatttatgaaattctccgagtttt
acactataagattttaataattcttttgtaaattattttgaatttaatcacaaaatttta
ttaaaattattggaatattttcctttttattaaattgcctaatttgattgataatggtaa
tttacttatgttagtaaaactataacaagtaagtaaatttttcaagttttatcgttttaa
taccatgaaataaatcgactgtgatgtgataaatcttcattaacttttattgctgacatt
agtttttctggaagagcttctcaagtcgttaattcaaatgatgcaattacatcagttata
cacgctattaattccactgatcttcagactagtatacaagcaATGGCTCAAATTGACGAA
GTTATTAGAAATCAAGATGTTCGTTCACAATTAATCAATTACATGGATAATCTTGTTATG
TCCATTACAATGCAGTTAAAAGCAACTTTAGGAAAATTGTCAGAAGAGAAATTCAGTGAA
TCTGAAATAACGGATATGTTTAAAGGAATGCTAGCTACTCTTGTTTCTATTTTCAATGCC
AAAGAATTGGCTAGTGAACTTAATCGAGACAGACTAATGAATTTGATGCAATCTATTTTA
AGAATTGTTATCAATGATCAATTAGTTTCTCTGTCTGAAGGTGGTACCATTATTCGTGGT
ATTAATATACTCAGTGTTCGCATACTGGAATCTTCTAATCAAACTAGAATGTTGAGTGCT

CTTCTTAGAATGATGAGTGATTGCTTGAAAAATGGGATACAAATAGCAAATATTTAAAT
CTGATATTCAAGTGCATTTGGTCAATGACTAAAAATTTGATTAATACCGTTAACTCTATC
GATTTGGTTGTGATCCTTAAAGATATTCATGCATTTTACACAGATTTTCCTCCTCATGTA
TGGGAGGCTAGAGAAAGTGGTAGAGATGTTCCATTAAGAACTGTGAAGACTTTAGTTTAT
TCCATTATTAATAACAGAAATGGGGGCGTTTTAGAAGCTCTTTATCAAATTCCTAATGCG
CCTCACAAAGATTTAGATACTTATATCAGAAAATCATATGCCAAACTCAATGTACCCATT
CCTACTGTGATGCCTTCACCATCAGATAACTCAAGTGCTACAGAAGCCCCAGCGCCTACT
TCAAATAAAACTAAATTCGCATTTAAATCAAATTCTCGGCCATATCTAGATGAATTGAAG
GAAATATTCGGTAAAATCGGCAATCCTGATCAAAATAAACAAGGATTGAATGATTTATTT
GACTTCAGACAGAAATATCCCTATGTTGATTTGTCACGATATTTGTCACAAAGCACACCT
TATTTCCAGAAAACTATTGAAACAGGATTGAAAAACATTGCTGACGAGCGAGGACAAGCT
ACTATATCTGTTTATGGTTCCAATCAGTTTAATAATAATAGTGGTGCTACCGCGTCTTTA
GAAAAAGACAATTTGGCTAATTGTAATATGGAGCCAGAAGCATTATTGCTTCGTTTGAAA
CAGTTGCGGAAAAATGCCGGTCTAGATAAATCTCATTCAGAAAAAATAGATCAGAATCCA
CCAACATCCCCAATAAGAGAAGAAATGGAAATCTCAGTAATATCAAAGCTGGTTCCGTCC
GGCGATGGGCCTGTCGAACCATCACCAAATAATCAGATGAATATGAAAAGAAAAATGTCA
GCTGTTGATCTAGCAGCAATCAAACAACGTTTTGAAGCGATCAAACGGCCTAATTGAgat
aattttatctatttatgaaattctccgagttttacactataaga
>DjScaffold1058:30152-31061 strand:+
ctaattaatattaatATGTATTTTATCTTAGTGTACACATTTTCTAATATGTCCAAAGAT
GCTTCAAAATTAGATCCATTTCGTGTTAAAAATGAATTAAAACTAAAAAAGCAGAAGAAA
AGTCAAGGTTCTTCACATTACAAGCCCAAAGCACCTACCGAATTAACTCCGTTATCATCC
TTTAAAGATACTCCAAATGCGGAACATAATGATTTATTTATTGGTAAGATGTCACAGTGT
TGCGTAGTGTTTGATTTTATGGATCCCATGAGTCATCTTAAAAGTAAAGAAATAAAACGA
GCTTGCTTGAATGAATTAGTGGAATTTATTTCAATAACAAGAGGCGTTCTAAATGAAGGA
ACTTACCAAGAAATCGTTAGAATGATTGCATGTAATATTTTTCGAAATTTGCCTCCAACA
GATAATGTCGACTTTGATCCAGAAGAAGACGAACCGGTACTTGAAGCTTCTTGGCCTCAT
CTTCAGATTGTATATGAATTTTTCCTACGTGTATTAGAATCTCAGGATTTCCAACCGAGT
ACAGCAAAGAAATTTATTGATCAAAAATTTGTATTACAGGTTCGATTTTAAttagttttt
gtaattatatatttattaattgaattgtctttaaacatttttctaatttaattttatt
ttatttaatttgattatatttgattatattaaatgtaaataacaaaatagaatttgttca
ttggctacaaattatgttttaaagttattgaaatattatagaat
>DjScaffold1058:52040-52406 strand:+
TGTGTTGTACAATTTCTTGAAAAAGATCCTACATTGACTGATTATGTTGTTAGAGGTTTA
TTAAAATTTTGGCCGAAAACTTCATCACCAAAAGAAGTGATGTTTCTGAATGAAATGGAA
GAAATACTGGATGTTCTTGAACCAGCACAATTTCAGAAAATTATGGTTGTTCTCTTTAAG
CAAATTGCAAAATGTGTTTCTAGTTCTCATTTTCAAGTGGCTGAAAGAGCTCTCTATTAT
TGGAATAATGAATATATTGTTTCATTAATTGAAGAAAACAATGAAATTTTAATGCCAATT
TTATTTCCGGCTTTTTAT
>DjScaffold2789:225-700 strand:+
TATAAAATATCAAATACAAATCGAGATATTGTTATCAAGAAAAGCAAAAATAAAGAAATT
GTTGACAAAAAAAATATTGAAGGAAATATAAATAAATTTTCATCTCAAATAATTCATAGA
AATATTGTTCGATATTTTGGTGCTTTGGAAAAATCTGGGAAACTTTTCATATTTATGGAG
TATATTTCTGGAAAATTCAATAATATATTATCTATTATCCATGTTAAATATATTTGTGGC
AATAATACCACTTTTAAAATAACATGAataagtaggtagatagtggaacactgaactgcc
tttttagtcgagcaaaggatttagtaatttagcttgaaatttggttcaaattttttcaa
ataggaatcaataaacattta
>DjScaffold2789:13535-14050 strand:+
attactttactatcagggctcactcaaaaatcttattgataaattaggaATGATAGAAAT
TAACGAAGTTATTCAATATAGCAAGCAAATATTAGTTGGACTACAATTCCTCCATTCCAA
AGCAATTATTCACAGAGACATCAAACCTTTAAATATTTTAGTAGATCACGATAAAACATT
AAAATTAGCAGATTTTGGTATCAGTAAATCATTAAAAAATTCATTTTCCGAATCCATTAA
GAAAGCTGATACTTTTTGCGGTTCTCCAAATTATTTGGCTCCAGAGATTTTCTCAAGTGA
ACCTTATGATTATCCATCTGATATTTGGTCAACTGGAGCAACAGTTATTCATATGATAAC
AGGTAATTGAgtaattattattgtgctatattaacagaactattgatcatcgatattgtt
attgatttgaccgaatataaatagatagaaatcaataataacaatg
>DjScaffold2684:39771-44287 strand:+

CGAACTTTGCTCAATACTTGTCCAGCATTTAGAGGTGCAAGTACAGGTTGCCTTAGGGCA
TTTGCTCTCAGATTTCGAACAACTCATATTCCTCCGGGTGATATGTTCATCCATCCGGGA
GATCTGCTAGACTCTGTCTACTTCATCTCGAGGGGATCAATAGAGATCATCTATGTCGGG
GAGCAGGCCTACCAGGACAAGCTGATTATGGGAATTTTAAATAAACATGACATTTTTGGT
GGATGTCCGACTGAATTTCTTCAAAACAAAGTCAATCAATCGAAATATGGAGTTCATGCT
ATGGGATTTGTTGATTTAAACAAAATCACATTGATTGATCTTAGAGATATTCTTTTCATG
TATCCAGAATGGTCTGCTTCATTTCTCAATTCTTTCCAAATAACATTCAAGCTCAATGAA
ATTGATCCCAAGCTCATTCAGAAATCTGCAATTCTCCAGAAAAATGACGTTCCTAATACA
GTCAATGAAGTCAGCCAAATAAAATCAAATCCTAATCAGCTGTGGACATCAGCTAAACCA
TTATCGCCGCAACCAAAATGCACCGACTCACTGGATGTTAACAAAGATAAAATCAATTTA
GAACATGATCTGGGATCAGTTATCAACAGAATGCAACATCTAAACAGCCGAGCGCAGGAG
ATGGAAAAGAATTACCAGGCAGCTTTCTCTGAGATTTTGCAGTTACTCACTACCAATTAA
aatgaaaacaaatatatttgtaatgtttatcgaaaaatatatttagttttcttatttcat
gattctcattccaaatttcaattttgtactttttacttgtaaacaaatgtgataaacag
cagaaaaagaatttttggaatatgttaatttgtgaaattattttcctttcgtattaatta
ttcattattttataacaattgatttcttcttatattgttctacaaatgtttgtatgcatg
tgtgtgtgttattcattaggtatgcaatttgtttgatggaaataaatgaagaaataatat
ttcgttgatgtacgaaaggagctgtcgataacagttgtgttatgataaacc
>DjScaffold3316:2455-22591 strand:+
AATCTGCTTTCTGCAAAATGGGTGCCTGACATAAATTTCCAACCTGATTTTGATCAATTT
CAATTTTACAAATCGAATTCTTTTAATAATATCTATAATTCTTCTGATGGAAAACTACCA
AAGGATTTTGAGTGGCATAGTTTAGCTTCAGCGTCACTGCATTCTGGAAATACTTCTTAC
ATTCTTTATGGAGGATCTCGGCATAAAAGTTTAGAAAATGACTCAGGATTTTCTAAAATC
ATTTCCAAAGTGATTCTGTGTGAAATTAAATTTGATAAGACATTATTTCTTGATGCAGTG
AAATGTTCACCTATAGAAACTGGCGATAATAATACATATCTTGGTGCATCTATGAAAGCT
ATATCTATTTCCAATAAAGAAGTGGTGATTGTATTTTGTGATCCTTTATGGCATGTGTCT
GATACAAAAGAACCCAAACCAAGTGGTAAATGTTATTTATTACTATTAAGTAGTACAAAT
ATACGAACAAATAAAAGTATTGAATTTTGTATGCTATCAACCAAGTCTTACAGCACTTGT
ATGGGTGGATTTTCTGTAGACCTAGTAAGCACAAATAATAGAGTAAATGGTATCGAAATG
ATTGTTGGGTTACCATATATGGTACCATATGGTGGAGTTAAATTAATTACCGATCCGTTT
GAATCGATTGGTACAAGTCAGCCGAGGAATGGAGTAGGAAAATATTATAATTTTGGCTAT
TCTGTTGCCATGACAAATAACGGTGATGGTTACGCTAGTTCTCCATTAAGTGATCAATAT
GGACCAAACGTTACAAACATCAGAGAAGATATTTATTTAACCAGTCCAGATAATGTGTTA
TTCAGTTCATTTGGAGTAACATTAACTACTTTATCAATAAATAAAGGACAAAATCATGCT
GTAGTCATTGGAGCTCCGTTTCATTCAATAAAACAAATTCAAAACGTTGGGAAAGTTTAT
ATATTTTGCTCATCGAATAACAATCGGAGTTCACCTTCAGCTTTTGTACACGGAGAGAAA
ATGGACGAATTCTTTGGATATTCATTAGCTAATATTGGAGATATAAATGGTGATGGTAAT
GATGACTTAGCCATAGGTTCGCCATACCTTAGAAATGATAACGCAATAGGTTTTGTAACT
TTGTACACGATCAATGGAAAATGTGAACTTAGCACTAAACCACATCAGATATTAAGAAGT
CAACAATCAAGTAAAACTGGAATATTTGGAATTGCTGTTACAGGAGGATCTGATATCGAT
TTTAATAGCTGGCCAGATGTAATAGTTTTAAGCACTCAATCAAATTCAATTGAAATGTAC
GCATCTCGTCAACAGTATGTTGCACAATGTTCGTTTAAATTTCATAATACCTATTTAATT
CAACCTCTTATGAAGGATGTGAACATTACTATCACATTGTCCGTTTCTTTATTAGATACT
AGAAAAAACAATTTTATTGATATTTCTTTTTTGAAGGAATTGAAACTTATATCCCAGGTT
AATCTTAATGAAAATTTAAAAATAAATCGATTTCAATTACATAATAATTCGATTCTTTTG
GATTATATAACTTCCAATAGTATTTCTGTAGAATTTAAAATTATGTCATTACAACATGTA
AAAGACATGAATCTTACGCTTCCAATCAATTTTGCTTATCGGTTTGTGGAAGACTGCAAC
AATCGTAAATGTAATAATTTATTATTTCCGTTAATTGATTGGACAAAATGCAACAGAGAA
TATTCAGTTAGAAAATACATTTGTGTAAAAGATCCAACTTGTGAAGCTGATATTTCTATT
GTTCTAGAAGATGAAATGGGTAATGAAGTATTTTTTGGCTATGATAAAAGCCATCATCGA
TCATTGAAAGTAAAAATTTTTAATAAGGGACCAACTGAGGGAAAAGATCTTAATCTTAGG
ATAACTACTTTTGAAAATTTAAGAATTAGCACTATATCCAGTAATGAAGATAGTAATATT
AAGCAAATAATCTTATCCAATCAAACTGAAGCAGTATGTAAATTAGAGAATGAAATTGAA
ATCAATGATATTATAATTGTTAACATCGAACTTTACTTTATTAATTTCACGGACGTTAGT
TATATGGTTTATGAAAATAATTCGGTCCTCGTTGAACTCTTTTCTGATGTCAAAGATTCA
AATCATACTAATAATAATGCTAGACTCGATTACAGTTTTATCTATCAACCAAATATTTCA
TTTTCAGTGTCGTCTAAGAAAGACACTGTTATTGATGCTAGGAATAGGTCACAACCTATA

AATCAAGGTCAGAGAGTTACTATTGATGTTATAGGACCAAGATTTTCTCAAACGTATATA
GTGGACAATATTGGTAAGTCAGTTGTTTATAACATGTTAGTAAAAATCAGTCTTCCGATT
CATACAAAGGATAAGTTGAATATTTTATATTTATTTGATAAAATCCGGGGAGTTAATCAG
TCTCGCGTTGGATGGATGTCAATATTGCCACAAATCACAATTGGTTCCGAAAAAATATCCA
TGTATTCTTCCGACAAAGTTCATAAATCCTTTAGATATTATTGGATTTGAAACTCACGAA
TCTCATCTTAAAGATATTGACAAAACTCCAATAGAAGGATTACGGTATTTCAGAAAGAAA
CGATCTGTTGAAAAATCCGAAATAAATAAATCAACAAAGTTTGAGAGGAATTCTAAAATG
ACCCAAGGAACAGTCCATCGAACACTGATTGAAGTAAGAATTAAATGAtttgcagtaaaa
tacatttattcaactaggacagcaaataaattgttatctatcgatttttactattgaaga
ttagtaagaaattttaatttgaacattagaaacaattttatacgtctaatagatcgagaa
atctttaaagttcgatcgaaaacaaacttcaatcttgctatcattatttttgcagctaat
agttgacgatttataaacttgtctagaaactggaataccattaataataattatagtaaa
ataagcagataaacatcataaattgaacgatgctacatgtttgtttggctgtggtacac
>DjScaffold4081:10928-23112 strand:+
ttaatgtttatgtagatttataaatcatttattatttgttttataatgaattttttaat
atttcttgaatgaataaaaataatagaattcaaagacaaaattgtgcatttgtagttgaa
aagtaaatgctgagtatttgttgtgttttaaatatgataaaaaataaaaaatctcatgaaa
attttgtaactaaatttttattagaccatcttttgttgttgaagacagacaaagttcta
ttgatagttgtacgactgtaaaatcatccacaataacggagggtcaaacgagcactttat
ccaatgaaagcaagaatagtagtggcagtcaaaatacaaattcaactgacaaaacatcaa
gcagtcagggatcagccgaaattgaagaaaaagaaagaacatttggaagcatcaatttac
atccattaccagacactcaaaacaaatcattatcagattgttatcgatacataaatcagc
gtattgtgaatgttcaagaaacaaatctgtcgaaaaatataagttttcgaaaattaccag
tctttgataaaccagtatcgccaccgaatattaatgcttcaaccactgatcaacaatcag
atattgctaagccaaaagttagaacgactagaattgctgaatatatattagaaagaacaa
taggtaaaggtaattttgcagtagtcaagttggctactcatagtctaatcaagactaaaa
ctgcaatcaaaattATGAACAAGTCATTATTGGGAATGAATAATTTGGAAAAAGTGACTC
GAGAAATTGAAGCTATGAAACGTTTAGAACACCCTCATATTATTAAACTTTACCAAGTTT
TGGAGACTGAAAGTAAAATTTTTATGGTAATGGAGTATGCTGAAAAAGGTGAAGTTTTCG
ATCATATTAGTCATGCTCATGCATTTACAGAAAAAGATGCTAGAGAGCTTTTCTGGCAAG
TCGTTTGTGCCATTAATTATTGCCATAAATCTAGTGTAGTTCATAGAGATTTAAAAGCAG
AAAATCTTTTGCTCAATGCCAATTATCAAATTAAGGTGGCTGATTTTGGATTCTGTAACT
TCTATGAATTAAATAAACTGCTATCAACTCATTGCGGAAGTCCACAATATGCTGCACCTG
AAGTCTTCAAAGGTGAATTGTATGATGGACCTTTAGCAGATGTATGGAGTTTAGGAGTTG
TTTTGTATATTATGGTTTGTGGCTCGTTCCCATTTCCCGGTGAAAGTTTGGGTGATATTA
GAAATCAAGTTTTAAGAGGCTTGGTGAGATTTCCATTTTTCTTATCCACTTCATGTGAGT
ATCTTGTAAGAGGAATGTTACAAGTTGATCCGACAAAGAGGTTCAGAATTGAACAAATCA
TTTCAACACCATGGATGCAGGCATCCAACAATGTTGATAAATATCGAGCTCTGATGGAGA
TTTATAATAAAAAAGCCATCGAAAATAAATTTGAAGAGATTTTCTACAGACAACATCCTG
AACATTCTGACATTATCAGAGAACAAATGTATCATTCTAGTAAAATCAAATTAGATTCAG
CTATTCAATTGGCTTTGCACATGAATGGGTTTGATGAAGACGAAATTCGAAATTCAGTTC
TCAATCGATTATTTGATCGAAATTACGCAGCATATCATTTATTAAAAGATAAAGTGTGCC
GATTTTTTAATAATCCATTACTTACTCTTTATCTCGAGGCAGCTCATTCTAATATTGTAA
ATAGTTCAAATGAAGAGATCGACTATCAAATTATGAATAATATTCCAGATATTTTATCAT
CTGTTGAAGATAGTACTCGACAGAATTTAGAAAATTTGAATAAAACTCAAGAGTCTTGTA
CTAAAGAAATGTACAGTTGCCGAAGAAATAGTTTATCATATTTGAAAAATAAATTTTATT
CAAATTCCGATGTAACGATAATGGCTAAACTTTACTGTTGGGTTTTGCAAACCGACGAAG
AATATTTGTTAAATAAAGAGCAAACCTCATTTATCGACCAAAATATGGCTCTACAGTTGA
ATGTTAGAAGGCATACAGTACAGTTAACAGAAATGCCTAATCAACTAAGAACTGTATCAA
ATTTGGCATCAATTTCTCCAGGATTTCCAAAAGATTCAGTTCAAAAGGTCAAAGAAGAAA
ATAAAGATGACTCT
>DjScaffold4081:39565-39621 strand:+
ATGCCAATTATCAAATTAAGGTGGCTGATTTTGGATTCTGTAACTTCTATGAATTAA
>DjScaffold3109:86191-95342 strand:+
ATTTACAGCCTCAAAATACCTCTCGATGTTACTAGCGGTGACTTTTCATTTTTAACCAAT
GTGATCAAATTATCCACAGTTCTTTCATTTATAAGCCTTGAGCTCAAATCTTTTTATAAT
TTTGAAGCAGTTATTAGCGAAATTAATGTTGAATCAGATAAAATGTCTTTATATATTGAA

ATTAGTGGACTACTACGTGAAATGGGTTGTCCATATTCAACTTTATTGGACGGTGATTTA
GTAAACAGATTTTCCAATGAAGCATCTATTGTTTTATTAATAAGGTTTTTAATATCCGAA
TTAGCAGCGGCCAAAATTATGGCAGTTGAATATCCTGATAATGATTATCTTGCAAAGGCA
AAAGTAATAACTTCTCAGGAAAGTAATTTAACAGCCATAATGAAACCTGCCTTAATGGCT
ATTGGTATTGGTCGTCCACCAGAAACTATGAACACCAGTTTGTTTTTTCTAAATTAGAA
AATACAATAAAAGAAAAATTACCAACAATTCCAAAATATTTCTTGGTAAGTCTTTAATA
AATAAAAAGTTATCTGAGACTCAATGGAACAAAGTCGAGGATTTCATAAGTGATATGAAT
AACGCTTACTATTCGCACAGACAAACTCTCATACAGCGCCTAAATGTAACCATTCAAAGC
TTCATGTGGTCCCGTAAAGCAAATAAAGAAAAGAACAAAATTGTTGAAAAATCCAGCTCA
AAGCGATCTAAATTGGAAGCACGTGCTTCTGTTAATCTTATAGACTTGCTTGCTGCTAGA
GATTATCCTTATGCATTAATAAATAAAACTAGCAGTGGAATTTCCCGTAAATTTAGTGCA
TGTCAATTAAACAAATACAAAATTGGGCCCGTACCTGATAGAGGTGGACGTACACTTGAA
ATG
>DjScaffold2491:18557-32115 strand:+
aaatatttagtgtttataaatataattccgcaatcaataaaggcgaaatataaaatcacc
tcatatttagctctatttctaaattactaaactcagttcATGGATAGTATTAAGAGCCTT
TCTTTGCACAATGAAAAATTTAGTAGATTACATTCGAATGTACCTAAAAGTGGTCATATC
CACAAAGGTCATTCTATAGTCTCTCATAATGCCACAATTCCTTCAAAATATGAAACATAT
GTAATTGAAAATGCTGACCATAAAGGATTTAATTCTACTGCCAAACGATTCAATTATGAA
TTAAATGAAACAGCTTTACCCGGAGTTGGTTATTATTATGGTGGACAGTCAGGGATGTCT
AATGATTCGCCTTCATTTTCAAAACGCGGAACAGGGTCTTTTGCATCTAAAAATCGATTT
ACAACATATAATCCTGCAGCCGAACTTCCAGCTCCAAATGTTTATTACGTAAAATCAGCT
GATTCCAAGTCAGATTTTAATCGATCAAACTTCTCAAAAAAATTTCCAACCGGATATTGCT
GTAAAAATCGAAGATAAAAATTCAAAGCTTCCAGCTCCAAATCTGTATAATATTTCATCA
ACAATCGGTGAGAAGAACAACATTTCTTCTTTTAAATCAAAATCGAATAGAGACACCAAT
TTAATGACGGACATATCGAAAAATCCAGGGCCGGGTCAATATTCTATAAATAAAGCTGAC
AATATCATGGGCCATAATCATACTGTAATTCGATCCGTTTTCAAATCAAATTCAAAAAGG
GATAATATTAAACCACCCGAGAAAGACTGGAATAAAAAAGTTTACTTCATATGCGAATCA
CAAATCTCAATTTTATATTCTAACTAGgtgaaatatttagtgtttataaatataattccg
caatcaataaaggcgaaatataaaatcacctcatatttagctctatttctaaattactaa
actcagttcATGGATAGTATTAAGAGCCTTTCTTTGCACAATGAAAAATTTAGTAGATTA
CATTCGAATGTACCTAAAAGTGGTCATATCCACAAAGGTCATTCTATAGTCTCTCATAAT
GCCACAATTCCTTCAAAATATGAAACATATGTAATTGAAAATGCTGACCATAAAGGATTT
AATTCTACTGCCAAACGATTCAATTATGAATTAAATGAAACAGCTTTACCCGGAGTTGGT
TATTATTATGGTGGACAGTCAGGGATGTCTAATGATTCGCCTTCATTTTCAAAACGCGGA
ACAGGGTCTTTTGCATCTAAAAATCGATTTACAACATATAATCCTGCAGCCGAACTTCCA
GCTCCAAATGTTTATTACGTAAAATCAGCTGATTCCAAGTCAGATTTTAATCGATCAAAC
TTCTCAAAAAAATTTCCAACCGGATATTGCTGTAAAAATCGAAGATAAAAATTCAAAGCTT
CCAGCTCCAAATCTGTATAATATTTCATCAACAATCGGTGAGAAGAACAACATTTCTTCT
TTTAAATCAAAATCGAATAGAGACACCAATTTAATGACGGACATATCGAAAAATCCAGGG
CCGGGTCAATATTCTATAAATAAAGCTGACAATATCATGGGCCATAATCATACTGTAATT
CGATCCGTTTTCAAATCAAATTCAAAAAGGGATAATATTAAACCACCCGAGGTAAAAATT
ATCCAATTTTTTATTTTAAAAAATCTTTAAaaaaatttaaaaaaattacattttatactg
gtttggacaaaaaagtttttcaggttt
>DjScaffold4493:42229-51133 strand:+
acctaaaaacacaaacgaatataaaatgtatttgaacgtttatttgattttgtaaagtgt
tatacgttaataaagtttctttaaaataaacgtgttattttagatgatatataattattt
attatatcattatttataataaataattcacgttcatgccaaataaagtttacttggagt
tagttctcttattattacgaaaaaagtttttttttattttctacaattgaaatacaatat
attatttcaatttaatacaaagaaataatgacatttgaaatattcagctccaatataaac
aggatgatttagaagctcaaaagttgttatcaaatcttggtgatcttgctgaaaaaatcg
aaATGGCAAAAACATATTATGAAAATAATAAATTTGACGAAGCCATTACATATCTGAATC
AAGTTATTGAAACAACAAGTCTCAGTGAAAGTTTACGAGAGATGCGAGCTAATAGTTATT
TGAAAACCGGAGATAATCAAAGAGCAATAAATGACTTTAGACATGCAGTGAAATTATCCA
ACGATGATCGAGAAGGAATTTTACGATTGAGTAAATTATTGTATGAAACAGGAAATGCCG
AGCAATCACTTATTGAAATTCGTGAATGTCTCAAACTTGACCCTGACCATAAAAATTGTT
TCCAACATTACAAATATATCAAAAAACTCGTCAAATTAATTAGCGTGATTCAAGAGTCTG

CTAAAGAAAATCGCTACAGTGACTGTATTGGAAAATCAGAAAAATTGAAATCAACCGAAC
CACTTTATTCTGGCTTAGCTAATCAGTATTTGTGCAGTTGTCATAGCAAGTCGAGTGATC
CGAAAATTGCTATCGAAATTTGTGAAATTGCACATGCAAATGAACCTCAAAATTGGGATG
TAATCTGTGATTTAGCTGAAGCGCTTATCGCTGATGAAAATTATGATTCAGCTATTAACT
ATTATCAAAAAGTCCTCGAATCTGATAGTGTCAATCAACGTGCAAAAGATGGAATGGCGA
GAGCTCAAAAATTACTGAAAAATTCAAAGAAGAGAAATTATTATAAAATTTTGAATGTAG
CTAGGACTGCATCTAAGAAAGAAATTCTTAAAGCTTACCGAAAATTAGCAGCTGAATATC
ATCCTGATCACTTTAAAGGTGAAAACAAAAAAATGGGAGAAAGAAAATTTATCGATATCT
CAGATGCCAAAGATGTTTTAACCGACTCAGAAAAACGTCAGAAATTCGACAATGGTGAAG
ATCCATTAGATCCAGAACAGCAACACCATGGCCCATCAGGAAATCCATTTTCTGGATTTA
CATTCCATAATTTCCACCCTTTTGAGAACGGTGGATTTGAGTTTCATTTCGGATAAaata
tatcaaataaatatttttcaatactttgtcttttgtgtgcctatttaaagtctgtcgggt
ttaatgtctgtcgtttctttttcttttttttgatctttttcaatgaataacagttttt
tgtttgcttgattatttaaatttataaatattattgtaacaatgaaaaatgtgatctgcg
atgttttatttgttaataaactgataatttcgaggtttaataatttagctattaactatt
atcaaaaagtcctcgaatctgatagtgtcaatcaacgtgcaaaagatggaATGGCGAGAG
CTCAAAAATTACTGAAAAATTCAAAGAAGAGAAATTATTATAAAATTTTGAATGTAGCTA
GGACTGCATCTAAGAAAGAAATTCTTAAAGCTTACCGAAAATTAGCAGCTGAATATCATC
CTGATCACTTTAAAGGTGAAAACAAAAAAATGGGAGAAAGAAAATTTATCGATATCTCAG
ATGCCAAAGATGTTTTAACCGACTCAGAAAAACGTCAGAAATTCGACAATGGTGAAGATC
CATTAGATCCAGAACAGCAACACCATGGCCCATCAGGAAATCCATTTTCTGGATTTACAT
TCCATAATTTCCACCCTTTTGAGAACGGTGGATTTGAGTTTCATTTCGGATAAaatatat
caaataaatatttttcaatactttgtcttttgtgtgcctatttaaagtctgtcgggttta
atgtctgtcgtttctttttcttttttttgatctttttcaatgaataacagttttttgt
ttgcttgattatttaaatttataaatattattgtaacaatgaaaaatgtgatctgcgatg
ttttatttgttaataaactgataatttcgaggtttaataa
>DjScaffold2045:332-3414 strand:+
cacagactggattcaatagaatatattaatattattattatacaatattctttctctcat
ttgaatttcggttctattcactATGGGGAATTTGATCATGCCTAGAAAGAAGTTACCAGT
TGAAGATTTGAACTTTCTTATACAAAATACCAACTTTACTAAAAGTCAAATCAAACAATG
GTATCGAGGCTTCATACGAGACTGTCCGAATGGTCAACTTAGTCAAAAGAAATTTATTGA
GGTTTATTCGAGTTTTTTTCCGGAAGGACGAGCTGAAGAATTTTGTTCTCATGTTTTCCG
AACATTTGATAAAGATAATTCTGGAAAAATAGATTTCAATGAGTTTCTATTAGCAATTAA
TATTACATCCGGCGGTAAACCGGAACAAAAACTTGAATGGGCTTTTCAAATGTACGATAT
AGATGGAAATGGAACTATAGAATGGAATGAAATGACAGAAATAATCAAGGCGATTTATAA
TATGCTTGGTGCTGAAGACACAACTGGGGATAATTCTCCAGAAGCAAGAACAAATGACAT
TTTCGCAAAAATGGATGAAAATAAAGACGGAGTTTTAAATAAAAAAGAATTTGTGAAAGG
TTGTATGAATGATAAACAACTTTATTCAATGCTCTTGGCCGATACATCTATGGAATAAat
taacagaagcttaaatatatttattcatcaaccaataatgacacagactggattcaatag
aatatattaatattattattatacaatattctttctctcatttgaatttcggttctattc
actATGGGGAATTTGATCATGCCTAGAAAGAAGTTACCAGTTGAAGATTTGAACTTTCTT
ATACAAAATACCAACTTTACTAAAAGTCAAATCAAACAATGGTATCGAGGCTTCATACGA
GACTGTCCGAATGGTCAACTTAGTCAAAAGAAATTTATTGAGGTTTATTCGAGTTTTTTT
CCGGAAGGACGAGCTGAAGAATTTTGTTCTCATGTTTTCCGAACATTTGATAAAGATAAT
TCTGGAAAAATAGATTTCAATGAGTTTCTATTAGCAATTAATATTACATCCGGCGGTAAA
CCGGAACAAAAACTTGAATGGGCTTTTCAAATGTACGATATAGATGGAAATGGAACTATA
GAATGGAATGAAATGACAGAAATAATCAAGGTGATTTTTACATTAATTTTAGTTTTTGTT
TTATTAATTGAATTTCGTGTATTTTCTGCAAAGGCGATTTATAATATGCTTGGTGCTGAA
GACACAACTGGGGATAATTCTCCAGAAGCAAGAACAAATGACATTTTCGCAAAAATGGAT
GAAAATAAAGACGGAGTTTTAAATAAAAAAGAATTTGTGAAAGGTTGTATGAATGATAAA
CAACTTTATTCAATGCTCTTGGCCGATACATCTATGGAATAAattaacagaagcttaaat
atatttattcatcaaccaataatga
>DjScaffold820:13059-13914 strand:+
tattatcttaatttgaaaatttttacagtcattagatttttcgttttgtgATGATTTGCT
TTGCGTTACTGATGATTTTATTAGTTACATTTGAAAATTGTAAAGGTGATATCGATCCAA
ATTCACTCTTCGTTACTAAAACTTTGGTGACATTCAATGAAGCTTTACAAACTTGTAAAT
CTCGAGGAATGAAATTAGTTCGAGTAGATAGTGCAGAAGATAATGCTGTTGTAAATGGAT

TCGCTAACAAAGTTATGGCTGATGCATACTGGATCGATGGTAATGATCATAATGCTGAAG
GAGTTTTTGTAGACTCAAAAGGAAAATTATTAAATTACAAAAATTTTGGAACTAATGAAC
CAAATGGTGGTCGAGCAGAGAATTGTATAAACGTTTATCGTGTACCCAGTGGATTTTGGA
ATGATATTTCATGTGTTGTCAGAATATGGGCTATATGTCAAAAAGATCCTAACCTAGTGA
CTTCTGACCAATTGGAAATTGAAGGCAGTATTTTCATGGCATCAAAAAGTAAGATATCAT
TTGCAGATGCTAAAGAATATTGCATAGTAAGAGGCTTACGAATGGTTCGAGTCAATCATG
TAATAGATAATTCAATAGTTCACAGTTTTGCAAGAGAAACCAACTCTGATTTATATTGGG
TAGATGGTAATGATATCGTAACTGAAGGACAATGGGTTGATGCAGATGGAAATGAATTAG
TTCACAAAAATTTCCATCCTGGAGAACCGAATGGTAACAGAATTGAAAACTGTGCTCATG
GGTTTCTAAATATAGATGGTCTTTGGAACGATATTCGTTGTGATTTACCATTGTGGGCTA
TTTGCTACAACGAATCGAGAAGAGGAGTAACAACCGGTTATTTACTTTTATCATTAATTA
CAATAATATTACAATACTAAttaagtttcaaaacttgaacaattaacccacacctaaaca
aaaggttttatagttaaaatatccaagaaaacccacttattaatcctatcatttatttac
atttagatatttatatcaaatgaattcgttcgtttatatttttcttcaaagaattgatct
tgtaaattattcaactttttttaataattttttttactacagataaagtacacaattaaaca
gaatttcttaatgtattgtattaaatgtataaggactatgaattgcttaactcaacttgc
ataggacgttttaaagagattcaatataattataatttttagaccaattggaaattgaag
gcagtattttcATGGCATCAAAAAGTAAGATATCATTTGCAGATGCTAAAGAATATTGCA
TAGTAAGAGGCTTACGAATGGTTCGAGTCAATCATGTAATAGATAATTCAATAGTTCACA
GTTTTGCAAGAGAAACCAACTCTGATTTATATTGGGTAGATGGTAATGATATCGTAACTG
AAGGACAATGGGTTGATGCAGATGGAAATGAATTAGTTCACAAAAATTTCCATCCTGGAG
AACCGAATGGTAACAGAATTGAAAACTGTGCTCATGGGTTTCTAAATATAGATGGTCTTT
GGAACGATATTCGTTGTGATTTACCATTGTGGGCTATTTGCTACAACGAATCGAGAAGAG
GAGTAACAACCGGTTATTTACTTTTATCATTAATTACAATAATATTACAATACTAAttaa
gtttcaaaacttgaacaattaacccacacctaaacaaaaggttttatagttaaaatatcc
aagaaaacccacttattaatcctatcatttatttacatttagatatttatatcaaatgaa
ttcgttcgtttatatttttcttcaaagaattgatcttgtaaattattcaactttttttaat
aattttttttactacagataaagtacacaattaaacagaatttcttaatgtattgtattaa
atgtataaggactatgaattgcttaactcaacttgcataggacgttttaaagagattcaa
tataattataatt
>DjScaffold820:48929-50040 strand:+
cagatcctaatgcattgattgcttcacatgaaagattaacatggaatgaagctcaagatt
attgcaaagcacaaggttataaaATGGTCAGAGTCGATAATGCTGGACAGAATGCTATTG
TTCATGGATTCACAAGAAGGATTTGGGCTGATCTTTATTGGCTTGATGGTAATGATGCCA
AAACTGAAGGTCGTTGGGTTGATTATAAAGGCGATGATTTAAAACATAAAGGATTTGCTC
TCGGTGAACCAAATGGAGGTGTAAATGAAAATTGTTTGAATGGTTATACTTATATTGATG
CTTTAATGAACGATTTTCCGTGCACTTACAAAAATCTATACGCAATTTGTGCAAGAGATT
CTTTATATATTAGATTTGCATTTGATTATGAACTCGATTCTGAAAAAATGTTGGTATCGA
CTGACAGGTACACTTACAATGAGGCTTTACAGTTTTGTCGAACTAGAGGAACACAACTAG
CACGTGTGAACAGTGCTGCTGATAACCAAGCTGTTGCTGCTTTTGCAAAACGAGTTGTGG
CTGACTACTATTGGATTGATGCAAACGATTATACAGAAGAAGGCAAGTGGGTTGATTCAA
ATGGAAAAGAGAAACCATACAAAAATTGGGCAGCCAATGAACCAAATGGAAATCGTGTAG
AAAATTGTGTGTTTTCTTTTTATTTAGAGGAAGGAACATGGAGAGATATTGTGTGCTCTT
ATAAATATCTTGCTATTTGCTCTTTTGCGGAATTTAGGAATCTGCCAATTATTGTTTAAt
gttccttgttaaaaaaaaaagaataacaatatcgatttaattctaaaatatcaattttgc
caaataaaaatttaatattgctg
>DjScaffold162:23463-29239 strand:+
ccattaatatttattatttgatacacttaATGAGTACAAAACAAACATTAAGTCTTAGAC
AACAGAAAATTAGAAACATTTTACAGTTTGAAAAAGAAAATGGAAATGATTTGACAAAGA
AAGAATTGATCGATAGAATCCGAAAAATTCTTGAGCAAGATTATCAGATGAATCAAAGTT
TAAAAGAAAAAGAACTTAGAGCCGAAGAAATACGAAGAATCCGAAAACTTCGTATGGATG
GTAAAATCTCTAGTGAAGAAGAAGAATATTTAACTCAGAGTTATCAAAATCAAAAGCCAG
CAGTTCAAGAAAAAGAAGAGAAAATCGTTTTTGAATCTTTTCAACCGAATCCAGCTCATT
CTAATCGGAATAAAGAAATTAAAAAAGCGAAAAATTGTAATGCAGATTCAACTGAAACTA
TTGAAAAAGAATCAGAAGTAGATCAAAAAGAAAAAGTAGACGAAGAGAAACCAACCACAA
CATCTGAAGAAGAAGTGGTTGAAACCAAGGAATCTGAAGAAAAAGAGGTTGTTGAGGAAA
ATGAAACTGAGGAAAAAAACATTCAAATATCGGAAACTGAAACAAATAAGGAAGAGAAAG

CAGAAAATGAAGATAGTCATGCATTTCAACGCGAGAAATCTGAATTAACATTAGCTCTAG
AAAATGAAACCAGCCTGATAAAATTATATGAGCTTGCAGAAAAGATGGTTTTTGTATCTC
CCAAGAAATGAatcaaaatgaaggtgccaaatattgaactacaattaaaacatgaaagat
tttattatgtttctattttgtatgttgttaacactcgattattttttatga
>DjScaffold162:3369-3764 strand:+
ATGAAAGTAAAGATTGCAGATTTCGGTTTAGCAACGCAATTAGAAATCGGAGTAGAACAT
TTCACAATGTGCGGCACTCCAAATTATATTTCACCGGAAGTTGTTAGTCGTTCCAAACAA
GGCCTGGAAGCTGATGTGTGGAGTGTCGGTTGTATGTTGTACACAATGTTAGTAGGGAAA
GCACCATTTGACACTAAAGAAATCAAAGGAACACTTAATCGTGTTCAAATCGGAAAATTT
CATTTGCCTTCAAACATCAGCGAAACTGCAAAGCAACTCATTACTGGTCTTTTGCAAACC
AATCCAGATAAACGCTTGAATCTCCAAGAATTGTCGGTGCATGGTTTTATGCAAATCAAT
AAAAGTTCAGATTTATTTCAAGTAAGTAATAAATAA
>DjScaffold2170:11503-17423 strand:+
caagaatttaaagacgcATGTCAGAACAAGAAGAAATTGAGCGCTTGAATGCGGCAATAG
AACATGAAAGAAATTATAAAGATATGATTGAACAGTCAACCACTGAAATCCGAAGTGCGC
TGAGTGATATTGAAAAACAATTAGCCAATATTGAAGATGAAAGTAATGAATGGAAAACAA
GATATGATATTCAATGTGAAATCAATGATCAGTATAACAAACAAATTCTCTTGTTACAAG
ATAAATTGCTTTCTTCTAAAAGTCAATTAAAAGAAGTTCAAAAATCTGTGAGAACAAATA
CTTTGCAAGCTGATTTTGGAGGTGAAATTACCACACATACTGTGCGATTATTGGAAAGAG
AAAAAGAAAATCTAATAAATCAACGTAAAGATATTGACTGGAGAATAGATCAAGAAGCGA
AAGCGTATCATAAAGCCTGTGAAGATAGGAAAACCTGTCAAATTGAATTGGCAGCTGCTC
AGCTCAATATGATTGAATTAAGAATTAAAAATCAAAAAGAAATGGATGCCCAACAAATGA
TATCTGCTCGACCTCACGCTTCTCATACTCGCCGCTCGCCTAAACGAACAATTGGTTTAA
GGAATATTCCTCAACACCAAAGAATAATCGATAGAAAATATGGACCGATTAACAAAACTG
CTGCAATTAAAGCATTACCGTCAATAAGAGATTAAtttttaaaatatatttgcacaaaac
ttattcaattctgctgattttattataatatttgctctgatattttattctattcaacat
ttttgttataaattacatttttactttttta
>DjScaffold2170:5498-5932 strand:+
ATGTCGTTTAAAATTGCCACAGCGCCATGTACATTGCAAAATGTAATGACAATTATTATA
GGAGACATGAATTGGAAAGAAATTTGGGTCTATCTGGATGATATTTTAATTTTTTCGAAA
AAAATTGATGAAAGTATTTCAATTTTGGACAAGCTATTTACTAGAATATCTAAATTTTGT
TTACGAATTAATCCTGATAAATGTAAATTTCTAGTATCAGAAATAAAATTTTTAGGTCAT
GTAATTAATACTGACGGAATACTGCCTGATAACGATAAAGTAGAATCTATAAAGACATTT
AAAATATCGGGTTGCGTTAAACACGTCAGAAGCTTTTTGGGTCTAACTAGCTACTATAAA
AAATTCATTGAAGGATATTCTCAGCTAGCAAAACTACTAAAATCTGTATTAAGTACTGTA
AGGATTAAAAATTGA
>DjScaffold1929:67820-74205 strand:+
TCTTGGTCTACTAGAAGTAAAAGGTTTATAGAAAATGAAGCACTAGATGTTAGTACTGCA
TCGAAAAATAATGAATCACAAGTCGCGAAGAATGATGGTAGTGTAATAAATGGAACAGAG
ACAGCAGAAAAGTCTCCAGAAGAAATAAAAGTTGAAAACATTCCCAGTGAAACCACATCC
ATGTCACTTAATGAAGACGATGATAATATTGATGTTGGAGGCATTTTGCCAGAAACAGAA
GATGAAGAAGAAGAAATTCCAGAAATTACAACTGCTGGAGAAAATGAATTTAAATTGATA
GCGCGAGATCTTGCTGGAATAGTGATGTTTAAGAATGAAATCCAACAGAAAATTGATAAC
AAAGAAGGCAATAAACAAGAACGAGAAGGATATCAAATTTTACTCCAGCAATTATGTAAT
ATCCAATTATTTTTCGAAGCCAACGAATCGAATCGTAAAGAAAAATGTAACCAAAGTCGT
CTTAGACTACGCTTAGAATTTGAAGTCTTTGAAAACAAATCGACATTTAATACAAAAGCA
GAAAAAGCCGCATCAAAATCTGATACAAGAGAATCATGTTCTGAATTCGAGTCAGAATCC
AGCAGTGAACGATATACTGAATCAGAAATTATGACATCCACTAAAATGAAGGCTACCAGA
GCAGAGCGATTGGCTCGTCGGCAAAAAAGAAAATTAAGTGACATCGATATTAATCAAACA
AAACGACAAAAACTAGAACGATCAACTCAAGAAGATTGTCAACAACATCAACAACAAGAA
CTGCAATCAGAAAATCTGTCGAACACTCCACAACAAGTGAAAAAGAAAGTCTATCGAATA
TGTGATGATTATTTCACTGAAGATCTTGAACCAGTTCAAATAAATGAAGATGGAACACTT
CTGCATTTGTCGAAAGACCTGATTAGTAGTGACCGCTATGAACTTGCGAGAAAAATGATA
AATATGTACAAAATGAAAACCAATACTCTCATGTCTTCGGCTCCAAATGGAACCATGCCT
CTTTTGAGTAGCAGCTCTTGAaaaccattcggatcaattttgtttctgatccagattatt
agatggattccaatttctgaaatttcgcgtcctgcatttaaacatgtatagattcacagt
actattaataaattgacgagtttttataactgtattg

>DjScaffold5257:106157-106561 strand:+
ATGCCTTTTAGAATTGCAGCAACACCAGGTACGTTCCAGGAAATTATGAATAAAGTGATC
GGGAAAATAAATGGAGCGATGGTTTATCTAGACGATGTTCTCATATTTACCGATACAAAG
GAGCAACACTATGAAATAGTCAATAAAGTATTGATAGAAATTAAAAATGCTGGTCTATTA
GGTAATCCGGAGAAATGTCAATTCATCCAAAATGAGATCAAATTTTTGGGACACGTTTTC
AATAAAGAAAGTGTCAAAACAGATCCAACTAAGATAGAGGCAATTAATAACTTTGGTAAA
CCAAAATGCGTTAAGAAGATGAGAAACCTTTTGGGAATTTGCAATTACTATCGCAAGTTT
AATAAAGGTTATTTAAAAAACAAGATTGCTGGAACAATTGTGTAG
>DjScaffold4501:13383-19650 strand:+
cttccatatttattattaacaaacaatatttgtttaacaaattattgaaatttattttat
ttttactattgcgaATGTCTGAGGTAGATTATAAAGATTTAGATCAATGGATCGAACAAT
TATATGATAATAAGCAATTAAGCGAAAGCCAAGTTAAAGTGCTATGTGAAACAGCAAAAG
AAATATTGGCTAAAGAATCCAATGTACAGCCAGTCAAATGTCCAGTTACAGTGTGTGGTG
ATGTTCATGGCCAATTTCATGACTTGATGGAATTGTTCAAAATCGGTGGTAAATCACCAG
ATACGAATTATTTATTTATGGGGGATTACGTTGACAGAGGTTATTACTCTGTTGAGACTG
TAACTCTACTTGTAGCTCTCAAAGTTCGATTCAAAGACAGAATTACTATATTACGTGGTA
ATCACGAGAGTCGACAAATTACTCAAGTTTACGGATTTTATGACGAATGTCTTCGGAAAT
ATACAAACGCCAATGTTTGGAAATATTTTACGGACCTATTTGATTATTTGCCTCTTACTG
CATTGGTTGAAAATTCAATATTTTGTTTGCACGGTGGTTTATCACCTTCAATCGATACAT
TAGACCACATAAGAGCTCTAGATCGCATCCAAGAAGTTCCACATGAAGGTCCTATGTGCG
ATTTGTTGTGGTCGGATCCCGATGACCGTAATGGTTGGGGAATAAGTCCTAGAGGAGCTG
GTTATACTTTTGGATTGGATATTTCGGAAACATTTAATCATAGTAATAATTTGACTTTGG
TTAGTCGAGCACATCAACTTGTCATGGAGGTAAATTATTTTTGTAAT
>DjScaffold4501:36493-36690 strand:+
ATGCGAAAGAAGATCAAATTTTTGGGAAAAGTTATCAGTAAAGAAGATGTCAAAACAGAT
CCAACTAAAATAGAAGCAATTAAGAACTTTGGTAAACCAAAATGCGTTAAGAGGATGACT
AGTTTTTTAGGAATTTGCAATTTTTATCGATCGGAAATTTTGAATTTCAATACTATAATT
TATTATCAGGATTTTTGA
>DjScaffold863:44670-47373 strand:+
attgttgttatcaccatatttaaATGACGCATTTTTTGAAACTTCGTGGTCTGCCCTATT
CTGCAACTGAAACTGATATTCTTACATTCCTTAACGGTCTTCCAATTTGGAATGAAAGTG
GAATTCTTATTACGTTGAATGACGACGGTCGACCTTCAGGAGAAGCGCTAGTTAAACTAG
ATAATGAAATTGGTTTAAATGACTTACGTGATTATAATGGTAAATATATGGGGACACGTT
TCATTGAAGTATTTCCTGCAACTCAAAAAGATTGGTTTGGTGAAGAAATGCGTATGAAAC
TGTCAGATATGACTTATGTAAGGTTGCGTGGGTTACCTTATGATGTAACAAAGGAGGACA
TATTTATTTTTTTTAGTCGCTTAATGATTTCCCGCGATGGGGTGGGTTTACTGTATGGGC
CTGACGATGTTCCTACCGGGGAAGCATTTGTTCAATTTATGACTAGAGAAGACGCTAATT
TGGCACTTCAGCGACATAATCAATGTATAAGGTCAAGATATATAGAAATTTATAAAAGCT
CTTTAACTGAAGTTTATAGAGCTATGGAAAAGCAATATGAAATTAATCGGGAAGAAATTC
TAAGGCGGTCTAGACATCGCCCAGCGAATTTTCCACCAAAATTCAATGCAGATCCTTATT
CACATGTTAACAGTTATTACACAGATGGGTATAGTTTCCCACAGAATAAAAGCTCCCAAG
GACCCATGAGGCGTAGTCGGGAAAGTAAAAATAGGTCTAATCCCTATGGGGGAAAAAATT
CGCACTCAAGTGTTAAAGTAGAAACTCAAAATTTTGGTGAGTTTTTGGGTCCTCTTTCAA
TACACATGCGGGGCCTCCCTTATACTGCAACCGAAAAAGACGTTCATGAATTTTTTGCTC
CCCTTCGAGTAGCGGAAGTAAAAATACAATTAGGCCCAGACGGGAAAAATACTGGTGAAG
CTGAAGCGGATTTTTATTCAGAAAATGATGCTGTGAAAGCAATGGAAAAGGATCGTTGTA
AAATGAGTTGGAGATACATTGAATTATTTAGGGGCTCATCGGACTAAggccaataatcga
cagctaattgtatttcaaattttactgttgtgttactatttaatataatttttatcttta
attgttgttatcaccatatttaaATGACGCATTTTTTGAAACTTCGTGGTCTGCCCTATT
CTGCAACTGAAACTGATATTCTTACATTCCTTAACGGTCTTCCAATTTGGAATGAAAGTG
GAATTCTTATTACGTTGAATGACGACGGTCGACCTTCAGGAGAAGCGCTAGTTAAACTAG
ATAATGAAATTGGTTTAAATGACTTACGTGATTATAATGGTAAATATATGGGGACACGTT
TCATTGAAGTATTTCCTGCAACTCAAAAAGATTGGTTTGGTGAAGAAATGCGTATGAAAC
TGTCAGATATGACTTATGTAAGGTTGCGTGGGTTACCTTATGATGTAACAAAGGAGGACA
TATTTATTTTTTTTAGTCGCTTAATGATTTCCCGCGATGGGGTGGGTTTACTGTATGGGC
CTGACGATGTTCCTACCGGGGAAGCATTTGTTCAATTTATGACTAGAGAAGACGCTAATT
TGGCACTTCAGCGACATAATCAATGTATAAGGTCAAGATATATAGAAATTTATAAAAGCT

CTTTAACTGAAGTTTATAGAGCTATGGAAAAGCAATATGAAATTAATCGGGAAGAAATTC
TAAGGCGGTCTAGACATCGCCCAGCGAATTTTCCACCAAAATTCAATGCAGATCCTTATT
CACATGTTAACAGTTATTACACAGATGGGTATAGTTTCCCACAGAATAAAAGCTCCCAAG
GACCCATGAGGCGTAGTCGGGAAAGTAAAAATAGGTCTAATCCCTATGGGGGAAAAAATT
CGCACTCAAGTGTTAAAGTAGAAACTCAAAATTTTGGTGAGTTTTTGGGTCCTCTTTCAA
TACACATGCGGGGCCTCCCTTATACTGCAACCGAAAAAGACGTTCATGAATTTTTTGCTC
CCCTTCGAGTAGCGGAAGTAAAAATACAATTAGGCCCAGACGGGAAAAATACTGGTGAAG
CTGAAGCGGATTTTTATTCAGAAAATGATGCTGTGAAAGCAATGGAAAAGGATCGTTGTA
AAATGAGTTGGAGATACATTGAATTATTTAGGGGCTCATCGGACTAAggtttattctgat
ttcaaataatttcattgttaatgtttgtaggccaataatcgacagctaattgtatttcaa
attttactgttgtgttactatttaatataattttatctttaattgttgttatcaccata
tttaaatgacgcatttttgaaacttcgtggtctgccctattctgcaactgaaactgata
ttcttacattccttaacggtcttccaatttggaatgaaagtggaattcttattacgttga
atgacgacggtcgaccttcaggagaagcgctagttaaactagataatgaaattggtttaa
atgacttacgtgattataatggtaaatatatggggacacgtttcattgaagtatttcctg
caactcaaaaagattggtttggtgaagaaatgcgtatgaaactgtcagatatgacttatg
taaggttgcgtgggttaccttatgatgtaacaaaggaggacatatttattttttttagtc
atatatagaaatttataaaagctctttaactgaagtttatagagctATGGAAAAGCAATA
TGAAATTAATCGGGAAGAAATTCTAAGGCGGTCTAGACATCGCCCAGCGAATTTTCCACC
AAAATTCAATGCAGATCCTTATTCACATGTTAACAGTTATTACACAGATGGGTATAGTTT
CCCACAGAATAAAAGCTCCCAAGGACCCATGAGGCGTAGTCGGGAAAGTAAAAATAGGTC
TAATCCCTATGGGGGAAAAAATTCGCACTCAAGTGTTAAAGTAGAAACTCAAAATTTTGG
TGAGTTTTTGGGTCCTCTTTCAATACACATGCGGGGCCTCCCTTATACTGCAACCGAAAA
AGACGTTCATGAATTTTTTGCTCCCCTTCGAGTAGCGGAAGTAAAAATACAATTAGGCCC
AGACGGGAAAAATACTGGTGAAGCTGAAGCGGATTTTTATTCAGAAAATGATGCTGTGAA
AGCAATGGAAAAGGATCGTTGTAAAATGAGTTGGAGATACATTGAATTATTTAGGGGCTC
ATCGGACTAAggccaataatcgacagctaattgtatttcaaattttactgttgtgttact
atttaatataattttatctttaa
>DjScaffold863:62519-67497 strand:+
acacatttattattttatgaactcaatagtattggtatgataatcaaaatttcaaataaa
ccaatgatgactgactactttaaagaaaatgatgtaatttttgtATGTGTATACGTTTTT
CTTTGTATAACTTTTATCAGATAGTTGATATAATCATTTTTAGAATCTTTTGGTTTAACT
TAAGCATTTTTGTATTATTTACGATGCAGTCAGTTCAAGACAAGGACTCTATTTCAGAGA
AATCGAGCAAAGGCTGTAGTTTAAATTTATCAGACGAAGATTGTTCTGGATATTTGTCAA
TGAAAAGTAGTGGCCATAAGAAGCTTAGTTATACTAAAACGTGGTGTGTATTGAAAAATA
AACTTCTCAGTGTTTACAAAAAACAGGATGGAAAGCCGTTAGAAGATATCAATGTTTCAG
TTTCAACAATAAAGCCAATTAAAAAAGATGGATCAAACAATTTTTCCTTTCGAATTATTA
ATAATTGGAATGATTACCTATTCTCAACTAAAAATGAAAATCAGTATGAAGTTTGGATGA
AAAAATTGCAATCTGTTGCATCTGATAATAATAATAATAATAATAATAATAATACAACAA
ATAATGCTGATAAAAACGGTTGTATTTTAAAACAGTTCAGCCGAGATGAATGTACCTCTT
CATTTGAAAAGTCTGATTCAGTTGACCAATTTGATCTAAAATCTCCAAATCACAATGGAT
TTGATGAAGTTGATGAAACATATAGTTCAATAAATGACATAAAAATAAATCCAGAAGAAA
AGATTACATCATCTGATACATTTTTCGATGACGTATATTTCATGGAAGATAATAAAAGAG
ATACAAACAAATTACAACCAATGAGAGATTTTGAAACTGAAAGTCAAGTAAATTATATTT
TGGAAATTATTTTAAATTAAtatctgaaattaaaatgaaattaatctaaataattaacta
ataaataatacttaattaattgtaattatattaaacttttaattatttcaaacagaatag
aaactaactaaaataatcaaatgataacataattaaataattagtaaatataaattaatt
aataataattcattttaattaaaatatttaaatgcaaattaatattagatttgttaggaa
ggtaaaattcaagactaaactgtttttaataaatgcattttcaattgacaatttgctgt
ctcttttaattcaaaa
>DjScaffold691:43508-48861 strand:+
TGTGTTCATGACAGACGTTATGACGATGCTTATACCATACAAAAAGTGTATGCAGAATAT
TATAGACCATTTAATTTTATAGCTGGAATTGTACCATTTATAAGAAATTTATTAGAAGAC
AGAGTAAAAGAAAAGTTTCTACACGCAAAAGAAGCATATCGGGTTTGCATGGCATTTCGA
GAAATTAAAGAATTTATGTTAGTTAATGGAAAATCTGTTAAAGCTCGACAAAAATTATTA
AGAAATCTACGCGTGATAGATTTGTTAGTAAAATTATTAAATATGCCTCTTTGTGGACAA
CCTGATGAGGCGAATTTTCTTAAGGTTCATTTAGAATGTTATGATATTCTATATACGTAT

ATGATTGGGTCTAGCAGAAAGAATGCTTTATATTTTGCTAAATATATTGAACTATTCGAA
TCACAAATGCTGGATAAATCGGAAATCAGTCTTAAAGTTGTTCAACTTATCGTTGAACTG
ATAAGAGACAACAGGAAAATAGTCGACCGTATTAGCAAAATTCAAATCAATCAATTCGTT
GAAGCTCTAAGAACCAATGAAAATTACCGATATTTAGAATTATTGAAAGTTCTATGTGTT
TGTAATGGAGTTGGGATAGTTGATAATCAAAGTTATATAGCACAAAGATGGTTACTGGAA
GATAGAAGAGGAGTTTATCTGACAGAAAGAGGTCAAAATATACATGAGCAACCGAATATT
TTATATGTAAGTACAGACTGTATGAAATCATGGATAGCTTTACATAAATTTCTTCAAATT
GATTATCCCGGTTATGAAGAAGAAAAAGTCTTATTTCTATTAGCACAACTTGATTTGTTT
AACGCAATATGTTATGGACGTAATGAATATTGTATTCGTCTTATAACTAAAGATCTTAGC
TACCTCTCATGGGAAGAAGGATTTCTTTCATTGCAATCCGAAATTCTTCCAGATGATGTT
CGAGCAAAATATTGCGAAATGATAATTGGACTATTTGTTGATGTCGGCAATAACTATTCT
GTTGTTGATCACCCT
>DjScaffold2086:11427-12119 strand:+
ATTCAGGCAGGATTAAGCAATTTAAGCAAAGGGATTTTATGTACGTTTGGCAGCTTTTTG
TTACTATTTTCCCTGGGCACTGTTTATACTTTCGGAAATATGAGTTCATATATTGCATCA
TACTTAAAATACCGAACAAATATGACTTCTGTAACTAATGGTGATACTGTATGGATATTC
TCTGTGTCAGTTCTTTCCAAAACAATAACTATGCCACTCGGTGGATTTCTCGAAAGATAT
GTTGGTGCTCGATCAACATGTCTAATCGGTGGTTTGATTATGAGTTTTGGAACCATTTTG
ACGTATATTTCTATAAAAGTTCATTTTTGGTTAGTAGTTTTCACATATGGATTTTTGTAT
GGCACTGGAATGGGATTAATTTATTCCGTAGCATTAGCAGCTTCTGTAAAATGGTTTCCT
TTACAAAGTGGATTAATCAGTGGTATCGTAGTAACTGGATATGGTTTAGGAGCGTTGATT
TTAAATTTGATTCAAACCAAATATATAAATCCATTAAATATATCCCCAAATGCCAGCATA
TTTACAGATAAATATTTTACAGATTCATCGGTTTTAGACAATATTCCGTCTTGCTTTATA
CTAATTGGAATAATATTTGCTATAATACAATTGATTGGTGTGAGTCTTTTGGTTACGGAT
AATGATAAAAAATCGTCCGATGAGGAGCATTATTTGATTGAGGAAGAAAACGACAAAACT
ATTGATTATACTCCCCAATACTTCACTGTGAAGGAAGCAATGACACAATGCAAATTTTAT
TTATTATGGATTACATTTTTTTTTAATGGTGTATTCATTGTAACCATCTCATCGCTTTAT
AAAAATTTTGGTCAAACTTTTATTAATGACGACCACTTTTTTTCAATTATTGGAGCAATA
GCTGCAATTTTTAATGCTGGAGGAAGAATGGTTTGGGGAAAACTATTCGATATTTTTAAA
TATAAAACAATAATGACTTTCCTTACATTTTCTATGGCTCTGGGATCGGTCACTTTTTAT
TTCACATGTCTTATTCAATCTGTTATTGGTGCTAAACTAATGTATTCGTTATGGGTGTTT
GCATTATTTTTTGCATTATCAGGTAGTTTTTCGGTAATTCCTGCTGCTACTTGCCATCAT
TTTGGAACGAAAAATTTAGCGATTCTTTATGGAATCATCTATTCAGGATATGTTAGTTTG
ATTTGAtttaatttaatatcagaaaacatattgattgtaagttattttataaagtaaatt
ttttaatataattttatgataatgtgaattattatttctctaaaaaaaaacatttctaatt
tgtcttcttttgtaaagttgcatttcataaaaattcttttttttgaagttgatattact
gaaagataaaattctgcctggataatttcggataatctcagaaataatctgttgaacgat
agataaacatgccttattttgaaaaaaagactaccaaaatttaaagaattttggtcaaac
ttttattaatgacgaccactttttttcaattattggagcaatagctgcaatttttaatgc
tggaggaagaATGGTTTGGGGAAAACTATTCGATATTTTTAAATATAAAACAATAATGAC
TTTCCTTACATTTTCTATGGCTCTGGGATCGGTCACTTTTTATTTCACATGTCTTATTCA
ATCTGTTATTGGTGCTAAACTAATGTATTCGTTATGGGTGTTTGCATTATTTTTTGCATT
ATCAGGTAGTTTTTCGGTAATTCCTGCTGCTACTTGCCATCATTTTGGAACGAAAAATTT
AGCGATTCTTTATGGAATCATCTATTCAGGATATGTTAGTTTGATTTGAtttaatttaat
atcagaaaacatattgattgtaagttattttataaagtaaatttttttaatataattttat
gataatgtgaattattatttctctaaaaaaaaacatttctaatttgtcttcttttgtaaag
ttgcatttcataaaaattcttttttttgaagttgatattactgaaagataaaattctgc
ctggataatttcggataatctcagaaataatctgttgaacgatagataaacatgccttat
tttgaaaaaaagactaccaaaa
>DjScaffold2086:82576-83447 strand:+
ataaagtagaatgtctattagatacagggtctcgaattaacgtgatgagtttgagttgtt
ttaataaactacgagaagtaaagttagaaatgacttATGCGAAAGGTTAACAGCAAAACG
GATGAAAAGGTAGAAAATTACATTTGTAATATTGAAGCGAAATTTGGGAGACAAATTAAA
AAAGGAGAGCGATTAACAAGAGCTTTAAATATTTTGAAGATTAAAAAGGATCAACGGTTA
TTGAAAATAATTGAAAGTAAAGAAGATGCTTTTATGGCAGATAATTTGGATATAGGACGT
ACACATTTAACAAGACACAAAATAATCACAAGTGGTGAACCAATCAATGTAAAACCGTAT
AGACAGCCAATGAACTTAGAATCTAAAATTGAAGAAGCTATCCAAAACCTTTTCGAGAAT

AATATTATCCGGAAATGTAACTCTCCTTGGGATACCCCATTGATTTGTGTTTGGAAAAAG
GAAAAGGAGGACATAAGAATATGTTCGGATTTCAGACAATTGAATGCTGTAACGAAAAGA
CAAGCCTCTCCTATGCATAATGTCGATGAAATGCTGGACAAGCTAGATGGAGCCAAATAT
TTCAGTTCAATAGATTTAGGAAATGCATACTATCAGGTAGAGCTTGAAGGAGAATCTCAA
GAAAAAACGGTGTTTTCAACAAAGCATGGACAATATTGTTTT
>DjScaffold1628:13459-17936 strand:+
TTTAACAGTGTATTCGAAACAAATTTAACGGATTCAATAAAGAGATATTACGGAATTAAT
ACTGCAATTCAGAGATATTCTGATTTAACCTCAGCGATTGATCAATTGCAGCAACAATTT
TCATGTTGTGGAGCCAGTGGTTCTACTAATACCTCTAAATCTTGGGAACTTTATAAATCA
TCTACCTGGTTTTTAACTCAAAATTATGGACCACCATATGTACCAGCAAGCTGTTGCAAA
CAAGATTTAATTAGTGAAACACTGGCTACTGATTATTGTCAAGGTAAAATATCAATAGGA
AGCCCAGCTGCTCCTCCGGTCTCGAATAGTTTCACACAAAACCAATACCTCAATACACAG
GGTTGTATAACTTCAATGTCCAGTTCAGTGAATTATTTTTCTTTAGCAATGTTCGGATTG
GCTCTCGGATCAGCACTTTATGTGATGATTTTGTTGATTCTTTCCTTCTGCTTGTTGAGA
AAAATTGAAGTTATAAACAAAGAATTAACCGCACCTCAATCGAACATTAGGACAAAAGAC
CTTAAGAAAATTACTCAACCCAATCAATCAAAAAAGAAACTTCCAGATCTTACTGAGAAT
TCTGAAACTAATTCTCCTGTAAAAGTTGTCTACAAAGATTCTCAAAAGATATTAAAATGA
ttatttagttaattattaaataattaactttgcttattttaaaataattttatctttttt
tataaaaaaaaaattcttgaaatttttaat
>DjScaffold1715:4093-4494 strand:+
ACGGTAAATTTTAATACTAATAAAGGTAGTCCTTATTCATTTGGTACTTGTGATCTTGAA
AAATCAAAATCTGTTACTCGAACTAGAAGAAGAAAATCGACTTCTGAAAAGGGTAACAGA
ACATGTGTAGTTTGTGGAGAAAAGGCATCTGGATTTAACTTTGACAGATTAACTTGTGAA
AGTTGTAAAGCATTTTTCCGAAGAAATGCAATGAAACCTAAAGAAAAGATGAAACCCTGT
CCTCATGGAGGAGGTTGCAATATATCAGGCAGCCAGCGCAAACATTGCCCTTACTGTAGA
TTGGAA
>DjScaffold1715:37814-44066 strand:+
aattcctttagcatcaggaATGGAGAAAGGTCTCTCCATAGATGACCAAGAATTATTGTC
AAGTTTATCTGAGTGTATTGCTAAGTTAAAAGAACCCTTTACACCCCAAGATCATCCTGA
CACTGAGACAGCACAAACCCTGGAAGAAGCTTACAATCGTATGGACACATGTATTCGACG
AATCATTCGTTTAGTGAAGTTATTACCCTGTTTCTTGGATATGGATAAACAAATGCAATT
AATGCTGTTAAGAACCAACATATATGGTCTAATCATCATTTACAGTTCATTCTTTTTTGA
AAAGGAAATCAGTAAATTATGTTATCCTGTTGCATCGGAAGATGGAAACGTCCATTTAAT
AACTGTTTCAGTGTCCCCTAATGATCTTGGATTTTCTGACTGTCAATCAAAT
>DjScaffold328:93601-107929 strand:+
TATTATCCTTATTATTTCATTGTTTATATTTTACAGGTTATAATAAATTACCTTGATACT
ACCTTATTTCCTCAAAGAAATGAGAAACGAAAATTGTTTACCATAGAAATTTATAAAAAG
CAATTGTATTCAGAATTTATCAAAAGATATATTAATTTGTATTTTGGTAATGAAATTCGT
CAGATAATTCACTTGCAATTCATAACATGGACTCATGTTTCAGTAACTAACGCTCAAGTT
ATATTAGAATTAAGAAGACAAATGAATTTAGAATGGTTTGATCATCGAGATCCCATAGTT
GTTCATTGCATTAGTGGATGTGGTAGATCTGGAACCTTTGTGGCAATATCAAATATGTGC
GATTTAATAGATAAATTAAAAGAATCAAGAGAAGACGAAAAAATTAGTATTCCTTATGAA
GTTATGGAAGTCAGAGAGTGTAGAATGTTTTCAGTAGCAAATCTTCATCAGTATTCTATG
ATATACGAAACTATAATTCATTATATTATCAGTGGAGTTACAACAATTATTTTAAAAGAC
CTCGAAATTGAAATGCAATTTCTTTCTATGACATCGTCTGTGACTGGAGCAACAAGATTT
GAGCGTCAATTCAAGAATGCAAATAGCAGAACCCGCAAATTATATCTTACTGATTGTGCT
GCAGGAATTCGTCAGGAAAATCAAAGTAAAAATCGAATTTTCAACATAATACCGAGAGAT
TACGATCGTCCATATTTGAAAAATATCATTCAAAAACACCGATCGGATTATATTAATGCA
GTTTATGTCAGTTCATTCAATTCTAAAGATCATTTTATCGTCACACAATGGCCCCTAACC
AATACGACTAATGATATTTGGAGATTGGTTTTCGATAAAAATATACACACGGTTGTTCTA
CTTGATGTTTTCGATTGTTCTAAGTCTTACCCTGTGTTTTGGCCAAACACAAAAAAATCAA
GCCGTAGGATGTGGTAGTATTGTTGTGAAACTTGTCAAGGATGAACTCATCAAAAGTTAT
ATTAGATGTCAGATTTTTCAACTTACGAAATTGACTTTTCGATTAAAAGAAGCGGATAGA
GCTATTCTGAAGACAAAAAATATAAAAATTACATCAAAAACCACGGATGAAAAGTGGGAA
GTGACATTTTTTCATCTACTAAATTGGAATAATAAAATGCCAGATAATTTAGAAAATATC
CTAGATTTTATTTATATAATTGAAGAATATAATACTTTACGATCTTGCTTAGCTCAAACT
AGCAAACCTCCGATTGTAATCATTTCAAAAGATGGAGTTGAACGAGCTGGTGTAATTTGT

GTAATGTCAAATTGTCTGCAGACAATTAGTAACTGTGGAGAAATTGATGTGTATAGTTCT
GTGTTGAATGTCAAAAGAAATAGACCTGAGCTTATTCCGAATTTGGAAGAATATTTATTA
TGTTATGAATTATGTCTTGCTTATCTACTTTCAAAAAAGAAAAACCAAGAATCACAAATT
GAAGATTATGCTGAATATCAGGATAATGACGAAAGCTTAAAAGAAAATGAAGACTATGAA
GGTGTTGATGAAATTGAATATTTAAGTGAAAATGTTGATATTAATGAAAATTTTGGCTCC
GAAACACGTTTTATCGTTGATGATGAAaatatgtaatgctatttcttttaattattatag
ttatcataattattttctatttattaataaattttccatccacttctatattcttcgctt
ttttaaatgtgttttaattttctaaccgaacatttaagtcatataattgaaatctttagt
atgatacaatttatttattcctttctttttgaaattattcaaaatataatattattat
tagtcttaccctgtgttttggccaaacacaaaaaatcaagccgtaggatgtggtagtatt
gttgtgaaacttgtcaaggatgaactcatcaaaagttatattagatgtcagatttttcaa
cttacgaaattgacttttcgattaaaagaagcggatagagctattctgaagacaaaaaat
ataaaaattacatcaaaaaccacggatgaaaagtgggaagtgacatttttttcatctacta
aattggaataataaaATGCCAGATAATTTAGAAAATATCCTAGATTTTATTTATATAATT
GAAGAATATAATACTTTACGATCTTGCTTAGCTCAAACTAGCAAACCTCCGATTGTAATC
ATTTCAAAAGATGGAGTTGAACGAGCTGGTGTAATTTGTGTAATGTCAAATTGTCTGCAG
ACAATTAGTAACTGTGGAGAAATTGATGTGTATAGTTCTGTGTTGAATGTCAAAAGAAAT
AGACCTGAGCTTATTCCGAATTTGGAAGAATATTTATTATGTTATGAATTATGTCTTGCT
TATCTACTTTCAAAAAAGAAAAACCAAGAATCACAAATTGAAGATTATGCTGAATATCAG
GATAATGACGAAAGCTTAAAAGAAAATGAAGACTATGAAGGTGTTGATGAAATTGAATAT
TTAAGTGAAAATGTTGATATTAATGAAAATTTTGGCTCCGAAACACGTTTTATCGTTGAT
GATGAA
>DjScaffold2942:10737-11531 strand:+
AATTTCATAACTGCGTCTATTAGCAACGCTTTCAGAGAACTTAATGTTGATCATCAACCA
GTTCTACTCAAGAAGACCAAAGAGAGCTCATTCCAACATCAAACAAACGGGAACGACTGT
GGACCTCTTGTATGTGGCTTTATTCTCAAAATTGCAAATGACTCTCCTCCAACAAACATA
GATATTAGAAATATTAGACAATGGGTTTTGGATAATTGCCGAGAAAGTGGCAGTGGACCA
AGCAAATATTCAAACAGCAAAACCAAGGGCTCTCTTATGAAACATATTCGAGAAAACCTC
TTCAATACTTGTGATCTCATATTTTTCAAGACAAAACATCTCATGCTCTTCTGTAATGAA
ATCATCAAAGGTTCCCGCTTTCCCCTTATGCACCCAGATACATCTCGTCGTATCGCTACT
AATGATGAAGCTTTTATCAAGGAACACATTAGCTTCCGTCAATTCATAAACAAAATACTC
GTTCTTTGTCCGATGCAAATTGATGATCTTCAATGGATTCTAATTGTTATTGATTAActc
aagaaagttgcctatattttggaccctagagaaattccggacactattaggaataatagt
gtagaaatacaactcactgactctataaatagtttcttctatgaaaacaggtaattttat
catttactattttattttctcaaaattattattgacggcaaaatgaaatcaatagatgtg
gaaattaaagattttggcgataacaaactttgaagttgaaaacgataattttc
>DjScaffold3453:34734-38106 strand:+
gtaatttcaATGAATTTCAATATTTTTTTACTTTTATTGACAGTTTTTATTGTTAGAGGA
GAAGTAAGACAAAATTTTAATACTACAATCGAAGAAGGATTAAATTCGCAGATTAATATC
GAATTGAAAGCGTTCTACATTTATGAATCAATGGCAGCTTTTTTCCAAAGACCCGACGTT
GGCTTAGAAGGATTTGAAAAGTTTTTCAGAAAATTAGCCGATGAAGAATTAAAACACGCT
CGGGACATGATTGAATACATGAACAAAAGAGGAGGAAAATACATCCCCAAACCAATTTCA
CTTTCTTCTAGTGACGCCAAAATTACCTGGGATGGAGCATTGGATGCTGTGAGGGGTGCC
TTGATAAAAGAAAAGGAAGTGAATGCTGCAATAATTGATTTGCATAAAAAAGCGGGAGAT
TTATCTGATCCAAATGTTCAAAACTTTTTGGATGATTATCTGATGGAGCAAGTCAATTCA
ATTGCATCGTTACAAGTTTTAATAAGTAGATTTGAAAAACTAAAGAATACAGAATTGGCT
CAATTTCTTATTGATCAAGAATTAAAGAAATGAttattagaaaaccatatgaaccgataa
aattatgttgaatgagttgaattaaatgaaaactgaaaacacactgtagtactatatgga
taatatttcttttctttattaatatttaaatgtttcataacaaatatttctatcgcaaat
ttcattataattgttatgtaaatttaatgctcatttgaataactacttttcagagatatt
ggaaacataactcgtgccctggcacaatacttcgtgcac
>DjScaffold3453:91123-91908 strand:+
tatctttactcatactttttagtaatgtctataaagttgatttaATGGAATGTTATGTAT
GCAATACTCAGGAAGGAAATAATGACAAATGCATTAAGACATCAATGCAGTGTTTGGAAA
ATGAAGATTTTTGCTTGACAGAAATAACTTATGAAACTCCACCATATTGGTTATATATTA
TGGAAAGACGTCATTTTATAAAGAAATCTTGTGTTTCTAAAGCACAATGTTTTAAAGTCA
AAGATCAATATGCATCGTCTTGCTATAGAGAATATTATATGGATTGGAAATGTGCGGAAT

GTTGTGAAGGAGAATTATGTAATTATTACGCCACATTATCGGGTAAACAAATGCGCTTGT
CGTGGATAATTTTGATAGCTCTGTCGACGATAGCTTGCGGATTTGTCTGGATTCATTGAa
tggaatgatttgtctttttggtcaaaaataggctgtgttccacgttttttcatatcagt
tgtatatttattccttattaaactgtttttatgacctgtacctatattgataataatat
tggttgtattt
>DjScaffold3453:14365-14415 strand:+
ATGTATAGAAAACTACGCAAGACAATCAAGAATCTTAGAACAGTTGTGTAG
>DjScaffold2844:1378-8770 strand:+
TCCACAAACAAATGTTCCACTGCCCCTACCAGTAATTTAGGTTCACCGAAATATCATAAC
AACAAGCCTTATAGTAAAGATAAAAAATCGAATTCCATGGAAAATAATTTTCATAAAGAA
AAAGATGTACAAATTAATAATTTTTATTTTTTCAATGAGAAATCTAATATTGACAATTCT
CAAAGTATTGAGGTGATTTTTGAAGAAAATAATAGTTTTTCAGAAATGATCAACAGTTTT
GTACAGTATGAAAATAATAAAGATAATAATGACGATCTACAACAATCAGTCTCGTCTGGA
ATTTTTGATTCGCCTAAACATGTTATTGGGAAACATTTTTTAAAACGTTCATTTTCATTC
AATGACATCAGTATAAGACATATTTCTTCATCAAAAAAGAAACACGGAAGTATTCAAACA
TTTATTTCAACTAATGCAACTTTATCTGTTGAAATCGAAAGCAAGCCAGATTATTTTGAA
GAAACCATATCCTCAGATCAAATCAAAAATGAAAACCAAGAAATCGTATTTAAATCTAAT
CAAGACTCCTTAATAAATATTAAAGAAAATTTTGAAAATAAATTAATTTTAAAAGGAGAT
GAAGGAAGTGAAAAAAAAGATATATTAATCAATAAAGAGGATGAAGGAAAACAACAGCCA
GTTGATATTATTCATGAAAGCTATTTTGCCATAATTGGGAAAGTCATTATTCATCCATTA
TTTCTCACTGGAGTTTTCATCACAGGCATAGTGATATTATTGCCATCAATCAACAATAAG
ATCTCAGAATGGTTATCAATTGAAGCACCTGGACCCATTGAAGTAGAGCCAATCCAAACT
CCAATAGAAACACAAACTATATCATTGAAACCTTTAAATCCAGAATTCTTATTTGTTCAA
CCATCGAGAGCTATTAGAAAATTACAATTAAAAATTTTGAATCACTATGATTTCAATAAA
GAAAACAATATCATTTTCCCATTTATCATGTTGAAAATCATGAAAGATCAAATAAGAAAA
GGAAGTGCTTTCTTGACAAAATTTTATAAAAACGACTGAtatatggatgttttatttgaa
attttatttttttgttataatttaccaccgattttagaaaattaaattccataaaaatg
cctcgataataatgcaaaacatgtaactgaataagaaactttcaagattcgacttattta
tttttttactaaaatatattttgactctttttataaaaaaacaTCCACAAACAAATGTTC
CACTGCCCCTACCAGTAATTTAGGTTCACCGAAATATCATAACAACAAGCCTTATAGTAA
AGATAAAAAATCGAATTCCATGGAAAATAATTTTCATAAAGAAAAAGATGTACAAATTAA
TAATTTTTATTTTTTCAATGAGAAATCTAATATTGACAATTCTCAAAGTATTGAGGTGAT
TTTTGAAGAAAATAATAGTTTTTCAGAAATGATCAACAGTTTTGTACAGTATGAAAATAA
TAAAGATAATAATGACGATCTACAACAATCAGTCTCGTCTGGAATTTTTGATTCGCCTAA
ACATGTTATTGGGAAACATTTTTTAAAACGTTCATTTTCATTCAATGACATCAGTATAAG
ACATATTTCTTCATCAAAAAAGAAACACGGAAGTATTCAAACATTTATTTCAACTAATGC
AACTTTATCTGTTGAAATCGAAAGCAAGCCAGATTATTTTGAAGAAACCATATCCTCAGA
AGATATATTAATCAATAAAGAGGATGAAGGAAAACAACAGCCAGTTGATATTATTCATGA
AAGCTATTTTGCCATAATTGGGAAAGTCATTATTCATCCATTATTTCTCACTGGAGTTTT
CATCACAGGCATAGTGATATTATTGCCATCAATCAACAATAAGATCTCAGAATGGTTATC
AATTGAAGCACCTGGACCCATTGAAGTAGAGCCAATCCAAACTCCAATAGAAACACAAAC
TATATCATTGAAACCTTTAAATCCAGAATTCTTATTTGTTCAACCATCGAGAGCTATTAG
AAAATTACAATTAAAAATTTTGAATCACTATGATTTCAATAAAGAAAACAATATCATTTT
CCCATTTATCATGTTGAAAATCATGAAAGATCAAATAAGAAAAGGAAGTGCTTTCTTGAC
AAAATTTTATAAAAACGACTGAtatatggatgttttatttgaaattttatttttttgtt
ataatttaccaccgattttagaaaattaaattccataaaaatgcctcgataataatgcaa
aacatgtaactgaataagaaactttcaagattcgacttatttatttttttactaaaatat
attttgactctttttataaaaaaaca
>DjScaffold4769:82003-82296 strand:+
ATGCGAAAGGAAATCAAATTTTTGGGACACGTTATCAGTAAAGAAGGTGTCAAAACAGAT
CCATCAAAAATAGAAACAATTAAGAACTTTGGTAGACCAAAATGCGTTAAGAAAATGAGT
AGCTTTTTGGAAATTTGCAATTACTATCTCAAGTTTATTAAAGATTATTCGAAAAAAGCT
AGATTGCTGGAACAGATGTGTAGTGGGCCAAGTAACAAGAAAATTGAATGGACGAACGAA
TTAGAAGAAGTTTTTAAGGGAATGAAGGAAGCACTAATGATGTCTCCAATATAA
>DjScaffold365:2638-3190 strand:+
tggaagcagggttgagagtcaacccagaaaaaccaagaaatcaaatttttggggcatatc
atcagtgcagaATGAACAAGAACAGACTCGGGAATACATTAGGAAGATCTAGTTGCGTGA

AGACGATGCGTAGTTTCTTGGGAATATGCAATTATTACAGAAAATTTATTAGCCAATATG
CGGTCAAAGCGAGGGCTCCGGAAACATTGTGCAGTGATTCGGGTAATACAAAATTAGAAT
GGAACAAGGAGTTAGAGGGCACATTTCAAAGACTGAAATCAGCATTGATGTCAACACCAA
TCTTAGCATTTCCCGACTTTGAAAAGGATTTGTTAATAGAGACAGACACTAGTTTTGATA
CAATCCGAGCAGTACTATCACAACGCGATGAGTGAaatagagaaagagttatagcatatg
gatcgcaagatatgactagtcacgagaaaggagtttctggctgtgtattttttgtaacc
atttcaaacattatttgtacggaaaacgatttacgctacggatcgaccagaag
>DjScaffold365:77599-78258 strand:+
tttaacaatcaatttaatgttgaatactttctaaattatATGAATTCCTTACACACTAAT
ATAAAATTCTCAACTGAAATGGAAACTCCTGGTAGCTTCTGTGAATCTCGTTATAAATTT
AATATGATTAAAGGATTAATATATAGATCATGGACTCTCTCTTCTTCTCATTTGGAGTCT
TGTAAAAATGTTCAAAATTTGATGTCTTTATTAAATAAAAATGGATATTCAAAATCAATT
GTAGAAACATTATCTTATAAAACGATTAATAAAATTGTTCTTAACAGTAATATTTATCCA
AATGATGAGAATGTTATTAAGAACCCAAATAAATCTAATAATATCGATAAAATTAGTTAT
GTTTTAAATATTCCTTATTCTGAGGGCTTTAAGGATTTTAAAAATGGTTTGTTAAAATTT
TCAGTAAATCTCCCAAATTTTAAAATTATAAGTGTGTTCAGTATTAAGTCTAAGACTCCT
TATGGATTGGTTTTTTAAtcttatttataaatatgagtgtaatgggtgtaatgccacata
cattggaaagacttcccg
>DjScaffold3117:50338-68955 strand:+
cttagatatagacaattgcaatgagtacaaatacaaatcaaacaccaaggcatatcaagc
tgttatgtggaaacctttagctgttgtggcgtttattctgtctattggtggactgctaac
tgtgttaaccctatatattattaacttgtcttctatatatagtgtaagcataatttgaaa
ttttttaatgaaaaatttaaagataaaaggaaaatttgtaataaaacaatagaaacattt
cataaaaaataatttttgaatgttagtaaaagcactcgttgacgtttgaatagagcctgg
gatatcatcatatgaatcagaacctaatcaatcatttgaactaataaaacgttgtattga
tactaaaataattcctaaaataccaccaaatATGAAAAATAGCACCTATATTTATCTAGG
AGCAACAGCTGGAATGAGAATGTTATCCTTAAATAATGCGAATGCTTCTAAGCAAATCAT
GCAAGTCATTCGAGATTCATTACAAAAATATTCTTTTAAAGTTAATAATTCCTTATCAGA
TATATCTATTATTTCTGGTTCTCAAGAAGGTCTTTATGGATGGACAACCATTAATTTTTT
ATTGCAAAATTTACCTAAAAAAATTGAAAAGAACCCAAATGTAATCGGTGCATTAGATAT
GGGTGGAGCGAGTACTCAAATTACTTTTATTGATAAAATTCCAAGCAATTCATCAACAAC
AATAAATTTAAAACTGTATGGAAATACATATCCAATTTATAGTCATAGTTATCTTTGCTA
TGGTAAATCTCAAGCTGAAATTCGATTTTTAGCTGAATTAATAGAAGCATCTAATTTTTC
TACTTCTATTTCTAATCCATGTATGTCTAAAAGTGAATCAGTTATTTATGGAGCTAATTA
TATTTTTAATGTACCCTGTGTTTGGGGCCAATTTGCAATAGATAATTATGGTCATCAAAT
TATTCCACCTAATGATTGGCTTACA
>DjScaffold3354:53785-55558 strand:+
gaatatacatatttgattattttatatcttaacattaaatttctgaaATGTCTGAAGAAT
TAAAAAATAAAGGTAATTTGGCCTTGCAGTCTGGTCAAATTCAGGAAGCTATTAAATATT
ATTCACAAGCTATTCAAGTTGATCCAAGTAATTATGTGCTTTACAGCAACAGATCAGCAG
CTTACTGTAAAGCAAATCGGTATCTAGAGGCACTGGCTGATGCTGATGACTGCATCAAAC
TTCAAGCTAATTGGTCTAAAGGTTATTTAAGGAAAGCAGCAGCTTTGGAGTTTTTGAATA
ATTATGAGGAAGCAATATCGACTTATGAGAAAATTTTAACATTTGAACCAAATAACGAGT
CTGTAATGAAAAATCTGAAAGATTTGAAAAATAGAATACCTGAAAATGGTGATCAACGTG
GATTATCATTGGCGGCTATTTTTAATGATCCAAATTTCTTACTCAAATTGGAGAATCATC
CAAAAACACGTGAATATTTAAAAGATCCGGCTTTCAAAATGAAATTAGAAATGATGAAAA
GAGATCCAAATCTATTAAATATGATGTTTCAAGACAAGCAAATTCTCGATGTGATTGCTG
TTGGTATGGGTGCTGAACCAGATAATTTTGAAATTAAACCACAAGATGAAGTTCAAGAAT
CTTCACTACCAAATATGAAGGACAATGAATGTAAAATGGATATTGATTTGTCAGATAAAG
AAAAAGCGTCATTAATCGAAAAAGTAAAAGGTAATGATGCATATAAAGCCAAAAATTTTG
CGGTAGCCGTTGATTGTTATAACAAAGCAATTGAATTGGATCCTACTAATTGTACTTACT
ATACTAATATGGCAGCAGTTTTTTTTGAACAACAGGAGTATGATAAATGCGTAGACATGT
GTAATAAATCCGTTGATATAGGAAGAGAAAACAGAGCTGATTATAAACTTATAGCAAAGG
CATATTCCCGAGCAGGAAATGCCTTTTTAAAAAAGAATGATTTAATTTCAGCAAAAAAAT
ATTTTGAAAAATCTCTATCTGAACATCGTGTTCCCGAAATTGTAGCTAAATGTAATGACG
TTGAAAGAAAAATTAAGGAACAAGAAAGATTAGCTTATATTAATCCCGAATTATCTGAGA
AAGAAAAGCAGTTGGGAAATTTGGAATTTCAGAAAGGAAAATATCCTGAAGCTATGAAAC

ATTACACTGAGGCAATTAAAAGAAACCCAGATGATCCAAAAATATATAGTAATAGAGCTG
CGTGTTATACTAAACTAATGGAGTTCAGTATGGCCCTTAAAGATTGTGAAAAATGTATTC
AACTGGATCCAAAATTTGTCAAGGGGTACCTTCGAAAAGCCGCTTGTTTAATGGCTATGA
AAGATTCGTCCAAAGCAGCTGATGCATACAGGAAGGCTTTAGAAGTTGATTCTAATTGTG
AGGAAGCGCGTGAAGGATTAAGACAATCTTATATGACTTCTGAGGACCCTGAACAGATGA
GGAAAAGAGCTATGCAAGATCCAGAAATACAAGAAATACTAAGTGATCCTGCCATGCAAA
TTATTCTTGGTCAAATGCAAAAAGATCCAGCAGCATTTAAAGAACATTTGCAAAATCCTC
AAATTGCTGCAAAATTGCAAAAATTAATGGAAAAAGGATTTATAGCAATTCGATAActtt
gttaattataataaaaatacattaattgttacacttcattttaaaatttaaa
>DjScaffold404:25714-26190 strand:+
caatttaattgtaaaatgtttgattgtacaaaactaattaattacaattgactaagaaag
caacaatctattgaactaaagaaataatttatttgagtacgcaaattaacatatttaaca
agattttattttagcagaaattgaataaaaaaattaaatcacaaATGCCAACTGTTATGA
TATGTATTTTAACTTTAATCAGTGTGATCCTAATTTGTGTCTCAGTCATTATTGTTCGAA
AGTATTTACGAATGAAGCGAAGATCAAAAACTTTGGTTAAAATTATTACAGAAGAAAACG
AAGGAAGTGCAACGTTTAATAACAGTAATATAAATTCAAAAAAAGGAAATTATTCGTTCT
CAGTTTATGATGACAACATTTACGAAATTTCTTGAttacagaaatatttaataattataa
ttaattaat
>DjScaffold404:19013-19252 strand:+
ATGATGAAACAAATGACAGGCCAAATAAATAGAGCCAATGTTTATCTCGATGACATTTTG
ATATTTTCGAACAATAAAGTTCAAAACTATGAAACAATTGGCAGAGTATTGGCGGAAATA
ATAAAATCTAGTTTAAGAATTAATCCAGAAAAGTGTCATTTTCTTCAAAGCAAAATCAAA
AAATTAAATTATATTGTATTTAGTGTAAATTATATTGTAATTAAATATACTACTCCCTAA
>DjScaffold2036:65870-67428 strand:+
tatatgtcatttttgttaattattaatttatattaaataaatttatttatatattttttt
attgtataatttataatttatttcataatttataaaattgtaaaatttagatattgtaat
atttattattgttgtcatcaggtgtatgcaatttgttagaaattaatatactaattattc
aattaattaaattacttaatatttttaaATGAAATTATATATTATAAATGGAGAAAACAA
TGATAAAAAAATAGAAATTAATATTAATGAAGATTCCTTGTTGCACGATGTAAAACATAA
ATTAGACGAGATTCTCCATGGGAACGGAAATTTTATAAAATATTATTTCACATTAATTCA
TAAAAATATTATTTTATCTATGTCGATAAAATATTACAAAATATGTGAAAATGATGAAAT
TTGGTTTGGTAAGATTGAGAATTATTTGAGAAAAGATGATGTCATTATTCATATATTTAT
TTGTCAACGCATATATGGTCGTATTTATTTGATAGTTAAAATGAATGATACAATTTCAAG
AATCAGGTCAATAATAGAAAGAATTTATTCTGAAATAATAAATATTTGGAATGCAGATGG
TAAACAAATGAAAAATGATCAAATTGCCACTGATATTGGCACAAATAAAAATTATAAATT
GATTATTAGATGTGATGATGGAAAAGGAAATGATATTGATAATACATTTGATGAATGCAG
CTATTTGCATGACAAACAGCGGGTAGATCGATATTTGTTTGCTGATCAGATTCGTGATGA
TAATAAAAATGAATCAACAAGAACAATATTGTTACGTCTTATTTATCAAATTAGACAAAT
AATCTCATCAAATAACACAATTGATGACAGATTGAATCAAAATTTGTCAGATTTGCCCTC
CTCATTTCTCAATTCGACAGATTTATTTTGCATCGATATAAAAAATCGTATTTTTGATAT
TTTTAATGATTATTGGACAGATTTGGAGAATCAATGTGATAATGATGTTGTGAGGATATT
TAGACAATATTTCCAATTTGCAATGCGTGCTGCATTGTGGGATTTTTCCAATATTAGACA
ACAAAACAACAATAAAACAAATAAGTTAGTGAGCGAAATCAAAATCGATGCTTTGGATTA
TTTTGTGAAATATTTGATTGATTCCAACAATGATTGGCGATATTGGCTGAGATTATTGAT
GAATCAATATTTATTGAGATCTTTCAGTGATTCTGATTTGAATGAGAGAATTGATAAATG
TGCAATCAATAAATTCACAATAAAAATAAGATTGAACGGAAAGAATAGAAATAATTTCAA
CCAATCCAAACCAGAAACAATTATCGAAGCAAAACAAGAAATATCAGCTGTACACAAAGT
GGACATTAAACATTTGTATTGCATCAAGCCAGGAGGAGACCACAAACTAAATGATGAAGA
CAAATTTGATGATGAATTTATCATTGAATTTCTGTTGAACATGAATGATATCGAGATATT
GAGATCGATAGGACGAGGAGGATTTGCTATAATTAATCTTTGTCGAGATAAAATATCCAA
CAAACAATTTGTTATGAAGATACCAATAAAATACGATGGAGATCTCTATCGAGATATAAA
TATTTATTCTAAACTCTATTCATCACGAAATATCATGAATTATTCTGGATTATTCAGATA
TGGAGAAAACATTTGCATTGGCATGGAATACATGTCCGGAGGATCTCTTAGATCTTATTT
GGACAAAGTGAAACATTTATCGGAACGAGAAATAGTGAAAATATCATATGAAATCTTGAC
ACAAATAAATGAATTGCATAAATTAGGAATTATTCATCGAGATATAAAATCTGATAATAT
TGTTTTCGATGAACACAATCGTGCAAAATTAATTGATTTTGGAATTTCAAAATATACAAT
TGGATTGACAAGTAAAGATCTATTAGATAAATGGAAAGGATGTGATATTGGAACAATTTG

TTACATGGCACCAGAATTGATCAAAGGAGAACCATATTCAGAGAAAATTGATATTTGGTC
ATTTGGATTGGTGATGGTTGAGATGGTGACAGGTAAATACATATTTTATATATTCAATTA
TTATTTCACTAGGTGGTTTATGAGCGGCAAGTCGCCACGTAATAAAAATGAAACTTAAaa
gataaaattaattgaacattttaaattatactgacgattcttgattatatgtcatttttg
ttaattattaatttatattaaataaatttatttatatattttttattgtataatttata
atttatttcataatttataaaattgtaaaatttagatattgtaatatttattattgttgt
catcaggtgtatgcaatttgttagaaattaatatactaattattcaattaattaaattac
ttaatatttttaaATGAAATTATATATTATAAATGGAGAAAACAATGATAAAAAAAATAGA
AATTAATATTAATGAAGATTCCTTGTTGCACGATGTAAAACATAAATTAGACGAGATTCT
CCATGGGAACGGAAATTTTATAAAATATTATTTCACATTAATTCATAAAAATATTATTTT
ATCTATGTCGATAAAATATTACAAAATATGTGAAAATGATGAAATTTGGTTTGGTAAGAT
TGAGAATTATTTGAGAAAAGATGATGTCATTATTCATATATTTATTTGTCAACGCATATA
TGGTCGTATTTATTTGATAGTTAAAATGAATGATACAATTTCAAGAATCAGGTCAATAAT
AGAAAGAATTTATTCTGAAATAATAAATATTTGGAATGCAGATGGTAAACAAATGAAAAA
TGATCAAATTGCCACTGATATTGGCACAAATAAAAATTATAAATTGATTATTAGATGTGA
TGATGGAAAAGGAAATGATATTGATAATACATTTGATGAATGCAGCTATTTGCATGACAA
ACAGCGGGTAGATCGATATTTGTTTGCTGATCAGATTCGTGATGATAATAAAAATGAATC
AACAAGAACAATATTGTTACGTCTTATTTATCAAATTAGACAAATAATCTCATCAAATAA
CACAATTGATGACAGATTGAATCAAAATTTGTCAGATTTGCCCTCCTCATTTCTCAATTC
GACAGATTTATTTTGCATCGATATAAAAAATCGTATTTTTGATATTTTTAATGATTATTG
GACAGATTTGGAGAATCAATGTGATAATGATGTTGTGAGGATATTTAGACAATATTTCCA
ATTTGCAATGCGTGCTGCATTGTGGGATTTTTCCAATATTAGACAACAAAACAACAATAA
AACAAATAAGTTAGTGAGCGAAATCAAAATCGATGCTTTGGATTATTTTGTGAAATATTT
GATTGATTCCAACAATGATTGGCGATATTGGCTGAGATTATTGATGAATCAATATTTATT
GAGATCTTTCAGTGATTCTGATTTGAATGAGAGAATTGATAAATGTGCAATCAATAAATT
CACAATAAAAATAAGATTGAACGGAAAGAATAGAAATAATTTCAACCAATCCAAACCAGA
AACAATTATCGAAGCAAAACAAGAAATATCAGCTGTACACAAAGTGGACATTAAACATTT
GTATTGCATCAAGCCAGGAGGAGACCACAAACTAAATGATGAAGACAAATTTGATGATGA
ATTTATCATTGAATTTCTGTTGAACATGAATGATATCGAGATATTGAGATCGATAGGACG
AGGAGGATTTGCTATAATTAATCTTTGTCGAGATAAAATATCCAACAAACAATTTGTTAT
GAAGATACCAATAAAATACGATGGAGATCTCTATCGAGATATAAATATTTATTCTAAACT
CTATTCATCACGAAATATCATGAATTATTCTGGATTATTCAGATATGGAGAAAACATTTG
CATTGGCATGGAATACATGTCCGGAGTTGATTTTATTTTACTATTGAAATTATTTTGAta
atattcgtatttgacattattcagggatctcttagatcttatttggacaaagtgaaacat
ttatcggaacgagaaatagtgaaaatatcatatgaaatcttgacacaaataaatgaattg
cataaattaggaattattcatcgagatataaaatctgataatattgttttcgatgaacac
aatcgtgcaaaattaattgattttggaatttcaaaatatacaattggattgacaagtaaa
gatctattagataaatggaaaggatgtgatattggaacaatttgttacatggcaccagaa
ttgatcaaaggagaaccatattcagagaaaattgatatttggtcatttggattggtgatg
gttgagatggtgacaggtaaatacatattttatatattcaattattatttcactaggtgg
tttatgagcggcaagtcgccacgtaataaaaatgaaacttaaaagataaaattaattgaa
cattttaaattatactgacgattcttgatatatttgtaactatgattgctctctctcatt
tgatattgtatttatcaattaaataaaaatataatttaattgaaaaaatattattttatt
aattgatttgtgtgattcaggcgtgctgcattgtgggatttttccaatattagacaacaa
aacaacaataaaacaaataagttagtgagcgaaatcaaaatcgatgctttggattatttt
gtgaaatatttgattgattccaacaatgattggcgatattggctgagattattgATGAAT
CAATATTTATTGAGATCTTTCAGTGATTCTGATTTGAATGAGAGAATTGATAAATGTGCA
ATCAATAAATTCACAATAAAAATAAGATTGAACGGAAAGAATAGAAATAATTTCAACCAA
TCCAAACCAGAAACAATTATCGAAGCAAAACAAGAAATATCAGCTGTACACAAAGTGGAC
ATTAAACATTTGTATTGCATCAAGCCAGGAGGAGACCACAAACTAAATGATGAAGACAAA
TTTGATGATGAATTTATCATTGAATTTCTGTTGAACATGAATGATATCGAGATATTGAGA
TCGATAGGACGAGGAGGATTTGCTATAATTAATCTTTGTCGAGATAAAATATCCAACAAA
CAATTTGTTATGAAGATACCAATAAAATACGATGGAGATCTCTATCGAGATATAAATATT
TATTCTAAACTCTATTCATCACGAAATATCATGAATTATTCTGGATTATTCAGATATGGA
GAAAACATTTGCATTGGCATGGAATACATGTCCGGAGGATCTCTTAGATCTTATTTGGAC
AAAGTGAAACATTTATCGGAACGAGAAATAGTGAAAATATCATATGAAATCTTGACACAA
ATAAATGAATTGCATAAATTAGGAATTATTCATCGAGATATAAAATCTGATAATATTGTT

TTCGATGAACACAATCGTGCAAAATTAATTGATTTTGGAATTTCAAAATATACAATTGGA
TTGACAAGTAAAGATCTATTAGATAAATGGAAAGGATGTGATATTGGAACAATTTGTTAC
ATGGCACCAGAATTGATCAAAGGAGAACCATATTCAGAGAAAATTGATATTTGGTCATTT
GGATTGGTGATGGTTGAGATGGTGACAGGTAAATACATATTTTATATATTCAATTATTAT
TTCACTAGGTGGTTTATGAGCGGCAAGTCGCCACGTAATAAAAATGAAACTTAAaagata
aaattaattgaacattttaaattatactgacgattcttgatcgtgctgcattgtgggatt
tttccaatattagacaacaaaacaacaataaaacaaataagttagtgagcgaaatcaagt
aaaataaaatgatttattgataaattattttgatattgtttgatagaatcgatgctttgg
attattttgtgaaatatttgattgattccaacaatgattggcgatattggctgagattat
tgATGAATCAATATTTATTGAGATCTTTCAGTGATTCTGATTTGAATGAGAGAATTGATA
AATGTGCAATCAATAAATTCACAATAAAAATAAGATTGAACGGAAAGAATAGAAATAATT
TCAACCAATCCAAACCAGAAACAATTATCGAAGCAAAACAAGAAATATCAGCTGTACACA
AAGTGGACATTAAACATTTGTATTGCATCAAGCCAGGAGGAGACCACAAACTAAATGATG
AAGACAAATTTGATGATGAATTTATCATTGAATTTCTGTTGAACATGAATGATATCGAGA
TATTGAGATCGATAGGACGAGGAGGATTTGCTATAATTAATCTTTGTCGAGATAAAATAT
CCAACAAACAATTTGTTATGAAGATACCAATAAAATACGATGGAGATCTCTATCGAGATA
TAAATATTTATTCTAAACTCTATTCATCACGAAATATCATGAATTATTCTGGATTATTCA
GATATGGAGAAAACATTTGCATTGGCATGGAATACATGTCCGGAGGATCTCTTAGATCTT
ATTTGGACAAAGTGAAACATTTATCGGAACGAGAAATAGTGAAAATATCATATGAAATCT
TGACACAAATAAATGAATTGCATAAATTAGGAATTATTCATCGAGATATAAAATCTGATA
ATATTGTTTTCGATGAACACAATCGTGCAAAATTAATTGATTTTGGAATTTCAAAATATA
CAATTGGATTGACAAGTAAAGATCTATTAGATAAATGGAAAGGATGTGATATTGGAACAA
TTTGTTACATGGCACCAGAATTGATCAAAGGAGAACCATATTCAGAGAAAATTGATATTT
GGTCATTTGGATTGGTGATGGTTGAGATGGTGACAGGTAAATACATATTTTATATATTCA
ATTATTATTTCACTAGGTGGTTTATGAGCGGCAAGTCGCCACGTAATAAAAATGAAACTT
AAaagataaaattaattgaacattttaaattatactgacgattcttgat
>DjScaffold1299:86782-87090 strand:+
ATGTTATTTGGCATAGCATCAGCACCAGGGACATTTCATGAGATGATGAAAAAATTGATA
GGTGAAATCAATGGAGCCATGGTTAATCTTGATGACATCCTGATATTTGCGAACAATAAA
GACCAATACCATGAAGTAATTGAAAGAGTATTAGCGGAAATAATGAATGGTGTTTTAAGA
ATTAATCCAGAAAATTGTCAGTTTCTTCAAAGCGAAATCAAATTTCTTGAGCACATAATC
AGCAGAGAAGGTGCTAAAACTGATCCGTCTAAAATAAAAGCAATAAGAACATTTGACACG
TCTAACTGA
>DjScaffold5071:84406-98512 strand:+
CTTATTGTTGGAAATAACGACTACAGAAATTCGGCTTACAAAAGATTCGAGTCATATCTA
GTAGTGAAAAGAATTAGAATAATACCAATAAGTTTGAAATCTCCAACCAAGGTTTGCTTG
AGAATCGGTTTATATGGGTGTGATTATAATGATGAAGTTATTGAATATCAAATGCCAAGA
GGCGACTTCATTGTTCGTGGTTCGAAATATATTTCCTTTGATGATGATAATTACGATGGT
AAACAATTTTCAAACCAAACATGGTTGCAAGGCGGAATAGGTAGACTTATGGACAATATT
GCTTTCACAAGTGATAAACCAATAAATACTATCCAAAATTATGTAGTAGCCTGGAAGCAT
ACATCAAATTCACTGGATCATATAAATATTTTCTTTAAATTTTTCAGAGTGAGACAGCTA
CAGTCAGTAACAATTTACTTTAATGAAGATCATACATATAATTTTTACACATTCAAATCA
TTAATTGTTGAATTTCTTGATGAATCTGATAAAGGATCCTTTCATATTTCTAATAAAATC
GAAATTCATGATCCAAATTTTATTAATGAAGAACCATTAACTGTGGATTTGAAGAAATAT
CGGTCGAAATTTGTGAGAATTAAATTATTTTTTAGAAATGAGTGGATAGCTCTCAATGAA
ATCGTGTTTTCAACAGAAGATATTCCACCAAATATTCCAATTGATATTACCTCTAGGAAT
AAAGAACAAATTAGTTATGTTTTGTTACCTATAACTTCCTCAACAATCAAAACTCCAGTT
TATGTTTCGCAAAATGTCATTTATTATATTATTTTAATAATATGTGTTATTGGATCGATT
ATTCTTCTGGGAGTGATATTTTTACTTTGCTTTTTATGGATGAAACAACGTCAAAGAAGA
CGATATAAAAACAAATTTTTCGATCACCGTTCAATGATTTTTAATGATCCTTACTCCAAT
ACATGCGGTCTCAGAACAATATCTGGAAATAATCCTCTTCTTTATCGTCCACTTACTCCT
CATTCTTATGTTCCCCCGATTACAAACAATGCTCAAAATATGGGATTCAGATTTGTACAT
TCACAATTTCCACCCTGCCCTGATTATTTCCCCCCATCTTTGAACAATGAATATGAAGCT
ATAGATTCATATTGGCCGAAAACATTAAGTCAGAGCATTTCATTTACCGATAATAAGTTG
GATCATCCAGATCGACAAACCCTTATAACAGCGCCTGCACCAGATATTACTTGTATCAAT
AAAAATGGAGCTGTATATTGTTCAGCATCATCATATTCTTCTAACATTTATTCACAACCG
GACAATGAAATCAATTTTCCTGCTCCAGATTTACTTCCATTAGCTGCATTGACTAATGAA

TCATTAAATGGAATCTTTAAAATCCATTTTAATAATCCACTGAAAGGACCTTTAGGCTCA
ACAATTTCAAATTTCCAACTAACTCCAGCGTCTCAGTCTTGGATTGAATCGGCTTTATCA
AGGATTCCCGAAATGCATGCTAGCCAAATAAATCTTGAAGCCTTGGTTACTGAAGGAGAA
ATTGCAGCTTTTGTTAGTGGTCATATGGGTCCAAGTAATACTGAAATCATAGTGGCTCGA
TACAAATCAAACAACCATAGAAAAGATTCCAAATCATTATTTTATCGCCAATGTTTTGCC
AAGTTAAAAGAAGATTTTTCAAGTCATCCTAATTTATTATCTATCATTGGACATTGTCTA
CTTGGTTTGGTCGGAGATAATAACACTCCTTCCATTGTATACGCTTATCCATCACAAATA
AATATTGTTAAATTTTTCAACAAACTTCATCTCATTCCCAGAACAAATGATATCAGTTTT
GCTTGCAGCTACGTGAGTCAGAGTAGTTTATTATATGTCGTGAAACAATTGGCTGGAGCT
TTGTCATATCTTTCTGAACTAGGGGTTGTTCATAAAGATGTTTCAGCAAGAAACTGTTCA
ATTGGAGAACATTTTCATTTAATTCTGAGTGATTTTGCCGTAGGAATGGAAGAATTTAGT
GACGATTATATTCCTTTATCAACTTCACAATTCAATGCTGATCCAAATTCACAATCATTA
ACTGTCCAATTACCCTTATTATGGATGCCATGGGAAGCTATTATAATGGTAATTCGATTT
TTATATTATACTTTTAACGTTTTAAGTATTTAAaaataataattaaattaaacttataaa
ttaatgaaaacttatatgtattaatataacaataataataaaaacaatatcaactaatcc
atttattattattttgtatttaataaagttttatttgtattttataaatttgacttgtga
agtttaaaattatcttctataaaagcattaaagcttgtatttttcaaacattaaatcat
gtttattaattatttaaataatctttgcaatcaattaaataaaaatatattgaatttcaa
tatcaaaattaatgatgatataacttaaatgatcttgtttgtttatcttacaccacattt
catatcac
>DjScaffold2858:1149-18235 strand:+
ctaaatcaatattatttgatacaATGGGATTTTTCCAGTGGCATCATTTACCATGGCAAA
TTTTCAATGACCCATTGAAACAATGGGAAAATTTACCAACATTTCTTGGAATAGAATACA
TGAGTTATTTTTTGGTTATTATTTCTTTTATTCATGCCGCAAAAAATGACAAAAATCATT
TGTTGATTTGGACTGCTTCTTTCGTTACCGGTTCCGCTAATGATATATTTTTTATGGCCT
TACCAATGGTTGACAATTTCTGGCAGGCTCAAGCTACAATTATGATTTCTCCGAGATTGC
CATTATATATTTGTTGTATATATAATATATTTTTGTATTATTCCACAATATCATCATGGA
GACTAAGTTTCGAGAACTTATGGCCGGAAGCTGCAGTAACTGGTGTCTTGGCATTGTTTA
TTTATTATCCGTACGATCTTATCGGTGTAAAACATTTATGGTGGACGTGGCATACGACCG
ATCCTGCAATATTGAATCGTGTTTTTGGTGTTCCCATTGGTAGTTCATTATGGATATTAA
CATTCTCGGGATGCTTTTCTTTTATTCTTTACATTTGTCGCGGAAAATTTAAATTGAATT
TCATTAAAACCATCCTTATCAGTGCCATAACTACTACGCCTTTGATGATTTTGATAATGA
CCATTTTACAATTTCTTTCTCTTGAACAACAAGGAGTACCGAGTCTTTCTGGATTTTCTG
TATGCCTATTTATCTACGTGACAATCATAATTCTTTACAGTACATTATTAATTGATACAA
GTCACTCATTCAAGTGGATTGAACATCGATGTTTAACAAAATTCGACATTTTACTTGGCT
TGTCTTTGATTATTTATTATACTTTCTTAATAATTGCAGGAAACCTAGGAAGACCAGAAT
TGTATAAAAGCATCGGAATTCATCAAACATTTGGTTCGTGTAATGCAACTGTCACAGATT
TTATGGGATTGACAAGAAATAAATATGCGTGCTCTAATAATATTCCTAGTGAATGGAATT
TTGCATGTTTGCCAAAGGACAATTCTCCATTGCAATCAAAAGAAGTTTTGGATTGGTACA
CTATTTGTGGCACCCATCATATCGAGGAATTAAAGTGGAAAAACTATCTAACTATATTTT
GCCTAATTGCGGGAGTTATATATGTTTCTATATTAATTTTGTTGACGATTTTTACCAAAA
GAATTTAT
>DjScaffold2057:17720-25609 strand:+
tatattcatattaaaaacatttaattaaaaatttaaataataaacataatATGGTTTCTT
TGGAAAAACTAAACGCAATAGCTGAGTTTATTAACAGTAAAATAACAATTCGACCTAAAA
TTGGTATAATTTGTGGTTCTGGTTTGGGTGGCCTTGCAGAATTAGTCACAAACAAATTAA
TAATCCCTTATTCTGAGATTAACGGTTTTCCCGAAAGCACTGTTGTTGGTCACAAAGGAA
ATTTGGTATTTGGTTATTTAGGAGGTAAAGAAGTGGTACTTATGCAAGGACGATTTCATC
CATATGAAGGTTATACTACTCAAACTTGCATTATCCCGGTGAGAGTGATGCATTTGATTG
GAGTGAAATATTTATTACTTACTAATGCTGCTGGTGGGCTGAATCCTTCTTATAAACTTG
GCGATTTGATGATAATTAAGGATCACATAAACTTTCCAGGATTATGCGGATTCAACCCTC
TTGTTGGTCCTAATGACGAAAGTTTAGGAGAAAGATTTATTCCATTGTCAGATGCATATG
ATAAGAATCTAAGGTTAAAAGCAAAGAAAATCATTAACAAACTAGGAGAAAGCAACAGAG
TACATGAAGGAGTTTATGCAATGCAAACTGGTCCAGCATTTGAAACGGTTTCAGAATGTC
GAATGATGCAGATTTGTGGAGGAGATGTTTGTGGAATGAGTACAGTCCATGAAGTGATCA
TAGCCCGGCATATGAACATGCATGTATTTGCAATGTCAATCGTTACAGATGAATGTATTT
TGGATTATGATTCAGAAAAAACCACTCTTCACGAAGAAATCCTAAAAGTTTCTGAGAACA

GTACGAAAATTGTACAGAAAATTATTACTGAATTGATATCGGAAATTACCGTTTAAcatt
taactataattttcaataatagttgaacaatctcatagtttagaatgaaatatgtgaaag
tttatgatttcttaaaatttatttgaaaatatgttatttgaataattttatttttagttt
tatataataaaatattttctaattaatcaaatatattcatattaaaaacatttaattaaa
aatttaaataataaacataatatggtttctttggaaaaactaaacgcaatagctgagttt
attaacagtaaaataacaattcgacctaaaattggtataatttgtggttctggtttgggt
ggccttgcagaattagtcacaaacaaattaataatcccttattctgagattaacggtttt
cccgaaagcactggtttttaaaactttttgaatttttaatattttaaatattttagttgt
tggtcacaaaggaaatttggtatttggttatttaggaggtaaagaagtggtacttATGCA
AGGACGATTTCATCCATATGAAGGTTATACTACTCAAACTTGCATTATCCCGGTGAGAGT
GATGCATTTGATTGGAGTGAAATATTTATTACTTACTAATGCTGCTGGTGGGCTGAATCC
TTCTTATAAACTTGGCGATTTGATGATAATTAAGGATCACATAAACTTTCCAGGATTATG
CGGATTCAACCCTCTTGTTGGTCCTAATGACGAAAGTTTAGGAGAAAGATTTATTCCATT
GTCAGATGCATATGATAAGAATCTAAGGTTAAAAGCAAAGAAAATCATTAACAAACTAGG
AGAAAGCAACAGAGTACATGAAGGAGTTTATGCAATGCAAACTGGTCCAGCATTTGAAAC
GGTTTCAGAATGTCGAATGATGCAGATTTGTGGAGGAGATGTTTGTGGAATGAGTACAGT
CCATGAAGTGATCATAGCCCGGCATATGAACATGCATGTATTTGCAATGTCAATCGTTAC
AGATGAATGTATTTTGGATTATGATTCAGAAAAAACCACTCTTCACGAAGAAATCCTAAA
AGTTTCTGAGAACAGTACGAAAATTGTACAGAAAATTATTACTGAATTGATATCGGAAAT
TACCGTTTAAcatttaactataattttcaataatagttgaacaatctcatagtttagaat
gaaatatgtgaaagtttatgatttcttaaaatttatttgaaaatatgttatttgaataat
tttatttttagttttatataataaaatattttctaattaatcaaatatattcatattaaa
aacatttaattaaaaatttaaataataaacataatatggtttctttggaaaaactaaacg
caatagctgagtttattaacagtaaaataacaattcgacctaaaattggtataatttgtg
gttctggtttgggtggccttgcagaattagtcacaaacaaattaataatcccttattctg
agattaacggttttcccgaaagcactggtttttaaaactttttgaatttttaatatttta
aatattttagttgttggtcacaaaggaaatttggtatttggttatttaggaggtaaagaa
gtggtacttATGCAAGGACGATTTCATCCATATGAAGGTTATACTACTCAAACTTGCATT
ATCCCGGTGAGAGTGATGCATTTGATTGGAGTGAAATATTTATTACTTACTAATGCTGCT
GGTGGGCTGAATCCTTCTTATAAACTTGGCGATTTGATGATAATTAAGGATCACATAAAC
TTTCCAGGATTATGCGGATTCAACCCTCTTGTTGGTCCTAATGACGAAAGTTTAGGAGAA
AGATTTATTCCATTGTCAGATGCATATGATAAGAATCTAAGGTTAAAAGCAAAGAAAATC
ATTAACAAACTAGGAGAAAGCAACAGAGTACATGAAGGAGTTTATGCAATGCAAACTGGT
CCAGCATTTGAAACGGTTTCAGAATGTCGAATGATGCAGATTTGTGGAGGAGATGTTTGT
GGTAGATTTTTATTATTTTTATAAtttatttagctgacagattcaaataaatatggcatt
ttattatatatatacagtctgttacattaaattgtccagtaaaaaaattcaatactttttt
caattaaataaagttgtttataaaatatcagattatttatagttgtcagttcttattaaaa
tttttgtatttcattttgtaaaaccacgtaagtgttttatgctttataattcagctcgaa
tattagtttagtttaatttccagtatattcgtcaaattatgggcggtatcgatttaacac
cttttgacaatttatgcaatgcaaactggtccagcatttgaaacggtttcagaatgtcga
atgatgcagatttgtggaggagatgtttgtggtagatttttattatttttataatttatt
tagctgacagattcaaataaatatggcattttattatatatatacagtctgttacattaa
attgtccagtaaaaaaattcaatactttttcaattaaataaagttgtttataaaatatcag
attatttatagtttaaaaaaattcaatactttttcaattaaaataaaatggtttatatat
atcagattatttatagttgtcaattcttattttaatttttgtatttcattttgttaaacc
atgtaagtgttttatgctttataattcagctcgaatattagtttagtttaatttccagta
tattcgtcaaattATGCGCGGTATCGATTTAACACCTTTTGACAAAGGACAGATTTATGC
ATTACGCAAACATGCCGAATGGCCATATGAACAGATAGCCAGTACTCTTAATTTGACACT
GTCTGCTGTCAGTAAATATTGCCAGCGAAACATCAATAATAATCTGCAACGTGAAGGAAA
CCGTATCAATTGTAAGAGACCGCGAAAAACAACAGATAGGTTTGACAGACATATTGTGAG
ATATGTCGAACAACATCCATTCAAAGGAACTCGGCAAGTAGCATCAGACTTATCTCCAAT
CCAAAATGTATCATATGGCACGATCAATCGCCGTTTGAATGAAACTGGTCTTCTTTCTTT
TTCTCCAGCACACAAGCCCTTCCTTTCAAAGGCTCACAAAGCAGCGCGTTTGTTCTGGGC
ACAACAGCATCGCCAAACAAACTGGTCAGCAGTCATCTTCAGTGATGAATCAAAGTTCCA
GATGAATAATAGACAACAGTTCGTACGGCGTAGGAAAAACACTAGGCTGCAGGATCGGTT
TGTTACTCATCTTCCAAACAGAAGTTCATGTCACTGCATGGTGTGGGGATCGTTTTGTAG
AGATGGTTACAGTGATTTGGTTAGAATCCAACATCGGCTTAATTCTGAAGGCTATGTTGA

ACTATTAGAACAGAAC
>DjScaffold2057:117532-120433 strand:+
gttttaattgttattattttataatttaaaattgaaagtaatatctaattagcttttAT
GAATACTGAAATTAATAGTTCTCAGATGGAGGTAGGATCAAATGAAATAGATATATTATC
AATGTTTATTGGAGAGAAATGGCGTAATACCAGAGGACAATTAGAGCTAGTAAATCCGTA
TATCCAAAAGGGCGAAACTCCAAAAGATATTCTTAAAAATCATTTCAAAGTTAATATCAA
CCATTTATCTGATGATGTTATAGAACATTGGTCTATTATTTTGCATCTTATTATGGAAGG
ATCTCAAAGAAAGCGAATTGAAAATATTAACACTTTTAAACAAGCTTGTGATCTTATTCG
AGACAGCAAAAATATTATTGTTTTAACTGGCGCCGGTATTTCAGTCCCTTGTGGGGTGCC
AGACTTTCGAAGTCGAAATGGAATTTATGCAAGATTAGCCGTTGATTTTCCAGATTTAGA
TGAACCTGAATCTATGTTTGATATAAATTATTTCTCAAAAAACCCAAAACCATTTTTTAA
ATTTGCAAAAGAAATTTATCCTGGAAAATTTACTCCATCACTTGGACACAAATTTATTAA
ACTACTTGAAGATAAAAATAAATTGTTAAGAAATTATACTCAAAATATTGATACACTTGA
ACAAGTTGCAGGGATTAAAAACATCATTCACTGCCATGGGTCATTTGCATTTGCTACTTG
TATGAGATGCGAGTTTAAAGTTAATGGGGATGCTATTGAAAATGATGTACTTAACCAGCG
TATTCCAATTTGTCCATCTTGTACTAGCGAAGAAGAAAAGGACCAGGAAGCGTTTCTTCG
TTCACTAGAGAATCGTGCTGAAATCGATACTGATATACCTATAATGAAACCAGATGTTGT
CTTTTTTGGCGAAGGTCTTCCGAAAGATTTTCATTCAAAATTATCTGAAGATAAAGATAA
AGCTGATTTGTTGATAGTGATTGGGTCGTCATTAAAAGTCAGACCAGTGGCACATATACC
GGGATGTTTACCACCGGATATTCCACAAATTCTTATCAATAAAGAAACATTGACTAGTCA
T
>DjScaffold2057:140353-144210 strand:+
gttttaattgatattattttaaaatttgaaattgaaagtaatctcccattagctttaAT
GAATACTGAAAGTAATAATTCTGAGATGGAGGTGGGATCAAATGAATTAGATATACTGTC
AATGCTTATTGGAGACGATGAAGAGAAATGGCGTAATACCAGAGGACAATTAGAGCTAGT
AAATCAGTGCATCCAAAAGGGTGAAACTCCAAAAGATATTCTTAAAAATCATTTCAAAGT
TAATATCAACCATTTATCTGATGATGCTCTAGAACATTGGTCTATTATTTTACATCTTAT
TATGGAAGGATCTCAAAGAAAGCGAATTGAAAATATTAACACTTTTAAACAAGCTTGTGA
TCTTATTCGAGACAGCAAAAATATTATTGTTTTAACTGGCGCCGGTATTTCAGTCCCTTG
TGGGGTGCCAGACTTTCGAAGTCGAAATGGAATTTATGCAAGATTAGCCGTTGATTTTCC
AGATTTAGATGAACCTCAATCTATGTTTGATATTCATTATTTTTCAAAAAACCCAAAGCC
ATTTTTTAAATTTGCAAAAGAAATTTATCCTGGAAAATTTACTCCATCACTTGGACACAA
ATTTATTAAACTACTTGAAGATAAAAATAAATTGTTAAGAAATTATACTCAAAATATTGA
TACACTTGAACAAGTTGCAGGGATTAAAAACATCATTCACTGCCATGGGTCATTTGCATT
TGCTACTTGTATGAGATGCGAGTTTAAAGTTAATGGGGATGCTATTGAAAATGATGTACT
TAACCAGCGTATTCCAATTTGTCCATCTTGTACTAGCGAAGAAGAAAAGGACCAGGAAGC
GTTTCTTCGTTCACTAGAGAATCGTGCTGAAATCGATACTGATATACCTATAATGAAACC
AGATGTTGTCTTTTTTGGCGAAGGTCTTCCGAAAGATTTTCATTCAAAATTATCTGAAGA
TAAAGATAAAGCTGATTTGTTGATAGTGATTGGGTCGTCATTAAAAGTCAGACCAGTGGC
ACATATACCGGGATGTTTACCACCGGATATTCCACAAATTCTTATCAATAAAGAAACATT
GACTAGTCAT
>DjScaffold3818:40777-60257 strand:+
ttaattaattaattataattaatttgttaacattgtttatcgactatgataaatattcgt
attttggcaaaaacgttaacttgaattgaagagtttaattttaaaaatctctttgaaatc
cacagatcttaattgataagagacggaaattgatgctcattttaatttaaaatgtatttt
ttatcattattttttctattattttgatatattacattttgaataattttagatttttt
ataagagataaaaatgtttgatttgtgccacagattaatttcacatttttatcaaattta
atttttcattatttttaaaaaaaaaattattttaatttttattatatttatgatttgattt
tgattaaatttttagagttcgttaactcacaagaatatactatcactaatcgactcgcaa
attaatgaacaacaaagtgatatttttgaagttctaATGCTACTTGCTTATTGTTCCAAA
AATGTTGCCCAATTAATGCAAAGTAAACAGAAAATGAATCAGCACTTGTCTGAAAATGTC
ATTATTAAGATTTTATGTGATGTTATTGAAGCTGTTGCTCGACTTCACTACTGCCAAACT
CCAGTTATCCATCGAGATCTAAAAGTAGAAAATATATTGATCGATGAAAGAAATAATTAT
GTCCTCTGTGATTTTGGAAGTGCCACTGCAAGACAAATCATTCCTACTGAATACGGAATC
GGGAAAACTGAAGAAGAAATTTCCAAATACACAACATTGGCATATCGGTCACCCGAAATG
GTTGAATTAGGAAGTGAAATAATCAATTGTAAATCTGACATTTGGGCTCTTGGATGTTTA
CTTTATTATTTATGCTTTTTTACATTGCCATTTGCCGATAAAGTTCTAGCTATACAATCT

GGAAAATTTATCATTCCTAAAACTGATGTCACCTACTCAGACAAAATTCTCAAATTAATA
CAATATATGTTAACAGTTAATCCAACAGATCGTCCT
>DjScaffold3818:64793-65727 strand:+
AAACCTTTAAAAGCAAATATTGATGCACGAATTCCAAGTGAAAATGGTGAGTTGGAGCAT
CATTTGAATGGCCTTCAAACTCACTATGTAAAACTCGATGTTGTCATTAAACAATTATCA
ACCGAAAATTTCATTCAGGATACCGGTGCTTTAAATAAACTTTTCAAAGAGCTTTTCACC
CCGTTTTTAAAAAATTATATTGATAATGAACTGAAATGGTTAAGAGAAAGATTGAATTTG
CATCTTTATCGTTATTATGATACTGTTAATCATCAAAAGAAAGTGTTGAATGAAGGGGGA
ATAGTTGATTTCTACCGTGATATTCAAGCGATATTTACTTCAGGAACAACGATTCCTCCT
GATGATAAAGAGGGCTATCAAAAATTATTATTGTCACAAGAAGTTGCAGTTAATATTCTC
GAAGATGTTAAATCTTCATGTAAACGTTGTAAAATAGTGAGATTTTTATTTTTTAAAATT
TTTATCTTTTATTTATTTTAAattgctaattaactcgtgttttggaaagttttataataa
tatgaatgtataatgctaagtattagatgagtttgaagcactcattaatgtcaaaactcg
tttccataaaagatttattcacagaattaaaactctaaatacaaatttgttttttcaaa
caaattaaaaaatttatattcgaaatggaaaatagcaaatatcagcattttttaaaatat
tttattttaaattttactaatttaaaattaaaaatacaatataaaacatattttattgac
aataatttacactttattttat
>DjScaffold3818:138750-146475 strand:+
accaaagtttgataattctaaaatATGTTAATTAATTCTTCATTATTCCTTTTTAAAAAA
AAATTGTCAGAATTATCTACTCCAACTGTCCAATCAGAAAATATTTGCAAAATTATGGAT
TGTTTACTAATTTATCTAATAGAGAATCATTTGAATTATGGAATCGAATTTTGTTTACAA
GGATTTATCAATATTGACACAAAACGTGAACCAAGTCTTTTGTTTTTCCAAGTGGTTCAT
GAAGCTAATAACATATTCCAATTTTTCGATAAGGTAGTTTCAGACAACATTTTACCATTA
ATATCAAATCCAGAAAGACATGCTGAACGTGTCACAAAACGTCAGCAAATTCTCAAAAAT
CTAGAAAATAAAATTAATCAAGGCCTAGAAAAACTAATGCATATTTGTTTGAATTATATT
CAACATATTTTGTCGAGTGAACAAAAGAAAACTGATTACAGACCCGAAAATGATATTCCT
ATTAATGTAACAACATCAATGACAGCCGTCAGAGTTACTTCATTTCTCAAAAAGATACCA
CCTCAAATAGATATTTATCTTGACGGTCAAAATAAGGAAATGTTTTGTCAAGAATTTGGT
CTTCAATTTCATAGATTATTAATGGATCATTTTTATTCATTTTCTTATTCCTGCGAAGGT
GGTTACGTTGCTATGCAAGATGTTAATTTATATGACGAGGTAATGCGAAAATTCAATTGT
CTATTGCTCAATCATTTATTTAATACTCTGCTTACACTCATGAATATTGTGTCAATGAAA
CAAGAAAACATTAAATCTATAATATCAAATCATTTAGCAGGTGGACTTCCGAAAGAATTA
TTGATGAATTTTGTTAGTCTGCGATCTGATTCAAAGATTACTAGGTCACTAGTGGACCTG
AAATAAaaaatctaataatatcaatttttatattccatgtgatgtgataaaaaatatcat
ttttgattttttcta
>DjScaffold2945:10306-17509 strand:+
GTGCCTGGACTAGGAGGATCTCAAATAATATCAAAATATATAGAAACCCCAAAATTAGTA
AGATGCAATGCTTTGAACAAATCCTTTTTATCATGGATTAATCCAATTTATTTTACCCCA
TTTACAGTGAATTGTATAATAAGTCTTCTTAAATTAGAATATAATACTTCTTCGCATACT
ACTTATCCGCCAAAATACTGTGAAACCTCGATACCTGGATTTGGAAATGTTGACACAATA
TCTTACTTATCAAATATCAAAATTCCTTTAATGTCGTATTATAGCGAAATTATTAAAGAT
CTTGTCAAAACACACAAGTATACTGCTAATATTGATATTCGAGGAGCACCTTATGATTTT
AGAAAAGGACCGAATGAACTAGGAATTTATCTAGATAATGTCAAATCTCTTATCGAGGAA
ATGTATTACAAAGGAAATGGGAAATCTGTCGTGATAATTTGTCATAGCTTAGGATGTTTG
TTGACTATACGAATACTAAATAAAATGCCACAAGAATGGAAAGATAAATATATACGTTCA
TTTGTAAGCATAAGCGCACCTTATGCTGGTTCAATGGTAGCTGTAGAAAGCGTATTGTCA
GGATTTAAATCTGCTACATTTTTTATCAATCCCTTAAGACTAAGAATTTTAGAAAGTTCT
TGGCCCAGTTTATTTTATCTCATGCCAATCAATGGAATCTGGCCAAATGAATCAATTGTT
TTAACTGCAGGCAAGAATTTTTCAGCGAATAATATATCTGAAATGTTAAAATTATCTCAA
GTGAAATTCGCATTTGACCATTACAATGACATTCTTGATGAAATGGACAAAAGACAGGCT
CCAAACGTAGAAGTTCACTGTGTGATCAGTTTCAATTTACCTACAACTGCGGTTTTGGAT
TATCGCAAAGTAAATTTTCCAGATTCGAAACCGCAAAAAATAAAGCAAAGTGGAGATGGA
ACAGTACCTTTTCACTCTTTAAATAAATGCTCTCAATGGAAAACCAAACAAGCCAAACCT
GTTGAATCATACATGATTTCAAATGTTAAACACTTGAAAATGATCAAGCATAAA
>DjScaffold2945:53731-56789 strand:+
ctcatatgtatttataaatactaacgattatttaaaaaaatattattATGAAGATTTTGC
TAATTTTATTATTGTCAATTATTATTCAGGATGTGTTGTGTAAACATCCCGTTTTATTAG

TTCCTGGTGATGGTGGTTCACAATTGATGGGAACGTTTTTAAAAACTCCTCCTCATTATC
TTTGCGGAAGACAAAACAAGTTGTTCATGCTATGGCTAAACCCTTTTTTTATGTTACCAT
TATCTTTGGATTGCTTTGTAGAAACTATCAAATTAGAGTACAATTCTTCTTCTCATCATA
CGTATTCTCCAGAGTTTTGTGAGGTTGACGTTCCTGGATTTGGATCTTTAGACACGATTA
CATACTTGAGTAACGTAAAAATTAGAAAAACTAGTTACTTCTATTATATAATCAATGATC
TTATCGAGAATCAAGGTTATGTTCCTGAAGTGAATATTCGCGGTGCTCCATACGACTTCA
GAAAATCTCCAAGTAATTATTTATTAATATTATTTTATATTATTGTTATGATCACATTGA
TAATAAGAATAATTTACAAAATCAGTAGAATTAAAATTCATATTTTCTAAaatataatta
ataaatttactacttaatcctactatttatcttttgaatcttctgaatcttaaattctta
attttcttgcaataataaattacttacaatgctccaataaaatctgcaatttacaattac
aattcaataaatttattattaccaaataaaaaagttaaattcactattattataaaaata
ttacaaactaaatagtactaacttgattcttcaaaaaattttgactactaattctaacat
tcaactacactactaagtttgtcaacactcattaaaaaaaatacacaaatt
>DjScaffold2945:74440-77294 strand:+
atcatcatcatgatcatcatgatcatcatgatcattatcatcatcatcatcactatttta
aacaattaataatcaaatattcatttcaggaaaaATGGATGATTTAAAAGGAAGCATACG
AAACCTAATAGAAAACATGTATATTGAAAACAGAAATTCCCCAGTCATTTTGATCGGACA
TAGTCTAGGCAATCTTTATTTGAATTTGATATTAAATTCAATGGAACAACAATGGAGAGA
TAAATATGTCAAAGCTTTTATCAGTATTAGTGGACCGTATTTAGGAGCTATAGATGCTTT
GAGAACAGCTTTATCAGGTAATAATGAACATATTCCAATTGTAAATCCAGTACGAGTAAG
AAAAGCTCAAAGTACTTGGCCAAGTACTTACTTTCTAATGCCAATTCCGGGATTGTGGTT
AAATCAAACTATAGTCTCGACTAACAAAAGGTATTATACTACAAACACAATTATAGACCT
TTTAAAAGATTCTGGAATTCCCAATGCTGTTGACCATTATAAAGTAATT
>DjScaffold2945:142093-154569 strand:+
cggctcttattagaatataccttttgtactgtgtacatagtgtacataagggaaaaaaa
ttgtatataatcgttcattgtcataaataaagacaaATGTCAGATTTTCATGAAAATACA
ACAGAAAGAAATCCATCTGGAATAAATCCAAATGTAGCTACTTGTCAAATTGCTAATTCA
TCGATATATAATCCCCTTGGTCAAGTATCAACTGTTATTTCGAATGTTCCTAATGGGCCT
TACTATTCTTCGAGTTTTGTTTATTCAGACGATTGGTCAATGCCGGGGTTATCTTTATCA
AATACTACTGAACAATTAACAAGACAAATTTCTCAACTGAGTTATGATGACCATAGTGCT
TACTGGCCACCTCATCTTAGTATGCCTTTGTCAGGTGATAATAGAACATGGGATACTTCT
GAGAATAGCATTTCACAACCGATTCCTATGCATCCAGGAAGACGAGATACTTTAGGAAAC
GAAAGTGGCTCTTTTATGTCACCTATATCAAAGGAGATGGTTGCTTTGGGGATGCAAGTT
GTAAATAAGGTTTTAAGCTCTTCTCCAGGAACTGGAGTGATGTGTAGATCGGATATTATG
GAGAACTTCTATAATAATCCATCAAGCAATTTCGGTTATTCCAATGGAAATGCCCAACCA
TTTCCTCAGTCGCCACCTCACTATTATTTGCCTTCTAGCCAGCTTTATCAACCGTCGACT
TTATGTGGTACACCAGAAAATTCTCAAGTTTTCGGTTGTAACAACCAGCAAAATCAATCA
ATTTCACAACAGCCTCAACAACAGAGCAGTGCCAACCAGCAGTTCTTTGACCCAGCAATT
ATCGCAGCTTCATTCTCGAATGCTGGCCAAGGCTATCCACCTCAACAACAAGCTCCTCAA
GGTTTACCACCTCCACCTGGATTAGCCCCATCTCATAGTGATTATATGACTGGTGAACCC
ACAAGTCCGTTACATAACAAACAACCTGATGAGGAACAAAATCAATATATCTGGTCTCCC
GGTACTAATAACGGTATGGTACATGATTTTATGGGCGGCAATCCGGGCCCTACTTATGGA
TATCATCAACAAGCGCCTCTTTATGATCGTCCGACATCAGCTTTACCAACACAACAACTA
TATGCTAGTCAAAACGGTACACTTCATCATGGAACACAAACATCTCAAATTCAAAATCAG
TCTGCTGCAGCTGCATTATTCGCACGCTCTCTATATCTGGCAGCTTTAGGTAGCGGGCAT
GTGCCTCCGGCAACACAACAAATGGCACCTCAACAGCAACCACCAAGTTTAGTTCCAGAC
GCATTGCATACCTTAACCAGTATGCTGGCACATGGTTCCAATTCTTATGGTCAGCAACCG
CAACAACAACCTAATTTTCCTAGTAGTCAAAATAATCTAATTGGTCAAAATCAGGCTGCT
TTATCGGCTTTATCAGCAGCGATGAGTGGTTACTTTCTATCACCACAAATCGGTACTCAT
GCTCCTCCACCAACACAACAGTTTTCCGTAATGCCTGGAAGTAATCCTACGATGCTTGGT
GCACCGCAACCGAGTCATACTTTAATGCCAGCAACCGGTTCACAGCTCATCTCACACCAA
GGTAATAAACCTATGCAATCAGGATACAAAGGAGTTGTAGGAAATCTTAAGGACGGAGTT
ACCAGGAGTCGATTATTAGAAGATTTTCGAGCTAATAGATTGACAACGCTCACATTACAA
GATATTTCCGGCCATGTTGTAGAATTTGCTCAAGATCAACATGGCTCTCGATTCATTCAA
CAGAAATTGCAAGAATCTAGCCATAATGAGAAAACAATGGTGTTTCGAGAAATACTTCCA
CAGTGCTATAGTTTAATGACGGATGTATTTGGAAATTATGTTATTCAAAGATTTTTTGAT
TTGGGAACACCTGAGCAAATTCAGATTCTCGGTGATAGAATTCGTAATCAAGTCCTTCAA

TTATCACTTCAGATGTATGGCTGCAGAGTTATACAGAAAGCTTTAGAAACTGTTAGTAAG
GTTACTCAAATTAATATTGTGCGTGAATTGGAGGGATCAGTAATTAAATGTGTAAAAGAT
CAAAATGGCAATCATGTTGTACAGAAATGTGTAGAATGTGTTCCTCCAGAACATCTCGAT
TTTATTATCGATGCCTTTAAAGACAACGTTTACTCTCTTTCAACTCATTCGTATGGTTGT
AGAGTTATACAACGCATTCTGGAACATTGTACACCAGAACAAACCGCTCCAATATTAGCC
GAATTACATCATTTCACTGAGGAATTGGTTAAGGATCAATATGGTAATTATGTTATTCAA
CATGTTCTTGAACACGGTAAAACCGAAGACAAAAGCAAAATTGTAAATCTCCTTAGAGGA
CGAATAGTTGAACTCAGCATTCACAAATTTGCCAGTAATGTAGTTGAAAAAGCTGTAGCA
CATGCAACTCGCCAAGAACGCCAAGCATTAATAAACGAAGTACTTCAAGATTCCATTCCA
GTTTCAGCAAGTAATGCAATAATGCGTACTGCTGATGTCAGCGGTGTAGTGTACGGATCA
GAAACCGACGGTAGTGATACCGATGGTGGAGGATCTGTACAAAGAGAATCTGTTCTTTAT
TGGATGATGAAAGATCAATTTGCAAATTACGTTATACAAAAAATGCTCGATGTCGCTGAA
CAGCCTATGAGAAAAGAGTTGATGACAAAAATTAAGCCTCATCTAGGTAGCTTAAGAAAA
TACACTTACGGAAAACATATAATAAACAAAATGGAAAAATATTATATGAAGAACAATCAA
GATCTTGGTATTCTGAGTAGTAATTCTGGTATGGCTTGTGTTGGTCATATGTGAaaacaa
aattggttgatgaagatggtgatgttgatgatgatgatgatgatttcattcgtatttgtg
cgtatttcgattttcatatttatcgtgtgtaatattacagaaatcgtttttagcatgtg
gttattaattatacataaatgtgtaaaatatatataaaatgtatattataattatttttt
aattggttttgcttctctcgacttgctgcaattatactttgttactttgtttaggatatt
tatcacttttattttatccacattttcaaccaacaacaataacaacaacaatccaaataa
tggcttcttcaatttggcgttattattattattattctttattattattattctttatta
ttattattctttttttatttttattgttgttattattattattattattattaatcatgag
taaatctttgattgattcaggcaactatacattatttgatcacaaaatgacggggagcta
tatttgtttgttatatgtgcatatatatattttatgtgtgaaaccacacgattgtgtttg
tacttattgtgaaaatgtattgattgacatatagtaacataatatagcaatacaacgtat
ttccattaataaccccactgtaatatatgtacttagttaatatatttatagttattgatt
tatgctttgattaatgcacattacacactatttttgattaaatatacatatttattaatg
ttaattaattttatgattggaatttatatatgtgtacattattataataattattttatt
ttgagtgtgtgtatacgagtctgtgtgtgtgtgcttgtatgttaggtaattgttattttt
aatttaacggttggggtttgtgtgtatcattttgattgtgttgtattatatatatatatc
tatacatatatactgtcattaattattaacttcggtaaatgtggtgatttttacttgctt
tattactactacttattgattaataagctgcttcgttgaatataattagtatgactaaca
ctgttttggaaaatttaaactacagttacaattttaaataagttaatttgttcatgtggt
tcaatgtaaagagattttgttagaaattgattttattaagaggagtaaaatactttaaaa
atatgattgtgaaattatataaataaattagttcacacccggctcttattagaatatacc
tttttgtactgtgtacatagtgtacataagggaaaaaaattgtatataatcgttcattgt
cataaataaagacaaATGTCAGATTTTCATGAAAATACAACAGAAAGAAATCCATCTGGA
ATAAATCCAAATGTAGCTACTTGTCAAATTGCTAATTCATCGATATATAATCCCCTTGGT
CAAGTATCAACTGTTATTTCGAATGTTCCTAATGGGCCTTACTATTCTTCGAGTTTTGTT
TATTCAGACGATTGGTCAATGCCGGGGTTATCTTTATCAAATACTACTGAACAATTAACA
AGACAAATTTCTCAACTGAGTTATGATGACCATAGTGCTTACTGGCCACCTCATCTTAGT
ATGCCTTTGTCAGGTGATAATAGAACATGGGATACTTCTGAGAATAGCATTTCACAACCG
ATTCCTATGCATCCAGGAAGACGAGATACTTTAGGAAACGAAAGTGGCTCTTTTATGTCA
CCTATATCAAAGGAGATGGTTGCTTTGGGGATGCAAGTTGTAAATAAGGTTTTAAGCTCT
TCTCCAGGAACTGGAGTGATGTGTAGATCGGATATTATGGAGAACTTCTATAATAATCCA
TCAAGCAATTTCGGTTATTCCAATGGAAATGCCCAACCATTTCCTCAGTCGCCACCTCAC
TATTATTTGCCTTCTAGCCAGCTTTATCAACCGTCGACTTTATGTGGTACACCAGAAAAT
TCTCAAGTTTTCGGTTGTAACAACCAGCAAAATCAATCAATTTCACAACAGCCTCAACAA
CAGAGCAGTGCCAACCAGCAGTTCTTTGACCCAGCAATTATCGCAGCTTCATTCTCGAAT
GCTGGCCAAGGCTATCCACCTCAACAACAAGCTCCTCAAGGTTTACCACCTCCACCTGGA
TTAGCCCCATCTCATAGTGATTATATGACTGGTGAACCCACAAGTCCGTTACATAACAAA
CAACCTGATGAGGAACAAAATCAATATATCTGGTCTCCCGGTACTAATAACGGTATGGTA
CATGATTTTATGGGCGGCAATCCGGGCCCTACTTATGGATATCATCAACAAGCGCCTCTT
TATGATCGTCCGACATCAGCTTTACCAACACAACAACTATATGCTAGTCAAAACGGTACA
CTTCATCATGGAACACAAACATCTCAAATTCAAAATCAGTCTGCTGCAGCTGCATTATTC
GCACGCTCTCTATATCTGGCAGCTTTAGGTAGCGGGCATGTGCCTCCGGCAACACAACAA
ATGGCACCTCAACAGCAACCACCAAGTTTAGTTCCAGACGCATTGCATACCTTAACCAGT

ATGCTGGCACATGGTTCCAATTCTTATGGTCAGCAACCGCAACAACAACCTAATTTTCCT
AGTAGTCAAAATAATCTAATTGGTCAAAATCAGGCTGCTTTATCGGCTTTATCAGCAGCG
ATGAGTGGTTACTTTCTATCACCACAAATCGGTACTCATGCTCCTCCACCAACACAACAG
TTTTCCGTAATGCCTGGAAGTAATCCTACGATGCTTGGTGCACCGCAACCGAGTCATACT
TTAATGCCAGCAACCGGTTCACAGCTCATCTCACACCAAGGTAATAAACCTATGCAATCA
GGATACAAAGGAGTTGTAGGAAATCTTAAGGACGGAGTTACCAGGAGTCGATTATTAGAA
GATTTTCGAGCTAATAGATTGACAACGCTCACATTACAAGATATTTCCGGCCATGTTGTA
GAATTTGCTCAAGATCAACATGGCTCTCGATTCATTCAACAGAAATTGCAAGAATCTAGC
CATAATGAGAAAACAATGGTGTTTCGAGAAATACTTCCACAGTGCTATAGTTTAATGACG
GATGTATTTGGAAATTATGTTATTCAAAGATTTTTTGATTTGGGAACACCTGAGCAAATT
CAGATTCTCGGTGATAGAATTCGTAATCAAGTCCTTCAATTATCACTTCAGATGTATGGC
TGCAGAGTTATACAGAAAGCTTTAGAAACTGTTAGTAAGGTTACTCAAATTAATATTGTG
CGTGAATTGGAGGGATCAGTAATTAAATGTGTAAAAGATCAAAATGGCAATCATGTTGTA
CAGAAATGTGTAGAATGTGTTCCTCCAGAACATCTCGATTTTATTATCGATGCCTTTAAA
GACAACGTAAGTTATTTTTGTATTGAT
>DjScaffold3724:33842-36932 strand:+
attcggggtATGGTTCAAAGATTAGCAGGAAAGAAAGGTCGATTTCGAGGAAATATTATG
GGAAAACGAACGAATTTCACTAGTCGAACAGTCATTTCACCAGATCCTTCTTTAAAAATC
AATGAGATTAGTATTCCATTATATTGTGCCTTGAGATTAACATTCACAGAAAAAGTAACA
CCTTACAATATAAATCATTTAAAGCAATTAGTACTAAATGGTGATAAATGTCATCCTGGT
GCAGTTGCCTATGCATCAGCTAATGATAACGAACCTCAACTGCCTATGTCTCATTTGAAA
TTAATGATGACAAAAATTTTATGGAATAGATTAGATAAAGAAGACCGTAGGCTTAACGCA
GCTAAGAGATTAAAAGTTGGAGATTATGTACAACGACACATCGATTGTGGAGACATAGTA
TTGTTCAACCGTCAGCCATCATTACATAGACTTAGTTTTCAAGCTTTTAATATAGTTGTT
AAAAAAGAAAAAACGTTTAGTTTTAATCCATGTTGCTGTAATCCATTTAATGCAGATTTT
GACGGTGATGAAATGAATGTTCATTTCCCACAAACCTATGAAGCAAAATCTGAAGCAATT
GAATTG
>DjScaffold3724:36963-39550 strand:+
AATAGTGGTGAACCTTTAACTTCACCACTTCAAGATATTCTTACAGCAGCTTATCTTCTT
ACATCCAAAGAAACTTTTTATACGGAAAACGAATTTCGGCATTTAATTTTACCAATTGTA
AAGCTTTGTCCACCTGACAAAATAATTATGATGCCACGTCCAGTGGTAATGAAACCAATA
AAGTTATATACGGGTAAACAACTCATGTCACTTGTTATTTCTCCGTATGGGAAATTGTCG
CAAAATAAAAAGATTATGATTCCTGATTCAAGAATCAACCTTGTTGTTGAAACTAGTGGG
AATCCTATTGATTATCTTAATCCCAATGATACTCTGGTCGTCATTCATAATAGTGAACTG
CTTATGGGTCAATTGCCTAAAAAGCTGTTAGGTGGTGGAAGCAAATCATCTATATTTTTT
CGTCTTCTTAAAGATGTTAGTGATTTGGCAAGTTGTGAAAGTATGGAAAGACTGTGTAAA
ATTGCTTTACCCGATTTGCTATCTCGAGGTTTTAGTATTGGTGTAACTGATGTTTCACCT
AGTCGAAAGCTTCGTGAAAAACAAAAGGAGCTCGTTTTTCAAAATTTTAATGTTTGCGAA
GATTTCATCAAATCTTATGAACGGAAAACACTAGTCTGTGATCCTGGTTGTAGTTTAGAA
GAAACATTAGAATCACGAATTAGTTCACAATTATCAAAGATTCGTGACGAAGGTGGTAAG
GAGTGTAAAATTACTTTACCAAAAACTAATGCGCCATTAATAATGGCATTATGTGGTTCT
AAAGGTTCTTTTATAAATATTTCTCAAATGGTATTTTGCGTTGGTCAACAGATTATTGAA
GGTAAACGAGTTTCGGATGTTAAAGGAACAAAACGAACCCTTATGTTTTCTCAGAAAAAT
TGTCGTAGTCCAATGTCTCGAGGATTCGTGTCAAATAGTTTTTATTCGGGTCTAAATGTT
GTTGAATTTCATTTCCATGCCATGAGTGGTAGAGAGGGTTTAATTGATACCGCAGTCAAA
ACATCAGAAACCGGATATTTGCAAAGAAGATTAATTAAGTTCTTAGAAGATTTAGCAATT
AGATATGATAAAACGGTTCGTGATTTCGCTGGTAATTTGGTGCAATTTCAATATGGGATT
GATGGATTCGACCCTTCAAAATTGGAGACTACTTATTGCCCATGTGATTTCACTAGAGAA
CTTTATCATGCATTACAAGATTACACGTATGATGAAAATCCAATAGGTTCTGAAATTAAA
TACGTTTTAAAAGAAATCTTGAGTTATCAGAAAAGAAAATTTGAGAACCTGGATGAAAGT
CTTGAAGAAACTATTACTGATTATTGTAATTCTAAAGAAGGTGCGGAATTTGGTATTACA
AGACCTCAATTGCGTTTATTTGTGGAAAATTGTTATAAGAAATATTTTAGAGCTGTTATT
GAACCAGGTACAGCGATCGGTGTGATTTGTGGACAAAGTGTAGGTGAACCGGCTACACAA
ATGACACTGAAAACTTTTCATTTTGCCGGTGTTGCAAGTATGAATATAACACGAGGAGTT
CCAAGAATGCAAGAGATTATGAATGCTGTTGCCACTATTAAAACTCCAATTATTACAGTT
TATTTAACAGATGATACAAGCGAAAGTTTTGCAAAAGAAATGCAATTAAAAATTCAACCC
ATCTTTCTCCGTGATATAATTTATCAATCTTACGAATATTTATGTCCTACTGGAATGTTT

TTTTACTTGAACCTAAATAACGTAAAAATGAACCAATTGTCTATCAAGACAGAAGATTTA
GAAATAGTTCTTAAATCAGTTAAAATCAGAAATTATGAATTTTCAGTAGTTTACAAAGAG
AACGGTCGTGTTTATATTGAGGTTTATTCTGCATACGAAATTAATTGTCAACTAGTTTTA
ACTGCTTTGGGTAAAATACAAGTAAAAGGTGTGAAAAACGTAAAACGAACAGTTATTCAA
AGAAATGAACAGGGAAAGCATAATATTTATGTCGAAGGAGGTAAATTACAAAACCTATTC
AATATCAAAGGATTGGATGGAATAAATACTCATAGTAATCATATTCTTGAAATCTACGAA
GTGTTTGGAATAGAAGCTGCTAAAGATATGATCATTTACGAATTGAACGCTGTTATGGGA
GCACACAACCTGACACCAGATATTCGACATGTCATGTTGCTTGCAGATGTCATGTGTTAT
ACTGGCCAAGTTTGTGGTTTCCAAAGAAGTGGAATTAGCAAATCTAAGAATAGTGTTATT
TGTTTGGCTTCGTTTGAAAAAACTCTCGATCATTTACTTGAGGCCTCTTATCATAATCAG
AAAGATAAACTTCATGGTATTAGTGATTGTATAACGTTTGGTAAAACCATTCCACTGGGT
ACTGGAATTCCAACACTTCTCATGGATGACAACAGTAAAAAGAATCTGAAGAGGCGTGAA
CCTATTGTTTTTTGTAAATAAa
>DjScaffold3724:48644-49139 strand:+
atactgatataaaacatgaacataattgtgttgtcataagaggaaaaactgatagttata
tgaaaaattatgaggaaataattattaattaataatttcagccaataagaattgagataa
ctaaatattattatttagttaattgtatcttataatcattttttatattaaatatagttt
gtagtagtaaaataaaaaATGTCTAACAATATGAGTTCTGATTTTCAAAATGAATCAACA
ATTGAAGATTTACCTAACATGGTGGCAGCGGTAAATACCGATTCTATTACTGAGAGGTTA
TCACCAAAAGTGTTCTGCAGAGGAGATGATATTGAGAAATTTATCAAGCAATGTGAATTG
TATTTTGAAGCTAAAAACATCACTCATGAGAAACAACAGGAGAGATTAGTCTCTTGTTTG
CTAGATGAAGATTTGTTGGAAGAA
>DjScaffold3724:50225-51096 strand:+
ataaagtagaatgtctattagatacaggggctcgaattaacgtgatgagtttgagttgtt
ttaataaactacgagaagtaaagttagaaatgacttATGCGAAAGGTCGATAGCAAAACG
AATGAAAAAGTCGAAAATTACATTTGCAATATTGAAGCAAATTTTGGAAGACAAATTAAA
AAAGGAGAGCGATTGACAAGAGCTTTAAATATTTTTAAGATTAAAGAGGATCAACGGTTA
TTGAAAATAATTGAAAGTAAAGAAGATGCTTTTATGGCAGATAAATGGGATGTAGGACGT
ACACATCTGACCAGACACAAAATAATCACAAGTGGTGAACCAATCAATGTAAAACCATAT
AGACAACCAATGAACTTAGAATCTAAAATTGAAGAAGCTATCCAAAACCTTTTCGAGAAT
AATATTATCAGGAAATGCAACTCTCCATGGAATACCCCATTGATATGTGTTTGGAAAAAG
GAAAAGAAAGACATAAGAATATGTTTGGATTTCAGACAATTGAATGCAGTAACGAAAAGA
CAAGCCTTTCCGATGCCTAATGTCGATGAAATGCTGGACAAGTTAGATGGAGCCAAATAT
TTCAGTTCAATAGATTTGGGAAATGCATACTATCAAGTAGAGCTCGAAGAAGAATCTCAA
GAAAAAACGGCGTTTTCAACGAAACACGGGCAATATTGTTTT
>DjScaffold3724:74270-76266 strand:+
taaattttcgaattattttcaatataaggattttctcaattaaatgttgcttattgggtt
catctagtttcataattgattcgacctttgaaacgcattctgacaatatattgtccacct
tgtccataattctaatttttaatttaaatcatgacaggcgtaatttattttttttatttc
actgattatattcttttcttctaacaaatgaagttttttgccaaatcatagcatccaca
ttgattacagctaggattaaagtatttctgtcaattttcattaattataattaatatttc
tataattaaatattaaaagtaattttaaaaaaaaatctctatatatatgttaattatataa
gttgattatatcaaatttggttttagatcggtgttgtcaaatttccagaaaATGTTATGT
TCAATCCTTGCTTTCTCACTGTTTTATATCTTTTTTGGTAAATTTGTAGCTGTATTTTTT
CTTATCTTTTCCTTCATCATTTATGTATTATTTTTGTTAATTTATGGGTTTTTAATTCGT
TGGTTAATTGGAATATTTCTTGAAAATCTCGTAATTTTATCTACGCAAAGCAGACAGATT
ATACTTCTTGTTCAAGAAATTCTCTCTCTTGATTATGGTTTTTCAAAAATTGTACATCTA
AAGGACAGTTCTATTTATGATTGTTCGAAATCTTTTAATGAAGATTTCCTAGAATATTTT
GTCAATAGAATTTTTTCAATTTTATTTGATCTTTTTTCTCACATGGAAAAATTCAACAAT
AATATTTTGACACATGTAAAAAACTCCAAATCTATCATTGAAGAGATTCCATGGATTCAA
GAAAAATTTGAAAATATTTTAGATGTTGACTTTCGAAATTTCAATTTTCGACAACCAATT
AGTCAACTGAAAAACATACACAATTCTGTGCAATTACAGTTTTCTGAACTTCTTAAAATG
ATAATTCTATTATTATATTATTTCAATAATCATCCAGACATCTTTTTATCAGTGAATATT
TTATGTTTATTGCAAAAATTTCATGAAATTCGCCAGCATTTTATGAAATTTAAAGAAATT
TTACAAATGAAAACAAAATCCAATTTATCCAAGTGTATTTCTGATGAAATTGTCGATAAT
AAAGTTAATAACGACAACTTTAAAAATGTCAAATCTACTATTCGACGATTAGATTTGCAT
TTATTTGAATTATCAGAATTTTCAAGTCAATTATCAGAAAGTTTAGAAAATAATAAAATA

CGCACTGAATCGTTATTGTGTGAATATTTAGAACCTCAACTTAACCTAATGGAGAAAAGT
AAAAAACATATTGACGGTATTAAAAATTGTTGGAGGGACTATGTGAAGATATTAGAGAA
ATCATAAGTTATAAAGATAGAAGCAACATTTCGATTTCTATTGAATCGAAAATTGTAGAA
CAAGATGAGAAATGTGAAGATGTTAATGAGATAGTAATTAACAATTTCCCAGAAATTTTA
GATGAAGTAATAGAAGGAATCCCTGAGGATAGAGAAAATGATTTTGAAAACGGTTTACAT
CAAAAGTTAATAGATCCATTTGAAGAAAGATATGCGACAAATGCTAAGGATTTATTAAAT
GAGTTACAATCAGTTATAGAAGTACGAGCAAAAGATTGGAAAAAACGGGAGAAATTAGCG
CTTAAACGCCGAGCTGCCGAAGGAAAATTTGTATTTAATAAAGAATCTCAACAAGTCAAA
GATTTCGATGATCTCAATTCTGAAGTAAATAGTGAAATTTGTGACGTAAAATGTTTGCCA
CCGACTGGCCAAGTAAATTTTGAGTTGGATTTCCGGAAGTCTTTAGGTGACATGATACTG
AAACAAAGACATGTCATGCTTTCAGATTCGAACAAATACGGAGAAACAGAAGAATGTTTT
GAGGACTAAatcaatcaattaattaaattta
>DjScaffold3724:51103-53103 strand:+
ATGCCATTTGGCATCGCAGCGGCACCAGGGACATTTCAAGAGATGATGACAAAAGTGGTA
GGCGGAATCAATGGAGCCATGGTTTATCTTGATGACATACTGATATTTACGAACAATAAA
GACCAACACTATGAAGTAATTGAAAGAGTATTGACGGAAATAATGAAGGCTGGTCTAAGA
ATTAATCCGGAAAAATGTCAGTTTCTTCAAAGTGAAATCAAGTTTCTTGGACACATAATT
AGCAGGGATGGTGTTAGAACTGATCCGTCTAAAATAGAAGCTATAAGAACATTTGGCAAA
CCTAACTGCGTGAAGAAGTTACGAAGTTTCTTGGGAATTTGTAATTATTACAGGAAATTT
ATAGAAAACTACGCAAGAAAATCAAAAATATTAGAGCAGTTGTGTAGCGGACCAAGCAAC
AAGAAATTAGAATGGACAGATGAAATAAACGATACTTTTGAGGGTCTAAAAAAATCTTTA
ATGACTGCACCAATATTGTGTTTTCCGGACATAAACAAAGAATTTATATTAGATACGGAT
GCCAGTTTTGATACGATTGGAGCAGTATTGTCACAGAGAGATAAAGAAGGAAGAGAAAGA
GTTGTTGCCTATGGATCACACTCAATGACAAGTCATGAAAAGGGATACTGCATTACACGA
AAGGAGTTATTGGCTGTTTATTATTTCTGTATCCATTTTAAACATTATTTGTATGGTAGA
AGATTTACTCTAAGAACAGATCACAAAGCTCTAACTTTCATGATGACGACGAAGAAACCA
ATTACGTCACAATTCCAGACATGGATTAATTATCTGAGCAGTTTAGATATGATTTTGGAA
TATAGAAAAGGAATCCAACATGCTAATGCAGATAAGTTATCTAGACCAAGATGTGAGGTA
TGTGTACAATGTCAAATGTCACATGAAGATGCCAAGAAAGGGAAGATTAAAACGAGAATA
CTGAATATAATTGAAAAAGAAAAAGAAGATCATTTAAACATTTCTGAGGAAACAGATTTA
GTCAAGAAAATGAAAGTTCCGGATGACAAACTACGTGAAAAAATAGAAGAAATACATAAA
TTATTATGTCATGCAGGAGTTGAAAAGGTGTACGAATATATGAGAAACAAATATATTGCA
AAGAATTTGTGTAGCCAAATACAGAAGATAGTTCGAAGTTGTGAAATTTGTCAGAAGACC
AAAGTAGTGACAACACCAACTAAAGAGAAGACAAAGAGTTTAGAAGCTACCAATTTATTT
GAAGTGATATTCACAGATATATGTGGGCCAATGCGGGAAACAAGTGGACGGAAAAAATAC
ATTCTTGGAATAATCGACCAATACAGTAAATATCTGGTTCTTATACCATTGCCTAGACAA
GACGAGGAAACGATACGAAAAGCTATTTTTGAAAAGTGGATATTGAGATTTGGATGTCCA
AGAGAAATAAGATTAGATTGTGGAAAAGCGTTTGAATCCAGAGTTATGAGAGAGTTTGCA
CAGAAGCTAGACATTAAACTTTGCTTTTCGAGTCCATATCATCATAATGCTAACGGACAA
ATAGAAAGACAGTTCAGGACCATTCGAGATTTGATTAATGCGACCCTTCAAGAAAGAAAA
TCAACAGATTGGGCAGATCTTTTACCGGAAATAGAATTTACAATGAACGCAACAAAGCAG
AAGACTACTGGAAAGAGCCCGGCTGAGATCGTTTTTGGAAAGAAAATTAGCAGAGAAAAG
TGGTACGAGTCAGACGTTTCAGATATAATTAAAGAAAACATTACAGACGAAATTACTAAA
AGAGAATTCAAAGTGGGCGATTATGTACTCATCAAGGTAGAAACAAGAAATAAAAGTCAA
GACAGATTTGAGGGACCTTATCGAATAATTGAGAAAATTCATGAAAGGCGGTATGCAGTA
CAAGGAGAAAATGGACAGTGTTTAGAAAGAAATGTGGAAAAACTTAGAAAGTTTCTTAAG
AAGGGGGATATGAGGAAATAA
>DjScaffold2281:37138-37698 strand:+
attagtttttaagtattcgaaggataatatattaaattgttaaattataagtATGGCCTT
TAGCGATAAAGATGATATGGAATTGAGGATGAAAGCAAGAAAAGAACTTTCAACTGCTAA
AGATCCTTTAACTAAATTAAGACTACAGTGCTTATGTAATGGTGCTAGTGGGATTAAAGG
ATTATCTAGACGCTTTCGAATCATGAACGATGATGATGATAAATTTTTATCACAAGCGGA
ATTTAAAAATGGTATTAAAGGTTTCGGATTGCAATTAACTGATTCAGAAATTGATGATTT
ATTTAAACTGATGGATAGAGACGGCAGTAACACTATTTGTATGACAGAATTCTTACGTGC
ATTAAGACCGCCATTGACTGAAAATAGATTAAATTTAATTAAGAAAGCCTTTACTTTAAT
GGATAAGACAGGAGATGGTGTTATTACTATTGAAGATCTAAAACATGTATATAATGCTAC
AAAACACCCAAAATGTCAAAACGGCGAATGGTCAGAAACTCGTGTTTTTCAAGAATTTCT

TAAAAGTTTCGAAGCTCCAAATGATGCTGATGGAATAGTAATAATTATTTTTATAATGT
TCTCTTTTATTATCATTTGTTATTATTATTATAAttattattattatttatattatattg
tttattctttaaaatatttaatataattaatttaataattttttttaatgcgtcagatat
ttacttataaatagtgaatcagttattcatttcttggaagattattgattagtttttaag
tattcgaaggataatatattaaattgttaaattataagtATGGCCTTTAGCGATAAAGAT
GATATGGAATTGAGGATGAAAGCAAGAAAAGAACTTTCAACTGCTAAAGATCCTTTAACT
AAATTAAGACTACAGTGCTTATGTAATGGTGCTAGTGGGATTAAAGGATTATCTAGACGC
TTTCGAATCATGAACGATGATGATGATAAATTTTTATCACAAGCGGAATTTAAAAATGGT
ATTAAAGGTTTCGGATTGCAATTAACTGATTCAGAAATTGATGATTTATTTAAACTGATG
GATAGAGACGGCAGTAACACTATTTGTATGACAGAATTCTTACGTGCATTAAGACCGCCA
TTGACTGAAAATAGATTAAATTTAATTAAGAAAGCCTTTACTTTAATGGATAAGACAGGA
GATGGTGTTATTACTGTAAACAGTTAAtaattatagatt
>DjScaffold1967:11653-12446 strand:+
gaaatcagatttaggacaatgcttatataaaagtcgctttggcaagtcaacaaaaaatat
taaaataaacacgagaaaacctATGAAATCAGTTTTAGAAGAGTCTGATGTTCAGAAAAT
ATTTTCTTTAGGTGAACTTTCCAATAAGGATTTTACTGCTATTAGGGTAATCAATGATAA
AGACAGAGAAGGTATTCTTTCACAGATTGAAAAAAAAATGTTTGATGAAGTTAAAAGTGA
ATATTCTAAACTTTTATCAGTTCCCAGAAGACCAAAAGGTGCCAGATTAATGAAAAAGGA
GCAACTATTAGCTGCAGAAAAAGATAATTTTTTAAAATGGAGACGTCAATTAGCAGAAAT
TGAGAACGATAAACGAGTTAAATTGACTCCTTACGAATTGAATTTAGATTTTTGGCGACA
ATTATGGCGTGTTATTGAACGAAGTGATGTTCTAATTCAAGTTGTTGACGCTAGAAATCC
ACTTTTATTTCGAAGTGAAGATTTGGAAAACTATGTTAAAGAAGTTGATCAAAATAAAGT
CAACGTTATTTTAATCAACAAAGCTGATTTATTGACAGAAAAGCAAAGAGAAATTTGGGC
AAGTTATTTTGATGAATTGAATTTGAGAGTAATTTTCTGGTCAGCAAAAATTGCTGAAGA
AAAACGAAAAGCAGAAAAGTTGGCAAGTATAAGTGAATCAGAAAATGATGAAATGGAGGA
GGAAATTAAAAGTGAAAAGGATAATCAATCAGAAGAAGAATCACATGAGGAAATAAGTGA
TGTTAATGTTGATTCTGAAGAACCGCAGTCGGATGGTATTTCCGAAGAGATGAATGTAAG
TGACCACGACGAGAGTGACGATGATGATCATTCTGAATCTGAAGAATCTTACGTTATGAG
CGACGACTCTGAAGAAGAAAAACCTGATTCGGATAATCACAGCACGAATGACCAAATTCC
TGAAGTTATAAAGAATTCTTCAGAGATGATTGGTGTGGATGAAATCATAAGTTTTTTGGA
ATCATTCGAAATCAGGAATCCTCACGAACCCGGTAAAATAACAACCGGACTTGTCGGATA
TCCAAATGTCGGTAAATCTAGCACAATCAATGCGATATTTGGTCGGAAAAAAGTGTCAGT
TTCTGCTACTCCCGGTCATACAAAGCGATTTCAAACATTAAATGTTACCGATAAATTGAT
ATTTTGTGATTGTCCTGGTCTTGTTATGCCATCCGTTGTTCATTCAAAGGGCCATTTAAT
GGTTTCTGGTATACTTCCAATCGATACCATCCGGGATTATATCGCACCAATCAATATTAT
TGCAGAACAATTTCCACGGGAAACCATAGAGTTGTTATATGGTATACAATTACCAGAAAC
CAGAGACGGTGAAGATCCATCAAGACCTCCCACAGCAAGAGAAGTTCTATCGTCTTATTC
ATATATGCACGGTATGATGACATCAAAAGGAAACCCTAATTTGGATCGGGCAGCAAGAAT
AATTCTAAAGGATTATGTTGATGGTCGTTTGTTGTACCGTCACCCTCCTCCTGGAATGGA
ACCAAAAGAATTTGAATCTGCAGCAGAATTACCAGAAGATAAAAAGAACAAAAACCGACC
GAAACCAAGTGGCGGAAAGAACACAAAAAATCCAATAATTCAAGAACCTGAAATGCCATT
GCACAGTGAAACTATTCAATCTATCAAGGGACCGAATTATTTGAAAATTAAAAGTTCAGC
AATGCATGACGGAATGAAAAGATTGACTGTTAACAATAGTAATCATAATGTTGTCAATGA
CGCTTGTGAAACGGAATCTGTTACCAGCAATGCTAAACCTTGGAGACAAATTAATAAACG
CGGGACAAAAGAGAAAATGCGGAATAAACATTCAGCATTAGATCAACACAGTTACGTTTA
CTTTTGAtcttcatcaaatataatcaatataaaatagaaatcagatttaggacaatgctt
atataaaagtcgctttggcaagtcaacaaaaaatattaaaataaacacgagaaaacctAT
GAAATCAGTTTTAGAAGAGTCTGATGTTCAGAAAATATTTTCTTTAGGTGAACTTTCCAA
TAAGGATTTTACTGCTATTAGGGTAATCAATGATAAAGACAGAGAAGGTATTCTTTCACA
GATTGAAAAAAAAATGTTTGATGAAGTTAAAAGTGAATATTCTAAACTTTTATCAGTTCC
CAGAAGACCAAAAGGTGCCAGATTAATGAAAAAGGAGCAACTATTAGCTGCAGAAAAAGA
TAATTTTTTAAAATGGAGACGTCAATTAGCAGAAATTGAGAACGATAAACGAGTTAAATT
GACTCCTTACGAATTGAATTTAGATTTTTGGCGACAATTATGGCGTGTTATTGAACGAAG
TGATGTTCTAATTCAAGTTGTTGACGCTAGAAATCCACTTTTATTTCGAAGTGAAGATTT
GGAAAACTATGTTAAAGAAGTTGATCAAAATAAAGTCAACGTTATTTTAATCAACAAAGC
TGATTTATTGACAGAAAAGCAAAGGTAAattcgaaaataattaatt
>DjScaffold3411:156979-175397 strand:+

gtaaaaatagattagaatcatatatatatcttttcagaataagagtgtatttatttttaa
aaaatattagtaattactactgtctttacttaatggaATGCTACTAAGAAATAATTCTAA
TATTGCATGTGTTACAGAGAAACAATATTTTCAGAATTCCAATTTACAAATGAGTAATCC
TAATAATATCAATAACAGTACTTTAACAGAGTTAAATCTTCAAGGAAATTTAGTATGCAA
TATTTATGATAATAATCCTCCTGAAAAAAGTTATTTTAATACTTATAATGTACCAGAAAG
ATCAAACCTGGAGTTAAATCAAATTAATTATAACACAAACGTTACGAATTTTCCTGCGTC
CAGCTTGTCTGATCAAATGTTAATTTCTAATAGTCAATCCACAAATTTATCGAATTCTGC
AACTTATCAAGATTACTGCAATATTGCATCATCTTCACCAGTCGCTTATCCCATAGAGAT
GAATTCTGTTATAAATCATATTTCCCCTGCTCCGACTTTGATATCTAAACCATCTAATTC
CTTTTCTCCTGATAATTCGCAGAATAATTTTCTTTCCCCTACCTACAACTACAATTGTAG
TAATACAATGAATTACATTCAACAAAAAAACCCTCTTCAAATCAACAGATCTACTAGCCA
CGTTGGGCTCGTACCAAACTCACAAAATTTGAATTATTCTCAATCTATACCAGAAACACA
AACCGTTAATCATCTCAGTCATTCCTTTAGTAATTTTACTATAAATAATTCACAACCAAA
TTCTCAGTGGAATTCTCAGAATAACTTTCAACAACCTAATAGATTACATCATCCATTTGA
TAACGCAAATAAAAATCTTTTGTATAATAATACCCAATTTAATGACACTAATCAAAATTC
ACTTAATCACTTGCATAGTTGGAATTCTAGTTCTCAAAGTTTACCACAATCTTGCTTAAG
TCCAAATAATTTCCAAAACAAAGATTTACAAAGTGTGCCAGTTTCAATGTCAATAAGCAC
ACCCACAGTTGTTCCTAATCCATCGAATCTTGGTAACATTAATAATTCAAAATCTAAACT
GACTCCACCTTTATTGGGTCATCAACAAAAACCTTCCTTTACTGGGTCAAATCCAAGCAA
TCTCACTCCATCAAGGCGAATGATAAATGTCATGGAAGAGAGGTTGCTAATTCCAGCGAC
AGGAATCATGAATCCTGTCCCTAATCTGATGCATATTACGAAAAAGCCAAATTGTGATCC
AAGTATAATGCGTTGCACAATGAATGCAGTACCAAATAGTGATGATATTCTCAACAAAAC
CAGATTGCCTTTCGTTTTGCTTATTCACCCGTTTAAAGATCTTGCTAATTTACAAGTCAT
AAGATCAGAGAAAGTAGTTCGTTGCAGAACAATATCATGTCGGGCTTATTTAAACCCATT
TGTTAGTTTTTCAGATCAAAAAAGATGGAAATGTAATATCTGCCAAAGACTTAATGAATT
ATCTGACGATTTTCTGTATGATGAATCTATTGGGGCTTATATAGAACCACAGAAGCGTGC
AGAAATTCGACTTGCCACTGTAGAGTATATTGCTCCGAAAGAATATTCCATGAAGAGTTT
TCAAGTGCCTGTCTATGTTTTTATGATAGATATCAGTGTAAATTCTGTTTCTTCTTTGTA
TTCTAATACTCTCAATCGTATACTCAATACTATTGATGAACTTCTCGATCGAATACCCGG
ACAAAAACATGCAAAATTTTCTTTGATAGGTTACGACAGTAATATTCATCTTTATCATAT
TAATGACAAAAAATACCAAGAAATAGTCTTGAGTGATTTATCCGAGCCAATAGAAGTTCC
TGTTACCACTGGCATTCTTGTTGACATACATAATTACAGAGATCAAATTCAAGAAGTTTT
GATTTCGTTATTGGATAATGTTGATCATGAATTATCCAACCAACCATCGATTTTGCTAAA
TTGTTTAGGATCAGCGCTTTTGCTTGCAAAACAATTAATCAATAATTCTGGTGGTCGTAT
CACAATATTCAATTCATCTTGTCCTAATATTGGAGAAGGAGTTTTGACTGTTAGAAATTG
TAAAAAGTCAAATAATGTTTTTCCACTTGCTCCAGATAAAGAATTTTATAAAACTCTTTC
TTTGGAATTTGCTCAAGCTCAAGTTAGATTTATTGCAATATTTTGAataatagaaacaac
ttttttatatcaatttacttaattttaccgattcttattattattgtaatatttttatgc
gaaattatagttcaaccaatcagaaattttaaaaattttcttatataaataattcaatgt
ttattaatgttagggaaatcttcaattggtaattcatttcccaaatcaaaactaatattt
tgagatatcatttattttactactacaactctgttatttgaaaaaaactataattaaaaa
gaactga
>DjScaffold739:19049-20066 strand:+
attattattagtataataattaaaatcagatctcatacaataaagggaaaataaaaaata
ataaatatattaatacttataaacaagtgtaaccattttagATGGATGCTACATTTGTAT
GGAAACTTGCTAAACTTGGTCGCTTGGGATCGAGTCATTTGAGATATGATGATGATTTTA
TTGACAGATTAAATTACCAAATAACTGGAATTCTACTTTTTCTGTTCATTGGAATAATTG
GAATACGGCAGTATGTTGGAAAACCTATTCAGTGTTGGAGTCCTCAAGAATTCACTCGTG
GTTGGGAGGAATATGCTGAAAATTATTGTTGGGTTTCAAATACTTACTATGCATCAGTGT
CGAACCGTCTTCCCGATAAACCTAATAGAAAAGATCTCATGATAGGTTACTACCAATGGG
CTTGGATATTTCTTGGTGTTCAAGCGCTAATGTTTTATATTCCTTGTATATTATGGAGAG
GACTTTACAATCAATCCCGTTTTAATATTCGTAGAATTATCCAAATGTCTGCTAGTGCCA
CTGATATCATAATGCCATTATCTGAAAAAAATGGAAACGCATCATCCAACACTAGTATCA
AATTCATTGCAAAATATTTGGACAGCTGTATTAGAAGAAGACATCTTGGAAGATGTAAAC
CCGATAAAAAACTAAACAACAACATTAAGCCTGGTCATCAAGTGTCCACGTCATGGTGCT
GTAGTCATAAGTCAGGAAACTGTCTTACTATTCTATATCTATTGATTAAACTGCTTTATA
TCCTTAATGCAATTAGTCAGGTCTACCTGATGGAAATATTTATTGGTACTAAGTATACAT

TCTATGGGGTATATGTACTTAAAGACCTACTCAGAGGACTTCATTGGGCAGACAGTGGTC
ATTTTCCTCGAGTTACATTCTGTGATTTCCAGGCTAAAAAGCTTGGTAAAAATCATTTGT
AAggtt
>DjScaffold5247:75441-76165 strand:+
TGCACCACACTAGGTTTAAGTACATATAATGAATTTTATAGGTTGCATGTACATCATTTG
ACATGTTTTATTATTGGTAAGAAACAATTACTATTGGAAATATTAAAAGAAATACCATGT
AAAAAAATGAAATTAGATTTAGAAGTGAATGTTATCGAGGAACCAGAAGTCATTAATGAA
CCAGATTGGAGTAATGTGGAAATAATCGAAGAAATGAATGAAAAAGAAGGAGATGAAGAT
TTACTTTTACAAAAGTTTTTAGATGAAGAAGATACGCAAGTTGGCGGTTCTCTTAATGAA
CCTACCATATAAagtgacctatataagtgtcatcaaatattgaaacttgacttgtccaaa
ttaaatatgaaacaaataattttaccggatagtttaatagaaaaattattgaagataata
gaagaaatgaatagtgtaaaaatacaatttggaaaacatacggaatttgtcaaggaataa
aaacatgtagattatcgaatagagcaggtcctttaagcgataagtttttcgaagaagg
>DjScaffold3997:36689-36891 strand:+
tgttgggtttttaggatttggtgttttaaaactctttctttgaactcattattctcgctt
ttaATGTCTAGGGGCATACCTAAAAACCACAAGTATTTATGTCACCTTTGTGACATGCCC
CTTTCTTTCCACGGGTTCAGCGCGACTTTGCGGTATCGCGTCTTACTCATAAAATCTATG
AAC
>DjScaffold5075:3839-4144 strand:+
aaacatatagtcaaatttcgtaataaatcaatgctgtttcacgaaattaaatctttagtt
gaatttttaggctgtggtaaatcatataATGCTAGCATTAAGTAGAATTTCCGATCCCAT
TTATTTTCTTGAAAGCGAATTAAAAATCCGTTCATATAAACTCGATATTAAAATATTTTT
CAAGTGAatacgaaccaatggtaactaat
>DjScaffold5075:72035-72906 strand:+
ataaagtagaatgtctattagatacaggggctcgaattaacgtgatgagtttgagttgtt
ttaataaactacgagaagtaaagttagaaatgacttATGCGAAAGGTCGATAGCAAAACG
AATGAAAAAGTCGAAAATTACATTTGCAATATTGAAGCAAATTTTGGAAGACAAATTAAA
AAAGGAGAGCGATTGACAAGAGCTTTAAATATTTTTAAGATTAAAGAGGATCAACGGTTA
TTGAAAATAATTGAAAGTAAAGAAGATGCTTTTATGGCAGATAAATGGGATGTAGGACGT
ACACATCTGACCAGACACAAAATAATCACAAGTGGTGAACCAATCAATGTAAAACCATAT
AGACAACCAATGAACTTAGAATCTAAAATTGAAGAAGCTATCCAAAACCTTTTCGAGAAT
AATATTATCAGGAAATGCAACTCTCCATGGAATACCCCATTGATATGTGTTTGGAAAAAG
GAAAAGAAAGACATAAGAATATGTTTGGATTTCAGACAATTGAATGCAGTAACGAAAAGA
CAAGCCTTTCCGATGCCTAATGTCGATGAAATGCTGGACAAGTTAGATGGAGCCAAATAT
TTCAGTTCAATAGATTTGGGAAATGCATACTATCAAGTAGAGCTCGAAGAAGAATCTCAA
GAAAAAACGGCGTTTTCAACGAAACACGGGCAATATTGTTTT
>DjScaffold5075:72913-74913 strand:+
ATGCCATTTGGCATCGCAGCGGCACCAGGGACATTTCAAGAGATGATGACAAAAGTGGTA
GGCGGAATCAATGGAGCCATGGTTTATCTTGATGACATACTGATATTTACGAACAATAAA
GACCAACACTATGAAGTAATTGAAAGAGTATTGACGGAAATAATGAAGGCTGGTCTAAGA
ATTAATCCGGAAAAATGTCAGTTTCTTCAAAGTGAAATCAAGTTTCTTGGACACATAATT
AGCAGGGATGGTGTTAGAACTGATCCGTCTAAAATAGAAGCTATAAGAACATTTGGCAAA
CCTAACTGCGTGAAGAAGTTACGAAGTTTCTTGGGAATTTGTAATTATTACAGGAAATTT
ATAGAAAACTACGCAAGAAAATCAAAAATATTAGAGCAGTTGTGTAGCGGACCAAGCAAC
AAGAAATTAGAATGGACAGATGAAATAAACGATACTTTTGAGGGTCTAAAAAAAATCTTTA
ATGACTGCACCAATATTGTGTTTTCCGGACATAAACAAAGAATTTATATTAGATACGGAT
GCCAGTTTTGATACGATTGGAGCAGTATTGTCACAGAGAGATAAAGAAGGAAGAGAAAGA
GTTGTTGCCTATGGATCACACTCAATGACAAGTCATGAAAAGGGATACTGCATTACACGA
AAGGAGTTATTGGCTGTTTATTATTTCTGTATCCATTTTAAACATTATTTGTATGGTAGA
AGATTTACTCTAAGAACAGATCACAAAGCTCTAACTTTCATGATGACGACGAAGAAACCA
ATTACGTCACAATTCCAGACATGGATTAATTATCTGAGCAGTTTAGATATGATTTTGGAA
TATAGAAAAGGAATCCAACATGCTAATGCAGATACGTTATCTAGACCAAGATGTGAGGTA
TGTGTACAATGTCAAATGTCACATGAAGATGCCAAGAAAGGGAAGATTAAAACGAGAATA
CTGAATATAATTGAAAAAGAAAAAGAAGATCATTTAAACATTTCTGAGGAAACAGATTTA
GTCAAGAAAATGAAAGTTCCGGATGACAAACTACGTGAAAAAATAGAAGAAATACATAAA
TTATTATGTCATGCAGGAGTTGAAAAGGTGTACGAATATATGAGAAACAAATATATTGCA
AAGAATTTGTGTAGCCAAATACAGAAGATAGTTCGAAGTTGTGAAATTTGTCAGAAGACC

AAAGTAGTGACAACACCAACTAAAGAGAAGACAAAGAGTTTAGAAGCTACCAATTTATTT
GAAGTGATATTCACAGATATATGTGGGCCAATGCGGGAAACAAGTGGACGGAAAAAATAC
ATTCTTGGAATAATCGACCAATACAGTAAATATCTGGTTCTTATACCATTGCCTAGACAA
GACGAGGAAACGATACGAAAAGCTATTTTTGAAAAGTGGATATTGAGATTTGGATGTCCA
AGAGAAATAAGATTAGATTGTGGAAAAGCGTTTGAATCCAGAGTTATGAGAGAGTTTGCA
CAGAAGCTAGACATTAAACTTTGCTTTTCGAGTCCATATCATCATAATGCTAACGGACAA
ATAGAAAGACAGTTCAGGACCATTCGAGATTTGATTAATGCGACCCTTCAAGAAAGAAAA
TCAACAGATTGGGCAGATCTTTTACCGGAAATAGAATTTACAATGAACGCAACAAAGCAG
AAGACTACTGGAAAGAGCCCGGCTGAGATCGTTTTTGGAAAGAAAATTAGCAGAGAAAAG
TGGTACGAGTCAGACGTTTCAGATATAATTAAAGAAAACATTTCAGACGAAATTACTAAA
AGAGAATTCAAAGTGGGCGATTATGTACTCATCAAGGTAGAAACAAGAAATAAAAGTCAA
GACAGATTTGAGGGACCTTATCGAATAATTGAGAAAATTCATGAAAGGCGGTATGCAGTA
CAAGGAGAAAATGGACAGTGTTTAGAAAGAAATGTGGAAAAACTTAGAAAGTTTCTTAAG
AAGGGGGATATGAGGAAATAA
>DjScaffold1209:93202-109410 strand:+
taccattttgaattctagttattttatttaaatcttatttatttttatttttttgtaatt
cataattgatATGGATTCTGAAAAAATTTCTAAATTGCTGAGTGAAAAAATAGTAATAGA
AAAGCCAACGATACCCCAGTGTTATTGTAATGCACAAAGAGACTTTAGTGTTCCGGATTT
TCAATGCAGTAGTTGTAATAAATGGTTTCATAAAAATTGTATAGGTTTTACAATAGGTAA
AGCAATTCCTTTTATGATAGCTTATCATTTTATGTGTAAAAAATGCAATGAAAGTTGTGA
AGAAGTATTTACGAGAAAACAAAGTAATTTTGCTAGTATGTGTCAAACTGCACTCGCTAA
TCTAATGTATATGAACAATGGAAAAATATATTTTTCAAAAGAAAAAGATATAGTACCATT
TTTGGAGGAAAATTGGGAGGCTTTAACAACTCAACCAAAGAAAACACTAGCTGCTCTTAA
TCCGAGTATCCATAAAACATTAATAAGTACCGATATTTTTAAGCATCAAATTTTTGGAGA
TGACGTCATTGTTTGTCTGAAGAACACGAACTTATATCAAATGAAACCAGGTTACGATCA
ATTTCGAATGCTTGCGAGTCAACTGAAAACCAAAGTAACAACTGGTGGCGGTATTTTGCC
ACAATCTGGTGGCTGGCATGCTAACAACAGAAAACGAAAAGCAACAAACAATAATGCAAC
GATAAATACAGGTGCGTCAACTCAGTCGACTACTAGAGTTGGTGAAATGGTTACTAATAC
AGGTAAACCGAAGCGTGGAATCGAAGAAACTAAAGTAAAACTTACTCAATACAATTATCC
GGTCGATCATCCATTTAACAAAGACAGCTACAGATACATTTATGTGGAGTCTGATCCTCA
CGCTAAAGGACGTCAGCTTTGGGAGGAATGTGAAGCTACAGCTGGCCGTATCATACCGGG
ATTCTTTTATCGTGTTGCTATTAGCCAACAAATTGCATTAGCTACAAACGATCGAGCTCA
TCAGTTGAAAATATCTGATAATCAAATGACTGTAACTGGCGATAAAGGTTATTGTATGAT
AAGAGCAACACAACCAATTTCAAGAGGGTGTTGGTATTTTGAAACTAAAATCGTAGATCA
ACCAGAAAATTCACACACACGAATTGGTTGGTCTCAATCACTCGGTAATTTGCAAGCCCC
TTGTGGTTTCGATAAGTTTAGCTACGCTTGTCGATCACGATTTGGTACCGCTTTTCATCA
GTCTAAAGGGAAACATTATATGGATAATGGTTACAAGAAAGGAGATGTTCTCGGTTGCAT
GTTTATTGCTGATATCCCAAATTTAAAACAAATCCCAAAACAACAGATTTCCGAAGCTTT
ATTTGGTAAACTGCCGGAATCGTTTAAAGACAGACCGCTTATTAAATTCAAAAATAGCTA
TTATTACGAAGAGAAAGAAGAAGCCGCCAAAGCCGAAAAGGAATTGAAACCGTTAGAAAA
TAGTAAAATTATATTCTACAAGAACGGAATTTCATATGGACCAGCTTTTACTGATATTTT
TGATGGGGCATATTATCCTGCAATTTCAATTTATAAACAAGCTTCCGTTTTAGTGAATTT
TGGTCCTAATTTTACATATCCTCCAAACGATATCGAAAATTGGAAACCTATTTTGGAACG
ACACACCGATTTGGAGATTGAACAAACAATATCTGATATGCTTTGGTTAACTGAAAATGA
TGATAGCATAGAGCAGATGATTATGAATCCTCAGTAGttccattttgtgcagttgtaaat
attttatgaatatatttatcgctatttgttattttgaattctagttattttatttaaatc
ttatttatttttatttttttgtaattcataattgatatggattctgaaaaaatttctaaa
ttgctgagtgaaaaaatagtaatagaaaagccaacgataccccagtgttattggttaatt
aaattttaaATGTTTCGATTCAATAAATTTGGTTTTAGTAATGCACAAAGAGACTTTAGT
GTTCCGGATTTTCAATGCAGTAGTTGTAATAAATGGTTTCATAAAAATTGTATAGGTTTT
ACAATAGGTAAAGCAATTCCTTTTATGATAGCTTATCATTTTATGTGTAAAAAATGCAAT
GAAAGTTGTGAAGAAGTATTTACGAGAAAACAAAGTAATTTTGCTAGTATGTGTCAAACT
GCACTCGCTAATCTAATGTATATGAACAATGGAAAAATATATTTTTCAAAAGAAAAAGAT
ATAGTACCATTTTTGGAGGAAAATTGGGAGGCTTTAACAACTCAACCAAAGAAAACACTA
GCTGCTCTTAATCCGAGTATCCATAAAACATTAATAAGTACCGATATTTTTAAGCATCAA
ATTTTTGGAGATGACGTCATTGTTTGTCTGAAGAACACGAACTTATATCAAATGAAACCA
GGTTACGATCAATTTCGAATGCTTGCGAGTCAACTGAAAACCAAAGTAACAACTGGTGGC

GGTATTTTGCCACAATCTGGTGGCTGGCATGCTAACAACAGAAAACGAAAAGCAACAAAC
AATAATGCAACGATAAATACAGGTGCGTCAACTCAGTCGACTACTAGAGTTGGTGAAATG
GTTACTAATACAGGTAAACCGAAGCGTGGAATCGAAGAAACTAAAGTAAAACTTACTCAA
TACAATTATCCGGTCGATCATCCATTTAACAAAGACAGCTACAGATACATTTATGTGGAG
TCTGATCCTCACGCTAAAGGACGTCAGCTTTGGGAGGAATGTGAAGCTACAGCTGGCCGT
ATCATACCGGGATTCTTTTATCGTGTTGCTATTAGCCAACAAATTGCATTAGCTACAAAC
GATCGAGCTCATCAGTTGAAAATATCTGATAATCAAATGACTGTAACTGGCGATAAAGGT
TATTGTATGATAAGAGCAACACAACCAATTTCAAGAGGGTGTTGGTATTTTGAAACTAAA
ATCGTAGATCAACCAGAAAATTCACACACACGAATTGGTTGGTCTCAATCACTCGGTAAT
TTGCAAGCCCCTTGTGGTTTCGATAAGTTTAGCTACGCTTGTCGATCACGATTTGGTACC
GCTTTTCATCAGTCTAAAGGGAAACATTATATGGATAATGGTTACAAGAAAGGAGATGTT
CTCGGTTGCATGTTTATTGCTGATATCCCAAATTTAAAACAAATCCCAAAACAACAGATT
TCCGAAGCTTTATTTGGTAAACTGCCGGAATCGTTTAAAGACAGACCGCTTATTAAATTC
AAAAATAGCTATTATTACGAAGAGAAAGAAGAAGCCGCCAAAGCCGAAAAGGAATTGAAA
CCGTTAGAAAATAGTAAAATTATATTCTACAAGAACGGAATTTCATATGGACCAGCTTTT
ACTGATATTTTTGATGGGGCATATTATCCTGCAATTTCAATTTATAAACAAGCTTCCGTT
TTAGTGAATTTTGGTCCTAATTTTACATATCCTCCAAACGATATCGAAAATTGGAAACCT
ATTTTGGAACGACACACCGATTTGGAGATTGAACAAACAATATCTGATATGCTTTGGTTA
ACTGAAAATGATGATAGCATAGAGCAGATGATTATGAATCCTCAGTAGttccattttgtg
cagttgtaaatattttatgaatatatttatcgctatttgtt
>DjScaffold1209:66645-68018 strand:+
ATGATAGATGAAATACACGAAAGATCAGTCGATTCTGATGTTTTATTAGGATTATTGAAA
CTAATAGCATCAGAAAGAAAAGATCTGAAAATTTTGATAACTTCAGCAACAATTAACGAT
GAAAAGTTTTCCAAATATTTTTCTCGAGCTCCTGTTATTTCATTTGAAGGTAAAATGTAT
GATGTTGAAGTAATTTACAAACCTATGTATACTTGGTCAATATATTTCATAAAGAACATA
ATTGAAAAAATACATTTTGAGCCTATTGATGGAGATATTTTAGTTTTTTTAACTGGTCAA
GAAGATATTGATAAATGTTGTGAAGGTCTAGAAAATGTAGAAAATCTTGTAGTTCTGCCG
TTATATGGTTCAATGGATTTTGACAATCAACAAAAAATATTTAATCCTGTCGAAAATGGA
AAAAGAAAAGTGATTGTAGCAACAAATATTGCTGAAACTAGTATAACTATTGATGATATT
CGATATGTTATCGATTGTGGTTATGTCAAGCAGAATCGATTTGATGTCAGATCTGGAATT
GACACCTTGCAAAATGTTATAATATCGAAAAATCAAGCTATTCAACGATATGGTCGAGCT
GGTAGAACTAATACAGGATGGTGTTATCGTTTATATAGTGAAAAGAATTTTGAATCTATG
AAAGTAGAAACTACTCCAGAGATTCATCGTAAAAATCTATGTGGAATATATTTGAAATTA
GTTGCGTTTGGACTTGATCCTATGAATTTCGATTTCATTGATTCACCAGATCCAGTTACA
CTTAAGAAATGTCTAGAAGAACTTCAAGATCTCGATTTACTTGATAGTAATAAAAAAATA
ACAACATTAGGAAGAATGATGCAAGATTTTCCAGTAGAACCGAAAATAGGTAAAGTTTTA
TTAAAGTCAGCTTCGTATTCTTGTTCATTAGATATTATTACAATACTAGCAATGTTGACT
GAACGAAATGTGTTTGCTCGACCAAAGAATCTAACAAATAAAGCAGATTCTGCAAAAGAA
CAATTTTTCAGTGAAGATGGTGATCAATTTATGTTGTTGAATGTGTTTAACAAATGGACG
GAAAACAATAAATCAAAACAGTGGTGCTATGATAATTTTGTTAACTCTAAAACAATGGAA
GAAATTGATTTGGTTAGAACACAATTATTGGATTTAATGAAACAAAAATTTCCCAAGCAA
GAAATGAAATCAAATAATATTTCCATAGATTCTATTAAAAAGTCTTTAATTTCTGGTTTT
TATAAAAATATTTGTTACCAAAGATCAAAAGGAGTTTACGTAAATTATAGCAATAAAGAA
GTTGTCTTCATGCATCCGGGTAGTTGTTTAATGACTCAGCTTCCACAAATGTAA
>DjScaffold3370:21834-23978 strand:+
ATGCCATTTGGCATTGCAGCAGCTCCTGCTACATTTCAAAATTTAATGACAAAAGTATTG
GGACCATTAAATTGGAAAGAAGCAATAGTGTATCTTGATGATATTCTTATTTTCGCTAAA
ACTGAAAATGAACATAATAAAAGATTAGAAAGAGTTTTTAAAAGAATTAAAGATGCCAAT
TTAAAGTTAAGTCCTGATAAATGTGAGTATCTAAAAAAATGAGATTAAGTTTTTAGGTTAC
ATTATAAATAAAAACGGAATTTGTACAGATAAGAATAAAATCCAAGCAGTAATGGATTTT
CAACCACCTAAGTGCATAAAGAAATTACGAAGCTTTTTAGGACTAACGAATTATTATAGA
AAATTTGTTAAAAATTATGCACAACATGCCAGAGTACTCGAAAAACTTTGTGGATCTAAC
AAAAGTAAATTGATTTGGACTGAGGAATGTCAAAATAGTTTTGATAATTTAAAAAAACAA
TTAACAACAACTCCAATTTTGAGTTTTGCTGACATCACTAAGGATTTCATTTTAGATACT
GATGCAAGTTTTGACACAATAGGTGCAGTTTTATCACAACGCGATGATCATGGATTCGAA
CGTGTTATTGCTTATGGATCTAAATCGATGACCAAACACGAGTTAGGGTATTGCATAACT
AGGAAAGAATTGTTGGCTATTTATTATTTTACTCAACATTTCAAGCACTATTTATATGGA

AAGAAATTTTTAATCAGAACGGATCATAAAGCAATTACTTTTATGATAAATACCAAAAAA
CCTGTAACGCCACAGTTTCAAACATGGCTGAACTTTTTGAGCAGTTTGGACATGAAAATG
GAATATCGAAAAGGAGAATTACATGGCAATGCAGATGCAATGTCTAGGAAAGAATGTCAG
TCTTGCAATCAGTGCCAAATGATTCACGAAGATCCTAAAAAAGGCAAAATTAAAACAAAG
GTTTTGGTAATAGAATCTTCAGAAAACGGATTAAAATGGCAAAACAACTCTGAAGAAATT
GAAAATATTAAAAAAGACATTTTTTCTAATAAAGTAGACTTTATTTTAGTCGATAACGTT
GTCAGAACAAAAGAAAACAAAATCTGGATTCCATTTGATAGAAAAGTTGAATTCATAAAA
GAAATTCATCATAGTTTATGTCATGCAGGGATTAAGAAAGTATATGATTTTGTTCGAGAA
AACTTTGACATGAAAAATACTTTAAAAGCAATTATGGATGTTATAGGGAGTTGCGAATTA
TGCCAAAAGCGAAAAACTCTTACAGGAAAGTCAAAAGAAGATAATAAGATATTATGTGCT
CAAGATACATTTGAGAATATTTTTATCGATTTCTGTGGCCCACTGAAAAGAACATTATCA
GGAAAAGTTTATATCATGGCTATTATTGATCAATTTAGTAGATATATTTTGTTAAATGCT
GTAAACCATCAAGATGAAAGAACGGCATCAAAAGTTATTGTTGAAAAATGGATATTGAAA
TATGGGGCTCCGAAAACTATAAATTGTGATCGGGGTAAGGTGTTTGAATCAAATGTAATA
CTTGAACTAGCTAAGAAATATGGAATAGAAATGCGATACTCTAGCCCATATCATCATCAG
ACTAACGGTATTATTGAGAGGCAATTTAGAACCATCCGAGATGCAATTTCAATAAAAATG
AGAGAGAATCAATTTAAAGATTGGACAGAGACACTACCTGATATTGAATTCATGATGAAT
TGTACAATACAACAAACTACTGGATATTCACCAGCAGAAATAGTATTTGGTAAAAGAATA
TTCAAAGAATGGTTTGATAAATATAAATTTTCGTCCAATGAAGACAATTATGAAAACCGA
TTTTCAAATATTAGAAGAGACGAAATACAACGAAATATGCACGAAAAGAATGATAGGCTA
CAAGAAAAGATTGTTAAAACTAATAGATCATTTGAAATTGGACAAAAGGTTTTGGTTAAA
ATTGATATTAGAAATAAAATGGATAATCGATATGAAGGTCCATTTATTATAATGGAAATA
TTACATGAAAGAAGTTACAAATTAAAAAATGAATTTGGAAAGATTATTATCCGAAATATT
GAATGGTTAAAGAATTATAAGGAGGGGGATGTGAGATGTGATTAA
>DjScaffold1344:78545-80041 strand:+
agtacagataaaagtttccaagtcttgattttaatcacgaatcttttggatagctactaa
aatacttggttcaatttatgaaataaaacaaatcatatgtataatttaaagttgataatt
tattgataaggtcatacacaccatcatgtttgaataaaatttataatattgatttttaaa
tttcgattactacaATGTTGGGCGTTTATCAAAAATTCAACGAGTACATTAAAAGATCAA
GAACTTCCAATGATAATATCACTCATATTGAAGATCTACCTAAAGACACAAAGTCAATCG
AAAAGTTACCTGAAGAAATATTGCTCAATATATTTAGTTATTTATCATTAGATGATCTCT
GTAATTGTTCATTGGTATCGAATACATGGAATCGAATATGTGATGATGTTTTGTTGTGGG
AATTTAAAATTCTTTTAGAATCATATAAATGGGTTGGAATTATATCAAAACAAGCACAGA
CTATTAAAATTACTCATCCTAAAGATATGTATACATCAATAAGTCCTATCTACAGTTTAT
CATCAAAAGAATTAATTCGATCAGGAATATTAGTGTATTTTAGTTTAATTTTAAAAAGTT
ACTGGGGTCATAAATCTCCACATATCGCTACTCTTGGCTCAGGCTTGGATAATTCTCGAA
CTCGCAAATTGTTTCATCGAATGCTTCGTAATAATTGTCCAGAATTTGAACCAACCGGGC
AATTTATGCCACCAGTCGGATCTGGTGTTACAATGAAATTAATATCGGATGATAATTATC
CTTATAAATTCCATTTTTATATGCTCTATTCTTTTCAACCAATGTCAAAATATTCACCAA
GTCTACAAGAAAATCGAATCAGAGAAAGTGTTTTATTTGAAAAACTGGATGATGATGAAC
CATTAAGTTCGTTGAAAATAAGGGAAAACGTCAAAGAAATATTTGTGAATTTAAACGGAC
TAATATACAGCATTGATTCAGAAGTTACAGATTTAGATTTTCAGTCATTAATATATGAAT
TTAAACTCGTTTCTAGTTTACTAAAAGATGACATCCCAGTTTTATTGATTTTAATAAACT
CCGAAAAATCACGACCAAACATTAAGACTACATTCCAAATTGCTCAATTATTTGAATTAT
ATAATTTACCAAATCCTTGGTGTGTTTTGAGTGCATCTGTCAAAGAATTATTGGTTGTAA
AACTCGGTATTATTTGGCTTATTCAAAAGACTTTTAAAATGTAAtcgttttgagaaaatt
cgtcttatacttcataatttatattaacttacgatttttatctattgtgaccgctttctt
tttttattattattttatttttccatttagatttggaattatcagtaaatttataatttc
agattgaagattatttttgactgatttgtaaaattaatacaattttattgtctatagatt
atttatt
>DjScaffold1344:133148-136313 strand:+
TTTGATCGAACTAAATGGGATGCGGCTGAAAGAAAATTGAAAGAATGGAAAGATTATCCA
AACAAACATCATATTTCATATTATTACAGTGATCAATATTTTGGATTTGCTTATACGAAA
TCTCTTTATCGATGTCGAGTTGAATCTTTCTATGAAGTATTGAAAATGATTTCATCAGTG
AGTGGAGTGAGCGAAATATTGAAGAAGAATTTCTCCATTGTTAGTGTTGGATGCGGACCA
GGACCTGAAATTGCAGCATATGTTGCTTTTATGCGAAATCAATTAAATGTGTATTCAACT
TTAAATTTCCTTGGTATTGATAGGGTAAATTATTGGCGAGAATTCTTGTTAGAAGCTATG

AAATGTGATAATAATATCAATATTTTCTTTGAGAATTGCGAATTCGACCAATATTATTTA
GAATCGAGTAATTTGAACGGAATATGTATTATGTCGTATCCTGAATCTGTTCTTTTCACA
AGCGATGTTTGGATAATGTTGAAAAGTAGATTTAATCTAATTATAGTTTTGGATCAATCA
AAACATGAACTACATTCGAAATTACGAAATGCTGGATTTACAATTCTCGGTTTTCATTAT
TTACAAGAGAAACGTTTTGCTTACTACATAAATAGTTGGATGAGAACAATAAATTATGGG
AATAACAATTACTATAATTTGTCGAATGGAAGATCATTTGCTTATTCTGGTAATCATGGA
AACAGAAATGATTATTATTCGAATTATAATACTGATGGTTATTTATATTCCAACGAGAAT
TTCCATACGAAAGCATCGTCAAATATCAAACGCGATTACAGATCACAAAATGGGATTGAA
GTTGGGAATAATAATCGTAATCAAGCAAATTGTCCATTGTCTATTATAATCACATTTTTT
ATAATACTTTTTATTATTTATTTTTTATACAATATCGTTTTCAAT
>DjScaffold3837:65400-74023 strand:+
aattggagtatttcttgtttcttttggtccattcatatttATGAATCAATTGACTCAAGT
ATTAAGTCGGTTATTTCCTTTTGATTCCAGAGGATTAACTCACGCGTATTGGGCACCCAA
TATTTGGGCTATTTACAACTCTTTAGACATGATTCTATTCAGGATAATTGGAAAATTTAT
TGGTGTGTCGCAATTGTCTAATGAAATGACCAAGGGACTCGTTGAAACATACAATCATTT
ACTGTTGCCTAATATCAAGCCTGTGACAACATTAATTTTAACTTTGTCCAGCATCATACC
AGCTTTAATCAAATGTCGGTTCACAAAATCTATCACTGATTTTCTCAAGGTGCTTATTAT
TTGTGGATGGTCTGCATTTTTATTCGGTTGGCATGTTCATGAAAAGGCAATTCTTATGAT
AACATTGCCATTTATGATTCTTGAAATTCTTGATCGGAAATGCTTGGCTTTTGGGTTTTA
TCTTACGACCATTAGTCATTATTCTTTATTACCATTGATCAATACAATACCAGAATATTC
CTTAAAAATGGCATTATTTCTGGCCTGTACGTGTACTCATTTTATCCAAATCAACCATAT
GCATGGTGGAAATGTATTAAATACTATTCCGTCTATATTTAGATGGCATCTTTCGATGCT
TATTCCTTTATGGATGATTACAGATGTTCTTATTCCAATATTTCTGCCAAGATACCAGTT
TTTGCCATTGATGCTCATTTCACTGTTTTGTGCGGCTGGTATTGTGTTTGCATATGTGAA
ATTCTATTTATACTATTGGACTGGTGATGATTCATGGGTGTTTTCCCAACAAAATTTAAA
AATTACTAAAGATAAAAAAGAATAAtaataatttaaatcggttttttattttggtaatat
atggagttagattttttata
>DjScaffold3837:90400-90927 strand:+
ATGCCGTTCGGATTATGCACCGCTCCTGCAACATTTCAAGAATTAATGCATAAAATTTTC
AACAAGATGTTATTTACTGTAGTGCTCGTTTATCTTGATGACATTTTAATTTTTGGTCAG
ACACAAGAAATTCATGATAAACATTTACAAAATGTGTTAGAAATAATCGAACAATCTGGT
CTCAAAATTAATCCTGAAAAATGTATATTTAGTGTATCAAAACTCAAATTTCTTGGTCAT
ACAATCAGTTCTAAAGGTGTTGAAACAAATAATGAATTTATTAATAAAGTAAAAGAAGCT
GTTATTCCAAAATGTGCTACCCAAATTCGAACTCTCTTTGGATTGACAAATTATTATCGT
AGATTTATTAAAGATTACGCAACAACTGCCAGTTCATTATATGCAGCAATAACTGGATCA
AAAGAAGTCGTAGAGTGGATTGAAGAATGTGACAGCAGGTTTAAACTACTGAAAGAAATA
TTATTTAGTTCTTTAATCTTGGCATTTTCAAATCCTCAAAGTAAATGA
>DjScaffold4169:1689-7189 strand:+
ACGAATATTAAAGATATTGAAAAATTGATTTCATTCCGAACTGAACAAGGCTTATTTTAT
TCATATTTTCAAGAGTTTATCGAACAGAATAATTTTATTAATGGCTTTTTAGCTTTATAT
CAAAATAATATGACAGAATATCCTCAAAAAATAAATGTTATTAATCGTATGAATATATGG
CAAGAAATAATTTTAGGTTTGATTTATCGCGTTGCAAATTATTTAAAAATAATTATTTTG
CCGATAGAATTTTATGTATACTCCGTTATTTTAATCCAAGCGATTTCAATCAGTTCGATA
TTCCTTTTATCATACTTGATCAATCATTCTCTTCTGGCTGGAATTTTATCAGTCGGATTT
TTTGCTCTAAATCGTCACGCAGTATCTCGAGTGGTTTACGTTTTGCCATTGAGGGAAAAC
TTTGCGTTACCTTTCTGGTGGTTACATGTTCTTACATTTCATTTGTTGATACAACCTAAG
AAACCAAGTGCCTCAATGCAGAAATTCATTCGAAATTGGTTTTATGTTTCCACTTTTCTA
TTTTGTCTACAATGGCAGTTTAGTCAATTTGTATTGTTATTAGAATGTGTCGTTATCTTT
GGTCTTGAAATGTTTGAATTTATTTCTCCAGCAATTGCTCGTGGATATTATTGGACAGGT
TTACTATGTTTGCTTGGTACCTGTGCTATGCAAATGGGAAATTTGATGTTACTCTCATCA
ATTACAGTTGCAGTGATGATATCTTCACTC
>DjScaffold1376:71673-78640 strand:+
AGATTTGATAATGCCAGTTTTAATACAATCAATAGCAATTGCACAGACACATTTTGTGTA
AATAAATGCAATCCAAATGTTTTCTCTCCAGTTTGCGATACGAATTTTGGAGTAACGTAC
TTTTCACCGTGTTATGCTGGTTGTTCAAATGTAAATACACATAAAAATGATACGAAGTAT
TATTATAACTGTACATGTACAGCAACTGTATTGAATTATGAGAATCCTCCAACGTACCTT
ACAGCTGGAGAATGTCCATTGAAATGCAATTCACTTATCACATTTGTATTGTTTCTTACT

ATGGCTCTTTTTTTGACTGGTGTAACCCAAAATCCACTTTTAATGGTAACAATGAGATCA
GTACAAAAACACGAGCGTGCGTTCGCTCTAGGATTTCAATTTGTCATTCTCCGACTCCTG
GCATATATACCATCACCTATCATTTATGGGACAGTTATTGATGCAGCCTGTGTCCATTGG
AAGAGAAATTGCGCTGAGCGTGGCGATTGCTTAGTGACTGATTTAAAGTTACTGAATCAG
AATTTAATCGGCCTTAGTTTCGCGGTGAAATTAGGTGCTGCAGTTTTCTATATCATTTTG
TTCTTTATTTTACCAAAACATGCACATATAACTAACCCAGACTCTATTAGTATAACTGAA
GAGCCAATAAAAGAGGAAATTGAATTTATTGAATCACATACTTCCCAGAATATCGAAGAA
AATATTACAATTGATCCAAAT
>DjScaffold1125:7825-8664 strand:+
atagattcatgtctcaatctttatctcaagttccaagtattttggagaaatataaatttg
atttttcaaagttacctgaacgattgaaatgcgttttgtgtcatgatattttaaaaaatg
ctaaacaaacttcatgcggttgtaaacatgtccaagcaatcagtcagattgttcagatgt
catattgaatattaacgaagatattcatcaggataatgctactaataaagaaattggaaa
tttagtggtgaaatgtccatttttttcatgcaaccatagttgtgctctgcgaaatATGGT
TGATCACATTGAATCTTGTGATTTACGTGAAATAACCTGTGAAAAGTGTGGAATTAAATT
TGGTGCGAAAGAAAAAGCCAATCATGATAAAAATAAATGTGAAATGAGAATGGTCAATTG
TGATTATTGTGGAATAAATATGAAATCTAAAGATTTGAATATTATTCATTTGAATCCTGA
TCGAGATGAATTGTGTTCCAAATATGTTGGCATTTGTTCAAATAATTGTAATGAAACTCG
TAGAGTTAATTTAAAAGAACATTTGAAAATATGTCCAAATAAAAAACTCGAATGTCCTTT
TCATCAATTTGATTGTAAAATAGATAAAATGACAAAAGAGAAATTGATGAAACATTTGGA
TGAGGAATATTTCAATCACACATCATCTCTGATAAACAAAGTTAAATATTTGGAA
>DjScaffold1125:28417-29166 strand:+
NNNNNNNCGCTGATAAACAAAGTTAAATATTCGGAAACGAAAATTAATGATTTGCAATTA
GAATTGCACAATCAAATTCTTATAACTAAAGAATTGTTTGAAATGAATAAGAAATCGAGT
GAATCGCATTTGGACATTGAAAAAACGATTGTTTTAGTTTCGAATNNNNNNNNNNNNNNN
NNNNNNNNNNNNNNTCGATAACAAAAATGTGATTTTCAGAAATGCATCTTTCATTTGGAAA
TTTACAGATTTCGAAGTAAGACGAGTAGATGCAGAGAGTGGAGGGCGCGTGTCGGTAACA
AGTGATGCGTTCTACACGAGCGAATTTGGATATAAAATGTGTTTGCGTGTTTATCCTGCT
GGGGATGGAGTCGGGAAAGGTACGCATCTGTCTGTTTTCTTTGCGATTATGAAGGGAGAG
TTTGATGATTTGTTGTCCTGGCCATTCAAACAGAAAGTAACTCTGAGCATATTGGACCAA
GAGAGTGGCAGCAAACACCATTCAGACACATTCAAACCGGATGCCAGATCAGCGTGTTAT
CAGAAACCAACAGATGAATGCAATATCGCATCTGGATCTCCTAAATTCATTTTGCTCTCC
TTAATAATGAATAACTCAATTTACTGCAAGAACGACACGATTTTCCTCAAGATTGCGGTC
GATACGATGGGCATTTCTAAACCTTAA
>DjScaffold2195:18089-19019 strand:+
atttatcattctaaattttttgttcaaaatttttcatgtttcatattttttgttcgag
attttttcattcgaaatttttcattctagatttttcattcgagatttttcattctagatt
tttgttcgaaatttttcatgtttcATGTACCCGCTTGGCGCCAATTTCATTCGAGATTTT
TCTTTCGAGATTTTTCATTCTAGATTTCGAGATTTTTTGTTCGAGATTTTTCATTCTATA
TTTTTCATGTTTCAAGTTTCATGTACCTGCTTGGCGCCAACTCGTATATTTACTATTACT
CATATATTTCTACCATCACAAATGCATTTGGGACCACAAAAGTTTTGTCTTGGTTTCTGT
GATTGGATGCCCAAAGGTGCTTGCGTTTTGAAAATAATTCTTGGATTGTGTTTGAGTTTA
GCATTTTTTATTATATTCTGCATGATTTGGCTACATTTCTCATTAATAAAAGATAATTTA
ATAACTGTGTTGGTTTTTGGTTTGATTTTATTTTATTTTGTATTTAAAGATAAATATTTC
AATATAATTTTATTTGGGTAGccgttatttacgaatttattttttatcaacttgagagaa
atcaatttcatttcttcatttgaagatagtctcgcagctcgaattctttaacctttaatt
atactaattttctggctgatggtagtattactactccaaggtaatatatgttcactgtga
aatggtttcatatataattgtgtagttaatatttcaccaggtttcttttgaattaaagtg
tccagaaaaagaatctgaaagtcaattgatttctctaaagtgaatttaatgttattatta
atactgttggctaattctagaatggtctctaaaggagaattagttaca
>DjScaffold2195:33976-35011 strand:+
ttgtcttgtaatattgaattactcgcgaaaacttaattgattaattttcacaatgttata
atggctctcgagtgaaaataatttggttaatgttggccatttagatgtccagtttgtcga
tatcctcaaactccacagatcATGAGTGACAAATATAAATGCGGAATGTGTGAGTTGCGT
GAAGATCTGTGGCTGTGTTTGATTTGTGCTCATATAGGATGTGGTAGACATGAAAATAAA
CACGCTGAATTACATTTCTATAATACCGGTCATCCATATGCCATGAAAATTGGCAATAAT
CTCGTTTGGGATTATACTACCGACTCTCATGTGCATCGGTTAGTAGCTAATACAAGTGAC

GGGAAACCAGTTCAAGTTGACTCAGAGGATCCAGAAGACAAGAAAATGTCCAATATTTGC
ATGGAATACTCTGCATTATTAGCCGATCAATTATCGTCACAACGAGCTTACTTGGAGAGT
GAGTTCTGTATGCGATTATCCAATGTGGAAAATGAGTTAGAACATCAAGCATTTATCGCA
AAAGAAACAGAAGACAGATATCAAACTGCCCTCAAAGAGAAACAAGCTCTTGAAAAGCGT
TTCAATCTAATGTGCAACAATTACCAAAAATTGCAGAAAGAACACAACGAAGAATTGATG
ATGAATAATTTACTGAGAGAAAAACAGAATATTTTCAATAAAACTCTGAAAATGAAAGAC
GAAGAACTGAAGAATGTCCAAGAGCAGTTGTCAGATTTGATGCTCAATCTACAAATGGGT
TCTAAAATCTCAGAAGCTCAAAACAGAGGAGAACTCAATGAAGAAGAAATACAAGGTGCA
AGTGTTTCGGTTGGTCCTCTCAGCAACCATGGGAAAAATCGACGAAGTAAAAGGAAATAA
agatgtcatttattttcaatttgttcatttatcaatcaatatattttgttctttattatt
attaaccaa
>DjScaffold2195:41741-45256 strand:+
taattttaacttgtaatattataaatgcaatctaacaaataataaatctaaattaacttt
attgctataactcaatatcattcagtaataatctATGTCAAAATATAAGTGCCCTGAATG
TAATAAATGTTTTAAAAATCGATCTGGCAAAAGTCTTTGTATGAGAAAACATAATAATAA
AGATTTGGTATTTCAATGTCCTTATTGTCCTCGTGTATATAAAGATCCCAGCAGTCTACG
GAAACACAAAAGACTTTTTGAGCATAGCAAAGATTTGAAAGAACTAGAATCTGAAGTTTC
GAATACTAAAACCGGTATATCTGAAACAGTAGTTATTCCAATGAATCTTAGTTGTCATGT
TTCTCAATCTATTGAAATCAATGAAAACCGCAAAGAAATTTTGCCAAAAACAACTCTTCC
ATCAATTGATACATTCGCATTTAATCATTCAGCGATAGATTGTCGAATTAATAATTCGAA
AGATAATCAACAAAATTTCAGTTTTCCCGAAAGAGCTCTTGATTTGACATCAACATTTCG
ACCAAAGGTTTTCATAAATCCGTGTTCACATTTGTCATACGTTCCGTCATATCAAACTGT
AGATGATAATCTGCAATACCAATATCCTATTAATCTTACAATTCAACACCAATCAAATGG
TTATTATGCGTACGATTCACAACTGCCTATTGATCTAAGTTATCACACGTTTTGGAATTC
TAACCACAATTCTCAAACTATGAATTTTACAAGTTTCCTCAACAGTGCTTGTCCTCTCGA
TTTGACATTGGACCAGCCATATTCTCATGAATACAATAACAATTCTCATATTGGATGGAT
TTGCTGAtattatttcattctaatatatttataatgtttcttctatcaatacttttcatt
tttattatcatacatttttatttttatattttgtttaaatattactattatc
>DjScaffold2195:46202-46759 strand:+
AATTTCTATAACAATTTCAACAATATCAACTTCAATAGTCTCGAATCTTCTGTTTGTCAT
TTAGCTTATGTGTCTCATGTTGAATCAATAGCTTCTTCCTCTAGTGCTGCGTTTCCGTTA
TCTGATTTAACCGAGTTACCCACGTGTCCAGTTTGTCTTGAGAGATTGGATGAGCCATTG
AACGGTATTCTCACCATCGTGTGTAATCACACATTCCACAGCAAATGCATACAACAGATG
AATGAATCTACGTAAggccggccatatttatcattaatctatagttcaatcaatgagcag
cgattataacattgtgaaattacctagattttacaactgcatttctctacaacattcac
attcgattagaggttaacataatgacataacaacatagcaattcaactgaactagttacc
cagctataataataataatcacttt
>DjScaffold12:36016-36964 strand:+
ttcttaatttctattattgaaatattgttcaatagaaattcctgaaattaaaggaaatca
aacaaatacataaattttacgtaaaccaactttaaaccatttttaattaaaacaaaaaa
atttgtgccctattttgatttatagtttcaaataaatttctatcgttaaaaatattcgat
ttaaaaaatttaacgtaaaaaaatttaattcttatttattggagctaatgatatctgtaca
atagtattacttgcattatgtttgaaatatatcgatttaaattcagaagaaaactgtatt
gtgccaaaatcaataatccaatcaaaaaacttctttggttattttaaagattcagtaatt
acaacaagtaaccaatcagaaatttattattgcttcgattcgaagtggaattctatattc
caaacagatcttccaaaagatttcgaaataataaatgaaaagaatttaaatATGAAATCA
ACTAAAATACCAGCATATAAACCGTTGCCAGTGGTTGAAATCATACGCGAGAATGAGCAG
TTAAACAATGAATCTGTTAAATTCGAATCTGAATTACAAAAACCTGAAGAAAATGATTTA
AAAAATCATTCTTTCTTATTTAAATTCAATTCTGGACCTCCAAAAATCTTTAATTTATTT
GAATTTGTACTTGTTTGTGTAATTATTTGTTTGTTTTTCTTTTTTCTTCTCGTTCTTCTC
ATTTTGTTGTCGAAATTTGTATTGATCAGTCAAGTGAGACGGAAATTTATCAAGCCTAAT
TTACTACTGATGGAATCGGAAACATTAGTCGTAAATAATAAAAAAGCGTTTGTAAATAAT
AAAGATTTTCCAGTGTTAAGCGAAAAACTGTCATTAAGTGAGTCTAGTGCTGAATCA
>DjScaffold12:70938-72014 strand:+
aattttagaaATGTTCGAAAATATTTGGAAAGTAATGAAAGAAAATAAATTACAACCGAT
AAGTGAAATAAGTAATGACTTAATTTGTAGTATTTGTAATAAATTAATGATCAATGCTTG
TAATGGTCCTTGTGGTTGCAGATATTGCTTTGATTGTATCACTAATTATTTAGCAGGAGA

AAATAAACTTTGTCCAAGTCAATCAAGATATTGTCAAATAAGATCGATAAATCGTGAAAA
CGATATTATTATAGATCGACCAATAAATATTAGAATTACAGAAACAGTTGTAAAATGTCC
AAATAAATTTTGTGAATATTCCAGTGAGTTGAATGTGATAGAAAATCACATGAGAACATG
TGACAAGAATCCAACAAGTTGTCCATATTATGAAATTGGATGTAAAAATGATGAAGTAAT
GATTGATGCAATGAAAGATCATCTTTGTACTGAGATTTTCTGTCACACTAAATTATTATT
AGATTTTATCAATAATTTACGAAATGAGATGGAATCAATAAAAAAAGAAAATTTTAAAAC
AAAAGAAATCAGTCAAGAAAATATCAAAAAATTCGCTCTAACTACTGATGCCCACAAATT
AGAATTAGAAGAATTAAATGAAGAAATGGTGGGATTGAAAAATGAAAATAGATCTCTAAG
AAGTGAAATGGAAAATATTAAAAAATCCTTAAATGTAATATTGAAGACAATCCAAACAAA
ATCAGAACAACAAACTGAAGAAATCAATGAAATCAAACAAAACTCTGAACTAACCAATCA
GAAACAAATTGAAATGAAAATTGTAATCGATAATTTAACAAATACAAATTCTGATTTATT
GGATAAATTAAATAATCACAATAAAATTATCGATGATATTATCAATGATAAATTGAAAAC
CATTGATAATACAATTTATCATATTGAAAATCAATTG
>DjScaffold12:81028-82003 strand:+
ATTGATCCTGAGGACGATGCAGTATATTCAGTCGATGCTTCACAGTGTGATGATAGGGTT
TGGTTGGTAAAAATTCCTAAATACTTGTCTAACGAATGGATGGTGTCACCAGATAATTCT
ATTGTTGCTAAAATTGTAGTTGAGCAGGATAAAGACAAAGATAAAGAAGCAGCTTATAAA
TTAATATGCAATCCAGATTTTATCAAAAATAAAGATATACCAACAGTTAATAAATTTATT
ATTCAAGGTATCACGGAAAAGGTTAACGTTAAAAGTGAAGAAAGAGCTAAAAGTTTATCT
ATTGGTTCACGTGATGGTAAAACATTTATACTGCAATCATCTGACATAGAAGGAGGATAT
AATCCATTAAAAAAGCCAAAGTATAAAAAAAAAGCTATTATAGGTCGTGTTAGTGTAAGA
TGTAATGTTATGCCTCCAGATGATAGTAGTTATTTTGCATTAAAAACTAAACAAATTAAA
TCATATAACACCCCGCTAAGAAAAACACAAGTTTGTGAAGAACCAGGTGTGGAATTTAAG
CCTAAATCTACTGGAACTATAAATAAGAAAAAAGATGCCAATGGAAGAGATAGTAATCGA
TCAGCAAGAATGGAACATTCAAAACTGATGGATCTCATTTGTTCACATTTTGAGAATCAC
CAATATTATAACATTAAAGATTTAATAGATTTAACTGGACAGCCACCGGGTTATGTCAAA
GAAATACTTAAAGAAGTGGCCACATTAAGTAAGGCTCCATCCCGTCGACACATGTGGGAG
TTGAAACCTGAATATAGACATTATTCATAAatactttgtaatgtttaatatttactcgtt
tactttgttttattgtaaataaaacaataaatttaa
>DjScaffold3432:1540-3156 strand:+
ATGAACACAGTGTTAACAGGGGTTGAACATGTAGTTGTATATATGGATGATATACTCATA
GCATCTGAAACATTTGAGCAACATTTGATTAACATACAGAATGTATTGTCAAGAATTAGA
AAAGCGAATATTAAGTTAAAACCCACAAAGTGCAAGTGGGCAGAGGAAAGTGTGTTATTT
CTCGGACATATTGTATCATCAAGAGGATTAGAGCCAAATCCAGAAAAGGTAAAAGCGGTC
CAAGATTTTCCATTGCCAAAGAGCGTACAACAATTACAAGGATTTAATGGACTAGTGGGA
TATTATCGTCGATTCATACAAGATTTTGCAAAAATATCAGCACCATTGGTAGAATTAACC
AAAGGCATGAAAACGAAAGGGGAAAGTAAAAGTGTCATGATTGTAGAAAAATTAGATGAA
AAATCGAAGAAGCCTCAGAAAAAATGGGGCCCAAAACAGCAAGAGGCTTTAGATACATTA
AAACAACGATTGCTAACAACTCCAATATTAAGATATCCTGACTTTACAAAACAATTCATA
GTAATGAAGGATGCAAGTGGATATGCTATAGGAGCAGTGCTTGGTCAGGAAGACGAGAAA
AATAAAGATTATGTAATTGCATATGCTAGTAGAATGTTGAAAGGAGCAGTGACAAGGTAT
TCAACAATTGAAAAAGAATGTTTAGCTATCATATTTGCGTTAAAAGAATTTAGACCGTTT
ATATATGGAACAGAAATAATCATCCGAACAGATCATAAATCTTTAGAGGGTCTATGGAAA
AATGAAGATGCATCCAGTAGAATACTGAAGTGGGCAATGAAAATTCAAGATATGAGCATA
CGTATTATATATAAGCCAGGTAGAGCAAACAAAAACGCCGATGCATTATCAAGAATTCCA
GAAGTGAATATATTAGAAATGATGTCAATTGTGATGACAGGAGCAAACCCAAAAACGTTC
TTAGAGGAACAAAATAAAGATAAACAAATACAAGAGATTAAAAAAATGATGCTTCAAGAA
AAGAATACTAAATATAAAATGTATAAGAATTTAGTTATGTACAATAATTTAGGGAAAAAT
CTGATGGTTGTACCAGATTCTTTAAAACAATTATTATTATTAAAATATCATGATGGAGCA
CTCGGAGGGCATCTATCTAGTAGAAAAACCAAGTCAAGATTACTACAAAAATACTATTGG
GAGAATATCAAAGAAGATGTAAAGAGGTGGTGTCAAGTATGTAGTATCTGTGCCACAAGA
AAGAATACAGGAACAAAAACCAGAGTTCCATTACAACCAATGCCAATACCCAGTTCACCA
ATGGAAACATGCGCAATGGATTTTTTGTGCAAATTACCAACGACTATAAAAGGCAATAAA
CATATATTAGTATTCAATGACTATTTCACAAAGTGGGCAGAGGCATTTCCTACAAAGGAT
GAAACAGCAGATACAGTAGCAAAGTTATTAGTTGAAAAAATAATATACAAATATGGAGCG
CCAAGAAAGCTGCTGTCCGATAGGGGAAAGAGTTTCCTCAATGAAGTCATACAAAGAGTG
AAAAATATGTTAGATATTAAAAAAAATAAATACGACGGCATATCACCCACAAACTGA

>DjScaffold1557:14526-14918 strand:+
ATGATGACAAAATTAGTAGGCAAAATTAAAGGAGTCGTGGTATATCTTGATGAAATTGCG
ATATTTACGAACAAAAAAGATCAACACTATGAAGTAATTGACAAAGTTTTGACAGAAATA
ATAAATGCTTGTTTAATAATTATTCCAGATAAATTTCAGTTTCTTCAAAGCAAATTTGAA
GTTCTTGGGCAACTAATTATCAGAGATGTTGTTAGAATTGATCTGCTTAAAATAGAAGTA
AAAAGATCATTTGAAAAATCTAACTTCGTTATAAAGTTACGAAGTTTCTTAAGAATTTGT
AATTACTACAGGAAGTTTATAGAAAACTACCCAAGAAAATCAAGGATCTCAGAACAGTTG
TGGAGGAAGTCAAGCAATAAGAAATTAGAATGA
>DjScaffold881:114539-115147 strand:+
CACGCGTATGCCGTTCGGAATCGCGGCGGCACCAGGAACTTTCCAAGAGTTAATGACGAA
AGTATTAGCCGGATCAAAGAAGACGATGGTATATCTAGATCACATAATGATATTCACAGA
AGATCTAAGAAGCATTATAAAATTTTGAACGAGGTTTTGGAAAAAATAAAGAAAGCCGGA
TTACGAATTAATCCAGAAAAGTGCCATTTGTTGAGACAGCAGGTGAAGTTTTTGGGACAC
GTAGTAGATAGAGAAGGAATCAGACCTGACATGACAAAGATAGAAGCTATAAAAGTGTAC
GAAAGACCGAAATGCATAAAGAAGCTACTAGGATTTCTAGGAATGTGCAACTATTACAGT
CGATTCATCAAGAATTATTCACAGAAATCAATGTGTTTAGAAGAACTCATTGGTATGAAC
AACGTAAAGTTAATTTGGACTGACAAGTGCGATAAAGCATTTACGGAGATGAAAGAAGCA
TTAATGAAAACGCCGGTATTGGGATTCTCGGACATCACGAAAACATTCATACTAGATACG
AATGCTAGTTTCGATACGATCGGAGCAGTTCTGGCACAACGAGACGAAAATGGGAAAATA
CATCAATAA
>DjScaffold665:15432-20350 strand:+
gataataacaaacaaaattattacgtttggATGAGATGGGCTCGTGTAGGTAAAATTGGC
CAAATGAAGTTGACAAAATTTCAGGATAATTTGTCTGCTGCCAAAGAATGTTTTATGAAG
AAATTTCTTGATAAAACTAGAAATGAATGGGAAAATCGAAATAAATTTGTTAAAGTTAAT
GGGAAATACGATCTTTTAGAAATAGATTATTCTAAAAAAGAAAATATTGAACCGATTAAA
AAGGAGACTTGTGTAAAATTGGAACCATTAGAATGCAAATTAGAAGAAAAACTTCAAGAT
CTTATGCGAATCATTTTTGATATAAAAGAAATGAATAATGCGGTTGTGGAAATGAATTAT
GATTCAAATAAAATACCATTGGGACGATTAACTGAAGCACAAATTGCTGCTGGCTACAAA
GCTCTTAGAGAAATAGAAGGCTTGCTGAATGAAGGTCATTCTCGTAAAACCCTAGTGGAA
GCATGTAATGCTTATTATACAAGAATACCTCATGATTTTGGAATGCGCATTCCTCCTCTC
ATTCAAACGGTTTCTGAAATAAAACGAGAACTAGAATTGTTGGAAACATTGAGTGATATC
AAAATTGCACTGAAACTTATCAATGAATCTGATGATATGCATGTTCATCCACTTGATCGT
AATTATCGCAATCTTCATTGTGACATTAATTCGATTTCTCCCGATAAACCAATCTACCAG
GTGATCATT
>DjScaffold665:38715-39574 strand:+
caggtatataaatgatattttctttgttactaattctcatttagaaaatatttgggaact
agccaatagtattaatgacaacatcaaattcactttaaagaagttaatcgactttcagat
tctttatttggacaccttaatccaaaaaaacctggtgaattattacttacccagttgcat
atgaaactatttcacagtgggcatgtattaccctggagtaaacatttgaataatcaaggg
tgaaagattaagaactaaaagactatctttaaatgaagaaataatagattgacttccctc
aggttaataaaatataaatttctaaataatggctatccaaataaaattatatctaagtat
ttattctcaaatacagaatttaataaaatcaaacaaaagacaaatacagttattaaatta
ccttttattaatgagaaatgtagccaaatcATGCAAAATATAATTAAAAAGTCGAAACTA
AAACACAATCCAAGAATTATATTTAAAACGCAAGCACCTTTGGGCATCCAATTACAGAAA
CCAAGACAAAATTTTTGTGGTCCCAAATGCATTTGTGATGGTAGAAATATATGCAATAAA
AAAAATATCACTTACAAAATAACATGCACCTTATGTAAGGATACATATATAGGAGAATCA
TTCAGAACCATCCGTACAAGAATTAAAGAACATCTTAAACAGAAAGAAAGTGAAGTCTTC
AAACACTTTCAAAACAAACATAATGTTTTACCAGAAAATCATCATATTACATGGAAAACA
TTAGGAACAGGCTACATTTCTACACTTCACAGAAAGAAACATGAACAAATTTTGATCAAG
>DjScaffold553:10577-11425 strand:+
ATCTTTCATTTGTTAACATTCAGAGTATTCCAAATTCGATCGTTTTTCAAAACTACAAGT
TCTTCACCAGCACTTATTGAGAATCCAAAGATATGGGATCGATCAAAGAATATTAAATTT
CCAGAATGCAAGAAACCATCATTTATCAGCAGCCAAGATCCAAAAACTAATGTTCCTGCA
AAAGCTGTTAGCAAAAGTGTTTTGATTATTTATAAAGAAGAATCATTGCGAATGTATGAT
ATATTTGAGCAAACAATGCATTTACAAATGGATGCGCTGTCTATTGAAGAACAAATTTAC
AAATCGGAAATTATTTTAGTTGTAGAACTGTGGTTTGGCGAGAAGAGAGATGAAATTATT
GAAGAAATGCGGTGTGTTTGTGAGCGCCAATTGAAGGTTCATCTCATTCCAAGTCAAGGA

TTGCATCATCATATCACGGTAATATTTGATTACTATTATCTTGCCGCAGTCGAACTGGTC
GTCCATGCATCTTTAGTCAGTATATCTCCATGTTATCTCAGGTTACATTAAttaatcaat
attatgtaattattaatattattattattattattattattattatttattatt
>DjScaffold3498:85917-88648 strand:+
TTGAAAAAGGGTTCTTCCTATCATTGGGACTTATTAGTTGTCGCATTGATTAACGGTGGT
TTATCTGTATTTGGCTTACCATGGGTTCATGGTGCACTTCCTCAATCTCCATTACATGTT
AAAGCATTGGCTGATTTAGAAGAAAGAATAGATACAGCTCATCATGTTCATCAAACGGTG
CGTGAAACAAGAATAACAAGCATTTTATCACATATTTTTATTGCAATTTCTCTCCAAATG
TTGCCAATACCATTGATCTATATTCCAGTAGCAGTACTTGACGGTCTGTTTGTTTATATG
GCTGTCACTACACTTTATGGTAATCAATTTTTTGAACGAATATTGCTGTTGTTTACTGAG
CAGTCTGCTTATCCACCATCACATTATTTACGCAGGGTTCCTCAGAGAAAGCTTCATATT
TTTACTGGCTTGCAGATATTTCAGCTGGCTGGACTCTGCTTGCTTGGATTTTCCCCTAGC
CCGTATTTGAAAATGGTTTTTCCTCTTTTGCTTCTATTTCACATGCTTTTCAGGTAA
>DjScaffold2533:6266-9816 strand:+
gtggcaagtctggtcataattttagatcttgttcgattttacagggttcgtctagtagca
ggaacaataaaattttgtgtattcaagaggaattggacgattcaaaaaaagacgatgaga
gcgtgacagatcatgatgtagtaatgggaaaaaccagaacgactccattgcttacatttc
gtcgatggagtcagtatagaaaaaaatgaggtaaatcgaaatgatgatgaaagaggagag
ATGTTTGAAAAGGAAGATGGACGAGAGTGGAGTAGAGTAGATGTTATAGGAAAATTAAGT
ATACATTTGAACAAGAAAGTCAGGAAAATTAAAGTCATCATAGTTAGACAGATCCACCCA
GAATTAATAGGCGGTATTGATACAATGCAAAGTTTTGGAGTGGAATTAACTGAAATAAAT
AACATCGAAAATGAGAGTCTGAAGATAGGATATTCTGATGAAAATAGAAGAAATAACGCG
TTGCAGTTGTTTAAAGAAATAGATAACAGAAAAATGGTTCAGTTGATCCAGATTTTCGGT
GATATATCCATGGGTTTAAAGTTCGATATTGGGCATACAAGAAAAGTAATACATGAAATA
AAAGTTAAAGAAGTTTCAATAGTAGAGCACCCTAGAAGACAGCCGATGCATTTAGAAAAT
AAAATTGAAGAAATGATTAAAGAATTAGCAAAAATTGGCGTAATCAGCACATGCAATAGT
CCTTGGAATTTTCCATTAGTGATTGTAGATAAAAAGGACGGCTTGATAAGAATGTGCGTG
AATTACAGATAAgtgaatttgaggacagaa
>DjScaffold642:54084-54780 strand:+
tttaccatacacatcatgtttagctatatgccaatcttcatagttggaattaaatctggA
TGATATTATTGAGTTTTGTAAAGTTTTCTATCTTTATCTCATTCCAAGACTCGCCAACCC
ACGAATTAACCAAAGATTGTGCTGCTGGTTTTGGATTGCCTGTTTTTGTATATTCAACAT
ATTCAGACTTTTTCCATTCATTAATGCGCAAAAATATTAAATCTTTTAATGAGCGGAAAA
TTCCTATATCTCCTGCTTGAagatatgtggcattgcttttctaccatcttcccaaacagt
aaaagaagaaagaagaagtatcgtttatttaatttgcttataaacgaagcagtattaaat
atgatttgtggtgggaataaaattttgagttatttgagtaatgatattatttaaatagta
tcaccaacattctgacactttagggatattatttaggcagtgatactatttgg
>DjScaffold5054:145-10079 strand:+
ttaactttttacttaatttttttagatttcgactagaaagATGGAATCCATCATTAATT
TTTGTGATAATCAATTTTACAACGGAAAGAATTATTGTGGAATTGGTTTCATAACAAAAG
ATAATAAAAGTTGTTTGAAATGGAAAAATTCCCTTGGAGAATTCGATAAACAGGTAGAAA
TGACAGCATTATGCAAAAAAGGAATTTTTTCTAATTGGTGTTGGACAAAAACTTACAAAA
GTAAATTTGGATTCGAATACAATTATGATTATTGTAGAAAACTAAATTGTGAACCTAATA
AAATTAATTATTGTCAATCAAATATTTTCTGGTCATCAACCATTTCAACTATTAATCTCT
ATTTCAGTAAATCTGCAAATAATTATCAACTGATTTTATTGAGATACGGTGACGAAGAAA
ATTGTTTTGTAAATAAGATTTGTAGAGATTGTCAAGCGGTCAACAAAATTTTGGAAAATC
AATTGAAACCTTGCCAAGTTATGAAAAATAAAAAACAAACAATTATTATAGAAAATCTTT
TAGATAATATGGTTTATTCTTTTTATTTAATTCCAGTAATAAATGAAGATACGATTGGAA
CACTAATTAAATTAGGTCTATACACAGAAATGAAAAAGCCACCAGATATTATTGTGTTGC
AGAAAACAAAAGACAGTATTTCTCTGAAATTTCCAAATCTTGGTCGAAATGAATTAGACA
TTACCATAGAAATCAAAGGAAAAAATTACAAACGACAACTGCACACATTCGTAACAACAA
TTGTTGTTGGAGATTTATTACCATGTAGAGAATATTGTTTTACTTATTGGATTATAGGCA
AACCG
>DjScaffold1123:11275-33383 strand:+
ttactaaatattacagttaatatctgttaatatgttatgtcatttaaATGACGTCTAAGT
TTTCACCAAGTACAAAGATTAAATCTAATTTTGGACAAGGAGTGTTACGTGGAACTGATG
CGTTTGATTCTGAACAACTTTATAGTCTTTTAGGGTTGCTCATTTTGGAAGGCAGAATAC

CAGTTCAATCTGAAAACGGTCGAAAACCCGGACCTCATGCTCATTATGCGTTCAATAACA
ATAATTTAAAGAATCATATTTGTGATCCAAATAATGCTTGTTGTATTATGGCTCAACACT
ATCGTGATAGATTGACAGAAATAGCTCGACGGAATCAGATGGTTTGTATTGAAGTATGGT
TATTACCCGAATGTGGTCATTATGACAATTTTCTGCGAACTGCTTATGATAGTGATTCTG
TTAATGAATGTATGCAGTTGGACTGTCCTATGCAAGATAGCTTTCTTTTAATTGAACAAT
GGATCATTAAAATGGTTCCTAAAAGAAACTTTCAAGGTCCAGTGCAAACAGTAACGTCAT
TGTTGAAAACCGTTACGTCATATCTACATAGTTCTATTCTCAGTAATGAATTAATTGAAA
GTCAAGGAAGATCACCCAGAAATATTTATTACAGAATATCAGCATTGCAGAATACATTAC
AACAAATTCAAGAAAATCAATTATATCTCTACAAAAGTCTTTCATTTCCAGCTATAGCAG
CTGGTGAAACCAATATTTTAAATATTTCTGTTTGTGCTATTAATATAGCATCTAAAATGA
AATCAATAAGTTGCGATATTTGTTTATGTGATTCAAAAATGAGCTTAGATTTATCAATTG
AACAAAGGCAAAATATGAATCTCGTTAAAAATAAAACTCAAAATACAAGAGTCAAGTTAA
GTTCCTCTTCCAGCGCATTCAGTGTTTCTGGAATGAGTAAAAGAGAAAAATCAATCTCTG
AGAGTCAAGATAAAAGGCGTGGGTTTACACTACCATTAAATAGTACACGATTTAAACAGC
AGATTATTAGTCCTATGGTAGTATGTACGGATCAGTATTCGCAACCACCACCTTCATTTC
TGCAAATTTTTTCTAATAAGCAATCAATCAAAGATTCTACACCTTATCCAAAATATAAAA
CTAATCAAGATATGATTTCAAGTTTTGCACAATCTTATAAAAACGATTCTATTCAACAAA
CAGATTCATCAACTCCTGTTAATAAATCTCATCCGAAATCAGAAAAGAAAATATTATTTA
CGGATTCTAGTAGCAGTGAATCTCTTGACAAGTCAAACAATCAAACTATATTTCCGATTT
CTTCAGAAAAAGAACCTTATCGACATTGTTCGGATAATTTAGAAAACAAAATTGGTATTT
CAACTAACTCAAGATTAACACAACCATTCTCATTTAGTAATTCCTCTTTTCAAAATCAAT
CTGAAATGTTTAATAAGAAAAGTGGTCTACCTTTGCAGTCTAGTCCGGCTCCTATAAAGA
AATATTCTTCAAATAATTTTTATTCAAATATGACGTCAAGTTTAGAAATTAAAAACATTA
AAAATACAACAATGATGAGCAGATTAGCAGTAACACCAGGTGTTGAATTGGAGAAAAATA
GAACGTTGTCAATTGATGCCTCCTCTGATAGAAATCTCAGTTCTAGTGCTCCAGTCGTCA
GTTCACAGAAATTGTTAGTGAATTTCGAGGTAAACTATTCTTTATACTTTATTATTTTAA
ATATTAATATTTAAataattgaaaataatttttaaatttaatccaaaacacgttcgattg
cctttaactacatatttctttcaactttcggaagacaatgcaccatctccgtatttgggt
tttgttgatcttcagactctatgtaataaacgaggctaccaagtacctaaaaaaggctca
gttcaattgacgctttttaatccgaatggagcagtaatcaaaatgtttgtggtgctatat
aatctttctgatatgcctccaaatcatcaaacatttcttcgacaacgaactatttatatg
cctattcaacaaaattcgtctattgagcattctcattctcaaaattctgataattgttgg
agtcattggacagttggtggtaattcaatgaattcttcaagcgagatgaatattcaagtt
aatctcccatcatatttacgctaccttgttcatttaaaattgcatacaagcaaaactgga
aaactttatcttcatactgatattagacttatttttctcgtgacaaatttgaatttgac
ccaacagttgctacttatgaattaagatcatttgttgatcctccaccggaacctagatat
tctcctaaaaattgaatagaaagaattaattatatcaaaactatgttttgtgatattttc
aattgtgtatttattttatatacttattttacttttattttgcttgtttgtatatgaa
taagtgtgtatataaaagttctttagattttaacttttagatattgttctataaatacaa
aattgattgaatataatttattttacagtaatgagtgtacaatagctattgttatttgtt
ggttatccttttcaatgtttctatttttataatttgtatttcgtcaaattcgataatta
tatactctgtgattatttgtgtaaccaaattcgattttattaatcagatattatagtgtt
ttgtcttttactttgtatttatgtagttgaatcagtataaatttatctatatatgacaa
>DjScaffold1123:61226-65863 strand:+
catttgaaaaggactatttgtaatcaggaaagtATGCTCAATATGAGAATACATCCTGTT
GGCGTCATAGATGGTTTTGAAATAGAATTGGGAGCTAGTGGCAGCTTTAATCCAAAACAC
GTTCGATTGCCTTTAACTACATATTTCTTTCAACTTTCGGAAGACAATGCACCATCTCCG
TATTTGGGTTTTGTTGATCTTCAGACTCTATGTAATAAACGAGGCTACCAAGTACCTAAA
AAAGGCTCAGTTCAATTGACGCTTTTTAATCCGAATGGAGCAGTAATCAAAATGTTTGTG
GTGCTATATAATCTTTCTGATATGCCTCCAAATCATCAAACATTTCTTCGACAACGAACT
ATTTATATGCCTATTCAACAAAATTCGTCTATTGAGCATTCTCATTCTCAAAATTCTGAT
AATTGTTGGAGTCATTGGACAGTTGGTGGTAATTCAATGAATTCTTCAAGCGAGATGAAT
ATTCAAGTTAATCTCCCATCATATTTACGCTACCTTGTTCATTTAAAATTGCATACAAGC
AAAACTGGAAAACTTTATCTTCATACTGATATTAGACTTATTTTTCTCGTGACAAATTT
GAATTTGACCCAACAGTTGCTACTTATGAATTAAGATCATTTGTTGATCCTCCACCGGAA
CCTAGATATTCTCCTAAAAATTGAatagaaagaattaattatatcaaaactatgttttgt
gatattttcaattgtgtatttattttatatacttattttacttttattttgcttgttt

gtatatgaataagtgtgtatataaaagttctttagattttaa
>DjScaffold1008:32402-36131 strand:+
attggtacaattcccaactaagtgtaattgaaaatccaaatttaaaaagttattttacta
caaagacaacaaatttaaactaattgtcactatatatatatatatatatatatatatata
taatgtgtgtgtgtgtgtgtttaatatttataaatatttataaatgcattttaggatgtc
ggttctgacgcaggtatagaaatatgggatatagatgaattttatcccagaaaaataaca
gaggaagttccagctggaacattttataatggtgactgttatatcattcttcatacacgc
caggatgagaaatctcacaattctctcatacattctatttattactggatcggaaatata
gcaacacttgacaaagtcacttctgcagctgttcatgctgtttaccttcgcaacttttta
tcaactgattgtagatgtgaacgaattgaacaaggagatgaaagcgatgaattcatctct
cttttcgaaggaaatATGATGGTTTGTGAAGGCTCTCGTGGTGAAAGTGGATTCATGAAA
ACTGAAGAAAAAGTGCATCGTAAGAAAATGTATAGATTATTTGGAGCTGACAAAAGTATA
TCAATATATTCTCTTGATCCCACTCCAGAATCTTTGGATACGAAATTTGTTTATTTATAT
GATGAATCTCCAACGCGTCTCTTTTTATGGATCGGCAGTAAAAGTCGTTTGATGCTTCAA
ACAAAAGGGAGATTACTTGCGGATAATATAAATCAAACAGAACGTAAAGGACAAAGTGAA
ATCATAGTTGAACGTGAAAATTACGAAAGTGATGAATTCAAGAAATCTTTACAAAGTTCA
GATACGGATTTAGTTGATTGGAGTTCCGTGACCAAAAGTGTGGTTCCTATTGATTGGAAA
CCACCGTCACCAATTCTTTATGACGTGAAATTAGGCAAAGGCTATCTGGAAATGCCACAG
ATTGAATTAGTGAATGGCCGTCTTGTAAAATCTTTACTAGAAGCTAAACATGTTTATATC
CTCGATTCCAATGGTGACATGTTTGTGTGGATGGGAGACATGTCGCCGAAATTTCTAAAA
TTTGCTGGCCTGAAATTATCATATGAATTACTCGAATTATTACCGAGATCATCACGTCGT
AGTCCGACAGTTTGTTGTCAATCATCTGAACCACAAATATTTAAAATGCAATTTGTCGAT
TGGGTAGATATGTGTGCTGTTGACTTTACAAGATCCGTAGAATCCGTACGCAAACGAGGT
GTAGATTTAAATGAAATCGTTGAAAATAATAAAATGAAAATCGATATGAGGGCTTTCTTT
ATGCCCAGAGAAAAACCAACGCCATGGGACGAAGCGAAAGCAATTATGGAAGAATGTAAT
GATGATTTAGTTGACAATGGTGATACAACAGATTCCAAAACATTCAGATTCAAACAATTT
GTTTATATTAATCGCAAATGGGTTCCTGTTGAAGATAAATGGATTGGACACTTTTTCAGT
CAAGATTGTTACATTGTTAATGCAGAATATTGGGAAATTCCAGAAGAGGACGATCCAGAT
ATTGAAGAAGACGGAACAGAAGAAGACACTTCTCGTACTCAAACCGTAATTTACTTTTGG
CAAGGCAGAGAAGCTAGTGATATTCCATGGTTGCAATTCAATTTTTCCATTAAAAAAGAG
ATGGAAATCAAATTCAGTGGTTCAGATAATACATCAATAACGAAAGATGCTTTGAGAAAA
GTAGAAACAgagaaaaaccaacgccatgggacgaagcgaaagcaattATGGAAGAATGTA
ATGATGATTTAGTTGACAATGGTGATACAACAGATTCCAAAACATTCAGATTCAAACAAT
TTGTTTATATTAATCGCAAATGGGTTCCTGTTGAAGATAAATGGATTGGACACTTTTTCA
GTCAAGATTGTTACATTGTTAATGCAGAATATTGGGAAATTCCAGAAGAGGACGATCCAG
ATATTGAAGAAGACGGAACAGAAGAAGACACTTCTCGTACTCAAACCGTAATTTACTTTT
GGCAAGGCAGAGAAGCTAGTGATATTCCATGGTTGCAATTCAATTTTTCCATTAAAAAAG
AGATGGAAATCAAATTCAGTGGTTCAGATAATACATCAATAACGAAAGATGCTTTGAGAA
AAGTAGAAACAAAACGTTTATTTCAACAGCAAGAAATCACAACATTTCTTGCGCATTTCC
AACACAGATTTGTCATTCATCTCGGTAAATATCAAGACAGATATAGTGAACAAAGGAAGA
ATCGTGTGGAATTATATTATTTAAGGGAAAATGGTAACGCTCTTTGCACCAGAACTATTG
AGGTGATGCCAATGATTGATTTCTTAAATTCTTGTTTTCATTATATCATGAAAATCCCAG
CTTCTATGTTGAACTGTGAAAAAGATCAAGTTATTTGTTGGATTGGTAGAAATTCCGATT
CTAATTATAATGACATATTAGAATCAATAGCTTTAGATACATTTTCCGAATCAAATATTG
TAACTGTGAAAGAAGGTGAAGAACCTGAAATATTTTGGAAGGTTTTCGGTGGAAAAAAGA
ATTATGACCACGAAGCTAATTTTATGAGTTTCTCACGTTTATTCCGGTTGAGTAATGAGC
AGGGCTTTTTCTGTGCTAGTGAAAAGTGTAGTGACTTTTGTCAAGATGATTTAGCAGATG
AAGATGTTATGCTTTTAGATACCGGCACACAGATTTATCTGTGGTTTGGAAATCGAACGT
CAGATGTCGAAAAGAAATTATCGTATAAAGCAGCGAAATTGTATCACGAACATATATCAA
GAATTCAACCTGACAACACAAGACAACTAAAAATCACTCAGAAAAAGAAAGAACCACATA
ATTTCCGGCGATGTTTCCATGGATGGGGTGGATTTAAAGAAGCAAAAGATTGGTCGGGCT
GAtttttataattctcccttagtcaattttgatattattgatttttatatacatttata
ataccaccaataaataataaatggcttgttctgtttgattgtgtcatgcttattttccat
atttatttatatttgattatgatggcaattatgtttagtcagtttggagggataattcgc
taattatcctcattgtttgtgtgtgtgcaggaatcaatgttatattgctctatattgatt
atgtgtatattacaatgtttttattgattttgttaaaattattattattttctttcgtgc
aaaatttttcacctataaaataatttaaattcattcaaaatgggttttaaaaatgaattc

agtattatttttcagctttcacaaattgtttaccagcacattatcgtaaattgatatgta
attattgtgtggtataaaattaataaaatggtttgcagtcttaaagagaaaaaccaacgc
catgggacgaagcgaaagcaattATGGAAGAATGTAATGATGATTTAGTTGACAATGGTG
ATACAACAGATTCCAAAACATTCAGATTCAAACAATTTGTTTATATTAATCGCAAATGGG
TTCCTGTTGAAGATAAATGGATTGGACACTTTTTCAGTCAAGATTGTTACATTGTTAATG
CAGAATATTGGGAAATTCCAGAAGAGGACGATCCAGATATTGAAGAAGACGGAACAGAAG
AAGACACTTCTCGTACTCAAACCGTAATTTACTTTTGGCAAGGCAGAGAAGCTAGTGATA
TTCCATGGTTGCAATTCAATTTTTCCATTAAAAAAGAGATGGAAATCAAATTCAGTGGTT
CAGATAATACATCAATAACGAAAGATGCTTTGAGAAAAGTAGAAACAAAACGTTTATTTC
AACAGCAAGAAATCACAACATTTCTTGCGCATTTCCAACACAGATTTGTCATTCATCTCG
GTAAATATCAAGACAGATATAGTGAACAAAGGAAGAATCGTGTGGAATTATATTATTTAA
GGGAAAATGGTAACGCTCTTTGCACCAGAACTATTGAGGTGATGCCAATGATTGATTTCT
TAAATTCTTGTTTTCATTATATCATGAAAATCCCAGCTTCTATGTTGAACTGTGAAAAAG
ATCAAGTTATTTGTTGGATTGGTAGAAATTCCGATTCTAATTATAATGACATATTAGAAT
CAATAGCTTTAGATACATTTTCCGTAACTAATTTTTGGTCTTGAaatgaa
>DjScaffold1008:67231-68805 strand:+
tcactgaatgcatagttatataaatagctattcgttgttattggataatttatatacagt
ttaataaaaggcatgtgacgcgtgtattttaataaatacagttaaatcatttaaataaat
gaacaaagcaattaaatttttttaattaatttgaagaagaaaatttgataaaaaaagaaaat
ctcatactaatatatattttcacacaaaataactaggataaaagaacccattatttttcc
aataggattttaattatttgaaatttggaattatttgtATGGGTAATTCTTTCACTGTT
GATAATCGTACATCAAATACAATTGAAGTTGCTTATTATCTTGCCGGATCCAAAGAAAAT
TGCAAAAATGAAATTATTGAATCGTTATCAAAAAAGAAGTATTATCTTTATTCGTTACAA
GGATCAAAAACTTTAATTGTATCTATTAATAATAAAGCCTTTCGTGCTGTTCGATTAAAT
CAATCCATTTGTATTGATCAATTAGAAAATGGACCAATAATAATAAACATTGCTTCTGAC
CCATTTCATATGGAATCAGATATTGAACAAATCAAGGAAATTGTAGAAAAGAATTTAAAT
TGGAATCTCATTGCTGATAATGATGTGAATAATGGAAATAGAAAAGTTGCAAATGAAATG
ATAAAAATGATGAATAATGTGACTTATTACCTTGGAAACGTGGTAAGTAATGTTGTTCGT
AGCATTCCTACTGATAAAAAATGCTAGTAATCTATTTGGAAAGATTGTATCAGATTTCATC
GGGAATTCCATGAAAACTTCAGAAATCAAATCAAAAAGTACGAAAGATTTTAATTCATTA
GAAGATATAAAACGAATTCAATATCAACTAATTGGGATTTCTAGAGCTATCGAAATTGAT
TATTCTTATCTCAAGGAAAAAATTAAAATCGATAATTCCAGAAGTTTTAAATTTCTACAC
AATCAGTTAATGAAAATAACTAATACAGATATGTTGATCTATCTAGAAAATTGTGGTTTG
GAAGAAGGTATTCCTGTTTACATCTATGCAGTAAATTTGGCACTGCAAATATTTAATGAA
ATTCGAAAATTAAATGAAATGGAAGTTACGGTATTAGAACAGAATTATATTTCAATATTT
ACAGACAACTTTGAATATATGTGGGGCAATATTATTGATAAAATATCGAACAACTGTTCT
ATTAAAACTAAAGGAGTTCTTATTAAAACTGCTGAATTTCGATACGAAGATCAACATATT
TATAAAGTCAAGAGCGTATGCAAATCTCGAATTAAAAAACGAATAAATGATATAAAAAAT
TCATTCATTGAAAATAGAAATTATCAAATGGGATATTTATTTAATCACCCAGATCAGTTG
ATTACTAAATGGAGAGAAATATCGAAACTAAATACTTGAacattttaactatttaatccc
aaattatttaattaccgtggattttttcaataaatgtt
>DjScaffold4747:18480-19473 strand:+
ccaaaattttgatcaaagcttttatattaaatgaataattaataataataataataatcc
tgcaagttctttgattgtttcgaagaaaatttagtatgtaaaaattcatctaaagcaagt
cgttttttcaaattgtagtctcgcatttaatatatataaaatatatatttgtatatttag
cattcaaagaagagcgtcgcATGAGATCATTTATTGAAAATCATTGGAAACAAAGTCAAG
AACAAATTAAAGAACTTGAATCAAGGTTAAGATCAACAGAAGATTTGGTTAATCAGGAAC
GTTACCAAATTAGAGATTTAGTCAATCAATCAAAAAGTGTAGAAGAATTAATGTTTTCAA
CCAAAAAAGATTCCTCGCTGCTCAATGATAGACACATATCAAAGTTAACACAATTAGAAC
GCGAAATGGAAATCCTAAATGACAATCGAAATAAACTAGAGAGGATATCATTTCAGTTAG
TTGATGAAATGAAAACCATCAAAACCAAACTAGAATCACAAGGATTTGACTTTACTGCCA
TAACTCATGAATTGAGAAATAAATCGAAAAAATTAGAAGATGAGTATCAGCAAAATGTAA
GTAAAAAGAAATATTTAGTTGATTTAAGATGGTTTTTATCATAGaaataaaactatgaaa
attcatacttttaactattatttaataattttctgggaaaaatcagtatacgtataaaaa
ttttaattaattcatttaaattaaattataaaaattgttaactaatctgttgtgtaaccg
gttaacagtttatgtcgtatatatgagtaaattataaatttgctaatcatatgaaatttt
ttttattgagaatttattttataaacaattacaaatacttcaaattgtaaaaataatgat

catgacaataaaattaatattttcagctatcaagaaaataa
>DjScaffold4747:40964-41608 strand:+
ATGCCAACCATTGATAGTATAAATACTGATAAATACACAAATTGTACAATTTTTCATTCT
TCTCTAAAAGGATTTGTAACTGAAGGATTTGATAAGAAGAATTCGATTAAAGTGATATTG
AAATCATTCATCAAAATCAATAATGAAAGTTCATTTATCAAAGATGAAACAACTAATGAA
ATTATTCCAAAAGAATTATATTTCCTCAAAAAATTAACTGGTCAAGAATATATTCCAAAA
TTAATAAATTATCATGATGGTGTAAACACATCAACTGTTGTTATGGAATTTCTTGATGAT
GAACATTGGATAGATTTATTTGAATTCATTAATGAGTGTAACGATGAAACTATTATAAAG
AAAATCATCAAAAATACAATCATAACAATGTATAAATTATCTGATATTGGATATTATCAT
CAAGATATCAAACCAGAAAATATCATGGTAAATAAATCAAGTTTAGAAATCAAATTTATC
GATTTTGAAGATATGTTTTACAGTGATTGTAATAATCCATCTTGTATTGCTCCTAAATCA
GGAACAATTGGATACAAAAGTCCAGAATCATTTCTATCAAGATCGTATTATGTAAAACCA
AGTTTTGTATTCAATATTGGATGTTTGGCATATTTCTGTTTAGAA
>DjScaffold5022:16800-17192 strand:+
ATGCAATTTGGCATCGCAGCGGCACCAGGGACATTTCAAGAGATGATGACAAAAGTGGTA
GGCGGAATCAATAAAGCCATGGTTTATCTTGATGACATACTGATATTTACGAACAATAAA
GACCAACACTATGAATTCATTGAAAAAGTATTGATGGAAATAATGAAGGCTGATCTAAGA
ATTAATCCGGAAAAAGGTCAGTTTCTTCAAAGTGAAATCAAGTTTCTTGGACACATAATT
AGCAGGGATGGTGTTAGAGCTGATCCGTCTAAAATAAAAGCTATAAGAACTTTTGACAAA
CCTAACTGCGTGAAGAAGTTACGAAGTTTCTTGCGAATTTGTAATTATTACAGGAAATTT
ATAGAAAACTACGCAAGAAAATCAAAAATATTA
>DjScaffold2824:38848-43120 strand:+
AATAAGCAGTGGCAAGAAGCTCAGCAAAATCTCCAAGATATTCTCTCATATGAAGTTATT
CCTGAAAATCCGGAAAAGAGTCGAACAGCAGCTTTTCAATTATTAGCAACAATGTATATA
AAATATATTCAAATTTTTCGTAAACTAGAGCAATGTTATGATCAAATAGTTCATCCTCAA
AAACGAAGAATTGTCAAAAAAGTATTAGATGGAGTCATAGGACGTATCATCGAAATAAAA
AATGAAATGGTCAATCTAGAACTTTCAGAATATCATTATTTTGATGATATTTTATCTGAT
TTAAAGCTTACTCCACAAGACGTTGAAATGCCCATACCCAAATACTTTGTTGTTGAAAGA
AAGAAAATCCTTAATGAACGAGAGAAATTATTAGGACAAATTTTAGTTAAAACAAAATTA
GACGATCTGAAGGATAAATCTGAAATAAAAATGCATTTGGATGAAGCTATAAGAATGATT
CAAATTCACGAAAGAGCCCGACAGGGTAGACTAAGGGCTAAGTTGATGCAAGAAATCCGA
TTACAAGAAGATAAAGAAAGAAATGCTCATACTAAAAATGCTCAAACAATGGATTTCAAC
GAAGCAGCTTTAAAAATTCAGCGAATGTGGCGTGGAATCCTAGCAAGACGTTATGTTAAG
AAAATCAAAGAAGAAGAGTTTATTTGGCTCGGAATGACAACACCCTCAGTATCTAACACA
TCTTTTAAGAGTGCACAACTTCAGAGAACTCTAAAAATTGAAAGTGATCGAAGAATCAAA
CAAGCCATGTTTGAACAAGAATATAACCAGGCGCTTGTTACAATAAAGGATAAAATTCGA
GAAACAGAAGGACCTGATATGAATGAATCGATGAAAGATCAAATAAGACAATGGTTTCTA
GAATCAAGAGATATTTTAGGAAAATTTCCAGATTTTCCCGACGAAGATGAAGGTGGATCG
TTAATTATTTTTAAGGAAAAGACTCCCGATGAATTAGCTGCAGAATTAGCTTTAAGAGAA
ACTGGAGAAGAAAAAGGTAAAAAAAAGGGTAAAGGGAAAGATGATAAAGCTAAAACTAAA
GATAAAAAAGGAAAAGGAAAAAAAGATGATGACGACGAAGAAGGTTGGAAGATGGCCGAA
TCATATTTTCTTCCTTCAATTAAAGAGGGGAATCATGTCTACGAAAGTATTTGGAAAAAT
AGAAACGAAGAGAATAATTTCAGTCAAGGTCATGATTCAGAAATTATTAAAGAAGAAAAA
CGAAGAGAAGTTGAAATAGAAATCAGAATCCAAGTTGATGAACTAATGAGGCAAGAATTG
AGAAATTTGAAAATGAGCGTGGATAAAGAAAAAGCAAAAAGAATAAATAAGAAAAAAACGA
AAGGGCAAGAAAGGTAAAAAGGGCAAAAAAGGAAAGAAAGAAAAGGATTTAACACCTGAA
AGAACTATTGAATCCCTTTATGAAGAATTAGTTATTCATGGAATTATTAAAAGAGTTCCA
TCGATATCACTTTCAGAATACGAAGGTGAGTACAGTTATCTCGGAACAACTCTCCGTCAA
GCTAGTATTGAACCACAACCTTCTTTAAGTGATGTGAGACGATTAGTTACAGAATTCTGC
ATTTTACCAATGGGATCACCTGAAGTCAGAGAGAAAGCTCCATTAATAAAATCGGTTTTG
TTAACTGGTCCTCGAGGAGTTGGAAAAACTATGTTAACACATGCAGTATGCACTGAATTG
GGAGCAAATCTTTTTGATTTAACATCTTCGAATTTACAAGGGAAATATCCTGGAAAAGAT
GGTCTAAAAATGTTATTACACTTAGTGTTTAAAGTATCACGATTACTTCAACCATCAATA
TTATTGGTTGATCAATGTGAAAAAATGTTTAAAAAGAAAATTCCTAAAACAGATTTGTCG
GATCCAAAAAGGTTGAAAAAAGACTTACCTAAACTCGTAAAAGGTATAAAACCTGAAGAT
CGAATCATGCTTATTGGTATTTCATCAGCACCTTTTGATGCTGAACTCAAACCATTTTGT
TCGCTTTATCAAAAAGTTATCTTAATTCCTAGACCTGATTATGCTTCTAGAAACATAATT

TGGAGAAAACTCATTCTTCGTCATAATGGAATAATAACCAATCAACTTGATATTAGTTCA
CTTACGAAAATATCAGACGGTTATACTATGAGTGATATAAATATGGTTTCACAGCAAGTC
CTTACTGAACGTAGAATTGCACAACTTACAAAGAAACCTCTTCAAGCAAATGAGTTCATT
GCACATTTAGCTCGTATTGACCCTGTTTACACTGAAGAAGAAGAAGCTTTTAAAGACTGG
TATAAAAAGACACCTTTAGGAAAAAAAAGAATTAAGGCTCAAGAAGCAGAATTAGAAGAA
GCAGCTAACGGTGGCAAAGGAAATAAGCAGTGGCAAGAAGCTCAGCAAAATCTCCAAGAT
ATTCTCTCATATGAAGTTATTCCTGAAAATCCGGAAAAGAGTCGAACAGCAGCTTTTCAA
TTATTAGCAACAATGTATATAAAATATATTCAAATTTTTCGTAAACTAGAGCAATGTTAT
GATCAAATAGTTCATCCTCAAAAACGAAGAATTGTCAAAAAAGTATTAGATGGAGTCATA
GGACGTATCATCGAAATAAAAAATGAAATGGTCAATCTAGAACTTTCAGAATATCATTAT
TTTGATGATATTTTATCTGATTTAAAGCTTACTCCACAAGACGTTGAAATGCCCATACCC
AAATACTTTGTTGTTGAAAGAAAGAAAATCCTTAATGAACGAGAGAAATTATTAGGACAA
ATTTTAGTTAAAACAAAATTAGACGATCTGAAGGATAAATCTGAAATAAAAATGCATTTG
GATGAAGCTATAAGAATGATTCAAATTCACGAAAGAGCCCGACAGGGTAGACTAAGGGCT
AAGTTGATGCAAGAAATCCGATTACAAGAAGATAAAGAAAGAAATGCTCATACTAAAAAT
GCTCAAACAATGGATTTCAACGAAGCAGCTTTAAAAATTCAGCGAATGTGGCGTGGAATC
CTAGCAAGACGTTATGTTAAGAAAATCAAAGAAGAAGAGTTTATTTGGCTCGGAATGACA
ACACCCTCAGTATCTAACACATCTTTTAAGAGTGCACAACTTCAGAGAACTCTAAAAATT
GAAAGTGATCGAAGAATCAAACAAGCCATGTTTGAACAAGAATATAACCAGGCGCTTGTT
ACAATAAAGGATAAAATTCGAGAAACAGAAGGACCTGATATGAATGAATCGATGAAAGAT
CAAATAAGACAATGGTTTCTAGAATCAAGAGATATTTTAGGAAAATTTCCAGATTTTCCC
GACGAAGATGAAGGTGGATCGTTAATTATTTTTAAGGAAAAGACTCCCGATGAATTAGCT
GCAGAATTAGCTTTAAGAGTAAAT
>DjScaffold2752:121657-122254 strand:+
AAAGAGAAAAAGATATGTCCAGGAAACGCAGATGAATGTAAAACTGAATTAATTTCTTTA
AATGAAAACATTGCTGTTGATCATGCAACAAATAATAAAATTTCGAAATTAATTGTAAAA
TGTCCAGAGACATCCTGTGAATTTAAAGATGAATTGAGAAACATTGAGGATCATATTCGA
ATTTGTAATTATCGATATGTGTGTTGTCCATATTTGAATATTGGATGCGATTCATCTAAT
ATACGTGATGGAAATGTAATTGATCATTTACAATTAGAAATTTTAAATCACACAAAATTA
TTTATTGATTGCATTGATAATTTGAGTAATGAAATTGGAAGATTGAAAATAATTGTTGAT
AAATTAGGAGAAGAAAATAAATTGTTGAAATGTGAAATAAATGAAATAAAATTAGATGAA
GAAAGAAAACAAGTCGAAAAAAACATCGATACATTAAATAAGCAATTTCAAGATATGAAA
TCGAAATTAGAAGTAAAAAGCAAATTATCACAATCAATTACTGAAAAGAAATTCGAGAAA
TTAAAATTGGATGGA
>DjScaffold2752:122438-123552 strand:+
AAATCGGATGTAGAGAAAAAAGAAAAAGCATTTGAAGAATTACAAAGAATATGTGACGAA
AAAACTCGTAAACTTGATTTGTTAAACAATGAAATTGTTAAGATAAAAAAATGTTGATAAA
TGCATGAAAGTAGATGTAGATAAATTAAAATTGAATGAAGAGAGAAAAGAGAAAACCTTT
GAAGAATTACAAAGGAAATGTGATGAGAAAACTCGTAAACTTGATTTATTTAACGATGCA
ATTGTAAAATTAAAAAATGTTGATGAATGTCTGATAGAACATGTAGATATATTGAAATCG
GATGGAGAGAGAAAAGAAAAAGCAATTAAAGAATTACAAAGGATATGTGACGAGAAAGCT
TGTAAACTTGATTTATTGAATAAAGAAATTGTAAATTTGAAAAATATTGATGGATACATG
AGAGAAGATCTCGACATATTGAAATCAGGTGGAGAGGAAAAAGAAAAATCATTTGAAAAA
TTACAAAGGAAATGCGATGAACACACTCAGAAAATTGATTTATTGAACAAAGAAATTGAA
AAACGGAAAGATGTTGACGAATGTCTTATAGAACATGTAGACAAATTGAAATTGGATAAA
CGCAGGAAAGTAAAGCGTCAAATAAAAACTTAGaatgatacacacaagagtgtaatatag
ttttcaataatttcatagtcagtctcaaatagcatattatctgatatatatttgaAAATC
GGATGTAGAGAAAAAAGAAAAAGCATTTGAAGAATTACAAAGAATATGTGACGAAAAAAC
TCGTAAACTTGATTTGTTAAACAATGAAATTGTTAAGATAAAAAAATGTTGATAAATGCAT
GAAAGTAGATGTAGATAAATTAAAATTGAATGAAGAGAGAAAAGAGAAAACCTTTGAAGA
ATTACAAAGGAAATGTGATGAGAAAACTCGTAAACTTGATTTATTTAACGATGCAATTGT
AAAATTAAAAAATGTTGATGAATGTCTGATAGAACATGTAGATATATTGAAATCGGATGG
AGAGAGAAAAGAAGTAGGATGGTTTATTGATAGTTATGACAATACTTATTAGgtagatag
taacgtatcaaacaacatcgccctactgataaagaagtgttaaaattggttgttttgaat
tagctatagcattttaattagttgatacacttcagttgaaatgttttcagatatttaact
tcattggtcgtaaatctgtccaaataaattcaaattttgaatttatatttaagtttgtct
cgaccaaagatgatcgcacggtactatttgacccagaaataaaagttgtctaatcattat

ctaaagcactatttgtcgaaaaac
>DjScaffold1294:50439-74251 strand:+
actaccaatttcggggattatgaagatcttatagaatctgatcaatttaatctggattca
tctgggatttatgaaatattcagatttatgacattaattgtgaatgttgatgatttctat
gaagaaaatatctggatcaaatgctttcatgatttcgttactgatttcattatttatatg
ccaaaaatgttgaaagatttgaaaaccgagagtttaaggacagcgtcaaatgttgtttat
ttttcttcattgatgggattaataggacatttgcacggattacctgagcattatttgagt
gggggtgctagtggtggtgaattcacctcacgttctgcaaataatgaatttctcgaaaca
ttatcattggaattctgggctccacaggaaataatgacaactacagatattattccgcca
tctcttgttagtaatatcgctggcaacaattacaacaaaaattggactagcaagttgaat
tattcatacaaacaagcaacacttttcaaattcattcgagactgtggagatctgatcggc
catccggaattgttggtggattatctatttatgttgaagggtttgtgcagcaatgaaaga
gtcgcggtttacatttacaatctattcaatagtaatcttaccggtcatcataataaacta
tcatgggaacatttttcaagttgcatgcgtaatcatgcgaaattttacatttccaattct
cataaaaatcaaataatggatccaatggaactttctggtttattaattgtactgcaattg
attaagcaagttacaaaaatgagttcgaaagtctgtgaatcgatgattaacaatgaatcg
ttgatgttagtgcagaacattattcttttgttgtcatgcggtgtcccaaaatcattgaaa
gctgaattattcaattgtctgtcggtattttgtcagaatacactagttgctaaatctatt
tggcaagcacttgaagataataaaatcattggaaattctcaggaaataaattcttgtagt
attcaagttgaactgagtgagaacgagccaaaattagaagagttttatattaccagagct
tttctgaattttctctgtgtcattacacctcatatagtgtgcgatgtttcgtatttggac
cagtctatgatgtcttacgataaactgaattcatcgataatctcgcctcctaaccattac
acggtattggaaaatttaacatgcgttttacaagtagtaatggacaatgtgttccttaaa
catccaatgaggacctacagaaatatagaagaaaagtttgccatatgtgaaagcttcttg
aaactgtgttgtaaatgtgtagaactatttctagcacaatctggaaatacaaaaactgct
aaaaataaaacaccttggtgtcaatacgatccgggatttttcattatcaatcaattgttg
ttcgagtctccatttttaagacgataataagtattatcgaaagtggatatttacaaatc
actgaaactttaagtaatgtcacgtttggtcctaagacttacacaggagaagctatagag
ttattaggaaattcaatttaccttgcaatggatttggtatcgattgttttgaagaacgaa
ggatttttagttgaattaagttcgaaatttaatagtcaattcaaaattgaaaatttcaga
tccaatgctcgtttttcgcgacccagaaattttataccccaagaacttagtagattattg
atgacaactggtggagttcagtcatttttaccaaatcttatcaaatacattagtatcgtc
gatgaaaatgagaaaattggtcgaaaagctttgactatattcaattattgtatccaattc
gtgcagccacacagcaatctcatgttaatgctcaccaataacaatgttgttgatgttgat
attctctcgatcatggtcaactgtattgataaatatggagatattgattcatctttcgtt
gattgtgtcgaaaatcaaaaattatctaggcttctttacaatgattacgtgaatgattcc
cctagaactttggttttgaagatattaataaactgtcttcaacagccaactacaaatctt
gatcaatatttaatgggattttcttgtggaagcacttttccgttaaaagaacttcattac
aagaagctggtgttggagattttccaaaaacatgtttgcattccctattaaatctaattc
agcgactagtgtcgggaatatctgcgaatcccATGTTTAAGGATGATCAAATACCTTTAG
AATATTTACCGGAATTGACTGCAGATATGATCGATCTTAGCCTTGGATATCACATTCTCT
ATTTATTGGTCAGCAATAGTAACACTTCAATGGCGGTATTGAGATTCATTAGAAGCAATT
ACCAGTTTATTTTCAAACAAATCGACCTACTAGCTTTTAGTACTTCGAAATTGAACAATA
TTCCAGAATTGAGAGCTCTTGTGTGGAATCAAAAGTCTTGGCTTCTAAAAATAATCACCA
TTGAAATTCGTTTATGTACCGCCAATCAGCAGAGATCTTACATTTCAAAGTTATTGAGAC
TTTTATTTGGAGAAGATTTGTATTCATCAAATAGCAACACATTTCTCACATGGTTATCAG
ATGAAAATACCGTTTATCAGGGCCTTATTTATCAAATTTTTGAAGATTTTGACATCACTG
AACCTGAAGAATTTATTGATTTTTCCGATTGTCAAAGATTGAAACCGGAAGTTTTAAAAG
AGTATTTAACCGATTGCCTTGAAGAGACTTATATGGGAATACAATTAATGAACACTGAGA
AATTCAAACAGATAATATTCTCAGATATTAATATGGTTGTCGATGAAGAACAAATTCGAA
TGTTACAATACGAAGTGGACAGTGTTTGTAAACTTGTAGACAAGTATAATACAAACGTTA
TATGGCTGCACTTTGGTAAATCGTCAGCAGTCGAAGCGTGGTCGCAACTAGTCGAAGTAG
TAATGGTTTCAGAATGTAGTGGGGAAGATTGCATTGATATTGAAATTAAAAAGAAAAAAA
TCTACGAAATCTTACAACTCGTTATTGAAAAAATACTCATTTCCAGTACACCAATTTGTT
TGATTACTCCCATCAGTCGAGTTTGTTTGATATTGAATGCGGTTATTGCTGGAATGTTTG
TGTGCAATCAGAGCGAGAATAATTTCATTTTACAAGTGATGCCGGACGATGAAGTTGGAA
ATACTGAAGAAAGACGTCTTGAAACGGTAGATGCTGATAATCTGTACACGCAATTGAGAA

TGCTAATTAAATTGACAACGCGATGCATCATGGAAATCAATTTCAAATATCAGAACACAA
GAATTAAATTTTATGGTGCACTTACATATCTGTTGGAAATTGTCTTGACTATTAATACTT
TAAATCCAAATTTTTCAAGCCTTGTAAATGAATGTTATTTAGTTATTAATAATCATTCGT
TGCTGTTGGAAAAGATTTCTGAGAATTCTCTTTATTCCACCGTGTGTTGTGACTGTATCA
GTGGAAACTTTCATCTTTCCTTATCGGCATTAACTCTTCTTGATTGCATTGCCGCTCTTG
ATAAAGACAGTGGTGGTTTGGTGTCATTTTTGACTTCTCATGGATATCTTAAACATTTCA
TAGTTAGTTTAATTAGAGATTGTGAAAATATACATGCAAATATGAAACATTCTCATGATA
CAAGTTACATGCGGGATTCTAGTAATCCAAATGCTATGTATTCTTATTATTTATATTTGT
CGAGAATGTCTTTATTGTGTAAATTATCGACTCAGCACAATAGTGCAAAGGCATTGGTTC
AATGTGGTGCTCTTACGAATTTGTCAAGTTGTGAATTGTTTCGATTAAGACCTCTTATTG
GTGATTTCTCTCAAAATTTACCAAGTTCTGACGAGGAAAACATTGAGGATATTCATGTTC
AATATAGAACCATTGTTTCATTAGCAGTTCAGTTGGCTAATAATATTTTAGCAACTTTGG
GACTTGACAAGAGCGATGCTATTAGAGAAGTGCTTGTTTTCATTGGAGAATCGATTGATA
GTTTCTGTGAGCCATTCCAATCTCCAATAAATGCTTGTAATAACATCTCTATCAAAGAAA
TGATTCAAATTAGTAATCTCTTCGTTCTTCTTGTACCAGGAATATTGAAATCCAAATCTT
TATCAGAAGTAGAACATAAATATCGAACTTTGGAGAAAATTAAACGAACTGCAGTTCATG
TGATGACATTTTTGTTGGACTTATTGAATAAACCAATACACAGCGATAATGAAATGATGC
TTCATTTTCTTCAAATACAATTGATGGGAAATCTCCTTTCAATCGCTTCCGGATTTGTTG
CTCCTTGTTCTAATGAAACTAATCGAATTTTCTTTTCTGCGTCTATCGATGGTTCAATTG
GTGGAAAAAATAATTGGAATTCTGCAATCAGGAAGATATCTCAGTCAGTTCATTCTGGCA
ATAAGCCACCAATGAGTTTAATTATCATAATATTATACAAAATGCAAGAATGCTTTGAAA
ATGTCAGCAAAAAACTTTCATTTTTCCAATCATTAGAATACGATATCAATATAACAAATA
GCGATTCGAAAAAATTGTTATCAACAAGAGAAATAGAAGATAAATTGATGCGAATGTCTA
GAGACCAATTGCAACTCCTATGGAACTCTGTGTTCGAAAATGTTTCAAGCATAGAAGATA
GAATGGATTTTGTCGAAGAAAATATTGCTTACCGAAATACCGAACGAGATATCGTCAAAG
ATCTTGTTGTTGTCTACAAAAGAATTACAAATGTAATTTCCTCGTTAAAATATTCCCTAG
AAAATTGTTGCTTTTTACTTTGGAATCACGCTTCAGCTTATAAATCAACCACAATTCTAG
ACAATAGCGAACTTTTAGTGTCCGATGTCAGCCTACATCCAATTCAACGGCTTCATAAAA
TTCAACAAATTAAAATGGAACTAGGCCAACCCAAAACAACA
>DjScaffold4362:12961-18348 strand:+
aaaacatattttgttaaactaacgaaaaaattttctataaagaatttgtttatctgtaat
aatttttattcatcaaaattctgtATGCCATTTGTGATTTCAGGACCGAATGAAGTATTG
GTGGTTTCGGGATGCTGCTTAGGTAAACCCTTGATGATTATTGGTGGAAAGAAGTTTGTA
TGGTCTGGTTTTCAAACATACCAAAAATTATCTTTAAATGTTAAAACAATTGTTATCGTA
AGTCCGAAGATATATACAAAATTAGGGGTAGCTCTTACAGTTACAGGAGTAGCGCAGATA
AAAATTGAAGGTAGAAATCGACACATGTTATTGTCTGCATGTGAACAGTTTCTTGGAAAG
CAAGATTTAGAAATATCAAATATTGTCAAGGAAACATTAGAAGCACATCAAAGAGCTATA
ATGGGTATTATGACTGTTGAAGAAATTTATCAAGATCGTAAAAAATTCTCAGAAGAAGTA
TTTAGGGTAGCTTCATCGGATTTGATGGGCATGGGGATCAGTGTAGTGAGCTATACAATA
AAAGATATTTCAGATGAACAAGGATATTTAAAAGCACTTGGGCAAACACGAACTGCACAA
GTGATTAGAGATGCACGAATCGGTGAAGCCGAAGCTATACGAGATGCTGAAATAAGAAAA
GCTCGAGCACAACAAGCAAAAATGCATGCAAAATTGGCCAATGATACTGCAATAGCAAAA
GCCGATAGAGATTATAAATTACAAAAAGCGGTTTTTGATAAAGAAGTCCATTCAGCAGAA
GCTATTGCGGAAATGGCTTATCGATTACAAGAGGCCAAGACGAATCAATTGATAATGGAA
GAGACGAAAGAAATCGATGTGATAGAGAGACAATGCAAAATTCATGTGCACGAATTAGAA
GTGTTACGTGTTGTGAAAAATATGGAAGCCACAATAAAAAATGAAGCTACTGCAGAGAAG
TATCGTATGGAAAAGTTGGCTGAAGCTGAAAAGATACAAATAATTAATGAAAGCGATGCA
TTGTCGGAATCTATTAAATTGAAGGGAGAAGCTGAAGCGTTTGCAATTGAAACTGTGGGC
AAGGCGGATGCTGCTCAAATGACGATGAAAGCTGAAGCATGGAAAGAATACGAGAAAGCA
GCGAAAATCGAAATGGTTTTGTCAATGTTGCCAAATATGGCTGCTGAAATTGCTCAACCT
CTGTCATGTTGTCGGAAAATAACAATGGTTTCCAATGGAGATGGAGAGATTGGTGTCTCC
AAATTGGCAGGAGAAGTGATAAATGTTATTAAAACATTGTCTGATGCTGTCAACAAAATG
TCTCGAGAGGAAATGAAAACTGTTTGAtttgcgcgcgcacattattaaatgttaaattaa
taaatttgatataaactagtcttgtccgttaaggatgctgcttaggtaaacccttgATGA
TTATTGGTGGAAAGAAGTTTGTATGGTCTGGTTTTCAAACATACCAAAAATTATCTTTAA
ATGTTAAAACAATTGTTATCGTAAGTCCGAAGATATATACAAAATTAGGGGTAGCTCTTA
CAGTTACAGGAGTAGCGCAGATAAAAATTGAAGGTAGAAATCGACACATGTTATTGTCTG

CATGTGAACAGTTTCTTGGAAAGCAAGATTTAGAAATATCAAATATTGTCAAGGAAACAT
TAGAAGCACATCAAAGAGCTATAATGGGTATTATGACTGTTGAAGAAATTTATCAAGATC
GTAAAAAATTCTCAGAAGAAGTATTTAGGGTAGCTTCATCGGATTTGATGGGCATGGGGA
TCAGTGTAGTGAGCTATACAATAAAAGATATTTCAGATGAACAAGGATATTTAAAAGCAC
TTGGGCAAACACGAACTGCACAAGTGATTAGAGATGCACGAATCGGTGAAGCCGAAGCTA
TACGAGATGCTGAAATAAGAAAAGCTCGAGCACAACAAGCAAAAATGCATGCAAAATTGG
CCAATGATACTGCAATAGCAAAAGCCGATAGAGATTATAAATTACAAAAAGCGGTTTTTG
ATAAAGAAGTCCATTCAGCAGAAGCTATTGCGGAAATGGCTTATCGATTACAAGAGGCCA
AGACGAATCAATTGATAATGGAAGAGACGAAAGAAATCGATGTGATAGAGAGACAATGCA
AAATTCATGTGCACGAATTAGAAGTGTTACGTGTTGTGAAAAATATGGAAGCCACAATAA
AAAATGAAGCTACTGCAGAGAAGTATCGTATGGAAAAGTTGGCTGAAGCTGAAAAGATAC
AAATAATTAATGAAAGCGATGCATTGTCGGAATCTATTAAATTGAAGGGAGAAGCTGAAG
CGTTTGCAATTGAAACTGTGGGCAAGGCGGATGCTGCTCAAATGACGATGAAAGCTGAAG
CATGGAAAGAATACGAGAAAGCAGCGAAAATCGAAATGGTTTTGTCAATGTTGCCAAATA
TGGCTGCTGAAATTGCTCAACCTCTGTCATGTTGTCGGAAAATAACAATGGTTTCCAATG
GAGATGGAGAGATTGGTGTCTCCAAATTGGCAGGAGAAGTGATAAATGTTATTAAAACAT
TGTCTGATGCTGTCAACAAAATGTCTCGAGAGGAAATGAAAACTGTTTGAtttgcgcgcg
cacattattaaatgttaaattaataaatttgatataaactagtcttgtccg
>DjScaffold3355:22799-27127 strand:+
aacttATGAATACTAATTTGATATTTATCACAAATATTCCAAAGTCCCAACGACCAATTT
TATTATTGAAAGAATTAAACAATAAATGGTCCAAGATGGTTTATATAGACAATCCTTATT
CGGAACTAGTGGATGCGTTTGTTTATAACCTTACACCAAATCTCGCAGTTTTTGATAAAG
GTATTGAAAAATTGCGAAGAAAGGAAATTGACGAAACAACACCTTTACAAGTTTATTATG
TATGGTTTCCTCCACCACATGTTTTCATGTCCGCTGTGATACCAGAAGGATCAATTATGC
TACCGTACATATTGGTGGATAGTTTACCTAAAGATATAGAGGTGCATGATGTTATTGGAG
ATTTGCCGTTCAAGGCAAATGACGTTAAAGAGTGGGATATTCCACAGAAAGATGACAAAT
GGATTGTGATAAGATTACCAATAACTCACGATGACGCAACAAGAAATTATGTGTTAAACT
ATTTGCGTAATAAACGATTAGCAAAAGGAATTTTTCCCAGAGTTACTGAGATGTTTGTGT
CGGAGGAATCACGTTTATCTCGGATTATGGTTCCTAAATCATTAAAGTCATTATCGATCA
CACATCCGTATAAATGCAATCTAATTTTCACATATGATTTACCTACAAGGCTTACTGGTC
ATCATATAGTCGATGCTTTGAAAGATCTCGATTATAAGCACTGTAAAGCTTATTTAATTG
ATATTACTGAGCAGCAAAGATTAGGATTTATTTCCGCTTCTAAATCGTTCCCCGATTACA
AATTTAACGAATTAATTCAGAAACTGAAGACATTAGAACTTGATGTTCCGTCAGTTAATA
AAGTTAAGTTAAATATCAGTGAGGTTTGGTTGCAACCTGTAAACTGTGAACCTCCACAAC
CAAAAAAGTCTGAAAGTGAAAATAAAATTGAATTCGTTTTGATAAATTACATCTATTTGC
ACAATCTCAGCTCAACTGTTCAACCGGCAGATTTAAACGTAAAATCTCCGGTTCCATTTC
GAGTACATATTTATCATAGTCAAGATTCCAATACCCAAATCGGGTTTGCATCTGTACAAG
ACATTCATGAATCTAAAGTATGTAAATTGATATCGTATCTGCGATCATTGGTATTCGATG
GTTTACCCGTGCTGGTTGATGAGATGTGGATCAAAGAACAAGATTTACCAGACATTGCTA
GTCGAAAACCTGTTCATATCTCGAGGAAGACTTCTACAAAAAATGAAATAATTCCCTTCA
AAAATAATGTAGAACCATTAGACAATTTAGATGATATTTGTGGGGTAAAAGAATTTGGCA
TCAAATTAGCTGAGGAACCGCCAACATCATCGAATTTAATGAAATCCCACATGATTTTTT
TGATGAATCTCCCACCAAATTTGAATAAAAGCAAAATTATTTTCGATATGAATCTTCCTT
GTGAAGGATTGTGCTTAATGCAAACCTCAGCTGAAAAAGTAGCTTTTCTTTATCATCGTC
AAATGTCGGATATAAAATGGCTGGAGATTTTGGATAAAATCTGTTATACCAACCTCAGTG
ACAGGAGAGTTGTCGCTAAGGAAATCTATTTAATGTCCGACGAAACAATGGAATTGATAC
AGTCAAAATACCCGTCAATCTATTTGAGCAGCTATGTTTTTATTTACAATATCGGATCGG
AAAGCGAACCGAATATGGACGAATTAGCTACCCATAATTACATCATCAGACATTGTCTTA
TGATCACAGACTCAAATTCCAGGTGTGCATTCGTTCAGCTGAGAGACGATTTAACGCCAC
CTCAAATTATTGAAGTATTGAATTATATTAGAACAATAAATATCGGTGGAAAAATCGTCT
GTGCAGACCTCATGTGGCTAGCAAATATTACATTGGTGGATCGACGATTCAATAATTGTG
AAGATCCCAGAAATCTTTGGATGAAAGAACCACCAGAAAAACGATTCAAAAACGATTAAa
acttaaatgtttataccggaatacaaatcaagaaatagttgttttcgttaatgataatat
atcgttacacaatttttctattatcattattgtgaaacatttatttttgaaattttttt
acaaataataataattataatataataataataataataataatttagtggtcattgctg
tttaattgattgactgatgttttctgatttgttttatcgttgtgatatcacctactttta
ttattattattgttattattattatcgtcattgttgtcactaatattgctgtccatatgg

aaccaatattttatagttgtaattatttatataattattatattcaataaattaacttAT
GAATACTAATTTGATATTTATCACAAATATTCCAAAGTCCCAACGACCAATTTTATTATT
GAAAGAATTAAACAATAAATGGTCCAAGATGGTTTATATAGACAATCCTTATTCGGAACT
AGTGGATGCGTTTGTTTATAACCTTACACCAAATCTCGCAGTTTTTGATAAAGGTATTGA
AAAATTGCGAAGAAAGGAAATTGACGAAACAACACCTTTACAAGTTTATTATGTATGGTT
TCCTCCACCACATGTTTTCATGTCCGCTGTGATACCAGAAGGATCAATTATGCTACCGTA
CATATTGGTGGATAGTTTACCTAAAGATATAGAGGTGCATGATGTTATTGGAGATTTGCC
GTTCAAGGCAAATGACGTTAAAGAGTGGGATATTCCACAGAAAGATGACAAATGGATTGT
GATAAGATTACCAATAACTCACGATGACGCAACAAGAAATTATGTGTTAAACTATTTGCG
TAATAAACGATTAGCAAAAGGAATTTTTCCCAGAGTTACTGAGATGTTTGTGTCGGAGGA
ATCACGTTTATCTCGGATTATGGTTCCTAAATCATTAAAGTCATTATCGATCACACATCC
GTATAAATGCAATCTAATTTTCACATATGATTTACCTACAAGGCTTACTGGTCATCATAT
AGTCGATGCTTTGAAAGATCTCGATTATAAGCACTGTAAAGCTTATTTAATTGATATTAC
TGAGCAGCAAAGATTAGGATTTATTTCCGCTTCTAAATCGTTCCCCGATTACAAATTTAA
CGAATTAATTCAGAAACTGAAGACATTAGAACTTGATGTTCCGTCAGTTAATAAAGTTAA
GTTAAATATCAGTGAGGTTTGGTTGCAACCTGTAAACTGTGAACCTCCACAACCAAAAAA
GTCTGAAAGTGAAAATAAAATTGAATTCGTTTTGATAAATTACATCTATTTGCACAATCT
CAGCTCAACTGTTCAACCGGCAGATTTAAACGTAAAATCTCCGGTTCCATTTCGAGTACA
TATTTATCATAGTCAAGATTCCAATACCCAAATCGGGTTTGCATCTGTACAAGACATTCA
TGAATCTAAAGTATGTAAATTGATATCGTATCTGCGATCATTGGTATTCGATGGTTTACC
CGTGCTGGTTGATGAGATGTGGATCAAAGAACAAGATTTACCAGACATTGCTAGTCGAAA
ACCTGTTCATATCTCGAGGAAGACTTCTACAAAAAATGAAATAATTCCCTTCAAAAATAA
TGTAGAACCATTAGACAATTTAGATGATATTTGTGGGGTAAAAGAATTTGGCATCAAATT
AGCTGAGGAACCGCCAACATCATCGAATTTAATGAAATCCCACATGATTTTTTTGATGAA
TCTCCCACCAAATTTGAATAAAAGCAAAATTATTTTCGATATGAATCTTCCTTGTGAAGG
ATTGTGCTTAATGCAAACCTCAGCTGAAAAGTAAttatttcattattcatatttcaatca
gattaaaaatccggataaatttcgatgattattacatttcaaatcgattttcaataagag
aactcaaaaaatctacacctaatgattataataccgttgcattttcaaataattataaca
taaggatagagagatattttctagttaaattgtatagctttattgatatgttttggcgta
tattagattcaaaaagtcctagtgaggccagcagattgatgtcaaacaaaacaaaacaga
aaaaagatgagaaaaaaatgccaagcagggaaagatggtaccgtagaaatcaagctccat
ttctgattatatgttatcgttgacgtggtcatcaaccacctcagctaataaagtctggcc
ggaatggaatcgtccgttcttagatgatcacgctatagatttttaacactatcaataac
gaacttcttaacagcgttagttttaagacgtacatgtttgagcacaggcttattttccag
acaccacgattacattattaaataataatgaatttacgtctattaagatattttaattga
ggtctattatattttaaatttgcaaatttcacactatttcataaattttcaggtatttga
aattatatatgaagaattcatatttttagtagaattattcaaataaattatttattttgt
gaaaatatttgaaaaataatttagaaagtattaggcatattttatgaaatagctactgtt
gtaataatttctacataattgtataattgcttatttgttacagtctgaaagtgaaaataa
aattgaattcgttttgataaattacatctatttgcacaatctcagctcaactgttcaacc
ggcagatttaaacgtaaaatctccggttccatttcgagtacatatttatcatagtcaaga
ttccaatacccaaatcgggtttgcatctgtacaagacattcatgaatctaaagtatgtaa
attgatatcgtatctgcgatcattggtattcgatggtttacccgtgctggttgatgagAT
GTGGATCAAAGAACAAGATTTACCAGACATTGCTAGTCGAAAACCTGTTCATATCTCGAG
GAAGACTTCTACAAAAAATGAAATAATTCCCTTCAAAAATAATGTAGAACCATTAGACAA
TTTAGATGATATTTGTGGGGTAAAAGAATTTGGCATCAAATTAGCTGAGGAACCGCCAAC
ATCATCGAATTTAATGAAATCCCACATGATTTTTTTGATGAATCTCCCACCAAATTTGAA
TAAAAGCAAAATTATTTTCGATATGAATCTTCCTTGTGAAGGATTGTGCTTAATGCAAAC
CTCAGCTGAAAAAGTAGCTTTTCTTTATCATCGTCAAATGTCGGATATAAAATGGCTGGA
GATTTTGGATAAAATCTGTTATACCAACCTCAGTGACAGGAGAGTTGTCGCTAAGGAAAT
CTATTTAATGTCCGACGAAACAATGGAATTGATACAGTCAAAATACCCGTCAATCTATTT
GAGCAGCTATGTTTTTATTTACAATATCGGATCGGAAAGCGAACCGAATATGGACGAATT
AGCTACCCATAATTACATCATCAGACATTGTCTTATGATCACAGACTCAAATTCCAGGTG
TGCATTCGTTCAGCTGAGAGACGATTTAACGCCACCTCAAATTATTGAAGTATTGAATTA
TATTAGAACAATAAATATCGGTGGAAAAATCGTCTGTGCAGACCTCATGTGGCTAGCAAA
TATTACATTGGTGGATCGACGATTCAATAATTGTGAAGATCCCAGAAATCTTTGGATGAA
AGAACCACCAGAAAAACGATTCAAAAACGATTAAaacttaaatgtttataccggaataca

aatcaagaaatagttgttttcgttaatgataatatatcgttacacaatttttctattatc
attattgtgaaacatttatttttgaaattttttacaaataataataattataatataa
taataataataataataatttagtggtcattgctgtttaattgattgactgatgttttct
gatttgttttatcgttgtgatatcacctacttttattattattattgttattattattat
cgtcattgttgtcactaatattgctgtccatatggaaccaatattttatagttgtaatta
tttatataattattatattcaataaatt
>DjScaffold941:42965-78699 strand:+
atataatttcttgtaaatttaatttaatcgtgttattttaaatttatataatgtaaatga
tatttataaatttatgtttatcatattatcacgaaacaaattttaaacttaaaatttcaa
tttatatgtgccttattatgtaagttttcctcaatttgaacataaacggtttatttctat
attttaattgagtagtccgataaatatttctctatttaataaaagttaaaacgttgaaat
tcgaggaagatatttttacttttattggaattcatgtactttattcaaacattgtaaatg
acaatttgctggtttacattaatttttgctatacattttatttttcccatatttgcaggt
tgctatagcattatttaggccgtatgaattacatcaacgtataaaacaaatcagaagaga
ttataagagatggtgtatacaaaatgctgaacttattaaggaacagaaacgaatcaaaaa
agaaaagaagcaatccctagcgttaATGAAAGAACAAAAACGTCGTGAAAAATTAGTTAA
AAAGAAACCAATAGTTATTAAAAGAGGCTCGATTTTTACTCGAATTATGGAAGAAAAATC
TCGTCAAAGAACAGTGTCTACTGGTAGTCTCGATGGAATGATGATTACTCAAATGAATCA
ATTAGTGAATGCATCTCAAGTAGATATGAATGGATTAATATTAGCTCATACGAATATTAA
TAATGGTCAAATGGAAAGTATGACATCAATAGCTGCATCATTATTACCTCCTTATGCAAT
CGATCGTGTAATAGAAGCCACACTAAAAAAAAATCAAATCTAATAAAAATATTTCTAAAAA
GAAAGGTTCCAGTGTTTGTGAGTCTATTTCAAAAAAATCGTCTGCAAATTCGATTTCTGA
AATGTTTTCTTATAATGAAAATAATATAGAAGATATACCTATATCATTGGTAAAAGAGTA
TGTAGCGAAACAAAGAACTATCATAAAAGATGAAAATACTGATTCAAATAATTTAGAAAT
TTCTTTCAAAGTTGGATCAACAACTCTTCTAAACGAAGTATCTGAAAATCAAAAAAAATAG
TACATTGTTTGTTAGTCAATCTTCCTTCAGTTCTCAATCTAACAAACCTCAAACATTGAC
GCCTAATACAGAAATTAATTCAGATCAAGTTTCTCTTAAAATGTCTTTGGAATTACCTCA
TGATATAAAAACCTTTGTTCATGCGGAAATTATGAAAGAGCTTGATCGCCCAATTAACAG
AAAAGATTTATATTTCACTGGCTCTTTACACCAATTTCTAAATAACAGAAATACTTCTAT
TGGCAATCGCTACCTCTCACTTAATAATTTCAGTCGTAATAATCTGCCAGCTTGTCCGTA
CAGTTGTCCAACAATGAATCAGCCAAATAAATTAGAAATTGAGTATACCGATTTACCTCA
AAATAATTTTCCCACAGATAGACGTGCTAGTTGGATAACTGATGTTTTTGATTTTAGTAT
TTTACAGAGTTGTGCTTTTCAAATGTTGACTGTATCTAGCATTTCAATGATGTTAGGATT
TTTTGTTCCGTTTTTCTTTGTACCACTTCTATTGAATTATGAATCAATTCAGTGGATATC
TGGAGCATTATTTGCCTTGGGTTTGTCAAATATTATTGGAAGATTGGTTGCAACTATTTA
TATTGAAAAAGTTGGGCCTTGAaatataaacaagctaatggtgctaatttaacagtgatt
tttcttggattttccagtgtttcaatattattaatgccttatgtccgcttgattcatgtc
agtttggttgttatactatcattggtgtatggattttgtattgcaatgattgtatcgatt
agaagtatgcttgttgttgagctgtttggagtagaaaatttaacgaagacttttactttt
ctcctcatatttcaaggcttagcggcaattttaggtcctttattagcaggtgttatctgc
gattatttttcagttttaatgaataaagcagaaccagctttcttattcagtggtattttg
tttttgatatcaactttttgtgcattaattttgaaaaaaactcaaatcaaaatttgattct
gaaattaaaaatcctaaaaacaactctacatcattttcaataatgtcgggacaggattcg
aatattaataatgaatacaatccacaaactactcaaaccaccaatcttatgataccatgc
gaataaggatgtgtatatatggatcaattagattgagcttgtatagatcatattt
>DjScaffold2838:9599-15510 strand:+
gtttttttcgctgttttcaacttaggagaaagttcagcaccagaatgtagtttaccaaag
gttgttggaATGTGCATGGCATATTTTCCTAGATTTTATTTCAATGCTGAATCAGGTAAA
TGCGAATCATTTGTCTATGGTGGATGTCAAGGAAATGGAAATAATTTCGAAAGAATTTCC
GATTGTCAAGCTCGCTGTGAAAATTAAtaatcaatttgtgatttaataaattattttact
tttgaaa
>DjScaffold2838:35799-36202 strand:+
catattgatatttatggaaattgtgtataattttgaaacaaatttataaATGTCAAATGA
AATATTTGAGAATATTTGGAAAATTATGAAAGAAAATAAATTTCAATTAATAAATGAAAT
TAATAATGATTTTATTTGTAATATTTGTAATAAATTAATGGTGAAGGCTTTTAATACCCC
TTGTGGTTGCAAATATTGCTGTGAGTGTATTGAAATTTATTTAGATGGTGAGGACAAATT
TTGTCCTGGAAAAGATGACTTCTGTCAAATGAAAATGATTAATTTTGAGAAAGATGTTTT

TATTAATTATCAAACAAATAGAAGGATTGCAGAAATTGTTGTGAAATGTTCAAATACAAA
TTGTGAATTCACAAGTGAGTTGAAGTTGATAGAAAATCACATGAGAACATGTAACAAGAA
TCCAACAAGTTGTCCATATTATGAAATTGGATGTAAGAATAATGAAGTGATGAATGATGA
AATGAAAGATCATCTTTGTACAGAGATTTACTGTCACACTAAATTATTATTAGATTTTAT
CAATAATTTACGAAATGAGATGGAATCAATGAAGAATGACATTGTAGAATTGAAATTAGA
AAATAAAAATTTGAAAACAGAATCAGATCGAATAAAAATCGAATCAAAAAATAATCAAAT
GAAAAATGAAGAATTTGAAGTGAAAATCAAGAATAATTTATCAGAAGTTGAAGAATTAAA
TGAAGAAATGGTTGTAATAAAGAATGAAAATAGATCAATGATGAATAAATTAGAGAATAT
TGAGAAATCACGAGTAATATTTGAGATAAATGAGGAGGTGATTGAGAGAATGCAGAGAAA
ATCAGAACAACAAACACGAGAAATTGATGAAATGAAAGAAAAATGTGAAGAGCAGAAAGA
TAATAAAGAAATTGAAATGAGGAGAATAATTGATGAAATATTTGATAAAATTGATTATTC
AGATTTGATGAAGAAATCAAATATTAATATTAATAATTATcatattgatatttatggaaa
ttgtgtataattttgaaacaaatttataaATGTCAAATGAAATATTTGAGAATATTTGGA
AAATTATGAAAGAAAATAAATTTCAATTAATAAATGAAATTAATAATGATTTTATTTGTA
ATATTTGTAATAAATTAATGGTGAAGGCTTTTAATACCCCTTGTGGTTGCAAATATTGCT
GTGAGTGTATTGAAATTTATTTAGATGGTGAGGACAAATTTTGTCCTGGAAAAGATGACT
TCTGTCAAATGAAAATGATTAATTTTGAGAAAGATGTTTTTATTAATTATCAAACAAATA
GAAGGATTGCAGAAATTGTTGTGAAATGTTCAAATACAAATTGTGAATTCACAAGTGAGT
TGAAGTTGATAGAAAATCACATGAGAACATGTAACAAGAATCCAACAAGTTGTCCATATT
ATGAAATTGGATGTAAGAATAATGAAGTGATGAATGATGAAATGAAAGATCATCTTTGTA
CAGAGATTTACTGTCACACTAAATTATTATTAGATTTTATCAATAATTTACGAAATGAGA
TGGAATCAATGAAGAATGACATTGTAGAATTGAAATTAGAAAATAAAAATTTGAAAACAG
AATCAGATCGAATAAAAATCGAATCAAAAAATAATCAAATGAAAAATGAAGAATTTGAAG
TGAAAATCAAGAATAATTTATCAGAAGTTGAAGAATTAAATGAAGAAATGGTTGTAATAA
AGAATGAAAATAGATCAATGATGAATAAATTAGAGAATATTGAGAAATCACGAATTGAAA
TGAGGAGAATAATTGATGAAATATTTGATAAAATTGATTATTCAGATTTGATGAAGAAAT
CAAATATTAATATTAATAATTATttctgtcaaATGAAAATGATTAATTATGAGAGAGATG
TTGTTATTAATAATCTAACAAATAGAAGGATTGCAGAAATTGTTGTGAAATGTTCAAATA
CAAATTGTGAATTCACAAGTGAGTTGAAGTTGATAGAAAATCACATGAGAACATGTAACA
AGAATCCAACAAGTTGTCCATATTATGAAATTGGATGTAAGAATAATGAAGTGATGAATG
ATGAAATGAAAGATCATCTTTGTACAGAGATTTACTGTCACACTAAATTATTATTAGATT
TTATCAATAATTTACGAAATGAGATGGAATCAATGAAGAATGACATTGTAGAATTGAAAT
TAGAAAATAAAAATTTGAAAACAGAATCAGATCGAATAAAAATCGAATCAAAAAATAATC
AAGTAAATTTATATATTGATTATTTGAATTTATTTATTTTGTTGTAGatgaaaaatgaag
aatttgaagtgaaaatcaagaataatttatcagaagttgaagaattaaatgaagaaatgg
ttgtaataaagaatgaaaatagatcaatgatgaataaattagagaatattgagaaatcac
gaataaatgaggaggtgattgagagaatgcagagaaaatcagaacaacaaacacgagaaa
ttgatgaaatgaaagaaaaatgtgaagagcagaaagataataaagaaattgaaatgagga
gaataattgatgaaatatttgataaaattgattattcagatttgatgaagaaatcaaata
ttaatattaataattagaataatttatcagaagttgaagaattaaatgaagaaatggttg
taataaagaatgaaaatagatcaatgatgaataaattagagaatattgagaaatcacgag
tgagaaatgagattatttgttgttttattgattattgattataatttgtgaggtaatatt
tgagataaatgaggaggtgattgagagaATGCAGAGAAAATCAGAACAACAAACACGAGA
AATTGATGAAATGAAAGAAAAATGTGAAGAGCAGAAAGATAATAAAGAAATTGAAATGAG
GAGAATAATTGATGAAATATTTGATAAAATTGATTATTCAGATTTGATGAAGAAATCAAA
TATTAATATTAATAAT
>DjScaffold3930:2235-12109 strand:+
ATGAGTAATGACTTGGTTCCAAAACTTAATCCATATCAATCAAATATTACAAGAGAGATT
GGATCCAATTTGAAAATCGAATGCCCCATTAGTTCTGCAAGTTCGGATGGAGGCACAAGC
AGTCTAATGATTAGTTGGTCAGTAAGAGATATTGATGAAAATTTAGTAGCCGCAGATTCA
CGATATTCTTTCACAAATGGAGGTCGTACTGCTCAGGTCACAAAAGTTACACCTGCTGAT
TCCGGTGTCTACAAATGTCGTGGAGTAACAGGGTTTGGTAGTCAAGAAGCGATTTTCTAT
GTAAACATTCTCGAACACAATTCATGTACGGCGTATGATTCAAATCAAAGAAAAAGAAAA
CAAAGACCGTGTTTTATCGACCGAGATATGATGTCTAGCACCAATAAAATAAAAACAATT
CATAAAAAGGTCAATTCATCAATAGAATTTCGATGTGACGCTGAAGGTAATCCGAAACCG
AATTACAAATGGTTCAACGGGAAATCGTCAGCGGATTGGATTATGGCAGACGTTAGAAAG
CCATATTTTAAAATTGATGTTTTAAGAAAAGAACACACTGGACATTATACATGTAAAGTG

TCTAATGATATCGGTGAAATCAGCTTTACATTTGCTTTAGTTGTGAAAGGTAAGAAGAAT
TTACGTTTTTATTTTTTAAAGAAATTTTAAAATTAACAATTTTACTAAAATTACCCGCT
TTAATAGTTGAAAATAAATTTCGTTGA
>DjScaffold782:3684-4654 strand:+
tggaataaatattatgaaattaaaatttataattagaactaaacaaaatggtagcagtat
ttagaaaacgtttactgggttatacttcttgtgtcagataacttgaatgatgaatatcag
gacaaaaataacaaccccattgtagtgagtacagtatgcattaaagaacttgaatgatac
ataatagtgcgaaaataacagccgatagaagctgtacattgtactgaacagtatgcaata
gaggtttacttgtaaattctatatttatttcaatttgtaatgcatttttattaaatttt
attggatattttaggtggtcattattgttgtttattcttattttcttgtacaaattATGA
CATCACAATTTCTTGATCCAAAAAGACATACCTTTATCTATTTTCCTTTGTTGAATATTT
TTGAATTTGTCTTTATTATGGGATGGTTAAAGGTTGCAATGTGTGTTATGAATCCATTTG
GAGAAGATGACGAAGATTTTCAAACGTCAGAAATGTTGGATTATAATTTGAATTTGTCTT
ATAGGTGTGCAACAGCTCATGATGATATGTATCCAGCATATTTAAAGCATCCAAATATTG
TTTATTCACTACCATTAGCATTTCAAAGTCAAAAAGCGGATGCCACTGAAACGAAAACGT
TTTTTGATTCATTAAATGCCACACAAACCGAACTTGCTAGGGAATATGCTGCTTTTCAGT
CTGAGATATGTAGTAACAACATTAATAATCAAAAATCATCTGCAAATTGCAATAAGATTT
TCAATCTATTAAGATCATTAAAAATGTGTATACCTATTGGAAGAAAACCAGAAAGTATGT
TTTTGTAAtttacgaaaaaatgtttttattg
>DjScaffold5356:63625-66683 strand:+
ataataaagatattgattaattattaattcatttaaaataaaagcatcgatcaacatttt
tgttttttattgatattggataaaaattcgatatttcgacttagtcttcttcaggaatt
gactgaattcttcaattcttcaattcctgtagaagactaagtcgaaatatcgaattgtta
tcaaatatcaataaaaaacaaaaatttattcgaagcttttattttaaatgaattctgta
aaacgatcaatggttatttccaacaattattctcgtaataattgcaattgtaaaataatt
attaaaatgaatgcatcgtataatatgaaatatagggtaatcatgtagaagcttgagcct
tccccaaagcctataaaaatttgcgctccacaatggacgaaaatcgattaaatgggtttg
ctttaccaaccttcctatccaaaatgaatataaaatatttaattcatttattatatatta
aaaatatttaaattgaactcgactcaatattattgttgttttatttgatccccctacgcg
aaaaatttgggtgcgcgtctgtgtagaagttaaccgaaatattacgtaatcatgcatgta
tttaatgtgcttgtaggatttatggttaaaagatacaaaagggtgaattcaaagtgtaaa
atcagtattattaatgtttatttattgtgatgtattgaaaaaaaaattcgatatttgtaaa
aattctcaaatcaaataataattattaaatcgatttggaaagtaaaaataaatatttatg
taaatctcaaattaaatcatcgattagaacccacaacccacaattagtgaagacagcgat
aattggagaactaaaaaacctcaaccagtgtctcaaccgtctccggataattatccgccg
agaaatgacagaccgaattggcgagataataaaccatctacaattcgcacagatgataat
aataaagccttatcagagaattctgaaaattggcgaaagggaacgagtagagacacaaca
aagagtggtaattttaatagacgaaacgaaactATGCAAGATAGTGGGAGTACTAAACCC
ATGTCGTATGCTGAGCGATCAGAACCGAAAGATCTGATTAAAGAAACGAAAAAAGTATCG
ATATTTGGAGATGCAAAACCGGTTGATACTCAATCTAAAGATTTGGAAATTGAAGAGAAA
ATTGCACAACAAAAGAAGCAAGTCGATGAAACTAGCAAAACTGATAATTTTTCACCGACT
TTTGCTCGGAATCCAAAGAAACCCGCAAATACCACCACGCAGAATTCTACAACAACAACA
ACAGTAACAACAGTAACAACAACTATTGATAAAGTAGAAAAATCCGACAGCCGAACGAAA
GTGATTGCGAATCCAACAAAACCCCAGTCGAATGTTAGTATTTTACAACGCAATGAACAA
CCCCTCGTGAGACCGTCGGAGTCCGCTAAAGCAGAACAACCACCACCAGCATCAGCAGCC
GCCACCGAACCTGTTAAAGTAGAACCACAACCTGCTGCGCCACCCACGGTAAATGTTTGG
AATGAAAGATTGAAAGCTAAAGGAATTTCTCCTCCGACTCCAATTAATCGTAACAATAGC
GCATCGAAACCCGGATTGGATAAATCTAGTTTGCTACAAAAATCACCGTCAACTGAGAAA
CTCGACCCGAACCCGACTGAGCATCAGGTTGTTGCCAATAGCAACACTGTTCCTCAATCG
AATGCGGCGGCGGCGACAGTAACGGCGCCAGCGGCGGTTACCACCGAATCCAAAGTAGCC
CAGAAAACACCGGTTGTGATTTCTGTGAACGGCCCATTCCTCGATCCGAAGTCTCGCACG
CCTCATCATAGCATGTCTGACAATTCATCCAATAAAACCCCAACAGCAGTTCCAATCAAC
AAGTCTTCTCCATCTGACAATATCGATTCTCGATCTGTATCAAAGCCTTTATCTTCCGCA
ATATCTTCGTCATCTTCGTTGGAACCGCAACAATCGAAACCAACAAATAGGCCTGTAAAT
GTATGTAGTCAACAAAGTTCATGGGCTAAAGGTCCCCCGAAAGGTTTAATAGAAAAGACC
GAATTCAATCAATAAtattgattgttctgatttcagaatcttacaaattaattgtatcat
cgatattattctgtgaattatcgcaggaatcaactaccaccgtttttcttcggatttctt

ctgttggatgatgatgtcttttattattgataataaaatagttgaattatcgtaatttga
ttggctgattataatttattatatatttatcgattgtgtgtgtatgagtgattcatattt
tccgtattgtgtttttaccgattaaaacttaaatgcaaacttgttgagagtgtgaattgt
tgaactggccaatcaaatttgtgagtttgtttaaatttctaccagttttgatgacgtttt
tttttggttttggctcagtattttatgaagattatttcatgatttggttttttttcggata
tttgtaaagtctatttatacatatttcgggttttgtgtttgcaaatttattttataactt
aacattctaaaatcggttttaatatttttattgttatcaaatcagttgcatattattgat
gtgtttgataacacttgaaataactattacttaactcacatggttcaaatgatttatatg
gtataaataaatccggcatatttttgttaaataaaacattttaaattaggaccttgattg
tatttcaatattgaacgattcatttatcaaaactaaaccatattgaatgagaatttcctt
gttggttactattttgccgatctgttgagttatgaatatcgggtttaataaggttctatc
agaagtaataatcaaatgcaatatgatcacttttattgatttagtatcaaatgatttaaa
ttgtttttaaaattgta
>DjScaffold5356:78550-79160 strand:+
ATGCCGTTCGGATTAGTTACAGCGCCAGCTAAATTCCAAAAATTGGTGCATGATATACTA
AAAGTTGTGATTTTCAAAGGAGTTATTGTATATTTAGACTATATATTGGTTCATGTGAGA
TTGATGGAGTACCATATTGACATGTTAGAGACAGTTTTTTCAGGATTNAGAAGACTCGGG
TTTCAGAATAAACCCAGAAAATGTAAATTTTATCAAAAGGAATTGGTGTTCTTAGGACAT
ACGGTGAGTGCAGCTGATATAAGAACGAATGAAGCTGGGATTCATGAAATAAAACAAGCC
ATAGTTACAAAACGTTTATCAAAACTTCGTCCGTTCCTCTTGTTAATCAATTATTACAGA
CGGTTTATAAAGGACTATACTAGATTTGCAGCTTCTTTATACAAAGCAATTTCAAGATAG
>DjScaffold2390:66718-67233 strand:+
ttttgaatatgaaatttatgctatttaataatattttttatgaaaatttattctctttt
atagtcattaacataaaatgcagaaagtttgaggacttctatttggaaggaaaatgaatt
attttaagtttaaaaaacaaaataaaatttaaaaataaaaattcataattaaaataaacc
aataaaattttgataaaatctaaagaattgagtaacaaagatctattgctttgaatcaat
caagaagtcatcATGTTCCTTAAGAAGACTATTGTAGAAATTTTGAATTTCTTTACAAAT
ACATTTTATTTTTATCGAAGCTTTTTCTTTATTTTAATGAAATCTCAAATGAGATCAATG
GTTAAGTCCAACAATTATTCTCACAATAATTGTAATTATATAACACAGATAAAAAACAAT
ACAACTAAAAAAATTAATAAATTAAACAAACTTAAGAATATAAATATTTTCAAATAAagt
tgaaatattataaa
>DjScaffold1553:53364-58937 strand:+
aatatgtaaaatcttacgacagacagaaaattaacgatttcataatatatatttctaaaa
ttatggaaatcaatgatataaatagtattatggatttaccaaattttattatttcattta
atcttttattgtttaaaaaacgtactagagcaaataatataataaaatagaattaataaa
tatttgaaaatagaaaaatttctaaaaaataaaatgatttttatcaaattctattttgta
gaatgaaagagcggaaATGTTAATTGGTCTTGAAGATTTCAAAATTTGTCCAATGGAGAG
TTTCAAATCAAAAAATTTTTATTTTAAAATTTATAATAGCAAAGTAAATTTCACATTTGC
TATTGAATCTCAAACTGGATTCATGAAATGGTTAAATAAATTACAAATGAGCGCCTCAAA
TATTGACCTTGAAAATATAGAAGGACAAATTGCCGGTTTCACTCCAGGTTATACGAGCAT
AATAAAACAGTTTTTACCAGTATCAAAACCATTAGAAACATTGCAAGAAACCGATTCTAA
CGAATCCTTAGGTTACAGTGAAAGCGAGGATAGTGATGTACAAAGTGATAAAGTTTCTTG
TGACACATCATCTGACAATAGAAGTGTAAAAGATGACATTAAAATGAAATCTGCTGAGCC
AATTAATAACCTCAATAGAACATCAGAAGACCTAAGTGAAATGACAATGAATGCTATTAA
ACGAGTCATTGAAATTGAAAGGCAGATGAGAGAAAAACGGCAGTTACTCATACAAATTGA
AACATTTCTGAAATATCCAGTGAATTCATACAGAATTCAAGAATTTTGGGATGATTATGG
GGACCAAATAACCAAACTCTAGaac
>DjScaffold2608:2269-3288 strand:+
aacatttaactgaataacatttcttttctgaaaaatgaagttttttatatataaaaagttt
tgttgaaaatcatttatgtaatcttcaattgtactggtatattcaATGATATTTTCTAGT
AAAAGTGACGAATATGAGTTTGATAACGTTAAATTCAATGGAGATAAATTTATTGAATTT
ATTAACGATGCTAATGAATTTATTCATCATATATTTGCTGGTTTCGACAACAAGTCTAAA
CAGTGGTTCAGATTGGATAAAGATATATGTAAAGTTCAATCGATGAAGAATGTTAGAATC
CTACCGAATGTTCTATTTACTAATGAATCAACATTGCTTGTTGTGCTGGAATTTCTCTAT
TCAGACAAGAGATATTTTTGGTTGTTTATCGTCACATTAATTCAGGAATACGAACGAACT
TATATCGTCGAATTCTTTCCCAATCTCATTGTTCTCTATCAGCTGCTAATCAATAAACCG
AGTGAACAGAACGCAGTGAAATTTCTCGTCAAATTATTCACGAGAAAGCAAGGTTGGATC

CAAATCTTATTGAAAAGTAAAATCACCGAACGAGTGACAGAATCTGTCGAGTTATATTTG
CAAAATGACGATAAGAAAAACATTCTCAAAGAACTCGTCAAACAAATGTCGAACAATCGT
GATTTCATAATGAAATACAATCGATATTTGCCGGTGTTGTCGGCGCTGACAATCGATAAT
TTCCAAATCGGCAGCACCAACGAGATCTATAATTGGGCAGATGTTGCGGATTTATCGATT
TCTGATGCGGAACAAGGCGGATCCGATCGTCGAGTTACTCTCGATATCGTGATAAATGAG
GATATTTTCTCTTCTCCAACTGTGCTCAGTTTGCCGATGATCGATCGAACTGAGGCGGAA
TCGATCGAGTTTCTCCTTTCTGCCCAGCTCGATCTCGTTCGTTCTCATCATTGTCGATCG
ATAAAACAACAA
>DjScaffold2332:22103-63693 strand:+
tattataatttctaggtattttaaaatATGGCATATATGAGCAATACGAAATCTATTGAA
GATTTAACAAAGAGATCTATTACTAAAAAGCCAGATGAAAATTATAATTCAATGAAAAAG
AAAAATTCCACAATGAGTTTTGTAGAGAGACGGAAAAATGTTTCTGGTAAATCACCACCC
AATCCATTTATGTCCCGTATTAATATATCATCATTCTTAGATCTTATGATGTCAGATAAA
AATGATCAATCATTTCCAAAAAGTCGAAAGAAAAACGAATCACTCTCTTTGTTTCTTACT
CAAGCCAAATCCACAAAGGATAATGTTCAATCTATGAACAATATTGTCGTATCGGATCAG
AATACTGGGTTAATAACTGAAAATCTTGATTCAACTCTAAATGAAAAGGTTTGCCCGGTT
TGCGCAAATGAATTGCCTGGAATGCTTTACAATTATACAAGCTGTGCTCATTACTCATGT
ATAGACTGTTGGCAGTGTTATGTGATCAGTCGAATAGAAAATTATGAAATTGGATTTGGA
TTTACTTGTTTAACATGTAGTAGTGAAATTCCGTATCACAATATTGTCGAAATTATCAAG
CAAGCAACAGATGCGGAATATTCCGCCAAACTCATCCACAGATTAGAAAGTTTTTATCTA
GAAAGAGCACTTGTTCAGGATATCAATTTTAGACGTTGTCCTAATCGAGGATGCGAGTAT
GGTATTTCTTTAAATCCTGAATTTTCCGGTTGTCCGAAATTAGCCAAATGTTCTTTGAGG
CCTAATTGCTCTAGTCAACTGTGCGGTGGTCAATGTGCACTATTTTGGAAATCAATAGCA
CTTCATGACTGTAAATATCAGAACCGAGAACCTTTAGATCCCCAATCAGATTTTCTCAAT
TCCCCAAACAAAACTTTACATCCATCAAAATCAGACAATAGTGATAAATTAAGTACTCAG
AATAATGTTCCTTCATCAATAAATGAGCCAAAAAAACCTGGAAATTCAAGAAGTTTGCTT
ACTCGATGTTTTCCGTGGAGACGTACAGGACAACATGAAGAATCAAAATTTCGAAAGTTA
AGAAATGATAAAAATAAAACCGAAACTGAAAAATCCCCATCAAAAGTGCCAATGGCTTCT
CATACAGATATCGATGATATAACGTCAATGGGATCGATAAAAACAAATTCAAGTCGAACT
AAATTCAATTATTCTACTGAACCACTACAAACAGAAGGGGAAATAAAAATGTGTCCGAAA
TGTCACACTTATGTGTTAAAAATGAATGACGGAAGTTGCAATCATATGGTTTGTGCAGTC
TGTGGTTGTGAATTTTGCTGGCTTTGCATGCGGCAAATAACAGATATGCATTATTTGAGT
CCTTCAGGGTGTACTTTTTGGGGTAGGCGACAGTGGAATGTCGAAAAACGTGCTCTTTGG
CAAATGGGAACATTAATAGGTGCTCCACTTGCTATTGGATTTTTAGCTGCTTTAGCTATA
CCTGCAATGCTTATTGGAATTCCGCTTTATGTTGGTCGCAAGATCCATGGTGCTTTAAAA
ACAAAAAACAAACTTCGCAAAAGAACTATAGCAGTTGCTGGAGGTGTTGGTGCCTTCATA
GTTTCTCCTGTTATTGCTGCTGTGGCAGTTGGATTAGGTGTTCCATTGATTCTGGCATAT
GTCTATGTAGTGATGCCTATCGCACTATGCAGAGCTTCAGCATGTAGTTGTGATGAAAAT
AATCCTCCTCAAGAAGAATTGGACTTCTCTGATGTTGAAAGTCCTTTTACACAACGAATT
TATGATCCAAATTTGGAATCTCCTGTGAAAGGAGATGCTGATTTGTCTATTGCTATACAT
GAAACAGCTAAAGGTAATAAACCCTCAGAATCACCCAATATTTCAATTGACAATGTCCAC
CGGCCTGTAGTTGCAAAATTCAAATTTGATGTACGATCAAGAAATTCAATAGCAACAACA
TCTAATAAAAATAATAAGTACAAATCCAATTTAATAGAGTCATTTTCTGTGGAAAATCTC
CGGTCTACACAAATCGATTCAGGTATTTATAAAACCCGGTACAGTGATCCGTTAAGTAGA
TCGACTGGTGGAACTGAAAGTGTGCATTCGTTTCAATAGgaaaaataattgacaaaacac
agccatcatgataaactcagaactcttgccttagttttgagcgagattacgctttctaaa
ttatagataaatatataaatattatatgcagagatcttgaataataataataataataat
aataataaatcattctcgcgtaatattttctgtaaaaatgcgttttgtcataataatgtt
caatttataaagttataaaaagacacatttgtagatttactattgctagtttccttttaa
aattggtaaattttgatgtcgatgaaaaccagtgttcagttggaattgatggatttcgga
ttttatgaaagttataatgatttttattgtttcagagatttgttattttcgtttatgaat
aatttgtggttctttcaatacatatacatattttggaattcaatatattatatattatat
tatattatatatattcagttcacgctgtgaatacgatggagatgtcgtatgtaaatgtta
catttgatgcttgtatttcaacagaataaatt
>DjScaffold4766:13-5816 strand:+
atcaatttctttttaaaaattcattcaaaaatgaactaattttacataaaattataaaat
taattttttttgaactaaaaattttaaaagtttctttcaaattcgtattaggacaacta

attaatatatttaattaatattattaatattatatttaattaatattatttcgttatgat
atttgttaaaatttcagttaatattagcctaaaaagaaaatattacatttttaatataaa
tttagtttactaatttttagtttcaaaattttttttttaaatctatatttcgcaatattg
ttttttatttgtgtcttttaggaactttgtctgctttgaaatcaATGAATTCTCAAGATC
TAGTTGACAGTATTTGTTGCGGAGACGATCCAAATAGTTTACCTAAACCAGATCCATTTT
TTTCAAAAATACTTTCTAACAATCTCAAAGTTCCTATTTCATCGATGGCAATGGTTGGTG
ATACTAGGACGGATATGGAATTCGGCAAAAATTCTGGAATGTCTGGTAGAATTGGAGTAC
TCACCGGAATTGGGGATAGTGAAATCCTTCAAAATGCGGGTGCGACGGATATAATTCGAT
CAGTTGAAGACATTTTACCACTTCTATTGCCCAGTTGTCCAATAAAATTGTCGAAGGATA
TTGAACTGATTACTTCACAAAACTGGAAACTTGTAATTATGGATAAAGATGGAACTTTAA
CTGATGTTTATTCAAAATATAAAAAATGGGCTTTG
>DjScaffold4766:17729-30690 strand:+
GTTGTTGCTGCAGAAGATTCTGATCCAAATTTTAAACCTTTGCCACATTCGATTTTGAAA
ATTTGTCACGAACTTCAAGTTTCTCCCAAAGAAACTTTAGTAATTGGCGATAGTCAAACT
GATATGCTTTGTGGCAGATCGGCTAAAGTTGGTGCATCTATTGGTGTATTATCAGGACTT
GGGAAATCTCAAAATTTAATTGGCCTTTCTCAGTTGTTAATTCCTAATATTAATTATTTG
CCACAGTTATTACATTTACGGAAATTTCATACTTCTAATTCAAAATATTCCCAAGAACGA
ACCGGCAACAAACAATCGTTCGATTACATCATAATTGGAGCAGGTTCAGCTGGTTGTGTT
TTAGCAAATAGGCTGTCTGCAAATAATAAACACACTGTGAATTTAATAGAAGCTGGACCA
AATGACAACACGTGGACTATAAAAATGCCAGCTGCCTTAACTTATAATTTATGCCATAAC
AAATACAACTGGTATTATACAACATCACCTCAAAAACACATGAATAATCGTCAAATGTAT
TGGCCAAGAGGTCGTGTTCTTGGCGGTAGTTCTTCATTGAATGCAATGGTCTATGTAAGA
GGTCACCCATTAGATTATGACAGATGGGAAACGGAAGGCGCTACTGGATGGTCGTATGCT
TCTTGTTTACCATATTTTAAAAAAGCTCAAACACATGAACTCGGAGAGAATGCTTATAGA
GGGGGTAATGGACCTCTTCATGTTAGTCGAAGTAAAGTGAATGGTATACTTCATCAAACA
TGGATAAAAGCTGGAGTCCAAGCTGGCTACGGTTTTACTGATGATATGAATGGTTATAAA
CAGGAAGGGTTTGGTGAGTTCGATCAAACAATCCACAATGGAGAAAGGTGGTCATCGTCT
TATGCATTTTTAAAACCAGCAATGAAAAATAGAAATTTAACAGTGTCAACCGAAACAATG
GTTACGAAGATTTTGTTTGATGGTAAAAAAGCAATTGGAGTTGAAATATTAAATAAAAGA
AAAGAAATAGAA
>DjScaffold4766:30825-36960 strand:+
TTGAAAAAGCATTCTATTGATGTTGTACAAGACATTCCTGGTGTTGGACAGAATTTACAA
GATCATCTAGAATTATATGTTCAATATAAATCGAAAAAACCAGTCACATTATATTCGGCT
ACTTGGAAATATCCACATAATATGATACGCATTGGCCTGCAATGGTTCTTATTCAGATCT
GGACTAGGAGCTACTAGTCATTTAGATTCAGGAGGATTCATTAGATCTGAACCGAGTATT
CCTCATCCAGACATACAATTTCATTTTTTACCTTCAATTGTTAAAGACCACGGTCGTATA
TTAGCAAATTGTCATGCATTTCAAGCTCATGTTGGACCAATGAGATCGCAATCTAAAGGC
ACCATAAAATTGCAATCAAAAGACCCTAGACAGAATCCAATTATCGATCCGAATTATAAT
GCCCTTGAACGTGATTTCTGGGAATTTCGACAATCAATTAGATTGACAAGAGAAATCTTT
GCACAGCCTGCATTTGATGAATTTCGTGGAGAAGAAATTCTTCCTGGTGTTAATTGTCAA
ACTGATTTACAATTAGATAACTTTATTAAAAAAGAGGCCGATTCCGCTTATCATCCATCA
TGCACTTGCAAAATGGGATCAACTACGAGTAATGAAACAGTAGTAGACCCAAAATGTCAA
GTGGTAGGATTGGAAAATCTACGAGTGGTAGATGCATCAATAATGCCAAGCATCATCAGC
GGTAATCTCAATGCTCCAACAATAATGATTGCAGAAAAGGCTGCGGATATAATATTAGGA
AATACTCCTTTGTCAAGTTTGGAAAACGTACCAATTTGGATTCCTAGACATTCGCACAGA
CAAAGAGAAAATGATTAAtaataaaatatttatttttttatatttcgattatttaaaaa
atttgtatatttcatcatcataaaaatgatgtttgcagcataataattaattttgattaa
ttcgtaaaatgctttgcataatttacaataaacattattggct
>DjScaffold1284:30255-34330 strand:+
aatgacgattaaccctacaagtgtaaagtccgaacggtcgttttcaactgcaggtttgat
gtattttattattatatttattattatttattaagtaatttactatttattaataaaatg
caaaatagttcagatgaacttactatgatataaattgattccgacagtgtcaaaaatact
ataattgaacaaggcatttgattcaattacgtgaattgggagttatagaaaaaaaagaat
tgaaagtagaaaaaacggacctcggtgagctgggcaggacaatttcgaccgtcttagttg
tgattatcatcacaaATGGACAATCTTACACCAAATATTGTTTCTAGTGTTTTGAAAAAT
TTCAGTTTTGTTGAAGAAATCTCCAACAAAGATATTATTTGCACCTTTTGTAAAAAATCTT
TTAAATAATGCTTACAGTGCACCTTGTGGATGTAGGTATTGCTATAAGTGTATATCAAAT

TATTTAGGTGACAATAAAAAACTTTGTCCGGGCAATACTGAAGATTGTAAATCAAAATTA
ATTGGATTGAAAGATAATGTTTTCGTTGATAATTGTATGAATAATAAAATATCAAGATTA
ATTGTGAAATGTCCAGAGAAATCGTGTGATTTTAAAGATGAGTTCAGAAATATCAAAAAT
CATTTTCGAATATGTAATGCTACAAATAGTGATCGGATATTACAATGTCCATATTCAAGG
ATTGGCTGCAATATTTCAGAAATCATTGATACTAATCTCAATGAACATTTACAAAACGCA
ACCGGTACTCATGCGACATTATTAATGGATTTTATTGATAATATAAATAATGAAACTAAT
ACACTAAAAGAAATTATCAGTAAATTAAAGAAGGAAAATGACAGTTTGAATAAAAATATT
TTAGAAAGAAATTTAATCGTTGAAAAATATCAAATAGAATTGAATAGAATAAATCATGAT
ATGGAATTATTGAAAATTTCGAAATCAGATGAAAATATAAAAGAAATAAGAATTCAAGAA
TTAGAAAATAAATACGAACAGCTAAATAAACAGTTTTCGGAAATCAATAATAATTATCAA
CAAATTAGAACAAATGTTCTCACAACGAAACAAGAAATAGAAAAAGAAAGAAATTTGATG
AGAAATATTGATGAAAAAGAAAAGGAAAACGACAAAACTATGCAGAATATGCAGTTGAAA
TTATTGGAAATAGAAGCTCTACAAAGTAAACTAAATTATGGAGAATTTGTTTGGAGAATT
GATGAATTCAATAATAAATTAAAAGATGCAAAAAATGGAATCATTACAAAACTTTCTAGT
AATACATTTTATTCACATAAACACGGTTATAAAATGTGTCTGAAAATTTATCCAAACGGA
TGGGCAGGGGGTAAAGGTACCCATCTATCGATTTATTTCTGTTTAATGAAAGGAGAGTAT
GATGATATTTTAGAATGGCCATTTAAATTTCTTGTTACATGTTCATTGATCAATCAACAA
ACTCGGTTTGAATACAACACTGGCAAATTTGAATGCTATAATTTATCGAACTGTATTGAT
TTGAATAAACCAAATTCAATGAAGAATAACGGATTAGGGTTTCCAAATTTTATAAAATTG
CAGGATTTACTAAATAATAAAGATCTTATATTCAATGATCAAATATTTGTTAAATGTACG
GTAACAGTTTTATCATGAttctatttattttcataaaatccgacttgtctaaattatttt
tattttcacttttgttattgatatttctatcaatcttg
>DjScaffold3506:43467-44643 strand:+
aacaaaatttccttttatcttaaattggaaaatatctataaaattaatcaaattaaaaaa
tggtgatgttttgataaatttagaaaaatgtttgtaatacagaattcatatttcctttt
tctgaaaatttgtgaatttaagttgtaccgaaaatttttattaaagacaaaaaccgttg
gttttaaaaaaagtcaattttgggaatattcactatacattgtaatttacggtttttaat
ttcgagattgcagcgatgtgtacataaacgattttgatatatataaaatggaaactggat
aatatttacgtaattcaccttgatgaatttaaataaactttcttatcaaaaatattgata
tggctaaatttgttaaaattctattatcaaaggtctcattccactcgaatatcatttgac
tcctagaATGAAAGGCTTGGAATTAGTTCTGGAAAATCTTGGAGATAAATTAGATGCATT
TTATAATATAACAATATTGTTTGAGGACAAAATCAATTACGATAAAGGCAAATCATGGAA
AAGATGTCTTCCGGGAATGTTTGAGTGGTATCGCAAAACGAGAGTGATTCACATTAACAT
TGAACGACTTAGTTTATCAGAAATACCAAGATCTGGAGAACCTCTGCGTAATTGGTTACA
CAATATATTTCTTGAGAAAGATGAATTAATGAGAAAAATCTTAACAAATGAAATAAGTTC
TACCGATTCGAATTGGCCATTGAAAAATGTTTATAGAAAAAATATAAGATTACTTGAAAC
ACTTCCGTGCTTTCTCTTTTTATTGGGTGTTTCTACTTTGTTCATGTTTACTTATTGGGG
TCGTTTATTGTACTTCGGAATAACTGTTGGATGTGGGATTTTTGGAATAGTTTATGCTTT
CCTTTACATTTAAtgaggttcaaattatatattattatactatctactactaaattatcg
atgtgtgtgtgtgtgttattgtgtgtgtgcgtgcgtgctgttcattgaagattgaatttg
atttcatatatatatatatttgtttgttgcttcattatataattgtgttatttttttttt
accaaata
>DjScaffold5226:94457-95472 strand:+
CATGAATCTTATCACGACGGATTCAGAAAACATGGTCCACCAATTACAAGTTTACGAAGT
AGATCCCCAAAGAATTCCTATAACAAAAGTTTTGCAATTACTAATGACACTAGTTTATAT
GGCAATAGTGCCTTTAATCAAGGGAAATTAAATCAATCTATTAGACAACCGAAGCCATAT
GAAAATTACAACAAAAAGAATCTTAATATTAGCATTAAAGAAAGTGATGCTTACACTTCA
TTTTTTACGAAAAGTGAAGCGATTTCTGAAACCGATAACGAATTTGAGAATTGGGTGACG
ATTTTCGGTTATGCACCGAGTGAAGCTAGATTTGTGTTGACTTACTTTGCAGAAATTGGA
AATATTATCGATCAAAAAATCTCCAACAGCGGCAACTGGATGCATATTCAATATCAAAAC
AAGATGCATGCCATTGCAGCGATAAATAAAAATGGTCGAATCATCAATTTGAATGTTATG
ATTGGTGTAAAACCTTGTGATGATAAAATTGTTGATGTACCTCCGAGCAATCGTTTCAAG
GATTTATCGATTTTGAATCGTACGGAAAATATGAATGCCAGTCGTATTATAGAAGAACCA
GCAAAGGTTAATGAATTCAACGCGTTACCAATGGAACCTGTTAAACTGAATGGCAGTCTC
ATGAATTTATCGATGCGATCACTGAACCACCAAAACAACAACATCAATAATCATTTTACT
CCGGAAAAAGTTGAAAATCGTCCTAAAGGTGGCTATTTGAATAGAGCAATGTCGATGGTG
TTTGGTCGACGATAGacgcga

>DjScaffold3861:8806-9553 strand:+
tatatattaatacattttagttaaatATGCAAATGAATATTGTTAATCGAAATAATGTCA
GTGTACTTTTAAATGTTTGTCGTAACTTAAAGAAAAAATCTGCATTAAAACCTACTTTTC
TGAAATACGGAGAAAAACTTGATGAATCAGATTCTCGATTATATTTTGATGCTGATCCCC
CAGAAAAAGTTAATAACAATTTAAGTATCAATCACCCCTTAATTACTAAGTTTATTGGGC
AAATGTTTCGGAAAGGGAAACGAGATATTGTTCGAACTGTTATGCAAAATACTGCAGCAG
AAATAAAATTTGTTCAATTAAATAAATACAAAAAAGCACAAACCGATAATGAAAAACGTG
AAATCGAAATGGATTATAGAATGATAATTGTAAAAGCTATAGAGAATTGTGCTCCGAAAA
TGTACATCACAGAGATTCCAAGAAGTGGAGTCATTTATTCAGTTCCTATTAGCACTTCAA
CGAAAACCGATTCACAATTACGAGGAGTCAATTTGTTGTTGGAAGTAGTCAGAAGCAAAG
ATAAACACGTTCGAATGTGGATCGTTTTAGCTAATGAATTGCTAAATGCTTACAATGGAG
AAGGAGTTACTATAAGAAAGAAGAAAGAAATTTATAAACTTTGTGAAGAAAATCGA
>DjScaffold3861:9715-26661 strand:+
atttttagattgATGCTTGTGATTTCCCGACGATTTTCCAAATCTCTCCGTAACGTCGGA
ATCATCGCGCATATTGATGCTGGAAAGACTACAACAACAGAACGGATGTTATTCAATTGC
GGCAAAATAAAGGTAGTTGGGGAAGTTGATAGTGGAACTACCATGACCGATTTTCTTGAT
CAAGAACGTGAACGAGGTATTAGTATTACTAGTGCTACAGCAACTATAAATTGGAAAGAC
CACCAGATCAATTTAATTGACACTCCGGGTCACGTAGATTTTACGTTGGAAGTTGAACGG
TCATTAGCTATTTTGGACGGCGCAGTTACTGTTCTTGATGGTAATAGAGGTGTAGAAGCT
CAAACTAACACTGTATGGAATCAAGCCAAAAATTACAAGATTCCAAACATTTTCTACATC
AATAAATTTGATAGAAAATCTGCTAAATTAAATTTCTGTTTGAAATCCATCAAGAATAAA
TTATCTAATACATTACCTTTATTGATTAATTTACCATTTTACTGCCGAGAAAGTGGTCAA
TTTTGTGGAATTGCAAATGTTGTCAATCTACAATTAGAATATTGGGATATTATGAATAAA
TCGCACAAGGATGTATTCACAGAAAGTTTTACAAGAAAAATAAGCGATTCTGAAGATTTA
TTCAAGTTATGTATTGAAGCTAGGCGAAAGCTTTTAGAAGATCTTATTGATTTGGATGAT
GGTATATTACGTGAAGAATTTTTGTTGCGTGAAAATGCAGCTTTTTTAATACCCCCAAAA
ATGATTCATGATGCAATCAGAAAAGTTACAATATCTGCTAAGGCATCTTCTGTGATCATT
GGTAGCAGTAAAATGAATATTGGTGTAAATAACATATTAGATTGTATTCTTTTATATTTG
CCCGATCCGATTGAAAAGGAAATGAGTTCTAAGTTTGCATCTTTAGCCTCTGACCCTGAT
CATTTAACCGCATTAGCTTTCAAAGTTTCTCATGATAAATTCAAAGGTCCTTTGACTTTT
TTACGCATTTACTCAGGACTTTTAATGCCTGGACTATTGTTAAAGAACTTGACGAGAAAT
AATAATGAAAAGGTTGGAAAGATAATTCAATTTACTGGTGACAGTTTTATAGAATTAGAC
AGTGCATCTACTGGAAACATTGTAGCTATATCTGGACTAAAAAATTGTTTTACTGGAGAT
TTATTAGTAGATTCCAACATGCCGAATATAACTGAAAACGATAGTAGATATTCAGATTTG
ATAAGTATTCCATCTCCAGTTATTTATGCTTCGATAGAAGCA
>DjScaffold2329:115922-123969 strand:+
tatttttatttttttagatttatgatccATGAAAGAAGACTGTAAAATTTTCATTTTGGG
CCTGGGATTGTCATATATTTTGGTTTTTATATTGAGTTTTATTAGTATTACTACAAACAT
TATATTTTTCACCGACCGTATTTCAGTTATCACTTTATCTAGTCATTCAGAAAACAAAGA
TATTTTTATAGAAAAGAAATGCTCAAATGACTATAAAAAGGATTACTTAAAATGGAAAAA
TTGTTCTCCTATAAAGTGCGGACGAGGTGTATCAGATCAAATAGTTAATGAAAACGAAGC
AGAAAAATTATTAGAATTAGCAAAATATGGATTTAGTTTTGGAGGTTCTAGTGGAGGCGC
GTCAATATTAGATTTACATTCTGGCGCGTTAAGCAAAGGGGAACATTTTGTTGATTTTTA
CAGTTTGTTGAAAAACAAATCCGATTTAATTAATTTGGAAATAATTACACTTTATAAATC
TGTTAAAGAACGAATTGCGAAAAAAATAATTGAAACGTTTCAACTGAAAACCAAAAATTT
ATATTTAACAAAGCCAACATTTTTTTCTCAAATGACACCAAAACCTGCAAGGACAATTCA
TGATGAATATTGGCATCGACATATTGATCGTGAAACCTACGGTTCATTCCATTATACGAG
TTTGCTTTATCTGACCACTTATTCTGTAAATTTTACAGGTGGTAGATTCATTTTTGTTGA
TGGGAATTATTCCCAACAAATAATTGAACCTAAAATAGGTTGGTAGtctgattttagttt
tttaataatttagattattaaataaataataaatgtagaatt
>DjScaffold3349:46249-47279 strand:+
taaaaatgtcgacatattcgataaggattttctgataattccagtgatagaatcccagca
ttggttccttgttattgtttgtttcccttggATGGCTGGGATGGTGGATTACACGAAATT
GTTGGGGAAATCAGAGTCGGAGTCAATATCAAATATTCCAGACAATCAGTCAGAATTTGC
CACCAAAACAAGGCATGAAAGGCGTGCACAGATGAAAGCCAAAGATCCAGGATTTGATCC
GTTACCTTGTATACTCGTATTTGACTCTTTGTCGTACCAAAAACGAACCGACAAAGTAAA
CATTATTCGGGATTATCTCAAATCTGAGTGGGACGCTAAAAGATTGGCAAAAGATGGCCC

ATTAATATTTGACAGGACGACTGTCCGAGGCTATTGTCCTAAAGTGCCTCAGCAGAAAAA
TTTAACCGATAGCGGAATTTTTTGCTCCATTACCTAGAAACATTTTGCAAGGAACCAAT
CAAGGCATACACCGTGGATTTTTTCCAGAAATTCTTCGCAGATTGGTTCGATCAGGATGA
TGCCATTAAGAAACGCACTAGTATCAAGAATATTGTTGAAAGAGTTATTGTCAAGTCAGA
ATCCTTCCAGCCAATGAACGATGATGATGATTAGtatttcaatcaaaaaatgggaaatgt
ctaatgatgtgtatataataatattttaattttaaatttc
>DjScaffold2338:7655-19480 strand:+
tgttgaatttattgatttagatATGAAATGCGACAAAGTAGCTATTGAAAAAGCTCTTTT
GAAAAACCATTTATTTATAAATATTATTAACTCTTCGACAATATCATCTTTAGATAAAGA
TTCTAAAAATGGTTATCTGAAGAAAATACAAAATCATATTCAGAATTCGTTATCGTTCCG
TTATTATAATCTGTTGAAACATGCATTTATTTCGAGAAATCGGAATGCTTTGTTTTTATT
GGTTGATGACGATGATTATAATGATGATGGAGGGGAATTTGGTATTGGCGAGGAGGAGTT
TGGAGGAAATAATTCAGACTTTGATAAATATTCTGGTCGGGGCTGTGAGATGAGCAGGAT
TGGTTTGACGTCTTTGATCATGCATGATCAACGCATCAGAATCTCAACAGGACATTCATT
TGAAAGAAAAATGCAAATTTGGTTATTGACTAAACTAATTTCGTTGGATGGTAATTTTCC
TTTCTTTTGTTTTCCAATTCTTTCCAAACTGATTTCCTTGTATAATGGTATTGTTCAAAT
GAAATTAAGTTCAATTGATAGCTGTGAATCGCCATTAAGTGAACAATATGCTTGTATGGT
GAGGGAGAGACTTTCTGTAATTTCCAATGAATTTCATTCCTACACTGTGCCTATTTTTAT
CCATGCCAAGCAAACAGTAGACTGGTCGATTATCAAGAAATGTATATATAAGAAATTCAA
TTCCAGTTCTACTTTCATTGGTCATTTCAAATCCCGAATACATCCAATTTGTCAGGATAT
CAATTTTCTGCTGGTGTCACTTCTAAAATATTTCAGAATTTATCCTAAGGATATTCAGTT
ACCCTTCACCACTCCACAAATGGCTACACTTGTCAATGATTGTTTGAGAGCAAAATCATC
TGTTCACGATGGCAGGTTTTTAATCATATTTATTGAAGATATTGATTGGATTGCCTCCTC
TTCACTCACTAATGATGCTTGCTCCTGGTTGCCTGAAAAATGCGCCCCAAATATACAAAT
TATAATAACTGCTGCGAAGGATTCCATTCATGAAAAAAGTTTAAAATGCCGTCTCAATCC
TCCGGTTTTTATTGATTTTTCTTATCAGGAATCTTCAGTATCTGTTGATAAAATTTGTAC
ATGTTGTCAATCTTGGTGTTTGAATAAACTGATCCACTTACCGACAAAATTCGATATTTT
AATTACCAGTTTTAATGACGACGTAAAACTTTATATCAGGGAAATATTATCTATTTTAAT
GTGCTCT
>DjScaffold2338:19929-22007 strand:+
TCTACAGCAATTCATGCATTGATTGATAATTATATTATTCAACAGATCAATCTTAATAGT
TTGGATGAACCTTGCTATTCCTTAACTGTTCCTCAATTGACTCTGTACATCAGTAATTAC
TTCCTGGATCGTATTGAACCGAAAGATATAGAATTGATCAAATGTCGACTTGGTGCATAT
TATGTGTACCAGGCTACTGATTTGCACATGGAGACGTTATTTGTTCCAAGTTTGCTACAG
TATTTATCAGAATTGAAAGACACGACTCTCAAAGATTGGTTACTTAATTGGCCTGTATTT
ATGAAGTTGAAGGATGAATTTTGTGTACCAGTGAAATTGCTATCTTTATGGCGGTCTCTC
CTTGGTCCAAATACAGATTATAAGCCCATGATTAATGCTTATATATCGACATATCTTCCA
GACAAAAGGGATCTTGGTGATTGCTGCAGTTGCAAAAGATCAATAAATGTAGAGTTGCAA
CAAGGTCCCATTCAGTTATCAGTTCTTCGTCAGTCTCCTTCTGATTTCTCTAAAGCATAT
AGTAAACATCGATATCGAGTTCTTCTCGATCTAGCGAGAACATCTATGCAAGTAGGTCAG
TATGCTGCCGCCAAAGAAATGGCTCGATCTCTTTTAAAACAATTGGATTACTTAACAATC
GATCGATCGTATTGTCCACCGTTGGATTCAAATAGGAACCTTTTAGCTTCATGTCTTCAT
TTATTATTTTGTACTTTGGATGAACGCTTGAAGACCTTTGAATTTGTAACAGAACAAACC
ACTCCAATATTCCGAAGAACTGTTCATTACGGAGCTCGAGCGTTGTTATTGCAGAATGAA
ATCGTCACTCAGATACAACGGGCAATAGAAAATAAAGATAATCAAGGGTTTGGAGATGTG
AGGATAATCGATTCTGCAAAACTTCAACAGCAGTTATGTCAGGCGGAAATACGTTTAGCT
CACATAATGACGCATTTCGTATTTTCGTTGGACGGAGTGGAACAAACAGTTATCAATCGT
CATTTTATGCAATCAGATATTCGTTCAGTTACTTATGCTTGCATGTGTCGTGCTCATGAT
ATCGCTACTCGATTGAAAGATCGACAGTTATTATTGGAAATCCAAATGACATTTGGAGTT
ATTCAACCACGTGAAAATTTCAAAGAAAAGATTTTACTCTACAAAACACCGTTACGGCAA
GCATTATGTATCTACGGAGAGAGTCACATATTTGTTTCAAGACTCTATATTAATATTGGT
ATTTCTTATGAAGAACAAAAGAAATATATCAAAGCTTTCGAATATTTTACTTTATGGTCA
CATGTTACTCATGAACTATTGGGACGGAATCATTGCAAATCCTTGCGTAGCTGCGGTGTT
TTTAAAGAACCAATATTTAAGTTAACTGCTGAATTTTTAAAGAATCTCAATGATGAAGAG
CCAAATGAAAACTTAGAAGAAGAATTGAGTGATTGTATTAACAACATCGACTCAGATAAT
CTGGATATCCAAGGAACTGGAATTAGTGTATCTGGAATAGGTATAGGAGGATCGGCGACT
AACCATTCCTCTGAAAATATATCATATGAAAACATTTTCATGATGCGTACTACCATGGAA

CATGAATCTGATTGGGAAATACATTCGAGTGACGACGAAACTCCTAATTATACAACAGCT
TTTGAAGAGGTCATTTGTAAGTTTAAGAAGAGACATTACAGTTCAATCGCAAAAGAGTTA
TTACAGTCTATTCTCAATAATTCATGGATTGATTCCGATGATTTATCCAAAGAAGATATC
GAATGTCTCGTCCGATCTCAAGAAATCCCTAATGAGACTGATTCCAGTTCTAGACTGAGT
GGATCGAACAACTTGGATAACAGTGTCAGAATCACACGAATGTTCCGTGATTTACGCGAA
CTCGCTCAGTGGATTCCTACTACAAATGTATCCCCATCAGATATCAGTCTTTCAAGGAGA
TTGTTGGAATTACAACGAGAATTAGAAGAATTTCTAGATAATTGAtatattttgtgaatt
atgtggtatggtTCTACAGCAATTCATGCATTGATTGATAATTATATTATTCAACAGATC
AATCTTAATAGTTTGGATGAACCTTGCTATTCCTTAACTGTTCCTCAATTGACTCTGTAC
ATCAGTAATTACTTCCTGGATCGTATTGAACCGAAAGATATAGAATTGATCAAATGTCGA
CTTGGTGCATATTATGTGTACCAGGCTACTGATTTGCACATGGAGACGTTATTTGTTCCA
AGTTTGCTACAGTATTTATCAGAATTGAAAGACACGACTCTCAAAGATTGGTTACTTAAT
TGGCCTGTATTTATGAAGTTGAAGGATGAATTTTGTGTACCAGTGAAATTGCTATCTTTA
TGGCGGTCTCTCCTTGGTCCAAATACAGATTATAAGCCCATGATTAATGCTTATATATCG
ACATATCTTCCAGACAAAAGGGATCTTGGTGATTGCTGCAGTTGCAAAAGATCAATAAAT
GTAGAGTTGCAACAAGGTCCCATTCAGTTATCAGTTCTTCGTCAGTCTCCTTCTGATTTC
TCTAAAGCATATAGTAAACATCGATATCGAGTTCTTCTCGATCTAGCGAGAACATCTATG
CAAGTAGGTCAGTATGCTGCCGCCAAAGAAATGGCTCGATCTCTTTTAAAACAATTGGAT
TACTTAACAATCGATCGATCGTATTGTCCACCGTTGGATTCAAATAGGAACCTTTTAGCT
TCATGTCTTCATTTATTATTTTGTACTTTGGATGAACGCTTGAAGACCTTTGAATTTGTA
ACAGAACAAACCACTCCAATATTCCGAAGAACTGTTCATTACGGAGCTCGAGCGTTGTTA
TTGCAGAATGAAATCGTCACTCAGATACAACGGGCAATAGAAAATAAAGATAATCAAGGG
TTTGGAGATGTGAGGATAATCGATTCTGCAAAACTTCAACAGCAGTTATGTCAGGCGGAA
ATACGTTTAGCTCACATAATGACGCATTTCGTATTTTCGTTGGACGGAGTGGAACAAACA
GTTATCAATCGTCATTTTATGCAATCAGATATTCGTTCAGTTACTTATGCTTGCATGTGT
CGTGCTCATGATATCGCTACTCGATTGAAAGATCGACAGTTATTATTGGAAATCCAAATG
ACATTTGGAGTTATTCAACCACGTGAAAATTTCAAAGAAAAGATTTTACTCTACAAAACA
CCGTTACGGCAAGCATTATGTATCTACGGAGAGAGTCACATATTTGTTTCAAGACTCTAT
ATTAATATTGGTATTTCTTATGAAGAACAAAAGAAATATATCAAAGCTTTCGAATATTTT
ACTTTATGGTCACATGTTACTCATGAACTATTGGGTAGTGTTTCA
>DjScaffold1765:4535-21139 strand:+
gttatttaggaattcactaataattaaattattttatttatttaaattgatggctgtgat
tcaagtatagacgttttatagtggctcgaattctactcgatttatatctttattaaaatc
tcttataaataaattgatgatagaataatctaacaatatatcaaataatttaatgttatg
cttttaatttaataaatatcctgagatgcatactaataaatatcatagacacgcccattg
gtgtaaggtccatatttcttacttcattgggttttatgctttaaataatacgtcattcca
agtttaaattattaacaattttgtagaacggacatcgggctattgatttggcaaagaatc
acaaagatataattgcaattctttcagataaaaattgtgtgtttgactttaaagaaaata
ctagttcggatgctgttgtagtggatttggaagatttacatacttatggcaatcagattt
tcaaatgcatcaaagaaaaaaacatcaatctatttaagaatatatatgatccacttaaac
caatcATGACAGATACACAGACCGAAACGACATTAATGGAGTTTGCCATTTCAGAAAAAG
CCTACGAATTCGTGGAAATGGTATTATCTAACAATATGAATAATGATAAATTCGAAAAAT
ATTTAATTCATTTTAAAGAAAAACATGCAGATTTATTCGATTTAATTCTTGAACAATATA
CAATTTATCGAGCCATCAGATTCAATTTGAAAACAGAATTAAAATCAATCAAACTTATTA
ATCAAAATTGCATACTCACGGGATCATCTTTTTTTGAATTAGCTGTAAAATACGGATCAC
TGGAATACTCTAATCTTATTATGATGAAATTAATCGATAAAAACGAGAATTTGTGGAACA
AAGCTAAACAGATTGCCATAGATAATCTAAATTATGAAATATGTAAGTTGATAACCAATC
GAGAAAAATATCTAGAAACCAGATATGAAGCCTTGTGGACTCGGGCGCATATCGCTGTTA
ATGATGATGATATTGAGATGGTTAAATATCTTATTAAATCCAGCCGCGATACTTTCAATA
CATTACCTTCGATTGGTTCTCCATTGAATTTAGCTGCTATTTCTGGACGGATTGAGTGTT
TCAAGTTATTACTGGGAATGTTTTCGAAAGAAAAAAACAGTTTCCATATTGCAATTAAAA
ACGGAATGAGTCGATATTGTATTGATAATATGCACGCTATATCTGACTTAAATGAAATGG
ATGAAGATGGAAATACATTACTTCATCTTTCTGCTAAATTTCTCTCAACTAAATTATGTG
GTGTTCTTCTTGAACAGAAGGCTGATCCAAATATTATAAATTGCTCTGGAAACACACCAT
TGCAAGAAATGCTGTCTTTCAATTTTCACCGATTTAGTCATATGGAAAAGGAGATATGTT
CTATTTCAAAATTGCTACTCGATAATAAAACTGATATCAATATTAAAAATAATAACGGAG
ATTCAATTTTTCAAAATTGTCTGAAATTGAACAATGTGAATTTTATTCTTCTTATTGCGG

ACAATCAAAACCATATCGAATTAACAAGAAATGATCTAAAAATCATTATGGATAATGATT
GGATTGATGTACTTGAAATTGTTTTACCGCAATGTTTACAAGCTAATCAATTTATCGATA
AGGAAATGTTGATTTATTTCCTGAAAAATCGCACATCTGAATCTTTCAATTCTGTTTGGT
TTTTAGCGGATCCAGGTACAAAGGAACAATGTGTAGCTACACTATTATACGAATGTGCAT
TGAGTAACAATAAAGAAACATTCACAATTCTCAGTGAAGAATCTAATTGTGATTCTATAC
AATTTCAAAATGGTGAAACTCTACTCACATCTTTGCTGAAATTACAGAAACAAGATTTCT
GTTGTATTTTAATCAACTCGAATCTTTCCTCCATTTTAGTCAATCAGTCTAATTCTTTTA
AAGAGACTCCTTTGCACATTGCAGCTAAAAATGAATATTTTAATTTATTGGAAAATATGC
TTTACGTTGCTGACGATATTAATAAGGAGGACTGTCAAGGATTTACTCCTTTCACAATAT
TGATTAATAGTATGAGCTCGTCCGAAGAGGTTTAGttattgatattatttattaaatatt
attagtaataaatacatttaattaaactcaaattttatgtgccattttcaaataattcaa
cttctaaattgataaaattcatttttatatgaaccaatcgaaaatatgcatgattaattt
tttatgatttttgatttttcagacgttatttaggaattcactaataattaaattatttt
atttatttaaattgatggctgtgattcaagtatagacgttttatagtggctcgaattcta
ctcgatttatatctttattaaaatctcttataaataaattgatgatagaataatctaaca
atatatcaaataatttaatgttatgcttttaatttaataaatatcctgagatgcatacta
ataaatatcatagacacgcccattggtgtaaggtccatatttcttacttcattgggtttt
atgctttaaataatacgtcattccaagtttaaattattaacaattttgtagaacggacat
cgggctattgatttggcaaagaatcacaaagatataattgcaattctttcagataaaaat
tgtgtgtttgactttaaagaaaatactagttcggatgctgttgtagtggatttggaagat
ttacatacttatggcaatcagattttcaaatgcatcaaagaaaaaaacatcaatctattt
aagaatatatatgatccacttaaaccaatcatgacagatacacagaccgaaacgacatta
atggagtttgccatttcagaaaaagcctacgaattcgtggaaatggtattatctaacaat
atgaataatgataaattcgaaaaatatttaattcattttaaagaaaaacatgcagattta
ttcgatttaattcttgaacaatatacaatttatcgagccatcagattcaatttgaaaaca
gaattaaaatcaatcaaacttattaatcaaaattgcatactcacgggatcatctttttt
gaattagctgtaaaatacggatcactggaatactctaatcttattatgatgaaattaatc
gataaaaacgagaatttgtggaacaaagctaaacagattgccatagataatctaaattat
gaaatatgtaagttgataaccaatcgagaaaaatatctagaaaccagatatgaagccttg
tggactcgggcgcatatcgctgttaatgatgatgatattgagatggttaaatatcttatt
aaatccagccgcgatactttcaatacattacctgttggtcaatacatttttttattttgtt
aatctcatttattaactttttattaactgcttttatagtcgattggttctccattgaat
ttagctgctatttctggacggattgagtgtttcaagttattactgggaATGTTTTCGAAA
GAAAAAAACAGTTTCCATATTGCAATTAAAAACGGAATGAGTCGATATTGTATTGATAAT
ATGCACGCTATATCTGACTTAAATGAAATGGATGAAGATGGAAATACATTACTTCATCTT
TCTGCTAAATTTCTCTCAACTAAATTATGTGGTGTTCTTCTTGAACAGAAGGCTGATCCA
AATATTATAAATTGCTCTGGAAACACACCATTGCAAGAAATGCTGTCTTTCAATTTTCAC
CGATTTAGTCATATGGAAAAGGAGATATGTTCTATTTCAAAATTGCTACTCGATAATAAA
ACTGATATCAATATTAAAAATAATAACGGAGATTCAATTTTTCAAAATTGTCTGAAATTG
AACAATGTGAATTTTATTCTTCTTATTGCGGACAATCAAAACCATATCGAATTAACAAGA
AATGATCTAAAAATCATTATGGATAATGATTGGATTGATGTACTTGAAATTGTTTTACCG
CAATGTTTACAAGCTAATCAATTTATCGATAAGGAAATGTTGATTTATTTCCTGAAAAAT
CGCACATCTGAATCTTTCAATTCTGTTTGGTTTTTAGCGGATCCAGGTACAAAGGAACAA
TGTGTAGCTACACTATTATACGAATGTGCATTGAGTAACAATAAAGAAACATTCACAATT
CTCAGTGAAGAATCTAATTGTGATTCTATACAATTTCAAAATGGTGAAACTCTACTCACA
TCTTTGCTGAAATTACAGAAACAAGATTTCTGTTGTATTTTAATCAACTCGAATCTTTCC
TCCATTTTAGTCAATCAGTCTAATTCTTTTAAAGAGACTCCTTTGCACATTGCAGCTAAA
AATGAATATTTTAATTTATTGGAAAATATGCTTTACGTTGCTGACGATATTAATAAGGAG
GACTGTCAAGGATTTACTCCTTTCACAATATTGATTAATAGTATGAGCTCGTCCGAAGAG
GTTTAGttattgatattatttattaaatattattagtaataaatacatttaattaaactc
aaattttatgtgccattttcaaataattcaacttctaaattgataaaattcatttttata
tgaaccaatcgaaaatatgcatgattaattttttatgatttttgatttttcagac
>DjScaffold1765:31899-32629 strand:+
attattgaatgttgtaccaacaactattgcaaatccattttagcgccaataaaataatta
tcatgatatatttaaacaaattttgtcctcaaatagctgacgcaccctgtatatacaaca
gtttatttaaaatttattaatttctttgttggatatatttataaaatcttacattgtcgc
aatttttaatatcgcatgtttttattttaatattttcaatggtttcaaaaacaatttgtg

attgaattgtaaaaaaaattagatgaaatttgtttactgctggatttttctatgaataa
atttttATGATTCAGGTCGAAAGAATTTCCAAGTTAATAATTTCCAAAAGTCTTTCTCTT
AATAACGAAAATCTTTCAATGTTGAAATCTATTGAAAATTGTCGAAATCAAGATTTAAAA
ACTAAACTTTTTAAATTGTATAAAACAGAAATAGAAAGAAAACTGAAGAATGAAGAAGTT
AAAAATATGTGTAATGTTTGCTTAGACAACATAATCAACATTTCCTTTGATTGTGGACAC
ACATCGTGTAATGCCTGTGCTATTCGGATGAAATCGTGTCCCATTTGCCGTAGCGTTATT
CGTAACAGAAATGCCTTGTTTTTATGAggatattttttaacttgaaaaattaaatgaaat
aaattattataaattgtta
>DjScaffold1306:35646-53256 strand:+
tttattcattattttagagtattcaagtaaacgtgaaagtacaaataccaATGCTGGATT
CAACAATTGCTCCTCTGAATATTATTTTTGATAATTTAAATGTTGAGGTTTTATCAATAG
ATTCTATTTCACAGATTAAAACGAAAATTCTGGACAAAGTAATCATGGAATTTGTAGAAT
CAGAAAATCTTGAAATCAATATTTATGATGACAATTGTACAATTGATTTAACAAATTTCA
ATAAATTTCTATTTTATTTTATATCAAATTCTGCAAAGATTTTACTCGATGATTTTGATA
ACACAAGCACAACAACTGTTTTAGACAAGGAATATACTCAAGTTAACACTTTAAAGCATT
ATGGAATAAGAAGTGGATCACAGTTCTTACTTGTTAGTAATTTCTATGATGAATTATCAT
ATGGTAATCGAGAAAGTATTTCATTAAAAAATAAAAATTTGATTCATTTACACACAATAG
GCGATTCAAAATCTCCAAATTTCGATTCAAAAGTTCAAAGCTCTTTAGAAAAAAAGAATT
CACCAGTGAAGATAATTACTAATGATTCAAATAAAGAGTCTTGTATCATGCTTTTACTAG
CTACAAAAAAAAGTATTAATCCATTTATTGATGGATTCTTCCAATGCATATTTAACAGAA
AATTAGAAAGTCAATTAATGACGTCATCAAATTCACTATTTGAATCAATTTCTGATAATC
AATTAGGTTATAATTTGAACGACAATTATTTAATGTTTACACAGTATTATTTTAATTTGC
TTAATGATATTGGATTGCAATTAAATATTGACGATCAAAGTACTATTCAAATATGGAAAA
GTAACAGGTATGATTTTGAATGActatattgtttaaatattgatattaatatctaatttt
aaataaattaactaatgcctttatcgatttatatgcaaccagtctttccaaatggaaact
attggctattgaattaaagatatgatttttcaaggactcacctattttcaagcaactat
ttcaagaagaagtgcagaataaatattctaattggtggcgaatcttttccaattctagaa
ttccagttgaggaagaaaatattatatcgatgatggatgctattagtgaagtaactataa
ttatgttaattataatattacgatagatattaatataattaaatttgtcaaaacgaatta
ttgtaaaaaaaaattattcaataataggtttcataaaaattgtagaattaagaaattagct
ttcattttaactttcaattagatttttatcaatattgtaaattttctaatagattaaaa
cgaaaattctggacaaagtaatcATGGAATTTGTAGAATCAGAAAATCTTGAAATCAATA
TTTATGATGACAATTGTACAATTGATTTAACAAATTTCAATAAATTTCTATTTTATTTTA
TATCAAATTCTGCAAAGATTTTACTCGATGATTTTGATAACACAAGCACAACAACTGTTT
TAGACAAGGAATATACTCAAGTTAACACTTTAAAGCATTATGGAATAAGAAGTGGATCAC
AGTTCTTACTTGTTAGTAATTTCTATGATGAATTATCATATGGTAATCGAGAAAGTATTT
CATTAAAAAATAAAAATTTGATTCATTTACACACAATAGGCGATTCAAAATCTCCAAATT
TCGATTCAAAAGTTCAAAGCTCTTTAGAAAAAAAGAATTCACCAGTGAAGATAATTACTA
ATGATTCAAATAAAGAGTCTTGTATCATGCTTTTACTAGCTACAAAAAAAAGTATTAATC
CATTTATTGATGGATTCTTCCAATGCATATTTAACAGAAAATTAGAAAGTCAATTAATGA
CGTCATCAAATTCACTATTTGAATCAATTTCTGATAATCAATTAGGTTATAATTTGAACG
ACAATTATTTAATGTTTACACAGTATTATTTTAATTTGCTTAATGATATTGGATTGCAAT
TAAATATTGACGATCAAAGTACTATTCAAATATGGAAAAGTAACAGGTATGATTTTGAAT
GActatattgtttaaatattgatattaatatctaattttaaataaattaactaatgcctt
tatcgatttatatgcaaccagtctttccaaatggaaactattggctattgaattaaagat
atgatttttcaaggactcacctattttcaagcaactatttcaagaagaagtgcagaata
aatattctaattggtggcgaatcttttccaattctagaattccagttgaggaagaaaata
ttatatcgatgatggatgctattagtgaagtaactataattatgttaattataatattac
gatagatattaatataattaaatttgtcaaaacgaattattgtaaaaaaaaattattcaat
aataggtttcataaaaattgtagaattaagaaattagctttcattttaactttcaattag
atttttta
>DjScaffold2368:47837-48106 strand:+
TCAGCCAAGCGTCATAGAAAAGTTCTAAGAGATAACATCCAAGGCATTAAAAAACCTGCT
ATCAGAAGACTAGCAAGGAGAGGTGGTGTCAAGCGAATTTCAGGACTTATCTATGAAGAA
ACTCGAGGTGTCTTGAAAGTCTTCCTTGAAAATGTTATCAGAGATGCTGTAACTTATACT
GAGCATGCAAAAAGAAAATCTGTTACTGCTATGGATGTTGTTTATGCTTTAAAACGACAA
GGACGTACATTGAACGGTTTTGGAGGTTAA

>DjScaffold5404:11072-12012 strand:+
GTAACTAATTCTCCTTTAGAGACCATTCTAGAATTAGCCAACAGTATTAATAATAACATT
AAATTCACTTTAGAGAAATCAATTGACTTTCAGATTCCTTTTTGGACACTTTAATTCTA
AAGAAACCTGGTGAAATATTAATTTCACAATTATATATGAAACCATTTCACAGTGGACAT
ATATTACCTTGGAGTAGTAATATTACTATCAGTCAAAAAATTGGTATAATTAAATGTGAA
AGAATTCGAGCTGAGAGGCTATCTTCAAATGAAGAAATGAAATTGATCTCTCTCAAGTTG
ATAAAAATTAAATTTTTAAATAATGGCTATCCAAATAAAATTATATCGAAATATTTATCT
TTAAATACAAAACCAAATAAAATCAAACCAAAAACAAAGACAGTTATTAAATTACCTTTT
ATTAATGAGAAATGTAGCCAAATCATGCAGAATATAATAAAAAATGCTAAACTCAAACAC
AATCCAAGAATTATTTTCAAAACCCAAGCACCTTTGGGCATTCAATTACAGAAACCAAGA
CAAAAAAATATATACCATAAAAAATGTCACTTATAAaataacatgcaccatttgtcaaga
aacatcaatataggtgaaacatgcagaaccatctgaacaagaattaaggaacatttgaaa
caaaaagaaagtgaagtattcaaacactttcaacacaaacataaaatttcaccagaacaa
catcacattacatggaaaacactaggaacaaactacatttctacactacatagaaaaaaa
catgaagaaaaactaatcaaagaaaaccatcctaaaataaatataatttacaaataatta
aattaaataattaaatataaaataagaattaaaattaaccaattaaaatttaataaaaat
gaattatt
>DjScaffold4153:23014-35164 strand:+
AAATTTGAAAGTTTTGAAAATGAAAAATTAACTATTGAAGATGAAATAAATGCTATTGAA
TATTTTAAGATTTGGAACAAAACAGAAGAAAAGATTTACAATTTGGAAAAAAAATTGGAA
ATAGCTAGAGAAAATCGACAATATGAAATCCCCGTTCTTCAAAAACCAATCAATATGAGA
GAATCATCCTCTAGAAAAGACAGAAAACCAAAACTGTCCTTTTTAACAAATAACTATCAA
GTAAACCAAGAAACTGATTCCTCGAAACCAAACTCAAAATGGTCTGATGTTAAATATGGA
ATCGCTGAAGAAGAAAAAGAAATTCTCAGAAAATTTCATTCTCGTTTGATATCAGAAGTG
CGACCAGATTCAATGATAGATCAATTATTGACAAAAAAAGTTTTAAATATCGAAGAAAGT
GGTGAAATATTCAGTAGTTTAAATAGTTCAGATCAAATGAGTTTTCTGTTGGATGTTCTC
GTAATGAAAACTGGACAAGCATTTACGGAATTTCTAAATGCTTTAAAAAGTCGATATTTT
GAATTATACTTGGATATTTATGAGGAATATCAGCATATGAAACAAGAAATTCTCGCAAAT
AAACCAGAGGGAGTATCATATTGTCATACTTATCTCAGCAATTATTATATAAAAATCTAC
AATCAATGGAATCCCATTTTATGGTACCCTGATTCATTTGTCAACATTCAATCATTTTAT
TGTCCACTCCAAATTCTTGATGTTAATGGATGTCATATAGAACCATCTGAAATTTTATCA
CATAGAACTCAAAAAGGTCCAACGCGAACTATCATAGTTGAAGGTGATCCAGGCACTGGT
AAATCTTTTCTATCTCATTTACTTGCTTCGAAATGGGGTAGTGATCCTCTGGTTTTCGGG
GAGGATTTTTGTATTAAGCTTGTTTTTCATCTTGATCTAAAAATCACCGCCCCAAATTTG
AAGTGGATGCTATTAGATCAACTTTTTACTGTTGATCTCAAATTAACTGAACGTGAACTT
TGGGAAGTTATTGATAAACACCAGGAAATTTGTTTATTTATTCTCGATGGATATGATGAT
AATATCAATGATGAAATACGTCAGTTAATATCTGGGGCATTATTTCCAAATTGTCATCTA
ATAATATTTAGTAATTCCTCTTCTACAAATAGTATTAAAGAGTTTTGCCAGGAAAAATTA
GTGCTTACTGGATTTATGTCTGGAGAACCAGTTAATTTTACTAAAAAATATATCAAACTT
ACTTCTCGGCCATACGAAGTGTTTGAAAGTCTCATACAAAAATTGTCTAAAACTTTTTTG
AAGAATAAAAATAACAAAACCCCTGATGATGAAGAGGTTCGTACTTCATCGTCCGTAATG
AGTTATTCTCAAAATGATCAATTGCCTGTAATTAATAACACCAGTTTAATTGGGCAAAAT
AAAGAAAGTCCAACCTGGATGGATCCAGAAGTTTCATTTCTTGGCAGACAACGAGCCGGA
AGTGTACTACCAGGTTCTTTGATGGGATTCAGTAGAACAATTACTAATAAAACTAGTAAA
CTATCCCCGATTGTTAATGAGACTGCAGATCCTAAAGAAATTGTAGAAGTTCAACAATCA
AATGATTCCGAATCAAGTTCTCAAGAAAGATTAATGGCTTTAAGATTTTATTCTCAATTT
CCATTGTATTGTTCGTTTTTCTGTGTGATGACCGATCTAAAATTGAGTATTGATTTGACA
TCCTGGTCATCAATGCTGGAACATTTTTTTACATCAATCAAAATGAAACTTATCGAACGA
GAGCGTGAAAGTCGATTTTCTGTTAATAAAATTTTTAACACTCTTATTGAATCATCTTTT
GAATCAGTAAAACAAGGATTATCTACAACTTTCGTTAAAGACTCGGATAAATTAACAAAA
TTCGGTATTTTAAAGAAAACGTCGTCATCGAATTCGAATTTTTCATCGAAATATGTTTTT
CTTAATGATGTTGTTCAAAACTTTTTAGCCGCAAAATGTTTGGATCTCTACGGTTTAGAA
GAAGCAAACAATTATTTTCAAAATATAACTATTTCAAAATCGATGTATACATTAATTATT
TTCTATTGTGGAATTCGAAGAAATGATTTTGAAACTGCCGCTTTACGGACTTTATTTCGG
AAAATCATTGATTTAAATTTACAGATTTTAATTAATGATTCCGATACGGTTGATTATTCG
AAATCAAAATCCCGTTCGGTGAATATAATGAATTTTCAGTTGAGTTTAGATTGTGTTGCA
GCAATGAATGGCAGAAATGATTTAGCATTGAAAGTTGGACAAACATTACCAGAAAGGTTG

CTTATTCAGCATACTTGTACAATTCCTGTATTAACTTTACAAGGACTTTCATTTATTATT
GACTTGAGTTGTAAAAATATTAAACAACTTGATATCCGTTTGAATTATCTCAGTCGACAA
ACTCCAGAAGCATTTCAAAACCTTGCGAATTCAATTCATCAAAATTCATCGATAAAAATT
CTACGAATTTATTGGGAACAGTTAGAATTGATGAGTGCTTTTTTCAGTAAAGTCTGTGAA
AAAAATCAATCAATTACCACAATTGAATGTTTTGACGAATCAAATCACAGCTATGACAAT
TTTACGCAATACGTAGCATTTAATATTCGAAATGCTTGTAGAAACATGAAAATTGTTGAA
GGATTTGCTTTTAATGATTGTGCTTTTCCTAACTTAACACATGCGATTATTAGAAATATT
CCGGAAAATTTGATTTCACTTGATCTAAGTGGTTGCAGTTTCACAATAATTGCTTGTAAA
GATCTTGCTGAAAAAATCAATCAAAGTAAATGTCTAAAAGTGTTGAAATTGGAAAAAGTC
CAAATTCAAAGTATGGCGTTTAATACGTTTATCAGATCGTTTTGTAATAATAGAACTGTA
TCCGAATTGTATTTAAATGGAGTTAAAATGGATAATGTTTGTTTTTTTTCTTTAGCTGAA
GCATTAAAGCTAAATAATCGTTTGGAAAAATTGTGTTTATTGGACATCATTATTAATGAA
CACAACTGTTCTGAGCTCGCTCAAGTTTTACGGGTTAATAATACCTTGAAAGAAATCTAT
CTTACTGAAAACAAGGTTTCTATTGTTGGCCAGATTGAATTGCATAAATATGAAAAATAT
ACGAAGATTATTGGTTTGAAAAGTCAGATTTTGAAAGCACCTGTTTTGGGAAAAAATGGA
GATTTTTAAaaattttgaa
>DjScaffold468:21234-26685 strand:+
GACAAAGGGAAAGAACTAATAGAAAATCATATAGAAGAAACTGTAGCTTTTTATATAAAA
CAAAGTACCGCTGAAAATCATGCTGTGAGAGAAACTTCCTGTTCCTGTATATCAGAGATT
GCATTGAAATTAAATAAAGATACAGTGCGACCTTATTTAGTTCAGCTACTTGATATTTTG
ATTATTCTCTTAAATGATCCTAATTGGTCTGTTCGTGATATGGCATGCTTGACATGCGGA
CGACTAATAGCTGCTTTTCCAATGGAAGCAAAACAGCATAGGCCTGATGTTTTGATTCCA
CTTATTTGTAATCATGTAACGGATAGTGTCATTTGTGGACGAGAAAGTGGAGCGTATTCT
ATTGCTAATATCGTTGGTGCTTTTAACCCCGAGGATACTAGCTATTTAAAGGACATTGTA
AGTTTTATATTGGAAAACTTGGAGAATATTAAAAATCAAAAAGCAGAATCATCCCTATTT
TCAGAAATAAAACAATCACCCCTATCAAAACCTATTAAATCCAATACTAATCAATGTGAT
ATTAGAATTATACATCAGGACAAATACCGATGTGAAATTTTATCTCCTACAAGACACATG
GGTATAATTTATGTTAATAAAATATGAttaagaacaatttaaattacatatta
>DjScaffold468:56079-56764 strand:+
TTTTTGTCACAATATATTTTGTCATGGAACCGCAAAATATATTTATATTTTATCATAAAT
TGGAAACAAGTTAAGAAAGTTGGCTTACATTTCAGCGAAATAAAATTTTTTGTTTATTTG
ATTATGTGCTCAACGTTATTAGGTTGTCAGAATATTGAAAATGATAGTGAATTCAAAATA
CAGATGAGCGAACCTTGGGAAAAGGCTGATGGGTGTGTTCTTCTTATTGCCGAATTAGCA
TTACGCTGTAAAGATTCGTATTCTTATATGGATGAATTAGTATCGGCTATGATAAAAGCC
TGTGGCTATCAACATTATCCTAACTATGTATATTTTTTAGCCACAGCTTGTAAAGCAATA
TCCGATTTGTGCGAATGTATGGATAAACCTCGATTTAAAAAATATTTATCCGATTTTTTA
CCTATTATTTATATTAGTTTATGCTGCGATTGCCAATATACCGTGTCAATAGCGGAGAAT
ACAATTAGAGTTTTAAGTTCTAAAGTTGGGTCAAATATTCTTAAAGGCCGAATTGAGATG
TATAATAGTGATTTATTATCAAAGACAGAGCACCTGTTTTCTATTTAAattataacgaga
aatcttggatattctagtgttcataaatatatattatttatca
>DjScaffold280:87166-88637 strand:+
aaattgcaattaattgtaaactataATGTCCAATGTCGTAAATCGTAAAGAAATAAGTAA
AATTGCAGAGAAAATTTACAAAGACACTAATTTTACTAAAGTGGAATGTGAAAATCTTTT
AAAAATTCTTGATTTTGAAAATTCAAAAAAACCTCACAAAAAATTGGATAGAAGCGGATT
TCGGGATATTTTACATAAGACATTTCGAATGACAGATGATATGTTGATGGATAGAATATT
TCGGGCATTCGATCAAGATAATGACGGAGTTATTAGTCCAACAGAATGGGTAAAAGGATT
ATCCGTATTTCTACTTGGATCATTAAAAGAGAAAATAAAGTTTGCTTTTACTGTTTATGA
TTTGAATTCTGATGGATTGATTTCGAGGGAAGAAATGTTTCAATTGTTAAAGCCAACACT
TGTCAGACAACCAACAGAAGAAGATCCAGATGAAGGTACAAAAGATTTGGTTGATATGAC
CTTTAAAAAAATTTGATGTTGACCATGATGGGAAATTAACTAGGGAAAATTTTGAAGAAGC
TGTTGAAAACGAGCCATTACTTTTAGAAGGCCTTGGACCTTGTCTTCCTGAAGAAAAGTC
AGCAGCTGCTTTCTTAATGACGTTTCAGGAATCGGAAATTTTCAATAGCATATCTTATTT
GGGTAAACAAGACAGAATTGTTGTCACTTCTGAACAATAAgattttataatatttcaaat
tttcagttgatttcattttcaaatataaataaataatattatttgtcaaatttttagcaa
ccaacagaagaagatccagatgaaggtacaaaagatttggttgatATGACCTTTAAAAAA
TTTGATGTTGACCATGATGGGAAATTAACTAGGGAAAATTTTGAAGAAGCTGTTGAAAAC
GAGCCATTACTTTTAGAAGGCCTTGGACCTTGTCTTCCTGAAGAAAAGTCAGCAGCTGCT

TTCTTAATGACGTTTCAGGAATCGGAAATTTTCAATAGCATATCTTATTTGGGTAAACAA
GACAGAATTGTTGTCACTTCTGAACAATAAgattttataatatttcaaattttcagttga
tttcattttcaaatataaataaataatattatttgtcaaa
>DjScaffold3189:21471-22304 strand:+
ATATTAGATACGGATGCCAGTTTTGATACTTTTAGAGCAGTATTGTCACATAGCGATAAA
GAAGGAAGAGAAAGAGTTGTTGCGAATGGATCACACTCAATGACAAGTCATGAAAAGGCA
TACTGCATTACACGAAAAGAGTTATTGACTGTTTATTATTTCTGTATGCATTTCAAACAT
TATTTGTACGGTAGAAGATTTACTCTTAGAACAGATCACAAAGCTCTAACGTTTAGGATG
ACGACAAAGAAACGAATTACATCGCAATTTCAGACATGGATTAATTATCTTACCAGTTTA
TACATGGTTTTAGAGTATAAAAAAGGAATTCAACATGCTAATGCAGATTTAGACCAAGAT
GTGATATGTGATGTACAGTGTCAAATGTCTCATGAAGATGCCAAGAAAGGGAATAGTAAA
TCGAGAATACTAAACATGATTGAAAAGGAAAAAGAAGATCATTTAAACATTTCCAAGGAA
ACAGATTTGATCAAGTAAatgaaaattccggatgacaaactacatgaaacaatagaagaa
atacataaattattatgccatgcaggagttgaaaaggtgtacgaatatatgagaaaccaa
tatatagcaaggaatttgtggagccaaatacagcagatagttcgtatttgtgaaatt
>DjScaffold1156:70410-87500 strand:+
ATGCCAAATGAAGTTAATTCTGTTGATTCAAAGATTTCAAGAAAACGTCCTTTACCGATG
ATTAATTCGAGTTCGAATAAAATTGAGCAGTCATCAGACAAACCTATTCCATTCAAAAAG
CTGAATTTGTTTGACTCACAACTATCAGTAATCAATAGAGACCACCACACAAATCAGTTA
GGATTTATGATCAATCCCCTAATGTCTTATCAAAATCAACTGGCATCATATCTATCATTG
AATAACCAAACTGGGTTGCAATCAAATTCTTTGAATTCTTTAAATTTACAAGCATTGTTA
GCTTCTAATAATGGAAATCTTATGTCTATGGGGCCAAGTGTGATTTGTCCTAATACTTTA
ATAAATGTCAACGGATTCCCAATGATTTCACCTGTGCCAACTCAGCAACAATTGGCTGCT
ACTACATTATTACAAGCTGCAAATGCCTTTCAATATAACAGAGGCTTAGCAGATTTTGGT
GGTTTTCAATAAaatacatttatatttgaaaaaaaactttttatacaccaaagaggacct
tgataattgtatcaattgaaattgaaagtttattttatactactactatttatcagtaga
tagtaatgcaactattttatttaaatattaatgtttataaaagttttcttatgtgcaatt
tgtaagcaaaaaatcaacattttaagtgccattattgattagagaaatcaagtaattatg
gtctttaattgaattattatatattgttatcccacatgaagtgccttgcgtttgatattt
gtttacaaaacaagatgatttcgtatttggttgaaacaataaattgttttcttttgtctt
gttatttgcacgtttgaacaagaataaagataattaaaattattaa
>DjScaffold5413:17712-20317 strand:+
CCTCCTTCATATGATGTTGCCATCAAATCACAACCAATCGCTCCTGGAAGTTACAATCAA
TATTCTGTAGGAGTACCACAACCAGCTGGTAACCCACAATATTTATACCAGCCAACTCAA
CCACAACCACTCGTTATGCAACCATCGCAGACCACGGTAATAATTCAATCTGGAGCTACT
GGAACCACAGAATGTCCAGATTGTCACAATTTCGTTAATCCAATTGTGGATTATAAAATA
GGATGTTTTGCCTGGTTGATGTGTTTTGTAATGTTATTATTTTTACCACTCGGTTGTTGC
TTCATTCCATTTTGCGTTGTCGGATGTAAAGACACTCATTATCGTTGCCCGACATGCATG
AAAACCATTTCGGTTACGTCTCGATGCTAAaaatctttacgaaattttatttcagtaaac
tacata
>DjScaffold4824:7268-34292 strand:+
aatttagaaaatttaataaaacgattaagatggttttttaaataaattatttcataaaaa
tagctattttaattataaaattttgaaattagagatttctgaatttcatgaaaataataa
ATGACTGAAAAATACCCGTATTTAAGTTCGGTGATTTTCGATAGCCCTAAATTGCCAAAT
GGGAAAAATGAAATTGAAAGAAAGTCTGAAATTTCTCAAAATTTTAGTTCTAGTAGCTTA
GTATTTAATGATGAACATTTTCAAGAAGTCGATTTAAATGGTTGTTCAGAAAATAGTTCA
GAATCTCCTATTAACAGTGAAAAAATGTATATGGAAACTAGGTACAGATGTAATGGAATTA
ATATCAGATGCTAATAATGTAGCATCAGCTGCTCAAACTTCAGACGTTTTCCTTTCGTCT
TTTTCAAATAACGTATTTAATTGTTCTTCAATTTCTAGTTTGTCTTCTATAACTAGCAAC
AACGATCTTTCAAATCAAATTAATCCATGTAATAAAGAAATTGCCAAACCAAATTTATTC
GACAAAAGTGATAATAAAACCAATAATAGAATCTCTGTGAACAATCAAGAAACTAAGACA
AATATTTTCTCTCAGATCAGTCAAATGATTTTTAATCGACCTCAAAAATTGAATAAATCA
TCAAGTGAAACTCCTCTTAAATCGACAACTTGCCTCATTTTGAATGATCGCCCTTTAGGT
CTTCCACCGAAATCAACCGATGAAATCAAACGTCACCAAGATGAATACAATCAACTTATA
TCAAATATCAAAAAGAAAGAAAGGCAACAAAATCATGCTAATAGAGCTCTGTTGAAATCC
AAAATTTATCAAGAAGAACGGCAATATCAATTGACTCATGTGTGGCGAAATTTTATACTT
CCTAATTGGTTTCATCAACAAACGTCACAATATGTTCGTCACTTATGGTGGGAAGGTATT

CCTACATCAGTCAGAGATAAGGTGTGGCGACAATGTATTCCAAATACTCTCAAATTAACC
AAAACTCATTACGACAATTTTTTATCACAAATGCAAGAACGTATTGAAAGATATCTGGAA
CCGGTGTCACAAGATGATCTAGCAACCAAAGCAACTGATAAACCAAATACTGATATCGAT
CTCAATAATAGGAATGGAACACATATTAGTCCAACAGTTGAATCGAAATCCTCAGCAGCA
TATGATTTATATTTTACAGAAACTGTCAGCATACACAATAAAAGATCTTCGTCGCTTAGT
TATTCAGCCAATGTTGGAAGCTCCAAGCAAACAGCTTCTTCATCAGATGTCGACATCAGA
CAAATTTTACTCAACACCATAAAACTTGATGTGTCTCGTACGTTTCCTCATTTAAATCTA
TTTCAAGACGGTGGTCCTCATCATGATTTACTCTACCAATTACTGGGAGCTTATGCATGT
TATCGACCAGATATTGGCTATGTACAGGGAATGTCATTTCTAGCAGCAGTTCTCATTTTG
GTGATGAATGTAGAAGATGCATTTATTTGTTTTGCAAATATACTCGAATTACCCTGCCAC
CATGCTTTCTATGCGTTAGATGAAAGTGAGTTTACTGTTTATTTGGAATCTTTCAAACTT
CTATTATTTACCCACTTACCTCAACTCGCTCAACATTTTATTCTACACAAGCTTGATGTC
AATATTTTTCTTTGTGATTGGCTGTTCACGATATATAGTCGAGCTTTACCTTTGGAAACG
GCTTTAAGAGTTTGGGATATCTTTTGTAGAGATGGTGAGGCTACTCTTTTTCGAGCCGGA
CTTGGAATTCTTAGGTGTTATGAAATGCAATTGCTCAACAGTGATTTTGAAGAGATGGCT
GGTTTTCTGACCGGTCAAATGCCGGAATCATTGACTGCATCAGTTTTGGTTGATGAAATG
CGCGAGATCAAATTATATGCTTCAAATTTTTTATCAACAATTGAAGAAGTTCGAACCAGA
TTATTAAAAGAAAAAGTCAGTTGAaatttagaaaaaattattattattgttgagattact
tactattactattattttacttgttttatatcaattgacgtttgtaaaatagtatgtata
ttattgtttaatgttgttgaatttcttcattcgtgttacactatcaaaccgttactatga
aaaaatattccatttcgtttgacgctaatttgtattgtttataatctttcatgtgatttt
attgttgagtttcactttctactgtggccaaatatctggctttaattttatttttgagta
ttatttcagatatttggttttattttataatttatgtgatgatttttttattattaatata
taaatatatata
>DjScaffold4824:43474-43971 strand:+
ATGACATTTTGTTTGAAAAATGCATCACACATATTTCAAAGACACATGGACAAGCGTCTA
AGTATATGTCAAGCTTTTTCTTCTGCTTCTATTGATGATATCCTGATCTCTTCCAAGGAT
GACAAAGAATACGAAATTCATTTAAAACAAGTCATGTCGGAACTTGAAAAACGTTTATTG
ATCATCAACTCGGACAAATGCCAACTCAATAAAATTGCAGTGGAATTTCTAGGATTCTCG
ATATCCAAGAAAGTTATAAAGTTTATCAATGAACGAATACAAGCTATCAAAAAGCTATCA
ACACCAAGGAATGCGATAACCCTACGAAGATTCTTAGGCACTATGGCATTCTATCAATGG
ATGATTCCACGACTATCAAAGACACTTGCTTCACTTAATGAACTTACAACATCATGTAAT
AAGAAGAAATTGTTGTTAGAAGAAACGAGTATATTAACAGCTTTGTGGAAGAAATTGACA
AAATGGTGGAGGTACTAA
>DjScaffold4824:45246-45677 strand:+
ATGCCGTTTGGAATTGCAGCAGCGTTAAGTACATTCCTTGAATTGATGACAAACGTACTG
GAAAGCACACCTGGAGCAATGGTATATATGTATGATATTTTAATTTATTCTGATAATGCA
ACGAACCATTTCAAGACGCTGGACACTGTGCTGAAAGAAGTAACAAAAGCTAAATTACGT
ATCAACTCCACAAAATGCAGTCTCCTGCGGAAAGAGATCAAGCATCTAGGATATATCTTG
AACGAAAATGGCATCCAAATAGATATTTCAAAAATAGAAAGTATACAGAACTTTGAGAGG
ACAAAATGTACAAAACAAGTTTTAAGCTTTTTATGTATGTGTTACTATTACGGGAAATTC
ATAGAGGGGTATTCTGGAAAAGCCAAAGCATTAGAAGAATTATGTGACAGAAACAAAGAC
AAGCTAATATAG
>DjScaffold3659:4221-12002 strand:+
atgatattttaattgtaaagtaatgttgttttagaaatttgctagttATGGAATCTCAGC
AACTTTTAGAAATATATCCATCAAATTCTATAAAATTTGAAGGTACTTTTAAAGAAGTAA
TTACCAAAAAGTTAACAATAAAAAATCCACTTGACAAAAATGTAGCATTTAAAATTAAAA
CTACCACACCAAAGCAGTATTGTGTCAGGCCAAGTGTTGGATTTGTAGCTCCTCAAGAAA
CTAAAGAAATTTCAATTATTTTGCAACCAATGCAATACGCAATTACAGAAAAGGCCAGAC
ATAAATTCATGATCCAGTCTTTAATTATATCTGAAGAAGAATCGAGCAACATTGATCATG
TGTGGAAAAAATGCAGCTCCTGGAAATATTATGTGTACCAAACTTCTTAGTGTTCTAGTTC
AAACTCCTGAATTGATCAAAGATTCTGAAGCTCTTATAGAACCTGTTAACTTAGTCACAC
CTGAAATGGTTTCAACCTTCGAATCATTAGAGTCACCAGTAGCTAGTCCAATTAACGGTT
TGTCAGAACATGGTGATGTAATTATGACTGACGAAACAAGCTCAGAAGAAAGTAAAGTAG
AGAAATTGACGGAAGAAAACAATAAATTGAAAGAACAAGTTATAACATTGAAAAAAATGG
TGTTTGACCAAACCTATAAATTGGAGTCTTTGCAGACTGAAACAAGTATCGACAAAGAAA
AAAAATTGTTGAAAGAAGAGGTCAGCCACCGAGTAGAATCCACAGGACAATTGGGATTAC

CTCTGATCATTTATCAATTGTTGGCGTTCTTCCTTGGTCTCATTATCGGGAAATTCTTTC
TATAAtgcatcattgattaattattgttattgttattattctggtcatggtagatgtgac
gatgctgtctgagattttcctgtgtctggttgttatctcacacacacatacacatac
>DjScaffold3659:63793-64568 strand:+
atcgtttagtttatttaatgcatcgatgtatattattctataaaattgtatatcattcga
tattattctatattaatttatatcaatgcataactaatctatttaaacaaatctgattca
ctaatattttattatcaataATGAATTCTCAAATACGACAAAATTTCCATGTTGAATCTG
AATCTGGAATTAATGATCAGATCAATAAAGAATTACATGCTAGTTTTGTCTATGAAGCAA
TGAGTTCATATTTCGATCGAGATGATGTTGCTCTTCAAGGTTTTAGTCATTTCTTTCACG
AACAATCTCATGAAGAAAGAGAACATGCAGAAAAATTAATTAAATACCAAAATAAACGAG
GTGGTCGAGTAGTTCTCAAAGATATTTCCAAACCATTAGTAAACGAATGGACAGCAGAAG
CAGCTTTTGAATCTGCATTAGCTTTGGAAAAAGAAGTAAATCAGTCATTGTTGGATTTGC
ATAAAATTGCAGAGAAACATGGAGATCCACAAATGATGGACTTTTTGGAAGGAGAATATT
TGGAAGAACAAGTAAAATCGATCAAAGAAATCGGTGATTATTTGACAAATATTCGTCGAA
ATGGTGCTGGAATTGGAGTTTATTTATTTGACAAACTTACTTTGAAAAAAATAAttgatgt
ttttataaatatttttgattttatattttttgttaaatctataattctaatctctca
>DjScaffold1863:24043-24743 strand:+
aaaatttcaaatttattgcaaaATGAAAGCAACTGGAACTTTGAAAGAATATAAAGTCAC
AGGTAGAAGATTGCCTACTGAAAAGGACCCAAATCCAACTCCTTATCAAATGCGAATTTT
TGCTCCCGATAATGTTACAGCTAAAAGTCGTTATTGGTATTTCATTAGACAATTACAAAA
GATGAAGAAAGGTAATGGTGAAATAGTTGAATGCAAGGAAATTCATGATAAGAAGCCAAC
TAATGTTAAGAATTTTGGTATTTGGATTAAATACTACAGTAGAAGTGGTATTCACAATAT
GTACAAAGAATACCGTGACTTAACCTGTGGTTCTGCTGTAACTCAATTATACCAAGAAAT
GGCTGCTCGTCACAGAGCAAGAGGTAGCTCTATTCACGTTATTGACGTAAGTGAAATTGC
CGCTAACAAATGTAGACGTCCACACATGAAAGCCATGCATGATTCAAAACTTGTCTTCCC
TGTCAAGAGAAGCCTCAACAAAAACTTGCACAAACCAAGGTTTTCAACAAGCAGACCAAC
CACATCATTTTAGatacattaataaatttttggatataatttaagttattgaattcaaaa
taaaacaaaa
>DjScaffold1863:31228-43219 strand:+
tttttttctctttttatgaATGTCTTTGAAATTAAATATCTATAGAGAGATATGTTCAA
AACTTTTCGATCCACTATACCAAGAGAATGTCTGGCGAATTCTCGTACTTGATGAATACA
GTACGAAAATAACTTCAAATATTCTTAAAATGCACGATCTTTTTGAAAAAGGAATAACTC
TCGTTGACGATCTCAACAAAAAACGACAACCCGTGCCTTTGGAAGCTATATATTTGATAT
CTTCACAACAAAATAAAATCGAGCTAATGCTTAATGATTTTATGTCAACACCAGTTTATA
GCAGAGCTCATGTGTTTTTCATTGATGAATGTAGTTCTGAACATTTGTCCTTAATTGCGG
AATCAGTGGCTGCTAATTACATATCAACTCTAAAAGAAATCAATTTAAAATTTCTTATTA
TTGAAAGTCAACTTTTTCATTTGGATTTTCAATTAGATGAAAACGCATTTACATCTTTCA
ATGATCTAGATGATGAGTTAGCATGGGAACTAGCTCGAGCTATTACTAGTCTTTGTGATA
TTTTAAGTGAAAATCCTTCTATATATTATCAGACAACTGAAATGGCTCAAAGATTCGCAA
TTAAAGTGCAACAAGAAATGTTCAAAAGTGAAACGGAAAACGGTTACGTGGGGCATACAA
TGCACAAAGATAGAAGCGTGCTATTAATATTAGATCGATCAATTGATCCTCTGACACCAC
TATTGCACGATCTTCATCTACAGTGTTTGGCTTATGATTTATTGGAAATCGAAAATAACA
CATATCAATATAAAAATTTTGAAGCTGACAAATTAATAAGTGTTGATTTATCGGAAAATG
ACGACTTATGGGTTCAGTTGCGCCACATTCATATTGCAGATGTTTCTAGAATTCTTCCTG
TAAAACTAAAACAATTAGCTAACGCTCAGAAATGCATTGATAACAAGGTAAATGGACCGG
AAAATATTAATAAAACTCCAGATTCTAGTTCTGATAAAACATCAAATAAAGACTTAGTAA
GAAAG
>DjScaffold1863:70274-71049 strand:+
aacaaacactaaacaaataattattaaatgcaaacaatacttttatcgattttatttcta
tttagttatcgaagatatctagcctcaaacagtttccatttcttattaaaaatagaaaaa
atattctaaatggttccagttatactttatcaagatggataccatttattgaagatctta
ttgcggatgtgattagaggcaatttcaaaactggattaatagcaaaacttgaccagccaa
gaacaaatccagattatttgaaacctgatcttaatggttatgtattacctcaaagacaaa
gttgggcaagattaaatgaaactgaaaatcacacaggacctcgattgattatatttatta
tcggtggtgtttcattagcggagtctcgagtggcatataactatacaaaattgtgccatt
ctATGAGGTCTGGAAATATTCCTGATGAAATTAAAAAAAATATTAATTTTCCAACAGAAA
ATGCTGGTGGTAGAGGCTGGAATTGGGAAGTACTAATCGGTGGAACATCAATACTGACTC

CAGAAAAATATTTAAATTTTTTGGCAAAAACAAAAACAACGACGCCTTGActgtttataa
attgattgtttttattttttgacaaatttaagagtttttgtaaatcacgaataagtaaat
tagtagttagattt
>DjScaffold3720:135728-135964 strand:+
ATGCCATTTGGCATCGCAGCGGCACCAGGGACATTTCAAGAGATGATGACAAAAGTGGTA
AGAGGAATCAATAGAGCCATGGTTTATCTTGATGACATACTGATATTTACGAACAATAAA
GACCAACACTATGAAGTAATTGAAAGAGTATTGACGGAAATAATGAAGGCTGGTCCGGAA
TTGATCCGGAAAAATGTCAGTTTCTTCAAAGTGAAATCAAGTTTCTTGGACACATAA
>DjScaffold4039:19189-23092 strand:+
ttttaaagttgtaatcacattgttagatattaaagtaatcattagaaaATGCCATTAGCA
GGTGGTGTAGCTGCACCAGTGGTTGCTCCACTAAAATTAAACAAATCCGGAAAGCCGAAA
AATCACATTGATACTGGAACATATATTGAATTAGATTCAGAAACACCGAATTGTGGAGTT
TATTTTACAACCGATGGAACTAAACCTAATCCGTGGAAACGGATTATAAATGGTAAAGAA
ATCACTTATAGATATAATGGACCATTCACATTGAAACCTGGAAAAAGAACATTGAAAGCT
GTTGCCGTTCATAAGGTTACAAAAGTCGAAAGTCATGTTGTAACGAAAACATTTTTCGTT
GATGATATTGTAGATGTTGAAAGCGAAGATGAAAAAAATGATGAATCGGATTCAAATCAT
TCATATAATTCAGATATTGAAGATAAGAAAGTTTCTAAACATTATTCTAAACCTCGTGAC
GCATGGAATGAAGATGCAGTTGACAAATTTCTTCATAGAACTGCAGATCAAGGATTTGCT
GCAACTAATGTCTCAGGCACTCAAATCAATTTGTGGGGAGGAAATCCTGGAATGGGCTGG
GATGTACGAACTCCAGGTAATACTACATTCTCTGGCAGTTATGCACCACAAAATCAATTT
CCACCGCAACCAATGTTTCCACAACCAGATATGTCCATGATACAGCAACATCAATTAAAC
GCTATTGCCAACCAATTAACTCAACAAATAGATCATAACCGACAAGCTACAGTTGCAGAA
GTTCGAAATGCATTAAAAGAATTAGAAAAAAGCAAAATGAAAGCTCTTCCTCCTTCAGCT
GAGCCAGTAAAAAAAAATACTGAACCTGAGGTTTTACCGGTCAGCCCCGGTAATGGAGAT
TTGAAATCTCAATTGGAACACATATATGGACACTTATTGCAGTATGCACGAAGTAATGGA
GATTTCAAAGCGGCGATTGCACCAGCAAAAATGGGGAAAGTAATATCA
>DjScaffold5095:19206-30078 strand:+
ggaaaaATGGATAAACGTTTTATATCTAGATATAGTATTAAAGATTTTAAAAGAAAACGT
AAAAGTGATGTTCAAACATATCGTTTTGAAGAACCATGTTTAGATGAAGACGTTAAACCA
TTTAAACGTCATATTACAGAAGAAGATTATGACACTTTTACAGACAGCTCAAATTCTACA
ACTCCATTGTATAGTAATAAATTCCAGGAATATGAATCTTCAACAAATGGCGGGTTATCT
AGTGATGAAAGTCAATATAAAGATAATTACAGTTATGATGGTAAATCAAATAGAAAAATT
AATGGTGATATACCATTACAAATTAAATCAGAAAAAATTTTACATCCTGGAGTTGAAACC
AGTGGAAAATCTACTCATAAATCATATGTATCAACTACTAATGAGAATCCTGTATCGTGT
GAACAAGATACTTTTCGTGTTGATATGAGTCGATTTCGATATTTCACTTCGTCTACTCAT
GGAAATAGTTTCCTGAATAATGACTTCACCAGCTCGTTAGAATCACCTACGTACAAAATT
AAAACTTCTTCAGCAACTCCTCCGAGATTGCAGCTGCCCAGGAAAAACAAATTACACATT
TATCAGCGACACAGACACAATAAAAGTGTTACAGTCAGTTTAGTGCCATGCAAAGGTCCA
GCTTGTACATCTCAAATATGCTGTGCGCATTTCTGTTCGCAGAAATGTTATGATAACCAG
AAATGGCCAATTGGATCCGGGAATAATTTCAAAACCTCAATACCAGATTATGATAGTTGT
GGCCAGCAAGAAGTTTGTGATTATGGTTTGCATTATCCTGATCACACGTATTGCAAGGAT
CACAAAAATATTTACTTCAATGCAGATCCTAATATTGATTGTCCACCCTTTTTCGATCTC
GTAGAATCGATATTTTACGACGAAAAAGAAATTAGAATCAAAAATCCAGTTCCAATTCCG
AATTGTAACGGTGATCCGATTTCATCGAACGGTTATGTTTCACAAACAACTAAACGCGGG
CGCCCAAAACGCTCAAATGTTGTCGGATTGCTCAATTCCTATATTTTCCATCCGAGTTCC
GATGTGATTTATACGCCGTTCATATTTAAAGAGTGTATTGTCGAAGCTGATAAAATGTGT
ACAGATATGCAATCTGCCGATCCGATTGTGTATGCATTACGGCAATGTAGTAATTTATAC
AAAGAAGTGAGAGCAGATATTCCGAAAGAAGTTCTCTACGGAAATGATAATTCAGTTGAA
AATTCAGATATCTATGATAAGATCAAACAAGAATGTAAAGATGATCTACCTTCAGGTCTT
GCTTTTGATGACTTGTTTGATCAGGTTGCTGATGACTTTTCCTTTAAATGAgctgagatg
tgatgtgctcgtctctctctctctatctctctgtgtctgtgtgtctgtgtgaccgg
agaaaaatggataaacgttttatatctagatatagtattaaagattttaaaagaaaacgt
aaaagtgatgttcaaacatatcgttttgaagaaccatgtttagatgaagacgttaaacca
tttaaacgtcatattacagaagaagattatgacacttttacagacaggttaacaattaat
tggtttcaagttcataagaatattttagctcaaattctacaactccattgtatagtaata
aattccaggaatatgaatcttcaacaaatggcgggttatctagtgatgaaagtcaatata
aagataattacagttatgatggtaaatcaaatagaaaaattaatggtgatataccattac

aaattaaatcagaaaaaatttacatcctggagttgaaaccagtggaaaatctactcata
aatcatatgtatcaactactaatgagaatcctgtatcgtgtgaacaagatacttttcgtg
ttgatATGAGTCGATTTCGATATTTCACTTCGTCTACTCATGGAAATAGTTTCCTGAATA
ATGACTTCACCAGCTCGTTAGAATCACCTACGTACAAAATTAAAACTTCTTCAGCAACTC
CTCCGAGATTGCAGCTGCCCAGGAAAAACAAATTACACATTTATCAGCGACACAGACACA
ATAAAAGTGTTACAGTCAGTTTAGTGCCATGCAAAGGTCCAGCTTGTACATCTCAAATAT
GCTGTGCGCATTTCTGTTCGCAGAAATGTTATGATAACCAGAAATGGCCAATTGGATCCG
GGAATAATTTCAAAACCTCAATACCAGATTATGATAGTTGTGGCCAGCAAGAAGTTTGTG
ATTATGGTTTGCATTATCCTGATCACACGTATTGCAAGGATCACAAAAATATTTACTTCA
ATGCAGATCCTAATATTGATTGTCCACCCTTTTTCGATCTCGTAGAATCGATATTTTACG
ACGAAAAAGAAATTAGAATCAAAAATCCAGTTCCAATTCCGAATTGTAACGGTGATCCGA
TTTCATCGAACGGTTATGTTTCACAAACAACTAAACGCGGGCGCCCAAAACGCTCAAATG
TTGTCGGATTGCTCAATTCCTATATTTTCCATCCGAGTTCCGATGTGATTTATACGCCGT
TCATATTTAAAGAGTGTATTGTCGAAGCTGATAAAATGTGTACAGATATGCAATCTGCCG
ATCCGATTGTGTATGCATTACGGCAATGTAGTAATTTATACAAAGAAGTGAGAGCAGATA
TTCCGAAAGAAGTTCTCTACGGAAATGATAATTCAGTTGAAAATTCAGATATCTATGATA
AGATCAAACAAGAATGTAAAGATGATCTACCTTCAGGTCTTGCTTTTGATGACTTGTTTG
ATCAGGTTGCTGATGACTTTTCCTTTAAATGAgctgagatgtgatgtgctcgtctctctc
tctctctatctctctctgtgtctgtgtgtctgtgtgaccgga
>DjScaffold75:52556-55928 strand:+
TCTTTGAAAGCAAAATTTAATTTAATTTTACTGCCTTATTTATATCAAACATTTTCAAAT
TTTTGTAGTTTATTATCACATTATTTTGTTTATAACATATTAGACGAAATGAACAAAATC
TCTAAATTTTGCTTTACAGTTAGAAATAATTCACTCTTAAAATTTAGTTCAATACCTTAT
GTATCATTAAAATGTTATCATAATCCTGTTCATTTCTTTTCTGAAGAAGAAAATATGATG
AAAGAGACTGTTTCGAAATTCGCCATTGAAAAAATTCAACCATTTGTTAAAAAAATGGAT
TCGCAAGGTTATATGGAAAAATCTGTTATTGATGGACTTTTTGAAAACGGTTTAATGGGA
ATAGAAGTTCCTGTCACTTACGGTGGAACAGAAGCTAGTTTTTTCTCATCTATTTTAGTT
ATCGAAGAGTTGTCTAAAGTGGATCCAAGTATTGCTATTCTGGTTGACATTCAAAACACA
CTTATTATCACGTTAATAAAACAATTGGGAAGTGATGAGCAAAAATCATATTGGTTACCT
AGACTTGCCAAAAATACAATTGGAAGTTTTGCTCTTTCTGAATCAGAATCAGGATCAGAC
GCATTTTCTTTGAAAACTGTAGCTATTGCCGATGGAAATGATTTCATATTAAATGGATCA
AAAATGTGGATTAGTAACTCTGTAGAAGCAGGAATGTTTCTTGTTATGGCAAATATTAAC
CCGTCTGATGGTTATAAAGGTATCACTGTTTTCATAGTTCCTAAAGATAGTCCGGGTTTA
TCGATTGGTAAAAAGGAAGATAAATTGGGATTACGAGCTTCTTCGACATGTCAA
>DjScaffold75:171653-172939 strand:+
aatatgaaaacccaataaaatctctactttcatcagttaaatgtcatgaaccagttccat
catcgATGTACTGTGTTCCCAAAGATCACAAAGAAATAATGAAAGGAAGACCTATTGTAA
ACGCTTCTGACACTCCTTCTACTGCATTATGCAAACTTTTAGCAGATATATTAAAATCAC
TATTAAATTTCATTCCGGCACATTTAAAGAACAGTTATGAGTTTATTGAACGAATTAAAA
ATTATGAATGGAAAAGAGATGATGAATTTGGTTCATTAGATGTAACAAATCTATATGGAT
CAATTCCAATTGAAGGACCTGAAAATGTCATCGATATTATCACCAAAAATGATTTCAAAT
GTCTTATCAATTTGTGTCTTTTCTCTGATAGCATTTTTATCGGTGAACAACTTTTTAAAC
AAAAAGGTGGTCTTGCAATGGGTAATAATTTATCTCCAATCCTAGCAATAATTTTCATGA
ACTTCATTGAAAATCAAATAATTCTTAAATCAAACAAGAAAATTCATGTTTGGCTTAGAT
ACATATATGATATTTTCTTTGTAACTAAATCTCCTTAAgagaacatattagtattagcca
acagtataaattataatattaaatgcactttagataaatcaatcgactttcagatttctt
ttctggacacttaacaaccatttcacagtggacaaatattaccttgcagtattaatacga
acatcattcagaaaatttgtataattaaagatgaaagaattcgagctgagagactatctt
caaatgaagaaatgatattgatttctctcaatttgataaaaaattaattcttaaataatg
gctactcaaaaaaattatatcaaatacatttgtgatgaaaaatgtaacttataaaataac
atgcaccatttttcaagaaacatatgtgagtgaaacatacagaaccatctgaacaaaaat
taaggaacctttgaaaccaaaagaaactgaagtcttcaaaatttttcaacacaaacataa
aacttcaccagaacaacgtcacattacacggaaaacactaggaacaggctacatttctac
actacatagaaaaaaaacataaggaattactaattaaataagacaatcataaaataaatg
taatttgtaataattaaatttaaataaaaatttaccaattaaccaattaaaattcaataa
aaataaattaattataaaa
>DjScaffold75:134496-134606 strand:+

ATGCCATTTGGAATAACTGCAGTACTAGCAACATTTCAAAAATTAATGACAAAAGTTTTA
GGACCCTTAAAAATTGGAAGGAAGCAATTGTTTATCTTGATGATATACTGA
>DjScaffold4079:9994-10599 strand:+
gatttttaatttttcaaaattggtttaaataatataaattaattttATGTCTTGTACAAA
AGTTCTCACTGAAACCAATTATACAATTGGTTCAAGTTATCAAGGAAATACCGGTTGTGA
TACCGGTATTTCCACTGTGGGTTTTCGAACTGGGAAATATTCACCAGAAGAATGGAATGC
ATTGAATTATGAAAAATATCACCAGTCTTTGATGGATAGAGAAGTGTCTCAAAGATTAAG
AGAAGAAAGTCGTTCTCTAATTAAAGAGACTGACGCAGTGACTGCAAAAAAATCAATTTGA
GTCAACAAAAAGACTTTTAGAAAGATTACATGATATCAACTTTTGGAAATCAGAATTGGA
AAGAGAAATTCAAGACATTACTGATGAAAATGAATTGCTGGTGGAACAAAAACGACGATT
AGATAATGCTTTAATCGCTACTGGGTTACCTTTGCATATAGCAACTGATAATATCAATTG
TAGAGATCAGCGATATGGAGTAGATAGAGTTGAGGACGATGTTGAAATTAATCTAATAAA
GGAAGTCGATATCATCAACAAAGTCCAGGAATTACTAAAGAATACAATTGAACATGCAAA
TGAACAAATTCAACGAAATCGAGATGCAAAAGAAATTCTCGAAATGGATTGGAGTAATAA
AAAAGAAGCTGATGAAAACGATTCGATTGCTGCTGCTTTGAAAAACGGTTCAACAAATAA
ACAGTTTTATGCTGGTGTTGCAAGATATCAAGAAAATATGTCAGATCCGGAAACATGGGC
TGCAGAATCACACAAGCATATTTTACATGCAGAAGGCGAGAGAATGGCGAGTGTGGAATT
GCGAACTTTAATCAACAATATTTTAACTGACACTTCACGAGACATGAGACAACAAGCTGA
TGTGGTTGAAGTTGAATTGAGTAAAAATATCGATCGTTTGAATGATTCTAGAAGTAAATT
GGAAGATAATCTGAAAAAAACAGTAGGAGAAATAGCTAAACAAGAAAAAAATATTGATGA
AATTAAGGCTGCAATTAGAGCTAAAGAAGATCCAATAAAAGTTGCTCAAACTAGACTTCA
TCAACGGAATTTCCGACCAAATATCGAATTATGTAAAGATCCGGCCCAGGACAAACTTTT
AGCAGAAGTTCAAGATCTACAATTGTCTCTTGATGCTTTAGAAGCACAAAAGATAAATGC
TGAAAATTCTTTAAAGAACCTTCTTGATACTCGAGTTACGCTTGAAAAAGAAATACAATT
AAAAAAGAATTCCATATTTATTGATCAACAAAAAGTACTACCAAGACGAAGTCGGTATCC
GAGTGTTTTGAAATTACAAGGCTACAATGCTTGAactaatttgtgcacatttttctacat
acataattgagtgttgtgacaaaattagaaaataattatatcaattgatctgtttctacg
gttttgaaatatcgtttgatttgattaaatacttcggatttgaaaattttattttctttt
ttttcatttgaaaatatgttttgttaacttaaatctaagatgttaatatattatcagatg
tagaaatataggaattgtatattagatttttaatttttcaaaattggtttaaataatata
aattaattttATGTCTTGTACAAAAGTTCTCACTGAAACCAATTATACAATTGGTTCAAG
TTATCAAGGAAATACCGGTTGTGATACCGGTATTTCCACTGTGGGTTTTCGAACTGGGAA
ATATTCACCAGAAGAATGGAATGCATTGAATTATGAAAAATATCACCAGTCTTTGATGGA
TAGAGAAGTGTCTCAAAGATTAAGAGAAGAAAGTCGTTCTCTAATTAAAGAGACTGACGC
AGTGACTGCAAAAAAATCAATTTGAGTCAACAAAAAGACTTTTAGAAAGATTACATGATAT
CAACTTTTGGAAATCAGAATTGGAAAGAGAAATTCAAGACATTACTGATGAAAATGAATT
GCTGGTGGAACAAAAACGACGATTAGATAATGCTTTAATCGCTACTGGGTTACCTTTGCA
TATAGCAACTGATAATATCAATTGTAGAGATCAGCGATATGGAGTAGATAGAGTTGAGGA
CGATGTTGAAATTAATCTAATAAAGGTTCGAATATACTCA
>DjScaffold1201:1167-2732 strand:+
ATGAGTATTTCCGTTCTTTCATCAAATAATCTTAACGACGGAGACAATAAAAAGTTGAAT
AATTCGATTGATTGTGACTGGAAAAAAAGTCTAAAGTTACCACCAAAAGATAATAGAATC
AAAACAAATGATGTTTTGGCAACAAAAGGAAGCACTTTTGAATCTTATCATTTGAATCGA
GATTTAATGAAGGCCATTTTTGAAAAAGGTTGGGATACACCTTCTCCAATACAAGAACAA
GGAATTCCAGCTGCAATGTCTGGGAGTGATATACTAGCAAGAGCGAAAAAATGGTACAGGA
AAAACTGGAGCGTATGCTCTTCCTTTATTAAATAAAATAAATCCTGACTTACAGGCAGTA
CAGGGTTTAATACTTGTTCCAACAAGAGAATTAGCACTACAAACCAGTCAAATATGCATC
GAATTATCAAAATATCTAAAACTTAAAGTAATGGTTACTACTGGTGGTACAGATCTCACA
GAAGATATTTGTCGACTTAATCGACCAGTCCATTTGGTTATTGGTACTCCGGGGAGAATT
CAAGACTTATTAAAGAGAGAAGTGATTAATGGTGAATATTGTAAAACATTAGTTTTGGAT
GAGGCCGATAAATTATTATCAAAGGATTTTAAAGAAATAATGAGTAATATATTGTCATGT
CTTCCTAAAGATCGTCAAATACTTCTTTACTCAGCTACGTATCCTGTTACTTTGCATGAA
TTTATGATGAAACATTTAAAATCACCAATTAAAATTAATTTAATGGAGGAATTAACCTTA
AAAGGAATAACTCAATATTATGCATATGTTAAAGAAAAGCATAAAGTACATTGTCTAAAT
ACATTATTTTCAAAACTTGCTGTTAATCAATCCATTATTTTTTGTAATACAACACAACGT
GTCGAGTTACTTGCAAAGAAAATCACTGATCTTGGTTATTCGTGCTATTATATTCATGCA
AGAATGTCACAGCATTTTAGAAATCGAGTTTTTCATGATTTTAGAGCTGGACATTGTCGC

AATCTCGTTTGCACAGATTTATTTACAAGGGGCATAGATATACCAACAGTGAATGTAGTA
ATAAATTTTGATTTTCCTAAATACGCAGAAACATATTTGCATCGTATCGGACGATCAGGA
AGATATGGTCATTTAGGAATAGCAATAAATTTAATAACATATGACGATAGATATGCATTA
AAAGAAATTGAATCTCAACTCGATACAAAAATTGAAGCGATACCAAAAGATATTGATAAA
CGACTTTATGTATCAGAATATCAAATTGAAAATAATTTCGATGAACCAACAAAAAAGGCT
ATTATTAGTGGCCGCGGACTTCCTCGTTCTAATAAGCCTAGTGATGAAATGAAACGAGAA
ACAACTGAAATAGGGACTGATATGGCAAATGGCAATACCAATTTAAATTAA
>DjScaffold768:29841-30536 strand:+
TATCCAGGTGAAAGACATTTATTTAAACATAATTACACTGGACCTGGTACAAGATTAGAT
ATAAGATTAGATGAAGATAATATTCCAAAACCTGGTGAAGAACCAATTAATAGAGTTGAT
AAATCTGCATTAAAACATGACATTGCATATAATAGTGAAAATATAAGAGATAGACATGTA
GCTGATGTTAATTTAATACATGATCTTAATAATATTGAAAATCCTACATTAGCAGAAAAA
TTTGAAAGAGCACTTGTAAAATTAGTTATGAAAGGTAAAATTCTTATTGGAAAGGGTGGA
ACTAAAAAAGATCCATTTAAAAAAAGTACAATTATAAAAGATGAATCTGATCATGAAATG
GAGGATAGACAAATCTTAAGTAAAGAACTTCATAAAGAATATAGAAAACCTAAACATTTT
AGAAAAGTAATTGTTTATGAAAAAGATGATATTTGGACTGCAGATTTAATATTTCAAGGT
ATAGAAAGAAAAGTTATAGATTTATACACTAGATTTGCATTTGTTAATCTTTGTAAAAAT
AAAACTGGACCAACAATAACAAAAGCATTTGAAAATATTCTAGAAAAAAATAATAGAGTT
CCAAAGAAATTATGGACAGATGAAGGATTAGAATTTTATAATTCATCATTTAAATCTTTC
TGTAAAAAAAATAAC
>DjScaffold2245:1991-2170 strand:+
ATGAATAATTTAAATATGGCGTTTGAAAATCTTCGAAGAAAAGTTCCAACGTTTGTGTTT
GAAAAACGCTTATCGAGAATCGAAACTTTACGTTTAGCAATTATTTATATAAATTTTATG
AATTGTATGCTTAACGGAATACAATCTGATAAAATACGTATTCAAAGAACAAAATTTTAA
>DjScaffold5266:37530-52006 strand:+
tacctagaatcagtcatcaaagcaaatcgagtatagataacattattaaagattccacga
gtaaacattcaaattatatcaataattctttcaagcaattcacaaatacaactcaaagaa
gaaaattatctgaaatatcttcgatagatagttataatagtaaatgtagtttttcaaaga
agcgacttttcgataataaaaaatgttccattatgagatctacaagtcatcgatcaatag
gatcatttgctaatatcattataggtaataatgaatattttatggtcaatgatatcattt
agaaataatttctttgaaaattttccagtattacaaattatgttattttcattgtaattt
gtagaaattaatagaatttcctttcatttaaggtgaagataccaaacaaccgccagatgg
aaaaataataaatgaaaatcaaaaatatagaaataattcatgtaatgaaaatgatattag
atcggaattgtatgaagaggatgtgttggatcaacctgtctttccgggaacatggaaaca
tttactactgacaattattccgcttgatttagatgaatggaaagaacaatctgttccttc
taaaatcttttctgtgatacaaatcccatatgtacttgtatttactttgacaattccagt
tgtcgatgaaaaagctctgATGAAAGGATGGTGCAAGATTTTGAATTGCATACATTGTTT
ATTATCTCCAATAACTTGGACTATAATGGCTAATGTTTTTCTTACCAAATTCGGAAATAG
TAACTTCATTTTAATATATTTATTTCTAATTATCGGAGTGATTGTTACAATTGTTGTTGC
GTTTACATCTACTGTTGATAAGCCACCAATTTATCATCCTGCTTTTGCATTTATTGGACT
TATCACATCAGTTTTGTGGATTTATGAATTATCAAATGAAATCGTTAATGTACTCTCCGT
TTTTGGAGTTGTCTTCTCAATTTCTGAAGTGATTCTTGGATTAAGTGTTCTGGCAATTGC
AAATTCTATAAGCGATCTGGTTTCTGATATAGCACTTGCTAGAAAGAATTATCCACGTAT
TGGTTACACAGGGTGTATTGGAAGTCCTTTGTTTGGATTATTAATTGGAATTGGTTTACC
GTTTTCAATAAAAACTGTCAGTGGTCCTGTACAGGTAAGTTGTTTTTTATGTATTGAATT
AGGTTTTTAAtatttttctattattagtaaatggtaataataataataataatagtaata
ataataataataataaatgataaaaataatataatagtaataat
>DjScaffold2316:52443-65339 strand:+
aaataaacgaatgtagataaaaaataactccaatatgtacgcttatttattatataagaa
aggtatacttttagaaaataaagtttggtagtaaagaatttatataatgttatttgtgtt
ctttttctacagaaattcaaaataatttttggcattaattaaatagaaatgttaaaaaga
tttgtagaaattagaatcggggtttgaatttttcagtaatttttgtttcattatttttt
cacacttgatttttaatttataattaaataattttcgtactgtaatattaatttcacttg
ttttttttaaaatttaaatatttcacaattcattatttgtattcctataggaatacatc
tttgattagtgatatcgttgatttttctgaatgctaccatgaattagtaaagtgctgtta
ttttctgactgaattaaaacaattaagtaacggtagtaatctatttccttcgtcagacAT
GAAATACAACGAAGAATATCAAAGACTTATTGATGAGTCTGATTCTTTAAATCAAGAAAA

GTTTTTCGGGAAATGTTTAGGATTTTACTACTGCAGTAGTGCTCAAAGAATTCTTCAAGT
ACTTGGATCAATAATGTCCGGATATGCACATTCATTTATTACAAATAAATCTGGTCTACG
TGCCATGTTTAATACTGTTTTACAAAGTGTAAGTTGTTTCTGGAATCCTGAAAATCGCGG
TCAATTAATGAGTTCTATTACTAAAAATGCTACCGTCGAATTCACAAAACAATTTTGGAA
TCTTGGTGAACATCCTTTAGTTTTGGAAGCTCCAAATATGATTTTGCCACCTGTTGAAGT
AGCGATAAATTTTGATTTGCCTCCAATTGCATTTAGCGTTCCTACCAAAGATTTCGATTC
TACAAAACCTAAAAATGTAACAATTTATCCTCCTACAGAAAAGAATTCTTCAAATTCTAC
TGCTAGTATAAAATTGAGATTAATCAGTTTGAAAATAAGGGAAGGTTTGAATTGGATAGA
TAGAAGACAATCCAATGATTATATTACCACCGATACATCATCATTTATTAAAGGTTTCAG
TACAAAACCCAATATATATAAAGAACGATCGAAACATCTATTAATTCACTGTCATGGAGG
TGGTTTTGTGGCACAATCTGCCAAATCTCATGACACATATTTACGACTATGGGCTGAACA
ATTGGATTGTCCTATATTGTCAATTGACTATTCACTAGCTCCGGAATTTCCATATCCTCG
TGCACTAGAAGAAGTTTTCTATGCAGTTTCTTGGGCAATTAGAAATCGTCGTCAGCTAGG
ATGCACAAATAATGCCAAAGTAGTTCTTTGCGGAGATTCTGCTGGTGCAAATTTGGCATT
ATGTTCCACTTTGAAATCTATTAGATGTGGAATTCCAGTTCCTGATGCTTTATTTCTTGC
ATATGTTCCAGTTTTGATCACCATGACACCTTCGCCTGCTAGACTTTTAGCATCTATTGA
TCCTTTGTTACCTGTTGGCATATTGGCTCGATGTTTATTAGCATATGTTGGAATCGATGA
GAAACAATTGATCAGTGAAATAGATCACAAAAATTCATTCCAACCCTCAACGAATTCCCT
GTCTGGAAATTCCATCAGAACCGATCAACCGACAAAACCAAGACACAGTTTAATTGAAAA
TAACGACACAAAACTTTTGATGGAAATCATTCAGAAATCCAATGAAAAATCGTCTCCGTT
AACAAAAGTTCGACTTGCGAAAATTCCCGAAGATCCTTTCATGTCTCCTTTATTGGCGGA
CGATGATATATTTTCTAAATTACCTCCTATTTCTGTGATT
>DjScaffold2316:76828-80907 strand:+
attctaacaatgagcaactttcaataATGGTTCATAAAGTGGCAGATACTTGGTTACTTG
ATGAATTTGATATTGATGCTCTCTTTCTTCGTCATCAGCATTATGCTGACTGGGATTGGC
TGCGTAATTTTATTAAACAAGAATGCTTTCAATTAGCAAAACCTGATTTCAGTCGGGCAT
CAATATCAATGAGAGATTTATATGCTAAATTTTTGTATCACAGTGTCAGTCAGCCGAGTA
TTGAAATGCCTAATACTGAAATGCAGAGTATATCTGGTGACCAATCACCATCACAATTAT
CAGTATTTAAACAACCAATACGATTGAAAATGGAATCCAAATTTTCCTCCACCAATCATC
GGTTTGATACAAAAAAATGAAGACTTTCTGAATCTTGTTGATTGGAAATTAGAAGATTTAA
GTTTTGTAGTTGGTTCAGATTTATCAATATTTGGAACCAAAAGTCATCCATGTATAAGTC
TTAGGTTAAGTCCTTGTCATGAACCAATAAATATTTTAACTGGGATGGATATGTGGTTGG
AGAACATGTTGAATGAAGTTCCTGAGATTGCCATGTGTTATCACGATCAGGGAATTGTTA
TGAGAGAGTATGAAATTCTTAAGACAGAAGACATTCCAACATTAGCTGATTTTAATCAAG
AAGAGGTTAAATCTATTGCAAAAAATTTGTCACATTTCTTGTCAACGAATGCCACAAAAG
AAGGACATACTTATTGGTTATTTAAGGAATCTGATAGTACAACGGTTAAATTGTATGATT
TAACAAGTATTTGGGAGAAAGACCAAAAAGAAATCCATAGAAACAAAACACAAAATCAGG
AAAATCTATATGACAATAATCCATTTATAATACCAGTAGCTATTTTGTGTTATAAAATGG
CTGAAAAACGCGTTAAT
>DjScaffold2316:113047-113945 strand:+
ACAGATTTACAAGATTTTAATAGTAATCTAGATGACAATCTTGTTGCTTTAGTATGGTCA
AATAAAGCTAAGTTATGCCGCTTAGCAAGTATATTATTATCTGAAATTCAATACGATTTT
ATTGCCCTCGAGGCATATACTAACGCAATTGATACTTTATCAAAACAAAATAAAAAGAAA
CCAACTCCAATGGCTCTCAATATCAAATGTGATCGTCTCGATTTATGTATCTTGACCCTC
GAGAAAGCATATAATTTTTATAAAACGATTCCGGAAATGAATCGAGCAGTCGATTGCGCA
GAAAAGTTTCATCGCATTGAATTATCTTTACAATCAATACCAATTATAAAATCAAAATTA
ACCAGCTTGCGTCTCCGAATCAATTTTATCCGCTATTACCTTAATCTCAAAGCAAATCGT
CCTCTGAATTACGATCAAATTTTAGGTGATCTGAAAAATGATCGTACATTAATTCTGCAA
CAAGCAAACGACTTGGCGAAATGTAAAGAAATATTATTGATTCAGTTCTACATGTTTAAA
ATCCAATCTGATAGATGTGGTAATTTTTGAatttcgctaatttttatagaatttcattag
ctcattatcacaattatctatttccaatgttattttcaactattaaatacttaggaattt
aaaattaatcaatttagatttatacgaattgcacttttaaaaaattgtatttttattcta
gcatattgattttatattaaatttatatcagatgttaaaagaaaagtaaaaatttaaatt
tttcattgattgtgttggcgcgtaatgttttgcggaatttttaagaaaaaattacaatatt
aattttt
>DjScaffold1919:52499-67936 strand:+
tgtttattcttgtcatacataaccaaccagcaaatgatgtttaaataattattttaattt

ctggaatttaaaaataatgttatagtgATGAGATTGGAAGAAAAAGAGAAAGAACAGAAA
AAAGAGTATCAAAAACTTTACGAACGTTACACAGACATTTTCAAATGTCATGCTGATTTA
TTGGAAAGGCTAAAGCTTTCTGGGCACGGAAATGTAGTTTCTTTACAGAAACATTCCATC
AGCAAATTCATGAAACCAATTGACATAGGAATGGAATCAAATGATGTTTGTAACGATGCC
AATTATAAAAGATATGCACAGATGGATAATTATGATTTGGAACTACTCGAACAAACTCTT
TCAACTGACTGGAAAGATACCGGCCGGCCTGAATGTGTCGCCGATATTATTAAGGATAAT
CCAGAAACCCAGTCAGAATCTAATGTATTAACTGAAAGATCCCAAGAAACCGACAGCAAC
ATATTCGATGAGTTGACATCTGATGATATGAAATCTCCTAAATCTCCTTCAAATTTTTTT
ATGAAAGGTGTTGGTAAAGAGATTGAAAATATGATAAAAGAAAATCAAGAGCTTGTGGAA
ACAAAAAACGCTTTGAACATAGTTAAGGATGATCTCATTAAAAAAGTGGATTATCTAACC
AGTGAAAATGATATGTTGAGAAGCGTATTGGAGGCCAACAGTAATCGCGAAATTGATTTA
AAAGTCCGAGTTACAAATATGGAGCAGGAAATTAAAGATCTCCGTCATCAAAATGAAGAT
TTTAAATCCAAACTAGAAAACGCAGAAGAGCCAAATGATAGTAAGAGATTCACAAGAGTT
GAAATGGCAATATTACTAAAAGAGCGAAATCAATTCAAGGAGAAATTATTGGAATTACAA
GATGCTCTAAGATATACAGAA
>DjScaffold1919:68315-76182 strand:+
agtcttccattttaagcaatgtacctattcctgttggttcgcattccattttagagccga
aagattcgatcatttgcgtcactaaatcaccaaatttggaaaattcagatcaatttttgt
ggataattgtagaaaggccagttcgagacggtagtgcatactatttggctcaattttcat
tgactagtagacatgaaataacgccaaacacaatttcttatttaaaaatttctaaaaaac
ctctcaatattttagcagttccaaaaacatctcaagttattaatgatcgcgctatgttaa
ctgaggacaataaaatatccccagagtattcaccatctaaacgaccaagttcaactattt
ctgataatgttaaagtcagccgagttcacaatactagggctaaaattaagcaaccatcac
tgatattacataccaaacattatggattaattatgatgatggaataatagtggtgtattc
agtaacagttaataatatatatgatggacgtcttgctacaatttttcttccgggatcagt
ttctacaATGAAATATTGCCAGGGAAGAGTGATAATTGCTCAAACTGATGGTTGTTTATG
TGTATTTGATCTGAAATCATGTCATGACAGCTGGGAAGTACAACAATGTCTGGTAGTAGA
AATGTCTCATAGTAGTCCAATATCAGCCTTGGTGGGTCATAGAAATTCAGAATCAATTTG
GTTTGGATCTGATCAGCAGTGCTACATTATCGGTGTTTCAAAATTAAATTTGATTAACAA
TTTTCATGTTAATGGTAATCTCACTGAAGAACACATGTGTCGCATAGAATCGATTTGCGC
ATCTCACGATTCAGAATTAATGTGGATAAGTATGAGTCAAGTTGGAATTCTCTATGCTTA
TGACAGTTATACT
>DjScaffold2955:9064-12618 strand:+
attattgcttttaaaatttcggttagttttatacagatattctaatcttctaatatctgt
tgatttgttttcaaatataatattttaacagaaATGAATTTTCTTAAACGTATGGCACCT
TGCTTTTCTATTAATGAAGCAAACATAAAAATTTTAAAAGAACCTAATGATTTTTTTCAG
TTTATCAAGTTGAAAGCAAGTTCGTGTAGTAAAAGAATTGTAATAGCATCTCTTTATTTA
GGATCAGGAATTAAAGAAAAATCATTGGTTGATGCAATATTCAACTCTATAAAAAACAAT
CTTAATAATAAAAATTTTCGAGTTCATCTTTTGTTTGATGCAACAAGAGGGTCGAGATCA
GTTAAAGATTTTGAAGGGAATAATTCAAGTTCTCGGTCAATGTGTTTGCCTTTATTAGGG
AATGAAAACACTTTATCGAGAATTAAGGTGTCATTTTATCACACTCCAAATTTGAGATGG
TATCTTTATAAATTGTTACCTGAAAAGTGGAATGAAATAATTGGATTACAACATATGAAG
ATTTATTTATTTGATGATTGTCTTGTTATATCAGGGGCAAATCTCAGTGAAGATTATTTT
ACCAATCGACAAGACCGATATGTTGTATTTGAAAATAGTTCACTACTCTGTGATTTTTAT
GAAGAATTAGTTGATATTGTAGGTTCGAGATCTTATAATATGAATTCAACAAATGTGTTA
GAATCACCGAAAATCGGTTGTGATCCGTTATCAGGCAATTTGGATGAATATATTCTTGAC
TTCAATAATGCCATTAATACTTTTATCAGCAATCAAATTATTATATCTCAAAATCAAGAA
AATGGGCCTTTATAAtattgtacaagatcaattagctctaaagaatttatttaaatcact
gagttctgatgataacaatcggagttctgtttttcttgcgtcgggatatttcaatctaga
ttgtgataatgagaacttattatttgaaatggctgaaagaaacataccaatcgaagtttt
aactgcttcgcctgaagtttgtattagtatattttcgaaatttgtgcaaattcaagagta
gagtgtcccataagttcatagacgttttgttcacaaaatttaaaaattataaagaaatac
aagcaaaataaatatccaaaatacaatcgattatggtccatgatttggttttctccattt
tccactcaattctaaaattcatggcttcattattgcttttaaaatttcggttagttttat
acagatattctaatcttctaatatctgttgatttgttttcaaatataatattttaacaga
aATGAATTTTCTTAAACGTATGGCACCTTGCTTTTCTATTAATGAAGCAAACATAAAAAT
TTTAAAAGAACCTAATGATTTTTTTCAGTTTATCAAGTTGAAAGCAAGTTCGTGTAGTAA

AAGAATTGTAATAGCATCTCTTTATTTAGGATCAGGAATTAAAGAAAAATCATTGGTTGA
TGCAATATTCAACTCTATAAAAAACAATCTTAATAATAAAAATTTTCGAGTTCATCTTTT
GTTTGATGCAACAAGAGGGTCGAGATCAGTTAAAGATTTTGAAGGAATAATTCAAGTTC
TCGGTCAATGTGTTTGCCTTTATTAGGGAATGAAAACACTTTATCGAGAATTAAGGTGTC
ATTTTATCACACTCCAAATTTGAGATGGTATCTTTATAAATTGTTACCTGAAAAGTGGAA
TGAAATAATTGGATTACAACATATGAAGATTTATTTATTTGATGATTGTCTTGTTATATC
AGGGGCAAATCTCAGTGAAGATTATTTTACCAATCGACAAGACCGATATGTTGTATTTGA
AAATAGTTCACTACTCTGTGATTTTTATGAAGAATTAGTTGATATTGTAGGTTCGAGATC
TTATAATATGAATTCAACAAATGTGTTAGAATCACCGAAAATCGGTTGTGATCCGTTATC
AGGCAATTTGGATGAATATATTCTTGACTTCAATAATGCCATTAATACTTTTATCAGCAA
TCAAATTATTATATCTCAAAATCAAGAAAGTACACGTTCTAGTGAATTGACTAGAGTTTA
TCCTCTAATTCAGATGGGCCTTTATAATATTGTACAAGATCAATTAGCTCTAAAGAATTT
ATTTAAATCACTGAGTTCTGATGATAACAATCGGAGTTCTGTTTTTCTTGCGTCGGGATA
TTTCAATCTAGATTGTGATAATGAGAACTTATTATTTGAAATGGCTGAAAGAAACATACC
AATCGAAGTTTTAACTGCTTCGCCTGAAGTTTGTATTAGTATATTTTCGAAATTTGTGCA
AATTCAAGAGTAGagtgtcccataagttcatagacgttttgttcacaaaatttaaaaatt
ataaagaaatacaagcaaaataaatatccaaaatacaatcgattatggtccatgatttgg
ttttctccattttccactcaattctaaaattcatggcttc
>DjScaffold336:9758-14741 strand:+
aatctatcaaaaATGTTCGACAAATTTCGTGTAGTTTCATTGATGAATGAAATTTTTAAT
ACCGGGGTTAAATGCTCTGCTCATTCAACTTGTGGTGCAACTTATTCAAAAAATCTGAAT
TCCAACTGTGATTATGCATTTGAAATGGCATGCTCTAATATTCGTTATGGGCCAGGAGTT
TCTAAAGAAATTGGAATGGATTTAATAAATTTTAATGCGAAAAAAACGCTTTTATTAACT
GATAAAAAATGTGGCAAAATTACCTAGTTTTAAAATTATTCTTGATTCATTGTCGAAATGT
GGAATTGATTTTGATTTATATGATGAAGTAAATATTGAACCAACTGATGTCAGTATGATG
CATGCTGTAAATTTTGCTAGAAGTAAATCAGTGCCTTATGACATCTATGTTGCTATTGGA
GGTGGTTCAGTAATAGACACTTGTAAGGTGGTTAATTTGCTGAATTCTGACAAGGAGGCC
GATATACTTGACTATGTAAATGCTCCAATCGGAAAAGGGAAATTCATCCGATCAATATTG
AAACCTATGATTGCAGTTCCTACAACATCCGGTACCGGTAGTGAAACTACGGGTGTAGCA
ATATTTGATTTGATTTCCATGAAAGCCAAAGTAGGAATTTCTCAAAGAGCATTGAAACCG
ACTTTAGGTATTGTAGATCCAGATTTTACAGCTTATTTGCCAAGAGATGTTGCATGCTTC
AGTGGATTCGATGTTTTGTGTCATGCGTTGGAATCGTATACAGCAATTCCGTATAATCAG
CGATCTCCGCGACCTTTGAATCCAATAGAAAGACCAGCTTATCAAGGAAGTAATCCAATA
AGCGATGTATGGTCGGTACATGCATTGAAAATATTAGCCAAATATTTTCGACGAAGTGTC
AATGATCAATCAGACATTGAAGCTCGCAGTCAAATGCATTTTGCAAGTGCTTTGGCTGGA
ATGGGATTTGGTAATGCTGGTGTTCATCTCTGTCACGGAATGAGTTATCCAATATCTGGT
CTAGCTAAACAAATGTATTCGAACTCCAATGATTATAAACCTGTCACAGAAAATTCAAAA
CCACTTATACCACATGGATTATCGGTTGTCATCACAGCCCCTGCAGTGTTTAAATTCACA
TCAACACTGTTTACTGAACGGCATTTATATGCAGCCAATATTCTTGACGAAACTACAACT
GAATCTGAGCACAATGTCAAAGATGGAGGCAATATTTTATCTGATGTGGTA
>DjScaffold2407:35699-36261 strand:+
taatttcaaacaaaaaaataaacaaaattagatgaaaatctgttctttacagataactcc
ggccccggacgtcagaaaatattcaaacaagaaaaatcgagaggaaaatagttaagtgca
attagaaccctaaaaagaaaaagcgtgctgctctaaaaatagttgccaatcATGGCACCG
AAATTTACAAAAATTTTCAGAAAATTGGTGCTAGCACCAATAAAATGGAAAAAGAGCTGG
TCTAAAAATACCAGCAAATCGAAACCTAGTTGGGGTGTCCAGAAAATCCTATATAAGGAC
CTCCAACTTTCAAGACAAAACCACCCAAAAAACAACATTATTGAAACTTATTGTACTAAG
TGCACCTACTCGACTACAACACTATTATTTTATCAATTCACCAGGTACAGATAAttgcca
acaaacttgtttaaagttcacagttactcaatccagtgaattttaaattgca
>DjScaffold5086:14828-18424 strand:+
gtaaaataaatgtcaacttttatcactacatgagaactaggtaattaattcggacgggta
taatattcgctcaactacttaaaaattcaaaaatattggttaatttaaacaaaaataagc
ttttccatcgattttctttgaaaatataaataaaaatcattaatatatttaatttaacca
aaaaaattaatttacttaaatttataataatgtattaattgcaaaccttcaaaaaacgtt
aaaatattttacagaaatcataaaagttaaatcaaaagtaaattattaaatttttataat
tatttataaattatatttaaataggcgttttctgtgaatagaactactttggaatttcta
gaatttattgatgtcaacATGATGAGTTGTGCCAGGTACATGGATGAATCGATGGTATCA

TATGTTGACGATATGAATAATCTTTATGTGGGGTCTGGTTGGGGTCTATATAGACCAGAC
GTTAAAGATCCAGATGTACCAGATAATAATTCAACAATGTTGTATGCCGATTGGAGACTA
AGTCGAAATTTGTTTACCCTTCCAGCTGCTATGAGCTGGGTTTTATCAAGTGACGTAAAT
TTTATCAATTTTGTCCAGGAAACCAAAAATCATTTGTATTTTTTTGGTCATGAAATAAAT
AATCAATATGGATTAAATAACCAAGAGGACCGGCATGCATGGCAGAGTTTCATGTTGAGG
ATTTGTAAAAATGATCTTGGATTCAAATATGTACCTATTGAGAACAACTACGAGTTAAGC
AAACTAGTTAATCATCGATATTATAGGATTACAGAAGGAAGTAAAGACTATGCTGCATCA
CTGATTCATAAAGATTCCATGAATGAGAAATGGTATAAACAAACTGTTCATTTACCAACC
AAGCATTTTGTAAATTCCCAGAAACTTAATTTCGGGTGCTATCTTGGGAAACGTCCTCGA
GCAATCAGTGATAACCATTATTGGGGTGATACTAATGTTGTTTACATAAATAAAATTGGT
AAATATTAAtatttttatc
>DjScaffold4789:54078-55397 strand:+
agcgtttatctttacaggtttaaaaacttcaatattttccttaaattttagaaaattttt
atttttctaaacaATGAGTCTTCAATGGGTAGCTATTGCTGGGTTTCTTTATTTAGAAA
TGTTCATAGCATTATTGTTCTCCATCCCTTTTATATCAGCTTCTAGATGGAGCAAAATTT
TTAAGTCAAGAATAATAAAAAAATTTAGTAGTTATTCAACTTTATATTTTAACTTTCTGC
TAATTGTACTCGCACTTCTATTTCTTGATGCTATTAGAGAAGTTATTAAGTACAATAAGA
TGGAACATAAAATTGGTTCTGGTCATGTGCATGATGGCGAAGCTACTATATTAATGAGAT
TGTTCCGTGCGCAAAGAAATCTTTATGTTTCTGGATTTTCACTTTTCATGTGGTTTGTTT
TGCGACGCATGGTTGTTCTGATAACAAACGTAGCACAGTTAGAGGCATCAAGTGCTGCAG
CTCTGAAACAAGCACAAAGTGCATCGGATGCTGCTCGAGCAATGCTGAAAAAGCCTGATG
TTACTGATAATAAACCCAAATTTGATGAAGAATTAAAAGAAAAACTGAAAACAGCAACTG
TTCAATTAGAGTTTTCAGAAAATGAAGTAAAACGTTTAGAACTGGATTTGGAAACGTTAA
AGAAGCAATATGTCAACCACAATAGAGCATACGACGATTTATTGAAGGAACATGACAATC
TACAAGTAATATAAagcgtttatctttacaggtttaaaaacttcaatattttccttaaat
tttagaaaatttttatttttctaaacaATGAGTCTTCAATGGGTAGCTATTGCTGGGTT
TCTTTATTTAGAAATGTTCATAGCATTATTGTTCTCCATCCCTTTTATATCAGCTTCTAG
ATGGAGCAAAATTTTTAAGTCAAGAATAATAAAAAAATTTAGTAGTTATTCAACTTTATA
TTTTAACTTTCTGCTAATTGTACTCGCACTTCTATTTCTTGATGCTATTAGAGAAGTTAT
TAAGTACAATAAGATGGAACATAAAATTGGTTCTGGTCATGTGCATGATGGCGAAGCTAC
TATATTAATGAGATTGTTCCGTGCGCAAAGAAATCTTTATGTTTCTGGATTTTCACTTTT
CATGTGGTTTGTTTTGCGACGCATGGTTGTTCTGATAACAAACGTAGCACAGTTAGAGGC
ATCAAGTGCTGCAGCTCTGAAACAAGCACAAAGTGCATCGGATGCTGCTCGAGCAATGCT
GAAAAAGCCTGATGTTACTGATAATAAACCCAAATTTGATGAAGAATTAAAAGAAAAACT
GAAAACAGCAACTGTTCAATTAGAGTTTTCAGAAAATGGTATATTTATTGAAACTGGATT
TGGAAACGTTAAAGAAGCAATATGTCAACCACAATAGagcatacgacgatttattgaagg
aacatgacaatctacaagtaatataatgataataccgtgaatttgcttattgttaatgat
ttttaactttatttataagaaaatgaaaatatttactaaaataattatcatt
>DjScaffold3565:41337-47714 strand:+
ATGTTAGATTTGGAGTCAAGTCTTCGGAAAATGTTTGACCCAGTTCCTACACAACATAGT
CAAAATAAATATAAAAATATCAAAAGACGTAATAGCGTCGGCGCTATCAAGTCGGAATCT
AAGACCAATTATCGTGATCCAAGTATATATCAATGGTCAGCTAGGAAACATTGTGTTAAT
GATGATATTGATTGCCGATCAATTATGATTGACGTAAACGAAGAGAATTTCTATAGTCAC
AATGGTCAACCATTTAGTCATCAAGAACAAGCATATGAACCATTTATCCAAAAATCGAGG
AATAAAACTAGACTTACTACTAATGATATAAAAGAAATTTTTCAACCTCTAAAACAAATG
CATGAAAATTCTGATAATTCCAGCGTTCTTCTGGGAGAAATGGAACTTGCATCCCTCTGT
TCCGACGCTGACTTAGATTCAGTGGCAAGTGATAGCACCAATAGTAATAATGTCCATAAA
CTCAAGACCACATCGATGGATCGTAATTACGGAATCTCACTCTGTACTAAAGCAAATTCA
TTCAATAGTAGTATCGATACTAATTCATCAACATCGAAGGAATTCAATAATAATAATTCT
CATAGAAAAAAAGTGCTATTTTCTAGTGTGCGCGAATCTAAAACAACAAGAAGTAATAGC
GATTGGGGATCTCTGTTTGAAACGGATCAAAAACCGGGAATCGTTAAAAGGTTCTTTAAA
GCATCAGCAAAATTGACAAAACGAGATAAAAACAAGCCTGAAATGTGTTCAATTGATAAC
GAAAATAATAATAAAGCAGCATCAATAGACGAACATTATTCATTTAAATCATCAACCAGT
AATTGTGATATCAGACGAAAAATTAACAAATCTAATTCCATTGATTACATTGGTAAACCA
CATTCTCTAATCCAAAAATCTAACTCCGGAAGGAAAAAAACCATTCCCCCTACACCTTGT
TTACTAGAAAACATTAAAACCAAAAATGATGAAGATCAAATAGAATCAAAAATAGATGAT
TCATATCCACCTATCAAACCGCCCAGACAATTGCCACAATTCAAAATCGAAATGTTCGAT

AAAATGAAATCAGCATCGAGTGATCGAATAAAAACGAATACCAGTTCTCAAACGAAATCT
CTTAATAACAATGAACTATCAAATAATACTAATAATAACAATAATTTGGCAAATTCCGAA
TCAGCCACCCTATCTAGGGTAAAGGATATGGTCTCATTATTCCAATCTGATCGATAGtct
ttctatggcgatgttgtgttattgacctcatgcatttgactgaattcctcctctattctc
attgacctaatcaattctcgtattgattgatggggttggttgagaacgtgataggaatat
taaaagcgcttttatcatctctgatcgttttataattaaaacccagatgtataaattaaa
ttgaataaaaataattttttgaattaaatatcttctacatatactgttatttatgtttt
attttttactaataaacgtactttcgtgattataaaattaactaaaataactcga
>DjScaffold3467:2865-14271 strand:+
GAAGCTTTATCAAATGTTGGAGAAGAAATAAAGTCGAAGTATCATAATGCAATAAAGTTT
TATTATTATTTAGCAGTATATTTGGGACCTGGTGCTGTGACATTATTGGAAAAAAGGCAA
AACGAATGGTCGCAAATCCATGATGAAAATATGGTTATAGCTACTAAAATTAAGGCAGTT
GATGGCGCTATTGGAGTTTTATATTCAGTCAATAGTCACAATTTCAATAAAATAACACAA
TTAGAAAAAGAGTTAAGCACTATTGCCACACTAAAAAATGACGTTGCTCAACTCACTAAT
ATTTGTGTTTCCTTAAAAGATACCTTTGTGCTTTTGGAAGAAGATTTAGCTGTTTTTGAA
AATCAAATAGAGGAAGGTAAACTCGTAAAATATCAGCAAAATATAAAACAAACAGTTTTG
GTTCATCAAAATGAATGGAATATAAAAATTGAGTTGCTTAAAAAACAATTATCTATGAAG
CAGTCGGTAAATGCTGCATTAAAAGAGAAAGATGTTCAGAAACAACTTACAGAACGGCAA
AAAATATTTGAAGAGGAGTATCAACAAGATTTAGCTGAATATTTAAGGAAAGGTCGTTTG
GAAAGACATGTTTCCGATCCACAAACTTTGCCAGTTTCAGTAAATGAAGATTTAGATATT
CAGATTGAACCAAACGAAAAACAAGAATTAGATGAATTTTTAAATTGAaattattatttt
acattagttatttatatattt
>DjScaffold4987:37009-37792 strand:+
aatataaatttgattctaatatttaATGGTTCAAGTAAGTGACTTCGTTAAAGGAATCTC
ATGTGTTTGTGGAGGATTTCTTATTCAGTTTTCCCTAGGGACAATCTATACATTTGGAAA
TATGAGCCCTTATATAGCATCGTATCTTAAATATCGCACTGGTAATACATGGGTTACGAA
TGGTGATATGGTCTGGATTTTTTCATTAGCAGTGTTCTCCCAAGCTTTTACTATGTCTCT
AGGTGGACTTCTAGAAAAGAAAATAGGGCCCAGATTAACTTGTTGGATCGGGGGTTGGAC
AATGAGCCTTGGCACTGTTTTAACATATTTTGCCATCAAATATAACATATGGACTGTCAT
ATTAACATATGGATTTATATTTGGTATAGGAACTGGATTGACATATTCAGTAGCAATAGC
AACACCAATGAAGTGGTTTCCACTTCATCGAGGATTAGTTGCAGGTTCTGTTGTTGCTGG
TTACGGATTAGGATCGTTGGTTTTTAATATTATACAGACTCAATATATCAATCCACTCAA
TATTTCTCCAAATTATACGCAAACAGTTGAAAGGTTATAAtaatttataataataataaa
ttattattttattattattattttattattattattattattttattattattattatta
atatttttattctaggttcaagtaagtgacttcgttaaaggaatctcatgtgtttgtgga
ggatttcttattcagttttccctagggacaatctatacatttggaaatATGAGCCCTTAT
ATAGCATCGTATCTTAAATATCGCACTGGTAATACATGGGTTACGAATGGTGATATGGTC
TGGATTTTTTCATTAGCAGTGTTCTCCCAAGCTTTTACTATGTCTCTAGGTGGACTTCTA
GAAAAGAAAATAGGGCCCAGATTAACTTGTTGGATCGGGGGTTGGACAATGAGCCTTGGC
ACTGTTTTAACATATTTTGCCATCAAATATAACATATGGACTGTCATATTAACATATGGA
TTTATATTTGGTATAGGAACTGGATTGACATATTCAGTAGCAATAGCAACACCAATGAAG
TGGTTTCCACTTCATCGAGGATTAGTTGCAGGTTCTGTTGTTGCTGGTTACGGATTAGGA
TCGTTGGTTTTTAATATTATACAGACTCAATATATCAATCCACTCAATATTTCTCCAAAT
TATACGCAAACAGTTGAAAGGTTATAAtaatttataataataataaattattattttatt
attattattttattattattattattattttattattattattattaatatt
>DjScaffold4987:88212-95491 strand:+
aataataacatcttaattatacagattatctattATGGCTGGACGAGATTATGCAGTATT
AGAATCTATCAAGGAATGTCGTTTTTGGATTCTTTGGAGTACATTTTTCTTCAATGGAAT
AGTCATAGTTACCATTTCTTCTTTGTATAAAAGTTTTGGGCAAACATTTATTAGTAATGA
TAAATTCTTCGCTATTGTTGGAGCTATTGCGGCAATTTTTAATTCTGGAGGCCGACTGAT
TTGGGGAAGATTGTTGGATACATATTCGTACAAAAAAAATAATGTCAACTCTAACAATGCT
AATGTCAATTGTAATGGGAACATTTTATTTTACTAGTTTGATGAAAAATGTTTTGTTTAC
GCAAATTGCATATGCAACGTGGGTATGCTTATTGTTTTTCTGTTTGGCTGGTAGTTTTGC
AATGCTTCCAACAACAACATCTCATTGTTTTGGTAGTAAGAATTTAGCTATAACCTATGG
TGTGGTTTTTACTGGTTATGCCATGTCTAGTATTTTGGGAGCAATACTTACAAGCAATCT
TTTAGAAATAATTGAATACTGGGGACAGTTCCTTTTAGCCACATTTGCAGCAACAATGAG
TTTGATTTTAACGCAATTTTTTCCAACACCTGATTCCGCTTCAAGCAAATAAgaattata

gattcacacgagaagcatttattaatttttggttgtagaattattgctttattgatcttg
gttattgctctattttgaatttttgataagcctaataattattaaatatagaaagcaaag
gttgttaaataataacatcttaattatacagattatctattATGGCTGGACGAGATTATG
CAGTATTAGAATCTATCAAGGAATGTCGTTTTTGGATTCTTTGGAGTACATTTTTCTTCA
ATGGAATAGTCATAGTTACCATTTCTTCTTTGTATAAAAGTTTTGGGCAAACATTTATTA
GTAATGATAAATTCTTCGCTATTGTTGGAGCTATTGCGGCAATTTTTAATTCTGGAGGCC
GACTGATTTGGGGAAGATTGTTGGATACATATTCGTACAAAAAAATAATGTCAACTCTAA
CAATGCTAATGTCAATTGTAATGGGAACATTTTATTTTACTAGTTTGATGAAAAATGTTT
TGTTTACGCAAATTGCATATGCAACGTGGGTATGCTTATTGTTTTTCTGTTTGGCTGGTA
GTTTTGCAATGCTTCCAACAACAACATCTCATTGTTTTGGTAGTAAGAATTTAGCTATAA
CCTATGGTGTGGTTTTTACTGGTTATGCCATGTCTAGTATTTTGGGAGCAATACTTACAA
GCAATCTTTTAGAAATAATTGAATACTGGGGACAGTTCCTTTTAGCCACATTTGCAGCAA
CAATGAGTTTGATTTTAACGCAATTTTTTCCAACACCTGATTCCGCTTCAAGCAAATAAg
aattatagattcacacgagaagcatttattaatttttggttgtagaattattgctttatt
gatcttggttattgctctattttggtttggtttttaactttattaaaagggaaatatct
tttgtagaatttttgataagcctaataattattaaatatagaaagcaaaggtt
>DjScaffold3590:24683-38319 strand:+
gtacgagataaagatcaaattttacacagaactcaagatttatatacgtctgagcaagat
gtgaagaatcgttatATGGACGAAAATCAGAAGAAATCTAGACAAATTGAAAAACTGGAA
AAAGCTGTGAAAAATTTGAATGAAGAAGTGTCGAAAGGCAACGATATCATACAGAAACTG
CAAAATGATATCAAACATTGCCATTCAAAGATTAAATTAAGAACTCAAATAACAGCCGAA
CAAGAAAAAGTGCTCGGAGAGCGAGAATCAGAAATTAAAAGGCTGTCTTCTGAAATGGAA
ATTTTGAAGAAAGAAGTTTGTAGTAAAGAAGAAGATGTTCAGAAAGCTAATTTGAATTTG
GAACAGACAACCAAGAAACTGGAAGAAGCTAATCACGTGATAAAAACCAATGAGAATGTA
ATTAATTGGCTCAATCGCCAAGTTACTGAATACGAGTTATCAAAACAAAAGCCTGGTTTC
AGTGGTTTGCACCCTCCGAAATCACAAACCGGAACGACATTTAGTTCCTCAACAGATACT
ACTATTAAATATAGACCGATGTCAGCATTATCTAATTTGAGTAACGCCGGCGATCATCCG
AATAATCCTGTATTGGGAGGTATTGTTAATCGGGGCAAGTTGTTACCGGGTTCAAATACA
AGTACCATTACTTCTCAGTCACAGGGATCGTACAACACTCAATTGCTGCCAACAAACAAG
TTTGATCCATCCAAGAATTTCTCTTCGTTACCATTTTCAATGACATCAGAAAAATTGCCA
AATTCATCTGAGACGGATAGTTTGGATCCAAAATATCTAATCAAATCATCGACTGTTACA
ACTTCT
>DjScaffold2313:1512-3549 strand:+
TTACAGAAGGATGTTGATTTAGAAACTTGTCGAATAGTTTCTTTGGAAGACATTTGGCAT
TGGTATTGTATCCGTAAAATCGATAGTCTTCAATGCAAATTAAAGAAAACCTATATTAAT
CAAAATCAAATGAAAGAAAAAGATGATGAACAAAATTTGCTTAATATTGAAATTATGACA
GATCCTGAAAAAGCAATGCTTAAATATATGAATGAAGAAAAATCACGGAATAATTTTCCA
ATGAATAAAAAAATTCTTAATTGTGCTCAGCGTTTATTATTACATACTAATGATGAAGCA
CTAATAAAATTTGCGCGTTTATTTTGTTCTGGATCTGAAGATTATTGGATGCTAAGAAAG
CAGCTGACAAATGATTTCGCTGTTTTAAACATGATACAATATCTTATGCAAATCTCTTCT
CTTAGACCATCGCAATATATTATCGATCTAAGAAAAGCTACATTACAAATTATCTCACCA
CAATTACACTTATCTGAGGATGCGCGTTTAGAAAATCTGTCAAATTTACCTTTTAGATTA
AGCCCGAATATTGTTAAATTTTTACAACCAAGTGGCCAAAGTGAAAGTTTATGGAAAACA
ACTAGTCAACCTTATACCGCTAGCATGATTGCCACTGGTCATGTATTATATTGGTGTAGG
AATAATTTGCTTGCTCTTATAAGAGCTTTACTGCGCGATTATTTTGTATTGTGGCATAAA
TATCTTATAAATAGTTATGAAAACAAAGAAATGTATCCAATTGAATTAGAAAACTGTATT
CTTGAAAAATGGTTGTCATCTGCTATCGAACAAATTCAGTATAGAATATCAAATGTTGGT
CGTGTTGACGATGATGCAATTATACGAGAAAAAATTCAAAGTTCCTTAGATCCATTGAAT
CAGGCTCTTATGAAACCTAGTTATCAATCATGGATTTAGgtgtaaattaaatataaatttc
aatttttttaaaa
>DjScaffold2313:34666-42198 strand:+
ctttattaagcaaaATGTCGACGTTTTTCAGTTTTGAAAATATCATAAATAGTGTATTTT
CAACTGATTTTCAAAAAATATGCTTTTACTGCTTCGGAATTTACTGTCCTGTTAAATTCTC
TATTTCAAATTTATAGTTCGAATCCGTTTTTGGCAAATGCTGATAATCGTAAAACAACAT
TTAATCAATGGAATAAAGATGTGAATTTGATCGAAGTAGCCAATCAAAATGGATTTTATT
ATACTGGTGTTGAGGATGAAACTATCTGCGTGGGTTGTGGATTGGGATTAAAGGCATGGG
CTCGAGATGATTGTCCAATGGCTTTACATGCTGTCCATTCTCCGCATTGTTCATTTGTAA

TGGGCATTGAAATTTTGGATCTTCATAAAGACCGATTAAATGTATTTAAACACATGAAAC
CTACAACATCCCAAATCGAAACCATTTCATCATCGTTTAAATCAATAATCGAATTCCTTA
ATCACCCTTTGGATGCAATTAACAATGCAAGAGGTATTGGGCATAGTGATATCATTATTG
TACTTGCTGCTTATCGGCATTTAAACGATTACAATACATTGCCATTCGACACGATTTCAT
TGCTTCGTAGTATTATGCATATTGATGACGAATTGGAAAATCACAACATGTCTGAGAGTG
ATTTACCGCCATTTATGGATTTTCTGAAAATGTGGAGTGAGGAATTTTAAacatggctac
atattctctcacccagtagatttaacattgaatttgaaattataatagagaattttcaat
tttgtcttatatatacatcgtagcatcaattttcagtttttcttttatttagaattttta
gatattttataattatgataacgtattaaaatatcttcaat
>DjScaffold2313:47473-48115 strand:+
ATGATGACAAAAGTGGTTGGCGGAATCAATGGGGCCATGGTTTATCTTGATGACATTCTG
ATATATGAATTCAATAAAGATCAACACTATGAAGTAATTGAAAAATTATTGACGTTAATA
ATGAAAGCTGTTTTAAGAAAATATCCAGAAAAGTATCTGTTTCTTCAAAGCGAAATCAGA
TTTCTTGGACACACAATTAGCAAGAATGGTGTCAGAACTGATCAGTCTAAAATAGAAGCA
ATAAAAACATTTGGAAAACCTAACTGTGTGAAGAAGTTGCGAAGTTTCTTAGGAATTTGT
AATTATTACAGAAAGTTTATAGAAAACTACAACATGAAAAGGGATATTGCATCACACAAA
ATGAATTATTGGCTGTGTATTATTTCTGATTGCATTTCAAACATTACTTGTACGGTAGAA
GATTTACTTTTAGAACAGATCATAAAGCTCTAA
>DjScaffold1478:69-302 strand:+
ATGATGACAAAATTGGTATGCGGAATCAATGGAGCCATGGTTTATCTTGATGACATACTG
ATATTTACGAACAATAAAGACCAACACTATGAAGTAATTGAAAGAGTATTGATGGAAATA
ATAAAGGCTGGTCTAAGAAATCATTCGGAAAAATGTCAGTTTCTTCAAAGTGAAATCAAG
TTTCTTGGACACATAATTAGCAGGGATGGTGTTAGAACTGATCCATCTAAATAG
>DjScaffold3868:58676-59104 strand:+
ATGCCGTTTGGGATAGCGGCGGCACCAGGAACGTTCCAAAAAATGATGATGAAAGTAATC
GGAAAATTAGAGGGAGCAATGGTATATTTACATGATATCTTAATATTTCCAAATGACAAA
AAACATCATTATGAGATTGTAGATACGGTCCTGGGTCGATTATTAGAAGCAATGTTGATA
GTCAACTTTGAGAAATGCGAATTTGTAAAGCGAGAAATCAAATTTTTGGGGAACATCATC
AGTGTAGACGGAATACGAACAAACACGGGGAAAATTGAAGCTATTAAAACATTTGAGAGA
CTTAGTTGTGTGAAGACGATGCGTAGTTTCTTGAGAATATGCAATTATTACAGGAAATTT
ATTAGTGGGTATGCGGTTAAAGCGAAAGCACTAGAAAAATTATGCAGTGGTCCGAGTAAT
AAAAATTAG
>DjScaffold585:3602-6527 strand:+
TCAGAAGAAATATCAGTGGATAAAAAATATAGAACACTTAAAGCTATACGAGAACTTGGT
GTGACACTTGACGTTTTGAAGGAGTCAAGAATAGGTAGAGCAGTTAAAAAATGTTCAAAA
CTACCAGGGAAACCTGGTTTATTAGCTAATAAAATAGTCCAGGAGTGGACAGAAATGATT
CAGGATAATGAGAAAAAAAATAAACATGATGTTGTAAAACCAAGTAAATCAGTAAAATAT
ACAGAAAATAAACCGGTTCAACAAACTCGTAGTTCTTCTGAAAGTGAATCTGAATATGCT
TACAGAAATAAATCTAAAAATAGTGATTATAAAAAATTAAAGGATGATCATGCTTCAGAT
ATTCAAACATCTAGTGACTCTCTAAGTGAAAATGAAAAGCCAATTAATCATAATAGGAAA
GAATTTGAAAATCATAATTCTGACATTGATGAAAATATAAATAAAAAATTTCAGCTGCAT
AATAAAACTTCAGAAAGCAGTAAAATGAATCAAATCAAATTAAATAGAAATAATAATGAC
AATTCAGATTTTTCTAGTGTTGATATCGATTCTGATGAATGTGATGAATTTGCCGAAAAT
CTAAAATCATCTAAAAAATCTGATAGAAAACATGAAGATAAGATTAATTCAAAAGTTCGG
TACGATGATGAAAATTCCGAGGAATATAATCATAGTGTTGTGCAGAAAAAAACAGATAGT
TCTACTAAATATACTCACAAGCAAATGAAACAAGAAAGTGATGATTCTGATTTAGATAGT
GACGAGGCACACTTTCAACGCCTCGAAGAAAGTAGAAAGAAATCTTCAAAAATGGATACA
ATAGATTTTCTAATGCCAATTAATGAAAATAGTAATAAAAATAACAAAAGGAGGGTTGAT
AAAGTGAGAAATGAAATAAAAGGTGATCTTTTGTTAAAATCTGATAAACATATTTTTCAA
GATGATAGCAAAAGGCCAATCAAAAGTGATCAAATAAGTATCGACAATAAAAAATTCCCA
GTTGACAAATCATCGGAATTAAAACCACCATCTAATGTAAAAATCTTTTCAAATTCAGAC
CAACAGCATAAAAATAAATCAAGAAAACCCGATATAAAGCAGAATGTAACGCCATTTGAA
ACGTTTCTTAAAGACAGTGCTTTGCCGAAACCTACTGAAAATCAAGGAAAAAAGAGAAAA
TCAGAGAACTTTCATAATATTGAAAGTAAAAAACCACATATACAATTGAAGAAAAGTCCA
GCGCATTTCGCTCCAACAGAACTATCGGATTTACCTGCGTTAAATTCTTCATTATCGAAT
TCTGATATGGACGAACAATCAGTTTATAATCGAACAGATTTAATTCTACAAAAGAAAGAA
AATAATATCAACTATGAAATTAAAGAAGATATCGATTGTACATCAAAATACTCATCAGAA

CGAAAAATATGGACTCCAAAAAGTAATATAGTAAAACATGATACAGTTGAAGAAAATCTC
AGAACGATAAACGGAGTTTTAGAAACTGAAATCGCTCAATTTAATTTTGAGAAATTGAAA
GCTTTTGATGGGGAAAGCAAGAAAATCCAGAGAAAAACCGAATTTTTCTGGGAAAAACTT
TTCAAAAAAAATCACAAATCAGCAAACAGACGTGACGGTGAAACATGGCGAGAAGCCTAT
ATAAGAAATCAGATCGCTCAAGATTATCGTTTGAAAGAAATATCAGAAAGATTGAAGAAA
AACACTGAAGAAAGTTTAAATGCCATTCCTAAAGCTAAACTACTTACAAACAGCAATTTA
GCTAAACCTAGGCGACGAATGACCGACATGAGGCGCTGAatgtcttgcaccatcaacaga
aaatttcaagaaataattataaaattctacttttgtctttaaacatgtgttaaattggtt
tgcttgaagctgtttgcgtgtaatttattattattattaattactaagatttgtcta
ttttgtgcgctttatttatgtgatttgcgttagtaggataatacagacgtgaaaaataga
gctgtgtatttttattgtatctcattttgtttgaaatgggaattatgtgtgttttttgc
gtgtgtgtatatttgtgtgtatttaatgtatgctgatttattattcttaggtttgttgtg
ttgcaaactattttcagaatttgtttcagtttaacattttcaggaacatctcatcacttt
atgattatgattttaaaactatttttaacaatattcaacataaacgaaagagtaaaaaat
ttgaattaggtgatgatggtgttgtttttattttaaatcattgaaaaattaaaatcggtc
tttgacttttgttatttgattagttgggattatatatatatttaattattttaattttta
ttaaaattataaactaatgataattaattattatttttatcaaaataatttttggtcagg
atttatactcccaactagtcgttcattttccgataattatttttagatatttatgtattt
aagatttttatttttggatttattactttctgttgacagtagatttat
>DjScaffold2626:168295-169064 strand:+
gATGGACCAATGGATTCCTCATTTATTGAATAATTCCCAAAAATTGCAAAAATTAAAAAT
TTGCCAAATGTTGCTGGATCGAAATAAATCGGAATCTTTTTTAGATCGCATTGTCACTTG
TGATGAAAAAATGGGTTATTCATCACAATCGTAAACGTTCTGCACAATGGTTAGATGCCGA
TCAACCTTCTTTACACTGTCCTAAACCGGATTTACACCCAAAAAAGACAATGCTGACAGT
TTGGTGCTCTATAGCTGGAGTCATACATTTTTCTTTGTTAGAAGAAGGTCAAACTATTAA
CTCAGACAAATACTGTTCTGAAATTGAATTAATGCACAAAAATTTATGCATTAAACAGCC
TTCATTGGTCAATAGGAAAGGAATCATCCTCTTGCATGATAATGTCCGACCACATGTGGC
AAAGAAGACGTCGCAAAAGTTGACAGATTTAAAAATTGAAACTTTGCCACATCCACCATA
TTCGCTCGACTTATCTCCGACAGATTATCTTTTTTCTAAGCATTTTGATAATTTTTTGGT
TAAAAAAATCATTGCTGATGGGGAGGATGTCAAAATGGCCAGAAAAACATTTCTTTGCTA
Atggtattttgaaactattttctagatagcaaaaagttgtagaatccgatggtgacaatt
ttacttaaataaaatttgattttattggacttttattgattttattgtctattatgtaat
cgaacatttcatatgtgacaacctaat
>DjScaffold4495:21539-22189 strand:+
ATGGTGATTATATGGATATTCTCAATGGCTGCTTCCTTACCAACTGTAATGTACACCGAG
ATTCGCCATCATGTTATTCCACTTAACGATACATGCCTGCAGCTGAGATTCTGTAGCAGT
GAGCATCCGTATTTCGATATTATTACATGGATTAGATTCTGTGTTTATTTTGTTGTTCCA
TTTTTTATTATAAGTTTTTGCTACATTGGAATTTCGACAACTCTTTGTAAGAGTCAAAAT
GTACCATTGCTTGTGGTCACCAATAATATGGATAACAAAACTCTTGCTATTCCAAGACGA
CAAATGGAAGCTAGAAGAAAATTATCTGTGGTCGTTTTAGCTTTAGTTGTCATTTTTGTG
GTTTGCAGACTACCTTATGAATCGCATTCGATATTCTTACTATTACAAAACGAAAGCATT
CATTCCTGGCTAGCAGCTGCAACGGTTTTGTTATATTTCTATTCATCGATCAACCCACTA
ATTTTATGCATTCTCAGTCCTACTTTTCGTCGGTTTATGCTAAAAATGTGGTGCAGATGT
TTTCTGAATTTGTCCACTGCTGTTCAGACTCACACTGCTCCAATTACATCAACAAAAATA
AGAACCAATGGAATCCCGTCTCCTGTTGGCATAAATATTAGACACGTCTAA
>DjScaffold2522:9242-12537 strand:+
ttatttactaatacttttatgactaaaataatATGAAATATGTAACATTACAAGTTCGAA
GAACTCGAACAATCAGTGATTCAGAAAGTACAGAATTACCAGATATTGTCAGTACTGTCA
GTGCTTCACAAAGTAATTTAGGAGAAGACAATATCAATTTATTAACAGCATTAGTTCTGT
GTACAATATGGTGTGTTATCAGTGTATTAAACAATATAATTAGTAAAAGTTTATTAAACA
ATTTCCCATTTCCATTAACATCAACATTGGCACAATTATTGGCTACTGCAATATGTGCTT
ATCCACTTGTTAAAATCTGGAAAATACCAGAAATAAATTGGAAAAATATATCATATCAAT
ACATTTTAAAAGTTATTATTCCACTAGGTTTAGGGAAGTTTATGGCTGCATGTGGAACTC
ATGTGAGTGTATCCAAACTTCCTGTATCATATGCACATACAGTGAAGGCATCTATGCCAA
TATTTGTTGTGATTTTGTCGTATATATTCTACAACGAAAGGTATTCAATTAAGATTTACA
TTTCGTTGGCAGTGATATTTACTGGCATAGCTATTGCTTCCATTACCGAACTGTCTTTCA
ATGCTGTCGGTCTTCTTTCAGCAGTTATTGCAACAGCGTCCTTTGCTTTTCTAAATATTC

TAACTAAAAGATGTCTAAATACAACTCATATGCATCAGTTCAGTCTTCTATTACTGTTGG
CAAGAATTTCTACAGTGATGTTTCTACCTATTTGGTTATACAACGATGGTGCCATATTTT
TATCTTCCAACATAAAAGTTTGTTTGAAATAAtttattattttgttacttgattaaaatg
atcaatcgctccaattttgaacatttagtggtgcgcgctgtaaaatcgtgaaattcgtct
tctgaaggatttaaacaatttaagaatggttggtaaaatatataaattactctttaaatt
agtttcacagtcttatgaaatcataaacatgtttagtaataggttcaaaacaccaattta
attgtgttctcttgtttacaattgtgaatgtaattggtgtaattaagaaa
>DjScaffold1681:71208-80812 strand:+
tattattattattattattagaaatccaccatttttgatgataacccaATGAACAT
TTATCAAAAAATTCTTAGCAATAAATTTGAATTTCCTCCTAATCTTGTAGAACCGGATGC
AAGAGATTTGATTAGAAAGCTGCTAGTTAGTGATCGCACAAAACGTCTCGGAAATATGAA
AGATGGCACTGATGACGTTCTTAATCACAAATGGTTTGCCAATATCAGTTGGAACGATGT
ATTGGAAAGGAAATTAAAGCCAGTCATTATTCCTCAAGTCAGTCACAATGGTGACACTCG
ATGCTATGATAAATACGATGAACAAGATTGGCGAAAAATTCCTTCTTGTTCTACAGCTGC
TCAGGAAATGTTTTCTGAATTTTAGaattcatgtatttcaaatttcgttcgaaacatgta
gataaaactgttgaataaaatttgattcattcatttt
>DjScaffold1681:10738-11107 strand:+
ATGGCTGTCCGCGAAAAAGAAACTAATACGTATTATGCTTTAAAAATTCTAATTATAGAT
GAAGTAATTAGAATGAAACAAGTTGAACATGTGAAAAATGAAAAATTTATCCTCGAAAAA
GTCAACCATCCTTTCATTGTTAATTTATTCTGGACAAGACATGATGTGAAATTTCTTTAT
ATGTTGCTGGAATATGTTTGTGGTGGAGAATTATTCACTTATTTACGAAATCAAGGCAAA
TTCACCAATGAAATCGGATTGTTTTTTTCATGTGAAATAATATCAGCTCTTACTTATCTT
CACAGGTGA
>DjScaffold2976:81436-82052 strand:+
attccaaaaggcagtacccaaaaggcaggactccaccagtagaggagtttgttgaagcca
tccattgttaaATGAAGTTAGTAATTATCCTCAAGAGGTCGAAAAGATGGGATGATATAG
GAAAAGTTACAGAAAAAGTATACAGGTTCTTAAAGAGGGAAAATTTTGCGGATGATAAAA
AAGTAGAATGCTTTGGAACAGTTGAAGCAGAAAAGAAAACTAATATTTTGTACAATAAAT
TTCAAGACAGACCAAGTGTTACAGTATCAATTCAAGAAAATAAAGTATGAtgcctattag
atacaggggcttgaattaacttgatgagtttgagttgttttaataaattgcgagaagtaa
atttagaaatgattgatgaaagattgcattgtgcaaataatagtacgcttggaattgttg
ggaaagttagaagtggagtgataattgatgaagtttgcaaagagattactttcttagttg
ctgaaagagtgata
>DjScaffold1355:28817-30236 strand:+
tatttttacggcaatagtATGAAAAAAACTGGAATTAAGAAAATGTCATAGAAATAATGAA
ATAAAATCCATACGCATTAATAAACTTGATACTTTTCTGTCTGTGAAGTATATGTTACAT
AAAAAAATTGATGTCGATGTAAAATCTTTGGTTATTTGTTCTGATATAAATGCTGTTGAT
GTGAAATTAAATAATGATTTTATTCCTGAACATGAATTAATTTTTTACTATAATATACTT
CAAGAACCACCACCTAACGATGTGATTCTTCAATTCAAAAGAAATTATTTCGATGTTCCA
AAATATGTTCAAATCTCAAAATTGGAAACAATACAACGTTTAAAAGAAATAATAAATTAT
ACAAACAGTGGTTTGAAATTGTTTCTGTTTGACAGCAATGGAATATTATTAGCTGATGAT
AATAAGTCCATAGAAGAGTGTGGTCTAAGTGATAATTCCAAGTTAATGGTTTGGCAAGGA
AATAATTTAAAAGTTCAATTAACAATTTGTGATCCACTAATTAAAATTAATGAACAATGT
CTGTCACAAATTATTTGTACGTTGGATTGTACGATTAATGATTCAGTTGAAAAAGTGAAA
AATGACATTTCAAAGTTCATGAAACTAGGGAAAGAAATGAATTTAGCTTTGTATTCAGAA
TTGTCTGTTTTGCACAATAAAAAAATTTCTAATCGCTTATCCCATTCAAAATTCAAATAAT
ATAATTTATTTTGTGGATTTAATTAAGTGAgatttcaataatgtataccaatttatgaat
tatgaataatatatcgaagttcaatcaattatttgatcaatgaaattaaatatacattat
tataagcaaagcttgaaactatggattgacttaataactattaatcagcaggtataatcg
attaaataattatggattttatagatccatctcataataatacttaaaacagtcatatta
aaagaactacttgattttttaattcaattttgagtagttgggacgattaactctgcataa
ttaatgaacacataattagcatgacatttg
>DjScaffold1355:49668-50806 strand:+
ttttttgaaaataatgtttccagaactgaaaaaatcgatcaagaagaggaaattagtaaa
ataaaatgtttcaaaATGCTCAAAGGTAAAATTATGAGCACTAATAAAACAGTTATTATT
AATAAAATATAAAACTGAAAATCAAAAGGAATTCGAAAGGAAATCATTGAAATATGAAATT
ATTACTCATTTGAAATTGACAGCATGTAAAAAATCTTTATTTGTTCAGTTGATATCGTTG

TATGAAGAAAAATCCAATTTTAATATAGTTGTAGAATATATGGAGCAAGGTTCATTAAAA
GACCTTTATTTGTCTAGAGAATTGTCAACAGACGAAATATTAAACTATTCTTATCAAATA
TTGGAGGCTCTTTCGTTCATGCATGATAATTTCATTATTCACAGAGCTGTTAAATGCTCC
AGTGTTCTACTACATGAAGATAAGAAAAAGATTAAACTAACTAATTTTCATTTATCCAAG
TTTTTATTTAATAAGACATTTGAGGATATGGAACTAGGATTTAAAGCTTCTGAACACGGT
ACAGATAGTGACGTGTTTTGGGTAGCGCCTGAAGTGTTGAGAAAGGAATTATATGGATTG
AAAGCTGATATTTGGTCGTTTGGAGCCACGGTTATTGAAATGTTAACCCGTGACCATCCA
TTCCCTGGATTTAATCCAGGTCGGGCAGTAAACATAATTGCTAATGAAGGGGCTATTCCT
TTTGATTTACATTTGGATCCAGCTATCAATACTCTTGTAGAAAGGTGTGTCACTAGCGAA
CAATTAGTTCGACCCGTTGCATCCCAGTTAATGAAGGACAGCATCATTTTCAATAAAATA
TTACGTAATCAGGATGTGCTCAATGTTTAGcgagactcttcattgaagcaaaataaagcc
gaatcatcaaaattcaagaagaatgttaattaatacattttatacattat
>DjScaffold2222:2014-2943 strand:+
gaatcagacgataacgaatcgtttgaagatATGGATGATGAGGAAATTAAGAAGATGCTC
GGCAATTCTGATAATAGTGACATGGAGTCTGATGATTTCTCAGAAGAAGAATTGGTTGAA
TCAAAGGAAGCAAAAGTTGGAAAGAAAAGAAAACTTGATACTGATGATGAAGATGATATT
AAAGATAGTAAAAAAAGTAAAATTTGTGAAAAAGGTGAATCAGATGATATCGAAGAGGAT
GAAGACGATGACGATGAAGAAGATGATGAAGATATGCTAGATGATAGCTTTATTGATGAT
GATATTGAAGAAGAAGAAAAAGAAGAAAAGGTTACTAAAAAACCAATTAAAAAGGTGGAA
AATGATAAAGAAAAACCAATTAAAGATGAAAAATCTGTAAACAATGAAGAGAAAAAAAAT
AAAGAAAAACTAATTAAAATTGAAAGAACTCAAGAAAACGAAGAATTCCAGAAAATTTAC
GAAGACACTGCTAAAGTTGAAAGTGAAAGTCTAAGAAAAGAATTGAAAGACAGAATTGTC
GACAGGAAAAACAAAACTCTAGTTTTATTAGGATTACCATTTAAGTTCAACCAAGCACAA
GCTAAAGAAATAATTCCGGACATGGTATCGATTATTCTCCCGTATAACGTGCATCGTCAC
CAAATCAATGGTAACGCTTATATCGATGTTTCCAAGCCGGAATTAGTCGCAAGCTATGTT
TCAAAATTGAAAGGATTTAAAATAAAAAATCAT
>DjScaffold2222:20485-21381 strand:+
gaatcagacgataacgaatcgtttgaagatATGGATGATGAGGAAATTAAGAAGATGCTC
GGCAATTCTGATAATAGTGACATGGAGTCTGATGATTTCTCAGAAGAAGAATTGGTTGAA
TCAAAGGAAGCAAAAGTCGGAAAGAAAAGAAAACTTGATACTGATGATGAAGAAGATATT
AAAGATAGTAAAAAAAGTAAAATTTGTGAAAAAGGTGAATCAGATGATATCGAAGAGGAT
GAAGACGAAGACGAGGAAGAAGATGATGAAGAAATGCTAGATGATAGCTTTATTGATGAT
GATATTGAAGAAGATGAAGAAGAAGAAAAAGTTACTAAAAAACCAATTAAAAAGGTGGAA
AATGATAAAGAAAAACCAATTAAAGATGAAAAATCTGTAAACAATGAAGAGAAAAAAAAT
AAAGAAAAACTAATTAAAATTGAAAGAACTCAAGAAAACGAAGAATTCCAGAAAATTTAC
GAAGACACTGCTAAAGTTGAAAGTGAAAGTCTAAGAAAAGAATTGAAAGACAGAATTGTC
GACAGGAAAAACAAAACTCTAGTTTTATTAGGATTACCATTTAAGTTCAACCAAGCACAA
GCTAAAGAAATAATTCCGGACATGGTATCGATTATTCTCCCGTATAACGTGCATCGTCAC
CAAATCAATGGTAACGCTTATATCGATGTTTCCAAGCCGGAATTAGTCGCAAGCTATGTT
TCAAAATTGAAAGGATTCAAAATAAAAAATCAT
>DjScaffold3418:140189-140904 strand:+
TCAAATTTAGATGCATTGTCCATAAGTCGTCCTTTGAATTTTTTAACCATTCCAGGTTCT
CATGATAGTTTTACTTGTGATATCTCTGAAACTTCAGAAATTGGTCCCGATGTGAGGGAT
GCATCCCTAAAACTTCTAAAAAATATTGGATACTATGGGAAATGTATTTTAAAGCCTTGG
TTAGTTACACAAATTATGAATCCAATAGAACAACTTAGAAGTGGTGTTCGTTATTTCGAT
CTTAGAATTGGAAAAAGAAATTCAGAAAATATTGAATTACTTATTACGCATCAATTATTT
TCTGGAAAATTAGATATTTATTTACAACAGATACAAGAATTTCTAAATAATCATCCAAAA
GAAGTTGTTTTATTGGATTTCAATCACATTTATTTAGATGAAATGGAACATGAATTATTA
ACCGAGTTAATTATAAAGGTTTGTGAAATCATTTTATTAATTTTCTTTGTTTCTTTTTTA
AAATTTCTTTAAaagtttctcattaatcgtttactttttagttttaaattacaaattgg
taaaaattaattaaaattataatcaaatgaaaaatgcaaacaattttatttaactgatac
aataatctcgaaaatcgataaatggtgctgaaattgctcacggatctaatccagccaaaa
ctatattttca
>DjScaffold3418:88588-88971 strand:+
ATGCCATTTGGTATCGCAGCGGCACCAGGGAAATTTCAAGAGATGATGACAAAAGTGGTA
GGCAGAATCAATGCACCCATGGTTTATCTTAATGACATACTGATATTTACGAACAATAAA
GACCAACACTATGAAGTAATTGAAAGAGTATTGACGGAAATAATGAAAGCTGGTCTAAGA

ATTAATCCAGAAAATTGTCAGATTCTTCAAAGCGAAATCAAATTTCTTAGACACATAATT
AGCAGGGACGGTTTCAAAACTGATCCGTCTAAAATAGAAGCAATAAGAACATTTAACAAA
CCTAACCGTGTGAAGAAGTTGCGAAGTTTGTTAGGAATATGTAATTATTACAGAAGGTTT
ATAGAAAACTACGCAAGAAAATGA
>DjScaffold1328:156790-160336 strand:+
attagttcgagaATGAGATATGAAACTATGTGCTTTGGTTCTATGAATCGTACTTCTGAT
AACGCTAGTGATAATGCTATTAAACGTTCTTCTTCCGATGGTAAATTAAACTATCGGAAT
TATTCAAGAAAAATGAGTTCAGAACCTCCTGAACGCCAGCCATTTCAATTGTTAAATAAT
GAAAAATTTATCCAAAATACAAATGAATCTATAAAACCAGTCGAAATAACAAACTTGATT
TGTCAGTCAAATCAATCCAACATTATTCGTCAAAGTCGAAGTCCAATGCCTTTTCTTATT
AATCAACAGTCAATAAGTCTTACACCTAAAAGACATCGATCTGGTGAGGCAAGCTCTACT
ATGTTCACTGGAAATTATCGATCAGATACTCCTATGCTTTTAAATGATTTAGTATCAACT
GCAACCAGTAGTAAAGGTTATGAAGCTGATTCAAGCCGGGCTTCTGAAAATGGAGAATTT
GATATTAATGATCATTTTTCTAGTTCTGCTGTTGACAATAAAATGGACAATATAAAAAGT
GAAATGGTTCCATTTTGGATGTTATTAAATATTCACGATTATCAGAAACAGATTGAAATT
TATTTTCATCACACGGATAATTGTTGGTCATCAGTTCCAAATAATCATGATTCTATTTGT
GATCATTGCAGTGCTTTCGAATCAATCTTTGAAAAATTCAATGAATGTGTTCATCAAGTT
AATCAGTGTTTGTTATTAGAAAAATTAGCTCAAACTCGAATTTGTCGGGATATACTGGTA
CCAGAAGATAGTACCGATCAGAAAATCTCTTCTGTTGCTCAATTAATGGATTATGATTAT
GACGATGAATCTATTGAATTTTCTAAAAAATTCGGTATCGGCGCTTTTGCCTGTCCAAAA
ATTTTGAAAATAACTTTTCGATTGCATTCACGGTTAAGAGCATCTACTGATAAAAATGCG
AAGGCTATTCCTTTGTTGAGAAGTGGATTGGAAAATATCGCCGTTATAAATCGTAAAAAC
ATGCTCATATTTGTTGATGATAATACAAATGGGATTTTTTATTGTGTTATGCGAGAAATC
ACAGGAAATTTAGATCAAGAAGACATAGAAAGACTGAACGAAAATTCAGTTTTGTTATAT
AATTCCAATAATCAACTTTCTAGAACAGTGAAAGTCGAAGAATTAATTCTACACAGAAAT
GTAGAAAATTTCGATAATCTATCAATCAAGTCT
>DjScaffold1328:160985-167951 strand:+
TTAACGGATTGTTACTTGAATTTTAATTTGTGGGTTCGTGGAGTAGTTAATGTGGATATT
ATTAAGAAAAAAATTGTAAATGCTATTCAATACTCACTGAATGATTTATTTATGGAATAT
GTTATTATTCCAAGTCCAATTTCATCTTTTGATGACGATCTCTTTGCCGAAATAAAATCT
AATCCAAAAGCCCTTGAAAACGTTGAAGATTTAGATTTAAAAATCATGGGCTACGAAAAT
GGTGACATTGGAAATTTAAACAACATTTTCGCTGAGAATATTTTCAACTGGATTGAAGAT
TTATCGAAAAACGATTGTCCTTTATTAAAATATCGTAGCTTTGAAATGTTTTCCGATTTC
AATGTAGAATTCTTAATTAACGATCTCGTCGAATCGATGAATGATTCTTTACATTTCAAC
ATTTTTAATTACGAAAATAATACTTGGAATCCGTGGAAAGTTAAATTTCCGAAAAGTGGT
AAAGAATATCCAGGTATGAAACGAAATTTTATTATAATCGGTCGTTTAGAAGAATTATGG
AAACGATTTGTCACGAAAAGATTTGTTACTGTAGCTGAGTCCGATGATATTTCATTAAAT
AATAGCCAAACATCTTTATCATTCAATGGTAATCCTTTGGGATCCAGTATAAATTCCGTT
TCTTCAAATCAACCAACGAACAATCTTAAAGAATTAAGAAAGGTTCGAACTTTATTGAAA
CAACCGCCTTACGATTACAATTTAATGAATTCTTCTAACATTCAATCGAATACAAGCATT
CTTAATTCTAGTAATTTGAACAATGGTATGCCAGAATTTTGCAATGTTCGCGTTTCTAAT
TCTATAATTATTCCACGACAAACTTTGTGTATTATTAAAATTCATGGGTGTCAAGTGGAT
ACAATTTCCTACAATTTGGCGAAAGAAATATTTGATCATTTGTATAGTTCTATTGATGCT
ATTATTAAGCACCATAAATCTAAATTCCAGATGCTGAATTTATTTATCTATCAAAAACTG
GGATTATTCTATGAATGTTCAAGTAAAAGTTGTTTGCAATCAATAAGTGTATCATCAGTA
AAAAGTTTTATTCTCAATTCCCCTGCTAATAAAAATAAATCTACAAATACTGGTATTAGC
GATTTAAATCGTGAAAGAAAGCCCAGTGGATCCGTTAGATCATTCTCCCCATCGAATCAA
ATGTCAAAAACTTTAACT
>DjScaffold1328:170436-193935 strand:+
AGACAATCGATTTCATTTTTGCAACCGTCATCTGGATTTAAACAATCTGAGTTATCTGAG
ATCGAATGTCCACTTCAGCAATTGGAAGTATTCTCTTGTTTGAAAAGAGCTTGTAGAATA
TTTCATTCCGTGGTGTGTCCAATTTTATTTATTCCAAATATTCGAAAAAAGATTTTGAAA
TCTTTAAACAATCAATTTTGTAGATGTGACAGCGACGACAACTATGCTAATGATGATTTG
ATTCATGAAATGGGTCGATCCATATTAGCCCATTATGCTGATTATGTGCAAAAAGAGCAT
GGATTTCATTTCATTAATCCTCCTCGTCCGAATATGTCACAAAATTCTGGTTCTCATGCA
TTATTTCAAAAATTATCAAATAATTCTGGTATTTATTTAATGGAGGTATTTCTTCTTAAA
GGTTGCTTATGTGTAAGAATAAACTTACTAGAAATCAGAAGATTGAACAATTCTCATCCT

GGTTTGACATCGTCATTTACAAGTTTCCAAAATGTTCAAACCATGCAAAAATCATTTGTA
AAAGAAGCGTGCAAACTCTGTGATCTGACTCACTTGGTTTCATTTATGTATGATGTATAT
ATAAAAATAACAGCAAAATCCATATCATATCCCGACGAAAATAAAAAACCATTCAATCCG
CTATCATTTCTACGTGATTTGGTTAAAATTTATGCTCAACCACCTCTTTATGCTACTAGT
CACATTTACTTAATGCAATTTCCCGTTGAGCATGATGTGATAAAACCAGATAATTTATTT
GACTTTCTTCAAAGACATTTTGAAGATTACAATTTTTCAATATGTAACAATTTTATTAAT
CAACCAAATGATGATGTGAGTTCTGTGTTTCTGTTTAATGTTATTGAAGAAAGAGGCACT
TGCCCGAAAGTAATGAGTTCATTAGCGTCATCACCATTGGACACATCCTCGTTTGATTCA
AAAATATTTTATAAATATGCAGATACTGGGAAGGAATGGGACTTAACTTCAATATTTTGC
CTTACTCAATCTTTACAGGATCCAGAATCATCATTTGATTTATTTATAATAACCTTTAAT
CAATCTAATTGTTTTCCTCATCAAAAATTGACTACACTTGGAAATAACACTTCGAAATCT
TCTAAATGTCTAGCTTCATATTTGACTATTTTACCTCATATAAGTACAGATCCTGTTAAT
TTATTGAATTTAAACCCGTGTCCTATTTCATTGACATATATTGATAGAAATTATATGCCA
ACAGTATGTGCTTGGCGAACATATGATAATCGATCAGTTGTACCAACACAAGCTCCTTTA
TTTTTAACCTTAAAAAGTCGTATTGAAAAATATTTAAAGGAATGCAATGAAAAGTTTTCA
AGAGCTATTACAGATTGTCATAAGGATTCATTGTGGCACAGAATGTATCAAAGATCAGAA
ACTTGTGATTCTAATAAATTCTCGTTGTCATTTGAAGAGTTTCAGGACTTGATAAATTCT
GGCAGTGTTGTCGATATTTTAGAATTAGATAATCGCCTTACAGATAGTATGAAAATTGCT
GCTTCTTATTTGAAGGAACTTAATGATTATATGGGGAAATTGTCTGATGATTGCTATACT
GTGCTTTATGAAAACACTAAATCCATTTATAAAATGATTTCTGGTAATATCTCTAGTCAT
CTAGTTTTGATGTCAAAGAAAACGAAAGATGGATTCTTATTACTTTCCAAAAATCAAGAA
ATCAATTCTAATCCAGATAATTATAGCGTACAGCTAAAAGCAGTTTTTCGAAGTCAAGAT
TGTAGAAATCGAATACCTCTTCAACATAAATATTTTTCTTCATCCTTACGAGATATTACT
GTTATGTTAGCGGAGACGGCTGCTTTCTGTATATGGAGAAAATTAGCTATGAAACCAGGA
AACTGTGGAAAATAAtgtttatatttcagatgatttttgataaaactttaatgtaatt
attttgttatatttgtaaatttaatatttaaagttctgagaaaataaattatgtttttta
a
>DjScaffold1144:76157-77031 strand:+
gtttcattcttataaaatttgagttcttttcacataatttttcaacagtgtttttttaa
tcgatacaaattcaatccattatctttttatccatttcaatgagctgagtatatcgctaa
tcatgttcaaaataatgcaagatacattataggtgcttgtgttggtggtattATGCGTAA
ATTAAATAATAATTTATTGAAAATGGAAGAATCTGGAAAAATTCCGAATGATAAATACAT
TAAAGGTCTTATTAATCAGTGTTATTTTGATTTGTTTGTTGTTGAAAGCTGTTTGTATTT
CACAACGAGATTTGCTGACAATTATTACTCGCCTGAAATTGAATTAGAAAGTGCTGCTGT
TAAGGTATGCAGTTGTGAATATTCTCTAAAAATGCTTGATAATTATTATAAAATACTTTT
ACTCATCGGTGAACCAATCACAGCCGAGTTTCAGAATTATTACAACCGAATAAATGCGAT
ACAAAATTTTATGGGATCGAATTTTTTCACAAAGCTCTATATTGGTGGTACAATGATACA
GTTATCTCTACTTAGACACATGGAATTTTCATCAAGAATGACCACACCGTTGGATAATTT
GCCTTATTTAAAGAAAACATTTAAAGAATCCCTGCATCCGATGGTCCTAACTAAAAATAC
CGGGGATCCAGCGATTCACAATATTATTAAATTCGATTTATATATGATTGATTTGAAAAG
AATATTCTCTAAAATTCAACGA
>DjScaffold1606:24782-39254 strand:+
ACTGTTATTTGGCAGCCATATCCAGGTCCCACAGTAGAATGTTACACTTTGGAAATTGAC
GATGGTGCCAACGGGTCATTCAGGAAAGTCTATCGAGGCTGTGAATCAATATGTACCATT
GAAGGACTTCATTTTAAATCAATTTATCGAATACGTGTCAAGGCCCACAATCGTCATGGA
GACAGTAATTACAGTGATCCAGTTTGTCTGCAAACCTCCAGTGTTCTCTGGTTCTGTATG
GATCAAAAATCAGCAAAAAATGACATTATTTTTTCCAACGATAATAGAACCATAACAACA
GAATCAATTGAAGAAAAAACTGTTCTTGCGACCACCAGTTTATCACGTGGAGTTCACTAC
TGGGAATATGTAGTTGATAAAATTGAACCTGGCGGGCAGCCAGCATTCGGCGTAGCAAAG
TCAGATTGCAATAAAGAAATGATGCTGGGCAAGGACAATATTGGGTGGGCAATGTATATT
GACTCTAAACGTAGTTGGTTCATAAATAACAATGATCACTTTGAAAGAACTGATGGCGGA
ATAAAATCAGGCTCAATTATTGGTATTCGTTTAGACTGTGACAAAGGAAATCTCAGTTAT
TATATAAATGATGAACCGCATGGTCCGATTGCTTTTACAAAATTAAAAGGTTCTCTTTAT
CCTGCTGTTAGTTTCAATAAATTCGTACAATTAACACTTGTTCTTGGAAGAGAAGCGCCT
AGTGAAAGTGAGGAAAGCGAAGAAGATGATGAAAATTTAGGAGCAAAATCTTCGGCTGGG
CAAAAGTAAataaaaacgaacatttaaatttagctcaaatggggaaaattttgttgattt
cttttatcaggttgaacaataatggatgagttgtaaatgttttcattgtaattattacat

gacttttttaacatgataaacagtcaccaaattatggttaatgtttgtgattatgagca
tttatctatggtgtattaaacatgggatagatataagtaactaacaaatcaactaataaa
ccgaataatctaagtgttttctcttatgttgagtagattttaatttgaaaattttattgg
ataattgatattatttatttgtttattaaataataaatatgaattgaataaa
>DjScaffold3661:10013-76411 strand:+
aacataatgaaagtgatggtaaaattattcaaagatttcctaataaagactggtcagatt
ataaatatgaagtacaagttgaaatgttttgtcaaccttgtggatggacattatcagatt
ctaaacgtcctcctacatttttttgtagcgtatcttacaaatactttgggagaaatttatt
atttagcatgtctttcttttcatgaagcaatttccgcgtctttgtttgatcacggaaaac
caattaatgccagttcagctcattcttcatattctaaaaaaattcaacattcaaacagta
gttcacactcacgaaatctcagtaccagtagtggaaatcagataaataaaattaacggtg
aatctattgggcacgaatctaatggtttggtagatcatgataatgacggttttcttgaag
atccgaatttagtaaaaccagctgataaatacgcaccaaaatcattgattttactttctc
gtcatggacatttcgatgtactcaaaaattgtctcagtataatttattcaatttatatgg
atcctcatcgagaaatatcactggaatacattgttggaaatattctaagcggtgtagaag
caccatctatcggtggctccagaatctcatttactattggagctggagataagcaagtta
ttcaaccttcagtatgtcccacgttaccagtgacaagaagtagtgtttcaatgttattca
agcatttaggtatgcaaaacgtattggcgattttctctagtttaataaatgatcaaaaga
tattactctattcacagagtctaaaccgattaacagaagcttgccacgcttttcgatcat
tgatgtatcccttaaaatatggaaatgtcttcattccaattattccaaaaactatcattg
acgtgttaagtacaccgactccatttttagcaggagttcacacatcttacaggccgatgt
atgatttgaccgacgtggttatggtggatctcgatggaggaagtgcaaaatatccagatg
gcataccattagtaccgctatgtgaaccttataatagtatcacaatcgatagtttaacca
aaattctcaatccagatttattaactgccgatgaagtttatccaccgtttccaagtccgc
ctccagatcctgtttggcaggataaacaattgcgagctgttttcattcgattattttgta
gactgtttgcaggctatagatcatgtctcacattgacaagaataagagttaaaccggtga
tccattttaatgagtcaatgtttttactgttgagaggattacgaaacgattccgatgaat
tcatttatcgtttacttggatgtatgagtttcggtcaatttattcaagatcgaggtactc
catatcgggtttgcgatgtttttgatgaagaatatgaatcaatgacagatctattacgat
caaaatctgaagtatttgatgatctgtcaactattgataaactagctaaaaaactgcatg
ataatgaaaataggggaccgcctgttgtgcatcaactgcctcaagaagctaaattagcac
atactagaatacacacacaacaatttcaattgctggatgaaaatctcgttacagatcata
ttaaaacaaatacccgaaatagatcaaggcgagagaaacaaaatcagcctgatctgtatg
attcaactagtgtaaataaactggaaactgtggaaccgagatttgttccacatggtttag
agttagataaacagattttcaaaactgaagccttatcagacaaaaataaagtcattagag
aatttgtggcaaatatatttaataatcatataaccgaagctcttaaaagacgaaatacga
ttagacaagatttaaaaaatagattaattcgtcgaatattcattgatgaattatatcgcc
aattatatccagatcagtctgatacatttggtggtcaatatgatctgaatatcgctcctt
ataattcggaaataagttgcgaacggaaagctttattgggatatcagcagtttgaattag
ttaatgatttaatgggtgaagcactaagacaagaaggtcaaaatgaagaaagtggagttg
tacctataataatggagttgagcactaaattctgcactcaattaaacaatattcgttatt
attccaatatgactcatcaaattcaagaacatccagtatgggagagtcaaaaattttgga
ttacagctttctgtgatcaagtcaataatgaaatacggcaactttaccttcatcattctg
aatcacaataccgtcaacgtaatgaacggatgaatctagctgaaaagcattcagcaaatt
tacatacatctggaggagacagaaaacgtcaattgattgatgttagtttcatataatttg
ttatgattgtctatgttgaaaacttcactttttctccaactttctgcacacactttcaa
tgcacacaaaatttcacaagaactatatataacattaggaaaacactaggaacatgttac
atttccatacttcacaggaagaaacatgaagaaatactaattaaaaacgataaacctaaa
atcaatgtaatttataataattaaaataaattaaccaattaaaattcattaaagtaaatg
ataaaattaattctatttcttctaataacttgaattaatcaaaaagtaaatttccgaaga
agacaattgtcgaaaattcggattattaacaaaaaacaaaaaacttcacttttatgtaaa
ctttttaatttattgaaacttgaaaatacgatcaatggtttaattcaacaattattctcg
caataattgtaattgtataactaatattcgtttgttcatctaatttttaattctttattg
ctgagatccattgaaaaactagctacaagggatatttaggaataattgagggtcatatat
ccaatttggaagcaatatatccggaagtccagaatattcctaaagtaaaaaaggttgaag
ttacaagtccggttttattaccaaatgagaaagttattataattaataataaggaaagtt
tgcgttgtcaccttttacttgatgggagacatgaaaaattggatATGCAACGTCAACGAG

AACAGGACTTTGAAGGATTGGGAGGGCAAACAGTGTCACAAGGTAACACACACAGATTTA
TTGATGACGAAGATGATTTCGACGCTCTTCGACCTCTTTACCAGCACAGGGTGCTTTAT
TTATTACAAATTATAGATTAGTGTTTCATGGTGCTCCAAAAGATCCTTATCAATCCAATA
AACTGGTTTGCAGGAGTTTCCCTATTGCTGCATTAAAGTATGTTAAAAAACTCGGAATGA
AATCTTTTGTTTTTGGTGCTTCAAATTCACAAGGAGTATCTTTTAATTTAATGAATAATC
GGACAAATAAAATACGTGGAGGTGAAGGCTGCTTGGATGTTATTAGAATTCGATCCAATA
CTTTCCAAATGCTTAAAATTGGATTGGAACAAGAAGATTCCTCACAAGAAATTAAGGAAG
AGCTGTGCATTAAATTGACATCACTTCGTTATCCACAAAATTTGAGTTTCTCATTCAACA
TATCAAATAAAATTGGAATTTCTCATCAGAAAATGAATTATTGTACTGGACTCCCTGACG
AAACATTAGAAACCCATAAACGCTGTCCTACAGAATCGATTAGTTCTCTTTATTCATCTA
GTCATAATTATTCCTTCTCCACTACCAGTCCATCTATCCATAGCAATCTTAAGCAAAAAA
ATAATTTTACTCGTAATACACTAAGATTGGCTAAGGAAAGCATTAATAAAATATCGAAAA
TTAAAGGTCGAACTTCTCCTTCATCTACGAGTAAATCCAAAGGTATTTCGGAATCCGAAT
CAGAAAGAAGCAGTGCTATTCATGATGACGAAAATAGAGATTTTGTATTGGATCTTAATT
ATCCTGCAACAGATTCTAAACTGTTGGAAAAGCTACGGTCTACTTTAGGTTACTCTGAAT
ATTCTAGATTGAATATTGGAAATTTCCCAGGAACATTTAGTATAAGTGACCGATCTAATA
ATCGTAATGAATTAGTCAGATTGATATATAACTTCAATGATAAATATCAAAACGTTAAAT
CATATCAATCAATGATGGTTGTACCCCAGATTTTTAATGAAGCATCTTTGAAAAAATTTG
CCAAAACTCACAGGAAAAATCGATTCCCAATAATTACTTGGAGAAGTATGAAAACAAAGG
CATATTTATTACGAGGAGCTGGATTTCAAGCTAAAGGAATAATGGGGAAATTGTTAAAGC
AAACCAGTGGTGGTAGCAGTCAAGGAAATCCAAGTTCAACTAGTTCACTTATGCTTCGAA
ATGTTACAACAACATTACAAAGAGGATTTCAAAATATGACTCTGAAACCCATATATAGTG
ACAGAAGCCTTGATGAAAAACCAGGCGATCCAGAAATTACAGCTGCCAAAGAACAAGTGC
GATATTTTCATGCCCTTGTTGAAATTTCACCAAGCTACGACGATATTCAACAGCATATTT
CTAAATTGGATCCTCTTGTTCATTGGACTGACAATTGTAGACAAAATATTTTACATCACA
AAAAAAATCCATTAGAAAAAGCTTACAAATCCGTAATGGCTGGAGGTGAAAAAGCAAGAA
CGTTATTAAAGAATGAGGTTTTCAAAATGTCCTCATCTCATGATGATTTGAATAGTACCA
ATTTTATTTCTTCTTTTCGAGGTAATCCATCTGTATTTACACCGTTGAACAATCTCTCAT
ATCCTGGTATTCGAAGATCTAGTAAAGAAAGTTTAACGAGTGGAGCACCCATTGCCGATG
AAATAATCGATGACGAGGAGAGCAGCATAGCTGCAAACAATGCGTCATCTCAAAGTGGAG
ATCCTAACGCTCATTTATCTGAACTACGAATAGTACCATTATATATTCTTGGAGAAAAGT
CCGTGAAGACCATGAGTAAATCCGACAGTTTTCCCGATATAGAATTCATTGCAGTAGAAA
TTACAAGCAAACAACAAATCAAAGAAGCATATGAACAGTTGTTGAAAGCCGCTTTACCTG
ATAACGTTAGTTCAGTTCCAATGGTTAATTTACATCCCAGCGGCTCTTCGAATCGTTTTC
AAGAACCACAACAAGGATCTGAATCAAGCTTGTCTTTAGCAGCTAACAAATGGATGCTTC
AAATTCAATCTTTATTAGAATTATCAGGAGCAGTAGTAGATTTGATTGATATTCAATCAG
CCTCTGTTGCAATTTGTCTTGAAGATGGCTGGGATATAACTAGTCAGATAGTTTCGCTTG
CACAAGTTATGCTTGATCCTCATTATCGAACTATAAACGGGTTTTCGGCATTAGTTGAAA
AAGATTGGCTGATGGCTGGTCATAAATTTCAATCTCGATTATGCATGAGTTCTAATCCTA
AGACAAACAACTTATTTCCAGTATTTTTGCAATTTCTCGATGCTGTTCATCAACTGTTAA
ATCAATTTCCAATGGCATTTGAATTCAATGATTTCTTTCTTAAATTTTTGGCTTATCATC
ATTTATCAAATCGTTTCCATAATTTTAAAATGGATAGTGAAGGTGAACGAATTCAGTGGA
TGCGGTTAATTGATGAAGATTGTGTCCCGGGATCCAAAATATATGAGCAGCATTCTATTT
GGAATTATATCCAGCATCATCATGAAGAATGGCCAGTATTTTTTAATTTTCTATATAATC
CAAGATTGTCACAGGAGATTCTTCGACCTTGTACAAATTTAGCAATGATTGATATTTGGG
AGTATTATTTAAATGAGGATTTATCATCAGGTTCTGTTTACGATCTTGATCATTTTTGTC
CTAGTTATAGAAAACGTAATACTCAACATTATGACCCTATACTCAATGGTGCTTTCAATA
ATAGTCATGTAGAGAGTGTTTATGCCATTCTTGGTTTACGTGAAGGTTGTGAAGTAAGAA
ATTGGCAGAAAATCTGGAAAGAATCGTGTGAAGAAAGTGAACAAGATCTACAAAAATTAG
AGTCGATTTTCATTAACTCAGAAGATGAAGATTTTGATGCTGATGCGACACTCAAATCTT
ATTCAAGCCCTACGAAAAGATCAAATAAATTGGCTAATAACTATCTTATATCAAATGCCA
AGCATCACAAGGAAGAAATTAAAAAAATCCGAACCATTGCTTATAATGAACACAAAAATA
CGTCGTTACTCAGATTAGAAAAAAAGCATCTGGAAAATCCACAAGAAAATCCCACATTGC
ATGGTGCATCGGATACTAATTATCAGTTACCAAATTCGGATCACAAATTACAGAAGCTAC
CTACACATCTTGAATTTCAAATGCTTTGTGAAATTTGTCGGAACAGAGCTACGTTAACTG
ATACAGTAATGTTGAAATGCACCGCATGCAATCTAGCATGTCATCACAAATGCATTTGTG
ATTTCCCACGTGAATGTCGAGCAAAAACTGACCATTCAGCAAAAGAATCATCTCCAAATT

TTTCAGAACCCGCAGATTCAAATCTCGATAATCCACCTCTTACACCAGATACAGTCACCC
CCATATCTGCCTTATCAAATCAGACAATTCAGCCAGGGGGAAATAAATTAAACGATTTGT
CCCATGACAAATTTTCTGATTTTGATACACCAAATAAAAAATCAGATGATCTCAATCAGC
AGACTTCAGAAACTTCTTTTTCTGGCGATCTCTACAAATTAAGTCAACACACATTATTTC
CAGGCTGGCAGAAAAAGTATTTCACTCTCGACATTATACGGCACGAATTGAGGTATTCCG
ATTCAAAAGATGTTCGTTCAAATAGTAAACACATTTTACTTCAAGATATAACTGGTGTCC
GTATTTTAACTACGCTCCAAAATCATCAAAAACGATTTCCGGAATCAGCATTTCTAGAAT
TACAAACAGAAGAAAGAACATATAAATTAGCCAGTGACAGTCCATCTGTAATAAATGAAT
GGATGGAAAAAATTCAAAACTCAATTCAATGAtgtactttgaaatataactgcattatt
ataatgccagattctttttattatatgtgcattgtaaataactaattgatttccctttta
gaattattatataaaatgacttactttctcatagatttttaaacaggcttcaaaaaaatt
aaactattaaatcatttgtcatgtttctgtttcttattttgcattaatttgaaattcgg
tccaaaaagtagagcttatcgattaatatttattggaatttctttcaatattattgaata
tatttcattgacttccatttttacatccgttaatcgatataataataatgctgatttatt
atttttattgataatctgatttatcgaactgttataatgtatagtgaatcaagtatgggg
ttatgttttgagtgccacataaacatatatatttatttcaatttatttttagacaatttt
gattttcttattttatttaacacttactagtaacacagtgtatttgcaagaattgagaaa
ttcatagtttattttgtttgatttttcatgtgtaacatatatttgtggcctttgatgaga
atcttttgtgattcagtatatatcttgctctgtttgagtttctgtgtctagacttgtttg
tgtatgtgtgttatgtataaataatgagtaataatttcgaaaatctcagtgtaccaatgt
acaaattattgtttttgtaaattgaaaataatagtaaatattatagatataattgaaatg
attatgattgaataatgatattattttgattggtgaatttaattcggatgctttagattt
tcattttacccagttggatgatgtgtaaagaaagatttatattgatttattaggtttatg
atgagctatctatcttgttttgctcttttataatacatcttttagtaacataatgaaagt
gatggtaaaattattcaaagatttcctaataaagactggtcagattataaatatgaagta
caagttgaaatgttttgtcaaccttgtggatggacattatcagattctaaacgtcctcct
acattttttgtagcgtatcttacaaatactttgggagaaatttattatttagcatgtctt
tcttttcatgaagcaatttccgcgtctttgtttgatcacggaaaaccaattaatgccagt
tcagctcattcttcatattctaaaaaaaattcaacattcaaacagtagttcacactcacga
aatctcagtaccagtagtggaaatcagataaataaaattaacggtgaatctattgggcac
gaatctaatggtttggtagatcatgataatgacggttttcttgaagatccgaatttagta
aaaccagctgataaatacgcaccaaaatcattgattttactttctcgtcatggacatttc
gatgtactcaaaaattgtctcagtataatttattcaatttatatggatcctcatcgagaa
atatcactggaatacattgttggaaatattctaagcggtgtagaagcaccatctatcggt
ggctccagaatctcatttactattggagctggagataagcaagttattcaaccttcagta
tgtcccacgttaccagtgacaagaagtagtgtttcaatgttattcaagcatttaggtatg
caaaacgtattggcgattttctctagtttaataaatgatcaaaagatattactctattca
cagagtctaaaccgattaacagaagcttgccacgcttttcgatcattgatgtatccctta
aaatatggaaatgtcttcattccaattattccaaaaactatcattgacgtgttaagtaca
ccgactccatttttagcaggagttcacacatcttacaggccgatgtatgatttgaccgac
gtggttatggtggatctcgatggaggaagtgcaaaatatccagatggcataccattagta
ccgctatgtgaaccttataatagtatcacaatcgatagtttaaccaaaattctcaatcca
gatttattaactgccgatgaagtttatccaccgtttccaagtccgcctccagatcctgtt
tggcaggataaacaattgcgagctgttttcattcgattattttgtagactgtttgcaggc
tatagatcatgtctcacattgacaagaataagagttaaaccggtgatccattttaatgag
tcaatgtttttactgttgagaggattacgaaacgattccgatgaattcatttatcgttta
cttggatgtatgagtttcggtcaatttattcaagatcgaggtactccatatcgggtttgc
gatgtttttgatgaagaatatgaatcaatgacagatctattacgatcaaaatctgaagta
tttgatgatctgtcaactattgataaactagctaaaaaactgcatgataatgaaaatagg
ggaccgcctgttgtgcatcaactgcctcaagaagctaaattagcacatactagaatacac
acacaacaatttcaattgctggatgaaaatctcgttacagatcatattaaaacaaatacc
cgaaatagatcaagtgtaaataaactggaaactgtggaaccgagatttgttccacatggt
ttagagttagataaacagattttcaaaactgaagccttatcagacaaaaataaagtcatt
agagaatttgtggcaaatatatttaataatcatataaccgaagctcttaaaagacgaaat
acgattagacaagatttaaaaaatagattaattcgtcgaatattcattgatgaattatat
cgccaattatatccagatcagtctgatacatttggtggtcaatatgatctgaatatcgct
ccttataattcggaaataagttgcgaacggaaagctttattgggatatcagcagtttgaa

ttagttaatgatttaatgggtgaagcactaagacaagaaggtcaaaatgaagaaagtgga
gttgtacctataataatggagttgagcactaaattctgcactcaattaaacaatattcgt
tattattccaatatgactcatcaaattcaagaacatccagtatgggagagtcaaaaattt
tggattacagctttctgtgatcaagtcaataatgaaatacggcaactttaccttcatcat
tctgaatcacaataccgtcaacgtaatgaacggatgaatctagctgaaaagcattcagca
aatttacatacatctggaggagacagaaaacgtcaattgattgatgttagtttcatataa
tttgttatgattgtctatgttgaaaacttcactttttctccaactttctgcacacactt
tcaatgcacacaaaatttcacaagaactatatataacattaggaaaacactaggaacatg
ttacatttccatacttcacaggaagaaacatgaagaaatactaattaaaaacgataaacc
taaaatcaatgtaatttataataattaaaataaattaaccaattaaaattcattaaagta
aatgataaaattaattctatttcttctaataacttgaattaatcaaaaagtaaatttccg
aagaagacaattgtcgaaaattcggattattaacaaaaaacaaaaaacttcacttttatg
taaactttttaatttattgaaacttgaaaatacgatcaatggtttaattcaacaattatt
ctcgcaataattgtaattgtataactaatattcgtttgttcatctaatttttaattcttt
attgctgagatccattgaaaaactagctacaagggatatttaggaataattgagggtcat
atatccaatttggaagcaatatatccggaagtccagaatattcctaaagtaaaaaaggtt
gaagttacaagtccggttttattaccaaatgagaaagttattataattaataataaggaa
agtttgcgttgtcaccttttacttgatgggagacatgaaaaattggatATGCAACGTCAA
CGAGAACAGGACTTTGAAGGATTGGGAGGGCAAACAGTGTCACAAGGTAACACACACAGA
TTTATTGATGACGAAGATGATTTCGACGCTCTTCGACCTCTTTTACCAGCACAGGGTGCT
TTATTTATTACAAATTATAGATTAGTGTTTCATGGTGCTCCAAAAGATCCTTATCAATCC
AATAAACTGGTTTGCAGGAGTTTCCCTATTGCTGCATTAAAGTATGTTAAAAAACTCGGA
ATGAAATCTTTTGTTTTTGGTGCTTCAAATTCACAAGGAGTATCTTTTAATTTAATGAAT
AATCGGACAAATAAAATACGTGGAGGTGAAGGCTGCTTGGATGTTATTAGAATTCGATCC
AATACTTTCCAAATGCTTAAAATTGGATTGGAACAAGAAGATTCCTCACAAGAAATTAAG
GAAGAGCTGTGCATTAAATTGACATCACTTCGTTATCCACAAAATTTGAGTTTCTCATTC
AACATATCAAATAAAATTGGAATTTCTCATCAGAAAATGAATTATTGTACTGGACTCCCT
GACGAAACATTAGAAACCCATAAACGCTGTCCTACAGAATCGATTAGTTCTCTTTATTCA
TCTAGTCATAATTATTCCTTCTCCACTACCAGTCCATCTATCCATAGCAATCTTAAGCAA
AAAAATAATTTTACTCGTAATACACTAAGATTGGCTAAGGAAAGCATTAATAAAATATCG
AAAATTAAAGGTCGAACTTCTCCTTCATCTACGAGTAAATCCAAAGGTATTTCGGAATCC
GAATCAGAAAGAAGCAGTGCTATTCATGATGACGAAAATAGAGATTTTGTATTGGATCTT
AATTATCCTGCAACAGATTCTAAACTGTTGGAAAAGCTACGGTCTACTTTAGGTTACTCT
GAATATTCTAGATTGAATATTGGAAATTTCCCAGGAACATTTAGTATAAGTGACCGATCT
AATAATCGTAATGAATTAGTCAGATTGATATATAACTTCAATGATAAATATCAAAACGTT
AAATCATATCAATCAATGATGGTTGTACCCCAGATTTTTAATGAAGCATCTTTGAAAAAA
TTTGCCAAAACTCACAGGAAAAATCGATTCCCAATAATTACTTGGAGAAGTATGAAAACA
AAGGCATATTTATTACGAGGAGCTGGATTTCAAGCTAAAGGAATAATGGGGAAATTGTTA
AAGCAAACCAGTGGTGGTAGCAGTCAAGGAAATCCAAGTTCAACTAGTTCACTTATGCTT
CGAAATGTTACAACAACATTACAAAGAGGATTTCAAAATATGACTCTGAAACCCATATAT
AGTGACAGAAGCCTTGATGAAAAACCAGGCGATCCAGAAATTACAGCTGCCAAAGAACAA
GTGCGATATTTTCATGCCCTTGTTGAAATTTCACCAAGCTACGACGATATTCAACAGCAT
ATTTCTAAATTGGATCCTCTTGTTCATTGGACTGACAATTGTAGACAAAATATTTTACAT
CACAAAAAAAATCCATTAGAAAAAGCTTACAAATCCGTAATGGCTGGAGGTGAAAAAGCA
AGAACGTTATTAAAGAATGAGGTTTTCAAAATGTCCTCATCTCATGATGATTTGAATAGT
ACCAATTTTATTTCTTCTTTTCGAGGTAATCCATCTGTATTTACACCGTTGAACAATCTC
TCATATCCTGGTATTCGAAGATCTAGTAAAGAAAGTTTAACGAGTGGAGCACCCATTGCC
GATGAAATAATCGATGACGAGGAGAGCAGCATAGCTGCAAACAATGCGTCATCTCAAAGT
GGAGATCCTAACGCTCATTTATCTGAACTACGAATAGTACCATTATATATTCTTGGAGAA
AAGTCCGTGAAGACCATGAGTAAATCCGACAGTTTTCCCGATATAGAATTCATTGCAGTA
GAAATTACAAGCAAACAACAAATCAAAGAAGCATATGAACAGTTGTTGAAAGCCGCTTTA
CCTGATAACGTTAGTTCAGTTCCAATGGTTAATTTACATCCCAGCGGCTCTTCGAATCGT
TTTCAAGAACCACAACAAGGATCTGAATCAAGCTTGTCTTTAGCAGCTAACAAATGGATG
CTTCAAATTCAATCTTTATTAGAATTATCAGGAGCAGTAGTAGATTTGATTGATATTCAA
TCAGCCTCTGTTGCAATTTGTCTTGAAGATGGCTGGGATATAACTAGTCAGATAGTTTCG
CTTGCACAAGTTATGCTTGATCCTCATTATCGAACTATAAACGGGTTTTCGGCATTAGTT
GAAAAAGATTGGCTGATGGCTGGTCATAAATTTCAATCTCGATTATGCATGAGTTCTAAT

CCTAAGACAAACAACTTATTTCCAGTATTTTTGCAATTTCTCGATGCTGTTCATCAACTG
TTAAATCAATTTCCAATGGCATTTGAATTCAATGATTTCTTTCTTAAATTTTTGGCTTAT
CATCATTTATCAAATCGTTTCCATAATTTTAAAATGGATAGTGAAGGTGAACGAATTCAG
TGGATGCGGTTAATTGATGAAGATTGTGTCCCGGGATCCAAAATATATGAGCAGCATTCT
ATTTGGAATTATATCCAGCATCATCATGAAGAATGGCCAGTATTTTTTAATTTTCTATAT
AATCCAAGATTGTCACAGGAGATTCTTCGACCTTGTACAAATTTAGCAATGATTGATATT
TGGGAGTATTATTTAAATGAGGATTTATCATCAGGTTCTGTTTACGATCTTGATCATTTT
TGTCCTAGTTATAGAAAACGTAATACTCAACATTATGACCCTATACTCAATGGTGCTTTC
AATAATAGTCATGTAGAGAGTGTTTATGCCATTCTTGGTTTACGTGAAGGTTGTGAAGTA
AGAAATTGGCAGAAAATCTGGAAAGAATCGTGTGAAGAAAGTGAACAAGATCTACAAAAA
TTAGAGTCGATTTTCATTAACTCAGAAGATGAAGATTTTGATGCTGATGCGACACTCAAA
TCTTATTCAAGCCCTACGAAAAGATCAAATAAATTGGCTAATAACTATCTTATATCAAAT
GCCAAGCATCACAAGGAAGAAATTAAAAAAATCCGAACCATTGCTTATAATGAACACAAA
AATACGTCGTTACTCAGATTAGAAAAAAAGCATCTGGAAAATCCACAAGAAAATCCCACA
TTGCATGGTGCATCGGATACTAATTATCAGTTACCAAATTCGGATCACAAATTACAGAAG
CTACCTACACATCTTGAATTTCAAATGCTTTGTGAAATTTGTCGGAACAGAGCTACGTTA
ACTGATACAGTAATGTTGAAATGCACCGCATGCAATCTAGCATGTCATCACAAATGCATT
TGTGATTTCCCACGTGAATGTCGAGCAAAAACTGACCATTCAGCAAAAGAATCATCTCCA
AATTTTTCAGAACCCGCAGATTCAAATCTCGATAATCCACCTCTTACACCAGATACAGTC
ACCCCCATATCTGCCTTATCAAATCAGACAATTCAGCCAGGGGGAAATAAATTAAACGAT
TTGTCCCATGACAAATTTTCTGATTTTGATACACCAAATAAAAAATCAGATGATCTCAAT
CAGCAGACTTCAGAAACTTCTTTTTCTGGCGATCTCTACAAATTAAGTCAACACACATTA
TTTCCAGGCTGGCAGAAAAAGTATTTCACTCTCGACATTATACGGCACGAATTGAGGTAT
TCCGATTCAAAAGATGTTCGTTCAAATAGTAAACACATTTTACTTCAAGATATAACTGGT
GTCCGTATTTTAACTACGCTCCAAAATCATCAAAAACGATTTCCGGAATCAGCATTTCTA
GAATTACAAACAGAAGAAAGAACATATAAATTAGCCAGTGACAGTCCATCTGTAATAAAT
GAATGGATGGAAAAAATTCAAAACTCAATTCAATGAtgtactttgaaatataactgcat
tattataatgccagattctttttattatatgtgcattgtaaataactaattgatttccct
tttagaattattatataaaatgacttactttctcatagatttttaaacaggcttcaaaaa
aattaaactattaaatcatttgtcatgtttctgttttcttattttgcattaatttgaaat
tcggtccaaaaagtagagcttatcgattaatatttattggaatttctttcaatattattg
aatatatttcattgacttccatttttacatccgttaatcgatataataataatgctgatt
tattatttttattgataatctgatttatcgaactgttataatgtatagtgaatcaagtat
ggggttatgttttgagtgccacataaacatatatatttatttcaatttatttttagacaa
ttttgattttcttattttatttaacacttactagtaacacagtgtatttgcaagaattga
gaaattcatagtttattttgtttgatttttcatgtgtaacatatatttgtggcctttgat
gagaatcttttgtgattcagtatatatcttgctctgtttgagtttctgtgtctagacttg
tttgtgtatgtgtgttatgtataaataatgagtaataatttcgaaaatctcagtgtacca
atgtacaaattattgtttttgtaaattgaaaataatagtaaatattatagatataattga
aatgattatgattgaataatgatattattttgattggtgaatttaattcggatgctttag
attttcattttacccagttggatgatgtgtaaagaaagatttatattgatttattaggtt
tatgatgagctatctatcttgttttgctcttttataatacatcttttagt
>DjScaffold3663:46458-47125 strand:+
AGTAACGTAAATATATCTTCAAATGATGAAAAAATAGATCCAAACAAATCATTGGAATTT
AGAGCTAATCTTGGAGGGAAAAATCTTATTTTCAAAAATGGAGAATGGATTTCAGAAGGA
GATGATTTCAATCAAAACTATCAAGATGGAAATGCATTGAATAATTTACGAAAAGAACGC
AAGAATCTCACAGAAGAAAATGCTTTGTTACGTATTAAATTGAATATTCTTATTGAAAGA
CTTGGTCGTAAAACTCTGGAATCTCATCAAGTATCTCAGAATATAGAAAAAATACGTGAC
AAAATAAGGAAAGCGGATAAACGATAAatttgctaaaattaaaataaatttcaatttct
ttttaatttataaataagttaaatataattttatatttatcttagttgattgttctaaa
tttgaattcatttaaatgatttctttatcaataaatgaatacgatgttttactttatatg
ccatattaatggtttgtttgacattgtacggaattataaatttaatgtaatactacttat
tatatatttatattagtaattagttatgattttgttatt
>DjScaffold3663:68115-68195 strand:+
ATGTCATCGATAAGAATGGAGTTCGAACCGATCCAACAAAAATTGAAGCGAAAAAAAATT
TCGGAAGACCTAAATGTGTAA
>DjScaffold602:3363-11919 strand:+

gtgacaaatatctttataATGGAAACTACCGAATTTTTACACAATCACTCACAACCTCAT
TCATTAGATATATCAAATATAATTTCCAAAAACTTTGGATCATTATTCATGACTGACCGT
GTTGACCCAGAAATTATTGAGAATTTAAAAGTATCTCAAGGAAGCACAGATTCAAGTCAC
GTAAAATATGTTACTGAGCTAATAAAGATAACAAATGATCGACAAGAAACATTTGAACTG
ACAAAACGACTTAAAGATCATATTTTATGGGCAAGAGATGAAGCTTTAAAAGAAGATGAA
AAGTTGAAGAAAGAAAATAAAGAATATGAATATCTTAATTTACCTCCAGAAGAATCAAAA
TTTTTCACATTAATCAATAATGATCTACTACGCGAAAATGGATTAATAGCCCCAGAAGAT
ATTTTCATGAACTTCAGAGAAGTGAACACCGAAAATCTTCATAAAAAGCATTCATTTTCA
GATTTAGATATTGATATACAAAATACTAATAGAAGAAACTATAGAATTCTTTCATCAAGC
AAGAAAGATTATCGGTTACCACCAATTGAAAAATCGTCTTCGAGTGAACAGTCAAAGAAA
AGAAAATCAGATAAGAAACGGAATCCAGTTGATATGATATTAGCTACAGAAAAAGATGAA
AACAAAATAGGACTAGAAATGATGGCTGCT
>DjScaffold602:29141-44159 strand:+
atttgttattattttaaattataaaatatcaccacaatgtgcttttataataagtataa
agataaatttacacgtacgccttttatatcatgtctgatgtatgataagattgtggaaa
agttgaaaaatatgaactgcggcctgcaatctcagaaagttttagcaatcctgatttaac
ttaaattttcgtattgcattttaaataattaataaattataattaattaatcatttaaaa
ttaaagttattttaactttcatgaattttctacttttctaatatttataaaaaaattgttt
tactttcaaagaattgtagaatgattattgataattttatatttacaacaattaatttga
ttaaataggttaaagcacaccatagtgtatttcatttggtgttacaaggaattgcagatt
ttaaacccgacctttcaaattcatcagcgttatgtaacgaaggagtaactaaacagaaac
catttattaaagcgactgatgaaggattaaccgattctcaatgttcttcatcatctccga
atatttttaaaATGGGCGGTGTTGTTTTAAATGATGTAACTAGTTTGGAAATCGAAGTCA
AAGGAATCAGTATTCCATTAAATGTTCGATTTAATACTCCATCAATAATTATACCAGGAG
AACTACTTATTAACACACCTGTTCGAAAACAATTACAAATTATTAATGAAAGCATGTGTC
CTGTTAGTTTAATTTGGGATAAACCAAAATCAGAAGACTACATTTTTGAAGTTGAACCTA
AATTGGGCATGATTAACACAAAAGAAATTGGCGTAGTCGATGTTTGTATCGTGGGAAATA
AACCTGGCTTTTATAGAGATACTTTGATTTGCCATGTTGAATGTTTGGATAATCCACTAG
AACTGATTGTTGAAGCTACTTTTAAAGGACCAGAACTCGAAATTGATAAAACAAACTTAG
ATTTTGGATTAGTGAAAGTGAATGATACTGTTAAGAAATCATTAACATTATATAATAAAA
GCTCTATTAACTGTTCGTGGAGATTAAGAATCGGTGGTACAGCAGACATTGCATTATTTG
ATGAAATAAATATTCATCCTAAAAACGGAATTATAGCTCCACTTCAGACTCAAAATGTTC
TTGTAAAGTTTACACCCAAGGAATCAAAATTTGTTAATTGTAGCATTGAAATCGGTTTAG
AAAACGGTTCCGCAAAATTTATGAATGTTCTGGCGTGTGTTCAAAGTCCCAGAGTAGTAT
TGAATTCAACAAATTTAAATGTGACCGGAATATTATTTGTTAATACTCCATCGGTATTCA
TCGTTACTTTATGCAACGAAAGTCATTTGGCTTCCAATTTTATTTGGAACTCTCCTGTTG
GCAAAGATGCAGATTTCATTTCAATTTTTACGGATCACAATTCGGGTATCATCGAACCAA
AAGGAAAGATTGATATTAAATTAACGATAACCTTTCAGTTACAGATGGAAATTACTGATT
TAATTTTACCATGTGAGATTGAAGGAATGGATGAACCAATTGGCCTTAGCATATTTGGTT
CTGTATATGGGCCTCAATTAACGTATTCTGTGTTTGATAATATCGATTCTGCGATAAATG
AATTAGATGAAACTAATCTTCAAATTCTTAATTTTGGAAATGAAGTTCCAATTTTTCAAA
ATATGAAAAGGTATTTATTGATTGAAAATCGAACATCAATTACAACAAATTTTGAAATAT
CAATTGATTATTTCAAAACATCTTTGAGTAACAATGATGCTGAAAAATCAATTAAAAAAC
GCCCAAATCTAGCAGATAGACAAAGTAAACCAGAAAAATTGGCTAATTTCGGTATT
>DjScaffold602:50430-58293 strand:+
AATGAATATGAATATTGTTTGTTAAAAACAAAAGCATTTACTAGTTATGCTACATTGCGA
AATTTAAAGGATGTTGAAATAAAATTCTCTCTAAAAACTCGATCTCCATTCGAAATAGAT
AATGGAAAAAATAAAGATGAAAATGCAATTAAAATTGGAGAAACTATTAAAGTGAATTTA
AAATTCATCCTTGACAAAGGTCATTTATCATTACTTAAAGATGAAAACCTCTACAACCAC
AATGATTCTCACTGTTTAAAAATTGAAAATCAACTAATACCGAATGAATCTAAGAAAACA
GCTAAAATGATTCAATTTCGAGATGTTATTTCAGTGGTTTATAAAAATACGAAAATTGAT
CAATTAATTCCAATAAGAGCTCAAATATTTTTCCCAGAATTTATAGTTTCTAAATCGGCA
[truncated: 485,233 more chars]
